# Genome-Scale Reconstruction of *Escherichia coli*'s Transcriptional and Translational Machinery: A Knowledge Base, Its Mathematical Formulation, and Its Functional Characterization

**DOI:** 10.1371/journal.pcbi.1000312

**Published:** 2009-03-13

**Authors:** Ines Thiele, Neema Jamshidi, Ronan M. T. Fleming, Bernhard Ø. Palsson

**Affiliations:** 1 Ph.D. Program in Bioinformatics, University of California San Diego, La Jolla, California, United States of America; 2 Systems Biology Research Group, Bioengineering Department, University of California San Diego, La Jolla, California, United States of America; King's College London, United Kingdom

## Abstract

Metabolic network reconstructions represent valuable scaffolds for ‘-omics’ data integration and are used to computationally interrogate network properties. However, they do not explicitly account for the synthesis of macromolecules (i.e., proteins and RNA). Here, we present the first genome-scale, fine-grained reconstruction of *Escherichia coli*'s transcriptional and translational machinery, which produces 423 functional gene products in a sequence-specific manner and accounts for all necessary chemical transformations. Legacy data from over 500 publications and three databases were reviewed, and many pathways were considered, including stable RNA maturation and modification, protein complex formation, and iron–sulfur cluster biogenesis. This reconstruction represents the most comprehensive knowledge base for these important cellular functions in *E. coli* and is unique in its scope. Furthermore, it was converted into a mathematical model and used to: (1) quantitatively integrate gene expression data as reaction constraints and (2) compute functional network states, which were compared to reported experimental data. For example, the model predicted accurately the ribosome production, without any parameterization. Also, *in silico* rRNA operon deletion suggested that a high RNA polymerase density on the remaining rRNA operons is needed to reproduce the reported experimental ribosome numbers. Moreover, functional protein modules were determined, and many were found to contain gene products from multiple subsystems, highlighting the functional interaction of these proteins. This genome-scale reconstruction of *E. coli*'s transcriptional and translational machinery presents a milestone in systems biology because it will enable quantitative integration of ‘-omics’ datasets and thus the study of the mechanistic principles underlying the genotype–phenotype relationship.

## Author Summary

Systems biology aims to understand the interactions of cellular components in a systemic manner. Mathematical modeling is critical to the integration and analysis of these components on a conceptual as well as mechanistic level. To date, detailed genome-scale reconstructions of metabolism have become available for a growing number of organisms. Although metabolism has an important role in cells, other cellular functions need to be considered as well, such as signaling, regulation, and macromolecular synthesis. For instance, the cellular machinery required for RNA and protein synthesis consists of a complex set of proteins. Here, we show that one can collect all of the necessary information for a prokaryotic organism to create a gene-specific, fine-grained representation of the macromolecular synthesis machinery. *E. coli* was chosen as a model organism because of the wealth of available information. The explicit representation of transcription and translation in terms of a mass-balanced network enables a detailed, quantitative accounting of the protein synthesis capabilities of *E. coli in silico*. Hence, this study demonstrates the feasibility of constructing very large networks and also represents a critical step toward building cellular models of growth that can account for gene-specific protein production in a stoichiometric fashion on the genome scale.

## Introduction

High-throughput experimental technologies enable the production of heterogeneous data, such as expression profiles and proteomic data, for almost any organism of interest. A detailed mathematical representation of the *in vivo* cellular network is required to obtain a holistic understanding of cellular processes from these data sets and to quantitatively integrate them into a biological context. One such approach is the bottom-up network reconstruction, which builds manually networks in a brick-by-brick manner using genome annotation and component-specific information (e.g., biochemical characterization of enzymes) [1],[2]. This reconstruction procedure is well established for metabolic reaction networks and has been applied to many organisms, including Human [3], *Saccharomyces cerevisiae* [4],[5], *Leishmani major* [6], *Escherichia coli* [7], *Helicobacter pylori* [8], *Pseudomonas aeruginosa* [9], and *Pseudomonas putida* [10],[11] (see http://systemsbiology.ucsd.edu/ for an continually updated table of metabolic reconstructions).

These bottom-up metabolic networks differ from other network reconstructions as they are tailored to the genomic content of the target organism and built manually using biochemical, physiological, and other experimental information in addition to the genome annotation. Hence, these reconstructions can be thought of as biochemically, genetically, and genomically structured (BiGG) knowledge bases [12]. The reconstruction and modeling procedure is a 4-step process: 1) obtaining a draft reaction list based on genome annotation and biochemical databases, 2) refinement of reaction list using experimental information (e.g., from literature), 3) conversion of the reaction list (reconstruction) into a computable format and application of systems boundaries to define condition-specific models, and 4) the evaluation and validation of the model content using various mathematical methods (see also [1],[2],[12],[13]). By iterating step 2 to 4, reconstructions that are self-consistent within their defined scope can be generated.

**Figure 1:**
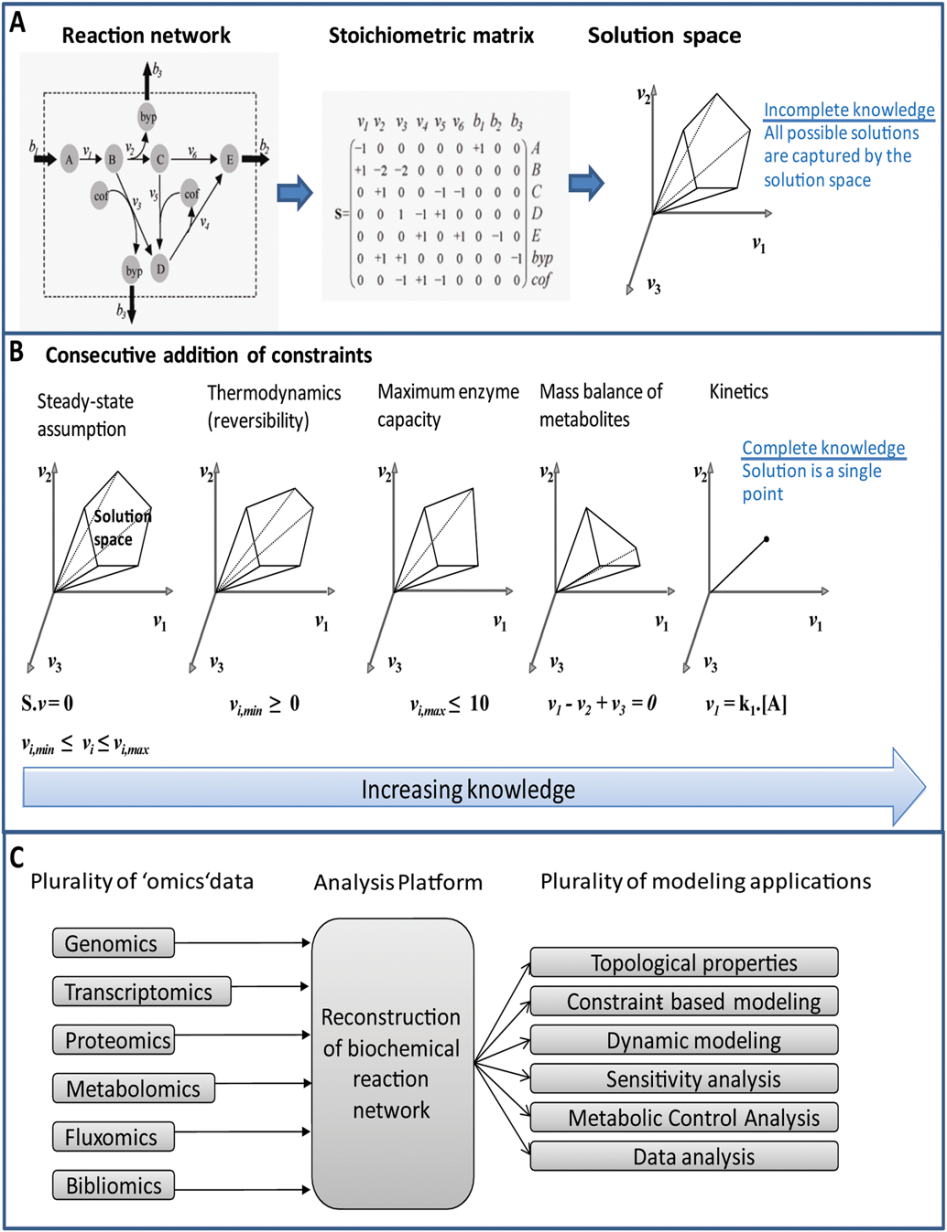
Overview of constraint-based reconstruction and analysis. (A) Schematic illustration of the conversion of a biochemical reaction network into a mathematical format (stoichiometric matrix, S). Since there are normally less columns (reactions) than rows (metabolites) there does not exist a single solution but rather a steady-state solution space containing all possible solutions. (B) The successive addition of constraints will shrink the solution space by eliminating biologically infeasible steady-state solutions. Complete knowledge would reduce the steady-state solution space to a single solution. Since complete knowledge is not available for the majority of biochemical reaction networks the investigation of properties and capabilities of the solution space is very useful. (C) This graphic illustrate the central role of reconstruction of biochemical networks to systems biology and how they serve as a foundation for many applications and problem-specific models.

Metabolic network reconstruction have demonstrated to be useful in at least 5 areas of applications [2]: (i) biological discovery [14], (ii) phenotypic behavior [15], (iii) bacterial evolution [16], (iv) network analysis [17], and (v) metabolic engineering [18]. This wide range of applications of the metabolic reconstructions is possible because they can be readily converted into predictive, condition-specific models. Unlike more traditional approaches to modeling metabolism, the constraint-based modeling approach (COBRA) requires few, if any, parameters [12],[19]. The stoichiometric information encoded in the reconstruction (i.e., reaction list) can be represented mathematically as a stoichiometric matrix, S, where the rows correspond to the components and the columns correspond to the reactions (Figure 1).

While the COBRA approach has been successfully applied to metabolic networks, the same principles and assumptions can be also employed to reconstruct and model other cellular functions, such as signaling [20]–[22], regulation [23], and protein synthesis [24]. In this study, we extended and refined earlier work by Allen *et al.*, which proposed a stoichiometric formalism to model protein synthesis and illustrated it on some *E. coli* genes and operons [24]. We created a more detailed, gene-specific representation of the transcriptional and translational processes, which explicitly accounts for the sequence-specific synthesis of DNA, mRNA, and proteins. This reconstruction enables quantitative integration of high-throughput data such as gene expression, proteomic, and mRNA degradation data. Moreover, proteins are produced in high copy numbers in growing cells; thus, any quantitative mechanistic modeling and analysis of high-throughput data needs to account for the synthesis cost associated with these molecules.

Numerous studies have been published that investigate protein synthesis using kinetic models [25]–[29]. These models are generally tailored to the questions they address making it difficult to readily apply them for modified problems. Since stoichiometric relationships are a common requisite for any type of mechanistic modeling, organism-specific BiGG knowledge bases can be used as templates to derive problem-specific, mechanistic models (Figure 1). In fact, network stoichiometry is a dominant feature of kinetic models as well [30]. Thus, network reconstruction serves as a platform for steady-state and kinetic modeling (Figure 1).

**Figure 2:**
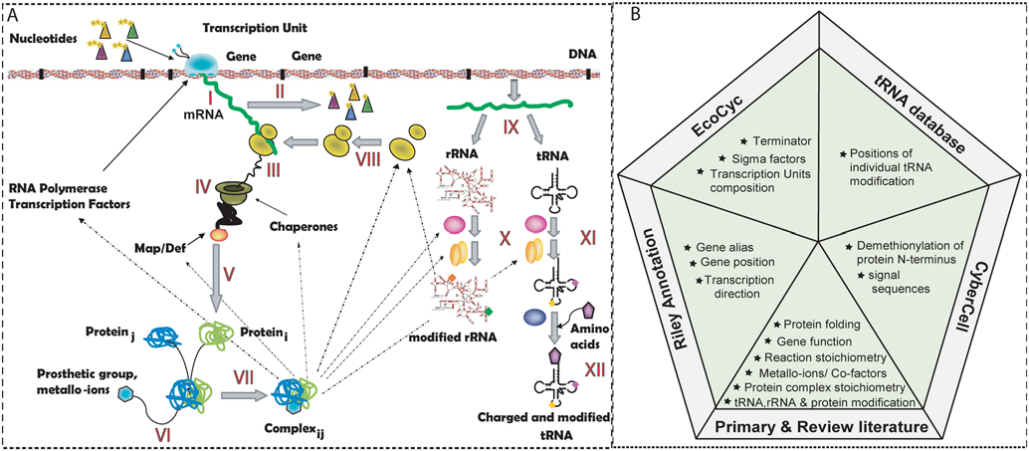
Content of the ‘E-matrix’. (A) Schematic representation of the network components and reactions is shown. In addition to the macromolecular synthesis of RNA and proteins, rRNA and tRNA processing reactions were included in the reconstruction. I: Transcription; II: mRNA degradation; III: translation; IV: protein maturation; V: protein folding; VI: metallo-ion binding; VII: protein complex formation; VIII: ribosome assembly; IX: RNA processing; X: rRNA modification; XI: tRNA modification; XII: tRNA charging (see Table 1 for complete list of subsystems and Figure S1 for a complete protein map). (B) The pentagram shows the five main data sources incorporated in the ‘E-matrix’: EcoCyc [36], CyberCell [70], and tRNA DB [71], the revised genome annotation [32], and the genome sequence (m56, [65]).

In this study, we present a new generation of network reconstructions, which directly account for the synthesis of individual mRNA and proteins (Figure 2A). We named the mathematical representation of this reconstruction the Expression matrix, or ‘E-matrix’, since it encodes the expression of mRNA and proteins. All network reactions were formulated to account for gene-specific and *E. coli*-specific details, such as nucleotide composition, operon association, and sigma factor usage. Furthermore, we used information from three databases and more than 500 scientific publications to formulate mechanistically detailed and accurate reactions. This reconstruction is the first comprehensive database detailing the available information for these cellular functions and can thus be deemed a knowledge base. After conversion of the ‘E-matrix’ reconstruction into condition-specific models corresponding to different doubling times, we were able to accurately predict the ribosome production reported in literature, without any parameterization. Furthermore, we show that the ‘E-matrix’ can be used to study the effect of rRNA operon deletion. Our results predict that a high density of RNA polymerases is required on the remaining rRNA operons, to achieve the reported ribosome numbers. Finally, we show that proteins used in the ‘E-matrix’ could be grouped into functional modules which lead to a more simplified view of the network.

## Results/Discussion

The ‘central dogma’ of molecular biology was first enunciated by Crick in 1958 and dealt with the transfer of sequential information from DNA to RNA to proteins [31]. The machinery necessary to conduct this information transfer was reconstructed in this study on a genome-scale, i.e., all known components in *E. coli* were considered. The ‘E-matrix’ encodes for all known reactions, which synthesize the components of the macromolecular synthesis machinery, in a mechanistically detailed fashion.

### Reconstruction of the Networks and Formulation of the ‘E-Matrix’

#### Legacy data

The ‘E-matrix’ reconstruction was based on *E. coli*-specific information derived from more than 500 primary and review publications, three databases, and the revised genome annotation [32] (Figure 2B). This detailed information enabled the sequence-specific formulation of synthesis reactions, at high resolution, for every network component, namely DNA, mRNA, proteins, protein complexes, and metabolites. The reconstructed network accurately represents all known reactions required to produce the active, functional components of the transcriptional and translational machinery in *E. coli* (Figure 2A).

#### Reconstruction approach

The manual reconstruction of the ‘E-matrix’ was performed in an algorithmic manner by first identifying key components in the genome annotation (Tables S1, S15, S16, and S17). The functional roles of these key components were determined and then translated into stoichiometrically accurate reactions using multiple data sources (Figure 2B). A total of 303 components (proteins and RNA) were found to be directly involved in one or more subsystems, which represent groups of functionally related transformation pathways (Table 1 and Tables S2, S4, and S10). In this reconstruction linear transformation steps, e.g., elongation of nascent mRNA during transcription, were combined into a single reaction, while key reactions and known rate limiting steps were kept as separate reactions, e.g., transcription initiation and elongation. This representation captures key events in cellular processes and can be directly used to understand their reaction mechanisms at a high resolution.

**Table 1:** Reactions per subsystems.

Number	Subsystem	Reactions
I	Transcription	783
II	mRNA degradation	628
III	Translation	6,812
IV	Protein maturation	628
IX	RNA processing	122
V	Protein folding	570
VI	Metallo-ion binding	128
VII	Protein complex formation	87
VIII	Ribosomal assembly	13
X	rRNA modification	864
XI	tRNA modification	1,597
XII	tRNA charging	177
XIII	Aminoacyl-tRNA synthetase charging	33
XIV	Charging EF-Tu	4
XV	Cleavage polycistronic mRNA	222
XVI	Demands	302
XVII	Exchange reactions	76
XVIII	Iron–sulfur cluster biosynthesis	6
XIX	Iron–sulfur cluster incorporation	6
XX	Protein modification	12
XXI	Protein recycling	148
XXII	Ribosomal protein modification	21
XXIII	rRNA formation	38
XXIV	Sinks	35
XXV	Transcription regulation	261
XXVI	Transport	76
XXVII	tRNA activation (EF-TU)	45
	Total number of reactions	13,694

The numbers I to XII correspond to the numbering shown in Figure 2A.

A comprehensive, iterative quality control/quality assurance (QC/QA) procedure ensured that the resulting network had similar properties and capabilities as *E. coli*. This QC/QA procedure included gap analysis, testing for the production of every network component, and mass- and charge-balancing of more than 99% of the network reactions (Tables S7 and S8). Hence, the ‘E-matrix’ reconstruction follows the quality control standards developed for metabolic network reconstructions [1].

#### Unique properties of the ‘E-matrix’

This reconstruction is unique in the depth and breadth of information included as well as an advancement of other transcriptional and translational networks currently available [25]–[29]. It is also the largest reconstructed network to date, with 11,991 components and 13,694 reactions (Table 2 and Tables S12 and S13). The ‘E-matrix’ accounts for all known gene products necessary to produce the active components of the machinery itself, and is therefore self-contained. Furthermore, sequence-dependent synthesis reactions were carefully formulated to incorporate known reaction stoichiometry including protein-substrate complex intermediates, metallo-ions and cofactors. Two recently published large-scale datasets [33],[34] were used for the assigning the folding pathway to the individual polypeptides (Tables S5 and S6). Necessary modifications of stable RNA and proteins were also considered (Tables S16 and S17). Additionally, the transcription reactions were formulated in terms of transcription units rather than genes (Table S9), providing a biologically accurate representation of operon organization in bacterial genomes. These reactions can be readily extended to account for the production of other gene products such as metabolic enzymes or transcription factors. Lastly, this framework facilitates future integration of the ‘E-matrix’ reconstruction with the metabolic and regulatory network of *E. coli*.

**Table 2:** Overview of the ‘E-matrix’ content.

Number of transcription units	249
Number of genes (involved*)	423 (303)
Number of genes with/without transcription unit	411/12
Number of components (with/without genes)	337 (303/34)
tRNA	86
rRNA	22
miscellaneous RNA	1
involved* proteins (with/without genes)	228 (194/34)
Number of subsystems	27
Number of reactions	13,694
Number of demand reactions	302
Number of exchange reactions	76
Number of network components	11,991
Number of references	+500

*: involved refers to those gene products that are functionally involved in ‘E-matrix’ processes compared to genes that were included because of co-transcription with involved genes.

#### ‘E-matrix’ versus available databases

The ‘E-matrix’ is distinguished from available online databases, such as KEGG [35] and EcoCyc [36], as all transcriptional, translational, and modification reactions were defined in a sequence dependent manner for every included *E. coli* gene. This task was achieved by determining the nucleotide and amino acid composition of each DNA, RNA and protein from the genome sequence, respectively. Furthermore, we determined the elemental composition of these macromolecules and mass balanced all network reactions. In contrast, KEGG [35] and EcoCyc [36] list mainly generic reactions using gene- and organism independent terms such as ‘DNA’, ‘protein’, and ‘RNA’. Subsequently, they contain only a subset of the synthesis reactions present in the ‘E-matrix’. Furthermore, neither of these databases can be directly converted into a comprehensive, self-consistent mathematical format that permits rigorous computational characterization of network fluxes. Another difference between the ‘E-matrix’ and these databases is the extent of mechanistic detail incorporated into the ‘E-matrix’, such as rRNA and tRNA modification reactions, iron–sulfur cluster formation, chaperone-dependent protein folding and protein complex formation.

#### Knowledge gaps

The transcriptional and translational machinery is essential for cellular growth. Considering the wealth of information available for *E. coli*, it was surprising to discover numerous knowledge gaps, or missing information, during the reconstruction process. For example, reaction mechanisms for some RNA modifications and iron–sulfur cluster biogenesis were either poorly understood or a general consensus on the mechanistic details was lacking. For instance, 15% of the included proteins had no gene annotation and their existence was suggested in the literature solely based on identification of modified proteins or stable RNA (Table S3). Furthermore, there are three metabolites with unknown metabolic transformations. One of these metabolites is preQ_0_, a precursor of preQ_1_, which is important for the queuosine formation in some tRNA (position G34). This precursor is formed from GTP and it has been suggested that two ribose units of two GTP molecules contribute to the formation of three carbons in preQ_0_ (C_5_,C_6_, and cyano carbon) but further information is missing [37],[38]. The two other missing metabolites are byproducts of the formation of uridine-5-oxyacetic-acid at position U34 in some tRNA. It has been suggested that chorismate acts as precursor for this nucleotide modification, however, such reaction would release two metabolites with formulae of C_10_H_8_O_5_ and C_9_H_9_O_4_, which have not been characterized yet [37],[38]. All of the knowledge gaps were highlighted in the reconstruction and associated with notes about currently available information (Tables S15, S16, and S17), which will hopefully promote their elucidation as it has been the case for some of the metabolic knowledge gaps in *E. coli* [14].

#### Network topology

The ‘E-matrix’ has a relatively ‘linear structure’ with only few components participating in multiple reactions since a majority of network components are only transferred from one reaction to another (Text S1, Figure D). This linearity is a dominant feature of the ‘E-matrix’ and it is less evident in metabolic reconstructions due to their much higher connectivity. Analysis of the component connectivity of the ‘E-matrix’ showed that the highest connected components are protons, water, and orthophosphate, which participate in 44%, 39%, and 32% of reactions, respectively. These compounds are also found to have the highest connectivity in metabolic networks [39]. In contrast to metabolic networks, ATP and ADP were not the next most highly connected but rather GTP and GDP, which participated in the numerous translational reactions. While the ATP requirement for cellular functions is accounted for in the biomass reaction of metabolic reconstructions, the high GTP requirement is not generally considered [7].

### Determining Network Capabilities

The conversion of a network reconstruction into a mathematical model can be achieved, analogously to metabolic networks [1], by defining system boundaries and applying condition-dependent constraints on exchange and intracellular reactions (Figure 1) [1],[40]. Therefore, experimental data can be used to constrain the set of feasible network fluxes in a physiologically relevant manner. In the following section, we will illustrate the use of condition-specific models that were derived from the ‘E-matrix’ reconstruction.

#### Validation of the ‘E-matrix’ functionality—ribosome production

**Figure 3:**
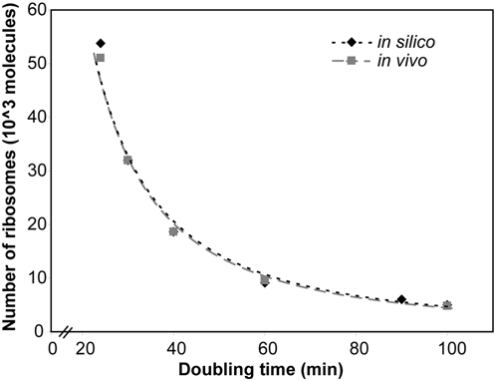
Comparison of *in vivo* [42] and *in silico* maximal number of ribosomes at different doubling times. Two sets of constraints were applied to the models: uptake rates for amino acids and NTPs, and maximal possible rates on stable RNA transcription initiation (see text for more details).

**Figure 4:**
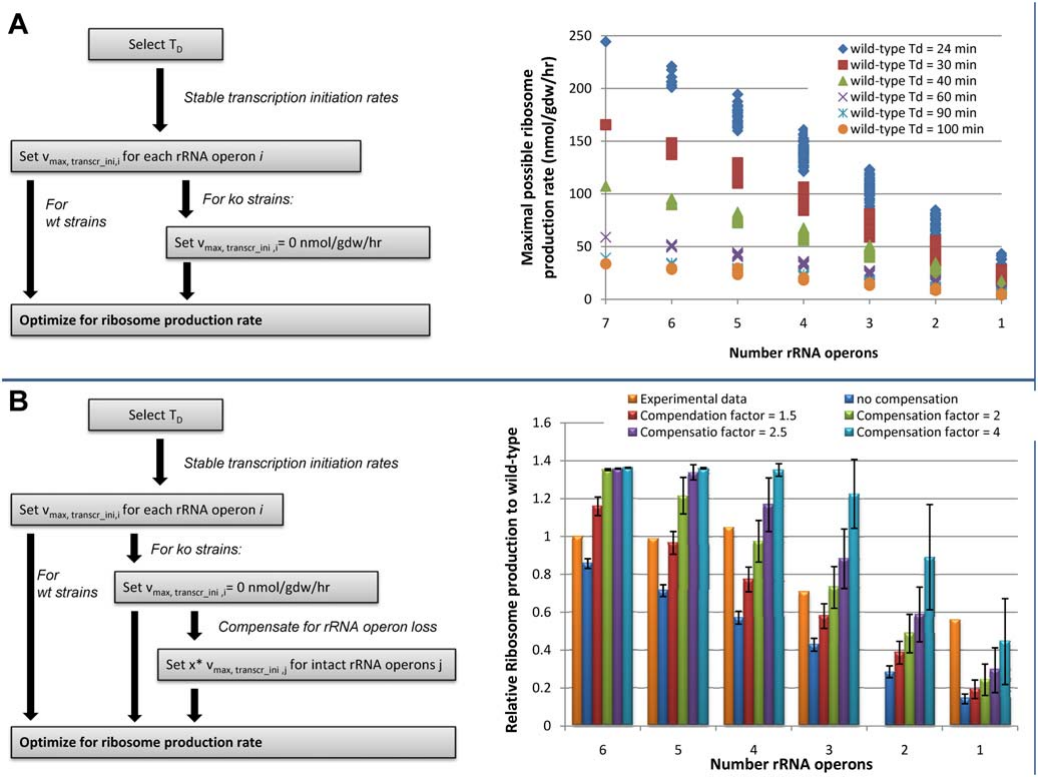
rRNA operon deletion study. (A) Analysis of the effect of rRNA operon deletion to the ribosome production capability of the network. As expected, the ribosome production rate decreased with decreasing number of available rRNA operons. All possible combinations of operon deletions were considered resulting in different maximal possible ribosome production rates for a given number of remaining rRNA operons. This is due the gene dosage effect since multiple replication forks are present at higher growth rates. (B) Experimental data (orange bars, [46],[48]) suggested much higher ribosome production than we determined in (A). This compensation is achieved by increasing the transcription rate of the remaining rRNA operon. We tested different possible compensation factors and compared the results with the experimental data. The error bars are again caused by different combination of rRNA operons.

Cell growth is directly correlated with the protein synthesis capacity and thus with the number of active ribosomes [41]. Accordingly, we used the model's ribosome production capability as an indicator of its ability to support growth. For every growth rate, the uptake rates for NTP and amino acids as well as the transcription initiation rates of the rRNA operons were quantitatively constrained based on experimental data [42]. The *in silico* computed ribosome production capabilities showed very good agreement with the reported *in vivo* ribosome production capabilities [42] for all investigated doubling times (Figure 3), indicating that the capabilities of the reconstruction were very similar to those of an *E. coli* cell. This overlap between experimental data and predictions was somewhat expected as the constraints used, i.e., stable RNA transcription initiation rates as upper constraints for the rRNA operons (see Material & Methods), were dominant (governing) constraints. Thus, these results validated the predictive capability of the reconstructed network. Moreover, our results show that: (i) the network is capable of reproducing experimentally reported ribosome number given the uptake constraints, and (ii) an increase in transcription initiation rate would lead to an increase of ribosome production (see also Figure 4B). This latter result implies that the regulation of rRNA synthesis, which is outside the scope of this reconstruction, plays a significant role in determining the transcription rate [43],[44].

#### The effect of *in silico* rRNA operon deletions on ribosome production

The *E. coli* genome contains seven rRNA operons, which have similar structures (16S rRNA, tRNA, 23S rRNA, tRNA, 5S rRNA, and, in some cases, tRNA). Generally, it is assumed that rRNA operon redundancy in *E. coli* and other species, has evolved to provide high levels of ribosomes and thus to support rapid growth rates [45]. However, there is experimental evidence that rRNA operon multiplicity is rather required for rapid adaptation to changes in physiological conditions [46],[47]. In fact, it has been shown that the presence of only one rRNA operon on the chromosome is sufficient for synthesis of 56% of the wild-type rRNA concentration [48] and the deletion of multiple rRNA operons had only small effect on growth rate and ribosome content [46],[48],[49]. Subsequently, it was experimentally observed that the remaining rRNA operons were able to compensate for the loss by increasing the transcriptional rate [46].

Since the early days of the development and application of COBRA methods, *in silico* gene deletion analysis has been productively used to evaluate the consequences of gene deletions to metabolism and cellular growth [8], [50]–[52]. Here, we used the same approach to evaluate the consequences of rRNA operon multiplicity to the ribosome production capabilities of the ‘E-matrix’ by *in silico* operon deletion analysis. First, we set the stable RNA transcription initiation rates based on doubling time as reported in Neidhardt *et al.* [42], and optimized for ribosome production using linear programming. Subsequently, we created single and multiple *in silico* knockout mutants by deleting the rRNA operons and optimized again for ribosome production (Figure 4). Since the maximal possible rRNA transcription rates were set to the reported rates, we observed a linear decrease in ribosome production for all tested doubling times (Figure 4). This result was expected as the stable RNA transcription initiation rates were found to be the governing constraints (see above). Therefore, this simulation setup did not allow for the compensation of rRNA operon loss.

To simulate this compensation, we multiplied the transcription initiation rate of each rRNA operon with various scaling factors and re-computed the maximal possible ribosome production rate (see Figure 4 and Materials and Methods). Comparison with experimental data [46],[48] showed that similar compensation could be obtained *in silico* by using a transcriptional compensation factor. The compensation factor had to be increased *in silico* when multiple rRNA operons were deleted. To compare the calculated compensation factor with experimental data, we converted the measured number of RNA polymerases (RNAP) per operon in rRNA operon deficient strains [46] into compensation factors by diving them with the reported RNAP binding frequency in the wild-type [53]. These experimental compensation factors in good agreement with our *in silico* results (data not shown). Surprisingly, it was found experimentally that strains with only one intact rRNA operon can still produce 56% of wild-type rRNA [48]. This situation would correspond to an *in silico* compensation factor of 4 and thus, to approx. 150 RNAP bound to the remaining rRNA operon. Since the average length of an rRNA operon is 5100 nucleotides, this high number of bound RNAP corresponds to a RNAP every 34 nucleotides. Such an increase in RNAP density on the operon could be achieved by increasing the transcription elongation rate and/or modulating the frequency of RNAP binding to the promoter [46]. It is not known which regulatory elements could lead to such an increase in rRNA transcription; however, Condon *et al.* found the ppGpp concentration, responsible for the stringent response under amino acid starvation, unaltered [46]. Gaal *et al.* showed that rRNA synthesis is regulated by NTPs, which stabilize the open complex of RNAP and P1 promoter of an rRNA operon. The formation of the open complex is necessary for successful transcription initiation [54]. Feedback inhibition is also controlling the rRNA synthesis, where an excess of ribosomes might regulate the transcriptional rate [43]. In agreement with our predictions, experimental data have shown an increase in ribosomal content for some rRNA deficient strains (Figure 4) [46]. Furthermore, different rRNA operon knockout combinations resulted in large differences in compensation due to different gene dosage depending on the positions of the various operons on the chromosome (Figure 4 and Table 3). We did not determine the growth rates of the knockout strains as such calculation would require to assume the same correlation between doubling time and ribosome production as is present in wild-type *E. coli* (Figure 2). Our results suggest that the transcriptional initiation rate, and thus ribosome production rate, will be limited by competition for precursors, especially NTPs (data not shown). This agrees with the experimental observation that an increase in rRNA operon number will reduce the overall transcription initiation rate and thus maintain a constant rRNA content in the cell [55]. However, many complex regulatory mechanisms, which are outside the scope of the current model, are known to control ribosome production [43],[54]. The incorporation of regulation with the current model should lend further insight into the nature of rRNA operon multiplicity.

**Table 3:** List of rRNA transcription units and their basic characteristics.

Transcription Unit^a^ (Promoter)	Gene Names	Gene Alias	Strand	Coordinates (in Base Pairs)	Genes/Cell at T_D_ = 30 min	Genes/Cell at T_D_ = 90 min	Genes/Cell at T_D_ = 100 min	Genes/Cell at T_D_ = 60 min	Genes/Cell at T_D_ = 40 min	Genes/Cell at T_D_ = 24 min
TU0-1181 (P1)	b3851–b3855	rrsA-ileT-alaT-rrlA-rrF	Forward	4,033,554–4,038,659	4.49	2.07	1.92	2.37	3.24	6.17
TU0-1182 (P1)	b3968–b3971	rrsB-gltT-rrlB-rrfB	Forward	4,164,682–4,169,779	4.24	2.01	1.87	2.29	3.10	5.77
TU0-1186 (P1)	b4007–b4010	rrsE-gltV-rrlE-rrfE	Forward	4,206,170–4,211,182	4.17	1.99	1.85	2.27	3.06	5.64
TU0-1189 (P1); TU0-1190 (P2)	b0201–b0205	rrsH-ileV-alaV-rrlH-rrfH	Forward	223,771–228,875	3.15	1.72	1.62	1.93	2.45	4.00
TU0-1187 (P1); TU0-1188 (P2)	b2588–b2591	rrsG-gltW-rrlG-rrfG	Complement	2,727,638–2,724,210	2.81	1.62	1.54	1.80	2.25	3.49
TU0-1191 (P1); TU0-1192 (P2)	b3272–b3278	rrsD-ileU-alaU-rrlD-rrfD-thrV-rrfF	Complement	3,425,243–3,421,564	3.79	1.90	1.77	2.15	2.84	5.02
TU0-1183 (P1); TU0-1184 (P2)	b3756–b3759	rrsC-gltU-rrlC-rrfC	Forward	3,939,831–3,944,842	4.67	2.12	1.95	2.42	3.35	6.48

This information was obtained from the most recent genome annotation [32].

a Transcription unit names are listed as given by EcoCyc [36]. The gene number per cell (gene dosage) was calculated as described in Text S1.

#### Integration of ‘-omics’ data into ‘E-matrix’

**Figure 5:**
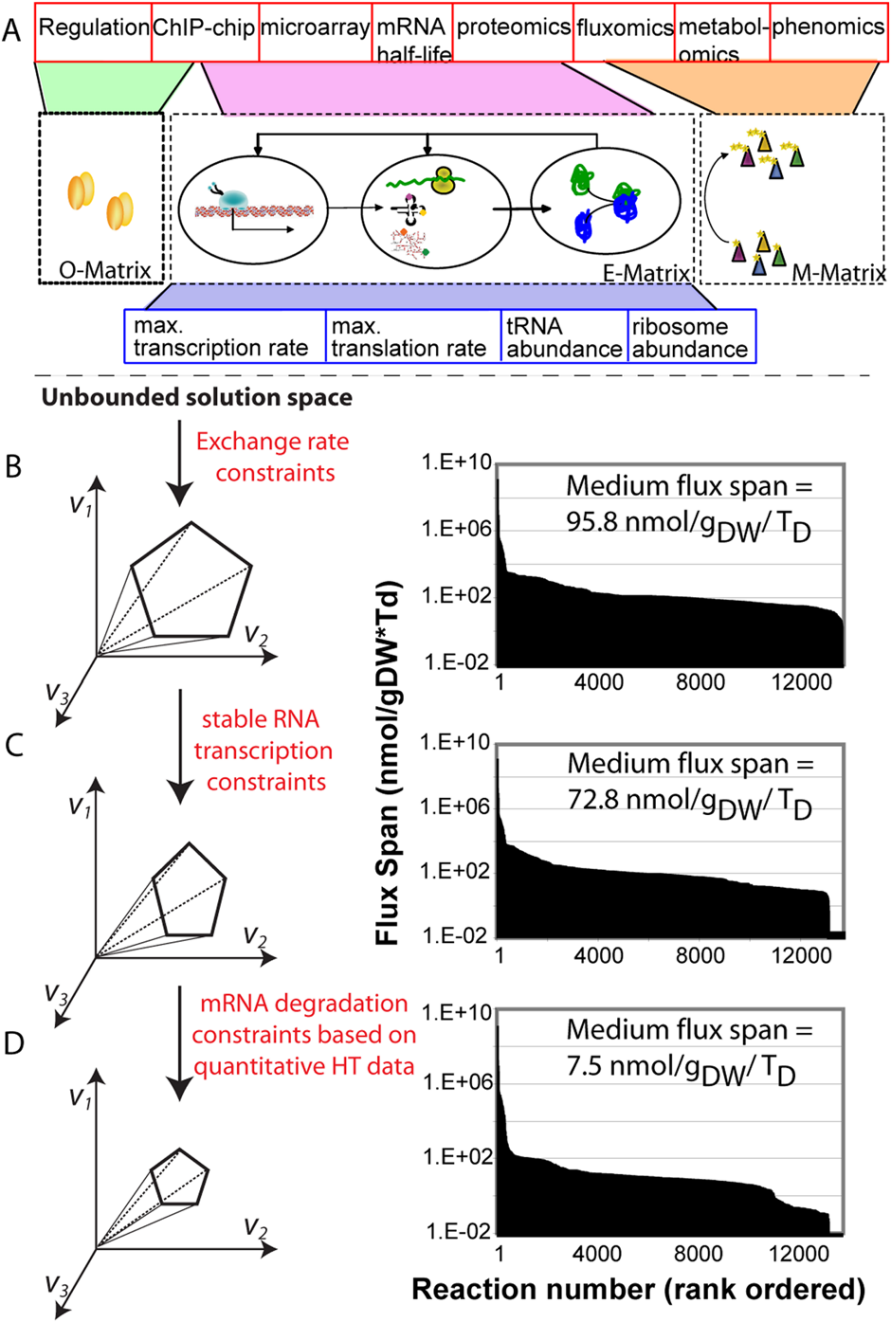
Integration of ‘-omics’ data into ‘E-matrix’ as reaction constraints. (A) This schema illustrates the types of high-throughput data (HT, red boxes) or low-throughput data (LT, blue boxes) that can be directly integrated with the ‘E-matrix’ as it accounts for the different macromolecules measured in these data sets. In contrast, the integration of regulatory information would require the formulation of the regulatory network in matrix format (‘Operon’ or ‘O’-matrix). Furthermore, the metabolic network, here represented as ‘M-matrix’, would enable the mapping of fluxomic, metabolomic and phenomic data. (B–D) Absolute flux span in ‘E-matrix’ while incorporating successively more complex constraints (see text for more details). (B) LB-medium specific constraints were applied on exchange reactions. (C) The upper bounds of stable RNA transcription initiation reactions were constrained. (D) Additional constraints on upper bound of mRNA degradation flux rates were applied.

An overall aim of this reconstruction effort was to create a stoichiometric representation of mRNA and protein synthesis machinery that allows the integration with experimental data. Interrogation of the data-constraint model would allow the investigation of the remaining network capabilities (Figure 5A). Here, we incorporated successively experimental data sets into the model as constraints, and investigated the resulting network capabilities. More specifically, we used the difference between minimal and maximal flux rate for each reaction (flux span) as a measure of constraint stringency.

We successively integrated three different datasets (Figure 5):

- First, we constrained the upper bounds of exchange reactions in the ‘E-matrix’ to uptake rates corresponding to LB-medium conditions (Figure 5B). This set of constraints was not sufficient to eliminate biologically irrelevant solutions since, for instance, the model was able to produce up to 45,000 ribosomes while approximately 30,000 ribosomes were observed experimentally [42].
- Second, further constraints were applied on the stable RNA transcription initiation rates based on low-throughput data [42] to exclude physiologically infeasible stable RNA transcription rates (Figure 5C). However, the maximal flux rates for synthesis reactions of most network mRNAs were still found to be too high when compared to expression data [56].
- Finally, we used high-throughput data, namely gene expression data from LB medium [56] and mRNA half life times [56], to further constrain the network. Numerical values for mRNA degradation rate, specific to each sequence of mRNA, were calculated based on these two data sets and applied as upper bounds on the mRNA degradation reactions in the network. This last set of constraints had a significant effect on the overall flux span, which highlights the importance of mRNA transcription constraints on the set of feasible solutions (Figure 5D).

A qualitative evaluation of mRNA expression in Boolean terms (on/off)—as used in metabolic modeling [52]—did not result in significant reduction of the size of the solution space (data not shown). Despite the mRNA degradation reaction constraints, many protein synthesis reactions still achieved high flux values. This result is consistent with the fact that low numbers of transcripts can be sufficient to synthesize high numbers of proteins and hence, the translation reactions can carry large flux rates. Thus, the application of quantitatively accurate proteomic data could greatly help to further constrain the set of feasible steady-state solutions.

#### Defining functional modules

**Figure 6:**
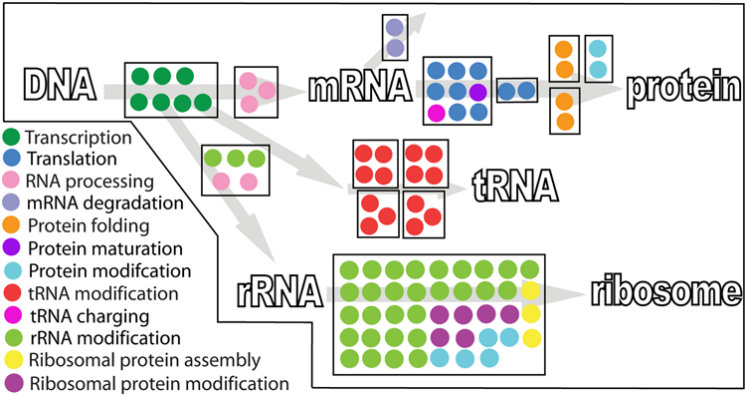
Schematic representation is shown of the calculated functional modules, the associated proteins and their canonical assignments. Functional modules that consist of one protein are not shown.

Correlated reaction sets (co-sets) have been calculated for metabolic networks to obtain insight into the network structure and properties [15],[57]. Here, we applied the same concept to the ‘E-matrix’ to identify functional coupling between proteins. In the reconstruction, every protein is associated with a recycling reaction representing its overall utilization rate in the cell. It can be expected that proteins whose utilization rates are perfectly correlated based on stoichiometry would show similar pattern of protein expression, but not necessarily of gene expression, under different environmental conditions. A total of 14 multi-protein modules (or co-sets) were identified accounting for 91 out of 153 proteins or protein complexes (Table S14). Interestingly, many modules contained proteins from different subsystems, which were assigned based on classical pathway designation (Figure 6). Hence, our calculations suggest that some canonical pathway assignments may not necessarily represent the functional relationships between the proteins in the cell (Figure 6). Furthermore, no direct correlation between the calculated functional modules and protein-protein interaction data [58],[59] could be observed (data not shown). In contrast, stoichiometrically coupled changes of translation initiation factor 1 (IF-1) and ribosomes [60] observed experimentally, suggest that our calculated functional modules are biologically relevant. As more accurate quantitative proteomic data becomes available the functional modules reported herein should be useful in interpretation of this data and help resolve missing gene annotations.

#### Integration with other cellular functions

The scope of the ‘E-matrix’ was limited to the reactions required for synthesis of *E. coli*'s transcriptional and translational machinery, which can account for 50% of the dry weight in fast growing cells [53]. Subsequently, the synthesis and maintenance of this machinery places significant material and energy demands for biosynthetic precursors from metabolism. In the ‘E-matrix’, these precursors are provided via exchange reactions. As a next step, one could imagine replacing these exchange reaction with the stoichiometric matrix for the metabolic network of *E. coli* [7] (‘M-matrix’, Figure 5A). This integration would allow the direct assessment of the metabolic demand that the transcriptional and translational machinery imposes on a cell. Moreover, integration of the transcriptional regulation of individual operons would enable a more accurate determination of the genotype – phenotype relationship (‘O-matrix’, Figure 5A). Thus the genome-scale integrated network, or ‘OME-matrix’, would account for three major cellular processes and may capture more than 2,000 of *E. coli*'s gene. Recently, two studies proposed approaches to integrate different cellular processes [61],[62] but no genome-scale representation is available yet.

### Conclusion

In this study, we present the first, mechanistically and chemically detailed, genome-scale network reconstruction of the transcriptional and translational machinery of *E. coli*. Biochemical components, reaction formulation, and quality control measures analogous to metabolic network reconstructions were used to incorporate bibliomic data from the last 50 years into one reconstruction (Figure 2). The corresponding knowledge base can be queried online (http://bigg.ucsd.edu/E-matrix). This stoichiometric reconstruction represents a first step towards modeling this complex cellular function, and will require iterative refinement as new data becomes available. By describing the stoichiometric relationships between the components involved in transcription and translation, this reconstruction enables the quantitative integration of disparate ‘-omics’ data into a computational model (Figure 5). We demonstrated that low- and high-throughput data can be readily integrated and used as constraints on model reactions and the subsequent reduction of the feasible set of reaction fluxes results in physiological relevant predictions (Figure 5B–D). Furthermore, we showed that the computational model can be used to accurately predict ribosome production under different growth conditions (Figure 3). The deletion of single or multiple rRNA operons from the ‘E-matrix’ predicted that a high density of RNA polymerases is required on the remaining rRNA operons to achieve the reported ribosome numbers (Figure 4B). Computational analysis of the ‘E-matrix’ can provide further insight into the topologically local and global relationship between proteins in terms of functional modules (Figure 6).

This ‘E-matrix’ reconstruction ushers in a new generation of cellular network models that account quantitatively for mRNA and proteins. The ‘E-matrix’ offers the potential to (i) serve as a platform for integrated, numerical analysis of heterogeneous, quantitative high-throughput datasets; (ii) increase our understanding of the relationship between mRNA and protein abundance; (iii) be integrated with metabolism by extending the transcriptional and translational reactions to metabolic genes; (iv) be integrated with regulatory events by formulating regulatory rules for the genes of the ‘E-matrix’ and extending the transcriptional and translational reactions to transcription factors; and (v) enable computation of the material and energetic cost of macromolecular synthesis. These capabilities are important milestones in moving towards a more comprehensive genome-scale *in silico* model of all cellular processes in *E. coli*. Furthermore, the underlying reconstruction methodology can be readily extended and applied to other prokaryotes. Such extension could lead to further insight into conserved and unique features of the transcriptional and translational machinery of prokaryotes.

The history of *E. coli* metabolic reconstructions now spans more than 17 years, with numerous iterative reconstruction refinements and applications superseding initial expectations [63]. The reconstruction of transcriptional and translational machinery *E. coli*, and other prokaryotes, will have the same impact on systems biology, especially when integrated with metabolism, regulation, and condition-specific high-throughput data sets (Figure 5 A). This work represents hence a crucial step towards the important and ambitious goal of whole cell modeling [64].

## Materials and Methods

### Reconstruction Procedure

The reconstruction of the transcriptional and translational machinery of *E. coli* was approached by first identifying the main components from genome annotation [32], *E. coli* specific primary and review literature, as well as multiple databases (Figure 2B). For each of these components the gene ID (b-number), gene position, necessary metallo-ions and cofactors, and protein stoichiometry were extracted. The synthesis reactions for every network component were created using template reactions, which was possible since reaction mechanisms are similar for all network components (see Text S1 for examples). These template reactions were carefully formulated and derived from primary and review literature (Tables S15, S16, S17). The template-based network reconstruction was performed using the scripting language, Perl (http://www.perl.com/). Each template reaction as well as protein complex formation reactions were generated manually based on legacy data (Tables S15, S16, S17, and S18). Every network reaction was mass- and charged balances assuming a physiological pH of 7.2[1].

The basis for the reconstruction was the genome sequence, m56 [65], the most current gene coordinates [32], and the transcription unit definitions provided by EcoCyc (version 10.6, [36]). This information was also used to (i) calculate the formula and charge for each mRNA and protein species; (ii) individually adjust the template reactions for, e.g., NTP requirement; and (iii) transcribe operons rather than genes. A complete list of all transcription units can be found in Table S9. The genetic code used for this reconstruction is listed in Table S11. Network gap analysis was performed after the initial reaction list was obtained. Multiple iterations of content refinement and evaluation ensured completeness of the network within its scope by including missing components and reactions (Text S1, Figure A–c). One network gap remained, which is the RNase PH that is annotated as pseudogene in Riley *et al.* [32].

The systems boundaries of the ‘E-matrix’ were defined by adding 76 exchange reactions for amino acids, NTP, and other metabolic components. Furthermore, demand reactions were added for each protein gene product (Tables S9 and S12). The ‘E-matrix’ model is available in Matlab format (Dataset S1).

### Constraint-Based Modeling

The mathematical model of the ‘E-matrix’ was represented by a stoichiometric matrix, **S** (*m* rows×*n* columns), where *m* is the number of components and *n* is the number of reactions [1]. Reactions within the network were mass-balanced and assumed to be at steady state such that 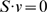, where 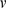 is flux vector. Additional constraints on upper, 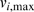, and lower, 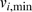, bounds were applied in form of 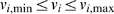 on each reaction *i*. The lower limits were set to zero for irreversible reactions. The unit for each reaction flux was defined to be 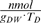, where the doubling time (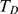) is given in minutes, if not stated differently.

### Simulation Constraints

The upper bounds on exchange reactions for NTPs and amino acids were constrained for all simulation conditions, while the lower bounds remained unconstrained. The fractional contribution of NTPs and amino acids were calculated based on experimental data [53] and scaled by RNA and protein content found at each doubling time (Text S1). The upper bounds of stable RNA transcription initiation reactions were constraint based on experimental data [42] using the following formula: 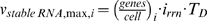 where 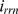 is the rRNA transcription initiation rate, 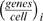 is the copy number of the stable RNA gene *i* per cell due to gene dosage (Table 3), and T_D_ the doubling time (see Text S1). The mRNA degradation rates were calculated using expression data in LB medium and mRNA half-life times [56] with 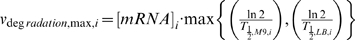 where 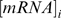 is the concentration of mRNA *i* in the cell, 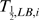 is the half-life time of mRNA *i* in LB medium, 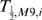 is the half-life time of mRNA *i* in M9 medium+glucose (refer to Text S1 for detailed calculation). A total number of 4,600 mRNA per cell at 30 min doubling time was assumed [42]. The lower bound (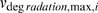) was set to be 0. Since the expression data as well as the total mRNA number have experimental errors, the upper bound on each reaction flux had to be relaxed by multiplying each mRNA concentration with a factor of 10. The upper bound on mRNA recycling, or CONV2 reactions, were constrained using the following formula: 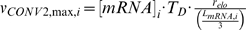 where 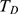 is the doubling time (s), 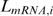 is the length of mRNA *i* , and 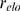 is the translation elongation rate at T_D_. This later set of reactions accounts for multiple translation rounds of an mRNA transcript between synthesis and degradation.

### Ribosome Production Rate

The exchange flux rates and the transcription initiation rates of ribosomal RNA operons were constrained as described above. At each doubling time, the ribosome production rate (DM_rib_50) was chosen as objective function, and the maximal possible production rate under the given set of constraints was calculated using linear programming.

### *In Silico* rRNA Operon Deletion

This analysis was carried out as illustrated in Figure 4. First, the transcription initiation rates were applied as constraints to all rRNA operons for the different doubling times (as described above). Using flux balance analysis (FBA) [66],[67], we optimized for ribosome production (DM_rib_50). For the strains deficient in one rRNA operon, we deleted each operon separately by setting the maximal possible transcription initiation rate to 0 (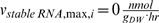), which corresponds the deletion of the reaction from the network. We optimized again for the ribosome production. For multiple rRNA operon deficient strains, all possible combinations of rRNA operon deletion were considered (Table 3), leading to the error bars in Figure 4. The compensation factors were chosen arbitrarily (1.5, 2, 2.5, and 4) and multiplied to all active rRNA operons in the mutant strains. Note that the unit for these simulations was 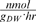.

### Flux Variability Analysis

Flux variability analysis was performed as described by Mahadevan [68] using linear programming. Briefly, for every network reaction the minimal and maximal solution was determined by successively defining each network reaction as objective function. The lower bound of the ribosome production rate (DM_rib_50) was constrained to 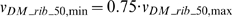.

### Correlation of Protein Utilization

The pair-wise correlations between protein component recycling reactions (PROT_RECYCL) were determined in LB-medium using linear programming. The maximal reaction flux for reaction A was determined and its upper and lower bound was set to be the maximal flux value. The minimal and maximal reaction flux for reaction B was determined under this new set of constraints. The same procedure was repeated for the minimal flux rate through reaction A. The same approach was repeated for reaction B with respect to reaction A. This method resulted in pair wise dependency plots for all recycling reactions. The area of feasible flux rates was determined using a convex hull algorithm [69] and scaled by the maximal flux rates for each reaction. The reaction correlation was defined to be 1 minus the area between two network reactions.

All calculation were performed using MatLab (The MathWorks, Inc, Natick, MA) and TomLab (TomLab Optimization, Inc, Pullman, WA) as linear programming solver.

### Availability

This knowledge base is freely available at http://bigg.ucsd.edu/E-matrix

## Supporting Information

Dataset S1 Compressed Matlab file containing E-matrix model
(1.49 MB ZIP)

Figure S1 Map of proteins included in the reconstruction.
(1.40 MB PDF)

Table S1 This table lists the network protein components included in the ‘E-matrix’ reconstruction by the subsystem in which they are mainly involved.
(0.03 MB DOC)

Table S2 Reactions per subsystem
(0.01 MB PDF)

Table S3 Proteins without gene annotation
(0.05 MB DOC)

Table S4 E-matrix proteins
(0.03 MB PDF)

Table S5 DnaK-dependent protein folding
(0.01 MB PDF)

Table S6 GroEL-dependent protein folding
(0.04 MB PDF)

Table S7 Unbalanced exchange reactions
(0.01 MB PDF)

Table S8 Unbalanced internal reactions
(0.01 MB PDF)

Table S9 E-matrix transcription units
(0.02 MB PDF)

Table S10 E-matrix genes
(0.07 MB PDF)

Table S11 Used genetic code
(0.04 MB PDF)

Table S12 Complete model reaction list and flux variability (FVA) results
(1.75 MB PDF)

Table S13 Component list
(0.76 MB PDF)

Table S14 List of functional modules
(0.04 MB PDF)

Table S15 Template reactions
(0.74 MB DOC)

Table S16 Template reactions for rRNA modification
(0.40 MB DOC)

Table S17 Template reactions for tRNA modification
(0.76 MB DOC)

Table S18 References for individual network reactions
(3.92 MB DOC)

Text S1 The supplemental text describes in detail the network content, reconstruction approach, and underlying assumptions.
(1.45 MB DOC)
