# Supplementary figures and images for: Genome-Scale Reconstruction of Escherichia coli's Transcriptional and Translational Machinery: A Knowledge Base, Its Mathematical Formulation, and Its Functional Characterization
Source: PLoS Comput Biol. 2009 Mar 13;5(3):e1000312. doi: 10.1371/journal.pcbi.1000312 (PMC2648898; doi:10.1371/journal.pcbi.1000312)

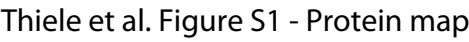

Thiele et al. Figure S1 - Protein map

Supplement: Figure S1 — Map of proteins included in the reconstruction. (1.40 MB PDF) [file pcbi.1000312.s002.pdf]
