# Supplementary material for: Genome-Scale Reconstruction of Escherichia coli's Transcriptional and Translational Machinery: A Knowledge Base, Its Mathematical Formulation, and Its Functional Characterization
Source: PLoS Comput Biol. 2009 Mar 13;5(3):e1000312. doi: 10.1371/journal.pcbi.1000312 (PMC2648898; doi:10.1371/journal.pcbi.1000312)
Supplement: Table S1 — This table lists the network protein components included in the ‘E-matrix’ reconstruction by the subsystem in which they are mainly involved. (0.03 MB DOC) [file pcbi.1000312.s003.doc]

*Thiele et al.:* " Genome-scale reconstruction of E. coli's transcriptional and translational machinery: A knowledge-base and its mathematical formulation";

**Table S1:** This table lists the network protein components included in the ‘E-matrix’ reconstruction by the subsystem in which they are mainly involved.
