# Supplementary material for: Genome-Scale Reconstruction of Escherichia coli's Transcriptional and Translational Machinery: A Knowledge Base, Its Mathematical Formulation, and Its Functional Characterization
Source: PLoS Comput Biol. 2009 Mar 13;5(3):e1000312. doi: 10.1371/journal.pcbi.1000312 (PMC2648898; doi:10.1371/journal.pcbi.1000312)
Supplement: Table S2 — Reactions per subsystem (0.01 MB PDF) [file pcbi.1000312.s004.pdf]

Table S2 - Reactions per subsystem

Reactions per Subsystem		
**Number**	**Subsystem**	**Reactions**
I	Transcription	783
II	mRNA degradation	628
III	Translation	6812
IV	Protein Maturation	628
IX	RNA processing	122
V	Protein Folding	570
VI	Metallo-ion Binding	128
VII	Protein complex formation	87
VIII	Ribosomal Assembly	13
X	rRNA modification	864
XII	tRNA charging	177
XI	tRNA modification	1597
XIII	Aminoacyl-tRNA synthetase char	33
XIV	Charging EF-Tu	4
XV	Cleavage polycistronic mRNA	222
XVI	Demands	302
XVII	Exchange reactions	76
XVIII	Iron-sulfur cluster biosynthesis	6
XIX	Iron-sulfur cluster incorporation	6
XX	Protein Modification	12
XXI	Protein Recycling	148
XXII	Ribosomal protein modification	21
XXIII	rRNA formation	38
XXIV	Sinks	35
XXV	Transcription Regulation	261
XXVI	Transport	76
XXVII	tRNA activation (EF-TU)	45
	Total Number of Reactions	13694

Components per Subsystem	
**Subsystem**	**Components**
Folding	597
Iron-sulfur cluster biosynthesis	12
Iron-sulfur cluster incorporation	3
Maturation	628
mRNA cleavage	111
mRNA degradation	324
Others	147
Protein Folding	14
Protein Modification	14
Ribosomal Assembly	44
Ribosomal protein modification	21
Ribosome maturation	2
RNA cleavage	18
RNA cutting	138
rRNA Modification	896
Transcription	1166
Translation	5885
tRNA activitation	1
tRNA charging	151
tRNA modification	1819
Total Number of Components	11991
