# Supplementary material for: Genome-Scale Reconstruction of Escherichia coli's Transcriptional and Translational Machinery: A Knowledge Base, Its Mathematical Formulation, and Its Functional Characterization
Source: PLoS Comput Biol. 2009 Mar 13;5(3):e1000312. doi: 10.1371/journal.pcbi.1000312 (PMC2648898; doi:10.1371/journal.pcbi.1000312)
Supplement: Table S3 — Proteins without gene annotation (0.05 MB DOC) [file pcbi.1000312.s005.doc]

*Thiele et al*.: " Genome-scale reconstruction of E. coli's transcriptional and translational machinery: A knowledge-base and its mathematical formulation";

**Table S3. Proteins without known gene annotation.**

| **Protein Abbreviation** | **Protein Function** | **Subsystem** |
| --- | --- | --- |
| MeT_EF-TU | unknown methyltransferase of EF-TU (Lys56) | Protein Modification |
| AcT_EF-TU | EF-TU acetyltransferase (Ser1) | Protein Modification |
| MeST_S12 | beta-methylthio-transferase on aspartic acid of S12 | Ribosomal protein modification |
| MeT_L16 | unknown Methyltransferase of ribosomal protein L16 | Ribosomal protein modification |
| MeT_L33 | unknown methyltransferase of ribosomal protein L33 | Ribosomal protein modification |
| MeT_L7/L12 | unknown Methyltransferase of ribosomal protein L7/L12 | Ribosomal protein modification |
| MeT_S11 | unknown methyltransferase of ribosomal protein S11 | Ribosomal protein modification |
| RNase_m16 | unknown ribonuclease for 16S rRNA cleavage | RNA Processing |
| RNase_m23 | unknown ribonuclease for 23S rRNA cleavage | RNA Processing |
| RNase_m5 | unknown ribonuclease for 5S rRNA cleavage | RNA Processing |
| DU_23S_2449 | dihydrouridine synthetase, 23S rRNA, position 2449 | rRNA Modification |
| MeT_16S_1402 | unknown Methyltransferase of 16S rRNA, position 1402 | rRNA Modification |
| MeT_16S_1407 | unknown Methyltransferase of 16S rRNA, position 1407 | rRNA Modification |
| MeT_16S_1516 | unknown Methyltransferase of 16S rRNA, position 1516 | rRNA Modification |
| MeT_16S_527 | unknown Methyltransferase of 16S rRNA, position 527 | rRNA Modification |
| MeT_16S_966 | unknown Methyltransferase of 16S rRNA, position 966 | rRNA Modification |
| MeT_23S_1618 | unknown Methyltransferase of 23S rRNA, position 1618 | rRNA Modification |
| MeT_23S_1835 | unknown Methyltransferase of 23S rRNA, position 1835 | rRNA Modification |
| MeT_23S_1962 | unknown Methyltransferase of 23S rRNA, position 1962 | rRNA Modification |
| MeT_23S_2030 | unknown Methyltransferase of 23S rRNA, position 2030 | rRNA Modification |
| MeT_23S_2069 | unknown Methyltransferase of 23S rRNA, position 2069 | rRNA Modification |
| MeT_23S_2445 | unknown Methyltransferase of 23S rRNA, position 2445 | rRNA Modification |
| MeT_23S_2498 | unknown Methyltransferase of 23S rRNA, position 2498 | rRNA Modification |
| MeT_23S_2503 | unknown Methyltransferase of 23S rRNA, position 2503 | rRNA Modification |
| EoR_tRNA_pos34_Q | Unknown epoxide reductase, tRNA, position (34 (Q) | tRNA Modification |
| HyL_tRNA_pos_34_ho5U | unknown hydroxylase, tRNA, position 34 (ho5U0 | tRNA Modification |
| MeT_tRNA_pos_32_Cm | unknown Methyltransferase, tRNA, position 32 (Cm) | tRNA Modification |
| MeT_tRNA_pos_32_Um | unknown Methyltransferase, tRNA, position 32 (Um) | tRNA Modification |
| MeT_tRNA_pos_37_m2A | unknown Methyltransferase, tRNA, position 37 (m2A) | tRNA Modification |
| MeT_tRNA_pos_37_m6A | unknown Methyltransferase, tRNA, position 37 (m6A) | tRNA Modification |
| MeT_tRNA_pos_37_m6t6A | unknown Methyltransferase, tRNA, position 37 (m6t6A) | tRNA Modification |
| Up_tRNA_pos_37_t6A | unknown protein, tRNA, position 37 (t6A) | tRNA Modification |
| AcpT_tRNA_pos_47_acp3U | unknown tRNA-uridine 3-(3-amino-3-carboxypropyl)transferase, tRNA, position 47 (acp3U) | tRNA Modification |
| AcT_tRNA_pos_34_ac4C | unknown acetyltransferase, tRNA, position 34 (ac4C) | tRNA Modification |
