# Supplementary material for: Genome-Scale Reconstruction of Escherichia coli's Transcriptional and Translational Machinery: A Knowledge Base, Its Mathematical Formulation, and Its Functional Characterization
Source: PLoS Comput Biol. 2009 Mar 13;5(3):e1000312. doi: 10.1371/journal.pcbi.1000312 (PMC2648898; doi:10.1371/journal.pcbi.1000312)
Supplement: Table S4 — E-matrix proteins (0.03 MB PDF) [file pcbi.1000312.s006.pdf]

Table S4 - E-matrix proteins

Gene	Gene Symbol	Protein Function	Subsystem
b2528	iscA	FeS cluster assembly protein	Iron-Sulfur Cluster
b2529	iscU	scaffold protein	Iron-Sulfur Cluster
b2530	iscS	cysteine desulfurase (tRNA sulfurtransferase), PLP-	Iron-Sulfur Cluster
b2779	eno	enolase	mRNA degradation
b3164	pnp	polynucleotide phosphorylase/polyadenylase	mRNA degradation
b3780	rhlB	ATP-dependent RNA helicase	mRNA degradation
b4162	orn	oligoribonuclease	mRNA degradation
b0014	dnaK	chaperone Hsp70, co-chaperone with DnaJ	Protein Folding
b0015	dnaJ	chaperone Hsp40, co-chaperone with DnaK	Protein Folding
b2614	grpE	heat shock protein	Protein Folding
b4142	groS	Cpn10 chaperonin GroES, small subunit of GroESL	Protein Folding
b4143	groL	Cpn60 chaperonin GroEL, large subunit of GroESL	Protein Folding
b0168	map	methionine aminopeptidase	Protein Maturation
b3287	def	peptide deformylase	Protein Maturation
b1212	prmC	N5-glutamine methyltransferase, modifies release factors RF-1 and RF-2	Protein Modification
unknown	unknown	unknown methyltransferase of EF-TU (Lys56)	Protein Modification
unknown	unknown	EF-TU acetyltransferase (Ser1)	Protein Modification
b2566	era	membrane-associated, 16S rRNA-binding GTPase	Ribosomal protein assembly
b2608	rimM	16S rRNA processing protein	Ribosomal protein assembly
b3167	rbfA	30s ribosome binding factor	Ribosomal protein assembly
b3282	yrdC	predicted ribosome maturation factor	Ribosomal protein assembly
b0852	rimK	ribosomal protein S6 modification protein	Ribosomal protein modification
b1066	rimJ	ribosomal-protein-S5-alanine N-acetyltransferase	Ribosomal protein modification
b1427	rimL	ribosomal-protein-L7/L12-serine acetyltransferase	Ribosomal protein modification
b2330	prmB	N5-glutamine methyltransferase	Ribosomal protein modification
b3259	prmA	methylase for 50S ribosomal subunit protein L11	Ribosomal protein modification
b4373	rimI	acetylase for 30S ribosomal subunit protein S18	Ribosomal protein modification

unknown	unknown	beta-methylthio-transferase on aspartic acid of S12	Ribosomal protein modification
unknown	unknown	unknown Methyltransferase of ribosomal protein L16	Ribosomal protein modification
unknown	unknown	unknown methyltransferase of ribosomal protein L33	Ribosomal protein modification
unknown	unknown	unknown Methyltransferase of ribosomal protein L7/L12	Ribosomal protein modification
unknown	unknown	unknown methyltransferase of ribosomal protein S11	Ribosomal protein modification
b1084	rne	fused ribonucleaseE: endoribonuclease -!- RNA-binding protein -!-RNA degradosome binding protein	RNA Processing
b1286	rnb	ribonuclease II	RNA Processing
b1652	rnt	ribonuclease T (RNase T)	RNA Processing
b1804	rnd	ribonuclease D	RNA Processing
b2268	elaC	binuclear zinc phosphodiesterase	RNA Processing
b2567	rnc	RNase III	RNA Processing
b3247	rng	ribonuclease G	RNA Processing
b3704	rnpA	protein C5 component of RNase P	RNA Processing
unknown	unknown	unknown ribonuclease for 16S rRNA cleavage	RNA Processing
unknown	unknown	unknown ribonuclease for 23S rRNA cleavage	RNA Processing
unknown	unknown	unknown ribonuclease for 5S rRNA cleavage	RNA Processing
b0051	ksgA	S-adenosylmethionine-6-N',N'-adenosyl (rRNA) dimethyltransferase	rRNA Modification
b0058	rluA	pseudouridine synthase for 23S rRNA (position 746) and tRNAphe(position 32)	rRNA Modification
b0859	rumB	23S rRNA m(5)U747 methyltransferase	rRNA Modification
b1086	rluC	23S rRNA pseudouridylate synthase	rRNA Modification
b1135	ymfC	23S rRNA pseudouridine synthase	rRNA Modification
b1269	rluB	23S rRNA pseudouridylate synthase	rRNA Modification
b1822	rrmA	23S rRNA m1G745 methyltransferase	rRNA Modification
b2183	rsuA	16S rRNA pseudouridylate 516 synthase	rRNA Modification
b2594	rluD	23S rRNA pseudouridine synthase	rRNA Modification
b2785	rumA	23S rRNA (uracil-5)-methyltransferase	rRNA Modification
b2946	yggJ	predicted protein	rRNA Modification
b3179	rrmJ	23S rRNA methyltransferase	rRNA Modification
b3289	rsmB	16S rRNA m5C967 methyltransferase, S-adenosyl-L-methionine-dependent	rRNA Modification
b4022	yjbC	23S rRNA pseudouridine synthase	rRNA Modification
b4180	rlmB	23S rRNA (Gm2251)-methyltransferase	rRNA Modification
b4371	rsmC	16S RNA m2G1207 methylase	rRNA Modification
unknown	unknown	dihydrouridine synthetase, 23S rRNA, position 2449	rRNA Modification

unknown	unknown	unknown Methyltransferase of 16S rRNA, position 1402	rRNA Modification
unknown	unknown	unknown Methyltransferase of 16S rRNA, position 1407	rRNA Modification
unknown	unknown	unknown Methyltransferase of 16S rRNA, position 1516	rRNA Modification
unknown	unknown	unknown Methyltransferase of 16S rRNA, position 527	rRNA Modification
unknown	unknown	unknown Methyltransferase of 16S rRNA, position 966	rRNA Modification
unknown	unknown	unknown Methyltransferase of 23S rRNA, position 1618	rRNA Modification
unknown	unknown	unknown Methyltransferase of 23S rRNA, position 1835	rRNA Modification
unknown	unknown	unknown Methyltransferase of 23S rRNA, position 1962	rRNA Modification
unknown	unknown	unknown Methyltransferase of 23S rRNA, position 2030	rRNA Modification
unknown	unknown	unknown Methyltransferase of 23S rRNA, position 2069	rRNA Modification
unknown	unknown	unknown Methyltransferase of 23S rRNA, position 2445	rRNA Modification
unknown	unknown	unknown Methyltransferase of 23S rRNA, position 2498	rRNA Modification
unknown	unknown	unknown Methyltransferase of 23S rRNA, position 2503	rRNA Modification
b0416	nusB	transcription antitermination protein	Transcription
b1114	mfd	transcription-repair coupling factor	Transcription
b1922	fliA	RNA polymerase, sigma 28 (sigma F) factor	Transcription
b2573	rpoE	RNA polymerase, sigma 24 (sigma E) factor	Transcription
b2741	rpoS	RNA polymerase, sigma S (sigma 38) factor	Transcription
b3067	rpoD	RNA polymerase, sigma 70 (sigma D) factor	Transcription
b3169	nusA	transcription termination/antitermination L factor	Transcription
b3181	greA	transcription elongation factor	Transcription
b3202	rpoN	RNA polymerase, sigma 54 (sigma N) factor	Transcription
b3295	rpoA	RNA polymerase, alpha subunit	Transcription
b3406	greB	transcription elongation factor	Transcription
b3461	rpoH	RNA polymerase, sigma 32 (sigma H) factor	Transcription
b3649	rpoZ	RNA polymerase, omega subunit	Transcription
b3783	rho	transcription termination factor	Transcription
b3982	nusG	transcription termination factor	Transcription
b3987	rpoB	RNA polymerase, beta subunit	Transcription
b3988	rpoC	RNA polymerase, beta prime subunit	Transcription
b4293	fecI	KpLE2 phage-like element; RNA polymerase, sigma 19	Transcription
b0023	rpsT	30S ribosomal subunit protein S20	Translation
b0169	rpsB	30S ribosomal subunit protein S2	Translation
b0170	tsf	protein chain elongation factor EF-Ts	Translation
b0172	rrf	ribosome recycling factor	Translation
b0436	tig	peptidyl-prolyl cis/trans isomerase (trigger factor)	Translation
b0884	infA	translation initiation factor IF-1	Translation

b0911	rpsA	30S ribosomal subunit protein S1	Translation
b1089	rpmF	50S ribosomal subunit protein L32	Translation
b1211	prfA	peptide chain release factor RF-1	Translation
b1480	sra	30S ribosomal subunit protein S22	Translation
b1716	rplT	50S ribosomal subunit protein L20	Translation
b1717	rpmI	50S ribosomal subunit protein L35	Translation
b1718	infC	protein chain initiation factor IF-3	Translation
b2185	rplY	50S ribosomal subunit protein L25	Translation
b2606	rplS	50S ribosomal subunit protein L19	Translation
b2609	rpsP	30S ribosomal subunit protein S16	Translation
b2891	prfB	peptide chain release factor RF-2	Translation
b3065	rpsU	30S ribosomal subunit protein S21	Translation
b3165	rpsO	30S ribosomal subunit protein S15	Translation
b3168	infB	fused protein chain initiation factor 2, IF2: membrane protein -!- conserved protein	Translation
b3185	rpmA	50S ribosomal subunit protein L27	Translation
b3186	rplU	50S ribosomal subunit protein L21	Translation
b3230	rpsI	30S ribosomal subunit protein S9	Translation
b3231	rplM	50S ribosomal subunit protein L13	Translation
b3294	rplQ	50S ribosomal subunit protein L17	Translation
b3296	rpsD	30S ribosomal subunit protein S4	Translation
b3297	rpsK	30S ribosomal subunit protein S11	Translation
b3298	rpsM	30S ribosomal subunit protein S13	Translation
b3299	rpmJ	50S ribosomal subunit protein L36	Translation
b3301	rplO	50S ribosomal subunit protein L15	Translation
b3302	rpmD	50S ribosomal subunit protein L30	Translation
b3303	rpsE	30S ribosomal subunit protein S5	Translation
b3304	rplR	50S ribosomal subunit protein L18	Translation
b3305	rplF	50S ribosomal subunit protein L6	Translation
b3306	rpsH	30S ribosomal subunit protein S8	Translation
b3307	rpsN	30S ribosomal subunit protein S14	Translation
b3308	rplE	50S ribosomal subunit protein L5	Translation
b3309	rplX	50S ribosomal subunit protein L24	Translation
b3310	rplN	50S ribosomal subunit protein L14	Translation
b3311	rpsQ	30S ribosomal subunit protein S17	Translation
b3312	rpmC	50S ribosomal subunit protein L29	Translation
b3313	rplP	50S ribosomal subunit protein L16	Translation

b3314	rpsC	30S ribosomal subunit protein S3	Translation
b3315	rplV	50S ribosomal subunit protein L22	Translation
b3316	rpsS	30S ribosomal subunit protein S19	Translation
b3317	rplB	50S ribosomal subunit protein L2	Translation
b3318	rplW	50S ribosomal subunit protein L23	Translation
b3319	rplD	50S ribosomal subunit protein L4	Translation
b3320	rplC	50S ribosomal subunit protein L3	Translation
b3321	rpsJ	30S ribosomal subunit protein S10	Translation
b3339	tufA	protein chain elongation factor EF-Tu (duplicate of tufB)	Translation
b3340	fusA	protein chain elongation factor EF-G, GTP-binding	Translation
b3341	rpsG	30S ribosomal subunit protein S7	Translation
b3342	rpsL	30S ribosomal subunit protein S12	Translation
b3636	rpmG	50S ribosomal subunit protein L33	Translation
b3637	rpmB	50S ribosomal subunit protein L28	Translation
b3703	rpmH	50S ribosomal subunit protein L34	Translation
b3936	rpmE	50S ribosomal subunit protein L31	Translation
b3980	tufB	protein chain elongation factor EF-Tu (duplicate of tufA)	Translation
b3983	rplK	50S ribosomal subunit protein L11	Translation
b3984	rplA	50S ribosomal subunit protein L1	Translation
b3985	rplJ	50S ribosomal subunit protein L10	Translation
b3986	rplL	50S ribosomal subunit protein L7/L12	Translation
b4200	rpsF	30S ribosomal subunit protein S6	Translation
b4202	rpsR	30S ribosomal subunit protein S18	Translation
b4203	rplI	50S ribosomal subunit protein L9	Translation
b4375	prfC	peptide chain release factor RF-3	Translation
b0026	ileS	isoleucyl-tRNA synthetase	tRNA charging
b0144	yadB	glutamyl-Q tRNA(Asp) synthetase	tRNA charging
b0194	proS	prolyl-tRNA synthetase	tRNA charging
b0526	cysS	cysteinyl-tRNA synthetase	tRNA charging
b0642	leuS	leucyl-tRNA synthetase	tRNA charging
b0680	glnS	glutamyl-tRNA synthetase	tRNA charging
b0893	serS	seryl-tRNA synthetase, also charges selenocysteinyl-tRNA with serine	tRNA charging
b0930	asnS	asparaginyl tRNA synthetase	tRNA charging
b1637	tyrS	tyrosyl-tRNA synthetase	tRNA charging
b1713	pheT	phenylalanine tRNA synthetase, beta subunit	tRNA charging
b1714	pheS	phenylalanine tRNA synthetase, alpha subunit	tRNA charging

b1719	thrS	threonyl-tRNA synthetase	tRNA charging
b1866	aspS	aspartyl-tRNA synthetase	tRNA charging
b1876	argS	arginyl-tRNA synthetase	tRNA charging
b2114	metG	methionyl-tRNA synthetase	tRNA charging
b2400	gltX	glutamyl-tRNA synthetase	tRNA charging
b2514	hisS	histidyl tRNA synthetase	tRNA charging
b2697	alaS	alanyl-tRNA synthetase	tRNA charging
b2890	lysS	lysine tRNA synthetase, constitutive	tRNA charging
b3288	fmt	10-formyltetrahydrofolate:L-methionyl-tRNA(fMet) N-formyltransferase	tRNA charging
b3384	trpS	tryptophanyl-tRNA synthetase	tRNA charging
b3559	glyS	glycine tRNA synthetase, beta subunit	tRNA charging
b3560	glyQ	glycine tRNA synthetase, alpha subunit	tRNA charging
b3590	selB	selenocysteinyl-tRNA-specific translation factor	tRNA charging
b3591	selA	selenocysteine synthase	tRNA charging
b3887	dtd	D-tyr-tRNA(Tyr) deacylase	tRNA charging
b4129	lysU	lysine tRNA synthetase, inducible	tRNA charging
b4258	valS	valyl-tRNA synthetase	tRNA charging
b0188	tilS	tRNA(Ile)-lysidine synthetase	tRNA Modification
b0405	queA	S-adenosylmethionine:tRNA ribosyltransferase-isomerase	tRNA Modification
b0406	tgt	tRNA-guanine transglycosylase	tRNA Modification
b0423	thiI	sulfurtransferase required for thiamine and 4-thiouridine biosynthesis	tRNA Modification
b0503	ybbB	tRNA 2-selenouridine synthase, selenophosphate-	tRNA Modification
b0661	miaB	isopentenyl-adenosine A37 tRNA methylthiolase	tRNA Modification
b0969	yccK	predicted sulfite reductase subunit	tRNA Modification
b1133	trmU	tRNA (5-methylaminomethyl-2-thiouridylate)-	tRNA Modification
b1344	ydaO	predicted C32 tRNA thiolase	tRNA Modification
b1870	yecO	predicted methyltransferase	tRNA Modification
b1871	yecP	predicted S-adenosyl-L-methionine-dependent methyltransferase	tRNA Modification
b2140	dusC	tRNA-dihydrouridine synthase C	tRNA Modification
b2318	truA	pseudouridylate synthase I	tRNA Modification
b2324	trmC	fused 5-methylaminomethyl-2-thiouridine-forming enzyme methyltransferase -!- FAD-dependent demodification	tRNA Modification
b2559	tadA	tRNA-specific adenosine deaminase	tRNA Modification
b2607	trmD	tRNA (guanine-1-)-methyltransferase	tRNA Modification

b2745	truD	pseudoruidine synthase	tRNA Modification
b2791	yqcB	tRNA pseudouridine synthase	tRNA Modification
b2794	queF	conserved protein	tRNA Modification
b2960	yggH	tRNA (m7G46) methyltransferase, SAM-dependent	tRNA Modification
b3166	truB	tRNA pseudouridine synthase	tRNA Modification
b3260	dusB	tRNA-dihydrouridine synthase B	tRNA Modification
b3343	yheL	predicted intracellular sulfur oxidation protein	tRNA Modification
b3344	yheM	predicted intraceullular sulfur oxidation protein	tRNA Modification
b3345	yheN	predicted intracellular sulfur oxidation protein	tRNA Modification
b3470	yhhP	conserved protein required for cell growth	tRNA Modification
b3651	trmH	tRNA (Guanosine-2'-O-)-methyltransferase	tRNA Modification
b3706	trmE	GTPase	tRNA Modification
b3741	gidA	glucose-inhibited cell-division protein	tRNA Modification
b3965	trmA	tRNA (uracil-5-)-methyltransferase	tRNA Modification
b4049	dusA	tRNA-dihydrouridine synthase A	tRNA Modification
b4171	miaA	delta(2)-isopentenylpyrophosphate tRNA-adenosine	tRNA Modification
unknown	unknown	Unknown epoxide reductase, tRNA, position (34 (Q)	tRNA Modification
unknown	unknown	unknown hydroxylase, tRNA, position 34 (ho5U0	tRNA Modification
unknown	unknown	unknown Methyltransferase, tRNA, position 32 (Cm)	tRNA Modification
unknown	unknown	unknown Methyltransferase, tRNA, position 32 (Um)	tRNA Modification
unknown	unknown	unknown Methyltransferase, tRNA, position 37 (m2A)	tRNA Modification
unknown	unknown	unknown Methyltransferase, tRNA, position 37 (m6A)	tRNA Modification
unknown	unknown	unknown Methyltransferase, tRNA, position 37 (m6t6A)	tRNA Modification
unknown	unknown	unknown protein, tRNA, position 37 (t6A)	tRNA Modification
unknown	unknown	unknown tRNA-uridine 3-(3-amino-3-carboxypropyl)transferase, tRNA, position 47 (acp3U)	tRNA Modification
unknown	unknown	unknown acetyltransferase, tRNA, position 34 (ac4C)	tRNA Modification

**Subsystem**	**Proteins per subsyste m**	**Unknown proteins per subsyste m**	**% protein per subsyste m of total proteins**
Iron-Sulfur Cluster	3		0.013
mRNA degradation	4		0.018
Protein Folding	5		0.022
Protein Maturation	2		0.009
Protein Modification	3	2	0.013
Ribosomal protein asser	4		0.018
Ribosomal protein modif	11	5	0.048
RNA Processing	11	3	0.048
rRNA Modification	30	14	0.132
Transcription	18		0.079
Translation	67		0.294
tRNA charging	28		0.123
tRNA Modification	42	10	0.184
	228	34	1
