# Supplementary material for: Genome-Scale Reconstruction of Escherichia coli's Transcriptional and Translational Machinery: A Knowledge Base, Its Mathematical Formulation, and Its Functional Characterization
Source: PLoS Comput Biol. 2009 Mar 13;5(3):e1000312. doi: 10.1371/journal.pcbi.1000312 (PMC2648898; doi:10.1371/journal.pcbi.1000312)
Supplement: Table S5 — DnaK-dependent protein folding (0.01 MB PDF) [file pcbi.1000312.s007.pdf]

Table S5 - DnaK-dependent Folding

taken from Deuerling et al. Mol Microbiol, 2003, 47(5),1317-1328, Trigger Factor and DnaK possess overlapping substrate pools and binding specificities

B number	Locus Name
b0008	talB
b0032	carA
b0033	carB
b0059	hepA
b0095	ftsZ
b0114	aceE
b0115	aceF
b0118	acnB
b0194	proS
b0438	clpX
b0439	lon
b0642	leuS
b0680	glnR
b0726	sucA
b0728	sucC
b0755	gpmA
b0893	serS
b0903	pflB
b0930	asnS
b0932	pepN
b1014	putA
b1095	fabF
b1114	mfd
b1136	icd
b1175	minD
b1224	narG
b1241	adhE
b1275	cysB
b1479	sfcA
b1612	fumA
b1614	ydgA
b1676	pykF
b1713	pheT
b1719	thrS
b1779	gapA
b1945	fliM
b2029	gnd
b2036	glf
b2114	metG
b2231	gyrA
b2234	nrdA
b2284	nuoF
b2287	nuoB
b2297	pta
b2463	

b2507	guaA
b2508	guaB
b2557	purL
b2697	alaS
b2699	recA
b2764	cysJ
b2780	pyrG
b2913	serA
b2925	fbaA
b2926	pgk
b2935	tktA
b3067	rpoD
b3189	murA
b3212	gltB
b3295	rpoA
b3339	tufA
b3340	fusA
b3384	trpS
b3686	ibpB
b3687	ibpA
b3708	tnaA
b3744	asnA
b3783	rho
b3829	metE
b3831	udp
b3870	glnA
b3893	fdoH
b3931	hslU
b3942	katG
b3980	tufB
b3987	rpoB
b3988	rpoC
b4019	metH
b4129	lysU
b4131	cadA
b4147	efp
b4177	purA
b4232	fbp
b4239	treC
b4258	valS
b4260	pepA
b4375	prfC
b4382	deoA
b4383	deoB
b4384	deoD
b4391	yjjK
