# Supplementary material for: Genome-Scale Reconstruction of Escherichia coli's Transcriptional and Translational Machinery: A Knowledge Base, Its Mathematical Formulation, and Its Functional Characterization
Source: PLoS Comput Biol. 2009 Mar 13;5(3):e1000312. doi: 10.1371/journal.pcbi.1000312 (PMC2648898; doi:10.1371/journal.pcbi.1000312)
Supplement: Table S6 — GroEL-dependent protein folding (0.04 MB PDF) [file pcbi.1000312.s008.pdf]

Table S6 - GroEL-dependent Folding

taken from Kerner et al. 2005, 'Proteome-wide Analysis of Chaperonin-dependent protein folding in Escherichia coli'

B number	SwissProtEntry Name	SwissProt Accession Number	Protein Description	Predicted GroEL Substrate	Moelcular weight	Essentiality	pI	Oligomeric State (SwissProt Entry	Subcellular Location (SwissProt Entry)	Subcellular Localization (PSORTb v2.0)	COG Functional Category
b0014	dnak_ecoli	P04475	Chaperone protein dnaK (Heat shock protein	II	69	1	4.8			Cytoplasmic	O
b0015	dnaj_ecoli	P08622	Chaperone protein dnaJ (Heat shock protein J)	III	41	0	8	Homodimer.	Cytoplasmic.	Cytoplasmic	O
b0061	arad_ecoli	P08203	L-ribulose-5-phosphate 4-epimerase (EC	II	25.5	0	5.7			Unknown	G
b0062	araa_ecoli	P08202	L-arabinose isomerase (EC 5.3.1.4).	III	56.1	0	6			Unknown	G
b0064	arac_ecoli	P03021	Arabinose operon regulatory protein.	III	33.4	0	6.5	Homodimer.	Cytoplasmic.	Cytoplasmic	K
b0114	odp1_ecoli	P06958	Pyruvate dehydrogenase E1 component (EC	I	99.5	0	5.5	Homodimer.		Unknown	C
b0115	odp2_ecoli	P06959	Dihydrolipoamide acetyltransferase component	I	66	1	5.1	Forms a 24-polypeptide		Cytoplasmic	C
b0116	dldh_ecoli	P00391	Dihydrolipoamide dehydrogenase (EC 1.8.1.4)	I or II	50.6	1	5.8	Homodimer.	Cytoplasmic.	Cytoplasmic	C
b0130	yade_ecoli	P31666	Hypothetical protein yadE precursor.	II	46.3	0	9.8			Unknown	G
b0134	panb_ecoli	P31057	3-methyl-2-oxobutanoate	I	28.2	0	5.2	Hexamer (Potential).		Unknown	H

b0143	pcnb_ecoli	P13685	Poly(A) polymerase (EC 2.7.7.19) (PAP)	II	54.7	0	9.7	Monomer. Interacts with		Cytoplasmic	J
b0144	yadb_ecoli	P27305	Hypothetical protein yadB.	II	33.6	0	6.4			Cytoplasmic	J
b0154	gsa_ecoli	P23893	Glutamate-1-semialdehyde 2,1-aminomutase	II	45.4	1	4.7	Homodimer.	Cytoplasmic	Cytoplasmic	H
b0167	glnd_ecoli	P27249	[Protein-PII] uridylyltransferase (EC 2.7.7.59)	II	102.4	1	6.2			Cytoplasmic	O
b0170	efts_ecoli	P02997	Elongation factor Ts (EF-Ts).	I	30.3	1	5.2	Heterotetramer composed	Cytoplasmic.	Cytoplasmic	J
b0172	rrf_ecoli	P16174	Ribosome recycling factor (Ribosome releasing	I	20.6	1	6.4		Cytoplasmic.	Cytoplasmic	J
b0181	lpxa_ecoli	P10440	Acyl-[acyl-carrier-protein]--UDP-N-	II	28.1	1	6.6	Homotrimer.	Cytoplasmic.	Cytoplasmic	M
b0185	acca_ecoli	P30867	Acetyl-coenzyme A carboxylase carboxyl	II	35.1	1	5.8	Acetyl-CoA carboxylase is		Cytoplasmic	I
b0209	yafd_ecoli	P30865	Hypothetical protein yafD.	III	30	0	9.6		Cytoplasmic	Unknown	S
b0259; and Name=insH2;	insh_ecoli	P03837	Transposase insH for insertion sequence	II	39.3	0	9.6			Cytoplasmic	L
b0369	hem2_ecoli	P15002	Delta-aminolevulinic acid dehydratase (EC	III	35.5	1	5.3	Homooctamer.		Cytoplasmic	H
b0404	yajb_ecoli	P21515	Hypothetical protein yajB.	III	23	0	5.9			Cytoplasmic	S
b0436	tig_ecoli	P22257	Trigger factor (TF).	I	48.2	0	4.8	Homodimer and monomer.		Cytoplasmic	O

b0439	lon_ecoli	P08177	ATP-dependent protease La (EC 3.4.21.53).	II	87.4	0	6	Homotetramer.	Cytoplasmic.	Cytoplasmic	O
b0503	ybbb_ecoli	P33667	Hypothetical protein ybbB.	II	41.1	0	5.9			Unknown	R
b0525	ppib_ecoli	P23869	Peptidyl-prolyl cis-trans isomerase B (EC	II	18.2	1	5.5		Cytoplasmic.	Cytoplasmic	O
b0593	entc_ecoli	P10377	Isochorismate synthase entC (EC 5.4.99.6)	II	42.9	0	5.5	Monomer.		Unknown	H
b0605	ahpc_ecoli	P26427	Alkyl hydroperoxide reductase subunit C (EC	I	20.6	0	5	Homodimer		Cytoplasmic	O
b0607	uspg_ecoli	P39177	Universal stress protein G.	II	15.9	0	6	Interacts with groEL.		Cytoplasmic	T
b0628	lipa_ecoli	P25845	Lipoic acid synthetase (Lip-syn) (Lipoate	III	36.1	0	8.1	Monomer or homodimer.	Cytoplasmic.	Cytoplasmic	H
b0660	phol_ecoli	P77349	PhoH-like protein.	III	40.7	0	6.2		Cytoplasmic	Cytoplasmic	T
b0687	seqa_ecoli	P36658	SeqA protein.	II	20.3	1	8.8			Cytoplasmic	L
b0720	cisy_ecoli	P00891	Citrate synthase (EC 2.3.3.1).	II	48	0	6.2	Homohexamer.		Cytoplasmic	C
b0723	dhsa_ecoli	P10444	Succinate dehydrogenase flavoprotein subunit	III	64.4	0	5.9	Part of an enzyme		Unknown (This	C
b0726	odo1_ecoli	P07015	2-oxoglutarate dehydrogenase E1 component	II	105.1	1	6	Homodimer.		Cytoplasmic	C
b0727	odo2_ecoli	P07016	Dihydrolipoamide succinyltransferase	II	43.9	1	5.6	Forms a 24-polypeptide		Cytoplasmic	C
b0755	gpma_ecoli	P31217	2,3-bisphosphoglycerate-dependent	I	28.4	0	5.9	Homodimer.		Unknown	G
b0776	biof_ecoli	P12998	8-amino-7-oxononanoate synthase (EC	III	41.6	0	6.6	Homodimer.		Unknown	H
b0781	moaa_ecoli	P30745	Molybdenum cofactor biosynthesis protein A.	II	37.3	0	8.2			Cytoplasmic	H

b0782	moab_ecoli	P30746	Molybdenum cofactor biosynthesis protein B.	II	18.5	0	5.7			Cytoplasmic	H
b0783	moac_ecoli	P30747	Molybdenum cofactor biosynthesis protein C.	II	17.3	0	6.6	Homohexamer.		Unknown	H
b0797	rhle_ecoli	P25888	Putative ATP-dependent RNA helicase rhlE.	III	50	0	10	Interacts with pcnB.		Cytoplasmic	L
b0812	dps_ecoli	P27430	DNA protection during starvation protein.	II	18.6	0	5.7	Associates into a complex		Unknown	L
b0870	ltae_ecoli	P75823	Low-specificity L-threonine aldolase (EC	III	36.5	0	5.8	Homotetramer (Probable).		Unknown	E
b0902	pfla_ecoli	P09374	Pyruvate formate-lyase 1 activating enzyme	III	28.1	0	6		Cytoplasmic.	Cytoplasmic	O
b0907	serc_ecoli	P23721	Phosphoserine aminotransferase (EC 2.6.1.52)	II	39.7	0	5.4	Homodimer.	Cytoplasmic.	Cytoplasmic	H
b0929	ompf_ecoli	P02931	Outer membrane protein F precursor (Porin	II	39.3	0	4.6	Homotrimer.	Integral	Outer Membrane	M
b0930	syn_ecoli	P17242	Asparaginyl-tRNA synthetase (EC 6.1.1.22)	I	52.4	1	5.2	Homodimer.	Cytoplasmic.	Cytoplasmic	J
b0954	faba_ecoli	P18391	3-hydroxydecanoyl-[acyl-carrier-protein]	I	18.8	1	6.2	Homodimer.	Cytoplasmic.	Cytoplasmic	I
b0957	ompa_ecoli	P02934	Outer membrane protein A precursor (Outer	I	37.2	0	5.6	Monomer (Probable).	Integral	Outer Membrane	M
b1062	pyrc_ecoli	P05020	Dihydroorotase (EC 3.5.2.3) (DHOase).	II	38.7	0	5.8	Homodimer.		Cytoplasmic	F
b1066	rimj_ecoli	P09454	Ribosomal-protein-alanine acetyltransferase	III	22.7	0	9.2		Cytoplasmic.	Cytoplasmic	J
b1086	rluc_ecoli	P23851	Ribosomal large subunit pseudouridine	III	36	0	9.9			Cytoplasmic	J
b1093	fabg_ecoli	P25716	3-oxoacyl-[acyl-carrier protein] reductase (EC	II	25.6	1	6.8			Cytoplasmic	Q

b1095	fabf_ecoli	P39435	3-oxoacyl-[acyl-carrier-protein] synthase II (EC	III	42.9	0	5.7	Homodimer.		Cytoplasmic	I
b1107	nagz_ecoli	P75949	Beta-hexosaminidase (EC 3.2.1.52) (N-acetyl-	III	37.6	0	5.9	Monomer (Potential).	Cytoplasmic.	Cytoplasmic	G
b1109	dhna_ecoli	P00393	NADH dehydrogenase (EC 1.6.99.3).	II	47.2	0	9		Membrane.	Unknown	C
b1130	phop_ecoli	P23836	Transcriptional regulatory protein phoP.	II	25.5	0	5.1		Cytoplasmic	Cytoplasmic	T
b1189	dada_ecoli	P29011	D-amino acid dehydrogenase small subunit (EC	III	47.6	1	6.2	Heterodimer of a small	Inner membrane-	Cytoplasmic	E
b1190	alr2_ecoli	P29012	Alanine racemase, catabolic (EC 5.1.1.1).	III	38.8	0	6.6			Cytoplasmic	M
b1204	pth_ecoli	P23932	Peptidyl-tRNA hydrolase (EC 3.1.1.29) (PTH).	II	21.1	1	9	Monomer.	Cytoplasmic.	Cytoplasmic	J
b1207	kprs_ecoli	P08330	Ribose-phosphate pyrophosphokinase (EC	II	34.1	1	5.2		Cytoplasmic.	Cytoplasmic	F
b1215	kdsa_ecoli	P17579	2-dehydro-3-deoxyphosphooctonate aldolase	II	30.8	0	6.3	Homotrimer.	Cytoplasmic.	Cytoplasmic	M
b1241	adhe_ecoli	P17547	Aldehyde-alcohol dehydrogenase [Includes:	II	96	0	6.3	Seems to form a rod		Cytoplasmic	C
b1243	oppa_ecoli	P23843	Periplasmic oligopeptide-binding protein	I	60.9	0	5.9		Periplasmic.	Periplasmic	E
b1269	rlub_ecoli	P37765	Ribosomal large subunit pseudouridine	II	32.7	1	10			Cytoplasmic	J
b1275	cysb_ecoli	P06613	HTH-type transcriptional regulator cysB (Cys	II	36.2	0	6.9	Homotetramer (By	Cytoplasmic.	Cytoplasmic	K
b1281	pyrf_ecoli	P08244	Orotidine 5'-phosphate decarboxylase (EC	II	26.4	1	5.8	Homodimer.		Unknown	F
b1324	tpx_ecoli	P37901	Thiol peroxidase (EC 1.11.1.-) (Scavengase	I	17.7	0	4.8		Periplasmic.	Unknown	O
b1370	inh5_ecoli	P76071	Transposase insH for insertion sequence	II	37.8	1	9.6			Cytoplasmic	L

b1398	paak_ecoli	P76085	Phenylacetate-coenzyme A ligase (EC 6.2.1.30)	II	49	0	6.2			Cytoplasmic	H
b1413	hrpa_ecoli	P43329	ATP-dependent helicase hrpA.	II	149	0	7.9			Cytoplasmic	L
b1427	riml_ecoli	P13857	Ribosomal-protein-serine acetyltransferase (EC	II	20.7	1	5.9		Cytoplasmic.	Cytoplasmic	J
b1430	tehb_ecoli	P25397	Tellurite resistance protein tehB.	II	22.5	0	6.8		Cytoplasmic	Cytoplasmic	Q
b1581	rspa_ecoli	P38104	Starvation sensing protein rspA.	III	46	0	5.7			Cytoplasmic	H
b1623	add_ecoli	P22333	Adenosine deaminase (EC 3.5.4.4) (Adenosine	III	36.4	0	5.4			Cytoplasmic	F
b1680	csdb_ecoli	P77444	Selenocysteine lyase (EC 4.4.1.16)	III	44.4	0	5.9	Homodimer.		Unknown	E
b1718	if3_ecoli	P02999	Translation initiation factor IF-3.	I or II	20.6	1	9.5	Monomer.	Cytoplasmic.	Cytoplasmic	J
b1719	syt_ecoli	P00955	Threonyl-tRNA synthetase (EC 6.1.1.3)	II	74	1	5.8	Homodimer.	Cytoplasmic.	Cytoplasmic	J
b1748	argm_ecoli	P77581	Succinylornithine transaminase (EC 2.6.1.-)	II	43.7	0	5.9			Unknown	E
b1779	g3p1_ecoli	P06977	Glyceraldehyde 3-phosphate dehydrogenase A	I	35.4	1	6.6	Homotetramer.	Cytoplasmic.	Cytoplasmic	G
b1831	proq_ecoli	P45577	ProP effector.	II	25.9		9.7		Cytoplasmic	Unknown	T
b1914	uvry_ecoli	P07027	Response regulator uvrY.	II	23.9	0	6.5		Cytoplasmic	Cytoplasmic	T
b2022	his7_ecoli	P06987	Histidine biosynthesis bifunctional protein hisB	III	40.3	0	5.8		Cytoplasmic.	Cytoplasmic	E
b2029	6pgd_ecoli	P00350	6-phosphogluconate dehydrogenase,	I	51.5	0	5.1			Unknown	G
b2038	rfbc_ecoli	P37745	dTDP-4-dehydrorhamnose 3,5-epimerase (EC	III	21.3	0	5.5	Homodimer (By		Cytoplasmic	M
b2091	gatd_ecoli	P37190	Galactitol-1-phosphate 5-dehydrogenase (EC	II	37.4	1	5.9			Cytoplasmic	E
b2095	gatz_ecoli	P37191	Putative tagatose 6-phosphate kinase gatZ (EC	III	47.1	0	5.5			Cytoplasmic	G

b2096	gaty_ecoli	P37192	Tagatose-1,6-bisphosphate aldolase gatY (EC	III	30.8	0	5.9			Cytoplasmic	G
b2097	alf1_ecoli	P71295	Fructose-bisphosphate aldolase class I (EC	III	38	0	6.2	Homooctamer or	Cytoplasmic	Unknown	G
b2140	dusc_ecoli	P33371	tRNA-dihydrouridine synthase C (EC 1.-.-.-).	III	35.2	0	6.1			Cytoplasmic	J
b2149	mgla_ecoli	P23199	Galactoside transport ATP-binding protein	II	56.4	0	7.2		Inner membrane-	Cytoplasmic	G
b2153	gch1_ecoli	P27511	GTP cyclohydrolase I (EC 3.5.4.16) (GTP-CH-	III	24.7	1	6.9	Homodecamer, composed		Unknown	H
b2155	cira_ecoli	P17315	Colicin I receptor precursor.	I	73.9	0	5		Outer membrane.	Outer Membrane	P
b2159	end4_ecoli	P12638	Endonuclease IV (EC 3.1.21.2)	III	31.5	0	5.4	Monomer.		Cytoplasmic	L
b2215	ompc_ecoli	P06996	Outer membrane protein C precursor (Porin	I	40.4	0	4.5	Homotrimer.	Integral	Outer Membrane	M
b2217	rcsb_ecoli	P14374	Capsular synthesis regulator component B.	II	23.7	0	6.9			Cytoplasmic	T
b2231	gyra_ecoli	P09097	DNA gyrase subunit A (EC 5.99.1.3).	II	97	1	5.1	Made up of two chains.		Cytoplasmic	L
b2237	inaa_ecoli	P27294	Protein inaA.	II	25.3	0	9.1			Cytoplasmic	S
b2242	glpb_ecoli	P13033	Anaerobic glycerol-3-phosphate dehydrogenase	II	45.4	0	5.8	Composed of a catalytic	Loosely bound to	Cytoplasmic	E
b2284	nuof_ecoli	P31979	NADH-quinone oxidoreductase chain F (EC	II	49.3	0	6.4	Composed of 13 different		Cytoplasmic	C
b2285	nuoe_ecoli	P33601	NADH-quinone oxidoreductase chain E (EC	II	18.6		5.4	Composed of 13 different		Cytoplasmic	C

b2286	nucd_ecoli	P33599	NADH-quinone oxidoreductase chain C/D (EC	II	68.7	0	6	Composed of 13 different		Cytoplasmic	C	
b2296	acka_ecoli	P15046	Acetate kinase (EC 2.7.2.1) (Acetokinase).	I	43.3	1	5.9	Homodimer.	Cytoplasmic.	Cytoplasmic	C	
b2323	fabb_ecoli	P14926	3-oxoacyl-[acyl-carrier-protein] synthase I (EC	I	42.6	1	5.4	Homodimer.	Cytoplasmic.	Cytoplasmic	I	
b2349	ints_ecoli	P37326	Putative prophage CPS-53 integrase.	III	44.1		9.4			Cytoplasmic	L	
b2417	ptga_ecoli	P08837	PTS system, glucose-specific IIA component	I	18.1	0	4.7		Cytoplasmic.	Cytoplasmic	G	
b2435	amia_ecoli	P36548	Probable N-acetylmuramoyl-L-alanine amidase	III	31.4	0	9.9			Unknown	M	
b2441	eutb_ecoli	P19635	Ethanolamine ammonia-lyase heavy chain (EC	III	49.4	0	4.8	Heterodimer of two		Unknown	C	
b2478	dapa_ecoli	P05640	Dihydrodipicolinate synthase (EC 4.2.1.52)	III	31.3	1	6	Homotetramer.	Cytoplasmic.	Unknown	E	
b2530	iscs_ecoli	P39171	Cysteine desulfurase (EC 4.4.1.-) (ThiI	II	45.1	1	5.9			Cytoplasmic	E	
b2533	suhb_ecoli	P22783	Inositol-1-monophosphatase (EC 3.1.3.25)	III	29.2	1	6.5	Monomer.		Cytoplasmic	G	
b2551	glya_ecoli	P00477	Serine hydroxymethyltransferase (EC 2.1.2.1)	I	45.3	1	6	Homotetramer.	Cytoplasmic.	Cytoplasmic	E	
b2557	pur4_ecoli	P15254	Phosphoribosylformylglycinamidine synthase	II	141.4	0	5.2	Monomer.	Cytoplasmic.	Unknown	F	
b2599	phea_ecoli	P07022	P-protein [Includes: Chorismate mutase (EC	III	43.1	0	6.2		Cytoplasmic.	Cytoplasmic	E	
b2607	trmd_ecoli	P07020	tRNA (Guanine-N(1)-)-methyltransferase (EC	III	28.4	1	5.5	Monomer.	Cytoplasmic	Cytoplasmic	J	
b2608	rimm_ecoli	P21504	16S rRNA processing protein rimM (21K).	II	20.6	1	4.6		Cytoplasmic	Cytoplasmic	J	
b2614	grpe_ecoli	P09372	GrpE protein (HSP-70 cofactor) (Heat shock	I	21.8	1	4.7			Cytoplasmic	O	

b2620	ssrp_ecoli	P32052	SsrA-binding protein (Small protein B).	III	18.1	0	9.9		Cytoplasmic	Cytoplasmic	O
b2687	luxs_ecoli	P45578	S-ribosylhomocysteinase (EC 3.13.1.-)	II	19.3		5.2	Homodimer (By		Cytoplasmic	T
b2779	eno_ecoli	P08324	Enolase (EC 4.2.1.11) (2-phosphoglycerate	I	45.5		5.3	Homodimer.	Cytoplasmic.	Cytoplasmic	G
b2805	fucr_ecoli	P11554	L-fucose operon activator.	III	27.4	0	7.8			Cytoplasmic	K
b2830	nudh_ecoli	Q46930	(Di)nucleoside polyphosphate hydrolase (EC	II	20.8	0	10	Monomer.		Unknown	L
b2834	tas_ecoli	Q46933	Tas protein.	II	38.5	0	6.3			Unknown	C
b2913	sera_ecoli	P08328	D-3-phosphoglycerate dehydrogenase (EC	I	44	0	5.9	Homotetramer.		Cytoplasmic	E
b2916	icia_ecoli	P24194	Chromosome initiation inhibitor (OriC	III	33.5	0	6.4	Behaves as an homodimer		Cytoplasmic	K
b2925	alf_ecoli	P11604	Fructose-bisphosphate aldolase class II (EC	II	39	1	5.5	Homodimer.		Unknown	G
b2926	pgk_ecoli	P11665	Phosphoglycerate kinase (EC 2.7.2.3).	I	41	1	5.1	Monomer.	Cytoplasmic.	Cytoplasmic	G
b2942	metk_ecoli	P04384	S-adenosylmethionine synthetase (EC 2.5.1.6)	III	41.8	1	5.1	Homotetramer.	Cytoplasmic.	Cytoplasmic	H
b2960	trmb_ecoli	P32049	tRNA (guanine-N(7)-)-methyltransferase (EC	III	27.3	0	6.4	Monomer.		Cytoplasmic	J
b2980	glcc_ecoli	P52072	Glc operon transcriptional activator.	III	28.8	0	9.2			Cytoplasmic	K
b3019	parc_ecoli	P20082	Topoisomerase IV subunit A (EC 5.99.1.-).	III	83.8	1	6.2	Composed of two	Membrane-	Cytoplasmic	L
b3068	mug_ecoli	P43342	G/U mismatch-specific DNA glycosylase (EC	II	18.7	0	9.2		Cytoplasmic	Cytoplasmic	L
b3084	rsmd_ecoli	P42596	Putative ribosomal RNA small subunit	III	42.3	0	6.3			Cytoplasmic	J
b3092	uxac_ecoli	P42607	Uronate isomerase (EC 5.3.1.12) (Glucuronate	III	54		5.4			Cytoplasmic	G

b3162	dead_ecoli	P23304	Cold-shock DEAD-box protein A (ATP-	III	70.4	0	8.8		Cytoplasmic	Cytoplasmic	L
b3164	pnp_ecoli	P05055	Polyribonucleotide nucleotidyltransferase (EC	I	77.1	1	5.1	Homotrimer.	Cytoplasmic.	Cytoplasmic	J
b3168	if2_ecoli	P02995	Translation initiation factor IF-2.	II	97.4	1	5.8		Cytoplasmic.	Cytoplasmic	J
b3181	grea_ecoli	P21346	Transcription elongation factor greA	II	17.6	0	4.7			Cytoplasmic	K
b3225	nana_ecoli	P06995	N-acetylneuraminate lyase (EC 4.1.3.3) (N-	III	32.5	0	5.6	Homotetramer.	Cytoplasmic.	Unknown	E
b3244	tldd_ecoli	P46473	TldD protein.	III	51.4	0	4.9			Cytoplasmic	O
b3251	mreb_ecoli	P13519	Rod shape-determining protein mreB.	II	37	1	5.2			Cytoplasmic	D
b3256	accc_ecoli	P24182	Biotin carboxylase (EC 6.3.4.14) (A subunit of	II	49.3		6.7	Acetyl-CoA carboxylase is		Cytoplasmic	I
b3260	dusb_ecoli	P25717	tRNA-dihydrouridine synthase B (EC 1.-.-.-).	III	35.9	0	6.3			Cytoplasmic	J
b3295	rpoa_ecoli	P00574	DNA-directed RNA polymerase alpha chain	I	36.5		5	Homodimer. The RNAP		Cytoplasmic	K
b3340	efg_ecoli	P02996	Elongation factor G (EF-G).	I	77.5	1	5.2		Cytoplasmic.	Cytoplasmic	J
b3357	crp_ecoli	P03020	Catabolite gene activator (cAMP receptor	III	23.6		8.4	Binds DNA as a dimer.		Cytoplasmic	T
b3390	arok_ecoli	P24167	Shikimate kinase I (EC 2.7.1.71) (SKI).	II	19.4	1	5.3		Cytoplasmic	Cytoplasmic	E
b3401	hslo_ecoli	P45803	33 kDa chaperonin (Heat shock protein 33)	II	32.5		4.4		Cytoplasmic.	Cytoplasmic	O
b3405	ompr_ecoli	P03025	Transcriptional regulatory protein ompR.	II	27.4	0	6	Monomer and multimer.	Cytoplasmic.	Cytoplasmic	T
b3417	phsm_ecoli	P00490	Maltodextrin phosphorylase (EC 2.4.1.1).	III	90.3	0	7.1	Homodimer.		Unknown	G

b3433	dhas_ecoli	P00353	Aspartate-semialdehyde dehydrogenase (EC	III	40	1	5.4	Homodimer.		Unknown	E
b3463	ftse_ecoli	P10115	Cell division ATP-binding protein ftsE.	III	24.4	1	9.4			Cytoplasmic	D
b3481	nikr_ecoli	P28910	Nickel responsive regulator.	II	15.1	0	5.8	Homotetrame r.		Cytoplasmic	K
b3517	dcea_ecoli	P80063	Glutamate decarboxylase alpha (EC 4.1.1.15)	II	52.7		5.2	Homohexam er.			E
b3565	xyla_ecoli	P00944	Xylose isomerase (EC 5.3.1.5) (D-xylulose	III	49.7	0	5.8	Homotetrame r.	Cytoplasmic.	Cytoplasmic	G
b3572	avta_ecoli	P09053	Valine--pyruvate aminotransferase (EC	II	46.7	0	5.7	Homodimer (By	Cytoplasmic (By	Cytoplasmic	E
b3588	aldb_ecoli	P37685	Aldehyde dehydrogenase B (EC 1.2.1.22)	III	56.3		5.4			Cytoplasmic	C
b3605	lldd_ecoli	P33232	L-lactate dehydrogenase (Cytochrome) (EC	III	42.7	0	6.3			Unknown	C
b3607	cyse_ecoli	P05796	Serine acetyltransferase (EC 2.3.1.30) (SAT).	II	29.3	1	6.1	Homohexam er. Dimer of a	Cytoplasmic.	Cytoplasmic	E
b3650	spot_ecoli	P17580	Guanosine-3',5'-bis(Diphosphate) 3'-	II	79.3		8.9			Cytoplasmic	T
b3651	trmh_ecoli	P19396	tRNA (Guanosine-2'-O-)-methyltransferase (EC	II	25.3	0	6.7		Cytoplasmic	Cytoplasmic	J
b3708	tnaa_ecoli	P00913	Tryptophanase (EC 4.1.99.1) (L-tryptophan	II	52.8	0	5.9	Homotetrame r.		Cytoplasmic	E
b3725	pstb_ecoli	P07655	Phosphate import ATP-binding protein pstB	II	28.9	0	6.1	The complex is composed	Inner membrane-	Cytoplasmic	P
b3741	gida_ecoli	P17112	Glucose inhibited division protein A.	II	69.5	0	6.2			Cytoplasmic	D
b3751	rbsb_ecoli	P02925	D-ribose-binding periplasmic protein precursor.	I	31	0	6		Periplasmic.	Periplasmic	G

b3775	ppic_ecoli	P39159	Peptidyl-prolyl cis-trans isomerase C (EC	II	10.1	0	9.2		Cytoplasmic.	Outer Membrane	O
b3780	rhlb_ecoli	P24229	ATP-dependent RNA helicase rhlB (EC 3.6.1.-	II	47	0	7.3	Component of the		Unknown	L
b3783	rho_ecoli	P03002	Transcription termination factor rho.	II	47		6.8	Homohexamer.		Cytoplasmic	K
b3845	thik_ecoli	P21151	3-ketoacyl-CoA thiolase (EC 2.3.1.16) (Fatty	III	40.9	0	6.3	Tetramer of two alpha	Cytoplasmic.	Cytoplasmic	I
b3847	pepq_ecoli	P21165	Xaa-Pro dipeptidase (EC 3.4.13.9) (X-Pro	III	50.2	0	5.6			Cytoplasmic	E
b3850	hemg_ecoli	P27863	Protoporphyrinogen oxidase (EC 1.3.3.4)	II	21.2	1	9.7	Belongs to a multi-protein		Unknown	C
b3865	engb_ecoli	P24253	Probable GTP-binding protein engB.	II	23.6	1	6.9			Unknown	D
b3871	typa_ecoli	P32132	GTP-binding protein typA/BipA (Tyrosine	II	65.4	0	5.1			Unknown (This	N
b3912	cpxr_ecoli	P16244	Transcriptional regulatory protein cpxR.	II	26.3	0	5.4		Cytoplasmic	Cytoplasmic	T
b3941	metf_ecoli	P00394	5,10-methylenetetrahydrofolate reductase (EC	III	33.1	0	6	Homotetramer.		Cytoplasmic	E
b3957	arge_ecoli	P23908	Acetylornithine deacetylase (EC 3.5.1.16)	III	42.3		5.5	Homodimer.	Cytoplasmic	Cytoplasmic	E
b3961	oxyr_ecoli	P11721	Hydrogen peroxide-inducible genes activator	II	34.3	0	6	Homodimer and		Cytoplasmic	K
b3962	stha_ecoli	P27306	Soluble pyridine nucleotide transhydrogenase	II	51.4	0	6.1	Homooligomer; probable	Cytoplasmic.	Cytoplasmic	C
b3965	trma_ecoli	P23003	tRNA (Uracil-5-)-methyltransferase (EC	III	42	0	5.7			Unknown	J
b3982	nusg_ecoli	P16921	Transcription antitermination protein nusG.	II	20.4	0	6.3			Cytoplasmic	K

b3987	rpob_ecoli	P00575	DNA-directed RNA polymerase beta chain (EC	II	150.6	1	5.2	The RNAP catalytic core		Cytoplasmic	K
b3988	rpoc_ecoli	P00577	DNA-directed RNA polymerase beta' chain (EC	II	155.2	1	6.7	The RNAP catalytic core		Cytoplasmic	K
b3990	thih_ecoli	P30140	Thiazole biosynthesis protein thiH.	III	43.2		6.6			Unknown	H
b3991	thig_ecoli	P30139	Thiazole biosynthesis protein thiG.	I	26.9		5.4		Cytoplasmic.	Cytoplasmic	F
b3993	thie_ecoli	P30137	Thiamine-phosphate pyrophosphorylase (EC	II	23	0	5.5			Unknown	H
b3995	rsd_ecoli	P31690	Regulator of sigma D.	III	18.2	1	5.7			Unknown	K
b3997	dcup_ecoli	P29680	Uroporphyrinogen decarboxylase (EC 4.1.1.37)	II	39.2	1	5.9		Cytoplasmic	Unknown	H
b4039	ubic_ecoli	P26602	Chorismate--pyruvate lyase (EC 4.-.-.-).	III	18.6	0	7.7	Monomer.	Cytoplasmic.	Cytoplasmic	H
b4049	dusa_ecoli	P32695	tRNA-dihydrouridine synthase A (EC 1.-.-.-).	II	36.8	0	6.1			Cytoplasmic	J
b4154	frda_ecoli	P00363	Fumarate reductase flavoprotein subunit (EC	III	65.8	0	5.9	Fumarate dehydrogenase		Periplasmic	C
b4177	pura_ecoli	P12283	Adenylosuccinate synthetase (EC 6.3.4.4)	I	47.2	0	5.3	Homodimer.	Cytoplasmic.	Cytoplasmic	F
b4179	rnr_ecoli	P21499	Ribonuclease R (EC 3.1.-.-) (RNase R) (VacB	II	92.1	0	8.8	Monomer.		Cytoplasmic	K
b4226	ipyr_ecoli	P17288	Inorganic pyrophosphatase (EC 3.6.1.1)	I	19.6	1	5	Homohexamer.	Cytoplasmic.	Cytoplasmic	C
b4235	pmba_ecoli	P24231	PmbA protein (TldE protein).	III	48.4	0	5.4		Cytoplasmic.	Cytoplasmic	O
b4260	ampa_ecoli	P11648	Cytosol aminopeptidase (EC 3.4.11.1) (Leucine	III	54.9	0	6.8	Homohexamer.		Cytoplasmic	E
b4371	rsmc_ecoli	P39406	Ribosomal RNA small subunit	II	37.5	0	6			Unknown	J

b4381	deoc_ecoli	P00882	Deoxyribose-phosphate aldolase (EC 4.1.2.4)	I	27.7	0	5.5	Monomer and homodimer.	Cytoplasmic.	Cytoplasmic	F
b4382	typh_ecoli	P07650	Thymidine phosphorylase (EC 2.4.2.4)	III	47.2	0	5.2	Homodimer.		Unknown	F
b4384	deod_ecoli	P09743	Purine nucleoside phosphorylase (EC 2.4.2.1)	I	25.8	0	5.4	Homohexamer.		Cytoplasmic	F
b4396	rob_ecoli	P27292	Right origin-binding protein.	II	33.1	0	6.7			Cytoplasmic	K
	blat_ecoli	P00810	Beta-lactamase TEM precursor (EC 3.5.2.6)	I	31.5		5.5			Periplasmic	M
	muli_ecoli	P02937	Major outer membrane lipoprotein precursor	I or II	8.3		8.1		Attached to the	Unknown	N
	ygea_ecoli	P03813	Hypothetical protein ygeA.	II	25.2	0	5.1			Cytoplasmic	M
	hlpa_ecoli	P11457	Histone-like protein HLP-1 precursor (DNA-	III	17.7	0	9.5	Homotetramer.	Either in the	Outer Membrane	M
	kpy1_ecoli	P14178	Pyruvate kinase I (EC 2.7.1.40) (PK-1).	I	50.7	0	5.8	Homotetramer.		Unknown	G
	ypt1_ecoli	P29368	Hypothetical 31.7 kDa protein in TRAX-FINO	III	31.8		5.5			Unknown	R
	ybib_ecoli	P30177	Hypothetical protein ybiB.	II	35	0	6.4			Cytoplasmic	E
	yihe_ecoli	P32127	Hypothetical protein yihE.	II	38.1	0	5			Cytoplasmic	R
	yihx_ecoli	P32145	Hypothetical protein yihX.	II	22.7	0	5.2			Cytoplasmic	R
	yijo_ecoli	P32677	Hypothetical transcriptional regulator yijO.	II	32.1	0	8.3			Cytoplasmic	K
	yjbq_ecoli	P32698	Hypothetical protein yjbQ.	II	15.7	0	6.5			Cytoplasmic	S
	yfif_ecoli	P33635	Hypothetical tRNA/rRNA methyltransferase	III	37.8	0	8.9			Unknown	J
	thi2_ecoli	P33636	Thioredoxin 2 (EC 1.8.1.8) (Protein-disulfide	I	15.6	1	5		Cytoplasmic.	Cytoplasmic	O

	yhbj_ecoli	P33995	Hypothetical UPF0042 protein yhbJ.	III	32.5	0	6.7			Cytoplasmic	R
	yadf_ecoli	P36857	Protein yadF.	II	25.1	1	6.2			Cytoplasmic	P
	yegd_ecoli	P36928	Hypothetical chaperone protein yegD.	II	49.4	0	5.1			Unknown	O
	ydhd_ecoli	P37010	Protein ydhD.	II	12.9	1	4.8			Unknown	O
	ybak_ecoli	P37175	Protein ybaK.	III	17.1	0	9			Cytoplasmic	S
	ygaf_ecoli	P37339	Hypothetical protein ygaF.	II	48.6		9.2			Unknown	R
	ycfh_ecoli	P37346	Putative deoxyribonuclease ycfH (EC 3.1.21.-).	III	29.8	0	5.2			Cytoplasmic	L
	yjju_ecoli	P39407	Hypothetical protein yjjU.	III	39.8	0	8.7			Unknown	R
	yjjv_ecoli	P39408	Putative deoxyribonuclease yjjV (EC 3.1.21.-).	II	28.9	1	6.1			Cytoplasmic	L
	glr2_ecoli	P39811	Glutaredoxin 2 (Grx2).	II	24.4	0	7.7			Unknown	O
	ycaj_ecoli	P45526	Hypothetical protein ycaJ.	II	49.6	0	6			Cytoplasmic	L
	ycio_ecoli	P45847	Protein yciO.	II	23.2	0	6			Cytoplasmic	J
	yhgi_ecoli	P46847	Protein yhgI.	I or II	21	0	4.5			Cytoplasmic	O
	ybff_ecoli	P75736	Putative esterase/lipase ybfF (EC 3.1.-.-).	II	28.4	0	5.9			Unknown	R
	ybjs_ecoli	P75821	Hypothetical protein ybjS.	III	38.1	1	8.8			Unknown	M
	ycbl_ecoli	P75849	Hypothetical protein ycbL.	II	23.8	0	5			Unknown	R
	ycby_ecoli	P75864	Hypothetical protein ycbY.	III	78.9	0	9			Unknown	L
	ycfp_ecoli	P75950	Hypothetical protein ycfP.	III	21.2	1	6.1			Cytoplasmic	R
	ydcp_ecoli	P76104	Putative protease ydcP precursor (EC 3.4.-.-).	II	72.7	0	6.7			Unknown	O
	ynce_ecoli	P76116	Hypothetical protein yncE precursor.	I	38.6	1	8.8			Unknown	S
	yneb_ecoli	P76143	Putative aldolase yneB (EC 4.2.1.-).	III	31.9	0	6.1			Unknown	G

	ydhf_ecoli	P76187	Hypothetical oxidoreductase ydhF (EC 1.-.-.-).	II	33.6	0	5.8			Cytoplasmic	R
	yeat_ecoli	P76250	Putative HTH-type transcriptional regulator	II	34.6		6.2			Unknown (This	K
	yoda_ecoli	P76344	Protein yodA.	II	24.8	0	5.7			Unknown	R
	yohl_ecoli	P76424	Hypothetical protein yohL.	II	10.1	1	8.8			Cytoplasmic	S
	yfbu_ecoli	P76492	Protein yfbU.	II	19.5	0	6.1			Cytoplasmic	S
	yffs_ecoli	P76550	Hypothetical protein yffS.	II	31	1	5.5			Cytoplasmic	S
	ycjz_ecoli	P77333	Putative HTH-type transcriptional regulator	II	33.5	0	9.1			Cytoplasmic	K
	ybbn_ecoli	P77395	Protein ybbN.	II	31.8	0	4.5			Cytoplasmic	O
	yqab_ecoli	P77475	Hypothetical protein yqaB.	III	20.8	0	5.5			Cytoplasmic	R
	yfhp_ecoli	P77484	Hypothetical protein yfhP.	II	17.3	0	6.8			Unknown	K
	yfbq_ecoli	P77727	Probable aminotransferase yfbQ (EC 2.6.1.-).	III	45.5		5.9	Homodimer (By	Cytoplasmic (By	Cytoplasmic	E
	ydcr_ecoli	P77730	Hypothetical protein ydcR.	II	52.8	0	8.9			Cytoplasmic	K
	yajo_ecoli	P77735	Hypothetical oxidoreductase yajO (EC 1.-.-.-).	III	36.4	0	5.2			Cytoplasmic	C
	ynia_ecoli	P77739	Hypothetical protein yniA.	II	32.5		5			Unknown	S
	yghz_ecoli	Q46851	Hypothetical protein yghZ.	II	38.8	0	6.7			Unknown	C
	yqji_ecoli	Q46872	Hypothetical protein yqjI.	III	23.4	0	6.3			Cytoplasmic	K
	ypt2_ecoli	Q99390	Hypothetical 31.7 kDa protein in TRAX-FINO	II	31.7		5.4			Unknown	R
