# Supplementary material for: Genome-Scale Reconstruction of Escherichia coli's Transcriptional and Translational Machinery: A Knowledge Base, Its Mathematical Formulation, and Its Functional Characterization
Source: PLoS Comput Biol. 2009 Mar 13;5(3):e1000312. doi: 10.1371/journal.pcbi.1000312 (PMC2648898; doi:10.1371/journal.pcbi.1000312)
Supplement: Table S7 — Unbalanced exchange reactions (0.01 MB PDF) [file pcbi.1000312.s009.pdf]

Table S7 - unbalanced exchange rxns

Reaction Abreviation							
Exch_10fthf[e]	N: -7	C: -20	O: -7	H: -21	charge: 2		
Exch_5fthf[e]	N: -7	C: -20	O: -7	H: -21	charge: 2		
Exch_5mta[e]	S: -1	N: -5	C: -11	O: -3	H: -15		
Exch_accoa[e]	S: -1	N: -7	C: -23	O: -17	P: -3	H: -34	charge: 4
Exch_ade[e]	N: -5	C: -5	H: -5				
Exch_adocbl[e]	N: -18	K: -1	C: -72	O: -17	P: -1	H: -100	
Exch_adp[e]	N: -5	C: -10	O: -10	P: -2	H: -12	charge: 3	
Exch_ahcys[e]	S: -1	N: -6	C: -14	O: -5	H: -20		
Exch_ala-L[e]	N: -1	C: -3	O: -2	H: -7			
Exch_amet[e]	S: -1	N: -6	C: -15	O: -5	H: -23	charge: -1	
Exch_amp[e]	N: -5	C: -10	O: -7	P: -1	H: -12	charge: 2	
Exch_arg-L[e]	N: -4	C: -6	O: -2	H: -15	charge: -1		
Exch_asn-L[e]	N: -2	C: -4	O: -3	H: -8			
Exch_asp-L[e]	N: -1	C: -4	O: -4	H: -6	charge: 1		
Exch_atp[e]	N: -5	C: -10	O: -13	P: -3	H: -12	charge: 4	
Exch_C10H8O5[e]	C: -10	O: -5	H: -8	charge: 2			
Exch_C9H9O4[e]	C: -9	O: -4	H: -9	charge: 1			
Exch_cdp[e]	N: -3	C: -9	O: -11	P: -2	H: -12	charge: 3	
Exch_chor[e]	C: -10	O: -6	H: -8	charge: 2			
Exch_cmp[e]	N: -3	C: -9	O: -8	P: -1	H: -12	charge: 2	
Exch_coa[e]	S: -1	N: -7	C: -21	O: -16	P: -3	H: -32	charge: 4
Exch_ctp[e]	N: -3	C: -9	O: -14	P: -3	H: -12	charge: 4	
Exch_cys-L[e]	S: -1	N: -1	C: -3	O: -2	H: -7		
Exch_dad-5[e]	N: -5	C: -10	O: -3	H: -13			
Exch_dmpp[e]	C: -5	O: -7	P: -2	H: -9	charge: 3		
Exch_fad[e]	N: -9	C: -27	O: -15	P: -2	H: -31	charge: 2	
Exch_fadh2[e]	N: -9	C: -27	O: -15	P: -2	H: -33	charge: 2	
Exch_Fe(2)[e]	F: -1	charge: -2					
Exch_Fe(3)[e]	F: -1	charge: -3					
Exch_fldox[e]	X: -1						
Exch_fldrd[e]	X: -1	H: -2					
Exch_for[e]	C: -1	O: -2	H: -1	charge: 1			
Exch_gdp[e]	N: -5	C: -10	O: -11	P: -2	H: -12	charge: 3	
Exch_gln-L[e]	N: -2	C: -5	O: -3	H: -10			
Exch_glu-L[e]	N: -1	C: -5	O: -4	H: -8	charge: 1		
Exch_glx[e]	C: -2	O: -3	H: -1	charge: 1			
Exch_gly[e]	N: -1	C: -2	O: -2	H: -5			
Exch_gmp[e]	N: -5	C: -10	O: -8	P: -1	H: -12	charge: 2	
Exch_gtp[e]	N: -5	C: -10	O: -14	P: -3	H: -12	charge: 4	
Exch_gua[e]	N: -5	C: -5	O: -1	H: -5			
Exch_h[e]	H: -1	charge: -1					
Exch_h2o[e]	O: -1	H: -2					
Exch_h2s[e]	S: -1	H: -2					
Exch_hco3[e]	C: -1	O: -3	H: -1	charge: 1			
Exch_his-L[e]	N: -3	C: -6	O: -2	H: -9			
Exch_ile-L[e]	N: -1	C: -6	O: -2	H: -13			
Exch_leu-L[e]	N: -1	C: -6	O: -2	H: -13			
Exch_lys-L[e]	N: -2	C: -6	O: -2	H: -15	charge: -1		
Exch_met-L[e]	S: -1	N: -1	C: -5	O: -2	H: -11		

Exch_mg2[e]	M: -1	charge: -2				
Exch_nad[e]	N: -7	C: -21	O: -14	P: -2	H: -26	charge: 1
Exch_nadh[e]	N: -7	C: -21	O: -14	P: -2	H: -27	charge: 2
Exch_nadp[e]	N: -7	C: -21	O: -17	P: -3	H: -25	charge: 3
Exch_nadph[e]	N: -7	C: -21	O: -17	P: -3	H: -26	charge: 4
Exch_nh3[e]	N: -1	H: -3				
Exch_phe-L[e]	N: -1	C: -9	O: -2	H: -11		
Exch_pi[e]	O: -4	P: -1	H: -1	charge: 2		
Exch_ppi[e]	O: -7	P: -2	H: -1	charge: 3		
Exch_pro-L[e]	N: -1	C: -5	O: -2	H: -9		
Exch_pydx5p	N: -1	C: -8	O: -6	P: -1	H: -8	charge: 2
Exch_selnp[e]	E: -1	O: -3	P: -1	H: -2	charge: 1	
Exch_ser-L[e]	N: -1	C: -3	O: -3	H: -7		
Exch_so4[e]	S: -1	O: -4	charge: 2			
Exch_sprm[e]	N: -4	C: -10	H: -30	charge: -4		
Exch_thf[e]	N: -7	C: -19	O: -6	H: -21	charge: 2	
Exch_thr-L[e]	N: -1	C: -4	O: -3	H: -9		
Exch_trdox[e]	X: -1					
Exch_trdrd[e]	X: -1	H: -2				
Exch_trp-L[e]	N: -2	C: -11	O: -2	H: -12		
Exch_tyr-D[e]	N: -1	C: -9	O: -3	H: -11		
Exch_tyr-L[e]	N: -1	C: -9	O: -3	H: -11		
Exch_udp[e]	N: -2	C: -9	O: -12	P: -2	H: -11	charge: 3
Exch_ump[e]	N: -2	C: -9	O: -9	P: -1	H: -11	charge: 2
Exch_utp[e]	N: -2	C: -9	O: -15	P: -3	H: -11	charge: 4
Exch_val-L[e]	N: -1	C: -5	O: -2	H: -11		
Exch_zn2[e]	Z: -1	charge: -2				
