# Supplementary material for: Genome-Scale Reconstruction of Escherichia coli's Transcriptional and Translational Machinery: A Knowledge Base, Its Mathematical Formulation, and Its Functional Characterization
Source: PLoS Comput Biol. 2009 Mar 13;5(3):e1000312. doi: 10.1371/journal.pcbi.1000312 (PMC2648898; doi:10.1371/journal.pcbi.1000312)
Supplement: Table S8 — Unbalanced internal reactions (0.01 MB PDF) [file pcbi.1000312.s010.pdf]

Table S8 - unbalanced internal rxns

Reaction Abreviation						
[2Fe-2S]_FORM	charge: -2					
[4Fe-4S]_FORM	charge: -2					
16S_rRNA_FORM1	N: 1	H: 1				
16S_rRNA_FORM2	N: -2	C: -1	O: 1			
16S_rRNA_FORM3	N: -3	C: -1	O: 3	H: -1		
16S_rRNA_FORM4	N: -1	O: 1	H: -1			
16S_rRNA_FORM5	N: -2	C: -1	O: 1			
16S_rRNA_FORM6	O: -2					
16S_rRNA_FORM7	N: 4	C: 1	O: -2	H: 2		
23S_rRNA_FORM1	N: -1	C: -8	O: -7	P: -1	H: -7	
23S_rRNA_FORM2	N: 1	C: 2	O: 1	H: 1		
23S_rRNA_FORM3	N: -1	C: 1	O: 4	H: 1		
23S_rRNA_FORM4	N: -1	C: 1	O: 3	H: 1		
23S_rRNA_FORM5	N: 1	C: 2	O: 1	H: 1		
23S_rRNA_FORM6	N: 1	C: 2	O: 1	H: 1		
23S_rRNA_FORM7	N: 2	C: 1	H: 4			
5S_rRNA_FORM1	H: 1					
5S_rRNA_FORM2	N: -1	O: 1				
5S_rRNA_FORM3	N: 1	C: 1				
5S_rRNA_FORM4	N: 1	C: 1				
5S_rRNA_FORM5	N: -1	O: 1				
5S_rRNA_FORM6	O: 1	H: 1				
5S_rRNA_FORM7	N: 1	C: 1				
5S_rRNA_FORM8	N: 1	C: 1				
Dus_gen_FORMa	S: -4	N: -7	C: -33	O: -13	H: -25	charge: 2
Dus_gen_FORMb	N: -2	C: 8	O: 1	H: 1	charge: -1	
Dus_gen_FORMc	S: 3	N: 10	C: 25	O: 12	H: 23	charge: -1
EF-TU_FORM5b	C: -1	O: -1	H: -2			
ileX_to_ile2	N: -1	H: -1				
leuP_to_leu1	C: -4	O: -5	H: -4			
leuQ_to_leu1	N: -1	C: -4	O: -4	H: -5		
leuT_to_leu1	N: -1	C: -4	O: -4	H: -5		
leuV_to_leu1	N: -1	C: -4	O: -4	H: -5		
leuW_to_leu1	N: 1	C: 14	O: 14	P: 2	H: 17	charge: -2

leuX_to_leu2	C: 8	O: 10	P: 1	H: 11	charge: -1			
leuZ_to_leu2	S: -1	C: -9	O: -11	P: -1	H: -11	charge: 1		
metY_to_fmet	C: 1	O: 1	H: 2					
proK_to_pro1	C: 1	O: 2						
proL_to_pro2	N: -1	C: 1	O: 2	H: -1				
proM_to_pro1	C: -1	O: -2	H: -1	charge: 1				
proM_to_pro2	N: 1	C: -1	O: -2	charge: 1				
RNase_Gen_FORMa	S: -1	N: 130	Z: 1	M: -1	C: 455	O: 129	H: 780	charge: -1
RNase_Gen_FORMb	S: 2	N: 183	Z: 1	M: -2	C: 616	O: 197	H: 961	charge: -9
RNase_Gen_FORMc	S: 19	N: 700	Z: 1	M: 3	C: 2537	O: 749	H: 3968	charge: -11
RNase_Gen_FORMd	S: -2	N: -205	Z: 1	M: 2	C: -682	O: -202	H: -1103	charge: 7
RNase_Gen_FORMe	S: 1	N: -106	Z: -1	M: 3	C: -387	O: -123	H: -636	charge: 3
RnpBb_RENAME	O: 6	P: 2	charge: -2					
serT_to_ser2	S: -1	C: -6	O: -1	H: -10	charge: 1			
serT_to_ser3	S: -1	N: 5	C: 8	O: 4	P: 1	H: 10		
serU_to_ser3	N: -6	C: -8	O: -4	P: -1	H: -10	charge: 1		
serW_to_ser2	S: 1	C: 3	H: 5					
serX_to_ser2	S: 1	C: 3	H: 5					
sink_preQ0	N: -5	C: -7	O: -1	H: -5				
TEMP_gltT_tRNA	S: 1	E: -1						
TEMP_gltU_tRNA	S: 1	E: -1						
TEMP_gltV_tRNA	S: 1	E: -1						
TEMP_gltW_tRNA	S: 1	E: -1						
TEMP_lysQ_tRNA	S: 1	E: -1						
TEMP_lysT_tRNA	S: 1	E: -1						
TEMP_lysV_tRNA	S: 1	E: -1						
TEMP_lysW_tRNA	S: 1	E: -1						
TEMP_lysY_tRNA	S: 1	E: -1						
TEMP_lysZ_tRNA	S: 1	E: -1						
thrT_to_thr1	N: -1	C: -1	O: 1					
thrT_to_thr2	N: -1	C: -1	H: -1					
thrU_to_thr2	N: 1	O: 2	H: 1					
thrU_to_thr3	N: -1	O: 1						
thrV_to_thr1	N: 1	O: -1						
thrV_to_thr2	N: 1	O: -2	H: -1					
thrW_to_thr3	O: -1	H: -1						
tyrT_to_tyr1	N: 1	O: -1						

tyrU_to_tyr1	N: -2	C: -1	O: 1	H: -1	
tyrV_to_tyr1	N: 1	O: 5	P: 2	charge: -2	
valT_to_val3	C: 3	O: 3	H: 5		
valU_to_val3	C: 3	O: 3	H: 5		
valV_to_val2	N: 1	H: 1			
valV_to_val3	N: 2	C: -7	O: -8	P: -1	H: -10
valW_to_val2	N: -2	H: -2			
valW_to_val3	N: -1	C: -7	O: -8	P: -1	H: -13
valX_to_val3	C: 3	O: 3	H: 5		
valY_to_val3	C: 3	O: 3	H: 5		
valZ_to_val3	C: 3	O: 3	H: 5		
