# Supplementary material for: Genome-Scale Reconstruction of Escherichia coli's Transcriptional and Translational Machinery: A Knowledge Base, Its Mathematical Formulation, and Its Functional Characterization
Source: PLoS Comput Biol. 2009 Mar 13;5(3):e1000312. doi: 10.1371/journal.pcbi.1000312 (PMC2648898; doi:10.1371/journal.pcbi.1000312)
Supplement: Table S9 — E-matrix transcription units (0.02 MB PDF) [file pcbi.1000312.s011.pdf]

**Table S9 E-matrix Transcription Units**

Taken from EcoCyc, version 10.6 (Dec 2006)

| Transcription Unit Name | Promotor Name | Genes | Left | Right | Transcription direction | Terminator | Number of genes per TU | Type |
|---|---|---|---|---|---|---|---|---|
| TU00021 | dusBp | b3260;b3261; | 3408302 | 3409589 | + | | 2 | CDS |
| TU00083 | rpoHp5 | b3461_v4; | 3597952 | 3598806 | - | Rho-Independent-Terminators | 1 | CDS |
| TU00084 | rpoHp4 | b3461_v3; | 3597952 | 3598806 | - | Rho-Independent-Terminators | 1 | CDS |
| TU00221 | lysUp1 | b4129_v1; | 4351223 | 4352740 | - | | 1 | CDS |
| TU00236 | yjeFp1 | b4168_v2;b4167_v2;b4169_v2;b4170_v2;b4171_v2;b4172_v2;b4173_v2;b4174_v2;b4175_v2; | 4392089 | 4402327 | + | | 9 | CDS |
| TU00258 | defp | b3287;b3288; | 3431712 | 3433183 | + | | 2 | CDS |
| TU00260 | dnaKp1 | b0014_v2;b0015_v2; | 12163 | 15298 | + | | 2 | CDS |
| TU00277 | rrmJp1 | b3178_v1;b3179_v1; | 3323023 | 3325686 | - | Rho-Independent-Terminators | 2 | CDS |
| TU00281 | glyQp | b3559;b3560; | 3720351 | 3723341 | - | Rho-Independent-Terminators | 2 | CDS |
| TU00291 | ribFp | b0025;b0026;b0027;b0028;b0029; | 21407 | 27227 | + | | 5 | CDS |
| TU00304 | mreBp | b3247;b3248;b3249;b3250;b3251; | 3394348 | 3399109 | - | | 5 | CDS |
| TU00309 | nlpDp1 | b2741_v2;b2742_v1; | 2864581 | 2866775 | - | | 2 | CDS |
| TU00311 | yhbCp | b3170_v2;b3168_v2;b3169_v2; | 3311364 | 3316028 | - | | 3 | CDS |
| TU00314 | yhdTp | b3257;b3258;b3259; | 3405397 | 3407973 | + | Rho-Independent-Terminators | 3 | CDS |
| TU00324 | prfBp | b2890;b2891; | 3031679 | 3034304 | - | | 2 | CDS |
| TU00332 | rncp | b2566_v1;b2567_v1; | 2700503 | 2702085 | - | | 2 | CDS |
| TU00333 | rntp | b1652;b1653; | 1726371 | 1731727 | + | | 2 | CDS |
| TU00335 | rplJp | b3985;b3986;b3987;b3988; | 4178019 | 4187596 | + | Rho-Independent-Terminators | 4 | CDS |
| TU00336 | rplMp | b3230;b3231; | 3375837 | 3376673 | - | Rho-Independent-Terminators | 2 | CDS |
| TU00337 | rplNp | b3299;b3300;b3301;b3302;b3303;b3304;b3305;b3306;b3307;b3308;b3309;b3310; | 3440640 | 3446171 | - | | 12 | CDS |
| TU00338 | rplUp | b3185;b3186; | 3330884 | 3331473 | - | | 2 | CDS |
| TU00339 | rpmBp | b3636;b3637; | 3809273 | 3809697 | - | Rho-Independent-Terminators | 2 | CDS |
| TU00340 | rpmHp1 | b3703_v2;b3704_v2; | 3882359 | 3882875 | + | | 2 | CDS |
| TU00342 | rpoEp2 | b2570_v2;b2571_v2;b2572_v2;b2573_v1; | 2705344 | 2708034 | - | | 4 | CDS |
| TU00343 | rpoNp | b3202_v1; | 3342739 | 3344172 | + | | 1 | CDS |

| | | | | | | | | |
|---|---|---|---|---|---|---|---|---|
| TU00344 | rpoZp | b3649;b3650;b3651;b3652; | 3820129 | 3825314 | + | | 4 | CDS |
| TU00345 | rpsBp | b0169;b0170; | 189874 | 191708 | + | | 2 | CDS |
| TU00346 | rpsFp | b4200;b4201;b4202;b4203; | 4423141 | 4424580 | + | | 4 | CDS |
| TU00347 | rpsJp | b3311;b3312;b3313;b3314;b3315;b3316;b3317;b3318;b3319;b3320;b3321; | 3446336 | 3451292 | - | Rho-Independent-Terminators | 11 | CDS |
| TU00348 | rpsLp | b3339;b3340;b3341;b3342; | 3468167 | 3472574 | - | | 4 | CDS |
| TU00349 | rpsMp | b3294;b3295;b3296;b3297;b3298; | 3437638 | 3440493 | - | Rho-Independent-Terminators | 5 | CDS |
| TU00351 | rpsPp | b2606;b2607;b2608;b2609; | 2742205 | 2744207 | - | | 4 | CDS |
| TU00352 | rpsUp1 | b3065_v3;b3066_v2;b3067_v5; | 3208803 | 3212910 | + | Rho-Independent-Terminators | 3 | CDS |
| TU00354 | secEp | b3981;b3982; | 4175381 | 4176311 | + | | 2 | CDS |
| TU00355 | selAp | b3590;b3591; | 3756040 | 3759272 | - | | 2 | CDS |
| TU00362 | surAp | b0049;b0050;b0051;b0052;b0053; | 50380 | 54702 | - | | 5 | CDS |
| TU00366 | thrSp | b1716;b1717;b1718;b1719; | 1797417 | 1800594 | - | Rho-Independent-Terminators | 4 | CDS |
| TU00411 | grpE | b2614; | 2748137 | 2748730 | - | | 1 | CDS |
| TU00414 | rrmJp2 | b3178;b3179; | 3323023 | 3325686 | - | Rho-Independent-Terminators | 2 | CDS |
| TU00415 | fliAp1 | b1920_v1;b1921_v1;b1922_v1; | 1997609 | 1999813 | - | Rho-Independent-Terminators | 3 | CDS |
| TU00417 | dnaKp2 | b0014_v1;b0015_v1; | 12163 | 15298 | + | | 2 | CDS |
| TU00418 | dnaKp3 | b0014;b0015; | 12163 | 15298 | + | | 2 | CDS |
| TU00426 | rpoHp1 | b3461_v2; | 3597952 | 3598806 | - | Rho-Independent-Terminators | 1 | CDS |
| TU00427 | rpoHp3 | b3461_v1; | 3597952 | 3598806 | - | Rho-Independent-Terminators | 1 | CDS |
| TU00429 | lysUp2 | b4129; | 4351223 | 4352740 | - | | 1 | CDS |
| TU00430 | trmAp | b3965; | 4160193 | 4161293 | - | | 1 | CDS |
| TU00434 | rpsUp2 | b3065_v2;b3066_v1;b3067_v4; | 3208803 | 3212910 | + | Rho-Independent-Terminators | 3 | CDS |
| TU00435 | rpsUp3 | b3065_v1;b3066;b3067_v3; | 3208803 | 3212910 | + | Rho-Independent-Terminators | 3 | CDS |
| TU00436 | rpoDp1 | b3067_v2; | 3211069 | 3212910 | + | Rho-Independent-Terminators | 1 | CDS |
| TU00437 | rpoDp2 | b3067_v1; | 3211069 | 3212910 | + | Rho-Independent-Terminators | 1 | CDS |
| TU00438 | rpoDp3 | b3067; | 3211069 | 3212910 | + | Rho-Independent-Terminators | 1 | CDS |
| TU00440 | nlpDp2 | b2741_v1;b2742; | 2864581 | 2866775 | - | | 2 | CDS |
| TU00442 | rpoSp | b2741; | 2864581 | 2865573 | - | | 1 | CDS |
| TU00460 | fecIp | b4292;b4293; | 4514787 | 4516258 | - | | 2 | CDS |
| TU00471 | fliAp2 | b1920;b1921;b1922; | 1997609 | 1999813 | - | Rho-Independent-Terminators | 3 | CDS |
| TU00472 | rpsUp1 | b3065; | 3208803 | 3209018 | + | Rho-Independent-Terminators | 1 | CDS |
| TU00487 | rpoNp | b3202;b3203;b3204;b3205;b3206; | 3342739 | 3346260 | + | Rho-Dependent-Terminators | 5 | CDS |
| TU00489 | aspV | b0216; | 236931 | 237007 | + | | 1 | tRNA |
| TU00490 | thrWp | b0244; | 262095 | 262170 | + | | 1 | tRNA |

| | | | | | | | | |
|---|---|---|---|---|---|---|---|---|
| TU00491 | argUp | b0536; | 563946 | 564022 | + | | 1 | tRNA |
| TU00492 | lysTp | b0743;b0744;b0745; | 779777 | 780141 | + | | 3 | tRNA |
| TU00493 | valVp | b1665;b1666; | 1744459 | 1744616 | + | | 2 | tRNA |
| TU00494 | proLp | b2189; | 2284233 | 2284309 | + | | 1 | tRNA |
| TU00495 | valUp | b2401;b2402;b2403;b2404; | 2518953 | 2519350 | + | | 4 | tRNA |
| TU00496 | metZp | b2814;b2815;b2816; | 2945409 | 2945705 | + | | 3 | tRNA |
| TU00497 | asnTp | b1977; | 2042573 | 2042648 | + | | 1 | tRNA |
| TU00498 | asnUp | b1986; | 2057875 | 2057950 | + | | 1 | tRNA |
| TU00499 | asnVp | b1989; | 2060284 | 2060359 | + | | 1 | tRNA |
| TU00500 | argWp | b2348; | 2464331 | 2464405 | + | | 1 | tRNA |
| TU00501 | pheVp | b2967; | 3108388 | 3108463 | + | | 1 | tRNA |
| TU00502 | ileXp | b3069; | 3213620 | 3213695 | + | | 1 | tRNA |
| TU00503 | argXp | b3796;b3797;b3798;b3799; | 3980398 | 3980834 | + | | 4 | tRNA |
| TU00504 | thrUp | b3976;b3977;b3978;b3979; | 4173411 | 4173852 | + | | 4 | tRNA |
| TU00505 | glyVp | b4163;b4164;b4165; | 4390383 | 4390681 | + | | 3 | tRNA |
| TU00506 | leuXp | b4270; | 4494428 | 4494512 | + | | 1 | tRNA |
| TU00507 | metTp | b0664;b0665;b0666;b0668;b0670;b0672;b0673; | 695653 | 696356 | - | | 7 | tRNA |
| TU00508 | serWp | b0883; | 925107 | 925194 | - | | 1 | tRNA |
| TU00509 | serTp | b0971; | 1030848 | 1030935 | - | | 1 | tRNA |
| TU00510 | serXp | b1032; | 1096788 | 1096875 | - | | 1 | tRNA |
| TU00511 | tyrTp | b1229;b1230;b1231; | 1286310 | 1286845 | - | | 3 | CDS |
| TU00512 | glyWp | b1909;b1910;b1911; | 1989839 | 1990141 | - | | 3 | tRNA |
| TU00513 | serUp | b1975; | 2041492 | 2041581 | - | | 1 | tRNA |
| TU00514 | alaWp | b2396;b2397; | 2516063 | 2516253 | - | | 2 | tRNA |
| TU00515 | serVp | b2691;b2692;b2693;b2694;b2695; | 2815806 | 2816667 | - | | 5 | tRNA |
| TU00516 | glyUp | b2864; | 2997006 | 2997079 | - | | 1 | tRNA |
| TU00517 | metYp1 | b3168_v1;b3169_v1;b3170_v1;b3171_v2; | 3311364 | 3316311 | - | | 4 | CDS |
| TU00518 | leuUp | b3174; | 3320094 | 3320180 | - | | 1 | tRNA |
| TU00519 | proKp | b3545; | 3706639 | 3706715 | - | | 1 | tRNA |
| TU00520 | pheUp | b4134; | 4360574 | 4360649 | - | | 1 | tRNA |
| TU00521 | leuQp | b4368;b4369;b4370; | 4604102 | 4604424 | - | | 3 | tRNA |
| TU0-1 | rpmFp | b1093_v4;b1092_v3;b1091_v2;b1090_v1;b1089_v2; | 1146590 | 1150627 | + | | 5 | CDS |
| TU0-1181 | rrnAp1 | b3855;b3854;b3853;b3852;b3851; | 4033554 | 4038659 | + | | 5 | rRNA |
| TU0-1182 | rrsBp1 | b3971_v1;b3970_v1;b3969_v1;b3968_v1; | 4164682 | 4169779 | + | | 4 | rRNA |
| TU0-1183 | rrnCp1 | b3759_v1;b3758_v1;b3757_v1;b3756_v1; | 3939831 | 3944842 | + | | 4 | rRNA |

| | | | | | | | | |
|---|---|---|---|---|---|---|---|---|
| TU0-1184 | rrnCp2 | b3759;b3758;b3757;b3756; | 3939831 | 3944842 | + | | 4 | rRNA |
| TU0-1186 | rrnEp | b4010;b4009;b4008;b4007; | 4206170 | 4211182 | + | | 4 | rRNA |
| TU0-1187 | rrsGp1 | b2588_v1;b2589_v1;b2590_v1;b2591_v1; | 2724091 | 2729179 | - | | 4 | rRNA |
| TU0-1188 | rrsGp2 | b2588;b2589;b2590;b2591; | 2724091 | 2729179 | - | | 4 | rRNA |
| TU0-1189 | rrsHp1 | b0205_v1;b0204_v1;b0203_v1;b0202_v1;b0201_v1; | 223771 | 228875 | + | | 5 | rRNA |
| TU0-1190 | rrsHp2 | b0205;b0204;b0203;b0202;b0201; | 223771 | 228875 | + | | 5 | rRNA |
| TU0-1191 | rrnDp1 | b3272_v1;b3273_v1;b3274_v1;b3275_v1;b3276_v1;b3277_v1;b3278_v1; | 3421445 | 3426784 | - | | 7 | rRNA |
| TU0-1192 | rrnDp2 | b3272;b3273;b3274;b3275;b3276;b3277;b3278; | 3421445 | 3426784 | - | | 7 | rRNA |
| TU0-12803 | | b0059_v1;b0058; | 59687 | 63264 | - | | 2 | CDS |
| TU0-12827 | | b0168;b0167;b0166_v1; | 185123 | 189506 | - | | 3 | CDS |
| TU0-12830 | | b0172; | 192872 | 193429 | + | | 1 | CDS |
| TU0-12833 | | b0188; | 212331 | 213629 | + | | 1 | CDS |
| TU0-12921 | | b0416;b0415;b0414;b0413; | 432226 | 434780 | + | | 4 | CDS |
| TU0-12928 | | b0436_v1; | 454357 | 455655 | + | | 1 | CDS |
| TU0-13010 | | b0642;b0641;b0640;b0639;b0638; | 668519 | 674006 | - | | 5 | CDS |
| TU0-13018 | | b0661; | 692754 | 694178 | - | | 1 | CDS |
| TU0-13020 | | b0680_v1; | 705316 | 706980 | + | | 1 | CDS |
| TU0-13034 | | b0747;b0746; | 780291 | 780445 | + | | 2 | tRNA |
| TU0-13035 | | b0748; | 780592 | 780667 | + | | 1 | tRNA |
| TU0-13036 | | b0749; | 780800 | 780875 | + | | 1 | tRNA |
| TU0-13072 | | b0859;b0858; | 897212 | 898868 | + | | 2 | CDS |
| TU0-13080 | | b0893; | 938651 | 939943 | + | | 1 | CDS |
| TU0-13093 | | b0930; | 986808 | 988208 | - | | 1 | CDS |
| TU0-13104 | | b0969; | 1029562 | 1029891 | - | | 1 | CDS |
| TU0-13134 | | b1086; | 1144163 | 1145122 | + | | 1 | CDS |
| TU0-13139 | | b1114_v2; | 1169741 | 1173187 | - | | 1 | CDS |
| TU0-13145 | | b1135;b1134; | 1193050 | 1194174 | - | | 2 | CDS |
| TU0-13195 | | b1269; | 1324876 | 1325751 | + | | 1 | CDS |
| TU0-13225 | | b1344; | 1409037 | 1409972 | - | | 1 | CDS |
| TU0-13421 | | b1822; | 1904275 | 1905084 | - | | 1 | CDS |
| TU0-13443 | | b1871;b1870;b1869; | 1950290 | 1952437 | + | | 3 | CDS |
| TU0-13444 | | b1876; | 1958086 | 1959819 | + | | 1 | CDS |
| TU0-13519 | | b2114; | 2192322 | 2194355 | + | | 1 | CDS |
| TU0-13534 | | b2140; | 2227460 | 2228407 | - | | 1 | CDS |
| TU0-13555 | | b2183; | 2277810 | 2278505 | - | | 1 | CDS |

| | | | | | | | | |
|---|---|---|---|---|---|---|---|---|
| TU0-13557 | | b2185; | 2280539 | 2280823 | + | | 1 | CDS |
| TU0-13573 | | b2268; | 2379630 | 2380547 | + | | 1 | CDS |
| TU0-13593 | | b2330;b2329;b2328;b2327;b2326;b2325; | 2441913 | 2446462 | - | | 6 | CDS |
| TU0-13667 | | b2560;b2559; | 2695376 | 2696572 | - | | 2 | CDS |
| TU0-13675 | | b2594;b2593; | 2732325 | 2734033 | - | | 2 | CDS |
| TU0-13704 | | b2652; | 2783784 | 2783859 | - | | 1 | tRNA |
| TU0-13717 | | b2697_v1; | 2817403 | 2820033 | - | | 1 | CDS |
| TU0-13734 | | b2780_v2;b2779; | 2904665 | 2907688 | - | | 2 | CDS |
| TU0-13735 | | b2785; | 2911721 | 2913022 | - | | 1 | CDS |
| TU0-13736 | | b2792;b2791;b2790; | 2920557 | 2922135 | - | | 3 | CDS |
| TU0-13738 | | b2794; | 2923370 | 2924218 | + | | 1 | CDS |
| TU0-13805 | | b2947;b2946; | 3089156 | 3090850 | + | | 2 | CDS |
| TU0-13809 | | b2960;b2959; | 3099829 | 3100874 | - | | 2 | CDS |
| TU0-13915 | | b3346;b3345;b3344;b3343; | 3472700 | 3474462 | - | | 4 | CDS |
| TU0-13931 | | b3406; | 3534834 | 3535310 | + | | 1 | CDS |
| TU0-14007 | | b3658; | 3834245 | 3834339 | + | | 1 | tRNA |
| TU0-14021 | | b3706; | 3884851 | 3886215 | + | | 1 | CDS |
| TU0-14029 | | b3741;b3740; | 3921080 | 3923656 | - | | 2 | CDS |
| TU0-14037 | | b3780; | 3962388 | 3963653 | - | | 1 | CDS |
| TU0-14038 | | b3783_v1;b3782_v1; | 3964254 | 3965699 | + | | 2 | CDS |
| TU0-14070 | | b3888;b3887;b3886;b3885; | 4073576 | 4076461 | + | | 4 | CDS |
| TU0-14100 | | b3980_v1; | 4173967 | 4175151 | + | | 1 | CDS |
| TU0-14118 | | b4049; | 4259692 | 4260729 | + | | 1 | CDS |
| TU0-14151 | | b4162; | 4389627 | 4390172 | + | | 1 | CDS |
| TU0-14215 | | b4371; | 4604692 | 4605723 | - | | 1 | CDS |
| TU0-14216 | | b4374;b4373;b4372; | 4605826 | 4607346 | + | | 3 | CDS |
| TU0-14217 | | b4375; | 4607437 | 4609026 | + | | 1 | CDS |
| TU0-14252 | tigp | b0436; | 454357 | 455655 | + | | 1 | CDS |
| TU0-1881 | iscRp | b2528;b2529;b2530;b2531; | 2657585 | 2660153 | - | | 4 | CDS |
| TU0-2081 | mfdp1 | b1114_v1; | 1169741 | 1173187 | - | | 1 | CDS |
| TU0-2082 | mfdp2 | b1114; | 1169741 | 1173187 | - | | 1 | CDS |
| TU0-2101 | ybjCp | b0853_v1;b0852_v1;b0851;b0850; | 890136 | 892656 | + | | 4 | CDS |
| TU0-2121 | rimKp | b0853;b0852; | 891190 | 892656 | + | | 2 | CDS |
| TU0-3304 | infCp | b1718_v2; | 1798120 | 1798662 | - | | 1 | CDS |
| TU0-3305 | infCp2 | b1718_v1; | 1798120 | 1798662 | - | | 1 | CDS |
| TU0-3364 | rrsBp2 | b3971;b3970;b3969;b3968; | 4164682 | 4169779 | + | | 4 | rRNA |
| TU0-3561 | asnWp | b1984; | 2056051 | 2056126 | - | | 1 | tRNA |
| TU0-3901 | srap | b1480_v1; | 1553850 | 1553987 | - | | 1 | CDS |
| TU0-4925 | tufBp | b3980; | 4173967 | 4175151 | + | | 1 | CDS |
| TU0-5003 | groSp | b4142; | 4368711 | 4369004 | + | | 1 | CDS |

| | | | | | | | | |
|---|---|---|---|---|---|---|---|---|
| TU0-5121 | fusAp | b3339_v1;b3340_v1; | 3468167 | 3471536 | - | | 2 | CDS |
| TU0-5182 | pdxBp | b2317_v1;b2318_v1;b2319_v1;b2320; | 2432104 | 2435873 | - | | 4 | CDS |
| TU0-5183 | usgp | b2317;b2318;b2319; | 2432104 | 2434671 | - | | 3 | CDS |
| TU0-5186 | rsmAp | b0049_v3;b0050_v3;b0051_v3; | 50380 | 52430 | - | | 3 | CDS |
| TU0-5201 | pdxAp | b0049_v2;b0050_v2;b0051_v2;b0052_v2; | 50380 | 53416 | - | | 4 | CDS |
| TU0-5221 | pdxAp | b0051_v1;b0052_v1; | 51609 | 53416 | - | | 2 | CDS |
| TU0-5522 | yicRp | b3635_v3;b3636_v3;b3637_v3;b3638_v1; | 3808366 | 3810582 | - | | 4 | CDS |
| TU0-5543 | rpmBp | b3635_v1;b3636_v2;b3637_v2; | 3808366 | 3809697 | - | | 3 | CDS |
| TU0-5601 | yicRp | b3636_v1;b3637_v1;b3638; | 3809273 | 3810582 | - | Rho-Independent-Terminators | 3 | CDS |
| TU0-6223 | metYp1 | b3164_v3;b3165_v3;b3166_v1;b3167_v1;b3168_v3;b3169_v3;b3170_v3;b3171_v4; | 3307055 | 3316311 | - | Rho-Independent-Terminators | 8 | CDS |
| TU0-6405 | valSp1 | b4258_v1; | 4479005 | 4481860 | - | Rho-Independent-Terminators | 1 | CDS |
| TU0-6409 | gltXp3 | b2400_v2; | 2517279 | 2518694 | - | | 1 | CDS |
| TU0-6423 | rimLp | b1427; | 1496962 | 1497501 | + | | 1 | CDS |
| TU0-6441 | alaSp | b2697; | 2817403 | 2820033 | - | | 1 | CDS |
| TU0-6504 | glnSp | b0680; | 705316 | 706980 | + | Rho-Independent-Terminators | 1 | CDS |
| TU0-6506 | hisSp | b2514; | 2637323 | 2638597 | - | | 1 | CDS |
| TU0-6512 | rplKp | b3988_v2;b3987_v2;b3986_v2;b3985_v1;b3984;b3983; | 4176470 | 4187596 | + | Rho-Independent-Terminators | 6 | CDS |
| TU0-6550 | gltXp2 | b2400_v1; | 2517279 | 2518694 | - | | 1 | CDS |
| TU0-6551 | gltXp1 | b2400; | 2517279 | 2518694 | - | | 1 | CDS |
| TU0-6562 | proSp | b0194; | 217057 | 218775 | - | Rho-Independent-Terminators | 1 | CDS |
| TU0-6626 | pheMp | b1712_v1;b1715_v2;b1713;b1714; | 1793277 | 1797294 | - | Rho-Independent-Terminators | 4 | CDS |
| TU0-6654 | pnpp | b3164_v2; | 3307055 | 3309259 | - | Rho-Independent-Terminators | 1 | CDS |
| TU0-6657 | rplTp | b1716_v2; | 1797417 | 1797773 | - | Rho-Independent-Terminators | 1 | CDS |
| TU0-6658 | rpoHp2 | b3461_v5; | 3597952 | 3598806 | - | Rho-Independent-Terminators | 1 | CDS |
| TU0-6660 | rpsOp | b3164_v1;b3165_v2; | 3307055 | 3309706 | - | Rho-Independent-Terminators | 2 | CDS |
| TU0-6687 | valSp2 | b4258; | 4479005 | 4481860 | - | Rho-Independent-Terminators | 1 | CDS |
| TU0-6941 | rhoLp | b3782;b3783; | 3964254 | 3965699 | + | | 2 | CDS |
| TU0-7002 | rpoBp | b3988_v1;b3987_v1; | 4179268 | 4187596 | + | Rho-Independent-Terminators | 2 | CDS |
| TU0-7141 | bdmp | b1480;b1481; | 1553850 | 1554304 | - | | 2 | CDS |
| TU0-7281 | yrdDp | b3280;b3281;b3282;b3283; | 3427788 | 3429984 | - | | 4 | CDS |
| TU0-7842 | rpsTp1 | b0023_v1; | 20815 | 21078 | - | Rho-Independent-Terminators | 1 | CDS |
| TU0-7844 | rpsTp2 | b0023; | 20815 | 21078 | - | Rho-Independent-Terminators | 1 | CDS |
| TU0-8084 | aspSp | b1866; | 1946774 | 1948546 | - | Rho-Independent-Terminators | 1 | CDS |

| | | | | | | | | | |
|---|---|---|---|---|---|---|---|---|---|
| TU0-8281 | rimJp | b1066; | 1124785 | 1125369 | + | | 1 | CDS |
| TU0-8464 | rplLp | b3986_v1; | 4178583 | 4178948 | + | | 1 | CDS |
| TU0-8474 | rpsOp | b3165_v1; | 3309437 | 3309706 | - | Rho-Independent-Terminators | 1 | CDS |
| TU0-8476 | metYp2 | b3171_v3; | 3316235 | 3316311 | - | Rho-Independent-Terminators | 1 | tRNA |
| TU0-8477 | cysSp | b0526; | 553834 | 555219 | + | | 1 | CDS |
| TU0-8502 | nsrRp | b4180;b4179;b4178; | 4404213 | 4408029 | + | | 3 | CDS |
| TU0-8505 | rluFp | b4022; | 4228377 | 4229249 | + | | 1 | CDS |
| TU0-8506 | rpmEp | b3936; | 4125036 | 4125248 | + | | 1 | CDS |
| TU0-8510 | tusAp | b3470; | 3606774 | 3607019 | - | | 1 | CDS |
| TU0-8529 | thiIp | b0423; | 440773 | 442221 | + | | 1 | CDS |
| TU0-8823 | trmCp | b2324; | 2439786 | 2441792 | + | | 1 | CDS |
| TU0-8855 | rplTp | b1715_v1;b1716_v1; | 1797250 | 1797773 | - | Rho-Independent-Terminators | 2 | CDS |
| TU0-8862 | trpSp | b3384_v6; | 3510656 | 3511660 | - | | 1 | CDS |
| TU0-8864 | pdxHp | b1636_v1;b1637_v1;b1638; | 1713050 | 1716031 | - | | 3 | CDS |
| TU0-8865 | tyrSp | b1636;b1637; | 1713050 | 1715246 | - | | 2 | CDS |
| TU221 | rpoHp6 | b3461; | 3597952 | 3598806 | - | Rho-Independent-Terminators | 1 | CDS |
| TU281 | rpoEp1 | b2570;b2571;b2572;b2573; | 2705344 | 2708034 | - | | 4 | CDS |
| TU341 | metYp2 | b3164;b3165;b3166;b3167;b3168;<br>b3169;b3170;b3171_v1; | 3307055 | 3316311 | - | Rho-Independent-Terminators | 8 | CDS |
| TU343 | metYp1 | b3171; | 3316235 | 3316311 | - | Rho-Independent-Terminators | 1 | tRNA |
| TU361 | cmkp | b0911_v2;b0910; | 960424 | 962891 | + | | 2 | CDS |
| TU482 | garPp | b3123_v1;b3124;b3125;b3126;b31<br>27; | 3268238 | 3272929 | - | Rho-Independent-Terminators | 5 | CDS |
| TU483 | rncp | b2563_v1;b2564_v1;b2565;b2566;<br>b2567; | 2698640 | 2702085 | - | | 5 | CDS |
| TU543 | rnpBp | b3123; | 3268238 | 3268614 | - | Rho-Independent-Terminators | 1 | misc_RNA |
| TU564 | rpsAp1 | b0912_v1;b0911_v1; | 961218 | 963335 | + | | 2 | CDS |
| TU565 | rpsAp3 | b0912;b0911; | 961218 | 963335 | + | | 2 | CDS |
| TU582 | rnbp1 | b1286_v1; | 1345002 | 1346936 | - | Rho-Independent-Terminators | 1 | CDS |
| TU583 | rnbp2 | b1286; | 1345002 | 1346936 | - | Rho-Independent-Terminators | 1 | CDS |
| TU601 | yceDp1 | b1089_v1;b1088_v1; | 1146017 | 1146763 | + | | 2 | CDS |
| TU602 | yceDp2 | b1089;b1088; | 1146017 | 1146763 | + | | 2 | CDS |
| TU682 | rpmHp2 | b3704_v1;b3703_v1; | 3882359 | 3882875 | + | | 2 | CDS |
| TU743 | rpmHp3 | b3704;b3703; | 3882359 | 3882875 | + | | 2 | CDS |
| TU801 | yjeFp2 | b4175_v1;b4174_v1;b4173_v1;b4<br>172_v1;b4171_v1;b4170_v1;b416<br>9_v1;b4168_v1;b4167_v1; | 4392089 | 4402327 | + | | 9 | CDS |
| TU802 | yjeFp3 | b4175;b4174;b4173;b4172;b4171;<br>b4170;b4169;b4168;b4167; | 4392089 | 4402327 | + | | 9 | CDS |
| TU-8389 | infAp2 | b0884_v1; | 925448 | 925666 | - | Rho-Independent-Terminators | 1 | CDS |

| | | | | | | | | |
|---|---|---|---|---|---|---|---|---|
| TU-8390 | infAp1 | b0884; | 925448 | 925666 | - | Rho-Independent-Terminators | 1 | CDS |
| TU-8392 | dsbCp2 | b2891_v1;b2892;b2893_v1; | 3033206 | 3036844 | - | | 3 | CDS |
| TU-8397 | greAp | b3181; | 3326261 | 3326737 | - | | 1 | CDS |
| TU-8398 | lptBp | b3205_v1;b3204_v1;b3203_v1;b3202_v2;b3201; | 3341966 | 3345991 | + | | 5 | CDS |
| TU-8407 | rnep | b1084; | 1140405 | 1143590 | - | | 1 | CDS |
| TU870 | tgtp | b0407;b0406; | 425361 | 426843 | + | Rho-Independent-Terminators | 2 | CDS |
| TU871 | queAp | b0405; | 424235 | 425305 | + | | 1 | CDS |
| TU872 | aroKp1 | b3384_v5;b3385_v5;b3386_v5;b3387_v4;b3388_v2;b3389_v1;b3390_v1; | 3510656 | 3517086 | - | | 7 | CDS |
| TU873 | aroKp2 | b3384_v4;b3385_v4;b3386_v4;b3387_v3;b3388_v1;b3389;b3390; | 3510656 | 3517086 | - | | 7 | CDS |
| TU874 | damXp | b3384_v3;b3385_v3;b3386_v3;b3387_v2;b3388; | 3510656 | 3515328 | - | | 5 | CDS |
| TU875 | damp1 | b3384_v2;b3385_v2;b3386_v2;b3387_v1; | 3510656 | 3513935 | - | | 4 | CDS |
| TU876 | damp2 | b3384_v1;b3385_v1;b3386_v1;b3387; | 3510656 | 3513935 | - | | 4 | CDS |
| TU877 | rpep | b3384;b3385;b3386; | 3510656 | 3513081 | - | | 3 | CDS |
| b0144 | | b0144 | 159186 | 160112 | - | | | CDS |
| b0206 | | b0206 | 228928 | 229004 | + | | | tRNA |
| b0503 | | b0503 | 529356 | 530450 | - | | | CDS |
| b1133 | | b1133 | 1191890 | 1192996 | - | | | CDS |
| b1211 | | b1211 | 1264235 | 1265317 | + | | | CDS |
| b1212 | | b1212 | 1265317 | 1266150 | + | | | CDS |
| b1804 | | b1804 | 1884888 | 1886015 | - | | | CDS |
| b2745 | | b2745 | 2868277 | 2869326 | - | | | CDS |
| b3289 | | b3289 | 3433229 | 3434518 | + | | | CDS |
| b3760 | | b3760 | 3944895 | 3944971 | + | | | tRNA |
| b3761 | | b3761 | 3944980 | 3945055 | + | | | tRNA |
| b4143 | | b4143 | 4369048 | 4370694 | + | | | CDS |
