# Supplementary material for: Genome-Scale Reconstruction of Escherichia coli's Transcriptional and Translational Machinery: A Knowledge Base, Its Mathematical Formulation, and Its Functional Characterization
Source: PLoS Comput Biol. 2009 Mar 13;5(3):e1000312. doi: 10.1371/journal.pcbi.1000312 (PMC2648898; doi:10.1371/journal.pcbi.1000312)
Supplement: Table S10 — E-matrix genes (0.07 MB PDF) [file pcbi.1000312.s012.pdf]

# Table S10 - E-matrix genes

| | | | | Information taken from Riley et al, 2006, PMID: 16397293 | | | | | | | |
|---|---|---|---|---|---|---|---|---|---|---|---|
| **Blattner Number** | **Gene Alias** | **Dogma Product** | **Product** | **Name** | **Annotation Evidence** | **Model status** | **Part of Transcription Unit** | **COG classification of gene product** | **Gene Ontology** | **Gene Ontology** | |
| | | | | | E = Experimental,C = Computational | Involved are those gene products which are directly involved in dogma functions.Not involved genes are included in the reconstruction because they share transcription units (co-transcribed) with involved genes | | | | | |
| b0014 | dnaK | DnaK_mono | Protein | chaperone Hsp70, co-chaperone with DnaJ | E | involved | TU00260;TU00417;TU00418 | COG0443;Molecular chaperone | GO:0005737 cytoplasm | GO:0006457 protein folding -!- GO:0006970 response to osmotic stress | |
| b0015 | dnaJ | DnaJ_mono | Protein | chaperone Hsp40, co-chaperone with DnaK | E | involved | TU00260;TU00417;TU00418 | COG0484;DnaJ-class molecular chaperone with C-terminal Zn finger domain | GO:0005737 cytoplasm | GO:0006457 protein folding | |
| b0023 | rpsT | RpsT_mono | Protein | 30S ribosomal subunit protein S20 | E | involved | TU0-7842;TU0-7844 | COG0268;Ribosomal protein S20 | GO:0009281 cytosolic ribosome (sensu Bacteria) -!- GO:0005737 cytoplasm | GO:0006412 protein biosynthesis | GO:0003735 structural constituent of ribosome |
| b0025 | ribF | RibF_mono | Protein | bifunctional riboflavin kinase and FAD synthetase | E | not involved | TU00291 | COG0196;FAD synthase | GO:0005737 cytoplasm | | |
| b0026 | ileS | IleS_mono | Protein | isoleucyl-tRNA synthetase | E | involved | TU00291 | COG0060;Isoleucyl-tRNA synthetase | GO:0005737 cytoplasm | GO:0006418 amino acid activation | |
| b0027 | lspA | LspA_mono | Protein | prolipoprotein signal peptidase (signal peptidase II) | E | not involved | TU0-14253;TU00291 | COG0597;Lipoprotein signal peptidase | GO:0009274 cell wall (sensu Bacteria) -!- GO:0019866 inner membrane | | |
| b0028 | fkpB | FkpB_mono | Protein | FKBP-type peptidyl-prolyl cis-trans isomerase (rotamase) | E | not involved | TU0-14253;TU00291 | COG1047;FKBP-type peptidyl-prolyl cis-trans isomerases 2 | GO:0005737 cytoplasm | GO:0006457 protein folding | |
| b0029 | ispH | IspH_mono | Protein | 1-hydroxy-2-methyl-2-(E)-butenyl 4-diphosphate reductase, 4Fe-4S protein | E | not involved | TU0-14253;TU00291 | COG0761;Penicillin tolerance protein | GO:0005737 cytoplasm | GO:0042493 response to drug | |
| b0049 | apaH | ApaH_mono | Protein | diadenosine tetraphosphatase | E | not involved | TU0-5186;TU0-5201;TU0-5202;TU00362 | COG0639;Diadenosine tetraphosphatase and related serine/threonine protein phosphatases | GO:0005737 cytoplasm | GO:0015949 nucleobase, nucleoside and nucleotide interconversion | |
| b0050 | apaG | ApaG_mono | Protein | protein associated with Co2+ and Mg2+ efflux | C | not involved | TU0-5186;TU0-5201;TU0-5202;TU00362 | COG2967;Uncharacterized protein affecting Mg2+/Co2+ transport | | | |
| b0051 | ksgA | KsgA_mono | Protein | S-adenosylmethionine-6-N',N'-adenosyl (rRNA) dimethyltransferase | E | involved | TU0-5186;TU0-5201;TU0-5221;TU00362 | COG0030;Dimethyladenosine transferase (rRNA methylation) | GO:0005737 cytoplasm | GO:0009451 RNA modification -!- GO:0042493 response to drug | |
| b0052 | pdxA | PdxA_mono | Protein | 4-hydroxy-L-threonine phosphate dehydrogenase, NAD-dependent | E | not involved | TU0-4287;TU0-5201;TU0-5221;TU00362 | COG1995;Pyridoxal phosphate biosynthesis protein | GO:0005737 cytoplasm | GO:0008615 pyridoxine biosynthesis | |
| b0053 | surA | SurA_mono | Protein | peptidyl-prolyl cis-trans isomerase (PPIase) | E | not involved | TU0-4287;TU00362 | COG0760;Parvulin-like peptidyl-prolyl isomerase | GO:0009279 external outer membrane (sensu Gram-negative Bacteria) | GO:0006457 protein folding | |
| b0058 | rluA | RluA_mono | Protein | pseudouridine synthase for 23S rRNA (position 746) and tRNAphe(position 32) | E | involved | TU0-12803 | COG0564;Pseudouridylate synthases, 23S RNA-specific | | GO:0009451 RNA modification | |

| | | | | | | | | | | | |
|---|---|---|---|---|---|---|---|---|---|---|---|
| b0059 | hepA | HepA_mono | Protein | RNA polymerase-associated helicase protein (ATPase and RNA polymerase recycling factor) | E | not involved | TU0-12803;TU0-3841 | COG0553;Superfamily II DNA/RNA helicases, SNF2 family | | GO:0009451 RNA modification | |
| b0144 | yadB | YadB_mono | Protein | glutamyl-Q tRNA(Asp) synthetase | E | involved | | COG0008;Glutamyl- and glutaminyl-tRNA synthetases | | GO:0006418 amino acid activation | |
| b0166 | dapD | DapD_mono | Protein | 2,3,4,5-tetrahydropyridine-2-carboxylate N-succinyltransferase | E | not involved | TU0-12827;TU0-6443 | COG2171;Tetrahydrodipicolinate N-succinyltransferase | GO:0005737 cytoplasm | GO:0009089 lysine biosynthesis via diaminopimelate | |
| b0167 | glnD | GlnD_mono | Protein | uridylyltransferase | E | not involved | TU0-12827 | COG2844;UTP:GlnB (protein PII) uridylyltransferase | GO:0005737 cytoplasm | GO:0006807 nitrogen metabolism -!- GO:0006542 glutamine biosynthesis -!- GO:0006464 protein modification | |
| b0168 | map | Map_mono | Protein | methionine aminopeptidase | E | involved | TU0-12827 | COG0024;Methionine aminopeptidase | | GO:0006457 protein folding | |
| b0169 | rpsB | RpsB_mono | Protein | 30S ribosomal subunit protein S2 | E | involved | TU00345 | COG0052;Ribosomal protein S2 | GO:0009281 cytosolic ribosome (sensu Bacteria) -!- GO:0005737 cytoplasm | GO:0006412 protein biosynthesis | GO:0003735 structural constituent of ribosome |
| b0170 | tsf | Tsf_mono | Protein | protein chain elongation factor EF-Ts | E | involved | TU00345 | COG0264;Translation elongation factor Ts | GO:0005737 cytoplasm | GO:0006412 protein biosynthesis | |
| b0172 | rrf | Rrf_mono | Protein | ribosome recycling factor | E | involved | TU0-12830 | COG0233;Ribosome recycling factor | GO:0005737 cytoplasm | GO:0006412 protein biosynthesis -!- GO:0009386 translational attenuation | |
| b0188 | tilS | TilS_mono | Protein | tRNA(Ile)-lysidine synthetase | E | involved | TU0-12833 | COG0037;Predicted ATPase of the PP-loop superfamily implicated in cell cycle control | | | |
| b0194 | proS | ProS_mono | Protein | prolyl-tRNA synthetase | E | involved | TU0-6562 | COG0442;Prolyl-tRNA synthetase | GO:0005737 cytoplasm | GO:0006418 amino acid activation | |
| b0201 | rrsH | rrsH | rRNA | 16S rRNA (rrsH) | E | involved | TU0-1189;TU0-1190 | | | | |
| b0202 | ileV | ileV_tRNA | tRNA | tRNA-Ile(GAU) (Isoleucine tRNA1) | E | involved | TU0-1189;TU0-1190 | | | | |
| b0203 | alaV | alaV_tRNA | tRNA | tRNA-Ala(UGC) (Alanine tRNA 1B) | E | involved | TU0-1189;TU0-1190 | | | | |
| b0204 | rrlH | rrlH | rRNA | 23S rRNA (rrlH) | E | involved | TU0-1189;TU0-1190 | | | | |
| b0205 | rrfH | rrfH | rRNA | 5S rRNA (rrfH) | E | involved | TU0-1189;TU0-1190 | | | | |
| b0206 | aspU | aspU_tRNA | tRNA | tRNA-Asp(GUC) (Aspartate tRNA1) | E | involved | | | | | |
| b0216 | aspV | aspV_tRNA | tRNA | tRNA-Asp(GUC) (Aspartate tRNA1) | E | involved | TU00489 | | | | |
| b0244 | thrW | thrW_tRNA | tRNA | tRNA-Thr(CGU) (Threonine tRNA2) | E | involved | TU00490 | | | | |
| b0405 | queA | QueA_mono | Protein | S-adenosylmethionine:tRNA ribosyltransferase-isomerase | E | involved | TU871 | COG0809;S-adenosylmethionine:tRNA-ribosyltransferase-isomerase (queuine synthetase) | GO:0005737 cytoplasm | GO:0009451 RNA modification | |
| b0406 | tgt | Tgt_mono | Protein | tRNA-guanine transglycosylase | E | involved | TU870 | COG0343;Queuine/archaeosine tRNA-ribosyltransferase | GO:0005737 cytoplasm | GO:0009451 RNA modification | |
| b0407 | yajC | YajC_mono | Protein | SecYEG protein translocase auxillary subunit | E | not involved | TU869;TU870 | COG1862;Preprotein translocase subunit YajC | GO:0009274 cell wall (sensu Bacteria) -!- GO:0019866 inner membrane | | |
| b0413 | ybaD | YbaD_mono | Protein | conserved protein | C | not involved | TU0-12921 | COG1327;Predicted transcriptional regulator, consists of a Zn-ribbon and ATP-cone domains | | | |

| | | | | | | | | | | | |
|---|---|---|---|---|---|---|---|---|---|---|---|
| b0414 | ribD | RibD_mono | Protein | fused diaminohydroxyphosphoribosylaminopyrimidine deaminase -!- 5-amino-6-(5-phosphoribosylamino) uracil reductase | E | not involved | TU0-12921 | COG0117;Pyrimidine deaminase -!- COG1985;Pyrimidine reductase, riboflavin biosynthesis | | | |
| b0415 | ribE | RibE_mono | Protein | riboflavin synthase beta chain | E | not involved | TU0-12921 | COG0054;Riboflavin synthase beta-chain | | | |
| b0416 | nusB | NusB_mono | Protein | transcription antitermination protein | E | involved | TU0-12921 | COG0781;Transcription termination factor | GO:0005737 cytoplasm | GO:0006350 transcription | |
| b0423 | thiI | ThiI_mono | Protein | sulfurtransferase required for thiamine and 4-thiouridine biosynthesis | E | involved | TU0-8529 | COG0301;Thiamine biosynthesis ATP pyrophosphatase -!- COG0607;Rhodanese-related sulfurtransferase | | GO:0009228 thiamin biosynthesis -!- GO:0009451 RNA modification | |
| b0436 | tig | Tig_mono | Protein | peptidyl-prolyl cis/trans isomerase (trigger factor) | E | involved | TU0-12928;TU0-14252 | COG0544;FKBP-type peptidyl-prolyl cis-trans isomerase (trigger factor) | GO:0005737 cytoplasm | GO:0006457 protein folding | |
| b0503 | ybbB | YbbB_mono | Protein | tRNA 2-selenouridine synthase, selenophosphate-dependent | E | involved | | COG2603;Predicted ATPase | | | |
| b0526 | cysS | CysS_mono | Protein | cysteinyl-tRNA synthetase | E | involved | TU0-8477 | COG0215;Cysteinyl-tRNA synthetase | GO:0005737 cytoplasm | GO:0006418 amino acid activation | |
| b0536 | argU | argU_tRNA | tRNA | tRNA-Arg(UCU) (Arginine tRNA4) | E | involved | TU00491 | | | | |
| b0638 | cobC | CobC_mono | Protein | predicted alpha-ribazole-5'-P phosphatase | C | not involved | TU0-13010 | COG0406;Fructose-2,6-bisphosphatase | | GO:0009236 vitamin B12 biosynthesis | |
| b0639 | nadD | NadD_mono | Protein | nicotinic acid mononucleotide adenylyltransferase, NAD(P)-dependent | E | not involved | TU0-13010 | COG1057;Nicotinic acid mononucleotide adenylyltransferase | | GO:0009435 nicotinamide adenine dinucleotide biosynthesis | |
| b0640 | holA | HolA_mono | Protein | DNA polymerase III, delta subunit | E | not involved | TU0-13010 | COG1466;DNA polymerase III, delta subunit | GO:0005737 cytoplasm | GO:0006261 DNA dependent DNA replication | |
| b0641 | rlpB | RlpB_mono | Protein | minor lipoprotein | E | not involved | TU0-13010 | COG2980;Rare lipoprotein B | GO:0019866 inner membrane | GO:0042158 lipoprotein biosynthesis | |
| b0642 | leuS | LeuS_mono | Protein | leucyl-tRNA synthetase | E | involved | TU0-13010 | COG0495;Leucyl-tRNA synthetase | GO:0005737 cytoplasm | GO:0006418 amino acid activation | |
| b0661 | miaB | MiaB_mono | Protein | isopentenyl-adenosine A37 tRNA methylthiolase | E | involved | TU0-13018 | COG0621;2-methylthioadenine synthetase | GO:0005737 cytoplasm | GO:0009451 RNA modification | |
| b0664 | glnX | glnX_tRNA | tRNA | tRNA-Gln(CUG) (Glutamine tRNA2) | E | involved | TU00507 | | | | |
| b0665 | glnV | glnV_tRNA | tRNA | tRNA-Gln(CUG) (Glutamine tRNA2) | E | involved | TU00507 | | | | |
| b0666 | metU | metU_tRNA | tRNA | tRNA-Met(CAU) (Methionine tRNAm) | E | involved | TU00507 | | | | |
| b0668 | glnW | glnW_tRNA | tRNA | tRNA-Gln(UUG) (Glutamine tRNA1) | E | involved | TU00507 | | | | |
| b0670 | glnU | glnU_tRNA | tRNA | tRNA-Gln(UUG) (Glutamine tRNA1) | E | involved | TU00507 | | | | |
| b0672 | leuW | leuW_tRNA | tRNA | tRNA-Leu(UAG) (Leucine tRNA3) | E | involved | TU00507 | | | | |
| b0673 | metT | metT_tRNA | tRNA | tRNA-Met(CAU) (Methionine tRNAm) | E | involved | TU00507 | | | | |
| b0680 | glnS | GlnS_mono | Protein | glutamyl-tRNA synthetase | E | involved | TU0-13020;TU0-6504 | COG0008;Glutamyl- and glutaminyl-tRNA synthetases | GO:0005737 cytoplasm | GO:0006418 amino acid activation | |
| b0743 | lysT | lysT_tRNA | tRNA | tRNA-Lys(UUU) (Lysine tRNA) | E | involved | TU00492 | | | | |
| b0744 | valT | valT_tRNA | tRNA | tRNA-Val(UAC) (Valine tRNA1) | E | involved | TU00492 | | | | |
| b0745 | lysW | lysW_tRNA | tRNA | tRNA-Lys(UUU) (Lysine tRNA) | E | involved | TU00492 | | | | |

| | | | | | | | | | | | |
|---|---|---|---|---|---|---|---|---|---|---|---|
| b0746 | valZ | valZ_tRNA | tRNA | tRNA-Val(UAC) (Valine tRNA1) | E | involved | TU0-13034 | | | | |
| b0747 | lysY | lysY_tRNA | tRNA | tRNA-Lys(UUU) (Lysine tRNA) | E | involved | TU0-13034 | | | | |
| b0748 | lysZ | lysZ_tRNA | tRNA | tRNA-Lys(UUU), (Lysine tRNA) | E | involved | TU0-13035 | | | | |
| b0749 | lysQ | lysQ_tRNA | tRNA | tRNA-Lys(UUU) (Lysine tRNA) | E | involved | TU0-13036 | | | | |
| b0850 | ybjC | YbjC_mono | Protein | predicted inner membrane protein | C | not involved | TU0-2101 | | | | |
| b0851 | nfsA | NfsA_mono | Protein | nitroreductase A, NADPH-dependent, FMN-dependent | E | not involved | TU0-2101 | COG0778;Nitroreductase | | GO:0009061 anaerobic respiration | |
| b0852 | rimK | RimK_mono | Protein | ribosomal protein S6 modification protein | E | involved | TU0-2101;TU0-2121 | COG0189;Glutathione synthase/Ribosomal protein S6 modification enzyme (glutaminyl transferase) | GO:0009281 cytosolic ribosome (sensu Bacteria) -!- GO:0005737 cytoplasm | GO:0006412 protein biosynthesis | GO:0003735 structural constituent of ribosome |
| b0853 | ybjN | YbjN_mono | Protein | predicted oxidoreductase | C | not involved | TU0-2101;TU0-2121 | | | | |
| b0858 | ybjO | YbjO_mono | Protein | predicted inner membrane protein | C | not involved | TU0-13072 | | | | |
| b0859 | rumB | RumB_mono | Protein | 23S rRNA m(5)U747 methyltransferase | E | involved | TU0-13072 | COG2265;SAM-dependent methyltransferases related to tRNA (uracil-5-)-methyltransferase | | GO:0009451 RNA modification | |
| b0883 | serW | serW_tRNA | tRNA | tRNA-Ser(GGA) (Serine tRNA5) | E | involved | TU00508 | | | | |
| b0884 | infA | InfA_mono | Protein | translation initiation factor IF-1 | E | involved | TU-8389;TU-8390 | COG0361;Translation initiation factor 1 (IF-1) | GO:0005737 cytoplasm | GO:0006412 protein biosynthesis | |
| b0893 | serS | SerS_mono | Protein | seryl-tRNA synthetase, also charges selenocysteinyl-tRNA with serine | E | involved | TU0-13080 | COG0172;Seryl-tRNA synthetase | GO:0005737 cytoplasm | GO:0006418 amino acid activation | |
| b0910 | cmk | Cmk_mono | Protein | cytidylate kinase | E | not involved | TU361 | COG0283;Cytidylate kinase | GO:0005737 cytoplasm | GO:0015949 nucleobase, nucleoside and nucleotide interconversion | |
| b0911 | rpsA | RpsA_mono | Protein | 30S ribosomal subunit protein S1 | E | involved | TU361;TU564;TU565 | COG0539;Ribosomal protein S1 | GO:0009281 cytosolic ribosome (sensu Bacteria) -!- GO:0005737 cytoplasm | GO:0006412 protein biosynthesis | GO:0003735 structural constituent of ribosome |
| b0912 | ihfB | IhfB_mono | Protein | integration host factor (IHF), DNA-binding protein, beta subunit | E | not involved | TU00175;TU564;TU565 | COG0776;Bacterial nucleoid DNA-binding protein | GO:0005737 cytoplasm | GO:0006310 DNA recombination | |
| b0930 | asnS | AsnS_mono | Protein | asparaginyl tRNA synthetase | E | involved | TU0-13093 | COG0017;Aspartyl/asparaginyl-tRNA synthetases | GO:0005737 cytoplasm | GO:0006418 amino acid activation | |
| b0969 | yccK | YccK_mono | Protein | predicted sulfite reductase subunit | C | involved | TU0-13104 | | | | |
| b0971 | serT | serT_tRNA | tRNA | tRNA-Ser(UGA) (Serine tRNA1) | E | involved | TU00509 | | | | |
| b1032 | serX | serX_tRNA | tRNA | tRNA-Ser(GGA) (Serine tRNA5) | E | involved | TU00510 | | | | |
| b1066 | rimJ | RimJ_mono | Protein | ribosomal-protein-S5-alanine N-acetyltransferase | E | involved | TU0-8281 | COG1670;Acetyltransferases, including N-acetylases of ribosomal proteins | GO:0009281 cytosolic ribosome (sensu Bacteria) -!- GO:0005737 cytoplasm | GO:0006464 protein modification | |
| b1084 | rne | Rne_mono | Protein | fused ribonucleaseE: endoribonuclease -!- RNA-binding protein -!-RNA degradosome binding protein | E | involved | TU-8407 | COG1530;Ribonucleases G and E | GO:0005737 cytoplasm | GO:0006401 RNA catabolism -!- GO:0006401 RNA catabolism | |
| b1086 | rluC | RluC_mono | Protein | 23S rRNA pseudouridylate synthase | E | involved | TU0-13134 | COG0564;Pseudouridylate synthases, 23S RNA-specific | GO:0005737 cytoplasm | GO:0009451 RNA modification | |

| | | | | | | | | | | | |
|---|---|---|---|---|---|---|---|---|---|---|---|
| b1088 | yceD | YceD_mono | Protein | conserved protein | C | not involved | TU601;TU602 | COG1399;Predicted metal-binding, possibly nucleic acid-binding protein | | | |
| b1089 | rpmF | RpmF_mono | Protein | 50S ribosomal subunit protein L32 | E | involved | TU0-1;TU601;TU602 | COG0333;Ribosomal protein L32 | GO:0009281 cytosolic ribosome (sensu Bacteria) -!- GO:0005737 cytoplasm | GO:0006412 protein biosynthesis | GO:0003735 structural constituent of ribosome |
| b1090 | plsX | PlsX_mono | Protein | fatty acid/phospholipid synthesis protein | E | not involved | TU0-1;TU0-5 | COG0416;Fatty acid/phospholipid biosynthesis enzyme | | GO:0008654 phospholipid biosynthesis | |
| b1091 | fabH | FabH_mono | Protein | 3-oxoacyl-[acyl-carrier-protein] synthase III | E | not involved | TU0-1;TU0-5;TU00266 | COG0332;3-oxoacyl-[acyl-carrier-protein] synthase III | GO:0005737 cytoplasm | | |
| b1092 | fabD | FabD_mono | Protein | malonyl-CoA-[acyl-carrier-protein] transacylase | E | not involved | TU0-1;TU0-5;TU00266;TU442 | COG0331;(acyl-carrier-protein) S-malonyltransferase | GO:0005737 cytoplasm | | |
| b1093 | fabG | FabG_mono | Protein | 3-oxoacyl-[acyl-carrier-protein] reductase | E | not involved | TU0-1;TU0-5;TU00266;TU442;TU443 | COG1028;Dehydrogenases with different specificities (related to short-chain alcohol dehydrogenases) | GO:0005737 cytoplasm | | |
| b1114 | mfd | Mfd_mono | Protein | transcription-repair coupling factor | E | involved | TU0-13139;TU0-2081;TU0-2082 | COG1197;Transcription-repair coupling factor (superfamily II helicase) | GO:0005737 cytoplasm | GO:0006350 transcription | |
| b1133 | trmU | TrmU_mono | Protein | tRNA (5-methylaminomethyl-2-thiouridylate)-methyltransferase | E | involved | | | GO:0005737 cytoplasm | GO:0009451 RNA modification | |
| b1134 | ymfB | YmfB_mono | Protein | bifunctional thiamin pyrimidine pyrophosphate hydrolase and thiamin pyrophosphate hydrolase | E | not involved | TU0-13145 | COG0494;NTP pyrophosphohydrolases including oxidative damage repair enzymes | | | |
| b1135 | ymfC | YmfC_mono | Protein | 23S rRNA pseudouridine synthase | E | involved | TU0-13145 | COG1187;16S rRNA uridine-516 pseudouridylate synthase and related pseudouridylate synthases | | GO:0009451 RNA modification | |
| b1211 | prfA | PrfA_mono | Protein | peptide chain release factor RF-1 | E | involved | | COG0216;Protein chain release factor A | GO:0005737 cytoplasm | GO:0006412 protein biosynthesis | |
| b1212 | prmC | PrmC_mono | Protein | N5-glutamine methyltransferase, modifies release factors RF-1 and RF-2 | E | involved | | COG2890;Methylase of polypeptide chain release factors | | | |
| b1229 | tpr | Tpr_mono | Protein | predicted protamine-like protein | C | not involved | TU00511 | | GO:0005737 cytoplasm | | |
| b1230 | tyrV | tyrV_tRNA | tRNA | tRNA-Tyr(GUA) (Tyrosine tRNA1) | E | involved | TU00511 | | | | |
| b1231 | tyrT | tyrT_tRNA | tRNA | tRNA-Tyr(GUA) (Tyrosine tRNA1) | E | involved | TU00511 | | | | |
| b1269 | rluB | RluB_mono | Protein | 23S rRNA pseudouridylate synthase | E | involved | TU0-13195 | COG1187;16S rRNA uridine-516 pseudouridylate synthase and related pseudouridylate synthases | | GO:0009451 RNA modification | |
| b1286 | rnb | Rnb_mono | Protein | ribonuclease II | E | involved | TU582;TU583 | COG4776;Exoribonuclease II | | GO:0006401 RNA catabolism -!- GO:0006401 RNA catabolism | |
| b1344 | ydaO | YdaO_mono | Protein | predicted C32 tRNA thiolase | C | involved | TU0-13225 | COG0037;Predicted ATPase of the PP-loop superfamily implicated in cell cycle control | | | |
| b1427 | rimL | RimL_mono | Protein | ribosomal-protein-L7/L12-serine acetyltransferase | E | involved | TU0-6423 | COG1670;Acetyltransferases, including N-acetylases of ribosomal proteins | GO:0009281 cytosolic ribosome (sensu Bacteria) -!- GO:0005737 cytoplasm | GO:0006464 protein modification | |

| | | | | | | | | | | | |
|---|---|---|---|---|---|---|---|---|---|---|---|
| b1480 | sra | Sra_mono | Protein | 30S ribosomal subunit protein S22 | E | involved | TU0-3901;TU0-7141 | | GO:0009281 cytosolic ribosome (sensu Bacteria) -!- GO:0005737 cytoplasm | GO:0006412 protein biosynthesis | GO:0003735 structural constituent of ribosome |
| b1481 | bdm | Bdm_mono | Protein | biofilm-dependent modulation protein | E | not involved | TU0-7141 | | | | |
| b1636 | pdxY | PdxY_mono | Protein | pyridoxal kinase 2/pyridoxine kinase | E | not involved | TU0-8864;TU0-8865 | COG2240;Pyridoxal/pyridoxine/pyridoxamine kinase | | GO:0009443 pyridoxal 5'-phosphate salvage | |
| b1637 | tyrS | TyrS_mono | Protein | tyrosyl-tRNA synthetase | E | involved | TU0-8864;TU0-8865 | COG0162;Tyrosyl-tRNA synthetase | GO:0005737 cytoplasm | GO:0006418 amino acid activation | |
| b1638 | pdxH | PdxH_mono | Protein | pyridoxine 5'-phosphate oxidase | E | not involved | TU0-8864 | COG0259;Pyridoxamine-phosphate oxidase | | GO:0008615 pyridoxine biosynthesis -!- GO:0009443 pyridoxal 5'-phosphate salvage | |
| b1652 | rnt | Rnt_mono | Protein | ribonuclease T (RNase T) | E | involved | TU00333 | COG0847;DNA polymerase III, epsilon subunit and related 3'-5' exonucleases | | GO:0006401 RNA catabolism -!- GO:0006308 DNA catabolism -!- GO:0006401 RNA catabolism -!- GO:0006308 DNA catabolism | |
| b1653 | lhr | Lhr_mono | Protein | predicted ATP-dependent helicase | C | not involved | TU-8430;TU00333 | COG1201;Lhr-like helicases | GO:0005737 cytoplasm | GO:0006261 DNA dependent DNA replication | |
| b1665 | valV | valV_tRNA | tRNA | tRNA-Val(GAC) (Valine tRNA2B) | E | involved | TU00493 | | | | |
| b1666 | valW | valW_tRNA | tRNA | tRNA-Val(GAC) (Valine tRNA2A) | E | involved | TU00493 | | | | |
| b1712 | ihfA | IhfA_mono | Protein | integration host factor (IHF), DNA-binding protein, alpha subunit | E | not involved | TU0-6626;TU00174 | COG0776;Bacterial nucleoid DNA-binding protein | GO:0005737 cytoplasm | GO:0006310 DNA recombination | |
| b1713 | pheT | PheT_mono | Protein | phenylalanine tRNA synthetase, beta subunit | E | involved | TU0-6626 | COG0073;EMAP domain -!- COG0072;Phenylalanyl-tRNA synthetase beta subunit | GO:0005737 cytoplasm | GO:0006418 amino acid activation | |
| b1714 | pheS | PheS_mono | Protein | phenylalanine tRNA synthetase, alpha subunit | E | involved | TU0-6626 | COG0016;Phenylalanyl-tRNA synthetase alpha subunit | GO:0005737 cytoplasm | GO:0006418 amino acid activation | |
| b1715 | pheM | PheM_mono | Protein | phenylalanyl-tRNA synthetase operon leader peptide | E | not involved | TU0-6626;TU0-8855;TU0-8856 | | GO:0005737 cytoplasm | GO:0006418 amino acid activation | |
| b1716 | rplT | RplT_mono | Protein | 50S ribosomal subunit protein L20 | E | involved | TU0-6657;TU0-8855;TU00366 | COG0292;Ribosomal protein L20 | GO:0009281 cytosolic ribosome (sensu Bacteria) -!- GO:0005737 cytoplasm | GO:0006412 protein biosynthesis | GO:0003735 structural constituent of ribosome |
| b1717 | rpmI | RpmI_mono | Protein | 50S ribosomal subunit protein L35 | E | involved | TU00366 | | GO:0009281 cytosolic ribosome (sensu Bacteria) -!- GO:0005737 cytoplasm | GO:0006412 protein biosynthesis | GO:0003735 structural constituent of ribosome |
| b1718 | infC | InfC_mono | Protein | protein chain initiation factor IF-3 | E | involved | TU0-3304;TU0-3305;TU00366 | COG0290;Translation initiation factor 3 (IF-3) | GO:0005737 cytoplasm | GO:0006412 protein biosynthesis | |
| b1719 | thrS | ThrS_mono | Protein | threonyl-tRNA synthetase | E | involved | TU00366 | COG0441;Threonyl-tRNA synthetase | GO:0005737 cytoplasm | GO:0006418 amino acid activation | |
| b1804 | rnd | Rnd_mono | Protein | ribonuclease D | E | involved | | COG0349;Ribonuclease D | GO:0005737 cytoplasm | GO:0006401 RNA catabolism -!- GO:0009451 RNA modification | |
| b1822 | rrmA | RrmA_mono | Protein | 23S rRNA m1G745 methyltransferase | E | involved | TU0-13421 | COG0500;SAM-dependent methyltransferases | GO:0005737 cytoplasm | GO:0009451 RNA modification | |
| b1866 | aspS | AspS_mono | Protein | aspartyl-tRNA synthetase | E | involved | TU0-8084 | COG0173;Aspartyl-tRNA synthetase | GO:0005737 cytoplasm | GO:0006418 amino acid activation | |
| b1869 | yecN | YecN_mono | Protein | predicted inner membrane protein | C | not involved | TU0-13443 | | | | |

| | | | | | | | | | | | |
|---|---|---|---|---|---|---|---|---|---|---|---|
| b1870 | yecO | YecO_mono | Protein | predicted methyltransferase | C | involved | TU0-13443 | COG0500;SAM-dependent methyltransferases | | | |
| b1871 | yecP | YecP_mono | Protein | predicted S-adenosyl-L-methionine-dependent methyltransferase | C | involved | TU0-13443 | COG0500;SAM-dependent methyltransferases | | | |
| b1876 | argS | ArgS_mono | Protein | arginyl-tRNA synthetase | E | involved | TU0-13444 | COG0018;Arginyl-tRNA synthetase | GO:0005737 cytoplasm | GO:0006418 amino acid activation | |
| b1909 | leuZ | leuZ_tRNA | tRNA | tRNA-Leu(UAA) (Leucine tRNA4) | E | involved | TU00512 | | | | |
| b1910 | cysT | cysT_tRNA | tRNA | tRNA-Cys(GCA) (Cysteine tRNA) | E | involved | TU00512 | | | | |
| b1911 | glyW | glyW_tRNA | tRNA | tRNA-Gly(GCC) (Glycine tRNA3) | E | involved | TU00512 | | | | |
| b1920 | fliY | FliY_mono | Protein | cystine transporter subunit -!- periplasmic-binding component of ABC superfamily | E | not involved | TU00415;TU00471 | COG0834;ABC-type amino acid transport/signal transduction systems, periplasmic component/domain | GO:0042597 periplasmic space | GO:0019344 cysteine biosynthesis -!- GO:0042883 L-cysteine transport | |
| b1921 | fliZ | FliZ_mono | Protein | predicted regulator of FliA activity | C | not involved | TU00415;TU00471 | | | | |
| b1922 | fliA | FliA_mono | Protein | RNA polymerase, sigma 28 (sigma F) factor | E | involved | TU00415;TU00471 | | GO:0019861 flagellum -!- GO:0005737 cytoplasm | GO:0006350 transcription -!- GO:0042330 taxis | |
| b1975 | serU | serU_tRNA | tRNA | tRNA-Ser(CGA) (Serine tRNA2) | E | involved | TU00513 | | | | |
| b1977 | asnT | asnT_tRNA | tRNA | tRNA-Asn(GUU) (Asparagine tRNA) | E | involved | TU00497 | | | | |
| b1984 | asnW | asnW_tRNA | tRNA | tRNA-Asn(GUU) (Asparagine tRNA) | E | involved | TU0-3561 | | | | |
| b1986 | asnU | asnU_tRNA | tRNA | tRNA-Asn(GUU) (Asparagine tRNA) | E | involved | TU00498 | | | | |
| b1989 | asnV | asnV_tRNA | tRNA | tRNA-Asn(GUU) (Asparagine tRNA) | E | involved | TU00499 | | | | |
| b2114 | metG | MetG_mono | Protein | methionyl-tRNA synthetase | E | involved | TU0-13519 | COG0143;Methionyl-tRNA synthetase -!- COG0073;EMAP domain | GO:0005737 cytoplasm | GO:0006418 amino acid activation | |
| b2140 | dusC | DusC_mono | Protein | tRNA-dihydrouridine synthase C | E | involved | TU0-13534 | COG0042;tRNA-dihydrouridine synthase | | | |
| b2183 | rsuA | RsuA_mono | Protein | 16S rRNA pseudouridylate 516 synthase | E | involved | TU0-13555 | COG1187;16S rRNA uridine-516 pseudouridylate synthase and related pseudouridylate synthases | GO:0009281 cytosolic ribosome (sensu Bacteria) -!- GO:0005737 cytoplasm | GO:0009451 RNA modification | |
| b2185 | rplY | RplY_mono | Protein | 50S ribosomal subunit protein L25 | E | involved | TU0-13557 | COG1825;Ribosomal protein L25 (general stress protein Ctc) | GO:0009281 cytosolic ribosome (sensu Bacteria) -!- GO:0005737 cytoplasm | GO:0006412 protein biosynthesis | GO:0003735 structural constituent of ribosome |
| b2189 | proL | proL_tRNA | tRNA | tRNA-Pro(GGG) (Proline tRNA2) | E | involved | TU00494 | | | | |
| b2268 | elaC | ElaC_mono | Protein | binuclear zinc phosphodiesterase | E | involved | TU0-13573 | | | | |
| b2317 | dedA | DedA_mono | Protein | conserved inner membrane protein | C | not involved | TU0-5182;TU0-5183 | COG0586;Uncharacterized membrane-associated protein | GO:0009274 cell wall (sensu Bacteria) | | |
| b2318 | truA | TruA_mono | Protein | pseudouridylate synthase I | E | involved | TU0-5182;TU0-5183 | COG0101;Pseudouridylate synthase | GO:0005737 cytoplasm | GO:0006418 amino acid activation | |
| b2319 | usg | Usg_mono | Protein | predicted semialdehyde dehydrogenase | C | not involved | TU0-5182;TU0-5183 | COG0136;Aspartate-semialdehyde dehydrogenase | | | |
| b2320 | pdxB | PdxB_mono | Protein | erythronate-4-phosphate dehydrogenase | E | not involved | TU0-5182 | COG0111;Phosphoglycerate dehydrogenase and related dehydrogenases | | GO:0008615 pyridoxine biosynthesis | |

| | | | | | | | | | | | |
|---|---|---|---|---|---|---|---|---|---|---|---|
| b2324 | trmC | TrmC_mono | Protein | fused 5-methylaminomethyl-2-thiouridine-forming enzyme methyltransferase !- FAD-dependent demodification enzyme | E | involved | TU0-8823 | | | | |
| b2325 | yfcL | YfcL_mono | Protein | predicted protein | C | not involved | TU0-13593 | | | | |
| b2326 | yfcM | YfcM_mono | Protein | conserved protein | C | not involved | TU0-13593 | COG3101;Uncharacterized protein conserved in bacteria | | | |
| b2327 | yfcA | YfcA_mono | Protein | conserved inner membrane protein | C | not involved | TU0-13593 | COG0730;Predicted permeases | | | |
| b2328 | mepA | MepA_mono | Protein | murein DD-endopeptidase | E | not involved | TU0-13593 | COG3770;Murein endopeptidase | GO:0009274 cell wall (sensu Bacteria) | GO:0009252 peptidoglycan biosynthesis -!- GO:0042493 response to drug | |
| b2329 | aroC | AroC_mono | Protein | chorismate synthase | E | not involved | TU0-13593 | COG0082;Chorismate synthase | | GO:0009423 chorismate biosynthesis | |
| b2330 | prmB | PrmB_mono | Protein | N5-glutamine methyltransferase | E | involved | TU0-13593 | | | | |
| b2348 | argW | argW_tRNA | tRNA | tRNA-Arg(CCU) (Arginine tRNA5) | E | involved | TU00500 | | | | |
| b2396 | alaX | alaX_tRNA | tRNA | tRNA-Ala(GGC) (Alanine tRNA 2) | E | involved | TU00514 | | | | |
| b2397 | alaW | alaW_tRNA | tRNA | tRNA-Ala(GGC), (Alanine tRNA 2) | E | involved | TU00514 | | | | |
| b2400 | gltX | GltX_mono | Protein | glutamyl-tRNA synthetase | E | involved | TU0-6409;TU0-6550;TU0-6551 | COG0008;Glutamyl- and glutaminyl-tRNA synthetases | GO:0005737 cytoplasm | GO:0006418 amino acid activation | |
| b2401 | valU | valU_tRNA | tRNA | tRNA-Val(UAC) (Valine tRNA1) | E | involved | TU00495 | | | | |
| b2402 | valX | valX_tRNA | tRNA | tRNA-Val(UAC), (Valine tRNA1) | E | involved | TU00495 | | | | |
| b2403 | valY | valY_tRNA | tRNA | tRNA-Val(UAC) (Valine tRNA1) | E | involved | TU00495 | | | | |
| b2404 | lysV | lysV_tRNA | tRNA | tRNA-Lys(UUU) (Lysine tRNA) | E | involved | TU00495 | | | | |
| b2514 | hisS | HisS_mono | Protein | histidyl tRNA synthetase | E | involved | TU0-6506 | COG0124;Histidyl-tRNA synthetase | GO:0005737 cytoplasm | GO:0006418 amino acid activation | |
| b2528 | iscA | IscA_mono | Protein | FeS cluster assembly protein | E | involved | TU0-1881 | COG0316;Uncharacterized conserved protein | | GO:0006457 protein folding | |
| b2529 | iscU | IscU_mono | Protein | scaffold protein | E | involved | TU0-1881 | COG0822;NifU homolog involved in Fe-S cluster formation | | GO:0006412 protein biosynthesis | |
| b2530 | iscS | IscS_mono | Protein | cysteine desulfurase (tRNA sulfurtransferase), PLP-dependent | E | involved | TU0-1881 | | | GO:0009451 RNA modification | |
| b2531 | iscR | IscR_mono | Protein | DNA-binding transcriptional repressor | E | not involved | TU0-1881 | COG1959;Predicted transcriptional regulator | | GO:0006412 protein biosynthesis | |
| b2559 | tadA | TadA_mono | Protein | tRNA-specific adenosine deaminase | E | involved | TU0-13667 | COG0590;Cytosine/adenosine deaminases | | GO:0009451 RNA modification | |
| b2560 | yfhB | YfhB_mono | Protein | conserved protein | C | not involved | TU0-13667 | | | | |
| b2563 | acpS | AcpS_mono | Protein | holo-[acyl-carrier-protein] synthase 1 | E | not involved | TU483;TU484 | COG0736;Phosphopantetheinyl transferase (holo-ACP synthase) | GO:0005737 cytoplasm | | |
| b2564 | pdxJ | PdxJ_mono | Protein | pyridoxine 5'-phosphate synthase | E | not involved | TU483;TU484 | COG0854;Pyridoxal phosphate biosynthesis protein | GO:0005737 cytoplasm | GO:0008615 pyridoxine biosynthesis | |
| b2565 | recO | RecO_mono | Protein | gap repair protein | E | not involved | TU483 | COG1381;Recombinational DNA repair protein (RecF pathway) | GO:0005737 cytoplasm | GO:0006281 DNA repair -!- GO:0006310 DNA recombination | |
| b2566 | era | Era_mono | Protein | membrane-associated, 16S rRNA-binding GTPase | E | involved | TU00332;TU483 | COG1159;GTPase | | GO:0007049 cell cycle | |

| | | | | | | | | | | | |
|---|---|---|---|---|---|---|---|---|---|---|---|
| b2567 | rnc | Rnc_mono | Protein | RNase III | E | involved | TU00332;TU483 | COG0571;dsRNA-specific ribonuclease | | GO:0006401 RNA catabolism -!- GO:0006401 RNA catabolism | |
| b2570 | rseC | RseC_mono | Protein | RseC protein involved in reduction of the SoxR iron-sulfur cluster | E | not involved | TU00342;TU00526;TU281 | COG3086;Positive regulator of sigma E activity | GO:0009274 cell wall (sensu Bacteria) -!- GO:0019866 inner membrane | GO:0006350 transcription -!- GO:0042594 response to starvation | |
| b2571 | rseB | RseB_mono | Protein | anti-sigma factor | E | not involved | TU00342;TU00526;TU281 | COG3026;Negative regulator of sigma E activity | GO:0005737 cytoplasm | GO:0006350 transcription -!- GO:0042594 response to starvation | |
| b2572 | rseA | RseA_mono | Protein | anti-sigma factor | E | not involved | TU00342;TU00526;TU281 | COG3073;Negative regulator of sigma E activity | GO:0005737 cytoplasm | GO:0006350 transcription -!- GO:0009266 response to temperature | |
| b2573 | rpoE | RpoE_mono | Protein | RNA polymerase, sigma 24 (sigma E) factor | E | involved | TU00342;TU281 | COG1595;DNA-directed RNA polymerase specialized sigma subunit, sigma24 homolog | GO:0005737 cytoplasm | GO:0006350 transcription -!- GO:0009266 response to temperature | |
| b2588 | rrfG | rrfG | rRNA | 5S rRNA (rrfG) | E | involved | TU0-1187;TU0-1188 | | | | |
| b2589 | rrlG | rrlG | rRNA | 23S rRNA (rrlG) | E | involved | TU0-1187;TU0-1188 | | | | |
| b2590 | gltW | gltW_tRNA | tRNA | tRNA-Glu(UUC) (Glutamate tRNA2) | E | involved | TU0-1187;TU0-1188 | | | | |
| b2591 | rrsG | rrsG | rRNA | 16S rRNA (rrnG) | E | involved | TU0-1187;TU0-1188 | | | | |
| b2593 | yfiH | YfiH_mono | Protein | conserved protein | C | not involved | TU0-13675 | COG1496;Uncharacterized conserved protein | | | |
| b2594 | rluD | RluD_mono | Protein | 23S rRNA pseudouridine synthase | E | involved | TU0-13675 | COG0564;Pseudouridylate synthases, 23S RNA-specific | GO:0005737 cytoplasm | GO:0009451 RNA modification | |
| b2606 | rplS | RplS_mono | Protein | 50S ribosomal subunit protein L19 | E | involved | TU00351 | COG0335;Ribosomal protein L19 | GO:0009281 cytosolic ribosome (sensu Bacteria) -!- GO:0005737 cytoplasm | GO:0006412 protein biosynthesis | GO:0003735 structural constituent of ribosome |
| b2607 | trmD | TrmD_mono | Protein | tRNA (guanine-1-)-methyltransferase | E | involved | TU00351 | COG0336;tRNA-(guanine-N1)-methyltransferase | GO:0005737 cytoplasm | GO:0009451 RNA modification | |
| b2608 | rimM | RimM_mono | Protein | 16S rRNA processing protein | E | involved | TU00351 | | | GO:0009451 RNA modification | |
| b2609 | rpsP | RpsP_mono | Protein | 30S ribosomal subunit protein S16 | E | involved | TU00351 | COG0228;Ribosomal protein S16 | GO:0009281 cytosolic ribosome (sensu Bacteria) -!- GO:0005737 cytoplasm | GO:0006412 protein biosynthesis | GO:0003735 structural constituent of ribosome |
| b2614 | grpE | GrpE_mono | Protein | heat shock protein | E | involved | TU00411 | COG0576;Molecular chaperone GrpE (heat shock protein) | GO:0005737 cytoplasm | GO:0006457 protein folding | |
| b2652 | ileY | ileY_tRNA | tRNA | tRNA-Ile(CAU) (Isoleucine tRNA2 variant) | E | involved | TU0-13704 | | | | |
| b2691 | argQ | argQ_tRNA | tRNA | tRNA-Arg(ACG) (Arginine tRNA2) | E | involved | TU00515 | | | | |
| b2692 | argZ | argZ_tRNA | tRNA | tRNA-Arg(ACG) (Arginine tRNA2) | E | involved | TU00515 | | | | |
| b2693 | argY | argY_tRNA | tRNA | tRNA-Arg(ACG) (Arginine tRNA2) | E | involved | TU00515 | | | | |
| b2694 | argV | argV_tRNA | tRNA | tRNA-Arg(ACG) (Arginine tRNA2) | E | involved | TU00515 | | | | |
| b2695 | serV | serV_tRNA | tRNA | tRNA-Ser(GCU) (Serine tRNA3) | E | involved | TU00515 | | | | |
| b2697 | alaS | AlaS_mono | Protein | alanyl-tRNA synthetase | E | involved | TU0-13717;TU0-6441 | COG0013;Alanyl-tRNA synthetase | GO:0005737 cytoplasm | GO:0006418 amino acid activation | |
| b2741 | rpoS | RpoS_mono | Protein | RNA polymerase, sigma S (sigma 38) factor | E | involved | TU00309;TU00440;TU00442 | COG0568;DNA-directed RNA polymerase, sigma subunit (sigma70/sigma32) | GO:0005737 cytoplasm | GO:0006350 transcription -!- GO:0006970 response to osmotic stress | |

| | | | | | | | | | | | |
|---|---|---|---|---|---|---|---|---|---|---|---|
| b2742 | nlpD | NlpD_mono | Protein | predicted outer membrane lipoprotein | C | not involved | TU00309;TU00440 | COG0739;Membrane proteins related to metalloendopeptidases | GO:0009274 cell wall (sensu Bacteria) -!- GO:0019866 inner membrane | | |
| b2745 | truD | TruD_mono | Protein | pseudoruidine synthase | E | involved | | COG0585;Uncharacterized conserved protein | | | |
| b2779 | eno | Eno_mono | Protein | enolase | E | involved | TU0-13734 | COG0148;Enolase | GO:0005737 cytoplasm | GO:0006096 glycolysis -!- GO:0009061 anaerobic respiration -!- GO:0006094 gluconeogenesis | |
| b2780 | pyrG | PyrG_mono | Protein | CTP synthetase | E | not involved | TU0-13734;TU0-6428;TU0-6557 | COG0504;CTP synthase (UTP-ammonia lyase) | | GO:0015949 nucleobase, nucleoside and nucleotide interconversion | |
| b2785 | rumA | RumA_mono | Protein | 23S rRNA (uracil-5)-methyltransferase | E | involved | TU0-13735 | COG2265;SAM-dependent methyltransferases related to tRNA (uracil-5-)-methyltransferase | | GO:0009451 RNA modification | |
| b2790 | yqcA | YqcA_mono | Protein | predicted flavoprotein | C | not involved | TU0-13736 | COG0716;Flavodoxins | | | |
| b2791 | yqcB | YqcB_mono | Protein | tRNA pseudouridine synthase | E | involved | TU0-13736 | COG0564;Pseudouridylate synthases, 23S RNA-specific | | | |
| b2792 | yqcC | YqcC_mono | Protein | conserved protein | C | not involved | TU0-13736 | COG3098;Uncharacterized protein conserved in bacteria | | | |
| b2794 | queF | QueF_mono | Protein | conserved protein | C | involved | TU0-13738 | COG2904;Uncharacterized protein conserved in bacteria -!- COG0780;Enzyme related to GTP cyclohydrolase I | | | |
| b2814 | metZ | metZ_tRNA | tRNA | tRNA-initiator Met(CAU) (Initiator methionine tRNAf1) | E | involved | TU00496 | | | | |
| b2815 | metW | metW_tRNA | tRNA | tRNA-initiator Met(CAU) (Initiator methionine tRNAf1) | E | involved | TU00496 | | | | |
| b2816 | metV | metV_tRNA | tRNA | tRNA-initiator Met(CAU) (Initiator methionine tRNAf1) | E | involved | TU00496 | | | | |
| b2864 | glyU | glyU_tRNA | tRNA | tRNA-Gly(CCC) (Glycine tRNA1) | E | involved | TU0-13770;TU00516 | | | | |
| b2890 | lysS | LysS_mono | Protein | lysine tRNA synthetase, constitutive | E | involved | TU00324 | COG1190;Lysyl-tRNA synthetase (class II) | GO:0005737 cytoplasm | GO:0006418 amino acid activation | |
| b2891 | prfB | PrfB_mono | Protein | peptide chain release factor RF-2 | E | involved | TU-8392;TU00324 | COG1186;Protein chain release factor B | GO:0005737 cytoplasm | GO:0006412 protein biosynthesis | |
| b2892 | recJ | RecJ_mono | Protein | ssDNA exonuclease, 5' --> 3'-specific | E | not involved | TU-8392 | COG0608;Single-stranded DNA-specific exonuclease | | GO:0006308 DNA catabolism -!- GO:0006308 DNA catabolism | |
| b2893 | dsbC | DsbC_mono | Protein | protein disulfide isomerase II | E | not involved | TU-8392;TU0-4141 | COG1651;Protein-disulfide isomerase | GO:0042597 periplasmic space | GO:0006457 protein folding | |
| b2946 | yggJ | YggJ_mono | Protein | predicted protein | C | involved | TU0-13805 | | | | |
| b2947 | gshB | GshB_mono | Protein | glutathione synthetase | E | not involved | TU0-13805 | COG0189;Glutathione synthase/Ribosomal protein S6 modification enzyme (glutaminyl transferase) | | GO:0006750 glutathione biosynthesis | |
| b2959 | yggL | YggL_mono | Protein | predicted protein | C | not involved | TU0-13809 | | | | |
| b2960 | yggH | YggH_mono | Protein | tRNA (m7G46) methyltransferase, SAM-dependent | E | involved | TU0-13809 | COG0220;Predicted S-adenosylmethionine-dependent methyltransferase | | | |
| b2967 | pheV | pheV_tRNA | tRNA | tRNA-Phe(GAA) (Phenylalanine tRNA) | E | involved | TU00501 | | | | |
| b3065 | rpsU | RpsU_mono | Protein | 30S ribosomal subunit protein S21 | E | involved | TU00352;TU00434;TU00435;TU00472 | COG0828;Ribosomal protein S21 | GO:0009281 cytosolic ribosome (sensu Bacteria) -!- GO:0005737 cytoplasm | GO:0006412 protein biosynthesis | GO:0003735 structural constituent of ribosome |

| | | | | | | | | | | | |
|---|---|---|---|---|---|---|---|---|---|---|---|
| b3066 | dnaG | DnaG_mono | Protein | DNA primase | E | not involved | TU00352;TU00434;TU00435 | COG0358;DNA primase (bacterial type) | GO:0005737 cytoplasm | GO:0006261 DNA dependent DNA replication | |
| b3067 | rpoD | RpoD_mono | Protein | RNA polymerase, sigma 70 (sigma D) factor | E | involved | TU00352;TU00434;TU00435;TU00436;TU00437;TU00438 | COG0568;DNA-directed RNA polymerase, sigma subunit (sigma70/sigma32) | GO:0005737 cytoplasm | GO:0006350 transcription | |
| b3069 | ileX | ileX_tRNA | tRNA | tRNA-Ile(CAU) (Isoleucine tRNA2) | E | involved | TU00502 | | | | |
| b3123 | rnpB | RnpB_RNA | RNA | RNase P, RNA component precursor RnpB | E | involved | TU482;TU543 | | | | |
| b3124 | garK | GarK_mono | Protein | glycerate kinase I | E | not involved | TU482 | | | GO:0016052 carbohydrate catabolism | |
| b3125 | garR | GarR_mono | Protein | tartronate semialdehyde reductase | E | not involved | TU482 | COG2084;3-hydroxyisobutyrate dehydrogenase and related beta-hydroxyacid dehydrogenases | | GO:0016052 carbohydrate catabolism -!- GO:0009441 glycolate metabolism | |
| b3126 | garL | GarL_mono | Protein | alpha-dehydro-beta-deoxy-D-glucarate aldolase | E | not involved | TU482 | COG3836;2,4-dihydroxyhept-2-ene-1,7-dioic acid aldolase | | GO:0016052 carbohydrate catabolism | |
| b3127 | garP | GarP_mono | Protein | predicted (D)-galactarate transporter | C | not involved | TU482 | COG0477;Permeases of the major facilitator superfamily | GO:0009274 cell wall (sensu Bacteria) -!- GO:0019866 inner membrane | GO:0016052 carbohydrate catabolism | |
| b3164 | pnp | Pnp_mono | Protein | polynucleotide phosphorylase/polyadenylase | E | involved | TU0-6223;TU0-6654;TU0-6660;TU341 | | GO:0005737 cytoplasm | GO:0006401 RNA catabolism -!- GO:0006401 RNA catabolism | |
| b3165 | rpsO | RpsO_mono | Protein | 30S ribosomal subunit protein S15 | E | involved | TU0-6223;TU0-6660;TU0-8474;TU341 | COG0184;Ribosomal protein S15P/S13E | GO:0009281 cytosolic ribosome (sensu Bacteria) -!- GO:0005737 cytoplasm | GO:0006412 protein biosynthesis | GO:0003735 structural constituent of ribosome |
| b3166 | truB | TruB_mono | Protein | tRNA pseudouridine synthase | E | involved | TU0-6223;TU341 | COG0130;Pseudouridine synthase | GO:0005737 cytoplasm | GO:0009451 RNA modification | |
| b3167 | rbfA | RbfA_mono | Protein | 30s ribosome binding factor | E | involved | TU0-6223;TU341 | COG0858;Ribosome-binding factor A | GO:0005737 cytoplasm | GO:0009451 RNA modification | |
| b3168 | infB | InfB_mono | Protein | fused protein chain initiation factor 2, IF2: membrane protein -!- conserved protein | E | involved | TU0-6223;TU00311;TU00517;TU341 | COG0532;Translation initiation factor 2 (IF-2; GTPase) | GO:0005737 cytoplasm | GO:0006412 protein biosynthesis | |
| b3169 | nusA | NusA_mono | Protein | transcription termination/antitermination L factor | E | involved | TU0-6223;TU00311;TU00517;TU341 | COG0195;Transcription elongation factor | GO:0005737 cytoplasm | GO:0006350 transcription -!- GO:0006355 regulation of transcription, DNA-dependent | |
| b3170 | yhbC | YhbC_mono | Protein | conserved protein | C | not involved | TU0-6223;TU00311;TU00517;TU341 | | | | |
| b3171 | metY | metY_tRNA | tRNA | tRNA-initiator Met(CAU) (Initiator methionine tRNAf2) | E | involved | TU0-6223;TU0-8476;TU00517;TU341;TU343 | | | | |
| b3174 | leuU | leuU_tRNA | tRNA | tRNA-Leu(GAG) (Leucine tRNA2) | E | involved | TU00518 | | | | |
| b3178 | ftsH | FtsH_mono | Protein | protease, ATP-dependent zinc-metallo | E | not involved | TU00277;TU00414 | COG0465;ATP-dependent Zn proteases | GO:0009274 cell wall (sensu Bacteria) -!- GO:0019866 inner membrane | | |
| b3179 | rrmJ | RrmJ_mono | Protein | 23S rRNA methyltransferase | E | involved | TU00277;TU00414 | COG0293;23S rRNA methylase | | | |
| b3181 | greA | GreA_mono | Protein | transcription elongation factor | E | involved | TU-8397 | | GO:0005737 cytoplasm | GO:0006350 transcription -!- GO:0006355 regulation of transcription, DNA-dependent | |

| | | | | | | | | | | | |
|---|---|---|---|---|---|---|---|---|---|---|---|
| b3185 | rpmA | RpmA_mono | Protein | 50S ribosomal subunit protein L27 | E | involved | TU00338 | COG0211;Ribosomal protein L27 | GO:0009281 cytosolic ribosome (sensu Bacteria) -!- GO:0005737 cytoplasm | GO:0006412 protein biosynthesis | GO:0003735 structural constituent of ribosome |
| b3186 | rplU | RplU_mono | Protein | 50S ribosomal subunit protein L21 | E | involved | TU00338 | COG0261;Ribosomal protein L21 | GO:0009281 cytosolic ribosome (sensu Bacteria) -!- GO:0005737 cytoplasm | GO:0006412 protein biosynthesis | GO:0003735 structural constituent of ribosome |
| b3201 | yhbG | YhbG_mono | Protein | predicted transporter subunit: ATP-binding component of ABC superfamily | C | not involved | TU-8398 | COG1137;ABC-type (unclassified) transport system, ATPase component | GO:0005737 cytoplasm | | |
| b3202 | rpoN | RpoN_mono | Protein | RNA polymerase, sigma 54 (sigma N) factor | E | involved | TU-8398;TU00343;TU00487 | COG1508;DNA-directed RNA polymerase specialized sigma subunit, sigma54 homolog | | GO:0006807 nitrogen metabolism -!- GO:0006350 transcription | |
| b3203 | yhbH | YhbH_mono | Protein | predicted ribosome-associated, sigma 54 modulation protein | C | not involved | TU-8398;TU00487 | COG1544;Ribosome-associated protein Y (PSrp-1) | | GO:0006350 transcription | |
| b3204 | ptsN | PtsN_mono | Protein | sugar-specific enzyme IIA component of PTS | E | not involved | TU-8398;TU00487 | COG1762;Phosphotransferase system mannitol/fructose-specific IIA domain (Ntr-type) | | | |
| b3205 | yhbJ | YhbJ_mono | Protein | predicted protein with nucleoside triphosphate hydrolase domain | C | not involved | TU-8398;TU00487 | COG1660;Predicted P-loop-containing kinase | | | |
| b3206 | npr | Npr_mono | Protein | phosphohistidinoprotein-hexose phosphotransferase component of N-regulated PTS system (Npr) | E | not involved | TU00487 | COG1925;Phosphotransferase system, HPr-related proteins | GO:0005737 cytoplasm | GO:0006464 protein modification | |
| b3230 | rpsI | RpsI_mono | Protein | 30S ribosomal subunit protein S9 | E | involved | TU00336 | COG0103;Ribosomal protein S9 | GO:0009281 cytosolic ribosome (sensu Bacteria) -!- GO:0005737 cytoplasm | GO:0006412 protein biosynthesis | GO:0003735 structural constituent of ribosome |
| b3231 | rplM | RplM_mono | Protein | 50S ribosomal subunit protein L13 | E | involved | TU00336 | COG0102;Ribosomal protein L13 | GO:0009281 cytosolic ribosome (sensu Bacteria) -!- GO:0005737 cytoplasm | GO:0006412 protein biosynthesis | GO:0003735 structural constituent of ribosome |
| b3247 | rng | Rng_mono | Protein | ribonuclease G | E | involved | TU00304 | COG1530;Ribonucleases G and E | GO:0005737 cytoplasm | GO:0009451 RNA modification | |
| b3248 | yhdE | YhdE_mono | Protein | conserved protein | C | not involved | TU00304 | COG0424;Nucleotide-binding protein implicated in inhibition of septum formation | | | |
| b3249 | mreD | MreD_mono | Protein | cell wall structural complex MreBCD transmembrane component MreD | E | not involved | TU00304 | COG2891;Cell shape-determining protein | GO:0009274 cell wall (sensu Bacteria) -!- GO:0009274 cell wall (sensu Bacteria) -!- GO:0019866 inner membrane | GO:0009252 peptidoglycan biosynthesis | |
| b3250 | mreC | MreC_mono | Protein | cell wall structural complex MreBCD transmembrane component MreC | E | not involved | TU00304 | COG1792;Cell shape-determining protein | GO:0009274 cell wall (sensu Bacteria) | GO:0009252 peptidoglycan biosynthesis | |
| b3251 | mreB | MreB_mono | Protein | cell wall structural complex MreBCD, actin-like component MreB | E | not involved | TU00304 | COG1077;Actin-like ATPase involved in cell morphogenesis | | GO:0042493 response to drug | |
| b3257 | yhdT | YhdT_mono | Protein | conserved inner membrane protein | C | not involved | TU00314 | COG3924;Predicted membrane protein | | | |

| | | | | | | | | | | | |
|---|---|---|---|---|---|---|---|---|---|---|---|
| b3258 | panF | PanF_mono | Protein | pantothenate:sodium symporter | E | not involved | TU00314 | | GO:0009274 cell wall (sensu Bacteria) -!- GO:0019866 inner membrane | GO:0015937 coenzyme A biosynthesis | |
| b3259 | prmA | PrmA_mono | Protein | methylase for 50S ribosomal subunit protein L11 | E | involved | TU00314 | COG2264;Ribosomal protein L11 methylase | GO:0009281 cytosolic ribosome (sensu Bacteria) -!- GO:0005737 cytoplasm | GO:0009451 RNA modification | |
| b3260 | dusB | DusB_mono | Protein | tRNA-dihydrouridine synthase B | E | involved | TU00021 | COG0042;tRNA-dihydrouridine synthase | | | |
| b3261 | fis | Fis_mono | Protein | global DNA-binding transcriptional dual regulator | E | not involved | TU00021 | COG2901;Factor for inversion stimulation Fis, transcriptional activator | GO:0005737 cytoplasm | GO:0006310 DNA recombination | |
| b3272 | rrfF | rrfF | rRNA | 5S rRNA (rrfF) | E | involved | TU0-1191;TU0-1192 | | | | |
| b3273 | thrV | thrV_tRNA | tRNA | tRNA-Thr(GGU) (Threonine tRNA1) | E | involved | TU0-1191;TU0-1192 | | | | |
| b3274 | rrfD | rrfD | rRNA | 5S rRNA (rrfD) | E | involved | TU0-1191;TU0-1192 | | | | |
| b3275 | rrlD | rrlD | rRNA | 23S rRNA (rrlD) | E | involved | TU0-1191;TU0-1192 | | | | |
| b3276 | alaU | alaU_tRNA | tRNA | tRNA-Ala(UGC) (Alanine tRNA 1B) | E | involved | TU0-1191;TU0-1192 | | | | |
| b3277 | ileU | ileU_tRNA | tRNA | tRNA-Ile(GAU) (Isoleucine tRNA1) | E | involved | TU0-1191;TU0-1192 | | | | |
| b3278 | rrsD | rrsD | rRNA | 16S rRNA (rrnD) | E | involved | TU0-1191;TU0-1192 | | | | |
| b3280 | yrdB | YrdB_mono | Protein | conserved protein | C | not involved | TU0-7281 | | | | |
| b3281 | aroE | AroE_mono | Protein | dehydroshikimate reductase, NAD(P)-binding | E | not involved | TU0-7281 | COG0169;Shikimate 5-dehydrogenase | | GO:0009423 chorismate biosynthesis | |
| b3282 | yrdC | YrdC_mono | Protein | predicted ribosome maturation factor | C | involved | TU0-7281 | COG0009;Putative translation factor (SUA5) | | | |
| b3283 | yrdD | YrdD_mono | Protein | predicted DNA topoisomerase | C | not involved | TU0-7281 | | | | |
| b3287 | def | Def_mono | Protein | peptide deformylase | E | involved | TU00258 | COG0242;N-formylmethionyl-tRNA deformylase | | GO:0006464 protein modification | |
| b3288 | fmt | Fmt_mono | Protein | 10-formyltetrahydrofolate:L-methionyl-tRNA(fMet) N-formyltransferase | E | involved | TU00258 | COG0223;Methionyl-tRNA formyltransferase | GO:0005737 cytoplasm | GO:0009451 RNA modification | |
| b3289 | rsmB | RsmB_mono | Protein | 16S rRNA m5C967 methyltransferase, S-adenosyl-L-methionine-dependent | E | involved | | COG0144;tRNA and rRNA cytosine-C5-methylases | GO:0005737 cytoplasm | GO:0009451 RNA modification | |
| b3294 | rplQ | RplQ_mono | Protein | 50S ribosomal subunit protein L17 | E | involved | TU00349 | COG0203;Ribosomal protein L17 | GO:0009281 cytosolic ribosome (sensu Bacteria) -!- GO:0005737 cytoplasm | GO:0006412 protein biosynthesis | GO:0003735 structural constituent of ribosome |
| b3295 | rpoA | RpoA_mono | Protein | RNA polymerase, alpha subunit | E | involved | TU00349 | COG0202;DNA-directed RNA polymerase, alpha subunit/40 kD subunit | GO:0005737 cytoplasm | GO:0006350 transcription | |
| b3296 | rpsD | RpsD_mono | Protein | 30S ribosomal subunit protein S4 | E | involved | TU00349 | COG0522;Ribosomal protein S4 and related proteins | GO:0009281 cytosolic ribosome (sensu Bacteria) -!- GO:0005737 cytoplasm | GO:0006412 protein biosynthesis | GO:0003735 structural constituent of ribosome |
| b3297 | rpsK | RpsK_mono | Protein | 30S ribosomal subunit protein S11 | E | involved | TU00349 | COG0100;Ribosomal protein S11 | GO:0009281 cytosolic ribosome (sensu Bacteria) -!- GO:0005737 cytoplasm | GO:0006412 protein biosynthesis | GO:0003735 structural constituent of ribosome |
| b3298 | rpsM | RpsM_mono | Protein | 30S ribosomal subunit protein S13 | E | involved | TU00349 | COG0099;Ribosomal protein S13 | GO:0009281 cytosolic ribosome (sensu Bacteria) -!- GO:0005737 cytoplasm | GO:0006412 protein biosynthesis | GO:0003735 structural constituent of ribosome |

| | | | | | | | | | | | |
|---|---|---|---|---|---|---|---|---|---|---|---|
| b3299 | rpmJ | RpmJ_mono | Protein | 50S ribosomal subunit protein L36 | E | involved | TU00337 | COG0257;Ribosomal protein L36 | GO:0009281 cytosolic ribosome (sensu Bacteria) -!- GO:0005737 cytoplasm | GO:0006412 protein biosynthesis | GO:0003735 structural constituent of ribosome |
| b3300 | secY | SecY_mono | Protein | preprotein translocase membrane subunit | E | not involved | TU00337 | COG0201;Preprotein translocase subunit SecY | GO:0009274 cell wall (sensu Bacteria) -!- GO:0019866 inner membrane | | |
| b3301 | rplO | RplO_mono | Protein | 50S ribosomal subunit protein L15 | E | involved | TU00337 | COG0200;Ribosomal protein L15 | GO:0009281 cytosolic ribosome (sensu Bacteria) -!- GO:0005737 cytoplasm | GO:0006412 protein biosynthesis | GO:0003735 structural constituent of ribosome |
| b3302 | rpmD | RpmD_mono | Protein | 50S ribosomal subunit protein L30 | E | involved | TU00337 | COG1841;Ribosomal protein L30/L7E | GO:0009281 cytosolic ribosome (sensu Bacteria) -!- GO:0005737 cytoplasm | GO:0006412 protein biosynthesis | GO:0003735 structural constituent of ribosome |
| b3303 | rpsE | RpsE_mono | Protein | 30S ribosomal subunit protein S5 | E | involved | TU00337 | COG0098;Ribosomal protein S5 | GO:0009281 cytosolic ribosome (sensu Bacteria) -!- GO:0005737 cytoplasm | GO:0006412 protein biosynthesis | GO:0003735 structural constituent of ribosome |
| b3304 | rplR | RplR_mono | Protein | 50S ribosomal subunit protein L18 | E | involved | TU00337 | COG0256;Ribosomal protein L18 | GO:0009281 cytosolic ribosome (sensu Bacteria) -!- GO:0005737 cytoplasm | GO:0006412 protein biosynthesis | GO:0003735 structural constituent of ribosome |
| b3305 | rplF | RplF_mono | Protein | 50S ribosomal subunit protein L6 | E | involved | TU00337 | COG0097;Ribosomal protein L6P/L9E | GO:0009281 cytosolic ribosome (sensu Bacteria) -!- GO:0005737 cytoplasm | GO:0006412 protein biosynthesis | GO:0003735 structural constituent of ribosome |
| b3306 | rpsH | RpsH_mono | Protein | 30S ribosomal subunit protein S8 | E | involved | TU00337 | COG0096;Ribosomal protein S8 | GO:0009281 cytosolic ribosome (sensu Bacteria) -!- GO:0005737 cytoplasm | GO:0006412 protein biosynthesis -!- GO:0009386 translational attenuation | GO:0003735 structural constituent of ribosome |
| b3307 | rpsN | RpsN_mono | Protein | 30S ribosomal subunit protein S14 | E | involved | TU00337 | COG0199;Ribosomal protein S14 | GO:0009281 cytosolic ribosome (sensu Bacteria) -!- GO:0005737 cytoplasm | GO:0006412 protein biosynthesis | GO:0003735 structural constituent of ribosome |
| b3308 | rplE | RplE_mono | Protein | 50S ribosomal subunit protein L5 | E | involved | TU00337 | COG0094;Ribosomal protein L5 | GO:0009281 cytosolic ribosome (sensu Bacteria) -!- GO:0005737 cytoplasm | GO:0006412 protein biosynthesis | GO:0003735 structural constituent of ribosome |
| b3309 | rplX | RplX_mono | Protein | 50S ribosomal subunit protein L24 | E | involved | TU00337 | COG0198;Ribosomal protein L24 | GO:0009281 cytosolic ribosome (sensu Bacteria) -!- GO:0005737 cytoplasm | GO:0006412 protein biosynthesis | GO:0003735 structural constituent of ribosome |
| b3310 | rplN | RplN_mono | Protein | 50S ribosomal subunit protein L14 | E | involved | TU00337 | COG0093;Ribosomal protein L14 | GO:0009281 cytosolic ribosome (sensu Bacteria) -!- GO:0005737 cytoplasm | GO:0006412 protein biosynthesis | GO:0003735 structural constituent of ribosome |
| b3311 | rpsQ | RpsQ_mono | Protein | 30S ribosomal subunit protein S17 | E | involved | TU00347 | COG0186;Ribosomal protein S17 | GO:0009281 cytosolic ribosome (sensu Bacteria) -!- GO:0005737 cytoplasm | GO:0006412 protein biosynthesis | GO:0003735 structural constituent of ribosome |

| | | | | | | | | | | | |
|---|---|---|---|---|---|---|---|---|---|---|---|
| b3312 | rpmC | RpmC_mono | Protein | 50S ribosomal subunit protein L29 | E | involved | TU00347 | COG0255;Ribosomal protein L29 | GO:0009281 cytosolic ribosome (sensu Bacteria) -!- GO:0005737 cytoplasm | GO:0006412 protein biosynthesis | GO:0003735 structural constituent of ribosome |
| b3313 | rplP | RplP_mono | Protein | 50S ribosomal subunit protein L16 | E | involved | TU00347 | COG0197;Ribosomal protein L16/L10E | GO:0009281 cytosolic ribosome (sensu Bacteria) -!- GO:0005737 cytoplasm | GO:0006412 protein biosynthesis | GO:0003735 structural constituent of ribosome |
| b3314 | rpsC | RpsC_mono | Protein | 30S ribosomal subunit protein S3 | E | involved | TU00347 | COG0092;Ribosomal protein S3 | GO:0009281 cytosolic ribosome (sensu Bacteria) -!- GO:0005737 cytoplasm | GO:0006412 protein biosynthesis | GO:0003735 structural constituent of ribosome |
| b3315 | rplV | RplV_mono | Protein | 50S ribosomal subunit protein L22 | E | involved | TU00347 | COG0091;Ribosomal protein L22 | GO:0009281 cytosolic ribosome (sensu Bacteria) -!- GO:0005737 cytoplasm | GO:0006412 protein biosynthesis | GO:0003735 structural constituent of ribosome |
| b3316 | rpsS | RpsS_mono | Protein | 30S ribosomal subunit protein S19 | E | involved | TU00347 | COG0185;Ribosomal protein S19 | GO:0009281 cytosolic ribosome (sensu Bacteria) -!- GO:0005737 cytoplasm | GO:0006412 protein biosynthesis | GO:0003735 structural constituent of ribosome |
| b3317 | rplB | RplB_mono | Protein | 50S ribosomal subunit protein L2 | E | involved | TU00347 | COG0090;Ribosomal protein L2 | GO:0009281 cytosolic ribosome (sensu Bacteria) -!- GO:0005737 cytoplasm | GO:0006412 protein biosynthesis | GO:0003735 structural constituent of ribosome |
| b3318 | rplW | RplW_mono | Protein | 50S ribosomal subunit protein L23 | E | involved | TU00347 | COG0089;Ribosomal protein L23 | GO:0009281 cytosolic ribosome (sensu Bacteria) -!- GO:0005737 cytoplasm | GO:0006412 protein biosynthesis | GO:0003735 structural constituent of ribosome |
| b3319 | rplD | RplD_mono | Protein | 50S ribosomal subunit protein L4 | E | involved | TU00347 | COG0088;Ribosomal protein L4 | GO:0009281 cytosolic ribosome (sensu Bacteria) -!- GO:0005737 cytoplasm | GO:0006412 protein biosynthesis -!- GO:0006355 regulation of transcription, DNA-dependent -!- GO:0009386 translational attenuation | GO:0003735 structural constituent of ribosome |
| b3320 | rplC | RplC_mono | Protein | 50S ribosomal subunit protein L3 | E | involved | TU00347 | COG0087;Ribosomal protein L3 | GO:0009281 cytosolic ribosome (sensu Bacteria) -!- GO:0005737 cytoplasm | GO:0006412 protein biosynthesis | GO:0003735 structural constituent of ribosome |
| b3321 | rpsJ | RpsJ_mono | Protein | 30S ribosomal subunit protein S10 | E | involved | TU00347 | COG0051;Ribosomal protein S10 | GO:0009281 cytosolic ribosome (sensu Bacteria) -!- GO:0005737 cytoplasm | GO:0006412 protein biosynthesis | GO:0003735 structural constituent of ribosome |
| b3339 | tufA | TufA_mono | Protein | protein chain elongation factor EF-Tu (duplicate of tufB) | E | involved | TU0-5121;TU00348 | COG0050;GTPases - translation elongation factors | GO:0005737 cytoplasm | GO:0006412 protein biosynthesis -!- GO:0006970 response to osmotic stress | |
| b3340 | fusA | FusA_mono | Protein | protein chain elongation factor EF-G, GTP-binding | E | involved | TU0-5121;TU00348 | COG0480;Translation elongation factors (GTPases) | GO:0005737 cytoplasm | GO:0006412 protein biosynthesis | |
| b3341 | rpsG | RpsG_mono | Protein | 30S ribosomal subunit protein S7 | E | involved | TU00348 | COG0049;Ribosomal protein S7 | GO:0009281 cytosolic ribosome (sensu Bacteria) -!- GO:0005737 cytoplasm | GO:0006412 protein biosynthesis | GO:0003735 structural constituent of ribosome |

| | | | | | | | | | | | |
|---|---|---|---|---|---|---|---|---|---|---|---|
| b3342 | rpsL | RpsL_mono | Protein | 30S ribosomal subunit protein S12 | E | involved | TU00348 | COG0048;Ribosomal protein S12 | GO:0009281 cytosolic ribosome (sensu Bacteria) -!- GO:0005737 cytoplasm | GO:0006412 protein biosynthesis | GO:0003735 structural constituent of ribosome |
| b3343 | yheL | YheL_mono | Protein | predicted intracellular sulfur oxidation protein | C | involved | TU0-13915 | COG2168;Uncharacterized conserved protein involved in oxidation of intracellular sulfur | | | |
| b3344 | yheM | YheM_mono | Protein | predicted intraceullular sulfur oxidation protein | C | involved | TU0-13915 | COG2923;Uncharacterized protein involved in the oxidation of intracellular sulfur | | | |
| b3345 | yheN | YheN_mono | Protein | predicted intracellular sulfur oxidation protein | C | involved | TU0-13915 | COG1553;Uncharacterized conserved protein involved in intracellular sulfur reduction | | | |
| b3346 | yheO | YheO_mono | Protein | predicted DNA-binding transcriptional regulator | C | not involved | TU0-13915 | | | | |
| b3384 | trpS | TrpS_mono | Protein | tryptophanyl-tRNA synthetase | E | involved | TU0-8862;TU872;TU873;TU874;TU875;TU876;TU877 | COG0180;Tryptophanyl-tRNA synthetase | GO:0005737 cytoplasm | GO:0006418 amino acid activation | |
| b3385 | gph | Gph_mono | Protein | phosphoglycolate phosphatase | E | not involved | TU872;TU873;TU874;TU875;TU876;TU877 | COG0546;Predicted phosphatases | | GO:0016052 carbohydrate catabolism | |
| b3386 | rpe | Rpe_mono | Protein | D-ribulose-5-phosphate 3-epimerase | E | not involved | TU872;TU873;TU874;TU875;TU876;TU877 | COG0036;Pentose-5-phosphate-3-epimerase | | GO:0016052 carbohydrate catabolism -!- GO:0009052 pentose-phosphate shunt, non-oxidative branch | |
| b3387 | dam | Dam_mono | Protein | DNA adenine methylase | E | not involved | TU872;TU873;TU874;TU875;TU876 | COG0338;Site-specific DNA methylase | GO:0005737 cytoplasm | GO:0006306 DNA methylation | |
| b3388 | damX | DamX_mono | Protein | predicted protein | C | not involved | TU872;TU873;TU874 | COG3266;Uncharacterized protein conserved in bacteria | GO:0009274 cell wall (sensu Bacteria) -!- GO:0019866 inner membrane | | |
| b3389 | aroB | AroB_mono | Protein | 3-dehydroquinate synthase | E | not involved | TU872;TU873 | COG0337;3-dehydroquinate synthetase | | GO:0009423 chorismate biosynthesis | |
| b3390 | aroK | AroK_mono | Protein | shikimate kinase I | E | not involved | TU872;TU873 | | | GO:0009423 chorismate biosynthesis | |
| b3406 | greB | GreB_mono | Protein | transcription elongation factor | E | involved | TU0-13931 | | GO:0005737 cytoplasm | GO:0006350 transcription -!- GO:0006355 regulation of transcription, DNA-dependent | |
| b3461 | rpoH | RpoH_mono | Protein | RNA polymerase, sigma 32 (sigma H) factor | E | involved | TU0-6658;TU00083;TU00084;TU00426;TU00427;TU221 | COG0568;DNA-directed RNA polymerase, sigma subunit (sigma70/sigma32) | GO:0005737 cytoplasm | GO:0006350 transcription -!- GO:0009266 response to temperature | |
| b3470 | yhhP | YhhP_mono | Protein | conserved protein required for cell growth | E | involved | TU0-8510 | COG0425;Predicted redox protein, regulator of disulfide bond formation | | | |
| b3545 | proK | proK_tRNA | tRNA | tRNA-Pro(CGG) (Proline tRNA1) | E | involved | TU00519 | | | | |
| b3559 | glyS | GlyS_mono | Protein | glycine tRNA synthetase, beta subunit | E | involved | TU00281 | COG0751;Glycyl-tRNA synthetase, beta subunit | GO:0005737 cytoplasm | GO:0006418 amino acid activation | |
| b3560 | glyQ | GlyQ_mono | Protein | glycine tRNA synthetase, alpha subunit | E | involved | TU00281 | COG0752;Glycyl-tRNA synthetase, alpha subunit | GO:0005737 cytoplasm | GO:0006418 amino acid activation | |
| b3590 | selB | SelB_mono | Protein | selenocysteinyl-tRNA-specific translation factor | E | involved | TU00355 | COG3276;Selenocysteine-specific translation elongation factor | GO:0005737 cytoplasm | GO:0006412 protein biosynthesis | |
| b3591 | selA | SelA_mono | Protein | selenocysteine synthase | E | involved | TU00355 | COG1921;Selenocysteine synthase [seryl-tRNASer selenium transferase] | GO:0005737 cytoplasm | GO:0008615 pyridoxine biosynthesis -!- GO:0009451 RNA modification | |

| | | | | | | | | | | | |
|---|---|---|---|---|---|---|---|---|---|---|---|
| b3635 | mutM | MutM_mono | Protein | formamidopyrimidine/5-formyluracil/ 5-hydroxymethyluracil DNA glycosylase | E | not involved | TU0-5522;TU0-5523;TU0-5543;TU0-8355 | COG0266;Formamidopyrimidine-DNA glycosylase | GO:0005737 cytoplasm | GO:0006281 DNA repair | |
| b3636 | rpmG | RpmG_mono | Protein | 50S ribosomal subunit protein L33 | E | involved | TU0-5522;TU0-5543;TU0-5601;TU00339 | COG0267;Ribosomal protein L33 | GO:0009281 cytosolic ribosome (sensu Bacteria) -!- GO:0005737 cytoplasm | GO:0006412 protein biosynthesis | GO:0003735 structural constituent of ribosome |
| b3637 | rpmB | RpmB_mono | Protein | 50S ribosomal subunit protein L28 | E | involved | TU0-5522;TU0-5543;TU0-5601;TU00339 | COG0227;Ribosomal protein L28 | GO:0009281 cytosolic ribosome (sensu Bacteria) -!- GO:0005737 cytoplasm | GO:0006412 protein biosynthesis | GO:0003735 structural constituent of ribosome |
| b3638 | yicR | YicR_mono | Protein | protein associated with replication fork, possible DNA repair protein | E | not involved | TU0-5522;TU0-5601 | COG2003;DNA repair proteins | GO:0005737 cytoplasm | GO:0006261 DNA dependent DNA replication -!- GO:0006281 DNA repair | |
| b3649 | rpoZ | RpoZ_mono | Protein | RNA polymerase, omega subunit | E | involved | TU00344 | COG1758;DNA-directed RNA polymerase, subunit K/omega | GO:0005737 cytoplasm | GO:0006350 transcription | |
| b3650 | spoT | SpoT_mono | Protein | bifunctional (p)ppGpp synthetase II and guanosine-3',5'-bis pyrophosphate 3'-pyrophosphohydrolase | E | not involved | TU00344 | COG0317;Guanosine polyphosphate pyrophosphohydrolases/synthetases | GO:0005737 cytoplasm | GO:0015949 nucleobase, nucleoside and nucleotide interconversion -!- GO:0042594 response to starvation | GO:0008233 peptidase activity |
| b3651 | trmH | TrmH_mono | Protein | tRNA (Guanosine-2'-O-)-methyltransferase | E | involved | TU00344 | COG0566;rRNA methylases | | GO:0009451 RNA modification | |
| b3652 | recG | RecG_mono | Protein | ATP-dependent DNA helicase | E | not involved | TU00344 | COG1200;RecG-like helicase | GO:0005737 cytoplasm | GO:0006261 DNA dependent DNA replication | |
| b3658 | selC | selC_tRNA | tRNA | tRNA-Sec(UCA) (Selenocysteyl tRNA-UCA, converted from serine tRNA) | E | involved | TU0-14007 | | | | |
| b3703 | rpmH | RpmH_mono | Protein | 50S ribosomal subunit protein L34 | E | involved | TU00340;TU682;TU743 | COG0230;Ribosomal protein L34 | GO:0009281 cytosolic ribosome (sensu Bacteria) -!- GO:0005737 cytoplasm | GO:0006412 protein biosynthesis | GO:0003735 structural constituent of ribosome |
| b3704 | rnpA | RnpA_mono | Protein | protein C5 component of RNase P | E | involved | TU00340;TU682;TU743 | COG0594;RNase P protein component | GO:0005737 cytoplasm | GO:0006401 RNA catabolism -!- GO:0009451 RNA modification | |
| b3706 | trmE | TrmE_mono | Protein | GTPase | E | involved | TU0-14021 | COG0486;Predicted GTPase | | GO:0009451 RNA modification -!- GO:0006805 xenobiotic metabolism | |
| b3740 | gidB | GidB_mono | Protein | methyltransferase, SAM-dependent methyltransferase, glucose-inhibited cell-division protein | E | not involved | TU0-14029 | COG0357;Predicted S-adenosylmethionine-dependent methyltransferase involved in bacterial cell division | | | |
| b3741 | gidA | GidA_mono | Protein | glucose-inhibited cell-division protein | E | involved | TU0-14029 | COG0445;NAD/FAD-utilizing enzyme apparently involved in cell division | | | |
| b3756 | rrsC | rrsC | rRNA | 16S rRNA (rrnC) | E | involved | TU0-1183;TU0-1184 | | | | |
| b3757 | gltU | gltU_tRNA | tRNA | tRNA-Glu(UUC) (Glutamate tRNA2) | E | involved | TU0-1183;TU0-1184 | | | | |
| b3758 | rrlC | rrlC | rRNA | 23S rRNA (rrlC) | E | involved | TU0-1183;TU0-1184 | | | | |
| b3759 | rrfC | rrfC | rRNA | 5S rRNA (rrfC) | E | involved | TU0-1183;TU0-1184 | | | | |
| b3760 | aspT | aspT_tRNA | tRNA | tRNA-Asp(GUC) (Aspartate tRNA1) | E | involved | | | | | |
| b3761 | trpT | trpT_tRNA | tRNA | tRNA-Trp(CCA) (Tryptophan tRNA) | E | involved | | | | | |
| b3780 | rhlB | RhlB_mono | Protein | ATP-dependent RNA helicase | E | involved | TU0-14037 | COG0513;Superfamily II DNA and RNA helicases | | | |

| | | | | | | | | | | | |
|---|---|---|---|---|---|---|---|---|---|---|---|
| b3782 | rhoL | RhoL_mono | Protein | rho operon leader peptide | E | not involved | TU0-14038;TU0-6941 | | GO:0005737 cytoplasm | GO:0006350 transcription | |
| b3783 | rho | Rho_mono | Protein | transcription termination factor | E | involved | TU0-14038;TU0-6941 | COG1158;Transcription termination factor | GO:0005737 cytoplasm | GO:0006350 transcription | GO:0016564 transcriptional repressor activity |
| b3796 | argX | argX_tRNA | tRNA | tRNA-Arg(CCG) (Arginine tRNA3) | E | involved | TU00503 | | | | |
| b3797 | hisR | hisR_tRNA | tRNA | tRNA-His(GUG) (Histidine tRNA) | E | involved | TU00503 | | | | |
| b3798 | leuT | leuT_tRNA | tRNA | tRNA-Leu(CAG) (Leucine tRNA10 | E | involved | TU00503 | | | | |
| b3799 | proM | proM_tRNA | tRNA | tRNA-Pro(UGG) (Proline tRNA3) | E | involved | TU00503 | | | | |
| b3851 | rrsA | rrsA | rRNA | 16S rRNA (rrnA) | E | involved | TU0-1181 | | | | |
| b3852 | ileT | ileT_tRNA | tRNA | tRNA-Ile(GAU) (Isoleucine tRNA1) | E | involved | TU0-1181 | | | | |
| b3853 | alaT | alaT_tRNA | tRNA | tRNA-Ala(UGC) (Alanine tRNA 1B) | E | involved | TU0-1181 | | | | |
| b3854 | rrlA | rrlA | rRNA | 23S rRNA (rrlA) | E | involved | TU0-1181 | | | | |
| b3855 | rrfA | rrfA | rRNA | 5S rRNA (rrfA) | E | involved | TU0-1181 | | | | |
| b3885 | yihX | YihX_mono | Protein | predicted hydrolase | C | not involved | TU0-14070 | | | | |
| b3886 | rbn | Rbn_mono | Protein | predicted inner membrane protein | C | not involved | TU0-14070 | COG1295;Predicted membrane protein | GO:0005737 cytoplasm | | |
| b3887 | dtd | Dtd_mono | Protein | D-tyr-tRNA(Tyr) deacylase | E | involved | TU0-14070 | COG1490;D-Tyr-tRNAtyr deacylase | GO:0005737 cytoplasm | GO:0009451 RNA modification -!- GO:0006805 xenobiotic metabolism | |
| b3888 | yiiD | YiiD_mono | Protein | predicted acetyltransferase | C | not involved | TU0-14070 | COG0454;Histone acetyltransferase HPA2 and related acetyltransferases | | | |
| b3936 | rpmE | RpmE_mono | Protein | 50S ribosomal subunit protein L31 | E | involved | TU0-8506 | COG0254;Ribosomal protein L31 | GO:0009281 cytosolic ribosome (sensu Bacteria) -!- GO:0005737 cytoplasm | GO:0006412 protein biosynthesis | GO:0003735 structural constituent of ribosome |
| b3965 | trmA | TrmA_mono | Protein | tRNA (uracil-5-)-methyltransferase | E | involved | TU00430 | COG2265;SAM-dependent methyltransferases related to tRNA (uracil-5-)-methyltransferase | GO:0005737 cytoplasm | GO:0009451 RNA modification | |
| b3968 | rrsB | rrsB | rRNA | 16S rRNA (rrnB) | E | involved | TU0-1182;TU0-3364 | | | | |
| b3969 | gltT | gltT_tRNA | tRNA | tRNA-Glu(UUC) (Glutamate tRNA2) | E | involved | TU0-1182;TU0-3364 | | | | |
| b3970 | rrlB | rrlB | rRNA | 23S rRNA (rrlB) | E | involved | TU0-1182;TU0-3364 | | | | |
| b3971 | rrfB | rrfB | rRNA | 5S rRNA (rrfB) | E | involved | TU0-1182;TU0-3364 | | | | |
| b3976 | thrU | thrU_tRNA | tRNA | tRNA-Thr(UGU) (Threonine tRNA4) | E | involved | TU00504 | | | | |
| b3977 | tyrU | tyrU_tRNA | tRNA | tRNA-Tyr(GUA) (Tyrosine tRNA2) | E | involved | TU00504 | | | | |
| b3978 | glyT | glyT_tRNA | tRNA | tRNA-Gly(UCC) (Glycine tRNA2) | E | involved | TU00504 | | | | |
| b3979 | thrT | thrT_tRNA | tRNA | tRNA-Thr(GGU) (Threonine tRNA3) | E | involved | TU00504 | | | | |
| b3980 | tufB | TufB_mono | Protein | protein chain elongation factor EF-Tu (duplicate of tufA) | E | involved | TU0-14100;TU0-4925 | COG0050;GTPases - translation elongation factors | GO:0005737 cytoplasm | GO:0006412 protein biosynthesis -!- GO:0006970 response to osmotic stress | |
| b3981 | secE | SecE_mono | Protein | preprotein translocase membrane subunit | E | not involved | TU00354 | COG0690;Preprotein translocase subunit SecE | GO:0009274 cell wall (sensu Bacteria) -!- GO:0019866 inner membrane | | |
| b3982 | nusG | NusG_mono | Protein | transcription termination factor | E | involved | TU00354 | COG0250;Transcription antiterminator | GO:0005737 cytoplasm | GO:0006350 transcription | |
| b3983 | rplK | RplK_mono | Protein | 50S ribosomal subunit protein L11 | E | involved | TU0-6512 | COG0080;Ribosomal protein L11 | GO:0009281 cytosolic ribosome (sensu Bacteria) -!- GO:0005737 cytoplasm | GO:0006412 protein biosynthesis | GO:0003735 structural constituent of ribosome |

| | | | | | | | | | | | |
|---|---|---|---|---|---|---|---|---|---|---|---|
| b3984 | rplA | RplA_mono | Protein | 50S ribosomal subunit protein L1 | E | involved | TU0-6512 | COG0081;Ribosomal protein L1 | GO:0009281 cytosolic ribosome (sensu Bacteria) -!- GO:0005737 cytoplasm | GO:0006412 protein biosynthesis -!- GO:0009386 translational attenuation | GO:0003735 structural constituent of ribosome |
| b3985 | rplJ | RplJ_mono | Protein | 50S ribosomal subunit protein L10 | E | involved | TU0-6512;TU00335 | COG0244;Ribosomal protein L10 | GO:0009281 cytosolic ribosome (sensu Bacteria) -!- GO:0005737 cytoplasm | GO:0006412 protein biosynthesis | GO:0003735 structural constituent of ribosome |
| b3986 | rplL | RplL_mono | Protein | 50S ribosomal subunit protein L7/L12 | E | involved | TU0-6512;TU0-8464;TU00335 | COG0222;Ribosomal protein L7/L12 | GO:0009281 cytosolic ribosome (sensu Bacteria) -!- GO:0005737 cytoplasm | GO:0006412 protein biosynthesis | GO:0003735 structural constituent of ribosome |
| b3987 | rpoB | RpoB_mono | Protein | RNA polymerase, beta subunit | E | involved | TU0-6512;TU0-7002;TU00335 | COG0085;DNA-directed RNA polymerase, beta subunit/140 kD subunit | GO:0005737 cytoplasm | GO:0006350 transcription | |
| b3988 | rpoC | RpoC_mono | Protein | RNA polymerase, beta prime subunit | E | involved | TU0-6512;TU0-7002;TU00335 | COG0086;DNA-directed RNA polymerase, beta' subunit/160 kD subunit | GO:0005737 cytoplasm | GO:0006350 transcription | |
| b4007 | rrsE | rrsE | rRNA | 16S rRNA (rrnE) | E | involved | TU0-1186 | | | | |
| b4008 | gltV | gltV_tRNA | tRNA | tRNA-Glu(UUC) (Glutamate tRNA2) | E | involved | TU0-1186 | | | | |
| b4009 | rrlE | rrlE | rRNA | 23S rRNA (rrlE) | E | involved | TU0-1186 | | | | |
| b4010 | rrfE | rrfE | rRNA | 5S rRNA (rrfE) | E | involved | TU0-1186 | | | | |
| b4022 | yjbC | YjbC_mono | Protein | 23S rRNA pseudouridine synthase | E | involved | TU0-8505 | COG1187;16S rRNA uridine-516 pseudouridylate synthase and related pseudouridylate synthases | | | |
| b4049 | dusA | DusA_mono | Protein | tRNA-dihydrouridine synthase A | E | involved | TU0-14118 | COG0042;tRNA-dihydrouridine synthase | | | |
| b4129 | lysU | LysU_mono | Protein | lysine tRNA synthetase, inducible | E | involved | TU00221;TU00429 | COG1190;Lysyl-tRNA synthetase (class II) | GO:0005737 cytoplasm | GO:0006418 amino acid activation | |
| b4134 | pheU | pheU_tRNA | tRNA | tRNA-Phe(GAA) (Phenylalanine tRNA) | E | involved | TU00520 | | | | |
| b4142 | groS | GroS_mono | Protein | Cpn10 chaperonin GroES, small subunit of GroESL | E | involved | TU0-5003 | COG0234;Co-chaperonin GroES (HSP10) | GO:0005737 cytoplasm | GO:0006457 protein folding | |
| b4143 | groL | GroL_mono | Protein | Cpn60 chaperonin GroEL, large subunit of GroESL | E | involved | | COG0459;Chaperonin GroEL (HSP60 family) | GO:0005737 cytoplasm | GO:0006457 protein folding | |
| b4162 | orn | Orn_mono | Protein | oligoribonuclease | E | involved | TU0-14151 | | | GO:0006401 RNA catabolism | |
| b4163 | glyV | glyV_tRNA | tRNA | tRNA-Gly(GCC) (Glycine tRNA3) | E | involved | TU00505 | | | | |
| b4164 | glyX | glyX_tRNA | tRNA | tRNA-Gly(GCC) (Glycine tRNA3) | E | involved | TU00505 | | | | |
| b4165 | glyY | glyY_tRNA | tRNA | tRNA-Gly(GCC) (Glycine tRNA3) | E | involved | TU00505 | | | | |
| b4167 | yjeF | YjeF_mono | Protein | predicted carbohydrate kinase | C | not involved | TU00236;TU801;TU802 | COG0062;Uncharacterized conserved protein -!- COG0063;Predicted sugar kinase | | | |
| b4168 | yjeE | YjeE_mono | Protein | ATPase with strong ADP affinity | E | not involved | TU00236;TU801;TU802 | COG0802;Predicted ATPase or kinase | | | |
| b4169 | amiB | AmiB_mono | Protein | N-acetylmuramoyl-l-alanine amidase II | E | not involved | TU00236;TU801;TU802 | COG0860;N-acetylmuramoyl-L-alanine amidase | GO:0009274 cell wall (sensu Bacteria) | GO:0009252 peptidoglycan biosynthesis | |
| b4170 | mutL | MutL_mono | Protein | methyl-directed mismatch repair protein | E | not involved | TU00236;TU801;TU802 | COG0323;DNA mismatch repair enzyme (predicted ATPase) | GO:0005737 cytoplasm | GO:0006281 DNA repair | |
| b4171 | miaA | MiaA_mono | Protein | delta(2)-isopentenylpyrophosphate tRNA-adenosine transferase | E | involved | TU00236;TU801;TU802 | COG0324;tRNA delta(2)-isopentenylpyrophosphate transferase | GO:0005737 cytoplasm | GO:0009451 RNA modification | |

| | | | | | | | | | | | |
|---|---|---|---|---|---|---|---|---|---|---|---|
| b4172 | hfq | Hfq_mono | Protein | HF-I, host factor for RNA phage Q beta replication | E | not involved | TU00236;TU801;TU802 | COG1923;Uncharacterized host factor I protein | | GO:0009386 translational attenuation | |
| b4173 | hflX | HflX_mono | Protein | predicted GTPase | C | not involved | TU00236;TU801;TU802 | COG2262;GTPases | | | |
| b4174 | hflK | HflK_mono | Protein | modulator for HflB protease specific for phage lambda cII repressor | E | not involved | TU00236;TU801;TU802 | COG0330;Membrane protease subunits, stomatin/prohibitin homologs | | | |
| b4175 | hflC | HflC_mono | Protein | modulator for HflB protease specific for phage lambda cII repressor | E | not involved | TU00236;TU801;TU802 | COG0330;Membrane protease subunits, stomatin/prohibitin homologs | | | |
| b4178 | yjeB | YjeB_mono | Protein | predicted DNA-binding transcriptional regulator | C | not involved | TU0-8502 | COG1959;Predicted transcriptional regulator | | | |
| b4179 | rnr | Rnr_mono | Protein | exoribonuclease R, RNase R | E | not involved | TU0-8502 | | | GO:0006401 RNA catabolism -!- GO:0006401 RNA catabolism | |
| b4180 | rlmB | RlmB_mono | Protein | 23S rRNA (Gm2251)-methyltransferase | E | involved | TU0-8502 | COG0566;rRNA methylases | | GO:0009451 RNA modification | |
| b4200 | rpsF | RpsF_mono | Protein | 30S ribosomal subunit protein S6 | E | involved | TU00346 | COG0360;Ribosomal protein S6 | GO:0009281 cytosolic ribosome (sensu Bacteria) -!- GO:0005737 cytoplasm | GO:0006412 protein biosynthesis | GO:0003735 structural constituent of ribosome |
| b4201 | priB | PriB_mono | Protein | primosomal protein N | E | not involved | TU00346 | COG2965;Primosomal replication protein N | GO:0005737 cytoplasm | GO:0006261 DNA dependent DNA replication | |
| b4202 | rpsR | RpsR_mono | Protein | 30S ribosomal subunit protein S18 | E | involved | TU00346 | COG0238;Ribosomal protein S18 | GO:0009281 cytosolic ribosome (sensu Bacteria) -!- GO:0005737 cytoplasm | GO:0006412 protein biosynthesis | GO:0003735 structural constituent of ribosome |
| b4203 | rplI | RplI_mono | Protein | 50S ribosomal subunit protein L9 | E | involved | TU00346 | COG0359;Ribosomal protein L9 | GO:0009281 cytosolic ribosome (sensu Bacteria) -!- GO:0005737 cytoplasm | GO:0006412 protein biosynthesis | GO:0003735 structural constituent of ribosome |
| b4258 | valS | ValS_mono | Protein | valyl-tRNA synthetase | E | involved | TU0-6405;TU0-6687 | COG0525;Valyl-tRNA synthetase | GO:0005737 cytoplasm | GO:0006418 amino acid activation | |
| b4270 | leuX | leuX_tRNA | tRNA | tRNA-Leu(CAA) (Leucine tRNA5) | E | involved | TU00506 | | | | |
| b4292 | fecR | FecR_mono | Protein | KpLE2 phage-like element; transmembrane signal transducer for ferric citrate transport | E | not involved | TU00460 | COG3712;Fe2+-dicitrate sensor, membrane component | GO:0019866 inner membrane | GO:0006350 transcription -!- GO:0006826 iron ion transport | |
| b4293 | fecI | FecI_mono | Protein | KpLE2 phage-like element; RNA polymerase, sigma 19 factor | E | involved | TU00460 | COG1595;DNA-directed RNA polymerase specialized sigma subunit, sigma24 homolog | GO:0005737 cytoplasm | GO:0006350 transcription | |
| b4368 | leuV | leuV_tRNA | tRNA | tRNA-Leu(CAG) (Leucine tRNA1) | E | involved | TU00521 | | | | |
| b4369 | leuP | leuP_tRNA | tRNA | tRNA-Leu(CAG) (Leucine tRNA1) | E | involved | TU00521 | | | | |
| b4370 | leuQ | leuQ_tRNA | tRNA | tRNA-Leu(CAG) (Leucine tRNA1) | E | involved | TU00521 | | | | |
| b4371 | rsmC | RsmC_mono | Protein | 16S RNA m2G1207 methylase | E | involved | TU0-14215 | COG2813;16S RNA G1207 methylase RsmC | GO:0005737 cytoplasm | GO:0009451 RNA modification | |
| b4372 | holD | HolD_mono | Protein | DNA polymerase III, psi subunit | E | not involved | TU0-14216 | COG3050;DNA polymerase III, psi subunit | GO:0005737 cytoplasm | GO:0006261 DNA dependent DNA replication | |

| | | | | | | | | | | | |
|---|---|---|---|---|---|---|---|---|---|---|---|
| b4373 | rimI | RimI_mono | Protein | acetylase for 30S ribosomal subunit protein S18 | E | involved | TU0-14216 | COG0456;Acetyltransferases | GO:0009281 cytosolic ribosome (sensu Bacteria) -!- GO:0005737 cytoplasm | GO:0006464 protein modification | |
| b4374 | yjjG | YjjG_mono | Protein | predicted hydrolase | C | not involved | TU0-14216 | COG1011;Predicted hydrolase (HAD superfamily) | | | |
| b4375 | prfC | PrfC_mono | Protein | peptide chain release factor RF-3 | E | involved | TU0-14217 | COG4108;Peptide chain release factor RF-3 | GO:0005737 cytoplasm | GO:0006412 protein biosynthesis | |
