# Supplementary material for: Genome-Scale Reconstruction of Escherichia coli's Transcriptional and Translational Machinery: A Knowledge Base, Its Mathematical Formulation, and Its Functional Characterization
Source: PLoS Comput Biol. 2009 Mar 13;5(3):e1000312. doi: 10.1371/journal.pcbi.1000312 (PMC2648898; doi:10.1371/journal.pcbi.1000312)
Supplement: Table S11 — Used genetic code (0.04 MB PDF) [file pcbi.1000312.s013.pdf]

Table S11 - Genetic code

generic tRNA	codon	Amino acid
ala1-tRNA	gct	ala-L
ala1-tRNA	gca	ala-L
ala1-tRNA	gcg	ala-L
ala2-tRNA	gcc	ala-L
arg1-tRNA	cgt	arg-L
arg1-tRNA	cgc	arg-L
arg1-tRNA	cga	arg-L
argU-tRNA	aga	arg-L
argW-tRNA	agg	arg-L
argX-tRNA	cgg	arg-L
asn1-tRNA	aac	asn-L
asn1-tRNA	aat	asn-L
asp1-tRNA	gac	asp-L
asp1-tRNA	gat	asp-L
cysT-tRNA	tgc	cys-L
cysT-tRNA	tgt	cys-L
gln1-tRNA	cag	gln-L
gln2-tRNA	caa	gln-L
glu1-tRNA	gaa	glu-L
glu1-tRNA	gag	glu-L
gly1-tRNA	ggc	gly
gly1-tRNA	ggt	gly
glyT-tRNA	gga	gly
glyU-tRNA	ggg	gly
hisR-tRNA	cac	his-L
hisR-tRNA	cat	his-L
ile1-tRNA	atc	ile-L
ile1-tRNA	att	ile-L
ile2-tRNA	ata	ile-L
leu1-tRNA	ctg	leu-L
leuU-tRNA	ctc	leu-L
leuU-tRNA	ctt	leu-L
leuW-tRNA	cta	leu-L
leu2-tRNA	ttg	leu-L
leuZ-tRNA	tta	leu-L
lys1-tRNA	aaa	lys-L
lys1-tRNA	aag	lys-L
met1-tRNA	atg	met-L
phe1-tRNA	ttc	phe-L
phe1-tRNA	ttt	phe-L
pro1-tRNA	ccg	pro-L
proL-tRNA	ccc	pro-L
pro2-tRNA	cct	pro-L
proM-tRNA	cca	pro-L
ser1-tRNA	tcc	ser-L
ser2-tRNA	tct	ser-L
serT-tRNA	tca	ser-L
ser3-tRNA	tcg	ser-L
serV-tRNA	agc	ser-L
serV-tRNA	agt	ser-L
thr1-tRNA	acc	thr-L
thr2-tRNA	act	thr-L
thrU-tRNA	aca	thr-L
thr3-tRNA	acg	thr-L
trpT-tRNA	tgg	trp-L
tyr1-tRNA	tac	tyr-L
tyr1-tRNA	tat	tyr-L
val1-tRNA	gta	val-L
val1-tRNA	gtg	val-L
val2-tRNA	gtc	val-L
val3-tRNA	gtt	val-L

tRNA sequences and modification were obtained from PMID: 15608164 - (Sprinzl and Vassilenko '05.D139-40)

Nucleotide Modification Abr.	Nucleotide Modification Name
ac4C	N4-acetylcytidine
acp3U	3-(3-amino-3-carboxypropyl)uridine
Cm	2'-O-methylcytidine
cmo5U	uridine-5-oxyacetic-acid
D	dihydrouridine
Gm	2'-O-methylguanosine
I	inosine
i6A	N6-isopentenyladenosine
k2C	lysidine
m1G	1-methylguanosine
m2A	2-methyladenosine
m5U	Ribosylthymine
m6A	N6-methyladenosine
m6t6A	N6-methyl-N6-threonylcarbamoylader
m7G	7-methylguanosine
mnm5s2U	5-methylaminomethyl-2-thiouridine
mnm5se2U	5-methylaminomethyl-2-selenouridine
mnm5U	5-methylaminomethyluridine
ms2i6A	2-methylthio-N6-isopentenyladenosin
psi	pseudouridine
Q	Queuosine
s2C	2-thiocytidine
s4U	4-thiouridine
t6A	N6-threonylcarbamoyladenosine
Um	2'-O-methyluridine

	Blattner Number	b0672	b4270	b0244	b3976	b2397	b2396	b3853	b3276	b0203	b1910	b3760	b0206	b0216	b3969	b3757	b4008	b2590	b4134	b2967	b2864	b4163	b1911	b4164	b4165	b3978	b3797	b3852	b3277	b0202	b3069	b2652	b0749	b0743	b2404	b0745	b0747
	Gene Name	leuW	leuX	thrW	thrU	alaW	alaX	alaT	alaU	alaV	cysT	aspT	aspU	aspV	gltT	gltU	gltV	gltW	pheU	pheV	glyU	glyV	glyW	glyX	glyY	glyT	hisR	ileT	ileU	ileV	ileX	ileY	lysQ	lysT	lysV	lysW	lysY
	generic tRNA in model	leuW-tRNA	leuX-tRNA	thrW-tRNA	trhU-tRNA	ala2-tRNA	ala2-tRNA	ala1-tRNA	ala1-tRNA	ala1-tRNA	cysT-tRNA	asp1-tRNA	asp1-tRNA	asp1-tRNA	glu1-tRNA	glu1-tRNA	glu1-tRNA	glu1-tRNA	phe1-tRNA	phe1-tRNA	gly2-tRNA	gly1-tRNA	gly1-tRNA	gly1-tRNA	gly1-tRNA	glyT-tRNA	hisR-tRNA	ile1-tRNA	ile1-tRNA	ile1-tRNA	ile2-tRNA	ile2-tRNA	lys1-tRNA	lys1-tRNA	lys1-tRNA	lys1-tRNA	lys1-tRNA
	tRNA DB ID	R1	R2	R3	R4	RA1660	RA1660	RA1662	RA1662	RA1662	RC1660	RD1660	RD1660	RD1660	RE1661	RE1661	RE1661	RE1661	RF1660	RF1660	RG1660	RG1661	RG1661	RG1661	RG1661	RG1662	RH1660	RI1661	RI1661	RI1661	RI1662	RI1662	RK1660	RK1660	RK1660	RK1660	RK1660
	Anticodon	UAG	CAA	CGU	UGU	GGC	GGC	VGC	VGC	VGC	GCA	QUC	QUC	QUC	SUC	SUC	SUC	SUC	GAA	GAA	CCC	GCC	GCC	GCC	GCC	NCC	QUG	GAU	GAU	GAU	k2CAU	k2CAU	SUU	SUU	SUU	SUU	SUU
Nucleotide position	0	-	-	-	-	-	-	-	-	-	-	-	-	-	-	-	-	-	-	-	-	-	-	-	-	-	G	-	-	-	-	-	-	-	-	-	-
	1	G	G	G	G	G	G	G	G	G	G	G	G	G	G	G	G	G	G	G	G	G	G	G	G	G	G	A	A	A	G	G	G	G	G	G	G
	2	C	C	C	C	G	G	G	G	G	G	G	G	G	U	U	U	U	C	C	C	C	C	C	C	C	U	G	G	G	G	G	G	G	G	G	G
	3	G	C	C	C	G	G	G	G	G	C	A	A	A	C	C	C	C	C	C	G	G	G	G	G	G	G	G	G	G	C	C	G	G	G	G	G
	4	G	G	G	G	G	G	G	G	G	G	G	G	G	C	C	C	C	C	C	G	G	G	G	G	G	G	C	C	C	C	C	U	U	U	U	U
	5	G	A	A	A	C	C	C	C	C	C	C	C	C	C	C	C	C	G	G	G	G	G	G	G	G	C	U	U	U	C	C	C	C	C	C	C
	6	A	A	U	C	U	U	U	U	U	G	G	G	G	C	C	C	C	G	G	C	A	A	A	A	C	U	U	U	U	C	C	G	G	G	G	G
	7	G	G	A	U	A	A	A	A	A	U	G	G	G	U	U	U	U	A	A	G	A	A	A	A	A	A	G	G	G	U	U	U	U	U	U	U
	8	U	U	U	U	U	U	U	U	U	s4U	s4U	s4U	s4U	U	U	U	U	s4U	s4U	s4U	U	U	U	U	U	s4U	U	U	U	s4U	s4U	U	U	U	U	U
	9	G	G	A	A	A	A	A	A	A	A	A	A	A	C	C	C	C	A	A	A	A	A	A	A	C	A	A	A	A	A	A	A	A	A	A	A
	10	G	G	G	G	G	G	G	G	G	A	G	G	G	G	G	G	G	G	G	G	G	G	G	G	G	G	G	G	G	G	G	G	G	G	G	G
	11	C	C	C	C	C	C	C	C	C	C	U	U	U	U	U	U	U	C	C	U	C	C	C	C	U	C	C	C	C	C	C	C	C	C	C	C
	12	G	G	U	U	U	U	U	U	U	A	U	U	U	C	C	C	C	U	U	U	U	U	U	U	A	U	U	U	U	U	U	U	U	U	U	U
	13	A	A	C	C	C	C	C	C	C	A	C	C	C	psi	psi	psi	psi	C	C	C	C	C	C	C	U	C	C	C	C	C	C	C	C	C	C	C
	14	A	A	A	A	A	A	A	A	A	A	A	A	A	A	A	A	A	A	A	A	A	A	A	A	A	A	A	A	A	A	A	A	A	A	A	A
	15	A	A	G	G	G	G	G	G	G	G	G	G	G	G	G	G	G	G	G	A	G	G	G	G	A	G	G	G	G	G	G	G	G	G	G	G
	16	D	D	D	D	C	C	C	C	C	C	D	D	D	A	A	A	A	D	D	U	D	D	D	D	U	D	G	G	G	U	U	D	D	D	D	D
	17	D	C	D	A	D	D	D	D	D	-	C	C	C	-	-	-	-	C	C	-	D	D	D	D	-	D	U	U	U	-	-	D	D	D	D	D
	17A	-	-	-	-	-	-	-	-	-	-	-	-	-	-	-	-	-	-	-	-	-	-	-	-	-	-	-	-	-	-	-	-	-	-	-	-
	18	Gm	Gm	G	G	G	G	G	G	G	G	G	G	G	G	G	G	G	G	G	G	G	G	G	G	G	G	G	G	G	Gm	Gm	G	G	G	G	G
	19	G	G	G	G	G	G	G	G	G	G	G	G	G	G	G	G	G	G	G	G	G	G	G	G	G	G	G	G	G	G	G	G	G	G	G	G
	20	D	D	D	D	G	G	G	G	G	D	D	D	D	C	C	C	C	D	D	D	D	D	D	D	C	D	D	D	D	D	D	D	D	D	D	D
	20A	A	A	-	-	-	-	-	-	-	-	D	D	D	C	C	C	C	-	-	-	-	-	-	-	U	-	D	D	D	D	D	-	-	-	-	-
	20B	-	-	-	-	-	-	-	-	-	-	-	-	-	C	C	C	C	-	-	-	-	-	-	-	-	-	-	-	-	-	-	-	-	-	-	-
	21	G	G	A	A	A	A	A	A	A	D	A	A	A	A	A	A	A	A	A	A	A	A	A	A	A	A	A	A	A	A	A	A	A	A	A	A
	22	A	A	G	G	G	G	G	G	G	A	G	G	G	G	G	G	G	G	G	G	G	G	G	G	U	G	G	G	G	G	G	G	G	G	G	G
	23	C	C	A	A	A	A	A	A	A	U	A	A	A	G	G	G	G	A	A	A	A	A	A	A	U	A	A	A	A	A	A	A	A	A	A	A
	24	G	G	G	G	G	G	G	G	G	G	A	A	A	A	A	A	A	G	G	A	G	G	G	G	A	G	G	G	G	G	G	G	G	G	G	G
	25	C	C	C	C	C	C	C	C	C	U	U	U	U	C	C	C	C	C	C	C	C	C	C	C	C	C	C	C	C	C	C	C	C	C	C	C
	26	A	A	A	A	G	G	G	G	G	A	A	A	A	A	A	A	A	A	A	G	A	A	A	A	C	C	G	G	G	A	A	A	A	A	A	A
	27	C	G	G	A	C	C	C	C	C	G	C	C	C	C	C	C	C	G	G	A	C	C	C	C	U	C	C	C	C	G	G	G	G	G	G	G
	28	C	U	C	C	U	U	C	C	C	C	C	C	C	C	C	C	C	G	G	G	G	G	G	G	C	U	A	A	A	G	G	U	U	U	U	U
	29	A	U	G	U	U	U	U	U	U	G	U	U	U	G	G	G	G	G	G	A	A	A	A	A	A	G	C	C	C	C	C	U	U	U	U	U
	30	G	G	C	G	G	G	G	G	G	G	G	G	G	C	C	C	C	G	G	G	C	C	C	C	G	G	C	C	C	G	G	G	G	G	G	G
	31	A	A	A	A	C	C	C	C	C	A	C	C	C	C	C	C	C	A	A	C	C	C	C	C	C	A	C	C	C	A	A	A	A	A	A	A
	32	U	U	U	C	A	A	U	U	U	psi	C	C	C	C	C	C	C	psi	psi	U	U	U	U	U	C	U	C	C	C	C	C	C	C	C	C	C
	33	U	U	U	U	U	U	U	U	U	U	U	U	U	U	U	U	U	U	U	U	U	U	U	U	U	U	U	U	U	U	U	U	U	U	U	U
	34	U	C	C	U	G	G	cmo5U	cmo5U	cmo5U	G	Q	Q	Q	mnm5s2U	mnm5s2U	mnm5s2U	mnm5s2U	G	G	C	G	G	G	G	U	Q	G	G	G	k2C	k2C	mnm5s2U	mnm5s2U	mnm5s2U	mnm5s2U	mnm5s2U
	35	A	A	G	G	G	G	G	G	G	C	U	U	U	U	U	U	U	A	A	C	C	C	C	C	C	U	A	A	A	A	A	U	U	U	U	U
	36	G	A	U	U	C	C	C	C	C	A	C	C	C	C	C	C	C	A	A	C	C	C	C	C	C	G	U	U	U	U	U	U	U	U	U	U
	37	G	ms2i6A	m6t6A	m6t6A	A	A	A	A	A	ms2i6A	m2A	m2A	m2A	m2A	m2A	m2A	m2A	ms2i6A	ms2i6A	A	A	A	A	A	A	m2A	t6A	t6A	t6A	t6A	t6A	t6A	t6A	t6A	t6A	t6A
	38	psi	A	A	A	U	U	C	C	C	A	C	C	C	C	C	C	C	A	A	A	A	A	A	A	A	psi	A	A	A	A	A	A	A	A	A	A
	39	U	psi	U	U	G	G	G	G	G	psi	G	G	G	G	G	G	G	psi	psi	G	G	G	G	G	G	psi	G	G	G	psi	psi	psi	psi	psi	psi	psi
	40	C	C	G	C	C	C	C	C	C	C	C	C	C	G	G	G	G	C	C	C	G	G	G	G	C	C	G	G	G	C	C	C	C	C	C	C
	41	U	A	C	A	A	A	A	A	A	C	A	A	A	C	C	C	C	C	C	U	U	U	U	U	U	C	G	G	G	G	G	A	A	A	A	A
	42	G	A	G	G	A	A	G	G	G	G	G	G	G	G	G	G	G	C	C	C	C	C	C	C	G	A	U	U	U	C	C	A	A	A	A	A
	43	G	C	A	U	G	G	G	G	G	U	G	G	G	G	G	G	G	C	C	U	G	G	G	G	A	G	G	G	G	U	U	U	U	U	U	U
	44	C	C	A	A	A	A	A	A	A	C	G	G	G	U	U	U	U	G	G	A	G	G	G	G	U	U	A	A	A	U	U	U	U	U	U	U
	45	-	-	G	G	G	G	G	G	G	U	G	G	G	A	A	A	A	U	U	U	G	G	G	G	G	U	G	G	G	G	G	G	G	G	G	G
	e11	G	G	-	-	-	-	-	-	-	-	-	-	-	-	-	-	-	-	-	-	-	-	-	-	-	-	-	-	-	-	-	-	-	-	-	-
	e12	C	U	-	-	-	-	-	-	-	-	-	-	-	-	-	-	-	-	-	-	-	-	-	-	-	-	-	-	-	-	-	-	-	-	-	-
	e13	C	A	-	-	-	-	-	-	-	-	-	-	-	-	-	-	-	-	-	-	-	-	-	-	-	-	-	-	-	-	-	-	-	-	-	-
	e14	-	-	-	-	-	-	-	-	-	-	-	-	-	-	-	-	-	-	-	-	-	-	-	-	-	-	-	-	-	-	-	-	-	-	-	-
	e15	-	-	-	-	-	-	-	-	-	-	-	-	-	-	-	-	-	-	-	-	-	-	-	-	-	-	-	-	-	-	-	-	-	-	-	-
	e16	-	-	-	-	-	-	-	-	-	-	-	-	-	-	-	-	-	-	-	-	-	-	-	-	-	-	-	-	-	-	-	-	-	-	-	-
	e17	-	-	-	-	-	-	-	-	-	-	-	-	-	-	-	-	-	-	-	-	-	-	-	-	-	-	-	-	-	-	-	-	-	-	-	-
	e1	G	G	-	-	-	-	-	-	-	-	-	-	-	-	-	-	-	-	-	-	-	-	-	-	-	-	-	-	-	-	-	-	-	-	-	-
	e2	C	A	-	-	-	-	-	-	-	-	-	-	-	-	-	-	-	-	-	-	-	-	-	-	-	-	-	-	-	-	-	-	-	-	-	-
	e3	A	A	-	-	-	-	-	-	-	-	-	-	-	-	-	-	-	-	-	-	-	-	-	-	-	-	-	-	-	-	-	-	-	-	-	-
	e4	A	A	-	-	-	-	-	-	-	-	-	-	-	-	-	-	-	-	-	-	-	-	-	-	-	-	-	-	-	-	-	-	-	-	-	-
	e5	-	-	-	-	-	-	-	-	-	-	-	-	-	-	-	-	-	-	-	-	-	-	-	-	-	-	-	-	-	-	-	-	-	-	-	-
	e27	-	-	-	-	-	-	-	-	-	-	-	-	-	-	-	-	-	-	-	-	-	-	-	-	-	-	-	-	-	-	-	-	-	-	-	-
	e26	-	-	-	-	-	-	-	-	-	-	-	-	-	-	-	-	-	-	-	-	-	-	-	-	-	-	-	-	-	-	-	-	-	-	-	-
	e25	-	-	-	-	-	-	-	-	-	-	-	-	-	-	-	-	-	-	-	-	-	-	-	-	-	-	-	-	-	-	-	-	-	-	-	-
	e24	-	-	-	-	-	-	-	-	-	-	-	-	-	-	-	-	-	-	-	-	-	-	-	-	-	-	-	-	-	-	-	-	-	-	-	-
	e23	G	U	-	-	-	-	-	-	-	-	-	-	-	-	-	-	-	-	-	-	-	-	-	-	-	-	-	-	-	-	-	-	-	-	-	-
	e22	G	A	-	-	-	-	-	-	-	-	-	-	-	-	-	-	-	-	-	-	-	-	-	-	-	-	-	-	-	-	-	-	-	-	-	-
	e21	U	C	-	-	-	-	-	-	-	-	-	-	-	-	-	-	-	-	-	-	-	-	-	-	-	-	-	-	-	-	-	-	-	-	-	-
	46	G	G	m7G	m7G	m7G	m7G	m7G	m7G	m7G	A	m7G	m7G	m7G	A	A	A	A	m7G	m7G	A	m7G	m7G	m7G	m7G	A	m7G	m7G	m7G	m7G	m7G	m7G	m7G	m7G	m7G	m7G	m7G
	47	-	-	U	U	U	U	U	U	U	-	U	U	U	-	-	-	-	acp3U	acp3U	-	U	U	U	U	-	U	acp3U	acp3U	acp3U	acp3U	acp3U	acp3U	acp3U	acp3U	acp3U	acp3U
	48	U	U	C	C	C	C	C	C	C	G	C	C	C	C	C	C	C	C	C	C	C	C	C	C	U	C	C	C	C	C	C	C	C	C	C	C
	49	G	G	G	A	A	A	U	U	U	U	G	G	G	A	A	A	A	C	C	G	G	G	G	G	G	G	G	G	G	G	G	G	G	G	G	G
	5	C	C	U	C	G	G	G	G	G	C	C	C	C	G	G	G	G	U	U	A	C	C	C	C	C	U	G	G	G	C	C	C	C	C	C	C
	51	G	C	A	C	C	C	C	C	C	C	G	G	G	G	G	G	G	U	U	G	G	G	G	G	G	G	U	U	U	U	U	A	A	A	A	A
	52	A	G	G	A	G	G	G	G	G	G	G	G	G	G	G	G	G	G	G	G	A	A	A	A	G	G	G	G	G	G	G	G	G	G	G	G
	53	G	G	G	G	G	G	G	G	G	G	G	G	G	G	G	G	G	G	G	G	G	G	G	G	G	G	G	G	G	G	G	G	G	G	G	G
	54	U	U	m5U	m5U	m5U	m5U	m5U	m5U	m5U	m5U	m5U	m5U	m5U	m5U	m5U	m5U	m5U	m5U	m5U	m5U	m5U	m5U	m5U	m5U	m5U	m5U	m5U	m5U	m5U	m5U	m5U	m5U	m5U	m5U	m5U	m5U
	55	psi	psi	psi	psi	psi	psi	psi	psi	psi	psi	psi	psi	psi	psi	psi	psi	psi	psi	psi	psi	psi	psi	psi	psi	psi	psi	psi	psi	psi	psi	psi	psi	psi	psi	psi	psi
	56	C	C	C	C	C	C	C	C	C	C	C	C	C	C	C	C	C	C	C	C	C	C	C	C	C	C	C	C	C	C	C	C	C	C	C	C
	57	A	G	G	G	G	G	G	G	G	G	G	G	G	G	G	G	G	G	G	G	G	G	G	G	G	G	A	A	A	A	A	G	G	G	G	G
	58	A	A	A	A	A	A	A	A	A	A	A	A	A	A	A	A	A	A	A	A	A	A	A	A	A	A	A	A	A	A	A	A	A	A	A	A
	59	G	G	C	U	U	U	U	U	U	C	G	G	G	A	A	A	A	U	U	U	G	G	G	G	U	A	G	G	G	G	G	A	A	A	A	A
	60	U	U	U	U	C	C	C	C	C	U	U	U	U	U	U	U	U	U	U	U	U	U	U	U	U	U	U	U	U	U	U	U	U	U	U	U
	61	C	C	C	C	C	C	C	C	C	C	C	C	C	C	C	C	C	C	C	C	C	C	C	C	C	C	C	C	C	C	C	C	C	C	C	C
	62	U	C	C	C	C	C	C	C	C	C	C	C	C	C	C	C	C	C	C	C	U	U	U	U	C	C	C	C	C	C	C	C	C	C	C	C
	63	C	G	U	G	G	G	G	G	G	G	C	C	C	C	C	C	C	G	G	C	C	C	C	C	C	C	A	A	A	A	A	U	U	U	U	U
	64	G	G	A	G	C	C	C	C	C	G	G	G	G	C	C	C	C	A	A	U	G	G	G	G	G	A	C	C	C	G	G	G	G	G	G	G
	65	C	C	U	U	U	U	A	A	A	A	psi	psi	psi	U	U	U	U	G	G	U	U	U	U	U	C	U	psi	psi	psi	C	C	C	C	C	C	C
	66	C	C	U	A	U	U	U	U	U	A	C	C	C	A	A	A	A	U	U	C	U	U	U	U	U	U	C	C	C	A	A	A	A	A	A	A
	67	U	U	A	G	A	A	A	A	A	C	C	C	C	G	G	G	G	C	C	G	U	U	U	U	G	A	A	A	A	G	G	C	C	C	C	C
	68	C	U	U	U	G	G	G	G	G	G	G	G	G	G	G	G	G	C	C	C	C	C	C	C	C	G	G	G	G	G	G	G	G	G	G	G
	69	C	C	C	C	C	C	C	C	C	C	U	U	U	G	G	G	G	G	G	C	C	C	C	C	C	C	G	G	G	G	G	A	A	A	A	A
	7	C	G	G	G	U	U	U	U	U	G	U	U	U	G	G	G	G	G	G	C	C	C	C	C	C	C	C	C	C	G	G	C	C	C	C	C
	71	G	G	G	G	C	C	C	C	C	C	C	C	C	A	A	A	A	G	G	G	G	G	G	G	G	A	C	C	C	C	C	C	C	C	C	C
	72	C	C	C	C	C	C	C	C	C	C	C	C	C	C	C	C	C	C	C	C	C	C	C	C	C	C	U	U	U	C	C	C	C	C	C	C
	73	A	A	A	A	A	A	A	A	A	U	G	G	G	G	G	G	G	A	A	U	U	U	U	U	U	C	A	A	A	A	A	A	A	A	A	A
	74	C	C	C	C	C	C	C	C	C	C	C	C	C	C	C	C	C	C	C	C	C	C	C	C	C	C	C	C	C	C	C	C	C	C	C	C
	75	C	C	C	C	C	C	C	C	C	C	C	C	C	C	C	C	C	C	C	C	C	C	C	C	C	C	C	C	C	C	C	C	C	C	C	C
	76	A	A	A	A	A	A	A	A	A	A	A	A	A	A	A	A	A	A	A	A	A	A	A	A	A	A	A	A	A	A	A	A	A	A	A	A

Nucleotide position

Blattner Number	b0748	b1909	b4369	b4370	b3798	b4368	b3174	b0673	b0666	b1977	b1986	b1989	b1984	b3545	b2189	b3799	b0670	b0668	b0665	b0664	b2691	b2694	b2693	b2692	b2348	b0536	b3796	b1975	b2695	b0883	b1032	b0971	b3979	b3273	b1666	b1665	b0744	b2401	b2402	b2403	b0746	b3761
Gene Name	lysZ	leuZ	leuP	leuQ	leuT	leuV	leuU	metT	metU	asnT	asnU	asnV	asnW	proK	proL	proM	glnU	glnW	glnV	glnX	argQ	argV	argY	argZ	argW	argU	argX	serU	serV	serW	serX	serT	thrT	thrV	valW	valV	valT	valU	valX	valY	valZ	trpT
generic tRNA in model	lys1-tRNA	leuZ-tRNA	leu1-tRNA	leu1-tRNA	leu1-tRNA	leu1-tRNA	leuU-tRNA	met1-tRNA	met1-tRNA	asn1-tRNA	asn1-tRNA	asn1-tRNA	asn1-tRNA	proK-tRNA	proL-tRNA	proM-tRNA	gln1-tRNA	gln1-tRNA	gln2-tRNA	gln2-tRNA	arg1-tRNA	arg1-tRNA	arg1-tRNA	arg1-tRNA	argW-tRNA	argU-tRNA	argX-tRNA	serU-tRNA	serV-tRNA	ser1-tRNA	serX-tRNA	serT-tRNA	thr1-tRNA	thr1-tRNA	val2-tRNA	val2-tRNA	val1-tRNA	val1-tRNA	val1-tRNA	val1-tRNA	val1-tRNA	trpT-tRNA
tRNA DB ID	RK1660	RL1660	RL1661	RL1661	RL1661	RL1661	RL1662	RM1660	RM1660	RN1660	RN1660	RN1660	RN1660	RP1700	RP1701	RP1702	RQ1660	RQ1660	RQ1661	RQ1661	RR1661	RR1661	RR1661	RR1661	RR1662	RR1663	RR1664	RS1660	RS1661	RS1662	RS1662	RS1664	RT1660	RT1660	RV1660	RV1661	RV1662	RV1662	RV1662	RV1662	RV1662	RW1660
Anticodon	SUU	HAA	CAG	CAG	CAG	CAG	GAG	MAU	MAU	QUU	QUU	QUU	QUU	CGG	GGG	VGG	CUG	CUG	NUG	NUG	ICG	ICG	ICG	ICG	mnm5UCU	mnm5UCU	CCG	CGA	GCU	GGA	GGA	VGA	GGU	GGU	GAC	GAC	VAC	VAC	VAC	VAC	VAC	CCA
0	-	-	-	-	-	-	-	-	-	-	-	-	-	-	-	-	-	-	-	-	-	-	-	-	-	-	-	-	-	-	-	-	-	-	-	-	-	-	-	-	-	-
1	G	G	G	G	G	G	G	G	G	U	U	U	U	C	C	C	U	U	U	U	G	G	G	G	G	G	G	G	G	G	G	G	G	G	G	G	G	G	G	G	G	A
2	G	C	C	C	C	C	C	G	G	C	C	C	C	G	G	G	G	G	G	G	C	C	C	C	U	C	C	G	G	G	G	G	C	C	C	C	G	G	G	G	G	G
3	G	C	G	G	G	G	C	C	C	C	C	C	C	G	G	G	G	G	G	G	A	A	A	A	C	G	G	A	U	U	U	A	U	U	G	G	G	G	G	G	G	G
4	U	C	A	A	A	A	G	U	U	U	U	U	U	U	C	C	G	G	G	G	U	U	U	U	C	C	C	G	G	G	G	A	G	G	U	U	U	U	U	U	U	G
5	C	G	A	A	A	A	A	A	A	C	C	C	C	G	A	G	G	G	G	G	C	C	C	C	U	C	C	A	A	A	A	G	A	A	C	U	G	G	G	G	G	G
6	G	G	G	G	G	G	G	C	C	U	U	U	U	A	C	A	U	U	U	U	C	C	C	C	C	C	C	G	G	G	G	U	U	U	C	C	A	A	A	A	A	C
7	U	A	G	G	G	G	G	G	G	G	G	G	G	U	G	G	A	A	A	A	G	G	G	G	U	U	G	A	G	G	G	G	U	U	G	A	U	U	U	U	U	G
8	U	s4U	U	U	U	U	U	s4U	s4U	s4U	s4U	s4U	s4U	s4U	s4U	s4U	s4U	s4U	s4U	s4U	s4U	s4U	s4U	s4U	U	U	U	U	s4U	U	U	s4U	U	U	s4U	s4U	s4U	s4U	s4U	s4U	s4U	s4U
9	A	G	G	G	G	G	G	A	A	A	A	A	A	G	A	A	C	C	C	C	A	A	A	A	A	A	A	G	G	G	G	G	A	A	A	A	A	A	A	A	A	A
10	G	G	G	G	G	G	G	G	G	G	G	G	G	G	G	G	G	G	G	G	G	G	G	G	G	G	G	C	G	U	U	G	G	G	G	G	G	G	G	G	G	G
11	C	U	C	C	C	C	U	C	C	U	U	U	U	C	C	C	C	C	C	C	C	C	C	C	U	C	C	C	C	C	C	C	C	C	C	C	C	C	C	C	C	U
12	U	G	G	G	G	G	G	U	U	U	U	U	U	G	G	G	C	C	C	C	U	U	U	U	U	U	U	G	C	C	C	C	U	U	U	U	U	U	U	U	U	U
13	C	G	G	G	G	G	G	C	C	C	C	C	C	C	C	C	A	A	A	A	C	C	C	C	A	C	C	G	G	G	G	G	C	C	C	C	C	C	C	C	C	C
14	A	A	A	A	A	A	A	A	A	A	A	A	A	A	A	A	A	A	A	A	A	A	A	A	A	A	A	A	A	A	A	A	A	A	A	A	A	A	A	A	A	A
15	G	A	A	A	A	A	A	G	G	G	G	G	G	G	G	G	G	G	G	G	G	G	G	G	A	G	G	G	G	G	G	G	G	G	G	G	G	G	G	G	G	A
16	D	D	D	D	D	D	D	D	D	D	D	D	D	C	C	C	C	C	C	C	C	C	C	C	U	U	C	C	A	U	U	C	D	D	D	D	C	C	C	C	C	D
17	D	C	D	D	D	D	D	D	D	C	C	C	C	C	C	U	-	-	-	-	D	D	D	D	-	U	D	-	-	-	-	-	D	D	D	D	D	D	D	D	D	D
17A	-	-	-	-	-	-	-	-	-	-	-	-	-	U	U	U	-	-	-	-	-	-	-	-	-	-	-	-	-	-	-	-	-	-	-	-	-	-	-	-	-	-
18	G	Gm	Gm	Gm	Gm	Gm	Gm	Gm	Gm	G	G	G	G	G	G	G	Gm	Gm	Gm	Gm	G	G	G	G	G	G	G	Gm	G	Gm	Gm	Gm	G	G	G	G	G	G	G	G	G	G
19	G	G	G	G	G	G	G	G	G	G	G	G	G	G	G	G	G	G	G	G	G	G	G	G	G	G	G	G	G	G	G	G	G	G	G	G	G	G	G	G	G	G
20	D	D	D	D	D	D	D	D	D	D	D	D	D	D	D	D	D	D	D	D	A	A	A	A	A	A	A	C	C	C	C	D	D	D	D	D	G	G	G	G	G	D
20A	-	A	A	A	A	A	A	D	D	-	-	-	-	-	-	-	-	-	-	-	D	D	D	D	D	U	D	D	D	D	D	D	-	-	D	D	-	-	-	-	-	-
20B	-	-	-	-	-	-	-	-	-	-	-	-	-	-	-	-	-	-	-	-	-	-	-	-	-	-	-	G	G	G	G	G	-	-	-	-	-	-	-	-	-	-
21	A	G	G	G	G	G	G	A	A	A	A	A	A	A	A	A	A	A	A	A	A	A	A	A	A	A	A	A	A	A	A	A	A	A	A	A	A	A	A	A	A	A
22	G	A	A	A	A	A	A	G	G	G	G	G	G	G	G	G	A	A	A	A	G	G	G	G	U	G	G	A	A	A	A	A	G	G	G	G	G	G	G	G	G	G
23	A	C	C	C	C	C	C	A	A	A	A	A	A	C	C	C	G	G	G	G	A	A	A	A	A	A	A	C	G	G	G	G	A	A	A	A	A	A	A	A	A	A
24	G	A	G	G	G	G	A	G	G	A	A	A	A	G	G	G	G	G	G	G	G	G	G	G	A	G	G	G	G	G	G	G	G	G	G	G	G	G	G	G	G	G
25	C	C	C	C	C	C	C	C	C	C	C	C	C	C	C	C	C	C	C	C	U	U	U	U	C	C	C	G	A	A	C	C	C	C	C	C	C	C	C	C	C	C
26	A	A	G	G	G	G	G	A	A	G	G	G	G	A	A	A	A	A	A	A	A	A	A	A	G	A	G	A	G	G	G	A	G	G	A	A	A	A	A	A	A	A
27	G	A	C	C	C	C	C	C	C	G	G	G	G	C	C	A	C	C	C	C	C	C	C	C	A	A	C	C	C	C	C	C	C	C	C	C	C	C	C	C	C	C
28	U	G	U	U	U	U	U	A	A	C	C	C	C	U	C	C	C	C	C	C	U	U	U	U	G	C	U	C	U	A	A	C	A	A	C	C	C	C	C	C	C	C
29	U	G	A	A	A	A	A	U	U	G	G	G	G	U	G	U	G	G	G	G	C	C	C	C	C	G	G	G	C	G	G	G	C	C	A	A	U	U	U	U	U	G
30	G	G	G	G	G	G	C	C	C	G	G	G	G	C	U	G	G	G	G	G	G	G	G	G	C	A	C	G	C	G	G	G	C	C	C	C	C	C	C	C	C	G
31	A	A	C	C	C	C	C	A	A	A	A	A	A	G	C	G	A	A	U	U	G	G	G	G	C	C	C	U	C	C	C	U	C	C	C	C	C	C	C	C	C	U
32	C	psi	U	U	U	U	U	C	C	C	C	C	C	Um	Cm	Um	Um	Um	Um	Um	C	C	C	C	s2C	s2C	s2C	C	s2C	C	C	Cm	U	U	U	U	C	C	C	C	C	Cm
33	U	U	U	U	U	U	U	U	U	U	U	U	U	U	U	U	U	U	U	U	U	U	U	U	U	U	U	U	U	U	U	U	U	U	U	U	U	U	U	U	U	U
34	mnm5s2U	A	C	C	C	C	G	ac4C	ac4C	Q	Q	Q	Q	C	G	cmo5U	C	C	U	U	I	I	I	I	mnm5U	mnm5U	C	C	G	G	G	cmo5U	G	G	G	G	cmo5U	cmo5U	cmo5U	cmo5U	cmo5U	C
35	U	A	A	A	A	A	A	A	A	U	U	U	U	G	G	G	U	U	U	U	C	C	C	C	C	C	C	G	C	G	G	G	G	G	A	A	A	A	A	A	A	C
36	U	A	G	G	G	G	G	U	U	U	U	U	U	G	G	G	G	G	G	G	G	G	G	G	U	U	G	A	U	A	A	A	U	U	C	C	C	C	C	C	C	A
37	t6A	ms2i6A	G	G	G	G	G	t6A	t6A	t6A	t6A	t6A	t6A	m1G	m1G	m1G	m2A	m2A	m2A	m2A	m2A	m2A	m2A	m2A	t6A	t6A	m1G	ms2i6A	t6A	A	A	ms2i6A	m6t6A	m6t6A	A	A	m6A	m6A	m6A	m6A	m6A	ms2i6A
38	A	A	psi	psi	psi	psi	psi	A	A	A	A	A	A	A	U	A	psi	psi	psi	psi	A	A	A	A	A	A	A	A	A	A	A	A	A	A	U	U	A	A	A	A	A	A
39	psi	psi	G	G	G	G	G	psi	psi	psi	psi	psi	psi	C	psi	C	psi	psi	A	A	C	C	C	C	G	G	G	A	G	G	G	A	G	G	G	G	G	G	G	G	G	A
40	C	C	psi	psi	psi	psi	G	G	G	C	C	C	C	G	G	C	C	C	C	C	C	C	C	C	G	psi	G	C	G	psi	psi	C	G	G	G	G	G	G	G	G	G	C
41	A	C	U	U	U	U	U	A	A	C	C	C	C	A	C	A	C	C	C	C	G	G	G	G	G	C	C	C	G	G	G	C	G	G	U	U	A	A	A	A	A	C
42	A	C	A	A	A	A	A	U	U	G	G	G	G	A	G	G	G	G	G	G	A	A	A	A	C	G	A	G	A	U	U	G	U	U	G	G	G	G	G	G	G	G
43	U	U	G	G	G	G	G	G	G	U	U	U	U	G	G	U	G	G	G	G	G	G	G	G	U	U	G	G	G	G	G	G	G	G	G	G	G	G	G	G	G	G
44	U	C	U	U	U	U	U	G	G	A	A	A	A	G	G	G	C	C	C	C	C	C	C	C	A	G	A	A	U	U	U	C	A	A	G	G	G	G	G	G	G	G
45	G	-	-	-	-	-	-	G	G	U	U	U	U	G	G	G	A	A	A	A	G	G	G	G	A	G	G	-	-	-	-	-	G	G	G	G	G	G	G	G	G	U
e11	-	G	G	G	G	G	G	-	-	-	-	-	-	-	-	-	-	-	-	-	-	-	-	-	-	-	-	G	A	A	A	G	-	-	-	-	-	-	-	-	-	-
e12	-	G	U	U	U	U	C	-	-	-	-	-	-	-	-	-	-	-	-	-	-	-	-	-	-	-	-	U	U	U	U	A	-	-	-	-	-	-	-	-	-	-
e13	-	C	C	C	C	C	C	-	-	-	-	-	-	-	-	-	-	-	-	-	-	-	-	-	-	-	-	A	G	A	A	C	-	-	-	-	-	-	-	-	-	-
e14	-	G	C	C	C	C	C	-	-	-	-	-	-	-	-	-	-	-	-	-	-	-	-	-	-	-	-	G	C	C	C	C	-	-	-	-	-	-	-	-	-	-
e15	-	-	-	-	-	-	-	-	-	-	-	-	-	-	-	-	-	-	-	-	-	-	-	-	-	-	-	G	G	G	G	C	-	-	-	-	-	-	-	-	-	-
e16	-	-	-	-	-	-	-	-	-	-	-	-	-	-	-	-	-	-	-	-	-	-	-	-	-	-	-	G	G	-	-	-	-	-	-	-	-	-	-	-	-	-
e17	-	-	-	-	-	-	-	-	-	-	-	-	-	-	-	-	-	-	-	-	-	-	-	-	-	-	-	-	U	-	-	-	-	-	-	-	-	-	-	-	-	-
e1	-	U	U	U	U	U	A	-	-	-	-	-	-	-	-	-	-	-	-	-	-	-	-	-	-	-	-	G	C	G	G	G	-	-	-	-	-	-	-	-	-	-
e2	-	U	U	U	U	U	A	-	-	-	-	-	-	-	-	-	-	-	-	-	-	-	-	-	-	-	-	C	A	C	C	A	-	-	-	-	-	-	-	-	-	-
e3	-	C	A	A	A	A	U	-	-	-	-	-	-	-	-	-	-	-	-	-	-	-	-	-	-	-	-	A	A	A	A	A	-	-	-	-	-	-	-	-	-	-
e4	-	G	C	C	C	C	A	-	-	-	-	-	-	-	-	-	-	-	-	-	-	-	-	-	-	-	-	A	A	A	A	A	-	-	-	-	-	-	-	-	-	-
e5	-	-	-	-	-	-	-	-	-	-	-	-	-	-	-	-	-	-	-	-	-	-	-	-	-	-	-	-	A	-	-	-	-	-	-	-	-	-	-	-	-	-
e27	-	-	-	-	-	-	-	-	-	-	-	-	-	-	-	-	-	-	-	-	-	-	-	-	-	-	-	-	G	-	-	-	-	-	-	-	-	-	-	-	-	-
e26	-	-	-	-	-	-	-	-	-	-	-	-	-	-	-	-	-	-	-	-	-	-	-	-	-	-	-	C	C	-	-	-	-	-	-	-	-	-	-	-	-	-
e25	-	-	-	-	-	-	-	-	-	-	-	-	-	-	-	-	-	-	-	-	-	-	-	-	-	-	-	U	U	C	C	G	-	-	-	-	-	-	-	-	-	-
e24	-	C	G	G	G	G	G	-	-	-	-	-	-	-	-	-	-	-	-	-	-	-	-	-	-	-	-	C	G	G	G	G	-	-	-	-	-	-	-	-	-	-
e23	-	G	G	G	G	G	G	-	-	-	-	-	-	-	-	-	-	-	-	-	-	-	-	-	-	-	-	U	C	U	U	G	-	-	-	-	-	-	-	-	-	-
e22	-	C	A	A	A	A	G	-	-	-	-	-	-	-	-	-	-	-	-	-	-	-	-	-	-	-	-	A	A	A	A	U	-	-	-	-	-	-	-	-	-	-
e21	-	U	C	C	C	C	C	-	-	-	-	-	-	-	-	-	-	-	-	-	-	-	-	-	-	-	-	C	U	U	U	U	-	-	-	-	-	-	-	-	-	-
46	m7G	G	G	G	G	G	U	m7G	m7G	m7G	m7G	m7G	m7G	m7G	m7G	m7G	U	U	U	U	m7G	m7G	m7G	m7G	U	G	m7G	-	-	-	-	-	m7G	m7G	m7G	m7G	m7G	m7G	m7G	m7G	m7G	m7G
47	acp3U	-	-	-	-	-	-	acp3U	acp3U	U	U	U	U	U	U	U	U	U	U	U	acp3U	acp3U	acp3U	acp3U	-	C	U	-	-	-	-	-	U	U	acp3U	acp3U	U	U	U	U	U	U
48	C	U	U	U	U	U	U	C	C	C	C	C	C	C	C	C	C	C	C	C	C	C	C	C	U	C	C	C	C	C	C	C	C	C	C	C	C	C	C	C	C	U
49	G	G	G	G	G	G	A	A	A	A	A	A	A	G	G	G	C	C	C	C	G	G	G	G	G	G	U	G	C	G	G	C	G	G	G	G	G	G	G	G	G	G
5	C	C	G	G	G	G	G	C	C	C	C	C	C	C	C	C	G	G	C	C	G	G	G	G	C	C	C	G	G	G	G	A	G	G	G	U	G	G	G	G	G	G
51	A	G	G	G	G	G	G	A	A	U	U	U	U	A	A	A	A	A	U	U	A	A	A	A	A	A	A	G	G	G	G	G	C	C	U	U	C	C	C	C	C	G
52	G	G	G	G	G	G	G	G	G	G	G	G	G	G	G	G	G	G	G	G	G	G	G	G	G	G	G	G	G	G	G	A	A	A	G	G	G	G	G	G	G	A
53	G	G	G	G	G	G	G	G	G	G	G	G	G	G	G	G	G	G	G	G	G	G	G	G	G	G	G	G	G	G	G	G	G	G	G	G	G	G	G	G	G	G
54	m5U	m5U	m5U	m5U	m5U	m5U	m5U	m5U	m5U	m5U	m5U	m5U	m5U	m5U	m5U	m5U	m5U	m5U	m5U	m5U	m5U	m5U	m5U	m5U	m5U	m5U	m5U	m5U	m5U	m5U	m5U	m5U	m5U	m5U	m5U	m5U	m5U	m5U	m5U	m5U	m5U	m5U
55	psi	psi	psi	psi	psi	psi	psi	psi	psi	psi	psi	psi	psi	psi	psi	psi	psi	psi	psi	psi	psi	psi	psi	psi	psi	psi	psi	psi	psi	psi	psi	psi	psi	psi	psi	psi	psi	psi	psi	psi	psi	psi
56	C	C	C	C	C	C	C	C	C	C	C	C	C	C	C	C	C	C	C	C	C	C	C	C	C	C	C	C	C	C	C	C	C	C	C	C	C	C	C	C	C	C
57	G	A	A	A	A	A	A	G	G	G	G	G	G	G	A	G	G	G	G	G	G	G	G	G	G	G	G	A	G	G	G	G	G	G	G	G	G	G	G	G	G	G
58	A	A	A	A	A	A	A	A	A	A	A	A	A	A	A	A	A	A	A	A	A	A	A	A	A	A	A	A	A	A	A	A	A	A	A	A	A	A	A	A	A	A
59	A	G	G	G	G	G	G	A	A	G	G	G	G	A	A	A	A	A	A	A	A	A	A	A	U	A	A	A	A	A	A	A	A	A	G	G	U	U	U	U	U	G
60	U	U	U	U	U	U	U	U	U	U	U	U	U	U	U	U	U	U	U	U	U	U	U	U	U	U	U	U	U	U	U	U	U	U	U	U	C	C	C	C	C	U
61	C	C	C	C	C	C	C	C	C	C	C	C	C	C	C	C	C	C	C	C	C	C	C	C	C	C	C	C	C	C	C	C	C	C	C	C	C	C	C	C	C	C
62	C	C	C	C	C	C	C	C	C	C	C	C	C	C	C	C	C	C	C	C	C	C	C	C	C	C	C	C	C	C	C	U	U	U	C	C	C	C	C	C	C	U
63	U	C	C	C	C	C	C	C	C	A	A	A	A	U	U	U	U	U	A	A	U	U	U	U	U	U	U	C	C	C	C	C	G	G	A	A	G	G	G	G	G	C
64	G	G	C	C	C	C	G	G	G	G	G	G	G	C	C	C	C	C	G	G	C	C	C	C	G	G	G	C	C	C	C	U	C	C	C	A	U	U	U	U	U	U
65	C	C	C	C	C	C	U	U	U	U	U	U	U	U	U	psi	G	G	G	G	C	C	C	C	C	C	U	C	G	C	C	G	C	C	U	U	C	C	C	C	C	C
66	A	U	C	C	C	C	C	C	C	C	C	C	C	A	C	C	U	U	U	U	C	C	C	C	A	A	C	U	C	C	C	C	U	U	C	U	A	A	A	A	A	C
67	C	C	C	C	C	C	C	G	G	A	A	A	A	U	G	U	A	A	A	A	G	G	G	G	G	G	G	C	C	C	C	G	A	A	G	G	U	U	U	U	U	G
68	G	C	C	C	C	C	U	U	U	G	G	G	G	C	U	C	C	C	C	C	G	G	G	G	G	G	G	U	U	U	U	C	U	U	G	A	C	C	C	C	C	C
69	A	G	U	U	U	U	C	A	A	A	A	A	A	A	G	G	C	C	C	C	A	A	A	A	G	G	G	C	C	C	C	U	C	C	A	A	A	A	A	A	A	C
7	C	G	C	C	C	C	G	G	G	G	G	G	G	C	C	C	C	C	C	C	U	U	U	U	G	C	C	U	A	A	A	U	A	A	C	C	C	C	C	C	C	C
71	C	G	G	G	G	G	G	C	C	G	G	G	G	C	C	C	C	C	C	C	G	G	G	G	A	G	G	C	C	C	C	C	G	G	G	G	C	C	C	C	C	C
72	C	U	C	C	C	C	U	C	C	A	A	A	A	G	G	G	A	A	A	A	C	C	C	C	C	C	C	C	C	C	C	C	C	C	C	C	C	C	C	C	C	U
73	A	A	A	A	A	A	A	A	A	G	G	G	G	A	A	A	G	G	G	G	A	A	A	A	A	G	G	G	G	G	G	G	A	A	A	A	A	A	A	A	A	G
74	C	C	C	C	C	C	C	C	C	C	C	C	C	C	C	C	C	C	C	C	C	C	C	C	C	C	C	C	C	C	C	C	C	C	C	C	C	C	C	C	C	C
75	C	C	C	C	C	C	C	C	C	C	C	C	C	C	C	C	C	C	C	C	C	C	C	C	C	C	C	C	C	C	C	C	C	C	C	C	C	C	C	C	C	C
76	A	A	A	A	A	A	A	A	A	A	A	A	A	A	A	A	A	A	A	A	A	A	A	A	A	A	A	A	A	A	A	A	A	A	A	A	A	A	A	A	A	A

	**Blattner Number**	b3171	b2816	b2815	b2814	b1231	b1230	b3977	b3658
	**Gene Name**	metY	metV	metW	metZ	tyrT	tyrV	tyrU	selC
	**generic tRNA in model**	met1-tRNA	met1-tRNA	met1-tRN/	met1-tR	tyr1-tRN	tyr1-tRN	tyr1-tRN	selC-tRNA
	**tRNA DB ID**	RX1660	RX1661	RX1661	RX1661	RY1660	RY1660	RY1661	RZ1665
	**Anticodon**	CAU	CAU	CAU	CAU	QUA	QUA	QUA	UCA
**Nucleotide position**	**0**	-	-	-	-	-	-	-	G
	**1**	C	C	C	C	G	G	G	G
	**2**	G	G	G	G	G	G	G	A
	**3**	C	C	C	C	U	U	U	A
	**4**	G	G	G	G	G	G	G	G
	**5**	G	G	G	G	G	G	G	A
	**6**	G	G	G	G	G	G	G	U
	**7**	G	G	G	G	G	G	G	C
	**8**	**s4U**	**s4U**	**s4U**	**s4U**	**s4U**	**s4U**	**s4U**	G
	**9**	G	G	G	G	U	U	U	U
	**10**	G	G	G	G	C	C	C	C
	**11**	A	A	A	A	C	C	C	G
	**12**	G	G	G	G	C	C	C	U
	**13**	C	C	C	C	G	G	G	C
	**14**	A	A	A	A	A	A	A	U
	**15**	G	G	G	G	G	G	G	C
	**16**	C	C	C	C	C	C	C	C
	**17**	C	C	C	C	-	-	-	-
	**17A**	U	U	U	U	-	-	-	-
	**18**	G	G	G	G	**Gm**	**Gm**	**Gm**	G
	**19**	G	G	G	G	G	G	G	G
	**20**	**D**	**D**	**D**	**D**	C	C	C	**D**
	**20A**	-	-	-	-	C	C	C	G
	**20B**	-	-	-	-	A	A	A	-
	**21**	A	A	A	A	A	A	A	A
	**22**	G	G	G	G	A	A	A	G
	**23**	C	C	C	C	G	G	G	G
	**24**	U	U	U	U	G	G	G	C
	**25**	C	C	C	C	G	G	G	G
	**26**	G	G	G	G	A	A	A	G
	**27**	U	U	U	U	G	G	G	C
	**28**	C	C	C	C	C	C	C	U
	**29**	G	G	G	G	A	A	A	G
	**30**	G	G	G	G	G	G	G	G
	**31**	G	G	G	G	A	A	A	A
	**32**	**Cm**	**Cm**	**Cm**	**Cm**	C	C	C	C
	**33**	U	U	U	U	U	U	U	U
	**34**	C	C	C	C	**Q**	**Q**	**Q**	U
	**35**	A	A	A	A	U	U	U	C
	**36**	U	U	U	U	A	A	A	A
	**37**	A	A	A	A	**ms2i6A**	**ms2i6A**	**ms2i6A**	**i6A**
	**38**	A	A	A	A	A	A	A	A
	**39**	C	C	C	C	**psi**	**psi**	**psi**	U
	**40**	C	C	C	C	C	C	C	C
	**41**	C	C	C	C	U	U	U	C
	**42**	G	G	G	G	G	G	G	A
	**43**	A	A	A	A	C	C	C	G
	**44**	A	A	A	A	C	C	C	U
	**45**	G	G	G	G	-	-	-	-
	**e11**	-	-	-	-	G	G	G	U
	**e12**	-	-	-	-	U	U	U	G
	**e13**	-	-	-	-	C	C	C	G
	**e14**	-	-	-	-	-	-	-	G
	**e15**	-	-	-	-	-	-	-	G
	**e16**	-	-	-	-	-	-	-	C
	**e17**	-	-	-	-	-	-	-	C
	**e1**	-	-	-	-	A	A	A	G
	**e2**	-	-	-	-	U	U	C	C
	**e3**	-	-	-	-	C	C	A	C
	**e4**	-	-	-	-	-	-	-	G
	**e5**	-	-	-	-	-	-	-	C
	**e27**	-	-	-	-	-	-	-	G
	**e26**	-	-	-	-	-	-	-	G
	**e25**	-	-	-	-	-	-	-	U
	**e24**	-	-	-	-	-	-	-	C
	**e23**	-	-	-	-	G	G	G	C
	**e22**	-	-	-	-	A	A	A	C
	**e21**	-	-	-	-	C	C	C	G
	**46**	A	**m7G**	**m7G**	**m7G**	U	U	U	-
	**47**	U	U	U	U	U	U	U	-
	**48**	C	C	C	C	C	C	C	G
	**49**	G	G	G	G	G	G	G	G
	**5**	U	U	U	U	A	A	A	C
	**51**	C	C	C	C	A	A	A	A
	**52**	G	G	G	G	G	G	G	G
	**53**	G	G	G	G	G	G	G	G
	**54**	**m5U**	**m5U**	**m5U**	**m5U**	**m5U**	**m5U**	**m5U**	**m5U**
	**55**	**psi**	**psi**	**psi**	**psi**	**psi**	**psi**	**psi**	**psi**
	**56**	C	C	C	C	C	C	C	C
	**57**	A	A	A	A	G	G	G	G
	**58**	A	A	A	A	A	A	A	A
	**59**	A	A	A	A	A	A	A	C
	**60**	U	U	U	U	U	U	U	U
	**61**	C	C	C	C	C	C	C	C
	**62**	C	C	C	C	C	C	C	C
	**63**	G	G	G	G	U	U	U	U
	**64**	G	G	G	G	U	U	U	G
	**65**	C	C	C	C	C	C	C	U
	**66**	C	C	C	C	C	C	C	G
	**67**	C	C	C	C	C	C	C	A
	**68**	C	C	C	C	C	C	C	U
	**69**	C	C	C	C	C	C	C	C
	**7**	G	G	G	G	A	A	A	U
	**71**	C	C	C	C	C	C	C	U
	**72**	A	A	A	A	C	C	C	C
	**73**	A	A	A	A	A	A	A	G
	**74**	C	C	C	C	C	C	C	C
	**75**	C	C	C	C	C	C	C	C
	**76**	A	A	A	A	A	A	A	A
									C
									A
