# Supplementary material for: Genome-Scale Reconstruction of Escherichia coli's Transcriptional and Translational Machinery: A Knowledge Base, Its Mathematical Formulation, and Its Functional Characterization
Source: PLoS Comput Biol. 2009 Mar 13;5(3):e1000312. doi: 10.1371/journal.pcbi.1000312 (PMC2648898; doi:10.1371/journal.pcbi.1000312)
Supplement: Table S13 — Component list (0.76 MB PDF) [file pcbi.1000312.s015.pdf]

Table S13 - Component List

Component Abbreviation	Component Name	Formula	Charge	Subsystem
10fthf	10-Formyltetrahydrofolate	C20H21N7O7	-2	tRNA modification
10fthf[e]	10-Formyltetrahydrofolate	C20H21N7O7	-2	Others
16S_rRNA	generic 16S rRNA (formulae is average of all 16S rRNA)	C14766H16675N6062O10721P1542	-1543	Ribosomal Assembly
1_sec_tRNA_ser_SelA_deca_cplx	complex of 1 SelA decamer and 1 sec_tRNA_ser	C23110H37243N6783O7394S160P100Mg2	-162	tRNA charging
23S_rRNA	generic 23S rRNA (formulae is average of all 23S rRNA)	C27826H31385N11470O20159P2904	-2905.14	Ribosomal Assembly
2_sec_tRNA_ser_SelA_deca_cplx	complex of 1 SelA decamer and 2 sec_tRNA_ser	C24020H38286N7141O8068S160P195Mg4	-254	tRNA charging
3_sec_tRNA_ser_SelA_deca_cplx	complex of 1 SelA decamer and 3 sec_tRNA_ser	C24930H39329N7499O8742S160P290Mg6	-346	tRNA charging
4_sec_tRNA_ser_SelA_deca_cplx	complex of 1 SelA decamer and 4 sec_tRNA_ser	C25840H40372N7857O9416S160P385Mg8	-438	tRNA charging
5S_rRNA	generic 5S rRNA (formulae is average of all 5S rRNA)	C1145H1301N469O838P120	-121	Ribosomal Assembly
5_sec_tRNA_ser_SelA_deca_cplx	complex of 1 SelA decamer and 5 sec_tRNA_ser	C26750H41415N8215O10090S160P480Mg10	-530	tRNA charging
5fthf	5-Formyltetrahydrofolate	C20H21N7O7	-2	Others
5fthf[e]	5-Formyltetrahydrofolate	C20H21N7O7	-2	Others
5mta	5-Methylthioadenosine	C11H15N5O3S1	0	Others
5mta[e]	5-Methylthioadenosine	C11H15N5O3S1	0	Others
AcT_EF-TU	EF-TU acetyltransferase (Ser1)		0	Protein Modification
AcT_EF-TU_cplx_a	Acetyltransferase/EF-TU/accoa complex (b3339)	C1940H3084N530O598S14P3	-19	Protein Modification
AcT_EF-TU_cplx_b	Acetyltransferase/EF-TU/aacoa complex (b3980)	C1941H3086N530O599S14P3	-19	Protein Modification
AcT_EF-TU_inact	EF-TU acetyltransferase (Ser1)		0	Protein Modification
AcT_tRNA_pos_34_ac4C	unknown acetyltransferase tRNA, position 34 ac4C	C0H0N0S0P0	0	tRNA Modification
AcT_tRNA_pos_34_ac4C_inact	unknown acetyltransferase tRNA, position 34 ac4C	C0H0N0S0P0	0	tRNA Modification
AcpS_mono	holo-[acyl-carrier-protein] synthase 1 (b2563, AcpS, monomer)	C627H1006N183O172S2	3	Folding
AcpT_tRNA_pos_47_acp3U	unknown tRNA-uridine 3-(3-amino-3-carboxypropyl)transferase, tRNA modification, position 47, acp3U		0	tRNA Modification
AcpT_tRNA_pos_47_acp3U_inact	unknown tRNA-uridine 3-(3-amino-3-carboxypropyl)transferase, tRNA modification, position 47, acp3U		0	tRNA Modification
AlaS_DnaK_GrpE_complex	AlaS (b2697) DnaK GrpE_dim complex - Deuerling et al. DnaKJ/GrpE dependent folding	C9078H14582O2905N2577S57P0Zn1	-81	Folding
AlaS_mono	alanyl-tRNA synthetase (b2697, AlaS, monomer)	C4210H6656N1207O1304Zn1S26	-21	Folding
Ala_RS_ala_amp	charged Alanyl-tRNA synthetase	C16853H26643N4834O5225S104P1Zn4	-86	tRNA charging
Ala_RS_tetra	Alanyl-tRNA synthetase	C16840H26624N4828O5216S104Zn4	-84	tRNA charging
Ala_RS_tetra_inact	Alanyl-tRNA synthetase	C16840H26624N4828O5216S104Zn4	-84	tRNA charging
AmiB_mono	N-acetylmuramoyl-l-alanine amidase II (b4169, AmiB, monomer)	C1983H3203N589O626S5	4	Folding
ApaG_mono	protein associated with Co2+ and Mg2+ efflux (b0050, ApaG, monomer)	C616H954N164O186S3	-6	Folding
ApaH_mono	diadenosine tetraphosphatase (b0049, ApaH, monomer)	C1406H2148N374O407S11	-10	Folding
ArgS_mono	arginyl-tRNA synthetase (b1876, ArgS, monomer)	C2874H4520N785O860S26	-19	Folding
ArgS_mono_inact	arginyl-tRNA synthetase (b1876, ArgS, monomer)	C2874H4520N785O860S26	-19	Folding
Arg_RS_arg_amp	charged Arginyl-tRNA synthetase	C2890H4547N794O869S26PZn	-18	tRNA charging
AroB_mono	3-dehydroquinate synthase (b3389, AroB, monomer)	C1724H2781N473O510S14	-6	Folding
AroC_mono	chorismate synthase (b2329, AroC, monomer)	C1705H2736N496O527S12	-6	Folding
AroE_mono	dehydroshikimate reductase, NAD(P)-binding (b3281, AroE, monomer)	C1319H2067N358O388S8	-9	Folding
AroK_DnaK_GrpE_complex	AroK (b3390) DnaK GrpE_dim complex - Kerner et al. class II can interact w/ GroEL/ES, cannot fold spontanously	C5709H9296O1872N1619S34P0	-65	Folding
AroK_GroEL.(7)ADP.transGroES	AroK (b3390) GroEL GroES complex - Kerner et al. class II can interact w/ GroEL/ES, cannot fold spontanously	C38753H64314O12584N10833S367P14Mg7	-299	Folding
AroK_mono	shikimate kinase I (b3390, AroK, monomer)	C841H1370N249O271S3	-5	Folding
AsnS_DnaK_GrpE_complex	AsnS (b0930) DnaK GrpE_dim complex - Kerner et al. class I can interact w/ GroEL/ES	C7217H11531O2299N2012S44P0	-75	Folding
AsnS_GroEL.(7)ADP.transGroES	AsnS (b0930) GroEL GroES complex - Kerner et al. class I can interact w/ GroEL/ES	C40261H66549O13011N11226S377P14Mg7	-309	Folding
AsnS_mono	asparaginyl tRNA synthetase (b0930, AsnS, monomer)	C2349H3605N642O698S13	-15	Folding
Asn_RS_asn_amp	charged Asparaginyl-tRNA synthetase	C4712H7230N1291O1406S26P	-32	tRNA charging

Asn_RS_dim	Asparaginyl-tRNA synthetase (uncharged)	C4698H7210N1284O1396S26	-30	tRNA charging
Asn_RS_dim_inact	Asparaginyl-tRNA synthetase (uncharged)	C4698H7210N1284O1396S26	-30	tRNA charging
AspS_mono	aspartyl-tRNA synthetase (b1866, AspS, monomer)	C2922H4626N812O871S26	-12	Folding
Asp_RS_2asp_2amp	charged Aapartyl-tRNA synthetase (2* aspartate, 2* amp)	C5872H9288N1636O1764S52P2	-30	tRNA charging
Asp_RS_asp_amp	charged Aapartyl-tRNA synthetase (1* aspartate, 1* amp)	C5858H9270N1630O1753S52P	-27	tRNA charging
Asp_RS_dim	Aspartyl-tRNA synthetase (uncharged) (Dimer)	C5844H9252N1624O1742S52	-24	tRNA charging
Asp_RS_dim_inact	Aspartyl-tRNA synthetase (uncharged) (Dimer)	C5844H9252N1624O1742S52	-24	tRNA charging
Bdm_mono	biofilm-dependent modulation protein (b1481, Bdm, monomer)	C347H524N89O117S1	-9	Folding
C10H8O5	C10H8O5	C10H8O5	-2	Others
C10H8O5[e]	C10H8O5[e]	C10H8O5	-2	Others
C9H9O4	C9H9O4	C9H9O4	-1	Others
C9H9O4[e]	C9H9O4[e]	C9H9O4	-1	Others
Cmk_mono	cytidylate kinase (b0910, Cmk, monomer)	C1085H1763N317O327S4	-6	Folding
CobC_mono	predicted alpha-ribazole-5'-P phosphatase (b0638, CobC, monomer)	C1031H1582N301O295S8	-7	Folding
CysS_mono	cysteinyl-tRNA synthetase (b0526, CysS, monomer)	C2300H3546N647O693Zn1S26	-17	Folding
CysS_mono_inact	cysteinyl-tRNA synthetase (b0526, CysS, monomer)	C2300H3546N647O693Zn1S26	-17	Folding
Cys_RS_cys_amp	Cysteinyl-tRNA synthetase (charged)	C2313H3565N653O702S27ZnP	-19	tRNA charging
DU_23S_2449	unknown Dihydrouridine synthetase, 23S rRNA modification, position 2449		0	rRNA Modification
DU_23S_2449_inact	unknown Dihydrouridine synthetase, 23S rRNA modification, position 2449		0	rRNA Modification
DamX_mono	predicted protein (b3388, DamX, monomer)	C1962H3197N590O680S3	-3	Folding
Dam_mono	DNA adenine methylase (b3387, Dam, monomer)	C1455H2217N390O409S8	5	Folding
DapD_mono	2,3,4,5-tetrahydropyridine-2-carboxylate N-succinyltransferase (b0166, DapD, monomer)	C1325H2114N368O402S8	-4	Folding
DedA_mono	conserved inner membrane protein (b2317, DedA, monomer)	C1156H1792N280O284S7	2	Folding
Def_mono	peptide deformylase (b3287, Def, monomer)	C844H1394N241O255Fe1S6	-5	Folding
Def_mono_inact	peptide deformylase (b3287, Def, monomer)	C844H1394N241O255Fe1S6	-5	Folding
DnaG_mono	DNA primase (b3066, DnaG, monomer)	C2893H4562N821O867S22	-11	Folding
DnaJ_GroEL.(7)ADP.transGroES	DnaJ (b0015) GroEL GroES complex - Kerner et al. class III needs GroEL/ES	C39691H65763O12862N11117S380P14Mg7Zn2	-288	Folding
DnaJ_dim	chaperone Hsp40, co-chaperone with DnaK (b0015, DnaJ, dimer)	C3558H5638N1066O1098S32Zn4	12	Protein Folding
DnaJ_dim_inact	chaperone Hsp40, co-chaperone with DnaK (b0015, DnaJ, dimer)	C3558H5638N1066O1098S32Zn4	12	Protein Folding
DnaJ_mono	chaperone Hsp40, co-chaperone with DnaK (b0015, DnaJ, monomer)	C1779H2819N533O549Zn2S16	6	Folding
DnaKJ_RI_30_cplx	DnaK(monomer)/RI_30 complex	C30231H42077N10744O15388S99P1545Zn4	-1404	Protein Folding
DnaK_DnaK_GrpE_complex	DnaK (b0014) DnaK GrpE_dim complex - Kerner et al. class II can interact w/ GroEL/ES, cannot fold spontanously	C7864H12790O2588N2212S46P0	-90	Folding
DnaK_GroEL.(7)ADP.transGroES	DnaK (b0014) GroEL GroES complex - Kerner et al. class II can interact w/ GroEL/ES, cannot fold spontanously	C40908H67808O13300N11426S379P14Mg7	-324	Folding
DnaK_GrpE_RI_30_complex	Complex DnaK/GrpE with ribosome subunit 30S assembly molecule 1	C28535H39489N10201O14891P1542S83	-1442	Ribosomal Assembly
DnaK_mono	chaperone Hsp70, co-chaperone with DnaJ (b0014, DnaK, monomer)	C2996H4864N842O987S15	-30	Folding
DnaK_mono.ATP	DnaK monomer complexed with ATP	C3006H4876N847O1000S15P3	-34	Protein Folding
DnaK_mono.ATP_inact	DnaK monomer complexed with ATP	C3006H4876N847O1000S15P3	-34	Protein Folding
DsbC_mono	protein disulfide isomerase II (b2893, DsbC, monomer)	C1035H1651N273O318S14	-4	Folding
Dtd_dim	D-tyr-tRNA_tyr deacylase	C1384H2214N396O440S14Zn12	14	tRNA charging
Dtd_dim_inact	D-tyr-tRNA_tyr deacylase	C1384H2214N396O440S14Zn12	14	tRNA charging
Dtd_dim_tyr	D-tyr-tRNA_tyr deacylase complex	C2225H3181N726O1049P88S16Zn12Mg2	-72	tRNA charging
Dtd_mono	D-tyr-tRNA(Tyr) deacylase (b3887, Dtd, monomer)	C692H1107N198O220Zn6S7	7	Folding
DusA_DnaK_GrpE_complex	DusA (b4049) DnaK GrpE_dim complex - Kerner et al. class II can interact w/ GroEL/ES, cannot fold spontanously	C6487H10463O2080N1829S49P0	-66	Folding
DusA_GroEL.(7)ADP.transGroES	DusA (b4049) GroEL GroES complex - Kerner et al. class II can interact w/ GroEL/ES, cannot fold spontanously	C39531H65481O12792N11043S382P14Mg7	-300	Folding
DusA_mono	tRNA-dihydrouridine synthase A (b4049, DusA, monomer)	C1619H2537N459O479S18	-6	Folding
DusB_GroEL.(7)ADP.transGroES	DusB (b3260) GroEL GroES complex - Kerner et al. class III needs GroEL/ES	C39490H65455O12778N11038S378P14Mg7	-297	Folding
DusB_mono	tRNA-dihydrouridine synthase B (b3260, DusB, monomer)	C1578H2511N454O465S14	-3	Folding

DusC_GroEL.(7)ADP.transGroES	DusC (b2140) GroEL GroES complex - Kerner et al. class III needs GroEL/ES	C39473H65433O12767N11026S375P14Mg7	-297	Folding
DusC_mono	tRNA-dihydrouridine synthase C (b2140, DusC, monomer)	C1561H2489N442O454S11	-3	Folding
Dus_gen	generic Dus protein (stands for DusA, DusB or DusC)	C1586H2512N452O466S14	-4	tRNA Modification
Dus_gen_inact	generic Dus protein (stands for DusA, DusB or DusC)	C1586H2512N452O466S14	-4	tRNA Modification
EF-G.GDP	translation elongation factor EF-G (GDP bound)	C3439H5430N947O1060P2S25Mg	-25	Translation
EF-G.GTP	translation elongation factor EF-G (GTP bound)	C3439H5430N947O1063P3S25Mg	-26	Translation
EF-Ts	protein chain elongation factor EF-Ts (b0170, Tsf, monomer)	C1331H2170N363O417S11	-9	Translation
EF-Ts_inact	protein chain elongation factor EF-Ts (b0170, Tsf, monomer)	C1331H2170N363O417S11	-9	Translation
EF-Tu.EF-Ts	binary complex of EF-Tu and EF-Ts	C3251H5224N886O999S24Mg	-22	Translation
EF-Tu.GDP	EF-Tu.GDP	C1930H3066N528O593P2S13Mg	-16	Translation
EF-Tu.GDP.EF-Ts	ternary complex of EF-Tu, GDP and EF-Ts	C3261H5236N891O1010S24MgP2	-25	Translation
EF-Tu.GTP	EF-Tu.GTP	C1930H3066N528O596P3S13Mg	-17	Translation
EF-Tu.GTP-EF-Ts	ternary complex of EF-Tu, GTP and EF-Ts	C3261H5236N891O1013S24P3Mg	-26	Translation
EF-Tu.GTP.ala1-tRNA	EF-Tu.GTP.ala1-tRNA	C2659H3902Mg3N818O1136P79S13	-91	Translation
EF-Tu.GTP.ala2-tRNA	EF-Tu.GTP.ala2-tRNA	C2659H3901Mg3N822O1131P79S13	-90	Translation
EF-Tu.GTP.arg1-tRNA	EF-Tu.GTP.arg1-tRNA	C2676H3931Mg3N829O1137P80S14	-90	Translation
EF-Tu.GTP.argU-tRNA	EF-Tu.GTP.argU-tRNA	C2676H3927Mg3N828O1141P80S14	-91	Translation
EF-Tu.GTP.argW-tRNA	EF-Tu.GTP.argW-tRNA	C2656H3903Mg3N814O1127P78S14	-89	Translation
EF-Tu.GTP.argX-tRNA	EF-Tu.GTP.argX-tRNA	C2671H3926Mg3N828O1138P80S14	-90	Translation
EF-Tu.GTP.asn1-tRNA	EF-Tu.GTP.asn1-tRNA	C2670H3915Mg3N815O1141P79S14	-91	Translation
EF-Tu.GTP.asp1-tRNA	EF-Tu.GTP.asp1-tRNA	C2675H3925Mg3N819O1147P80S14	-92	Translation
EF-Tu.GTP.cysT-tRNA	EF-Tu.GTP.cysT-tRNA	C2643H3888Mg3N809O1116P77S16	-88	Translation
EF-Tu.GTP.gln1-tRNA	EF-Tu.GTP.gln1-tRNA	C2650H3895Mg3N810O1126P78S14	-89	Translation
EF-Tu.GTP.gln2-tRNA	EF-Tu.GTP.gln2-tRNA	C2650H3896Mg3N811O1125P78S14	-89	Translation
EF-Tu.GTP.glu1-tRNA	EF-Tu.GTP.glu1-tRNA	C2659H3907Mg3N817O1131P79S14	-91	Translation
EF-Tu.GTP.gly1-tRNA	EF-Tu.GTP.gly1-tRNA	C2656H3901Mg3N816O1135P79S13	-90	Translation
EF-Tu.GTP.glyT-tRNA	EF-Tu.GTP.glyT-tRNA	C2640H3880Mg3N799O1130P78S13	-89	Translation
EF-Tu.GTP.glyU-tRNA	EF-Tu.GTP.glyU-tRNA	C2634H3873Mg3N804O1118P77S14	-88	Translation
EF-Tu.GTP.hisR-tRNA	EF-Tu.GTP.hisR-tRNA	C2675H3923Mg3N812O1147P80S14	-91	Translation
EF-Tu.GTP.ile1-tRNA	EF-Tu.GTP.ile1-tRNA	C2681H3931Mg3N827O1145P80S13	-92	Translation
EF-Tu.GTP.ile2-tRNA	EF-Tu.GTP.ile2-tRNA	C2676H3934Mg3N820O1138P79S14	-91	Translation
EF-Tu.GTP.leu1-tRNA	EF-Tu.GTP.leu1-tRNA	C2761H4023Mg3N858O1208P90S13	-101	Translation
EF-Tu.GTP.leu2-tRNA	EF-Tu.GTP.leu2-tRNA	C2763H4026Mg3N862O1197P89S14	-100	Translation
EF-Tu.GTP.leuU-tRNA	EF-Tu.GTP.leuU-tRNA	C2767H4027Mg3N862O1211P90S13	-101	Translation
EF-Tu.GTP.leuW-tRNA	EF-Tu.GTP.leuW-tRNA	C2747H4006Mg3N857O1194P88S13	-99	Translation
EF-Tu.GTP.leuZ-tRNA	EF-Tu.GTP.leuZ-tRNA	C2772H4037Mg3N862O1208P90S15	-101	Translation
EF-Tu.GTP.lys1-tRNA	EF-Tu.GTP.lys1-tRNA	C2670H3925Mg3N814O1140P79S14	-90	Translation
EF-Tu.GTP.met1-tRNA	EF-Tu.GTP.met1-tRNA	C2681H3936Mg3N822O1143P80S15	-92	Translation
EF-Tu.GTP.phe1-tRNA	EF-Tu.GTP.phe1-tRNA	C2674H3923Mg3N820O1133P79S15	-90	Translation
EF-Tu.GTP.pro1-tRNA	EF-Tu.GTP.pro1-tRNA	C2673H3917Mg3N824O1141P80S14	-91	Translation
EF-Tu.GTP.pro2-tRNA	EF-Tu.GTP.pro2-tRNA	C2673H3918Mg3N825O1141P80S14	-91	Translation
EF-Tu.GTP.proL-tRNA	EF-Tu.GTP.proL-tRNA	C2672H3919Mg3N826O1139P80S14	-91	Translation
EF-Tu.GTP.proM-tRNA	EF-Tu.GTP.proM-tRNA	C2674H3918Mg3N824O1143P80S14	-92	Translation
EF-Tu.GTP.ser1-tRNA	EF-Tu.GTP.ser1-tRNA	C2775H4032Mg3N873O1214P91S13	-102	Translation
EF-Tu.GTP.ser2-tRNA	EF-Tu.GTP.ser2-tRNA	C2778H4037Mg3N873O1214P91S14	-102	Translation
EF-Tu.GTP.ser3-tRNA	EF-Tu.GTP.ser3-tRNA	C2792H4057Mg3N878O1219P92S14	-103	Translation
EF-Tu.GTP.serT-tRNA	EF-Tu.GTP.serT-tRNA	C2784H4047Mg3N873O1215P91S15	-103	Translation
EF-Tu.GTP.serV-tRNA	EF-Tu.GTP.serV-tRNA	C2826H4092Mg3N894O1250P96S15	-108	Translation
EF-Tu.GTP.thr1-tRNA	EF-Tu.GTP.thr1-tRNA	C2665H3912Mg3N819O1138P79S13	-91	Translation
EF-Tu.GTP.thr2-tRNA	EF-Tu.GTP.thr2-tRNA	C2665H3911Mg3N819O1137P79S13	-91	Translation
EF-Tu.GTP.thr3-tRNA	EF-Tu.GTP.thr3-tRNA	C2665H3910Mg3N817O1136P79S13	-91	Translation
EF-Tu.GTP.thrU-tRNA	EF-Tu.GTP.thrU-tRNA	C2665H3910Mg3N818O1135P79S13	-91	Translation
EF-Tu.GTP.trpT-tRNA	EF-Tu.GTP.trpT-tRNA	C2672H3920Mg3N817O1133P79S15	-90	Translation
EF-Tu.GTP.tyr1-tRNA	EF-Tu.GTP.tyr1-tRNA	C2761H4021Mg3N853O1192P88S15	-99	Translation
EF-Tu.GTP.val1-tRNA	EF-Tu.GTP.val1-tRNA	C2663H3909Mg3N821O1132P79S14	-91	Translation
EF-Tu.GTP.val2-tRNA	EF-Tu.GTP.val2-tRNA	C2673H3925Mg3N820O1143P80S14	-91	Translation
EF-Tu.GTP.val3-tRNA	EF-Tu.GTP.val3-tRNA	C2666H3914Mg3N821O1135P79S14	-91	Translation

ElaC_mono	binuclear zinc phosphodiesterase (b2268, ElaC, monomer)	C1462H2302N403O436Zn1S9	-7	Folding
Eno_DnaK_GrpE_complex	Eno (b2779) DnaK GrpE_dim complex - Kerner et al. class I can interact w/ GroEL/ES	C6871H11147O2235N1916S44P0Mg2	-67	Folding
Eno_GroEL.(7)ADP.transGroES	Eno (b2779) GroEL GroES complex - Kerner et al. class I can interact w/ GroEL/ES	C39915H66165O12947N11130S377P14Mg9	-301	Folding
Eno_dim	enolase (b2779, Eno, dimer)	C4006H6442N1092O1268S26Mg4	-14	mRNA degradation
Eno_mono	enolase (b2779, Eno, monomer)	C2003H3221N546O634Mg2S13	-7	Folding
EoR_tRNA_pos34_Q	unknown epoxide reductase, tRNA modification, position 34, Q		0	tRNA Modification
EoR_tRNA_pos34_Q_inact	unknown epoxide reductase, tRNA modification, position 34, Q		0	tRNA Modification
Era_dim.GDP	dimer of Era_mono (membrane-associated, 16S rRNA-binding GTPase) bound to GDP	C3018H4870N846O904S20P4	-8	Ribosome maturation
Era_dim.GTP	dimer of Era_mono (membrane-associated, 16S rRNA-binding GTPase) bound to GTP	C3018H4870N846O910S20P6	-10	Ribosome maturation
Era_mono	membrane-associated, 16S rRNA-binding GTPase (b2566, Era, monomer)	C1499H2423N418O441S10	-1	Folding
FabD_mono	malonyl-CoA-[acyl-carrier-protein] transacylase (b1092, FabD, monomer)	C1431H2265N383O435S15	-10	Folding
FabG_DnaK_GrpE_complex	FabG (b1093) DnaK GrpE_dim complex - Kerner et al. class II can interact w/ GroEL/ES, cannot fold spontanously	C5971H9742O1946N1692S41P0	-60	Folding
FabG_GroEL.(7)ADP.transGroES	FabG (b1093) GroEL GroES complex - Kerner et al. class II can interact w/ GroEL/ES, cannot fold spontanously	C39015H64760O12658N10906S374P14Mg7	-294	Folding
FabG_mono	3-oxoacyl-[acyl-carrier-protein] reductase (b1093, FabG, monomer)	C1103H1816N322O345S10	0	Folding
FabH_mono	3-oxoacyl-[acyl-carrier-protein] synthase III (b1091, FabH, monomer)	C1472H2343N407O459S13	-12	Folding
FecI_mono	KpLE2 phage-like element; RNA polymerase, sigma 19 factor (b4293, FecI, monomer)	C861H1374N231O260S7	-3	Folding
FecR_mono	KpLE2 phage-like element; transmembrane signal transducer for ferric citrate transport (b4292, FecR, monomer)	C1565H2486N463O472S2	3	Folding
Fis_mono	global DNA-binding transcriptional dual regulator (b3261, Fis, monomer)	C487H804N141O151S6	3	Folding
FkpB_mono	FKBP-type peptidyl-prolyl cis-trans isomerase (rotamase) (b0028, FkpB, monomer)	C704H1084N184O230S4	-16	Folding
FliA_mono	RNA polymerase, sigma 28 (sigma F) factor (b1922, FliA, monomer)	C1195H1928N355O374S4	-9	Folding
FliY_mono	cystine transporter subunit -!- periplasmic-binding component of ABC superfamily (b1920, FliY, monomer)	C1154H1857N315O366S2	-4	Folding
FliZ_mono	predicted regulator of FliA activity (b1921, FliZ, monomer)	C953H1513N280O274S8	7	Folding
Fmt_fmet_tRNA_met_1_cplx	methionyl-tRNA formyltransferase/fmet_tRNA_met_1 complex	C2277H3318N719O996S13P77Mg2	-80	tRNA charging
Fmt_mono	10-formyltetrahydrofolate:L-methionyl-tRNA(fMet) N-formyltransferase (b3288, Fmt, monomer)	C1517H2442N414O450S11	-4	Folding
Fmt_mono_inact	10-formyltetrahydrofolate:L-methionyl-tRNA(fMet) N-formyltransferase (b3288, Fmt, monomer)	C1517H2442N414O450S11	-4	Folding
FtsH_mono	protease, ATP-dependent zinc-metallo (b3178, FtsH, monomer)	C3115H4997N880O935S26	-7	Folding
FusA_DnaK_GrpE_complex	FusA (b3340) DnaK GrpE_dim complex - Kerner et al. class I can interact w/ GroEL/ES	C8297H13344O2650N2312S56P0	-84	Folding
FusA_GroEL.(7)ADP.transGroES	FusA (b3340) GroEL GroES complex - Kerner et al. class I can interact w/ GroEL/ES	C41341H68362O13362N11526S389P14Mg7	-318	Folding
FusA_mono	protein chain elongation factor EF-G, GTP-binding (b3340, FusA, monomer)	C3429H5418N942O1049S25	-24	Folding
GarK_mono	glycerate kinase I (b3124, GarK, monomer)	C1701H2734N477O544S12	-17	Folding
GarL_mono	alpha-dehydro-beta-deoxy-D-glucarate aldolase (b3126, GarL, monomer)	C1234H1926N333O364S4	-5	Folding
GarP_mono	predicted (D)-galactarate transporter (b3127, GarP, monomer)	C2309H3532N542O579S23	8	Folding
GarR_mono	tartronate semialdehyde reductase (b3125, GarR, monomer)	C1333H2194N359O408S16	-5	Folding
GidA_DnaK_GrpE_complex	GidA (b3741) DnaK GrpE_dim complex - Kerner et al. class II can interact w/ GroEL/ES, cannot fold spontanously	C7924H12814O2524N2249S52P0	-65	Folding
GidA_GroEL.(7)ADP.transGroES	GidA (b3741) GroEL GroES complex - Kerner et al. class II can interact w/ GroEL/ES, cannot fold spontanously	C40968H67832O13236N11463S385P14Mg7	-299	Folding
GidA_mono	glucose-inhibited cell-division protein (b3741, GidA, monomer)	C3056H4888N879O923S21	-5	Folding
GidA_mono_inact	glucose-inhibited cell-division protein (b3741, GidA, monomer)	C3056H4888N879O923S21	-5	Folding

GidB_mono	methyltransferase, SAM-dependent methyltransferase, glucose-inhibited cell-division protein (b3740, GidB, monomer)	C1053H1671N287O299S5	-4	Folding
GlnD_DnaK_GrpE_complex	GlnD (b0167) DnaK GrpE_dim complex - Kerner et al. class II can interact w/ GroEL/ES, cannot fold spontanously	C9432H15101O2907N2686S58P0	-75	Folding
GlnD_GroEL.(7)ADP.transGroES	GlnD (b0167) GroEL GroES complex - Kerner et al. class II can interact w/ GroEL/ES, cannot fold spontanously	C42476H70119O13619N11900S391P14Mg7	-309	Folding
GlnD_mono	uridylyltransferase (b0167, GlnD, monomer)	C4564H7175N1316O1306S27	-15	Folding
GlnS_DnaK_GrpE_complex	GlnS (b0680) DnaK GrpE_dim complex - Deuerling et al. DnaKJ/GrpE dependent folding	C7684H12284O2440N2157S52P0	-71	Folding
GlnS_mono	glutamyl-tRNA synthetase (b0680, GlnS, monomer)	C2816H4358N787O839S21	-11	Folding
GlnS_mono_inact	glutamyl-tRNA synthetase (b0680, GlnS, monomer)	C2816H4358N787O839S21	-11	Folding
Gln_RS_gln_amp	Glutaminyl-tRNA synthetase (charged)	C2831H4380N794O849S21P	-13	tRNA charging
GltX_mono	glutamyl-tRNA synthetase (b2400, GltX, monomer)	C2374H3667N674O715Zn1S18	-15	Folding
GltX_mono_inact	glutamyl-tRNA synthetase (b2400, GltX, monomer)	C2374H3667N674O715Zn1S18	-15	Folding
Glu_RS_glu_amp	Glutamyl-tRNA synthetase (charged)	C2389H3687N680O726S18P1Zn	-18	tRNA charging
GlyQ_mono	glycine tRNA synthetase, alpha subunit (b3560, GlyQ, monomer)	C1562H2359N408O462S12	-13	Folding
GlyS_mono	glycine tRNA synthetase, beta subunit (b3559, GlyS, monomer)	C3419H5440N946O1018S18	-18	Folding
Gly_RS_2gly_2amp	Glycyl-tRNA synthetase (charged, 2*glycine, 2*amp)	C9986H15632N2720O2978S60P2	-66	tRNA charging
Gly_RS_gly_amp	Glycyl-tRNA synthetase (charged)	C9974H15615N2714O2969S60P	-64	tRNA charging
Gly_RS_tetra	Glycyl-tRNA synthetase, tetramer (uncharged)	C9962H15598N2708O2960S60	-62	tRNA charging
Gly_RS_tetra_inact	Glycyl-tRNA synthetase, tetramer (uncharged)	C9962H15598N2708O2960S60	-62	tRNA charging
Gph_mono	phosphoglycolate phosphatase (b3385, Gph, monomer)	C1220H1906N323O372S6	-15	Folding
GreA_DnaK_GrpE_complex	GreA (b3181) DnaK GrpE_dim complex - Kerner et al. class II can interact w/ GroEL/ES, cannot fold spontanously	C5640H9166O1849N1585S35P0	-71	Folding
GreA_GroEL.(7)ADP.transGroES	GreA (b3181) GroEL GroES complex - Kerner et al. class II can interact w/ GroEL/ES, cannot fold spontanously	C38684H64184O12561N10799S368P14Mg7	-305	Folding
GreA_mono	transcription elongation factor (b3181, GreA, monomer)	C772H1240N215O248S4	-11	Folding
GreA_mono_inact	transcription elongation factor (b3181, GreA, monomer)	C772H1240N215O248S4	-11	Folding
GreB_mono	transcription elongation factor (b3406, GreB, monomer)	C833H1303N228O245S3	1	Folding
GreB_mono_inact	transcription elongation factor (b3406, GreB, monomer)	C833H1303N228O245S3	1	Folding
GroEL.(7)ADP.cisGroES	ternary complex GroEL (14-mer), GroES (7-mer) and 7 ADP	C37912H62944N10584O12313S364Mg7P14	-294	Protein Folding
GroEL.(7)ADP.transGroES	ternary complex GroEL (14-mer), GroES (7-mer) and 7 ADP	C37912H62944N10584O12313S364Mg7P14	-294	Protein Folding
GroEL.(7)ADP.transGroES_RI_50_cplx	Complex GroEL/GroES (ADP) with ribosome subunit 50S assembly molecule 1	C81791H120579N26911O37700S438P3038Mg7	-3143	Ribosomal Assembly
GroEL.(7)ATP.transGroES_RI_50_cplx	Complex GroEL/GroES (ATP) with ribosome subunit 50S assembly molecule 1	C81791H120579N26911O37721P3045S438Mg7	-3150	Ribosomal Assembly
GroL_(14)	Cpn60 chaperonin GroEL, large subunit of GroESL (b4143, GroL, 14-mer)	C34664H57526N9660O11228S350	-266	Protein Folding
GroL_hepta	Cpn60 chaperonin GroEL, large subunit of GroESL (b4143, GroL, helptamer)	C17332H28763N4830O5614S175	-133	Protein Folding
GroL_mono	Cpn60 chaperonin GroEL, large subunit of GroESL (b4143, GroL, monomer)	C2476H4109N690O802S25	-19	Folding
GroS_hepta	Cpn10 chaperonin GroES, small subunit of GroESL (b4142, GroS, heptamer)	C3178H5334N889O1015S14	-21	Protein Folding
GroS_mono	Cpn10 chaperonin GroES, small subunit of GroESL (b4142, GroS, monomer)	C454H762N127O145S2	-3	Folding
GrpE_DnaK_GrpE_complex	GrpE (b2614) DnaK GrpE_dim complex - Kerner et al. class I can interact w/ GroEL/ES	C5804H9457O1908N1634S39P0	-75	Folding
GrpE_GroEL.(7)ADP.transGroES	GrpE (b2614) GroEL GroES complex - Kerner et al. class I can interact w/ GroEL/ES	C38848H64475O12620N10848S372P14Mg7	-309	Folding
GrpE_dim	heat shock protein (b2614, GrpE, dimer)	C1872H3062N528O614S16	-30	Protein Folding
GrpE_dim_inact	heat shock protein (b2614, GrpE, dimer)	C1872H3062N528O614S16	-30	Protein Folding
GrpE_mono	heat shock protein (b2614, GrpE, monomer)	C936H1531N264O307S8	-15	Folding
GshB_mono	glutathione synthetase (b2947, GshB, monomer)	C1586H2514N426O472S14	-10	Folding
HepA_DnaK_GrpE_complex	HepA (b0059) DnaK GrpE_dim complex - Deuerling et al. DnaKJ/GrpE dependent folding	C9699H15511O3078N2752S60P0	-109	Folding
HepA_mono	RNA polymerase-associated helicase protein (ATPase and RNA polymerase recycling factor) (b0059, HepA, monomer)	C4831H7585N1382O1477S29	-49	Folding

HflC_mono	modulator for HflB protease specific for phage lambda cII repressor (b4175, HflC, monomer)	C1649H2663N472O508S9	-1	Folding
HflK_mono	modulator for HflB protease specific for phage lambda cII repressor (b4174, HflK, monomer)	C1963H3156N587O636S8	-1	Folding
HflX_mono	predicted GTPase (b4173, HflX, monomer)	C2124H3425N623O640S8	-10	Folding
Hfq_mono	HF-I, host factor for RNA phage Q beta replication (b4172, Hfq, monomer)	C478H757N143O156S1	0	Folding
HisS_mono	histidyl tRNA synthetase (b2514, HisS, monomer)	C2073H3275N593O623Mg2S13	-4	Folding
His_RS_2his_2amp	Histidyl-tRNA synthetase (charged, 2*histidine, 2*amp)	C4178H6592N1202O1264S26P2Mg4	-12	tRNA charging
His_RS_dim	Histidyl-tRNA synthetase (uncharged)	C4146H6550N1186O1246S26Mg4	-8	tRNA charging
His_RS_dim_inact	Histidyl-tRNA synthetase (uncharged)	C4146H6550N1186O1246S26Mg4	-8	tRNA charging
His_RS_his_amp	Histidyl-tRNA synthetase (charged, 1*histidine, 1*amp)	C4162H6571N1194O1255S26Mg4P	-10	tRNA charging
HolA_mono	DNA polymerase III, delta subunit (b0640, HolA, monomer)	C1728H2779N493O495S10	-2	Folding
HolD_mono	DNA polymerase III, psi subunit (b4372, HolD, monomer)	C665H1059N192O198S4	-3	Folding
HyL_tRNA_pos_34_ho5U	unknown hydroxylase, tRNA modification, position 35, ho5U		0	tRNA Modification
HyL_tRNA_pos_34_ho5U_inact	unknown hydroxylase, tRNA modification, position 35, ho5U		0	tRNA Modification
IF1	translation initiation factor IF-1 (b0884, InfA, monomer)	C356H589N103O107S3	2	Translation
IF1_inact	translation initiation factor IF-1 (b0884, InfA, monomer)	C356H589N103O107S3	2	Translation
IF2-GDP	translation initiation factor IF2 - GDP bound	C4190H6902N1269O1368S24P2	-17	Translation
IF2-GTP	translation initiation factor IF2 - GTP bound	C4190H6902N1269O1371S24P3	-18	Translation
IF3	translation initiation factor IF3 (monomer, no modification)	C902H1516N264O268S7	8	Translation
IF3_inact	translation initiation factor IF3 (monomer, no modification)	C902H1516N264O268S7	8	Translation
IhfA_mono	integration host factor (IHF), DNA-binding protein, alpha subunit (b1712, IhfA, monomer)	C493H805N146O151S1	3	Folding
IhfB_mono	integration host factor (IHF), DNA-binding protein, beta subunit (b0912, IhfB, monomer)	C467H756N139O140S3	3	Folding
IleS_mono	isoleucyl-tRNA synthetase (b0026, IleS, monomer)	C4664H7219N1261O1375Zn2S37	-18	Folding
IleS_mono_inact	isoleucyl-tRNA synthetase (b0026, IleS, monomer)	C4664H7219N1261O1375Zn2S37	-18	Folding
Ile_RS_ile_Amp	Isoleucyl-tRNA synthetase (charged)	C4680H7244N1267O1384S37Zn2P	-20	tRNA charging
InfA_mono	translation initiation factor IF-1 (b0884, InfA, monomer)	C356H589N103O107S3	2	Folding
InfB_DnaK_GrpE_complex	InfB (b3168) DnaK GrpE_dim complex - Kerner et al. class II can interact w/ GroEL/ES, cannot fold spontanously	C9048H14816O2958N2634S55P0	-74	Folding
InfB_GroEL.(7)ADP.transGroES	InfB (b3168) GroEL GroES complex - Kerner et al. class II can interact w/ GroEL/ES, cannot fold spontanously	C42092H69834O13670N11848S388P14Mg7	-308	Folding
InfB_mono	fused protein chain initiation factor 2, IF2: membrane protein -!- conserved protein (b3168, InfB, monomer)	C4180H6890N1264O1357S24	-14	Folding
InfC_DnaK_GrpE_complex	InfC (b1718) DnaK GrpE_dim complex - Kerner et al. class I or II can interact w/ GroEL/ES, behaves here as class I	C5770H9442O1869N1634S38P0	-52	Folding
InfC_GroEL.(7)ADP.transGroES	InfC (b1718) GroEL GroES complex - Kerner et al. class I or II can interact w/ GroEL/ES, behaves here as class I	C38814H64460O12581N10848S371P14Mg7	-286	Folding
InfC_mono	protein chain initiation factor IF-3 (b1718, InfC, monomer)	C902H1516N264O268S7	8	Folding
IscA_mono	FeS cluster assembly protein (b2528, IscA, monomer)	C505H783N134O160S4	-5	Folding
IscA_tetra	FeS cluster assembly protein, tetramer	C2020H3132N536O640S16	-20	Iron-sulfur cluster biosynthesis
IscA_tetra_Fe(2)	FeS cluster assembly protein, tetramer, bound to Fe(2)	C2020H3132N536O640S16Fe2	-16	Iron-sulfur cluster biosynthesis
IscR_mono	DNA-binding transcriptional repressor (b2531, IscR, monomer)	C738H1214N226O239S4	0	Folding
IscS_DnaK_GrpE_complex	IscS (b2530) DnaK GrpE_dim complex - Kerner et al. class II can interact w/ GroEL/ES, cannot fold spontanously	C6845H11075O2199N1933S49P0	-68	Folding
IscS_GroEL.(7)ADP.transGroES	IscS (b2530) GroEL GroES complex - Kerner et al. class II can interact w/ GroEL/ES, cannot fold spontanously	C39889H66093O12911N11147S382P14Mg7	-302	Folding
IscS_IscU_cplx	complex of cysteine desulfurase (IscS) and scaffold protein dimer	C5162H8208N1452O1604S47P2	-34	Iron-sulfur cluster biosynthesis
IscS_dim_S-H	cysteine desulfurase (tRNA sulfurtransferase), PLP-dependent (b2530, IscS, dimer)	C3970H6314N1128O1208S36P2	-20	tRNA Modification
IscS_dim_S-SH	cysteine desulfurase S-SH	C3970H6314N1128O1208S37P2	-20	tRNA Modification
IscS_mono	cysteine desulfurase (tRNA sulfurtransferase), PLP-dependent (b2530, IscS, monomer)	C1977H3149N563O598S18	-8	Folding

IscU_dim_2Fe(2)-2S	scaffold protein, dimer, bound to Fe(2)	C1192H1894N324O396S12Fe2	-12	Iron-sulfur cluster biosynthesis
IscU_dim_SH	scaffold protein, dimer	C1192H1894N324O396S10	-14	Iron-sulfur cluster biosynthesis
IscU_dim_[2Fe-2S]2	scaffold protein, dimer, bound to [2Fe-2S] cluster	C1192H1894N324O396S14Fe4	-10	Iron-sulfur cluster biosynthesis
IscU_dim_[4Fe-4S]	scaffold protein, dimer, bound to [4Fe-4S] cluster	C1192H1894N324O396S14Fe4	-12	Iron-sulfur cluster biosynthesis
IscU_mono	scaffold protein (b2529, IscU, monomer)	C596H947N162O198S5	-7	Folding
IscU_mono_S-SH	scaffold protein	C596H947N162O198S6	-7	Iron-sulfur cluster biosynthesis
IspH_mono	1-hydroxy-2-methyl-2-(E)-butenyl 4-diphosphate reductase, 4Fe-4S protein (b0029, IspH, monomer)	C1517H2445N436O472S9	-11	Folding
KsgA_mono	S-adenosylmethionine-6-N',N'-adenosyl (rRNA) dimethyltransferase (b0051, KsgA, monomer)	C1352H2143N376O388S13	1	Folding
KsgA_mono_inact	S-adenosylmethionine-6-N',N'-adenosyl (rRNA) dimethyltransferase (b0051, KsgA, monomer)	C1352H2143N376O388S13	1	Folding
LeuS_DnaK_GrpE_complex	LeuS (b0642) DnaK GrpE_dim complex - Deuerling et al. DnaKJ/GrpE dependent folding	C9202H14605O2892N2528S75P0Zn1	-87	Folding
LeuS_mono	leucyl-tRNA synthetase (b0642, LeuS, monomer)	C4334H6679N1158O1291Zn1S44	-27	Folding
LeuS_mono_inact	leucyl-tRNA synthetase (b0642, LeuS, monomer)	C4334H6679N1158O1291Zn1S44	-27	Folding
Leu_RS_leu_amp	Leucyl-tRNA synthetase (charged	C4350H6704N1164O1300S44ZnP	-29	tRNA charging
Lhr_mono	predicted ATP-dependent helicase (b1653, Lhr, monomer)	C7480H12000N2189O2224S33	-11	Folding
LspA_mono	prolipoprotein signal peptidase (signal peptidase II) (b0027, LspA, monomer)	C848H1290N210O215S5	2	Folding
LysII_RS_2lys_2amp	Lysyl-tRNA synthetase II (charged, 1*lysine, 1*amp)	C5156H8024N1438O1560S36P2Mg6	-32	tRNA charging
LysII_RS_dim	Lysyl-tRNA synthetase II, inducible (uncharged)	C5124H7970N1424O1542S36Mg6	-30	tRNA charging
LysII_RS_dim_inact	Lysyl-tRNA synthetase II, inducible (uncharged)	C5124H7970N1424O1542S36Mg6	-30	tRNA charging
LysII_RS_lys_amp	Lysyl-tRNA synthetase II (charged, 1*lysine, 1*amp)	C5140H7997N1431O1551S36PMg6	-31	tRNA charging
LysI_RS_2lys_2amp	Lysyl-tRNA synthetase I, inducible (charged, 2*lysine, 2*amp)	C5136H8020N1428O1556S36P2	-46	tRNA charging
LysI_RS_dim	Lysyl-tRNA synthetase I , constitutive (uncharged)	C5104H7966N1414O1538S36	-44	tRNA charging
LysI_RS_dim_inact	Lysyl-tRNA synthetase I , constitutive (uncharged)	C5104H7966N1414O1538S36	-44	tRNA charging
LysI_RS_lys_amp	Lysyl-tRNA synthetase I, inducible (charged, 1*lysine, 1*amp)	C5120H7993N1421O1547S36P	-45	tRNA charging
LysS_mono	lysine tRNA synthetase, constitutive (b2890, LysS, monomer)	C2552H3983N707O769S18	-22	Folding
LysU_DnaK_GrpE_complex	LysU (b4129) DnaK GrpE_dim complex - Deuerling et al. DnaKJ/GrpE dependent folding	C7430H11911O2372N2082S49P0Mg3	-75	Folding
LysU_mono	lysine tRNA synthetase, inducible (b4129, LysU, monomer)	C2562H3985N712O771Mg3S18	-15	Folding
Map_mono	methionine aminopeptidase (b0168, Map, monomer)	C1286H2062N352O391Fe2S15	-4	Folding
Map_mono_inact	methionine aminopeptidase (b0168, Map, monomer)	C1286H2062N352O391Fe2S15	-4	Folding
MeST_S12	beta-methylthio-transferase on aspartic acid of S12		0	Ribosomal protein modification
MeST_S12_inact	beta-methylthio-transferase on aspartic acid of S12		0	Ribosomal protein modification
MeST_rpS12_cplx	beta-methylthio-transferase/ rpS12/ amet/cys L/Fe2+ complex	C608H1049N203O172S6Fe	24	Ribosomal protein modification
MeT_16S_1402	unknown Methyltransferase 16S rRNA, position 1402		0	rRNA Modification
MeT_16S_1402_inact	unknown Methyltransferase 16S rRNA, position 1402		0	rRNA Modification
MeT_16S_1407	unknown Methyltransferase 16S rRNA, position 1407		0	rRNA Modification
MeT_16S_1407_inact	unknown Methyltransferase 16S rRNA, position 1407		0	rRNA Modification
MeT_16S_1516	unknown Methyltransferase 16S rRNA, position 1516		0	rRNA Modification
MeT_16S_1516_inact	unknown Methyltransferase 16S rRNA, position 1516		0	rRNA Modification
MeT_16S_527	unknown Methyltransferase 16S rRNA, position 527		0	rRNA Modification
MeT_16S_527_inact	unknown Methyltransferase 16S rRNA, position 527		0	rRNA Modification
MeT_16S_966	unknown Methyltransferase 16S rRNA, position 966		0	rRNA Modification
MeT_16S_966_inact	unknown Methyltransferase 16S rRNA, position 966		0	rRNA Modification
MeT_23S_1618	unknown Methyltransferase 23S rRNA, position 1618		0	rRNA Modification
MeT_23S_1618_inact	unknown Methyltransferase 23S rRNA, position 1618		0	rRNA Modification
MeT_23S_1835	unknown Methyltransferase 23S rRNA, position 1835		0	rRNA Modification
MeT_23S_1835_inact	unknown Methyltransferase 23S rRNA, position 1835		0	rRNA Modification

MeT_23S_1962	unknown Methyltransferase 23S rRNA, position 1962		0	rRNA Modification
MeT_23S_1962_inact	unknown Methyltransferase 23S rRNA, position 1962		0	rRNA Modification
MeT_23S_2030	unknown Methyltransferase 23S rRNA, position 2030		0	rRNA Modification
MeT_23S_2030_inact	unknown Methyltransferase 23S rRNA, position 2030		0	rRNA Modification
MeT_23S_2069	unknown Methyltransferase 23S rRNA, position 2069		0	rRNA Modification
MeT_23S_2069_inact	unknown Methyltransferase 23S rRNA, position 2069		0	rRNA Modification
MeT_23S_2445	unknown Methyltransferase 23S rRNA, position 2445		0	rRNA Modification
MeT_23S_2445_inact	unknown Methyltransferase 23S rRNA, position 2445		0	rRNA Modification
MeT_23S_2498	unknown Methyltransferase 23S rRNA, position 2498		0	rRNA Modification
MeT_23S_2498_inact	unknown Methyltransferase 23S rRNA, position 2498		0	rRNA Modification
MeT_23S_2503	unknown Methyltransferase 23S rRNA, position 2503		0	rRNA Modification
MeT_23S_2503_inact	unknown Methyltransferase 23S rRNA, position 2503		0	rRNA Modification
MeT_EF-TU	methyltransferase of EF-TU (Lys56)		0	Protein Modification
MeT_EF-TU_cplx_a	Methyltransferase/pEF-TU_ac/amet complex (b3339)	C1934H3075N529O587S14	-14	Protein Modification
MeT_EF-TU_cplx_b	Methyltransferase/pEF-TU_ac/ amet complex (b3980)	C1935H3077N529O588S14	-14	Protein Modification
MeT_EF-TU_inact	methyltransferase of EF-TU (Lys56)		0	Protein Modification
MeT_L16	unknown Methyltransferase of L16		0	Ribosomal protein modification
MeT_L16_inact	unknown Methyltransferase of L16		0	Ribosomal protein modification
MeT_L33	unknown methyltransferase of L33		0	Ribosomal protein modification
MeT_L33_inact	unknown methyltransferase of L33		0	Ribosomal protein modification
MeT_L7/L12	unknown Methyltransferase ribosomal protein L7/L12		0	Ribosomal protein modification
MeT_L7/L12_inact	unknown Methyltransferase ribosomal protein L7/L12		0	Ribosomal protein modification
MeT_L7/L12_rpL12_cplx	complex of unknown Methyltransferase ribosomal protein L7/L12 and L7/L12	C549H913N144O181S4	-7	Ribosomal protein modification
MeT_S11	Methyltransferase (ribosomal protein S11) (unknown gene)		0	Ribosomal protein modification
MeT_S11_inact	Methyltransferase (ribosomal protein S11) (unknown gene)		0	Ribosomal protein modification
MeT_rpL16_cplx	Methyltransferase/ L16/ amet complex	C701H1180N211O182S7	21	Ribosomal protein modification
MeT_rpL33_cplx	methyltransferase/L33/amet complex	C299H508N87O81S	11	Ribosomal protein modification
MeT_rpS11_cplx	Methyltransferase/ S11/ amet complex	C610H1020N202O176S4	16	Ribosomal protein modification
MeT_tRNA_pos_32_Cm	unknown MethyltransferasetRNA, position 32, Cm		0	tRNA Modification
MeT_tRNA_pos_32_Cm_inact	unknown MethyltransferasetRNA, position 32, Cm		0	tRNA Modification
MeT_tRNA_pos_32_Um	unknown MethyltransferasetRNA, position 32, Um		0	tRNA Modification
MeT_tRNA_pos_32_Um_inact	unknown MethyltransferasetRNA, position 32, Um		0	tRNA Modification
MeT_tRNA_pos_37_m2A	unknown MethyltransferasetRNA, position 37, m2A		0	tRNA Modification
MeT_tRNA_pos_37_m2A_inact	unknown MethyltransferasetRNA, position 37, m2A		0	tRNA Modification
MeT_tRNA_pos_37_m6A	unknown MethyltransferasetRNA, position 37, m6A		0	tRNA Modification
MeT_tRNA_pos_37_m6A_inact	unknown MethyltransferasetRNA, position 37, m6A		0	tRNA Modification
MeT_tRNA_pos_37_m6t6A	unknown MethyltransferasetRNA, position 37, m6t6A		0	tRNA Modification
MeT_tRNA_pos_37_m6t6A_inact	unknown MethyltransferasetRNA, position 37, m6t6A		0	tRNA Modification
MepA_mono	murein DD-endopeptidase (b2328, MepA, monomer)	C1257H1979N359O362S12	2	Folding
MetG_DnaK_GrpE_complex	MetG (b2114) DnaK GrpE_dim complex - Deuerling et al. DnaKJ/GrpE dependent folding	C8284H13169O2606N2285S59P0Zn1	-74	Folding
MetG_mono	methionyl-tRNA synthetase (b2114, MetG, monomer)	C3416H5243N915O1005Zn1S28	-14	Folding
Met_RS_2met_2amp	Methionyl-tRNA synthetase (charged: 2*methionine, 2*amp)	C6862H10532N1842O2028P2S58Zn2	-32	tRNA charging
Met_RS_dim	Methionyl-tRNA synthetase (uncharged) (dimer, 2 Zn2+)	C6832H10486N1830O2010S56Zn2	-28	tRNA charging
Met_RS_dim_inact	Methionyl-tRNA synthetase (uncharged) (dimer, 2 Zn2+)	C6832H10486N1830O2010S56Zn2	-28	tRNA charging
Met_RS_met_amp	Methionyl-tRNA synthetase (charged: 2*methionine, 2*amp)	C6847H10509N1836O2019S57PZn2	-30	tRNA charging
Mfd_DnaK_GrpE_complex	Mfd (b1114) DnaK GrpE_dim complex - Deuerling et al. DnaKJ/GrpE dependent folding	C10642H17081O3297N3009S67P0Mg1	-84	Folding

Mfd_mono	transcription-repair coupling factor (b1114, Mfd, monomer)	C5774H9155N1639O1696Mg1S36	-24	Folding
Mfd_mono_inact	transcription-repair coupling factor (b1114, Mfd, monomer)	C5774H9155N1639O1696Mg1S36	-24	Folding
MiaA_dim	delta(2)-isopentenylpyrophosphate tRNA-adenosine transferase (b4171, MiaA, dimer)	C3112H4934N876O916S18Mg2	-10	tRNA Modification
MiaA_dim_inact	delta(2)-isopentenylpyrophosphate tRNA-adenosine transferase (b4171, MiaA, dimer)	C3112H4934N876O916S18Mg2	-10	tRNA Modification
MiaA_mono	delta(2)-isopentenylpyrophosphate tRNA-adenosine transferase (b4171, MiaA, monomer)	C1556H2467N438O458Mg1S9	-5	Folding
MiaB_mono	isopentenyl-adenosine A37 tRNA methylthiolase (b0661, MiaB, monomer)	C2358H3725N659O725Fe4S23	-14	Folding
MiaB_mono_inact	isopentenyl-adenosine A37 tRNA methylthiolase (b0661, MiaB, monomer)	C2358H3725N659O725Fe4S23	-14	Folding
MreB_DnaK_GrpE_complex	MreB (b3251) DnaK GrpE_dim complex - Kerner et al. class II can interact w/ GroEL/ES, cannot fold spontanously	C6481H10550O2099N1830S47P0	-70	Folding
MreB_GroEL.(7)ADP.transGroES	MreB (b3251) GroEL GroES complex - Kerner et al. class II can interact w/ GroEL/ES, cannot fold spontanously	C39525H65568O12811N11044S380P14Mg7	-304	Folding
MreB_mono	cell wall structural complex MreBCD, actin-like component MreB (b3251, MreB, monomer)	C1613H2624N460O498S16	-10	Folding
MreC_mono	cell wall structural complex MreBCD transmembrane component MreC (b3250, MreC, monomer)	C1731H2836N502O525S10	2	Folding
MreD_mono	cell wall structural complex MreBCD transmembrane component MreD (b3249, MreD, monomer)	C902H1400N216O202S5	6	Folding
MutL_mono	methyl-directed mismatch repair protein (b4170, MutL, monomer)	C3008H4827N871O886S13	-6	Folding
MutM_mono	formamidopyrimidine/5-formyluracil/ 5-hydroxymethyluracil DNA glycosylase (b3635, MutM, monomer)	C1353H2160N382O381S9	4	Folding
NadD_mono	nicotinic acid mononucleotide adenylyltransferase, NAD(P)-dependent (b0639, NadD, monomer)	C1106H1699N297O319S4	-8	Folding
NfsA_mono	nitroreductase A, NADPH-dependent, FMN-dependent (b0851, NfsA, monomer)	C1187H1883N337O348S7	-2	Folding
NlpD_mono	predicted outer membrane lipoprotein (b2742, NlpD, monomer)	C1631H2609N469O533S7	6	Folding
Npr_mono	phosphohistidinoprotein-hexose phosphotransferase component of N-regulated PTS system (Npr) (b3206, Npr, monomer)	C421H670N112O140S4	-10	Folding
NusA_mono	transcription termination/antitermination L factor (b3169, NusA, monomer)	C2396H3835N669O775S13	-40	Folding
NusA_mono_inact	transcription termination/antitermination L factor (b3169, NusA, monomer)	C2396H3835N669O775S13	-40	Folding
NusB_mono	transcription antitermination protein (b0416, NusB, monomer)	C700H1120N190O206S2	0	Folding
NusB_mono_inact	transcription antitermination protein (b0416, NusB, monomer)	C700H1120N190O206S2	0	Folding
NusG_DnaK_GrpE_complex	NusG (b3982) DnaK GrpE_dim complex - Kerner et al. class II can interact w/ GroEL/ES, cannot fold spontanously	C5776H9356O1867N1625S38P0	-61	Folding
NusG_GroEL.(7)ADP.transGroES	NusG (b3982) GroEL GroES complex - Kerner et al. class II can interact w/ GroEL/ES, cannot fold spontanously	C38820H64374O12579N10839S371P14Mg7	-295	Folding
NusG_mono	transcription termination factor (b3982, NusG, monomer)	C908H1430N255O266S7	-1	Folding
NusG_mono_inact	transcription termination factor (b3982, NusG, monomer)	C908H1430N255O266S7	-1	Folding
Orn_dim	oligoribonuclease dimer	C1842H2858N502O558S12Mg2	-16	mRNA degradation
Orn_dim_inact	oligoribonuclease dimer	C1842H2858N502O558S12Mg2	-16	mRNA degradation
Orn_mono	oligoribonuclease (b4162, Orn, monomer)	C921H1429N251O279Mg1S6	-8	Folding
PanF_mono	pantothenate:sodium symporter (b3258, PanF, monomer)	C2407H3862N590O618S20	10	Folding
PdxA_mono	4-hydroxy-L-threonine phosphate dehydrogenase, NAD-dependent (b0052, PdxA, monomer)	C1566H2523N430O453S11	-7	Folding
PdxB_mono	erythronate-4-phosphate dehydrogenase (b2320, PdxB, monomer)	C1846H2955N513O538S9	-4	Folding
PdxH_mono	pyridoxine 5'-phosphate oxidase (b1638, PdxH, monomer)	C1140H1779N331O321S5	4	Folding
PdxJ_mono	pyridoxine 5'-phosphate synthase (b2564, PdxJ, monomer)	C1138H1857N337O350S12	-8	Folding
PdxY_mono	pyridoxal kinase 2/pyridoxine kinase (b1636, PdxY, monomer)	C1395H2202N385O400S13	-7	Folding
PheM_mono	phenylalanyl-tRNA synthetase operon leader peptide (b1715, PheM, monomer)	C83H108N17O18S0	1	Folding
PheS_mono	phenylalanine tRNA synthetase, alpha subunit (b1714, PheS, monomer)	C1634H2541N461O483S10	-8	Folding
PheT_DnaK_GrpE_complex	PheT (b1713) DnaK GrpE_dim complex - Deuerling et al. DnaKJ/GrpE dependent folding	C8722H14118O2767N2455S57P0	-89	Folding

PheT_mono	phenylalanine tRNA synthetase, beta subunit (b1713, PheT, monomer)	C3854H6192N1085O1166S26	-29	Folding
Phe_RS_phe_amp	Phenyl-tRNA synthetase (charged)	C10995H17489N3098O3307S72Zn2P1	-72	tRNA charging
Phe_RS_tetra	Phenyl-tRNA synthetase (uncharged)	C10976H17466N3092O3298S72Zn2	-70	tRNA charging
Phe_RS_tetra_inact	Phenyl-tRNA synthetase (uncharged)	C10976H17466N3092O3298S72Zn2	-70	tRNA charging
PlsX_mono	fatty acid/phospholipid synthesis protein (b1090, PlsX, monomer)	C1684H2796N479O499S11	7	Folding
Pnp_DnaK_GrpE_complex	Pnp (b3164) DnaK GrpE_dim complex - Kerner et al. class I can interact w/ GroEL/ES	C8253H13401O2654N2323S53P0	-84	Folding
Pnp_GroEL.(7)ADP.transGroES	Pnp (b3164) GroEL GroES complex - Kerner et al. class I can interact w/ GroEL/ES	C41297H68419O13366N11537S386P14Mg7	-318	Folding
Pnp_mono	polynucleotide phosphorylase/polyadenylase (b3164, Pnp, monomer)	C3385H5475N953O1053S22	-24	Folding
Pnp_trim	Polynucleotide phosphorylase trimer	C10155H16425N2859O3159S66	-72	mRNA degradation
PrfA_mono	peptide chain release factor RF-1 (b1211, PrfA, monomer)	C1732H2765N525O563S13	-18	Folding
PrfB_mono	peptide chain release factor RF-2 (b2891, PrfB, monomer)	C1783H2796N507O596S11	-27	Folding
PrfC_DnaK_GrpE_complex	PrfC (b4375) DnaK GrpE_dim complex - Deuerling et al. DnaKJ/GrpE dependent folding	C7505H12091O2393N2097S53P0	-72	Folding
PrfC_mono	peptide chain release factor RF-3 (b4375, PrfC, monomer)	C2637H4165N727O792S22	-12	Folding
PriB_mono	primosomal protein N (b4201, PriB, monomer)	C491H796N150O145S6	2	Folding
PrmA_mono	methylase for 50S ribosomal subunit protein L11 (b3259, PrmA, monomer)	C1410H2179N372O439S11	-27	Folding
PrmA_mono_inact	methylase for 50S ribosomal subunit protein L11 (b3259, PrmA, monomer)	C1410H2179N372O439S11	-27	Folding
PrmA_rpL11_cplx	methyltransferase/ L11/ amet complex	C2196H3474N605O680S26	-11	Ribosomal protein modification
PrmB_mono	N5-glutamine methyltransferase (b2330, PrmB, monomer)	C1560H2389N415O469S12	-24	Folding
PrmB_mono_inact	N5-glutamine methyltransferase (b2330, PrmB, monomer)	C1560H2389N415O469S12	-24	Folding
PrmB_rpL3_cplx	protein-(glutamine-N5) methyltransferase/ rpL3/ amet complex	C2554H4028N709O768S17	-13	Ribosomal protein modification
PrmC_RF1_cplx	PrmC/ RF1/ amet complex	C3109H4910N920O982S22	-34	Protein Modification
PrmC_RF2_cplx	PrmC/ RF2/ amet complex	C3160H4941N902O1015S20	-43	Protein Modification
PrmC_mono	N5-glutamine methyltransferase, modifies release factors RF-1 and RF-2 (b1212, PrmC, monomer)	C1362H2122N389O414S8	-17	Folding
PrmC_mono_inact	N5-glutamine methyltransferase, modifies release factors RF-1 and RF-2 (b1212, PrmC, monomer)	C1362H2122N389O414S8	-17	Folding
ProS_DnaK_GrpE_complex	ProS (b0194) DnaK GrpE_dim complex - Deuerling et al. DnaKJ/GrpE dependent folding	C7700H12403O2459N2144S49P0	-79	Folding
ProS_mono	prolyl-tRNA synthetase (b0194, ProS, monomer)	C2832H4477N774O858S18	-19	Folding
Pro_RS_2pro_2amp	Prolyl-tRNA synthetase (charged, 2*prolne, 2*amp)	C5694H8996N1560O1734S36P2	-42	tRNA charging
Pro_RS_dim	Prolyl-tRNA synthetase (uncharged)	C5664H8954N1548O1716S36	-38	tRNA charging
Pro_RS_dim_inact	Prolyl-tRNA synthetase (uncharged)	C5664H8954N1548O1716S36	-38	tRNA charging
Pro_RS_pro_amp	Prolyl-tRNA synthetase (charged)	C5679H8975N1554O1725S36P	-40	tRNA charging
PtsN_mono	sugar-specific enzyme IIA component of PTS (b3204, PtsN, monomer)	C779H1284N222O246S4	-4	Folding
PyrG_DnaK_GrpE_complex	PyrG (b2780) DnaK GrpE_dim complex - Deuerling et al. DnaKJ/GrpE dependent folding	C7540H12179O2402N2112S51P0	-73	Folding
PyrG_mono	CTP synthetase (b2780, PyrG, monomer)	C2672H4253N742O801S20	-13	Folding
QueA_mono	S-adenosylmethionine:tRNA ribosyltransferase-isomerase (b0405, QueA, monomer)	C1762H2732N471O528S10	-17	Folding
QueA_mono_inact	S-adenosylmethionine:tRNA ribosyltransferase-isomerase (b0405, QueA, monomer)	C1762H2732N471O528S10	-17	Folding
QueF_dim	7-cyano-7-deazaguanine reductase (b2794, yqcD)	C2880H4434N812O880S12	-14	tRNA Modification
QueF_dim_inact	7-cyano-7-deazaguanine reductase (b2794, yqcD)	C2880H4434N812O880S12	-14	tRNA Modification
QueF_mono	conserved protein (b2794, QueF, monomer)	C1440H2217N406O440S6	-7	Folding
RF1_mono	peptide chain release factor RF-1 (monomer, methylated)	C1733H2767N525O563S13	-18	Translation
RF1_mono_inact	peptide chain release factor RF-1 (monomer, methylated)	C1733H2767N525O563S13	-18	Translation
RF2_mono	release factor 2 (translation termination) (monomer, Gln 252 methylated)	C1784H2798N507O596S11	-27	Translation
RF2_mono_inact	release factor 2 (translation termination) (monomer, Gln 252 methylated)	C1784H2798N507O596S11	-27	Translation
RF3_mono.GDP	protein release factor RF3 (monomer, no modification) GDP bound	C2647H4177N732O803S22P2	-15	Translation
RF3_mono.GDP_inact	protein release factor RF3 (monomer, no modification) GDP bound	C2647H4177N732O803S22P2	-15	Translation

RI_30	ribosome subunit 30S assembly molecule 1 (21S)	C29179H40379S91N10353O14959P1548	-1408	Ribosomal Assembly
RI_30*	ribosome subunit 30S assembly molecule 1* (acitvated)	C23667H31563N8831O13290S52P1542	-1382	Ribosomal Assembly
RI_50	ribosome subunit 50S assembly molecule 1	C43879H57635N16327O25387S74P3024	-2849.14	Ribosomal Assembly
RI_50*	ribosome subunit 50S assembly molecule 1 (activated)	C43879H57635N16327O25387S74P3024	-2849.14	Ribosomal Assembly
RNAP_19	RNA polymerase, sigma 19	C17532H28315N4914O5393S120ZnMg2	-81	Transcription
RNAP_24	RNA polymerase, sigma 24	C17626H28470N4953O5423S117Zn1Mg2	-81	Transcription
RNAP_28	RNA polymerase, sigma 28	C17866H28869N5038O5507S117ZnMg2	-87	Transcription
RNAP_32	RNA polymerase, sigma 32	C18089H29190N5096O5566S123ZnMg2	-84	Transcription
RNAP_38	RNA polymerase, sigma 38	C18327H29605N5165O5656S117ZnMg2	-96	Transcription
RNAP_54	RNA polymerase, sigma 54	C19031H30690N5331O5898S125ZnMg2	-111	Transcription
RNAP_70	RNAP_70	C19713H31808N5539O6126S141ZnMg2	-123	Transcription
RNase_BN_dim	RNase BN (alias RNase Z) (b2268, ElaC, dimer)	C2924H4604N806O872S18Zn2	-14	RNA cleavage
RNase_E_tetra	RNase E (tetrahomomer)	C20508H33028N6216O6480S72Zn2	-120	RNA cleavage
RNase_E_tetra_inact	RNase E (tetrahomomer)	C20508H33028N6216O6480S72Zn2	-120	RNA cleavage
RNase_G_dim	ribonuclease G (b3247, Rng, dimer)	C4850H7786N1388O1484S36	-26	RNA cleavage
RNase_G_dim_inact	ribonuclease G (b3247, Rng, dimer)	C4850H7786N1388O1484S36	-26	RNA cleavage
RNase_Gen	generic RNase	C2537H3968N700O749S19Mg3Zn1	-11	RNA cleavage
RNase_Gen_inact	generic RNase	C2537H3968N700O749S19Mg3Zn1	-11	RNA cleavage
RNase_III_dim	RNaseIII (homodimer)	C2238H3600N656O680S8Mg2	0	RNA cleavage
RNase_III_dim_inact	RNaseIII (homodimer)	C2238H3600N656O680S8Mg2	0	RNA cleavage
RNase_PH	ribonuclease PH, pseudogene based on Riley annotation		0	RNA cleavage
RNase_P_cplx	RNase P (RnpA_mono + RnpB_RNA)	C4218H5113N1710O2768S2P377Mg2	-358	mRNA degradation
RNase_P_cplx_inact	RNase P (RnpA_mono + RnpB_RNA)	C4218H5113N1710O2768S2P377Mg2	-358	mRNA degradation
RNase_T_dim	ribonuclease T (RNase T) (b1652, Rnt, dimer)	C2082H3188N570O620S20Mg4	-10	RNA cleavage
RNase_T_dim_inact	ribonuclease T (RNase T) (b1652, Rnt, dimer)	C2082H3188N570O620S20Mg4	-10	RNA cleavage
RNase_m16	unknown ribonuclease 16S rRNA		0	RNA cleavage
RNase_m16_inact	unknown ribonuclease 16S rRNA		0	RNA cleavage
RNase_m23	unknown ribonuclease 23S rRNA		0	RNA cleavage
RNase_m23_inact	unknown ribonuclease 23S rRNA		0	RNA cleavage
RNase_m5	unknown ribonuclease 5S rRNA		0	RNA cleavage
RNase_m5_inact	unknown ribonuclease 5S rRNA		0	RNA cleavage
RbfA_mono	30s ribosome binding factor (b3167, RbfA, monomer)	C656H1073N186O202S7	-1	Folding
RbfA_mono_inact	30s ribosome binding factor (b3167, RbfA, monomer)	C656H1073N186O202S7	-1	Folding
Rbn_mono	predicted inner membrane protein (b3886, Rbn, monomer)	C1544H2395N373O392S8	4	Folding
RecG_mono	ATP-dependent DNA helicase (b3652, RecG, monomer)	C3380H5478N971O984S26	-3	Folding
RecJ_mono	ssDNA exonuclease, 5' --> 3'-specific (b2892, RecJ, monomer)	C2808H4480N799O823S20	-15	Folding
RecO_mono	gap repair protein (b2565, RecO, monomer)	C1231H1955N344O344S6	11	Folding
RhlB_DnaK_GrpE_complex	RhlB (b3780) DnaK GrpE_dim complex - Kerner et al. class II can interact w/ GroEL/ES, cannot fold spontanously	C6954H11249O2211N1973S43P0	-60	Folding
RhlB_GroEL.(7)ADP.transGroES	RhlB (b3780) GroEL GroES complex - Kerner et al. class II can interact w/ GroEL/ES, cannot fold spontanously	C39998H66267O12923N11187S376P14Mg7	-294	Folding
RhlB_dim	RNA helicase RhlB dimer	C4172H6646N1206O1220S24	0	mRNA degradation
RhlB_mono	ATP-dependent RNA helicase (b3780, RhlB, monomer)	C2086H3323N603O610S12	0	Folding
RhoL_mono	rho operon leader peptide (b3782, RhoL, monomer)	C148H249N51O51S2	4	Folding
Rho_DnaK_GrpE_complex	Rho (b3783) DnaK GrpE_dim complex - Kerner et al. class II can interact w/ GroEL/ES, cannot fold spontanously	C6942H11303O2222N1956S48P0	-61	Folding
Rho_GroEL.(7)ADP.transGroES	Rho (b3783) GroEL GroES complex - Kerner et al. class II can interact w/ GroEL/ES, cannot fold spontanously	C39986H66321O12934N11170S381P14Mg7	-295	Folding
Rho_hexa	transcription termination factor, hexamer	C12474H20298N3531O3765S102P9Mg3	-12	Transcription
Rho_hexa_inact	transcription termination factor, hexamer	C12474H20298N3531O3765S102P9Mg3	-12	Transcription
Rho_mono	transcription termination factor (b3783, Rho, monomer)	C2074H3377N586O621S17	-1	Folding
RibD_mono	fused diaminohydroxyphosphoribosylaminopyrimidine deaminase -!- 5-amino-6-(5-phosphoribosylamino) uracil reductase (b0414, RibD, monomer)	C1771H2843N519O520S15	0	Folding
RibE_mono	riboflavin synthase beta chain (b0415, RibE, monomer)	C715H1157N195O219S1	-4	Folding

RibF_mono	bifunctional riboflavin kinase and FAD synthetase (b0025, RibF, monomer)	C1546H2504N451O434S8	7	Folding
RimI_mono	acetylase for 30S ribosomal subunit protein S18 (b4373, RimI, monomer)	C735H1137N200O225S3	-7	Folding
RimI_mono_inact	acetylase for 30S ribosomal subunit protein S18 (b4373, RimI, monomer)	C735H1137N200O225S3	-7	Folding
RimI_rps18_cplx	rps18 (b4202_m) rimI acetyl-Coa complex	C1153H1822N330O349S5P3	1	Ribosomal protein modification
RimJ_GroEL.(7)ADP.transGroES	RimJ (b1066) GroEL GroES complex - Kerner et al. class III needs GroEL/ES	C38934H64486O12590N10875S371P14Mg7	-289	Folding
RimJ_mono	ribosomal-protein-S5-alanine N-acetyltransferase (b1066, RimJ, monomer)	C1022H1542N291O277S7	5	Folding
RimJ_mono_inact	ribosomal-protein-S5-alanine N-acetyltransferase (b1066, RimJ, monomer)	C1022H1542N291O277S7	5	Folding
RimJ_rps5_cplx	rps5 (b3296_m) rimJ acetyl-Coa complex	C1806H2849N530O520S14P3	10	Ribosomal protein modification
RimK_mono	ribosomal protein S6 modification protein (b0852, RimK, monomer)	C1416H2329N414O423S12	1	Folding
RimK_mono_inact	ribosomal protein S6 modification protein (b0852, RimK, monomer)	C1416H2329N414O423S12	1	Folding
RimL_DnaK_GrpE_complex	RimL (b1427) DnaK GrpE_dim complex - Kerner et al. class II can interact w/ GroEL/ES, cannot fold spontanously	C5784H9356O1873N1624S37P0	-64	Folding
RimL_GroEL.(7)ADP.transGroES	RimL (b1427) GroEL GroES complex - Kerner et al. class II can interact w/ GroEL/ES, cannot fold spontanously	C38828H64374O12585N10838S370P14Mg7	-298	Folding
RimL_mono	ribosomal-protein-L7/L12-serine acetyltransferase (b1427, RimL, monomer)	C916H1430N254O272S6	-4	Folding
RimL_mono_inact	ribosomal-protein-L7/L12-serine acetyltransferase (b1427, RimL, monomer)	C916H1430N254O272S6	-4	Folding
RimL_rpL7_cplx	rpL7 (b3986) rimL acetyl-CoA complex	C1473H2354N399O465S10P3	-16	Ribosomal protein modification
RimM_DnaK_GrpE_complex	RimM (b2608) DnaK GrpE_dim complex - Kerner et al. class II can interact w/ GroEL/ES, cannot fold spontanously	C5785H9341O1881N1607S38P0	-70	Folding
RimM_GroEL.(7)ADP.transGroES	RimM (b2608) GroEL GroES complex - Kerner et al. class II can interact w/ GroEL/ES, cannot fold spontanously	C38829H64359O12593N10821S371P14Mg7	-304	Folding
RimM_mono	16S rRNA processing protein (b2608, RimM, monomer)	C917H1415N237O280S7	-10	Folding
RimM_mono_inact	16S rRNA processing protein (b2608, RimM, monomer)	C917H1415N237O280S7	-10	Folding
RlmB_dim	23S rRNA (Gm2251)-methyltransferase (b4180, RlmB, dimer)	C2306H3780N678O696S22	-6	tRNA Modification
RlmB_dim_inact	23S rRNA (Gm2251)-methyltransferase (b4180, RlmB, dimer)	C2306H3780N678O696S22	-6	tRNA Modification
RlmB_mono	23S rRNA (Gm2251)-methyltransferase (b4180, RlmB, monomer)	C1153H1890N339O348S11	-3	Folding
RlpB_mono	minor lipoprotein (b0641, RlpB, monomer)	C840H1380N245O268S8	1	Folding
RluA_mono	pseudouridine synthase for 23S rRNA (position 746) and tRNAphe(position 32) (b0058, RluA, monomer)	C1103H1740N313O314S10	1	Folding
RluA_mono_inact	pseudouridine synthase for 23S rRNA (position 746) and tRNAphe(position 32) (b0058, RluA, monomer)	C1103H1740N313O314S10	1	Folding
RluB_DnaK_GrpE_complex	RluB (b1269) DnaK GrpE_dim complex - Kerner et al. class II can interact w/ GroEL/ES, cannot fold spontanously	C6286H10274O2021N1821S36P0	-45	Folding
RluB_GroEL.(7)ADP.transGroES	RluB (b1269) GroEL GroES complex - Kerner et al. class II can interact w/ GroEL/ES, cannot fold spontanously	C39330H65292O12733N11035S369P14Mg7	-279	Folding
RluB_mono	23S rRNA pseudouridylate synthase (b1269, RluB, monomer)	C1418H2348N451O420S5	15	Folding
RluB_mono_inact	23S rRNA pseudouridylate synthase (b1269, RluB, monomer)	C1418H2348N451O420S5	15	Folding
RluC_GroEL.(7)ADP.transGroES	RluC (b1086) GroEL GroES complex - Kerner et al. class III needs GroEL/ES	C39497H65557O12769N11066S370P14Mg7	-279	Folding
RluC_mono	23S rRNA pseudouridylate synthase (b1086, RluC, monomer)	C1585H2613N482O456S6	15	Folding
RluC_mono_inact	23S rRNA pseudouridylate synthase (b1086, RluC, monomer)	C1585H2613N482O456S6	15	Folding
RluD_mono	23S rRNA pseudouridine synthase (b2594, RluD, monomer)	C1637H2610N480O474Mg1S12	-4	Folding
RluD_mono_inact	23S rRNA pseudouridine synthase (b2594, RluD, monomer)	C1637H2610N480O474Mg1S12	-4	Folding
Rnb_mono	ribonuclease II (b1286, Rnb, monomer)	C3219H5071N905O951Mg1S21	-18	Folding
Rnc_mono	RNase III (b2567, Rnc, monomer)	C1119H1800N328O340Mg1S4	0	Folding
Rnd_mono	ribonuclease D (b1804, Rnd, monomer)	C1921H3007N517O552Mg5S17	-2	Folding
Rne_mono	fused ribonucleaseE: endoribonuclease -!-RNA-binding protein -!-RNA degradosome binding protein (b1084, Rne, monomer)	C5127H8257N1554O1620S18	-31	Folding
Rng_mono	ribonuclease G (b3247, Rng, monomer)	C2425H3893N694O742S18	-13	Folding
RnpA_mono	protein C5 component of RNase P (b3704, RnpA, monomer)	C606H1022N198O158S2	16	Folding

RnpB_RNA	RnpB RNA; catalytic subunit of RNAse P (formulae of b3123_1_RNA - 5'-mono-p)	C3612H4091N1512O2610P377	-378	mRNA degradation
Rnr_DnaK_GrpE_complex	Rnr (b4179) DnaK GrpE_dim complex - Kerner et al. class II can interact w/ GroEL/ES, cannot fold spontanously	C8915H14426O2809N2553S60P0	-51	Folding
Rnr_GroEL.(7)ADP.transGroES	Rnr (b4179) GroEL GroES complex - Kerner et al. class II can interact w/ GroEL/ES, cannot fold spontanously	C41959H69444O13521N11767S393P14Mg7	-285	Folding
Rnr_mono	exoribonuclease R, RNase R (b4179, Rnr, monomer)	C4047H6500N1183O1208S29	9	Folding
Rnt_mono	ribonuclease T (RNase T) (b1652, Rnt, monomer)	C1041H1594N285O310Mg2S10	-5	Folding
Rpe_mono	D-ribulose-5-phosphate 3-epimerase (b3386, Rpe, monomer)	C1094H1728N291O325S8	-9	Folding
RplA_mono	50S ribosomal subunit protein L1 (b3984, RplA, monomer)	C1076H1815N314O329S6	9	Folding
RplB_mono	50S ribosomal subunit protein L2 (b3317, RplB, monomer)	C1294H2170N425O366S7	33	Folding
RplB_mono_spmd	50S ribosomal subunit protein L2 (b3317, RplB, monomer) (+ 1 spermidine)	C1301H2192N428O366S7	36	Ribosomal Assembly
RplC_mono	50S ribosomal subunit protein L3 (b3320, RplC, monomer)	C979H1616N288O294S4	10	Folding
RplC_mono_me_spmd	50S ribosomal subunit protein L3 (b3320, RplC, monomer) (Q150 methylated) (+ 1 spermidine)	C987H1640N291O294S4	13	Ribosomal Assembly
RplD_mono	50S ribosomal subunit protein L4 (b3319, RplD, monomer)	C974H1619N283O290S5	8	Folding
RplE_mono	50S ribosomal subunit protein L5 (b3308, RplE, monomer)	C905H1460N251O258S6	7	Folding
RplF_mono	50S ribosomal subunit protein L6 (b3305, RplF, monomer)	C832H1374N243O246S2	9	Folding
RplF_mono_spmd	50S ribosomal subunit protein L6 (b3305, RplF, monomer) (+ 1 spermidine)	C839H1396N246O246S2	12	Ribosomal Assembly
RplI_mono	50S ribosomal subunit protein L9 (b4203, RplI, monomer)	C699H1148N197O214S1	-1	Folding
RplJ_mono	50S ribosomal subunit protein L10 (b3985, RplJ, monomer)	C776H1283N220O231S6	3	Folding
RplK_mono	50S ribosomal subunit protein L11 (b3983, RplK, monomer)	C651H1088N179O196S6	7	Folding
RplK_mono_me3	50S ribosomal subunit protein L11 (b3983, RplK, monomer) (3 trimethylations: 2*lys,1*ala)	C660H1106N179O196S6	7	Ribosomal Assembly
RplL_mono	50S ribosomal subunit protein L7/L12 (b3986, RplL, monomer)	C534H890N138O176S3	-8	Folding
RplL_mono_ac	50S ribosomal subunit protein L7/L12 (b3986, RplL, monomer) (acetylated)	C536H892N138O177S3	-8	Ribosomal Assembly
RplL_mono_me	50S ribosomal subunit protein L7/L12 (b3986, RplL, monomer) (methylated)	C535H892N138O176S3	-8	Ribosomal Assembly
RplM_mono	50S ribosomal subunit protein L13 (b3231, RplM, monomer)	C714H1162N212O199S4	10	Folding
RplN_mono	50S ribosomal subunit protein L14 (b3310, RplN, monomer)	C593H1023N181O167S6	12	Folding
RplO_mono	50S ribosomal subunit protein L15 (b3301, RplO, monomer)	C654H1129N207O190S2	16	Folding
RplO_mono_spmd	50S ribosomal subunit protein L15 (b3301, RplO, monomer) (+ 1 spermidine)	C661H1151N210O190S2	19	Ribosomal Assembly
RplP_mono	50S ribosomal subunit protein L16 (b3313, RplP, monomer)	C686H1157N205O177S6	20	Folding
RplP_mono_me	50S ribosomal subunit protein L16 (b3313, RplP, monomer) (methylated)	C687H1159N205O177S6	20	Ribosomal Assembly
RplQ_mono	50S ribosomal subunit protein L17 (b3294, RplQ, monomer)	C621H1045N204O178S5	13	Folding
RplQ_mono_spmd	50S ribosomal subunit protein L17 (b3294, RplQ, monomer) (+ 1 spermidine)	C628H1067N207O178S5	16	Ribosomal Assembly
RplR_mono	50S ribosomal subunit protein L18 (b3304, RplR, monomer)	C557H935N179O163S1	10	Folding
RplR_mono_spmd	50S ribosomal subunit protein L18 (b3304, RplR, monomer) (+ 1 spermidine)	C564H957N182O163S1	13	Ribosomal Assembly
RplS_mono	50S ribosomal subunit protein L19 (b2606, RplS, monomer)	C574H965N179O163S1	12	Folding
RplT_mono	50S ribosomal subunit protein L20 (b1716, RplT, monomer)	C604H1022N192O151S0	24	Folding
RplU_mono	50S ribosomal subunit protein L21 (b3186, RplU, monomer)	C516H839N153O145S2	6	Folding
RplV_mono	50S ribosomal subunit protein L22 (b3315, RplV, monomer)	C532H922N166O156S3	10	Folding
RplW_mono	50S ribosomal subunit protein L23 (b3318, RplW, monomer)	C496H846N146O143S2	8	Folding
RplX_mono	50S ribosomal subunit protein L24 (b3309, RplX, monomer)	C498H847N148O143S0	11	Folding
RplY_mono	50S ribosomal subunit protein L25 (b2185, RplY, monomer)	C479H780N137O134S3	5	Folding
RpmA_mono	50S ribosomal subunit protein L27 (b3185, RpmA, monomer)	C391H656N129O113S1	11	Folding
RpmB_mono	50S ribosomal subunit protein L28 (b3637, RpmB, monomer)	C388H655N129O106S2	12	Folding
RpmC_mono	50S ribosomal subunit protein L29 (b3312, RpmC, monomer)	C313H543N99O95S2	4	Folding

RpmD_mono	50S ribosomal subunit protein L30 (b3302, RpmD, monomer)	C281H491N87O79S2	6	Folding
RpmE_mono	50S ribosomal subunit protein L31 (b3936, RpmE, monomer)	C339H552N104O100S6	6	Folding
RpmF_mono	50S ribosomal subunit protein L32 (b1089, RpmF, monomer)	C269H461N94O80S1	9	Folding
RpmG_mono	50S ribosomal subunit protein L33 (b3636, RpmG, monomer)	C284H485N81O76S0	10	Folding
RpmG_mono_me	50S ribosomal subunit protein L33 (b3636, RpmG, monomer) (methylated)	C285H487N81O76S0	10	Ribosomal Assembly
RpmH_mono	50S ribosomal subunit protein L34 (b3703, RpmH, monomer)	C228H418N90O57S2	16	Folding
RpmI_mono	50S ribosomal subunit protein L35 (b1717, RpmI, monomer)	C323H574N105O74S2	19	Folding
RpmJ_mono	50S ribosomal subunit protein L36 (b3299, RpmJ, monomer)	C185H343N65O48S4	10	Folding
RpoA_DnaK_GrpE_complex	RpoA (b3295) DnaK GrpE_dim complex - Kerner et al. class I can interact w/ GroEL/ES	C6467H10538O2105N1820S40P0	-78	Folding
RpoA_GroEL.(7)ADP.transGroES	RpoA (b3295) GroEL GroES complex - Kerner et al. class I can interact w/ GroEL/ES	C39511H65556O12817N11034S373P14Mg7	-312	Folding
RpoA_mono	RNA polymerase, alpha subunit (b3295, RpoA, monomer)	C1599H2612N450O504S9	-18	Folding
RpoB_DnaK_GrpE_complex	RpoB (b3987) DnaK GrpE_dim complex - Kerner et al. class II can interact w/ GroEL/ES, cannot fold spontanously	C11509H18529O3658N3213S75P0Zn1	-102	Folding
RpoB_GroEL.(7)ADP.transGroES	RpoB (b3987) GroEL GroES complex - Kerner et al. class II can interact w/ GroEL/ES, cannot fold spontanously	C44553H73547O14370N12427S408P14Mg7Zn1	-336	Folding
RpoB_mono	RNA polymerase, beta subunit (b3987, RpoB, monomer)	C6641H10603N1843O2057Zn1S44	-42	Folding
RpoC_DnaK_GrpE_complex	RpoC (b3988) DnaK GrpE_dim complex - Kerner et al. class II can interact w/ GroEL/ES, cannot fold spontanously	C11700H19040O3669N3310S82P0Mg2	-60	Folding
RpoC_GroEL.(7)ADP.transGroES	RpoC (b3988) GroEL GroES complex - Kerner et al. class II can interact w/ GroEL/ES, cannot fold spontanously	C44744H74058O14381N12524S415P14Mg9	-294	Folding
RpoC_mono	RNA polymerase, beta prime subunit (b3988, RpoC, monomer)	C6832H11114N1940O2068Mg2S51	0	Folding
RpoD_DnaK_GrpE_complex	RpoD (b3067) DnaK GrpE_dim complex - Deuerling et al. DnaKJ/GrpE dependent folding	C7910H12793O2594N2226S59P0	-105	Folding
RpoD_mono	RNA polymerase, sigma 70 (sigma D) factor (b3067, RpoD, monomer)	C3042H4867N856O993S28	-45	Folding
RpoD_mono_inact	RNA polymerase, sigma 70 (sigma D) factor (b3067, RpoD, monomer)	C3042H4867N856O993S28	-45	Folding
RpoE_mono	RNA polymerase, sigma 24 (sigma E) factor (b2573, RpoE, monomer)	C955H1529N270O290S4	-3	Folding
RpoE_mono_inact	RNA polymerase, sigma 24 (sigma E) factor (b2573, RpoE, monomer)	C955H1529N270O290S4	-3	Folding
RpoH_mono	RNA polymerase, sigma 32 (sigma H) factor (b3461, RpoH, monomer)	C1418H2249N413O433S10	-6	Folding
RpoH_mono_inact	RNA polymerase, sigma 32 (sigma H) factor (b3461, RpoH, monomer)	C1418H2249N413O433S10	-6	Folding
RpoN_mono	RNA polymerase, sigma 54 (sigma N) factor (b3202, RpoN, monomer)	C2360H3749N648O765S12	-33	Folding
RpoN_mono_inact	RNA polymerase, sigma 54 (sigma N) factor (b3202, RpoN, monomer)	C2360H3749N648O765S12	-33	Folding
RpoS_mono	RNA polymerase, sigma S (sigma 38) factor (b2741, RpoS, monomer)	C1656H2664N482O523S4	-18	Folding
RpoS_mono_inact	RNA polymerase, sigma S (sigma 38) factor (b2741, RpoS, monomer)	C1656H2664N482O523S4	-18	Folding
RpoZ_mono	RNA polymerase, omega subunit (b3649, RpoZ, monomer)	C430H722N136O142Mg1S1	-2	Folding
RpoZ_mono_inact	RNA polymerase, omega subunit (b3649, RpoZ, monomer)	C430H722N136O142Mg1S1	-2	Folding
RpsA_mono	30S ribosomal subunit protein S1 (b0911, RpsA, monomer)	C2690H4314N751O856S8	-29	Folding
RpsB_mono	30S ribosomal subunit protein S2 (b0169, RpsB, monomer)	C1180H1885N332O352S8	-1	Folding
RpsC_mono	30S ribosomal subunit protein S3 (b3314, RpsC, monomer)	C1149H1913N346O323S4	19	Folding
RpsC_mono_spmd	30S ribosomal subunit protein S3 (b3314, RpsC, monomer) (+ 1 spermidine)	C1156H1935N349O323S4	22	Ribosomal Assembly
RpsD_mono	30S ribosomal subunit protein S4 (b3296, RpsD, monomer)	C1026H1710N315O298S4	17	Folding
RpsD_mono_spmd	30S ribosomal subunit protein S4 (b3296, RpsD, monomer) (+ 1 spermidine)	C1033H1732N318O298S4	20	Ribosomal Assembly
RpsE_mono	30S ribosomal subunit protein S5 (b3303, RpsE, monomer)	C761H1273N232O226S6	9	Folding
RpsE_mono_ac	30S ribosomal subunit protein S5 (b3303, RpsE, monomer) (Alanine N-acetylated)	C763H1275N232O227S6	9	Ribosomal Assembly
RpsF_mono	30S ribosomal subunit protein S6 (b4200, RpsF, monomer)	C657H1026N194O207S7	-8	Folding
RpsF_mono_glu4	30S ribosomal subunit protein S6 (b4200, RpsF, monomer) (4 glutamyl residues)	C677H1050N198O219S7	-12	Ribosomal Assembly

RpsG_mono	30S ribosomal subunit protein S7 (b3341, RpsG, monomer)	C874H1449N269O253S4	14	Folding
RpsH_mono	30S ribosomal subunit protein S8 (b3306, RpsH, monomer)	C616H1034N173O184S6	5	Folding
RpsI_mono	30S ribosomal subunit protein S9 (b3230, RpsI, monomer)	C642H1084N208O183S3	16	Folding
RpsJ_mono	30S ribosomal subunit protein S10 (b3321, RpsJ, monomer)	C514H865N158O151S2	3	Folding
RpsK_mono	30S ribosomal subunit protein S11 (b3297, RpsK, monomer)	C595H997N196O171S3	15	Folding
RpsK_mono_me	30S ribosomal subunit protein S11 (b3297, RpsK, monomer) (A1 methylated)	C596H999N196O171S3	15	Ribosomal Assembly
RpsL_mono	30S ribosomal subunit protein S12 (b3342, RpsL, monomer)	C590H1019N196O165S4	21	Folding
RpsL_mono_me	30S ribosomal subunit protein S12 (b3342, RpsL, monomer) (methylated)	C591H1021N196O165S5	21	Ribosomal Assembly
RpsM_mono	30S ribosomal subunit protein S13 (b3298, RpsM, monomer)	C564H981N183O160S3	14	Folding
RpsN_mono	30S ribosomal subunit protein S14 (b3307, RpsN, monomer)	C499H847N164O139S3	15	Folding
RpsO_mono	30S ribosomal subunit protein S15 (b3165, RpsO, monomer)	C439H737N144O130S1	7	Folding
RpsP_mono	30S ribosomal subunit protein S16 (b2609, RpsP, monomer)	C406H666N128O114S1	6	Folding
RpsQ_mono	30S ribosomal subunit protein S17 (b3311, RpsQ, monomer)	C425H716N124O120S3	6	Folding
RpsR_mono	30S ribosomal subunit protein S18 (b4202, RpsR, monomer)	C395H651N123O107S1	12	Folding
RpsR_mono_ac	30S ribosomal subunit protein S18 (b4202, RpsR, monomer) (acetylated)	C397H653N123O108S1	12	Ribosomal Assembly
RpsS_mono	30S ribosomal subunit protein S19 (b3316, RpsS, monomer)	C464H769N139O122S2	12	Folding
RpsT_mono	30S ribosomal subunit protein S20 (b0023, RpsT, monomer)	C414H722N138O115S3	16	Folding
RpsU_mono	30S ribosomal subunit protein S21 (b3065, RpsU, monomer)	C366H631N125O98S1	14	Folding
Rrf_DnaK_GrpE_complex	Rrf (b0172) DnaK GrpE_dim complex - Kerner et al. class I can interact w/ GroEL/ES	C5756H9419O1888N1633S37P0	-60	Folding
Rrf_GroEL.(7)ADP.transGroES	Rrf (b0172) GroEL GroES complex - Kerner et al. class I can interact w/ GroEL/ES	C38800H64437O12600N10847S370P14Mg7	-294	Folding
Rrf_mono	ribosome recycling factor (b0172, Rrf, monomer)	C888H1493N263O287S6	0	Folding
Rrf_mono_inact	ribosome recycling factor (b0172, Rrf, monomer)	C888H1493N263O287S6	0	Folding
RrmA_dim	23S rRNA m1G745 methyltransferase (b1822, RrmA, dimer, 2 Zn2+)	C2702H4188N758O770S30Zn2	2	tRNA Modification
RrmA_dim_inact	23S rRNA m1G745 methyltransferase (b1822, RrmA, dimer, 2 Zn2+)	C2702H4188N758O770S30Zn2	2	tRNA Modification
RrmA_mono	23S rRNA m1G745 methyltransferase (b1822, RrmA, monomer)	C1351H2094N379O385Zn1S15	1	Folding
RrmJ_mono	23S rRNA methyltransferase (b3179, RrmJ, monomer)	C1032H1666N289O298S10	7	Folding
RrmJ_mono_inact	23S rRNA methyltransferase (b3179, RrmJ, monomer)	C1032H1666N289O298S10	7	Folding
RseA_mono	anti-sigma factor (b2572, RseA, monomer)	C1050H1652N305O332S10	-9	Folding
RseB_mono	anti-sigma factor (b2571, RseB, monomer)	C1472H2341N414O448S9	1	Folding
RseC_mono	RseC protein involved in reduction of the SoxR iron-sulfur cluster (b2570, RseC, monomer)	C742H1191N195O215S7	0	Folding
RsmB_mono	16S rRNA m5C967 methyltransferase, S-adenosyl-L-methionine-dependent (b3289, RsmB, monomer)	C2152H3420N612O623S16	0	Folding
RsmB_mono_inact	16S rRNA m5C967 methyltransferase, S-adenosyl-L-methionine-dependent (b3289, RsmB, monomer)	C2152H3420N612O623S16	0	Folding
RsmC_DnaK_GrpE_complex	RsmC (b4371) DnaK GrpE_dim complex - Kerner et al. class II can interact w/ GroEL/ES, cannot fold spontanously	C6537H10534O2098N1837S41P0	-65	Folding
RsmC_GroEL.(7)ADP.transGroES	RsmC (b4371) GroEL GroES complex - Kerner et al. class II can interact w/ GroEL/ES, cannot fold spontanously	C39581H65552O12810N11051S374P14Mg7	-299	Folding
RsmC_mono	16S RNA m2G1207 methylase (b4371, RsmC, monomer)	C1669H2608N467O497S10	-5	Folding
RsmC_mono_inact	16S RNA m2G1207 methylase (b4371, RsmC, monomer)	C1669H2608N467O497S10	-5	Folding
RsuA_mono	16S rRNA pseudouridylate 516 synthase (b2183, RsuA, monomer)	C1145H1800N328O344S6	-8	Folding
RsuA_mono_inact	16S rRNA pseudouridylate 516 synthase (b2183, RsuA, monomer)	C1145H1800N328O344S6	-8	Folding
RumA_mono	23S rRNA (uracil-5)-methyltransferase (b2785, RumA, monomer)	C2113H3403N612O623Fe4S22	7	Folding
RumA_mono_inact	23S rRNA (uracil-5)-methyltransferase (b2785, RumA, monomer)	C2113H3403N612O623Fe4S22	7	Folding
RumB_mono	23S rRNA m(5)U747 methyltransferase (b0859, RumB, monomer)	C1878H2955N506O529Fe4S27	1	Folding
RumB_mono_inact	23S rRNA m(5)U747 methyltransferase (b0859, RumB, monomer)	C1878H2955N506O529Fe4S27	1	Folding
SecE_mono	preprotein translocase membrane subunit (b3981, SecE, monomer)	C621H1038N166O162S3	6	Folding

SecY_mono	preprotein translocase membrane subunit (b3300, SecY, monomer)	C2254H3574N565O579S17	17	Folding
SelA_deca	selenocysteine synthase, decamer	C22200H36200N6425O6720S160P5	-70	tRNA charging
SelA_deca_inact	selenocysteine synthase, decamer	C22200H36200N6425O6720S160P5	-70	tRNA charging
SelA_mono	selenocysteine synthase (b3591, SelA, monomer)	C2216H3616N642O669S16	-6	Folding
SelB.GTP.sec_tRNA	activated selenocysteinyl-tRNA-specific translation factor bound to selecnocysteine tRNA	C3963H5889N1264O1580S17P98Se1Mg2	-107	tRNA activitation
SelB_mono	selenocysteinyl-tRNA-specific translation factor (b3590, SelB, monomer)	C3043H4832N901O892S17	-11	Folding
SerS_DnaK_GrpE_complex	SerS (b0893) DnaK GrpE_dim complex - Deuerling et al. DnaKJ/GrpE dependent folding	C6981H11292O2253N1976S49P0	-74	Folding
SerS_mono	seryl-tRNA synthetase, also charges selenocysteinyl-tRNA with serine (b0893, SerS, monomer)	C2113H3366N606O652S18	-14	Folding
Ser_RS_2ser_2amp	Serinyl-tRNA synthetase (charged, 2*serine, 2*amp)	C4252H6770N1224O1324S36P2	-32	tRNA charging
Ser_RS_dim	Seryl-tRNA synthetase (uncharged) (Dimer)	C4226H6732N1212O1304S36	-28	tRNA charging
Ser_RS_dim_inact	Seryl-tRNA synthetase (uncharged) (Dimer)	C4226H6732N1212O1304S36	-28	tRNA charging
Ser_RS_ser_amp	Serinyl-tRNA synthetase (charged, 1*serine, 1*amp)	C4239H6751N1218O1314S36P	-30	tRNA charging
SpoT_DnaK_GrpE_complex	SpoT (b3650) DnaK GrpE_dim complex - Kerner et al. class II can interact w/ GroEL/ES, cannot fold spontanously	C8368H13575O2623N2382S61P0	-51	Folding
SpoT_GroEL.(7)ADP.transGroES	SpoT (b3650) GroEL GroES complex - Kerner et al. class II can interact w/ GroEL/ES, cannot fold spontanously	C41412H68593O13335N11596S394P14Mg7	-285	Folding
SpoT_mono	bifunctional (p)ppGpp synthetase II and guanosine-3',5'-bis pyrophosphate 3'-pyrophosphohydrolase (b3650, SpoT, monomer)	C3500H5649N1012O1022S30	9	Folding
Sra_mono	30S ribosomal subunit protein S22 (b1480, Sra, monomer)	C214H377N76O66S1	7	Folding
SurA_mono	peptidyl-prolyl cis-trans isomerase (PPIase) (b0053, SurA, monomer)	C2012H3263N598O628S16	-3	Folding
TU-8389_DNA_act	DNA transcription unit TU-8389 (activated form)	C2135H2457N841O1297P219	-219	Transcription
TU-8389_DNA_neu	DNA transcription unit TU-8389 (inactive form)	C2135H2457N841O1297P219	-219	Transcription
TU-8390_DNA_act	DNA transcription unit TU-8390 (activated form)	C2135H2457N841O1297P219	-219	Transcription
TU-8390_DNA_neu	DNA transcription unit TU-8390 (inactive form)	C2135H2457N841O1297P219	-219	Transcription
TU-8392_DNA_act	DNA transcription unit TU-8392 (activated form)	C35487H40831N13983O21753P3639	-3639	Transcription
TU-8392_DNA_neu	DNA transcription unit TU-8392 (inactive form)	C35487H40831N13983O21753P3639	-3639	Transcription
TU-8392_mRNA	mRNA TU-8392	C34685H39228N13983O25399P3641	-3642	Transcription
TU-8392_mRNA_cleav_cplx	TU-8392 mRNA /RNase III cleavage complex	C36923H42828N14639O26079S8P3641Mg2Zn0Fe0	-3642	mRNA cleavage
TU-8397_DNA_act	DNA transcription unit TU-8397 (activated form)	C4666H5357N1847O2836P477	-477	Transcription
TU-8397_DNA_neu	DNA transcription unit TU-8397 (inactive form)	C4666H5357N1847O2836P477	-477	Transcription
TU-8398_DNA_act	DNA transcription unit TU-8398 (activated form)	C39211H45191N15317O24035P4026	-4026	Transcription
TU-8398_DNA_neu	DNA transcription unit TU-8398 (inactive form)	C39211H45191N15317O24035P4026	-4026	Transcription
TU-8398_mRNA	mRNA TU-8398	C38306H43382N15317O28068P4028	-4029	Transcription
TU-8398_mRNA_cleav_cplx	TU-8398 mRNA /RNase III cleavage complex	C40544H46982N15973O28748S8P4028Mg2Zn0Fe0	-4029	mRNA cleavage
TU-8407_DNA_act	DNA transcription unit TU-8407 (activated form)	C30973H35661N12311O18933P3186	-3186	Transcription
TU-8407_DNA_neu	DNA transcription unit TU-8407 (inactive form)	C30973H35661N12311O18933P3186	-3186	Transcription
TU0-1181_DNA_act	DNA transcription unit TU0-1181 (activated form)	C49931H57235N20065O30366P5106	-5106	Transcription
TU0-1181_DNA_neu	DNA transcription unit TU0-1181 (inactive form)	C49931H57235N20065O30366P5106	-5106	Transcription
TU0-1181_RNA	RNA TU0-1181	C48862H55098N20065O35479P5108	-5109	RNA cutting
TU0-1181_RNA_cut_cplx	TU0-1181 RNA /RNase III, RNAse m16, RNase m23, RNase m5, RNase P, RNase F, RNase E cutting complex	C85295H111781N31305O48260S157P5485Mg11Zn3Fe0	-5634	RNA cutting
TU0-1182_DNA_act	DNA transcription unit TU0-1182 (activated form)	C49844H57138N20038O30314P5098	-5098	Transcription
TU0-1182_DNA_neu	DNA transcription unit TU0-1182 (inactive form)	C49844H57138N20038O30314P5098	-5098	Transcription
TU0-1182_RNA	RNA TU0-1182	C48784H55019N20038O35419P5100	-5101	RNA cutting
TU0-1182_RNA_cut_cplx	TU0-1182 RNA /RNase III, RNAse m16, RNase m23, RNase m5, RNase P, RNase F, RNase E cutting complex	C85217H111702N31278O48200S157P5477Mg11Zn3Fe0	-5626	RNA cutting
TU0-1183_DNA_act	DNA transcription unit TU0-1183 (activated form)	C49000H56169N19709O29799P5012	-5012	Transcription
TU0-1183_DNA_neu	DNA transcription unit TU0-1183 (inactive form)	C49000H56169N19709O29799P5012	-5012	Transcription

TU0-1183_RNA	RNA TU0-1183	C47963H54096N19709O34818P5014	-5015	RNA cutting
TU0-1183_RNA_cut_cplx	TU0-1183 RNA /RNase III, RNAse m16, RNase m23, RNase m5, RNase P, RNase F, RNase E cutting complex	C84396H110779N30949O47599S157P5391Mg11Zn3Fe0	-5540	RNA cutting
TU0-1184_DNA_act	DNA transcription unit TU0-1184 (activated form)	C49000H56169N19709O29799P5012	-5012	Transcription
TU0-1184_DNA_neu	DNA transcription unit TU0-1184 (inactive form)	C49000H56169N19709O29799P5012	-5012	Transcription
TU0-1184_RNA	RNA TU0-1184	C47963H54096N19709O34818P5014	-5015	RNA cutting
TU0-1184_RNA_cut_cplx	TU0-1184 RNA /RNase III, RNAse m16, RNase m23, RNase m5, RNase P, RNase F, RNase E cutting complex	C84396H110779N30949O47599S157P5391Mg11Zn3Fe0	-5540	RNA cutting
TU0-1186_DNA_act	DNA transcription unit TU0-1186 (activated form)	C49011H56183N19707O29811P5013	-5013	Transcription
TU0-1186_DNA_neu	DNA transcription unit TU0-1186 (inactive form)	C49011H56183N19707O29811P5013	-5013	Transcription
TU0-1186_RNA	RNA TU0-1186	C47971H54104N19707O34831P5015	-5016	RNA cutting
TU0-1186_RNA_cut_cplx	TU0-1186 RNA /RNase III, RNAse m16, RNase m23, RNase m5, RNase P, RNase F, RNase E cutting complex	C84404H110787N30947O47612S157P5392Mg11Zn3Fe0	-5541	RNA cutting
TU0-1187_DNA_act	DNA transcription unit TU0-1187 (activated form)	C49753H57040N19988O30269P5089	-5089	Transcription
TU0-1187_DNA_neu	DNA transcription unit TU0-1187 (inactive form)	C49753H57040N19988O30269P5089	-5089	Transcription
TU0-1187_RNA	RNA TU0-1187	C48692H54919N19988O35365P5091	-5092	RNA cutting
TU0-1187_RNA_cut_cplx	TU0-1187 RNA /RNase III, RNAse m16, RNase m23, RNase m5, RNase P, RNase F, RNase E cutting complex	C85125H111602N31228O48146S157P5468Mg11Zn3Fe0	-5617	RNA cutting
TU0-1188_DNA_act	DNA transcription unit TU0-1188 (activated form)	C49753H57040N19988O30269P5089	-5089	Transcription
TU0-1188_DNA_neu	DNA transcription unit TU0-1188 (inactive form)	C49753H57040N19988O30269P5089	-5089	Transcription
TU0-1188_RNA	RNA TU0-1188	C48692H54919N19988O35365P5091	-5092	RNA cutting
TU0-1188_RNA_cut_cplx	TU0-1188 RNA /RNase III, RNAse m16, RNase m23, RNase m5, RNase P, RNase F, RNase E cutting complex	C85125H111602N31228O48146S157P5468Mg11Zn3Fe0	-5617	RNA cutting
TU0-1189_DNA_act	DNA transcription unit TU0-1189 (activated form)	C49918H57224N20054O30365P5105	-5105	Transcription
TU0-1189_DNA_neu	DNA transcription unit TU0-1189 (inactive form)	C49918H57224N20054O30365P5105	-5105	Transcription
TU0-1189_RNA	RNA TU0-1189	C48849H55087N20054O35477P5107	-5108	RNA cutting
TU0-1189_RNA_cut_cplx	TU0-1189 RNA /RNase III, RNAse m16, RNase m23, RNase m5, RNase P, RNase F, RNase E cutting complex	C85282H111770N31294O48258S157P5484Mg11Zn3Fe0	-5633	RNA cutting
TU0-1190_DNA_act	DNA transcription unit TU0-1190 (activated form)	C49918H57224N20054O30365P5105	-5105	Transcription
TU0-1190_DNA_neu	DNA transcription unit TU0-1190 (inactive form)	C49918H57224N20054O30365P5105	-5105	Transcription
TU0-1190_RNA	RNA TU0-1190	C48849H55087N20054O35477P5107	-5108	RNA cutting
TU0-1190_RNA_cut_cplx	TU0-1190 RNA /RNase III, RNAse m16, RNase m23, RNase m5, RNase P, RNase F, RNase E cutting complex	C85282H111770N31294O48258S157P5484Mg11Zn3Fe0	-5633	RNA cutting
TU0-1191_DNA_act	DNA transcription unit TU0-1191 (activated form)	C52206H59857N20961O31769P5340	-5340	Transcription
TU0-1191_DNA_neu	DNA transcription unit TU0-1191 (inactive form)	C52206H59857N20961O31769P5340	-5340	Transcription
TU0-1191_RNA	RNA TU0-1191	C51089H57624N20961O37116P5342	-5343	RNA cutting
TU0-1191_RNA_cut_cplx	TU0-1191 RNA /RNase III, RNAse m16, RNase m23, RNase m5, RNase P, RNase F, RNase E cutting complex	C87522H114307N32201O49897S157P5719Mg11Zn3Fe0	-5868	RNA cutting
TU0-1192_DNA_act	DNA transcription unit TU0-1192 (activated form)	C52206H59857N20961O31769P5340	-5340	Transcription
TU0-1192_DNA_neu	DNA transcription unit TU0-1192 (inactive form)	C52206H59857N20961O31769P5340	-5340	Transcription
TU0-1192_RNA	RNA TU0-1192	C51089H57624N20961O37116P5342	-5343	RNA cutting
TU0-1192_RNA_cut_cplx	TU0-1192 RNA /RNase III, RNAse m16, RNase m23, RNase m5, RNase P, RNase F, RNase E cutting complex	C87522H114307N32201O49897S157P5719Mg11Zn3Fe0	-5868	RNA cutting
TU0-12803_DNA_act	DNA transcription unit TU0-12803 (activated form)	C34846H40135N13691O21403P3578	-3578	Transcription
TU0-12803_DNA_neu	DNA transcription unit TU0-12803 (inactive form)	C34846H40135N13691O21403P3578	-3578	Transcription
TU0-12803_mRNA	mRNA TU0-12803	C34069H38582N13691O24988P3580	-3581	Transcription
TU0-12803_mRNA_cleav_cplx	TU0-12803 mRNA /RNase III cleavage complex	C36307H42182N14347O25668S8P3580Mg2Zn0Fe0	-3581	mRNA cleavage
TU0-12827_DNA_act	DNA transcription unit TU0-12827 (activated form)	C42680H49225N16597O26239P4384	-4384	Transcription
TU0-12827_DNA_neu	DNA transcription unit TU0-12827 (inactive form)	C42680H49225N16597O26239P4384	-4384	Transcription
TU0-12827_mRNA	mRNA TU0-12827	C41679H47224N16597O30630P4386	-4387	Transcription
TU0-12827_mRNA_cleav_cplx	TU0-12827 mRNA /RNase III cleavage complex	C43917H50824N17253O31310S8P4386Mg2Zn0Fe0	-4387	mRNA cleavage
TU0-12830_DNA_act	DNA transcription unit TU0-12830 (activated form)	C5450H6249N2197O3296P558	-558	Transcription
TU0-12830_DNA_neu	DNA transcription unit TU0-12830 (inactive form)	C5450H6249N2197O3296P558	-558	Transcription
TU0-12833_DNA_act	DNA transcription unit TU0-12833 (activated form)	C12653H14569N4981O7809P1299	-1299	Transcription

TU0-12833_DNA_neu	DNA transcription unit TU0-12833 (inactive form)	C12653H14569N4981O7809P1299	-1299	Transcription
TU0-12921_DNA_act	DNA transcription unit TU0-12921 (activated form)	C24906H28661N9819O15260P2555	-2555	Transcription
TU0-12921_DNA_neu	DNA transcription unit TU0-12921 (inactive form)	C24906H28661N9819O15260P2555	-2555	Transcription
TU0-12921_mRNA	mRNA TU0-12921	C24350H27550N9819O17822P2557	-2558	Transcription
TU0-12921_mRNA_cleav_cplx	TU0-12921 mRNA /RNase III cleavage complex	C26588H31150N10475O18502S8P2557Mg2Zn0Fe0	-2558	mRNA cleavage
TU0-12928_DNA_act	DNA transcription unit TU0-12928 (activated form)	C12692H14548N5122O7678P1299	-1299	Transcription
TU0-12928_DNA_neu	DNA transcription unit TU0-12928 (inactive form)	C12692H14548N5122O7678P1299	-1299	Transcription
TU0-13010_DNA_act	DNA transcription unit TU0-13010 (activated form)	C53484H61607N20931O32798P5488	-5488	Transcription
TU0-13010_DNA_neu	DNA transcription unit TU0-13010 (inactive form)	C53484H61607N20931O32798P5488	-5488	Transcription
TU0-13010_mRNA	mRNA TU0-13010	C52245H59130N20931O38293P5490	-5491	Transcription
TU0-13010_mRNA_cleav_cplx	TU0-13010 mRNA /RNase III cleavage complex	C54483H62730N21587O38973S8P5490Mg2Zn0Fe0	-5491	mRNA cleavage
TU0-13018_DNA_act	DNA transcription unit TU0-13018 (activated form)	C13876H15983N5453O8513P1425	-1425	Transcription
TU0-13018_DNA_neu	DNA transcription unit TU0-13018 (inactive form)	C13876H15983N5453O8513P1425	-1425	Transcription
TU0-13020_DNA_act	DNA transcription unit TU0-13020 (activated form)	C16227H18691N6351O9951P1665	-1665	Transcription
TU0-13020_DNA_neu	DNA transcription unit TU0-13020 (inactive form)	C16227H18691N6351O9951P1665	-1665	Transcription
TU0-13034_DNA_act	DNA transcription unit TU0-13034 (activated form)	C1507H1741N581O934P155	-155	Transcription
TU0-13034_DNA_neu	DNA transcription unit TU0-13034 (inactive form)	C1507H1741N581O934P155	-155	Transcription
TU0-13034_RNA	RNA TU0-13034	C1471H1670N581O1096P157	-158	RNA cutting
TU0-13034_RNA_cut_cplx	TU0-13034 RNA /RNase III, RNAse m16, RNase m23, RNase m5, RNase P, RNase F, RNase E cutting complex	C37904H58353N11821O13877S157P534Mg11Zn3Fe0	-683	RNA cutting
TU0-13035_DNA_act	DNA transcription unit TU0-13035 (activated form)	C742H857N281O460P76	-76	Transcription
TU0-13035_DNA_neu	DNA transcription unit TU0-13035 (inactive form)	C742H857N281O460P76	-76	Transcription
TU0-13035_RNA	tRNA	C721H816N281O543P78	-79	RNA cutting
TU0-13035_RNA_cut_cplx	TU0-13035 RNA /RNase P (5' trimming), RNase_Gen (T, PH, II, D, or BN), RNase E (3' trimming) cutting complex	C27984H42925N8907O10540S93P455Mg5Zn3Fe0	-568	RNA cutting
TU0-13036_DNA_act	DNA transcription unit TU0-13036 (activated form)	C742H857N281O460P76	-76	Transcription
TU0-13036_DNA_neu	DNA transcription unit TU0-13036 (inactive form)	C742H857N281O460P76	-76	Transcription
TU0-13036_RNA	tRNA	C721H816N281O543P78	-79	RNA cutting
TU0-13036_RNA_cut_cplx	TU0-13036 RNA /RNase P (5' trimming), RNase_Gen (T, PH, II, D, or BN), RNase E (3' trimming) cutting complex	C27984H42925N8907O10540S93P455Mg5Zn3Fe0	-568	RNA cutting
TU0-13072_DNA_act	DNA transcription unit TU0-13072 (activated form)	C16149H18644N6192O10008P1657	-1657	Transcription
TU0-13072_DNA_neu	DNA transcription unit TU0-13072 (inactive form)	C16149H18644N6192O10008P1657	-1657	Transcription
TU0-13072_mRNA	mRNA TU0-13072	C15732H17811N6192O11672P1659	-1660	Transcription
TU0-13072_mRNA_cleav_cplx	TU0-13072 mRNA /RNase III cleavage complex	C17970H21411N6848O12352S8P1659Mg2Zn0Fe0	-1660	mRNA cleavage
TU0-13080_DNA_act	DNA transcription unit TU0-13080 (activated form)	C12610H14512N4958O7724P1293	-1293	Transcription
TU0-13080_DNA_neu	DNA transcription unit TU0-13080 (inactive form)	C12610H14512N4958O7724P1293	-1293	Transcription
TU0-13093_DNA_act	DNA transcription unit TU0-13093 (activated form)	C13659H15759N5259O8435P1401	-1401	Transcription
TU0-13093_DNA_neu	DNA transcription unit TU0-13093 (inactive form)	C13659H15759N5259O8435P1401	-1401	Transcription
TU0-13104_DNA_act	DNA transcription unit TU0-13104 (activated form)	C3236H3708N1288O1960P330	-330	Transcription
TU0-13104_DNA_neu	DNA transcription unit TU0-13104 (inactive form)	C3236H3708N1288O1960P330	-330	Transcription
TU0-13134_DNA_act	DNA transcription unit TU0-13134 (activated form)	C9388H10784N3704O5742P960	-960	Transcription
TU0-13134_DNA_neu	DNA transcription unit TU0-13134 (inactive form)	C9388H10784N3704O5742P960	-960	Transcription
TU0-13139_DNA_act	DNA transcription unit TU0-13139 (activated form)	C33565H38669N13169O20627P3447	-3447	Transcription
TU0-13139_DNA_neu	DNA transcription unit TU0-13139 (inactive form)	C33565H38669N13169O20627P3447	-3447	Transcription
TU0-13145_DNA_act	DNA transcription unit TU0-13145 (activated form)	C10968H12639N4269O6724P1125	-1125	Transcription
TU0-13145_DNA_neu	DNA transcription unit TU0-13145 (inactive form)	C10968H12639N4269O6724P1125	-1125	Transcription
TU0-13145_mRNA	mRNA TU0-13145	C10704H12112N4269O7856P1127	-1128	Transcription
TU0-13145_mRNA_cleav_cplx	TU0-13145 mRNA /RNase III cleavage complex	C12942H15712N4925O8536S8P1127Mg2Zn0Fe0	-1128	mRNA cleavage
TU0-13195_DNA_act	DNA transcription unit TU0-13195 (activated form)	C8555H9840N3358O5259P876	-876	Transcription

TU0-13195_DNA_neu	DNA transcription unit TU0-13195 (inactive form)	C8555H9840N3358O5259P876	-876	Transcription
TU0-13225_DNA_act	DNA transcription unit TU0-13225 (activated form)	C9151H10519N3593O5581P936	-936	Transcription
TU0-13225_DNA_neu	DNA transcription unit TU0-13225 (inactive form)	C9151H10519N3593O5581P936	-936	Transcription
TU0-13421_DNA_act	DNA transcription unit TU0-13421 (activated form)	C7903H9110N3056O4866P810	-810	Transcription
TU0-13421_DNA_neu	DNA transcription unit TU0-13421 (inactive form)	C7903H9110N3056O4866P810	-810	Transcription
TU0-13443_DNA_act	DNA transcription unit TU0-13443 (activated form)	C20959H24200N7982O12984P2148	-2148	Transcription
TU0-13443_DNA_neu	DNA transcription unit TU0-13443 (inactive form)	C20959H24200N7982O12984P2148	-2148	Transcription
TU0-13443_mRNA	mRNA TU0-13443	C20387H23057N7982O15139P2150	-2151	Transcription
TU0-13443_mRNA_cleav_cplx	TU0-13443 mRNA /RNase III cleavage complex	C22625H26657N8638O15819S8P2150Mg2Zn0Fe0	-2151	mRNA cleavage
TU0-13444_DNA_act	DNA transcription unit TU0-13444 (activated form)	C16904H19439N6703O10324P1734	-1734	Transcription
TU0-13444_DNA_neu	DNA transcription unit TU0-13444 (inactive form)	C16904H19439N6703O10324P1734	-1734	Transcription
TU0-13519_DNA_act	DNA transcription unit TU0-13519 (activated form)	C19806H22836N7716O12173P2034	-2034	Transcription
TU0-13519_DNA_neu	DNA transcription unit TU0-13519 (inactive form)	C19806H22836N7716O12173P2034	-2034	Transcription
TU0-13534_DNA_act	DNA transcription unit TU0-13534 (activated form)	C9266H10641N3673O5680P948	-948	Transcription
TU0-13534_DNA_neu	DNA transcription unit TU0-13534 (inactive form)	C9266H10641N3673O5680P948	-948	Transcription
TU0-13555_DNA_act	DNA transcription unit TU0-13555 (activated form)	C6781H7822N2624O4180P696	-696	Transcription
TU0-13555_DNA_neu	DNA transcription unit TU0-13555 (inactive form)	C6781H7822N2624O4180P696	-696	Transcription
TU0-13557_DNA_act	DNA transcription unit TU0-13557 (activated form)	C2778H3191N1113O1681P285	-285	Transcription
TU0-13557_DNA_neu	DNA transcription unit TU0-13557 (inactive form)	C2778H3191N1113O1681P285	-285	Transcription
TU0-13573_DNA_act	DNA transcription unit TU0-13573 (activated form)	C8947H10323N3449O5506P918	-918	Transcription
TU0-13573_DNA_neu	DNA transcription unit TU0-13573 (inactive form)	C8947H10323N3449O5506P918	-918	Transcription
TU0-13593_DNA_act	DNA transcription unit TU0-13593 (activated form)	C44318H51120N17176O27335P4550	-4550	Transcription
TU0-13593_DNA_neu	DNA transcription unit TU0-13593 (inactive form)	C44318H51120N17176O27335P4550	-4550	Transcription
TU0-13593_mRNA	mRNA TU0-13593	C43248H48981N17176O31892P4552	-4553	Transcription
TU0-13593_mRNA_cleav_cplx	TU0-13593 mRNA /RNase III cleavage complex	C45486H52581N17832O32572S8P4552Mg2Zn0Fe0	-4553	mRNA cleavage
TU0-13667_DNA_act	DNA transcription unit TU0-13667 (activated form)	C11685H13476N4488O7236P1197	-1197	Transcription
TU0-13667_DNA_neu	DNA transcription unit TU0-13667 (inactive form)	C11685H13476N4488O7236P1197	-1197	Transcription
TU0-13667_mRNA	mRNA TU0-13667	C11376H12859N4488O8440P1199	-1200	Transcription
TU0-13667_mRNA_cleav_cplx	TU0-13667 mRNA /RNase III cleavage complex	C13614H16459N5144O9120S8P1199Mg2Zn0Fe0	-1200	mRNA cleavage
TU0-13675_DNA_act	DNA transcription unit TU0-13675 (activated form)	C16662H19200N6486O10265P1709	-1709	Transcription
TU0-13675_DNA_neu	DNA transcription unit TU0-13675 (inactive form)	C16662H19200N6486O10265P1709	-1709	Transcription
TU0-13675_mRNA	mRNA TU0-13675	C16261H18399N6486O11981P1711	-1712	Transcription
TU0-13675_mRNA_cleav_cplx	TU0-13675 mRNA /RNase III cleavage complex	C18499H21999N7142O12661S8P1711Mg2Zn0Fe0	-1712	mRNA cleavage
TU0-13704_DNA_act	DNA transcription unit TU0-13704 (activated form)	C739H853N287O457P76	-76	Transcription
TU0-13704_DNA_neu	DNA transcription unit TU0-13704 (inactive form)	C739H853N287O457P76	-76	Transcription
TU0-13704_RNA	tRNA	C722H820N287O540P78	-79	RNA cutting
TU0-13704_RNA_cut_cplx	TU0-13704 RNA /RNase P (5' trimming), RNase_Gen (T, PH, II, D, or BN), RNase E (3' trimming) cutting complex	C27985H42929N8913O10537S93P455Mg5Zn3Fe0	-568	RNA cutting
TU0-13717_DNA_act	DNA transcription unit TU0-13717 (activated form)	C25673H29534N10102O15752P2631	-2631	Transcription
TU0-13717_DNA_neu	DNA transcription unit TU0-13717 (inactive form)	C25673H29534N10102O15752P2631	-2631	Transcription
TU0-13734_DNA_act	DNA transcription unit TU0-13734 (activated form)	C29499H33969N11523O18073P3024	-3024	Transcription
TU0-13734_DNA_neu	DNA transcription unit TU0-13734 (inactive form)	C29499H33969N11523O18073P3024	-3024	Transcription
TU0-13734_mRNA	mRNA TU0-13734	C28794H32560N11523O21104P3026	-3027	Transcription
TU0-13734_mRNA_cleav_cplx	TU0-13734 mRNA /RNase III cleavage complex	C31032H36160N12179O21784S8P3026Mg2Zn0Fe0	-3027	mRNA cleavage
TU0-13735_DNA_act	DNA transcription unit TU0-13735 (activated form)	C12699H14623N4965O7782P1302	-1302	Transcription
TU0-13735_DNA_neu	DNA transcription unit TU0-13735 (inactive form)	C12699H14623N4965O7782P1302	-1302	Transcription
TU0-13736_DNA_act	DNA transcription unit TU0-13736 (activated form)	C15404H17731N6037O9461P1579	-1579	Transcription
TU0-13736_DNA_neu	DNA transcription unit TU0-13736 (inactive form)	C15404H17731N6037O9461P1579	-1579	Transcription

TU0-13736_mRNA	mRNA TU0-13736	C15042H17008N6037O11047P1581	-1582	Transcription
TU0-13736_mRNA_cleav_cplx	TU0-13736 mRNA /RNase III cleavage complex	C17280H20608N6693O11727S8P1581Mg2Zn0Fe0	-1582	mRNA cleavage
TU0-13738_DNA_act	DNA transcription unit TU0-13738 (activated form)	C8282H9553N3187O5088P849	-849	Transcription
TU0-13738_DNA_neu	DNA transcription unit TU0-13738 (inactive form)	C8282H9553N3187O5088P849	-849	Transcription
TU0-13805_DNA_act	DNA transcription unit TU0-13805 (activated form)	C16522H19018N6500O10123P1695	-1695	Transcription
TU0-13805_DNA_neu	DNA transcription unit TU0-13805 (inactive form)	C16522H19018N6500O10123P1695	-1695	Transcription
TU0-13805_mRNA	mRNA TU0-13805	C16149H18273N6500O11825P1697	-1698	Transcription
TU0-13805_mRNA_cleav_cplx	TU0-13805 mRNA /RNase III cleavage complex	C18387H21873N7156O12505S8P1697Mg2Zn0Fe0	-1698	mRNA cleavage
TU0-13809_DNA_act	DNA transcription unit TU0-13809 (activated form)	C10229H11752N4030O6260P1046	-1046	Transcription
TU0-13809_DNA_neu	DNA transcription unit TU0-13809 (inactive form)	C10229H11752N4030O6260P1046	-1046	Transcription
TU0-13809_mRNA	mRNA TU0-13809	C9983H11261N4030O7313P1048	-1049	Transcription
TU0-13809_mRNA_cleav_cplx	TU0-13809 mRNA /RNase III cleavage complex	C12221H14861N4686O7993S8P1048Mg2Zn0Fe0	-1049	mRNA cleavage
TU0-13915_DNA_act	DNA transcription unit TU0-13915 (activated form)	C17200H19860N6554O10635P1763	-1763	Transcription
TU0-13915_DNA_neu	DNA transcription unit TU0-13915 (inactive form)	C17200H19860N6554O10635P1763	-1763	Transcription
TU0-13915_mRNA	mRNA TU0-13915	C16733H18927N6554O12405P1765	-1766	Transcription
TU0-13915_mRNA_cleav_cplx	TU0-13915 mRNA /RNase III cleavage complex	C18971H22527N7210O13085S8P1765Mg2Zn0Fe0	-1766	mRNA cleavage
TU0-13931_DNA_act	DNA transcription unit TU0-13931 (activated form)	C4660H5353N1847O2835P477	-477	Transcription
TU0-13931_DNA_neu	DNA transcription unit TU0-13931 (inactive form)	C4660H5353N1847O2835P477	-477	Transcription
TU0-14007_DNA_act	DNA transcription unit TU0-14007 (activated form)	C921H1065N357O576P95	-95	Transcription
TU0-14007_DNA_neu	DNA transcription unit TU0-14007 (inactive form)	C921H1065N357O576P95	-95	Transcription
TU0-14007_RNA	tRNA	C901H1026N357O678P97	-98	RNA cutting
TU0-14007_RNA_cut_cplx	TU0-14007 RNA /RNase P (5' trimming), RNase_Gen (T, PH, II, D, or BN), RNase E (3' trimming) cutting complex	C28164H43135N8983O10675S93P474Mg5Zn3Fe0	-587	RNA cutting
TU0-14021_DNA_act	DNA transcription unit TU0-14021 (activated form)	C13284H15303N5229O8170P1365	-1365	Transcription
TU0-14021_DNA_neu	DNA transcription unit TU0-14021 (inactive form)	C13284H15303N5229O8170P1365	-1365	Transcription
TU0-14029_DNA_act	DNA transcription unit TU0-14029 (activated form)	C25105H28941N9773O15436P2577	-2577	Transcription
TU0-14029_DNA_neu	DNA transcription unit TU0-14029 (inactive form)	C25105H28941N9773O15436P2577	-2577	Transcription
TU0-14029_mRNA	mRNA TU0-14029	C24511H27754N9773O18020P2579	-2580	Transcription
TU0-14029_mRNA_cleav_cplx	TU0-14029 mRNA /RNase III cleavage complex	C26749H31354N10429O18700S8P2579Mg2Zn0Fe0	-2580	mRNA cleavage
TU0-14037_DNA_act	DNA transcription unit TU0-14037 (activated form)	C12331H14221N4787O7585P1266	-1266	Transcription
TU0-14037_DNA_neu	DNA transcription unit TU0-14037 (inactive form)	C12331H14221N4787O7585P1266	-1266	Transcription
TU0-14038_DNA_act	DNA transcription unit TU0-14038 (activated form)	C14092H16252N5456O8642P1446	-1446	Transcription
TU0-14038_DNA_neu	DNA transcription unit TU0-14038 (inactive form)	C14092H16252N5456O8642P1446	-1446	Transcription
TU0-14038_mRNA	mRNA TU0-14038	C13746H15561N5456O10095P1448	-1449	Transcription
TU0-14038_mRNA_cleav_cplx	TU0-14038 mRNA /RNase III cleavage complex	C15984H19161N6112O10775S8P1448Mg2Zn0Fe0	-1449	mRNA cleavage
TU0-14070_DNA_act	DNA transcription unit TU0-14070 (activated form)	C28139H32447N10885O17361P2886	-2886	Transcription
TU0-14070_DNA_neu	DNA transcription unit TU0-14070 (inactive form)	C28139H32447N10885O17361P2886	-2886	Transcription
TU0-14070_mRNA	mRNA TU0-14070	C27438H31046N10885O20254P2888	-2889	Transcription
TU0-14070_mRNA_cleav_cplx	TU0-14070 mRNA /RNase III cleavage complex	C29676H34646N11541O20934S8P2888Mg2Zn0Fe0	-2889	mRNA cleavage
TU0-14100_DNA_act	DNA transcription unit TU0-14100 (activated form)	C11543H13297N4525O7082P1185	-1185	Transcription
TU0-14100_DNA_neu	DNA transcription unit TU0-14100 (inactive form)	C11543H13297N4525O7082P1185	-1185	Transcription
TU0-14118_DNA_act	DNA transcription unit TU0-14118 (activated form)	C10138H11655N3995O6221P1038	-1038	Transcription
TU0-14118_DNA_neu	DNA transcription unit TU0-14118 (inactive form)	C10138H11655N3995O6221P1038	-1038	Transcription
TU0-14151_DNA_act	DNA transcription unit TU0-14151 (activated form)	C5327H6123N2113O3259P546	-546	Transcription
TU0-14151_DNA_neu	DNA transcription unit TU0-14151 (inactive form)	C5327H6123N2113O3259P546	-546	Transcription
TU0-14215_DNA_act	DNA transcription unit TU0-14215 (activated form)	C10048H11593N3893O6217P1032	-1032	Transcription
TU0-14215_DNA_neu	DNA transcription unit TU0-14215 (inactive form)	C10048H11593N3893O6217P1032	-1032	Transcription
TU0-14216_DNA_act	DNA transcription unit TU0-14216 (activated form)	C14798H17069N5767O9108P1521	-1521	Transcription
TU0-14216_DNA_neu	DNA transcription unit TU0-14216 (inactive form)	C14798H17069N5767O9108P1521	-1521	Transcription

TU0-14216_mRNA	mRNA TU0-14216	C14460H16394N5767O10636P1523	-1524	Transcription
TU0-14216_mRNA_cleav_cplx	TU0-14216 mRNA /RNase III cleavage complex	C16698H19994N6423O11316S8P1523Mg2Zn0Fe0	-1524	mRNA cleavage
TU0-14217_DNA_act	DNA transcription unit TU0-14217 (activated form)	C15489H17837N6087O9503P1590	-1590	Transcription
TU0-14217_DNA_neu	DNA transcription unit TU0-14217 (inactive form)	C15489H17837N6087O9503P1590	-1590	Transcription
TU0-14252_DNA_act	DNA transcription unit TU0-14252 (activated form)	C12692H14548N5122O7678P1299	-1299	Transcription
TU0-14252_DNA_neu	DNA transcription unit TU0-14252 (inactive form)	C12692H14548N5122O7678P1299	-1299	Transcription
TU0-1881_DNA_act	DNA transcription unit TU0-1881 (activated form)	C25058H28840N9838O15345P2569	-2569	Transcription
TU0-1881_DNA_neu	DNA transcription unit TU0-1881 (inactive form)	C25058H28840N9838O15345P2569	-2569	Transcription
TU0-1881_mRNA	mRNA TU0-1881	C24477H27679N9838O17921P2571	-2572	Transcription
TU0-1881_mRNA_cleav_cplx	TU0-1881 mRNA /RNase III cleavage complex	C26715H31279N10494O18601S8P2571Mg2Zn0Fe0	-2572	mRNA cleavage
TU0-1_DNA_act	DNA transcription unit TU0-1 (activated form)	C39403H45348N15446O24204P4038	-4038	Transcription
TU0-1_DNA_neu	DNA transcription unit TU0-1 (inactive form)	C39403H45348N15446O24204P4038	-4038	Transcription
TU0-1_mRNA	mRNA TU0-1	C38473H43489N15446O28249P4040	-4041	Transcription
TU0-1_mRNA_cleav_cplx	TU0-1 mRNA /RNase III cleavage complex	C40711H47089N16102O28929S8P4040Mg2Zn0Fe0	-4041	mRNA cleavage
TU0-2081_DNA_act	DNA transcription unit TU0-2081 (activated form)	C33565H38669N13169O20627P3447	-3447	Transcription
TU0-2081_DNA_neu	DNA transcription unit TU0-2081 (inactive form)	C33565H38669N13169O20627P3447	-3447	Transcription
TU0-2082_DNA_act	DNA transcription unit TU0-2082 (activated form)	C33565H38669N13169O20627P3447	-3447	Transcription
TU0-2082_DNA_neu	DNA transcription unit TU0-2082 (inactive form)	C33565H38669N13169O20627P3447	-3447	Transcription
TU0-2101_DNA_act	DNA transcription unit TU0-2101 (activated form)	C24601H28363N9491O15195P2521	-2521	Transcription
TU0-2101_DNA_neu	DNA transcription unit TU0-2101 (inactive form)	C24601H28363N9491O15195P2521	-2521	Transcription
TU0-2101_mRNA	mRNA TU0-2101	C23969H27100N9491O17723P2523	-2524	Transcription
TU0-2101_mRNA_cleav_cplx	TU0-2101 mRNA /RNase III cleavage complex	C26207H30700N10147O18403S8P2523Mg2Zn0Fe0	-2524	mRNA cleavage
TU0-2121_DNA_act	DNA transcription unit TU0-2121 (activated form)	C14322H16509N5523O8854P1467	-1467	Transcription
TU0-2121_DNA_neu	DNA transcription unit TU0-2121 (inactive form)	C14322H16509N5523O8854P1467	-1467	Transcription
TU0-2121_mRNA	mRNA TU0-2121	C13950H15766N5523O10328P1469	-1470	Transcription
TU0-2121_mRNA_cleav_cplx	TU0-2121 mRNA /RNase III cleavage complex	C16188H19366N6179O11008S8P1469Mg2Zn0Fe0	-1470	mRNA cleavage
TU0-3304_DNA_act	DNA transcription unit TU0-3304 (activated form)	C5313H6083N2151O3199P543	-543	Transcription
TU0-3304_DNA_neu	DNA transcription unit TU0-3304 (inactive form)	C5313H6083N2151O3199P543	-543	Transcription
TU0-3305_DNA_act	DNA transcription unit TU0-3305 (activated form)	C5313H6083N2151O3199P543	-543	Transcription
TU0-3305_DNA_neu	DNA transcription unit TU0-3305 (inactive form)	C5313H6083N2151O3199P543	-543	Transcription
TU0-3364_DNA_act	DNA transcription unit TU0-3364 (activated form)	C49844H57138N20038O30314P5098	-5098	Transcription
TU0-3364_DNA_neu	DNA transcription unit TU0-3364 (inactive form)	C49844H57138N20038O30314P5098	-5098	Transcription
TU0-3364_RNA	RNA TU0-3364	C48784H55019N20038O35419P5100	-5101	RNA cutting
TU0-3364_RNA_cut_cplx	TU0-3364 RNA /RNase III, RNAse m16, RNase m23, RNase m5, RNase P, RNase F, RNase E cutting complex	C85217H111702N31278O48200S157P5477Mg11Zn3Fe0	-5626	RNA cutting
TU0-3561_DNA_act	DNA transcription unit TU0-3561 (activated form)	C742H856N284O460P76	-76	Transcription
TU0-3561_DNA_neu	DNA transcription unit TU0-3561 (inactive form)	C742H856N284O460P76	-76	Transcription
TU0-3561_RNA	tRNA	C722H817N284O543P78	-79	RNA cutting
TU0-3561_RNA_cut_cplx	TU0-3561 RNA /RNase P (5' trimming), RNase_Gen (T, PH, II, D, or BN), RNase E (3' trimming) cutting complex	C27985H42926N8910O10540S93P455Mg5Zn3Fe0	-568	RNA cutting
TU0-3901_DNA_act	DNA transcription unit TU0-3901 (activated form)	C1346H1543N547O801P138	-138	Transcription
TU0-3901_DNA_neu	DNA transcription unit TU0-3901 (inactive form)	C1346H1543N547O801P138	-138	Transcription
TU0-4925_DNA_act	DNA transcription unit TU0-4925 (activated form)	C11543H13297N4525O7082P1185	-1185	Transcription
TU0-4925_DNA_neu	DNA transcription unit TU0-4925 (inactive form)	C11543H13297N4525O7082P1185	-1185	Transcription
TU0-5003_DNA_act	DNA transcription unit TU0-5003 (activated form)	C2880H3306N1134O1757P294	-294	Transcription
TU0-5003_DNA_neu	DNA transcription unit TU0-5003 (inactive form)	C2880H3306N1134O1757P294	-294	Transcription
TU0-5121_DNA_act	DNA transcription unit TU0-5121 (activated form)	C32863H37842N12860O20134P3370	-3370	Transcription
TU0-5121_DNA_neu	DNA transcription unit TU0-5121 (inactive form)	C32863H37842N12860O20134P3370	-3370	Transcription
TU0-5121_mRNA	mRNA TU0-5121	C32091H36299N12860O23511P3372	-3373	Transcription
TU0-5121_mRNA_cleav_cplx	TU0-5121 mRNA /RNase III cleavage complex	C34329H39899N13516O24191S8P3372Mg2Zn0Fe0	-3373	mRNA cleavage

TU0-5182_DNA_act	DNA transcription unit TU0-5182 (activated form)	C36788H42394N14254O22698P3770	-3770	Transcription
TU0-5182_DNA_neu	DNA transcription unit TU0-5182 (inactive form)	C36788H42394N14254O22698P3770	-3770	Transcription
TU0-5182_mRNA	mRNA TU0-5182	C35864H40547N14254O26475P3772	-3773	Transcription
TU0-5182_mRNA_cleav_cplx	TU0-5182 mRNA /RNase III cleavage complex	C38102H44147N14910O27155S8P3772Mg2Zn0Fe0	-3773	mRNA cleavage
TU0-5183_DNA_act	DNA transcription unit TU0-5183 (activated form)	C25053H28866N9732O15443P2568	-2568	Transcription
TU0-5183_DNA_neu	DNA transcription unit TU0-5183 (inactive form)	C25053H28866N9732O15443P2568	-2568	Transcription
TU0-5183_mRNA	mRNA TU0-5183	C24435H27631N9732O18018P2570	-2571	Transcription
TU0-5183_mRNA_cleav_cplx	TU0-5183 mRNA /RNase III cleavage complex	C26673H31231N10388O18698S8P2570Mg2Zn0Fe0	-2571	mRNA cleavage
TU0-5186_DNA_act	DNA transcription unit TU0-5186 (activated form)	C19969H23029N7769O12296P2051	-2051	Transcription
TU0-5186_DNA_neu	DNA transcription unit TU0-5186 (inactive form)	C19969H23029N7769O12296P2051	-2051	Transcription
TU0-5186_mRNA	mRNA TU0-5186	C19501H22094N7769O14354P2053	-2054	Transcription
TU0-5186_mRNA_cleav_cplx	TU0-5186 mRNA /RNase III cleavage complex	C21739H25694N8425O15034S8P2053Mg2Zn0Fe0	-2054	mRNA cleavage
TU0-5201_DNA_act	DNA transcription unit TU0-5201 (activated form)	C29552H34101N11467O18235P3037	-3037	Transcription
TU0-5201_DNA_neu	DNA transcription unit TU0-5201 (inactive form)	C29552H34101N11467O18235P3037	-3037	Transcription
TU0-5201_mRNA	mRNA TU0-5201	C28858H32714N11467O21279P3039	-3040	Transcription
TU0-5201_mRNA_cleav_cplx	TU0-5201 mRNA /RNase III cleavage complex	C31096H36314N12123O21959S8P3039Mg2Zn0Fe0	-3040	mRNA cleavage
TU0-5221_DNA_act	DNA transcription unit TU0-5221 (activated form)	C17583H20303N6801O10863P1808	-1808	Transcription
TU0-5221_DNA_neu	DNA transcription unit TU0-5221 (inactive form)	C17583H20303N6801O10863P1808	-1808	Transcription
TU0-5221_mRNA	mRNA TU0-5221	C17168H19474N6801O12678P1810	-1811	Transcription
TU0-5221_mRNA_cleav_cplx	TU0-5221 mRNA /RNase III cleavage complex	C19406H23074N7457O13358S8P1810Mg2Zn0Fe0	-1811	mRNA cleavage
TU0-5522_DNA_act	DNA transcription unit TU0-5522 (activated form)	C21663H24937N8421O13299P2217	-2217	Transcription
TU0-5522_DNA_neu	DNA transcription unit TU0-5522 (inactive form)	C21663H24937N8421O13299P2217	-2217	Transcription
TU0-5522_mRNA	mRNA TU0-5522	C21113H23838N8421O15523P2219	-2220	Transcription
TU0-5522_mRNA_cleav_cplx	TU0-5522 mRNA /RNase III cleavage complex	C23351H27438N9077O16203S8P2219Mg2Zn0Fe0	-2220	mRNA cleavage
TU0-5543_DNA_act	DNA transcription unit TU0-5543 (activated form)	C13010H14972N5080O7981P1332	-1332	Transcription
TU0-5543_DNA_neu	DNA transcription unit TU0-5543 (inactive form)	C13010H14972N5080O7981P1332	-1332	Transcription
TU0-5543_mRNA	mRNA TU0-5543	C12690H14333N5080O9320P1334	-1335	Transcription
TU0-5543_mRNA_cleav_cplx	TU0-5543 mRNA /RNase III cleavage complex	C14928H17933N5736O10000S8P1334Mg2Zn0Fe0	-1335	mRNA cleavage
TU0-5601_DNA_act	DNA transcription unit TU0-5601 (activated form)	C12802H14738N4970O7839P1310	-1310	Transcription
TU0-5601_DNA_neu	DNA transcription unit TU0-5601 (inactive form)	C12802H14738N4970O7839P1310	-1310	Transcription
TU0-5601_mRNA	mRNA TU0-5601	C12474H14083N4970O9156P1312	-1313	Transcription
TU0-5601_mRNA_cleav_cplx	TU0-5601 mRNA /RNase III cleavage complex	C14712H17683N5626O9836S8P1312Mg2Zn0Fe0	-1313	mRNA cleavage
TU0-6223_DNA_act	DNA transcription unit TU0-6223 (activated form)	C90330H103877N35655O55242P9257	-9257	Transcription
TU0-6223_DNA_neu	DNA transcription unit TU0-6223 (inactive form)	C90330H103877N35655O55242P9257	-9257	Transcription
TU0-6223_mRNA	mRNA TU0-6223	C88280H99778N35655O64506P9259	-9260	Transcription
TU0-6223_mRNA_cleav_cplx	TU0-6223 mRNA /RNase III cleavage complex	C90518H103378N36311O65186S8P9259Mg2Zn0Fe0	-9260	mRNA cleavage
TU0-6405_DNA_act	DNA transcription unit TU0-6405 (activated form)	C27801H32005N10995O17017P2856	-2856	Transcription
TU0-6405_DNA_neu	DNA transcription unit TU0-6405 (inactive form)	C27801H32005N10995O17017P2856	-2856	Transcription
TU0-6409_DNA_act	DNA transcription unit TU0-6409 (activated form)	C13806H15899N5403O8470P1416	-1416	Transcription
TU0-6409_DNA_neu	DNA transcription unit TU0-6409 (inactive form)	C13806H15899N5403O8470P1416	-1416	Transcription
TU0-6423_DNA_act	DNA transcription unit TU0-6423 (activated form)	C5291H6083N2053O3220P540	-540	Transcription
TU0-6423_DNA_neu	DNA transcription unit TU0-6423 (inactive form)	C5291H6083N2053O3220P540	-540	Transcription
TU0-6441_DNA_act	DNA transcription unit TU0-6441 (activated form)	C25673H29534N10102O15752P2631	-2631	Transcription
TU0-6441_DNA_neu	DNA transcription unit TU0-6441 (inactive form)	C25673H29534N10102O15752P2631	-2631	Transcription
TU0-6504_DNA_act	DNA transcription unit TU0-6504 (activated form)	C16227H18691N6351O9951P1665	-1665	Transcription
TU0-6504_DNA_neu	DNA transcription unit TU0-6504 (inactive form)	C16227H18691N6351O9951P1665	-1665	Transcription
TU0-6506_DNA_act	DNA transcription unit TU0-6506 (activated form)	C12437H14320N4864O7663P1275	-1275	Transcription
TU0-6506_DNA_neu	DNA transcription unit TU0-6506 (inactive form)	C12437H14320N4864O7663P1275	-1275	Transcription
TU0-6512_DNA_act	DNA transcription unit TU0-6512 (activated form)	C108473H124903N42523O66475P11127	-11127	Transcription

TU0-6512_DNA_neu	DNA transcription unit TU0-6512 (inactive form)	C108473H124903N42523O66475P11127	-11127	Transcription
TU0-6512_mRNA	mRNA TU0-6512	C105967H119892N42523O77609P11129	-11130	Transcription
TU0-6512_mRNA_cleav_cplx	TU0-6512 mRNA /RNase III cleavage complex	C108205H123492N43179O78289S8P11129Mg2Zn0Fe0	-11130	mRNA cleavage
TU0-6550_DNA_act	DNA transcription unit TU0-6550 (activated form)	C13806H15899N5403O8470P1416	-1416	Transcription
TU0-6550_DNA_neu	DNA transcription unit TU0-6550 (inactive form)	C13806H15899N5403O8470P1416	-1416	Transcription
TU0-6551_DNA_act	DNA transcription unit TU0-6551 (activated form)	C13806H15899N5403O8470P1416	-1416	Transcription
TU0-6551_DNA_neu	DNA transcription unit TU0-6551 (inactive form)	C13806H15899N5403O8470P1416	-1416	Transcription
TU0-6562_DNA_act	DNA transcription unit TU0-6562 (activated form)	C16747H19279N6599O10272P1719	-1719	Transcription
TU0-6562_DNA_neu	DNA transcription unit TU0-6562 (inactive form)	C16747H19279N6599O10272P1719	-1719	Transcription
TU0-6626_DNA_act	DNA transcription unit TU0-6626 (activated form)	C39183H45106N15372O24049P4018	-4018	Transcription
TU0-6626_DNA_neu	DNA transcription unit TU0-6626 (inactive form)	C39183H45106N15372O24049P4018	-4018	Transcription
TU0-6626_mRNA	mRNA TU0-6626	C38275H43291N15372O28074P4020	-4021	Transcription
TU0-6626_mRNA_cleav_cplx	TU0-6626 mRNA /RNase III cleavage complex	C40513H46891N16028O28754S8P4020Mg2Zn0Fe0	-4021	mRNA cleavage
TU0-6654_DNA_act	DNA transcription unit TU0-6654 (activated form)	C21491H24745N8437O13189P2205	-2205	Transcription
TU0-6654_DNA_neu	DNA transcription unit TU0-6654 (inactive form)	C21491H24745N8437O13189P2205	-2205	Transcription
TU0-6657_DNA_act	DNA transcription unit TU0-6657 (activated form)	C3484H4015N1349O2133P357	-357	Transcription
TU0-6657_DNA_neu	DNA transcription unit TU0-6657 (inactive form)	C3484H4015N1349O2133P357	-357	Transcription
TU0-6658_DNA_act	DNA transcription unit TU0-6658 (activated form)	C8333H9585N3301O5098P855	-855	Transcription
TU0-6658_DNA_neu	DNA transcription unit TU0-6658 (inactive form)	C8333H9585N3301O5098P855	-855	Transcription
TU0-6660_DNA_act	DNA transcription unit TU0-6660 (activated form)	C25850H29772N10120O15871P2652	-2652	Transcription
TU0-6660_DNA_neu	DNA transcription unit TU0-6660 (inactive form)	C25850H29772N10120O15871P2652	-2652	Transcription
TU0-6660_mRNA	mRNA TU0-6660	C25250H28573N10120O18530P2654	-2655	Transcription
TU0-6660_mRNA_cleav_cplx	TU0-6660 mRNA /RNase III cleavage complex	C27488H32173N10776O19210S8P2654Mg2Zn0Fe0	-2655	mRNA cleavage
TU0-6687_DNA_act	DNA transcription unit TU0-6687 (activated form)	C27801H32005N10995O17017P2856	-2856	Transcription
TU0-6687_DNA_neu	DNA transcription unit TU0-6687 (inactive form)	C27801H32005N10995O17017P2856	-2856	Transcription
TU0-6941_DNA_act	DNA transcription unit TU0-6941 (activated form)	C14092H16252N5456O8642P1446	-1446	Transcription
TU0-6941_DNA_neu	DNA transcription unit TU0-6941 (inactive form)	C14092H16252N5456O8642P1446	-1446	Transcription
TU0-6941_mRNA	mRNA TU0-6941	C13746H15561N5456O10095P1448	-1449	Transcription
TU0-6941_mRNA_cleav_cplx	TU0-6941 mRNA /RNase III cleavage complex	C15984H19161N6112O10775S8P1448Mg2Zn0Fe0	-1449	mRNA cleavage
TU0-7002_DNA_act	DNA transcription unit TU0-7002 (activated form)	C81147H93465N31821O49773P8329	-8329	Transcription
TU0-7002_DNA_neu	DNA transcription unit TU0-7002 (inactive form)	C81147H93465N31821O49773P8329	-8329	Transcription
TU0-7002_mRNA	mRNA TU0-7002	C79301H89774N31821O58109P8331	-8332	Transcription
TU0-7002_mRNA_cleav_cplx	TU0-7002 mRNA /RNase III cleavage complex	C81539H93374N32477O58789S8P8331Mg2Zn0Fe0	-8332	mRNA cleavage
TU0-7141_DNA_act	DNA transcription unit TU0-7141 (activated form)	C4448H5118N1732O2705P455	-455	Transcription
TU0-7141_DNA_neu	DNA transcription unit TU0-7141 (inactive form)	C4448H5118N1732O2705P455	-455	Transcription
TU0-7141_mRNA	mRNA TU0-7141	C4335H4893N1732O3167P457	-458	Transcription
TU0-7141_mRNA_cleav_cplx	TU0-7141 mRNA /RNase III cleavage complex	C6573H8493N2388O3847S8P457Mg2Zn0Fe0	-458	mRNA cleavage
TU0-7281_DNA_act	DNA transcription unit TU0-7281 (activated form)	C21461H24741N8245O13230P2197	-2197	Transcription
TU0-7281_DNA_neu	DNA transcription unit TU0-7281 (inactive form)	C21461H24741N8245O13230P2197	-2197	Transcription
TU0-7281_mRNA	mRNA TU0-7281	C20887H23594N8245O15434P2199	-2200	Transcription
TU0-7281_mRNA_cleav_cplx	TU0-7281 mRNA /RNase III cleavage complex	C23125H27194N8901O16114S8P2199Mg2Zn0Fe0	-2200	mRNA cleavage
TU0-7842_DNA_act	DNA transcription unit TU0-7842 (activated form)	C2574H2956N1032O1547P264	-264	Transcription
TU0-7842_DNA_neu	DNA transcription unit TU0-7842 (inactive form)	C2574H2956N1032O1547P264	-264	Transcription
TU0-7844_DNA_act	DNA transcription unit TU0-7844 (activated form)	C2574H2956N1032O1547P264	-264	Transcription
TU0-7844_DNA_neu	DNA transcription unit TU0-7844 (inactive form)	C2574H2956N1032O1547P264	-264	Transcription
TU0-8084_DNA_act	DNA transcription unit TU0-8084 (activated form)	C17279H19906N6754O10624P1773	-1773	Transcription
TU0-8084_DNA_neu	DNA transcription unit TU0-8084 (inactive form)	C17279H19906N6754O10624P1773	-1773	Transcription
TU0-8281_DNA_act	DNA transcription unit TU0-8281 (activated form)	C5710H6586N2192O3516P585	-585	Transcription

TU0-8281_DNA_neu	DNA transcription unit TU0-8281 (inactive form)	C5710H6586N2192O3516P585	-585	Transcription
TU0-8464_DNA_act	DNA transcription unit TU0-8464 (activated form)	C3590H4111N1435O2169P366	-366	Transcription
TU0-8464_DNA_neu	DNA transcription unit TU0-8464 (inactive form)	C3590H4111N1435O2169P366	-366	Transcription
TU0-8474_DNA_act	DNA transcription unit TU0-8474 (activated form)	C2624H3029N1021O1608P270	-270	Transcription
TU0-8474_DNA_neu	DNA transcription unit TU0-8474 (inactive form)	C2624H3029N1021O1608P270	-270	Transcription
TU0-8476_DNA_act	DNA transcription unit TU0-8476 (activated form)	C744H859N297O459P77	-77	Transcription
TU0-8476_DNA_neu	DNA transcription unit TU0-8476 (inactive form)	C744H859N297O459P77	-77	Transcription
TU0-8476_RNA	tRNA	C732H836N297O543P79	-80	RNA cutting
TU0-8476_RNA_cut_cplx	TU0-8476 RNA /RNase P (5' trimming), RNase_Gen (T, PH, II, D, or BN), RNase E (3' trimming) cutting complex	C27995H42945N8923O10540S93P456Mg5Zn3Fe0	-569	RNA cutting
TU0-8477_DNA_act	DNA transcription unit TU0-8477 (activated form)	C13512H15541N5349O8275P1386	-1386	Transcription
TU0-8477_DNA_neu	DNA transcription unit TU0-8477 (inactive form)	C13512H15541N5349O8275P1386	-1386	Transcription
TU0-8502_DNA_act	DNA transcription unit TU0-8502 (activated form)	C37219H42812N14708O22798P3817	-3817	Transcription
TU0-8502_DNA_neu	DNA transcription unit TU0-8502 (inactive form)	C37219H42812N14708O22798P3817	-3817	Transcription
TU0-8502_mRNA	mRNA TU0-8502	C36394H41163N14708O26622P3819	-3820	Transcription
TU0-8502_mRNA_cleav_cplx	TU0-8502 mRNA /RNase III cleavage complex	C38632H44763N15364O27302S8P3819Mg2Zn0Fe0	-3820	mRNA cleavage
TU0-8505_DNA_act	DNA transcription unit TU0-8505 (activated form)	C8522H9784N3406O5177P873	-873	Transcription
TU0-8505_DNA_neu	DNA transcription unit TU0-8505 (inactive form)	C8522H9784N3406O5177P873	-873	Transcription
TU0-8506_DNA_act	DNA transcription unit TU0-8506 (activated form)	C2073H2391N807O1266P213	-213	Transcription
TU0-8506_DNA_neu	DNA transcription unit TU0-8506 (inactive form)	C2073H2391N807O1266P213	-213	Transcription
TU0-8510_DNA_act	DNA transcription unit TU0-8510 (activated form)	C2393H2767N913O1484P246	-246	Transcription
TU0-8510_DNA_neu	DNA transcription unit TU0-8510 (inactive form)	C2393H2767N913O1484P246	-246	Transcription
TU0-8529_DNA_act	DNA transcription unit TU0-8529 (activated form)	C14132H16283N5497O8689P1449	-1449	Transcription
TU0-8529_DNA_neu	DNA transcription unit TU0-8529 (inactive form)	C14132H16283N5497O8689P1449	-1449	Transcription
TU0-8823_DNA_act	DNA transcription unit TU0-8823 (activated form)	C19590H22563N7617O12068P2007	-2007	Transcription
TU0-8823_DNA_neu	DNA transcription unit TU0-8823 (inactive form)	C19590H22563N7617O12068P2007	-2007	Transcription
TU0-8855_DNA_act	DNA transcription unit TU0-8855 (activated form)	C5116H5907N1943O3146P524	-524	Transcription
TU0-8855_DNA_neu	DNA transcription unit TU0-8855 (inactive form)	C5116H5907N1943O3146P524	-524	Transcription
TU0-8855_mRNA	mRNA TU0-8855	C4973H5622N1943O3677P526	-527	Transcription
TU0-8855_mRNA_cleav_cplx	TU0-8855 mRNA /RNase III cleavage complex	C7211H9222N2599O4357S8P526Mg2Zn0Fe0	-527	mRNA cleavage
TU0-8862_DNA_act	DNA transcription unit TU0-8862 (activated form)	C9805H11282N3854O6002P1005	-1005	Transcription
TU0-8862_DNA_neu	DNA transcription unit TU0-8862 (inactive form)	C9805H11282N3854O6002P1005	-1005	Transcription
TU0-8864_DNA_act	DNA transcription unit TU0-8864 (activated form)	C29104H33518N11330O17878P2982	-2982	Transcription
TU0-8864_DNA_neu	DNA transcription unit TU0-8864 (inactive form)	C29104H33518N11330O17878P2982	-2982	Transcription
TU0-8864_mRNA	mRNA TU0-8864	C28388H32087N11330O20867P2984	-2985	Transcription
TU0-8864_mRNA_cleav_cplx	TU0-8864 mRNA /RNase III cleavage complex	C30626H35687N11986O21547S8P2984Mg2Zn0Fe0	-2985	mRNA cleavage
TU0-8865_DNA_act	DNA transcription unit TU0-8865 (activated form)	C21446H24679N8401O13151P2197	-2197	Transcription
TU0-8865_DNA_neu	DNA transcription unit TU0-8865 (inactive form)	C21446H24679N8401O13151P2197	-2197	Transcription
TU0-8865_mRNA	mRNA TU0-8865	C20934H23656N8401O15355P2199	-2200	Transcription
TU0-8865_mRNA_cleav_cplx	TU0-8865 mRNA /RNase III cleavage complex	C23172H27256N9057O16035S8P2199Mg2Zn0Fe0	-2200	mRNA cleavage
TU00021_DNA_act	DNA transcription unit TU00021 (activated form)	C12567H14462N4932O7682P1288	-1288	Transcription
TU00021_DNA_neu	DNA transcription unit TU00021 (inactive form)	C12567H14462N4932O7682P1288	-1288	Transcription
TU00021_mRNA	mRNA TU00021	C12273H13875N4932O8977P1290	-1291	Transcription
TU00021_mRNA_cleav_cplx	TU00021 mRNA /RNase III cleavage complex	C14511H17475N5588O9657S8P1290Mg2Zn0Fe0	-1291	mRNA cleavage
TU00083_DNA_act	DNA transcription unit TU00083 (activated form)	C8333H9585N3301O5098P855	-855	Transcription
TU00083_DNA_neu	DNA transcription unit TU00083 (inactive form)	C8333H9585N3301O5098P855	-855	Transcription
TU00084_DNA_act	DNA transcription unit TU00084 (activated form)	C8333H9585N3301O5098P855	-855	Transcription
TU00084_DNA_neu	DNA transcription unit TU00084 (inactive form)	C8333H9585N3301O5098P855	-855	Transcription
TU00221_DNA_act	DNA transcription unit TU00221 (activated form)	C14840H17095N5719O9104P1518	-1518	Transcription

TU00221_DNA_neu	DNA transcription unit TU00221 (inactive form)	C14840H17095N5719O9104P1518	-1518	Transcription
TU00236_DNA_act	DNA transcription unit TU00236 (activated form)	C99775H114849N39305O61243P10239	-10239	Transcription
TU00236_DNA_neu	DNA transcription unit TU00236 (inactive form)	C99775H114849N39305O61243P10239	-10239	Transcription
TU00236_mRNA	mRNA TU00236	C97555H110410N39305O71489P10241	-10242	Transcription
TU00236_mRNA_cleav_cplx	TU00236 mRNA /RNase III cleavage complex	C99793H114010N39961O72169S8P10241Mg2Zn0Fe0	-10242	mRNA cleavage
TU00258_DNA_act	DNA transcription unit TU00258 (activated form)	C14368H16537N5621O8792P1472	-1472	Transcription
TU00258_DNA_neu	DNA transcription unit TU00258 (inactive form)	C14368H16537N5621O8792P1472	-1472	Transcription
TU00258_mRNA	mRNA TU00258	C14023H15848N5621O10271P1474	-1475	Transcription
TU00258_mRNA_cleav_cplx	TU00258 mRNA /RNase III cleavage complex	C16261H19448N6277O10951S8P1474Mg2Zn0Fe0	-1475	mRNA cleavage
TU00260_DNA_act	DNA transcription unit TU00260 (activated form)	C30586H35142N12194O18618P3136	-3136	Transcription
TU00260_DNA_neu	DNA transcription unit TU00260 (inactive form)	C30586H35142N12194O18618P3136	-3136	Transcription
TU00260_mRNA	mRNA TU00260	C29940H33851N12194O21761P3138	-3139	Transcription
TU00260_mRNA_cleav_cplx	TU00260 mRNA /RNase III cleavage complex	C32178H37451N12850O22441S8P3138Mg2Zn0Fe0	-3139	mRNA cleavage
TU00277_DNA_act	DNA transcription unit TU00277 (activated form)	C26012H29930N10186O15967P2664	-2664	Transcription
TU00277_DNA_neu	DNA transcription unit TU00277 (inactive form)	C26012H29930N10186O15967P2664	-2664	Transcription
TU00277_mRNA	mRNA TU00277	C25386H28679N10186O18638P2666	-2667	Transcription
TU00277_mRNA_cleav_cplx	TU00277 mRNA /RNase III cleavage complex	C27624H32279N10842O19318S8P2666Mg2Zn0Fe0	-2667	mRNA cleavage
TU00281_DNA_act	DNA transcription unit TU00281 (activated form)	C29116H33543N11441O17905P2991	-2991	Transcription
TU00281_DNA_neu	DNA transcription unit TU00281 (inactive form)	C29116H33543N11441O17905P2991	-2991	Transcription
TU00281_mRNA	mRNA TU00281	C28474H32260N11441O20903P2993	-2994	Transcription
TU00281_mRNA_cleav_cplx	TU00281 mRNA /RNase III cleavage complex	C30712H35860N12097O21583S8P2993Mg2Zn0Fe0	-2994	mRNA cleavage
TU00291_DNA_act	DNA transcription unit TU00291 (activated form)	C56720H65333N22219O34901P5821	-5821	Transcription
TU00291_DNA_neu	DNA transcription unit TU00291 (inactive form)	C56720H65333N22219O34901P5821	-5821	Transcription
TU00291_mRNA	mRNA TU00291	C55418H62730N22219O40729P5823	-5824	Transcription
TU00291_mRNA_cleav_cplx	TU00291 mRNA /RNase III cleavage complex	C57656H66330N22875O41409S8P5823Mg2Zn0Fe0	-5824	mRNA cleavage
TU00304_DNA_act	DNA transcription unit TU00304 (activated form)	C46462H53537N18029O28670P4762	-4762	Transcription
TU00304_DNA_neu	DNA transcription unit TU00304 (inactive form)	C46462H53537N18029O28670P4762	-4762	Transcription
TU00304_mRNA	mRNA TU00304	C45307H51228N18029O33439P4764	-4765	Transcription
TU00304_mRNA_cleav_cplx	TU00304 mRNA /RNase III cleavage complex	C47545H54828N18685O34119S8P4764Mg2Zn0Fe0	-4765	mRNA cleavage
TU00309_DNA_act	DNA transcription unit TU00309 (activated form)	C21402H24611N8481O13056P2195	-2195	Transcription
TU00309_DNA_neu	DNA transcription unit TU00309 (inactive form)	C21402H24611N8481O13056P2195	-2195	Transcription
TU00309_mRNA	mRNA TU00309	C20936H23680N8481O15258P2197	-2198	Transcription
TU00309_mRNA_cleav_cplx	TU00309 mRNA /RNase III cleavage complex	C23174H27280N9137O15938S8P2197Mg2Zn0Fe0	-2198	mRNA cleavage
TU00311_DNA_act	DNA transcription unit TU00311 (activated form)	C45541H52273N18233O27700P4665	-4665	Transcription
TU00311_DNA_neu	DNA transcription unit TU00311 (inactive form)	C45541H52273N18233O27700P4665	-4665	Transcription
TU00311_mRNA	mRNA TU00311	C44583H50358N18233O32372P4667	-4668	Transcription
TU00311_mRNA_cleav_cplx	TU00311 mRNA /RNase III cleavage complex	C46821H53958N18889O33052S8P4667Mg2Zn0Fe0	-4668	mRNA cleavage
TU00314_DNA_act	DNA transcription unit TU00314 (activated form)	C25115H29015N9571O15621P2577	-2577	Transcription
TU00314_DNA_neu	DNA transcription unit TU00314 (inactive form)	C25115H29015N9571O15621P2577	-2577	Transcription
TU00314_mRNA	mRNA TU00314	C24447H27680N9571O18205P2579	-2580	Transcription
TU00314_mRNA_cleav_cplx	TU00314 mRNA /RNase III cleavage complex	C26685H31280N10227O18885S8P2579Mg2Zn0Fe0	-2580	mRNA cleavage
TU00324_DNA_act	DNA transcription unit TU00324 (activated form)	C25581H29446N10092O15649P2626	-2626	Transcription
TU00324_DNA_neu	DNA transcription unit TU00324 (inactive form)	C25581H29446N10092O15649P2626	-2626	Transcription
TU00324_mRNA	mRNA TU00324	C25021H28327N10092O18282P2628	-2629	Transcription
TU00324_mRNA_cleav_cplx	TU00324 mRNA /RNase III cleavage complex	C27259H31927N10748O18962S8P2628Mg2Zn0Fe0	-2629	mRNA cleavage
TU00332_DNA_act	DNA transcription unit TU00332 (activated form)	C15440H17767N6073O9441P1583	-1583	Transcription
TU00332_DNA_neu	DNA transcription unit TU00332 (inactive form)	C15440H17767N6073O9441P1583	-1583	Transcription
TU00332_mRNA	mRNA TU00332	C15086H17060N6073O11031P1585	-1586	Transcription
TU00332_mRNA_cleav_cplx	TU00332 mRNA /RNase III cleavage complex	C17324H20660N6729O11711S8P1585Mg2Zn0Fe0	-1586	mRNA cleavage
TU00333_DNA_act	DNA transcription unit TU00333 (activated form)	C52081H60119N20231O32190P5357	-5357	Transcription

TU00333_DNA_neu	DNA transcription unit TU00333 (inactive form)	C52081H60119N20231O32190P5357	-5357	Transcription
TU00333_mRNA	mRNA TU00333	C50889H57736N20231O37554P5359	-5360	Transcription
TU00333_mRNA_cleav_cplx	TU00333 mRNA /RNase III cleavage complex	C53127H61336N20887O38234S8P5359Mg2Zn0Fe0	-5360	mRNA cleavage
TU00335_DNA_act	DNA transcription unit TU00335 (activated form)	C93358H107521N36557O57256P9578	-9578	Transcription
TU00335_DNA_neu	DNA transcription unit TU00335 (inactive form)	C93358H107521N36557O57256P9578	-9578	Transcription
TU00335_mRNA	mRNA TU00335	C91195H103196N36557O66841P9580	-9581	Transcription
TU00335_mRNA_cleav_cplx	TU00335 mRNA /RNase III cleavage complex	C93433H106796N37213O67521S8P9580Mg2Zn0Fe0	-9581	mRNA cleavage
TU00336_DNA_act	DNA transcription unit TU00336 (activated form)	C8156H9396N3190O4993P837	-837	Transcription
TU00336_DNA_neu	DNA transcription unit TU00336 (inactive form)	C8156H9396N3190O4993P837	-837	Transcription
TU00336_mRNA	mRNA TU00336	C7967H9019N3190O5837P839	-840	Transcription
TU00336_mRNA_cleav_cplx	TU00336 mRNA /RNase III cleavage complex	C10205H12619N3846O6517S8P839Mg2Zn0Fe0	-840	mRNA cleavage
TU00337_DNA_act	DNA transcription unit TU00337 (activated form)	C54022H62227N20939O33205P5532	-5532	Transcription
TU00337_DNA_neu	DNA transcription unit TU00337 (inactive form)	C54022H62227N20939O33205P5532	-5532	Transcription
TU00337_mRNA	mRNA TU00337	C52647H59478N20939O38744P5534	-5535	Transcription
TU00337_mRNA_cleav_cplx	TU00337 mRNA /RNase III cleavage complex	C54885H63078N21595O39424S8P5534Mg2Zn0Fe0	-5535	mRNA cleavage
TU00338_DNA_act	DNA transcription unit TU00338 (activated form)	C5773H6626N2288O3511P590	-590	Transcription
TU00338_DNA_neu	DNA transcription unit TU00338 (inactive form)	C5773H6626N2288O3511P590	-590	Transcription
TU00338_mRNA	mRNA TU00338	C5637H6355N2288O4108P592	-593	Transcription
TU00338_mRNA_cleav_cplx	TU00338 mRNA /RNase III cleavage complex	C7875H9955N2944O4788S8P592Mg2Zn0Fe0	-593	mRNA cleavage
TU00339_DNA_act	DNA transcription unit TU00339 (activated form)	C4149H4773N1629O2521P425	-425	Transcription
TU00339_DNA_neu	DNA transcription unit TU00339 (inactive form)	C4149H4773N1629O2521P425	-425	Transcription
TU00339_mRNA	mRNA TU00339	C4051H4578N1629O2953P427	-428	Transcription
TU00339_mRNA_cleav_cplx	TU00339 mRNA /RNase III cleavage complex	C6289H8178N2285O3633S8P427Mg2Zn0Fe0	-428	mRNA cleavage
TU00340_DNA_act	DNA transcription unit TU00340 (activated form)	C5020H5809N1919O3110P517	-517	Transcription
TU00340_DNA_neu	DNA transcription unit TU00340 (inactive form)	C5020H5809N1919O3110P517	-517	Transcription
TU00340_mRNA	mRNA TU00340	C4898H5566N1919O3634P519	-520	Transcription
TU00340_mRNA_cleav_cplx	TU00340 mRNA /RNase III cleavage complex	C7136H9166N2575O4314S8P519Mg2Zn0Fe0	-520	mRNA cleavage
TU00342_DNA_act	DNA transcription unit TU00342 (activated form)	C26244H30252N10170O16142P2691	-2691	Transcription
TU00342_DNA_neu	DNA transcription unit TU00342 (inactive form)	C26244H30252N10170O16142P2691	-2691	Transcription
TU00342_mRNA	mRNA TU00342	C25593H28951N10170O18840P2693	-2694	Transcription
TU00342_mRNA_cleav_cplx	TU00342 mRNA /RNase III cleavage complex	C27831H32551N10826O19520S8P2693Mg2Zn0Fe0	-2694	mRNA cleavage
TU00343_DNA_act	DNA transcription unit TU00343 (activated form)	C13958H16073N5509O8526P1434	-1434	Transcription
TU00343_DNA_neu	DNA transcription unit TU00343 (inactive form)	C13958H16073N5509O8526P1434	-1434	Transcription
TU00344_DNA_act	DNA transcription unit TU00344 (activated form)	C50438H58112N19888O30932P5186	-5186	Transcription
TU00344_DNA_neu	DNA transcription unit TU00344 (inactive form)	C50438H58112N19888O30932P5186	-5186	Transcription
TU00344_mRNA	mRNA TU00344	C49372H55981N19888O36125P5188	-5189	Transcription
TU00344_mRNA_cleav_cplx	TU00344 mRNA /RNase III cleavage complex	C51610H59581N20544O36805S8P5188Mg2Zn0Fe0	-5189	mRNA cleavage
TU00345_DNA_act	DNA transcription unit TU00345 (activated form)	C17924H20592N7102O10916P1835	-1835	Transcription
TU00345_DNA_neu	DNA transcription unit TU00345 (inactive form)	C17924H20592N7102O10916P1835	-1835	Transcription
TU00345_mRNA	mRNA TU00345	C17517H19779N7102O12758P1837	-1838	Transcription
TU00345_mRNA_cleav_cplx	TU00345 mRNA /RNase III cleavage complex	C19755H23379N7758O13438S8P1837Mg2Zn0Fe0	-1838	mRNA cleavage
TU00346_DNA_act	DNA transcription unit TU00346 (activated form)	C14039H16169N5491O8597P1440	-1440	Transcription
TU00346_DNA_neu	DNA transcription unit TU00346 (inactive form)	C14039H16169N5491O8597P1440	-1440	Transcription
TU00346_mRNA	mRNA TU00346	C13710H15512N5491O10044P1442	-1443	Transcription
TU00346_mRNA_cleav_cplx	TU00346 mRNA /RNase III cleavage complex	C15948H19112N6147O10724S8P1442Mg2Zn0Fe0	-1443	mRNA cleavage
TU00347_DNA_act	DNA transcription unit TU00347 (activated form)	C48382H55635N19085O29554P4957	-4957	Transcription
TU00347_DNA_neu	DNA transcription unit TU00347 (inactive form)	C48382H55635N19085O29554P4957	-4957	Transcription
TU00347_mRNA	mRNA TU00347	C47274H53420N19085O34518P4959	-4960	Transcription
TU00347_mRNA_cleav_cplx	TU00347 mRNA /RNase III cleavage complex	C49512H57020N19741O35198S8P4959Mg2Zn0Fe0	-4960	mRNA cleavage
TU00348_DNA_act	DNA transcription unit TU00348 (activated form)	C42990H49513N16785O26346P4408	-4408	Transcription

TU00348_DNA_neu	DNA transcription unit TU00348 (inactive form)	C42990H49513N16785O26346P4408	-4408	Transcription
TU00348_mRNA	mRNA TU00348	C41965H47464N16785O30761P4410	-4411	Transcription
TU00348_mRNA_cleav_cplx	TU00348 mRNA /RNase III cleavage complex	C44203H51064N17441O31441S8P4410Mg2Zn0Fe0	-4411	mRNA cleavage
TU00349_DNA_act	DNA transcription unit TU00349 (activated form)	C27867H32087N10881O17097P2856	-2856	Transcription
TU00349_DNA_neu	DNA transcription unit TU00349 (inactive form)	C27867H32087N10881O17097P2856	-2856	Transcription
TU00349_mRNA	mRNA TU00349	C27196H30746N10881O19960P2858	-2859	Transcription
TU00349_mRNA_cleav_cplx	TU00349 mRNA /RNase III cleavage complex	C29434H34346N11537O20640S8P2858Mg2Zn0Fe0	-2859	mRNA cleavage
TU00351_DNA_act	DNA transcription unit TU00351 (activated form)	C19567H22504N7676O11975P2003	-2003	Transcription
TU00351_DNA_neu	DNA transcription unit TU00351 (inactive form)	C19567H22504N7676O11975P2003	-2003	Transcription
TU00351_mRNA	mRNA TU00351	C19096H21563N7676O13985P2005	-2006	Transcription
TU00351_mRNA_cleav_cplx	TU00351 mRNA /RNase III cleavage complex	C21334H25163N8332O14665S8P2005Mg2Zn0Fe0	-2006	mRNA cleavage
TU00352_DNA_act	DNA transcription unit TU00352 (activated form)	C40025H46063N15805O24445P4108	-4108	Transcription
TU00352_DNA_neu	DNA transcription unit TU00352 (inactive form)	C40025H46063N15805O24445P4108	-4108	Transcription
TU00352_mRNA	mRNA TU00352	C39150H44314N15805O28560P4110	-4111	Transcription
TU00352_mRNA_cleav_cplx	TU00352 mRNA /RNase III cleavage complex	C41388H47914N16461O29240S8P4110Mg2Zn0Fe0	-4111	mRNA cleavage
TU00354_DNA_act	DNA transcription unit TU00354 (activated form)	C9096H10477N3519O5621P931	-931	Transcription
TU00354_DNA_neu	DNA transcription unit TU00354 (inactive form)	C9096H10477N3519O5621P931	-931	Transcription
TU00354_mRNA	mRNA TU00354	C8860H10006N3519O6559P933	-934	Transcription
TU00354_mRNA_cleav_cplx	TU00354 mRNA /RNase III cleavage complex	C11098H13606N4175O7239S8P933Mg2Zn0Fe0	-934	mRNA cleavage
TU00355_DNA_act	DNA transcription unit TU00355 (activated form)	C31495H36263N12395O19384P3233	-3233	Transcription
TU00355_DNA_neu	DNA transcription unit TU00355 (inactive form)	C31495H36263N12395O19384P3233	-3233	Transcription
TU00355_mRNA	mRNA TU00355	C30795H34864N12395O22624P3235	-3236	Transcription
TU00355_mRNA_cleav_cplx	TU00355 mRNA /RNase III cleavage complex	C33033H38464N13051O23304S8P3235Mg2Zn0Fe0	-3236	mRNA cleavage
TU00362_DNA_act	DNA transcription unit TU00362 (activated form)	C42068H48504N16438O25872P4323	-4323	Transcription
TU00362_DNA_neu	DNA transcription unit TU00362 (inactive form)	C42068H48504N16438O25872P4323	-4323	Transcription
TU00362_mRNA	mRNA TU00362	C41117H46603N16438O30202P4325	-4326	Transcription
TU00362_mRNA_cleav_cplx	TU00362 mRNA /RNase III cleavage complex	C43355H50203N17094O30882S8P4325Mg2Zn0Fe0	-4326	mRNA cleavage
TU00366_DNA_act	DNA transcription unit TU00366 (activated form)	C31046H35700N12196O18926P3178	-3178	Transcription
TU00366_DNA_neu	DNA transcription unit TU00366 (inactive form)	C31046H35700N12196O18926P3178	-3178	Transcription
TU00366_mRNA	mRNA TU00366	C30304H34217N12196O22111P3180	-3181	Transcription
TU00366_mRNA_cleav_cplx	TU00366 mRNA /RNase III cleavage complex	C32542H37817N12852O22791S8P3180Mg2Zn0Fe0	-3181	mRNA cleavage
TU00411_DNA_act	DNA transcription unit TU00411 (activated form)	C5819H6663N2341O3527P594	-594	Transcription
TU00411_DNA_neu	DNA transcription unit TU00411 (inactive form)	C5819H6663N2341O3527P594	-594	Transcription
TU00414_DNA_act	DNA transcription unit TU00414 (activated form)	C26012H29930N10186O15967P2664	-2664	Transcription
TU00414_DNA_neu	DNA transcription unit TU00414 (inactive form)	C26012H29930N10186O15967P2664	-2664	Transcription
TU00414_mRNA	mRNA TU00414	C25386H28679N10186O18638P2666	-2667	Transcription
TU00414_mRNA_cleav_cplx	TU00414 mRNA /RNase III cleavage complex	C27624H32279N10842O19318S8P2666Mg2Zn0Fe0	-2667	mRNA cleavage
TU00415_DNA_act	DNA transcription unit TU00415 (activated form)	C21547H24762N8498O13136P2205	-2205	Transcription
TU00415_DNA_neu	DNA transcription unit TU00415 (inactive form)	C21547H24762N8498O13136P2205	-2205	Transcription
TU00415_mRNA	mRNA TU00415	C21040H23749N8498O15348P2207	-2208	Transcription
TU00415_mRNA_cleav_cplx	TU00415 mRNA /RNase III cleavage complex	C23278H27349N9154O16028S8P2207Mg2Zn0Fe0	-2208	mRNA cleavage
TU00417_DNA_act	DNA transcription unit TU00417 (activated form)	C30586H35142N12194O18618P3136	-3136	Transcription
TU00417_DNA_neu	DNA transcription unit TU00417 (inactive form)	C30586H35142N12194O18618P3136	-3136	Transcription
TU00417_mRNA	mRNA TU00417	C29940H33851N12194O21761P3138	-3139	Transcription
TU00417_mRNA_cleav_cplx	TU00417 mRNA /RNase III cleavage complex	C32178H37451N12850O22441S8P3138Mg2Zn0Fe0	-3139	mRNA cleavage
TU00418_DNA_act	DNA transcription unit TU00418 (activated form)	C30586H35142N12194O18618P3136	-3136	Transcription
TU00418_DNA_neu	DNA transcription unit TU00418 (inactive form)	C30586H35142N12194O18618P3136	-3136	Transcription
TU00418_mRNA	mRNA TU00418	C29940H33851N12194O21761P3138	-3139	Transcription
TU00418_mRNA_cleav_cplx	TU00418 mRNA /RNase III cleavage complex	C32178H37451N12850O22441S8P3138Mg2Zn0Fe0	-3139	mRNA cleavage
TU00426_DNA_act	DNA transcription unit TU00426 (activated form)	C8333H9585N3301O5098P855	-855	Transcription

TU00426_DNA_neu	DNA transcription unit TU00426 (inactive form)	C8333H9585N3301O5098P855	-855	Transcription
TU00427_DNA_act	DNA transcription unit TU00427 (activated form)	C8333H9585N3301O5098P855	-855	Transcription
TU00427_DNA_neu	DNA transcription unit TU00427 (inactive form)	C8333H9585N3301O5098P855	-855	Transcription
TU00429_DNA_act	DNA transcription unit TU00429 (activated form)	C14840H17095N5719O9104P1518	-1518	Transcription
TU00429_DNA_neu	DNA transcription unit TU00429 (inactive form)	C14840H17095N5719O9104P1518	-1518	Transcription
TU00430_DNA_act	DNA transcription unit TU00430 (activated form)	C10741H12363N4211O6569P1101	-1101	Transcription
TU00430_DNA_neu	DNA transcription unit TU00430 (inactive form)	C10741H12363N4211O6569P1101	-1101	Transcription
TU00434_DNA_act	DNA transcription unit TU00434 (activated form)	C40025H46063N15805O24445P4108	-4108	Transcription
TU00434_DNA_neu	DNA transcription unit TU00434 (inactive form)	C40025H46063N15805O24445P4108	-4108	Transcription
TU00434_mRNA	mRNA TU00434	C39150H44314N15805O28560P4110	-4111	Transcription
TU00434_mRNA_cleav_cplx	TU00434 mRNA /RNase III cleavage complex	C41388H47914N16461O29240S8P4110Mg2Zn0Fe0	-4111	mRNA cleavage
TU00435_DNA_act	DNA transcription unit TU00435 (activated form)	C40025H46063N15805O24445P4108	-4108	Transcription
TU00435_DNA_neu	DNA transcription unit TU00435 (inactive form)	C40025H46063N15805O24445P4108	-4108	Transcription
TU00435_mRNA	mRNA TU00435	C39150H44314N15805O28560P4110	-4111	Transcription
TU00435_mRNA_cleav_cplx	TU00435 mRNA /RNase III cleavage complex	C41388H47914N16461O29240S8P4110Mg2Zn0Fe0	-4111	mRNA cleavage
TU00436_DNA_act	DNA transcription unit TU00436 (activated form)	C17943H20626N7164O10914P1842	-1842	Transcription
TU00436_DNA_neu	DNA transcription unit TU00436 (inactive form)	C17943H20626N7164O10914P1842	-1842	Transcription
TU00437_DNA_act	DNA transcription unit TU00437 (activated form)	C17943H20626N7164O10914P1842	-1842	Transcription
TU00437_DNA_neu	DNA transcription unit TU00437 (inactive form)	C17943H20626N7164O10914P1842	-1842	Transcription
TU00438_DNA_act	DNA transcription unit TU00438 (activated form)	C17943H20626N7164O10914P1842	-1842	Transcription
TU00438_DNA_neu	DNA transcription unit TU00438 (inactive form)	C17943H20626N7164O10914P1842	-1842	Transcription
TU00440_DNA_act	DNA transcription unit TU00440 (activated form)	C21402H24611N8481O13056P2195	-2195	Transcription
TU00440_DNA_neu	DNA transcription unit TU00440 (inactive form)	C21402H24611N8481O13056P2195	-2195	Transcription
TU00440_mRNA	mRNA TU00440	C20936H23680N8481O15258P2197	-2198	Transcription
TU00440_mRNA_cleav_cplx	TU00440 mRNA /RNase III cleavage complex	C23174H27280N9137O15938S8P2197Mg2Zn0Fe0	-2198	mRNA cleavage
TU00442_DNA_act	DNA transcription unit TU00442 (activated form)	C9705H11145N3849O5926P993	-993	Transcription
TU00442_DNA_neu	DNA transcription unit TU00442 (inactive form)	C9705H11145N3849O5926P993	-993	Transcription
TU00460_DNA_act	DNA transcription unit TU00460 (activated form)	C14289H16492N5598O8807P1472	-1472	Transcription
TU00460_DNA_neu	DNA transcription unit TU00460 (inactive form)	C14289H16492N5598O8807P1472	-1472	Transcription
TU00460_mRNA	mRNA TU00460	C13989H15893N5598O10286P1474	-1475	Transcription
TU00460_mRNA_cleav_cplx	TU00460 mRNA /RNase III cleavage complex	C16227H19493N6254O10966S8P1474Mg2Zn0Fe0	-1475	mRNA cleavage
TU00471_DNA_act	DNA transcription unit TU00471 (activated form)	C21547H24762N8498O13136P2205	-2205	Transcription
TU00471_DNA_neu	DNA transcription unit TU00471 (inactive form)	C21547H24762N8498O13136P2205	-2205	Transcription
TU00471_mRNA	mRNA TU00471	C21040H23749N8498O15348P2207	-2208	Transcription
TU00471_mRNA_cleav_cplx	TU00471 mRNA /RNase III cleavage complex	C23278H27349N9154O16028S8P2207Mg2Zn0Fe0	-2208	mRNA cleavage
TU00472_DNA_act	DNA transcription unit TU00472 (activated form)	C2105H2421N835O1282P216	-216	Transcription
TU00472_DNA_neu	DNA transcription unit TU00472 (inactive form)	C2105H2421N835O1282P216	-216	Transcription
TU00487_DNA_act	DNA transcription unit TU00487 (activated form)	C34313H39537N13411O21012P3522	-3522	Transcription
TU00487_DNA_neu	DNA transcription unit TU00487 (inactive form)	C34313H39537N13411O21012P3522	-3522	Transcription
TU00487_mRNA	mRNA TU00487	C33518H37948N13411O24541P3524	-3525	Transcription
TU00487_mRNA_cleav_cplx	TU00487 mRNA /RNase III cleavage complex	C35756H41548N14067O25221S8P3524Mg2Zn0Fe0	-3525	mRNA cleavage
TU00489_DNA_act	DNA transcription unit TU00489 (activated form)	C748H864N290O468P77	-77	Transcription
TU00489_DNA_neu	DNA transcription unit TU00489 (inactive form)	C748H864N290O468P77	-77	Transcription
TU00489_RNA	tRNA	C731H831N290O552P79	-80	RNA cutting
TU00489_RNA_cut_cplx	TU00489 RNA /RNase P (5' trimming), RNase_Gen (T, PH, II, D, or BN), RNase E (3' trimming) cutting complex	C27994H42940N8916O10549S93P456Mg5Zn3Fe0	-569	RNA cutting
TU00490_DNA_act	DNA transcription unit TU00490 (activated form)	C742H855N287O457P76	-76	Transcription
TU00490_DNA_neu	DNA transcription unit TU00490 (inactive form)	C742H855N287O457P76	-76	Transcription
TU00490_RNA	tRNA	C723H818N287O540P78	-79	RNA cutting

TU00490_RNA_cut_cplx	TU00490 RNA /RNase P (5' trimming), RNase_Gen (T, PH, II, D, or BN), RNase E (3' trimming) cutting complex	C27986H42927N8913O10537S93P455Mg5Zn3Fe0	-568	RNA cutting
TU00491_DNA_act	DNA transcription unit TU00491 (activated form)	C747H862N294O462P77	-77	Transcription
TU00491_DNA_neu	DNA transcription unit TU00491 (inactive form)	C747H862N294O462P77	-77	Transcription
TU00491_RNA	tRNA	C732H833N294O546P79	-80	RNA cutting
TU00491_RNA_cut_cplx	TU00491 RNA /RNase P (5' trimming), RNase_Gen (T, PH, II, D, or BN), RNase E (3' trimming) cutting complex	C27995H42942N8920O10543S93P456Mg5Zn3Fe0	-569	RNA cutting
TU00492_DNA_act	DNA transcription unit TU00492 (activated form)	C3559H4097N1397O2182P365	-365	Transcription
TU00492_DNA_neu	DNA transcription unit TU00492 (inactive form)	C3559H4097N1397O2182P365	-365	Transcription
TU00492_RNA	RNA TU00492	C3477H3934N1397O2554P367	-368	RNA cutting
TU00492_RNA_cut_cplx	TU00492 RNA /RNase III, RNAse m16, RNase m23, RNase m5, RNase P, RNase F, RNase E cutting complex	C39910H60617N12637O15335S157P744Mg11Zn3Fe0	-893	RNA cutting
TU00493_DNA_act	DNA transcription unit TU00493 (activated form)	C1539H1777N591O958P158	-158	Transcription
TU00493_DNA_neu	DNA transcription unit TU00493 (inactive form)	C1539H1777N591O958P158	-158	Transcription
TU00493_RNA	RNA TU00493	C1500H1700N591O1123P160	-161	RNA cutting
TU00493_RNA_cut_cplx	TU00493 RNA /RNase III, RNAse m16, RNase m23, RNase m5, RNase P, RNase F, RNase E cutting complex	C37933H58383N11831O13904S157P537Mg11Zn3Fe0	-686	RNA cutting
TU00494_DNA_act	DNA transcription unit TU00494 (activated form)	C747H861N297O464P77	-77	Transcription
TU00494_DNA_neu	DNA transcription unit TU00494 (inactive form)	C747H861N297O464P77	-77	Transcription
TU00494_RNA	tRNA	C733H834N297O548P79	-80	RNA cutting
TU00494_RNA_cut_cplx	TU00494 RNA /RNase P (5' trimming), RNase_Gen (T, PH, II, D, or BN), RNase E (3' trimming) cutting complex	C27996H42943N8923O10545S93P456Mg5Zn3Fe0	-569	RNA cutting
TU00495_DNA_act	DNA transcription unit TU00495 (activated form)	C3874H4471N1499O2392P398	-398	Transcription
TU00495_DNA_neu	DNA transcription unit TU00495 (inactive form)	C3874H4471N1499O2392P398	-398	Transcription
TU00495_RNA	RNA TU00495	C3781H4286N1499O2797P400	-401	RNA cutting
TU00495_RNA_cut_cplx	TU00495 RNA /RNase III, RNAse m16, RNase m23, RNase m5, RNase P, RNase F, RNase E cutting complex	C40214H60969N12739O15578S157P777Mg11Zn3Fe0	-926	RNA cutting
TU00496_DNA_act	DNA transcription unit TU00496 (activated form)	C2887H3320N1160O1765P297	-297	Transcription
TU00496_DNA_neu	DNA transcription unit TU00496 (inactive form)	C2887H3320N1160O1765P297	-297	Transcription
TU00496_RNA	RNA TU00496	C2834H3215N1160O2069P299	-300	RNA cutting
TU00496_RNA_cut_cplx	TU00496 RNA /RNase III, RNAse m16, RNase m23, RNase m5, RNase P, RNase F, RNase E cutting complex	C39267H59898N12400O14850S157P676Mg11Zn3Fe0	-825	RNA cutting
TU00497_DNA_act	DNA transcription unit TU00497 (activated form)	C742H856N284O460P76	-76	Transcription
TU00497_DNA_neu	DNA transcription unit TU00497 (inactive form)	C742H856N284O460P76	-76	Transcription
TU00497_RNA	tRNA	C722H817N284O543P78	-79	RNA cutting
TU00497_RNA_cut_cplx	TU00497 RNA /RNase P (5' trimming), RNase_Gen (T, PH, II, D, or BN), RNase E (3' trimming) cutting complex	C27985H42926N8910O10540S93P455Mg5Zn3Fe0	-568	RNA cutting
TU00498_DNA_act	DNA transcription unit TU00498 (activated form)	C742H856N284O460P76	-76	Transcription
TU00498_DNA_neu	DNA transcription unit TU00498 (inactive form)	C742H856N284O460P76	-76	Transcription
TU00498_RNA	tRNA	C722H817N284O543P78	-79	RNA cutting
TU00498_RNA_cut_cplx	TU00498 RNA /RNase P (5' trimming), RNase_Gen (T, PH, II, D, or BN), RNase E (3' trimming) cutting complex	C27985H42926N8910O10540S93P455Mg5Zn3Fe0	-568	RNA cutting
TU00499_DNA_act	DNA transcription unit TU00499 (activated form)	C742H856N284O460P76	-76	Transcription
TU00499_DNA_neu	DNA transcription unit TU00499 (inactive form)	C742H856N284O460P76	-76	Transcription
TU00499_RNA	tRNA	C722H817N284O543P78	-79	RNA cutting
TU00499_RNA_cut_cplx	TU00499 RNA /RNase P (5' trimming), RNase_Gen (T, PH, II, D, or BN), RNase E (3' trimming) cutting complex	C27985H42926N8910O10540S93P455Mg5Zn3Fe0	-568	RNA cutting
TU00500_DNA_act	DNA transcription unit TU00500 (activated form)	C731H844N280O450P75	-75	Transcription
TU00500_DNA_neu	DNA transcription unit TU00500 (inactive form)	C731H844N280O450P75	-75	Transcription
TU00500_RNA	tRNA	C712H807N280O532P77	-78	RNA cutting
TU00500_RNA_cut_cplx	TU00500 RNA /RNase P (5' trimming), RNase_Gen (T, PH, II, D, or BN), RNase E (3' trimming) cutting complex	C27975H42916N8906O10529S93P454Mg5Zn3Fe0	-567	RNA cutting
TU00501_DNA_act	DNA transcription unit TU00501 (activated form)	C739H852N290O457P76	-76	Transcription
TU00501_DNA_neu	DNA transcription unit TU00501 (inactive form)	C739H852N290O457P76	-76	Transcription
TU00501_RNA	tRNA	C723H821N290O540P78	-79	RNA cutting

TU00501_RNA_cut_cplx	TU00501 RNA /RNase P (5' trimming), RNase_Gen (T, PH, II, D, or BN), RNase E (3' trimming) cutting complex	C27986H42930N8916O10537S93P455Mg5Zn3Fe0	-568	RNA cutting
TU00502_DNA_act	DNA transcription unit TU00502 (activated form)	C738H852N288O457P76	-76	Transcription
TU00502_DNA_neu	DNA transcription unit TU00502 (inactive form)	C738H852N288O457P76	-76	Transcription
TU00502_RNA	tRNA	C722H821N288O540P78	-79	RNA cutting
TU00502_RNA_cut_cplx	TU00502 RNA /RNase P (5' trimming), RNase_Gen (T, PH, II, D, or BN), RNase E (3' trimming) cutting complex	C27985H42930N8914O10537S93P455Mg5Zn3Fe0	-568	RNA cutting
TU00503_DNA_act	DNA transcription unit TU00503 (activated form)	C4264H4914N1652O2636P437	-437	Transcription
TU00503_DNA_neu	DNA transcription unit TU00503 (inactive form)	C4264H4914N1652O2636P437	-437	Transcription
TU00503_RNA	RNA TU00503	C4157H4701N1652O3080P439	-440	RNA cutting
TU00503_RNA_cut_cplx	TU00503 RNA /RNase III, RNAse m16, RNase m23, RNase m5, RNase P, RNase F, RNase E cutting complex	C40590H61384N12892O15861S157P816Mg11Zn3Fe0	-965	RNA cutting
TU00504_DNA_act	DNA transcription unit TU00504 (activated form)	C4302H4962N1674O2647P442	-442	Transcription
TU00504_DNA_neu	DNA transcription unit TU00504 (inactive form)	C4302H4962N1674O2647P442	-442	Transcription
TU00504_RNA	RNA TU00504	C4202H4763N1674O3096P444	-445	RNA cutting
TU00504_RNA_cut_cplx	TU00504 RNA /RNase III, RNAse m16, RNase m23, RNase m5, RNase P, RNase F, RNase E cutting complex	C40635H61446N12914O15877S157P821Mg11Zn3Fe0	-970	RNA cutting
TU00505_DNA_act	DNA transcription unit TU00505 (activated form)	C2910H3351N1149O1787P299	-299	Transcription
TU00505_DNA_neu	DNA transcription unit TU00505 (inactive form)	C2910H3351N1149O1787P299	-299	Transcription
TU00505_RNA	RNA TU00505	C2848H3228N1149O2093P301	-302	RNA cutting
TU00505_RNA_cut_cplx	TU00505 RNA /RNase III, RNAse m16, RNase m23, RNase m5, RNase P, RNase F, RNase E cutting complex	C39281H59911N12389O14874S157P678Mg11Zn3Fe0	-827	RNA cutting
TU00506_DNA_act	DNA transcription unit TU00506 (activated form)	C828H951N333O503P85	-85	Transcription
TU00506_DNA_neu	DNA transcription unit TU00506 (inactive form)	C828H951N333O503P85	-85	Transcription
TU00506_RNA	tRNA	C812H920N333O595P87	-88	RNA cutting
TU00506_RNA_cut_cplx	TU00506 RNA /RNase P (5' trimming), RNase_Gen (T, PH, II, D, or BN), RNase E (3' trimming) cutting complex	C28075H43029N8959O10592S93P464Mg5Zn3Fe0	-577	RNA cutting
TU00507_DNA_act	DNA transcription unit TU00507 (activated form)	C6852H7918N2622O4237P704	-704	Transcription
TU00507_DNA_neu	DNA transcription unit TU00507 (inactive form)	C6852H7918N2622O4237P704	-704	Transcription
TU00507_RNA	RNA TU00507	C6678H7571N2622O4948P706	-707	RNA cutting
TU00507_RNA_cut_cplx	TU00507 RNA /RNase III, RNAse m16, RNase m23, RNase m5, RNase P, RNase F, RNase E cutting complex	C43111H64254N13862O17729S157P1083Mg11Zn3Fe0	-1232	RNA cutting
TU00508_DNA_act	DNA transcription unit TU00508 (activated form)	C856H984N344O526P88	-88	Transcription
TU00508_DNA_neu	DNA transcription unit TU00508 (inactive form)	C856H984N344O526P88	-88	Transcription
TU00508_RNA	tRNA	C840H953N344O621P90	-91	RNA cutting
TU00508_RNA_cut_cplx	TU00508 RNA /RNase P (5' trimming), RNase_Gen (T, PH, II, D, or BN), RNase E (3' trimming) cutting complex	C28103H43062N8970O10618S93P467Mg5Zn3Fe0	-580	RNA cutting
TU00509_DNA_act	DNA transcription unit TU00509 (activated form)	C856H984N344O525P88	-88	Transcription
TU00509_DNA_neu	DNA transcription unit TU00509 (inactive form)	C856H984N344O525P88	-88	Transcription
TU00509_RNA	tRNA	C840H953N344O620P90	-91	RNA cutting
TU00509_RNA_cut_cplx	TU00509 RNA /RNase P (5' trimming), RNase_Gen (T, PH, II, D, or BN), RNase E (3' trimming) cutting complex	C28103H43062N8970O10617S93P467Mg5Zn3Fe0	-580	RNA cutting
TU00510_DNA_act	DNA transcription unit TU00510 (activated form)	C856H984N344O526P88	-88	Transcription
TU00510_DNA_neu	DNA transcription unit TU00510 (inactive form)	C856H984N344O526P88	-88	Transcription
TU00510_RNA	tRNA	C840H953N344O621P90	-91	RNA cutting
TU00510_RNA_cut_cplx	TU00510 RNA /RNase P (5' trimming), RNase_Gen (T, PH, II, D, or BN), RNase E (3' trimming) cutting complex	C28103H43062N8970O10618S93P467Mg5Zn3Fe0	-580	RNA cutting
TU00511_DNA_act	DNA transcription unit TU00511 (activated form)	C5209H5996N2078O3174P536	-536	Transcription
TU00511_DNA_neu	DNA transcription unit TU00511 (inactive form)	C5209H5996N2078O3174P536	-536	Transcription
TU00511_mRNA	mRNA TU00511	C5109H5797N2078O3717P538	-539	Transcription
TU00511_mRNA_cleav_cplx	TU00511 mRNA /RNase III cleavage complex	C7347H9397N2734O4397S8P538Mg2Zn0Fe0	-539	mRNA cleavage
TU00512_DNA_act	DNA transcription unit TU00512 (activated form)	C2950H3402N1148O1824P303	-303	Transcription
TU00512_DNA_neu	DNA transcription unit TU00512 (inactive form)	C2950H3402N1148O1824P303	-303	Transcription
TU00512_RNA	RNA TU00512	C2881H3265N1148O2134P305	-306	RNA cutting

TU00512_RNA_cut_cplx	TU00512 RNA /RNase III, RNAse m16, RNase m23, RNase m5, RNase P, RNase F, RNase E cutting complex	C39314H59948N12388O14915S157P682Mg11Zn3Fe0	-831	RNA cutting
TU00513_DNA_act	DNA transcription unit TU00513 (activated form)	C872H1003N355O533P90	-90	Transcription
TU00513_DNA_neu	DNA transcription unit TU00513 (inactive form)	C872H1003N355O533P90	-90	Transcription
TU00513_RNA	tRNA	C859H978N355O630P92	-93	RNA cutting
TU00513_RNA_cut_cplx	TU00513 RNA /RNase P (5' trimming), RNase_Gen (T, PH, II, D, or BN), RNase E (3' trimming) cutting complex	C28122H43087N8981O10627S93P469Mg5Zn3Fe0	-582	RNA cutting
TU00514_DNA_act	DNA transcription unit TU00514 (activated form)	C1858H2141N731O1144P191	-191	Transcription
TU00514_DNA_neu	DNA transcription unit TU00514 (inactive form)	C1858H2141N731O1144P191	-191	Transcription
TU00514_RNA	RNA TU00514	C1818H2062N731O1342P193	-194	RNA cutting
TU00514_RNA_cut_cplx	TU00514 RNA /RNase III, RNAse m16, RNase m23, RNase m5, RNase P, RNase F, RNase E cutting complex	C38251H58745N11971O14123S157P570Mg11Zn3Fe0	-719	RNA cutting
TU00515_DNA_act	DNA transcription unit TU00515 (activated form)	C8402H9671N3307O5144P862	-862	Transcription
TU00515_DNA_neu	DNA transcription unit TU00515 (inactive form)	C8402H9671N3307O5144P862	-862	Transcription
TU00515_RNA	RNA TU00515	C8213H9294N3307O6013P864	-865	RNA cutting
TU00515_RNA_cut_cplx	TU00515 RNA /RNase III, RNAse m16, RNase m23, RNase m5, RNase P, RNase F, RNase E cutting complex	C44646H65977N14547O18794S157P1241Mg11Zn3Fe0	-1390	RNA cutting
TU00516_DNA_act	DNA transcription unit TU00516 (activated form)	C718H831N275O446P74	-74	Transcription
TU00516_DNA_neu	DNA transcription unit TU00516 (inactive form)	C718H831N275O446P74	-74	Transcription
TU00516_RNA	tRNA	C701H798N275O527P76	-77	RNA cutting
TU00516_RNA_cut_cplx	TU00516 RNA /RNase P (5' trimming), RNase_Gen (T, PH, II, D, or BN), RNase E (3' trimming) cutting complex	C27964H42907N8901O10524S93P453Mg5Zn3Fe0	-566	RNA cutting
TU00517_DNA_act	DNA transcription unit TU00517 (activated form)	C48306H55471N19263O29424P4948	-4948	Transcription
TU00517_DNA_neu	DNA transcription unit TU00517 (inactive form)	C48306H55471N19263O29424P4948	-4948	Transcription
TU00517_mRNA	mRNA TU00517	C47263H53386N19263O34379P4950	-4951	Transcription
TU00517_mRNA_cleav_cplx	TU00517 mRNA /RNase III cleavage complex	C49501H56986N19919O35059S8P4950Mg2Zn0Fe0	-4951	mRNA cleavage
TU00518_DNA_act	DNA transcription unit TU00518 (activated form)	C849H977N333O525P87	-87	Transcription
TU00518_DNA_neu	DNA transcription unit TU00518 (inactive form)	C849H977N333O525P87	-87	Transcription
TU00518_RNA	tRNA	C829H938N333O619P89	-90	RNA cutting
TU00518_RNA_cut_cplx	TU00518 RNA /RNase P (5' trimming), RNase_Gen (T, PH, II, D, or BN), RNase E (3' trimming) cutting complex	C28092H43047N8959O10616S93P466Mg5Zn3Fe0	-579	RNA cutting
TU00519_DNA_act	DNA transcription unit TU00519 (activated form)	C749H863N295O464P77	-77	Transcription
TU00519_DNA_neu	DNA transcription unit TU00519 (inactive form)	C749H863N295O464P77	-77	Transcription
TU00519_RNA	tRNA	C733H832N295O548P79	-80	RNA cutting
TU00519_RNA_cut_cplx	TU00519 RNA /RNase P (5' trimming), RNase_Gen (T, PH, II, D, or BN), RNase E (3' trimming) cutting complex	C27996H42941N8921O10545S93P456Mg5Zn3Fe0	-569	RNA cutting
TU00520_DNA_act	DNA transcription unit TU00520 (activated form)	C739H852N290O457P76	-76	Transcription
TU00520_DNA_neu	DNA transcription unit TU00520 (inactive form)	C739H852N290O457P76	-76	Transcription
TU00520_RNA	tRNA	C723H821N290O540P78	-79	RNA cutting
TU00520_RNA_cut_cplx	TU00520 RNA /RNase P (5' trimming), RNase_Gen (T, PH, II, D, or BN), RNase E (3' trimming) cutting complex	C27986H42930N8916O10537S93P455Mg5Zn3Fe0	-568	RNA cutting
TU00521_DNA_act	DNA transcription unit TU00521 (activated form)	C3148H3624N1238O1940P323	-323	Transcription
TU00521_DNA_neu	DNA transcription unit TU00521 (inactive form)	C3148H3624N1238O1940P323	-323	Transcription
TU00521_RNA	RNA TU00521	C3077H3483N1238O2270P325	-326	RNA cutting
TU00521_RNA_cut_cplx	TU00521 RNA /RNase III, RNAse m16, RNase m23, RNase m5, RNase P, RNase F, RNase E cutting complex	C39510H60166N12478O15051S157P702Mg11Zn3Fe0	-851	RNA cutting
TU221_DNA_act	DNA transcription unit TU221 (activated form)	C8333H9585N3301O5098P855	-855	Transcription
TU221_DNA_neu	DNA transcription unit TU221 (inactive form)	C8333H9585N3301O5098P855	-855	Transcription
TU281_DNA_act	DNA transcription unit TU281 (activated form)	C26244H30252N10170O16142P2691	-2691	Transcription
TU281_DNA_neu	DNA transcription unit TU281 (inactive form)	C26244H30252N10170O16142P2691	-2691	Transcription
TU281_mRNA	mRNA TU281	C25593H28951N10170O18840P2693	-2694	Transcription
TU281_mRNA_cleav_cplx	TU281 mRNA /RNase III cleavage complex	C27831H32551N10826O19520S8P2693Mg2Zn0Fe0	-2694	mRNA cleavage
TU341_DNA_act	DNA transcription unit TU341 (activated form)	C90330H103877N35655O55242P9257	-9257	Transcription
TU341_DNA_neu	DNA transcription unit TU341 (inactive form)	C90330H103877N35655O55242P9257	-9257	Transcription

TU341_mRNA	mRNA TU341	C88280H99778N35655O64506P9259	-9260	Transcription
TU341_mRNA_cleav_cplx	TU341 mRNA /RNase III cleavage complex	C90518H103378N36311O65186S8P9259Mg2Zn0Fe0	-9260	mRNA cleavage
TU343_DNA_act	DNA transcription unit TU343 (activated form)	C744H859N297O459P77	-77	Transcription
TU343_DNA_neu	DNA transcription unit TU343 (inactive form)	C744H859N297O459P77	-77	Transcription
TU343_RNA	tRNA	C732H836N297O543P79	-80	RNA cutting
TU343_RNA_cut_cplx	TU343 RNA /RNase P (5' trimming), RNase_Gen (T, PH, II, D, or BN), RNase E (3' trimming) cutting complex	C27995H42945N8923O10540S93P456Mg5Zn3Fe0	-569	RNA cutting
TU361_DNA_act	DNA transcription unit TU361 (activated form)	C24087H27699N9501O14719P2468	-2468	Transcription
TU361_DNA_neu	DNA transcription unit TU361 (inactive form)	C24087H27699N9501O14719P2468	-2468	Transcription
TU361_mRNA	mRNA TU361	C23536H26598N9501O17194P2470	-2471	Transcription
TU361_mRNA_cleav_cplx	TU361 mRNA /RNase III cleavage complex	C25774H30198N10157O17874S8P2470Mg2Zn0Fe0	-2471	mRNA cleavage
TU482_DNA_act	DNA transcription unit TU482 (activated form)	C45779H52715N17869O28177P4692	-4692	Transcription
TU482_DNA_neu	DNA transcription unit TU482 (inactive form)	C45779H52715N17869O28177P4692	-4692	Transcription
TU482_mRNA	mRNA TU482	C44676H50510N17869O32876P4694	-4695	Transcription
TU482_mRNA_cleav_cplx	TU482 mRNA /RNase III cleavage complex	C46914H54110N18525O33556S8P4694Mg2Zn0Fe0	-4695	mRNA cleavage
TU483_DNA_act	DNA transcription unit TU483 (activated form)	C33597H38669N13215O20588P3446	-3446	Transcription
TU483_DNA_neu	DNA transcription unit TU483 (inactive form)	C33597H38669N13215O20588P3446	-3446	Transcription
TU483_mRNA	mRNA TU483	C32834H37144N13215O24041P3448	-3449	Transcription
TU483_mRNA_cleav_cplx	TU483 mRNA /RNase III cleavage complex	C35072H40744N13871O24721S8P3448Mg2Zn0Fe0	-3449	mRNA cleavage
TU543_DNA_act	DNA transcription unit TU543 (activated form)	C3669H4204N1512O2232P377	-377	Transcription
TU543_DNA_neu	DNA transcription unit TU543 (inactive form)	C3669H4204N1512O2232P377	-377	Transcription
TU543_RNA	misc_RNA	C3612H4091N1512O2616P379	-380	RNA cutting
TU543_RNA_cleav_cplx	TU543 RNA RNase II complex	C5850H7691N2168O3296S8P379Mg2Zn0Fe0	-380	RNA cutting
TU564_DNA_act	DNA transcription unit TU564 (activated form)	C20675H23776N8146O12617P2118	-2118	Transcription
TU564_DNA_neu	DNA transcription unit TU564 (inactive form)	C20675H23776N8146O12617P2118	-2118	Transcription
TU564_mRNA	mRNA TU564	C20197H22821N8146O14742P2120	-2121	Transcription
TU564_mRNA_cleav_cplx	TU564 mRNA /RNase III cleavage complex	C22435H26421N8802O15422S8P2120Mg2Zn0Fe0	-2121	mRNA cleavage
TU565_DNA_act	DNA transcription unit TU565 (activated form)	C20675H23776N8146O12617P2118	-2118	Transcription
TU565_DNA_neu	DNA transcription unit TU565 (inactive form)	C20675H23776N8146O12617P2118	-2118	Transcription
TU565_mRNA	mRNA TU565	C20197H22821N8146O14742P2120	-2121	Transcription
TU565_mRNA_cleav_cplx	TU565 mRNA /RNase III cleavage complex	C22435H26421N8802O15422S8P2120Mg2Zn0Fe0	-2121	mRNA cleavage
TU582_DNA_act	DNA transcription unit TU582 (activated form)	C18829H21719N7331O11573P1935	-1935	Transcription
TU582_DNA_neu	DNA transcription unit TU582 (inactive form)	C18829H21719N7331O11573P1935	-1935	Transcription
TU583_DNA_act	DNA transcription unit TU583 (activated form)	C18829H21719N7331O11573P1935	-1935	Transcription
TU583_DNA_neu	DNA transcription unit TU583 (inactive form)	C18829H21719N7331O11573P1935	-1935	Transcription
TU601_DNA_act	DNA transcription unit TU601 (activated form)	C7283H8391N2839O4463P747	-747	Transcription
TU601_DNA_neu	DNA transcription unit TU601 (inactive form)	C7283H8391N2839O4463P747	-747	Transcription
TU601_mRNA	mRNA TU601	C7109H8044N2839O5217P749	-750	Transcription
TU601_mRNA_cleav_cplx	TU601 mRNA /RNase III cleavage complex	C9347H11644N3495O5897S8P749Mg2Zn0Fe0	-750	mRNA cleavage
TU602_DNA_act	DNA transcription unit TU602 (activated form)	C7283H8391N2839O4463P747	-747	Transcription
TU602_DNA_neu	DNA transcription unit TU602 (inactive form)	C7283H8391N2839O4463P747	-747	Transcription
TU602_mRNA	mRNA TU602	C7109H8044N2839O5217P749	-750	Transcription
TU602_mRNA_cleav_cplx	TU602 mRNA /RNase III cleavage complex	C9347H11644N3495O5897S8P749Mg2Zn0Fe0	-750	mRNA cleavage
TU682_DNA_act	DNA transcription unit TU682 (activated form)	C5020H5809N1919O3110P517	-517	Transcription
TU682_DNA_neu	DNA transcription unit TU682 (inactive form)	C5020H5809N1919O3110P517	-517	Transcription
TU682_mRNA	mRNA TU682	C4898H5566N1919O3634P519	-520	Transcription
TU682_mRNA_cleav_cplx	TU682 mRNA /RNase III cleavage complex	C7136H9166N2575O4314S8P519Mg2Zn0Fe0	-520	mRNA cleavage
TU743_DNA_act	DNA transcription unit TU743 (activated form)	C5020H5809N1919O3110P517	-517	Transcription
TU743_DNA_neu	DNA transcription unit TU743 (inactive form)	C5020H5809N1919O3110P517	-517	Transcription
TU743_mRNA	mRNA TU743	C4898H5566N1919O3634P519	-520	Transcription

TU743_mRNA_cleav_cplx	TU743 mRNA /RNase III cleavage complex	C7136H9166N2575O4314S8P519Mg2Zn0Fe0	-520	mRNA cleavage
TU801_DNA_act	DNA transcription unit TU801 (activated form)	C99775H114849N39305O61243P10239	-10239	Transcription
TU801_DNA_neu	DNA transcription unit TU801 (inactive form)	C99775H114849N39305O61243P10239	-10239	Transcription
TU801_mRNA	mRNA TU801	C97555H110410N39305O71489P10241	-10242	Transcription
TU801_mRNA_cleav_cplx	TU801 mRNA /RNase III cleavage complex	C99793H114010N39961O72169S8P10241Mg2Zn0Fe0	-10242	mRNA cleavage
TU802_DNA_act	DNA transcription unit TU802 (activated form)	C99775H114849N39305O61243P10239	-10239	Transcription
TU802_DNA_neu	DNA transcription unit TU802 (inactive form)	C99775H114849N39305O61243P10239	-10239	Transcription
TU802_mRNA	mRNA TU802	C97555H110410N39305O71489P10241	-10242	Transcription
TU802_mRNA_cleav_cplx	TU802 mRNA /RNase III cleavage complex	C99793H114010N39961O72169S8P10241Mg2Zn0Fe0	-10242	mRNA cleavage
TU870_DNA_act	DNA transcription unit TU870 (activated form)	C14481H16686N5598O8909P1483	-1483	Transcription
TU870_DNA_neu	DNA transcription unit TU870 (inactive form)	C14481H16686N5598O8909P1483	-1483	Transcription
TU870_mRNA	mRNA TU870	C14108H15941N5598O10399P1485	-1486	Transcription
TU870_mRNA_cleav_cplx	TU870 mRNA /RNase III cleavage complex	C16346H19541N6254O11079S8P1485Mg2Zn0Fe0	-1486	mRNA cleavage
TU871_DNA_act	DNA transcription unit TU871 (activated form)	C10425H12026N4050O6425P1071	-1071	Transcription
TU871_DNA_neu	DNA transcription unit TU871 (inactive form)	C10425H12026N4050O6425P1071	-1071	Transcription
TU872_DNA_act	DNA transcription unit TU872 (activated form)	C62725H72234N24506O38500P6431	-6431	Transcription
TU872_DNA_neu	DNA transcription unit TU872 (inactive form)	C62725H72234N24506O38500P6431	-6431	Transcription
TU872_mRNA	mRNA TU872	C61232H69249N24506O44938P6433	-6434	Transcription
TU872_mRNA_cleav_cplx	TU872 mRNA /RNase III cleavage complex	C63470H72849N25162O45618S8P6433Mg2Zn0Fe0	-6434	mRNA cleavage
TU873_DNA_act	DNA transcription unit TU873 (activated form)	C62725H72234N24506O38500P6431	-6431	Transcription
TU873_DNA_neu	DNA transcription unit TU873 (inactive form)	C62725H72234N24506O38500P6431	-6431	Transcription
TU873_mRNA	mRNA TU873	C61232H69249N24506O44938P6433	-6434	Transcription
TU873_mRNA_cleav_cplx	TU873 mRNA /RNase III cleavage complex	C63470H72849N25162O45618S8P6433Mg2Zn0Fe0	-6434	mRNA cleavage
TU874_DNA_act	DNA transcription unit TU874 (activated form)	C45551H52456N17848O27919P4673	-4673	Transcription
TU874_DNA_neu	DNA transcription unit TU874 (inactive form)	C45551H52456N17848O27919P4673	-4673	Transcription
TU874_mRNA	mRNA TU874	C44498H50351N17848O32599P4675	-4676	Transcription
TU874_mRNA_cleav_cplx	TU874 mRNA /RNase III cleavage complex	C46736H53951N18504O33279S8P4675Mg2Zn0Fe0	-4676	mRNA cleavage
TU875_DNA_act	DNA transcription unit TU875 (activated form)	C31997H36870N12424O19671P3280	-3280	Transcription
TU875_DNA_neu	DNA transcription unit TU875 (inactive form)	C31997H36870N12424O19671P3280	-3280	Transcription
TU875_mRNA	mRNA TU875	C31207H35291N12424O22958P3282	-3283	Transcription
TU875_mRNA_cleav_cplx	TU875 mRNA /RNase III cleavage complex	C33445H38891N13080O23638S8P3282Mg2Zn0Fe0	-3283	mRNA cleavage
TU876_DNA_act	DNA transcription unit TU876 (activated form)	C31997H36870N12424O19671P3280	-3280	Transcription
TU876_DNA_neu	DNA transcription unit TU876 (inactive form)	C31997H36870N12424O19671P3280	-3280	Transcription
TU876_mRNA	mRNA TU876	C31207H35291N12424O22958P3282	-3283	Transcription
TU876_mRNA_cleav_cplx	TU876 mRNA /RNase III cleavage complex	C33445H38891N13080O23638S8P3282Mg2Zn0Fe0	-3283	mRNA cleavage
TU877_DNA_act	DNA transcription unit TU877 (activated form)	C23664H27259N9219O14543P2426	-2426	Transcription
TU877_DNA_neu	DNA transcription unit TU877 (inactive form)	C23664H27259N9219O14543P2426	-2426	Transcription
TU877_mRNA	mRNA TU877	C23091H26114N9219O16976P2428	-2429	Transcription
TU877_mRNA_cleav_cplx	TU877 mRNA /RNase III cleavage complex	C25329H29714N9875O17656S8P2428Mg2Zn0Fe0	-2429	mRNA cleavage
TadA_dim	tRNA-specific adenosine deaminase (b2559, TadA, dimer)	C1750H2780N528O492S22Zn2	8	tRNA Modification
TadA_dim_inact	tRNA-specific adenosine deaminase (b2559, TadA, dimer)	C1750H2780N528O492S22Zn2	8	tRNA Modification
TadA_mono	tRNA-specific adenosine deaminase (b2559, TadA, monomer)	C875H1390N264O246Zn1S11	4	Folding
Tgt_hexa	tRNA-guanine transglycosylase (b0406, Tgt, hexamer)	C11316H17622N3186O3312S132Zn6	-24	tRNA Modification
Tgt_hexa_inact	tRNA-guanine transglycosylase (b0406, Tgt, hexamer)	C11316H17622N3186O3312S132Zn6	-24	tRNA Modification
Tgt_mono	tRNA-guanine transglycosylase (b0406, Tgt, monomer)	C1886H2937N531O552Zn1S22	-4	Folding
Tgt_trim	tRNA-guanine transglycosylase (b0406, Tgt, trimer)	C5658H8811N1593O1656S66Zn3	-12	tRNA Modification
ThiI_mono	sulfurtransferase required for thiamine and 4-thiouridine biosynthesis (b0423, ThiI, monomer)	C2448H3903N685O718S13	-6	Folding
ThiI_mono_inact	sulfurtransferase required for thiamine and 4-thiouridine biosynthesis (b0423, ThiI, monomer)	C2448H3903N685O718S13	-6	Folding

ThrS_DnaK_GrpE_complex	ThrS (b1719) DnaK GrpE_dim complex - Kerner et al. class II can interact w/ GroEL/ES, cannot fold spontanously	C8142H13002O2569N2285S66P0Zn1	-75	Folding
ThrS_GroEL.(7)ADP.transGroES	ThrS (b1719) GroEL GroES complex - Kerner et al. class II can interact w/ GroEL/ES, cannot fold spontanously	C41186H68020O13281N11499S399P14Mg7Zn1	-309	Folding
ThrS_mono	threonyl-tRNA synthetase (b1719, ThrS, monomer)	C3274H5076N915O968Zn1S35	-15	Folding
Thr_RS_2thr_2amp	Threonyl-tRNA synthetase (charged) (2*threonine, 2*amp)	C6576H10194N1842O1956P2S70Zn2	-34	tRNA charging
Thr_RS_dim	Threonyl-tRNA synthetase (uncharged) (dimer, 2 Zn2+)	C6548H10152N1830O1936S70Zn2	-30	tRNA charging
Thr_RS_dim_inact	Threonyl-tRNA synthetase (uncharged) (dimer, 2 Zn2+)	C6548H10152N1830O1936S70Zn2	-30	tRNA charging
Thr_RS_thr_amp	Threonyl-tRNA synthetase (charged) (1*threonine, 1*amp)	C6562H10173N1836O1946S70Zn2P	-32	tRNA charging
Tig_DnaK_GrpE_complex	Tig (b0436) DnaK GrpE_dim complex - Kerner et al. class I can interact w/ GroEL/ES	C6987H11329O2275N1952S42P0	-83	Folding
Tig_GroEL.(7)ADP.transGroES	Tig (b0436) GroEL GroES complex - Kerner et al. class I can interact w/ GroEL/ES	C40031H66347O12987N11166S375P14Mg7	-317	Folding
Tig_mono	peptidyl-prolyl cis/trans isomerase (trigger factor) (b0436, Tig, monomer)	C2119H3403N582O674S11	-23	Translation
TilS_mono	tRNA(Ile)-lysidine synthetase (b0188, TilS, monomer)	C2145H3396N624O614S10	-2	Folding
TilS_mono_inact	tRNA(Ile)-lysidine synthetase (b0188, TilS, monomer)	C2145H3396N624O614S10	-2	Folding
Tpr_mono	predicted protamine-like protein (b1229, Tpr, monomer)	C150H268N68O46S2	8	Folding
TrmA_GroEL.(7)ADP.transGroES	TrmA (b3965) GroEL GroES complex - Kerner et al. class III needs GroEL/ES	C39771H65868O12867N11101S382P14Mg7	-303	Folding
TrmA_mono	tRNA (uracil-5-)-methyltransferase (b3965, TrmA, monomer)	C1859H2924N517O554S18	-9	Folding
TrmA_mono_inact	tRNA (uracil-5-)-methyltransferase (b3965, TrmA, monomer)	C1859H2924N517O554S18	-9	Folding
TrmC_mono	fused 5-methylaminomethyl-2-thiouridine-forming enzyme methyltransferase -!- FAD-dependent demodification enzyme (b2324, TrmC, monomer)	C3315H5058N919O977S27	-19	Folding
TrmC_mono_inact	fused 5-methylaminomethyl-2-thiouridine-forming enzyme methyltransferase -!- FAD-dependent demodification enzyme (b2324, TrmC, monomer)	C3315H5058N919O977S27	-19	Folding
TrmD_GroEL.(7)ADP.transGroES	TrmD (b2607) GroEL GroES complex - Kerner et al. class III needs GroEL/ES	C39168H64921O12688N10942S374P14Mg7	-303	Folding
TrmD_dim	tRNA (guanine-1-)-methyltransferase (active protein - homodimer)	C2512H3954N716O750S20	-18	tRNA Modification
TrmD_dim_inact	tRNA (guanine-1-)-methyltransferase (active protein - homodimer)	C2512H3954N716O750S20	-18	tRNA Modification
TrmD_mono	tRNA (guanine-1-)-methyltransferase (b2607, TrmD, monomer)	C1256H1977N358O375S10	-9	Folding
TrmE_dim	GTPase (b3706, TrmE, dimer)	C4332H6930N1236O1336S14	-46	tRNA Modification
TrmE_dim_inact	GTPase (b3706, TrmE, dimer)	C4332H6930N1236O1336S14	-46	tRNA Modification
TrmE_mono	GTPase (b3706, TrmE, monomer)	C2166H3465N618O668S7	-23	Folding
TrmH_DnaK_GrpE_complex	TrmH (b3651) DnaK GrpE_dim complex - Kerner et al. class II can interact w/ GroEL/ES, cannot fold spontanously	C5966H9686O1929N1695S45P0	-61	Folding
TrmH_GroEL.(7)ADP.transGroES	TrmH (b3651) GroEL GroES complex - Kerner et al. class II can interact w/ GroEL/ES, cannot fold spontanously	C39010H64704O12641N10909S378P14Mg7	-295	Folding
TrmH_dim	tRNA (Guanosine-2'-O-)-methyltransferase (b3651, TrmH, dimer)	C2196H3520N650O656S28	-2	tRNA Modification
TrmH_dim_inact	tRNA (Guanosine-2'-O-)-methyltransferase (b3651, TrmH, dimer)	C2196H3520N650O656S28	-2	tRNA Modification
TrmH_mono	tRNA (Guanosine-2'-O-)-methyltransferase (b3651, TrmH, monomer)	C1098H1760N325O328S14	-1	Folding
TrmU_mono	tRNA (5-methylaminomethyl-2-thiouridylate)-methyltransferase (b1133, TrmU, monomer)	C1822H2820N488O554S12	-18	tRNA Modification
TrmU_mono_inact	tRNA (5-methylaminomethyl-2-thiouridylate)-methyltransferase (b1133, TrmU, monomer)	C1822H2820N488O554S12	-18	tRNA Modification
TrpS_DnaK_GrpE_complex	TrpS (b3384) DnaK GrpE_dim complex - Deuerling et al. DnaKJ/GrpE dependent folding	C6530H10547O2098N1822S44P0	-63	Folding
TrpS_mono	tryptophanyl-tRNA synthetase (b3384, TrpS, monomer)	C1662H2621N452O497S13	-3	Folding
Trp_RS_2trp_2amp	Tryptophyl-tRNA synthetase (charged, 2*tryptophane 2*amp)	C3366H5290N918O1012S26P2	-10	tRNA charging
Trp_RS_dim	Tryptophyl-tRNA synthetase (uncharged)	C3324H5242N904O994S26	-6	tRNA charging
Trp_RS_dim_inact	Tryptophyl-tRNA synthetase (uncharged)	C3324H5242N904O994S26	-6	tRNA charging
Trp_RS_trp_amp	Tryptophyl-tRNA synthetase (charged)	C3345H5266N911O1003S26P	-8	tRNA charging
TruA_dim	pseudouridylate synthase I (b2318, TruA, dimer)	C2710H4202N780O770S16	6	rRNA Modification
TruA_dim_inact	pseudouridylate synthase I (b2318, TruA, dimer)	C2710H4202N780O770S16	6	rRNA Modification
TruA_mono	pseudouridylate synthase I (b2318, TruA, monomer)	C1355H2101N390O385S8	3	Folding

TruB_mono	tRNA pseudouridine synthase (b3166, TruB, monomer)	C1540H2475N438O469S10	-5	Folding
TruB_mono_inact	tRNA pseudouridine synthase (b3166, TruB, monomer)	C1540H2475N438O469S10	-5	Folding
TruD_mono	pseudoruidine synthase (b2745, TruD, monomer)	C1743H2742N499O509S8	-3	Folding
TruD_mono_inact	pseudoruidine synthase (b2745, TruD, monomer)	C1743H2742N499O509S8	-3	Folding
Tsf_DnaK_GrpE_complex	Tsf (b0170) DnaK GrpE_dim complex - Kerner et al. class I can interact w/ GroEL/ES	C6199H10096O2018N1733S42P0	-69	Folding
Tsf_GroEL.(7)ADP.transGroES	Tsf (b0170) GroEL GroES complex - Kerner et al. class I can interact w/ GroEL/ES	C39243H65114O12730N10947S375P14Mg7	-303	Folding
Tsf_mono	protein chain elongation factor EF-Ts (b0170, Tsf, monomer)	C1331H2170N363O417S11	-9	Folding
TufA_DnaK_GrpE_complex	TufA (b3339) DnaK GrpE_dim complex - Deuerling et al. DnaKJ/GrpE dependent folding	C6785H10976O2182N1893S44P0	-75	Folding
TufA_mono	protein chain elongation factor EF-Tu (duplicate of tufB) (b3339, TufA, monomer)	C1917H3050N523O581S13	-15	Folding
TufB_DnaK_GrpE_complex	TufB (b3980) DnaK GrpE_dim complex - Deuerling et al. DnaKJ/GrpE dependent folding	C6786H10978O2183N1893S44P0	-75	Folding
TufB_mono	protein chain elongation factor EF-Tu (duplicate of tufA) (b3980, TufB, monomer)	C1918H3052N523O582S13	-15	Folding
TyrS_mono	tyrosyl-tRNA synthetase (b1637, TyrS, monomer)	C2118H3295N576O634S13	-7	Folding
Tyr_RS_2tyr-D_2amp	Tyrosinyl-tRNA synthetase (charged,2*D-tyrosine, 1*amp)	C4274H6636N1164O1288S26P2	-18	tRNA charging
Tyr_RS_2tyr_2amp	Tyrosinyl-tRNA synthetase (charged,2*L-tyrosine, 2*amp)	C4274H6636N1164O1288S26P2	-18	tRNA charging
Tyr_RS_dim	tyrosyl-tRNA synthetase (dimer) (uncharged)	C4236H6590N1152O1268S26	-14	tRNA charging
Tyr_RS_dim_inact	tyrosyl-tRNA synthetase (dimer) (uncharged)	C4236H6590N1152O1268S26	-14	tRNA charging
Tyr_RS_tyr-D_amp	Tyrosinyl-tRNA synthetase (charged - 1*D-tyrosine, 1*amp)	C4255H6613N1158O1278S26P	-16	tRNA charging
Tyr_RS_tyr_amp	Tyrosinyl-tRNA synthetase (charged,1*L-tyrosine, 1*amp)	C4255H6613N1158O1278S26P	-16	tRNA charging
Up_tRNA_pos_37_t6A	unknown protein, tRNA modification, position 37, t6A		0	tRNA Modification
Up_tRNA_pos_37_t6A_inact	unknown protein, tRNA modification, position 37, t6A		0	tRNA Modification
Usg_mono	predicted semialdehyde dehydrogenase (b2319, Usg, monomer)	C1622H2525N429O503S8	-24	Folding
ValS_DnaK_GrpE_complex	ValS (b4258) DnaK GrpE_dim complex - Deuerling et al. DnaKJ/GrpE dependent folding	C9692H15438O3030N2691S71P0	-91	Folding
ValS_mono	valyl-tRNA synthetase (b4258, ValS, monomer)	C4824H7512N1321O1429S40	-31	Folding
ValS_mono_inact	valyl-tRNA synthetase (b4258, ValS, monomer)	C4824H7512N1321O1429S40	-31	Folding
Val_RS_val_amp	Valyl-tRNA synthetase (charged)	C4839H7535N1327O1438S40P	-33	tRNA charging
YadB_DnaK_GrpE_complex	YadB (b0144) DnaK GrpE_dim complex - Kerner et al. class II can interact w/ GroEL/ES, cannot fold spontanously	C6371H10238O2029N1806S35P0	-64	Folding
YadB_GroEL.(7)ADP.transGroES	YadB (b0144) GroEL GroES complex - Kerner et al. class II can interact w/ GroEL/ES, cannot fold spontanously	C39415H65256O12741N11020S368P14Mg7	-298	Folding
YadB_mono	glutamyl-Q tRNA(Asp) synthetase (b0144, YadB, monomer)	C1503H2312N436O428S4	-4	Folding
YajC_mono	SecYEG protein translocase auxillary subunit (b0407, YajC, monomer)	C534H878N138O148S5	4	Folding
YbaD_mono	conserved protein (b0413, YbaD, monomer)	C745H1215N218O224S9	1	Folding
YbbB_DnaK_GrpE_complex	YbbB (b0503) DnaK GrpE_dim complex - Kerner et al. class II can interact w/ GroEL/ES, cannot fold spontanously	C6675H10765O2135N1897S46P0	-68	Folding
YbbB_GroEL.(7)ADP.transGroES	YbbB (b0503) GroEL GroES complex - Kerner et al. class II can interact w/ GroEL/ES, cannot fold spontanously	C39719H65783O12847N11111S379P14Mg7	-302	Folding
YbbB_dim	dimer of tRNA 2-selenouridine synthase, selenophosphate-dependent	C3614H5678N1054O1068S30	-16	tRNA modification
YbbB_dim_inact	dimer of tRNA 2-selenouridine synthase, selenophosphate-dependent	C3614H5678N1054O1068S30	-16	tRNA modification
YbbB_mono	tRNA 2-selenouridine synthase, selenophosphate-dependent (b0503, YbbB, monomer)	C1807H2839N527O534S15	-8	Folding
YbjC_mono	predicted inner membrane protein (b0850, YbjC, monomer)	C472H789N125O126S5	2	Folding
YbjN_mono	predicted oxidoreductase (b0853, YbjN, monomer)	C780H1193N199O242S9	-16	Folding
YbjO_mono	predicted inner membrane protein (b0858, YbjO, monomer)	C862H1352N215O217S6	5	Folding
YccK_mono	predicted sulfite reductase subunit (b0969, YccK, monomer)	C561H875N143O160S3	0	Folding
YccK_mono_inact	predicted sulfite reductase subunit (b0969, YccK, monomer)	C561H875N143O160S3	0	Folding

YceD_mono	conserved protein (b1088, YceD, monomer)	C846H1322N220O271S8	-16	Folding
YdaO_mono	predicted C32 tRNA thiolase (b1344, YdaO, monomer)	C1562H2491N437O466S18	-4	Folding
YdaO_mono_inact	predicted C32 tRNA thiolase (b1344, YdaO, monomer)	C1562H2491N437O466S18	-4	Folding
YecN_mono	predicted inner membrane protein (b1869, YecN, monomer)	C701H1079N181O167S12	4	Folding
YecO_mono	predicted methyltransferase (b1870, YecO, monomer)	C1225H1904N340O366S12	-10	Folding
YecO_mono_inact	predicted methyltransferase (b1870, YecO, monomer)	C1225H1904N340O366S12	-10	Folding
YecP_mono	predicted S-adenosyl-L-methionine-dependent methyltransferase (b1871, YecP, monomer)	C1673H2590N455O467S10	-5	Folding
YecP_mono_inact	predicted S-adenosyl-L-methionine-dependent methyltransferase (b1871, YecP, monomer)	C1673H2590N455O467S10	-5	Folding
YfcA_mono	conserved inner membrane protein (b2327, YfcA, monomer)	C1316H2107N330O339S17	9	Folding
YfcL_mono	predicted protein (b2325, YfcL, monomer)	C428H658N118O146S2	-12	Folding
YfcM_mono	conserved protein (b2326, YfcM, monomer)	C954H1375N251O278S6	-14	Folding
YfhB_mono	conserved protein (b2560, YfhB, monomer)	C1113H1732N306O295S6	4	Folding
YfiH_mono	conserved protein (b2593, YfiH, monomer)	C1163H1800N326O344S11	-2	Folding
YggH_GroEL.(7)ADP.transGroES	YggH (b2960) GroEL GroES complex - Kerner et al. class III needs GroEL/ES	C39122H64820O12655N10931S377P14Mg7	-297	Folding
YggH_mono	tRNA (m7G46) methyltransferase, SAM-dependent (b2960, YggH, monomer)	C1210H1876N347O342S13	-3	Folding
YggH_mono_inact	tRNA (m7G46) methyltransferase, SAM-dependent (b2960, YggH, monomer)	C1210H1876N347O342S13	-3	Folding
YggJ_mono	predicted protein (b2946, YggJ, monomer)	C1177H1917N342O354S10	-3	Folding
YggJ_mono_inact	predicted protein (b2946, YggJ, monomer)	C1177H1917N342O354S10	-3	Folding
YggL_mono	predicted protein (b2959, YggL, monomer)	C569H866N155O171S4	-7	Folding
YhbC_mono	conserved protein (b3170, YhbC, monomer)	C685H1077N183O211S3	-10	Folding
YhbG_mono	predicted transporter subunit: ATP-binding component of ABC superfamily (b3201, YhbG, monomer)	C1174H1902N343O355S5	-7	Folding
YhbH_mono	predicted ribosome-associated, sigma 54 modulation protein (b3203, YhbH, monomer)	C476H765N134O145S2	-1	Folding
YhbJ_mono	predicted protein with nucleoside triphosphate hydrolase domain (b3205, YhbJ, monomer)	C1435H2287N408O422S11	-1	Folding
YhdE_mono	conserved protein (b3248, YhdE, monomer)	C937H1515N270O293S4	-5	Folding
YhdT_mono	conserved inner membrane protein (b3257, YhdT, monomer)	C426H634N100O105S4	-2	Folding
YheLMN_cplx	YheLMN complex (2*YheL,2*YheM,2*YheN)	C3330H5196N902O988S10	-26	tRNA Modification
YheLMN_cplx_inact	YheLMN complex (2*YheL,2*YheM,2*YheN)	C3330H5196N902O988S10	-26	tRNA Modification
YheL_mono	predicted intracellular sulfur oxidation protein (b3343, YheL, monomer)	C478H757N129O140S1	-4	Folding
YheM_mono	predicted intraceullular sulfur oxidation protein (b3344, YheM, monomer)	C589H916N153O169S2	-5	Folding
YheN_mono	predicted intracellular sulfur oxidation protein (b3345, YheN, monomer)	C598H925N169O185S2	-4	Folding
YheO_mono	predicted DNA-binding transcriptional regulator (b3346, YheO, monomer)	C1168H1879N325O368S10	-6	Folding
YhhP_mono	conserved protein required for cell growth (b3470, YhhP, monomer)	C394H628N107O119S6	-3	Folding
YhhP_mono_inact	conserved protein required for cell growth (b3470, YhhP, monomer)	C394H628N107O119S6	-3	Folding
YicR_mono	protein associated with replication fork, possible DNA repair protein (b3638, YicR, monomer)	C1127H1815N317O320S9	2	Folding
YihX_mono	predicted hydrolase (b3885, YihX, monomer)	C1021H1545N273O297S6	-12	Folding
YiiD_mono	predicted acetyltransferase (b3888, YiiD, monomer)	C1644H2559N463O481S14	-8	Folding
YjbC_mono	23S rRNA pseudouridine synthase (b4022, YjbC, monomer)	C1427H2371N418O422S7	15	Folding
YjbC_mono_inact	23S rRNA pseudouridine synthase (b4022, YjbC, monomer)	C1427H2371N418O422S7	15	Folding
YjeB_mono	predicted DNA-binding transcriptional regulator (b4178, YjeB, monomer)	C684H1117N188O206S6	1	Folding
YjeE_mono	ATPase with strong ADP affinity (b4168, YjeE, monomer)	C747H1146N197O229S5	-11	Folding
YjeF_mono	predicted carbohydrate kinase (b4167, YjeF, monomer)	C2398H3840N701O721S15	-11	Folding
YjjG_mono	predicted hydrolase (b4374, YjjG, monomer)	C1134H1723N301O337S6	-12	Folding
YmfB_mono	bifunctional thiamin pyrimidine pyrophosphate hydrolase and thiamin pyrophosphate hydrolase (b1134, YmfB, monomer)	C781H1181N208O224S7	-9	Folding

YmfC_mono	23S rRNA pseudouridine synthase (b1135, YmfC, monomer)	C1098H1761N327O320S3	10	Folding
YmfC_mono_inact	23S rRNA pseudouridine synthase (b1135, YmfC, monomer)	C1098H1761N327O320S3	10	Folding
YqcA_mono	predicted flavoprotein (b2790, YqcA, monomer)	C724H1091N179O233S3	-16	Folding
YqcB_mono	tRNA pseudouridine synthase (b2791, YqcB, monomer)	C1314H2056N387O379S6	-3	Folding
YqcB_mono_inact	tRNA pseudouridine synthase (b2791, YqcB, monomer)	C1314H2056N387O379S6	-3	Folding
YqcC_mono	conserved protein (b2792, YqcC, monomer)	C571H858N155O164S4	-9	Folding
YrdB_mono	conserved protein (b3280, YrdB, monomer)	C436H632N117O139S4	-11	Folding
YrdC_mono	predicted ribosome maturation factor (b3282, YrdC, monomer)	C921H1454N253O275S5	-5	Folding
YrdC_mono_inact	predicted ribosome maturation factor (b3282, YrdC, monomer)	C921H1454N253O275S5	-5	Folding
YrdD_mono	predicted DNA topoisomerase (b3283, YrdD, monomer)	C861H1361N249O250S17	6	Folding
accoa	Acetyl-CoA	C23H34N7O17P3S	-4	Others
accoa[e]	Acetyl-CoA	C23H34N7O17P3S	-4	Others
ade	Adenine	C5H5N5	0	Others
ade[e]	Adenine	C5H5N5	0	Others
adocbl	Adenosylcobalamin	C72H100Co1N18O17P1	0	Others
adocbl[e]	Adenosylcobalamin	C72H100Co1N18O17P1	0	Others
adp	ADP	C10H12N5O10P2	-3	Others
adp[e]	ADP	C10H12N5O10P2	-3	Others
ahcys	S-Adenosyl-L-homocysteine	C14H20N6O5S	0	Others
ahcys[e]	S-Adenosyl-L-homocysteine	C14H20N6O5S	0	Others
ala-L	L-Alanine	C3H7NO2	0	Others
ala-L[e]	L-Alanine	C3H7NO2	0	Others
ala1_tRNA	generic tRNA for:alaT_tRNA,alaU_tRNA,alaV_tRNA (uncharged tRNA)	C726H829Mg2N289O538P76S0	-74	tRNA charging
ala1_tRNA_ala	ala1_tRNA (charged tRNA)	C729H836N290O540P76S0Se0Mg2	-74	tRNA charging
ala2_tRNA	ala2_tRNA (uncharged tRNA)	C726H828N293O533P76S0Se0Mg2	-73	tRNA charging
ala2_tRNA_ala	ala2_tRNA (charged tRNA)	C729H835Mg2N294O535P76S0	-73	tRNA charging
alaT_tRNA	alaT tRNA (b3853, uncharged tRNA)	C726H829Mg2N289O538P76	-74	tRNA Modification
alaU_tRNA	alaU_tRNA (b3276,uncharged tRNA)	C726H829Mg2N289O538P76	-74	tRNA Modification
alaV_tRNA	alaV_tRNA (b0203, uncharged tRNA)	C726H829Mg2N289O538P76	-74	tRNA Modification
alaW_tRNA	alaW_tRNA (b2397, uncharged tRNA)	C726H828Mg2N293O533P76	-73	tRNA Modification
alaX_tRNA	alaX_tRNA (b2396, uncharged tRNA)	C726H828Mg2N293O533P76	-73	tRNA Modification
amet	S-Adenosyl-L-methionine	C15H23N6O5S	1	Others
amet[e]	S-Adenosyl-L-methionine	C15H23N6O5S	1	Others
amp	AMP	C10H12N5O7P	-2	Others
amp[e]	AMP	C10H12N5O7P	-2	Others
arg-L	L-Arginine	C6H15N4O2	1	Others
arg-L[e]	L-Arginine	C6H15N4O2	1	Others
arg1_tRNA	arg1_tRNA (uncharged tRNA)	C740H850N297O539P77S1Se0Mg2	-74	tRNA charging
arg1_tRNA_ala	arg1_tRNA (charged tRNA)	C746H865N301O541P77S1Se0Mg2	-73	tRNA charging
argQ_tRNA	argQ_tRNA (b2691, uncharged tRNA)	C740H850Mg2N297O539P77S1	-74	tRNA Modification
argU_tRNA	argU_tRNA (b0536, uncharged tRNA)	C740H846Mg2N296O543P77S1	-75	tRNA Modification
argU_tRNA_ala	argU_tRNA (charged tRNA)	C746H861Mg2N300O545P77S1	-74	tRNA charging
argV_tRNA	argV_tRNA (b2694, uncharged tRNA)	C740H850Mg2N297O539P77S1	-74	tRNA Modification
argW_tRNA	argW_tRNA (uncharged tRNA)	C720H822Mg2N282O529P75S1	-73	tRNA Modification
argW_tRNA_arg	argW_tRNA (charged tRNA)	C726H837N286O531P75S1Se0Mg2	-72	tRNA charging
argX_tRNA	argX_tRNA (b3796, uncharged tRNA)	C735H845Mg2N296O540P77S1	-74	tRNA Modification
argX_tRNA_arg	argX_tRNA (charged tRNA)	C741H860Mg2N300O542P77S1	-73	tRNA charging
argY_tRNA	argY_tRNA (b2693, uncharged tRNA)	C740H850Mg2N297O539P77S1	-74	tRNA Modification
argZ_tRNA	argZ_tRNA (b2692, uncharged tRNA)	C740H850Mg2N297O539P77S1	-74	tRNA Modification
asn-L	L-Asparagine	C4H8N2O3	0	Others
asn-L[e]	L-Asparagine	C4H8N2O3	0	Others
asn1_tRNA	asn1_tRNA (uncharged tRNA)	C736H841N285O542P76S1Mg2	-74	tRNA charging
asn1_tRNA_asn	asn1_tRNA (charged tRNA)	C740H849N287O545P76S1Mg2	-74	tRNA charging
asnT_tRNA	asnT_tRNA (b1977, uncharged tRNA)	C736H841Mg2N285O542P76S1	-74	tRNA Modification
asnU_tRNA	asnU_tRNA (b1986, uncharged tRNA)	C736H841Mg2N285O542P76S1	-74	tRNA Modification
asnV_tRNA	asnV_tRNA (b1989, uncharged tRNA)	C736H841Mg2N285O542P76S1	-74	tRNA Modification
asnW_tRNA	asnW_tRNA (b1984, uncharged tRNA)	C736H841Mg2N285O542P76S1	-74	tRNA Modification

asp-L	L-Aspartate	C4H6NO4	-1	Others
asp-L[e]	L-Aspartate	C4H6NO4	-1	Others
asp1_tRNA	asp1_tRNA (uncharged tRNA)	C741H853N290O547P77S1Mg2	-74	tRNA charging
asp1_tRNA_asp	asp1_tRNA (charged tRNA)	C745H859Mg2N291O551P77S1	-75	tRNA charging
aspT_tRNA	aspT_tRNA (b3760, uncharged tRNA)	C741H853Mg2N290O547P77S1	-74	tRNA Modification
aspU_tRNA	aspU_tRNA (b0206, uncharged tRNA)	C741H853Mg2N290O547P77S1	-74	tRNA Modification
aspV_tRNA	aspV_tRNA (b0216, uncharged tRNA)	C741H853Mg2N290O547P77S1	-74	tRNA Modification
atp	ATP	C10H12N5O13P3	-4	Others
atp[e]	ATP	C10H12N5O13P3	-4	Others
b0014_aa	polypeptide b0014_v2	C3002H4872N843O989S16	-31	Translation
b0014_def_map_cplx	Polypeptide b0014 peptide deformylase and methionine aminopeptidase complex	C5132H8328N1436O1635S37Mg0Zn0Fe3	-40	Maturation
b0014_m	Matured polypeptide b0014	C2996H4864N842O987S15	-30	Maturation
b0014_mRNA	mRNA b0014	C18301H20694N7453O13277P1919	-1920	Translation
b0014_mRNA_1	mRNA b0014	C18301H20694N7453O13277P1919	-1920	Translation
b0014_mRNA_2	mRNA b0014	C18301H20694N7453O13277P1919	-1920	Translation
b0014_mRNA_2_degr	mRNA b0014 degradation complex	C58984H86093N19328O25962S200P1919Mg6Zn2Fe0	-2142	mRNA degradation
b0014_m_DnaKJ_complex	b0014 DnaK DnaJ_dim complex - Kerner et al. class II can interact w/ GroEL/ES, cannot fold spontanously	C9560H15378O3085N2755S62P3Zn4	-52	Folding
b0014_m_GroEL.(7)ATP.transGroES	b0014 GroEL GroES complex - Kerner et al. class II can interact w/ GroEL/ES, cannot fold spontanously	C40908H67808O13321N11426S379P21Mg7	-331	Folding
b0014_v1_mRNA	mRNA b0014_v1	C18301H20694N7453O13277P1919	-1920	Translation
b0014_v2_mRNA	mRNA b0014_v2	C18301H20694N7453O13277P1919	-1920	Translation
b0015_aa	polypeptide b0015_v2	C1785H2827N534O551S17	1	Translation
b0015_def_map_cplx	Polypeptide b0015 peptide deformylase and methionine aminopeptidase complex	C3915H6283N1127O1197S38Mg0Zn0Fe3	-8	Maturation
b0015_m	Matured polypeptide b0015	C1779H2819N533O549S16	2	Maturation
b0015_mRNA	mRNA b0015	C10801H12211N4406O7873P1131	-1132	Translation
b0015_mRNA_1	mRNA b0015	C10801H12211N4406O7873P1131	-1132	Translation
b0015_mRNA_2	mRNA b0015	C10801H12211N4406O7873P1131	-1132	Translation
b0015_mRNA_2_degr	mRNA b0015 degradation complex	C51484H77610N16281O20558S200P1131Mg6Zn2Fe0	-1354	mRNA degradation
b0015_m_GroEL.(7)ATP.transGroES	b0015 GroEL GroES complex - Kerner et al. class III needs GroEL/ES	C39691H65763O12883N11117S380P21Mg7Zn2	-295	Folding
b0015_m_Zn	b0015 plus _Zn	C1779H2819N533O549S16Zn2	6	Folding
b0015_v1_mRNA	mRNA b0015_v1	C10801H12211N4406O7873P1131	-1132	Translation
b0015_v2_mRNA	mRNA b0015_v2	C10801H12211N4406O7873P1131	-1132	Translation
b0023_aa	polypeptide b0023_v1	C420H730N139O117S4	15	Translation
b0023_def_map_cplx	Polypeptide b0023 peptide deformylase and methionine aminopeptidase complex	C2550H4186N732O763S25Mg0Zn0Fe3	6	Maturation
b0023_m	Matured polypeptide b0023	C414H722N138O115S3	16	Maturation
b0023_mRNA	mRNA b0023	C2522H2853N1032O1818P266	-267	Translation
b0023_mRNA_1	mRNA b0023	C2522H2853N1032O1818P266	-267	Translation
b0023_mRNA_2	mRNA b0023	C2522H2853N1032O1818P266	-267	Translation
b0023_mRNA_2_degr	mRNA b0023 degradation complex	C43205H68252N12907O14503S200P266Mg6Zn2Fe0	-489	mRNA degradation
b0023_v1_mRNA	mRNA b0023_v1	C2522H2853N1032O1818P266	-267	Translation
b0025_aa	polypeptide b0025	C1552H2512N452O436S9	6	Translation
b0025_def_map_cplx	Polypeptide b0025 peptide deformylase and methionine aminopeptidase complex	C3682H5968N1045O1082S30Mg0Zn0Fe3	-3	Maturation
b0025_m	Matured polypeptide b0025	C1546H2504N451O434S8	7	Maturation
b0025_mRNA	mRNA b0025	C8967H10137N3578O6616P944	-945	Translation
b0025_mRNA_1	mRNA b0025	C8967H10137N3578O6616P944	-945	Translation
b0025_mRNA_2	mRNA b0025	C8967H10137N3578O6616P944	-945	Translation
b0025_mRNA_2_degr	mRNA b0025 degradation complex	C49650H75536N15453O19301S200P944Mg6Zn2Fe0	-1167	mRNA degradation
b0026_aa	polypeptide b0026	C4670H7227N1262O1377S38	-23	Translation
b0026_def_map_cplx	Polypeptide b0026 peptide deformylase and methionine aminopeptidase complex	C6800H10683N1855O2023S59Mg0Zn0Fe3	-32	Maturation
b0026_m	Matured polypeptide b0026	C4664H7219N1261O1375S37	-22	Maturation
b0026_mRNA	mRNA b0026	C26832H30397N10818O19656P2817	-2818	Translation
b0026_mRNA_1	mRNA b0026	C26832H30397N10818O19656P2817	-2818	Translation
b0026_mRNA_2	mRNA b0026	C26832H30397N10818O19656P2817	-2818	Translation
b0026_mRNA_2_degr	mRNA b0026 degradation complex	C67515H95796N22693O32341S200P2817Mg6Zn2Fe0	-3040	mRNA degradation
b0026_m_Zn	b0026 plus _Zn	C4664H7219N1261O1375S37Zn2	-18	Folding

b0027_aa	polypeptide b0027_v1	C854H1298N211O217S6	1	Translation
b0027_def_map_cplx	Polypeptide b0027 peptide deformylase and methionine aminopeptidase complex	C2984H4754N804O863S27Mg0Zn0Fe3	-8	Maturation
b0027_m	Matured polypeptide b0027	C848H1290N210O215S5	2	Maturation
b0027_mRNA	mRNA b0027 (1 nt short)	C4678H5297N1808O3506P494	-495	Translation
b0027_mRNA_1	mRNA b0027 (1 nt short)	C4678H5297N1808O3506P494	-495	Translation
b0027_mRNA_2	mRNA b0027 (1 nt short)	C4678H5297N1808O3506P494	-495	Translation
b0027_mRNA_2_degr	mRNA b0027 degradation complex	C45361H70696N13683O16191S200P494Mg6Zn2Fe0	-717	mRNA degradation
b0028_aa	polypeptide b0028_v1	C710H1092N185O232S5	-17	Translation
b0028_def_map_cplx	Polypeptide b0028 peptide deformylase and methionine aminopeptidase complex	C2840H4548N778O878S26Mg0Zn0Fe3	-26	Maturation
b0028_m	Matured polypeptide b0028	C704H1084N184O230S4	-16	Maturation
b0028_mRNA	mRNA b0028	C4275H4846N1695O3156P450	-451	Translation
b0028_mRNA_1	mRNA b0028	C4275H4846N1695O3156P450	-451	Translation
b0028_mRNA_2	mRNA b0028	C4275H4846N1695O3156P450	-451	Translation
b0028_mRNA_2_degr	mRNA b0028 degradation complex	C44958H70245N13570O15841S200P450Mg6Zn2Fe0	-673	mRNA degradation
b0029_aa	polypeptide b0029_v1	C1523H2453N437O474S10	-12	Translation
b0029_def_map_cplx	Polypeptide b0029 peptide deformylase and methionine aminopeptidase complex	C3653H5909N1030O1120S31Mg0Zn0Fe3	-21	Maturation
b0029_m	Matured polypeptide b0029	C1517H2445N436O472S9	-11	Maturation
b0029_mRNA	mRNA b0029	C9075H10255N3678O6645P951	-952	Translation
b0029_mRNA_1	mRNA b0029	C9075H10255N3678O6645P951	-952	Translation
b0029_mRNA_2	mRNA b0029	C9075H10255N3678O6645P951	-952	Translation
b0029_mRNA_2_degr	mRNA b0029 degradation complex	C49758H75654N15553O19330S200P951Mg6Zn2Fe0	-1174	mRNA degradation
b0049_aa	polypeptide b0049_v3	C1412H2156N375O409S12	-11	Translation
b0049_def_map_cplx	Polypeptide b0049 peptide deformylase and methionine aminopeptidase complex	C3542H5612N968O1055S33Mg0Zn0Fe3	-20	Maturation
b0049_m	Matured polypeptide b0049	C1406H2148N374O407S11	-10	Maturation
b0049_mRNA	mRNA b0049	C8024H9086N3215O5905P843	-844	Translation
b0049_mRNA_1	mRNA b0049	C8024H9086N3215O5905P843	-844	Translation
b0049_mRNA_2	mRNA b0049	C8024H9086N3215O5905P843	-844	Translation
b0049_mRNA_2_degr	mRNA b0049 degradation complex	C48707H74485N15090O18590S200P843Mg6Zn2Fe0	-1066	mRNA degradation
b0049_v2_mRNA	mRNA b0049_v2	C8024H9086N3215O5905P843	-844	Translation
b0049_v3_mRNA	mRNA b0049_v3	C8024H9086N3215O5905P843	-844	Translation
b0050_aa	polypeptide b0050_v3	C622H962N165O188S4	-7	Translation
b0050_def_map_cplx	Polypeptide b0050 peptide deformylase and methionine aminopeptidase complex	C2752H4418N758O834S25Mg0Zn0Fe3	-16	Maturation
b0050_m	Matured polypeptide b0050	C616H954N164O186S3	-6	Maturation
b0050_mRNA	mRNA b0050	C3588H4069N1416O2645P378	-379	Translation
b0050_mRNA_1	mRNA b0050	C3588H4069N1416O2645P378	-379	Translation
b0050_mRNA_2	mRNA b0050	C3588H4069N1416O2645P378	-379	Translation
b0050_mRNA_2_degr	mRNA b0050 degradation complex	C44271H69468N13291O15330S200P378Mg6Zn2Fe0	-601	mRNA degradation
b0050_v2_mRNA	mRNA b0050_v2	C3588H4069N1416O2645P378	-379	Translation
b0050_v3_mRNA	mRNA b0050_v3	C3588H4069N1416O2645P378	-379	Translation
b0051_aa	polypeptide b0051_v3	C1358H2151N377O390S14	0	Translation
b0051_def_map_cplx	Polypeptide b0051 peptide deformylase and methionine aminopeptidase complex	C3488H5607N970O1036S35Mg0Zn0Fe3	-9	Maturation
b0051_m	Matured polypeptide b0051	C1352H2143N376O388S13	1	Maturation
b0051_mRNA	mRNA b0051 (4 nt short)	C7772H8811N3086O5720P818	-819	Translation
b0051_mRNA_1	mRNA b0051 (4 nt short)	C7772H8811N3086O5720P818	-819	Translation
b0051_mRNA_2	mRNA b0051 (4 nt short)	C7772H8811N3086O5720P818	-819	Translation
b0051_mRNA_2_degr	mRNA b0051 degradation complex	C48455H74210N14961O18405S200P818Mg6Zn2Fe0	-1041	mRNA degradation
b0051_v1_mRNA	mRNA b0051_v1 (4 nt short)	C7772H8811N3086O5720P818	-819	Translation
b0051_v2_mRNA	mRNA b0051_v2 (4 nt short)	C7772H8811N3086O5720P818	-819	Translation
b0051_v3_mRNA	mRNA b0051_v3	C7811H8854N3103O5753P824	-825	Translation
b0052_aa	polypeptide b0052_v3	C1572H2531N431O455S12	-8	Translation
b0052_def_map_cplx	Polypeptide b0052 peptide deformylase and methionine aminopeptidase complex	C3702H5987N1024O1101S33Mg0Zn0Fe3	-17	Maturation
b0052_m	Matured polypeptide b0052	C1566H2523N430O453S11	-7	Maturation
b0052_mRNA	mRNA b0052 (1 nt short)	C9386H10653N3710O6947P989	-990	Translation

b0052_mRNA_1	mRNA b0052 (1 nt short)	C9386H10653N3710O6947P989	-990	Translation
b0052_mRNA_2	mRNA b0052 (1 nt short)	C9386H10653N3710O6947P989	-990	Translation
b0052_mRNA_2_degr	mRNA b0052 degradation complex	C50069H76052N15585O19632S200P989Mg6Zn2Fe0	-1212	mRNA degradation
b0052_v1_mRNA	mRNA b0052_v1	C9396H10664N3715O6959P992	-993	Translation
b0052_v2_mRNA	mRNA b0052_v2	C9396H10664N3715O6959P992	-993	Translation
b0053_aa	polypeptide b0053_v1	C2055H3331N608O640S16	-3	Translation
b0053_def_cplx	Polypeptide b0053 peptide deformylase complex	C2899H4725N849O895S22Mg0Zn0Fe1	-8	Maturation
b0053_m	Matured polypeptide b0053	C2012H3263N598O628S16	-3	Maturation
b0053_mRNA	mRNA b0053	C12269H13901N4976O8936P1289	-1290	Translation
b0053_mRNA_1	mRNA b0053	C12269H13901N4976O8936P1289	-1290	Translation
b0053_mRNA_2	mRNA b0053	C12269H13901N4976O8936P1289	-1290	Translation
b0053_mRNA_2_degr	mRNA b0053 degradation complex	C52952H79300N16851O21621S200P1289Mg6Zn2Fe0	-1512	mRNA degradation
b0058_aa	polypeptide b0058	C1109H1748N314O316S11	0	Translation
b0058_def_map_cplx	Polypeptide b0058 peptide deformylase and methionine aminopeptidase complex	C3239H5204N907O962S32Mg0Zn0Fe3	-9	Maturation
b0058_m	Matured polypeptide b0058	C1103H1740N313O314S10	1	Maturation
b0058_mRNA	mRNA b0058	C6303H7118N2563O4603P660	-661	Translation
b0058_mRNA_1	mRNA b0058	C6303H7118N2563O4603P660	-661	Translation
b0058_mRNA_2	mRNA b0058	C6303H7118N2563O4603P660	-661	Translation
b0058_mRNA_2_degr	mRNA b0058 degradation complex	C46986H72517N14438O17288S200P660Mg6Zn2Fe0	-883	mRNA degradation
b0059_aa	polypeptide b0059_v1	C4837H7593N1383O1479S30	-50	Translation
b0059_def_map_cplx	Polypeptide b0059 peptide deformylase and methionine aminopeptidase complex	C6967H11049N1976O2125S51Mg0Zn0Fe3	-59	Maturation
b0059_m	Matured polypeptide b0059	C4831H7585N1382O1477S29	-49	Maturation
b0059_mRNA_1	mRNA b0059	C27659H31344N11079O20313P2909	-2910	Translation
b0059_mRNA_2	mRNA b0059	C27659H31344N11079O20313P2909	-2910	Translation
b0059_mRNA_2_degr	mRNA b0059 degradation complex	C68342H96743N22954O32998S200P2909Mg6Zn2Fe0	-3132	mRNA degradation
b0059_m_DnaKJ_complex	b0059 DnaK DnaJ_dim complex - Deuerling et al. DnaKJ/GrpE dependent folding	C11395H18099O3575N3295S76P3Zn4	-71	Folding
b0059_v1_mRNA	mRNA b0059_v1	C27659H31344N11079O20313P2909	-2910	Translation
b0144_DNA_act	DNA b0144 (activated form)	C9011H10418N3454O5574P927	-927	Transcription
b0144_DNA_neu	DNA b0144 (inactivate form)	C9011H10418N3454O5574P927	-927	Transcription
b0144_aa	polypeptide b0144	C1574H2411N448O443S6	-5	Translation
b0144_def_map_cplx	Polypeptide b0144 peptide deformylase and methionine aminopeptidase complex	C3704H5867N1041O1089S27Mg0Zn0Fe3	-14	Maturation
b0144_m	Matured polypeptide b0144	C1503H2312N436O428S4	-4	Maturation
b0144_mRNA	mRNA b0144	C8790H9977N3454O6508P929	-930	Translation
b0144_mRNA_1	mRNA b0144	C8790H9977N3454O6508P929	-930	Translation
b0144_mRNA_2	mRNA b0144	C8790H9977N3454O6508P929	-930	Translation
b0144_mRNA_2_degr	mRNA b0144 degradation complex	C49473H75376N15329O19193S200P929Mg6Zn2Fe0	-1152	mRNA degradation
b0144_m_DnaKJ_complex	b0144 DnaK DnaJ_dim complex - Kerner et al. class II can interact w/ GroEL/ES, cannot fold spontanously	C8067H12826O2526N2349S51P3Zn4	-26	Folding
b0144_m_GroEL.(7)ATP.transGroES	b0144 GroEL GroES complex - Kerner et al. class II can interact w/ GroEL/ES, cannot fold spontanously	C39415H65256O12762N11020S368P21Mg7	-305	Folding
b0166_aa	polypeptide b0166_v1	C1326H2113N368O403S8	-5	Translation
b0166_def_cplx	Polypeptide b0166 peptide deformylase complex	C2170H3507N609O658S14Mg0Zn0Fe1	-10	Maturation
b0166_m	Matured polypeptide b0166	C1325H2114N368O402S8	-4	Maturation
b0166_mRNA_1	mRNA b0166	C7858H8888N3153O5771P827	-828	Translation
b0166_mRNA_2	mRNA b0166	C7858H8888N3153O5771P827	-828	Translation
b0166_mRNA_2_degr	mRNA b0166 degradation complex	C48541H74287N15028O18456S200P827Mg6Zn2Fe0	-1050	mRNA degradation
b0166_v1_mRNA	mRNA b0166_v1	C7858H8888N3153O5765P825	-826	Translation
b0167_aa	polypeptide b0167	C4570H7183N1317O1308S28	-16	Translation
b0167_def_map_cplx	Polypeptide b0167 peptide deformylase and methionine aminopeptidase complex	C6700H10639N1910O1954S49Mg0Zn0Fe3	-25	Maturation
b0167_m	Matured polypeptide b0167	C4564H7175N1316O1306S27	-15	Maturation
b0167_mRNA	mRNA b0167	C25375H28796N10047O18697P2673	-2674	Translation
b0167_mRNA_1	mRNA b0167	C25375H28796N10047O18697P2673	-2674	Translation
b0167_mRNA_2	mRNA b0167	C25375H28796N10047O18697P2673	-2674	Translation
b0167_mRNA_2_degr	mRNA b0167 degradation complex	C66058H94195N21922O31382S200P2673Mg6Zn2Fe0	-2896	mRNA degradation

b0167_m_DnaKJ_complex	b0167 DnaK DnaJ_dim complex - Kerner et al. class II can interact w/ GroEL/ES, cannot fold spontanously	C11128H17689O3404N3229S74P3Zn4	-37	Folding
b0167_m_GroEL.(7)ATP.transGroES	b0167 GroEL GroES complex - Kerner et al. class II can interact w/ GroEL/ES, cannot fold spontanously	C42476H70119O13640N11900S391P21Mg7	-316	Folding
b0168_aa	polypeptide b0168	C1292H2070N353O393S16	-9	Translation
b0168_def_map_cplx	Polypeptide b0168 peptide deformylase and methionine aminopeptidase complex	C3422H5526N946O1039S37Mg0Zn0Fe3	-18	Maturation
b0168_m	Matured polypeptide b0168	C1286H2062N352O391S15	-8	Maturation
b0168_mRNA	mRNA b0168	C7578H8570N3055O5530P797	-798	Translation
b0168_mRNA_1	mRNA b0168	C7578H8570N3055O5530P797	-798	Translation
b0168_mRNA_2	mRNA b0168	C7578H8570N3055O5530P797	-798	Translation
b0168_mRNA_2_degr	mRNA b0168 degradation complex	C48261H73969N14930O18215S200P797Mg6Zn2Fe0	-1020	mRNA degradation
b0168_m_Fe	b0168 plus _Fe	C1286H2062N352O391S15Fe2	-4	Folding
b0169_aa	polypeptide b0169	C1186H1893N333O354S9	-2	Translation
b0169_def_cplx	Polypeptide b0169 peptide deformylase complex	C2030H3287N574O609S15Mg0Zn0Fe1	-7	Maturation
b0169_m	Matured polypeptide b0169	C1180H1885N332O352S8	-1	Maturation
b0169_mRNA	mRNA b0169	C6910H7821N2764O5068P728	-729	Translation
b0169_mRNA_1	mRNA b0169	C6910H7821N2764O5068P728	-729	Translation
b0169_mRNA_2	mRNA b0169	C6910H7821N2764O5068P728	-729	Translation
b0169_mRNA_2_degr	mRNA b0169 degradation complex	C47593H73220N14639O17753S200P728Mg6Zn2Fe0	-951	mRNA degradation
b0170_aa	polypeptide b0170	C1337H2178N364O419S12	-10	Translation
b0170_def_map_cplx	Polypeptide b0170 peptide deformylase and methionine aminopeptidase complex	C3467H5634N957O1065S33Mg0Zn0Fe3	-19	Maturation
b0170_m	Matured polypeptide b0170	C1331H2170N363O417S11	-9	Maturation
b0170_mRNA	mRNA b0170	C8154H9193N3348O5898P852	-853	Translation
b0170_mRNA_1	mRNA b0170	C8154H9193N3348O5898P852	-853	Translation
b0170_mRNA_2	mRNA b0170	C8154H9193N3348O5898P852	-853	Translation
b0170_mRNA_2_degr	mRNA b0170 degradation complex	C48837H74592N15223O18583S200P852Mg6Zn2Fe0	-1075	mRNA degradation
b0170_m_DnaKJ_complex	b0170 DnaK DnaJ_dim complex - Kerner et al. class I can interact w/ GroEL/ES	C7895H12684O2515N2276S58P3Zn4	-31	Folding
b0170_m_GroEL.(7)ATP.transGroES	b0170 GroEL GroES complex - Kerner et al. class I can interact w/ GroEL/ES	C39243H65114O12751N10947S375P21Mg7	-310	Folding
b0172_aa	polypeptide b0172	C889H1492N263O288S6	-1	Translation
b0172_def_cplx	Polypeptide b0172 peptide deformylase complex	C1733H2886N504O543S12Mg0Zn0Fe1	-6	Maturation
b0172_m	Matured polypeptide b0172	C888H1493N263O287S6	0	Maturation
b0172_mRNA	mRNA b0172	C5339H6028N2197O3861P560	-561	Translation
b0172_mRNA_1	mRNA b0172	C5339H6028N2197O3861P560	-561	Translation
b0172_mRNA_2	mRNA b0172	C5339H6028N2197O3861P560	-561	Translation
b0172_mRNA_2_degr	mRNA b0172 degradation complex	C46022H71427N14072O16546S200P560Mg6Zn2Fe0	-783	mRNA degradation
b0172_m_DnaKJ_complex	b0172 DnaK DnaJ_dim complex - Kerner et al. class I can interact w/ GroEL/ES	C7452H12007O2385N2176S53P3Zn4	-22	Folding
b0172_m_GroEL.(7)ATP.transGroES	b0172 GroEL GroES complex - Kerner et al. class I can interact w/ GroEL/ES	C38800H64437O12621N10847S370P21Mg7	-301	Folding
b0188_aa	polypeptide b0188	C2151H3404N625O616S11	-3	Translation
b0188_def_map_cplx	Polypeptide b0188 peptide deformylase and methionine aminopeptidase complex	C4281H6860N1218O1262S32Mg0Zn0Fe3	-12	Maturation
b0188_m	Matured polypeptide b0188	C2145H3396N624O614S10	-2	Maturation
b0188_mRNA	mRNA b0188	C12373H14010N4981O9115P1301	-1302	Translation
b0188_mRNA_1	mRNA b0188	C12373H14010N4981O9115P1301	-1302	Translation
b0188_mRNA_2	mRNA b0188	C12373H14010N4981O9115P1301	-1302	Translation
b0188_mRNA_2_degr	mRNA b0188 degradation complex	C53056H79409N16856O21800S200P1301Mg6Zn2Fe0	-1524	mRNA degradation
b0194_aa	polypeptide b0194	C2833H4476N774O859S18	-20	Translation
b0194_def_cplx	Polypeptide b0194 peptide deformylase complex	C3677H5870N1015O1114S24Mg0Zn0Fe1	-25	Maturation
b0194_m	matured polypeptide b0194 _ error CCDB	C2832H4477N774O858S18	-19	Maturation
b0194_mRNA	mRNA b0194	C16377H18540N6599O11998P1721	-1722	Translation
b0194_mRNA_1	mRNA b0194	C16377H18540N6599O11998P1721	-1722	Translation
b0194_mRNA_2	mRNA b0194	C16377H18540N6599O11998P1721	-1722	Translation
b0194_mRNA_2_degr	mRNA b0194 degradation complex	C57060H83939N18474O24683S200P1721Mg6Zn2Fe0	-1944	mRNA degradation
b0194_m_DnaKJ_complex	b0194 DnaK DnaJ_dim complex - Deuerling et al. DnaKJ/GrpE dependent folding	C9396H14991O2956N2687S65P3Zn4	-41	Folding
b0201_RNA	rRNA	C14752H16647N6058O10723P1542	-1543	rRNA Modification
b0201_RNA_1		C14753H16649N6058O10723P1542S0	-1543	rRNA Modification
b0201_RNA_10		C14764H16671N6058O10723P1542S0	-1543	rRNA Modification

b0201_RNA_10_RsmB_mono	b0201_RNA_10, RsmB_mono	C16931H20114N6676O11351S17P1542Mg0Zn0Fe0	-1542	rRNA Modification
b0201_RNA_1_MeT_16S_1402	b0201_RNA_1, MeT_16S_1402	C14783H16695N6070O10733S2P1542Mg0Zn0Fe0	-1541	rRNA Modification
b0201_RNA_2		C14755H16653N6058O10723P1542S0	-1543	rRNA Modification
b0201_RNA_2_MeT_16S_1407	b0201_RNA_2, MeT_16S_1407	C14770H16676N6064O10728S1P1542Mg0Zn0Fe0	-1542	rRNA Modification
b0201_RNA_3		C14756H16655N6058O10723P1542S0	-1543	rRNA Modification
b0201_RNA_3_YggJ_mono	b0201_RNA_3, YggJ_mono	C15948H18595N6406O11082S11P1542Mg0Zn0Fe0	-1545	rRNA Modification
b0201_RNA_4		C14757H16657N6058O10723P1542S0	-1543	rRNA Modification
b0201_RNA_4_MeT_16S_1516	b0201_RNA_4, MeT_16S_1516	C14772H16680N6064O10728S1P1542Mg0Zn0Fe0	-1542	rRNA Modification
b0201_RNA_5		C14758H16659N6058O10723P1542S0	-1543	rRNA Modification
b0201_RNA_5_KsgA_mono	b0201_RNA_5, KsgA_mono	C16140H18848N6446O11121S15P1542Mg0Zn0Fe0	-1540	rRNA Modification
b0201_RNA_6		C14760H16663N6058O10723P1542S0	-1543	rRNA Modification
b0201_RNA_6_KsgA_mono	b0201_RNA_6, KsgA_mono	C16142H18852N6446O11121S15P1542Mg0Zn0Fe0	-1540	rRNA Modification
b0201_RNA_7		C14762H16667N6058O10723P1542S0	-1543	rRNA Modification
b0201_RNA_7_RsuA_mono	b0201_RNA_7, RsuA_mono	C15907H18467N6386O11067S6P1542Mg0Zn0Fe0	-1551	rRNA Modification
b0201_RNA_8		C14762H16667N6058O10723P1542S0	-1543	rRNA Modification
b0201_RNA_8_MeT_16S_527	b0201_RNA_8, MeT_16S_527	C14777H16690N6064O10728S1P1542Mg0Zn0Fe0	-1542	rRNA Modification
b0201_RNA_9		C14763H16669N6058O10723P1542S0	-1543	rRNA Modification
b0201_RNA_9_MeT_16S_966	b0201_RNA_9, MeT_16S_966	C14778H16692N6064O10728S1P1542Mg0Zn0Fe0	-1542	rRNA Modification
b0201_RNA_RsmC_mono	b0201_RNA, RsmC_mono	C16436H19278N6531O11225S11P1542Mg0Zn0Fe0	-1547	rRNA Modification
b0201_v1_RNA	rRNA	C14752H16647N6058O10723P1542	-1543	rRNA Modification
b0201_v1_RNA_1		C14753H16649N6058O10723P1542S0	-1543	rRNA Modification
b0201_v1_RNA_10		C14764H16671N6058O10723P1542S0	-1543	rRNA Modification
b0201_v1_RNA_10_RsmB_mono	b0201_v1_RNA_10, RsmB_mono	C16931H20114N6676O11351S17P1542Mg0Zn0Fe0	-1542	rRNA Modification
b0201_v1_RNA_1_MeT_16S_1402	b0201_v1_RNA_1, MeT_16S_1402	C14783H16695N6070O10733S2P1542Mg0Zn0Fe0	-1541	rRNA Modification
b0201_v1_RNA_2		C14755H16653N6058O10723P1542S0	-1543	rRNA Modification
b0201_v1_RNA_2_MeT_16S_1407	b0201_v1_RNA_2, MeT_16S_1407	C14770H16676N6064O10728S1P1542Mg0Zn0Fe0	-1542	rRNA Modification
b0201_v1_RNA_3		C14756H16655N6058O10723P1542S0	-1543	rRNA Modification
b0201_v1_RNA_3_YggJ_mono	b0201_v1_RNA_3, YggJ_mono	C15948H18595N6406O11082S11P1542Mg0Zn0Fe0	-1545	rRNA Modification
b0201_v1_RNA_4		C14757H16657N6058O10723P1542S0	-1543	rRNA Modification
b0201_v1_RNA_4_MeT_16S_1516	b0201_v1_RNA_4, MeT_16S_1516	C14772H16680N6064O10728S1P1542Mg0Zn0Fe0	-1542	rRNA Modification
b0201_v1_RNA_5		C14758H16659N6058O10723P1542S0	-1543	rRNA Modification
b0201_v1_RNA_5_KsgA_mono	b0201_v1_RNA_5, KsgA_mono	C16140H18848N6446O11121S15P1542Mg0Zn0Fe0	-1540	rRNA Modification
b0201_v1_RNA_6		C14760H16663N6058O10723P1542S0	-1543	rRNA Modification
b0201_v1_RNA_6_KsgA_mono	b0201_v1_RNA_6, KsgA_mono	C16142H18852N6446O11121S15P1542Mg0Zn0Fe0	-1540	rRNA Modification
b0201_v1_RNA_7		C14762H16667N6058O10723P1542S0	-1543	rRNA Modification
b0201_v1_RNA_7_RsuA_mono	b0201_v1_RNA_7, RsuA_mono	C15907H18467N6386O11067S6P1542Mg0Zn0Fe0	-1551	rRNA Modification
b0201_v1_RNA_8		C14762H16667N6058O10723P1542S0	-1543	rRNA Modification
b0201_v1_RNA_8_MeT_16S_527	b0201_v1_RNA_8, MeT_16S_527	C14777H16690N6064O10728S1P1542Mg0Zn0Fe0	-1542	rRNA Modification
b0201_v1_RNA_9		C14763H16669N6058O10723P1542S0	-1543	rRNA Modification
b0201_v1_RNA_9_MeT_16S_966	b0201_v1_RNA_9, MeT_16S_966	C14778H16692N6064O10728S1P1542Mg0Zn0Fe0	-1542	rRNA Modification
b0201_v1_RNA_RsmC_mono	b0201_v1_RNA, RsmC_mono	C16436H19278N6531O11225S11P1542Mg0Zn0Fe0	-1547	rRNA Modification
b0202_RNA	tRNA (ileV)	C734H831N296O541P77	-78	tRNA Modification
b0202_tRNA_1	b0202_tRNA_1 (ileV)	C734H833Mg2N296O541P77	-74	tRNA Modification
b0202_tRNA_1_Dus_gen_cplx	b0202_tRNA_1 (ileV), Dus_gen	C2341H3372Mg2N755O1024P80S14	-81	tRNA Modification
b0202_tRNA_2	b0202_tRNA_2 (ileV)	C734H835Mg2N296O541P77	-74	tRNA Modification
b0202_tRNA_2_Up_tRNA_pos_37_t6A_cplx	b0202_tRNA_2 (ileV), Up_tRNA_pos_37_t6A	C749H857Mg3N302O560P80	-77	tRNA Modification
b0202_tRNA_3	b0202_tRNA_3 (ileV)	C739H841Mg2N297O545P77	-75	tRNA Modification
b0202_tRNA_3_YggH_mono_cplx	b0202_tRNA_3 (ileV), YggH_mono	C1964H2740Mg2N650O892P77S14	-77	tRNA Modification
b0202_tRNA_4	b0202_tRNA_4 (ileV)	C740H843Mg2N297O545P77	-75	tRNA Modification
b0202_tRNA_4_AcpT_tRNA_pos_47_acp3U_cplx	b0202_tRNA_4 (ileV), AcpT_tRNA_pos_47_acp3U	C755H866Mg2N303O550P77S1	-74	tRNA Modification
b0202_tRNA_5	b0202_tRNA_5 (ileV)	C744H850Mg2N298O547P77	-75	tRNA Modification
b0202_tRNA_5_TrmA_mono_cplx	b0202_tRNA_5 (ileV), TrmA_mono	C2618H3797Mg2N821O1106P77S19	-83	tRNA Modification
b0202_tRNA_6	b0202_tRNA_6 (ileV)	C745H852Mg2N298O547P77	-75	tRNA Modification
b0202_tRNA_6_TruB_mono_cplx	b0202_tRNA_6 (ileV), TruB_mono	C2285H3327Mg2N736O1016P77S10	-80	tRNA Modification

b0202_tRNA_7	b0202_tRNA_7 (ileV)	C745H852Mg2N298O547P77	-75	tRNA Modification
b0202_tRNA_7_YqcB_mono_cplx	b0202_tRNA_7 (ileV), YqcB_mono	C2059H2908Mg2N685O926P77S6	-78	tRNA Modification
b0202_tRNA_Mg2	tRNA (ileV) bound two Mg2 ions	C734H831Mg2N296O541P77	-74	tRNA Modification
b0202_tRNA_Mg2_Dus_gen_cplx	b0202_tRNA (ileV), Dus_gen	C2341H3370Mg2N755O1024P80S14	-81	tRNA Modification
b0202_v1_RNA	tRNA (ileV)	C734H831N296O541P77	-78	tRNA Modification
b0202_v1_tRNA_1	b0202_v1_tRNA_1 (ileV)	C734H833Mg2N296O541P77	-74	tRNA Modification
b0202_v1_tRNA_1_Dus_gen_cplx	b0202_v1_tRNA_1 (ileV), Dus_gen	C2341H3372Mg2N755O1024P80S14	-81	tRNA Modification
b0202_v1_tRNA_2	b0202_v1_tRNA_2 (ileV)	C734H835Mg2N296O541P77	-74	tRNA Modification
b0202_v1_tRNA_2_Up_tRNA_pos_37_t6A_cplx	b0202_v1_tRNA_2 (ileV), Up_tRNA_pos_37_t6A	C749H857Mg3N302O560P80	-77	tRNA Modification
b0202_v1_tRNA_3	b0202_v1_tRNA_3 (ileV)	C739H841Mg2N297O545P77	-75	tRNA Modification
b0202_v1_tRNA_3_YggH_mono_cplx	b0202_v1_tRNA_3 (ileV), YggH_mono	C1964H2740Mg2N650O892P77S14	-77	tRNA Modification
b0202_v1_tRNA_4	b0202_v1_tRNA_4 (ileV)	C740H843Mg2N297O545P77	-75	tRNA Modification
b0202_v1_tRNA_4_AcpT_tRNA_pos_47_acp3U_cplx	b0202_v1_tRNA_4 (ileV), AcpT_tRNA_pos_47_acp3U	C755H866Mg2N303O550P77S1	-74	tRNA Modification
b0202_v1_tRNA_5	b0202_v1_tRNA_5 (ileV)	C744H850Mg2N298O547P77	-75	tRNA Modification
b0202_v1_tRNA_5_TrmA_mono_cplx	b0202_v1_tRNA_5 (ileV), TrmA_mono	C2618H3797Mg2N821O1106P77S19	-83	tRNA Modification
b0202_v1_tRNA_6	b0202_v1_tRNA_6 (ileV)	C745H852Mg2N298O547P77	-75	tRNA Modification
b0202_v1_tRNA_6_TruB_mono_cplx	b0202_v1_tRNA_6 (ileV), TruB_mono	C2285H3327Mg2N736O1016P77S10	-80	tRNA Modification
b0202_v1_tRNA_7	b0202_v1_tRNA_7 (ileV)	C745H852Mg2N298O547P77	-75	tRNA Modification
b0202_v1_tRNA_7_YqcB_mono_cplx	b0202_v1_tRNA_7 (ileV), YqcB_mono	C2059H2908Mg2N685O926P77S6	-78	tRNA Modification
b0202_v1_tRNA_Mg2	tRNA (ileV) bound two Mg2 ions	C734H831Mg2N296O541P77	-74	tRNA Modification
b0202_v1_tRNA_Mg2_Dus_gen_cplx	b0202_v1_tRNA (ileV), Dus_gen	C2341H3370Mg2N755O1024P80S14	-81	tRNA Modification
b0203_RNA	tRNA (alaV)	C722H822N289O535P76	-77	tRNA Modification
b0203_tRNA_1	b0203_tRNA_1 (alaV)	C722H824Mg2N289O535P76	-73	tRNA Modification
b0203_tRNA_1_YecO_mono-YecP_mono-HyL_tRNA_pos_34_ho5U_cplx	b0203_tRNA_1 (alaV), YecO_mono, YecP_mono, HyL_tRNA_pos_34_ho5U	C3655H5357Mg2N1090O1385P76S23	-91	tRNA Modification
b0203_tRNA_2	b0203_tRNA_2 (alaV)	C724H825Mg2N289O538P76	-74	tRNA Modification
b0203_tRNA_2_YggH_mono_cplx	b0203_tRNA_2 (alaV), YggH_mono	C1949H2724Mg2N642O885P76S14	-76	tRNA Modification
b0203_tRNA_3	b0203_tRNA_3 (alaV)	C725H827Mg2N289O538P76	-74	tRNA Modification
b0203_tRNA_3_TrmA_mono_cplx	b0203_tRNA_3 (alaV), TrmA_mono	C2599H3774Mg2N812O1097P76S19	-82	tRNA Modification
b0203_tRNA_4	b0203_tRNA_4 (alaV)	C726H829Mg2N289O538P76	-74	tRNA Modification
b0203_tRNA_4_TruB_mono_cplx	b0203_tRNA_4 (alaV), TruB_mono	C2266H3304Mg2N727O1007P76S10	-79	tRNA Modification
b0203_tRNA_Mg2	tRNA (alaV) bound two Mg2 ions	C722H822Mg2N289O535P76	-73	tRNA Modification
b0203_tRNA_Mg2_Dus_gen_cplx	b0203_tRNA (alaV), Dus_gen	C2329H3361Mg2N748O1018P79S14	-80	tRNA Modification
b0203_v1_RNA	tRNA (alaV)	C722H822N289O535P76	-77	tRNA Modification
b0203_v1_tRNA_1	b0203_v1_tRNA_1 (alaV)	C722H824Mg2N289O535P76	-73	tRNA Modification
b0203_v1_tRNA_1_YecO_mono-YecP_mono-HyL_tRNA_pos_34_ho5U_cplx	b0203_v1_tRNA_1 (alaV), YecO_mono, YecP_mono, HyL_tRNA_pos_34_ho5U	C3655H5357Mg2N1090O1385P76S23	-91	tRNA Modification
b0203_v1_tRNA_2	b0203_v1_tRNA_2 (alaV)	C724H825Mg2N289O538P76	-74	tRNA Modification
b0203_v1_tRNA_2_YggH_mono_cplx	b0203_v1_tRNA_2 (alaV), YggH_mono	C1949H2724Mg2N642O885P76S14	-76	tRNA Modification
b0203_v1_tRNA_3	b0203_v1_tRNA_3 (alaV)	C725H827Mg2N289O538P76	-74	tRNA Modification
b0203_v1_tRNA_3_TrmA_mono_cplx	b0203_v1_tRNA_3 (alaV), TrmA_mono	C2599H3774Mg2N812O1097P76S19	-82	tRNA Modification
b0203_v1_tRNA_4	b0203_v1_tRNA_4 (alaV)	C726H829Mg2N289O538P76	-74	tRNA Modification
b0203_v1_tRNA_4_TruB_mono_cplx	b0203_v1_tRNA_4 (alaV), TruB_mono	C2266H3304Mg2N727O1007P76S10	-79	tRNA Modification
b0203_v1_tRNA_Mg2	tRNA (alaV) bound two Mg2 ions	C722H822Mg2N289O535P76	-73	tRNA Modification
b0203_v1_tRNA_Mg2_Dus_gen_cplx	b0203_v1_tRNA (alaV), Dus_gen	C2329H3361Mg2N748O1018P79S14	-80	tRNA Modification
b0204_RNA	rRNA	C27811H31351N11468O20159P2904	-2905	rRNA Modification
b0204_RNA_1		C27812H31353N11468O20159P2904S0	-2905	rRNA Modification
b0204_RNA_10		C27819H31367N11468O20159P2904S0	-2905	rRNA Modification
b0204_RNA_10_MeT_23S_2445	b0204_RNA_10, MeT_23S_2445	C27834H31390N11474O20164S1P2904Mg0Zn0Fe0	-2904	rRNA Modification
b0204_RNA_11		C27820H31369N11468O20159P2904S0	-2905	rRNA Modification
b0204_RNA_11_DU_23S_2449_a	b0204_RNA_11, DU_23S_2449 (NADH)	C27841H31397N11475O20173S0P2906Mg0Zn0Fe0	-2906	rRNA Modification
b0204_RNA_11_DU_23S_2449_b	b0204_RNA_11, DU_23S_2449 (NADPH)	C27841H31396N11475O20176S0P2907Mg0Zn0Fe0	-2908	rRNA Modification
b0204_RNA_12		C27820H31371N11468O20159P2904S0	-2905	rRNA Modification
b0204_RNA_12_YmfC_mono	b0204_RNA_12, YmfC_mono	C28918H33132N11795O20479S3P2904Mg0Zn0Fe0	-2895	rRNA Modification
b0204_RNA_13		C27820H31371N11468O20159P2904S0	-2905	rRNA Modification
b0204_RNA_13_MeT_23S_2498	b0204_RNA_13, MeT_23S_2498	C27835H31394N11474O20164S1P2904Mg0Zn0Fe0	-2904	rRNA Modification

b0204_RNA_14		C27821H31373N11468O20159P2904S0	-2905	rRNA Modification
b0204_RNA_14_MeT_23S_2503	b0204_RNA_14, MeT_23S_2503	C27836H31396N11474O20164S1P2904Mg0Zn0Fe0	-2904	rRNA Modification
b0204_RNA_15		C27822H31375N11468O20159P2904S0	-2905	rRNA Modification
b0204_RNA_15_RluC_mono	b0204_RNA_15, RluC_mono	C29407H33988N11950O20615S6P2904Mg0Zn0Fe0	-2890	rRNA Modification
b0204_RNA_16		C27822H31375N11468O20159P2904S0	-2905	rRNA Modification
b0204_RNA_16_RrmJ_mono	b0204_RNA_16, RrmJ_mono	C28869H33064N11763O20462S11P2904Mg0Zn0Fe0	-2897	rRNA Modification
b0204_RNA_17		C27823H31377N11468O20159P2904S0	-2905	rRNA Modification
b0204_RNA_17_RluC_mono	b0204_RNA_17, RluC_mono	C29408H33990N11950O20615S6P2904Mg0Zn0Fe0	-2890	rRNA Modification
b0204_RNA_18		C27823H31377N11468O20159P2904S0	-2905	rRNA Modification
b0204_RNA_18_YjbC_mono	b0204_RNA_18, YjbC_mono	C29250H33748N11886O20581S7P2904Mg0Zn0Fe0	-2890	rRNA Modification
b0204_RNA_19		C27823H31377N11468O20159P2904S0	-2905	rRNA Modification
b0204_RNA_19_RluB_mono	b0204_RNA_19, RluB_mono	C29241H33725N11919O20579S5P2904Mg0Zn0Fe0	-2890	rRNA Modification
b0204_RNA_1_MeT_23S_1835	b0204_RNA_1, MeT_23S_1835	C27827H31376N11474O20164S1P2904Mg0Zn0Fe0	-2904	rRNA Modification
b0204_RNA_2		C27813H31355N11468O20159P2904S0	-2905	rRNA Modification
b0204_RNA_20		C27823H31377N11468O20159P2904S0	-2905	rRNA Modification
b0204_RNA_20_RrmA_dim	b0204_RNA_20, RrmA_dim	C30540H35588N12232O20934S31P2904Mg0Zn2Fe0	-2902	rRNA Modification
b0204_RNA_21		C27824H31379N11468O20159P2904S0	-2905	rRNA Modification
b0204_RNA_21_RluA_mono	b0204_RNA_21, RluA_mono	C28927H33119N11781O20473S10P2904Mg0Zn0Fe0	-2904	rRNA Modification
b0204_RNA_22		C27824H31379N11468O20159P2904S0	-2905	rRNA Modification
b0204_RNA_22_RumB_mono	b0204_RNA_22, RumB_mono	C29717H34357N11980O20693S28P2904Mg0Zn0Fe4	-2903	rRNA Modification
b0204_RNA_23		C27825H31381N11468O20159P2904S0	-2905	rRNA Modification
b0204_RNA_23_RluC_mono	b0204_RNA_23, RluC_mono	C29410H33994N11950O20615S6P2904Mg0Zn0Fe0	-2890	rRNA Modification
b0204_RNA_2_RluD_mono	b0204_RNA_2, RluD_mono	C29450H33965N11948O20633S12P2904Mg1Zn0Fe0	-2909	rRNA Modification
b0204_RNA_3		C27813H31355N11468O20159P2904S0	-2905	rRNA Modification
b0204_RNA_3_RluD_mono	b0204_RNA_3, RluD_mono	C29465H33988N11954O20638S13P2904Mg1Zn0Fe0	-2908	rRNA Modification
b0204_RNA_4		C27814H31357N11468O20159P2904S0	-2905	rRNA Modification
b0204_RNA_4_RluD_mono	b0204_RNA_4, RluD_mono	C29451H33967N11948O20633S12P2904Mg1Zn0Fe0	-2909	rRNA Modification
b0204_RNA_5		C27814H31357N11468O20159P2904S0	-2905	rRNA Modification
b0204_RNA_5_RumA_mono	b0204_RNA_5, RumA_mono	C29942H34783N12086O20787S23P2904Mg0Zn0Fe4	-2897	rRNA Modification
b0204_RNA_6		C27815H31359N11468O20159P2904S0	-2905	rRNA Modification
b0204_RNA_6_MeT_23S_1962	b0204_RNA_6, MeT_23S_1962	C27830H31382N11474O20164S1P2904Mg0Zn0Fe0	-2904	rRNA Modification
b0204_RNA_7		C27816H31361N11468O20159P2904S0	-2905	rRNA Modification
b0204_RNA_7_MeT_23S_2030	b0204_RNA_7, MeT_23S_2030	C27831H31384N11474O20164S1P2904Mg0Zn0Fe0	-2904	rRNA Modification
b0204_RNA_8		C27817H31363N11468O20159P2904S0	-2905	rRNA Modification
b0204_RNA_8_MeT_23S_2069	b0204_RNA_8, MeT_23S_2069	C27832H31386N11474O20164S1P2904Mg0Zn0Fe0	-2904	rRNA Modification
b0204_RNA_9		C27818H31365N11468O20159P2904S0	-2905	rRNA Modification
b0204_RNA_9_RlmB_dim	b0204_RNA_9, RlmB_dim	C30139H35168N12152O20860S23P2904Mg0Zn0Fe0	-2910	rRNA Modification
b0204_RNA_MeT_23S_1618	b0204_RNA, MeT_23S_1618	C27826H31374N11474O20164S1P2904Mg0Zn0Fe0	-2904	rRNA Modification
b0204_v1_RNA	rRNA	C27811H31351N11468O20159P2904	-2905	rRNA Modification
b0204_v1_RNA_1		C27812H31353N11468O20159P2904S0	-2905	rRNA Modification
b0204_v1_RNA_10		C27819H31367N11468O20159P2904S0	-2905	rRNA Modification
b0204_v1_RNA_10_MeT_23S_2445	b0204_v1_RNA_10, MeT_23S_2445	C27834H31390N11474O20164S1P2904Mg0Zn0Fe0	-2904	rRNA Modification
b0204_v1_RNA_11		C27820H31369N11468O20159P2904S0	-2905	rRNA Modification
b0204_v1_RNA_11_DU_23S_2449_a	b0204_v1_RNA_11, DU_23S_2449 (NADH)	C27841H31397N11475O20173S0P2906Mg0Zn0Fe0	-2906	rRNA Modification
b0204_v1_RNA_11_DU_23S_2449_b	b0204_v1_RNA_11, DU_23S_2449 (NADPH)	C27841H31396N11475O20176S0P2907Mg0Zn0Fe0	-2908	rRNA Modification
b0204_v1_RNA_12		C27820H31371N11468O20159P2904S0	-2905	rRNA Modification
b0204_v1_RNA_12_YmfC_mono	b0204_v1_RNA_12, YmfC_mono	C28918H33132N11795O20479S3P2904Mg0Zn0Fe0	-2895	rRNA Modification
b0204_v1_RNA_13		C27820H31371N11468O20159P2904S0	-2905	rRNA Modification
b0204_v1_RNA_13_MeT_23S_2498	b0204_v1_RNA_13, MeT_23S_2498	C27835H31394N11474O20164S1P2904Mg0Zn0Fe0	-2904	rRNA Modification
b0204_v1_RNA_14		C27821H31373N11468O20159P2904S0	-2905	rRNA Modification
b0204_v1_RNA_14_MeT_23S_2503	b0204_v1_RNA_14, MeT_23S_2503	C27836H31396N11474O20164S1P2904Mg0Zn0Fe0	-2904	rRNA Modification
b0204_v1_RNA_15		C27822H31375N11468O20159P2904S0	-2905	rRNA Modification
b0204_v1_RNA_15_RluC_mono	b0204_v1_RNA_15, RluC_mono	C29407H33988N11950O20615S6P2904Mg0Zn0Fe0	-2890	rRNA Modification

b0204_v1_RNA_16		C27822H31375N11468O20159P2904S0	-2905	rRNA Modification
b0204_v1_RNA_16_RrmJ_mono	b0204_v1_RNA_16, RrmJ_mono	C28869H33064N11763O20462S11P2904Mg0Zn0Fe0	-2897	rRNA Modification
b0204_v1_RNA_17		C27823H31377N11468O20159P2904S0	-2905	rRNA Modification
b0204_v1_RNA_17_RluC_mono	b0204_v1_RNA_17, RluC_mono	C29408H33990N11950O20615S6P2904Mg0Zn0Fe0	-2890	rRNA Modification
b0204_v1_RNA_18		C27823H31377N11468O20159P2904S0	-2905	rRNA Modification
b0204_v1_RNA_18_YjbC_mono	b0204_v1_RNA_18, YjbC_mono	C29250H33748N11886O20581S7P2904Mg0Zn0Fe0	-2890	rRNA Modification
b0204_v1_RNA_19		C27823H31377N11468O20159P2904S0	-2905	rRNA Modification
b0204_v1_RNA_19_RluB_mono	b0204_v1_RNA_19, RluB_mono	C29241H33725N11919O20579S5P2904Mg0Zn0Fe0	-2890	rRNA Modification
b0204_v1_RNA_1_MeT_23S_1835	b0204_v1_RNA_1, MeT_23S_1835	C27827H31376N11474O20164S1P2904Mg0Zn0Fe0	-2904	rRNA Modification
b0204_v1_RNA_2		C27813H31355N11468O20159P2904S0	-2905	rRNA Modification
b0204_v1_RNA_20		C27823H31377N11468O20159P2904S0	-2905	rRNA Modification
b0204_v1_RNA_20_RrmA_dim	b0204_v1_RNA_20, RrmA_dim	C30540H35588N12232O20934S31P2904Mg0Zn2Fe0	-2902	rRNA Modification
b0204_v1_RNA_21		C27824H31379N11468O20159P2904S0	-2905	rRNA Modification
b0204_v1_RNA_21_RluA_mono	b0204_v1_RNA_21, RluA_mono	C28927H33119N11781O20473S10P2904Mg0Zn0Fe0	-2904	rRNA Modification
b0204_v1_RNA_22		C27824H31379N11468O20159P2904S0	-2905	rRNA Modification
b0204_v1_RNA_22_RumB_mono	b0204_v1_RNA_22, RumB_mono	C29717H34357N11980O20693S28P2904Mg0Zn0Fe4	-2903	rRNA Modification
b0204_v1_RNA_23		C27825H31381N11468O20159P2904S0	-2905	rRNA Modification
b0204_v1_RNA_23_RluC_mono	b0204_v1_RNA_23, RluC_mono	C29410H33994N11950O20615S6P2904Mg0Zn0Fe0	-2890	rRNA Modification
b0204_v1_RNA_2_RluD_mono	b0204_v1_RNA_2, RluD_mono	C29450H33965N11948O20633S12P2904Mg1Zn0Fe0	-2909	rRNA Modification
b0204_v1_RNA_3		C27813H31355N11468O20159P2904S0	-2905	rRNA Modification
b0204_v1_RNA_3_RluD_mono	b0204_v1_RNA_3, RluD_mono	C29465H33988N11954O20638S13P2904Mg1Zn0Fe0	-2908	rRNA Modification
b0204_v1_RNA_4		C27814H31357N11468O20159P2904S0	-2905	rRNA Modification
b0204_v1_RNA_4_RluD_mono	b0204_v1_RNA_4, RluD_mono	C29451H33967N11948O20633S12P2904Mg1Zn0Fe0	-2909	rRNA Modification
b0204_v1_RNA_5		C27814H31357N11468O20159P2904S0	-2905	rRNA Modification
b0204_v1_RNA_5_RumA_mono	b0204_v1_RNA_5, RumA_mono	C29942H34783N12086O20787S23P2904Mg0Zn0Fe4	-2897	rRNA Modification
b0204_v1_RNA_6		C27815H31359N11468O20159P2904S0	-2905	rRNA Modification
b0204_v1_RNA_6_MeT_23S_1962	b0204_v1_RNA_6, MeT_23S_1962	C27830H31382N11474O20164S1P2904Mg0Zn0Fe0	-2904	rRNA Modification
b0204_v1_RNA_7		C27816H31361N11468O20159P2904S0	-2905	rRNA Modification
b0204_v1_RNA_7_MeT_23S_2030	b0204_v1_RNA_7, MeT_23S_2030	C27831H31384N11474O20164S1P2904Mg0Zn0Fe0	-2904	rRNA Modification
b0204_v1_RNA_8		C27817H31363N11468O20159P2904S0	-2905	rRNA Modification
b0204_v1_RNA_8_MeT_23S_2069	b0204_v1_RNA_8, MeT_23S_2069	C27832H31386N11474O20164S1P2904Mg0Zn0Fe0	-2904	rRNA Modification
b0204_v1_RNA_9		C27818H31365N11468O20159P2904S0	-2905	rRNA Modification
b0204_v1_RNA_9_RlmB_dim	b0204_v1_RNA_9, RlmB_dim	C30139H35168N12152O20860S23P2904Mg0Zn0Fe0	-2910	rRNA Modification
b0204_v1_RNA_MeT_23S_1618	b0204_v1_RNA, MeT_23S_1618	C27826H31374N11474O20164S1P2904Mg0Zn0Fe0	-2904	rRNA Modification
b0205_RNA	rRNA	C1144H1301N468O838P120	-121	RNA cutting
b0205_v1_RNA	rRNA	C1144H1301N468O838P120	-121	RNA cutting
b0206_DNA_act	DNA b0206 (activated form)	C748H864N290O468P77	-77	Transcription
b0206_DNA_neu	DNA b0206 (inactivate form)	C748H864N290O468P77	-77	Transcription
b0206_RNA	tRNA (aspU)	C731H831N290O546P77	-78	tRNA Modification
b0206_RNA_cut_cplx	b0206 RNA /RNase P (5' trimming), RNase_Gen (T, PH, II, D, or BN), RNase E (3' trimming) cutting complex	C27994H42940N8916O10549S93P456Mg5Zn3Fe0	-569	RNA cutting
b0206_RNA_pre	tRNA pre	C731H831N290O552P79	-80	RNA cutting
b0206_tRNA_1	b0206_tRNA_1 (aspU)	C731H833Mg2N290O546P77	-74	tRNA Modification
b0206_tRNA_1_Dus_gen_cplx	b0206_tRNA_1 (aspU), Dus_gen	C2338H3372Mg2N749O1029P80S14	-81	tRNA Modification
b0206_tRNA_2	b0206_tRNA_2 (aspU)	C731H835Mg2N290O546P77	-74	tRNA Modification
b0206_tRNA_2_Dus_gen_cplx	b0206_tRNA_2 (aspU), Dus_gen	C2338H3374Mg2N749O1029P80S14	-81	tRNA Modification
b0206_tRNA_3	b0206_tRNA_3 (aspU)	C731H837Mg2N290O546P77	-74	tRNA Modification
b0206_tRNA_3_Tgt_hexa-QueA_mono-EoR_tRNA_pos34_Q_cplx	b0206_tRNA_3 (aspU), Tgt_hexa, QueA_mono, EoR_tRNA_pos34_Q	C13924Co1H21352Mg2N3983O4423P80S143Zn6	-114	tRNA Modification
b0206_tRNA_4	b0206_tRNA_4 (aspU)	C738H847Mg2N290O548P77	-74	tRNA Modification
b0206_tRNA_4_MeT_tRNA_pos_37_m2A_cplx	b0206_tRNA_4 (aspU), MeT_tRNA_pos_37_m2A	C753H870Mg2N296O553P77S1	-73	tRNA Modification
b0206_tRNA_5	b0206_tRNA_5 (aspU)	C739H849Mg2N290O548P77	-74	tRNA Modification
b0206_tRNA_5_YggH_mono_cplx	b0206_tRNA_5 (aspU), YggH_mono	C1964H2748Mg2N643O895P77S14	-76	tRNA Modification
b0206_tRNA_6	b0206_tRNA_6 (aspU)	C740H851Mg2N290O548P77	-74	tRNA Modification
b0206_tRNA_6_TrmA_mono_cplx	b0206_tRNA_6 (aspU), TrmA_mono	C2614H3798Mg2N813O1107P77S19	-82	tRNA Modification

b0206_tRNA_7	b0206_tRNA_7 (aspU)	C741H853Mg2N290O548P77	-74	tRNA Modification
b0206_tRNA_7_TruB_mono_cplx	b0206_tRNA_7 (aspU), TruB_mono	C2281H3328Mg2N728O1017P77S10	-79	tRNA Modification
b0206_tRNA_8	b0206_tRNA_8 (aspU)	C741H853Mg2N290O548P77	-74	tRNA Modification
b0206_tRNA_8_YqcB_mono_cplx	b0206_tRNA_8 (aspU), YqcB_mono	C2055H2909Mg2N677O927P77S6	-77	tRNA Modification
b0206_tRNA_9	b0206_tRNA_9 (aspU)	C741H853Mg2N290O548P77	-74	tRNA Modification
b0206_tRNA_9_ThiI_mono_cplx	b0206_tRNA_9 (aspU), ThiI_mono	C7169H11084Mg3N2108O2487P82S50X1	-102	tRNA Modification
b0206_tRNA_Mg2	tRNA (aspU) bound two Mg2 ions	C731H831Mg2N290O546P77	-74	tRNA Modification
b0206_tRNA_Mg2_Dus_gen_cplx	b0206_tRNA (aspU), Dus_gen	C2338H3370Mg2N749O1029P80S14	-81	tRNA Modification
b0216_RNA	tRNA (aspV)	C731H831N290O546P77	-78	tRNA Modification
b0216_tRNA_1	b0216_tRNA_1 (aspV)	C731H833Mg2N290O546P77	-74	tRNA Modification
b0216_tRNA_1_Dus_gen_cplx	b0216_tRNA_1 (aspV), Dus_gen	C2338H3372Mg2N749O1029P80S14	-81	tRNA Modification
b0216_tRNA_2	b0216_tRNA_2 (aspV)	C731H835Mg2N290O546P77	-74	tRNA Modification
b0216_tRNA_2_Dus_gen_cplx	b0216_tRNA_2 (aspV), Dus_gen	C2338H3374Mg2N749O1029P80S14	-81	tRNA Modification
b0216_tRNA_3	b0216_tRNA_3 (aspV)	C731H837Mg2N290O546P77	-74	tRNA Modification
b0216_tRNA_3_Tgt_hexa-QueA_mono-EoR_tRNA_pos34_Q_cplx	b0216_tRNA_3 (aspV), Tgt_hexa, QueA_mono, EoR_tRNA_pos34_Q	C13924Co1H21352Mg2N3983O4423P80S143Zn6	-114	tRNA Modification
b0216_tRNA_4	b0216_tRNA_4 (aspV)	C738H847Mg2N290O548P77	-74	tRNA Modification
b0216_tRNA_4_MeT_tRNA_pos_37_m2A_cplx	b0216_tRNA_4 (aspV), MeT_tRNA_pos_37_m2A	C753H870Mg2N296O553P77S1	-73	tRNA Modification
b0216_tRNA_5	b0216_tRNA_5 (aspV)	C739H849Mg2N290O548P77	-74	tRNA Modification
b0216_tRNA_5_YggH_mono_cplx	b0216_tRNA_5 (aspV), YggH_mono	C1964H2748Mg2N643O895P77S14	-76	tRNA Modification
b0216_tRNA_6	b0216_tRNA_6 (aspV)	C740H851Mg2N290O548P77	-74	tRNA Modification
b0216_tRNA_6_TrmA_mono_cplx	b0216_tRNA_6 (aspV), TrmA_mono	C2614H3798Mg2N813O1107P77S19	-82	tRNA Modification
b0216_tRNA_7	b0216_tRNA_7 (aspV)	C741H853Mg2N290O548P77	-74	tRNA Modification
b0216_tRNA_7_TruB_mono_cplx	b0216_tRNA_7 (aspV), TruB_mono	C2281H3328Mg2N728O1017P77S10	-79	tRNA Modification
b0216_tRNA_8	b0216_tRNA_8 (aspV)	C741H853Mg2N290O548P77	-74	tRNA Modification
b0216_tRNA_8_YqcB_mono_cplx	b0216_tRNA_8 (aspV), YqcB_mono	C2055H2909Mg2N677O927P77S6	-77	tRNA Modification
b0216_tRNA_9	b0216_tRNA_9 (aspV)	C741H853Mg2N290O548P77	-74	tRNA Modification
b0216_tRNA_9_ThiI_mono_cplx	b0216_tRNA_9 (aspV), ThiI_mono	C7169H11084Mg3N2108O2487P82S50X1	-102	tRNA Modification
b0216_tRNA_Mg2	tRNA (aspV) bound two Mg2 ions	C731H831Mg2N290O546P77	-74	tRNA Modification
b0216_tRNA_Mg2_Dus_gen_cplx	b0216_tRNA (aspV), Dus_gen	C2338H3370Mg2N749O1029P80S14	-81	tRNA Modification
b0244_RNA	tRNA (thrW)	C723H818N287O534P76	-77	tRNA Modification
b0244_tRNA_1	b0244_tRNA_1 (thrW)	C723H820Mg2N287O534P76	-73	tRNA Modification
b0244_tRNA_1_Dus_gen_cplx	b0244_tRNA_1 (thrW), Dus_gen	C2330H3359Mg2N746O1017P79S14	-80	tRNA Modification
b0244_tRNA_2	b0244_tRNA_2 (thrW)	C723H822Mg2N287O534P76	-73	tRNA Modification
b0244_tRNA_2_Dus_gen_cplx	b0244_tRNA_2 (thrW), Dus_gen	C2330H3361Mg2N746O1017P79S14	-80	tRNA Modification
b0244_tRNA_3	b0244_tRNA_3 (thrW)	C723H824Mg2N287O534P76	-73	tRNA Modification
b0244_tRNA_3_Up_tRNA_pos_37_t6A-MeT_tRNA_pos_37_m6t6A_cplx	b0244_tRNA_3 (thrW), Up_tRNA_pos_37_t6A, MeT_tRNA_pos_37_m6t6A	C753H869Mg3N299O558P79S1	-75	tRNA Modification
b0244_tRNA_4	b0244_tRNA_4 (thrW)	C729H832Mg2N288O538P76	-74	tRNA Modification
b0244_tRNA_4_YggH_mono_cplx	b0244_tRNA_4 (thrW), YggH_mono	C1954H2731Mg2N641O885P76S14	-76	tRNA Modification
b0244_tRNA_5	b0244_tRNA_5 (thrW)	C730H834Mg2N288O538P76	-74	tRNA Modification
b0244_tRNA_5_TrmA_mono_cplx	b0244_tRNA_5 (thrW), TrmA_mono	C2604H3781Mg2N811O1097P76S19	-82	tRNA Modification
b0244_tRNA_6	b0244_tRNA_6 (thrW)	C731H836Mg2N288O538P76	-74	tRNA Modification
b0244_tRNA_6_TruB_mono_cplx	b0244_tRNA_6 (thrW), TruB_mono	C2271H3311Mg2N726O1007P76S10	-79	tRNA Modification
b0244_tRNA_Mg2	tRNA (thrW) bound two Mg2 ions	C723H818Mg2N287O534P76	-73	tRNA Modification
b0244_tRNA_Mg2_Dus_gen_cplx	b0244_tRNA (thrW), Dus_gen	C2330H3357Mg2N746O1017P79S14	-80	tRNA Modification
b0405_aa	polypeptide b0405	C1768H2740N472O530S11	-18	Translation
b0405_def_map_cplx	Polypeptide b0405 peptide deformylase and methionine aminopeptidase complex	C3898H6196N1065O1176S32Mg0Zn0Fe3	-27	Maturation
b0405_m	Matured polypeptide b0405	C1762H2732N471O528S10	-17	Maturation
b0405_mRNA	mRNA b0405	C10180H11537N4050O7503P1073	-1074	Translation
b0405_mRNA_1	mRNA b0405	C10180H11537N4050O7503P1073	-1074	Translation
b0405_mRNA_2	mRNA b0405	C10180H11537N4050O7503P1073	-1074	Translation
b0405_mRNA_2_degr	mRNA b0405 degradation complex	C50863H76936N15925O20188S200P1073Mg6Zn2Fe0	-1296	mRNA degradation
b0406_aa	polypeptide b0406	C1887H2936N531O553S22	-7	Translation
b0406_def_cplx	Polypeptide b0406 peptide deformylase complex	C2731H4330N772O808S28Mg0Zn0Fe1	-12	Maturation
b0406_m	matured polypeptide b0406 _ error CCDB	C1886H2937N531O552S22	-6	Maturation
b0406_mRNA	mRNA b0406	C10725H12128N4249O7916P1130	-1131	Translation
b0406_mRNA_1	mRNA b0406	C10725H12128N4249O7916P1130	-1131	Translation
b0406_mRNA_2	mRNA b0406	C10725H12128N4249O7916P1130	-1131	Translation

b0406_mRNA_2_degr	mRNA b0406 degradation complex	C51408H77527N16124O20601S200P1130Mg6Zn2Fe0	-1353	mRNA degradation
b0406_m_Zn	b0406 plus _Zn	C1886H2937N531O552S22Zn1	-4	Folding
b0407_aa	polypeptide b0407_v1	C540H886N139O150S6	3	Translation
b0407_def_map_cplx	Polypeptide b0407 peptide deformylase and methionine aminopeptidase complex	C2670H4342N732O796S27Mg0Zn0Fe3	-6	Maturation
b0407_m	Matured polypeptide b0407	C534H878N138O148S5	4	Maturation
b0407_mRNA	mRNA b0407	C3171H3580N1263O2332P333	-334	Translation
b0407_mRNA_1	mRNA b0407	C3171H3580N1263O2332P333	-334	Translation
b0407_mRNA_2	mRNA b0407	C3171H3580N1263O2332P333	-334	Translation
b0407_mRNA_2_degr	mRNA b0407 degradation complex	C43854H68979N13138O15017S200P333Mg6Zn2Fe0	-556	mRNA degradation
b0413_aa	polypeptide b0413	C751H1223N219O226S10	0	Translation
b0413_def_map_cplx	Polypeptide b0413 peptide deformylase and methionine aminopeptidase complex	C2881H4679N812O872S31Mg0Zn0Fe3	-9	Maturation
b0413_m	Matured polypeptide b0413	C745H1215N218O224S9	1	Maturation
b0413_mRNA	mRNA b0413	C4292H4843N1726O3154P452	-453	Translation
b0413_mRNA_1	mRNA b0413	C4292H4843N1726O3154P452	-453	Translation
b0413_mRNA_2	mRNA b0413	C4292H4843N1726O3154P452	-453	Translation
b0413_mRNA_2_degr	mRNA b0413 degradation complex	C44975H70242N13601O15839S200P452Mg6Zn2Fe0	-675	mRNA degradation
b0414_aa	polypeptide b0414	C1777H2851N520O522S16	-1	Translation
b0414_def_map_cplx	Polypeptide b0414 peptide deformylase and methionine aminopeptidase complex	C3907H6307N1113O1168S37Mg0Zn0Fe3	-10	Maturation
b0414_m	Matured polypeptide b0414	C1771H2843N519O520S15	0	Maturation
b0414_mRNA	mRNA b0414	C10514H11908N4231O7710P1104	-1105	Translation
b0414_mRNA_1	mRNA b0414	C10514H11908N4231O7710P1104	-1105	Translation
b0414_mRNA_2	mRNA b0414	C10514H11908N4231O7710P1104	-1105	Translation
b0414_mRNA_2_degr	mRNA b0414 degradation complex	C51197H77307N16106O20395S200P1104Mg6Zn2Fe0	-1327	mRNA degradation
b0415_aa	polypeptide b0415	C721H1165N196O221S2	-5	Translation
b0415_def_map_cplx	Polypeptide b0415 peptide deformylase and methionine aminopeptidase complex	C2851H4621N789O867S23Mg0Zn0Fe3	-14	Maturation
b0415_m	Matured polypeptide b0415	C715H1157N195O219S1	-4	Maturation
b0415_mRNA	mRNA b0415	C4489H5074N1805O3289P471	-472	Translation
b0415_mRNA_1	mRNA b0415	C4489H5074N1805O3289P471	-472	Translation
b0415_mRNA_2	mRNA b0415	C4489H5074N1805O3289P471	-472	Translation
b0415_mRNA_2_degr	mRNA b0415 degradation complex	C45172H70473N13680O15974S200P471Mg6Zn2Fe0	-694	mRNA degradation
b0416_aa	polypeptide b0416	C706H1128N191O208S3	-1	Translation
b0416_def_map_cplx	Polypeptide b0416 peptide deformylase and methionine aminopeptidase complex	C2836H4584N784O854S24Mg0Zn0Fe3	-10	Maturation
b0416_m	Matured polypeptide b0416	C700H1120N190O206S2	0	Maturation
b0416_mRNA	mRNA b0416	C4002H4534N1617O2921P420	-421	Translation
b0416_mRNA_1	mRNA b0416	C4002H4534N1617O2921P420	-421	Translation
b0416_mRNA_2	mRNA b0416	C4002H4534N1617O2921P420	-421	Translation
b0416_mRNA_2_degr	mRNA b0416 degradation complex	C44685H69933N13492O15606S200P420Mg6Zn2Fe0	-643	mRNA degradation
b0423_aa	polypeptide b0423	C2454H3911N686O720S14	-7	Translation
b0423_def_map_cplx	Polypeptide b0423 peptide deformylase and methionine aminopeptidase complex	C4584H7367N1279O1366S35Mg0Zn0Fe3	-16	Maturation
b0423_m	Matured polypeptide b0423	C2448H3903N685O718S13	-6	Maturation
b0423_mRNA	mRNA b0423	C13788H15596N5497O10145P1451	-1452	Translation
b0423_mRNA_1	mRNA b0423	C13788H15596N5497O10145P1451	-1452	Translation
b0423_mRNA_2	mRNA b0423	C13788H15596N5497O10145P1451	-1452	Translation
b0423_mRNA_2_degr	mRNA b0423 degradation complex	C54471H80995N17372O22830S200P1451Mg6Zn2Fe0	-1674	mRNA degradation
b0436_aa	polypeptide b0436_v1	C2120H3402N582O675S11	-24	Translation
b0436_def_cplx	Polypeptide b0436 peptide deformylase complex	C2964H4796N823O930S17Mg0Zn0Fe1	-29	Maturation
b0436_m	Matured polypeptide b0436	C2119H3403N582O674S11	-23	Maturation
b0436_mRNA	mRNA b0436	C12433H14031N5122O8984P1301	-1302	Translation
b0436_mRNA_1	mRNA b0436	C12433H14031N5122O8984P1301	-1302	Translation
b0436_mRNA_2	mRNA b0436	C12433H14031N5122O8984P1301	-1302	Translation
b0436_mRNA_2_degr	mRNA b0436 degradation complex	C53116H79430N16997O21669S200P1301Mg6Zn2Fe0	-1524	mRNA degradation
b0436_m_DnaKJ_complex	b0436 DnaK DnaJ_dim complex - Kerner et al. class I can interact w/ GroEL/ES	C8683H13917O2772N2495S58P3Zn4	-45	Folding
b0436_m_GroEL.(7)ATP.transGroES	b0436 GroEL GroES complex - Kerner et al. class I can interact w/ GroEL/ES	C40031H66347O13008N11166S375P21Mg7	-324	Folding
b0436_v1_mRNA	mRNA b0436_v1	C12433H14031N5122O8984P1301	-1302	Translation

b0503_DNA_act	DNA b0503 (activated form)	C10677H12274N4242O6542P1095	-1095	Transcription
b0503_DNA_neu	DNA b0503 (inactivate form)	C10677H12274N4242O6542P1095	-1095	Transcription
b0503_aa	polypeptide b0503	C1813H2847N528O536S16	-9	Translation
b0503_def_map_cplx	Polypeptide b0503 peptide deformylase and methionine aminopeptidase complex	C3943H6303N1121O1182S37Mg0Zn0Fe3	-18	Maturation
b0503_m	Matured polypeptide b0503	C1807H2839N527O534S15	-8	Maturation
b0503_mRNA	mRNA b0503	C10448H11817N4242O7644P1097	-1098	Translation
b0503_mRNA_1	mRNA b0503	C10448H11817N4242O7644P1097	-1098	Translation
b0503_mRNA_2	mRNA b0503	C10448H11817N4242O7644P1097	-1098	Translation
b0503_mRNA_2_degr	mRNA b0503 degradation complex	C51131H77216N16117O20329S200P1097Mg6Zn2Fe0	-1320	mRNA degradation
b0503_m_DnaKJ_complex	b0503 DnaK DnaJ_dim complex - Kerner et al. class II can interact w/ GroEL/ES, cannot fold spontanously	C8371H13353O2632N2440S62P3Zn4	-30	Folding
b0503_m_GroEL.(7)ATP.transGroES	b0503 GroEL GroES complex - Kerner et al. class II can interact w/ GroEL/ES, cannot fold spontanously	C39719H65783O12868N11111S379P21Mg7	-309	Folding
b0526_aa	polypeptide b0526	C2301H3545N647O694S26	-20	Translation
b0526_def_cplx	Polypeptide b0526 peptide deformylase complex	C3145H4939N888O949S32Mg0Zn0Fe1	-25	Maturation
b0526_m	Matured polypeptide b0526	C2300H3546N647O693S26	-19	Maturation
b0526_mRNA	mRNA b0526	C13217H14952N5349O9668P1388	-1389	Translation
b0526_mRNA_1	mRNA b0526	C13217H14952N5349O9668P1388	-1389	Translation
b0526_mRNA_2	mRNA b0526	C13217H14952N5349O9668P1388	-1389	Translation
b0526_mRNA_2_degr	mRNA b0526 degradation complex	C53900H80351N17224O22353S200P1388Mg6Zn2Fe0	-1611	mRNA degradation
b0526_m_Zn	b0526 plus _Zn	C2300H3546N647O693S26Zn1	-17	Folding
b0536_RNA	tRNA (argU)	C732H833N294O540P77	-78	tRNA Modification
b0536_tRNA_1	b0536_tRNA_1 (argU)	C732H833Mg2N294O539P77S1	-74	tRNA Modification
b0536_tRNA_1_TrmE_dim-GidA_mono-TrmC_mono_cplx	b0536_tRNA_1 (argU), TrmE_dim, GidA_mono, TrmC_mono	C11536H17836Mg2N3365O3834P84S64	-153	tRNA Modification
b0536_tRNA_2	b0536_tRNA_2 (argU)	C734H838Mg2N295O539P77S1	-74	tRNA Modification
b0536_tRNA_2_Up_tRNA_pos_37_t6A_cplx	b0536_tRNA_2 (argU), Up_tRNA_pos_37_t6A	C749H860Mg3N301O558P80S1	-77	tRNA Modification
b0536_tRNA_3	b0536_tRNA_3 (argU)	C739H844Mg2N296O543P77S1	-75	tRNA Modification
b0536_tRNA_3_TruA_dim_cplx	b0536_tRNA_3 (argU), TruA_dim	C3449H5046Mg2N1076O1313P77S17	-69	tRNA Modification
b0536_tRNA_4	b0536_tRNA_4 (argU)	C739H844Mg2N296O543P77S1	-75	tRNA Modification
b0536_tRNA_4_TrmA_mono_cplx	b0536_tRNA_4 (argU), TrmA_mono	C2613H3791Mg2N819O1102P77S20	-83	tRNA Modification
b0536_tRNA_5	b0536_tRNA_5 (argU)	C740H846Mg2N296O543P77S1	-75	tRNA Modification
b0536_tRNA_5_TruB_mono_cplx	b0536_tRNA_5 (argU), TruB_mono	C2280H3321Mg2N734O1012P77S11	-80	tRNA Modification
b0536_tRNA_Mg2	tRNA (argU) bound two Mg2 ions	C732H833Mg2N294O540P77	-74	tRNA Modification
b0536_tRNA_Mg2_YdaO_mono_cplx	b0536_tRNA (argU), YdaO_mono	C6274H9652Mg3N1864O2227P82S55X1	-100	tRNA Modification
b0638_aa	polypeptide b0638	C1037H1590N302O297S9	-8	Translation
b0638_def_map_cplx	Polypeptide b0638 peptide deformylase and methionine aminopeptidase complex	C3167H5046N895O943S30Mg0Zn0Fe3	-17	Maturation
b0638_m	Matured polypeptide b0638	C1031H1582N301O295S8	-7	Maturation
b0638_mRNA	mRNA b0638	C5816H6582N2301O4288P612	-613	Translation
b0638_mRNA_1	mRNA b0638	C5816H6582N2301O4288P612	-613	Translation
b0638_mRNA_2	mRNA b0638	C5816H6582N2301O4288P612	-613	Translation
b0638_mRNA_2_degr	mRNA b0638 degradation complex	C46499H71981N14176O16973S200P612Mg6Zn2Fe0	-835	mRNA degradation
b0639_aa	polypeptide b0639	C1112H1707N298O321S5	-9	Translation
b0639_def_map_cplx	Polypeptide b0639 peptide deformylase and methionine aminopeptidase complex	C3242H5163N891O967S26Mg0Zn0Fe3	-18	Maturation
b0639_m	Matured polypeptide b0639	C1106H1699N297O319S4	-8	Maturation
b0639_mRNA	mRNA b0639	C6098H6910N2413O4480P642	-643	Translation
b0639_mRNA_1	mRNA b0639	C6098H6910N2413O4480P642	-643	Translation
b0639_mRNA_2	mRNA b0639	C6098H6910N2413O4480P642	-643	Translation
b0639_mRNA_2_degr	mRNA b0639 degradation complex	C46781H72309N14288O17165S200P642Mg6Zn2Fe0	-865	mRNA degradation
b0640_aa	polypeptide b0640	C1729H2778N493O496S10	-3	Translation
b0640_def_cplx	Polypeptide b0640 peptide deformylase complex	C2573H4172N734O751S16Mg0Zn0Fe1	-8	Maturation
b0640_m	Matured polypeptide b0640	C1728H2779N493O495S10	-2	Maturation
b0640_mRNA	mRNA b0640 (1 nt short)	C9797H11091N3878O7229P1031	-1032	Translation
b0640_mRNA_1	mRNA b0640 (1 nt short)	C9797H11091N3878O7229P1031	-1032	Translation
b0640_mRNA_2	mRNA b0640 (1 nt short)	C9797H11091N3878O7229P1031	-1032	Translation
b0640_mRNA_2_degr	mRNA b0640 degradation complex	C50480H76490N15753O19914S200P1031Mg6Zn2Fe0	-1254	mRNA degradation

b0641_aa	polypeptide b0641	C930H1533N266O291S9	1	Translation
b0641_def_map_cplx	Polypeptide b0641 peptide deformylase and methionine aminopeptidase complex	C3060H4989N859O937S30Mg0Zn0Fe3	-8	Maturation
b0641_m	Matured polypeptide b0641	C840H1380N245O268S8	1	Maturation
b0641_mRNA	mRNA b0641	C5537H6270N2211O4063P582	-583	Translation
b0641_mRNA_1	mRNA b0641	C5537H6270N2211O4063P582	-583	Translation
b0641_mRNA_2	mRNA b0641	C5537H6270N2211O4063P582	-583	Translation
b0641_mRNA_2_degr	mRNA b0641 degradation complex	C46220H71669N14086O16748S200P582Mg6Zn2Fe0	-805	mRNA degradation
b0642_aa	polypeptide b0642	C4340H6687N1159O1293S45	-30	Translation
b0642_def_map_cplx	Polypeptide b0642 peptide deformylase and methionine aminopeptidase complex	C6470H10143N1752O1939S66Mg0Zn0Fe3	-39	Maturation
b0642_m	Matured polypeptide b0642	C4334H6679N1158O1291S44	-29	Maturation
b0642_mRNA	mRNA b0642	C24632H27870N9975O17971P2585	-2586	Translation
b0642_mRNA_1	mRNA b0642	C24632H27870N9975O17971P2585	-2586	Translation
b0642_mRNA_2	mRNA b0642	C24632H27870N9975O17971P2585	-2586	Translation
b0642_mRNA_2_degr	mRNA b0642 degradation complex	C65315H93269N21850O30656S200P2585Mg6Zn2Fe0	-2808	mRNA degradation
b0642_m_DnaKJ_complex	b0642 DnaK DnaJ_dim complex - Deuerling et al. DnaKJ/GrpE dependent folding	C10898H17193O3389N3071S91P3Zn5	-49	Folding
b0642_m_Zn	b0642 plus _Zn	C4334H6679N1158O1291S44Zn1	-27	Folding
b0661_aa	polypeptide b0661	C2364H3733N660O727S20	-17	Translation
b0661_def_map_cplx	Polypeptide b0661 peptide deformylase and methionine aminopeptidase complex	C4494H7189N1253O1373S41Mg0Zn0Fe3	-26	Maturation
b0661_m	Matured polypeptide b0661	C2358H3725N659O725S19	-16	Maturation
b0661_mRNA	mRNA b0661	C13568H15368N5453O9945P1427	-1428	Translation
b0661_mRNA_1	mRNA b0661	C13568H15368N5453O9945P1427	-1428	Translation
b0661_mRNA_2	mRNA b0661	C13568H15368N5453O9945P1427	-1428	Translation
b0661_mRNA_2_degr	mRNA b0661 degradation complex	C54251H80767N17328O22630S200P1427Mg6Zn2Fe0	-1650	mRNA degradation
b0661_m_FeS	b0661 plus _FeS	C2358H3725N659O725S23Fe4	-14	Folding
b0661_m_IscU_cplx	b0661_m_IscU_cplx	C3550H5619N983O1121S33Fe4	-28	Iron-sulfur cluster incorporation
b0664_RNA	tRNA (glnX)	C711H810N281O527P75	-76	tRNA Modification
b0664_tRNA_1	b0664_tRNA_1 (glnX)	C712H812Mg2N281O527P75	-72	tRNA Modification
b0664_tRNA_1_Dus_gen_cplx	b0664_tRNA_1 (glnX), Dus_gen	C2319H3351Mg2N740O1010P78S14	-79	tRNA Modification
b0664_tRNA_2	b0664_tRNA_2 (glnX)	C712H814Mg2N281O527P75	-72	tRNA Modification
b0664_tRNA_2_MeT_tRNA_pos_32_Um_cplx	b0664_tRNA_2 (glnX), MeT_tRNA_pos_32_Um	C727H837Mg2N287O532P75S1	-71	tRNA Modification
b0664_tRNA_3	b0664_tRNA_3 (glnX)	C713H816Mg2N281O527P75	-72	tRNA Modification
b0664_tRNA_3_MeT_tRNA_pos_37_m2A_cplx	b0664_tRNA_3 (glnX), MeT_tRNA_pos_37_m2A	C728H839Mg2N287O532P75S1	-71	tRNA Modification
b0664_tRNA_4	b0664_tRNA_4 (glnX)	C714H818Mg2N281O527P75	-72	tRNA Modification
b0664_tRNA_4_TruA_dim_cplx	b0664_tRNA_4 (glnX), TruA_dim	C3424H5020Mg2N1061O1297P75S16	-66	tRNA Modification
b0664_tRNA_5	b0664_tRNA_5 (glnX)	C714H818Mg2N281O527P75	-72	tRNA Modification
b0664_tRNA_5_TrmA_mono_cplx	b0664_tRNA_5 (glnX), TrmA_mono	C2588H3765Mg2N804O1086P75S19	-80	tRNA Modification
b0664_tRNA_6	b0664_tRNA_6 (glnX)	C715H820Mg2N281O527P75	-72	tRNA Modification
b0664_tRNA_6_TruB_mono_cplx	b0664_tRNA_6 (glnX), TruB_mono	C2255H3295Mg2N719O996P75S10	-77	tRNA Modification
b0664_tRNA_7	b0664_tRNA_7 (glnX)	C715H820Mg2N281O527P75	-72	tRNA Modification
b0664_tRNA_7_ThiI_mono_cplx	b0664_tRNA_7 (glnX), ThiI_mono	C7143H11051Mg3N2099O2466P80S50X1	-100	tRNA Modification
b0664_tRNA_Mg2	tRNA (glnX) bound two Mg2 ions	C711H810Mg2N281O527P75	-72	tRNA Modification
b0664_tRNA_Mg2_TrmH_dim_cplx	b0664_tRNA (glnX), TrmH_dim	C2922H4353Mg2N937O1188P75S29	-73	tRNA Modification
b0665_RNA	tRNA (glnV)	C711H810N281O527P75	-76	tRNA Modification
b0665_tRNA_1	b0665_tRNA_1 (glnV)	C712H812Mg2N281O527P75	-72	tRNA Modification
b0665_tRNA_1_Dus_gen_cplx	b0665_tRNA_1 (glnV), Dus_gen	C2319H3351Mg2N740O1010P78S14	-79	tRNA Modification
b0665_tRNA_2	b0665_tRNA_2 (glnV)	C712H814Mg2N281O527P75	-72	tRNA Modification
b0665_tRNA_2_MeT_tRNA_pos_32_Um_cplx	b0665_tRNA_2 (glnV), MeT_tRNA_pos_32_Um	C727H837Mg2N287O532P75S1	-71	tRNA Modification
b0665_tRNA_3	b0665_tRNA_3 (glnV)	C713H816Mg2N281O527P75	-72	tRNA Modification
b0665_tRNA_3_MeT_tRNA_pos_37_m2A_cplx	b0665_tRNA_3 (glnV), MeT_tRNA_pos_37_m2A	C728H839Mg2N287O532P75S1	-71	tRNA Modification
b0665_tRNA_4	b0665_tRNA_4 (glnV)	C714H818Mg2N281O527P75	-72	tRNA Modification
b0665_tRNA_4_TruA_dim_cplx	b0665_tRNA_4 (glnV), TruA_dim	C3424H5020Mg2N1061O1297P75S16	-66	tRNA Modification
b0665_tRNA_5	b0665_tRNA_5 (glnV)	C714H818Mg2N281O527P75	-72	tRNA Modification
b0665_tRNA_5_TrmA_mono_cplx	b0665_tRNA_5 (glnV), TrmA_mono	C2588H3765Mg2N804O1086P75S19	-80	tRNA Modification
b0665_tRNA_6	b0665_tRNA_6 (glnV)	C715H820Mg2N281O527P75	-72	tRNA Modification
b0665_tRNA_6_TruB_mono_cplx	b0665_tRNA_6 (glnV), TruB_mono	C2255H3295Mg2N719O996P75S10	-77	tRNA Modification
b0665_tRNA_7	b0665_tRNA_7 (glnV)	C715H820Mg2N281O527P75	-72	tRNA Modification

b0665_tRNA_7_ThiI_mono_cplx	b0665_tRNA_7 (glnV), ThiI_mono	C7143H11051Mg3N2099O2466P80S50X1	-100	tRNA Modification
b0665_tRNA_Mg2	tRNA (glnV) bound two Mg2 ions	C711H810Mg2N281O527P75	-72	tRNA Modification
b0665_tRNA_Mg2_TrmH_dim_cplx	b0665_tRNA (glnV), TrmH_dim	C2922H4353Mg2N937O1188P75S29	-73	tRNA Modification
b0666_RNA	tRNA (metU)	C732H830N291O539P77	-78	tRNA Modification
b0666_tRNA_1	b0666_tRNA_1 (metU)	C732H832Mg2N291O539P77	-74	tRNA Modification
b0666_tRNA_10	b0666_tRNA_10 (metU)	C745H857Mg2N293O546P77	-75	tRNA Modification
b0666_tRNA_10_TrmA_mono_cplx	b0666_tRNA_10 (metU), TrmA_mono	C2619H3804Mg2N816O1105P77S19	-83	tRNA Modification
b0666_tRNA_11	b0666_tRNA_11 (metU)	C746H859Mg2N293O546P77	-75	tRNA Modification
b0666_tRNA_11_TruB_mono_cplx	b0666_tRNA_11 (metU), TruB_mono	C2286H3334Mg2N731O1015P77S10	-80	tRNA Modification
b0666_tRNA_12	b0666_tRNA_12 (metU)	C746H859Mg2N293O546P77	-75	tRNA Modification
b0666_tRNA_12_ThiI_mono_cplx	b0666_tRNA_12 (metU), ThiI_mono	C7174H11090Mg3N2111O2485P82S50X1	-103	tRNA Modification
b0666_tRNA_1_DusA_mono_cplx	b0666_tRNA_1 (metU), DusA_mono	C2372H3396Mg2N757O1035P80S18	-83	tRNA Modification
b0666_tRNA_2	b0666_tRNA_2 (metU)	C732H834Mg2N291O539P77	-74	tRNA Modification
b0666_tRNA_2_TrmH_dim_cplx	b0666_tRNA_2 (metU), TrmH_dim	C2943H4377Mg2N947O1200P77S29	-75	tRNA Modification
b0666_tRNA_3	b0666_tRNA_3 (metU)	C733H836Mg2N291O539P77	-74	tRNA Modification
b0666_tRNA_3_DusA_mono_cplx	b0666_tRNA_3 (metU), DusA_mono	C2373H3400Mg2N757O1035P80S18	-83	tRNA Modification
b0666_tRNA_4	b0666_tRNA_4 (metU)	C733H838Mg2N291O539P77	-74	tRNA Modification
b0666_tRNA_4_DusA_mono_cplx	b0666_tRNA_4 (metU), DusA_mono	C2373H3402Mg2N757O1035P80S18	-83	tRNA Modification
b0666_tRNA_5	b0666_tRNA_5 (metU)	C733H840Mg2N291O539P77	-74	tRNA Modification
b0666_tRNA_5_AcT_tRNA_pos_34_ac4C_cplx	b0666_tRNA_5 (metU), AcT_tRNA_pos_34_ac4C	C756H874Mg2N298O556P80S1	-78	tRNA Modification
b0666_tRNA_6	b0666_tRNA_6 (metU)	C735H842Mg2N291O540P77	-74	tRNA Modification
b0666_tRNA_6_Up_tRNA_pos_37_t6A_cplx	b0666_tRNA_6 (metU), Up_tRNA_pos_37_t6A	C750H864Mg3N297O559P80	-77	tRNA Modification
b0666_tRNA_7	b0666_tRNA_7 (metU)	C740H848Mg2N292O544P77	-75	tRNA Modification
b0666_tRNA_7_TruA_dim_cplx	b0666_tRNA_7 (metU), TruA_dim	C3450H5050Mg2N1072O1314P77S16	-69	tRNA Modification
b0666_tRNA_8	b0666_tRNA_8 (metU)	C740H848Mg2N292O544P77	-75	tRNA Modification
b0666_tRNA_8_YggH_mono_cplx	b0666_tRNA_8 (metU), YggH_mono	C1965H2747Mg2N645O891P77S14	-77	tRNA Modification
b0666_tRNA_9	b0666_tRNA_9 (metU)	C741H850Mg2N292O544P77	-75	tRNA Modification
b0666_tRNA_9_AcpT_tRNA_pos_47_acp3U_cplx	b0666_tRNA_9 (metU), AcpT_tRNA_pos_47_acp3U	C756H873Mg2N298O549P77S1	-74	tRNA Modification
b0666_tRNA_Mg2	tRNA (metU) bound two Mg2 ions	C732H830Mg2N291O539P77	-74	tRNA Modification
b0666_tRNA_Mg2_DusA_mono_cplx	b0666_tRNA (metU), DusA_mono	C2372H3394Mg2N757O1035P80S18	-83	tRNA Modification
b0668_RNA	tRNA (glnW)	C711H809N280O528P75	-76	tRNA Modification
b0668_tRNA_1	b0668_tRNA_1 (glnW)	C712H811Mg2N280O528P75	-72	tRNA Modification
b0668_tRNA_1_Dus_gen_cplx	b0668_tRNA_1 (glnW), Dus_gen	C2319H3350Mg2N739O1011P78S14	-79	tRNA Modification
b0668_tRNA_2	b0668_tRNA_2 (glnW)	C712H813Mg2N280O528P75	-72	tRNA Modification
b0668_tRNA_2_MeT_tRNA_pos_32_Um_cplx	b0668_tRNA_2 (glnW), MeT_tRNA_pos_32_Um	C727H836Mg2N286O533P75S1	-71	tRNA Modification
b0668_tRNA_3	b0668_tRNA_3 (glnW)	C713H815Mg2N280O528P75	-72	tRNA Modification
b0668_tRNA_3_MeT_tRNA_pos_37_m2A_cplx	b0668_tRNA_3 (glnW), MeT_tRNA_pos_37_m2A	C728H838Mg2N286O533P75S1	-71	tRNA Modification
b0668_tRNA_4	b0668_tRNA_4 (glnW)	C714H817Mg2N280O528P75	-72	tRNA Modification
b0668_tRNA_4_TruA_dim_cplx	b0668_tRNA_4 (glnW), TruA_dim	C3424H5019Mg2N1060O1298P75S16	-66	tRNA Modification
b0668_tRNA_5	b0668_tRNA_5 (glnW)	C714H817Mg2N280O528P75	-72	tRNA Modification
b0668_tRNA_5_TruA_dim_cplx	b0668_tRNA_5 (glnW), TruA_dim	C3424H5019Mg2N1060O1298P75S16	-66	tRNA Modification
b0668_tRNA_6	b0668_tRNA_6 (glnW)	C714H817Mg2N280O528P75	-72	tRNA Modification
b0668_tRNA_6_TrmA_mono_cplx	b0668_tRNA_6 (glnW), TrmA_mono	C2588H3764Mg2N803O1087P75S19	-80	tRNA Modification
b0668_tRNA_7	b0668_tRNA_7 (glnW)	C715H819Mg2N280O528P75	-72	tRNA Modification
b0668_tRNA_7_TruB_mono_cplx	b0668_tRNA_7 (glnW), TruB_mono	C2255H3294Mg2N718O997P75S10	-77	tRNA Modification
b0668_tRNA_8	b0668_tRNA_8 (glnW)	C715H819Mg2N280O528P75	-72	tRNA Modification
b0668_tRNA_8_ThiI_mono_cplx	b0668_tRNA_8 (glnW), ThiI_mono	C7143H11050Mg3N2098O2467P80S50X1	-100	tRNA Modification
b0668_tRNA_Mg2	tRNA (glnW) bound two Mg2 ions	C711H809Mg2N280O528P75	-72	tRNA Modification
b0668_tRNA_Mg2_TrmH_dim_cplx	b0668_tRNA (glnW), TrmH_dim	C2922H4352Mg2N936O1189P75S29	-73	tRNA Modification
b0670_RNA	tRNA (glnU)	C711H809N280O528P75	-76	tRNA Modification
b0670_tRNA_1	b0670_tRNA_1 (glnU)	C712H811Mg2N280O528P75	-72	tRNA Modification
b0670_tRNA_1_Dus_gen_cplx	b0670_tRNA_1 (glnU), Dus_gen	C2319H3350Mg2N739O1011P78S14	-79	tRNA Modification
b0670_tRNA_2	b0670_tRNA_2 (glnU)	C712H813Mg2N280O528P75	-72	tRNA Modification
b0670_tRNA_2_MeT_tRNA_pos_32_Um_cplx	b0670_tRNA_2 (glnU), MeT_tRNA_pos_32_Um	C727H836Mg2N286O533P75S1	-71	tRNA Modification
b0670_tRNA_3	b0670_tRNA_3 (glnU)	C713H815Mg2N280O528P75	-72	tRNA Modification
b0670_tRNA_3_MeT_tRNA_pos_37_m2A_cplx	b0670_tRNA_3 (glnU), MeT_tRNA_pos_37_m2A	C728H838Mg2N286O533P75S1	-71	tRNA Modification
b0670_tRNA_4	b0670_tRNA_4 (glnU)	C714H817Mg2N280O528P75	-72	tRNA Modification
b0670_tRNA_4_TruA_dim_cplx	b0670_tRNA_4 (glnU), TruA_dim	C3424H5019Mg2N1060O1298P75S16	-66	tRNA Modification

b0670_tRNA_5	b0670_tRNA_5 (glnU)	C714H817Mg2N280O528P75	-72	tRNA Modification
b0670_tRNA_5_TruA_dim_cplx	b0670_tRNA_5 (glnU), TruA_dim	C3424H5019Mg2N1060O1298P75S16	-66	tRNA Modification
b0670_tRNA_6	b0670_tRNA_6 (glnU)	C714H817Mg2N280O528P75	-72	tRNA Modification
b0670_tRNA_6_TrmA_mono_cplx	b0670_tRNA_6 (glnU), TrmA_mono	C2588H3764Mg2N803O1087P75S19	-80	tRNA Modification
b0670_tRNA_7	b0670_tRNA_7 (glnU)	C715H819Mg2N280O528P75	-72	tRNA Modification
b0670_tRNA_7_TruB_mono_cplx	b0670_tRNA_7 (glnU), TruB_mono	C2255H3294Mg2N718O997P75S10	-77	tRNA Modification
b0670_tRNA_8	b0670_tRNA_8 (glnU)	C715H819Mg2N280O528P75	-72	tRNA Modification
b0670_tRNA_8_ThiI_mono_cplx	b0670_tRNA_8 (glnU), ThiI_mono	C7143H11050Mg3N2098O2467P80S50X1	-100	tRNA Modification
b0670_tRNA_Mg2	tRNA (glnU) bound two Mg2 ions	C711H809Mg2N280O528P75	-72	tRNA Modification
b0670_tRNA_Mg2_TrmH_dim_cplx	b0670_tRNA (glnU), TrmH_dim	C2922H4352Mg2N936O1189P75S29	-73	tRNA Modification
b0672_RNA	tRNA (leuW)	C810H919N328O596P85	-86	tRNA Modification
b0672_tRNA_1	b0672_tRNA_1 (leuW)	C810H921Mg2N328O596P85	-82	tRNA Modification
b0672_tRNA_1_Dus_gen_cplx	b0672_tRNA_1 (leuW), Dus_gen	C2417H3460Mg2N787O1079P88S14	-89	tRNA Modification
b0672_tRNA_2	b0672_tRNA_2 (leuW)	C810H923Mg2N328O596P85	-82	tRNA Modification
b0672_tRNA_2_TrmH_dim_cplx	b0672_tRNA_2 (leuW), TrmH_dim	C3021H4466Mg2N984O1257P85S29	-83	tRNA Modification
b0672_tRNA_3	b0672_tRNA_3 (leuW)	C811H925Mg2N328O596P85	-82	tRNA Modification
b0672_tRNA_3_Dus_gen_cplx	b0672_tRNA_3 (leuW), Dus_gen	C2418H3464Mg2N787O1079P88S14	-89	tRNA Modification
b0672_tRNA_4	b0672_tRNA_4 (leuW)	C811H927Mg2N328O596P85	-82	tRNA Modification
b0672_tRNA_4_TruA_dim_cplx	b0672_tRNA_4 (leuW), TruA_dim	C3521H5129Mg2N1108O1366P85S16	-76	tRNA Modification
b0672_tRNA_5	b0672_tRNA_5 (leuW)	C811H927Mg2N328O596P85	-82	tRNA Modification
b0672_tRNA_5_TruB_mono_cplx	b0672_tRNA_5 (leuW), TruB_mono	C2351H3402Mg2N766O1065P85S10	-87	tRNA Modification
b0672_tRNA_Mg2	tRNA (leuW) bound two Mg2 ions	C810H919Mg2N328O596P85	-82	tRNA Modification
b0672_tRNA_Mg2_Dus_gen_cplx	b0672_tRNA (leuW), Dus_gen	C2417H3458Mg2N787O1079P88S14	-89	tRNA Modification
b0673_RNA	tRNA (metT)	C732H830N291O539P77	-78	tRNA Modification
b0673_tRNA_1	b0673_tRNA_1 (metT)	C732H832Mg2N291O539P77	-74	tRNA Modification
b0673_tRNA_10	b0673_tRNA_10 (metT)	C745H857Mg2N293O546P77	-75	tRNA Modification
b0673_tRNA_10_TrmA_mono_cplx	b0673_tRNA_10 (metT), TrmA_mono	C2619H3804Mg2N816O1105P77S19	-83	tRNA Modification
b0673_tRNA_11	b0673_tRNA_11 (metT)	C746H859Mg2N293O546P77	-75	tRNA Modification
b0673_tRNA_11_TruB_mono_cplx	b0673_tRNA_11 (metT), TruB_mono	C2286H3334Mg2N731O1015P77S10	-80	tRNA Modification
b0673_tRNA_12	b0673_tRNA_12 (metT)	C746H859Mg2N293O546P77	-75	tRNA Modification
b0673_tRNA_12_ThiI_mono_cplx	b0673_tRNA_12 (metT), ThiI_mono	C7174H11090Mg3N2111O2485P82S50X1	-103	tRNA Modification
b0673_tRNA_1_DusA_mono_cplx	b0673_tRNA_1 (metT), DusA_mono	C2372H3396Mg2N757O1035P80S18	-83	tRNA Modification
b0673_tRNA_2	b0673_tRNA_2 (metT)	C732H834Mg2N291O539P77	-74	tRNA Modification
b0673_tRNA_2_TrmH_dim_cplx	b0673_tRNA_2 (metT), TrmH_dim	C2943H4377Mg2N947O1200P77S29	-75	tRNA Modification
b0673_tRNA_3	b0673_tRNA_3 (metT)	C733H836Mg2N291O539P77	-74	tRNA Modification
b0673_tRNA_3_DusA_mono_cplx	b0673_tRNA_3 (metT), DusA_mono	C2373H3400Mg2N757O1035P80S18	-83	tRNA Modification
b0673_tRNA_4	b0673_tRNA_4 (metT)	C733H838Mg2N291O539P77	-74	tRNA Modification
b0673_tRNA_4_DusA_mono_cplx	b0673_tRNA_4 (metT), DusA_mono	C2373H3402Mg2N757O1035P80S18	-83	tRNA Modification
b0673_tRNA_5	b0673_tRNA_5 (metT)	C733H840Mg2N291O539P77	-74	tRNA Modification
b0673_tRNA_5_AcT_tRNA_pos_34_ac4C_cplx	b0673_tRNA_5 (metT), AcT_tRNA_pos_34_ac4C	C756H874Mg2N298O556P80S1	-78	tRNA Modification
b0673_tRNA_6	b0673_tRNA_6 (metT)	C735H842Mg2N291O540P77	-74	tRNA Modification
b0673_tRNA_6_Up_tRNA_pos_37_t6A_cplx	b0673_tRNA_6 (metT), Up_tRNA_pos_37_t6A	C750H864Mg3N297O559P80	-77	tRNA Modification
b0673_tRNA_7	b0673_tRNA_7 (metT)	C740H848Mg2N292O544P77	-75	tRNA Modification
b0673_tRNA_7_TruA_dim_cplx	b0673_tRNA_7 (metT), TruA_dim	C3450H5050Mg2N1072O1314P77S16	-69	tRNA Modification
b0673_tRNA_8	b0673_tRNA_8 (metT)	C740H848Mg2N292O544P77	-75	tRNA Modification
b0673_tRNA_8_YggH_mono_cplx	b0673_tRNA_8 (metT), YggH_mono	C1965H2747Mg2N645O891P77S14	-77	tRNA Modification
b0673_tRNA_9	b0673_tRNA_9 (metT)	C741H850Mg2N292O544P77	-75	tRNA Modification
b0673_tRNA_9_AcpT_tRNA_pos_47_acp3U_cplx	b0673_tRNA_9 (metT), AcpT_tRNA_pos_47_acp3U	C756H873Mg2N298O549P77S1	-74	tRNA Modification
b0673_tRNA_Mg2	tRNA (metT) bound two Mg2 ions	C732H830Mg2N291O539P77	-74	tRNA Modification
b0673_tRNA_Mg2_DusA_mono_cplx	b0673_tRNA (metT), DusA_mono	C2372H3394Mg2N757O1035P80S18	-83	tRNA Modification
b0680_aa	polypeptide b0680_v1	C2822H4366N788O841S22	-12	Translation
b0680_def_map_cplx	Polypeptide b0680 peptide deformylase and methionine aminopeptidase complex	C4952H7822N1381O1487S43Mg0Zn0Fe3	-21	Maturation
b0680_m	Matured polypeptide b0680	C2816H4358N787O839S21	-11	Maturation
b0680_mRNA	mRNA b0680	C15851H17940N6351O11623P1667	-1668	Translation
b0680_mRNA_1	mRNA b0680	C15851H17940N6351O11623P1667	-1668	Translation
b0680_mRNA_2	mRNA b0680	C15851H17940N6351O11623P1667	-1668	Translation
b0680_mRNA_2_degr	mRNA b0680 degradation complex	C56534H83339N18226O24308S200P1667Mg6Zn2Fe0	-1890	mRNA degradation

b0680_m_DnaKJ_complex	b0680 DnaK DnaJ_dim complex - Deuerling et al. DnaKJ/GrpE dependent folding	C9380H14872O2937N2700S68P3Zn4	-33	Folding
b0680_v1_mRNA	mRNA b0680_v1	C15851H17940N6351O11623P1667	-1668	Translation
b0743_RNA	tRNA (lysT)	C721H816N281O537P76	-77	tRNA Modification
b0743_tRNA_1	b0743_tRNA_1 (lysT)	C721H818Mg2N281O537P76	-73	tRNA Modification
b0743_tRNA_1_Dus_gen_cplx	b0743_tRNA_1 (lysT), Dus_gen	C2328H3357Mg2N740O1020P79S14	-80	tRNA Modification
b0743_tRNA_2	b0743_tRNA_2 (lysT)	C721H820Mg2N281O537P76	-73	tRNA Modification
b0743_tRNA_2_Dus_gen_cplx	b0743_tRNA_2 (lysT), Dus_gen	C2328H3359Mg2N740O1020P79S14	-80	tRNA Modification
b0743_tRNA_3	b0743_tRNA_3 (lysT)	C721H822Mg2N281O537P76	-73	tRNA Modification
b0743_tRNA_3_TrmU_mono-YhhP_mono-YheLMN_cplx-YccK_mono-TrmE_dim-GidA_mono-TrmC_mono_cplx	b0743_tRNA_3 (lysT), TrmU_mono, YhhP_mono, YheLMN_cplx, YccK_mono, TrmE_dim, GidA_mono, TrmC_mono	C21612H33672Mg3N6125O6874P88S131X1	-221	tRNA Modification
b0743_tRNA_4	b0743_tRNA_4 (lysT)	C723H827Mg2N282O536P76S1	-73	tRNA Modification
b0743_tRNA_4_Se	b0743_tRNA_4_Se - contains mnm5se2U instead of mnm5s2U,b0743_tRNA_4 (lysT)	C723H827Mg2N282O536P76S0Se1	-73	tRNA Modification
b0743_tRNA_4_Se_Up_tRNA_pos_37_t6A_cplx	b0743_tRNA_4_Se_Up_tRNA_pos_37_t6A_cplx - contains mnm5se2U instead of mnm5s2U,b0743_tRNA_4 (lysT), Up_tRNA_pos_37_t6A	C738H849Mg3N288O555P79S0Se1	-76	tRNA Modification
b0743_tRNA_4_Up_tRNA_pos_37_t6A_cplx	b0743_tRNA_4 (lysT), Up_tRNA_pos_37_t6A	C738H849Mg3N288O555P79S1	-76	tRNA Modification
b0743_tRNA_4_YbbB_dim_cplx	b0743_tRNA_4_YbbB_dim_cplx	C4337H6509N1336O1608S31P77Mg2Zn0Fe0Se1	-90	tRNA Modification
b0743_tRNA_5	b0743_tRNA_5 (lysT)	C728H833Mg2N283O540P76S1	-74	tRNA Modification
b0743_tRNA_5_Se	b0743_tRNA_5_Se - contains mnm5se2U instead of mnm5s2U,b0743_tRNA_5 (lysT)	C728H833Mg2N283O540P76S0Se1	-74	tRNA Modification
b0743_tRNA_5_Se_TruA_dim_cplx	b0743_tRNA_5_Se_TruA_dim_cplx - contains mnm5se2U instead of mnm5s2U,b0743_tRNA_5 (lysT), TruA_dim	C3438H5035Mg2N1063O1310P76S16Se1	-68	tRNA Modification
b0743_tRNA_5_TruA_dim_cplx	b0743_tRNA_5 (lysT), TruA_dim	C3438H5035Mg2N1063O1310P76S17	-68	tRNA Modification
b0743_tRNA_6	b0743_tRNA_6 (lysT)	C728H833Mg2N283O540P76S1	-74	tRNA Modification
b0743_tRNA_6_Se	b0743_tRNA_6_Se - contains mnm5se2U instead of mnm5s2U,b0743_tRNA_6 (lysT)	C728H833Mg2N283O540P76S0Se1	-74	tRNA Modification
b0743_tRNA_6_Se_YggH_mono_cplx	b0743_tRNA_6_Se_YggH_mono_cplx - contains mnm5se2U instead of mnm5s2U,b0743_tRNA_6 (lysT), YggH_mono	C1953H2732Mg2N636O887P76S14Se1	-76	tRNA Modification
b0743_tRNA_6_YggH_mono_cplx	b0743_tRNA_6 (lysT), YggH_mono	C1953H2732Mg2N636O887P76S15	-76	tRNA Modification
b0743_tRNA_7	b0743_tRNA_7 (lysT)	C729H835Mg2N283O540P76S1	-74	tRNA Modification
b0743_tRNA_7_AcpT_tRNA_pos_47_acp3U_cplx	b0743_tRNA_7 (lysT), AcpT_tRNA_pos_47_acp3U	C744H858Mg2N289O545P76S2	-73	tRNA Modification
b0743_tRNA_7_Se	b0743_tRNA_7_Se - contains mnm5se2U instead of mnm5s2U,b0743_tRNA_7 (lysT)	C729H835Mg2N283O540P76S0Se1	-74	tRNA Modification
b0743_tRNA_7_Se_AcpT_tRNA_pos_47_acp3U_cplx	b0743_tRNA_7_Se_AcpT_tRNA_pos_47_acp3U_cplx - contains mnm5se2U instead of mnm5s2U,b0743_tRNA_7 (lysT), AcpT_tRNA_pos_47_acp3U	C744H858Mg2N289O545P76S1Se1	-73	tRNA Modification
b0743_tRNA_8	b0743_tRNA_8 (lysT)	C733H842Mg2N284O542P76S1	-74	tRNA Modification
b0743_tRNA_8_Se	b0743_tRNA_8_Se - contains mnm5se2U instead of mnm5s2U,b0743_tRNA_8 (lysT)	C733H842Mg2N284O542P76S0Se1	-74	tRNA Modification
b0743_tRNA_8_Se_TrmA_mono_cplx	b0743_tRNA_8_Se_TrmA_mono_cplx - contains mnm5se2U instead of mnm5s2U,b0743_tRNA_8 (lysT), TrmA_mono	C2607H3789Mg2N807O1101P76S19Se1	-82	tRNA Modification
b0743_tRNA_8_TrmA_mono_cplx	b0743_tRNA_8 (lysT), TrmA_mono	C2607H3789Mg2N807O1101P76S20	-82	tRNA Modification
b0743_tRNA_9	b0743_tRNA_9 (lysT)	C734H844Mg2N284O542P76S1	-74	tRNA Modification
b0743_tRNA_9_Se	b0743_tRNA_9_Se - contains mnm5se2U instead of mnm5s2U,b0743_tRNA_9 (lysT)	C734H844Mg2N284O542P76S0Se1	-74	tRNA Modification
b0743_tRNA_9_Se_TruB_mono_cplx	b0743_tRNA_9_Se_TruB_mono_cplx - contains mnm5se2U instead of mnm5s2U,b0743_tRNA_9 (lysT), TruB_mono	C2274H3319Mg2N722O1011P76S10Se1	-79	tRNA Modification
b0743_tRNA_9_TruB_mono_cplx	b0743_tRNA_9 (lysT), TruB_mono	C2274H3319Mg2N722O1011P76S11	-79	tRNA Modification
b0743_tRNA_Mg2	tRNA (lysT) bound two Mg2 ions	C721H816Mg2N281O537P76	-73	tRNA Modification
b0743_tRNA_Mg2_Dus_gen_cplx	b0743_tRNA (lysT), Dus_gen	C2328H3355Mg2N740O1020P79S14	-80	tRNA Modification
b0744_RNA	tRNA (valT)	C723H823N292O532P76	-77	tRNA Modification
b0744_tRNA_1	b0744_tRNA_1 (valT)	C723H825Mg2N292O532P76	-73	tRNA Modification
b0744_tRNA_1_YecO_mono-YecP_mono-HyL_tRNA_pos_34_ho5U_cplx	b0744_tRNA_1 (valT), YecO_mono, YecP_mono, HyL_tRNA_pos_34_ho5U	C3656H5358Mg2N1093O1382P76S23	-91	tRNA Modification
b0744_tRNA_2	b0744_tRNA_2 (valT)	C725H826Mg2N292O535P76	-74	tRNA Modification
b0744_tRNA_2_MeT_tRNA_pos_37_m6A_cplx	b0744_tRNA_2 (valT), MeT_tRNA_pos_37_m6A	C740H849Mg2N298O540P76S1	-73	tRNA Modification

b0744_tRNA_3	b0744_tRNA_3 (valT)	C726H828Mg2N292O535P76	-74	tRNA Modification
b0744_tRNA_3_YggH_mono_cplx	b0744_tRNA_3 (valT), YggH_mono	C1951H2727Mg2N645O882P76S14	-76	tRNA Modification
b0744_tRNA_4	b0744_tRNA_4 (valT)	C727H830Mg2N292O535P76	-74	tRNA Modification
b0744_tRNA_4_TrmA_mono_cplx	b0744_tRNA_4 (valT), TrmA_mono	C2601H3777Mg2N815O1094P76S19	-82	tRNA Modification
b0744_tRNA_5	b0744_tRNA_5 (valT)	C728H832Mg2N292O535P76	-74	tRNA Modification
b0744_tRNA_5_TruB_mono_cplx	b0744_tRNA_5 (valT), TruB_mono	C2268H3307Mg2N730O1004P76S10	-79	tRNA Modification
b0744_tRNA_6	b0744_tRNA_6 (valT)	C728H832Mg2N292O535P76	-74	tRNA Modification
b0744_tRNA_6_ThiI_mono_cplx	b0744_tRNA_6 (valT), ThiI_mono	C7156H11063Mg3N2110O2474P81S50X1	-102	tRNA Modification
b0744_tRNA_Mg2	tRNA (valT) bound two Mg2 ions	C723H823Mg2N292O532P76	-73	tRNA Modification
b0744_tRNA_Mg2_Dus_gen_cplx	b0744_tRNA (valT), Dus_gen	C2330H3362Mg2N751O1015P79S14	-80	tRNA Modification
b0745_RNA	tRNA (lysW)	C721H816N281O537P76	-77	tRNA Modification
b0745_tRNA_1	b0745_tRNA_1 (lysW)	C721H818Mg2N281O537P76	-73	tRNA Modification
b0745_tRNA_1_Dus_gen_cplx	b0745_tRNA_1 (lysW), Dus_gen	C2328H3357Mg2N740O1020P79S14	-80	tRNA Modification
b0745_tRNA_2	b0745_tRNA_2 (lysW)	C721H820Mg2N281O537P76	-73	tRNA Modification
b0745_tRNA_2_Dus_gen_cplx	b0745_tRNA_2 (lysW), Dus_gen	C2328H3359Mg2N740O1020P79S14	-80	tRNA Modification
b0745_tRNA_3	b0745_tRNA_3 (lysW)	C721H822Mg2N281O537P76	-73	tRNA Modification
b0745_tRNA_3_TrmU_mono-YhhP_mono-YheLMN_cplx-YccK_mono-TrmE_dim-GidA_mono-TrmC_mono_cplx	b0745_tRNA_3 (lysW), TrmU_mono, YhhP_mono, YheLMN_cplx, YccK_mono, TrmE_dim, GidA_mono, TrmC_mono	C21612H33672Mg3N6125O6874P88S131X1	-221	tRNA Modification
b0745_tRNA_4	b0745_tRNA_4 (lysW)	C723H827Mg2N282O536P76S1	-73	tRNA Modification
b0745_tRNA_4_Se	b0745_tRNA_4_Se - contains mnm5se2U instead of mnm5s2U,b0745_tRNA_4 (lysW)	C723H827Mg2N282O536P76S0Se1	-73	tRNA Modification
b0745_tRNA_4_Se_Up_tRNA_pos_37_t6A_cplx	b0745_tRNA_4_Se_Up_tRNA_pos_37_t6A_cplx - contains mnm5se2U instead of mnm5s2U,b0745_tRNA_4 (lysW), Up_tRNA_pos_37_t6A	C738H849Mg3N288O555P79S0Se1	-76	tRNA Modification
b0745_tRNA_4_Up_tRNA_pos_37_t6A_cplx	b0745_tRNA_4 (lysW), Up_tRNA_pos_37_t6A	C738H849Mg3N288O555P79S1	-76	tRNA Modification
b0745_tRNA_4_YbbB_dim_cplx	b0745_tRNA_4_YbbB_dim_cplx	C4337H6509N1336O1608S31P77Mg2Zn0Fe0Se1	-90	tRNA Modification
b0745_tRNA_5	b0745_tRNA_5 (lysW)	C728H833Mg2N283O540P76S1	-74	tRNA Modification
b0745_tRNA_5_Se	b0745_tRNA_5_Se - contains mnm5se2U instead of mnm5s2U,b0745_tRNA_5 (lysW)	C728H833Mg2N283O540P76S0Se1	-74	tRNA Modification
b0745_tRNA_5_Se_TruA_dim_cplx	b0745_tRNA_5_Se_TruA_dim_cplx - contains mnm5se2U instead of mnm5s2U,b0745_tRNA_5 (lysW), TruA_dim	C3438H5035Mg2N1063O1310P76S16Se1	-68	tRNA Modification
b0745_tRNA_5_TruA_dim_cplx	b0745_tRNA_5 (lysW), TruA_dim	C3438H5035Mg2N1063O1310P76S17	-68	tRNA Modification
b0745_tRNA_6	b0745_tRNA_6 (lysW)	C728H833Mg2N283O540P76S1	-74	tRNA Modification
b0745_tRNA_6_Se	b0745_tRNA_6_Se - contains mnm5se2U instead of mnm5s2U,b0745_tRNA_6 (lysW)	C728H833Mg2N283O540P76S0Se1	-74	tRNA Modification
b0745_tRNA_6_Se_YggH_mono_cplx	b0745_tRNA_6_Se_YggH_mono_cplx - contains mnm5se2U instead of mnm5s2U,b0745_tRNA_6 (lysW), YggH_mono	C1953H2732Mg2N636O887P76S14Se1	-76	tRNA Modification
b0745_tRNA_6_YggH_mono_cplx	b0745_tRNA_6 (lysW), YggH_mono	C1953H2732Mg2N636O887P76S15	-76	tRNA Modification
b0745_tRNA_7	b0745_tRNA_7 (lysW)	C729H835Mg2N283O540P76S1	-74	tRNA Modification
b0745_tRNA_7_AcpT_tRNA_pos_47_acp3U_cplx	b0745_tRNA_7 (lysW), AcpT_tRNA_pos_47_acp3U	C744H858Mg2N289O545P76S2	-73	tRNA Modification
b0745_tRNA_7_Se	b0745_tRNA_7_Se - contains mnm5se2U instead of mnm5s2U,b0745_tRNA_7 (lysW)	C729H835Mg2N283O540P76S0Se1	-74	tRNA Modification
b0745_tRNA_7_Se_AcpT_tRNA_pos_47_acp3U_cplx	b0745_tRNA_7_Se_AcpT_tRNA_pos_47_acp3U_cplx - contains mnm5se2U instead of mnm5s2U,b0745_tRNA_7 (lysW), AcpT_tRNA_pos_47_acp3U	C744H858Mg2N289O545P76S1Se1	-73	tRNA Modification
b0745_tRNA_8	b0745_tRNA_8 (lysW)	C733H842Mg2N284O542P76S1	-74	tRNA Modification
b0745_tRNA_8_Se	b0745_tRNA_8_Se - contains mnm5se2U instead of mnm5s2U,b0745_tRNA_8 (lysW)	C733H842Mg2N284O542P76S0Se1	-74	tRNA Modification
b0745_tRNA_8_Se_TrmA_mono_cplx	b0745_tRNA_8_Se_TrmA_mono_cplx - contains mnm5se2U instead of mnm5s2U,b0745_tRNA_8 (lysW), TrmA_mono	C2607H3789Mg2N807O1101P76S19Se1	-82	tRNA Modification
b0745_tRNA_8_TrmA_mono_cplx	b0745_tRNA_8 (lysW), TrmA_mono	C2607H3789Mg2N807O1101P76S20	-82	tRNA Modification
b0745_tRNA_9	b0745_tRNA_9 (lysW)	C734H844Mg2N284O542P76S1	-74	tRNA Modification
b0745_tRNA_9_Se	b0745_tRNA_9_Se - contains mnm5se2U instead of mnm5s2U,b0745_tRNA_9 (lysW)	C734H844Mg2N284O542P76S0Se1	-74	tRNA Modification
b0745_tRNA_9_Se_TruB_mono_cplx	b0745_tRNA_9_Se_TruB_mono_cplx - contains mnm5se2U instead of mnm5s2U,b0745_tRNA_9 (lysW), TruB_mono	C2274H3319Mg2N722O1011P76S10Se1	-79	tRNA Modification
b0745_tRNA_9_TruB_mono_cplx	b0745_tRNA_9 (lysW), TruB_mono	C2274H3319Mg2N722O1011P76S11	-79	tRNA Modification
b0745_tRNA_Mg2	tRNA (lysW) bound two Mg2 ions	C721H816Mg2N281O537P76	-73	tRNA Modification

b0745_tRNA_Mg2_Dus_gen_cplx	b0745_tRNA (lysW), Dus_gen	C2328H3355Mg2N740O1020P79S14	-80	tRNA Modification
b0746_RNA	tRNA (valZ)	C723H823N292O532P76	-77	tRNA Modification
b0746_tRNA_1	b0746_tRNA_1 (valZ)	C723H825Mg2N292O532P76	-73	tRNA Modification
b0746_tRNA_1_YecO_mono-YecP_mono-HyL_tRNA_pos_34_ho5U_cplx	b0746_tRNA_1 (valZ), YecO_mono, YecP_mono, HyL_tRNA_pos_34_ho5U	C3656H5358Mg2N1093O1382P76S23	-91	tRNA Modification
b0746_tRNA_2	b0746_tRNA_2 (valZ)	C725H826Mg2N292O535P76	-74	tRNA Modification
b0746_tRNA_2_MeT_tRNA_pos_37_m6A_cplx	b0746_tRNA_2 (valZ), MeT_tRNA_pos_37_m6A	C740H849Mg2N298O540P76S1	-73	tRNA Modification
b0746_tRNA_3	b0746_tRNA_3 (valZ)	C726H828Mg2N292O535P76	-74	tRNA Modification
b0746_tRNA_3_YggH_mono_cplx	b0746_tRNA_3 (valZ), YggH_mono	C1951H2727Mg2N645O882P76S14	-76	tRNA Modification
b0746_tRNA_4	b0746_tRNA_4 (valZ)	C727H830Mg2N292O535P76	-74	tRNA Modification
b0746_tRNA_4_TrmA_mono_cplx	b0746_tRNA_4 (valZ), TrmA_mono	C2601H3777Mg2N815O1094P76S19	-82	tRNA Modification
b0746_tRNA_5	b0746_tRNA_5 (valZ)	C728H832Mg2N292O535P76	-74	tRNA Modification
b0746_tRNA_5_TruB_mono_cplx	b0746_tRNA_5 (valZ), TruB_mono	C2268H3307Mg2N730O1004P76S10	-79	tRNA Modification
b0746_tRNA_6	b0746_tRNA_6 (valZ)	C728H832Mg2N292O535P76	-74	tRNA Modification
b0746_tRNA_6_ThiI_mono_cplx	b0746_tRNA_6 (valZ), ThiI_mono	C7156H11063Mg3N2110O2474P81S50X1	-102	tRNA Modification
b0746_tRNA_Mg2	tRNA (valZ) bound two Mg2 ions	C723H823Mg2N292O532P76	-73	tRNA Modification
b0746_tRNA_Mg2_Dus_gen_cplx	b0746_tRNA (valZ), Dus_gen	C2330H3362Mg2N751O1015P79S14	-80	tRNA Modification
b0747_RNA	tRNA (lysY)	C721H816N281O537P76	-77	tRNA Modification
b0747_tRNA_1	b0747_tRNA_1 (lysY)	C721H818Mg2N281O537P76	-73	tRNA Modification
b0747_tRNA_1_Dus_gen_cplx	b0747_tRNA_1 (lysY), Dus_gen	C2328H3357Mg2N740O1020P79S14	-80	tRNA Modification
b0747_tRNA_2	b0747_tRNA_2 (lysY)	C721H820Mg2N281O537P76	-73	tRNA Modification
b0747_tRNA_2_Dus_gen_cplx	b0747_tRNA_2 (lysY), Dus_gen	C2328H3359Mg2N740O1020P79S14	-80	tRNA Modification
b0747_tRNA_3	b0747_tRNA_3 (lysY)	C721H822Mg2N281O537P76	-73	tRNA Modification
b0747_tRNA_3_TrmU_mono-YhhP_mono-YheLMN_cplx-YccK_mono-TrmE_dim-GidA_mono-TrmC_mono_cplx	b0747_tRNA_3 (lysY), TrmU_mono, YhhP_mono, YheLMN_cplx, YccK_mono, TrmE_dim, GidA_mono, TrmC_mono	C21612H33672Mg3N6125O6874P88S131X1	-221	tRNA Modification
b0747_tRNA_4	b0747_tRNA_4 (lysY)	C723H827Mg2N282O536P76S1	-73	tRNA Modification
b0747_tRNA_4_Se	b0747_tRNA_4_Se - contains mnm5se2U instead of mnm5s2U,b0747_tRNA_4 (lysY)	C723H827Mg2N282O536P76S0Se1	-73	tRNA Modification
b0747_tRNA_4_Se_Up_tRNA_pos_37_t6A_cplx	b0747_tRNA_4_Se_Up_tRNA_pos_37_t6A_cplx - contains mnm5se2U instead of mnm5s2U,b0747_tRNA_4 (lysY), Up_tRNA_pos_37_t6A	C738H849Mg3N288O555P79S0Se1	-76	tRNA Modification
b0747_tRNA_4_Up_tRNA_pos_37_t6A_cplx	b0747_tRNA_4 (lysY), Up_tRNA_pos_37_t6A	C738H849Mg3N288O555P79S1	-76	tRNA Modification
b0747_tRNA_4_YbbB_dim_cplx	b0747_tRNA_4_YbbB_dim_cplx	C4337H6509N1336O1608S31P77Mg2Zn0Fe0Se1	-90	tRNA Modification
b0747_tRNA_5	b0747_tRNA_5 (lysY)	C728H833Mg2N283O540P76S1	-74	tRNA Modification
b0747_tRNA_5_Se	b0747_tRNA_5_Se - contains mnm5se2U instead of mnm5s2U,b0747_tRNA_5 (lysY)	C728H833Mg2N283O540P76S0Se1	-74	tRNA Modification
b0747_tRNA_5_Se_TruA_dim_cplx	b0747_tRNA_5_Se_TruA_dim_cplx - contains mnm5se2U instead of mnm5s2U,b0747_tRNA_5 (lysY), TruA_dim	C3438H5035Mg2N1063O1310P76S16Se1	-68	tRNA Modification
b0747_tRNA_5_TruA_dim_cplx	b0747_tRNA_5 (lysY), TruA_dim	C3438H5035Mg2N1063O1310P76S17	-68	tRNA Modification
b0747_tRNA_6	b0747_tRNA_6 (lysY)	C728H833Mg2N283O540P76S1	-74	tRNA Modification
b0747_tRNA_6_Se	b0747_tRNA_6_Se - contains mnm5se2U instead of mnm5s2U,b0747_tRNA_6 (lysY)	C728H833Mg2N283O540P76S0Se1	-74	tRNA Modification
b0747_tRNA_6_Se_YggH_mono_cplx	b0747_tRNA_6_Se_YggH_mono_cplx - contains mnm5se2U instead of mnm5s2U,b0747_tRNA_6 (lysY), YggH_mono	C1953H2732Mg2N636O887P76S14Se1	-76	tRNA Modification
b0747_tRNA_6_YggH_mono_cplx	b0747_tRNA_6 (lysY), YggH_mono	C1953H2732Mg2N636O887P76S15	-76	tRNA Modification
b0747_tRNA_7	b0747_tRNA_7 (lysY)	C729H835Mg2N283O540P76S1	-74	tRNA Modification
b0747_tRNA_7_AcpT_tRNA_pos_47_acp3U_cplx	b0747_tRNA_7 (lysY), AcpT_tRNA_pos_47_acp3U	C744H858Mg2N289O545P76S2	-73	tRNA Modification
b0747_tRNA_7_Se	b0747_tRNA_7_Se - contains mnm5se2U instead of mnm5s2U,b0747_tRNA_7 (lysY)	C729H835Mg2N283O540P76S0Se1	-74	tRNA Modification
b0747_tRNA_7_Se_AcpT_tRNA_pos_47_acp3U_cplx	b0747_tRNA_7_Se_AcpT_tRNA_pos_47_acp3U_cplx - contains mnm5se2U instead of mnm5s2U,b0747_tRNA_7 (lysY), AcpT_tRNA_pos_47_acp3U	C744H858Mg2N289O545P76S1Se1	-73	tRNA Modification
b0747_tRNA_8	b0747_tRNA_8 (lysY)	C733H842Mg2N284O542P76S1	-74	tRNA Modification
b0747_tRNA_8_Se	b0747_tRNA_8_Se - contains mnm5se2U instead of mnm5s2U,b0747_tRNA_8 (lysY)	C733H842Mg2N284O542P76S0Se1	-74	tRNA Modification
b0747_tRNA_8_Se_TrmA_mono_cplx	b0747_tRNA_8_Se_TrmA_mono_cplx - contains mnm5se2U instead of mnm5s2U,b0747_tRNA_8 (lysY), TrmA_mono	C2607H3789Mg2N807O1101P76S19Se1	-82	tRNA Modification
b0747_tRNA_8_TrmA_mono_cplx	b0747_tRNA_8 (lysY), TrmA_mono	C2607H3789Mg2N807O1101P76S20	-82	tRNA Modification

b0747_tRNA_9	b0747_tRNA_9 (lysY)	C734H844Mg2N284O542P76S1	-74	tRNA Modification
b0747_tRNA_9_Se	b0747_tRNA_9_Se - contains mnm5se2U instead of mnm5s2U,b0747_tRNA_9 (lysY)	C734H844Mg2N284O542P76S0Se1	-74	tRNA Modification
b0747_tRNA_9_Se_TruB_mono_cplx	b0747_tRNA_9_Se_TruB_mono_cplx - contains mnm5se2U instead of mnm5s2U,b0747_tRNA_9 (lysY), TruB_mono	C2274H3319Mg2N722O1011P76S10Se1	-79	tRNA Modification
b0747_tRNA_9_TruB_mono_cplx	b0747_tRNA_9 (lysY), TruB_mono	C2274H3319Mg2N722O1011P76S11	-79	tRNA Modification
b0747_tRNA_Mg2	tRNA (lysY) bound two Mg2 ions	C721H816Mg2N281O537P76	-73	tRNA Modification
b0747_tRNA_Mg2_Dus_gen_cplx	b0747_tRNA (lysY), Dus_gen	C2328H3355Mg2N740O1020P79S14	-80	tRNA Modification
b0748_RNA	tRNA (lysZ)	C721H816N281O537P76	-77	tRNA Modification
b0748_tRNA_1	b0748_tRNA_1 (lysZ)	C721H818Mg2N281O537P76	-73	tRNA Modification
b0748_tRNA_1_Dus_gen_cplx	b0748_tRNA_1 (lysZ), Dus_gen	C2328H3357Mg2N740O1020P79S14	-80	tRNA Modification
b0748_tRNA_2	b0748_tRNA_2 (lysZ)	C721H820Mg2N281O537P76	-73	tRNA Modification
b0748_tRNA_2_Dus_gen_cplx	b0748_tRNA_2 (lysZ), Dus_gen	C2328H3359Mg2N740O1020P79S14	-80	tRNA Modification
b0748_tRNA_3	b0748_tRNA_3 (lysZ)	C721H822Mg2N281O537P76	-73	tRNA Modification
b0748_tRNA_3_TrmU_mono-YhhP_mono-YheLMN_cplx-YccK_mono-TrmE_dim-GidA_mono-TrmC_mono_cplx	b0748_tRNA_3 (lysZ), TrmU_mono, YhhP_mono, YheLMN_cplx, YccK_mono, TrmE_dim, GidA_mono, TrmC_mono	C21612H33672Mg3N6125O6874P88S131X1	-221	tRNA Modification
b0748_tRNA_4	b0748_tRNA_4 (lysZ)	C723H827Mg2N282O536P76S1	-73	tRNA Modification
b0748_tRNA_4_Se	b0748_tRNA_4_Se - contains mnm5se2U instead of mnm5s2U,b0748_tRNA_4 (lysZ)	C723H827Mg2N282O536P76S0Se1	-73	tRNA Modification
b0748_tRNA_4_Se_Up_tRNA_pos_37_t6A_cplx	b0748_tRNA_4_Se_Up_tRNA_pos_37_t6A_cplx - contains mnm5se2U instead of mnm5s2U,b0748_tRNA_4 (lysZ), Up_tRNA_pos_37_t6A	C738H849Mg3N288O555P79S0Se1	-76	tRNA Modification
b0748_tRNA_4_Up_tRNA_pos_37_t6A_cplx	b0748_tRNA_4 (lysZ), Up_tRNA_pos_37_t6A	C738H849Mg3N288O555P79S1	-76	tRNA Modification
b0748_tRNA_4_YbbB_dim_cplx	b0748_tRNA_4_YbbB_dim_cplx	C4337H6509N1336O1608S31P77Mg2Zn0Fe0Se1	-90	tRNA Modification
b0748_tRNA_5	b0748_tRNA_5 (lysZ)	C728H833Mg2N283O540P76S1	-74	tRNA Modification
b0748_tRNA_5_Se	b0748_tRNA_5_Se - contains mnm5se2U instead of mnm5s2U,b0748_tRNA_5 (lysZ)	C728H833Mg2N283O540P76S0Se1	-74	tRNA Modification
b0748_tRNA_5_Se_TruA_dim_cplx	b0748_tRNA_5_Se_TruA_dim_cplx - contains mnm5se2U instead of mnm5s2U,b0748_tRNA_5 (lysZ), TruA_dim	C3438H5035Mg2N1063O1310P76S16Se1	-68	tRNA Modification
b0748_tRNA_5_TruA_dim_cplx	b0748_tRNA_5 (lysZ), TruA_dim	C3438H5035Mg2N1063O1310P76S17	-68	tRNA Modification
b0748_tRNA_6	b0748_tRNA_6 (lysZ)	C728H833Mg2N283O540P76S1	-74	tRNA Modification
b0748_tRNA_6_Se	b0748_tRNA_6_Se - contains mnm5se2U instead of mnm5s2U,b0748_tRNA_6 (lysZ)	C728H833Mg2N283O540P76S0Se1	-74	tRNA Modification
b0748_tRNA_6_Se_YggH_mono_cplx	b0748_tRNA_6_Se_YggH_mono_cplx - contains mnm5se2U instead of mnm5s2U,b0748_tRNA_6 (lysZ), YggH_mono	C1953H2732Mg2N636O887P76S14Se1	-76	tRNA Modification
b0748_tRNA_6_YggH_mono_cplx	b0748_tRNA_6 (lysZ), YggH_mono	C1953H2732Mg2N636O887P76S15	-76	tRNA Modification
b0748_tRNA_7	b0748_tRNA_7 (lysZ)	C729H835Mg2N283O540P76S1	-74	tRNA Modification
b0748_tRNA_7_AcpT_tRNA_pos_47_acp3U_cplx	b0748_tRNA_7 (lysZ), AcpT_tRNA_pos_47_acp3U	C744H858Mg2N289O545P76S2	-73	tRNA Modification
b0748_tRNA_7_Se	b0748_tRNA_7_Se - contains mnm5se2U instead of mnm5s2U,b0748_tRNA_7 (lysZ)	C729H835Mg2N283O540P76S0Se1	-74	tRNA Modification
b0748_tRNA_7_Se_AcpT_tRNA_pos_47_acp3U_cplx	b0748_tRNA_7_Se_AcpT_tRNA_pos_47_acp3U_cplx - contains mnm5se2U instead of mnm5s2U,b0748_tRNA_7 (lysZ), AcpT_tRNA_pos_47_acp3U	C744H858Mg2N289O545P76S1Se1	-73	tRNA Modification
b0748_tRNA_8	b0748_tRNA_8 (lysZ)	C733H842Mg2N284O542P76S1	-74	tRNA Modification
b0748_tRNA_8_Se	b0748_tRNA_8_Se - contains mnm5se2U instead of mnm5s2U,b0748_tRNA_8 (lysZ)	C733H842Mg2N284O542P76S0Se1	-74	tRNA Modification
b0748_tRNA_8_Se_TrmA_mono_cplx	b0748_tRNA_8_Se_TrmA_mono_cplx - contains mnm5se2U instead of mnm5s2U,b0748_tRNA_8 (lysZ), TrmA_mono	C2607H3789Mg2N807O1101P76S19Se1	-82	tRNA Modification
b0748_tRNA_8_TrmA_mono_cplx	b0748_tRNA_8 (lysZ), TrmA_mono	C2607H3789Mg2N807O1101P76S20	-82	tRNA Modification
b0748_tRNA_9	b0748_tRNA_9 (lysZ)	C734H844Mg2N284O542P76S1	-74	tRNA Modification
b0748_tRNA_9_Se	b0748_tRNA_9_Se - contains mnm5se2U instead of mnm5s2U,b0748_tRNA_9 (lysZ)	C734H844Mg2N284O542P76S0Se1	-74	tRNA Modification
b0748_tRNA_9_Se_TruB_mono_cplx	b0748_tRNA_9_Se_TruB_mono_cplx - contains mnm5se2U instead of mnm5s2U,b0748_tRNA_9 (lysZ), TruB_mono	C2274H3319Mg2N722O1011P76S10Se1	-79	tRNA Modification
b0748_tRNA_9_TruB_mono_cplx	b0748_tRNA_9 (lysZ), TruB_mono	C2274H3319Mg2N722O1011P76S11	-79	tRNA Modification
b0748_tRNA_Mg2	tRNA (lysZ) bound two Mg2 ions	C721H816Mg2N281O537P76	-73	tRNA Modification
b0748_tRNA_Mg2_Dus_gen_cplx	b0748_tRNA (lysZ), Dus_gen	C2328H3355Mg2N740O1020P79S14	-80	tRNA Modification

b0749_RNA	tRNA (lysQ)	C721H816N281O537P76	-77	tRNA Modification
b0749_tRNA_1	b0749_tRNA_1 (lysQ)	C721H818Mg2N281O537P76	-73	tRNA Modification
b0749_tRNA_1_Dus_gen_cplx	b0749_tRNA_1 (lysQ), Dus_gen	C2328H3357Mg2N740O1020P79S14	-80	tRNA Modification
b0749_tRNA_2	b0749_tRNA_2 (lysQ)	C721H820Mg2N281O537P76	-73	tRNA Modification
b0749_tRNA_2_Dus_gen_cplx	b0749_tRNA_2 (lysQ), Dus_gen	C2328H3359Mg2N740O1020P79S14	-80	tRNA Modification
b0749_tRNA_3	b0749_tRNA_3 (lysQ)	C721H822Mg2N281O537P76	-73	tRNA Modification
b0749_tRNA_3_TrmU_mono-YhhP_mono-YheLMN_cplx-YccK_mono-TrmE_dim-GidA_mono-TrmC_mono_cplx	b0749_tRNA_3 (lysQ), TrmU_mono, YhhP_mono, YheLMN_cplx, YccK_mono, TrmE_dim, GidA_mono, TrmC_mono	C21612H33672Mg3N6125O6874P88S131X1	-221	tRNA Modification
b0749_tRNA_4	b0749_tRNA_4 (lysQ)	C723H827Mg2N282O536P76S1	-73	tRNA Modification
b0749_tRNA_4_Se	b0749_tRNA_4_Se - contains mnm5se2U instead of mnm5s2U,b0749_tRNA_4 (lysQ)	C723H827Mg2N282O536P76S0Se1	-73	tRNA Modification
b0749_tRNA_4_Se_Up_tRNA_pos_37_t6A_cplx	b0749_tRNA_4_Se_Up_tRNA_pos_37_t6A_cplx - contains mnm5se2U instead of mnm5s2U,b0749_tRNA_4 (lysQ), Up_tRNA_pos_37_t6A	C738H849Mg3N288O555P79S0Se1	-76	tRNA Modification
b0749_tRNA_4_Up_tRNA_pos_37_t6A_cplx	b0749_tRNA_4 (lysQ), Up_tRNA_pos_37_t6A	C738H849Mg3N288O555P79S1	-76	tRNA Modification
b0749_tRNA_4_YbbB_dim_cplx	b0749_tRNA_4_YbbB_dim_cplx	C4337H6509N1336O1608S31P77Mg2Zn0Fe0Se1	-90	tRNA Modification
b0749_tRNA_5	b0749_tRNA_5 (lysQ)	C728H833Mg2N283O540P76S1	-74	tRNA Modification
b0749_tRNA_5_Se	b0749_tRNA_5_Se - contains mnm5se2U instead of mnm5s2U,b0749_tRNA_5 (lysQ)	C728H833Mg2N283O540P76S0Se1	-74	tRNA Modification
b0749_tRNA_5_Se_TruA_dim_cplx	b0749_tRNA_5_Se_TruA_dim_cplx - contains mnm5se2U instead of mnm5s2U,b0749_tRNA_5 (lysQ), TruA_dim	C3438H5035Mg2N1063O1310P76S16Se1	-68	tRNA Modification
b0749_tRNA_5_TruA_dim_cplx	b0749_tRNA_5 (lysQ), TruA_dim	C3438H5035Mg2N1063O1310P76S17	-68	tRNA Modification
b0749_tRNA_6	b0749_tRNA_6 (lysQ)	C728H833Mg2N283O540P76S1	-74	tRNA Modification
b0749_tRNA_6_Se	b0749_tRNA_6_Se - contains mnm5se2U instead of mnm5s2U,b0749_tRNA_6 (lysQ)	C728H833Mg2N283O540P76S0Se1	-74	tRNA Modification
b0749_tRNA_6_Se_YggH_mono_cplx	b0749_tRNA_6_Se_YggH_mono_cplx - contains mnm5se2U instead of mnm5s2U,b0749_tRNA_6 (lysQ), YggH_mono	C1953H2732Mg2N636O887P76S14Se1	-76	tRNA Modification
b0749_tRNA_6_YggH_mono_cplx	b0749_tRNA_6 (lysQ), YggH_mono	C1953H2732Mg2N636O887P76S15	-76	tRNA Modification
b0749_tRNA_7	b0749_tRNA_7 (lysQ)	C729H835Mg2N283O540P76S1	-74	tRNA Modification
b0749_tRNA_7_AcpT_tRNA_pos_47_acp3U_cplx	b0749_tRNA_7 (lysQ), AcpT_tRNA_pos_47_acp3U	C744H858Mg2N289O545P76S2	-73	tRNA Modification
b0749_tRNA_7_Se	b0749_tRNA_7_Se - contains mnm5se2U instead of mnm5s2U,b0749_tRNA_7 (lysQ)	C729H835Mg2N283O540P76S0Se1	-74	tRNA Modification
b0749_tRNA_7_Se_AcpT_tRNA_pos_47_acp3U_cplx	b0749_tRNA_7_Se_AcpT_tRNA_pos_47_acp3U_cplx - contains mnm5se2U instead of mnm5s2U,b0749_tRNA_7 (lysQ), AcpT_tRNA_pos_47_acp3U	C744H858Mg2N289O545P76S1Se1	-73	tRNA Modification
b0749_tRNA_8	b0749_tRNA_8 (lysQ)	C733H842Mg2N284O542P76S1	-74	tRNA Modification
b0749_tRNA_8_Se	b0749_tRNA_8_Se - contains mnm5se2U instead of mnm5s2U,b0749_tRNA_8 (lysQ)	C733H842Mg2N284O542P76S0Se1	-74	tRNA Modification
b0749_tRNA_8_Se_TrmA_mono_cplx	b0749_tRNA_8_Se_TrmA_mono_cplx - contains mnm5se2U instead of mnm5s2U,b0749_tRNA_8 (lysQ), TrmA_mono	C2607H3789Mg2N807O1101P76S19Se1	-82	tRNA Modification
b0749_tRNA_8_TrmA_mono_cplx	b0749_tRNA_8 (lysQ), TrmA_mono	C2607H3789Mg2N807O1101P76S20	-82	tRNA Modification
b0749_tRNA_9	b0749_tRNA_9 (lysQ)	C734H844Mg2N284O542P76S1	-74	tRNA Modification
b0749_tRNA_9_Se	b0749_tRNA_9_Se - contains mnm5se2U instead of mnm5s2U,b0749_tRNA_9 (lysQ)	C734H844Mg2N284O542P76S0Se1	-74	tRNA Modification
b0749_tRNA_9_Se_TruB_mono_cplx	b0749_tRNA_9_Se_TruB_mono_cplx - contains mnm5se2U instead of mnm5s2U,b0749_tRNA_9 (lysQ), TruB_mono	C2274H3319Mg2N722O1011P76S10Se1	-79	tRNA Modification
b0749_tRNA_9_TruB_mono_cplx	b0749_tRNA_9 (lysQ), TruB_mono	C2274H3319Mg2N722O1011P76S11	-79	tRNA Modification
b0749_tRNA_Mg2	tRNA (lysQ) bound two Mg2 ions	C721H816Mg2N281O537P76	-73	tRNA Modification
b0749_tRNA_Mg2_Dus_gen_cplx	b0749_tRNA (lysQ), Dus_gen	C2328H3355Mg2N740O1020P79S14	-80	tRNA Modification
b0850_aa	polypeptide b0850	C478H797N126O128S6	1	Translation
b0850_def_map_cplx	Polypeptide b0850 peptide deformylase and methionine aminopeptidase complex	C2608H4253N719O774S27Mg0Zn0Fe3	-8	Maturation
b0850_m	Matured polypeptide b0850	C472H789N125O126S5	2	Maturation
b0850_mRNA	mRNA b0850	C2732H3090N1065O2038P290	-291	Translation
b0850_mRNA_1	mRNA b0850	C2732H3090N1065O2038P290	-291	Translation
b0850_mRNA_2	mRNA b0850	C2732H3090N1065O2038P290	-291	Translation
b0850_mRNA_2_degr	mRNA b0850 degradation complex	C43415H68489N12940O14723S200P290Mg6Zn2Fe0	-513	mRNA degradation
b0851_aa	polypeptide b0851	C1193H1891N338O350S8	-3	Translation

b0851_def_map_cplx	Polypeptide b0851 peptide deformylase and methionine aminopeptidase complex	C3323H5347N931O996S29Mg0Zn0Fe3	-12	Maturation
b0851_m	Matured polypeptide b0851	C1187H1883N337O348S7	-2	Maturation
b0851_mRNA	mRNA b0851 (17 nt short)	C6713H7598N2667O4947P706	-707	Translation
b0851_mRNA_1	mRNA b0851 (17 nt short)	C6713H7598N2667O4947P706	-707	Translation
b0851_mRNA_2	mRNA b0851 (17 nt short)	C6713H7598N2667O4947P706	-707	Translation
b0851_mRNA_2_degr	mRNA b0851 degradation complex	C47396H72997N14542O17632S200P706Mg6Zn2Fe0	-929	mRNA degradation
b0852_aa	polypeptide b0852_v1	C1422H2337N415O425S13	0	Translation
b0852_def_map_cplx	Polypeptide b0852 peptide deformylase and methionine aminopeptidase complex	C3552H5793N1008O1071S34Mg0Zn0Fe3	-9	Maturation
b0852_m	Matured polypeptide b0852	C1416H2329N414O423S12	1	Maturation
b0852_mRNA	mRNA b0852	C8607H9731N3466O6339P905	-906	Translation
b0852_mRNA_1	mRNA b0852	C8607H9731N3466O6339P905	-906	Translation
b0852_mRNA_2	mRNA b0852	C8607H9731N3466O6339P905	-906	Translation
b0852_mRNA_2_degr	mRNA b0852 degradation complex	C49290H75130N15341O19024S200P905Mg6Zn2Fe0	-1128	mRNA degradation
b0852_v1_mRNA	mRNA b0852_v1	C8607H9731N3466O6333P903	-904	Translation
b0853_aa	polypeptide b0853_v1	C786H1201N200O244S10	-17	Translation
b0853_def_map_cplx	Polypeptide b0853 peptide deformylase and methionine aminopeptidase complex	C2916H4657N793O890S31Mg0Zn0Fe3	-26	Maturation
b0853_m	Matured polypeptide b0853	C780H1193N199O242S9	-16	Maturation
b0853_mRNA	mRNA b0853	C4522H5111N1752O3368P477	-478	Translation
b0853_mRNA_1	mRNA b0853	C4522H5111N1752O3368P477	-478	Translation
b0853_mRNA_2	mRNA b0853	C4522H5111N1752O3368P477	-478	Translation
b0853_mRNA_2_degr	mRNA b0853 degradation complex	C45205H70510N13627O16053S200P477Mg6Zn2Fe0	-700	mRNA degradation
b0853_v1_mRNA	mRNA b0853_v1	C4522H5111N1752O3368P477	-478	Translation
b0858_aa	polypeptide b0858	C868H1360N216O219S7	4	Translation
b0858_def_map_cplx	Polypeptide b0858 peptide deformylase and methionine aminopeptidase complex	C2998H4816N809O865S28Mg0Zn0Fe3	-5	Maturation
b0858_m	Matured polypeptide b0858	C862H1352N215O217S6	5	Maturation
b0858_mRNA	mRNA b0858	C4633H5240N1791O3476P491	-492	Translation
b0858_mRNA_1	mRNA b0858	C4633H5240N1791O3476P491	-492	Translation
b0858_mRNA_2	mRNA b0858	C4633H5240N1791O3476P491	-492	Translation
b0858_mRNA_2_degr	mRNA b0858 degradation complex	C45316H70639N13666O16161S200P491Mg6Zn2Fe0	-714	mRNA degradation
b0859_aa	polypeptide b0859	C1884H2963N507O531S24	-2	Translation
b0859_def_map_cplx	Polypeptide b0859 peptide deformylase and methionine aminopeptidase complex	C4014H6419N1100O1177S45Mg0Zn0Fe3	-11	Maturation
b0859_m	Matured polypeptide b0859	C1878H2955N506O529S23	-1	Maturation
b0859_mRNA	mRNA b0859	C10719H12145N4254O7913P1128	-1129	Translation
b0859_mRNA_1	mRNA b0859	C10719H12145N4254O7913P1128	-1129	Translation
b0859_mRNA_2	mRNA b0859	C10719H12145N4254O7913P1128	-1129	Translation
b0859_mRNA_2_degr	mRNA b0859 degradation complex	C51402H77544N16129O20598S200P1128Mg6Zn2Fe0	-1351	mRNA degradation
b0859_m_FeS	b0859 plus _FeS	C1878H2955N506O529S27Fe4	1	Folding
b0859_m_IscU_cplx	b0859_m_IscU_cplx	C3070H4849N830O925S37Fe4	-13	Iron-sulfur cluster incorporation
b0883_RNA	tRNA (serW)	C840H953N344O615P88	-89	tRNA Modification
b0883_tRNA_1	b0883_tRNA_1 (serW)	C841H955Mg2N344O615P88	-85	tRNA Modification
b0883_tRNA_1_Dus_gen_cplx	b0883_tRNA_1 (serW), Dus_gen	C2448H3494Mg2N803O1098P91S14	-92	tRNA Modification
b0883_tRNA_2	b0883_tRNA_2 (serW)	C841H957Mg2N344O615P88	-85	tRNA Modification
b0883_tRNA_2_TruA_dim_cplx	b0883_tRNA_2 (serW), TruA_dim	C3551H5159Mg2N1124O1385P88S16	-79	tRNA Modification
b0883_tRNA_3	b0883_tRNA_3 (serW)	C841H957Mg2N344O615P88	-85	tRNA Modification
b0883_tRNA_3_TrmA_mono_cplx	b0883_tRNA_3 (serW), TrmA_mono	C2715H3904Mg2N867O1174P88S19	-93	tRNA Modification
b0883_tRNA_4	b0883_tRNA_4 (serW)	C842H959Mg2N344O615P88	-85	tRNA Modification
b0883_tRNA_4_TruB_mono_cplx	b0883_tRNA_4 (serW), TruB_mono	C2382H3434Mg2N782O1084P88S10	-90	tRNA Modification
b0883_tRNA_Mg2	tRNA (serW) bound two Mg2 ions	C840H953Mg2N344O615P88	-85	tRNA Modification
b0883_tRNA_Mg2_TrmH_dim_cplx	b0883_tRNA (serW), TrmH_dim	C3051H4496Mg2N1000O1276P88S29	-86	tRNA Modification
b0884_aa	polypeptide b0884_v1	C362H597N104O109S4	1	Translation
b0884_def_map_cplx	Polypeptide b0884 peptide deformylase and methionine aminopeptidase complex	C2492H4053N697O755S25Mg0Zn0Fe3	-8	Maturation
b0884_m	Matured polypeptide b0884	C356H589N103O107S3	2	Maturation
b0884_mRNA	mRNA b0884	C2087H2362N841O1523P221	-222	Translation
b0884_mRNA_1	mRNA b0884	C2087H2362N841O1523P221	-222	Translation

b0884_mRNA_2	mRNA b0884	C2087H2362N841O1523P221	-222	Translation
b0884_mRNA_2_degr	mRNA b0884 degradation complex	C42770H67761N12716O14208S200P221Mg6Zn2Fe0	-444	mRNA degradation
b0884_v1_mRNA	mRNA b0884_v1	C2087H2362N841O1523P221	-222	Translation
b0893_aa	polypeptide b0893	C2119H3374N607O654S19	-15	Translation
b0893_def_map_cplx	Polypeptide b0893 peptide deformylase and methionine aminopeptidase complex	C4249H6830N1200O1300S40Mg0Zn0Fe3	-24	Maturation
b0893_m	Matured polypeptide b0893	C2113H3366N606O652S18	-14	Maturation
b0893_mRNA	mRNA b0893	C12321H13935N4958O9024P1295	-1296	Translation
b0893_mRNA_1	mRNA b0893	C12321H13935N4958O9024P1295	-1296	Translation
b0893_mRNA_2	mRNA b0893	C12321H13935N4958O9024P1295	-1296	Translation
b0893_mRNA_2_degr	mRNA b0893 degradation complex	C53004H79334N16833O21709S200P1295Mg6Zn2Fe0	-1518	mRNA degradation
b0893_m_DnaKJ_complex	b0893 DnaK DnaJ_dim complex - Deuerling et al. DnaKJ/GrpE dependent folding	C8677H13880O2750N2519S65P3Zn4	-36	Folding
b0910_aa	polypeptide b0910	C1091H1771N318O329S5	-7	Translation
b0910_def_map_cplx	Polypeptide b0910 peptide deformylase and methionine aminopeptidase complex	C3221H5227N911O975S26Mg0Zn0Fe3	-16	Maturation
b0910_m	Matured polypeptide b0910	C1085H1763N317O327S4	-6	Maturation
b0910_mRNA	mRNA b0910	C6511H7365N2602O4798P686	-687	Translation
b0910_mRNA_1	mRNA b0910	C6511H7365N2602O4798P686	-687	Translation
b0910_mRNA_2	mRNA b0910	C6511H7365N2602O4798P686	-687	Translation
b0910_mRNA_2_degr	mRNA b0910 degradation complex	C47194H72764N14477O17483S200P686Mg6Zn2Fe0	-909	mRNA degradation
b0911_aa	polypeptide b0911_v2	C2691H4313N751O857S8	-30	Translation
b0911_def_cplx	Polypeptide b0911 peptide deformylase complex	C3535H5707N992O1112S14Mg0Zn0Fe1	-35	Maturation
b0911_m	Matured polypeptide b0911	C2690H4314N751O856S8	-29	Maturation
b0911_mRNA	mRNA b0911	C15973H18046N6467O11646P1676	-1677	Translation
b0911_mRNA_1	mRNA b0911	C15973H18046N6467O11646P1676	-1677	Translation
b0911_mRNA_2	mRNA b0911	C15973H18046N6467O11646P1676	-1677	Translation
b0911_mRNA_2_degr	mRNA b0911 degradation complex	C56656H83445N18342O24331S200P1676Mg6Zn2Fe0	-1899	mRNA degradation
b0911_v1_mRNA	mRNA b0911_v1	C15973H18046N6467O11646P1676	-1677	Translation
b0911_v2_mRNA	mRNA b0911_v2	C15973H18046N6467O11640P1674	-1675	Translation
b0912_aa	polypeptide b0912_v2	C468H755N139O141S3	2	Translation
b0912_def_cplx	Polypeptide b0912 peptide deformylase complex	C1312H2149N380O396S9Mg0Zn0Fe1	-3	Maturation
b0912_m	Matured polypeptide b0912	C467H756N139O140S3	3	Maturation
b0912_mRNA	mRNA b0912	C2719H3075N1102O1973P285	-286	Translation
b0912_mRNA_1	mRNA b0912	C2719H3075N1102O1973P285	-286	Translation
b0912_mRNA_2	mRNA b0912	C2719H3075N1102O1973P285	-286	Translation
b0912_mRNA_2_degr	mRNA b0912 degradation complex	C43402H68474N12977O14658S200P285Mg6Zn2Fe0	-508	mRNA degradation
b0912_v1_mRNA	mRNA b0912_v1	C2719H3075N1102O1973P285	-286	Translation
b0930_aa	polypeptide b0930	C2355H3613N643O700S14	-16	Translation
b0930_def_map_cplx	Polypeptide b0930 peptide deformylase and methionine aminopeptidase complex	C4485H7069N1236O1346S35Mg0Zn0Fe3	-25	Maturation
b0930_m	Matured polypeptide b0930	C2349H3605N642O698S13	-15	Maturation
b0930_mRNA	mRNA b0930	C13311H15064N5259O9843P1403	-1404	Translation
b0930_mRNA_1	mRNA b0930	C13311H15064N5259O9843P1403	-1404	Translation
b0930_mRNA_2	mRNA b0930	C13311H15064N5259O9843P1403	-1404	Translation
b0930_mRNA_2_degr	mRNA b0930 degradation complex	C53994H80463N17134O22528S200P1403Mg6Zn2Fe0	-1626	mRNA degradation
b0930_m_DnaKJ_complex	b0930 DnaK DnaJ_dim complex - Kerner et al. class I can interact w/ GroEL/ES	C8913H14119O2796N2555S60P3Zn4	-37	Folding
b0930_m_GroEL.(7)ATP.transGroES	b0930 GroEL GroES complex - Kerner et al. class I can interact w/ GroEL/ES	C40261H66549O13032N11226S377P21Mg7	-316	Folding
b0969_aa	polypeptide b0969	C567H883N144O162S4	-1	Translation
b0969_def_map_cplx	Polypeptide b0969 peptide deformylase and methionine aminopeptidase complex	C2697H4339N737O808S25Mg0Zn0Fe3	-10	Maturation
b0969_m	Matured polypeptide b0969	C561H875N143O160S3	0	Maturation
b0969_mRNA	mRNA b0969	C3158H3553N1288O2297P332	-333	Translation
b0969_mRNA_1	mRNA b0969	C3158H3553N1288O2297P332	-333	Translation
b0969_mRNA_2	mRNA b0969	C3158H3553N1288O2297P332	-333	Translation
b0969_mRNA_2_degr	mRNA b0969 degradation complex	C43841H68952N13163O14982S200P332Mg6Zn2Fe0	-555	mRNA degradation
b0971_RNA	tRNA (serT)	C840H953N344O614P88	-89	tRNA Modification
b0971_tRNA_1	b0971_tRNA_1 (serT)	C841H955Mg2N344O614P88	-85	tRNA Modification

b0971_tRNA_1_Dus_gen_cplx	b0971_tRNA_1 (serT), Dus_gen	C2448H3494Mg2N803O1097P91S14	-92	tRNA Modification
b0971_tRNA_2	b0971_tRNA_2 (serT)	C841H957Mg2N344O614P88	-85	tRNA Modification
b0971_tRNA_2_Dus_gen_cplx	b0971_tRNA_2 (serT), Dus_gen	C2448H3496Mg2N803O1097P91S14	-92	tRNA Modification
b0971_tRNA_3	b0971_tRNA_3 (serT)	C841H959Mg2N344O614P88	-85	tRNA Modification
b0971_tRNA_3_MeT_tRNA_pos_32_Cm_cplx	b0971_tRNA_3 (serT), MeT_tRNA_pos_32_Cm	C856H982Mg2N350O619P88S1	-84	tRNA Modification
b0971_tRNA_4	b0971_tRNA_4 (serT)	C842H961Mg2N344O614P88	-85	tRNA Modification
b0971_tRNA_4_YecO_mono-YecP_mono-HyL_tRNA_pos_34_ho5U_cplx	b0971_tRNA_4 (serT), YecO_mono, YecP_mono, HyL_tRNA_pos_34_ho5U	C3775H5494Mg2N1145O1464P88S23	-103	tRNA Modification
b0971_tRNA_5	b0971_tRNA_5 (serT)	C844H962Mg2N344O617P88	-86	tRNA Modification
b0971_tRNA_5_MiaA_dim-MiaB_mono_cplx	b0971_tRNA_5 (serT), MiaA_dim, MiaB_mono	C10319Fe4H15992Mg4N3019O3483P92S80X1	-131	tRNA Modification
b0971_tRNA_6	b0971_tRNA_6 (serT)	C850H972Mg2N344O617P88S1	-86	tRNA Modification
b0971_tRNA_6_TrmA_mono_cplx	b0971_tRNA_6 (serT), TrmA_mono	C2724H3919Mg2N867O1176P88S20	-94	tRNA Modification
b0971_tRNA_7	b0971_tRNA_7 (serT)	C851H974Mg2N344O617P88S1	-86	tRNA Modification
b0971_tRNA_7_TruB_mono_cplx	b0971_tRNA_7 (serT), TruB_mono	C2391H3449Mg2N782O1086P88S11	-91	tRNA Modification
b0971_tRNA_8	b0971_tRNA_8 (serT)	C851H974Mg2N344O617P88S1	-86	tRNA Modification
b0971_tRNA_8_ThiI_mono_cplx	b0971_tRNA_8 (serT), ThiI_mono	C7279H11205Mg3N2162O2556P93S51X1	-114	tRNA Modification
b0971_tRNA_Mg2	tRNA (serT) bound two Mg2 ions	C840H953Mg2N344O614P88	-85	tRNA Modification
b0971_tRNA_Mg2_TrmH_dim_cplx	b0971_tRNA (serT), TrmH_dim	C3051H4496Mg2N1000O1275P88S29	-86	tRNA Modification
b1032_RNA	tRNA (serX)	C840H953N344O615P88	-89	tRNA Modification
b1032_tRNA_1	b1032_tRNA_1 (serX)	C841H955Mg2N344O615P88	-85	tRNA Modification
b1032_tRNA_1_Dus_gen_cplx	b1032_tRNA_1 (serX), Dus_gen	C2448H3494Mg2N803O1098P91S14	-92	tRNA Modification
b1032_tRNA_2	b1032_tRNA_2 (serX)	C841H957Mg2N344O615P88	-85	tRNA Modification
b1032_tRNA_2_TruA_dim_cplx	b1032_tRNA_2 (serX), TruA_dim	C3551H5159Mg2N1124O1385P88S16	-79	tRNA Modification
b1032_tRNA_3	b1032_tRNA_3 (serX)	C841H957Mg2N344O615P88	-85	tRNA Modification
b1032_tRNA_3_TrmA_mono_cplx	b1032_tRNA_3 (serX), TrmA_mono	C2715H3904Mg2N867O1174P88S19	-93	tRNA Modification
b1032_tRNA_4	b1032_tRNA_4 (serX)	C842H959Mg2N344O615P88	-85	tRNA Modification
b1032_tRNA_4_TruB_mono_cplx	b1032_tRNA_4 (serX), TruB_mono	C2382H3434Mg2N782O1084P88S10	-90	tRNA Modification
b1032_tRNA_Mg2	tRNA (serX) bound two Mg2 ions	C840H953Mg2N344O615P88	-85	tRNA Modification
b1032_tRNA_Mg2_TrmH_dim_cplx	b1032_tRNA (serX), TrmH_dim	C3051H4496Mg2N1000O1276P88S29	-86	tRNA Modification
b1066_aa	polypeptide b1066	C1028H1550N292O279S8	4	Translation
b1066_def_map_cplx	Polypeptide b1066 peptide deformylase and methionine aminopeptidase complex	C3158H5006N885O925S29Mg0Zn0Fe3	-5	Maturation
b1066_m	Matured polypeptide b1066	C1022H1542N291O277S7	5	Maturation
b1066_mRNA	mRNA b1066	C5559H6285N2192O4108P587	-588	Translation
b1066_mRNA_1	mRNA b1066	C5559H6285N2192O4108P587	-588	Translation
b1066_mRNA_2	mRNA b1066	C5559H6285N2192O4108P587	-588	Translation
b1066_mRNA_2_degr	mRNA b1066 degradation complex	C46242H71684N14067O16793S200P587Mg6Zn2Fe0	-810	mRNA degradation
b1066_m_GroEL.(7)ATP.transGroES	b1066 GroEL GroES complex - Kerner et al. class III needs GroEL/ES	C38934H64486O12611N10875S371P21Mg7	-296	Folding
b1084_aa	polypeptide b1084	C5128H8256N1554O1621S18	-32	Translation
b1084_def_cplx	Polypeptide b1084 peptide deformylase complex	C5972H9650N1795O1876S24Mg0Zn0Fe1	-37	Maturation
b1084_m	matured polypeptide b1084 _ error CCDB	C5127H8257N1554O1620S18	-31	Maturation
b1084_mRNA	mRNA b1084	C30358H34432N12311O22126P3188	-3189	Translation
b1084_mRNA_1	mRNA b1084	C30358H34432N12311O22126P3188	-3189	Translation
b1084_mRNA_2	mRNA b1084	C30358H34432N12311O22126P3188	-3189	Translation
b1084_mRNA_2_degr	mRNA b1084 degradation complex	C71041H99831N24186O34811S200P3188Mg6Zn2Fe0	-3411	mRNA degradation
b1086_aa	polypeptide b1086	C1591H2621N483O458S7	14	Translation
b1086_def_map_cplx	Polypeptide b1086 peptide deformylase and methionine aminopeptidase complex	C3721H6077N1076O1104S28Mg0Zn0Fe3	5	Maturation
b1086_m	Matured polypeptide b1086	C1585H2613N482O456S6	15	Maturation
b1086_mRNA	mRNA b1086	C9164H10337N3704O6709P962	-963	Translation
b1086_mRNA_1	mRNA b1086	C9164H10337N3704O6709P962	-963	Translation
b1086_mRNA_2	mRNA b1086	C9164H10337N3704O6709P962	-963	Translation
b1086_mRNA_2_degr	mRNA b1086 degradation complex	C49847H75736N15579O19394S200P962Mg6Zn2Fe0	-1185	mRNA degradation
b1086_m_GroEL.(7)ATP.transGroES	b1086 GroEL GroES complex - Kerner et al. class III needs GroEL/ES	C39497H65557O12790N11066S370P21Mg7	-286	Folding
b1088_aa	polypeptide b1088_v1	C852H1330N221O273S9	-17	Translation
b1088_def_map_cplx	Polypeptide b1088 peptide deformylase and methionine aminopeptidase complex	C2982H4786N814O919S30Mg0Zn0Fe3	-26	Maturation
b1088_m	Matured polypeptide b1088	C846H1322N220O271S8	-16	Maturation
b1088_mRNA	mRNA b1088	C4970H5614N1981O3656P524	-525	Translation
b1088_mRNA_1	mRNA b1088	C4970H5614N1981O3656P524	-525	Translation

b1088_mRNA_2	mRNA b1088	C4970H5614N1981O3656P524	-525	Translation
b1088_mRNA_2_degr	mRNA b1088 degradation complex	C45653H71013N13856O16341S200P524Mg6Zn2Fe0	-747	mRNA degradation
b1088_v1_mRNA	mRNA b1088_v1	C4970H5614N1981O3656P524	-525	Translation
b1089_aa	polypeptide b1089_v2	C275H469N95O82S2	8	Translation
b1089_def_map_cplx	Polypeptide b1089 peptide deformylase and methionine aminopeptidase complex	C2405H3925N688O728S23Mg0Zn0Fe3	-1	Maturation
b1089_m	Matured polypeptide b1089	C269H461N94O80S1	9	Maturation
b1089_mRNA	mRNA b1089	C1652H1883N662O1203P174	-175	Translation
b1089_mRNA_1	mRNA b1089	C1652H1883N662O1203P174	-175	Translation
b1089_mRNA_2	mRNA b1089	C1652H1883N662O1203P174	-175	Translation
b1089_mRNA_2_degr	mRNA b1089 degradation complex	C42335H67282N12537O13888S200P174Mg6Zn2Fe0	-397	mRNA degradation
b1089_v1_mRNA	mRNA b1089_v1	C1652H1883N662O1203P174	-175	Translation
b1089_v2_mRNA	mRNA b1089_v2	C1652H1883N662O1209P176	-177	Translation
b1090_aa	polypeptide b1090_v1	C1690H2804N480O501S12	6	Translation
b1090_def_map_cplx	Polypeptide b1090 peptide deformylase and methionine aminopeptidase complex	C3820H6260N1073O1147S33Mg0Zn0Fe3	-3	Maturation
b1090_m	Matured polypeptide b1090	C1684H2796N479O499S11	7	Maturation
b1090_mRNA_1	mRNA b1090	C10198H11519N4068O7517P1073	-1074	Translation
b1090_mRNA_2	mRNA b1090	C10198H11519N4068O7517P1073	-1074	Translation
b1090_mRNA_2_degr	mRNA b1090 degradation complex	C50881H76918N15943O20202S200P1073Mg6Zn2Fe0	-1296	mRNA degradation
b1090_v1_mRNA	mRNA b1090_v1	C10198H11519N4068O7511P1071	-1072	Translation
b1091_aa	polypeptide b1091_v2	C1473H2342N407O460S13	-13	Translation
b1091_def_cplx	Polypeptide b1091 peptide deformylase complex	C2317H3736N648O715S19Mg0Zn0Fe1	-18	Maturation
b1091_m	Matured polypeptide b1091	C1472H2343N407O459S13	-12	Maturation
b1091_mRNA_1	mRNA b1091	C9074H10269N3612O6705P956	-957	Translation
b1091_mRNA_2	mRNA b1091	C9074H10269N3612O6705P956	-957	Translation
b1091_mRNA_2_degr	mRNA b1091 degradation complex	C49757H75668N15487O19390S200P956Mg6Zn2Fe0	-1179	mRNA degradation
b1091_v2_mRNA	mRNA b1091_v2	C9074H10269N3612O6699P954	-955	Translation
b1092_aa	polypeptide b1092_v3	C1437H2273N384O437S16	-11	Translation
b1092_def_map_cplx	Polypeptide b1092 peptide deformylase and methionine aminopeptidase complex	C3567H5729N977O1083S37Mg0Zn0Fe3	-20	Maturation
b1092_m	Matured polypeptide b1092	C1431H2265N383O435S15	-10	Maturation
b1092_mRNA_1	mRNA b1092	C8867H10026N3579O6510P932	-933	Translation
b1092_mRNA_2	mRNA b1092	C8867H10026N3579O6510P932	-933	Translation
b1092_mRNA_2_degr	mRNA b1092 degradation complex	C49550H75425N15454O19195S200P932Mg6Zn2Fe0	-1155	mRNA degradation
b1092_v3_mRNA	mRNA b1092_v3	C8867H10026N3579O6504P930	-931	Translation
b1093_aa	polypeptide b1093_v4	C1109H1824N323O347S11	-1	Translation
b1093_def_map_cplx	Polypeptide b1093 peptide deformylase and methionine aminopeptidase complex	C3239H5280N916O993S32Mg0Zn0Fe3	-10	Maturation
b1093_m	Matured polypeptide b1093	C1103H1816N322O345S10	0	Maturation
b1093_mRNA_1	mRNA b1093	C7011H7912N2823O5144P737	-738	Translation
b1093_mRNA_2	mRNA b1093	C7011H7912N2823O5144P737	-738	Translation
b1093_mRNA_2_degr	mRNA b1093 degradation complex	C47694H73311N14698O17829S200P737Mg6Zn2Fe0	-960	mRNA degradation
b1093_m_DnaKJ_complex	b1093 DnaK DnaJ_dim complex - Kerner et al. class II can interact w/ GroEL/ES, cannot fold spontanously	C7667H12330O2443N2235S57P3Zn4	-22	Folding
b1093_m_GroEL.(7)ATP.transGroES	b1093 GroEL GroES complex - Kerner et al. class II can interact w/ GroEL/ES, cannot fold spontanously	C39015H64760O12679N10906S374P21Mg7	-301	Folding
b1093_v4_mRNA	mRNA b1093_v4	C7011H7912N2823O5138P735	-736	Translation
b1114_aa	polypeptide b1114_v2	C5780H9163N1640O1698S37	-27	Translation
b1114_def_map_cplx	Polypeptide b1114 peptide deformylase and methionine aminopeptidase complex	C7910H12619N2233O2344S58Mg0Zn0Fe3	-36	Maturation
b1114_m	Matured polypeptide b1114	C5774H9155N1639O1696S36	-26	Maturation
b1114_mRNA	mRNA b1114	C32813H37166N13169O24081P3449	-3450	Translation
b1114_mRNA_1	mRNA b1114	C32813H37166N13169O24081P3449	-3450	Translation
b1114_mRNA_2	mRNA b1114	C32813H37166N13169O24081P3449	-3450	Translation
b1114_mRNA_2_degr	mRNA b1114 degradation complex	C73496H102565N25044O36766S200P3449Mg6Zn2Fe0	-3672	mRNA degradation
b1114_m_DnaKJ_complex	b1114 DnaK DnaJ_dim complex - Deuerling et al. DnaKJ/GrpE dependent folding	C12338H19669O3794N3552S83P3Mg1Zn4	-46	Folding
b1114_m_Mg	b1114 plus _Mg	C5774H9155N1639O1696S36Mg1	-24	Folding
b1114_v1_mRNA	mRNA b1114_v1	C32813H37166N13169O24081P3449	-3450	Translation
b1114_v2_mRNA	mRNA b1114_v2	C32813H37166N13169O24081P3449	-3450	Translation
b1133_DNA_act	DNA b1133 (activated form)	C10787H12427N4219O6630P1107	-1107	Transcription

b1133_DNA_neu	DNA b1133 (inactivate form)	C10787H12427N4219O6630P1107	-1107	Transcription
b1133_aa	polypeptide b1133	C1828H2828N489O556S13	-19	Translation
b1133_def_map_cplx	Polypeptide b1133 peptide deformylase and methionine aminopeptidase complex	C3958H6284N1082O1202S34Mg0Zn0Fe3	-28	Maturation
b1133_m	Matured polypeptide b1133	C1822H2820N488O554S12	-18	Maturation
b1133_mRNA	mRNA b1133	C10537H11928N4219O7744P1109	-1110	Translation
b1133_mRNA_1	mRNA b1133	C10537H11928N4219O7744P1109	-1110	Translation
b1133_mRNA_2	mRNA b1133	C10537H11928N4219O7744P1109	-1110	Translation
b1133_mRNA_2_degr	mRNA b1133 degradation complex	C51220H77327N16094O20429S200P1109Mg6Zn2Fe0	-1332	mRNA degradation
b1134_aa	polypeptide b1134	C787H1189N209O226S8	-10	Translation
b1134_def_map_cplx	Polypeptide b1134 peptide deformylase and methionine aminopeptidase complex	C2917H4645N802O872S29Mg0Zn0Fe3	-19	Maturation
b1134_m	Matured polypeptide b1134	C781H1181N208O224S7	-9	Maturation
b1134_mRNA	mRNA b1134	C4394H4966N1741O3239P462	-463	Translation
b1134_mRNA_1	mRNA b1134	C4394H4966N1741O3239P462	-463	Translation
b1134_mRNA_2	mRNA b1134	C4394H4966N1741O3239P462	-463	Translation
b1134_mRNA_2_degr	mRNA b1134 degradation complex	C45077H70365N13616O15924S200P462Mg6Zn2Fe0	-685	mRNA degradation
b1135_aa	polypeptide b1135	C1104H1769N328O322S4	9	Translation
b1135_def_map_cplx	Polypeptide b1135 peptide deformylase and methionine aminopeptidase complex	C3234H5225N921O968S25Mg0Zn0Fe3	0	Maturation
b1135_m	Matured polypeptide b1135	C1098H1761N327O320S3	10	Maturation
b1135_mRNA	mRNA b1135	C6222H7049N2488O4558P656	-657	Translation
b1135_mRNA_1	mRNA b1135	C6222H7049N2488O4558P656	-657	Translation
b1135_mRNA_2	mRNA b1135	C6222H7049N2488O4558P656	-657	Translation
b1135_mRNA_2_degr	mRNA b1135 degradation complex	C46905H72448N14363O17243S200P656Mg6Zn2Fe0	-879	mRNA degradation
b1211_DNA_act	DNA b1211 (activated form)	C10554H12146N4164O6455P1083	-1083	Transcription
b1211_DNA_neu	DNA b1211 (inactivate form)	C10554H12146N4164O6455P1083	-1083	Transcription
b1211_aa	polypeptide b1211	C1738H2773N526O565S14	-19	Translation
b1211_def_map_cplx	Polypeptide b1211 peptide deformylase and methionine aminopeptidase complex	C3868H6229N1119O1211S35Mg0Zn0Fe3	-28	Maturation
b1211_m	Matured polypeptide b1211	C1732H2765N525O563S13	-18	Maturation
b1211_mRNA	mRNA b1211	C10321H11681N4164O7545P1085	-1086	Translation
b1211_mRNA_1	mRNA b1211	C10321H11681N4164O7545P1085	-1086	Translation
b1211_mRNA_2	mRNA b1211	C10321H11681N4164O7545P1085	-1086	Translation
b1211_mRNA_2_degr	mRNA b1211 degradation complex	C51004H77080N16039O20230S200P1085Mg6Zn2Fe0	-1308	mRNA degradation
b1212_DNA_act	DNA b1212 (activated form)	C8121H9367N3153O5016P834	-834	Transcription
b1212_DNA_neu	DNA b1212 (inactivate form)	C8121H9367N3153O5016P834	-834	Transcription
b1212_aa	polypeptide b1212	C1368H2130N390O416S9	-18	Translation
b1212_def_map_cplx	Polypeptide b1212 peptide deformylase and methionine aminopeptidase complex	C3498H5586N983O1062S30Mg0Zn0Fe3	-27	Maturation
b1212_m	Matured polypeptide b1212	C1362H2122N389O414S8	-17	Maturation
b1212_mRNA	mRNA b1212	C7928H8982N3153O5857P836	-837	Translation
b1212_mRNA_1	mRNA b1212	C7928H8982N3153O5857P836	-837	Translation
b1212_mRNA_2	mRNA b1212	C7928H8982N3153O5857P836	-837	Translation
b1212_mRNA_2_degr	mRNA b1212 degradation complex	C48611H74381N15028O18542S200P836Mg6Zn2Fe0	-1059	mRNA degradation
b1229_aa	polypeptide b1229	C156H276N69O48S3	7	Translation
b1229_def_map_cplx	Polypeptide b1229 peptide deformylase and methionine aminopeptidase complex	C2286H3732N662O694S24Mg0Zn0Fe3	-2	Maturation
b1229_m	Matured polypeptide b1229	C150H268N68O46S2	8	Maturation
b1229_mRNA	mRNA b1229	C983H1113N426O699P102	-103	Translation
b1229_mRNA_1	mRNA b1229	C983H1113N426O699P102	-103	Translation
b1229_mRNA_2	mRNA b1229	C983H1113N426O699P102	-103	Translation
b1229_mRNA_2_degr	mRNA b1229 degradation complex	C41666H66512N12301O13384S200P102Mg6Zn2Fe0	-325	mRNA degradation
b1230_RNA	tRNA (tyrV)	C807H920N323O587P83	-84	tRNA Modification
b1230_RNA_cut_cplx	b1230 RNA /RNase P (5' trimming), RNase_Gen (T, PH, II, D, or BN), RNase E (3' trimming) cutting complex	C28070H43029N8949O10590S93P462Mg5Zn3Fe0	-575	RNA cutting
b1230_RNA_pre	tRNA pre	C807H920N323O593P85	-86	RNA cutting
b1230_tRNA_1	b1230_tRNA_1 (tyrV)	C808H922Mg2N323O587P83	-80	tRNA Modification
b1230_tRNA_1_Tgt_hexa-QueA_mono-EoR_tRNA_pos34_Q_cplx	b1230_tRNA_1 (tyrV), Tgt_hexa, QueA_mono, EoR_tRNA_pos34_Q	C14001Co1H21437Mg2N4016O4464P86S143Zn6	-120	tRNA Modification
b1230_tRNA_2	b1230_tRNA_2 (tyrV)	C815H932Mg2N323O589P83	-80	tRNA Modification
b1230_tRNA_2_MiaA_dim-MiaB_mono_cplx	b1230_tRNA_2 (tyrV), MiaA_dim, MiaB_mono	C10290Fe4H15962Mg4N2998O3455P87S80X1	-125	tRNA Modification
b1230_tRNA_3	b1230_tRNA_3 (tyrV)	C821H942Mg2N323O589P83S1	-80	tRNA Modification

b1230_tRNA_3_TruA_dim_cplx	b1230_tRNA_3 (tyrV), TruA_dim	C3531H5144Mg2N1103O1359P83S17	-74	tRNA Modification
b1230_tRNA_4	b1230_tRNA_4 (tyrV)	C821H942Mg2N323O589P83S1	-80	tRNA Modification
b1230_tRNA_4_TrmA_mono_cplx	b1230_tRNA_4 (tyrV), TrmA_mono	C2695H3889Mg2N846O1148P83S20	-88	tRNA Modification
b1230_tRNA_5	b1230_tRNA_5 (tyrV)	C822H944Mg2N323O589P83S1	-80	tRNA Modification
b1230_tRNA_5_TruB_mono_cplx	b1230_tRNA_5 (tyrV), TruB_mono	C2362H3419Mg2N761O1058P83S11	-85	tRNA Modification
b1230_tRNA_6	b1230_tRNA_6 (tyrV)	C822H944Mg2N323O589P83S1	-80	tRNA Modification
b1230_tRNA_6_ThiI_mono_cplx	b1230_tRNA_6 (tyrV), ThiI_mono	C7250H11175Mg3N2141O2528P88S51X1	-108	tRNA Modification
b1230_tRNA_Mg2	tRNA (tyrV) bound two Mg2 ions	C807H920Mg2N323O587P83	-80	tRNA Modification
b1230_tRNA_Mg2_TrmH_dim_cplx	b1230_tRNA (tyrV), TrmH_dim	C3018H4463Mg2N979O1248P83S29	-81	tRNA Modification
b1231_RNA	tRNA (tyrT)	C807H920N323O593P85	-86	tRNA Modification
b1231_RNA_cut_cplx	b1231 RNA /RNase P (5' trimming), RNase_Gen (T, PH, II, D, or BN), RNase E (3' trimming) cutting complex	C28070H43029N8949O10596S93P464Mg5Zn3Fe0	-577	RNA cutting
b1231_RNA_pre	tRNA pre	C807H920N323O599P87	-88	RNA cutting
b1231_tRNA_1	b1231_tRNA_1 (tyrT)	C808H922Mg2N323O593P85	-82	tRNA Modification
b1231_tRNA_1_Tgt_hexa-QueA_mono-EoR_tRNA_pos34_Q_cplx	b1231_tRNA_1 (tyrT), Tgt_hexa, QueA_mono, EoR_tRNA_pos34_Q	C14001Co1H21437Mg2N4016O4470P88S143Zn6	-122	tRNA Modification
b1231_tRNA_2	b1231_tRNA_2 (tyrT)	C815H932Mg2N323O595P85	-82	tRNA Modification
b1231_tRNA_2_MiaA_dim-MiaB_mono_cplx	b1231_tRNA_2 (tyrT), MiaA_dim, MiaB_mono	C10290Fe4H15962Mg4N2998O3461P89S80X1	-127	tRNA Modification
b1231_tRNA_3	b1231_tRNA_3 (tyrT)	C821H942Mg2N323O595P85S1	-82	tRNA Modification
b1231_tRNA_3_TruA_dim_cplx	b1231_tRNA_3 (tyrT), TruA_dim	C3531H5144Mg2N1103O1365P85S17	-76	tRNA Modification
b1231_tRNA_4	b1231_tRNA_4 (tyrT)	C821H942Mg2N323O595P85S1	-82	tRNA Modification
b1231_tRNA_4_TrmA_mono_cplx	b1231_tRNA_4 (tyrT), TrmA_mono	C2695H3889Mg2N846O1154P85S20	-90	tRNA Modification
b1231_tRNA_5	b1231_tRNA_5 (tyrT)	C822H944Mg2N323O595P85S1	-82	tRNA Modification
b1231_tRNA_5_TruB_mono_cplx	b1231_tRNA_5 (tyrT), TruB_mono	C2362H3419Mg2N761O1064P85S11	-87	tRNA Modification
b1231_tRNA_6	b1231_tRNA_6 (tyrT)	C822H944Mg2N323O595P85S1	-82	tRNA Modification
b1231_tRNA_6_ThiI_mono_cplx	b1231_tRNA_6 (tyrT), ThiI_mono	C7250H11175Mg3N2141O2534P90S51X1	-110	tRNA Modification
b1231_tRNA_Mg2	tRNA (tyrT) bound two Mg2 ions	C807H920Mg2N323O593P85	-82	tRNA Modification
b1231_tRNA_Mg2_TrmH_dim_cplx	b1231_tRNA (tyrT), TrmH_dim	C3018H4463Mg2N979O1254P85S29	-83	tRNA Modification
b1269_aa	polypeptide b1269	C1424H2356N452O422S6	14	Translation
b1269_def_map_cplx	Polypeptide b1269 peptide deformylase and methionine aminopeptidase complex	C3554H5812N1045O1068S27Mg0Zn0Fe3	5	Maturation
b1269_m	Matured polypeptide b1269	C1418H2348N451O420S5	15	Maturation
b1269_mRNA	mRNA b1269	C8351H9433N3358O6142P878	-879	Translation
b1269_mRNA_1	mRNA b1269	C8351H9433N3358O6142P878	-879	Translation
b1269_mRNA_2	mRNA b1269	C8351H9433N3358O6142P878	-879	Translation
b1269_mRNA_2_degr	mRNA b1269 degradation complex	C49034H74832N15233O18827S200P878Mg6Zn2Fe0	-1101	mRNA degradation
b1269_m_DnaKJ_complex	b1269 DnaK DnaJ_dim complex - Kerner et al. class II can interact w/ GroEL/ES, cannot fold spontanously	C7982H12862O2518N2364S52P3Zn4	-7	Folding
b1269_m_GroEL.(7)ATP.transGroES	b1269 GroEL GroES complex - Kerner et al. class II can interact w/ GroEL/ES, cannot fold spontanously	C39330H65292O12754N11035S369P21Mg7	-286	Folding
b1286_aa	polypeptide b1286_v1	C3225H5079N906O953S22	-21	Translation
b1286_def_map_cplx	Polypeptide b1286 peptide deformylase and methionine aminopeptidase complex	C5355H8535N1499O1599S43Mg0Zn0Fe3	-30	Maturation
b1286_m	Matured polypeptide b1286	C3219H5071N905O951S21	-20	Maturation
b1286_mRNA	mRNA b1286	C18395H20852N7331O13515P1937	-1938	Translation
b1286_mRNA_1	mRNA b1286	C18395H20852N7331O13515P1937	-1938	Translation
b1286_mRNA_2	mRNA b1286	C18395H20852N7331O13515P1937	-1938	Translation
b1286_mRNA_2_degr	mRNA b1286 degradation complex	C59078H86251N19206O26200S200P1937Mg6Zn2Fe0	-2160	mRNA degradation
b1286_m_Mg	b1286 plus _Mg	C3219H5071N905O951S21Mg1	-18	Folding
b1286_v1_mRNA	mRNA b1286_v1	C18395H20852N7331O13515P1937	-1938	Translation
b1344_aa	polypeptide b1344	C1568H2499N438O468S19	-5	Translation
b1344_def_map_cplx	Polypeptide b1344 peptide deformylase and methionine aminopeptidase complex	C3698H5955N1031O1114S40Mg0Zn0Fe3	-14	Maturation
b1344_m	Matured polypeptide b1344	C1562H2491N437O466S18	-4	Maturation
b1344_mRNA	mRNA b1344	C8928H10074N3593O6524P938	-939	Translation
b1344_mRNA_1	mRNA b1344	C8928H10074N3593O6524P938	-939	Translation
b1344_mRNA_2	mRNA b1344	C8928H10074N3593O6524P938	-939	Translation
b1344_mRNA_2_degr	mRNA b1344 degradation complex	C49611H75473N15468O19209S200P938Mg6Zn2Fe0	-1161	mRNA degradation
b1427_aa	polypeptide b1427	C922H1438N255O274S7	-5	Translation
b1427_def_map_cplx	Polypeptide b1427 peptide deformylase and methionine aminopeptidase complex	C3052H4894N848O920S28Mg0Zn0Fe3	-14	Maturation
b1427_m	Matured polypeptide b1427	C916H1430N254O272S6	-4	Maturation

b1427_mRNA	mRNA b1427	C5148H5798N2053O3767P542	-543	Translation
b1427_mRNA_1	mRNA b1427	C5148H5798N2053O3767P542	-543	Translation
b1427_mRNA_2	mRNA b1427	C5148H5798N2053O3767P542	-543	Translation
b1427_mRNA_2_degr	mRNA b1427 degradation complex	C45831H71197N13928O16452S200P542Mg6Zn2Fe0	-765	mRNA degradation
b1427_m_DnaKJ_complex	b1427 DnaK DnaJ_dim complex - Kerner et al. class II can interact w/ GroEL/ES, cannot fold spontanously	C7480H11944O2370N2167S53P3Zn4	-26	Folding
b1427_m_GroEL.(7)ATP.transGroES	b1427 GroEL GroES complex - Kerner et al. class II can interact w/ GroEL/ES, cannot fold spontanously	C38828H64374O12606N10838S370P21Mg7	-305	Folding
b1480_aa	polypeptide b1480_v1	C215H376N76O67S1	6	Translation
b1480_def_cplx	Polypeptide b1480 peptide deformylase complex	C1059H1770N317O322S7Mg0Zn0Fe1	1	Maturation
b1480_m	Matured polypeptide b1480	C214H377N76O66S1	7	Maturation
b1480_mRNA	mRNA b1480	C1321H1494N547O940P138	-139	Translation
b1480_mRNA_1	mRNA b1480	C1321H1494N547O940P138	-139	Translation
b1480_mRNA_2	mRNA b1480	C1321H1494N547O940P138	-139	Translation
b1480_mRNA_2_degr	mRNA b1480 degradation complex	C42004H66893N12422O13625S200P138Mg6Zn2Fe0	-361	mRNA degradation
b1480_v1_mRNA	mRNA b1480_v1	C1321H1494N547O946P140	-141	Translation
b1481_aa	polypeptide b1481	C353H532N90O119S2	-10	Translation
b1481_def_map_cplx	Polypeptide b1481 peptide deformylase and methionine aminopeptidase complex	C2483H3988N683O765S23Mg0Zn0Fe3	-19	Maturation
b1481_m	Matured polypeptide b1481 _ error CCDB	C347H524N89O117S1	-9	Maturation
b1481_mRNA	mRNA b1481	C2054H2321N812O1513P218	-219	Translation
b1481_mRNA_1	mRNA b1481	C2054H2321N812O1513P218	-219	Translation
b1481_mRNA_2	mRNA b1481	C2054H2321N812O1513P218	-219	Translation
b1481_mRNA_2_degr	mRNA b1481 degradation complex	C42737H67720N12687O14198S200P218Mg6Zn2Fe0	-441	mRNA degradation
b1636_aa	polypeptide b1636_v1	C1401H2210N386O402S14	-8	Translation
b1636_def_map_cplx	Polypeptide b1636 peptide deformylase and methionine aminopeptidase complex	C3531H5666N979O1048S35Mg0Zn0Fe3	-17	Maturation
b1636_m	Matured polypeptide b1636	C1395H2202N385O400S13	-7	Maturation
b1636_mRNA	mRNA b1636	C8230H9303N3298O6047P864	-865	Translation
b1636_mRNA_1	mRNA b1636	C8230H9303N3298O6047P864	-865	Translation
b1636_mRNA_2	mRNA b1636	C8230H9303N3298O6047P864	-865	Translation
b1636_mRNA_2_degr	mRNA b1636 degradation complex	C48913H74702N15173O18732S200P864Mg6Zn2Fe0	-1087	mRNA degradation
b1636_v1_mRNA	mRNA b1636_v1	C8230H9303N3298O6047P864	-865	Translation
b1637_aa	polypeptide b1637_v1	C2124H3303N577O636S14	-8	Translation
b1637_def_map_cplx	Polypeptide b1637 peptide deformylase and methionine aminopeptidase complex	C4254H6759N1170O1282S35Mg0Zn0Fe3	-17	Maturation
b1637_m	Matured polypeptide b1637	C2118H3295N576O634S13	-7	Maturation
b1637_mRNA	mRNA b1637	C12148H13730N4875O8906P1277	-1278	Translation
b1637_mRNA_1	mRNA b1637	C12148H13730N4875O8906P1277	-1278	Translation
b1637_mRNA_2	mRNA b1637	C12148H13730N4875O8906P1277	-1278	Translation
b1637_mRNA_2_degr	mRNA b1637 degradation complex	C52831H79129N16750O21591S200P1277Mg6Zn2Fe0	-1500	mRNA degradation
b1637_v1_mRNA	mRNA b1637_v1	C12148H13730N4875O8900P1275	-1276	Translation
b1638_aa	polypeptide b1638	C1146H1787N332O323S6	3	Translation
b1638_def_map_cplx	Polypeptide b1638 peptide deformylase and methionine aminopeptidase complex	C3276H5243N925O969S27Mg0Zn0Fe3	-6	Maturation
b1638_m	Matured polypeptide b1638	C1140H1779N331O321S5	4	Maturation
b1638_mRNA	mRNA b1638	C6242H7067N2468O4612P659	-660	Translation
b1638_mRNA_1	mRNA b1638	C6242H7067N2468O4612P659	-660	Translation
b1638_mRNA_2	mRNA b1638	C6242H7067N2468O4612P659	-660	Translation
b1638_mRNA_2_degr	mRNA b1638 degradation complex	C46925H72466N14343O17297S200P659Mg6Zn2Fe0	-882	mRNA degradation
b1652_aa	polypeptide b1652	C1047H1602N286O312S11	-10	Translation
b1652_def_map_cplx	Polypeptide b1652 peptide deformylase and methionine aminopeptidase complex	C3177H5058N879O958S32Mg0Zn0Fe3	-19	Maturation
b1652_m	Matured polypeptide b1652	C1041H1594N285O310S10	-9	Maturation
b1652_mRNA	mRNA b1652	C6148H6983N2430O4538P650	-651	Translation
b1652_mRNA_1	mRNA b1652	C6148H6983N2430O4538P650	-651	Translation
b1652_mRNA_2	mRNA b1652	C6148H6983N2430O4538P650	-651	Translation
b1652_mRNA_2_degr	mRNA b1652 degradation complex	C46831H72382N14305O17223S200P650Mg6Zn2Fe0	-873	mRNA degradation
b1652_m_Mg	b1652 plus _Mg	C1041H1594N285O310S10Mg2	-5	Folding
b1653_aa	polypeptide b1653_v1	C7486H12008N2190O2226S34	-12	Translation
b1653_def_map_cplx	Polypeptide b1653 peptide deformylase and methionine aminopeptidase complex	C9616H15464N2783O2872S55Mg0Zn0Fe3	-21	Maturation

b1653_m	Matured polypeptide b1653	C7480H12000N2189O2224S33	-11	Maturation
b1653_mRNA	mRNA b1653	C43866H49767N17456O32372P4617	-4618	Translation
b1653_mRNA_1	mRNA b1653	C43866H49767N17456O32372P4617	-4618	Translation
b1653_mRNA_2	mRNA b1653	C43866H49767N17456O32372P4617	-4618	Translation
b1653_mRNA_2_degr	mRNA b1653 degradation complex	C84549H115166N29331O45057S200P4617Mg6Zn2Fe0	-4840	mRNA degradation
b1665_RNA	tRNA (valV)	C732H828N289O544P77	-78	tRNA Modification
b1665_tRNA_1	b1665_tRNA_1 (valV)	C732H830Mg2N289O544P77	-74	tRNA Modification
b1665_tRNA_1_Dus_gen_cplx	b1665_tRNA_1 (valV), Dus_gen	C2339H3369Mg2N748O1027P80S14	-81	tRNA Modification
b1665_tRNA_2	b1665_tRNA_2 (valV)	C732H832Mg2N289O544P77	-74	tRNA Modification
b1665_tRNA_2_Dus_gen_cplx	b1665_tRNA_2 (valV), Dus_gen	C2339H3371Mg2N748O1027P80S14	-81	tRNA Modification
b1665_tRNA_3	b1665_tRNA_3 (valV)	C732H834Mg2N289O544P77	-74	tRNA Modification
b1665_tRNA_3_Dus_gen_cplx	b1665_tRNA_3 (valV), Dus_gen	C2339H3373Mg2N748O1027P80S14	-81	tRNA Modification
b1665_tRNA_4	b1665_tRNA_4 (valV)	C732H836Mg2N289O544P77	-74	tRNA Modification
b1665_tRNA_4_YggH_mono_cplx	b1665_tRNA_4 (valV), YggH_mono	C1957H2735Mg2N642O891P77S14	-76	tRNA Modification
b1665_tRNA_5	b1665_tRNA_5 (valV)	C733H838Mg2N289O544P77	-74	tRNA Modification
b1665_tRNA_5_AcpT_tRNA_pos_47_acp3U_cplx	b1665_tRNA_5 (valV), AcpT_tRNA_pos_47_acp3U	C748H861Mg2N295O549P77S1	-73	tRNA Modification
b1665_tRNA_6	b1665_tRNA_6 (valV)	C737H845Mg2N290O546P77	-74	tRNA Modification
b1665_tRNA_6_TrmA_mono_cplx	b1665_tRNA_6 (valV), TrmA_mono	C2611H3792Mg2N813O1105P77S19	-82	tRNA Modification
b1665_tRNA_7	b1665_tRNA_7 (valV)	C738H847Mg2N290O546P77	-74	tRNA Modification
b1665_tRNA_7_TruB_mono_cplx	b1665_tRNA_7 (valV), TruB_mono	C2278H3322Mg2N728O1015P77S10	-79	tRNA Modification
b1665_tRNA_8	b1665_tRNA_8 (valV)	C738H847Mg2N290O546P77	-74	tRNA Modification
b1665_tRNA_8_ThiI_mono_cplx	b1665_tRNA_8 (valV), ThiI_mono	C7166H11078Mg3N2108O2485P82S50X1	-102	tRNA Modification
b1665_tRNA_Mg2	tRNA (valV) bound two Mg2 ions	C732H828Mg2N289O544P77	-74	tRNA Modification
b1665_tRNA_Mg2_Dus_gen_cplx	b1665_tRNA (valV), Dus_gen	C2339H3367Mg2N748O1027P80S14	-81	tRNA Modification
b1666_RNA	tRNA (valW)	C732H831N292O544P77	-78	tRNA Modification
b1666_tRNA_1	b1666_tRNA_1 (valW)	C732H833Mg2N292O544P77	-74	tRNA Modification
b1666_tRNA_1_Dus_gen_cplx	b1666_tRNA_1 (valW), Dus_gen	C2339H3372Mg2N751O1027P80S14	-81	tRNA Modification
b1666_tRNA_2	b1666_tRNA_2 (valW)	C732H835Mg2N292O544P77	-74	tRNA Modification
b1666_tRNA_2_Dus_gen_cplx	b1666_tRNA_2 (valW), Dus_gen	C2339H3374Mg2N751O1027P80S14	-81	tRNA Modification
b1666_tRNA_3	b1666_tRNA_3 (valW)	C732H837Mg2N292O544P77	-74	tRNA Modification
b1666_tRNA_3_Dus_gen_cplx	b1666_tRNA_3 (valW), Dus_gen	C2339H3376Mg2N751O1027P80S14	-81	tRNA Modification
b1666_tRNA_4	b1666_tRNA_4 (valW)	C732H839Mg2N292O544P77	-74	tRNA Modification
b1666_tRNA_4_YggH_mono_cplx	b1666_tRNA_4 (valW), YggH_mono	C1957H2738Mg2N645O891P77S14	-76	tRNA Modification
b1666_tRNA_5	b1666_tRNA_5 (valW)	C733H841Mg2N292O544P77	-74	tRNA Modification
b1666_tRNA_5_AcpT_tRNA_pos_47_acp3U_cplx	b1666_tRNA_5 (valW), AcpT_tRNA_pos_47_acp3U	C748H864Mg2N298O549P77S1	-73	tRNA Modification
b1666_tRNA_6	b1666_tRNA_6 (valW)	C737H848Mg2N293O546P77	-74	tRNA Modification
b1666_tRNA_6_TrmA_mono_cplx	b1666_tRNA_6 (valW), TrmA_mono	C2611H3795Mg2N816O1105P77S19	-82	tRNA Modification
b1666_tRNA_7	b1666_tRNA_7 (valW)	C738H850Mg2N293O546P77	-74	tRNA Modification
b1666_tRNA_7_TruB_mono_cplx	b1666_tRNA_7 (valW), TruB_mono	C2278H3325Mg2N731O1015P77S10	-79	tRNA Modification
b1666_tRNA_8	b1666_tRNA_8 (valW)	C738H850Mg2N293O546P77	-74	tRNA Modification
b1666_tRNA_8_ThiI_mono_cplx	b1666_tRNA_8 (valW), ThiI_mono	C7166H11081Mg3N2111O2485P82S50X1	-102	tRNA Modification
b1666_tRNA_Mg2	tRNA (valW) bound two Mg2 ions	C732H831Mg2N292O544P77	-74	tRNA Modification
b1666_tRNA_Mg2_Dus_gen_cplx	b1666_tRNA (valW), Dus_gen	C2339H3370Mg2N751O1027P80S14	-81	tRNA Modification
b1712_aa	polypeptide b1712_v1	C499H813N147O153S2	2	Translation
b1712_def_map_cplx	Polypeptide b1712 peptide deformylase and methionine aminopeptidase complex	C2629H4269N740O799S23Mg0Zn0Fe3	-7	Maturation
b1712_m	Matured polypeptide b1712	C493H805N146O151S1	3	Maturation
b1712_mRNA_1	mRNA b1712	C2870H3238N1177O2083P302	-303	Translation
b1712_mRNA_2	mRNA b1712	C2870H3238N1177O2083P302	-303	Translation
b1712_mRNA_2_degr	mRNA b1712 degradation complex	C43553H68637N13052O14768S200P302Mg6Zn2Fe0	-525	mRNA degradation
b1712_v1_mRNA	mRNA b1712_v1	C2870H3238N1177O2077P300	-301	Translation
b1713_aa	polypeptide b1713	C3860H6200N1086O1168S27	-30	Translation
b1713_def_map_cplx	Polypeptide b1713 peptide deformylase and methionine aminopeptidase complex	C5990H9656N1679O1814S48Mg0Zn0Fe3	-39	Maturation
b1713_m	Matured polypeptide b1713	C3854H6192N1085O1166S26	-29	Maturation
b1713_mRNA	mRNA b1713	C22755H25732N9153O16701P2388	-2389	Translation
b1713_mRNA_1	mRNA b1713	C22755H25732N9153O16701P2388	-2389	Translation
b1713_mRNA_2	mRNA b1713	C22755H25732N9153O16701P2388	-2389	Translation
b1713_mRNA_2_degr	mRNA b1713 degradation complex	C63438H91131N21028O29386S200P2388Mg6Zn2Fe0	-2611	mRNA degradation
b1713_m_DnaKJ_complex	b1713 DnaK DnaJ_dim complex - Deuerling et al. DnaKJ/GrpE dependent folding	C10418H16706O3264N2998S73P3Zn4	-51	Folding

b1714_aa	polypeptide b1714	C1640H2549N462O485S11	-9	Translation
b1714_def_map_cplx	Polypeptide b1714 peptide deformylase and methionine aminopeptidase complex	C3770H6005N1055O1131S32Mg0Zn0Fe3	-18	Maturation
b1714_m	Matured polypeptide b1714	C1634H2541N461O483S10	-8	Maturation
b1714_mRNA	mRNA b1714	C9344H10594N3697O6897P984	-985	Translation
b1714_mRNA_1	mRNA b1714	C9344H10594N3697O6897P984	-985	Translation
b1714_mRNA_2	mRNA b1714	C9344H10594N3697O6897P984	-985	Translation
b1714_mRNA_2_degr	mRNA b1714 degradation complex	C50027H75993N15572O19582S200P984Mg6Zn2Fe0	-1207	mRNA degradation
b1715_aa	polypeptide b1715_v2	C89H116N18O20S1	0	Translation
b1715_def_map_cplx	Polypeptide b1715 peptide deformylase and methionine aminopeptidase complex	C2219H3572N611O666S22Mg0Zn0Fe3	-9	Maturation
b1715_m	Matured polypeptide b1715	C83H108N17O18S0	1	Maturation
b1715_mRNA_1	mRNA b1715	C419H475N142O335P47	-48	Translation
b1715_mRNA_2	mRNA b1715	C419H475N142O335P47	-48	Translation
b1715_mRNA_2_degr	mRNA b1715 degradation complex	C41102H65874N12017O13020S200P47Mg6Zn2Fe0	-270	mRNA degradation
b1715_v1_mRNA	mRNA b1715_v1	C419H475N142O329P45	-46	Translation
b1715_v2_mRNA	mRNA b1715_v2	C419H475N142O335P47	-48	Translation
b1716_aa	polypeptide b1716_v2	C610H1030N193O153S1	23	Translation
b1716_def_map_cplx	Polypeptide b1716 peptide deformylase and methionine aminopeptidase complex	C2740H4486N786O799S22Mg0Zn0Fe3	14	Maturation
b1716_m	Matured polypeptide b1716	C604H1022N192O151S0	24	Maturation
b1716_mRNA	mRNA b1716	C3396H3840N1349O2491P357	-358	Translation
b1716_mRNA_1	mRNA b1716	C3396H3840N1349O2491P357	-358	Translation
b1716_mRNA_2	mRNA b1716	C3396H3840N1349O2491P357	-358	Translation
b1716_mRNA_2_degr	mRNA b1716 degradation complex	C44079H69239N13224O15176S200P357Mg6Zn2Fe0	-580	mRNA degradation
b1716_v1_mRNA	mRNA b1716_v1	C3396H3840N1349O2497P359	-360	Translation
b1716_v2_mRNA	mRNA b1716_v2	C3396H3840N1349O2497P359	-360	Translation
b1717_aa	polypeptide b1717	C329H582N106O76S3	18	Translation
b1717_def_map_cplx	Polypeptide b1717 peptide deformylase and methionine aminopeptidase complex	C2459H4038N699O722S24Mg0Zn0Fe3	9	Maturation
b1717_m	Matured polypeptide b1717	C323H574N105O74S2	19	Maturation
b1717_mRNA	mRNA b1717	C1889H2141N770O1364P198	-199	Translation
b1717_mRNA_1	mRNA b1717	C1889H2141N770O1364P198	-199	Translation
b1717_mRNA_2	mRNA b1717	C1889H2141N770O1364P198	-199	Translation
b1717_mRNA_2_degr	mRNA b1717 degradation complex	C42572H67540N12645O14049S200P198Mg6Zn2Fe0	-421	mRNA degradation
b1718_aa	polypeptide b1718_v2	C903H1515N264O269S7	7	Translation
b1718_def_cplx	Polypeptide b1718 peptide deformylase complex	C1747H2909N505O524S13Mg0Zn0Fe1	2	Maturation
b1718_m	Matured polypeptide b1718	C902H1516N264O268S7	8	Maturation
b1718_mRNA	mRNA b1718	C5203H5864N2151O3743P543	-544	Translation
b1718_mRNA_1	mRNA b1718	C5203H5864N2151O3743P543	-544	Translation
b1718_mRNA_2	mRNA b1718	C5203H5864N2151O3743P543	-544	Translation
b1718_mRNA_2_degr	mRNA b1718 degradation complex	C45886H71263N14026O16428S200P543Mg6Zn2Fe0	-766	mRNA degradation
b1718_m_DnaKJ_complex	b1718 DnaK DnaJ_dim complex - Kerner et al. class I or II can interact w/ GroEL/ES, behaves here as class I	C7466H12030O2366N2177S54P3Zn4	-14	Folding
b1718_m_GroEL.(7)ATP.transGroES	b1718 GroEL GroES complex - Kerner et al. class I or II can interact w/ GroEL/ES, behaves here as class I	C38814H64460O12602N10848S371P21Mg7	-293	Folding
b1718_v1_mRNA	mRNA b1718_v1	C5203H5864N2151O3749P545	-546	Translation
b1718_v2_mRNA	mRNA b1718_v2	C5203H5864N2151O3749P545	-546	Translation
b1719_aa	polypeptide b1719	C3280H5084N916O970S36	-18	Translation
b1719_def_map_cplx	Polypeptide b1719 peptide deformylase and methionine aminopeptidase complex	C5410H8540N1509O1616S57Mg0Zn0Fe3	-27	Maturation
b1719_m	Matured polypeptide b1719	C3274H5076N915O968S35	-17	Maturation
b1719_mRNA	mRNA b1719	C18384H20766N7379O13445P1931	-1932	Translation
b1719_mRNA_1	mRNA b1719	C18384H20766N7379O13445P1931	-1932	Translation
b1719_mRNA_2	mRNA b1719	C18384H20766N7379O13445P1931	-1932	Translation
b1719_mRNA_2_degr	mRNA b1719 degradation complex	C59067H86165N19254O26130S200P1931Mg6Zn2Fe0	-2154	mRNA degradation
b1719_m_DnaKJ_complex	b1719 DnaK DnaJ_dim complex - Kerner et al. class II can interact w/ GroEL/ES, cannot fold spontanously	C9838H15590O3066N2828S82P3Zn5	-37	Folding
b1719_m_GroEL.(7)ATP.transGroES	b1719 GroEL GroES complex - Kerner et al. class II can interact w/ GroEL/ES, cannot fold spontanously	C41186H68020O13302N11499S399P21Mg7Zn1	-316	Folding
b1719_m_Zn	b1719 plus _Zn	C3274H5076N915O968S35Zn1	-15	Folding
b1804_DNA_act	DNA b1804 (activated form)	C10991H12668N4282O6770P1128	-1128	Transcription

b1804_DNA_neu	DNA b1804 (inactivate form)	C10991H12668N4282O6770P1128	-1128	Transcription
b1804_aa	polypeptide b1804	C1922H3006N517O553S17	-13	Translation
b1804_def_cplx	Polypeptide b1804 peptide deformylase complex	C2766H4400N758O808S23Mg0Zn0Fe1	-18	Maturation
b1804_m	Matured polypeptide b1804	C1921H3007N517O552S17	-12	Maturation
b1804_mRNA	mRNA b1804	C10731H12149N4282O7905P1130	-1131	Translation
b1804_mRNA_1	mRNA b1804	C10731H12149N4282O7905P1130	-1131	Translation
b1804_mRNA_2	mRNA b1804	C10731H12149N4282O7905P1130	-1131	Translation
b1804_mRNA_2_degr	mRNA b1804 degradation complex	C51414H77548N16157O20590S200P1130Mg6Zn2Fe0	-1353	mRNA degradation
b1804_m_Mg	b1804 plus _Mg	C1921H3007N517O552S17Mg5	-2	Folding
b1822_aa	polypeptide b1822	C1357H2102N380O387S16	-2	Translation
b1822_def_map_cplx	Polypeptide b1822 peptide deformylase and methionine aminopeptidase complex	C3487H5558N973O1033S37Mg0Zn0Fe3	-11	Maturation
b1822_m	Matured polypeptide b1822	C1351H2094N379O385S15	-1	Maturation
b1822_mRNA	mRNA b1822	C7703H8711N3056O5683P812	-813	Translation
b1822_mRNA_1	mRNA b1822	C7703H8711N3056O5683P812	-813	Translation
b1822_mRNA_2	mRNA b1822	C7703H8711N3056O5683P812	-813	Translation
b1822_mRNA_2_degr	mRNA b1822 degradation complex	C48386H74110N14931O18368S200P812Mg6Zn2Fe0	-1035	mRNA degradation
b1822_m_Zn	b1822 plus _Zn	C1351H2094N379O385S15Zn1	1	Folding
b1866_aa	polypeptide b1866	C2923H4625N812O872S26	-13	Translation
b1866_def_cplx	Polypeptide b1866 peptide deformylase complex	C3767H6019N1053O1127S32Mg0Zn0Fe1	-18	Maturation
b1866_m	Matured polypeptide b1866	C2922H4626N812O871S26	-12	Maturation
b1866_mRNA	mRNA b1866	C16876H19101N6754O12404P1775	-1776	Translation
b1866_mRNA_1	mRNA b1866	C16876H19101N6754O12404P1775	-1776	Translation
b1866_mRNA_2	mRNA b1866	C16876H19101N6754O12404P1775	-1776	Translation
b1866_mRNA_2_degr	mRNA b1866 degradation complex	C57559H84500N18629O25089S200P1775Mg6Zn2Fe0	-1998	mRNA degradation
b1869_aa	polypeptide b1869	C707H1087N182O169S13	3	Translation
b1869_def_map_cplx	Polypeptide b1869 peptide deformylase and methionine aminopeptidase complex	C2837H4543N775O815S34Mg0Zn0Fe3	-6	Maturation
b1869_m	Matured polypeptide b1869	C701H1079N181O167S12	4	Maturation
b1869_mRNA	mRNA b1869	C3746H4229N1424O2840P398	-399	Translation
b1869_mRNA_1	mRNA b1869	C3746H4229N1424O2840P398	-399	Translation
b1869_mRNA_2	mRNA b1869	C3746H4229N1424O2840P398	-399	Translation
b1869_mRNA_2_degr	mRNA b1869 degradation complex	C44429H69628N13299O15525S200P398Mg6Zn2Fe0	-621	mRNA degradation
b1870_aa	polypeptide b1870	C1231H1912N341O368S13	-11	Translation
b1870_def_map_cplx	Polypeptide b1870 peptide deformylase and methionine aminopeptidase complex	C3361H5368N934O1014S34Mg0Zn0Fe3	-20	Maturation
b1870_m	Matured polypeptide b1870	C1225H1904N340O366S12	-10	Maturation
b1870_mRNA	mRNA b1870	C7062H7995N2774O5222P744	-745	Translation
b1870_mRNA_1	mRNA b1870	C7062H7995N2774O5222P744	-745	Translation
b1870_mRNA_2	mRNA b1870	C7062H7995N2774O5222P744	-745	Translation
b1870_mRNA_2_degr	mRNA b1870 degradation complex	C47745H73394N14649O17907S200P744Mg6Zn2Fe0	-967	mRNA degradation
b1871_aa	polypeptide b1871	C1679H2598N456O469S11	-6	Translation
b1871_def_map_cplx	Polypeptide b1871 peptide deformylase and methionine aminopeptidase complex	C3809H6054N1049O1115S32Mg0Zn0Fe3	-15	Maturation
b1871_m	Matured polypeptide b1871	C1673H2590N455O467S10	-5	Maturation
b1871_mRNA	mRNA b1871 (4 nt short)	C9207H10411N3656O6790P968	-969	Translation
b1871_mRNA_1	mRNA b1871 (4 nt short)	C9207H10411N3656O6790P968	-969	Translation
b1871_mRNA_2	mRNA b1871 (4 nt short)	C9207H10411N3656O6790P968	-969	Translation
b1871_mRNA_2_degr	mRNA b1871 degradation complex	C49890H75810N15531O19475S200P968Mg6Zn2Fe0	-1191	mRNA degradation
b1876_aa	polypeptide b1876	C2875H4519N785O861S26	-20	Translation
b1876_def_cplx	Polypeptide b1876 peptide deformylase complex	C3719H5913N1026O1116S32Mg0Zn0Fe1	-25	Maturation
b1876_m	Matured polypeptide b1876	C2874H4520N785O860S26	-19	Maturation
b1876_mRNA	mRNA b1876	C16539H18710N6703O12065P1736	-1737	Translation
b1876_mRNA_1	mRNA b1876	C16539H18710N6703O12065P1736	-1737	Translation
b1876_mRNA_2	mRNA b1876	C16539H18710N6703O12065P1736	-1737	Translation
b1876_mRNA_2_degr	mRNA b1876 degradation complex	C57222H84109N18578O24750S200P1736Mg6Zn2Fe0	-1959	mRNA degradation
b1909_RNA	tRNA (leuZ)	C828H940N333O611P87	-88	tRNA Modification
b1909_tRNA_1	b1909_tRNA_1 (leuZ)	C828H942Mg2N333O611P87	-84	tRNA Modification
b1909_tRNA_1_TrmH_dim_cplx	b1909_tRNA_1 (leuZ), TrmH_dim	C3039H4485Mg2N989O1272P87S29	-85	tRNA Modification
b1909_tRNA_2	b1909_tRNA_2 (leuZ)	C829H944Mg2N333O611P87	-84	tRNA Modification
b1909_tRNA_2_Dus_gen_cplx	b1909_tRNA_2 (leuZ), Dus_gen	C2436H3483Mg2N792O1094P90S14	-91	tRNA Modification

b1909_tRNA_3	b1909_tRNA_3 (leuZ)	C829H946Mg2N333O611P87	-84	tRNA Modification
b1909_tRNA_3_RluA_mono_cplx	b1909_tRNA_3 (leuZ), RluA_mono	C1932H2686Mg2N646O925P87S10	-83	tRNA Modification
b1909_tRNA_4	b1909_tRNA_4 (leuZ)	C829H946Mg2N333O611P87	-84	tRNA Modification
b1909_tRNA_4_MiaA_dim-MiaB_mono_cplx	b1909_tRNA_4 (leuZ), MiaA_dim, MiaB_mono	C10304Fe4H15976Mg4N3008O3477P91S80X1	-129	tRNA Modification
b1909_tRNA_5	b1909_tRNA_5 (leuZ)	C835H956Mg2N333O611P87S1	-84	tRNA Modification
b1909_tRNA_5_TruA_dim_cplx	b1909_tRNA_5 (leuZ), TruA_dim	C3545H5158Mg2N1113O1381P87S17	-78	tRNA Modification
b1909_tRNA_6	b1909_tRNA_6 (leuZ)	C835H956Mg2N333O611P87S1	-84	tRNA Modification
b1909_tRNA_6_TrmA_mono_cplx	b1909_tRNA_6 (leuZ), TrmA_mono	C2709H3903Mg2N856O1170P87S20	-92	tRNA Modification
b1909_tRNA_7	b1909_tRNA_7 (leuZ)	C836H958Mg2N333O611P87S1	-84	tRNA Modification
b1909_tRNA_7_TruB_mono_cplx	b1909_tRNA_7 (leuZ), TruB_mono	C2376H3433Mg2N771O1080P87S11	-89	tRNA Modification
b1909_tRNA_8	b1909_tRNA_8 (leuZ)	C836H958Mg2N333O611P87S1	-84	tRNA Modification
b1909_tRNA_8_ThiI_mono_cplx	b1909_tRNA_8 (leuZ), ThiI_mono	C7264H11189Mg3N2151O2550P92S51X1	-112	tRNA Modification
b1909_tRNA_Mg2	tRNA (leuZ) bound two Mg2 ions	C828H940Mg2N333O611P87	-84	tRNA Modification
b1909_tRNA_Mg2_Dus_gen_cplx	b1909_tRNA (leuZ), Dus_gen	C2435H3479Mg2N792O1094P90S14	-91	tRNA Modification
b1910_RNA	tRNA (cysT)	C703H799N280O519P74	-75	tRNA Modification
b1910_tRNA_1	b1910_tRNA_1 (cysT)	C703H801Mg2N280O519P74	-71	tRNA Modification
b1910_tRNA_1_Dus_gen_cplx	b1910_tRNA_1 (cysT), Dus_gen	C2310H3340Mg2N739O1002P77S14	-78	tRNA Modification
b1910_tRNA_2	b1910_tRNA_2 (cysT)	C703H803Mg2N280O519P74	-71	tRNA Modification
b1910_tRNA_2_RluA_mono_cplx	b1910_tRNA_2 (cysT), RluA_mono	C1806H2543Mg2N593O833P74S10	-70	tRNA Modification
b1910_tRNA_3	b1910_tRNA_3 (cysT)	C703H803Mg2N280O519P74	-71	tRNA Modification
b1910_tRNA_3_MiaA_dim-MiaB_mono_cplx	b1910_tRNA_3 (cysT), MiaA_dim, MiaB_mono	C10178Fe4H15833Mg4N2955O3385P78S80X1	-116	tRNA Modification
b1910_tRNA_4	b1910_tRNA_4 (cysT)	C709H813Mg2N280O519P74S1	-71	tRNA Modification
b1910_tRNA_4_TruA_dim_cplx	b1910_tRNA_4 (cysT), TruA_dim	C3419H5015Mg2N1060O1289P74S17	-65	tRNA Modification
b1910_tRNA_5	b1910_tRNA_5 (cysT)	C709H813Mg2N280O519P74S1	-71	tRNA Modification
b1910_tRNA_5_TrmA_mono_cplx	b1910_tRNA_5 (cysT), TrmA_mono	C2583H3760Mg2N803O1078P74S20	-79	tRNA Modification
b1910_tRNA_6	b1910_tRNA_6 (cysT)	C710H815Mg2N280O519P74S1	-71	tRNA Modification
b1910_tRNA_6_TruB_mono_cplx	b1910_tRNA_6 (cysT), TruB_mono	C2250H3290Mg2N718O988P74S11	-76	tRNA Modification
b1910_tRNA_7	b1910_tRNA_7 (cysT)	C710H815Mg2N280O519P74S1	-71	tRNA Modification
b1910_tRNA_7_ThiI_mono_cplx	b1910_tRNA_7 (cysT), ThiI_mono	C7138H11046Mg3N2098O2458P79S51X1	-99	tRNA Modification
b1910_tRNA_Mg2	tRNA (cysT) bound two Mg2 ions	C703H799Mg2N280O519P74	-71	tRNA Modification
b1910_tRNA_Mg2_Dus_gen_cplx	b1910_tRNA (cysT), Dus_gen	C2310H3338Mg2N739O1002P77S14	-78	tRNA Modification
b1911_RNA	tRNA (glyW)	C722H820N287O537P76	-77	tRNA Modification
b1911_tRNA_1	b1911_tRNA_1 (glyW)	C722H822Mg2N287O537P76	-73	tRNA Modification
b1911_tRNA_1_Dus_gen_cplx	b1911_tRNA_1 (glyW), Dus_gen	C2329H3361Mg2N746O1020P79S14	-80	tRNA Modification
b1911_tRNA_2	b1911_tRNA_2 (glyW)	C722H824Mg2N287O537P76	-73	tRNA Modification
b1911_tRNA_2_Dus_gen_cplx	b1911_tRNA_2 (glyW), Dus_gen	C2329H3363Mg2N746O1020P79S14	-80	tRNA Modification
b1911_tRNA_3	b1911_tRNA_3 (glyW)	C722H826Mg2N287O537P76	-73	tRNA Modification
b1911_tRNA_3_YggH_mono_cplx	b1911_tRNA_3 (glyW), YggH_mono	C1947H2725Mg2N640O884P76S14	-75	tRNA Modification
b1911_tRNA_4	b1911_tRNA_4 (glyW)	C723H828Mg2N287O537P76	-73	tRNA Modification
b1911_tRNA_4_TrmA_mono_cplx	b1911_tRNA_4 (glyW), TrmA_mono	C2597H3775Mg2N810O1096P76S19	-81	tRNA Modification
b1911_tRNA_5	b1911_tRNA_5 (glyW)	C724H830Mg2N287O537P76	-73	tRNA Modification
b1911_tRNA_5_TruB_mono_cplx	b1911_tRNA_5 (glyW), TruB_mono	C2264H3305Mg2N725O1006P76S10	-78	tRNA Modification
b1911_tRNA_Mg2	tRNA (glyW) bound two Mg2 ions	C722H820Mg2N287O537P76	-73	tRNA Modification
b1911_tRNA_Mg2_Dus_gen_cplx	b1911_tRNA (glyW), Dus_gen	C2329H3359Mg2N746O1020P79S14	-80	tRNA Modification
b1920_aa	polypeptide b1920_v1	C1287H2084N352O399S6	-2	Translation
b1920_def_map_cplx	Polypeptide b1920 peptide deformylase and methionine aminopeptidase complex	C3417H5540N945O1045S27Mg0Zn0Fe3	-11	Maturation
b1920_m	Matured polypeptide b1920	C1154H1857N315O366S2	-4	Maturation
b1920_mRNA	mRNA b1920	C7666H8630N3135O5570P801	-802	Translation
b1920_mRNA_1	mRNA b1920	C7666H8630N3135O5570P801	-802	Translation
b1920_mRNA_2	mRNA b1920	C7666H8630N3135O5570P801	-802	Translation
b1920_mRNA_2_degr	mRNA b1920 degradation complex	C48349H74029N15010O18255S200P801Mg6Zn2Fe0	-1024	mRNA degradation
b1920_v1_mRNA	mRNA b1920_v1	C7666H8630N3135O5570P801	-802	Translation
b1921_aa	polypeptide b1921_v1	C959H1521N281O276S9	6	Translation
b1921_def_map_cplx	Polypeptide b1921 peptide deformylase and methionine aminopeptidase complex	C3089H4977N874O922S30Mg0Zn0Fe3	-3	Maturation
b1921_m	Matured polypeptide b1921	C953H1513N280O274S8	7	Maturation
b1921_mRNA	mRNA b1921	C5240H5931N2058O3854P552	-553	Translation
b1921_mRNA_1	mRNA b1921	C5240H5931N2058O3854P552	-553	Translation
b1921_mRNA_2	mRNA b1921	C5240H5931N2058O3854P552	-553	Translation
b1921_mRNA_2_degr	mRNA b1921 degradation complex	C45923H71330N13933O16539S200P552Mg6Zn2Fe0	-775	mRNA degradation

b1921_v1_mRNA	mRNA b1921_v1	C5240H5931N2058O3854P552	-553	Translation
b1922_aa	polypeptide b1922_v1	C1201H1936N356O376S5	-10	Translation
b1922_def_map_cplx	Polypeptide b1922 peptide deformylase and methionine aminopeptidase complex	C3331H5392N949O1022S26Mg0Zn0Fe3	-19	Maturation
b1922_m	Matured polypeptide b1922	C1195H1928N355O374S4	-9	Maturation
b1922_mRNA	mRNA b1922	C6876H7776N2807O5007P722	-723	Translation
b1922_mRNA_1	mRNA b1922	C6876H7776N2807O5007P722	-723	Translation
b1922_mRNA_2	mRNA b1922	C6876H7776N2807O5007P722	-723	Translation
b1922_mRNA_2_degr	mRNA b1922 degradation complex	C47559H73175N14682O17692S200P722Mg6Zn2Fe0	-945	mRNA degradation
b1922_v1_mRNA	mRNA b1922_v1	C6876H7776N2807O5007P722	-723	Translation
b1975_RNA	tRNA (serU)	C859H978N355O624P90	-91	tRNA Modification
b1975_tRNA_1	b1975_tRNA_1 (serU)	C860H980Mg2N355O624P90	-87	tRNA Modification
b1975_tRNA_1_Dus_gen_cplx	b1975_tRNA_1 (serU), Dus_gen	C2467H3519Mg2N814O1107P93S14	-94	tRNA Modification
b1975_tRNA_2	b1975_tRNA_2 (serU)	C860H982Mg2N355O624P90	-87	tRNA Modification
b1975_tRNA_2_MiaA_dim-MiaB_mono_cplx	b1975_tRNA_2 (serU), MiaA_dim, MiaB_mono	C10335Fe4H16012Mg4N3030O3490P94S80X1	-132	tRNA Modification
b1975_tRNA_3	b1975_tRNA_3 (serU)	C866H992Mg2N355O624P90S1	-87	tRNA Modification
b1975_tRNA_3_TrmA_mono_cplx	b1975_tRNA_3 (serU), TrmA_mono	C2740H3939Mg2N878O1183P90S20	-95	tRNA Modification
b1975_tRNA_4	b1975_tRNA_4 (serU)	C867H994Mg2N355O624P90S1	-87	tRNA Modification
b1975_tRNA_4_TruB_mono_cplx	b1975_tRNA_4 (serU), TruB_mono	C2407H3469Mg2N793O1093P90S11	-92	tRNA Modification
b1975_tRNA_Mg2	tRNA (serU) bound two Mg2 ions	C859H978Mg2N355O624P90	-87	tRNA Modification
b1975_tRNA_Mg2_TrmH_dim_cplx	b1975_tRNA (serU), TrmH_dim	C3070H4521Mg2N1011O1285P90S29	-88	tRNA Modification
b1977_RNA	tRNA (asnT)	C722H817N284O537P76	-77	tRNA Modification
b1977_tRNA_1	b1977_tRNA_1 (asnT)	C722H819Mg2N284O537P76	-73	tRNA Modification
b1977_tRNA_1_Dus_gen_cplx	b1977_tRNA_1 (asnT), Dus_gen	C2329H3358Mg2N743O1020P79S14	-80	tRNA Modification
b1977_tRNA_2	b1977_tRNA_2 (asnT)	C722H821Mg2N284O537P76	-73	tRNA Modification
b1977_tRNA_2_Tgt_hexa-QueA_mono-EoR_tRNA_pos34_Q_cplx	b1977_tRNA_2 (asnT), Tgt_hexa, QueA_mono, EoR_tRNA_pos34_Q	C13915Co1H21336Mg2N3977O4414P79S143Zn6	-113	tRNA Modification
b1977_tRNA_3	b1977_tRNA_3 (asnT)	C729H831Mg2N284O539P76	-73	tRNA Modification
b1977_tRNA_3_Up_tRNA_pos_37_t6A_cplx	b1977_tRNA_3 (asnT), Up_tRNA_pos_37_t6A	C744H853Mg3N290O558P79	-76	tRNA Modification
b1977_tRNA_4	b1977_tRNA_4 (asnT)	C734H837Mg2N285O543P76	-74	tRNA Modification
b1977_tRNA_4_TruA_dim_cplx	b1977_tRNA_4 (asnT), TruA_dim	C3444H5039Mg2N1065O1313P76S16	-68	tRNA Modification
b1977_tRNA_5	b1977_tRNA_5 (asnT)	C734H837Mg2N285O543P76	-74	tRNA Modification
b1977_tRNA_5_YggH_mono_cplx	b1977_tRNA_5 (asnT), YggH_mono	C1959H2736Mg2N638O890P76S14	-76	tRNA Modification
b1977_tRNA_6	b1977_tRNA_6 (asnT)	C735H839Mg2N285O543P76	-74	tRNA Modification
b1977_tRNA_6_TrmA_mono_cplx	b1977_tRNA_6 (asnT), TrmA_mono	C2609H3786Mg2N808O1102P76S19	-82	tRNA Modification
b1977_tRNA_7	b1977_tRNA_7 (asnT)	C736H841Mg2N285O543P76	-74	tRNA Modification
b1977_tRNA_7_TruB_mono_cplx	b1977_tRNA_7 (asnT), TruB_mono	C2276H3316Mg2N723O1012P76S10	-79	tRNA Modification
b1977_tRNA_8	b1977_tRNA_8 (asnT)	C736H841Mg2N285O543P76	-74	tRNA Modification
b1977_tRNA_8_ThiI_mono_cplx	b1977_tRNA_8 (asnT), ThiI_mono	C7164H11072Mg3N2103O2482P81S50X1	-102	tRNA Modification
b1977_tRNA_Mg2	tRNA (asnT) bound two Mg2 ions	C722H817Mg2N284O537P76	-73	tRNA Modification
b1977_tRNA_Mg2_Dus_gen_cplx	b1977_tRNA (asnT), Dus_gen	C2329H3356Mg2N743O1020P79S14	-80	tRNA Modification
b1984_RNA	tRNA (asnW)	C722H817N284O537P76	-77	tRNA Modification
b1984_tRNA_1	b1984_tRNA_1 (asnW)	C722H819Mg2N284O537P76	-73	tRNA Modification
b1984_tRNA_1_Dus_gen_cplx	b1984_tRNA_1 (asnW), Dus_gen	C2329H3358Mg2N743O1020P79S14	-80	tRNA Modification
b1984_tRNA_2	b1984_tRNA_2 (asnW)	C722H821Mg2N284O537P76	-73	tRNA Modification
b1984_tRNA_2_Tgt_hexa-QueA_mono-EoR_tRNA_pos34_Q_cplx	b1984_tRNA_2 (asnW), Tgt_hexa, QueA_mono, EoR_tRNA_pos34_Q	C13915Co1H21336Mg2N3977O4414P79S143Zn6	-113	tRNA Modification
b1984_tRNA_3	b1984_tRNA_3 (asnW)	C729H831Mg2N284O539P76	-73	tRNA Modification
b1984_tRNA_3_Up_tRNA_pos_37_t6A_cplx	b1984_tRNA_3 (asnW), Up_tRNA_pos_37_t6A	C744H853Mg3N290O558P79	-76	tRNA Modification
b1984_tRNA_4	b1984_tRNA_4 (asnW)	C734H837Mg2N285O543P76	-74	tRNA Modification
b1984_tRNA_4_TruA_dim_cplx	b1984_tRNA_4 (asnW), TruA_dim	C3444H5039Mg2N1065O1313P76S16	-68	tRNA Modification
b1984_tRNA_5	b1984_tRNA_5 (asnW)	C734H837Mg2N285O543P76	-74	tRNA Modification
b1984_tRNA_5_YggH_mono_cplx	b1984_tRNA_5 (asnW), YggH_mono	C1959H2736Mg2N638O890P76S14	-76	tRNA Modification
b1984_tRNA_6	b1984_tRNA_6 (asnW)	C735H839Mg2N285O543P76	-74	tRNA Modification
b1984_tRNA_6_TrmA_mono_cplx	b1984_tRNA_6 (asnW), TrmA_mono	C2609H3786Mg2N808O1102P76S19	-82	tRNA Modification
b1984_tRNA_7	b1984_tRNA_7 (asnW)	C736H841Mg2N285O543P76	-74	tRNA Modification
b1984_tRNA_7_TruB_mono_cplx	b1984_tRNA_7 (asnW), TruB_mono	C2276H3316Mg2N723O1012P76S10	-79	tRNA Modification
b1984_tRNA_8	b1984_tRNA_8 (asnW)	C736H841Mg2N285O543P76	-74	tRNA Modification
b1984_tRNA_8_ThiI_mono_cplx	b1984_tRNA_8 (asnW), ThiI_mono	C7164H11072Mg3N2103O2482P81S50X1	-102	tRNA Modification
b1984_tRNA_Mg2	tRNA (asnW) bound two Mg2 ions	C722H817Mg2N284O537P76	-73	tRNA Modification

b1984_tRNA_Mg2_Dus_gen_cplx	b1984_tRNA (asnW), Dus_gen	C2329H3356Mg2N743O1020P79S14	-80	tRNA Modification
b1986_RNA	tRNA (asnU)	C722H817N284O537P76	-77	tRNA Modification
b1986_tRNA_1	b1986_tRNA_1 (asnU)	C722H819Mg2N284O537P76	-73	tRNA Modification
b1986_tRNA_1_Dus_gen_cplx	b1986_tRNA_1 (asnU), Dus_gen	C2329H3358Mg2N743O1020P79S14	-80	tRNA Modification
b1986_tRNA_2	b1986_tRNA_2 (asnU)	C722H821Mg2N284O537P76	-73	tRNA Modification
b1986_tRNA_2_Tgt_hexa-QueA_mono-EoR_tRNA_pos34_Q_cplx	b1986_tRNA_2 (asnU), Tgt_hexa, QueA_mono, EoR_tRNA_pos34_Q	C13915Co1H21336Mg2N3977O4414P79S143Zn6	-113	tRNA Modification
b1986_tRNA_3	b1986_tRNA_3 (asnU)	C729H831Mg2N284O539P76	-73	tRNA Modification
b1986_tRNA_3_Up_tRNA_pos_37_t6A_cplx	b1986_tRNA_3 (asnU), Up_tRNA_pos_37_t6A	C744H853Mg3N290O558P79	-76	tRNA Modification
b1986_tRNA_4	b1986_tRNA_4 (asnU)	C734H837Mg2N285O543P76	-74	tRNA Modification
b1986_tRNA_4_TruA_dim_cplx	b1986_tRNA_4 (asnU), TruA_dim	C3444H5039Mg2N1065O1313P76S16	-68	tRNA Modification
b1986_tRNA_5	b1986_tRNA_5 (asnU)	C734H837Mg2N285O543P76	-74	tRNA Modification
b1986_tRNA_5_YggH_mono_cplx	b1986_tRNA_5 (asnU), YggH_mono	C1959H2736Mg2N638O890P76S14	-76	tRNA Modification
b1986_tRNA_6	b1986_tRNA_6 (asnU)	C735H839Mg2N285O543P76	-74	tRNA Modification
b1986_tRNA_6_TrmA_mono_cplx	b1986_tRNA_6 (asnU), TrmA_mono	C2609H3786Mg2N808O1102P76S19	-82	tRNA Modification
b1986_tRNA_7	b1986_tRNA_7 (asnU)	C736H841Mg2N285O543P76	-74	tRNA Modification
b1986_tRNA_7_TruB_mono_cplx	b1986_tRNA_7 (asnU), TruB_mono	C2276H3316Mg2N723O1012P76S10	-79	tRNA Modification
b1986_tRNA_8	b1986_tRNA_8 (asnU)	C736H841Mg2N285O543P76	-74	tRNA Modification
b1986_tRNA_8_ThiI_mono_cplx	b1986_tRNA_8 (asnU), ThiI_mono	C7164H11072Mg3N2103O2482P81S50X1	-102	tRNA Modification
b1986_tRNA_Mg2	tRNA (asnU) bound two Mg2 ions	C722H817Mg2N284O537P76	-73	tRNA Modification
b1986_tRNA_Mg2_Dus_gen_cplx	b1986_tRNA (asnU), Dus_gen	C2329H3356Mg2N743O1020P79S14	-80	tRNA Modification
b1989_RNA	tRNA (asnV)	C722H817N284O537P76	-77	tRNA Modification
b1989_tRNA_1	b1989_tRNA_1 (asnV)	C722H819Mg2N284O537P76	-73	tRNA Modification
b1989_tRNA_1_Dus_gen_cplx	b1989_tRNA_1 (asnV), Dus_gen	C2329H3358Mg2N743O1020P79S14	-80	tRNA Modification
b1989_tRNA_2	b1989_tRNA_2 (asnV)	C722H821Mg2N284O537P76	-73	tRNA Modification
b1989_tRNA_2_Tgt_hexa-QueA_mono-EoR_tRNA_pos34_Q_cplx	b1989_tRNA_2 (asnV), Tgt_hexa, QueA_mono, EoR_tRNA_pos34_Q	C13915Co1H21336Mg2N3977O4414P79S143Zn6	-113	tRNA Modification
b1989_tRNA_3	b1989_tRNA_3 (asnV)	C729H831Mg2N284O539P76	-73	tRNA Modification
b1989_tRNA_3_Up_tRNA_pos_37_t6A_cplx	b1989_tRNA_3 (asnV), Up_tRNA_pos_37_t6A	C744H853Mg3N290O558P79	-76	tRNA Modification
b1989_tRNA_4	b1989_tRNA_4 (asnV)	C734H837Mg2N285O543P76	-74	tRNA Modification
b1989_tRNA_4_TruA_dim_cplx	b1989_tRNA_4 (asnV), TruA_dim	C3444H5039Mg2N1065O1313P76S16	-68	tRNA Modification
b1989_tRNA_5	b1989_tRNA_5 (asnV)	C734H837Mg2N285O543P76	-74	tRNA Modification
b1989_tRNA_5_YggH_mono_cplx	b1989_tRNA_5 (asnV), YggH_mono	C1959H2736Mg2N638O890P76S14	-76	tRNA Modification
b1989_tRNA_6	b1989_tRNA_6 (asnV)	C735H839Mg2N285O543P76	-74	tRNA Modification
b1989_tRNA_6_TrmA_mono_cplx	b1989_tRNA_6 (asnV), TrmA_mono	C2609H3786Mg2N808O1102P76S19	-82	tRNA Modification
b1989_tRNA_7	b1989_tRNA_7 (asnV)	C736H841Mg2N285O543P76	-74	tRNA Modification
b1989_tRNA_7_TruB_mono_cplx	b1989_tRNA_7 (asnV), TruB_mono	C2276H3316Mg2N723O1012P76S10	-79	tRNA Modification
b1989_tRNA_8	b1989_tRNA_8 (asnV)	C736H841Mg2N285O543P76	-74	tRNA Modification
b1989_tRNA_8_ThiI_mono_cplx	b1989_tRNA_8 (asnV), ThiI_mono	C7164H11072Mg3N2103O2482P81S50X1	-102	tRNA Modification
b1989_tRNA_Mg2	tRNA (asnV) bound two Mg2 ions	C722H817Mg2N284O537P76	-73	tRNA Modification
b1989_tRNA_Mg2_Dus_gen_cplx	b1989_tRNA (asnV), Dus_gen	C2329H3356Mg2N743O1020P79S14	-80	tRNA Modification
b2114_aa	polypeptide b2114	C3422H5251N916O1007S29	-17	Translation
b2114_def_map_cplx	Polypeptide b2114 peptide deformylase and methionine aminopeptidase complex	C5552H8707N1509O1653S50Mg0Zn0Fe3	-26	Maturation
b2114_m	Matured polypeptide b2114	C3416H5243N915O1005S28	-16	Maturation
b2114_mRNA	mRNA b2114	C19344H21913N7716O14214P2036	-2037	Translation
b2114_mRNA_1	mRNA b2114	C19344H21913N7716O14214P2036	-2037	Translation
b2114_mRNA_2	mRNA b2114	C19344H21913N7716O14214P2036	-2037	Translation
b2114_mRNA_2_degr	mRNA b2114 degradation complex	C60027H87312N19591O26899S200P2036Mg6Zn2Fe0	-2259	mRNA degradation
b2114_m_DnaKJ_complex	b2114 DnaK DnaJ_dim complex - Deuerling et al. DnaKJ/GrpE dependent folding	C9980H15757O3103N2828S75P3Zn5	-36	Folding
b2114_m_Zn	b2114 plus _Zn	C3416H5243N915O1005S28Zn1	-14	Folding
b2140_aa	polypeptide b2140	C1567H2497N443O456S12	-4	Translation
b2140_def_map_cplx	Polypeptide b2140 peptide deformylase and methionine aminopeptidase complex	C3697H5953N1036O1102S33Mg0Zn0Fe3	-13	Maturation
b2140_m	Matured polypeptide b2140	C1561H2489N442O454S11	-3	Maturation
b2140_mRNA	mRNA b2140	C9053H10216N3673O6635P950	-951	Translation
b2140_mRNA_1	mRNA b2140	C9053H10216N3673O6635P950	-951	Translation
b2140_mRNA_2	mRNA b2140	C9053H10216N3673O6635P950	-951	Translation
b2140_mRNA_2_degr	mRNA b2140 degradation complex	C49736H75615N15548O19320S200P950Mg6Zn2Fe0	-1173	mRNA degradation
b2140_m_GroEL.(7)ATP.transGroES	b2140 GroEL GroES complex - Kerner et al. class III needs GroEL/ES	C39473H65433O12788N11026S375P21Mg7	-304	Folding

b2183_aa	polypeptide b2183	C1146H1799N328O345S6	-9	Translation
b2183_def_cplx	Polypeptide b2183 peptide deformylase complex	C1990H3193N569O600S12Mg0Zn0Fe1	-14	Maturation
b2183_m	Matured polypeptide b2183	C1145H1800N328O344S6	-8	Maturation
b2183_mRNA	mRNA b2183	C6615H7491N2624O4883P698	-699	Translation
b2183_mRNA_1	mRNA b2183	C6615H7491N2624O4883P698	-699	Translation
b2183_mRNA_2	mRNA b2183	C6615H7491N2624O4883P698	-699	Translation
b2183_mRNA_2_degr	mRNA b2183 degradation complex	C47298H72890N14499O17568S200P698Mg6Zn2Fe0	-921	mRNA degradation
b2185_aa	polypeptide b2185	C480H779N137O135S3	4	Translation
b2185_def_cplx	Polypeptide b2185 peptide deformylase complex	C1324H2173N378O390S9Mg0Zn0Fe1	-1	Maturation
b2185_m	Matured polypeptide b2185	C479H780N137O134S3	5	Maturation
b2185_mRNA	mRNA b2185	C2722H3080N1113O1973P287	-288	Translation
b2185_mRNA_1	mRNA b2185	C2722H3080N1113O1973P287	-288	Translation
b2185_mRNA_2	mRNA b2185	C2722H3080N1113O1973P287	-288	Translation
b2185_mRNA_2_degr	mRNA b2185 degradation complex	C43405H68479N12988O14658S200P287Mg6Zn2Fe0	-510	mRNA degradation
b2189_RNA	tRNA (proL)	C733H834N297O542P77	-78	tRNA Modification
b2189_tRNA_1	b2189_tRNA_1 (proL)	C733H836Mg2N297O542P77	-74	tRNA Modification
b2189_tRNA_1_MeT_tRNA_pos_32_Cm_cplx	b2189_tRNA_1 (proL), MeT_tRNA_pos_32_Cm	C748H859Mg2N303O547P77S1	-73	tRNA Modification
b2189_tRNA_2	b2189_tRNA_2 (proL)	C734H838Mg2N297O542P77	-74	tRNA Modification
b2189_tRNA_2_TrmD_dim_cplx	b2189_tRNA_2 (proL), TrmD_dim	C3261H4815Mg2N1019O1297P77S21	-91	tRNA Modification
b2189_tRNA_3	b2189_tRNA_3 (proL)	C735H840Mg2N297O542P77	-74	tRNA Modification
b2189_tRNA_3_TruA_dim_cplx	b2189_tRNA_3 (proL), TruA_dim	C3445H5042Mg2N1077O1312P77S16	-68	tRNA Modification
b2189_tRNA_4	b2189_tRNA_4 (proL)	C735H840Mg2N297O542P77	-74	tRNA Modification
b2189_tRNA_4_YggH_mono_cplx	b2189_tRNA_4 (proL), YggH_mono	C1960H2739Mg2N650O889P77S14	-76	tRNA Modification
b2189_tRNA_5	b2189_tRNA_5 (proL)	C736H842Mg2N297O542P77	-74	tRNA Modification
b2189_tRNA_5_TrmA_mono_cplx	b2189_tRNA_5 (proL), TrmA_mono	C2610H3789Mg2N820O1101P77S19	-82	tRNA Modification
b2189_tRNA_6	b2189_tRNA_6 (proL)	C737H844Mg2N297O542P77	-74	tRNA Modification
b2189_tRNA_6_TruB_mono_cplx	b2189_tRNA_6 (proL), TruB_mono	C2277H3319Mg2N735O1011P77S10	-79	tRNA Modification
b2189_tRNA_7	b2189_tRNA_7 (proL)	C737H844Mg2N297O542P77	-74	tRNA Modification
b2189_tRNA_7_ThiI_mono_cplx	b2189_tRNA_7 (proL), ThiI_mono	C7165H11075Mg3N2115O2481P82S50X1	-102	tRNA Modification
b2189_tRNA_Mg2	tRNA (proL) bound two Mg2 ions	C733H834Mg2N297O542P77	-74	tRNA Modification
b2189_tRNA_Mg2_Dus_gen_cplx	b2189_tRNA (proL), Dus_gen	C2340H3373Mg2N756O1025P80S14	-81	tRNA Modification
b2268_aa	polypeptide b2268	C1468H2310N404O438S10	-10	Translation
b2268_def_map_cplx	Polypeptide b2268 peptide deformylase and methionine aminopeptidase complex	C3598H5766N997O1084S31Mg0Zn0Fe3	-19	Maturation
b2268_m	Matured polypeptide b2268	C1462H2302N403O436S9	-9	Maturation
b2268_mRNA	mRNA b2268	C8722H9874N3449O6431P920	-921	Translation
b2268_mRNA_1	mRNA b2268	C8722H9874N3449O6431P920	-921	Translation
b2268_mRNA_2	mRNA b2268	C8722H9874N3449O6431P920	-921	Translation
b2268_mRNA_2_degr	mRNA b2268 degradation complex	C49405H75273N15324O19116S200P920Mg6Zn2Fe0	-1143	mRNA degradation
b2268_m_Zn	b2268 plus _Zn	C1462H2302N403O436S9Zn1	-7	Folding
b2317_aa	polypeptide b2317_v1	C1162H1800N281O286S8	1	Translation
b2317_def_map_cplx	Polypeptide b2317 peptide deformylase and methionine aminopeptidase complex	C3292H5256N874O932S29Mg0Zn0Fe3	-8	Maturation
b2317_m	Matured polypeptide b2317	C1156H1792N280O284S7	2	Maturation
b2317_mRNA	mRNA b2317	C6244H7059N2386O4688P660	-661	Translation
b2317_mRNA_1	mRNA b2317	C6244H7059N2386O4688P660	-661	Translation
b2317_mRNA_2	mRNA b2317	C6244H7059N2386O4688P660	-661	Translation
b2317_mRNA_2_degr	mRNA b2317 degradation complex	C46927H72458N14261O17373S200P660Mg6Zn2Fe0	-883	mRNA degradation
b2317_v1_mRNA	mRNA b2317_v1	C6244H7059N2386O4688P660	-661	Translation
b2318_aa	polypeptide b2318_v1	C1361H2109N391O387S9	2	Translation
b2318_def_map_cplx	Polypeptide b2318 peptide deformylase and methionine aminopeptidase complex	C3491H5565N984O1033S30Mg0Zn0Fe3	-7	Maturation
b2318_m	Matured polypeptide b2318	C1355H2101N390O385S8	3	Maturation
b2318_mRNA	mRNA b2318 (1 nt short)	C7738H8759N3122O5671P812	-813	Translation
b2318_mRNA_1	mRNA b2318 (1 nt short)	C7738H8759N3122O5671P812	-813	Translation
b2318_mRNA_2	mRNA b2318 (1 nt short)	C7738H8759N3122O5671P812	-813	Translation
b2318_mRNA_2_degr	mRNA b2318 degradation complex	C48421H74158N14997O18356S200P812Mg6Zn2Fe0	-1035	mRNA degradation
b2318_v1_mRNA	mRNA b2318_v1 (1 nt short)	C7738H8759N3122O5671P812	-813	Translation
b2319_aa	polypeptide b2319_v1	C1623H2524N429O504S8	-25	Translation

b2319_def_cplx	Polypeptide b2319 peptide deformylase complex	C2467H3918N670O759S14Mg0Zn0Fe1	-30	Maturation
b2319_m	Matured polypeptide b2319	C1622H2525N429O503S8	-24	Maturation
b2319_mRNA	mRNA b2319	C9665H10929N3894O7100P1016	-1017	Translation
b2319_mRNA_1	mRNA b2319	C9665H10929N3894O7100P1016	-1017	Translation
b2319_mRNA_2	mRNA b2319	C9665H10929N3894O7100P1016	-1017	Translation
b2319_mRNA_2_degr	mRNA b2319 degradation complex	C50348H76328N15769O19785S200P1016Mg6Zn2Fe0	-1239	mRNA degradation
b2319_v1_mRNA	mRNA b2319_v1	C9665H10929N3894O7094P1014	-1015	Translation
b2320_aa	polypeptide b2320	C1852H2963N514O540S10	-5	Translation
b2320_def_map_cplx	Polypeptide b2320 peptide deformylase and methionine aminopeptidase complex	C3982H6419N1107O1186S31Mg0Zn0Fe3	-14	Maturation
b2320_m	Matured polypeptide b2320	C1846H2955N513O538S9	-4	Maturation
b2320_mRNA	mRNA b2320	C10819H12225N4300O7998P1139	-1140	Translation
b2320_mRNA_1	mRNA b2320	C10819H12225N4300O7998P1139	-1140	Translation
b2320_mRNA_2	mRNA b2320	C10819H12225N4300O7998P1139	-1140	Translation
b2320_mRNA_2_degr	mRNA b2320 degradation complex	C51502H77624N16175O20683S200P1139Mg6Zn2Fe0	-1362	mRNA degradation
b2324_aa	polypeptide b2324	C3321H5066N920O979S28	-20	Translation
b2324_def_map_cplx	Polypeptide b2324 peptide deformylase and methionine aminopeptidase complex	C5451H8522N1513O1625S49Mg0Zn0Fe3	-29	Maturation
b2324_m	Matured polypeptide b2324	C3315H5058N919O977S27	-19	Maturation
b2324_mRNA	mRNA b2324	C19104H21592N7617O14082P2009	-2010	Translation
b2324_mRNA_1	mRNA b2324	C19104H21592N7617O14082P2009	-2010	Translation
b2324_mRNA_2	mRNA b2324	C19104H21592N7617O14082P2009	-2010	Translation
b2324_mRNA_2_degr	mRNA b2324 degradation complex	C59787H86991N19492O26767S200P2009Mg6Zn2Fe0	-2232	mRNA degradation
b2325_aa	polypeptide b2325	C434H666N119O148S3	-13	Translation
b2325_def_map_cplx	Polypeptide b2325 peptide deformylase and methionine aminopeptidase complex	C2564H4122N712O794S24Mg0Zn0Fe3	-22	Maturation
b2325_m	Matured polypeptide b2325	C428H658N118O146S2	-12	Maturation
b2325_mRNA	mRNA b2325	C2661H3003N1070O1956P279	-280	Translation
b2325_mRNA_1	mRNA b2325	C2661H3003N1070O1956P279	-280	Translation
b2325_mRNA_2	mRNA b2325	C2661H3003N1070O1956P279	-280	Translation
b2325_mRNA_2_degr	mRNA b2325 degradation complex	C43344H68402N12945O14641S200P279Mg6Zn2Fe0	-502	mRNA degradation
b2326_aa	polypeptide b2326	C960H1383N252O280S7	-15	Translation
b2326_def_map_cplx	Polypeptide b2326 peptide deformylase and methionine aminopeptidase complex	C3090H4839N845O926S28Mg0Zn0Fe3	-24	Maturation
b2326_m	Matured polypeptide b2326	C954H1375N251O278S6	-14	Maturation
b2326_mRNA	mRNA b2326 (1 nt short)	C5214H5890N2069O3843P548	-549	Translation
b2326_mRNA_1	mRNA b2326 (1 nt short)	C5214H5890N2069O3843P548	-549	Translation
b2326_mRNA_2	mRNA b2326 (1 nt short)	C5214H5890N2069O3843P548	-549	Translation
b2326_mRNA_2_degr	mRNA b2326 degradation complex	C45897H71289N13944O16528S200P548Mg6Zn2Fe0	-771	mRNA degradation
b2327_aa	polypeptide b2327	C1322H2115N331O341S18	8	Translation
b2327_def_map_cplx	Polypeptide b2327 peptide deformylase and methionine aminopeptidase complex	C3452H5571N924O987S39Mg0Zn0Fe3	-1	Maturation
b2327_m	Matured polypeptide b2327	C1316H2107N330O339S17	9	Maturation
b2327_mRNA	mRNA b2327 (1 nt short)	C7662H8674N2963O5734P809	-810	Translation
b2327_mRNA_1	mRNA b2327 (1 nt short)	C7662H8674N2963O5734P809	-810	Translation
b2327_mRNA_2	mRNA b2327 (1 nt short)	C7662H8674N2963O5734P809	-810	Translation
b2327_mRNA_2_degr	mRNA b2327 degradation complex	C48345H74073N14838O18419S200P809Mg6Zn2Fe0	-1032	mRNA degradation
b2328_aa	polypeptide b2328	C1340H2124N380O387S13	2	Translation
b2328_def_map_cplx	Polypeptide b2328 peptide deformylase and methionine aminopeptidase complex	C3470H5580N973O1033S34Mg0Zn0Fe3	-7	Maturation
b2328_m	Matured polypeptide b2328	C1257H1979N359O362S12	2	Maturation
b2328_mRNA	mRNA b2328	C7837H8894N3117O5772P825	-826	Translation
b2328_mRNA_1	mRNA b2328	C7837H8894N3117O5772P825	-826	Translation
b2328_mRNA_2	mRNA b2328	C7837H8894N3117O5772P825	-826	Translation
b2328_mRNA_2_degr	mRNA b2328 degradation complex	C48520H74293N14992O18457S200P825Mg6Zn2Fe0	-1048	mRNA degradation
b2329_aa	polypeptide b2329	C1711H2744N497O529S13	-7	Translation
b2329_def_map_cplx	Polypeptide b2329 peptide deformylase and methionine aminopeptidase complex	C3841H6200N1090O1175S34Mg0Zn0Fe3	-16	Maturation
b2329_m	Matured polypeptide b2329	C1705H2736N496O527S12	-6	Maturation

b2329_mRNA	mRNA b2329	C10332H11724N4151O7579P1086	-1087	Translation
b2329_mRNA_1	mRNA b2329	C10332H11724N4151O7579P1086	-1087	Translation
b2329_mRNA_2	mRNA b2329	C10332H11724N4151O7579P1086	-1087	Translation
b2329_mRNA_2_degr	mRNA b2329 degradation complex	C51015H77123N16026O20264S200P1086Mg6Zn2Fe0	-1309	mRNA degradation
b2330_aa	polypeptide b2330	C1566H2397N416O471S13	-25	Translation
b2330_def_map_cplx	Polypeptide b2330 peptide deformylase and methionine aminopeptidase complex	C3696H5853N1009O1117S34Mg0Zn0Fe3	-34	Maturation
b2330_m	Matured polypeptide b2330 _ error CCDB	C1560H2389N415O469S12	-24	Maturation
b2330_mRNA	mRNA b2330	C8866H10039N3512O6545P935	-936	Translation
b2330_mRNA_1	mRNA b2330	C8866H10039N3512O6545P935	-936	Translation
b2330_mRNA_2	mRNA b2330	C8866H10039N3512O6545P935	-936	Translation
b2330_mRNA_2_degr	mRNA b2330 degradation complex	C49549H75438N15387O19230S200P935Mg6Zn2Fe0	-1158	mRNA degradation
b2348_RNA	tRNA (argW)	C712H807N280O526P75	-76	tRNA Modification
b2348_tRNA_1	b2348_tRNA_1 (argW)	C712H809Mg2N280O526P75	-72	tRNA Modification
b2348_tRNA_1_YdaO_mono_cplx	b2348_tRNA_1 (argW), YdaO_mono	C6254H9628Mg3N1850O2213P80S55X1	-98	tRNA Modification
b2348_tRNA_2	b2348_tRNA_2 (argW)	C712H809Mg2N280O525P75S1	-72	tRNA Modification
b2348_tRNA_2_TrmE_dim-GidA_mono-TrmC_mono_cplx	b2348_tRNA_2 (argW), TrmE_dim, GidA_mono, TrmC_mono	C11516H17812Mg2N3351O3820P82S64	-151	tRNA Modification
b2348_tRNA_3	b2348_tRNA_3 (argW)	C714H814Mg2N281O525P75S1	-72	tRNA Modification
b2348_tRNA_3_Up_tRNA_pos_37_t6A_cplx	b2348_tRNA_3 (argW), Up_tRNA_pos_37_t6A	C729H836Mg3N287O544P78S1	-75	tRNA Modification
b2348_tRNA_4	b2348_tRNA_4 (argW)	C719H820Mg2N282O529P75S1	-73	tRNA Modification
b2348_tRNA_4_TrmA_mono_cplx	b2348_tRNA_4 (argW), TrmA_mono	C2593H3767Mg2N805O1088P75S20	-81	tRNA Modification
b2348_tRNA_5	b2348_tRNA_5 (argW)	C720H822Mg2N282O529P75S1	-73	tRNA Modification
b2348_tRNA_5_TruB_mono_cplx	b2348_tRNA_5 (argW), TruB_mono	C2260H3297Mg2N720O998P75S11	-78	tRNA Modification
b2348_tRNA_Mg2	tRNA (argW) bound two Mg2 ions	C712H807Mg2N280O526P75	-72	tRNA Modification
b2348_tRNA_Mg2_Dus_gen_cplx	b2348_tRNA (argW), Dus_gen	C2319H3346Mg2N739O1009P78S14	-79	tRNA Modification
b2396_RNA	tRNA (alaX)	C724H822N293O533P76	-77	tRNA Modification
b2396_tRNA_1	b2396_tRNA_1 (alaX)	C724H824Mg2N293O533P76	-73	tRNA Modification
b2396_tRNA_1_YggH_mono_cplx	b2396_tRNA_1 (alaX), YggH_mono	C1949H2723Mg2N646O880P76S14	-75	tRNA Modification
b2396_tRNA_2	b2396_tRNA_2 (alaX)	C725H826Mg2N293O533P76	-73	tRNA Modification
b2396_tRNA_2_TrmA_mono_cplx	b2396_tRNA_2 (alaX), TrmA_mono	C2599H3773Mg2N816O1092P76S19	-81	tRNA Modification
b2396_tRNA_3	b2396_tRNA_3 (alaX)	C726H828Mg2N293O533P76	-73	tRNA Modification
b2396_tRNA_3_TruB_mono_cplx	b2396_tRNA_3 (alaX), TruB_mono	C2266H3303Mg2N731O1002P76S10	-78	tRNA Modification
b2396_tRNA_Mg2	tRNA (alaX) bound two Mg2 ions	C724H822Mg2N293O533P76	-73	tRNA Modification
b2396_tRNA_Mg2_Dus_gen_cplx	b2396_tRNA (alaX), Dus_gen	C2331H3361Mg2N752O1016P79S14	-80	tRNA Modification
b2397_RNA	tRNA (alaW)	C724H822N293O533P76	-77	tRNA Modification
b2397_tRNA_1	b2397_tRNA_1 (alaW)	C724H824Mg2N293O533P76	-73	tRNA Modification
b2397_tRNA_1_YggH_mono_cplx	b2397_tRNA_1 (alaW), YggH_mono	C1949H2723Mg2N646O880P76S14	-75	tRNA Modification
b2397_tRNA_2	b2397_tRNA_2 (alaW)	C725H826Mg2N293O533P76	-73	tRNA Modification
b2397_tRNA_2_TrmA_mono_cplx	b2397_tRNA_2 (alaW), TrmA_mono	C2599H3773Mg2N816O1092P76S19	-81	tRNA Modification
b2397_tRNA_3	b2397_tRNA_3 (alaW)	C726H828Mg2N293O533P76	-73	tRNA Modification
b2397_tRNA_3_TruB_mono_cplx	b2397_tRNA_3 (alaW), TruB_mono	C2266H3303Mg2N731O1002P76S10	-78	tRNA Modification
b2397_tRNA_Mg2	tRNA (alaW) bound two Mg2 ions	C724H822Mg2N293O533P76	-73	tRNA Modification
b2397_tRNA_Mg2_Dus_gen_cplx	b2397_tRNA (alaW), Dus_gen	C2331H3361Mg2N752O1016P79S14	-80	tRNA Modification
b2400_aa	polypeptide b2400_v2	C2380H3675N675O717S19	-18	Translation
b2400_def_map_cplx	Polypeptide b2400 peptide deformylase and methionine aminopeptidase complex	C4510H7131N1268O1363S40Mg0Zn0Fe3	-27	Maturation
b2400_m	Matured polypeptide b2400	C2374H3667N674O715S18	-17	Maturation
b2400_mRNA	mRNA b2400	C13483H15254N5403O9893P1418	-1419	Translation
b2400_mRNA_1	mRNA b2400	C13483H15254N5403O9893P1418	-1419	Translation
b2400_mRNA_2	mRNA b2400	C13483H15254N5403O9893P1418	-1419	Translation
b2400_mRNA_2_degr	mRNA b2400 degradation complex	C54166H80653N17278O22578S200P1418Mg6Zn2Fe0	-1641	mRNA degradation
b2400_m_Zn	b2400 plus _Zn	C2374H3667N674O715S18Zn1	-15	Folding
b2400_v1_mRNA	mRNA b2400_v1	C13483H15254N5403O9893P1418	-1419	Translation
b2400_v2_mRNA	mRNA b2400_v2	C13483H15254N5403O9893P1418	-1419	Translation
b2401_RNA	tRNA (valU)	C723H823N292O532P76	-77	tRNA Modification
b2401_tRNA_1	b2401_tRNA_1 (valU)	C723H825Mg2N292O532P76	-73	tRNA Modification
b2401_tRNA_1_YecO_mono-YecP_mono-HyL_tRNA_pos_34_ho5U_cplx	b2401_tRNA_1 (valU), YecO_mono, YecP_mono, HyL_tRNA_pos_34_ho5U	C3656H5358Mg2N1093O1382P76S23	-91	tRNA Modification
b2401_tRNA_2	b2401_tRNA_2 (valU)	C725H826Mg2N292O535P76	-74	tRNA Modification

b2401_tRNA_2_MeT_tRNA_pos_37_m6A_cplx	b2401_tRNA_2 (valU), MeT_tRNA_pos_37_m6A	C740H849Mg2N298O540P76S1	-73	tRNA Modification
b2401_tRNA_3	b2401_tRNA_3 (valU)	C726H828Mg2N292O535P76	-74	tRNA Modification
b2401_tRNA_3_YggH_mono_cplx	b2401_tRNA_3 (valU), YggH_mono	C1951H2727Mg2N645O882P76S14	-76	tRNA Modification
b2401_tRNA_4	b2401_tRNA_4 (valU)	C727H830Mg2N292O535P76	-74	tRNA Modification
b2401_tRNA_4_TrmA_mono_cplx	b2401_tRNA_4 (valU), TrmA_mono	C2601H3777Mg2N815O1094P76S19	-82	tRNA Modification
b2401_tRNA_5	b2401_tRNA_5 (valU)	C728H832Mg2N292O535P76	-74	tRNA Modification
b2401_tRNA_5_TruB_mono_cplx	b2401_tRNA_5 (valU), TruB_mono	C2268H3307Mg2N730O1004P76S10	-79	tRNA Modification
b2401_tRNA_6	b2401_tRNA_6 (valU)	C728H832Mg2N292O535P76	-74	tRNA Modification
b2401_tRNA_6_ThiI_mono_cplx	b2401_tRNA_6 (valU), ThiI_mono	C7156H11063Mg3N2110O2474P81S50X1	-102	tRNA Modification
b2401_tRNA_Mg2	tRNA (valU) bound two Mg2 ions	C723H823Mg2N292O532P76	-73	tRNA Modification
b2401_tRNA_Mg2_Dus_gen_cplx	b2401_tRNA (valU), Dus_gen	C2330H3362Mg2N751O1015P79S14	-80	tRNA Modification
b2402_RNA	tRNA (valX)	C723H823N292O532P76	-77	tRNA Modification
b2402_tRNA_1	b2402_tRNA_1 (valX)	C723H825Mg2N292O532P76	-73	tRNA Modification
b2402_tRNA_1_YecO_mono-YecP_mono-HyL_tRNA_pos_34_ho5U_cplx	b2402_tRNA_1 (valX), YecO_mono, YecP_mono, HyL_tRNA_pos_34_ho5U	C3656H5358Mg2N1093O1382P76S23	-91	tRNA Modification
b2402_tRNA_2	b2402_tRNA_2 (valX)	C725H826Mg2N292O535P76	-74	tRNA Modification
b2402_tRNA_2_MeT_tRNA_pos_37_m6A_cplx	b2402_tRNA_2 (valX), MeT_tRNA_pos_37_m6A	C740H849Mg2N298O540P76S1	-73	tRNA Modification
b2402_tRNA_3	b2402_tRNA_3 (valX)	C726H828Mg2N292O535P76	-74	tRNA Modification
b2402_tRNA_3_YggH_mono_cplx	b2402_tRNA_3 (valX), YggH_mono	C1951H2727Mg2N645O882P76S14	-76	tRNA Modification
b2402_tRNA_4	b2402_tRNA_4 (valX)	C727H830Mg2N292O535P76	-74	tRNA Modification
b2402_tRNA_4_TrmA_mono_cplx	b2402_tRNA_4 (valX), TrmA_mono	C2601H3777Mg2N815O1094P76S19	-82	tRNA Modification
b2402_tRNA_5	b2402_tRNA_5 (valX)	C728H832Mg2N292O535P76	-74	tRNA Modification
b2402_tRNA_5_TruB_mono_cplx	b2402_tRNA_5 (valX), TruB_mono	C2268H3307Mg2N730O1004P76S10	-79	tRNA Modification
b2402_tRNA_6	b2402_tRNA_6 (valX)	C728H832Mg2N292O535P76	-74	tRNA Modification
b2402_tRNA_6_ThiI_mono_cplx	b2402_tRNA_6 (valX), ThiI_mono	C7156H11063Mg3N2110O2474P81S50X1	-102	tRNA Modification
b2402_tRNA_Mg2	tRNA (valX) bound two Mg2 ions	C723H823Mg2N292O532P76	-73	tRNA Modification
b2402_tRNA_Mg2_Dus_gen_cplx	b2402_tRNA (valX), Dus_gen	C2330H3362Mg2N751O1015P79S14	-80	tRNA Modification
b2403_RNA	tRNA (valY)	C723H823N292O532P76	-77	tRNA Modification
b2403_tRNA_1	b2403_tRNA_1 (valY)	C723H825Mg2N292O532P76	-73	tRNA Modification
b2403_tRNA_1_YecO_mono-YecP_mono-HyL_tRNA_pos_34_ho5U_cplx	b2403_tRNA_1 (valY), YecO_mono, YecP_mono, HyL_tRNA_pos_34_ho5U	C3656H5358Mg2N1093O1382P76S23	-91	tRNA Modification
b2403_tRNA_2	b2403_tRNA_2 (valY)	C725H826Mg2N292O535P76	-74	tRNA Modification
b2403_tRNA_2_MeT_tRNA_pos_37_m6A_cplx	b2403_tRNA_2 (valY), MeT_tRNA_pos_37_m6A	C740H849Mg2N298O540P76S1	-73	tRNA Modification
b2403_tRNA_3	b2403_tRNA_3 (valY)	C726H828Mg2N292O535P76	-74	tRNA Modification
b2403_tRNA_3_YggH_mono_cplx	b2403_tRNA_3 (valY), YggH_mono	C1951H2727Mg2N645O882P76S14	-76	tRNA Modification
b2403_tRNA_4	b2403_tRNA_4 (valY)	C727H830Mg2N292O535P76	-74	tRNA Modification
b2403_tRNA_4_TrmA_mono_cplx	b2403_tRNA_4 (valY), TrmA_mono	C2601H3777Mg2N815O1094P76S19	-82	tRNA Modification
b2403_tRNA_5	b2403_tRNA_5 (valY)	C728H832Mg2N292O535P76	-74	tRNA Modification
b2403_tRNA_5_TruB_mono_cplx	b2403_tRNA_5 (valY), TruB_mono	C2268H3307Mg2N730O1004P76S10	-79	tRNA Modification
b2403_tRNA_6	b2403_tRNA_6 (valY)	C728H832Mg2N292O535P76	-74	tRNA Modification
b2403_tRNA_6_ThiI_mono_cplx	b2403_tRNA_6 (valY), ThiI_mono	C7156H11063Mg3N2110O2474P81S50X1	-102	tRNA Modification
b2403_tRNA_Mg2	tRNA (valY) bound two Mg2 ions	C723H823Mg2N292O532P76	-73	tRNA Modification
b2403_tRNA_Mg2_Dus_gen_cplx	b2403_tRNA (valY), Dus_gen	C2330H3362Mg2N751O1015P79S14	-80	tRNA Modification
b2404_RNA	tRNA (lysV)	C721H816N281O537P76	-77	tRNA Modification
b2404_tRNA_1	b2404_tRNA_1 (lysV)	C721H818Mg2N281O537P76	-73	tRNA Modification
b2404_tRNA_1_Dus_gen_cplx	b2404_tRNA_1 (lysV), Dus_gen	C2328H3357Mg2N740O1020P79S14	-80	tRNA Modification
b2404_tRNA_2	b2404_tRNA_2 (lysV)	C721H820Mg2N281O537P76	-73	tRNA Modification
b2404_tRNA_2_Dus_gen_cplx	b2404_tRNA_2 (lysV), Dus_gen	C2328H3359Mg2N740O1020P79S14	-80	tRNA Modification
b2404_tRNA_3	b2404_tRNA_3 (lysV)	C721H822Mg2N281O537P76	-73	tRNA Modification
b2404_tRNA_3_TrmU_mono-YhhP_mono-YheLMN_cplx-YccK_mono-TrmE_dim-GidA_mono-TrmC_mono_cplx	b2404_tRNA_3 (lysV), TrmU_mono, YhhP_mono, YheLMN_cplx, YccK_mono, TrmE_dim, GidA_mono, TrmC_mono	C21612H33672Mg3N6125O6874P88S131X1	-221	tRNA Modification
b2404_tRNA_4	b2404_tRNA_4 (lysV)	C723H827Mg2N282O536P76S1	-73	tRNA Modification
b2404_tRNA_4_Se	b2404_tRNA_4_Se - contains mnm5se2U instead of mnm5s2U,b2404_tRNA_4 (lysV)	C723H827Mg2N282O536P76S0Se1	-73	tRNA Modification
b2404_tRNA_4_Se_Up_tRNA_pos_37_t6A_cplx	b2404_tRNA_4_Se_Up_tRNA_pos_37_t6A_cplx - contains mnm5se2U instead of mnm5s2U,b2404_tRNA_4 (lysV), Up_tRNA_pos_37_t6A	C738H849Mg3N288O555P79S0Se1	-76	tRNA Modification
b2404_tRNA_4_Up_tRNA_pos_37_t6A_cplx	b2404_tRNA_4 (lysV), Up_tRNA_pos_37_t6A	C738H849Mg3N288O555P79S1	-76	tRNA Modification
b2404_tRNA_4_YbbB_dim_cplx	b2404_tRNA_4_YbbB_dim_cplx	C4337H6509N1336O1608S31P77Mg2Zn0Fe0Se1	-90	tRNA Modification

b2404_tRNA_5	b2404_tRNA_5 (lysV)	C728H833Mg2N283O540P76S1	-74	tRNA Modification
b2404_tRNA_5_Se	b2404_tRNA_5_Se - contains mnm5se2U instead of mnm5s2U,b2404_tRNA_5 (lysV)	C728H833Mg2N283O540P76S0Se1	-74	tRNA Modification
b2404_tRNA_5_Se_TruA_dim_cplx	b2404_tRNA_5_Se_TruA_dim_cplx - contains mnm5se2U instead of mnm5s2U,b2404_tRNA_5 (lysV), TruA_dim	C3438H5035Mg2N1063O1310P76S16Se1	-68	tRNA Modification
b2404_tRNA_5_TruA_dim_cplx	b2404_tRNA_5 (lysV), TruA_dim	C3438H5035Mg2N1063O1310P76S17	-68	tRNA Modification
b2404_tRNA_6	b2404_tRNA_6 (lysV)	C728H833Mg2N283O540P76S1	-74	tRNA Modification
b2404_tRNA_6_Se	b2404_tRNA_6_Se - contains mnm5se2U instead of mnm5s2U,b2404_tRNA_6 (lysV)	C728H833Mg2N283O540P76S0Se1	-74	tRNA Modification
b2404_tRNA_6_Se_YggH_mono_cplx	b2404_tRNA_6_Se_YggH_mono_cplx - contains mnm5se2U instead of mnm5s2U,b2404_tRNA_6 (lysV), YggH_mono	C1953H2732Mg2N636O887P76S14Se1	-76	tRNA Modification
b2404_tRNA_6_YggH_mono_cplx	b2404_tRNA_6 (lysV), YggH_mono	C1953H2732Mg2N636O887P76S15	-76	tRNA Modification
b2404_tRNA_7	b2404_tRNA_7 (lysV)	C729H835Mg2N283O540P76S1	-74	tRNA Modification
b2404_tRNA_7_AcpT_tRNA_pos_47_acp3U_cplx	b2404_tRNA_7 (lysV), AcpT_tRNA_pos_47_acp3U	C744H858Mg2N289O545P76S2	-73	tRNA Modification
b2404_tRNA_7_Se	b2404_tRNA_7_Se - contains mnm5se2U instead of mnm5s2U,b2404_tRNA_7 (lysV)	C729H835Mg2N283O540P76S0Se1	-74	tRNA Modification
b2404_tRNA_7_Se_AcpT_tRNA_pos_47_acp3U_cplx	b2404_tRNA_7_Se_AcpT_tRNA_pos_47_acp3U_cplx - contains mnm5se2U instead of mnm5s2U,b2404_tRNA_7 (lysV), AcpT_tRNA_pos_47_acp3U	C744H858Mg2N289O545P76S1Se1	-73	tRNA Modification
b2404_tRNA_8	b2404_tRNA_8 (lysV)	C733H842Mg2N284O542P76S1	-74	tRNA Modification
b2404_tRNA_8_Se	b2404_tRNA_8_Se - contains mnm5se2U instead of mnm5s2U,b2404_tRNA_8 (lysV)	C733H842Mg2N284O542P76S0Se1	-74	tRNA Modification
b2404_tRNA_8_Se_TrmA_mono_cplx	b2404_tRNA_8_Se_TrmA_mono_cplx - contains mnm5se2U instead of mnm5s2U,b2404_tRNA_8 (lysV), TrmA_mono	C2607H3789Mg2N807O1101P76S19Se1	-82	tRNA Modification
b2404_tRNA_8_TrmA_mono_cplx	b2404_tRNA_8 (lysV), TrmA_mono	C2607H3789Mg2N807O1101P76S20	-82	tRNA Modification
b2404_tRNA_9	b2404_tRNA_9 (lysV)	C734H844Mg2N284O542P76S1	-74	tRNA Modification
b2404_tRNA_9_Se	b2404_tRNA_9_Se - contains mnm5se2U instead of mnm5s2U,b2404_tRNA_9 (lysV)	C734H844Mg2N284O542P76S0Se1	-74	tRNA Modification
b2404_tRNA_9_Se_TruB_mono_cplx	b2404_tRNA_9_Se_TruB_mono_cplx - contains mnm5se2U instead of mnm5s2U,b2404_tRNA_9 (lysV), TruB_mono	C2274H3319Mg2N722O1011P76S10Se1	-79	tRNA Modification
b2404_tRNA_9_TruB_mono_cplx	b2404_tRNA_9 (lysV), TruB_mono	C2274H3319Mg2N722O1011P76S11	-79	tRNA Modification
b2404_tRNA_Mg2	tRNA (lysV) bound two Mg2 ions	C721H816Mg2N281O537P76	-73	tRNA Modification
b2404_tRNA_Mg2_Dus_gen_cplx	b2404_tRNA (lysV), Dus_gen	C2328H3355Mg2N740O1020P79S14	-80	tRNA Modification
b2514_aa	polypeptide b2514	C2079H3283N594O625S14	-9	Translation
b2514_def_map_cplx	Polypeptide b2514 peptide deformylase and methionine aminopeptidase complex	C4209H6739N1187O1271S35Mg0Zn0Fe3	-18	Maturation
b2514_m	Matured polypeptide b2514	C2073H3275N593O623S13	-8	Maturation
b2514_mRNA	mRNA b2514	C12142H13731N4864O8945P1277	-1278	Translation
b2514_mRNA_1	mRNA b2514	C12142H13731N4864O8945P1277	-1278	Translation
b2514_mRNA_2	mRNA b2514	C12142H13731N4864O8945P1277	-1278	Translation
b2514_mRNA_2_degr	mRNA b2514 degradation complex	C52825H79130N16739O21630S200P1277Mg6Zn2Fe0	-1500	mRNA degradation
b2514_m_Mg	b2514 plus _Mg	C2073H3275N593O623S13Mg2	-4	Folding
b2528_aa	polypeptide b2528	C511H791N135O162S5	-6	Translation
b2528_def_map_cplx	Polypeptide b2528 peptide deformylase and methionine aminopeptidase complex	C2641H4247N728O808S26Mg0Zn0Fe3	-15	Maturation
b2528_m	Matured polypeptide b2528	C505H783N134O160S4	-5	Maturation
b2528_mRNA	mRNA b2528	C3098H3491N1262O2260P324	-325	Translation
b2528_mRNA_1	mRNA b2528	C3098H3491N1262O2260P324	-325	Translation
b2528_mRNA_2	mRNA b2528	C3098H3491N1262O2260P324	-325	Translation
b2528_mRNA_2_degr	mRNA b2528 degradation complex	C43781H68890N13137O14945S200P324Mg6Zn2Fe0	-547	mRNA degradation
b2529_aa	polypeptide b2529	C602H955N163O200S6	-8	Translation
b2529_def_map_cplx	Polypeptide b2529 peptide deformylase and methionine aminopeptidase complex	C2732H4411N756O846S27Mg0Zn0Fe3	-17	Maturation
b2529_m	Matured polypeptide b2529	C596H947N162O198S5	-7	Maturation
b2529_mRNA	mRNA b2529	C3702H4179N1520O2675P387	-388	Translation
b2529_mRNA_1	mRNA b2529	C3702H4179N1520O2675P387	-388	Translation
b2529_mRNA_2	mRNA b2529	C3702H4179N1520O2675P387	-388	Translation
b2529_mRNA_2_degr	mRNA b2529 degradation complex	C44385H69578N13395O15360S200P387Mg6Zn2Fe0	-610	mRNA degradation
b2530_aa	polypeptide b2530	C1983H3157N564O600S19	-9	Translation

b2530_def_map_cplx	Polypeptide b2530 peptide deformylase and methionine aminopeptidase complex	C4113H6613N1157O1246S40Mg0Zn0Fe3	-18	Maturation
b2530_m	Matured polypeptide b2530	C1977H3149N563O598S18	-8	Maturation
b2530_mRNA	mRNA b2530	C11559H13097N4624O8477P1215	-1216	Translation
b2530_mRNA_1	mRNA b2530	C11559H13097N4624O8477P1215	-1216	Translation
b2530_mRNA_2	mRNA b2530	C11559H13097N4624O8477P1215	-1216	Translation
b2530_mRNA_2_degr	mRNA b2530 degradation complex	C52242H78496N16499O21162S200P1215Mg6Zn2Fe0	-1438	mRNA degradation
b2530_m_DnaKJ_complex	b2530 DnaK DnaJ_dim complex - Kerner et al. class II can interact w/ GroEL/ES, cannot fold spontanously	C8541H13663O2696N2476S65P3Zn4	-30	Folding
b2530_m_GroEL.(7)ATP.transGroES	b2530 GroEL GroES complex - Kerner et al. class II can interact w/ GroEL/ES, cannot fold spontanously	C39889H66093O12932N11147S382P21Mg7	-309	Folding
b2531_aa	polypeptide b2531	C744H1222N227O241S5	-1	Translation
b2531_def_map_cplx	Polypeptide b2531 peptide deformylase and methionine aminopeptidase complex	C2874H4678N820O887S26Mg0Zn0Fe3	-10	Maturation
b2531_m	Matured polypeptide b2531	C738H1214N226O239S4	0	Maturation
b2531_mRNA	mRNA b2531	C4646H5263N1840O3437P491	-492	Translation
b2531_mRNA_1	mRNA b2531	C4646H5263N1840O3437P491	-492	Translation
b2531_mRNA_2	mRNA b2531	C4646H5263N1840O3437P491	-492	Translation
b2531_mRNA_2_degr	mRNA b2531 degradation complex	C45329H70662N13715O16122S200P491Mg6Zn2Fe0	-714	mRNA degradation
b2559_aa	polypeptide b2559	C881H1398N265O248S12	1	Translation
b2559_def_map_cplx	Polypeptide b2559 peptide deformylase and methionine aminopeptidase complex	C3011H4854N858O894S33Mg0Zn0Fe3	-8	Maturation
b2559_m	Matured polypeptide b2559	C875H1390N264O246S11	2	Maturation
b2559_mRNA	mRNA b2559	C5119H5783N2058O3764P537	-538	Translation
b2559_mRNA_1	mRNA b2559	C5119H5783N2058O3764P537	-538	Translation
b2559_mRNA_2	mRNA b2559	C5119H5783N2058O3764P537	-538	Translation
b2559_mRNA_2_degr	mRNA b2559 degradation complex	C45802H71182N13933O16449S200P537Mg6Zn2Fe0	-760	mRNA degradation
b2559_m_Zn	b2559 plus _Zn	C875H1390N264O246S11Zn1	4	Folding
b2560_aa	polypeptide b2560	C1119H1740N307O297S7	3	Translation
b2560_def_map_cplx	Polypeptide b2560 peptide deformylase and methionine aminopeptidase complex	C3249H5196N900O943S28Mg0Zn0Fe3	-6	Maturation
b2560_m	Matured polypeptide b2560	C1113H1732N306O295S6	4	Maturation
b2560_mRNA	mRNA b2560	C6027H6819N2336O4511P638	-639	Translation
b2560_mRNA_1	mRNA b2560	C6027H6819N2336O4511P638	-639	Translation
b2560_mRNA_2	mRNA b2560	C6027H6819N2336O4511P638	-639	Translation
b2560_mRNA_2_degr	mRNA b2560 degradation complex	C46710H72218N14211O17196S200P638Mg6Zn2Fe0	-861	mRNA degradation
b2563_aa	polypeptide b2563_v1	C633H1014N184O174S3	2	Translation
b2563_def_map_cplx	Polypeptide b2563 peptide deformylase and methionine aminopeptidase complex	C2763H4470N777O820S24Mg0Zn0Fe3	-7	Maturation
b2563_m	Matured polypeptide b2563	C627H1006N183O172S2	3	Maturation
b2563_mRNA_1	mRNA b2563 (1 nt short)	C3631H4094N1475O2651P380	-381	Translation
b2563_mRNA_2	mRNA b2563 (1 nt short)	C3631H4094N1475O2651P380	-381	Translation
b2563_mRNA_2_degr	mRNA b2563 degradation complex	C44314H69493N13350O15336S200P380Mg6Zn2Fe0	-603	mRNA degradation
b2563_v1_mRNA	mRNA b2563_v1 (1 nt short)	C3631H4094N1475O2651P380	-381	Translation
b2564_aa	polypeptide b2564_v1	C1144H1865N338O352S13	-9	Translation
b2564_def_map_cplx	Polypeptide b2564 peptide deformylase and methionine aminopeptidase complex	C3274H5321N931O998S34Mg0Zn0Fe3	-18	Maturation
b2564_m	Matured polypeptide b2564	C1138H1857N337O350S12	-8	Maturation
b2564_mRNA_1	mRNA b2564	C6982H7907N2838O5104P734	-735	Translation
b2564_mRNA_2	mRNA b2564	C6982H7907N2838O5104P734	-735	Translation
b2564_mRNA_2_degr	mRNA b2564 degradation complex	C47665H73306N14713O17789S200P734Mg6Zn2Fe0	-957	mRNA degradation
b2564_v1_mRNA	mRNA b2564_v1	C6982H7907N2838O5098P732	-733	Translation
b2565_aa	polypeptide b2565	C1237H1963N345O346S7	10	Translation
b2565_def_map_cplx	Polypeptide b2565 peptide deformylase and methionine aminopeptidase complex	C3367H5419N938O992S28Mg0Zn0Fe3	1	Maturation
b2565_m	Matured polypeptide b2565	C1231H1955N344O344S6	11	Maturation
b2565_mRNA	mRNA b2565	C6925H7850N2745O5109P729	-730	Translation
b2565_mRNA_1	mRNA b2565	C6925H7850N2745O5109P729	-730	Translation
b2565_mRNA_2	mRNA b2565	C6925H7850N2745O5109P729	-730	Translation
b2565_mRNA_2_degr	mRNA b2565 degradation complex	C47608H73249N14620O17794S200P729Mg6Zn2Fe0	-952	mRNA degradation
b2566_aa	polypeptide b2566_v1	C1505H2431N419O443S11	-2	Translation

b2566_def_map_cplx	Polypeptide b2566 peptide deformylase and methionine aminopeptidase complex	C3635H5887N1012O1089S32Mg0Zn0Fe3	-11	Maturation
b2566_m	Matured polypeptide b2566	C1499H2423N418O441S10	-1	Maturation
b2566_mRNA	mRNA b2566 (4 nt short)	C8598H9730N3473O6272P902	-903	Translation
b2566_mRNA_1	mRNA b2566 (4 nt short)	C8598H9730N3473O6272P902	-903	Translation
b2566_mRNA_2	mRNA b2566 (4 nt short)	C8598H9730N3473O6272P902	-903	Translation
b2566_mRNA_2_degr	mRNA b2566 degradation complex	C49281H75129N15348O18957S200P902Mg6Zn2Fe0	-1125	mRNA degradation
b2566_v1_mRNA	mRNA b2566_v1 (4 nt short)	C8598H9730N3473O6272P902	-903	Translation
b2567_aa	polypeptide b2567_v1	C1125H1808N329O342S5	-3	Translation
b2567_def_map_cplx	Polypeptide b2567 peptide deformylase and methionine aminopeptidase complex	C3255H5264N922O988S26Mg0Zn0Fe3	-12	Maturation
b2567_m	Matured polypeptide b2567	C1119H1800N328O340S4	-2	Maturation
b2567_mRNA	mRNA b2567	C6488H7331N2600O4760P683	-684	Translation
b2567_mRNA_1	mRNA b2567	C6488H7331N2600O4760P683	-684	Translation
b2567_mRNA_2	mRNA b2567	C6488H7331N2600O4760P683	-684	Translation
b2567_mRNA_2_degr	mRNA b2567 degradation complex	C47171H72730N14475O17445S200P683Mg6Zn2Fe0	-906	mRNA degradation
b2567_m_Mg	b2567 plus _Mg	C1119H1800N328O340S4Mg1	0	Folding
b2567_v1_mRNA	mRNA b2567_v1	C6488H7331N2600O4760P683	-684	Translation
b2570_aa	polypeptide b2570_v2	C748H1199N196O217S8	-1	Translation
b2570_def_map_cplx	Polypeptide b2570 peptide deformylase and methionine aminopeptidase complex	C2878H4655N789O863S29Mg0Zn0Fe3	-10	Maturation
b2570_m	Matured polypeptide b2570	C742H1191N195O215S7	0	Maturation
b2570_mRNA	mRNA b2570 (4 nt short)	C4514H5113N1764O3367P476	-477	Translation
b2570_mRNA_1	mRNA b2570 (4 nt short)	C4514H5113N1764O3367P476	-477	Translation
b2570_mRNA_2	mRNA b2570 (4 nt short)	C4514H5113N1764O3367P476	-477	Translation
b2570_mRNA_2_degr	mRNA b2570 degradation complex	C45197H70512N13639O16052S200P476Mg6Zn2Fe0	-699	mRNA degradation
b2570_v2_mRNA	mRNA b2570_v2 (4 nt short)	C4514H5113N1764O3367P476	-477	Translation
b2571_aa	polypeptide b2571_v2	C1586H2516N441O479S11	1	Translation
b2571_def_map_cplx	Polypeptide b2571 peptide deformylase and methionine aminopeptidase complex	C3716H5972N1034O1125S32Mg0Zn0Fe3	-8	Maturation
b2571_m	Matured polypeptide b2571	C1472H2341N414O448S9	1	Maturation
b2571_mRNA	mRNA b2571 (1 nt short)	C9068H10274N3553O6709P956	-957	Translation
b2571_mRNA_1	mRNA b2571 (1 nt short)	C9068H10274N3553O6709P956	-957	Translation
b2571_mRNA_2	mRNA b2571 (1 nt short)	C9068H10274N3553O6709P956	-957	Translation
b2571_mRNA_2_degr	mRNA b2571 degradation complex	C49751H75673N15428O19394S200P956Mg6Zn2Fe0	-1179	mRNA degradation
b2571_v2_mRNA	mRNA b2571_v2 (1 nt short)	C9068H10274N3553O6709P956	-957	Translation
b2572_aa	polypeptide b2572_v2	C1056H1660N306O334S11	-10	Translation
b2572_def_map_cplx	Polypeptide b2572 peptide deformylase and methionine aminopeptidase complex	C3186H5116N899O980S32Mg0Zn0Fe3	-19	Maturation
b2572_m	Matured polypeptide b2572	C1050H1652N305O332S10	-9	Maturation
b2572_mRNA	mRNA b2572	C6209H7034N2525O4503P651	-652	Translation
b2572_mRNA_1	mRNA b2572	C6209H7034N2525O4503P651	-652	Translation
b2572_mRNA_2	mRNA b2572	C6209H7034N2525O4503P651	-652	Translation
b2572_mRNA_2_degr	mRNA b2572 degradation complex	C46892H72433N14400O17188S200P651Mg6Zn2Fe0	-874	mRNA degradation
b2572_v2_mRNA	mRNA b2572_v2	C6209H7034N2525O4503P651	-652	Translation
b2573_aa	polypeptide b2573_v1	C961H1537N271O292S5	-4	Translation
b2573_def_map_cplx	Polypeptide b2573 peptide deformylase and methionine aminopeptidase complex	C3091H4993N864O938S26Mg0Zn0Fe3	-13	Maturation
b2573_m	Matured polypeptide b2573	C955H1529N270O290S4	-3	Maturation
b2573_mRNA	mRNA b2573	C5493H6188N2197O4042P578	-579	Translation
b2573_mRNA_1	mRNA b2573	C5493H6188N2197O4042P578	-579	Translation
b2573_mRNA_2	mRNA b2573	C5493H6188N2197O4042P578	-579	Translation
b2573_mRNA_2_degr	mRNA b2573 degradation complex	C46176H71587N14072O16727S200P578Mg6Zn2Fe0	-801	mRNA degradation
b2573_v1_mRNA	mRNA b2573_v1	C5493H6188N2197O4042P578	-579	Translation
b2588_RNA	rRNA	C1144H1301N468O838P120	-121	RNA cutting
b2588_v1_RNA	rRNA	C1144H1301N468O838P120	-121	RNA cutting
b2589_RNA	rRNA	C27810H31354N11469O20158P2904	-2905	rRNA Modification
b2589_RNA_1		C27811H31356N11469O20158P2904S0	-2905	rRNA Modification
b2589_RNA_10		C27818H31370N11469O20158P2904S0	-2905	rRNA Modification
b2589_RNA_10_MeT_23S_2445	b2589_RNA_10, MeT_23S_2445	C27833H31393N11475O20163S1P2904Mg0Zn0Fe0	-2904	rRNA Modification
b2589_RNA_11		C27819H31372N11469O20158P2904S0	-2905	rRNA Modification
b2589_RNA_11_DU_23S_2449_a	b2589_RNA_11, DU_23S_2449 (NADH)	C27840H31400N11476O20172S0P2906Mg0Zn0Fe0	-2906	rRNA Modification

b2589_RNA_11_DU_23S_2449_b	b2589_RNA_11, DU_23S_2449 (NADPH)	C27840H31399N11476O20175S0P2907Mg0Zn0Fe0	-2908	rRNA Modification
b2589_RNA_12		C27819H31374N11469O20158P2904S0	-2905	rRNA Modification
b2589_RNA_12_YmfC_mono	b2589_RNA_12, YmfC_mono	C28917H33135N11796O20478S3P2904Mg0Zn0Fe0	-2895	rRNA Modification
b2589_RNA_13		C27819H31374N11469O20158P2904S0	-2905	rRNA Modification
b2589_RNA_13_MeT_23S_2498	b2589_RNA_13, MeT_23S_2498	C27834H31397N11475O20163S1P2904Mg0Zn0Fe0	-2904	rRNA Modification
b2589_RNA_14		C27820H31376N11469O20158P2904S0	-2905	rRNA Modification
b2589_RNA_14_MeT_23S_2503	b2589_RNA_14, MeT_23S_2503	C27835H31399N11475O20163S1P2904Mg0Zn0Fe0	-2904	rRNA Modification
b2589_RNA_15		C27821H31378N11469O20158P2904S0	-2905	rRNA Modification
b2589_RNA_15_RluC_mono	b2589_RNA_15, RluC_mono	C29406H33991N11951O20614S6P2904Mg0Zn0Fe0	-2890	rRNA Modification
b2589_RNA_16		C27821H31378N11469O20158P2904S0	-2905	rRNA Modification
b2589_RNA_16_RrmJ_mono	b2589_RNA_16, RrmJ_mono	C28868H33067N11764O20461S11P2904Mg0Zn0Fe0	-2897	rRNA Modification
b2589_RNA_17		C27822H31380N11469O20158P2904S0	-2905	rRNA Modification
b2589_RNA_17_RluC_mono	b2589_RNA_17, RluC_mono	C29407H33993N11951O20614S6P2904Mg0Zn0Fe0	-2890	rRNA Modification
b2589_RNA_18		C27822H31380N11469O20158P2904S0	-2905	rRNA Modification
b2589_RNA_18_YjbC_mono	b2589_RNA_18, YjbC_mono	C29249H33751N11887O20580S7P2904Mg0Zn0Fe0	-2890	rRNA Modification
b2589_RNA_19		C27822H31380N11469O20158P2904S0	-2905	rRNA Modification
b2589_RNA_19_RluB_mono	b2589_RNA_19, RluB_mono	C29240H33728N11920O20578S5P2904Mg0Zn0Fe0	-2890	rRNA Modification
b2589_RNA_1_MeT_23S_1835	b2589_RNA_1, MeT_23S_1835	C27826H31379N11475O20163S1P2904Mg0Zn0Fe0	-2904	rRNA Modification
b2589_RNA_2		C27812H31358N11469O20158P2904S0	-2905	rRNA Modification
b2589_RNA_20		C27822H31380N11469O20158P2904S0	-2905	rRNA Modification
b2589_RNA_20_RrmA_dim	b2589_RNA_20, RrmA_dim	C30539H35591N12233O20933S31P2904Mg0Zn2Fe0	-2902	rRNA Modification
b2589_RNA_21		C27823H31382N11469O20158P2904S0	-2905	rRNA Modification
b2589_RNA_21_RluA_mono	b2589_RNA_21, RluA_mono	C28926H33122N11782O20472S10P2904Mg0Zn0Fe0	-2904	rRNA Modification
b2589_RNA_22		C27823H31382N11469O20158P2904S0	-2905	rRNA Modification
b2589_RNA_22_RumB_mono	b2589_RNA_22, RumB_mono	C29716H34360N11981O20692S28P2904Mg0Zn0Fe4	-2903	rRNA Modification
b2589_RNA_23		C27824H31384N11469O20158P2904S0	-2905	rRNA Modification
b2589_RNA_23_RluC_mono	b2589_RNA_23, RluC_mono	C29409H33997N11951O20614S6P2904Mg0Zn0Fe0	-2890	rRNA Modification
b2589_RNA_2_RluD_mono	b2589_RNA_2, RluD_mono	C29449H33968N11949O20632S12P2904Mg1Zn0Fe0	-2909	rRNA Modification
b2589_RNA_3		C27812H31358N11469O20158P2904S0	-2905	rRNA Modification
b2589_RNA_3_RluD_mono	b2589_RNA_3, RluD_mono	C29464H33991N11955O20637S13P2904Mg1Zn0Fe0	-2908	rRNA Modification
b2589_RNA_4		C27813H31360N11469O20158P2904S0	-2905	rRNA Modification
b2589_RNA_4_RluD_mono	b2589_RNA_4, RluD_mono	C29450H33970N11949O20632S12P2904Mg1Zn0Fe0	-2909	rRNA Modification
b2589_RNA_5		C27813H31360N11469O20158P2904S0	-2905	rRNA Modification
b2589_RNA_5_RumA_mono	b2589_RNA_5, RumA_mono	C29941H34786N12087O20786S23P2904Mg0Zn0Fe4	-2897	rRNA Modification
b2589_RNA_6		C27814H31362N11469O20158P2904S0	-2905	rRNA Modification
b2589_RNA_6_MeT_23S_1962	b2589_RNA_6, MeT_23S_1962	C27829H31385N11475O20163S1P2904Mg0Zn0Fe0	-2904	rRNA Modification
b2589_RNA_7		C27815H31364N11469O20158P2904S0	-2905	rRNA Modification
b2589_RNA_7_MeT_23S_2030	b2589_RNA_7, MeT_23S_2030	C27830H31387N11475O20163S1P2904Mg0Zn0Fe0	-2904	rRNA Modification
b2589_RNA_8		C27816H31366N11469O20158P2904S0	-2905	rRNA Modification
b2589_RNA_8_MeT_23S_2069	b2589_RNA_8, MeT_23S_2069	C27831H31389N11475O20163S1P2904Mg0Zn0Fe0	-2904	rRNA Modification
b2589_RNA_9		C27817H31368N11469O20158P2904S0	-2905	rRNA Modification
b2589_RNA_9_RlmB_dim	b2589_RNA_9, RlmB_dim	C30138H35171N12153O20859S23P2904Mg0Zn0Fe0	-2910	rRNA Modification
b2589_RNA_MeT_23S_1618	b2589_RNA, MeT_23S_1618	C27825H31377N11475O20163S1P2904Mg0Zn0Fe0	-2904	rRNA Modification
b2589_v1_RNA	rRNA	C27810H31354N11469O20158P2904	-2905	rRNA Modification
b2589_v1_RNA_1		C27811H31356N11469O20158P2904S0	-2905	rRNA Modification
b2589_v1_RNA_10		C27818H31370N11469O20158P2904S0	-2905	rRNA Modification
b2589_v1_RNA_10_MeT_23S_2445	b2589_v1_RNA_10, MeT_23S_2445	C27833H31393N11475O20163S1P2904Mg0Zn0Fe0	-2904	rRNA Modification
b2589_v1_RNA_11		C27819H31372N11469O20158P2904S0	-2905	rRNA Modification
b2589_v1_RNA_11_DU_23S_2449_a	b2589_v1_RNA_11, DU_23S_2449 (NADH)	C27840H31400N11476O20172S0P2906Mg0Zn0Fe0	-2906	rRNA Modification
b2589_v1_RNA_11_DU_23S_2449_b	b2589_v1_RNA_11, DU_23S_2449 (NADPH)	C27840H31399N11476O20175S0P2907Mg0Zn0Fe0	-2908	rRNA Modification
b2589_v1_RNA_12		C27819H31374N11469O20158P2904S0	-2905	rRNA Modification
b2589_v1_RNA_12_YmfC_mono	b2589_v1_RNA_12, YmfC_mono	C28917H33135N11796O20478S3P2904Mg0Zn0Fe0	-2895	rRNA Modification
b2589_v1_RNA_13		C27819H31374N11469O20158P2904S0	-2905	rRNA Modification

b2589_v1_RNA_13_MeT_23S_2498	b2589_v1_RNA_13, MeT_23S_2498	C27834H31397N11475O20163S1P2904Mg0Zn0Fe0	-2904	rRNA Modification
b2589_v1_RNA_14		C27820H31376N11469O20158P2904S0	-2905	rRNA Modification
b2589_v1_RNA_14_MeT_23S_2503	b2589_v1_RNA_14, MeT_23S_2503	C27835H31399N11475O20163S1P2904Mg0Zn0Fe0	-2904	rRNA Modification
b2589_v1_RNA_15		C27821H31378N11469O20158P2904S0	-2905	rRNA Modification
b2589_v1_RNA_15_RluC_mono	b2589_v1_RNA_15, RluC_mono	C29406H33991N11951O20614S6P2904Mg0Zn0Fe0	-2890	rRNA Modification
b2589_v1_RNA_16		C27821H31378N11469O20158P2904S0	-2905	rRNA Modification
b2589_v1_RNA_16_RrmJ_mono	b2589_v1_RNA_16, RrmJ_mono	C28868H33067N11764O20461S11P2904Mg0Zn0Fe0	-2897	rRNA Modification
b2589_v1_RNA_17		C27822H31380N11469O20158P2904S0	-2905	rRNA Modification
b2589_v1_RNA_17_RluC_mono	b2589_v1_RNA_17, RluC_mono	C29407H33993N11951O20614S6P2904Mg0Zn0Fe0	-2890	rRNA Modification
b2589_v1_RNA_18		C27822H31380N11469O20158P2904S0	-2905	rRNA Modification
b2589_v1_RNA_18_YjbC_mono	b2589_v1_RNA_18, YjbC_mono	C29249H33751N11887O20580S7P2904Mg0Zn0Fe0	-2890	rRNA Modification
b2589_v1_RNA_19		C27822H31380N11469O20158P2904S0	-2905	rRNA Modification
b2589_v1_RNA_19_RluB_mono	b2589_v1_RNA_19, RluB_mono	C29240H33728N11920O20578S5P2904Mg0Zn0Fe0	-2890	rRNA Modification
b2589_v1_RNA_1_MeT_23S_1835	b2589_v1_RNA_1, MeT_23S_1835	C27826H31379N11475O20163S1P2904Mg0Zn0Fe0	-2904	rRNA Modification
b2589_v1_RNA_2		C27812H31358N11469O20158P2904S0	-2905	rRNA Modification
b2589_v1_RNA_20		C27822H31380N11469O20158P2904S0	-2905	rRNA Modification
b2589_v1_RNA_20_RrmA_dim	b2589_v1_RNA_20, RrmA_dim	C30539H35591N12233O20933S31P2904Mg0Zn2Fe0	-2902	rRNA Modification
b2589_v1_RNA_21		C27823H31382N11469O20158P2904S0	-2905	rRNA Modification
b2589_v1_RNA_21_RluA_mono	b2589_v1_RNA_21, RluA_mono	C28926H33122N11782O20472S10P2904Mg0Zn0Fe0	-2904	rRNA Modification
b2589_v1_RNA_22		C27823H31382N11469O20158P2904S0	-2905	rRNA Modification
b2589_v1_RNA_22_RumB_mono	b2589_v1_RNA_22, RumB_mono	C29716H34360N11981O20692S28P2904Mg0Zn0Fe4	-2903	rRNA Modification
b2589_v1_RNA_23		C27824H31384N11469O20158P2904S0	-2905	rRNA Modification
b2589_v1_RNA_23_RluC_mono	b2589_v1_RNA_23, RluC_mono	C29409H33997N11951O20614S6P2904Mg0Zn0Fe0	-2890	rRNA Modification
b2589_v1_RNA_2_RluD_mono	b2589_v1_RNA_2, RluD_mono	C29449H33968N11949O20632S12P2904Mg1Zn0Fe0	-2909	rRNA Modification
b2589_v1_RNA_3		C27812H31358N11469O20158P2904S0	-2905	rRNA Modification
b2589_v1_RNA_3_RluD_mono	b2589_v1_RNA_3, RluD_mono	C29464H33991N11955O20637S13P2904Mg1Zn0Fe0	-2908	rRNA Modification
b2589_v1_RNA_4		C27813H31360N11469O20158P2904S0	-2905	rRNA Modification
b2589_v1_RNA_4_RluD_mono	b2589_v1_RNA_4, RluD_mono	C29450H33970N11949O20632S12P2904Mg1Zn0Fe0	-2909	rRNA Modification
b2589_v1_RNA_5		C27813H31360N11469O20158P2904S0	-2905	rRNA Modification
b2589_v1_RNA_5_RumA_mono	b2589_v1_RNA_5, RumA_mono	C29941H34786N12087O20786S23P2904Mg0Zn0Fe4	-2897	rRNA Modification
b2589_v1_RNA_6		C27814H31362N11469O20158P2904S0	-2905	rRNA Modification
b2589_v1_RNA_6_MeT_23S_1962	b2589_v1_RNA_6, MeT_23S_1962	C27829H31385N11475O20163S1P2904Mg0Zn0Fe0	-2904	rRNA Modification
b2589_v1_RNA_7		C27815H31364N11469O20158P2904S0	-2905	rRNA Modification
b2589_v1_RNA_7_MeT_23S_2030	b2589_v1_RNA_7, MeT_23S_2030	C27830H31387N11475O20163S1P2904Mg0Zn0Fe0	-2904	rRNA Modification
b2589_v1_RNA_8		C27816H31366N11469O20158P2904S0	-2905	rRNA Modification
b2589_v1_RNA_8_MeT_23S_2069	b2589_v1_RNA_8, MeT_23S_2069	C27831H31389N11475O20163S1P2904Mg0Zn0Fe0	-2904	rRNA Modification
b2589_v1_RNA_9		C27817H31368N11469O20158P2904S0	-2905	rRNA Modification
b2589_v1_RNA_9_RlmB_dim	b2589_v1_RNA_9, RlmB_dim	C30138H35171N12153O20859S23P2904Mg0Zn0Fe0	-2910	rRNA Modification
b2589_v1_RNA_MeT_23S_1618	b2589_v1_RNA, MeT_23S_1618	C27825H31377N11475O20163S1P2904Mg0Zn0Fe0	-2904	rRNA Modification
b2590_RNA	tRNA (gltW)	C720H824N287O532P76	-77	tRNA Modification
b2590_tRNA_1	b2590_tRNA_1 (gltW)	C720H824Mg2N287O532P76	-73	tRNA Modification
b2590_tRNA_1_TrmU_mono-YhhP_mono-YheLMN_cplx-YccK_mono-TrmE_dim-GidA_mono-TrmC_mono_cplx	b2590_tRNA_1 (gltW), TrmU_mono, YhhP_mono, YheLMN_cplx, YccK_mono, TrmE_dim, GidA_mono, TrmC_mono	C21611H33674Mg3N6131O6869P88S131X1	-221	tRNA Modification
b2590_tRNA_2	b2590_tRNA_2 (gltW)	C722H829Mg2N288O531P76S1	-73	tRNA Modification
b2590_tRNA_2_MeT_tRNA_pos_37_m2A_cplx	b2590_tRNA_2 (gltW), MeT_tRNA_pos_37_m2A	C737H852Mg2N294O536P76S2	-72	tRNA Modification
b2590_tRNA_2_Se	b2590_tRNA_2_Se - contains mnm5se2U instead of mnm5s2U,b2590_tRNA_2 (gltW)	C722H829Mg2N288O531P76S0Se1	-73	tRNA Modification
b2590_tRNA_2_Se_MeT_tRNA_pos_37_m2A_cplx	b2590_tRNA_2_Se_MeT_tRNA_pos_37_m2A_cplx - contains mnm5se2U instead of mnm5s2U,b2590_tRNA_2 (gltW), MeT_tRNA_pos_37_m2A	C737H852Mg2N294O536P76S1Se1	-72	tRNA Modification
b2590_tRNA_2_YbbB_dim_cplx	b2590_tRNA_2_YbbB_dim_cplx	C4336H6511N1342O1603S31P77Mg2Zn0Fe0Se1	-90	tRNA Modification
b2590_tRNA_3	b2590_tRNA_3 (gltW)	C723H831Mg2N288O531P76S1	-73	tRNA Modification
b2590_tRNA_3_Se	b2590_tRNA_3_Se - contains mnm5se2U instead of mnm5s2U,b2590_tRNA_3 (gltW)	C723H831Mg2N288O531P76S0Se1	-73	tRNA Modification

b2590_tRNA_3_Se_TrmA_mono_cplx	b2590_tRNA_3_Se_TrmA_mono_cplx - contains mnm5se2U instead of mnm5s2U,b2590_tRNA_3 (gltW), TrmA_mono	C2597H3778Mg2N811O1090P76S19Se1	-81	tRNA Modification
b2590_tRNA_3_TrmA_mono_cplx	b2590_tRNA_3 (gltW), TrmA_mono	C2597H3778Mg2N811O1090P76S20	-81	tRNA Modification
b2590_tRNA_4	b2590_tRNA_4 (gltW)	C724H833Mg2N288O531P76S1	-73	tRNA Modification
b2590_tRNA_4_Se	b2590_tRNA_4_Se - contains mnm5se2U instead of mnm5s2U,b2590_tRNA_4 (gltW)	C724H833Mg2N288O531P76S0Se1	-73	tRNA Modification
b2590_tRNA_4_Se_TruB_mono_cplx	b2590_tRNA_4_Se_TruB_mono_cplx - contains mnm5se2U instead of mnm5s2U,b2590_tRNA_4 (gltW), TruB_mono	C2264H3308Mg2N726O1000P76S10Se1	-78	tRNA Modification
b2590_tRNA_4_TruB_mono_cplx	b2590_tRNA_4 (gltW), TruB_mono	C2264H3308Mg2N726O1000P76S11	-78	tRNA Modification
b2590_tRNA_Mg2	tRNA (gltW) bound two Mg2 ions	C720H824Mg2N287O532P76	-73	tRNA Modification
b2590_tRNA_Mg2_TruD_mono_cplx	b2590_tRNA (gltW), TruD_mono	C2463H3566Mg2N786O1041P76S8	-76	tRNA Modification
b2590_v1_RNA	tRNA (gltW)	C720H824N287O532P76	-77	tRNA Modification
b2590_v1_tRNA_1	b2590_v1_tRNA_1 (gltW)	C720H824Mg2N287O532P76	-73	tRNA Modification
b2590_v1_tRNA_1_TrmU_mono-YhhP_mono-YheLMN_cplx-YccK_mono-TrmE_dim-GidA_mono-TrmC_mono_cplx	b2590_v1_tRNA_1 (gltW), TrmU_mono, YhhP_mono, YheLMN_cplx, YccK_mono, TrmE_dim, GidA_mono, TrmC_mono	C21611H33674Mg3N6131O6869P88S131X1	-221	tRNA Modification
b2590_v1_tRNA_2	b2590_v1_tRNA_2 (gltW)	C722H829Mg2N288O531P76S1	-73	tRNA Modification
b2590_v1_tRNA_2_MeT_tRNA_pos_37_m2A_cplx	b2590_v1_tRNA_2 (gltW), MeT_tRNA_pos_37_m2A	C737H852Mg2N294O536P76S2	-72	tRNA Modification
b2590_v1_tRNA_2_Se	b2590_v1_tRNA_2_Se - contains mnm5se2U instead of mnm5s2U,b2590_v1_tRNA_2 (gltW)	C722H829Mg2N288O531P76S0Se1	-73	tRNA Modification
b2590_v1_tRNA_2_Se_MeT_tRNA_pos_37_m2A_cplx	b2590_v1_tRNA_2_Se_MeT_tRNA_pos_37_m2A_cplx - contains mnm5se2U instead of mnm5s2U,b2590_v1_tRNA_2 (gltW), MeT_tRNA_pos_37_m2A	C737H852Mg2N294O536P76S1Se1	-72	tRNA Modification
b2590_v1_tRNA_2_YbbB_dim_cplx	b2590_tRNA_2_YbbB_dim_cplx	C4336H6511N1342O1603S31P77Mg2Zn0Fe0Se1	-90	tRNA Modification
b2590_v1_tRNA_3	b2590_v1_tRNA_3 (gltW)	C723H831Mg2N288O531P76S1	-73	tRNA Modification
b2590_v1_tRNA_3_Se	b2590_v1_tRNA_3_Se - contains mnm5se2U instead of mnm5s2U,b2590_v1_tRNA_3 (gltW)	C723H831Mg2N288O531P76S0Se1	-73	tRNA Modification
b2590_v1_tRNA_3_Se_TrmA_mono_cplx	b2590_v1_tRNA_3_Se_TrmA_mono_cplx - contains mnm5se2U instead of mnm5s2U,b2590_v1_tRNA_3 (gltW), TrmA_mono	C2597H3778Mg2N811O1090P76S19Se1	-81	tRNA Modification
b2590_v1_tRNA_3_TrmA_mono_cplx	b2590_v1_tRNA_3 (gltW), TrmA_mono	C2597H3778Mg2N811O1090P76S20	-81	tRNA Modification
b2590_v1_tRNA_4	b2590_v1_tRNA_4 (gltW)	C724H833Mg2N288O531P76S1	-73	tRNA Modification
b2590_v1_tRNA_4_Se	b2590_v1_tRNA_4_Se - contains mnm5se2U instead of mnm5s2U,b2590_v1_tRNA_4 (gltW)	C724H833Mg2N288O531P76S0Se1	-73	tRNA Modification
b2590_v1_tRNA_4_Se_TruB_mono_cplx	b2590_v1_tRNA_4_Se_TruB_mono_cplx - contains mnm5se2U instead of mnm5s2U,b2590_v1_tRNA_4 (gltW), TruB_mono	C2264H3308Mg2N726O1000P76S10Se1	-78	tRNA Modification
b2590_v1_tRNA_4_TruB_mono_cplx	b2590_v1_tRNA_4 (gltW), TruB_mono	C2264H3308Mg2N726O1000P76S11	-78	tRNA Modification
b2590_v1_tRNA_Mg2	tRNA (gltW) bound two Mg2 ions	C720H824Mg2N287O532P76	-73	tRNA Modification
b2590_v1_tRNA_Mg2_TruD_mono_cplx	b2590_v1_tRNA (gltW), TruD_mono	C2463H3566Mg2N786O1041P76S8	-76	tRNA Modification
b2591_RNA	rRNA	C14753H16649N6062O10723P1542	-1543	rRNA Modification
b2591_RNA_1		C14754H16651N6062O10723P1542S0	-1543	rRNA Modification
b2591_RNA_10		C14765H16673N6062O10723P1542S0	-1543	rRNA Modification
b2591_RNA_10_RsmB_mono	b2591_RNA_10, RsmB_mono	C16932H20116N6680O11351S17P1542Mg0Zn0Fe0	-1542	rRNA Modification
b2591_RNA_1_MeT_16S_1402	b2591_RNA_1, MeT_16S_1402	C14784H16697N6074O10733S2P1542Mg0Zn0Fe0	-1541	rRNA Modification
b2591_RNA_2		C14756H16655N6062O10723P1542S0	-1543	rRNA Modification
b2591_RNA_2_MeT_16S_1407	b2591_RNA_2, MeT_16S_1407	C14771H16678N6068O10728S1P1542Mg0Zn0Fe0	-1542	rRNA Modification
b2591_RNA_3		C14757H16657N6062O10723P1542S0	-1543	rRNA Modification
b2591_RNA_3_YggJ_mono	b2591_RNA_3, YggJ_mono	C15949H18597N6410O11082S11P1542Mg0Zn0Fe0	-1545	rRNA Modification
b2591_RNA_4		C14758H16659N6062O10723P1542S0	-1543	rRNA Modification
b2591_RNA_4_MeT_16S_1516	b2591_RNA_4, MeT_16S_1516	C14773H16682N6068O10728S1P1542Mg0Zn0Fe0	-1542	rRNA Modification
b2591_RNA_5		C14759H16661N6062O10723P1542S0	-1543	rRNA Modification
b2591_RNA_5_KsgA_mono	b2591_RNA_5, KsgA_mono	C16141H18850N6450O11121S15P1542Mg0Zn0Fe0	-1540	rRNA Modification
b2591_RNA_6		C14761H16665N6062O10723P1542S0	-1543	rRNA Modification
b2591_RNA_6_KsgA_mono	b2591_RNA_6, KsgA_mono	C16143H18854N6450O11121S15P1542Mg0Zn0Fe0	-1540	rRNA Modification
b2591_RNA_7		C14763H16669N6062O10723P1542S0	-1543	rRNA Modification
b2591_RNA_7_RsuA_mono	b2591_RNA_7, RsuA_mono	C15908H18469N6390O11067S6P1542Mg0Zn0Fe0	-1551	rRNA Modification

b2591_RNA_8		C14763H16669N6062O10723P1542S0	-1543	rRNA Modification
b2591_RNA_8_MeT_16S_527	b2591_RNA_8, MeT_16S_527	C14778H16692N6068O10728S1P1542Mg0Zn0Fe0	-1542	rRNA Modification
b2591_RNA_9		C14764H16671N6062O10723P1542S0	-1543	rRNA Modification
b2591_RNA_9_MeT_16S_966	b2591_RNA_9, MeT_16S_966	C14779H16694N6068O10728S1P1542Mg0Zn0Fe0	-1542	rRNA Modification
b2591_RNA_RsmC_mono	b2591_RNA, RsmC_mono	C16437H19280N6535O11225S11P1542Mg0Zn0Fe0	-1547	rRNA Modification
b2591_v1_RNA	rRNA	C14753H16649N6062O10723P1542	-1543	rRNA Modification
b2591_v1_RNA_1		C14754H16651N6062O10723P1542S0	-1543	rRNA Modification
b2591_v1_RNA_10		C14765H16673N6062O10723P1542S0	-1543	rRNA Modification
b2591_v1_RNA_10_RsmB_mono	b2591_v1_RNA_10, RsmB_mono	C16932H20116N6680O11351S17P1542Mg0Zn0Fe0	-1542	rRNA Modification
b2591_v1_RNA_1_MeT_16S_1402	b2591_v1_RNA_1, MeT_16S_1402	C14784H16697N6074O10733S2P1542Mg0Zn0Fe0	-1541	rRNA Modification
b2591_v1_RNA_2		C14756H16655N6062O10723P1542S0	-1543	rRNA Modification
b2591_v1_RNA_2_MeT_16S_1407	b2591_v1_RNA_2, MeT_16S_1407	C14771H16678N6068O10728S1P1542Mg0Zn0Fe0	-1542	rRNA Modification
b2591_v1_RNA_3		C14757H16657N6062O10723P1542S0	-1543	rRNA Modification
b2591_v1_RNA_3_YggJ_mono	b2591_v1_RNA_3, YggJ_mono	C15949H18597N6410O11082S11P1542Mg0Zn0Fe0	-1545	rRNA Modification
b2591_v1_RNA_4		C14758H16659N6062O10723P1542S0	-1543	rRNA Modification
b2591_v1_RNA_4_MeT_16S_1516	b2591_v1_RNA_4, MeT_16S_1516	C14773H16682N6068O10728S1P1542Mg0Zn0Fe0	-1542	rRNA Modification
b2591_v1_RNA_5		C14759H16661N6062O10723P1542S0	-1543	rRNA Modification
b2591_v1_RNA_5_KsgA_mono	b2591_v1_RNA_5, KsgA_mono	C16141H18850N6450O11121S15P1542Mg0Zn0Fe0	-1540	rRNA Modification
b2591_v1_RNA_6		C14761H16665N6062O10723P1542S0	-1543	rRNA Modification
b2591_v1_RNA_6_KsgA_mono	b2591_v1_RNA_6, KsgA_mono	C16143H18854N6450O11121S15P1542Mg0Zn0Fe0	-1540	rRNA Modification
b2591_v1_RNA_7		C14763H16669N6062O10723P1542S0	-1543	rRNA Modification
b2591_v1_RNA_7_RsuA_mono	b2591_v1_RNA_7, RsuA_mono	C15908H18469N6390O11067S6P1542Mg0Zn0Fe0	-1551	rRNA Modification
b2591_v1_RNA_8		C14763H16669N6062O10723P1542S0	-1543	rRNA Modification
b2591_v1_RNA_8_MeT_16S_527	b2591_v1_RNA_8, MeT_16S_527	C14778H16692N6068O10728S1P1542Mg0Zn0Fe0	-1542	rRNA Modification
b2591_v1_RNA_9		C14764H16671N6062O10723P1542S0	-1543	rRNA Modification
b2591_v1_RNA_9_MeT_16S_966	b2591_v1_RNA_9, MeT_16S_966	C14779H16694N6068O10728S1P1542Mg0Zn0Fe0	-1542	rRNA Modification
b2591_v1_RNA_RsmC_mono	b2591_v1_RNA, RsmC_mono	C16437H19280N6535O11225S11P1542Mg0Zn0Fe0	-1547	rRNA Modification
b2593_aa	polypeptide b2593	C1169H1808N327O346S12	-3	Translation
b2593_def_map_cplx	Polypeptide b2593 peptide deformylase and methionine aminopeptidase complex	C3299H5264N920O992S33Mg0Zn0Fe3	-12	Maturation
b2593_m	Matured polypeptide b2593	C1163H1800N326O344S11	-2	Maturation
b2593_mRNA	mRNA b2593 (4 nt short)	C6919H7829N2738O5129P728	-729	Translation
b2593_mRNA_1	mRNA b2593 (4 nt short)	C6919H7829N2738O5129P728	-729	Translation
b2593_mRNA_2	mRNA b2593 (4 nt short)	C6919H7829N2738O5129P728	-729	Translation
b2593_mRNA_2_degr	mRNA b2593 degradation complex	C47602H73228N14613O17814S200P728Mg6Zn2Fe0	-951	mRNA degradation
b2594_aa	polypeptide b2594	C1643H2618N481O476S13	-7	Translation
b2594_def_map_cplx	Polypeptide b2594 peptide deformylase and methionine aminopeptidase complex	C3773H6074N1074O1122S34Mg0Zn0Fe3	-16	Maturation
b2594_m	Matured polypeptide b2594	C1637H2610N480O474S12	-6	Maturation
b2594_mRNA	mRNA b2594	C9342H10571N3748O6853P983	-984	Translation
b2594_mRNA_1	mRNA b2594	C9342H10571N3748O6853P983	-984	Translation
b2594_mRNA_2	mRNA b2594	C9342H10571N3748O6853P983	-984	Translation
b2594_mRNA_2_degr	mRNA b2594 degradation complex	C50025H75970N15623O19538S200P983Mg6Zn2Fe0	-1206	mRNA degradation
b2594_m_Mg	b2594 plus _Mg	C1637H2610N480O474S12Mg1	-4	Folding
b2606_aa	polypeptide b2606	C580H973N180O165S2	11	Translation
b2606_def_map_cplx	Polypeptide b2606 peptide deformylase and methionine aminopeptidase complex	C2710H4429N773O811S23Mg0Zn0Fe3	2	Maturation
b2606_m	Matured polypeptide b2606	C574H965N179O163S1	12	Maturation
b2606_mRNA	mRNA b2606	C3310H3737N1308O2441P348	-349	Translation
b2606_mRNA_1	mRNA b2606	C3310H3737N1308O2441P348	-349	Translation
b2606_mRNA_2	mRNA b2606	C3310H3737N1308O2441P348	-349	Translation
b2606_mRNA_2_degr	mRNA b2606 degradation complex	C43993H69136N13183O15126S200P348Mg6Zn2Fe0	-571	mRNA degradation
b2607_aa	polypeptide b2607	C1257H1976N358O376S10	-10	Translation
b2607_def_cplx	Polypeptide b2607 peptide deformylase complex	C2101H3370N599O631S16Mg0Zn0Fe1	-15	Maturation
b2607_m	Matured polypeptide b2607	C1256H1977N358O375S10	-9	Maturation
b2607_mRNA	mRNA b2607	C7335H8283N2984O5351P768	-769	Translation

b2607_mRNA_1	mRNA b2607	C7335H8283N2984O5351P768	-769	Translation
b2607_mRNA_2	mRNA b2607	C7335H8283N2984O5351P768	-769	Translation
b2607_mRNA_2_degr	mRNA b2607 degradation complex	C48018H73682N14859O18036S200P768Mg6Zn2Fe0	-991	mRNA degradation
b2607_m_GroEL.(7)ATP.transGroES	b2607 GroEL GroES complex - Kerner et al. class III needs GroEL/ES	C39168H64921O12709N10942S374P21Mg7	-310	Folding
b2608_aa	polypeptide b2608	C923H1423N238O282S8	-11	Translation
b2608_def_map_cplx	Polypeptide b2608 peptide deformylase and methionine aminopeptidase complex	C3053H4879N831O928S29Mg0Zn0Fe3	-20	Maturation
b2608_m	Matured polypeptide b2608	C917H1415N237O280S7	-10	Maturation
b2608_mRNA	mRNA b2608	C5234H5905N2098O3834P549	-550	Translation
b2608_mRNA_1	mRNA b2608	C5234H5905N2098O3834P549	-550	Translation
b2608_mRNA_2	mRNA b2608	C5234H5905N2098O3834P549	-550	Translation
b2608_mRNA_2_degr	mRNA b2608 degradation complex	C45917H71304N13973O16519S200P549Mg6Zn2Fe0	-772	mRNA degradation
b2608_m_DnaKJ_complex	b2608 DnaK DnaJ_dim complex - Kerner et al. class II can interact w/ GroEL/ES, cannot fold spontanously	C7481H11929O2378N2150S54P3Zn4	-32	Folding
b2608_m_GroEL.(7)ATP.transGroES	b2608 GroEL GroES complex - Kerner et al. class II can interact w/ GroEL/ES, cannot fold spontanously	C38829H64359O12614N10821S371P21Mg7	-311	Folding
b2609_aa	polypeptide b2609	C407H665N128O115S1	5	Translation
b2609_def_cplx	Polypeptide b2609 peptide deformylase complex	C1251H2059N369O370S7Mg0Zn0Fe1	0	Maturation
b2609_m	Matured polypeptide b2609	C406H666N128O114S1	6	Maturation
b2609_mRNA	mRNA b2609	C2367H2681N940O1747P251	-252	Translation
b2609_mRNA_1	mRNA b2609	C2367H2681N940O1747P251	-252	Translation
b2609_mRNA_2	mRNA b2609	C2367H2681N940O1747P251	-252	Translation
b2609_mRNA_2_degr	mRNA b2609 degradation complex	C43050H68080N12815O14432S200P251Mg6Zn2Fe0	-474	mRNA degradation
b2614_aa	polypeptide b2614	C942H1539N265O309S9	-16	Translation
b2614_def_map_cplx	Polypeptide b2614 peptide deformylase and methionine aminopeptidase complex	C3072H4995N858O955S30Mg0Zn0Fe3	-25	Maturation
b2614_m	Matured polypeptide b2614	C936H1531N264O307S8	-15	Maturation
b2614_mRNA	mRNA b2614	C5690H6406N2341O4128P596	-597	Translation
b2614_mRNA_1	mRNA b2614	C5690H6406N2341O4128P596	-597	Translation
b2614_mRNA_2	mRNA b2614	C5690H6406N2341O4128P596	-597	Translation
b2614_mRNA_2_degr	mRNA b2614 degradation complex	C46373H71805N14216O16813S200P596Mg6Zn2Fe0	-819	mRNA degradation
b2614_m_DnaKJ_complex	b2614 DnaK DnaJ_dim complex - Kerner et al. class I can interact w/ GroEL/ES	C7500H12045O2405N2177S55P3Zn4	-37	Folding
b2614_m_GroEL.(7)ATP.transGroES	b2614 GroEL GroES complex - Kerner et al. class I can interact w/ GroEL/ES	C38848H64475O12641N10848S372P21Mg7	-316	Folding
b2652_RNA	tRNA (ileY)	C722H820N287O534P76	-77	tRNA Modification
b2652_tRNA_1	b2652_tRNA_1 (ileY)	C723H822Mg2N287O534P76	-73	tRNA Modification
b2652_tRNA_10	b2652_tRNA_10 (ileY)	C740H855Mg2N291O541P76	-74	tRNA Modification
b2652_tRNA_10_ThiI_mono_cplx	b2652_tRNA_10 (ileY), ThiI_mono	C7168H11086Mg3N2109O2480P81S50X1	-102	tRNA Modification
b2652_tRNA_1_Dus_gen_cplx	b2652_tRNA_1 (ileY), Dus_gen	C2330H3361Mg2N746O1017P79S14	-80	tRNA Modification
b2652_tRNA_2	b2652_tRNA_2 (ileY)	C723H824Mg2N287O534P76	-73	tRNA Modification
b2652_tRNA_2_Dus_gen_cplx	b2652_tRNA_2 (ileY), Dus_gen	C2330H3363Mg2N746O1017P79S14	-80	tRNA Modification
b2652_tRNA_3	b2652_tRNA_3 (ileY)	C723H826Mg2N287O534P76	-73	tRNA Modification
b2652_tRNA_3_TilS_mono_cplx	b2652_tRNA_3 (ileY), TilS_mono	C2884H4249Mg2N918O1163P79S10	-78	tRNA Modification
b2652_tRNA_4	b2652_tRNA_4 (ileY)	C729H838Mg2N289O535P76	-73	tRNA Modification
b2652_tRNA_4_Up_tRNA_pos_37_t6A_cplx	b2652_tRNA_4 (ileY), Up_tRNA_pos_37_t6A	C744H860Mg3N295O554P79	-76	tRNA Modification
b2652_tRNA_5	b2652_tRNA_5 (ileY)	C734H844Mg2N290O539P76	-74	tRNA Modification
b2652_tRNA_5_TruA_dim_cplx	b2652_tRNA_5 (ileY), TruA_dim	C3444H5046Mg2N1070O1309P76S16	-68	tRNA Modification
b2652_tRNA_6	b2652_tRNA_6 (ileY)	C734H844Mg2N290O539P76	-74	tRNA Modification
b2652_tRNA_6_YggH_mono_cplx	b2652_tRNA_6 (ileY), YggH_mono	C1959H2743Mg2N643O886P76S14	-76	tRNA Modification
b2652_tRNA_7	b2652_tRNA_7 (ileY)	C735H846Mg2N290O539P76	-74	tRNA Modification
b2652_tRNA_7_AcpT_tRNA_pos_47_acp3U_cplx	b2652_tRNA_7 (ileY), AcpT_tRNA_pos_47_acp3U	C750H869Mg2N296O544P76S1	-73	tRNA Modification
b2652_tRNA_8	b2652_tRNA_8 (ileY)	C739H853Mg2N291O541P76	-74	tRNA Modification
b2652_tRNA_8_TrmA_mono_cplx	b2652_tRNA_8 (ileY), TrmA_mono	C2613H3800Mg2N814O1100P76S19	-82	tRNA Modification
b2652_tRNA_9	b2652_tRNA_9 (ileY)	C740H855Mg2N291O541P76	-74	tRNA Modification
b2652_tRNA_9_TruB_mono_cplx	b2652_tRNA_9 (ileY), TruB_mono	C2280H3330Mg2N729O1010P76S10	-79	tRNA Modification
b2652_tRNA_Mg2	tRNA (ileY) bound two Mg2 ions	C722H820Mg2N287O534P76	-73	tRNA Modification
b2652_tRNA_Mg2_TrmH_dim_cplx	b2652_tRNA (ileY), TrmH_dim	C2933H4363Mg2N943O1195P76S29	-74	tRNA Modification
b2691_RNA	tRNA (argQ)	C733H834N297O537P77	-78	tRNA Modification
b2691_tRNA_1	b2691_tRNA_1 (argQ)	C733H836Mg2N297O537P77	-74	tRNA Modification
b2691_tRNA_1_Dus_gen_cplx	b2691_tRNA_1 (argQ), Dus_gen	C2340H3375Mg2N756O1020P80S14	-81	tRNA Modification

b2691_tRNA_2	b2691_tRNA_2 (argQ)	C733H838Mg2N297O537P77	-74	tRNA Modification
b2691_tRNA_2_TadA_dim_cplx	b2691_tRNA_2 (argQ), TadA_dim	C2483H3620Mg2N825O1030P77S22Zn2	-66	tRNA Modification
b2691_tRNA_3	b2691_tRNA_3 (argQ)	C733H837Mg2N296O538P77	-74	tRNA Modification
b2691_tRNA_3_MeT_tRNA_pos_37_m2A_cplx	b2691_tRNA_3 (argQ), MeT_tRNA_pos_37_m2A	C748H860Mg2N302O543P77S1	-73	tRNA Modification
b2691_tRNA_4	b2691_tRNA_4 (argQ)	C734H839Mg2N296O538P77	-74	tRNA Modification
b2691_tRNA_4_YggH_mono_cplx	b2691_tRNA_4 (argQ), YggH_mono	C1959H2738Mg2N649O885P77S14	-76	tRNA Modification
b2691_tRNA_5	b2691_tRNA_5 (argQ)	C735H841Mg2N296O538P77	-74	tRNA Modification
b2691_tRNA_5_AcpT_tRNA_pos_47_acp3U_cplx	b2691_tRNA_5 (argQ), AcpT_tRNA_pos_47_acp3U	C750H864Mg2N302O543P77S1	-73	tRNA Modification
b2691_tRNA_6	b2691_tRNA_6 (argQ)	C739H848Mg2N297O540P77	-74	tRNA Modification
b2691_tRNA_6_TrmA_mono_cplx	b2691_tRNA_6 (argQ), TrmA_mono	C2613H3795Mg2N820O1099P77S19	-82	tRNA Modification
b2691_tRNA_7	b2691_tRNA_7 (argQ)	C740H850Mg2N297O540P77	-74	tRNA Modification
b2691_tRNA_7_TruB_mono_cplx	b2691_tRNA_7 (argQ), TruB_mono	C2280H3325Mg2N735O1009P77S10	-79	tRNA Modification
b2691_tRNA_8	b2691_tRNA_8 (argQ)	C740H850Mg2N297O540P77	-74	tRNA Modification
b2691_tRNA_8_ThiI_mono_cplx	b2691_tRNA_8 (argQ), ThiI_mono	C7168H11081Mg3N2115O2479P82S50X1	-102	tRNA Modification
b2691_tRNA_Mg2	tRNA (argQ) bound two Mg2 ions	C733H834Mg2N297O537P77	-74	tRNA Modification
b2691_tRNA_Mg2_Dus_gen_cplx	b2691_tRNA (argQ), Dus_gen	C2340H3373Mg2N756O1020P80S14	-81	tRNA Modification
b2692_RNA	tRNA (argZ)	C733H834N297O537P77	-78	tRNA Modification
b2692_tRNA_1	b2692_tRNA_1 (argZ)	C733H836Mg2N297O537P77	-74	tRNA Modification
b2692_tRNA_1_Dus_gen_cplx	b2692_tRNA_1 (argZ), Dus_gen	C2340H3375Mg2N756O1020P80S14	-81	tRNA Modification
b2692_tRNA_2	b2692_tRNA_2 (argZ)	C733H838Mg2N297O537P77	-74	tRNA Modification
b2692_tRNA_2_TadA_dim_cplx	b2692_tRNA_2 (argZ), TadA_dim	C2483H3620Mg2N825O1030P77S22Zn2	-66	tRNA Modification
b2692_tRNA_3	b2692_tRNA_3 (argZ)	C733H837Mg2N296O538P77	-74	tRNA Modification
b2692_tRNA_3_MeT_tRNA_pos_37_m2A_cplx	b2692_tRNA_3 (argZ), MeT_tRNA_pos_37_m2A	C748H860Mg2N302O543P77S1	-73	tRNA Modification
b2692_tRNA_4	b2692_tRNA_4 (argZ)	C734H839Mg2N296O538P77	-74	tRNA Modification
b2692_tRNA_4_YggH_mono_cplx	b2692_tRNA_4 (argZ), YggH_mono	C1959H2738Mg2N649O885P77S14	-76	tRNA Modification
b2692_tRNA_5	b2692_tRNA_5 (argZ)	C735H841Mg2N296O538P77	-74	tRNA Modification
b2692_tRNA_5_AcpT_tRNA_pos_47_acp3U_cplx	b2692_tRNA_5 (argZ), AcpT_tRNA_pos_47_acp3U	C750H864Mg2N302O543P77S1	-73	tRNA Modification
b2692_tRNA_6	b2692_tRNA_6 (argZ)	C739H848Mg2N297O540P77	-74	tRNA Modification
b2692_tRNA_6_TrmA_mono_cplx	b2692_tRNA_6 (argZ), TrmA_mono	C2613H3795Mg2N820O1099P77S19	-82	tRNA Modification
b2692_tRNA_7	b2692_tRNA_7 (argZ)	C740H850Mg2N297O540P77	-74	tRNA Modification
b2692_tRNA_7_TruB_mono_cplx	b2692_tRNA_7 (argZ), TruB_mono	C2280H3325Mg2N735O1009P77S10	-79	tRNA Modification
b2692_tRNA_8	b2692_tRNA_8 (argZ)	C740H850Mg2N297O540P77	-74	tRNA Modification
b2692_tRNA_8_ThiI_mono_cplx	b2692_tRNA_8 (argZ), ThiI_mono	C7168H11081Mg3N2115O2479P82S50X1	-102	tRNA Modification
b2692_tRNA_Mg2	tRNA (argZ) bound two Mg2 ions	C733H834Mg2N297O537P77	-74	tRNA Modification
b2692_tRNA_Mg2_Dus_gen_cplx	b2692_tRNA (argZ), Dus_gen	C2340H3373Mg2N756O1020P80S14	-81	tRNA Modification
b2693_RNA	tRNA (argY)	C733H834N297O537P77	-78	tRNA Modification
b2693_tRNA_1	b2693_tRNA_1 (argY)	C733H836Mg2N297O537P77	-74	tRNA Modification
b2693_tRNA_1_Dus_gen_cplx	b2693_tRNA_1 (argY), Dus_gen	C2340H3375Mg2N756O1020P80S14	-81	tRNA Modification
b2693_tRNA_2	b2693_tRNA_2 (argY)	C733H838Mg2N297O537P77	-74	tRNA Modification
b2693_tRNA_2_TadA_dim_cplx	b2693_tRNA_2 (argY), TadA_dim	C2483H3620Mg2N825O1030P77S22Zn2	-66	tRNA Modification
b2693_tRNA_3	b2693_tRNA_3 (argY)	C733H837Mg2N296O538P77	-74	tRNA Modification
b2693_tRNA_3_MeT_tRNA_pos_37_m2A_cplx	b2693_tRNA_3 (argY), MeT_tRNA_pos_37_m2A	C748H860Mg2N302O543P77S1	-73	tRNA Modification
b2693_tRNA_4	b2693_tRNA_4 (argY)	C734H839Mg2N296O538P77	-74	tRNA Modification
b2693_tRNA_4_YggH_mono_cplx	b2693_tRNA_4 (argY), YggH_mono	C1959H2738Mg2N649O885P77S14	-76	tRNA Modification
b2693_tRNA_5	b2693_tRNA_5 (argY)	C735H841Mg2N296O538P77	-74	tRNA Modification
b2693_tRNA_5_AcpT_tRNA_pos_47_acp3U_cplx	b2693_tRNA_5 (argY), AcpT_tRNA_pos_47_acp3U	C750H864Mg2N302O543P77S1	-73	tRNA Modification
b2693_tRNA_6	b2693_tRNA_6 (argY)	C739H848Mg2N297O540P77	-74	tRNA Modification
b2693_tRNA_6_TrmA_mono_cplx	b2693_tRNA_6 (argY), TrmA_mono	C2613H3795Mg2N820O1099P77S19	-82	tRNA Modification
b2693_tRNA_7	b2693_tRNA_7 (argY)	C740H850Mg2N297O540P77	-74	tRNA Modification
b2693_tRNA_7_TruB_mono_cplx	b2693_tRNA_7 (argY), TruB_mono	C2280H3325Mg2N735O1009P77S10	-79	tRNA Modification
b2693_tRNA_8	b2693_tRNA_8 (argY)	C740H850Mg2N297O540P77	-74	tRNA Modification
b2693_tRNA_8_ThiI_mono_cplx	b2693_tRNA_8 (argY), ThiI_mono	C7168H11081Mg3N2115O2479P82S50X1	-102	tRNA Modification
b2693_tRNA_Mg2	tRNA (argY) bound two Mg2 ions	C733H834Mg2N297O537P77	-74	tRNA Modification
b2693_tRNA_Mg2_Dus_gen_cplx	b2693_tRNA (argY), Dus_gen	C2340H3373Mg2N756O1020P80S14	-81	tRNA Modification
b2694_RNA	tRNA (argV)	C733H834N297O537P77	-78	tRNA Modification
b2694_tRNA_1	b2694_tRNA_1 (argV)	C733H836Mg2N297O537P77	-74	tRNA Modification
b2694_tRNA_1_Dus_gen_cplx	b2694_tRNA_1 (argV), Dus_gen	C2340H3375Mg2N756O1020P80S14	-81	tRNA Modification
b2694_tRNA_2	b2694_tRNA_2 (argV)	C733H838Mg2N297O537P77	-74	tRNA Modification
b2694_tRNA_2_TadA_dim_cplx	b2694_tRNA_2 (argV), TadA_dim	C2483H3620Mg2N825O1030P77S22Zn2	-66	tRNA Modification

b2694_tRNA_3	b2694_tRNA_3 (argV)	C733H837Mg2N296O538P77	-74	tRNA Modification
b2694_tRNA_3_MeT_tRNA_pos_37_m2A_cplx	b2694_tRNA_3 (argV), MeT_tRNA_pos_37_m2A	C748H860Mg2N302O543P77S1	-73	tRNA Modification
b2694_tRNA_4	b2694_tRNA_4 (argV)	C734H839Mg2N296O538P77	-74	tRNA Modification
b2694_tRNA_4_YggH_mono_cplx	b2694_tRNA_4 (argV), YggH_mono	C1959H2738Mg2N649O885P77S14	-76	tRNA Modification
b2694_tRNA_5	b2694_tRNA_5 (argV)	C735H841Mg2N296O538P77	-74	tRNA Modification
b2694_tRNA_5_AcpT_tRNA_pos_47_acp3U_cplx	b2694_tRNA_5 (argV), AcpT_tRNA_pos_47_acp3U	C750H864Mg2N302O543P77S1	-73	tRNA Modification
b2694_tRNA_6	b2694_tRNA_6 (argV)	C739H848Mg2N297O540P77	-74	tRNA Modification
b2694_tRNA_6_TrmA_mono_cplx	b2694_tRNA_6 (argV), TrmA_mono	C2613H3795Mg2N820O1099P77S19	-82	tRNA Modification
b2694_tRNA_7	b2694_tRNA_7 (argV)	C740H850Mg2N297O540P77	-74	tRNA Modification
b2694_tRNA_7_TruB_mono_cplx	b2694_tRNA_7 (argV), TruB_mono	C2280H3325Mg2N735O1009P77S10	-79	tRNA Modification
b2694_tRNA_8	b2694_tRNA_8 (argV)	C740H850Mg2N297O540P77	-74	tRNA Modification
b2694_tRNA_8_ThiI_mono_cplx	b2694_tRNA_8 (argV), ThiI_mono	C7168H11081Mg3N2115O2479P82S50X1	-102	tRNA Modification
b2694_tRNA_Mg2	tRNA (argV) bound two Mg2 ions	C733H834Mg2N297O537P77	-74	tRNA Modification
b2694_tRNA_Mg2_Dus_gen_cplx	b2694_tRNA (argV), Dus_gen	C2340H3373Mg2N756O1020P80S14	-81	tRNA Modification
b2695_RNA	tRNA (serV)	C887H1009N364O649P93	-94	tRNA Modification
b2695_tRNA_1	b2695_tRNA_1 (serV)	C887H1011Mg2N364O649P93	-90	tRNA Modification
b2695_tRNA_1_YdaO_mono_cplx	b2695_tRNA_1 (serV), YdaO_mono	C6429H9830Mg3N1934O2336P98S55X1	-116	tRNA Modification
b2695_tRNA_2	b2695_tRNA_2 (serV)	C887H1011Mg2N364O648P93S1	-90	tRNA Modification
b2695_tRNA_2_Up_tRNA_pos_37_t6A_cplx	b2695_tRNA_2 (serV), Up_tRNA_pos_37_t6A	C902H1033Mg3N370O667P96S1	-93	tRNA Modification
b2695_tRNA_3	b2695_tRNA_3 (serV)	C892H1017Mg2N365O652P93S1	-91	tRNA Modification
b2695_tRNA_3_TrmA_mono_cplx	b2695_tRNA_3 (serV), TrmA_mono	C2766H3964Mg2N888O1211P93S20	-99	tRNA Modification
b2695_tRNA_4	b2695_tRNA_4 (serV)	C893H1019Mg2N365O652P93S1	-91	tRNA Modification
b2695_tRNA_4_TruB_mono_cplx	b2695_tRNA_4 (serV), TruB_mono	C2433H3494Mg2N803O1121P93S11	-96	tRNA Modification
b2695_tRNA_5	b2695_tRNA_5 (serV)	C893H1019Mg2N365O652P93S1	-91	tRNA Modification
b2695_tRNA_5_ThiI_mono_cplx	b2695_tRNA_5 (serV), ThiI_mono	C7321H11250Mg3N2183O2591P98S51X1	-119	tRNA Modification
b2695_tRNA_Mg2	tRNA (serV) bound two Mg2 ions	C887H1009Mg2N364O649P93	-90	tRNA Modification
b2695_tRNA_Mg2_Dus_gen_cplx	b2695_tRNA (serV), Dus_gen	C2494H3548Mg2N823O1132P96S14	-97	tRNA Modification
b2697_aa	polypeptide b2697_v1	C4216H6664N1208O1306S27	-24	Translation
b2697_def_map_cplx	Polypeptide b2697 peptide deformylase and methionine aminopeptidase complex	C6346H10120N1801O1952S48Mg0Zn0Fe3	-33	Maturation
b2697_m	Matured polypeptide b2697	C4210H6656N1207O1304S26	-23	Maturation
b2697_mRNA	mRNA b2697	C25080H28349N10102O18390P2633	-2634	Translation
b2697_mRNA_1	mRNA b2697	C25080H28349N10102O18390P2633	-2634	Translation
b2697_mRNA_2	mRNA b2697	C25080H28349N10102O18390P2633	-2634	Translation
b2697_mRNA_2_degr	mRNA b2697 degradation complex	C65763H93748N21977O31075S200P2633Mg6Zn2Fe0	-2856	mRNA degradation
b2697_m_DnaKJ_complex	b2697 DnaK DnaJ_dim complex - Deuerling et al. DnaKJ/GrpE dependent folding	C10774H17170O3402N3120S73P3Zn5	-43	Folding
b2697_m_Zn	b2697 plus _Zn	C4210H6656N1207O1304S26Zn1	-21	Folding
b2697_v1_mRNA	mRNA b2697_v1	C25080H28349N10102O18390P2633	-2634	Translation
b2741_aa	polypeptide b2741_v2	C1662H2672N483O525S5	-19	Translation
b2741_def_map_cplx	Polypeptide b2741 peptide deformylase and methionine aminopeptidase complex	C3792H6128N1076O1171S26Mg0Zn0Fe3	-28	Maturation
b2741_m	Matured polypeptide b2741	C1656H2664N482O523S4	-18	Maturation
b2741_mRNA	mRNA b2741	C9483H10702N3849O6926P995	-996	Translation
b2741_mRNA_1	mRNA b2741	C9483H10702N3849O6926P995	-996	Translation
b2741_mRNA_2	mRNA b2741	C9483H10702N3849O6926P995	-996	Translation
b2741_mRNA_2_degr	mRNA b2741 degradation complex	C50166H76101N15724O19611S200P995Mg6Zn2Fe0	-1218	mRNA degradation
b2741_v1_mRNA	mRNA b2741_v1	C9483H10702N3849O6920P993	-994	Translation
b2741_v2_mRNA	mRNA b2741_v2	C9483H10702N3849O6920P993	-994	Translation
b2742_aa	polypeptide b2742_v1	C1752H2808N502O564S8	8	Translation
b2742_def_map_cplx	Polypeptide b2742 peptide deformylase and methionine aminopeptidase complex	C3882H6264N1095O1210S29Mg0Zn0Fe3	-1	Maturation
b2742_m	Matured polypeptide b2742	C1631H2609N469O533S7	6	Maturation
b2742_mRNA	mRNA b2742	C10863H12312N4397O7903P1142	-1143	Translation
b2742_mRNA_1	mRNA b2742	C10863H12312N4397O7903P1142	-1143	Translation
b2742_mRNA_2	mRNA b2742	C10863H12312N4397O7903P1142	-1143	Translation
b2742_mRNA_2_degr	mRNA b2742 degradation complex	C51546H77711N16272O20588S200P1142Mg6Zn2Fe0	-1365	mRNA degradation
b2742_v1_mRNA	mRNA b2742_v1	C10863H12312N4397O7903P1142	-1143	Translation
b2745_DNA_act	DNA b2745 (activated form)	C10262H11790N4054O6278P1050	-1050	Transcription
b2745_DNA_neu	DNA b2745 (inactivate form)	C10262H11790N4054O6278P1050	-1050	Transcription

b2745_aa	polypeptide b2745	C1744H2741N499O510S8	-4	Translation
b2745_def_cplx	Polypeptide b2745 peptide deformylase complex	C2588H4135N740O765S14Mg0Zn0Fe1	-9	Maturation
b2745_m	Matured polypeptide b2745	C1743H2742N499O509S8	-3	Maturation
b2745_mRNA	mRNA b2745	C10022H11311N4054O7335P1052	-1053	Translation
b2745_mRNA_1	mRNA b2745	C10022H11311N4054O7335P1052	-1053	Translation
b2745_mRNA_2	mRNA b2745	C10022H11311N4054O7335P1052	-1053	Translation
b2745_mRNA_2_degr	mRNA b2745 degradation complex	C50705H76710N15929O20020S200P1052Mg6Zn2Fe0	-1275	mRNA degradation
b2779_aa	polypeptide b2779	C2009H3229N547O636S14	-12	Translation
b2779_def_map_cplx	Polypeptide b2779 peptide deformylase and methionine aminopeptidase complex	C4139H6685N1140O1282S35Mg0Zn0Fe3	-21	Maturation
b2779_m	Matured polypeptide b2779	C2003H3221N546O634S13	-11	Maturation
b2779_mRNA	mRNA b2779	C12379H13999N4982O9031P1299	-1300	Translation
b2779_mRNA_1	mRNA b2779	C12379H13999N4982O9031P1299	-1300	Translation
b2779_mRNA_2	mRNA b2779	C12379H13999N4982O9031P1299	-1300	Translation
b2779_mRNA_2_degr	mRNA b2779 degradation complex	C53062H79398N16857O21716S200P1299Mg6Zn2Fe0	-1522	mRNA degradation
b2779_m_DnaKJ_complex	b2779 DnaK DnaJ_dim complex - Kerner et al. class I can interact w/ GroEL/ES	C8567H13735O2732N2459S60P3Mg2Zn4	-29	Folding
b2779_m_GroEL.(7)ATP.transGroES	b2779 GroEL GroES complex - Kerner et al. class I can interact w/ GroEL/ES	C39915H66165O12968N11130S377P21Mg9	-308	Folding
b2779_m_Mg	b2779 plus _Mg	C2003H3221N546O634S13Mg2	-7	Folding
b2780_aa	polypeptide b2780_v2	C2678H4261N743O803S21	-14	Translation
b2780_def_map_cplx	Polypeptide b2780 peptide deformylase and methionine aminopeptidase complex	C4808H7717N1336O1449S42Mg0Zn0Fe3	-23	Maturation
b2780_m	Matured polypeptide b2780	C2672H4253N742O801S20	-13	Maturation
b2780_mRNA_1	mRNA b2780	C15584H17629N6208O11465P1640	-1641	Translation
b2780_mRNA_2	mRNA b2780	C15584H17629N6208O11465P1640	-1641	Translation
b2780_mRNA_2_degr	mRNA b2780 degradation complex	C56267H83028N18083O24150S200P1640Mg6Zn2Fe0	-1863	mRNA degradation
b2780_m_DnaKJ_complex	b2780 DnaK DnaJ_dim complex - Deuerling et al. DnaKJ/GrpE dependent folding	C9236H14767O2899N2655S67P3Zn4	-35	Folding
b2780_v2_mRNA	mRNA b2780_v2	C15584H17629N6208O11465P1640	-1641	Translation
b2785_aa	polypeptide b2785	C2119H3411N613O625S19	4	Translation
b2785_def_map_cplx	Polypeptide b2785 peptide deformylase and methionine aminopeptidase complex	C4249H6867N1206O1271S40Mg0Zn0Fe3	-5	Maturation
b2785_m	Matured polypeptide b2785	C2113H3403N612O623S18	5	Maturation
b2785_mRNA	mRNA b2785	C12398H14022N4965O9091P1304	-1305	Translation
b2785_mRNA_1	mRNA b2785	C12398H14022N4965O9091P1304	-1305	Translation
b2785_mRNA_2	mRNA b2785	C12398H14022N4965O9091P1304	-1305	Translation
b2785_mRNA_2_degr	mRNA b2785 degradation complex	C53081H79421N16840O21776S200P1304Mg6Zn2Fe0	-1527	mRNA degradation
b2785_m_FeS	b2785 plus _FeS	C2113H3403N612O623S22Fe4	7	Folding
b2785_m_IscU_cplx	b2785_m_IscU_cplx	C3305H5297N936O1019S32Fe4	-7	Iron-sulfur cluster incorporation
b2790_aa	polypeptide b2790	C730H1099N180O235S4	-17	Translation
b2790_def_map_cplx	Polypeptide b2790 peptide deformylase and methionine aminopeptidase complex	C2860H4555N773O881S25Mg0Zn0Fe3	-26	Maturation
b2790_m	Matured polypeptide b2790	C724H1091N179O233S3	-16	Maturation
b2790_mRNA	mRNA b2790	C4294H4845N1732O3150P450	-451	Translation
b2790_mRNA_1	mRNA b2790	C4294H4845N1732O3150P450	-451	Translation
b2790_mRNA_2	mRNA b2790	C4294H4845N1732O3150P450	-451	Translation
b2790_mRNA_2_degr	mRNA b2790 degradation complex	C44977H70244N13607O15835S200P450Mg6Zn2Fe0	-673	mRNA degradation
b2791_aa	polypeptide b2791	C1320H2064N388O381S7	-4	Translation
b2791_def_map_cplx	Polypeptide b2791 peptide deformylase and methionine aminopeptidase complex	C3450H5520N981O1027S28Mg0Zn0Fe3	-13	Maturation
b2791_m	Matured polypeptide b2791	C1314H2056N387O379S6	-3	Maturation
b2791_mRNA	mRNA b2791 (1 nt short)	C7449H8428N2993O5466P782	-783	Translation
b2791_mRNA_1	mRNA b2791 (1 nt short)	C7449H8428N2993O5466P782	-783	Translation
b2791_mRNA_2	mRNA b2791 (1 nt short)	C7449H8428N2993O5466P782	-783	Translation
b2791_mRNA_2_degr	mRNA b2791 degradation complex	C48132H73827N14868O18151S200P782Mg6Zn2Fe0	-1005	mRNA degradation
b2792_aa	polypeptide b2792	C577H866N156O166S5	-10	Translation
b2792_def_map_cplx	Polypeptide b2792 peptide deformylase and methionine aminopeptidase complex	C2707H4322N749O812S26Mg0Zn0Fe3	-19	Maturation
b2792_m	Matured polypeptide b2792	C571H858N155O164S4	-9	Maturation
b2792_mRNA	mRNA b2792	C3134H3552N1239O2318P332	-333	Translation
b2792_mRNA_1	mRNA b2792	C3134H3552N1239O2318P332	-333	Translation

b2792_mRNA_2	mRNA b2792	C3134H3552N1239O2318P332	-333	Translation
b2792_mRNA_2_degr	mRNA b2792 degradation complex	C43817H68951N13114O15003S200P332Mg6Zn2Fe0	-555	mRNA degradation
b2794_aa	polypeptide b2794	C1446H2225N407O442S7	-8	Translation
b2794_def_map_cplx	Polypeptide b2794 peptide deformylase and methionine aminopeptidase complex	C3576H5681N1000O1088S28Mg0Zn0Fe3	-17	Maturation
b2794_m	Matured polypeptide b2794	C1440H2217N406O440S6	-7	Maturation
b2794_mRNA	mRNA b2794	C8068H9126N3187O5944P851	-852	Translation
b2794_mRNA_1	mRNA b2794	C8068H9126N3187O5944P851	-852	Translation
b2794_mRNA_2	mRNA b2794	C8068H9126N3187O5944P851	-852	Translation
b2794_mRNA_2_degr	mRNA b2794 degradation complex	C48751H74525N15062O18629S200P851Mg6Zn2Fe0	-1074	mRNA degradation
b2814_RNA	tRNA (metZ)	C732H836N297O538P77	-78	tRNA Modification
b2814_tRNA_1	b2814_tRNA_1 (metZ)	C732H838Mg2N297O538P77	-74	tRNA Modification
b2814_tRNA_1_MeT_tRNA_pos_32_Cm_cplx	b2814_tRNA_1 (metZ), MeT_tRNA_pos_32_Cm	C747H861Mg2N303O543P77S1	-73	tRNA Modification
b2814_tRNA_2	b2814_tRNA_2 (metZ)	C733H840Mg2N297O538P77	-74	tRNA Modification
b2814_tRNA_2_YggH_mono_cplx	b2814_tRNA_2 (metZ), YggH_mono	C1958H2739Mg2N650O885P77S14	-76	tRNA Modification
b2814_tRNA_3	b2814_tRNA_3 (metZ)	C734H842Mg2N297O538P77	-74	tRNA Modification
b2814_tRNA_3_TrmA_mono_cplx	b2814_tRNA_3 (metZ), TrmA_mono	C2608H3789Mg2N820O1097P77S19	-82	tRNA Modification
b2814_tRNA_4	b2814_tRNA_4 (metZ)	C735H844Mg2N297O538P77	-74	tRNA Modification
b2814_tRNA_4_TruB_mono_cplx	b2814_tRNA_4 (metZ), TruB_mono	C2275H3319Mg2N735O1007P77S10	-79	tRNA Modification
b2814_tRNA_5	b2814_tRNA_5 (metZ)	C735H844Mg2N297O538P77	-74	tRNA Modification
b2814_tRNA_5_ThiI_mono_cplx	b2814_tRNA_5 (metZ), ThiI_mono	C7163H11075Mg3N2115O2477P82S50X1	-102	tRNA Modification
b2814_tRNA_Mg2	tRNA (metZ) bound two Mg2 ions	C732H836Mg2N297O538P77	-74	tRNA Modification
b2814_tRNA_Mg2_DusA_mono_cplx	b2814_tRNA (metZ), DusA_mono	C2372H3400Mg2N763O1034P80S18	-83	tRNA Modification
b2815_RNA	tRNA (metW)	C732H836N297O538P77	-78	tRNA Modification
b2815_tRNA_1	b2815_tRNA_1 (metW)	C732H838Mg2N297O538P77	-74	tRNA Modification
b2815_tRNA_1_MeT_tRNA_pos_32_Cm_cplx	b2815_tRNA_1 (metW), MeT_tRNA_pos_32_Cm	C747H861Mg2N303O543P77S1	-73	tRNA Modification
b2815_tRNA_2	b2815_tRNA_2 (metW)	C733H840Mg2N297O538P77	-74	tRNA Modification
b2815_tRNA_2_YggH_mono_cplx	b2815_tRNA_2 (metW), YggH_mono	C1958H2739Mg2N650O885P77S14	-76	tRNA Modification
b2815_tRNA_3	b2815_tRNA_3 (metW)	C734H842Mg2N297O538P77	-74	tRNA Modification
b2815_tRNA_3_TrmA_mono_cplx	b2815_tRNA_3 (metW), TrmA_mono	C2608H3789Mg2N820O1097P77S19	-82	tRNA Modification
b2815_tRNA_4	b2815_tRNA_4 (metW)	C735H844Mg2N297O538P77	-74	tRNA Modification
b2815_tRNA_4_TruB_mono_cplx	b2815_tRNA_4 (metW), TruB_mono	C2275H3319Mg2N735O1007P77S10	-79	tRNA Modification
b2815_tRNA_5	b2815_tRNA_5 (metW)	C735H844Mg2N297O538P77	-74	tRNA Modification
b2815_tRNA_5_ThiI_mono_cplx	b2815_tRNA_5 (metW), ThiI_mono	C7163H11075Mg3N2115O2477P82S50X1	-102	tRNA Modification
b2815_tRNA_Mg2	tRNA (metW) bound two Mg2 ions	C732H836Mg2N297O538P77	-74	tRNA Modification
b2815_tRNA_Mg2_DusA_mono_cplx	b2815_tRNA (metW), DusA_mono	C2372H3400Mg2N763O1034P80S18	-83	tRNA Modification
b2816_RNA	tRNA (metV)	C732H836N297O538P77	-78	tRNA Modification
b2816_tRNA_1	b2816_tRNA_1 (metV)	C732H838Mg2N297O538P77	-74	tRNA Modification
b2816_tRNA_1_MeT_tRNA_pos_32_Cm_cplx	b2816_tRNA_1 (metV), MeT_tRNA_pos_32_Cm	C747H861Mg2N303O543P77S1	-73	tRNA Modification
b2816_tRNA_2	b2816_tRNA_2 (metV)	C733H840Mg2N297O538P77	-74	tRNA Modification
b2816_tRNA_2_YggH_mono_cplx	b2816_tRNA_2 (metV), YggH_mono	C1958H2739Mg2N650O885P77S14	-76	tRNA Modification
b2816_tRNA_3	b2816_tRNA_3 (metV)	C734H842Mg2N297O538P77	-74	tRNA Modification
b2816_tRNA_3_TrmA_mono_cplx	b2816_tRNA_3 (metV), TrmA_mono	C2608H3789Mg2N820O1097P77S19	-82	tRNA Modification
b2816_tRNA_4	b2816_tRNA_4 (metV)	C735H844Mg2N297O538P77	-74	tRNA Modification
b2816_tRNA_4_TruB_mono_cplx	b2816_tRNA_4 (metV), TruB_mono	C2275H3319Mg2N735O1007P77S10	-79	tRNA Modification
b2816_tRNA_5	b2816_tRNA_5 (metV)	C735H844Mg2N297O538P77	-74	tRNA Modification
b2816_tRNA_5_ThiI_mono_cplx	b2816_tRNA_5 (metV), ThiI_mono	C7163H11075Mg3N2115O2477P82S50X1	-102	tRNA Modification
b2816_tRNA_Mg2	tRNA (metV) bound two Mg2 ions	C732H836Mg2N297O538P77	-74	tRNA Modification
b2816_tRNA_Mg2_DusA_mono_cplx	b2816_tRNA (metV), DusA_mono	C2372H3400Mg2N763O1034P80S18	-83	tRNA Modification
b2864_RNA	tRNA (glyU)	C701H798N275O521P74	-75	tRNA Modification
b2864_tRNA_1	b2864_tRNA_1 (glyU)	C701H800Mg2N275O521P74	-71	tRNA Modification
b2864_tRNA_1_TrmA_mono_cplx	b2864_tRNA_1 (glyU), TrmA_mono	C2575H3747Mg2N798O1080P74S19	-79	tRNA Modification
b2864_tRNA_2	b2864_tRNA_2 (glyU)	C702H802Mg2N275O521P74	-71	tRNA Modification
b2864_tRNA_2_TruB_mono_cplx	b2864_tRNA_2 (glyU), TruB_mono	C2242H3277Mg2N713O990P74S10	-76	tRNA Modification
b2864_tRNA_3	b2864_tRNA_3 (glyU)	C702H802Mg2N275O521P74	-71	tRNA Modification
b2864_tRNA_3_ThiI_mono_cplx	b2864_tRNA_3 (glyU), ThiI_mono	C7130H11033Mg3N2093O2460P79S50X1	-99	tRNA Modification
b2864_tRNA_Mg2	tRNA (glyU) bound two Mg2 ions	C701H798Mg2N275O521P74	-71	tRNA Modification
b2864_tRNA_Mg2_Dus_gen_cplx	b2864_tRNA (glyU), Dus_gen	C2308H3337Mg2N734O1004P77S14	-78	tRNA Modification

b2864_v1_RNA	tRNA (glyU)	C701H798N275O521P74	-75	tRNA Modification
b2864_v1_tRNA_1	b2864_v1_tRNA_1 (glyU)	C701H800Mg2N275O521P74	-71	tRNA Modification
b2864_v1_tRNA_1_TrmA_mono_cplx	b2864_v1_tRNA_1 (glyU), TrmA_mono	C2575H3747Mg2N798O1080P74S19	-79	tRNA Modification
b2864_v1_tRNA_2	b2864_v1_tRNA_2 (glyU)	C702H802Mg2N275O521P74	-71	tRNA Modification
b2864_v1_tRNA_2_TruB_mono_cplx	b2864_v1_tRNA_2 (glyU), TruB_mono	C2242H3277Mg2N713O990P74S10	-76	tRNA Modification
b2864_v1_tRNA_3	b2864_v1_tRNA_3 (glyU)	C702H802Mg2N275O521P74	-71	tRNA Modification
b2864_v1_tRNA_3_ThiI_mono_cplx	b2864_v1_tRNA_3 (glyU), ThiI_mono	C7130H11033Mg3N2093O2460P79S50X1	-99	tRNA Modification
b2864_v1_tRNA_Mg2	tRNA (glyU) bound two Mg2 ions	C701H798Mg2N275O521P74	-71	tRNA Modification
b2864_v1_tRNA_Mg2_Dus_gen_cplx	b2864_v1_tRNA (glyU), Dus_gen	C2308H3337Mg2N734O1004P77S14	-78	tRNA Modification
b2890_aa	polypeptide b2890	C2558H3991N708O771S19	-23	Translation
b2890_def_map_cplx	Polypeptide b2890 peptide deformylase and methionine aminopeptidase complex	C4688H7447N1301O1417S40Mg0Zn0Fe3	-32	Maturation
b2890_m	Matured polypeptide b2890	C2552H3983N707O769S18	-22	Maturation
b2890_mRNA	mRNA b2890	C14442H16363N5778O10587P1518	-1519	Translation
b2890_mRNA_1	mRNA b2890	C14442H16363N5778O10587P1518	-1519	Translation
b2890_mRNA_2	mRNA b2890	C14442H16363N5778O10587P1518	-1519	Translation
b2890_mRNA_2_degr	mRNA b2890 degradation complex	C55125H81762N17653O23272S200P1518Mg6Zn2Fe0	-1741	mRNA degradation
b2891_aa	polypeptide b2891_v1	C1784H2795N507O597S11	-28	Translation
b2891_def_cplx	Polypeptide b2891 peptide deformylase complex	C2628H4189N748O852S17Mg0Zn0Fe1	-33	Maturation
b2891_m	Matured polypeptide b2891	C1783H2796N507O596S11	-27	Maturation
b2891_mRNA	mRNA b2891	C10492H11866N4275O7637P1101	-1102	Translation
b2891_mRNA_1	mRNA b2891	C10492H11866N4275O7637P1101	-1102	Translation
b2891_mRNA_2	mRNA b2891	C10492H11866N4275O7637P1101	-1102	Translation
b2891_mRNA_2_degr	mRNA b2891 degradation complex	C51175H77265N16150O20322S200P1101Mg6Zn2Fe0	-1324	mRNA degradation
b2891_v1_mRNA	mRNA b2891_v1	C10492H11866N4275O7631P1099	-1100	Translation
b2892_aa	polypeptide b2892	C2814H4488N800O825S21	-16	Translation
b2892_def_map_cplx	Polypeptide b2892 peptide deformylase and methionine aminopeptidase complex	C4944H7944N1393O1471S42Mg0Zn0Fe3	-25	Maturation
b2892_m	Matured polypeptide b2892	C2808H4480N799O823S20	-15	Maturation
b2892_mRNA	mRNA b2892	C16503H18687N6608O12163P1734	-1735	Translation
b2892_mRNA_1	mRNA b2892	C16503H18687N6608O12163P1734	-1735	Translation
b2892_mRNA_2	mRNA b2892	C16503H18687N6608O12163P1734	-1735	Translation
b2892_mRNA_2_degr	mRNA b2892 degradation complex	C57186H84086N18483O24848S200P1734Mg6Zn2Fe0	-1957	mRNA degradation
b2893_aa	polypeptide b2893_v1	C1140H1809N296O342S16	-3	Translation
b2893_def_map_cplx	Polypeptide b2893 peptide deformylase and methionine aminopeptidase complex	C3270H5265N889O988S37Mg0Zn0Fe3	-12	Maturation
b2893_m	Matured polypeptide b2893	C1035H1651N273O318S14	-4	Maturation
b2893_mRNA_1	mRNA b2893	C6790H7658N2751O4945P713	-714	Translation
b2893_mRNA_2	mRNA b2893	C6790H7658N2751O4945P713	-714	Translation
b2893_mRNA_2_degr	mRNA b2893 degradation complex	C47473H73057N14626O17630S200P713Mg6Zn2Fe0	-936	mRNA degradation
b2893_v1_mRNA	mRNA b2893_v1	C6790H7658N2751O4945P713	-714	Translation
b2946_aa	polypeptide b2946	C1183H1925N343O356S11	-4	Translation
b2946_def_map_cplx	Polypeptide b2946 peptide deformylase and methionine aminopeptidase complex	C3313H5381N936O1002S32Mg0Zn0Fe3	-13	Maturation
b2946_m	Matured polypeptide b2946	C1177H1917N342O354S10	-3	Maturation
b2946_mRNA	mRNA b2946	C6965H7886N2783O5122P734	-735	Translation
b2946_mRNA_1	mRNA b2946	C6965H7886N2783O5122P734	-735	Translation
b2946_mRNA_2	mRNA b2946	C6965H7886N2783O5122P734	-735	Translation
b2946_mRNA_2_degr	mRNA b2946 degradation complex	C47648H73285N14658O17807S200P734Mg6Zn2Fe0	-957	mRNA degradation
b2947_aa	polypeptide b2947	C1587H2513N426O473S14	-11	Translation
b2947_def_cplx	Polypeptide b2947 peptide deformylase complex	C2431H3907N667O728S20Mg0Zn0Fe1	-16	Maturation
b2947_m	Matured polypeptide b2947	C1586H2514N426O472S14	-10	Maturation
b2947_mRNA	mRNA b2947	C9066H10257N3662O6625P951	-952	Translation
b2947_mRNA_1	mRNA b2947	C9066H10257N3662O6625P951	-952	Translation
b2947_mRNA_2	mRNA b2947	C9066H10257N3662O6625P951	-952	Translation
b2947_mRNA_2_degr	mRNA b2947 degradation complex	C49749H75656N15537O19310S200P951Mg6Zn2Fe0	-1174	mRNA degradation
b2959_aa	polypeptide b2959	C575H874N156O173S5	-8	Translation

b2959_def_map_cplx	Polypeptide b2959 peptide deformylase and methionine aminopeptidase complex	C2705H4330N749O819S26Mg0Zn0Fe3	-17	Maturation
b2959_m	Matured polypeptide b2959	C569H866N155O171S4	-7	Maturation
b2959_mRNA	mRNA b2959 (1 nt short)	C3123H3515N1284O2260P326	-327	Translation
b2959_mRNA_1	mRNA b2959 (1 nt short)	C3123H3515N1284O2260P326	-327	Translation
b2959_mRNA_2	mRNA b2959 (1 nt short)	C3123H3515N1284O2260P326	-327	Translation
b2959_mRNA_2_degr	mRNA b2959 degradation complex	C43806H68914N13159O14945S200P326Mg6Zn2Fe0	-549	mRNA degradation
b2960_aa	polypeptide b2960	C1216H1884N348O344S14	-4	Translation
b2960_def_map_cplx	Polypeptide b2960 peptide deformylase and methionine aminopeptidase complex	C3346H5340N941O990S35Mg0Zn0Fe3	-13	Maturation
b2960_m	Matured polypeptide b2960	C1210H1876N347O342S13	-3	Maturation
b2960_mRNA	mRNA b2960	C6860H7747N2746O5054P722	-723	Translation
b2960_mRNA_1	mRNA b2960	C6860H7747N2746O5054P722	-723	Translation
b2960_mRNA_2	mRNA b2960	C6860H7747N2746O5054P722	-723	Translation
b2960_mRNA_2_degr	mRNA b2960 degradation complex	C47543H73146N14621O17739S200P722Mg6Zn2Fe0	-945	mRNA degradation
b2960_m_GroEL.(7)ATP.transGroES	b2960 GroEL GroES complex - Kerner et al. class III needs GroEL/ES	C39122H64820O12676N10931S377P21Mg7	-304	Folding
b2967_RNA	tRNA (pheV)	C723H821N290O534P76	-77	tRNA Modification
b2967_tRNA_1	b2967_tRNA_1 (pheV)	C723H823Mg2N290O534P76	-73	tRNA Modification
b2967_tRNA_1_Dus_gen_cplx	b2967_tRNA_1 (pheV), Dus_gen	C2330H3362Mg2N749O1017P79S14	-80	tRNA Modification
b2967_tRNA_2	b2967_tRNA_2 (pheV)	C723H825Mg2N290O534P76	-73	tRNA Modification
b2967_tRNA_2_RluA_mono_cplx	b2967_tRNA_2 (pheV), RluA_mono	C1826H2565Mg2N603O848P76S10	-72	tRNA Modification
b2967_tRNA_3	b2967_tRNA_3 (pheV)	C723H825Mg2N290O534P76	-73	tRNA Modification
b2967_tRNA_3_MiaA_dim-MiaB_mono_cplx	b2967_tRNA_3 (pheV), MiaA_dim, MiaB_mono	C10198Fe4H15855Mg4N2965O3400P80S80X1	-118	tRNA Modification
b2967_tRNA_4	b2967_tRNA_4 (pheV)	C729H835Mg2N290O534P76S1	-73	tRNA Modification
b2967_tRNA_4_TruA_dim_cplx	b2967_tRNA_4 (pheV), TruA_dim	C3439H5037Mg2N1070O1304P76S17	-67	tRNA Modification
b2967_tRNA_5	b2967_tRNA_5 (pheV)	C729H835Mg2N290O534P76S1	-73	tRNA Modification
b2967_tRNA_5_YggH_mono_cplx	b2967_tRNA_5 (pheV), YggH_mono	C1954H2734Mg2N643O881P76S15	-75	tRNA Modification
b2967_tRNA_6	b2967_tRNA_6 (pheV)	C730H837Mg2N290O534P76S1	-73	tRNA Modification
b2967_tRNA_6_AcpT_tRNA_pos_47_acp3U_cplx	b2967_tRNA_6 (pheV), AcpT_tRNA_pos_47_acp3U	C745H860Mg2N296O539P76S2	-72	tRNA Modification
b2967_tRNA_7	b2967_tRNA_7 (pheV)	C734H844Mg2N291O536P76S1	-73	tRNA Modification
b2967_tRNA_7_TrmA_mono_cplx	b2967_tRNA_7 (pheV), TrmA_mono	C2608H3791Mg2N814O1095P76S20	-81	tRNA Modification
b2967_tRNA_8	b2967_tRNA_8 (pheV)	C735H846Mg2N291O536P76S1	-73	tRNA Modification
b2967_tRNA_8_TruB_mono_cplx	b2967_tRNA_8 (pheV), TruB_mono	C2275H3321Mg2N729O1005P76S11	-78	tRNA Modification
b2967_tRNA_9	b2967_tRNA_9 (pheV)	C735H846Mg2N291O536P76S1	-73	tRNA Modification
b2967_tRNA_9_ThiI_mono_cplx	b2967_tRNA_9 (pheV), ThiI_mono	C7163H11077Mg3N2109O2475P81S51X1	-101	tRNA Modification
b2967_tRNA_Mg2	tRNA (pheV) bound two Mg2 ions	C723H821Mg2N290O534P76	-73	tRNA Modification
b2967_tRNA_Mg2_Dus_gen_cplx	b2967_tRNA (pheV), Dus_gen	C2330H3360Mg2N749O1017P79S14	-80	tRNA Modification
b3065_aa	polypeptide b3065_v3	C372H639N126O100S2	13	Translation
b3065_def_map_cplx	Polypeptide b3065 peptide deformylase and methionine aminopeptidase complex	C2502H4095N719O746S23Mg0Zn0Fe3	4	Maturation
b3065_m	Matured polypeptide b3065	C366H631N125O98S1	14	Maturation
b3065_mRNA	mRNA b3065	C2060H2332N835O1505P218	-219	Translation
b3065_mRNA_1	mRNA b3065	C2060H2332N835O1505P218	-219	Translation
b3065_mRNA_2	mRNA b3065	C2060H2332N835O1505P218	-219	Translation
b3065_mRNA_2_degr	mRNA b3065 degradation complex	C42743H67731N12710O14190S200P218Mg6Zn2Fe0	-441	mRNA degradation
b3065_v1_mRNA	mRNA b3065_v1	C2060H2332N835O1505P218	-219	Translation
b3065_v2_mRNA	mRNA b3065_v2	C2060H2332N835O1505P218	-219	Translation
b3065_v3_mRNA	mRNA b3065_v3	C2060H2332N835O1505P218	-219	Translation
b3066_aa	polypeptide b3066_v2	C2899H4570N822O869S23	-12	Translation
b3066_def_map_cplx	Polypeptide b3066 peptide deformylase and methionine aminopeptidase complex	C5029H8026N1415O1515S44Mg0Zn0Fe3	-21	Maturation
b3066_m	Matured polypeptide b3066	C2893H4562N821O867S22	-11	Maturation
b3066_mRNA	mRNA b3066	C16617H18813N6650O12178P1746	-1747	Translation
b3066_mRNA_1	mRNA b3066	C16617H18813N6650O12178P1746	-1747	Translation
b3066_mRNA_2	mRNA b3066	C16617H18813N6650O12178P1746	-1747	Translation
b3066_mRNA_2_degr	mRNA b3066 degradation complex	C57300H84212N18525O24863S200P1746Mg6Zn2Fe0	-1969	mRNA degradation
b3066_v1_mRNA	mRNA b3066_v1	C16617H18813N6650O12178P1746	-1747	Translation
b3066_v2_mRNA	mRNA b3066_v2	C16617H18813N6650O12178P1746	-1747	Translation
b3067_aa	polypeptide b3067_v5	C3043H4866N856O994S28	-46	Translation

b3067_def_cplx	Polypeptide b3067 peptide deformylase complex	C3887H6260N1097O1249S34Mg0Zn0Fe1	-51	Maturation
b3067_m	Matured polypeptide b3067	C3042H4867N856O993S28	-45	Maturation
b3067_mRNA	mRNA b3067	C17579H19899N7164O12763P1844	-1845	Translation
b3067_mRNA_1	mRNA b3067	C17579H19899N7164O12763P1844	-1845	Translation
b3067_mRNA_2	mRNA b3067	C17579H19899N7164O12763P1844	-1845	Translation
b3067_mRNA_2_degr	mRNA b3067 degradation complex	C58262H85298N19039O25448S200P1844Mg6Zn2Fe0	-2067	mRNA degradation
b3067_m_DnaKJ_complex	b3067 DnaK DnaJ_dim complex - Deuerling et al. DnaKJ/GrpE dependent folding	C9606H15381O3091N2769S75P3Zn4	-67	Folding
b3067_v1_mRNA	mRNA b3067_v1	C17579H19899N7164O12763P1844	-1845	Translation
b3067_v2_mRNA	mRNA b3067_v2	C17579H19899N7164O12763P1844	-1845	Translation
b3067_v3_mRNA	mRNA b3067_v3	C17579H19899N7164O12757P1842	-1843	Translation
b3067_v4_mRNA	mRNA b3067_v4	C17579H19899N7164O12757P1842	-1843	Translation
b3067_v5_mRNA	mRNA b3067_v5	C17579H19899N7164O12757P1842	-1843	Translation
b3069_RNA	tRNA (ileX)	C722H821N288O534P76	-77	tRNA Modification
b3069_tRNA_1	b3069_tRNA_1 (ileX)	C723H823Mg2N288O534P76	-73	tRNA Modification
b3069_tRNA_10	b3069_tRNA_10 (ileX)	C740H856Mg2N292O541P76	-74	tRNA Modification
b3069_tRNA_10_ThiI_mono_cplx	b3069_tRNA_10 (ileX), ThiI_mono	C7168H11087Mg3N2110O2480P81S50X1	-102	tRNA Modification
b3069_tRNA_1_Dus_gen_cplx	b3069_tRNA_1 (ileX), Dus_gen	C2330H3362Mg2N747O1017P79S14	-80	tRNA Modification
b3069_tRNA_2	b3069_tRNA_2 (ileX)	C723H825Mg2N288O534P76	-73	tRNA Modification
b3069_tRNA_2_Dus_gen_cplx	b3069_tRNA_2 (ileX), Dus_gen	C2330H3364Mg2N747O1017P79S14	-80	tRNA Modification
b3069_tRNA_3	b3069_tRNA_3 (ileX)	C723H827Mg2N288O534P76	-73	tRNA Modification
b3069_tRNA_3_TilS_mono_cplx	b3069_tRNA_3 (ileX), TilS_mono	C2884H4250Mg2N919O1163P79S10	-78	tRNA Modification
b3069_tRNA_4	b3069_tRNA_4 (ileX)	C729H839Mg2N290O535P76	-73	tRNA Modification
b3069_tRNA_4_Up_tRNA_pos_37_t6A_cplx	b3069_tRNA_4 (ileX), Up_tRNA_pos_37_t6A	C744H861Mg3N296O554P79	-76	tRNA Modification
b3069_tRNA_5	b3069_tRNA_5 (ileX)	C734H845Mg2N291O539P76	-74	tRNA Modification
b3069_tRNA_5_TruA_dim_cplx	b3069_tRNA_5 (ileX), TruA_dim	C3444H5047Mg2N1071O1309P76S16	-68	tRNA Modification
b3069_tRNA_6	b3069_tRNA_6 (ileX)	C734H845Mg2N291O539P76	-74	tRNA Modification
b3069_tRNA_6_YggH_mono_cplx	b3069_tRNA_6 (ileX), YggH_mono	C1959H2744Mg2N644O886P76S14	-76	tRNA Modification
b3069_tRNA_7	b3069_tRNA_7 (ileX)	C735H847Mg2N291O539P76	-74	tRNA Modification
b3069_tRNA_7_AcpT_tRNA_pos_47_acp3U_cplx	b3069_tRNA_7 (ileX), AcpT_tRNA_pos_47_acp3U	C750H870Mg2N297O544P76S1	-73	tRNA Modification
b3069_tRNA_8	b3069_tRNA_8 (ileX)	C739H854Mg2N292O541P76	-74	tRNA Modification
b3069_tRNA_8_TrmA_mono_cplx	b3069_tRNA_8 (ileX), TrmA_mono	C2613H3801Mg2N815O1100P76S19	-82	tRNA Modification
b3069_tRNA_9	b3069_tRNA_9 (ileX)	C740H856Mg2N292O541P76	-74	tRNA Modification
b3069_tRNA_9_TruB_mono_cplx	b3069_tRNA_9 (ileX), TruB_mono	C2280H3331Mg2N730O1010P76S10	-79	tRNA Modification
b3069_tRNA_Mg2	tRNA (ileX) bound two Mg2 ions	C722H821Mg2N288O534P76	-73	tRNA Modification
b3069_tRNA_Mg2_TrmH_dim_cplx	b3069_tRNA (ileX), TrmH_dim	C2933H4364Mg2N944O1195P76S29	-74	tRNA Modification
b3123_RNA	misc_RNA	C3612H4091N1512O2610P377	-378	RNA cutting
b3123_v1_RNA	misc_RNA	C3612H4091N1512O2604P375	-376	RNA cutting
b3123_v1_RNA_cut_cplx	b3123_v1 RNA /RNase P (5' trimming), RNase_Gen (T, PH, II, D, or BN), RNase E (3' trimming) cutting complex	C30875H46200N10138O12607S93P754Mg5Zn3Fe0	-867	RNA cutting
b3123_v1_RNA_pre	misc_RNA pre	C3612H4091N1512O2610P377	-378	RNA cutting
b3124_aa	polypeptide b3124	C1834H2957N523O581S15	-14	Translation
b3124_def_map_cplx	Polypeptide b3124 peptide deformylase and methionine aminopeptidase complex	C3964H6413N1116O1227S36Mg0Zn0Fe3	-23	Maturation
b3124_m	Matured polypeptide b3124	C1701H2734N477O544S12	-17	Maturation
b3124_mRNA	mRNA b3124	C11723H13226N4769O8568P1227	-1228	Translation
b3124_mRNA_1	mRNA b3124	C11723H13226N4769O8568P1227	-1228	Translation
b3124_mRNA_2	mRNA b3124	C11723H13226N4769O8568P1227	-1228	Translation
b3124_mRNA_2_degr	mRNA b3124 degradation complex	C52406H78625N16644O21253S200P1227Mg6Zn2Fe0	-1450	mRNA degradation
b3125_aa	polypeptide b3125	C1348H2218N362O413S18	-6	Translation
b3125_def_map_cplx	Polypeptide b3125 peptide deformylase and methionine aminopeptidase complex	C3478H5674N955O1059S39Mg0Zn0Fe3	-15	Maturation
b3125_m	Matured polypeptide b3125	C1333H2194N359O408S16	-5	Maturation
b3125_mRNA	mRNA b3125	C8516H9606N3471O6210P891	-892	Translation
b3125_mRNA_1	mRNA b3125	C8516H9606N3471O6210P891	-892	Translation
b3125_mRNA_2	mRNA b3125	C8516H9606N3471O6210P891	-892	Translation
b3125_mRNA_2_degr	mRNA b3125 degradation complex	C49199H75005N15346O18895S200P891Mg6Zn2Fe0	-1114	mRNA degradation
b3126_aa	polypeptide b3126	C1235H1925N333O365S4	-6	Translation
b3126_def_cplx	Polypeptide b3126 peptide deformylase complex	C2079H3319N574O620S10Mg0Zn0Fe1	-11	Maturation
b3126_m	Matured polypeptide b3126	C1234H1926N333O364S4	-5	Maturation

b3126_mRNA	mRNA b3126	C7324H8302N2903O5395P771	-772	Translation
b3126_mRNA_1	mRNA b3126	C7324H8302N2903O5395P771	-772	Translation
b3126_mRNA_2	mRNA b3126	C7324H8302N2903O5395P771	-772	Translation
b3126_mRNA_2_degr	mRNA b3126 degradation complex	C48007H73701N14778O18080S200P771Mg6Zn2Fe0	-994	mRNA degradation
b3127_aa	polypeptide b3127	C2315H3540N543O581S24	7	Translation
b3127_def_map_cplx	Polypeptide b3127 peptide deformylase and methionine aminopeptidase complex	C4445H6996N1136O1227S45Mg0Zn0Fe3	-2	Maturation
b3127_m	Matured polypeptide b3127	C2309H3532N542O579S23	8	Maturation
b3127_mRNA	mRNA b3127	C12632H14301N4854O9472P1337	-1338	Translation
b3127_mRNA_1	mRNA b3127	C12632H14301N4854O9472P1337	-1338	Translation
b3127_mRNA_2	mRNA b3127	C12632H14301N4854O9472P1337	-1338	Translation
b3127_mRNA_2_degr	mRNA b3127 degradation complex	C53315H79700N16729O22157S200P1337Mg6Zn2Fe0	-1560	mRNA degradation
b3164_aa	polypeptide b3164_v3	C3502H5687N997O1085S23	-18	Translation
b3164_def_cplx	Polypeptide b3164 peptide deformylase complex	C4346H7081N1238O1340S29Mg0Zn0Fe1	-23	Maturation
b3164_m	Matured polypeptide b3164	C3385H5475N953O1053S22	-24	Maturation
b3164_mRNA	mRNA b3164	C21001H23766N8437O15395P2205	-2206	Translation
b3164_mRNA_1	mRNA b3164	C21001H23766N8437O15395P2205	-2206	Translation
b3164_mRNA_2	mRNA b3164	C21001H23766N8437O15395P2205	-2206	Translation
b3164_mRNA_2_degr	mRNA b3164 degradation complex	C61684H89165N20312O28080S200P2205Mg6Zn2Fe0	-2428	mRNA degradation
b3164_m_DnaKJ_complex	b3164 DnaK DnaJ_dim complex - Kerner et al. class I can interact w/ GroEL/ES	C9949H15989O3151N2866S69P3Zn4	-46	Folding
b3164_m_GroEL.(7)ATP.transGroES	b3164 GroEL GroES complex - Kerner et al. class I can interact w/ GroEL/ES	C41297H68419O13387N11537S386P21Mg7	-325	Folding
b3164_v1_mRNA	mRNA b3164_v1	C21001H23766N8437O15395P2205	-2206	Translation
b3164_v2_mRNA	mRNA b3164_v2	C21001H23766N8437O15401P2207	-2208	Translation
b3164_v3_mRNA	mRNA b3164_v3	C21001H23766N8437O15395P2205	-2206	Translation
b3165_aa	polypeptide b3165_v3	C445H745N145O132S2	6	Translation
b3165_def_map_cplx	Polypeptide b3165 peptide deformylase and methionine aminopeptidase complex	C2575H4201N738O778S23Mg0Zn0Fe3	-3	Maturation
b3165_m	Matured polypeptide b3165	C439H737N144O130S1	7	Maturation
b3165_mRNA	mRNA b3165	C2565H2912N1021O1879P270	-271	Translation
b3165_mRNA_1	mRNA b3165	C2565H2912N1021O1879P270	-271	Translation
b3165_mRNA_2	mRNA b3165	C2565H2912N1021O1879P270	-271	Translation
b3165_mRNA_2_degr	mRNA b3165 degradation complex	C43248H68311N12896O14564S200P270Mg6Zn2Fe0	-493	mRNA degradation
b3165_v1_mRNA	mRNA b3165_v1	C2565H2912N1021O1885P272	-273	Translation
b3165_v2_mRNA	mRNA b3165_v2	C2565H2912N1021O1885P272	-273	Translation
b3165_v3_mRNA	mRNA b3165_v3	C2565H2912N1021O1879P270	-271	Translation
b3166_aa	polypeptide b3166_v1	C1546H2483N439O471S11	-6	Translation
b3166_def_map_cplx	Polypeptide b3166 peptide deformylase and methionine aminopeptidase complex	C3676H5939N1032O1117S32Mg0Zn0Fe3	-15	Maturation
b3166_m	Matured polypeptide b3166	C1540H2475N438O469S10	-5	Maturation
b3166_mRNA	mRNA b3166 (1 nt short)	C8981H10157N3574O6617P944	-945	Translation
b3166_mRNA_1	mRNA b3166 (1 nt short)	C8981H10157N3574O6617P944	-945	Translation
b3166_mRNA_2	mRNA b3166 (1 nt short)	C8981H10157N3574O6617P944	-945	Translation
b3166_mRNA_2_degr	mRNA b3166 degradation complex	C49664H75556N15449O19302S200P944Mg6Zn2Fe0	-1167	mRNA degradation
b3166_v1_mRNA	mRNA b3166_v1 (1 nt short)	C8981H10157N3574O6617P944	-945	Translation
b3167_aa	polypeptide b3167_v1	C662H1081N187O204S8	-2	Translation
b3167_def_map_cplx	Polypeptide b3167 peptide deformylase and methionine aminopeptidase complex	C2792H4537N780O850S29Mg0Zn0Fe3	-11	Maturation
b3167_m	Matured polypeptide b3167	C656H1073N186O202S7	-1	Maturation
b3167_mRNA	mRNA b3167	C3838H4341N1564O2790P402	-403	Translation
b3167_mRNA_1	mRNA b3167	C3838H4341N1564O2790P402	-403	Translation
b3167_mRNA_2	mRNA b3167	C3838H4341N1564O2790P402	-403	Translation
b3167_mRNA_2_degr	mRNA b3167 degradation complex	C44521H69740N13439O15475S200P402Mg6Zn2Fe0	-625	mRNA degradation
b3167_v1_mRNA	mRNA b3167_v1	C3838H4341N1564O2790P402	-403	Translation
b3168_aa	polypeptide b3168_v3	C4181H6889N1264O1358S24	-15	Translation
b3168_def_cplx	Polypeptide b3168 peptide deformylase complex	C5025H8283N1505O1613S30Mg0Zn0Fe1	-20	Maturation
b3168_m	matured polypeptide b3168 _ error CCDB	C4180H6890N1264O1357S24	-14	Maturation
b3168_mRNA	mRNA b3168	C25568H28889N10526O18497P2673	-2674	Translation
b3168_mRNA_1	mRNA b3168	C25568H28889N10526O18497P2673	-2674	Translation
b3168_mRNA_2	mRNA b3168	C25568H28889N10526O18497P2673	-2674	Translation
b3168_mRNA_2_degr	mRNA b3168 degradation complex	C66251H94288N22401O31182S200P2673Mg6Zn2Fe0	-2896	mRNA degradation

b3168_m_DnaKJ_complex	b3168 DnaK DnaJ_dim complex - Kerner et al. class II can interact w/ GroEL/ES, cannot fold spontanously	C10744H17404O3455N3177S71P3Zn4	-36	Folding
b3168_m_GroEL.(7)ATP.transGroES	b3168 GroEL GroES complex - Kerner et al. class II can interact w/ GroEL/ES, cannot fold spontanously	C42092H69834O13691N11848S388P21Mg7	-315	Folding
b3168_v1_mRNA	mRNA b3168_v1	C25568H28889N10526O18497P2673	-2674	Translation
b3168_v2_mRNA	mRNA b3168_v2	C25568H28889N10526O18497P2673	-2674	Translation
b3168_v3_mRNA	mRNA b3168_v3	C25568H28889N10526O18497P2673	-2674	Translation
b3169_aa	polypeptide b3169_v3	C2397H3834N669O776S13	-41	Translation
b3169_def_cplx	Polypeptide b3169 peptide deformylase complex	C3241H5228N910O1031S19Mg0Zn0Fe1	-46	Maturation
b3169_m	Matured polypeptide b3169	C2396H3835N669O775S13	-40	Maturation
b3169_mRNA	mRNA b3169	C14212H16053N5788O10346P1488	-1489	Translation
b3169_mRNA_1	mRNA b3169	C14212H16053N5788O10346P1488	-1489	Translation
b3169_mRNA_2	mRNA b3169	C14212H16053N5788O10346P1488	-1489	Translation
b3169_mRNA_2_degr	mRNA b3169 degradation complex	C54895H81452N17663O23031S200P1488Mg6Zn2Fe0	-1711	mRNA degradation
b3169_v1_mRNA	mRNA b3169_v1	C14212H16053N5788O10346P1488	-1489	Translation
b3169_v2_mRNA	mRNA b3169_v2	C14212H16053N5788O10346P1488	-1489	Translation
b3169_v3_mRNA	mRNA b3169_v3	C14212H16053N5788O10346P1488	-1489	Translation
b3170_aa	polypeptide b3170_v3	C740H1168N196O231S5	-12	Translation
b3170_def_map_cplx	Polypeptide b3170 peptide deformylase and methionine aminopeptidase complex	C2870H4624N789O877S26Mg0Zn0Fe3	-21	Maturation
b3170_m	Matured polypeptide b3170	C685H1077N183O211S3	-10	Maturation
b3170_mRNA	mRNA b3170	C4310H4865N1706O3177P453	-454	Translation
b3170_mRNA_1	mRNA b3170	C4310H4865N1706O3177P453	-454	Translation
b3170_mRNA_2	mRNA b3170	C4310H4865N1706O3177P453	-454	Translation
b3170_mRNA_2_degr	mRNA b3170 degradation complex	C44993H70264N13581O15862S200P453Mg6Zn2Fe0	-676	mRNA degradation
b3170_v1_mRNA	mRNA b3170_v1	C4310H4865N1706O3177P453	-454	Translation
b3170_v2_mRNA	mRNA b3170_v2	C4310H4865N1706O3183P455	-456	Translation
b3170_v3_mRNA	mRNA b3170_v3	C4310H4865N1706O3177P453	-454	Translation
b3171_RNA	tRNA (metY)	C732H836N297O537P77	-78	tRNA Modification
b3171_tRNA_1	b3171_tRNA_1 (metY)	C732H838Mg2N297O537P77	-74	tRNA Modification
b3171_tRNA_1_MeT_tRNA_pos_32_Cm_cplx	b3171_tRNA_1 (metY), MeT_tRNA_pos_32_Cm	C747H861Mg2N303O542P77S1	-73	tRNA Modification
b3171_tRNA_2	b3171_tRNA_2 (metY)	C733H840Mg2N297O537P77	-74	tRNA Modification
b3171_tRNA_2_TrmA_mono_cplx	b3171_tRNA_2 (metY), TrmA_mono	C2607H3787Mg2N820O1096P77S19	-82	tRNA Modification
b3171_tRNA_3	b3171_tRNA_3 (metY)	C734H842Mg2N297O537P77	-74	tRNA Modification
b3171_tRNA_3_TruB_mono_cplx	b3171_tRNA_3 (metY), TruB_mono	C2274H3317Mg2N735O1006P77S10	-79	tRNA Modification
b3171_tRNA_4	b3171_tRNA_4 (metY)	C734H842Mg2N297O537P77	-74	tRNA Modification
b3171_tRNA_4_ThiI_mono_cplx	b3171_tRNA_4 (metY), ThiI_mono	C7162H11073Mg3N2115O2476P82S50X1	-102	tRNA Modification
b3171_tRNA_Mg2	tRNA (metY) bound two Mg2 ions	C732H836Mg2N297O537P77	-74	tRNA Modification
b3171_tRNA_Mg2_DusA_mono_cplx	b3171_tRNA (metY), DusA_mono	C2372H3400Mg2N763O1033P80S18	-83	tRNA Modification
b3171_v1_RNA	tRNA (metY)	C732H836N297O537P77	-78	tRNA Modification
b3171_v1_RNA_cut_cplx	b3171_v1 RNA /RNase P (5' trimming), RNase_Gen (T, PH, II, D, or BN), RNase E (3' trimming) cutting complex	C27995H42945N8923O10540S93P456Mg5Zn3Fe0	-569	RNA cutting
b3171_v1_RNA_pre	tRNA pre	C732H836N297O543P79	-80	RNA cutting
b3171_v1_tRNA_1	b3171_v1_tRNA_1 (metY)	C732H838Mg2N297O537P77	-74	tRNA Modification
b3171_v1_tRNA_1_MeT_tRNA_pos_32_Cm_cplx	b3171_v1_tRNA_1 (metY), MeT_tRNA_pos_32_Cm	C747H861Mg2N303O542P77S1	-73	tRNA Modification
b3171_v1_tRNA_2	b3171_v1_tRNA_2 (metY)	C733H840Mg2N297O537P77	-74	tRNA Modification
b3171_v1_tRNA_2_TrmA_mono_cplx	b3171_v1_tRNA_2 (metY), TrmA_mono	C2607H3787Mg2N820O1096P77S19	-82	tRNA Modification
b3171_v1_tRNA_3	b3171_v1_tRNA_3 (metY)	C734H842Mg2N297O537P77	-74	tRNA Modification
b3171_v1_tRNA_3_TruB_mono_cplx	b3171_v1_tRNA_3 (metY), TruB_mono	C2274H3317Mg2N735O1006P77S10	-79	tRNA Modification
b3171_v1_tRNA_4	b3171_v1_tRNA_4 (metY)	C734H842Mg2N297O537P77	-74	tRNA Modification
b3171_v1_tRNA_4_ThiI_mono_cplx	b3171_v1_tRNA_4 (metY), ThiI_mono	C7162H11073Mg3N2115O2476P82S50X1	-102	tRNA Modification
b3171_v1_tRNA_Mg2	tRNA (metY) bound two Mg2 ions	C732H836Mg2N297O537P77	-74	tRNA Modification
b3171_v1_tRNA_Mg2_DusA_mono_cplx	b3171_v1_tRNA (metY), DusA_mono	C2372H3400Mg2N763O1033P80S18	-83	tRNA Modification
b3171_v2_RNA	tRNA (metY)	C732H836N297O537P77	-78	tRNA Modification
b3171_v2_RNA_cut_cplx	b3171_v2 RNA /RNase P (5' trimming), RNase_Gen (T, PH, II, D, or BN), RNase E (3' trimming) cutting complex	C27995H42945N8923O10540S93P456Mg5Zn3Fe0	-569	RNA cutting
b3171_v2_RNA_pre	tRNA pre	C732H836N297O543P79	-80	RNA cutting
b3171_v2_tRNA_1	b3171_v2_tRNA_1 (metY)	C732H838Mg2N297O537P77	-74	tRNA Modification
b3171_v2_tRNA_1_MeT_tRNA_pos_32_Cm_cplx	b3171_v2_tRNA_1 (metY), MeT_tRNA_pos_32_Cm	C747H861Mg2N303O542P77S1	-73	tRNA Modification

b3171_v2_tRNA_2	b3171_v2_tRNA_2 (metY)	C733H840Mg2N297O537P77	-74	tRNA Modification
b3171_v2_tRNA_2_TrmA_mono_cplx	b3171_v2_tRNA_2 (metY), TrmA_mono	C2607H3787Mg2N820O1096P77S19	-82	tRNA Modification
b3171_v2_tRNA_3	b3171_v2_tRNA_3 (metY)	C734H842Mg2N297O537P77	-74	tRNA Modification
b3171_v2_tRNA_3_TruB_mono_cplx	b3171_v2_tRNA_3 (metY), TruB_mono	C2274H3317Mg2N735O1006P77S10	-79	tRNA Modification
b3171_v2_tRNA_4	b3171_v2_tRNA_4 (metY)	C734H842Mg2N297O537P77	-74	tRNA Modification
b3171_v2_tRNA_4_ThiI_mono_cplx	b3171_v2_tRNA_4 (metY), ThiI_mono	C7162H11073Mg3N2115O2476P82S50X1	-102	tRNA Modification
b3171_v2_tRNA_Mg2	tRNA (metY) bound two Mg2 ions	C732H836Mg2N297O537P77	-74	tRNA Modification
b3171_v2_tRNA_Mg2_DusA_mono_cplx	b3171_v2_tRNA (metY), DusA_mono	C2372H3400Mg2N763O1033P80S18	-83	tRNA Modification
b3171_v3_RNA	tRNA (metY)	C732H836N297O537P77	-78	tRNA Modification
b3171_v3_tRNA_1	b3171_v3_tRNA_1 (metY)	C732H838Mg2N297O537P77	-74	tRNA Modification
b3171_v3_tRNA_1_MeT_tRNA_pos_32_Cm_cplx	b3171_v3_tRNA_1 (metY), MeT_tRNA_pos_32_Cm	C747H861Mg2N303O542P77S1	-73	tRNA Modification
b3171_v3_tRNA_2	b3171_v3_tRNA_2 (metY)	C733H840Mg2N297O537P77	-74	tRNA Modification
b3171_v3_tRNA_2_TrmA_mono_cplx	b3171_v3_tRNA_2 (metY), TrmA_mono	C2607H3787Mg2N820O1096P77S19	-82	tRNA Modification
b3171_v3_tRNA_3	b3171_v3_tRNA_3 (metY)	C734H842Mg2N297O537P77	-74	tRNA Modification
b3171_v3_tRNA_3_TruB_mono_cplx	b3171_v3_tRNA_3 (metY), TruB_mono	C2274H3317Mg2N735O1006P77S10	-79	tRNA Modification
b3171_v3_tRNA_4	b3171_v3_tRNA_4 (metY)	C734H842Mg2N297O537P77	-74	tRNA Modification
b3171_v3_tRNA_4_ThiI_mono_cplx	b3171_v3_tRNA_4 (metY), ThiI_mono	C7162H11073Mg3N2115O2476P82S50X1	-102	tRNA Modification
b3171_v3_tRNA_Mg2	tRNA (metY) bound two Mg2 ions	C732H836Mg2N297O537P77	-74	tRNA Modification
b3171_v3_tRNA_Mg2_DusA_mono_cplx	b3171_v3_tRNA (metY), DusA_mono	C2372H3400Mg2N763O1033P80S18	-83	tRNA Modification
b3171_v4_RNA	tRNA (metY)	C732H836N297O537P77	-78	tRNA Modification
b3171_v4_RNA_cut_cplx	b3171_v4 RNA /RNase P (5' trimming), RNase_Gen (T, PH, II, D, or BN), RNase E (3' trimming) cutting complex	C27995H42945N8923O10540S93P456Mg5Zn3Fe0	-569	RNA cutting
b3171_v4_RNA_pre	tRNA pre	C732H836N297O543P79	-80	RNA cutting
b3171_v4_tRNA_1	b3171_v4_tRNA_1 (metY)	C732H838Mg2N297O537P77	-74	tRNA Modification
b3171_v4_tRNA_1_MeT_tRNA_pos_32_Cm_cplx	b3171_v4_tRNA_1 (metY), MeT_tRNA_pos_32_Cm	C747H861Mg2N303O542P77S1	-73	tRNA Modification
b3171_v4_tRNA_2	b3171_v4_tRNA_2 (metY)	C733H840Mg2N297O537P77	-74	tRNA Modification
b3171_v4_tRNA_2_TrmA_mono_cplx	b3171_v4_tRNA_2 (metY), TrmA_mono	C2607H3787Mg2N820O1096P77S19	-82	tRNA Modification
b3171_v4_tRNA_3	b3171_v4_tRNA_3 (metY)	C734H842Mg2N297O537P77	-74	tRNA Modification
b3171_v4_tRNA_3_TruB_mono_cplx	b3171_v4_tRNA_3 (metY), TruB_mono	C2274H3317Mg2N735O1006P77S10	-79	tRNA Modification
b3171_v4_tRNA_4	b3171_v4_tRNA_4 (metY)	C734H842Mg2N297O537P77	-74	tRNA Modification
b3171_v4_tRNA_4_ThiI_mono_cplx	b3171_v4_tRNA_4 (metY), ThiI_mono	C7162H11073Mg3N2115O2476P82S50X1	-102	tRNA Modification
b3171_v4_tRNA_Mg2	tRNA (metY) bound two Mg2 ions	C732H836Mg2N297O537P77	-74	tRNA Modification
b3171_v4_tRNA_Mg2_DusA_mono_cplx	b3171_v4_tRNA (metY), DusA_mono	C2372H3400Mg2N763O1033P80S18	-83	tRNA Modification
b3174_RNA	tRNA (leuU)	C829H938N333O613P87	-88	tRNA Modification
b3174_tRNA_1	b3174_tRNA_1 (leuU)	C829H940Mg2N333O613P87	-84	tRNA Modification
b3174_tRNA_1_Dus_gen_cplx	b3174_tRNA_1 (leuU), Dus_gen	C2436H3479Mg2N792O1096P90S14	-91	tRNA Modification
b3174_tRNA_2	b3174_tRNA_2 (leuU)	C829H942Mg2N333O613P87	-84	tRNA Modification
b3174_tRNA_2_TrmH_dim_cplx	b3174_tRNA_2 (leuU), TrmH_dim	C3040H4485Mg2N989O1274P87S29	-85	tRNA Modification
b3174_tRNA_3	b3174_tRNA_3 (leuU)	C830H944Mg2N333O613P87	-84	tRNA Modification
b3174_tRNA_3_Dus_gen_cplx	b3174_tRNA_3 (leuU), Dus_gen	C2437H3483Mg2N792O1096P90S14	-91	tRNA Modification
b3174_tRNA_4	b3174_tRNA_4 (leuU)	C830H946Mg2N333O613P87	-84	tRNA Modification
b3174_tRNA_4_TruA_dim_cplx	b3174_tRNA_4 (leuU), TruA_dim	C3540H5148Mg2N1113O1383P87S16	-78	tRNA Modification
b3174_tRNA_5	b3174_tRNA_5 (leuU)	C830H946Mg2N333O613P87	-84	tRNA Modification
b3174_tRNA_5_TrmA_mono_cplx	b3174_tRNA_5 (leuU), TrmA_mono	C2704H3893Mg2N856O1172P87S19	-92	tRNA Modification
b3174_tRNA_6	b3174_tRNA_6 (leuU)	C831H948Mg2N333O613P87	-84	tRNA Modification
b3174_tRNA_6_TruB_mono_cplx	b3174_tRNA_6 (leuU), TruB_mono	C2371H3423Mg2N771O1082P87S10	-89	tRNA Modification
b3174_tRNA_Mg2	tRNA (leuU) bound two Mg2 ions	C829H938Mg2N333O613P87	-84	tRNA Modification
b3174_tRNA_Mg2_Dus_gen_cplx	b3174_tRNA (leuU), Dus_gen	C2436H3477Mg2N792O1096P90S14	-91	tRNA Modification
b3178_aa	polypeptide b3178_v1	C3121H5005N881O937S27	-8	Translation
b3178_def_map_cplx	Polypeptide b3178 peptide deformylase and methionine aminopeptidase complex	C5251H8461N1474O1583S48Mg0Zn0Fe3	-17	Maturation
b3178_m	Matured polypeptide b3178	C3115H4997N880O935S26	-7	Maturation
b3178_mRNA	mRNA b3178	C18438H20850N7415O13520P1935	-1936	Translation
b3178_mRNA_1	mRNA b3178	C18438H20850N7415O13520P1935	-1936	Translation
b3178_mRNA_2	mRNA b3178	C18438H20850N7415O13520P1935	-1936	Translation
b3178_mRNA_2_degr	mRNA b3178 degradation complex	C59121H86249N19290O26205S200P1935Mg6Zn2Fe0	-2158	mRNA degradation
b3178_v1_mRNA	mRNA b3178_v1	C18438H20850N7415O13520P1935	-1936	Translation
b3179_aa	polypeptide b3179_v1	C1038H1674N290O300S11	6	Translation

b3179_def_map_cplx	Polypeptide b3179 peptide deformylase and methionine aminopeptidase complex	C3168H5130N883O946S32Mg0Zn0Fe3	-3	Maturation
b3179_m	Matured polypeptide b3179	C1032H1666N289O298S10	7	Maturation
b3179_mRNA	mRNA b3179	C6002H6770N2393O4427P632	-633	Translation
b3179_mRNA_1	mRNA b3179	C6002H6770N2393O4427P632	-633	Translation
b3179_mRNA_2	mRNA b3179	C6002H6770N2393O4427P632	-633	Translation
b3179_mRNA_2_degr	mRNA b3179 degradation complex	C46685H72169N14268O17112S200P632Mg6Zn2Fe0	-855	mRNA degradation
b3179_v1_mRNA	mRNA b3179_v1	C6002H6770N2393O4427P632	-633	Translation
b3181_aa	polypeptide b3181	C773H1239N215O249S4	-12	Translation
b3181_def_cplx	Polypeptide b3181 peptide deformylase complex	C1617H2633N456O504S10Mg0Zn0Fe1	-17	Maturation
b3181_m	Matured polypeptide b3181	C772H1240N215O248S4	-11	Maturation
b3181_mRNA	mRNA b3181	C4556H5138N1847O3320P479	-480	Translation
b3181_mRNA_1	mRNA b3181	C4556H5138N1847O3320P479	-480	Translation
b3181_mRNA_2	mRNA b3181	C4556H5138N1847O3320P479	-480	Translation
b3181_mRNA_2_degr	mRNA b3181 degradation complex	C45239H70537N13722O16005S200P479Mg6Zn2Fe0	-702	mRNA degradation
b3181_m_DnaKJ_complex	b3181 DnaK DnaJ_dim complex - Kerner et al. class II can interact w/ GroEL/ES, cannot fold spontanously	C7336H11754O2346N2128S51P3Zn4	-33	Folding
b3181_m_GroEL.(7)ATP.transGroES	b3181 GroEL GroES complex - Kerner et al. class II can interact w/ GroEL/ES, cannot fold spontanously	C38684H64184O12582N10799S368P21Mg7	-312	Folding
b3185_aa	polypeptide b3185	C397H664N130O115S2	10	Translation
b3185_def_map_cplx	Polypeptide b3185 peptide deformylase and methionine aminopeptidase complex	C2527H4120N723O761S23Mg0Zn0Fe3	1	Maturation
b3185_m	Matured polypeptide b3185	C391H656N129O113S1	11	Maturation
b3185_mRNA	mRNA b3185	C2463H2782N999O1792P258	-259	Translation
b3185_mRNA_1	mRNA b3185	C2463H2782N999O1792P258	-259	Translation
b3185_mRNA_2	mRNA b3185	C2463H2782N999O1792P258	-259	Translation
b3185_mRNA_2_degr	mRNA b3185 degradation complex	C43146H68181N12874O14477S200P258Mg6Zn2Fe0	-481	mRNA degradation
b3186_aa	polypeptide b3186	C517H838N153O146S2	5	Translation
b3186_def_cplx	Polypeptide b3186 peptide deformylase complex	C1361H2232N394O401S8Mg0Zn0Fe1	0	Maturation
b3186_m	Matured polypeptide b3186	C516H839N153O145S2	6	Maturation
b3186_mRNA	mRNA b3186	C2981H3358N1207O2180P314	-315	Translation
b3186_mRNA_1	mRNA b3186	C2981H3358N1207O2180P314	-315	Translation
b3186_mRNA_2	mRNA b3186	C2981H3358N1207O2180P314	-315	Translation
b3186_mRNA_2_degr	mRNA b3186 degradation complex	C43664H68757N13082O14865S200P314Mg6Zn2Fe0	-537	mRNA degradation
b3201_aa	polypeptide b3201	C1180H1910N344O357S6	-8	Translation
b3201_def_map_cplx	Polypeptide b3201 peptide deformylase and methionine aminopeptidase complex	C3310H5366N937O1003S27Mg0Zn0Fe3	-17	Maturation
b3201_m	Matured polypeptide b3201 _ error CCDB	C1174H1902N343O355S5	-7	Maturation
b3201_mRNA	mRNA b3201	C6904H7826N2757O5080P728	-729	Translation
b3201_mRNA_1	mRNA b3201	C6904H7826N2757O5080P728	-729	Translation
b3201_mRNA_2	mRNA b3201	C6904H7826N2757O5080P728	-729	Translation
b3201_mRNA_2_degr	mRNA b3201 degradation complex	C47587H73225N14632O17765S200P728Mg6Zn2Fe0	-951	mRNA degradation
b3202_aa	polypeptide b3202_v2	C2366H3757N649O767S13	-34	Translation
b3202_def_map_cplx	Polypeptide b3202 peptide deformylase and methionine aminopeptidase complex	C4496H7213N1242O1413S34Mg0Zn0Fe3	-43	Maturation
b3202_m	Matured polypeptide b3202	C2360H3749N648O765S12	-33	Maturation
b3202_mRNA	mRNA b3202	C13659H15476N5509O9967P1436	-1437	Translation
b3202_mRNA_1	mRNA b3202	C13659H15476N5509O9967P1436	-1437	Translation
b3202_mRNA_2	mRNA b3202	C13659H15476N5509O9967P1436	-1437	Translation
b3202_mRNA_2_degr	mRNA b3202 degradation complex	C54342H80875N17384O22652S200P1436Mg6Zn2Fe0	-1659	mRNA degradation
b3202_v1_mRNA	mRNA b3202_v1	C13659H15476N5509O9967P1436	-1437	Translation
b3202_v2_mRNA	mRNA b3202_v2	C13659H15476N5509O9961P1434	-1435	Translation
b3203_aa	polypeptide b3203_v1	C477H764N134O146S2	-2	Translation
b3203_def_cplx	Polypeptide b3203 peptide deformylase complex	C1321H2158N375O401S8Mg0Zn0Fe1	-7	Maturation
b3203_m	Matured polypeptide b3203	C476H765N134O145S2	-1	Maturation
b3203_mRNA	mRNA b3203	C2751H3106N1119O1984P288	-289	Translation
b3203_mRNA_1	mRNA b3203	C2751H3106N1119O1984P288	-289	Translation
b3203_mRNA_2	mRNA b3203	C2751H3106N1119O1984P288	-289	Translation
b3203_mRNA_2_degr	mRNA b3203 degradation complex	C43434H68505N12994O14669S200P288Mg6Zn2Fe0	-511	mRNA degradation
b3203_v1_mRNA	mRNA b3203_v1	C2751H3106N1119O1984P288	-289	Translation

b3204_aa	polypeptide b3204_v1	C785H1292N223O248S5	-5	Translation
b3204_def_map_cplx	Polypeptide b3204 peptide deformylase and methionine aminopeptidase complex	C2915H4748N816O894S26Mg0Zn0Fe3	-14	Maturation
b3204_m	Matured polypeptide b3204	C779H1284N222O246S4	-4	Maturation
b3204_mRNA	mRNA b3204	C4686H5309N1888O3418P492	-493	Translation
b3204_mRNA_1	mRNA b3204	C4686H5309N1888O3418P492	-493	Translation
b3204_mRNA_2	mRNA b3204	C4686H5309N1888O3418P492	-493	Translation
b3204_mRNA_2_degr	mRNA b3204 degradation complex	C45369H70708N13763O16103S200P492Mg6Zn2Fe0	-715	mRNA degradation
b3204_v1_mRNA	mRNA b3204_v1	C4686H5309N1888O3418P492	-493	Translation
b3205_aa	polypeptide b3205_v1	C1441H2295N409O424S12	-2	Translation
b3205_def_map_cplx	Polypeptide b3205 peptide deformylase and methionine aminopeptidase complex	C3571H5751N1002O1070S33Mg0Zn0Fe3	-11	Maturation
b3205_m	Matured polypeptide b3205	C1435H2287N408O422S11	-1	Maturation
b3205_mRNA	mRNA b3205	C8106H9195N3176O5999P855	-856	Translation
b3205_mRNA_1	mRNA b3205	C8106H9195N3176O5999P855	-856	Translation
b3205_mRNA_2	mRNA b3205	C8106H9195N3176O5999P855	-856	Translation
b3205_mRNA_2_degr	mRNA b3205 degradation complex	C48789H74594N15051O18684S200P855Mg6Zn2Fe0	-1078	mRNA degradation
b3205_v1_mRNA	mRNA b3205_v1	C8106H9195N3176O5999P855	-856	Translation
b3206_aa	polypeptide b3206	C427H678N113O142S5	-11	Translation
b3206_def_map_cplx	Polypeptide b3206 peptide deformylase and methionine aminopeptidase complex	C2557H4134N706O788S26Mg0Zn0Fe3	-20	Maturation
b3206_m	Matured polypeptide b3206	C421H670N112O140S4	-10	Maturation
b3206_mRNA	mRNA b3206 (4 nt short)	C2567H2896N1035O1876P269	-270	Translation
b3206_mRNA_1	mRNA b3206 (4 nt short)	C2567H2896N1035O1876P269	-270	Translation
b3206_mRNA_2	mRNA b3206 (4 nt short)	C2567H2896N1035O1876P269	-270	Translation
b3206_mRNA_2_degr	mRNA b3206 degradation complex	C43250H68295N12910O14561S200P269Mg6Zn2Fe0	-492	mRNA degradation
b3230_aa	polypeptide b3230	C648H1092N209O185S4	15	Translation
b3230_def_map_cplx	Polypeptide b3230 peptide deformylase and methionine aminopeptidase complex	C2778H4548N802O831S25Mg0Zn0Fe3	6	Maturation
b3230_m	Matured polypeptide b3230	C642H1084N208O183S3	16	Maturation
b3230_mRNA	mRNA b3230	C3734H4230N1479O2753P393	-394	Translation
b3230_mRNA_1	mRNA b3230	C3734H4230N1479O2753P393	-394	Translation
b3230_mRNA_2	mRNA b3230	C3734H4230N1479O2753P393	-394	Translation
b3230_mRNA_2_degr	mRNA b3230 degradation complex	C44417H69629N13354O15438S200P393Mg6Zn2Fe0	-616	mRNA degradation
b3231_aa	polypeptide b3231	C715H1161N212O200S4	9	Translation
b3231_def_cplx	Polypeptide b3231 peptide deformylase complex	C1559H2555N453O455S10Mg0Zn0Fe1	4	Maturation
b3231_m	Matured polypeptide b3231	C714H1162N212O199S4	10	Maturation
b3231_mRNA	mRNA b3231	C4089H4629N1652O2980P431	-432	Translation
b3231_mRNA_1	mRNA b3231	C4089H4629N1652O2980P431	-432	Translation
b3231_mRNA_2	mRNA b3231	C4089H4629N1652O2980P431	-432	Translation
b3231_mRNA_2_degr	mRNA b3231 degradation complex	C44772H70028N13527O15665S200P431Mg6Zn2Fe0	-654	mRNA degradation
b3247_aa	polypeptide b3247	C2431H3901N695O744S19	-14	Translation
b3247_def_map_cplx	Polypeptide b3247 peptide deformylase and methionine aminopeptidase complex	C4561H7357N1288O1390S40Mg0Zn0Fe3	-23	Maturation
b3247_m	Matured polypeptide b3247	C2425H3893N694O742S18	-13	Maturation
b3247_mRNA	mRNA b3247 (11 nt short)	C13909H15716N5599O10183P1459	-1460	Translation
b3247_mRNA_1	mRNA b3247 (11 nt short)	C13909H15716N5599O10183P1459	-1460	Translation
b3247_mRNA_2	mRNA b3247 (11 nt short)	C13909H15716N5599O10183P1459	-1460	Translation
b3247_mRNA_2_degr	mRNA b3247 degradation complex	C54592H81115N17474O22868S200P1459Mg6Zn2Fe0	-1682	mRNA degradation
b3248_aa	polypeptide b3248	C943H1523N271O295S5	-6	Translation
b3248_def_map_cplx	Polypeptide b3248 peptide deformylase and methionine aminopeptidase complex	C3073H4979N864O941S26Mg0Zn0Fe3	-15	Maturation
b3248_m	Matured polypeptide b3248	C937H1515N270O293S4	-5	Maturation
b3248_mRNA	mRNA b3248	C5673H6401N2302O4152P594	-595	Translation
b3248_mRNA_1	mRNA b3248	C5673H6401N2302O4152P594	-595	Translation
b3248_mRNA_2	mRNA b3248	C5673H6401N2302O4152P594	-595	Translation
b3248_mRNA_2_degr	mRNA b3248 degradation complex	C46356H71800N14177O16837S200P594Mg6Zn2Fe0	-817	mRNA degradation
b3249_aa	polypeptide b3249	C908H1408N217O204S6	5	Translation

b3249_def_map_cplx	Polypeptide b3249 peptide deformylase and methionine aminopeptidase complex	C3038H4864N810O850S27Mg0Zn0Fe3	-4	Maturation
b3249_m	Matured polypeptide b3249	C902H1400N216O202S5	6	Maturation
b3249_mRNA	mRNA b3249 (1 nt short)	C4618H5216N1763O3485P488	-489	Translation
b3249_mRNA_1	mRNA b3249 (1 nt short)	C4618H5216N1763O3485P488	-489	Translation
b3249_mRNA_2	mRNA b3249 (1 nt short)	C4618H5216N1763O3485P488	-489	Translation
b3249_mRNA_2_degr	mRNA b3249 degradation complex	C45301H70615N13638O16170S200P488Mg6Zn2Fe0	-711	mRNA degradation
b3250_aa	polypeptide b3250	C1737H2844N503O527S11	1	Translation
b3250_def_map_cplx	Polypeptide b3250 peptide deformylase and methionine aminopeptidase complex	C3867H6300N1096O1173S32Mg0Zn0Fe3	-8	Maturation
b3250_m	Matured polypeptide b3250	C1731H2836N502O525S10	2	Maturation
b3250_mRNA	mRNA b3250	C10491H11886N4163O7761P1104	-1105	Translation
b3250_mRNA_1	mRNA b3250	C10491H11886N4163O7761P1104	-1105	Translation
b3250_mRNA_2	mRNA b3250	C10491H11886N4163O7761P1104	-1105	Translation
b3250_mRNA_2_degr	mRNA b3250 degradation complex	C51174H77285N16038O20446S200P1104Mg6Zn2Fe0	-1327	mRNA degradation
b3251_aa	polypeptide b3251	C1614H2623N460O499S16	-11	Translation
b3251_def_cplx	Polypeptide b3251 peptide deformylase complex	C2458H4017N701O754S22Mg0Zn0Fe1	-16	Maturation
b3251_m	Matured polypeptide b3251	C1613H2624N460O498S16	-10	Maturation
b3251_mRNA	mRNA b3251	C9922H11231N3930O7346P1046	-1047	Translation
b3251_mRNA_1	mRNA b3251	C9922H11231N3930O7346P1046	-1047	Translation
b3251_mRNA_2	mRNA b3251	C9922H11231N3930O7346P1046	-1047	Translation
b3251_mRNA_2_degr	mRNA b3251 degradation complex	C50605H76630N15805O20031S200P1046Mg6Zn2Fe0	-1269	mRNA degradation
b3251_m_DnaKJ_complex	b3251 DnaK DnaJ_dim complex - Kerner et al. class II can interact w/ GroEL/ES, cannot fold spontanously	C8177H13138O2596N2373S63P3Zn4	-32	Folding
b3251_m_GroEL.(7)ATP.transGroES	b3251 GroEL GroES complex - Kerner et al. class II can interact w/ GroEL/ES, cannot fold spontanously	C39525H65568O12832N11044S380P21Mg7	-311	Folding
b3257_aa	polypeptide b3257	C432H642N101O107S5	-3	Translation
b3257_def_map_cplx	Polypeptide b3257 peptide deformylase and methionine aminopeptidase complex	C2562H4098N694O753S26Mg0Zn0Fe3	-12	Maturation
b3257_m	Matured polypeptide b3257	C426H634N100O105S4	-2	Maturation
b3257_mRNA	mRNA b3257	C2298H2601N878O1743P245	-246	Translation
b3257_mRNA_1	mRNA b3257	C2298H2601N878O1743P245	-246	Translation
b3257_mRNA_2	mRNA b3257	C2298H2601N878O1743P245	-246	Translation
b3257_mRNA_2_degr	mRNA b3257 degradation complex	C42981H68000N12753O14428S200P245Mg6Zn2Fe0	-468	mRNA degradation
b3258_aa	polypeptide b3258	C2413H3870N591O620S21	9	Translation
b3258_def_map_cplx	Polypeptide b3258 peptide deformylase and methionine aminopeptidase complex	C4543H7326N1184O1266S42Mg0Zn0Fe3	0	Maturation
b3258_m	Matured polypeptide b3258	C2407H3862N590O618S20	10	Maturation
b3258_mRNA	mRNA b3258 (11 nt short)	C13645H15456N5279O10223P1441	-1442	Translation
b3258_mRNA_1	mRNA b3258 (11 nt short)	C13645H15456N5279O10223P1441	-1442	Translation
b3258_mRNA_2	mRNA b3258 (11 nt short)	C13645H15456N5279O10223P1441	-1442	Translation
b3258_mRNA_2_degr	mRNA b3258 degradation complex	C54328H80855N17154O22908S200P1441Mg6Zn2Fe0	-1664	mRNA degradation
b3259_aa	polypeptide b3259	C1416H2187N373O441S12	-28	Translation
b3259_def_map_cplx	Polypeptide b3259 peptide deformylase and methionine aminopeptidase complex	C3546H5643N966O1087S33Mg0Zn0Fe3	-37	Maturation
b3259_m	Matured polypeptide b3259 _ error CCDB	C1410H2179N372O439S11	-27	Maturation
b3259_mRNA	mRNA b3259	C8400H9508N3375O6162P882	-883	Translation
b3259_mRNA_1	mRNA b3259	C8400H9508N3375O6162P882	-883	Translation
b3259_mRNA_2	mRNA b3259	C8400H9508N3375O6162P882	-883	Translation
b3259_mRNA_2_degr	mRNA b3259 degradation complex	C49083H74907N15250O18847S200P882Mg6Zn2Fe0	-1105	mRNA degradation
b3260_aa	polypeptide b3260	C1584H2519N455O467S15	-4	Translation
b3260_def_map_cplx	Polypeptide b3260 peptide deformylase and methionine aminopeptidase complex	C3714H5975N1048O1113S36Mg0Zn0Fe3	-13	Maturation
b3260_m	Matured polypeptide b3260	C1578H2511N454O465S14	-3	Maturation
b3260_mRNA	mRNA b3260	C9198H10405N3684O6752P968	-969	Translation
b3260_mRNA_1	mRNA b3260	C9198H10405N3684O6752P968	-969	Translation
b3260_mRNA_2	mRNA b3260	C9198H10405N3684O6752P968	-969	Translation
b3260_mRNA_2_degr	mRNA b3260 degradation complex	C49881H75804N15559O19437S200P968Mg6Zn2Fe0	-1191	mRNA degradation
b3260_m_GroEL.(7)ATP.transGroES	b3260 GroEL GroES complex - Kerner et al. class III needs GroEL/ES	C39490H65455O12799N11038S378P21Mg7	-304	Folding

b3261_aa	polypeptide b3261	C488H803N141O152S6	2	Translation
b3261_def_cplx	Polypeptide b3261 peptide deformylase complex	C1332H2197N382O407S12Mg0Zn0Fe1	-3	Maturation
b3261_m	Matured polypeptide b3261	C487H804N141O151S6	3	Maturation
b3261_mRNA	mRNA b3261	C2832H3199N1140O2061P297	-298	Translation
b3261_mRNA_1	mRNA b3261	C2832H3199N1140O2061P297	-298	Translation
b3261_mRNA_2	mRNA b3261	C2832H3199N1140O2061P297	-298	Translation
b3261_mRNA_2_degr	mRNA b3261 degradation complex	C43515H68598N13015O14746S200P297Mg6Zn2Fe0	-520	mRNA degradation
b3272_RNA	rRNA	C1145H1300N469O837P120	-121	RNA cutting
b3272_v1_RNA	rRNA	C1145H1300N469O837P120	-121	RNA cutting
b3273_RNA	tRNA (thrV)	C723H819N288O536P76	-77	tRNA Modification
b3273_tRNA_1	b3273_tRNA_1 (thrV)	C723H821Mg2N288O536P76	-73	tRNA Modification
b3273_tRNA_1_Dus_gen_cplx	b3273_tRNA_1 (thrV), Dus_gen	C2330H3360Mg2N747O1019P79S14	-80	tRNA Modification
b3273_tRNA_2	b3273_tRNA_2 (thrV)	C723H823Mg2N288O536P76	-73	tRNA Modification
b3273_tRNA_2_Dus_gen_cplx	b3273_tRNA_2 (thrV), Dus_gen	C2330H3362Mg2N747O1019P79S14	-80	tRNA Modification
b3273_tRNA_3	b3273_tRNA_3 (thrV)	C723H825Mg2N288O536P76	-73	tRNA Modification
b3273_tRNA_3_Up_tRNA_pos_37_t6A-MeT_tRNA_pos_37_m6t6A_cplx	b3273_tRNA_3 (thrV), Up_tRNA_pos_37_t6A, MeT_tRNA_pos_37_m6t6A	C753H870Mg3N300O560P79S1	-75	tRNA Modification
b3273_tRNA_4	b3273_tRNA_4 (thrV)	C729H833Mg2N289O540P76	-74	tRNA Modification
b3273_tRNA_4_YggH_mono_cplx	b3273_tRNA_4 (thrV), YggH_mono	C1954H2732Mg2N642O887P76S14	-76	tRNA Modification
b3273_tRNA_5	b3273_tRNA_5 (thrV)	C730H835Mg2N289O540P76	-74	tRNA Modification
b3273_tRNA_5_TrmA_mono_cplx	b3273_tRNA_5 (thrV), TrmA_mono	C2604H3782Mg2N812O1099P76S19	-82	tRNA Modification
b3273_tRNA_6	b3273_tRNA_6 (thrV)	C731H837Mg2N289O540P76	-74	tRNA Modification
b3273_tRNA_6_TruB_mono_cplx	b3273_tRNA_6 (thrV), TruB_mono	C2271H3312Mg2N727O1009P76S10	-79	tRNA Modification
b3273_tRNA_Mg2	tRNA (thrV) bound two Mg2 ions	C723H819Mg2N288O536P76	-73	tRNA Modification
b3273_tRNA_Mg2_Dus_gen_cplx	b3273_tRNA (thrV), Dus_gen	C2330H3358Mg2N747O1019P79S14	-80	tRNA Modification
b3273_v1_RNA	tRNA (thrV)	C723H819N288O536P76	-77	tRNA Modification
b3273_v1_tRNA_1	b3273_v1_tRNA_1 (thrV)	C723H821Mg2N288O536P76	-73	tRNA Modification
b3273_v1_tRNA_1_Dus_gen_cplx	b3273_v1_tRNA_1 (thrV), Dus_gen	C2330H3360Mg2N747O1019P79S14	-80	tRNA Modification
b3273_v1_tRNA_2	b3273_v1_tRNA_2 (thrV)	C723H823Mg2N288O536P76	-73	tRNA Modification
b3273_v1_tRNA_2_Dus_gen_cplx	b3273_v1_tRNA_2 (thrV), Dus_gen	C2330H3362Mg2N747O1019P79S14	-80	tRNA Modification
b3273_v1_tRNA_3	b3273_v1_tRNA_3 (thrV)	C723H825Mg2N288O536P76	-73	tRNA Modification
b3273_v1_tRNA_3_Up_tRNA_pos_37_t6A-MeT_tRNA_pos_37_m6t6A_cplx	b3273_v1_tRNA_3 (thrV), Up_tRNA_pos_37_t6A, MeT_tRNA_pos_37_m6t6A	C753H870Mg3N300O560P79S1	-75	tRNA Modification
b3273_v1_tRNA_4	b3273_v1_tRNA_4 (thrV)	C729H833Mg2N289O540P76	-74	tRNA Modification
b3273_v1_tRNA_4_YggH_mono_cplx	b3273_v1_tRNA_4 (thrV), YggH_mono	C1954H2732Mg2N642O887P76S14	-76	tRNA Modification
b3273_v1_tRNA_5	b3273_v1_tRNA_5 (thrV)	C730H835Mg2N289O540P76	-74	tRNA Modification
b3273_v1_tRNA_5_TrmA_mono_cplx	b3273_v1_tRNA_5 (thrV), TrmA_mono	C2604H3782Mg2N812O1099P76S19	-82	tRNA Modification
b3273_v1_tRNA_6	b3273_v1_tRNA_6 (thrV)	C731H837Mg2N289O540P76	-74	tRNA Modification
b3273_v1_tRNA_6_TruB_mono_cplx	b3273_v1_tRNA_6 (thrV), TruB_mono	C2271H3312Mg2N727O1009P76S10	-79	tRNA Modification
b3273_v1_tRNA_Mg2	tRNA (thrV) bound two Mg2 ions	C723H819Mg2N288O536P76	-73	tRNA Modification
b3273_v1_tRNA_Mg2_Dus_gen_cplx	b3273_v1_tRNA (thrV), Dus_gen	C2330H3358Mg2N747O1019P79S14	-80	tRNA Modification
b3274_RNA	rRNA	C1144H1301N468O838P120	-121	RNA cutting
b3274_v1_RNA	rRNA	C1144H1301N468O838P120	-121	RNA cutting
b3275_RNA	rRNA	C27811H31354N11471O20156P2904	-2905	rRNA Modification
b3275_RNA_1		C27812H31356N11471O20156P2904S0	-2905	rRNA Modification
b3275_RNA_10		C27819H31370N11471O20156P2904S0	-2905	rRNA Modification
b3275_RNA_10_MeT_23S_2445	b3275_RNA_10, MeT_23S_2445	C27834H31393N11477O20161S1P2904Mg0Zn0Fe0	-2904	rRNA Modification
b3275_RNA_11		C27820H31372N11471O20156P2904S0	-2905	rRNA Modification
b3275_RNA_11_DU_23S_2449_a	b3275_RNA_11, DU_23S_2449 (NADH)	C27841H31400N11478O20170S0P2906Mg0Zn0Fe0	-2906	rRNA Modification
b3275_RNA_11_DU_23S_2449_b	b3275_RNA_11, DU_23S_2449 (NADPH)	C27841H31399N11478O20173S0P2907Mg0Zn0Fe0	-2908	rRNA Modification
b3275_RNA_12		C27820H31374N11471O20156P2904S0	-2905	rRNA Modification
b3275_RNA_12_YmfC_mono	b3275_RNA_12, YmfC_mono	C28918H33135N11798O20476S3P2904Mg0Zn0Fe0	-2895	rRNA Modification
b3275_RNA_13		C27820H31374N11471O20156P2904S0	-2905	rRNA Modification
b3275_RNA_13_MeT_23S_2498	b3275_RNA_13, MeT_23S_2498	C27835H31397N11477O20161S1P2904Mg0Zn0Fe0	-2904	rRNA Modification
b3275_RNA_14		C27821H31376N11471O20156P2904S0	-2905	rRNA Modification
b3275_RNA_14_MeT_23S_2503	b3275_RNA_14, MeT_23S_2503	C27836H31399N11477O20161S1P2904Mg0Zn0Fe0	-2904	rRNA Modification
b3275_RNA_15		C27822H31378N11471O20156P2904S0	-2905	rRNA Modification

b3275_RNA_15_RluC_mono	b3275_RNA_15, RluC_mono	C29407H33991N11953O20612S6P2904Mg0Zn0Fe0	-2890	rRNA Modification
b3275_RNA_16		C27822H31378N11471O20156P2904S0	-2905	rRNA Modification
b3275_RNA_16_RrmJ_mono	b3275_RNA_16, RrmJ_mono	C28869H33067N11766O20459S11P2904Mg0Zn0Fe0	-2897	rRNA Modification
b3275_RNA_17		C27823H31380N11471O20156P2904S0	-2905	rRNA Modification
b3275_RNA_17_RluC_mono	b3275_RNA_17, RluC_mono	C29408H33993N11953O20612S6P2904Mg0Zn0Fe0	-2890	rRNA Modification
b3275_RNA_18		C27823H31380N11471O20156P2904S0	-2905	rRNA Modification
b3275_RNA_18_YjbC_mono	b3275_RNA_18, YjbC_mono	C29250H33751N11889O20578S7P2904Mg0Zn0Fe0	-2890	rRNA Modification
b3275_RNA_19		C27823H31380N11471O20156P2904S0	-2905	rRNA Modification
b3275_RNA_19_RluB_mono	b3275_RNA_19, RluB_mono	C29241H33728N11922O20576S5P2904Mg0Zn0Fe0	-2890	rRNA Modification
b3275_RNA_1_MeT_23S_1835	b3275_RNA_1, MeT_23S_1835	C27827H31379N11477O20161S1P2904Mg0Zn0Fe0	-2904	rRNA Modification
b3275_RNA_2		C27813H31358N11471O20156P2904S0	-2905	rRNA Modification
b3275_RNA_20		C27823H31380N11471O20156P2904S0	-2905	rRNA Modification
b3275_RNA_20_RrmA_dim	b3275_RNA_20, RrmA_dim	C30540H35591N12235O20931S31P2904Mg0Zn2Fe0	-2902	rRNA Modification
b3275_RNA_21		C27824H31382N11471O20156P2904S0	-2905	rRNA Modification
b3275_RNA_21_RluA_mono	b3275_RNA_21, RluA_mono	C28927H33122N11784O20470S10P2904Mg0Zn0Fe0	-2904	rRNA Modification
b3275_RNA_22		C27824H31382N11471O20156P2904S0	-2905	rRNA Modification
b3275_RNA_22_RumB_mono	b3275_RNA_22, RumB_mono	C29717H34360N11983O20690S28P2904Mg0Zn0Fe4	-2903	rRNA Modification
b3275_RNA_23		C27825H31384N11471O20156P2904S0	-2905	rRNA Modification
b3275_RNA_23_RluC_mono	b3275_RNA_23, RluC_mono	C29410H33997N11953O20612S6P2904Mg0Zn0Fe0	-2890	rRNA Modification
b3275_RNA_2_RluD_mono	b3275_RNA_2, RluD_mono	C29450H33968N11951O20630S12P2904Mg1Zn0Fe0	-2909	rRNA Modification
b3275_RNA_3		C27813H31358N11471O20156P2904S0	-2905	rRNA Modification
b3275_RNA_3_RluD_mono	b3275_RNA_3, RluD_mono	C29465H33991N11957O20635S13P2904Mg1Zn0Fe0	-2908	rRNA Modification
b3275_RNA_4		C27814H31360N11471O20156P2904S0	-2905	rRNA Modification
b3275_RNA_4_RluD_mono	b3275_RNA_4, RluD_mono	C29451H33970N11951O20630S12P2904Mg1Zn0Fe0	-2909	rRNA Modification
b3275_RNA_5		C27814H31360N11471O20156P2904S0	-2905	rRNA Modification
b3275_RNA_5_RumA_mono	b3275_RNA_5, RumA_mono	C29942H34786N12089O20784S23P2904Mg0Zn0Fe4	-2897	rRNA Modification
b3275_RNA_6		C27815H31362N11471O20156P2904S0	-2905	rRNA Modification
b3275_RNA_6_MeT_23S_1962	b3275_RNA_6, MeT_23S_1962	C27830H31385N11477O20161S1P2904Mg0Zn0Fe0	-2904	rRNA Modification
b3275_RNA_7		C27816H31364N11471O20156P2904S0	-2905	rRNA Modification
b3275_RNA_7_MeT_23S_2030	b3275_RNA_7, MeT_23S_2030	C27831H31387N11477O20161S1P2904Mg0Zn0Fe0	-2904	rRNA Modification
b3275_RNA_8		C27817H31366N11471O20156P2904S0	-2905	rRNA Modification
b3275_RNA_8_MeT_23S_2069	b3275_RNA_8, MeT_23S_2069	C27832H31389N11477O20161S1P2904Mg0Zn0Fe0	-2904	rRNA Modification
b3275_RNA_9		C27818H31368N11471O20156P2904S0	-2905	rRNA Modification
b3275_RNA_9_RlmB_dim	b3275_RNA_9, RlmB_dim	C30139H35171N12155O20857S23P2904Mg0Zn0Fe0	-2910	rRNA Modification
b3275_RNA_MeT_23S_1618	b3275_RNA, MeT_23S_1618	C27826H31377N11477O20161S1P2904Mg0Zn0Fe0	-2904	rRNA Modification
b3275_v1_RNA	rRNA	C27811H31354N11471O20156P2904	-2905	rRNA Modification
b3275_v1_RNA_1		C27812H31356N11471O20156P2904S0	-2905	rRNA Modification
b3275_v1_RNA_10		C27819H31370N11471O20156P2904S0	-2905	rRNA Modification
b3275_v1_RNA_10_MeT_23S_2445	b3275_v1_RNA_10, MeT_23S_2445	C27834H31393N11477O20161S1P2904Mg0Zn0Fe0	-2904	rRNA Modification
b3275_v1_RNA_11		C27820H31372N11471O20156P2904S0	-2905	rRNA Modification
b3275_v1_RNA_11_DU_23S_2449_a	b3275_v1_RNA_11, DU_23S_2449 (NADH)	C27841H31400N11478O20170S0P2906Mg0Zn0Fe0	-2906	rRNA Modification
b3275_v1_RNA_11_DU_23S_2449_b	b3275_v1_RNA_11, DU_23S_2449 (NADPH)	C27841H31399N11478O20173S0P2907Mg0Zn0Fe0	-2908	rRNA Modification
b3275_v1_RNA_12		C27820H31374N11471O20156P2904S0	-2905	rRNA Modification
b3275_v1_RNA_12_YmfC_mono	b3275_v1_RNA_12, YmfC_mono	C28918H33135N11798O20476S3P2904Mg0Zn0Fe0	-2895	rRNA Modification
b3275_v1_RNA_13		C27820H31374N11471O20156P2904S0	-2905	rRNA Modification
b3275_v1_RNA_13_MeT_23S_2498	b3275_v1_RNA_13, MeT_23S_2498	C27835H31397N11477O20161S1P2904Mg0Zn0Fe0	-2904	rRNA Modification
b3275_v1_RNA_14		C27821H31376N11471O20156P2904S0	-2905	rRNA Modification
b3275_v1_RNA_14_MeT_23S_2503	b3275_v1_RNA_14, MeT_23S_2503	C27836H31399N11477O20161S1P2904Mg0Zn0Fe0	-2904	rRNA Modification
b3275_v1_RNA_15		C27822H31378N11471O20156P2904S0	-2905	rRNA Modification
b3275_v1_RNA_15_RluC_mono	b3275_v1_RNA_15, RluC_mono	C29407H33991N11953O20612S6P2904Mg0Zn0Fe0	-2890	rRNA Modification
b3275_v1_RNA_16		C27822H31378N11471O20156P2904S0	-2905	rRNA Modification
b3275_v1_RNA_16_RrmJ_mono	b3275_v1_RNA_16, RrmJ_mono	C28869H33067N11766O20459S11P2904Mg0Zn0Fe0	-2897	rRNA Modification
b3275_v1_RNA_17		C27823H31380N11471O20156P2904S0	-2905	rRNA Modification

b3275_v1_RNA_17_RluC_mono	b3275_v1_RNA_17, RluC_mono	C29408H33993N11953O20612S6P2904Mg0Zn0Fe0	-2890	rRNA Modification
b3275_v1_RNA_18		C27823H31380N11471O20156P2904S0	-2905	rRNA Modification
b3275_v1_RNA_18_YjbC_mono	b3275_v1_RNA_18, YjbC_mono	C29250H33751N11889O20578S7P2904Mg0Zn0Fe0	-2890	rRNA Modification
b3275_v1_RNA_19		C27823H31380N11471O20156P2904S0	-2905	rRNA Modification
b3275_v1_RNA_19_RluB_mono	b3275_v1_RNA_19, RluB_mono	C29241H33728N11922O20576S5P2904Mg0Zn0Fe0	-2890	rRNA Modification
b3275_v1_RNA_1_MeT_23S_1835	b3275_v1_RNA_1, MeT_23S_1835	C27827H31379N11477O20161S1P2904Mg0Zn0Fe0	-2904	rRNA Modification
b3275_v1_RNA_2		C27813H31358N11471O20156P2904S0	-2905	rRNA Modification
b3275_v1_RNA_20		C27823H31380N11471O20156P2904S0	-2905	rRNA Modification
b3275_v1_RNA_20_RrmA_dim	b3275_v1_RNA_20, RrmA_dim	C30540H35591N12235O20931S31P2904Mg0Zn2Fe0	-2902	rRNA Modification
b3275_v1_RNA_21		C27824H31382N11471O20156P2904S0	-2905	rRNA Modification
b3275_v1_RNA_21_RluA_mono	b3275_v1_RNA_21, RluA_mono	C28927H33122N11784O20470S10P2904Mg0Zn0Fe0	-2904	rRNA Modification
b3275_v1_RNA_22		C27824H31382N11471O20156P2904S0	-2905	rRNA Modification
b3275_v1_RNA_22_RumB_mono	b3275_v1_RNA_22, RumB_mono	C29717H34360N11983O20690S28P2904Mg0Zn0Fe4	-2903	rRNA Modification
b3275_v1_RNA_23		C27825H31384N11471O20156P2904S0	-2905	rRNA Modification
b3275_v1_RNA_23_RluC_mono	b3275_v1_RNA_23, RluC_mono	C29410H33997N11953O20612S6P2904Mg0Zn0Fe0	-2890	rRNA Modification
b3275_v1_RNA_2_RluD_mono	b3275_v1_RNA_2, RluD_mono	C29450H33968N11951O20630S12P2904Mg1Zn0Fe0	-2909	rRNA Modification
b3275_v1_RNA_3		C27813H31358N11471O20156P2904S0	-2905	rRNA Modification
b3275_v1_RNA_3_RluD_mono	b3275_v1_RNA_3, RluD_mono	C29465H33991N11957O20635S13P2904Mg1Zn0Fe0	-2908	rRNA Modification
b3275_v1_RNA_4		C27814H31360N11471O20156P2904S0	-2905	rRNA Modification
b3275_v1_RNA_4_RluD_mono	b3275_v1_RNA_4, RluD_mono	C29451H33970N11951O20630S12P2904Mg1Zn0Fe0	-2909	rRNA Modification
b3275_v1_RNA_5		C27814H31360N11471O20156P2904S0	-2905	rRNA Modification
b3275_v1_RNA_5_RumA_mono	b3275_v1_RNA_5, RumA_mono	C29942H34786N12089O20784S23P2904Mg0Zn0Fe4	-2897	rRNA Modification
b3275_v1_RNA_6		C27815H31362N11471O20156P2904S0	-2905	rRNA Modification
b3275_v1_RNA_6_MeT_23S_1962	b3275_v1_RNA_6, MeT_23S_1962	C27830H31385N11477O20161S1P2904Mg0Zn0Fe0	-2904	rRNA Modification
b3275_v1_RNA_7		C27816H31364N11471O20156P2904S0	-2905	rRNA Modification
b3275_v1_RNA_7_MeT_23S_2030	b3275_v1_RNA_7, MeT_23S_2030	C27831H31387N11477O20161S1P2904Mg0Zn0Fe0	-2904	rRNA Modification
b3275_v1_RNA_8		C27817H31366N11471O20156P2904S0	-2905	rRNA Modification
b3275_v1_RNA_8_MeT_23S_2069	b3275_v1_RNA_8, MeT_23S_2069	C27832H31389N11477O20161S1P2904Mg0Zn0Fe0	-2904	rRNA Modification
b3275_v1_RNA_9		C27818H31368N11471O20156P2904S0	-2905	rRNA Modification
b3275_v1_RNA_9_RlmB_dim	b3275_v1_RNA_9, RlmB_dim	C30139H35171N12155O20857S23P2904Mg0Zn0Fe0	-2910	rRNA Modification
b3275_v1_RNA_MeT_23S_1618	b3275_v1_RNA, MeT_23S_1618	C27826H31377N11477O20161S1P2904Mg0Zn0Fe0	-2904	rRNA Modification
b3276_RNA	tRNA (alaU)	C722H822N289O535P76	-77	tRNA Modification
b3276_tRNA_1	b3276_tRNA_1 (alaU)	C722H824Mg2N289O535P76	-73	tRNA Modification
b3276_tRNA_1_YecO_mono-YecP_mono-HyL_tRNA_pos_34_ho5U_cplx	b3276_tRNA_1 (alaU), YecO_mono, YecP_mono, HyL_tRNA_pos_34_ho5U	C3655H5357Mg2N1090O1385P76S23	-91	tRNA Modification
b3276_tRNA_2	b3276_tRNA_2 (alaU)	C724H825Mg2N289O538P76	-74	tRNA Modification
b3276_tRNA_2_YggH_mono_cplx	b3276_tRNA_2 (alaU), YggH_mono	C1949H2724Mg2N642O885P76S14	-76	tRNA Modification
b3276_tRNA_3	b3276_tRNA_3 (alaU)	C725H827Mg2N289O538P76	-74	tRNA Modification
b3276_tRNA_3_TrmA_mono_cplx	b3276_tRNA_3 (alaU), TrmA_mono	C2599H3774Mg2N812O1097P76S19	-82	tRNA Modification
b3276_tRNA_4	b3276_tRNA_4 (alaU)	C726H829Mg2N289O538P76	-74	tRNA Modification
b3276_tRNA_4_TruB_mono_cplx	b3276_tRNA_4 (alaU), TruB_mono	C2266H3304Mg2N727O1007P76S10	-79	tRNA Modification
b3276_tRNA_Mg2	tRNA (alaU) bound two Mg2 ions	C722H822Mg2N289O535P76	-73	tRNA Modification
b3276_tRNA_Mg2_Dus_gen_cplx	b3276_tRNA (alaU), Dus_gen	C2329H3361Mg2N748O1018P79S14	-80	tRNA Modification
b3276_v1_RNA	tRNA (alaU)	C722H822N289O535P76	-77	tRNA Modification
b3276_v1_tRNA_1	b3276_v1_tRNA_1 (alaU)	C722H824Mg2N289O535P76	-73	tRNA Modification
b3276_v1_tRNA_1_YecO_mono-YecP_mono-HyL_tRNA_pos_34_ho5U_cplx	b3276_v1_tRNA_1 (alaU), YecO_mono, YecP_mono, HyL_tRNA_pos_34_ho5U	C3655H5357Mg2N1090O1385P76S23	-91	tRNA Modification
b3276_v1_tRNA_2	b3276_v1_tRNA_2 (alaU)	C724H825Mg2N289O538P76	-74	tRNA Modification
b3276_v1_tRNA_2_YggH_mono_cplx	b3276_v1_tRNA_2 (alaU), YggH_mono	C1949H2724Mg2N642O885P76S14	-76	tRNA Modification
b3276_v1_tRNA_3	b3276_v1_tRNA_3 (alaU)	C725H827Mg2N289O538P76	-74	tRNA Modification
b3276_v1_tRNA_3_TrmA_mono_cplx	b3276_v1_tRNA_3 (alaU), TrmA_mono	C2599H3774Mg2N812O1097P76S19	-82	tRNA Modification
b3276_v1_tRNA_4	b3276_v1_tRNA_4 (alaU)	C726H829Mg2N289O538P76	-74	tRNA Modification
b3276_v1_tRNA_4_TruB_mono_cplx	b3276_v1_tRNA_4 (alaU), TruB_mono	C2266H3304Mg2N727O1007P76S10	-79	tRNA Modification
b3276_v1_tRNA_Mg2	tRNA (alaU) bound two Mg2 ions	C722H822Mg2N289O535P76	-73	tRNA Modification
b3276_v1_tRNA_Mg2_Dus_gen_cplx	b3276_v1_tRNA (alaU), Dus_gen	C2329H3361Mg2N748O1018P79S14	-80	tRNA Modification

b3277_RNA	tRNA (ileU)	C734H831N296O541P77	-78	tRNA Modification
b3277_tRNA_1	b3277_tRNA_1 (ileU)	C734H833Mg2N296O541P77	-74	tRNA Modification
b3277_tRNA_1_Dus_gen_cplx	b3277_tRNA_1 (ileU), Dus_gen	C2341H3372Mg2N755O1024P80S14	-81	tRNA Modification
b3277_tRNA_2	b3277_tRNA_2 (ileU)	C734H835Mg2N296O541P77	-74	tRNA Modification
b3277_tRNA_2_Up_tRNA_pos_37_t6A_cplx	b3277_tRNA_2 (ileU), Up_tRNA_pos_37_t6A	C749H857Mg3N302O560P80	-77	tRNA Modification
b3277_tRNA_3	b3277_tRNA_3 (ileU)	C739H841Mg2N297O545P77	-75	tRNA Modification
b3277_tRNA_3_YggH_mono_cplx	b3277_tRNA_3 (ileU), YggH_mono	C1964H2740Mg2N650O892P77S14	-77	tRNA Modification
b3277_tRNA_4	b3277_tRNA_4 (ileU)	C740H843Mg2N297O545P77	-75	tRNA Modification
b3277_tRNA_4_AcpT_tRNA_pos_47_acp3U_cplx	b3277_tRNA_4 (ileU), AcpT_tRNA_pos_47_acp3U	C755H866Mg2N303O550P77S1	-74	tRNA Modification
b3277_tRNA_5	b3277_tRNA_5 (ileU)	C744H850Mg2N298O547P77	-75	tRNA Modification
b3277_tRNA_5_TrmA_mono_cplx	b3277_tRNA_5 (ileU), TrmA_mono	C2618H3797Mg2N821O1106P77S19	-83	tRNA Modification
b3277_tRNA_6	b3277_tRNA_6 (ileU)	C745H852Mg2N298O547P77	-75	tRNA Modification
b3277_tRNA_6_TruB_mono_cplx	b3277_tRNA_6 (ileU), TruB_mono	C2285H3327Mg2N736O1016P77S10	-80	tRNA Modification
b3277_tRNA_7	b3277_tRNA_7 (ileU)	C745H852Mg2N298O547P77	-75	tRNA Modification
b3277_tRNA_7_YqcB_mono_cplx	b3277_tRNA_7 (ileU), YqcB_mono	C2059H2908Mg2N685O926P77S6	-78	tRNA Modification
b3277_tRNA_Mg2	tRNA (ileU) bound two Mg2 ions	C734H831Mg2N296O541P77	-74	tRNA Modification
b3277_tRNA_Mg2_Dus_gen_cplx	b3277_tRNA (ileU), Dus_gen	C2341H3370Mg2N755O1024P80S14	-81	tRNA Modification
b3277_v1_RNA	tRNA (ileU)	C734H831N296O541P77	-78	tRNA Modification
b3277_v1_tRNA_1	b3277_v1_tRNA_1 (ileU)	C734H833Mg2N296O541P77	-74	tRNA Modification
b3277_v1_tRNA_1_Dus_gen_cplx	b3277_v1_tRNA_1 (ileU), Dus_gen	C2341H3372Mg2N755O1024P80S14	-81	tRNA Modification
b3277_v1_tRNA_2	b3277_v1_tRNA_2 (ileU)	C734H835Mg2N296O541P77	-74	tRNA Modification
b3277_v1_tRNA_2_Up_tRNA_pos_37_t6A_cplx	b3277_v1_tRNA_2 (ileU), Up_tRNA_pos_37_t6A	C749H857Mg3N302O560P80	-77	tRNA Modification
b3277_v1_tRNA_3	b3277_v1_tRNA_3 (ileU)	C739H841Mg2N297O545P77	-75	tRNA Modification
b3277_v1_tRNA_3_YggH_mono_cplx	b3277_v1_tRNA_3 (ileU), YggH_mono	C1964H2740Mg2N650O892P77S14	-77	tRNA Modification
b3277_v1_tRNA_4	b3277_v1_tRNA_4 (ileU)	C740H843Mg2N297O545P77	-75	tRNA Modification
b3277_v1_tRNA_4_AcpT_tRNA_pos_47_acp3U_cplx	b3277_v1_tRNA_4 (ileU), AcpT_tRNA_pos_47_acp3U	C755H866Mg2N303O550P77S1	-74	tRNA Modification
b3277_v1_tRNA_5	b3277_v1_tRNA_5 (ileU)	C744H850Mg2N298O547P77	-75	tRNA Modification
b3277_v1_tRNA_5_TrmA_mono_cplx	b3277_v1_tRNA_5 (ileU), TrmA_mono	C2618H3797Mg2N821O1106P77S19	-83	tRNA Modification
b3277_v1_tRNA_6	b3277_v1_tRNA_6 (ileU)	C745H852Mg2N298O547P77	-75	tRNA Modification
b3277_v1_tRNA_6_TruB_mono_cplx	b3277_v1_tRNA_6 (ileU), TruB_mono	C2285H3327Mg2N736O1016P77S10	-80	tRNA Modification
b3277_v1_tRNA_7	b3277_v1_tRNA_7 (ileU)	C745H852Mg2N298O547P77	-75	tRNA Modification
b3277_v1_tRNA_7_YqcB_mono_cplx	b3277_v1_tRNA_7 (ileU), YqcB_mono	C2059H2908Mg2N685O926P77S6	-78	tRNA Modification
b3277_v1_tRNA_Mg2	tRNA (ileU) bound two Mg2 ions	C734H831Mg2N296O541P77	-74	tRNA Modification
b3277_v1_tRNA_Mg2_Dus_gen_cplx	b3277_v1_tRNA (ileU), Dus_gen	C2341H3370Mg2N755O1024P80S14	-81	tRNA Modification
b3278_RNA	rRNA	C14753H16650N6063O10720P1542	-1543	rRNA Modification
b3278_RNA_1		C14754H16652N6063O10720P1542S0	-1543	rRNA Modification
b3278_RNA_10		C14765H16674N6063O10720P1542S0	-1543	rRNA Modification
b3278_RNA_10_RsmB_mono	b3278_RNA_10, RsmB_mono	C16932H20117N6681O11348S17P1542Mg0Zn0Fe0	-1542	rRNA Modification
b3278_RNA_1_MeT_16S_1402	b3278_RNA_1, MeT_16S_1402	C14784H16698N6075O10730S2P1542Mg0Zn0Fe0	-1541	rRNA Modification
b3278_RNA_2		C14756H16656N6063O10720P1542S0	-1543	rRNA Modification
b3278_RNA_2_MeT_16S_1407	b3278_RNA_2, MeT_16S_1407	C14771H16679N6069O10725S1P1542Mg0Zn0Fe0	-1542	rRNA Modification
b3278_RNA_3		C14757H16658N6063O10720P1542S0	-1543	rRNA Modification
b3278_RNA_3_YggJ_mono	b3278_RNA_3, YggJ_mono	C15949H18598N6411O11079S11P1542Mg0Zn0Fe0	-1545	rRNA Modification
b3278_RNA_4		C14758H16660N6063O10720P1542S0	-1543	rRNA Modification
b3278_RNA_4_MeT_16S_1516	b3278_RNA_4, MeT_16S_1516	C14773H16683N6069O10725S1P1542Mg0Zn0Fe0	-1542	rRNA Modification
b3278_RNA_5		C14759H16662N6063O10720P1542S0	-1543	rRNA Modification
b3278_RNA_5_KsgA_mono	b3278_RNA_5, KsgA_mono	C16141H18851N6451O11118S15P1542Mg0Zn0Fe0	-1540	rRNA Modification
b3278_RNA_6		C14761H16666N6063O10720P1542S0	-1543	rRNA Modification
b3278_RNA_6_KsgA_mono	b3278_RNA_6, KsgA_mono	C16143H18855N6451O11118S15P1542Mg0Zn0Fe0	-1540	rRNA Modification
b3278_RNA_7		C14763H16670N6063O10720P1542S0	-1543	rRNA Modification
b3278_RNA_7_RsuA_mono	b3278_RNA_7, RsuA_mono	C15908H18470N6391O11064S6P1542Mg0Zn0Fe0	-1551	rRNA Modification
b3278_RNA_8		C14763H16670N6063O10720P1542S0	-1543	rRNA Modification
b3278_RNA_8_MeT_16S_527	b3278_RNA_8, MeT_16S_527	C14778H16693N6069O10725S1P1542Mg0Zn0Fe0	-1542	rRNA Modification
b3278_RNA_9		C14764H16672N6063O10720P1542S0	-1543	rRNA Modification
b3278_RNA_9_MeT_16S_966	b3278_RNA_9, MeT_16S_966	C14779H16695N6069O10725S1P1542Mg0Zn0Fe0	-1542	rRNA Modification

b3278_RNA_RsmC_mono	b3278_RNA, RsmC_mono	C16437H19281N6536O11222S11P1542Mg0Zn0Fe0	-1547	rRNA Modification
b3278_v1_RNA	rRNA	C14753H16650N6063O10720P1542	-1543	rRNA Modification
b3278_v1_RNA_1		C14754H16652N6063O10720P1542S0	-1543	rRNA Modification
b3278_v1_RNA_10		C14765H16674N6063O10720P1542S0	-1543	rRNA Modification
b3278_v1_RNA_10_RsmB_mono	b3278_v1_RNA_10, RsmB_mono	C16932H20117N6681O11348S17P1542Mg0Zn0Fe0	-1542	rRNA Modification
b3278_v1_RNA_1_MeT_16S_1402	b3278_v1_RNA_1, MeT_16S_1402	C14784H16698N6075O10730S2P1542Mg0Zn0Fe0	-1541	rRNA Modification
b3278_v1_RNA_2		C14756H16656N6063O10720P1542S0	-1543	rRNA Modification
b3278_v1_RNA_2_MeT_16S_1407	b3278_v1_RNA_2, MeT_16S_1407	C14771H16679N6069O10725S1P1542Mg0Zn0Fe0	-1542	rRNA Modification
b3278_v1_RNA_3		C14757H16658N6063O10720P1542S0	-1543	rRNA Modification
b3278_v1_RNA_3_YggJ_mono	b3278_v1_RNA_3, YggJ_mono	C15949H18598N6411O11079S11P1542Mg0Zn0Fe0	-1545	rRNA Modification
b3278_v1_RNA_4		C14758H16660N6063O10720P1542S0	-1543	rRNA Modification
b3278_v1_RNA_4_MeT_16S_1516	b3278_v1_RNA_4, MeT_16S_1516	C14773H16683N6069O10725S1P1542Mg0Zn0Fe0	-1542	rRNA Modification
b3278_v1_RNA_5		C14759H16662N6063O10720P1542S0	-1543	rRNA Modification
b3278_v1_RNA_5_KsgA_mono	b3278_v1_RNA_5, KsgA_mono	C16141H18851N6451O11118S15P1542Mg0Zn0Fe0	-1540	rRNA Modification
b3278_v1_RNA_6		C14761H16666N6063O10720P1542S0	-1543	rRNA Modification
b3278_v1_RNA_6_KsgA_mono	b3278_v1_RNA_6, KsgA_mono	C16143H18855N6451O11118S15P1542Mg0Zn0Fe0	-1540	rRNA Modification
b3278_v1_RNA_7		C14763H16670N6063O10720P1542S0	-1543	rRNA Modification
b3278_v1_RNA_7_RsuA_mono	b3278_v1_RNA_7, RsuA_mono	C15908H18470N6391O11064S6P1542Mg0Zn0Fe0	-1551	rRNA Modification
b3278_v1_RNA_8		C14763H16670N6063O10720P1542S0	-1543	rRNA Modification
b3278_v1_RNA_8_MeT_16S_527	b3278_v1_RNA_8, MeT_16S_527	C14778H16693N6069O10725S1P1542Mg0Zn0Fe0	-1542	rRNA Modification
b3278_v1_RNA_9		C14764H16672N6063O10720P1542S0	-1543	rRNA Modification
b3278_v1_RNA_9_MeT_16S_966	b3278_v1_RNA_9, MeT_16S_966	C14779H16695N6069O10725S1P1542Mg0Zn0Fe0	-1542	rRNA Modification
b3278_v1_RNA_RsmC_mono	b3278_v1_RNA, RsmC_mono	C16437H19281N6536O11222S11P1542Mg0Zn0Fe0	-1547	rRNA Modification
b3280_aa	polypeptide b3280	C442H640N118O141S5	-12	Translation
b3280_def_map_cplx	Polypeptide b3280 peptide deformylase and methionine aminopeptidase complex	C2572H4096N711O787S26Mg0Zn0Fe3	-21	Maturation
b3280_m	Matured polypeptide b3280	C436H632N117O139S4	-11	Maturation
b3280_mRNA	mRNA b3280 (4 nt short)	C2431H2736N993O1768P254	-255	Translation
b3280_mRNA_1	mRNA b3280 (4 nt short)	C2431H2736N993O1768P254	-255	Translation
b3280_mRNA_2	mRNA b3280 (4 nt short)	C2431H2736N993O1768P254	-255	Translation
b3280_mRNA_2_degr	mRNA b3280 degradation complex	C43114H68135N12868O14453S200P254Mg6Zn2Fe0	-477	mRNA degradation
b3281_aa	polypeptide b3281	C1320H2066N358O389S8	-10	Translation
b3281_def_cplx	Polypeptide b3281 peptide deformylase complex	C2164H3460N599O644S14Mg0Zn0Fe1	-15	Maturation
b3281_m	Matured polypeptide b3281	C1319H2067N358O388S8	-9	Maturation
b3281_mRNA	mRNA b3281	C7777H8780N3039O5776P819	-820	Translation
b3281_mRNA_1	mRNA b3281	C7777H8780N3039O5776P819	-820	Translation
b3281_mRNA_2	mRNA b3281	C7777H8780N3039O5776P819	-820	Translation
b3281_mRNA_2_degr	mRNA b3281 degradation complex	C48460H74179N14914O18461S200P819Mg6Zn2Fe0	-1042	mRNA degradation
b3282_aa	polypeptide b3282	C927H1462N254O277S6	-6	Translation
b3282_def_map_cplx	Polypeptide b3282 peptide deformylase and methionine aminopeptidase complex	C3057H4918N847O923S27Mg0Zn0Fe3	-15	Maturation
b3282_m	Matured polypeptide b3282	C921H1454N253O275S5	-5	Maturation
b3282_mRNA	mRNA b3282	C5442H6156N2141O4034P573	-574	Translation
b3282_mRNA_1	mRNA b3282	C5442H6156N2141O4034P573	-574	Translation
b3282_mRNA_2	mRNA b3282	C5442H6156N2141O4034P573	-574	Translation
b3282_mRNA_2_degr	mRNA b3282 degradation complex	C46125H71555N14016O16719S200P573Mg6Zn2Fe0	-796	mRNA degradation
b3283_aa	polypeptide b3283	C867H1369N250O252S18	5	Translation
b3283_def_map_cplx	Polypeptide b3283 peptide deformylase and methionine aminopeptidase complex	C2997H4825N843O898S39Mg0Zn0Fe3	-4	Maturation
b3283_m	Matured polypeptide b3283 _ error CCDB	C861H1361N249O250S17	6	Maturation
b3283_mRNA	mRNA b3283	C5161H5839N2042O3805P545	-546	Translation
b3283_mRNA_1	mRNA b3283	C5161H5839N2042O3805P545	-546	Translation
b3283_mRNA_2	mRNA b3283	C5161H5839N2042O3805P545	-546	Translation
b3283_mRNA_2_degr	mRNA b3283 degradation complex	C45844H71238N13917O16490S200P545Mg6Zn2Fe0	-768	mRNA degradation
b3287_aa	polypeptide b3287	C850H1402N242O257S7	-8	Translation

b3287_def_map_cplx	Polypeptide b3287 peptide deformylase and methionine aminopeptidase complex	C2980H4858N835O903S28Mg0Zn0Fe3	-17	Maturation
b3287_m	Matured polypeptide b3287	C844H1394N241O255S6	-7	Maturation
b3287_mRNA	mRNA b3287	C4870H5486N1965O3553P512	-513	Translation
b3287_mRNA_1	mRNA b3287	C4870H5486N1965O3553P512	-513	Translation
b3287_mRNA_2	mRNA b3287	C4870H5486N1965O3553P512	-513	Translation
b3287_mRNA_2_degr	mRNA b3287 degradation complex	C45553H70885N13840O16238S200P512Mg6Zn2Fe0	-735	mRNA degradation
b3287_m_Fe	b3287 plus _Fe	C844H1394N241O255S6Fe1	-5	Folding
b3288_aa	polypeptide b3288	C1523H2450N415O452S12	-5	Translation
b3288_def_map_cplx	Polypeptide b3288 peptide deformylase and methionine aminopeptidase complex	C3653H5906N1008O1098S33Mg0Zn0Fe3	-14	Maturation
b3288_m	Matured polypeptide b3288	C1517H2442N414O450S11	-4	Maturation
b3288_mRNA	mRNA b3288	C9017H10211N3596O6626P948	-949	Translation
b3288_mRNA_1	mRNA b3288	C9017H10211N3596O6626P948	-949	Translation
b3288_mRNA_2	mRNA b3288	C9017H10211N3596O6626P948	-949	Translation
b3288_mRNA_2_degr	mRNA b3288 degradation complex	C49700H75610N15471O19311S200P948Mg6Zn2Fe0	-1171	mRNA degradation
b3289_DNA_act	DNA b3289 (activated form)	C12583H14506N4868O7723P1290	-1290	Transcription
b3289_DNA_neu	DNA b3289 (inactivate form)	C12583H14506N4868O7723P1290	-1290	Transcription
b3289_aa	polypeptide b3289	C2153H3419N612O624S16	-1	Translation
b3289_def_cplx	Polypeptide b3289 peptide deformylase complex	C2997H4813N853O879S22Mg0Zn0Fe1	-6	Maturation
b3289_m	Matured polypeptide b3289	C2152H3420N612O623S16	0	Maturation
b3289_mRNA	mRNA b3289	C12267H13875N4868O9020P1292	-1293	Translation
b3289_mRNA_1	mRNA b3289	C12267H13875N4868O9020P1292	-1293	Translation
b3289_mRNA_2	mRNA b3289	C12267H13875N4868O9020P1292	-1293	Translation
b3289_mRNA_2_degr	mRNA b3289 degradation complex	C52950H79274N16743O21705S200P1292Mg6Zn2Fe0	-1515	mRNA degradation
b3294_aa	polypeptide b3294	C622H1044N204O179S5	12	Translation
b3294_def_cplx	Polypeptide b3294 peptide deformylase complex	C1466H2438N445O434S11Mg0Zn0Fe1	7	Maturation
b3294_m	Matured polypeptide b3294	C621H1045N204O178S5	13	Maturation
b3294_mRNA	mRNA b3294	C3653H4137N1458O2687P384	-385	Translation
b3294_mRNA_1	mRNA b3294	C3653H4137N1458O2687P384	-385	Translation
b3294_mRNA_2	mRNA b3294	C3653H4137N1458O2687P384	-385	Translation
b3294_mRNA_2_degr	mRNA b3294 degradation complex	C44336H69536N13333O15372S200P384Mg6Zn2Fe0	-607	mRNA degradation
b3295_aa	polypeptide b3295	C1600H2611N450O505S9	-19	Translation
b3295_def_cplx	Polypeptide b3295 peptide deformylase complex	C2444H4005N691O760S15Mg0Zn0Fe1	-24	Maturation
b3295_m	Matured polypeptide b3295	C1599H2612N450O504S9	-18	Maturation
b3295_mRNA	mRNA b3295	C9425H10662N3771O6916P990	-991	Translation
b3295_mRNA_1	mRNA b3295	C9425H10662N3771O6916P990	-991	Translation
b3295_mRNA_2	mRNA b3295	C9425H10662N3771O6916P990	-991	Translation
b3295_mRNA_2_degr	mRNA b3295 degradation complex	C50108H76061N15646O19601S200P990Mg6Zn2Fe0	-1213	mRNA degradation
b3295_m_DnaKJ_complex	b3295 DnaK DnaJ_dim complex - Kerner et al. class I can interact w/ GroEL/ES	C8163H13126O2602N2363S56P3Zn4	-40	Folding
b3295_m_GroEL.(7)ATP.transGroES	b3295 GroEL GroES complex - Kerner et al. class I can interact w/ GroEL/ES	C39511H65556O12838N11034S373P21Mg7	-319	Folding
b3296_aa	polypeptide b3296	C1032H1718N316O300S5	16	Translation
b3296_def_map_cplx	Polypeptide b3296 peptide deformylase and methionine aminopeptidase complex	C3162H5174N909O946S26Mg0Zn0Fe3	7	Maturation
b3296_m	Matured polypeptide b3296	C1026H1710N315O298S4	17	Maturation
b3296_mRNA	mRNA b3296	C5923H6684N2383O4338P621	-622	Translation
b3296_mRNA_1	mRNA b3296	C5923H6684N2383O4338P621	-622	Translation
b3296_mRNA_2	mRNA b3296	C5923H6684N2383O4338P621	-622	Translation
b3296_mRNA_2_degr	mRNA b3296 degradation complex	C46606H72083N14258O17023S200P621Mg6Zn2Fe0	-844	mRNA degradation
b3297_aa	polypeptide b3297	C601H1005N197O173S4	14	Translation
b3297_def_map_cplx	Polypeptide b3297 peptide deformylase and methionine aminopeptidase complex	C2731H4461N790O819S25Mg0Zn0Fe3	5	Maturation
b3297_m	Matured polypeptide b3297	C595H997N196O171S3	15	Maturation
b3297_mRNA	mRNA b3297	C3704H4197N1464O2730P390	-391	Translation
b3297_mRNA_1	mRNA b3297	C3704H4197N1464O2730P390	-391	Translation
b3297_mRNA_2	mRNA b3297	C3704H4197N1464O2730P390	-391	Translation
b3297_mRNA_2_degr	mRNA b3297 degradation complex	C44387H69596N13339O15415S200P390Mg6Zn2Fe0	-613	mRNA degradation

b3298_aa	polypeptide b3298	C570H989N184O162S4	13	Translation
b3298_def_map_cplx	Polypeptide b3298 peptide deformylase and methionine aminopeptidase complex	C2700H4445N777O808S25Mg0Zn0Fe3	4	Maturation
b3298_m	Matured polypeptide b3298	C564H981N183O160S3	14	Maturation
b3298_mRNA	mRNA b3298	C3397H3844N1355O2502P359	-360	Translation
b3298_mRNA_1	mRNA b3298	C3397H3844N1355O2502P359	-360	Translation
b3298_mRNA_2	mRNA b3298	C3397H3844N1355O2502P359	-360	Translation
b3298_mRNA_2_degr	mRNA b3298 degradation complex	C44080H69243N13230O15187S200P359Mg6Zn2Fe0	-582	mRNA degradation
b3299_aa	polypeptide b3299	C186H342N65O49S4	9	Translation
b3299_def_cplx	Polypeptide b3299 peptide deformylase complex	C1030H1736N306O304S10Mg0Zn0Fe1	4	Maturation
b3299_m	Matured polypeptide b3299	C185H343N65O48S4	10	Maturation
b3299_mRNA	mRNA b3299	C1117H1260N451O815P117	-118	Translation
b3299_mRNA_1	mRNA b3299	C1117H1260N451O815P117	-118	Translation
b3299_mRNA_2	mRNA b3299	C1117H1260N451O815P117	-118	Translation
b3299_mRNA_2_degr	mRNA b3299 degradation complex	C41800H66659N12326O13500S200P117Mg6Zn2Fe0	-340	mRNA degradation
b3300_aa	polypeptide b3300	C2260H3582N566O581S18	16	Translation
b3300_def_map_cplx	Polypeptide b3300 peptide deformylase and methionine aminopeptidase complex	C4390H7038N1159O1227S39Mg0Zn0Fe3	7	Maturation
b3300_m	Matured polypeptide b3300	C2254H3574N565O579S17	17	Maturation
b3300_mRNA	mRNA b3300	C12624H14269N4884O9423P1332	-1333	Translation
b3300_mRNA_1	mRNA b3300	C12624H14269N4884O9423P1332	-1333	Translation
b3300_mRNA_2	mRNA b3300	C12624H14269N4884O9423P1332	-1333	Translation
b3300_mRNA_2_degr	mRNA b3300 degradation complex	C53307H79668N16759O22108S200P1332Mg6Zn2Fe0	-1555	mRNA degradation
b3301_aa	polypeptide b3301	C655H1128N207O191S2	15	Translation
b3301_def_cplx	Polypeptide b3301 peptide deformylase complex	C1499H2522N448O446S8Mg0Zn0Fe1	10	Maturation
b3301_m	Matured polypeptide b3301	C654H1129N207O190S2	16	Maturation
b3301_mRNA	mRNA b3301	C4143H4681N1656O3054P435	-436	Translation
b3301_mRNA_1	mRNA b3301	C4143H4681N1656O3054P435	-436	Translation
b3301_mRNA_2	mRNA b3301	C4143H4681N1656O3054P435	-436	Translation
b3301_mRNA_2_degr	mRNA b3301 degradation complex	C44826H70080N13531O15739S200P435Mg6Zn2Fe0	-658	mRNA degradation
b3302_aa	polypeptide b3302	C287H499N88O81S3	5	Translation
b3302_def_map_cplx	Polypeptide b3302 peptide deformylase and methionine aminopeptidase complex	C2417H3955N681O727S24Mg0Zn0Fe3	-4	Maturation
b3302_m	Matured polypeptide b3302	C281H491N87O79S2	6	Maturation
b3302_mRNA	mRNA b3302	C1713H1937N682O1259P180	-181	Translation
b3302_mRNA_1	mRNA b3302	C1713H1937N682O1259P180	-181	Translation
b3302_mRNA_2	mRNA b3302	C1713H1937N682O1259P180	-181	Translation
b3302_mRNA_2_degr	mRNA b3302 degradation complex	C42396H67336N12557O13944S200P180Mg6Zn2Fe0	-403	mRNA degradation
b3303_aa	polypeptide b3303	C767H1281N233O228S7	8	Translation
b3303_def_map_cplx	Polypeptide b3303 peptide deformylase and methionine aminopeptidase complex	C2897H4737N826O874S28Mg0Zn0Fe3	-1	Maturation
b3303_m	Matured polypeptide b3303	C761H1273N232O226S6	9	Maturation
b3303_mRNA	mRNA b3303	C4802H5424N1923O3520P504	-505	Translation
b3303_mRNA_1	mRNA b3303	C4802H5424N1923O3520P504	-505	Translation
b3303_mRNA_2	mRNA b3303	C4802H5424N1923O3520P504	-505	Translation
b3303_mRNA_2_degr	mRNA b3303 degradation complex	C45485H70823N13798O16205S200P504Mg6Zn2Fe0	-727	mRNA degradation
b3304_aa	polypeptide b3304	C558H934N179O164S1	9	Translation
b3304_def_cplx	Polypeptide b3304 peptide deformylase complex	C1402H2328N420O419S7Mg0Zn0Fe1	4	Maturation
b3304_m	Matured polypeptide b3304	C557H935N179O163S1	10	Maturation
b3304_mRNA	mRNA b3304	C3366H3816N1343O2473P354	-355	Translation
b3304_mRNA_1	mRNA b3304	C3366H3816N1343O2473P354	-355	Translation
b3304_mRNA_2	mRNA b3304	C3366H3816N1343O2473P354	-355	Translation
b3304_mRNA_2_degr	mRNA b3304 degradation complex	C44049H69215N13218O15158S200P354Mg6Zn2Fe0	-577	mRNA degradation
b3305_aa	polypeptide b3305	C838H1382N244O248S3	8	Translation
b3305_def_map_cplx	Polypeptide b3305 peptide deformylase and methionine aminopeptidase complex	C2968H4838N837O894S24Mg0Zn0Fe3	-1	Maturation
b3305_m	Matured polypeptide b3305	C832H1374N243O246S2	9	Maturation
b3305_mRNA	mRNA b3305	C5083H5744N2025O3745P534	-535	Translation

b3305_mRNA_1	mRNA b3305	C5083H5744N2025O3745P534	-535	Translation
b3305_mRNA_2	mRNA b3305	C5083H5744N2025O3745P534	-535	Translation
b3305_mRNA_2_degr	mRNA b3305 degradation complex	C45766H71143N13900O16430S200P534Mg6Zn2Fe0	-757	mRNA degradation
b3306_aa	polypeptide b3306	C622H1042N174O186S7	4	Translation
b3306_def_map_cplx	Polypeptide b3306 peptide deformylase and methionine aminopeptidase complex	C2752H4498N767O832S28Mg0Zn0Fe3	-5	Maturation
b3306_m	Matured polypeptide b3306	C616H1034N173O184S6	5	Maturation
b3306_mRNA	mRNA b3306	C3746H4230N1503O2744P393	-394	Translation
b3306_mRNA_1	mRNA b3306	C3746H4230N1503O2744P393	-394	Translation
b3306_mRNA_2	mRNA b3306	C3746H4230N1503O2744P393	-394	Translation
b3306_mRNA_2_degr	mRNA b3306 degradation complex	C44429H69629N13378O15429S200P393Mg6Zn2Fe0	-616	mRNA degradation
b3307_aa	polypeptide b3307	C505H855N165O141S4	14	Translation
b3307_def_cplx	Polypeptide b3307 peptide deformylase complex	C1349H2249N406O396S10Mg0Zn0Fe1	9	Maturation
b3307_m	Matured polypeptide b3307	C499H847N164O139S3	15	Maturation
b3307_mRNA	mRNA b3307	C2915H3299N1172O2134P306	-307	Translation
b3307_mRNA_1	mRNA b3307	C2915H3299N1172O2134P306	-307	Translation
b3307_mRNA_2	mRNA b3307	C2915H3299N1172O2134P306	-307	Translation
b3307_mRNA_2_degr	mRNA b3307 degradation complex	C43598H68698N13047O14819S200P306Mg6Zn2Fe0	-529	mRNA degradation
b3308_aa	polypeptide b3308	C911H1468N252O260S7	6	Translation
b3308_def_map_cplx	Polypeptide b3308 peptide deformylase and methionine aminopeptidase complex	C3041H4924N845O906S28Mg0Zn0Fe3	-3	Maturation
b3308_m	Matured polypeptide b3308	C905H1460N251O258S6	7	Maturation
b3308_mRNA	mRNA b3308	C5133H5811N2036O3770P540	-541	Translation
b3308_mRNA_1	mRNA b3308	C5133H5811N2036O3770P540	-541	Translation
b3308_mRNA_2	mRNA b3308	C5133H5811N2036O3770P540	-541	Translation
b3308_mRNA_2_degr	mRNA b3308 degradation complex	C45816H71210N13911O16455S200P540Mg6Zn2Fe0	-763	mRNA degradation
b3309_aa	polypeptide b3309	C504H855N149O145S1	10	Translation
b3309_def_map_cplx	Polypeptide b3309 peptide deformylase and methionine aminopeptidase complex	C2634H4311N742O791S22Mg0Zn0Fe3	1	Maturation
b3309_m	Matured polypeptide b3309	C498H847N148O143S0	11	Maturation
b3309_mRNA	mRNA b3309	C3012H3394N1227O2180P315	-316	Translation
b3309_mRNA_1	mRNA b3309	C3012H3394N1227O2180P315	-316	Translation
b3309_mRNA_2	mRNA b3309	C3012H3394N1227O2180P315	-316	Translation
b3309_mRNA_2_degr	mRNA b3309 degradation complex	C43695H68793N13102O14865S200P315Mg6Zn2Fe0	-538	mRNA degradation
b3310_aa	polypeptide b3310	C594H1022N181O168S6	11	Translation
b3310_def_cplx	Polypeptide b3310 peptide deformylase complex	C1438H2416N422O423S12Mg0Zn0Fe1	6	Maturation
b3310_m	Matured polypeptide b3310	C593H1023N181O167S6	12	Maturation
b3310_mRNA	mRNA b3310	C3544H4004N1419O2607P374	-375	Translation
b3310_mRNA_1	mRNA b3310	C3544H4004N1419O2607P374	-375	Translation
b3310_mRNA_2	mRNA b3310	C3544H4004N1419O2607P374	-375	Translation
b3310_mRNA_2_degr	mRNA b3310 degradation complex	C44227H69403N13294O15292S200P374Mg6Zn2Fe0	-597	mRNA degradation
b3311_aa	polypeptide b3311	C431H724N125O122S4	5	Translation
b3311_def_map_cplx	Polypeptide b3311 peptide deformylase and methionine aminopeptidase complex	C2561H4180N718O768S25Mg0Zn0Fe3	-4	Maturation
b3311_m	Matured polypeptide b3311	C425H716N124O120S3	6	Maturation
b3311_mRNA	mRNA b3311 (1 nt short)	C2422H2734N973O1768P254	-255	Translation
b3311_mRNA_1	mRNA b3311 (1 nt short)	C2422H2734N973O1768P254	-255	Translation
b3311_mRNA_2	mRNA b3311 (1 nt short)	C2422H2734N973O1768P254	-255	Translation
b3311_mRNA_2_degr	mRNA b3311 degradation complex	C43105H68133N12848O14453S200P254Mg6Zn2Fe0	-477	mRNA degradation
b3312_aa	polypeptide b3312	C314H542N99O96S2	3	Translation
b3312_def_cplx	Polypeptide b3312 peptide deformylase complex	C1158H1936N340O351S8Mg0Zn0Fe1	-2	Maturation
b3312_m	Matured polypeptide b3312	C313H543N99O95S2	4	Maturation
b3312_mRNA	mRNA b3312 (1 nt short)	C1830H2066N759O1323P191	-192	Translation
b3312_mRNA_1	mRNA b3312 (1 nt short)	C1830H2066N759O1323P191	-192	Translation
b3312_mRNA_2	mRNA b3312 (1 nt short)	C1830H2066N759O1323P191	-192	Translation
b3312_mRNA_2_degr	mRNA b3312 degradation complex	C42513H67465N12634O14008S200P191Mg6Zn2Fe0	-414	mRNA degradation
b3313_aa	polypeptide b3313	C687H1156N205O178S6	19	Translation
b3313_def_cplx	Polypeptide b3313 peptide deformylase complex	C1531H2550N446O433S12Mg0Zn0Fe1	14	Maturation

b3313_m	Matured polypeptide b3313	C686H1157N205O177S6	20	Maturation
b3313_mRNA	mRNA b3313	C3927H4431N1598O2862P411	-412	Translation
b3313_mRNA_1	mRNA b3313	C3927H4431N1598O2862P411	-412	Translation
b3313_mRNA_2	mRNA b3313	C3927H4431N1598O2862P411	-412	Translation
b3313_mRNA_2_degr	mRNA b3313 degradation complex	C44610H69830N13473O15547S200P411Mg6Zn2Fe0	-634	mRNA degradation
b3314_aa	polypeptide b3314	C1155H1921N347O325S5	18	Translation
b3314_def_map_cplx	Polypeptide b3314 peptide deformylase and methionine aminopeptidase complex	C3285H5377N940O971S26Mg0Zn0Fe3	9	Maturation
b3314_m	Matured polypeptide b3314	C1149H1913N346O323S4	19	Maturation
b3314_mRNA	mRNA b3314	C6694H7568N2703O4881P702	-703	Translation
b3314_mRNA_1	mRNA b3314	C6694H7568N2703O4881P702	-703	Translation
b3314_mRNA_2	mRNA b3314	C6694H7568N2703O4881P702	-703	Translation
b3314_mRNA_2_degr	mRNA b3314 degradation complex	C47377H72967N14578O17566S200P702Mg6Zn2Fe0	-925	mRNA degradation
b3315_aa	polypeptide b3315	C533H921N166O157S3	9	Translation
b3315_def_cplx	Polypeptide b3315 peptide deformylase complex	C1377H2315N407O412S9Mg0Zn0Fe1	4	Maturation
b3315_m	Matured polypeptide b3315	C532H922N166O156S3	10	Maturation
b3315_mRNA	mRNA b3315	C3169H3585N1264O2323P333	-334	Translation
b3315_mRNA_1	mRNA b3315	C3169H3585N1264O2323P333	-334	Translation
b3315_mRNA_2	mRNA b3315	C3169H3585N1264O2323P333	-334	Translation
b3315_mRNA_2_degr	mRNA b3315 degradation complex	C43852H68984N13139O15008S200P333Mg6Zn2Fe0	-556	mRNA degradation
b3316_aa	polypeptide b3316	C470H777N140O124S3	11	Translation
b3316_def_map_cplx	Polypeptide b3316 peptide deformylase and methionine aminopeptidase complex	C2600H4233N733O770S24Mg0Zn0Fe3	2	Maturation
b3316_m	Matured polypeptide b3316	C464H769N139O122S2	12	Maturation
b3316_mRNA	mRNA b3316	C2657H3004N1063O1943P279	-280	Translation
b3316_mRNA_1	mRNA b3316	C2657H3004N1063O1943P279	-280	Translation
b3316_mRNA_2	mRNA b3316	C2657H3004N1063O1943P279	-280	Translation
b3316_mRNA_2_degr	mRNA b3316 degradation complex	C43340H68403N12938O14628S200P279Mg6Zn2Fe0	-502	mRNA degradation
b3317_aa	polypeptide b3317	C1300H2178N426O368S8	32	Translation
b3317_def_map_cplx	Polypeptide b3317 peptide deformylase and methionine aminopeptidase complex	C3430H5634N1019O1014S29Mg0Zn0Fe3	23	Maturation
b3317_m	Matured polypeptide b3317	C1294H2170N425O366S7	33	Maturation
b3317_mRNA	mRNA b3317	C7825H8857N3134O5738P822	-823	Translation
b3317_mRNA_1	mRNA b3317	C7825H8857N3134O5738P822	-823	Translation
b3317_mRNA_2	mRNA b3317	C7825H8857N3134O5738P822	-823	Translation
b3317_mRNA_2_degr	mRNA b3317 degradation complex	C48508H74256N15009O18423S200P822Mg6Zn2Fe0	-1045	mRNA degradation
b3318_aa	polypeptide b3318	C497H845N146O144S2	7	Translation
b3318_def_cplx	Polypeptide b3318 peptide deformylase complex	C1341H2239N387O399S8Mg0Zn0Fe1	2	Maturation
b3318_m	Matured polypeptide b3318	C496H846N146O143S2	8	Maturation
b3318_mRNA	mRNA b3318 (4 nt short)	C2862H3228N1177O2064P299	-300	Translation
b3318_mRNA_1	mRNA b3318 (4 nt short)	C2862H3228N1177O2064P299	-300	Translation
b3318_mRNA_2	mRNA b3318 (4 nt short)	C2862H3228N1177O2064P299	-300	Translation
b3318_mRNA_2_degr	mRNA b3318 degradation complex	C43545H68627N13052O14749S200P299Mg6Zn2Fe0	-522	mRNA degradation
b3319_aa	polypeptide b3319	C975H1618N283O291S5	7	Translation
b3319_def_cplx	Polypeptide b3319 peptide deformylase complex	C1819H3012N524O546S11Mg0Zn0Fe1	2	Maturation
b3319_m	Matured polypeptide b3319	C974H1619N283O290S5	8	Maturation
b3319_mRNA	mRNA b3319	C5774H6527N2318O4238P606	-607	Translation
b3319_mRNA_1	mRNA b3319	C5774H6527N2318O4238P606	-607	Translation
b3319_mRNA_2	mRNA b3319	C5774H6527N2318O4238P606	-607	Translation
b3319_mRNA_2_degr	mRNA b3319 degradation complex	C46457H71926N14193O16923S200P606Mg6Zn2Fe0	-829	mRNA degradation
b3320_aa	polypeptide b3320	C980H1615N288O295S4	9	Translation
b3320_def_cplx	Polypeptide b3320 peptide deformylase complex	C1824H3009N529O550S10Mg0Zn0Fe1	4	Maturation
b3320_m	Matured polypeptide b3320	C979H1616N288O294S4	10	Maturation
b3320_mRNA	mRNA b3320	C6008H6787N2422O4393P630	-631	Translation
b3320_mRNA_1	mRNA b3320	C6008H6787N2422O4393P630	-631	Translation
b3320_mRNA_2	mRNA b3320	C6008H6787N2422O4393P630	-631	Translation
b3320_mRNA_2_degr	mRNA b3320 degradation complex	C46691H72186N14297O17078S200P630Mg6Zn2Fe0	-853	mRNA degradation
b3321_aa	polypeptide b3321	C515H864N158O152S2	2	Translation
b3321_def_cplx	Polypeptide b3321 peptide deformylase complex	C1359H2258N399O407S8Mg0Zn0Fe1	-3	Maturation

b3321_m	Matured polypeptide b3321	C514H865N158O151S2	3	Maturation
b3321_mRNA	mRNA b3321	C2965H3368N1185O2182P314	-315	Translation
b3321_mRNA_1	mRNA b3321	C2965H3368N1185O2182P314	-315	Translation
b3321_mRNA_2	mRNA b3321	C2965H3368N1185O2182P314	-315	Translation
b3321_mRNA_2_degr	mRNA b3321 degradation complex	C43648H68767N13060O14867S200P314Mg6Zn2Fe0	-537	mRNA degradation
b3339_aa	polypeptide b3339_v1	C1923H3058N524O583S14	-16	Translation
b3339_def_map_cplx	Polypeptide b3339 peptide deformylase and methionine aminopeptidase complex	C4053H6514N1117O1229S35Mg0Zn0Fe3	-25	Maturation
b3339_m	Matured polypeptide b3339	C1917H3050N523O581S13	-15	Maturation
b3339_mRNA	mRNA b3339	C11276H12768N4509O8278P1185	-1186	Translation
b3339_mRNA_1	mRNA b3339	C11276H12768N4509O8278P1185	-1186	Translation
b3339_mRNA_2	mRNA b3339	C11276H12768N4509O8278P1185	-1186	Translation
b3339_mRNA_2_degr	mRNA b3339 degradation complex	C51959H78167N16384O20963S200P1185Mg6Zn2Fe0	-1408	mRNA degradation
b3339_m_DnaKJ_complex	b3339 DnaK DnaJ_dim complex - Deuerling et al. DnaKJ/GrpE dependent folding	C8481H13564O2679N2436S60P3Zn4	-37	Folding
b3339_v1_mRNA	mRNA b3339_v1	C11276H12768N4509O8278P1185	-1186	Translation
b3340_aa	polypeptide b3340_v1	C3435H5426N943O1051S26	-25	Translation
b3340_def_map_cplx	Polypeptide b3340 peptide deformylase and methionine aminopeptidase complex	C5565H8882N1536O1697S47Mg0Zn0Fe3	-34	Maturation
b3340_m	Matured polypeptide b3340	C3429H5418N942O1049S25	-24	Maturation
b3340_mRNA	mRNA b3340	C20146H22777N8078O14744P2115	-2116	Translation
b3340_mRNA_1	mRNA b3340	C20146H22777N8078O14744P2115	-2116	Translation
b3340_mRNA_2	mRNA b3340	C20146H22777N8078O14744P2115	-2116	Translation
b3340_mRNA_2_degr	mRNA b3340 degradation complex	C60829H88176N19953O27429S200P2115Mg6Zn2Fe0	-2338	mRNA degradation
b3340_m_DnaKJ_complex	b3340 DnaK DnaJ_dim complex - Kerner et al. class I can interact w/ GroEL/ES	C9993H15932O3147N2855S72P3Zn4	-46	Folding
b3340_m_GroEL.(7)ATP.transGroES	b3340 GroEL GroES complex - Kerner et al. class I can interact w/ GroEL/ES	C41341H68362O13383N11526S389P21Mg7	-325	Folding
b3340_v1_mRNA	mRNA b3340_v1	C20146H22777N8078O14750P2117	-2118	Translation
b3341_aa	polypeptide b3341	C880H1457N270O255S5	13	Translation
b3341_def_map_cplx	Polypeptide b3341 peptide deformylase and methionine aminopeptidase complex	C3010H4913N863O901S26Mg0Zn0Fe3	4	Maturation
b3341_m	Matured polypeptide b3341	C874H1449N269O253S4	14	Maturation
b3341_mRNA	mRNA b3341	C5134H5804N2031O3786P540	-541	Translation
b3341_mRNA_1	mRNA b3341	C5134H5804N2031O3786P540	-541	Translation
b3341_mRNA_2	mRNA b3341	C5134H5804N2031O3786P540	-541	Translation
b3341_mRNA_2_degr	mRNA b3341 degradation complex	C45817H71203N13906O16471S200P540Mg6Zn2Fe0	-763	mRNA degradation
b3342_aa	polypeptide b3342	C596H1027N197O167S5	20	Translation
b3342_def_map_cplx	Polypeptide b3342 peptide deformylase and methionine aminopeptidase complex	C2726H4483N790O813S26Mg0Zn0Fe3	11	Maturation
b3342_m	Matured polypeptide b3342	C590H1019N196O165S4	21	Maturation
b3342_mRNA	mRNA b3342	C3565H4042N1421O2625P377	-378	Translation
b3342_mRNA_1	mRNA b3342	C3565H4042N1421O2625P377	-378	Translation
b3342_mRNA_2	mRNA b3342	C3565H4042N1421O2625P377	-378	Translation
b3342_mRNA_2_degr	mRNA b3342 degradation complex	C44248H69441N13296O15310S200P377Mg6Zn2Fe0	-600	mRNA degradation
b3343_aa	polypeptide b3343	C484H765N130O142S2	-5	Translation
b3343_def_map_cplx	Polypeptide b3343 peptide deformylase and methionine aminopeptidase complex	C2614H4221N723O788S23Mg0Zn0Fe3	-14	Maturation
b3343_m	Matured polypeptide b3343	C478H757N129O140S1	-4	Maturation
b3343_mRNA	mRNA b3343	C2727H3093N1058O2026P288	-289	Translation
b3343_mRNA_1	mRNA b3343	C2727H3093N1058O2026P288	-289	Translation
b3343_mRNA_2	mRNA b3343	C2727H3093N1058O2026P288	-289	Translation
b3343_mRNA_2_degr	mRNA b3343 degradation complex	C43410H68492N12933O14711S200P288Mg6Zn2Fe0	-511	mRNA degradation
b3344_aa	polypeptide b3344	C595H924N154O171S3	-6	Translation
b3344_def_map_cplx	Polypeptide b3344 peptide deformylase and methionine aminopeptidase complex	C2725H4380N747O817S24Mg0Zn0Fe3	-15	Maturation
b3344_m	Matured polypeptide b3344	C589H916N153O169S2	-5	Maturation
b3344_mRNA	mRNA b3344 (1 nt short)	C3398H3845N1306O2546P359	-360	Translation
b3344_mRNA_1	mRNA b3344 (1 nt short)	C3398H3845N1306O2546P359	-360	Translation
b3344_mRNA_2	mRNA b3344 (1 nt short)	C3398H3845N1306O2546P359	-360	Translation

b3344_mRNA_2_degr	mRNA b3344 degradation complex	C44081H69244N13181O15231S200P359Mg6Zn2Fe0	-582	mRNA degradation
b3345_aa	polypeptide b3345	C604H933N170O187S3	-5	Translation
b3345_def_map_cplx	Polypeptide b3345 peptide deformylase and methionine aminopeptidase complex	C2734H4389N763O833S24Mg0Zn0Fe3	-14	Maturation
b3345_m	Matured polypeptide b3345	C598H925N169O185S2	-4	Maturation
b3345_mRNA	mRNA b3345 (1 nt short)	C3679H4157N1478O2711P386	-387	Translation
b3345_mRNA_1	mRNA b3345 (1 nt short)	C3679H4157N1478O2711P386	-387	Translation
b3345_mRNA_2	mRNA b3345 (1 nt short)	C3679H4157N1478O2711P386	-387	Translation
b3345_mRNA_2_degr	mRNA b3345 degradation complex	C44362H69556N13353O15396S200P386Mg6Zn2Fe0	-609	mRNA degradation
b3346_aa	polypeptide b3346	C1174H1887N326O370S11	-7	Translation
b3346_def_map_cplx	Polypeptide b3346 peptide deformylase and methionine aminopeptidase complex	C3304H5343N919O1016S32Mg0Zn0Fe3	-16	Maturation
b3346_m	Matured polypeptide b3346	C1168H1879N325O368S10	-6	Maturation
b3346_mRNA	mRNA b3346	C6863H7761N2688O5073P725	-726	Translation
b3346_mRNA_1	mRNA b3346	C6863H7761N2688O5073P725	-726	Translation
b3346_mRNA_2	mRNA b3346	C6863H7761N2688O5073P725	-726	Translation
b3346_mRNA_2_degr	mRNA b3346 degradation complex	C47546H73160N14563O17758S200P725Mg6Zn2Fe0	-948	mRNA degradation
b3384_aa	polypeptide b3384_v6	C1668H2629N453O499S14	-4	Translation
b3384_def_map_cplx	Polypeptide b3384 peptide deformylase and methionine aminopeptidase complex	C3798H6085N1046O1145S35Mg0Zn0Fe3	-13	Maturation
b3384_m	Matured polypeptide b3384 _ error CCDB	C1662H2621N452O497S13	-3	Maturation
b3384_mRNA	mRNA b3384 (8 nt short)	C9501H10743N3822O6954P997	-998	Translation
b3384_mRNA_1	mRNA b3384 (8 nt short)	C9501H10743N3822O6954P997	-998	Translation
b3384_mRNA_2	mRNA b3384 (8 nt short)	C9501H10743N3822O6954P997	-998	Translation
b3384_mRNA_2_degr	mRNA b3384 degradation complex	C50184H76142N15697O19639S200P997Mg6Zn2Fe0	-1220	mRNA degradation
b3384_m_DnaKJ_complex	b3384 DnaK DnaJ_dim complex - Deuerling et al. DnaKJ/GrpE dependent folding	C8226H13135O2595N2365S60P3Zn4	-25	Folding
b3384_v1_mRNA	mRNA b3384_v1 (8 nt short)	C9501H10743N3822O6954P997	-998	Translation
b3384_v2_mRNA	mRNA b3384_v2 (8 nt short)	C9501H10743N3822O6954P997	-998	Translation
b3384_v3_mRNA	mRNA b3384_v3 (8 nt short)	C9501H10743N3822O6954P997	-998	Translation
b3384_v4_mRNA	mRNA b3384_v4 (8 nt short)	C9501H10743N3822O6954P997	-998	Translation
b3384_v5_mRNA	mRNA b3384_v5 (8 nt short)	C9501H10743N3822O6954P997	-998	Translation
b3384_v6_mRNA	mRNA b3384_v6	C9578H10829N3854O7014P1007	-1008	Translation
b3385_aa	polypeptide b3385_v5	C1226H1914N324O374S7	-16	Translation
b3385_def_map_cplx	Polypeptide b3385 peptide deformylase and methionine aminopeptidase complex	C3356H5370N917O1020S28Mg0Zn0Fe3	-25	Maturation
b3385_m	Matured polypeptide b3385	C1220H1906N323O372S6	-15	Maturation
b3385_mRNA	mRNA b3385 (8 nt short)	C7149H8084N2855O5263P751	-752	Translation
b3385_mRNA_1	mRNA b3385 (8 nt short)	C7149H8084N2855O5263P751	-752	Translation
b3385_mRNA_2	mRNA b3385 (8 nt short)	C7149H8084N2855O5263P751	-752	Translation
b3385_mRNA_2_degr	mRNA b3385 degradation complex	C47832H73483N14730O17948S200P751Mg6Zn2Fe0	-974	mRNA degradation
b3385_v1_mRNA	mRNA b3385_v1 (8 nt short)	C7149H8084N2855O5263P751	-752	Translation
b3385_v2_mRNA	mRNA b3385_v2 (8 nt short)	C7149H8084N2855O5263P751	-752	Translation
b3385_v3_mRNA	mRNA b3385_v3 (8 nt short)	C7149H8084N2855O5263P751	-752	Translation
b3385_v4_mRNA	mRNA b3385_v4 (8 nt short)	C7149H8084N2855O5263P751	-752	Translation
b3385_v5_mRNA	mRNA b3385_v5 (8 nt short)	C7149H8084N2855O5263P751	-752	Translation
b3386_aa	polypeptide b3386_v5	C1100H1736N292O327S9	-10	Translation
b3386_def_map_cplx	Polypeptide b3386 peptide deformylase and methionine aminopeptidase complex	C3230H5192N885O973S30Mg0Zn0Fe3	-19	Maturation
b3386_m	Matured polypeptide b3386	C1094H1728N291O325S8	-9	Maturation
b3386_mRNA	mRNA b3386	C6441H7289N2542O4761P680	-681	Translation
b3386_mRNA_1	mRNA b3386	C6441H7289N2542O4761P680	-681	Translation
b3386_mRNA_2	mRNA b3386	C6441H7289N2542O4761P680	-681	Translation
b3386_mRNA_2_degr	mRNA b3386 degradation complex	C47124H72688N14417O17446S200P680Mg6Zn2Fe0	-903	mRNA degradation
b3386_v1_mRNA	mRNA b3386_v1	C6441H7289N2542O4755P678	-679	Translation
b3386_v2_mRNA	mRNA b3386_v2	C6441H7289N2542O4755P678	-679	Translation
b3386_v3_mRNA	mRNA b3386_v3	C6441H7289N2542O4755P678	-679	Translation
b3386_v4_mRNA	mRNA b3386_v4	C6441H7289N2542O4755P678	-679	Translation
b3386_v5_mRNA	mRNA b3386_v5	C6441H7289N2542O4755P678	-679	Translation

b3387_aa	polypeptide b3387_v4	C1461H2225N391O411S9	4	Translation
b3387_def_map_cplx	Polypeptide b3387 peptide deformylase and methionine aminopeptidase complex	C3591H5681N984O1057S30Mg0Zn0Fe3	-5	Maturation
b3387_m	Matured polypeptide b3387	C1455H2217N390O409S8	5	Maturation
b3387_mRNA	mRNA b3387	C7952H8994N3135O5872P839	-840	Translation
b3387_mRNA_1	mRNA b3387	C7952H8994N3135O5872P839	-840	Translation
b3387_mRNA_2	mRNA b3387	C7952H8994N3135O5872P839	-840	Translation
b3387_mRNA_2_degr	mRNA b3387 degradation complex	C48635H74393N15010O18557S200P839Mg6Zn2Fe0	-1062	mRNA degradation
b3387_v1_mRNA	mRNA b3387_v1	C7952H8994N3135O5872P839	-840	Translation
b3387_v2_mRNA	mRNA b3387_v2	C7952H8994N3135O5866P837	-838	Translation
b3387_v3_mRNA	mRNA b3387_v3	C7952H8994N3135O5866P837	-838	Translation
b3387_v4_mRNA	mRNA b3387_v4	C7952H8994N3135O5866P837	-838	Translation
b3388_aa	polypeptide b3388_v2	C1968H3205N591O682S4	-4	Translation
b3388_def_map_cplx	Polypeptide b3388 peptide deformylase and methionine aminopeptidase complex	C4098H6661N1184O1328S25Mg0Zn0Fe3	-13	Maturation
b3388_m	Matured polypeptide b3388 _ error CCDB	C1962H3197N590O680S3	-3	Maturation
b3388_mRNA	mRNA b3388	C12284H13925N5030O8897P1289	-1290	Translation
b3388_mRNA_1	mRNA b3388	C12284H13925N5030O8897P1289	-1290	Translation
b3388_mRNA_2	mRNA b3388	C12284H13925N5030O8897P1289	-1290	Translation
b3388_mRNA_2_degr	mRNA b3388 degradation complex	C52967H79324N16905O21582S200P1289Mg6Zn2Fe0	-1512	mRNA degradation
b3388_v1_mRNA	mRNA b3388_v1	C12284H13925N5030O8891P1287	-1288	Translation
b3388_v2_mRNA	mRNA b3388_v2	C12284H13925N5030O8891P1287	-1288	Translation
b3389_aa	polypeptide b3389_v1	C1730H2789N474O512S15	-7	Translation
b3389_def_map_cplx	Polypeptide b3389 peptide deformylase and methionine aminopeptidase complex	C3860H6245N1067O1158S36Mg0Zn0Fe3	-16	Maturation
b3389_m	Matured polypeptide b3389	C1724H2781N473O510S14	-6	Maturation
b3389_mRNA	mRNA b3389	C10349H11704N4087O7677P1089	-1090	Translation
b3389_mRNA_1	mRNA b3389	C10349H11704N4087O7677P1089	-1090	Translation
b3389_mRNA_2	mRNA b3389	C10349H11704N4087O7677P1089	-1090	Translation
b3389_mRNA_2_degr	mRNA b3389 degradation complex	C51032H77103N15962O20362S200P1089Mg6Zn2Fe0	-1312	mRNA degradation
b3389_v1_mRNA	mRNA b3389_v1	C10349H11704N4087O7677P1089	-1090	Translation
b3390_aa	polypeptide b3390_v1	C847H1378N250O273S4	-6	Translation
b3390_def_map_cplx	Polypeptide b3390 peptide deformylase and methionine aminopeptidase complex	C2977H4834N843O919S25Mg0Zn0Fe3	-15	Maturation
b3390_m	Matured polypeptide b3390	C841H1370N249O271S3	-5	Maturation
b3390_mRNA	mRNA b3390	C4987H5625N2026O3632P524	-525	Translation
b3390_mRNA_1	mRNA b3390	C4987H5625N2026O3632P524	-525	Translation
b3390_mRNA_2	mRNA b3390	C4987H5625N2026O3632P524	-525	Translation
b3390_mRNA_2_degr	mRNA b3390 degradation complex	C45670H71024N13901O16317S200P524Mg6Zn2Fe0	-747	mRNA degradation
b3390_m_DnaKJ_complex	b3390 DnaK DnaJ_dim complex - Kerner et al. class II can interact w/ GroEL/ES, cannot fold spontanously	C7405H11884O2369N2162S50P3Zn4	-27	Folding
b3390_m_GroEL.(7)ATP.transGroES	b3390 GroEL GroES complex - Kerner et al. class II can interact w/ GroEL/ES, cannot fold spontanously	C38753H64314O12605N10833S367P21Mg7	-306	Folding
b3390_v1_mRNA	mRNA b3390_v1	C4987H5625N2026O3632P524	-525	Translation
b3406_aa	polypeptide b3406	C834H1302N228O246S3	0	Translation
b3406_def_cplx	Polypeptide b3406 peptide deformylase complex	C1678H2696N469O501S9Mg0Zn0Fe1	-5	Maturation
b3406_m	Matured polypeptide b3406	C833H1303N228O245S3	1	Maturation
b3406_mRNA	mRNA b3406	C4554H5142N1847O3319P479	-480	Translation
b3406_mRNA_1	mRNA b3406	C4554H5142N1847O3319P479	-480	Translation
b3406_mRNA_2	mRNA b3406	C4554H5142N1847O3319P479	-480	Translation
b3406_mRNA_2_degr	mRNA b3406 degradation complex	C45237H70541N13722O16004S200P479Mg6Zn2Fe0	-702	mRNA degradation
b3461_aa	polypeptide b3461_v5	C1424H2257N414O435S11	-7	Translation
b3461_def_map_cplx	Polypeptide b3461 peptide deformylase and methionine aminopeptidase complex	C3554H5713N1007O1081S32Mg0Zn0Fe3	-16	Maturation
b3461_m	Matured polypeptide b3461	C1418H2249N413O433S10	-6	Maturation
b3461_mRNA	mRNA b3461	C8153H9226N3301O5960P857	-858	Translation
b3461_mRNA_1	mRNA b3461	C8153H9226N3301O5960P857	-858	Translation
b3461_mRNA_2	mRNA b3461	C8153H9226N3301O5960P857	-858	Translation
b3461_mRNA_2_degr	mRNA b3461 degradation complex	C48836H74625N15176O18645S200P857Mg6Zn2Fe0	-1080	mRNA degradation

b3461_v1_mRNA	mRNA b3461_v1	C8153H9226N3301O5960P857	-858	Translation
b3461_v2_mRNA	mRNA b3461_v2	C8153H9226N3301O5960P857	-858	Translation
b3461_v3_mRNA	mRNA b3461_v3	C8153H9226N3301O5960P857	-858	Translation
b3461_v4_mRNA	mRNA b3461_v4	C8153H9226N3301O5960P857	-858	Translation
b3461_v5_mRNA	mRNA b3461_v5	C8153H9226N3301O5960P857	-858	Translation
b3470_aa	polypeptide b3470	C400H636N108O121S7	-4	Translation
b3470_def_map_cplx	Polypeptide b3470 peptide deformylase and methionine aminopeptidase complex	C2530H4092N701O767S28Mg0Zn0Fe3	-13	Maturation
b3470_m	Matured polypeptide b3470	C394H628N107O119S6	-3	Maturation
b3470_mRNA	mRNA b3470	C2332H2646N913O1737P248	-249	Translation
b3470_mRNA_1	mRNA b3470	C2332H2646N913O1737P248	-249	Translation
b3470_mRNA_2	mRNA b3470	C2332H2646N913O1737P248	-249	Translation
b3470_mRNA_2_degr	mRNA b3470 degradation complex	C43015H68045N12788O14422S200P248Mg6Zn2Fe0	-471	mRNA degradation
b3545_RNA	tRNA (proK)	C733H832N295O542P77	-78	tRNA Modification
b3545_tRNA_1	b3545_tRNA_1 (proK)	C733H834Mg2N295O542P77	-74	tRNA Modification
b3545_tRNA_1_MeT_tRNA_pos_32_Um_cplx	b3545_tRNA_1 (proK), MeT_tRNA_pos_32_Um	C748H857Mg2N301O547P77S1	-73	tRNA Modification
b3545_tRNA_2	b3545_tRNA_2 (proK)	C734H836Mg2N295O542P77	-74	tRNA Modification
b3545_tRNA_2_TrmD_dim_cplx	b3545_tRNA_2 (proK), TrmD_dim	C3261H4813Mg2N1017O1297P77S21	-91	tRNA Modification
b3545_tRNA_3	b3545_tRNA_3 (proK)	C735H838Mg2N295O542P77	-74	tRNA Modification
b3545_tRNA_3_YggH_mono_cplx	b3545_tRNA_3 (proK), YggH_mono	C1960H2737Mg2N648O889P77S14	-76	tRNA Modification
b3545_tRNA_4	b3545_tRNA_4 (proK)	C736H840Mg2N295O542P77	-74	tRNA Modification
b3545_tRNA_4_TrmA_mono_cplx	b3545_tRNA_4 (proK), TrmA_mono	C2610H3787Mg2N818O1101P77S19	-82	tRNA Modification
b3545_tRNA_5	b3545_tRNA_5 (proK)	C737H842Mg2N295O542P77	-74	tRNA Modification
b3545_tRNA_5_TruB_mono_cplx	b3545_tRNA_5 (proK), TruB_mono	C2277H3317Mg2N733O1011P77S10	-79	tRNA Modification
b3545_tRNA_6	b3545_tRNA_6 (proK)	C737H842Mg2N295O542P77	-74	tRNA Modification
b3545_tRNA_6_ThiI_mono_cplx	b3545_tRNA_6 (proK), ThiI_mono	C7165H11073Mg3N2113O2481P82S50X1	-102	tRNA Modification
b3545_tRNA_Mg2	tRNA (proK) bound two Mg2 ions	C733H832Mg2N295O542P77	-74	tRNA Modification
b3545_tRNA_Mg2_Dus_gen_cplx	b3545_tRNA (proK), Dus_gen	C2340H3371Mg2N754O1025P80S14	-81	tRNA Modification
b3559_aa	polypeptide b3559	C3425H5448N947O1020S19	-19	Translation
b3559_def_map_cplx	Polypeptide b3559 peptide deformylase and methionine aminopeptidase complex	C5555H8904N1540O1666S40Mg0Zn0Fe3	-28	Maturation
b3559_m	Matured polypeptide b3559	C3419H5440N946O1018S18	-18	Maturation
b3559_mRNA	mRNA b3559	C19716H22320N7931O14470P2070	-2071	Translation
b3559_mRNA_1	mRNA b3559	C19716H22320N7931O14470P2070	-2071	Translation
b3559_mRNA_2	mRNA b3559	C19716H22320N7931O14470P2070	-2071	Translation
b3559_mRNA_2_degr	mRNA b3559 degradation complex	C60399H87719N19806O27155S200P2070Mg6Zn2Fe0	-2293	mRNA degradation
b3560_aa	polypeptide b3560	C1568H2367N409O464S13	-14	Translation
b3560_def_map_cplx	Polypeptide b3560 peptide deformylase and methionine aminopeptidase complex	C3698H5823N1002O1110S34Mg0Zn0Fe3	-23	Maturation
b3560_m	Matured polypeptide b3560	C1562H2359N408O462S12	-13	Maturation
b3560_mRNA	mRNA b3560	C8671H9843N3472O6371P914	-915	Translation
b3560_mRNA_1	mRNA b3560	C8671H9843N3472O6371P914	-915	Translation
b3560_mRNA_2	mRNA b3560	C8671H9843N3472O6371P914	-915	Translation
b3560_mRNA_2_degr	mRNA b3560 degradation complex	C49354H75242N15347O19056S200P914Mg6Zn2Fe0	-1137	mRNA degradation
b3590_aa	polypeptide b3590	C3044H4831N901O893S17	-12	Translation
b3590_def_cplx	Polypeptide b3590 peptide deformylase complex	C3888H6225N1142O1148S23Mg0Zn0Fe1	-17	Maturation
b3590_m	Matured polypeptide b3590	C3043H4832N901O892S17	-11	Maturation
b3590_mRNA	mRNA b3590 (4 nt short)	C17538H19864N7073O12862P1841	-1842	Translation
b3590_mRNA_1	mRNA b3590 (4 nt short)	C17538H19864N7073O12862P1841	-1842	Translation
b3590_mRNA_2	mRNA b3590 (4 nt short)	C17538H19864N7073O12862P1841	-1842	Translation
b3590_mRNA_2_degr	mRNA b3590 degradation complex	C58221H85263N18948O25547S200P1841Mg6Zn2Fe0	-2064	mRNA degradation
b3591_aa	polypeptide b3591	C2222H3624N643O671S17	-7	Translation
b3591_def_map_cplx	Polypeptide b3591 peptide deformylase and methionine aminopeptidase complex	C4352H7080N1236O1317S38Mg0Zn0Fe3	-16	Maturation
b3591_m	Matured polypeptide b3591 _ error CCDB	C2216H3616N642O669S16	-6	Maturation
b3591_mRNA	mRNA b3591	C13257H15001N5322O9763P1394	-1395	Translation
b3591_mRNA_1	mRNA b3591	C13257H15001N5322O9763P1394	-1395	Translation
b3591_mRNA_2	mRNA b3591	C13257H15001N5322O9763P1394	-1395	Translation
b3591_mRNA_2_degr	mRNA b3591 degradation complex	C53940H80400N17197O22448S200P1394Mg6Zn2Fe0	-1617	mRNA degradation
b3635_aa	polypeptide b3635_v3	C1359H2168N383O383S10	3	Translation

b3635_def_map_cplx	Polypeptide b3635 peptide deformylase and methionine aminopeptidase complex	C3489H5624N976O1029S31Mg0Zn0Fe3	-6	Maturation
b3635_m	Matured polypeptide b3635	C1353H2160N382O381S9	4	Maturation
b3635_mRNA_1	mRNA b3635	C7729H8724N3121O5677P812	-813	Translation
b3635_mRNA_2	mRNA b3635	C7729H8724N3121O5677P812	-813	Translation
b3635_mRNA_2_degr	mRNA b3635 degradation complex	C48412H74123N14996O18362S200P812Mg6Zn2Fe0	-1035	mRNA degradation
b3635_v1_mRNA	mRNA b3635_v1	C7729H8724N3121O5671P810	-811	Translation
b3635_v3_mRNA	mRNA b3635_v3	C7729H8724N3121O5671P810	-811	Translation
b3636_aa	polypeptide b3636_v3	C290H493N82O78S1	9	Translation
b3636_def_map_cplx	Polypeptide b3636 peptide deformylase and methionine aminopeptidase complex	C2420H3949N675O724S22Mg0Zn0Fe3	0	Maturation
b3636_m	Matured polypeptide b3636	C284H485N81O76S0	10	Maturation
b3636_mRNA	mRNA b3636	C1607H1810N655O1155P168	-169	Translation
b3636_mRNA_1	mRNA b3636	C1607H1810N655O1155P168	-169	Translation
b3636_mRNA_2	mRNA b3636	C1607H1810N655O1155P168	-169	Translation
b3636_mRNA_2_degr	mRNA b3636 degradation complex	C42290H67209N12530O13840S200P168Mg6Zn2Fe0	-391	mRNA degradation
b3636_v1_mRNA	mRNA b3636_v1	C1607H1810N655O1155P168	-169	Translation
b3636_v2_mRNA	mRNA b3636_v2	C1607H1810N655O1155P168	-169	Translation
b3636_v3_mRNA	mRNA b3636_v3	C1607H1810N655O1155P168	-169	Translation
b3637_aa	polypeptide b3637_v3	C394H663N130O108S3	11	Translation
b3637_def_map_cplx	Polypeptide b3637 peptide deformylase and methionine aminopeptidase complex	C2524H4119N723O754S24Mg0Zn0Fe3	2	Maturation
b3637_m	Matured polypeptide b3637	C388H655N129O106S2	12	Maturation
b3637_mRNA	mRNA b3637	C2251H2554N893O1663P239	-240	Translation
b3637_mRNA_1	mRNA b3637	C2251H2554N893O1663P239	-240	Translation
b3637_mRNA_2	mRNA b3637	C2251H2554N893O1663P239	-240	Translation
b3637_mRNA_2_degr	mRNA b3637 degradation complex	C42934H67953N12768O14348S200P239Mg6Zn2Fe0	-462	mRNA degradation
b3637_v1_mRNA	mRNA b3637_v1	C2251H2554N893O1657P237	-238	Translation
b3637_v2_mRNA	mRNA b3637_v2	C2251H2554N893O1663P239	-240	Translation
b3637_v3_mRNA	mRNA b3637_v3	C2251H2554N893O1657P237	-238	Translation
b3638_aa	polypeptide b3638_v1	C1133H1823N318O322S10	1	Translation
b3638_def_map_cplx	Polypeptide b3638 peptide deformylase and methionine aminopeptidase complex	C3263H5279N911O968S31Mg0Zn0Fe3	-8	Maturation
b3638_m	Matured polypeptide b3638	C1127H1815N317O320S9	2	Maturation
b3638_mRNA	mRNA b3638	C6372H7185N2534O4692P671	-672	Translation
b3638_mRNA_1	mRNA b3638	C6372H7185N2534O4692P671	-672	Translation
b3638_mRNA_2	mRNA b3638	C6372H7185N2534O4692P671	-672	Translation
b3638_mRNA_2_degr	mRNA b3638 degradation complex	C47055H72584N14409O17377S200P671Mg6Zn2Fe0	-894	mRNA degradation
b3638_v1_mRNA	mRNA b3638_v1	C6372H7185N2534O4692P671	-672	Translation
b3649_aa	polypeptide b3649	C436H730N137O144S2	-5	Translation
b3649_def_map_cplx	Polypeptide b3649 peptide deformylase and methionine aminopeptidase complex	C2566H4186N730O790S23Mg0Zn0Fe3	-14	Maturation
b3649_m	Matured polypeptide b3649	C430H722N136O142S1	-4	Maturation
b3649_mRNA	mRNA b3649	C2640H2985N1088O1914P278	-279	Translation
b3649_mRNA_1	mRNA b3649	C2640H2985N1088O1914P278	-279	Translation
b3649_mRNA_2	mRNA b3649	C2640H2985N1088O1914P278	-279	Translation
b3649_mRNA_2_degr	mRNA b3649 degradation complex	C43323H68384N12963O14599S200P278Mg6Zn2Fe0	-501	mRNA degradation
b3649_m_Mg	b3649 plus _Mg	C430H722N136O142S1Mg1	-2	Folding
b3650_aa	polypeptide b3650	C3506H5657N1013O1024S31	8	Translation
b3650_def_map_cplx	Polypeptide b3650 peptide deformylase and methionine aminopeptidase complex	C5636H9113N1606O1670S52Mg0Zn0Fe3	-1	Maturation
b3650_m	Matured polypeptide b3650	C3500H5649N1012O1022S30	9	Maturation
b3650_mRNA	mRNA b3650	C20071H22747N8054O14694P2109	-2110	Translation
b3650_mRNA_1	mRNA b3650	C20071H22747N8054O14694P2109	-2110	Translation
b3650_mRNA_2	mRNA b3650	C20071H22747N8054O14694P2109	-2110	Translation
b3650_mRNA_2_degr	mRNA b3650 degradation complex	C60754H88146N19929O27379S200P2109Mg6Zn2Fe0	-2332	mRNA degradation
b3650_m_DnaKJ_complex	b3650 DnaK DnaJ_dim complex - Kerner et al. class II can interact w/ GroEL/ES, cannot fold spontanously	C10064H16163O3120N2925S77P3Zn4	-13	Folding

b3650_m_GroEL.(7)ATP.transGroES	b3650 GroEL GroES complex - Kerner et al. class II can interact w/ GroEL/ES, cannot fold spontanously	C41412H68593O13356N11596S394P21Mg7	-292	Folding
b3651_aa	polypeptide b3651	C1104H1768N326O330S15	-2	Translation
b3651_def_map_cplx	Polypeptide b3651 peptide deformylase and methionine aminopeptidase complex	C3234H5224N919O976S36Mg0Zn0Fe3	-11	Maturation
b3651_m	Matured polypeptide b3651	C1098H1760N325O328S14	-1	Maturation
b3651_mRNA	mRNA b3651	C6565H7448N2637O4809P690	-691	Translation
b3651_mRNA_1	mRNA b3651	C6565H7448N2637O4809P690	-691	Translation
b3651_mRNA_2	mRNA b3651	C6565H7448N2637O4809P690	-691	Translation
b3651_mRNA_2_degr	mRNA b3651 degradation complex	C47248H72847N14512O17494S200P690Mg6Zn2Fe0	-913	mRNA degradation
b3651_m_DnaKJ_complex	b3651 DnaK DnaJ_dim complex - Kerner et al. class II can interact w/ GroEL/ES, cannot fold spontanously	C7662H12274O2426N2238S61P3Zn4	-23	Folding
b3651_m_GroEL.(7)ATP.transGroES	b3651 GroEL GroES complex - Kerner et al. class II can interact w/ GroEL/ES, cannot fold spontanously	C39010H64704O12662N10909S378P21Mg7	-302	Folding
b3652_aa	polypeptide b3652	C3386H5486N972O986S27	-4	Translation
b3652_def_map_cplx	Polypeptide b3652 peptide deformylase and methionine aminopeptidase complex	C5516H8942N1565O1632S48Mg0Zn0Fe3	-13	Maturation
b3652_m	Matured polypeptide b3652	C3380H5478N971O984S26	-3	Maturation
b3652_mRNA	mRNA b3652	C19823H22493N8006O14504P2082	-2083	Translation
b3652_mRNA_1	mRNA b3652	C19823H22493N8006O14504P2082	-2083	Translation
b3652_mRNA_2	mRNA b3652	C19823H22493N8006O14504P2082	-2083	Translation
b3652_mRNA_2_degr	mRNA b3652 degradation complex	C60506H87892N19881O27189S200P2082Mg6Zn2Fe0	-2305	mRNA degradation
b3658_RNA	tRNA (selC)	C901H1026N357O672P95	-96	tRNA Modification
b3658_tRNA_1	b3658_tRNA_1 (selC)	C901H1028Mg2N357O672P95	-92	tRNA Modification
b3658_tRNA_1_MiaA_dim_cplx	b3658_tRNA_1 (selC), MiaA_dim	C4018H5971Mg4N1233O1595P97S18	-105	tRNA Modification
b3658_tRNA_2	b3658_tRNA_2 (selC)	C906H1036Mg2N357O672P95	-92	tRNA Modification
b3658_tRNA_2_TrmA_mono_cplx	b3658_tRNA_2 (selC), TrmA_mono	C2780H3983Mg2N880O1231P95S19	-100	tRNA Modification
b3658_tRNA_3	b3658_tRNA_3 (selC)	C907H1038Mg2N357O672P95	-92	tRNA Modification
b3658_tRNA_3_TruB_mono_cplx	b3658_tRNA_3 (selC), TruB_mono	C2447H3513Mg2N795O1141P95S10	-97	tRNA Modification
b3658_tRNA_Mg2	tRNA (selC) bound two Mg2 ions	C901H1026Mg2N357O672P95	-92	tRNA Modification
b3658_tRNA_Mg2_Dus_gen_cplx	b3658_tRNA (selC), Dus_gen	C2508H3565Mg2N816O1155P98S14	-99	tRNA Modification
b3703_aa	polypeptide b3703_v2	C229H417N90O58S2	15	Translation
b3703_def_cplx	Polypeptide b3703 peptide deformylase complex	C1073H1811N331O313S8Mg0Zn0Fe1	10	Maturation
b3703_m	Matured polypeptide b3703	C228H418N90O57S2	16	Maturation
b3703_mRNA	mRNA b3703	C1333H1514N513O1002P143	-144	Translation
b3703_mRNA_1	mRNA b3703	C1333H1514N513O1002P143	-144	Translation
b3703_mRNA_2	mRNA b3703	C1333H1514N513O1002P143	-144	Translation
b3703_mRNA_2_degr	mRNA b3703 degradation complex	C42016H66913N12388O13687S200P143Mg6Zn2Fe0	-366	mRNA degradation
b3703_v1_mRNA	mRNA b3703_v1	C1333H1514N513O1002P143	-144	Translation
b3703_v2_mRNA	mRNA b3703_v2	C1333H1514N513O1002P143	-144	Translation
b3704_aa	polypeptide b3704_v2	C612H1030N199O160S3	15	Translation
b3704_def_map_cplx	Polypeptide b3704 peptide deformylase and methionine aminopeptidase complex	C2742H4486N792O806S24Mg0Zn0Fe3	6	Maturation
b3704_m	Matured polypeptide b3704	C606H1022N198O158S2	16	Maturation
b3704_mRNA	mRNA b3704	C3413H3880N1345O2524P360	-361	Translation
b3704_mRNA_1	mRNA b3704	C3413H3880N1345O2524P360	-361	Translation
b3704_mRNA_2	mRNA b3704	C3413H3880N1345O2524P360	-361	Translation
b3704_mRNA_2_degr	mRNA b3704 degradation complex	C44096H69279N13220O15209S200P360Mg6Zn2Fe0	-583	mRNA degradation
b3704_v1_mRNA	mRNA b3704_v1	C3413H3880N1345O2524P360	-361	Translation
b3704_v2_mRNA	mRNA b3704_v2	C3413H3880N1345O2524P360	-361	Translation
b3706_aa	polypeptide b3706	C2172H3473N619O670S8	-24	Translation
b3706_def_map_cplx	Polypeptide b3706 peptide deformylase and methionine aminopeptidase complex	C4302H6929N1212O1316S29Mg0Zn0Fe3	-33	Maturation
b3706_m	Matured polypeptide b3706	C2166H3465N618O668S7	-23	Maturation
b3706_mRNA	mRNA b3706	C12996H14728N5229O9542P1367	-1368	Translation
b3706_mRNA_1	mRNA b3706	C12996H14728N5229O9542P1367	-1368	Translation
b3706_mRNA_2	mRNA b3706	C12996H14728N5229O9542P1367	-1368	Translation
b3706_mRNA_2_degr	mRNA b3706 degradation complex	C53679H80127N17104O22227S200P1367Mg6Zn2Fe0	-1590	mRNA degradation
b3740_aa	polypeptide b3740	C1059H1679N288O301S6	-5	Translation

b3740_def_map_cplx	Polypeptide b3740 peptide deformylase and methionine aminopeptidase complex	C3189H5135N881O947S27Mg0Zn0Fe3	-14	Maturation
b3740_m	Matured polypeptide b3740	C1053H1671N287O299S5	-4	Maturation
b3740_mRNA	mRNA b3740	C5931H6705N2342O4369P624	-625	Translation
b3740_mRNA_1	mRNA b3740	C5931H6705N2342O4369P624	-625	Translation
b3740_mRNA_2	mRNA b3740	C5931H6705N2342O4369P624	-625	Translation
b3740_mRNA_2_degr	mRNA b3740 degradation complex	C46614H72104N14217O17054S200P624Mg6Zn2Fe0	-847	mRNA degradation
b3741_aa	polypeptide b3741	C3062H4896N880O925S22	-6	Translation
b3741_def_map_cplx	Polypeptide b3741 peptide deformylase and methionine aminopeptidase complex	C5192H8352N1473O1571S43Mg0Zn0Fe3	-15	Maturation
b3741_m	Matured polypeptide b3741	C3056H4888N879O923S21	-5	Maturation
b3741_mRNA	mRNA b3741	C17979H20367N7184O13223P1892	-1893	Translation
b3741_mRNA_1	mRNA b3741	C17979H20367N7184O13223P1892	-1893	Translation
b3741_mRNA_2	mRNA b3741	C17979H20367N7184O13223P1892	-1893	Translation
b3741_mRNA_2_degr	mRNA b3741 degradation complex	C58662H85766N19059O25908S200P1892Mg6Zn2Fe0	-2115	mRNA degradation
b3741_m_DnaKJ_complex	b3741 DnaK DnaJ_dim complex - Kerner et al. class II can interact w/ GroEL/ES, cannot fold spontanously	C9620H15402O3021N2792S68P3Zn4	-27	Folding
b3741_m_GroEL.(7)ATP.transGroES	b3741 GroEL GroES complex - Kerner et al. class II can interact w/ GroEL/ES, cannot fold spontanously	C40968H67832O13257N11463S385P21Mg7	-306	Folding
b3756_RNA	rRNA	C14754H16650N6065O10718P1542	-1543	rRNA Modification
b3756_RNA_1		C14755H16652N6065O10718P1542S0	-1543	rRNA Modification
b3756_RNA_10		C14766H16674N6065O10718P1542S0	-1543	rRNA Modification
b3756_RNA_10_RsmB_mono	b3756_RNA_10, RsmB_mono	C16933H20117N6683O11346S17P1542Mg0Zn0Fe0	-1542	rRNA Modification
b3756_RNA_1_MeT_16S_1402	b3756_RNA_1, MeT_16S_1402	C14785H16698N6077O10728S2P1542Mg0Zn0Fe0	-1541	rRNA Modification
b3756_RNA_2		C14757H16656N6065O10718P1542S0	-1543	rRNA Modification
b3756_RNA_2_MeT_16S_1407	b3756_RNA_2, MeT_16S_1407	C14772H16679N6071O10723S1P1542Mg0Zn0Fe0	-1542	rRNA Modification
b3756_RNA_3		C14758H16658N6065O10718P1542S0	-1543	rRNA Modification
b3756_RNA_3_YggJ_mono	b3756_RNA_3, YggJ_mono	C15950H18598N6413O11077S11P1542Mg0Zn0Fe0	-1545	rRNA Modification
b3756_RNA_4		C14759H16660N6065O10718P1542S0	-1543	rRNA Modification
b3756_RNA_4_MeT_16S_1516	b3756_RNA_4, MeT_16S_1516	C14774H16683N6071O10723S1P1542Mg0Zn0Fe0	-1542	rRNA Modification
b3756_RNA_5		C14760H16662N6065O10718P1542S0	-1543	rRNA Modification
b3756_RNA_5_KsgA_mono	b3756_RNA_5, KsgA_mono	C16142H18851N6453O11116S15P1542Mg0Zn0Fe0	-1540	rRNA Modification
b3756_RNA_6		C14762H16666N6065O10718P1542S0	-1543	rRNA Modification
b3756_RNA_6_KsgA_mono	b3756_RNA_6, KsgA_mono	C16144H18855N6453O11116S15P1542Mg0Zn0Fe0	-1540	rRNA Modification
b3756_RNA_7		C14764H16670N6065O10718P1542S0	-1543	rRNA Modification
b3756_RNA_7_RsuA_mono	b3756_RNA_7, RsuA_mono	C15909H18470N6393O11062S6P1542Mg0Zn0Fe0	-1551	rRNA Modification
b3756_RNA_8		C14764H16670N6065O10718P1542S0	-1543	rRNA Modification
b3756_RNA_8_MeT_16S_527	b3756_RNA_8, MeT_16S_527	C14779H16693N6071O10723S1P1542Mg0Zn0Fe0	-1542	rRNA Modification
b3756_RNA_9		C14765H16672N6065O10718P1542S0	-1543	rRNA Modification
b3756_RNA_9_MeT_16S_966	b3756_RNA_9, MeT_16S_966	C14780H16695N6071O10723S1P1542Mg0Zn0Fe0	-1542	rRNA Modification
b3756_RNA_RsmC_mono	b3756_RNA, RsmC_mono	C16438H19281N6538O11220S11P1542Mg0Zn0Fe0	-1547	rRNA Modification
b3756_v1_RNA	rRNA	C14754H16650N6065O10718P1542	-1543	rRNA Modification
b3756_v1_RNA_1		C14755H16652N6065O10718P1542S0	-1543	rRNA Modification
b3756_v1_RNA_10		C14766H16674N6065O10718P1542S0	-1543	rRNA Modification
b3756_v1_RNA_10_RsmB_mono	b3756_v1_RNA_10, RsmB_mono	C16933H20117N6683O11346S17P1542Mg0Zn0Fe0	-1542	rRNA Modification
b3756_v1_RNA_1_MeT_16S_1402	b3756_v1_RNA_1, MeT_16S_1402	C14785H16698N6077O10728S2P1542Mg0Zn0Fe0	-1541	rRNA Modification
b3756_v1_RNA_2		C14757H16656N6065O10718P1542S0	-1543	rRNA Modification
b3756_v1_RNA_2_MeT_16S_1407	b3756_v1_RNA_2, MeT_16S_1407	C14772H16679N6071O10723S1P1542Mg0Zn0Fe0	-1542	rRNA Modification
b3756_v1_RNA_3		C14758H16658N6065O10718P1542S0	-1543	rRNA Modification
b3756_v1_RNA_3_YggJ_mono	b3756_v1_RNA_3, YggJ_mono	C15950H18598N6413O11077S11P1542Mg0Zn0Fe0	-1545	rRNA Modification
b3756_v1_RNA_4		C14759H16660N6065O10718P1542S0	-1543	rRNA Modification
b3756_v1_RNA_4_MeT_16S_1516	b3756_v1_RNA_4, MeT_16S_1516	C14774H16683N6071O10723S1P1542Mg0Zn0Fe0	-1542	rRNA Modification
b3756_v1_RNA_5		C14760H16662N6065O10718P1542S0	-1543	rRNA Modification
b3756_v1_RNA_5_KsgA_mono	b3756_v1_RNA_5, KsgA_mono	C16142H18851N6453O11116S15P1542Mg0Zn0Fe0	-1540	rRNA Modification
b3756_v1_RNA_6		C14762H16666N6065O10718P1542S0	-1543	rRNA Modification
b3756_v1_RNA_6_KsgA_mono	b3756_v1_RNA_6, KsgA_mono	C16144H18855N6453O11116S15P1542Mg0Zn0Fe0	-1540	rRNA Modification

b3756_v1_RNA_7		C14764H16670N6065O10718P1542S0	-1543	rRNA Modification
b3756_v1_RNA_7_RsuA_mono	b3756_v1_RNA_7, RsuA_mono	C15909H18470N6393O11062S6P1542Mg0Zn0Fe0	-1551	rRNA Modification
b3756_v1_RNA_8		C14764H16670N6065O10718P1542S0	-1543	rRNA Modification
b3756_v1_RNA_8_MeT_16S_527	b3756_v1_RNA_8, MeT_16S_527	C14779H16693N6071O10723S1P1542Mg0Zn0Fe0	-1542	rRNA Modification
b3756_v1_RNA_9		C14765H16672N6065O10718P1542S0	-1543	rRNA Modification
b3756_v1_RNA_9_MeT_16S_966	b3756_v1_RNA_9, MeT_16S_966	C14780H16695N6071O10723S1P1542Mg0Zn0Fe0	-1542	rRNA Modification
b3756_v1_RNA_RsmC_mono	b3756_v1_RNA, RsmC_mono	C16438H19281N6538O11220S11P1542Mg0Zn0Fe0	-1547	rRNA Modification
b3757_RNA	tRNA (gltU)	C720H824N287O532P76	-77	tRNA Modification
b3757_tRNA_1	b3757_tRNA_1 (gltU)	C720H824Mg2N287O532P76	-73	tRNA Modification
b3757_tRNA_1_TrmU_mono-YhhP_mono-YheLMN_cplx-YccK_mono-TrmE_dim-GidA_mono-TrmC_mono_cplx	b3757_tRNA_1 (gltU), TrmU_mono, YhhP_mono, YheLMN_cplx, YccK_mono, TrmE_dim, GidA_mono, TrmC_mono	C21611H33674Mg3N6131O6869P88S131X1	-221	tRNA Modification
b3757_tRNA_2	b3757_tRNA_2 (gltU)	C722H829Mg2N288O531P76S1	-73	tRNA Modification
b3757_tRNA_2_MeT_tRNA_pos_37_m2A_cplx	b3757_tRNA_2 (gltU), MeT_tRNA_pos_37_m2A	C737H852Mg2N294O536P76S2	-72	tRNA Modification
b3757_tRNA_2_Se	b3757_tRNA_2_Se - contains mnm5se2U instead of mnm5s2U,b3757_tRNA_2 (gltU)	C722H829Mg2N288O531P76S0Se1	-73	tRNA Modification
b3757_tRNA_2_Se_MeT_tRNA_pos_37_m2A_cplx	b3757_tRNA_2_Se_MeT_tRNA_pos_37_m2A_cplx - contains mnm5se2U instead of mnm5s2U,b3757_tRNA_2 (gltU), MeT_tRNA_pos_37_m2A	C737H852Mg2N294O536P76S1Se1	-72	tRNA Modification
b3757_tRNA_2_YbbB_dim_cplx	b3757_tRNA_2_YbbB_dim_cplx	C4336H6511N1342O1603S31P77Mg2Zn0Fe0Se1	-90	tRNA Modification
b3757_tRNA_3	b3757_tRNA_3 (gltU)	C723H831Mg2N288O531P76S1	-73	tRNA Modification
b3757_tRNA_3_Se	b3757_tRNA_3_Se - contains mnm5se2U instead of mnm5s2U,b3757_tRNA_3 (gltU)	C723H831Mg2N288O531P76S0Se1	-73	tRNA Modification
b3757_tRNA_3_Se_TrmA_mono_cplx	b3757_tRNA_3_Se_TrmA_mono_cplx - contains mnm5se2U instead of mnm5s2U,b3757_tRNA_3 (gltU), TrmA_mono	C2597H3778Mg2N811O1090P76S19Se1	-81	tRNA Modification
b3757_tRNA_3_TrmA_mono_cplx	b3757_tRNA_3 (gltU), TrmA_mono	C2597H3778Mg2N811O1090P76S20	-81	tRNA Modification
b3757_tRNA_4	b3757_tRNA_4 (gltU)	C724H833Mg2N288O531P76S1	-73	tRNA Modification
b3757_tRNA_4_Se	b3757_tRNA_4_Se - contains mnm5se2U instead of mnm5s2U,b3757_tRNA_4 (gltU)	C724H833Mg2N288O531P76S0Se1	-73	tRNA Modification
b3757_tRNA_4_Se_TruB_mono_cplx	b3757_tRNA_4_Se_TruB_mono_cplx - contains mnm5se2U instead of mnm5s2U,b3757_tRNA_4 (gltU), TruB_mono	C2264H3308Mg2N726O1000P76S10Se1	-78	tRNA Modification
b3757_tRNA_4_TruB_mono_cplx	b3757_tRNA_4 (gltU), TruB_mono	C2264H3308Mg2N726O1000P76S11	-78	tRNA Modification
b3757_tRNA_Mg2	tRNA (gltU) bound two Mg2 ions	C720H824Mg2N287O532P76	-73	tRNA Modification
b3757_tRNA_Mg2_TruD_mono_cplx	b3757_tRNA (gltU), TruD_mono	C2463H3566Mg2N786O1041P76S8	-76	tRNA Modification
b3757_v1_RNA	tRNA (gltU)	C720H824N287O532P76	-77	tRNA Modification
b3757_v1_tRNA_1	b3757_v1_tRNA_1 (gltU)	C720H824Mg2N287O532P76	-73	tRNA Modification
b3757_v1_tRNA_1_TrmU_mono-YhhP_mono-YheLMN_cplx-YccK_mono-TrmE_dim-GidA_mono-TrmC_mono_cplx	b3757_v1_tRNA_1 (gltU), TrmU_mono, YhhP_mono, YheLMN_cplx, YccK_mono, TrmE_dim, GidA_mono, TrmC_mono	C21611H33674Mg3N6131O6869P88S131X1	-221	tRNA Modification
b3757_v1_tRNA_2	b3757_v1_tRNA_2 (gltU)	C722H829Mg2N288O531P76S1	-73	tRNA Modification
b3757_v1_tRNA_2_MeT_tRNA_pos_37_m2A_cplx	b3757_v1_tRNA_2 (gltU), MeT_tRNA_pos_37_m2A	C737H852Mg2N294O536P76S2	-72	tRNA Modification
b3757_v1_tRNA_2_Se	b3757_v1_tRNA_2_Se - contains mnm5se2U instead of mnm5s2U,b3757_v1_tRNA_2 (gltU)	C722H829Mg2N288O531P76S0Se1	-73	tRNA Modification
b3757_v1_tRNA_2_Se_MeT_tRNA_pos_37_m2A_cplx	b3757_v1_tRNA_2_Se_MeT_tRNA_pos_37_m2A_cplx - contains mnm5se2U instead of mnm5s2U,b3757_v1_tRNA_2 (gltU), MeT_tRNA_pos_37_m2A	C737H852Mg2N294O536P76S1Se1	-72	tRNA Modification
b3757_v1_tRNA_2_YbbB_dim_cplx	b3757_tRNA_2_YbbB_dim_cplx	C4336H6511N1342O1603S31P77Mg2Zn0Fe0Se1	-90	tRNA Modification
b3757_v1_tRNA_3	b3757_v1_tRNA_3 (gltU)	C723H831Mg2N288O531P76S1	-73	tRNA Modification
b3757_v1_tRNA_3_Se	b3757_v1_tRNA_3_Se - contains mnm5se2U instead of mnm5s2U,b3757_v1_tRNA_3 (gltU)	C723H831Mg2N288O531P76S0Se1	-73	tRNA Modification
b3757_v1_tRNA_3_Se_TrmA_mono_cplx	b3757_v1_tRNA_3_Se_TrmA_mono_cplx - contains mnm5se2U instead of mnm5s2U,b3757_v1_tRNA_3 (gltU), TrmA_mono	C2597H3778Mg2N811O1090P76S19Se1	-81	tRNA Modification
b3757_v1_tRNA_3_TrmA_mono_cplx	b3757_v1_tRNA_3 (gltU), TrmA_mono	C2597H3778Mg2N811O1090P76S20	-81	tRNA Modification
b3757_v1_tRNA_4	b3757_v1_tRNA_4 (gltU)	C724H833Mg2N288O531P76S1	-73	tRNA Modification
b3757_v1_tRNA_4_Se	b3757_v1_tRNA_4_Se - contains mnm5se2U instead of mnm5s2U,b3757_v1_tRNA_4 (gltU)	C724H833Mg2N288O531P76S0Se1	-73	tRNA Modification

b3757_v1_tRNA_4_Se_TruB_mono_cplx	b3757_v1_tRNA_4_Se_TruB_mono_cplx - contains mnm5se2U instead of mnm5s2U,b3757_v1_tRNA_4 (gltU), TruB_mono	C2264H3308Mg2N726O1000P76S10Se1	-78	tRNA Modification
b3757_v1_tRNA_4_TruB_mono_cplx	b3757_v1_tRNA_4 (gltU), TruB_mono	C2264H3308Mg2N726O1000P76S11	-78	tRNA Modification
b3757_v1_tRNA_Mg2	tRNA (gltU) bound two Mg2 ions	C720H824Mg2N287O532P76	-73	tRNA Modification
b3757_v1_tRNA_Mg2_TruD_mono_cplx	b3757_v1_tRNA (gltU), TruD_mono	C2463H3566Mg2N786O1041P76S8	-76	tRNA Modification
b3758_RNA	rRNA	C27811H31354N11471O20155P2904	-2905	rRNA Modification
b3758_RNA_1		C27812H31356N11471O20155P2904S0	-2905	rRNA Modification
b3758_RNA_10		C27819H31370N11471O20155P2904S0	-2905	rRNA Modification
b3758_RNA_10_MeT_23S_2445	b3758_RNA_10, MeT_23S_2445	C27834H31393N11477O20160S1P2904Mg0Zn0Fe0	-2904	rRNA Modification
b3758_RNA_11		C27820H31372N11471O20155P2904S0	-2905	rRNA Modification
b3758_RNA_11_DU_23S_2449_a	b3758_RNA_11, DU_23S_2449 (NADH)	C27841H31400N11478O20169S0P2906Mg0Zn0Fe0	-2906	rRNA Modification
b3758_RNA_11_DU_23S_2449_b	b3758_RNA_11, DU_23S_2449 (NADPH)	C27841H31399N11478O20172S0P2907Mg0Zn0Fe0	-2908	rRNA Modification
b3758_RNA_12		C27820H31374N11471O20155P2904S0	-2905	rRNA Modification
b3758_RNA_12_YmfC_mono	b3758_RNA_12, YmfC_mono	C28918H33135N11798O20475S3P2904Mg0Zn0Fe0	-2895	rRNA Modification
b3758_RNA_13		C27820H31374N11471O20155P2904S0	-2905	rRNA Modification
b3758_RNA_13_MeT_23S_2498	b3758_RNA_13, MeT_23S_2498	C27835H31397N11477O20160S1P2904Mg0Zn0Fe0	-2904	rRNA Modification
b3758_RNA_14		C27821H31376N11471O20155P2904S0	-2905	rRNA Modification
b3758_RNA_14_MeT_23S_2503	b3758_RNA_14, MeT_23S_2503	C27836H31399N11477O20160S1P2904Mg0Zn0Fe0	-2904	rRNA Modification
b3758_RNA_15		C27822H31378N11471O20155P2904S0	-2905	rRNA Modification
b3758_RNA_15_RluC_mono	b3758_RNA_15, RluC_mono	C29407H33991N11953O20611S6P2904Mg0Zn0Fe0	-2890	rRNA Modification
b3758_RNA_16		C27822H31378N11471O20155P2904S0	-2905	rRNA Modification
b3758_RNA_16_RrmJ_mono	b3758_RNA_16, RrmJ_mono	C28869H33067N11766O20458S11P2904Mg0Zn0Fe0	-2897	rRNA Modification
b3758_RNA_17		C27823H31380N11471O20155P2904S0	-2905	rRNA Modification
b3758_RNA_17_RluC_mono	b3758_RNA_17, RluC_mono	C29408H33993N11953O20611S6P2904Mg0Zn0Fe0	-2890	rRNA Modification
b3758_RNA_18		C27823H31380N11471O20155P2904S0	-2905	rRNA Modification
b3758_RNA_18_YjbC_mono	b3758_RNA_18, YjbC_mono	C29250H33751N11889O20577S7P2904Mg0Zn0Fe0	-2890	rRNA Modification
b3758_RNA_19		C27823H31380N11471O20155P2904S0	-2905	rRNA Modification
b3758_RNA_19_RluB_mono	b3758_RNA_19, RluB_mono	C29241H33728N11922O20575S5P2904Mg0Zn0Fe0	-2890	rRNA Modification
b3758_RNA_1_MeT_23S_1835	b3758_RNA_1, MeT_23S_1835	C27827H31379N11477O20160S1P2904Mg0Zn0Fe0	-2904	rRNA Modification
b3758_RNA_2		C27813H31358N11471O20155P2904S0	-2905	rRNA Modification
b3758_RNA_20		C27823H31380N11471O20155P2904S0	-2905	rRNA Modification
b3758_RNA_20_RrmA_dim	b3758_RNA_20, RrmA_dim	C30540H35591N12235O20930S31P2904Mg0Zn2Fe0	-2902	rRNA Modification
b3758_RNA_21		C27824H31382N11471O20155P2904S0	-2905	rRNA Modification
b3758_RNA_21_RluA_mono	b3758_RNA_21, RluA_mono	C28927H33122N11784O20469S10P2904Mg0Zn0Fe0	-2904	rRNA Modification
b3758_RNA_22		C27824H31382N11471O20155P2904S0	-2905	rRNA Modification
b3758_RNA_22_RumB_mono	b3758_RNA_22, RumB_mono	C29717H34360N11983O20689S28P2904Mg0Zn0Fe4	-2903	rRNA Modification
b3758_RNA_23		C27825H31384N11471O20155P2904S0	-2905	rRNA Modification
b3758_RNA_23_RluC_mono	b3758_RNA_23, RluC_mono	C29410H33997N11953O20611S6P2904Mg0Zn0Fe0	-2890	rRNA Modification
b3758_RNA_2_RluD_mono	b3758_RNA_2, RluD_mono	C29450H33968N11951O20629S12P2904Mg1Zn0Fe0	-2909	rRNA Modification
b3758_RNA_3		C27813H31358N11471O20155P2904S0	-2905	rRNA Modification
b3758_RNA_3_RluD_mono	b3758_RNA_3, RluD_mono	C29465H33991N11957O20634S13P2904Mg1Zn0Fe0	-2908	rRNA Modification
b3758_RNA_4		C27814H31360N11471O20155P2904S0	-2905	rRNA Modification
b3758_RNA_4_RluD_mono	b3758_RNA_4, RluD_mono	C29451H33970N11951O20629S12P2904Mg1Zn0Fe0	-2909	rRNA Modification
b3758_RNA_5		C27814H31360N11471O20155P2904S0	-2905	rRNA Modification
b3758_RNA_5_RumA_mono	b3758_RNA_5, RumA_mono	C29942H34786N12089O20783S23P2904Mg0Zn0Fe4	-2897	rRNA Modification
b3758_RNA_6		C27815H31362N11471O20155P2904S0	-2905	rRNA Modification
b3758_RNA_6_MeT_23S_1962	b3758_RNA_6, MeT_23S_1962	C27830H31385N11477O20160S1P2904Mg0Zn0Fe0	-2904	rRNA Modification
b3758_RNA_7		C27816H31364N11471O20155P2904S0	-2905	rRNA Modification
b3758_RNA_7_MeT_23S_2030	b3758_RNA_7, MeT_23S_2030	C27831H31387N11477O20160S1P2904Mg0Zn0Fe0	-2904	rRNA Modification
b3758_RNA_8		C27817H31366N11471O20155P2904S0	-2905	rRNA Modification
b3758_RNA_8_MeT_23S_2069	b3758_RNA_8, MeT_23S_2069	C27832H31389N11477O20160S1P2904Mg0Zn0Fe0	-2904	rRNA Modification
b3758_RNA_9		C27818H31368N11471O20155P2904S0	-2905	rRNA Modification

b3758_RNA_9_RlmB_dim	b3758_RNA_9, RlmB_dim	C30139H35171N12155O20856S23P2904Mg0Zn0Fe0	-2910	rRNA Modification
b3758_RNA_MeT_23S_1618	b3758_RNA, MeT_23S_1618	C27826H31377N11477O20160S1P2904Mg0Zn0Fe0	-2904	rRNA Modification
b3758_v1_RNA	rRNA	C27811H31354N11471O20155P2904	-2905	rRNA Modification
b3758_v1_RNA_1		C27812H31356N11471O20155P2904S0	-2905	rRNA Modification
b3758_v1_RNA_10		C27819H31370N11471O20155P2904S0	-2905	rRNA Modification
b3758_v1_RNA_10_MeT_23S_2445	b3758_v1_RNA_10, MeT_23S_2445	C27834H31393N11477O20160S1P2904Mg0Zn0Fe0	-2904	rRNA Modification
b3758_v1_RNA_11		C27820H31372N11471O20155P2904S0	-2905	rRNA Modification
b3758_v1_RNA_11_DU_23S_2449_a	b3758_v1_RNA_11, DU_23S_2449 (NADH)	C27841H31400N11478O20169S0P2906Mg0Zn0Fe0	-2906	rRNA Modification
b3758_v1_RNA_11_DU_23S_2449_b	b3758_v1_RNA_11, DU_23S_2449 (NADPH)	C27841H31399N11478O20172S0P2907Mg0Zn0Fe0	-2908	rRNA Modification
b3758_v1_RNA_12		C27820H31374N11471O20155P2904S0	-2905	rRNA Modification
b3758_v1_RNA_12_YmfC_mono	b3758_v1_RNA_12, YmfC_mono	C28918H33135N11798O20475S3P2904Mg0Zn0Fe0	-2895	rRNA Modification
b3758_v1_RNA_13		C27820H31374N11471O20155P2904S0	-2905	rRNA Modification
b3758_v1_RNA_13_MeT_23S_2498	b3758_v1_RNA_13, MeT_23S_2498	C27835H31397N11477O20160S1P2904Mg0Zn0Fe0	-2904	rRNA Modification
b3758_v1_RNA_14		C27821H31376N11471O20155P2904S0	-2905	rRNA Modification
b3758_v1_RNA_14_MeT_23S_2503	b3758_v1_RNA_14, MeT_23S_2503	C27836H31399N11477O20160S1P2904Mg0Zn0Fe0	-2904	rRNA Modification
b3758_v1_RNA_15		C27822H31378N11471O20155P2904S0	-2905	rRNA Modification
b3758_v1_RNA_15_RluC_mono	b3758_v1_RNA_15, RluC_mono	C29407H33991N11953O20611S6P2904Mg0Zn0Fe0	-2890	rRNA Modification
b3758_v1_RNA_16		C27822H31378N11471O20155P2904S0	-2905	rRNA Modification
b3758_v1_RNA_16_RrmJ_mono	b3758_v1_RNA_16, RrmJ_mono	C28869H33067N11766O20458S11P2904Mg0Zn0Fe0	-2897	rRNA Modification
b3758_v1_RNA_17		C27823H31380N11471O20155P2904S0	-2905	rRNA Modification
b3758_v1_RNA_17_RluC_mono	b3758_v1_RNA_17, RluC_mono	C29408H33993N11953O20611S6P2904Mg0Zn0Fe0	-2890	rRNA Modification
b3758_v1_RNA_18		C27823H31380N11471O20155P2904S0	-2905	rRNA Modification
b3758_v1_RNA_18_YjbC_mono	b3758_v1_RNA_18, YjbC_mono	C29250H33751N11889O20577S7P2904Mg0Zn0Fe0	-2890	rRNA Modification
b3758_v1_RNA_19		C27823H31380N11471O20155P2904S0	-2905	rRNA Modification
b3758_v1_RNA_19_RluB_mono	b3758_v1_RNA_19, RluB_mono	C29241H33728N11922O20575S5P2904Mg0Zn0Fe0	-2890	rRNA Modification
b3758_v1_RNA_1_MeT_23S_1835	b3758_v1_RNA_1, MeT_23S_1835	C27827H31379N11477O20160S1P2904Mg0Zn0Fe0	-2904	rRNA Modification
b3758_v1_RNA_2		C27813H31358N11471O20155P2904S0	-2905	rRNA Modification
b3758_v1_RNA_20		C27823H31380N11471O20155P2904S0	-2905	rRNA Modification
b3758_v1_RNA_20_RrmA_dim	b3758_v1_RNA_20, RrmA_dim	C30540H35591N12235O20930S31P2904Mg0Zn2Fe0	-2902	rRNA Modification
b3758_v1_RNA_21		C27824H31382N11471O20155P2904S0	-2905	rRNA Modification
b3758_v1_RNA_21_RluA_mono	b3758_v1_RNA_21, RluA_mono	C28927H33122N11784O20469S10P2904Mg0Zn0Fe0	-2904	rRNA Modification
b3758_v1_RNA_22		C27824H31382N11471O20155P2904S0	-2905	rRNA Modification
b3758_v1_RNA_22_RumB_mono	b3758_v1_RNA_22, RumB_mono	C29717H34360N11983O20689S28P2904Mg0Zn0Fe4	-2903	rRNA Modification
b3758_v1_RNA_23		C27825H31384N11471O20155P2904S0	-2905	rRNA Modification
b3758_v1_RNA_23_RluC_mono	b3758_v1_RNA_23, RluC_mono	C29410H33997N11953O20611S6P2904Mg0Zn0Fe0	-2890	rRNA Modification
b3758_v1_RNA_2_RluD_mono	b3758_v1_RNA_2, RluD_mono	C29450H33968N11951O20629S12P2904Mg1Zn0Fe0	-2909	rRNA Modification
b3758_v1_RNA_3		C27813H31358N11471O20155P2904S0	-2905	rRNA Modification
b3758_v1_RNA_3_RluD_mono	b3758_v1_RNA_3, RluD_mono	C29465H33991N11957O20634S13P2904Mg1Zn0Fe0	-2908	rRNA Modification
b3758_v1_RNA_4		C27814H31360N11471O20155P2904S0	-2905	rRNA Modification
b3758_v1_RNA_4_RluD_mono	b3758_v1_RNA_4, RluD_mono	C29451H33970N11951O20629S12P2904Mg1Zn0Fe0	-2909	rRNA Modification
b3758_v1_RNA_5		C27814H31360N11471O20155P2904S0	-2905	rRNA Modification
b3758_v1_RNA_5_RumA_mono	b3758_v1_RNA_5, RumA_mono	C29942H34786N12089O20783S23P2904Mg0Zn0Fe4	-2897	rRNA Modification
b3758_v1_RNA_6		C27815H31362N11471O20155P2904S0	-2905	rRNA Modification
b3758_v1_RNA_6_MeT_23S_1962	b3758_v1_RNA_6, MeT_23S_1962	C27830H31385N11477O20160S1P2904Mg0Zn0Fe0	-2904	rRNA Modification
b3758_v1_RNA_7		C27816H31364N11471O20155P2904S0	-2905	rRNA Modification
b3758_v1_RNA_7_MeT_23S_2030	b3758_v1_RNA_7, MeT_23S_2030	C27831H31387N11477O20160S1P2904Mg0Zn0Fe0	-2904	rRNA Modification
b3758_v1_RNA_8		C27817H31366N11471O20155P2904S0	-2905	rRNA Modification
b3758_v1_RNA_8_MeT_23S_2069	b3758_v1_RNA_8, MeT_23S_2069	C27832H31389N11477O20160S1P2904Mg0Zn0Fe0	-2904	rRNA Modification
b3758_v1_RNA_9		C27818H31368N11471O20155P2904S0	-2905	rRNA Modification
b3758_v1_RNA_9_RlmB_dim	b3758_v1_RNA_9, RlmB_dim	C30139H35171N12155O20856S23P2904Mg0Zn0Fe0	-2910	rRNA Modification
b3758_v1_RNA_MeT_23S_1618	b3758_v1_RNA, MeT_23S_1618	C27826H31377N11477O20160S1P2904Mg0Zn0Fe0	-2904	rRNA Modification
b3759_RNA	rRNA	C1144H1301N468O838P120	-121	RNA cutting
b3759_v1_RNA	rRNA	C1144H1301N468O838P120	-121	RNA cutting

b3760_DNA_act	DNA b3760 (activated form)	C748H864N290O468P77	-77	Transcription
b3760_DNA_neu	DNA b3760 (inactivate form)	C748H864N290O468P77	-77	Transcription
b3760_RNA	tRNA (aspT)	C731H831N290O546P77	-78	tRNA Modification
b3760_RNA_cut_cplx	b3760 RNA /RNase P (5' trimming), RNase_Gen (T, PH, II, D, or BN), RNase E (3' trimming) cutting complex	C27994H42940N8916O10549S93P456Mg5Zn3Fe0	-569	RNA cutting
b3760_RNA_pre	tRNA pre	C731H831N290O552P79	-80	RNA cutting
b3760_tRNA_1	b3760_tRNA_1 (aspT)	C731H833Mg2N290O546P77	-74	tRNA Modification
b3760_tRNA_1_Dus_gen_cplx	b3760_tRNA_1 (aspT), Dus_gen	C2338H3372Mg2N749O1029P80S14	-81	tRNA Modification
b3760_tRNA_2	b3760_tRNA_2 (aspT)	C731H835Mg2N290O546P77	-74	tRNA Modification
b3760_tRNA_2_Dus_gen_cplx	b3760_tRNA_2 (aspT), Dus_gen	C2338H3374Mg2N749O1029P80S14	-81	tRNA Modification
b3760_tRNA_3	b3760_tRNA_3 (aspT)	C731H837Mg2N290O546P77	-74	tRNA Modification
b3760_tRNA_3_Tgt_hexa-QueA_mono-EoR_tRNA_pos34_Q_cplx	b3760_tRNA_3 (aspT), Tgt_hexa, QueA_mono, EoR_tRNA_pos34_Q	C13924Co1H21352Mg2N3983O4423P80S143Zn6	-114	tRNA Modification
b3760_tRNA_4	b3760_tRNA_4 (aspT)	C738H847Mg2N290O548P77	-74	tRNA Modification
b3760_tRNA_4_MeT_tRNA_pos_37_m2A_cplx	b3760_tRNA_4 (aspT), MeT_tRNA_pos_37_m2A	C753H870Mg2N296O553P77S1	-73	tRNA Modification
b3760_tRNA_5	b3760_tRNA_5 (aspT)	C739H849Mg2N290O548P77	-74	tRNA Modification
b3760_tRNA_5_YggH_mono_cplx	b3760_tRNA_5 (aspT), YggH_mono	C1964H2748Mg2N643O895P77S14	-76	tRNA Modification
b3760_tRNA_6	b3760_tRNA_6 (aspT)	C740H851Mg2N290O548P77	-74	tRNA Modification
b3760_tRNA_6_TrmA_mono_cplx	b3760_tRNA_6 (aspT), TrmA_mono	C2614H3798Mg2N813O1107P77S19	-82	tRNA Modification
b3760_tRNA_7	b3760_tRNA_7 (aspT)	C741H853Mg2N290O548P77	-74	tRNA Modification
b3760_tRNA_7_TruB_mono_cplx	b3760_tRNA_7 (aspT), TruB_mono	C2281H3328Mg2N728O1017P77S10	-79	tRNA Modification
b3760_tRNA_8	b3760_tRNA_8 (aspT)	C741H853Mg2N290O548P77	-74	tRNA Modification
b3760_tRNA_8_YqcB_mono_cplx	b3760_tRNA_8 (aspT), YqcB_mono	C2055H2909Mg2N677O927P77S6	-77	tRNA Modification
b3760_tRNA_9	b3760_tRNA_9 (aspT)	C741H853Mg2N290O548P77	-74	tRNA Modification
b3760_tRNA_9_ThiI_mono_cplx	b3760_tRNA_9 (aspT), ThiI_mono	C7169H11084Mg3N2108O2487P82S50X1	-102	tRNA Modification
b3760_tRNA_Mg2	tRNA (aspT) bound two Mg2 ions	C731H831Mg2N290O546P77	-74	tRNA Modification
b3760_tRNA_Mg2_Dus_gen_cplx	b3760_tRNA (aspT), Dus_gen	C2338H3370Mg2N749O1029P80S14	-81	tRNA Modification
b3761_DNA_act	DNA b3761 (activated form)	C739H853N287O459P76	-76	Transcription
b3761_DNA_neu	DNA b3761 (inactivate form)	C739H853N287O459P76	-76	Transcription
b3761_RNA	tRNA (trpT)	C722H820N287O536P76	-77	tRNA Modification
b3761_RNA_cut_cplx	b3761 RNA /RNase P (5' trimming), RNase_Gen (T, PH, II, D, or BN), RNase E (3' trimming) cutting complex	C27985H42929N8913O10539S93P455Mg5Zn3Fe0	-568	RNA cutting
b3761_RNA_pre	tRNA pre	C722H820N287O542P78	-79	RNA cutting
b3761_tRNA_1	b3761_tRNA_1 (trpT)	C722H822Mg2N287O536P76	-73	tRNA Modification
b3761_tRNA_1_Dus_gen_cplx	b3761_tRNA_1 (trpT), Dus_gen	C2329H3361Mg2N746O1019P79S14	-80	tRNA Modification
b3761_tRNA_2	b3761_tRNA_2 (trpT)	C722H824Mg2N287O536P76	-73	tRNA Modification
b3761_tRNA_2_Dus_gen_cplx	b3761_tRNA_2 (trpT), Dus_gen	C2329H3363Mg2N746O1019P79S14	-80	tRNA Modification
b3761_tRNA_3	b3761_tRNA_3 (trpT)	C722H826Mg2N287O536P76	-73	tRNA Modification
b3761_tRNA_3_MeT_tRNA_pos_32_Cm_cplx	b3761_tRNA_3 (trpT), MeT_tRNA_pos_32_Cm	C737H849Mg2N293O541P76S1	-72	tRNA Modification
b3761_tRNA_4	b3761_tRNA_4 (trpT)	C723H828Mg2N287O536P76	-73	tRNA Modification
b3761_tRNA_4_MiaA_dim-MiaB_mono_cplx	b3761_tRNA_4 (trpT), MiaA_dim, MiaB_mono	C10198Fe4H15858Mg4N2962O3402P80S80X1	-118	tRNA Modification
b3761_tRNA_5	b3761_tRNA_5 (trpT)	C729H838Mg2N287O536P76S1	-73	tRNA Modification
b3761_tRNA_5_YggH_mono_cplx	b3761_tRNA_5 (trpT), YggH_mono	C1954H2737Mg2N640O883P76S15	-75	tRNA Modification
b3761_tRNA_6	b3761_tRNA_6 (trpT)	C730H840Mg2N287O536P76S1	-73	tRNA Modification
b3761_tRNA_6_TrmA_mono_cplx	b3761_tRNA_6 (trpT), TrmA_mono	C2604H3787Mg2N810O1095P76S20	-81	tRNA Modification
b3761_tRNA_7	b3761_tRNA_7 (trpT)	C731H842Mg2N287O536P76S1	-73	tRNA Modification
b3761_tRNA_7_TruB_mono_cplx	b3761_tRNA_7 (trpT), TruB_mono	C2271H3317Mg2N725O1005P76S11	-78	tRNA Modification
b3761_tRNA_8	b3761_tRNA_8 (trpT)	C731H842Mg2N287O536P76S1	-73	tRNA Modification
b3761_tRNA_8_ThiI_mono_cplx	b3761_tRNA_8 (trpT), ThiI_mono	C7159H11073Mg3N2105O2475P81S51X1	-101	tRNA Modification
b3761_tRNA_Mg2	tRNA (trpT) bound two Mg2 ions	C722H820Mg2N287O536P76	-73	tRNA Modification
b3761_tRNA_Mg2_Dus_gen_cplx	b3761_tRNA (trpT), Dus_gen	C2329H3359Mg2N746O1019P79S14	-80	tRNA Modification
b3780_aa	polypeptide b3780	C2092H3331N604O612S13	-1	Translation
b3780_def_map_cplx	Polypeptide b3780 peptide deformylase and methionine aminopeptidase complex	C4222H6787N1197O1258S34Mg0Zn0Fe3	-10	Maturation
b3780_m	Matured polypeptide b3780	C2086H3323N603O610S12	0	Maturation
b3780_mRNA	mRNA b3780	C12036H13632N4787O8858P1268	-1269	Translation
b3780_mRNA_1	mRNA b3780	C12036H13632N4787O8858P1268	-1269	Translation
b3780_mRNA_2	mRNA b3780	C12036H13632N4787O8858P1268	-1269	Translation
b3780_mRNA_2_degr	mRNA b3780 degradation complex	C52719H79031N16662O21543S200P1268Mg6Zn2Fe0	-1491	mRNA degradation

b3780_m_DnaKJ_complex	b3780 DnaK DnaJ_dim complex - Kerner et al. class II can interact w/ GroEL/ES, cannot fold spontanously	C8650H13837O2708N2516S59P3Zn4	-22	Folding
b3780_m_GroEL.(7)ATP.transGroES	b3780 GroEL GroES complex - Kerner et al. class II can interact w/ GroEL/ES, cannot fold spontanously	C39998H66267O12944N11187S376P21Mg7	-301	Folding
b3782_aa	polypeptide b3782_v1	C154H257N52O53S3	3	Translation
b3782_def_map_cplx	Polypeptide b3782 peptide deformylase and methionine aminopeptidase complex	C2284H3713N645O699S24Mg0Zn0Fe3	-6	Maturation
b3782_m	Matured polypeptide b3782	C148H249N51O51S2	4	Maturation
b3782_mRNA	mRNA b3782	C967H1095N376O725P104	-105	Translation
b3782_mRNA_1	mRNA b3782	C967H1095N376O725P104	-105	Translation
b3782_mRNA_2	mRNA b3782	C967H1095N376O725P104	-105	Translation
b3782_mRNA_2_degr	mRNA b3782 degradation complex	C41650H66494N12251O13410S200P104Mg6Zn2Fe0	-327	mRNA degradation
b3782_v1_mRNA	mRNA b3782_v1	C967H1095N376O725P104	-105	Translation
b3783_aa	polypeptide b3783_v1	C2075H3376N586O622S17	-2	Translation
b3783_def_cplx	Polypeptide b3783 peptide deformylase complex	C2919H4770N827O877S23Mg0Zn0Fe1	-7	Maturation
b3783_m	Matured polypeptide b3783	C2074H3377N586O621S17	-1	Maturation
b3783_mRNA	mRNA b3783	C11983H13563N4768O8791P1260	-1261	Translation
b3783_mRNA_1	mRNA b3783	C11983H13563N4768O8791P1260	-1261	Translation
b3783_mRNA_2	mRNA b3783	C11983H13563N4768O8791P1260	-1261	Translation
b3783_mRNA_2_degr	mRNA b3783 degradation complex	C52666H78962N16643O21476S200P1260Mg6Zn2Fe0	-1483	mRNA degradation
b3783_m_DnaKJ_complex	b3783 DnaK DnaJ_dim complex - Kerner et al. class II can interact w/ GroEL/ES, cannot fold spontanously	C8638H13891O2719N2499S64P3Zn4	-23	Folding
b3783_m_GroEL.(7)ATP.transGroES	b3783 GroEL GroES complex - Kerner et al. class II can interact w/ GroEL/ES, cannot fold spontanously	C39986H66321O12955N11170S381P21Mg7	-302	Folding
b3783_v1_mRNA	mRNA b3783_v1	C11983H13563N4768O8791P1260	-1261	Translation
b3796_RNA	tRNA (argX)	C732H835N296O541P77	-78	tRNA Modification
b3796_tRNA_1	b3796_tRNA_1 (argX)	C732H837Mg2N296O541P77	-74	tRNA Modification
b3796_tRNA_1_Dus_gen_cplx	b3796_tRNA_1 (argX), Dus_gen	C2339H3376Mg2N755O1024P80S14	-81	tRNA Modification
b3796_tRNA_2	b3796_tRNA_2 (argX)	C732H839Mg2N296O541P77	-74	tRNA Modification
b3796_tRNA_2_YdaO_mono_cplx	b3796_tRNA_2 (argX), YdaO_mono	C6274H9658Mg3N1866O2228P82S55X1	-100	tRNA Modification
b3796_tRNA_3	b3796_tRNA_3 (argX)	C732H839Mg2N296O540P77S1	-74	tRNA Modification
b3796_tRNA_3_TrmD_dim_cplx	b3796_tRNA_3 (argX), TrmD_dim	C3259H4816Mg2N1018O1295P77S22	-91	tRNA Modification
b3796_tRNA_4	b3796_tRNA_4 (argX)	C733H841Mg2N296O540P77S1	-74	tRNA Modification
b3796_tRNA_4_YggH_mono_cplx	b3796_tRNA_4 (argX), YggH_mono	C1958H2740Mg2N649O887P77S15	-76	tRNA Modification
b3796_tRNA_5	b3796_tRNA_5 (argX)	C734H843Mg2N296O540P77S1	-74	tRNA Modification
b3796_tRNA_5_TrmA_mono_cplx	b3796_tRNA_5 (argX), TrmA_mono	C2608H3790Mg2N819O1099P77S20	-82	tRNA Modification
b3796_tRNA_6	b3796_tRNA_6 (argX)	C735H845Mg2N296O540P77S1	-74	tRNA Modification
b3796_tRNA_6_TruB_mono_cplx	b3796_tRNA_6 (argX), TruB_mono	C2275H3320Mg2N734O1009P77S11	-79	tRNA Modification
b3796_tRNA_Mg2	tRNA (argX) bound two Mg2 ions	C732H835Mg2N296O541P77	-74	tRNA Modification
b3796_tRNA_Mg2_Dus_gen_cplx	b3796_tRNA (argX), Dus_gen	C2339H3374Mg2N755O1024P80S14	-81	tRNA Modification
b3797_RNA	tRNA (hisR)	C729H826N281O548P77	-78	tRNA Modification
b3797_tRNA_1	b3797_tRNA_1 (hisR)	C729H828Mg2N281O548P77	-74	tRNA Modification
b3797_tRNA_10	b3797_tRNA_10 (hisR)	C739H848Mg2N281O550P77	-74	tRNA Modification
b3797_tRNA_10_ThiI_mono_cplx	b3797_tRNA_10 (hisR), ThiI_mono	C7167H11079Mg3N2099O2489P82S50X1	-102	tRNA Modification
b3797_tRNA_1_Dus_gen_cplx	b3797_tRNA_1 (hisR), Dus_gen	C2336H3367Mg2N740O1031P80S14	-81	tRNA Modification
b3797_tRNA_2	b3797_tRNA_2 (hisR)	C729H830Mg2N281O548P77	-74	tRNA Modification
b3797_tRNA_2_Dus_gen_cplx	b3797_tRNA_2 (hisR), Dus_gen	C2336H3369Mg2N740O1031P80S14	-81	tRNA Modification
b3797_tRNA_3	b3797_tRNA_3 (hisR)	C729H832Mg2N281O548P77	-74	tRNA Modification
b3797_tRNA_3_Tgt_hexa-QueA_mono-EoR_tRNA_pos34_Q_cplx	b3797_tRNA_3 (hisR), Tgt_hexa, QueA_mono, EoR_tRNA_pos34_Q	C13922Co1H21347Mg2N3974O4425P80S143Zn6	-114	tRNA Modification
b3797_tRNA_4	b3797_tRNA_4 (hisR)	C736H842Mg2N281O550P77	-74	tRNA Modification
b3797_tRNA_4_MeT_tRNA_pos_37_m2A_cplx	b3797_tRNA_4 (hisR), MeT_tRNA_pos_37_m2A	C751H865Mg2N287O555P77S1	-73	tRNA Modification
b3797_tRNA_5	b3797_tRNA_5 (hisR)	C737H844Mg2N281O550P77	-74	tRNA Modification
b3797_tRNA_5_TruA_dim_cplx	b3797_tRNA_5 (hisR), TruA_dim	C3447H5046Mg2N1061O1320P77S16	-68	tRNA Modification
b3797_tRNA_6	b3797_tRNA_6 (hisR)	C737H844Mg2N281O550P77	-74	tRNA Modification
b3797_tRNA_6_TruA_dim_cplx	b3797_tRNA_6 (hisR), TruA_dim	C3447H5046Mg2N1061O1320P77S16	-68	tRNA Modification
b3797_tRNA_7	b3797_tRNA_7 (hisR)	C737H844Mg2N281O550P77	-74	tRNA Modification
b3797_tRNA_7_YggH_mono_cplx	b3797_tRNA_7 (hisR), YggH_mono	C1962H2743Mg2N634O897P77S14	-76	tRNA Modification
b3797_tRNA_8	b3797_tRNA_8 (hisR)	C738H846Mg2N281O550P77	-74	tRNA Modification
b3797_tRNA_8_TrmA_mono_cplx	b3797_tRNA_8 (hisR), TrmA_mono	C2612H3793Mg2N804O1109P77S19	-82	tRNA Modification

b3797_tRNA_9	b3797_tRNA_9 (hisR)	C739H848Mg2N281O550P77	-74	tRNA Modification
b3797_tRNA_9_TruB_mono_cplx	b3797_tRNA_9 (hisR), TruB_mono	C2279H3323Mg2N719O1019P77S10	-79	tRNA Modification
b3797_tRNA_Mg2	tRNA (hisR) bound two Mg2 ions	C729H826Mg2N281O548P77	-74	tRNA Modification
b3797_tRNA_Mg2_Dus_gen_cplx	b3797_tRNA (hisR), Dus_gen	C2336H3365Mg2N740O1031P80S14	-81	tRNA Modification
b3798_RNA	tRNA (leuT)	C827H939N330O614P87	-88	tRNA Modification
b3798_tRNA_1	b3798_tRNA_1 (leuT)	C827H941Mg2N330O614P87	-84	tRNA Modification
b3798_tRNA_1_Dus_gen_cplx	b3798_tRNA_1 (leuT), Dus_gen	C2434H3480Mg2N789O1097P90S14	-91	tRNA Modification
b3798_tRNA_2	b3798_tRNA_2 (leuT)	C827H943Mg2N330O614P87	-84	tRNA Modification
b3798_tRNA_2_TrmH_dim_cplx	b3798_tRNA_2 (leuT), TrmH_dim	C3038H4486Mg2N986O1275P87S29	-85	tRNA Modification
b3798_tRNA_3	b3798_tRNA_3 (leuT)	C828H945Mg2N330O614P87	-84	tRNA Modification
b3798_tRNA_3_Dus_gen_cplx	b3798_tRNA_3 (leuT), Dus_gen	C2435H3484Mg2N789O1097P90S14	-91	tRNA Modification
b3798_tRNA_4	b3798_tRNA_4 (leuT)	C828H947Mg2N330O614P87	-84	tRNA Modification
b3798_tRNA_4_TruA_dim_cplx	b3798_tRNA_4 (leuT), TruA_dim	C3538H5149Mg2N1110O1384P87S16	-78	tRNA Modification
b3798_tRNA_5	b3798_tRNA_5 (leuT)	C828H947Mg2N330O614P87	-84	tRNA Modification
b3798_tRNA_5_TruA_dim_cplx	b3798_tRNA_5 (leuT), TruA_dim	C3538H5149Mg2N1110O1384P87S16	-78	tRNA Modification
b3798_tRNA_6	b3798_tRNA_6 (leuT)	C828H947Mg2N330O614P87	-84	tRNA Modification
b3798_tRNA_6_TrmA_mono_cplx	b3798_tRNA_6 (leuT), TrmA_mono	C2702H3894Mg2N853O1173P87S19	-92	tRNA Modification
b3798_tRNA_7	b3798_tRNA_7 (leuT)	C829H949Mg2N330O614P87	-84	tRNA Modification
b3798_tRNA_7_TruB_mono_cplx	b3798_tRNA_7 (leuT), TruB_mono	C2369H3424Mg2N768O1083P87S10	-89	tRNA Modification
b3798_tRNA_Mg2	tRNA (leuT) bound two Mg2 ions	C827H939Mg2N330O614P87	-84	tRNA Modification
b3798_tRNA_Mg2_Dus_gen_cplx	b3798_tRNA (leuT), Dus_gen	C2434H3478Mg2N789O1097P90S14	-91	tRNA Modification
b3799_RNA	tRNA (proM)	C733H832N295O543P77	-78	tRNA Modification
b3799_tRNA_1	b3799_tRNA_1 (proM)	C733H834Mg2N295O543P77	-74	tRNA Modification
b3799_tRNA_1_MeT_tRNA_pos_32_Um_cplx	b3799_tRNA_1 (proM), MeT_tRNA_pos_32_Um	C748H857Mg2N301O548P77S1	-73	tRNA Modification
b3799_tRNA_2	b3799_tRNA_2 (proM)	C734H836Mg2N295O543P77	-74	tRNA Modification
b3799_tRNA_2_YecO_mono-YecP_mono-HyL_tRNA_pos_34_ho5U_cplx	b3799_tRNA_2 (proM), YecO_mono, YecP_mono, HyL_tRNA_pos_34_ho5U	C3667H5369Mg2N1096O1393P77S23	-92	tRNA Modification
b3799_tRNA_3	b3799_tRNA_3 (proM)	C736H837Mg2N295O546P77	-75	tRNA Modification
b3799_tRNA_3_TrmD_dim_cplx	b3799_tRNA_3 (proM), TrmD_dim	C3263H4814Mg2N1017O1301P77S21	-92	tRNA Modification
b3799_tRNA_4	b3799_tRNA_4 (proM)	C737H839Mg2N295O546P77	-75	tRNA Modification
b3799_tRNA_4_YggH_mono_cplx	b3799_tRNA_4 (proM), YggH_mono	C1962H2738Mg2N648O893P77S14	-77	tRNA Modification
b3799_tRNA_5	b3799_tRNA_5 (proM)	C738H841Mg2N295O546P77	-75	tRNA Modification
b3799_tRNA_5_TrmA_mono_cplx	b3799_tRNA_5 (proM), TrmA_mono	C2612H3788Mg2N818O1105P77S19	-83	tRNA Modification
b3799_tRNA_6	b3799_tRNA_6 (proM)	C739H843Mg2N295O546P77	-75	tRNA Modification
b3799_tRNA_6_TruB_mono_cplx	b3799_tRNA_6 (proM), TruB_mono	C2279H3318Mg2N733O1015P77S10	-80	tRNA Modification
b3799_tRNA_7	b3799_tRNA_7 (proM)	C739H843Mg2N295O546P77	-75	tRNA Modification
b3799_tRNA_7_YqcB_mono_cplx	b3799_tRNA_7 (proM), YqcB_mono	C2053H2899Mg2N682O925P77S6	-78	tRNA Modification
b3799_tRNA_8	b3799_tRNA_8 (proM)	C739H843Mg2N295O546P77	-75	tRNA Modification
b3799_tRNA_8_ThiI_mono_cplx	b3799_tRNA_8 (proM), ThiI_mono	C7167H11074Mg3N2113O2485P82S50X1	-103	tRNA Modification
b3799_tRNA_Mg2	tRNA (proM) bound two Mg2 ions	C733H832Mg2N295O543P77	-74	tRNA Modification
b3799_tRNA_Mg2_Dus_gen_cplx	b3799_tRNA (proM), Dus_gen	C2340H3371Mg2N754O1026P80S14	-81	tRNA Modification
b3851_RNA	rRNA	C14753H16648N6061O10721P1542	-1543	rRNA Modification
b3851_RNA_1		C14754H16650N6061O10721P1542S0	-1543	rRNA Modification
b3851_RNA_10		C14765H16672N6061O10721P1542S0	-1543	rRNA Modification
b3851_RNA_10_RsmB_mono	b3851_RNA_10, RsmB_mono	C16932H20115N6679O11349S17P1542Mg0Zn0Fe0	-1542	rRNA Modification
b3851_RNA_1_MeT_16S_1402	b3851_RNA_1, MeT_16S_1402	C14784H16696N6073O10731S2P1542Mg0Zn0Fe0	-1541	rRNA Modification
b3851_RNA_2		C14756H16654N6061O10721P1542S0	-1543	rRNA Modification
b3851_RNA_2_MeT_16S_1407	b3851_RNA_2, MeT_16S_1407	C14771H16677N6067O10726S1P1542Mg0Zn0Fe0	-1542	rRNA Modification
b3851_RNA_3		C14757H16656N6061O10721P1542S0	-1543	rRNA Modification
b3851_RNA_3_YggJ_mono	b3851_RNA_3, YggJ_mono	C15949H18596N6409O11080S11P1542Mg0Zn0Fe0	-1545	rRNA Modification
b3851_RNA_4		C14758H16658N6061O10721P1542S0	-1543	rRNA Modification
b3851_RNA_4_MeT_16S_1516	b3851_RNA_4, MeT_16S_1516	C14773H16681N6067O10726S1P1542Mg0Zn0Fe0	-1542	rRNA Modification
b3851_RNA_5		C14759H16660N6061O10721P1542S0	-1543	rRNA Modification
b3851_RNA_5_KsgA_mono	b3851_RNA_5, KsgA_mono	C16141H18849N6449O11119S15P1542Mg0Zn0Fe0	-1540	rRNA Modification
b3851_RNA_6		C14761H16664N6061O10721P1542S0	-1543	rRNA Modification
b3851_RNA_6_KsgA_mono	b3851_RNA_6, KsgA_mono	C16143H18853N6449O11119S15P1542Mg0Zn0Fe0	-1540	rRNA Modification
b3851_RNA_7		C14763H16668N6061O10721P1542S0	-1543	rRNA Modification
b3851_RNA_7_RsuA_mono	b3851_RNA_7, RsuA_mono	C15908H18468N6389O11065S6P1542Mg0Zn0Fe0	-1551	rRNA Modification

b3851_RNA_8		C14763H16668N6061O10721P1542S0	-1543	rRNA Modification
b3851_RNA_8_MeT_16S_527	b3851_RNA_8, MeT_16S_527	C14778H16691N6067O10726S1P1542Mg0Zn0Fe0	-1542	rRNA Modification
b3851_RNA_9		C14764H16670N6061O10721P1542S0	-1543	rRNA Modification
b3851_RNA_9_MeT_16S_966	b3851_RNA_9, MeT_16S_966	C14779H16693N6067O10726S1P1542Mg0Zn0Fe0	-1542	rRNA Modification
b3851_RNA_RsmC_mono	b3851_RNA, RsmC_mono	C16437H19279N6534O11223S11P1542Mg0Zn0Fe0	-1547	rRNA Modification
b3852_RNA	tRNA (ileT)	C734H831N296O541P77	-78	tRNA Modification
b3852_tRNA_1	b3852_tRNA_1 (ileT)	C734H833Mg2N296O541P77	-74	tRNA Modification
b3852_tRNA_1_Dus_gen_cplx	b3852_tRNA_1 (ileT), Dus_gen	C2341H3372Mg2N755O1024P80S14	-81	tRNA Modification
b3852_tRNA_2	b3852_tRNA_2 (ileT)	C734H835Mg2N296O541P77	-74	tRNA Modification
b3852_tRNA_2_Up_tRNA_pos_37_t6A_cplx	b3852_tRNA_2 (ileT), Up_tRNA_pos_37_t6A	C749H857Mg3N302O560P80	-77	tRNA Modification
b3852_tRNA_3	b3852_tRNA_3 (ileT)	C739H841Mg2N297O545P77	-75	tRNA Modification
b3852_tRNA_3_YggH_mono_cplx	b3852_tRNA_3 (ileT), YggH_mono	C1964H2740Mg2N650O892P77S14	-77	tRNA Modification
b3852_tRNA_4	b3852_tRNA_4 (ileT)	C740H843Mg2N297O545P77	-75	tRNA Modification
b3852_tRNA_4_AcpT_tRNA_pos_47_acp3U_cplx	b3852_tRNA_4 (ileT), AcpT_tRNA_pos_47_acp3U	C755H866Mg2N303O550P77S1	-74	tRNA Modification
b3852_tRNA_5	b3852_tRNA_5 (ileT)	C744H850Mg2N298O547P77	-75	tRNA Modification
b3852_tRNA_5_TrmA_mono_cplx	b3852_tRNA_5 (ileT), TrmA_mono	C2618H3797Mg2N821O1106P77S19	-83	tRNA Modification
b3852_tRNA_6	b3852_tRNA_6 (ileT)	C745H852Mg2N298O547P77	-75	tRNA Modification
b3852_tRNA_6_TruB_mono_cplx	b3852_tRNA_6 (ileT), TruB_mono	C2285H3327Mg2N736O1016P77S10	-80	tRNA Modification
b3852_tRNA_7	b3852_tRNA_7 (ileT)	C745H852Mg2N298O547P77	-75	tRNA Modification
b3852_tRNA_7_YqcB_mono_cplx	b3852_tRNA_7 (ileT), YqcB_mono	C2059H2908Mg2N685O926P77S6	-78	tRNA Modification
b3852_tRNA_Mg2	tRNA (ileT) bound two Mg2 ions	C734H831Mg2N296O541P77	-74	tRNA Modification
b3852_tRNA_Mg2_Dus_gen_cplx	b3852_tRNA (ileT), Dus_gen	C2341H3370Mg2N755O1024P80S14	-81	tRNA Modification
b3853_RNA	tRNA (alaT)	C722H822N289O535P76	-77	tRNA Modification
b3853_tRNA_1	b3853_tRNA_1 (alaT)	C722H824Mg2N289O535P76	-73	tRNA Modification
b3853_tRNA_1_YecO_mono-YecP_mono-HyL_tRNA_pos_34_ho5U_cplx	b3853_tRNA_1 (alaT), YecO_mono, YecP_mono, HyL_tRNA_pos_34_ho5U	C3655H5357Mg2N1090O1385P76S23	-91	tRNA Modification
b3853_tRNA_2	b3853_tRNA_2 (alaT)	C724H825Mg2N289O538P76	-74	tRNA Modification
b3853_tRNA_2_YggH_mono_cplx	b3853_tRNA_2 (alaT), YggH_mono	C1949H2724Mg2N642O885P76S14	-76	tRNA Modification
b3853_tRNA_3	b3853_tRNA_3 (alaT)	C725H827Mg2N289O538P76	-74	tRNA Modification
b3853_tRNA_3_TrmA_mono_cplx	b3853_tRNA_3 (alaT), TrmA_mono	C2599H3774Mg2N812O1097P76S19	-82	tRNA Modification
b3853_tRNA_4	b3853_tRNA_4 (alaT)	C726H829Mg2N289O538P76	-74	tRNA Modification
b3853_tRNA_4_TruB_mono_cplx	b3853_tRNA_4 (alaT), TruB_mono	C2266H3304Mg2N727O1007P76S10	-79	tRNA Modification
b3853_tRNA_Mg2	tRNA (alaT) bound two Mg2 ions	C722H822Mg2N289O535P76	-73	tRNA Modification
b3853_tRNA_Mg2_Dus_gen_cplx	b3853_tRNA (alaT), Dus_gen	C2329H3361Mg2N748O1018P79S14	-80	tRNA Modification
b3854_RNA	rRNA	C27820H31362N11471O20166P2905	-2906	rRNA Modification
b3854_RNA_1		C27821H31364N11471O20166P2905S0	-2906	rRNA Modification
b3854_RNA_10		C27828H31378N11471O20166P2905S0	-2906	rRNA Modification
b3854_RNA_10_MeT_23S_2445	b3854_RNA_10, MeT_23S_2445	C27843H31401N11477O20171S1P2905Mg0Zn0Fe0	-2905	rRNA Modification
b3854_RNA_11		C27829H31380N11471O20166P2905S0	-2906	rRNA Modification
b3854_RNA_11_DU_23S_2449_a	b3854_RNA_11, DU_23S_2449 (NADH)	C27850H31408N11478O20180S0P2907Mg0Zn0Fe0	-2907	rRNA Modification
b3854_RNA_11_DU_23S_2449_b	b3854_RNA_11, DU_23S_2449 (NADPH)	C27850H31407N11478O20183S0P2908Mg0Zn0Fe0	-2909	rRNA Modification
b3854_RNA_12		C27829H31382N11471O20166P2905S0	-2906	rRNA Modification
b3854_RNA_12_YmfC_mono	b3854_RNA_12, YmfC_mono	C28927H33143N11798O20486S3P2905Mg0Zn0Fe0	-2896	rRNA Modification
b3854_RNA_13		C27829H31382N11471O20166P2905S0	-2906	rRNA Modification
b3854_RNA_13_MeT_23S_2498	b3854_RNA_13, MeT_23S_2498	C27844H31405N11477O20171S1P2905Mg0Zn0Fe0	-2905	rRNA Modification
b3854_RNA_14		C27830H31384N11471O20166P2905S0	-2906	rRNA Modification
b3854_RNA_14_MeT_23S_2503	b3854_RNA_14, MeT_23S_2503	C27845H31407N11477O20171S1P2905Mg0Zn0Fe0	-2905	rRNA Modification
b3854_RNA_15		C27831H31386N11471O20166P2905S0	-2906	rRNA Modification
b3854_RNA_15_RluC_mono	b3854_RNA_15, RluC_mono	C29416H33999N11953O20622S6P2905Mg0Zn0Fe0	-2891	rRNA Modification
b3854_RNA_16		C27831H31386N11471O20166P2905S0	-2906	rRNA Modification
b3854_RNA_16_RrmJ_mono	b3854_RNA_16, RrmJ_mono	C28878H33075N11766O20469S11P2905Mg0Zn0Fe0	-2898	rRNA Modification
b3854_RNA_17		C27832H31388N11471O20166P2905S0	-2906	rRNA Modification
b3854_RNA_17_RluC_mono	b3854_RNA_17, RluC_mono	C29417H34001N11953O20622S6P2905Mg0Zn0Fe0	-2891	rRNA Modification
b3854_RNA_18		C27832H31388N11471O20166P2905S0	-2906	rRNA Modification
b3854_RNA_18_YjbC_mono	b3854_RNA_18, YjbC_mono	C29259H33759N11889O20588S7P2905Mg0Zn0Fe0	-2891	rRNA Modification
b3854_RNA_19		C27832H31388N11471O20166P2905S0	-2906	rRNA Modification

b3854_RNA_19_RluB_mono	b3854_RNA_19, RluB_mono	C29250H33736N11922O20586S5P2905Mg0Zn0Fe0	-2891	rRNA Modification
b3854_RNA_1_MeT_23S_1835	b3854_RNA_1, MeT_23S_1835	C27836H31387N11477O20171S1P2905Mg0Zn0Fe0	-2905	rRNA Modification
b3854_RNA_2		C27822H31366N11471O20166P2905S0	-2906	rRNA Modification
b3854_RNA_20		C27832H31388N11471O20166P2905S0	-2906	rRNA Modification
b3854_RNA_20_RrmA_dim	b3854_RNA_20, RrmA_dim	C30549H35599N12235O20941S31P2905Mg0Zn2Fe0	-2903	rRNA Modification
b3854_RNA_21		C27833H31390N11471O20166P2905S0	-2906	rRNA Modification
b3854_RNA_21_RluA_mono	b3854_RNA_21, RluA_mono	C28936H33130N11784O20480S10P2905Mg0Zn0Fe0	-2905	rRNA Modification
b3854_RNA_22		C27833H31390N11471O20166P2905S0	-2906	rRNA Modification
b3854_RNA_22_RumB_mono	b3854_RNA_22, RumB_mono	C29726H34368N11983O20700S28P2905Mg0Zn0Fe4	-2904	rRNA Modification
b3854_RNA_23		C27834H31392N11471O20166P2905S0	-2906	rRNA Modification
b3854_RNA_23_RluC_mono	b3854_RNA_23, RluC_mono	C29419H34005N11953O20622S6P2905Mg0Zn0Fe0	-2891	rRNA Modification
b3854_RNA_2_RluD_mono	b3854_RNA_2, RluD_mono	C29459H33976N11951O20640S12P2905Mg1Zn0Fe0	-2910	rRNA Modification
b3854_RNA_3		C27822H31366N11471O20166P2905S0	-2906	rRNA Modification
b3854_RNA_3_RluD_mono	b3854_RNA_3, RluD_mono	C29474H33999N11957O20645S13P2905Mg1Zn0Fe0	-2909	rRNA Modification
b3854_RNA_4		C27823H31368N11471O20166P2905S0	-2906	rRNA Modification
b3854_RNA_4_RluD_mono	b3854_RNA_4, RluD_mono	C29460H33978N11951O20640S12P2905Mg1Zn0Fe0	-2910	rRNA Modification
b3854_RNA_5		C27823H31368N11471O20166P2905S0	-2906	rRNA Modification
b3854_RNA_5_RumA_mono	b3854_RNA_5, RumA_mono	C29951H34794N12089O20794S23P2905Mg0Zn0Fe4	-2898	rRNA Modification
b3854_RNA_6		C27824H31370N11471O20166P2905S0	-2906	rRNA Modification
b3854_RNA_6_MeT_23S_1962	b3854_RNA_6, MeT_23S_1962	C27839H31393N11477O20171S1P2905Mg0Zn0Fe0	-2905	rRNA Modification
b3854_RNA_7		C27825H31372N11471O20166P2905S0	-2906	rRNA Modification
b3854_RNA_7_MeT_23S_2030	b3854_RNA_7, MeT_23S_2030	C27840H31395N11477O20171S1P2905Mg0Zn0Fe0	-2905	rRNA Modification
b3854_RNA_8		C27826H31374N11471O20166P2905S0	-2906	rRNA Modification
b3854_RNA_8_MeT_23S_2069	b3854_RNA_8, MeT_23S_2069	C27841H31397N11477O20171S1P2905Mg0Zn0Fe0	-2905	rRNA Modification
b3854_RNA_9		C27827H31376N11471O20166P2905S0	-2906	rRNA Modification
b3854_RNA_9_RlmB_dim	b3854_RNA_9, RlmB_dim	C30148H35179N12155O20867S23P2905Mg0Zn0Fe0	-2911	rRNA Modification
b3854_RNA_MeT_23S_1618	b3854_RNA, MeT_23S_1618	C27835H31385N11477O20171S1P2905Mg0Zn0Fe0	-2905	rRNA Modification
b3855_RNA	rRNA	C1145H1300N469O838P120	-121	RNA cutting
b3885_aa	polypeptide b3885	C1027H1553N274O299S7	-13	Translation
b3885_def_map_cplx	Polypeptide b3885 peptide deformylase and methionine aminopeptidase complex	C3157H5009N867O945S28Mg0Zn0Fe3	-22	Maturation
b3885_m	Matured polypeptide b3885	C1021H1545N273O297S6	-12	Maturation
b3885_mRNA	mRNA b3885	C5708H6447N2262O4218P602	-603	Translation
b3885_mRNA_1	mRNA b3885	C5708H6447N2262O4218P602	-603	Translation
b3885_mRNA_2	mRNA b3885	C5708H6447N2262O4218P602	-603	Translation
b3885_mRNA_2_degr	mRNA b3885 degradation complex	C46391H71846N14137O16903S200P602Mg6Zn2Fe0	-825	mRNA degradation
b3886_aa	polypeptide b3886	C1550H2403N374O394S9	3	Translation
b3886_def_map_cplx	Polypeptide b3886 peptide deformylase and methionine aminopeptidase complex	C3680H5859N967O1040S30Mg0Zn0Fe3	-6	Maturation
b3886_m	Matured polypeptide b3886	C1544H2395N373O392S8	4	Maturation
b3886_mRNA	mRNA b3886 (7 nt short)	C8184H9280N3131O6133P866	-867	Translation
b3886_mRNA_1	mRNA b3886 (7 nt short)	C8184H9280N3131O6133P866	-867	Translation
b3886_mRNA_2	mRNA b3886 (7 nt short)	C8184H9280N3131O6133P866	-867	Translation
b3886_mRNA_2_degr	mRNA b3886 degradation complex	C48867H74679N15006O18818S200P866Mg6Zn2Fe0	-1089	mRNA degradation
b3887_aa	polypeptide b3887	C693H1106N198O221S7	-6	Translation
b3887_def_cplx	Polypeptide b3887 peptide deformylase complex	C1537H2500N439O476S13Mg0Zn0Fe1	-11	Maturation
b3887_m	Matured polypeptide b3887	C692H1107N198O220S7	-5	Maturation
b3887_mRNA	mRNA b3887 (4 nt short)	C4143H4683N1684O3029P434	-435	Translation
b3887_mRNA_1	mRNA b3887 (4 nt short)	C4143H4683N1684O3029P434	-435	Translation
b3887_mRNA_2	mRNA b3887 (4 nt short)	C4143H4683N1684O3029P434	-435	Translation
b3887_mRNA_2_degr	mRNA b3887 degradation complex	C44826H70082N13559O15714S200P434Mg6Zn2Fe0	-657	mRNA degradation
b3887_m_Zn	b3887 plus _Zn	C692H1107N198O220S7Zn6	7	Folding
b3888_aa	polypeptide b3888	C1650H2567N464O483S15	-9	Translation
b3888_def_map_cplx	Polypeptide b3888 peptide deformylase and methionine aminopeptidase complex	C3780H6023N1057O1129S36Mg0Zn0Fe3	-18	Maturation

b3888_m	Matured polypeptide b3888	C1644H2559N463O481S14	-8	Maturation
b3888_mRNA	mRNA b3888 (4 nt short)	C9403H10639N3808O6877P986	-987	Translation
b3888_mRNA_1	mRNA b3888 (4 nt short)	C9403H10639N3808O6877P986	-987	Translation
b3888_mRNA_2	mRNA b3888 (4 nt short)	C9403H10639N3808O6877P986	-987	Translation
b3888_mRNA_2_degr	mRNA b3888 degradation complex	C50086H76038N15683O19562S200P986Mg6Zn2Fe0	-1209	mRNA degradation
b3936_aa	polypeptide b3936	C340H551N104O101S6	5	Translation
b3936_def_cplx	Polypeptide b3936 peptide deformylase complex	C1184H1945N345O356S12Mg0Zn0Fe1	0	Maturation
b3936_m	Matured polypeptide b3936	C339H552N104O100S6	6	Maturation
b3936_mRNA	mRNA b3936	C2025H2296N807O1486P215	-216	Translation
b3936_mRNA_1	mRNA b3936	C2025H2296N807O1486P215	-216	Translation
b3936_mRNA_2	mRNA b3936	C2025H2296N807O1486P215	-216	Translation
b3936_mRNA_2_degr	mRNA b3936 degradation complex	C42708H67695N12682O14171S200P215Mg6Zn2Fe0	-438	mRNA degradation
b3965_aa	polypeptide b3965	C1860H2923N517O555S18	-10	Translation
b3965_def_cplx	Polypeptide b3965 peptide deformylase complex	C2704H4317N758O810S24Mg0Zn0Fe1	-15	Maturation
b3965_m	Matured polypeptide b3965	C1859H2924N517O554S18	-9	Maturation
b3965_mRNA	mRNA b3965	C10489H11860N4211O7677P1103	-1104	Translation
b3965_mRNA_1	mRNA b3965	C10489H11860N4211O7677P1103	-1104	Translation
b3965_mRNA_2	mRNA b3965	C10489H11860N4211O7677P1103	-1104	Translation
b3965_mRNA_2_degr	mRNA b3965 degradation complex	C51172H77259N16086O20362S200P1103Mg6Zn2Fe0	-1326	mRNA degradation
b3965_m_GroEL.(7)ATP.transGroES	b3965 GroEL GroES complex - Kerner et al. class III needs GroEL/ES	C39771H65868O12888N11101S382P21Mg7	-310	Folding
b3968_RNA	rRNA	C14754H16649N6064O10720P1542	-1543	rRNA Modification
b3968_RNA_1		C14755H16651N6064O10720P1542S0	-1543	rRNA Modification
b3968_RNA_10		C14766H16673N6064O10720P1542S0	-1543	rRNA Modification
b3968_RNA_10_RsmB_mono	b3968_RNA_10, RsmB_mono	C16933H20116N6682O11348S17P1542Mg0Zn0Fe0	-1542	rRNA Modification
b3968_RNA_1_MeT_16S_1402	b3968_RNA_1, MeT_16S_1402	C14785H16697N6076O10730S2P1542Mg0Zn0Fe0	-1541	rRNA Modification
b3968_RNA_2		C14757H16655N6064O10720P1542S0	-1543	rRNA Modification
b3968_RNA_2_MeT_16S_1407	b3968_RNA_2, MeT_16S_1407	C14772H16678N6070O10725S1P1542Mg0Zn0Fe0	-1542	rRNA Modification
b3968_RNA_3		C14758H16657N6064O10720P1542S0	-1543	rRNA Modification
b3968_RNA_3_YggJ_mono	b3968_RNA_3, YggJ_mono	C15950H18597N6412O11079S11P1542Mg0Zn0Fe0	-1545	rRNA Modification
b3968_RNA_4		C14759H16659N6064O10720P1542S0	-1543	rRNA Modification
b3968_RNA_4_MeT_16S_1516	b3968_RNA_4, MeT_16S_1516	C14774H16682N6070O10725S1P1542Mg0Zn0Fe0	-1542	rRNA Modification
b3968_RNA_5		C14760H16661N6064O10720P1542S0	-1543	rRNA Modification
b3968_RNA_5_KsgA_mono	b3968_RNA_5, KsgA_mono	C16142H18850N6452O11118S15P1542Mg0Zn0Fe0	-1540	rRNA Modification
b3968_RNA_6		C14762H16665N6064O10720P1542S0	-1543	rRNA Modification
b3968_RNA_6_KsgA_mono	b3968_RNA_6, KsgA_mono	C16144H18854N6452O11118S15P1542Mg0Zn0Fe0	-1540	rRNA Modification
b3968_RNA_7		C14764H16669N6064O10720P1542S0	-1543	rRNA Modification
b3968_RNA_7_RsuA_mono	b3968_RNA_7, RsuA_mono	C15909H18469N6392O11064S6P1542Mg0Zn0Fe0	-1551	rRNA Modification
b3968_RNA_8		C14764H16669N6064O10720P1542S0	-1543	rRNA Modification
b3968_RNA_8_MeT_16S_527	b3968_RNA_8, MeT_16S_527	C14779H16692N6070O10725S1P1542Mg0Zn0Fe0	-1542	rRNA Modification
b3968_RNA_9		C14765H16671N6064O10720P1542S0	-1543	rRNA Modification
b3968_RNA_9_MeT_16S_966	b3968_RNA_9, MeT_16S_966	C14780H16694N6070O10725S1P1542Mg0Zn0Fe0	-1542	rRNA Modification
b3968_RNA_RsmC_mono	b3968_RNA, RsmC_mono	C16438H19280N6537O11222S11P1542Mg0Zn0Fe0	-1547	rRNA Modification
b3968_v1_RNA	rRNA	C14754H16649N6064O10720P1542	-1543	rRNA Modification
b3968_v1_RNA_1		C14755H16651N6064O10720P1542S0	-1543	rRNA Modification
b3968_v1_RNA_10		C14766H16673N6064O10720P1542S0	-1543	rRNA Modification
b3968_v1_RNA_10_RsmB_mono	b3968_v1_RNA_10, RsmB_mono	C16933H20116N6682O11348S17P1542Mg0Zn0Fe0	-1542	rRNA Modification
b3968_v1_RNA_1_MeT_16S_1402	b3968_v1_RNA_1, MeT_16S_1402	C14785H16697N6076O10730S2P1542Mg0Zn0Fe0	-1541	rRNA Modification
b3968_v1_RNA_2		C14757H16655N6064O10720P1542S0	-1543	rRNA Modification
b3968_v1_RNA_2_MeT_16S_1407	b3968_v1_RNA_2, MeT_16S_1407	C14772H16678N6070O10725S1P1542Mg0Zn0Fe0	-1542	rRNA Modification
b3968_v1_RNA_3		C14758H16657N6064O10720P1542S0	-1543	rRNA Modification
b3968_v1_RNA_3_YggJ_mono	b3968_v1_RNA_3, YggJ_mono	C15950H18597N6412O11079S11P1542Mg0Zn0Fe0	-1545	rRNA Modification
b3968_v1_RNA_4		C14759H16659N6064O10720P1542S0	-1543	rRNA Modification
b3968_v1_RNA_4_MeT_16S_1516	b3968_v1_RNA_4, MeT_16S_1516	C14774H16682N6070O10725S1P1542Mg0Zn0Fe0	-1542	rRNA Modification
b3968_v1_RNA_5		C14760H16661N6064O10720P1542S0	-1543	rRNA Modification

b3968_v1_RNA_5_KsgA_mono	b3968_v1_RNA_5, KsgA_mono	C16142H18850N6452O11118S15P1542Mg0Zn0Fe0	-1540	rRNA Modification
b3968_v1_RNA_6		C14762H16665N6064O10720P1542S0	-1543	rRNA Modification
b3968_v1_RNA_6_KsgA_mono	b3968_v1_RNA_6, KsgA_mono	C16144H18854N6452O11118S15P1542Mg0Zn0Fe0	-1540	rRNA Modification
b3968_v1_RNA_7		C14764H16669N6064O10720P1542S0	-1543	rRNA Modification
b3968_v1_RNA_7_RsuA_mono	b3968_v1_RNA_7, RsuA_mono	C15909H18469N6392O11064S6P1542Mg0Zn0Fe0	-1551	rRNA Modification
b3968_v1_RNA_8		C14764H16669N6064O10720P1542S0	-1543	rRNA Modification
b3968_v1_RNA_8_MeT_16S_527	b3968_v1_RNA_8, MeT_16S_527	C14779H16692N6070O10725S1P1542Mg0Zn0Fe0	-1542	rRNA Modification
b3968_v1_RNA_9		C14765H16671N6064O10720P1542S0	-1543	rRNA Modification
b3968_v1_RNA_9_MeT_16S_966	b3968_v1_RNA_9, MeT_16S_966	C14780H16694N6070O10725S1P1542Mg0Zn0Fe0	-1542	rRNA Modification
b3968_v1_RNA_RsmC_mono	b3968_v1_RNA, RsmC_mono	C16438H19280N6537O11222S11P1542Mg0Zn0Fe0	-1547	rRNA Modification
b3969_RNA	tRNA (gltT)	C720H824N287O532P76	-77	tRNA Modification
b3969_tRNA_1	b3969_tRNA_1 (gltT)	C720H824Mg2N287O532P76	-73	tRNA Modification
b3969_tRNA_1_TrmU_mono-YhhP_mono-YheLMN_cplx-YccK_mono-TrmE_dim-GidA_mono-TrmC_mono_cplx	b3969_tRNA_1 (gltT), TrmU_mono, YhhP_mono, YheLMN_cplx, YccK_mono, TrmE_dim, GidA_mono, TrmC_mono	C21611H33674Mg3N6131O6869P88S131X1	-221	tRNA Modification
b3969_tRNA_2	b3969_tRNA_2 (gltT)	C722H829Mg2N288O531P76S1	-73	tRNA Modification
b3969_tRNA_2_MeT_tRNA_pos_37_m2A_cplx	b3969_tRNA_2 (gltT), MeT_tRNA_pos_37_m2A	C737H852Mg2N294O536P76S2	-72	tRNA Modification
b3969_tRNA_2_Se	b3969_tRNA_2_Se - contains mnm5se2U instead of mnm5s2U,b3969_tRNA_2 (gltT)	C722H829Mg2N288O531P76S0Se1	-73	tRNA Modification
b3969_tRNA_2_Se_MeT_tRNA_pos_37_m2A_cplx	b3969_tRNA_2_Se_MeT_tRNA_pos_37_m2A_cplx - contains mnm5se2U instead of mnm5s2U,b3969_tRNA_2 (gltT), MeT_tRNA_pos_37_m2A	C737H852Mg2N294O536P76S1Se1	-72	tRNA Modification
b3969_tRNA_2_YbbB_dim_cplx	b3969_tRNA_2_YbbB_dim_cplx	C4336H6511N1342O1603S31P77Mg2Zn0Fe0Se1	-90	tRNA Modification
b3969_tRNA_3	b3969_tRNA_3 (gltT)	C723H831Mg2N288O531P76S1	-73	tRNA Modification
b3969_tRNA_3_Se	b3969_tRNA_3_Se - contains mnm5se2U instead of mnm5s2U,b3969_tRNA_3 (gltT)	C723H831Mg2N288O531P76S0Se1	-73	tRNA Modification
b3969_tRNA_3_Se_TrmA_mono_cplx	b3969_tRNA_3_Se_TrmA_mono_cplx - contains mnm5se2U instead of mnm5s2U,b3969_tRNA_3 (gltT), TrmA_mono	C2597H3778Mg2N811O1090P76S19Se1	-81	tRNA Modification
b3969_tRNA_3_TrmA_mono_cplx	b3969_tRNA_3 (gltT), TrmA_mono	C2597H3778Mg2N811O1090P76S20	-81	tRNA Modification
b3969_tRNA_4	b3969_tRNA_4 (gltT)	C724H833Mg2N288O531P76S1	-73	tRNA Modification
b3969_tRNA_4_Se	b3969_tRNA_4_Se - contains mnm5se2U instead of mnm5s2U,b3969_tRNA_4 (gltT)	C724H833Mg2N288O531P76S0Se1	-73	tRNA Modification
b3969_tRNA_4_Se_TruB_mono_cplx	b3969_tRNA_4_Se_TruB_mono_cplx - contains mnm5se2U instead of mnm5s2U,b3969_tRNA_4 (gltT), TruB_mono	C2264H3308Mg2N726O1000P76S10Se1	-78	tRNA Modification
b3969_tRNA_4_TruB_mono_cplx	b3969_tRNA_4 (gltT), TruB_mono	C2264H3308Mg2N726O1000P76S11	-78	tRNA Modification
b3969_tRNA_Mg2	tRNA (gltT) bound two Mg2 ions	C720H824Mg2N287O532P76	-73	tRNA Modification
b3969_tRNA_Mg2_TruD_mono_cplx	b3969_tRNA (gltT), TruD_mono	C2463H3566Mg2N786O1041P76S8	-76	tRNA Modification
b3969_v1_RNA	tRNA (gltT)	C720H824N287O532P76	-77	tRNA Modification
b3969_v1_tRNA_1	b3969_v1_tRNA_1 (gltT)	C720H824Mg2N287O532P76	-73	tRNA Modification
b3969_v1_tRNA_1_TrmU_mono-YhhP_mono-YheLMN_cplx-YccK_mono-TrmE_dim-GidA_mono-TrmC_mono_cplx	b3969_v1_tRNA_1 (gltT), TrmU_mono, YhhP_mono, YheLMN_cplx, YccK_mono, TrmE_dim, GidA_mono, TrmC_mono	C21611H33674Mg3N6131O6869P88S131X1	-221	tRNA Modification
b3969_v1_tRNA_2	b3969_v1_tRNA_2 (gltT)	C722H829Mg2N288O531P76S1	-73	tRNA Modification
b3969_v1_tRNA_2_MeT_tRNA_pos_37_m2A_cplx	b3969_v1_tRNA_2 (gltT), MeT_tRNA_pos_37_m2A	C737H852Mg2N294O536P76S2	-72	tRNA Modification
b3969_v1_tRNA_2_Se	b3969_v1_tRNA_2_Se - contains mnm5se2U instead of mnm5s2U,b3969_v1_tRNA_2 (gltT)	C722H829Mg2N288O531P76S0Se1	-73	tRNA Modification
b3969_v1_tRNA_2_Se_MeT_tRNA_pos_37_m2A_cplx	b3969_v1_tRNA_2_Se_MeT_tRNA_pos_37_m2A_cplx - contains mnm5se2U instead of mnm5s2U,b3969_v1_tRNA_2 (gltT), MeT_tRNA_pos_37_m2A	C737H852Mg2N294O536P76S1Se1	-72	tRNA Modification
b3969_v1_tRNA_2_YbbB_dim_cplx	b3969_tRNA_2_YbbB_dim_cplx	C4336H6511N1342O1603S31P77Mg2Zn0Fe0Se1	-90	tRNA Modification
b3969_v1_tRNA_3	b3969_v1_tRNA_3 (gltT)	C723H831Mg2N288O531P76S1	-73	tRNA Modification
b3969_v1_tRNA_3_Se	b3969_v1_tRNA_3_Se - contains mnm5se2U instead of mnm5s2U,b3969_v1_tRNA_3 (gltT)	C723H831Mg2N288O531P76S0Se1	-73	tRNA Modification
b3969_v1_tRNA_3_Se_TrmA_mono_cplx	b3969_v1_tRNA_3_Se_TrmA_mono_cplx - contains mnm5se2U instead of mnm5s2U,b3969_v1_tRNA_3 (gltT), TrmA_mono	C2597H3778Mg2N811O1090P76S19Se1	-81	tRNA Modification
b3969_v1_tRNA_3_TrmA_mono_cplx	b3969_v1_tRNA_3 (gltT), TrmA_mono	C2597H3778Mg2N811O1090P76S20	-81	tRNA Modification
b3969_v1_tRNA_4	b3969_v1_tRNA_4 (gltT)	C724H833Mg2N288O531P76S1	-73	tRNA Modification

b3969_v1_tRNA_4_Se	b3969_v1_tRNA_4_Se - contains mnm5se2U instead of mnm5s2U,b3969_v1_tRNA_4 (gltT)	C724H833Mg2N288O531P76S0Se1	-73	tRNA Modification
b3969_v1_tRNA_4_Se_TruB_mono_cplx	b3969_v1_tRNA_4_Se_TruB_mono_cplx - contains mnm5se2U instead of mnm5s2U,b3969_v1_tRNA_4 (gltT), TruB_mono	C2264H3308Mg2N726O1000P76S10Se1	-78	tRNA Modification
b3969_v1_tRNA_4_TruB_mono_cplx	b3969_v1_tRNA_4 (gltT), TruB_mono	C2264H3308Mg2N726O1000P76S11	-78	tRNA Modification
b3969_v1_tRNA_Mg2	tRNA (gltT) bound two Mg2 ions	C720H824Mg2N287O532P76	-73	tRNA Modification
b3969_v1_tRNA_Mg2_TruD_mono_cplx	b3969_v1_tRNA (gltT), TruD_mono	C2463H3566Mg2N786O1041P76S8	-76	tRNA Modification
b3970_RNA	rRNA	C27810H31354N11469O20158P2904	-2905	rRNA Modification
b3970_RNA_1		C27811H31356N11469O20158P2904S0	-2905	rRNA Modification
b3970_RNA_10		C27818H31370N11469O20158P2904S0	-2905	rRNA Modification
b3970_RNA_10_MeT_23S_2445	b3970_RNA_10, MeT_23S_2445	C27833H31393N11475O20163S1P2904Mg0Zn0Fe0	-2904	rRNA Modification
b3970_RNA_11		C27819H31372N11469O20158P2904S0	-2905	rRNA Modification
b3970_RNA_11_DU_23S_2449_a	b3970_RNA_11, DU_23S_2449 (NADH)	C27840H31400N11476O20172S0P2906Mg0Zn0Fe0	-2906	rRNA Modification
b3970_RNA_11_DU_23S_2449_b	b3970_RNA_11, DU_23S_2449 (NADPH)	C27840H31399N11476O20175S0P2907Mg0Zn0Fe0	-2908	rRNA Modification
b3970_RNA_12		C27819H31374N11469O20158P2904S0	-2905	rRNA Modification
b3970_RNA_12_YmfC_mono	b3970_RNA_12, YmfC_mono	C28917H33135N11796O20478S3P2904Mg0Zn0Fe0	-2895	rRNA Modification
b3970_RNA_13		C27819H31374N11469O20158P2904S0	-2905	rRNA Modification
b3970_RNA_13_MeT_23S_2498	b3970_RNA_13, MeT_23S_2498	C27834H31397N11475O20163S1P2904Mg0Zn0Fe0	-2904	rRNA Modification
b3970_RNA_14		C27820H31376N11469O20158P2904S0	-2905	rRNA Modification
b3970_RNA_14_MeT_23S_2503	b3970_RNA_14, MeT_23S_2503	C27835H31399N11475O20163S1P2904Mg0Zn0Fe0	-2904	rRNA Modification
b3970_RNA_15		C27821H31378N11469O20158P2904S0	-2905	rRNA Modification
b3970_RNA_15_RluC_mono	b3970_RNA_15, RluC_mono	C29406H33991N11951O20614S6P2904Mg0Zn0Fe0	-2890	rRNA Modification
b3970_RNA_16		C27821H31378N11469O20158P2904S0	-2905	rRNA Modification
b3970_RNA_16_RrmJ_mono	b3970_RNA_16, RrmJ_mono	C28868H33067N11764O20461S11P2904Mg0Zn0Fe0	-2897	rRNA Modification
b3970_RNA_17		C27822H31380N11469O20158P2904S0	-2905	rRNA Modification
b3970_RNA_17_RluC_mono	b3970_RNA_17, RluC_mono	C29407H33993N11951O20614S6P2904Mg0Zn0Fe0	-2890	rRNA Modification
b3970_RNA_18		C27822H31380N11469O20158P2904S0	-2905	rRNA Modification
b3970_RNA_18_YjbC_mono	b3970_RNA_18, YjbC_mono	C29249H33751N11887O20580S7P2904Mg0Zn0Fe0	-2890	rRNA Modification
b3970_RNA_19		C27822H31380N11469O20158P2904S0	-2905	rRNA Modification
b3970_RNA_19_RluB_mono	b3970_RNA_19, RluB_mono	C29240H33728N11920O20578S5P2904Mg0Zn0Fe0	-2890	rRNA Modification
b3970_RNA_1_MeT_23S_1835	b3970_RNA_1, MeT_23S_1835	C27826H31379N11475O20163S1P2904Mg0Zn0Fe0	-2904	rRNA Modification
b3970_RNA_2		C27812H31358N11469O20158P2904S0	-2905	rRNA Modification
b3970_RNA_20		C27822H31380N11469O20158P2904S0	-2905	rRNA Modification
b3970_RNA_20_RrmA_dim	b3970_RNA_20, RrmA_dim	C30539H35591N12233O20933S31P2904Mg0Zn2Fe0	-2902	rRNA Modification
b3970_RNA_21		C27823H31382N11469O20158P2904S0	-2905	rRNA Modification
b3970_RNA_21_RluA_mono	b3970_RNA_21, RluA_mono	C28926H33122N11782O20472S10P2904Mg0Zn0Fe0	-2904	rRNA Modification
b3970_RNA_22		C27823H31382N11469O20158P2904S0	-2905	rRNA Modification
b3970_RNA_22_RumB_mono	b3970_RNA_22, RumB_mono	C29716H34360N11981O20692S28P2904Mg0Zn0Fe4	-2903	rRNA Modification
b3970_RNA_23		C27824H31384N11469O20158P2904S0	-2905	rRNA Modification
b3970_RNA_23_RluC_mono	b3970_RNA_23, RluC_mono	C29409H33997N11951O20614S6P2904Mg0Zn0Fe0	-2890	rRNA Modification
b3970_RNA_2_RluD_mono	b3970_RNA_2, RluD_mono	C29449H33968N11949O20632S12P2904Mg1Zn0Fe0	-2909	rRNA Modification
b3970_RNA_3		C27812H31358N11469O20158P2904S0	-2905	rRNA Modification
b3970_RNA_3_RluD_mono	b3970_RNA_3, RluD_mono	C29464H33991N11955O20637S13P2904Mg1Zn0Fe0	-2908	rRNA Modification
b3970_RNA_4		C27813H31360N11469O20158P2904S0	-2905	rRNA Modification
b3970_RNA_4_RluD_mono	b3970_RNA_4, RluD_mono	C29450H33970N11949O20632S12P2904Mg1Zn0Fe0	-2909	rRNA Modification
b3970_RNA_5		C27813H31360N11469O20158P2904S0	-2905	rRNA Modification
b3970_RNA_5_RumA_mono	b3970_RNA_5, RumA_mono	C29941H34786N12087O20786S23P2904Mg0Zn0Fe4	-2897	rRNA Modification
b3970_RNA_6		C27814H31362N11469O20158P2904S0	-2905	rRNA Modification
b3970_RNA_6_MeT_23S_1962	b3970_RNA_6, MeT_23S_1962	C27829H31385N11475O20163S1P2904Mg0Zn0Fe0	-2904	rRNA Modification
b3970_RNA_7		C27815H31364N11469O20158P2904S0	-2905	rRNA Modification
b3970_RNA_7_MeT_23S_2030	b3970_RNA_7, MeT_23S_2030	C27830H31387N11475O20163S1P2904Mg0Zn0Fe0	-2904	rRNA Modification
b3970_RNA_8		C27816H31366N11469O20158P2904S0	-2905	rRNA Modification
b3970_RNA_8_MeT_23S_2069	b3970_RNA_8, MeT_23S_2069	C27831H31389N11475O20163S1P2904Mg0Zn0Fe0	-2904	rRNA Modification

b3970_RNA_9		C27817H31368N11469O20158P2904S0	-2905	rRNA Modification
b3970_RNA_9_RlmB_dim	b3970_RNA_9, RlmB_dim	C30138H35171N12153O20859S23P2904Mg0Zn0Fe0	-2910	rRNA Modification
b3970_RNA_MeT_23S_1618	b3970_RNA, MeT_23S_1618	C27825H31377N11475O20163S1P2904Mg0Zn0Fe0	-2904	rRNA Modification
b3970_v1_RNA	rRNA	C27810H31354N11469O20158P2904	-2905	rRNA Modification
b3970_v1_RNA_1		C27811H31356N11469O20158P2904S0	-2905	rRNA Modification
b3970_v1_RNA_10		C27818H31370N11469O20158P2904S0	-2905	rRNA Modification
b3970_v1_RNA_10_MeT_23S_2445	b3970_v1_RNA_10, MeT_23S_2445	C27833H31393N11475O20163S1P2904Mg0Zn0Fe0	-2904	rRNA Modification
b3970_v1_RNA_11		C27819H31372N11469O20158P2904S0	-2905	rRNA Modification
b3970_v1_RNA_11_DU_23S_2449_a	b3970_v1_RNA_11, DU_23S_2449 (NADH)	C27840H31400N11476O20172S0P2906Mg0Zn0Fe0	-2906	rRNA Modification
b3970_v1_RNA_11_DU_23S_2449_b	b3970_v1_RNA_11, DU_23S_2449 (NADPH)	C27840H31399N11476O20175S0P2907Mg0Zn0Fe0	-2908	rRNA Modification
b3970_v1_RNA_12		C27819H31374N11469O20158P2904S0	-2905	rRNA Modification
b3970_v1_RNA_12_YmfC_mono	b3970_v1_RNA_12, YmfC_mono	C28917H33135N11796O20478S3P2904Mg0Zn0Fe0	-2895	rRNA Modification
b3970_v1_RNA_13		C27819H31374N11469O20158P2904S0	-2905	rRNA Modification
b3970_v1_RNA_13_MeT_23S_2498	b3970_v1_RNA_13, MeT_23S_2498	C27834H31397N11475O20163S1P2904Mg0Zn0Fe0	-2904	rRNA Modification
b3970_v1_RNA_14		C27820H31376N11469O20158P2904S0	-2905	rRNA Modification
b3970_v1_RNA_14_MeT_23S_2503	b3970_v1_RNA_14, MeT_23S_2503	C27835H31399N11475O20163S1P2904Mg0Zn0Fe0	-2904	rRNA Modification
b3970_v1_RNA_15		C27821H31378N11469O20158P2904S0	-2905	rRNA Modification
b3970_v1_RNA_15_RluC_mono	b3970_v1_RNA_15, RluC_mono	C29406H33991N11951O20614S6P2904Mg0Zn0Fe0	-2890	rRNA Modification
b3970_v1_RNA_16		C27821H31378N11469O20158P2904S0	-2905	rRNA Modification
b3970_v1_RNA_16_RrmJ_mono	b3970_v1_RNA_16, RrmJ_mono	C28868H33067N11764O20461S11P2904Mg0Zn0Fe0	-2897	rRNA Modification
b3970_v1_RNA_17		C27822H31380N11469O20158P2904S0	-2905	rRNA Modification
b3970_v1_RNA_17_RluC_mono	b3970_v1_RNA_17, RluC_mono	C29407H33993N11951O20614S6P2904Mg0Zn0Fe0	-2890	rRNA Modification
b3970_v1_RNA_18		C27822H31380N11469O20158P2904S0	-2905	rRNA Modification
b3970_v1_RNA_18_YjbC_mono	b3970_v1_RNA_18, YjbC_mono	C29249H33751N11887O20580S7P2904Mg0Zn0Fe0	-2890	rRNA Modification
b3970_v1_RNA_19		C27822H31380N11469O20158P2904S0	-2905	rRNA Modification
b3970_v1_RNA_19_RluB_mono	b3970_v1_RNA_19, RluB_mono	C29240H33728N11920O20578S5P2904Mg0Zn0Fe0	-2890	rRNA Modification
b3970_v1_RNA_1_MeT_23S_1835	b3970_v1_RNA_1, MeT_23S_1835	C27826H31379N11475O20163S1P2904Mg0Zn0Fe0	-2904	rRNA Modification
b3970_v1_RNA_2		C27812H31358N11469O20158P2904S0	-2905	rRNA Modification
b3970_v1_RNA_20		C27822H31380N11469O20158P2904S0	-2905	rRNA Modification
b3970_v1_RNA_20_RrmA_dim	b3970_v1_RNA_20, RrmA_dim	C30539H35591N12233O20933S31P2904Mg0Zn2Fe0	-2902	rRNA Modification
b3970_v1_RNA_21		C27823H31382N11469O20158P2904S0	-2905	rRNA Modification
b3970_v1_RNA_21_RluA_mono	b3970_v1_RNA_21, RluA_mono	C28926H33122N11782O20472S10P2904Mg0Zn0Fe0	-2904	rRNA Modification
b3970_v1_RNA_22		C27823H31382N11469O20158P2904S0	-2905	rRNA Modification
b3970_v1_RNA_22_RumB_mono	b3970_v1_RNA_22, RumB_mono	C29716H34360N11981O20692S28P2904Mg0Zn0Fe4	-2903	rRNA Modification
b3970_v1_RNA_23		C27824H31384N11469O20158P2904S0	-2905	rRNA Modification
b3970_v1_RNA_23_RluC_mono	b3970_v1_RNA_23, RluC_mono	C29409H33997N11951O20614S6P2904Mg0Zn0Fe0	-2890	rRNA Modification
b3970_v1_RNA_2_RluD_mono	b3970_v1_RNA_2, RluD_mono	C29449H33968N11949O20632S12P2904Mg1Zn0Fe0	-2909	rRNA Modification
b3970_v1_RNA_3		C27812H31358N11469O20158P2904S0	-2905	rRNA Modification
b3970_v1_RNA_3_RluD_mono	b3970_v1_RNA_3, RluD_mono	C29464H33991N11955O20637S13P2904Mg1Zn0Fe0	-2908	rRNA Modification
b3970_v1_RNA_4		C27813H31360N11469O20158P2904S0	-2905	rRNA Modification
b3970_v1_RNA_4_RluD_mono	b3970_v1_RNA_4, RluD_mono	C29450H33970N11949O20632S12P2904Mg1Zn0Fe0	-2909	rRNA Modification
b3970_v1_RNA_5		C27813H31360N11469O20158P2904S0	-2905	rRNA Modification
b3970_v1_RNA_5_RumA_mono	b3970_v1_RNA_5, RumA_mono	C29941H34786N12087O20786S23P2904Mg0Zn0Fe4	-2897	rRNA Modification
b3970_v1_RNA_6		C27814H31362N11469O20158P2904S0	-2905	rRNA Modification
b3970_v1_RNA_6_MeT_23S_1962	b3970_v1_RNA_6, MeT_23S_1962	C27829H31385N11475O20163S1P2904Mg0Zn0Fe0	-2904	rRNA Modification
b3970_v1_RNA_7		C27815H31364N11469O20158P2904S0	-2905	rRNA Modification
b3970_v1_RNA_7_MeT_23S_2030	b3970_v1_RNA_7, MeT_23S_2030	C27830H31387N11475O20163S1P2904Mg0Zn0Fe0	-2904	rRNA Modification
b3970_v1_RNA_8		C27816H31366N11469O20158P2904S0	-2905	rRNA Modification
b3970_v1_RNA_8_MeT_23S_2069	b3970_v1_RNA_8, MeT_23S_2069	C27831H31389N11475O20163S1P2904Mg0Zn0Fe0	-2904	rRNA Modification
b3970_v1_RNA_9		C27817H31368N11469O20158P2904S0	-2905	rRNA Modification
b3970_v1_RNA_9_RlmB_dim	b3970_v1_RNA_9, RlmB_dim	C30138H35171N12153O20859S23P2904Mg0Zn0Fe0	-2910	rRNA Modification
b3970_v1_RNA_MeT_23S_1618	b3970_v1_RNA, MeT_23S_1618	C27825H31377N11475O20163S1P2904Mg0Zn0Fe0	-2904	rRNA Modification
b3971_RNA	rRNA	C1145H1301N470O837P120	-121	RNA cutting

b3971_v1_RNA	rRNA	C1145H1301N470O837P120	-121	RNA cutting
b3976_RNA	tRNA (thrU)	C723H819N288O532P76	-77	tRNA Modification
b3976_tRNA_1	b3976_tRNA_1 (thrU)	C723H821Mg2N288O532P76	-73	tRNA Modification
b3976_tRNA_1_Dus_gen_cplx	b3976_tRNA_1 (thrU), Dus_gen	C2330H3360Mg2N747O1015P79S14	-80	tRNA Modification
b3976_tRNA_2	b3976_tRNA_2 (thrU)	C723H823Mg2N288O532P76	-73	tRNA Modification
b3976_tRNA_2_Up_tRNA_pos_37_t6A-MeT_tRNA_pos_37_m6t6A_cplx	b3976_tRNA_2 (thrU), Up_tRNA_pos_37_t6A, MeT_tRNA_pos_37_m6t6A	C753H868Mg3N300O556P79S1	-75	tRNA Modification
b3976_tRNA_3	b3976_tRNA_3 (thrU)	C729H831Mg2N289O536P76	-74	tRNA Modification
b3976_tRNA_3_YggH_mono_cplx	b3976_tRNA_3 (thrU), YggH_mono	C1954H2730Mg2N642O883P76S14	-76	tRNA Modification
b3976_tRNA_4	b3976_tRNA_4 (thrU)	C730H833Mg2N289O536P76	-74	tRNA Modification
b3976_tRNA_4_TrmA_mono_cplx	b3976_tRNA_4 (thrU), TrmA_mono	C2604H3780Mg2N812O1095P76S19	-82	tRNA Modification
b3976_tRNA_5	b3976_tRNA_5 (thrU)	C731H835Mg2N289O536P76	-74	tRNA Modification
b3976_tRNA_5_TruB_mono_cplx	b3976_tRNA_5 (thrU), TruB_mono	C2271H3310Mg2N727O1005P76S10	-79	tRNA Modification
b3976_tRNA_Mg2	tRNA (thrU) bound two Mg2 ions	C723H819Mg2N288O532P76	-73	tRNA Modification
b3976_tRNA_Mg2_Dus_gen_cplx	b3976_tRNA (thrU), Dus_gen	C2330H3358Mg2N747O1015P79S14	-80	tRNA Modification
b3977_RNA	tRNA (tyrU)	C808H921N326O591P85	-86	tRNA Modification
b3977_tRNA_1	b3977_tRNA_1 (tyrU)	C809H923Mg2N326O591P85	-82	tRNA Modification
b3977_tRNA_1_Tgt_hexa-QueA_mono-EoR_tRNA_pos34_Q_cplx	b3977_tRNA_1 (tyrU), Tgt_hexa, QueA_mono, EoR_tRNA_pos34_Q	C14002Co1H21438Mg2N4019O4468P88S143Zn6	-122	tRNA Modification
b3977_tRNA_2	b3977_tRNA_2 (tyrU)	C816H933Mg2N326O593P85	-82	tRNA Modification
b3977_tRNA_2_MiaA_dim-MiaB_mono_cplx	b3977_tRNA_2 (tyrU), MiaA_dim, MiaB_mono	C10291Fe4H15963Mg4N3001O3459P89S80X1	-127	tRNA Modification
b3977_tRNA_3	b3977_tRNA_3 (tyrU)	C822H943Mg2N326O593P85S1	-82	tRNA Modification
b3977_tRNA_3_TruA_dim_cplx	b3977_tRNA_3 (tyrU), TruA_dim	C3532H5145Mg2N1106O1363P85S17	-76	tRNA Modification
b3977_tRNA_4	b3977_tRNA_4 (tyrU)	C822H943Mg2N326O593P85S1	-82	tRNA Modification
b3977_tRNA_4_TrmA_mono_cplx	b3977_tRNA_4 (tyrU), TrmA_mono	C2696H3890Mg2N849O1152P85S20	-90	tRNA Modification
b3977_tRNA_5	b3977_tRNA_5 (tyrU)	C823H945Mg2N326O593P85S1	-82	tRNA Modification
b3977_tRNA_5_TruB_mono_cplx	b3977_tRNA_5 (tyrU), TruB_mono	C2363H3420Mg2N764O1062P85S11	-87	tRNA Modification
b3977_tRNA_6	b3977_tRNA_6 (tyrU)	C823H945Mg2N326O593P85S1	-82	tRNA Modification
b3977_tRNA_6_ThiI_mono_cplx	b3977_tRNA_6 (tyrU), ThiI_mono	C7251H11176Mg3N2144O2532P90S51X1	-110	tRNA Modification
b3977_tRNA_Mg2	tRNA (tyrU) bound two Mg2 ions	C808H921Mg2N326O591P85	-82	tRNA Modification
b3977_tRNA_Mg2_TrmH_dim_cplx	b3977_tRNA (tyrU), TrmH_dim	C3019H4464Mg2N982O1252P85S29	-83	tRNA Modification
b3978_RNA	tRNA (glyT)	C707H807N270O532P75	-76	tRNA Modification
b3978_tRNA_1	b3978_tRNA_1 (glyT)	C708H809Mg2N270O532P75	-72	tRNA Modification
b3978_tRNA_1_TruB_mono_cplx	b3978_tRNA_1 (glyT), TruB_mono	C2248H3284Mg2N708O1001P75S10	-77	tRNA Modification
b3978_tRNA_Mg2	tRNA (glyT) bound two Mg2 ions	C707H807Mg2N270O532P75	-72	tRNA Modification
b3978_tRNA_Mg2_TrmA_mono_cplx	b3978_tRNA (glyT), TrmA_mono	C2581H3754Mg2N793O1091P75S19	-80	tRNA Modification
b3979_RNA	tRNA (thrT)	C724H819N290O534P76	-77	tRNA Modification
b3979_tRNA_1	b3979_tRNA_1 (thrT)	C724H821Mg2N290O534P76	-73	tRNA Modification
b3979_tRNA_1_Dus_gen_cplx	b3979_tRNA_1 (thrT), Dus_gen	C2331H3360Mg2N749O1017P79S14	-80	tRNA Modification
b3979_tRNA_2	b3979_tRNA_2 (thrT)	C724H823Mg2N290O534P76	-73	tRNA Modification
b3979_tRNA_2_Dus_gen_cplx	b3979_tRNA_2 (thrT), Dus_gen	C2331H3362Mg2N749O1017P79S14	-80	tRNA Modification
b3979_tRNA_3	b3979_tRNA_3 (thrT)	C724H825Mg2N290O534P76	-73	tRNA Modification
b3979_tRNA_3_Up_tRNA_pos_37_t6A-MeT_tRNA_pos_37_m6t6A_cplx	b3979_tRNA_3 (thrT), Up_tRNA_pos_37_t6A, MeT_tRNA_pos_37_m6t6A	C754H870Mg3N302O558P79S1	-75	tRNA Modification
b3979_tRNA_4	b3979_tRNA_4 (thrT)	C730H833Mg2N291O538P76	-74	tRNA Modification
b3979_tRNA_4_YggH_mono_cplx	b3979_tRNA_4 (thrT), YggH_mono	C1955H2732Mg2N644O885P76S14	-76	tRNA Modification
b3979_tRNA_5	b3979_tRNA_5 (thrT)	C731H835Mg2N291O538P76	-74	tRNA Modification
b3979_tRNA_5_TrmA_mono_cplx	b3979_tRNA_5 (thrT), TrmA_mono	C2605H3782Mg2N814O1097P76S19	-82	tRNA Modification
b3979_tRNA_6	b3979_tRNA_6 (thrT)	C732H837Mg2N291O538P76	-74	tRNA Modification
b3979_tRNA_6_TruB_mono_cplx	b3979_tRNA_6 (thrT), TruB_mono	C2272H3312Mg2N729O1007P76S10	-79	tRNA Modification
b3979_tRNA_Mg2	tRNA (thrT) bound two Mg2 ions	C724H819Mg2N290O534P76	-73	tRNA Modification
b3979_tRNA_Mg2_Dus_gen_cplx	b3979_tRNA (thrT), Dus_gen	C2331H3358Mg2N749O1017P79S14	-80	tRNA Modification
b3980_aa	polypeptide b3980_v1	C1924H3060N524O584S14	-16	Translation
b3980_def_map_cplx	Polypeptide b3980 peptide deformylase and methionine aminopeptidase complex	C4054H6516N1117O1230S35Mg0Zn0Fe3	-25	Maturation
b3980_m	Matured polypeptide b3980	C1918H3052N523O582S13	-15	Maturation
b3980_mRNA	mRNA b3980	C11281H12774N4525O8274P1187	-1188	Translation
b3980_mRNA_1	mRNA b3980	C11281H12774N4525O8274P1187	-1188	Translation
b3980_mRNA_2	mRNA b3980	C11281H12774N4525O8274P1187	-1188	Translation
b3980_mRNA_2_degr	mRNA b3980 degradation complex	C51964H78173N16400O20959S200P1187Mg6Zn2Fe0	-1410	mRNA degradation

b3980_m_DnaKJ_complex	b3980 DnaK DnaJ_dim complex - Deuerling et al. DnaKJ/GrpE dependent folding	C8482H13566O2680N2436S60P3Zn4	-37	Folding
b3980_v1_mRNA	mRNA b3980_v1	C11281H12774N4525O8274P1187	-1188	Translation
b3981_aa	polypeptide b3981	C627H1046N167O164S4	5	Translation
b3981_def_map_cplx	Polypeptide b3981 peptide deformylase and methionine aminopeptidase complex	C2757H4502N760O810S25Mg0Zn0Fe3	-4	Maturation
b3981_m	Matured polypeptide b3981	C621H1038N166O162S3	6	Maturation
b3981_mRNA	mRNA b3981	C3650H4125N1440O2722P386	-387	Translation
b3981_mRNA_1	mRNA b3981	C3650H4125N1440O2722P386	-387	Translation
b3981_mRNA_2	mRNA b3981	C3650H4125N1440O2722P386	-387	Translation
b3981_mRNA_2_degr	mRNA b3981 degradation complex	C44333H69524N13315O15407S200P386Mg6Zn2Fe0	-609	mRNA degradation
b3982_aa	polypeptide b3982	C914H1438N256O268S8	-2	Translation
b3982_def_map_cplx	Polypeptide b3982 peptide deformylase and methionine aminopeptidase complex	C3044H4894N849O914S29Mg0Zn0Fe3	-11	Maturation
b3982_m	Matured polypeptide b3982	C908H1430N255O266S7	-1	Maturation
b3982_mRNA	mRNA b3982	C5200H5871N2074O3831P546	-547	Translation
b3982_mRNA_1	mRNA b3982	C5200H5871N2074O3831P546	-547	Translation
b3982_mRNA_2	mRNA b3982	C5200H5871N2074O3831P546	-547	Translation
b3982_mRNA_2_degr	mRNA b3982 degradation complex	C45883H71270N13949O16516S200P546Mg6Zn2Fe0	-769	mRNA degradation
b3982_m_DnaKJ_complex	b3982 DnaK DnaJ_dim complex - Kerner et al. class II can interact w/ GroEL/ES, cannot fold spontanously	C7472H11944O2364N2168S54P3Zn4	-23	Folding
b3982_m_GroEL.(7)ATP.transGroES	b3982 GroEL GroES complex - Kerner et al. class II can interact w/ GroEL/ES, cannot fold spontanously	C38820H64374O12600N10839S371P21Mg7	-302	Folding
b3983_aa	polypeptide b3983	C657H1096N180O198S7	6	Translation
b3983_def_map_cplx	Polypeptide b3983 peptide deformylase and methionine aminopeptidase complex	C2787H4552N773O844S28Mg0Zn0Fe3	-3	Maturation
b3983_m	Matured polypeptide b3983	C651H1088N179O196S6	7	Maturation
b3983_mRNA	mRNA b3983	C4089H4629N1652O2987P431	-432	Translation
b3983_mRNA_1	mRNA b3983	C4089H4629N1652O2987P431	-432	Translation
b3983_mRNA_2	mRNA b3983	C4089H4629N1652O2987P431	-432	Translation
b3983_mRNA_2_degr	mRNA b3983 degradation complex	C44772H70028N13527O15672S200P431Mg6Zn2Fe0	-654	mRNA degradation
b3984_aa	polypeptide b3984	C1082H1823N315O331S7	8	Translation
b3984_def_map_cplx	Polypeptide b3984 peptide deformylase and methionine aminopeptidase complex	C3212H5279N908O977S28Mg0Zn0Fe3	-1	Maturation
b3984_m	Matured polypeptide b3984	C1076H1815N314O329S6	9	Maturation
b3984_mRNA	mRNA b3984	C6732H7611N2744O4885P705	-706	Translation
b3984_mRNA_1	mRNA b3984	C6732H7611N2744O4885P705	-706	Translation
b3984_mRNA_2	mRNA b3984	C6732H7611N2744O4885P705	-706	Translation
b3984_mRNA_2_degr	mRNA b3984 degradation complex	C47415H73010N14619O17570S200P705Mg6Zn2Fe0	-928	mRNA degradation
b3985_aa	polypeptide b3985_v1	C782H1291N221O233S7	2	Translation
b3985_def_map_cplx	Polypeptide b3985 peptide deformylase and methionine aminopeptidase complex	C2912H4747N814O879S28Mg0Zn0Fe3	-7	Maturation
b3985_m	Matured polypeptide b3985	C776H1283N220O231S6	3	Maturation
b3985_mRNA	mRNA b3985	C4742H5363N1898O3488P500	-501	Translation
b3985_mRNA_1	mRNA b3985	C4742H5363N1898O3488P500	-501	Translation
b3985_mRNA_2	mRNA b3985	C4742H5363N1898O3488P500	-501	Translation
b3985_mRNA_2_degr	mRNA b3985 degradation complex	C45425H70762N13773O16173S200P500Mg6Zn2Fe0	-723	mRNA degradation
b3985_v1_mRNA	mRNA b3985_v1	C4742H5363N1898O3482P498	-499	Translation
b3986_aa	polypeptide b3986_v2	C540H898N139O178S4	-9	Translation
b3986_def_map_cplx	Polypeptide b3986 peptide deformylase and methionine aminopeptidase complex	C2670H4354N732O824S25Mg0Zn0Fe3	-18	Maturation
b3986_m	Matured polypeptide b3986	C534H890N138O176S3	-8	Maturation
b3986_mRNA	mRNA b3986	C3505H3942N1435O2536P366	-367	Translation
b3986_mRNA_1	mRNA b3986	C3505H3942N1435O2536P366	-367	Translation
b3986_mRNA_2	mRNA b3986	C3505H3942N1435O2536P366	-367	Translation
b3986_mRNA_2_degr	mRNA b3986 degradation complex	C44188H69341N13310O15221S200P366Mg6Zn2Fe0	-589	mRNA degradation
b3986_v1_mRNA	mRNA b3986_v1	C3505H3942N1435O2542P368	-369	Translation
b3986_v2_mRNA	mRNA b3986_v2	C3505H3942N1435O2536P366	-367	Translation
b3987_aa	polypeptide b3987_v2	C6642H10602N1843O2058S44	-45	Translation
b3987_def_cplx	Polypeptide b3987 peptide deformylase complex	C7486H11996N2084O2313S50Mg0Zn0Fe1	-50	Maturation

b3987_m	Matured polypeptide b3987	C6641H10603N1843O2057S44	-44	Maturation
b3987_mRNA	mRNA b3987	C38365H43428N15403O28079P4029	-4030	Translation
b3987_mRNA_1	mRNA b3987	C38365H43428N15403O28079P4029	-4030	Translation
b3987_mRNA_2	mRNA b3987	C38365H43428N15403O28079P4029	-4030	Translation
b3987_mRNA_2_degr	mRNA b3987 degradation complex	C79048H108827N27278O40764S200P4029Mg6Zn2Fe0	-4252	mRNA degradation
b3987_m_DnaKJ_complex	b3987 DnaK DnaJ_dim complex - Kerner et al. class II can interact w/ GroEL/ES, cannot fold spontanously	C13205H21117O4155N3756S91P3Zn5	-64	Folding
b3987_m_GroEL.(7)ATP.transGroES	b3987 GroEL GroES complex - Kerner et al. class II can interact w/ GroEL/ES, cannot fold spontanously	C44553H73547O14391N12427S408P21Mg7Zn1	-343	Folding
b3987_m_Zn	b3987 plus _Zn	C6641H10603N1843O2057S44Zn1	-42	Folding
b3987_v1_mRNA	mRNA b3987_v1	C38365H43428N15403O28085P4031	-4032	Translation
b3987_v2_mRNA	mRNA b3987_v2	C38365H43428N15403O28079P4029	-4030	Translation
b3988_aa	polypeptide b3988_v2	C6833H11113N1940O2069S51	-5	Translation
b3988_def_cplx	Polypeptide b3988 peptide deformylase complex	C7677H12507N2181O2324S57Mg0Zn0Fe1	-10	Maturation
b3988_m	Matured polypeptide b3988	C6832H11114N1940O2068S51	-4	Maturation
b3988_mRNA	mRNA b3988	C40214H45527N16130O29495P4224	-4225	Translation
b3988_mRNA_1	mRNA b3988	C40214H45527N16130O29495P4224	-4225	Translation
b3988_mRNA_2	mRNA b3988	C40214H45527N16130O29495P4224	-4225	Translation
b3988_mRNA_2_degr	mRNA b3988 degradation complex	C80897H110926N28005O42180S200P4224Mg6Zn2Fe0	-4447	mRNA degradation
b3988_m_DnaKJ_complex	b3988 DnaK DnaJ_dim complex - Kerner et al. class II can interact w/ GroEL/ES, cannot fold spontanously	C13396H21628O4166N3853S98P3Mg2Zn4	-22	Folding
b3988_m_GroEL.(7)ATP.transGroES	b3988 GroEL GroES complex - Kerner et al. class II can interact w/ GroEL/ES, cannot fold spontanously	C44744H74058O14402N12524S415P21Mg9	-301	Folding
b3988_m_Mg	b3988 plus _Mg	C6832H11114N1940O2068S51Mg2	0	Folding
b3988_v1_mRNA	mRNA b3988_v1	C40214H45527N16130O29495P4224	-4225	Translation
b3988_v2_mRNA	mRNA b3988_v2	C40214H45527N16130O29495P4224	-4225	Translation
b4007_RNA	rRNA	C14754H16649N6064O10720P1542	-1543	rRNA Modification
b4007_RNA_1		C14755H16651N6064O10720P1542S0	-1543	rRNA Modification
b4007_RNA_10		C14766H16673N6064O10720P1542S0	-1543	rRNA Modification
b4007_RNA_10_RsmB_mono	b4007_RNA_10, RsmB_mono	C16933H20116N6682O11348S17P1542Mg0Zn0Fe0	-1542	rRNA Modification
b4007_RNA_1_MeT_16S_1402	b4007_RNA_1, MeT_16S_1402	C14785H16697N6076O10730S2P1542Mg0Zn0Fe0	-1541	rRNA Modification
b4007_RNA_2		C14757H16655N6064O10720P1542S0	-1543	rRNA Modification
b4007_RNA_2_MeT_16S_1407	b4007_RNA_2, MeT_16S_1407	C14772H16678N6070O10725S1P1542Mg0Zn0Fe0	-1542	rRNA Modification
b4007_RNA_3		C14758H16657N6064O10720P1542S0	-1543	rRNA Modification
b4007_RNA_3_YggJ_mono	b4007_RNA_3, YggJ_mono	C15950H18597N6412O11079S11P1542Mg0Zn0Fe0	-1545	rRNA Modification
b4007_RNA_4		C14759H16659N6064O10720P1542S0	-1543	rRNA Modification
b4007_RNA_4_MeT_16S_1516	b4007_RNA_4, MeT_16S_1516	C14774H16682N6070O10725S1P1542Mg0Zn0Fe0	-1542	rRNA Modification
b4007_RNA_5		C14760H16661N6064O10720P1542S0	-1543	rRNA Modification
b4007_RNA_5_KsgA_mono	b4007_RNA_5, KsgA_mono	C16142H18850N6452O11118S15P1542Mg0Zn0Fe0	-1540	rRNA Modification
b4007_RNA_6		C14762H16665N6064O10720P1542S0	-1543	rRNA Modification
b4007_RNA_6_KsgA_mono	b4007_RNA_6, KsgA_mono	C16144H18854N6452O11118S15P1542Mg0Zn0Fe0	-1540	rRNA Modification
b4007_RNA_7		C14764H16669N6064O10720P1542S0	-1543	rRNA Modification
b4007_RNA_7_RsuA_mono	b4007_RNA_7, RsuA_mono	C15909H18469N6392O11064S6P1542Mg0Zn0Fe0	-1551	rRNA Modification
b4007_RNA_8		C14764H16669N6064O10720P1542S0	-1543	rRNA Modification
b4007_RNA_8_MeT_16S_527	b4007_RNA_8, MeT_16S_527	C14779H16692N6070O10725S1P1542Mg0Zn0Fe0	-1542	rRNA Modification
b4007_RNA_9		C14765H16671N6064O10720P1542S0	-1543	rRNA Modification
b4007_RNA_9_MeT_16S_966	b4007_RNA_9, MeT_16S_966	C14780H16694N6070O10725S1P1542Mg0Zn0Fe0	-1542	rRNA Modification
b4007_RNA_RsmC_mono	b4007_RNA, RsmC_mono	C16438H19280N6537O11222S11P1542Mg0Zn0Fe0	-1547	rRNA Modification
b4008_RNA	tRNA (gltV)	C720H824N287O532P76	-77	tRNA Modification
b4008_tRNA_1	b4008_tRNA_1 (gltV)	C720H824Mg2N287O532P76	-73	tRNA Modification
b4008_tRNA_1_TrmU_mono-YhhP_mono-YheLMN_cplx-YccK_mono-TrmE_dim-GidA_mono-TrmC_mono_cplx	b4008_tRNA_1 (gltV), TrmU_mono, YhhP_mono, YheLMN_cplx, YccK_mono, TrmE_dim, GidA_mono, TrmC_mono	C21611H33674Mg3N6131O6869P88S131X1	-221	tRNA Modification
b4008_tRNA_2	b4008_tRNA_2 (gltV)	C722H829Mg2N288O531P76S1	-73	tRNA Modification
b4008_tRNA_2_MeT_tRNA_pos_37_m2A_cplx	b4008_tRNA_2 (gltV), MeT_tRNA_pos_37_m2A	C737H852Mg2N294O536P76S2	-72	tRNA Modification
b4008_tRNA_2_Se	b4008_tRNA_2_Se - contains mnm5se2U instead of mnm5s2U,b4008_tRNA_2 (gltV)	C722H829Mg2N288O531P76S0Se1	-73	tRNA Modification

b4008_tRNA_2_Se_MeT_tRNA_pos_37_m2A_cplx	b4008_tRNA_2_Se_MeT_tRNA_pos_37_m2A_cplx - contains mnm5se2U instead of mnm5s2U,b4008_tRNA_2 (gltV), MeT_tRNA_pos_37_m2A	C737H852Mg2N294O536P76S1Se1	-72	tRNA Modification
b4008_tRNA_2_YbbB_dim_cplx	b4008_tRNA_2_YbbB_dim_cplx	C4336H6511N1342O1603S31P77Mg2Zn0Fe0Se1	-90	tRNA Modification
b4008_tRNA_3	b4008_tRNA_3 (gltV)	C723H831Mg2N288O531P76S1	-73	tRNA Modification
b4008_tRNA_3_Se	b4008_tRNA_3_Se - contains mnm5se2U instead of mnm5s2U,b4008_tRNA_3 (gltV)	C723H831Mg2N288O531P76S0Se1	-73	tRNA Modification
b4008_tRNA_3_Se_TrmA_mono_cplx	b4008_tRNA_3_Se_TrmA_mono_cplx - contains mnm5se2U instead of mnm5s2U,b4008_tRNA_3 (gltV), TrmA_mono	C2597H3778Mg2N811O1090P76S19Se1	-81	tRNA Modification
b4008_tRNA_3_TrmA_mono_cplx	b4008_tRNA_3 (gltV), TrmA_mono	C2597H3778Mg2N811O1090P76S20	-81	tRNA Modification
b4008_tRNA_4	b4008_tRNA_4 (gltV)	C724H833Mg2N288O531P76S1	-73	tRNA Modification
b4008_tRNA_4_Se	b4008_tRNA_4_Se - contains mnm5se2U instead of mnm5s2U,b4008_tRNA_4 (gltV)	C724H833Mg2N288O531P76S0Se1	-73	tRNA Modification
b4008_tRNA_4_Se_TruB_mono_cplx	b4008_tRNA_4_Se_TruB_mono_cplx - contains mnm5se2U instead of mnm5s2U,b4008_tRNA_4 (gltV), TruB_mono	C2264H3308Mg2N726O1000P76S10Se1	-78	tRNA Modification
b4008_tRNA_4_TruB_mono_cplx	b4008_tRNA_4 (gltV), TruB_mono	C2264H3308Mg2N726O1000P76S11	-78	tRNA Modification
b4008_tRNA_Mg2	tRNA (gltV) bound two Mg2 ions	C720H824Mg2N287O532P76	-73	tRNA Modification
b4008_tRNA_Mg2_TruD_mono_cplx	b4008_tRNA (gltV), TruD_mono	C2463H3566Mg2N786O1041P76S8	-76	tRNA Modification
b4009_RNA	rRNA	C27810H31354N11469O20158P2904	-2905	rRNA Modification
b4009_RNA_1		C27811H31356N11469O20158P2904S0	-2905	rRNA Modification
b4009_RNA_10		C27818H31370N11469O20158P2904S0	-2905	rRNA Modification
b4009_RNA_10_MeT_23S_2445	b4009_RNA_10, MeT_23S_2445	C27833H31393N11475O20163S1P2904Mg0Zn0Fe0	-2904	rRNA Modification
b4009_RNA_11		C27819H31372N11469O20158P2904S0	-2905	rRNA Modification
b4009_RNA_11_DU_23S_2449_a	b4009_RNA_11, DU_23S_2449 (NADH)	C27840H31400N11476O20172S0P2906Mg0Zn0Fe0	-2906	rRNA Modification
b4009_RNA_11_DU_23S_2449_b	b4009_RNA_11, DU_23S_2449 (NADPH)	C27840H31399N11476O20175S0P2907Mg0Zn0Fe0	-2908	rRNA Modification
b4009_RNA_12		C27819H31374N11469O20158P2904S0	-2905	rRNA Modification
b4009_RNA_12_YmfC_mono	b4009_RNA_12, YmfC_mono	C28917H33135N11796O20478S3P2904Mg0Zn0Fe0	-2895	rRNA Modification
b4009_RNA_13		C27819H31374N11469O20158P2904S0	-2905	rRNA Modification
b4009_RNA_13_MeT_23S_2498	b4009_RNA_13, MeT_23S_2498	C27834H31397N11475O20163S1P2904Mg0Zn0Fe0	-2904	rRNA Modification
b4009_RNA_14		C27820H31376N11469O20158P2904S0	-2905	rRNA Modification
b4009_RNA_14_MeT_23S_2503	b4009_RNA_14, MeT_23S_2503	C27835H31399N11475O20163S1P2904Mg0Zn0Fe0	-2904	rRNA Modification
b4009_RNA_15		C27821H31378N11469O20158P2904S0	-2905	rRNA Modification
b4009_RNA_15_RluC_mono	b4009_RNA_15, RluC_mono	C29406H33991N11951O20614S6P2904Mg0Zn0Fe0	-2890	rRNA Modification
b4009_RNA_16		C27821H31378N11469O20158P2904S0	-2905	rRNA Modification
b4009_RNA_16_RrmJ_mono	b4009_RNA_16, RrmJ_mono	C28868H33067N11764O20461S11P2904Mg0Zn0Fe0	-2897	rRNA Modification
b4009_RNA_17		C27822H31380N11469O20158P2904S0	-2905	rRNA Modification
b4009_RNA_17_RluC_mono	b4009_RNA_17, RluC_mono	C29407H33993N11951O20614S6P2904Mg0Zn0Fe0	-2890	rRNA Modification
b4009_RNA_18		C27822H31380N11469O20158P2904S0	-2905	rRNA Modification
b4009_RNA_18_YjbC_mono	b4009_RNA_18, YjbC_mono	C29249H33751N11887O20580S7P2904Mg0Zn0Fe0	-2890	rRNA Modification
b4009_RNA_19		C27822H31380N11469O20158P2904S0	-2905	rRNA Modification
b4009_RNA_19_RluB_mono	b4009_RNA_19, RluB_mono	C29240H33728N11920O20578S5P2904Mg0Zn0Fe0	-2890	rRNA Modification
b4009_RNA_1_MeT_23S_1835	b4009_RNA_1, MeT_23S_1835	C27826H31379N11475O20163S1P2904Mg0Zn0Fe0	-2904	rRNA Modification
b4009_RNA_2		C27812H31358N11469O20158P2904S0	-2905	rRNA Modification
b4009_RNA_20		C27822H31380N11469O20158P2904S0	-2905	rRNA Modification
b4009_RNA_20_RrmA_dim	b4009_RNA_20, RrmA_dim	C30539H35591N12233O20933S31P2904Mg0Zn2Fe0	-2902	rRNA Modification
b4009_RNA_21		C27823H31382N11469O20158P2904S0	-2905	rRNA Modification
b4009_RNA_21_RluA_mono	b4009_RNA_21, RluA_mono	C28926H33122N11782O20472S10P2904Mg0Zn0Fe0	-2904	rRNA Modification
b4009_RNA_22		C27823H31382N11469O20158P2904S0	-2905	rRNA Modification
b4009_RNA_22_RumB_mono	b4009_RNA_22, RumB_mono	C29716H34360N11981O20692S28P2904Mg0Zn0Fe4	-2903	rRNA Modification
b4009_RNA_23		C27824H31384N11469O20158P2904S0	-2905	rRNA Modification
b4009_RNA_23_RluC_mono	b4009_RNA_23, RluC_mono	C29409H33997N11951O20614S6P2904Mg0Zn0Fe0	-2890	rRNA Modification
b4009_RNA_2_RluD_mono	b4009_RNA_2, RluD_mono	C29449H33968N11949O20632S12P2904Mg1Zn0Fe0	-2909	rRNA Modification
b4009_RNA_3		C27812H31358N11469O20158P2904S0	-2905	rRNA Modification
b4009_RNA_3_RluD_mono	b4009_RNA_3, RluD_mono	C29464H33991N11955O20637S13P2904Mg1Zn0Fe0	-2908	rRNA Modification

b4009_RNA_4		C27813H31360N11469O20158P2904S0	-2905	rRNA Modification
b4009_RNA_4_RluD_mono	b4009_RNA_4, RluD_mono	C29450H33970N11949O20632S12P2904Mg1Zn0Fe0	-2909	rRNA Modification
b4009_RNA_5		C27813H31360N11469O20158P2904S0	-2905	rRNA Modification
b4009_RNA_5_RumA_mono	b4009_RNA_5, RumA_mono	C29941H34786N12087O20786S23P2904Mg0Zn0Fe4	-2897	rRNA Modification
b4009_RNA_6		C27814H31362N11469O20158P2904S0	-2905	rRNA Modification
b4009_RNA_6_MeT_23S_1962	b4009_RNA_6, MeT_23S_1962	C27829H31385N11475O20163S1P2904Mg0Zn0Fe0	-2904	rRNA Modification
b4009_RNA_7		C27815H31364N11469O20158P2904S0	-2905	rRNA Modification
b4009_RNA_7_MeT_23S_2030	b4009_RNA_7, MeT_23S_2030	C27830H31387N11475O20163S1P2904Mg0Zn0Fe0	-2904	rRNA Modification
b4009_RNA_8		C27816H31366N11469O20158P2904S0	-2905	rRNA Modification
b4009_RNA_8_MeT_23S_2069	b4009_RNA_8, MeT_23S_2069	C27831H31389N11475O20163S1P2904Mg0Zn0Fe0	-2904	rRNA Modification
b4009_RNA_9		C27817H31368N11469O20158P2904S0	-2905	rRNA Modification
b4009_RNA_9_RlmB_dim	b4009_RNA_9, RlmB_dim	C30138H35171N12153O20859S23P2904Mg0Zn0Fe0	-2910	rRNA Modification
b4009_RNA_MeT_23S_1618	b4009_RNA, MeT_23S_1618	C27825H31377N11475O20163S1P2904Mg0Zn0Fe0	-2904	rRNA Modification
b4010_RNA	rRNA	C1145H1301N470O837P120	-121	RNA cutting
b4022_aa	polypeptide b4022	C1433H2379N419O424S8	14	Translation
b4022_def_map_cplx	Polypeptide b4022 peptide deformylase and methionine aminopeptidase complex	C3563H5835N1012O1070S29Mg0Zn0Fe3	5	Maturation
b4022_m	Matured polypeptide b4022	C1427H2371N418O422S7	15	Maturation
b4022_mRNA	mRNA b4022	C8341H9423N3406O6057P875	-876	Translation
b4022_mRNA_1	mRNA b4022	C8341H9423N3406O6057P875	-876	Translation
b4022_mRNA_2	mRNA b4022	C8341H9423N3406O6057P875	-876	Translation
b4022_mRNA_2_degr	mRNA b4022 degradation complex	C49024H74822N15281O18742S200P875Mg6Zn2Fe0	-1098	mRNA degradation
b4049_aa	polypeptide b4049	C1691H2653N482O505S21	-7	Translation
b4049_def_map_cplx	Polypeptide b4049 peptide deformylase and methionine aminopeptidase complex	C3821H6109N1075O1151S42Mg0Zn0Fe3	-16	Maturation
b4049_m	Matured polypeptide b4049	C1619H2537N459O479S18	-6	Maturation
b4049_mRNA	mRNA b4049	C9901H11182N3995O7266P1040	-1041	Translation
b4049_mRNA_1	mRNA b4049	C9901H11182N3995O7266P1040	-1041	Translation
b4049_mRNA_2	mRNA b4049	C9901H11182N3995O7266P1040	-1041	Translation
b4049_mRNA_2_degr	mRNA b4049 degradation complex	C50584H76581N15870O19951S200P1040Mg6Zn2Fe0	-1263	mRNA degradation
b4049_m_DnaKJ_complex	b4049 DnaK DnaJ_dim complex - Kerner et al. class II can interact w/ GroEL/ES, cannot fold spontanously	C8183H13051O2577N2372S65P3Zn4	-28	Folding
b4049_m_GroEL.(7)ATP.transGroES	b4049 GroEL GroES complex - Kerner et al. class II can interact w/ GroEL/ES, cannot fold spontanously	C39531H65481O12813N11043S382P21Mg7	-307	Folding
b4129_aa	polypeptide b4129_v1	C2568H3993N713O773S19	-22	Translation
b4129_def_map_cplx	Polypeptide b4129 peptide deformylase and methionine aminopeptidase complex	C4698H7449N1306O1419S40Mg0Zn0Fe3	-31	Maturation
b4129_m	Matured polypeptide b4129	C2562H3985N712O771S18	-21	Maturation
b4129_mRNA	mRNA b4129	C14443H16302N5719O10629P1520	-1521	Translation
b4129_mRNA_1	mRNA b4129	C14443H16302N5719O10629P1520	-1521	Translation
b4129_mRNA_2	mRNA b4129	C14443H16302N5719O10629P1520	-1521	Translation
b4129_mRNA_2_degr	mRNA b4129 degradation complex	C55126H81701N17594O23314S200P1520Mg6Zn2Fe0	-1743	mRNA degradation
b4129_m_DnaKJ_complex	b4129 DnaK DnaJ_dim complex - Deuerling et al. DnaKJ/GrpE dependent folding	C9126H14499O2869N2625S65P3Mg3Zn4	-37	Folding
b4129_m_Mg	b4129 plus _Mg	C2562H3985N712O771S18Mg3	-15	Folding
b4129_v1_mRNA	mRNA b4129_v1	C14443H16302N5719O10629P1520	-1521	Translation
b4134_RNA	tRNA (pheU)	C723H821N290O534P76	-77	tRNA Modification
b4134_tRNA_1	b4134_tRNA_1 (pheU)	C723H823Mg2N290O534P76	-73	tRNA Modification
b4134_tRNA_1_Dus_gen_cplx	b4134_tRNA_1 (pheU), Dus_gen	C2330H3362Mg2N749O1017P79S14	-80	tRNA Modification
b4134_tRNA_2	b4134_tRNA_2 (pheU)	C723H825Mg2N290O534P76	-73	tRNA Modification
b4134_tRNA_2_RluA_mono_cplx	b4134_tRNA_2 (pheU), RluA_mono	C1826H2565Mg2N603O848P76S10	-72	tRNA Modification
b4134_tRNA_3	b4134_tRNA_3 (pheU)	C723H825Mg2N290O534P76	-73	tRNA Modification
b4134_tRNA_3_MiaA_dim-MiaB_mono_cplx	b4134_tRNA_3 (pheU), MiaA_dim, MiaB_mono	C10198Fe4H15855Mg4N2965O3400P80S80X1	-118	tRNA Modification
b4134_tRNA_4	b4134_tRNA_4 (pheU)	C729H835Mg2N290O534P76S1	-73	tRNA Modification
b4134_tRNA_4_TruA_dim_cplx	b4134_tRNA_4 (pheU), TruA_dim	C3439H5037Mg2N1070O1304P76S17	-67	tRNA Modification
b4134_tRNA_5	b4134_tRNA_5 (pheU)	C729H835Mg2N290O534P76S1	-73	tRNA Modification
b4134_tRNA_5_YggH_mono_cplx	b4134_tRNA_5 (pheU), YggH_mono	C1954H2734Mg2N643O881P76S15	-75	tRNA Modification
b4134_tRNA_6	b4134_tRNA_6 (pheU)	C730H837Mg2N290O534P76S1	-73	tRNA Modification

b4134_tRNA_6_AcpT_tRNA_pos_47_acp3U_cplx	b4134_tRNA_6 (pheU), AcpT_tRNA_pos_47_acp3U	C745H860Mg2N296O539P76S2	-72	tRNA Modification
b4134_tRNA_7	b4134_tRNA_7 (pheU)	C734H844Mg2N291O536P76S1	-73	tRNA Modification
b4134_tRNA_7_TrmA_mono_cplx	b4134_tRNA_7 (pheU), TrmA_mono	C2608H3791Mg2N814O1095P76S20	-81	tRNA Modification
b4134_tRNA_8	b4134_tRNA_8 (pheU)	C735H846Mg2N291O536P76S1	-73	tRNA Modification
b4134_tRNA_8_TruB_mono_cplx	b4134_tRNA_8 (pheU), TruB_mono	C2275H3321Mg2N729O1005P76S11	-78	tRNA Modification
b4134_tRNA_9	b4134_tRNA_9 (pheU)	C735H846Mg2N291O536P76S1	-73	tRNA Modification
b4134_tRNA_9_ThiI_mono_cplx	b4134_tRNA_9 (pheU), ThiI_mono	C7163H11077Mg3N2109O2475P81S51X1	-101	tRNA Modification
b4134_tRNA_Mg2	tRNA (pheU) bound two Mg2 ions	C723H821Mg2N290O534P76	-73	tRNA Modification
b4134_tRNA_Mg2_Dus_gen_cplx	b4134_tRNA (pheU), Dus_gen	C2330H3360Mg2N749O1017P79S14	-80	tRNA Modification
b4142_aa	polypeptide b4142	C455H761N127O146S2	-4	Translation
b4142_def_cplx	Polypeptide b4142 peptide deformylase complex	C1299H2155N368O401S8Mg0Zn0Fe1	-9	Maturation
b4142_m	Matured polypeptide b4142	C454H762N127O145S2	-3	Maturation
b4142_mRNA	mRNA b4142	C2808H3163N1134O2058P296	-297	Translation
b4142_mRNA_1	mRNA b4142	C2808H3163N1134O2058P296	-297	Translation
b4142_mRNA_2	mRNA b4142	C2808H3163N1134O2058P296	-297	Translation
b4142_mRNA_2_degr	mRNA b4142 degradation complex	C43491H68562N13009O14743S200P296Mg6Zn2Fe0	-519	mRNA degradation
b4143_DNA_act	DNA b4143 (activated form)	C16076H18456N6430O9784P1647	-1647	Transcription
b4143_DNA_neu	DNA b4143 (inactivate form)	C16076H18456N6430O9784P1647	-1647	Transcription
b4143_aa	polypeptide b4143	C2482H4117N691O804S26	-20	Translation
b4143_def_map_cplx	Polypeptide b4143 peptide deformylase and methionine aminopeptidase complex	C4612H7573N1284O1450S47Mg0Zn0Fe3	-29	Maturation
b4143_m	Matured polypeptide b4143 _ error CCDB	C2476H4109N690O802S25	-19	Maturation
b4143_mRNA	mRNA b4143	C15737H17779N6430O11438P1649	-1650	Translation
b4143_mRNA_1	mRNA b4143	C15737H17779N6430O11438P1649	-1650	Translation
b4143_mRNA_2	mRNA b4143	C15737H17779N6430O11438P1649	-1650	Translation
b4143_mRNA_2_degr	mRNA b4143 degradation complex	C56420H83178N18305O24123S200P1649Mg6Zn2Fe0	-1872	mRNA degradation
b4162_aa	polypeptide b4162	C927H1437N252O281S7	-11	Translation
b4162_def_map_cplx	Polypeptide b4162 peptide deformylase and methionine aminopeptidase complex	C3057H4893N845O927S28Mg0Zn0Fe3	-20	Maturation
b4162_m	Matured polypeptide b4162	C921H1429N251O279S6	-10	Maturation
b4162_mRNA	mRNA b4162	C5210H5890N2113O3812P548	-549	Translation
b4162_mRNA_1	mRNA b4162	C5210H5890N2113O3812P548	-549	Translation
b4162_mRNA_2	mRNA b4162	C5210H5890N2113O3812P548	-549	Translation
b4162_mRNA_2_degr	mRNA b4162 degradation complex	C45893H71289N13988O16497S200P548Mg6Zn2Fe0	-771	mRNA degradation
b4162_m_Mg	b4162 plus _Mg	C921H1429N251O279S6Mg1	-8	Folding
b4163_RNA	tRNA (glyV)	C722H820N287O537P76	-77	tRNA Modification
b4163_tRNA_1	b4163_tRNA_1 (glyV)	C722H822Mg2N287O537P76	-73	tRNA Modification
b4163_tRNA_1_Dus_gen_cplx	b4163_tRNA_1 (glyV), Dus_gen	C2329H3361Mg2N746O1020P79S14	-80	tRNA Modification
b4163_tRNA_2	b4163_tRNA_2 (glyV)	C722H824Mg2N287O537P76	-73	tRNA Modification
b4163_tRNA_2_Dus_gen_cplx	b4163_tRNA_2 (glyV), Dus_gen	C2329H3363Mg2N746O1020P79S14	-80	tRNA Modification
b4163_tRNA_3	b4163_tRNA_3 (glyV)	C722H826Mg2N287O537P76	-73	tRNA Modification
b4163_tRNA_3_YggH_mono_cplx	b4163_tRNA_3 (glyV), YggH_mono	C1947H2725Mg2N640O884P76S14	-75	tRNA Modification
b4163_tRNA_4	b4163_tRNA_4 (glyV)	C723H828Mg2N287O537P76	-73	tRNA Modification
b4163_tRNA_4_TrmA_mono_cplx	b4163_tRNA_4 (glyV), TrmA_mono	C2597H3775Mg2N810O1096P76S19	-81	tRNA Modification
b4163_tRNA_5	b4163_tRNA_5 (glyV)	C724H830Mg2N287O537P76	-73	tRNA Modification
b4163_tRNA_5_TruB_mono_cplx	b4163_tRNA_5 (glyV), TruB_mono	C2264H3305Mg2N725O1006P76S10	-78	tRNA Modification
b4163_tRNA_Mg2	tRNA (glyV) bound two Mg2 ions	C722H820Mg2N287O537P76	-73	tRNA Modification
b4163_tRNA_Mg2_Dus_gen_cplx	b4163_tRNA (glyV), Dus_gen	C2329H3359Mg2N746O1020P79S14	-80	tRNA Modification
b4164_RNA	tRNA (glyX)	C722H820N287O537P76	-77	tRNA Modification
b4164_tRNA_1	b4164_tRNA_1 (glyX)	C722H822Mg2N287O537P76	-73	tRNA Modification
b4164_tRNA_1_Dus_gen_cplx	b4164_tRNA_1 (glyX), Dus_gen	C2329H3361Mg2N746O1020P79S14	-80	tRNA Modification
b4164_tRNA_2	b4164_tRNA_2 (glyX)	C722H824Mg2N287O537P76	-73	tRNA Modification
b4164_tRNA_2_Dus_gen_cplx	b4164_tRNA_2 (glyX), Dus_gen	C2329H3363Mg2N746O1020P79S14	-80	tRNA Modification
b4164_tRNA_3	b4164_tRNA_3 (glyX)	C722H826Mg2N287O537P76	-73	tRNA Modification
b4164_tRNA_3_YggH_mono_cplx	b4164_tRNA_3 (glyX), YggH_mono	C1947H2725Mg2N640O884P76S14	-75	tRNA Modification
b4164_tRNA_4	b4164_tRNA_4 (glyX)	C723H828Mg2N287O537P76	-73	tRNA Modification
b4164_tRNA_4_TrmA_mono_cplx	b4164_tRNA_4 (glyX), TrmA_mono	C2597H3775Mg2N810O1096P76S19	-81	tRNA Modification
b4164_tRNA_5	b4164_tRNA_5 (glyX)	C724H830Mg2N287O537P76	-73	tRNA Modification
b4164_tRNA_5_TruB_mono_cplx	b4164_tRNA_5 (glyX), TruB_mono	C2264H3305Mg2N725O1006P76S10	-78	tRNA Modification
b4164_tRNA_Mg2	tRNA (glyX) bound two Mg2 ions	C722H820Mg2N287O537P76	-73	tRNA Modification

b4164_tRNA_Mg2_Dus_gen_cplx	b4164_tRNA (glyX), Dus_gen	C2329H3359Mg2N746O1020P79S14	-80	tRNA Modification
b4165_RNA	tRNA (glyY)	C722H820N287O537P76	-77	tRNA Modification
b4165_tRNA_1	b4165_tRNA_1 (glyY)	C722H822Mg2N287O537P76	-73	tRNA Modification
b4165_tRNA_1_Dus_gen_cplx	b4165_tRNA_1 (glyY), Dus_gen	C2329H3361Mg2N746O1020P79S14	-80	tRNA Modification
b4165_tRNA_2	b4165_tRNA_2 (glyY)	C722H824Mg2N287O537P76	-73	tRNA Modification
b4165_tRNA_2_Dus_gen_cplx	b4165_tRNA_2 (glyY), Dus_gen	C2329H3363Mg2N746O1020P79S14	-80	tRNA Modification
b4165_tRNA_3	b4165_tRNA_3 (glyY)	C722H826Mg2N287O537P76	-73	tRNA Modification
b4165_tRNA_3_YggH_mono_cplx	b4165_tRNA_3 (glyY), YggH_mono	C1947H2725Mg2N640O884P76S14	-75	tRNA Modification
b4165_tRNA_4	b4165_tRNA_4 (glyY)	C723H828Mg2N287O537P76	-73	tRNA Modification
b4165_tRNA_4_TrmA_mono_cplx	b4165_tRNA_4 (glyY), TrmA_mono	C2597H3775Mg2N810O1096P76S19	-81	tRNA Modification
b4165_tRNA_5	b4165_tRNA_5 (glyY)	C724H830Mg2N287O537P76	-73	tRNA Modification
b4165_tRNA_5_TruB_mono_cplx	b4165_tRNA_5 (glyY), TruB_mono	C2264H3305Mg2N725O1006P76S10	-78	tRNA Modification
b4165_tRNA_Mg2	tRNA (glyY) bound two Mg2 ions	C722H820Mg2N287O537P76	-73	tRNA Modification
b4165_tRNA_Mg2_Dus_gen_cplx	b4165_tRNA (glyY), Dus_gen	C2329H3359Mg2N746O1020P79S14	-80	tRNA Modification
b4167_aa	polypeptide b4167_v2	C2404H3848N702O723S16	-12	Translation
b4167_def_cplx	Polypeptide b4167 peptide deformylase complex	C3248H5242N943O978S22Mg0Zn0Fe1	-17	Maturation
b4167_m	Matured polypeptide b4167	C2398H3840N701O721S15	-11	Maturation
b4167_mRNA	mRNA b4167	C14743H16694N5931O10849P1550	-1551	Translation
b4167_mRNA_1	mRNA b4167	C14743H16694N5931O10849P1550	-1551	Translation
b4167_mRNA_2	mRNA b4167	C14743H16694N5931O10849P1550	-1551	Translation
b4167_mRNA_2_degr	mRNA b4167 degradation complex	C55426H82093N17806O23534S200P1550Mg6Zn2Fe0	-1773	mRNA degradation
b4167_v1_mRNA	mRNA b4167_v1	C14743H16694N5931O10849P1550	-1551	Translation
b4167_v2_mRNA	mRNA b4167_v2	C14743H16694N5931O10849P1550	-1551	Translation
b4168_aa	polypeptide b4168_v2	C753H1154N198O231S6	-12	Translation
b4168_def_map_cplx	Polypeptide b4168 peptide deformylase and methionine aminopeptidase complex	C2883H4610N791O877S27Mg0Zn0Fe3	-21	Maturation
b4168_m	Matured polypeptide b4168	C747H1146N197O229S5	-11	Maturation
b4168_mRNA	mRNA b4168 (29 nt short)	C4116H4655N1628O3046P433	-434	Translation
b4168_mRNA_1	mRNA b4168 (29 nt short)	C4116H4655N1628O3046P433	-434	Translation
b4168_mRNA_2	mRNA b4168 (29 nt short)	C4116H4655N1628O3046P433	-434	Translation
b4168_mRNA_2_degr	mRNA b4168 degradation complex	C44799H70054N13503O15731S200P433Mg6Zn2Fe0	-656	mRNA degradation
b4168_v1_mRNA	mRNA b4168_v1 (29 nt short)	C4116H4655N1628O3046P433	-434	Translation
b4168_v2_mRNA	mRNA b4168_v2 (29 nt short)	C4116H4655N1628O3046P433	-434	Translation
b4169_aa	polypeptide b4169_v2	C2100H3394N619O653S8	5	Translation
b4169_def_cplx	Polypeptide b4169 peptide deformylase complex	C2944H4788N860O908S14Mg0Zn0Fe1	0	Maturation
b4169_m	Matured polypeptide b4169	C1983H3203N589O626S5	4	Maturation
b4169_mRNA	mRNA b4169	C12741H14436N5129O9332P1338	-1339	Translation
b4169_mRNA_1	mRNA b4169	C12741H14436N5129O9332P1338	-1339	Translation
b4169_mRNA_2	mRNA b4169	C12741H14436N5129O9332P1338	-1339	Translation
b4169_mRNA_2_degr	mRNA b4169 degradation complex	C53424H79835N17004O22017S200P1338Mg6Zn2Fe0	-1561	mRNA degradation
b4169_v1_mRNA	mRNA b4169_v1	C12741H14436N5129O9332P1338	-1339	Translation
b4169_v2_mRNA	mRNA b4169_v2	C12741H14436N5129O9332P1338	-1339	Translation
b4170_aa	polypeptide b4170_v2	C3014H4835N872O888S14	-7	Translation
b4170_def_map_cplx	Polypeptide b4170 peptide deformylase and methionine aminopeptidase complex	C5144H8291N1465O1534S35Mg0Zn0Fe3	-16	Maturation
b4170_m	Matured polypeptide b4170	C3008H4827N871O886S13	-6	Maturation
b4170_mRNA	mRNA b4170	C17592H19957N7092O12873P1848	-1849	Translation
b4170_mRNA_1	mRNA b4170	C17592H19957N7092O12873P1848	-1849	Translation
b4170_mRNA_2	mRNA b4170	C17592H19957N7092O12873P1848	-1849	Translation
b4170_mRNA_2_degr	mRNA b4170 degradation complex	C58275H85356N18967O25558S200P1848Mg6Zn2Fe0	-2071	mRNA degradation
b4170_v1_mRNA	mRNA b4170_v1	C17592H19957N7092O12873P1848	-1849	Translation
b4170_v2_mRNA	mRNA b4170_v2	C17592H19957N7092O12873P1848	-1849	Translation
b4171_aa	polypeptide b4171_v2	C1562H2475N439O460S10	-8	Translation
b4171_def_map_cplx	Polypeptide b4171 peptide deformylase and methionine aminopeptidase complex	C3692H5931N1032O1106S31Mg0Zn0Fe3	-17	Maturation
b4171_m	Matured polypeptide b4171	C1556H2467N438O458S9	-7	Maturation
b4171_mRNA	mRNA b4171 (8 nt short)	C8984H10147N3596O6618P943	-944	Translation
b4171_mRNA_1	mRNA b4171 (8 nt short)	C8984H10147N3596O6618P943	-944	Translation
b4171_mRNA_2	mRNA b4171 (8 nt short)	C8984H10147N3596O6618P943	-944	Translation
b4171_mRNA_2_degr	mRNA b4171 degradation complex	C49667H75546N15471O19303S200P943Mg6Zn2Fe0	-1166	mRNA degradation
b4171_m_Mg	b4171 plus _Mg	C1556H2467N438O458S9Mg1	-5	Folding

b4171_v1_mRNA	mRNA b4171_v1 (8 nt short)	C8984H10147N3596O6618P943	-944	Translation
b4171_v2_mRNA	mRNA b4171_v2 (8 nt short)	C8984H10147N3596O6618P943	-944	Translation
b4172_aa	polypeptide b4172_v2	C484H765N144O158S2	-1	Translation
b4172_def_map_cplx	Polypeptide b4172 peptide deformylase and methionine aminopeptidase complex	C2614H4221N737O804S23Mg0Zn0Fe3	-10	Maturation
b4172_m	Matured polypeptide b4172	C478H757N143O156S1	0	Maturation
b4172_mRNA	mRNA b4172	C2938H3323N1164O2160P309	-310	Translation
b4172_mRNA_1	mRNA b4172	C2938H3323N1164O2160P309	-310	Translation
b4172_mRNA_2	mRNA b4172	C2938H3323N1164O2160P309	-310	Translation
b4172_mRNA_2_degr	mRNA b4172 degradation complex	C43621H68722N13039O14845S200P309Mg6Zn2Fe0	-532	mRNA degradation
b4172_v1_mRNA	mRNA b4172_v1	C2938H3323N1164O2160P309	-310	Translation
b4172_v2_mRNA	mRNA b4172_v2	C2938H3323N1164O2160P309	-310	Translation
b4173_aa	polypeptide b4173_v2	C2130H3433N624O642S9	-11	Translation
b4173_def_map_cplx	Polypeptide b4173 peptide deformylase and methionine aminopeptidase complex	C4260H6889N1217O1288S30Mg0Zn0Fe3	-20	Maturation
b4173_m	Matured polypeptide b4173	C2124H3425N623O640S8	-10	Maturation
b4173_mRNA	mRNA b4173	C12205H13799N4902O8963P1281	-1282	Translation
b4173_mRNA_1	mRNA b4173	C12205H13799N4902O8963P1281	-1282	Translation
b4173_mRNA_2	mRNA b4173	C12205H13799N4902O8963P1281	-1282	Translation
b4173_mRNA_2_degr	mRNA b4173 degradation complex	C52888H79198N16777O21648S200P1281Mg6Zn2Fe0	-1504	mRNA degradation
b4173_v1_mRNA	mRNA b4173_v1	C12205H13799N4902O8963P1281	-1282	Translation
b4173_v2_mRNA	mRNA b4173_v2	C12205H13799N4902O8963P1281	-1282	Translation
b4174_aa	polypeptide b4174_v2	C1969H3164N588O638S9	-2	Translation
b4174_def_map_cplx	Polypeptide b4174 peptide deformylase and methionine aminopeptidase complex	C4099H6620N1181O1284S30Mg0Zn0Fe3	-11	Maturation
b4174_m	Matured polypeptide b4174	C1963H3156N587O636S8	-1	Maturation
b4174_mRNA	mRNA b4174	C12038H13626N4941O8739P1260	-1261	Translation
b4174_mRNA_1	mRNA b4174	C12038H13626N4941O8739P1260	-1261	Translation
b4174_mRNA_2	mRNA b4174	C12038H13626N4941O8739P1260	-1261	Translation
b4174_mRNA_2_degr	mRNA b4174 degradation complex	C52721H79025N16816O21424S200P1260Mg6Zn2Fe0	-1483	mRNA degradation
b4174_v1_mRNA	mRNA b4174_v1	C12038H13626N4941O8739P1260	-1261	Translation
b4174_v2_mRNA	mRNA b4174_v2	C12038H13626N4941O8739P1260	-1261	Translation
b4175_aa	polypeptide b4175_v2	C1655H2671N473O510S10	-2	Translation
b4175_def_map_cplx	Polypeptide b4175 peptide deformylase and methionine aminopeptidase complex	C3785H6127N1066O1156S31Mg0Zn0Fe3	-11	Maturation
b4175_m	Matured polypeptide b4175	C1649H2663N472O508S9	-1	Maturation
b4175_mRNA	mRNA b4175	C9577H10832N3855O7012P1005	-1006	Translation
b4175_mRNA_1	mRNA b4175	C9577H10832N3855O7012P1005	-1006	Translation
b4175_mRNA_2	mRNA b4175	C9577H10832N3855O7012P1005	-1006	Translation
b4175_mRNA_2_degr	mRNA b4175 degradation complex	C50260H76231N15730O19697S200P1005Mg6Zn2Fe0	-1228	mRNA degradation
b4175_v1_mRNA	mRNA b4175_v1	C9577H10832N3855O7012P1005	-1006	Translation
b4175_v2_mRNA	mRNA b4175_v2	C9577H10832N3855O7012P1005	-1006	Translation
b4178_aa	polypeptide b4178	C690H1125N189O208S7	0	Translation
b4178_def_map_cplx	Polypeptide b4178 peptide deformylase and methionine aminopeptidase complex	C2820H4581N782O854S28Mg0Zn0Fe3	-9	Maturation
b4178_m	Matured polypeptide b4178	C684H1117N188O206S6	1	Maturation
b4178_mRNA	mRNA b4178	C4054H4570N1601O3003P428	-429	Translation
b4178_mRNA_1	mRNA b4178	C4054H4570N1601O3003P428	-429	Translation
b4178_mRNA_2	mRNA b4178	C4054H4570N1601O3003P428	-429	Translation
b4178_mRNA_2_degr	mRNA b4178 degradation complex	C44737H69969N13476O15688S200P428Mg6Zn2Fe0	-651	mRNA degradation
b4179_aa	polypeptide b4179	C4053H6508N1184O1210S30	8	Translation
b4179_def_map_cplx	Polypeptide b4179 peptide deformylase and methionine aminopeptidase complex	C6183H9964N1777O1856S51Mg0Zn0Fe3	-1	Maturation
b4179_m	Matured polypeptide b4179	C4047H6500N1183O1208S29	9	Maturation
b4179_mRNA	mRNA b4179	C23295H26360N9457O17009P2442	-2443	Translation
b4179_mRNA_1	mRNA b4179	C23295H26360N9457O17009P2442	-2443	Translation
b4179_mRNA_2	mRNA b4179	C23295H26360N9457O17009P2442	-2443	Translation
b4179_mRNA_2_degr	mRNA b4179 degradation complex	C63978H91759N21332O29694S200P2442Mg6Zn2Fe0	-2665	mRNA degradation
b4179_m_DnaKJ_complex	b4179 DnaK DnaJ_dim complex - Kerner et al. class II can interact w/ GroEL/ES, cannot fold spontanously	C10611H17014O3306N3096S76P3Zn4	-13	Folding
b4179_m_GroEL.(7)ATP.transGroES	b4179 GroEL GroES complex - Kerner et al. class II can interact w/ GroEL/ES, cannot fold spontanously	C41959H69444O13542N11767S393P21Mg7	-292	Folding
b4180_aa	polypeptide b4180	C1159H1898N340O350S12	-4	Translation

b4180_def_map_cplx	Polypeptide b4180 peptide deformylase and methionine aminopeptidase complex	C3289H5354N933O996S33Mg0Zn0Fe3	-13	Maturation
b4180_m	Matured polypeptide b4180	C1153H1890N339O348S11	-3	Maturation
b4180_mRNA	mRNA b4180	C6969H7896N2801O5119P732	-733	Translation
b4180_mRNA_1	mRNA b4180	C6969H7896N2801O5119P732	-733	Translation
b4180_mRNA_2	mRNA b4180	C6969H7896N2801O5119P732	-733	Translation
b4180_mRNA_2_degr	mRNA b4180 degradation complex	C47652H73295N14676O17804S200P732Mg6Zn2Fe0	-955	mRNA degradation
b4200_aa	polypeptide b4200	C658H1025N194O208S7	-9	Translation
b4200_def_cplx	Polypeptide b4200 peptide deformylase complex	C1502H2419N435O463S13Mg0Zn0Fe1	-14	Maturation
b4200_m	Matured polypeptide b4200	C657H1026N194O207S7	-8	Maturation
b4200_mRNA	mRNA b4200	C3774H4273N1524O2759P398	-399	Translation
b4200_mRNA_1	mRNA b4200	C3774H4273N1524O2759P398	-399	Translation
b4200_mRNA_2	mRNA b4200	C3774H4273N1524O2759P398	-399	Translation
b4200_mRNA_2_degr	mRNA b4200 degradation complex	C44457H69672N13399O15444S200P398Mg6Zn2Fe0	-621	mRNA degradation
b4201_aa	polypeptide b4201	C497H804N151O147S7	1	Translation
b4201_def_map_cplx	Polypeptide b4201 peptide deformylase and methionine aminopeptidase complex	C2627H4260N744O793S28Mg0Zn0Fe3	-8	Maturation
b4201_m	Matured polypeptide b4201	C491H796N150O145S6	2	Maturation
b4201_mRNA	mRNA b4201	C2998H3392N1197O2204P315	-316	Translation
b4201_mRNA_1	mRNA b4201	C2998H3392N1197O2204P315	-316	Translation
b4201_mRNA_2	mRNA b4201	C2998H3392N1197O2204P315	-316	Translation
b4201_mRNA_2_degr	mRNA b4201 degradation complex	C43681H68791N13072O14889S200P315Mg6Zn2Fe0	-538	mRNA degradation
b4202_aa	polypeptide b4202	C401H659N124O109S2	11	Translation
b4202_def_map_cplx	Polypeptide b4202 peptide deformylase and methionine aminopeptidase complex	C2531H4115N717O755S23Mg0Zn0Fe3	2	Maturation
b4202_m	Matured polypeptide b4202	C395H651N123O107S1	12	Maturation
b4202_mRNA	mRNA b4202	C2161H2459N852O1589P228	-229	Translation
b4202_mRNA_1	mRNA b4202	C2161H2459N852O1589P228	-229	Translation
b4202_mRNA_2	mRNA b4202	C2161H2459N852O1589P228	-229	Translation
b4202_mRNA_2_degr	mRNA b4202 degradation complex	C42844H67858N12727O14274S200P228Mg6Zn2Fe0	-451	mRNA degradation
b4203_aa	polypeptide b4203	C700H1147N197O215S1	-2	Translation
b4203_def_cplx	Polypeptide b4203 peptide deformylase complex	C1544H2541N438O470S7Mg0Zn0Fe1	-7	Maturation
b4203_m	Matured polypeptide b4203	C699H1148N197O214S1	-1	Maturation
b4203_mRNA	mRNA b4203	C4293H4846N1731O3135P450	-451	Translation
b4203_mRNA_1	mRNA b4203	C4293H4846N1731O3135P450	-451	Translation
b4203_mRNA_2	mRNA b4203	C4293H4846N1731O3135P450	-451	Translation
b4203_mRNA_2_degr	mRNA b4203 degradation complex	C44976H70245N13606O15820S200P450Mg6Zn2Fe0	-673	mRNA degradation
b4258_aa	polypeptide b4258_v1	C4825H7511N1321O1430S40	-32	Translation
b4258_def_cplx	Polypeptide b4258 peptide deformylase complex	C5669H8905N1562O1685S46Mg0Zn0Fe1	-37	Maturation
b4258_m	Matured polypeptide b4258	C4824H7512N1321O1429S40	-31	Maturation
b4258_mRNA	mRNA b4258	C27212H30828N10995O19880P2858	-2859	Translation
b4258_mRNA_1	mRNA b4258	C27212H30828N10995O19880P2858	-2859	Translation
b4258_mRNA_2	mRNA b4258	C27212H30828N10995O19880P2858	-2859	Translation
b4258_mRNA_2_degr	mRNA b4258 degradation complex	C67895H96227N22870O32565S200P2858Mg6Zn2Fe0	-3081	mRNA degradation
b4258_m_DnaKJ_complex	b4258 DnaK DnaJ_dim complex - Deuerling et al. DnaKJ/GrpE dependent folding	C11388H18026O3527N3234S87P3Zn4	-53	Folding
b4258_v1_mRNA	mRNA b4258_v1	C27212H30828N10995O19880P2858	-2859	Translation
b4270_RNA	tRNA (leuX)	C812H920N333O589P85	-86	tRNA Modification
b4270_tRNA_1	b4270_tRNA_1 (leuX)	C812H922Mg2N333O589P85	-82	tRNA Modification
b4270_tRNA_1_TrmH_dim_cplx	b4270_tRNA_1 (leuX), TrmH_dim	C3023H4465Mg2N989O1250P85S29	-83	tRNA Modification
b4270_tRNA_2	b4270_tRNA_2 (leuX)	C813H924Mg2N333O589P85	-82	tRNA Modification
b4270_tRNA_2_Dus_gen_cplx	b4270_tRNA_2 (leuX), Dus_gen	C2420H3463Mg2N792O1072P88S14	-89	tRNA Modification
b4270_tRNA_3	b4270_tRNA_3 (leuX)	C813H926Mg2N333O589P85	-82	tRNA Modification
b4270_tRNA_3_MiaA_dim-MiaB_mono_cplx	b4270_tRNA_3 (leuX), MiaA_dim, MiaB_mono	C10288Fe4H15956Mg4N3008O3455P89S80X1	-127	tRNA Modification
b4270_tRNA_4	b4270_tRNA_4 (leuX)	C819H936Mg2N333O589P85S1	-82	tRNA Modification
b4270_tRNA_4_TruA_dim_cplx	b4270_tRNA_4 (leuX), TruA_dim	C3529H5138Mg2N1113O1359P85S17	-76	tRNA Modification
b4270_tRNA_5	b4270_tRNA_5 (leuX)	C819H936Mg2N333O589P85S1	-82	tRNA Modification
b4270_tRNA_5_TruB_mono_cplx	b4270_tRNA_5 (leuX), TruB_mono	C2359H3411Mg2N771O1058P85S11	-87	tRNA Modification
b4270_tRNA_Mg2	tRNA (leuX) bound two Mg2 ions	C812H920Mg2N333O589P85	-82	tRNA Modification

b4270_tRNA_Mg2_Dus_gen_cplx	b4270_tRNA (leuX), Dus_gen	C2419H3459Mg2N792O1072P88S14	-89	tRNA Modification
b4292_aa	polypeptide b4292	C1571H2494N464O474S3	2	Translation
b4292_def_map_cplx	Polypeptide b4292 peptide deformylase and methionine aminopeptidase complex	C3701H5950N1057O1120S24Mg0Zn0Fe3	-7	Maturation
b4292_m	Matured polypeptide b4292	C1565H2486N463O472S2	3	Maturation
b4292_mRNA	mRNA b4292 (4 nt short)	C9031H10268N3629O6629P950	-951	Translation
b4292_mRNA_1	mRNA b4292 (4 nt short)	C9031H10268N3629O6629P950	-951	Translation
b4292_mRNA_2	mRNA b4292 (4 nt short)	C9031H10268N3629O6629P950	-951	Translation
b4292_mRNA_2_degr	mRNA b4292 degradation complex	C49714H75667N15504O19314S200P950Mg6Zn2Fe0	-1173	mRNA degradation
b4293_aa	polypeptide b4293	C867H1382N232O262S8	-4	Translation
b4293_def_map_cplx	Polypeptide b4293 peptide deformylase and methionine aminopeptidase complex	C2997H4838N825O908S29Mg0Zn0Fe3	-13	Maturation
b4293_m	Matured polypeptide b4293	C861H1374N231O260S7	-3	Maturation
b4293_mRNA	mRNA b4293	C4958H5626N1969O3658P524	-525	Translation
b4293_mRNA_1	mRNA b4293	C4958H5626N1969O3658P524	-525	Translation
b4293_mRNA_2	mRNA b4293	C4958H5626N1969O3658P524	-525	Translation
b4293_mRNA_2_degr	mRNA b4293 degradation complex	C45641H71025N13844O16343S200P524Mg6Zn2Fe0	-747	mRNA degradation
b4368_RNA	tRNA (leuV)	C827H939N330O614P87	-88	tRNA Modification
b4368_tRNA_1	b4368_tRNA_1 (leuV)	C827H941Mg2N330O614P87	-84	tRNA Modification
b4368_tRNA_1_Dus_gen_cplx	b4368_tRNA_1 (leuV), Dus_gen	C2434H3480Mg2N789O1097P90S14	-91	tRNA Modification
b4368_tRNA_2	b4368_tRNA_2 (leuV)	C827H943Mg2N330O614P87	-84	tRNA Modification
b4368_tRNA_2_TrmH_dim_cplx	b4368_tRNA_2 (leuV), TrmH_dim	C3038H4486Mg2N986O1275P87S29	-85	tRNA Modification
b4368_tRNA_3	b4368_tRNA_3 (leuV)	C828H945Mg2N330O614P87	-84	tRNA Modification
b4368_tRNA_3_Dus_gen_cplx	b4368_tRNA_3 (leuV), Dus_gen	C2435H3484Mg2N789O1097P90S14	-91	tRNA Modification
b4368_tRNA_4	b4368_tRNA_4 (leuV)	C828H947Mg2N330O614P87	-84	tRNA Modification
b4368_tRNA_4_TruA_dim_cplx	b4368_tRNA_4 (leuV), TruA_dim	C3538H5149Mg2N1110O1384P87S16	-78	tRNA Modification
b4368_tRNA_5	b4368_tRNA_5 (leuV)	C828H947Mg2N330O614P87	-84	tRNA Modification
b4368_tRNA_5_TruA_dim_cplx	b4368_tRNA_5 (leuV), TruA_dim	C3538H5149Mg2N1110O1384P87S16	-78	tRNA Modification
b4368_tRNA_6	b4368_tRNA_6 (leuV)	C828H947Mg2N330O614P87	-84	tRNA Modification
b4368_tRNA_6_TrmA_mono_cplx	b4368_tRNA_6 (leuV), TrmA_mono	C2702H3894Mg2N853O1173P87S19	-92	tRNA Modification
b4368_tRNA_7	b4368_tRNA_7 (leuV)	C829H949Mg2N330O614P87	-84	tRNA Modification
b4368_tRNA_7_TruB_mono_cplx	b4368_tRNA_7 (leuV), TruB_mono	C2369H3424Mg2N768O1083P87S10	-89	tRNA Modification
b4368_tRNA_Mg2	tRNA (leuV) bound two Mg2 ions	C827H939Mg2N330O614P87	-84	tRNA Modification
b4368_tRNA_Mg2_Dus_gen_cplx	b4368_tRNA (leuV), Dus_gen	C2434H3478Mg2N789O1097P90S14	-91	tRNA Modification
b4369_RNA	tRNA (leuP)	C827H938N329O615P87	-88	tRNA Modification
b4369_tRNA_1	b4369_tRNA_1 (leuP)	C827H940Mg2N329O615P87	-84	tRNA Modification
b4369_tRNA_1_Dus_gen_cplx	b4369_tRNA_1 (leuP), Dus_gen	C2434H3479Mg2N788O1098P90S14	-91	tRNA Modification
b4369_tRNA_2	b4369_tRNA_2 (leuP)	C827H942Mg2N329O615P87	-84	tRNA Modification
b4369_tRNA_2_TrmH_dim_cplx	b4369_tRNA_2 (leuP), TrmH_dim	C3038H4485Mg2N985O1276P87S29	-85	tRNA Modification
b4369_tRNA_3	b4369_tRNA_3 (leuP)	C828H944Mg2N329O615P87	-84	tRNA Modification
b4369_tRNA_3_Dus_gen_cplx	b4369_tRNA_3 (leuP), Dus_gen	C2435H3483Mg2N788O1098P90S14	-91	tRNA Modification
b4369_tRNA_4	b4369_tRNA_4 (leuP)	C828H946Mg2N329O615P87	-84	tRNA Modification
b4369_tRNA_4_TruA_dim_cplx	b4369_tRNA_4 (leuP), TruA_dim	C3538H5148Mg2N1109O1385P87S16	-78	tRNA Modification
b4369_tRNA_5	b4369_tRNA_5 (leuP)	C828H946Mg2N329O615P87	-84	tRNA Modification
b4369_tRNA_5_TruA_dim_cplx	b4369_tRNA_5 (leuP), TruA_dim	C3538H5148Mg2N1109O1385P87S16	-78	tRNA Modification
b4369_tRNA_6	b4369_tRNA_6 (leuP)	C828H946Mg2N329O615P87	-84	tRNA Modification
b4369_tRNA_6_TrmA_mono_cplx	b4369_tRNA_6 (leuP), TrmA_mono	C2702H3893Mg2N852O1174P87S19	-92	tRNA Modification
b4369_tRNA_7	b4369_tRNA_7 (leuP)	C829H948Mg2N329O615P87	-84	tRNA Modification
b4369_tRNA_7_TruB_mono_cplx	b4369_tRNA_7 (leuP), TruB_mono	C2369H3423Mg2N767O1084P87S10	-89	tRNA Modification
b4369_tRNA_Mg2	tRNA (leuP) bound two Mg2 ions	C827H938Mg2N329O615P87	-84	tRNA Modification
b4369_tRNA_Mg2_Dus_gen_cplx	b4369_tRNA (leuP), Dus_gen	C2434H3477Mg2N788O1098P90S14	-91	tRNA Modification
b4370_RNA	tRNA (leuQ)	C827H939N330O614P87	-88	tRNA Modification
b4370_tRNA_1	b4370_tRNA_1 (leuQ)	C827H941Mg2N330O614P87	-84	tRNA Modification
b4370_tRNA_1_Dus_gen_cplx	b4370_tRNA_1 (leuQ), Dus_gen	C2434H3480Mg2N789O1097P90S14	-91	tRNA Modification
b4370_tRNA_2	b4370_tRNA_2 (leuQ)	C827H943Mg2N330O614P87	-84	tRNA Modification
b4370_tRNA_2_TrmH_dim_cplx	b4370_tRNA_2 (leuQ), TrmH_dim	C3038H4486Mg2N986O1275P87S29	-85	tRNA Modification
b4370_tRNA_3	b4370_tRNA_3 (leuQ)	C828H945Mg2N330O614P87	-84	tRNA Modification
b4370_tRNA_3_Dus_gen_cplx	b4370_tRNA_3 (leuQ), Dus_gen	C2435H3484Mg2N789O1097P90S14	-91	tRNA Modification
b4370_tRNA_4	b4370_tRNA_4 (leuQ)	C828H947Mg2N330O614P87	-84	tRNA Modification
b4370_tRNA_4_TruA_dim_cplx	b4370_tRNA_4 (leuQ), TruA_dim	C3538H5149Mg2N1110O1384P87S16	-78	tRNA Modification

b4370_tRNA_5	b4370_tRNA_5 (leuQ)	C828H947Mg2N330O614P87	-84	tRNA Modification
b4370_tRNA_5_TruA_dim_cplx	b4370_tRNA_5 (leuQ), TruA_dim	C3538H5149Mg2N1110O1384P87S16	-78	tRNA Modification
b4370_tRNA_6	b4370_tRNA_6 (leuQ)	C828H947Mg2N330O614P87	-84	tRNA Modification
b4370_tRNA_6_TrmA_mono_cplx	b4370_tRNA_6 (leuQ), TrmA_mono	C2702H3894Mg2N853O1173P87S19	-92	tRNA Modification
b4370_tRNA_7	b4370_tRNA_7 (leuQ)	C829H949Mg2N330O614P87	-84	tRNA Modification
b4370_tRNA_7_TruB_mono_cplx	b4370_tRNA_7 (leuQ), TruB_mono	C2369H3424Mg2N768O1083P87S10	-89	tRNA Modification
b4370_tRNA_Mg2	tRNA (leuQ) bound two Mg2 ions	C827H939Mg2N330O614P87	-84	tRNA Modification
b4370_tRNA_Mg2_Dus_gen_cplx	b4370_tRNA (leuQ), Dus_gen	C2434H3478Mg2N789O1097P90S14	-91	tRNA Modification
b4371_aa	polypeptide b4371	C1675H2616N468O499S11	-6	Translation
b4371_def_map_cplx	Polypeptide b4371 peptide deformylase and methionine aminopeptidase complex	C3805H6072N1061O1145S32Mg0Zn0Fe3	-15	Maturation
b4371_m	Matured polypeptide b4371	C1669H2608N467O497S10	-5	Maturation
b4371_mRNA	mRNA b4371	C9807H11112N3893O7256P1034	-1035	Translation
b4371_mRNA_1	mRNA b4371	C9807H11112N3893O7256P1034	-1035	Translation
b4371_mRNA_2	mRNA b4371	C9807H11112N3893O7256P1034	-1035	Translation
b4371_mRNA_2_degr	mRNA b4371 degradation complex	C50490H76511N15768O19941S200P1034Mg6Zn2Fe0	-1257	mRNA degradation
b4371_m_DnaKJ_complex	b4371 DnaK DnaJ_dim complex - Kerner et al. class II can interact w/ GroEL/ES, cannot fold spontanously	C8233H13122O2595N2380S57P3Zn4	-27	Folding
b4371_m_GroEL.(7)ATP.transGroES	b4371 GroEL GroES complex - Kerner et al. class II can interact w/ GroEL/ES, cannot fold spontanously	C39581H65552O12831N11051S374P21Mg7	-306	Folding
b4372_aa	polypeptide b4372	C671H1067N193O200S5	-4	Translation
b4372_def_map_cplx	Polypeptide b4372 peptide deformylase and methionine aminopeptidase complex	C2801H4523N786O846S26Mg0Zn0Fe3	-13	Maturation
b4372_m	Matured polypeptide b4372	C665H1059N192O198S4	-3	Maturation
b4372_mRNA	mRNA b4372	C3934H4473N1576O2894P416	-417	Translation
b4372_mRNA_1	mRNA b4372	C3934H4473N1576O2894P416	-417	Translation
b4372_mRNA_2	mRNA b4372	C3934H4473N1576O2894P416	-417	Translation
b4372_mRNA_2_degr	mRNA b4372 degradation complex	C44617H69872N13451O15579S200P416Mg6Zn2Fe0	-639	mRNA degradation
b4373_aa	polypeptide b4373	C741H1145N201O227S4	-8	Translation
b4373_def_map_cplx	Polypeptide b4373 peptide deformylase and methionine aminopeptidase complex	C2871H4601N794O873S25Mg0Zn0Fe3	-17	Maturation
b4373_m	Matured polypeptide b4373	C735H1137N200O225S3	-7	Maturation
b4373_mRNA	mRNA b4373 (32 nt short)	C3952H4476N1589O2891P415	-416	Translation
b4373_mRNA_1	mRNA b4373 (32 nt short)	C3952H4476N1589O2891P415	-416	Translation
b4373_mRNA_2	mRNA b4373 (32 nt short)	C3952H4476N1589O2891P415	-416	Translation
b4373_mRNA_2_degr	mRNA b4373 degradation complex	C44635H69875N13464O15576S200P415Mg6Zn2Fe0	-638	mRNA degradation
b4374_aa	polypeptide b4374	C1140H1731N302O339S7	-13	Translation
b4374_def_map_cplx	Polypeptide b4374 peptide deformylase and methionine aminopeptidase complex	C3270H5187N895O985S28Mg0Zn0Fe3	-22	Maturation
b4374_m	Matured polypeptide b4374	C1134H1723N301O337S6	-12	Maturation
b4374_mRNA	mRNA b4374	C6438H7296N2543O4758P678	-679	Translation
b4374_mRNA_1	mRNA b4374	C6438H7296N2543O4758P678	-679	Translation
b4374_mRNA_2	mRNA b4374	C6438H7296N2543O4758P678	-679	Translation
b4374_mRNA_2_degr	mRNA b4374 degradation complex	C47121H72695N14418O17443S200P678Mg6Zn2Fe0	-901	mRNA degradation
b4375_aa	polypeptide b4375	C2643H4173N728O794S23	-13	Translation
b4375_def_map_cplx	Polypeptide b4375 peptide deformylase and methionine aminopeptidase complex	C4773H7629N1321O1440S44Mg0Zn0Fe3	-22	Maturation
b4375_m	Matured polypeptide b4375	C2637H4165N727O792S22	-12	Maturation
b4375_mRNA	mRNA b4375	C15142H17144N6087O11100P1592	-1593	Translation
b4375_mRNA_1	mRNA b4375	C15142H17144N6087O11100P1592	-1593	Translation
b4375_mRNA_2	mRNA b4375	C15142H17144N6087O11100P1592	-1593	Translation
b4375_mRNA_2_degr	mRNA b4375 degradation complex	C55825H82543N17962O23785S200P1592Mg6Zn2Fe0	-1815	mRNA degradation
b4375_m_DnaKJ_complex	b4375 DnaK DnaJ_dim complex - Deuerling et al. DnaKJ/GrpE dependent folding	C9201H14679O2890N2640S69P3Zn4	-34	Folding
cdp	CDP	C9H12N3O11P2	-3	Others
cdp[e]	CDP	C9H12N3O11P2	-3	Others
chor	Chorismate	C10H8O6	-2	Others
chor[e]	Chorismate	C10H8O6	-2	Others
cisGroES_hepta	cis version of Cpn10 chaperonin GroES, small subunit of GroESL (b4142, GroS, heptamer)	C3178H5334N889O1015S14	-21	Protein Folding
cmp	CMP	C9H12N3O8P	-2	Others

cmp[e]	CMP	C9H12N3O8P	-2	Others
coa	Coenzyme A	C21H32N7O16P3S	-4	Others
coa[e]	Coenzyme A	C21H32N7O16P3S	-4	Others
ctp	CTP	C9H12N3O14P3	-4	Others
ctp[e]	CTP	C9H12N3O14P3	-4	Others
cys-L	L-Cysteine	C3H7NO2S	0	Others
cys-L[e]	L-Cysteine	C3H7NO2S	0	Others
cysT_tRNA	cysT_tRNA (b1910, uncharged tRNA)	C710H815Mg2N280O518P74S2	-71	tRNA Modification
cysT_tRNA_cys	cysT_tRNA (charged tRNA)	C713H822Mg2N281O520P74S3	-71	tRNA charging
dad-5	5'-Deoxyadenosine	C10H13N5O3	0	Others
dad-5[e]	5'-Deoxyadenosine	C10H13N5O3	0	Others
degradosome	degradosome (1*RNase E, 1*Pnp, 1*RhlB, 1*Eno)	C38841H62541N11373O12127S188Zn2Mg4	-206	mRNA degradation
degradosome_inact	degradosome (1*RNase E, 1*Pnp, 1*RhlB, 1*Eno)	C38841H62541N11373O12127S188Zn2Mg4	-206	mRNA degradation
dmpp	Dimethylallyl diphosphate	C5H9O7P2	-3	Others
dmpp[e]	Dimethylallyl diphosphate	C5H9O7P2	-3	Others
fad	Flavin adenine dinucleotide oxidized	C27H31N9O15P2	-2	Others
fad[e]	Flavin adenine dinucleotide oxidized	C27H31N9O15P2	-2	Others
fadh2	Flavin adenine dinucleotide reduced	C27H33N9O15P2	-2	Others
fadh2[e]	Flavin adenine dinucleotide reduced	C27H33N9O15P2	-2	Others
fe2	Fe2+	Fe	2	Iron-sulfur cluster biosynthesis
fe2[e]	Iron(II)	Fe	2	Iron-sulfur cluster biosynthesis
fe3	Iron(III)	Fe	3	Iron-sulfur cluster biosynthesis
fe3[e]	Iron(III)	Fe	3	Iron-sulfur cluster biosynthesis
fldox	flavodoxin (oxidized)	X1	0	Others
fldox[e]	flavodoxin (oxidized)	X1	0	Others
fldrd	flavodoxin (reduced)	X1H2	0	Others
fldrd[e]	flavodoxin (reduced)	X1H2	0	Others
fmet_tRNA	fmet_tRNA (uncharged tRNA)	C735H844N297O537P77S1Mg2	-74	tRNA charging
fmet_tRNA_met	fmet_tRNA (charged and formylated)	C741H854Mg2N298O540P77S2	-75	tRNA charging
fmet_tRNA_met_1	fmet_tRNA (charged tRNA, not formylated)	C740H855Mg2N298O539P77S2	-74	tRNA charging
for	Formate	CH1O2	-1	Others
for[e]	Formate	CH1O2	-1	Others
gdp	GDP	C10H12N5O11P2	-3	Others
gdp[e]	GDP	C10H12N5O11P2	-3	Others
gln-L	L-Glutamine	C5H10N2O3	0	Others
gln-L[e]	L-Glutamine	C5H10N2O3	0	Others
gln1_tRNA	gln1_tRNA (uncharged tRNA)	C715H819N280O527P75S1Mg2	-72	tRNA charging
gln1_tRNA_gln	gln1_tRNA (charged tRNA)	C720H829Mg2N282O530P75S1	-72	tRNA charging
gln2_tRNA	gln2_tRNA (uncharged tRNA)	C715H820N281O526P75S1Mg2	-72	tRNA charging
gln2_tRNA_gln	gln2_tRNA (charged tRNA)	C720H830Mg2N283O529P75S1	-72	tRNA charging
glnU_tRNA	glnU_tRNA (uncharged tRNA)	C715H819Mg2N280O527P75S1	-72	tRNA Modification
glnV_tRNA	glnV_tRNA (uncharged tRNA)	C715H820Mg2N281O526P75S1	-72	tRNA Modification
glnW_tRNA	glnW_tRNA (uncharged tRNA)	C715H819Mg2N280O527P75S1	-72	tRNA Modification
glnX_tRNA	glnX_tRNA (uncharged tRNA)	C715H820Mg2N281O526P75S1	-72	tRNA Modification
gltT_tRNA	gltT_tRNA (b3969, uncharged tRNA)	C724H833Mg2N288O531P76S1	-73	tRNA Modification
gltT_tRNA_Se	b3969 tRNA (modified, uncharged) - contains mnm5se2U instead of mnm5s2U	C724H833Mg2N288O531P76S0Se1	-73	tRNA Modification
gltU_tRNA	gltU_tRNA (b3757, uncharged tRNA)	C724H833Mg2N288O531P76S1	-73	tRNA Modification
gltU_tRNA_Se	b3757 tRNA (modified, uncharged) - contains mnm5se2U instead of mnm5s2U	C724H833Mg2N288O531P76S0Se1	-73	tRNA Modification
gltV_tRNA	gltV_tRNA (b4008, uncharged tRNA)	C724H833Mg2N288O531P76S1	-73	tRNA Modification
gltV_tRNA_Se	b4008 tRNA (modified, uncharged) - contains mnm5se2U instead of mnm5s2U	C724H833Mg2N288O531P76S0Se1	-73	tRNA Modification
gltW_tRNA	gltW_tRNA (b2590, tRNA)	C724H833Mg2N288O531P76S1	-73	tRNA Modification
gltW_tRNA_Se	b2590 tRNA (modified, uncharged) - contains mnm5se2U instead of mnm5s2U	C724H833Mg2N288O531P76S0Se1	-73	tRNA Modification
glu-L	L-Glutamate	C5H8NO4	-1	Others
glu-L[e]	L-Glutamate	C5H8NO4	-1	Others
glu1_tRNA	glu1_tRNA (uncharged tRNA)	C724H833N288O531P76S1Mg2	-73	tRNA charging

glu1_tRNA_glu	glu1_tRNA (charged tRNA)	C729H841Mg2N289O535P76S1	-74	tRNA charging
glx	Glyoxylate	C2H1O3	-1	Others
glx[e]	Glyoxylate	C2H1O3	-1	Others
gly	Glycine	C2H5NO2	0	Others
gly1_tRNA	gly1_tRNA (uncharged tRNA)	C724H830N287O537P76S0Mg2	-73	tRNA charging
gly1_tRNA_gly	gly1_tRNA (charged tRNA)	C726H835Mg2N288O539P76S0	-73	tRNA charging
glyT_tRNA	glyT_tRNA (uncharged tRNA)	C708H809Mg2N270O532P75	-72	tRNA Modification
glyT_tRNA_gly	glyT_tRNA (charged tRNA)	C710H814Mg2N271O534P75S0	-72	tRNA charging
glyU_tRNA	glyU_tRNA (uncharged tRNA)	C702H802Mg2N275O520P74S1	-71	tRNA Modification
glyU_tRNA_gly	glyU_tRNA (charged tRNA)	C704H807Mg2N276O522P74S1	-71	tRNA charging
glyV_tRNA	glyV_tRNA (uncharged tRNA)	C724H830Mg2N287O537P76	-73	tRNA Modification
glyW_tRNA	glyW_tRNA (uncharged tRNA)	C724H830Mg2N287O537P76	-73	tRNA Modification
glyX_tRNA	glyX_tRNA (uncharged tRNA)	C724H830Mg2N287O537P76	-73	tRNA Modification
glyY_tRNA	glyY_tRNA (uncharged tRNA)	C724H830Mg2N287O537P76	-73	tRNA Modification
gly[e]	Glycine	C2H5NO2	0	Others
gmp	GMP	C10H12N5O8P	-2	Others
gmp[e]	GMP	C10H12N5O8P	-2	Others
gtp	GTP	C10H12N5O14P3	-4	Others
gtp[e]	GTP	C10H12N5O14P3	-4	Others
gua	Guanine	C5H5N5O1	0	Others
gua[e]	Guanine	C5H5N5O1	0	Others
h	H+	H	1	Others
h2o	H2O	H2O	0	Others
h2o[e]	H2O	H2O	0	Others
h2s	h2s	H2S1	0	Others
h2s[e]	h2s	H2S1	0	Others
hRNAP	holo-RNA polymerase	C16671H26941N4683O5133S113ZnMg2	-78	Transcription
hRNAP_inact	holo-RNA polymerase	C16671H26941N4683O5133S113ZnMg2	-78	Transcription
h[e]	H+	H1	1	Others
hco3	Bicarbonate	C1H1O3	-1	Others
hco3[e]	Bicarbonate	C1H1O3	-1	Others
his-L	L-Histidine	C6H9N3O2	0	Others
his-L[e]	L-Histidine	C6H9N3O2	0	Others
hisR_tRNA	hisR_tRNA (b3797, uncharged tRNA)	C739H848Mg2N281O549P77S1	-74	tRNA Modification
hisR_tRNA_his	hisR_tRNA (charged tRNA)	C745H857Mg2N284O551P77S1	-74	tRNA charging
ile-L	L-Isoleucine	C6H13NO2	0	Others
ile-L[e]	L-Isoleucine	C6H13NO2	0	Others
ile1_tRNA	ile1_tRNA (uncharged tRNA)	C745H852N298O547P77S0Mg2	-75	tRNA charging
ile1_tRNA_ile	ile1_tRNA (charged tRNA)	C751H865N299O549P77S0Mg2	-75	tRNA charging
ile2_tRNA	ile2_tRNA (uncharged tRNA)	C740H855N291O540P76S1Mg2	-74	tRNA charging
ile2_tRNA_ile	ile2_tRNA (charged tRNA)	C746H868Mg2N292O542P76S1	-74	tRNA charging
ileT_tRNA	ileT_tRNA (b3852, uncharged tRNA)	C745H852Mg2N298O547P77	-75	tRNA Modification
ileU_tRNA	ileU_tRNA (b3277, uncharged tRNA)	C745H852Mg2N298O547P77	-75	tRNA Modification
ileV_tRNA	ileV_tRNA (b0202, uncharged tRNA)	C745H852Mg2N298O547P77	-75	tRNA Modification
ileX_tRNA	ileX_tRNA (b3069, uncharged tRNA)	C740H856Mg2N292O540P76S1	-74	tRNA Modification
ileY_tRNA	ileY_tRNA (b2652, uncharged tRNA)	C740H855Mg2N291O540P76S1	-74	tRNA Modification
leu-L	L-Leucine	C6H13NO2	0	Others
leu-L[e]	L-Leucine	C6H13NO2	0	Others
leu1_tRNA	leu1_tRNA (uncharged tRNA)	C825H944N329O610P87S0Mg2	-84	tRNA charging
leu1_tRNA_leu	leu1_tRNA (charged tRNA)	C831H957Mg2N330O612P87S0	-84	tRNA charging
leu2_tRNA	leu2_tRNA (uncharged tRNA)	C827H947N333O599P86S1Mg2	-83	tRNA charging
leu2_tRNA_leu	leu2_tRNA (charged tRNA)	C833H960Mg2N334O601P86S1	-83	tRNA charging
leuP_tRNA	leuP_tRNA (b4369, uncharged tRNA)	C829H948Mg2N329O615P87	-84	tRNA Modification
leuQ_tRNA	leuQ_tRNA (b4370, uncharged tRNA)	C829H949Mg2N330O614P87	-84	tRNA Modification
leuT_tRNA	leuT_tRNA (b3798, uncharged tRNA)	C829H949Mg2N330O614P87	-84	tRNA Modification
leuU_tRNA	leuU_tRNA (uncharged tRNA)	C831H948Mg2N333O613P87	-84	tRNA Modification
leuU_tRNA_leu	leuU_tRNA (charged tRNA)	C837H961Mg2N334O615P87S0	-84	tRNA charging
leuV_tRNA	leuV_tRNA (b4368, uncharged tRNA)	C829H949Mg2N330O614P87	-84	tRNA Modification
leuW_tRNA	leuW_tRNA (b0672, uncharged tRNA)	C811H927Mg2N328O596P85	-82	tRNA Modification
leuW_tRNA_leu	leuW_tRNA (charged tRNA)	C817H940Mg2N329O598P85S0	-82	tRNA charging
leuX_tRNA	leuX_tRNA (b4270, uncharged tRNA)	C819H936Mg2N333O589P85S1	-82	tRNA Modification

leuZ_tRNA	leuZ_tRNA (uncharged tRNA)	C836H958Mg2N333O610P87S2	-84	tRNA Modification
leuZ_tRNA_leu	leuZ_tRNA (charged tRNA)	C842H971Mg2N334O612P87S2	-84	tRNA charging
lys-L	L-Lysine	C6H15N2O2	1	Others
lys-L[e]	L-Lysine	C6H15N2O2	1	Others
lys1_tRNA	lys1_tRNA (uncharged tRNA)	C734H844Mg2N284O542P76S1	-74	tRNA charging
lys1_tRNA_lys	lys1_tRNA (charged tRNA)	C740H859Mg2N286O544P76S1	-73	tRNA charging
lysQ_tRNA	lysQ_tRNA (b0749, uncharged tRNA)	C734H844Mg2N284O542P76S1	-74	tRNA Modification
lysQ_tRNA_Se	b0749 tRNA (modified, uncharged) - contains mnm5se2U instead of mnm5s2U	C734H844Mg2N284O542P76S0Se1	-74	tRNA Modification
lysT_tRNA	lysT_tRNA (b0743, uncharged tRNA)	C734H844Mg2N284O542P76S1	-74	tRNA Modification
lysT_tRNA_Se	b0743 tRNA (modified, uncharged) - contains mnm5se2U instead of mnm5s2U	C734H844Mg2N284O542P76S0Se1	-74	tRNA Modification
lysV_tRNA	lysV_tRNA (b2404, uncharged tRNA)	C734H844Mg2N284O542P76S1	-74	tRNA Modification
lysV_tRNA_Se	b2404 tRNA (modified, uncharged) - contains mnm5se2U instead of mnm5s2U	C734H844Mg2N284O542P76S0Se1	-74	tRNA Modification
lysW_tRNA	lysW_tRNA (b0745, uncharged tRNA)	C734H844Mg2N284O542P76S1	-74	tRNA Modification
lysW_tRNA_Se	b0745 tRNA (modified, uncharged) - contains mnm5se2U instead of mnm5s2U	C734H844Mg2N284O542P76S0Se1	-74	tRNA Modification
lysY_tRNA	lysY_tRNA (b0747, uncharged tRNA)	C734H844Mg2N284O542P76S1	-74	tRNA Modification
lysY_tRNA_Se	b0747 tRNA (modified, uncharged) - contains mnm5se2U instead of mnm5s2U	C734H844Mg2N284O542P76S0Se1	-74	tRNA Modification
lysZ_tRNA	lysZ_tRNA (b0748, uncharged tRNA)	C734H844Mg2N284O542P76S1	-74	tRNA Modification
lysZ_tRNA_Se	b0748 tRNA (modified, uncharged) - contains mnm5se2U instead of mnm5s2U	C734H844Mg2N284O542P76S0Se1	-74	tRNA Modification
met-L	L-Methionine	C5H11NO2S	0	Others
met-L[e]	L-Methionine	C5H11NO2S	0	Others
met1_tRNA	met1_tRNA (uncharged tRNA)	C746H859N293O545P77S1Se0Mg2	-75	tRNA charging
met1_tRNA_met	met1_tRNA (charged tRNA)	C751H870Mg2N294O547P77S2	-75	tRNA charging
metT_tRNA	metT_tRNA (b0673, uncharged tRNA)	C746H859Mg2N293O545P77S1	-75	tRNA Modification
metU_tRNA	metU_tRNA (b0673, uncharged tRNA)	C746H859Mg2N293O545P77S1	-75	tRNA Modification
metV_tRNA	metV_tRNA (b2816, uncharged tRNA)	C735H844Mg2N297O537P77S1	-74	tRNA Modification
metW_tRNA	metW_tRNA (b2815, uncharged tRNA)	C735H844Mg2N297O537P77S1	-74	tRNA Modification
metY_tRNA	metY_tRNA (b3171, uncharged tRNA)	C734H842Mg2N297O536P77S1	-74	tRNA Modification
metZ_tRNA	metZ_tRNA (b2814, uncharged tRNA)	C735H844Mg2N297O537P77S1	-74	tRNA Modification
mg2	magnesium	Mg	2	Others
mg2[e]	magnesium	Mg	2	Others
nad	Nicotinamide adenine dinucleotide	C21H26N7O14P2	-1	Others
nad[e]	Nicotinamide adenine dinucleotide	C21H26N7O14P2	-1	Others
nadh	Nicotinamide adenine dinucleotide - reduced	C21H27N7O14P2	-2	Others
nadh[e]	Nicotinamide adenine dinucleotide - reduced	C21H27N7O14P2	-2	Others
nadp	Nicotinamide adenine dinucleotide phosphate	C21H25N7O17P3	-3	Others
nadp[e]	Nicotinamide adenine dinucleotide phosphate	C21H25N7O17P3	-3	Others
nadph	Nicotinamide adenine dinucleotide phosphate - reduced	C21H26N7O17P3	-4	Others
nadph[e]	Nicotinamide adenine dinucleotide phosphate - reduced	C21H26N7O17P3	-4	Others
nh3	Ammonium	H3N1	0	Others
nh3[e]	Ammonium	H3N1	0	Others
pEF-TU_ac_a	precursor EF-TU (acetylated) (b3339)	C1919H3052N523O582S13	-15	Protein Modification
pEF-TU_ac_b	presursorEF-TU (acetylated) (b3980)	C1920H3054N523O583S13	-15	Protein Modification
pEF-TU_me_a	methylated and acetylated precursor of EF-TU (b3339)	C1920H3054N523O582S13	-15	Protein Modification
pEF-TU_me_b	methylated and acetylated precursor of EF-TU (b3980)	C1921H3056N523O583S13	-15	Protein Modification
phe-L	L-Phenylalanine	C9H11NO2	0	Others
phe-L[e]	L-Phenylalanine	C9H11NO2	0	Others
phe1_tRNA	phe1_tRNA (uncharged tRNA)	C735H846N291O535P76S2Mg2	-73	tRNA charging
phe1_tRNA_phe	phe1_tRNA (charged tRNA)	C744H857Mg2N292O537P76S2	-73	tRNA charging
pheU_tRNA	pheU_tRNA (b4134, uncharged tRNA)	C735H846Mg2N291O535P76S2	-73	tRNA Modification
pheV_tRNA	pheV_tRNA (b2967, uncharged tRNA)	C735H846Mg2N291O535P76S2	-73	tRNA Modification
pi	Phosphate	HO4P	-2	Others
pi[e]	Orthophosphate	HO4P	-2	Others
ppi	Diphosphate	HO7P2	-3	Others

ppi[e]	Diphosphate	H1O7P2	-3	Others
pre_Q0	7-cyano-7-deazaguanine	C7H5N5O1	0	tRNA Modification
pre_Q1	7-aminomethyl-7-deazaguanine	C7H10N5O1	1	tRNA Modification
pre_Q1_QueF_dim_cplx	pre_Q1_QueF_dim_cplx	C2929H4494N831O915P6S12	-19	tRNA Modification
pro-L	L-Proline	C5H9NO2	0	Others
pro-L[e]	L-Proline	C5H9NO2	0	Others
pro1_tRNA	pro1_tRNA (uncharged tRNA)	C738H842N295O543P77S1Mg2	-74	tRNA charging
pro1_tRNA_pro	pro1_tRNA (charged tRNA)	C743H851Mg2N296O545P77S1	-74	tRNA charging
pro2_tRNA	pro2_tRNA (uncharged tRNA)	C738H843N296O543P77S1Mg2	-74	tRNA charging
pro2_tRNA_pro	pro2_tRNA (charged tRNA)	C743H852Mg2N297O545P77S1	-74	tRNA charging
proK_tRNA	proK_tRNA (b3545, uncharged tRNA)	C737H842Mg2N295O541P77S1	-74	tRNA Modification
proL_tRNA	proL_tRNA (b2189, uncharged tRNA)	C737H844Mg2N297O541P77S1	-74	tRNA Modification
proL_tRNA_pro	proL_tRNA (charged tRNA)	C742H853Mg2N298O543P77S1	-74	tRNA charging
proM_tRNA	proM_tRNA (b3799, uncharged tRNA)	C739H843Mg2N295O545P77S1	-75	tRNA Modification
proM_tRNA_pro	proM_tRNA (charged tRNA)	C744H852Mg2N296O547P77S1	-75	tRNA charging
pydx5p	Pyridoxal 5'-phosphate	C8H8NO6P	-2	Others
pydx5p[e]	Pyridoxal 5'-phosphate	C8H8NO6P	-2	Others
rib_30	Ribosomal subunit 30	C30286H42417N10786O15275S79P1542Mg60	-1231	Ribosomal Assembly
rib_30_IF1_IF3	30S ribosomal subunit/ IF1/ IF3 complex	C31544H44522N11153O15650S89P1542Mg60	-1221	Ribosomal Assembly
rib_30_ini	ribosome initiation complex (subunit 30)	C35734H51424N12422O17021S113P1545Mg60	-1239	Ribosomal Assembly
rib_30_ini_inact	ribosome initiation complex (subunit 30)	C35734H51424N12422O17021S113P1545Mg60	-1239	Ribosomal Assembly
rib_50	ribosomal subunit 50S	C51997H71236N18783O27738Mg111S123P3024	-2496.14	Ribosomal Assembly
rib_50_inact	ribosomal subunit 50S	C51997H71236N18783O27738Mg111S123P3024	-2496.14	Ribosomal Assembly
rib_70	rib_70	C82283H113653N29569O43013S202P4566Mg171	-3727.14	Ribosomal Assembly
rib_70_elo1_b0014_18_cplx	Translation elongation complex: 18 * ribosome 70S/b0014/EF-TU-tRNA's/EF-G	C71665123H109390728N20854693O26133311S447012P1044461Mg106308Zn0Fe0	-1310306.52	Translation
rib_70_elo1_b0014_1_cplx	Translation elongation complex: 1 * ribosome 70S/b0014/EF-TU-tRNA's/EF-G	C3998680H6096807N1165633O1464390S24834P59838Mg5906Zn0Fe0	-74608.14	Translation
rib_70_elo1_b0014_37_cplx	Translation elongation complex: 37 * ribosome 70S/b0014/EF-TU-tRNA's/EF-G	C147292324H224836875N42860113O53704458S918858P2144922Mg218522Zn0Fe0	-2691381.18	Translation
rib_70_elo1_b0015_11_cplx	Translation elongation complex: 11 * ribosome 70S/b0015/EF-TU-tRNA's/EF-G	C26155590H39870557N7636635O9594956S162349P396647Mg39028Zn0Fe0	-487762.54	Translation
rib_70_elo1_b0015_1_cplx	Translation elongation complex: 1 * ribosome 70S/b0015/EF-TU-tRNA's/EF-G	C2387600H3635697N698245O879426S14759P37087Mg3548Zn0Fe0	-45371.14	Translation
rib_70_elo1_b0015_22_cplx	Translation elongation complex: 22 * ribosome 70S/b0015/EF-TU-tRNA's/EF-G	C52300379H79728903N15268864O19182039S324698P792163Mg78056Zn0Fe0	-974393.08	Translation
rib_70_elo1_b0023_1_cplx	Translation elongation complex: 1 * ribosome 70S/b0023/EF-TU-tRNA's/EF-G	C611564H922083N183123O235258S3533P12074Mg947Zn0Fe0	-13336.14	Translation
rib_70_elo1_b0023_2_cplx	Translation elongation complex: 2 * ribosome 70S/b0023/EF-TU-tRNA's/EF-G	C1220606H1841313N365214O468698S7066P23882Mg1894Zn0Fe0	-26405.28	Translation
rib_70_elo1_b0023_5_cplx	Translation elongation complex: 5 * ribosome 70S/b0023/EF-TU-tRNA's/EF-G	C3047732H4599003N911487O1169018S17665P59306Mg4735Zn0Fe0	-65612.7	Translation
rib_70_elo1_b0025_18_cplx	Translation elongation complex: 18 * ribosome 70S/b0025/EF-TU-tRNA's/EF-G	C35892597H54664401N10503104O13214170S221760P557252Mg53658Zn0Fe0	-678701.52	Translation
rib_70_elo1_b0025_1_cplx	Translation elongation complex: 1 * ribosome 70S/b0025/EF-TU-tRNA's/EF-G	C2002502H3046485N586885O740369S12320P31850Mg2981Zn0Fe0	-38598.14	Translation
rib_70_elo1_b0025_9_cplx	Translation elongation complex: 9 * ribosome 70S/b0025/EF-TU-tRNA's/EF-G	C17950782H27337269N5253341O6610393S110880P279098Mg26829Zn0Fe0	-339823.26	Translation
rib_70_elo1_b0026_1_cplx	Translation elongation complex: 1 * ribosome 70S/b0026/EF-TU-tRNA's/EF-G	C5847329H8919416N1702293O2136135S36564P86264Mg8606Zn0Fe0	-108289.14	Translation
rib_70_elo1_b0026_27_cplx	Translation elongation complex: 27 * ribosome 70S/b0026/EF-TU-tRNA's/EF-G	C157180251H240033910N45680643O57164589S987228P2255886Mg232362Zn0Fe0	-2850538.78	Translation
rib_70_elo1_b0026_55_cplx	Translation elongation complex: 55 * ribosome 70S/b0026/EF-TU-tRNA's/EF-G	C320154167H488926442N93041943O116426001S2011020P4592402Mg473330Zn0Fe0	-5803730.7	Translation
rib_70_elo1_b0027_1_cplx	Translation elongation complex: 1 * ribosome 70S/b0027/EF-TU-tRNA's/EF-G	C1087057H1647592N321281O408495S6527P18971Mg1640Zn0Fe0	-22101.14	Translation
rib_70_elo1_b0027_4_cplx	Translation elongation complex: 4 * ribosome 70S/b0027/EF-TU-tRNA's/EF-G	C4334194H6574477N1279700O1623462S26108P74402Mg6560Zn0Fe0	-86919.56	Translation
rib_70_elo1_b0027_9_cplx	Translation elongation complex: 9 * ribosome 70S/b0027/EF-TU-tRNA's/EF-G	C9746089H14785952N2877065O3648407S58743P166787Mg14760Zn0Fe0	-194950.26	Translation

rib_70_elo1_b0028_1_cplx	Translation elongation complex: 1 * ribosome 70S/b0028/EF-TU-tRNA's/EF-G	C994049H1505807N294310O374543S5939P17602Mg1505Zn0Fe0	-20383.14	Translation
rib_70_elo1_b0028_4_cplx	Translation elongation complex: 4 * ribosome 70S/b0028/EF-TU-tRNA's/EF-G	C3963371H6008690N1172155O1488704S23756P69058Mg6020Zn0Fe0	-80179.56	Translation
rib_70_elo1_b0028_8_cplx	Translation elongation complex: 8 * ribosome 70S/b0028/EF-TU-tRNA's/EF-G	C7922467H12012534N2342615O2974252S47512P137666Mg12040Zn0Fe0	-159908.12	Translation
rib_70_elo1_b0029_18_cplx	Translation elongation complex: 18 * ribosome 70S/b0029/EF-TU-tRNA's/EF-G	C36212043H55156135N10595526O13325655S223614P560535Mg54144Zn0Fe0	-683766.52	Translation
rib_70_elo1_b0029_1_cplx	Translation elongation complex: 1 * ribosome 70S/b0029/EF-TU-tRNA's/EF-G	C2020351H3073915N592114O746590S12423P32039Mg3008Zn0Fe0	-38886.14	Translation
rib_70_elo1_b0029_9_cplx	Translation elongation complex: 9 * ribosome 70S/b0029/EF-TU-tRNA's/EF-G	C18110559H27583195N5299602O6666150S111807P280743Mg27072Zn0Fe0	-342359.26	Translation
rib_70_elo1_b0049_16_cplx	Translation elongation complex: 16 * ribosome 70S/b0049/EF-TU-tRNA's/EF-G	C28690040H43665838N8405567O10591953S176656P452827Mg42944Zn0Fe0	-547998.24	Translation
rib_70_elo1_b0049_1_cplx	Translation elongation complex: 1 * ribosome 70S/b0049/EF-TU-tRNA's/EF-G	C1800650H2737633N528362O667533S11041P29092Mg2684Zn0Fe0	-35041.14	Translation
rib_70_elo1_b0049_8_cplx	Translation elongation complex: 8 * ribosome 70S/b0049/EF-TU-tRNA's/EF-G	C14349032H21837462N4204391O5298929S88328P226835Mg21472Zn0Fe0	-274421.12	Translation
rib_70_elo1_b0050_1_cplx	Translation elongation complex: 1 * ribosome 70S/b0050/EF-TU-tRNA's/EF-G	C845913H1279751N251253O320458S5018P15449Mg1289Zn0Fe0	-17639.14	Translation
rib_70_elo1_b0050_3_cplx	Translation elongation complex: 3 * ribosome 70S/b0050/EF-TU-tRNA's/EF-G	C2530563H3831115N750927O956084S15054P45591Mg3867Zn0Fe0	-52159.42	Translation
rib_70_elo1_b0050_7_cplx	Translation elongation complex: 7 * ribosome 70S/b0050/EF-TU-tRNA's/EF-G	C5899863H8933843N1750275O2227336S35126P105875Mg9023Zn0Fe0	-121199.98	Translation
rib_70_elo1_b0051_16_cplx	Translation elongation complex: 16 * ribosome 70S/b0051/EF-TU-tRNA's/EF-G	C27992972H42603707N8202542O10334824S172448P441890Mg41936Zn0Fe0	-534581.24	Translation
rib_70_elo1_b0051_1_cplx	Translation elongation complex: 1 * ribosome 70S/b0051/EF-TU-tRNA's/EF-G	C1756847H2670992N515552O651289S10778P28385Mg2621Zn0Fe0	-34179.14	Translation
rib_70_elo1_b0051_8_cplx	Translation elongation complex: 8 * ribosome 70S/b0051/EF-TU-tRNA's/EF-G	C14000372H21306259N4102814O5170272S86224P221354Mg20968Zn0Fe0	-267700.12	Translation
rib_70_elo1_b0052_19_cplx	Translation elongation complex: 19 * ribosome 70S/b0052/EF-TU-tRNA's/EF-G	C39731869H60525843N11618239O14613862S244929P612257Mg59375Zn0Fe0	-748338.66	Translation
rib_70_elo1_b0052_1_cplx	Translation elongation complex: 1 * ribosome 70S/b0052/EF-TU-tRNA's/EF-G	C2100043H3195663N615001O775732S12891P33161Mg3125Zn0Fe0	-40324.14	Translation
rib_70_elo1_b0052_9_cplx	Translation elongation complex: 9 * ribosome 70S/b0052/EF-TU-tRNA's/EF-G	C18825299H28675743N5505329O6926012S116019P290537Mg28125Zn0Fe0	-354997.26	Translation
rib_70_elo1_b0053_12_cplx	Translation elongation complex: 12 * ribosome 70S/b0053/EF-TU-tRNA's/EF-G	C32386901H49374353N9451532O11871344S201048P488321Mg48192Zn0Fe0	-603415.68	Translation
rib_70_elo1_b0053_1_cplx	Translation elongation complex: 1 * ribosome 70S/b0053/EF-TU-tRNA's/EF-G	C2710155H4127272N792189O997470S16754P41875Mg4016Zn0Fe0	-51467.14	Translation
rib_70_elo1_b0053_25_cplx	Translation elongation complex: 25 * ribosome 70S/b0053/EF-TU-tRNA's/EF-G	C67459419H102848176N19685301O24722286S418850P1015939Mg100400Zn0Fe0	-1255718.5	Translation
rib_70_elo1_b0058_12_cplx	Translation elongation complex: 12 * ribosome 70S/b0058/EF-TU-tRNA's/EF-G	C17023575H25881506N5001391O6314875S104208P276600Mg25620Zn0Fe0	-330362.68	Translation
rib_70_elo1_b0058_1_cplx	Translation elongation complex: 1 * ribosome 70S/b0058/EF-TU-tRNA's/EF-G	C1424409H2163317N419132O530459S8684P23655Mg2135Zn0Fe0	-28136.14	Translation
rib_70_elo1_b0058_6_cplx	Translation elongation complex: 6 * ribosome 70S/b0058/EF-TU-tRNA's/EF-G	C8514939H12944312N2501977O3159739S52104P138630Mg12810Zn0Fe0	-165511.84	Translation
rib_70_elo1_b0059_1_cplx	Translation elongation complex: 1 * ribosome 70S/b0059/EF-TU-tRNA's/EF-G	C6034786H9204602N1757299O2205250S37714P89237Mg8876Zn0Fe0	-111980.14	Translation
rib_70_elo1_b0059_28_cplx	Translation elongation complex: 28 * ribosome 70S/b0059/EF-TU-tRNA's/EF-G	C168227215H256882568N48905239O61198549S1055992P2420093Mg248528Zn0Fe0	-3056873.92	Translation
rib_70_elo1_b0059_56_cplx	Translation elongation complex: 56 * ribosome 70S/b0059/EF-TU-tRNA's/EF-G	C336426771H513733792N97799399O122376785S2111984P4837277Mg497056Zn0Fe0	-6110837.84	Translation
rib_70_elo1_b0144_18_cplx	Translation elongation complex: 18 * ribosome 70S/b0144/EF-TU-tRNA's/EF-G	C35348838H53827151N10345246O13019536S218160P550217Mg52848Zn0Fe0	-669470.52	Translation
rib_70_elo1_b0144_1_cplx	Translation elongation complex: 1 * ribosome 70S/b0144/EF-TU-tRNA's/EF-G	C1972126H2999820N577998O729454S12120P31445Mg2936Zn0Fe0	-38071.14	Translation
rib_70_elo1_b0144_9_cplx	Translation elongation complex: 9 * ribosome 70S/b0144/EF-TU-tRNA's/EF-G	C17678814H26918564N5174350O6513022S109080P275573Mg26424Zn0Fe0	-335200.26	Translation
rib_70_elo1_b0166_16_cplx	Translation elongation complex: 16 * ribosome 70S/b0166/EF-TU-tRNA's/EF-G	C28067954H42727656N8222961O10355179S172592P440939Mg42080Zn0Fe0	-534302.24	Translation

rib_70_elo1_b0166_1_cplx	Translation elongation complex: 1 * ribosome 70S/b0166/EF-TU-tRNA's/EF-G	C1761614H2678811N516891O652609S10787P28334Mg2630Zn0Fe0	-34170.14	Translation
rib_70_elo1_b0166_8_cplx	Translation elongation complex: 8 * ribosome 70S/b0166/EF-TU-tRNA's/EF-G	C14037906H21368272N4113057O5180475S86296P220883Mg21040Zn0Fe0	-267565.12	Translation
rib_70_elo1_b0167_1_cplx	Translation elongation complex: 1 * ribosome 70S/b0167/EF-TU-tRNA's/EF-G	C5555828H8472845N1618206O2031379S34747P82499Mg8174Zn0Fe0	-103273.14	Translation
rib_70_elo1_b0167_26_cplx	Translation elongation complex: 26 * ribosome 70S/b0167/EF-TU-tRNA's/EF-G	C143817153H219574070N41822181O52348429S903422P2078149Mg212524Zn0Fe0	-2618251.64	Translation
rib_70_elo1_b0167_52_cplx	Translation elongation complex: 52 * ribosome 70S/b0167/EF-TU-tRNA's/EF-G	C287608931H439119344N83634315O104678161S1806844P4153625Mg425048Zn0Fe0	-5233829.28	Translation
rib_70_elo1_b0168_15_cplx	Translation elongation complex: 15 * ribosome 70S/b0168/EF-TU-tRNA's/EF-G	C25399248H38659130N7442350O9379300S156120P401027Mg38100Zn0Fe0	-484880.1	Translation
rib_70_elo1_b0168_1_cplx	Translation elongation complex: 1 * ribosome 70S/b0168/EF-TU-tRNA's/EF-G	C1700356H2585274N499008O630448S10408P27479Mg2540Zn0Fe0	-33070.14	Translation
rib_70_elo1_b0168_7_cplx	Translation elongation complex: 7 * ribosome 70S/b0168/EF-TU-tRNA's/EF-G	C11857024H18045498N3474726O4379956S72856P187571Mg17780Zn0Fe0	-226702.98	Translation
rib_70_elo1_b0169_14_cplx	Translation elongation complex: 14 * ribosome 70S/b0169/EF-TU-tRNA's/EF-G	C21737038H33068779N6376348O8045352S133014P347634Mg32662Zn0Fe0	-418070.96	Translation
rib_70_elo1_b0169_1_cplx	Translation elongation complex: 1 * ribosome 70S/b0169/EF-TU-tRNA's/EF-G	C1559062H2369318N458020O579374S9501P25507Mg2333Zn0Fe0	-30539.14	Translation
rib_70_elo1_b0169_7_cplx	Translation elongation complex: 7 * ribosome 70S/b0169/EF-TU-tRNA's/EF-G	C10871974H16538300N3189556O4025210S66507P174181Mg16331Zn0Fe0	-209399.98	Translation
rib_70_elo1_b0170_16_cplx	Translation elongation complex: 16 * ribosome 70S/b0170/EF-TU-tRNA's/EF-G	C28929674H44055065N8468372O10663018S177872P451188Mg43376Zn0Fe0	-548519.24	Translation
rib_70_elo1_b0170_1_cplx	Translation elongation complex: 1 * ribosome 70S/b0170/EF-TU-tRNA's/EF-G	C1815749H2762060N532412O671968S11117P28998Mg2711Zn0Fe0	-35082.14	Translation
rib_70_elo1_b0170_8_cplx	Translation elongation complex: 8 * ribosome 70S/b0170/EF-TU-tRNA's/EF-G	C14468914H22032129N4235860O5334458S88936P226020Mg21688Zn0Fe0	-274686.12	Translation
rib_70_elo1_b0172_10_cplx	Translation elongation complex: 10 * ribosome 70S/b0172/EF-TU-tRNA's/EF-G	C12105419H18386008N3566297O4512511S73370P202070Mg18290Zn0Fe0	-238682.4	Translation
rib_70_elo1_b0172_1_cplx	Translation elongation complex: 1 * ribosome 70S/b0172/EF-TU-tRNA's/EF-G	C1215347H1844026N358607O454726S7337P20711Mg1829Zn0Fe0	-24373.14	Translation
rib_70_elo1_b0172_5_cplx	Translation elongation complex: 5 * ribosome 70S/b0172/EF-TU-tRNA's/EF-G	C6055379H9196018N1784247O2258186S36685P101315Mg9145Zn0Fe0	-119621.7	Translation
rib_70_elo1_b0188_12_cplx	Translation elongation complex: 12 * ribosome 70S/b0188/EF-TU-tRNA's/EF-G	C32688649H49831374N9542749O11980891S203136P493709Mg48624Zn0Fe0	-609391.68	Translation
rib_70_elo1_b0188_1_cplx	Translation elongation complex: 1 * ribosome 70S/b0188/EF-TU-tRNA's/EF-G	C2735396H4165457N799795O1006763S16928P42335Mg4052Zn0Fe0	-51976.14	Translation
rib_70_elo1_b0188_25_cplx	Translation elongation complex: 25 * ribosome 70S/b0188/EF-TU-tRNA's/EF-G	C68087948H103800185N19875331O24950315S423200P1027151Mg101300Zn0Fe0	-1268155.5	Translation
rib_70_elo1_b0194_16_cplx	Translation elongation complex: 16 * ribosome 70S/b0194/EF-TU-tRNA's/EF-G	C57292921H87410796N16692103O20924254S357536P845241Mg84992Zn0Fe0	-1054988.24	Translation
rib_70_elo1_b0194_1_cplx	Translation elongation complex: 1 * ribosome 70S/b0194/EF-TU-tRNA's/EF-G	C3596161H5480556N1049443O1319014S22346P54441Mg5312Zn0Fe0	-67551.14	Translation
rib_70_elo1_b0194_33_cplx	Translation elongation complex: 33 * ribosome 70S/b0194/EF-TU-tRNA's/EF-G	C118149249H180265068N34420451O43143526S737418P1741481Mg175296Zn0Fe0	-2174083.62	Translation
rib_70_elo1_b0405_10_cplx	Translation elongation complex: 10 * ribosome 70S/b0405/EF-TU-tRNA's/EF-G	C22587660H34411017N6603160O8302793S139780P347033Mg33680Zn0Fe0	-425355.4	Translation
rib_70_elo1_b0405_1_cplx	Translation elongation complex: 1 * ribosome 70S/b0405/EF-TU-tRNA's/EF-G	C2267928H3451485N663961O837032S13978P35669Mg3368Zn0Fe0	-43502.14	Translation
rib_70_elo1_b0405_20_cplx	Translation elongation complex: 20 * ribosome 70S/b0405/EF-TU-tRNA's/EF-G	C45165140H68810497N13202270O16598083S279560P692993Mg67360Zn0Fe0	-849636.8	Translation
rib_70_elo1_b0406_11_cplx	Translation elongation complex: 11 * ribosome 70S/b0406/EF-TU-tRNA's/EF-G	C26113054H39796180N7627205O9585209S162272P397746Mg38929Zn0Fe0	-488740.54	Translation
rib_70_elo1_b0406_1_cplx	Translation elongation complex: 1 * ribosome 70S/b0406/EF-TU-tRNA's/EF-G	C2383664H3628860N697245O878579S14752P37186Mg3539Zn0Fe0	-45459.14	Translation
rib_70_elo1_b0406_22_cplx	Translation elongation complex: 22 * ribosome 70S/b0406/EF-TU-tRNA's/EF-G	C52215383H79580232N15250161O19162502S324544P794362Mg77858Zn0Fe0	-976350.08	Translation
rib_70_elo1_b0407_1_cplx	Translation elongation complex: 1 * ribosome 70S/b0407/EF-TU-tRNA's/EF-G	C753675H1138879N224474O287067S4424P14146Mg1154Zn0Fe0	-15973.14	Translation
rib_70_elo1_b0407_3_cplx	Translation elongation complex: 3 * ribosome 70S/b0407/EF-TU-tRNA's/EF-G	C2254683H3409477N670896O856537S13272P41772Mg3462Zn0Fe0	-47251.42	Translation

rib_70_elo1_b0407_6_cplx	Translation elongation complex: 6 * ribosome 70S/b0407/EF-TU-tRNA's/EF-G	C4506195H6815374N1340529O1710742S26544P83211Mg6924Zn0Fe0	-94168.84	Translation
rib_70_elo1_b0413_1_cplx	Translation elongation complex: 1 * ribosome 70S/b0413/EF-TU-tRNA's/EF-G	C993766H1505628N294258O374140S5969P17560Mg1505Zn0Fe0	-20326.14	Translation
rib_70_elo1_b0413_4_cplx	Translation elongation complex: 4 * ribosome 70S/b0413/EF-TU-tRNA's/EF-G	C3962188H6007983N1171854O1487098S23876P68884Mg6020Zn0Fe0	-79945.56	Translation
rib_70_elo1_b0413_8_cplx	Translation elongation complex: 8 * ribosome 70S/b0413/EF-TU-tRNA's/EF-G	C7920084H12011123N2341982O2971042S47752P137316Mg12040Zn0Fe0	-159438.12	Translation
rib_70_elo1_b0414_10_cplx	Translation elongation complex: 10 * ribosome 70S/b0414/EF-TU-tRNA's/EF-G	C23253184H35432168N6795191O8541440S144110P355774Mg34670Zn0Fe0	-436456.4	Translation
rib_70_elo1_b0414_1_cplx	Translation elongation complex: 1 * ribosome 70S/b0414/EF-TU-tRNA's/EF-G	C2334781H3553934N683327O861083S14411P36571Mg3467Zn0Fe0	-44640.14	Translation
rib_70_elo1_b0414_21_cplx	Translation elongation complex: 21 * ribosome 70S/b0414/EF-TU-tRNA's/EF-G	C48820121H74394454N14265247O17928543S302631P745911Mg72807Zn0Fe0	-915342.94	Translation
rib_70_elo1_b0415_1_cplx	Translation elongation complex: 1 * ribosome 70S/b0415/EF-TU-tRNA's/EF-G	C1035849H1570115N306298O389343S6183P18080Mg1568Zn0Fe0	-21045.14	Translation
rib_70_elo1_b0415_4_cplx	Translation elongation complex: 4 * ribosome 70S/b0415/EF-TU-tRNA's/EF-G	C4129929H6265238N1219777O1547505S24732P70907Mg6272Zn0Fe0	-82764.56	Translation
rib_70_elo1_b0415_9_cplx	Translation elongation complex: 9 * ribosome 70S/b0415/EF-TU-tRNA's/EF-G	C9286729H14090443N2742242O3477775S55647P158952Mg14112Zn0Fe0	-185630.26	Translation
rib_70_elo1_b0416_1_cplx	Translation elongation complex: 1 * ribosome 70S/b0416/EF-TU-tRNA's/EF-G	C932679H1412079N276485O352235S5555P16744Mg1415Zn0Fe0	-19269.14	Translation
rib_70_elo1_b0416_4_cplx	Translation elongation complex: 4 * ribosome 70S/b0416/EF-TU-tRNA's/EF-G	C3718710H5634714N1101089O1400177S22220P65716Mg5660Zn0Fe0	-75813.56	Translation
rib_70_elo1_b0416_8_cplx	Translation elongation complex: 8 * ribosome 70S/b0416/EF-TU-tRNA's/EF-G	C7433418H11264894N2200561O2797433S44440P131012Mg11320Zn0Fe0	-151206.12	Translation
rib_70_elo1_b0423_14_cplx	Translation elongation complex: 14 * ribosome 70S/b0423/EF-TU-tRNA's/EF-G	C42417562H64689716N12369457O15520577S264250P633313Mg63028Zn0Fe0	-785761.96	Translation
rib_70_elo1_b0423_1_cplx	Translation elongation complex: 1 * ribosome 70S/b0423/EF-TU-tRNA's/EF-G	C3042629H4635176N888637O1118033S18875P46584Mg4502Zn0Fe0	-57474.14	Translation
rib_70_elo1_b0423_28_cplx	Translation elongation complex: 28 * ribosome 70S/b0423/EF-TU-tRNA's/EF-G	C84821336H129363836N24733417O31031009S528500P1265175Mg126056Zn0Fe0	-1570071.92	Translation
rib_70_elo1_b0436_12_cplx	Translation elongation complex: 12 * ribosome 70S/b0436/EF-TU-tRNA's/EF-G	C32652025H49793547N9525742O11956184S202884P489473Mg48624Zn0Fe0	-606103.68	Translation
rib_70_elo1_b0436_1_cplx	Translation elongation complex: 1 * ribosome 70S/b0436/EF-TU-tRNA's/EF-G	C2732399H4162324N798507O1004584S16907P41982Mg4052Zn0Fe0	-51702.14	Translation
rib_70_elo1_b0436_25_cplx	Translation elongation complex: 25 * ribosome 70S/b0436/EF-TU-tRNA's/EF-G	C68011583H103721356N19839747O24898984S422675P1018326Mg101300Zn0Fe0	-1261305.5	Translation
rib_70_elo1_b0503_10_cplx	Translation elongation complex: 10 * ribosome 70S/b0503/EF-TU-tRNA's/EF-G	C23068868H35150797N6741012O8474284S142880P353217Mg34400Zn0Fe0	-433209.4	Translation
rib_70_elo1_b0503_1_cplx	Translation elongation complex: 1 * ribosome 70S/b0503/EF-TU-tRNA's/EF-G	C2316290H3525715N677919O854308S14288P36309Mg3440Zn0Fe0	-44309.14	Translation
rib_70_elo1_b0503_21_cplx	Translation elongation complex: 21 * ribosome 70S/b0503/EF-TU-tRNA's/EF-G	C48433130H73803675N14151459O17787588S300048P740549Mg72240Zn0Fe0	-908531.94	Translation
rib_70_elo1_b0526_13_cplx	Translation elongation complex: 13 * ribosome 70S/b0526/EF-TU-tRNA's/EF-G	C37703168H57496571N10994509O13799873S234962P563872Mg56069Zn0Fe0	-698996.82	Translation
rib_70_elo1_b0526_1_cplx	Translation elongation complex: 1 * ribosome 70S/b0526/EF-TU-tRNA's/EF-G	C2912444H4436615N850669O1070453S18074P44656Mg4313Zn0Fe0	-55051.14	Translation
rib_70_elo1_b0526_27_cplx	Translation elongation complex: 27 * ribosome 70S/b0526/EF-TU-tRNA's/EF-G	C78292346H119399853N22828989O28650863S487998P1169624Mg116451Zn0Fe0	-1450266.78	Translation
rib_70_elo1_b0638_11_cplx	Translation elongation complex: 11 * ribosome 70S/b0638/EF-TU-tRNA's/EF-G	C14531393H22081041N4274063O5403220S88649P239257Mg21901Zn0Fe0	-284139.54	Translation
rib_70_elo1_b0638_1_cplx	Translation elongation complex: 1 * ribosome 70S/b0638/EF-TU-tRNA's/EF-G	C1326323H2013351N390643O495100S8059P22307Mg1991Zn0Fe0	-26388.14	Translation
rib_70_elo1_b0638_5_cplx	Translation elongation complex: 5 * ribosome 70S/b0638/EF-TU-tRNA's/EF-G	C6608351H10040427N1944011O2458348S40295P109087Mg9955Zn0Fe0	-129488.7	Translation
rib_70_elo1_b0639_12_cplx	Translation elongation complex: 12 * ribosome 70S/b0639/EF-TU-tRNA's/EF-G	C16586726H25211410N4874713O6158764S101220P270978Mg24972Zn0Fe0	-322856.68	Translation
rib_70_elo1_b0639_1_cplx	Translation elongation complex: 1 * ribosome 70S/b0639/EF-TU-tRNA's/EF-G	C1387817H2107285N408438O517337S8435P23170Mg2081Zn0Fe0	-27494.14	Translation
rib_70_elo1_b0639_6_cplx	Translation elongation complex: 6 * ribosome 70S/b0639/EF-TU-tRNA's/EF-G	C8296412H12609160N2438563O3081622S50610P135810Mg12486Zn0Fe0	-161749.84	Translation

rib_70_elo1_b0640_10_cplx	Translation elongation complex: 10 * ribosome 70S/b0640/EF-TU-tRNA's/EF-G	C21809497H33213911N6379598O8028359S134830P338251Mg32510Zn0Fe0	-413073.4	Translation
rib_70_elo1_b0640_1_cplx	Translation elongation complex: 1 * ribosome 70S/b0640/EF-TU-tRNA's/EF-G	C2189767H3331373N641450O809342S13483P34753Mg3251Zn0Fe0	-42236.14	Translation
rib_70_elo1_b0640_20_cplx	Translation elongation complex: 20 * ribosome 70S/b0640/EF-TU-tRNA's/EF-G	C43609197H66416731N12755318O16049489S269660P675471Mg65020Zn0Fe0	-825114.8	Translation
rib_70_elo1_b0641_11_cplx	Translation elongation complex: 11 * ribosome 70S/b0641/EF-TU-tRNA's/EF-G	C13859872H21053802N4080890O5160775S84183P230086Mg20911Zn0Fe0	-272438.54	Translation
rib_70_elo1_b0641_1_cplx	Translation elongation complex: 1 * ribosome 70S/b0641/EF-TU-tRNA's/EF-G	C1265022H1919682N373000O472855S7653P21446Mg1901Zn0Fe0	-25297.14	Translation
rib_70_elo1_b0641_5_cplx	Translation elongation complex: 5 * ribosome 70S/b0641/EF-TU-tRNA's/EF-G	C6302962H9573330N1856156O2348023S38265P104902Mg9505Zn0Fe0	-124153.7	Translation
rib_70_elo1_b0642_1_cplx	Translation elongation complex: 1 * ribosome 70S/b0642/EF-TU-tRNA's/EF-G	C5365018H8183646N1561858O1960011S33555P79185Mg7904Zn0Fe0	-99347.14	Translation
rib_70_elo1_b0642_25_cplx	Translation elongation complex: 25 * ribosome 70S/b0642/EF-TU-tRNA's/EF-G	C133534282H203922270N38807050O48568971S838875P1917585Mg197600Zn0Fe0	-2421614.5	Translation
rib_70_elo1_b0642_50_cplx	Translation elongation complex: 50 * ribosome 70S/b0642/EF-TU-tRNA's/EF-G	C267043932H407816670N77604125O97119971S1677750P3832585Mg395200Zn0Fe0	-4840643	Translation
rib_70_elo1_b0661_13_cplx	Translation elongation complex: 13 * ribosome 70S/b0661/EF-TU-tRNA's/EF-G	C38748719H59091632N11301803O14180335S241371P579238Mg57590Zn0Fe0	-718366.82	Translation
rib_70_elo1_b0661_1_cplx	Translation elongation complex: 1 * ribosome 70S/b0661/EF-TU-tRNA's/EF-G	C2993195H4559696N874403O1099975S18567P45874Mg4430Zn0Fe0	-56577.14	Translation
rib_70_elo1_b0661_27_cplx	Translation elongation complex: 27 * ribosome 70S/b0661/EF-TU-tRNA's/EF-G	C80463497H122712224N23467103O29440755S501309P1201496Mg119610Zn0Fe0	-1490454.78	Translation
rib_70_elo1_b0680_16_cplx	Translation elongation complex: 16 * ribosome 70S/b0680/EF-TU-tRNA's/EF-G	C55527899H84713716N16177023O20282055S347152P820115Mg82400Zn0Fe0	-1022486.24	Translation
rib_70_elo1_b0680_1_cplx	Translation elongation complex: 1 * ribosome 70S/b0680/EF-TU-tRNA's/EF-G	C3485354H5311426N1017018O1278525S21697P52820Mg5150Zn0Fe0	-65469.14	Translation
rib_70_elo1_b0680_32_cplx	Translation elongation complex: 32 * ribosome 70S/b0680/EF-TU-tRNA's/EF-G	C111039947H169409492N32347695O40552487S694304P1638563Mg164800Zn0Fe0	-2043304.48	Translation
rib_70_elo1_b0850_1_cplx	Translation elongation complex: 1 * ribosome 70S/b0850/EF-TU-tRNA's/EF-G	C662357H999016N198139O254229S3850P12946Mg1019Zn0Fe0	-14401.14	Translation
rib_70_elo1_b0850_2_cplx	Translation elongation complex: 2 * ribosome 70S/b0850/EF-TU-tRNA's/EF-G	C1321982H1994942N395213O506420S7700P25602Mg2038Zn0Fe0	-28511.28	Translation
rib_70_elo1_b0850_5_cplx	Translation elongation complex: 5 * ribosome 70S/b0850/EF-TU-tRNA's/EF-G	C3300857H4982720N986435O1262993S19250P63570Mg5095Zn0Fe0	-70841.7	Translation
rib_70_elo1_b0851_14_cplx	Translation elongation complex: 14 * ribosome 70S/b0851/EF-TU-tRNA's/EF-G	C21676963H32965362N6363777O8030601S132664P349236Mg32536Zn0Fe0	-419042.96	Translation
rib_70_elo1_b0851_1_cplx	Translation elongation complex: 1 * ribosome 70S/b0851/EF-TU-tRNA's/EF-G	C1554588H2361724N457032O578208S9476P25601Mg2324Zn0Fe0	-30588.14	Translation
rib_70_elo1_b0851_7_cplx	Translation elongation complex: 7 * ribosome 70S/b0851/EF-TU-tRNA's/EF-G	C10841838H16486480N3183222O4017774S66332P174971Mg16268Zn0Fe0	-209874.98	Translation
rib_70_elo1_b0852_17_cplx	Translation elongation complex: 17 * ribosome 70S/b0852/EF-TU-tRNA's/EF-G	C32541983H49553698N9526713O11990523S200311P507301Mg48688Zn0Fe0	-616988.38	Translation
rib_70_elo1_b0852_1_cplx	Translation elongation complex: 1 * ribosome 70S/b0852/EF-TU-tRNA's/EF-G	C1922335H2924082N563657O711291S11783P30693Mg2864Zn0Fe0	-37146.14	Translation
rib_70_elo1_b0852_8_cplx	Translation elongation complex: 8 * ribosome 70S/b0852/EF-TU-tRNA's/EF-G	C15318431H23324539N4484994O5645955S94264P239209Mg22912Zn0Fe0	-290827.12	Translation
rib_70_elo1_b0853_1_cplx	Translation elongation complex: 1 * ribosome 70S/b0853/EF-TU-tRNA's/EF-G	C1049857H1590890N310414O394922S6311P18425Mg1586Zn0Fe0	-21421.14	Translation
rib_70_elo1_b0853_4_cplx	Translation elongation complex: 4 * ribosome 70S/b0853/EF-TU-tRNA's/EF-G	C4185862H6348227N1236400O1569584S25244P72269Mg6344Zn0Fe0	-84250.56	Translation
rib_70_elo1_b0853_9_cplx	Translation elongation complex: 9 * ribosome 70S/b0853/EF-TU-tRNA's/EF-G	C9412537H14277122N2779710O3527354S56799P162009Mg14274Zn0Fe0	-188966.26	Translation
rib_70_elo1_b0858_1_cplx	Translation elongation complex: 1 * ribosome 70S/b0858/EF-TU-tRNA's/EF-G	C1075815H1630069N318246O404691S6464P18912Mg1622Zn0Fe0	-21993.14	Translation
rib_70_elo1_b0858_4_cplx	Translation elongation complex: 4 * ribosome 70S/b0858/EF-TU-tRNA's/EF-G	C4289361H6504556N1267611O1608336S25856P74175Mg6488Zn0Fe0	-86496.56	Translation
rib_70_elo1_b0858_9_cplx	Translation elongation complex: 9 * ribosome 70S/b0858/EF-TU-tRNA's/EF-G	C9645271H14628701N2849886O3614411S58176P166280Mg14598Zn0Fe0	-194002.26	Translation
rib_70_elo1_b0859_11_cplx	Translation elongation complex: 11 * ribosome 70S/b0859/EF-TU-tRNA's/EF-G	C26119164H39801114N7631302O9589804S162206P398767Mg38929Zn0Fe0	-489717.54	Translation

rib_70_elo1_b0859_1_cplx	Translation elongation complex: 1 * ribosome 70S/b0859/EF-TU-tRNA's/EF-G	C2384214H3629324N697622O878994S14746P37277Mg3539Zn0Fe0	-45546.14	Translation
rib_70_elo1_b0859_22_cplx	Translation elongation complex: 22 * ribosome 70S/b0859/EF-TU-tRNA's/EF-G	C52227609H79590083N15258350O19171695S324412P796406Mg77858Zn0Fe0	-978306.08	Translation
rib_70_elo1_b0884_1_cplx	Translation elongation complex: 1 * ribosome 70S/b0884/EF-TU-tRNA's/EF-G	C519934H781870N156655O202167S2959P10836Mg812Zn0Fe0	-11731.14	Translation
rib_70_elo1_b0884_2_cplx	Translation elongation complex: 2 * ribosome 70S/b0884/EF-TU-tRNA's/EF-G	C1037781H1561378N312469O402811S5918P21451Mg1624Zn0Fe0	-23240.28	Translation
rib_70_elo1_b0884_4_cplx	Translation elongation complex: 4 * ribosome 70S/b0884/EF-TU-tRNA's/EF-G	C2073475H3120394N624097O804099S11836P42681Mg3248Zn0Fe0	-46258.56	Translation
rib_70_elo1_b0893_12_cplx	Translation elongation complex: 12 * ribosome 70S/b0893/EF-TU-tRNA's/EF-G	C32533761H49600539N9494222O11923140S202116P490487Mg48408Zn0Fe0	-606049.68	Translation
rib_70_elo1_b0893_1_cplx	Translation elongation complex: 1 * ribosome 70S/b0893/EF-TU-tRNA's/EF-G	C2722441H4146152N795730O1001867S16843P42061Mg4034Zn0Fe0	-51692.14	Translation
rib_70_elo1_b0893_25_cplx	Translation elongation complex: 25 * ribosome 70S/b0893/EF-TU-tRNA's/EF-G	C67765321H103319360N19774258O24830099S421075P1020445Mg100850Zn0Fe0	-1261199.5	Translation
rib_70_elo1_b0910_13_cplx	Translation elongation complex: 13 * ribosome 70S/b0910/EF-TU-tRNA's/EF-G	C19081320H29014616N5605927O7074211S116506P308864Mg28691Zn0Fe0	-369706.82	Translation
rib_70_elo1_b0910_1_cplx	Translation elongation complex: 1 * ribosome 70S/b0910/EF-TU-tRNA's/EF-G	C1473804H2238692N433627O548599S8962P24392Mg2207Zn0Fe0	-29073.14	Translation
rib_70_elo1_b0910_6_cplx	Translation elongation complex: 6 * ribosome 70S/b0910/EF-TU-tRNA's/EF-G	C8810269H13395327N2588752O3267604S53772P142922Mg13242Zn0Fe0	-171003.84	Translation
rib_70_elo1_b0911_16_cplx	Translation elongation complex: 16 * ribosome 70S/b0911/EF-TU-tRNA's/EF-G	C55783269H85120254N16248307O20369950S347616P821052Mg82832Zn0Fe0	-1025423.24	Translation
rib_70_elo1_b0911_1_cplx	Translation elongation complex: 1 * ribosome 70S/b0911/EF-TU-tRNA's/EF-G	C3501429H5336934N1021582O1284040S21726P52887Mg5177Zn0Fe0	-65661.14	Translation
rib_70_elo1_b0911_32_cplx	Translation elongation complex: 32 * ribosome 70S/b0911/EF-TU-tRNA's/EF-G	C111550565H170222462N32490147O40728254S695232P1640428Mg165664Zn0Fe0	-2049169.48	Translation
rib_70_elo1_b0912_1_cplx	Translation elongation complex: 1 * ribosome 70S/b0912/EF-TU-tRNA's/EF-G	C655167H988476N195884O251204S3811P12744Mg1010Zn0Fe0	-14167.14	Translation
rib_70_elo1_b0912_2_cplx	Translation elongation complex: 2 * ribosome 70S/b0912/EF-TU-tRNA's/EF-G	C1307615H1973877N390666O500435S7622P25203Mg2020Zn0Fe0	-28048.28	Translation
rib_70_elo1_b0912_5_cplx	Translation elongation complex: 5 * ribosome 70S/b0912/EF-TU-tRNA's/EF-G	C3264959H4930080N975012O1248128S19055P62580Mg5050Zn0Fe0	-69691.7	Translation
rib_70_elo1_b0930_13_cplx	Translation elongation complex: 13 * ribosome 70S/b0930/EF-TU-tRNA's/EF-G	C38105963H58109776N11114110O13945817S237458P569893Mg56654Zn0Fe0	-706473.82	Translation
rib_70_elo1_b0930_1_cplx	Translation elongation complex: 1 * ribosome 70S/b0930/EF-TU-tRNA's/EF-G	C2943515H4483888N859786O1081841S18266P45133Mg4358Zn0Fe0	-55640.14	Translation
rib_70_elo1_b0930_27_cplx	Translation elongation complex: 27 * ribosome 70S/b0930/EF-TU-tRNA's/EF-G	C79128819H120673312N23077488O28953789S493182P1182113Mg117666Zn0Fe0	-1465779.78	Translation
rib_70_elo1_b0969_1_cplx	Translation elongation complex: 1 * ribosome 70S/b0969/EF-TU-tRNA's/EF-G	C747824H1129818N222836O284936S4406P14088Mg1145Zn0Fe0	-15887.14	Translation
rib_70_elo1_b0969_3_cplx	Translation elongation complex: 3 * ribosome 70S/b0969/EF-TU-tRNA's/EF-G	C2237156H3382348N665932O850214S13218P41600Mg3435Zn0Fe0	-46995.42	Translation
rib_70_elo1_b0969_6_cplx	Translation elongation complex: 6 * ribosome 70S/b0969/EF-TU-tRNA's/EF-G	C4471154H6761143N1330576O1698131S26436P82868Mg6870Zn0Fe0	-93657.84	Translation
rib_70_elo1_b1066_11_cplx	Translation elongation complex: 11 * ribosome 70S/b1066/EF-TU-tRNA's/EF-G	C13923639H21152751N4096821O5181709S84909P230564Mg21010Zn0Fe0	-272916.54	Translation
rib_70_elo1_b1066_1_cplx	Translation elongation complex: 1 * ribosome 70S/b1066/EF-TU-tRNA's/EF-G	C1270839H1928691N374431O474799S7719P21494Mg1910Zn0Fe0	-25345.14	Translation
rib_70_elo1_b1066_5_cplx	Translation elongation complex: 5 * ribosome 70S/b1066/EF-TU-tRNA's/EF-G	C6331959H9618315N1863387O2357563S38595P105122Mg9550Zn0Fe0	-124373.7	Translation
rib_70_elo1_b1084_1_cplx	Translation elongation complex: 1 * ribosome 70S/b1084/EF-TU-tRNA's/EF-G	C6599892H10070393N1921288O2407640S41318P96631Mg9713Zn0Fe0	-121674.14	Translation
rib_70_elo1_b1084_31_cplx	Translation elongation complex: 31 * ribosome 70S/b1084/EF-TU-tRNA's/EF-G	C203685912H311149223N59190598O73973060S1280858P2899921Mg301103Zn0Fe0	-3676228.34	Translation
rib_70_elo1_b1084_62_cplx	Translation elongation complex: 62 * ribosome 70S/b1084/EF-TU-tRNA's/EF-G	C407341466H622264014N118368885O147923994S2561716P5796654Mg602206Zn0Fe0	-7349267.68	Translation
rib_70_elo1_b1086_18_cplx	Translation elongation complex: 18 * ribosome 70S/b1086/EF-TU-tRNA's/EF-G	C36558830H55682627N10697252O13455661S225864P566666Mg54630Zn0Fe0	-690887.52	Translation
rib_70_elo1_b1086_1_cplx	Translation elongation complex: 1 * ribosome 70S/b1086/EF-TU-tRNA's/EF-G	C2039701H3103242N597790O753873S12548P32390Mg3035Zn0Fe0	-39292.14	Translation

rib_70_elo1_b1086_9_cplx	Translation elongation complex: 9 * ribosome 70S/b1086/EF-TU-tRNA's/EF-G	C18283997H27846482N5350478O6731185S112932P283814Mg27315Zn0Fe0	-345925.26	Translation
rib_70_elo1_b1088_10_cplx	Translation elongation complex: 10 * ribosome 70S/b1088/EF-TU-tRNA's/EF-G	C11368290H17258504N3352051O4244336S69000P191754Mg17210Zn0Fe0	-225416.4	Translation
rib_70_elo1_b1088_1_cplx	Translation elongation complex: 1 * ribosome 70S/b1088/EF-TU-tRNA's/EF-G	C1141302H1730903N336988O427724S6900P19647Mg1721Zn0Fe0	-23014.14	Translation
rib_70_elo1_b1088_5_cplx	Translation elongation complex: 5 * ribosome 70S/b1088/EF-TU-tRNA's/EF-G	C5686630H8632059N1677016O2123996S34500P96139Mg8605Zn0Fe0	-112970.7	Translation
rib_70_elo1_b1089_1_cplx	Translation elongation complex: 1 * ribosome 70S/b1089/EF-TU-tRNA's/EF-G	C427736H641012N129911O168773S2371P9543Mg677Zn0Fe0	-10060.14	Translation
rib_70_elo1_b1089_3_cplx	Translation elongation complex: 3 * ribosome 70S/b1089/EF-TU-tRNA's/EF-G	C1279904H1919270N388409O503913S7113P28281Mg2031Zn0Fe0	-29830.42	Translation
rib_70_elo1_b1090_10_cplx	Translation elongation complex: 10 * ribosome 70S/b1090/EF-TU-tRNA's/EF-G	C22598008H34422649N6608848O8311227S139740P348463Mg33680Zn0Fe0	-426545.4	Translation
rib_70_elo1_b1090_1_cplx	Translation elongation complex: 1 * ribosome 70S/b1090/EF-TU-tRNA's/EF-G	C2268979H3452632N664546O837888S13974P35812Mg3368Zn0Fe0	-43621.14	Translation
rib_70_elo1_b1090_20_cplx	Translation elongation complex: 20 * ribosome 70S/b1090/EF-TU-tRNA's/EF-G	C45185818H68833779N13213628O16614937S279480P695853Mg67360Zn0Fe0	-852016.8	Translation
rib_70_elo1_b1091_18_cplx	Translation elongation complex: 18 * ribosome 70S/b1091/EF-TU-tRNA's/EF-G	C36327260H55327491N10628454O13372875S223848P562682Mg54306Zn0Fe0	-686813.52	Translation
rib_70_elo1_b1091_1_cplx	Translation elongation complex: 1 * ribosome 70S/b1091/EF-TU-tRNA's/EF-G	C2026751H3083448N593881O749270S12436P32163Mg3017Zn0Fe0	-39060.14	Translation
rib_70_elo1_b1091_9_cplx	Translation elongation complex: 9 * ribosome 70S/b1091/EF-TU-tRNA's/EF-G	C18168167H27668880N5316033O6689790S111924P281819Mg27153Zn0Fe0	-343885.26	Translation
rib_70_elo1_b1092_18_cplx	Translation elongation complex: 18 * ribosome 70S/b1092/EF-TU-tRNA's/EF-G	C35429645H53960508N10365873O13043604S218466P549212Mg53010Zn0Fe0	-669851.52	Translation
rib_70_elo1_b1092_1_cplx	Translation elongation complex: 1 * ribosome 70S/b1092/EF-TU-tRNA's/EF-G	C1976688H3007275N579262O730793S12137P31392Mg2945Zn0Fe0	-38095.14	Translation
rib_70_elo1_b1092_9_cplx	Translation elongation complex: 9 * ribosome 70S/b1092/EF-TU-tRNA's/EF-G	C17719256H26985267N5184726O6525057S109233P275072Mg26505Zn0Fe0	-335392.26	Translation
rib_70_elo1_b1093_14_cplx	Translation elongation complex: 14 * ribosome 70S/b1093/EF-TU-tRNA's/EF-G	C21995019H33461752N6453505O8138626S134428P351367Mg33040Zn0Fe0	-422965.96	Translation
rib_70_elo1_b1093_1_cplx	Translation elongation complex: 1 * ribosome 70S/b1093/EF-TU-tRNA's/EF-G	C1577583H2397472N463586O586107S9602P25782Mg2360Zn0Fe0	-30897.14	Translation
rib_70_elo1_b1093_7_cplx	Translation elongation complex: 7 * ribosome 70S/b1093/EF-TU-tRNA's/EF-G	C11001015H16734832N3228164O4071885S67214P176052Mg16520Zn0Fe0	-211851.98	Translation
rib_70_elo1_b1114_1_cplx	Translation elongation complex: 1 * ribosome 70S/b1114/EF-TU-tRNA's/EF-G	C7141768H10895725N2078501O2606778S44695P104870Mg10496Zn0Fe0	-131978.14	Translation
rib_70_elo1_b1114_33_cplx	Translation elongation complex: 33 * ribosome 70S/b1114/EF-TU-tRNA's/EF-G	C234628328H358369613N68169125O85253082S1474935P3350342Mg346368Zn0Fe0	-4244878.62	Translation
rib_70_elo1_b1114_67_cplx	Translation elongation complex: 67 * ribosome 70S/b1114/EF-TU-tRNA's/EF-G	C476332798H727560619N138390413O173064780S2994565P6798656Mg703232Zn0Fe0	-8614835.38	Translation
rib_70_elo1_b1133_10_cplx	Translation elongation complex: 10 * ribosome 70S/b1133/EF-TU-tRNA's/EF-G	C23307487H35518628N6808409O8558794S144430P355749Mg34760Zn0Fe0	-436681.4	Translation
rib_70_elo1_b1133_1_cplx	Translation elongation complex: 1 * ribosome 70S/b1133/EF-TU-tRNA's/EF-G	C2340232H3562598N684638O862849S14443P36573Mg3476Zn0Fe0	-44667.14	Translation
rib_70_elo1_b1133_21_cplx	Translation elongation complex: 21 * ribosome 70S/b1133/EF-TU-tRNA's/EF-G	C48934132H74575998N14293018O17964949S303303P745853Mg72996Zn0Fe0	-915809.94	Translation
rib_70_elo1_b1134_1_cplx	Translation elongation complex: 1 * ribosome 70S/b1134/EF-TU-tRNA's/EF-G	C1017811H1542458N301046O382707S6126P17849Mg1541Zn0Fe0	-20716.14	Translation
rib_70_elo1_b1134_4_cplx	Translation elongation complex: 4 * ribosome 70S/b1134/EF-TU-tRNA's/EF-G	C4058062H6154934N1198961O1521111S24504P70010Mg6164Zn0Fe0	-81475.56	Translation
rib_70_elo1_b1134_9_cplx	Translation elongation complex: 9 * ribosome 70S/b1134/EF-TU-tRNA's/EF-G	C9125147H13842394N2695486O3418451S55134P156945Mg13869Zn0Fe0	-182741.26	Translation
rib_70_elo1_b1135_12_cplx	Translation elongation complex: 12 * ribosome 70S/b1135/EF-TU-tRNA's/EF-G	C16875810H25656329N4959292O6261022S103116P274460Mg25404Zn0Fe0	-327430.68	Translation
rib_70_elo1_b1135_1_cplx	Translation elongation complex: 1 * ribosome 70S/b1135/EF-TU-tRNA's/EF-G	C1412021H2144489N415555O525930S8593P23473Mg2117Zn0Fe0	-27888.14	Translation
rib_70_elo1_b1135_6_cplx	Translation elongation complex: 6 * ribosome 70S/b1135/EF-TU-tRNA's/EF-G	C8441016H12831689N2480890O3132790S51558P137558Mg12702Zn0Fe0	-164043.84	Translation
rib_70_elo1_b1211_10_cplx	Translation elongation complex: 10 * ribosome 70S/b1211/EF-TU-tRNA's/EF-G	C22823211H34776131N6671084O8385195S141380P349675Mg34040Zn0Fe0	-428827.4	Translation

rib_70_elo1_b1211_1_cplx	Translation elongation complex: 1 * ribosome 70S/b1211/EF-TU-tRNA's/EF-G	C2291610H3488126N670856O845310S14138P35944Mg3404Zn0Fe0	-43860.14	Translation
rib_70_elo1_b1211_21_cplx	Translation elongation complex: 21 * ribosome 70S/b1211/EF-TU-tRNA's/EF-G	C47917390H73017026N14004696O17600610S296898P733124Mg71484Zn0Fe0	-899342.94	Translation
rib_70_elo1_b1212_16_cplx	Translation elongation complex: 16 * ribosome 70S/b1212/EF-TU-tRNA's/EF-G	C28383784H43201462N8317441O10477569S174592P447652Mg42512Zn0Fe0	-541831.24	Translation
rib_70_elo1_b1212_1_cplx	Translation elongation complex: 1 * ribosome 70S/b1212/EF-TU-tRNA's/EF-G	C1781419H2708512N522796O660339S10912P28762Mg2657Zn0Fe0	-34649.14	Translation
rib_70_elo1_b1212_8_cplx	Translation elongation complex: 8 * ribosome 70S/b1212/EF-TU-tRNA's/EF-G	C14195856H21605222N4160297O5241713S87296P224244Mg21256Zn0Fe0	-271334.12	Translation
rib_70_elo1_b1229_1_cplx	Translation elongation complex: 1 * ribosome 70S/b1229/EF-TU-tRNA's/EF-G	C279749H415074N87027O114870S1452P7419Mg461Zn0Fe0	-7351.14	Translation
rib_70_elo1_b1269_17_cplx	Translation elongation complex: 17 * ribosome 70S/b1269/EF-TU-tRNA's/EF-G	C31610433H48128154N9259654O11653981S194667P495017Mg47311Zn0Fe0	-600607.38	Translation
rib_70_elo1_b1269_1_cplx	Translation elongation complex: 1 * ribosome 70S/b1269/EF-TU-tRNA's/EF-G	C1867297H2839946N547846O691309S11451P29945Mg2783Zn0Fe0	-36157.14	Translation
rib_70_elo1_b1269_8_cplx	Translation elongation complex: 8 * ribosome 70S/b1269/EF-TU-tRNA's/EF-G	C14879919H22653537N4359262O5487478S91608P233414Mg22264Zn0Fe0	-283104.12	Translation
rib_70_elo1_b1286_18_cplx	Translation elongation complex: 18 * ribosome 70S/b1286/EF-TU-tRNA's/EF-G	C72361241H110442986N21064199O26385981S452538P1056611Mg107280Zn0Fe0	-1323788.52	Translation
rib_70_elo1_b1286_1_cplx	Translation elongation complex: 1 * ribosome 70S/b1286/EF-TU-tRNA's/EF-G	C4037442H6155415N1177157O1478652S25141P60530Mg5960Zn0Fe0	-75374.14	Translation
rib_70_elo1_b1286_37_cplx	Translation elongation complex: 37 * ribosome 70S/b1286/EF-TU-tRNA's/EF-G	C148723134H226999683N43290893O54223584S930217P2169878Mg220520Zn0Fe0	-2719075.18	Translation
rib_70_elo1_b1344_18_cplx	Translation elongation complex: 18 * ribosome 70S/b1344/EF-TU-tRNA's/EF-G	C35669502H54325020N10434827O13131584S220698P553394Mg53334Zn0Fe0	-674429.52	Translation
rib_70_elo1_b1344_1_cplx	Translation elongation complex: 1 * ribosome 70S/b1344/EF-TU-tRNA's/EF-G	C1990071H3027571N583106O735694S12261P31630Mg2963Zn0Fe0	-38355.14	Translation
rib_70_elo1_b1344_9_cplx	Translation elongation complex: 9 * ribosome 70S/b1344/EF-TU-tRNA's/EF-G	C17839215H27167547N5219210O6569054S110349P277166Mg26667Zn0Fe0	-337684.26	Translation
rib_70_elo1_b1427_10_cplx	Translation elongation complex: 10 * ribosome 70S/b1427/EF-TU-tRNA's/EF-G	C11738678H17824298N3458233O4378787S71330P196942Mg17750Zn0Fe0	-231974.4	Translation
rib_70_elo1_b1427_1_cplx	Translation elongation complex: 1 * ribosome 70S/b1427/EF-TU-tRNA's/EF-G	C1178501H1787648N347671O441269S7133P20182Mg1775Zn0Fe0	-23686.14	Translation
rib_70_elo1_b1427_5_cplx	Translation elongation complex: 5 * ribosome 70S/b1427/EF-TU-tRNA's/EF-G	C5871913H8915048N1730143O2191277S35665P98742Mg8875Zn0Fe0	-116258.7	Translation
rib_70_elo1_b1480_1_cplx	Translation elongation complex: 1 * ribosome 70S/b1480/EF-TU-tRNA's/EF-G	C353734H528025N108416O141833S1909P8475Mg569Zn0Fe0	-8707.14	Translation
rib_70_elo1_b1480_2_cplx	Translation elongation complex: 2 * ribosome 70S/b1480/EF-TU-tRNA's/EF-G	C706147H1054556N216285O282726S3818P16812Mg1138Zn0Fe0	-17275.28	Translation
rib_70_elo1_b1481_1_cplx	Translation elongation complex: 1 * ribosome 70S/b1481/EF-TU-tRNA's/EF-G	C513952H772580N154902O200116S2918P10773Mg803Zn0Fe0	-11651.14	Translation
rib_70_elo1_b1481_2_cplx	Translation elongation complex: 2 * ribosome 70S/b1481/EF-TU-tRNA's/EF-G	C1025850H1542839N308992O398719S5836P21328Mg1606Zn0Fe0	-23083.28	Translation
rib_70_elo1_b1481_4_cplx	Translation elongation complex: 4 * ribosome 70S/b1481/EF-TU-tRNA's/EF-G	C2049646H3083357N617172O795925S11672P42438Mg3212Zn0Fe0	-45947.56	Translation
rib_70_elo1_b1636_16_cplx	Translation elongation complex: 16 * ribosome 70S/b1636/EF-TU-tRNA's/EF-G	C29353638H44688583N8595058O10825231S180864P459952Mg43952Zn0Fe0	-558307.24	Translation
rib_70_elo1_b1636_1_cplx	Translation elongation complex: 1 * ribosome 70S/b1636/EF-TU-tRNA's/EF-G	C1842318H2801758N540283O682246S11304P29557Mg2747Zn0Fe0	-35705.14	Translation
rib_70_elo1_b1636_8_cplx	Translation elongation complex: 8 * ribosome 70S/b1636/EF-TU-tRNA's/EF-G	C14680934H22348943N4299178O5415639S90432P230408Mg21976Zn0Fe0	-279586.12	Translation
rib_70_elo1_b1637_12_cplx	Translation elongation complex: 12 * ribosome 70S/b1637/EF-TU-tRNA's/EF-G	C32093308H48925622N9366063O11762726S199584P484277Mg47760Zn0Fe0	-597943.68	Translation
rib_70_elo1_b1637_1_cplx	Translation elongation complex: 1 * ribosome 70S/b1637/EF-TU-tRNA's/EF-G	C2685578H4089721N784974O988391S16632P41527Mg3980Zn0Fe0	-51000.14	Translation
rib_70_elo1_b1637_24_cplx	Translation elongation complex: 24 * ribosome 70S/b1637/EF-TU-tRNA's/EF-G	C64174468H97837514N18727251O23516546S399168P967277Mg95520Zn0Fe0	-1194609.36	Translation
rib_70_elo1_b1638_12_cplx	Translation elongation complex: 12 * ribosome 70S/b1638/EF-TU-tRNA's/EF-G	C16961306H25782695N4985192O6296164S103752P276743Mg25512Zn0Fe0	-329905.68	Translation
rib_70_elo1_b1638_1_cplx	Translation elongation complex: 1 * ribosome 70S/b1638/EF-TU-tRNA's/EF-G	C1419164H2155036N417695O528908S8646P23666Mg2126Zn0Fe0	-28097.14	Translation

rib_70_elo1_b1638_6_cplx	Translation elongation complex: 6 * ribosome 70S/b1638/EF-TU-tRNA's/EF-G	C8483774H12894881N2493830O3150388S51876P138701Mg12756Zn0Fe0	-165282.84	Translation
rib_70_elo1_b1652_12_cplx	Translation elongation complex: 12 * ribosome 70S/b1652/EF-TU-tRNA's/EF-G	C16720600H25420787N4912458O6203978S102096P271730Mg25188Zn0Fe0	-324316.68	Translation
rib_70_elo1_b1652_1_cplx	Translation elongation complex: 1 * ribosome 70S/b1652/EF-TU-tRNA's/EF-G	C1399019H2124800N411599O521158S8508P23240Mg2099Zn0Fe0	-27623.14	Translation
rib_70_elo1_b1652_6_cplx	Translation elongation complex: 6 * ribosome 70S/b1652/EF-TU-tRNA's/EF-G	C8363374H12713885N2457444O3104258S51048P136190Mg12594Zn0Fe0	-162483.84	Translation
rib_70_elo1_b1653_1_cplx	Translation elongation complex: 1 * ribosome 70S/b1653/EF-TU-tRNA's/EF-G	C9544833H14564664N2777201O3480575S59795P139319Mg14006Zn0Fe0	-175886.14	Translation
rib_70_elo1_b1653_45_cplx	Translation elongation complex: 45 * ribosome 70S/b1653/EF-TU-tRNA's/EF-G	C427587381H653220132N124205981O155201507S2690775P6066207Mg630270Zn0Fe0	-7711684.3	Translation
rib_70_elo1_b1653_90_cplx	Translation elongation complex: 90 * ribosome 70S/b1653/EF-TU-tRNA's/EF-G	C855130896H1306390497N248394506O310370642S5381550P12127797Mg1260540Zn0Fe0	-15418750.6	Translation
rib_70_elo1_b1712_1_cplx	Translation elongation complex: 1 * ribosome 70S/b1712/EF-TU-tRNA's/EF-G	C685976H1035527N204856O262434S4011P13189Mg1055Zn0Fe0	-14734.14	Translation
rib_70_elo1_b1712_2_cplx	Translation elongation complex: 2 * ribosome 70S/b1712/EF-TU-tRNA's/EF-G	C1369082H2067816N408535O522785S8022P26076Mg2110Zn0Fe0	-29165.28	Translation
rib_70_elo1_b1712_5_cplx	Translation elongation complex: 5 * ribosome 70S/b1712/EF-TU-tRNA's/EF-G	C3418400H5164683N1019572O1303838S20055P64737Mg5275Zn0Fe0	-72458.7	Translation
rib_70_elo1_b1713_1_cplx	Translation elongation complex: 1 * ribosome 70S/b1713/EF-TU-tRNA's/EF-G	C4967273H7575204N1447228O1816762S30984P73812Mg7319Zn0Fe0	-92376.14	Translation
rib_70_elo1_b1713_23_cplx	Translation elongation complex: 23 * ribosome 70S/b1713/EF-TU-tRNA's/EF-G	C113746669H173663588N33084878O41418104S712632P1645140Mg168337Zn0Fe0	-2072093.22	Translation
rib_70_elo1_b1713_46_cplx	Translation elongation complex: 46 * ribosome 70S/b1713/EF-TU-tRNA's/EF-G	C227470583H347301444N66160603O82819507S1425264P3287892Mg336674Zn0Fe0	-4141797.44	Translation
rib_70_elo1_b1714_19_cplx	Translation elongation complex: 19 * ribosome 70S/b1714/EF-TU-tRNA's/EF-G	C39509622H60184316N11553816O14533176S244302P609573Mg59033Zn0Fe0	-744742.66	Translation
rib_70_elo1_b1714_1_cplx	Translation elongation complex: 1 * ribosome 70S/b1714/EF-TU-tRNA's/EF-G	C2088306H3177632N611598O771438S12858P33015Mg3107Zn0Fe0	-40130.14	Translation
rib_70_elo1_b1714_9_cplx	Translation elongation complex: 9 * ribosome 70S/b1714/EF-TU-tRNA's/EF-G	C18720002H28513936N5474806O6887766S115722P289263Mg27963Zn0Fe0	-353291.26	Translation
rib_70_elo1_b1715_1_cplx	Translation elongation complex: 1 * ribosome 70S/b1715/EF-TU-tRNA's/EF-G	C163117H236793N53093O72647S714P5784Mg290Zn0Fe0	-5261.14	Translation
rib_70_elo1_b1716_1_cplx	Translation elongation complex: 1 * ribosome 70S/b1716/EF-TU-tRNA's/EF-G	C802459H1213634N238593O304528S4736P14790Mg1226Zn0Fe0	-16792.14	Translation
rib_70_elo1_b1716_3_cplx	Translation elongation complex: 3 * ribosome 70S/b1716/EF-TU-tRNA's/EF-G	C2400585H3633222N713081O908602S14208P43656Mg3678Zn0Fe0	-49660.42	Translation
rib_70_elo1_b1716_6_cplx	Translation elongation complex: 6 * ribosome 70S/b1716/EF-TU-tRNA's/EF-G	C4797774H7262604N1424813O1814713S28416P86955Mg7356Zn0Fe0	-98962.84	Translation
rib_70_elo1_b1717_1_cplx	Translation elongation complex: 1 * ribosome 70S/b1717/EF-TU-tRNA's/EF-G	C476528H715741N143960O186354S2679P10192Mg749Zn0Fe0	-10902.14	Translation
rib_70_elo1_b1717_3_cplx	Translation elongation complex: 3 * ribosome 70S/b1717/EF-TU-tRNA's/EF-G	C1425806H2142941N430340O556334S8037P30180Mg2247Zn0Fe0	-32308.42	Translation
rib_70_elo1_b1718_10_cplx	Translation elongation complex: 10 * ribosome 70S/b1718/EF-TU-tRNA's/EF-G	C11791283H17909384N3473421O4395483S71530P197023Mg17840Zn0Fe0	-232205.4	Translation
rib_70_elo1_b1718_1_cplx	Translation elongation complex: 1 * ribosome 70S/b1718/EF-TU-tRNA's/EF-G	C1183811H1796216N349278O442917S7153P20191Mg1784Zn0Fe0	-23710.14	Translation
rib_70_elo1_b1718_5_cplx	Translation elongation complex: 5 * ribosome 70S/b1718/EF-TU-tRNA's/EF-G	C5898243H8957624N1737786O2199613S35765P98783Mg8920Zn0Fe0	-116374.7	Translation
rib_70_elo1_b1719_18_cplx	Translation elongation complex: 18 * ribosome 70S/b1719/EF-TU-tRNA's/EF-G	C72168954H110140338N21004847O26322425S452610P1055903Mg106956Zn0Fe0	-1322162.52	Translation
rib_70_elo1_b1719_1_cplx	Translation elongation complex: 1 * ribosome 70S/b1719/EF-TU-tRNA's/EF-G	C4026749H6138520N1173905O1475055S25145P60485Mg5942Zn0Fe0	-75278.14	Translation
rib_70_elo1_b1719_37_cplx	Translation elongation complex: 37 * ribosome 70S/b1719/EF-TU-tRNA's/EF-G	C148327889H226377664N43168841O54093015S930365P2168429Mg219854Zn0Fe0	-2715739.18	Translation
rib_70_elo1_b1804_11_cplx	Translation elongation complex: 11 * ribosome 70S/b1804/EF-TU-tRNA's/EF-G	C26141132H39826946N7640724O9604954S162008P401035Mg38929Zn0Fe0	-491941.54	Translation
rib_70_elo1_b1804_1_cplx	Translation elongation complex: 1 * ribosome 70S/b1804/EF-TU-tRNA's/EF-G	C2386222H3631676N698504O880364S14728P37485Mg3539Zn0Fe0	-45750.14	Translation
rib_70_elo1_b1804_22_cplx	Translation elongation complex: 22 * ribosome 70S/b1804/EF-TU-tRNA's/EF-G	C52271533H79641743N15277166O19202003S324016P800940Mg77858Zn0Fe0	-982752.08	Translation

rib_70_elo1_b1822_15_cplx	Translation elongation complex: 15 * ribosome 70S/b1822/EF-TU-tRNA's/EF-G	C25871663H39375026N7581086O9552538S159495P408872Mg38775Zn0Fe0	-494030.1	Translation
rib_70_elo1_b1822_1_cplx	Translation elongation complex: 1 * ribosome 70S/b1822/EF-TU-tRNA's/EF-G	C1731967H2633132N508258O642140S10633P28016Mg2585Zn0Fe0	-33694.14	Translation
rib_70_elo1_b1822_7_cplx	Translation elongation complex: 7 * ribosome 70S/b1822/EF-TU-tRNA's/EF-G	C12077551H18379658N3539470O4460882S74431P191240Mg18095Zn0Fe0	-230980.98	Translation
rib_70_elo1_b1866_17_cplx	Translation elongation complex: 17 * ribosome 70S/b1866/EF-TU-tRNA's/EF-G	C62729944H95721808N18267678O22898008S391714P921815Mg93058Zn0Fe0	-1151964.38	Translation
rib_70_elo1_b1866_1_cplx	Translation elongation complex: 1 * ribosome 70S/b1866/EF-TU-tRNA's/EF-G	C3705880H5648672N1080926O1358616S23042P55895Mg5474Zn0Fe0	-69434.14	Translation
rib_70_elo1_b1866_34_cplx	Translation elongation complex: 34 * ribosome 70S/b1866/EF-TU-tRNA's/EF-G	C125443012H191424515N36528602O45783612S783428P1841855Mg186116Zn0Fe0	-2302152.76	Translation
rib_70_elo1_b1869_1_cplx	Translation elongation complex: 1 * ribosome 70S/b1869/EF-TU-tRNA's/EF-G	C884403H1337983N262554O334946S5270P16132Mg1343Zn0Fe0	-18455.14	Translation
rib_70_elo1_b1869_3_cplx	Translation elongation complex: 3 * ribosome 70S/b1869/EF-TU-tRNA's/EF-G	C2645717H4005491N784814O999158S15810P47600Mg4029Zn0Fe0	-54567.42	Translation
rib_70_elo1_b1869_7_cplx	Translation elongation complex: 7 * ribosome 70S/b1869/EF-TU-tRNA's/EF-G	C6168345H9340507N1829334O2327582S36890P110536Mg9401Zn0Fe0	-126791.98	Translation
rib_70_elo1_b1870_14_cplx	Translation elongation complex: 14 * ribosome 70S/b1870/EF-TU-tRNA's/EF-G	C22269330H33875059N6534320O8240918S136626P356512Mg33418Zn0Fe0	-428754.96	Translation
rib_70_elo1_b1870_1_cplx	Translation elongation complex: 1 * ribosome 70S/b1870/EF-TU-tRNA's/EF-G	C1597224H2427071N469313O593486S9759P26156Mg2387Zn0Fe0	-31317.14	Translation
rib_70_elo1_b1870_7_cplx	Translation elongation complex: 7 * ribosome 70S/b1870/EF-TU-tRNA's/EF-G	C11138196H16941527N3268547O4123070S68313P178628Mg16709Zn0Fe0	-214749.98	Translation
rib_70_elo1_b1871_19_cplx	Translation elongation complex: 19 * ribosome 70S/b1871/EF-TU-tRNA's/EF-G	C39071326H59504009N11429268O14383482S241547P606023Mg58349Zn0Fe0	-739083.66	Translation
rib_70_elo1_b1871_1_cplx	Translation elongation complex: 1 * ribosome 70S/b1871/EF-TU-tRNA's/EF-G	C2065108H3141653N605004O763458S12713P32813Mg3071Zn0Fe0	-39817.14	Translation
rib_70_elo1_b1871_9_cplx	Translation elongation complex: 9 * ribosome 70S/b1871/EF-TU-tRNA's/EF-G	C18512316H28191589N5415788O6816802S114417P287573Mg27639Zn0Fe0	-350602.26	Translation
rib_70_elo1_b1876_16_cplx	Translation elongation complex: 16 * ribosome 70S/b1876/EF-TU-tRNA's/EF-G	C57770971H88147686N16824351O21094673S360512P850792Mg85712Zn0Fe0	-1062507.24	Translation
rib_70_elo1_b1876_1_cplx	Translation elongation complex: 1 * ribosome 70S/b1876/EF-TU-tRNA's/EF-G	C3626191H5526771N1057806O1329728S22532P54802Mg5357Zn0Fe0	-68035.14	Translation
rib_70_elo1_b1876_33_cplx	Translation elongation complex: 33 * ribosome 70S/b1876/EF-TU-tRNA's/EF-G	C119135055H181784723N34693102O43494944S743556P1752914Mg176781Zn0Fe0	-2189575.62	Translation
rib_70_elo1_b1920_15_cplx	Translation elongation complex: 15 * ribosome 70S/b1920/EF-TU-tRNA's/EF-G	C25593391H38951555N7499190O9455960S157005P405051Mg38370Zn0Fe0	-489504.1	Translation
rib_70_elo1_b1920_1_cplx	Translation elongation complex: 1 * ribosome 70S/b1920/EF-TU-tRNA's/EF-G	C1713381H2604825N502872O635596S10467P27751Mg2558Zn0Fe0	-33382.14	Translation
rib_70_elo1_b1920_7_cplx	Translation elongation complex: 7 * ribosome 70S/b1920/EF-TU-tRNA's/EF-G	C11947671H18181995N3501294O4415752S73269P189451Mg17906Zn0Fe0	-228862.98	Translation
rib_70_elo1_b1921_10_cplx	Translation elongation complex: 10 * ribosome 70S/b1921/EF-TU-tRNA's/EF-G	C11993700H18210921N3533758O4474284S72950P201342Mg18110Zn0Fe0	-237164.4	Translation
rib_70_elo1_b1921_1_cplx	Translation elongation complex: 1 * ribosome 70S/b1921/EF-TU-tRNA's/EF-G	C1204086H1826430N355228O450897S7295P20631Mg1811Zn0Fe0	-24214.14	Translation
rib_70_elo1_b1921_5_cplx	Translation elongation complex: 5 * ribosome 70S/b1921/EF-TU-tRNA's/EF-G	C5999470H9108426N1767908O2239069S36475P100947Mg9055Zn0Fe0	-118858.7	Translation
rib_70_elo1_b1922_14_cplx	Translation elongation complex: 14 * ribosome 70S/b1922/EF-TU-tRNA's/EF-G	C21593672H32838952N6340663O8000729S132216P348482Mg32410Zn0Fe0	-417840.96	Translation
rib_70_elo1_b1922_1_cplx	Translation elongation complex: 1 * ribosome 70S/b1922/EF-TU-tRNA's/EF-G	C1548790H2352860N455511O576130S9444P25562Mg2315Zn0Fe0	-30517.14	Translation
rib_70_elo1_b1922_7_cplx	Translation elongation complex: 7 * ribosome 70S/b1922/EF-TU-tRNA's/EF-G	C10800274H16423364N3171735O4002868S66108P174602Mg16205Zn0Fe0	-209281.98	Translation
rib_70_elo1_b2114_19_cplx	Translation elongation complex: 19 * ribosome 70S/b2114/EF-TU-tRNA's/EF-G	C80270879H122504729N23363010O29272257S502854P1172892Mg118883Zn0Fe0	-1470226.66	Translation
rib_70_elo1_b2114_1_cplx	Translation elongation complex: 1 * ribosome 70S/b2114/EF-TU-tRNA's/EF-G	C4243109H6468377N1236942O1554111S26466P63660Mg6257Zn0Fe0	-79310.14	Translation
rib_70_elo1_b2114_39_cplx	Translation elongation complex: 39 * ribosome 70S/b2114/EF-TU-tRNA's/EF-G	C164746179H251434009N47947530O60070197S1032174P2405372Mg244023Zn0Fe0	-3015689.46	Translation
rib_70_elo1_b2140_18_cplx	Translation elongation complex: 18 * ribosome 70S/b2140/EF-TU-tRNA's/EF-G	C36112643H54999478N10566649O13293641S223254P560156Mg53982Zn0Fe0	-682901.52	Translation

rib_70_elo1_b2140_1_cplx	Translation elongation complex: 1 * ribosome 70S/b2140/EF-TU-tRNA's/EF-G	C2014808H3065175N590505O744802S12403P32017Mg2999Zn0Fe0	-38837.14	Translation
rib_70_elo1_b2140_9_cplx	Translation elongation complex: 9 * ribosome 70S/b2140/EF-TU-tRNA's/EF-G	C18060848H27504847N5285161O6650138S111627P280553Mg26991Zn0Fe0	-341926.26	Translation
rib_70_elo1_b2183_13_cplx	Translation elongation complex: 13 * ribosome 70S/b2183/EF-TU-tRNA's/EF-G	C19390733H29491452N5691632O7183730S118599P312230Mg29159Zn0Fe0	-374216.82	Translation
rib_70_elo1_b2183_1_cplx	Translation elongation complex: 1 * ribosome 70S/b2183/EF-TU-tRNA's/EF-G	C1497701H2275488N440240O557102S9123P24662Mg2243Zn0Fe0	-29431.14	Translation
rib_70_elo1_b2183_6_cplx	Translation elongation complex: 6 * ribosome 70S/b2183/EF-TU-tRNA's/EF-G	C8953131H13615473N2628320O3318197S54738P144482Mg13458Zn0Fe0	-173091.84	Translation
rib_70_elo1_b2185_1_cplx	Translation elongation complex: 1 * ribosome 70S/b2185/EF-TU-tRNA's/EF-G	C654717H987975N195658O250928S3812P12695Mg1010Zn0Fe0	-14126.14	Translation
rib_70_elo1_b2185_2_cplx	Translation elongation complex: 2 * ribosome 70S/b2185/EF-TU-tRNA's/EF-G	C1306712H1972870N390203O499883S7624P25103Mg2020Zn0Fe0	-27964.28	Translation
rib_70_elo1_b2185_5_cplx	Translation elongation complex: 5 * ribosome 70S/b2185/EF-TU-tRNA's/EF-G	C3262697H4927555N973838O1246748S19060P62327Mg5050Zn0Fe0	-69478.7	Translation
rib_70_elo1_b2268_17_cplx	Translation elongation complex: 17 * ribosome 70S/b2268/EF-TU-tRNA's/EF-G	C33076204H50363602N9680461O12189787S203847P515884Mg49453Zn0Fe0	-627679.38	Translation
rib_70_elo1_b2268_1_cplx	Translation elongation complex: 1 * ribosome 70S/b2268/EF-TU-tRNA's/EF-G	C1953868H2971858N572685O723099S11991P31212Mg2909Zn0Fe0	-37789.14	Translation
rib_70_elo1_b2268_8_cplx	Translation elongation complex: 8 * ribosome 70S/b2268/EF-TU-tRNA's/EF-G	C15569890H23705746N4557337O5739775S95928P243256Mg23272Zn0Fe0	-295866.12	Translation
rib_70_elo1_b2317_12_cplx	Translation elongation complex: 12 * ribosome 70S/b2317/EF-TU-tRNA's/EF-G	C17034676H25894119N5005306O6322100S103980P277524Mg25620Zn0Fe0	-331226.68	Translation
rib_70_elo1_b2317_1_cplx	Translation elongation complex: 1 * ribosome 70S/b2317/EF-TU-tRNA's/EF-G	C1425280H2164314N419296O531139S8665P23732Mg2135Zn0Fe0	-28208.14	Translation
rib_70_elo1_b2317_6_cplx	Translation elongation complex: 6 * ribosome 70S/b2317/EF-TU-tRNA's/EF-G	C8520460H12950589N2503846O3163394S51990P139092Mg12810Zn0Fe0	-165943.84	Translation
rib_70_elo1_b2318_15_cplx	Translation elongation complex: 15 * ribosome 70S/b2318/EF-TU-tRNA's/EF-G	C25954648H39504629N7604027O9580846S159825P409217Mg38910Zn0Fe0	-495020.1	Translation
rib_70_elo1_b2318_1_cplx	Translation elongation complex: 1 * ribosome 70S/b2318/EF-TU-tRNA's/EF-G	C1737532H2641817N509849O644016S10655P28039Mg2594Zn0Fe0	-33760.14	Translation
rib_70_elo1_b2318_7_cplx	Translation elongation complex: 7 * ribosome 70S/b2318/EF-TU-tRNA's/EF-G	C12116296H18440165N3550211O4474086S74585P191401Mg18158Zn0Fe0	-231442.98	Translation
rib_70_elo1_b2319_19_cplx	Translation elongation complex: 19 * ribosome 70S/b2319/EF-TU-tRNA's/EF-G	C40687943H61978156N11900953O14965876S251560P627807Mg60743Zn0Fe0	-767897.66	Translation
rib_70_elo1_b2319_1_cplx	Translation elongation complex: 1 * ribosome 70S/b2319/EF-TU-tRNA's/EF-G	C2150627H3272362N630055O794404S13240P34005Mg3197Zn0Fe0	-41379.14	Translation
rib_70_elo1_b2319_9_cplx	Translation elongation complex: 9 * ribosome 70S/b2319/EF-TU-tRNA's/EF-G	C19278323H29363826N5639343O7092836S119160P297917Mg28773Zn0Fe0	-364276.26	Translation
rib_70_elo1_b2320_11_cplx	Translation elongation complex: 11 * ribosome 70S/b2320/EF-TU-tRNA's/EF-G	C26327362H40117884N7691573O9669309S163075P402276Mg39226Zn0Fe0	-494062.54	Translation
rib_70_elo1_b2320_1_cplx	Translation elongation complex: 1 * ribosome 70S/b2320/EF-TU-tRNA's/EF-G	C2403232H3658194N703143O886299S14825P37606Mg3566Zn0Fe0	-45951.14	Translation
rib_70_elo1_b2320_22_cplx	Translation elongation complex: 22 * ribosome 70S/b2320/EF-TU-tRNA's/EF-G	C52643905H80223543N15378846O19330620S326150P803413Mg78452Zn0Fe0	-986985.08	Translation
rib_70_elo1_b2324_19_cplx	Translation elongation complex: 19 * ribosome 70S/b2324/EF-TU-tRNA's/EF-G	C79225069H120901321N23062008O28893759S497078P1159356Mg117344Zn0Fe0	-1452111.66	Translation
rib_70_elo1_b2324_1_cplx	Translation elongation complex: 1 * ribosome 70S/b2324/EF-TU-tRNA's/EF-G	C4187839H6383683N1221006O1534065S26162P62922Mg6176Zn0Fe0	-78331.14	Translation
rib_70_elo1_b2324_39_cplx	Translation elongation complex: 39 * ribosome 70S/b2324/EF-TU-tRNA's/EF-G	C162599769H248143141N47329788O59293419S1020318P2377616Mg240864Zn0Fe0	-2978534.46	Translation
rib_70_elo1_b2325_1_cplx	Translation elongation complex: 1 * ribosome 70S/b2325/EF-TU-tRNA's/EF-G	C643623H970461N192667O247358S3736P12659Mg992Zn0Fe0	-14047.14	Translation
rib_70_elo1_b2325_2_cplx	Translation elongation complex: 2 * ribosome 70S/b2325/EF-TU-tRNA's/EF-G	C1284585H1937919N384264O492760S7472P25039Mg1984Zn0Fe0	-27814.28	Translation
rib_70_elo1_b2325_5_cplx	Translation elongation complex: 5 * ribosome 70S/b2325/EF-TU-tRNA's/EF-G	C3207471H4840293N959055O1228966S18680P62179Mg4960Zn0Fe0	-69115.7	Translation
rib_70_elo1_b2326_10_cplx	Translation elongation complex: 10 * ribosome 70S/b2326/EF-TU-tRNA's/EF-G	C11915534H18096320N3508629O4439943S72580P198758Mg18020Zn0Fe0	-234500.4	Translation
rib_70_elo1_b2326_1_cplx	Translation elongation complex: 1 * ribosome 70S/b2326/EF-TU-tRNA's/EF-G	C1196246H1814933N352725O447453S7258P20369Mg1802Zn0Fe0	-23944.14	Translation

rib_70_elo1_b2326_5_cplx	Translation elongation complex: 5 * ribosome 70S/b2326/EF-TU-tRNA's/EF-G	C5960374H9051105N1755349O2221893S36290P99653Mg9010Zn0Fe0	-117524.7	Translation
rib_70_elo1_b2327_15_cplx	Translation elongation complex: 15 * ribosome 70S/b2327/EF-TU-tRNA's/EF-G	C25900602H39407179N7594283O9574054S159135P411929Mg38775Zn0Fe0	-497177.1	Translation
rib_70_elo1_b2327_1_cplx	Translation elongation complex: 1 * ribosome 70S/b2327/EF-TU-tRNA's/EF-G	C1733858H2635241N509051O643622S10609P28217Mg2585Zn0Fe0	-33901.14	Translation
rib_70_elo1_b2327_7_cplx	Translation elongation complex: 7 * ribosome 70S/b2327/EF-TU-tRNA's/EF-G	C12091034H18394643N3545579O4470950S74263P192665Mg18095Zn0Fe0	-232447.98	Translation
rib_70_elo1_b2328_16_cplx	Translation elongation complex: 16 * ribosome 70S/b2328/EF-TU-tRNA's/EF-G	C28098493H42762270N8234877O10377676S172976P444329Mg42080Zn0Fe0	-537148.24	Translation
rib_70_elo1_b2328_1_cplx	Translation elongation complex: 1 * ribosome 70S/b2328/EF-TU-tRNA's/EF-G	C1763503H2680980N517602O654016S10811P28544Mg2630Zn0Fe0	-34346.14	Translation
rib_70_elo1_b2328_8_cplx	Translation elongation complex: 8 * ribosome 70S/b2328/EF-TU-tRNA's/EF-G	C14053165H21385582N4118997O5191724S86488P222577Mg21040Zn0Fe0	-268987.12	Translation
rib_70_elo1_b2329_10_cplx	Translation elongation complex: 10 * ribosome 70S/b2329/EF-TU-tRNA's/EF-G	C22877332H34860944N6685451O8403529S141510P349676Mg34130Zn0Fe0	-429058.4	Translation
rib_70_elo1_b2329_1_cplx	Translation elongation complex: 1 * ribosome 70S/b2329/EF-TU-tRNA's/EF-G	C2297032H3496646N672281O847174S14151P35945Mg3413Zn0Fe0	-43884.14	Translation
rib_70_elo1_b2329_21_cplx	Translation elongation complex: 21 * ribosome 70S/b2329/EF-TU-tRNA's/EF-G	C48031032H73195086N14034881O17639074S297171P733125Mg71673Zn0Fe0	-899826.94	Translation
rib_70_elo1_b2330_18_cplx	Translation elongation complex: 18 * ribosome 70S/b2330/EF-TU-tRNA's/EF-G	C35573518H54170059N10410194O13103183S219636P553445Mg53172Zn0Fe0	-674174.52	Translation
rib_70_elo1_b2330_1_cplx	Translation elongation complex: 1 * ribosome 70S/b2330/EF-TU-tRNA's/EF-G	C1984680H3018929N581661O734136S12202P31630Mg2954Zn0Fe0	-38338.14	Translation
rib_70_elo1_b2330_9_cplx	Translation elongation complex: 9 * ribosome 70S/b2330/EF-TU-tRNA's/EF-G	C17791192H27090049N5206853O6554864S109818P277190Mg26586Zn0Fe0	-337555.26	Translation
rib_70_elo1_b2400_13_cplx	Translation elongation complex: 13 * ribosome 70S/b2400/EF-TU-tRNA's/EF-G	C38503389H58718223N11226691O14091064S239902P575290Mg57239Zn0Fe0	-713534.82	Translation
rib_70_elo1_b2400_1_cplx	Translation elongation complex: 1 * ribosome 70S/b2400/EF-TU-tRNA's/EF-G	C2974245H4530867N868579O1093060S18454P45562Mg4403Zn0Fe0	-56197.14	Translation
rib_70_elo1_b2400_27_cplx	Translation elongation complex: 27 * ribosome 70S/b2400/EF-TU-tRNA's/EF-G	C79954057H121936805N23311155O29255402S498258P1193306Mg118881Zn0Fe0	-1480428.78	Translation
rib_70_elo1_b2514_12_cplx	Translation elongation complex: 12 * ribosome 70S/b2514/EF-TU-tRNA's/EF-G	C32094382H48926535N9368620O11763437S199356P484661Mg47760Zn0Fe0	-598219.68	Translation
rib_70_elo1_b2514_1_cplx	Translation elongation complex: 1 * ribosome 70S/b2514/EF-TU-tRNA's/EF-G	C2685662H4089798N785177O988486S16613P41559Mg3980Zn0Fe0	-51023.14	Translation
rib_70_elo1_b2514_24_cplx	Translation elongation complex: 24 * ribosome 70S/b2514/EF-TU-tRNA's/EF-G	C64176622H97839339N18732376O23517929S398712P968045Mg95520Zn0Fe0	-1195161.36	Translation
rib_70_elo1_b2528_1_cplx	Translation elongation complex: 1 * ribosome 70S/b2528/EF-TU-tRNA's/EF-G	C735108H1110541N219070O280291S4327P13880Mg1127Zn0Fe0	-15630.14	Translation
rib_70_elo1_b2528_3_cplx	Translation elongation complex: 3 * ribosome 70S/b2528/EF-TU-tRNA's/EF-G	C2199128H3324641N654686O836353S12981P40992Mg3381Zn0Fe0	-46240.42	Translation
rib_70_elo1_b2528_6_cplx	Translation elongation complex: 6 * ribosome 70S/b2528/EF-TU-tRNA's/EF-G	C4395158H6645791N1308110O1670446S25962P81660Mg6762Zn0Fe0	-92155.84	Translation
rib_70_elo1_b2529_1_cplx	Translation elongation complex: 1 * ribosome 70S/b2529/EF-TU-tRNA's/EF-G	C864363H1307927N256606O327225S5131P15708Mg1316Zn0Fe0	-17980.14	Translation
rib_70_elo1_b2529_3_cplx	Translation elongation complex: 3 * ribosome 70S/b2529/EF-TU-tRNA's/EF-G	C2585685H3915423N766778O976325S15393P46350Mg3948Zn0Fe0	-53164.42	Translation
rib_70_elo1_b2529_7_cplx	Translation elongation complex: 7 * ribosome 70S/b2529/EF-TU-tRNA's/EF-G	C6028329H9130415N1787122O2274525S35917P107634Mg9212Zn0Fe0	-123532.98	Translation
rib_70_elo1_b2530_11_cplx	Translation elongation complex: 11 * ribosome 70S/b2530/EF-TU-tRNA's/EF-G	C28081370H42799577N8200614O10303399S174086P426409Mg41800Zn0Fe0	-525323.54	Translation
rib_70_elo1_b2530_1_cplx	Translation elongation complex: 1 * ribosome 70S/b2530/EF-TU-tRNA's/EF-G	C2563360H3902777N749714O944379S15826P39869Mg3800Zn0Fe0	-48862.14	Translation
rib_70_elo1_b2530_23_cplx	Translation elongation complex: 23 * ribosome 70S/b2530/EF-TU-tRNA's/EF-G	C58702982H89475737N17141694O21534223S363998P890257Mg87400Zn0Fe0	-1097077.22	Translation
rib_70_elo1_b2531_1_cplx	Translation elongation complex: 1 * ribosome 70S/b2531/EF-TU-tRNA's/EF-G	C1074508H1628528N317767O403953S6440P18796Mg1622Zn0Fe0	-21877.14	Translation
rib_70_elo1_b2531_4_cplx	Translation elongation complex: 4 * ribosome 70S/b2531/EF-TU-tRNA's/EF-G	C4284094H6498323N1265548O1605501S25760P73711Mg6488Zn0Fe0	-86032.56	Translation
rib_70_elo1_b2531_9_cplx	Translation elongation complex: 9 * ribosome 70S/b2531/EF-TU-tRNA's/EF-G	C9633404H14614648N2845183O3608081S57960P165236Mg14598Zn0Fe0	-192958.26	Translation

rib_70_elo1_b2559_10_cplx	Translation elongation complex: 10 * ribosome 70S/b2559/EF-TU-tRNA's/EF-G	C11668479H17720673N3437568O4351094S70790P195327Mg17660Zn0Fe0	-230039.4	Translation
rib_70_elo1_b2559_1_cplx	Translation elongation complex: 1 * ribosome 70S/b2559/EF-TU-tRNA's/EF-G	C1171455H1777272N345609O438497S7079P20016Mg1766Zn0Fe0	-23488.14	Translation
rib_70_elo1_b2559_5_cplx	Translation elongation complex: 5 * ribosome 70S/b2559/EF-TU-tRNA's/EF-G	C5836799H8863228N1719813O2177429S35395P97932Mg8830Zn0Fe0	-115288.7	Translation
rib_70_elo1_b2560_12_cplx	Translation elongation complex: 12 * ribosome 70S/b2560/EF-TU-tRNA's/EF-G	C16455219H25004943N4839788O6115787S100524P270602Mg24756Zn0Fe0	-321652.68	Translation
rib_70_elo1_b2560_1_cplx	Translation elongation complex: 1 * ribosome 70S/b2560/EF-TU-tRNA's/EF-G	C1376793H2089996N405457O513784S8377P23135Mg2063Zn0Fe0	-27390.14	Translation
rib_70_elo1_b2560_6_cplx	Translation elongation complex: 6 * ribosome 70S/b2560/EF-TU-tRNA's/EF-G	C8230623H12505881N2421062O3060149S50262P135620Mg12378Zn0Fe0	-161145.84	Translation
rib_70_elo1_b2563_1_cplx	Translation elongation complex: 1 * ribosome 70S/b2563/EF-TU-tRNA's/EF-G	C851612H1288660N252874O322421S5048P15492Mg1298Zn0Fe0	-17704.14	Translation
rib_70_elo1_b2563_3_cplx	Translation elongation complex: 3 * ribosome 70S/b2563/EF-TU-tRNA's/EF-G	C2547574H3857792N755672O961961S15144P45716Mg3894Zn0Fe0	-52350.42	Translation
rib_70_elo1_b2563_7_cplx	Translation elongation complex: 7 * ribosome 70S/b2563/EF-TU-tRNA's/EF-G	C5939498H8996056N1761268O2241041S35336P106164Mg9086Zn0Fe0	-121642.98	Translation
rib_70_elo1_b2564_14_cplx	Translation elongation complex: 14 * ribosome 70S/b2564/EF-TU-tRNA's/EF-G	C21889192H33308573N6418982O8093786S133896P348298Mg32914Zn0Fe0	-419308.96	Translation
rib_70_elo1_b2564_1_cplx	Translation elongation complex: 1 * ribosome 70S/b2564/EF-TU-tRNA's/EF-G	C1569997H2386526N461134O582867S9564P25560Mg2351Zn0Fe0	-30633.14	Translation
rib_70_elo1_b2564_7_cplx	Translation elongation complex: 7 * ribosome 70S/b2564/EF-TU-tRNA's/EF-G	C10948087H16658240N3210910O4049445S66948P174516Mg16457Zn0Fe0	-210021.98	Translation
rib_70_elo1_b2565_14_cplx	Translation elongation complex: 14 * ribosome 70S/b2565/EF-TU-tRNA's/EF-G	C21856487H33238348N6416929O8096997S133952P352437Mg32788Zn0Fe0	-422579.96	Translation
rib_70_elo1_b2565_1_cplx	Translation elongation complex: 1 * ribosome 70S/b2565/EF-TU-tRNA's/EF-G	C1567608H2381457N460901O583101S9568P25851Mg2342Zn0Fe0	-30862.14	Translation
rib_70_elo1_b2565_7_cplx	Translation elongation complex: 7 * ribosome 70S/b2565/EF-TU-tRNA's/EF-G	C10931706H16623099N3209837O4051053S66976P176583Mg16394Zn0Fe0	-211654.98	Translation
rib_70_elo1_b2566_17_cplx	Translation elongation complex: 17 * ribosome 70S/b2566/EF-TU-tRNA's/EF-G	C32652287H49720008N9557405O12031681S201195P509015Mg48841Zn0Fe0	-619280.38	Translation
rib_70_elo1_b2566_1_cplx	Translation elongation complex: 1 * ribosome 70S/b2566/EF-TU-tRNA's/EF-G	C1928815H2933864N565469O713649S11835P30791Mg2873Zn0Fe0	-37278.14	Translation
rib_70_elo1_b2566_8_cplx	Translation elongation complex: 8 * ribosome 70S/b2566/EF-TU-tRNA's/EF-G	C15370334H23402802N4499441O5665288S94680P240014Mg22984Zn0Fe0	-291904.12	Translation
rib_70_elo1_b2567_13_cplx	Translation elongation complex: 13 * ribosome 70S/b2567/EF-TU-tRNA's/EF-G	C19017389H28911661N5588011O7056129S116350P309381Mg28574Zn0Fe0	-369612.82	Translation
rib_70_elo1_b2567_1_cplx	Translation elongation complex: 1 * ribosome 70S/b2567/EF-TU-tRNA's/EF-G	C1468865H2230741N432247O547173S8950P24429Mg2198Zn0Fe0	-29063.14	Translation
rib_70_elo1_b2567_6_cplx	Translation elongation complex: 6 * ribosome 70S/b2567/EF-TU-tRNA's/EF-G	C8780750H13347791N2580482O3259238S53700P143159Mg13188Zn0Fe0	-170958.84	Translation
rib_70_elo1_b2570_1_cplx	Translation elongation complex: 1 * ribosome 70S/b2570/EF-TU-tRNA's/EF-G	C1056582H1600905N312668O397537S6348P18584Mg1595Zn0Fe0	-21592.14	Translation
rib_70_elo1_b2570_4_cplx	Translation elongation complex: 4 * ribosome 70S/b2570/EF-TU-tRNA's/EF-G	C4212786H6388281N1245380O1580047S25392P72908Mg6380Zn0Fe0	-84937.56	Translation
rib_70_elo1_b2570_9_cplx	Translation elongation complex: 9 * ribosome 70S/b2570/EF-TU-tRNA's/EF-G	C9473126H14367241N2799900O3550897S57132P163448Mg14355Zn0Fe0	-190513.26	Translation
rib_70_elo1_b2571_18_cplx	Translation elongation complex: 18 * ribosome 70S/b2571/EF-TU-tRNA's/EF-G	C36506426H55576076N10691467O13454203S225666P570710Mg54468Zn0Fe0	-694697.52	Translation
rib_70_elo1_b2571_1_cplx	Translation elongation complex: 1 * ribosome 70S/b2571/EF-TU-tRNA's/EF-G	C2036699H3097263N597326O753792S12537P32609Mg3026Zn0Fe0	-39498.14	Translation
rib_70_elo1_b2571_9_cplx	Translation elongation complex: 9 * ribosome 70S/b2571/EF-TU-tRNA's/EF-G	C18257747H27793175N5347510O6730456S112833P285833Mg27234Zn0Fe0	-347827.26	Translation
rib_70_elo1_b2572_12_cplx	Translation elongation complex: 12 * ribosome 70S/b2572/EF-TU-tRNA's/EF-G	C16795673H25534790N4934837O6231207S102684P273075Mg25296Zn0Fe0	-325937.68	Translation
rib_70_elo1_b2572_1_cplx	Translation elongation complex: 1 * ribosome 70S/b2572/EF-TU-tRNA's/EF-G	C1405331H2134347N413551O523395S8557P23353Mg2108Zn0Fe0	-27759.14	Translation
rib_70_elo1_b2572_6_cplx	Translation elongation complex: 6 * ribosome 70S/b2572/EF-TU-tRNA's/EF-G	C8400941H12770912N2468681O3117855S51342P136863Mg12648Zn0Fe0	-163294.84	Translation
rib_70_elo1_b2573_11_cplx	Translation elongation complex: 11 * ribosome 70S/b2573/EF-TU-tRNA's/EF-G	C13726662H20849626N4042761O5112519S83523P228454Mg20713Zn0Fe0	-270234.54	Translation

rib_70_elo1_b2573_1_cplx	Translation elongation complex: 1 * ribosome 70S/b2573/EF-TU-tRNA's/EF-G	C1252872H1901046N369521O468449S7593P21294Mg1883Zn0Fe0	-25093.14	Translation
rib_70_elo1_b2573_5_cplx	Translation elongation complex: 5 * ribosome 70S/b2573/EF-TU-tRNA's/EF-G	C6242388H9480478N1838817O2326077S37965P104158Mg9415Zn0Fe0	-123149.7	Translation
rib_70_elo1_b2593_14_cplx	Translation elongation complex: 14 * ribosome 70S/b2593/EF-TU-tRNA's/EF-G	C21910325H33330867N6428458O8106621S134470P350322Mg32914Zn0Fe0	-421150.96	Translation
rib_70_elo1_b2593_1_cplx	Translation elongation complex: 1 * ribosome 70S/b2593/EF-TU-tRNA's/EF-G	C1571448H2388046N461718O583807S9605P25699Mg2351Zn0Fe0	-30759.14	Translation
rib_70_elo1_b2593_7_cplx	Translation elongation complex: 7 * ribosome 70S/b2593/EF-TU-tRNA's/EF-G	C10958622H16669348N3215598O4055875S67235P175525Mg16457Zn0Fe0	-210939.98	Translation
rib_70_elo1_b2594_19_cplx	Translation elongation complex: 19 * ribosome 70S/b2594/EF-TU-tRNA's/EF-G	C39376867H59989087N11513815O14478887S243352P606247Mg58862Zn0Fe0	-740941.66	Translation
rib_70_elo1_b2594_1_cplx	Translation elongation complex: 1 * ribosome 70S/b2594/EF-TU-tRNA's/EF-G	C2081317H3167335N609541O768539S12808P32839Mg3098Zn0Fe0	-39929.14	Translation
rib_70_elo1_b2594_9_cplx	Translation elongation complex: 9 * ribosome 70S/b2594/EF-TU-tRNA's/EF-G	C18657117H28421447N5455885O6862027S115272P287687Mg27882Zn0Fe0	-351490.26	Translation
rib_70_elo1_b2606_1_cplx	Translation elongation complex: 1 * ribosome 70S/b2606/EF-TU-tRNA's/EF-G	C784177H1185623N233332O298001S4625P14554Mg1199Zn0Fe0	-16487.14	Translation
rib_70_elo1_b2606_3_cplx	Translation elongation complex: 3 * ribosome 70S/b2606/EF-TU-tRNA's/EF-G	C2345911H3549395N697380O889121S13875P42966Mg3597Zn0Fe0	-48763.42	Translation
rib_70_elo1_b2606_6_cplx	Translation elongation complex: 6 * ribosome 70S/b2606/EF-TU-tRNA's/EF-G	C4688512H7095053N1393452O1775801S27750P85584Mg7194Zn0Fe0	-97177.84	Translation
rib_70_elo1_b2607_15_cplx	Translation elongation complex: 15 * ribosome 70S/b2607/EF-TU-tRNA's/EF-G	C24589725H37413123N7211414O9092831S150945P391878Mg36885Zn0Fe0	-472191.1	Translation
rib_70_elo1_b2607_1_cplx	Translation elongation complex: 1 * ribosome 70S/b2607/EF-TU-tRNA's/EF-G	C1646161H2501939N483546O611183S10063P26842Mg2459Zn0Fe0	-32197.14	Translation
rib_70_elo1_b2607_7_cplx	Translation elongation complex: 7 * ribosome 70S/b2607/EF-TU-tRNA's/EF-G	C11479117H17463875N3366918O4246175S70441P183286Mg17213Zn0Fe0	-220765.98	Translation
rib_70_elo1_b2608_10_cplx	Translation elongation complex: 10 * ribosome 70S/b2608/EF-TU-tRNA's/EF-G	C11920744H18103495N3510598O4445294S72400P199369Mg18020Zn0Fe0	-235161.4	Translation
rib_70_elo1_b2608_1_cplx	Translation elongation complex: 1 * ribosome 70S/b2608/EF-TU-tRNA's/EF-G	C1196785H1815664N352948O447980S7240P20431Mg1802Zn0Fe0	-24011.14	Translation
rib_70_elo1_b2608_5_cplx	Translation elongation complex: 5 * ribosome 70S/b2608/EF-TU-tRNA's/EF-G	C5962989H9054700N1756348O2224564S36200P99959Mg9010Zn0Fe0	-117855.7	Translation
rib_70_elo1_b2609_1_cplx	Translation elongation complex: 1 * ribosome 70S/b2609/EF-TU-tRNA's/EF-G	C580941H875239N174314O224182S3343P11658Mg902Zn0Fe0	-12795.14	Translation
rib_70_elo1_b2609_2_cplx	Translation elongation complex: 2 * ribosome 70S/b2609/EF-TU-tRNA's/EF-G	C1159515H1747797N347688O446617S6686P23065Mg1804Zn0Fe0	-25338.28	Translation
rib_70_elo1_b2609_4_cplx	Translation elongation complex: 4 * ribosome 70S/b2609/EF-TU-tRNA's/EF-G	C2316663H3492913N694436O891487S13372P45879Mg3608Zn0Fe0	-50424.56	Translation
rib_70_elo1_b2614_11_cplx	Translation elongation complex: 11 * ribosome 70S/b2614/EF-TU-tRNA's/EF-G	C14110858H21444955N4150969O5246607S85778P232091Mg21307Zn0Fe0	-275741.54	Translation
rib_70_elo1_b2614_1_cplx	Translation elongation complex: 1 * ribosome 70S/b2614/EF-TU-tRNA's/EF-G	C1287978H1955365N379489O480717S7798P21641Mg1937Zn0Fe0	-25610.14	Translation
rib_70_elo1_b2614_5_cplx	Translation elongation complex: 5 * ribosome 70S/b2614/EF-TU-tRNA's/EF-G	C6417130H9751201N1888081O2387073S38990P105821Mg9685Zn0Fe0	-125662.7	Translation
rib_70_elo1_b2697_1_cplx	Translation elongation complex: 1 * ribosome 70S/b2697/EF-TU-tRNA's/EF-G	C5465976H8336795N1591841O1997940S34135P80904Mg8048Zn0Fe0	-101432.14	Translation
rib_70_elo1_b2697_25_cplx	Translation elongation complex: 25 * ribosome 70S/b2697/EF-TU-tRNA's/EF-G	C136047480H207739499N39553577O49507140S853375P1959408Mg201200Zn0Fe0	-2472587.5	Translation
rib_70_elo1_b2697_51_cplx	Translation elongation complex: 51 * ribosome 70S/b2697/EF-TU-tRNA's/EF-G	C277510776H423759095N80678791O100975440S1740885P3994454Mg410448Zn0Fe0	-5041339.14	Translation
rib_70_elo1_b2741_19_cplx	Translation elongation complex: 19 * ribosome 70S/b2741/EF-TU-tRNA's/EF-G	C39879729H60744829N11666638O14673102S246259P616937Mg59546Zn0Fe0	-753398.66	Translation
rib_70_elo1_b2741_1_cplx	Translation elongation complex: 1 * ribosome 70S/b2741/EF-TU-tRNA's/EF-G	C2107917H3207235N617680O778830S12961P33413Mg3134Zn0Fe0	-40596.14	Translation
rib_70_elo1_b2741_9_cplx	Translation elongation complex: 9 * ribosome 70S/b2741/EF-TU-tRNA's/EF-G	C18895389H28779499N5528328O6954062S116649P292757Mg28206Zn0Fe0	-357397.26	Translation
rib_70_elo1_b2742_11_cplx	Translation elongation complex: 11 * ribosome 70S/b2742/EF-TU-tRNA's/EF-G	C26385981H40207148N7708742O9687320S163361P402081Mg39325Zn0Fe0	-494637.54	Translation
rib_70_elo1_b2742_1_cplx	Translation elongation complex: 1 * ribosome 70S/b2742/EF-TU-tRNA's/EF-G	C2408601H3666388N704792O887850S14851P37591Mg3575Zn0Fe0	-46006.14	Translation

rib_70_elo1_b2742_22_cplx	Translation elongation complex: 22 * ribosome 70S/b2742/EF-TU-tRNA's/EF-G	C52761099H80401984N15413087O19366737S326722P803020Mg78650Zn0Fe0	-988132.08	Translation
rib_70_elo1_b2745_10_cplx	Translation elongation complex: 10 * ribosome 70S/b2745/EF-TU-tRNA's/EF-G	C22144322H33739391N6472854O8137875S137210P339802Mg33050Zn0Fe0	-416244.4	Translation
rib_70_elo1_b2745_1_cplx	Translation elongation complex: 1 * ribosome 70S/b2745/EF-TU-tRNA's/EF-G	C2223452H3384119N650934O820389S13721P34927Mg3305Zn0Fe0	-42572.14	Translation
rib_70_elo1_b2745_20_cplx	Translation elongation complex: 20 * ribosome 70S/b2745/EF-TU-tRNA's/EF-G	C44278622H67467471N12941654O16268415S274420P678552Mg66100Zn0Fe0	-831435.8	Translation
rib_70_elo1_b2779_12_cplx	Translation elongation complex: 12 * ribosome 70S/b2779/EF-TU-tRNA's/EF-G	C32662267H49802491N9528602O11966923S202260P490551Mg48624Zn0Fe0	-607193.68	Translation
rib_70_elo1_b2779_1_cplx	Translation elongation complex: 1 * ribosome 70S/b2779/EF-TU-tRNA's/EF-G	C2733203H4163040N798617O1005522S16855P42070Mg4052Zn0Fe0	-51791.14	Translation
rib_70_elo1_b2779_25_cplx	Translation elongation complex: 25 * ribosome 70S/b2779/EF-TU-tRNA's/EF-G	C68032979H103740024N19845857O24921306S421375P1020574Mg101300Zn0Fe0	-1263578.5	Translation
rib_70_elo1_b2780_16_cplx	Translation elongation complex: 16 * ribosome 70S/b2780/EF-TU-tRNA's/EF-G	C54640768H83357773N15921520O19963193S340912P807912Mg81104Zn0Fe0	-1007035.24	Translation
rib_70_elo1_b2780_1_cplx	Translation elongation complex: 1 * ribosome 70S/b2780/EF-TU-tRNA's/EF-G	C3429658H5226388N1000915O1258448S21307P52032Mg5069Zn0Fe0	-64478.14	Translation
rib_70_elo1_b2780_32_cplx	Translation elongation complex: 32 * ribosome 70S/b2780/EF-TU-tRNA's/EF-G	C109265952H166697917N31836832O39914921S681824P1614184Mg162208Zn0Fe0	-2012429.48	Translation
rib_70_elo1_b2785_12_cplx	Translation elongation complex: 12 * ribosome 70S/b2785/EF-TU-tRNA's/EF-G	C32770766H49955970N9565977O12012571S204024P495128Mg48732Zn0Fe0	-611302.68	Translation
rib_70_elo1_b2785_1_cplx	Translation elongation complex: 1 * ribosome 70S/b2785/EF-TU-tRNA's/EF-G	C2742262H4175851N801716O1009381S17002P42456Mg4061Zn0Fe0	-52138.14	Translation
rib_70_elo1_b2785_25_cplx	Translation elongation complex: 25 * ribosome 70S/b2785/EF-TU-tRNA's/EF-G	C68258998H104059747N19923740O25016341S425050P1030104Mg101525Zn0Fe0	-1272133.5	Translation
rib_70_elo1_b2790_1_cplx	Translation elongation complex: 1 * ribosome 70S/b2790/EF-TU-tRNA's/EF-G	C994416H1506180N294454O374725S5955P17638Mg1505Zn0Fe0	-20419.14	Translation
rib_70_elo1_b2790_4_cplx	Translation elongation complex: 4 * ribosome 70S/b2790/EF-TU-tRNA's/EF-G	C3964782H6010185N1172620O1489450S23820P69202Mg6020Zn0Fe0	-80323.56	Translation
rib_70_elo1_b2790_8_cplx	Translation elongation complex: 8 * ribosome 70S/b2790/EF-TU-tRNA's/EF-G	C7925270H12015525N2343508O2975750S47640P137954Mg12040Zn0Fe0	-160196.12	Translation
rib_70_elo1_b2791_15_cplx	Translation elongation complex: 15 * ribosome 70S/b2791/EF-TU-tRNA's/EF-G	C25055184H38122978N7346048O9263001S153915P398807Mg37560Zn0Fe0	-480710.1	Translation
rib_70_elo1_b2791_1_cplx	Translation elongation complex: 1 * ribosome 70S/b2791/EF-TU-tRNA's/EF-G	C1677298H2549398N492530O622635S10261P27317Mg2504Zn0Fe0	-32778.14	Translation
rib_70_elo1_b2791_7_cplx	Translation elongation complex: 7 * ribosome 70S/b2791/EF-TU-tRNA's/EF-G	C11696392H17795218N3429752O4325649S71827P186527Mg17528Zn0Fe0	-224748.98	Translation
rib_70_elo1_b2792_1_cplx	Translation elongation complex: 1 * ribosome 70S/b2792/EF-TU-tRNA's/EF-G	C747809H1129798N222726O285027S4404P14094Mg1145Zn0Fe0	-15888.14	Translation
rib_70_elo1_b2792_3_cplx	Translation elongation complex: 3 * ribosome 70S/b2792/EF-TU-tRNA's/EF-G	C2237159H3382290N665700O850445S13212P41618Mg3435Zn0Fe0	-46998.42	Translation
rib_70_elo1_b2792_6_cplx	Translation elongation complex: 6 * ribosome 70S/b2792/EF-TU-tRNA's/EF-G	C4471184H6761028N1330161O1698572S26424P82904Mg6870Zn0Fe0	-93663.84	Translation
rib_70_elo1_b2794_16_cplx	Translation elongation complex: 16 * ribosome 70S/b2794/EF-TU-tRNA's/EF-G	C28907860H43990742N8470323O10677416S178112P457443Mg43232Zn0Fe0	-553558.24	Translation
rib_70_elo1_b2794_1_cplx	Translation elongation complex: 1 * ribosome 70S/b2794/EF-TU-tRNA's/EF-G	C1814305H2757977N532383O672911S11132P29388Mg2702Zn0Fe0	-35396.14	Translation
rib_70_elo1_b2794_8_cplx	Translation elongation complex: 8 * ribosome 70S/b2794/EF-TU-tRNA's/EF-G	C14457964H21999934N4236755O5341680S89056P229147Mg21616Zn0Fe0	-277205.12	Translation
rib_70_elo1_b2890_14_cplx	Translation elongation complex: 14 * ribosome 70S/b2890/EF-TU-tRNA's/EF-G	C44375360H67688961N12933602O16224393S276486P659042Mg65926Zn0Fe0	-819498.96	Translation
rib_70_elo1_b2890_1_cplx	Translation elongation complex: 1 * ribosome 70S/b2890/EF-TU-tRNA's/EF-G	C3183079H4850120N929194O1168716S19749P48484Mg4709Zn0Fe0	-59946.14	Translation
rib_70_elo1_b2890_29_cplx	Translation elongation complex: 29 * ribosome 70S/b2890/EF-TU-tRNA's/EF-G	C91904915H140195316N26784842O33596328S572721P1363532Mg136561Zn0Fe0	-1695906.06	Translation
rib_70_elo1_b2891_10_cplx	Translation elongation complex: 10 * ribosome 70S/b2891/EF-TU-tRNA's/EF-G	C23135332H35250586N6761595O8500167S143220P354411Mg34490Zn0Fe0	-434873.4	Translation
rib_70_elo1_b2891_1_cplx	Translation elongation complex: 1 * ribosome 70S/b2891/EF-TU-tRNA's/EF-G	C2322976H3535738N680007O856890S14322P36432Mg3449Zn0Fe0	-44479.14	Translation
rib_70_elo1_b2891_21_cplx	Translation elongation complex: 21 * ribosome 70S/b2891/EF-TU-tRNA's/EF-G	C48572656H74013178N14194647O17841950S300762P743052Mg72429Zn0Fe0	-912021.94	Translation

rib_70_elo1_b2892_16_cplx	Translation elongation complex: 16 * ribosome 70S/b2892/EF-TU-tRNA's/EF-G	C57801175H88181551N16842032O21116035S360624P854374Mg85712Zn0Fe0	-1065417.24	Translation
rib_70_elo1_b2892_1_cplx	Translation elongation complex: 1 * ribosome 70S/b2892/EF-TU-tRNA's/EF-G	C3628045H5528866N1058822O1331155S22539P55024Mg5357Zn0Fe0	-68215.14	Translation
rib_70_elo1_b2892_33_cplx	Translation elongation complex: 33 * ribosome 70S/b2892/EF-TU-tRNA's/EF-G	C119197389H181854594N34729670O43538899S743787P1760304Mg176781Zn0Fe0	-2195579.62	Translation
rib_70_elo1_b2893_13_cplx	Translation elongation complex: 13 * ribosome 70S/b2893/EF-TU-tRNA's/EF-G	C19794441H30106805N5807758O7331771S121433P318043Mg29744Zn0Fe0	-381940.82	Translation
rib_70_elo1_b2893_1_cplx	Translation elongation complex: 1 * ribosome 70S/b2893/EF-TU-tRNA's/EF-G	C1528917H2322977N449290O568547S9341P25123Mg2288Zn0Fe0	-30039.14	Translation
rib_70_elo1_b2893_6_cplx	Translation elongation complex: 6 * ribosome 70S/b2893/EF-TU-tRNA's/EF-G	C9139552H13899572N2681985O3386557S56046P147173Mg13728Zn0Fe0	-176664.84	Translation
rib_70_elo1_b2946_14_cplx	Translation elongation complex: 14 * ribosome 70S/b2946/EF-TU-tRNA's/EF-G	C21925617H33349838N6435937O8117086S134316P351952Mg32914Zn0Fe0	-422710.96	Translation
rib_70_elo1_b2946_1_cplx	Translation elongation complex: 1 * ribosome 70S/b2946/EF-TU-tRNA's/EF-G	C1572583H2389454N462294O584548S9594P25821Mg2351Zn0Fe0	-30876.14	Translation
rib_70_elo1_b2946_7_cplx	Translation elongation complex: 7 * ribosome 70S/b2946/EF-TU-tRNA's/EF-G	C10966291H16678862N3219360O4061104S67158P176343Mg16457Zn0Fe0	-211722.98	Translation
rib_70_elo1_b2947_18_cplx	Translation elongation complex: 18 * ribosome 70S/b2947/EF-TU-tRNA's/EF-G	C36221898H55168179N10596572O13333087S223560P561309Mg54144Zn0Fe0	-684558.52	Translation
rib_70_elo1_b2947_1_cplx	Translation elongation complex: 1 * ribosome 70S/b2947/EF-TU-tRNA's/EF-G	C2020890H3074586N592157O746984S12420P32082Mg3008Zn0Fe0	-38930.14	Translation
rib_70_elo1_b2947_9_cplx	Translation elongation complex: 9 * ribosome 70S/b2947/EF-TU-tRNA's/EF-G	C18115482H27589218N5300117O6669856S111780P281130Mg27072Zn0Fe0	-342755.26	Translation
rib_70_elo1_b2959_1_cplx	Translation elongation complex: 1 * ribosome 70S/b2959/EF-TU-tRNA's/EF-G	C741088H1119814N220785O282282S4370P13937Mg1136Zn0Fe0	-15708.14	Translation
rib_70_elo1_b2959_3_cplx	Translation elongation complex: 3 * ribosome 70S/b2959/EF-TU-tRNA's/EF-G	C2217018H3352412N659787O842326S13110P41159Mg3408Zn0Fe0	-46470.42	Translation
rib_70_elo1_b2959_6_cplx	Translation elongation complex: 6 * ribosome 70S/b2959/EF-TU-tRNA's/EF-G	C4430913H6701309N1318290O1682392S26220P81992Mg6816Zn0Fe0	-92613.84	Translation
rib_70_elo1_b2960_14_cplx	Translation elongation complex: 14 * ribosome 70S/b2960/EF-TU-tRNA's/EF-G	C21574644H32816929N6330956O7986650S132650P346186Mg32410Zn0Fe0	-415642.96	Translation
rib_70_elo1_b2960_1_cplx	Translation elongation complex: 1 * ribosome 70S/b2960/EF-TU-tRNA's/EF-G	C1547416H2351260N454761O575168S9475P25398Mg2315Zn0Fe0	-30360.14	Translation
rib_70_elo1_b2960_7_cplx	Translation elongation complex: 7 * ribosome 70S/b2960/EF-TU-tRNA's/EF-G	C10790752H16412338N3166851O3995852S66325P173454Mg16205Zn0Fe0	-208182.98	Translation
rib_70_elo1_b3065_1_cplx	Translation elongation complex: 1 * ribosome 70S/b3065/EF-TU-tRNA's/EF-G	C513320H771991N154734O199566S2922P10705Mg803Zn0Fe0	-11561.14	Translation
rib_70_elo1_b3065_2_cplx	Translation elongation complex: 2 * ribosome 70S/b3065/EF-TU-tRNA's/EF-G	C1024580H1541650N308633O397627S5844P21192Mg1606Zn0Fe0	-22903.28	Translation
rib_70_elo1_b3065_4_cplx	Translation elongation complex: 4 * ribosome 70S/b3065/EF-TU-tRNA's/EF-G	C2047100H3080968N616431O793749S11688P42166Mg3212Zn0Fe0	-45587.56	Translation
rib_70_elo1_b3066_17_cplx	Translation elongation complex: 17 * ribosome 70S/b3066/EF-TU-tRNA's/EF-G	C61831031H94332212N18010840O22583435S386546P912844Mg91681Zn0Fe0	-1138811.38	Translation
rib_70_elo1_b3066_1_cplx	Translation elongation complex: 1 * ribosome 70S/b3066/EF-TU-tRNA's/EF-G	C3652759H5566660N1065720O1339899S22738P55340Mg5393Zn0Fe0	-68633.14	Translation
rib_70_elo1_b3066_34_cplx	Translation elongation complex: 34 * ribosome 70S/b3066/EF-TU-tRNA's/EF-G	C123645445H188645611N36015030O45154692S773092P1823942Mg183362Zn0Fe0	-2275875.76	Translation
rib_70_elo1_b3067_18_cplx	Translation elongation complex: 18 * ribosome 70S/b3067/EF-TU-tRNA's/EF-G	C68961107H105239781N20080404O25164235S430866P1011446Mg102258Zn0Fe0	-1265753.52	Translation
rib_70_elo1_b3067_1_cplx	Translation elongation complex: 1 * ribosome 70S/b3067/EF-TU-tRNA's/EF-G	C3847775H5865448N1122344O1410067S23937P57933Mg5681Zn0Fe0	-72062.14	Translation
rib_70_elo1_b3067_36_cplx	Translation elongation complex: 36 * ribosome 70S/b3067/EF-TU-tRNA's/EF-G	C137904635H210459663N40153644O50315707S861732P2021048Mg204516Zn0Fe0	-2529662.04	Translation
rib_70_elo1_b3124_12_cplx	Translation elongation complex: 12 * ribosome 70S/b3124/EF-TU-tRNA's/EF-G	C30904295H47109974N9021737O11333160S191520P467079Mg46032Zn0Fe0	-576569.68	Translation
rib_70_elo1_b3124_1_cplx	Translation elongation complex: 1 * ribosome 70S/b3124/EF-TU-tRNA's/EF-G	C2586104H3937955N756183O952284S15960P40048Mg3836Zn0Fe0	-49173.14	Translation
rib_70_elo1_b3124_24_cplx	Translation elongation complex: 24 * ribosome 70S/b3124/EF-TU-tRNA's/EF-G	C61796867H94206722N18038705O22657752S383040P932931Mg92064Zn0Fe0	-1151911.36	Translation
rib_70_elo1_b3125_17_cplx	Translation elongation complex: 17 * ribosome 70S/b3125/EF-TU-tRNA's/EF-G	C32128843H48918963N9405321O11846523S197472P501983Mg48076Zn0Fe0	-610752.38	Translation

rib_70_elo1_b3125_1_cplx	Translation elongation complex: 1 * ribosome 70S/b3125/EF-TU-tRNA's/EF-G	C1897947H2886627N556521O702699S11616P30367Mg2828Zn0Fe0	-36766.14	Translation
rib_70_elo1_b3125_8_cplx	Translation elongation complex: 8 * ribosome 70S/b3125/EF-TU-tRNA's/EF-G	C15123964H23025774N4427871O5578122S92928P236699Mg22624Zn0Fe0	-287885.12	Translation
rib_70_elo1_b3126_15_cplx	Translation elongation complex: 15 * ribosome 70S/b3126/EF-TU-tRNA's/EF-G	C24676519H37545637N7236578O9122995S151350P392361Mg37020Zn0Fe0	-473244.1	Translation
rib_70_elo1_b3126_1_cplx	Translation elongation complex: 1 * ribosome 70S/b3126/EF-TU-tRNA's/EF-G	C1651937H2510791N485148O613235S10090P26877Mg2468Zn0Fe0	-32270.14	Translation
rib_70_elo1_b3126_7_cplx	Translation elongation complex: 7 * ribosome 70S/b3126/EF-TU-tRNA's/EF-G	C11519615H17525725N3378618O4260275S70630P183513Mg17276Zn0Fe0	-221258.98	Translation
rib_70_elo1_b3127_13_cplx	Translation elongation complex: 13 * ribosome 70S/b3127/EF-TU-tRNA's/EF-G	C36391325H55472613N10620823O13337683S226252P549378Mg54080Zn0Fe0	-678795.82	Translation
rib_70_elo1_b3127_1_cplx	Translation elongation complex: 1 * ribosome 70S/b3127/EF-TU-tRNA's/EF-G	C2810993H4280325N821467O1034719S17404P43494Mg4160Zn0Fe0	-53450.14	Translation
rib_70_elo1_b3127_26_cplx	Translation elongation complex: 26 * ribosome 70S/b3127/EF-TU-tRNA's/EF-G	C72770018H110930925N21236792O26665894S452504P1097419Mg108160Zn0Fe0	-1356253.64	Translation
rib_70_elo1_b3164_1_cplx	Translation elongation complex: 1 * ribosome 70S/b3164/EF-TU-tRNA's/EF-G	C4589941H6999679N1337604O1679078S28567P68277Mg6770Zn0Fe0	-85365.14	Translation
rib_70_elo1_b3164_21_cplx	Translation elongation complex: 21 * ribosome 70S/b3164/EF-TU-tRNA's/EF-G	C95968741H146517939N27920944O34952738S599907P1389717Mg142170Zn0Fe0	-1748547.94	Translation
rib_70_elo1_b3164_43_cplx	Translation elongation complex: 43 * ribosome 70S/b3164/EF-TU-tRNA's/EF-G	C196485421H299988025N57162618O71553764S1228381P2843301Mg291110Zn0Fe0	-3578049.02	Translation
rib_70_elo1_b3165_1_cplx	Translation elongation complex: 1 * ribosome 70S/b3165/EF-TU-tRNA's/EF-G	C625007H942193N187203O240551S3610P12379Mg965Zn0Fe0	-13676.14	Translation
rib_70_elo1_b3165_2_cplx	Translation elongation complex: 2 * ribosome 70S/b3165/EF-TU-tRNA's/EF-G	C1247449H1881474N373385O479223S7220P24488Mg1930Zn0Fe0	-27081.28	Translation
rib_70_elo1_b3165_5_cplx	Translation elongation complex: 5 * ribosome 70S/b3165/EF-TU-tRNA's/EF-G	C3114775H4699317N931931O1195239S18050P60815Mg4825Zn0Fe0	-67296.7	Translation
rib_70_elo1_b3166_18_cplx	Translation elongation complex: 18 * ribosome 70S/b3166/EF-TU-tRNA's/EF-G	C36023921H54856553N10543996O13269431S222210P560852Mg53820Zn0Fe0	-683039.52	Translation
rib_70_elo1_b3166_1_cplx	Translation elongation complex: 1 * ribosome 70S/b3166/EF-TU-tRNA's/EF-G	C2009811H3057179N589153O743440S12345P32050Mg2990Zn0Fe0	-38839.14	Translation
rib_70_elo1_b3166_9_cplx	Translation elongation complex: 9 * ribosome 70S/b3166/EF-TU-tRNA's/EF-G	C18016451H27433355N5273785O6638024S111105P280898Mg26910Zn0Fe0	-341992.26	Translation
rib_70_elo1_b3167_1_cplx	Translation elongation complex: 1 * ribosome 70S/b3167/EF-TU-tRNA's/EF-G	C895375H1355319N265654O338507S5333P16170Mg1361Zn0Fe0	-18554.14	Translation
rib_70_elo1_b3167_3_cplx	Translation elongation complex: 3 * ribosome 70S/b3167/EF-TU-tRNA's/EF-G	C2678449H4057275N793834O1009941S15999P47706Mg4083Zn0Fe0	-54856.42	Translation
rib_70_elo1_b3167_7_cplx	Translation elongation complex: 7 * ribosome 70S/b3167/EF-TU-tRNA's/EF-G	C6244597H9461187N1850194O2352809S37331P110778Mg9527Zn0Fe0	-127460.98	Translation
rib_70_elo1_b3168_1_cplx	Translation elongation complex: 1 * ribosome 70S/b3168/EF-TU-tRNA's/EF-G	C5547443H8463254N1615386O2025805S34616P81658Mg8174Zn0Fe0	-102555.14	Translation
rib_70_elo1_b3168_26_cplx	Translation elongation complex: 26 * ribosome 70S/b3168/EF-TU-tRNA's/EF-G	C143594318H219322379N41736886O52208505S900016P2056283Mg212524Zn0Fe0	-2599583.64	Translation
rib_70_elo1_b3168_52_cplx	Translation elongation complex: 52 * ribosome 70S/b3168/EF-TU-tRNA's/EF-G	C287163068H438615869N83463246O104398513S1800032P4109893Mg425048Zn0Fe0	-5196493.28	Translation
rib_70_elo1_b3169_14_cplx	Translation elongation complex: 14 * ribosome 70S/b3169/EF-TU-tRNA's/EF-G	C43494684H66350111N12679232O15900080S270690P645292Mg64666Zn0Fe0	-802878.96	Translation
rib_70_elo1_b3169_1_cplx	Translation elongation complex: 1 * ribosome 70S/b3169/EF-TU-tRNA's/EF-G	C3119960H4754200N911034O1145327S19335P47474Mg4619Zn0Fe0	-58731.14	Translation
rib_70_elo1_b3169_29_cplx	Translation elongation complex: 29 * ribosome 70S/b3169/EF-TU-tRNA's/EF-G	C90080904H137422316N26257922O32924795S560715P1335082Mg133951Zn0Fe0	-1661511.06	Translation
rib_70_elo1_b3170_1_cplx	Translation elongation complex: 1 * ribosome 70S/b3170/EF-TU-tRNA's/EF-G	C999745H1514726N295884O376390S5983P17634Mg1514Zn0Fe0	-20446.14	Translation
rib_70_elo1_b3170_4_cplx	Translation elongation complex: 4 * ribosome 70S/b3170/EF-TU-tRNA's/EF-G	C3986050H6044309N1178418O1496029S23932P69177Mg6056Zn0Fe0	-80422.56	Translation
rib_70_elo1_b3170_8_cplx	Translation elongation complex: 8 * ribosome 70S/b3170/EF-TU-tRNA's/EF-G	C7967790H12083753N2355130O2988881S47864P137901Mg12112Zn0Fe0	-160391.12	Translation
rib_70_elo1_b3178_18_cplx	Translation elongation complex: 18 * ribosome 70S/b3178/EF-TU-tRNA's/EF-G	C72364650H110446314N21066461O26389964S452412P1057041Mg107280Zn0Fe0	-1324632.52	Translation
rib_70_elo1_b3178_1_cplx	Translation elongation complex: 1 * ribosome 70S/b3178/EF-TU-tRNA's/EF-G	C4037672H6155598N1177362O1478878S25134P60552Mg5960Zn0Fe0	-75419.14	Translation

rib_70_elo1_b3178_37_cplx	Translation elongation complex: 37 * ribosome 70S/b3178/EF-TU-tRNA's/EF-G	C148730096H227006526N43295454O54231766S929958P2170764Mg220520Zn0Fe0	-2720812.18	Translation
rib_70_elo1_b3179_12_cplx	Translation elongation complex: 12 * ribosome 70S/b3179/EF-TU-tRNA's/EF-G	C16292678H24763178N4791053O6052883S99540P266960Mg24540Zn0Fe0	-317626.68	Translation
rib_70_elo1_b3179_1_cplx	Translation elongation complex: 1 * ribosome 70S/b3179/EF-TU-tRNA's/EF-G	C1363225H2069804N401448O508465S8295P22826Mg2045Zn0Fe0	-27049.14	Translation
rib_70_elo1_b3179_6_cplx	Translation elongation complex: 6 * ribosome 70S/b3179/EF-TU-tRNA's/EF-G	C8149340H12384974N2396723O3028655S49770P133796Mg12270Zn0Fe0	-159129.84	Translation
rib_70_elo1_b3181_1_cplx	Translation elongation complex: 1 * ribosome 70S/b3181/EF-TU-tRNA's/EF-G	C1048096H1588969N309824O393614S6288P18242Mg1586Zn0Fe0	-21244.14	Translation
rib_70_elo1_b3181_4_cplx	Translation elongation complex: 4 * ribosome 70S/b3181/EF-TU-tRNA's/EF-G	C4178716H6340462N1233755O1564496S25152P71531Mg6344Zn0Fe0	-83536.56	Translation
rib_70_elo1_b3181_9_cplx	Translation elongation complex: 9 * ribosome 70S/b3181/EF-TU-tRNA's/EF-G	C9396416H14259617N2773640O3515966S56592P160346Mg14274Zn0Fe0	-187357.26	Translation
rib_70_elo1_b3185_1_cplx	Translation elongation complex: 1 * ribosome 70S/b3185/EF-TU-tRNA's/EF-G	C599269H903298N179608O230820S3456P11908Mg929Zn0Fe0	-13112.14	Translation
rib_70_elo1_b3185_2_cplx	Translation elongation complex: 2 * ribosome 70S/b3185/EF-TU-tRNA's/EF-G	C1196075H1803814N358217O459848S6912P23558Mg1858Zn0Fe0	-25965.28	Translation
rib_70_elo1_b3185_5_cplx	Translation elongation complex: 5 * ribosome 70S/b3185/EF-TU-tRNA's/EF-G	C2986493H4505362N894044O1146932S17280P58508Mg4645Zn0Fe0	-64524.7	Translation
rib_70_elo1_b3186_1_cplx	Translation elongation complex: 1 * ribosome 70S/b3186/EF-TU-tRNA's/EF-G	C709692H1072101N211600O270752S4160P13442Mg1091Zn0Fe0	-15090.14	Translation
rib_70_elo1_b3186_3_cplx	Translation elongation complex: 3 * ribosome 70S/b3186/EF-TU-tRNA's/EF-G	C2123114H3209587N632386O807896S12480P39698Mg3273Zn0Fe0	-44640.42	Translation
rib_70_elo1_b3186_6_cplx	Translation elongation complex: 6 * ribosome 70S/b3186/EF-TU-tRNA's/EF-G	C4243247H6415816N1263565O1613612S24960P79082Mg6546Zn0Fe0	-88965.84	Translation
rib_70_elo1_b3201_14_cplx	Translation elongation complex: 14 * ribosome 70S/b3201/EF-TU-tRNA's/EF-G	C21753398H33087012N6385385O8055374S133028P349468Mg32662Zn0Fe0	-419680.96	Translation
rib_70_elo1_b3201_1_cplx	Translation elongation complex: 1 * ribosome 70S/b3201/EF-TU-tRNA's/EF-G	C1560225H2370625N458659O580101S9502P25638Mg2333Zn0Fe0	-30654.14	Translation
rib_70_elo1_b3201_7_cplx	Translation elongation complex: 7 * ribosome 70S/b3201/EF-TU-tRNA's/EF-G	C10880151H16547419N3194071O4030227S66514P175098Mg16331Zn0Fe0	-210204.98	Translation
rib_70_elo1_b3202_14_cplx	Translation elongation complex: 14 * ribosome 70S/b3202/EF-TU-tRNA's/EF-G	C42027155H64075626N12262887O15394637S261380P631870Mg62398Zn0Fe0	-782820.96	Translation
rib_70_elo1_b3202_1_cplx	Translation elongation complex: 1 * ribosome 70S/b3202/EF-TU-tRNA's/EF-G	C3014623H4591201N881036O1108872S18670P46467Mg4457Zn0Fe0	-57250.14	Translation
rib_70_elo1_b3202_28_cplx	Translation elongation complex: 28 * ribosome 70S/b3202/EF-TU-tRNA's/EF-G	C84040651H128135776N24520265O30779307S522760P1262304Mg124796Zn0Fe0	-1564204.92	Translation
rib_70_elo1_b3203_1_cplx	Translation elongation complex: 1 * ribosome 70S/b3203/EF-TU-tRNA's/EF-G	C661136H997650N197492O253375S3846P12810Mg1019Zn0Fe0	-14267.14	Translation
rib_70_elo1_b3203_2_cplx	Translation elongation complex: 2 * ribosome 70S/b3203/EF-TU-tRNA's/EF-G	C1319521H1992194N393865O504766S7692P25332Mg2038Zn0Fe0	-28245.28	Translation
rib_70_elo1_b3203_5_cplx	Translation elongation complex: 5 * ribosome 70S/b3203/EF-TU-tRNA's/EF-G	C3294676H4975826N982984O1258939S19230P62898Mg5095Zn0Fe0	-70179.7	Translation
rib_70_elo1_b3204_1_cplx	Translation elongation complex: 1 * ribosome 70S/b3204/EF-TU-tRNA's/EF-G	C1080271H1637521N319371O405851S6478P18832Mg1631Zn0Fe0	-21951.14	Translation
rib_70_elo1_b3204_4_cplx	Translation elongation complex: 4 * ribosome 70S/b3204/EF-TU-tRNA's/EF-G	C4307026H6534157N1271820O1613150S25912P73852Mg6524Zn0Fe0	-86325.56	Translation
rib_70_elo1_b3204_9_cplx	Translation elongation complex: 9 * ribosome 70S/b3204/EF-TU-tRNA's/EF-G	C9684951H14695217N2859235O3625315S58302P165552Mg14679Zn0Fe0	-193616.26	Translation
rib_70_elo1_b3205_16_cplx	Translation elongation complex: 16 * ribosome 70S/b3205/EF-TU-tRNA's/EF-G	C29110602H44299963N8532920O10751375S179344P460807Mg43520Zn0Fe0	-557530.24	Translation
rib_70_elo1_b3205_1_cplx	Translation elongation complex: 1 * ribosome 70S/b3205/EF-TU-tRNA's/EF-G	C1827012H2777368N536285O677585S11209P29602Mg2720Zn0Fe0	-35648.14	Translation
rib_70_elo1_b3205_8_cplx	Translation elongation complex: 8 * ribosome 70S/b3205/EF-TU-tRNA's/EF-G	C14559354H22154579N4268048O5378687S89672P230831Mg21760Zn0Fe0	-279193.12	Translation
rib_70_elo1_b3206_1_cplx	Translation elongation complex: 1 * ribosome 70S/b3206/EF-TU-tRNA's/EF-G	C630270H950510N188641O242159S3652P12375Mg974Zn0Fe0	-13715.14	Translation
rib_70_elo1_b3206_2_cplx	Translation elongation complex: 2 * ribosome 70S/b3206/EF-TU-tRNA's/EF-G	C1257973H1898124N376247O482442S7304P24481Mg1948Zn0Fe0	-27160.28	Translation
rib_70_elo1_b3206_5_cplx	Translation elongation complex: 5 * ribosome 70S/b3206/EF-TU-tRNA's/EF-G	C3141082H4740966N939065O1203291S18260P60799Mg4870Zn0Fe0	-67495.7	Translation

rib_70_elo1_b3230_1_cplx	Translation elongation complex: 1 * ribosome 70S/b3230/EF-TU-tRNA's/EF-G	C876574H1326764N260179O331575S5201P15879Mg1334Zn0Fe0	-18165.14	Translation
rib_70_elo1_b3230_3_cplx	Translation elongation complex: 3 * ribosome 70S/b3230/EF-TU-tRNA's/EF-G	C2622254H3971832N777579O989219S15603P46851Mg4002Zn0Fe0	-53707.42	Translation
rib_70_elo1_b3230_7_cplx	Translation elongation complex: 7 * ribosome 70S/b3230/EF-TU-tRNA's/EF-G	C6113614H9261968N1812379O2304507S36407P108795Mg9338Zn0Fe0	-124791.98	Translation
rib_70_elo1_b3231_1_cplx	Translation elongation complex: 1 * ribosome 70S/b3231/EF-TU-tRNA's/EF-G	C949533H1438502N281092O357818S5662P16826Mg1442Zn0Fe0	-19429.14	Translation
rib_70_elo1_b3231_4_cplx	Translation elongation complex: 4 * ribosome 70S/b3231/EF-TU-tRNA's/EF-G	C3785865H5740121N1119412O1422332S22648P66011Mg5768Zn0Fe0	-76420.56	Translation
rib_70_elo1_b3231_8_cplx	Translation elongation complex: 8 * ribosome 70S/b3231/EF-TU-tRNA's/EF-G	C7567641H11475613N2237172O2841684S45296P131591Mg11536Zn0Fe0	-152409.12	Translation
rib_70_elo1_b3247_14_cplx	Translation elongation complex: 14 * ribosome 70S/b3247/EF-TU-tRNA's/EF-G	C43005207H65592962N12539183O15728417S267988P640615Mg63910Zn0Fe0	-795639.96	Translation
rib_70_elo1_b3247_1_cplx	Translation elongation complex: 1 * ribosome 70S/b3247/EF-TU-tRNA's/EF-G	C3084716H4699805N900855O1132914S19142P47113Mg4565Zn0Fe0	-58187.14	Translation
rib_70_elo1_b3247_28_cplx	Translation elongation complex: 28 * ribosome 70S/b3247/EF-TU-tRNA's/EF-G	C85996505H131170208N25072767O31446651S535976P1279771Mg127820Zn0Fe0	-1589819.92	Translation
rib_70_elo1_b3248_11_cplx	Translation elongation complex: 11 * ribosome 70S/b3248/EF-TU-tRNA's/EF-G	C14124591H21460108N4156254O5256289S85888P233574Mg21307Zn0Fe0	-276927.54	Translation
rib_70_elo1_b3248_1_cplx	Translation elongation complex: 1 * ribosome 70S/b3248/EF-TU-tRNA's/EF-G	C1289211H1956738N379934O481619S7808P21774Mg1937Zn0Fe0	-25716.14	Translation
rib_70_elo1_b3248_5_cplx	Translation elongation complex: 5 * ribosome 70S/b3248/EF-TU-tRNA's/EF-G	C6423363H9758086N1890462O2391487S39040P106494Mg9685Zn0Fe0	-126200.7	Translation
rib_70_elo1_b3249_1_cplx	Translation elongation complex: 1 * ribosome 70S/b3249/EF-TU-tRNA's/EF-G	C1075489H1629662N318070O404403S6470P18864Mg1622Zn0Fe0	-21945.14	Translation
rib_70_elo1_b3249_4_cplx	Translation elongation complex: 4 * ribosome 70S/b3249/EF-TU-tRNA's/EF-G	C4288102H6503000N1266991O1607157S25880P73992Mg6488Zn0Fe0	-86313.56	Translation
rib_70_elo1_b3249_9_cplx	Translation elongation complex: 9 * ribosome 70S/b3249/EF-TU-tRNA's/EF-G	C9642457H14625230N2848526O3611747S58230P165872Mg14598Zn0Fe0	-193594.26	Translation
rib_70_elo1_b3250_10_cplx	Translation elongation complex: 10 * ribosome 70S/b3250/EF-TU-tRNA's/EF-G	C23260181H35438586N6799493O8547191S143820P356584Mg34670Zn0Fe0	-437436.4	Translation
rib_70_elo1_b3250_1_cplx	Translation elongation complex: 1 * ribosome 70S/b3250/EF-TU-tRNA's/EF-G	C2335460H3554556N683696O861704S14382P36652Mg3467Zn0Fe0	-44738.14	Translation
rib_70_elo1_b3250_21_cplx	Translation elongation complex: 21 * ribosome 70S/b3250/EF-TU-tRNA's/EF-G	C48834840H74407956N14274356O17940564S302022P747612Mg72807Zn0Fe0	-917400.94	Translation
rib_70_elo1_b3251_10_cplx	Translation elongation complex: 10 * ribosome 70S/b3251/EF-TU-tRNA's/EF-G	C22024392H33554171N6441140O8096316S135970P338466Mg32870Zn0Fe0	-414618.4	Translation
rib_70_elo1_b3251_1_cplx	Translation elongation complex: 1 * ribosome 70S/b3251/EF-TU-tRNA's/EF-G	C2211369H3365525N647651O816243S13597P34788Mg3287Zn0Fe0	-42404.14	Translation
rib_70_elo1_b3251_20_cplx	Translation elongation complex: 20 * ribosome 70S/b3251/EF-TU-tRNA's/EF-G	C44038862H67097111N12878350O16185286S271940P675886Mg65740Zn0Fe0	-828189.8	Translation
rib_70_elo1_b3257_1_cplx	Translation elongation complex: 1 * ribosome 70S/b3257/EF-TU-tRNA's/EF-G	C569218H857067N170914O220119S3274P11548Mg884Zn0Fe0	-12631.14	Translation
rib_70_elo1_b3257_2_cplx	Translation elongation complex: 2 * ribosome 70S/b3257/EF-TU-tRNA's/EF-G	C1136138H1711533N340950O438495S6548P22851Mg1768Zn0Fe0	-25016.28	Translation
rib_70_elo1_b3257_4_cplx	Translation elongation complex: 4 * ribosome 70S/b3257/EF-TU-tRNA's/EF-G	C2269978H3420465N681022O875247S13096P45457Mg3536Zn0Fe0	-49786.56	Translation
rib_70_elo1_b3258_14_cplx	Translation elongation complex: 14 * ribosome 70S/b3258/EF-TU-tRNA's/EF-G	C42553065H64872122N12418827O15587799S264110P640149Mg63154Zn0Fe0	-793073.96	Translation
rib_70_elo1_b3258_1_cplx	Translation elongation complex: 1 * ribosome 70S/b3258/EF-TU-tRNA's/EF-G	C3052175H4648075N891961O1122907S18865P47063Mg4511Zn0Fe0	-57987.14	Translation
rib_70_elo1_b3258_28_cplx	Translation elongation complex: 28 * ribosome 70S/b3258/EF-TU-tRNA's/EF-G	C85092485H129728788N24832375O31165375S528220P1278857Mg126308Zn0Fe0	-1584705.92	Translation
rib_70_elo1_b3259_17_cplx	Translation elongation complex: 17 * ribosome 70S/b3259/EF-TU-tRNA's/EF-G	C31822693H48447948N9318321O11736196S195942P498336Mg47617Zn0Fe0	-605473.38	Translation
rib_70_elo1_b3259_1_cplx	Translation elongation complex: 1 * ribosome 70S/b3259/EF-TU-tRNA's/EF-G	C1879829H2858828N551313O696164S11526P30144Mg2801Zn0Fe0	-36447.14	Translation
rib_70_elo1_b3259_8_cplx	Translation elongation complex: 8 * ribosome 70S/b3259/EF-TU-tRNA's/EF-G	C14979832H22804068N4386879O5526178S92208P234978Mg22408Zn0Fe0	-285396.12	Translation
rib_70_elo1_b3260_18_cplx	Translation elongation complex: 18 * ribosome 70S/b3260/EF-TU-tRNA's/EF-G	C36765450H56000953N10754274O13526534S227196P568130Mg54954Zn0Fe0	-693575.52	Translation

rib_70_elo1_b3260_1_cplx	Translation elongation complex: 1 * ribosome 70S/b3260/EF-TU-tRNA's/EF-G	C2051212H3120991N600939O757851S12622P32477Mg3053Zn0Fe0	-39447.14	Translation
rib_70_elo1_b3260_9_cplx	Translation elongation complex: 9 * ribosome 70S/b3260/EF-TU-tRNA's/EF-G	C18387324H28005679N5378979O6766643S113598P284549Mg27477Zn0Fe0	-347272.26	Translation
rib_70_elo1_b3261_1_cplx	Translation elongation complex: 1 * ribosome 70S/b3261/EF-TU-tRNA's/EF-G	C680025H1026352N203082O260337S3971P13119Mg1046Zn0Fe0	-14646.14	Translation
rib_70_elo1_b3261_2_cplx	Translation elongation complex: 2 * ribosome 70S/b3261/EF-TU-tRNA's/EF-G	C1357218H2049505N405024O518613S7942P25941Mg2092Zn0Fe0	-28994.28	Translation
rib_70_elo1_b3261_5_cplx	Translation elongation complex: 5 * ribosome 70S/b3261/EF-TU-tRNA's/EF-G	C3388797H5118964N1010850O1293441S19855P64407Mg5230Zn0Fe0	-72038.7	Translation
rib_70_elo1_b3280_1_cplx	Translation elongation complex: 1 * ribosome 70S/b3280/EF-TU-tRNA's/EF-G	C599548H903549N179693O230931S3483P11937Mg929Zn0Fe0	-13149.14	Translation
rib_70_elo1_b3280_2_cplx	Translation elongation complex: 2 * ribosome 70S/b3280/EF-TU-tRNA's/EF-G	C1196665H1804362N358393O460094S6966P23620Mg1858Zn0Fe0	-26043.28	Translation
rib_70_elo1_b3280_5_cplx	Translation elongation complex: 5 * ribosome 70S/b3280/EF-TU-tRNA's/EF-G	C2988016H4506801N894493O1147583S17415P58669Mg4645Zn0Fe0	-64725.7	Translation
rib_70_elo1_b3281_16_cplx	Translation elongation complex: 16 * ribosome 70S/b3281/EF-TU-tRNA's/EF-G	C27905793H42465372N8179759O10309248S171344P441939Mg41792Zn0Fe0	-534406.24	Translation
rib_70_elo1_b3281_1_cplx	Translation elongation complex: 1 * ribosome 70S/b3281/EF-TU-tRNA's/EF-G	C1751403H2662317N514084O649743S10709P28389Mg2612Zn0Fe0	-34169.14	Translation
rib_70_elo1_b3281_8_cplx	Translation elongation complex: 8 * ribosome 70S/b3281/EF-TU-tRNA's/EF-G	C13956785H21237076N4091399O5157512S85672P221379Mg20896Zn0Fe0	-267613.12	Translation
rib_70_elo1_b3282_11_cplx	Translation elongation complex: 11 * ribosome 70S/b3282/EF-TU-tRNA's/EF-G	C13645970H20730409N4017636O5079258S83006P226194Mg20614Zn0Fe0	-267688.54	Translation
rib_70_elo1_b3282_1_cplx	Translation elongation complex: 1 * ribosome 70S/b3282/EF-TU-tRNA's/EF-G	C1245490H1890179N367186O465418S7546P21084Mg1874Zn0Fe0	-24857.14	Translation
rib_70_elo1_b3282_5_cplx	Translation elongation complex: 5 * ribosome 70S/b3282/EF-TU-tRNA's/EF-G	C6205682H9426271N1827366O2310954S37730P103128Mg9370Zn0Fe0	-121989.7	Translation
rib_70_elo1_b3283_10_cplx	Translation elongation complex: 10 * ribosome 70S/b3283/EF-TU-tRNA's/EF-G	C11787681H17903689N3470992O4392835S71840P196705Mg17840Zn0Fe0	-231777.4	Translation
rib_70_elo1_b3283_1_cplx	Translation elongation complex: 1 * ribosome 70S/b3283/EF-TU-tRNA's/EF-G	C1183413H1795624N348937O442708S7184P20161Mg1784Zn0Fe0	-23669.14	Translation
rib_70_elo1_b3283_5_cplx	Translation elongation complex: 5 * ribosome 70S/b3283/EF-TU-tRNA's/EF-G	C5896421H8954764N1736517O2198320S35920P98625Mg8920Zn0Fe0	-116161.7	Translation
rib_70_elo1_b3287_1_cplx	Translation elongation complex: 1 * ribosome 70S/b3287/EF-TU-tRNA's/EF-G	C1116520H1693264N329780O418687S6725P19278Mg1685Zn0Fe0	-22539.14	Translation
rib_70_elo1_b3287_4_cplx	Translation elongation complex: 4 * ribosome 70S/b3287/EF-TU-tRNA's/EF-G	C4451470H6756598N1313225O1664089S26900P75576Mg6740Zn0Fe0	-88617.56	Translation
rib_70_elo1_b3287_9_cplx	Translation elongation complex: 9 * ribosome 70S/b3287/EF-TU-tRNA's/EF-G	C10009720H15195488N2952300O3739759S60525P169406Mg15165Zn0Fe0	-198748.26	Translation
rib_70_elo1_b3288_18_cplx	Translation elongation complex: 18 * ribosome 70S/b3288/EF-TU-tRNA's/EF-G	C36126287H55014899N10571972O13306934S222678P561702Mg53982Zn0Fe0	-684555.52	Translation
rib_70_elo1_b3288_1_cplx	Translation elongation complex: 1 * ribosome 70S/b3288/EF-TU-tRNA's/EF-G	C2015532H3066027N590728O745532S12371P32101Mg2999Zn0Fe0	-38927.14	Translation
rib_70_elo1_b3288_9_cplx	Translation elongation complex: 9 * ribosome 70S/b3288/EF-TU-tRNA's/EF-G	C18067652H27512555N5287784O6656780S111339P281325Mg26991Zn0Fe0	-342752.26	Translation
rib_70_elo1_b3289_12_cplx	Translation elongation complex: 12 * ribosome 70S/b3289/EF-TU-tRNA's/EF-G	C32462667H49489779N9473900O11896484S201948P489584Mg48300Zn0Fe0	-604486.68	Translation
rib_70_elo1_b3289_1_cplx	Translation elongation complex: 1 * ribosome 70S/b3289/EF-TU-tRNA's/EF-G	C2716467H4136867N793954O999642S16829P41983Mg4025Zn0Fe0	-51559.14	Translation
rib_70_elo1_b3289_25_cplx	Translation elongation complex: 25 * ribosome 70S/b3289/EF-TU-tRNA's/EF-G	C67617267H103088675N19732018O24774570S420725P1018567Mg100625Zn0Fe0	-1257946.5	Translation
rib_70_elo1_b3294_1_cplx	Translation elongation complex: 1 * ribosome 70S/b3294/EF-TU-tRNA's/EF-G	C858371H1298806N254938O325076S5090P15641Mg1307Zn0Fe0	-17869.14	Translation
rib_70_elo1_b3294_3_cplx	Translation elongation complex: 3 * ribosome 70S/b3294/EF-TU-tRNA's/EF-G	C2567807H3888144N761898O969854S15270P46155Mg3921Zn0Fe0	-52837.42	Translation
rib_70_elo1_b3294_7_cplx	Translation elongation complex: 7 * ribosome 70S/b3294/EF-TU-tRNA's/EF-G	C5986679H9066820N1775818O2259410S35630P107183Mg9149Zn0Fe0	-122773.98	Translation
rib_70_elo1_b3295_19_cplx	Translation elongation complex: 19 * ribosome 70S/b3295/EF-TU-tRNA's/EF-G	C39757843H60558950N11630897O14630836S245214P614842Mg59375Zn0Fe0	-751189.66	Translation
rib_70_elo1_b3295_1_cplx	Translation elongation complex: 1 * ribosome 70S/b3295/EF-TU-tRNA's/EF-G	C2101447H3197414N615725O776596S12906P33298Mg3125Zn0Fe0	-40475.14	Translation

rib_70_elo1_b3295_9_cplx	Translation elongation complex: 9 * ribosome 70S/b3295/EF-TU-tRNA's/EF-G	C18837623H28691430N5511357O6934036S116154P291762Mg28125Zn0Fe0	-356348.26	Translation
rib_70_elo1_b3296_12_cplx	Translation elongation complex: 12 * ribosome 70S/b3296/EF-TU-tRNA's/EF-G	C16072219H24426588N4726999O5973426S97884P263961Mg24216Zn0Fe0	-313691.68	Translation
rib_70_elo1_b3296_1_cplx	Translation elongation complex: 1 * ribosome 70S/b3296/EF-TU-tRNA's/EF-G	C1344781H2041676N396101O501762S8157P22566Mg2018Zn0Fe0	-26711.14	Translation
rib_70_elo1_b3296_6_cplx	Translation elongation complex: 6 * ribosome 70S/b3296/EF-TU-tRNA's/EF-G	C8039071H12216636N2364691O2988882S48942P132291Mg12108Zn0Fe0	-157156.84	Translation
rib_70_elo1_b3297_1_cplx	Translation elongation complex: 1 * ribosome 70S/b3297/EF-TU-tRNA's/EF-G	C869338H1315998N257941O328636S5155P15675Mg1325Zn0Fe0	-17953.14	Translation
rib_70_elo1_b3297_3_cplx	Translation elongation complex: 3 * ribosome 70S/b3297/EF-TU-tRNA's/EF-G	C2600606H3939600N770895O980448S15465P46245Mg3975Zn0Fe0	-53077.42	Translation
rib_70_elo1_b3297_7_cplx	Translation elongation complex: 7 * ribosome 70S/b3297/EF-TU-tRNA's/EF-G	C6063142H9186804N1796803O2284072S36085P107385Mg9275Zn0Fe0	-123325.98	Translation
rib_70_elo1_b3298_1_cplx	Translation elongation complex: 1 * ribosome 70S/b3298/EF-TU-tRNA's/EF-G	C802835H1214083N238780O304882S4732P14838Mg1226Zn0Fe0	-16840.14	Translation
rib_70_elo1_b3298_3_cplx	Translation elongation complex: 3 * ribosome 70S/b3298/EF-TU-tRNA's/EF-G	C2401711H3634561N713630O909642S14196P43796Mg3678Zn0Fe0	-49800.42	Translation
rib_70_elo1_b3298_6_cplx	Translation elongation complex: 6 * ribosome 70S/b3298/EF-TU-tRNA's/EF-G	C4800025H7265278N1425905O1816782S28392P87233Mg7356Zn0Fe0	-99240.84	Translation
rib_70_elo1_b3299_1_cplx	Translation elongation complex: 1 * ribosome 70S/b3299/EF-TU-tRNA's/EF-G	C310422H461995N95820O126002S1647P7839Mg506Zn0Fe0	-7893.14	Translation
rib_70_elo1_b3299_2_cplx	Translation elongation complex: 2 * ribosome 70S/b3299/EF-TU-tRNA's/EF-G	C619727H922730N191189O251189S3294P15561Mg1012Zn0Fe0	-15668.28	Translation
rib_70_elo1_b3300_13_cplx	Translation elongation complex: 13 * ribosome 70S/b3300/EF-TU-tRNA's/EF-G	C36292751H55328749N10592266O13295553S225433P546435Mg53963Zn0Fe0	-675683.82	Translation
rib_70_elo1_b3300_1_cplx	Translation elongation complex: 1 * ribosome 70S/b3300/EF-TU-tRNA's/EF-G	C2803403H4269229N819298O1031433S17341P43263Mg4151Zn0Fe0	-53206.14	Translation
rib_70_elo1_b3300_26_cplx	Translation elongation complex: 26 * ribosome 70S/b3300/EF-TU-tRNA's/EF-G	C72572878H110643229N21179648O26581683S450866P1091538Mg107926Zn0Fe0	-1350034.64	Translation
rib_70_elo1_b3301_1_cplx	Translation elongation complex: 1 * ribosome 70S/b3301/EF-TU-tRNA's/EF-G	C962165H1457621N284996O362617S5716P17058Mg1460Zn0Fe0	-19692.14	Translation
rib_70_elo1_b3301_4_cplx	Translation elongation complex: 4 * ribosome 70S/b3301/EF-TU-tRNA's/EF-G	C3836231H5816441N1135016O1441306S22864P66927Mg5840Zn0Fe0	-77460.56	Translation
rib_70_elo1_b3301_8_cplx	Translation elongation complex: 8 * ribosome 70S/b3301/EF-TU-tRNA's/EF-G	C7668319H11628201N2268376O2879558S45728P133419Mg11680Zn0Fe0	-154485.12	Translation
rib_70_elo1_b3302_1_cplx	Translation elongation complex: 1 * ribosome 70S/b3302/EF-TU-tRNA's/EF-G	C439692H659397N133358O173012S2442P9681Mg695Zn0Fe0	-10257.14	Translation
rib_70_elo1_b3302_3_cplx	Translation elongation complex: 3 * ribosome 70S/b3302/EF-TU-tRNA's/EF-G	C1315650H1974317N398710O516518S7326P28683Mg2085Zn0Fe0	-30409.42	Translation
rib_70_elo1_b3303_1_cplx	Translation elongation complex: 1 * ribosome 70S/b3303/EF-TU-tRNA's/EF-G	C1103075H1673039N325719O413552S6620P18991Mg1667Zn0Fe0	-22206.14	Translation
rib_70_elo1_b3303_4_cplx	Translation elongation complex: 4 * ribosome 70S/b3303/EF-TU-tRNA's/EF-G	C4397894H6675884N1297107O1643648S26480P74452Mg6668Zn0Fe0	-87309.56	Translation
rib_70_elo1_b3303_9_cplx	Translation elongation complex: 9 * ribosome 70S/b3303/EF-TU-tRNA's/EF-G	C9889259H15013959N2916087O3693808S59580P166887Mg15003Zn0Fe0	-195815.26	Translation
rib_70_elo1_b3304_1_cplx	Translation elongation complex: 1 * ribosome 70S/b3304/EF-TU-tRNA's/EF-G	C796127H1203972N236765O302309S4685P14706Mg1217Zn0Fe0	-16690.14	Translation
rib_70_elo1_b3304_3_cplx	Translation elongation complex: 3 * ribosome 70S/b3304/EF-TU-tRNA's/EF-G	C2381649H3604284N707609O901981S14055P43410Mg3651Zn0Fe0	-49360.42	Translation
rib_70_elo1_b3304_6_cplx	Translation elongation complex: 6 * ribosome 70S/b3304/EF-TU-tRNA's/EF-G	C4759932H7204752N1413875O1801489S28110P86466Mg7302Zn0Fe0	-98365.84	Translation
rib_70_elo1_b3305_10_cplx	Translation elongation complex: 10 * ribosome 70S/b3305/EF-TU-tRNA's/EF-G	C11600713H17619274N3417175O4325825S70100P193854Mg17570Zn0Fe0	-228406.4	Translation
rib_70_elo1_b3305_1_cplx	Translation elongation complex: 1 * ribosome 70S/b3305/EF-TU-tRNA's/EF-G	C1164646H1767097N343540O435953S7010P19866Mg1757Zn0Fe0	-23322.14	Translation
rib_70_elo1_b3305_5_cplx	Translation elongation complex: 5 * ribosome 70S/b3305/EF-TU-tRNA's/EF-G	C5802898H8812509N1709600O2164785S35050P97194Mg8785Zn0Fe0	-114470.7	Translation
rib_70_elo1_b3306_1_cplx	Translation elongation complex: 1 * ribosome 70S/b3306/EF-TU-tRNA's/EF-G	C876570H1326684N260165O331647S5199P15878Mg1334Zn0Fe0	-18189.14	Translation
rib_70_elo1_b3306_3_cplx	Translation elongation complex: 3 * ribosome 70S/b3306/EF-TU-tRNA's/EF-G	C2622218H3971592N777489O989453S15597P46848Mg4002Zn0Fe0	-53779.42	Translation

rib_70_elo1_b3306_7_cplx	Translation elongation complex: 7 * ribosome 70S/b3306/EF-TU-tRNA's/EF-G	C6113514H9261408N1812137O2305065S36393P108788Mg9338Zn0Fe0	-124959.98	Translation
rib_70_elo1_b3307_1_cplx	Translation elongation complex: 1 * ribosome 70S/b3307/EF-TU-tRNA's/EF-G	C698470H1054555N208533O267019S4089P13379Mg1073Zn0Fe0	-14965.14	Translation
rib_70_elo1_b3307_2_cplx	Translation elongation complex: 2 * ribosome 70S/b3307/EF-TU-tRNA's/EF-G	C1394025H2105811N415894O531904S8178P26452Mg2146Zn0Fe0	-29623.28	Translation
rib_70_elo1_b3307_5_cplx	Translation elongation complex: 5 * ribosome 70S/b3307/EF-TU-tRNA's/EF-G	C3480690H5259579N1037977O1326559S20445P65671Mg5365Zn0Fe0	-73597.7	Translation
rib_70_elo1_b3308_10_cplx	Translation elongation complex: 10 * ribosome 70S/b3308/EF-TU-tRNA's/EF-G	C11732463H17818181N3456476O4375140S71050P196310Mg17750Zn0Fe0	-231282.4	Translation
rib_70_elo1_b3308_1_cplx	Translation elongation complex: 1 * ribosome 70S/b3308/EF-TU-tRNA's/EF-G	C1177866H1787048N347480O440907S7105P20117Mg1775Zn0Fe0	-23615.14	Translation
rib_70_elo1_b3308_5_cplx	Translation elongation complex: 5 * ribosome 70S/b3308/EF-TU-tRNA's/EF-G	C5868798H8911996N1729256O2189455S35525P98425Mg8875Zn0Fe0	-115911.7	Translation
rib_70_elo1_b3309_1_cplx	Translation elongation complex: 1 * ribosome 70S/b3309/EF-TU-tRNA's/EF-G	C715983H1081650N213429O273112S4193P13536Mg1100Zn0Fe0	-15209.14	Translation
rib_70_elo1_b3309_3_cplx	Translation elongation complex: 3 * ribosome 70S/b3309/EF-TU-tRNA's/EF-G	C2141925H3238162N637833O814976S12579P39978Mg3300Zn0Fe0	-44995.42	Translation
rib_70_elo1_b3309_6_cplx	Translation elongation complex: 6 * ribosome 70S/b3309/EF-TU-tRNA's/EF-G	C4280838H6472930N1274439O1627772S25158P79641Mg6600Zn0Fe0	-89674.84	Translation
rib_70_elo1_b3310_1_cplx	Translation elongation complex: 1 * ribosome 70S/b3310/EF-TU-tRNA's/EF-G	C833222H1260602N247549O315780S4931P15233Mg1271Zn0Fe0	-17366.14	Translation
rib_70_elo1_b3310_3_cplx	Translation elongation complex: 3 * ribosome 70S/b3310/EF-TU-tRNA's/EF-G	C2492578H3773798N739809O942126S14793P44951Mg3813Zn0Fe0	-51348.42	Translation
rib_70_elo1_b3310_7_cplx	Translation elongation complex: 7 * ribosome 70S/b3310/EF-TU-tRNA's/EF-G	C5811290H8800190N1724329O2194818S34517P104387Mg8897Zn0Fe0	-119312.98	Translation
rib_70_elo1_b3311_1_cplx	Translation elongation complex: 1 * ribosome 70S/b3311/EF-TU-tRNA's/EF-G	C593372H894217N177908O228699S3424P11839Mg920Zn0Fe0	-13026.14	Translation
rib_70_elo1_b3311_2_cplx	Translation elongation complex: 2 * ribosome 70S/b3311/EF-TU-tRNA's/EF-G	C1184322H1785700N354843O455630S6848P23424Mg1840Zn0Fe0	-25797.28	Translation
rib_70_elo1_b3311_4_cplx	Translation elongation complex: 4 * ribosome 70S/b3311/EF-TU-tRNA's/EF-G	C2366222H3568666N708713O909492S13696P46594Mg3680Zn0Fe0	-51339.56	Translation
rib_70_elo1_b3312_1_cplx	Translation elongation complex: 1 * ribosome 70S/b3312/EF-TU-tRNA's/EF-G	C464885H697670N140738O182354S2607P10099Mg731Zn0Fe0	-10767.14	Translation
rib_70_elo1_b3312_3_cplx	Translation elongation complex: 3 * ribosome 70S/b3312/EF-TU-tRNA's/EF-G	C1390995H2088878N420696O544416S7821P29915Mg2193Zn0Fe0	-31917.42	Translation
rib_70_elo1_b3313_1_cplx	Translation elongation complex: 1 * ribosome 70S/b3313/EF-TU-tRNA's/EF-G	C912748H1382290N270554O344438S5437P16317Mg1388Zn0Fe0	-18759.14	Translation
rib_70_elo1_b3313_4_cplx	Translation elongation complex: 4 * ribosome 70S/b3313/EF-TU-tRNA's/EF-G	C3639211H5515867N1077422O1369166S21748P64035Mg5552Zn0Fe0	-73800.56	Translation
rib_70_elo1_b3313_8_cplx	Translation elongation complex: 8 * ribosome 70S/b3313/EF-TU-tRNA's/EF-G	C7274495H11027303N2153246O2735470S43496P127659Mg11104Zn0Fe0	-147189.12	Translation
rib_70_elo1_b3314_13_cplx	Translation elongation complex: 13 * ribosome 70S/b3314/EF-TU-tRNA's/EF-G	C19538635H29723527N5734299O7232985S119431P313118Mg29393Zn0Fe0	-375780.82	Translation
rib_70_elo1_b3314_1_cplx	Translation elongation complex: 1 * ribosome 70S/b3314/EF-TU-tRNA's/EF-G	C1509151H2293411N443595O560889S9187P24734Mg2261Zn0Fe0	-29555.14	Translation
rib_70_elo1_b3314_6_cplx	Translation elongation complex: 6 * ribosome 70S/b3314/EF-TU-tRNA's/EF-G	C9021436H13722626N2648055O3340929S55122P144894Mg13566Zn0Fe0	-173815.84	Translation
rib_70_elo1_b3315_1_cplx	Translation elongation complex: 1 * ribosome 70S/b3315/EF-TU-tRNA's/EF-G	C753655H1138918N224460O287077S4421P14140Mg1154Zn0Fe0	-15963.14	Translation
rib_70_elo1_b3315_3_cplx	Translation elongation complex: 3 * ribosome 70S/b3315/EF-TU-tRNA's/EF-G	C2254627H3409584N670852O856585S13263P41754Mg3462Zn0Fe0	-47221.42	Translation
rib_70_elo1_b3315_6_cplx	Translation elongation complex: 6 * ribosome 70S/b3315/EF-TU-tRNA's/EF-G	C4506085H6815583N1340440O1710847S26526P83175Mg6924Zn0Fe0	-94108.84	Translation
rib_70_elo1_b3316_1_cplx	Translation elongation complex: 1 * ribosome 70S/b3316/EF-TU-tRNA's/EF-G	C642703H969520N192178O246682S3734P12548Mg992Zn0Fe0	-13922.14	Translation
rib_70_elo1_b3316_2_cplx	Translation elongation complex: 2 * ribosome 70S/b3316/EF-TU-tRNA's/EF-G	C1282749H1936036N383293O491421S7468P24817Mg1984Zn0Fe0	-27564.28	Translation
rib_70_elo1_b3316_5_cplx	Translation elongation complex: 5 * ribosome 70S/b3316/EF-TU-tRNA's/EF-G	C3202887H4835584N956638O1225638S18670P61624Mg4960Zn0Fe0	-68490.7	Translation
rib_70_elo1_b3317_16_cplx	Translation elongation complex: 16 * ribosome 70S/b3317/EF-TU-tRNA's/EF-G	C27961985H42570729N8190126O10315866S171824P438774Mg41936Zn0Fe0	-531177.24	Translation

rib_70_elo1_b3317_1_cplx	Translation elongation complex: 1 * ribosome 70S/b3317/EF-TU-tRNA's/EF-G	C1754960H2668974N514821O650121S10739P28194Mg2621Zn0Fe0	-33970.14	Translation
rib_70_elo1_b3317_8_cplx	Translation elongation complex: 8 * ribosome 70S/b3317/EF-TU-tRNA's/EF-G	C13984905H21289793N4096630O5160802S85912P219798Mg20968Zn0Fe0	-266000.12	Translation
rib_70_elo1_b3318_1_cplx	Translation elongation complex: 1 * ribosome 70S/b3318/EF-TU-tRNA's/EF-G	C691647H1044399N206399O264380S4038P13226Mg1064Zn0Fe0	-14802.14	Translation
rib_70_elo1_b3318_2_cplx	Translation elongation complex: 2 * ribosome 70S/b3318/EF-TU-tRNA's/EF-G	C1380432H2085570N411621O526696S8076P26153Mg2128Zn0Fe0	-29304.28	Translation
rib_70_elo1_b3318_5_cplx	Translation elongation complex: 5 * ribosome 70S/b3318/EF-TU-tRNA's/EF-G	C3446787H5209083N1027287O1313644S20190P64934Mg5320Zn0Fe0	-72810.7	Translation
rib_70_elo1_b3319_11_cplx	Translation elongation complex: 11 * ribosome 70S/b3319/EF-TU-tRNA's/EF-G	C14390342H21868818N4233171O5351525S87428P236842Mg21703Zn0Fe0	-281317.54	Translation
rib_70_elo1_b3319_1_cplx	Translation elongation complex: 1 * ribosome 70S/b3319/EF-TU-tRNA's/EF-G	C1313462H1994008N386941O490355S7948P22082Mg1973Zn0Fe0	-26126.14	Translation
rib_70_elo1_b3319_5_cplx	Translation elongation complex: 5 * ribosome 70S/b3319/EF-TU-tRNA's/EF-G	C6544214H9943932N1925433O2434823S39740P107986Mg9865Zn0Fe0	-128202.7	Translation
rib_70_elo1_b3320_12_cplx	Translation elongation complex: 12 * ribosome 70S/b3320/EF-TU-tRNA's/EF-G	C16268348H24734923N4780582O6037933S99072P264522Mg24540Zn0Fe0	-315476.68	Translation
rib_70_elo1_b3320_1_cplx	Translation elongation complex: 1 * ribosome 70S/b3320/EF-TU-tRNA's/EF-G	C1361203H2067465N400602O507188S8256P22621Mg2045Zn0Fe0	-26868.14	Translation
rib_70_elo1_b3320_6_cplx	Translation elongation complex: 6 * ribosome 70S/b3320/EF-TU-tRNA's/EF-G	C8137178H12370855N2391502O3021163S49536P132576Mg12270Zn0Fe0	-158053.84	Translation
rib_70_elo1_b3321_1_cplx	Translation elongation complex: 1 * ribosome 70S/b3321/EF-TU-tRNA's/EF-G	C710230H1072725N211845O271178S4147P13503Mg1091Zn0Fe0	-15149.14	Translation
rib_70_elo1_b3321_3_cplx	Translation elongation complex: 3 * ribosome 70S/b3321/EF-TU-tRNA's/EF-G	C2124760H3211439N633165O809170S12441P39881Mg3273Zn0Fe0	-44817.42	Translation
rib_70_elo1_b3321_6_cplx	Translation elongation complex: 6 * ribosome 70S/b3321/EF-TU-tRNA's/EF-G	C4246555H6419510N1265145O1616158S24882P79448Mg6546Zn0Fe0	-89319.84	Translation
rib_70_elo1_b3339_11_cplx	Translation elongation complex: 11 * ribosome 70S/b3339/EF-TU-tRNA's/EF-G	C27372445H41729663N7990553O10036120S169565P413553Mg40810Zn0Fe0	-510014.54	Translation
rib_70_elo1_b3339_1_cplx	Translation elongation complex: 1 * ribosome 70S/b3339/EF-TU-tRNA's/EF-G	C2498655H3805213N730513O919900S15415P38673Mg3710Zn0Fe0	-47443.14	Translation
rib_70_elo1_b3339_23_cplx	Translation elongation complex: 23 * ribosome 70S/b3339/EF-TU-tRNA's/EF-G	C57220993H87239003N16702601O20975584S354545P863409Mg85330Zn0Fe0	-1065100.22	Translation
rib_70_elo1_b3340_1_cplx	Translation elongation complex: 1 * ribosome 70S/b3340/EF-TU-tRNA's/EF-G	C4404938H6717235N1283505O1611629S27441P65580Mg6500Zn0Fe0	-81947.14	Translation
rib_70_elo1_b3340_20_cplx	Translation elongation complex: 20 * ribosome 70S/b3340/EF-TU-tRNA's/EF-G	C87715986H133911937N25516618O31952444S548820P1271415Mg130000Zn0Fe0	-1598738.8	Translation
rib_70_elo1_b3340_41_cplx	Translation elongation complex: 41 * ribosome 70S/b3340/EF-TU-tRNA's/EF-G	C179796618H274495555N52300585O65487029S1125081P2604180Mg266500Zn0Fe0	-3275192.74	Translation
rib_70_elo1_b3341_10_cplx	Translation elongation complex: 10 * ribosome 70S/b3341/EF-TU-tRNA's/EF-G	C11741564H17827734N3461521O4381706S71100P197430Mg17750Zn0Fe0	-232362.4	Translation
rib_70_elo1_b3341_1_cplx	Translation elongation complex: 1 * ribosome 70S/b3341/EF-TU-tRNA's/EF-G	C1178777H1787997N347980O441578S7110P20229Mg1775Zn0Fe0	-23723.14	Translation
rib_70_elo1_b3341_5_cplx	Translation elongation complex: 5 * ribosome 70S/b3341/EF-TU-tRNA's/EF-G	C5873349H8916769N1731776O2192746S35550P98985Mg8875Zn0Fe0	-116451.7	Translation
rib_70_elo1_b3342_1_cplx	Translation elongation complex: 1 * ribosome 70S/b3342/EF-TU-tRNA's/EF-G	C839348H1269959N249318O317998S4971P15326Mg1280Zn0Fe0	-17472.14	Translation
rib_70_elo1_b3342_3_cplx	Translation elongation complex: 3 * ribosome 70S/b3342/EF-TU-tRNA's/EF-G	C2510914H3801793N745112O948744S14913P45224Mg3840Zn0Fe0	-51660.42	Translation
rib_70_elo1_b3342_7_cplx	Translation elongation complex: 7 * ribosome 70S/b3342/EF-TU-tRNA's/EF-G	C5854046H8865461N1736700O2210236S34797P105020Mg8960Zn0Fe0	-120036.98	Translation
rib_70_elo1_b3343_1_cplx	Translation elongation complex: 1 * ribosome 70S/b3343/EF-TU-tRNA's/EF-G	C662523H999200N198138O254372S3845P12961Mg1019Zn0Fe0	-14411.14	Translation
rib_70_elo1_b3343_2_cplx	Translation elongation complex: 2 * ribosome 70S/b3343/EF-TU-tRNA's/EF-G	C1322319H1995307N395218O506718S7690P25634Mg2038Zn0Fe0	-28533.28	Translation
rib_70_elo1_b3343_5_cplx	Translation elongation complex: 5 * ribosome 70S/b3343/EF-TU-tRNA's/EF-G	C3301707H4983628N986458O1263756S19225P63653Mg5095Zn0Fe0	-70899.7	Translation
rib_70_elo1_b3344_1_cplx	Translation elongation complex: 1 * ribosome 70S/b3344/EF-TU-tRNA's/EF-G	C809093H1223488N240535O307199S4783P14943Mg1235Zn0Fe0	-16978.14	Translation
rib_70_elo1_b3344_3_cplx	Translation elongation complex: 3 * ribosome 70S/b3344/EF-TU-tRNA's/EF-G	C2420483H3662774N718993O916505S14349P44111Mg3705Zn0Fe0	-50214.42	Translation

rib_70_elo1_b3344_7_cplx	Translation elongation complex: 7 * ribosome 70S/b3344/EF-TU-tRNA's/EF-G	C5643263H8541346N1675909O2135117S33481P102447Mg8645Zn0Fe0	-116686.98	Translation
rib_70_elo1_b3345_1_cplx	Translation elongation complex: 1 * ribosome 70S/b3345/EF-TU-tRNA's/EF-G	C864733H1308271N256784O327521S5138P15767Mg1316Zn0Fe0	-18028.14	Translation
rib_70_elo1_b3345_3_cplx	Translation elongation complex: 3 * ribosome 70S/b3345/EF-TU-tRNA's/EF-G	C2586841H3916499N767396O977141S15414P46529Mg3948Zn0Fe0	-53310.42	Translation
rib_70_elo1_b3345_7_cplx	Translation elongation complex: 7 * ribosome 70S/b3345/EF-TU-tRNA's/EF-G	C6031057H9132955N1788620O2276381S35966P108053Mg9212Zn0Fe0	-123874.98	Translation
rib_70_elo1_b3346_14_cplx	Translation elongation complex: 14 * ribosome 70S/b3346/EF-TU-tRNA's/EF-G	C21680599H32970481N6364876O8032547S132846P349311Mg32536Zn0Fe0	-419313.96	Translation
rib_70_elo1_b3346_1_cplx	Translation elongation complex: 1 * ribosome 70S/b3346/EF-TU-tRNA's/EF-G	C1554987H2362241N457130O578464S9489P25624Mg2324Zn0Fe0	-30625.14	Translation
rib_70_elo1_b3346_7_cplx	Translation elongation complex: 7 * ribosome 70S/b3346/EF-TU-tRNA's/EF-G	C10843731H16489121N3183782O4018810S66423P175018Mg16268Zn0Fe0	-210019.98	Translation
rib_70_elo1_b3384_19_cplx	Translation elongation complex: 19 * ribosome 70S/b3384/EF-TU-tRNA's/EF-G	C40344012H61452791N11797407O14841280S249508P622848Mg60230Zn0Fe0	-761323.66	Translation
rib_70_elo1_b3384_1_cplx	Translation elongation complex: 1 * ribosome 70S/b3384/EF-TU-tRNA's/EF-G	C2132370H3244535N624537O787708S13132P33726Mg3170Zn0Fe0	-41015.14	Translation
rib_70_elo1_b3384_9_cplx	Translation elongation complex: 9 * ribosome 70S/b3384/EF-TU-tRNA's/EF-G	C19115322H29114871N5590257O7033740S118188P295558Mg28530Zn0Fe0	-361152.26	Translation
rib_70_elo1_b3385_14_cplx	Translation elongation complex: 14 * ribosome 70S/b3385/EF-TU-tRNA's/EF-G	C22689305H34520436N6653891O8391753S139160P361699Mg34048Zn0Fe0	-435747.96	Translation
rib_70_elo1_b3385_1_cplx	Translation elongation complex: 1 * ribosome 70S/b3385/EF-TU-tRNA's/EF-G	C1627303H2473252N477929O604298S9940P26533Mg2432Zn0Fe0	-31823.14	Translation
rib_70_elo1_b3385_7_cplx	Translation elongation complex: 7 * ribosome 70S/b3385/EF-TU-tRNA's/EF-G	C11348227H17264260N3328373O4198508S69580P181225Mg17024Zn0Fe0	-218249.98	Translation
rib_70_elo1_b3386_13_cplx	Translation elongation complex: 13 * ribosome 70S/b3386/EF-TU-tRNA's/EF-G	C18921129H28770244N5557338O7016545S115531P306388Mg28457Zn0Fe0	-366619.82	Translation
rib_70_elo1_b3386_1_cplx	Translation elongation complex: 1 * ribosome 70S/b3386/EF-TU-tRNA's/EF-G	C1461417H2219824N429834O544129S8887P24196Mg2189Zn0Fe0	-28830.14	Translation
rib_70_elo1_b3386_6_cplx	Translation elongation complex: 6 * ribosome 70S/b3386/EF-TU-tRNA's/EF-G	C8736297H13282499N2566294O3240969S53322P141776Mg13134Zn0Fe0	-169575.84	Translation
rib_70_elo1_b3387_16_cplx	Translation elongation complex: 16 * ribosome 70S/b3387/EF-TU-tRNA's/EF-G	C28500752H43374354N8350895O10521248S175920P450199Mg42656Zn0Fe0	-544602.24	Translation
rib_70_elo1_b3387_1_cplx	Translation elongation complex: 1 * ribosome 70S/b3387/EF-TU-tRNA's/EF-G	C1788752H2719329N524870O663083S10995P28924Mg2666Zn0Fe0	-34825.14	Translation
rib_70_elo1_b3387_8_cplx	Translation elongation complex: 8 * ribosome 70S/b3387/EF-TU-tRNA's/EF-G	C14254352H21691674N4177015O5263560S87960P225519Mg21328Zn0Fe0	-272721.12	Translation
rib_70_elo1_b3388_12_cplx	Translation elongation complex: 12 * ribosome 70S/b3388/EF-TU-tRNA's/EF-G	C32374592H49358033N9449834O11862413S200784P487313Mg48192Zn0Fe0	-602527.68	Translation
rib_70_elo1_b3388_1_cplx	Translation elongation complex: 1 * ribosome 70S/b3388/EF-TU-tRNA's/EF-G	C2709143H4125934N792097O996690S16732P41791Mg4016Zn0Fe0	-51393.14	Translation
rib_70_elo1_b3388_25_cplx	Translation elongation complex: 25 * ribosome 70S/b3388/EF-TU-tRNA's/EF-G	C67433759H102814150N19681705O24703722S418300P1013839Mg100400Zn0Fe0	-1253868.5	Translation
rib_70_elo1_b3389_10_cplx	Translation elongation complex: 10 * ribosome 70S/b3389/EF-TU-tRNA's/EF-G	C22961709H34980544N6712627O8441617S142080P353049Mg34220Zn0Fe0	-432641.4	Translation
rib_70_elo1_b3389_1_cplx	Translation elongation complex: 1 * ribosome 70S/b3389/EF-TU-tRNA's/EF-G	C2305485H3508588N674941O851071S14208P36285Mg3422Zn0Fe0	-44245.14	Translation
rib_70_elo1_b3389_21_cplx	Translation elongation complex: 21 * ribosome 70S/b3389/EF-TU-tRNA's/EF-G	C48208205H73446268N14092021O17718951S298368P740205Mg71862Zn0Fe0	-907347.94	Translation
rib_70_elo1_b3390_10_cplx	Translation elongation complex: 10 * ribosome 70S/b3390/EF-TU-tRNA's/EF-G	C11362597H17252785N3349606O4240872S68710P191184Mg17210Zn0Fe0	-224766.4	Translation
rib_70_elo1_b3390_1_cplx	Translation elongation complex: 1 * ribosome 70S/b3390/EF-TU-tRNA's/EF-G	C1140748H1730341N336784O427356S6871P19590Mg1721Zn0Fe0	-22949.14	Translation
rib_70_elo1_b3390_5_cplx	Translation elongation complex: 5 * ribosome 70S/b3390/EF-TU-tRNA's/EF-G	C5683792H8629205N1675816O2122252S34355P95854Mg8605Zn0Fe0	-112645.7	Translation
rib_70_elo1_b3406_1_cplx	Translation elongation complex: 1 * ribosome 70S/b3406/EF-TU-tRNA's/EF-G	C1049139H1590178N310210O394241S6306P18338Mg1586Zn0Fe0	-21321.14	Translation
rib_70_elo1_b3406_4_cplx	Translation elongation complex: 4 * ribosome 70S/b3406/EF-TU-tRNA's/EF-G	C4182894H6345286N1235299O1567007S25224P71915Mg6344Zn0Fe0	-83844.56	Translation
rib_70_elo1_b3406_9_cplx	Translation elongation complex: 9 * ribosome 70S/b3406/EF-TU-tRNA's/EF-G	C9405819H14270466N2777114O3521617S56754P161210Mg14274Zn0Fe0	-188050.26	Translation

rib_70_elo1_b3461_16_cplx	Translation elongation complex: 16 * ribosome 70S/b3461/EF-TU-tRNA's/EF-G	C29072345H44256090N8515477O10726904S178960P457001Mg43520Zn0Fe0	-554092.24	Translation
rib_70_elo1_b3461_1_cplx	Translation elongation complex: 1 * ribosome 70S/b3461/EF-TU-tRNA's/EF-G	C1824665H2774655N535312O676019S11185P29366Mg2720Zn0Fe0	-35435.14	Translation
rib_70_elo1_b3461_8_cplx	Translation elongation complex: 8 * ribosome 70S/b3461/EF-TU-tRNA's/EF-G	C14540249H22132658N4259389O5366432S89480P228929Mg21760Zn0Fe0	-277475.12	Translation
rib_70_elo1_b3470_1_cplx	Translation elongation complex: 1 * ribosome 70S/b3470/EF-TU-tRNA's/EF-G	C575202H866300N172662O222243S3309P11617Mg893Zn0Fe0	-12727.14	Translation
rib_70_elo1_b3470_2_cplx	Translation elongation complex: 2 * ribosome 70S/b3470/EF-TU-tRNA's/EF-G	C1148072H1729954N344411O442749S6618P22986Mg1786Zn0Fe0	-25205.28	Translation
rib_70_elo1_b3470_4_cplx	Translation elongation complex: 4 * ribosome 70S/b3470/EF-TU-tRNA's/EF-G	C2293812H3457262N687909O883761S13236P45724Mg3572Zn0Fe0	-50161.56	Translation
rib_70_elo1_b3559_1_cplx	Translation elongation complex: 1 * ribosome 70S/b3559/EF-TU-tRNA's/EF-G	C4314742H6578776N1257672O1579692S26854P64523Mg6365Zn0Fe0	-80484.14	Translation
rib_70_elo1_b3559_20_cplx	Translation elongation complex: 20 * ribosome 70S/b3559/EF-TU-tRNA's/EF-G	C85920236H131151440N25002751O31318910S537080P1251130Mg127300Zn0Fe0	-1570333.8	Translation
rib_70_elo1_b3559_40_cplx	Translation elongation complex: 40 * ribosome 70S/b3559/EF-TU-tRNA's/EF-G	C171820756H262280560N49997571O62623350S1074160P2500190Mg254600Zn0Fe0	-3138596.6	Translation
rib_70_elo1_b3560_17_cplx	Translation elongation complex: 17 * ribosome 70S/b3560/EF-TU-tRNA's/EF-G	C32863296H50040537N9617261O12107158S202946P512359Mg49147Zn0Fe0	-623151.38	Translation
rib_70_elo1_b3560_1_cplx	Translation elongation complex: 1 * ribosome 70S/b3560/EF-TU-tRNA's/EF-G	C1941296H2952825N568989O718182S11938P30999Mg2891Zn0Fe0	-37517.14	Translation
rib_70_elo1_b3560_8_cplx	Translation elongation complex: 8 * ribosome 70S/b3560/EF-TU-tRNA's/EF-G	C15469671H23553699N4527608O5700859S95504P241594Mg23128Zn0Fe0	-293732.12	Translation
rib_70_elo1_b3590_18_cplx	Translation elongation complex: 18 * ribosome 70S/b3590/EF-TU-tRNA's/EF-G	C69065340H105396994N20109041O25201900S431406P1012991Mg102420Zn0Fe0	-1266614.52	Translation
rib_70_elo1_b3590_1_cplx	Translation elongation complex: 1 * ribosome 70S/b3590/EF-TU-tRNA's/EF-G	C3853527H5874149N1123849O1412253S23967P58016Mg5690Zn0Fe0	-72107.14	Translation
rib_70_elo1_b3590_36_cplx	Translation elongation complex: 36 * ribosome 70S/b3590/EF-TU-tRNA's/EF-G	C138113142H210774124N40211009O50390938S862812P2024141Mg204840Zn0Fe0	-2531387.04	Translation
rib_70_elo1_b3591_13_cplx	Translation elongation complex: 13 * ribosome 70S/b3591/EF-TU-tRNA's/EF-G	C37904149H57785974N11063473O13887705S235664P571067Mg56303Zn0Fe0	-706568.82	Translation
rib_70_elo1_b3591_1_cplx	Translation elongation complex: 1 * ribosome 70S/b3591/EF-TU-tRNA's/EF-G	C2927941H4458922N855949O1077297S18128P45215Mg4331Zn0Fe0	-55639.14	Translation
rib_70_elo1_b3591_27_cplx	Translation elongation complex: 27 * ribosome 70S/b3591/EF-TU-tRNA's/EF-G	C78709725H120000868N22972251O28833181S489456P1184561Mg116937Zn0Fe0	-1465986.78	Translation
rib_70_elo1_b3635_15_cplx	Translation elongation complex: 15 * ribosome 70S/b3635/EF-TU-tRNA's/EF-G	C25887559H39393474N7588741O9565027S159375P410732Mg38775Zn0Fe0	-495995.1	Translation
rib_70_elo1_b3635_1_cplx	Translation elongation complex: 1 * ribosome 70S/b3635/EF-TU-tRNA's/EF-G	C1733051H2634374N508829O642967S10625P28140Mg2585Zn0Fe0	-33825.14	Translation
rib_70_elo1_b3635_7_cplx	Translation elongation complex: 7 * ribosome 70S/b3635/EF-TU-tRNA's/EF-G	C12084983H18388274N3543077O4466707S74375P192108Mg18095Zn0Fe0	-231897.98	Translation
rib_70_elo1_b3636_1_cplx	Translation elongation complex: 1 * ribosome 70S/b3636/EF-TU-tRNA's/EF-G	C415097H621844N126152O164034S2296P9325Mg659Zn0Fe0	-9801.14	Translation
rib_70_elo1_b3636_3_cplx	Translation elongation complex: 3 * ribosome 70S/b3636/EF-TU-tRNA's/EF-G	C1242077H1861912N377146O489792S6888P27639Mg1977Zn0Fe0	-29065.42	Translation
rib_70_elo1_b3637_1_cplx	Translation elongation complex: 1 * ribosome 70S/b3637/EF-TU-tRNA's/EF-G	C556763H838177N167340O215533S3191P11356Mg866Zn0Fe0	-12382.14	Translation
rib_70_elo1_b3637_2_cplx	Translation elongation complex: 2 * ribosome 70S/b3637/EF-TU-tRNA's/EF-G	C1111275H1673800N333787O429403S6382P22473Mg1732Zn0Fe0	-24524.28	Translation
rib_70_elo1_b3637_4_cplx	Translation elongation complex: 4 * ribosome 70S/b3637/EF-TU-tRNA's/EF-G	C2220299H3345046N666681O857143S12764P44707Mg3464Zn0Fe0	-48808.56	Translation
rib_70_elo1_b3638_13_cplx	Translation elongation complex: 13 * ribosome 70S/b3638/EF-TU-tRNA's/EF-G	C18695536H28421363N5493591O6937878S114348P304429Mg28106Zn0Fe0	-363490.82	Translation
rib_70_elo1_b3638_1_cplx	Translation elongation complex: 1 * ribosome 70S/b3638/EF-TU-tRNA's/EF-G	C1444000H2192891N424923O538014S8796P24037Mg2162Zn0Fe0	-28581.14	Translation
rib_70_elo1_b3638_6_cplx	Translation elongation complex: 6 * ribosome 70S/b3638/EF-TU-tRNA's/EF-G	C8632140H13121421N2536868O3204624S52776P140867Mg12972Zn0Fe0	-168126.84	Translation
rib_70_elo1_b3649_1_cplx	Translation elongation complex: 1 * ribosome 70S/b3649/EF-TU-tRNA's/EF-G	C635710H959131N190192O243858S3685P12393Mg983Zn0Fe0	-13752.14	Translation
rib_70_elo1_b3649_2_cplx	Translation elongation complex: 2 * ribosome 70S/b3649/EF-TU-tRNA's/EF-G	C1268780H1915277N379296O485802S7370P24508Mg1966Zn0Fe0	-27225.28	Translation

rib_70_elo1_b3649_5_cplx	Translation elongation complex: 5 * ribosome 70S/b3649/EF-TU-tRNA's/EF-G	C3167990H4783715N946608O1211634S18425P60853Mg4915Zn0Fe0	-67644.7	Translation
rib_70_elo1_b3650_1_cplx	Translation elongation complex: 1 * ribosome 70S/b3650/EF-TU-tRNA's/EF-G	C4396876H6703457N1281752O1609884S27406P65840Mg6482Zn0Fe0	-82090.14	Translation
rib_70_elo1_b3650_20_cplx	Translation elongation complex: 20 * ribosome 70S/b3650/EF-TU-tRNA's/EF-G	C87556171H133636947N25482014O31918494S548120P1276729Mg129640Zn0Fe0	-1601712.8	Translation
rib_70_elo1_b3650_41_cplx	Translation elongation complex: 41 * ribosome 70S/b3650/EF-TU-tRNA's/EF-G	C179469076H273931857N52229672O65417484S1123646P2615080Mg265762Zn0Fe0	-3281295.74	Translation
rib_70_elo1_b3651_13_cplx	Translation elongation complex: 13 * ribosome 70S/b3651/EF-TU-tRNA's/EF-G	C19227793H29243577N5644715O7123011S117611P309635Mg28925Zn0Fe0	-371010.82	Translation
rib_70_elo1_b3651_1_cplx	Translation elongation complex: 1 * ribosome 70S/b3651/EF-TU-tRNA's/EF-G	C1485121H2256381N436643O552363S9047P24455Mg2225Zn0Fe0	-29177.14	Translation
rib_70_elo1_b3651_6_cplx	Translation elongation complex: 6 * ribosome 70S/b3651/EF-TU-tRNA's/EF-G	C8877901H13501046N2606673O3290133S54282P143280Mg13350Zn0Fe0	-171607.84	Translation
rib_70_elo1_b3652_1_cplx	Translation elongation complex: 1 * ribosome 70S/b3652/EF-TU-tRNA's/EF-G	C4340885H6617978N1265607O1589590S27008P65059Mg6401Zn0Fe0	-81083.14	Translation
rib_70_elo1_b3652_20_cplx	Translation elongation complex: 20 * ribosome 70S/b3652/EF-TU-tRNA's/EF-G	C86441063H131932193N25160026O31516224S540160P1261622Mg128020Zn0Fe0	-1582085.8	Translation
rib_70_elo1_b3652_40_cplx	Translation elongation complex: 40 * ribosome 70S/b3652/EF-TU-tRNA's/EF-G	C172862303H263841893N50312046O63017944S1080320P2521162Mg256040Zn0Fe0	-3162088.6	Translation
rib_70_elo1_b3703_1_cplx	Translation elongation complex: 1 * ribosome 70S/b3703/EF-TU-tRNA's/EF-G	C359785H537344N110184O143992S1948P8557Mg578Zn0Fe0	-8800.14	Translation
rib_70_elo1_b3703_2_cplx	Translation elongation complex: 2 * ribosome 70S/b3703/EF-TU-tRNA's/EF-G	C718237H1073174N219855O286982S3896P16971Mg1156Zn0Fe0	-17456.28	Translation
rib_70_elo1_b3704_1_cplx	Translation elongation complex: 1 * ribosome 70S/b3704/EF-TU-tRNA's/EF-G	C809169H1223671N240633O307176S4786P14947Mg1235Zn0Fe0	-16962.14	Translation
rib_70_elo1_b3704_3_cplx	Translation elongation complex: 3 * ribosome 70S/b3704/EF-TU-tRNA's/EF-G	C2420681H3663253N719209O916480S14358P44121Mg3705Zn0Fe0	-50164.42	Translation
rib_70_elo1_b3704_7_cplx	Translation elongation complex: 7 * ribosome 70S/b3704/EF-TU-tRNA's/EF-G	C5643705H8542417N1676361O2135088S33502P102469Mg8645Zn0Fe0	-116568.98	Translation
rib_70_elo1_b3706_13_cplx	Translation elongation complex: 13 * ribosome 70S/b3706/EF-TU-tRNA's/EF-G	C37156362H56653973N10842679O13609102S230438P558027Mg55250Zn0Fe0	-690707.82	Translation
rib_70_elo1_b3706_1_cplx	Translation elongation complex: 1 * ribosome 70S/b3706/EF-TU-tRNA's/EF-G	C2870178H4371593N838879O1055662S17726P44187Mg4250Zn0Fe0	-54394.14	Translation
rib_70_elo1_b3706_26_cplx	Translation elongation complex: 26 * ribosome 70S/b3706/EF-TU-tRNA's/EF-G	C74299728H113293218N21680129O27208662S460876P1114687Mg110500Zn0Fe0	-1380047.64	Translation
rib_70_elo1_b3740_12_cplx	Translation elongation complex: 12 * ribosome 70S/b3740/EF-TU-tRNA's/EF-G	C16153851H24546525N4750886O6006229S98436P265824Mg24324Zn0Fe0	-316034.68	Translation
rib_70_elo1_b3740_1_cplx	Translation elongation complex: 1 * ribosome 70S/b3740/EF-TU-tRNA's/EF-G	C1351591H2051690N398054O504524S8203P22724Mg2027Zn0Fe0	-26909.14	Translation
rib_70_elo1_b3740_6_cplx	Translation elongation complex: 6 * ribosome 70S/b3740/EF-TU-tRNA's/EF-G	C8079891H12276615N2376614O3005299S49218P133224Mg12162Zn0Fe0	-158329.84	Translation
rib_70_elo1_b3741_18_cplx	Translation elongation complex: 18 * ribosome 70S/b3741/EF-TU-tRNA's/EF-G	C70731969H107938215N20596178O25807061S441648P1036874Mg104850Zn0Fe0	-1297301.52	Translation
rib_70_elo1_b3741_1_cplx	Translation elongation complex: 1 * ribosome 70S/b3741/EF-TU-tRNA's/EF-G	C3946534H6015803N1151017O1446214S24536P59391Mg5825Zn0Fe0	-73860.14	Translation
rib_70_elo1_b3741_37_cplx	Translation elongation complex: 37 * ribosome 70S/b3741/EF-TU-tRNA's/EF-G	C145374514H221851499N42329005O53033890S907832P2129355Mg215525Zn0Fe0	-2664677.18	Translation
rib_70_elo1_b3780_12_cplx	Translation elongation complex: 12 * ribosome 70S/b3780/EF-TU-tRNA's/EF-G	C31875132H48590796N9303995O11684786S197796P481484Mg47436Zn0Fe0	-594130.68	Translation
rib_70_elo1_b3780_1_cplx	Translation elongation complex: 1 * ribosome 70S/b3780/EF-TU-tRNA's/EF-G	C2667294H4061729N779721O981852S16483P41286Mg3953Zn0Fe0	-50674.14	Translation
rib_70_elo1_b3780_24_cplx	Translation elongation complex: 24 * ribosome 70S/b3780/EF-TU-tRNA's/EF-G	C63738228H97167960N18603203O23360714S395592P961700Mg94872Zn0Fe0	-1186992.36	Translation
rib_70_elo1_b3782_1_cplx	Translation elongation complex: 1 * ribosome 70S/b3782/EF-TU-tRNA's/EF-G	C280735H416164N87403O115583S1456P7519Mg461Zn0Fe0	-7455.14	Translation
rib_70_elo1_b3783_12_cplx	Translation elongation complex: 12 * ribosome 70S/b3783/EF-TU-tRNA's/EF-G	C31729543H48369303N9262360O11633323S196836P479448Mg47220Zn0Fe0	-591842.68	Translation
rib_70_elo1_b3783_1_cplx	Translation elongation complex: 1 * ribosome 70S/b3783/EF-TU-tRNA's/EF-G	C2655113H4043208N776234O977502S16403P41109Mg3935Zn0Fe0	-50476.14	Translation
rib_70_elo1_b3783_24_cplx	Translation elongation complex: 24 * ribosome 70S/b3783/EF-TU-tRNA's/EF-G	C63447103H96725043N18519952O23257855S393672P957636Mg94440Zn0Fe0	-1182424.36	Translation

rib_70_elo1_b3885_11_cplx	Translation elongation complex: 11 * ribosome 70S/b3885/EF-TU-tRNA's/EF-G	C14260960H21667867N4194967O5304843S86955P235342Mg21505Zn0Fe0	-279278.54	Translation
rib_70_elo1_b3885_1_cplx	Translation elongation complex: 1 * ribosome 70S/b3885/EF-TU-tRNA's/EF-G	C1301640H1975667N383417O486093S7905P21942Mg1955Zn0Fe0	-25937.14	Translation
rib_70_elo1_b3885_5_cplx	Translation elongation complex: 5 * ribosome 70S/b3885/EF-TU-tRNA's/EF-G	C6485368H9852547N1908037O2413593S39525P107302Mg9775Zn0Fe0	-127273.7	Translation
rib_70_elo1_b3886_17_cplx	Translation elongation complex: 17 * ribosome 70S/b3886/EF-TU-tRNA's/EF-G	C31551650H48017399N9246320O11644809S194191P497606Mg47158Zn0Fe0	-603060.38	Translation
rib_70_elo1_b3886_1_cplx	Translation elongation complex: 1 * ribosome 70S/b3886/EF-TU-tRNA's/EF-G	C1863682H2833287N546848O690761S11423P30086Mg2774Zn0Fe0	-36290.14	Translation
rib_70_elo1_b3886_8_cplx	Translation elongation complex: 8 * ribosome 70S/b3886/EF-TU-tRNA's/EF-G	C14852168H22601336N4352867O5483157S91384P234626Mg22192Zn0Fe0	-284252.12	Translation
rib_70_elo1_b3887_1_cplx	Translation elongation complex: 1 * ribosome 70S/b3887/EF-TU-tRNA's/EF-G	C968802H1467544N286950O365035S5800P17185Mg1469Zn0Fe0	-19859.14	Translation
rib_70_elo1_b3887_4_cplx	Translation elongation complex: 4 * ribosome 70S/b3887/EF-TU-tRNA's/EF-G	C3862779H5856127N1142748O1451053S23200P67438Mg5876Zn0Fe0	-78131.56	Translation
rib_70_elo1_b3887_8_cplx	Translation elongation complex: 8 * ribosome 70S/b3887/EF-TU-tRNA's/EF-G	C7721415H11707571N2283812O2899077S46400P134442Mg11752Zn0Fe0	-155828.12	Translation
rib_70_elo1_b3888_19_cplx	Translation elongation complex: 19 * ribosome 70S/b3888/EF-TU-tRNA's/EF-G	C39743248H60541181N11621225O14618086S245879P613128Mg59375Zn0Fe0	-749152.66	Translation
rib_70_elo1_b3888_1_cplx	Translation elongation complex: 1 * ribosome 70S/b3888/EF-TU-tRNA's/EF-G	C2100658H3196457N615251O775888S12941P33204Mg3125Zn0Fe0	-40364.14	Translation
rib_70_elo1_b3888_9_cplx	Translation elongation complex: 9 * ribosome 70S/b3888/EF-TU-tRNA's/EF-G	C18830698H28683001N5506795O6927976S116469P290948Mg28125Zn0Fe0	-355381.26	Translation
rib_70_elo1_b3936_1_cplx	Translation elongation complex: 1 * ribosome 70S/b3936/EF-TU-tRNA's/EF-G	C507378H762778N152941O197551S2893P10634Mg794Zn0Fe0	-11475.14	Translation
rib_70_elo1_b3936_2_cplx	Translation elongation complex: 2 * ribosome 70S/b3936/EF-TU-tRNA's/EF-G	C1012731H1523260N305075O393616S5786P21053Mg1588Zn0Fe0	-22734.28	Translation
rib_70_elo1_b3936_4_cplx	Translation elongation complex: 4 * ribosome 70S/b3936/EF-TU-tRNA's/EF-G	C2023437H3044224N609343O785746S11572P41891Mg3176Zn0Fe0	-45252.56	Translation
rib_70_elo1_b3965_10_cplx	Translation elongation complex: 10 * ribosome 70S/b3965/EF-TU-tRNA's/EF-G	C23203019H35351780N6780611O8525357S144000P355673Mg34580Zn0Fe0	-436425.4	Translation
rib_70_elo1_b3965_1_cplx	Translation elongation complex: 1 * ribosome 70S/b3965/EF-TU-tRNA's/EF-G	C2329742H3545852N681851O859445S14400P36560Mg3458Zn0Fe0	-44636.14	Translation
rib_70_elo1_b3965_21_cplx	Translation elongation complex: 21 * ribosome 70S/b3965/EF-TU-tRNA's/EF-G	C48714802H74225692N14234651O17894805S302400P745700Mg72618Zn0Fe0	-915278.94	Translation
rib_70_elo1_b3980_11_cplx	Translation elongation complex: 11 * ribosome 70S/b3980/EF-TU-tRNA's/EF-G	C27374364H41731803N7991383O10037392S169587P413753Mg40810Zn0Fe0	-510214.54	Translation
rib_70_elo1_b3980_1_cplx	Translation elongation complex: 1 * ribosome 70S/b3980/EF-TU-tRNA's/EF-G	C2498834H3805413N730603O920012S15417P38693Mg3710Zn0Fe0	-47463.14	Translation
rib_70_elo1_b3980_23_cplx	Translation elongation complex: 23 * ribosome 70S/b3980/EF-TU-tRNA's/EF-G	C57225000H87243471N16704319O20978248S354591P863825Mg85330Zn0Fe0	-1065516.22	Translation
rib_70_elo1_b3981_1_cplx	Translation elongation complex: 1 * ribosome 70S/b3981/EF-TU-tRNA's/EF-G	C858483H1298861N254948O325266S5069P15667Mg1307Zn0Fe0	-17906.14	Translation
rib_70_elo1_b3981_3_cplx	Translation elongation complex: 3 * ribosome 70S/b3981/EF-TU-tRNA's/EF-G	C2568149H3888333N761964O970354S15207P46229Mg3921Zn0Fe0	-52944.42	Translation
rib_70_elo1_b3981_7_cplx	Translation elongation complex: 7 * ribosome 70S/b3981/EF-TU-tRNA's/EF-G	C5987481H9067277N1775996O2260530S35483P107353Mg9149Zn0Fe0	-123020.98	Translation
rib_70_elo1_b3982_10_cplx	Translation elongation complex: 10 * ribosome 70S/b3982/EF-TU-tRNA's/EF-G	C11854820H18004871N3492694O4418621S72100P198036Mg17930Zn0Fe0	-233518.4	Translation
rib_70_elo1_b3982_1_cplx	Translation elongation complex: 1 * ribosome 70S/b3982/EF-TU-tRNA's/EF-G	C1190162H1805771N351136O445310S7210P20295Mg1793Zn0Fe0	-23844.14	Translation
rib_70_elo1_b3982_5_cplx	Translation elongation complex: 5 * ribosome 70S/b3982/EF-TU-tRNA's/EF-G	C5930010H9005371N1747384O2211226S36050P99291Mg8965Zn0Fe0	-117032.7	Translation
rib_70_elo1_b3983_1_cplx	Translation elongation complex: 1 * ribosome 70S/b3983/EF-TU-tRNA's/EF-G	C949715H1438610N281301O358012S5655P16857Mg1442Zn0Fe0	-19467.14	Translation
rib_70_elo1_b3983_4_cplx	Translation elongation complex: 4 * ribosome 70S/b3983/EF-TU-tRNA's/EF-G	C3786593H5740553N1120248O1423087S22620P66135Mg5768Zn0Fe0	-76572.56	Translation
rib_70_elo1_b3983_8_cplx	Translation elongation complex: 8 * ribosome 70S/b3983/EF-TU-tRNA's/EF-G	C7569097H11476477N2238844O2843187S45240P131839Mg11536Zn0Fe0	-152713.12	Translation
rib_70_elo1_b3984_13_cplx	Translation elongation complex: 13 * ribosome 70S/b3984/EF-TU-tRNA's/EF-G	C19616660H29842286N5755933O7263188S119743P314213Mg29510Zn0Fe0	-377551.82	Translation

rib_70_elo1_b3984_1_cplx	Translation elongation complex: 1 * ribosome 70S/b3984/EF-TU-tRNA's/EF-G	C1515188H2302586N445297O563216S9211P24821Mg2270Zn0Fe0	-29694.14	Translation
rib_70_elo1_b3984_6_cplx	Translation elongation complex: 6 * ribosome 70S/b3984/EF-TU-tRNA's/EF-G	C9057468H13777461N2658062O3354871S55266P145401Mg13620Zn0Fe0	-174634.84	Translation
rib_70_elo1_b3985_1_cplx	Translation elongation complex: 1 * ribosome 70S/b3985/EF-TU-tRNA's/EF-G	C1091410H1655013N322476O409521S6547P18892Mg1649Zn0Fe0	-22063.14	Translation
rib_70_elo1_b3985_4_cplx	Translation elongation complex: 4 * ribosome 70S/b3985/EF-TU-tRNA's/EF-G	C4351414H6603963N1284210O1627620S26188P74068Mg6596Zn0Fe0	-86749.56	Translation
rib_70_elo1_b3985_9_cplx	Translation elongation complex: 9 * ribosome 70S/b3985/EF-TU-tRNA's/EF-G	C9784754H14852213N2887100O3657785S58923P166028Mg14841Zn0Fe0	-194560.26	Translation
rib_70_elo1_b3986_1_cplx	Translation elongation complex: 1 * ribosome 70S/b3986/EF-TU-tRNA's/EF-G	C820460H1241189N243829O311116S4836P15031Mg1253Zn0Fe0	-17150.14	Translation
rib_70_elo1_b3986_3_cplx	Translation elongation complex: 3 * ribosome 70S/b3986/EF-TU-tRNA's/EF-G	C2454370H3715683N728617O928276S14508P44361Mg3759Zn0Fe0	-50716.42	Translation
rib_70_elo1_b3986_7_cplx	Translation elongation complex: 7 * ribosome 70S/b3986/EF-TU-tRNA's/EF-G	C5722190H8664671N1698193O2162596S33852P103021Mg8771Zn0Fe0	-117848.98	Translation
rib_70_elo1_b3987_1_cplx	Translation elongation complex: 1 * ribosome 70S/b3987/EF-TU-tRNA's/EF-G	C8334813H12718403N2424615O3039878S52184P121702Mg12242Zn0Fe0	-153594.14	Translation
rib_70_elo1_b3987_39_cplx	Translation elongation complex: 39 * ribosome 70S/b3987/EF-TU-tRNA's/EF-G	C323599837H494367453N93974671O117488240S2035176P4593276Mg477438Zn0Fe0	-5837031.46	Translation
rib_70_elo1_b3987_78_cplx	Translation elongation complex: 78 * ribosome 70S/b3987/EF-TU-tRNA's/EF-G	C647161309H988691478N187933939O234948401S4070352P9182523Mg954876Zn0Fe0	-11670032.92	Translation
rib_70_elo1_b3988_1_cplx	Translation elongation complex: 1 * ribosome 70S/b3988/EF-TU-tRNA's/EF-G	C8732970H13327198N2540071O3184260S54631P127228Mg12827Zn0Fe0	-160713.14	Translation
rib_70_elo1_b3988_41_cplx	Translation elongation complex: 41 * ribosome 70S/b3988/EF-TU-tRNA's/EF-G	C356443210H544594038N103497711O129374860S2239871P5047388Mg525907Zn0Fe0	-6420238.74	Translation
rib_70_elo1_b3988_82_cplx	Translation elongation complex: 82 * ribosome 70S/b3988/EF-TU-tRNA's/EF-G	C712846206H1089142549N206979292O258720225S4479742P10090552Mg1051814Zn0Fe0	-12836252.48	Translation
rib_70_elo1_b4022_17_cplx	Translation elongation complex: 17 * ribosome 70S/b4022/EF-TU-tRNA's/EF-G	C31500314H47962870N9223033O11612620S194276P492464Mg47158Zn0Fe0	-597935.38	Translation
rib_70_elo1_b4022_1_cplx	Translation elongation complex: 1 * ribosome 70S/b4022/EF-TU-tRNA's/EF-G	C1860810H2830214N545737O688796S11428P29792Mg2774Zn0Fe0	-35997.14	Translation
rib_70_elo1_b4022_8_cplx	Translation elongation complex: 8 * ribosome 70S/b4022/EF-TU-tRNA's/EF-G	C14828093H22575751N4342054O5467969S91424P232211Mg22192Zn0Fe0	-281845.12	Translation
rib_70_elo1_b4049_10_cplx	Translation elongation complex: 10 * ribosome 70S/b4049/EF-TU-tRNA's/EF-G	C21911491H33378152N6406355O8057836S135690P337560Mg32690Zn0Fe0	-413072.4	Translation
rib_70_elo1_b4049_1_cplx	Translation elongation complex: 1 * ribosome 70S/b4049/EF-TU-tRNA's/EF-G	C2200060H3347879N644231O812323S13569P34692Mg3269Zn0Fe0	-42244.14	Translation
rib_70_elo1_b4049_20_cplx	Translation elongation complex: 20 * ribosome 70S/b4049/EF-TU-tRNA's/EF-G	C43813081H66745122N12808715O16108406S271380P674080Mg65380Zn0Fe0	-825103.8	Translation
rib_70_elo1_b4129_14_cplx	Translation elongation complex: 14 * ribosome 70S/b4129/EF-TU-tRNA's/EF-G	C44373569H67686758N12933221O16221663S276724P658806Mg65926Zn0Fe0	-819304.96	Translation
rib_70_elo1_b4129_1_cplx	Translation elongation complex: 1 * ribosome 70S/b4129/EF-TU-tRNA's/EF-G	C3182952H4849906N929112O1168560S19766P48469Mg4709Zn0Fe0	-59934.14	Translation
rib_70_elo1_b4129_29_cplx	Translation elongation complex: 29 * ribosome 70S/b4129/EF-TU-tRNA's/EF-G	C91901204H140190818N26784116O33590628S573214P1363041Mg136561Zn0Fe0	-1695502.06	Translation
rib_70_elo1_b4142_1_cplx	Translation elongation complex: 1 * ribosome 70S/b4142/EF-TU-tRNA's/EF-G	C673523H1016519N201245O257935S3915P12999Mg1037Zn0Fe0	-14513.14	Translation
rib_70_elo1_b4142_2_cplx	Translation elongation complex: 2 * ribosome 70S/b4142/EF-TU-tRNA's/EF-G	C1344238H2029875N401356O513812S7830P25702Mg2074Zn0Fe0	-28729.28	Translation
rib_70_elo1_b4142_5_cplx	Translation elongation complex: 5 * ribosome 70S/b4142/EF-TU-tRNA's/EF-G	C3356383H5069943N1001689O1281443S19575P63811Mg5185Zn0Fe0	-71377.7	Translation
rib_70_elo1_b4143_16_cplx	Translation elongation complex: 16 * ribosome 70S/b4143/EF-TU-tRNA's/EF-G	C54879177H83745203N15984702O20039598S341408P807025Mg81536Zn0Fe0	-1008356.24	Translation
rib_70_elo1_b4143_1_cplx	Translation elongation complex: 1 * ribosome 70S/b4143/EF-TU-tRNA's/EF-G	C3444702H5250743N1005072O1263198S21338P51985Mg5096Zn0Fe0	-64569.14	Translation
rib_70_elo1_b4143_32_cplx	Translation elongation complex: 32 * ribosome 70S/b4143/EF-TU-tRNA's/EF-G	C109742617H167472627N31962974O40067758S682816P1612401Mg163072Zn0Fe0	-2015062.48	Translation
rib_70_elo1_b4162_10_cplx	Translation elongation complex: 10 * ribosome 70S/b4162/EF-TU-tRNA's/EF-G	C11862660H18013260N3494963O4425102S71890P198938Mg17930Zn0Fe0	-234490.4	Translation
rib_70_elo1_b4162_1_cplx	Translation elongation complex: 1 * ribosome 70S/b4162/EF-TU-tRNA's/EF-G	C1190955H1806627N351398O445941S7189P20387Mg1793Zn0Fe0	-23943.14	Translation

rib_70_elo1_b4162_5_cplx	Translation elongation complex: 5 * ribosome 70S/b4162/EF-TU-tRNA's/EF-G	C5933935H9009575N1748538O2214457S35945P99743Mg8965Zn0Fe0	-117519.7	Translation
rib_70_elo1_b4167_15_cplx	Translation elongation complex: 15 * ribosome 70S/b4167/EF-TU-tRNA's/EF-G	C48472708H73934234N14130441O17727364S301440P720725Mg71985Zn0Fe0	-896153.1	Translation
rib_70_elo1_b4167_1_cplx	Translation elongation complex: 1 * ribosome 70S/b4167/EF-TU-tRNA's/EF-G	C3245274H4944530N947565O1191950S20096P49495Mg4799Zn0Fe0	-61191.14	Translation
rib_70_elo1_b4167_30_cplx	Translation elongation complex: 30 * ribosome 70S/b4167/EF-TU-tRNA's/EF-G	C96930673H147851774N28254951O35443879S602880P1439900Mg143970Zn0Fe0	-1790755.2	Translation
rib_70_elo1_b4168_1_cplx	Translation elongation complex: 1 * ribosome 70S/b4168/EF-TU-tRNA's/EF-G	C1018424H1543171N301311O383225S6108P17923Mg1541Zn0Fe0	-20787.14	Translation
rib_70_elo1_b4168_4_cplx	Translation elongation complex: 4 * ribosome 70S/b4168/EF-TU-tRNA's/EF-G	C4061348H6158719N1200360O1523762S24432P70393Mg6164Zn0Fe0	-81846.56	Translation
rib_70_elo1_b4168_9_cplx	Translation elongation complex: 9 * ribosome 70S/b4168/EF-TU-tRNA's/EF-G	C9132888H13851299N2698775O3424657S54972P157843Mg13869Zn0Fe0	-183612.26	Translation
rib_70_elo1_b4169_13_cplx	Translation elongation complex: 13 * ribosome 70S/b4169/EF-TU-tRNA's/EF-G	C36456486H55575695N10641742O13360371S226460P549457Mg54197Zn0Fe0	-679511.82	Translation
rib_70_elo1_b4169_1_cplx	Translation elongation complex: 1 * ribosome 70S/b4169/EF-TU-tRNA's/EF-G	C2816106H4288379N823330O1036335S17420P43501Mg4169Zn0Fe0	-53506.14	Translation
rib_70_elo1_b4169_26_cplx	Translation elongation complex: 26 * ribosome 70S/b4169/EF-TU-tRNA's/EF-G	C72900231H111136954N21278355O26711410S452920P1097576Mg108394Zn0Fe0	-1357684.64	Translation
rib_70_elo1_b4170_18_cplx	Translation elongation complex: 18 * ribosome 70S/b4170/EF-TU-tRNA's/EF-G	C69191142H105580021N20152458O25250295S432198P1016202Mg102582Zn0Fe0	-1270437.52	Translation
rib_70_elo1_b4170_1_cplx	Translation elongation complex: 1 * ribosome 70S/b4170/EF-TU-tRNA's/EF-G	C3860567H5884405N1126279O1414952S24011P58201Mg5699Zn0Fe0	-72326.14	Translation
rib_70_elo1_b4170_36_cplx	Translation elongation complex: 36 * ribosome 70S/b4170/EF-TU-tRNA's/EF-G	C138364692H211140085N40297824O50487717S864396P2030556Mg205164Zn0Fe0	-2539026.04	Translation
rib_70_elo1_b4171_18_cplx	Translation elongation complex: 18 * ribosome 70S/b4171/EF-TU-tRNA's/EF-G	C36242570H55189957N10608440O13346040S223776P563677Mg54144Zn0Fe0	-686800.52	Translation
rib_70_elo1_b4171_1_cplx	Translation elongation complex: 1 * ribosome 70S/b4171/EF-TU-tRNA's/EF-G	C2021961H3075692N592754O747697S12432P32206Mg3008Zn0Fe0	-39047.14	Translation
rib_70_elo1_b4171_9_cplx	Translation elongation complex: 9 * ribosome 70S/b4171/EF-TU-tRNA's/EF-G	C18125777H27600052N5306018O6676329S111888P282310Mg27072Zn0Fe0	-343872.26	Translation
rib_70_elo1_b4172_1_cplx	Translation elongation complex: 1 * ribosome 70S/b4172/EF-TU-tRNA's/EF-G	C705395H1064696N210590O269856S4132P13550Mg1082Zn0Fe0	-15176.14	Translation
rib_70_elo1_b4172_3_cplx	Translation elongation complex: 3 * ribosome 70S/b4172/EF-TU-tRNA's/EF-G	C2110309H3187442N629442O805248S12396P40032Mg3246Zn0Fe0	-44908.42	Translation
rib_70_elo1_b4172_6_cplx	Translation elongation complex: 6 * ribosome 70S/b4172/EF-TU-tRNA's/EF-G	C4217680H6371561N1257720O1608336S24792P79755Mg6492Zn0Fe0	-89506.84	Translation
rib_70_elo1_b4173_12_cplx	Translation elongation complex: 12 * ribosome 70S/b4173/EF-TU-tRNA's/EF-G	C32233057H49143431N9408066O11810591S200184P485745Mg47976Zn0Fe0	-599939.68	Translation
rib_70_elo1_b4173_1_cplx	Translation elongation complex: 1 * ribosome 70S/b4173/EF-TU-tRNA's/EF-G	C2697276H4107935N788499O992432S16682P41653Mg3998Zn0Fe0	-51170.14	Translation
rib_70_elo1_b4173_25_cplx	Translation elongation complex: 25 * ribosome 70S/b4173/EF-TU-tRNA's/EF-G	C67138980H102367199N19594827O24595688S417050P1010581Mg99950Zn0Fe0	-1248485.5	Translation
rib_70_elo1_b4174_12_cplx	Translation elongation complex: 12 * ribosome 70S/b4174/EF-TU-tRNA's/EF-G	C31724174H48360594N9261597O11630859S196836P479340Mg47220Zn0Fe0	-591626.68	Translation
rib_70_elo1_b4174_1_cplx	Translation elongation complex: 1 * ribosome 70S/b4174/EF-TU-tRNA's/EF-G	C2654716H4042540N776329O977249S16403P41100Mg3935Zn0Fe0	-50458.14	Translation
rib_70_elo1_b4174_24_cplx	Translation elongation complex: 24 * ribosome 70S/b4174/EF-TU-tRNA's/EF-G	C63436310H96707562N18518253O23252979S393672P957420Mg94440Zn0Fe0	-1181992.36	Translation
rib_70_elo1_b4175_19_cplx	Translation elongation complex: 19 * ribosome 70S/b4175/EF-TU-tRNA's/EF-G	C40335576H61445375N11798029O14834118S249261P621811Mg60230Zn0Fe0	-760324.66	Translation
rib_70_elo1_b4175_1_cplx	Translation elongation complex: 1 * ribosome 70S/b4175/EF-TU-tRNA's/EF-G	C2131998H3244229N624601O787386S13119P33679Mg3170Zn0Fe0	-40970.14	Translation
rib_70_elo1_b4175_9_cplx	Translation elongation complex: 9 * ribosome 70S/b4175/EF-TU-tRNA's/EF-G	C19111366H29111405N5590569O7030378S118071P295071Mg28530Zn0Fe0	-360683.26	Translation
rib_70_elo1_b4178_1_cplx	Translation elongation complex: 1 * ribosome 70S/b4178/EF-TU-tRNA's/EF-G	C945888H1431870N280458O357292S5650P17006Mg1433Zn0Fe0	-19581.14	Translation
rib_70_elo1_b4178_4_cplx	Translation elongation complex: 4 * ribosome 70S/b4178/EF-TU-tRNA's/EF-G	C3771390H5713770N1117029O1420159S22600P66740Mg5732Zn0Fe0	-77037.56	Translation
rib_70_elo1_b4178_8_cplx	Translation elongation complex: 8 * ribosome 70S/b4178/EF-TU-tRNA's/EF-G	C7538726H11422970N2232457O2837315S45200P133052Mg11464Zn0Fe0	-153646.12	Translation

rib_70_elo1_b4179_1_cplx	Translation elongation complex: 1 * ribosome 70S/b4179/EF-TU-tRNA's/EF-G	C5078860H7745765N1479659O1857092S31739P75436Mg7481Zn0Fe0	-94363.14	Translation
rib_70_elo1_b4179_23_cplx	Translation elongation complex: 23 * ribosome 70S/b4179/EF-TU-tRNA's/EF-G	C116301290H177572675N33824103O42338918S729997P1681304Mg172063Zn0Fe0	-2116606.22	Translation
rib_70_elo1_b4179_47_cplx	Translation elongation complex: 47 * ribosome 70S/b4179/EF-TU-tRNA's/EF-G	C237634850H362838395N69108951O86500910S1491733P3433160Mg351607Zn0Fe0	-4322689.58	Translation
rib_70_elo1_b4180_14_cplx	Translation elongation complex: 14 * ribosome 70S/b4180/EF-TU-tRNA's/EF-G	C21924081H33347804N6434919O8117489S134162P351908Mg32914Zn0Fe0	-422792.96	Translation
rib_70_elo1_b4180_1_cplx	Translation elongation complex: 1 * ribosome 70S/b4180/EF-TU-tRNA's/EF-G	C1572477H2389318N462238O584574S9583P25816Mg2351Zn0Fe0	-30880.14	Translation
rib_70_elo1_b4180_7_cplx	Translation elongation complex: 7 * ribosome 70S/b4180/EF-TU-tRNA's/EF-G	C10965525H16677850N3218860O4061304S67081P176320Mg16457Zn0Fe0	-211762.98	Translation
rib_70_elo1_b4200_1_cplx	Translation elongation complex: 1 * ribosome 70S/b4200/EF-TU-tRNA's/EF-G	C882070H1335360N261612O333351S5254P15891Mg1343Zn0Fe0	-18232.14	Translation
rib_70_elo1_b4200_3_cplx	Translation elongation complex: 3 * ribosome 70S/b4200/EF-TU-tRNA's/EF-G	C2638662H3997534N781788O994535S15762P46877Mg4029Zn0Fe0	-53898.42	Translation
rib_70_elo1_b4200_7_cplx	Translation elongation complex: 7 * ribosome 70S/b4200/EF-TU-tRNA's/EF-G	C6151846H9321882N1822140O2316903S36778P108849Mg9401Zn0Fe0	-125230.98	Translation
rib_70_elo1_b4201_1_cplx	Translation elongation complex: 1 * ribosome 70S/b4201/EF-TU-tRNA's/EF-G	C716768H1082492N213742O273651S4214P13628Mg1100Zn0Fe0	-15300.14	Translation
rib_70_elo1_b4201_3_cplx	Translation elongation complex: 3 * ribosome 70S/b4201/EF-TU-tRNA's/EF-G	C2144308H3240692N638832O816545S12642P40254Mg3300Zn0Fe0	-45268.42	Translation
rib_70_elo1_b4201_6_cplx	Translation elongation complex: 6 * ribosome 70S/b4201/EF-TU-tRNA's/EF-G	C4285618H6477992N1276467O1630886S25284P80193Mg6600Zn0Fe0	-90220.84	Translation
rib_70_elo1_b4202_1_cplx	Translation elongation complex: 1 * ribosome 70S/b4202/EF-TU-tRNA's/EF-G	C538556H810283N162080O208948S3075P11113Mg839Zn0Fe0	-12067.14	Translation
rib_70_elo1_b4202_2_cplx	Translation elongation complex: 2 * ribosome 70S/b4202/EF-TU-tRNA's/EF-G	C1074951H1618107N323308O416307S6150P21998Mg1678Zn0Fe0	-23905.28	Translation
rib_70_elo1_b4202_4_cplx	Translation elongation complex: 4 * ribosome 70S/b4202/EF-TU-tRNA's/EF-G	C2147741H3233755N645764O831025S12300P43768Mg3356Zn0Fe0	-47581.56	Translation
rib_70_elo1_b4203_1_cplx	Translation elongation complex: 1 * ribosome 70S/b4203/EF-TU-tRNA's/EF-G	C992863H1504464N293846O373740S5925P17482Mg1505Zn0Fe0	-20277.14	Translation
rib_70_elo1_b4203_4_cplx	Translation elongation complex: 4 * ribosome 70S/b4203/EF-TU-tRNA's/EF-G	C3958573H6003318N1170191O1485555S23700P68578Mg6020Zn0Fe0	-79755.56	Translation
rib_70_elo1_b4203_8_cplx	Translation elongation complex: 8 * ribosome 70S/b4203/EF-TU-tRNA's/EF-G	C7912853H12001790N2338651O2967975S47400P136706Mg12040Zn0Fe0	-159060.12	Translation
rib_70_elo1_b4258_1_cplx	Translation elongation complex: 1 * ribosome 70S/b4258/EF-TU-tRNA's/EF-G	C5925437H9039634N1724747O2163699S37054P87182Mg8723Zn0Fe0	-109538.14	Translation
rib_70_elo1_b4258_27_cplx	Translation elongation complex: 27 * ribosome 70S/b4258/EF-TU-tRNA's/EF-G	C159279287H243268590N46282299O57902993S1000458P2279606Mg235521Zn0Fe0	-2883195.78	Translation
rib_70_elo1_b4258_55_cplx	Translation elongation complex: 55 * ribosome 70S/b4258/EF-TU-tRNA's/EF-G	C324429587H495515158N94267355O117929925S2037970P4640678Mg479765Zn0Fe0	-5870211.7	Translation
rib_70_elo1_b4292_18_cplx	Translation elongation complex: 18 * ribosome 70S/b4292/EF-TU-tRNA's/EF-G	C36356737H55362878N10641611O13391573S224208P565880Mg54306Zn0Fe0	-689111.52	Translation
rib_70_elo1_b4292_1_cplx	Translation elongation complex: 1 * ribosome 70S/b4292/EF-TU-tRNA's/EF-G	C2028348H3085413N594628O750237S12456P32335Mg3017Zn0Fe0	-39182.14	Translation
rib_70_elo1_b4292_9_cplx	Translation elongation complex: 9 * ribosome 70S/b4292/EF-TU-tRNA's/EF-G	C18182884H27686573N5322620O6699101S112104P283415Mg27153Zn0Fe0	-345031.26	Translation
rib_70_elo1_b4293_10_cplx	Translation elongation complex: 10 * ribosome 70S/b4293/EF-TU-tRNA's/EF-G	C11389088H17282496N3360819O4259408S68780P193964Mg17210Zn0Fe0	-227436.4	Translation
rib_70_elo1_b4293_1_cplx	Translation elongation complex: 1 * ribosome 70S/b4293/EF-TU-tRNA's/EF-G	C1143371H1733313N337854O429233S6878P19868Mg1721Zn0Fe0	-23216.14	Translation
rib_70_elo1_b4293_5_cplx	Translation elongation complex: 5 * ribosome 70S/b4293/EF-TU-tRNA's/EF-G	C5697023H8644061N1681394O2131533S34390P97244Mg8605Zn0Fe0	-113980.7	Translation
rib_70_elo1_b4371_10_cplx	Translation elongation complex: 10 * ribosome 70S/b4371/EF-TU-tRNA's/EF-G	C21797097H33199672N6375253O8019416S134950P336814Mg32510Zn0Fe0	-411676.4	Translation
rib_70_elo1_b4371_1_cplx	Translation elongation complex: 1 * ribosome 70S/b4371/EF-TU-tRNA's/EF-G	C2188536H3329968N641029O808472S13495P34612Mg3251Zn0Fe0	-42099.14	Translation
rib_70_elo1_b4371_20_cplx	Translation elongation complex: 20 * ribosome 70S/b4371/EF-TU-tRNA's/EF-G	C43584387H66388232N12746613O16031576S269900P672594Mg65020Zn0Fe0	-822317.8	Translation
rib_70_elo1_b4372_1_cplx	Translation elongation complex: 1 * ribosome 70S/b4372/EF-TU-tRNA's/EF-G	C920307H1393168N272967O347734S5484P16568Mg1397Zn0Fe0	-19043.14	Translation

rib_70_elo1_b4372_4_cplx	Translation elongation complex: 4 * ribosome 70S/b4372/EF-TU-tRNA's/EF-G	C3669426H5559253N1087140O1382254S21936P65024Mg5588Zn0Fe0	-74921.56	Translation
rib_70_elo1_b4372_8_cplx	Translation elongation complex: 8 * ribosome 70S/b4372/EF-TU-tRNA's/EF-G	C7334918H11114033N2172704O2761614S43872P129632Mg11176Zn0Fe0	-149426.12	Translation
rib_70_elo1_b4373_1_cplx	Translation elongation complex: 1 * ribosome 70S/b4373/EF-TU-tRNA's/EF-G	C987567H1496028N292366O371926S5903P17469Mg1496Zn0Fe0	-20222.14	Translation
rib_70_elo1_b4373_4_cplx	Translation elongation complex: 4 * ribosome 70S/b4373/EF-TU-tRNA's/EF-G	C3938412H5970684N1164697O1479031S23612P68631Mg5984Zn0Fe0	-79640.56	Translation
rib_70_elo1_b4373_8_cplx	Translation elongation complex: 8 * ribosome 70S/b4373/EF-TU-tRNA's/EF-G	C7872872H11936892N2327805O2955171S47224P136847Mg11968Zn0Fe0	-158865.12	Translation
rib_70_elo1_b4374_13_cplx	Translation elongation complex: 13 * ribosome 70S/b4374/EF-TU-tRNA's/EF-G	C18920359H28768678N5556650O7015229S115661P306308Mg28457Zn0Fe0	-366435.82	Translation
rib_70_elo1_b4374_1_cplx	Translation elongation complex: 1 * ribosome 70S/b4374/EF-TU-tRNA's/EF-G	C1461355H2219710N429782O544025S8897P24188Mg2189Zn0Fe0	-28814.14	Translation
rib_70_elo1_b4374_6_cplx	Translation elongation complex: 6 * ribosome 70S/b4374/EF-TU-tRNA's/EF-G	C8735940H13281780N2565977O3240360S53382P141738Mg13134Zn0Fe0	-169489.84	Translation
rib_70_elo1_b4375_15_cplx	Translation elongation complex: 15 * ribosome 70S/b4375/EF-TU-tRNA's/EF-G	C49742647H75886934N14492472O18175995S310320P735842Mg73875Zn0Fe0	-916565.1	Translation
rib_70_elo1_b4375_1_cplx	Translation elongation complex: 1 * ribosome 70S/b4375/EF-TU-tRNA's/EF-G	C3330309H5075130N971846O1222093S20688P50542Mg4925Zn0Fe0	-62591.14	Translation
rib_70_elo1_b4375_31_cplx	Translation elongation complex: 31 * ribosome 70S/b4375/EF-TU-tRNA's/EF-G	C102785319H156814710N29944616O37551883S641328P1519042Mg152675Zn0Fe0	-1892535.34	Translation
rib_70_elo2_b0014_18_cplx	Translation elongation complex: 18 * ribosome 70S/b0014/second last codon EF-TU-tRNA/1 EF-G	C1663285H2322300N586531O844949S4626P85583Mg3150Zn0Fe0	-71672.52	Translation
rib_70_elo2_b0014_1_cplx	Translation elongation complex: 1 * ribosome 70S/b0014/second last codon EF-TU-tRNA/1 EF-G	C109689H148561N39624O59481S257P6567Mg175Zn0Fe0	-5795.14	Translation
rib_70_elo2_b0014_37_cplx	Translation elongation complex: 37 * ribosome 70S/b0014/second last codon EF-TU-tRNA/1 EF-G	C3399657H4751773N1197780O1722825S9509P173895Mg6475Zn0Fe0	-145300.18	Translation
rib_70_elo2_b0015_11_cplx	Translation elongation complex: 11 * ribosome 70S/b0015/second last codon EF-TU-tRNA/1 EF-G	C1002748H1396319N355031O511266S2838P52270Mg1925Zn0Fe0	-43406.54	Translation
rib_70_elo2_b0015_1_cplx	Translation elongation complex: 1 * ribosome 70S/b0015/second last codon EF-TU-tRNA/1 EF-G	C100978H138039N36281O53636S258P5780Mg175Zn0Fe0	-4975.14	Translation
rib_70_elo2_b0015_22_cplx	Translation elongation complex: 22 * ribosome 70S/b0015/second last codon EF-TU-tRNA/1 EF-G	C1994695H2780427N705656O1014659S5676P103409Mg3850Zn0Fe0	-85681.08	Translation
rib_70_elo2_b0023_1_cplx	Translation elongation complex: 1 * ribosome 70S/b0023/second last codon EF-TU-tRNA/1 EF-G	C91320H126563N32504O47146S244P4914Mg175Zn0Fe0	-4096.14	Translation
rib_70_elo2_b0023_2_cplx	Translation elongation complex: 2 * ribosome 70S/b0023/second last codon EF-TU-tRNA/1 EF-G	C180118H250273N63976O92474S488P9562Mg350Zn0Fe0	-7925.28	Translation
rib_70_elo2_b0023_5_cplx	Translation elongation complex: 5 * ribosome 70S/b0023/second last codon EF-TU-tRNA/1 EF-G	C446512H621403N158392O228458S1220P23506Mg875Zn0Fe0	-19412.7	Translation
rib_70_elo2_b0025_18_cplx	Translation elongation complex: 18 * ribosome 70S/b0025/second last codon EF-TU-tRNA/1 EF-G	C1627707H2268993N575708O828262S4482P84608Mg3150Zn0Fe0	-70031.52	Translation
rib_70_elo2_b0025_1_cplx	Translation elongation complex: 1 * ribosome 70S/b0025/second last codon EF-TU-tRNA/1 EF-G	C98897H135629N35363O52263S249P5592Mg175Zn0Fe0	-4783.14	Translation
rib_70_elo2_b0025_9_cplx	Translation elongation complex: 9 * ribosome 70S/b0025/second last codon EF-TU-tRNA/1 EF-G	C818337H1139565N289643O417439S2241P42776Mg1575Zn0Fe0	-35488.26	Translation
rib_70_elo2_b0026_1_cplx	Translation elongation complex: 1 * ribosome 70S/b0026/second last codon EF-TU-tRNA/1 EF-G	C119880H160603N43417O66239S278P7465Mg175Zn0Fe0	-6684.14	Translation
rib_70_elo2_b0026_27_cplx	Translation elongation complex: 27 * ribosome 70S/b0026/second last codon EF-TU-tRNA/1 EF-G	C2539128H3545959N890991O1277397S7506P128313Mg4725Zn0Fe0	-107203.78	Translation
rib_70_elo2_b0026_55_cplx	Translation elongation complex: 55 * ribosome 70S/b0026/second last codon EF-TU-tRNA/1 EF-G	C5144472H7191727N1803763O2581721S15290P258457Mg9625Zn0Fe0	-215455.7	Translation
rib_70_elo2_b0027_1_cplx	Translation elongation complex: 1 * ribosome 70S/b0027/second last codon EF-TU-tRNA/1 EF-G	C93899H129566N33344O48922S247P5141Mg175Zn0Fe0	-4336.14	Translation
rib_70_elo2_b0027_4_cplx	Translation elongation complex: 4 * ribosome 70S/b0027/second last codon EF-TU-tRNA/1 EF-G	C361562H502373N127952O185170S988P19082Mg700Zn0Fe0	-15859.56	Translation
rib_70_elo2_b0027_9_cplx	Translation elongation complex: 9 * ribosome 70S/b0027/second last codon EF-TU-tRNA/1 EF-G	C807667H1123718N285632O412250S2223P42317Mg1575Zn0Fe0	-35065.26	Translation
rib_70_elo2_b0028_1_cplx	Translation elongation complex: 1 * ribosome 70S/b0028/second last codon EF-TU-tRNA/1 EF-G	C93363H128918N33213O48599S245P5098Mg175Zn0Fe0	-4312.14	Translation
rib_70_elo2_b0028_4_cplx	Translation elongation complex: 4 * ribosome 70S/b0028/second last codon EF-TU-tRNA/1 EF-G	C360627H501134N127767O184928S980P19042Mg700Zn0Fe0	-15895.56	Translation

rib_70_elo2_b0028_8_cplx	Translation elongation complex: 8 * ribosome 70S/b0028/second last codon EF-TU-tRNA/1 EF-G	C716979H997422N253839O366700S1960P37634Mg1400Zn0Fe0	-31340.12	Translation
rib_70_elo2_b0029_18_cplx	Translation elongation complex: 18 * ribosome 70S/b0029/second last codon EF-TU-tRNA/1 EF-G	C1627563H2268481N575556O829137S4518P84633Mg3150Zn0Fe0	-70362.52	Translation
rib_70_elo2_b0029_1_cplx	Translation elongation complex: 1 * ribosome 70S/b0029/second last codon EF-TU-tRNA/1 EF-G	C98991H135712N35449O52339S251P5600Mg175Zn0Fe0	-4808.14	Translation
rib_70_elo2_b0029_9_cplx	Translation elongation complex: 9 * ribosome 70S/b0029/second last codon EF-TU-tRNA/1 EF-G	C818319H1139368N289617O417891S2259P42792Mg1575Zn0Fe0	-35657.26	Translation
rib_70_elo2_b0049_16_cplx	Translation elongation complex: 16 * ribosome 70S/b0049/second last codon EF-TU-tRNA/1 EF-G	C1446568H2013422N511423O737057S4048P75403Mg2800Zn0Fe0	-62702.24	Translation
rib_70_elo2_b0049_1_cplx	Translation elongation complex: 1 * ribosome 70S/b0049/second last codon EF-TU-tRNA/1 EF-G	C97933H134357N34978O51602S253P5503Mg175Zn0Fe0	-4710.14	Translation
rib_70_elo2_b0049_8_cplx	Translation elongation complex: 8 * ribosome 70S/b0049/second last codon EF-TU-tRNA/1 EF-G	C727296H1011254N257319O371481S2024P38123Mg1400Zn0Fe0	-31773.12	Translation
rib_70_elo2_b0050_1_cplx	Translation elongation complex: 1 * ribosome 70S/b0050/second last codon EF-TU-tRNA/1 EF-G	C92601H128030N32906O48055S245P5027Mg175Zn0Fe0	-4230.14	Translation
rib_70_elo2_b0050_3_cplx	Translation elongation complex: 3 * ribosome 70S/b0050/second last codon EF-TU-tRNA/1 EF-G	C270627H375952N95886O138875S735P14325Mg525Zn0Fe0	-11932.42	Translation
rib_70_elo2_b0050_7_cplx	Translation elongation complex: 7 * ribosome 70S/b0050/second last codon EF-TU-tRNA/1 EF-G	C626679H871796N221846O320515S1715P32921Mg1225Zn0Fe0	-27336.98	Translation
rib_70_elo2_b0051_16_cplx	Translation elongation complex: 16 * ribosome 70S/b0051/second last codon EF-TU-tRNA/1 EF-G	C1446220H2013947N511662O737144S4096P75458Mg2800Zn0Fe0	-62597.24	Translation
rib_70_elo2_b0051_1_cplx	Translation elongation complex: 1 * ribosome 70S/b0051/second last codon EF-TU-tRNA/1 EF-G	C97675H134132N34872O51434S256P5483Mg175Zn0Fe0	-4680.14	Translation
rib_70_elo2_b0051_8_cplx	Translation elongation complex: 8 * ribosome 70S/b0051/second last codon EF-TU-tRNA/1 EF-G	C726996H1011379N257374O371432S2048P38138Mg1400Zn0Fe0	-31708.12	Translation
rib_70_elo2_b0052_19_cplx	Translation elongation complex: 19 * ribosome 70S/b0052/second last codon EF-TU-tRNA/1 EF-G	C1718227H2395191N607074O874373S4807P89282Mg3325Zn0Fe0	-74142.66	Translation
rib_70_elo2_b0052_1_cplx	Translation elongation complex: 1 * ribosome 70S/b0052/second last codon EF-TU-tRNA/1 EF-G	C99325H136155N35466O52601S253P5636Mg175Zn0Fe0	-4840.14	Translation
rib_70_elo2_b0052_9_cplx	Translation elongation complex: 9 * ribosome 70S/b0052/second last codon EF-TU-tRNA/1 EF-G	C818837H1140171N289514O417833S2277P42812Mg1575Zn0Fe0	-35641.26	Translation
rib_70_elo2_b0053_12_cplx	Translation elongation complex: 12 * ribosome 70S/b0053/second last codon EF-TU-tRNA/1 EF-G	C1097585H1529777N388220O559196S3084P57065Mg2100Zn0Fe0	-47455.68	Translation
rib_70_elo2_b0053_1_cplx	Translation elongation complex: 1 * ribosome 70S/b0053/second last codon EF-TU-tRNA/1 EF-G	C102712H140224N36913O54791S257P5937Mg175Zn0Fe0	-5137.14	Translation
rib_70_elo2_b0053_25_cplx	Translation elongation complex: 25 * ribosome 70S/b0053/second last codon EF-TU-tRNA/1 EF-G	C2273344H3171976N803401O1155311S6425P117489Mg4375Zn0Fe0	-97468.5	Translation
rib_70_elo2_b0058_12_cplx	Translation elongation complex: 12 * ribosome 70S/b0058/second last codon EF-TU-tRNA/1 EF-G	C1080255H1504058N382351O550891S3036P56436Mg2100Zn0Fe0	-46778.68	Translation
rib_70_elo2_b0058_1_cplx	Translation elongation complex: 1 * ribosome 70S/b0058/second last codon EF-TU-tRNA/1 EF-G	C95799H131863N34212O50127S253P5308Mg175Zn0Fe0	-4504.14	Translation
rib_70_elo2_b0058_6_cplx	Translation elongation complex: 6 * ribosome 70S/b0058/second last codon EF-TU-tRNA/1 EF-G	C543279H755588N192457O277747S1518P28548Mg1050Zn0Fe0	-23719.84	Translation
rib_70_elo2_b0059_1_cplx	Translation elongation complex: 1 * ribosome 70S/b0059/second last codon EF-TU-tRNA/1 EF-G	C120863H161907N43786O66992S271P7556Mg175Zn0Fe0	-6802.14	Translation
rib_70_elo2_b0059_28_cplx	Translation elongation complex: 28 * ribosome 70S/b0059/second last codon EF-TU-tRNA/1 EF-G	C2637371H3687108N926875O1327325S7588P133025Mg4900Zn0Fe0	-111889.92	Translation
rib_70_elo2_b0059_56_cplx	Translation elongation complex: 56 * ribosome 70S/b0059/second last codon EF-TU-tRNA/1 EF-G	C5247083H7342872N1842671O2634337S15176P263141Mg9800Zn0Fe0	-220869.84	Translation
rib_70_elo2_b0144_18_cplx	Translation elongation complex: 18 * ribosome 70S/b0144/second last codon EF-TU-tRNA/1 EF-G	C1627638H2266763N575350O827920S4464P84557Mg3150Zn0Fe0	-70160.52	Translation
rib_70_elo2_b0144_1_cplx	Translation elongation complex: 1 * ribosome 70S/b0144/second last codon EF-TU-tRNA/1 EF-G	C98726H135354N35226O52142S248P5575Mg175Zn0Fe0	-4776.14	Translation
rib_70_elo2_b0144_9_cplx	Translation elongation complex: 9 * ribosome 70S/b0144/second last codon EF-TU-tRNA/1 EF-G	C818214H1138370N289402O417214S2232P42743Mg1575Zn0Fe0	-35545.26	Translation
rib_70_elo2_b0166_16_cplx	Translation elongation complex: 16 * ribosome 70S/b0166/second last codon EF-TU-tRNA/1 EF-G	C1443362H2010760N510385O735739S3984P75211Mg2800Zn0Fe0	-62414.24	Translation
rib_70_elo2_b0166_1_cplx	Translation elongation complex: 1 * ribosome 70S/b0166/second last codon EF-TU-tRNA/1 EF-G	C97577H134005N34855O51394S249P5476Mg175Zn0Fe0	-4677.14	Translation
rib_70_elo2_b0166_8_cplx	Translation elongation complex: 8 * ribosome 70S/b0166/second last codon EF-TU-tRNA/1 EF-G	C725610H1009824N256769O370755S1992P38019Mg1400Zn0Fe0	-31621.12	Translation

rib_70_elo2_b0167_1_cplx	Translation elongation complex: 1 * ribosome 70S/b0167/second last codon EF-TU-tRNA/1 EF-G	C118299H158932N42683O65198S269P7319Mg175Zn0Fe0	-6531.14	Translation
rib_70_elo2_b0167_26_cplx	Translation elongation complex: 26 * ribosome 70S/b0167/second last codon EF-TU-tRNA/1 EF-G	C2441399H3412332N858583O1227723S6994P123469Mg4550Zn0Fe0	-102959.64	Translation
rib_70_elo2_b0167_52_cplx	Translation elongation complex: 52 * ribosome 70S/b0167/second last codon EF-TU-tRNA/1 EF-G	C4857423H6795868N1707119O2436749S13988P244265Mg9100Zn0Fe0	-203245.28	Translation
rib_70_elo2_b0168_15_cplx	Translation elongation complex: 15 * ribosome 70S/b0168/second last codon EF-TU-tRNA/1 EF-G	C1352598H1884380N478330O689485S3855P70517Mg2625Zn0Fe0	-58580.1	Translation
rib_70_elo2_b0168_1_cplx	Translation elongation complex: 1 * ribosome 70S/b0168/second last codon EF-TU-tRNA/1 EF-G	C97246H133624N34740O51127S257P5445Mg175Zn0Fe0	-4650.14	Translation
rib_70_elo2_b0168_7_cplx	Translation elongation complex: 7 * ribosome 70S/b0168/second last codon EF-TU-tRNA/1 EF-G	C635254H883948N224850O324709S1799P33333Mg1225Zn0Fe0	-27762.98	Translation
rib_70_elo2_b0169_14_cplx	Translation elongation complex: 14 * ribosome 70S/b0169/second last codon EF-TU-tRNA/1 EF-G	C1260778H1756099N446074O642880S3500P65800Mg2450Zn0Fe0	-54560.96	Translation
rib_70_elo2_b0169_1_cplx	Translation elongation complex: 1 * ribosome 70S/b0169/second last codon EF-TU-tRNA/1 EF-G	C96472H132698N34429O50626S250P5376Mg175Zn0Fe0	-4574.14	Translation
rib_70_elo2_b0169_7_cplx	Translation elongation complex: 7 * ribosome 70S/b0169/second last codon EF-TU-tRNA/1 EF-G	C633844H881960N224419O323974S1750P33264Mg1225Zn0Fe0	-27644.98	Translation
rib_70_elo2_b0170_16_cplx	Translation elongation complex: 16 * ribosome 70S/b0170/second last codon EF-TU-tRNA/1 EF-G	C1445498H2013881N511380O737210S4048P75412Mg2800Zn0Fe0	-62695.24	Translation
rib_70_elo2_b0170_1_cplx	Translation elongation complex: 1 * ribosome 70S/b0170/second last codon EF-TU-tRNA/1 EF-G	C97988H134486N35100O51605S253P5512Mg175Zn0Fe0	-4718.14	Translation
rib_70_elo2_b0170_8_cplx	Translation elongation complex: 8 * ribosome 70S/b0170/second last codon EF-TU-tRNA/1 EF-G	C726826H1011537N257364O371554S2024P38132Mg1400Zn0Fe0	-31774.12	Translation
rib_70_elo2_b0172_10_cplx	Translation elongation complex: 10 * ribosome 70S/b0172/second last codon EF-TU-tRNA/1 EF-G	C898099H1250918N318177O458821S2480P47040Mg1750Zn0Fe0	-39002.4	Translation
rib_70_elo2_b0172_1_cplx	Translation elongation complex: 1 * ribosome 70S/b0172/second last codon EF-TU-tRNA/1 EF-G	C94615H130517N33795O49357S248P5208Mg175Zn0Fe0	-4405.14	Translation
rib_70_elo2_b0172_5_cplx	Translation elongation complex: 5 * ribosome 70S/b0172/second last codon EF-TU-tRNA/1 EF-G	C451719H628473N160187O231341S1240P23800Mg875Zn0Fe0	-19781.7	Translation
rib_70_elo2_b0188_12_cplx	Translation elongation complex: 12 * ribosome 70S/b0188/second last codon EF-TU-tRNA/1 EF-G	C1100725H1532898N389389O560395S3036P57281Mg2100Zn0Fe0	-47671.68	Translation
rib_70_elo2_b0188_1_cplx	Translation elongation complex: 1 * ribosome 70S/b0188/second last codon EF-TU-tRNA/1 EF-G	C103069H140584N37015O55055S253P5966Mg175Zn0Fe0	-5166.14	Translation
rib_70_elo2_b0188_25_cplx	Translation elongation complex: 25 * ribosome 70S/b0188/second last codon EF-TU-tRNA/1 EF-G	C2279773H3178360N805831O1157615S6325P117926Mg4375Zn0Fe0	-97905.5	Translation
rib_70_elo2_b0194_16_cplx	Translation elongation complex: 16 * ribosome 70S/b0194/second last codon EF-TU-tRNA/1 EF-G	C1475721H2057852N520279O749102S4128P76089Mg2800Zn0Fe0	-63532.24	Translation
rib_70_elo2_b0194_1_cplx	Translation elongation complex: 1 * ribosome 70S/b0194/second last codon EF-TU-tRNA/1 EF-G	C107586H145997N38704O58067S258P6369Mg175Zn0Fe0	-5585.14	Translation
rib_70_elo2_b0194_33_cplx	Translation elongation complex: 33 * ribosome 70S/b0194/second last codon EF-TU-tRNA/1 EF-G	C3026274H4224621N1066064O1532275S8514P155105Mg5775Zn0Fe0	-129205.62	Translation
rib_70_elo2_b0405_10_cplx	Translation elongation complex: 10 * ribosome 70S/b0405/second last codon EF-TU-tRNA/1 EF-G	C911620H1268777N322090O464843S2520P47553Mg1750Zn0Fe0	-39685.4	Translation
rib_70_elo2_b0405_1_cplx	Translation elongation complex: 1 * ribosome 70S/b0405/second last codon EF-TU-tRNA/1 EF-G	C100324H137261N35854O53237S252P5721Mg175Zn0Fe0	-4935.14	Translation
rib_70_elo2_b0405_20_cplx	Translation elongation complex: 20 * ribosome 70S/b0405/second last codon EF-TU-tRNA/1 EF-G	C1813060H2526017N640130O922183S5040P94033Mg3500Zn0Fe0	-78296.8	Translation
rib_70_elo2_b0406_11_cplx	Translation elongation complex: 11 * ribosome 70S/b0406/second last codon EF-TU-tRNA/1 EF-G	C1003805H1397468N354764O511419S2893P52269Mg1925Zn0Fe0	-43493.54	Translation
rib_70_elo2_b0406_1_cplx	Translation elongation complex: 1 * ribosome 70S/b0406/second last codon EF-TU-tRNA/1 EF-G	C101005H138068N36114O53689S263P5779Mg175Zn0Fe0	-4982.14	Translation
rib_70_elo2_b0406_22_cplx	Translation elongation complex: 22 * ribosome 70S/b0406/second last codon EF-TU-tRNA/1 EF-G	C1996885H2782808N705279O1014922S5786P103408Mg3850Zn0Fe0	-85856.08	Translation
rib_70_elo2_b0407_1_cplx	Translation elongation complex: 1 * ribosome 70S/b0407/second last codon EF-TU-tRNA/1 EF-G	C92188H127561N32775O47765S246P4992Mg175Zn0Fe0	-4185.14	Translation
rib_70_elo2_b0407_3_cplx	Translation elongation complex: 3 * ribosome 70S/b0407/second last codon EF-TU-tRNA/1 EF-G	C270222H375523N95799O138631S738P14310Mg525Zn0Fe0	-11887.42	Translation
rib_70_elo2_b0407_6_cplx	Translation elongation complex: 6 * ribosome 70S/b0407/second last codon EF-TU-tRNA/1 EF-G	C537273H747466N190335O274930S1476P28287Mg1050Zn0Fe0	-23440.84	Translation
rib_70_elo2_b0413_1_cplx	Translation elongation complex: 1 * ribosome 70S/b0413/second last codon EF-TU-tRNA/1 EF-G	C93436H129070N33279O48600S251P5101Mg175Zn0Fe0	-4297.14	Translation

rib_70_elo2_b0413_4_cplx	Translation elongation complex: 4 * ribosome 70S/b0413/second last codon EF-TU-tRNA/1 EF-G	C360868H501751N127938O184938S1004P19048Mg700Zn0Fe0	-15829.56	Translation
rib_70_elo2_b0413_8_cplx	Translation elongation complex: 8 * ribosome 70S/b0413/second last codon EF-TU-tRNA/1 EF-G	C717444H998659N254150O366722S2008P37644Mg1400Zn0Fe0	-31206.12	Translation
rib_70_elo2_b0414_10_cplx	Translation elongation complex: 10 * ribosome 70S/b0414/second last codon EF-TU-tRNA/1 EF-G	C912064H1270218N322761O465040S2560P47584Mg1750Zn0Fe0	-39556.4	Translation
rib_70_elo2_b0414_1_cplx	Translation elongation complex: 1 * ribosome 70S/b0414/second last codon EF-TU-tRNA/1 EF-G	C100669H137739N36084O53443S256P5752Mg175Zn0Fe0	-4950.14	Translation
rib_70_elo2_b0414_21_cplx	Translation elongation complex: 21 * ribosome 70S/b0414/second last codon EF-TU-tRNA/1 EF-G	C1903769H2654359N673144O968103S5376P98712Mg3675Zn0Fe0	-81852.94	Translation
rib_70_elo2_b0415_1_cplx	Translation elongation complex: 1 * ribosome 70S/b0415/second last codon EF-TU-tRNA/1 EF-G	C93588H129218N33338O48716S242P5119Mg175Zn0Fe0	-4320.14	Translation
rib_70_elo2_b0415_4_cplx	Translation elongation complex: 4 * ribosome 70S/b0415/second last codon EF-TU-tRNA/1 EF-G	C360885H501650N127937O184997S968P19063Mg700Zn0Fe0	-15864.56	Translation
rib_70_elo2_b0415_9_cplx	Translation elongation complex: 9 * ribosome 70S/b0415/second last codon EF-TU-tRNA/1 EF-G	C806380H1122370N285602O412132S2178P42303Mg1575Zn0Fe0	-35105.26	Translation
rib_70_elo2_b0416_1_cplx	Translation elongation complex: 1 * ribosome 70S/b0416/second last codon EF-TU-tRNA/1 EF-G	C93094H128657N33136O48344S244P5068Mg175Zn0Fe0	-4266.14	Translation
rib_70_elo2_b0416_4_cplx	Translation elongation complex: 4 * ribosome 70S/b0416/second last codon EF-TU-tRNA/1 EF-G	C360370H501026N127693O184613S976P19012Mg700Zn0Fe0	-15801.56	Translation
rib_70_elo2_b0416_8_cplx	Translation elongation complex: 8 * ribosome 70S/b0416/second last codon EF-TU-tRNA/1 EF-G	C716738H997518N253769O366305S1952P37604Mg1400Zn0Fe0	-31182.12	Translation
rib_70_elo2_b0423_14_cplx	Translation elongation complex: 14 * ribosome 70S/b0423/second last codon EF-TU-tRNA/1 EF-G	C1285604H1792252N453847O653249S3570P66537Mg2450Zn0Fe0	-55367.96	Translation
rib_70_elo2_b0423_1_cplx	Translation elongation complex: 1 * ribosome 70S/b0423/second last codon EF-TU-tRNA/1 EF-G	C104632H142500N37522O56081S255P6100Mg175Zn0Fe0	-5303.14	Translation
rib_70_elo2_b0423_28_cplx	Translation elongation complex: 28 * ribosome 70S/b0423/second last codon EF-TU-tRNA/1 EF-G	C2557420H3568908N902197O1296353S7140P131623Mg4900Zn0Fe0	-109283.92	Translation
rib_70_elo2_b0436_12_cplx	Translation elongation complex: 12 * ribosome 70S/b0436/second last codon EF-TU-tRNA/1 EF-G	C1098409H1530615N388102O559616S3012P57077Mg2100Zn0Fe0	-47719.68	Translation
rib_70_elo2_b0436_1_cplx	Translation elongation complex: 1 * ribosome 70S/b0436/second last codon EF-TU-tRNA/1 EF-G	C102931H140413N37037O54870S251P5949Mg175Zn0Fe0	-5170.14	Translation
rib_70_elo2_b0436_25_cplx	Translation elongation complex: 25 * ribosome 70S/b0436/second last codon EF-TU-tRNA/1 EF-G	C2274883H3173581N802997O1156134S6275P117501Mg4375Zn0Fe0	-98005.5	Translation
rib_70_elo2_b0503_10_cplx	Translation elongation complex: 10 * ribosome 70S/b0503/second last codon EF-TU-tRNA/1 EF-G	C912448H1270247N322922O465134S2570P47587Mg1750Zn0Fe0	-39629.4	Translation
rib_70_elo2_b0503_1_cplx	Translation elongation complex: 1 * ribosome 70S/b0503/second last codon EF-TU-tRNA/1 EF-G	C100648H137660N36110O53393S257P5746Mg175Zn0Fe0	-4951.14	Translation
rib_70_elo2_b0503_21_cplx	Translation elongation complex: 21 * ribosome 70S/b0503/second last codon EF-TU-tRNA/1 EF-G	C1904648H2654520N673470O968373S5397P98726Mg3675Zn0Fe0	-82013.94	Translation
rib_70_elo2_b0526_13_cplx	Translation elongation complex: 13 * ribosome 70S/b0526/second last codon EF-TU-tRNA/1 EF-G	C1192148H1659972N421024O606485S3471P61812Mg2275Zn0Fe0	-51622.82	Translation
rib_70_elo2_b0526_1_cplx	Translation elongation complex: 1 * ribosome 70S/b0526/second last codon EF-TU-tRNA/1 EF-G	C103904H141492N37324O55577S267P6036Mg175Zn0Fe0	-5253.14	Translation
rib_70_elo2_b0526_27_cplx	Translation elongation complex: 27 * ribosome 70S/b0526/second last codon EF-TU-tRNA/1 EF-G	C2461766H3431532N868674O1249211S7209P126884Mg4725Zn0Fe0	-105720.78	Translation
rib_70_elo2_b0638_11_cplx	Translation elongation complex: 11 * ribosome 70S/b0638/second last codon EF-TU-tRNA/1 EF-G	C989381H1376852N350286O504876S2739P51740Mg1925Zn0Fe0	-42986.54	Translation
rib_70_elo2_b0638_1_cplx	Translation elongation complex: 1 * ribosome 70S/b0638/second last codon EF-TU-tRNA/1 EF-G	C95231H131152N33936O49796S249P5260Mg175Zn0Fe0	-4465.14	Translation
rib_70_elo2_b0638_5_cplx	Translation elongation complex: 5 * ribosome 70S/b0638/second last codon EF-TU-tRNA/1 EF-G	C452891H629432N160476O231828S1245P23852Mg875Zn0Fe0	-19873.7	Translation
rib_70_elo2_b0639_12_cplx	Translation elongation complex: 12 * ribosome 70S/b0639/second last codon EF-TU-tRNA/1 EF-G	C1080146H1503406N382081O550876S2952P56430Mg2100Zn0Fe0	-46880.68	Translation
rib_70_elo2_b0639_1_cplx	Translation elongation complex: 1 * ribosome 70S/b0639/second last codon EF-TU-tRNA/1 EF-G	C95602H131618N34052O50013S246P5291Mg175Zn0Fe0	-4496.14	Translation
rib_70_elo2_b0639_6_cplx	Translation elongation complex: 6 * ribosome 70S/b0639/second last codon EF-TU-tRNA/1 EF-G	C543122H755158N192247O277678S1476P28536Mg1050Zn0Fe0	-23761.84	Translation
rib_70_elo2_b0640_10_cplx	Translation elongation complex: 10 * ribosome 70S/b0640/second last codon EF-TU-tRNA/1 EF-G	C910847H1268681N322118O464289S2500P47511Mg1750Zn0Fe0	-39493.4	Translation
rib_70_elo2_b0640_1_cplx	Translation elongation complex: 1 * ribosome 70S/b0640/second last codon EF-TU-tRNA/1 EF-G	C99902H136850N35702O52935S250P5679Mg175Zn0Fe0	-4878.14	Translation

rib_70_elo2_b0640_20_cplx	Translation elongation complex: 20 * ribosome 70S/b0640/second last codon EF-TU-tRNA/1 EF-G	C1811897H2526271N640358O921349S5000P93991Mg3500Zn0Fe0	-77954.8	Translation
rib_70_elo2_b0641_11_cplx	Translation elongation complex: 11 * ribosome 70S/b0641/second last codon EF-TU-tRNA/1 EF-G	C988035H1376045N349756O504629S2750P51710Mg1925Zn0Fe0	-42857.54	Translation
rib_70_elo2_b0641_1_cplx	Translation elongation complex: 1 * ribosome 70S/b0641/second last codon EF-TU-tRNA/1 EF-G	C94855H130795N33806O49569S250P5230Mg175Zn0Fe0	-4426.14	Translation
rib_70_elo2_b0641_5_cplx	Translation elongation complex: 5 * ribosome 70S/b0641/second last codon EF-TU-tRNA/1 EF-G	C452127H628895N160186O231593S1250P23822Mg875Zn0Fe0	-19798.7	Translation
rib_70_elo2_b0642_1_cplx	Translation elongation complex: 1 * ribosome 70S/b0642/second last codon EF-TU-tRNA/1 EF-G	C117348H157538N42465O64474S285P7233Mg175Zn0Fe0	-6459.14	Translation
rib_70_elo2_b0642_25_cplx	Translation elongation complex: 25 * ribosome 70S/b0642/second last codon EF-TU-tRNA/1 EF-G	C2342532H3269570N822225O1180546S7125P118785Mg4375Zn0Fe0	-99414.5	Translation
rib_70_elo2_b0642_50_cplx	Translation elongation complex: 50 * ribosome 70S/b0642/second last codon EF-TU-tRNA/1 EF-G	C4660432H6511270N1634475O2343121S14250P234985Mg8750Zn0Fe0	-196243	Translation
rib_70_elo2_b0661_13_cplx	Translation elongation complex: 13 * ribosome 70S/b0661/second last codon EF-TU-tRNA/1 EF-G	C1193370H1662806N421440O607204S3393P61864Mg2275Zn0Fe0	-51622.82	Translation
rib_70_elo2_b0661_1_cplx	Translation elongation complex: 1 * ribosome 70S/b0661/second last codon EF-TU-tRNA/1 EF-G	C104322H142094N37452O55888S261P6076Mg175Zn0Fe0	-5289.14	Translation
rib_70_elo2_b0661_27_cplx	Translation elongation complex: 27 * ribosome 70S/b0661/second last codon EF-TU-tRNA/1 EF-G	C2463926H3436970N869426O1250406S7047P126950Mg4725Zn0Fe0	-105678.78	Translation
rib_70_elo2_b0680_16_cplx	Translation elongation complex: 16 * ribosome 70S/b0680/second last codon EF-TU-tRNA/1 EF-G	C1475019H2055540N520271O748343S4208P76035Mg2800Zn0Fe0	-63350.24	Translation
rib_70_elo2_b0680_1_cplx	Translation elongation complex: 1 * ribosome 70S/b0680/second last codon EF-TU-tRNA/1 EF-G	C107049H145290N38471O57668S263P6315Mg175Zn0Fe0	-5523.14	Translation
rib_70_elo2_b0680_32_cplx	Translation elongation complex: 32 * ribosome 70S/b0680/second last codon EF-TU-tRNA/1 EF-G	C2934187H4093140N1034191O1485063S8416P150403Mg5600Zn0Fe0	-125032.48	Translation
rib_70_elo2_b0850_1_cplx	Translation elongation complex: 1 * ribosome 70S/b0850/second last codon EF-TU-tRNA/1 EF-G	C91601H126886N32516O47388S247P4939Mg175Zn0Fe0	-4134.14	Translation
rib_70_elo2_b0850_2_cplx	Translation elongation complex: 2 * ribosome 70S/b0850/second last codon EF-TU-tRNA/1 EF-G	C180470H250682N63967O92738S494P9588Mg350Zn0Fe0	-7977.28	Translation
rib_70_elo2_b0850_5_cplx	Translation elongation complex: 5 * ribosome 70S/b0850/second last codon EF-TU-tRNA/1 EF-G	C447077H622070N158320O228788S1235P23535Mg875Zn0Fe0	-19506.7	Translation
rib_70_elo2_b0851_14_cplx	Translation elongation complex: 14 * ribosome 70S/b0851/second last codon EF-TU-tRNA/1 EF-G	C1260903H1756086N446173O642815S3486P65792Mg2450Zn0Fe0	-54566.96	Translation
rib_70_elo2_b0851_1_cplx	Translation elongation complex: 1 * ribosome 70S/b0851/second last codon EF-TU-tRNA/1 EF-G	C96298H132490N34346O50509S249P5355Mg175Zn0Fe0	-4554.14	Translation
rib_70_elo2_b0851_7_cplx	Translation elongation complex: 7 * ribosome 70S/b0851/second last codon EF-TU-tRNA/1 EF-G	C633808H881842N224420O323881S1743P33249Mg1225Zn0Fe0	-27636.98	Translation
rib_70_elo2_b0852_17_cplx	Translation elongation complex: 17 * ribosome 70S/b0852/second last codon EF-TU-tRNA/1 EF-G	C1535173H2140137N543148O782134S4301P79921Mg2975Zn0Fe0	-66239.38	Translation
rib_70_elo2_b0852_1_cplx	Translation elongation complex: 1 * ribosome 70S/b0852/second last codon EF-TU-tRNA/1 EF-G	C98405H135049N35212O51974S253P5553Mg175Zn0Fe0	-4749.14	Translation
rib_70_elo2_b0852_8_cplx	Translation elongation complex: 8 * ribosome 70S/b0852/second last codon EF-TU-tRNA/1 EF-G	C726991H1012275N257434O371419S2024P38089Mg1400Zn0Fe0	-31651.12	Translation
rib_70_elo2_b0853_1_cplx	Translation elongation complex: 1 * ribosome 70S/b0853/second last codon EF-TU-tRNA/1 EF-G	C93699H129311N33277O48834S251P5126Mg175Zn0Fe0	-4339.14	Translation
rib_70_elo2_b0853_4_cplx	Translation elongation complex: 4 * ribosome 70S/b0853/second last codon EF-TU-tRNA/1 EF-G	C361230H501911N127852O185232S1004P19073Mg700Zn0Fe0	-15922.56	Translation
rib_70_elo2_b0853_9_cplx	Translation elongation complex: 9 * ribosome 70S/b0853/second last codon EF-TU-tRNA/1 EF-G	C807115H1122911N285477O412562S2259P42318Mg1575Zn0Fe0	-35228.26	Translation
rib_70_elo2_b0858_1_cplx	Translation elongation complex: 1 * ribosome 70S/b0858/second last codon EF-TU-tRNA/1 EF-G	C93868H129571N33332O48894S248P5138Mg175Zn0Fe0	-4330.14	Translation
rib_70_elo2_b0858_4_cplx	Translation elongation complex: 4 * ribosome 70S/b0858/second last codon EF-TU-tRNA/1 EF-G	C361573H502564N127955O185148S992P19079Mg700Zn0Fe0	-15844.56	Translation
rib_70_elo2_b0858_9_cplx	Translation elongation complex: 9 * ribosome 70S/b0858/second last codon EF-TU-tRNA/1 EF-G	C807748H1124219N285660O412238S2232P42314Mg1575Zn0Fe0	-35035.26	Translation
rib_70_elo2_b0859_11_cplx	Translation elongation complex: 11 * ribosome 70S/b0859/second last codon EF-TU-tRNA/1 EF-G	C1003480H1397419N354406O510943S2915P52245Mg1925Zn0Fe0	-43414.54	Translation
rib_70_elo2_b0859_1_cplx	Translation elongation complex: 1 * ribosome 70S/b0859/second last codon EF-TU-tRNA/1 EF-G	C100970H138079N36086O53643S265P5775Mg175Zn0Fe0	-4973.14	Translation
rib_70_elo2_b0859_22_cplx	Translation elongation complex: 22 * ribosome 70S/b0859/second last codon EF-TU-tRNA/1 EF-G	C1996241H2782693N704558O1013973S5830P103362Mg3850Zn0Fe0	-85700.08	Translation

rib_70_elo2_b0884_1_cplx	Translation elongation complex: 1 * ribosome 70S/b0884/second last codon EF-TU-tRNA/1 EF-G	C90841H125960N32286O46844S245P4870Mg175Zn0Fe0	-4065.14	Translation
rib_70_elo2_b0884_2_cplx	Translation elongation complex: 2 * ribosome 70S/b0884/second last codon EF-TU-tRNA/1 EF-G	C179595H249558N63731O92165S490P9519Mg350Zn0Fe0	-7908.28	Translation
rib_70_elo2_b0884_4_cplx	Translation elongation complex: 4 * ribosome 70S/b0884/second last codon EF-TU-tRNA/1 EF-G	C357103H496754N126621O182807S980P18817Mg700Zn0Fe0	-15594.56	Translation
rib_70_elo2_b0893_12_cplx	Translation elongation complex: 12 * ribosome 70S/b0893/second last codon EF-TU-tRNA/1 EF-G	C1098261H1530195N388214O559392S3108P57071Mg2100Zn0Fe0	-47593.68	Translation
rib_70_elo2_b0893_1_cplx	Translation elongation complex: 1 * ribosome 70S/b0893/second last codon EF-TU-tRNA/1 EF-G	C102816H140290N36896O54888S259P5943Mg175Zn0Fe0	-5154.14	Translation
rib_70_elo2_b0893_25_cplx	Translation elongation complex: 25 * ribosome 70S/b0893/second last codon EF-TU-tRNA/1 EF-G	C2274696H3172810N803408O1155624S6475P117495Mg4375Zn0Fe0	-97749.5	Translation
rib_70_elo2_b0910_13_cplx	Translation elongation complex: 13 * ribosome 70S/b0910/second last codon EF-TU-tRNA/1 EF-G	C1169608H1629128N414065O596818S3185P61110Mg2275Zn0Fe0	-50751.82	Translation
rib_70_elo2_b0910_1_cplx	Translation elongation complex: 1 * ribosome 70S/b0910/second last codon EF-TU-tRNA/1 EF-G	C95980H132116N34253O50338S245P5334Mg175Zn0Fe0	-4538.14	Translation
rib_70_elo2_b0910_6_cplx	Translation elongation complex: 6 * ribosome 70S/b0910/second last codon EF-TU-tRNA/1 EF-G	C543325H755871N192508O278038S1470P28574Mg1050Zn0Fe0	-23793.84	Translation
rib_70_elo2_b0911_16_cplx	Translation elongation complex: 16 * ribosome 70S/b0911/second last codon EF-TU-tRNA/1 EF-G	C1473045H2054798N519795O748622S3984P76044Mg2800Zn0Fe0	-63647.24	Translation
rib_70_elo2_b0911_1_cplx	Translation elongation complex: 1 * ribosome 70S/b0911/second last codon EF-TU-tRNA/1 EF-G	C107040H145343N38550O57707S249P6324Mg175Zn0Fe0	-5550.14	Translation
rib_70_elo2_b0911_32_cplx	Translation elongation complex: 32 * ribosome 70S/b0911/second last codon EF-TU-tRNA/1 EF-G	C2930117H4091550N1033123O1485598S7968P150412Mg5600Zn0Fe0	-125617.48	Translation
rib_70_elo2_b0912_1_cplx	Translation elongation complex: 1 * ribosome 70S/b0912/second last codon EF-TU-tRNA/1 EF-G	C91563H126811N32572O47324S243P4933Mg175Zn0Fe0	-4127.14	Translation
rib_70_elo2_b0912_2_cplx	Translation elongation complex: 2 * ribosome 70S/b0912/second last codon EF-TU-tRNA/1 EF-G	C180407H250547N64042O92675S486P9581Mg350Zn0Fe0	-7968.28	Translation
rib_70_elo2_b0912_5_cplx	Translation elongation complex: 5 * ribosome 70S/b0912/second last codon EF-TU-tRNA/1 EF-G	C446939H621755N158452O228728S1215P23525Mg875Zn0Fe0	-19491.7	Translation
rib_70_elo2_b0930_13_cplx	Translation elongation complex: 13 * ribosome 70S/b0930/second last codon EF-TU-tRNA/1 EF-G	C1192957H1660994N420973O606647S3328P61827Mg2275Zn0Fe0	-51572.82	Translation
rib_70_elo2_b0930_1_cplx	Translation elongation complex: 1 * ribosome 70S/b0930/second last codon EF-TU-tRNA/1 EF-G	C104053H141674N37237O55751S256P6051Mg175Zn0Fe0	-5263.14	Translation
rib_70_elo2_b0930_27_cplx	Translation elongation complex: 27 * ribosome 70S/b0930/second last codon EF-TU-tRNA/1 EF-G	C2463345H3433534N868665O1249359S6912P126899Mg4725Zn0Fe0	-105600.78	Translation
rib_70_elo2_b0969_1_cplx	Translation elongation complex: 1 * ribosome 70S/b0969/second last codon EF-TU-tRNA/1 EF-G	C92122H127439N32774O47679S244P4981Mg175Zn0Fe0	-4179.14	Translation
rib_70_elo2_b0969_3_cplx	Translation elongation complex: 3 * ribosome 70S/b0969/second last codon EF-TU-tRNA/1 EF-G	C270050H375211N95746O138443S732P14279Mg525Zn0Fe0	-11871.42	Translation
rib_70_elo2_b0969_6_cplx	Translation elongation complex: 6 * ribosome 70S/b0969/second last codon EF-TU-tRNA/1 EF-G	C536942H746869N190204O274589S1464P28226Mg1050Zn0Fe0	-23409.84	Translation
rib_70_elo2_b1066_11_cplx	Translation elongation complex: 11 * ribosome 70S/b1066/second last codon EF-TU-tRNA/1 EF-G	C989179H1376346N350155O504509S2739P51726Mg1925Zn0Fe0	-42829.54	Translation
rib_70_elo2_b1066_1_cplx	Translation elongation complex: 1 * ribosome 70S/b1066/second last codon EF-TU-tRNA/1 EF-G	C94979H130836N33825O49599S249P5236Mg175Zn0Fe0	-4428.14	Translation
rib_70_elo2_b1066_5_cplx	Translation elongation complex: 5 * ribosome 70S/b1066/second last codon EF-TU-tRNA/1 EF-G	C452659H629040N160357O231563S1245P23832Mg875Zn0Fe0	-19788.7	Translation
rib_70_elo2_b1084_1_cplx	Translation elongation complex: 1 * ribosome 70S/b1084/second last codon EF-TU-tRNA/1 EF-G	C123862H165672N45197O68951S259P7836Mg175Zn0Fe0	-7064.14	Translation
rib_70_elo2_b1084_31_cplx	Translation elongation complex: 31 * ribosome 70S/b1084/second last codon EF-TU-tRNA/1 EF-G	C2928982H4102872N1031777O1473701S8029P147276Mg5425Zn0Fe0	-123318.34	Translation
rib_70_elo2_b1084_62_cplx	Translation elongation complex: 62 * ribosome 70S/b1084/second last codon EF-TU-tRNA/1 EF-G	C5827606H8171312N2051243O2925276S16058P291364Mg10850Zn0Fe0	-243447.68	Translation
rib_70_elo2_b1086_18_cplx	Translation elongation complex: 18 * ribosome 70S/b1086/second last codon EF-TU-tRNA/1 EF-G	C1628858H2271533N576536O828769S4464P84644Mg3150Zn0Fe0	-69905.52	Translation
rib_70_elo2_b1086_1_cplx	Translation elongation complex: 1 * ribosome 70S/b1086/second last codon EF-TU-tRNA/1 EF-G	C99147H135959N35528O52379S248P5611Mg175Zn0Fe0	-4793.14	Translation
rib_70_elo2_b1086_9_cplx	Translation elongation complex: 9 * ribosome 70S/b1086/second last codon EF-TU-tRNA/1 EF-G	C819011H1140935N290120O417739S2232P42803Mg1575Zn0Fe0	-35434.26	Translation
rib_70_elo2_b1088_10_cplx	Translation elongation complex: 10 * ribosome 70S/b1088/second last codon EF-TU-tRNA/1 EF-G	C897350H1248864N317471O458536S2500P47004Mg1750Zn0Fe0	-39136.4	Translation

rib_70_elo2_b1088_1_cplx	Translation elongation complex: 1 * ribosome 70S/b1088/second last codon EF-TU-tRNA/1 EF-G	C94208H129939N33530O49144S250P5172Mg175Zn0Fe0	-4386.14	Translation
rib_70_elo2_b1088_5_cplx	Translation elongation complex: 5 * ribosome 70S/b1088/second last codon EF-TU-tRNA/1 EF-G	C451160H627239N159726O231096S1250P23764Mg875Zn0Fe0	-19830.7	Translation
rib_70_elo2_b1089_1_cplx	Translation elongation complex: 1 * ribosome 70S/b1089/second last codon EF-TU-tRNA/1 EF-G	C90313H125347N32085O46500S243P4822Mg175Zn0Fe0	-4011.14	Translation
rib_70_elo2_b1089_3_cplx	Translation elongation complex: 3 * ribosome 70S/b1089/second last codon EF-TU-tRNA/1 EF-G	C267635H372275N94931O137094S729P14118Mg525Zn0Fe0	-11683.42	Translation
rib_70_elo2_b1090_10_cplx	Translation elongation complex: 10 * ribosome 70S/b1090/second last codon EF-TU-tRNA/1 EF-G	C910968H1269519N322268O464657S2530P47563Mg1750Zn0Fe0	-39455.4	Translation
rib_70_elo2_b1090_1_cplx	Translation elongation complex: 1 * ribosome 70S/b1090/second last codon EF-TU-tRNA/1 EF-G	C100275H137319N35888O53231S253P5722Mg175Zn0Fe0	-4912.14	Translation
rib_70_elo2_b1090_20_cplx	Translation elongation complex: 20 * ribosome 70S/b1090/second last codon EF-TU-tRNA/1 EF-G	C1811738H2527519N640468O921797S5060P94053Mg3500Zn0Fe0	-77836.8	Translation
rib_70_elo2_b1091_18_cplx	Translation elongation complex: 18 * ribosome 70S/b1091/second last codon EF-TU-tRNA/1 EF-G	C1626554H2266371N574968O828729S4590P84620Mg3150Zn0Fe0	-70367.52	Translation
rib_70_elo2_b1091_1_cplx	Translation elongation complex: 1 * ribosome 70S/b1091/second last codon EF-TU-tRNA/1 EF-G	C98934H135608N35354O52373S255P5604Mg175Zn0Fe0	-4813.14	Translation
rib_70_elo2_b1091_9_cplx	Translation elongation complex: 9 * ribosome 70S/b1091/second last codon EF-TU-tRNA/1 EF-G	C817814H1138320N289290O417717S2295P42788Mg1575Zn0Fe0	-35662.26	Translation
rib_70_elo2_b1092_18_cplx	Translation elongation complex: 18 * ribosome 70S/b1092/second last codon EF-TU-tRNA/1 EF-G	C1627427H2266722N575277O829524S4608P84794Mg3150Zn0Fe0	-70505.52	Translation
rib_70_elo2_b1092_1_cplx	Translation elongation complex: 1 * ribosome 70S/b1092/second last codon EF-TU-tRNA/1 EF-G	C98787H135398N35340O52233S256P5591Mg175Zn0Fe0	-4798.14	Translation
rib_70_elo2_b1092_9_cplx	Translation elongation complex: 9 * ribosome 70S/b1092/second last codon EF-TU-tRNA/1 EF-G	C818147H1138374N289428O418017S2304P42863Mg1575Zn0Fe0	-35719.26	Translation
rib_70_elo2_b1093_14_cplx	Translation elongation complex: 14 * ribosome 70S/b1093/second last codon EF-TU-tRNA/1 EF-G	C1259997H1755434N446035O643054S3528P65823Mg2450Zn0Fe0	-54569.96	Translation
rib_70_elo2_b1093_1_cplx	Translation elongation complex: 1 * ribosome 70S/b1093/second last codon EF-TU-tRNA/1 EF-G	C96510H132735N34481O50709S252P5386Mg175Zn0Fe0	-4583.14	Translation
rib_70_elo2_b1093_7_cplx	Translation elongation complex: 7 * ribosome 70S/b1093/second last codon EF-TU-tRNA/1 EF-G	C633504H881673N224429O324099S1764P33280Mg1225Zn0Fe0	-27653.98	Translation
rib_70_elo2_b1114_1_cplx	Translation elongation complex: 1 * ribosome 70S/b1114/second last codon EF-TU-tRNA/1 EF-G	C126971H169309N46142O70990S277P8097Mg175Zn0Fe0	-7321.14	Translation
rib_70_elo2_b1114_33_cplx	Translation elongation complex: 33 * ribosome 70S/b1114/second last codon EF-TU-tRNA/1 EF-G	C3140027H4397885N1101278O1572078S9141P156833Mg5775Zn0Fe0	-131197.62	Translation
rib_70_elo2_b1114_67_cplx	Translation elongation complex: 67 * ribosome 70S/b1114/second last codon EF-TU-tRNA/1 EF-G	C6341399H8890747N2222360O3166984S18559P314865Mg11725Zn0Fe0	-262816.38	Translation
rib_70_elo2_b1133_10_cplx	Translation elongation complex: 10 * ribosome 70S/b1133/second last codon EF-TU-tRNA/1 EF-G	C912717H1270198N322459O465484S2540P47599Mg1750Zn0Fe0	-39741.4	Translation
rib_70_elo2_b1133_1_cplx	Translation elongation complex: 1 * ribosome 70S/b1133/second last codon EF-TU-tRNA/1 EF-G	C100755H137755N36043O53518S254P5758Mg175Zn0Fe0	-4973.14	Translation
rib_70_elo2_b1133_21_cplx	Translation elongation complex: 21 * ribosome 70S/b1133/second last codon EF-TU-tRNA/1 EF-G	C1905115H2654295N672523O968998S5334P98738Mg3675Zn0Fe0	-82235.94	Translation
rib_70_elo2_b1134_1_cplx	Translation elongation complex: 1 * ribosome 70S/b1134/second last codon EF-TU-tRNA/1 EF-G	C93578H129158N33292O48685S248P5111Mg175Zn0Fe0	-4318.14	Translation
rib_70_elo2_b1134_4_cplx	Translation elongation complex: 4 * ribosome 70S/b1134/second last codon EF-TU-tRNA/1 EF-G	C361130H501734N127945O185023S992P19058Mg700Zn0Fe0	-15883.56	Translation
rib_70_elo2_b1134_9_cplx	Translation elongation complex: 9 * ribosome 70S/b1134/second last codon EF-TU-tRNA/1 EF-G	C807050H1122694N285700O412253S2232P42303Mg1575Zn0Fe0	-35159.26	Translation
rib_70_elo2_b1135_12_cplx	Translation elongation complex: 12 * ribosome 70S/b1135/second last codon EF-TU-tRNA/1 EF-G	C1080186H1504325N382432O551062S2940P56444Mg2100Zn0Fe0	-46678.68	Translation
rib_70_elo2_b1135_1_cplx	Translation elongation complex: 1 * ribosome 70S/b1135/second last codon EF-TU-tRNA/1 EF-G	C95719H131822N34150O50100S245P5305Mg175Zn0Fe0	-4492.14	Translation
rib_70_elo2_b1135_6_cplx	Translation elongation complex: 6 * ribosome 70S/b1135/second last codon EF-TU-tRNA/1 EF-G	C543204H755687N192460O277810S1470P28550Mg1050Zn0Fe0	-23667.84	Translation
rib_70_elo2_b1211_10_cplx	Translation elongation complex: 10 * ribosome 70S/b1211/second last codon EF-TU-tRNA/1 EF-G	C911461H1269251N322744O465235S2550P47565Mg1750Zn0Fe0	-39707.4	Translation
rib_70_elo2_b1211_1_cplx	Translation elongation complex: 1 * ribosome 70S/b1211/second last codon EF-TU-tRNA/1 EF-G	C100435H137438N36022O53314S255P5733Mg175Zn0Fe0	-4948.14	Translation
rib_70_elo2_b1211_21_cplx	Translation elongation complex: 21 * ribosome 70S/b1211/second last codon EF-TU-tRNA/1 EF-G	C1902715H2652578N673182O968694S5355P98693Mg3675Zn0Fe0	-82190.94	Translation

rib_70_elo2_b1212_16_cplx	Translation elongation complex: 16 * ribosome 70S/b1212/second last codon EF-TU-tRNA/1 EF-G	C1443688H2010598N510593O735697S4000P75188Mg2800Zn0Fe0	-62599.24	Translation
rib_70_elo2_b1212_1_cplx	Translation elongation complex: 1 * ribosome 70S/b1212/second last codon EF-TU-tRNA/1 EF-G	C97663H134083N34868O51472S250P5483Mg175Zn0Fe0	-4697.14	Translation
rib_70_elo2_b1212_8_cplx	Translation elongation complex: 8 * ribosome 70S/b1212/second last codon EF-TU-tRNA/1 EF-G	C725808H1009790N256873O370777S2000P38012Mg1400Zn0Fe0	-31718.12	Translation
rib_70_elo2_b1229_1_cplx	Translation elongation complex: 1 * ribosome 70S/b1229/second last codon EF-TU-tRNA/1 EF-G	C89531H124390N31836O45959S244P4751Mg175Zn0Fe0	-3940.14	Translation
rib_70_elo2_b1269_17_cplx	Translation elongation complex: 17 * ribosome 70S/b1269/second last codon EF-TU-tRNA/1 EF-G	C1534951H2140162N543669O781886S4182P79894Mg2975Zn0Fe0	-65974.38	Translation
rib_70_elo2_b1269_1_cplx	Translation elongation complex: 1 * ribosome 70S/b1269/second last codon EF-TU-tRNA/1 EF-G	C98151H134770N35141O51774S246P5526Mg175Zn0Fe0	-4708.14	Translation
rib_70_elo2_b1269_8_cplx	Translation elongation complex: 8 * ribosome 70S/b1269/second last codon EF-TU-tRNA/1 EF-G	C726751H1012129N257622O371198S1968P38062Mg1400Zn0Fe0	-31512.12	Translation
rib_70_elo2_b1286_18_cplx	Translation elongation complex: 18 * ribosome 70S/b1286/second last codon EF-TU-tRNA/1 EF-G	C1667249H2325914N587633O844467S4716P85601Mg3150Zn0Fe0	-71510.52	Translation
rib_70_elo2_b1286_1_cplx	Translation elongation complex: 1 * ribosome 70S/b1286/second last codon EF-TU-tRNA/1 EF-G	C109998H148911N39570O59679S262P6585Mg175Zn0Fe0	-5803.14	Translation
rib_70_elo2_b1286_37_cplx	Translation elongation complex: 37 * ribosome 70S/b1286/second last codon EF-TU-tRNA/1 EF-G	C3407706H4759035N1200174O1721583S9694P173913Mg6475Zn0Fe0	-144948.18	Translation
rib_70_elo2_b1344_18_cplx	Translation elongation complex: 18 * ribosome 70S/b1344/second last codon EF-TU-tRNA/1 EF-G	C1628100H2268966N575381O828818S4680P84602Mg3150Zn0Fe0	-70223.52	Translation
rib_70_elo2_b1344_1_cplx	Translation elongation complex: 1 * ribosome 70S/b1344/second last codon EF-TU-tRNA/1 EF-G	C98882H135568N35359O52207S260P5586Mg175Zn0Fe0	-4788.14	Translation
rib_70_elo2_b1344_9_cplx	Translation elongation complex: 9 * ribosome 70S/b1344/second last codon EF-TU-tRNA/1 EF-G	C818514H1139520N289487O417671S2340P42770Mg1575Zn0Fe0	-35581.26	Translation
rib_70_elo2_b1427_10_cplx	Translation elongation complex: 10 * ribosome 70S/b1427/second last codon EF-TU-tRNA/1 EF-G	C898038H1249888N317853O458497S2480P47012Mg1750Zn0Fe0	-39014.4	Translation
rib_70_elo2_b1427_1_cplx	Translation elongation complex: 1 * ribosome 70S/b1427/second last codon EF-TU-tRNA/1 EF-G	C94437H130207N33633O49240S248P5189Mg175Zn0Fe0	-4390.14	Translation
rib_70_elo2_b1427_5_cplx	Translation elongation complex: 5 * ribosome 70S/b1427/second last codon EF-TU-tRNA/1 EF-G	C451593H627843N159953O231132S1240P23777Mg875Zn0Fe0	-19778.7	Translation
rib_70_elo2_b1480_1_cplx	Translation elongation complex: 1 * ribosome 70S/b1480/second last codon EF-TU-tRNA/1 EF-G	C89922H124865N31951O46222S242P4786Mg175Zn0Fe0	-3977.14	Translation
rib_70_elo2_b1480_2_cplx	Translation elongation complex: 2 * ribosome 70S/b1480/second last codon EF-TU-tRNA/1 EF-G	C178523H248236N63355O91504S484P9434Mg350Zn0Fe0	-7815.28	Translation
rib_70_elo2_b1481_1_cplx	Translation elongation complex: 1 * ribosome 70S/b1481/second last codon EF-TU-tRNA/1 EF-G	C90890H125952N32279O46918S242P4877Mg175Zn0Fe0	-4083.14	Translation
rib_70_elo2_b1481_2_cplx	Translation elongation complex: 2 * ribosome 70S/b1481/second last codon EF-TU-tRNA/1 EF-G	C179726H249583N63746O92323S484P9536Mg350Zn0Fe0	-7947.28	Translation
rib_70_elo2_b1481_4_cplx	Translation elongation complex: 4 * ribosome 70S/b1481/second last codon EF-TU-tRNA/1 EF-G	C357398H496845N126680O183133S968P18854Mg700Zn0Fe0	-15675.56	Translation
rib_70_elo2_b1636_16_cplx	Translation elongation complex: 16 * ribosome 70S/b1636/second last codon EF-TU-tRNA/1 EF-G	C1446374H2014247N511506O737055S4064P75408Mg2800Zn0Fe0	-62659.24	Translation
rib_70_elo2_b1636_1_cplx	Translation elongation complex: 1 * ribosome 70S/b1636/second last codon EF-TU-tRNA/1 EF-G	C98114H134612N35061O51735S254P5523Mg175Zn0Fe0	-4727.14	Translation
rib_70_elo2_b1636_8_cplx	Translation elongation complex: 8 * ribosome 70S/b1636/second last codon EF-TU-tRNA/1 EF-G	C727302H1011775N257402O371551S2032P38136Mg1400Zn0Fe0	-31762.12	Translation
rib_70_elo2_b1637_12_cplx	Translation elongation complex: 12 * ribosome 70S/b1637/second last codon EF-TU-tRNA/1 EF-G	C1098268H1529306N387735O559118S3060P57053Mg2100Zn0Fe0	-47503.68	Translation
rib_70_elo2_b1637_1_cplx	Translation elongation complex: 1 * ribosome 70S/b1637/second last codon EF-TU-tRNA/1 EF-G	C102658H140028N36780O54757S255P5925Mg175Zn0Fe0	-5130.14	Translation
rib_70_elo2_b1637_24_cplx	Translation elongation complex: 24 * ribosome 70S/b1637/second last codon EF-TU-tRNA/1 EF-G	C2184388H3044882N770595O1109330S6120P112829Mg4200Zn0Fe0	-93729.36	Translation
rib_70_elo2_b1638_12_cplx	Translation elongation complex: 12 * ribosome 70S/b1638/second last codon EF-TU-tRNA/1 EF-G	C1080662H1504451N382544O551056S2964P56447Mg2100Zn0Fe0	-46753.68	Translation
rib_70_elo2_b1638_1_cplx	Translation elongation complex: 1 * ribosome 70S/b1638/second last codon EF-TU-tRNA/1 EF-G	C95777H131849N34141O50149S247P5308Mg175Zn0Fe0	-4501.14	Translation
rib_70_elo2_b1638_6_cplx	Translation elongation complex: 6 * ribosome 70S/b1638/second last codon EF-TU-tRNA/1 EF-G	C543452H755759N192506O277834S1482P28553Mg1050Zn0Fe0	-23706.84	Translation
rib_70_elo2_b1652_12_cplx	Translation elongation complex: 12 * ribosome 70S/b1652/second last codon EF-TU-tRNA/1 EF-G	C1079272H1502003N381894O550766S3024P56426Mg2100Zn0Fe0	-46900.68	Translation

rib_70_elo2_b1652_1_cplx	Translation elongation complex: 1 * ribosome 70S/b1652/second last codon EF-TU-tRNA/1 EF-G	C95575H131568N34052O50057S252P5298Mg175Zn0Fe0	-4505.14	Translation
rib_70_elo2_b1652_6_cplx	Translation elongation complex: 6 * ribosome 70S/b1652/second last codon EF-TU-tRNA/1 EF-G	C542710H754493N192162O277652S1512P28538Mg1050Zn0Fe0	-23775.84	Translation
rib_70_elo2_b1653_1_cplx	Translation elongation complex: 1 * ribosome 70S/b1653/second last codon EF-TU-tRNA/1 EF-G	C139712H184735N50960O79803S274P9264Mg175Zn0Fe0	-8472.14	Translation
rib_70_elo2_b1653_45_cplx	Translation elongation complex: 45 * ribosome 70S/b1653/second last codon EF-TU-tRNA/1 EF-G	C4356936H6123327N1525136O2166767S12330P213732Mg7875Zn0Fe0	-178054.3	Translation
rib_70_elo2_b1653_90_cplx	Translation elongation complex: 90 * ribosome 70S/b1653/second last codon EF-TU-tRNA/1 EF-G	C8670006H12196887N3032816O4301162S24660P422847Mg15750Zn0Fe0	-351490.6	Translation
rib_70_elo2_b1712_1_cplx	Translation elongation complex: 1 * ribosome 70S/b1712/second last codon EF-TU-tRNA/1 EF-G	C91745H127035N32656O47440S243P4950Mg175Zn0Fe0	-4144.14	Translation
rib_70_elo2_b1712_2_cplx	Translation elongation complex: 2 * ribosome 70S/b1712/second last codon EF-TU-tRNA/1 EF-G	C180620H250832N64135O92797S486P9598Mg350Zn0Fe0	-7985.28	Translation
rib_70_elo2_b1712_5_cplx	Translation elongation complex: 5 * ribosome 70S/b1712/second last codon EF-TU-tRNA/1 EF-G	C447245H622223N158572O228868S1215P23542Mg875Zn0Fe0	-19508.7	Translation
rib_70_elo2_b1713_1_cplx	Translation elongation complex: 1 * ribosome 70S/b1713/second last codon EF-TU-tRNA/1 EF-G	C115008H154936N41573O63089S268P7037Mg175Zn0Fe0	-6263.14	Translation
rib_70_elo2_b1713_23_cplx	Translation elongation complex: 23 * ribosome 70S/b1713/second last codon EF-TU-tRNA/1 EF-G	C2144574H2997424N754813O1083625S6164P109315Mg4025Zn0Fe0	-91494.22	Translation
rib_70_elo2_b1713_46_cplx	Translation elongation complex: 46 * ribosome 70S/b1713/second last codon EF-TU-tRNA/1 EF-G	C4266393H5969116N1500473O2150549S12328P216242Mg8050Zn0Fe0	-180599.44	Translation
rib_70_elo2_b1714_19_cplx	Translation elongation complex: 19 * ribosome 70S/b1714/second last codon EF-TU-tRNA/1 EF-G	C1719838H2395930N607707O875197S4788P89296Mg3325Zn0Fe0	-74194.66	Translation
rib_70_elo2_b1714_1_cplx	Translation elongation complex: 1 * ribosome 70S/b1714/second last codon EF-TU-tRNA/1 EF-G	C99370H136138N35487O52597S252P5632Mg175Zn0Fe0	-4838.14	Translation
rib_70_elo2_b1714_9_cplx	Translation elongation complex: 9 * ribosome 70S/b1714/second last codon EF-TU-tRNA/1 EF-G	C819578H1140490N289807O418197S2268P42816Mg1575Zn0Fe0	-35663.26	Translation
rib_70_elo2_b1715_1_cplx	Translation elongation complex: 1 * ribosome 70S/b1715/second last codon EF-TU-tRNA/1 EF-G	C88891H123579N31494O45567S241P4695Mg175Zn0Fe0	-3892.14	Translation
rib_70_elo2_b1716_1_cplx	Translation elongation complex: 1 * ribosome 70S/b1716/second last codon EF-TU-tRNA/1 EF-G	C92384H127850N32875O47855S241P5005Mg175Zn0Fe0	-4179.14	Translation
rib_70_elo2_b1716_3_cplx	Translation elongation complex: 3 * ribosome 70S/b1716/second last codon EF-TU-tRNA/1 EF-G	C270360H375870N95927O138583S723P14301Mg525Zn0Fe0	-11821.42	Translation
rib_70_elo2_b1716_6_cplx	Translation elongation complex: 6 * ribosome 70S/b1716/second last codon EF-TU-tRNA/1 EF-G	C537324H747900N190505O274675S1446P28245Mg1050Zn0Fe0	-23284.84	Translation
rib_70_elo2_b1717_1_cplx	Translation elongation complex: 1 * ribosome 70S/b1717/second last codon EF-TU-tRNA/1 EF-G	C90596H125703N32209O46651S243P4846Mg175Zn0Fe0	-4025.14	Translation
rib_70_elo2_b1717_3_cplx	Translation elongation complex: 3 * ribosome 70S/b1717/second last codon EF-TU-tRNA/1 EF-G	C268010H372827N95087O137225S729P14142Mg525Zn0Fe0	-11677.42	Translation
rib_70_elo2_b1718_10_cplx	Translation elongation complex: 10 * ribosome 70S/b1718/second last codon EF-TU-tRNA/1 EF-G	C897903H1250714N318031O458433S2480P47013Mg1750Zn0Fe0	-38895.4	Translation
rib_70_elo2_b1718_1_cplx	Translation elongation complex: 1 * ribosome 70S/b1718/second last codon EF-TU-tRNA/1 EF-G	C94473H130349N33739O49212S248P5190Mg175Zn0Fe0	-4379.14	Translation
rib_70_elo2_b1718_5_cplx	Translation elongation complex: 5 * ribosome 70S/b1718/second last codon EF-TU-tRNA/1 EF-G	C451553H628289N160091O231088S1240P23778Mg875Zn0Fe0	-19719.7	Translation
rib_70_elo2_b1719_18_cplx	Translation elongation complex: 18 * ribosome 70S/b1719/second last codon EF-TU-tRNA/1 EF-G	C1668192H2325990N587843O844577S4986P85595Mg3150Zn0Fe0	-71432.52	Translation
rib_70_elo2_b1719_1_cplx	Translation elongation complex: 1 * ribosome 70S/b1719/second last codon EF-TU-tRNA/1 EF-G	C110040H148834N39627O59619S277P6579Mg175Zn0Fe0	-5793.14	Translation
rib_70_elo2_b1719_37_cplx	Translation elongation complex: 37 * ribosome 70S/b1719/second last codon EF-TU-tRNA/1 EF-G	C3409656H4759282N1200555O1721883S10249P173907Mg6475Zn0Fe0	-144794.18	Translation
rib_70_elo2_b1804_11_cplx	Translation elongation complex: 11 * ribosome 70S/b1804/second last codon EF-TU-tRNA/1 EF-G	C1003910H1397885N354533O511188S2838P52247Mg1925Zn0Fe0	-43537.54	Translation
rib_70_elo2_b1804_1_cplx	Translation elongation complex: 1 * ribosome 70S/b1804/second last codon EF-TU-tRNA/1 EF-G	C101020H138125N36123O53658S258P5777Mg175Zn0Fe0	-4986.14	Translation
rib_70_elo2_b1804_22_cplx	Translation elongation complex: 22 * ribosome 70S/b1804/second last codon EF-TU-tRNA/1 EF-G	C1997089H2783621N704784O1014471S5676P103364Mg3850Zn0Fe0	-85944.08	Translation
rib_70_elo2_b1822_15_cplx	Translation elongation complex: 15 * ribosome 70S/b1822/second last codon EF-TU-tRNA/1 EF-G	C1355168H1886666N479276O690478S3870P70667Mg2625Zn0Fe0	-58625.1	Translation
rib_70_elo2_b1822_1_cplx	Translation elongation complex: 1 * ribosome 70S/b1822/second last codon EF-TU-tRNA/1 EF-G	C97534H133908N34804O51336S258P5469Mg175Zn0Fe0	-4667.14	Translation

rib_70_elo2_b1822_7_cplx	Translation elongation complex: 7 * ribosome 70S/b1822/second last codon EF-TU-tRNA/1 EF-G	C636520H885090N225292O325254S1806P33411Mg1225Zn0Fe0	-27791.98	Translation
rib_70_elo2_b1866_17_cplx	Translation elongation complex: 17 * ribosome 70S/b1866/second last codon EF-TU-tRNA/1 EF-G	C1569163H2188590N553151O795883S4539P80791Mg2975Zn0Fe0	-67347.38	Translation
rib_70_elo2_b1866_1_cplx	Translation elongation complex: 1 * ribosome 70S/b1866/second last codon EF-TU-tRNA/1 EF-G	C108187H146718N38895O58491S267P6423Mg175Zn0Fe0	-5633.14	Translation
rib_70_elo2_b1866_34_cplx	Translation elongation complex: 34 * ribosome 70S/b1866/second last codon EF-TU-tRNA/1 EF-G	C3121450H4358079N1099548O1579362S9078P159807Mg5950Zn0Fe0	-132918.76	Translation
rib_70_elo2_b1869_1_cplx	Translation elongation complex: 1 * ribosome 70S/b1869/second last codon EF-TU-tRNA/1 EF-G	C92845H128317N32947O48221S254P5047Mg175Zn0Fe0	-4240.14	Translation
rib_70_elo2_b1869_3_cplx	Translation elongation complex: 3 * ribosome 70S/b1869/second last codon EF-TU-tRNA/1 EF-G	C271043H376493N95993O138983S762P14345Mg525Zn0Fe0	-11922.42	Translation
rib_70_elo2_b1869_7_cplx	Translation elongation complex: 7 * ribosome 70S/b1869/second last codon EF-TU-tRNA/1 EF-G	C627439H872845N222085O320507S1778P32941Mg1225Zn0Fe0	-27286.98	Translation
rib_70_elo2_b1870_14_cplx	Translation elongation complex: 14 * ribosome 70S/b1870/second last codon EF-TU-tRNA/1 EF-G	C1261588H1756483N446210O643328S3542P65816Mg2450Zn0Fe0	-54716.96	Translation
rib_70_elo2_b1870_1_cplx	Translation elongation complex: 1 * ribosome 70S/b1870/second last codon EF-TU-tRNA/1 EF-G	C96671H132887N34448O50801S253P5392Mg175Zn0Fe0	-4600.14	Translation
rib_70_elo2_b1870_7_cplx	Translation elongation complex: 7 * ribosome 70S/b1870/second last codon EF-TU-tRNA/1 EF-G	C634325H882239N224492O324275S1771P33280Mg1225Zn0Fe0	-27730.98	Translation
rib_70_elo2_b1871_19_cplx	Translation elongation complex: 19 * ribosome 70S/b1871/second last codon EF-TU-tRNA/1 EF-G	C1720518H2396640N607761O874805S4788P89299Mg3325Zn0Fe0	-74121.66	Translation
rib_70_elo2_b1871_1_cplx	Translation elongation complex: 1 * ribosome 70S/b1871/second last codon EF-TU-tRNA/1 EF-G	C99276H136002N35451O52475S252P5617Mg175Zn0Fe0	-4819.14	Translation
rib_70_elo2_b1871_9_cplx	Translation elongation complex: 9 * ribosome 70S/b1871/second last codon EF-TU-tRNA/1 EF-G	C819828H1140730N289811O417955S2268P42809Mg1575Zn0Fe0	-35620.26	Translation
rib_70_elo2_b1876_16_cplx	Translation elongation complex: 16 * ribosome 70S/b1876/second last codon EF-TU-tRNA/1 EF-G	C1476907H2059174N520655O749329S4272P76120Mg2800Zn0Fe0	-63579.24	Translation
rib_70_elo2_b1876_1_cplx	Translation elongation complex: 1 * ribosome 70S/b1876/second last codon EF-TU-tRNA/1 EF-G	C107812H146239N38825O58144S267P6385Mg175Zn0Fe0	-5602.14	Translation
rib_70_elo2_b1876_33_cplx	Translation elongation complex: 33 * ribosome 70S/b1876/second last codon EF-TU-tRNA/1 EF-G	C3028548H4227167N1066729O1532672S8811P155153Mg5775Zn0Fe0	-129286.62	Translation
rib_70_elo2_b1920_15_cplx	Translation elongation complex: 15 * ribosome 70S/b1920/second last codon EF-TU-tRNA/1 EF-G	C1352761H1884815N478335O689780S3705P70521Mg2625Zn0Fe0	-58494.1	Translation
rib_70_elo2_b1920_1_cplx	Translation elongation complex: 1 * ribosome 70S/b1920/second last codon EF-TU-tRNA/1 EF-G	C97339H133709N34815O51184S247P5449Mg175Zn0Fe0	-4648.14	Translation
rib_70_elo2_b1920_7_cplx	Translation elongation complex: 7 * ribosome 70S/b1920/second last codon EF-TU-tRNA/1 EF-G	C635377H884183N224895O324868S1729P33337Mg1225Zn0Fe0	-27724.98	Translation
rib_70_elo2_b1921_10_cplx	Translation elongation complex: 10 * ribosome 70S/b1921/second last codon EF-TU-tRNA/1 EF-G	C899570H1252091N318548O459274S2510P47122Mg1750Zn0Fe0	-39014.4	Translation
rib_70_elo2_b1921_1_cplx	Translation elongation complex: 1 * ribosome 70S/b1921/second last codon EF-TU-tRNA/1 EF-G	C94673H130547N33707O49396S251P5209Mg175Zn0Fe0	-4399.14	Translation
rib_70_elo2_b1921_5_cplx	Translation elongation complex: 5 * ribosome 70S/b1921/second last codon EF-TU-tRNA/1 EF-G	C452405H629011N160303O231564S1255P23837Mg875Zn0Fe0	-19783.7	Translation
rib_70_elo2_b1922_14_cplx	Translation elongation complex: 14 * ribosome 70S/b1922/second last codon EF-TU-tRNA/1 EF-G	C1262522H1758406N447069O644233S3458P65948Mg2450Zn0Fe0	-54820.96	Translation
rib_70_elo2_b1922_1_cplx	Translation elongation complex: 1 * ribosome 70S/b1922/second last codon EF-TU-tRNA/1 EF-G	C96565H132821N34540O50666S247P5381Mg175Zn0Fe0	-4587.14	Translation
rib_70_elo2_b1922_7_cplx	Translation elongation complex: 7 * ribosome 70S/b1922/second last codon EF-TU-tRNA/1 EF-G	C634699H883091N224938O324620S1729P33335Mg1225Zn0Fe0	-27771.98	Translation
rib_70_elo2_b2114_19_cplx	Translation elongation complex: 19 * ribosome 70S/b2114/second last codon EF-TU-tRNA/1 EF-G	C1763696H2458587N620352O892432S5130P90348Mg3325Zn0Fe0	-75398.66	Translation
rib_70_elo2_b2114_1_cplx	Translation elongation complex: 1 * ribosome 70S/b2114/second last codon EF-TU-tRNA/1 EF-G	C111152H150159N39960O60436S270P6684Mg175Zn0Fe0	-5898.14	Translation
rib_70_elo2_b2114_39_cplx	Translation elongation complex: 39 * ribosome 70S/b2114/second last codon EF-TU-tRNA/1 EF-G	C3599856H5023507N1265232O1816872S10530P183308Mg6825Zn0Fe0	-152621.46	Translation
rib_70_elo2_b2140_18_cplx	Translation elongation complex: 18 * ribosome 70S/b2140/second last codon EF-TU-tRNA/1 EF-G	C1629953H2270944N576433O829991S4536P84812Mg3150Zn0Fe0	-70397.52	Translation
rib_70_elo2_b2140_1_cplx	Translation elongation complex: 1 * ribosome 70S/b2140/second last codon EF-TU-tRNA/1 EF-G	C99103H135812N35493O52377S252P5609Mg175Zn0Fe0	-4809.14	Translation
rib_70_elo2_b2140_9_cplx	Translation elongation complex: 9 * ribosome 70S/b2140/second last codon EF-TU-tRNA/1 EF-G	C819503H1140580N290053O418313S2268P42881Mg1575Zn0Fe0	-35674.26	Translation

rib_70_elo2_b2183_13_cplx	Translation elongation complex: 13 * ribosome 70S/b2183/second last codon EF-TU-tRNA/1 EF-G	C1170583H1629865N414243O597202S3211P61135Mg2275Zn0Fe0	-50789.82	Translation
rib_70_elo2_b2183_1_cplx	Translation elongation complex: 1 * ribosome 70S/b2183/second last codon EF-TU-tRNA/1 EF-G	C96151H132289N34287O50446S247P5347Mg175Zn0Fe0	-4552.14	Translation
rib_70_elo2_b2183_6_cplx	Translation elongation complex: 6 * ribosome 70S/b2183/second last codon EF-TU-tRNA/1 EF-G	C543831H756279N192602O278261S1482P28592Mg1050Zn0Fe0	-23817.84	Translation
rib_70_elo2_b2185_1_cplx	Translation elongation complex: 1 * ribosome 70S/b2185/second last codon EF-TU-tRNA/1 EF-G	C91580H126839N32583O47319S243P4935Mg175Zn0Fe0	-4128.14	Translation
rib_70_elo2_b2185_2_cplx	Translation elongation complex: 2 * ribosome 70S/b2185/second last codon EF-TU-tRNA/1 EF-G	C180438H250598N64053O92665S486P9583Mg350Zn0Fe0	-7968.28	Translation
rib_70_elo2_b2185_5_cplx	Translation elongation complex: 5 * ribosome 70S/b2185/second last codon EF-TU-tRNA/1 EF-G	C447012H621875N158463O228703S1215P23527Mg875Zn0Fe0	-19488.7	Translation
rib_70_elo2_b2268_17_cplx	Translation elongation complex: 17 * ribosome 70S/b2268/second last codon EF-TU-tRNA/1 EF-G	C1536189H2139940N543029O782447S4267P79936Mg2975Zn0Fe0	-66441.38	Translation
rib_70_elo2_b2268_1_cplx	Translation elongation complex: 1 * ribosome 70S/b2268/second last codon EF-TU-tRNA/1 EF-G	C98573H135172N35189O52079S251P5568Mg175Zn0Fe0	-4775.14	Translation
rib_70_elo2_b2268_8_cplx	Translation elongation complex: 8 * ribosome 70S/b2268/second last codon EF-TU-tRNA/1 EF-G	C727530H1012258N257369O371615S2008P38104Mg1400Zn0Fe0	-31754.12	Translation
rib_70_elo2_b2317_12_cplx	Translation elongation complex: 12 * ribosome 70S/b2317/second last codon EF-TU-tRNA/1 EF-G	C1080820H1504599N381694O550700S2988P56436Mg2100Zn0Fe0	-46778.68	Translation
rib_70_elo2_b2317_1_cplx	Translation elongation complex: 1 * ribosome 70S/b2317/second last codon EF-TU-tRNA/1 EF-G	C95792H131854N33995O50189S249P5308Mg175Zn0Fe0	-4504.14	Translation
rib_70_elo2_b2317_6_cplx	Translation elongation complex: 6 * ribosome 70S/b2317/second last codon EF-TU-tRNA/1 EF-G	C543532H755829N192040O277694S1494P28548Mg1050Zn0Fe0	-23719.84	Translation
rib_70_elo2_b2318_15_cplx	Translation elongation complex: 15 * ribosome 70S/b2318/second last codon EF-TU-tRNA/1 EF-G	C1354048H1885454N478997O689776S3750P70547Mg2625Zn0Fe0	-58445.1	Translation
rib_70_elo2_b2318_1_cplx	Translation elongation complex: 1 * ribosome 70S/b2318/second last codon EF-TU-tRNA/1 EF-G	C97492H133872N34847O51278S250P5461Mg175Zn0Fe0	-4655.14	Translation
rib_70_elo2_b2318_7_cplx	Translation elongation complex: 7 * ribosome 70S/b2318/second last codon EF-TU-tRNA/1 EF-G	C636016H884550N225197O324920S1750P33355Mg1225Zn0Fe0	-27707.98	Translation
rib_70_elo2_b2319_19_cplx	Translation elongation complex: 19 * ribosome 70S/b2319/second last codon EF-TU-tRNA/1 EF-G	C1721508H2397690N608037O876730S4750P89499Mg3325Zn0Fe0	-74682.66	Translation
rib_70_elo2_b2319_1_cplx	Translation elongation complex: 1 * ribosome 70S/b2319/second last codon EF-TU-tRNA/1 EF-G	C99762H136548N35691O52870S250P5673Mg175Zn0Fe0	-4894.14	Translation
rib_70_elo2_b2319_9_cplx	Translation elongation complex: 9 * ribosome 70S/b2319/second last codon EF-TU-tRNA/1 EF-G	C820538H1141500N290067O419030S2250P42929Mg1575Zn0Fe0	-35911.26	Translation
rib_70_elo2_b2320_11_cplx	Translation elongation complex: 11 * ribosome 70S/b2320/second last codon EF-TU-tRNA/1 EF-G	C1003503H1397829N354705O511270S2761P52278Mg1925Zn0Fe0	-43480.54	Translation
rib_70_elo2_b2320_1_cplx	Translation elongation complex: 1 * ribosome 70S/b2320/second last codon EF-TU-tRNA/1 EF-G	C101063H138189N36155O53750S251P5788Mg175Zn0Fe0	-4989.14	Translation
rib_70_elo2_b2320_22_cplx	Translation elongation complex: 22 * ribosome 70S/b2320/second last codon EF-TU-tRNA/1 EF-G	C1996187H2783433N705110O1014542S5522P103417Mg3850Zn0Fe0	-85821.08	Translation
rib_70_elo2_b2324_19_cplx	Translation elongation complex: 19 * ribosome 70S/b2324/second last codon EF-TU-tRNA/1 EF-G	C1760929H2453953N620158O891350S5111P90283Mg3325Zn0Fe0	-75371.66	Translation
rib_70_elo2_b2324_1_cplx	Translation elongation complex: 1 * ribosome 70S/b2324/second last codon EF-TU-tRNA/1 EF-G	C110779H149611N39856O60254S269P6655Mg175Zn0Fe0	-5871.14	Translation
rib_70_elo2_b2324_39_cplx	Translation elongation complex: 39 * ribosome 70S/b2324/second last codon EF-TU-tRNA/1 EF-G	C3594429H5014333N1264938O1814790S10491P183203Mg6825Zn0Fe0	-152594.46	Translation
rib_70_elo2_b2325_1_cplx	Translation elongation complex: 1 * ribosome 70S/b2325/second last codon EF-TU-tRNA/1 EF-G	C91462H126639N32513O47304S244P4926Mg175Zn0Fe0	-4135.14	Translation
rib_70_elo2_b2325_2_cplx	Translation elongation complex: 2 * ribosome 70S/b2325/second last codon EF-TU-tRNA/1 EF-G	C180263H250275N63956O92652S488P9573Mg350Zn0Fe0	-7990.28	Translation
rib_70_elo2_b2325_5_cplx	Translation elongation complex: 5 * ribosome 70S/b2325/second last codon EF-TU-tRNA/1 EF-G	C446666H621183N158285O228696S1220P23514Mg875Zn0Fe0	-19555.7	Translation
rib_70_elo2_b2326_10_cplx	Translation elongation complex: 10 * ribosome 70S/b2326/second last codon EF-TU-tRNA/1 EF-G	C898694H1249640N317879O458793S2480P47028Mg1750Zn0Fe0	-39140.4	Translation
rib_70_elo2_b2326_1_cplx	Translation elongation complex: 1 * ribosome 70S/b2326/second last codon EF-TU-tRNA/1 EF-G	C94562H130265N33650O49338S248P5196Mg175Zn0Fe0	-4408.14	Translation
rib_70_elo2_b2326_5_cplx	Translation elongation complex: 5 * ribosome 70S/b2326/second last codon EF-TU-tRNA/1 EF-G	C451954H627765N159974O231318S1240P23788Mg875Zn0Fe0	-19844.7	Translation
rib_70_elo2_b2327_15_cplx	Translation elongation complex: 15 * ribosome 70S/b2327/second last codon EF-TU-tRNA/1 EF-G	C1353312H1885279N477863O689074S3885P70529Mg2625Zn0Fe0	-58352.1	Translation

rib_70_elo2_b2327_1_cplx	Translation elongation complex: 1 * ribosome 70S/b2327/second last codon EF-TU-tRNA/1 EF-G	C97372H133781N34623O51290S259P5457Mg175Zn0Fe0	-4646.14	Translation
rib_70_elo2_b2327_7_cplx	Translation elongation complex: 7 * ribosome 70S/b2327/second last codon EF-TU-tRNA/1 EF-G	C635632H884423N224583O324626S1813P33345Mg1225Zn0Fe0	-27662.98	Translation
rib_70_elo2_b2328_16_cplx	Translation elongation complex: 16 * ribosome 70S/b2328/second last codon EF-TU-tRNA/1 EF-G	C1443629H2010926N510669O735484S4048P75209Mg2800Zn0Fe0	-62316.24	Translation
rib_70_elo2_b2328_1_cplx	Translation elongation complex: 1 * ribosome 70S/b2328/second last codon EF-TU-tRNA/1 EF-G	C97574H134021N34839O51379S253P5474Mg175Zn0Fe0	-4669.14	Translation
rib_70_elo2_b2328_8_cplx	Translation elongation complex: 8 * ribosome 70S/b2328/second last codon EF-TU-tRNA/1 EF-G	C725733H1009910N256893O370628S2024P38017Mg1400Zn0Fe0	-31571.12	Translation
rib_70_elo2_b2329_10_cplx	Translation elongation complex: 10 * ribosome 70S/b2329/second last codon EF-TU-tRNA/1 EF-G	C911272H1269094N322431O464949S2550P47566Mg1750Zn0Fe0	-39588.4	Translation
rib_70_elo2_b2329_1_cplx	Translation elongation complex: 1 * ribosome 70S/b2329/second last codon EF-TU-tRNA/1 EF-G	C100426H137461N35979O53316S255P5734Mg175Zn0Fe0	-4937.14	Translation
rib_70_elo2_b2329_21_cplx	Translation elongation complex: 21 * ribosome 70S/b2329/second last codon EF-TU-tRNA/1 EF-G	C1902306H2652201N672539O968056S5355P98694Mg3675Zn0Fe0	-81939.94	Translation
rib_70_elo2_b2330_18_cplx	Translation elongation complex: 18 * ribosome 70S/b2330/second last codon EF-TU-tRNA/1 EF-G	C1628128H2267257N575012O828983S4572P84617Mg3150Zn0Fe0	-70580.52	Translation
rib_70_elo2_b2330_1_cplx	Translation elongation complex: 1 * ribosome 70S/b2330/second last codon EF-TU-tRNA/1 EF-G	C98825H135440N35262O52236S254P5584Mg175Zn0Fe0	-4805.14	Translation
rib_70_elo2_b2330_9_cplx	Translation elongation complex: 9 * ribosome 70S/b2330/second last codon EF-TU-tRNA/1 EF-G	C818497H1138648N289262O417764S2286P42776Mg1575Zn0Fe0	-35758.26	Translation
rib_70_elo2_b2400_13_cplx	Translation elongation complex: 13 * ribosome 70S/b2400/second last codon EF-TU-tRNA/1 EF-G	C1193194H1661639N421390O606814S3380P61829Mg2275Zn0Fe0	-51600.82	Translation
rib_70_elo2_b2400_1_cplx	Translation elongation complex: 1 * ribosome 70S/b2400/second last codon EF-TU-tRNA/1 EF-G	C104230H141899N37402O55810S260P6065Mg175Zn0Fe0	-5279.14	Translation
rib_70_elo2_b2400_27_cplx	Translation elongation complex: 27 * ribosome 70S/b2400/second last codon EF-TU-tRNA/1 EF-G	C2463652H3434669N869376O1249652S7020P126887Mg4725Zn0Fe0	-105642.78	Translation
rib_70_elo2_b2514_12_cplx	Translation elongation complex: 12 * ribosome 70S/b2514/second last codon EF-TU-tRNA/1 EF-G	C1097602H1528899N387964O558965S3048P57053Mg2100Zn0Fe0	-47503.68	Translation
rib_70_elo2_b2514_1_cplx	Translation elongation complex: 1 * ribosome 70S/b2514/second last codon EF-TU-tRNA/1 EF-G	C102597H139995N36789O54780S254P5925Mg175Zn0Fe0	-5130.14	Translation
rib_70_elo2_b2514_24_cplx	Translation elongation complex: 24 * ribosome 70S/b2514/second last codon EF-TU-tRNA/1 EF-G	C2183062H3044067N771064O1108985S6096P112829Mg4200Zn0Fe0	-93729.36	Translation
rib_70_elo2_b2528_1_cplx	Translation elongation complex: 1 * ribosome 70S/b2528/second last codon EF-TU-tRNA/1 EF-G	C91992H127270N32733O47632S246P4972Mg175Zn0Fe0	-4175.14	Translation
rib_70_elo2_b2528_3_cplx	Translation elongation complex: 3 * ribosome 70S/b2528/second last codon EF-TU-tRNA/1 EF-G	C269780H374828N95675O138376S738P14268Mg525Zn0Fe0	-11875.42	Translation
rib_70_elo2_b2528_6_cplx	Translation elongation complex: 6 * ribosome 70S/b2528/second last codon EF-TU-tRNA/1 EF-G	C536462H746165N190088O274492S1476P28212Mg1050Zn0Fe0	-23425.84	Translation
rib_70_elo2_b2529_1_cplx	Translation elongation complex: 1 * ribosome 70S/b2529/second last codon EF-TU-tRNA/1 EF-G	C92690H128129N33011O48090S247P5035Mg175Zn0Fe0	-4240.14	Translation
rib_70_elo2_b2529_3_cplx	Translation elongation complex: 3 * ribosome 70S/b2529/second last codon EF-TU-tRNA/1 EF-G	C270666H376029N95993O138920S741P14331Mg525Zn0Fe0	-11944.42	Translation
rib_70_elo2_b2529_7_cplx	Translation elongation complex: 7 * ribosome 70S/b2529/second last codon EF-TU-tRNA/1 EF-G	C626618H871829N221957O320580S1729P32923Mg1225Zn0Fe0	-27352.98	Translation
rib_70_elo2_b2530_11_cplx	Translation elongation complex: 11 * ribosome 70S/b2530/second last codon EF-TU-tRNA/1 EF-G	C1005673H1400813N355403O512519S2860P52354Mg1925Zn0Fe0	-43600.54	Translation
rib_70_elo2_b2530_1_cplx	Translation elongation complex: 1 * ribosome 70S/b2530/second last codon EF-TU-tRNA/1 EF-G	C101933H139253N36513O54299S260P5864Mg175Zn0Fe0	-5069.14	Translation
rib_70_elo2_b2530_23_cplx	Translation elongation complex: 23 * ribosome 70S/b2530/second last codon EF-TU-tRNA/1 EF-G	C2090161H2914685N738071O1062383S5980P108142Mg4025Zn0Fe0	-89838.22	Translation
rib_70_elo2_b2531_1_cplx	Translation elongation complex: 1 * ribosome 70S/b2531/second last codon EF-TU-tRNA/1 EF-G	C93768H129465N33400O48889S245P5139Mg175Zn0Fe0	-4337.14	Translation
rib_70_elo2_b2531_4_cplx	Translation elongation complex: 4 * ribosome 70S/b2531/second last codon EF-TU-tRNA/1 EF-G	C361134H502071N128080O185245S980P19083Mg700Zn0Fe0	-15872.56	Translation
rib_70_elo2_b2531_9_cplx	Translation elongation complex: 9 * ribosome 70S/b2531/second last codon EF-TU-tRNA/1 EF-G	C806744H1123081N285880O412505S2205P42323Mg1575Zn0Fe0	-35098.26	Translation
rib_70_elo2_b2559_10_cplx	Translation elongation complex: 10 * ribosome 70S/b2559/second last codon EF-TU-tRNA/1 EF-G	C897859H1249803N318048O458444S2530P47027Mg1750Zn0Fe0	-38969.4	Translation
rib_70_elo2_b2559_1_cplx	Translation elongation complex: 1 * ribosome 70S/b2559/second last codon EF-TU-tRNA/1 EF-G	C94393H130185N33657O49232S253P5186Mg175Zn0Fe0	-4381.14	Translation

rib_70_elo2_b2559_5_cplx	Translation elongation complex: 5 * ribosome 70S/b2559/second last codon EF-TU-tRNA/1 EF-G	C451489H627793N160053O231104S1265P23782Mg875Zn0Fe0	-19753.7	Translation
rib_70_elo2_b2560_12_cplx	Translation elongation complex: 12 * ribosome 70S/b2560/second last codon EF-TU-tRNA/1 EF-G	C1079967H1503507N382004O550523S2976P56414Mg2100Zn0Fe0	-46720.68	Translation
rib_70_elo2_b2560_1_cplx	Translation elongation complex: 1 * ribosome 70S/b2560/second last codon EF-TU-tRNA/1 EF-G	C95522H131543N33975O50012S248P5286Mg175Zn0Fe0	-4479.14	Translation
rib_70_elo2_b2560_6_cplx	Translation elongation complex: 6 * ribosome 70S/b2560/second last codon EF-TU-tRNA/1 EF-G	C542997H755163N192170O277517S1488P28526Mg1050Zn0Fe0	-23679.84	Translation
rib_70_elo2_b2563_1_cplx	Translation elongation complex: 1 * ribosome 70S/b2563/second last codon EF-TU-tRNA/1 EF-G	C92809H128278N33068O48149S245P5045Mg175Zn0Fe0	-4240.14	Translation
rib_70_elo2_b2563_3_cplx	Translation elongation complex: 3 * ribosome 70S/b2563/second last codon EF-TU-tRNA/1 EF-G	C271165H376646N96254O139145S735P14375Mg525Zn0Fe0	-11958.42	Translation
rib_70_elo2_b2563_7_cplx	Translation elongation complex: 7 * ribosome 70S/b2563/second last codon EF-TU-tRNA/1 EF-G	C627877H873382N222626O321137S1715P33035Mg1225Zn0Fe0	-27394.98	Translation
rib_70_elo2_b2564_14_cplx	Translation elongation complex: 14 * ribosome 70S/b2564/second last codon EF-TU-tRNA/1 EF-G	C1260262H1755751N446204O642972S3542P65806Mg2450Zn0Fe0	-54664.96	Translation
rib_70_elo2_b2564_1_cplx	Translation elongation complex: 1 * ribosome 70S/b2564/second last codon EF-TU-tRNA/1 EF-G	C96502H132753N34507O50666S253P5382Mg175Zn0Fe0	-4587.14	Translation
rib_70_elo2_b2564_7_cplx	Translation elongation complex: 7 * ribosome 70S/b2564/second last codon EF-TU-tRNA/1 EF-G	C633622H881829N224521O324038S1771P33270Mg1225Zn0Fe0	-27699.98	Translation
rib_70_elo2_b2565_14_cplx	Translation elongation complex: 14 * ribosome 70S/b2565/second last codon EF-TU-tRNA/1 EF-G	C1261507H1757108N446223O642809S3472P65801Mg2450Zn0Fe0	-54393.96	Translation
rib_70_elo2_b2565_1_cplx	Translation elongation complex: 1 * ribosome 70S/b2565/second last codon EF-TU-tRNA/1 EF-G	C96538H132797N34422O50659S248P5377Mg175Zn0Fe0	-4563.14	Translation
rib_70_elo2_b2565_7_cplx	Translation elongation complex: 7 * ribosome 70S/b2565/second last codon EF-TU-tRNA/1 EF-G	C634216H882479N224484O323959S1736P33265Mg1225Zn0Fe0	-27561.98	Translation
rib_70_elo2_b2566_17_cplx	Translation elongation complex: 17 * ribosome 70S/b2566/second last codon EF-TU-tRNA/1 EF-G	C1538394H2143740N544005O783665S4267P80105Mg2975Zn0Fe0	-66457.38	Translation
rib_70_elo2_b2566_1_cplx	Translation elongation complex: 1 * ribosome 70S/b2566/second last codon EF-TU-tRNA/1 EF-G	C98586H135260N35269O52001S251P5561Mg175Zn0Fe0	-4759.14	Translation
rib_70_elo2_b2566_8_cplx	Translation elongation complex: 8 * ribosome 70S/b2566/second last codon EF-TU-tRNA/1 EF-G	C728502H1013970N257841O372104S2008P38174Mg1400Zn0Fe0	-31752.12	Translation
rib_70_elo2_b2567_13_cplx	Translation elongation complex: 13 * ribosome 70S/b2567/second last codon EF-TU-tRNA/1 EF-G	C1170001H1629627N414193O596858S3198P61107Mg2275Zn0Fe0	-50683.82	Translation
rib_70_elo2_b2567_1_cplx	Translation elongation complex: 1 * ribosome 70S/b2567/second last codon EF-TU-tRNA/1 EF-G	C95989H132123N34261O50306S246P5331Mg175Zn0Fe0	-4530.14	Translation
rib_70_elo2_b2567_6_cplx	Translation elongation complex: 6 * ribosome 70S/b2567/second last codon EF-TU-tRNA/1 EF-G	C543494H756083N192566O278036S1476P28571Mg1050Zn0Fe0	-23760.84	Translation
rib_70_elo2_b2570_1_cplx	Translation elongation complex: 1 * ribosome 70S/b2570/second last codon EF-TU-tRNA/1 EF-G	C93629H129282N33284O48784S249P5123Mg175Zn0Fe0	-4320.14	Translation
rib_70_elo2_b2570_4_cplx	Translation elongation complex: 4 * ribosome 70S/b2570/second last codon EF-TU-tRNA/1 EF-G	C360974H501789N127844O185035S996P19064Mg700Zn0Fe0	-15849.56	Translation
rib_70_elo2_b2570_9_cplx	Translation elongation complex: 9 * ribosome 70S/b2570/second last codon EF-TU-tRNA/1 EF-G	C806549H1122634N285444O412120S2241P42299Mg1575Zn0Fe0	-35065.26	Translation
rib_70_elo2_b2571_18_cplx	Translation elongation complex: 18 * ribosome 70S/b2571/second last codon EF-TU-tRNA/1 EF-G	C1628222H2269040N575341O828913S4536P84602Mg3150Zn0Fe0	-70097.52	Translation
rib_70_elo2_b2571_1_cplx	Translation elongation complex: 1 * ribosome 70S/b2571/second last codon EF-TU-tRNA/1 EF-G	C99021H135761N35319O52387S252P5603Mg175Zn0Fe0	-4798.14	Translation
rib_70_elo2_b2571_9_cplx	Translation elongation complex: 9 * ribosome 70S/b2571/second last codon EF-TU-tRNA/1 EF-G	C818645H1139657N289447O417811S2268P42779Mg1575Zn0Fe0	-35527.26	Translation
rib_70_elo2_b2572_12_cplx	Translation elongation complex: 12 * ribosome 70S/b2572/second last codon EF-TU-tRNA/1 EF-G	C1079285H1502594N382085O550911S3024P56415Mg2100Zn0Fe0	-46877.68	Translation
rib_70_elo2_b2572_1_cplx	Translation elongation complex: 1 * ribosome 70S/b2572/second last codon EF-TU-tRNA/1 EF-G	C95632H131664N34155O50037S252P5298Mg175Zn0Fe0	-4504.14	Translation
rib_70_elo2_b2572_6_cplx	Translation elongation complex: 6 * ribosome 70S/b2572/second last codon EF-TU-tRNA/1 EF-G	C542747H754814N192305O277707S1512P28533Mg1050Zn0Fe0	-23764.84	Translation
rib_70_elo2_b2573_11_cplx	Translation elongation complex: 11 * ribosome 70S/b2573/second last codon EF-TU-tRNA/1 EF-G	C988376H1376106N349929O504586S2706P51717Mg1925Zn0Fe0	-42908.54	Translation
rib_70_elo2_b2573_1_cplx	Translation elongation complex: 1 * ribosome 70S/b2573/second last codon EF-TU-tRNA/1 EF-G	C94846H130726N33809O49546S246P5227Mg175Zn0Fe0	-4427.14	Translation
rib_70_elo2_b2573_5_cplx	Translation elongation complex: 5 * ribosome 70S/b2573/second last codon EF-TU-tRNA/1 EF-G	C452258H628878N160257O231562S1230P23823Mg875Zn0Fe0	-19819.7	Translation

rib_70_elo2_b2593_14_cplx	Translation elongation complex: 14 * ribosome 70S/b2593/second last codon EF-TU-tRNA/1 EF-G	C1260773H1755225N446006O642955S3542P65800Mg2450Zn0Fe0	-54588.96	Translation
rib_70_elo2_b2593_1_cplx	Translation elongation complex: 1 * ribosome 70S/b2593/second last codon EF-TU-tRNA/1 EF-G	C96480H132643N34400O50688S253P5376Mg175Zn0Fe0	-4576.14	Translation
rib_70_elo2_b2593_7_cplx	Translation elongation complex: 7 * ribosome 70S/b2593/second last codon EF-TU-tRNA/1 EF-G	C633846H881527N224372O324042S1771P33264Mg1225Zn0Fe0	-27658.98	Translation
rib_70_elo2_b2594_19_cplx	Translation elongation complex: 19 * ribosome 70S/b2594/second last codon EF-TU-tRNA/1 EF-G	C1721831H2399384N609050O876274S4845P89504Mg3325Zn0Fe0	-74345.66	Translation
rib_70_elo2_b2594_1_cplx	Translation elongation complex: 1 * ribosome 70S/b2594/second last codon EF-TU-tRNA/1 EF-G	C99473H136298N35606O52612S255P5642Mg175Zn0Fe0	-4845.14	Translation
rib_70_elo2_b2594_9_cplx	Translation elongation complex: 9 * ribosome 70S/b2594/second last codon EF-TU-tRNA/1 EF-G	C820521H1142114N290470O418684S2295P42914Mg1575Zn0Fe0	-35734.26	Translation
rib_70_elo2_b2606_1_cplx	Translation elongation complex: 1 * ribosome 70S/b2606/second last codon EF-TU-tRNA/1 EF-G	C92278H127702N32817O47821S243P4996Mg175Zn0Fe0	-4182.14	Translation
rib_70_elo2_b2606_3_cplx	Translation elongation complex: 3 * ribosome 70S/b2606/second last codon EF-TU-tRNA/1 EF-G	C270214H375632N95835O138581S729P14292Mg525Zn0Fe0	-11848.42	Translation
rib_70_elo2_b2606_6_cplx	Translation elongation complex: 6 * ribosome 70S/b2606/second last codon EF-TU-tRNA/1 EF-G	C537118H747527N190362O274721S1458P28236Mg1050Zn0Fe0	-23347.84	Translation
rib_70_elo2_b2607_15_cplx	Translation elongation complex: 15 * ribosome 70S/b2607/second last codon EF-TU-tRNA/1 EF-G	C1351860H1882623N478349O689156S3750P70488Mg2625Zn0Fe0	-58581.1	Translation
rib_70_elo2_b2607_1_cplx	Translation elongation complex: 1 * ribosome 70S/b2607/second last codon EF-TU-tRNA/1 EF-G	C96970H133239N34675O50938S250P5416Mg175Zn0Fe0	-4623.14	Translation
rib_70_elo2_b2607_7_cplx	Translation elongation complex: 7 * ribosome 70S/b2607/second last codon EF-TU-tRNA/1 EF-G	C634780H882975N224821O324460S1750P33304Mg1225Zn0Fe0	-27747.98	Translation
rib_70_elo2_b2608_10_cplx	Translation elongation complex: 10 * ribosome 70S/b2608/second last codon EF-TU-tRNA/1 EF-G	C898334H1250105N317828O458734S2500P47029Mg1750Zn0Fe0	-39091.4	Translation
rib_70_elo2_b2608_1_cplx	Translation elongation complex: 1 * ribosome 70S/b2608/second last codon EF-TU-tRNA/1 EF-G	C94544H130325N33671O49324S250P5197Mg175Zn0Fe0	-4404.14	Translation
rib_70_elo2_b2608_5_cplx	Translation elongation complex: 5 * ribosome 70S/b2608/second last codon EF-TU-tRNA/1 EF-G	C451784H628005N159963O231284S1250P23789Mg875Zn0Fe0	-19820.7	Translation
rib_70_elo2_b2609_1_cplx	Translation elongation complex: 1 * ribosome 70S/b2609/second last codon EF-TU-tRNA/1 EF-G	C91152H126326N32401O47073S241P4899Mg175Zn0Fe0	-4091.14	Translation
rib_70_elo2_b2609_2_cplx	Translation elongation complex: 2 * ribosome 70S/b2609/second last codon EF-TU-tRNA/1 EF-G	C179937H249971N63862O92399S482P9547Mg350Zn0Fe0	-7930.28	Translation
rib_70_elo2_b2609_4_cplx	Translation elongation complex: 4 * ribosome 70S/b2609/second last codon EF-TU-tRNA/1 EF-G	C357507H497261N126784O183051S964P18843Mg700Zn0Fe0	-15608.56	Translation
rib_70_elo2_b2614_11_cplx	Translation elongation complex: 11 * ribosome 70S/b2614/second last codon EF-TU-tRNA/1 EF-G	C988210H1376115N349919O504848S2739P51724Mg1925Zn0Fe0	-43058.54	Translation
rib_70_elo2_b2614_1_cplx	Translation elongation complex: 1 * ribosome 70S/b2614/second last codon EF-TU-tRNA/1 EF-G	C95010H130925N33939O49648S249P5244Mg175Zn0Fe0	-4457.14	Translation
rib_70_elo2_b2614_5_cplx	Translation elongation complex: 5 * ribosome 70S/b2614/second last codon EF-TU-tRNA/1 EF-G	C452290H629001N160331O231728S1245P23836Mg875Zn0Fe0	-19897.7	Translation
rib_70_elo2_b2697_1_cplx	Translation elongation complex: 1 * ribosome 70S/b2697/second last codon EF-TU-tRNA/1 EF-G	C117663H157984N42635O64895S268P7280Mg175Zn0Fe0	-6500.14	Translation
rib_70_elo2_b2697_25_cplx	Translation elongation complex: 25 * ribosome 70S/b2697/second last codon EF-TU-tRNA/1 EF-G	C2339655H3269224N823427O1181015S6700P118808Mg4375Zn0Fe0	-99287.5	Translation
rib_70_elo2_b2697_51_cplx	Translation elongation complex: 51 * ribosome 70S/b2697/second last codon EF-TU-tRNA/1 EF-G	C4746813H6639734N1669285O2390145S13668P239630Mg8925Zn0Fe0	-199807.14	Translation
rib_70_elo2_b2741_19_cplx	Translation elongation complex: 19 * ribosome 70S/b2741/second last codon EF-TU-tRNA/1 EF-G	C1720205H2398166N608334O875777S4674P89307Mg3325Zn0Fe0	-74376.66	Translation
rib_70_elo2_b2741_1_cplx	Translation elongation complex: 1 * ribosome 70S/b2741/second last codon EF-TU-tRNA/1 EF-G	C99521H136358N35664O52655S246P5643Mg175Zn0Fe0	-4858.14	Translation
rib_70_elo2_b2741_9_cplx	Translation elongation complex: 9 * ribosome 70S/b2741/second last codon EF-TU-tRNA/1 EF-G	C819825H1141606N290184O418487S2214P42827Mg1575Zn0Fe0	-35755.26	Translation
rib_70_elo2_b2742_11_cplx	Translation elongation complex: 11 * ribosome 70S/b2742/second last codon EF-TU-tRNA/1 EF-G	C1002447H1396211N354670O511439S2739P52281Mg1925Zn0Fe0	-43340.54	Translation
rib_70_elo2_b2742_1_cplx	Translation elongation complex: 1 * ribosome 70S/b2742/second last codon EF-TU-tRNA/1 EF-G	C101007H138121N36240O53679S249P5791Mg175Zn0Fe0	-4979.14	Translation
rib_70_elo2_b2742_22_cplx	Translation elongation complex: 22 * ribosome 70S/b2742/second last codon EF-TU-tRNA/1 EF-G	C1994031H2780110N704943O1014975S5478P103420Mg3850Zn0Fe0	-85538.08	Translation
rib_70_elo2_b2745_10_cplx	Translation elongation complex: 10 * ribosome 70S/b2745/second last codon EF-TU-tRNA/1 EF-G	C911222H1268561N322364O464475S2490P47532Mg1750Zn0Fe0	-39524.4	Translation

rib_70_elo2_b2745_1_cplx	Translation elongation complex: 1 * ribosome 70S/b2745/second last codon EF-TU-tRNA/1 EF-G	C100142H137036N35885O53049S249P5700Mg175Zn0Fe0	-4900.14	Translation	
rib_70_elo2_b2745_20_cplx	Translation elongation complex: 20 * ribosome 70S/b2745/second last codon EF-TU-tRNA/1 EF-G	C1812422H2525811N640674O921615S4980P94012Mg3500Zn0Fe0	-77995.8	Translation	
rib_70_elo2_b2779_12_cplx	Translation elongation complex: 12 * ribosome 70S/b2779/second last codon EF-TU-tRNA/1 EF-G	C1097023H1528507N387542O559195S3048P57075Mg2100Zn0Fe0	-47573.68	Translation	
rib_70_elo2_b2779_1_cplx	Translation elongation complex: 1 * ribosome 70S/b2779/second last codon EF-TU-tRNA/1 EF-G	C102766H140208N36862O54878S254P5947Mg175Zn0Fe0	-5156.14	Translation	
rib_70_elo2_b2779_25_cplx	Translation elongation complex: 25 * ribosome 70S/b2779/second last codon EF-TU-tRNA/1 EF-G	C2272054H3169224N801982O1155206S6350P117499Mg4375Zn0Fe0	-97703.5	Translation	
rib_70_elo2_b2780_16_cplx	Translation elongation complex: 16 * ribosome 70S/b2780/second last codon EF-TU-tRNA/1 EF-G	C1472608H2053725N519344O747753S4192P76008Mg2800Zn0Fe0	-63371.24	Translation	
rib_70_elo2_b2780_1_cplx	Translation elongation complex: 1 * ribosome 70S/b2780/second last codon EF-TU-tRNA/1 EF-G	C106648H144885N38279O57483S262P6288Mg175Zn0Fe0	-5499.14	Translation	
rib_70_elo2_b2780_32_cplx	Translation elongation complex: 32 * ribosome 70S/b2780/second last codon EF-TU-tRNA/1 EF-G	C2929632H4089821N1032480O1484041S8384P150376Mg5600Zn0Fe0	-125101.48	Translation	
rib_70_elo2_b2785_12_cplx	Translation elongation complex: 12 * ribosome 70S/b2785/second last codon EF-TU-tRNA/1 EF-G	C1098458H1530894N388257O559171S3120P57080Mg2100Zn0Fe0	-47386.68	Translation	
rib_70_elo2_b2785_1_cplx	Translation elongation complex: 1 * ribosome 70S/b2785/second last codon EF-TU-tRNA/1 EF-G	C102903H140428N36906O54931S260P5952Mg175Zn0Fe0	-5145.14	Translation	
rib_70_elo2_b2785_25_cplx	Translation elongation complex: 25 * ribosome 70S/b2785/second last codon EF-TU-tRNA/1 EF-G	C2275023H3174172N803490O1155091S6500P117504Mg4375Zn0Fe0	-97308.5	Translation	
rib_70_elo2_b2790_1_cplx	Translation elongation complex: 1 * ribosome 70S/b2790/second last codon EF-TU-tRNA/1 EF-G	C93518H129054N33300O48673S244P5110Mg175Zn0Fe0	-4323.14	Translation	
rib_70_elo2_b2790_4_cplx	Translation elongation complex: 4 * ribosome 70S/b2790/second last codon EF-TU-tRNA/1 EF-G	C361190H501681N128004O185242S976P19090Mg700Zn0Fe0	-15939.56	Translation	
rib_70_elo2_b2790_8_cplx	Translation elongation complex: 8 * ribosome 70S/b2790/second last codon EF-TU-tRNA/1 EF-G	C718086H998517N254276O367334S1952P37730Mg1400Zn0Fe0	-31428.12	Translation	
rib_70_elo2_b2791_15_cplx	Translation elongation complex: 15 * ribosome 70S/b2791/second last codon EF-TU-tRNA/1 EF-G	C1354659H1886038N479633O690501S3705P70682Mg2625Zn0Fe0	-58670.1	Translation	
rib_70_elo2_b2791_1_cplx	Translation elongation complex: 1 * ribosome 70S/b2791/second last codon EF-TU-tRNA/1 EF-G	C97263H133602N34769O51135S247P5442Mg175Zn0Fe0	-4642.14	Translation	
rib_70_elo2_b2791_7_cplx	Translation elongation complex: 7 * ribosome 70S/b2791/second last codon EF-TU-tRNA/1 EF-G	C636147H884646N225425O325149S1729P33402Mg1225Zn0Fe0	-27796.98	Translation	
rib_70_elo2_b2792_1_cplx	Translation elongation complex: 1 * ribosome 70S/b2792/second last codon EF-TU-tRNA/1 EF-G	C92256H127588N32804O47808S247P4997Mg175Zn0Fe0	-4204.14	Translation	
rib_70_elo2_b2792_3_cplx	Translation elongation complex: 3 * ribosome 70S/b2792/second last codon EF-TU-tRNA/1 EF-G	C270500H375660N95934O138788S741P14327Mg525Zn0Fe0	-11946.42	Translation	
rib_70_elo2_b2792_6_cplx	Translation elongation complex: 6 * ribosome 70S/b2792/second last codon EF-TU-tRNA/1 EF-G	C537866H747768N190629O275258S1482P28322Mg1050Zn0Fe0	-23559.84	Translation	
rib_70_elo2_b2794_16_cplx	Translation elongation complex: 16 * ribosome 70S/b2794/second last codon EF-TU-tRNA/1 EF-G	C1445076H2012262N510899O736200S3968P75203Mg2800Zn0Fe0	-62454.24	Translation	
rib_70_elo2_b2794_1_cplx	Translation elongation complex: 1 * ribosome 70S/b2794/second last codon EF-TU-tRNA/1 EF-G	C97881H134322N34919O51585S248P5498Mg175Zn0Fe0	-4702.14	Translation	
rib_70_elo2_b2794_8_cplx	Translation elongation complex: 8 * ribosome 70S/b2794/second last codon EF-TU-tRNA/1 EF-G	C726572H1010694N257043O371072S1984P38027Mg1400Zn0Fe0	-31653.12	Translation	
rib_70_elo2_b2890_14_cplx	Translation elongation complex: 14 * ribosome 70S/b2890/second last codon EF-TU-tRNA/1 EF-G	C1287658H1794167N454282O654391S3640P66590Mg2450Zn0Fe0	-55658.96	Translation	
rib_70_elo2_b2890_1_cplx	Translation elongation complex: 1 * ribosome 70S/b2890/second last codon EF-TU-tRNA/1 EF-G	C105386H143349N37814O56573S260P6166Mg175Zn0Fe0	-5386.14	Translation	
rib_70_elo2_b2890_29_cplx	Translation elongation complex: 29 * ribosome 70S/b2890/second last codon EF-TU-tRNA/1 EF-G	C2651818H3698957N934822O1344181S7540P136310Mg5075Zn0Fe0	-113666.06	Translation	
rib_70_elo2_b2891_10_cplx	Translation elongation complex: 10 * ribosome 70S/b2891/second last codon EF-TU-tRNA/1 EF-G	C913212H1270906N323115O466437S2530P47691Mg1750Zn0Fe0	-39923.4	Translation	
rib_70_elo2_b2891_1_cplx	Translation elongation complex: 1 * ribosome 70S/b2891/second last codon EF-TU-tRNA/1 EF-G	C100764H137770N36159O53517S253P5760Mg175Zn0Fe0	-4984.14	Translation	
rib_70_elo2_b2891_21_cplx	Translation elongation complex: 21 * ribosome 70S/b2891/second last codon EF-TU-tRNA/1 EF-G	C1906204H2655850N673839O971117S5313P98940Mg3675Zn0Fe0	-82626.94	Translation	
rib_70_elo2_b2892_16_cplx	Translation elongation complex: 16 * ribosome 70S/b2892/second last codon EF-TU-tRNA/1 EF-G	C1475879H2058543N520880O748883S4176P76118Mg2800Zn0Fe0	-63513.24	Translation	
rib_70_elo2_b2892_1_cplx	Translation elongation complex: 1 * ribosome 70S/b2892/second last codon EF-TU-tRNA/1 EF-G	C107714H146178N38750O58208S261P6383Mg175Zn0Fe0	-5596.14	Translation	

rib_70_elo2_b2892_33_cplx	Translation elongation complex: 33 * ribosome 70S/b2892/second last codon EF-TU-tRNA/1 EF-G	C3026466H4225890N1067294O1531648S8613P155151Mg5775Zn0Fe0	-129152.62	Translation
rib_70_elo2_b2893_13_cplx	Translation elongation complex: 13 * ribosome 70S/b2893/second last codon EF-TU-tRNA/1 EF-G	C1170628H1630110N413863O597186S3341P61137Mg2275Zn0Fe0	-50726.82	Translation
rib_70_elo2_b2893_1_cplx	Translation elongation complex: 1 * ribosome 70S/b2893/second last codon EF-TU-tRNA/1 EF-G	C96316H132462N34375O50502S257P5361Mg175Zn0Fe0	-4561.14	Translation
rib_70_elo2_b2893_6_cplx	Translation elongation complex: 6 * ribosome 70S/b2893/second last codon EF-TU-tRNA/1 EF-G	C543946H756482N192495O278287S1542P28601Mg1050Zn0Fe0	-23796.84	Translation
rib_70_elo2_b2946_14_cplx	Translation elongation complex: 14 * ribosome 70S/b2946/second last codon EF-TU-tRNA/1 EF-G	C1260791H1756570N446219O643046S3514P65806Mg2450Zn0Fe0	-54594.96	Translation
rib_70_elo2_b2946_1_cplx	Translation elongation complex: 1 * ribosome 70S/b2946/second last codon EF-TU-tRNA/1 EF-G	C96524H132792N34457O50688S251P5382Mg175Zn0Fe0	-4582.14	Translation
rib_70_elo2_b2946_7_cplx	Translation elongation complex: 7 * ribosome 70S/b2946/second last codon EF-TU-tRNA/1 EF-G	C633878H882228N224501O324084S1757P33270Mg1225Zn0Fe0	-27664.98	Translation
rib_70_elo2_b2947_18_cplx	Translation elongation complex: 18 * ribosome 70S/b2947/second last codon EF-TU-tRNA/1 EF-G	C1628238H2268951N575162O828739S4590P84597Mg3150Zn0Fe0	-70308.52	Translation
rib_70_elo2_b2947_1_cplx	Translation elongation complex: 1 * ribosome 70S/b2947/second last codon EF-TU-tRNA/1 EF-G	C99020H135740N35412O52298S255P5598Mg175Zn0Fe0	-4805.14	Translation
rib_70_elo2_b2947_9_cplx	Translation elongation complex: 9 * ribosome 70S/b2947/second last codon EF-TU-tRNA/1 EF-G	C818652H1139604N289412O417682S2295P42774Mg1575Zn0Fe0	-35630.26	Translation
rib_70_elo2_b2959_1_cplx	Translation elongation complex: 1 * ribosome 70S/b2959/second last codon EF-TU-tRNA/1 EF-G	C92091H127393N32774O47653S246P4975Mg175Zn0Fe0	-4179.14	Translation
rib_70_elo2_b2959_3_cplx	Translation elongation complex: 3 * ribosome 70S/b2959/second last codon EF-TU-tRNA/1 EF-G	C270027H375149N95754O138439S738P14273Mg525Zn0Fe0	-11883.42	Translation
rib_70_elo2_b2959_6_cplx	Translation elongation complex: 6 * ribosome 70S/b2959/second last codon EF-TU-tRNA/1 EF-G	C536931H746783N190224O274618S1476P28220Mg1050Zn0Fe0	-23439.84	Translation
rib_70_elo2_b2960_14_cplx	Translation elongation complex: 14 * ribosome 70S/b2960/second last codon EF-TU-tRNA/1 EF-G	C1261288H1756053N446210O642880S3570P65794Mg2450Zn0Fe0	-54596.96	Translation
rib_70_elo2_b2960_1_cplx	Translation elongation complex: 1 * ribosome 70S/b2960/second last codon EF-TU-tRNA/1 EF-G	C96462H132626N34422O50613S255P5370Mg175Zn0Fe0	-4571.14	Translation
rib_70_elo2_b2960_7_cplx	Translation elongation complex: 7 * ribosome 70S/b2960/second last codon EF-TU-tRNA/1 EF-G	C634074H881900N224478O323967S1785P33258Mg1225Zn0Fe0	-27659.98	Translation
rib_70_elo2_b3065_1_cplx	Translation elongation complex: 1 * ribosome 70S/b3065/second last codon EF-TU-tRNA/1 EF-G	C90906H126066N32329O46871S244P4875Mg175Zn0Fe0	-4058.14	Translation
rib_70_elo2_b3065_2_cplx	Translation elongation complex: 2 * ribosome 70S/b3065/second last codon EF-TU-tRNA/1 EF-G	C179752H249800N63823O92237S488P9532Mg350Zn0Fe0	-7897.28	Translation
rib_70_elo2_b3065_4_cplx	Translation elongation complex: 4 * ribosome 70S/b3065/second last codon EF-TU-tRNA/1 EF-G	C357444H497268N126811O182969S976P18846Mg700Zn0Fe0	-15575.56	Translation
rib_70_elo2_b3066_17_cplx	Translation elongation complex: 17 * ribosome 70S/b3066/second last codon EF-TU-tRNA/1 EF-G	C1568462H2187418N553200O795606S4488P80762Mg2975Zn0Fe0	-67301.38	Translation
rib_70_elo2_b3066_1_cplx	Translation elongation complex: 1 * ribosome 70S/b3066/second last codon EF-TU-tRNA/1 EF-G	C107902H146378N38800O58262S264P6394Mg175Zn0Fe0	-5603.14	Translation
rib_70_elo2_b3066_34_cplx	Translation elongation complex: 34 * ribosome 70S/b3066/second last codon EF-TU-tRNA/1 EF-G	C3120307H4356023N1099750O1579034S8976P159778Mg5950Zn0Fe0	-132855.76	Translation
rib_70_elo2_b3067_18_cplx	Translation elongation complex: 18 * ribosome 70S/b3067/second last codon EF-TU-tRNA/1 EF-G	C1663427H2321559N586584O844615S4842P85526Mg3150Zn0Fe0	-71867.52	Translation
rib_70_elo2_b3067_1_cplx	Translation elongation complex: 1 * ribosome 70S/b3067/second last codon EF-TU-tRNA/1 EF-G	C109015H147769N39354O58977S269P6493Mg175Zn0Fe0	-5735.14	Translation
rib_70_elo2_b3067_36_cplx	Translation elongation complex: 36 * ribosome 70S/b3067/second last codon EF-TU-tRNA/1 EF-G	C3309275H4623219N1166004O1676467S9684P169208Mg6300Zn0Fe0	-141890.04	Translation
rib_70_elo2_b3124_12_cplx	Translation elongation complex: 12 * ribosome 70S/b3124/second last codon EF-TU-tRNA/1 EF-G	C1093979H1524146N386873O557856S3072P56979Mg2100Zn0Fe0	-47489.68	Translation
rib_70_elo2_b3124_1_cplx	Translation elongation complex: 1 * ribosome 70S/b3124/second last codon EF-TU-tRNA/1 EF-G	C101911H139136N36611O54342S256P5873Mg175Zn0Fe0	-5083.14	Translation
rib_70_elo2_b3124_24_cplx	Translation elongation complex: 24 * ribosome 70S/b3124/second last codon EF-TU-tRNA/1 EF-G	C2176235H3035066N768977O1107144S6144P112731Mg4200Zn0Fe0	-93751.36	Translation
rib_70_elo2_b3125_17_cplx	Translation elongation complex: 17 * ribosome 70S/b3125/second last codon EF-TU-tRNA/1 EF-G	C1534096H2138329N542422O781835S4403P79924Mg2975Zn0Fe0	-66344.38	Translation
rib_70_elo2_b3125_1_cplx	Translation elongation complex: 1 * ribosome 70S/b3125/second last codon EF-TU-tRNA/1 EF-G	C98256H134825N35174O51835S259P5540Mg175Zn0Fe0	-4742.14	Translation
rib_70_elo2_b3125_8_cplx	Translation elongation complex: 8 * ribosome 70S/b3125/second last codon EF-TU-tRNA/1 EF-G	C726436H1011358N257095O371210S2072P38083Mg1400Zn0Fe0	-31693.12	Translation

rib_70_elo2_b3126_15_cplx	Translation elongation complex: 15 * ribosome 70S/b3126/second last codon EF-TU-tRNA/1 EF-G	C1351639H1882102N477818O689095S3675P70491Mg2625Zn0Fe0	-58524.1	Translation
rib_70_elo2_b3126_1_cplx	Translation elongation complex: 1 * ribosome 70S/b3126/second last codon EF-TU-tRNA/1 EF-G	C96945H133222N34564O50975S245P5419Mg175Zn0Fe0	-4622.14	Translation
rib_70_elo2_b3126_7_cplx	Translation elongation complex: 7 * ribosome 70S/b3126/second last codon EF-TU-tRNA/1 EF-G	C634671H882742N224530O324455S1715P33307Mg1225Zn0Fe0	-27722.98	Translation
rib_70_elo2_b3127_13_cplx	Translation elongation complex: 13 * ribosome 70S/b3127/second last codon EF-TU-tRNA/1 EF-G	C1191745H1659256N419177O604820S3445P61761Mg2275Zn0Fe0	-51220.82	Translation
rib_70_elo2_b3127_1_cplx	Translation elongation complex: 1 * ribosome 70S/b3127/second last codon EF-TU-tRNA/1 EF-G	C103333H140836N36725O55268S265P5985Mg175Zn0Fe0	-5175.14	Translation
rib_70_elo2_b3127_26_cplx	Translation elongation complex: 26 * ribosome 70S/b3127/second last codon EF-TU-tRNA/1 EF-G	C2370858H3304211N833500O1200168S6890P122185Mg4550Zn0Fe0	-101103.64	Translation
rib_70_elo2_b3164_1_cplx	Translation elongation complex: 1 * ribosome 70S/b3164/second last codon EF-TU-tRNA/1 EF-G	C112879H152437N40766O61684S264P6853Mg175Zn0Fe0	-6067.14	Translation
rib_70_elo2_b3164_21_cplx	Translation elongation complex: 21 * ribosome 70S/b3164/second last codon EF-TU-tRNA/1 EF-G	C1950439H2725857N687346O987464S5544P99813Mg3675Zn0Fe0	-83289.94	Translation
rib_70_elo2_b3164_43_cplx	Translation elongation complex: 43 * ribosome 70S/b3164/second last codon EF-TU-tRNA/1 EF-G	C3971755H5556619N1398584O2005822S11352P202069Mg7525Zn0Fe0	-168235.02	Translation
rib_70_elo2_b3165_1_cplx	Translation elongation complex: 1 * ribosome 70S/b3165/second last codon EF-TU-tRNA/1 EF-G	C91402H126658N32507O47223S243P4919Mg175Zn0Fe0	-4109.14	Translation
rib_70_elo2_b3165_2_cplx	Translation elongation complex: 2 * ribosome 70S/b3165/second last codon EF-TU-tRNA/1 EF-G	C180239H250404N63993O92567S486P9568Mg350Zn0Fe0	-7947.28	Translation
rib_70_elo2_b3165_5_cplx	Translation elongation complex: 5 * ribosome 70S/b3165/second last codon EF-TU-tRNA/1 EF-G	C446750H621642N158451O228599S1215P23515Mg875Zn0Fe0	-19461.7	Translation
rib_70_elo2_b3166_18_cplx	Translation elongation complex: 18 * ribosome 70S/b3166/second last codon EF-TU-tRNA/1 EF-G	C1627613H2268491N575470O828893S4518P84608Mg3150Zn0Fe0	-70247.52	Translation
rib_70_elo2_b3166_1_cplx	Translation elongation complex: 1 * ribosome 70S/b3166/second last codon EF-TU-tRNA/1 EF-G	C98905H135620N35346O52299S251P5592Mg175Zn0Fe0	-4795.14	Translation
rib_70_elo2_b3166_9_cplx	Translation elongation complex: 9 * ribosome 70S/b3166/second last codon EF-TU-tRNA/1 EF-G	C818297H1139324N289522O417755S2259P42776Mg1575Zn0Fe0	-35596.26	Translation
rib_70_elo2_b3167_1_cplx	Translation elongation complex: 1 * ribosome 70S/b3167/second last codon EF-TU-tRNA/1 EF-G	C92893H128426N33085O48214S249P5051Mg175Zn0Fe0	-4249.14	Translation
rib_70_elo2_b3167_3_cplx	Translation elongation complex: 3 * ribosome 70S/b3167/second last codon EF-TU-tRNA/1 EF-G	C271003H376596N96127O139062S747P14349Mg525Zn0Fe0	-11941.42	Translation
rib_70_elo2_b3167_7_cplx	Translation elongation complex: 7 * ribosome 70S/b3167/second last codon EF-TU-tRNA/1 EF-G	C627223H872936N222211O320758S1743P32945Mg1225Zn0Fe0	-27325.98	Translation
rib_70_elo2_b3168_1_cplx	Translation elongation complex: 1 * ribosome 70S/b3168/second last codon EF-TU-tRNA/1 EF-G	C118127H158758N43123O65066S264P7321Mg175Zn0Fe0	-6533.14	Translation
rib_70_elo2_b3168_26_cplx	Translation elongation complex: 26 * ribosome 70S/b3168/second last codon EF-TU-tRNA/1 EF-G	C2432102H3405483N858048O1229291S6864P123521Mg4550Zn0Fe0	-103011.64	Translation
rib_70_elo2_b3168_52_cplx	Translation elongation complex: 52 * ribosome 70S/b3168/second last codon EF-TU-tRNA/1 EF-G	C4838636H6782077N1705570O2440085S13728P244369Mg9100Zn0Fe0	-203349.28	Translation
rib_70_elo2_b3169_14_cplx	Translation elongation complex: 14 * ribosome 70S/b3169/second last codon EF-TU-tRNA/1 EF-G	C1285062H1791449N453816O654164S3542P66560Mg2450Zn0Fe0	-55880.96	Translation
rib_70_elo2_b3169_1_cplx	Translation elongation complex: 1 * ribosome 70S/b3169/second last codon EF-TU-tRNA/1 EF-G	C104987H142867N37790O56333S253P6136Mg175Zn0Fe0	-5374.14	Translation
rib_70_elo2_b3169_29_cplx	Translation elongation complex: 29 * ribosome 70S/b3169/second last codon EF-TU-tRNA/1 EF-G	C2646687H3693659N933846O1343969S7337P136280Mg5075Zn0Fe0	-114158.06	Translation
rib_70_elo2_b3170_1_cplx	Translation elongation complex: 1 * ribosome 70S/b3170/second last codon EF-TU-tRNA/1 EF-G	C93437H129030N33237O48616S247P5101Mg175Zn0Fe0	-4309.14	Translation
rib_70_elo2_b3170_4_cplx	Translation elongation complex: 4 * ribosome 70S/b3170/second last codon EF-TU-tRNA/1 EF-G	C360818H501525N127830O184933S988P19045Mg700Zn0Fe0	-15874.56	Translation
rib_70_elo2_b3170_8_cplx	Translation elongation complex: 8 * ribosome 70S/b3170/second last codon EF-TU-tRNA/1 EF-G	C717326H998185N253954O366689S1976P37637Mg1400Zn0Fe0	-31295.12	Translation
rib_70_elo2_b3178_18_cplx	Translation elongation complex: 18 * ribosome 70S/b3178/second last codon EF-TU-tRNA/1 EF-G	C1665564H2324850N587177O844256S4824P85599Mg3150Zn0Fe0	-71274.52	Translation
rib_70_elo2_b3178_1_cplx	Translation elongation complex: 1 * ribosome 70S/b3178/second last codon EF-TU-tRNA/1 EF-G	C109945H148850N39624O59672S268P6583Mg175Zn0Fe0	-5788.14	Translation
rib_70_elo2_b3178_37_cplx	Translation elongation complex: 37 * ribosome 70S/b3178/second last codon EF-TU-tRNA/1 EF-G	C3404197H4756850N1199148O1721144S9916P173911Mg6475Zn0Fe0	-144465.18	Translation
rib_70_elo2_b3179_12_cplx	Translation elongation complex: 12 * ribosome 70S/b3179/second last codon EF-TU-tRNA/1 EF-G	C1079126H1502798N381965O550595S3024P56420Mg2100Zn0Fe0	-46690.68	Translation

rib_70_elo2_b3179_1_cplx	Translation elongation complex: 1 * ribosome 70S/b3179/second last codon EF-TU-tRNA/1 EF-G	C95429H131439N34024O49941S252P5281Mg175Zn0Fe0	-4471.14	Translation
rib_70_elo2_b3179_6_cplx	Translation elongation complex: 6 * ribosome 70S/b3179/second last codon EF-TU-tRNA/1 EF-G	C542564H754784N192179O277511S1512P28526Mg1050Zn0Fe0	-23661.84	Translation
rib_70_elo2_b3181_1_cplx	Translation elongation complex: 1 * ribosome 70S/b3181/second last codon EF-TU-tRNA/1 EF-G	C93806H129472N33435O48852S244P5138Mg175Zn0Fe0	-4346.14	Translation
rib_70_elo2_b3181_4_cplx	Translation elongation complex: 4 * ribosome 70S/b3181/second last codon EF-TU-tRNA/1 EF-G	C361556H502474N128199O185448S976P19115Mg700Zn0Fe0	-15944.56	Translation
rib_70_elo2_b3181_9_cplx	Translation elongation complex: 9 * ribosome 70S/b3181/second last codon EF-TU-tRNA/1 EF-G	C807806H1124144N286139O413108S2196P42410Mg1575Zn0Fe0	-35275.26	Translation
rib_70_elo2_b3185_1_cplx	Translation elongation complex: 1 * ribosome 70S/b3185/second last codon EF-TU-tRNA/1 EF-G	C91236H126430N32461O47111S243P4906Mg175Zn0Fe0	-4092.14	Translation
rib_70_elo2_b3185_2_cplx	Translation elongation complex: 2 * ribosome 70S/b3185/second last codon EF-TU-tRNA/1 EF-G	C180009H250078N63923O92430S486P9554Mg350Zn0Fe0	-7925.28	Translation
rib_70_elo2_b3185_5_cplx	Translation elongation complex: 5 * ribosome 70S/b3185/second last codon EF-TU-tRNA/1 EF-G	C446328H621022N158309O228387S1215P23498Mg875Zn0Fe0	-19424.7	Translation
rib_70_elo2_b3186_1_cplx	Translation elongation complex: 1 * ribosome 70S/b3186/second last codon EF-TU-tRNA/1 EF-G	C91876H127175N32697O47532S242P4962Mg175Zn0Fe0	-4153.14	Translation
rib_70_elo2_b3186_3_cplx	Translation elongation complex: 3 * ribosome 70S/b3186/second last codon EF-TU-tRNA/1 EF-G	C269666H374809N95677O138236S726P14258Mg525Zn0Fe0	-11829.42	Translation
rib_70_elo2_b3186_6_cplx	Translation elongation complex: 6 * ribosome 70S/b3186/second last codon EF-TU-tRNA/1 EF-G	C536351H746260N190147O274292S1452P28202Mg1050Zn0Fe0	-23343.84	Translation
rib_70_elo2_b3201_14_cplx	Translation elongation complex: 14 * ribosome 70S/b3201/second last codon EF-TU-tRNA/1 EF-G	C1262186H1757952N446851O644082S3444P65954Mg2450Zn0Fe0	-54798.96	Translation
rib_70_elo2_b3201_1_cplx	Translation elongation complex: 1 * ribosome 70S/b3201/second last codon EF-TU-tRNA/1 EF-G	C96567H132835N34478O50723S246P5387Mg175Zn0Fe0	-4591.14	Translation
rib_70_elo2_b3201_7_cplx	Translation elongation complex: 7 * ribosome 70S/b3201/second last codon EF-TU-tRNA/1 EF-G	C634545H882889N224804O324581S1722P33341Mg1225Zn0Fe0	-27763.98	Translation
rib_70_elo2_b3202_14_cplx	Translation elongation complex: 14 * ribosome 70S/b3202/second last codon EF-TU-tRNA/1 EF-G	C1284145H1789906N453299O653645S3556P66508Mg2450Zn0Fe0	-55730.96	Translation
rib_70_elo2_b3202_1_cplx	Translation elongation complex: 1 * ribosome 70S/b3202/second last codon EF-TU-tRNA/1 EF-G	C104408H142221N37494O55944S254P6084Mg175Zn0Fe0	-5315.14	Translation
rib_70_elo2_b3202_28_cplx	Translation elongation complex: 28 * ribosome 70S/b3202/second last codon EF-TU-tRNA/1 EF-G	C2554631H3564336N901089O1297323S7112P131580Mg4900Zn0Fe0	-110024.92	Translation
rib_70_elo2_b3203_1_cplx	Translation elongation complex: 1 * ribosome 70S/b3203/second last codon EF-TU-tRNA/1 EF-G	C91619H126869N32578O47352S243P4937Mg175Zn0Fe0	-4135.14	Translation
rib_70_elo2_b3203_2_cplx	Translation elongation complex: 2 * ribosome 70S/b3203/second last codon EF-TU-tRNA/1 EF-G	C180487H250632N64037O92720S486P9586Mg350Zn0Fe0	-7981.28	Translation
rib_70_elo2_b3203_5_cplx	Translation elongation complex: 5 * ribosome 70S/b3203/second last codon EF-TU-tRNA/1 EF-G	C447091H621921N158414O228824S1215P23533Mg875Zn0Fe0	-19519.7	Translation
rib_70_elo2_b3204_1_cplx	Translation elongation complex: 1 * ribosome 70S/b3204/second last codon EF-TU-tRNA/1 EF-G	C93849H129581N33444O48877S245P5140Mg175Zn0Fe0	-4342.14	Translation
rib_70_elo2_b3204_4_cplx	Translation elongation complex: 4 * ribosome 70S/b3204/second last codon EF-TU-tRNA/1 EF-G	C361338H502397N128112O185254S980P19084Mg700Zn0Fe0	-15889.56	Translation
rib_70_elo2_b3204_9_cplx	Translation elongation complex: 9 * ribosome 70S/b3204/second last codon EF-TU-tRNA/1 EF-G	C807153H1123757N285892O412549S2205P42324Mg1575Zn0Fe0	-35135.26	Translation
rib_70_elo2_b3205_16_cplx	Translation elongation complex: 16 * ribosome 70S/b3205/second last codon EF-TU-tRNA/1 EF-G	C1445418H2013819N511144O736271S4048P75239Mg2800Zn0Fe0	-62410.24	Translation
rib_70_elo2_b3205_1_cplx	Translation elongation complex: 1 * ribosome 70S/b3205/second last codon EF-TU-tRNA/1 EF-G	C97938H134484N34924O51641S253P5504Mg175Zn0Fe0	-4703.14	Translation
rib_70_elo2_b3205_8_cplx	Translation elongation complex: 8 * ribosome 70S/b3205/second last codon EF-TU-tRNA/1 EF-G	C726762H1011507N257160O371135S2024P38047Mg1400Zn0Fe0	-31633.12	Translation
rib_70_elo2_b3206_1_cplx	Translation elongation complex: 1 * ribosome 70S/b3206/second last codon EF-TU-tRNA/1 EF-G	C91387H126578N32482O47238S246P4918Mg175Zn0Fe0	-4125.14	Translation
rib_70_elo2_b3206_2_cplx	Translation elongation complex: 2 * ribosome 70S/b3206/second last codon EF-TU-tRNA/1 EF-G	C180207H250260N63929O92600S492P9567Mg350Zn0Fe0	-7980.28	Translation
rib_70_elo2_b3206_5_cplx	Translation elongation complex: 5 * ribosome 70S/b3206/second last codon EF-TU-tRNA/1 EF-G	C446667H621306N158270O228686S1230P23514Mg875Zn0Fe0	-19545.7	Translation
rib_70_elo2_b3230_1_cplx	Translation elongation complex: 1 * ribosome 70S/b3230/second last codon EF-TU-tRNA/1 EF-G	C92774H128323N33029O48150S245P5042Mg175Zn0Fe0	-4223.14	Translation
rib_70_elo2_b3230_3_cplx	Translation elongation complex: 3 * ribosome 70S/b3230/second last codon EF-TU-tRNA/1 EF-G	C270854H376509N96129O138944S735P14340Mg525Zn0Fe0	-11881.42	Translation

rib_70_elo2_b3230_7_cplx	Translation elongation complex: 7 * ribosome 70S/b3230/second last codon EF-TU-tRNA/1 EF-G	C627014H872881N222329O320532S1715P32936Mg1225Zn0Fe0	-27197.98	Translation
rib_70_elo2_b3231_1_cplx	Translation elongation complex: 1 * ribosome 70S/b3231/second last codon EF-TU-tRNA/1 EF-G	C93201H128793N33206O48400S244P5080Mg175Zn0Fe0	-4268.14	Translation
rib_70_elo2_b3231_4_cplx	Translation elongation complex: 4 * ribosome 70S/b3231/second last codon EF-TU-tRNA/1 EF-G	C360537H501285N127868O184660S976P19027Mg700Zn0Fe0	-15776.56	Translation
rib_70_elo2_b3231_8_cplx	Translation elongation complex: 8 * ribosome 70S/b3231/second last codon EF-TU-tRNA/1 EF-G	C716985H997941N254084O366340S1952P37623Mg1400Zn0Fe0	-31121.12	Translation
rib_70_elo2_b3247_14_cplx	Translation elongation complex: 14 * ribosome 70S/b3247/second last codon EF-TU-tRNA/1 EF-G	C1285515H1792470N454047O653651S3640P66545Mg2450Zn0Fe0	-55487.96	Translation
rib_70_elo2_b3247_1_cplx	Translation elongation complex: 1 * ribosome 70S/b3247/second last codon EF-TU-tRNA/1 EF-G	C104738H142627N37631O56145S260P6108Mg175Zn0Fe0	-5319.14	Translation
rib_70_elo2_b3247_28_cplx	Translation elongation complex: 28 * ribosome 70S/b3247/second last codon EF-TU-tRNA/1 EF-G	C2557121H3569224N902495O1297119S7280P131631Mg4900Zn0Fe0	-109515.92	Translation
rib_70_elo2_b3248_11_cplx	Translation elongation complex: 11 * ribosome 70S/b3248/second last codon EF-TU-tRNA/1 EF-G	C988182H1375945N349924O504707S2695P51722Mg1925Zn0Fe0	-42935.54	Translation
rib_70_elo2_b3248_1_cplx	Translation elongation complex: 1 * ribosome 70S/b3248/second last codon EF-TU-tRNA/1 EF-G	C94992H130905N33904O49657S245P5242Mg175Zn0Fe0	-4444.14	Translation
rib_70_elo2_b3248_5_cplx	Translation elongation complex: 5 * ribosome 70S/b3248/second last codon EF-TU-tRNA/1 EF-G	C452268H628921N160312O231677S1225P23834Mg875Zn0Fe0	-19840.7	Translation
rib_70_elo2_b3249_1_cplx	Translation elongation complex: 1 * ribosome 70S/b3249/second last codon EF-TU-tRNA/1 EF-G	C93893H129595N33305O48888S247P5135Mg175Zn0Fe0	-4326.14	Translation
rib_70_elo2_b3249_4_cplx	Translation elongation complex: 4 * ribosome 70S/b3249/second last codon EF-TU-tRNA/1 EF-G	C361718H502732N127931O185097S988P19076Mg700Zn0Fe0	-15837.56	Translation
rib_70_elo2_b3249_9_cplx	Translation elongation complex: 9 * ribosome 70S/b3249/second last codon EF-TU-tRNA/1 EF-G	C808093H1124627N285641O412112S2223P42311Mg1575Zn0Fe0	-35023.26	Translation
rib_70_elo2_b3250_10_cplx	Translation elongation complex: 10 * ribosome 70S/b3250/second last codon EF-TU-tRNA/1 EF-G	C911531H1270036N322443O465021S2520P47574Mg1750Zn0Fe0	-39516.4	Translation
rib_70_elo2_b3250_1_cplx	Translation elongation complex: 1 * ribosome 70S/b3250/second last codon EF-TU-tRNA/1 EF-G	C100595H137701N35991O53487S252P5751Mg175Zn0Fe0	-4946.14	Translation
rib_70_elo2_b3250_21_cplx	Translation elongation complex: 21 * ribosome 70S/b3250/second last codon EF-TU-tRNA/1 EF-G	C1902675H2654001N672551O968007S5292P98691Mg3675Zn0Fe0	-81768.94	Translation
rib_70_elo2_b3251_10_cplx	Translation elongation complex: 10 * ribosome 70S/b3251/second last codon EF-TU-tRNA/1 EF-G	C909822H1267301N321850O464376S2570P47526Mg1750Zn0Fe0	-39588.4	Translation
rib_70_elo2_b3251_1_cplx	Translation elongation complex: 1 * ribosome 70S/b3251/second last codon EF-TU-tRNA/1 EF-G	C99912H136838N35722O53049S257P5694Mg175Zn0Fe0	-4901.14	Translation
rib_70_elo2_b3251_20_cplx	Translation elongation complex: 20 * ribosome 70S/b3251/second last codon EF-TU-tRNA/1 EF-G	C1809722H2523371N639770O921406S5140P94006Mg3500Zn0Fe0	-78129.8	Translation
rib_70_elo2_b3257_1_cplx	Translation elongation complex: 1 * ribosome 70S/b3257/second last codon EF-TU-tRNA/1 EF-G	C91108H126223N32312O47061S245P4893Mg175Zn0Fe0	-4093.14	Translation
rib_70_elo2_b3257_2_cplx	Translation elongation complex: 2 * ribosome 70S/b3257/second last codon EF-TU-tRNA/1 EF-G	C179918H249845N63746O92379S490P9541Mg350Zn0Fe0	-7940.28	Translation
rib_70_elo2_b3257_4_cplx	Translation elongation complex: 4 * ribosome 70S/b3257/second last codon EF-TU-tRNA/1 EF-G	C357538H497089N126614O183015S980P18837Mg700Zn0Fe0	-15634.56	Translation
rib_70_elo2_b3258_14_cplx	Translation elongation complex: 14 * ribosome 70S/b3258/second last codon EF-TU-tRNA/1 EF-G	C1284831H1791566N452145O651913S3668P66513Mg2450Zn0Fe0	-55133.96	Translation
rib_70_elo2_b3258_1_cplx	Translation elongation complex: 1 * ribosome 70S/b3258/second last codon EF-TU-tRNA/1 EF-G	C104444H142321N37198O56058S262P6089Mg175Zn0Fe0	-5277.14	Translation
rib_70_elo2_b3258_28_cplx	Translation elongation complex: 28 * ribosome 70S/b3258/second last codon EF-TU-tRNA/1 EF-G	C2556017H3567676N899011O1293603S7336P131585Mg4900Zn0Fe0	-108825.92	Translation
rib_70_elo2_b3259_17_cplx	Translation elongation complex: 17 * ribosome 70S/b3259/second last codon EF-TU-tRNA/1 EF-G	C1535068H2137551N542309O782314S4301P79898Mg2975Zn0Fe0	-66709.38	Translation
rib_70_elo2_b3259_1_cplx	Translation elongation complex: 1 * ribosome 70S/b3259/second last codon EF-TU-tRNA/1 EF-G	C98204H134687N35077O51818S253P5530Mg175Zn0Fe0	-4755.14	Translation
rib_70_elo2_b3259_8_cplx	Translation elongation complex: 8 * ribosome 70S/b3259/second last codon EF-TU-tRNA/1 EF-G	C726832H1010940N256991O371410S2024P38066Mg1400Zn0Fe0	-31860.12	Translation
rib_70_elo2_b3260_18_cplx	Translation elongation complex: 18 * ribosome 70S/b3260/second last codon EF-TU-tRNA/1 EF-G	C1628514H2269387N575868O828956S4590P84632Mg3150Zn0Fe0	-70235.52	Translation
rib_70_elo2_b3260_1_cplx	Translation elongation complex: 1 * ribosome 70S/b3260/second last codon EF-TU-tRNA/1 EF-G	C99160H135904N35472O52430S255P5616Mg175Zn0Fe0	-4817.14	Translation
rib_70_elo2_b3260_9_cplx	Translation elongation complex: 9 * ribosome 70S/b3260/second last codon EF-TU-tRNA/1 EF-G	C818856H1139896N289776O417854S2295P42800Mg1575Zn0Fe0	-35602.26	Translation

rib_70_elo2_b3261_1_cplx	Translation elongation complex: 1 * ribosome 70S/b3261/second last codon EF-TU-tRNA/1 EF-G	C91708H126994N32610O47428S247P4945Mg175Zn0Fe0	-4140.14	Translation
rib_70_elo2_b3261_2_cplx	Translation elongation complex: 2 * ribosome 70S/b3261/second last codon EF-TU-tRNA/1 EF-G	C180584H250789N64080O92795S494P9593Mg350Zn0Fe0	-7982.28	Translation
rib_70_elo2_b3261_5_cplx	Translation elongation complex: 5 * ribosome 70S/b3261/second last codon EF-TU-tRNA/1 EF-G	C447212H622174N158490O228896S1235P23537Mg875Zn0Fe0	-19508.7	Translation
rib_70_elo2_b3280_1_cplx	Translation elongation complex: 1 * ribosome 70S/b3280/second last codon EF-TU-tRNA/1 EF-G	C91262H126371N32452O47123S246P4903Mg175Zn0Fe0	-4111.14	Translation
rib_70_elo2_b3280_2_cplx	Translation elongation complex: 2 * ribosome 70S/b3280/second last codon EF-TU-tRNA/1 EF-G	C180093H250006N63911O92478S492P9552Mg350Zn0Fe0	-7967.28	Translation
rib_70_elo2_b3280_5_cplx	Translation elongation complex: 5 * ribosome 70S/b3280/second last codon EF-TU-tRNA/1 EF-G	C446586H620911N158288O228543S1230P23499Mg875Zn0Fe0	-19535.7	Translation
rib_70_elo2_b3281_16_cplx	Translation elongation complex: 16 * ribosome 70S/b3281/second last codon EF-TU-tRNA/1 EF-G	C1442945H2009516N510095O735376S3968P75187Mg2800Zn0Fe0	-62486.24	Translation
rib_70_elo2_b3281_1_cplx	Translation elongation complex: 1 * ribosome 70S/b3281/second last codon EF-TU-tRNA/1 EF-G	C97475H133826N34730O51376S248P5467Mg175Zn0Fe0	-4674.14	Translation
rib_70_elo2_b3281_8_cplx	Translation elongation complex: 8 * ribosome 70S/b3281/second last codon EF-TU-tRNA/1 EF-G	C725361H1009148N256567O370576S1984P38003Mg1400Zn0Fe0	-31653.12	Translation
rib_70_elo2_b3282_11_cplx	Translation elongation complex: 11 * ribosome 70S/b3282/second last codon EF-TU-tRNA/1 EF-G	C987533H1374721N349444O504204S2717P51679Mg1925Zn0Fe0	-42892.54	Translation
rib_70_elo2_b3282_1_cplx	Translation elongation complex: 1 * ribosome 70S/b3282/second last codon EF-TU-tRNA/1 EF-G	C94723H130571N33714O49504S247P5219Mg175Zn0Fe0	-4421.14	Translation
rib_70_elo2_b3282_5_cplx	Translation elongation complex: 5 * ribosome 70S/b3282/second last codon EF-TU-tRNA/1 EF-G	C451847H628231N160006O231384S1235P23803Mg875Zn0Fe0	-19809.7	Translation
rib_70_elo2_b3283_10_cplx	Translation elongation complex: 10 * ribosome 70S/b3283/second last codon EF-TU-tRNA/1 EF-G	C897591H1249369N317862O458365S2590P47025Mg1750Zn0Fe0	-38927.4	Translation
rib_70_elo2_b3283_1_cplx	Translation elongation complex: 1 * ribosome 70S/b3283/second last codon EF-TU-tRNA/1 EF-G	C94404H130192N33624O49261S259P5193Mg175Zn0Fe0	-4384.14	Translation
rib_70_elo2_b3283_5_cplx	Translation elongation complex: 5 * ribosome 70S/b3283/second last codon EF-TU-tRNA/1 EF-G	C451376H627604N159952O231085S1295P23785Mg875Zn0Fe0	-19736.7	Translation
rib_70_elo2_b3287_1_cplx	Translation elongation complex: 1 * ribosome 70S/b3287/second last codon EF-TU-tRNA/1 EF-G	C94098H129868N33540O49021S247P5160Mg175Zn0Fe0	-4365.14	Translation
rib_70_elo2_b3287_4_cplx	Translation elongation complex: 4 * ribosome 70S/b3287/second last codon EF-TU-tRNA/1 EF-G	C361782H503014N128265O185425S988P19104Mg700Zn0Fe0	-15921.56	Translation
rib_70_elo2_b3287_9_cplx	Translation elongation complex: 9 * ribosome 70S/b3287/second last codon EF-TU-tRNA/1 EF-G	C807922H1124924N286140O412765S2223P42344Mg1575Zn0Fe0	-35182.26	Translation
rib_70_elo2_b3288_18_cplx	Translation elongation complex: 18 * ribosome 70S/b3288/second last codon EF-TU-tRNA/1 EF-G	C1627451H2268293N575096O828686S4554P84630Mg3150Zn0Fe0	-70233.52	Translation
rib_70_elo2_b3288_1_cplx	Translation elongation complex: 1 * ribosome 70S/b3288/second last codon EF-TU-tRNA/1 EF-G	C98930H135660N35346O52296S253P5597Mg175Zn0Fe0	-4798.14	Translation
rib_70_elo2_b3288_9_cplx	Translation elongation complex: 9 * ribosome 70S/b3288/second last codon EF-TU-tRNA/1 EF-G	C818234H1139252N289346O417656S2277P42789Mg1575Zn0Fe0	-35591.26	Translation
rib_70_elo2_b3289_12_cplx	Translation elongation complex: 12 * ribosome 70S/b3289/second last codon EF-TU-tRNA/1 EF-G	C1098735H1530843N388148O559088S3084P57068Mg2100Zn0Fe0	-47434.68	Translation
rib_70_elo2_b3289_1_cplx	Translation elongation complex: 1 * ribosome 70S/b3289/second last codon EF-TU-tRNA/1 EF-G	C102806H140289N36808O54859S257P5940Mg175Zn0Fe0	-5138.14	Translation
rib_70_elo2_b3289_25_cplx	Translation elongation complex: 25 * ribosome 70S/b3289/second last codon EF-TU-tRNA/1 EF-G	C2275742H3174225N803368O1154995S6425P117492Mg4375Zn0Fe0	-97421.5	Translation
rib_70_elo2_b3294_1_cplx	Translation elongation complex: 1 * ribosome 70S/b3294/second last codon EF-TU-tRNA/1 EF-G	C92651H128165N32994O48070S246P5032Mg175Zn0Fe0	-4216.14	Translation
rib_70_elo2_b3294_3_cplx	Translation elongation complex: 3 * ribosome 70S/b3294/second last codon EF-TU-tRNA/1 EF-G	C270647H376221N96066O138836S738P14328Mg525Zn0Fe0	-11878.42	Translation
rib_70_elo2_b3294_7_cplx	Translation elongation complex: 7 * ribosome 70S/b3294/second last codon EF-TU-tRNA/1 EF-G	C626639H872333N222210O320368S1722P32920Mg1225Zn0Fe0	-27202.98	Translation
rib_70_elo2_b3295_19_cplx	Translation elongation complex: 19 * ribosome 70S/b3295/second last codon EF-TU-tRNA/1 EF-G	C1718969H2396967N607629O875387S4750P89302Mg3325Zn0Fe0	-74371.66	Translation
rib_70_elo2_b3295_1_cplx	Translation elongation complex: 1 * ribosome 70S/b3295/second last codon EF-TU-tRNA/1 EF-G	C99401H136257N35553O52625S250P5638Mg175Zn0Fe0	-4853.14	Translation
rib_70_elo2_b3295_9_cplx	Translation elongation complex: 9 * ribosome 70S/b3295/second last codon EF-TU-tRNA/1 EF-G	C819209H1141017N289809O418297S2250P42822Mg1575Zn0Fe0	-35750.26	Translation
rib_70_elo2_b3296_12_cplx	Translation elongation complex: 12 * ribosome 70S/b3296/second last codon EF-TU-tRNA/1 EF-G	C1078939H1503240N382111O550518S2952P56397Mg2100Zn0Fe0	-46559.68	Translation

rib_70_elo2_b3296_1_cplx	Translation elongation complex: 1 * ribosome 70S/b3296/second last codon EF-TU-tRNA/1 EF-G	C95341H131397N34027O49853S246P5269Mg175Zn0Fe0	-4450.14	Translation
rib_70_elo2_b3296_6_cplx	Translation elongation complex: 6 * ribosome 70S/b3296/second last codon EF-TU-tRNA/1 EF-G	C542431H754962N192247O277428S1476P28509Mg1050Zn0Fe0	-23590.84	Translation
rib_70_elo2_b3297_1_cplx	Translation elongation complex: 1 * ribosome 70S/b3297/second last codon EF-TU-tRNA/1 EF-G	C92685H128185N32997O48110S245P5038Mg175Zn0Fe0	-4221.14	Translation
rib_70_elo2_b3297_3_cplx	Translation elongation complex: 3 * ribosome 70S/b3297/second last codon EF-TU-tRNA/1 EF-G	C270647H376161N96063O138870S735P14334Mg525Zn0Fe0	-11881.42	Translation
rib_70_elo2_b3297_7_cplx	Translation elongation complex: 7 * ribosome 70S/b3297/second last codon EF-TU-tRNA/1 EF-G	C626571H872113N222195O320390S1715P32926Mg1225Zn0Fe0	-27201.98	Translation
rib_70_elo2_b3298_1_cplx	Translation elongation complex: 1 * ribosome 70S/b3298/second last codon EF-TU-tRNA/1 EF-G	C92353H127828N32867O47879S245P5007Mg175Zn0Fe0	-4191.14	Translation
rib_70_elo2_b3298_3_cplx	Translation elongation complex: 3 * ribosome 70S/b3298/second last codon EF-TU-tRNA/1 EF-G	C270265H375796N95891O138633S735P14303Mg525Zn0Fe0	-11853.42	Translation
rib_70_elo2_b3298_6_cplx	Translation elongation complex: 6 * ribosome 70S/b3298/second last codon EF-TU-tRNA/1 EF-G	C537133H747748N190427O274764S1470P28247Mg1050Zn0Fe0	-23346.84	Translation
rib_70_elo2_b3299_1_cplx	Translation elongation complex: 1 * ribosome 70S/b3299/second last codon EF-TU-tRNA/1 EF-G	C89679H124583N31847O46074S244P4765Mg175Zn0Fe0	-3952.14	Translation
rib_70_elo2_b3299_2_cplx	Translation elongation complex: 2 * ribosome 70S/b3299/second last codon EF-TU-tRNA/1 EF-G	C178241H247906N63243O91333S488P9413Mg350Zn0Fe0	-7786.28	Translation
rib_70_elo2_b3300_13_cplx	Translation elongation complex: 13 * ribosome 70S/b3300/second last codon EF-TU-tRNA/1 EF-G	C1191100H1659848N419675O604732S3367P61769Mg2275Zn0Fe0	-51098.82	Translation
rib_70_elo2_b3300_1_cplx	Translation elongation complex: 1 * ribosome 70S/b3300/second last codon EF-TU-tRNA/1 EF-G	C103276H140852N36791O55216S259P5981Mg175Zn0Fe0	-5161.14	Translation
rib_70_elo2_b3300_26_cplx	Translation elongation complex: 26 * ribosome 70S/b3300/second last codon EF-TU-tRNA/1 EF-G	C2369576H3305427N834466O1200041S6734P122206Mg4550Zn0Fe0	-100864.64	Translation
rib_70_elo2_b3301_1_cplx	Translation elongation complex: 1 * ribosome 70S/b3301/second last codon EF-TU-tRNA/1 EF-G	C93174H128793N33195O48449S243P5083Mg175Zn0Fe0	-4264.14	Translation
rib_70_elo2_b3301_4_cplx	Translation elongation complex: 4 * ribosome 70S/b3301/second last codon EF-TU-tRNA/1 EF-G	C360267H501129N127812O184634S972P19027Mg700Zn0Fe0	-15748.56	Translation
rib_70_elo2_b3301_8_cplx	Translation elongation complex: 8 * ribosome 70S/b3301/second last codon EF-TU-tRNA/1 EF-G	C716391H997577N253968O366214S1944P37619Mg1400Zn0Fe0	-31061.12	Translation
rib_70_elo2_b3302_1_cplx	Translation elongation complex: 1 * ribosome 70S/b3302/second last codon EF-TU-tRNA/1 EF-G	C90376H125420N32102O46544S244P4828Mg175Zn0Fe0	-4019.14	Translation
rib_70_elo2_b3302_3_cplx	Translation elongation complex: 3 * ribosome 70S/b3302/second last codon EF-TU-tRNA/1 EF-G	C267702H372386N94942O137114S732P14124Mg525Zn0Fe0	-11695.42	Translation
rib_70_elo2_b3303_1_cplx	Translation elongation complex: 1 * ribosome 70S/b3303/second last codon EF-TU-tRNA/1 EF-G	C93955H129700N33484O48963S248P5152Mg175Zn0Fe0	-4341.14	Translation
rib_70_elo2_b3303_4_cplx	Translation elongation complex: 4 * ribosome 70S/b3303/second last codon EF-TU-tRNA/1 EF-G	C361414H502528N128167O185292S992P19096Mg700Zn0Fe0	-15849.56	Translation
rib_70_elo2_b3303_9_cplx	Translation elongation complex: 9 * ribosome 70S/b3303/second last codon EF-TU-tRNA/1 EF-G	C807179H1123908N285972O412507S2232P42336Mg1575Zn0Fe0	-35030.26	Translation
rib_70_elo2_b3304_1_cplx	Translation elongation complex: 1 * ribosome 70S/b3304/second last codon EF-TU-tRNA/1 EF-G	C92311H127747N32857O47845S243P5002Mg175Zn0Fe0	-4189.14	Translation
rib_70_elo2_b3304_3_cplx	Translation elongation complex: 3 * ribosome 70S/b3304/second last codon EF-TU-tRNA/1 EF-G	C270201H375609N95885O138589S729P14298Mg525Zn0Fe0	-11857.42	Translation
rib_70_elo2_b3304_6_cplx	Translation elongation complex: 6 * ribosome 70S/b3304/second last codon EF-TU-tRNA/1 EF-G	C537036H747402N190427O274705S1458P28242Mg1050Zn0Fe0	-23359.84	Translation
rib_70_elo2_b3305_10_cplx	Translation elongation complex: 10 * ribosome 70S/b3305/second last codon EF-TU-tRNA/1 EF-G	C897323H1249514N317745O458375S2440P47014Mg1750Zn0Fe0	-38896.4	Translation
rib_70_elo2_b3305_1_cplx	Translation elongation complex: 1 * ribosome 70S/b3305/second last codon EF-TU-tRNA/1 EF-G	C94307H130121N33597O49208S244P5182Mg175Zn0Fe0	-4371.14	Translation
rib_70_elo2_b3305_5_cplx	Translation elongation complex: 5 * ribosome 70S/b3305/second last codon EF-TU-tRNA/1 EF-G	C451203H627629N159885O231060S1220P23774Mg875Zn0Fe0	-19715.7	Translation
rib_70_elo2_b3306_1_cplx	Translation elongation complex: 1 * ribosome 70S/b3306/second last codon EF-TU-tRNA/1 EF-G	C92746H128251N33014O48136S247P5041Mg175Zn0Fe0	-4233.14	Translation
rib_70_elo2_b3306_3_cplx	Translation elongation complex: 3 * ribosome 70S/b3306/second last codon EF-TU-tRNA/1 EF-G	C270746H376293N96036O138920S741P14337Mg525Zn0Fe0	-11911.42	Translation
rib_70_elo2_b3306_7_cplx	Translation elongation complex: 7 * ribosome 70S/b3306/second last codon EF-TU-tRNA/1 EF-G	C626746H872377N222080O320488S1729P32929Mg1225Zn0Fe0	-27267.98	Translation
rib_70_elo2_b3307_1_cplx	Translation elongation complex: 1 * ribosome 70S/b3307/second last codon EF-TU-tRNA/1 EF-G	C91803H127147N32668O47483S246P4954Mg175Zn0Fe0	-4136.14	Translation

rib_70_elo2_b3307_2_cplx	Translation elongation complex: 2 * ribosome 70S/b3307/second last codon EF-TU-tRNA/1 EF-G	C180691H250995N64164O92832S492P9602Mg350Zn0Fe0	-7965.28	Translation
rib_70_elo2_b3307_5_cplx	Translation elongation complex: 5 * ribosome 70S/b3307/second last codon EF-TU-tRNA/1 EF-G	C447355H622539N158652O228879S1230P23546Mg875Zn0Fe0	-19452.7	Translation
rib_70_elo2_b3308_10_cplx	Translation elongation complex: 10 * ribosome 70S/b3308/second last codon EF-TU-tRNA/1 EF-G	C898103H1250441N317836O458520S2480P47020Mg1750Zn0Fe0	-38922.4	Translation
rib_70_elo2_b3308_1_cplx	Translation elongation complex: 1 * ribosome 70S/b3308/second last codon EF-TU-tRNA/1 EF-G	C94430H130274N33616O49245S248P5188Mg175Zn0Fe0	-4379.14	Translation
rib_70_elo2_b3308_5_cplx	Translation elongation complex: 5 * ribosome 70S/b3308/second last codon EF-TU-tRNA/1 EF-G	C451618H628126N159936O231145S1240P23780Mg875Zn0Fe0	-19731.7	Translation
rib_70_elo2_b3309_1_cplx	Translation elongation complex: 1 * ribosome 70S/b3309/second last codon EF-TU-tRNA/1 EF-G	C91902H127244N32704O47540S242P4963Mg175Zn0Fe0	-4150.14	Translation
rib_70_elo2_b3309_3_cplx	Translation elongation complex: 3 * ribosome 70S/b3309/second last codon EF-TU-tRNA/1 EF-G	C269682H374944N95658O138260S726P14259Mg525Zn0Fe0	-11818.42	Translation
rib_70_elo2_b3309_6_cplx	Translation elongation complex: 6 * ribosome 70S/b3309/second last codon EF-TU-tRNA/1 EF-G	C536352H746494N190089O274340S1452P28203Mg1050Zn0Fe0	-23320.84	Translation
rib_70_elo2_b3310_1_cplx	Translation elongation complex: 1 * ribosome 70S/b3310/second last codon EF-TU-tRNA/1 EF-G	C92621H128125N32977O48061S246P5033Mg175Zn0Fe0	-4218.14	Translation
rib_70_elo2_b3310_3_cplx	Translation elongation complex: 3 * ribosome 70S/b3310/second last codon EF-TU-tRNA/1 EF-G	C270775H376367N96093O138969S738P14351Mg525Zn0Fe0	-11904.42	Translation
rib_70_elo2_b3310_7_cplx	Translation elongation complex: 7 * ribosome 70S/b3310/second last codon EF-TU-tRNA/1 EF-G	C627083H872851N222325O320785S1722P32987Mg1225Zn0Fe0	-27276.98	Translation
rib_70_elo2_b3311_1_cplx	Translation elongation complex: 1 * ribosome 70S/b3311/second last codon EF-TU-tRNA/1 EF-G	C91330H126553N32471O47173S244P4913Mg175Zn0Fe0	-4104.14	Translation
rib_70_elo2_b3311_2_cplx	Translation elongation complex: 2 * ribosome 70S/b3311/second last codon EF-TU-tRNA/1 EF-G	C180238H250372N63969O92578S488P9572Mg350Zn0Fe0	-7953.28	Translation
rib_70_elo2_b3311_4_cplx	Translation elongation complex: 4 * ribosome 70S/b3311/second last codon EF-TU-tRNA/1 EF-G	C358054H498010N126965O183388S976P18890Mg700Zn0Fe0	-15651.56	Translation
rib_70_elo2_b3312_1_cplx	Translation elongation complex: 1 * ribosome 70S/b3312/second last codon EF-TU-tRNA/1 EF-G	C90522H125588N32191O46630S242P4839Mg175Zn0Fe0	-4033.14	Translation
rib_70_elo2_b3312_3_cplx	Translation elongation complex: 3 * ribosome 70S/b3312/second last codon EF-TU-tRNA/1 EF-G	C267906H372632N95055O137244S726P14135Mg525Zn0Fe0	-11715.42	Translation
rib_70_elo2_b3313_1_cplx	Translation elongation complex: 1 * ribosome 70S/b3313/second last codon EF-TU-tRNA/1 EF-G	C93012H128597N33140O48258S247P5060Mg175Zn0Fe0	-4238.14	Translation
rib_70_elo2_b3313_4_cplx	Translation elongation complex: 4 * ribosome 70S/b3313/second last codon EF-TU-tRNA/1 EF-G	C360267H501095N127766O184446S988P19007Mg700Zn0Fe0	-15716.56	Translation
rib_70_elo2_b3313_8_cplx	Translation elongation complex: 8 * ribosome 70S/b3313/second last codon EF-TU-tRNA/1 EF-G	C716607H997759N253934O366030S1976P37603Mg1400Zn0Fe0	-31021.12	Translation
rib_70_elo2_b3314_13_cplx	Translation elongation complex: 13 * ribosome 70S/b3314/second last codon EF-TU-tRNA/1 EF-G	C1170727H1631476N414478O596901S3198P61126Mg2275Zn0Fe0	-50442.82	Translation
rib_70_elo2_b3314_1_cplx	Translation elongation complex: 1 * ribosome 70S/b3314/second last codon EF-TU-tRNA/1 EF-G	C96235H132484N34378O50421S246P5350Mg175Zn0Fe0	-4529.14	Translation
rib_70_elo2_b3314_6_cplx	Translation elongation complex: 6 * ribosome 70S/b3314/second last codon EF-TU-tRNA/1 EF-G	C543940H757064N192753O278121S1476P28590Mg1050Zn0Fe0	-23659.84	Translation
rib_70_elo2_b3315_1_cplx	Translation elongation complex: 1 * ribosome 70S/b3315/second last codon EF-TU-tRNA/1 EF-G	C92094H127507N32771O47692S244P4982Mg175Zn0Fe0	-4169.14	Translation
rib_70_elo2_b3315_3_cplx	Translation elongation complex: 3 * ribosome 70S/b3315/second last codon EF-TU-tRNA/1 EF-G	C269944H375351N95785O138430S732P14280Mg525Zn0Fe0	-11839.42	Translation
rib_70_elo2_b3315_6_cplx	Translation elongation complex: 6 * ribosome 70S/b3315/second last codon EF-TU-tRNA/1 EF-G	C536719H747117N190306O274537S1464P28227Mg1050Zn0Fe0	-23344.84	Translation
rib_70_elo2_b3316_1_cplx	Translation elongation complex: 1 * ribosome 70S/b3316/second last codon EF-TU-tRNA/1 EF-G	C91513H126776N32531O47282S244P4927Mg175Zn0Fe0	-4113.14	Translation
rib_70_elo2_b3316_2_cplx	Translation elongation complex: 2 * ribosome 70S/b3316/second last codon EF-TU-tRNA/1 EF-G	C180369H250548N63999O92621S488P9575Mg350Zn0Fe0	-7946.28	Translation
rib_70_elo2_b3316_5_cplx	Translation elongation complex: 5 * ribosome 70S/b3316/second last codon EF-TU-tRNA/1 EF-G	C446937H621864N158403O228638S1220P23519Mg875Zn0Fe0	-19445.7	Translation
rib_70_elo2_b3317_16_cplx	Translation elongation complex: 16 * ribosome 70S/b3317/second last codon EF-TU-tRNA/1 EF-G	C1442801H2011625N511198O735066S3984P75190Mg2800Zn0Fe0	-61817.24	Translation
rib_70_elo2_b3317_1_cplx	Translation elongation complex: 1 * ribosome 70S/b3317/second last codon EF-TU-tRNA/1 EF-G	C97511H134030N34888O51321S249P5470Mg175Zn0Fe0	-4635.14	Translation
rib_70_elo2_b3317_8_cplx	Translation elongation complex: 8 * ribosome 70S/b3317/second last codon EF-TU-tRNA/1 EF-G	C725313H1010241N257166O370402S1992P38006Mg1400Zn0Fe0	-31320.12	Translation

rib_70_elo2_b3318_1_cplx	Translation elongation complex: 1 * ribosome 70S/b3318/second last codon EF-TU-tRNA/1 EF-G	C91735H127057N32655O47412S243P4947Mg175Zn0Fe0	-4136.14	Translation
rib_70_elo2_b3318_2_cplx	Translation elongation complex: 2 * ribosome 70S/b3318/second last codon EF-TU-tRNA/1 EF-G	C180608H250886N64133O92760S486P9595Mg350Zn0Fe0	-7972.28	Translation
rib_70_elo2_b3318_5_cplx	Translation elongation complex: 5 * ribosome 70S/b3318/second last codon EF-TU-tRNA/1 EF-G	C447227H622373N158567O228804S1215P23539Mg875Zn0Fe0	-19480.7	Translation
rib_70_elo2_b3319_11_cplx	Translation elongation complex: 11 * ribosome 70S/b3319/second last codon EF-TU-tRNA/1 EF-G	C988657H1377105N350094O504760S2695P51734Mg1925Zn0Fe0	-42815.54	Translation
rib_70_elo2_b3319_1_cplx	Translation elongation complex: 1 * ribosome 70S/b3319/second last codon EF-TU-tRNA/1 EF-G	C95127H131125N33934O49740S245P5254Mg175Zn0Fe0	-4444.14	Translation
rib_70_elo2_b3319_5_cplx	Translation elongation complex: 5 * ribosome 70S/b3319/second last codon EF-TU-tRNA/1 EF-G	C452539H629517N160398O231748S1225P23846Mg875Zn0Fe0	-19792.7	Translation
rib_70_elo2_b3320_12_cplx	Translation elongation complex: 12 * ribosome 70S/b3320/second last codon EF-TU-tRNA/1 EF-G	C1078304H1501927N381874O550465S2928P56406Mg2100Zn0Fe0	-46652.68	Translation
rib_70_elo2_b3320_1_cplx	Translation elongation complex: 1 * ribosome 70S/b3320/second last codon EF-TU-tRNA/1 EF-G	C95366H131382N34043O49899S244P5278Mg175Zn0Fe0	-4466.14	Translation
rib_70_elo2_b3320_6_cplx	Translation elongation complex: 6 * ribosome 70S/b3320/second last codon EF-TU-tRNA/1 EF-G	C542156H754357N192148O277429S1464P28518Mg1050Zn0Fe0	-23641.84	Translation
rib_70_elo2_b3321_1_cplx	Translation elongation complex: 1 * ribosome 70S/b3321/second last codon EF-TU-tRNA/1 EF-G	C91856H127213N32674O47544S242P4962Mg175Zn0Fe0	-4156.14	Translation
rib_70_elo2_b3321_3_cplx	Translation elongation complex: 3 * ribosome 70S/b3321/second last codon EF-TU-tRNA/1 EF-G	C269638H374903N95652O138268S726P14258Mg525Zn0Fe0	-11838.42	Translation
rib_70_elo2_b3321_6_cplx	Translation elongation complex: 6 * ribosome 70S/b3321/second last codon EF-TU-tRNA/1 EF-G	C536311H746438N190119O274354S1452P28202Mg1050Zn0Fe0	-23361.84	Translation
rib_70_elo2_b3339_11_cplx	Translation elongation complex: 11 * ribosome 70S/b3339/second last codon EF-TU-tRNA/1 EF-G	C1004565H1399197N354914O512001S2794P52313Mg1925Zn0Fe0	-43636.54	Translation
rib_70_elo2_b3339_1_cplx	Translation elongation complex: 1 * ribosome 70S/b3339/second last codon EF-TU-tRNA/1 EF-G	C101575H138807N36364O54071S254P5833Mg175Zn0Fe0	-5045.14	Translation
rib_70_elo2_b3339_23_cplx	Translation elongation complex: 23 * ribosome 70S/b3339/second last codon EF-TU-tRNA/1 EF-G	C2088153H2911665N737174O1061517S5842P108089Mg4025Zn0Fe0	-89946.22	Translation
rib_70_elo2_b3340_1_cplx	Translation elongation complex: 1 * ribosome 70S/b3340/second last codon EF-TU-tRNA/1 EF-G	C111967H151198N40349O61010S267P6763Mg175Zn0Fe0	-5985.14	Translation
rib_70_elo2_b3340_20_cplx	Translation elongation complex: 20 * ribosome 70S/b3340/second last codon EF-TU-tRNA/1 EF-G	C1856566H2591197N653498O940064S5340P95075Mg3500Zn0Fe0	-79498.8	Translation
rib_70_elo2_b3340_41_cplx	Translation elongation complex: 41 * ribosome 70S/b3340/second last codon EF-TU-tRNA/1 EF-G	C3784807H5288038N1331189O1911650S10947P192683Mg7175Zn0Fe0	-160750.74	Translation
rib_70_elo2_b3341_10_cplx	Translation elongation complex: 10 * ribosome 70S/b3341/second last codon EF-TU-tRNA/1 EF-G	C897814H1250294N318021O458486S2460P47020Mg1750Zn0Fe0	-38852.4	Translation
rib_70_elo2_b3341_1_cplx	Translation elongation complex: 1 * ribosome 70S/b3341/second last codon EF-TU-tRNA/1 EF-G	C94402H130253N33630O49256S246P5188Mg175Zn0Fe0	-4372.14	Translation
rib_70_elo2_b3341_5_cplx	Translation elongation complex: 5 * ribosome 70S/b3341/second last codon EF-TU-tRNA/1 EF-G	C451474H628049N160026O231136S1230P23780Mg875Zn0Fe0	-19696.7	Translation
rib_70_elo2_b3342_1_cplx	Translation elongation complex: 1 * ribosome 70S/b3342/second last codon EF-TU-tRNA/1 EF-G	C92539H128049N32951O48003S245P5025Mg175Zn0Fe0	-4202.14	Translation
rib_70_elo2_b3342_3_cplx	Translation elongation complex: 3 * ribosome 70S/b3342/second last codon EF-TU-tRNA/1 EF-G	C270487H376063N96011O138759S735P14321Mg525Zn0Fe0	-11850.42	Translation
rib_70_elo2_b3342_7_cplx	Translation elongation complex: 7 * ribosome 70S/b3342/second last codon EF-TU-tRNA/1 EF-G	C626383H872091N222131O320271S1715P32913Mg1225Zn0Fe0	-27146.98	Translation
rib_70_elo2_b3343_1_cplx	Translation elongation complex: 1 * ribosome 70S/b3343/second last codon EF-TU-tRNA/1 EF-G	C91594H126851N32519O47376S244P4936Mg175Zn0Fe0	-4137.14	Translation
rib_70_elo2_b3343_2_cplx	Translation elongation complex: 2 * ribosome 70S/b3343/second last codon EF-TU-tRNA/1 EF-G	C180461H250609N63980O92726S488P9584Mg350Zn0Fe0	-7985.28	Translation
rib_70_elo2_b3343_5_cplx	Translation elongation complex: 5 * ribosome 70S/b3343/second last codon EF-TU-tRNA/1 EF-G	C447062H621883N158363O228776S1220P23528Mg875Zn0Fe0	-19529.7	Translation
rib_70_elo2_b3344_1_cplx	Translation elongation complex: 1 * ribosome 70S/b3344/second last codon EF-TU-tRNA/1 EF-G	C92380H127766N32795O47925S245P5007Mg175Zn0Fe0	-4209.14	Translation
rib_70_elo2_b3344_3_cplx	Translation elongation complex: 3 * ribosome 70S/b3344/second last codon EF-TU-tRNA/1 EF-G	C270344H375608N95773O138683S735P14303Mg525Zn0Fe0	-11907.42	Translation
rib_70_elo2_b3344_7_cplx	Translation elongation complex: 7 * ribosome 70S/b3344/second last codon EF-TU-tRNA/1 EF-G	C626272H871292N221729O320199S1715P32895Mg1225Zn0Fe0	-27303.98	Translation
rib_70_elo2_b3345_1_cplx	Translation elongation complex: 1 * ribosome 70S/b3345/second last codon EF-TU-tRNA/1 EF-G	C92670H128087N32983O48106S245P5034Mg175Zn0Fe0	-4235.14	Translation

rib_70_elo2_b3345_3_cplx	Translation elongation complex: 3 * ribosome 70S/b3345/second last codon EF-TU-tRNA/1 EF-G	C270652H375947N95993O138896S735P14330Mg525Zn0Fe0	-11931.42	Translation
rib_70_elo2_b3345_7_cplx	Translation elongation complex: 7 * ribosome 70S/b3345/second last codon EF-TU-tRNA/1 EF-G	C626616H871667N222013O320476S1715P32922Mg1225Zn0Fe0	-27323.98	Translation
rib_70_elo2_b3346_14_cplx	Translation elongation complex: 14 * ribosome 70S/b3346/second last codon EF-TU-tRNA/1 EF-G	C1260703H1756109N445844O643263S3528P65797Mg2450Zn0Fe0	-54641.96	Translation
rib_70_elo2_b3346_1_cplx	Translation elongation complex: 1 * ribosome 70S/b3346/second last codon EF-TU-tRNA/1 EF-G	C96423H132643N34342O50658S252P5373Mg175Zn0Fe0	-4577.14	Translation
rib_70_elo2_b3346_7_cplx	Translation elongation complex: 7 * ribosome 70S/b3346/second last codon EF-TU-tRNA/1 EF-G	C633783H881935N224266O324168S1764P33261Mg1225Zn0Fe0	-27683.98	Translation
rib_70_elo2_b3384_19_cplx	Translation elongation complex: 19 * ribosome 70S/b3384/second last codon EF-TU-tRNA/1 EF-G	C1720603H2397561N607870O875539S4845P89328Mg3325Zn0Fe0	-74112.66	Translation
rib_70_elo2_b3384_1_cplx	Translation elongation complex: 1 * ribosome 70S/b3384/second last codon EF-TU-tRNA/1 EF-G	C99559H136365N35614O52669S255P5646Mg175Zn0Fe0	-4846.14	Translation
rib_70_elo2_b3384_9_cplx	Translation elongation complex: 9 * ribosome 70S/b3384/second last codon EF-TU-tRNA/1 EF-G	C820023H1141341N289950O418389S2295P42838Mg1575Zn0Fe0	-35631.26	Translation
rib_70_elo2_b3385_14_cplx	Translation elongation complex: 14 * ribosome 70S/b3385/second last codon EF-TU-tRNA/1 EF-G	C1261815H1756936N446067O643579S3472P65837Mg2450Zn0Fe0	-54793.96	Translation
rib_70_elo2_b3385_1_cplx	Translation elongation complex: 1 * ribosome 70S/b3385/second last codon EF-TU-tRNA/1 EF-G	C96768H133002N34513O50857S248P5400Mg175Zn0Fe0	-4612.14	Translation
rib_70_elo2_b3385_7_cplx	Translation elongation complex: 7 * ribosome 70S/b3385/second last codon EF-TU-tRNA/1 EF-G	C634482H882510N224461O324421S1736P33294Mg1225Zn0Fe0	-27772.98	Translation
rib_70_elo2_b3386_13_cplx	Translation elongation complex: 13 * ribosome 70S/b3386/second last codon EF-TU-tRNA/1 EF-G	C1169629H1628649N413654O596664S3250P61104Mg2275Zn0Fe0	-50771.82	Translation
rib_70_elo2_b3386_1_cplx	Translation elongation complex: 1 * ribosome 70S/b3386/second last codon EF-TU-tRNA/1 EF-G	C95917H132009N34166O50292S250P5328Mg175Zn0Fe0	-4534.14	Translation
rib_70_elo2_b3386_6_cplx	Translation elongation complex: 6 * ribosome 70S/b3386/second last codon EF-TU-tRNA/1 EF-G	C543297H755609N192286O277947S1500P28568Mg1050Zn0Fe0	-23799.84	Translation
rib_70_elo2_b3387_16_cplx	Translation elongation complex: 16 * ribosome 70S/b3387/second last codon EF-TU-tRNA/1 EF-G	C1445504H2012514N510639O735888S4000P75207Mg2800Zn0Fe0	-62282.24	Translation
rib_70_elo2_b3387_1_cplx	Translation elongation complex: 1 * ribosome 70S/b3387/second last codon EF-TU-tRNA/1 EF-G	C97799H134214N34854O51498S250P5487Mg175Zn0Fe0	-4680.14	Translation
rib_70_elo2_b3387_8_cplx	Translation elongation complex: 8 * ribosome 70S/b3387/second last codon EF-TU-tRNA/1 EF-G	C726728H1010754N256887O370880S2000P38023Mg1400Zn0Fe0	-31561.12	Translation
rib_70_elo2_b3388_12_cplx	Translation elongation complex: 12 * ribosome 70S/b3388/second last codon EF-TU-tRNA/1 EF-G	C1096532H1528325N388058O559661S2940P57065Mg2100Zn0Fe0	-47467.68	Translation
rib_70_elo2_b3388_1_cplx	Translation elongation complex: 1 * ribosome 70S/b3388/second last codon EF-TU-tRNA/1 EF-G	C102638H140125N36949O54794S245P5937Mg175Zn0Fe0	-5138.14	Translation
rib_70_elo2_b3388_25_cplx	Translation elongation complex: 25 * ribosome 70S/b3388/second last codon EF-TU-tRNA/1 EF-G	C2271134H3168925N803005O1156322S6125P117489Mg4375Zn0Fe0	-97493.5	Translation
rib_70_elo2_b3389_10_cplx	Translation elongation complex: 10 * ribosome 70S/b3389/second last codon EF-TU-tRNA/1 EF-G	C911429H1269394N322157O464907S2550P47569Mg1750Zn0Fe0	-39601.4	Translation
rib_70_elo2_b3389_1_cplx	Translation elongation complex: 1 * ribosome 70S/b3389/second last codon EF-TU-tRNA/1 EF-G	C100457H137473N35894O53400S255P5737Mg175Zn0Fe0	-4941.14	Translation
rib_70_elo2_b3389_21_cplx	Translation elongation complex: 21 * ribosome 70S/b3389/second last codon EF-TU-tRNA/1 EF-G	C1902617H2652853N672034O967860S5355P98697Mg3675Zn0Fe0	-81963.94	Translation
rib_70_elo2_b3390_10_cplx	Translation elongation complex: 10 * ribosome 70S/b3390/second last codon EF-TU-tRNA/1 EF-G	C897337H1249325N317816O458512S2450P47004Mg1750Zn0Fe0	-39026.4	Translation
rib_70_elo2_b3390_1_cplx	Translation elongation complex: 1 * ribosome 70S/b3390/second last codon EF-TU-tRNA/1 EF-G	C94222H129995N33605O49120S245P5172Mg175Zn0Fe0	-4375.14	Translation
rib_70_elo2_b3390_5_cplx	Translation elongation complex: 5 * ribosome 70S/b3390/second last codon EF-TU-tRNA/1 EF-G	C451162H627475N159921O231072S1225P23764Mg875Zn0Fe0	-19775.7	Translation
rib_70_elo2_b3406_1_cplx	Translation elongation complex: 1 * ribosome 70S/b3406/second last codon EF-TU-tRNA/1 EF-G	C93778H129437N33414O48781S244P5128Mg175Zn0Fe0	-4324.14	Translation
rib_70_elo2_b3406_4_cplx	Translation elongation complex: 4 * ribosome 70S/b3406/second last codon EF-TU-tRNA/1 EF-G	C361450H502322N128115O185167S976P19075Mg700Zn0Fe0	-15856.56	Translation
rib_70_elo2_b3406_9_cplx	Translation elongation complex: 9 * ribosome 70S/b3406/second last codon EF-TU-tRNA/1 EF-G	C807570H1123797N285950O412477S2196P42320Mg1575Zn0Fe0	-35077.26	Translation
rib_70_elo2_b3461_16_cplx	Translation elongation complex: 16 * ribosome 70S/b3461/second last codon EF-TU-tRNA/1 EF-G	C1444985H2013018N511253O736296S4016P75225Mg2800Zn0Fe0	-62476.24	Translation
rib_70_elo2_b3461_1_cplx	Translation elongation complex: 1 * ribosome 70S/b3461/second last codon EF-TU-tRNA/1 EF-G	C97955H134463N35048O51606S251P5505Mg175Zn0Fe0	-4709.14	Translation

rib_70_elo2_b3461_8_cplx	Translation elongation complex: 8 * ribosome 70S/b3461/second last codon EF-TU-tRNA/1 EF-G	C726569H1011122N257277O371128S2008P38041Mg1400Zn0Fe0	-31667.12	Translation
rib_70_elo2_b3470_1_cplx	Translation elongation complex: 1 * ribosome 70S/b3470/second last codon EF-TU-tRNA/1 EF-G	C91108H126263N32352O47068S247P4896Mg175Zn0Fe0	-4096.14	Translation
rib_70_elo2_b3470_2_cplx	Translation elongation complex: 2 * ribosome 70S/b3470/second last codon EF-TU-tRNA/1 EF-G	C179884H249880N63791O92399S494P9544Mg350Zn0Fe0	-7943.28	Translation
rib_70_elo2_b3470_4_cplx	Translation elongation complex: 4 * ribosome 70S/b3470/second last codon EF-TU-tRNA/1 EF-G	C357436H497114N126669O183061S988P18840Mg700Zn0Fe0	-15637.56	Translation
rib_70_elo2_b3559_1_cplx	Translation elongation complex: 1 * ribosome 70S/b3559/second last codon EF-TU-tRNA/1 EF-G	C111508H150739N40203O60689S260P6717Mg175Zn0Fe0	-5932.14	Translation
rib_70_elo2_b3559_20_cplx	Translation elongation complex: 20 * ribosome 70S/b3559/second last codon EF-TU-tRNA/1 EF-G	C1855556H2590700N653371O938850S5200P95010Mg3500Zn0Fe0	-79293.8	Translation
rib_70_elo2_b3559_40_cplx	Translation elongation complex: 40 * ribosome 70S/b3559/second last codon EF-TU-tRNA/1 EF-G	C3691396H5159080N1298811O1863230S10400P187950Mg7000Zn0Fe0	-156516.6	Translation
rib_70_elo2_b3560_17_cplx	Translation elongation complex: 17 * ribosome 70S/b3560/second last codon EF-TU-tRNA/1 EF-G	C1537889H2140997N543001O782914S4318P79930Mg2975Zn0Fe0	-66503.38	Translation
rib_70_elo2_b3560_1_cplx	Translation elongation complex: 1 * ribosome 70S/b3560/second last codon EF-TU-tRNA/1 EF-G	C98625H135205N35209O52050S254P5562Mg175Zn0Fe0	-4773.14	Translation
rib_70_elo2_b3560_8_cplx	Translation elongation complex: 8 * ribosome 70S/b3560/second last codon EF-TU-tRNA/1 EF-G	C728303H1012739N257368O371803S2032P38098Mg1400Zn0Fe0	-31780.12	Translation
rib_70_elo2_b3590_18_cplx	Translation elongation complex: 18 * ribosome 70S/b3590/second last codon EF-TU-tRNA/1 EF-G	C1663278H2320732N587195O842806S4644P85505Mg3150Zn0Fe0	-71252.52	Translation
rib_70_elo2_b3590_1_cplx	Translation elongation complex: 1 * ribosome 70S/b3590/second last codon EF-TU-tRNA/1 EF-G	C108968H147690N39302O58970S258P6489Mg175Zn0Fe0	-5698.14	Translation
rib_70_elo2_b3590_36_cplx	Translation elongation complex: 36 * ribosome 70S/b3590/second last codon EF-TU-tRNA/1 EF-G	C3309018H4621600N1167317O1672750S9288P169169Mg6300Zn0Fe0	-140663.04	Translation
rib_70_elo2_b3591_13_cplx	Translation elongation complex: 13 * ribosome 70S/b3591/second last codon EF-TU-tRNA/1 EF-G	C1191161H1661048N420945O606281S3354P61818Mg2275Zn0Fe0	-51459.82	Translation
rib_70_elo2_b3591_1_cplx	Translation elongation complex: 1 * ribosome 70S/b3591/second last codon EF-TU-tRNA/1 EF-G	C103865H141620N37293O55649S258P6042Mg175Zn0Fe0	-5246.14	Translation
rib_70_elo2_b3591_27_cplx	Translation elongation complex: 27 * ribosome 70S/b3591/second last codon EF-TU-tRNA/1 EF-G	C2459673H3433714N868539O1248685S6966P126890Mg4725Zn0Fe0	-105375.78	Translation
rib_70_elo2_b3635_15_cplx	Translation elongation complex: 15 * ribosome 70S/b3635/second last codon EF-TU-tRNA/1 EF-G	C1353904H1886169N478786O689647S3765P70532Mg2625Zn0Fe0	-58430.1	Translation
rib_70_elo2_b3635_1_cplx	Translation elongation complex: 1 * ribosome 70S/b3635/second last codon EF-TU-tRNA/1 EF-G	C97474H133887N34832O51275S251P5460Mg175Zn0Fe0	-4654.14	Translation
rib_70_elo2_b3635_7_cplx	Translation elongation complex: 7 * ribosome 70S/b3635/second last codon EF-TU-tRNA/1 EF-G	C635944H884865N225098O324863S1757P33348Mg1225Zn0Fe0	-27700.98	Translation
rib_70_elo2_b3636_1_cplx	Translation elongation complex: 1 * ribosome 70S/b3636/second last codon EF-TU-tRNA/1 EF-G	C90283H125298N32065O46448S242P4816Mg175Zn0Fe0	-4004.14	Translation
rib_70_elo2_b3636_3_cplx	Translation elongation complex: 3 * ribosome 70S/b3636/second last codon EF-TU-tRNA/1 EF-G	C267635H372274N94885O137034S726P14112Mg525Zn0Fe0	-11674.42	Translation
rib_70_elo2_b3637_1_cplx	Translation elongation complex: 1 * ribosome 70S/b3637/second last codon EF-TU-tRNA/1 EF-G	C91119H126312N32391O47037S245P4896Mg175Zn0Fe0	-4081.14	Translation
rib_70_elo2_b3637_2_cplx	Translation elongation complex: 2 * ribosome 70S/b3637/second last codon EF-TU-tRNA/1 EF-G	C179987H250070N63889O92411S490P9553Mg350Zn0Fe0	-7922.28	Translation
rib_70_elo2_b3637_4_cplx	Translation elongation complex: 4 * ribosome 70S/b3637/second last codon EF-TU-tRNA/1 EF-G	C357723H497586N126885O183159S980P18867Mg700Zn0Fe0	-15604.56	Translation
rib_70_elo2_b3638_13_cplx	Translation elongation complex: 13 * ribosome 70S/b3638/second last codon EF-TU-tRNA/1 EF-G	C1170262H1629923N414114O596738S3250P61108Mg2275Zn0Fe0	-50645.82	Translation
rib_70_elo2_b3638_1_cplx	Translation elongation complex: 1 * ribosome 70S/b3638/second last codon EF-TU-tRNA/1 EF-G	C95902H132011N34194O50234S250P5320Mg175Zn0Fe0	-4516.14	Translation
rib_70_elo2_b3638_6_cplx	Translation elongation complex: 6 * ribosome 70S/b3638/second last codon EF-TU-tRNA/1 EF-G	C543552H756141N192494O277944S1500P28565Mg1050Zn0Fe0	-23736.84	Translation
rib_70_elo2_b3649_1_cplx	Translation elongation complex: 1 * ribosome 70S/b3649/second last codon EF-TU-tRNA/1 EF-G	C91468H126716N32566O47270S243P4927Mg175Zn0Fe0	-4128.14	Translation
rib_70_elo2_b3649_2_cplx	Translation elongation complex: 2 * ribosome 70S/b3649/second last codon EF-TU-tRNA/1 EF-G	C180296H250447N64044O92626S486P9576Mg350Zn0Fe0	-7977.28	Translation
rib_70_elo2_b3649_5_cplx	Translation elongation complex: 5 * ribosome 70S/b3649/second last codon EF-TU-tRNA/1 EF-G	C446780H621640N158478O228694S1215P23523Mg875Zn0Fe0	-19524.7	Translation
rib_70_elo2_b3650_1_cplx	Translation elongation complex: 1 * ribosome 70S/b3650/second last codon EF-TU-tRNA/1 EF-G	C111965H151396N40396O60933S272P6757Mg175Zn0Fe0	-5946.14	Translation

rib_70_elo2_b3650_20_cplx	Translation elongation complex: 20 * ribosome 70S/b3650/second last codon EF-TU-tRNA/1 EF-G	C1857951H2595727N654894O939474S5440P95069Mg3500Zn0Fe0	-78832.8	Translation
rib_70_elo2_b3650_41_cplx	Translation elongation complex: 41 * ribosome 70S/b3650/second last codon EF-TU-tRNA/1 EF-G	C3787725H5297356N1334076O1910493S11152P192677Mg7175Zn0Fe0	-159391.74	Translation
rib_70_elo2_b3651_13_cplx	Translation elongation complex: 13 * ribosome 70S/b3651/second last codon EF-TU-tRNA/1 EF-G	C1169519H1628821N414022O596608S3328P61088Mg2275Zn0Fe0	-50651.82	Translation
rib_70_elo2_b3651_1_cplx	Translation elongation complex: 1 * ribosome 70S/b3651/second last codon EF-TU-tRNA/1 EF-G	C96023H132169N34282O50332S256P5336Mg175Zn0Fe0	-4534.14	Translation
rib_70_elo2_b3651_6_cplx	Translation elongation complex: 6 * ribosome 70S/b3651/second last codon EF-TU-tRNA/1 EF-G	C543313H755774N192507O277947S1536P28566Mg1050Zn0Fe0	-23749.84	Translation
rib_70_elo2_b3652_1_cplx	Translation elongation complex: 1 * ribosome 70S/b3652/second last codon EF-TU-tRNA/1 EF-G	C111587H150959N40311O60701S267P6730Mg175Zn0Fe0	-5931.14	Translation
rib_70_elo2_b3652_20_cplx	Translation elongation complex: 20 * ribosome 70S/b3652/second last codon EF-TU-tRNA/1 EF-G	C1855103H2591813N654106O938444S5340P95042Mg3500Zn0Fe0	-79045.8	Translation
rib_70_elo2_b3652_40_cplx	Translation elongation complex: 40 * ribosome 70S/b3652/second last codon EF-TU-tRNA/1 EF-G	C3690383H5161133N1300206O1862384S10680P188002Mg7000Zn0Fe0	-156008.6	Translation
rib_70_elo2_b3703_1_cplx	Translation elongation complex: 1 * ribosome 70S/b3703/second last codon EF-TU-tRNA/1 EF-G	C89948H124926N31931O46275S243P4791Mg175Zn0Fe0	-3973.14	Translation
rib_70_elo2_b3703_2_cplx	Translation elongation complex: 2 * ribosome 70S/b3703/second last codon EF-TU-tRNA/1 EF-G	C178563H248338N63349O91548S486P9439Mg350Zn0Fe0	-7802.28	Translation
rib_70_elo2_b3704_1_cplx	Translation elongation complex: 1 * ribosome 70S/b3704/second last codon EF-TU-tRNA/1 EF-G	C92519H128020N32932O47972S243P5020Mg175Zn0Fe0	-4201.14	Translation
rib_70_elo2_b3704_3_cplx	Translation elongation complex: 3 * ribosome 70S/b3704/second last codon EF-TU-tRNA/1 EF-G	C270731H376300N96106O138868S729P14340Mg525Zn0Fe0	-11881.42	Translation
rib_70_elo2_b3704_7_cplx	Translation elongation complex: 7 * ribosome 70S/b3704/second last codon EF-TU-tRNA/1 EF-G	C627155H872860N222454O320660S1701P32980Mg1225Zn0Fe0	-27241.98	Translation
rib_70_elo2_b3706_13_cplx	Translation elongation complex: 13 * ribosome 70S/b3706/second last codon EF-TU-tRNA/1 EF-G	C1190250H1658812N420540O606047S3237P61791Mg2275Zn0Fe0	-51653.82	Translation
rib_70_elo2_b3706_1_cplx	Translation elongation complex: 1 * ribosome 70S/b3706/second last codon EF-TU-tRNA/1 EF-G	C103554H141196N37176O55427S249P6015Mg175Zn0Fe0	-5236.14	Translation
rib_70_elo2_b3706_26_cplx	Translation elongation complex: 26 * ribosome 70S/b3706/second last codon EF-TU-tRNA/1 EF-G	C2367504H3302896N835851O1202552S6474P122215Mg4550Zn0Fe0	-101939.64	Translation
rib_70_elo2_b3740_12_cplx	Translation elongation complex: 12 * ribosome 70S/b3740/second last codon EF-TU-tRNA/1 EF-G	C1079403H1502889N381902O550621S2952P56412Mg2100Zn0Fe0	-46826.68	Translation
rib_70_elo2_b3740_1_cplx	Translation elongation complex: 1 * ribosome 70S/b3740/second last codon EF-TU-tRNA/1 EF-G	C95387H131387N33972O49890S246P5273Mg175Zn0Fe0	-4475.14	Translation
rib_70_elo2_b3740_6_cplx	Translation elongation complex: 6 * ribosome 70S/b3740/second last codon EF-TU-tRNA/1 EF-G	C542667H754797N192122O277495S1476P28518Mg1050Zn0Fe0	-23725.84	Translation
rib_70_elo2_b3741_18_cplx	Translation elongation complex: 18 * ribosome 70S/b3741/second last codon EF-TU-tRNA/1 EF-G	C1663899H2322135N587018O843671S4716P85556Mg3150Zn0Fe0	-71195.52	Translation
rib_70_elo2_b3741_1_cplx	Translation elongation complex: 1 * ribosome 70S/b3741/second last codon EF-TU-tRNA/1 EF-G	C109419H148243N39397O59359S262P6540Mg175Zn0Fe0	-5743.14	Translation
rib_70_elo2_b3741_37_cplx	Translation elongation complex: 37 * ribosome 70S/b3741/second last codon EF-TU-tRNA/1 EF-G	C3401259H4751779N1199065O1720255S9694P173868Mg6475Zn0Fe0	-144348.18	Translation
rib_70_elo2_b3780_12_cplx	Translation elongation complex: 12 * ribosome 70S/b3780/second last codon EF-TU-tRNA/1 EF-G	C1097652H1529376N388007O558722S3036P57044Mg2100Zn0Fe0	-47398.68	Translation
rib_70_elo2_b3780_1_cplx	Translation elongation complex: 1 * ribosome 70S/b3780/second last codon EF-TU-tRNA/1 EF-G	C102504H139944N36722O54680S253P5916Mg175Zn0Fe0	-5113.14	Translation
rib_70_elo2_b3780_24_cplx	Translation elongation complex: 24 * ribosome 70S/b3780/second last codon EF-TU-tRNA/1 EF-G	C2183268H3045120N771227O1108586S6072P112820Mg4200Zn0Fe0	-93528.36	Translation
rib_70_elo2_b3782_1_cplx	Translation elongation complex: 1 * ribosome 70S/b3782/second last codon EF-TU-tRNA/1 EF-G	C89513H124353N31769O45990S244P4753Mg175Zn0Fe0	-3946.14	Translation
rib_70_elo2_b3783_12_cplx	Translation elongation complex: 12 * ribosome 70S/b3783/second last codon EF-TU-tRNA/1 EF-G	C1098919H1531575N388456O559723S3108P57180Mg2100Zn0Fe0	-47558.68	Translation
rib_70_elo2_b3783_1_cplx	Translation elongation complex: 1 * ribosome 70S/b3783/second last codon EF-TU-tRNA/1 EF-G	C102561H140064N36742O54702S259P5920Mg175Zn0Fe0	-5119.14	Translation
rib_70_elo2_b3783_24_cplx	Translation elongation complex: 24 * ribosome 70S/b3783/second last codon EF-TU-tRNA/1 EF-G	C2185855H3049587N772144O1110655S6216P113100Mg4200Zn0Fe0	-93856.36	Translation
rib_70_elo2_b3885_11_cplx	Translation elongation complex: 11 * ribosome 70S/b3885/second last codon EF-TU-tRNA/1 EF-G	C988987H1376156N349840O504608S2739P51708Mg1925Zn0Fe0	-42998.54	Translation
rib_70_elo2_b3885_1_cplx	Translation elongation complex: 1 * ribosome 70S/b3885/second last codon EF-TU-tRNA/1 EF-G	C95097H130966N33860O49708S249P5248Mg175Zn0Fe0	-4457.14	Translation

rib_70_elo2_b3885_5_cplx	Translation elongation complex: 5 * ribosome 70S/b3885/second last codon EF-TU-tRNA/1 EF-G	C452653H629042N160252O231668S1245P23832Mg875Zn0Fe0	-19873.7	Translation
rib_70_elo2_b3886_17_cplx	Translation elongation complex: 17 * ribosome 70S/b3886/second last codon EF-TU-tRNA/1 EF-G	C1537181H2141029N542252O781537S4250P79899Mg2975Zn0Fe0	-66183.38	Translation
rib_70_elo2_b3886_1_cplx	Translation elongation complex: 1 * ribosome 70S/b3886/second last codon EF-TU-tRNA/1 EF-G	C98125H134677N34844O51745S250P5515Mg175Zn0Fe0	-4709.14	Translation
rib_70_elo2_b3886_8_cplx	Translation elongation complex: 8 * ribosome 70S/b3886/second last codon EF-TU-tRNA/1 EF-G	C727712H1012456N256835O371029S2000P38058Mg1400Zn0Fe0	-31604.12	Translation
rib_70_elo2_b3887_1_cplx	Translation elongation complex: 1 * ribosome 70S/b3887/second last codon EF-TU-tRNA/1 EF-G	C93216H128772N33218O48457S248P5082Mg175Zn0Fe0	-4285.14	Translation
rib_70_elo2_b3887_4_cplx	Translation elongation complex: 4 * ribosome 70S/b3887/second last codon EF-TU-tRNA/1 EF-G	C360435H501039N127820O184741S992P19026Mg700Zn0Fe0	-15835.56	Translation
rib_70_elo2_b3887_8_cplx	Translation elongation complex: 8 * ribosome 70S/b3887/second last codon EF-TU-tRNA/1 EF-G	C716727H997395N253956O366453S1984P37618Mg1400Zn0Fe0	-31236.12	Translation
rib_70_elo2_b3888_19_cplx	Translation elongation complex: 19 * ribosome 70S/b3888/second last codon EF-TU-tRNA/1 EF-G	C1719897H2396108N607932O874930S4864P89298Mg3325Zn0Fe0	-74177.66	Translation
rib_70_elo2_b3888_1_cplx	Translation elongation complex: 1 * ribosome 70S/b3888/second last codon EF-TU-tRNA/1 EF-G	C99429H136190N35604O52564S256P5634Mg175Zn0Fe0	-4839.14	Translation
rib_70_elo2_b3888_9_cplx	Translation elongation complex: 9 * ribosome 70S/b3888/second last codon EF-TU-tRNA/1 EF-G	C819637H1140598N289972O418060S2304P42818Mg1575Zn0Fe0	-35656.26	Translation
rib_70_elo2_b3936_1_cplx	Translation elongation complex: 1 * ribosome 70S/b3936/second last codon EF-TU-tRNA/1 EF-G	C90751H125842N32239O46802S247P4863Mg175Zn0Fe0	-4055.14	Translation
rib_70_elo2_b3936_2_cplx	Translation elongation complex: 2 * ribosome 70S/b3936/second last codon EF-TU-tRNA/1 EF-G	C179477H249388N63671O92118S494P9511Mg350Zn0Fe0	-7894.28	Translation
rib_70_elo2_b3936_4_cplx	Translation elongation complex: 4 * ribosome 70S/b3936/second last codon EF-TU-tRNA/1 EF-G	C356929H496480N126535O182750S988P18807Mg700Zn0Fe0	-15572.56	Translation
rib_70_elo2_b3965_10_cplx	Translation elongation complex: 10 * ribosome 70S/b3965/second last codon EF-TU-tRNA/1 EF-G	C912949H1271040N322661O465377S2590P47583Mg1750Zn0Fe0	-39645.4	Translation
rib_70_elo2_b3965_1_cplx	Translation elongation complex: 1 * ribosome 70S/b3965/second last codon EF-TU-tRNA/1 EF-G	C100735H137778N36056O53447S259P5751Mg175Zn0Fe0	-4958.14	Translation
rib_70_elo2_b3965_21_cplx	Translation elongation complex: 21 * ribosome 70S/b3965/second last codon EF-TU-tRNA/1 EF-G	C1905655H2656138N672956O968847S5439P98711Mg3675Zn0Fe0	-82040.94	Translation
rib_70_elo2_b3980_11_cplx	Translation elongation complex: 11 * ribosome 70S/b3980/second last codon EF-TU-tRNA/1 EF-G	C1006440H1401304N355788O513262S2816P52502Mg1925Zn0Fe0	-43836.54	Translation
rib_70_elo2_b3980_1_cplx	Translation elongation complex: 1 * ribosome 70S/b3980/second last codon EF-TU-tRNA/1 EF-G	C101750H139004N36458O54182S256P5852Mg175Zn0Fe0	-5065.14	Translation
rib_70_elo2_b3980_23_cplx	Translation elongation complex: 23 * ribosome 70S/b3980/second last codon EF-TU-tRNA/1 EF-G	C2092068H2916064N738984O1064158S5888P108482Mg4025Zn0Fe0	-90362.22	Translation
rib_70_elo2_b3981_1_cplx	Translation elongation complex: 1 * ribosome 70S/b3981/second last codon EF-TU-tRNA/1 EF-G	C92664H128168N32942O48094S246P5034Mg175Zn0Fe0	-4225.14	Translation
rib_70_elo2_b3981_3_cplx	Translation elongation complex: 3 * ribosome 70S/b3981/second last codon EF-TU-tRNA/1 EF-G	C270692H376254N95946O138838S738P14330Mg525Zn0Fe0	-11901.42	Translation
rib_70_elo2_b3981_7_cplx	Translation elongation complex: 7 * ribosome 70S/b3981/second last codon EF-TU-tRNA/1 EF-G	C626748H872426N221954O320326S1722P32922Mg1225Zn0Fe0	-27253.98	Translation
rib_70_elo2_b3982_10_cplx	Translation elongation complex: 10 * ribosome 70S/b3982/second last codon EF-TU-tRNA/1 EF-G	C898120H1250041N318004O458571S2480P47026Mg1750Zn0Fe0	-38998.4	Translation
rib_70_elo2_b3982_1_cplx	Translation elongation complex: 1 * ribosome 70S/b3982/second last codon EF-TU-tRNA/1 EF-G	C94492H130288N33667O49305S248P5194Mg175Zn0Fe0	-4392.14	Translation
rib_70_elo2_b3982_5_cplx	Translation elongation complex: 5 * ribosome 70S/b3982/second last codon EF-TU-tRNA/1 EF-G	C451660H627956N160039O231201S1240P23786Mg875Zn0Fe0	-19772.7	Translation
rib_70_elo2_b3983_1_cplx	Translation elongation complex: 1 * ribosome 70S/b3983/second last codon EF-TU-tRNA/1 EF-G	C93139H128729N33166O48405S248P5080Mg175Zn0Fe0	-4270.14	Translation
rib_70_elo2_b3983_4_cplx	Translation elongation complex: 4 * ribosome 70S/b3983/second last codon EF-TU-tRNA/1 EF-G	C360289H501029N127708O184659S992P19027Mg700Zn0Fe0	-15784.56	Translation
rib_70_elo2_b3983_8_cplx	Translation elongation complex: 8 * ribosome 70S/b3983/second last codon EF-TU-tRNA/1 EF-G	C716489H997429N253764O366331S1984P37623Mg1400Zn0Fe0	-31137.12	Translation
rib_70_elo2_b3984_13_cplx	Translation elongation complex: 13 * ribosome 70S/b3984/second last codon EF-TU-tRNA/1 EF-G	C1169842H1630206N414116O596983S3224P61129Mg2275Zn0Fe0	-50575.82	Translation
rib_70_elo2_b3984_1_cplx	Translation elongation complex: 1 * ribosome 70S/b3984/second last codon EF-TU-tRNA/1 EF-G	C96202H132426N34388O50431S248P5353Mg175Zn0Fe0	-4542.14	Translation
rib_70_elo2_b3984_6_cplx	Translation elongation complex: 6 * ribosome 70S/b3984/second last codon EF-TU-tRNA/1 EF-G	C543552H756501N192608O278161S1488P28593Mg1050Zn0Fe0	-23722.84	Translation

rib_70_elo2_b3985_1_cplx	Translation elongation complex: 1 * ribosome 70S/b3985/second last codon EF-TU-tRNA/1 EF-G	C93902H129634N33452O48932S247P5148Mg175Zn0Fe0	-4343.14	Translation
rib_70_elo2_b3985_4_cplx	Translation elongation complex: 4 * ribosome 70S/b3985/second last codon EF-TU-tRNA/1 EF-G	C361382H502447N128114O185264S988P19092Mg700Zn0Fe0	-15869.56	Translation
rib_70_elo2_b3985_9_cplx	Translation elongation complex: 9 * ribosome 70S/b3985/second last codon EF-TU-tRNA/1 EF-G	C807182H1123802N285884O412484S2223P42332Mg1575Zn0Fe0	-35080.26	Translation
rib_70_elo2_b3986_1_cplx	Translation elongation complex: 1 * ribosome 70S/b3986/second last codon EF-TU-tRNA/1 EF-G	C92431H127835N32902O47929S245P5014Mg175Zn0Fe0	-4220.14	Translation
rib_70_elo2_b3986_3_cplx	Translation elongation complex: 3 * ribosome 70S/b3986/second last codon EF-TU-tRNA/1 EF-G	C270283H375621N95836O138715S735P14310Mg525Zn0Fe0	-11926.42	Translation
rib_70_elo2_b3986_7_cplx	Translation elongation complex: 7 * ribosome 70S/b3986/second last codon EF-TU-tRNA/1 EF-G	C625987H871193N221704O320287S1715P32902Mg1225Zn0Fe0	-27338.98	Translation
rib_70_elo2_b3987_1_cplx	Translation elongation complex: 1 * ribosome 70S/b3987/second last codon EF-TU-tRNA/1 EF-G	C133383H177014N48578O75341S285P8677Mg175Zn0Fe0	-7918.14	Translation
rib_70_elo2_b3987_39_cplx	Translation elongation complex: 39 * ribosome 70S/b3987/second last codon EF-TU-tRNA/1 EF-G	C3744067H5253282N1309228O1871297S11115P185301Mg6825Zn0Fe0	-155667.46	Translation
rib_70_elo2_b3987_78_cplx	Translation elongation complex: 78 * ribosome 70S/b3987/second last codon EF-TU-tRNA/1 EF-G	C7449769H10463136N2603053O3714515S22230P366573Mg13650Zn0Fe0	-307304.92	Translation
rib_70_elo2_b3988_1_cplx	Translation elongation complex: 1 * ribosome 70S/b3988/second last codon EF-TU-tRNA/1 EF-G	C135423H179624N49402O76768S292P8872Mg175Zn0Fe0	-8073.14	Translation
rib_70_elo2_b3988_41_cplx	Translation elongation complex: 41 * ribosome 70S/b3988/second last codon EF-TU-tRNA/1 EF-G	C3943783H5543504N1380282O1967688S11972P194792Mg7175Zn0Fe0	-161998.74	Translation
rib_70_elo2_b3988_82_cplx	Translation elongation complex: 82 * ribosome 70S/b3988/second last codon EF-TU-tRNA/1 EF-G	C7847352H11041481N2744434O3905881S23944P385360Mg14350Zn0Fe0	-319772.48	Translation
rib_70_elo2_b4022_17_cplx	Translation elongation complex: 17 * ribosome 70S/b4022/second last codon EF-TU-tRNA/1 EF-G	C1535366H2140883N543326O781869S4233P79908Mg2975Zn0Fe0	-65988.38	Translation
rib_70_elo2_b4022_1_cplx	Translation elongation complex: 1 * ribosome 70S/b4022/second last codon EF-TU-tRNA/1 EF-G	C98166H134803N35166O51693S249P5524Mg175Zn0Fe0	-4706.14	Translation
rib_70_elo2_b4022_8_cplx	Translation elongation complex: 8 * ribosome 70S/b4022/second last codon EF-TU-tRNA/1 EF-G	C726941H1012463N257486O371145S1992P38067Mg1400Zn0Fe0	-31517.12	Translation
rib_70_elo2_b4049_10_cplx	Translation elongation complex: 10 * ribosome 70S/b4049/second last codon EF-TU-tRNA/1 EF-G	C910731H1267722N322225O464436S2620P47530Mg1750Zn0Fe0	-39552.4	Translation
rib_70_elo2_b4049_1_cplx	Translation elongation complex: 1 * ribosome 70S/b4049/second last codon EF-TU-tRNA/1 EF-G	C99984H136836N35818O52983S262P5689Mg175Zn0Fe0	-4892.14	Translation
rib_70_elo2_b4049_20_cplx	Translation elongation complex: 20 * ribosome 70S/b4049/second last codon EF-TU-tRNA/1 EF-G	C1811561H2524262N640455O921606S5240P94020Mg3500Zn0Fe0	-78063.8	Translation
rib_70_elo2_b4129_14_cplx	Translation elongation complex: 14 * ribosome 70S/b4129/second last codon EF-TU-tRNA/1 EF-G	C1287799H1794134N454293O654461S3640P66592Mg2450Zn0Fe0	-55646.96	Translation
rib_70_elo2_b4129_1_cplx	Translation elongation complex: 1 * ribosome 70S/b4129/second last codon EF-TU-tRNA/1 EF-G	C105397H143290N37760O56617S260P6168Mg175Zn0Fe0	-5387.14	Translation
rib_70_elo2_b4129_29_cplx	Translation elongation complex: 29 * ribosome 70S/b4129/second last codon EF-TU-tRNA/1 EF-G	C2652109H3698954N934908O1344281S7540P136312Mg5075Zn0Fe0	-113639.06	Translation
rib_70_elo2_b4142_1_cplx	Translation elongation complex: 1 * ribosome 70S/b4142/second last codon EF-TU-tRNA/1 EF-G	C91641H126904N32594O47415S242P4944Mg175Zn0Fe0	-4145.14	Translation
rib_70_elo2_b4142_2_cplx	Translation elongation complex: 2 * ribosome 70S/b4142/second last codon EF-TU-tRNA/1 EF-G	C180474H250645N64054O92772S484P9592Mg350Zn0Fe0	-7993.28	Translation
rib_70_elo2_b4142_5_cplx	Translation elongation complex: 5 * ribosome 70S/b4142/second last codon EF-TU-tRNA/1 EF-G	C446973H621868N158434O228843S1210P23536Mg875Zn0Fe0	-19537.7	Translation
rib_70_elo2_b4143_16_cplx	Translation elongation complex: 16 * ribosome 70S/b4143/second last codon EF-TU-tRNA/1 EF-G	C1469817H2051811N518878O747790S4272P76033Mg2800Zn0Fe0	-63492.24	Translation
rib_70_elo2_b4143_1_cplx	Translation elongation complex: 1 * ribosome 70S/b4143/second last codon EF-TU-tRNA/1 EF-G	C106617H144906N38458O57460S267P6298Mg175Zn0Fe0	-5515.14	Translation
rib_70_elo2_b4143_32_cplx	Translation elongation complex: 32 * ribosome 70S/b4143/second last codon EF-TU-tRNA/1 EF-G	C2923897H4085843N1031326O1484142S8544P150417Mg5600Zn0Fe0	-125334.48	Translation
rib_70_elo2_b4162_10_cplx	Translation elongation complex: 10 * ribosome 70S/b4162/second last codon EF-TU-tRNA/1 EF-G	C899250H1251210N318363O459452S2470P47138Mg1750Zn0Fe0	-39200.4	Translation
rib_70_elo2_b4162_1_cplx	Translation elongation complex: 1 * ribosome 70S/b4162/second last codon EF-TU-tRNA/1 EF-G	C94614H130422N33738O49376S247P5207Mg175Zn0Fe0	-4414.14	Translation
rib_70_elo2_b4162_5_cplx	Translation elongation complex: 5 * ribosome 70S/b4162/second last codon EF-TU-tRNA/1 EF-G	C452230H628550N160238O231632S1235P23843Mg875Zn0Fe0	-19874.7	Translation
rib_70_elo2_b4167_15_cplx	Translation elongation complex: 15 * ribosome 70S/b4167/second last codon EF-TU-tRNA/1 EF-G	C1376638H1919339N486576O699904S3855P71285Mg2625Zn0Fe0	-59393.1	Translation

rib_70_elo2_b4167_1_cplx	Translation elongation complex: 1 * ribosome 70S/b4167/second last codon EF-TU-tRNA/1 EF-G	C105536H143537N37974O56786S257P6199Mg175Zn0Fe0	-5407.14	Translation
rib_70_elo2_b4167_30_cplx	Translation elongation complex: 30 * ribosome 70S/b4167/second last codon EF-TU-tRNA/1 EF-G	C2738533H3821984N967221O1388959S7710P141020Mg5250Zn0Fe0	-117235.2	Translation
rib_70_elo2_b4168_1_cplx	Translation elongation complex: 1 * ribosome 70S/b4168/second last codon EF-TU-tRNA/1 EF-G	C93245H128790N33157O48487S246P5081Mg175Zn0Fe0	-4289.14	Translation
rib_70_elo2_b4168_4_cplx	Translation elongation complex: 4 * ribosome 70S/b4168/second last codon EF-TU-tRNA/1 EF-G	C360632H501195N127744O184810S984P19025Mg700Zn0Fe0	-15854.56	Translation
rib_70_elo2_b4168_9_cplx	Translation elongation complex: 9 * ribosome 70S/b4168/second last codon EF-TU-tRNA/1 EF-G	C806277H1121870N285389O412015S2214P42265Mg1575Zn0Fe0	-35130.26	Translation
rib_70_elo2_b4169_13_cplx	Translation elongation complex: 13 * ribosome 70S/b4169/second last codon EF-TU-tRNA/1 EF-G	C1189085H1657454N420453O605616S3237P61762Mg2275Zn0Fe0	-51247.82	Translation
rib_70_elo2_b4169_1_cplx	Translation elongation complex: 1 * ribosome 70S/b4169/second last codon EF-TU-tRNA/1 EF-G	C103229H140822N37077O55200S249P5986Mg175Zn0Fe0	-5178.14	Translation
rib_70_elo2_b4169_26_cplx	Translation elongation complex: 26 * ribosome 70S/b4169/second last codon EF-TU-tRNA/1 EF-G	C2365429H3300472N835777O1201900S6474P122186Mg4550Zn0Fe0	-101156.64	Translation
rib_70_elo2_b4170_18_cplx	Translation elongation complex: 18 * ribosome 70S/b4170/second last codon EF-TU-tRNA/1 EF-G	C1662612H2320699N586764O842529S4590P85512Mg3150Zn0Fe0	-71151.52	Translation
rib_70_elo2_b4170_1_cplx	Translation elongation complex: 1 * ribosome 70S/b4170/second last codon EF-TU-tRNA/1 EF-G	C108982H147776N39296O58965S255P6496Mg175Zn0Fe0	-5699.14	Translation
rib_70_elo2_b4170_36_cplx	Translation elongation complex: 36 * ribosome 70S/b4170/second last codon EF-TU-tRNA/1 EF-G	C3307632H4621441N1166436O1672185S9180P169176Mg6300Zn0Fe0	-140454.04	Translation
rib_70_elo2_b4171_18_cplx	Translation elongation complex: 18 * ribosome 70S/b4171/second last codon EF-TU-tRNA/1 EF-G	C1627868H2268355N575456O828678S4500P84607Mg3150Zn0Fe0	-70264.52	Translation
rib_70_elo2_b4171_1_cplx	Translation elongation complex: 1 * ribosome 70S/b4171/second last codon EF-TU-tRNA/1 EF-G	C98922H135603N35366O52288S250P5591Mg175Zn0Fe0	-4795.14	Translation
rib_70_elo2_b4171_9_cplx	Translation elongation complex: 9 * ribosome 70S/b4171/second last codon EF-TU-tRNA/1 EF-G	C818426H1139251N289526O417648S2250P42775Mg1575Zn0Fe0	-35604.26	Translation
rib_70_elo2_b4172_1_cplx	Translation elongation complex: 1 * ribosome 70S/b4172/second last codon EF-TU-tRNA/1 EF-G	C91798H127072N32640O47522S243P4957Mg175Zn0Fe0	-4154.14	Translation
rib_70_elo2_b4172_3_cplx	Translation elongation complex: 3 * ribosome 70S/b4172/second last codon EF-TU-tRNA/1 EF-G	C269518H374570N95592O138246S729P14253Mg525Zn0Fe0	-11842.42	Translation
rib_70_elo2_b4172_6_cplx	Translation elongation complex: 6 * ribosome 70S/b4172/second last codon EF-TU-tRNA/1 EF-G	C536098H745817N190020O274332S1458P28197Mg1050Zn0Fe0	-23374.84	Translation
rib_70_elo2_b4173_12_cplx	Translation elongation complex: 12 * ribosome 70S/b4173/second last codon EF-TU-tRNA/1 EF-G	C1098529H1531031N388494O559307S2988P57069Mg2100Zn0Fe0	-47555.68	Translation
rib_70_elo2_b4173_1_cplx	Translation elongation complex: 1 * ribosome 70S/b4173/second last codon EF-TU-tRNA/1 EF-G	C102732H140235N36868O54825S249P5930Mg175Zn0Fe0	-5138.14	Translation
rib_70_elo2_b4173_25_cplx	Translation elongation complex: 25 * ribosome 70S/b4173/second last codon EF-TU-tRNA/1 EF-G	C2275380H3174699N804052O1155513S6225P117506Mg4375Zn0Fe0	-97685.5	Translation
rib_70_elo2_b4174_12_cplx	Translation elongation complex: 12 * ribosome 70S/b4174/second last codon EF-TU-tRNA/1 EF-G	C1096178H1527402N387981O558843S3000P57036Mg2100Zn0Fe0	-47402.68	Translation
rib_70_elo2_b4174_1_cplx	Translation elongation complex: 1 * ribosome 70S/b4174/second last codon EF-TU-tRNA/1 EF-G	C102383H139774N36861O54581S250P5908Mg175Zn0Fe0	-5106.14	Translation
rib_70_elo2_b4174_24_cplx	Translation elongation complex: 24 * ribosome 70S/b4174/second last codon EF-TU-tRNA/1 EF-G	C2180318H3041178N771021O1108947S6000P112812Mg4200Zn0Fe0	-93544.36	Translation
rib_70_elo2_b4175_19_cplx	Translation elongation complex: 19 * ribosome 70S/b4175/second last codon EF-TU-tRNA/1 EF-G	C1720470H2398600N608321O875730S4769P89336Mg3325Zn0Fe0	-74082.66	Translation
rib_70_elo2_b4175_1_cplx	Translation elongation complex: 1 * ribosome 70S/b4175/second last codon EF-TU-tRNA/1 EF-G	C99624H136504N35669O52734S251P5654Mg175Zn0Fe0	-4852.14	Translation
rib_70_elo2_b4175_9_cplx	Translation elongation complex: 9 * ribosome 70S/b4175/second last codon EF-TU-tRNA/1 EF-G	C820000H1141880N290181O418510S2259P42846Mg1575Zn0Fe0	-35621.26	Translation
rib_70_elo2_b4178_1_cplx	Translation elongation complex: 1 * ribosome 70S/b4178/second last codon EF-TU-tRNA/1 EF-G	C93120H128679N33122O48415S248P5076Mg175Zn0Fe0	-4272.14	Translation
rib_70_elo2_b4178_4_cplx	Translation elongation complex: 4 * ribosome 70S/b4178/second last codon EF-TU-tRNA/1 EF-G	C360318H501006N127685O184651S992P19020Mg700Zn0Fe0	-15801.56	Translation
rib_70_elo2_b4178_8_cplx	Translation elongation complex: 8 * ribosome 70S/b4178/second last codon EF-TU-tRNA/1 EF-G	C716582H997442N253769O366299S1984P37612Mg1400Zn0Fe0	-31174.12	Translation
rib_70_elo2_b4179_1_cplx	Translation elongation complex: 1 * ribosome 70S/b4179/second last codon EF-TU-tRNA/1 EF-G	C115724H155852N41973O63423S271P7090Mg175Zn0Fe0	-6278.14	Translation
rib_70_elo2_b4179_23_cplx	Translation elongation complex: 23 * ribosome 70S/b4179/second last codon EF-TU-tRNA/1 EF-G	C2149162H3004676N757325O1084531S6233P109346Mg4025Zn0Fe0	-90651.22	Translation

rib_70_elo2_b4179_47_cplx	Translation elongation complex: 47 * ribosome 70S/b4179/second last codon EF-TU-tRNA/1 EF-G	C4367458H6112484N1537709O2198467S12737P220898Mg8225Zn0Fe0	-182694.58	Translation
rib_70_elo2_b4180_14_cplx	Translation elongation complex: 14 * ribosome 70S/b4180/second last codon EF-TU-tRNA/1 EF-G	C1262825H1758848N447287O644555S3556P66042Mg2450Zn0Fe0	-54844.96	Translation
rib_70_elo2_b4180_1_cplx	Translation elongation complex: 1 * ribosome 70S/b4180/second last codon EF-TU-tRNA/1 EF-G	C96673H132964N34550O50793S254P5397Mg175Zn0Fe0	-4598.14	Translation
rib_70_elo2_b4180_7_cplx	Translation elongation complex: 7 * ribosome 70S/b4180/second last codon EF-TU-tRNA/1 EF-G	C634897H883372N225044O324837S1778P33387Mg1225Zn0Fe0	-27788.98	Translation
rib_70_elo2_b4200_1_cplx	Translation elongation complex: 1 * ribosome 70S/b4200/second last codon EF-TU-tRNA/1 EF-G	C92808H128282N33050O48171S248P5046Mg175Zn0Fe0	-4251.14	Translation
rib_70_elo2_b4200_3_cplx	Translation elongation complex: 3 * ribosome 70S/b4200/second last codon EF-TU-tRNA/1 EF-G	C270876H376300N96102O138995S744P14342Mg525Zn0Fe0	-11955.42	Translation
rib_70_elo2_b4200_7_cplx	Translation elongation complex: 7 * ribosome 70S/b4200/second last codon EF-TU-tRNA/1 EF-G	C627012H872336N222206O320643S1736P32934Mg1225Zn0Fe0	-27363.98	Translation
rib_70_elo2_b4201_1_cplx	Translation elongation complex: 1 * ribosome 70S/b4201/second last codon EF-TU-tRNA/1 EF-G	C91888H127200N32682O47571S248P4964Mg175Zn0Fe0	-4159.14	Translation
rib_70_elo2_b4201_3_cplx	Translation elongation complex: 3 * ribosome 70S/b4201/second last codon EF-TU-tRNA/1 EF-G	C269668H374816N95652O138305S744P14262Mg525Zn0Fe0	-11845.42	Translation
rib_70_elo2_b4201_6_cplx	Translation elongation complex: 6 * ribosome 70S/b4201/second last codon EF-TU-tRNA/1 EF-G	C536338H746240N190107O274406S1488P28209Mg1050Zn0Fe0	-23374.84	Translation
rib_70_elo2_b4202_1_cplx	Translation elongation complex: 1 * ribosome 70S/b4202/second last codon EF-TU-tRNA/1 EF-G	C90929H126088N32300O46898S243P4875Mg175Zn0Fe0	-4060.14	Translation
rib_70_elo2_b4202_2_cplx	Translation elongation complex: 2 * ribosome 70S/b4202/second last codon EF-TU-tRNA/1 EF-G	C179697H249717N63748O92207S486P9522Mg350Zn0Fe0	-7891.28	Translation
rib_70_elo2_b4202_4_cplx	Translation elongation complex: 4 * ribosome 70S/b4202/second last codon EF-TU-tRNA/1 EF-G	C357233H496975N126644O182825S972P18816Mg700Zn0Fe0	-15553.56	Translation
rib_70_elo2_b4203_1_cplx	Translation elongation complex: 1 * ribosome 70S/b4203/second last codon EF-TU-tRNA/1 EF-G	C93369H128977N33260O48554S242P5098Mg175Zn0Fe0	-4296.14	Translation
rib_70_elo2_b4203_4_cplx	Translation elongation complex: 4 * ribosome 70S/b4203/second last codon EF-TU-tRNA/1 EF-G	C360597H501370N127847O184811S968P19042Mg700Zn0Fe0	-15831.56	Translation
rib_70_elo2_b4203_8_cplx	Translation elongation complex: 8 * ribosome 70S/b4203/second last codon EF-TU-tRNA/1 EF-G	C716901H997894N253963O366487S1936P37634Mg1400Zn0Fe0	-31212.12	Translation
rib_70_elo2_b4258_1_cplx	Translation elongation complex: 1 * ribosome 70S/b4258/second last codon EF-TU-tRNA/1 EF-G	C120514H161434N43689O66593S280P7517Mg175Zn0Fe0	-6745.14	Translation
rib_70_elo2_b4258_27_cplx	Translation elongation complex: 27 * ribosome 70S/b4258/second last codon EF-TU-tRNA/1 EF-G	C2546366H3557190N893733O1281131S7560P128651Mg4725Zn0Fe0	-107784.78	Translation
rib_70_elo2_b4258_55_cplx	Translation elongation complex: 55 * ribosome 70S/b4258/second last codon EF-TU-tRNA/1 EF-G	C5158822H7214158N1809165O2589095S15400P259103Mg9625Zn0Fe0	-216596.7	Translation
rib_70_elo2_b4292_18_cplx	Translation elongation complex: 18 * ribosome 70S/b4292/second last codon EF-TU-tRNA/1 EF-G	C1629895H2270870N576695O830255S4374P84812Mg3150Zn0Fe0	-70289.52	Translation
rib_70_elo2_b4292_1_cplx	Translation elongation complex: 1 * ribosome 70S/b4292/second last codon EF-TU-tRNA/1 EF-G	C99079H135857N35466O52386S243P5609Mg175Zn0Fe0	-4803.14	Translation
rib_70_elo2_b4292_9_cplx	Translation elongation complex: 9 * ribosome 70S/b4292/second last codon EF-TU-tRNA/1 EF-G	C819463H1140569N290162O418442S2187P42881Mg1575Zn0Fe0	-35620.26	Translation
rib_70_elo2_b4293_10_cplx	Translation elongation complex: 10 * ribosome 70S/b4293/second last codon EF-TU-tRNA/1 EF-G	C898508H1250536N318059O459108S2500P47114Mg1750Zn0Fe0	-39106.4	Translation
rib_70_elo2_b4293_1_cplx	Translation elongation complex: 1 * ribosome 70S/b4293/second last codon EF-TU-tRNA/1 EF-G	C94313H130117N33578O49203S250P5183Mg175Zn0Fe0	-4383.14	Translation
rib_70_elo2_b4293_5_cplx	Translation elongation complex: 5 * ribosome 70S/b4293/second last codon EF-TU-tRNA/1 EF-G	C451733H628081N160014O231383S1250P23819Mg875Zn0Fe0	-19815.7	Translation
rib_70_elo2_b4371_10_cplx	Translation elongation complex: 10 * ribosome 70S/b4371/second last codon EF-TU-tRNA/1 EF-G	C910317H1267082N321883O464346S2510P47514Mg1750Zn0Fe0	-39526.4	Translation
rib_70_elo2_b4371_1_cplx	Translation elongation complex: 1 * ribosome 70S/b4371/second last codon EF-TU-tRNA/1 EF-G	C99858H136709N35692O52965S251P5682Mg175Zn0Fe0	-4884.14	Translation
rib_70_elo2_b4371_20_cplx	Translation elongation complex: 20 * ribosome 70S/b4371/second last codon EF-TU-tRNA/1 EF-G	C1810827H2523052N639873O921436S5020P93994Mg3500Zn0Fe0	-78017.8	Translation
rib_70_elo2_b4372_1_cplx	Translation elongation complex: 1 * ribosome 70S/b4372/second last codon EF-TU-tRNA/1 EF-G	C92998H128544N33103O48314S246P5065Mg175Zn0Fe0	-4265.14	Translation
rib_70_elo2_b4372_4_cplx	Translation elongation complex: 4 * ribosome 70S/b4372/second last codon EF-TU-tRNA/1 EF-G	C360190H500757N127684O184574S984P19012Mg700Zn0Fe0	-15809.56	Translation
rib_70_elo2_b4372_8_cplx	Translation elongation complex: 8 * ribosome 70S/b4372/second last codon EF-TU-tRNA/1 EF-G	C716446H997041N253792O366254S1968P37608Mg1400Zn0Fe0	-31202.12	Translation

rib_70_elo2_b4373_1_cplx	Translation elongation complex: 1 * ribosome 70S/b4373/second last codon EF-TU-tRNA/1 EF-G	C93091H128631N33127O48336S245P5064Mg175Zn0Fe0	-4269.14	Translation
rib_70_elo2_b4373_4_cplx	Translation elongation complex: 4 * ribosome 70S/b4373/second last codon EF-TU-tRNA/1 EF-G	C360508H501096N127741O184671S980P19011Mg700Zn0Fe0	-15828.56	Translation
rib_70_elo2_b4373_8_cplx	Translation elongation complex: 8 * ribosome 70S/b4373/second last codon EF-TU-tRNA/1 EF-G	C717064H997716N253893O366451S1960P37607Mg1400Zn0Fe0	-31241.12	Translation
rib_70_elo2_b4374_13_cplx	Translation elongation complex: 13 * ribosome 70S/b4374/second last codon EF-TU-tRNA/1 EF-G	C1170341H1628786N413694O597051S3224P61115Mg2275Zn0Fe0	-50821.82	Translation
rib_70_elo2_b4374_1_cplx	Translation elongation complex: 1 * ribosome 70S/b4374/second last codon EF-TU-tRNA/1 EF-G	C95969H132026N34170O50319S248P5327Mg175Zn0Fe0	-4536.14	Translation
rib_70_elo2_b4374_6_cplx	Translation elongation complex: 6 * ribosome 70S/b4374/second last codon EF-TU-tRNA/1 EF-G	C543624H755676N192305O278124S1488P28572Mg1050Zn0Fe0	-23821.84	Translation
rib_70_elo2_b4375_15_cplx	Translation elongation complex: 15 * ribosome 70S/b4375/second last codon EF-TU-tRNA/1 EF-G	C1380652H1924724N486882O701340S3960P71327Mg2625Zn0Fe0	-59450.1	Translation
rib_70_elo2_b4375_1_cplx	Translation elongation complex: 1 * ribosome 70S/b4375/second last codon EF-TU-tRNA/1 EF-G	C106176H144316N38140O57116S264P6241Mg175Zn0Fe0	-5450.14	Translation
rib_70_elo2_b4375_31_cplx	Translation elongation complex: 31 * ribosome 70S/b4375/second last codon EF-TU-tRNA/1 EF-G	C2837196H3959476N999730O1437596S8184P145711Mg5425Zn0Fe0	-121164.34	Translation
rib_ini_b0014_1	b0014 complexed with 1* rib_30/fmet-tRNA/Mg	C101325H135201N37320O56830S204Se0Mg173P6562Fe0Zn0	-5722.14	Translation
rib_ini_b0014_18	b0014 complexed with 18* rib_30/fmet-tRNA/Mg	C1512733H2081820N545059O797231S3672Se0Mg3114P85493Fe0Zn0	-70358.52	Translation
rib_ini_b0014_37	b0014 complexed with 37* rib_30/fmet-tRNA/Mg	C3090189H4257453N1112532O1624738S7548Se0Mg6401P173710Fe0Zn0	-142599.18	Translation
rib_ini_b0015_1	b0015 complexed with 1* rib_30/fmet-tRNA/Mg	C93825H126718N34273O51426S204Se0Mg173P5774Fe0Zn0	-4934.14	Translation
rib_ini_b0015_11	b0015 complexed with 11* rib_30/fmet-tRNA/Mg	C924065H1271788N332943O486956S2244Se0Mg1903P52204Fe0Zn0	-42955.54	Translation
rib_ini_b0015_22	b0015 complexed with 22* rib_30/fmet-tRNA/Mg	C1837329H2531365N661480O966039S4488Se0Mg3806P103277Fe0Zn0	-84779.08	Translation
rib_ini_b0023_1	b0023 complexed with 1* rib_30/fmet-tRNA/Mg	C85546H117360N30899O45371S204Se0Mg173P4909Fe0Zn0	-4069.14	Translation
rib_ini_b0023_2	b0023 complexed with 2* rib_30/fmet-tRNA/Mg	C168570H231867N60766O88924S408Se0Mg346P9552Fe0Zn0	-7871.28	Translation
rib_ini_b0023_5	b0023 complexed with 5* rib_30/fmet-tRNA/Mg	C417642H575388N150367O219583S1020Se0Mg865P23481Fe0Zn0	-19277.7	Translation
rib_ini_b0025_1	b0025 complexed with 1* rib_30/fmet-tRNA/Mg	C91991H124644N33445O50169S204Se0Mg173P5587Fe0Zn0	-4747.14	Translation
rib_ini_b0025_18	b0025 complexed with 18* rib_30/fmet-tRNA/Mg	C1503399H2071263N541184O790570S3672Se0Mg3114P84518Fe0Zn0	-69383.52	Translation
rib_ini_b0025_9	b0025 complexed with 9* rib_30/fmet-tRNA/Mg	C756183H1040700N272381O398593S1836Se0Mg1557P42731Fe0Zn0	-35164.26	Translation
rib_ini_b0026_1	b0026 complexed with 1* rib_30/fmet-tRNA/Mg	C109856H144904N40685O63209S204Se0Mg173P7460Fe0Zn0	-6620.14	Translation
rib_ini_b0026_27	b0026 complexed with 27* rib_30/fmet-tRNA/Mg	C2268480H3122086N817227O1195587S5508Se0Mg4671P128178Fe0Zn0	-105475.78	Translation
rib_ini_b0026_55	b0026 complexed with 55* rib_30/fmet-tRNA/Mg	C4593152H6328282N1653503O2415071S11220Se0Mg9515P258182Fe0Zn0	-211935.7	Translation
rib_ini_b0027_1	b0027 complexed with 1* rib_30/fmet-tRNA/Mg	C87702H119804N31675O47059S204Se0Mg173P5137Fe0Zn0	-4297.14	Translation
rib_ini_b0027_4	b0027 complexed with 4* rib_30/fmet-tRNA/Mg	C336774H463325N121276O177718S816Se0Mg692P19066Fe0Zn0	-15703.56	Translation
rib_ini_b0027_9	b0027 complexed with 9* rib_30/fmet-tRNA/Mg	C751894H1035860N270611O395483S1836Se0Mg1557P42281Fe0Zn0	-34714.26	Translation
rib_ini_b0028_1	b0028 complexed with 1* rib_30/fmet-tRNA/Mg	C87299H119353N31562O46709S204Se0Mg173P5093Fe0Zn0	-4253.14	Translation
rib_ini_b0028_4	b0028 complexed with 4* rib_30/fmet-tRNA/Mg	C336371H462874N121163O177368S816Se0Mg692P19022Fe0Zn0	-15659.56	Translation
rib_ini_b0028_8	b0028 complexed with 8* rib_30/fmet-tRNA/Mg	C668467H920902N240631O351580S1632Se0Mg1384P37594Fe0Zn0	-30868.12	Translation
rib_ini_b0029_1	b0029 complexed with 1* rib_30/fmet-tRNA/Mg	C92099H124762N33545O50198S204Se0Mg173P5594Fe0Zn0	-4754.14	Translation
rib_ini_b0029_18	b0029 complexed with 18* rib_30/fmet-tRNA/Mg	C1503507H2071381N541284O790599S3672Se0Mg3114P84525Fe0Zn0	-69390.52	Translation
rib_ini_b0029_9	b0029 complexed with 9* rib_30/fmet-tRNA/Mg	C756291H1040818N272481O398622S1836Se0Mg1557P42738Fe0Zn0	-35171.26	Translation
rib_ini_b0049_1	b0049 complexed with 1* rib_30/fmet-tRNA/Mg	C91048H123593N33082O49458S204Se0Mg173P5486Fe0Zn0	-4646.14	Translation
rib_ini_b0049_16	b0049 complexed with 16* rib_30/fmet-tRNA/Mg	C1336408H1841198N481087O702753S3264Se0Mg2768P75131Fe0Zn0	-61678.24	Translation
rib_ini_b0049_8	b0049 complexed with 8* rib_30/fmet-tRNA/Mg	C672216H925142N242151O354329S1632Se0Mg1384P37987Fe0Zn0	-31261.12	Translation
rib_ini_b0050_1	b0050 complexed with 1* rib_30/fmet-tRNA/Mg	C86612H118576N31283O46198S204Se0Mg173P5021Fe0Zn0	-4181.14	Translation
rib_ini_b0050_3	b0050 complexed with 3* rib_30/fmet-tRNA/Mg	C252660H347590N91017O133304S612Se0Mg519P14307Fe0Zn0	-11785.42	Translation
rib_ini_b0050_7	b0050 complexed with 7* rib_30/fmet-tRNA/Mg	C584756H805618N210485O307516S1428Se0Mg1211P32879Fe0Zn0	-26993.98	Translation
rib_ini_b0051_1	b0051 complexed with 1* rib_30/fmet-tRNA/Mg	C90796H123318N32953O49273S204Se0Mg173P5461Fe0Zn0	-4621.14	Translation
rib_ini_b0051_16	b0051 complexed with 16* rib_30/fmet-tRNA/Mg	C1336156H1840923N480958O702568S3264Se0Mg2768P75106Fe0Zn0	-61653.24	Translation
rib_ini_b0051_8	b0051 complexed with 8* rib_30/fmet-tRNA/Mg	C671964H924867N242022O354144S1632Se0Mg1384P37962Fe0Zn0	-31236.12	Translation

rib_ini_b0052_1	b0052 complexed with 1* rib_30/fmet-tRNA/Mg	C92410H125160N33577O50500S204Se0Mg173P5632Fe0Zn0	-4792.14	Translation
rib_ini_b0052_19	b0052 complexed with 19* rib_30/fmet-tRNA/Mg	C1586842H2186286N571183O834454S3876Se0Mg3287P89206Fe0Zn0	-73230.66	Translation
rib_ini_b0052_9	b0052 complexed with 9* rib_30/fmet-tRNA/Mg	C756602H1041216N272513O398924S1836Se0Mg1557P42776Fe0Zn0	-35209.26	Translation
rib_ini_b0053_1	b0053 complexed with 1* rib_30/fmet-tRNA/Mg	C95293H128408N34843O52489S204Se0Mg173P5932Fe0Zn0	-5092.14	Translation
rib_ini_b0053_12	b0053 complexed with 12* rib_30/fmet-tRNA/Mg	C1008557H1387985N363380O531572S2448Se0Mg2076P57005Fe0Zn0	-46915.68	Translation
rib_ini_b0053_25	b0053 complexed with 25* rib_30/fmet-tRNA/Mg	C2087869H2876576N751651O1097761S5100Se0Mg4325P117364Fe0Zn0	-96343.5	Translation
rib_ini_b0058_1	b0058 complexed with 1* rib_30/fmet-tRNA/Mg	C89327H121625N32430O48156S204Se0Mg173P5303Fe0Zn0	-4463.14	Translation
rib_ini_b0058_12	b0058 complexed with 12* rib_30/fmet-tRNA/Mg	C1002591H1381202N360967O527239S2448Se0Mg2076P56376Fe0Zn0	-46286.68	Translation
rib_ini_b0058_6	b0058 complexed with 6* rib_30/fmet-tRNA/Mg	C504447H694160N181765O265921S1224Se0Mg1038P28518Fe0Zn0	-23473.84	Translation
rib_ini_b0059_1	b0059 complexed with 1* rib_30/fmet-tRNA/Mg	C110683H145851N40946O63866S204Se0Mg173P7552Fe0Zn0	-6712.14	Translation
rib_ini_b0059_28	b0059 complexed with 28* rib_30/fmet-tRNA/Mg	C2352331H3237540N847355O1239797S5712Se0Mg4844P132913Fe0Zn0	-109369.92	Translation
rib_ini_b0059_56	b0059 complexed with 56* rib_30/fmet-tRNA/Mg	C4677003H6443736N1683631O2459281S11424Se0Mg9688P262917Fe0Zn0	-215829.84	Translation
rib_ini_b0144_1	b0144 complexed with 1* rib_30/fmet-tRNA/Mg	C91814H124484N33321O50061S204Se0Mg173P5572Fe0Zn0	-4732.14	Translation
rib_ini_b0144_18	b0144 complexed with 18* rib_30/fmet-tRNA/Mg	C1503222H2071103N541060O790462S3672Se0Mg3114P84503Fe0Zn0	-69368.52	Translation
rib_ini_b0144_9	b0144 complexed with 9* rib_30/fmet-tRNA/Mg	C756006H1040540N272257O398485S1836Se0Mg1557P42716Fe0Zn0	-35149.26	Translation
rib_ini_b0166_1	b0166 complexed with 1* rib_30/fmet-tRNA/Mg	C90882H123395N33020O49324S204Se0Mg173P5470Fe0Zn0	-4630.14	Translation
rib_ini_b0166_16	b0166 complexed with 16* rib_30/fmet-tRNA/Mg	C1336242H1841000N481025O702619S3264Se0Mg2768P75115Fe0Zn0	-61662.24	Translation
rib_ini_b0166_8	b0166 complexed with 8* rib_30/fmet-tRNA/Mg	C672050H924944N242089O354195S1632Se0Mg1384P37971Fe0Zn0	-31245.12	Translation
rib_ini_b0167_1	b0167 complexed with 1* rib_30/fmet-tRNA/Mg	C108399H143303N39914O62250S204Se0Mg173P7316Fe0Zn0	-6476.14	Translation
rib_ini_b0167_26	b0167 complexed with 26* rib_30/fmet-tRNA/Mg	C2183999H3005978N786589O1151075S5304Se0Mg4498P123391Fe0Zn0	-101529.64	Translation
rib_ini_b0167_52	b0167 complexed with 52* rib_30/fmet-tRNA/Mg	C4342623H5983160N1563131O2283453S10608Se0Mg8996P244109Fe0Zn0	-200385.28	Translation
rib_ini_b0168_1	b0168 complexed with 1* rib_30/fmet-tRNA/Mg	C90602H123077N32922O49083S204Se0Mg173P5440Fe0Zn0	-4600.14	Translation
rib_ini_b0168_15	b0168 complexed with 15* rib_30/fmet-tRNA/Mg	C1252938H1726175N451060O658825S3060Se0Mg2595P70442Fe0Zn0	-57830.1	Translation
rib_ini_b0168_7	b0168 complexed with 7* rib_30/fmet-tRNA/Mg	C588746H810119N212124O310401S1428Se0Mg1211P33298Fe0Zn0	-27412.98	Translation
rib_ini_b0169_1	b0169 complexed with 1* rib_30/fmet-tRNA/Mg	C89934H122328N32631O48621S204Se0Mg173P5371Fe0Zn0	-4531.14	Translation
rib_ini_b0169_14	b0169 complexed with 14* rib_30/fmet-tRNA/Mg	C1169246H1610919N420902O614810S2856Se0Mg2422P65730Fe0Zn0	-53958.96	Translation
rib_ini_b0169_7	b0169 complexed with 7* rib_30/fmet-tRNA/Mg	C588078H809370N211833O309939S1428Se0Mg1211P33229Fe0Zn0	-27343.98	Translation
rib_ini_b0170_1	b0170 complexed with 1* rib_30/fmet-tRNA/Mg	C91178H123700N33215O49451S204Se0Mg173P5495Fe0Zn0	-4655.14	Translation
rib_ini_b0170_16	b0170 complexed with 16* rib_30/fmet-tRNA/Mg	C1336538H1841305N481220O702746S3264Se0Mg2768P75140Fe0Zn0	-61687.24	Translation
rib_ini_b0170_8	b0170 complexed with 8* rib_30/fmet-tRNA/Mg	C672346H925249N242284O354322S1632Se0Mg1384P37996Fe0Zn0	-31270.12	Translation
rib_ini_b0172_1	b0172 complexed with 1* rib_30/fmet-tRNA/Mg	C88363H120535N32064O47414S204Se0Mg173P5203Fe0Zn0	-4363.14	Translation
rib_ini_b0172_10	b0172 complexed with 10* rib_30/fmet-tRNA/Mg	C835579H1151098N300867O439391S2040Se0Mg1730P46990Fe0Zn0	-38582.4	Translation
rib_ini_b0172_5	b0172 complexed with 5* rib_30/fmet-tRNA/Mg	C420459H578563N151532O221626S1020Se0Mg865P23775Fe0Zn0	-19571.7	Translation
rib_ini_b0188_1	b0188 complexed with 1* rib_30/fmet-tRNA/Mg	C95397H128517N34848O52668S204Se0Mg173P5944Fe0Zn0	-5104.14	Translation
rib_ini_b0188_12	b0188 complexed with 12* rib_30/fmet-tRNA/Mg	C1008661H1388094N363385O531751S2448Se0Mg2076P57017Fe0Zn0	-46927.68	Translation
rib_ini_b0188_25	b0188 complexed with 25* rib_30/fmet-tRNA/Mg	C2087973H2876685N751656O1097940S5100Se0Mg4325P117376Fe0Zn0	-96355.5	Translation
rib_ini_b0194_1	b0194 complexed with 1* rib_30/fmet-tRNA/Mg	C99401H133047N36466O55551S204Se0Mg173P6364Fe0Zn0	-5524.14	Translation
rib_ini_b0194_16	b0194 complexed with 16* rib_30/fmet-tRNA/Mg	C1344761H1850652N484471O708846S3264Se0Mg2768P76009Fe0Zn0	-62556.24	Translation
rib_ini_b0194_33	b0194 complexed with 33* rib_30/fmet-tRNA/Mg	C2756169H3797271N992210O1449247S6732Se0Mg5709P154940Fe0Zn0	-127192.62	Translation
rib_ini_b0405_1	b0405 complexed with 1* rib_30/fmet-tRNA/Mg	C93204H126044N33917O51056S204Se0Mg173P5716Fe0Zn0	-4876.14	Translation
rib_ini_b0405_10	b0405 complexed with 10* rib_30/fmet-tRNA/Mg	C840420H1156607N302720O443033S2040Se0Mg1730P47503Fe0Zn0	-39095.4	Translation
rib_ini_b0405_20	b0405 complexed with 20* rib_30/fmet-tRNA/Mg	C1670660H2301677N601390O878563S4080Se0Mg3460P93933Fe0Zn0	-77116.8	Translation
rib_ini_b0406_1	b0406 complexed with 1* rib_30/fmet-tRNA/Mg	C93749H126635N34116O51469S204Se0Mg173P5773Fe0Zn0	-4933.14	Translation
rib_ini_b0406_11	b0406 complexed with 11* rib_30/fmet-tRNA/Mg	C923989H1271705N332786O486999S2244Se0Mg1903P52203Fe0Zn0	-42954.54	Translation
rib_ini_b0406_22	b0406 complexed with 22* rib_30/fmet-tRNA/Mg	C1837253H2531282N661323O966082S4488Se0Mg3806P103276Fe0Zn0	-84778.08	Translation
rib_ini_b0407_1	b0407 complexed with 1* rib_30/fmet-tRNA/Mg	C86195H118087N31130O45885S204Se0Mg173P4976Fe0Zn0	-4136.14	Translation
rib_ini_b0407_3	b0407 complexed with 3* rib_30/fmet-tRNA/Mg	C252243H347101N90864O132991S612Se0Mg519P14262Fe0Zn0	-11740.42	Translation

rib_ini_b0407_6	b0407 complexed with 6* rib_30/fmet-tRNA/Mg	C501315H690622N180465O263650S1224Se0Mg1038P28191Fe0Zn0	-23146.84	Translation
rib_ini_b0413_1	b0413 complexed with 1* rib_30/fmet-tRNA/Mg	C87316H119350N31593O46707S204Se0Mg173P5095Fe0Zn0	-4255.14	Translation
rib_ini_b0413_4	b0413 complexed with 4* rib_30/fmet-tRNA/Mg	C336388H462871N121194O177366S816Se0Mg692P19024Fe0Zn0	-15661.56	Translation
rib_ini_b0413_8	b0413 complexed with 8* rib_30/fmet-tRNA/Mg	C668484H920899N240662O351578S1632Se0Mg1384P37596Fe0Zn0	-30870.12	Translation
rib_ini_b0414_1	b0414 complexed with 1* rib_30/fmet-tRNA/Mg	C93538H126415N34098O51263S204Se0Mg173P5747Fe0Zn0	-4907.14	Translation
rib_ini_b0414_10	b0414 complexed with 10* rib_30/fmet-tRNA/Mg	C840754H1156978N302901O443240S2040Se0Mg1730P47534Fe0Zn0	-39126.4	Translation
rib_ini_b0414_21	b0414 complexed with 21* rib_30/fmet-tRNA/Mg	C1754018H2416555N631438O922323S4284Se0Mg3633P98607Fe0Zn0	-80949.94	Translation
rib_ini_b0415_1	b0415 complexed with 1* rib_30/fmet-tRNA/Mg	C87513H119581N31672O46842S204Se0Mg173P5114Fe0Zn0	-4274.14	Translation
rib_ini_b0415_4	b0415 complexed with 4* rib_30/fmet-tRNA/Mg	C336585H463102N121273O177501S816Se0Mg692P19043Fe0Zn0	-15680.56	Translation
rib_ini_b0415_9	b0415 complexed with 9* rib_30/fmet-tRNA/Mg	C751705H1035637N270608O395266S1836Se0Mg1557P42258Fe0Zn0	-34691.26	Translation
rib_ini_b0416_1	b0416 complexed with 1* rib_30/fmet-tRNA/Mg	C87026H119041N31484O46474S204Se0Mg173P5063Fe0Zn0	-4223.14	Translation
rib_ini_b0416_4	b0416 complexed with 4* rib_30/fmet-tRNA/Mg	C336098H462562N121085O177133S816Se0Mg692P18992Fe0Zn0	-15629.56	Translation
rib_ini_b0416_8	b0416 complexed with 8* rib_30/fmet-tRNA/Mg	C668194H920590N240553O351345S1632Se0Mg1384P37564Fe0Zn0	-30838.12	Translation
rib_ini_b0423_1	b0423 complexed with 1* rib_30/fmet-tRNA/Mg	C96812H130103N35364O53698S204Se0Mg173P6094Fe0Zn0	-5254.14	Translation
rib_ini_b0423_14	b0423 complexed with 14* rib_30/fmet-tRNA/Mg	C1176124H1618694N423635O619887S2856Se0Mg2422P66453Fe0Zn0	-54681.96	Translation
rib_ini_b0423_28	b0423 complexed with 28* rib_30/fmet-tRNA/Mg	C2338460H3221792N841773O1229629S5712Se0Mg4844P131455Fe0Zn0	-107911.92	Translation
rib_ini_b0436_1	b0436 complexed with 1* rib_30/fmet-tRNA/Mg	C95457H128538N34989O52537S204Se0Mg173P5944Fe0Zn0	-5104.14	Translation
rib_ini_b0436_12	b0436 complexed with 12* rib_30/fmet-tRNA/Mg	C1008721H1388115N363526O531620S2448Se0Mg2076P57017Fe0Zn0	-46927.68	Translation
rib_ini_b0436_25	b0436 complexed with 25* rib_30/fmet-tRNA/Mg	C2088033H2876706N751797O1097809S5100Se0Mg4325P117376Fe0Zn0	-96355.5	Translation
rib_ini_b0503_1	b0503 complexed with 1* rib_30/fmet-tRNA/Mg	C93472H126324N34109O51197S204Se0Mg173P5740Fe0Zn0	-4900.14	Translation
rib_ini_b0503_10	b0503 complexed with 10* rib_30/fmet-tRNA/Mg	C840688H1156887N302912O443174S2040Se0Mg1730P47527Fe0Zn0	-39119.4	Translation
rib_ini_b0503_21	b0503 complexed with 21* rib_30/fmet-tRNA/Mg	C1753952H2416464N631449O922257S4284Se0Mg3633P98600Fe0Zn0	-80942.94	Translation
rib_ini_b0526_1	b0526 complexed with 1* rib_30/fmet-tRNA/Mg	C96241H129459N35216O53221S204Se0Mg173P6031Fe0Zn0	-5191.14	Translation
rib_ini_b0526_13	b0526 complexed with 13* rib_30/fmet-tRNA/Mg	C1092529H1503543N393620O575857S2652Se0Mg2249P61747Fe0Zn0	-50816.82	Translation
rib_ini_b0526_27	b0526 complexed with 27* rib_30/fmet-tRNA/Mg	C2254865H3106641N811758O1185599S5508Se0Mg4671P126749Fe0Zn0	-104046.78	Translation
rib_ini_b0638_1	b0638 complexed with 1* rib_30/fmet-tRNA/Mg	C88840H121089N32168O47841S204Se0Mg173P5255Fe0Zn0	-4415.14	Translation
rib_ini_b0638_11	b0638 complexed with 11* rib_30/fmet-tRNA/Mg	C919080H1266159N330838O483371S2244Se0Mg1903P51685Fe0Zn0	-42436.54	Translation
rib_ini_b0638_5	b0638 complexed with 5* rib_30/fmet-tRNA/Mg	C420936H579117N151636O222053S1020Se0Mg865P23827Fe0Zn0	-19623.7	Translation
rib_ini_b0639_1	b0639 complexed with 1* rib_30/fmet-tRNA/Mg	C89122H121417N32280O48033S204Se0Mg173P5285Fe0Zn0	-4445.14	Translation
rib_ini_b0639_12	b0639 complexed with 12* rib_30/fmet-tRNA/Mg	C1002386H1380994N360817O527116S2448Se0Mg2076P56358Fe0Zn0	-46268.68	Translation
rib_ini_b0639_6	b0639 complexed with 6* rib_30/fmet-tRNA/Mg	C504242H693952N181615O265798S1224Se0Mg1038P28500Fe0Zn0	-23455.84	Translation
rib_ini_b0640_1	b0640 complexed with 1* rib_30/fmet-tRNA/Mg	C92821H125598N33745O50782S204Se0Mg173P5674Fe0Zn0	-4834.14	Translation
rib_ini_b0640_10	b0640 complexed with 10* rib_30/fmet-tRNA/Mg	C840037H1156161N302548O442759S2040Se0Mg1730P47461Fe0Zn0	-39053.4	Translation
rib_ini_b0640_20	b0640 complexed with 20* rib_30/fmet-tRNA/Mg	C1670277H2301231N601218O878289S4080Se0Mg3460P93891Fe0Zn0	-77074.8	Translation
rib_ini_b0641_1	b0641 complexed with 1* rib_30/fmet-tRNA/Mg	C88561H120777N32078O47616S204Se0Mg173P5225Fe0Zn0	-4385.14	Translation
rib_ini_b0641_11	b0641 complexed with 11* rib_30/fmet-tRNA/Mg	C918801H1265847N330748O483146S2244Se0Mg1903P51655Fe0Zn0	-42406.54	Translation
rib_ini_b0641_5	b0641 complexed with 5* rib_30/fmet-tRNA/Mg	C420657H578805N151546O221828S1020Se0Mg865P23797Fe0Zn0	-19593.7	Translation
rib_ini_b0642_1	b0642 complexed with 1* rib_30/fmet-tRNA/Mg	C107656H142377N39842O61524S204Se0Mg173P7228Fe0Zn0	-6388.14	Translation
rib_ini_b0642_25	b0642 complexed with 25* rib_30/fmet-tRNA/Mg	C2100232H2890545N756650O1106796S5100Se0Mg4325P118660Fe0Zn0	-97639.5	Translation
rib_ini_b0642_50	b0642 complexed with 50* rib_30/fmet-tRNA/Mg	C4175832H5753220N1503325O2195621S10200Se0Mg8650P234735Fe0Zn0	-192693	Translation
rib_ini_b0661_1	b0661 complexed with 1* rib_30/fmet-tRNA/Mg	C96592H129875N35320O53498S204Se0Mg173P6070Fe0Zn0	-5230.14	Translation
rib_ini_b0661_13	b0661 complexed with 13* rib_30/fmet-tRNA/Mg	C1092880H1503959N393724O576134S2652Se0Mg2249P61786Fe0Zn0	-50855.82	Translation
rib_ini_b0661_27	b0661 complexed with 27* rib_30/fmet-tRNA/Mg	C2255216H3107057N811862O1185876S5508Se0Mg4671P126788Fe0Zn0	-104085.78	Translation
rib_ini_b0680_1	b0680 complexed with 1* rib_30/fmet-tRNA/Mg	C98875H132447N36218O55176S204Se0Mg173P6310Fe0Zn0	-5470.14	Translation
rib_ini_b0680_16	b0680 complexed with 16* rib_30/fmet-tRNA/Mg	C1344235H1850052N484223O708471S3264Se0Mg2768P75955Fe0Zn0	-62502.24	Translation
rib_ini_b0680_32	b0680 complexed with 32* rib_30/fmet-tRNA/Mg	C2672619H3682164N962095O1405319S6528Se0Mg5536P150243Fe0Zn0	-123336.48	Translation
rib_ini_b0850_1	b0850 complexed with 1* rib_30/fmet-tRNA/Mg	C85756H117597N30932O45591S204Se0Mg173P4933Fe0Zn0	-4093.14	Translation

rib_ini_b0850_2	b0850 complexed with 2* rib_30/fmet-tRNA/Mg	C168780H232104N60799O89144S408Se0Mg346P9576Fe0Zn0	-7895.28	Translation
rib_ini_b0850_5	b0850 complexed with 5* rib_30/fmet-tRNA/Mg	C417852H575625N150400O219803S1020Se0Mg865P23505Fe0Zn0	-19301.7	Translation
rib_ini_b0851_1	b0851 complexed with 1* rib_30/fmet-tRNA/Mg	C89737H122105N32534O48500S204Se0Mg173P5349Fe0Zn0	-4509.14	Translation
rib_ini_b0851_14	b0851 complexed with 14* rib_30/fmet-tRNA/Mg	C1169049H1610696N420805O614689S2856Se0Mg2422P65708Fe0Zn0	-53936.96	Translation
rib_ini_b0851_7	b0851 complexed with 7* rib_30/fmet-tRNA/Mg	C587881H809147N211736O309818S1428Se0Mg1211P33207Fe0Zn0	-27321.98	Translation
rib_ini_b0852_1	b0852 complexed with 1* rib_30/fmet-tRNA/Mg	C91631H124238N33333O49892S204Se0Mg173P5548Fe0Zn0	-4708.14	Translation
rib_ini_b0852_17	b0852 complexed with 17* rib_30/fmet-tRNA/Mg	C1420015H1956350N511205O746740S3468Se0Mg2941P79836Fe0Zn0	-65542.38	Translation
rib_ini_b0852_8	b0852 complexed with 8* rib_30/fmet-tRNA/Mg	C672799H925787N242402O354763S1632Se0Mg1384P38049Fe0Zn0	-31323.12	Translation
rib_ini_b0853_1	b0853 complexed with 1* rib_30/fmet-tRNA/Mg	C87546H119618N31619O46921S204Se0Mg173P5120Fe0Zn0	-4280.14	Translation
rib_ini_b0853_4	b0853 complexed with 4* rib_30/fmet-tRNA/Mg	C336618H463139N121220O177580S816Se0Mg692P19049Fe0Zn0	-15686.56	Translation
rib_ini_b0853_9	b0853 complexed with 9* rib_30/fmet-tRNA/Mg	C751738H1035674N270555O395345S1836Se0Mg1557P42264Fe0Zn0	-34697.26	Translation
rib_ini_b0858_1	b0858 complexed with 1* rib_30/fmet-tRNA/Mg	C87657H119747N31658O47029S204Se0Mg173P5134Fe0Zn0	-4294.14	Translation
rib_ini_b0858_4	b0858 complexed with 4* rib_30/fmet-tRNA/Mg	C336729H463268N121259O177688S816Se0Mg692P19063Fe0Zn0	-15700.56	Translation
rib_ini_b0858_9	b0858 complexed with 9* rib_30/fmet-tRNA/Mg	C751849H1035803N270594O395453S1836Se0Mg1557P42278Fe0Zn0	-34711.26	Translation
rib_ini_b0859_1	b0859 complexed with 1* rib_30/fmet-tRNA/Mg	C93743H126652N34121O51466S204Se0Mg173P5771Fe0Zn0	-4931.14	Translation
rib_ini_b0859_11	b0859 complexed with 11* rib_30/fmet-tRNA/Mg	C923983H1271722N332791O486996S2244Se0Mg1903P52201Fe0Zn0	-42952.54	Translation
rib_ini_b0859_22	b0859 complexed with 22* rib_30/fmet-tRNA/Mg	C1837247H2531299N661328O966079S4488Se0Mg3806P103274Fe0Zn0	-84776.08	Translation
rib_ini_b0884_1	b0884 complexed with 1* rib_30/fmet-tRNA/Mg	C85111H116869N30708O45076S204Se0Mg173P4864Fe0Zn0	-4024.14	Translation
rib_ini_b0884_2	b0884 complexed with 2* rib_30/fmet-tRNA/Mg	C168135H231376N60575O88629S408Se0Mg346P9507Fe0Zn0	-7826.28	Translation
rib_ini_b0884_4	b0884 complexed with 4* rib_30/fmet-tRNA/Mg	C334183H460390N120309O175735S816Se0Mg692P18793Fe0Zn0	-15430.56	Translation
rib_ini_b0893_1	b0893 complexed with 1* rib_30/fmet-tRNA/Mg	C95345H128442N34825O52577S204Se0Mg173P5938Fe0Zn0	-5098.14	Translation
rib_ini_b0893_12	b0893 complexed with 12* rib_30/fmet-tRNA/Mg	C1008609H1388019N363362O531660S2448Se0Mg2076P57011Fe0Zn0	-46921.68	Translation
rib_ini_b0893_25	b0893 complexed with 25* rib_30/fmet-tRNA/Mg	C2087921H2876610N751633O1097849S5100Se0Mg4325P117370Fe0Zn0	-96349.5	Translation
rib_ini_b0910_1	b0910 complexed with 1* rib_30/fmet-tRNA/Mg	C89535H121872N32469O48351S204Se0Mg173P5329Fe0Zn0	-4489.14	Translation
rib_ini_b0910_13	b0910 complexed with 13* rib_30/fmet-tRNA/Mg	C1085823H1495956N390873O570987S2652Se0Mg2249P61045Fe0Zn0	-50114.82	Translation
rib_ini_b0910_6	b0910 complexed with 6* rib_30/fmet-tRNA/Mg	C504655H694407N181804O266116S1224Se0Mg1038P28544Fe0Zn0	-23499.84	Translation
rib_ini_b0911_1	b0911 complexed with 1* rib_30/fmet-tRNA/Mg	C98997H132553N36334O55199S204Se0Mg173P6319Fe0Zn0	-5479.14	Translation
rib_ini_b0911_16	b0911 complexed with 16* rib_30/fmet-tRNA/Mg	C1344357H1850158N484339O708494S3264Se0Mg2768P75964Fe0Zn0	-62511.24	Translation
rib_ini_b0911_32	b0911 complexed with 32* rib_30/fmet-tRNA/Mg	C2672741H3682270N962211O1405342S6528Se0Mg5536P150252Fe0Zn0	-123345.48	Translation
rib_ini_b0912_1	b0912 complexed with 1* rib_30/fmet-tRNA/Mg	C85743H117582N30969O45526S204Se0Mg173P4928Fe0Zn0	-4088.14	Translation
rib_ini_b0912_2	b0912 complexed with 2* rib_30/fmet-tRNA/Mg	C168767H232089N60836O89079S408Se0Mg346P9571Fe0Zn0	-7890.28	Translation
rib_ini_b0912_5	b0912 complexed with 5* rib_30/fmet-tRNA/Mg	C417839H575610N150437O219738S1020Se0Mg865P23500Fe0Zn0	-19296.7	Translation
rib_ini_b0930_1	b0930 complexed with 1* rib_30/fmet-tRNA/Mg	C96335H129571N35126O53396S204Se0Mg173P6046Fe0Zn0	-5206.14	Translation
rib_ini_b0930_13	b0930 complexed with 13* rib_30/fmet-tRNA/Mg	C1092623H1503655N393530O576032S2652Se0Mg2249P61762Fe0Zn0	-50831.82	Translation
rib_ini_b0930_27	b0930 complexed with 27* rib_30/fmet-tRNA/Mg	C2254959H3106753N811668O1185774S5508Se0Mg4671P126764Fe0Zn0	-104061.78	Translation
rib_ini_b0969_1	b0969 complexed with 1* rib_30/fmet-tRNA/Mg	C86182H118060N31155O45850S204Se0Mg173P4975Fe0Zn0	-4135.14	Translation
rib_ini_b0969_3	b0969 complexed with 3* rib_30/fmet-tRNA/Mg	C252230H347074N90889O132956S612Se0Mg519P14261Fe0Zn0	-11739.42	Translation
rib_ini_b0969_6	b0969 complexed with 6* rib_30/fmet-tRNA/Mg	C501302H690595N180490O263615S1224Se0Mg1038P28190Fe0Zn0	-23145.84	Translation
rib_ini_b1066_1	b1066 complexed with 1* rib_30/fmet-tRNA/Mg	C88583H120792N32059O47661S204Se0Mg173P5230Fe0Zn0	-4390.14	Translation
rib_ini_b1066_11	b1066 complexed with 11* rib_30/fmet-tRNA/Mg	C918823H1265862N330729O483191S2244Se0Mg1903P51660Fe0Zn0	-42411.54	Translation
rib_ini_b1066_5	b1066 complexed with 5* rib_30/fmet-tRNA/Mg	C420679H578820N151527O221873S1020Se0Mg865P23802Fe0Zn0	-19598.7	Translation
rib_ini_b1084_1	b1084 complexed with 1* rib_30/fmet-tRNA/Mg	C113382H148939N42178O65679S204Se0Mg173P7831Fe0Zn0	-6991.14	Translation
rib_ini_b1084_31	b1084 complexed with 31* rib_30/fmet-tRNA/Mg	C2604102H3584149N938188O1372269S6324Se0Mg5363P147121Fe0Zn0	-121055.34	Translation
rib_ini_b1084_62	b1084 complexed with 62* rib_30/fmet-tRNA/Mg	C5177846H7133866N1864065O2722412S12648Se0Mg10726P291054Fe0Zn0	-238921.68	Translation
rib_ini_b1086_1	b1086 complexed with 1* rib_30/fmet-tRNA/Mg	C92188H124844N33571O50262S204Se0Mg173P5605Fe0Zn0	-4765.14	Translation
rib_ini_b1086_18	b1086 complexed with 18* rib_30/fmet-tRNA/Mg	C1503596H2071463N541310O790663S3672Se0Mg3114P84536Fe0Zn0	-69401.52	Translation
rib_ini_b1086_9	b1086 complexed with 9* rib_30/fmet-tRNA/Mg	C756380H1040900N272507O398686S1836Se0Mg1557P42749Fe0Zn0	-35182.26	Translation

rib_ini_b1088_1	b1088 complexed with 1* rib_30/fmet-tRNA/Mg	C87994H120121N31848O47209S204Se0Mg173P5167Fe0Zn0	-4327.14	Translation
rib_ini_b1088_10	b1088 complexed with 10* rib_30/fmet-tRNA/Mg	C835210H1150684N300651O439186S2040Se0Mg1730P46954Fe0Zn0	-38546.4	Translation
rib_ini_b1088_5	b1088 complexed with 5* rib_30/fmet-tRNA/Mg	C420090H578149N151316O221421S1020Se0Mg865P23739Fe0Zn0	-19535.7	Translation
rib_ini_b1089_1	b1089 complexed with 1* rib_30/fmet-tRNA/Mg	C84676H116390N30529O44756S204Se0Mg173P4817Fe0Zn0	-3977.14	Translation
rib_ini_b1089_3	b1089 complexed with 3* rib_30/fmet-tRNA/Mg	C250724H345404N90263O131862S612Se0Mg519P14103Fe0Zn0	-11581.42	Translation
rib_ini_b1090_1	b1090 complexed with 1* rib_30/fmet-tRNA/Mg	C93222H126026N33935O51070S204Se0Mg173P5716Fe0Zn0	-4876.14	Translation
rib_ini_b1090_10	b1090 complexed with 10* rib_30/fmet-tRNA/Mg	C840438H1156589N302738O443047S2040Se0Mg1730P47503Fe0Zn0	-39095.4	Translation
rib_ini_b1090_20	b1090 complexed with 20* rib_30/fmet-tRNA/Mg	C1670678H2301659N601408O878577S4080Se0Mg3460P93933Fe0Zn0	-77116.8	Translation
rib_ini_b1091_1	b1091 complexed with 1* rib_30/fmet-tRNA/Mg	C92098H124776N33479O50258S204Se0Mg173P5599Fe0Zn0	-4759.14	Translation
rib_ini_b1091_18	b1091 complexed with 18* rib_30/fmet-tRNA/Mg	C1503506H2071395N541218O790659S3672Se0Mg3114P84530Fe0Zn0	-69395.52	Translation
rib_ini_b1091_9	b1091 complexed with 9* rib_30/fmet-tRNA/Mg	C756290H1040832N272415O398682S1836Se0Mg1557P42743Fe0Zn0	-35176.26	Translation
rib_ini_b1092_1	b1092 complexed with 1* rib_30/fmet-tRNA/Mg	C91891H124533N33446O50063S204Se0Mg173P5575Fe0Zn0	-4735.14	Translation
rib_ini_b1092_18	b1092 complexed with 18* rib_30/fmet-tRNA/Mg	C1503299H2071152N541185O790464S3672Se0Mg3114P84506Fe0Zn0	-69371.52	Translation
rib_ini_b1092_9	b1092 complexed with 9* rib_30/fmet-tRNA/Mg	C756083H1040589N272382O398487S1836Se0Mg1557P42719Fe0Zn0	-35152.26	Translation
rib_ini_b1093_1	b1093 complexed with 1* rib_30/fmet-tRNA/Mg	C90035H122419N32690O48697S204Se0Mg173P5380Fe0Zn0	-4540.14	Translation
rib_ini_b1093_14	b1093 complexed with 14* rib_30/fmet-tRNA/Mg	C1169347H1611010N420961O614886S2856Se0Mg2422P65739Fe0Zn0	-53967.96	Translation
rib_ini_b1093_7	b1093 complexed with 7* rib_30/fmet-tRNA/Mg	C588179H809461N211892O310015S1428Se0Mg1211P33238Fe0Zn0	-27352.98	Translation
rib_ini_b1114_1	b1114 complexed with 1* rib_30/fmet-tRNA/Mg	C115837H151673N43036O67634S204Se0Mg173P8092Fe0Zn0	-7252.14	Translation
rib_ini_b1114_33	b1114 complexed with 33* rib_30/fmet-tRNA/Mg	C2772605H3815897N998780O1461330S6732Se0Mg5709P156668Fe0Zn0	-128920.62	Translation
rib_ini_b1114_67	b1114 complexed with 67* rib_30/fmet-tRNA/Mg	C5595421H7709135N2014258O2942132S13668Se0Mg11591P314530Fe0Zn0	-258193.38	Translation
rib_ini_b1133_1	b1133 complexed with 1* rib_30/fmet-tRNA/Mg	C93561H126435N34086O51297S204Se0Mg173P5752Fe0Zn0	-4912.14	Translation
rib_ini_b1133_10	b1133 complexed with 10* rib_30/fmet-tRNA/Mg	C840777H1156998N302889O443274S2040Se0Mg1730P47539Fe0Zn0	-39131.4	Translation
rib_ini_b1133_21	b1133 complexed with 21* rib_30/fmet-tRNA/Mg	C1754041H2416575N631426O922357S4284Se0Mg3633P98612Fe0Zn0	-80954.94	Translation
rib_ini_b1134_1	b1134 complexed with 1* rib_30/fmet-tRNA/Mg	C87418H119473N31608O46792S204Se0Mg173P5105Fe0Zn0	-4265.14	Translation
rib_ini_b1134_4	b1134 complexed with 4* rib_30/fmet-tRNA/Mg	C336490H462994N121209O177451S816Se0Mg692P19034Fe0Zn0	-15671.56	Translation
rib_ini_b1134_9	b1134 complexed with 9* rib_30/fmet-tRNA/Mg	C751610H1035529N270544O395216S1836Se0Mg1557P42249Fe0Zn0	-34682.26	Translation
rib_ini_b1135_1	b1135 complexed with 1* rib_30/fmet-tRNA/Mg	C89246H121556N32355O48111S204Se0Mg173P5299Fe0Zn0	-4459.14	Translation
rib_ini_b1135_12	b1135 complexed with 12* rib_30/fmet-tRNA/Mg	C1002510H1381133N360892O527194S2448Se0Mg2076P56372Fe0Zn0	-46282.68	Translation
rib_ini_b1135_6	b1135 complexed with 6* rib_30/fmet-tRNA/Mg	C504366H694091N181690O265876S1224Se0Mg1038P28514Fe0Zn0	-23469.84	Translation
rib_ini_b1211_1	b1211 complexed with 1* rib_30/fmet-tRNA/Mg	C93345H126188N34031O51098S204Se0Mg173P5728Fe0Zn0	-4888.14	Translation
rib_ini_b1211_10	b1211 complexed with 10* rib_30/fmet-tRNA/Mg	C840561H1156751N302834O443075S2040Se0Mg1730P47515Fe0Zn0	-39107.4	Translation
rib_ini_b1211_21	b1211 complexed with 21* rib_30/fmet-tRNA/Mg	C1753825H2416328N631371O922158S4284Se0Mg3633P98588Fe0Zn0	-80930.94	Translation
rib_ini_b1212_1	b1212 complexed with 1* rib_30/fmet-tRNA/Mg	C90952H123489N33020O49410S204Se0Mg173P5479Fe0Zn0	-4639.14	Translation
rib_ini_b1212_16	b1212 complexed with 16* rib_30/fmet-tRNA/Mg	C1336312H1841094N481025O702705S3264Se0Mg2768P75124Fe0Zn0	-61671.24	Translation
rib_ini_b1212_8	b1212 complexed with 8* rib_30/fmet-tRNA/Mg	C672120H925038N242089O354281S1632Se0Mg1384P37980Fe0Zn0	-31254.12	Translation
rib_ini_b1229_1	b1229 complexed with 1* rib_30/fmet-tRNA/Mg	C84007H115620N30293O44252S204Se0Mg173P4745Fe0Zn0	-3905.14	Translation
rib_ini_b1269_1	b1269 complexed with 1* rib_30/fmet-tRNA/Mg	C91375H123940N33225O49695S204Se0Mg173P5521Fe0Zn0	-4681.14	Translation
rib_ini_b1269_17	b1269 complexed with 17* rib_30/fmet-tRNA/Mg	C1419759H1956052N511097O746543S3468Se0Mg2941P79809Fe0Zn0	-65515.38	Translation
rib_ini_b1269_8	b1269 complexed with 8* rib_30/fmet-tRNA/Mg	C672543H925489N242294O354566S1632Se0Mg1384P38022Fe0Zn0	-31296.12	Translation
rib_ini_b1286_1	b1286 complexed with 1* rib_30/fmet-tRNA/Mg	C101419H135359N37198O57068S204Se0Mg173P6580Fe0Zn0	-5740.14	Translation
rib_ini_b1286_18	b1286 complexed with 18* rib_30/fmet-tRNA/Mg	C1512827H2081978N544937O797469S3672Se0Mg3114P85511Fe0Zn0	-70376.52	Translation
rib_ini_b1286_37	b1286 complexed with 37* rib_30/fmet-tRNA/Mg	C3090283H4257611N1112410O1624976S7548Se0Mg6401P173728Fe0Zn0	-142617.18	Translation
rib_ini_b1344_1	b1344 complexed with 1* rib_30/fmet-tRNA/Mg	C91952H124581N33460O50077S204Se0Mg173P5581Fe0Zn0	-4741.14	Translation
rib_ini_b1344_18	b1344 complexed with 18* rib_30/fmet-tRNA/Mg	C1503360H2071200N541199O790478S3672Se0Mg3114P84512Fe0Zn0	-69377.52	Translation
rib_ini_b1344_9	b1344 complexed with 9* rib_30/fmet-tRNA/Mg	C756144H1040637N272396O398501S1836Se0Mg1557P42725Fe0Zn0	-35158.26	Translation
rib_ini_b1427_1	b1427 complexed with 1* rib_30/fmet-tRNA/Mg	C88172H120305N31920O47320S204Se0Mg173P5185Fe0Zn0	-4345.14	Translation
rib_ini_b1427_10	b1427 complexed with 10* rib_30/fmet-tRNA/Mg	C835388H1150868N300723O439297S2040Se0Mg1730P46972Fe0Zn0	-38564.4	Translation

rib_ini_b1427_5	b1427 complexed with 5* rib_30/fmet-tRNA/Mg	C420268H578333N151388O221532S1020Se0Mg865P23757Fe0Zn0	-19553.7	Translation
rib_ini_b1480_1	b1480 complexed with 1* rib_30/fmet-tRNA/Mg	C84345H116001N30414O44493S204Se0Mg173P4781Fe0Zn0	-3941.14	Translation
rib_ini_b1480_2	b1480 complexed with 2* rib_30/fmet-tRNA/Mg	C167369H230508N60281O88046S408Se0Mg346P9424Fe0Zn0	-7743.28	Translation
rib_ini_b1481_1	b1481 complexed with 1* rib_30/fmet-tRNA/Mg	C85078H116828N30679O45066S204Se0Mg173P4861Fe0Zn0	-4021.14	Translation
rib_ini_b1481_2	b1481 complexed with 2* rib_30/fmet-tRNA/Mg	C168102H231335N60546O88619S408Se0Mg346P9504Fe0Zn0	-7823.28	Translation
rib_ini_b1481_4	b1481 complexed with 4* rib_30/fmet-tRNA/Mg	C334150H460349N120280O175725S816Se0Mg692P18790Fe0Zn0	-15427.56	Translation
rib_ini_b1636_1	b1636 complexed with 1* rib_30/fmet-tRNA/Mg	C91254H123810N33165O49600S204Se0Mg173P5507Fe0Zn0	-4667.14	Translation
rib_ini_b1636_16	b1636 complexed with 16* rib_30/fmet-tRNA/Mg	C1336614H1841415N481170O702895S3264Se0Mg2768P75152Fe0Zn0	-61699.24	Translation
rib_ini_b1636_8	b1636 complexed with 8* rib_30/fmet-tRNA/Mg	C672422H925359N242234O354471S1632Se0Mg1384P38008Fe0Zn0	-31282.12	Translation
rib_ini_b1637_1	b1637 complexed with 1* rib_30/fmet-tRNA/Mg	C95172H128237N34742O52459S204Se0Mg173P5920Fe0Zn0	-5080.14	Translation
rib_ini_b1637_12	b1637 complexed with 12* rib_30/fmet-tRNA/Mg	C1008436H1387814N363279O531542S2448Se0Mg2076P56993Fe0Zn0	-46903.68	Translation
rib_ini_b1637_24	b1637 complexed with 24* rib_30/fmet-tRNA/Mg	C2004724H2761898N721683O1054178S4896Se0Mg4152P112709Fe0Zn0	-92529.36	Translation
rib_ini_b1638_1	b1638 complexed with 1* rib_30/fmet-tRNA/Mg	C89266H121574N32335O48165S204Se0Mg173P5302Fe0Zn0	-4462.14	Translation
rib_ini_b1638_12	b1638 complexed with 12* rib_30/fmet-tRNA/Mg	C1002530H1381151N360872O527248S2448Se0Mg2076P56375Fe0Zn0	-46285.68	Translation
rib_ini_b1638_6	b1638 complexed with 6* rib_30/fmet-tRNA/Mg	C504386H694109N181670O265930S1224Se0Mg1038P28517Fe0Zn0	-23472.84	Translation
rib_ini_b1652_1	b1652 complexed with 1* rib_30/fmet-tRNA/Mg	C89172H121490N32297O48091S204Se0Mg173P5293Fe0Zn0	-4453.14	Translation
rib_ini_b1652_12	b1652 complexed with 12* rib_30/fmet-tRNA/Mg	C1002436H1381067N360834O527174S2448Se0Mg2076P56366Fe0Zn0	-46276.68	Translation
rib_ini_b1652_6	b1652 complexed with 6* rib_30/fmet-tRNA/Mg	C504292H694025N181632O265856S1224Se0Mg1038P28508Fe0Zn0	-23463.84	Translation
rib_ini_b1653_1	b1653 complexed with 1* rib_30/fmet-tRNA/Mg	C126890H164274N47323O75925S204Se0Mg173P9260Fe0Zn0	-8420.14	Translation
rib_ini_b1653_45	b1653 complexed with 45* rib_30/fmet-tRNA/Mg	C3779946H5202582N1361471O1992257S9180Se0Mg7785P213552Fe0Zn0	-175714.3	Translation
rib_ini_b1653_90	b1653 complexed with 90* rib_30/fmet-tRNA/Mg	C7516026H10355397N2705486O3952142S18360Se0Mg15570P422487Fe0Zn0	-346810.6	Translation
rib_ini_b1712_1	b1712 complexed with 1* rib_30/fmet-tRNA/Mg	C85894H117745N31044O45636S204Se0Mg173P4945Fe0Zn0	-4105.14	Translation
rib_ini_b1712_2	b1712 complexed with 2* rib_30/fmet-tRNA/Mg	C168918H232252N60911O89189S408Se0Mg346P9588Fe0Zn0	-7907.28	Translation
rib_ini_b1712_5	b1712 complexed with 5* rib_30/fmet-tRNA/Mg	C417990H575773N150512O219848S1020Se0Mg865P23517Fe0Zn0	-19313.7	Translation
rib_ini_b1713_1	b1713 complexed with 1* rib_30/fmet-tRNA/Mg	C105779H140239N39020O60254S204Se0Mg173P7031Fe0Zn0	-6191.14	Translation
rib_ini_b1713_23	b1713 complexed with 23* rib_30/fmet-tRNA/Mg	C1932307H2659393N696094O1018420S4692Se0Mg3979P109177Fe0Zn0	-89838.22	Translation
rib_ini_b1713_46	b1713 complexed with 46* rib_30/fmet-tRNA/Mg	C3841859H5293054N1383035O2020139S9384Se0Mg7958P215966Fe0Zn0	-177287.44	Translation
rib_ini_b1714_1	b1714 complexed with 1* rib_30/fmet-tRNA/Mg	C92368H125101N33564O50450S204Se0Mg173P5627Fe0Zn0	-4787.14	Translation
rib_ini_b1714_19	b1714 complexed with 19* rib_30/fmet-tRNA/Mg	C1586800H2186227N571170O834404S3876Se0Mg3287P89201Fe0Zn0	-73225.66	Translation
rib_ini_b1714_9	b1714 complexed with 9* rib_30/fmet-tRNA/Mg	C756560H1041157N272500O398874S1836Se0Mg1557P42771Fe0Zn0	-35204.26	Translation
rib_ini_b1715_1	b1715 complexed with 1* rib_30/fmet-tRNA/Mg	C83443H114982N30009O43888S204Se0Mg173P4690Fe0Zn0	-3850.14	Translation
rib_ini_b1716_1	b1716 complexed with 1* rib_30/fmet-tRNA/Mg	C86420H118347N31216O46044S204Se0Mg173P5000Fe0Zn0	-4160.14	Translation
rib_ini_b1716_3	b1716 complexed with 3* rib_30/fmet-tRNA/Mg	C252468H347361N90950O133150S612Se0Mg519P14286Fe0Zn0	-11764.42	Translation
rib_ini_b1716_6	b1716 complexed with 6* rib_30/fmet-tRNA/Mg	C501540H690882N180551O263809S1224Se0Mg1038P28215Fe0Zn0	-23170.84	Translation
rib_ini_b1717_1	b1717 complexed with 1* rib_30/fmet-tRNA/Mg	C84913H116648N30637O44917S204Se0Mg173P4841Fe0Zn0	-4001.14	Translation
rib_ini_b1717_3	b1717 complexed with 3* rib_30/fmet-tRNA/Mg	C250961H345662N90371O132023S612Se0Mg519P14127Fe0Zn0	-11605.42	Translation
rib_ini_b1718_1	b1718 complexed with 1* rib_30/fmet-tRNA/Mg	C88227H120371N32018O47296S204Se0Mg173P5186Fe0Zn0	-4346.14	Translation
rib_ini_b1718_10	b1718 complexed with 10* rib_30/fmet-tRNA/Mg	C835443H1150934N300821O439273S2040Se0Mg1730P46973Fe0Zn0	-38565.4	Translation
rib_ini_b1718_5	b1718 complexed with 5* rib_30/fmet-tRNA/Mg	C420323H578399N151486O221508S1020Se0Mg865P23758Fe0Zn0	-19554.7	Translation
rib_ini_b1719_1	b1719 complexed with 1* rib_30/fmet-tRNA/Mg	C101408H135273N37246O56998S204Se0Mg173P6574Fe0Zn0	-5734.14	Translation
rib_ini_b1719_18	b1719 complexed with 18* rib_30/fmet-tRNA/Mg	C1512816H2081892N544985O797399S3672Se0Mg3114P85505Fe0Zn0	-70370.52	Translation
rib_ini_b1719_37	b1719 complexed with 37* rib_30/fmet-tRNA/Mg	C3090272H4257525N1112458O1624906S7548Se0Mg6401P173722Fe0Zn0	-142611.18	Translation
rib_ini_b1804_1	b1804 complexed with 1* rib_30/fmet-tRNA/Mg	C93755H126656N34149O51458S204Se0Mg173P5773Fe0Zn0	-4933.14	Translation
rib_ini_b1804_11	b1804 complexed with 11* rib_30/fmet-tRNA/Mg	C923995H1271726N332819O486988S2244Se0Mg1903P52203Fe0Zn0	-42954.54	Translation
rib_ini_b1804_22	b1804 complexed with 22* rib_30/fmet-tRNA/Mg	C1837259H2531303N661356O966071S4488Se0Mg3806P103276Fe0Zn0	-84778.08	Translation
rib_ini_b1822_1	b1822 complexed with 1* rib_30/fmet-tRNA/Mg	C90727H123218N32923O49236S204Se0Mg173P5455Fe0Zn0	-4615.14	Translation
rib_ini_b1822_15	b1822 complexed with 15* rib_30/fmet-tRNA/Mg	C1253063H1726316N451061O658978S3060Se0Mg2595P70457Fe0Zn0	-57845.1	Translation

rib_ini_b1822_7	b1822 complexed with 7* rib_30/fmet-tRNA/Mg	C588871H810260N212125O310554S1428Se0Mg1211P33313Fe0Zn0	-27427.98	Translation
rib_ini_b1866_1	b1866 complexed with 1* rib_30/fmet-tRNA/Mg	C99900H133608N36621O55957S204Se0Mg173P6418Fe0Zn0	-5578.14	Translation
rib_ini_b1866_17	b1866 complexed with 17* rib_30/fmet-tRNA/Mg	C1428284H1965720N514493O752805S3468Se0Mg2941P80706Fe0Zn0	-66412.38	Translation
rib_ini_b1866_34	b1866 complexed with 34* rib_30/fmet-tRNA/Mg	C2839692H3912339N1022232O1493206S6936Se0Mg5882P159637Fe0Zn0	-131048.76	Translation
rib_ini_b1869_1	b1869 complexed with 1* rib_30/fmet-tRNA/Mg	C86770H118736N31291O46393S204Se0Mg173P5041Fe0Zn0	-4201.14	Translation
rib_ini_b1869_3	b1869 complexed with 3* rib_30/fmet-tRNA/Mg	C252818H347750N91025O133499S612Se0Mg519P14327Fe0Zn0	-11805.42	Translation
rib_ini_b1869_7	b1869 complexed with 7* rib_30/fmet-tRNA/Mg	C584914H805778N210493O307711S1428Se0Mg1211P32899Fe0Zn0	-27013.98	Translation
rib_ini_b1870_1	b1870 complexed with 1* rib_30/fmet-tRNA/Mg	C90086H122502N32641O48775S204Se0Mg173P5387Fe0Zn0	-4547.14	Translation
rib_ini_b1870_14	b1870 complexed with 14* rib_30/fmet-tRNA/Mg	C1169398H1611093N420912O614964S2856Se0Mg2422P65746Fe0Zn0	-53974.96	Translation
rib_ini_b1870_7	b1870 complexed with 7* rib_30/fmet-tRNA/Mg	C588230H809544N211843O310093S1428Se0Mg1211P33245Fe0Zn0	-27359.98	Translation
rib_ini_b1871_1	b1871 complexed with 1* rib_30/fmet-tRNA/Mg	C92231H124918N33523O50343S204Se0Mg173P5611Fe0Zn0	-4771.14	Translation
rib_ini_b1871_19	b1871 complexed with 19* rib_30/fmet-tRNA/Mg	C1586663H2186044N571129O834297S3876Se0Mg3287P89185Fe0Zn0	-73209.66	Translation
rib_ini_b1871_9	b1871 complexed with 9* rib_30/fmet-tRNA/Mg	C756423H1040974N272459O398767S1836Se0Mg1557P42755Fe0Zn0	-35188.26	Translation
rib_ini_b1876_1	b1876 complexed with 1* rib_30/fmet-tRNA/Mg	C99563H133217N36570O55618S204Se0Mg173P6379Fe0Zn0	-5539.14	Translation
rib_ini_b1876_16	b1876 complexed with 16* rib_30/fmet-tRNA/Mg	C1344923H1850822N484575O708913S3264Se0Mg2768P76024Fe0Zn0	-62571.24	Translation
rib_ini_b1876_33	b1876 complexed with 33* rib_30/fmet-tRNA/Mg	C2756331H3797441N992314O1449314S6732Se0Mg5709P154955Fe0Zn0	-127207.62	Translation
rib_ini_b1920_1	b1920 complexed with 1* rib_30/fmet-tRNA/Mg	C90690H123137N33002O49123S204Se0Mg173P5444Fe0Zn0	-4604.14	Translation
rib_ini_b1920_15	b1920 complexed with 15* rib_30/fmet-tRNA/Mg	C1253026H1726235N451140O658865S3060Se0Mg2595P70446Fe0Zn0	-57834.1	Translation
rib_ini_b1920_7	b1920 complexed with 7* rib_30/fmet-tRNA/Mg	C588834H810179N212204O310441S1428Se0Mg1211P33302Fe0Zn0	-27416.98	Translation
rib_ini_b1921_1	b1921 complexed with 1* rib_30/fmet-tRNA/Mg	C88264H120438N31925O47407S204Se0Mg173P5195Fe0Zn0	-4355.14	Translation
rib_ini_b1921_10	b1921 complexed with 10* rib_30/fmet-tRNA/Mg	C835480H1151001N300728O439384S2040Se0Mg1730P46982Fe0Zn0	-38574.4	Translation
rib_ini_b1921_5	b1921 complexed with 5* rib_30/fmet-tRNA/Mg	C420360H578466N151393O221619S1020Se0Mg865P23767Fe0Zn0	-19563.7	Translation
rib_ini_b1922_1	b1922 complexed with 1* rib_30/fmet-tRNA/Mg	C89900H122283N32674O48560S204Se0Mg173P5365Fe0Zn0	-4525.14	Translation
rib_ini_b1922_14	b1922 complexed with 14* rib_30/fmet-tRNA/Mg	C1169212H1610874N420945O614749S2856Se0Mg2422P65724Fe0Zn0	-53952.96	Translation
rib_ini_b1922_7	b1922 complexed with 7* rib_30/fmet-tRNA/Mg	C588044H809325N211876O309878S1428Se0Mg1211P33223Fe0Zn0	-27337.98	Translation
rib_ini_b2114_1	b2114 complexed with 1* rib_30/fmet-tRNA/Mg	C102368H136420N37583O57767S204Se0Mg173P6679Fe0Zn0	-5839.14	Translation
rib_ini_b2114_19	b2114 complexed with 19* rib_30/fmet-tRNA/Mg	C1596800H2197546N575189O841721S3876Se0Mg3287P90253Fe0Zn0	-74277.66	Translation
rib_ini_b2114_39	b2114 complexed with 39* rib_30/fmet-tRNA/Mg	C3257280H4487686N1172529O1712781S7956Se0Mg6747P183113Fe0Zn0	-150320.46	Translation
rib_ini_b2140_1	b2140 complexed with 1* rib_30/fmet-tRNA/Mg	C92077H124723N33540O50188S204Se0Mg173P5593Fe0Zn0	-4753.14	Translation
rib_ini_b2140_18	b2140 complexed with 18* rib_30/fmet-tRNA/Mg	C1503485H2071342N541279O790589S3672Se0Mg3114P84524Fe0Zn0	-69389.52	Translation
rib_ini_b2140_9	b2140 complexed with 9* rib_30/fmet-tRNA/Mg	C756269H1040779N272476O398612S1836Se0Mg1557P42737Fe0Zn0	-35170.26	Translation
rib_ini_b2183_1	b2183 complexed with 1* rib_30/fmet-tRNA/Mg	C89639H121998N32491O48436S204Se0Mg173P5341Fe0Zn0	-4501.14	Translation
rib_ini_b2183_13	b2183 complexed with 13* rib_30/fmet-tRNA/Mg	C1085927H1496082N390895O571072S2652Se0Mg2249P61057Fe0Zn0	-50126.82	Translation
rib_ini_b2183_6	b2183 complexed with 6* rib_30/fmet-tRNA/Mg	C504759H694533N181826O266201S1224Se0Mg1038P28556Fe0Zn0	-23511.84	Translation
rib_ini_b2185_1	b2185 complexed with 1* rib_30/fmet-tRNA/Mg	C85746H117587N30980O45526S204Se0Mg173P4930Fe0Zn0	-4090.14	Translation
rib_ini_b2185_2	b2185 complexed with 2* rib_30/fmet-tRNA/Mg	C168770H232094N60847O89079S408Se0Mg346P9573Fe0Zn0	-7892.28	Translation
rib_ini_b2185_5	b2185 complexed with 5* rib_30/fmet-tRNA/Mg	C417842H575615N150448O219738S1020Se0Mg865P23502Fe0Zn0	-19298.7	Translation
rib_ini_b2268_1	b2268 complexed with 1* rib_30/fmet-tRNA/Mg	C91746H124381N33316O49984S204Se0Mg173P5563Fe0Zn0	-4723.14	Translation
rib_ini_b2268_17	b2268 complexed with 17* rib_30/fmet-tRNA/Mg	C1420130H1956493N511188O746832S3468Se0Mg2941P79851Fe0Zn0	-65557.38	Translation
rib_ini_b2268_8	b2268 complexed with 8* rib_30/fmet-tRNA/Mg	C672914H925930N242385O354855S1632Se0Mg1384P38064Fe0Zn0	-31338.12	Translation
rib_ini_b2317_1	b2317 complexed with 1* rib_30/fmet-tRNA/Mg	C89268H121566N32253O48241S204Se0Mg173P5303Fe0Zn0	-4463.14	Translation
rib_ini_b2317_12	b2317 complexed with 12* rib_30/fmet-tRNA/Mg	C1002532H1381143N360790O527324S2448Se0Mg2076P56376Fe0Zn0	-46286.68	Translation
rib_ini_b2317_6	b2317 complexed with 6* rib_30/fmet-tRNA/Mg	C504388H694101N181588O266006S1224Se0Mg1038P28518Fe0Zn0	-23473.84	Translation
rib_ini_b2318_1	b2318 complexed with 1* rib_30/fmet-tRNA/Mg	C90762H123266N32989O49224S204Se0Mg173P5455Fe0Zn0	-4615.14	Translation
rib_ini_b2318_15	b2318 complexed with 15* rib_30/fmet-tRNA/Mg	C1253098H1726364N451127O658966S3060Se0Mg2595P70457Fe0Zn0	-57845.1	Translation
rib_ini_b2318_7	b2318 complexed with 7* rib_30/fmet-tRNA/Mg	C588906H810308N212191O310542S1428Se0Mg1211P33313Fe0Zn0	-27427.98	Translation
rib_ini_b2319_1	b2319 complexed with 1* rib_30/fmet-tRNA/Mg	C92689H125436N33761O50653S204Se0Mg173P5659Fe0Zn0	-4819.14	Translation

rib_ini_b2319_19	b2319 complexed with 19* rib_30/fmet-tRNA/Mg	C1587121H2186562N571367O834607S3876Se0Mg3287P89233Fe0Zn0	-73257.66	Translation
rib_ini_b2319_9	b2319 complexed with 9* rib_30/fmet-tRNA/Mg	C756881H1041492N272697O399077S1836Se0Mg1557P42803Fe0Zn0	-35236.26	Translation
rib_ini_b2320_1	b2320 complexed with 1* rib_30/fmet-tRNA/Mg	C93843H126732N34167O51551S204Se0Mg173P5782Fe0Zn0	-4942.14	Translation
rib_ini_b2320_11	b2320 complexed with 11* rib_30/fmet-tRNA/Mg	C924083H1271802N332837O487081S2244Se0Mg1903P52212Fe0Zn0	-42963.54	Translation
rib_ini_b2320_22	b2320 complexed with 22* rib_30/fmet-tRNA/Mg	C1837347H2531379N661374O966164S4488Se0Mg3806P103285Fe0Zn0	-84787.08	Translation
rib_ini_b2324_1	b2324 complexed with 1* rib_30/fmet-tRNA/Mg	C102128H136099N37484O57635S204Se0Mg173P6652Fe0Zn0	-5812.14	Translation
rib_ini_b2324_19	b2324 complexed with 19* rib_30/fmet-tRNA/Mg	C1596560H2197225N575090O841589S3876Se0Mg3287P90226Fe0Zn0	-74250.66	Translation
rib_ini_b2324_39	b2324 complexed with 39* rib_30/fmet-tRNA/Mg	C3257040H4487365N1172430O1712649S7956Se0Mg6747P183086Fe0Zn0	-150293.46	Translation
rib_ini_b2325_1	b2325 complexed with 1* rib_30/fmet-tRNA/Mg	C85685H117510N30937O45509S204Se0Mg173P4922Fe0Zn0	-4082.14	Translation
rib_ini_b2325_2	b2325 complexed with 2* rib_30/fmet-tRNA/Mg	C168709H232017N60804O89062S408Se0Mg346P9565Fe0Zn0	-7884.28	Translation
rib_ini_b2325_5	b2325 complexed with 5* rib_30/fmet-tRNA/Mg	C417781H575538N150405O219721S1020Se0Mg865P23494Fe0Zn0	-19290.7	Translation
rib_ini_b2326_1	b2326 complexed with 1* rib_30/fmet-tRNA/Mg	C88238H120397N31936O47396S204Se0Mg173P5191Fe0Zn0	-4351.14	Translation
rib_ini_b2326_10	b2326 complexed with 10* rib_30/fmet-tRNA/Mg	C835454H1150960N300739O439373S2040Se0Mg1730P46978Fe0Zn0	-38570.4	Translation
rib_ini_b2326_5	b2326 complexed with 5* rib_30/fmet-tRNA/Mg	C420334H578425N151404O221608S1020Se0Mg865P23763Fe0Zn0	-19559.7	Translation
rib_ini_b2327_1	b2327 complexed with 1* rib_30/fmet-tRNA/Mg	C90686H123181N32830O49287S204Se0Mg173P5452Fe0Zn0	-4612.14	Translation
rib_ini_b2327_15	b2327 complexed with 15* rib_30/fmet-tRNA/Mg	C1253022H1726279N450968O659029S3060Se0Mg2595P70454Fe0Zn0	-57842.1	Translation
rib_ini_b2327_7	b2327 complexed with 7* rib_30/fmet-tRNA/Mg	C588830H810223N212032O310605S1428Se0Mg1211P33310Fe0Zn0	-27424.98	Translation
rib_ini_b2328_1	b2328 complexed with 1* rib_30/fmet-tRNA/Mg	C90861H123401N32984O49325S204Se0Mg173P5468Fe0Zn0	-4628.14	Translation
rib_ini_b2328_16	b2328 complexed with 16* rib_30/fmet-tRNA/Mg	C1336221H1841006N480989O702620S3264Se0Mg2768P75113Fe0Zn0	-61660.24	Translation
rib_ini_b2328_8	b2328 complexed with 8* rib_30/fmet-tRNA/Mg	C672029H924950N242053O354196S1632Se0Mg1384P37969Fe0Zn0	-31243.12	Translation
rib_ini_b2329_1	b2329 complexed with 1* rib_30/fmet-tRNA/Mg	C93356H126231N34018O51132S204Se0Mg173P5729Fe0Zn0	-4889.14	Translation
rib_ini_b2329_10	b2329 complexed with 10* rib_30/fmet-tRNA/Mg	C840572H1156794N302821O443109S2040Se0Mg1730P47516Fe0Zn0	-39108.4	Translation
rib_ini_b2329_21	b2329 complexed with 21* rib_30/fmet-tRNA/Mg	C1753836H2416371N631358O922192S4284Se0Mg3633P98589Fe0Zn0	-80931.94	Translation
rib_ini_b2330_1	b2330 complexed with 1* rib_30/fmet-tRNA/Mg	C91890H124546N33379O50098S204Se0Mg173P5578Fe0Zn0	-4738.14	Translation
rib_ini_b2330_18	b2330 complexed with 18* rib_30/fmet-tRNA/Mg	C1503298H2071165N541118O790499S3672Se0Mg3114P84509Fe0Zn0	-69374.52	Translation
rib_ini_b2330_9	b2330 complexed with 9* rib_30/fmet-tRNA/Mg	C756082H1040602N272315O398522S1836Se0Mg1557P42722Fe0Zn0	-35155.26	Translation
rib_ini_b2400_1	b2400 complexed with 1* rib_30/fmet-tRNA/Mg	C96507H129761N35270O53446S204Se0Mg173P6061Fe0Zn0	-5221.14	Translation
rib_ini_b2400_13	b2400 complexed with 13* rib_30/fmet-tRNA/Mg	C1092795H1503845N393674O576082S2652Se0Mg2249P61777Fe0Zn0	-50846.82	Translation
rib_ini_b2400_27	b2400 complexed with 27* rib_30/fmet-tRNA/Mg	C2255131H3106943N811812O1185824S5508Se0Mg4671P126779Fe0Zn0	-104076.78	Translation
rib_ini_b2514_1	b2514 complexed with 1* rib_30/fmet-tRNA/Mg	C95166H128238N34731O52498S204Se0Mg173P5920Fe0Zn0	-5080.14	Translation
rib_ini_b2514_12	b2514 complexed with 12* rib_30/fmet-tRNA/Mg	C1008430H1387815N363268O531581S2448Se0Mg2076P56993Fe0Zn0	-46903.68	Translation
rib_ini_b2514_24	b2514 complexed with 24* rib_30/fmet-tRNA/Mg	C2004718H2761899N721672O1054217S4896Se0Mg4152P112709Fe0Zn0	-92529.36	Translation
rib_ini_b2528_1	b2528 complexed with 1* rib_30/fmet-tRNA/Mg	C86122H117998N31129O45813S204Se0Mg173P4967Fe0Zn0	-4127.14	Translation
rib_ini_b2528_3	b2528 complexed with 3* rib_30/fmet-tRNA/Mg	C252170H347012N90863O132919S612Se0Mg519P14253Fe0Zn0	-11731.42	Translation
rib_ini_b2528_6	b2528 complexed with 6* rib_30/fmet-tRNA/Mg	C501242H690533N180464O263578S1224Se0Mg1038P28182Fe0Zn0	-23137.84	Translation
rib_ini_b2529_1	b2529 complexed with 1* rib_30/fmet-tRNA/Mg	C86726H118686N31387O46228S204Se0Mg173P5030Fe0Zn0	-4190.14	Translation
rib_ini_b2529_3	b2529 complexed with 3* rib_30/fmet-tRNA/Mg	C252774H347700N91121O133334S612Se0Mg519P14316Fe0Zn0	-11794.42	Translation
rib_ini_b2529_7	b2529 complexed with 7* rib_30/fmet-tRNA/Mg	C584870H805728N210589O307546S1428Se0Mg1211P32888Fe0Zn0	-27002.98	Translation
rib_ini_b2530_1	b2530 complexed with 1* rib_30/fmet-tRNA/Mg	C94583H127604N34491O52030S204Se0Mg173P5858Fe0Zn0	-5018.14	Translation
rib_ini_b2530_11	b2530 complexed with 11* rib_30/fmet-tRNA/Mg	C924823H1272674N333161O487560S2244Se0Mg1903P52288Fe0Zn0	-43039.54	Translation
rib_ini_b2530_23	b2530 complexed with 23* rib_30/fmet-tRNA/Mg	C1921111H2646758N691565O1010196S4692Se0Mg3979P108004Fe0Zn0	-88665.22	Translation
rib_ini_b2531_1	b2531 complexed with 1* rib_30/fmet-tRNA/Mg	C87670H119770N31707O46990S204Se0Mg173P5134Fe0Zn0	-4294.14	Translation
rib_ini_b2531_4	b2531 complexed with 4* rib_30/fmet-tRNA/Mg	C336742H463291N121308O177649S816Se0Mg692P19063Fe0Zn0	-15700.56	Translation
rib_ini_b2531_9	b2531 complexed with 9* rib_30/fmet-tRNA/Mg	C751862H1035826N270643O395414S1836Se0Mg1557P42278Fe0Zn0	-34711.26	Translation
rib_ini_b2559_1	b2559 complexed with 1* rib_30/fmet-tRNA/Mg	C88143H120290N31925O47317S204Se0Mg173P5180Fe0Zn0	-4340.14	Translation
rib_ini_b2559_10	b2559 complexed with 10* rib_30/fmet-tRNA/Mg	C835359H1150853N300728O439294S2040Se0Mg1730P46967Fe0Zn0	-38559.4	Translation
rib_ini_b2559_5	b2559 complexed with 5* rib_30/fmet-tRNA/Mg	C420239H578318N151393O221529S1020Se0Mg865P23752Fe0Zn0	-19548.7	Translation

rib_ini_b2560_1	b2560 complexed with 1* rib_30/fmet-tRNA/Mg	C89051H121326N32203O48064S204Se0Mg173P5281Fe0Zn0	-4441.14	Translation
rib_ini_b2560_12	b2560 complexed with 12* rib_30/fmet-tRNA/Mg	C1002315H1380903N360740O527147S2448Se0Mg2076P56354Fe0Zn0	-46264.68	Translation
rib_ini_b2560_6	b2560 complexed with 6* rib_30/fmet-tRNA/Mg	C504171H693861N181538O265829S1224Se0Mg1038P28496Fe0Zn0	-23451.84	Translation
rib_ini_b2563_1	b2563 complexed with 1* rib_30/fmet-tRNA/Mg	C86655H118601N31342O46204S204Se0Mg173P5023Fe0Zn0	-4183.14	Translation
rib_ini_b2563_3	b2563 complexed with 3* rib_30/fmet-tRNA/Mg	C252703H347615N91076O133310S612Se0Mg519P14309Fe0Zn0	-11787.42	Translation
rib_ini_b2563_7	b2563 complexed with 7* rib_30/fmet-tRNA/Mg	C584799H805643N210544O307522S1428Se0Mg1211P32881Fe0Zn0	-26995.98	Translation
rib_ini_b2564_1	b2564 complexed with 1* rib_30/fmet-tRNA/Mg	C90006H122414N32705O48657S204Se0Mg173P5377Fe0Zn0	-4537.14	Translation
rib_ini_b2564_14	b2564 complexed with 14* rib_30/fmet-tRNA/Mg	C1169318H1611005N420976O614846S2856Se0Mg2422P65736Fe0Zn0	-53964.96	Translation
rib_ini_b2564_7	b2564 complexed with 7* rib_30/fmet-tRNA/Mg	C588150H809456N211907O309975S1428Se0Mg1211P33235Fe0Zn0	-27349.98	Translation
rib_ini_b2565_1	b2565 complexed with 1* rib_30/fmet-tRNA/Mg	C89949H122357N32612O48662S204Se0Mg173P5372Fe0Zn0	-4532.14	Translation
rib_ini_b2565_14	b2565 complexed with 14* rib_30/fmet-tRNA/Mg	C1169261H1610948N420883O614851S2856Se0Mg2422P65731Fe0Zn0	-53959.96	Translation
rib_ini_b2565_7	b2565 complexed with 7* rib_30/fmet-tRNA/Mg	C588093H809399N211814O309980S1428Se0Mg1211P33230Fe0Zn0	-27344.98	Translation
rib_ini_b2566_1	b2566 complexed with 1* rib_30/fmet-tRNA/Mg	C91622H124237N33340O49825S204Se0Mg173P5545Fe0Zn0	-4705.14	Translation
rib_ini_b2566_17	b2566 complexed with 17* rib_30/fmet-tRNA/Mg	C1420006H1956349N511212O746673S3468Se0Mg2941P79833Fe0Zn0	-65539.38	Translation
rib_ini_b2566_8	b2566 complexed with 8* rib_30/fmet-tRNA/Mg	C672790H925786N242409O354696S1632Se0Mg1384P38046Fe0Zn0	-31320.12	Translation
rib_ini_b2567_1	b2567 complexed with 1* rib_30/fmet-tRNA/Mg	C89512H121838N32467O48313S204Se0Mg173P5326Fe0Zn0	-4486.14	Translation
rib_ini_b2567_13	b2567 complexed with 13* rib_30/fmet-tRNA/Mg	C1085800H1495922N390871O570949S2652Se0Mg2249P61042Fe0Zn0	-50111.82	Translation
rib_ini_b2567_6	b2567 complexed with 6* rib_30/fmet-tRNA/Mg	C504632H694373N181802O266078S1224Se0Mg1038P28541Fe0Zn0	-23496.84	Translation
rib_ini_b2570_1	b2570 complexed with 1* rib_30/fmet-tRNA/Mg	C87538H119620N31631O46920S204Se0Mg173P5119Fe0Zn0	-4279.14	Translation
rib_ini_b2570_4	b2570 complexed with 4* rib_30/fmet-tRNA/Mg	C336610H463141N121232O177579S816Se0Mg692P19048Fe0Zn0	-15685.56	Translation
rib_ini_b2570_9	b2570 complexed with 9* rib_30/fmet-tRNA/Mg	C751730H1035676N270567O395344S1836Se0Mg1557P42263Fe0Zn0	-34696.26	Translation
rib_ini_b2571_1	b2571 complexed with 1* rib_30/fmet-tRNA/Mg	C92092H124781N33420O50262S204Se0Mg173P5599Fe0Zn0	-4759.14	Translation
rib_ini_b2571_18	b2571 complexed with 18* rib_30/fmet-tRNA/Mg	C1503500H2071400N541159O790663S3672Se0Mg3114P84530Fe0Zn0	-69395.52	Translation
rib_ini_b2571_9	b2571 complexed with 9* rib_30/fmet-tRNA/Mg	C756284H1040837N272356O398686S1836Se0Mg1557P42743Fe0Zn0	-35176.26	Translation
rib_ini_b2572_1	b2572 complexed with 1* rib_30/fmet-tRNA/Mg	C89233H121541N32392O48056S204Se0Mg173P5294Fe0Zn0	-4454.14	Translation
rib_ini_b2572_12	b2572 complexed with 12* rib_30/fmet-tRNA/Mg	C1002497H1381118N360929O527139S2448Se0Mg2076P56367Fe0Zn0	-46277.68	Translation
rib_ini_b2572_6	b2572 complexed with 6* rib_30/fmet-tRNA/Mg	C504353H694076N181727O265821S1224Se0Mg1038P28509Fe0Zn0	-23464.84	Translation
rib_ini_b2573_1	b2573 complexed with 1* rib_30/fmet-tRNA/Mg	C88517H120695N32064O47595S204Se0Mg173P5221Fe0Zn0	-4381.14	Translation
rib_ini_b2573_11	b2573 complexed with 11* rib_30/fmet-tRNA/Mg	C918757H1265765N330734O483125S2244Se0Mg1903P51651Fe0Zn0	-42402.54	Translation
rib_ini_b2573_5	b2573 complexed with 5* rib_30/fmet-tRNA/Mg	C420613H578723N151532O221807S1020Se0Mg865P23793Fe0Zn0	-19589.7	Translation
rib_ini_b2593_1	b2593 complexed with 1* rib_30/fmet-tRNA/Mg	C89943H122336N32605O48682S204Se0Mg173P5371Fe0Zn0	-4531.14	Translation
rib_ini_b2593_14	b2593 complexed with 14* rib_30/fmet-tRNA/Mg	C1169255H1610927N420876O614871S2856Se0Mg2422P65730Fe0Zn0	-53958.96	Translation
rib_ini_b2593_7	b2593 complexed with 7* rib_30/fmet-tRNA/Mg	C588087H809378N211807O310000S1428Se0Mg1211P33229Fe0Zn0	-27343.98	Translation
rib_ini_b2594_1	b2594 complexed with 1* rib_30/fmet-tRNA/Mg	C92366H125078N33615O50406S204Se0Mg173P5626Fe0Zn0	-4786.14	Translation
rib_ini_b2594_19	b2594 complexed with 19* rib_30/fmet-tRNA/Mg	C1586798H2186204N571221O834360S3876Se0Mg3287P89200Fe0Zn0	-73224.66	Translation
rib_ini_b2594_9	b2594 complexed with 9* rib_30/fmet-tRNA/Mg	C756558H1041134N272551O398830S1836Se0Mg1557P42770Fe0Zn0	-35203.26	Translation
rib_ini_b2606_1	b2606 complexed with 1* rib_30/fmet-tRNA/Mg	C86334H118244N31175O45994S204Se0Mg173P4991Fe0Zn0	-4151.14	Translation
rib_ini_b2606_3	b2606 complexed with 3* rib_30/fmet-tRNA/Mg	C252382H347258N90909O133100S612Se0Mg519P14277Fe0Zn0	-11755.42	Translation
rib_ini_b2606_6	b2606 complexed with 6* rib_30/fmet-tRNA/Mg	C501454H690779N180510O263759S1224Se0Mg1038P28206Fe0Zn0	-23161.84	Translation
rib_ini_b2607_1	b2607 complexed with 1* rib_30/fmet-tRNA/Mg	C90359H122790N32851O48904S204Se0Mg173P5411Fe0Zn0	-4571.14	Translation
rib_ini_b2607_15	b2607 complexed with 15* rib_30/fmet-tRNA/Mg	C1252695H1725888N450989O658646S3060Se0Mg2595P70413Fe0Zn0	-57801.1	Translation
rib_ini_b2607_7	b2607 complexed with 7* rib_30/fmet-tRNA/Mg	C588503H809832N212053O310222S1428Se0Mg1211P33269Fe0Zn0	-27383.98	Translation
rib_ini_b2608_1	b2608 complexed with 1* rib_30/fmet-tRNA/Mg	C88258H120412N31965O47387S204Se0Mg173P5192Fe0Zn0	-4352.14	Translation
rib_ini_b2608_10	b2608 complexed with 10* rib_30/fmet-tRNA/Mg	C835474H1150975N300768O439364S2040Se0Mg1730P46979Fe0Zn0	-38571.4	Translation
rib_ini_b2608_5	b2608 complexed with 5* rib_30/fmet-tRNA/Mg	C420354H578440N151433O221599S1020Se0Mg865P23764Fe0Zn0	-19560.7	Translation
rib_ini_b2609_1	b2609 complexed with 1* rib_30/fmet-tRNA/Mg	C85391H117188N30807O45300S204Se0Mg173P4894Fe0Zn0	-4054.14	Translation
rib_ini_b2609_2	b2609 complexed with 2* rib_30/fmet-tRNA/Mg	C168415H231695N60674O88853S408Se0Mg346P9537Fe0Zn0	-7856.28	Translation

rib_ini_b2609_4	b2609 complexed with 4* rib_30/fmet-tRNA/Mg	C334463H460709N120408O175959S816Se0Mg692P18823Fe0Zn0	-15460.56	Translation
rib_ini_b2614_1	b2614 complexed with 1* rib_30/fmet-tRNA/Mg	C88714H120913N32208O47681S204Se0Mg173P5239Fe0Zn0	-4399.14	Translation
rib_ini_b2614_11	b2614 complexed with 11* rib_30/fmet-tRNA/Mg	C918954H1265983N330878O483211S2244Se0Mg1903P51669Fe0Zn0	-42420.54	Translation
rib_ini_b2614_5	b2614 complexed with 5* rib_30/fmet-tRNA/Mg	C420810H578941N151676O221893S1020Se0Mg865P23811Fe0Zn0	-19607.7	Translation
rib_ini_b2697_1	b2697 complexed with 1* rib_30/fmet-tRNA/Mg	C108104H142856N39969O61943S204Se0Mg173P7276Fe0Zn0	-6436.14	Translation
rib_ini_b2697_25	b2697 complexed with 25* rib_30/fmet-tRNA/Mg	C2100680H2891024N756777O1107215S5100Se0Mg4325P118708Fe0Zn0	-97687.5	Translation
rib_ini_b2697_51	b2697 complexed with 51* rib_30/fmet-tRNA/Mg	C4259304H5868206N1533319O2239593S10404Se0Mg8823P239426Fe0Zn0	-196543.14	Translation
rib_ini_b2741_1	b2741 complexed with 1* rib_30/fmet-tRNA/Mg	C92507H125209N33716O50479S204Se0Mg173P5638Fe0Zn0	-4798.14	Translation
rib_ini_b2741_19	b2741 complexed with 19* rib_30/fmet-tRNA/Mg	C1586939H2186335N571322O834433S3876Se0Mg3287P89212Fe0Zn0	-73236.66	Translation
rib_ini_b2741_9	b2741 complexed with 9* rib_30/fmet-tRNA/Mg	C756699H1041265N272652O398903S1836Se0Mg1557P42782Fe0Zn0	-35215.26	Translation
rib_ini_b2742_1	b2742 complexed with 1* rib_30/fmet-tRNA/Mg	C93887H126819N34264O51456S204Se0Mg173P5785Fe0Zn0	-4945.14	Translation
rib_ini_b2742_11	b2742 complexed with 11* rib_30/fmet-tRNA/Mg	C924127H1271889N332934O486986S2244Se0Mg1903P52215Fe0Zn0	-42966.54	Translation
rib_ini_b2742_22	b2742 complexed with 22* rib_30/fmet-tRNA/Mg	C1837391H2531466N661471O966069S4488Se0Mg3806P103288Fe0Zn0	-84790.08	Translation
rib_ini_b2745_1	b2745 complexed with 1* rib_30/fmet-tRNA/Mg	C93046H125818N33921O50888S204Se0Mg173P5695Fe0Zn0	-4855.14	Translation
rib_ini_b2745_10	b2745 complexed with 10* rib_30/fmet-tRNA/Mg	C840262H1156381N302724O442865S2040Se0Mg1730P47482Fe0Zn0	-39074.4	Translation
rib_ini_b2745_20	b2745 complexed with 20* rib_30/fmet-tRNA/Mg	C1670502H2301451N601394O878395S4080Se0Mg3460P93912Fe0Zn0	-77095.8	Translation
rib_ini_b2779_1	b2779 complexed with 1* rib_30/fmet-tRNA/Mg	C95403H128506N34849O52584S204Se0Mg173P5942Fe0Zn0	-5102.14	Translation
rib_ini_b2779_12	b2779 complexed with 12* rib_30/fmet-tRNA/Mg	C1008667H1388083N363386O531667S2448Se0Mg2076P57015Fe0Zn0	-46925.68	Translation
rib_ini_b2779_25	b2779 complexed with 25* rib_30/fmet-tRNA/Mg	C2087979H2876674N751657O1097856S5100Se0Mg4325P117374Fe0Zn0	-96353.5	Translation
rib_ini_b2780_1	b2780 complexed with 1* rib_30/fmet-tRNA/Mg	C98608H132136N36075O55018S204Se0Mg173P6283Fe0Zn0	-5443.14	Translation
rib_ini_b2780_16	b2780 complexed with 16* rib_30/fmet-tRNA/Mg	C1343968H1849741N484080O708313S3264Se0Mg2768P75928Fe0Zn0	-62475.24	Translation
rib_ini_b2780_32	b2780 complexed with 32* rib_30/fmet-tRNA/Mg	C2672352H3681853N961952O1405161S6528Se0Mg5536P150216Fe0Zn0	-123309.48	Translation
rib_ini_b2785_1	b2785 complexed with 1* rib_30/fmet-tRNA/Mg	C95422H128529N34832O52644S204Se0Mg173P5947Fe0Zn0	-5107.14	Translation
rib_ini_b2785_12	b2785 complexed with 12* rib_30/fmet-tRNA/Mg	C1008686H1388106N363369O531727S2448Se0Mg2076P57020Fe0Zn0	-46930.68	Translation
rib_ini_b2785_25	b2785 complexed with 25* rib_30/fmet-tRNA/Mg	C2087998H2876697N751640O1097916S5100Se0Mg4325P117379Fe0Zn0	-96358.5	Translation
rib_ini_b2790_1	b2790 complexed with 1* rib_30/fmet-tRNA/Mg	C87318H119352N31599O46703S204Se0Mg173P5093Fe0Zn0	-4253.14	Translation
rib_ini_b2790_4	b2790 complexed with 4* rib_30/fmet-tRNA/Mg	C336390H462873N121200O177362S816Se0Mg692P19022Fe0Zn0	-15659.56	Translation
rib_ini_b2790_8	b2790 complexed with 8* rib_30/fmet-tRNA/Mg	C668486H920901N240668O351574S1632Se0Mg1384P37594Fe0Zn0	-30868.12	Translation
rib_ini_b2791_1	b2791 complexed with 1* rib_30/fmet-tRNA/Mg	C90473H122935N32860O49019S204Se0Mg173P5425Fe0Zn0	-4585.14	Translation
rib_ini_b2791_15	b2791 complexed with 15* rib_30/fmet-tRNA/Mg	C1252809H1726033N450998O658761S3060Se0Mg2595P70427Fe0Zn0	-57815.1	Translation
rib_ini_b2791_7	b2791 complexed with 7* rib_30/fmet-tRNA/Mg	C588617H809977N212062O310337S1428Se0Mg1211P33283Fe0Zn0	-27397.98	Translation
rib_ini_b2792_1	b2792 complexed with 1* rib_30/fmet-tRNA/Mg	C86158H118059N31106O45871S204Se0Mg173P4975Fe0Zn0	-4135.14	Translation
rib_ini_b2792_3	b2792 complexed with 3* rib_30/fmet-tRNA/Mg	C252206H347073N90840O132977S612Se0Mg519P14261Fe0Zn0	-11739.42	Translation
rib_ini_b2792_6	b2792 complexed with 6* rib_30/fmet-tRNA/Mg	C501278H690594N180441O263636S1224Se0Mg1038P28190Fe0Zn0	-23145.84	Translation
rib_ini_b2794_1	b2794 complexed with 1* rib_30/fmet-tRNA/Mg	C91092H123633N33054O49497S204Se0Mg173P5494Fe0Zn0	-4654.14	Translation
rib_ini_b2794_16	b2794 complexed with 16* rib_30/fmet-tRNA/Mg	C1336452H1841238N481059O702792S3264Se0Mg2768P75139Fe0Zn0	-61686.24	Translation
rib_ini_b2794_8	b2794 complexed with 8* rib_30/fmet-tRNA/Mg	C672260H925182N242123O354368S1632Se0Mg1384P37995Fe0Zn0	-31269.12	Translation
rib_ini_b2890_1	b2890 complexed with 1* rib_30/fmet-tRNA/Mg	C97466H130870N35645O54140S204Se0Mg173P6161Fe0Zn0	-5321.14	Translation
rib_ini_b2890_14	b2890 complexed with 14* rib_30/fmet-tRNA/Mg	C1176778H1619461N423916O620329S2856Se0Mg2422P66520Fe0Zn0	-54748.96	Translation
rib_ini_b2890_29	b2890 complexed with 29* rib_30/fmet-tRNA/Mg	C2422138H3337066N871921O1273624S5916Se0Mg5017P136165Fe0Zn0	-111781.06	Translation
rib_ini_b2891_1	b2891 complexed with 1* rib_30/fmet-tRNA/Mg	C93516H126373N34142O51190S204Se0Mg173P5744Fe0Zn0	-4904.14	Translation
rib_ini_b2891_10	b2891 complexed with 10* rib_30/fmet-tRNA/Mg	C840732H1156936N302945O443167S2040Se0Mg1730P47531Fe0Zn0	-39123.4	Translation
rib_ini_b2891_21	b2891 complexed with 21* rib_30/fmet-tRNA/Mg	C1753996H2416513N631482O922250S4284Se0Mg3633P98604Fe0Zn0	-80946.94	Translation
rib_ini_b2892_1	b2892 complexed with 1* rib_30/fmet-tRNA/Mg	C99527H133194N36475O55716S204Se0Mg173P6377Fe0Zn0	-5537.14	Translation
rib_ini_b2892_16	b2892 complexed with 16* rib_30/fmet-tRNA/Mg	C1344887H1850799N484480O709011S3264Se0Mg2768P76022Fe0Zn0	-62569.24	Translation
rib_ini_b2892_33	b2892 complexed with 33* rib_30/fmet-tRNA/Mg	C2756295H3797418N992219O1449412S6732Se0Mg5709P154953Fe0Zn0	-127205.62	Translation
rib_ini_b2893_1	b2893 complexed with 1* rib_30/fmet-tRNA/Mg	C89814H122165N32618O48498S204Se0Mg173P5356Fe0Zn0	-4516.14	Translation

rib_ini_b2893_13	b2893 complexed with 13* rib_30/fmet-tRNA/Mg	C1086102H1496249N391022O571134S2652Se0Mg2249P61072Fe0Zn0	-50141.82	Translation
rib_ini_b2893_6	b2893 complexed with 6* rib_30/fmet-tRNA/Mg	C504934H694700N181953O266263S1224Se0Mg1038P28571Fe0Zn0	-23526.84	Translation
rib_ini_b2946_1	b2946 complexed with 1* rib_30/fmet-tRNA/Mg	C89989H122393N32650O48675S204Se0Mg173P5377Fe0Zn0	-4537.14	Translation
rib_ini_b2946_14	b2946 complexed with 14* rib_30/fmet-tRNA/Mg	C1169301H1610984N420921O614864S2856Se0Mg2422P65736Fe0Zn0	-53964.96	Translation
rib_ini_b2946_7	b2946 complexed with 7* rib_30/fmet-tRNA/Mg	C588133H809435N211852O309993S1428Se0Mg1211P33235Fe0Zn0	-27349.98	Translation
rib_ini_b2947_1	b2947 complexed with 1* rib_30/fmet-tRNA/Mg	C92090H124764N33529O50178S204Se0Mg173P5594Fe0Zn0	-4754.14	Translation
rib_ini_b2947_18	b2947 complexed with 18* rib_30/fmet-tRNA/Mg	C1503498H2071383N541268O790579S3672Se0Mg3114P84525Fe0Zn0	-69390.52	Translation
rib_ini_b2947_9	b2947 complexed with 9* rib_30/fmet-tRNA/Mg	C756282H1040820N272465O398602S1836Se0Mg1557P42738Fe0Zn0	-35171.26	Translation
rib_ini_b2959_1	b2959 complexed with 1* rib_30/fmet-tRNA/Mg	C86147H118022N31151O45813S204Se0Mg173P4969Fe0Zn0	-4129.14	Translation
rib_ini_b2959_3	b2959 complexed with 3* rib_30/fmet-tRNA/Mg	C252195H347036N90885O132919S612Se0Mg519P14255Fe0Zn0	-11733.42	Translation
rib_ini_b2959_6	b2959 complexed with 6* rib_30/fmet-tRNA/Mg	C501267H690557N180486O263578S1224Se0Mg1038P28184Fe0Zn0	-23139.84	Translation
rib_ini_b2960_1	b2960 complexed with 1* rib_30/fmet-tRNA/Mg	C89884H122254N32613O48607S204Se0Mg173P5365Fe0Zn0	-4525.14	Translation
rib_ini_b2960_14	b2960 complexed with 14* rib_30/fmet-tRNA/Mg	C1169196H1610845N420884O614796S2856Se0Mg2422P65724Fe0Zn0	-53952.96	Translation
rib_ini_b2960_7	b2960 complexed with 7* rib_30/fmet-tRNA/Mg	C588028H809296N211815O309925S1428Se0Mg1211P33223Fe0Zn0	-27337.98	Translation
rib_ini_b3065_1	b3065 complexed with 1* rib_30/fmet-tRNA/Mg	C85084H116839N30702O45058S204Se0Mg173P4861Fe0Zn0	-4021.14	Translation
rib_ini_b3065_2	b3065 complexed with 2* rib_30/fmet-tRNA/Mg	C168108H231346N60569O88611S408Se0Mg346P9504Fe0Zn0	-7823.28	Translation
rib_ini_b3065_4	b3065 complexed with 4* rib_30/fmet-tRNA/Mg	C334156H460360N120303O175717S816Se0Mg692P18790Fe0Zn0	-15427.56	Translation
rib_ini_b3066_1	b3066 complexed with 1* rib_30/fmet-tRNA/Mg	C99641H133320N36517O55731S204Se0Mg173P6389Fe0Zn0	-5549.14	Translation
rib_ini_b3066_17	b3066 complexed with 17* rib_30/fmet-tRNA/Mg	C1428025H1965432N514389O752579S3468Se0Mg2941P80677Fe0Zn0	-66383.38	Translation
rib_ini_b3066_34	b3066 complexed with 34* rib_30/fmet-tRNA/Mg	C2839433H3912051N1022128O1492980S6936Se0Mg5882P159608Fe0Zn0	-131019.76	Translation
rib_ini_b3067_1	b3067 complexed with 1* rib_30/fmet-tRNA/Mg	C100603H134406N37031O56316S204Se0Mg173P6487Fe0Zn0	-5647.14	Translation
rib_ini_b3067_18	b3067 complexed with 18* rib_30/fmet-tRNA/Mg	C1512011H2081025N544770O796717S3672Se0Mg3114P85418Fe0Zn0	-70283.52	Translation
rib_ini_b3067_36	b3067 complexed with 36* rib_30/fmet-tRNA/Mg	C3006443H4142151N1082376O1580671S7344Se0Mg6228P168992Fe0Zn0	-138722.04	Translation
rib_ini_b3124_1	b3124 complexed with 1* rib_30/fmet-tRNA/Mg	C94747H127733N34636O52121S204Se0Mg173P5870Fe0Zn0	-5030.14	Translation
rib_ini_b3124_12	b3124 complexed with 12* rib_30/fmet-tRNA/Mg	C1008011H1387310N363173O531204S2448Se0Mg2076P56943Fe0Zn0	-46853.68	Translation
rib_ini_b3124_24	b3124 complexed with 24* rib_30/fmet-tRNA/Mg	C2004299H2761394N721577O1053840S4896Se0Mg4152P112659Fe0Zn0	-92479.36	Translation
rib_ini_b3125_1	b3125 complexed with 1* rib_30/fmet-tRNA/Mg	C91540H124113N33338O49763S204Se0Mg173P5534Fe0Zn0	-4694.14	Translation
rib_ini_b3125_17	b3125 complexed with 17* rib_30/fmet-tRNA/Mg	C1419924H1956225N511210O746611S3468Se0Mg2941P79822Fe0Zn0	-65528.38	Translation
rib_ini_b3125_8	b3125 complexed with 8* rib_30/fmet-tRNA/Mg	C672708H925662N242407O354634S1632Se0Mg1384P38035Fe0Zn0	-31309.12	Translation
rib_ini_b3126_1	b3126 complexed with 1* rib_30/fmet-tRNA/Mg	C90348H122809N32770O48948S204Se0Mg173P5414Fe0Zn0	-4574.14	Translation
rib_ini_b3126_15	b3126 complexed with 15* rib_30/fmet-tRNA/Mg	C1252684H1725907N450908O658690S3060Se0Mg2595P70416Fe0Zn0	-57804.1	Translation
rib_ini_b3126_7	b3126 complexed with 7* rib_30/fmet-tRNA/Mg	C588492H809851N211972O310266S1428Se0Mg1211P33272Fe0Zn0	-27386.98	Translation
rib_ini_b3127_1	b3127 complexed with 1* rib_30/fmet-tRNA/Mg	C95656H128808N34721O53025S204Se0Mg173P5980Fe0Zn0	-5140.14	Translation
rib_ini_b3127_13	b3127 complexed with 13* rib_30/fmet-tRNA/Mg	C1091944H1502892N393125O575661S2652Se0Mg2249P61696Fe0Zn0	-50765.82	Translation
rib_ini_b3127_26	b3127 complexed with 26* rib_30/fmet-tRNA/Mg	C2171256H2991483N781396O1141850S5304Se0Mg4498P122055Fe0Zn0	-100193.64	Translation
rib_ini_b3164_1	b3164 complexed with 1* rib_30/fmet-tRNA/Mg	C104025H138273N38304O58948S204Se0Mg173P6848Fe0Zn0	-6008.14	Translation
rib_ini_b3164_21	b3164 complexed with 21* rib_30/fmet-tRNA/Mg	C1764505H2428413N635644O930008S4284Se0Mg3633P99708Fe0Zn0	-82050.94	Translation
rib_ini_b3164_43	b3164 complexed with 43* rib_30/fmet-tRNA/Mg	C3591033H4947567N1292718O1888174S8772Se0Mg7439P201854Fe0Zn0	-165698.02	Translation
rib_ini_b3165_1	b3165 complexed with 1* rib_30/fmet-tRNA/Mg	C85589H117419N30888O45432S204Se0Mg173P4913Fe0Zn0	-4073.14	Translation
rib_ini_b3165_2	b3165 complexed with 2* rib_30/fmet-tRNA/Mg	C168613H231926N60755O88985S408Se0Mg346P9556Fe0Zn0	-7875.28	Translation
rib_ini_b3165_5	b3165 complexed with 5* rib_30/fmet-tRNA/Mg	C417685H575447N150356O219644S1020Se0Mg865P23485Fe0Zn0	-19281.7	Translation
rib_ini_b3166_1	b3166 complexed with 1* rib_30/fmet-tRNA/Mg	C92005H124664N33441O50170S204Se0Mg173P5587Fe0Zn0	-4747.14	Translation
rib_ini_b3166_18	b3166 complexed with 18* rib_30/fmet-tRNA/Mg	C1503413H2071283N541180O790571S3672Se0Mg3114P84518Fe0Zn0	-69383.52	Translation
rib_ini_b3166_9	b3166 complexed with 9* rib_30/fmet-tRNA/Mg	C756197H1040720N272377O398594S1836Se0Mg1557P42731Fe0Zn0	-35164.26	Translation
rib_ini_b3167_1	b3167 complexed with 1* rib_30/fmet-tRNA/Mg	C86862H118848N31431O46343S204Se0Mg173P5045Fe0Zn0	-4205.14	Translation
rib_ini_b3167_3	b3167 complexed with 3* rib_30/fmet-tRNA/Mg	C252910H347862N91165O133449S612Se0Mg519P14331Fe0Zn0	-11809.42	Translation
rib_ini_b3167_7	b3167 complexed with 7* rib_30/fmet-tRNA/Mg	C585006H805890N210633O307661S1428Se0Mg1211P32903Fe0Zn0	-27017.98	Translation

rib_ini_b3168_1	b3168 complexed with 1* rib_30/fmet-tRNA/Mg	C108592H143396N40393O62050S204Se0Mg173P7316Fe0Zn0	-6476.14	Translation
rib_ini_b3168_26	b3168 complexed with 26* rib_30/fmet-tRNA/Mg	C2184192H3006071N787068O1150875S5304Se0Mg4498P123391Fe0Zn0	-101529.64	Translation
rib_ini_b3168_52	b3168 complexed with 52* rib_30/fmet-tRNA/Mg	C4342816H5983253N1563610O2283253S10608Se0Mg8996P244109Fe0Zn0	-200385.28	Translation
rib_ini_b3169_1	b3169 complexed with 1* rib_30/fmet-tRNA/Mg	C97236H130560N35655O53899S204Se0Mg173P6131Fe0Zn0	-5291.14	Translation
rib_ini_b3169_14	b3169 complexed with 14* rib_30/fmet-tRNA/Mg	C1176548H1619151N423926O620088S2856Se0Mg2422P66490Fe0Zn0	-54718.96	Translation
rib_ini_b3169_29	b3169 complexed with 29* rib_30/fmet-tRNA/Mg	C2421908H3336756N871931O1273383S5916Se0Mg5017P136135Fe0Zn0	-111751.06	Translation
rib_ini_b3170_1	b3170 complexed with 1* rib_30/fmet-tRNA/Mg	C87334H119372N31573O46730S204Se0Mg173P5096Fe0Zn0	-4256.14	Translation
rib_ini_b3170_4	b3170 complexed with 4* rib_30/fmet-tRNA/Mg	C336406H462893N121174O177389S816Se0Mg692P19025Fe0Zn0	-15662.56	Translation
rib_ini_b3170_8	b3170 complexed with 8* rib_30/fmet-tRNA/Mg	C668502H920921N240642O351601S1632Se0Mg1384P37597Fe0Zn0	-30871.12	Translation
rib_ini_b3178_1	b3178 complexed with 1* rib_30/fmet-tRNA/Mg	C101462H135357N37282O57073S204Se0Mg173P6578Fe0Zn0	-5738.14	Translation
rib_ini_b3178_18	b3178 complexed with 18* rib_30/fmet-tRNA/Mg	C1512870H2081976N545021O797474S3672Se0Mg3114P85509Fe0Zn0	-70374.52	Translation
rib_ini_b3178_37	b3178 complexed with 37* rib_30/fmet-tRNA/Mg	C3090326H4257609N1112494O1624981S7548Se0Mg6401P173726Fe0Zn0	-142615.18	Translation
rib_ini_b3179_1	b3179 complexed with 1* rib_30/fmet-tRNA/Mg	C89026H121277N32260O47980S204Se0Mg173P5275Fe0Zn0	-4435.14	Translation
rib_ini_b3179_12	b3179 complexed with 12* rib_30/fmet-tRNA/Mg	C1002290H1380854N360797O527063S2448Se0Mg2076P56348Fe0Zn0	-46258.68	Translation
rib_ini_b3179_6	b3179 complexed with 6* rib_30/fmet-tRNA/Mg	C504146H693812N181595O265745S1224Se0Mg1038P28490Fe0Zn0	-23445.84	Translation
rib_ini_b3181_1	b3181 complexed with 1* rib_30/fmet-tRNA/Mg	C87580H119645N31714O46873S204Se0Mg173P5122Fe0Zn0	-4282.14	Translation
rib_ini_b3181_4	b3181 complexed with 4* rib_30/fmet-tRNA/Mg	C336652H463166N121315O177532S816Se0Mg692P19051Fe0Zn0	-15688.56	Translation
rib_ini_b3181_9	b3181 complexed with 9* rib_30/fmet-tRNA/Mg	C751772H1035701N270650O395297S1836Se0Mg1557P42266Fe0Zn0	-34699.26	Translation
rib_ini_b3185_1	b3185 complexed with 1* rib_30/fmet-tRNA/Mg	C85487H117289N30866O45345S204Se0Mg173P4901Fe0Zn0	-4061.14	Translation
rib_ini_b3185_2	b3185 complexed with 2* rib_30/fmet-tRNA/Mg	C168511H231796N60733O88898S408Se0Mg346P9544Fe0Zn0	-7863.28	Translation
rib_ini_b3185_5	b3185 complexed with 5* rib_30/fmet-tRNA/Mg	C417583H575317N150334O219557S1020Se0Mg865P23473Fe0Zn0	-19269.7	Translation
rib_ini_b3186_1	b3186 complexed with 1* rib_30/fmet-tRNA/Mg	C86005H117865N31074O45733S204Se0Mg173P4957Fe0Zn0	-4117.14	Translation
rib_ini_b3186_3	b3186 complexed with 3* rib_30/fmet-tRNA/Mg	C252053H346879N90808O132839S612Se0Mg519P14243Fe0Zn0	-11721.42	Translation
rib_ini_b3186_6	b3186 complexed with 6* rib_30/fmet-tRNA/Mg	C501125H690400N180409O263498S1224Se0Mg1038P28172Fe0Zn0	-23127.84	Translation
rib_ini_b3201_1	b3201 complexed with 1* rib_30/fmet-tRNA/Mg	C89928H122333N32624O48633S204Se0Mg173P5371Fe0Zn0	-4531.14	Translation
rib_ini_b3201_14	b3201 complexed with 14* rib_30/fmet-tRNA/Mg	C1169240H1610924N420895O614822S2856Se0Mg2422P65730Fe0Zn0	-53958.96	Translation
rib_ini_b3201_7	b3201 complexed with 7* rib_30/fmet-tRNA/Mg	C588072H809375N211826O309951S1428Se0Mg1211P33229Fe0Zn0	-27343.98	Translation
rib_ini_b3202_1	b3202 complexed with 1* rib_30/fmet-tRNA/Mg	C96683H129983N35376O53520S204Se0Mg173P6079Fe0Zn0	-5239.14	Translation
rib_ini_b3202_14	b3202 complexed with 14* rib_30/fmet-tRNA/Mg	C1175995H1618574N423647O619709S2856Se0Mg2422P66438Fe0Zn0	-54666.96	Translation
rib_ini_b3202_28	b3202 complexed with 28* rib_30/fmet-tRNA/Mg	C2338331H3221672N841785O1229451S5712Se0Mg4844P131440Fe0Zn0	-107896.92	Translation
rib_ini_b3203_1	b3203 complexed with 1* rib_30/fmet-tRNA/Mg	C85775H117613N30986O45537S204Se0Mg173P4931Fe0Zn0	-4091.14	Translation
rib_ini_b3203_2	b3203 complexed with 2* rib_30/fmet-tRNA/Mg	C168799H232120N60853O89090S408Se0Mg346P9574Fe0Zn0	-7893.28	Translation
rib_ini_b3203_5	b3203 complexed with 5* rib_30/fmet-tRNA/Mg	C417871H575641N150454O219749S1020Se0Mg865P23503Fe0Zn0	-19299.7	Translation
rib_ini_b3204_1	b3204 complexed with 1* rib_30/fmet-tRNA/Mg	C87710H119816N31755O46971S204Se0Mg173P5135Fe0Zn0	-4295.14	Translation
rib_ini_b3204_4	b3204 complexed with 4* rib_30/fmet-tRNA/Mg	C336782H463337N121356O177630S816Se0Mg692P19064Fe0Zn0	-15701.56	Translation
rib_ini_b3204_9	b3204 complexed with 9* rib_30/fmet-tRNA/Mg	C751902H1035872N270691O395395S1836Se0Mg1557P42279Fe0Zn0	-34712.26	Translation
rib_ini_b3205_1	b3205 complexed with 1* rib_30/fmet-tRNA/Mg	C91130H123702N33043O49552S204Se0Mg173P5498Fe0Zn0	-4658.14	Translation
rib_ini_b3205_16	b3205 complexed with 16* rib_30/fmet-tRNA/Mg	C1336490H1841307N481048O702847S3264Se0Mg2768P75143Fe0Zn0	-61690.24	Translation
rib_ini_b3205_8	b3205 complexed with 8* rib_30/fmet-tRNA/Mg	C672298H925251N242112O354423S1632Se0Mg1384P37999Fe0Zn0	-31273.12	Translation
rib_ini_b3206_1	b3206 complexed with 1* rib_30/fmet-tRNA/Mg	C85591H117403N30902O45429S204Se0Mg173P4912Fe0Zn0	-4072.14	Translation
rib_ini_b3206_2	b3206 complexed with 2* rib_30/fmet-tRNA/Mg	C168615H231910N60769O88982S408Se0Mg346P9555Fe0Zn0	-7874.28	Translation
rib_ini_b3206_5	b3206 complexed with 5* rib_30/fmet-tRNA/Mg	C417687H575431N150370O219641S1020Se0Mg865P23484Fe0Zn0	-19280.7	Translation
rib_ini_b3230_1	b3230 complexed with 1* rib_30/fmet-tRNA/Mg	C86758H118737N31346O46306S204Se0Mg173P5036Fe0Zn0	-4196.14	Translation
rib_ini_b3230_3	b3230 complexed with 3* rib_30/fmet-tRNA/Mg	C252806H347751N91080O133412S612Se0Mg519P14322Fe0Zn0	-11800.42	Translation
rib_ini_b3230_7	b3230 complexed with 7* rib_30/fmet-tRNA/Mg	C584902H805779N210548O307624S1428Se0Mg1211P32894Fe0Zn0	-27008.98	Translation
rib_ini_b3231_1	b3231 complexed with 1* rib_30/fmet-tRNA/Mg	C87113H119136N31519O46533S204Se0Mg173P5074Fe0Zn0	-4234.14	Translation
rib_ini_b3231_4	b3231 complexed with 4* rib_30/fmet-tRNA/Mg	C336185H462657N121120O177192S816Se0Mg692P19003Fe0Zn0	-15640.56	Translation

rib_ini_b3231_8	b3231 complexed with 8* rib_30/fmet-tRNA/Mg	C668281H920685N240588O351404S1632Se0Mg1384P37575Fe0Zn0	-30849.12	Translation
rib_ini_b3247_1	b3247 complexed with 1* rib_30/fmet-tRNA/Mg	C96933H130223N35466O53736S204Se0Mg173P6102Fe0Zn0	-5262.14	Translation
rib_ini_b3247_14	b3247 complexed with 14* rib_30/fmet-tRNA/Mg	C1176245H1618814N423737O619925S2856Se0Mg2422P66461Fe0Zn0	-54689.96	Translation
rib_ini_b3247_28	b3247 complexed with 28* rib_30/fmet-tRNA/Mg	C2338581H3221912N841875O1229667S5712Se0Mg4844P131463Fe0Zn0	-107919.92	Translation
rib_ini_b3248_1	b3248 complexed with 1* rib_30/fmet-tRNA/Mg	C88697H120908N32169O47705S204Se0Mg173P5237Fe0Zn0	-4397.14	Translation
rib_ini_b3248_11	b3248 complexed with 11* rib_30/fmet-tRNA/Mg	C918937H1265978N330839O483235S2244Se0Mg1903P51667Fe0Zn0	-42418.54	Translation
rib_ini_b3248_5	b3248 complexed with 5* rib_30/fmet-tRNA/Mg	C420793H578936N151637O221917S1020Se0Mg865P23809Fe0Zn0	-19605.7	Translation
rib_ini_b3249_1	b3249 complexed with 1* rib_30/fmet-tRNA/Mg	C87642H119723N31630O47038S204Se0Mg173P5131Fe0Zn0	-4291.14	Translation
rib_ini_b3249_4	b3249 complexed with 4* rib_30/fmet-tRNA/Mg	C336714H463244N121231O177697S816Se0Mg692P19060Fe0Zn0	-15697.56	Translation
rib_ini_b3249_9	b3249 complexed with 9* rib_30/fmet-tRNA/Mg	C751834H1035779N270566O395462S1836Se0Mg1557P42275Fe0Zn0	-34708.26	Translation
rib_ini_b3250_1	b3250 complexed with 1* rib_30/fmet-tRNA/Mg	C93515H126393N34030O51314S204Se0Mg173P5747Fe0Zn0	-4907.14	Translation
rib_ini_b3250_10	b3250 complexed with 10* rib_30/fmet-tRNA/Mg	C840731H1156956N302833O443291S2040Se0Mg1730P47534Fe0Zn0	-39126.4	Translation
rib_ini_b3250_21	b3250 complexed with 21* rib_30/fmet-tRNA/Mg	C1753995H2416533N631370O922374S4284Se0Mg3633P98607Fe0Zn0	-80949.94	Translation
rib_ini_b3251_1	b3251 complexed with 1* rib_30/fmet-tRNA/Mg	C92946H125738N33797O50899S204Se0Mg173P5689Fe0Zn0	-4849.14	Translation
rib_ini_b3251_10	b3251 complexed with 10* rib_30/fmet-tRNA/Mg	C840162H1156301N302600O442876S2040Se0Mg1730P47476Fe0Zn0	-39068.4	Translation
rib_ini_b3251_20	b3251 complexed with 20* rib_30/fmet-tRNA/Mg	C1670402H2301371N601270O878406S4080Se0Mg3460P93906Fe0Zn0	-77089.8	Translation
rib_ini_b3257_1	b3257 complexed with 1* rib_30/fmet-tRNA/Mg	C85322H117108N30745O45296S204Se0Mg173P4888Fe0Zn0	-4048.14	Translation
rib_ini_b3257_2	b3257 complexed with 2* rib_30/fmet-tRNA/Mg	C168346H231615N60612O88849S408Se0Mg346P9531Fe0Zn0	-7850.28	Translation
rib_ini_b3257_4	b3257 complexed with 4* rib_30/fmet-tRNA/Mg	C334394H460629N120346O175955S816Se0Mg692P18817Fe0Zn0	-15454.56	Translation
rib_ini_b3258_1	b3258 complexed with 1* rib_30/fmet-tRNA/Mg	C96669H129963N35146O53776S204Se0Mg173P6084Fe0Zn0	-5244.14	Translation
rib_ini_b3258_14	b3258 complexed with 14* rib_30/fmet-tRNA/Mg	C1175981H1618554N423417O619965S2856Se0Mg2422P66443Fe0Zn0	-54671.96	Translation
rib_ini_b3258_28	b3258 complexed with 28* rib_30/fmet-tRNA/Mg	C2338317H3221652N841555O1229707S5712Se0Mg4844P131445Fe0Zn0	-107901.92	Translation
rib_ini_b3259_1	b3259 complexed with 1* rib_30/fmet-tRNA/Mg	C91424H124015N33242O49715S204Se0Mg173P5525Fe0Zn0	-4685.14	Translation
rib_ini_b3259_17	b3259 complexed with 17* rib_30/fmet-tRNA/Mg	C1419808H1956127N511114O746563S3468Se0Mg2941P79813Fe0Zn0	-65519.38	Translation
rib_ini_b3259_8	b3259 complexed with 8* rib_30/fmet-tRNA/Mg	C672592H925564N242311O354586S1632Se0Mg1384P38026Fe0Zn0	-31300.12	Translation
rib_ini_b3260_1	b3260 complexed with 1* rib_30/fmet-tRNA/Mg	C92222H124912N33551O50305S204Se0Mg173P5611Fe0Zn0	-4771.14	Translation
rib_ini_b3260_18	b3260 complexed with 18* rib_30/fmet-tRNA/Mg	C1503630H2071531N541290O790706S3672Se0Mg3114P84542Fe0Zn0	-69407.52	Translation
rib_ini_b3260_9	b3260 complexed with 9* rib_30/fmet-tRNA/Mg	C756414H1040968N272487O398729S1836Se0Mg1557P42755Fe0Zn0	-35188.26	Translation
rib_ini_b3261_1	b3261 complexed with 1* rib_30/fmet-tRNA/Mg	C85856H117706N31007O45614S204Se0Mg173P4940Fe0Zn0	-4100.14	Translation
rib_ini_b3261_2	b3261 complexed with 2* rib_30/fmet-tRNA/Mg	C168880H232213N60874O89167S408Se0Mg346P9583Fe0Zn0	-7902.28	Translation
rib_ini_b3261_5	b3261 complexed with 5* rib_30/fmet-tRNA/Mg	C417952H575734N150475O219826S1020Se0Mg865P23512Fe0Zn0	-19308.7	Translation
rib_ini_b3280_1	b3280 complexed with 1* rib_30/fmet-tRNA/Mg	C85455H117243N30860O45321S204Se0Mg173P4897Fe0Zn0	-4057.14	Translation
rib_ini_b3280_2	b3280 complexed with 2* rib_30/fmet-tRNA/Mg	C168479H231750N60727O88874S408Se0Mg346P9540Fe0Zn0	-7859.28	Translation
rib_ini_b3280_5	b3280 complexed with 5* rib_30/fmet-tRNA/Mg	C417551H575271N150328O219533S1020Se0Mg865P23469Fe0Zn0	-19265.7	Translation
rib_ini_b3281_1	b3281 complexed with 1* rib_30/fmet-tRNA/Mg	C90801H123287N32906O49329S204Se0Mg173P5462Fe0Zn0	-4622.14	Translation
rib_ini_b3281_16	b3281 complexed with 16* rib_30/fmet-tRNA/Mg	C1336161H1840892N480911O702624S3264Se0Mg2768P75107Fe0Zn0	-61654.24	Translation
rib_ini_b3281_8	b3281 complexed with 8* rib_30/fmet-tRNA/Mg	C671969H924836N241975O354200S1632Se0Mg1384P37963Fe0Zn0	-31237.12	Translation
rib_ini_b3282_1	b3282 complexed with 1* rib_30/fmet-tRNA/Mg	C88466H120663N32008O47587S204Se0Mg173P5216Fe0Zn0	-4376.14	Translation
rib_ini_b3282_11	b3282 complexed with 11* rib_30/fmet-tRNA/Mg	C918706H1265733N330678O483117S2244Se0Mg1903P51646Fe0Zn0	-42397.54	Translation
rib_ini_b3282_5	b3282 complexed with 5* rib_30/fmet-tRNA/Mg	C420562H578691N151476O221799S1020Se0Mg865P23788Fe0Zn0	-19584.7	Translation
rib_ini_b3283_1	b3283 complexed with 1* rib_30/fmet-tRNA/Mg	C88185H120346N31909O47358S204Se0Mg173P5188Fe0Zn0	-4348.14	Translation
rib_ini_b3283_10	b3283 complexed with 10* rib_30/fmet-tRNA/Mg	C835401H1150909N300712O439335S2040Se0Mg1730P46975Fe0Zn0	-38567.4	Translation
rib_ini_b3283_5	b3283 complexed with 5* rib_30/fmet-tRNA/Mg	C420281H578374N151377O221570S1020Se0Mg865P23760Fe0Zn0	-19556.7	Translation
rib_ini_b3287_1	b3287 complexed with 1* rib_30/fmet-tRNA/Mg	C87894H119993N31832O47106S204Se0Mg173P5155Fe0Zn0	-4315.14	Translation
rib_ini_b3287_4	b3287 complexed with 4* rib_30/fmet-tRNA/Mg	C336966H463514N121433O177765S816Se0Mg692P19084Fe0Zn0	-15721.56	Translation
rib_ini_b3287_9	b3287 complexed with 9* rib_30/fmet-tRNA/Mg	C752086H1036049N270768O395530S1836Se0Mg1557P42299Fe0Zn0	-34732.26	Translation
rib_ini_b3288_1	b3288 complexed with 1* rib_30/fmet-tRNA/Mg	C92041H124718N33463O50179S204Se0Mg173P5591Fe0Zn0	-4751.14	Translation

rib_ini_b3288_18	b3288 complexed with 18* rib_30/fmet-tRNA/Mg	C1503449H2071337N541202O790580S3672Se0Mg3114P84522Fe0Zn0	-69387.52	Translation
rib_ini_b3288_9	b3288 complexed with 9* rib_30/fmet-tRNA/Mg	C756233H1040774N272399O398603S1836Se0Mg1557P42735Fe0Zn0	-35168.26	Translation
rib_ini_b3289_1	b3289 complexed with 1* rib_30/fmet-tRNA/Mg	C95291H128382N34735O52573S204Se0Mg173P5935Fe0Zn0	-5095.14	Translation
rib_ini_b3289_12	b3289 complexed with 12* rib_30/fmet-tRNA/Mg	C1008555H1387959N363272O531656S2448Se0Mg2076P57008Fe0Zn0	-46918.68	Translation
rib_ini_b3289_25	b3289 complexed with 25* rib_30/fmet-tRNA/Mg	C2087867H2876550N751543O1097845S5100Se0Mg4325P117367Fe0Zn0	-96346.5	Translation
rib_ini_b3294_1	b3294 complexed with 1* rib_30/fmet-tRNA/Mg	C86677H118644N31325O46240S204Se0Mg173P5027Fe0Zn0	-4187.14	Translation
rib_ini_b3294_3	b3294 complexed with 3* rib_30/fmet-tRNA/Mg	C252725H347658N91059O133346S612Se0Mg519P14313Fe0Zn0	-11791.42	Translation
rib_ini_b3294_7	b3294 complexed with 7* rib_30/fmet-tRNA/Mg	C584821H805686N210527O307558S1428Se0Mg1211P32885Fe0Zn0	-26999.98	Translation
rib_ini_b3295_1	b3295 complexed with 1* rib_30/fmet-tRNA/Mg	C92449H125169N33638O50469S204Se0Mg173P5633Fe0Zn0	-4793.14	Translation
rib_ini_b3295_19	b3295 complexed with 19* rib_30/fmet-tRNA/Mg	C1586881H2186295N571244O834423S3876Se0Mg3287P89207Fe0Zn0	-73231.66	Translation
rib_ini_b3295_9	b3295 complexed with 9* rib_30/fmet-tRNA/Mg	C756641H1041225N272574O398893S1836Se0Mg1557P42777Fe0Zn0	-35210.26	Translation
rib_ini_b3296_1	b3296 complexed with 1* rib_30/fmet-tRNA/Mg	C88947H121191N32250O47891S204Se0Mg173P5264Fe0Zn0	-4424.14	Translation
rib_ini_b3296_12	b3296 complexed with 12* rib_30/fmet-tRNA/Mg	C1002211H1380768N360787O526974S2448Se0Mg2076P56337Fe0Zn0	-46247.68	Translation
rib_ini_b3296_6	b3296 complexed with 6* rib_30/fmet-tRNA/Mg	C504067H693726N181585O265656S1224Se0Mg1038P28479Fe0Zn0	-23434.84	Translation
rib_ini_b3297_1	b3297 complexed with 1* rib_30/fmet-tRNA/Mg	C86728H118704N31331O46283S204Se0Mg173P5033Fe0Zn0	-4193.14	Translation
rib_ini_b3297_3	b3297 complexed with 3* rib_30/fmet-tRNA/Mg	C252776H347718N91065O133389S612Se0Mg519P14319Fe0Zn0	-11797.42	Translation
rib_ini_b3297_7	b3297 complexed with 7* rib_30/fmet-tRNA/Mg	C584872H805746N210533O307601S1428Se0Mg1211P32891Fe0Zn0	-27005.98	Translation
rib_ini_b3298_1	b3298 complexed with 1* rib_30/fmet-tRNA/Mg	C86421H118351N31222O46055S204Se0Mg173P5002Fe0Zn0	-4162.14	Translation
rib_ini_b3298_3	b3298 complexed with 3* rib_30/fmet-tRNA/Mg	C252469H347365N90956O133161S612Se0Mg519P14288Fe0Zn0	-11766.42	Translation
rib_ini_b3298_6	b3298 complexed with 6* rib_30/fmet-tRNA/Mg	C501541H690886N180557O263820S1224Se0Mg1038P28217Fe0Zn0	-23172.84	Translation
rib_ini_b3299_1	b3299 complexed with 1* rib_30/fmet-tRNA/Mg	C84141H115767N30318O44368S204Se0Mg173P4760Fe0Zn0	-3920.14	Translation
rib_ini_b3299_2	b3299 complexed with 2* rib_30/fmet-tRNA/Mg	C167165H230274N60185O87921S408Se0Mg346P9403Fe0Zn0	-7722.28	Translation
rib_ini_b3300_1	b3300 complexed with 1* rib_30/fmet-tRNA/Mg	C95648H128776N34751O52976S204Se0Mg173P5975Fe0Zn0	-5135.14	Translation
rib_ini_b3300_13	b3300 complexed with 13* rib_30/fmet-tRNA/Mg	C1091936H1502860N393155O575612S2652Se0Mg2249P61691Fe0Zn0	-50760.82	Translation
rib_ini_b3300_26	b3300 complexed with 26* rib_30/fmet-tRNA/Mg	C2171248H2991451N781426O1141801S5304Se0Mg4498P122050Fe0Zn0	-100188.64	Translation
rib_ini_b3301_1	b3301 complexed with 1* rib_30/fmet-tRNA/Mg	C87167H119188N31523O46607S204Se0Mg173P5078Fe0Zn0	-4238.14	Translation
rib_ini_b3301_4	b3301 complexed with 4* rib_30/fmet-tRNA/Mg	C336239H462709N121124O177266S816Se0Mg692P19007Fe0Zn0	-15644.56	Translation
rib_ini_b3301_8	b3301 complexed with 8* rib_30/fmet-tRNA/Mg	C668335H920737N240592O351478S1632Se0Mg1384P37579Fe0Zn0	-30853.12	Translation
rib_ini_b3302_1	b3302 complexed with 1* rib_30/fmet-tRNA/Mg	C84737H116444N30549O44812S204Se0Mg173P4823Fe0Zn0	-3983.14	Translation
rib_ini_b3302_3	b3302 complexed with 3* rib_30/fmet-tRNA/Mg	C250785H345458N90283O131918S612Se0Mg519P14109Fe0Zn0	-11587.42	Translation
rib_ini_b3303_1	b3303 complexed with 1* rib_30/fmet-tRNA/Mg	C87826H119931N31790O47073S204Se0Mg173P5147Fe0Zn0	-4307.14	Translation
rib_ini_b3303_4	b3303 complexed with 4* rib_30/fmet-tRNA/Mg	C336898H463452N121391O177732S816Se0Mg692P19076Fe0Zn0	-15713.56	Translation
rib_ini_b3303_9	b3303 complexed with 9* rib_30/fmet-tRNA/Mg	C752018H1035987N270726O395497S1836Se0Mg1557P42291Fe0Zn0	-34724.26	Translation
rib_ini_b3304_1	b3304 complexed with 1* rib_30/fmet-tRNA/Mg	C86390H118323N31210O46026S204Se0Mg173P4997Fe0Zn0	-4157.14	Translation
rib_ini_b3304_3	b3304 complexed with 3* rib_30/fmet-tRNA/Mg	C252438H347337N90944O133132S612Se0Mg519P14283Fe0Zn0	-11761.42	Translation
rib_ini_b3304_6	b3304 complexed with 6* rib_30/fmet-tRNA/Mg	C501510H690858N180545O263791S1224Se0Mg1038P28212Fe0Zn0	-23167.84	Translation
rib_ini_b3305_1	b3305 complexed with 1* rib_30/fmet-tRNA/Mg	C88107H120251N31892O47298S204Se0Mg173P5177Fe0Zn0	-4337.14	Translation
rib_ini_b3305_10	b3305 complexed with 10* rib_30/fmet-tRNA/Mg	C835323H1150814N300695O439275S2040Se0Mg1730P46964Fe0Zn0	-38556.4	Translation
rib_ini_b3305_5	b3305 complexed with 5* rib_30/fmet-tRNA/Mg	C420203H578279N151360O221510S1020Se0Mg865P23749Fe0Zn0	-19545.7	Translation
rib_ini_b3306_1	b3306 complexed with 1* rib_30/fmet-tRNA/Mg	C86770H118737N31370O46297S204Se0Mg173P5036Fe0Zn0	-4196.14	Translation
rib_ini_b3306_3	b3306 complexed with 3* rib_30/fmet-tRNA/Mg	C252818H347751N91104O133403S612Se0Mg519P14322Fe0Zn0	-11800.42	Translation
rib_ini_b3306_7	b3306 complexed with 7* rib_30/fmet-tRNA/Mg	C584914H805779N210572O307615S1428Se0Mg1211P32894Fe0Zn0	-27008.98	Translation
rib_ini_b3307_1	b3307 complexed with 1* rib_30/fmet-tRNA/Mg	C85939H117806N31039O45687S204Se0Mg173P4949Fe0Zn0	-4109.14	Translation
rib_ini_b3307_2	b3307 complexed with 2* rib_30/fmet-tRNA/Mg	C168963H232313N60906O89240S408Se0Mg346P9592Fe0Zn0	-7911.28	Translation
rib_ini_b3307_5	b3307 complexed with 5* rib_30/fmet-tRNA/Mg	C418035H575834N150507O219899S1020Se0Mg865P23521Fe0Zn0	-19317.7	Translation
rib_ini_b3308_1	b3308 complexed with 1* rib_30/fmet-tRNA/Mg	C88157H120318N31903O47323S204Se0Mg173P5183Fe0Zn0	-4343.14	Translation
rib_ini_b3308_10	b3308 complexed with 10* rib_30/fmet-tRNA/Mg	C835373H1150881N300706O439300S2040Se0Mg1730P46970Fe0Zn0	-38562.4	Translation

rib_ini_b3308_5	b3308 complexed with 5* rib_30/fmet-tRNA/Mg	C420253H578346N151371O221535S1020Se0Mg865P23755Fe0Zn0	-19551.7	Translation
rib_ini_b3309_1	b3309 complexed with 1* rib_30/fmet-tRNA/Mg	C86036H117901N31094O45733S204Se0Mg173P4958Fe0Zn0	-4118.14	Translation
rib_ini_b3309_3	b3309 complexed with 3* rib_30/fmet-tRNA/Mg	C252084H346915N90828O132839S612Se0Mg519P14244Fe0Zn0	-11722.42	Translation
rib_ini_b3309_6	b3309 complexed with 6* rib_30/fmet-tRNA/Mg	C501156H690436N180429O263498S1224Se0Mg1038P28173Fe0Zn0	-23128.84	Translation
rib_ini_b3310_1	b3310 complexed with 1* rib_30/fmet-tRNA/Mg	C86568H118511N31286O46160S204Se0Mg173P5017Fe0Zn0	-4177.14	Translation
rib_ini_b3310_3	b3310 complexed with 3* rib_30/fmet-tRNA/Mg	C252616H347525N91020O133266S612Se0Mg519P14303Fe0Zn0	-11781.42	Translation
rib_ini_b3310_7	b3310 complexed with 7* rib_30/fmet-tRNA/Mg	C584712H805553N210488O307478S1428Se0Mg1211P32875Fe0Zn0	-26989.98	Translation
rib_ini_b3311_1	b3311 complexed with 1* rib_30/fmet-tRNA/Mg	C85446H117241N30840O45321S204Se0Mg173P4897Fe0Zn0	-4057.14	Translation
rib_ini_b3311_2	b3311 complexed with 2* rib_30/fmet-tRNA/Mg	C168470H231748N60707O88874S408Se0Mg346P9540Fe0Zn0	-7859.28	Translation
rib_ini_b3311_4	b3311 complexed with 4* rib_30/fmet-tRNA/Mg	C334518H460762N120441O175980S816Se0Mg692P18826Fe0Zn0	-15463.56	Translation
rib_ini_b3312_1	b3312 complexed with 1* rib_30/fmet-tRNA/Mg	C84854H116573N30626O44876S204Se0Mg173P4834Fe0Zn0	-3994.14	Translation
rib_ini_b3312_3	b3312 complexed with 3* rib_30/fmet-tRNA/Mg	C250902H345587N90360O131982S612Se0Mg519P14120Fe0Zn0	-11598.42	Translation
rib_ini_b3313_1	b3313 complexed with 1* rib_30/fmet-tRNA/Mg	C86951H118938N31465O46415S204Se0Mg173P5054Fe0Zn0	-4214.14	Translation
rib_ini_b3313_4	b3313 complexed with 4* rib_30/fmet-tRNA/Mg	C336023H462459N121066O177074S816Se0Mg692P18983Fe0Zn0	-15620.56	Translation
rib_ini_b3313_8	b3313 complexed with 8* rib_30/fmet-tRNA/Mg	C668119H920487N240534O351286S1632Se0Mg1384P37555Fe0Zn0	-30829.12	Translation
rib_ini_b3314_1	b3314 complexed with 1* rib_30/fmet-tRNA/Mg	C89718H122075N32570O48434S204Se0Mg173P5345Fe0Zn0	-4505.14	Translation
rib_ini_b3314_13	b3314 complexed with 13* rib_30/fmet-tRNA/Mg	C1086006H1496159N390974O571070S2652Se0Mg2249P61061Fe0Zn0	-50130.82	Translation
rib_ini_b3314_6	b3314 complexed with 6* rib_30/fmet-tRNA/Mg	C504838H694610N181905O266199S1224Se0Mg1038P28560Fe0Zn0	-23515.84	Translation
rib_ini_b3315_1	b3315 complexed with 1* rib_30/fmet-tRNA/Mg	C86193H118092N31131O45876S204Se0Mg173P4976Fe0Zn0	-4136.14	Translation
rib_ini_b3315_3	b3315 complexed with 3* rib_30/fmet-tRNA/Mg	C252241H347106N90865O132982S612Se0Mg519P14262Fe0Zn0	-11740.42	Translation
rib_ini_b3315_6	b3315 complexed with 6* rib_30/fmet-tRNA/Mg	C501313H690627N180466O263641S1224Se0Mg1038P28191Fe0Zn0	-23146.84	Translation
rib_ini_b3316_1	b3316 complexed with 1* rib_30/fmet-tRNA/Mg	C85681H117511N30930O45496S204Se0Mg173P4922Fe0Zn0	-4082.14	Translation
rib_ini_b3316_2	b3316 complexed with 2* rib_30/fmet-tRNA/Mg	C168705H232018N60797O89049S408Se0Mg346P9565Fe0Zn0	-7884.28	Translation
rib_ini_b3316_5	b3316 complexed with 5* rib_30/fmet-tRNA/Mg	C417777H575539N150398O219708S1020Se0Mg865P23494Fe0Zn0	-19290.7	Translation
rib_ini_b3317_1	b3317 complexed with 1* rib_30/fmet-tRNA/Mg	C90849H123364N33001O49291S204Se0Mg173P5465Fe0Zn0	-4625.14	Translation
rib_ini_b3317_16	b3317 complexed with 16* rib_30/fmet-tRNA/Mg	C1336209H1840969N481006O702586S3264Se0Mg2768P75110Fe0Zn0	-61657.24	Translation
rib_ini_b3317_8	b3317 complexed with 8* rib_30/fmet-tRNA/Mg	C672017H924913N242070O354162S1632Se0Mg1384P37966Fe0Zn0	-31240.12	Translation
rib_ini_b3318_1	b3318 complexed with 1* rib_30/fmet-tRNA/Mg	C85886H117735N31044O45617S204Se0Mg173P4942Fe0Zn0	-4102.14	Translation
rib_ini_b3318_2	b3318 complexed with 2* rib_30/fmet-tRNA/Mg	C168910H232242N60911O89170S408Se0Mg346P9585Fe0Zn0	-7904.28	Translation
rib_ini_b3318_5	b3318 complexed with 5* rib_30/fmet-tRNA/Mg	C417982H575763N150512O219829S1020Se0Mg865P23514Fe0Zn0	-19310.7	Translation
rib_ini_b3319_1	b3319 complexed with 1* rib_30/fmet-tRNA/Mg	C88798H121034N32185O47791S204Se0Mg173P5249Fe0Zn0	-4409.14	Translation
rib_ini_b3319_11	b3319 complexed with 11* rib_30/fmet-tRNA/Mg	C919038H1266104N330855O483321S2244Se0Mg1903P51679Fe0Zn0	-42430.54	Translation
rib_ini_b3319_5	b3319 complexed with 5* rib_30/fmet-tRNA/Mg	C420894H579062N151653O222003S1020Se0Mg865P23821Fe0Zn0	-19617.7	Translation
rib_ini_b3320_1	b3320 complexed with 1* rib_30/fmet-tRNA/Mg	C89032H121294N32289O47946S204Se0Mg173P5273Fe0Zn0	-4433.14	Translation
rib_ini_b3320_12	b3320 complexed with 12* rib_30/fmet-tRNA/Mg	C1002296H1380871N360826O527029S2448Se0Mg2076P56346Fe0Zn0	-46256.68	Translation
rib_ini_b3320_6	b3320 complexed with 6* rib_30/fmet-tRNA/Mg	C504152H693829N181624O265711S1224Se0Mg1038P28488Fe0Zn0	-23443.84	Translation
rib_ini_b3321_1	b3321 complexed with 1* rib_30/fmet-tRNA/Mg	C85989H117875N31052O45735S204Se0Mg173P4957Fe0Zn0	-4117.14	Translation
rib_ini_b3321_3	b3321 complexed with 3* rib_30/fmet-tRNA/Mg	C252037H346889N90786O132841S612Se0Mg519P14243Fe0Zn0	-11721.42	Translation
rib_ini_b3321_6	b3321 complexed with 6* rib_30/fmet-tRNA/Mg	C501109H690410N180387O263500S1224Se0Mg1038P28172Fe0Zn0	-23127.84	Translation
rib_ini_b3339_1	b3339 complexed with 1* rib_30/fmet-tRNA/Mg	C94300H127275N34376O51831S204Se0Mg173P5828Fe0Zn0	-4988.14	Translation
rib_ini_b3339_11	b3339 complexed with 11* rib_30/fmet-tRNA/Mg	C924540H1272345N333046O487361S2244Se0Mg1903P52258Fe0Zn0	-43009.54	Translation
rib_ini_b3339_23	b3339 complexed with 23* rib_30/fmet-tRNA/Mg	C1920828H2646429N691450O1009997S4692Se0Mg3979P107974Fe0Zn0	-88635.22	Translation
rib_ini_b3340_1	b3340 complexed with 1* rib_30/fmet-tRNA/Mg	C103170H137284N37945O58297S204Se0Mg173P6758Fe0Zn0	-5918.14	Translation
rib_ini_b3340_20	b3340 complexed with 20* rib_30/fmet-tRNA/Mg	C1680626H2312917N605418O885804S4080Se0Mg3460P94975Fe0Zn0	-78158.8	Translation
rib_ini_b3340_41	b3340 complexed with 41* rib_30/fmet-tRNA/Mg	C3424130H4717564N1232625O1800417S8364Se0Mg7093P192478Fe0Zn0	-158003.74	Translation
rib_ini_b3341_1	b3341 complexed with 1* rib_30/fmet-tRNA/Mg	C88158H120311N31898O47339S204Se0Mg173P5183Fe0Zn0	-4343.14	Translation
rib_ini_b3341_10	b3341 complexed with 10* rib_30/fmet-tRNA/Mg	C835374H1150874N300701O439316S2040Se0Mg1730P46970Fe0Zn0	-38562.4	Translation

rib_ini_b3341_5	b3341 complexed with 5* rib_30/fmet-tRNA/Mg	C420254H578339N151366O221551S1020Se0Mg865P23755Fe0Zn0	-19551.7	Translation
rib_ini_b3342_1	b3342 complexed with 1* rib_30/fmet-tRNA/Mg	C86589H118549N31288O46178S204Se0Mg173P5020Fe0Zn0	-4180.14	Translation
rib_ini_b3342_3	b3342 complexed with 3* rib_30/fmet-tRNA/Mg	C252637H347563N91022O133284S612Se0Mg519P14306Fe0Zn0	-11784.42	Translation
rib_ini_b3342_7	b3342 complexed with 7* rib_30/fmet-tRNA/Mg	C584733H805591N210490O307496S1428Se0Mg1211P32878Fe0Zn0	-26992.98	Translation
rib_ini_b3343_1	b3343 complexed with 1* rib_30/fmet-tRNA/Mg	C85751H117600N30925O45579S204Se0Mg173P4931Fe0Zn0	-4091.14	Translation
rib_ini_b3343_2	b3343 complexed with 2* rib_30/fmet-tRNA/Mg	C168775H232107N60792O89132S408Se0Mg346P9574Fe0Zn0	-7893.28	Translation
rib_ini_b3343_5	b3343 complexed with 5* rib_30/fmet-tRNA/Mg	C417847H575628N150393O219791S1020Se0Mg865P23503Fe0Zn0	-19299.7	Translation
rib_ini_b3344_1	b3344 complexed with 1* rib_30/fmet-tRNA/Mg	C86422H118352N31173O46099S204Se0Mg173P5002Fe0Zn0	-4162.14	Translation
rib_ini_b3344_3	b3344 complexed with 3* rib_30/fmet-tRNA/Mg	C252470H347366N90907O133205S612Se0Mg519P14288Fe0Zn0	-11766.42	Translation
rib_ini_b3344_7	b3344 complexed with 7* rib_30/fmet-tRNA/Mg	C584566H805394N210375O307417S1428Se0Mg1211P32860Fe0Zn0	-26974.98	Translation
rib_ini_b3345_1	b3345 complexed with 1* rib_30/fmet-tRNA/Mg	C86703H118664N31345O46264S204Se0Mg173P5029Fe0Zn0	-4189.14	Translation
rib_ini_b3345_3	b3345 complexed with 3* rib_30/fmet-tRNA/Mg	C252751H347678N91079O133370S612Se0Mg519P14315Fe0Zn0	-11793.42	Translation
rib_ini_b3345_7	b3345 complexed with 7* rib_30/fmet-tRNA/Mg	C584847H805706N210547O307582S1428Se0Mg1211P32887Fe0Zn0	-27001.98	Translation
rib_ini_b3346_1	b3346 complexed with 1* rib_30/fmet-tRNA/Mg	C89887H122268N32555O48626S204Se0Mg173P5368Fe0Zn0	-4528.14	Translation
rib_ini_b3346_14	b3346 complexed with 14* rib_30/fmet-tRNA/Mg	C1169199H1610859N420826O614815S2856Se0Mg2422P65727Fe0Zn0	-53955.96	Translation
rib_ini_b3346_7	b3346 complexed with 7* rib_30/fmet-tRNA/Mg	C588031H809310N211757O309944S1428Se0Mg1211P33226Fe0Zn0	-27340.98	Translation
rib_ini_b3384_1	b3384 complexed with 1* rib_30/fmet-tRNA/Mg	C92525H125250N33689O50507S204Se0Mg173P5640Fe0Zn0	-4800.14	Translation
rib_ini_b3384_19	b3384 complexed with 19* rib_30/fmet-tRNA/Mg	C1586957H2186376N571295O834461S3876Se0Mg3287P89214Fe0Zn0	-73238.66	Translation
rib_ini_b3384_9	b3384 complexed with 9* rib_30/fmet-tRNA/Mg	C756717H1041306N272625O398931S1836Se0Mg1557P42784Fe0Zn0	-35217.26	Translation
rib_ini_b3385_1	b3385 complexed with 1* rib_30/fmet-tRNA/Mg	C90173H122591N32722O48816S204Se0Mg173P5394Fe0Zn0	-4554.14	Translation
rib_ini_b3385_14	b3385 complexed with 14* rib_30/fmet-tRNA/Mg	C1169485H1611182N420993O615005S2856Se0Mg2422P65753Fe0Zn0	-53981.96	Translation
rib_ini_b3385_7	b3385 complexed with 7* rib_30/fmet-tRNA/Mg	C588317H809633N211924O310134S1428Se0Mg1211P33252Fe0Zn0	-27366.98	Translation
rib_ini_b3386_1	b3386 complexed with 1* rib_30/fmet-tRNA/Mg	C89465H121796N32409O48314S204Se0Mg173P5323Fe0Zn0	-4483.14	Translation
rib_ini_b3386_13	b3386 complexed with 13* rib_30/fmet-tRNA/Mg	C1085753H1495880N390813O570950S2652Se0Mg2249P61039Fe0Zn0	-50108.82	Translation
rib_ini_b3386_6	b3386 complexed with 6* rib_30/fmet-tRNA/Mg	C504585H694331N181744O266079S1224Se0Mg1038P28538Fe0Zn0	-23493.84	Translation
rib_ini_b3387_1	b3387 complexed with 1* rib_30/fmet-tRNA/Mg	C90976H123501N33002O49425S204Se0Mg173P5482Fe0Zn0	-4642.14	Translation
rib_ini_b3387_16	b3387 complexed with 16* rib_30/fmet-tRNA/Mg	C1336336H1841106N481007O702720S3264Se0Mg2768P75127Fe0Zn0	-61674.24	Translation
rib_ini_b3387_8	b3387 complexed with 8* rib_30/fmet-tRNA/Mg	C672144H925050N242071O354296S1632Se0Mg1384P37983Fe0Zn0	-31257.12	Translation
rib_ini_b3388_1	b3388 complexed with 1* rib_30/fmet-tRNA/Mg	C95308H128432N34897O52450S204Se0Mg173P5932Fe0Zn0	-5092.14	Translation
rib_ini_b3388_12	b3388 complexed with 12* rib_30/fmet-tRNA/Mg	C1008572H1388009N363434O531533S2448Se0Mg2076P57005Fe0Zn0	-46915.68	Translation
rib_ini_b3388_25	b3388 complexed with 25* rib_30/fmet-tRNA/Mg	C2087884H2876600N751705O1097722S5100Se0Mg4325P117364Fe0Zn0	-96343.5	Translation
rib_ini_b3389_1	b3389 complexed with 1* rib_30/fmet-tRNA/Mg	C93373H126211N33954O51230S204Se0Mg173P5732Fe0Zn0	-4892.14	Translation
rib_ini_b3389_10	b3389 complexed with 10* rib_30/fmet-tRNA/Mg	C840589H1156774N302757O443207S2040Se0Mg1730P47519Fe0Zn0	-39111.4	Translation
rib_ini_b3389_21	b3389 complexed with 21* rib_30/fmet-tRNA/Mg	C1753853H2416351N631294O922290S4284Se0Mg3633P98592Fe0Zn0	-80934.94	Translation
rib_ini_b3390_1	b3390 complexed with 1* rib_30/fmet-tRNA/Mg	C88011H120132N31893O47185S204Se0Mg173P5167Fe0Zn0	-4327.14	Translation
rib_ini_b3390_10	b3390 complexed with 10* rib_30/fmet-tRNA/Mg	C835227H1150695N300696O439162S2040Se0Mg1730P46954Fe0Zn0	-38546.4	Translation
rib_ini_b3390_5	b3390 complexed with 5* rib_30/fmet-tRNA/Mg	C420107H578160N151361O221397S1020Se0Mg865P23739Fe0Zn0	-19535.7	Translation
rib_ini_b3406_1	b3406 complexed with 1* rib_30/fmet-tRNA/Mg	C87578H119649N31714O46872S204Se0Mg173P5122Fe0Zn0	-4282.14	Translation
rib_ini_b3406_4	b3406 complexed with 4* rib_30/fmet-tRNA/Mg	C336650H463170N121315O177531S816Se0Mg692P19051Fe0Zn0	-15688.56	Translation
rib_ini_b3406_9	b3406 complexed with 9* rib_30/fmet-tRNA/Mg	C751770H1035705N270650O395296S1836Se0Mg1557P42266Fe0Zn0	-34699.26	Translation
rib_ini_b3461_1	b3461 complexed with 1* rib_30/fmet-tRNA/Mg	C91177H123733N33168O49513S204Se0Mg173P5500Fe0Zn0	-4660.14	Translation
rib_ini_b3461_16	b3461 complexed with 16* rib_30/fmet-tRNA/Mg	C1336537H1841338N481173O702808S3264Se0Mg2768P75145Fe0Zn0	-61692.24	Translation
rib_ini_b3461_8	b3461 complexed with 8* rib_30/fmet-tRNA/Mg	C672345H925282N242237O354384S1632Se0Mg1384P38001Fe0Zn0	-31275.12	Translation
rib_ini_b3470_1	b3470 complexed with 1* rib_30/fmet-tRNA/Mg	C85356H117153N30780O45290S204Se0Mg173P4891Fe0Zn0	-4051.14	Translation
rib_ini_b3470_2	b3470 complexed with 2* rib_30/fmet-tRNA/Mg	C168380H231660N60647O88843S408Se0Mg346P9534Fe0Zn0	-7853.28	Translation
rib_ini_b3470_4	b3470 complexed with 4* rib_30/fmet-tRNA/Mg	C334428H460674N120381O175949S816Se0Mg692P18820Fe0Zn0	-15457.56	Translation
rib_ini_b3559_1	b3559 complexed with 1* rib_30/fmet-tRNA/Mg	C102740H136827N37798O58023S204Se0Mg173P6713Fe0Zn0	-5873.14	Translation

rib_ini_b3559_20	b3559 complexed with 20* rib_30/fmet-tRNA/Mg	C1680196H2312460N605271O885530S4080Se0Mg3460P94930Fe0Zn0	-78113.8	Translation
rib_ini_b3559_40	b3559 complexed with 40* rib_30/fmet-tRNA/Mg	C3340676H4602600N1202611O1756590S8160Se0Mg6920P187790Fe0Zn0	-154156.6	Translation
rib_ini_b3560_1	b3560 complexed with 1* rib_30/fmet-tRNA/Mg	C91695H124350N33339O49924S204Se0Mg173P5557Fe0Zn0	-4717.14	Translation
rib_ini_b3560_17	b3560 complexed with 17* rib_30/fmet-tRNA/Mg	C1420079H1956462N511211O746772S3468Se0Mg2941P79845Fe0Zn0	-65551.38	Translation
rib_ini_b3560_8	b3560 complexed with 8* rib_30/fmet-tRNA/Mg	C672863H925899N242408O354795S1632Se0Mg1384P38058Fe0Zn0	-31332.12	Translation
rib_ini_b3590_1	b3590 complexed with 1* rib_30/fmet-tRNA/Mg	C100562H134371N36940O56415S204Se0Mg173P6484Fe0Zn0	-5644.14	Translation
rib_ini_b3590_18	b3590 complexed with 18* rib_30/fmet-tRNA/Mg	C1511970H2080990N544679O796816S3672Se0Mg3114P85415Fe0Zn0	-70280.52	Translation
rib_ini_b3590_36	b3590 complexed with 36* rib_30/fmet-tRNA/Mg	C3006402H4142116N1082285O1580770S7344Se0Mg6228P168989Fe0Zn0	-138719.04	Translation
rib_ini_b3591_1	b3591 complexed with 1* rib_30/fmet-tRNA/Mg	C96281H129508N35189O53316S204Se0Mg173P6037Fe0Zn0	-5197.14	Translation
rib_ini_b3591_13	b3591 complexed with 13* rib_30/fmet-tRNA/Mg	C1092569H1503592N393593O575952S2652Se0Mg2249P61753Fe0Zn0	-50822.82	Translation
rib_ini_b3591_27	b3591 complexed with 27* rib_30/fmet-tRNA/Mg	C2254905H3106690N811731O1185694S5508Se0Mg4671P126755Fe0Zn0	-104052.78	Translation
rib_ini_b3635_1	b3635 complexed with 1* rib_30/fmet-tRNA/Mg	C90753H123231N32988O49230S204Se0Mg173P5455Fe0Zn0	-4615.14	Translation
rib_ini_b3635_15	b3635 complexed with 15* rib_30/fmet-tRNA/Mg	C1253089H1726329N451126O658972S3060Se0Mg2595P70457Fe0Zn0	-57845.1	Translation
rib_ini_b3635_7	b3635 complexed with 7* rib_30/fmet-tRNA/Mg	C588897H810273N212190O310548S1428Se0Mg1211P33313Fe0Zn0	-27427.98	Translation
rib_ini_b3636_1	b3636 complexed with 1* rib_30/fmet-tRNA/Mg	C84631H116317N30522O44708S204Se0Mg173P4811Fe0Zn0	-3971.14	Translation
rib_ini_b3636_3	b3636 complexed with 3* rib_30/fmet-tRNA/Mg	C250679H345331N90256O131814S612Se0Mg519P14097Fe0Zn0	-11575.42	Translation
rib_ini_b3637_1	b3637 complexed with 1* rib_30/fmet-tRNA/Mg	C85275H117061N30760O45216S204Se0Mg173P4882Fe0Zn0	-4042.14	Translation
rib_ini_b3637_2	b3637 complexed with 2* rib_30/fmet-tRNA/Mg	C168299H231568N60627O88769S408Se0Mg346P9525Fe0Zn0	-7844.28	Translation
rib_ini_b3637_4	b3637 complexed with 4* rib_30/fmet-tRNA/Mg	C334347H460582N120361O175875S816Se0Mg692P18811Fe0Zn0	-15448.56	Translation
rib_ini_b3638_1	b3638 complexed with 1* rib_30/fmet-tRNA/Mg	C89396H121692N32401O48245S204Se0Mg173P5314Fe0Zn0	-4474.14	Translation
rib_ini_b3638_13	b3638 complexed with 13* rib_30/fmet-tRNA/Mg	C1085684H1495776N390805O570881S2652Se0Mg2249P61030Fe0Zn0	-50099.82	Translation
rib_ini_b3638_6	b3638 complexed with 6* rib_30/fmet-tRNA/Mg	C504516H694227N181736O266010S1224Se0Mg1038P28529Fe0Zn0	-23484.84	Translation
rib_ini_b3649_1	b3649 complexed with 1* rib_30/fmet-tRNA/Mg	C85664H117492N30955O45467S204Se0Mg173P4921Fe0Zn0	-4081.14	Translation
rib_ini_b3649_2	b3649 complexed with 2* rib_30/fmet-tRNA/Mg	C168688H231999N60822O89020S408Se0Mg346P9564Fe0Zn0	-7883.28	Translation
rib_ini_b3649_5	b3649 complexed with 5* rib_30/fmet-tRNA/Mg	C417760H575520N150423O219679S1020Se0Mg865P23493Fe0Zn0	-19289.7	Translation
rib_ini_b3650_1	b3650 complexed with 1* rib_30/fmet-tRNA/Mg	C103095H137254N37921O58247S204Se0Mg173P6752Fe0Zn0	-5912.14	Translation
rib_ini_b3650_20	b3650 complexed with 20* rib_30/fmet-tRNA/Mg	C1680551H2312887N605394O885754S4080Se0Mg3460P94969Fe0Zn0	-78152.8	Translation
rib_ini_b3650_41	b3650 complexed with 41* rib_30/fmet-tRNA/Mg	C3424055H4717534N1232601O1800367S8364Se0Mg7093P192472Fe0Zn0	-157997.74	Translation
rib_ini_b3651_1	b3651 complexed with 1* rib_30/fmet-tRNA/Mg	C89589H121955N32504O48362S204Se0Mg173P5333Fe0Zn0	-4493.14	Translation
rib_ini_b3651_13	b3651 complexed with 13* rib_30/fmet-tRNA/Mg	C1085877H1496039N390908O570998S2652Se0Mg2249P61049Fe0Zn0	-50118.82	Translation
rib_ini_b3651_6	b3651 complexed with 6* rib_30/fmet-tRNA/Mg	C504709H694490N181839O266127S1224Se0Mg1038P28548Fe0Zn0	-23503.84	Translation
rib_ini_b3652_1	b3652 complexed with 1* rib_30/fmet-tRNA/Mg	C102847H137000N37873O58057S204Se0Mg173P6725Fe0Zn0	-5885.14	Translation
rib_ini_b3652_20	b3652 complexed with 20* rib_30/fmet-tRNA/Mg	C1680303H2312633N605346O885564S4080Se0Mg3460P94942Fe0Zn0	-78125.8	Translation
rib_ini_b3652_40	b3652 complexed with 40* rib_30/fmet-tRNA/Mg	C3340783H4602773N1202686O1756624S8160Se0Mg6920P187802Fe0Zn0	-154168.6	Translation
rib_ini_b3703_1	b3703 complexed with 1* rib_30/fmet-tRNA/Mg	C84357H116021N30380O44555S204Se0Mg173P4786Fe0Zn0	-3946.14	Translation
rib_ini_b3703_2	b3703 complexed with 2* rib_30/fmet-tRNA/Mg	C167381H230528N60247O88108S408Se0Mg346P9429Fe0Zn0	-7748.28	Translation
rib_ini_b3704_1	b3704 complexed with 1* rib_30/fmet-tRNA/Mg	C86437H118387N31212O46077S204Se0Mg173P5003Fe0Zn0	-4163.14	Translation
rib_ini_b3704_3	b3704 complexed with 3* rib_30/fmet-tRNA/Mg	C252485H347401N90946O133183S612Se0Mg519P14289Fe0Zn0	-11767.42	Translation
rib_ini_b3704_7	b3704 complexed with 7* rib_30/fmet-tRNA/Mg	C584581H805429N210414O307395S1428Se0Mg1211P32861Fe0Zn0	-26975.98	Translation
rib_ini_b3706_1	b3706 complexed with 1* rib_30/fmet-tRNA/Mg	C96020H129235N35096O53095S204Se0Mg173P6010Fe0Zn0	-5170.14	Translation
rib_ini_b3706_13	b3706 complexed with 13* rib_30/fmet-tRNA/Mg	C1092308H1503319N393500O575731S2652Se0Mg2249P61726Fe0Zn0	-50795.82	Translation
rib_ini_b3706_26	b3706 complexed with 26* rib_30/fmet-tRNA/Mg	C2171620H2991910N781771O1141920S5304Se0Mg4498P122085Fe0Zn0	-100223.64	Translation
rib_ini_b3740_1	b3740 complexed with 1* rib_30/fmet-tRNA/Mg	C88955H121212N32209O47922S204Se0Mg173P5267Fe0Zn0	-4427.14	Translation
rib_ini_b3740_12	b3740 complexed with 12* rib_30/fmet-tRNA/Mg	C1002219H1380789N360746O527005S2448Se0Mg2076P56340Fe0Zn0	-46250.68	Translation
rib_ini_b3740_6	b3740 complexed with 6* rib_30/fmet-tRNA/Mg	C504075H693747N181544O265687S1224Se0Mg1038P28482Fe0Zn0	-23437.84	Translation
rib_ini_b3741_1	b3741 complexed with 1* rib_30/fmet-tRNA/Mg	C101003H134874N37051O56776S204Se0Mg173P6535Fe0Zn0	-5695.14	Translation
rib_ini_b3741_18	b3741 complexed with 18* rib_30/fmet-tRNA/Mg	C1512411H2081493N544790O797177S3672Se0Mg3114P85466Fe0Zn0	-70331.52	Translation

rib_ini_b3741_37	b3741 complexed with 37* rib_30/fmet-tRNA/Mg	C3089867H4257126N1112263O1624684S7548Se0Mg6401P173683Fe0Zn0	-142572.18	Translation
rib_ini_b3780_1	b3780 complexed with 1* rib_30/fmet-tRNA/Mg	C95060H128139N34654O52411S204Se0Mg173P5911Fe0Zn0	-5071.14	Translation
rib_ini_b3780_12	b3780 complexed with 12* rib_30/fmet-tRNA/Mg	C1008324H1387716N363191O531494S2448Se0Mg2076P56984Fe0Zn0	-46894.68	Translation
rib_ini_b3780_24	b3780 complexed with 24* rib_30/fmet-tRNA/Mg	C2004612H2761800N721595O1054130S4896Se0Mg4152P112700Fe0Zn0	-92520.36	Translation
rib_ini_b3782_1	b3782 complexed with 1* rib_30/fmet-tRNA/Mg	C83991H115602N30243O44278S204Se0Mg173P4747Fe0Zn0	-3907.14	Translation
rib_ini_b3783_1	b3783 complexed with 1* rib_30/fmet-tRNA/Mg	C95007H128070N34635O52344S204Se0Mg173P5903Fe0Zn0	-5063.14	Translation
rib_ini_b3783_12	b3783 complexed with 12* rib_30/fmet-tRNA/Mg	C1008271H1387647N363172O531427S2448Se0Mg2076P56976Fe0Zn0	-46886.68	Translation
rib_ini_b3783_24	b3783 complexed with 24* rib_30/fmet-tRNA/Mg	C2004559H2761731N721576O1054063S4896Se0Mg4152P112692Fe0Zn0	-92512.36	Translation
rib_ini_b3885_1	b3885 complexed with 1* rib_30/fmet-tRNA/Mg	C88732H120954N32129O47771S204Se0Mg173P5245Fe0Zn0	-4405.14	Translation
rib_ini_b3885_11	b3885 complexed with 11* rib_30/fmet-tRNA/Mg	C918972H1266024N330799O483301S2244Se0Mg1903P51675Fe0Zn0	-42426.54	Translation
rib_ini_b3885_5	b3885 complexed with 5* rib_30/fmet-tRNA/Mg	C420828H578982N151597O221983S1020Se0Mg865P23817Fe0Zn0	-19613.7	Translation
rib_ini_b3886_1	b3886 complexed with 1* rib_30/fmet-tRNA/Mg	C91208H123787N32998O49686S204Se0Mg173P5509Fe0Zn0	-4669.14	Translation
rib_ini_b3886_17	b3886 complexed with 17* rib_30/fmet-tRNA/Mg	C1419592H1955899N510870O746534S3468Se0Mg2941P79797Fe0Zn0	-65503.38	Translation
rib_ini_b3886_8	b3886 complexed with 8* rib_30/fmet-tRNA/Mg	C672376H925336N242067O354557S1632Se0Mg1384P38010Fe0Zn0	-31284.12	Translation
rib_ini_b3887_1	b3887 complexed with 1* rib_30/fmet-tRNA/Mg	C87167H119190N31551O46582S204Se0Mg173P5077Fe0Zn0	-4237.14	Translation
rib_ini_b3887_4	b3887 complexed with 4* rib_30/fmet-tRNA/Mg	C336239H462711N121152O177241S816Se0Mg692P19006Fe0Zn0	-15643.56	Translation
rib_ini_b3887_8	b3887 complexed with 8* rib_30/fmet-tRNA/Mg	C668335H920739N240620O351453S1632Se0Mg1384P37578Fe0Zn0	-30852.12	Translation
rib_ini_b3888_1	b3888 complexed with 1* rib_30/fmet-tRNA/Mg	C92427H125146N33675O50430S204Se0Mg173P5629Fe0Zn0	-4789.14	Translation
rib_ini_b3888_19	b3888 complexed with 19* rib_30/fmet-tRNA/Mg	C1586859H2186272N571281O834384S3876Se0Mg3287P89203Fe0Zn0	-73227.66	Translation
rib_ini_b3888_9	b3888 complexed with 9* rib_30/fmet-tRNA/Mg	C756619H1041202N272611O398854S1836Se0Mg1557P42773Fe0Zn0	-35206.26	Translation
rib_ini_b3936_1	b3936 complexed with 1* rib_30/fmet-tRNA/Mg	C85049H116803N30674O45039S204Se0Mg173P4858Fe0Zn0	-4018.14	Translation
rib_ini_b3936_2	b3936 complexed with 2* rib_30/fmet-tRNA/Mg	C168073H231310N60541O88592S408Se0Mg346P9501Fe0Zn0	-7820.28	Translation
rib_ini_b3936_4	b3936 complexed with 4* rib_30/fmet-tRNA/Mg	C334121H460324N120275O175698S816Se0Mg692P18787Fe0Zn0	-15424.56	Translation
rib_ini_b3965_1	b3965 complexed with 1* rib_30/fmet-tRNA/Mg	C93513H126367N34078O51230S204Se0Mg173P5746Fe0Zn0	-4906.14	Translation
rib_ini_b3965_10	b3965 complexed with 10* rib_30/fmet-tRNA/Mg	C840729H1156930N302881O443207S2040Se0Mg1730P47533Fe0Zn0	-39125.4	Translation
rib_ini_b3965_21	b3965 complexed with 21* rib_30/fmet-tRNA/Mg	C1753993H2416507N631418O922290S4284Se0Mg3633P98606Fe0Zn0	-80948.94	Translation
rib_ini_b3980_1	b3980 complexed with 1* rib_30/fmet-tRNA/Mg	C94305H127281N34392O51827S204Se0Mg173P5830Fe0Zn0	-4990.14	Translation
rib_ini_b3980_11	b3980 complexed with 11* rib_30/fmet-tRNA/Mg	C924545H1272351N333062O487357S2244Se0Mg1903P52260Fe0Zn0	-43011.54	Translation
rib_ini_b3980_23	b3980 complexed with 23* rib_30/fmet-tRNA/Mg	C1920833H2646435N691466O1009993S4692Se0Mg3979P107976Fe0Zn0	-88637.22	Translation
rib_ini_b3981_1	b3981 complexed with 1* rib_30/fmet-tRNA/Mg	C86674H118632N31307O46275S204Se0Mg173P5029Fe0Zn0	-4189.14	Translation
rib_ini_b3981_3	b3981 complexed with 3* rib_30/fmet-tRNA/Mg	C252722H347646N91041O133381S612Se0Mg519P14315Fe0Zn0	-11793.42	Translation
rib_ini_b3981_7	b3981 complexed with 7* rib_30/fmet-tRNA/Mg	C584818H805674N210509O307593S1428Se0Mg1211P32887Fe0Zn0	-27001.98	Translation
rib_ini_b3982_1	b3982 complexed with 1* rib_30/fmet-tRNA/Mg	C88224H120378N31941O47384S204Se0Mg173P5189Fe0Zn0	-4349.14	Translation
rib_ini_b3982_10	b3982 complexed with 10* rib_30/fmet-tRNA/Mg	C835440H1150941N300744O439361S2040Se0Mg1730P46976Fe0Zn0	-38568.4	Translation
rib_ini_b3982_5	b3982 complexed with 5* rib_30/fmet-tRNA/Mg	C420320H578406N151409O221596S1020Se0Mg865P23761Fe0Zn0	-19557.7	Translation
rib_ini_b3983_1	b3983 complexed with 1* rib_30/fmet-tRNA/Mg	C87113H119136N31519O46540S204Se0Mg173P5074Fe0Zn0	-4234.14	Translation
rib_ini_b3983_4	b3983 complexed with 4* rib_30/fmet-tRNA/Mg	C336185H462657N121120O177199S816Se0Mg692P19003Fe0Zn0	-15640.56	Translation
rib_ini_b3983_8	b3983 complexed with 8* rib_30/fmet-tRNA/Mg	C668281H920685N240588O351411S1632Se0Mg1384P37575Fe0Zn0	-30849.12	Translation
rib_ini_b3984_1	b3984 complexed with 1* rib_30/fmet-tRNA/Mg	C89756H122118N32611O48438S204Se0Mg173P5348Fe0Zn0	-4508.14	Translation
rib_ini_b3984_13	b3984 complexed with 13* rib_30/fmet-tRNA/Mg	C1086044H1496202N391015O571074S2652Se0Mg2249P61064Fe0Zn0	-50133.82	Translation
rib_ini_b3984_6	b3984 complexed with 6* rib_30/fmet-tRNA/Mg	C504876H694653N181946O266203S1224Se0Mg1038P28563Fe0Zn0	-23518.84	Translation
rib_ini_b3985_1	b3985 complexed with 1* rib_30/fmet-tRNA/Mg	C87766H119870N31765O47041S204Se0Mg173P5143Fe0Zn0	-4303.14	Translation
rib_ini_b3985_4	b3985 complexed with 4* rib_30/fmet-tRNA/Mg	C336838H463391N121366O177700S816Se0Mg692P19072Fe0Zn0	-15709.56	Translation
rib_ini_b3985_9	b3985 complexed with 9* rib_30/fmet-tRNA/Mg	C751958H1035926N270701O395465S1836Se0Mg1557P42287Fe0Zn0	-34720.26	Translation
rib_ini_b3986_1	b3986 complexed with 1* rib_30/fmet-tRNA/Mg	C86529H118449N31302O46089S204Se0Mg173P5009Fe0Zn0	-4169.14	Translation
rib_ini_b3986_3	b3986 complexed with 3* rib_30/fmet-tRNA/Mg	C252577H347463N91036O133195S612Se0Mg519P14295Fe0Zn0	-11773.42	Translation
rib_ini_b3986_7	b3986 complexed with 7* rib_30/fmet-tRNA/Mg	C584673H805491N210504O307407S1428Se0Mg1211P32867Fe0Zn0	-26981.98	Translation

rib_ini_b3987_1	b3987 complexed with 1* rib_30/fmet-tRNA/Mg	C121389H157935N45270O71632S204Se0Mg173P8672Fe0Zn0	-7832.14	Translation
rib_ini_b3987_39	b3987 complexed with 39* rib_30/fmet-tRNA/Mg	C3276301H4509201N1180216O1726646S7956Se0Mg6747P185106Fe0Zn0	-152313.46	Translation
rib_ini_b3987_78	b3987 complexed with 78* rib_30/fmet-tRNA/Mg	C6514237H8974974N2345029O3425213S15912Se0Mg13494P366183Fe0Zn0	-300596.92	Translation
rib_ini_b3988_1	b3988 complexed with 1* rib_30/fmet-tRNA/Mg	C123238H160034N45997O73048S204Se0Mg173P8867Fe0Zn0	-8027.14	Translation
rib_ini_b3988_41	b3988 complexed with 41* rib_30/fmet-tRNA/Mg	C3444198H4740314N1240677O1815168S8364Se0Mg7093P194587Fe0Zn0	-160112.74	Translation
rib_ini_b3988_82	b3988 complexed with 82* rib_30/fmet-tRNA/Mg	C6848182H9435101N2465224O3600841S16728Se0Mg14186P384950Fe0Zn0	-316000.48	Translation
rib_ini_b4022_1	b4022 complexed with 1* rib_30/fmet-tRNA/Mg	C91365H123930N33273O49610S204Se0Mg173P5518Fe0Zn0	-4678.14	Translation
rib_ini_b4022_17	b4022 complexed with 17* rib_30/fmet-tRNA/Mg	C1419749H1956042N511145O746458S3468Se0Mg2941P79806Fe0Zn0	-65512.38	Translation
rib_ini_b4022_8	b4022 complexed with 8* rib_30/fmet-tRNA/Mg	C672533H925479N242342O354481S1632Se0Mg1384P38019Fe0Zn0	-31293.12	Translation
rib_ini_b4049_1	b4049 complexed with 1* rib_30/fmet-tRNA/Mg	C92925H125689N33862O50819S204Se0Mg173P5683Fe0Zn0	-4843.14	Translation
rib_ini_b4049_10	b4049 complexed with 10* rib_30/fmet-tRNA/Mg	C840141H1156252N302665O442796S2040Se0Mg1730P47470Fe0Zn0	-39062.4	Translation
rib_ini_b4049_20	b4049 complexed with 20* rib_30/fmet-tRNA/Mg	C1670381H2301322N601335O878326S4080Se0Mg3460P93900Fe0Zn0	-77083.8	Translation
rib_ini_b4129_1	b4129 complexed with 1* rib_30/fmet-tRNA/Mg	C97467H130809N35586O54182S204Se0Mg173P6163Fe0Zn0	-5323.14	Translation
rib_ini_b4129_14	b4129 complexed with 14* rib_30/fmet-tRNA/Mg	C1176779H1619400N423857O620371S2856Se0Mg2422P66522Fe0Zn0	-54750.96	Translation
rib_ini_b4129_29	b4129 complexed with 29* rib_30/fmet-tRNA/Mg	C2422139H3337005N871862O1273666S5916Se0Mg5017P136167Fe0Zn0	-111783.06	Translation
rib_ini_b4142_1	b4142 complexed with 1* rib_30/fmet-tRNA/Mg	C85832H117670N31001O45611S204Se0Mg173P4939Fe0Zn0	-4099.14	Translation
rib_ini_b4142_2	b4142 complexed with 2* rib_30/fmet-tRNA/Mg	C168856H232177N60868O89164S408Se0Mg346P9582Fe0Zn0	-7901.28	Translation
rib_ini_b4142_5	b4142 complexed with 5* rib_30/fmet-tRNA/Mg	C417928H575698N150469O219823S1020Se0Mg865P23511Fe0Zn0	-19307.7	Translation
rib_ini_b4143_1	b4143 complexed with 1* rib_30/fmet-tRNA/Mg	C98761H132286N36297O54991S204Se0Mg173P6292Fe0Zn0	-5452.14	Translation
rib_ini_b4143_16	b4143 complexed with 16* rib_30/fmet-tRNA/Mg	C1344121H1849891N484302O708286S3264Se0Mg2768P75937Fe0Zn0	-62484.24	Translation
rib_ini_b4143_32	b4143 complexed with 32* rib_30/fmet-tRNA/Mg	C2672505H3682003N962174O1405134S6528Se0Mg5536P150225Fe0Zn0	-123318.48	Translation
rib_ini_b4162_1	b4162 complexed with 1* rib_30/fmet-tRNA/Mg	C88234H120397N31980O47365S204Se0Mg173P5191Fe0Zn0	-4351.14	Translation
rib_ini_b4162_10	b4162 complexed with 10* rib_30/fmet-tRNA/Mg	C835450H1150960N300783O439342S2040Se0Mg1730P46978Fe0Zn0	-38570.4	Translation
rib_ini_b4162_5	b4162 complexed with 5* rib_30/fmet-tRNA/Mg	C420330H578425N151448O221577S1020Se0Mg865P23763Fe0Zn0	-19559.7	Translation
rib_ini_b4167_1	b4167 complexed with 1* rib_30/fmet-tRNA/Mg	C97767H131201N35798O54402S204Se0Mg173P6193Fe0Zn0	-5353.14	Translation
rib_ini_b4167_15	b4167 complexed with 15* rib_30/fmet-tRNA/Mg	C1260103H1734299N453936O664144S3060Se0Mg2595P71195Fe0Zn0	-58583.1	Translation
rib_ini_b4167_30	b4167 complexed with 30* rib_30/fmet-tRNA/Mg	C2505463H3451904N901941O1317439S6120Se0Mg5190P140840Fe0Zn0	-115615.2	Translation
rib_ini_b4168_1	b4168 complexed with 1* rib_30/fmet-tRNA/Mg	C87140H119162N31495O46599S204Se0Mg173P5076Fe0Zn0	-4236.14	Translation
rib_ini_b4168_4	b4168 complexed with 4* rib_30/fmet-tRNA/Mg	C336212H462683N121096O177258S816Se0Mg692P19005Fe0Zn0	-15642.56	Translation
rib_ini_b4168_9	b4168 complexed with 9* rib_30/fmet-tRNA/Mg	C751332H1035218N270431O395023S1836Se0Mg1557P42220Fe0Zn0	-34653.26	Translation
rib_ini_b4169_1	b4169 complexed with 1* rib_30/fmet-tRNA/Mg	C95765H128943N34996O52885S204Se0Mg173P5981Fe0Zn0	-5141.14	Translation
rib_ini_b4169_13	b4169 complexed with 13* rib_30/fmet-tRNA/Mg	C1092053H1503027N393400O575521S2652Se0Mg2249P61697Fe0Zn0	-50766.82	Translation
rib_ini_b4169_26	b4169 complexed with 26* rib_30/fmet-tRNA/Mg	C2171365H2991618N781671O1141710S5304Se0Mg4498P122056Fe0Zn0	-100194.64	Translation
rib_ini_b4170_1	b4170 complexed with 1* rib_30/fmet-tRNA/Mg	C100616H134464N36959O56426S204Se0Mg173P6491Fe0Zn0	-5651.14	Translation
rib_ini_b4170_18	b4170 complexed with 18* rib_30/fmet-tRNA/Mg	C1512024H2081083N544698O796827S3672Se0Mg3114P85422Fe0Zn0	-70287.52	Translation
rib_ini_b4170_36	b4170 complexed with 36* rib_30/fmet-tRNA/Mg	C3006456H4142209N1082304O1580781S7344Se0Mg6228P168996Fe0Zn0	-138726.04	Translation
rib_ini_b4171_1	b4171 complexed with 1* rib_30/fmet-tRNA/Mg	C92008H124654N33463O50171S204Se0Mg173P5586Fe0Zn0	-4746.14	Translation
rib_ini_b4171_18	b4171 complexed with 18* rib_30/fmet-tRNA/Mg	C1503416H2071273N541202O790572S3672Se0Mg3114P84517Fe0Zn0	-69382.52	Translation
rib_ini_b4171_9	b4171 complexed with 9* rib_30/fmet-tRNA/Mg	C756200H1040710N272399O398595S1836Se0Mg1557P42730Fe0Zn0	-35163.26	Translation
rib_ini_b4172_1	b4172 complexed with 1* rib_30/fmet-tRNA/Mg	C85962H117830N31031O45713S204Se0Mg173P4952Fe0Zn0	-4112.14	Translation
rib_ini_b4172_3	b4172 complexed with 3* rib_30/fmet-tRNA/Mg	C252010H346844N90765O132819S612Se0Mg519P14238Fe0Zn0	-11716.42	Translation
rib_ini_b4172_6	b4172 complexed with 6* rib_30/fmet-tRNA/Mg	C501082H690365N180366O263478S1224Se0Mg1038P28167Fe0Zn0	-23122.84	Translation
rib_ini_b4173_1	b4173 complexed with 1* rib_30/fmet-tRNA/Mg	C95229H128306N34769O52516S204Se0Mg173P5924Fe0Zn0	-5084.14	Translation
rib_ini_b4173_12	b4173 complexed with 12* rib_30/fmet-tRNA/Mg	C1008493H1387883N363306O531599S2448Se0Mg2076P56997Fe0Zn0	-46907.68	Translation
rib_ini_b4173_25	b4173 complexed with 25* rib_30/fmet-tRNA/Mg	C2087805H2876474N751577O1097788S5100Se0Mg4325P117356Fe0Zn0	-96335.5	Translation
rib_ini_b4174_1	b4174 complexed with 1* rib_30/fmet-tRNA/Mg	C95062H128133N34808O52292S204Se0Mg173P5903Fe0Zn0	-5063.14	Translation
rib_ini_b4174_12	b4174 complexed with 12* rib_30/fmet-tRNA/Mg	C1008326H1387710N363345O531375S2448Se0Mg2076P56976Fe0Zn0	-46886.68	Translation

rib_ini_b4174_24	b4174 complexed with 24* rib_30/fmet-tRNA/Mg	C2004614H2761794N721749O1054011S4896Se0Mg4152P112692Fe0Zn0	-92512.36	Translation
rib_ini_b4175_1	b4175 complexed with 1* rib_30/fmet-tRNA/Mg	C92601H125339N33722O50565S204Se0Mg173P5648Fe0Zn0	-4808.14	Translation
rib_ini_b4175_19	b4175 complexed with 19* rib_30/fmet-tRNA/Mg	C1587033H2186465N571328O834519S3876Se0Mg3287P89222Fe0Zn0	-73246.66	Translation
rib_ini_b4175_9	b4175 complexed with 9* rib_30/fmet-tRNA/Mg	C756793H1041395N272658O398989S1836Se0Mg1557P42792Fe0Zn0	-35225.26	Translation
rib_ini_b4178_1	b4178 complexed with 1* rib_30/fmet-tRNA/Mg	C87078H119077N31468O46556S204Se0Mg173P5071Fe0Zn0	-4231.14	Translation
rib_ini_b4178_4	b4178 complexed with 4* rib_30/fmet-tRNA/Mg	C336150H462598N121069O177215S816Se0Mg692P19000Fe0Zn0	-15637.56	Translation
rib_ini_b4178_8	b4178 complexed with 8* rib_30/fmet-tRNA/Mg	C668246H920626N240537O351427S1632Se0Mg1384P37572Fe0Zn0	-30846.12	Translation
rib_ini_b4179_1	b4179 complexed with 1* rib_30/fmet-tRNA/Mg	C106319H140867N39324O60562S204Se0Mg173P7085Fe0Zn0	-6245.14	Translation
rib_ini_b4179_23	b4179 complexed with 23* rib_30/fmet-tRNA/Mg	C1932847H2660021N696398O1018728S4692Se0Mg3979P109231Fe0Zn0	-89892.22	Translation
rib_ini_b4179_47	b4179 complexed with 47* rib_30/fmet-tRNA/Mg	C3925423H5408189N1413206O2064000S9588Se0Mg8131P220663Fe0Zn0	-181143.58	Translation
rib_ini_b4180_1	b4180 complexed with 1* rib_30/fmet-tRNA/Mg	C89993H122403N32668O48672S204Se0Mg173P5375Fe0Zn0	-4535.14	Translation
rib_ini_b4180_14	b4180 complexed with 14* rib_30/fmet-tRNA/Mg	C1169305H1610994N420939O614861S2856Se0Mg2422P65734Fe0Zn0	-53962.96	Translation
rib_ini_b4180_7	b4180 complexed with 7* rib_30/fmet-tRNA/Mg	C588137H809445N211870O309990S1428Se0Mg1211P33233Fe0Zn0	-27347.98	Translation
rib_ini_b4200_1	b4200 complexed with 1* rib_30/fmet-tRNA/Mg	C86798H118780N31391O46312S204Se0Mg173P5041Fe0Zn0	-4201.14	Translation
rib_ini_b4200_3	b4200 complexed with 3* rib_30/fmet-tRNA/Mg	C252846H347794N91125O133418S612Se0Mg519P14327Fe0Zn0	-11805.42	Translation
rib_ini_b4200_7	b4200 complexed with 7* rib_30/fmet-tRNA/Mg	C584942H805822N210593O307630S1428Se0Mg1211P32899Fe0Zn0	-27013.98	Translation
rib_ini_b4201_1	b4201 complexed with 1* rib_30/fmet-tRNA/Mg	C86022H117899N31064O45757S204Se0Mg173P4958Fe0Zn0	-4118.14	Translation
rib_ini_b4201_3	b4201 complexed with 3* rib_30/fmet-tRNA/Mg	C252070H346913N90798O132863S612Se0Mg519P14244Fe0Zn0	-11722.42	Translation
rib_ini_b4201_6	b4201 complexed with 6* rib_30/fmet-tRNA/Mg	C501142H690434N180399O263522S1224Se0Mg1038P28173Fe0Zn0	-23128.84	Translation
rib_ini_b4202_1	b4202 complexed with 1* rib_30/fmet-tRNA/Mg	C85185H116966N30719O45142S204Se0Mg173P4871Fe0Zn0	-4031.14	Translation
rib_ini_b4202_2	b4202 complexed with 2* rib_30/fmet-tRNA/Mg	C168209H231473N60586O88695S408Se0Mg346P9514Fe0Zn0	-7833.28	Translation
rib_ini_b4202_4	b4202 complexed with 4* rib_30/fmet-tRNA/Mg	C334257H460487N120320O175801S816Se0Mg692P18800Fe0Zn0	-15437.56	Translation
rib_ini_b4203_1	b4203 complexed with 1* rib_30/fmet-tRNA/Mg	C87317H119353N31598O46688S204Se0Mg173P5093Fe0Zn0	-4253.14	Translation
rib_ini_b4203_4	b4203 complexed with 4* rib_30/fmet-tRNA/Mg	C336389H462874N121199O177347S816Se0Mg692P19022Fe0Zn0	-15659.56	Translation
rib_ini_b4203_8	b4203 complexed with 8* rib_30/fmet-tRNA/Mg	C668485H920902N240667O351559S1632Se0Mg1384P37594Fe0Zn0	-30868.12	Translation
rib_ini_b4258_1	b4258 complexed with 1* rib_30/fmet-tRNA/Mg	C110236H145335N40862O63433S204Se0Mg173P7501Fe0Zn0	-6661.14	Translation
rib_ini_b4258_27	b4258 complexed with 27* rib_30/fmet-tRNA/Mg	C2268860H3122517N817404O1195811S5508Se0Mg4671P128219Fe0Zn0	-105516.78	Translation
rib_ini_b4258_55	b4258 complexed with 55* rib_30/fmet-tRNA/Mg	C4593532H6328713N1653680O2415295S11220Se0Mg9515P258223Fe0Zn0	-211976.7	Translation
rib_ini_b4292_1	b4292 complexed with 1* rib_30/fmet-tRNA/Mg	C92055H124775N33496O50182S204Se0Mg173P5593Fe0Zn0	-4753.14	Translation
rib_ini_b4292_18	b4292 complexed with 18* rib_30/fmet-tRNA/Mg	C1503463H2071394N541235O790583S3672Se0Mg3114P84524Fe0Zn0	-69389.52	Translation
rib_ini_b4292_9	b4292 complexed with 9* rib_30/fmet-tRNA/Mg	C756247H1040831N272432O398606S1836Se0Mg1557P42737Fe0Zn0	-35170.26	Translation
rib_ini_b4293_1	b4293 complexed with 1* rib_30/fmet-tRNA/Mg	C87982H120133N31836O47211S204Se0Mg173P5167Fe0Zn0	-4327.14	Translation
rib_ini_b4293_10	b4293 complexed with 10* rib_30/fmet-tRNA/Mg	C835198H1150696N300639O439188S2040Se0Mg1730P46954Fe0Zn0	-38546.4	Translation
rib_ini_b4293_5	b4293 complexed with 5* rib_30/fmet-tRNA/Mg	C420078H578161N151304O221423S1020Se0Mg865P23739Fe0Zn0	-19535.7	Translation
rib_ini_b4371_1	b4371 complexed with 1* rib_30/fmet-tRNA/Mg	C92831H125619N33760O50809S204Se0Mg173P5677Fe0Zn0	-4837.14	Translation
rib_ini_b4371_10	b4371 complexed with 10* rib_30/fmet-tRNA/Mg	C840047H1156182N302563O442786S2040Se0Mg1730P47464Fe0Zn0	-39056.4	Translation
rib_ini_b4371_20	b4371 complexed with 20* rib_30/fmet-tRNA/Mg	C1670287H2301252N601233O878316S4080Se0Mg3460P93894Fe0Zn0	-77077.8	Translation
rib_ini_b4372_1	b4372 complexed with 1* rib_30/fmet-tRNA/Mg	C86958H118980N31443O46447S204Se0Mg173P5059Fe0Zn0	-4219.14	Translation
rib_ini_b4372_4	b4372 complexed with 4* rib_30/fmet-tRNA/Mg	C336030H462501N121044O177106S816Se0Mg692P18988Fe0Zn0	-15625.56	Translation
rib_ini_b4372_8	b4372 complexed with 8* rib_30/fmet-tRNA/Mg	C668126H920529N240512O351318S1632Se0Mg1384P37560Fe0Zn0	-30834.12	Translation
rib_ini_b4373_1	b4373 complexed with 1* rib_30/fmet-tRNA/Mg	C86976H118983N31456O46444S204Se0Mg173P5058Fe0Zn0	-4218.14	Translation
rib_ini_b4373_4	b4373 complexed with 4* rib_30/fmet-tRNA/Mg	C336048H462504N121057O177103S816Se0Mg692P18987Fe0Zn0	-15624.56	Translation
rib_ini_b4373_8	b4373 complexed with 8* rib_30/fmet-tRNA/Mg	C668144H920532N240525O351315S1632Se0Mg1384P37559Fe0Zn0	-30833.12	Translation
rib_ini_b4374_1	b4374 complexed with 1* rib_30/fmet-tRNA/Mg	C89462H121803N32410O48311S204Se0Mg173P5321Fe0Zn0	-4481.14	Translation
rib_ini_b4374_13	b4374 complexed with 13* rib_30/fmet-tRNA/Mg	C1085750H1495887N390814O570947S2652Se0Mg2249P61037Fe0Zn0	-50106.82	Translation
rib_ini_b4374_6	b4374 complexed with 6* rib_30/fmet-tRNA/Mg	C504582H694338N181745O266076S1224Se0Mg1038P28536Fe0Zn0	-23491.84	Translation
rib_ini_b4375_1	b4375 complexed with 1* rib_30/fmet-tRNA/Mg	C98166H131651N35954O54653S204Se0Mg173P6235Fe0Zn0	-5395.14	Translation

rib_ini_b4375_15	b4375 complexed with 15* rib_30/fmet-tRNA/Mg	C1260502H1734749N454092O664395S3060Se0Mg2595P71237Fe0Zn0	-58625.1	Translation
rib_ini_b4375_31	b4375 complexed with 31* rib_30/fmet-tRNA/Mg	C2588886H3566861N931964O1361243S6324Se0Mg5363P145525Fe0Zn0	-119459.34	Translation
rpL7/12	dimer L7/L12	C1071H1784N276O353S6	-16	Ribosomal Assembly
rps6_rimK_cplx	ribosomal protein S6/ rimK/ ATP/glu-L complex (first step for addition of gluatmyl residues)	C2133H3435N632O698S19P12	-27	Ribosomal protein modification
rrfA	5S rRNA (b3855)	C1145H1300N469O838P120	-121	Ribosomal Assembly
rrfB	5S rRNA (b3971)	C1145H1301N470O837P120	-121	Ribosomal Assembly
rrfC	5S rRNA (b3759)	C1144H1301N468O838P120	-121	Ribosomal Assembly
rrfD	5S rRNA (b3274)	C1144H1301N468O838P120	-121	Ribosomal Assembly
rrfE	5S rRNA (b4010)	C1145H1301N470O837P120	-121	Ribosomal Assembly
rrfF	5S rRNA (b3272)	C1145H1300N469O837P120	-121	Ribosomal Assembly
rrfG	5S rRNA (b2588)	C1144H1301N468O838P120	-121	Ribosomal Assembly
rrfH	5S rRNA (b0205)	C1144H1301N468O838P120	-121	Ribosomal Assembly
rrlA	23S_rRNA (b3854, modified: 9*pseudouridine, 14*methylation,1*Dihydrouridine)	C27834H31392N11471O20166P2905	-2906	rRNA Modification
rrlB	23S_rRNA (b3970, modified: 9*pseudouridine, 14*methylation,1*Dihydrouridine)	C27824H31384N11469O20158P2904	-2905	rRNA Modification
rrlC	23S_rRNA (b3758, modified: 9*pseudouridine, 14*methylation,1*Dihydrouridine)	C27825H31384N11471O20155P2904	-2905	rRNA Modification
rrlD	23S_rRNA (b3275, modified: 9*pseudouridine, 14*methylation,1*Dihydrouridine)	C27825H31384N11471O20156P2904	-2905	rRNA Modification
rrlE	23S_rRNA (b4009, modified: 9*pseudouridine, 14*methylation,1*Dihydrouridine)	C27824H31384N11469O20158P2904	-2905	rRNA Modification
rrlG	23S_rRNA (b2589, modified: 9*pseudouridine, 14*methylation,1*Dihydrouridine)	C27824H31384N11469O20158P2904	-2905	rRNA Modification
rrlH	23S_rRNA (b0204, modified: 9*pseudouridine, 14*methylation,1*Dihydrouridine)	C27825H31381N11468O20159P2904	-2905	rRNA Modification
rrsA	16S_rRNA (b3851, modified: 1*pseudouridine, 7*methylation,3*di-methylation)	C14766H16674N6061O10721P1542	-1543	rRNA Modification
rrsB	16S_rRNA (b3968, modified: 1*pseudouridine, 7*methylation,3*di-methylation)	C14767H16675N6064O10720P1542	-1543	rRNA Modification
rrsC	16S_rRNA (b3756, modified: 1*pseudouridine, 7*methylation,3*di-methylation)	C14767H16676N6065O10718P1542	-1543	rRNA Modification
rrsD	16S_rRNA (b3278, modified: 1*pseudouridine, 7*methylation,3*di-methylation)	C14766H16676N6063O10720P1542	-1543	rRNA Modification
rrsE	16S_rRNA (b4007, modified: 1*pseudouridine, 7*methylation,3*di-methylation)	C14767H16675N6064O10720P1542	-1543	rRNA Modification
rrsG	16S_rRNA (b2591, modified: 1*pseudouridine, 7*methylation,3*di-methylation)	C14766H16675N6062O10723P1542	-1543	rRNA Modification
rrsH	16S_rRNA (b0201, modified: 1*pseudouridine, 7*methylation,3*di-methylation)	C14765H16673N6058O10723P1542	-1543	rRNA Modification
sec_tRNA_sec	SelC_tRNA tRNA (b3658, charged with selenocysteine)	C910H1045Mg2N358O674P95Se1	-92	tRNA charging
sec_tRNA_ser	SelC_tRNA tRNA (b3658, charged with serine)	C910H1045Mg2N358O675P95	-92	tRNA charging
selC_tRNA	b3658 tRNA (modified, uncharged)	C907H1038Mg2N357O672P95	-92	tRNA Modification
selnp	Selenophosphate	H2O3PSe	-1	Others
selnp[e]	Selenophosphate	H2O3PSe	-1	Others
ser-L	L-Serine	C3H7NO3	0	Others
ser-L[e]	L-Serine	C3H7NO3	0	Others
ser1_tRNA	ser1_tRNA (uncharged tRNA)	C842H959N344O615P88S0Mg2	-85	tRNA charging
ser1_tRNA_ser	ser1_tRNA (charged tRNA)	C845H966Mg2N345O618P88S0	-85	tRNA charging
ser2_tRNA	ser2_tRNA (uncharged tRNA)	C845H964N344O615P88S1Mg2	-85	tRNA charging
ser2_tRNA_ser	ser2_tRNA (charged tRNA)	C848H971Mg2N345O618P88S1	-85	tRNA charging
ser3_tRNA	ser3_tRNA (uncharged tRNA)	C859H984N349O620P89S1Mg2	-86	tRNA charging
ser3_tRNA_ser	ser3_tRNA (charged tRNA)	C862H991Mg2N350O623P89S1	-86	tRNA charging
serT_tRNA	serT_tRNA (b0971, uncharged tRNA)	C851H974Mg2N344O616P88S2	-86	tRNA Modification
serT_tRNA_ser	serT_tRNA (charged tRNA)	C854H981Mg2N345O619P88S2	-86	tRNA charging
serU_tRNA	serU_tRNA (b1975, uncharged tRNA)	C867H994Mg2N355O624P90S1	-87	tRNA Modification
serV_tRNA	serV_tRNA (b2695, uncharged tRNA)	C893H1019Mg2N365O651P93S2	-91	tRNA Modification
serV_tRNA_ser	serV_tRNA (charged tRNA)	C896H1026Mg2N366O654P93S2	-91	tRNA charging
serW_tRNA	serW_tRNA (b0883, uncharged tRNA)	C842H959Mg2N344O615P88	-85	tRNA Modification

serX_tRNA	serX_tRNA (b1032, uncharged tRNA)	C842H959Mg2N344O615P88	-85	tRNA Modification
so4	Sulfate	O4S	-2	Others
so4[e]	Sulfate	O4S	-2	Others
spmd	Spermidine	C7H22N3	3	Others
spmd[e]	Spermidine	C7H22N3	3	Others
thf	5,6,7,8-Tetrahydrofolate	C19H21N7O6	-2	Others
thf[e]	5,6,7,8-Tetrahydrofolate	C19H21N7O6	-2	Others
thr-L	L-Threonine	C4H9NO3	0	Others
thr-L[e]	L-Threonine	C4H9NO3	0	Others
thr1_tRNA	thr1_tRNA (uncharged tRNA)	C731H837N290O539P76S0Mg2	-74	tRNA charging
thr1_tRNA_thr	thr1_tRNA (charged tRNA)	C735H846Mg2N291O542P76S0	-74	tRNA charging
thr2_tRNA	thr2_tRNA (uncharge tRNA)	C731H836N290O538P76S0Mg2	-74	tRNA charging
thr2_tRNA_thr	thr2_tRNA (charged tRNA)	C735H845Mg2N291O541P76S0	-74	tRNA charging
thr3_tRNA	thr3_tRNA (uncharge tRNA)	C731H835N288O537P76S0Mg2	-74	tRNA charging
thr3_tRNA_thr	thr3_tRNA (charged tRNA)	C735H844Mg2N289O540P76S0	-74	tRNA charging
thrT_tRNA	thrT_tRNA (b3979, uncharged tRNA)	C732H837Mg2N291O538P76	-74	tRNA Modification
thrU_tRNA	thrU_tRNA (b3976, uncharged tRNA)	C731H835Mg2N289O536P76	-74	tRNA Modification
thrU_tRNA_thr	thrU_tRNA (charged tRNA)	C735H844Mg2N290O539P76S0	-74	tRNA charging
thrV_tRNA	thrV_tRNA (b3273, uncharged tRNA)	C731H837Mg2N289O540P76	-74	tRNA Modification
thrW_tRNA	thrW_tRNA (b0224, uncharged tRNA)	C731H836Mg2N288O538P76	-74	tRNA Modification
tl_term_b0014_18_rib_cplx	Translation termination complex b0014 18 ribosomes	C1758109H2474166N613891O874703S5364P85619Mg3150Zn0Fe0	-72266.52	Translation
tl_term_b0014_18_rib_cplx2	Translation termination complex b0014 18 ribosomes 2 (RF2_mono)	C1759027H2474724N613567O875297S5328P85619Mg3150Zn0Fe0	-72428.52	Translation
tl_term_b0014_1_rib_cplx	Translation termination complex b0014 1 ribosomes	C114957H156998N41144O61134S298P6569Mg175Zn0Fe0	-5828.14	Translation
tl_term_b0014_1_rib_cplx2	Translation termination complex b0014 1 ribosomes 2 (RF2_mono)	C115008H157029N41126O61167S296P6569Mg175Zn0Fe0	-5837.14	Translation
tl_term_b0014_37_rib_cplx	Translation termination complex b0014 37 ribosomes	C3594573H5063942N1254020O1783986S11026P173969Mg6475Zn0Fe0	-146521.18	Translation
tl_term_b0014_37_rib_cplx2	Translation termination complex b0014 37 ribosomes 2 (RF2_mono)	C3596460H5065089N1253354O1785207S10952P173969Mg6475Zn0Fe0	-146854.18	Translation
tl_term_b0015_11_rib_cplx	Translation termination complex b0015 11 ribosomes	C1060696H1489126N371751O529449S3289P52292Mg1925Zn0Fe0	-43769.54	Translation
tl_term_b0015_11_rib_cplx2	Translation termination complex b0015 11 ribosomes 2 (RF2_mono)	C1061257H1489467N371553O529812S3267P52292Mg1925Zn0Fe0	-43868.54	Translation
tl_term_b0015_1_rib_cplx	Translation termination complex b0015 1 ribosomes	C106246H146476N37801O55289S299P5782Mg175Zn0Fe0	-5008.14	Translation
tl_term_b0015_1_rib_cplx2	Translation termination complex b0015 1 ribosomes 2 (RF2_mono)	C106297H146507N37783O55322S297P5782Mg175Zn0Fe0	-5017.14	Translation
tl_term_b0015_22_rib_cplx	Translation termination complex b0015 22 ribosomes	C2110591H2966041N739096O1051025S6578P103453Mg3850Zn0Fe0	-86407.08	Translation
tl_term_b0015_22_rib_cplx2	Translation termination complex b0015 22 ribosomes 2 (RF2_mono)	C2111713H2966723N738700O1051751S6534P103453Mg3850Zn0Fe0	-86605.08	Translation
tl_term_b0023_1_rib_cplx	Translation termination complex b0023 1 ribosomes	C96588H135000N34024O48799S285P4916Mg175Zn0Fe0	-4129.14	Translation
tl_term_b0023_1_rib_cplx2	Translation termination complex b0023 1 ribosomes 2 (RF2_mono)	C96639H135031N34006O48832S283P4916Mg175Zn0Fe0	-4138.14	Translation
tl_term_b0023_2_rib_cplx	Translation termination complex b0023 2 ribosomes	C190654H267147N67016O95780S570P9566Mg350Zn0Fe0	-7991.28	Translation
tl_term_b0023_2_rib_cplx2	Translation termination complex b0023 2 ribosomes 2 (RF2_mono)	C190756H267209N66980O95846S566P9566Mg350Zn0Fe0	-8009.28	Translation
tl_term_b0023_5_rib_cplx	Translation termination complex b0023 5 ribosomes	C472852H663588N165992O236723S1425P23516Mg875Zn0Fe0	-19577.7	Translation
tl_term_b0023_5_rib_cplx2	Translation termination complex b0023 5 ribosomes 2 (RF2_mono)	C473107H663743N165902O236888S1415P23516Mg875Zn0Fe0	-19622.7	Translation
tl_term_b0025_18_rib_cplx	Translation termination complex b0025 18 ribosomes	C1722531H2420859N603068O858016S5220P84644Mg3150Zn0Fe0	-70625.52	Translation
tl_term_b0025_18_rib_cplx2	Translation termination complex b0025 18 ribosomes 2 (RF2_mono)	C1723449H2421417N602744O858610S5184P84644Mg3150Zn0Fe0	-70787.52	Translation
tl_term_b0025_1_rib_cplx	Translation termination complex b0025 1 ribosomes	C104165H144066N36883O53916S290P5594Mg175Zn0Fe0	-4816.14	Translation
tl_term_b0025_1_rib_cplx2	Translation termination complex b0025 1 ribosomes 2 (RF2_mono)	C104216H144097N36865O53949S288P5594Mg175Zn0Fe0	-4825.14	Translation
tl_term_b0025_9_rib_cplx	Translation termination complex b0025 9 ribosomes	C865749H1215498N303323O432316S2610P42794Mg1575Zn0Fe0	-35785.26	Translation
tl_term_b0025_9_rib_cplx2	Translation termination complex b0025 9 ribosomes 2 (RF2_mono)	C866208H1215777N303161O432613S2592P42794Mg1575Zn0Fe0	-35866.26	Translation
tl_term_b0026_1_rib_cplx	Translation termination complex b0026 1 ribosomes	C125148H169040N44937O67892S319P7467Mg175Zn0Fe0	-6717.14	Translation
tl_term_b0026_27_rib_cplx	Translation termination complex b0026 27 ribosomes	C2681364H3773758N932031O1322028S8613P128367Mg4725Zn0Fe0	-108094.78	Translation
tl_term_b0026_55_rib_cplx	Translation termination complex b0026 55 ribosomes	C5434212H7655762N1887363O2672636S17545P258567Mg9625Zn0Fe0	-217270.7	Translation
tl_term_b0027_1_rib_cplx	Translation termination complex b0027 1 ribosomes	C99167H138003N34864O50575S288P5143Mg175Zn0Fe0	-4369.14	Translation
tl_term_b0027_1_rib_cplx2	Translation termination complex b0027 1 ribosomes 2 (RF2_mono)	C99218H138034N34846O50608S286P5143Mg175Zn0Fe0	-4378.14	Translation
tl_term_b0027_4_rib_cplx	Translation termination complex b0027 4 ribosomes	C382634H536121N134032O191782S1152P19090Mg700Zn0Fe0	-15991.56	Translation
tl_term_b0027_4_rib_cplx2	Translation termination complex b0027 4 ribosomes 2 (RF2_mono)	C382838H536245N133960O191914S1144P19090Mg700Zn0Fe0	-16027.56	Translation

tl_term_b0027_9_rib_cplx	Translation termination complex b0027 9 ribosomes	C855079H1199651N299312O427127S2592P42335Mg1575Zn0Fe0	-35362.26	Translation
tl_term_b0027_9_rib_cplx2	Translation termination complex b0027 9 ribosomes 2 (RF2_mono)	C855538H1199930N299150O427424S2574P42335Mg1575Zn0Fe0	-35443.26	Translation
tl_term_b0028_1_rib_cplx	Translation termination complex b0028 1 ribosomes	C98631H137355N34733O50252S286P5100Mg175Zn0Fe0	-4345.14	Translation
tl_term_b0028_1_rib_cplx2	Translation termination complex b0028 1 ribosomes 2 (RF2_mono)	C98682H137386N34715O50285S284P5100Mg175Zn0Fe0	-4354.14	Translation
tl_term_b0028_4_rib_cplx	Translation termination complex b0028 4 ribosomes	C381699H534882N133847O191540S1144P19050Mg700Zn0Fe0	-16027.56	Translation
tl_term_b0028_4_rib_cplx2	Translation termination complex b0028 4 ribosomes 2 (RF2_mono)	C381903H535006N133775O191672S1136P19050Mg700Zn0Fe0	-16063.56	Translation
tl_term_b0028_8_rib_cplx	Translation termination complex b0028 8 ribosomes	C759123H1064918N265999O379924S2288P37650Mg1400Zn0Fe0	-31604.12	Translation
tl_term_b0028_8_rib_cplx2	Translation termination complex b0028 8 ribosomes 2 (RF2_mono)	C759531H1065166N265855O380188S2272P37650Mg1400Zn0Fe0	-31676.12	Translation
tl_term_b0029_18_rib_cplx	Translation termination complex b0029 18 ribosomes	C1722387H2420347N602916O858891S5256P84669Mg3150Zn0Fe0	-70956.52	Translation
tl_term_b0029_18_rib_cplx2	Translation termination complex b0029 18 ribosomes 2 (RF2_mono)	C1723305H2420905N602592O859485S5220P84669Mg3150Zn0Fe0	-71118.52	Translation
tl_term_b0029_1_rib_cplx	Translation termination complex b0029 1 ribosomes	C104259H144149N36969O53992S292P5602Mg175Zn0Fe0	-4841.14	Translation
tl_term_b0029_1_rib_cplx2	Translation termination complex b0029 1 ribosomes 2 (RF2_mono)	C104310H144180N36951O54025S290P5602Mg175Zn0Fe0	-4850.14	Translation
tl_term_b0029_9_rib_cplx	Translation termination complex b0029 9 ribosomes	C865731H1215301N303297O432768S2628P42810Mg1575Zn0Fe0	-35954.26	Translation
tl_term_b0029_9_rib_cplx2	Translation termination complex b0029 9 ribosomes 2 (RF2_mono)	C866190H1215580N303135O433065S2610P42810Mg1575Zn0Fe0	-36035.26	Translation
tl_term_b0049_16_rib_cplx	Translation termination complex b0049 16 ribosomes	C1530856H2148414N535743O763505S4704P75435Mg2800Zn0Fe0	-63230.24	Translation
tl_term_b0049_16_rib_cplx2	Translation termination complex b0049 16 ribosomes 2 (RF2_mono)	C1531672H2148910N535455O764033S4672P75435Mg2800Zn0Fe0	-63374.24	Translation
tl_term_b0049_1_rib_cplx	Translation termination complex b0049 1 ribosomes	C103201H142794N36498O53255S294P5505Mg175Zn0Fe0	-4743.14	Translation
tl_term_b0049_1_rib_cplx2	Translation termination complex b0049 1 ribosomes 2 (RF2_mono)	C103252H142825N36480O53288S292P5505Mg175Zn0Fe0	-4752.14	Translation
tl_term_b0049_8_rib_cplx	Translation termination complex b0049 8 ribosomes	C769440H1078750N269479O384705S2352P38139Mg1400Zn0Fe0	-32037.12	Translation
tl_term_b0049_8_rib_cplx2	Translation termination complex b0049 8 ribosomes 2 (RF2_mono)	C769848H1078998N269335O384969S2336P38139Mg1400Zn0Fe0	-32109.12	Translation
tl_term_b0050_1_rib_cplx	Translation termination complex b0050 1 ribosomes	C97869H136467N34426O49708S286P5029Mg175Zn0Fe0	-4263.14	Translation
tl_term_b0050_1_rib_cplx2	Translation termination complex b0050 1 ribosomes 2 (RF2_mono)	C97920H136498N34408O49741S284P5029Mg175Zn0Fe0	-4272.14	Translation
tl_term_b0050_3_rib_cplx	Translation termination complex b0050 3 ribosomes	C286431H401263N100446O143834S858P14331Mg525Zn0Fe0	-12031.42	Translation
tl_term_b0050_3_rib_cplx2	Translation termination complex b0050 3 ribosomes 2 (RF2_mono)	C286584H401356N100392O143933S852P14331Mg525Zn0Fe0	-12058.42	Translation
tl_term_b0050_7_rib_cplx	Translation termination complex b0050 7 ribosomes	C663555H930855N232486O332086S2002P32935Mg1225Zn0Fe0	-27567.98	Translation
tl_term_b0050_7_rib_cplx2	Translation termination complex b0050 7 ribosomes 2 (RF2_mono)	C663912H931072N232360O332317S1988P32935Mg1225Zn0Fe0	-27630.98	Translation
tl_term_b0051_16_rib_cplx	Translation termination complex b0051 16 ribosomes	C1530508H2148939N535982O763592S4752P75490Mg2800Zn0Fe0	-63125.24	Translation
tl_term_b0051_16_rib_cplx2	Translation termination complex b0051 16 ribosomes 2 (RF2_mono)	C1531324H2149435N535694O764120S4720P75490Mg2800Zn0Fe0	-63269.24	Translation
tl_term_b0051_1_rib_cplx	Translation termination complex b0051 1 ribosomes	C102943H142569N36392O53087S297P5485Mg175Zn0Fe0	-4713.14	Translation
tl_term_b0051_1_rib_cplx2	Translation termination complex b0051 1 ribosomes 2 (RF2_mono)	C102994H142600N36374O53120S295P5485Mg175Zn0Fe0	-4722.14	Translation
tl_term_b0051_8_rib_cplx	Translation termination complex b0051 8 ribosomes	C769140H1078875N269534O384656S2376P38154Mg1400Zn0Fe0	-31972.12	Translation
tl_term_b0051_8_rib_cplx2	Translation termination complex b0051 8 ribosomes 2 (RF2_mono)	C769548H1079123N269390O384920S2360P38154Mg1400Zn0Fe0	-32044.12	Translation
tl_term_b0052_19_rib_cplx	Translation termination complex b0052 19 ribosomes	C1818319H2555494N635954O905780S5586P89320Mg3325Zn0Fe0	-74769.66	Translation
tl_term_b0052_1_rib_cplx	Translation termination complex b0052 1 ribosomes	C104593H144592N36986O54254S294P5638Mg175Zn0Fe0	-4873.14	Translation
tl_term_b0052_9_rib_cplx	Translation termination complex b0052 9 ribosomes	C866249H1216104N303194O432710S2646P42830Mg1575Zn0Fe0	-35938.26	Translation
tl_term_b0053_12_rib_cplx	Translation termination complex b0053 12 ribosomes	C1160801H1631021N406460O579032S3576P57089Mg2100Zn0Fe0	-47851.68	Translation
tl_term_b0053_12_rib_cplx2	Translation termination complex b0053 12 ribosomes 2 (RF2_mono)	C1161413H1631393N406244O579428S3552P57089Mg2100Zn0Fe0	-47959.68	Translation
tl_term_b0053_1_rib_cplx	Translation termination complex b0053 1 ribosomes	C107980H148661N38433O56444S298P5939Mg175Zn0Fe0	-5170.14	Translation
tl_term_b0053_1_rib_cplx2	Translation termination complex b0053 1 ribosomes 2 (RF2_mono)	C108031H148692N38415O56477S296P5939Mg175Zn0Fe0	-5179.14	Translation
tl_term_b0053_25_rib_cplx	Translation termination complex b0053 25 ribosomes	C2405044H3382901N841401O1196636S7450P117539Mg4375Zn0Fe0	-98293.5	Translation
tl_term_b0053_25_rib_cplx2	Translation termination complex b0053 25 ribosomes 2 (RF2_mono)	C2406319H3383676N840951O1197461S7400P117539Mg4375Zn0Fe0	-98518.5	Translation
tl_term_b0058_12_rib_cplx	Translation termination complex b0058 12 ribosomes	C1143471H1605302N400591O570727S3528P56460Mg2100Zn0Fe0	-47174.68	Translation
tl_term_b0058_12_rib_cplx2	Translation termination complex b0058 12 ribosomes 2 (RF2_mono)	C1144083H1605674N400375O571123S3504P56460Mg2100Zn0Fe0	-47282.68	Translation
tl_term_b0058_1_rib_cplx	Translation termination complex b0058 1 ribosomes	C101067H140300N35732O51780S294P5310Mg175Zn0Fe0	-4537.14	Translation
tl_term_b0058_1_rib_cplx2	Translation termination complex b0058 1 ribosomes 2 (RF2_mono)	C101118H140331N35714O51813S292P5310Mg175Zn0Fe0	-4546.14	Translation
tl_term_b0058_6_rib_cplx	Translation termination complex b0058 6 ribosomes	C574887H806210N201577O287665S1764P28560Mg1050Zn0Fe0	-23917.84	Translation
tl_term_b0058_6_rib_cplx2	Translation termination complex b0058 6 ribosomes 2 (RF2_mono)	C575193H806396N201469O287863S1752P28560Mg1050Zn0Fe0	-23971.84	Translation

tl_term_b0059_1_rib_cplx	Translation termination complex b0059 1 ribosomes	C126131H170344N45306O68645S312P7558Mg175Zn0Fe0	-6835.14	Translation
tl_term_b0059_1_rib_cplx2	Translation termination complex b0059 1 ribosomes 2 (RF2_mono)	C126182H170375N45288O68678S310P7558Mg175Zn0Fe0	-6844.14	Translation
tl_term_b0059_28_rib_cplx	Translation termination complex b0059 28 ribosomes	C2784875H3923344N969435O1373609S8736P133081Mg4900Zn0Fe0	-112813.92	Translation
tl_term_b0059_28_rib_cplx2	Translation termination complex b0059 28 ribosomes 2 (RF2_mono)	C2786303H3924212N968931O1374533S8680P133081Mg4900Zn0Fe0	-113065.92	Translation
tl_term_b0059_56_rib_cplx	Translation termination complex b0059 56 ribosomes	C5542091H7815344N1927791O2726905S17472P263253Mg9800Zn0Fe0	-222717.84	Translation
tl_term_b0059_56_rib_cplx2	Translation termination complex b0059 56 ribosomes 2 (RF2_mono)	C5544947H7817080N1926783O2728753S17360P263253Mg9800Zn0Fe0	-223221.84	Translation
tl_term_b0144_18_rib_cplx	Translation termination complex b0144 18 ribosomes	C1722462H2418629N602710O857674S5202P84593Mg3150Zn0Fe0	-70754.52	Translation
tl_term_b0144_1_rib_cplx	Translation termination complex b0144 1 ribosomes	C103994H143791N36746O53795S289P5577Mg175Zn0Fe0	-4809.14	Translation
tl_term_b0144_9_rib_cplx	Translation termination complex b0144 9 ribosomes	C865626H1214303N303082O432091S2601P42761Mg1575Zn0Fe0	-35842.26	Translation
tl_term_b0166_16_rib_cplx	Translation termination complex b0166 16 ribosomes	C1527650H2145752N534705O762187S4640P75243Mg2800Zn0Fe0	-62942.24	Translation
tl_term_b0166_16_rib_cplx2	Translation termination complex b0166 16 ribosomes 2 (RF2_mono)	C1528466H2146248N534417O762715S4608P75243Mg2800Zn0Fe0	-63086.24	Translation
tl_term_b0166_1_rib_cplx	Translation termination complex b0166 1 ribosomes	C102845H142442N36375O53047S290P5478Mg175Zn0Fe0	-4710.14	Translation
tl_term_b0166_1_rib_cplx2	Translation termination complex b0166 1 ribosomes 2 (RF2_mono)	C102896H142473N36357O53080S288P5478Mg175Zn0Fe0	-4719.14	Translation
tl_term_b0166_8_rib_cplx	Translation termination complex b0166 8 ribosomes	C767754H1077320N268929O383979S2320P38035Mg1400Zn0Fe0	-31885.12	Translation
tl_term_b0166_8_rib_cplx2	Translation termination complex b0166 8 ribosomes 2 (RF2_mono)	C768162H1077568N268785O384243S2304P38035Mg1400Zn0Fe0	-31957.12	Translation
tl_term_b0167_1_rib_cplx	Translation termination complex b0167 1 ribosomes	C123567H167369N44203O66851S310P7321Mg175Zn0Fe0	-6564.14	Translation
tl_term_b0167_26_rib_cplx	Translation termination complex b0167 26 ribosomes	C2578367H3631694N898103O1270701S8060P123521Mg4550Zn0Fe0	-103817.64	Translation
tl_term_b0167_52_rib_cplx	Translation termination complex b0167 52 ribosomes	C5131359H7234592N1786159O2522705S16120P244369Mg9100Zn0Fe0	-204961.28	Translation
tl_term_b0168_15_rib_cplx	Translation termination complex b0168 15 ribosomes	C1431618H2010935N501130O714280S4470P70547Mg2625Zn0Fe0	-59075.1	Translation
tl_term_b0168_15_rib_cplx2	Translation termination complex b0168 15 ribosomes 2 (RF2_mono)	C1432383H2011400N500860O714775S4440P70547Mg2625Zn0Fe0	-59210.1	Translation
tl_term_b0168_1_rib_cplx	Translation termination complex b0168 1 ribosomes	C102514H142061N36260O52780S298P5447Mg175Zn0Fe0	-4683.14	Translation
tl_term_b0168_1_rib_cplx2	Translation termination complex b0168 1 ribosomes 2 (RF2_mono)	C102565H142092N36242O52813S296P5447Mg175Zn0Fe0	-4692.14	Translation
tl_term_b0168_7_rib_cplx	Translation termination complex b0168 7 ribosomes	C672130H943007N235490O336280S2086P33347Mg1225Zn0Fe0	-27993.98	Translation
tl_term_b0168_7_rib_cplx2	Translation termination complex b0168 7 ribosomes 2 (RF2_mono)	C672487H943224N235364O336511S2072P33347Mg1225Zn0Fe0	-28056.98	Translation
tl_term_b0169_14_rib_cplx	Translation termination complex b0169 14 ribosomes	C1334530H1874217N467354O666022S4074P65828Mg2450Zn0Fe0	-55022.96	Translation
tl_term_b0169_14_rib_cplx2	Translation termination complex b0169 14 ribosomes 2 (RF2_mono)	C1335244H1874651N467102O666484S4046P65828Mg2450Zn0Fe0	-55148.96	Translation
tl_term_b0169_1_rib_cplx	Translation termination complex b0169 1 ribosomes	C101740H141135N35949O52279S291P5378Mg175Zn0Fe0	-4607.14	Translation
tl_term_b0169_1_rib_cplx2	Translation termination complex b0169 1 ribosomes 2 (RF2_mono)	C101791H141166N35931O52312S289P5378Mg175Zn0Fe0	-4616.14	Translation
tl_term_b0169_7_rib_cplx	Translation termination complex b0169 7 ribosomes	C670720H941019N235059O335545S2037P33278Mg1225Zn0Fe0	-27875.98	Translation
tl_term_b0169_7_rib_cplx2	Translation termination complex b0169 7 ribosomes 2 (RF2_mono)	C671077H941236N234933O335776S2023P33278Mg1225Zn0Fe0	-27938.98	Translation
tl_term_b0170_16_rib_cplx	Translation termination complex b0170 16 ribosomes	C1529786H2148873N535700O763658S4704P75444Mg2800Zn0Fe0	-63223.24	Translation
tl_term_b0170_16_rib_cplx2	Translation termination complex b0170 16 ribosomes 2 (RF2_mono)	C1530602H2149369N535412O764186S4672P75444Mg2800Zn0Fe0	-63367.24	Translation
tl_term_b0170_1_rib_cplx	Translation termination complex b0170 1 ribosomes	C103256H142923N36620O53258S294P5514Mg175Zn0Fe0	-4751.14	Translation
tl_term_b0170_1_rib_cplx2	Translation termination complex b0170 1 ribosomes 2 (RF2_mono)	C103307H142954N36602O53291S292P5514Mg175Zn0Fe0	-4760.14	Translation
tl_term_b0170_8_rib_cplx	Translation termination complex b0170 8 ribosomes	C768970H1079033N269524O384778S2352P38148Mg1400Zn0Fe0	-32038.12	Translation
tl_term_b0170_8_rib_cplx2	Translation termination complex b0170 8 ribosomes 2 (RF2_mono)	C769378H1079281N269380O385042S2336P38148Mg1400Zn0Fe0	-32110.12	Translation
tl_term_b0172_10_rib_cplx	Translation termination complex b0172 10 ribosomes	C950779H1335288N333377O475351S2890P47060Mg1750Zn0Fe0	-39332.4	Translation
tl_term_b0172_1_rib_cplx	Translation termination complex b0172 1 ribosomes	C99883H138954N35315O51010S289P5210Mg175Zn0Fe0	-4438.14	Translation
tl_term_b0172_5_rib_cplx	Translation termination complex b0172 5 ribosomes	C478059H670658N167787O239606S1445P23810Mg875Zn0Fe0	-19946.7	Translation
tl_term_b0188_12_rib_cplx	Translation termination complex b0188 12 ribosomes	C1163941H1634142N407629O580231S3528P57305Mg2100Zn0Fe0	-48067.68	Translation
tl_term_b0188_12_rib_cplx2	Translation termination complex b0188 12 ribosomes 2 (RF2_mono)	C1164553H1634514N407413O580627S3504P57305Mg2100Zn0Fe0	-48175.68	Translation
tl_term_b0188_1_rib_cplx	Translation termination complex b0188 1 ribosomes	C108337H149021N38535O56708S294P5968Mg175Zn0Fe0	-5199.14	Translation
tl_term_b0188_1_rib_cplx2	Translation termination complex b0188 1 ribosomes 2 (RF2_mono)	C108388H149052N38517O56741S292P5968Mg175Zn0Fe0	-5208.14	Translation
tl_term_b0188_25_rib_cplx	Translation termination complex b0188 25 ribosomes	C2411473H3389285N843831O1198940S7350P117976Mg4375Zn0Fe0	-98730.5	Translation
tl_term_b0188_25_rib_cplx2	Translation termination complex b0188 25 ribosomes 2 (RF2_mono)	C2412748H3390060N843381O1199765S7300P117976Mg4375Zn0Fe0	-98955.5	Translation
tl_term_b0194_16_rib_cplx	Translation termination complex b0194 16 ribosomes	C1560009H2192844N544599O775550S4784P76121Mg2800Zn0Fe0	-64060.24	Translation
tl_term_b0194_1_rib_cplx	Translation termination complex b0194 1 ribosomes	C112854H154434N40224O59720S299P6371Mg175Zn0Fe0	-5618.14	Translation

tl_term_b0194_33_rib_cplx	Translation termination complex b0194 33 ribosomes	C3200118H4503042N1116224O1586824S9867P155171Mg5775Zn0Fe0	-130294.62	Translation
tl_term_b0405_10_rib_cplx	Translation termination complex b0405 10 ribosomes	C964300H1353147N337290O481373S2930P47573Mg1750Zn0Fe0	-40015.4	Translation
tl_term_b0405_10_rib_cplx2	Translation termination complex b0405 10 ribosomes 2 (RF2_mono)	C964810H1353457N337110O481703S2910P47573Mg1750Zn0Fe0	-40105.4	Translation
tl_term_b0405_1_rib_cplx	Translation termination complex b0405 1 ribosomes	C105592H145698N37374O54890S293P5723Mg175Zn0Fe0	-4968.14	Translation
tl_term_b0405_1_rib_cplx2	Translation termination complex b0405 1 ribosomes 2 (RF2_mono)	C105643H145729N37356O54923S291P5723Mg175Zn0Fe0	-4977.14	Translation
tl_term_b0405_20_rib_cplx	Translation termination complex b0405 20 ribosomes	C1918420H2694757N670530O955243S5860P94073Mg3500Zn0Fe0	-78956.8	Translation
tl_term_b0405_20_rib_cplx2	Translation termination complex b0405 20 ribosomes 2 (RF2_mono)	C1919440H2695377N670170O955903S5820P94073Mg3500Zn0Fe0	-79136.8	Translation
tl_term_b0406_11_rib_cplx	Translation termination complex b0406 11 ribosomes	C1061753H1490275N371484O529602S3344P52291Mg1925Zn0Fe0	-43856.54	Translation
tl_term_b0406_11_rib_cplx2	Translation termination complex b0406 11 ribosomes 2 (RF2_mono)	C1062314H1490616N371286O529965S3322P52291Mg1925Zn0Fe0	-43955.54	Translation
tl_term_b0406_1_rib_cplx	Translation termination complex b0406 1 ribosomes	C106273H146505N37634O55342S304P5781Mg175Zn0Fe0	-5015.14	Translation
tl_term_b0406_1_rib_cplx2	Translation termination complex b0406 1 ribosomes 2 (RF2_mono)	C106324H146536N37616O55375S302P5781Mg175Zn0Fe0	-5024.14	Translation
tl_term_b0406_22_rib_cplx	Translation termination complex b0406 22 ribosomes	C2112781H2968422N738719O1051288S6688P103452Mg3850Zn0Fe0	-86582.08	Translation
tl_term_b0406_22_rib_cplx2	Translation termination complex b0406 22 ribosomes 2 (RF2_mono)	C2113903H2969104N738323O1052014S6644P103452Mg3850Zn0Fe0	-86780.08	Translation
tl_term_b0407_1_rib_cplx	Translation termination complex b0407 1 ribosomes	C97456H135998N34295O49418S287P4994Mg175Zn0Fe0	-4218.14	Translation
tl_term_b0407_1_rib_cplx2	Translation termination complex b0407 1 ribosomes 2 (RF2_mono)	C97507H136029N34277O49451S285P4994Mg175Zn0Fe0	-4227.14	Translation
tl_term_b0407_3_rib_cplx	Translation termination complex b0407 3 ribosomes	C286026H400834N100359O143590S861P14316Mg525Zn0Fe0	-11986.42	Translation
tl_term_b0407_3_rib_cplx2	Translation termination complex b0407 3 ribosomes 2 (RF2_mono)	C286179H400927N100305O143689S855P14316Mg525Zn0Fe0	-12013.42	Translation
tl_term_b0407_6_rib_cplx	Translation termination complex b0407 6 ribosomes	C568881H798088N199455O284848S1722P28299Mg1050Zn0Fe0	-23638.84	Translation
tl_term_b0407_6_rib_cplx2	Translation termination complex b0407 6 ribosomes 2 (RF2_mono)	C569187H798274N199347O285046S1710P28299Mg1050Zn0Fe0	-23692.84	Translation
tl_term_b0413_1_rib_cplx	Translation termination complex b0413 1 ribosomes	C98704H137507N34799O50253S292P5103Mg175Zn0Fe0	-4330.14	Translation
tl_term_b0413_1_rib_cplx2	Translation termination complex b0413 1 ribosomes 2 (RF2_mono)	C98755H137538N34781O50286S290P5103Mg175Zn0Fe0	-4339.14	Translation
tl_term_b0413_4_rib_cplx	Translation termination complex b0413 4 ribosomes	C381940H535499N134018O191550S1168P19056Mg700Zn0Fe0	-15961.56	Translation
tl_term_b0413_4_rib_cplx2	Translation termination complex b0413 4 ribosomes 2 (RF2_mono)	C382144H535623N133946O191682S1160P19056Mg700Zn0Fe0	-15997.56	Translation
tl_term_b0413_8_rib_cplx	Translation termination complex b0413 8 ribosomes	C759588H1066155N266310O379946S2336P37660Mg1400Zn0Fe0	-31470.12	Translation
tl_term_b0413_8_rib_cplx2	Translation termination complex b0413 8 ribosomes 2 (RF2_mono)	C759996H1066403N266166O380210S2320P37660Mg1400Zn0Fe0	-31542.12	Translation
tl_term_b0414_10_rib_cplx	Translation termination complex b0414 10 ribosomes	C964744H1354588N337961O481570S2970P47604Mg1750Zn0Fe0	-39886.4	Translation
tl_term_b0414_1_rib_cplx	Translation termination complex b0414 1 ribosomes	C105937H146176N37604O55096S297P5754Mg175Zn0Fe0	-4983.14	Translation
tl_term_b0414_21_rib_cplx	Translation termination complex b0414 21 ribosomes	C2014397H2831536N705064O1002816S6237P98754Mg3675Zn0Fe0	-82545.94	Translation
tl_term_b0415_1_rib_cplx	Translation termination complex b0415 1 ribosomes	C98856H137655N34858O50369S283P5121Mg175Zn0Fe0	-4353.14	Translation
tl_term_b0415_4_rib_cplx	Translation termination complex b0415 4 ribosomes	C381957H535398N134017O191609S1132P19071Mg700Zn0Fe0	-15996.56	Translation
tl_term_b0415_9_rib_cplx	Translation termination complex b0415 9 ribosomes	C853792H1198303N299282O427009S2547P42321Mg1575Zn0Fe0	-35402.26	Translation
tl_term_b0416_1_rib_cplx	Translation termination complex b0416 1 ribosomes	C98362H137094N34656O49997S285P5070Mg175Zn0Fe0	-4299.14	Translation
tl_term_b0416_4_rib_cplx	Translation termination complex b0416 4 ribosomes	C381442H534774N133773O191225S1140P19020Mg700Zn0Fe0	-15933.56	Translation
tl_term_b0416_8_rib_cplx	Translation termination complex b0416 8 ribosomes	C758882H1065014N265929O379529S2280P37620Mg1400Zn0Fe0	-31446.12	Translation
tl_term_b0423_14_rib_cplx	Translation termination complex b0423 14 ribosomes	C1359356H1910370N475127O676391S4144P66565Mg2450Zn0Fe0	-55829.96	Translation
tl_term_b0423_14_rib_cplx2	Translation termination complex b0423 14 ribosomes 2 (RF2_mono)	C1360070H1910804N474875O676853S4116P66565Mg2450Zn0Fe0	-55955.96	Translation
tl_term_b0423_1_rib_cplx	Translation termination complex b0423 1 ribosomes	C109900H150937N39042O57734S296P6102Mg175Zn0Fe0	-5336.14	Translation
tl_term_b0423_1_rib_cplx2	Translation termination complex b0423 1 ribosomes 2 (RF2_mono)	C109951H150968N39024O57767S294P6102Mg175Zn0Fe0	-5345.14	Translation
tl_term_b0423_28_rib_cplx	Translation termination complex b0423 28 ribosomes	C2704924H3805144N944757O1342637S8288P131679Mg4900Zn0Fe0	-110207.92	Translation
tl_term_b0423_28_rib_cplx2	Translation termination complex b0423 28 ribosomes 2 (RF2_mono)	C2706352H3806012N944253O1343561S8232P131679Mg4900Zn0Fe0	-110459.92	Translation
tl_term_b0436_12_rib_cplx	Translation termination complex b0436 12 ribosomes	C1161625H1631859N406342O579452S3504P57101Mg2100Zn0Fe0	-48115.68	Translation
tl_term_b0436_12_rib_cplx2	Translation termination complex b0436 12 ribosomes 2 (RF2_mono)	C1162237H1632231N406126O579848S3480P57101Mg2100Zn0Fe0	-48223.68	Translation
tl_term_b0436_1_rib_cplx	Translation termination complex b0436 1 ribosomes	C108199H148850N38557O56523S292P5951Mg175Zn0Fe0	-5203.14	Translation
tl_term_b0436_1_rib_cplx2	Translation termination complex b0436 1 ribosomes 2 (RF2_mono)	C108250H148881N38539O56556S290P5951Mg175Zn0Fe0	-5212.14	Translation
tl_term_b0436_25_rib_cplx	Translation termination complex b0436 25 ribosomes	C2406583H3384506N840997O1197459S7300P117551Mg4375Zn0Fe0	-98830.5	Translation
tl_term_b0436_25_rib_cplx2	Translation termination complex b0436 25 ribosomes 2 (RF2_mono)	C2407858H3385281N840547O1198284S7250P117551Mg4375Zn0Fe0	-99055.5	Translation
tl_term_b0503_10_rib_cplx	Translation termination complex b0503 10 ribosomes	C965128H1354617N338122O481664S2980P47607Mg1750Zn0Fe0	-39959.4	Translation

tl_term_b0503_10_rib_cplx2	Translation termination complex b0503 10 ribosomes 2 (RF2_mono)	C965638H1354927N337942O481994S2960P47607Mg1750Zn0Fe0	-40049.4	Translation
tl_term_b0503_1_rib_cplx	Translation termination complex b0503 1 ribosomes	C105916H146097N37630O55046S298P5748Mg175Zn0Fe0	-4984.14	Translation
tl_term_b0503_1_rib_cplx2	Translation termination complex b0503 1 ribosomes 2 (RF2_mono)	C105967H146128N37612O55079S296P5748Mg175Zn0Fe0	-4993.14	Translation
tl_term_b0503_21_rib_cplx	Translation termination complex b0503 21 ribosomes	C2015276H2831697N705390O1003086S6258P98768Mg3675Zn0Fe0	-82706.94	Translation
tl_term_b0503_21_rib_cplx2	Translation termination complex b0503 21 ribosomes 2 (RF2_mono)	C2016347H2832348N705012O1003779S6216P98768Mg3675Zn0Fe0	-82895.94	Translation
tl_term_b0526_13_rib_cplx	Translation termination complex b0526 13 ribosomes	C1260632H1769653N440784O627974S4004P61838Mg2275Zn0Fe0	-52051.82	Translation
tl_term_b0526_13_rib_cplx2	Translation termination complex b0526 13 ribosomes 2 (RF2_mono)	C1261295H1770056N440550O628403S3978P61838Mg2275Zn0Fe0	-52168.82	Translation
tl_term_b0526_1_rib_cplx	Translation termination complex b0526 1 ribosomes	C109172H149929N38844O57230S308P6038Mg175Zn0Fe0	-5286.14	Translation
tl_term_b0526_1_rib_cplx2	Translation termination complex b0526 1 ribosomes 2 (RF2_mono)	C109223H149960N38826O57263S306P6038Mg175Zn0Fe0	-5295.14	Translation
tl_term_b0526_27_rib_cplx	Translation termination complex b0526 27 ribosomes	C2604002H3659331N909714O1293842S8316P126938Mg4725Zn0Fe0	-106611.78	Translation
tl_term_b0526_27_rib_cplx2	Translation termination complex b0526 27 ribosomes 2 (RF2_mono)	C2605379H3660168N909228O1294733S8262P126938Mg4725Zn0Fe0	-106854.78	Translation
tl_term_b0638_11_rib_cplx	Translation termination complex b0638 11 ribosomes	C1047329H1469659N367006O523059S3190P51762Mg1925Zn0Fe0	-43349.54	Translation
tl_term_b0638_1_rib_cplx	Translation termination complex b0638 1 ribosomes	C100499H139589N35456O51449S290P5262Mg175Zn0Fe0	-4498.14	Translation
tl_term_b0638_5_rib_cplx	Translation termination complex b0638 5 ribosomes	C479231H671617N168076O240093S1450P23862Mg875Zn0Fe0	-20038.7	Translation
tl_term_b0639_12_rib_cplx	Translation termination complex b0639 12 ribosomes	C1143362H1604650N400321O570712S3444P56454Mg2100Zn0Fe0	-47276.68	Translation
tl_term_b0639_1_rib_cplx	Translation termination complex b0639 1 ribosomes	C100870H140055N35572O51666S287P5293Mg175Zn0Fe0	-4529.14	Translation
tl_term_b0639_6_rib_cplx	Translation termination complex b0639 6 ribosomes	C574730H805780N201367O287596S1722P28548Mg1050Zn0Fe0	-23959.84	Translation
tl_term_b0640_10_rib_cplx	Translation termination complex b0640 10 ribosomes	C963527H1353051N337318O480819S2910P47531Mg1750Zn0Fe0	-39823.4	Translation
tl_term_b0640_1_rib_cplx	Translation termination complex b0640 1 ribosomes	C105170H145287N37222O54588S291P5681Mg175Zn0Fe0	-4911.14	Translation
tl_term_b0640_20_rib_cplx	Translation termination complex b0640 20 ribosomes	C1917257H2695011N670758O954409S5820P94031Mg3500Zn0Fe0	-78614.8	Translation
tl_term_b0641_11_rib_cplx	Translation termination complex b0641 11 ribosomes	C1045983H1468852N366476O522812S3201P51732Mg1925Zn0Fe0	-43220.54	Translation
tl_term_b0641_1_rib_cplx	Translation termination complex b0641 1 ribosomes	C100123H139232N35326O51222S291P5232Mg175Zn0Fe0	-4459.14	Translation
tl_term_b0641_5_rib_cplx	Translation termination complex b0641 5 ribosomes	C478467H671080N167786O239858S1455P23832Mg875Zn0Fe0	-19963.7	Translation
tl_term_b0642_1_rib_cplx	Translation termination complex b0642 1 ribosomes	C122616H165975N43985O66127S326P7235Mg175Zn0Fe0	-6492.14	Translation
tl_term_b0642_1_rib_cplx2	Translation termination complex b0642 1 ribosomes 2 (RF2_mono)	C122667H166006N43967O66160S324P7235Mg175Zn0Fe0	-6501.14	Translation
tl_term_b0642_25_rib_cplx	Translation termination complex b0642 25 ribosomes	C2474232H3480495N860225O1221871S8150P118835Mg4375Zn0Fe0	-100239.5	Translation
tl_term_b0642_25_rib_cplx2	Translation termination complex b0642 25 ribosomes 2 (RF2_mono)	C2475507H3481270N859775O1222696S8100P118835Mg4375Zn0Fe0	-100464.5	Translation
tl_term_b0642_50_rib_cplx	Translation termination complex b0642 50 ribosomes	C4923832H6933120N1710475O2425771S16300P235085Mg8750Zn0Fe0	-197893	Translation
tl_term_b0642_50_rib_cplx2	Translation termination complex b0642 50 ribosomes 2 (RF2_mono)	C4926382H6934670N1709575O2427421S16200P235085Mg8750Zn0Fe0	-198343	Translation
tl_term_b0661_13_rib_cplx	Translation termination complex b0661 13 ribosomes	C1261854H1772487N441200O628693S3926P61890Mg2275Zn0Fe0	-52051.82	Translation
tl_term_b0661_13_rib_cplx2	Translation termination complex b0661 13 ribosomes 2 (RF2_mono)	C1262517H1772890N440966O629122S3900P61890Mg2275Zn0Fe0	-52168.82	Translation
tl_term_b0661_1_rib_cplx	Translation termination complex b0661 1 ribosomes	C109590H150531N38972O57541S302P6078Mg175Zn0Fe0	-5322.14	Translation
tl_term_b0661_1_rib_cplx2	Translation termination complex b0661 1 ribosomes 2 (RF2_mono)	C109641H150562N38954O57574S300P6078Mg175Zn0Fe0	-5331.14	Translation
tl_term_b0661_27_rib_cplx	Translation termination complex b0661 27 ribosomes	C2606162H3664769N910466O1295037S8154P127004Mg4725Zn0Fe0	-106569.78	Translation
tl_term_b0661_27_rib_cplx2	Translation termination complex b0661 27 ribosomes 2 (RF2_mono)	C2607539H3665606N909980O1295928S8100P127004Mg4725Zn0Fe0	-106812.78	Translation
tl_term_b0680_16_rib_cplx	Translation termination complex b0680 16 ribosomes	C1559307H2190532N544591O774791S4864P76067Mg2800Zn0Fe0	-63878.24	Translation
tl_term_b0680_16_rib_cplx2	Translation termination complex b0680 16 ribosomes 2 (RF2_mono)	C1560123H2191028N544303O775319S4832P76067Mg2800Zn0Fe0	-64022.24	Translation
tl_term_b0680_1_rib_cplx	Translation termination complex b0680 1 ribosomes	C112317H153727N39991O59321S304P6317Mg175Zn0Fe0	-5556.14	Translation
tl_term_b0680_1_rib_cplx2	Translation termination complex b0680 1 ribosomes 2 (RF2_mono)	C112368H153758N39973O59354S302P6317Mg175Zn0Fe0	-5565.14	Translation
tl_term_b0680_32_rib_cplx	Translation termination complex b0680 32 ribosomes	C3102763H4363124N1082831O1537959S9728P150467Mg5600Zn0Fe0	-126088.48	Translation
tl_term_b0680_32_rib_cplx2	Translation termination complex b0680 32 ribosomes 2 (RF2_mono)	C3104395H4364116N1082255O1539015S9664P150467Mg5600Zn0Fe0	-126376.48	Translation
tl_term_b0850_1_rib_cplx	Translation termination complex b0850 1 ribosomes	C96869H135323N34036O49041S288P4941Mg175Zn0Fe0	-4167.14	Translation
tl_term_b0850_2_rib_cplx	Translation termination complex b0850 2 ribosomes	C191006H267556N67007O96044S576P9592Mg350Zn0Fe0	-8043.28	Translation
tl_term_b0850_5_rib_cplx	Translation termination complex b0850 5 ribosomes	C473417H664255N165920O237053S1440P23545Mg875Zn0Fe0	-19671.7	Translation
tl_term_b0851_14_rib_cplx	Translation termination complex b0851 14 ribosomes	C1334655H1874204N467453O665957S4060P65820Mg2450Zn0Fe0	-55028.96	Translation
tl_term_b0851_14_rib_cplx2	Translation termination complex b0851 14 ribosomes 2 (RF2_mono)	C1335369H1874638N467201O666419S4032P65820Mg2450Zn0Fe0	-55154.96	Translation
tl_term_b0851_1_rib_cplx	Translation termination complex b0851 1 ribosomes	C101566H140927N35866O52162S290P5357Mg175Zn0Fe0	-4587.14	Translation

tl_term_b0851_1_rib_cplx2	Translation termination complex b0851 1 ribosomes 2 (RF2_mono)	C101617H140958N35848O52195S288P5357Mg175Zn0Fe0	-4596.14	Translation
tl_term_b0851_7_rib_cplx	Translation termination complex b0851 7 ribosomes	C670684H940901N235060O335452S2030P33263Mg1225Zn0Fe0	-27867.98	Translation
tl_term_b0851_7_rib_cplx2	Translation termination complex b0851 7 ribosomes 2 (RF2_mono)	C671041H941118N234934O335683S2016P33263Mg1225Zn0Fe0	-27930.98	Translation
tl_term_b0852_17_rib_cplx	Translation termination complex b0852 17 ribosomes	C1625596H2284093N568682O810796S4964P79955Mg2975Zn0Fe0	-66953.38	Translation
tl_term_b0852_1_rib_cplx	Translation termination complex b0852 1 ribosomes	C103724H143517N36714O53660S292P5555Mg175Zn0Fe0	-4791.14	Translation
tl_term_b0852_8_rib_cplx	Translation termination complex b0852 8 ribosomes	C769543H1080019N269450O384907S2336P38105Mg1400Zn0Fe0	-31987.12	Translation
tl_term_b0853_1_rib_cplx	Translation termination complex b0853 1 ribosomes	C98967H137748N34797O50487S292P5128Mg175Zn0Fe0	-4372.14	Translation
tl_term_b0853_4_rib_cplx	Translation termination complex b0853 4 ribosomes	C382302H535659N133932O191844S1168P19081Mg700Zn0Fe0	-16054.56	Translation
tl_term_b0853_9_rib_cplx	Translation termination complex b0853 9 ribosomes	C854527H1198844N299157O427439S2628P42336Mg1575Zn0Fe0	-35525.26	Translation
tl_term_b0858_1_rib_cplx	Translation termination complex b0858 1 ribosomes	C99136H138008N34852O50547S289P5140Mg175Zn0Fe0	-4363.14	Translation
tl_term_b0858_1_rib_cplx2	Translation termination complex b0858 1 ribosomes 2 (RF2_mono)	C99187H138039N34834O50580S287P5140Mg175Zn0Fe0	-4372.14	Translation
tl_term_b0858_4_rib_cplx	Translation termination complex b0858 4 ribosomes	C382645H536312N134035O191760S1156P19087Mg700Zn0Fe0	-15976.56	Translation
tl_term_b0858_4_rib_cplx2	Translation termination complex b0858 4 ribosomes 2 (RF2_mono)	C382849H536436N133963O191892S1148P19087Mg700Zn0Fe0	-16012.56	Translation
tl_term_b0858_9_rib_cplx	Translation termination complex b0858 9 ribosomes	C855160H1200152N299340O427115S2601P42332Mg1575Zn0Fe0	-35332.26	Translation
tl_term_b0858_9_rib_cplx2	Translation termination complex b0858 9 ribosomes 2 (RF2_mono)	C855619H1200431N299178O427412S2583P42332Mg1575Zn0Fe0	-35413.26	Translation
tl_term_b0859_11_rib_cplx	Translation termination complex b0859 11 ribosomes	C1061428H1490226N371126O529126S3366P52267Mg1925Zn0Fe0	-43777.54	Translation
tl_term_b0859_11_rib_cplx2	Translation termination complex b0859 11 ribosomes 2 (RF2_mono)	C1061989H1490567N370928O529489S3344P52267Mg1925Zn0Fe0	-43876.54	Translation
tl_term_b0859_1_rib_cplx	Translation termination complex b0859 1 ribosomes	C106238H146516N37606O55296S306P5777Mg175Zn0Fe0	-5006.14	Translation
tl_term_b0859_1_rib_cplx2	Translation termination complex b0859 1 ribosomes 2 (RF2_mono)	C106289H146547N37588O55329S304P5777Mg175Zn0Fe0	-5015.14	Translation
tl_term_b0859_22_rib_cplx	Translation termination complex b0859 22 ribosomes	C2112137H2968307N737998O1050339S6732P103406Mg3850Zn0Fe0	-86426.08	Translation
tl_term_b0859_22_rib_cplx2	Translation termination complex b0859 22 ribosomes 2 (RF2_mono)	C2113259H2968989N737602O1051065S6688P103406Mg3850Zn0Fe0	-86624.08	Translation
tl_term_b0884_1_rib_cplx	Translation termination complex b0884 1 ribosomes	C96109H134397N33806O48497S286P4872Mg175Zn0Fe0	-4098.14	Translation
tl_term_b0884_2_rib_cplx	Translation termination complex b0884 2 ribosomes	C190131H266432N66771O95471S572P9523Mg350Zn0Fe0	-7974.28	Translation
tl_term_b0884_4_rib_cplx	Translation termination complex b0884 4 ribosomes	C378175H530502N132701O189419S1144P18825Mg700Zn0Fe0	-15726.56	Translation
tl_term_b0893_12_rib_cplx	Translation termination complex b0893 12 ribosomes	C1161477H1631439N406454O579228S3600P57095Mg2100Zn0Fe0	-47989.68	Translation
tl_term_b0893_12_rib_cplx2	Translation termination complex b0893 12 ribosomes 2 (RF2_mono)	C1162089H1631811N406238O579624S3576P57095Mg2100Zn0Fe0	-48097.68	Translation
tl_term_b0893_1_rib_cplx	Translation termination complex b0893 1 ribosomes	C108084H148727N38416O56541S300P5945Mg175Zn0Fe0	-5187.14	Translation
tl_term_b0893_1_rib_cplx2	Translation termination complex b0893 1 ribosomes 2 (RF2_mono)	C108135H148758N38398O56574S298P5945Mg175Zn0Fe0	-5196.14	Translation
tl_term_b0893_25_rib_cplx	Translation termination complex b0893 25 ribosomes	C2406396H3383735N841408O1196949S7500P117545Mg4375Zn0Fe0	-98574.5	Translation
tl_term_b0893_25_rib_cplx2	Translation termination complex b0893 25 ribosomes 2 (RF2_mono)	C2407671H3384510N840958O1197774S7450P117545Mg4375Zn0Fe0	-98799.5	Translation
tl_term_b0910_13_rib_cplx	Translation termination complex b0910 13 ribosomes	C1238092H1738809N433825O618307S3718P61136Mg2275Zn0Fe0	-51180.82	Translation
tl_term_b0910_13_rib_cplx2	Translation termination complex b0910 13 ribosomes 2 (RF2_mono)	C1238755H1739212N433591O618736S3692P61136Mg2275Zn0Fe0	-51297.82	Translation
tl_term_b0910_1_rib_cplx	Translation termination complex b0910 1 ribosomes	C101248H140553N35773O51991S286P5336Mg175Zn0Fe0	-4571.14	Translation
tl_term_b0910_1_rib_cplx2	Translation termination complex b0910 1 ribosomes 2 (RF2_mono)	C101299H140584N35755O52024S284P5336Mg175Zn0Fe0	-4580.14	Translation
tl_term_b0910_6_rib_cplx	Translation termination complex b0910 6 ribosomes	C574933H806493N201628O287956S1716P28586Mg1050Zn0Fe0	-23991.84	Translation
tl_term_b0910_6_rib_cplx2	Translation termination complex b0910 6 ribosomes 2 (RF2_mono)	C575239H806679N201520O288154S1704P28586Mg1050Zn0Fe0	-24045.84	Translation
tl_term_b0911_16_rib_cplx	Translation termination complex b0911 16 ribosomes	C1557333H2189790N544115O775070S4640P76076Mg2800Zn0Fe0	-64175.24	Translation
tl_term_b0911_16_rib_cplx2	Translation termination complex b0911 16 ribosomes 2 (RF2_mono)	C1558149H2190286N543827O775598S4608P76076Mg2800Zn0Fe0	-64319.24	Translation
tl_term_b0911_1_rib_cplx	Translation termination complex b0911 1 ribosomes	C112308H153780N40070O59360S290P6326Mg175Zn0Fe0	-5583.14	Translation
tl_term_b0911_1_rib_cplx2	Translation termination complex b0911 1 ribosomes 2 (RF2_mono)	C112359H153811N40052O59393S288P6326Mg175Zn0Fe0	-5592.14	Translation
tl_term_b0911_32_rib_cplx	Translation termination complex b0911 32 ribosomes	C3098693H4361534N1081763O1538494S9280P150476Mg5600Zn0Fe0	-126673.48	Translation
tl_term_b0911_32_rib_cplx2	Translation termination complex b0911 32 ribosomes 2 (RF2_mono)	C3100325H4362526N1081187O1539550S9216P150476Mg5600Zn0Fe0	-126961.48	Translation
tl_term_b0912_1_rib_cplx	Translation termination complex b0912 1 ribosomes	C96831H135248N34092O48977S284P4935Mg175Zn0Fe0	-4160.14	Translation
tl_term_b0912_1_rib_cplx2	Translation termination complex b0912 1 ribosomes 2 (RF2_mono)	C96882H135279N34074O49010S282P4935Mg175Zn0Fe0	-4169.14	Translation
tl_term_b0912_2_rib_cplx	Translation termination complex b0912 2 ribosomes	C190943H267421N67082O95981S568P9585Mg350Zn0Fe0	-8034.28	Translation
tl_term_b0912_2_rib_cplx2	Translation termination complex b0912 2 ribosomes 2 (RF2_mono)	C191045H267483N67046O96047S564P9585Mg350Zn0Fe0	-8052.28	Translation
tl_term_b0912_5_rib_cplx	Translation termination complex b0912 5 ribosomes	C473279H663940N166052O236993S1420P23535Mg875Zn0Fe0	-19656.7	Translation

tl_term_b0912_5_rib_cplx2	Translation termination complex b0912 5 ribosomes 2 (RF2_mono)	C473534H664095N165962O237158S1410P23535Mg875Zn0Fe0	-19701.7	Translation
tl_term_b0930_13_rib_cplx	Translation termination complex b0930 13 ribosomes	C1261441H1770675N440733O628136S3861P61853Mg2275Zn0Fe0	-52001.82	Translation
tl_term_b0930_13_rib_cplx2	Translation termination complex b0930 13 ribosomes 2 (RF2_mono)	C1262104H1771078N440499O628565S3835P61853Mg2275Zn0Fe0	-52118.82	Translation
tl_term_b0930_1_rib_cplx	Translation termination complex b0930 1 ribosomes	C109321H150111N38757O57404S297P6053Mg175Zn0Fe0	-5296.14	Translation
tl_term_b0930_1_rib_cplx2	Translation termination complex b0930 1 ribosomes 2 (RF2_mono)	C109372H150142N38739O57437S295P6053Mg175Zn0Fe0	-5305.14	Translation
tl_term_b0930_27_rib_cplx	Translation termination complex b0930 27 ribosomes	C2605581H3661333N909705O1293990S8019P126953Mg4725Zn0Fe0	-106491.78	Translation
tl_term_b0930_27_rib_cplx2	Translation termination complex b0930 27 ribosomes 2 (RF2_mono)	C2606958H3662170N909219O1294881S7965P126953Mg4725Zn0Fe0	-106734.78	Translation
tl_term_b0969_1_rib_cplx	Translation termination complex b0969 1 ribosomes	C97390H135876N34294O49332S285P4983Mg175Zn0Fe0	-4212.14	Translation
tl_term_b0969_1_rib_cplx2	Translation termination complex b0969 1 ribosomes 2 (RF2_mono)	C97441H135907N34276O49365S283P4983Mg175Zn0Fe0	-4221.14	Translation
tl_term_b0969_3_rib_cplx	Translation termination complex b0969 3 ribosomes	C285854H400522N100306O143402S855P14285Mg525Zn0Fe0	-11970.42	Translation
tl_term_b0969_3_rib_cplx2	Translation termination complex b0969 3 ribosomes 2 (RF2_mono)	C286007H400615N100252O143501S849P14285Mg525Zn0Fe0	-11997.42	Translation
tl_term_b0969_6_rib_cplx	Translation termination complex b0969 6 ribosomes	C568550H797491N199324O284507S1710P28238Mg1050Zn0Fe0	-23607.84	Translation
tl_term_b0969_6_rib_cplx2	Translation termination complex b0969 6 ribosomes 2 (RF2_mono)	C568856H797677N199216O284705S1698P28238Mg1050Zn0Fe0	-23661.84	Translation
tl_term_b1066_11_rib_cplx	Translation termination complex b1066 11 ribosomes	C1047127H1469153N366875O522692S3190P51748Mg1925Zn0Fe0	-43192.54	Translation
tl_term_b1066_11_rib_cplx2	Translation termination complex b1066 11 ribosomes 2 (RF2_mono)	C1047688H1469494N366677O523055S3168P51748Mg1925Zn0Fe0	-43291.54	Translation
tl_term_b1066_1_rib_cplx	Translation termination complex b1066 1 ribosomes	C100247H139273N35345O51252S290P5238Mg175Zn0Fe0	-4461.14	Translation
tl_term_b1066_1_rib_cplx2	Translation termination complex b1066 1 ribosomes 2 (RF2_mono)	C100298H139304N35327O51285S288P5238Mg175Zn0Fe0	-4470.14	Translation
tl_term_b1066_5_rib_cplx	Translation termination complex b1066 5 ribosomes	C478999H671225N167957O239828S1450P23842Mg875Zn0Fe0	-19953.7	Translation
tl_term_b1066_5_rib_cplx2	Translation termination complex b1066 5 ribosomes 2 (RF2_mono)	C479254H671380N167867O239993S1440P23842Mg875Zn0Fe0	-19998.7	Translation
tl_term_b1084_1_rib_cplx	Translation termination complex b1084 1 ribosomes	C129130H174109N46717O70604S300P7838Mg175Zn0Fe0	-7097.14	Translation
tl_term_b1084_1_rib_cplx2	Translation termination complex b1084 1 ribosomes 2 (RF2_mono)	C129181H174140N46699O70637S298P7838Mg175Zn0Fe0	-7106.14	Translation
tl_term_b1084_31_rib_cplx	Translation termination complex b1084 31 ribosomes	C3092290H4364419N1078897O1524944S9300P147338Mg5425Zn0Fe0	-124341.34	Translation
tl_term_b1084_31_rib_cplx2	Translation termination complex b1084 31 ribosomes 2 (RF2_mono)	C3093871H4365380N1078339O1525967S9238P147338Mg5425Zn0Fe0	-124620.34	Translation
tl_term_b1084_62_rib_cplx	Translation termination complex b1084 62 ribosomes	C6154222H8694406N2145483O3027762S18600P291488Mg10850Zn0Fe0	-245493.68	Translation
tl_term_b1084_62_rib_cplx2	Translation termination complex b1084 62 ribosomes 2 (RF2_mono)	C6157384H8696328N2144367O3029808S18476P291488Mg10850Zn0Fe0	-246051.68	Translation
tl_term_b1086_18_rib_cplx	Translation termination complex b1086 18 ribosomes	C1723682H2423399N603896O858523S5202P84680Mg3150Zn0Fe0	-70499.52	Translation
tl_term_b1086_18_rib_cplx2	Translation termination complex b1086 18 ribosomes 2 (RF2_mono)	C1724600H2423957N603572O859117S5166P84680Mg3150Zn0Fe0	-70661.52	Translation
tl_term_b1086_1_rib_cplx	Translation termination complex b1086 1 ribosomes	C104415H144396N37048O54032S289P5613Mg175Zn0Fe0	-4826.14	Translation
tl_term_b1086_1_rib_cplx2	Translation termination complex b1086 1 ribosomes 2 (RF2_mono)	C104466H144427N37030O54065S287P5613Mg175Zn0Fe0	-4835.14	Translation
tl_term_b1086_9_rib_cplx	Translation termination complex b1086 9 ribosomes	C866423H1216868N303800O432616S2601P42821Mg1575Zn0Fe0	-35731.26	Translation
tl_term_b1086_9_rib_cplx2	Translation termination complex b1086 9 ribosomes 2 (RF2_mono)	C866882H1217147N303638O432913S2583P42821Mg1575Zn0Fe0	-35812.26	Translation
tl_term_b1088_10_rib_cplx	Translation termination complex b1088 10 ribosomes	C950030H1333234N332671O475066S2910P47024Mg1750Zn0Fe0	-39466.4	Translation
tl_term_b1088_10_rib_cplx2	Translation termination complex b1088 10 ribosomes 2 (RF2_mono)	C950540H1333544N332491O475396S2890P47024Mg1750Zn0Fe0	-39556.4	Translation
tl_term_b1088_1_rib_cplx	Translation termination complex b1088 1 ribosomes	C99476H138376N35050O50797S291P5174Mg175Zn0Fe0	-4419.14	Translation
tl_term_b1088_1_rib_cplx2	Translation termination complex b1088 1 ribosomes 2 (RF2_mono)	C99527H138407N35032O50830S289P5174Mg175Zn0Fe0	-4428.14	Translation
tl_term_b1088_5_rib_cplx	Translation termination complex b1088 5 ribosomes	C477500H669424N167326O239361S1455P23774Mg875Zn0Fe0	-19995.7	Translation
tl_term_b1088_5_rib_cplx2	Translation termination complex b1088 5 ribosomes 2 (RF2_mono)	C477755H669579N167236O239526S1445P23774Mg875Zn0Fe0	-20040.7	Translation
tl_term_b1089_1_rib_cplx	Translation termination complex b1089 1 ribosomes	C95581H133784N33605O48153S284P4824Mg175Zn0Fe0	-4044.14	Translation
tl_term_b1089_1_rib_cplx2	Translation termination complex b1089 1 ribosomes 2 (RF2_mono)	C95632H133815N33587O48186S282P4824Mg175Zn0Fe0	-4053.14	Translation
tl_term_b1089_3_rib_cplx	Translation termination complex b1089 3 ribosomes	C283439H397586N99491O142053S852P14124Mg525Zn0Fe0	-11782.42	Translation
tl_term_b1089_3_rib_cplx2	Translation termination complex b1089 3 ribosomes 2 (RF2_mono)	C283592H397679N99437O142152S846P14124Mg525Zn0Fe0	-11809.42	Translation
tl_term_b1090_10_rib_cplx	Translation termination complex b1090 10 ribosomes	C964158H1354199N337288O481517S2920P47583Mg1750Zn0Fe0	-39875.4	Translation
tl_term_b1090_1_rib_cplx	Translation termination complex b1090 1 ribosomes	C105594H145787N37390O54917S292P5724Mg175Zn0Fe0	-4954.14	Translation
tl_term_b1090_20_rib_cplx	Translation termination complex b1090 20 ribosomes	C1918118H2696879N670508O955517S5840P94093Mg3500Zn0Fe0	-78676.8	Translation
tl_term_b1091_18_rib_cplx	Translation termination complex b1091 18 ribosomes	C1722296H2418795N602004O859077S5292P84656Mg3150Zn0Fe0	-71123.52	Translation
tl_term_b1091_1_rib_cplx	Translation termination complex b1091 1 ribosomes	C104253H144076N36856O54059S294P5606Mg175Zn0Fe0	-4855.14	Translation
tl_term_b1091_9_rib_cplx	Translation termination complex b1091 9 ribosomes	C865685H1214532N302808O432891S2646P42806Mg1575Zn0Fe0	-36040.26	Translation

tl_term_b1092_18_rib_cplx	Translation termination complex b1092 18 ribosomes	C1722251H2418588N602637O859278S5346P84830Mg3150Zn0Fe0	-71099.52	Translation
tl_term_b1092_18_rib_cplx2	Translation termination complex b1092 18 ribosomes 2 (RF2_mono)	C1723169H2419146N602313O859872S5310P84830Mg3150Zn0Fe0	-71261.52	Translation
tl_term_b1092_1_rib_cplx	Translation termination complex b1092 1 ribosomes	C104055H143835N36860O53886S297P5593Mg175Zn0Fe0	-4831.14	Translation
tl_term_b1092_1_rib_cplx2	Translation termination complex b1092 1 ribosomes 2 (RF2_mono)	C104106H143866N36842O53919S295P5593Mg175Zn0Fe0	-4840.14	Translation
tl_term_b1092_9_rib_cplx	Translation termination complex b1092 9 ribosomes	C865559H1214307N303108O432894S2673P42881Mg1575Zn0Fe0	-36016.26	Translation
tl_term_b1092_9_rib_cplx2	Translation termination complex b1092 9 ribosomes 2 (RF2_mono)	C866018H1214586N302946O433191S2655P42881Mg1575Zn0Fe0	-36097.26	Translation
tl_term_b1093_14_rib_cplx	Translation termination complex b1093 14 ribosomes	C1333749H1873552N467315O666196S4102P65851Mg2450Zn0Fe0	-55031.96	Translation
tl_term_b1093_1_rib_cplx	Translation termination complex b1093 1 ribosomes	C101778H141172N36001O52362S293P5388Mg175Zn0Fe0	-4616.14	Translation
tl_term_b1093_7_rib_cplx	Translation termination complex b1093 7 ribosomes	C670380H940732N235069O335670S2051P33294Mg1225Zn0Fe0	-27884.98	Translation
tl_term_b1114_1_rib_cplx	Translation termination complex b1114 1 ribosomes	C132239H177746N47662O72643S318P8099Mg175Zn0Fe0	-7354.14	Translation
tl_term_b1114_1_rib_cplx2	Translation termination complex b1114 1 ribosomes 2 (RF2_mono)	C132290H177777N47644O72676S316P8099Mg175Zn0Fe0	-7363.14	Translation
tl_term_b1114_33_rib_cplx	Translation termination complex b1114 33 ribosomes	C3313871H4676306N1151438O1626627S10494P156899Mg5775Zn0Fe0	-132286.62	Translation
tl_term_b1114_33_rib_cplx2	Translation termination complex b1114 33 ribosomes 2 (RF2_mono)	C3315554H4677329N1150844O1627716S10428P156899Mg5775Zn0Fe0	-132583.62	Translation
tl_term_b1114_67_rib_cplx	Translation termination complex b1114 67 ribosomes	C6694355H9456026N2324200O3277735S21306P314999Mg11725Zn0Fe0	-265027.38	Translation
tl_term_b1114_67_rib_cplx2	Translation termination complex b1114 67 ribosomes 2 (RF2_mono)	C6697772H9458103N2322994O3279946S21172P314999Mg11725Zn0Fe0	-265630.38	Translation
tl_term_b1133_10_rib_cplx	Translation termination complex b1133 10 ribosomes	C965397H1354568N337659O482014S2950P47619Mg1750Zn0Fe0	-40071.4	Translation
tl_term_b1133_1_rib_cplx	Translation termination complex b1133 1 ribosomes	C106023H146192N37563O55171S295P5760Mg175Zn0Fe0	-5006.14	Translation
tl_term_b1133_21_rib_cplx	Translation termination complex b1133 21 ribosomes	C2015743H2831472N704443O1003711S6195P98780Mg3675Zn0Fe0	-82928.94	Translation
tl_term_b1134_1_rib_cplx	Translation termination complex b1134 1 ribosomes	C98846H137595N34812O50338S289P5113Mg175Zn0Fe0	-4351.14	Translation
tl_term_b1134_1_rib_cplx2	Translation termination complex b1134 1 ribosomes 2 (RF2_mono)	C98897H137626N34794O50371S287P5113Mg175Zn0Fe0	-4360.14	Translation
tl_term_b1134_4_rib_cplx	Translation termination complex b1134 4 ribosomes	C382202H535482N134025O191635S1156P19066Mg700Zn0Fe0	-16015.56	Translation
tl_term_b1134_4_rib_cplx2	Translation termination complex b1134 4 ribosomes 2 (RF2_mono)	C382406H535606N133953O191767S1148P19066Mg700Zn0Fe0	-16051.56	Translation
tl_term_b1134_9_rib_cplx	Translation termination complex b1134 9 ribosomes	C854462H1198627N299380O427130S2601P42321Mg1575Zn0Fe0	-35456.26	Translation
tl_term_b1134_9_rib_cplx2	Translation termination complex b1134 9 ribosomes 2 (RF2_mono)	C854921H1198906N299218O427427S2583P42321Mg1575Zn0Fe0	-35537.26	Translation
tl_term_b1135_12_rib_cplx	Translation termination complex b1135 12 ribosomes	C1143402H1605569N400672O570898S3432P56468Mg2100Zn0Fe0	-47074.68	Translation
tl_term_b1135_12_rib_cplx2	Translation termination complex b1135 12 ribosomes 2 (RF2_mono)	C1144014H1605941N400456O571294S3408P56468Mg2100Zn0Fe0	-47182.68	Translation
tl_term_b1135_1_rib_cplx	Translation termination complex b1135 1 ribosomes	C100987H140259N35670O51753S286P5307Mg175Zn0Fe0	-4525.14	Translation
tl_term_b1135_1_rib_cplx2	Translation termination complex b1135 1 ribosomes 2 (RF2_mono)	C101038H140290N35652O51786S284P5307Mg175Zn0Fe0	-4534.14	Translation
tl_term_b1135_6_rib_cplx	Translation termination complex b1135 6 ribosomes	C574812H806309N201580O287728S1716P28562Mg1050Zn0Fe0	-23865.84	Translation
tl_term_b1135_6_rib_cplx2	Translation termination complex b1135 6 ribosomes 2 (RF2_mono)	C575118H806495N201472O287926S1704P28562Mg1050Zn0Fe0	-23919.84	Translation
tl_term_b1211_10_rib_cplx	Translation termination complex b1211 10 ribosomes	C964141H1353621N337944O481765S2960P47585Mg1750Zn0Fe0	-40037.4	Translation
tl_term_b1211_10_rib_cplx2	Translation termination complex b1211 10 ribosomes 2 (RF2_mono)	C964651H1353931N337764O482095S2940P47585Mg1750Zn0Fe0	-40127.4	Translation
tl_term_b1211_1_rib_cplx	Translation termination complex b1211 1 ribosomes	C105703H145875N37542O54967S296P5735Mg175Zn0Fe0	-4981.14	Translation
tl_term_b1211_1_rib_cplx2	Translation termination complex b1211 1 ribosomes 2 (RF2_mono)	C105754H145906N37524O55000S294P5735Mg175Zn0Fe0	-4990.14	Translation
tl_term_b1211_21_rib_cplx	Translation termination complex b1211 21 ribosomes	C2013343H2829755N705102O1003407S6216P98735Mg3675Zn0Fe0	-82883.94	Translation
tl_term_b1211_21_rib_cplx2	Translation termination complex b1211 21 ribosomes 2 (RF2_mono)	C2014414H2830406N704724O1004100S6174P98735Mg3675Zn0Fe0	-83072.94	Translation
tl_term_b1212_16_rib_cplx	Translation termination complex b1212 16 ribosomes	C1527976H2145590N534913O762145S4656P75220Mg2800Zn0Fe0	-63127.24	Translation
tl_term_b1212_1_rib_cplx	Translation termination complex b1212 1 ribosomes	C102931H142520N36388O53125S291P5485Mg175Zn0Fe0	-4730.14	Translation
tl_term_b1212_8_rib_cplx	Translation termination complex b1212 8 ribosomes	C767952H1077286N269033O384001S2328P38028Mg1400Zn0Fe0	-31982.12	Translation
tl_term_b1229_1_rib_cplx	Translation termination complex b1229 1 ribosomes	C94799H132827N33356O47612S285P4753Mg175Zn0Fe0	-3973.14	Translation
tl_term_b1269_17_rib_cplx	Translation termination complex b1269 17 ribosomes	C1624507H2283591N569509O809987S4879P79928Mg2975Zn0Fe0	-66535.38	Translation
tl_term_b1269_17_rib_cplx2	Translation termination complex b1269 17 ribosomes 2 (RF2_mono)	C1625374H2284118N569203O810548S4845P79928Mg2975Zn0Fe0	-66688.38	Translation
tl_term_b1269_1_rib_cplx	Translation termination complex b1269 1 ribosomes	C103419H143207N36661O53427S287P5528Mg175Zn0Fe0	-4741.14	Translation
tl_term_b1269_1_rib_cplx2	Translation termination complex b1269 1 ribosomes 2 (RF2_mono)	C103470H143238N36643O53460S285P5528Mg175Zn0Fe0	-4750.14	Translation
tl_term_b1269_8_rib_cplx	Translation termination complex b1269 8 ribosomes	C768895H1079625N269782O384422S2296P38078Mg1400Zn0Fe0	-31776.12	Translation
tl_term_b1269_8_rib_cplx2	Translation termination complex b1269 8 ribosomes 2 (RF2_mono)	C769303H1079873N269638O384686S2280P38078Mg1400Zn0Fe0	-31848.12	Translation
tl_term_b1286_18_rib_cplx	Translation termination complex b1286 18 ribosomes	C1762073H2477780N614993O874221S5454P85637Mg3150Zn0Fe0	-72104.52	Translation

tl_term_b1286_18_rib_cplx2	Translation termination complex b1286 18 ribosomes 2 (RF2_mono)	C1762991H2478338N614669O874815S5418P85637Mg3150Zn0Fe0	-72266.52	Translation
tl_term_b1286_1_rib_cplx	Translation termination complex b1286 1 ribosomes	C115266H157348N41090O61332S303P6587Mg175Zn0Fe0	-5836.14	Translation
tl_term_b1286_1_rib_cplx2	Translation termination complex b1286 1 ribosomes 2 (RF2_mono)	C115317H157379N41072O61365S301P6587Mg175Zn0Fe0	-5845.14	Translation
tl_term_b1286_37_rib_cplx	Translation termination complex b1286 37 ribosomes	C3602622H5071204N1256414O1782744S11211P173987Mg6475Zn0Fe0	-146169.18	Translation
tl_term_b1286_37_rib_cplx2	Translation termination complex b1286 37 ribosomes 2 (RF2_mono)	C3604509H5072351N1255748O1783965S11137P173987Mg6475Zn0Fe0	-146502.18	Translation
tl_term_b1344_18_rib_cplx	Translation termination complex b1344 18 ribosomes	C1722924H2420832N602741O858572S5418P84638Mg3150Zn0Fe0	-70817.52	Translation
tl_term_b1344_18_rib_cplx2	Translation termination complex b1344 18 ribosomes 2 (RF2_mono)	C1723842H2421390N602417O859166S5382P84638Mg3150Zn0Fe0	-70979.52	Translation
tl_term_b1344_1_rib_cplx	Translation termination complex b1344 1 ribosomes	C104150H144005N36879O53860S301P5588Mg175Zn0Fe0	-4821.14	Translation
tl_term_b1344_1_rib_cplx2	Translation termination complex b1344 1 ribosomes 2 (RF2_mono)	C104201H144036N36861O53893S299P5588Mg175Zn0Fe0	-4830.14	Translation
tl_term_b1344_9_rib_cplx	Translation termination complex b1344 9 ribosomes	C865926H1215453N303167O432548S2709P42788Mg1575Zn0Fe0	-35878.26	Translation
tl_term_b1344_9_rib_cplx2	Translation termination complex b1344 9 ribosomes 2 (RF2_mono)	C866385H1215732N303005O432845S2691P42788Mg1575Zn0Fe0	-35959.26	Translation
tl_term_b1427_10_rib_cplx	Translation termination complex b1427 10 ribosomes	C950718H1334258N333053O475027S2890P47032Mg1750Zn0Fe0	-39344.4	Translation
tl_term_b1427_10_rib_cplx2	Translation termination complex b1427 10 ribosomes 2 (RF2_mono)	C951228H1334568N332873O475357S2870P47032Mg1750Zn0Fe0	-39434.4	Translation
tl_term_b1427_1_rib_cplx	Translation termination complex b1427 1 ribosomes	C99705H138644N35153O50893S289P5191Mg175Zn0Fe0	-4423.14	Translation
tl_term_b1427_1_rib_cplx2	Translation termination complex b1427 1 ribosomes 2 (RF2_mono)	C99756H138675N35135O50926S287P5191Mg175Zn0Fe0	-4432.14	Translation
tl_term_b1427_5_rib_cplx	Translation termination complex b1427 5 ribosomes	C477933H670028N167553O239397S1445P23787Mg875Zn0Fe0	-19943.7	Translation
tl_term_b1427_5_rib_cplx2	Translation termination complex b1427 5 ribosomes 2 (RF2_mono)	C478188H670183N167463O239562S1435P23787Mg875Zn0Fe0	-19988.7	Translation
tl_term_b1480_1_rib_cplx	Translation termination complex b1480 1 ribosomes	C95190H133302N33471O47875S283P4788Mg175Zn0Fe0	-4010.14	Translation
tl_term_b1480_1_rib_cplx2	Translation termination complex b1480 1 ribosomes 2 (RF2_mono)	C95241H133333N33453O47908S281P4788Mg175Zn0Fe0	-4019.14	Translation
tl_term_b1480_2_rib_cplx	Translation termination complex b1480 2 ribosomes	C189059H265110N66395O94810S566P9438Mg350Zn0Fe0	-7881.28	Translation
tl_term_b1480_2_rib_cplx2	Translation termination complex b1480 2 ribosomes 2 (RF2_mono)	C189161H265172N66359O94876S562P9438Mg350Zn0Fe0	-7899.28	Translation
tl_term_b1481_1_rib_cplx	Translation termination complex b1481 1 ribosomes	C96158H134389N33799O48571S283P4879Mg175Zn0Fe0	-4116.14	Translation
tl_term_b1481_1_rib_cplx2	Translation termination complex b1481 1 ribosomes 2 (RF2_mono)	C96209H134420N33781O48604S281P4879Mg175Zn0Fe0	-4125.14	Translation
tl_term_b1481_2_rib_cplx	Translation termination complex b1481 2 ribosomes	C190262H266457N66786O95629S566P9540Mg350Zn0Fe0	-8013.28	Translation
tl_term_b1481_2_rib_cplx2	Translation termination complex b1481 2 ribosomes 2 (RF2_mono)	C190364H266519N66750O95695S562P9540Mg350Zn0Fe0	-8031.28	Translation
tl_term_b1481_4_rib_cplx	Translation termination complex b1481 4 ribosomes	C378470H530593N132760O189745S1132P18862Mg700Zn0Fe0	-15807.56	Translation
tl_term_b1481_4_rib_cplx2	Translation termination complex b1481 4 ribosomes 2 (RF2_mono)	C378674H530717N132688O189877S1124P18862Mg700Zn0Fe0	-15843.56	Translation
tl_term_b1636_16_rib_cplx	Translation termination complex b1636 16 ribosomes	C1530662H2149239N535826O763503S4720P75440Mg2800Zn0Fe0	-63187.24	Translation
tl_term_b1636_1_rib_cplx	Translation termination complex b1636 1 ribosomes	C103382H143049N36581O53388S295P5525Mg175Zn0Fe0	-4760.14	Translation
tl_term_b1636_8_rib_cplx	Translation termination complex b1636 8 ribosomes	C769446H1079271N269562O384775S2360P38152Mg1400Zn0Fe0	-32026.12	Translation
tl_term_b1637_12_rib_cplx	Translation termination complex b1637 12 ribosomes	C1161484H1630550N405975O578954S3552P57077Mg2100Zn0Fe0	-47899.68	Translation
tl_term_b1637_12_rib_cplx2	Translation termination complex b1637 12 ribosomes 2 (RF2_mono)	C1162096H1630922N405759O579350S3528P57077Mg2100Zn0Fe0	-48007.68	Translation
tl_term_b1637_1_rib_cplx	Translation termination complex b1637 1 ribosomes	C107926H148465N38300O56410S296P5927Mg175Zn0Fe0	-5163.14	Translation
tl_term_b1637_1_rib_cplx2	Translation termination complex b1637 1 ribosomes 2 (RF2_mono)	C107977H148496N38282O56443S294P5927Mg175Zn0Fe0	-5172.14	Translation
tl_term_b1637_24_rib_cplx	Translation termination complex b1637 24 ribosomes	C2310820H3247370N807075O1149002S7104P112877Mg4200Zn0Fe0	-94521.36	Translation
tl_term_b1637_24_rib_cplx2	Translation termination complex b1637 24 ribosomes 2 (RF2_mono)	C2312044H3248114N806643O1149794S7056P112877Mg4200Zn0Fe0	-94737.36	Translation
tl_term_b1638_12_rib_cplx	Translation termination complex b1638 12 ribosomes	C1143878H1605695N400784O570892S3456P56471Mg2100Zn0Fe0	-47149.68	Translation
tl_term_b1638_1_rib_cplx	Translation termination complex b1638 1 ribosomes	C101045H140286N35661O51802S288P5310Mg175Zn0Fe0	-4534.14	Translation
tl_term_b1638_6_rib_cplx	Translation termination complex b1638 6 ribosomes	C575060H806381N201626O287752S1728P28565Mg1050Zn0Fe0	-23904.84	Translation
tl_term_b1652_12_rib_cplx	Translation termination complex b1652 12 ribosomes	C1142488H1603247N400134O570602S3516P56450Mg2100Zn0Fe0	-47296.68	Translation
tl_term_b1652_12_rib_cplx2	Translation termination complex b1652 12 ribosomes 2 (RF2_mono)	C1143100H1603619N399918O570998S3492P56450Mg2100Zn0Fe0	-47404.68	Translation
tl_term_b1652_1_rib_cplx	Translation termination complex b1652 1 ribosomes	C100843H140005N35572O51710S293P5300Mg175Zn0Fe0	-4538.14	Translation
tl_term_b1652_1_rib_cplx2	Translation termination complex b1652 1 ribosomes 2 (RF2_mono)	C100894H140036N35554O51743S291P5300Mg175Zn0Fe0	-4547.14	Translation
tl_term_b1652_6_rib_cplx	Translation termination complex b1652 6 ribosomes	C574318H805115N201282O287570S1758P28550Mg1050Zn0Fe0	-23973.84	Translation
tl_term_b1652_6_rib_cplx2	Translation termination complex b1652 6 ribosomes 2 (RF2_mono)	C574624H805301N201174O287768S1746P28550Mg1050Zn0Fe0	-24027.84	Translation
tl_term_b1653_1_rib_cplx	Translation termination complex b1653 1 ribosomes	C145031H193203N52462O81489S313P9266Mg175Zn0Fe0	-8514.14	Translation
tl_term_b1653_45_rib_cplx	Translation termination complex b1653 45 ribosomes	C4596291H6504387N1592726O2242637S14085P213822Mg7875Zn0Fe0	-179944.3	Translation

tl_term_b1653_90_rib_cplx	Translation termination complex b1653 90 ribosomes	C9148716H12959007N3167996O4452902S28170P423027Mg15750Zn0Fe0	-355270.6	Translation
tl_term_b1712_1_rib_cplx	Translation termination complex b1712 1 ribosomes	C97013H135472N34176O49093S284P4952Mg175Zn0Fe0	-4177.14	Translation
tl_term_b1712_1_rib_cplx2	Translation termination complex b1712 1 ribosomes 2 (RF2_mono)	C97064H135503N34158O49126S282P4952Mg175Zn0Fe0	-4186.14	Translation
tl_term_b1712_2_rib_cplx	Translation termination complex b1712 2 ribosomes	C191156H267706N67175O96103S568P9602Mg350Zn0Fe0	-8051.28	Translation
tl_term_b1712_2_rib_cplx2	Translation termination complex b1712 2 ribosomes 2 (RF2_mono)	C191258H267768N67139O96169S564P9602Mg350Zn0Fe0	-8069.28	Translation
tl_term_b1712_5_rib_cplx	Translation termination complex b1712 5 ribosomes	C473585H664408N166172O237133S1420P23552Mg875Zn0Fe0	-19673.7	Translation
tl_term_b1712_5_rib_cplx2	Translation termination complex b1712 5 ribosomes 2 (RF2_mono)	C473840H664563N166082O237298S1410P23552Mg875Zn0Fe0	-19718.7	Translation
tl_term_b1713_1_rib_cplx	Translation termination complex b1713 1 ribosomes	C120276H163373N43093O64742S309P7039Mg175Zn0Fe0	-6296.14	Translation
tl_term_b1713_23_rib_cplx	Translation termination complex b1713 23 ribosomes	C2265738H3191475N789773O1121644S7107P109361Mg4025Zn0Fe0	-92253.22	Translation
tl_term_b1713_46_rib_cplx	Translation termination complex b1713 46 ribosomes	C4508721H6357218N1570393O2226587S14214P216334Mg8050Zn0Fe0	-182117.44	Translation
tl_term_b1714_19_rib_cplx	Translation termination complex b1714 19 ribosomes	C1819930H2556233N636587O906604S5567P89334Mg3325Zn0Fe0	-74821.66	Translation
tl_term_b1714_19_rib_cplx2	Translation termination complex b1714 19 ribosomes 2 (RF2_mono)	C1820899H2556822N636245O907231S5529P89334Mg3325Zn0Fe0	-74992.66	Translation
tl_term_b1714_1_rib_cplx	Translation termination complex b1714 1 ribosomes	C104638H144575N37007O54250S293P5634Mg175Zn0Fe0	-4871.14	Translation
tl_term_b1714_1_rib_cplx2	Translation termination complex b1714 1 ribosomes 2 (RF2_mono)	C104689H144606N36989O54283S291P5634Mg175Zn0Fe0	-4880.14	Translation
tl_term_b1714_9_rib_cplx	Translation termination complex b1714 9 ribosomes	C866990H1216423N303487O433074S2637P42834Mg1575Zn0Fe0	-35960.26	Translation
tl_term_b1714_9_rib_cplx2	Translation termination complex b1714 9 ribosomes 2 (RF2_mono)	C867449H1216702N303325O433371S2619P42834Mg1575Zn0Fe0	-36041.26	Translation
tl_term_b1715_1_rib_cplx	Translation termination complex b1715 1 ribosomes	C94159H132016N33014O47220S282P4697Mg175Zn0Fe0	-3925.14	Translation
tl_term_b1716_1_rib_cplx	Translation termination complex b1716 1 ribosomes	C97652H136287N34395O49508S282P5007Mg175Zn0Fe0	-4212.14	Translation
tl_term_b1716_1_rib_cplx2	Translation termination complex b1716 1 ribosomes 2 (RF2_mono)	C97703H136318N34377O49541S280P5007Mg175Zn0Fe0	-4221.14	Translation
tl_term_b1716_3_rib_cplx	Translation termination complex b1716 3 ribosomes	C286164H401181N100487O143542S846P14307Mg525Zn0Fe0	-11920.42	Translation
tl_term_b1716_3_rib_cplx2	Translation termination complex b1716 3 ribosomes 2 (RF2_mono)	C286317H401274N100433O143641S840P14307Mg525Zn0Fe0	-11947.42	Translation
tl_term_b1716_6_rib_cplx	Translation termination complex b1716 6 ribosomes	C568932H798522N199625O284593S1692P28257Mg1050Zn0Fe0	-23482.84	Translation
tl_term_b1716_6_rib_cplx2	Translation termination complex b1716 6 ribosomes 2 (RF2_mono)	C569238H798708N199517O284791S1680P28257Mg1050Zn0Fe0	-23536.84	Translation
tl_term_b1717_1_rib_cplx	Translation termination complex b1717 1 ribosomes	C95864H134140N33729O48304S284P4848Mg175Zn0Fe0	-4058.14	Translation
tl_term_b1717_1_rib_cplx2	Translation termination complex b1717 1 ribosomes 2 (RF2_mono)	C95915H134171N33711O48337S282P4848Mg175Zn0Fe0	-4067.14	Translation
tl_term_b1717_3_rib_cplx	Translation termination complex b1717 3 ribosomes	C283814H398138N99647O142184S852P14148Mg525Zn0Fe0	-11776.42	Translation
tl_term_b1717_3_rib_cplx2	Translation termination complex b1717 3 ribosomes 2 (RF2_mono)	C283967H398231N99593O142283S846P14148Mg525Zn0Fe0	-11803.42	Translation
tl_term_b1718_10_rib_cplx	Translation termination complex b1718 10 ribosomes	C950583H1335084N333231O474963S2890P47033Mg1750Zn0Fe0	-39225.4	Translation
tl_term_b1718_10_rib_cplx2	Translation termination complex b1718 10 ribosomes 2 (RF2_mono)	C951093H1335394N333051O475293S2870P47033Mg1750Zn0Fe0	-39315.4	Translation
tl_term_b1718_1_rib_cplx	Translation termination complex b1718 1 ribosomes	C99741H138786N35259O50865S289P5192Mg175Zn0Fe0	-4412.14	Translation
tl_term_b1718_1_rib_cplx2	Translation termination complex b1718 1 ribosomes 2 (RF2_mono)	C99792H138817N35241O50898S287P5192Mg175Zn0Fe0	-4421.14	Translation
tl_term_b1718_5_rib_cplx	Translation termination complex b1718 5 ribosomes	C477893H670474N167691O239353S1445P23788Mg875Zn0Fe0	-19884.7	Translation
tl_term_b1718_5_rib_cplx2	Translation termination complex b1718 5 ribosomes 2 (RF2_mono)	C478148H670629N167601O239518S1435P23788Mg875Zn0Fe0	-19929.7	Translation
tl_term_b1719_18_rib_cplx	Translation termination complex b1719 18 ribosomes	C1763016H2477856N615203O874331S5724P85631Mg3150Zn0Fe0	-72026.52	Translation
tl_term_b1719_18_rib_cplx2	Translation termination complex b1719 18 ribosomes 2 (RF2_mono)	C1763934H2478414N614879O874925S5688P85631Mg3150Zn0Fe0	-72188.52	Translation
tl_term_b1719_1_rib_cplx	Translation termination complex b1719 1 ribosomes	C115308H157271N41147O61272S318P6581Mg175Zn0Fe0	-5826.14	Translation
tl_term_b1719_1_rib_cplx2	Translation termination complex b1719 1 ribosomes 2 (RF2_mono)	C115359H157302N41129O61305S316P6581Mg175Zn0Fe0	-5835.14	Translation
tl_term_b1719_37_rib_cplx	Translation termination complex b1719 37 ribosomes	C3604572H5071451N1256795O1783044S11766P173981Mg6475Zn0Fe0	-146015.18	Translation
tl_term_b1719_37_rib_cplx2	Translation termination complex b1719 37 ribosomes 2 (RF2_mono)	C3606459H5072598N1256129O1784265S11692P173981Mg6475Zn0Fe0	-146348.18	Translation
tl_term_b1804_11_rib_cplx	Translation termination complex b1804 11 ribosomes	C1061858H1490692N371253O529371S3289P52269Mg1925Zn0Fe0	-43900.54	Translation
tl_term_b1804_11_rib_cplx2	Translation termination complex b1804 11 ribosomes 2 (RF2_mono)	C1062419H1491033N371055O529734S3267P52269Mg1925Zn0Fe0	-43999.54	Translation
tl_term_b1804_1_rib_cplx	Translation termination complex b1804 1 ribosomes	C106288H146562N37643O55311S299P5779Mg175Zn0Fe0	-5019.14	Translation
tl_term_b1804_1_rib_cplx2	Translation termination complex b1804 1 ribosomes 2 (RF2_mono)	C106339H146593N37625O55344S297P5779Mg175Zn0Fe0	-5028.14	Translation
tl_term_b1804_22_rib_cplx	Translation termination complex b1804 22 ribosomes	C2112985H2969235N738224O1050837S6578P103408Mg3850Zn0Fe0	-86670.08	Translation
tl_term_b1804_22_rib_cplx2	Translation termination complex b1804 22 ribosomes 2 (RF2_mono)	C2114107H2969917N737828O1051563S6534P103408Mg3850Zn0Fe0	-86868.08	Translation
tl_term_b1822_15_rib_cplx	Translation termination complex b1822 15 ribosomes	C1434188H2013221N502076O715273S4485P70697Mg2625Zn0Fe0	-59120.1	Translation
tl_term_b1822_15_rib_cplx2	Translation termination complex b1822 15 ribosomes 2 (RF2_mono)	C1434953H2013686N501806O715768S4455P70697Mg2625Zn0Fe0	-59255.1	Translation

tl_term_b1822_1_rib_cplx	Translation termination complex b1822 1 ribosomes	C102802H142345N36324O52989S299P5471Mg175Zn0Fe0	-4700.14	Translation
tl_term_b1822_1_rib_cplx2	Translation termination complex b1822 1 ribosomes 2 (RF2_mono)	C102853H142376N36306O53022S297P5471Mg175Zn0Fe0	-4709.14	Translation
tl_term_b1822_7_rib_cplx	Translation termination complex b1822 7 ribosomes	C673396H944149N235932O336825S2093P33425Mg1225Zn0Fe0	-28022.98	Translation
tl_term_b1822_7_rib_cplx2	Translation termination complex b1822 7 ribosomes 2 (RF2_mono)	C673753H944366N235806O337056S2079P33425Mg1225Zn0Fe0	-28085.98	Translation
tl_term_b1866_17_rib_cplx	Translation termination complex b1866 17 ribosomes	C1658719H2332019N578991O823984S5236P80825Mg2975Zn0Fe0	-67908.38	Translation
tl_term_b1866_1_rib_cplx	Translation termination complex b1866 1 ribosomes	C113455H155155N40415O60144S308P6425Mg175Zn0Fe0	-5666.14	Translation
tl_term_b1866_34_rib_cplx	Translation termination complex b1866 34 ribosomes	C3300562H4644937N1151228O1635564S10472P159875Mg5950Zn0Fe0	-134040.76	Translation
tl_term_b1869_1_rib_cplx	Translation termination complex b1869 1 ribosomes	C98164H136785N34449O49907S293P5049Mg175Zn0Fe0	-4282.14	Translation
tl_term_b1869_3_rib_cplx	Translation termination complex b1869 3 ribosomes	C287000H401897N100499O144041S879P14351Mg525Zn0Fe0	-12048.42	Translation
tl_term_b1869_7_rib_cplx	Translation termination complex b1869 7 ribosomes	C664672H932121N232599O332309S2051P32955Mg1225Zn0Fe0	-27580.98	Translation
tl_term_b1870_14_rib_cplx	Translation termination complex b1870 14 ribosomes	C1335340H1874601N467490O666470S4116P65844Mg2450Zn0Fe0	-55178.96	Translation
tl_term_b1870_1_rib_cplx	Translation termination complex b1870 1 ribosomes	C101939H141324N35968O52454S294P5394Mg175Zn0Fe0	-4633.14	Translation
tl_term_b1870_7_rib_cplx	Translation termination complex b1870 7 ribosomes	C671201H941298N235132O335846S2058P33294Mg1225Zn0Fe0	-27961.98	Translation
tl_term_b1871_19_rib_cplx	Translation termination complex b1871 19 ribosomes	C1820610H2556943N636641O906212S5567P89337Mg3325Zn0Fe0	-74748.66	Translation
tl_term_b1871_19_rib_cplx2	Translation termination complex b1871 19 ribosomes 2 (RF2_mono)	C1821579H2557532N636299O906839S5529P89337Mg3325Zn0Fe0	-74919.66	Translation
tl_term_b1871_1_rib_cplx	Translation termination complex b1871 1 ribosomes	C104544H144439N36971O54128S293P5619Mg175Zn0Fe0	-4852.14	Translation
tl_term_b1871_1_rib_cplx2	Translation termination complex b1871 1 ribosomes 2 (RF2_mono)	C104595H144470N36953O54161S291P5619Mg175Zn0Fe0	-4861.14	Translation
tl_term_b1871_9_rib_cplx	Translation termination complex b1871 9 ribosomes	C867240H1216663N303491O432832S2637P42827Mg1575Zn0Fe0	-35917.26	Translation
tl_term_b1871_9_rib_cplx2	Translation termination complex b1871 9 ribosomes 2 (RF2_mono)	C867699H1216942N303329O433129S2619P42827Mg1575Zn0Fe0	-35998.26	Translation
tl_term_b1876_16_rib_cplx	Translation termination complex b1876 16 ribosomes	C1561195H2194166N544975O775777S4928P76152Mg2800Zn0Fe0	-64107.24	Translation
tl_term_b1876_16_rib_cplx2	Translation termination complex b1876 16 ribosomes 2 (RF2_mono)	C1562011H2194662N544687O776305S4896P76152Mg2800Zn0Fe0	-64251.24	Translation
tl_term_b1876_1_rib_cplx	Translation termination complex b1876 1 ribosomes	C113080H154676N40345O59797S308P6387Mg175Zn0Fe0	-5635.14	Translation
tl_term_b1876_1_rib_cplx2	Translation termination complex b1876 1 ribosomes 2 (RF2_mono)	C113131H154707N40327O59830S306P6387Mg175Zn0Fe0	-5644.14	Translation
tl_term_b1876_33_rib_cplx	Translation termination complex b1876 33 ribosomes	C3202392H4505588N1116889O1587221S10164P155219Mg5775Zn0Fe0	-130375.62	Translation
tl_term_b1876_33_rib_cplx2	Translation termination complex b1876 33 ribosomes 2 (RF2_mono)	C3204075H4506611N1116295O1588310S10098P155219Mg5775Zn0Fe0	-130672.62	Translation
tl_term_b1920_15_rib_cplx	Translation termination complex b1920 15 ribosomes	C1431781H2011370N501135O714575S4320P70551Mg2625Zn0Fe0	-58989.1	Translation
tl_term_b1920_15_rib_cplx2	Translation termination complex b1920 15 ribosomes 2 (RF2_mono)	C1432546H2011835N500865O715070S4290P70551Mg2625Zn0Fe0	-59124.1	Translation
tl_term_b1920_1_rib_cplx	Translation termination complex b1920 1 ribosomes	C102607H142146N36335O52837S288P5451Mg175Zn0Fe0	-4681.14	Translation
tl_term_b1920_1_rib_cplx2	Translation termination complex b1920 1 ribosomes 2 (RF2_mono)	C102658H142177N36317O52870S286P5451Mg175Zn0Fe0	-4690.14	Translation
tl_term_b1920_7_rib_cplx	Translation termination complex b1920 7 ribosomes	C672253H943242N235535O336439S2016P33351Mg1225Zn0Fe0	-27955.98	Translation
tl_term_b1920_7_rib_cplx2	Translation termination complex b1920 7 ribosomes 2 (RF2_mono)	C672610H943459N235409O336670S2002P33351Mg1225Zn0Fe0	-28018.98	Translation
tl_term_b1921_10_rib_cplx	Translation termination complex b1921 10 ribosomes	C952250H1336461N333748O475804S2920P47142Mg1750Zn0Fe0	-39344.4	Translation
tl_term_b1921_10_rib_cplx2	Translation termination complex b1921 10 ribosomes 2 (RF2_mono)	C952760H1336771N333568O476134S2900P47142Mg1750Zn0Fe0	-39434.4	Translation
tl_term_b1921_1_rib_cplx	Translation termination complex b1921 1 ribosomes	C99941H138984N35227O51049S292P5211Mg175Zn0Fe0	-4432.14	Translation
tl_term_b1921_1_rib_cplx2	Translation termination complex b1921 1 ribosomes 2 (RF2_mono)	C99992H139015N35209O51082S290P5211Mg175Zn0Fe0	-4441.14	Translation
tl_term_b1921_5_rib_cplx	Translation termination complex b1921 5 ribosomes	C478745H671196N167903O239829S1460P23847Mg875Zn0Fe0	-19948.7	Translation
tl_term_b1921_5_rib_cplx2	Translation termination complex b1921 5 ribosomes 2 (RF2_mono)	C479000H671351N167813O239994S1450P23847Mg875Zn0Fe0	-19993.7	Translation
tl_term_b1922_14_rib_cplx	Translation termination complex b1922 14 ribosomes	C1336274H1876524N468349O667375S4032P65976Mg2450Zn0Fe0	-55282.96	Translation
tl_term_b1922_14_rib_cplx2	Translation termination complex b1922 14 ribosomes 2 (RF2_mono)	C1336988H1876958N468097O667837S4004P65976Mg2450Zn0Fe0	-55408.96	Translation
tl_term_b1922_1_rib_cplx	Translation termination complex b1922 1 ribosomes	C101833H141258N36060O52319S288P5383Mg175Zn0Fe0	-4620.14	Translation
tl_term_b1922_1_rib_cplx2	Translation termination complex b1922 1 ribosomes 2 (RF2_mono)	C101884H141289N36042O52352S286P5383Mg175Zn0Fe0	-4629.14	Translation
tl_term_b1922_7_rib_cplx	Translation termination complex b1922 7 ribosomes	C671575H942150N235578O336191S2016P33349Mg1225Zn0Fe0	-28002.98	Translation
tl_term_b1922_7_rib_cplx2	Translation termination complex b1922 7 ribosomes 2 (RF2_mono)	C671932H942367N235452O336422S2002P33349Mg1225Zn0Fe0	-28065.98	Translation
tl_term_b2114_19_rib_cplx	Translation termination complex b2114 19 ribosomes	C1863788H2618890N649232O923839S5909P90386Mg3325Zn0Fe0	-76025.66	Translation
tl_term_b2114_19_rib_cplx2	Translation termination complex b2114 19 ribosomes 2 (RF2_mono)	C1864757H2619479N648890O924466S5871P90386Mg3325Zn0Fe0	-76196.66	Translation
tl_term_b2114_1_rib_cplx	Translation termination complex b2114 1 ribosomes	C116420H158596N41480O62089S311P6686Mg175Zn0Fe0	-5931.14	Translation
tl_term_b2114_1_rib_cplx2	Translation termination complex b2114 1 ribosomes 2 (RF2_mono)	C116471H158627N41462O62122S309P6686Mg175Zn0Fe0	-5940.14	Translation

tl_term_b2114_39_rib_cplx	Translation termination complex b2114 39 ribosomes	C3805308H5352550N1324512O1881339S12129P183386Mg6825Zn0Fe0	-153908.46	Translation
tl_term_b2114_39_rib_cplx2	Translation termination complex b2114 39 ribosomes 2 (RF2_mono)	C3807297H5353759N1323810O1882626S12051P183386Mg6825Zn0Fe0	-154259.46	Translation
tl_term_b2140_18_rib_cplx	Translation termination complex b2140 18 ribosomes	C1724777H2422810N603793O859745S5274P84848Mg3150Zn0Fe0	-70991.52	Translation
tl_term_b2140_18_rib_cplx2	Translation termination complex b2140 18 ribosomes 2 (RF2_mono)	C1725695H2423368N603469O860339S5238P84848Mg3150Zn0Fe0	-71153.52	Translation
tl_term_b2140_1_rib_cplx	Translation termination complex b2140 1 ribosomes	C104371H144249N37013O54030S293P5611Mg175Zn0Fe0	-4842.14	Translation
tl_term_b2140_1_rib_cplx2	Translation termination complex b2140 1 ribosomes 2 (RF2_mono)	C104422H144280N36995O54063S291P5611Mg175Zn0Fe0	-4851.14	Translation
tl_term_b2140_9_rib_cplx	Translation termination complex b2140 9 ribosomes	C866915H1216513N303733O433190S2637P42899Mg1575Zn0Fe0	-35971.26	Translation
tl_term_b2140_9_rib_cplx2	Translation termination complex b2140 9 ribosomes 2 (RF2_mono)	C867374H1216792N303571O433487S2619P42899Mg1575Zn0Fe0	-36052.26	Translation
tl_term_b2183_13_rib_cplx	Translation termination complex b2183 13 ribosomes	C1239067H1739546N434003O618691S3744P61161Mg2275Zn0Fe0	-51218.82	Translation
tl_term_b2183_13_rib_cplx2	Translation termination complex b2183 13 ribosomes 2 (RF2_mono)	C1239730H1739949N433769O619120S3718P61161Mg2275Zn0Fe0	-51335.82	Translation
tl_term_b2183_1_rib_cplx	Translation termination complex b2183 1 ribosomes	C101419H140726N35807O52099S288P5349Mg175Zn0Fe0	-4585.14	Translation
tl_term_b2183_1_rib_cplx2	Translation termination complex b2183 1 ribosomes 2 (RF2_mono)	C101470H140757N35789O52132S286P5349Mg175Zn0Fe0	-4594.14	Translation
tl_term_b2183_6_rib_cplx	Translation termination complex b2183 6 ribosomes	C575439H806901N201722O288179S1728P28604Mg1050Zn0Fe0	-24015.84	Translation
tl_term_b2183_6_rib_cplx2	Translation termination complex b2183 6 ribosomes 2 (RF2_mono)	C575745H807087N201614O288377S1716P28604Mg1050Zn0Fe0	-24069.84	Translation
tl_term_b2185_1_rib_cplx	Translation termination complex b2185 1 ribosomes	C96848H135276N34103O48972S284P4937Mg175Zn0Fe0	-4161.14	Translation
tl_term_b2185_1_rib_cplx2	Translation termination complex b2185 1 ribosomes 2 (RF2_mono)	C96899H135307N34085O49005S282P4937Mg175Zn0Fe0	-4170.14	Translation
tl_term_b2185_2_rib_cplx	Translation termination complex b2185 2 ribosomes	C190974H267472N67093O95971S568P9587Mg350Zn0Fe0	-8034.28	Translation
tl_term_b2185_2_rib_cplx2	Translation termination complex b2185 2 ribosomes 2 (RF2_mono)	C191076H267534N67057O96037S564P9587Mg350Zn0Fe0	-8052.28	Translation
tl_term_b2185_5_rib_cplx	Translation termination complex b2185 5 ribosomes	C473352H664060N166063O236968S1420P23537Mg875Zn0Fe0	-19653.7	Translation
tl_term_b2185_5_rib_cplx2	Translation termination complex b2185 5 ribosomes 2 (RF2_mono)	C473607H664215N165973O237133S1410P23537Mg875Zn0Fe0	-19698.7	Translation
tl_term_b2268_17_rib_cplx	Translation termination complex b2268 17 ribosomes	C1625745H2283369N568869O810548S4964P79970Mg2975Zn0Fe0	-67002.38	Translation
tl_term_b2268_17_rib_cplx2	Translation termination complex b2268 17 ribosomes 2 (RF2_mono)	C1626612H2283896N568563O811109S4930P79970Mg2975Zn0Fe0	-67155.38	Translation
tl_term_b2268_1_rib_cplx	Translation termination complex b2268 1 ribosomes	C103841H143609N36709O53732S292P5570Mg175Zn0Fe0	-4808.14	Translation
tl_term_b2268_1_rib_cplx2	Translation termination complex b2268 1 ribosomes 2 (RF2_mono)	C103892H143640N36691O53765S290P5570Mg175Zn0Fe0	-4817.14	Translation
tl_term_b2268_8_rib_cplx	Translation termination complex b2268 8 ribosomes	C769674H1079754N269529O384839S2336P38120Mg1400Zn0Fe0	-32018.12	Translation
tl_term_b2268_8_rib_cplx2	Translation termination complex b2268 8 ribosomes 2 (RF2_mono)	C770082H1080002N269385O385103S2320P38120Mg1400Zn0Fe0	-32090.12	Translation
tl_term_b2317_12_rib_cplx	Translation termination complex b2317 12 ribosomes	C1144036H1605843N399934O570536S3480P56460Mg2100Zn0Fe0	-47174.68	Translation
tl_term_b2317_12_rib_cplx2	Translation termination complex b2317 12 ribosomes 2 (RF2_mono)	C1144648H1606215N399718O570932S3456P56460Mg2100Zn0Fe0	-47282.68	Translation
tl_term_b2317_1_rib_cplx	Translation termination complex b2317 1 ribosomes	C101060H140291N35515O51842S290P5310Mg175Zn0Fe0	-4537.14	Translation
tl_term_b2317_1_rib_cplx2	Translation termination complex b2317 1 ribosomes 2 (RF2_mono)	C101111H140322N35497O51875S288P5310Mg175Zn0Fe0	-4546.14	Translation
tl_term_b2317_6_rib_cplx	Translation termination complex b2317 6 ribosomes	C575140H806451N201160O287612S1740P28560Mg1050Zn0Fe0	-23917.84	Translation
tl_term_b2317_6_rib_cplx2	Translation termination complex b2317 6 ribosomes 2 (RF2_mono)	C575446H806637N201052O287810S1728P28560Mg1050Zn0Fe0	-23971.84	Translation
tl_term_b2318_15_rib_cplx	Translation termination complex b2318 15 ribosomes	C1433068H2012009N501797O714571S4365P70577Mg2625Zn0Fe0	-58940.1	Translation
tl_term_b2318_15_rib_cplx2	Translation termination complex b2318 15 ribosomes 2 (RF2_mono)	C1433833H2012474N501527O715066S4335P70577Mg2625Zn0Fe0	-59075.1	Translation
tl_term_b2318_1_rib_cplx	Translation termination complex b2318 1 ribosomes	C102760H142309N36367O52931S291P5463Mg175Zn0Fe0	-4688.14	Translation
tl_term_b2318_1_rib_cplx2	Translation termination complex b2318 1 ribosomes 2 (RF2_mono)	C102811H142340N36349O52964S289P5463Mg175Zn0Fe0	-4697.14	Translation
tl_term_b2318_7_rib_cplx	Translation termination complex b2318 7 ribosomes	C672892H943609N235837O336491S2037P33369Mg1225Zn0Fe0	-27938.98	Translation
tl_term_b2318_7_rib_cplx2	Translation termination complex b2318 7 ribosomes 2 (RF2_mono)	C673249H943826N235711O336722S2023P33369Mg1225Zn0Fe0	-28001.98	Translation
tl_term_b2319_19_rib_cplx	Translation termination complex b2319 19 ribosomes	C1821600H2557993N636917O908137S5529P89537Mg3325Zn0Fe0	-75309.66	Translation
tl_term_b2319_19_rib_cplx2	Translation termination complex b2319 19 ribosomes 2 (RF2_mono)	C1822569H2558582N636575O908764S5491P89537Mg3325Zn0Fe0	-75480.66	Translation
tl_term_b2319_1_rib_cplx	Translation termination complex b2319 1 ribosomes	C105030H144985N37211O54523S291P5675Mg175Zn0Fe0	-4927.14	Translation
tl_term_b2319_1_rib_cplx2	Translation termination complex b2319 1 ribosomes 2 (RF2_mono)	C105081H145016N37193O54556S289P5675Mg175Zn0Fe0	-4936.14	Translation
tl_term_b2319_9_rib_cplx	Translation termination complex b2319 9 ribosomes	C867950H1217433N303747O433907S2619P42947Mg1575Zn0Fe0	-36208.26	Translation
tl_term_b2319_9_rib_cplx2	Translation termination complex b2319 9 ribosomes 2 (RF2_mono)	C868409H1217712N303585O434204S2601P42947Mg1575Zn0Fe0	-36289.26	Translation
tl_term_b2320_11_rib_cplx	Translation termination complex b2320 11 ribosomes	C1061451H1490636N371425O529453S3212P52300Mg1925Zn0Fe0	-43843.54	Translation
tl_term_b2320_11_rib_cplx2	Translation termination complex b2320 11 ribosomes 2 (RF2_mono)	C1062012H1490977N371227O529816S3190P52300Mg1925Zn0Fe0	-43942.54	Translation
tl_term_b2320_1_rib_cplx	Translation termination complex b2320 1 ribosomes	C106331H146626N37675O55403S292P5790Mg175Zn0Fe0	-5022.14	Translation

tl_term_b2320_1_rib_cplx2	Translation termination complex b2320 1 ribosomes 2 (RF2_mono)	C106382H146657N37657O55436S290P5790Mg175Zn0Fe0	-5031.14	Translation
tl_term_b2320_22_rib_cplx	Translation termination complex b2320 22 ribosomes	C2112083H2969047N738550O1050908S6424P103461Mg3850Zn0Fe0	-86547.08	Translation
tl_term_b2320_22_rib_cplx2	Translation termination complex b2320 22 ribosomes 2 (RF2_mono)	C2113205H2969729N738154O1051634S6380P103461Mg3850Zn0Fe0	-86745.08	Translation
tl_term_b2324_19_rib_cplx	Translation termination complex b2324 19 ribosomes	C1861021H2614256N649038O922757S5890P90321Mg3325Zn0Fe0	-75998.66	Translation
tl_term_b2324_19_rib_cplx2	Translation termination complex b2324 19 ribosomes 2 (RF2_mono)	C1861990H2614845N648696O923384S5852P90321Mg3325Zn0Fe0	-76169.66	Translation
tl_term_b2324_1_rib_cplx	Translation termination complex b2324 1 ribosomes	C116047H158048N41376O61907S310P6657Mg175Zn0Fe0	-5904.14	Translation
tl_term_b2324_1_rib_cplx2	Translation termination complex b2324 1 ribosomes 2 (RF2_mono)	C116098H158079N41358O61940S308P6657Mg175Zn0Fe0	-5913.14	Translation
tl_term_b2324_39_rib_cplx	Translation termination complex b2324 39 ribosomes	C3799881H5343376N1324218O1879257S12090P183281Mg6825Zn0Fe0	-153881.46	Translation
tl_term_b2324_39_rib_cplx2	Translation termination complex b2324 39 ribosomes 2 (RF2_mono)	C3801870H5344585N1323516O1880544S12012P183281Mg6825Zn0Fe0	-154232.46	Translation
tl_term_b2325_1_rib_cplx	Translation termination complex b2325 1 ribosomes	C96730H135076N34033O48957S285P4928Mg175Zn0Fe0	-4168.14	Translation
tl_term_b2325_1_rib_cplx2	Translation termination complex b2325 1 ribosomes 2 (RF2_mono)	C96781H135107N34015O48990S283P4928Mg175Zn0Fe0	-4177.14	Translation
tl_term_b2325_2_rib_cplx	Translation termination complex b2325 2 ribosomes	C190799H267149N66996O95958S570P9577Mg350Zn0Fe0	-8056.28	Translation
tl_term_b2325_2_rib_cplx2	Translation termination complex b2325 2 ribosomes 2 (RF2_mono)	C190901H267211N66960O96024S566P9577Mg350Zn0Fe0	-8074.28	Translation
tl_term_b2325_5_rib_cplx	Translation termination complex b2325 5 ribosomes	C473006H663368N165885O236961S1425P23524Mg875Zn0Fe0	-19720.7	Translation
tl_term_b2325_5_rib_cplx2	Translation termination complex b2325 5 ribosomes 2 (RF2_mono)	C473261H663523N165795O237126S1415P23524Mg875Zn0Fe0	-19765.7	Translation
tl_term_b2326_10_rib_cplx	Translation termination complex b2326 10 ribosomes	C951374H1334010N333079O475323S2890P47048Mg1750Zn0Fe0	-39470.4	Translation
tl_term_b2326_1_rib_cplx	Translation termination complex b2326 1 ribosomes	C99830H138702N35170O50991S289P5198Mg175Zn0Fe0	-4441.14	Translation
tl_term_b2326_5_rib_cplx	Translation termination complex b2326 5 ribosomes	C478294H669950N167574O239583S1445P23798Mg875Zn0Fe0	-20009.7	Translation
tl_term_b2327_15_rib_cplx	Translation termination complex b2327 15 ribosomes	C1432332H2011834N500663O713869S4500P70559Mg2625Zn0Fe0	-58847.1	Translation
tl_term_b2327_15_rib_cplx2	Translation termination complex b2327 15 ribosomes 2 (RF2_mono)	C1433097H2012299N500393O714364S4470P70559Mg2625Zn0Fe0	-58982.1	Translation
tl_term_b2327_1_rib_cplx	Translation termination complex b2327 1 ribosomes	C102640H142218N36143O52943S300P5459Mg175Zn0Fe0	-4679.14	Translation
tl_term_b2327_1_rib_cplx2	Translation termination complex b2327 1 ribosomes 2 (RF2_mono)	C102691H142249N36125O52976S298P5459Mg175Zn0Fe0	-4688.14	Translation
tl_term_b2327_7_rib_cplx	Translation termination complex b2327 7 ribosomes	C672508H943482N235223O336197S2100P33359Mg1225Zn0Fe0	-27893.98	Translation
tl_term_b2327_7_rib_cplx2	Translation termination complex b2327 7 ribosomes 2 (RF2_mono)	C672865H943699N235097O336428S2086P33359Mg1225Zn0Fe0	-27956.98	Translation
tl_term_b2328_16_rib_cplx	Translation termination complex b2328 16 ribosomes	C1527917H2145918N534989O761932S4704P75241Mg2800Zn0Fe0	-62844.24	Translation
tl_term_b2328_16_rib_cplx2	Translation termination complex b2328 16 ribosomes 2 (RF2_mono)	C1528733H2146414N534701O762460S4672P75241Mg2800Zn0Fe0	-62988.24	Translation
tl_term_b2328_1_rib_cplx	Translation termination complex b2328 1 ribosomes	C102842H142458N36359O53032S294P5476Mg175Zn0Fe0	-4702.14	Translation
tl_term_b2328_1_rib_cplx2	Translation termination complex b2328 1 ribosomes 2 (RF2_mono)	C102893H142489N36341O53065S292P5476Mg175Zn0Fe0	-4711.14	Translation
tl_term_b2328_8_rib_cplx	Translation termination complex b2328 8 ribosomes	C767877H1077406N269053O383852S2352P38033Mg1400Zn0Fe0	-31835.12	Translation
tl_term_b2328_8_rib_cplx2	Translation termination complex b2328 8 ribosomes 2 (RF2_mono)	C768285H1077654N268909O384116S2336P38033Mg1400Zn0Fe0	-31907.12	Translation
tl_term_b2329_10_rib_cplx	Translation termination complex b2329 10 ribosomes	C963952H1353464N337631O481479S2960P47586Mg1750Zn0Fe0	-39918.4	Translation
tl_term_b2329_10_rib_cplx2	Translation termination complex b2329 10 ribosomes 2 (RF2_mono)	C964462H1353774N337451O481809S2940P47586Mg1750Zn0Fe0	-40008.4	Translation
tl_term_b2329_1_rib_cplx	Translation termination complex b2329 1 ribosomes	C105694H145898N37499O54969S296P5736Mg175Zn0Fe0	-4970.14	Translation
tl_term_b2329_1_rib_cplx2	Translation termination complex b2329 1 ribosomes 2 (RF2_mono)	C105745H145929N37481O55002S294P5736Mg175Zn0Fe0	-4979.14	Translation
tl_term_b2329_21_rib_cplx	Translation termination complex b2329 21 ribosomes	C2012934H2829378N704459O1002769S6216P98736Mg3675Zn0Fe0	-82632.94	Translation
tl_term_b2329_21_rib_cplx2	Translation termination complex b2329 21 ribosomes 2 (RF2_mono)	C2014005H2830029N704081O1003462S6174P98736Mg3675Zn0Fe0	-82821.94	Translation
tl_term_b2330_18_rib_cplx	Translation termination complex b2330 18 ribosomes	C1722952H2419123N602372O858737S5310P84653Mg3150Zn0Fe0	-71174.52	Translation
tl_term_b2330_18_rib_cplx2	Translation termination complex b2330 18 ribosomes 2 (RF2_mono)	C1723870H2419681N602048O859331S5274P84653Mg3150Zn0Fe0	-71336.52	Translation
tl_term_b2330_1_rib_cplx	Translation termination complex b2330 1 ribosomes	C104093H143877N36782O53889S295P5586Mg175Zn0Fe0	-4838.14	Translation
tl_term_b2330_1_rib_cplx2	Translation termination complex b2330 1 ribosomes 2 (RF2_mono)	C104144H143908N36764O53922S293P5586Mg175Zn0Fe0	-4847.14	Translation
tl_term_b2330_9_rib_cplx	Translation termination complex b2330 9 ribosomes	C865909H1214581N302942O432641S2655P42794Mg1575Zn0Fe0	-36055.26	Translation
tl_term_b2330_9_rib_cplx2	Translation termination complex b2330 9 ribosomes 2 (RF2_mono)	C866368H1214860N302780O432938S2637P42794Mg1575Zn0Fe0	-36136.26	Translation
tl_term_b2400_13_rib_cplx	Translation termination complex b2400 13 ribosomes	C1261678H1771320N441150O628303S3913P61855Mg2275Zn0Fe0	-52029.82	Translation
tl_term_b2400_13_rib_cplx2	Translation termination complex b2400 13 ribosomes 2 (RF2_mono)	C1262341H1771723N440916O628732S3887P61855Mg2275Zn0Fe0	-52146.82	Translation
tl_term_b2400_1_rib_cplx	Translation termination complex b2400 1 ribosomes	C109498H150336N38922O57463S301P6067Mg175Zn0Fe0	-5312.14	Translation
tl_term_b2400_1_rib_cplx2	Translation termination complex b2400 1 ribosomes 2 (RF2_mono)	C109549H150367N38904O57496S299P6067Mg175Zn0Fe0	-5321.14	Translation
tl_term_b2400_27_rib_cplx	Translation termination complex b2400 27 ribosomes	C2605888H3662468N910416O1294283S8127P126941Mg4725Zn0Fe0	-106533.78	Translation

tl_term_b2400_27_rib_cplx2	Translation termination complex b2400 27 ribosomes 2 (RF2_mono)	C2607265H3663305N909930O1295174S8073P126941Mg4725Zn0Fe0	-106776.78	Translation
tl_term_b2514_12_rib_cplx	Translation termination complex b2514 12 ribosomes	C1160818H1630143N406204O578801S3540P57077Mg2100Zn0Fe0	-47899.68	Translation
tl_term_b2514_12_rib_cplx2	Translation termination complex b2514 12 ribosomes 2 (RF2_mono)	C1161430H1630515N405988O579197S3516P57077Mg2100Zn0Fe0	-48007.68	Translation
tl_term_b2514_1_rib_cplx	Translation termination complex b2514 1 ribosomes	C107865H148432N38309O56433S295P5927Mg175Zn0Fe0	-5163.14	Translation
tl_term_b2514_1_rib_cplx2	Translation termination complex b2514 1 ribosomes 2 (RF2_mono)	C107916H148463N38291O56466S293P5927Mg175Zn0Fe0	-5172.14	Translation
tl_term_b2514_24_rib_cplx	Translation termination complex b2514 24 ribosomes	C2309494H3246555N807544O1148657S7080P112877Mg4200Zn0Fe0	-94521.36	Translation
tl_term_b2514_24_rib_cplx2	Translation termination complex b2514 24 ribosomes 2 (RF2_mono)	C2310718H3247299N807112O1149449S7032P112877Mg4200Zn0Fe0	-94737.36	Translation
tl_term_b2528_1_rib_cplx	Translation termination complex b2528 1 ribosomes	C97260H135707N34253O49285S287P4974Mg175Zn0Fe0	-4208.14	Translation
tl_term_b2528_3_rib_cplx	Translation termination complex b2528 3 ribosomes	C285584H400139N100235O143335S861P14274Mg525Zn0Fe0	-11974.42	Translation
tl_term_b2528_6_rib_cplx	Translation termination complex b2528 6 ribosomes	C568070H796787N199208O284410S1722P28224Mg1050Zn0Fe0	-23623.84	Translation
tl_term_b2529_1_rib_cplx	Translation termination complex b2529 1 ribosomes	C97958H136566N34531O49743S288P5037Mg175Zn0Fe0	-4273.14	Translation
tl_term_b2529_1_rib_cplx2	Translation termination complex b2529 1 ribosomes 2 (RF2_mono)	C98009H136597N34513O49776S286P5037Mg175Zn0Fe0	-4282.14	Translation
tl_term_b2529_3_rib_cplx	Translation termination complex b2529 3 ribosomes	C286470H401340N100553O143879S864P14337Mg525Zn0Fe0	-12043.42	Translation
tl_term_b2529_3_rib_cplx2	Translation termination complex b2529 3 ribosomes 2 (RF2_mono)	C286623H401433N100499O143978S858P14337Mg525Zn0Fe0	-12070.42	Translation
tl_term_b2529_7_rib_cplx	Translation termination complex b2529 7 ribosomes	C663494H930888N232597O332151S2016P32937Mg1225Zn0Fe0	-27583.98	Translation
tl_term_b2529_7_rib_cplx2	Translation termination complex b2529 7 ribosomes 2 (RF2_mono)	C663851H931105N232471O332382S2002P32937Mg1225Zn0Fe0	-27646.98	Translation
tl_term_b2530_11_rib_cplx	Translation termination complex b2530 11 ribosomes	C1063621H1493620N372123O530702S3311P52376Mg1925Zn0Fe0	-43963.54	Translation
tl_term_b2530_11_rib_cplx2	Translation termination complex b2530 11 ribosomes 2 (RF2_mono)	C1064182H1493961N371925O531065S3289P52376Mg1925Zn0Fe0	-44062.54	Translation
tl_term_b2530_1_rib_cplx	Translation termination complex b2530 1 ribosomes	C107201H147690N38033O55952S301P5866Mg175Zn0Fe0	-5102.14	Translation
tl_term_b2530_1_rib_cplx2	Translation termination complex b2530 1 ribosomes 2 (RF2_mono)	C107252H147721N38015O55985S299P5866Mg175Zn0Fe0	-5111.14	Translation
tl_term_b2530_23_rib_cplx	Translation termination complex b2530 23 ribosomes	C2211325H3108736N773031O1100402S6923P108188Mg4025Zn0Fe0	-90597.22	Translation
tl_term_b2530_23_rib_cplx2	Translation termination complex b2530 23 ribosomes 2 (RF2_mono)	C2212498H3109449N772617O1101161S6877P108188Mg4025Zn0Fe0	-90804.22	Translation
tl_term_b2531_1_rib_cplx	Translation termination complex b2531 1 ribosomes	C99036H137902N34920O50542S286P5141Mg175Zn0Fe0	-4370.14	Translation
tl_term_b2531_1_rib_cplx2	Translation termination complex b2531 1 ribosomes 2 (RF2_mono)	C99087H137933N34902O50575S284P5141Mg175Zn0Fe0	-4379.14	Translation
tl_term_b2531_4_rib_cplx	Translation termination complex b2531 4 ribosomes	C382206H535819N134160O191857S1144P19091Mg700Zn0Fe0	-16004.56	Translation
tl_term_b2531_4_rib_cplx2	Translation termination complex b2531 4 ribosomes 2 (RF2_mono)	C382410H535943N134088O191989S1136P19091Mg700Zn0Fe0	-16040.56	Translation
tl_term_b2531_9_rib_cplx	Translation termination complex b2531 9 ribosomes	C854156H1199014N299560O427382S2574P42341Mg1575Zn0Fe0	-35395.26	Translation
tl_term_b2531_9_rib_cplx2	Translation termination complex b2531 9 ribosomes 2 (RF2_mono)	C854615H1199293N299398O427679S2556P42341Mg1575Zn0Fe0	-35476.26	Translation
tl_term_b2559_10_rib_cplx	Translation termination complex b2559 10 ribosomes	C950539H1334173N333248O474974S2940P47047Mg1750Zn0Fe0	-39299.4	Translation
tl_term_b2559_10_rib_cplx2	Translation termination complex b2559 10 ribosomes 2 (RF2_mono)	C951049H1334483N333068O475304S2920P47047Mg1750Zn0Fe0	-39389.4	Translation
tl_term_b2559_1_rib_cplx	Translation termination complex b2559 1 ribosomes	C99661H138622N35177O50885S294P5188Mg175Zn0Fe0	-4414.14	Translation
tl_term_b2559_1_rib_cplx2	Translation termination complex b2559 1 ribosomes 2 (RF2_mono)	C99712H138653N35159O50918S292P5188Mg175Zn0Fe0	-4423.14	Translation
tl_term_b2559_5_rib_cplx	Translation termination complex b2559 5 ribosomes	C477829H669978N167653O239369S1470P23792Mg875Zn0Fe0	-19918.7	Translation
tl_term_b2559_5_rib_cplx2	Translation termination complex b2559 5 ribosomes 2 (RF2_mono)	C478084H670133N167563O239534S1460P23792Mg875Zn0Fe0	-19963.7	Translation
tl_term_b2560_12_rib_cplx	Translation termination complex b2560 12 ribosomes	C1143795H1605123N400028O570755S3444P56438Mg2100Zn0Fe0	-47224.68	Translation
tl_term_b2560_1_rib_cplx	Translation termination complex b2560 1 ribosomes	C100841H140011N35477O51698S287P5288Mg175Zn0Fe0	-4521.14	Translation
tl_term_b2560_6_rib_cplx	Translation termination complex b2560 6 ribosomes	C574911H805971N201182O287633S1722P28538Mg1050Zn0Fe0	-23931.84	Translation
tl_term_b2563_1_rib_cplx	Translation termination complex b2563 1 ribosomes	C98077H136715N34588O49802S286P5047Mg175Zn0Fe0	-4273.14	Translation
tl_term_b2563_1_rib_cplx2	Translation termination complex b2563 1 ribosomes 2 (RF2_mono)	C98128H136746N34570O49835S284P5047Mg175Zn0Fe0	-4282.14	Translation
tl_term_b2563_3_rib_cplx	Translation termination complex b2563 3 ribosomes	C286969H401957N100814O144104S858P14381Mg525Zn0Fe0	-12057.42	Translation
tl_term_b2563_3_rib_cplx2	Translation termination complex b2563 3 ribosomes 2 (RF2_mono)	C287122H402050N100760O144203S852P14381Mg525Zn0Fe0	-12084.42	Translation
tl_term_b2563_7_rib_cplx	Translation termination complex b2563 7 ribosomes	C664753H932441N233266O332708S2002P33049Mg1225Zn0Fe0	-27625.98	Translation
tl_term_b2563_7_rib_cplx2	Translation termination complex b2563 7 ribosomes 2 (RF2_mono)	C665110H932658N233140O332939S1988P33049Mg1225Zn0Fe0	-27688.98	Translation
tl_term_b2564_14_rib_cplx	Translation termination complex b2564 14 ribosomes	C1334014H1873869N467484O666114S4116P65834Mg2450Zn0Fe0	-55126.96	Translation
tl_term_b2564_14_rib_cplx2	Translation termination complex b2564 14 ribosomes 2 (RF2_mono)	C1334728H1874303N467232O666576S4088P65834Mg2450Zn0Fe0	-55252.96	Translation
tl_term_b2564_1_rib_cplx	Translation termination complex b2564 1 ribosomes	C101770H141190N36027O52319S294P5384Mg175Zn0Fe0	-4620.14	Translation
tl_term_b2564_1_rib_cplx2	Translation termination complex b2564 1 ribosomes 2 (RF2_mono)	C101821H141221N36009O52352S292P5384Mg175Zn0Fe0	-4629.14	Translation

tl_term_b2564_7_rib_cplx	Translation termination complex b2564 7 ribosomes	C670498H940888N235161O335609S2058P33284Mg1225Zn0Fe0	-27930.98	Translation
tl_term_b2564_7_rib_cplx2	Translation termination complex b2564 7 ribosomes 2 (RF2_mono)	C670855H941105N235035O335840S2044P33284Mg1225Zn0Fe0	-27993.98	Translation
tl_term_b2565_14_rib_cplx	Translation termination complex b2565 14 ribosomes	C1335259H1875226N467503O665951S4046P65829Mg2450Zn0Fe0	-54855.96	Translation
tl_term_b2565_1_rib_cplx	Translation termination complex b2565 1 ribosomes	C101806H141234N35942O52312S289P5379Mg175Zn0Fe0	-4596.14	Translation
tl_term_b2565_7_rib_cplx	Translation termination complex b2565 7 ribosomes	C671092H941538N235124O335530S2023P33279Mg1225Zn0Fe0	-27792.98	Translation
tl_term_b2566_17_rib_cplx	Translation termination complex b2566 17 ribosomes	C1627950H2287169N569845O811766S4964P80139Mg2975Zn0Fe0	-67018.38	Translation
tl_term_b2566_17_rib_cplx2	Translation termination complex b2566 17 ribosomes 2 (RF2_mono)	C1628817H2287696N569539O812327S4930P80139Mg2975Zn0Fe0	-67171.38	Translation
tl_term_b2566_1_rib_cplx	Translation termination complex b2566 1 ribosomes	C103854H143697N36789O53654S292P5563Mg175Zn0Fe0	-4792.14	Translation
tl_term_b2566_1_rib_cplx2	Translation termination complex b2566 1 ribosomes 2 (RF2_mono)	C103905H143728N36771O53687S290P5563Mg175Zn0Fe0	-4801.14	Translation
tl_term_b2566_8_rib_cplx	Translation termination complex b2566 8 ribosomes	C770646H1081466N270001O385328S2336P38190Mg1400Zn0Fe0	-32016.12	Translation
tl_term_b2566_8_rib_cplx2	Translation termination complex b2566 8 ribosomes 2 (RF2_mono)	C771054H1081714N269857O385592S2320P38190Mg1400Zn0Fe0	-32088.12	Translation
tl_term_b2567_13_rib_cplx	Translation termination complex b2567 13 ribosomes	C1238485H1739308N433953O618347S3731P61133Mg2275Zn0Fe0	-51112.82	Translation
tl_term_b2567_1_rib_cplx	Translation termination complex b2567 1 ribosomes	C101257H140560N35781O51959S287P5333Mg175Zn0Fe0	-4563.14	Translation
tl_term_b2567_6_rib_cplx	Translation termination complex b2567 6 ribosomes	C575102H806705N201686O287954S1722P28583Mg1050Zn0Fe0	-23958.84	Translation
tl_term_b2570_1_rib_cplx	Translation termination complex b2570 1 ribosomes	C98897H137719N34804O50437S290P5125Mg175Zn0Fe0	-4353.14	Translation
tl_term_b2570_4_rib_cplx	Translation termination complex b2570 4 ribosomes	C382046H535537N133924O191647S1160P19072Mg700Zn0Fe0	-15981.56	Translation
tl_term_b2570_9_rib_cplx	Translation termination complex b2570 9 ribosomes	C853961H1198567N299124O426997S2610P42317Mg1575Zn0Fe0	-35362.26	Translation
tl_term_b2571_18_rib_cplx	Translation termination complex b2571 18 ribosomes	C1723046H2420906N602701O858667S5274P84638Mg3150Zn0Fe0	-70691.52	Translation
tl_term_b2571_1_rib_cplx	Translation termination complex b2571 1 ribosomes	C104289H144198N36839O54040S293P5605Mg175Zn0Fe0	-4831.14	Translation
tl_term_b2571_9_rib_cplx	Translation termination complex b2571 9 ribosomes	C866057H1215590N303127O432688S2637P42797Mg1575Zn0Fe0	-35824.26	Translation
tl_term_b2572_12_rib_cplx	Translation termination complex b2572 12 ribosomes	C1142501H1603838N400325O570747S3516P56439Mg2100Zn0Fe0	-47273.68	Translation
tl_term_b2572_12_rib_cplx2	Translation termination complex b2572 12 ribosomes 2 (RF2_mono)	C1143113H1604210N400109O571143S3492P56439Mg2100Zn0Fe0	-47381.68	Translation
tl_term_b2572_1_rib_cplx	Translation termination complex b2572 1 ribosomes	C100900H140101N35675O51690S293P5300Mg175Zn0Fe0	-4537.14	Translation
tl_term_b2572_1_rib_cplx2	Translation termination complex b2572 1 ribosomes 2 (RF2_mono)	C100951H140132N35657O51723S291P5300Mg175Zn0Fe0	-4546.14	Translation
tl_term_b2572_6_rib_cplx	Translation termination complex b2572 6 ribosomes	C574355H805436N201425O287625S1758P28545Mg1050Zn0Fe0	-23962.84	Translation
tl_term_b2572_6_rib_cplx2	Translation termination complex b2572 6 ribosomes 2 (RF2_mono)	C574661H805622N201317O287823S1746P28545Mg1050Zn0Fe0	-24016.84	Translation
tl_term_b2573_11_rib_cplx	Translation termination complex b2573 11 ribosomes	C1046324H1468913N366649O522769S3157P51739Mg1925Zn0Fe0	-43271.54	Translation
tl_term_b2573_1_rib_cplx	Translation termination complex b2573 1 ribosomes	C100114H139163N35329O51199S287P5229Mg175Zn0Fe0	-4460.14	Translation
tl_term_b2573_5_rib_cplx	Translation termination complex b2573 5 ribosomes	C478598H671063N167857O239827S1435P23833Mg875Zn0Fe0	-19984.7	Translation
tl_term_b2593_14_rib_cplx	Translation termination complex b2593 14 ribosomes	C1334525H1873343N467286O666097S4116P65828Mg2450Zn0Fe0	-55050.96	Translation
tl_term_b2593_14_rib_cplx2	Translation termination complex b2593 14 ribosomes 2 (RF2_mono)	C1335239H1873777N467034O666559S4088P65828Mg2450Zn0Fe0	-55176.96	Translation
tl_term_b2593_1_rib_cplx	Translation termination complex b2593 1 ribosomes	C101748H141080N35920O52341S294P5378Mg175Zn0Fe0	-4609.14	Translation
tl_term_b2593_1_rib_cplx2	Translation termination complex b2593 1 ribosomes 2 (RF2_mono)	C101799H141111N35902O52374S292P5378Mg175Zn0Fe0	-4618.14	Translation
tl_term_b2593_7_rib_cplx	Translation termination complex b2593 7 ribosomes	C670722H940586N235012O335613S2058P33278Mg1225Zn0Fe0	-27889.98	Translation
tl_term_b2593_7_rib_cplx2	Translation termination complex b2593 7 ribosomes 2 (RF2_mono)	C671079H940803N234886O335844S2044P33278Mg1225Zn0Fe0	-27952.98	Translation
tl_term_b2594_19_rib_cplx	Translation termination complex b2594 19 ribosomes	C1821923H2559687N637930O907681S5624P89542Mg3325Zn0Fe0	-74972.66	Translation
tl_term_b2594_1_rib_cplx	Translation termination complex b2594 1 ribosomes	C104741H144735N37126O54265S296P5644Mg175Zn0Fe0	-4878.14	Translation
tl_term_b2594_9_rib_cplx	Translation termination complex b2594 9 ribosomes	C867933H1218047N304150O433561S2664P42932Mg1575Zn0Fe0	-36031.26	Translation
tl_term_b2606_1_rib_cplx	Translation termination complex b2606 1 ribosomes	C97546H136139N34337O49474S284P4998Mg175Zn0Fe0	-4215.14	Translation
tl_term_b2606_1_rib_cplx2	Translation termination complex b2606 1 ribosomes 2 (RF2_mono)	C97597H136170N34319O49507S282P4998Mg175Zn0Fe0	-4224.14	Translation
tl_term_b2606_3_rib_cplx	Translation termination complex b2606 3 ribosomes	C286018H400943N100395O143540S852P14298Mg525Zn0Fe0	-11947.42	Translation
tl_term_b2606_3_rib_cplx2	Translation termination complex b2606 3 ribosomes 2 (RF2_mono)	C286171H401036N100341O143639S846P14298Mg525Zn0Fe0	-11974.42	Translation
tl_term_b2606_6_rib_cplx	Translation termination complex b2606 6 ribosomes	C568726H798149N199482O284639S1704P28248Mg1050Zn0Fe0	-23545.84	Translation
tl_term_b2606_6_rib_cplx2	Translation termination complex b2606 6 ribosomes 2 (RF2_mono)	C569032H798335N199374O284837S1692P28248Mg1050Zn0Fe0	-23599.84	Translation
tl_term_b2607_15_rib_cplx	Translation termination complex b2607 15 ribosomes	C1430880H2009178N501149O713951S4365P70518Mg2625Zn0Fe0	-59076.1	Translation
tl_term_b2607_15_rib_cplx2	Translation termination complex b2607 15 ribosomes 2 (RF2_mono)	C1431645H2009643N500879O714446S4335P70518Mg2625Zn0Fe0	-59211.1	Translation
tl_term_b2607_1_rib_cplx	Translation termination complex b2607 1 ribosomes	C102238H141676N36195O52591S291P5418Mg175Zn0Fe0	-4656.14	Translation

tl_term_b2607_1_rib_cplx2	Translation termination complex b2607 1 ribosomes 2 (RF2_mono)	C102289H141707N36177O52624S289P5418Mg175Zn0Fe0	-4665.14	Translation
tl_term_b2607_7_rib_cplx	Translation termination complex b2607 7 ribosomes	C671656H942034N235461O336031S2037P33318Mg1225Zn0Fe0	-27978.98	Translation
tl_term_b2607_7_rib_cplx2	Translation termination complex b2607 7 ribosomes 2 (RF2_mono)	C672013H942251N235335O336262S2023P33318Mg1225Zn0Fe0	-28041.98	Translation
tl_term_b2608_10_rib_cplx	Translation termination complex b2608 10 ribosomes	C951014H1334475N333028O475264S2910P47049Mg1750Zn0Fe0	-39421.4	Translation
tl_term_b2608_10_rib_cplx2	Translation termination complex b2608 10 ribosomes 2 (RF2_mono)	C951524H1334785N332848O475594S2890P47049Mg1750Zn0Fe0	-39511.4	Translation
tl_term_b2608_1_rib_cplx	Translation termination complex b2608 1 ribosomes	C99812H138762N35191O50977S291P5199Mg175Zn0Fe0	-4437.14	Translation
tl_term_b2608_1_rib_cplx2	Translation termination complex b2608 1 ribosomes 2 (RF2_mono)	C99863H138793N35173O51010S289P5199Mg175Zn0Fe0	-4446.14	Translation
tl_term_b2608_5_rib_cplx	Translation termination complex b2608 5 ribosomes	C478124H670190N167563O239549S1455P23799Mg875Zn0Fe0	-19985.7	Translation
tl_term_b2608_5_rib_cplx2	Translation termination complex b2608 5 ribosomes 2 (RF2_mono)	C478379H670345N167473O239714S1445P23799Mg875Zn0Fe0	-20030.7	Translation
tl_term_b2609_1_rib_cplx	Translation termination complex b2609 1 ribosomes	C96420H134763N33921O48726S282P4901Mg175Zn0Fe0	-4124.14	Translation
tl_term_b2609_1_rib_cplx2	Translation termination complex b2609 1 ribosomes 2 (RF2_mono)	C96471H134794N33903O48759S280P4901Mg175Zn0Fe0	-4133.14	Translation
tl_term_b2609_2_rib_cplx	Translation termination complex b2609 2 ribosomes	C190473H266845N66902O95705S564P9551Mg350Zn0Fe0	-7996.28	Translation
tl_term_b2609_2_rib_cplx2	Translation termination complex b2609 2 ribosomes 2 (RF2_mono)	C190575H266907N66866O95771S560P9551Mg350Zn0Fe0	-8014.28	Translation
tl_term_b2609_4_rib_cplx	Translation termination complex b2609 4 ribosomes	C378579H531009N132864O189663S1128P18851Mg700Zn0Fe0	-15740.56	Translation
tl_term_b2609_4_rib_cplx2	Translation termination complex b2609 4 ribosomes 2 (RF2_mono)	C378783H531133N132792O189795S1120P18851Mg700Zn0Fe0	-15776.56	Translation
tl_term_b2614_11_rib_cplx	Translation termination complex b2614 11 ribosomes	C1046158H1468922N366639O523031S3190P51746Mg1925Zn0Fe0	-43421.54	Translation
tl_term_b2614_11_rib_cplx2	Translation termination complex b2614 11 ribosomes 2 (RF2_mono)	C1046719H1469263N366441O523394S3168P51746Mg1925Zn0Fe0	-43520.54	Translation
tl_term_b2614_1_rib_cplx	Translation termination complex b2614 1 ribosomes	C100278H139362N35459O51301S290P5246Mg175Zn0Fe0	-4490.14	Translation
tl_term_b2614_1_rib_cplx2	Translation termination complex b2614 1 ribosomes 2 (RF2_mono)	C100329H139393N35441O51334S288P5246Mg175Zn0Fe0	-4499.14	Translation
tl_term_b2614_5_rib_cplx	Translation termination complex b2614 5 ribosomes	C478630H671186N167931O239993S1450P23846Mg875Zn0Fe0	-20062.7	Translation
tl_term_b2614_5_rib_cplx2	Translation termination complex b2614 5 ribosomes 2 (RF2_mono)	C478885H671341N167841O240158S1440P23846Mg875Zn0Fe0	-20107.7	Translation
tl_term_b2697_1_rib_cplx	Translation termination complex b2697 1 ribosomes	C122931H166421N44155O66548S309P7282Mg175Zn0Fe0	-6533.14	Translation
tl_term_b2697_1_rib_cplx2	Translation termination complex b2697 1 ribosomes 2 (RF2_mono)	C122982H166452N44137O66581S307P7282Mg175Zn0Fe0	-6542.14	Translation
tl_term_b2697_25_rib_cplx	Translation termination complex b2697 25 ribosomes	C2471355H3480149N861427O1222340S7725P118858Mg4375Zn0Fe0	-100112.5	Translation
tl_term_b2697_25_rib_cplx2	Translation termination complex b2697 25 ribosomes 2 (RF2_mono)	C2472630H3480924N860977O1223165S7675P118858Mg4375Zn0Fe0	-100337.5	Translation
tl_term_b2697_51_rib_cplx	Translation termination complex b2697 51 ribosomes	C5015481H7070021N1746805O2474448S15759P239732Mg8925Zn0Fe0	-201490.14	Translation
tl_term_b2697_51_rib_cplx2	Translation termination complex b2697 51 ribosomes 2 (RF2_mono)	C5018082H7071602N1745887O2476131S15657P239732Mg8925Zn0Fe0	-201949.14	Translation
tl_term_b2741_19_rib_cplx	Translation termination complex b2741 19 ribosomes	C1820297H2558469N637214O907184S5453P89345Mg3325Zn0Fe0	-75003.66	Translation
tl_term_b2741_19_rib_cplx2	Translation termination complex b2741 19 ribosomes 2 (RF2_mono)	C1821266H2559058N636872O907811S5415P89345Mg3325Zn0Fe0	-75174.66	Translation
tl_term_b2741_1_rib_cplx	Translation termination complex b2741 1 ribosomes	C104789H144795N37184O54308S287P5645Mg175Zn0Fe0	-4891.14	Translation
tl_term_b2741_1_rib_cplx2	Translation termination complex b2741 1 ribosomes 2 (RF2_mono)	C104840H144826N37166O54341S285P5645Mg175Zn0Fe0	-4900.14	Translation
tl_term_b2741_9_rib_cplx	Translation termination complex b2741 9 ribosomes	C867237H1217539N303864O433364S2583P42845Mg1575Zn0Fe0	-36052.26	Translation
tl_term_b2741_9_rib_cplx2	Translation termination complex b2741 9 ribosomes 2 (RF2_mono)	C867696H1217818N303702O433661S2565P42845Mg1575Zn0Fe0	-36133.26	Translation
tl_term_b2742_11_rib_cplx	Translation termination complex b2742 11 ribosomes	C1060395H1489018N371390O529622S3190P52303Mg1925Zn0Fe0	-43703.54	Translation
tl_term_b2742_11_rib_cplx2	Translation termination complex b2742 11 ribosomes 2 (RF2_mono)	C1060956H1489359N371192O529985S3168P52303Mg1925Zn0Fe0	-43802.54	Translation
tl_term_b2742_1_rib_cplx	Translation termination complex b2742 1 ribosomes	C106275H146558N37760O55332S290P5793Mg175Zn0Fe0	-5012.14	Translation
tl_term_b2742_1_rib_cplx2	Translation termination complex b2742 1 ribosomes 2 (RF2_mono)	C106326H146589N37742O55365S288P5793Mg175Zn0Fe0	-5021.14	Translation
tl_term_b2742_22_rib_cplx	Translation termination complex b2742 22 ribosomes	C2109927H2965724N738383O1051341S6380P103464Mg3850Zn0Fe0	-86264.08	Translation
tl_term_b2742_22_rib_cplx2	Translation termination complex b2742 22 ribosomes 2 (RF2_mono)	C2111049H2966406N737987O1052067S6336P103464Mg3850Zn0Fe0	-86462.08	Translation
tl_term_b2745_10_rib_cplx	Translation termination complex b2745 10 ribosomes	C963902H1352931N337564O481005S2900P47552Mg1750Zn0Fe0	-39854.4	Translation
tl_term_b2745_10_rib_cplx2	Translation termination complex b2745 10 ribosomes 2 (RF2_mono)	C964412H1353241N337384O481335S2880P47552Mg1750Zn0Fe0	-39944.4	Translation
tl_term_b2745_1_rib_cplx	Translation termination complex b2745 1 ribosomes	C105410H145473N37405O54702S290P5702Mg175Zn0Fe0	-4933.14	Translation
tl_term_b2745_1_rib_cplx2	Translation termination complex b2745 1 ribosomes 2 (RF2_mono)	C105461H145504N37387O54735S288P5702Mg175Zn0Fe0	-4942.14	Translation
tl_term_b2745_20_rib_cplx	Translation termination complex b2745 20 ribosomes	C1917782H2694551N671074O954675S5800P94052Mg3500Zn0Fe0	-78655.8	Translation
tl_term_b2745_20_rib_cplx2	Translation termination complex b2745 20 ribosomes 2 (RF2_mono)	C1918802H2695171N670714O955335S5760P94052Mg3500Zn0Fe0	-78835.8	Translation
tl_term_b2779_12_rib_cplx	Translation termination complex b2779 12 ribosomes	C1160239H1629751N405782O579031S3540P57099Mg2100Zn0Fe0	-47969.68	Translation
tl_term_b2779_12_rib_cplx2	Translation termination complex b2779 12 ribosomes 2 (RF2_mono)	C1160851H1630123N405566O579427S3516P57099Mg2100Zn0Fe0	-48077.68	Translation

tl_term_b2779_1_rib_cplx	Translation termination complex b2779 1 ribosomes	C108034H148645N38382O56531S295P5949Mg175Zn0Fe0	-5189.14	Translation
tl_term_b2779_1_rib_cplx2	Translation termination complex b2779 1 ribosomes 2 (RF2_mono)	C108085H148676N38364O56564S293P5949Mg175Zn0Fe0	-5198.14	Translation
tl_term_b2779_25_rib_cplx	Translation termination complex b2779 25 ribosomes	C2403754H3380149N839982O1196531S7375P117549Mg4375Zn0Fe0	-98528.5	Translation
tl_term_b2779_25_rib_cplx2	Translation termination complex b2779 25 ribosomes 2 (RF2_mono)	C2405029H3380924N839532O1197356S7325P117549Mg4375Zn0Fe0	-98753.5	Translation
tl_term_b2780_16_rib_cplx	Translation termination complex b2780 16 ribosomes	C1556896H2188717N543664O774201S4848P76040Mg2800Zn0Fe0	-63899.24	Translation
tl_term_b2780_16_rib_cplx2	Translation termination complex b2780 16 ribosomes 2 (RF2_mono)	C1557712H2189213N543376O774729S4816P76040Mg2800Zn0Fe0	-64043.24	Translation
tl_term_b2780_1_rib_cplx	Translation termination complex b2780 1 ribosomes	C111916H153322N39799O59136S303P6290Mg175Zn0Fe0	-5532.14	Translation
tl_term_b2780_1_rib_cplx2	Translation termination complex b2780 1 ribosomes 2 (RF2_mono)	C111967H153353N39781O59169S301P6290Mg175Zn0Fe0	-5541.14	Translation
tl_term_b2780_32_rib_cplx	Translation termination complex b2780 32 ribosomes	C3098208H4359805N1081120O1536937S9696P150440Mg5600Zn0Fe0	-126157.48	Translation
tl_term_b2780_32_rib_cplx2	Translation termination complex b2780 32 ribosomes 2 (RF2_mono)	C3099840H4360797N1080544O1537993S9632P150440Mg5600Zn0Fe0	-126445.48	Translation
tl_term_b2785_12_rib_cplx	Translation termination complex b2785 12 ribosomes	C1162286H1632510N406281O579403S3588P57104Mg2100Zn0Fe0	-47890.68	Translation
tl_term_b2785_1_rib_cplx	Translation termination complex b2785 1 ribosomes	C108222H148896N38408O56617S299P5954Mg175Zn0Fe0	-5187.14	Translation
tl_term_b2785_25_rib_cplx	Translation termination complex b2785 25 ribosomes	C2407998H3385872N841040O1197241S7475P117554Mg4375Zn0Fe0	-98358.5	Translation
tl_term_b2790_1_rib_cplx	Translation termination complex b2790 1 ribosomes	C98786H137491N34820O50326S285P5112Mg175Zn0Fe0	-4356.14	Translation
tl_term_b2790_4_rib_cplx	Translation termination complex b2790 4 ribosomes	C382262H535429N134084O191854S1140P19098Mg700Zn0Fe0	-16071.56	Translation
tl_term_b2790_8_rib_cplx	Translation termination complex b2790 8 ribosomes	C760230H1066013N266436O380558S2280P37746Mg1400Zn0Fe0	-31692.12	Translation
tl_term_b2791_15_rib_cplx	Translation termination complex b2791 15 ribosomes	C1433679H2012593N502433O715296S4320P70712Mg2625Zn0Fe0	-59165.1	Translation
tl_term_b2791_15_rib_cplx2	Translation termination complex b2791 15 ribosomes 2 (RF2_mono)	C1434444H2013058N502163O715791S4290P70712Mg2625Zn0Fe0	-59300.1	Translation
tl_term_b2791_1_rib_cplx	Translation termination complex b2791 1 ribosomes	C102531H142039N36289O52788S288P5444Mg175Zn0Fe0	-4675.14	Translation
tl_term_b2791_1_rib_cplx2	Translation termination complex b2791 1 ribosomes 2 (RF2_mono)	C102582H142070N36271O52821S286P5444Mg175Zn0Fe0	-4684.14	Translation
tl_term_b2791_7_rib_cplx	Translation termination complex b2791 7 ribosomes	C673023H943705N236065O336720S2016P33416Mg1225Zn0Fe0	-28027.98	Translation
tl_term_b2791_7_rib_cplx2	Translation termination complex b2791 7 ribosomes 2 (RF2_mono)	C673380H943922N235939O336951S2002P33416Mg1225Zn0Fe0	-28090.98	Translation
tl_term_b2792_1_rib_cplx	Translation termination complex b2792 1 ribosomes	C97524H136025N34324O49461S288P4999Mg175Zn0Fe0	-4237.14	Translation
tl_term_b2792_1_rib_cplx2	Translation termination complex b2792 1 ribosomes 2 (RF2_mono)	C97575H136056N34306O49494S286P4999Mg175Zn0Fe0	-4246.14	Translation
tl_term_b2792_3_rib_cplx	Translation termination complex b2792 3 ribosomes	C286304H400971N100494O143747S864P14333Mg525Zn0Fe0	-12045.42	Translation
tl_term_b2792_3_rib_cplx2	Translation termination complex b2792 3 ribosomes 2 (RF2_mono)	C286457H401064N100440O143846S858P14333Mg525Zn0Fe0	-12072.42	Translation
tl_term_b2792_6_rib_cplx	Translation termination complex b2792 6 ribosomes	C569474H798390N199749O285176S1728P28334Mg1050Zn0Fe0	-23757.84	Translation
tl_term_b2792_6_rib_cplx2	Translation termination complex b2792 6 ribosomes 2 (RF2_mono)	C569780H798576N199641O285374S1716P28334Mg1050Zn0Fe0	-23811.84	Translation
tl_term_b2794_16_rib_cplx	Translation termination complex b2794 16 ribosomes	C1529364H2147254N535219O762648S4624P75235Mg2800Zn0Fe0	-62982.24	Translation
tl_term_b2794_16_rib_cplx2	Translation termination complex b2794 16 ribosomes 2 (RF2_mono)	C1530180H2147750N534931O763176S4592P75235Mg2800Zn0Fe0	-63126.24	Translation
tl_term_b2794_1_rib_cplx	Translation termination complex b2794 1 ribosomes	C103149H142759N36439O53238S289P5500Mg175Zn0Fe0	-4735.14	Translation
tl_term_b2794_1_rib_cplx2	Translation termination complex b2794 1 ribosomes 2 (RF2_mono)	C103200H142790N36421O53271S287P5500Mg175Zn0Fe0	-4744.14	Translation
tl_term_b2794_8_rib_cplx	Translation termination complex b2794 8 ribosomes	C768716H1078190N269203O384296S2312P38043Mg1400Zn0Fe0	-31917.12	Translation
tl_term_b2794_8_rib_cplx2	Translation termination complex b2794 8 ribosomes 2 (RF2_mono)	C769124H1078438N269059O384560S2296P38043Mg1400Zn0Fe0	-31989.12	Translation
tl_term_b2890_14_rib_cplx	Translation termination complex b2890 14 ribosomes	C1361410H1912285N475562O677533S4214P66618Mg2450Zn0Fe0	-56120.96	Translation
tl_term_b2890_14_rib_cplx2	Translation termination complex b2890 14 ribosomes 2 (RF2_mono)	C1362124H1912719N475310O677995S4186P66618Mg2450Zn0Fe0	-56246.96	Translation
tl_term_b2890_1_rib_cplx	Translation termination complex b2890 1 ribosomes	C110654H151786N39334O58226S301P6168Mg175Zn0Fe0	-5419.14	Translation
tl_term_b2890_1_rib_cplx2	Translation termination complex b2890 1 ribosomes 2 (RF2_mono)	C110705H151817N39316O58259S299P6168Mg175Zn0Fe0	-5428.14	Translation
tl_term_b2890_29_rib_cplx	Translation termination complex b2890 29 ribosomes	C2804590H3943630N978902O1392118S8729P136368Mg5075Zn0Fe0	-114623.06	Translation
tl_term_b2890_29_rib_cplx2	Translation termination complex b2890 29 ribosomes 2 (RF2_mono)	C2806069H3944529N978380O1393075S8671P136368Mg5075Zn0Fe0	-114884.06	Translation
tl_term_b2891_10_rib_cplx	Translation termination complex b2891 10 ribosomes	C965892H1355276N338315O482967S2940P47711Mg1750Zn0Fe0	-40253.4	Translation
tl_term_b2891_1_rib_cplx	Translation termination complex b2891 1 ribosomes	C106032H146207N37679O55170S294P5762Mg175Zn0Fe0	-5017.14	Translation
tl_term_b2891_21_rib_cplx	Translation termination complex b2891 21 ribosomes	C2016832H2833027N705759O1005830S6174P98982Mg3675Zn0Fe0	-83319.94	Translation
tl_term_b2892_16_rib_cplx	Translation termination complex b2892 16 ribosomes	C1560983H2194031N544912O775859S4800P76150Mg2800Zn0Fe0	-64185.24	Translation
tl_term_b2892_1_rib_cplx	Translation termination complex b2892 1 ribosomes	C113033H154646N40252O59894S300P6385Mg175Zn0Fe0	-5638.14	Translation
tl_term_b2892_33_rib_cplx	Translation termination complex b2892 33 ribosomes	C3201993H4505334N1116860O1587286S9900P155217Mg5775Zn0Fe0	-130538.62	Translation
tl_term_b2893_13_rib_cplx	Translation termination complex b2893 13 ribosomes	C1239112H1739791N433623O618675S3874P61163Mg2275Zn0Fe0	-51155.82	Translation

tl_term_b2893_13_rib_cplx2	Translation termination complex b2893 13 ribosomes 2 (RF2_mono)	C1239775H1740194N433389O619104S3848P61163Mg2275Zn0Fe0	-51272.82	Translation
tl_term_b2893_1_rib_cplx	Translation termination complex b2893 1 ribosomes	C101584H140899N35895O52155S298P5363Mg175Zn0Fe0	-4594.14	Translation
tl_term_b2893_1_rib_cplx2	Translation termination complex b2893 1 ribosomes 2 (RF2_mono)	C101635H140930N35877O52188S296P5363Mg175Zn0Fe0	-4603.14	Translation
tl_term_b2893_6_rib_cplx	Translation termination complex b2893 6 ribosomes	C575554H807104N201615O288205S1788P28613Mg1050Zn0Fe0	-23994.84	Translation
tl_term_b2893_6_rib_cplx2	Translation termination complex b2893 6 ribosomes 2 (RF2_mono)	C575860H807290N201507O288403S1776P28613Mg1050Zn0Fe0	-24048.84	Translation
tl_term_b2946_14_rib_cplx	Translation termination complex b2946 14 ribosomes	C1334543H1874688N467499O666188S4088P65834Mg2450Zn0Fe0	-55056.96	Translation
tl_term_b2946_14_rib_cplx2	Translation termination complex b2946 14 ribosomes 2 (RF2_mono)	C1335257H1875122N467247O666650S4060P65834Mg2450Zn0Fe0	-55182.96	Translation
tl_term_b2946_1_rib_cplx	Translation termination complex b2946 1 ribosomes	C101792H141229N35977O52341S292P5384Mg175Zn0Fe0	-4615.14	Translation
tl_term_b2946_1_rib_cplx2	Translation termination complex b2946 1 ribosomes 2 (RF2_mono)	C101843H141260N35959O52374S290P5384Mg175Zn0Fe0	-4624.14	Translation
tl_term_b2946_7_rib_cplx	Translation termination complex b2946 7 ribosomes	C670754H941287N235141O335655S2044P33284Mg1225Zn0Fe0	-27895.98	Translation
tl_term_b2946_7_rib_cplx2	Translation termination complex b2946 7 ribosomes 2 (RF2_mono)	C671111H941504N235015O335886S2030P33284Mg1225Zn0Fe0	-27958.98	Translation
tl_term_b2947_18_rib_cplx	Translation termination complex b2947 18 ribosomes	C1723062H2420817N602522O858493S5328P84633Mg3150Zn0Fe0	-70902.52	Translation
tl_term_b2947_18_rib_cplx2	Translation termination complex b2947 18 ribosomes 2 (RF2_mono)	C1723980H2421375N602198O859087S5292P84633Mg3150Zn0Fe0	-71064.52	Translation
tl_term_b2947_1_rib_cplx	Translation termination complex b2947 1 ribosomes	C104288H144177N36932O53951S296P5600Mg175Zn0Fe0	-4838.14	Translation
tl_term_b2947_1_rib_cplx2	Translation termination complex b2947 1 ribosomes 2 (RF2_mono)	C104339H144208N36914O53984S294P5600Mg175Zn0Fe0	-4847.14	Translation
tl_term_b2947_9_rib_cplx	Translation termination complex b2947 9 ribosomes	C866064H1215537N303092O432559S2664P42792Mg1575Zn0Fe0	-35927.26	Translation
tl_term_b2947_9_rib_cplx2	Translation termination complex b2947 9 ribosomes 2 (RF2_mono)	C866523H1215816N302930O432856S2646P42792Mg1575Zn0Fe0	-36008.26	Translation
tl_term_b2959_1_rib_cplx	Translation termination complex b2959 1 ribosomes	C97359H135830N34294O49306S287P4977Mg175Zn0Fe0	-4212.14	Translation
tl_term_b2959_1_rib_cplx2	Translation termination complex b2959 1 ribosomes 2 (RF2_mono)	C97410H135861N34276O49339S285P4977Mg175Zn0Fe0	-4221.14	Translation
tl_term_b2959_3_rib_cplx	Translation termination complex b2959 3 ribosomes	C285831H400460N100314O143398S861P14279Mg525Zn0Fe0	-11982.42	Translation
tl_term_b2959_3_rib_cplx2	Translation termination complex b2959 3 ribosomes 2 (RF2_mono)	C285984H400553N100260O143497S855P14279Mg525Zn0Fe0	-12009.42	Translation
tl_term_b2959_6_rib_cplx	Translation termination complex b2959 6 ribosomes	C568539H797405N199344O284536S1722P28232Mg1050Zn0Fe0	-23637.84	Translation
tl_term_b2959_6_rib_cplx2	Translation termination complex b2959 6 ribosomes 2 (RF2_mono)	C568845H797591N199236O284734S1710P28232Mg1050Zn0Fe0	-23691.84	Translation
tl_term_b2960_14_rib_cplx	Translation termination complex b2960 14 ribosomes	C1335040H1874171N467490O666022S4144P65822Mg2450Zn0Fe0	-55058.96	Translation
tl_term_b2960_14_rib_cplx2	Translation termination complex b2960 14 ribosomes 2 (RF2_mono)	C1335754H1874605N467238O666484S4116P65822Mg2450Zn0Fe0	-55184.96	Translation
tl_term_b2960_1_rib_cplx	Translation termination complex b2960 1 ribosomes	C101730H141063N35942O52266S296P5372Mg175Zn0Fe0	-4604.14	Translation
tl_term_b2960_1_rib_cplx2	Translation termination complex b2960 1 ribosomes 2 (RF2_mono)	C101781H141094N35924O52299S294P5372Mg175Zn0Fe0	-4613.14	Translation
tl_term_b2960_7_rib_cplx	Translation termination complex b2960 7 ribosomes	C670950H940959N235118O335538S2072P33272Mg1225Zn0Fe0	-27890.98	Translation
tl_term_b2960_7_rib_cplx2	Translation termination complex b2960 7 ribosomes 2 (RF2_mono)	C671307H941176N234992O335769S2058P33272Mg1225Zn0Fe0	-27953.98	Translation
tl_term_b3065_1_rib_cplx	Translation termination complex b3065 1 ribosomes	C96174H134503N33849O48524S285P4877Mg175Zn0Fe0	-4091.14	Translation
tl_term_b3065_1_rib_cplx2	Translation termination complex b3065 1 ribosomes 2 (RF2_mono)	C96225H134534N33831O48557S283P4877Mg175Zn0Fe0	-4100.14	Translation
tl_term_b3065_2_rib_cplx	Translation termination complex b3065 2 ribosomes	C190288H266674N66863O95543S570P9536Mg350Zn0Fe0	-7963.28	Translation
tl_term_b3065_2_rib_cplx2	Translation termination complex b3065 2 ribosomes 2 (RF2_mono)	C190390H266736N66827O95609S566P9536Mg350Zn0Fe0	-7981.28	Translation
tl_term_b3065_4_rib_cplx	Translation termination complex b3065 4 ribosomes	C378516H531016N132891O189581S1140P18854Mg700Zn0Fe0	-15707.56	Translation
tl_term_b3065_4_rib_cplx2	Translation termination complex b3065 4 ribosomes 2 (RF2_mono)	C378720H531140N132819O189713S1132P18854Mg700Zn0Fe0	-15743.56	Translation
tl_term_b3066_17_rib_cplx	Translation termination complex b3066 17 ribosomes	C1658018H2330847N579040O823707S5185P80796Mg2975Zn0Fe0	-67862.38	Translation
tl_term_b3066_1_rib_cplx	Translation termination complex b3066 1 ribosomes	C113170H154815N40320O59915S305P6396Mg175Zn0Fe0	-5636.14	Translation
tl_term_b3066_34_rib_cplx	Translation termination complex b3066 34 ribosomes	C3299419H4642881N1151430O1635236S10370P159846Mg5950Zn0Fe0	-133977.76	Translation
tl_term_b3067_18_rib_cplx	Translation termination complex b3067 18 ribosomes	C1758251H2473425N613944O874369S5580P85562Mg3150Zn0Fe0	-72461.52	Translation
tl_term_b3067_18_rib_cplx2	Translation termination complex b3067 18 ribosomes 2 (RF2_mono)	C1759169H2473983N613620O874963S5544P85562Mg3150Zn0Fe0	-72623.52	Translation
tl_term_b3067_1_rib_cplx	Translation termination complex b3067 1 ribosomes	C114283H156206N40874O60630S310P6495Mg175Zn0Fe0	-5768.14	Translation
tl_term_b3067_1_rib_cplx2	Translation termination complex b3067 1 ribosomes 2 (RF2_mono)	C114334H156237N40856O60663S308P6495Mg175Zn0Fe0	-5777.14	Translation
tl_term_b3067_36_rib_cplx	Translation termination complex b3067 36 ribosomes	C3498923H4926951N1220724O1735975S11160P169280Mg6300Zn0Fe0	-143078.04	Translation
tl_term_b3067_36_rib_cplx2	Translation termination complex b3067 36 ribosomes 2 (RF2_mono)	C3500759H4928067N1220076O1737163S11088P169280Mg6300Zn0Fe0	-143402.04	Translation
tl_term_b3124_12_rib_cplx	Translation termination complex b3124 12 ribosomes	C1157195H1625390N405113O577692S3564P57003Mg2100Zn0Fe0	-47885.68	Translation
tl_term_b3124_1_rib_cplx	Translation termination complex b3124 1 ribosomes	C107179H147573N38131O55995S297P5875Mg175Zn0Fe0	-5116.14	Translation
tl_term_b3124_24_rib_cplx	Translation termination complex b3124 24 ribosomes	C2302667H3237554N805457O1146816S7128P112779Mg4200Zn0Fe0	-94543.36	Translation

tl_term_b3125_17_rib_cplx	Translation termination complex b3125 17 ribosomes	C1623652H2281758N568262O809936S5100P79958Mg2975Zn0Fe0	-66905.38	Translation
tl_term_b3125_17_rib_cplx2	Translation termination complex b3125 17 ribosomes 2 (RF2_mono)	C1624519H2282285N567956O810497S5066P79958Mg2975Zn0Fe0	-67058.38	Translation
tl_term_b3125_1_rib_cplx	Translation termination complex b3125 1 ribosomes	C103524H143262N36694O53488S300P5542Mg175Zn0Fe0	-4775.14	Translation
tl_term_b3125_1_rib_cplx2	Translation termination complex b3125 1 ribosomes 2 (RF2_mono)	C103575H143293N36676O53521S298P5542Mg175Zn0Fe0	-4784.14	Translation
tl_term_b3125_8_rib_cplx	Translation termination complex b3125 8 ribosomes	C768580H1078854N269255O384434S2400P38099Mg1400Zn0Fe0	-31957.12	Translation
tl_term_b3125_8_rib_cplx2	Translation termination complex b3125 8 ribosomes 2 (RF2_mono)	C768988H1079102N269111O384698S2384P38099Mg1400Zn0Fe0	-32029.12	Translation
tl_term_b3126_15_rib_cplx	Translation termination complex b3126 15 ribosomes	C1430659H2008657N500618O713890S4290P70521Mg2625Zn0Fe0	-59019.1	Translation
tl_term_b3126_15_rib_cplx2	Translation termination complex b3126 15 ribosomes 2 (RF2_mono)	C1431424H2009122N500348O714385S4260P70521Mg2625Zn0Fe0	-59154.1	Translation
tl_term_b3126_1_rib_cplx	Translation termination complex b3126 1 ribosomes	C102213H141659N36084O52628S286P5421Mg175Zn0Fe0	-4655.14	Translation
tl_term_b3126_1_rib_cplx2	Translation termination complex b3126 1 ribosomes 2 (RF2_mono)	C102264H141690N36066O52661S284P5421Mg175Zn0Fe0	-4664.14	Translation
tl_term_b3126_7_rib_cplx	Translation termination complex b3126 7 ribosomes	C671547H941801N235170O336026S2002P33321Mg1225Zn0Fe0	-27953.98	Translation
tl_term_b3126_7_rib_cplx2	Translation termination complex b3126 7 ribosomes 2 (RF2_mono)	C671904H942018N235044O336257S1988P33321Mg1225Zn0Fe0	-28016.98	Translation
tl_term_b3127_13_rib_cplx	Translation termination complex b3127 13 ribosomes	C1260229H1768937N438937O626309S3978P61787Mg2275Zn0Fe0	-51649.82	Translation
tl_term_b3127_13_rib_cplx2	Translation termination complex b3127 13 ribosomes 2 (RF2_mono)	C1260892H1769340N438703O626738S3952P61787Mg2275Zn0Fe0	-51766.82	Translation
tl_term_b3127_1_rib_cplx	Translation termination complex b3127 1 ribosomes	C108601H149273N38245O56921S306P5987Mg175Zn0Fe0	-5208.14	Translation
tl_term_b3127_1_rib_cplx2	Translation termination complex b3127 1 ribosomes 2 (RF2_mono)	C108652H149304N38227O56954S304P5987Mg175Zn0Fe0	-5217.14	Translation
tl_term_b3127_26_rib_cplx	Translation termination complex b3127 26 ribosomes	C2507826H3523573N873020O1243146S7956P122237Mg4550Zn0Fe0	-101961.64	Translation
tl_term_b3127_26_rib_cplx2	Translation termination complex b3127 26 ribosomes 2 (RF2_mono)	C2509152H3524379N872552O1244004S7904P122237Mg4550Zn0Fe0	-102195.64	Translation
tl_term_b3164_1_rib_cplx	Translation termination complex b3164 1 ribosomes	C118147H160874N42286O63337S305P6855Mg175Zn0Fe0	-6100.14	Translation
tl_term_b3164_1_rib_cplx2	Translation termination complex b3164 1 ribosomes 2 (RF2_mono)	C118198H160905N42268O63370S303P6855Mg175Zn0Fe0	-6109.14	Translation
tl_term_b3164_21_rib_cplx	Translation termination complex b3164 21 ribosomes	C2061067H2903034N719266O1022177S6405P99855Mg3675Zn0Fe0	-83982.94	Translation
tl_term_b3164_21_rib_cplx2	Translation termination complex b3164 21 ribosomes 2 (RF2_mono)	C2062138H2903685N718888O1022870S6363P99855Mg3675Zn0Fe0	-84171.94	Translation
tl_term_b3164_43_rib_cplx	Translation termination complex b3164 43 ribosomes	C4198279H5919410N1463944O2076901S13115P202155Mg7525Zn0Fe0	-169654.02	Translation
tl_term_b3164_43_rib_cplx2	Translation termination complex b3164 43 ribosomes 2 (RF2_mono)	C4200472H5920743N1463170O2078320S13029P202155Mg7525Zn0Fe0	-170041.02	Translation
tl_term_b3165_1_rib_cplx	Translation termination complex b3165 1 ribosomes	C96670H135095N34027O48876S284P4921Mg175Zn0Fe0	-4142.14	Translation
tl_term_b3165_1_rib_cplx2	Translation termination complex b3165 1 ribosomes 2 (RF2_mono)	C96721H135126N34009O48909S282P4921Mg175Zn0Fe0	-4151.14	Translation
tl_term_b3165_2_rib_cplx	Translation termination complex b3165 2 ribosomes	C190775H267278N67033O95873S568P9572Mg350Zn0Fe0	-8013.28	Translation
tl_term_b3165_2_rib_cplx2	Translation termination complex b3165 2 ribosomes 2 (RF2_mono)	C190877H267340N66997O95939S564P9572Mg350Zn0Fe0	-8031.28	Translation
tl_term_b3165_5_rib_cplx	Translation termination complex b3165 5 ribosomes	C473090H663827N166051O236864S1420P23525Mg875Zn0Fe0	-19626.7	Translation
tl_term_b3165_5_rib_cplx2	Translation termination complex b3165 5 ribosomes 2 (RF2_mono)	C473345H663982N165961O237029S1410P23525Mg875Zn0Fe0	-19671.7	Translation
tl_term_b3166_18_rib_cplx	Translation termination complex b3166 18 ribosomes	C1722437H2420357N602830O858647S5256P84644Mg3150Zn0Fe0	-70841.52	Translation
tl_term_b3166_18_rib_cplx2	Translation termination complex b3166 18 ribosomes 2 (RF2_mono)	C1723355H2420915N602506O859241S5220P84644Mg3150Zn0Fe0	-71003.52	Translation
tl_term_b3166_1_rib_cplx	Translation termination complex b3166 1 ribosomes	C104173H144057N36866O53952S292P5594Mg175Zn0Fe0	-4828.14	Translation
tl_term_b3166_1_rib_cplx2	Translation termination complex b3166 1 ribosomes 2 (RF2_mono)	C104224H144088N36848O53985S290P5594Mg175Zn0Fe0	-4837.14	Translation
tl_term_b3166_9_rib_cplx	Translation termination complex b3166 9 ribosomes	C865709H1215257N303202O432632S2628P42794Mg1575Zn0Fe0	-35893.26	Translation
tl_term_b3166_9_rib_cplx2	Translation termination complex b3166 9 ribosomes 2 (RF2_mono)	C866168H1215536N303040O432929S2610P42794Mg1575Zn0Fe0	-35974.26	Translation
tl_term_b3167_1_rib_cplx	Translation termination complex b3167 1 ribosomes	C98161H136863N34605O49867S290P5053Mg175Zn0Fe0	-4282.14	Translation
tl_term_b3167_1_rib_cplx2	Translation termination complex b3167 1 ribosomes 2 (RF2_mono)	C98212H136894N34587O49900S288P5053Mg175Zn0Fe0	-4291.14	Translation
tl_term_b3167_3_rib_cplx	Translation termination complex b3167 3 ribosomes	C286807H401907N100687O144021S870P14355Mg525Zn0Fe0	-12040.42	Translation
tl_term_b3167_3_rib_cplx2	Translation termination complex b3167 3 ribosomes 2 (RF2_mono)	C286960H402000N100633O144120S864P14355Mg525Zn0Fe0	-12067.42	Translation
tl_term_b3167_7_rib_cplx	Translation termination complex b3167 7 ribosomes	C664099H931995N232851O332329S2030P32959Mg1225Zn0Fe0	-27556.98	Translation
tl_term_b3167_7_rib_cplx2	Translation termination complex b3167 7 ribosomes 2 (RF2_mono)	C664456H932212N232725O332560S2016P32959Mg1225Zn0Fe0	-27619.98	Translation
tl_term_b3168_1_rib_cplx	Translation termination complex b3168 1 ribosomes	C123395H167195N44643O66719S305P7323Mg175Zn0Fe0	-6566.14	Translation
tl_term_b3168_1_rib_cplx2	Translation termination complex b3168 1 ribosomes 2 (RF2_mono)	C123446H167226N44625O66752S303P7323Mg175Zn0Fe0	-6575.14	Translation
tl_term_b3168_26_rib_cplx	Translation termination complex b3168 26 ribosomes	C2569070H3624845N897568O1272269S7930P123573Mg4550Zn0Fe0	-103869.64	Translation
tl_term_b3168_26_rib_cplx2	Translation termination complex b3168 26 ribosomes 2 (RF2_mono)	C2570396H3625651N897100O1273127S7878P123573Mg4550Zn0Fe0	-104103.64	Translation
tl_term_b3168_52_rib_cplx	Translation termination complex b3168 52 ribosomes	C5112572H7220801N1784610O2526041S15860P244473Mg9100Zn0Fe0	-205065.28	Translation

tl_term_b3168_52_rib_cplx2	Translation termination complex b3168 52 ribosomes 2 (RF2_mono)	C5115224H7222413N1783674O2527757S15756P244473Mg9100Zn0Fe0	-205533.28	Translation
tl_term_b3169_14_rib_cplx	Translation termination complex b3169 14 ribosomes	C1358814H1909567N475096O677306S4116P66588Mg2450Zn0Fe0	-56342.96	Translation
tl_term_b3169_14_rib_cplx2	Translation termination complex b3169 14 ribosomes 2 (RF2_mono)	C1359528H1910001N474844O677768S4088P66588Mg2450Zn0Fe0	-56468.96	Translation
tl_term_b3169_1_rib_cplx	Translation termination complex b3169 1 ribosomes	C110255H151304N39310O57986S294P6138Mg175Zn0Fe0	-5407.14	Translation
tl_term_b3169_1_rib_cplx2	Translation termination complex b3169 1 ribosomes 2 (RF2_mono)	C110306H151335N39292O58019S292P6138Mg175Zn0Fe0	-5416.14	Translation
tl_term_b3169_29_rib_cplx	Translation termination complex b3169 29 ribosomes	C2799459H3938332N977926O1391906S8526P136338Mg5075Zn0Fe0	-115115.06	Translation
tl_term_b3169_29_rib_cplx2	Translation termination complex b3169 29 ribosomes 2 (RF2_mono)	C2800938H3939231N977404O1392863S8468P136338Mg5075Zn0Fe0	-115376.06	Translation
tl_term_b3170_1_rib_cplx	Translation termination complex b3170 1 ribosomes	C98705H137467N34757O50269S288P5103Mg175Zn0Fe0	-4342.14	Translation
tl_term_b3170_1_rib_cplx2	Translation termination complex b3170 1 ribosomes 2 (RF2_mono)	C98756H137498N34739O50302S286P5103Mg175Zn0Fe0	-4351.14	Translation
tl_term_b3170_4_rib_cplx	Translation termination complex b3170 4 ribosomes	C381890H535273N133910O191545S1152P19053Mg700Zn0Fe0	-16006.56	Translation
tl_term_b3170_4_rib_cplx2	Translation termination complex b3170 4 ribosomes 2 (RF2_mono)	C382094H535397N133838O191677S1144P19053Mg700Zn0Fe0	-16042.56	Translation
tl_term_b3170_8_rib_cplx	Translation termination complex b3170 8 ribosomes	C759470H1065681N266114O379913S2304P37653Mg1400Zn0Fe0	-31559.12	Translation
tl_term_b3170_8_rib_cplx2	Translation termination complex b3170 8 ribosomes 2 (RF2_mono)	C759878H1065929N265970O380177S2288P37653Mg1400Zn0Fe0	-31631.12	Translation
tl_term_b3178_18_rib_cplx	Translation termination complex b3178 18 ribosomes	C1760388H2476716N614537O874010S5562P85635Mg3150Zn0Fe0	-71868.52	Translation
tl_term_b3178_18_rib_cplx2	Translation termination complex b3178 18 ribosomes 2 (RF2_mono)	C1761306H2477274N614213O874604S5526P85635Mg3150Zn0Fe0	-72030.52	Translation
tl_term_b3178_1_rib_cplx	Translation termination complex b3178 1 ribosomes	C115213H157287N41144O61325S309P6585Mg175Zn0Fe0	-5821.14	Translation
tl_term_b3178_1_rib_cplx2	Translation termination complex b3178 1 ribosomes 2 (RF2_mono)	C115264H157318N41126O61358S307P6585Mg175Zn0Fe0	-5830.14	Translation
tl_term_b3178_37_rib_cplx	Translation termination complex b3178 37 ribosomes	C3599113H5069019N1255388O1782305S11433P173985Mg6475Zn0Fe0	-145686.18	Translation
tl_term_b3178_37_rib_cplx2	Translation termination complex b3178 37 ribosomes 2 (RF2_mono)	C3601000H5070166N1254722O1783526S11359P173985Mg6475Zn0Fe0	-146019.18	Translation
tl_term_b3179_12_rib_cplx	Translation termination complex b3179 12 ribosomes	C1142342H1604042N400205O570431S3516P56444Mg2100Zn0Fe0	-47086.68	Translation
tl_term_b3179_12_rib_cplx2	Translation termination complex b3179 12 ribosomes 2 (RF2_mono)	C1142954H1604414N399989O570827S3492P56444Mg2100Zn0Fe0	-47194.68	Translation
tl_term_b3179_1_rib_cplx	Translation termination complex b3179 1 ribosomes	C100697H139876N35544O51594S293P5283Mg175Zn0Fe0	-4504.14	Translation
tl_term_b3179_1_rib_cplx2	Translation termination complex b3179 1 ribosomes 2 (RF2_mono)	C100748H139907N35526O51627S291P5283Mg175Zn0Fe0	-4513.14	Translation
tl_term_b3179_6_rib_cplx	Translation termination complex b3179 6 ribosomes	C574172H805406N201299O287429S1758P28538Mg1050Zn0Fe0	-23859.84	Translation
tl_term_b3179_6_rib_cplx2	Translation termination complex b3179 6 ribosomes 2 (RF2_mono)	C574478H805592N201191O287627S1746P28538Mg1050Zn0Fe0	-23913.84	Translation
tl_term_b3181_1_rib_cplx	Translation termination complex b3181 1 ribosomes	C99074H137909N34955O50505S285P5140Mg175Zn0Fe0	-4379.14	Translation
tl_term_b3181_1_rib_cplx2	Translation termination complex b3181 1 ribosomes 2 (RF2_mono)	C99125H137940N34937O50538S283P5140Mg175Zn0Fe0	-4388.14	Translation
tl_term_b3181_4_rib_cplx	Translation termination complex b3181 4 ribosomes	C382628H536222N134279O192060S1140P19123Mg700Zn0Fe0	-16076.56	Translation
tl_term_b3181_4_rib_cplx2	Translation termination complex b3181 4 ribosomes 2 (RF2_mono)	C382832H536346N134207O192192S1132P19123Mg700Zn0Fe0	-16112.56	Translation
tl_term_b3181_9_rib_cplx	Translation termination complex b3181 9 ribosomes	C855218H1200077N299819O427985S2565P42428Mg1575Zn0Fe0	-35572.26	Translation
tl_term_b3181_9_rib_cplx2	Translation termination complex b3181 9 ribosomes 2 (RF2_mono)	C855677H1200356N299657O428282S2547P42428Mg1575Zn0Fe0	-35653.26	Translation
tl_term_b3185_1_rib_cplx	Translation termination complex b3185 1 ribosomes	C96504H134867N33981O48764S284P4908Mg175Zn0Fe0	-4125.14	Translation
tl_term_b3185_1_rib_cplx2	Translation termination complex b3185 1 ribosomes 2 (RF2_mono)	C96555H134898N33963O48797S282P4908Mg175Zn0Fe0	-4134.14	Translation
tl_term_b3185_2_rib_cplx	Translation termination complex b3185 2 ribosomes	C190545H266952N66963O95736S568P9558Mg350Zn0Fe0	-7991.28	Translation
tl_term_b3185_2_rib_cplx2	Translation termination complex b3185 2 ribosomes 2 (RF2_mono)	C190647H267014N66927O95802S564P9558Mg350Zn0Fe0	-8009.28	Translation
tl_term_b3185_5_rib_cplx	Translation termination complex b3185 5 ribosomes	C472668H663207N165909O236652S1420P23508Mg875Zn0Fe0	-19589.7	Translation
tl_term_b3185_5_rib_cplx2	Translation termination complex b3185 5 ribosomes 2 (RF2_mono)	C472923H663362N165819O236817S1410P23508Mg875Zn0Fe0	-19634.7	Translation
tl_term_b3186_1_rib_cplx	Translation termination complex b3186 1 ribosomes	C97144H135612N34217O49185S283P4964Mg175Zn0Fe0	-4186.14	Translation
tl_term_b3186_1_rib_cplx2	Translation termination complex b3186 1 ribosomes 2 (RF2_mono)	C97195H135643N34199O49218S281P4964Mg175Zn0Fe0	-4195.14	Translation
tl_term_b3186_3_rib_cplx	Translation termination complex b3186 3 ribosomes	C285470H400120N100237O143195S849P14264Mg525Zn0Fe0	-11928.42	Translation
tl_term_b3186_3_rib_cplx2	Translation termination complex b3186 3 ribosomes 2 (RF2_mono)	C285623H400213N100183O143294S843P14264Mg525Zn0Fe0	-11955.42	Translation
tl_term_b3186_6_rib_cplx	Translation termination complex b3186 6 ribosomes	C567959H796882N199267O284210S1698P28214Mg1050Zn0Fe0	-23541.84	Translation
tl_term_b3186_6_rib_cplx2	Translation termination complex b3186 6 ribosomes 2 (RF2_mono)	C568265H797068N199159O284408S1686P28214Mg1050Zn0Fe0	-23595.84	Translation
tl_term_b3201_14_rib_cplx	Translation termination complex b3201 14 ribosomes	C1335938H1876070N468131O667224S4018P65982Mg2450Zn0Fe0	-55260.96	Translation
tl_term_b3201_1_rib_cplx	Translation termination complex b3201 1 ribosomes	C101835H141272N35998O52376S287P5389Mg175Zn0Fe0	-4624.14	Translation
tl_term_b3201_7_rib_cplx	Translation termination complex b3201 7 ribosomes	C671421H941948N235444O336152S2009P33355Mg1225Zn0Fe0	-27994.98	Translation
tl_term_b3202_14_rib_cplx	Translation termination complex b3202 14 ribosomes	C1357897H1908024N474579O676787S4130P66536Mg2450Zn0Fe0	-56192.96	Translation

tl_term_b3202_1_rib_cplx	Translation termination complex b3202 1 ribosomes	C109676H150658N39014O57597S295P6086Mg175Zn0Fe0	-5348.14	Translation
tl_term_b3202_28_rib_cplx	Translation termination complex b3202 28 ribosomes	C2702135H3800572N943649O1343607S8260P131636Mg4900Zn0Fe0	-110948.92	Translation
tl_term_b3203_1_rib_cplx	Translation termination complex b3203 1 ribosomes	C96887H135306N34098O49005S284P4939Mg175Zn0Fe0	-4168.14	Translation
tl_term_b3203_1_rib_cplx2	Translation termination complex b3203 1 ribosomes 2 (RF2_mono)	C96938H135337N34080O49038S282P4939Mg175Zn0Fe0	-4177.14	Translation
tl_term_b3203_2_rib_cplx	Translation termination complex b3203 2 ribosomes	C191023H267506N67077O96026S568P9590Mg350Zn0Fe0	-8047.28	Translation
tl_term_b3203_2_rib_cplx2	Translation termination complex b3203 2 ribosomes 2 (RF2_mono)	C191125H267568N67041O96092S564P9590Mg350Zn0Fe0	-8065.28	Translation
tl_term_b3203_5_rib_cplx	Translation termination complex b3203 5 ribosomes	C473431H664106N166014O237089S1420P23543Mg875Zn0Fe0	-19684.7	Translation
tl_term_b3203_5_rib_cplx2	Translation termination complex b3203 5 ribosomes 2 (RF2_mono)	C473686H664261N165924O237254S1410P23543Mg875Zn0Fe0	-19729.7	Translation
tl_term_b3204_1_rib_cplx	Translation termination complex b3204 1 ribosomes	C99168H138049N34946O50563S284P5142Mg175Zn0Fe0	-4384.14	Translation
tl_term_b3204_4_rib_cplx	Translation termination complex b3204 4 ribosomes	C382614H536269N134120O191998S1136P19092Mg700Zn0Fe0	-16057.56	Translation
tl_term_b3204_9_rib_cplx	Translation termination complex b3204 9 ribosomes	C855024H1199969N299410O427723S2556P42342Mg1575Zn0Fe0	-35513.26	Translation
tl_term_b3205_16_rib_cplx	Translation termination complex b3205 16 ribosomes	C1529706H2148811N535464O762719S4704P75271Mg2800Zn0Fe0	-62938.24	Translation
tl_term_b3205_1_rib_cplx	Translation termination complex b3205 1 ribosomes	C103206H142921N36444O53294S294P5506Mg175Zn0Fe0	-4736.14	Translation
tl_term_b3205_8_rib_cplx	Translation termination complex b3205 8 ribosomes	C768906H1079003N269320O384359S2352P38063Mg1400Zn0Fe0	-31897.12	Translation
tl_term_b3206_1_rib_cplx	Translation termination complex b3206 1 ribosomes	C96655H135015N34002O48891S287P4920Mg175Zn0Fe0	-4158.14	Translation
tl_term_b3206_1_rib_cplx2	Translation termination complex b3206 1 ribosomes 2 (RF2_mono)	C96706H135046N33984O48924S285P4920Mg175Zn0Fe0	-4167.14	Translation
tl_term_b3206_2_rib_cplx	Translation termination complex b3206 2 ribosomes	C190743H267134N66969O95906S574P9571Mg350Zn0Fe0	-8046.28	Translation
tl_term_b3206_2_rib_cplx2	Translation termination complex b3206 2 ribosomes 2 (RF2_mono)	C190845H267196N66933O95972S570P9571Mg350Zn0Fe0	-8064.28	Translation
tl_term_b3206_5_rib_cplx	Translation termination complex b3206 5 ribosomes	C473007H663491N165870O236951S1435P23524Mg875Zn0Fe0	-19710.7	Translation
tl_term_b3206_5_rib_cplx2	Translation termination complex b3206 5 ribosomes 2 (RF2_mono)	C473262H663646N165780O237116S1425P23524Mg875Zn0Fe0	-19755.7	Translation
tl_term_b3230_1_rib_cplx	Translation termination complex b3230 1 ribosomes	C98042H136760N34549O49803S286P5044Mg175Zn0Fe0	-4256.14	Translation
tl_term_b3230_1_rib_cplx2	Translation termination complex b3230 1 ribosomes 2 (RF2_mono)	C98093H136791N34531O49836S284P5044Mg175Zn0Fe0	-4265.14	Translation
tl_term_b3230_3_rib_cplx	Translation termination complex b3230 3 ribosomes	C286658H401820N100689O143903S858P14346Mg525Zn0Fe0	-11980.42	Translation
tl_term_b3230_3_rib_cplx2	Translation termination complex b3230 3 ribosomes 2 (RF2_mono)	C286811H401913N100635O144002S852P14346Mg525Zn0Fe0	-12007.42	Translation
tl_term_b3230_7_rib_cplx	Translation termination complex b3230 7 ribosomes	C663890H931940N232969O332103S2002P32950Mg1225Zn0Fe0	-27428.98	Translation
tl_term_b3230_7_rib_cplx2	Translation termination complex b3230 7 ribosomes 2 (RF2_mono)	C664247H932157N232843O332334S1988P32950Mg1225Zn0Fe0	-27491.98	Translation
tl_term_b3231_1_rib_cplx	Translation termination complex b3231 1 ribosomes	C98469H137230N34726O50053S285P5082Mg175Zn0Fe0	-4301.14	Translation
tl_term_b3231_1_rib_cplx2	Translation termination complex b3231 1 ribosomes 2 (RF2_mono)	C98520H137261N34708O50086S283P5082Mg175Zn0Fe0	-4310.14	Translation
tl_term_b3231_4_rib_cplx	Translation termination complex b3231 4 ribosomes	C381609H535033N133948O191272S1140P19035Mg700Zn0Fe0	-15908.56	Translation
tl_term_b3231_4_rib_cplx2	Translation termination complex b3231 4 ribosomes 2 (RF2_mono)	C381813H535157N133876O191404S1132P19035Mg700Zn0Fe0	-15944.56	Translation
tl_term_b3231_8_rib_cplx	Translation termination complex b3231 8 ribosomes	C759129H1065437N266244O379564S2280P37639Mg1400Zn0Fe0	-31385.12	Translation
tl_term_b3231_8_rib_cplx2	Translation termination complex b3231 8 ribosomes 2 (RF2_mono)	C759537H1065685N266100O379828S2264P37639Mg1400Zn0Fe0	-31457.12	Translation
tl_term_b3247_14_rib_cplx	Translation termination complex b3247 14 ribosomes	C1359267H1910588N475327O676793S4214P66573Mg2450Zn0Fe0	-55949.96	Translation
tl_term_b3247_14_rib_cplx2	Translation termination complex b3247 14 ribosomes 2 (RF2_mono)	C1359981H1911022N475075O677255S4186P66573Mg2450Zn0Fe0	-56075.96	Translation
tl_term_b3247_1_rib_cplx	Translation termination complex b3247 1 ribosomes	C110006H151064N39151O57798S301P6110Mg175Zn0Fe0	-5352.14	Translation
tl_term_b3247_1_rib_cplx2	Translation termination complex b3247 1 ribosomes 2 (RF2_mono)	C110057H151095N39133O57831S299P6110Mg175Zn0Fe0	-5361.14	Translation
tl_term_b3247_28_rib_cplx	Translation termination complex b3247 28 ribosomes	C2704625H3805460N945055O1343403S8428P131687Mg4900Zn0Fe0	-110439.92	Translation
tl_term_b3247_28_rib_cplx2	Translation termination complex b3247 28 ribosomes 2 (RF2_mono)	C2706053H3806328N944551O1344327S8372P131687Mg4900Zn0Fe0	-110691.92	Translation
tl_term_b3248_11_rib_cplx	Translation termination complex b3248 11 ribosomes	C1046130H1468752N366644O522890S3146P51744Mg1925Zn0Fe0	-43298.54	Translation
tl_term_b3248_1_rib_cplx	Translation termination complex b3248 1 ribosomes	C100260H139342N35424O51310S286P5244Mg175Zn0Fe0	-4477.14	Translation
tl_term_b3248_5_rib_cplx	Translation termination complex b3248 5 ribosomes	C478608H671106N167912O239942S1430P23844Mg875Zn0Fe0	-20005.7	Translation
tl_term_b3249_1_rib_cplx	Translation termination complex b3249 1 ribosomes	C99161H138032N34825O50541S288P5137Mg175Zn0Fe0	-4359.14	Translation
tl_term_b3249_1_rib_cplx2	Translation termination complex b3249 1 ribosomes 2 (RF2_mono)	C99212H138063N34807O50574S286P5137Mg175Zn0Fe0	-4368.14	Translation
tl_term_b3249_4_rib_cplx	Translation termination complex b3249 4 ribosomes	C382790H536480N134011O191709S1152P19084Mg700Zn0Fe0	-15969.56	Translation
tl_term_b3249_4_rib_cplx2	Translation termination complex b3249 4 ribosomes 2 (RF2_mono)	C382994H536604N133939O191841S1144P19084Mg700Zn0Fe0	-16005.56	Translation
tl_term_b3249_9_rib_cplx	Translation termination complex b3249 9 ribosomes	C855505H1200560N299321O426989S2592P42329Mg1575Zn0Fe0	-35320.26	Translation
tl_term_b3249_9_rib_cplx2	Translation termination complex b3249 9 ribosomes 2 (RF2_mono)	C855964H1200839N299159O427286S2574P42329Mg1575Zn0Fe0	-35401.26	Translation

tl_term_b3250_10_rib_cplx	Translation termination complex b3250 10 ribosomes	C964721H1354716N337463O481881S2910P47594Mg1750Zn0Fe0	-39936.4	Translation
tl_term_b3250_1_rib_cplx	Translation termination complex b3250 1 ribosomes	C105914H146169N37493O55173S291P5753Mg175Zn0Fe0	-4988.14	Translation
tl_term_b3250_21_rib_cplx	Translation termination complex b3250 21 ribosomes	C2014374H2831829N704093O1003413S6111P98733Mg3675Zn0Fe0	-82650.94	Translation
tl_term_b3251_10_rib_cplx	Translation termination complex b3251 10 ribosomes	C962502H1351671N337050O480906S2980P47546Mg1750Zn0Fe0	-39918.4	Translation
tl_term_b3251_10_rib_cplx2	Translation termination complex b3251 10 ribosomes 2 (RF2_mono)	C963012H1351981N336870O481236S2960P47546Mg1750Zn0Fe0	-40008.4	Translation
tl_term_b3251_1_rib_cplx	Translation termination complex b3251 1 ribosomes	C105180H145275N37242O54702S298P5696Mg175Zn0Fe0	-4934.14	Translation
tl_term_b3251_1_rib_cplx2	Translation termination complex b3251 1 ribosomes 2 (RF2_mono)	C105231H145306N37224O54735S296P5696Mg175Zn0Fe0	-4943.14	Translation
tl_term_b3251_20_rib_cplx	Translation termination complex b3251 20 ribosomes	C1915082H2692111N670170O954466S5960P94046Mg3500Zn0Fe0	-78789.8	Translation
tl_term_b3251_20_rib_cplx2	Translation termination complex b3251 20 ribosomes 2 (RF2_mono)	C1916102H2692731N669810O955126S5920P94046Mg3500Zn0Fe0	-78969.8	Translation
tl_term_b3257_1_rib_cplx	Translation termination complex b3257 1 ribosomes	C96376H134660N33832O48714S286P4895Mg175Zn0Fe0	-4126.14	Translation
tl_term_b3257_2_rib_cplx	Translation termination complex b3257 2 ribosomes	C190454H266719N66786O95685S572P9545Mg350Zn0Fe0	-8006.28	Translation
tl_term_b3257_4_rib_cplx	Translation termination complex b3257 4 ribosomes	C378610H530837N132694O189627S1144P18845Mg700Zn0Fe0	-15766.56	Translation
tl_term_b3258_14_rib_cplx	Translation termination complex b3258 14 ribosomes	C1358583H1909684N473425O675055S4242P66541Mg2450Zn0Fe0	-55595.96	Translation
tl_term_b3258_14_rib_cplx2	Translation termination complex b3258 14 ribosomes 2 (RF2_mono)	C1359297H1910118N473173O675517S4214P66541Mg2450Zn0Fe0	-55721.96	Translation
tl_term_b3258_1_rib_cplx	Translation termination complex b3258 1 ribosomes	C109712H150758N38718O57711S303P6091Mg175Zn0Fe0	-5310.14	Translation
tl_term_b3258_1_rib_cplx2	Translation termination complex b3258 1 ribosomes 2 (RF2_mono)	C109763H150789N38700O57744S301P6091Mg175Zn0Fe0	-5319.14	Translation
tl_term_b3258_28_rib_cplx	Translation termination complex b3258 28 ribosomes	C2703521H3803912N941571O1339887S8484P131641Mg4900Zn0Fe0	-109749.92	Translation
tl_term_b3258_28_rib_cplx2	Translation termination complex b3258 28 ribosomes 2 (RF2_mono)	C2704949H3804780N941067O1340811S8428P131641Mg4900Zn0Fe0	-110001.92	Translation
tl_term_b3259_17_rib_cplx	Translation termination complex b3259 17 ribosomes	C1624624H2280980N568149O810415S4998P79932Mg2975Zn0Fe0	-67270.38	Translation
tl_term_b3259_17_rib_cplx2	Translation termination complex b3259 17 ribosomes 2 (RF2_mono)	C1625491H2281507N567843O810976S4964P79932Mg2975Zn0Fe0	-67423.38	Translation
tl_term_b3259_1_rib_cplx	Translation termination complex b3259 1 ribosomes	C103472H143124N36597O53471S294P5532Mg175Zn0Fe0	-4788.14	Translation
tl_term_b3259_1_rib_cplx2	Translation termination complex b3259 1 ribosomes 2 (RF2_mono)	C103523H143155N36579O53504S292P5532Mg175Zn0Fe0	-4797.14	Translation
tl_term_b3259_8_rib_cplx	Translation termination complex b3259 8 ribosomes	C768976H1078436N269151O384634S2352P38082Mg1400Zn0Fe0	-32124.12	Translation
tl_term_b3259_8_rib_cplx2	Translation termination complex b3259 8 ribosomes 2 (RF2_mono)	C769384H1078684N269007O384898S2336P38082Mg1400Zn0Fe0	-32196.12	Translation
tl_term_b3260_18_rib_cplx	Translation termination complex b3260 18 ribosomes	C1723338H2421253N603228O858710S5328P84668Mg3150Zn0Fe0	-70829.52	Translation
tl_term_b3260_18_rib_cplx2	Translation termination complex b3260 18 ribosomes 2 (RF2_mono)	C1724256H2421811N602904O859304S5292P84668Mg3150Zn0Fe0	-70991.52	Translation
tl_term_b3260_1_rib_cplx	Translation termination complex b3260 1 ribosomes	C104428H144341N36992O54083S296P5618Mg175Zn0Fe0	-4850.14	Translation
tl_term_b3260_1_rib_cplx2	Translation termination complex b3260 1 ribosomes 2 (RF2_mono)	C104479H144372N36974O54116S294P5618Mg175Zn0Fe0	-4859.14	Translation
tl_term_b3260_9_rib_cplx	Translation termination complex b3260 9 ribosomes	C866268H1215829N303456O432731S2664P42818Mg1575Zn0Fe0	-35899.26	Translation
tl_term_b3260_9_rib_cplx2	Translation termination complex b3260 9 ribosomes 2 (RF2_mono)	C866727H1216108N303294O433028S2646P42818Mg1575Zn0Fe0	-35980.26	Translation
tl_term_b3261_1_rib_cplx	Translation termination complex b3261 1 ribosomes	C96976H135431N34130O49081S288P4947Mg175Zn0Fe0	-4173.14	Translation
tl_term_b3261_1_rib_cplx2	Translation termination complex b3261 1 ribosomes 2 (RF2_mono)	C97027H135462N34112O49114S286P4947Mg175Zn0Fe0	-4182.14	Translation
tl_term_b3261_2_rib_cplx	Translation termination complex b3261 2 ribosomes	C191120H267663N67120O96101S576P9597Mg350Zn0Fe0	-8048.28	Translation
tl_term_b3261_2_rib_cplx2	Translation termination complex b3261 2 ribosomes 2 (RF2_mono)	C191222H267725N67084O96167S572P9597Mg350Zn0Fe0	-8066.28	Translation
tl_term_b3261_5_rib_cplx	Translation termination complex b3261 5 ribosomes	C473552H664359N166090O237161S1440P23547Mg875Zn0Fe0	-19673.7	Translation
tl_term_b3261_5_rib_cplx2	Translation termination complex b3261 5 ribosomes 2 (RF2_mono)	C473807H664514N166000O237326S1430P23547Mg875Zn0Fe0	-19718.7	Translation
tl_term_b3280_1_rib_cplx	Translation termination complex b3280 1 ribosomes	C96530H134808N33972O48776S287P4905Mg175Zn0Fe0	-4144.14	Translation
tl_term_b3280_2_rib_cplx	Translation termination complex b3280 2 ribosomes	C190629H266880N66951O95784S574P9556Mg350Zn0Fe0	-8033.28	Translation
tl_term_b3280_5_rib_cplx	Translation termination complex b3280 5 ribosomes	C472926H663096N165888O236808S1435P23509Mg875Zn0Fe0	-19700.7	Translation
tl_term_b3281_16_rib_cplx	Translation termination complex b3281 16 ribosomes	C1527233H2144508N534415O761824S4624P75219Mg2800Zn0Fe0	-63014.24	Translation
tl_term_b3281_1_rib_cplx	Translation termination complex b3281 1 ribosomes	C102743H142263N36250O53029S289P5469Mg175Zn0Fe0	-4707.14	Translation
tl_term_b3281_8_rib_cplx	Translation termination complex b3281 8 ribosomes	C767505H1076644N268727O383800S2312P38019Mg1400Zn0Fe0	-31917.12	Translation
tl_term_b3282_11_rib_cplx	Translation termination complex b3282 11 ribosomes	C1045481H1467528N366164O522387S3168P51701Mg1925Zn0Fe0	-43255.54	Translation
tl_term_b3282_11_rib_cplx2	Translation termination complex b3282 11 ribosomes 2 (RF2_mono)	C1046042H1467869N365966O522750S3146P51701Mg1925Zn0Fe0	-43354.54	Translation
tl_term_b3282_1_rib_cplx	Translation termination complex b3282 1 ribosomes	C99991H139008N35234O51157S288P5221Mg175Zn0Fe0	-4454.14	Translation
tl_term_b3282_1_rib_cplx2	Translation termination complex b3282 1 ribosomes 2 (RF2_mono)	C100042H139039N35216O51190S286P5221Mg175Zn0Fe0	-4463.14	Translation
tl_term_b3282_5_rib_cplx	Translation termination complex b3282 5 ribosomes	C478187H670416N167606O239649S1440P23813Mg875Zn0Fe0	-19974.7	Translation

tl_term_b3282_5_rib_cplx2	Translation termination complex b3282 5 ribosomes 2 (RF2_mono)	C478442H670571N167516O239814S1430P23813Mg875Zn0Fe0	-20019.7	Translation
tl_term_b3283_10_rib_cplx	Translation termination complex b3283 10 ribosomes	C950271H1333739N333062O474895S3000P47045Mg1750Zn0Fe0	-39257.4	Translation
tl_term_b3283_10_rib_cplx2	Translation termination complex b3283 10 ribosomes 2 (RF2_mono)	C950781H1334049N332882O475225S2980P47045Mg1750Zn0Fe0	-39347.4	Translation
tl_term_b3283_1_rib_cplx	Translation termination complex b3283 1 ribosomes	C99672H138629N35144O50914S300P5195Mg175Zn0Fe0	-4417.14	Translation
tl_term_b3283_1_rib_cplx2	Translation termination complex b3283 1 ribosomes 2 (RF2_mono)	C99723H138660N35126O50947S298P5195Mg175Zn0Fe0	-4426.14	Translation
tl_term_b3283_5_rib_cplx	Translation termination complex b3283 5 ribosomes	C477716H669789N167552O239350S1500P23795Mg875Zn0Fe0	-19901.7	Translation
tl_term_b3283_5_rib_cplx2	Translation termination complex b3283 5 ribosomes 2 (RF2_mono)	C477971H669944N167462O239515S1490P23795Mg875Zn0Fe0	-19946.7	Translation
tl_term_b3287_1_rib_cplx	Translation termination complex b3287 1 ribosomes	C99366H138305N35060O50674S288P5162Mg175Zn0Fe0	-4398.14	Translation
tl_term_b3287_1_rib_cplx2	Translation termination complex b3287 1 ribosomes 2 (RF2_mono)	C99417H138336N35042O50707S286P5162Mg175Zn0Fe0	-4407.14	Translation
tl_term_b3287_4_rib_cplx	Translation termination complex b3287 4 ribosomes	C382854H536762N134345O192037S1152P19112Mg700Zn0Fe0	-16053.56	Translation
tl_term_b3287_4_rib_cplx2	Translation termination complex b3287 4 ribosomes 2 (RF2_mono)	C383058H536886N134273O192169S1144P19112Mg700Zn0Fe0	-16089.56	Translation
tl_term_b3287_9_rib_cplx	Translation termination complex b3287 9 ribosomes	C855334H1200857N299820O427642S2592P42362Mg1575Zn0Fe0	-35479.26	Translation
tl_term_b3287_9_rib_cplx2	Translation termination complex b3287 9 ribosomes 2 (RF2_mono)	C855793H1201136N299658O427939S2574P42362Mg1575Zn0Fe0	-35560.26	Translation
tl_term_b3288_18_rib_cplx	Translation termination complex b3288 18 ribosomes	C1722275H2420159N602456O858440S5292P84666Mg3150Zn0Fe0	-70827.52	Translation
tl_term_b3288_1_rib_cplx	Translation termination complex b3288 1 ribosomes	C104198H144097N36866O53949S294P5599Mg175Zn0Fe0	-4831.14	Translation
tl_term_b3288_9_rib_cplx	Translation termination complex b3288 9 ribosomes	C865646H1215185N303026O432533S2646P42807Mg1575Zn0Fe0	-35888.26	Translation
tl_term_b3289_12_rib_cplx	Translation termination complex b3289 12 ribosomes	C1161951H1632087N406388O578924S3576P57092Mg2100Zn0Fe0	-47830.68	Translation
tl_term_b3289_1_rib_cplx	Translation termination complex b3289 1 ribosomes	C108074H148726N38328O56512S298P5942Mg175Zn0Fe0	-5171.14	Translation
tl_term_b3289_25_rib_cplx	Translation termination complex b3289 25 ribosomes	C2407442H3385150N841368O1196320S7450P117542Mg4375Zn0Fe0	-98246.5	Translation
tl_term_b3294_1_rib_cplx	Translation termination complex b3294 1 ribosomes	C97919H136602N34514O49723S287P5034Mg175Zn0Fe0	-4249.14	Translation
tl_term_b3294_1_rib_cplx2	Translation termination complex b3294 1 ribosomes 2 (RF2_mono)	C97970H136633N34496O49756S285P5034Mg175Zn0Fe0	-4258.14	Translation
tl_term_b3294_3_rib_cplx	Translation termination complex b3294 3 ribosomes	C286451H401532N100626O143795S861P14334Mg525Zn0Fe0	-11977.42	Translation
tl_term_b3294_3_rib_cplx2	Translation termination complex b3294 3 ribosomes 2 (RF2_mono)	C286604H401625N100572O143894S855P14334Mg525Zn0Fe0	-12004.42	Translation
tl_term_b3294_7_rib_cplx	Translation termination complex b3294 7 ribosomes	C663515H931392N232850O331939S2009P32934Mg1225Zn0Fe0	-27433.98	Translation
tl_term_b3294_7_rib_cplx2	Translation termination complex b3294 7 ribosomes 2 (RF2_mono)	C663872H931609N232724O332170S1995P32934Mg1225Zn0Fe0	-27496.98	Translation
tl_term_b3295_19_rib_cplx	Translation termination complex b3295 19 ribosomes	C1819061H2557270N636509O906794S5529P89340Mg3325Zn0Fe0	-74998.66	Translation
tl_term_b3295_19_rib_cplx2	Translation termination complex b3295 19 ribosomes 2 (RF2_mono)	C1820030H2557859N636167O907421S5491P89340Mg3325Zn0Fe0	-75169.66	Translation
tl_term_b3295_1_rib_cplx	Translation termination complex b3295 1 ribosomes	C104669H144694N37073O54278S291P5640Mg175Zn0Fe0	-4886.14	Translation
tl_term_b3295_1_rib_cplx2	Translation termination complex b3295 1 ribosomes 2 (RF2_mono)	C104720H144725N37055O54311S289P5640Mg175Zn0Fe0	-4895.14	Translation
tl_term_b3295_9_rib_cplx	Translation termination complex b3295 9 ribosomes	C866621H1216950N303489O433174S2619P42840Mg1575Zn0Fe0	-36047.26	Translation
tl_term_b3295_9_rib_cplx2	Translation termination complex b3295 9 ribosomes 2 (RF2_mono)	C867080H1217229N303327O433471S2601P42840Mg1575Zn0Fe0	-36128.26	Translation
tl_term_b3296_12_rib_cplx	Translation termination complex b3296 12 ribosomes	C1142155H1604484N400351O570354S3444P56421Mg2100Zn0Fe0	-46955.68	Translation
tl_term_b3296_12_rib_cplx2	Translation termination complex b3296 12 ribosomes 2 (RF2_mono)	C1142767H1604856N400135O570750S3420P56421Mg2100Zn0Fe0	-47063.68	Translation
tl_term_b3296_1_rib_cplx	Translation termination complex b3296 1 ribosomes	C100609H139834N35547O51506S287P5271Mg175Zn0Fe0	-4483.14	Translation
tl_term_b3296_1_rib_cplx2	Translation termination complex b3296 1 ribosomes 2 (RF2_mono)	C100660H139865N35529O51539S285P5271Mg175Zn0Fe0	-4492.14	Translation
tl_term_b3296_6_rib_cplx	Translation termination complex b3296 6 ribosomes	C574039H805584N201367O287346S1722P28521Mg1050Zn0Fe0	-23788.84	Translation
tl_term_b3296_6_rib_cplx2	Translation termination complex b3296 6 ribosomes 2 (RF2_mono)	C574345H805770N201259O287544S1710P28521Mg1050Zn0Fe0	-23842.84	Translation
tl_term_b3297_1_rib_cplx	Translation termination complex b3297 1 ribosomes	C97953H136622N34517O49763S286P5040Mg175Zn0Fe0	-4254.14	Translation
tl_term_b3297_1_rib_cplx2	Translation termination complex b3297 1 ribosomes 2 (RF2_mono)	C98004H136653N34499O49796S284P5040Mg175Zn0Fe0	-4263.14	Translation
tl_term_b3297_3_rib_cplx	Translation termination complex b3297 3 ribosomes	C286451H401472N100623O143829S858P14340Mg525Zn0Fe0	-11980.42	Translation
tl_term_b3297_3_rib_cplx2	Translation termination complex b3297 3 ribosomes 2 (RF2_mono)	C286604H401565N100569O143928S852P14340Mg525Zn0Fe0	-12007.42	Translation
tl_term_b3297_7_rib_cplx	Translation termination complex b3297 7 ribosomes	C663447H931172N232835O331961S2002P32940Mg1225Zn0Fe0	-27432.98	Translation
tl_term_b3297_7_rib_cplx2	Translation termination complex b3297 7 ribosomes 2 (RF2_mono)	C663804H931389N232709O332192S1988P32940Mg1225Zn0Fe0	-27495.98	Translation
tl_term_b3298_1_rib_cplx	Translation termination complex b3298 1 ribosomes	C97621H136265N34387O49532S286P5009Mg175Zn0Fe0	-4224.14	Translation
tl_term_b3298_1_rib_cplx2	Translation termination complex b3298 1 ribosomes 2 (RF2_mono)	C97672H136296N34369O49565S284P5009Mg175Zn0Fe0	-4233.14	Translation
tl_term_b3298_3_rib_cplx	Translation termination complex b3298 3 ribosomes	C286069H401107N100451O143592S858P14309Mg525Zn0Fe0	-11952.42	Translation
tl_term_b3298_3_rib_cplx2	Translation termination complex b3298 3 ribosomes 2 (RF2_mono)	C286222H401200N100397O143691S852P14309Mg525Zn0Fe0	-11979.42	Translation

tl_term_b3298_6_rib_cplx	Translation termination complex b3298 6 ribosomes	C568741H798370N199547O284682S1716P28259Mg1050Zn0Fe0	-23544.84	Translation
tl_term_b3298_6_rib_cplx2	Translation termination complex b3298 6 ribosomes 2 (RF2_mono)	C569047H798556N199439O284880S1704P28259Mg1050Zn0Fe0	-23598.84	Translation
tl_term_b3299_1_rib_cplx	Translation termination complex b3299 1 ribosomes	C94947H133020N33367O47727S285P4767Mg175Zn0Fe0	-3985.14	Translation
tl_term_b3299_2_rib_cplx	Translation termination complex b3299 2 ribosomes	C188777H264780N66283O94639S570P9417Mg350Zn0Fe0	-7852.28	Translation
tl_term_b3300_13_rib_cplx	Translation termination complex b3300 13 ribosomes	C1259584H1769529N439435O626221S3900P61795Mg2275Zn0Fe0	-51527.82	Translation
tl_term_b3300_13_rib_cplx2	Translation termination complex b3300 13 ribosomes 2 (RF2_mono)	C1260247H1769932N439201O626650S3874P61795Mg2275Zn0Fe0	-51644.82	Translation
tl_term_b3300_1_rib_cplx	Translation termination complex b3300 1 ribosomes	C108544H149289N38311O56869S300P5983Mg175Zn0Fe0	-5194.14	Translation
tl_term_b3300_1_rib_cplx2	Translation termination complex b3300 1 ribosomes 2 (RF2_mono)	C108595H149320N38293O56902S298P5983Mg175Zn0Fe0	-5203.14	Translation
tl_term_b3300_26_rib_cplx	Translation termination complex b3300 26 ribosomes	C2506544H3524789N873986O1243019S7800P122258Mg4550Zn0Fe0	-101722.64	Translation
tl_term_b3300_26_rib_cplx2	Translation termination complex b3300 26 ribosomes 2 (RF2_mono)	C2507870H3525595N873518O1243877S7748P122258Mg4550Zn0Fe0	-101956.64	Translation
tl_term_b3301_1_rib_cplx	Translation termination complex b3301 1 ribosomes	C98442H137230N34715O50102S284P5085Mg175Zn0Fe0	-4297.14	Translation
tl_term_b3301_1_rib_cplx2	Translation termination complex b3301 1 ribosomes 2 (RF2_mono)	C98493H137261N34697O50135S282P5085Mg175Zn0Fe0	-4306.14	Translation
tl_term_b3301_4_rib_cplx	Translation termination complex b3301 4 ribosomes	C381339H534877N133892O191246S1136P19035Mg700Zn0Fe0	-15880.56	Translation
tl_term_b3301_4_rib_cplx2	Translation termination complex b3301 4 ribosomes 2 (RF2_mono)	C381543H535001N133820O191378S1128P19035Mg700Zn0Fe0	-15916.56	Translation
tl_term_b3301_8_rib_cplx	Translation termination complex b3301 8 ribosomes	C758535H1065073N266128O379438S2272P37635Mg1400Zn0Fe0	-31325.12	Translation
tl_term_b3301_8_rib_cplx2	Translation termination complex b3301 8 ribosomes 2 (RF2_mono)	C758943H1065321N265984O379702S2256P37635Mg1400Zn0Fe0	-31397.12	Translation
tl_term_b3302_1_rib_cplx	Translation termination complex b3302 1 ribosomes	C95644H133857N33622O48197S285P4830Mg175Zn0Fe0	-4052.14	Translation
tl_term_b3302_1_rib_cplx2	Translation termination complex b3302 1 ribosomes 2 (RF2_mono)	C95695H133888N33604O48230S283P4830Mg175Zn0Fe0	-4061.14	Translation
tl_term_b3302_3_rib_cplx	Translation termination complex b3302 3 ribosomes	C283506H397697N99502O142073S855P14130Mg525Zn0Fe0	-11794.42	Translation
tl_term_b3302_3_rib_cplx2	Translation termination complex b3302 3 ribosomes 2 (RF2_mono)	C283659H397790N99448O142172S849P14130Mg525Zn0Fe0	-11821.42	Translation
tl_term_b3303_1_rib_cplx	Translation termination complex b3303 1 ribosomes	C99223H138137N35004O50616S289P5154Mg175Zn0Fe0	-4374.14	Translation
tl_term_b3303_1_rib_cplx2	Translation termination complex b3303 1 ribosomes 2 (RF2_mono)	C99274H138168N34986O50649S287P5154Mg175Zn0Fe0	-4383.14	Translation
tl_term_b3303_4_rib_cplx	Translation termination complex b3303 4 ribosomes	C382486H536276N134247O191904S1156P19104Mg700Zn0Fe0	-15981.56	Translation
tl_term_b3303_4_rib_cplx2	Translation termination complex b3303 4 ribosomes 2 (RF2_mono)	C382690H536400N134175O192036S1148P19104Mg700Zn0Fe0	-16017.56	Translation
tl_term_b3303_9_rib_cplx	Translation termination complex b3303 9 ribosomes	C854591H1199841N299652O427384S2601P42354Mg1575Zn0Fe0	-35327.26	Translation
tl_term_b3303_9_rib_cplx2	Translation termination complex b3303 9 ribosomes 2 (RF2_mono)	C855050H1200120N299490O427681S2583P42354Mg1575Zn0Fe0	-35408.26	Translation
tl_term_b3304_1_rib_cplx	Translation termination complex b3304 1 ribosomes	C97579H136184N34377O49498S284P5004Mg175Zn0Fe0	-4222.14	Translation
tl_term_b3304_1_rib_cplx2	Translation termination complex b3304 1 ribosomes 2 (RF2_mono)	C97630H136215N34359O49531S282P5004Mg175Zn0Fe0	-4231.14	Translation
tl_term_b3304_3_rib_cplx	Translation termination complex b3304 3 ribosomes	C286005H400920N100445O143548S852P14304Mg525Zn0Fe0	-11956.42	Translation
tl_term_b3304_3_rib_cplx2	Translation termination complex b3304 3 ribosomes 2 (RF2_mono)	C286158H401013N100391O143647S846P14304Mg525Zn0Fe0	-11983.42	Translation
tl_term_b3304_6_rib_cplx	Translation termination complex b3304 6 ribosomes	C568644H798024N199547O284623S1704P28254Mg1050Zn0Fe0	-23557.84	Translation
tl_term_b3304_6_rib_cplx2	Translation termination complex b3304 6 ribosomes 2 (RF2_mono)	C568950H798210N199439O284821S1692P28254Mg1050Zn0Fe0	-23611.84	Translation
tl_term_b3305_10_rib_cplx	Translation termination complex b3305 10 ribosomes	C950003H1333884N332945O474905S2850P47034Mg1750Zn0Fe0	-39226.4	Translation
tl_term_b3305_10_rib_cplx2	Translation termination complex b3305 10 ribosomes 2 (RF2_mono)	C950513H1334194N332765O475235S2830P47034Mg1750Zn0Fe0	-39316.4	Translation
tl_term_b3305_1_rib_cplx	Translation termination complex b3305 1 ribosomes	C99575H138558N35117O50861S285P5184Mg175Zn0Fe0	-4404.14	Translation
tl_term_b3305_1_rib_cplx2	Translation termination complex b3305 1 ribosomes 2 (RF2_mono)	C99626H138589N35099O50894S283P5184Mg175Zn0Fe0	-4413.14	Translation
tl_term_b3305_5_rib_cplx	Translation termination complex b3305 5 ribosomes	C477543H669814N167485O239325S1425P23784Mg875Zn0Fe0	-19880.7	Translation
tl_term_b3305_5_rib_cplx2	Translation termination complex b3305 5 ribosomes 2 (RF2_mono)	C477798H669969N167395O239490S1415P23784Mg875Zn0Fe0	-19925.7	Translation
tl_term_b3306_1_rib_cplx	Translation termination complex b3306 1 ribosomes	C98014H136688N34534O49789S288P5043Mg175Zn0Fe0	-4266.14	Translation
tl_term_b3306_1_rib_cplx2	Translation termination complex b3306 1 ribosomes 2 (RF2_mono)	C98065H136719N34516O49822S286P5043Mg175Zn0Fe0	-4275.14	Translation
tl_term_b3306_3_rib_cplx	Translation termination complex b3306 3 ribosomes	C286550H401604N100596O143879S864P14343Mg525Zn0Fe0	-12010.42	Translation
tl_term_b3306_3_rib_cplx2	Translation termination complex b3306 3 ribosomes 2 (RF2_mono)	C286703H401697N100542O143978S858P14343Mg525Zn0Fe0	-12037.42	Translation
tl_term_b3306_7_rib_cplx	Translation termination complex b3306 7 ribosomes	C663622H931436N232720O332059S2016P32943Mg1225Zn0Fe0	-27498.98	Translation
tl_term_b3306_7_rib_cplx2	Translation termination complex b3306 7 ribosomes 2 (RF2_mono)	C663979H931653N232594O332290S2002P32943Mg1225Zn0Fe0	-27561.98	Translation
tl_term_b3307_1_rib_cplx	Translation termination complex b3307 1 ribosomes	C97071H135584N34188O49136S287P4956Mg175Zn0Fe0	-4169.14	Translation
tl_term_b3307_1_rib_cplx2	Translation termination complex b3307 1 ribosomes 2 (RF2_mono)	C97122H135615N34170O49169S285P4956Mg175Zn0Fe0	-4178.14	Translation
tl_term_b3307_2_rib_cplx	Translation termination complex b3307 2 ribosomes	C191227H267869N67204O96138S574P9606Mg350Zn0Fe0	-8031.28	Translation

tl_term_b3307_2_rib_cplx2	Translation termination complex b3307 2 ribosomes 2 (RF2_mono)	C191329H267931N67168O96204S570P9606Mg350Zn0Fe0	-8049.28	Translation
tl_term_b3307_5_rib_cplx	Translation termination complex b3307 5 ribosomes	C473695H664724N166252O237144S1435P23556Mg875Zn0Fe0	-19617.7	Translation
tl_term_b3307_5_rib_cplx2	Translation termination complex b3307 5 ribosomes 2 (RF2_mono)	C473950H664879N166162O237309S1425P23556Mg875Zn0Fe0	-19662.7	Translation
tl_term_b3308_10_rib_cplx	Translation termination complex b3308 10 ribosomes	C950783H1334811N333036O475050S2890P47040Mg1750Zn0Fe0	-39252.4	Translation
tl_term_b3308_10_rib_cplx2	Translation termination complex b3308 10 ribosomes 2 (RF2_mono)	C951293H1335121N332856O475380S2870P47040Mg1750Zn0Fe0	-39342.4	Translation
tl_term_b3308_1_rib_cplx	Translation termination complex b3308 1 ribosomes	C99698H138711N35136O50898S289P5190Mg175Zn0Fe0	-4412.14	Translation
tl_term_b3308_1_rib_cplx2	Translation termination complex b3308 1 ribosomes 2 (RF2_mono)	C99749H138742N35118O50931S287P5190Mg175Zn0Fe0	-4421.14	Translation
tl_term_b3308_5_rib_cplx	Translation termination complex b3308 5 ribosomes	C477958H670311N167536O239410S1445P23790Mg875Zn0Fe0	-19896.7	Translation
tl_term_b3308_5_rib_cplx2	Translation termination complex b3308 5 ribosomes 2 (RF2_mono)	C478213H670466N167446O239575S1435P23790Mg875Zn0Fe0	-19941.7	Translation
tl_term_b3309_1_rib_cplx	Translation termination complex b3309 1 ribosomes	C97170H135681N34224O49193S283P4965Mg175Zn0Fe0	-4183.14	Translation
tl_term_b3309_1_rib_cplx2	Translation termination complex b3309 1 ribosomes 2 (RF2_mono)	C97221H135712N34206O49226S281P4965Mg175Zn0Fe0	-4192.14	Translation
tl_term_b3309_3_rib_cplx	Translation termination complex b3309 3 ribosomes	C285486H400255N100218O143219S849P14265Mg525Zn0Fe0	-11917.42	Translation
tl_term_b3309_3_rib_cplx2	Translation termination complex b3309 3 ribosomes 2 (RF2_mono)	C285639H400348N100164O143318S843P14265Mg525Zn0Fe0	-11944.42	Translation
tl_term_b3309_6_rib_cplx	Translation termination complex b3309 6 ribosomes	C567960H797116N199209O284258S1698P28215Mg1050Zn0Fe0	-23518.84	Translation
tl_term_b3309_6_rib_cplx2	Translation termination complex b3309 6 ribosomes 2 (RF2_mono)	C568266H797302N199101O284456S1686P28215Mg1050Zn0Fe0	-23572.84	Translation
tl_term_b3310_1_rib_cplx	Translation termination complex b3310 1 ribosomes	C97889H136562N34497O49714S287P5035Mg175Zn0Fe0	-4251.14	Translation
tl_term_b3310_1_rib_cplx2	Translation termination complex b3310 1 ribosomes 2 (RF2_mono)	C97940H136593N34479O49747S285P5035Mg175Zn0Fe0	-4260.14	Translation
tl_term_b3310_3_rib_cplx	Translation termination complex b3310 3 ribosomes	C286579H401678N100653O143928S861P14357Mg525Zn0Fe0	-12003.42	Translation
tl_term_b3310_3_rib_cplx2	Translation termination complex b3310 3 ribosomes 2 (RF2_mono)	C286732H401771N100599O144027S855P14357Mg525Zn0Fe0	-12030.42	Translation
tl_term_b3310_7_rib_cplx	Translation termination complex b3310 7 ribosomes	C663959H931910N232965O332356S2009P33001Mg1225Zn0Fe0	-27507.98	Translation
tl_term_b3310_7_rib_cplx2	Translation termination complex b3310 7 ribosomes 2 (RF2_mono)	C664316H932127N232839O332587S1995P33001Mg1225Zn0Fe0	-27570.98	Translation
tl_term_b3311_1_rib_cplx	Translation termination complex b3311 1 ribosomes	C96598H134990N33991O48826S285P4915Mg175Zn0Fe0	-4137.14	Translation
tl_term_b3311_1_rib_cplx2	Translation termination complex b3311 1 ribosomes 2 (RF2_mono)	C96649H135021N33973O48859S283P4915Mg175Zn0Fe0	-4146.14	Translation
tl_term_b3311_2_rib_cplx	Translation termination complex b3311 2 ribosomes	C190774H267246N67009O95884S570P9576Mg350Zn0Fe0	-8019.28	Translation
tl_term_b3311_2_rib_cplx2	Translation termination complex b3311 2 ribosomes 2 (RF2_mono)	C190876H267308N66973O95950S566P9576Mg350Zn0Fe0	-8037.28	Translation
tl_term_b3311_4_rib_cplx	Translation termination complex b3311 4 ribosomes	C379126H531758N133045O190000S1140P18898Mg700Zn0Fe0	-15783.56	Translation
tl_term_b3311_4_rib_cplx2	Translation termination complex b3311 4 ribosomes 2 (RF2_mono)	C379330H531882N132973O190132S1132P18898Mg700Zn0Fe0	-15819.56	Translation
tl_term_b3312_1_rib_cplx	Translation termination complex b3312 1 ribosomes	C95790H134025N33711O48283S283P4841Mg175Zn0Fe0	-4066.14	Translation
tl_term_b3312_1_rib_cplx2	Translation termination complex b3312 1 ribosomes 2 (RF2_mono)	C95841H134056N33693O48316S281P4841Mg175Zn0Fe0	-4075.14	Translation
tl_term_b3312_3_rib_cplx	Translation termination complex b3312 3 ribosomes	C283710H397943N99615O142203S849P14141Mg525Zn0Fe0	-11814.42	Translation
tl_term_b3312_3_rib_cplx2	Translation termination complex b3312 3 ribosomes 2 (RF2_mono)	C283863H398036N99561O142302S843P14141Mg525Zn0Fe0	-11841.42	Translation
tl_term_b3313_1_rib_cplx	Translation termination complex b3313 1 ribosomes	C98280H137034N34660O49911S288P5062Mg175Zn0Fe0	-4271.14	Translation
tl_term_b3313_1_rib_cplx2	Translation termination complex b3313 1 ribosomes 2 (RF2_mono)	C98331H137065N34642O49944S286P5062Mg175Zn0Fe0	-4280.14	Translation
tl_term_b3313_4_rib_cplx	Translation termination complex b3313 4 ribosomes	C381339H534843N133846O191058S1152P19015Mg700Zn0Fe0	-15848.56	Translation
tl_term_b3313_4_rib_cplx2	Translation termination complex b3313 4 ribosomes 2 (RF2_mono)	C381543H534967N133774O191190S1144P19015Mg700Zn0Fe0	-15884.56	Translation
tl_term_b3313_8_rib_cplx	Translation termination complex b3313 8 ribosomes	C758751H1065255N266094O379254S2304P37619Mg1400Zn0Fe0	-31285.12	Translation
tl_term_b3313_8_rib_cplx2	Translation termination complex b3313 8 ribosomes 2 (RF2_mono)	C759159H1065503N265950O379518S2288P37619Mg1400Zn0Fe0	-31357.12	Translation
tl_term_b3314_13_rib_cplx	Translation termination complex b3314 13 ribosomes	C1239211H1741157N434238O618390S3731P61152Mg2275Zn0Fe0	-50871.82	Translation
tl_term_b3314_13_rib_cplx2	Translation termination complex b3314 13 ribosomes 2 (RF2_mono)	C1239874H1741560N434004O618819S3705P61152Mg2275Zn0Fe0	-50988.82	Translation
tl_term_b3314_1_rib_cplx	Translation termination complex b3314 1 ribosomes	C101503H140921N35898O52074S287P5352Mg175Zn0Fe0	-4562.14	Translation
tl_term_b3314_1_rib_cplx2	Translation termination complex b3314 1 ribosomes 2 (RF2_mono)	C101554H140952N35880O52107S285P5352Mg175Zn0Fe0	-4571.14	Translation
tl_term_b3314_6_rib_cplx	Translation termination complex b3314 6 ribosomes	C575548H807686N201873O288039S1722P28602Mg1050Zn0Fe0	-23857.84	Translation
tl_term_b3314_6_rib_cplx2	Translation termination complex b3314 6 ribosomes 2 (RF2_mono)	C575854H807872N201765O288237S1710P28602Mg1050Zn0Fe0	-23911.84	Translation
tl_term_b3315_1_rib_cplx	Translation termination complex b3315 1 ribosomes	C97362H135944N34291O49345S285P4984Mg175Zn0Fe0	-4202.14	Translation
tl_term_b3315_3_rib_cplx	Translation termination complex b3315 3 ribosomes	C285748H400662N100345O143389S855P14286Mg525Zn0Fe0	-11938.42	Translation
tl_term_b3315_6_rib_cplx	Translation termination complex b3315 6 ribosomes	C568327H797739N199426O284455S1710P28239Mg1050Zn0Fe0	-23542.84	Translation
tl_term_b3316_1_rib_cplx	Translation termination complex b3316 1 ribosomes	C96781H135213N34051O48935S285P4929Mg175Zn0Fe0	-4146.14	Translation

tl_term_b3316_1_rib_cplx2	Translation termination complex b3316 1 ribosomes 2 (RF2_mono)	C96832H135244N34033O48968S283P4929Mg175Zn0Fe0	-4155.14	Translation
tl_term_b3316_2_rib_cplx	Translation termination complex b3316 2 ribosomes	C190905H267422N67039O95927S570P9579Mg350Zn0Fe0	-8012.28	Translation
tl_term_b3316_2_rib_cplx2	Translation termination complex b3316 2 ribosomes 2 (RF2_mono)	C191007H267484N67003O95993S566P9579Mg350Zn0Fe0	-8030.28	Translation
tl_term_b3316_5_rib_cplx	Translation termination complex b3316 5 ribosomes	C473277H664049N166003O236903S1425P23529Mg875Zn0Fe0	-19610.7	Translation
tl_term_b3316_5_rib_cplx2	Translation termination complex b3316 5 ribosomes 2 (RF2_mono)	C473532H664204N165913O237068S1415P23529Mg875Zn0Fe0	-19655.7	Translation
tl_term_b3317_16_rib_cplx	Translation termination complex b3317 16 ribosomes	C1527089H2146617N535518O761514S4640P75222Mg2800Zn0Fe0	-62345.24	Translation
tl_term_b3317_16_rib_cplx2	Translation termination complex b3317 16 ribosomes 2 (RF2_mono)	C1527905H2147113N535230O762042S4608P75222Mg2800Zn0Fe0	-62489.24	Translation
tl_term_b3317_1_rib_cplx	Translation termination complex b3317 1 ribosomes	C102779H142467N36408O52974S290P5472Mg175Zn0Fe0	-4668.14	Translation
tl_term_b3317_1_rib_cplx2	Translation termination complex b3317 1 ribosomes 2 (RF2_mono)	C102830H142498N36390O53007S288P5472Mg175Zn0Fe0	-4677.14	Translation
tl_term_b3317_8_rib_cplx	Translation termination complex b3317 8 ribosomes	C767457H1077737N269326O383626S2320P38022Mg1400Zn0Fe0	-31584.12	Translation
tl_term_b3317_8_rib_cplx2	Translation termination complex b3317 8 ribosomes 2 (RF2_mono)	C767865H1077985N269182O383890S2304P38022Mg1400Zn0Fe0	-31656.12	Translation
tl_term_b3318_1_rib_cplx	Translation termination complex b3318 1 ribosomes	C97003H135494N34175O49065S284P4949Mg175Zn0Fe0	-4169.14	Translation
tl_term_b3318_1_rib_cplx2	Translation termination complex b3318 1 ribosomes 2 (RF2_mono)	C97054H135525N34157O49098S282P4949Mg175Zn0Fe0	-4178.14	Translation
tl_term_b3318_2_rib_cplx	Translation termination complex b3318 2 ribosomes	C191144H267760N67173O96066S568P9599Mg350Zn0Fe0	-8038.28	Translation
tl_term_b3318_2_rib_cplx2	Translation termination complex b3318 2 ribosomes 2 (RF2_mono)	C191246H267822N67137O96132S564P9599Mg350Zn0Fe0	-8056.28	Translation
tl_term_b3318_5_rib_cplx	Translation termination complex b3318 5 ribosomes	C473567H664558N166167O237069S1420P23549Mg875Zn0Fe0	-19645.7	Translation
tl_term_b3318_5_rib_cplx2	Translation termination complex b3318 5 ribosomes 2 (RF2_mono)	C473822H664713N166077O237234S1410P23549Mg875Zn0Fe0	-19690.7	Translation
tl_term_b3319_11_rib_cplx	Translation termination complex b3319 11 ribosomes	C1046605H1469912N366814O522943S3146P51756Mg1925Zn0Fe0	-43178.54	Translation
tl_term_b3319_1_rib_cplx	Translation termination complex b3319 1 ribosomes	C100395H139562N35454O51393S286P5256Mg175Zn0Fe0	-4477.14	Translation
tl_term_b3319_5_rib_cplx	Translation termination complex b3319 5 ribosomes	C478879H671702N167998O240013S1430P23856Mg875Zn0Fe0	-19957.7	Translation
tl_term_b3320_12_rib_cplx	Translation termination complex b3320 12 ribosomes	C1141520H1603171N400114O570301S3420P56430Mg2100Zn0Fe0	-47048.68	Translation
tl_term_b3320_12_rib_cplx2	Translation termination complex b3320 12 ribosomes 2 (RF2_mono)	C1142132H1603543N399898O570697S3396P56430Mg2100Zn0Fe0	-47156.68	Translation
tl_term_b3320_1_rib_cplx	Translation termination complex b3320 1 ribosomes	C100634H139819N35563O51552S285P5280Mg175Zn0Fe0	-4499.14	Translation
tl_term_b3320_1_rib_cplx2	Translation termination complex b3320 1 ribosomes 2 (RF2_mono)	C100685H139850N35545O51585S283P5280Mg175Zn0Fe0	-4508.14	Translation
tl_term_b3320_6_rib_cplx	Translation termination complex b3320 6 ribosomes	C573764H804979N201268O287347S1710P28530Mg1050Zn0Fe0	-23839.84	Translation
tl_term_b3320_6_rib_cplx2	Translation termination complex b3320 6 ribosomes 2 (RF2_mono)	C574070H805165N201160O287545S1698P28530Mg1050Zn0Fe0	-23893.84	Translation
tl_term_b3321_1_rib_cplx	Translation termination complex b3321 1 ribosomes	C97124H135650N34194O49197S283P4964Mg175Zn0Fe0	-4189.14	Translation
tl_term_b3321_1_rib_cplx2	Translation termination complex b3321 1 ribosomes 2 (RF2_mono)	C97175H135681N34176O49230S281P4964Mg175Zn0Fe0	-4198.14	Translation
tl_term_b3321_3_rib_cplx	Translation termination complex b3321 3 ribosomes	C285442H400214N100212O143227S849P14264Mg525Zn0Fe0	-11937.42	Translation
tl_term_b3321_3_rib_cplx2	Translation termination complex b3321 3 ribosomes 2 (RF2_mono)	C285595H400307N100158O143326S843P14264Mg525Zn0Fe0	-11964.42	Translation
tl_term_b3321_6_rib_cplx	Translation termination complex b3321 6 ribosomes	C567919H797060N199239O284272S1698P28214Mg1050Zn0Fe0	-23559.84	Translation
tl_term_b3321_6_rib_cplx2	Translation termination complex b3321 6 ribosomes 2 (RF2_mono)	C568225H797246N199131O284470S1686P28214Mg1050Zn0Fe0	-23613.84	Translation
tl_term_b3339_11_rib_cplx	Translation termination complex b3339 11 ribosomes	C1062513H1492004N371634O530184S3245P52335Mg1925Zn0Fe0	-43999.54	Translation
tl_term_b3339_11_rib_cplx2	Translation termination complex b3339 11 ribosomes 2 (RF2_mono)	C1063074H1492345N371436O530547S3223P52335Mg1925Zn0Fe0	-44098.54	Translation
tl_term_b3339_1_rib_cplx	Translation termination complex b3339 1 ribosomes	C106843H147244N37884O55724S295P5835Mg175Zn0Fe0	-5078.14	Translation
tl_term_b3339_1_rib_cplx2	Translation termination complex b3339 1 ribosomes 2 (RF2_mono)	C106894H147275N37866O55757S293P5835Mg175Zn0Fe0	-5087.14	Translation
tl_term_b3339_23_rib_cplx	Translation termination complex b3339 23 ribosomes	C2209317H3105716N772134O1099536S6785P108135Mg4025Zn0Fe0	-90705.22	Translation
tl_term_b3339_23_rib_cplx2	Translation termination complex b3339 23 ribosomes 2 (RF2_mono)	C2210490H3106429N771720O1100295S6739P108135Mg4025Zn0Fe0	-90912.22	Translation
tl_term_b3340_1_rib_cplx	Translation termination complex b3340 1 ribosomes	C117235H159635N41869O62663S308P6765Mg175Zn0Fe0	-6018.14	Translation
tl_term_b3340_1_rib_cplx2	Translation termination complex b3340 1 ribosomes 2 (RF2_mono)	C117286H159666N41851O62696S306P6765Mg175Zn0Fe0	-6027.14	Translation
tl_term_b3340_20_rib_cplx	Translation termination complex b3340 20 ribosomes	C1961926H2759937N683898O973124S6160P95115Mg3500Zn0Fe0	-80158.8	Translation
tl_term_b3340_20_rib_cplx2	Translation termination complex b3340 20 ribosomes 2 (RF2_mono)	C1962946H2760557N683538O973784S6120P95115Mg3500Zn0Fe0	-80338.8	Translation
tl_term_b3340_41_rib_cplx	Translation termination complex b3340 41 ribosomes	C4000795H5633955N1393509O1979423S12628P192765Mg7175Zn0Fe0	-162103.74	Translation
tl_term_b3340_41_rib_cplx2	Translation termination complex b3340 41 ribosomes 2 (RF2_mono)	C4002886H5635226N1392771O1980776S12546P192765Mg7175Zn0Fe0	-162472.74	Translation
tl_term_b3341_10_rib_cplx	Translation termination complex b3341 10 ribosomes	C950494H1334664N333221O475016S2870P47040Mg1750Zn0Fe0	-39182.4	Translation
tl_term_b3341_1_rib_cplx	Translation termination complex b3341 1 ribosomes	C99670H138690N35150O50909S287P5190Mg175Zn0Fe0	-4405.14	Translation
tl_term_b3341_5_rib_cplx	Translation termination complex b3341 5 ribosomes	C477814H670234N167626O239401S1435P23790Mg875Zn0Fe0	-19861.7	Translation

tl_term_b3342_1_rib_cplx	Translation termination complex b3342 1 ribosomes	C97807H136486N34471O49656S286P5027Mg175Zn0Fe0	-4235.14	Translation
tl_term_b3342_1_rib_cplx2	Translation termination complex b3342 1 ribosomes 2 (RF2_mono)	C97858H136517N34453O49689S284P5027Mg175Zn0Fe0	-4244.14	Translation
tl_term_b3342_3_rib_cplx	Translation termination complex b3342 3 ribosomes	C286291H401374N100571O143718S858P14327Mg525Zn0Fe0	-11949.42	Translation
tl_term_b3342_3_rib_cplx2	Translation termination complex b3342 3 ribosomes 2 (RF2_mono)	C286444H401467N100517O143817S852P14327Mg525Zn0Fe0	-11976.42	Translation
tl_term_b3342_7_rib_cplx	Translation termination complex b3342 7 ribosomes	C663259H931150N232771O331842S2002P32927Mg1225Zn0Fe0	-27377.98	Translation
tl_term_b3342_7_rib_cplx2	Translation termination complex b3342 7 ribosomes 2 (RF2_mono)	C663616H931367N232645O332073S1988P32927Mg1225Zn0Fe0	-27440.98	Translation
tl_term_b3343_1_rib_cplx	Translation termination complex b3343 1 ribosomes	C96862H135288N34039O49029S285P4938Mg175Zn0Fe0	-4170.14	Translation
tl_term_b3343_2_rib_cplx	Translation termination complex b3343 2 ribosomes	C190997H267483N67020O96032S570P9588Mg350Zn0Fe0	-8051.28	Translation
tl_term_b3343_5_rib_cplx	Translation termination complex b3343 5 ribosomes	C473402H664068N165963O237041S1425P23538Mg875Zn0Fe0	-19694.7	Translation
tl_term_b3344_1_rib_cplx	Translation termination complex b3344 1 ribosomes	C97648H136203N34315O49578S286P5009Mg175Zn0Fe0	-4242.14	Translation
tl_term_b3344_3_rib_cplx	Translation termination complex b3344 3 ribosomes	C286148H400919N100333O143642S858P14309Mg525Zn0Fe0	-12006.42	Translation
tl_term_b3344_7_rib_cplx	Translation termination complex b3344 7 ribosomes	C663148H930351N232369O331770S2002P32909Mg1225Zn0Fe0	-27534.98	Translation
tl_term_b3345_1_rib_cplx	Translation termination complex b3345 1 ribosomes	C97938H136524N34503O49759S286P5036Mg175Zn0Fe0	-4268.14	Translation
tl_term_b3345_3_rib_cplx	Translation termination complex b3345 3 ribosomes	C286456H401258N100553O143855S858P14336Mg525Zn0Fe0	-12030.42	Translation
tl_term_b3345_7_rib_cplx	Translation termination complex b3345 7 ribosomes	C663492H930726N232653O332047S2002P32936Mg1225Zn0Fe0	-27554.98	Translation
tl_term_b3346_14_rib_cplx	Translation termination complex b3346 14 ribosomes	C1334455H1874227N467124O666405S4102P65825Mg2450Zn0Fe0	-55103.96	Translation
tl_term_b3346_14_rib_cplx2	Translation termination complex b3346 14 ribosomes 2 (RF2_mono)	C1335169H1874661N466872O666867S4074P65825Mg2450Zn0Fe0	-55229.96	Translation
tl_term_b3346_1_rib_cplx	Translation termination complex b3346 1 ribosomes	C101691H141080N35862O52311S293P5375Mg175Zn0Fe0	-4610.14	Translation
tl_term_b3346_1_rib_cplx2	Translation termination complex b3346 1 ribosomes 2 (RF2_mono)	C101742H141111N35844O52344S291P5375Mg175Zn0Fe0	-4619.14	Translation
tl_term_b3346_7_rib_cplx	Translation termination complex b3346 7 ribosomes	C670659H940994N234906O335739S2051P33275Mg1225Zn0Fe0	-27914.98	Translation
tl_term_b3346_7_rib_cplx2	Translation termination complex b3346 7 ribosomes 2 (RF2_mono)	C671016H941211N234780O335970S2037P33275Mg1225Zn0Fe0	-27977.98	Translation
tl_term_b3384_19_rib_cplx	Translation termination complex b3384 19 ribosomes	C1820695H2557864N636750O906946S5624P89366Mg3325Zn0Fe0	-74739.66	Translation
tl_term_b3384_19_rib_cplx2	Translation termination complex b3384 19 ribosomes 2 (RF2_mono)	C1821664H2558453N636408O907573S5586P89366Mg3325Zn0Fe0	-74910.66	Translation
tl_term_b3384_1_rib_cplx	Translation termination complex b3384 1 ribosomes	C104827H144802N37134O54322S296P5648Mg175Zn0Fe0	-4879.14	Translation
tl_term_b3384_1_rib_cplx2	Translation termination complex b3384 1 ribosomes 2 (RF2_mono)	C104878H144833N37116O54355S294P5648Mg175Zn0Fe0	-4888.14	Translation
tl_term_b3384_9_rib_cplx	Translation termination complex b3384 9 ribosomes	C867435H1217274N303630O433266S2664P42856Mg1575Zn0Fe0	-35928.26	Translation
tl_term_b3384_9_rib_cplx2	Translation termination complex b3384 9 ribosomes 2 (RF2_mono)	C867894H1217553N303468O433563S2646P42856Mg1575Zn0Fe0	-36009.26	Translation
tl_term_b3385_14_rib_cplx	Translation termination complex b3385 14 ribosomes	C1335567H1875054N467347O666721S4046P65865Mg2450Zn0Fe0	-55255.96	Translation
tl_term_b3385_14_rib_cplx2	Translation termination complex b3385 14 ribosomes 2 (RF2_mono)	C1336281H1875488N467095O667183S4018P65865Mg2450Zn0Fe0	-55381.96	Translation
tl_term_b3385_1_rib_cplx	Translation termination complex b3385 1 ribosomes	C102036H141439N36033O52510S289P5402Mg175Zn0Fe0	-4645.14	Translation
tl_term_b3385_1_rib_cplx2	Translation termination complex b3385 1 ribosomes 2 (RF2_mono)	C102087H141470N36015O52543S287P5402Mg175Zn0Fe0	-4654.14	Translation
tl_term_b3385_7_rib_cplx	Translation termination complex b3385 7 ribosomes	C671358H941569N235101O335992S2023P33308Mg1225Zn0Fe0	-28003.98	Translation
tl_term_b3385_7_rib_cplx2	Translation termination complex b3385 7 ribosomes 2 (RF2_mono)	C671715H941786N234975O336223S2009P33308Mg1225Zn0Fe0	-28066.98	Translation
tl_term_b3386_13_rib_cplx	Translation termination complex b3386 13 ribosomes	C1238113H1738330N433414O618153S3783P61130Mg2275Zn0Fe0	-51200.82	Translation
tl_term_b3386_13_rib_cplx2	Translation termination complex b3386 13 ribosomes 2 (RF2_mono)	C1238776H1738733N433180O618582S3757P61130Mg2275Zn0Fe0	-51317.82	Translation
tl_term_b3386_1_rib_cplx	Translation termination complex b3386 1 ribosomes	C101185H140446N35686O51945S291P5330Mg175Zn0Fe0	-4567.14	Translation
tl_term_b3386_1_rib_cplx2	Translation termination complex b3386 1 ribosomes 2 (RF2_mono)	C101236H140477N35668O51978S289P5330Mg175Zn0Fe0	-4576.14	Translation
tl_term_b3386_6_rib_cplx	Translation termination complex b3386 6 ribosomes	C574905H806231N201406O287865S1746P28580Mg1050Zn0Fe0	-23997.84	Translation
tl_term_b3386_6_rib_cplx2	Translation termination complex b3386 6 ribosomes 2 (RF2_mono)	C575211H806417N201298O288063S1734P28580Mg1050Zn0Fe0	-24051.84	Translation
tl_term_b3387_16_rib_cplx	Translation termination complex b3387 16 ribosomes	C1529792H2147506N534959O762336S4656P75239Mg2800Zn0Fe0	-62810.24	Translation
tl_term_b3387_16_rib_cplx2	Translation termination complex b3387 16 ribosomes 2 (RF2_mono)	C1530608H2148002N534671O762864S4624P75239Mg2800Zn0Fe0	-62954.24	Translation
tl_term_b3387_1_rib_cplx	Translation termination complex b3387 1 ribosomes	C103067H142651N36374O53151S291P5489Mg175Zn0Fe0	-4713.14	Translation
tl_term_b3387_1_rib_cplx2	Translation termination complex b3387 1 ribosomes 2 (RF2_mono)	C103118H142682N36356O53184S289P5489Mg175Zn0Fe0	-4722.14	Translation
tl_term_b3387_8_rib_cplx	Translation termination complex b3387 8 ribosomes	C768872H1078250N269047O384104S2328P38039Mg1400Zn0Fe0	-31825.12	Translation
tl_term_b3387_8_rib_cplx2	Translation termination complex b3387 8 ribosomes 2 (RF2_mono)	C769280H1078498N268903O384368S2312P38039Mg1400Zn0Fe0	-31897.12	Translation
tl_term_b3388_12_rib_cplx	Translation termination complex b3388 12 ribosomes	C1159748H1629569N406298O579497S3432P57089Mg2100Zn0Fe0	-47863.68	Translation
tl_term_b3388_12_rib_cplx2	Translation termination complex b3388 12 ribosomes 2 (RF2_mono)	C1160360H1629941N406082O579893S3408P57089Mg2100Zn0Fe0	-47971.68	Translation

tl_term_b3388_1_rib_cplx	Translation termination complex b3388 1 ribosomes	C107906H148562N38469O56447S286P5939Mg175Zn0Fe0	-5171.14	Translation
tl_term_b3388_1_rib_cplx2	Translation termination complex b3388 1 ribosomes 2 (RF2_mono)	C107957H148593N38451O56480S284P5939Mg175Zn0Fe0	-5180.14	Translation
tl_term_b3388_25_rib_cplx	Translation termination complex b3388 25 ribosomes	C2402834H3379850N841005O1197647S7150P117539Mg4375Zn0Fe0	-98318.5	Translation
tl_term_b3388_25_rib_cplx2	Translation termination complex b3388 25 ribosomes 2 (RF2_mono)	C2404109H3380625N840555O1198472S7100P117539Mg4375Zn0Fe0	-98543.5	Translation
tl_term_b3389_10_rib_cplx	Translation termination complex b3389 10 ribosomes	C964109H1353764N337357O481437S2960P47589Mg1750Zn0Fe0	-39931.4	Translation
tl_term_b3389_10_rib_cplx2	Translation termination complex b3389 10 ribosomes 2 (RF2_mono)	C964619H1354074N337177O481767S2940P47589Mg1750Zn0Fe0	-40021.4	Translation
tl_term_b3389_1_rib_cplx	Translation termination complex b3389 1 ribosomes	C105725H145910N37414O55053S296P5739Mg175Zn0Fe0	-4974.14	Translation
tl_term_b3389_1_rib_cplx2	Translation termination complex b3389 1 ribosomes 2 (RF2_mono)	C105776H145941N37396O55086S294P5739Mg175Zn0Fe0	-4983.14	Translation
tl_term_b3389_21_rib_cplx	Translation termination complex b3389 21 ribosomes	C2013245H2830030N703954O1002573S6216P98739Mg3675Zn0Fe0	-82656.94	Translation
tl_term_b3389_21_rib_cplx2	Translation termination complex b3389 21 ribosomes 2 (RF2_mono)	C2014316H2830681N703576O1003266S6174P98739Mg3675Zn0Fe0	-82845.94	Translation
tl_term_b3390_10_rib_cplx	Translation termination complex b3390 10 ribosomes	C950017H1333695N333016O475042S2860P47024Mg1750Zn0Fe0	-39356.4	Translation
tl_term_b3390_10_rib_cplx2	Translation termination complex b3390 10 ribosomes 2 (RF2_mono)	C950527H1334005N332836O475372S2840P47024Mg1750Zn0Fe0	-39446.4	Translation
tl_term_b3390_1_rib_cplx	Translation termination complex b3390 1 ribosomes	C99490H138432N35125O50773S286P5174Mg175Zn0Fe0	-4408.14	Translation
tl_term_b3390_1_rib_cplx2	Translation termination complex b3390 1 ribosomes 2 (RF2_mono)	C99541H138463N35107O50806S284P5174Mg175Zn0Fe0	-4417.14	Translation
tl_term_b3390_5_rib_cplx	Translation termination complex b3390 5 ribosomes	C477502H669660N167521O239337S1430P23774Mg875Zn0Fe0	-19940.7	Translation
tl_term_b3390_5_rib_cplx2	Translation termination complex b3390 5 ribosomes 2 (RF2_mono)	C477757H669815N167431O239502S1420P23774Mg875Zn0Fe0	-19985.7	Translation
tl_term_b3406_1_rib_cplx	Translation termination complex b3406 1 ribosomes	C99046H137874N34934O50434S285P5130Mg175Zn0Fe0	-4357.14	Translation
tl_term_b3406_1_rib_cplx2	Translation termination complex b3406 1 ribosomes 2 (RF2_mono)	C99097H137905N34916O50467S283P5130Mg175Zn0Fe0	-4366.14	Translation
tl_term_b3406_4_rib_cplx	Translation termination complex b3406 4 ribosomes	C382522H536070N134195O191779S1140P19083Mg700Zn0Fe0	-15988.56	Translation
tl_term_b3406_4_rib_cplx2	Translation termination complex b3406 4 ribosomes 2 (RF2_mono)	C382726H536194N134123O191911S1132P19083Mg700Zn0Fe0	-16024.56	Translation
tl_term_b3406_9_rib_cplx	Translation termination complex b3406 9 ribosomes	C854982H1199730N299630O427354S2565P42338Mg1575Zn0Fe0	-35374.26	Translation
tl_term_b3406_9_rib_cplx2	Translation termination complex b3406 9 ribosomes 2 (RF2_mono)	C855441H1200009N299468O427651S2547P42338Mg1575Zn0Fe0	-35455.26	Translation
tl_term_b3461_16_rib_cplx	Translation termination complex b3461 16 ribosomes	C1529273H2148010N535573O762744S4672P75257Mg2800Zn0Fe0	-63004.24	Translation
tl_term_b3461_16_rib_cplx2	Translation termination complex b3461 16 ribosomes 2 (RF2_mono)	C1530089H2148506N535285O763272S4640P75257Mg2800Zn0Fe0	-63148.24	Translation
tl_term_b3461_1_rib_cplx	Translation termination complex b3461 1 ribosomes	C103223H142900N36568O53259S292P5507Mg175Zn0Fe0	-4742.14	Translation
tl_term_b3461_1_rib_cplx2	Translation termination complex b3461 1 ribosomes 2 (RF2_mono)	C103274H142931N36550O53292S290P5507Mg175Zn0Fe0	-4751.14	Translation
tl_term_b3461_8_rib_cplx	Translation termination complex b3461 8 ribosomes	C768713H1078618N269437O384352S2336P38057Mg1400Zn0Fe0	-31931.12	Translation
tl_term_b3461_8_rib_cplx2	Translation termination complex b3461 8 ribosomes 2 (RF2_mono)	C769121H1078866N269293O384616S2320P38057Mg1400Zn0Fe0	-32003.12	Translation
tl_term_b3470_1_rib_cplx	Translation termination complex b3470 1 ribosomes	C96376H134700N33872O48721S288P4898Mg175Zn0Fe0	-4129.14	Translation
tl_term_b3470_2_rib_cplx	Translation termination complex b3470 2 ribosomes	C190420H266754N66831O95705S576P9548Mg350Zn0Fe0	-8009.28	Translation
tl_term_b3470_4_rib_cplx	Translation termination complex b3470 4 ribosomes	C378508H530862N132749O189673S1152P18848Mg700Zn0Fe0	-15769.56	Translation
tl_term_b3559_1_rib_cplx	Translation termination complex b3559 1 ribosomes	C116776H159176N41723O62342S301P6719Mg175Zn0Fe0	-5965.14	Translation
tl_term_b3559_1_rib_cplx2	Translation termination complex b3559 1 ribosomes 2 (RF2_mono)	C116827H159207N41705O62375S299P6719Mg175Zn0Fe0	-5974.14	Translation
tl_term_b3559_20_rib_cplx	Translation termination complex b3559 20 ribosomes	C1960916H2759440N683771O971910S6020P95050Mg3500Zn0Fe0	-79953.8	Translation
tl_term_b3559_20_rib_cplx2	Translation termination complex b3559 20 ribosomes 2 (RF2_mono)	C1961936H2760060N683411O972570S5980P95050Mg3500Zn0Fe0	-80133.8	Translation
tl_term_b3559_40_rib_cplx	Translation termination complex b3559 40 ribosomes	C3902116H5496560N1359611O1929350S12040P188030Mg7000Zn0Fe0	-157836.6	Translation
tl_term_b3559_40_rib_cplx2	Translation termination complex b3559 40 ribosomes 2 (RF2_mono)	C3904156H5497800N1358891O1930670S11960P188030Mg7000Zn0Fe0	-158196.6	Translation
tl_term_b3560_17_rib_cplx	Translation termination complex b3560 17 ribosomes	C1627445H2284426N568841O811015S5015P79964Mg2975Zn0Fe0	-67064.38	Translation
tl_term_b3560_17_rib_cplx2	Translation termination complex b3560 17 ribosomes 2 (RF2_mono)	C1628312H2284953N568535O811576S4981P79964Mg2975Zn0Fe0	-67217.38	Translation
tl_term_b3560_1_rib_cplx	Translation termination complex b3560 1 ribosomes	C103893H143642N36729O53703S295P5564Mg175Zn0Fe0	-4806.14	Translation
tl_term_b3560_1_rib_cplx2	Translation termination complex b3560 1 ribosomes 2 (RF2_mono)	C103944H143673N36711O53736S293P5564Mg175Zn0Fe0	-4815.14	Translation
tl_term_b3560_8_rib_cplx	Translation termination complex b3560 8 ribosomes	C770447H1080235N269528O385027S2360P38114Mg1400Zn0Fe0	-32044.12	Translation
tl_term_b3560_8_rib_cplx2	Translation termination complex b3560 8 ribosomes 2 (RF2_mono)	C770855H1080483N269384O385291S2344P38114Mg1400Zn0Fe0	-32116.12	Translation
tl_term_b3590_18_rib_cplx	Translation termination complex b3590 18 ribosomes	C1758102H2472598N614555O872560S5382P85541Mg3150Zn0Fe0	-71846.52	Translation
tl_term_b3590_18_rib_cplx2	Translation termination complex b3590 18 ribosomes 2 (RF2_mono)	C1759020H2473156N614231O873154S5346P85541Mg3150Zn0Fe0	-72008.52	Translation
tl_term_b3590_1_rib_cplx	Translation termination complex b3590 1 ribosomes	C114236H156127N40822O60623S299P6491Mg175Zn0Fe0	-5731.14	Translation
tl_term_b3590_1_rib_cplx2	Translation termination complex b3590 1 ribosomes 2 (RF2_mono)	C114287H156158N40804O60656S297P6491Mg175Zn0Fe0	-5740.14	Translation

tl_term_b3590_36_rib_cplx	Translation termination complex b3590 36 ribosomes	C3498666H4925332N1222037O1732258S10764P169241Mg6300Zn0Fe0	-141851.04	Translation
tl_term_b3590_36_rib_cplx2	Translation termination complex b3590 36 ribosomes 2 (RF2_mono)	C3500502H4926448N1221389O1733446S10692P169241Mg6300Zn0Fe0	-142175.04	Translation
tl_term_b3591_13_rib_cplx	Translation termination complex b3591 13 ribosomes	C1259645H1770729N440705O627770S3887P61844Mg2275Zn0Fe0	-51888.82	Translation
tl_term_b3591_1_rib_cplx	Translation termination complex b3591 1 ribosomes	C109133H150057N38813O57302S299P6044Mg175Zn0Fe0	-5279.14	Translation
tl_term_b3591_27_rib_cplx	Translation termination complex b3591 27 ribosomes	C2601909H3661513N909579O1293316S8073P126944Mg4725Zn0Fe0	-106266.78	Translation
tl_term_b3635_15_rib_cplx	Translation termination complex b3635 15 ribosomes	C1432924H2012724N501586O714442S4380P70562Mg2625Zn0Fe0	-58925.1	Translation
tl_term_b3635_15_rib_cplx2	Translation termination complex b3635 15 ribosomes 2 (RF2_mono)	C1433689H2013189N501316O714937S4350P70562Mg2625Zn0Fe0	-59060.1	Translation
tl_term_b3635_1_rib_cplx	Translation termination complex b3635 1 ribosomes	C102742H142324N36352O52928S292P5462Mg175Zn0Fe0	-4687.14	Translation
tl_term_b3635_1_rib_cplx2	Translation termination complex b3635 1 ribosomes 2 (RF2_mono)	C102793H142355N36334O52961S290P5462Mg175Zn0Fe0	-4696.14	Translation
tl_term_b3635_7_rib_cplx	Translation termination complex b3635 7 ribosomes	C672820H943924N235738O336434S2044P33362Mg1225Zn0Fe0	-27931.98	Translation
tl_term_b3635_7_rib_cplx2	Translation termination complex b3635 7 ribosomes 2 (RF2_mono)	C673177H944141N235612O336665S2030P33362Mg1225Zn0Fe0	-27994.98	Translation
tl_term_b3636_1_rib_cplx	Translation termination complex b3636 1 ribosomes	C95551H133735N33585O48101S283P4818Mg175Zn0Fe0	-4037.14	Translation
tl_term_b3636_1_rib_cplx2	Translation termination complex b3636 1 ribosomes 2 (RF2_mono)	C95602H133766N33567O48134S281P4818Mg175Zn0Fe0	-4046.14	Translation
tl_term_b3636_3_rib_cplx	Translation termination complex b3636 3 ribosomes	C283439H397585N99445O141993S849P14118Mg525Zn0Fe0	-11773.42	Translation
tl_term_b3636_3_rib_cplx2	Translation termination complex b3636 3 ribosomes 2 (RF2_mono)	C283592H397678N99391O142092S843P14118Mg525Zn0Fe0	-11800.42	Translation
tl_term_b3637_1_rib_cplx	Translation termination complex b3637 1 ribosomes	C96387H134749N33911O48690S286P4898Mg175Zn0Fe0	-4114.14	Translation
tl_term_b3637_1_rib_cplx2	Translation termination complex b3637 1 ribosomes 2 (RF2_mono)	C96438H134780N33893O48723S284P4898Mg175Zn0Fe0	-4123.14	Translation
tl_term_b3637_2_rib_cplx	Translation termination complex b3637 2 ribosomes	C190523H266944N66929O95717S572P9557Mg350Zn0Fe0	-7988.28	Translation
tl_term_b3637_2_rib_cplx2	Translation termination complex b3637 2 ribosomes 2 (RF2_mono)	C190625H267006N66893O95783S568P9557Mg350Zn0Fe0	-8006.28	Translation
tl_term_b3637_4_rib_cplx	Translation termination complex b3637 4 ribosomes	C378795H531334N132965O189771S1144P18875Mg700Zn0Fe0	-15736.56	Translation
tl_term_b3637_4_rib_cplx2	Translation termination complex b3637 4 ribosomes 2 (RF2_mono)	C378999H531458N132893O189903S1136P18875Mg700Zn0Fe0	-15772.56	Translation
tl_term_b3638_13_rib_cplx	Translation termination complex b3638 13 ribosomes	C1238746H1739604N433874O618227S3783P61134Mg2275Zn0Fe0	-51074.82	Translation
tl_term_b3638_13_rib_cplx2	Translation termination complex b3638 13 ribosomes 2 (RF2_mono)	C1239409H1740007N433640O618656S3757P61134Mg2275Zn0Fe0	-51191.82	Translation
tl_term_b3638_1_rib_cplx	Translation termination complex b3638 1 ribosomes	C101170H140448N35714O51887S291P5322Mg175Zn0Fe0	-4549.14	Translation
tl_term_b3638_1_rib_cplx2	Translation termination complex b3638 1 ribosomes 2 (RF2_mono)	C101221H140479N35696O51920S289P5322Mg175Zn0Fe0	-4558.14	Translation
tl_term_b3638_6_rib_cplx	Translation termination complex b3638 6 ribosomes	C575160H806763N201614O287862S1746P28577Mg1050Zn0Fe0	-23934.84	Translation
tl_term_b3638_6_rib_cplx2	Translation termination complex b3638 6 ribosomes 2 (RF2_mono)	C575466H806949N201506O288060S1734P28577Mg1050Zn0Fe0	-23988.84	Translation
tl_term_b3649_1_rib_cplx	Translation termination complex b3649 1 ribosomes	C96736H135153N34086O48923S284P4929Mg175Zn0Fe0	-4161.14	Translation
tl_term_b3649_1_rib_cplx2	Translation termination complex b3649 1 ribosomes 2 (RF2_mono)	C96787H135184N34068O48956S282P4929Mg175Zn0Fe0	-4170.14	Translation
tl_term_b3649_2_rib_cplx	Translation termination complex b3649 2 ribosomes	C190832H267321N67084O95932S568P9580Mg350Zn0Fe0	-8043.28	Translation
tl_term_b3649_2_rib_cplx2	Translation termination complex b3649 2 ribosomes 2 (RF2_mono)	C190934H267383N67048O95998S564P9580Mg350Zn0Fe0	-8061.28	Translation
tl_term_b3649_5_rib_cplx	Translation termination complex b3649 5 ribosomes	C473120H663825N166078O236959S1420P23533Mg875Zn0Fe0	-19689.7	Translation
tl_term_b3649_5_rib_cplx2	Translation termination complex b3649 5 ribosomes 2 (RF2_mono)	C473375H663980N165988O237124S1410P23533Mg875Zn0Fe0	-19734.7	Translation
tl_term_b3650_1_rib_cplx	Translation termination complex b3650 1 ribosomes	C117233H159833N41916O62586S313P6759Mg175Zn0Fe0	-5979.14	Translation
tl_term_b3650_1_rib_cplx2	Translation termination complex b3650 1 ribosomes 2 (RF2_mono)	C117284H159864N41898O62619S311P6759Mg175Zn0Fe0	-5988.14	Translation
tl_term_b3650_20_rib_cplx	Translation termination complex b3650 20 ribosomes	C1963311H2764467N685294O972534S6260P95109Mg3500Zn0Fe0	-79492.8	Translation
tl_term_b3650_20_rib_cplx2	Translation termination complex b3650 20 ribosomes 2 (RF2_mono)	C1964331H2765087N684934O973194S6220P95109Mg3500Zn0Fe0	-79672.8	Translation
tl_term_b3650_41_rib_cplx	Translation termination complex b3650 41 ribosomes	C4003713H5643273N1396396O1978266S12833P192759Mg7175Zn0Fe0	-160744.74	Translation
tl_term_b3650_41_rib_cplx2	Translation termination complex b3650 41 ribosomes 2 (RF2_mono)	C4005804H5644544N1395658O1979619S12751P192759Mg7175Zn0Fe0	-161113.74	Translation
tl_term_b3651_13_rib_cplx	Translation termination complex b3651 13 ribosomes	C1238003H1738502N433782O618097S3861P61114Mg2275Zn0Fe0	-51080.82	Translation
tl_term_b3651_13_rib_cplx2	Translation termination complex b3651 13 ribosomes 2 (RF2_mono)	C1238666H1738905N433548O618526S3835P61114Mg2275Zn0Fe0	-51197.82	Translation
tl_term_b3651_1_rib_cplx	Translation termination complex b3651 1 ribosomes	C101291H140606N35802O51985S297P5338Mg175Zn0Fe0	-4567.14	Translation
tl_term_b3651_1_rib_cplx2	Translation termination complex b3651 1 ribosomes 2 (RF2_mono)	C101342H140637N35784O52018S295P5338Mg175Zn0Fe0	-4576.14	Translation
tl_term_b3651_6_rib_cplx	Translation termination complex b3651 6 ribosomes	C574921H806396N201627O287865S1782P28578Mg1050Zn0Fe0	-23947.84	Translation
tl_term_b3651_6_rib_cplx2	Translation termination complex b3651 6 ribosomes 2 (RF2_mono)	C575227H806582N201519O288063S1770P28578Mg1050Zn0Fe0	-24001.84	Translation
tl_term_b3652_1_rib_cplx	Translation termination complex b3652 1 ribosomes	C116855H159396N41831O62354S308P6732Mg175Zn0Fe0	-5964.14	Translation
tl_term_b3652_1_rib_cplx2	Translation termination complex b3652 1 ribosomes 2 (RF2_mono)	C116906H159427N41813O62387S306P6732Mg175Zn0Fe0	-5973.14	Translation

tl_term_b3652_20_rib_cplx	Translation termination complex b3652 20 ribosomes	C1960463H2760553N684506O971504S6160P95082Mg3500Zn0Fe0	-79705.8	Translation
tl_term_b3652_20_rib_cplx2	Translation termination complex b3652 20 ribosomes 2 (RF2_mono)	C1961483H2761173N684146O972164S6120P95082Mg3500Zn0Fe0	-79885.8	Translation
tl_term_b3652_40_rib_cplx	Translation termination complex b3652 40 ribosomes	C3901103H5498613N1361006O1928504S12320P188082Mg7000Zn0Fe0	-157328.6	Translation
tl_term_b3652_40_rib_cplx2	Translation termination complex b3652 40 ribosomes 2 (RF2_mono)	C3903143H5499853N1360286O1929824S12240P188082Mg7000Zn0Fe0	-157688.6	Translation
tl_term_b3703_1_rib_cplx	Translation termination complex b3703 1 ribosomes	C95216H133363N33451O47928S284P4793Mg175Zn0Fe0	-4006.14	Translation
tl_term_b3703_1_rib_cplx2	Translation termination complex b3703 1 ribosomes 2 (RF2_mono)	C95267H133394N33433O47961S282P4793Mg175Zn0Fe0	-4015.14	Translation
tl_term_b3703_2_rib_cplx	Translation termination complex b3703 2 ribosomes	C189099H265212N66389O94854S568P9443Mg350Zn0Fe0	-7868.28	Translation
tl_term_b3703_2_rib_cplx2	Translation termination complex b3703 2 ribosomes 2 (RF2_mono)	C189201H265274N66353O94920S564P9443Mg350Zn0Fe0	-7886.28	Translation
tl_term_b3704_1_rib_cplx	Translation termination complex b3704 1 ribosomes	C97787H136457N34452O49625S284P5022Mg175Zn0Fe0	-4234.14	Translation
tl_term_b3704_3_rib_cplx	Translation termination complex b3704 3 ribosomes	C286535H401611N100666O143827S852P14346Mg525Zn0Fe0	-11980.42	Translation
tl_term_b3704_7_rib_cplx	Translation termination complex b3704 7 ribosomes	C664031H931919N233094O332231S1988P32994Mg1225Zn0Fe0	-27472.98	Translation
tl_term_b3706_13_rib_cplx	Translation termination complex b3706 13 ribosomes	C1258734H1768493N440300O627536S3770P61817Mg2275Zn0Fe0	-52082.82	Translation
tl_term_b3706_13_rib_cplx2	Translation termination complex b3706 13 ribosomes 2 (RF2_mono)	C1259397H1768896N440066O627965S3744P61817Mg2275Zn0Fe0	-52199.82	Translation
tl_term_b3706_1_rib_cplx	Translation termination complex b3706 1 ribosomes	C108822H149633N38696O57080S290P6017Mg175Zn0Fe0	-5269.14	Translation
tl_term_b3706_1_rib_cplx2	Translation termination complex b3706 1 ribosomes 2 (RF2_mono)	C108873H149664N38678O57113S288P6017Mg175Zn0Fe0	-5278.14	Translation
tl_term_b3706_26_rib_cplx	Translation termination complex b3706 26 ribosomes	C2504472H3522258N875371O1245530S7540P122267Mg4550Zn0Fe0	-102797.64	Translation
tl_term_b3706_26_rib_cplx2	Translation termination complex b3706 26 ribosomes 2 (RF2_mono)	C2505798H3523064N874903O1246388S7488P122267Mg4550Zn0Fe0	-103031.64	Translation
tl_term_b3740_12_rib_cplx	Translation termination complex b3740 12 ribosomes	C1142619H1604133N400142O570457S3444P56436Mg2100Zn0Fe0	-47222.68	Translation
tl_term_b3740_12_rib_cplx2	Translation termination complex b3740 12 ribosomes 2 (RF2_mono)	C1143231H1604505N399926O570853S3420P56436Mg2100Zn0Fe0	-47330.68	Translation
tl_term_b3740_1_rib_cplx	Translation termination complex b3740 1 ribosomes	C100655H139824N35492O51543S287P5275Mg175Zn0Fe0	-4508.14	Translation
tl_term_b3740_1_rib_cplx2	Translation termination complex b3740 1 ribosomes 2 (RF2_mono)	C100706H139855N35474O51576S285P5275Mg175Zn0Fe0	-4517.14	Translation
tl_term_b3740_6_rib_cplx	Translation termination complex b3740 6 ribosomes	C574275H805419N201242O287413S1722P28530Mg1050Zn0Fe0	-23923.84	Translation
tl_term_b3740_6_rib_cplx2	Translation termination complex b3740 6 ribosomes 2 (RF2_mono)	C574581H805605N201134O287611S1710P28530Mg1050Zn0Fe0	-23977.84	Translation
tl_term_b3741_18_rib_cplx	Translation termination complex b3741 18 ribosomes	C1758723H2474001N614378O873425S5454P85592Mg3150Zn0Fe0	-71789.52	Translation
tl_term_b3741_18_rib_cplx2	Translation termination complex b3741 18 ribosomes 2 (RF2_mono)	C1759641H2474559N614054O874019S5418P85592Mg3150Zn0Fe0	-71951.52	Translation
tl_term_b3741_1_rib_cplx	Translation termination complex b3741 1 ribosomes	C114687H156680N40917O61012S303P6542Mg175Zn0Fe0	-5776.14	Translation
tl_term_b3741_1_rib_cplx2	Translation termination complex b3741 1 ribosomes 2 (RF2_mono)	C114738H156711N40899O61045S301P6542Mg175Zn0Fe0	-5785.14	Translation
tl_term_b3741_37_rib_cplx	Translation termination complex b3741 37 ribosomes	C3596175H5063948N1255305O1781416S11211P173942Mg6475Zn0Fe0	-145569.18	Translation
tl_term_b3741_37_rib_cplx2	Translation termination complex b3741 37 ribosomes 2 (RF2_mono)	C3598062H5065095N1254639O1782637S11137P173942Mg6475Zn0Fe0	-145902.18	Translation
tl_term_b3780_12_rib_cplx	Translation termination complex b3780 12 ribosomes	C1160868H1630620N406247O578558S3528P57068Mg2100Zn0Fe0	-47794.68	Translation
tl_term_b3780_12_rib_cplx2	Translation termination complex b3780 12 ribosomes 2 (RF2_mono)	C1161480H1630992N406031O578954S3504P57068Mg2100Zn0Fe0	-47902.68	Translation
tl_term_b3780_1_rib_cplx	Translation termination complex b3780 1 ribosomes	C107772H148381N38242O56333S294P5918Mg175Zn0Fe0	-5146.14	Translation
tl_term_b3780_1_rib_cplx2	Translation termination complex b3780 1 ribosomes 2 (RF2_mono)	C107823H148412N38224O56366S292P5918Mg175Zn0Fe0	-5155.14	Translation
tl_term_b3780_24_rib_cplx	Translation termination complex b3780 24 ribosomes	C2309700H3247608N807707O1148258S7056P112868Mg4200Zn0Fe0	-94320.36	Translation
tl_term_b3780_24_rib_cplx2	Translation termination complex b3780 24 ribosomes 2 (RF2_mono)	C2310924H3248352N807275O1149050S7008P112868Mg4200Zn0Fe0	-94536.36	Translation
tl_term_b3782_1_rib_cplx	Translation termination complex b3782 1 ribosomes	C94781H132790N33289O47643S285P4755Mg175Zn0Fe0	-3979.14	Translation
tl_term_b3783_12_rib_cplx	Translation termination complex b3783 12 ribosomes	C1162135H1632819N406696O579559S3600P57204Mg2100Zn0Fe0	-47954.68	Translation
tl_term_b3783_12_rib_cplx2	Translation termination complex b3783 12 ribosomes 2 (RF2_mono)	C1162747H1633191N406480O579955S3576P57204Mg2100Zn0Fe0	-48062.68	Translation
tl_term_b3783_1_rib_cplx	Translation termination complex b3783 1 ribosomes	C107829H148501N38262O56355S300P5922Mg175Zn0Fe0	-5152.14	Translation
tl_term_b3783_1_rib_cplx2	Translation termination complex b3783 1 ribosomes 2 (RF2_mono)	C107880H148532N38244O56388S298P5922Mg175Zn0Fe0	-5161.14	Translation
tl_term_b3783_24_rib_cplx	Translation termination complex b3783 24 ribosomes	C2312287H3252075N808624O1150327S7200P113148Mg4200Zn0Fe0	-94648.36	Translation
tl_term_b3783_24_rib_cplx2	Translation termination complex b3783 24 ribosomes 2 (RF2_mono)	C2313511H3252819N808192O1151119S7152P113148Mg4200Zn0Fe0	-94864.36	Translation
tl_term_b3885_11_rib_cplx	Translation termination complex b3885 11 ribosomes	C1046935H1468963N366560O522791S3190P51730Mg1925Zn0Fe0	-43361.54	Translation
tl_term_b3885_11_rib_cplx2	Translation termination complex b3885 11 ribosomes 2 (RF2_mono)	C1047496H1469304N366362O523154S3168P51730Mg1925Zn0Fe0	-43460.54	Translation
tl_term_b3885_1_rib_cplx	Translation termination complex b3885 1 ribosomes	C100365H139403N35380O51361S290P5250Mg175Zn0Fe0	-4490.14	Translation
tl_term_b3885_1_rib_cplx2	Translation termination complex b3885 1 ribosomes 2 (RF2_mono)	C100416H139434N35362O51394S288P5250Mg175Zn0Fe0	-4499.14	Translation
tl_term_b3885_5_rib_cplx	Translation termination complex b3885 5 ribosomes	C478993H671227N167852O239933S1450P23842Mg875Zn0Fe0	-20038.7	Translation

tl_term_b3885_5_rib_cplx2	Translation termination complex b3885 5 ribosomes 2 (RF2_mono)	C479248H671382N167762O240098S1440P23842Mg875Zn0Fe0	-20083.7	Translation
tl_term_b3886_17_rib_cplx	Translation termination complex b3886 17 ribosomes	C1626737H2284458N568092O809638S4947P79933Mg2975Zn0Fe0	-66744.38	Translation
tl_term_b3886_1_rib_cplx	Translation termination complex b3886 1 ribosomes	C103393H143114N36364O53398S291P5517Mg175Zn0Fe0	-4742.14	Translation
tl_term_b3886_8_rib_cplx	Translation termination complex b3886 8 ribosomes	C769856H1079952N268995O384253S2328P38074Mg1400Zn0Fe0	-31868.12	Translation
tl_term_b3887_1_rib_cplx	Translation termination complex b3887 1 ribosomes	C98484H137209N34738O50110S289P5084Mg175Zn0Fe0	-4318.14	Translation
tl_term_b3887_4_rib_cplx	Translation termination complex b3887 4 ribosomes	C381507H534787N133900O191353S1156P19034Mg700Zn0Fe0	-15967.56	Translation
tl_term_b3887_8_rib_cplx	Translation termination complex b3887 8 ribosomes	C758871H1064891N266116O379677S2312P37634Mg1400Zn0Fe0	-31500.12	Translation
tl_term_b3888_19_rib_cplx	Translation termination complex b3888 19 ribosomes	C1820958H2557000N636470O906964S5605P89336Mg3325Zn0Fe0	-74975.66	Translation
tl_term_b3888_1_rib_cplx	Translation termination complex b3888 1 ribosomes	C104748H144658N37106O54250S295P5636Mg175Zn0Fe0	-4881.14	Translation
tl_term_b3888_9_rib_cplx	Translation termination complex b3888 9 ribosomes	C867508H1216810N303490O433234S2655P42836Mg1575Zn0Fe0	-36034.26	Translation
tl_term_b3936_1_rib_cplx	Translation termination complex b3936 1 ribosomes	C96019H134279N33759O48455S288P4865Mg175Zn0Fe0	-4088.14	Translation
tl_term_b3936_1_rib_cplx2	Translation termination complex b3936 1 ribosomes 2 (RF2_mono)	C96070H134310N33741O48488S286P4865Mg175Zn0Fe0	-4097.14	Translation
tl_term_b3936_2_rib_cplx	Translation termination complex b3936 2 ribosomes	C190013H266262N66711O95424S576P9515Mg350Zn0Fe0	-7960.28	Translation
tl_term_b3936_2_rib_cplx2	Translation termination complex b3936 2 ribosomes 2 (RF2_mono)	C190115H266324N66675O95490S572P9515Mg350Zn0Fe0	-7978.28	Translation
tl_term_b3936_4_rib_cplx	Translation termination complex b3936 4 ribosomes	C378001H530228N132615O189362S1152P18815Mg700Zn0Fe0	-15704.56	Translation
tl_term_b3936_4_rib_cplx2	Translation termination complex b3936 4 ribosomes 2 (RF2_mono)	C378205H530352N132543O189494S1144P18815Mg700Zn0Fe0	-15740.56	Translation
tl_term_b3965_10_rib_cplx	Translation termination complex b3965 10 ribosomes	C965629H1355410N337861O481907S3000P47603Mg1750Zn0Fe0	-39975.4	Translation
tl_term_b3965_10_rib_cplx2	Translation termination complex b3965 10 ribosomes 2 (RF2_mono)	C966139H1355720N337681O482237S2980P47603Mg1750Zn0Fe0	-40065.4	Translation
tl_term_b3965_1_rib_cplx	Translation termination complex b3965 1 ribosomes	C106003H146215N37576O55100S300P5753Mg175Zn0Fe0	-4991.14	Translation
tl_term_b3965_1_rib_cplx2	Translation termination complex b3965 1 ribosomes 2 (RF2_mono)	C106054H146246N37558O55133S298P5753Mg175Zn0Fe0	-5000.14	Translation
tl_term_b3965_21_rib_cplx	Translation termination complex b3965 21 ribosomes	C2016283H2833315N704876O1003560S6300P98753Mg3675Zn0Fe0	-82733.94	Translation
tl_term_b3965_21_rib_cplx2	Translation termination complex b3965 21 ribosomes 2 (RF2_mono)	C2017354H2833966N704498O1004253S6258P98753Mg3675Zn0Fe0	-82922.94	Translation
tl_term_b3980_11_rib_cplx	Translation termination complex b3980 11 ribosomes	C1064388H1494111N372508O531445S3267P52524Mg1925Zn0Fe0	-44199.54	Translation
tl_term_b3980_11_rib_cplx2	Translation termination complex b3980 11 ribosomes 2 (RF2_mono)	C1064949H1494452N372310O531808S3245P52524Mg1925Zn0Fe0	-44298.54	Translation
tl_term_b3980_1_rib_cplx	Translation termination complex b3980 1 ribosomes	C107018H147441N37978O55835S297P5854Mg175Zn0Fe0	-5098.14	Translation
tl_term_b3980_1_rib_cplx2	Translation termination complex b3980 1 ribosomes 2 (RF2_mono)	C107069H147472N37960O55868S295P5854Mg175Zn0Fe0	-5107.14	Translation
tl_term_b3980_23_rib_cplx	Translation termination complex b3980 23 ribosomes	C2213232H3110115N773944O1102177S6831P108528Mg4025Zn0Fe0	-91121.22	Translation
tl_term_b3980_23_rib_cplx2	Translation termination complex b3980 23 ribosomes 2 (RF2_mono)	C2214405H3110828N773530O1102936S6785P108528Mg4025Zn0Fe0	-91328.22	Translation
tl_term_b3981_1_rib_cplx	Translation termination complex b3981 1 ribosomes	C97932H136605N34462O49747S287P5036Mg175Zn0Fe0	-4258.14	Translation
tl_term_b3981_3_rib_cplx	Translation termination complex b3981 3 ribosomes	C286496H401565N100506O143797S861P14336Mg525Zn0Fe0	-12000.42	Translation
tl_term_b3981_7_rib_cplx	Translation termination complex b3981 7 ribosomes	C663624H931485N232594O331897S2009P32936Mg1225Zn0Fe0	-27484.98	Translation
tl_term_b3982_10_rib_cplx	Translation termination complex b3982 10 ribosomes	C950800H1334411N333204O475101S2890P47046Mg1750Zn0Fe0	-39328.4	Translation
tl_term_b3982_10_rib_cplx2	Translation termination complex b3982 10 ribosomes 2 (RF2_mono)	C951310H1334721N333024O475431S2870P47046Mg1750Zn0Fe0	-39418.4	Translation
tl_term_b3982_1_rib_cplx	Translation termination complex b3982 1 ribosomes	C99760H138725N35187O50958S289P5196Mg175Zn0Fe0	-4425.14	Translation
tl_term_b3982_1_rib_cplx2	Translation termination complex b3982 1 ribosomes 2 (RF2_mono)	C99811H138756N35169O50991S287P5196Mg175Zn0Fe0	-4434.14	Translation
tl_term_b3982_5_rib_cplx	Translation termination complex b3982 5 ribosomes	C478000H670141N167639O239466S1445P23796Mg875Zn0Fe0	-19937.7	Translation
tl_term_b3982_5_rib_cplx2	Translation termination complex b3982 5 ribosomes 2 (RF2_mono)	C478255H670296N167549O239631S1435P23796Mg875Zn0Fe0	-19982.7	Translation
tl_term_b3983_1_rib_cplx	Translation termination complex b3983 1 ribosomes	C98407H137166N34686O50058S289P5082Mg175Zn0Fe0	-4303.14	Translation
tl_term_b3983_1_rib_cplx2	Translation termination complex b3983 1 ribosomes 2 (RF2_mono)	C98458H137197N34668O50091S287P5082Mg175Zn0Fe0	-4312.14	Translation
tl_term_b3983_4_rib_cplx	Translation termination complex b3983 4 ribosomes	C381361H534777N133788O191271S1156P19035Mg700Zn0Fe0	-15916.56	Translation
tl_term_b3983_4_rib_cplx2	Translation termination complex b3983 4 ribosomes 2 (RF2_mono)	C381565H534901N133716O191403S1148P19035Mg700Zn0Fe0	-15952.56	Translation
tl_term_b3983_8_rib_cplx	Translation termination complex b3983 8 ribosomes	C758633H1064925N265924O379555S2312P37639Mg1400Zn0Fe0	-31401.12	Translation
tl_term_b3983_8_rib_cplx2	Translation termination complex b3983 8 ribosomes 2 (RF2_mono)	C759041H1065173N265780O379819S2296P37639Mg1400Zn0Fe0	-31473.12	Translation
tl_term_b3984_13_rib_cplx	Translation termination complex b3984 13 ribosomes	C1238326H1739887N433876O618472S3757P61155Mg2275Zn0Fe0	-51004.82	Translation
tl_term_b3984_13_rib_cplx2	Translation termination complex b3984 13 ribosomes 2 (RF2_mono)	C1238989H1740290N433642O618901S3731P61155Mg2275Zn0Fe0	-51121.82	Translation
tl_term_b3984_1_rib_cplx	Translation termination complex b3984 1 ribosomes	C101470H140863N35908O52084S289P5355Mg175Zn0Fe0	-4575.14	Translation
tl_term_b3984_1_rib_cplx2	Translation termination complex b3984 1 ribosomes 2 (RF2_mono)	C101521H140894N35890O52117S287P5355Mg175Zn0Fe0	-4584.14	Translation

tl_term_b3984_6_rib_cplx	Translation termination complex b3984 6 ribosomes	C575160H807123N201728O288079S1734P28605Mg1050Zn0Fe0	-23920.84	Translation
tl_term_b3984_6_rib_cplx2	Translation termination complex b3984 6 ribosomes 2 (RF2_mono)	C575466H807309N201620O288277S1722P28605Mg1050Zn0Fe0	-23974.84	Translation
tl_term_b3985_1_rib_cplx	Translation termination complex b3985 1 ribosomes	C99170H138071N34972O50585S288P5150Mg175Zn0Fe0	-4376.14	Translation
tl_term_b3985_1_rib_cplx2	Translation termination complex b3985 1 ribosomes 2 (RF2_mono)	C99221H138102N34954O50618S286P5150Mg175Zn0Fe0	-4385.14	Translation
tl_term_b3985_4_rib_cplx	Translation termination complex b3985 4 ribosomes	C382454H536195N134194O191876S1152P19100Mg700Zn0Fe0	-16001.56	Translation
tl_term_b3985_4_rib_cplx2	Translation termination complex b3985 4 ribosomes 2 (RF2_mono)	C382658H536319N134122O192008S1144P19100Mg700Zn0Fe0	-16037.56	Translation
tl_term_b3985_9_rib_cplx	Translation termination complex b3985 9 ribosomes	C854594H1199735N299564O427361S2592P42350Mg1575Zn0Fe0	-35377.26	Translation
tl_term_b3985_9_rib_cplx2	Translation termination complex b3985 9 ribosomes 2 (RF2_mono)	C855053H1200014N299402O427658S2574P42350Mg1575Zn0Fe0	-35458.26	Translation
tl_term_b3986_1_rib_cplx	Translation termination complex b3986 1 ribosomes	C97699H136272N34422O49582S286P5016Mg175Zn0Fe0	-4253.14	Translation
tl_term_b3986_1_rib_cplx2	Translation termination complex b3986 1 ribosomes 2 (RF2_mono)	C97750H136303N34404O49615S284P5016Mg175Zn0Fe0	-4262.14	Translation
tl_term_b3986_3_rib_cplx	Translation termination complex b3986 3 ribosomes	C286087H400932N100396O143674S858P14316Mg525Zn0Fe0	-12025.42	Translation
tl_term_b3986_3_rib_cplx2	Translation termination complex b3986 3 ribosomes 2 (RF2_mono)	C286240H401025N100342O143773S852P14316Mg525Zn0Fe0	-12052.42	Translation
tl_term_b3986_7_rib_cplx	Translation termination complex b3986 7 ribosomes	C662863H930252N232344O331858S2002P32916Mg1225Zn0Fe0	-27569.98	Translation
tl_term_b3986_7_rib_cplx2	Translation termination complex b3986 7 ribosomes 2 (RF2_mono)	C663220H930469N232218O332089S1988P32916Mg1225Zn0Fe0	-27632.98	Translation
tl_term_b3987_1_rib_cplx	Translation termination complex b3987 1 ribosomes	C138651H185451N50098O76994S326P8679Mg175Zn0Fe0	-7951.14	Translation
tl_term_b3987_1_rib_cplx2	Translation termination complex b3987 1 ribosomes 2 (RF2_mono)	C138702H185482N50080O77027S324P8679Mg175Zn0Fe0	-7960.14	Translation
tl_term_b3987_39_rib_cplx	Translation termination complex b3987 39 ribosomes	C3949519H5582325N1368508O1935764S12714P185379Mg6825Zn0Fe0	-156954.46	Translation
tl_term_b3987_39_rib_cplx2	Translation termination complex b3987 39 ribosomes 2 (RF2_mono)	C3951508H5583534N1367806O1937051S12636P185379Mg6825Zn0Fe0	-157305.46	Translation
tl_term_b3987_78_rib_cplx	Translation termination complex b3987 78 ribosomes	C7860673H11121222N2721613O3843449S25428P366729Mg13650Zn0Fe0	-309878.92	Translation
tl_term_b3987_78_rib_cplx2	Translation termination complex b3987 78 ribosomes 2 (RF2_mono)	C7864651H11123640N2720209O3846023S25272P366729Mg13650Zn0Fe0	-310580.92	Translation
tl_term_b3988_1_rib_cplx	Translation termination complex b3988 1 ribosomes	C140691H188061N50922O78421S333P8874Mg175Zn0Fe0	-8106.14	Translation
tl_term_b3988_1_rib_cplx2	Translation termination complex b3988 1 ribosomes 2 (RF2_mono)	C140742H188092N50904O78454S331P8874Mg175Zn0Fe0	-8115.14	Translation
tl_term_b3988_41_rib_cplx	Translation termination complex b3988 41 ribosomes	C4159771H5889421N1442602O2035461S13653P194874Mg7175Zn0Fe0	-163351.74	Translation
tl_term_b3988_41_rib_cplx2	Translation termination complex b3988 41 ribosomes 2 (RF2_mono)	C4161862H5890692N1441864O2036814S13571P194874Mg7175Zn0Fe0	-163720.74	Translation
tl_term_b3988_82_rib_cplx	Translation termination complex b3988 82 ribosomes	C8279328H11733315N2869074O4041427S27306P385524Mg14350Zn0Fe0	-322478.48	Translation
tl_term_b3988_82_rib_cplx2	Translation termination complex b3988 82 ribosomes 2 (RF2_mono)	C8283510H11735857N2867598O4044133S27142P385524Mg14350Zn0Fe0	-323216.48	Translation
tl_term_b4022_17_rib_cplx	Translation termination complex b4022 17 ribosomes	C1624922H2284312N569166O809970S4930P79942Mg2975Zn0Fe0	-66549.38	Translation
tl_term_b4022_1_rib_cplx	Translation termination complex b4022 1 ribosomes	C103434H143240N36686O53346S290P5526Mg175Zn0Fe0	-4739.14	Translation
tl_term_b4022_8_rib_cplx	Translation termination complex b4022 8 ribosomes	C769085H1079959N269646O384369S2320P38083Mg1400Zn0Fe0	-31781.12	Translation
tl_term_b4049_10_rib_cplx	Translation termination complex b4049 10 ribosomes	C963411H1352092N337425O480966S3030P47550Mg1750Zn0Fe0	-39882.4	Translation
tl_term_b4049_10_rib_cplx2	Translation termination complex b4049 10 ribosomes 2 (RF2_mono)	C963921H1352402N337245O481296S3010P47550Mg1750Zn0Fe0	-39972.4	Translation
tl_term_b4049_1_rib_cplx	Translation termination complex b4049 1 ribosomes	C105252H145273N37338O54636S303P5691Mg175Zn0Fe0	-4925.14	Translation
tl_term_b4049_1_rib_cplx2	Translation termination complex b4049 1 ribosomes 2 (RF2_mono)	C105303H145304N37320O54669S301P5691Mg175Zn0Fe0	-4934.14	Translation
tl_term_b4049_20_rib_cplx	Translation termination complex b4049 20 ribosomes	C1916921H2693002N670855O954666S6060P94060Mg3500Zn0Fe0	-78723.8	Translation
tl_term_b4049_20_rib_cplx2	Translation termination complex b4049 20 ribosomes 2 (RF2_mono)	C1917941H2693622N670495O955326S6020P94060Mg3500Zn0Fe0	-78903.8	Translation
tl_term_b4129_14_rib_cplx	Translation termination complex b4129 14 ribosomes	C1361551H1912252N475573O677603S4214P66620Mg2450Zn0Fe0	-56108.96	Translation
tl_term_b4129_14_rib_cplx2	Translation termination complex b4129 14 ribosomes 2 (RF2_mono)	C1362265H1912686N475321O678065S4186P66620Mg2450Zn0Fe0	-56234.96	Translation
tl_term_b4129_1_rib_cplx	Translation termination complex b4129 1 ribosomes	C110665H151727N39280O58270S301P6170Mg175Zn0Fe0	-5420.14	Translation
tl_term_b4129_1_rib_cplx2	Translation termination complex b4129 1 ribosomes 2 (RF2_mono)	C110716H151758N39262O58303S299P6170Mg175Zn0Fe0	-5429.14	Translation
tl_term_b4129_29_rib_cplx	Translation termination complex b4129 29 ribosomes	C2804881H3943627N978988O1392218S8729P136370Mg5075Zn0Fe0	-114596.06	Translation
tl_term_b4129_29_rib_cplx2	Translation termination complex b4129 29 ribosomes 2 (RF2_mono)	C2806360H3944526N978466O1393175S8671P136370Mg5075Zn0Fe0	-114857.06	Translation
tl_term_b4142_1_rib_cplx	Translation termination complex b4142 1 ribosomes	C96909H135341N34114O49068S283P4946Mg175Zn0Fe0	-4178.14	Translation
tl_term_b4142_1_rib_cplx2	Translation termination complex b4142 1 ribosomes 2 (RF2_mono)	C96960H135372N34096O49101S281P4946Mg175Zn0Fe0	-4187.14	Translation
tl_term_b4142_2_rib_cplx	Translation termination complex b4142 2 ribosomes	C191010H267519N67094O96078S566P9596Mg350Zn0Fe0	-8059.28	Translation
tl_term_b4142_2_rib_cplx2	Translation termination complex b4142 2 ribosomes 2 (RF2_mono)	C191112H267581N67058O96144S562P9596Mg350Zn0Fe0	-8077.28	Translation
tl_term_b4142_5_rib_cplx	Translation termination complex b4142 5 ribosomes	C473313H664053N166034O237108S1415P23546Mg875Zn0Fe0	-19702.7	Translation
tl_term_b4142_5_rib_cplx2	Translation termination complex b4142 5 ribosomes 2 (RF2_mono)	C473568H664208N165944O237273S1405P23546Mg875Zn0Fe0	-19747.7	Translation

tl_term_b4143_16_rib_cplx	Translation termination complex b4143 16 ribosomes	C1554105H2186803N543198O774238S4928P76065Mg2800Zn0Fe0	-64020.24	Translation
tl_term_b4143_16_rib_cplx2	Translation termination complex b4143 16 ribosomes 2 (RF2_mono)	C1554921H2187299N542910O774766S4896P76065Mg2800Zn0Fe0	-64164.24	Translation
tl_term_b4143_1_rib_cplx	Translation termination complex b4143 1 ribosomes	C111885H153343N39978O59113S308P6300Mg175Zn0Fe0	-5548.14	Translation
tl_term_b4143_1_rib_cplx2	Translation termination complex b4143 1 ribosomes 2 (RF2_mono)	C111936H153374N39960O59146S306P6300Mg175Zn0Fe0	-5557.14	Translation
tl_term_b4143_32_rib_cplx	Translation termination complex b4143 32 ribosomes	C3092473H4355827N1079966O1537038S9856P150481Mg5600Zn0Fe0	-126390.48	Translation
tl_term_b4143_32_rib_cplx2	Translation termination complex b4143 32 ribosomes 2 (RF2_mono)	C3094105H4356819N1079390O1538094S9792P150481Mg5600Zn0Fe0	-126678.48	Translation
tl_term_b4162_10_rib_cplx	Translation termination complex b4162 10 ribosomes	C951930H1335580N333563O475982S2880P47158Mg1750Zn0Fe0	-39530.4	Translation
tl_term_b4162_10_rib_cplx2	Translation termination complex b4162 10 ribosomes 2 (RF2_mono)	C952440H1335890N333383O476312S2860P47158Mg1750Zn0Fe0	-39620.4	Translation
tl_term_b4162_1_rib_cplx	Translation termination complex b4162 1 ribosomes	C99882H138859N35258O51029S288P5209Mg175Zn0Fe0	-4447.14	Translation
tl_term_b4162_1_rib_cplx2	Translation termination complex b4162 1 ribosomes 2 (RF2_mono)	C99933H138890N35240O51062S286P5209Mg175Zn0Fe0	-4456.14	Translation
tl_term_b4162_5_rib_cplx	Translation termination complex b4162 5 ribosomes	C478570H670735N167838O239897S1440P23853Mg875Zn0Fe0	-20039.7	Translation
tl_term_b4162_5_rib_cplx2	Translation termination complex b4162 5 ribosomes 2 (RF2_mono)	C478825H670890N167748O240062S1430P23853Mg875Zn0Fe0	-20084.7	Translation
tl_term_b4167_15_rib_cplx	Translation termination complex b4167 15 ribosomes	C1455658H2045894N509376O724699S4470P71315Mg2625Zn0Fe0	-59888.1	Translation
tl_term_b4167_1_rib_cplx	Translation termination complex b4167 1 ribosomes	C110804H151974N39494O58439S298P6201Mg175Zn0Fe0	-5440.14	Translation
tl_term_b4167_30_rib_cplx	Translation termination complex b4167 30 ribosomes	C2896573H4075094N1012821O1438549S8940P141080Mg5250Zn0Fe0	-118225.2	Translation
tl_term_b4168_1_rib_cplx	Translation termination complex b4168 1 ribosomes	C98513H137227N34677O50140S287P5083Mg175Zn0Fe0	-4322.14	Translation
tl_term_b4168_1_rib_cplx2	Translation termination complex b4168 1 ribosomes 2 (RF2_mono)	C98564H137258N34659O50173S285P5083Mg175Zn0Fe0	-4331.14	Translation
tl_term_b4168_4_rib_cplx	Translation termination complex b4168 4 ribosomes	C381704H534943N133824O191422S1148P19033Mg700Zn0Fe0	-15986.56	Translation
tl_term_b4168_4_rib_cplx2	Translation termination complex b4168 4 ribosomes 2 (RF2_mono)	C381908H535067N133752O191554S1140P19033Mg700Zn0Fe0	-16022.56	Translation
tl_term_b4168_9_rib_cplx	Translation termination complex b4168 9 ribosomes	C853689H1197803N299069O426892S2583P42283Mg1575Zn0Fe0	-35427.26	Translation
tl_term_b4168_9_rib_cplx2	Translation termination complex b4168 9 ribosomes 2 (RF2_mono)	C854148H1198082N298907O427189S2565P42283Mg1575Zn0Fe0	-35508.26	Translation
tl_term_b4169_13_rib_cplx	Translation termination complex b4169 13 ribosomes	C1257569H1767135N440213O627105S3770P61788Mg2275Zn0Fe0	-51676.82	Translation
tl_term_b4169_13_rib_cplx2	Translation termination complex b4169 13 ribosomes 2 (RF2_mono)	C1258232H1767538N439979O627534S3744P61788Mg2275Zn0Fe0	-51793.82	Translation
tl_term_b4169_1_rib_cplx	Translation termination complex b4169 1 ribosomes	C108497H149259N38597O56853S290P5988Mg175Zn0Fe0	-5211.14	Translation
tl_term_b4169_1_rib_cplx2	Translation termination complex b4169 1 ribosomes 2 (RF2_mono)	C108548H149290N38579O56886S288P5988Mg175Zn0Fe0	-5220.14	Translation
tl_term_b4169_26_rib_cplx	Translation termination complex b4169 26 ribosomes	C2502397H3519834N875297O1244878S7540P122238Mg4550Zn0Fe0	-102014.64	Translation
tl_term_b4169_26_rib_cplx2	Translation termination complex b4169 26 ribosomes 2 (RF2_mono)	C2503723H3520640N874829O1245736S7488P122238Mg4550Zn0Fe0	-102248.64	Translation
tl_term_b4170_18_rib_cplx	Translation termination complex b4170 18 ribosomes	C1757436H2472565N614124O872283S5328P85548Mg3150Zn0Fe0	-71745.52	Translation
tl_term_b4170_1_rib_cplx	Translation termination complex b4170 1 ribosomes	C114250H156213N40816O60618S296P6498Mg175Zn0Fe0	-5732.14	Translation
tl_term_b4170_36_rib_cplx	Translation termination complex b4170 36 ribosomes	C3497280H4925173N1221156O1731693S10656P169248Mg6300Zn0Fe0	-141642.04	Translation
tl_term_b4171_18_rib_cplx	Translation termination complex b4171 18 ribosomes	C1722692H2420221N602816O858432S5238P84643Mg3150Zn0Fe0	-70858.52	Translation
tl_term_b4171_1_rib_cplx	Translation termination complex b4171 1 ribosomes	C104190H144040N36886O53941S291P5593Mg175Zn0Fe0	-4828.14	Translation
tl_term_b4171_9_rib_cplx	Translation termination complex b4171 9 ribosomes	C865838H1215184N303206O432525S2619P42793Mg1575Zn0Fe0	-35901.26	Translation
tl_term_b4172_1_rib_cplx	Translation termination complex b4172 1 ribosomes	C97066H135509N34160O49175S284P4959Mg175Zn0Fe0	-4187.14	Translation
tl_term_b4172_1_rib_cplx2	Translation termination complex b4172 1 ribosomes 2 (RF2_mono)	C97117H135540N34142O49208S282P4959Mg175Zn0Fe0	-4196.14	Translation
tl_term_b4172_3_rib_cplx	Translation termination complex b4172 3 ribosomes	C285322H399881N100152O143205S852P14259Mg525Zn0Fe0	-11941.42	Translation
tl_term_b4172_3_rib_cplx2	Translation termination complex b4172 3 ribosomes 2 (RF2_mono)	C285475H399974N100098O143304S846P14259Mg525Zn0Fe0	-11968.42	Translation
tl_term_b4172_6_rib_cplx	Translation termination complex b4172 6 ribosomes	C567706H796439N199140O284250S1704P28209Mg1050Zn0Fe0	-23572.84	Translation
tl_term_b4172_6_rib_cplx2	Translation termination complex b4172 6 ribosomes 2 (RF2_mono)	C568012H796625N199032O284448S1692P28209Mg1050Zn0Fe0	-23626.84	Translation
tl_term_b4173_12_rib_cplx	Translation termination complex b4173 12 ribosomes	C1161745H1632275N406734O579143S3480P57093Mg2100Zn0Fe0	-47951.68	Translation
tl_term_b4173_12_rib_cplx2	Translation termination complex b4173 12 ribosomes 2 (RF2_mono)	C1162357H1632647N406518O579539S3456P57093Mg2100Zn0Fe0	-48059.68	Translation
tl_term_b4173_1_rib_cplx	Translation termination complex b4173 1 ribosomes	C108000H148672N38388O56478S290P5932Mg175Zn0Fe0	-5171.14	Translation
tl_term_b4173_1_rib_cplx2	Translation termination complex b4173 1 ribosomes 2 (RF2_mono)	C108051H148703N38370O56511S288P5932Mg175Zn0Fe0	-5180.14	Translation
tl_term_b4173_25_rib_cplx	Translation termination complex b4173 25 ribosomes	C2407080H3385624N842052O1196838S7250P117556Mg4375Zn0Fe0	-98510.5	Translation
tl_term_b4173_25_rib_cplx2	Translation termination complex b4173 25 ribosomes 2 (RF2_mono)	C2408355H3386399N841602O1197663S7200P117556Mg4375Zn0Fe0	-98735.5	Translation
tl_term_b4174_12_rib_cplx	Translation termination complex b4174 12 ribosomes	C1159394H1628646N406221O578679S3492P57060Mg2100Zn0Fe0	-47798.68	Translation
tl_term_b4174_12_rib_cplx2	Translation termination complex b4174 12 ribosomes 2 (RF2_mono)	C1160006H1629018N406005O579075S3468P57060Mg2100Zn0Fe0	-47906.68	Translation

tl_term_b4174_1_rib_cplx	Translation termination complex b4174 1 ribosomes	C107651H148211N38381O56234S291P5910Mg175Zn0Fe0	-5139.14	Translation
tl_term_b4174_1_rib_cplx2	Translation termination complex b4174 1 ribosomes 2 (RF2_mono)	C107702H148242N38363O56267S289P5910Mg175Zn0Fe0	-5148.14	Translation
tl_term_b4174_24_rib_cplx	Translation termination complex b4174 24 ribosomes	C2306750H3243666N807501O1148619S6984P112860Mg4200Zn0Fe0	-94336.36	Translation
tl_term_b4174_24_rib_cplx2	Translation termination complex b4174 24 ribosomes 2 (RF2_mono)	C2307974H3244410N807069O1149411S6936P112860Mg4200Zn0Fe0	-94552.36	Translation
tl_term_b4175_19_rib_cplx	Translation termination complex b4175 19 ribosomes	C1820562H2558903N637201O907137S5548P89374Mg3325Zn0Fe0	-74709.66	Translation
tl_term_b4175_19_rib_cplx2	Translation termination complex b4175 19 ribosomes 2 (RF2_mono)	C1821531H2559492N636859O907764S5510P89374Mg3325Zn0Fe0	-74880.66	Translation
tl_term_b4175_1_rib_cplx	Translation termination complex b4175 1 ribosomes	C104892H144941N37189O54387S292P5656Mg175Zn0Fe0	-4885.14	Translation
tl_term_b4175_1_rib_cplx2	Translation termination complex b4175 1 ribosomes 2 (RF2_mono)	C104943H144972N37171O54420S290P5656Mg175Zn0Fe0	-4894.14	Translation
tl_term_b4175_9_rib_cplx	Translation termination complex b4175 9 ribosomes	C867412H1217813N303861O433387S2628P42864Mg1575Zn0Fe0	-35918.26	Translation
tl_term_b4175_9_rib_cplx2	Translation termination complex b4175 9 ribosomes 2 (RF2_mono)	C867871H1218092N303699O433684S2610P42864Mg1575Zn0Fe0	-35999.26	Translation
tl_term_b4178_1_rib_cplx	Translation termination complex b4178 1 ribosomes	C98388H137116N34642O50068S289P5078Mg175Zn0Fe0	-4305.14	Translation
tl_term_b4178_4_rib_cplx	Translation termination complex b4178 4 ribosomes	C381390H534754N133765O191263S1156P19028Mg700Zn0Fe0	-15933.56	Translation
tl_term_b4178_8_rib_cplx	Translation termination complex b4178 8 ribosomes	C758726H1064938N265929O379523S2312P37628Mg1400Zn0Fe0	-31438.12	Translation
tl_term_b4179_1_rib_cplx	Translation termination complex b4179 1 ribosomes	C120992H164289N43493O65076S312P7092Mg175Zn0Fe0	-6311.14	Translation
tl_term_b4179_23_rib_cplx	Translation termination complex b4179 23 ribosomes	C2270326H3198727N792285O1122550S7176P109392Mg4025Zn0Fe0	-91410.22	Translation
tl_term_b4179_47_rib_cplx	Translation termination complex b4179 47 ribosomes	C4615054H6509023N1609149O2276158S14664P220992Mg8225Zn0Fe0	-184245.58	Translation
tl_term_b4180_14_rib_cplx	Translation termination complex b4180 14 ribosomes	C1336577H1876966N468567O667697S4130P66070Mg2450Zn0Fe0	-55306.96	Translation
tl_term_b4180_14_rib_cplx2	Translation termination complex b4180 14 ribosomes 2 (RF2_mono)	C1337291H1877400N468315O668159S4102P66070Mg2450Zn0Fe0	-55432.96	Translation
tl_term_b4180_1_rib_cplx	Translation termination complex b4180 1 ribosomes	C101941H141401N36070O52446S295P5399Mg175Zn0Fe0	-4631.14	Translation
tl_term_b4180_1_rib_cplx2	Translation termination complex b4180 1 ribosomes 2 (RF2_mono)	C101992H141432N36052O52479S293P5399Mg175Zn0Fe0	-4640.14	Translation
tl_term_b4180_7_rib_cplx	Translation termination complex b4180 7 ribosomes	C671773H942431N235684O336408S2065P33401Mg1225Zn0Fe0	-28019.98	Translation
tl_term_b4180_7_rib_cplx2	Translation termination complex b4180 7 ribosomes 2 (RF2_mono)	C672130H942648N235558O336639S2051P33401Mg1225Zn0Fe0	-28082.98	Translation
tl_term_b4200_1_rib_cplx	Translation termination complex b4200 1 ribosomes	C98076H136719N34570O49824S289P5048Mg175Zn0Fe0	-4284.14	Translation
tl_term_b4200_1_rib_cplx2	Translation termination complex b4200 1 ribosomes 2 (RF2_mono)	C98127H136750N34552O49857S287P5048Mg175Zn0Fe0	-4293.14	Translation
tl_term_b4200_3_rib_cplx	Translation termination complex b4200 3 ribosomes	C286680H401611N100662O143954S867P14348Mg525Zn0Fe0	-12054.42	Translation
tl_term_b4200_3_rib_cplx2	Translation termination complex b4200 3 ribosomes 2 (RF2_mono)	C286833H401704N100608O144053S861P14348Mg525Zn0Fe0	-12081.42	Translation
tl_term_b4200_7_rib_cplx	Translation termination complex b4200 7 ribosomes	C663888H931395N232846O332214S2023P32948Mg1225Zn0Fe0	-27594.98	Translation
tl_term_b4200_7_rib_cplx2	Translation termination complex b4200 7 ribosomes 2 (RF2_mono)	C664245H931612N232720O332445S2009P32948Mg1225Zn0Fe0	-27657.98	Translation
tl_term_b4201_1_rib_cplx	Translation termination complex b4201 1 ribosomes	C97207H135668N34184O49257S287P4966Mg175Zn0Fe0	-4201.14	Translation
tl_term_b4201_3_rib_cplx	Translation termination complex b4201 3 ribosomes	C285625H400220N100158O143363S861P14268Mg525Zn0Fe0	-11971.42	Translation
tl_term_b4201_6_rib_cplx	Translation termination complex b4201 6 ribosomes	C568252H797048N199119O284522S1722P28221Mg1050Zn0Fe0	-23626.84	Translation
tl_term_b4202_1_rib_cplx	Translation termination complex b4202 1 ribosomes	C96197H134525N33820O48551S284P4877Mg175Zn0Fe0	-4093.14	Translation
tl_term_b4202_1_rib_cplx2	Translation termination complex b4202 1 ribosomes 2 (RF2_mono)	C96248H134556N33802O48584S282P4877Mg175Zn0Fe0	-4102.14	Translation
tl_term_b4202_2_rib_cplx	Translation termination complex b4202 2 ribosomes	C190233H266591N66788O95513S568P9526Mg350Zn0Fe0	-7957.28	Translation
tl_term_b4202_2_rib_cplx2	Translation termination complex b4202 2 ribosomes 2 (RF2_mono)	C190335H266653N66752O95579S564P9526Mg350Zn0Fe0	-7975.28	Translation
tl_term_b4202_4_rib_cplx	Translation termination complex b4202 4 ribosomes	C378305H530723N132724O189437S1136P18824Mg700Zn0Fe0	-15685.56	Translation
tl_term_b4202_4_rib_cplx2	Translation termination complex b4202 4 ribosomes 2 (RF2_mono)	C378509H530847N132652O189569S1128P18824Mg700Zn0Fe0	-15721.56	Translation
tl_term_b4203_1_rib_cplx	Translation termination complex b4203 1 ribosomes	C98637H137414N34780O50207S283P5100Mg175Zn0Fe0	-4329.14	Translation
tl_term_b4203_1_rib_cplx2	Translation termination complex b4203 1 ribosomes 2 (RF2_mono)	C98688H137445N34762O50240S281P5100Mg175Zn0Fe0	-4338.14	Translation
tl_term_b4203_4_rib_cplx	Translation termination complex b4203 4 ribosomes	C381669H535118N133927O191423S1132P19050Mg700Zn0Fe0	-15963.56	Translation
tl_term_b4203_4_rib_cplx2	Translation termination complex b4203 4 ribosomes 2 (RF2_mono)	C381873H535242N133855O191555S1124P19050Mg700Zn0Fe0	-15999.56	Translation
tl_term_b4203_8_rib_cplx	Translation termination complex b4203 8 ribosomes	C759045H1065390N266123O379711S2264P37650Mg1400Zn0Fe0	-31476.12	Translation
tl_term_b4203_8_rib_cplx2	Translation termination complex b4203 8 ribosomes 2 (RF2_mono)	C759453H1065638N265979O379975S2248P37650Mg1400Zn0Fe0	-31548.12	Translation
tl_term_b4258_1_rib_cplx	Translation termination complex b4258 1 ribosomes	C125782H169871N45209O68246S321P7519Mg175Zn0Fe0	-6778.14	Translation
tl_term_b4258_1_rib_cplx2	Translation termination complex b4258 1 ribosomes 2 (RF2_mono)	C125833H169902N45191O68279S319P7519Mg175Zn0Fe0	-6787.14	Translation
tl_term_b4258_27_rib_cplx	Translation termination complex b4258 27 ribosomes	C2688602H3784989N934773O1325762S8667P128705Mg4725Zn0Fe0	-108675.78	Translation
tl_term_b4258_27_rib_cplx2	Translation termination complex b4258 27 ribosomes 2 (RF2_mono)	C2689979H3785826N934287O1326653S8613P128705Mg4725Zn0Fe0	-108918.78	Translation

tl_term_b4258_55_rib_cplx	Translation termination complex b4258 55 ribosomes	C5448562H7678193N1892765O2680010S17655P259213Mg9625Zn0Fe0	-218411.7	Translation
tl_term_b4258_55_rib_cplx2	Translation termination complex b4258 55 ribosomes 2 (RF2_mono)	C5451367H7679898N1891775O2681825S17545P259213Mg9625Zn0Fe0	-218906.7	Translation
tl_term_b4292_18_rib_cplx	Translation termination complex b4292 18 ribosomes	C1724719H2422736N604055O860009S5112P84848Mg3150Zn0Fe0	-70883.52	Translation
tl_term_b4292_18_rib_cplx2	Translation termination complex b4292 18 ribosomes 2 (RF2_mono)	C1725637H2423294N603731O860603S5076P84848Mg3150Zn0Fe0	-71045.52	Translation
tl_term_b4292_1_rib_cplx	Translation termination complex b4292 1 ribosomes	C104347H144294N36986O54039S284P5611Mg175Zn0Fe0	-4836.14	Translation
tl_term_b4292_1_rib_cplx2	Translation termination complex b4292 1 ribosomes 2 (RF2_mono)	C104398H144325N36968O54072S282P5611Mg175Zn0Fe0	-4845.14	Translation
tl_term_b4292_9_rib_cplx	Translation termination complex b4292 9 ribosomes	C866875H1216502N303842O433319S2556P42899Mg1575Zn0Fe0	-35917.26	Translation
tl_term_b4292_9_rib_cplx2	Translation termination complex b4292 9 ribosomes 2 (RF2_mono)	C867334H1216781N303680O433616S2538P42899Mg1575Zn0Fe0	-35998.26	Translation
tl_term_b4293_10_rib_cplx	Translation termination complex b4293 10 ribosomes	C951188H1334906N333259O475638S2910P47134Mg1750Zn0Fe0	-39436.4	Translation
tl_term_b4293_1_rib_cplx	Translation termination complex b4293 1 ribosomes	C99581H138554N35098O50856S291P5185Mg175Zn0Fe0	-4416.14	Translation
tl_term_b4293_5_rib_cplx	Translation termination complex b4293 5 ribosomes	C478073H670266N167614O239648S1455P23829Mg875Zn0Fe0	-19980.7	Translation
tl_term_b4371_10_rib_cplx	Translation termination complex b4371 10 ribosomes	C962997H1351452N337083O480876S2920P47534Mg1750Zn0Fe0	-39856.4	Translation
tl_term_b4371_10_rib_cplx2	Translation termination complex b4371 10 ribosomes 2 (RF2_mono)	C963507H1351762N336903O481206S2900P47534Mg1750Zn0Fe0	-39946.4	Translation
tl_term_b4371_1_rib_cplx	Translation termination complex b4371 1 ribosomes	C105126H145146N37212O54618S292P5684Mg175Zn0Fe0	-4917.14	Translation
tl_term_b4371_1_rib_cplx2	Translation termination complex b4371 1 ribosomes 2 (RF2_mono)	C105177H145177N37194O54651S290P5684Mg175Zn0Fe0	-4926.14	Translation
tl_term_b4371_20_rib_cplx	Translation termination complex b4371 20 ribosomes	C1916187H2691792N670273O954496S5840P94034Mg3500Zn0Fe0	-78677.8	Translation
tl_term_b4371_20_rib_cplx2	Translation termination complex b4371 20 ribosomes 2 (RF2_mono)	C1917207H2692412N669913O955156S5800P94034Mg3500Zn0Fe0	-78857.8	Translation
tl_term_b4372_1_rib_cplx	Translation termination complex b4372 1 ribosomes	C98266H136981N34623O49967S287P5067Mg175Zn0Fe0	-4298.14	Translation
tl_term_b4372_4_rib_cplx	Translation termination complex b4372 4 ribosomes	C381262H534505N133764O191186S1148P19020Mg700Zn0Fe0	-15941.56	Translation
tl_term_b4372_8_rib_cplx	Translation termination complex b4372 8 ribosomes	C758590H1064537N265952O379478S2296P37624Mg1400Zn0Fe0	-31466.12	Translation
tl_term_b4373_1_rib_cplx	Translation termination complex b4373 1 ribosomes	C98359H137068N34647O49989S286P5066Mg175Zn0Fe0	-4302.14	Translation
tl_term_b4373_1_rib_cplx2	Translation termination complex b4373 1 ribosomes 2 (RF2_mono)	C98410H137099N34629O50022S284P5066Mg175Zn0Fe0	-4311.14	Translation
tl_term_b4373_4_rib_cplx	Translation termination complex b4373 4 ribosomes	C381580H534844N133821O191283S1144P19019Mg700Zn0Fe0	-15960.56	Translation
tl_term_b4373_4_rib_cplx2	Translation termination complex b4373 4 ribosomes 2 (RF2_mono)	C381784H534968N133749O191415S1136P19019Mg700Zn0Fe0	-15996.56	Translation
tl_term_b4373_8_rib_cplx	Translation termination complex b4373 8 ribosomes	C759208H1065212N266053O379675S2288P37623Mg1400Zn0Fe0	-31505.12	Translation
tl_term_b4373_8_rib_cplx2	Translation termination complex b4373 8 ribosomes 2 (RF2_mono)	C759616H1065460N265909O379939S2272P37623Mg1400Zn0Fe0	-31577.12	Translation
tl_term_b4374_13_rib_cplx	Translation termination complex b4374 13 ribosomes	C1238825H1738467N433454O618540S3757P61141Mg2275Zn0Fe0	-51250.82	Translation
tl_term_b4374_1_rib_cplx	Translation termination complex b4374 1 ribosomes	C101237H140463N35690O51972S289P5329Mg175Zn0Fe0	-4569.14	Translation
tl_term_b4374_6_rib_cplx	Translation termination complex b4374 6 ribosomes	C575232H806298N201425O288042S1734P28584Mg1050Zn0Fe0	-24019.84	Translation
tl_term_b4375_15_rib_cplx	Translation termination complex b4375 15 ribosomes	C1459672H2051279N509682O726135S4575P71357Mg2625Zn0Fe0	-59945.1	Translation
tl_term_b4375_15_rib_cplx2	Translation termination complex b4375 15 ribosomes 2 (RF2_mono)	C1460437H2051744N509412O726630S4545P71357Mg2625Zn0Fe0	-60080.1	Translation
tl_term_b4375_1_rib_cplx	Translation termination complex b4375 1 ribosomes	C111444H152753N39660O58769S305P6243Mg175Zn0Fe0	-5483.14	Translation
tl_term_b4375_1_rib_cplx2	Translation termination complex b4375 1 ribosomes 2 (RF2_mono)	C111495H152784N39642O58802S303P6243Mg175Zn0Fe0	-5492.14	Translation
tl_term_b4375_31_rib_cplx	Translation termination complex b4375 31 ribosomes	C3000504H4221023N1046850O1488839S9455P145773Mg5425Zn0Fe0	-122187.34	Translation
tl_term_b4375_31_rib_cplx2	Translation termination complex b4375 31 ribosomes 2 (RF2_mono)	C3002085H4221984N1046292O1489862S9393P145773Mg5425Zn0Fe0	-122466.34	Translation
transGroES_hepta	trans version" of Cpn10 chaperonin GroES, small subunit of GroESL (b4142, GroS, heptamer)	C3178H5334N889O1015S14	-21	Protein Folding
transcr_elo_TU-8389_cplx	transcription elongation complex TU-8389 (RHO INDEPENDENT TERMINATION)	C30075H47259N8737O9914S177P237Mg4Zn1Fe0	-393	Transcription
transcr_elo_TU-8390_cplx	transcription elongation complex TU-8390 (RHO INDEPENDENT TERMINATION)	C30075H47259N8737O9914S177P237Mg4Zn1Fe0	-393	Transcription
transcr_elo_TU-8392_cplx	transcription elongation complex TU-8392 (RHO DEPENDENT TERMINATION)	C75900H105927N25404O34140S279P3666Mg7Zn1Fe0	-3825	Transcription
transcr_elo_TU-8397_cplx	transcription elongation complex TU-8397 (RHO DEPENDENT TERMINATION)	C45076H70454N13263O15224S279P504Mg7Zn1Fe0	-663	Transcription
transcr_elo_TU-8398_cplx	transcription elongation complex TU-8398 (RHO DEPENDENT TERMINATION)	C79622H110288N26735O36422S279P4053Mg7Zn1Fe0	-4212	Transcription
transcr_elo_TU-8407_cplx	transcription elongation complex TU-8407 (RHO DEPENDENT TERMINATION)	C71387H100758N23735O31317S279P3213Mg7Zn1Fe0	-3372	Transcription
transcr_elo_TU0-1181_cplx	transcription elongation complex TU0-1181	C95266H130469N32938O44191S300P5133Mg7Zn1Fe0	-5238	Transcription

transcr_elo_TU0-1182_cplx	transcription elongation complex TU0-1182	C95179H130372N32911O44139S300P5125Mg7Zn1Fe0	-5230	Transcription
transcr_elo_TU0-1183_cplx	transcription elongation complex TU0-1183	C94335H129403N32582O43624S300P5039Mg7Zn1Fe0	-5144	Transcription
transcr_elo_TU0-1184_cplx	transcription elongation complex TU0-1184	C94335H129403N32582O43624S300P5039Mg7Zn1Fe0	-5144	Transcription
transcr_elo_TU0-1186_cplx	transcription elongation complex TU0-1186	C94346H129417N32580O43636S300P5040Mg7Zn1Fe0	-5145	Transcription
transcr_elo_TU0-1187_cplx	transcription elongation complex TU0-1187	C95088H130274N32861O44094S300P5116Mg7Zn1Fe0	-5221	Transcription
transcr_elo_TU0-1188_cplx	transcription elongation complex TU0-1188	C95088H130274N32861O44094S300P5116Mg7Zn1Fe0	-5221	Transcription
transcr_elo_TU0-1189_cplx	transcription elongation complex TU0-1189	C95253H130458N32927O44190S300P5132Mg7Zn1Fe0	-5237	Transcription
transcr_elo_TU0-1190_cplx	transcription elongation complex TU0-1190	C95253H130458N32927O44190S300P5132Mg7Zn1Fe0	-5237	Transcription
transcr_elo_TU0-1191_cplx	transcription elongation complex TU0-1191	C97541H133091N33834O45594S300P5367Mg7Zn1Fe0	-5472	Transcription
transcr_elo_TU0-1192_cplx	transcription elongation complex TU0-1192	C97541H133091N33834O45594S300P5367Mg7Zn1Fe0	-5472	Transcription
transcr_elo_TU0-12803_cplx	transcription elongation complex TU0-12803 (RHO DEPENDENT TERMINATION)	C75253H105229N25098O33796S279P3605Mg7Zn1Fe0	-3764	Transcription
transcr_elo_TU0-12827_cplx	transcription elongation complex TU0-12827 (RHO DEPENDENT TERMINATION)	C83089H114321N28010O38628S279P4411Mg7Zn1Fe0	-4570	Transcription
transcr_elo_TU0-12830_cplx	transcription elongation complex TU0-12830 (RHO DEPENDENT TERMINATION)	C45861H71345N13614O15685S279P585Mg7Zn1Fe0	-744	Transcription
transcr_elo_TU0-12833_cplx	transcription elongation complex TU0-12833 (RHO DEPENDENT TERMINATION)	C53062H79667N16396O20196S279P1326Mg7Zn1Fe0	-1485	Transcription
transcr_elo_TU0-12921_cplx	transcription elongation complex TU0-12921 (RHO DEPENDENT TERMINATION)	C65313H93756N21227O27652S279P2582Mg7Zn1Fe0	-2741	Transcription
transcr_elo_TU0-12928_cplx	transcription elongation complex TU0-12928 (RHO DEPENDENT TERMINATION)	C53102H79643N16536O20069S279P1326Mg7Zn1Fe0	-1485	Transcription
transcr_elo_TU0-13010_cplx	transcription elongation complex TU0-13010 (RHO DEPENDENT TERMINATION)	C93896H126706N32353O45182S279P5515Mg7Zn1Fe0	-5674	Transcription
transcr_elo_TU0-13018_cplx	transcription elongation complex TU0-13018 (RHO DEPENDENT TERMINATION)	C54287H81082N16873O20896S279P1452Mg7Zn1Fe0	-1611	Transcription
transcr_elo_TU0-13020_cplx	transcription elongation complex TU0-13020 (RHO DEPENDENT TERMINATION)	C56642H83790N17779O22336S279P1692Mg7Zn1Fe0	-1851	Transcription
transcr_elo_TU0-13034_cplx	transcription elongation complex TU0-13034	C46840H74976N13451O14762S300P182Mg7Zn1Fe0	-287	Transcription
transcr_elo_TU0-13035_cplx	transcription elongation complex TU0-13035	C46074H74091N13148O14290S300P103Mg7Zn1Fe0	-208	Transcription
transcr_elo_TU0-13036_cplx	transcription elongation complex TU0-13036	C46074H74091N13148O14290S300P103Mg7Zn1Fe0	-208	Transcription
transcr_elo_TU0-13072_cplx	transcription elongation complex TU0-13072 (RHO DEPENDENT TERMINATION)	C56563H83743N17618O22392S279P1684Mg7Zn1Fe0	-1843	Transcription
transcr_elo_TU0-13080_cplx	transcription elongation complex TU0-13080 (RHO DEPENDENT TERMINATION)	C53018H79609N16370O20113S279P1320Mg7Zn1Fe0	-1479	Transcription
transcr_elo_TU0-13093_cplx	transcription elongation complex TU0-13093 (RHO DEPENDENT TERMINATION)	C54069H80855N16674O20827S279P1428Mg7Zn1Fe0	-1587	Transcription
transcr_elo_TU0-13104_cplx	transcription elongation complex TU0-13104 (RHO DEPENDENT TERMINATION)	C43646H68804N12703O14350S279P357Mg7Zn1Fe0	-516	Transcription
transcr_elo_TU0-13134_cplx	transcription elongation complex TU0-13134 (RHO DEPENDENT TERMINATION)	C49801H75883N15128O18125S279P987Mg7Zn1Fe0	-1146	Transcription
transcr_elo_TU0-13139_cplx	transcription elongation complex TU0-13139 (RHO DEPENDENT TERMINATION)	C73975H103766N24585O33014S279P3474Mg7Zn1Fe0	-3633	Transcription
transcr_elo_TU0-13145_cplx	transcription elongation complex TU0-13145 (RHO DEPENDENT TERMINATION)	C51379H77736N15687O19111S279P1152Mg7Zn1Fe0	-1311	Transcription
transcr_elo_TU0-13195_cplx	transcription elongation complex TU0-13195 (RHO DEPENDENT TERMINATION)	C48968H74939N14782O17643S279P903Mg7Zn1Fe0	-1062	Transcription
transcr_elo_TU0-13225_cplx	transcription elongation complex TU0-13225 (RHO DEPENDENT TERMINATION)	C49564H75618N15017O17963S279P963Mg7Zn1Fe0	-1122	Transcription
transcr_elo_TU0-13421_cplx	transcription elongation complex TU0-13421 (RHO DEPENDENT TERMINATION)	C48308H74202N14457O17263S279P837Mg7Zn1Fe0	-996	Transcription
transcr_elo_TU0-13443_cplx	transcription elongation complex TU0-13443 (RHO DEPENDENT TERMINATION)	C61370H89297N19400O25374S279P2175Mg7Zn1Fe0	-2334	Transcription
transcr_elo_TU0-13444_cplx	transcription elongation complex TU0-13444 (RHO DEPENDENT TERMINATION)	C57314H84535N18118O22714S279P1761Mg7Zn1Fe0	-1920	Transcription
transcr_elo_TU0-13519_cplx	transcription elongation complex TU0-13519 (RHO DEPENDENT TERMINATION)	C60217H87934N19135O24560S279P2061Mg7Zn1Fe0	-2220	Transcription

transcr_elo_TU0-13534_cplx	transcription elongation complex TU0-13534 (RHO DEPENDENT TERMINATION)	C49676H75736N15087O18073S279P975Mg7Zn1Fe0	-1134	Transcription
transcr_elo_TU0-13555_cplx	transcription elongation complex TU0-13555 (RHO DEPENDENT TERMINATION)	C47192H72918N14041O16568S279P723Mg7Zn1Fe0	-882	Transcription
transcr_elo_TU0-13557_cplx	transcription elongation complex TU0-13557 (RHO DEPENDENT TERMINATION)	C43187H68286N12525O14071S279P312Mg7Zn1Fe0	-471	Transcription
transcr_elo_TU0-13573_cplx	transcription elongation complex TU0-13573 (RHO DEPENDENT TERMINATION)	C49356H75415N14858O17899S279P945Mg7Zn1Fe0	-1104	Transcription
transcr_elo_TU0-13593_cplx	transcription elongation complex TU0-13593 (RHO DEPENDENT TERMINATION)	C84729H116215N28592O39725S279P4577Mg7Zn1Fe0	-4736	Transcription
transcr_elo_TU0-13667_cplx	transcription elongation complex TU0-13667 (RHO DEPENDENT TERMINATION)	C52095H78574N15905O19624S279P1224Mg7Zn1Fe0	-1383	Transcription
transcr_elo_TU0-13675_cplx	transcription elongation complex TU0-13675 (RHO DEPENDENT TERMINATION)	C57074H84299N17908O22650S279P1736Mg7Zn1Fe0	-1895	Transcription
transcr_elo_TU0-13704_cplx	transcription elongation complex TU0-13704	C46069H74087N13150O14287S300P103Mg7Zn1Fe0	-208	Transcription
transcr_elo_TU0-13717_cplx	transcription elongation complex TU0-13717 (RHO DEPENDENT TERMINATION)	C66086H94634N21527O28136S279P2658Mg7Zn1Fe0	-2817	Transcription
transcr_elo_TU0-13734_cplx	transcription elongation complex TU0-13734 (RHO DEPENDENT TERMINATION)	C69911H99067N22944O30457S279P3051Mg7Zn1Fe0	-3210	Transcription
transcr_elo_TU0-13735_cplx	transcription elongation complex TU0-13735 (RHO DEPENDENT TERMINATION)	C53108H79719N16378O20172S279P1329Mg7Zn1Fe0	-1488	Transcription
transcr_elo_TU0-13736_cplx	transcription elongation complex TU0-13736 (RHO DEPENDENT TERMINATION)	C55813H82829N17452O21848S279P1606Mg7Zn1Fe0	-1765	Transcription
transcr_elo_TU0-13738_cplx	transcription elongation complex TU0-13738 (RHO DEPENDENT TERMINATION)	C48690H74647N14596O17480S279P876Mg7Zn1Fe0	-1035	Transcription
transcr_elo_TU0-13805_cplx	transcription elongation complex TU0-13805 (RHO DEPENDENT TERMINATION)	C56930H84116N17913O22512S279P1722Mg7Zn1Fe0	-1881	Transcription
transcr_elo_TU0-13809_cplx	transcription elongation complex TU0-13809 (RHO DEPENDENT TERMINATION)	C50642H76851N15454O18643S279P1073Mg7Zn1Fe0	-1232	Transcription
transcr_elo_TU0-13915_cplx	transcription elongation complex TU0-13915 (RHO DEPENDENT TERMINATION)	C57608H84955N17964O23028S279P1790Mg7Zn1Fe0	-1949	Transcription
transcr_elo_TU0-13931_cplx	transcription elongation complex TU0-13931 (RHO DEPENDENT TERMINATION)	C45071H70452N13267O15221S279P504Mg7Zn1Fe0	-663	Transcription
transcr_elo_TU0-14007_cplx	transcription elongation complex TU0-14007	C46253H74300N13225O14404S300P122Mg7Zn1Fe0	-227	Transcription
transcr_elo_TU0-14021_cplx	transcription elongation complex TU0-14021 (RHO DEPENDENT TERMINATION)	C53697H80401N16652O20555S279P1392Mg7Zn1Fe0	-1551	Transcription
transcr_elo_TU0-14029_cplx	transcription elongation complex TU0-14029 (RHO DEPENDENT TERMINATION)	C65513H94035N21182O27829S279P2604Mg7Zn1Fe0	-2763	Transcription
transcr_elo_TU0-14037_cplx	transcription elongation complex TU0-14037 (RHO DEPENDENT TERMINATION)	C52743H79319N16208O19969S279P1293Mg7Zn1Fe0	-1452	Transcription
transcr_elo_TU0-14038_cplx	transcription elongation complex TU0-14038 (RHO DEPENDENT TERMINATION)	C54506H81351N16882O21026S279P1473Mg7Zn1Fe0	-1632	Transcription
transcr_elo_TU0-14070_cplx	transcription elongation complex TU0-14070 (RHO DEPENDENT TERMINATION)	C68546H97540N22291O29755S279P2913Mg7Zn1Fe0	-3072	Transcription
transcr_elo_TU0-14100_cplx	transcription elongation complex TU0-14100 (RHO DEPENDENT TERMINATION)	C51956H78394N15947O19467S279P1212Mg7Zn1Fe0	-1371	Transcription
transcr_elo_TU0-14118_cplx	transcription elongation complex TU0-14118 (RHO DEPENDENT TERMINATION)	C50548H76751N15410O18611S279P1065Mg7Zn1Fe0	-1224	Transcription
transcr_elo_TU0-14151_cplx	transcription elongation complex TU0-14151 (RHO DEPENDENT TERMINATION)	C45740H71221N13536O15644S279P573Mg7Zn1Fe0	-732	Transcription
transcr_elo_TU0-14215_cplx	transcription elongation complex TU0-14215 (RHO DEPENDENT TERMINATION)	C50455H76688N15301O18609S279P1059Mg7Zn1Fe0	-1218	Transcription
transcr_elo_TU0-14216_cplx	transcription elongation complex TU0-14216 (RHO DEPENDENT TERMINATION)	C55209H82168N17187O21494S279P1548Mg7Zn1Fe0	-1707	Transcription
transcr_elo_TU0-14217_cplx	transcription elongation complex TU0-14217 (RHO DEPENDENT TERMINATION)	C55896H82931N17494O21897S279P1617Mg7Zn1Fe0	-1776	Transcription
transcr_elo_TU0-14252_cplx	transcription elongation complex TU0-14252 (RHO DEPENDENT TERMINATION)	C53102H79643N16536O20069S279P1326Mg7Zn1Fe0	-1485	Transcription
transcr_elo_TU0-1881_cplx	transcription elongation complex TU0-1881 (RHO DEPENDENT TERMINATION)	C65469H93937N21256O27732S279P2596Mg7Zn1Fe0	-2755	Transcription
transcr_elo_TU0-1_cplx	transcription elongation complex TU0-1 (RHO DEPENDENT TERMINATION)	C79815H110447N26868O36589S279P4065Mg7Zn1Fe0	-4224	Transcription

transcr_elo_TU0-2081_cplx	transcription elongation complex TU0-2081 (RHO DEPENDENT TERMINATION)	C73975H103766N24585O33014S279P3474Mg7Zn1Fe0	-3633	Transcription
transcr_elo_TU0-2082_cplx	transcription elongation complex TU0-2082 (RHO DEPENDENT TERMINATION)	C73975H103766N24585O33014S279P3474Mg7Zn1Fe0	-3633	Transcription
transcr_elo_TU0-2101_cplx	transcription elongation complex TU0-2101 (RHO DEPENDENT TERMINATION)	C65012H93461N20910O27584S279P2548Mg7Zn1Fe0	-2707	Transcription
transcr_elo_TU0-2121_cplx	transcription elongation complex TU0-2121 (RHO DEPENDENT TERMINATION)	C54733H81605N16940O21242S279P1494Mg7Zn1Fe0	-1653	Transcription
transcr_elo_TU0-3304_cplx	transcription elongation complex TU0-3304 (RHO DEPENDENT TERMINATION)	C45727H71182N13577O15582S279P570Mg7Zn1Fe0	-729	Transcription
transcr_elo_TU0-3305_cplx	transcription elongation complex TU0-3305 (RHO DEPENDENT TERMINATION)	C45727H71182N13577O15582S279P570Mg7Zn1Fe0	-729	Transcription
transcr_elo_TU0-3364_cplx	transcription elongation complex TU0-3364	C95179H130372N32911O44139S300P5125Mg7Zn1Fe0	-5230	Transcription
transcr_elo_TU0-3561_cplx	transcription elongation complex TU0-3561	C46071H74088N13143O14292S300P103Mg7Zn1Fe0	-208	Transcription
transcr_elo_TU0-3901_cplx	transcription elongation complex TU0-3901 (RHO DEPENDENT TERMINATION)	C41757H66641N11966O13187S279P165Mg7Zn1Fe0	-324	Transcription
transcr_elo_TU0-4925_cplx	transcription elongation complex TU0-4925 (RHO DEPENDENT TERMINATION)	C51956H78394N15947O19467S279P1212Mg7Zn1Fe0	-1371	Transcription
transcr_elo_TU0-5003_cplx	transcription elongation complex TU0-5003 (RHO DEPENDENT TERMINATION)	C43289H68401N12546O14147S279P321Mg7Zn1Fe0	-480	Transcription
transcr_elo_TU0-5121_cplx	transcription elongation complex TU0-5121 (RHO DEPENDENT TERMINATION)	C73273H102940N24277O32521S279P3397Mg7Zn1Fe0	-3556	Transcription
transcr_elo_TU0-5182_cplx	transcription elongation complex TU0-5182 (RHO DEPENDENT TERMINATION)	C77198H107489N25668O35089S279P3797Mg7Zn1Fe0	-3956	Transcription
transcr_elo_TU0-5183_cplx	transcription elongation complex TU0-5183 (RHO DEPENDENT TERMINATION)	C65465H93963N21152O27832S279P2595Mg7Zn1Fe0	-2754	Transcription
transcr_elo_TU0-5186_cplx	transcription elongation complex TU0-5186 (RHO DEPENDENT TERMINATION)	C60380H88126N19187O24683S279P2078Mg7Zn1Fe0	-2237	Transcription
transcr_elo_TU0-5201_cplx	transcription elongation complex TU0-5201 (RHO DEPENDENT TERMINATION)	C69963H99199N22886O30620S279P3064Mg7Zn1Fe0	-3223	Transcription
transcr_elo_TU0-5221_cplx	transcription elongation complex TU0-5221 (RHO DEPENDENT TERMINATION)	C57994H85401N18220O23248S279P1835Mg7Zn1Fe0	-1994	Transcription
transcr_elo_TU0-5522_cplx	transcription elongation complex TU0-5522 (RHO DEPENDENT TERMINATION)	C62075H90035N19842O25683S279P2244Mg7Zn1Fe0	-2403	Transcription
transcr_elo_TU0-5543_cplx	transcription elongation complex TU0-5543 (RHO DEPENDENT TERMINATION)	C53418H80069N16492O20372S279P1359Mg7Zn1Fe0	-1518	Transcription
transcr_elo_TU0-5601_cplx	transcription elongation complex TU0-5601 (RHO INDEPENDENT TERMINATION)	C40740H59538N12860O16458S177P1328Mg4Zn1Fe0	-1484	Transcription
transcr_elo_TU0-6223_cplx	transcription elongation complex TU0-6223 (RHO INDEPENDENT TERMINATION)	C118269H148679N43549O63865S177P9275Mg4Zn1Fe0	-9431	Transcription
transcr_elo_TU0-6405_cplx	transcription elongation complex TU0-6405 (RHO INDEPENDENT TERMINATION)	C55741H76805N18889O25635S177P2874Mg4Zn1Fe0	-3030	Transcription
transcr_elo_TU0-6409_cplx	transcription elongation complex TU0-6409 (RHO DEPENDENT TERMINATION)	C54218H80997N16824O20853S279P1443Mg7Zn1Fe0	-1602	Transcription
transcr_elo_TU0-6423_cplx	transcription elongation complex TU0-6423 (RHO DEPENDENT TERMINATION)	C45704H71181N13476O15604S279P567Mg7Zn1Fe0	-726	Transcription
transcr_elo_TU0-6441_cplx	transcription elongation complex TU0-6441 (RHO DEPENDENT TERMINATION)	C66086H94634N21527O28136S279P2658Mg7Zn1Fe0	-2817	Transcription
transcr_elo_TU0-6504_cplx	transcription elongation complex TU0-6504 (RHO INDEPENDENT TERMINATION)	C44168H63492N14248O18571S177P1683Mg4Zn1Fe0	-1839	Transcription
transcr_elo_TU0-6506_cplx	transcription elongation complex TU0-6506 (RHO DEPENDENT TERMINATION)	C52849H79418N16285O20048S279P1302Mg7Zn1Fe0	-1461	Transcription
transcr_elo_TU0-6512_cplx	transcription elongation complex TU0-6512 (RHO INDEPENDENT TERMINATION)	C136412H169703N50415O75095S177P11145Mg4Zn1Fe0	-11301	Transcription
transcr_elo_TU0-6550_cplx	transcription elongation complex TU0-6550 (RHO DEPENDENT TERMINATION)	C54218H80997N16824O20853S279P1443Mg7Zn1Fe0	-1602	Transcription
transcr_elo_TU0-6551_cplx	transcription elongation complex TU0-6551 (RHO DEPENDENT TERMINATION)	C54218H80997N16824O20853S279P1443Mg7Zn1Fe0	-1602	Transcription
transcr_elo_TU0-6562_cplx	transcription elongation complex TU0-6562 (RHO INDEPENDENT TERMINATION)	C44683H64078N14484O18895S177P1737Mg4Zn1Fe0	-1893	Transcription
transcr_elo_TU0-6626_cplx	transcription elongation complex TU0-6626 (RHO INDEPENDENT TERMINATION)	C67118H89902N23252O32676S177P4036Mg4Zn1Fe0	-4192	Transcription

transcr_elo_TU0-6654_cplx	transcription elongation complex TU0-6654 (RHO INDEPENDENT TERMINATION)	C49430H69546N16330O21810S177P2223Mg4Zn1Fe0	-2379	Transcription
transcr_elo_TU0-6657_cplx	transcription elongation complex TU0-6657 (RHO INDEPENDENT TERMINATION)	C31421H48815N9237O10755S177P375Mg4Zn1Fe0	-531	Transcription
transcr_elo_TU0-6658_cplx	transcription elongation complex TU0-6658 (RHO INDEPENDENT TERMINATION)	C36271H54385N11191O13718S177P873Mg4Zn1Fe0	-1029	Transcription
transcr_elo_TU0-6660_cplx	transcription elongation complex TU0-6660 (RHO INDEPENDENT TERMINATION)	C53785H74569N18001O24497S177P2670Mg4Zn1Fe0	-2826	Transcription
transcr_elo_TU0-6687_cplx	transcription elongation complex TU0-6687 (RHO INDEPENDENT TERMINATION)	C55741H76805N18889O25635S177P2874Mg4Zn1Fe0	-3030	Transcription
transcr_elo_TU0-6941_cplx	transcription elongation complex TU0-6941 (RHO DEPENDENT TERMINATION)	C54506H81351N16882O21026S279P1473Mg7Zn1Fe0	-1632	Transcription
transcr_elo_TU0-7002_cplx	transcription elongation complex TU0-7002 (RHO INDEPENDENT TERMINATION)	C109081H138261N39699O58400S177P8347Mg4Zn1Fe0	-8503	Transcription
transcr_elo_TU0-7141_cplx	transcription elongation complex TU0-7141 (RHO DEPENDENT TERMINATION)	C44855H70210N13137O15099S279P482Mg7Zn1Fe0	-641	Transcription
transcr_elo_TU0-7281_cplx	transcription elongation complex TU0-7281 (RHO DEPENDENT TERMINATION)	C61873H89840N19667O25615S279P2224Mg7Zn1Fe0	-2383	Transcription
transcr_elo_TU0-7842_cplx	transcription elongation complex TU0-7842 (RHO INDEPENDENT TERMINATION)	C30510H47753N8915O10171S177P282Mg4Zn1Fe0	-438	Transcription
transcr_elo_TU0-7844_cplx	transcription elongation complex TU0-7844 (RHO INDEPENDENT TERMINATION)	C30510H47753N8915O10171S177P282Mg4Zn1Fe0	-438	Transcription
transcr_elo_TU0-8084_cplx	transcription elongation complex TU0-8084 (RHO INDEPENDENT TERMINATION)	C45216H64704N14640O19247S177P1791Mg4Zn1Fe0	-1947	Transcription
transcr_elo_TU0-8281_cplx	transcription elongation complex TU0-8281 (RHO DEPENDENT TERMINATION)	C46119H71681N13604O15908S279P612Mg7Zn1Fe0	-771	Transcription
transcr_elo_TU0-8464_cplx	transcription elongation complex TU0-8464 (RHO DEPENDENT TERMINATION)	C44000H69207N12850O14557S279P393Mg7Zn1Fe0	-552	Transcription
transcr_elo_TU0-8474_cplx	transcription elongation complex TU0-8474 (RHO INDEPENDENT TERMINATION)	C30559H47826N8902O10234S177P288Mg4Zn1Fe0	-444	Transcription
transcr_elo_TU0-8476_cplx	transcription elongation complex TU0-8476	C46079H74097N13174O14285S300P104Mg7Zn1Fe0	-209	Transcription
transcr_elo_TU0-8477_cplx	transcription elongation complex TU0-8477 (RHO DEPENDENT TERMINATION)	C53922H80637N16764O20662S279P1413Mg7Zn1Fe0	-1572	Transcription
transcr_elo_TU0-8502_cplx	transcription elongation complex TU0-8502 (RHO DEPENDENT TERMINATION)	C77630H107908N26125O35188S279P3844Mg7Zn1Fe0	-4003	Transcription
transcr_elo_TU0-8505_cplx	transcription elongation complex TU0-8505 (RHO DEPENDENT TERMINATION)	C48930H74881N14818O17567S279P900Mg7Zn1Fe0	-1059	Transcription
transcr_elo_TU0-8506_cplx	transcription elongation complex TU0-8506 (RHO DEPENDENT TERMINATION)	C42486H67488N12229O13650S279P240Mg7Zn1Fe0	-399	Transcription
transcr_elo_TU0-8510_cplx	transcription elongation complex TU0-8510 (RHO DEPENDENT TERMINATION)	C42800H67861N12320O13877S279P273Mg7Zn1Fe0	-432	Transcription
transcr_elo_TU0-8529_cplx	transcription elongation complex TU0-8529 (RHO DEPENDENT TERMINATION)	C54542H81377N16910O21079S279P1476Mg7Zn1Fe0	-1635	Transcription
transcr_elo_TU0-8823_cplx	transcription elongation complex TU0-8823 (RHO DEPENDENT TERMINATION)	C60000H87661N19034O24454S279P2034Mg7Zn1Fe0	-2193	Transcription
transcr_elo_TU0-8855_cplx	transcription elongation complex TU0-8855 (RHO INDEPENDENT TERMINATION)	C33053H50707N9831O11768S177P542Mg4Zn1Fe0	-698	Transcription
transcr_elo_TU0-8862_cplx	transcription elongation complex TU0-8862 (RHO DEPENDENT TERMINATION)	C50215H76380N15271O18389S279P1032Mg7Zn1Fe0	-1191	Transcription
transcr_elo_TU0-8864_cplx	transcription elongation complex TU0-8864 (RHO DEPENDENT TERMINATION)	C69515H98615N22748O30266S279P3009Mg7Zn1Fe0	-3168	Transcription
transcr_elo_TU0-8865_cplx	transcription elongation complex TU0-8865 (RHO DEPENDENT TERMINATION)	C61858H89777N19822O25537S279P2224Mg7Zn1Fe0	-2383	Transcription
transcr_elo_TU00021_cplx	transcription elongation complex TU00021 (RHO DEPENDENT TERMINATION)	C52978H79560N16351O20069S279P1315Mg7Zn1Fe0	-1474	Transcription
transcr_elo_TU00083_cplx	transcription elongation complex TU00083 (RHO INDEPENDENT TERMINATION)	C36271H54385N11191O13718S177P873Mg4Zn1Fe0	-1029	Transcription
transcr_elo_TU00084_cplx	transcription elongation complex TU00084 (RHO INDEPENDENT TERMINATION)	C36271H54385N11191O13718S177P873Mg4Zn1Fe0	-1029	Transcription
transcr_elo_TU00221_cplx	transcription elongation complex TU00221 (RHO DEPENDENT TERMINATION)	C55253H82193N17142O21488S279P1545Mg7Zn1Fe0	-1704	Transcription

transcr_elo_TU00236_cplx	transcription elongation complex TU00236 (RHO DEPENDENT TERMINATION)	C140187H179948N50727O73627S279P10266Mg7Zn1Fe0	-10425	Transcription
transcr_elo_TU00258_cplx	transcription elongation complex TU00258 (RHO DEPENDENT TERMINATION)	C54778H81632N17035O21183S279P1499Mg7Zn1Fe0	-1658	Transcription
transcr_elo_TU00260_cplx	transcription elongation complex TU00260 (RHO DEPENDENT TERMINATION)	C70999H100238N23615O31005S279P3163Mg7Zn1Fe0	-3322	Transcription
transcr_elo_TU00277_cplx	transcription elongation complex TU00277 (RHO INDEPENDENT TERMINATION)	C53952H74731N18081O24586S177P2682Mg4Zn1Fe0	-2838	Transcription
transcr_elo_TU00281_cplx	transcription elongation complex TU00281 (RHO INDEPENDENT TERMINATION)	C57054H78341N19329O26527S177P3009Mg4Zn1Fe0	-3165	Transcription
transcr_elo_TU00291_cplx	transcription elongation complex TU00291 (RHO DEPENDENT TERMINATION)	C97132H130431N33640O47288S279P5848Mg7Zn1Fe0	-6007	Transcription
transcr_elo_TU00304_cplx	transcription elongation complex TU00304 (RHO DEPENDENT TERMINATION)	C86873H118632N29445O41058S279P4789Mg7Zn1Fe0	-4948	Transcription
transcr_elo_TU00309_cplx	transcription elongation complex TU00309 (RHO DEPENDENT TERMINATION)	C61815H89711N19906O25442S279P2222Mg7Zn1Fe0	-2381	Transcription
transcr_elo_TU00311_cplx	transcription elongation complex TU00311 (RHO DEPENDENT TERMINATION)	C85950H117369N29646O40090S279P4692Mg7Zn1Fe0	-4851	Transcription
transcr_elo_TU00314_cplx	transcription elongation complex TU00314 (RHO INDEPENDENT TERMINATION)	C53050H73812N17452O24248S177P2595Mg4Zn1Fe0	-2751	Transcription
transcr_elo_TU00324_cplx	transcription elongation complex TU00324 (RHO DEPENDENT TERMINATION)	C65991H94540N21505O28039S279P2653Mg7Zn1Fe0	-2812	Transcription
transcr_elo_TU00332_cplx	transcription elongation complex TU00332 (RHO DEPENDENT TERMINATION)	C55850H82865N17490O21827S279P1610Mg7Zn1Fe0	-1769	Transcription
transcr_elo_TU00333_cplx	transcription elongation complex TU00333 (RHO DEPENDENT TERMINATION)	C92490H125216N31645O44579S279P5384Mg7Zn1Fe0	-5543	Transcription
transcr_elo_TU00335_cplx	transcription elongation complex TU00335 (RHO INDEPENDENT TERMINATION)	C121292H152317N44435O65883S177P9596Mg4Zn1Fe0	-9752	Transcription
transcr_elo_TU00336_cplx	transcription elongation complex TU00336 (RHO INDEPENDENT TERMINATION)	C36093H54194N11076O13615S177P855Mg4Zn1Fe0	-1011	Transcription
transcr_elo_TU00337_cplx	transcription elongation complex TU00337 (RHO DEPENDENT TERMINATION)	C94435H127326N32363O45588S279P5559Mg7Zn1Fe0	-5718	Transcription
transcr_elo_TU00338_cplx	transcription elongation complex TU00338 (RHO DEPENDENT TERMINATION)	C46181H71721N13698O15904S279P617Mg7Zn1Fe0	-776	Transcription
transcr_elo_TU00339_cplx	transcription elongation complex TU00339 (RHO INDEPENDENT TERMINATION)	C32083H49572N9510O11147S177P443Mg4Zn1Fe0	-599	Transcription
transcr_elo_TU00340_cplx	transcription elongation complex TU00340 (RHO DEPENDENT TERMINATION)	C45429H70905N13332O15499S279P544Mg7Zn1Fe0	-703	Transcription
transcr_elo_TU00342_cplx	transcription elongation complex TU00342 (RHO DEPENDENT TERMINATION)	C66657H95350N21593O28528S279P2718Mg7Zn1Fe0	-2877	Transcription
transcr_elo_TU00343_cplx	transcription elongation complex TU00343 (RHO DEPENDENT TERMINATION)	C54370H81170N16929O20914S279P1461Mg7Zn1Fe0	-1620	Transcription
transcr_elo_TU00344_cplx	transcription elongation complex TU00344 (RHO DEPENDENT TERMINATION)	C90849H123210N31307O43320S279P5213Mg7Zn1Fe0	-5372	Transcription
transcr_elo_TU00345_cplx	transcription elongation complex TU00345 (RHO DEPENDENT TERMINATION)	C58333H85688N18515O23306S279P1862Mg7Zn1Fe0	-2021	Transcription
transcr_elo_TU00346_cplx	transcription elongation complex TU00346 (RHO DEPENDENT TERMINATION)	C54450H81266N16909O20984S279P1467Mg7Zn1Fe0	-1626	Transcription
transcr_elo_TU00347_cplx	transcription elongation complex TU00347 (RHO INDEPENDENT TERMINATION)	C76322H100437N26981O38170S177P4975Mg4Zn1Fe0	-5131	Transcription
transcr_elo_TU00348_cplx	transcription elongation complex TU00348 (RHO DEPENDENT TERMINATION)	C83401H114611N28204O38732S279P4435Mg7Zn1Fe0	-4594	Transcription
transcr_elo_TU00349_cplx	transcription elongation complex TU00349 (RHO INDEPENDENT TERMINATION)	C55804H76887N18769O25721S177P2874Mg4Zn1Fe0	-3030	Transcription
transcr_elo_TU00351_cplx	transcription elongation complex TU00351 (RHO DEPENDENT TERMINATION)	C59976H87598N19087O24367S279P2030Mg7Zn1Fe0	-2189	Transcription
transcr_elo_TU00352_cplx	transcription elongation complex TU00352 (RHO INDEPENDENT TERMINATION)	C67963H90862N23694O33067S177P4126Mg4Zn1Fe0	-4282	Transcription
transcr_elo_TU00354_cplx	transcription elongation complex TU00354 (RHO DEPENDENT TERMINATION)	C49508H75575N14940O18008S279P958Mg7Zn1Fe0	-1117	Transcription
transcr_elo_TU00355_cplx	transcription elongation complex TU00355 (RHO DEPENDENT TERMINATION)	C71907H101363N23818O31767S279P3260Mg7Zn1Fe0	-3419	Transcription

transcr_elo_TU00362_cplx	transcription elongation complex TU00362 (RHO DEPENDENT TERMINATION)	C82483H113603N27866O38254S279P4350Mg7Zn1Fe0	-4509	Transcription
transcr_elo_TU00366_cplx	transcription elongation complex TU00366 (RHO INDEPENDENT TERMINATION)	C58980H80497N20075O27552S177P3196Mg4Zn1Fe0	-3352	Transcription
transcr_elo_TU00411_cplx	transcription elongation complex TU00411 (RHO DEPENDENT TERMINATION)	C46233H71761N13766O15911S279P621Mg7Zn1Fe0	-780	Transcription
transcr_elo_TU00414_cplx	transcription elongation complex TU00414 (RHO INDEPENDENT TERMINATION)	C53952H74731N18081O24586S177P2682Mg4Zn1Fe0	-2838	Transcription
transcr_elo_TU00415_cplx	transcription elongation complex TU00415 (RHO INDEPENDENT TERMINATION)	C49482H69559N16379O21761S177P2223Mg4Zn1Fe0	-2379	Transcription
transcr_elo_TU00417_cplx	transcription elongation complex TU00417 (RHO DEPENDENT TERMINATION)	C70999H100238N23615O31005S279P3163Mg7Zn1Fe0	-3322	Transcription
transcr_elo_TU00418_cplx	transcription elongation complex TU00418 (RHO DEPENDENT TERMINATION)	C70999H100238N23615O31005S279P3163Mg7Zn1Fe0	-3322	Transcription
transcr_elo_TU00426_cplx	transcription elongation complex TU00426 (RHO INDEPENDENT TERMINATION)	C36271H54385N11191O13718S177P873Mg4Zn1Fe0	-1029	Transcription
transcr_elo_TU00427_cplx	transcription elongation complex TU00427 (RHO INDEPENDENT TERMINATION)	C36271H54385N11191O13718S177P873Mg4Zn1Fe0	-1029	Transcription
transcr_elo_TU00429_cplx	transcription elongation complex TU00429 (RHO DEPENDENT TERMINATION)	C55253H82193N17142O21488S279P1545Mg7Zn1Fe0	-1704	Transcription
transcr_elo_TU00430_cplx	transcription elongation complex TU00430 (RHO DEPENDENT TERMINATION)	C51150H77463N15628O18954S279P1128Mg7Zn1Fe0	-1287	Transcription
transcr_elo_TU00434_cplx	transcription elongation complex TU00434 (RHO INDEPENDENT TERMINATION)	C67963H90862N23694O33067S177P4126Mg4Zn1Fe0	-4282	Transcription
transcr_elo_TU00435_cplx	transcription elongation complex TU00435 (RHO INDEPENDENT TERMINATION)	C67963H90862N23694O33067S177P4126Mg4Zn1Fe0	-4282	Transcription
transcr_elo_TU00436_cplx	transcription elongation complex TU00436 (RHO INDEPENDENT TERMINATION)	C45881H65428N15056O19533S177P1860Mg4Zn1Fe0	-2016	Transcription
transcr_elo_TU00437_cplx	transcription elongation complex TU00437 (RHO INDEPENDENT TERMINATION)	C45881H65428N15056O19533S177P1860Mg4Zn1Fe0	-2016	Transcription
transcr_elo_TU00438_cplx	transcription elongation complex TU00438 (RHO INDEPENDENT TERMINATION)	C45881H65428N15056O19533S177P1860Mg4Zn1Fe0	-2016	Transcription
transcr_elo_TU00440_cplx	transcription elongation complex TU00440 (RHO DEPENDENT TERMINATION)	C61815H89711N19906O25442S279P2222Mg7Zn1Fe0	-2381	Transcription
transcr_elo_TU00442_cplx	transcription elongation complex TU00442 (RHO DEPENDENT TERMINATION)	C50117H76243N15270O18312S279P1020Mg7Zn1Fe0	-1179	Transcription
transcr_elo_TU00460_cplx	transcription elongation complex TU00460 (RHO DEPENDENT TERMINATION)	C54698H81590N17013O21196S279P1499Mg7Zn1Fe0	-1658	Transcription
transcr_elo_TU00471_cplx	transcription elongation complex TU00471 (RHO INDEPENDENT TERMINATION)	C49482H69559N16379O21761S177P2223Mg4Zn1Fe0	-2379	Transcription
transcr_elo_TU00472_cplx	transcription elongation complex TU00472 (RHO INDEPENDENT TERMINATION)	C30043H47220N8724O9904S177P234Mg4Zn1Fe0	-390	Transcription
transcr_elo_TU00487_cplx	transcription elongation complex TU00487 (RHO DEPENDENT TERMINATION)	C74725H104634N24831O33400S279P3549Mg7Zn1Fe0	-3708	Transcription
transcr_elo_TU00489_cplx	transcription elongation complex TU00489	C46082H74099N13162O14296S300P104Mg7Zn1Fe0	-209	Transcription
transcr_elo_TU00490_cplx	transcription elongation complex TU00490	C46074H74090N13155O14284S300P103Mg7Zn1Fe0	-208	Transcription
transcr_elo_TU00491_cplx	transcription elongation complex TU00491	C46077H74097N13158O14291S300P104Mg7Zn1Fe0	-209	Transcription
transcr_elo_TU00492_cplx	transcription elongation complex TU00492	C48891H77331N14264O16012S300P392Mg7Zn1Fe0	-497	Transcription
transcr_elo_TU00493_cplx	transcription elongation complex TU00493	C46870H75011N13456O14787S300P185Mg7Zn1Fe0	-290	Transcription
transcr_elo_TU00494_cplx	transcription elongation complex TU00494	C46080H74099N13170O14289S300P104Mg7Zn1Fe0	-209	Transcription
transcr_elo_TU00495_cplx	transcription elongation complex TU00495	C49207H77706N14369O16220S300P425Mg7Zn1Fe0	-530	Transcription
transcr_elo_TU00496_cplx	transcription elongation complex TU00496	C48222H76558N14037O15591S300P324Mg7Zn1Fe0	-429	Transcription
transcr_elo_TU00497_cplx	transcription elongation complex TU00497	C46071H74088N13143O14292S300P103Mg7Zn1Fe0	-208	Transcription
transcr_elo_TU00498_cplx	transcription elongation complex TU00498	C46071H74088N13143O14292S300P103Mg7Zn1Fe0	-208	Transcription
transcr_elo_TU00499_cplx	transcription elongation complex TU00499	C46071H74088N13143O14292S300P103Mg7Zn1Fe0	-208	Transcription
transcr_elo_TU00500_cplx	transcription elongation complex TU00500	C46061H74076N13141O14280S300P102Mg7Zn1Fe0	-207	Transcription
transcr_elo_TU00501_cplx	transcription elongation complex TU00501	C46071H74088N13159O14284S300P103Mg7Zn1Fe0	-208	Transcription
transcr_elo_TU00502_cplx	transcription elongation complex TU00502	C46068H74087N13152O14286S300P103Mg7Zn1Fe0	-208	Transcription

transcr_elo_TU00503_cplx	transcription elongation complex TU00503	C49595H78151N14520O16463S300P464Mg7Zn1Fe0	-569	Transcription
transcr_elo_TU00504_cplx	transcription elongation complex TU00504	C49633H78197N14540O16475S300P469Mg7Zn1Fe0	-574	Transcription
transcr_elo_TU00505_cplx	transcription elongation complex TU00505	C48244H76587N14022O15613S300P326Mg7Zn1Fe0	-431	Transcription
transcr_elo_TU00506_cplx	transcription elongation complex TU00506	C46163H74188N13209O14327S300P112Mg7Zn1Fe0	-217	Transcription
transcr_elo_TU00507_cplx	transcription elongation complex TU00507	C52184H81153N15490O18065S300P731Mg7Zn1Fe0	-836	Transcription
transcr_elo_TU00508_cplx	transcription elongation complex TU00508	C46190H74219N13216O14355S300P115Mg7Zn1Fe0	-220	Transcription
transcr_elo_TU00509_cplx	transcription elongation complex TU00509	C46191H74221N13220O14351S300P115Mg7Zn1Fe0	-220	Transcription
transcr_elo_TU00510_cplx	transcription elongation complex TU00510	C46190H74219N13216O14355S300P115Mg7Zn1Fe0	-220	Transcription
transcr_elo_TU00511_cplx	transcription elongation complex TU00511 (RHO DEPENDENT TERMINATION)	C45620H71094N13497O15565S279P563Mg7Zn1Fe0	-722	Transcription
transcr_elo_TU00512_cplx	transcription elongation complex TU00512	C48284H76638N14021O15650S300P330Mg7Zn1Fe0	-435	Transcription
transcr_elo_TU00513_cplx	transcription elongation complex TU00513	C46208H74241N13234O14357S300P117Mg7Zn1Fe0	-222	Transcription
transcr_elo_TU00514_cplx	transcription elongation complex TU00514	C47191H75377N13602O14971S300P218Mg7Zn1Fe0	-323	Transcription
transcr_elo_TU00515_cplx	transcription elongation complex TU00515	C53738H82908N16185O18970S300P889Mg7Zn1Fe0	-994	Transcription
transcr_elo_TU00516_cplx	transcription elongation complex TU00516	C46051H74066N13145O14274S300P101Mg7Zn1Fe0	-206	Transcription
transcr_elo_TU00517_cplx	transcription elongation complex TU00517 (RHO DEPENDENT TERMINATION)	C88719H120571N30688O41812S279P4975Mg7Zn1Fe0	-5134	Transcription
transcr_elo_TU00518_cplx	transcription elongation complex TU00518	C46184H74213N13208O14352S300P114Mg7Zn1Fe0	-219	Transcription
transcr_elo_TU00519_cplx	transcription elongation complex TU00519	C46082H74099N13166O14292S300P104Mg7Zn1Fe0	-209	Transcription
transcr_elo_TU00520_cplx	transcription elongation complex TU00520	C46071H74088N13159O14284S300P103Mg7Zn1Fe0	-208	Transcription
transcr_elo_TU00521_cplx	transcription elongation complex TU00521	C48484H76861N14116O15765S300P350Mg7Zn1Fe0	-455	Transcription
transcr_elo_TU221_cplx	transcription elongation complex TU221 (RHO INDEPENDENT TERMINATION)	C36271H54385N11191O13718S177P873Mg4Zn1Fe0	-1029	Transcription
transcr_elo_TU281_cplx	transcription elongation complex TU281 (RHO DEPENDENT TERMINATION)	C66657H95350N21593O28528S279P2718Mg7Zn1Fe0	-2877	Transcription
transcr_elo_TU341_cplx	transcription elongation complex TU341 (RHO INDEPENDENT TERMINATION)	C118269H148679N43549O63865S177P9275Mg4Zn1Fe0	-9431	Transcription
transcr_elo_TU343_cplx	transcription elongation complex TU343	C46079H74097N13174O14285S300P104Mg7Zn1Fe0	-209	Transcription
transcr_elo_TU361_cplx	transcription elongation complex TU361 (RHO DEPENDENT TERMINATION)	C64497H92797N20918O27107S279P2495Mg7Zn1Fe0	-2654	Transcription
transcr_elo_TU482_cplx	transcription elongation complex TU482 (RHO INDEPENDENT TERMINATION)	C73715H97514N25754O36801S177P4710Mg4Zn1Fe0	-4866	Transcription
transcr_elo_TU483_cplx	transcription elongation complex TU483 (RHO DEPENDENT TERMINATION)	C74007H103767N24632O32974S279P3473Mg7Zn1Fe0	-3632	Transcription
transcr_elo_TU543_cplx	transcription elongation complex TU543	C49004H77442N14389O16054S300P404Mg7Zn1Fe0	-509	Transcription
transcr_elo_TU564_cplx	transcription elongation complex TU564 (RHO DEPENDENT TERMINATION)	C61084H88870N19557O25009S279P2145Mg7Zn1Fe0	-2304	Transcription
transcr_elo_TU565_cplx	transcription elongation complex TU565 (RHO DEPENDENT TERMINATION)	C61084H88870N19557O25009S279P2145Mg7Zn1Fe0	-2304	Transcription
transcr_elo_TU582_cplx	transcription elongation complex TU582 (RHO INDEPENDENT TERMINATION)	C46765H66518N15216O20196S177P1953Mg4Zn1Fe0	-2109	Transcription
transcr_elo_TU583_cplx	transcription elongation complex TU583 (RHO INDEPENDENT TERMINATION)	C46765H66518N15216O20196S177P1953Mg4Zn1Fe0	-2109	Transcription
transcr_elo_TU601_cplx	transcription elongation complex TU601 (RHO DEPENDENT TERMINATION)	C47697H73489N14264O16846S279P774Mg7Zn1Fe0	-933	Transcription
transcr_elo_TU602_cplx	transcription elongation complex TU602 (RHO DEPENDENT TERMINATION)	C47697H73489N14264O16846S279P774Mg7Zn1Fe0	-933	Transcription
transcr_elo_TU682_cplx	transcription elongation complex TU682 (RHO DEPENDENT TERMINATION)	C45429H70905N13332O15499S279P544Mg7Zn1Fe0	-703	Transcription
transcr_elo_TU743_cplx	transcription elongation complex TU743 (RHO DEPENDENT TERMINATION)	C45429H70905N13332O15499S279P544Mg7Zn1Fe0	-703	Transcription
transcr_elo_TU801_cplx	transcription elongation complex TU801 (RHO DEPENDENT TERMINATION)	C140187H179948N50727O73627S279P10266Mg7Zn1Fe0	-10425	Transcription
transcr_elo_TU802_cplx	transcription elongation complex TU802 (RHO DEPENDENT TERMINATION)	C140187H179948N50727O73627S279P10266Mg7Zn1Fe0	-10425	Transcription
transcr_elo_TU870_cplx	transcription elongation complex TU870 (RHO INDEPENDENT TERMINATION)	C42419H61484N13486O17532S177P1501Mg4Zn1Fe0	-1657	Transcription
transcr_elo_TU871_cplx	transcription elongation complex TU871 (RHO DEPENDENT TERMINATION)	C50834H77122N15463O18816S279P1098Mg7Zn1Fe0	-1257	Transcription
transcr_elo_TU872_cplx	transcription elongation complex TU872 (RHO DEPENDENT TERMINATION)	C103139H137334N35933O50883S279P6458Mg7Zn1Fe0	-6617	Transcription
transcr_elo_TU873_cplx	transcription elongation complex TU873 (RHO DEPENDENT TERMINATION)	C103139H137334N35933O50883S279P6458Mg7Zn1Fe0	-6617	Transcription
transcr_elo_TU874_cplx	transcription elongation complex TU874 (RHO DEPENDENT TERMINATION)	C85963H117553N29268O40305S279P4700Mg7Zn1Fe0	-4859	Transcription
transcr_elo_TU875_cplx	transcription elongation complex TU875 (RHO DEPENDENT TERMINATION)	C72411H101969N23850O32054S279P3307Mg7Zn1Fe0	-3466	Transcription

transcr_elo_TU876_cplx	transcription elongation complex TU876 (RHO DEPENDENT TERMINATION)	C72411H101969N23850O32054S279P3307Mg7Zn1Fe0	-3466	Transcription
transcr_elo_TU877_cplx	transcription elongation complex TU877 (RHO DEPENDENT TERMINATION)	C64076H92355N20638O26930S279P2453Mg7Zn1Fe0	-2612	Transcription
transcr_elo_b0144_cplx	transcription elongation complex b0144 (RHO DEPENDENT TERMINATION)	C49417H75513N14860O17967S279P954Mg7Zn1Fe0	-1113	Transcription
transcr_elo_b0206_cplx	transcription elongation complex b0206	C46082H74099N13162O14296S300P104Mg7Zn1Fe0	-209	Transcription
transcr_elo_b0503_cplx	transcription elongation complex b0503 (RHO DEPENDENT TERMINATION)	C51091H77374N15669O18924S279P1122Mg7Zn1Fe0	-1281	Transcription
transcr_elo_b1133_cplx	transcription elongation complex b1133 (RHO DEPENDENT TERMINATION)	C51198H77525N15638O19016S279P1134Mg7Zn1Fe0	-1293	Transcription
transcr_elo_b1211_cplx	transcription elongation complex b1211 (RHO DEPENDENT TERMINATION)	C50963H77242N15577O18845S279P1110Mg7Zn1Fe0	-1269	Transcription
transcr_elo_b1212_cplx	transcription elongation complex b1212 (RHO DEPENDENT TERMINATION)	C48532H74464N14571O17402S279P861Mg7Zn1Fe0	-1020	Transcription
transcr_elo_b1804_cplx	transcription elongation complex b1804 (RHO DEPENDENT TERMINATION)	C51402H77764N15699O19157S279P1155Mg7Zn1Fe0	-1314	Transcription
transcr_elo_b2745_cplx	transcription elongation complex b2745 (RHO DEPENDENT TERMINATION)	C50673H76884N15469O18669S279P1077Mg7Zn1Fe0	-1236	Transcription
transcr_elo_b3289_cplx	transcription elongation complex b3289 (RHO DEPENDENT TERMINATION)	C52997H79605N16294O20104S279P1317Mg7Zn1Fe0	-1476	Transcription
transcr_elo_b3760_cplx	transcription elongation complex b3760	C46082H74099N13162O14296S300P104Mg7Zn1Fe0	-209	Transcription
transcr_elo_b3761_cplx	transcription elongation complex b3761	C46073H74088N13159O14286S300P103Mg7Zn1Fe0	-208	Transcription
transcr_elo_b4143_cplx	transcription elongation complex b4143 (RHO DEPENDENT TERMINATION)	C56489H83555N17854O22169S279P1674Mg7Zn1Fe0	-1833	Transcription
transcr_ini_TU-8389_cplx	transcription initiation complex TU-8389	C18962H29574N5595O6542S113P237Mg2Zn1Fe0	-316	Transcription
transcr_ini_TU-8390_cplx	transcription initiation complex TU-8390	C18962H29574N5595O6542S113P237Mg2Zn1Fe0	-316	Transcription
transcr_ini_TU-8392_cplx	transcription initiation complex TU-8392	C52313H67944N18731O27003S113P3657Mg2Zn1Fe0	-3736	Transcription
transcr_ini_TU-8397_cplx	transcription initiation complex TU-8397	C21489H32471N6590O8087S113P495Mg2Zn1Fe0	-574	Transcription
transcr_ini_TU-8398_cplx	transcription initiation complex TU-8398	C56035H72305N20062O29285S113P4044Mg2Zn1Fe0	-4123	Transcription
transcr_ini_TU-8407_cplx	transcription initiation complex TU-8407	C47800H62775N17062O24180S113P3204Mg2Zn1Fe0	-3283	Transcription
transcr_ini_TU0-1181_cplx	transcription initiation complex TU0-1181	C66757H84348N24813O35616S113P5124Mg2Zn1Fe0	-5203	Transcription
transcr_ini_TU0-1182_cplx	transcription initiation complex TU0-1182	C66670H84251N24786O35564S113P5116Mg2Zn1Fe0	-5195	Transcription
transcr_ini_TU0-1183_cplx	transcription initiation complex TU0-1183	C65826H83282N24457O35049S113P5030Mg2Zn1Fe0	-5109	Transcription
transcr_ini_TU0-1184_cplx	transcription initiation complex TU0-1184	C65826H83282N24457O35049S113P5030Mg2Zn1Fe0	-5109	Transcription
transcr_ini_TU0-1186_cplx	transcription initiation complex TU0-1186	C65837H83296N24455O35061S113P5031Mg2Zn1Fe0	-5110	Transcription
transcr_ini_TU0-1187_cplx	transcription initiation complex TU0-1187	C66579H84153N24736O35519S113P5107Mg2Zn1Fe0	-5186	Transcription
transcr_ini_TU0-1188_cplx	transcription initiation complex TU0-1188	C66579H84153N24736O35519S113P5107Mg2Zn1Fe0	-5186	Transcription
transcr_ini_TU0-1189_cplx	transcription initiation complex TU0-1189	C66744H84337N24802O35615S113P5123Mg2Zn1Fe0	-5202	Transcription
transcr_ini_TU0-1190_cplx	transcription initiation complex TU0-1190	C66744H84337N24802O35615S113P5123Mg2Zn1Fe0	-5202	Transcription
transcr_ini_TU0-1191_cplx	transcription initiation complex TU0-1191	C69032H86970N25709O37019S113P5358Mg2Zn1Fe0	-5437	Transcription
transcr_ini_TU0-1192_cplx	transcription initiation complex TU0-1192	C69032H86970N25709O37019S113P5358Mg2Zn1Fe0	-5437	Transcription
transcr_ini_TU0-12803_cplx	transcription initiation complex TU0-12803	C51666H67246N18425O26659S113P3596Mg2Zn1Fe0	-3675	Transcription
transcr_ini_TU0-12827_cplx	transcription initiation complex TU0-12827	C59502H76338N21337O31491S113P4402Mg2Zn1Fe0	-4481	Transcription
transcr_ini_TU0-12830_cplx	transcription initiation complex TU0-12830	C22274H33362N6941O8548S113P576Mg2Zn1Fe0	-655	Transcription
transcr_ini_TU0-12833_cplx	transcription initiation complex TU0-12833	C29475H41684N9723O13059S113P1317Mg2Zn1Fe0	-1396	Transcription
transcr_ini_TU0-12921_cplx	transcription initiation complex TU0-12921	C41726H55773N14554O20515S113P2573Mg2Zn1Fe0	-2652	Transcription
transcr_ini_TU0-12928_cplx	transcription initiation complex TU0-12928	C29515H41660N9863O12932S113P1317Mg2Zn1Fe0	-1396	Transcription
transcr_ini_TU0-13010_cplx	transcription initiation complex TU0-13010	C70309H88723N25680O38045S113P5506Mg2Zn1Fe0	-5585	Transcription
transcr_ini_TU0-13018_cplx	transcription initiation complex TU0-13018	C30700H43099N10200O13759S113P1443Mg2Zn1Fe0	-1522	Transcription
transcr_ini_TU0-13020_cplx	transcription initiation complex TU0-13020	C33055H45807N11106O15199S113P1683Mg2Zn1Fe0	-1762	Transcription
transcr_ini_TU0-13034_cplx	transcription initiation complex TU0-13034	C18331H28855N5326O6187S113P173Mg2Zn1Fe0	-252	Transcription
transcr_ini_TU0-13035_cplx	transcription initiation complex TU0-13035	C17565H27970N5023O5715S113P94Mg2Zn1Fe0	-173	Transcription
transcr_ini_TU0-13036_cplx	transcription initiation complex TU0-13036	C17565H27970N5023O5715S113P94Mg2Zn1Fe0	-173	Transcription
transcr_ini_TU0-13072_cplx	transcription initiation complex TU0-13072	C32976H45760N10945O15255S113P1675Mg2Zn1Fe0	-1754	Transcription
transcr_ini_TU0-13080_cplx	transcription initiation complex TU0-13080	C29431H41626N9697O12976S113P1311Mg2Zn1Fe0	-1390	Transcription
transcr_ini_TU0-13093_cplx	transcription initiation complex TU0-13093	C30482H42872N10001O13690S113P1419Mg2Zn1Fe0	-1498	Transcription
transcr_ini_TU0-13104_cplx	transcription initiation complex TU0-13104	C20059H30821N6030O7213S113P348Mg2Zn1Fe0	-427	Transcription

transcr_ini_TU0-13134_cplx	transcription initiation complex TU0-13134	C26214H37900N8455O10988S113P978Mg2Zn1Fe0	-1057	Transcription
transcr_ini_TU0-13139_cplx	transcription initiation complex TU0-13139	C50388H65783N17912O25877S113P3465Mg2Zn1Fe0	-3544	Transcription
transcr_ini_TU0-13145_cplx	transcription initiation complex TU0-13145	C27792H39753N9014O11974S113P1143Mg2Zn1Fe0	-1222	Transcription
transcr_ini_TU0-13195_cplx	transcription initiation complex TU0-13195	C25381H36956N8109O10506S113P894Mg2Zn1Fe0	-973	Transcription
transcr_ini_TU0-13225_cplx	transcription initiation complex TU0-13225	C25977H37635N8344O10826S113P954Mg2Zn1Fe0	-1033	Transcription
transcr_ini_TU0-13421_cplx	transcription initiation complex TU0-13421	C24721H36219N7784O10126S113P828Mg2Zn1Fe0	-907	Transcription
transcr_ini_TU0-13443_cplx	transcription initiation complex TU0-13443	C37783H51314N12727O18237S113P2166Mg2Zn1Fe0	-2245	Transcription
transcr_ini_TU0-13444_cplx	transcription initiation complex TU0-13444	C33727H46552N11445O15577S113P1752Mg2Zn1Fe0	-1831	Transcription
transcr_ini_TU0-13519_cplx	transcription initiation complex TU0-13519	C36630H49951N12462O17423S113P2052Mg2Zn1Fe0	-2131	Transcription
transcr_ini_TU0-13534_cplx	transcription initiation complex TU0-13534	C26089H37753N8414O10936S113P966Mg2Zn1Fe0	-1045	Transcription
transcr_ini_TU0-13555_cplx	transcription initiation complex TU0-13555	C23605H34935N7368O9431S113P714Mg2Zn1Fe0	-793	Transcription
transcr_ini_TU0-13557_cplx	transcription initiation complex TU0-13557	C19600H30303N5852O6934S113P303Mg2Zn1Fe0	-382	Transcription
transcr_ini_TU0-13573_cplx	transcription initiation complex TU0-13573	C25769H37432N8185O10762S113P936Mg2Zn1Fe0	-1015	Transcription
transcr_ini_TU0-13593_cplx	transcription initiation complex TU0-13593	C61142H78232N21919O32588S113P4568Mg2Zn1Fe0	-4647	Transcription
transcr_ini_TU0-13667_cplx	transcription initiation complex TU0-13667	C28508H40591N9232O12487S113P1215Mg2Zn1Fe0	-1294	Transcription
transcr_ini_TU0-13675_cplx	transcription initiation complex TU0-13675	C33487H46316N11235O15513S113P1727Mg2Zn1Fe0	-1806	Transcription
transcr_ini_TU0-13704_cplx	transcription initiation complex TU0-13704	C17560H27966N5025O5712S113P94Mg2Zn1Fe0	-173	Transcription
transcr_ini_TU0-13717_cplx	transcription initiation complex TU0-13717	C42499H56651N14854O20999S113P2649Mg2Zn1Fe0	-2728	Transcription
transcr_ini_TU0-13734_cplx	transcription initiation complex TU0-13734	C46324H61084N16271O23320S113P3042Mg2Zn1Fe0	-3121	Transcription
transcr_ini_TU0-13735_cplx	transcription initiation complex TU0-13735	C29521H41736N9705O13035S113P1320Mg2Zn1Fe0	-1399	Transcription
transcr_ini_TU0-13736_cplx	transcription initiation complex TU0-13736	C32226H44846N10779O14711S113P1597Mg2Zn1Fe0	-1676	Transcription
transcr_ini_TU0-13738_cplx	transcription initiation complex TU0-13738	C25103H36664N7923O10343S113P867Mg2Zn1Fe0	-946	Transcription
transcr_ini_TU0-13805_cplx	transcription initiation complex TU0-13805	C33343H46133N11240O15375S113P1713Mg2Zn1Fe0	-1792	Transcription
transcr_ini_TU0-13809_cplx	transcription initiation complex TU0-13809	C27055H38868N8781O11506S113P1064Mg2Zn1Fe0	-1143	Transcription
transcr_ini_TU0-13915_cplx	transcription initiation complex TU0-13915	C34021H46972N11291O15891S113P1781Mg2Zn1Fe0	-1860	Transcription
transcr_ini_TU0-13931_cplx	transcription initiation complex TU0-13931	C21484H32469N6594O8084S113P495Mg2Zn1Fe0	-574	Transcription
transcr_ini_TU0-14007_cplx	transcription initiation complex TU0-14007	C17744H28179N5100O5829S113P113Mg2Zn1Fe0	-192	Transcription
transcr_ini_TU0-14021_cplx	transcription initiation complex TU0-14021	C30110H42418N9979O13418S113P1383Mg2Zn1Fe0	-1462	Transcription
transcr_ini_TU0-14029_cplx	transcription initiation complex TU0-14029	C41926H56052N14509O20692S113P2595Mg2Zn1Fe0	-2674	Transcription
transcr_ini_TU0-14037_cplx	transcription initiation complex TU0-14037	C29156H41336N9535O12832S113P1284Mg2Zn1Fe0	-1363	Transcription
transcr_ini_TU0-14038_cplx	transcription initiation complex TU0-14038	C30919H43368N10209O13889S113P1464Mg2Zn1Fe0	-1543	Transcription
transcr_ini_TU0-14070_cplx	transcription initiation complex TU0-14070	C44959H59557N15618O22618S113P2904Mg2Zn1Fe0	-2983	Transcription
transcr_ini_TU0-14100_cplx	transcription initiation complex TU0-14100	C28369H40411N9274O12330S113P1203Mg2Zn1Fe0	-1282	Transcription
transcr_ini_TU0-14118_cplx	transcription initiation complex TU0-14118	C26961H38768N8737O11474S113P1056Mg2Zn1Fe0	-1135	Transcription
transcr_ini_TU0-14151_cplx	transcription initiation complex TU0-14151	C22153H33238N6863O8507S113P564Mg2Zn1Fe0	-643	Transcription
transcr_ini_TU0-14215_cplx	transcription initiation complex TU0-14215	C26868H38705N8628O11472S113P1050Mg2Zn1Fe0	-1129	Transcription
transcr_ini_TU0-14216_cplx	transcription initiation complex TU0-14216	C31622H44185N10514O14357S113P1539Mg2Zn1Fe0	-1618	Transcription
transcr_ini_TU0-14217_cplx	transcription initiation complex TU0-14217	C32309H44948N10821O14760S113P1608Mg2Zn1Fe0	-1687	Transcription
transcr_ini_TU0-14252_cplx	transcription initiation complex TU0-14252	C29515H41660N9863O12932S113P1317Mg2Zn1Fe0	-1396	Transcription
transcr_ini_TU0-1881_cplx	transcription initiation complex TU0-1881	C41882H55954N14583O20595S113P2587Mg2Zn1Fe0	-2666	Transcription
transcr_ini_TU0-1_cplx	transcription initiation complex TU0-1	C56228H72464N20195O29452S113P4056Mg2Zn1Fe0	-4135	Transcription
transcr_ini_TU0-2081_cplx	transcription initiation complex TU0-2081	C50388H65783N17912O25877S113P3465Mg2Zn1Fe0	-3544	Transcription
transcr_ini_TU0-2082_cplx	transcription initiation complex TU0-2082	C50388H65783N17912O25877S113P3465Mg2Zn1Fe0	-3544	Transcription
transcr_ini_TU0-2101_cplx	transcription initiation complex TU0-2101	C41425H55478N14237O20447S113P2539Mg2Zn1Fe0	-2618	Transcription
transcr_ini_TU0-2121_cplx	transcription initiation complex TU0-2121	C31146H43622N10267O14105S113P1485Mg2Zn1Fe0	-1564	Transcription
transcr_ini_TU0-3304_cplx	transcription initiation complex TU0-3304	C22140H33199N6904O8445S113P561Mg2Zn1Fe0	-640	Transcription
transcr_ini_TU0-3305_cplx	transcription initiation complex TU0-3305	C22140H33199N6904O8445S113P561Mg2Zn1Fe0	-640	Transcription

transcr_ini_TU0-3364_cplx	transcription initiation complex TU0-3364	C66670H84251N24786O35564S113P5116Mg2Zn1Fe0	-5195	Transcription
transcr_ini_TU0-3561_cplx	transcription initiation complex TU0-3561	C17562H27967N5018O5717S113P94Mg2Zn1Fe0	-173	Transcription
transcr_ini_TU0-3901_cplx	transcription initiation complex TU0-3901	C18170H28658N5293O6050S113P156Mg2Zn1Fe0	-235	Transcription
transcr_ini_TU0-4925_cplx	transcription initiation complex TU0-4925	C28369H40411N9274O12330S113P1203Mg2Zn1Fe0	-1282	Transcription
transcr_ini_TU0-5003_cplx	transcription initiation complex TU0-5003	C19702H30418N5873O7010S113P312Mg2Zn1Fe0	-391	Transcription
transcr_ini_TU0-5121_cplx	transcription initiation complex TU0-5121	C49686H64957N17604O25384S113P3388Mg2Zn1Fe0	-3467	Transcription
transcr_ini_TU0-5182_cplx	transcription initiation complex TU0-5182	C53611H69506N18995O27952S113P3788Mg2Zn1Fe0	-3867	Transcription
transcr_ini_TU0-5183_cplx	transcription initiation complex TU0-5183	C41878H55980N14479O20695S113P2586Mg2Zn1Fe0	-2665	Transcription
transcr_ini_TU0-5186_cplx	transcription initiation complex TU0-5186	C36793H50143N12514O17546S113P2069Mg2Zn1Fe0	-2148	Transcription
transcr_ini_TU0-5201_cplx	transcription initiation complex TU0-5201	C46376H61216N16213O23483S113P3055Mg2Zn1Fe0	-3134	Transcription
transcr_ini_TU0-5221_cplx	transcription initiation complex TU0-5221	C34407H47418N11547O16111S113P1826Mg2Zn1Fe0	-1905	Transcription
transcr_ini_TU0-5522_cplx	transcription initiation complex TU0-5522	C38488H52052N13169O18546S113P2235Mg2Zn1Fe0	-2314	Transcription
transcr_ini_TU0-5543_cplx	transcription initiation complex TU0-5543	C29831H42086N9819O13235S113P1350Mg2Zn1Fe0	-1429	Transcription
transcr_ini_TU0-5601_cplx	transcription initiation complex TU0-5601	C29627H41853N9718O13086S113P1328Mg2Zn1Fe0	-1407	Transcription
transcr_ini_TU0-6223_cplx	transcription initiation complex TU0-6223	C107156H130994N40407O60493S113P9275Mg2Zn1Fe0	-9354	Transcription
transcr_ini_TU0-6405_cplx	transcription initiation complex TU0-6405	C44628H59120N15747O22263S113P2874Mg2Zn1Fe0	-2953	Transcription
transcr_ini_TU0-6409_cplx	transcription initiation complex TU0-6409	C30631H43014N10151O13716S113P1434Mg2Zn1Fe0	-1513	Transcription
transcr_ini_TU0-6423_cplx	transcription initiation complex TU0-6423	C22117H33198N6803O8467S113P558Mg2Zn1Fe0	-637	Transcription
transcr_ini_TU0-6441_cplx	transcription initiation complex TU0-6441	C42499H56651N14854O20999S113P2649Mg2Zn1Fe0	-2728	Transcription
transcr_ini_TU0-6504_cplx	transcription initiation complex TU0-6504	C33055H45807N11106O15199S113P1683Mg2Zn1Fe0	-1762	Transcription
transcr_ini_TU0-6506_cplx	transcription initiation complex TU0-6506	C29262H41435N9612O12911S113P1293Mg2Zn1Fe0	-1372	Transcription
transcr_ini_TU0-6512_cplx	transcription initiation complex TU0-6512	C125299H152018N47273O71723S113P11145Mg2Zn1Fe0	-11224	Transcription
transcr_ini_TU0-6550_cplx	transcription initiation complex TU0-6550	C30631H43014N10151O13716S113P1434Mg2Zn1Fe0	-1513	Transcription
transcr_ini_TU0-6551_cplx	transcription initiation complex TU0-6551	C30631H43014N10151O13716S113P1434Mg2Zn1Fe0	-1513	Transcription
transcr_ini_TU0-6562_cplx	transcription initiation complex TU0-6562	C33570H46393N11342O15523S113P1737Mg2Zn1Fe0	-1816	Transcription
transcr_ini_TU0-6626_cplx	transcription initiation complex TU0-6626	C56005H72217N20110O29304S113P4036Mg2Zn1Fe0	-4115	Transcription
transcr_ini_TU0-6654_cplx	transcription initiation complex TU0-6654	C38317H51861N13188O18438S113P2223Mg2Zn1Fe0	-2302	Transcription
transcr_ini_TU0-6657_cplx	transcription initiation complex TU0-6657	C20308H31130N6095O7383S113P375Mg2Zn1Fe0	-454	Transcription
transcr_ini_TU0-6658_cplx	transcription initiation complex TU0-6658	C25158H36700N8049O10346S113P873Mg2Zn1Fe0	-952	Transcription
transcr_ini_TU0-6660_cplx	transcription initiation complex TU0-6660	C42672H56884N14859O21125S113P2670Mg2Zn1Fe0	-2749	Transcription
transcr_ini_TU0-6687_cplx	transcription initiation complex TU0-6687	C44628H59120N15747O22263S113P2874Mg2Zn1Fe0	-2953	Transcription
transcr_ini_TU0-6941_cplx	transcription initiation complex TU0-6941	C30919H43368N10209O13889S113P1464Mg2Zn1Fe0	-1543	Transcription
transcr_ini_TU0-7002_cplx	transcription initiation complex TU0-7002	C97968H120576N36557O55028S113P8347Mg2Zn1Fe0	-8426	Transcription
transcr_ini_TU0-7141_cplx	transcription initiation complex TU0-7141	C21268H32227N6464O7962S113P473Mg2Zn1Fe0	-552	Transcription
transcr_ini_TU0-7281_cplx	transcription initiation complex TU0-7281	C38286H51857N12994O18478S113P2215Mg2Zn1Fe0	-2294	Transcription
transcr_ini_TU0-7842_cplx	transcription initiation complex TU0-7842	C19397H30068N5773O6799S113P282Mg2Zn1Fe0	-361	Transcription
transcr_ini_TU0-7844_cplx	transcription initiation complex TU0-7844	C19397H30068N5773O6799S113P282Mg2Zn1Fe0	-361	Transcription
transcr_ini_TU0-8084_cplx	transcription initiation complex TU0-8084	C34103H47019N11498O15875S113P1791Mg2Zn1Fe0	-1870	Transcription
transcr_ini_TU0-8281_cplx	transcription initiation complex TU0-8281	C22532H33698N6931O8771S113P603Mg2Zn1Fe0	-682	Transcription
transcr_ini_TU0-8464_cplx	transcription initiation complex TU0-8464	C20413H31224N6177O7420S113P384Mg2Zn1Fe0	-463	Transcription
transcr_ini_TU0-8474_cplx	transcription initiation complex TU0-8474	C19446H30141N5760O6862S113P288Mg2Zn1Fe0	-367	Transcription
transcr_ini_TU0-8476_cplx	transcription initiation complex TU0-8476	C17570H27976N5049O5710S113P95Mg2Zn1Fe0	-174	Transcription
transcr_ini_TU0-8477_cplx	transcription initiation complex TU0-8477	C30335H42654N10091O13525S113P1404Mg2Zn1Fe0	-1483	Transcription
transcr_ini_TU0-8502_cplx	transcription initiation complex TU0-8502	C54043H69925N19452O28051S113P3835Mg2Zn1Fe0	-3914	Transcription
transcr_ini_TU0-8505_cplx	transcription initiation complex TU0-8505	C25343H36898N8145O10430S113P891Mg2Zn1Fe0	-970	Transcription
transcr_ini_TU0-8506_cplx	transcription initiation complex TU0-8506	C18899H29505N5556O6513S113P231Mg2Zn1Fe0	-310	Transcription
transcr_ini_TU0-8510_cplx	transcription initiation complex TU0-8510	C19213H29878N5647O6740S113P264Mg2Zn1Fe0	-343	Transcription

transcr_ini_TU0-8529_cplx	transcription initiation complex TU0-8529	C30955H43394N10237O13942S113P1467Mg2Zn1Fe0	-1546	Transcription
transcr_ini_TU0-8823_cplx	transcription initiation complex TU0-8823	C36413H49678N12361O17317S113P2025Mg2Zn1Fe0	-2104	Transcription
transcr_ini_TU0-8855_cplx	transcription initiation complex TU0-8855	C21940H33022N6689O8396S113P542Mg2Zn1Fe0	-621	Transcription
transcr_ini_TU0-8862_cplx	transcription initiation complex TU0-8862	C26628H38397N8598O11252S113P1023Mg2Zn1Fe0	-1102	Transcription
transcr_ini_TU0-8864_cplx	transcription initiation complex TU0-8864	C45928H60632N16075O23129S113P3000Mg2Zn1Fe0	-3079	Transcription
transcr_ini_TU0-8865_cplx	transcription initiation complex TU0-8865	C38271H51794N13149O18400S113P2215Mg2Zn1Fe0	-2294	Transcription
transcr_ini_TU00021_cplx	transcription initiation complex TU00021	C29391H41577N9678O12932S113P1306Mg2Zn1Fe0	-1385	Transcription
transcr_ini_TU00083_cplx	transcription initiation complex TU00083	C25158H36700N8049O10346S113P873Mg2Zn1Fe0	-952	Transcription
transcr_ini_TU00084_cplx	transcription initiation complex TU00084	C25158H36700N8049O10346S113P873Mg2Zn1Fe0	-952	Transcription
transcr_ini_TU00221_cplx	transcription initiation complex TU00221	C31666H44210N10469O14351S113P1536Mg2Zn1Fe0	-1615	Transcription
transcr_ini_TU00236_cplx	transcription initiation complex TU00236	C116600H141965N44054O66490S113P10257Mg2Zn1Fe0	-10336	Transcription
transcr_ini_TU00258_cplx	transcription initiation complex TU00258	C31191H43649N10362O14046S113P1490Mg2Zn1Fe0	-1569	Transcription
transcr_ini_TU00260_cplx	transcription initiation complex TU00260	C47412H62255N16942O23868S113P3154Mg2Zn1Fe0	-3233	Transcription
transcr_ini_TU00277_cplx	transcription initiation complex TU00277	C42839H57046N14939O21214S113P2682Mg2Zn1Fe0	-2761	Transcription
transcr_ini_TU00281_cplx	transcription initiation complex TU00281	C45941H60656N16187O23155S113P3009Mg2Zn1Fe0	-3088	Transcription
transcr_ini_TU00291_cplx	transcription initiation complex TU00291	C73545H92448N26967O40151S113P5839Mg2Zn1Fe0	-5918	Transcription
transcr_ini_TU00304_cplx	transcription initiation complex TU00304	C63286H80649N22772O33921S113P4780Mg2Zn1Fe0	-4859	Transcription
transcr_ini_TU00309_cplx	transcription initiation complex TU00309	C38228H51728N13233O18305S113P2213Mg2Zn1Fe0	-2292	Transcription
transcr_ini_TU00311_cplx	transcription initiation complex TU00311	C62363H79386N22973O32953S113P4683Mg2Zn1Fe0	-4762	Transcription
transcr_ini_TU00314_cplx	transcription initiation complex TU00314	C41937H56127N14310O20876S113P2595Mg2Zn1Fe0	-2674	Transcription
transcr_ini_TU00324_cplx	transcription initiation complex TU00324	C42404H56557N14832O20902S113P2644Mg2Zn1Fe0	-2723	Transcription
transcr_ini_TU00332_cplx	transcription initiation complex TU00332	C32263H44882N10817O14690S113P1601Mg2Zn1Fe0	-1680	Transcription
transcr_ini_TU00333_cplx	transcription initiation complex TU00333	C68903H87233N24972O37442S113P5375Mg2Zn1Fe0	-5454	Transcription
transcr_ini_TU00335_cplx	transcription initiation complex TU00335	C110179H134632N41293O62511S113P9596Mg2Zn1Fe0	-9675	Transcription
transcr_ini_TU00336_cplx	transcription initiation complex TU00336	C24980H36509N7934O10243S113P855Mg2Zn1Fe0	-934	Transcription
transcr_ini_TU00337_cplx	transcription initiation complex TU00337	C70848H89343N25690O38451S113P5550Mg2Zn1Fe0	-5629	Transcription
transcr_ini_TU00338_cplx	transcription initiation complex TU00338	C22594H33738N7025O8767S113P608Mg2Zn1Fe0	-687	Transcription
transcr_ini_TU00339_cplx	transcription initiation complex TU00339	C20970H31887N6368O7775S113P443Mg2Zn1Fe0	-522	Transcription
transcr_ini_TU00340_cplx	transcription initiation complex TU00340	C21842H32922N6659O8362S113P535Mg2Zn1Fe0	-614	Transcription
transcr_ini_TU00342_cplx	transcription initiation complex TU00342	C43070H57367N14920O21391S113P2709Mg2Zn1Fe0	-2788	Transcription
transcr_ini_TU00343_cplx	transcription initiation complex TU00343	C30783H43187N10256O13777S113P1452Mg2Zn1Fe0	-1531	Transcription
transcr_ini_TU00344_cplx	transcription initiation complex TU00344	C67262H85227N24634O36183S113P5204Mg2Zn1Fe0	-5283	Transcription
transcr_ini_TU00345_cplx	transcription initiation complex TU00345	C34746H47705N11842O16169S113P1853Mg2Zn1Fe0	-1932	Transcription
transcr_ini_TU00346_cplx	transcription initiation complex TU00346	C30863H43283N10236O13847S113P1458Mg2Zn1Fe0	-1537	Transcription
transcr_ini_TU00347_cplx	transcription initiation complex TU00347	C65209H82752N23839O34798S113P4975Mg2Zn1Fe0	-5054	Transcription
transcr_ini_TU00348_cplx	transcription initiation complex TU00348	C59814H76628N21531O31595S113P4426Mg2Zn1Fe0	-4505	Transcription
transcr_ini_TU00349_cplx	transcription initiation complex TU00349	C44691H59202N15627O22349S113P2874Mg2Zn1Fe0	-2953	Transcription
transcr_ini_TU00351_cplx	transcription initiation complex TU00351	C36389H49615N12414O17230S113P2021Mg2Zn1Fe0	-2100	Transcription
transcr_ini_TU00352_cplx	transcription initiation complex TU00352	C56850H73177N20552O29695S113P4126Mg2Zn1Fe0	-4205	Transcription
transcr_ini_TU00354_cplx	transcription initiation complex TU00354	C25921H37592N8267O10871S113P949Mg2Zn1Fe0	-1028	Transcription
transcr_ini_TU00355_cplx	transcription initiation complex TU00355	C48320H63380N17145O24630S113P3251Mg2Zn1Fe0	-3330	Transcription
transcr_ini_TU00362_cplx	transcription initiation complex TU00362	C58896H75620N21193O31117S113P4341Mg2Zn1Fe0	-4420	Transcription
transcr_ini_TU00366_cplx	transcription initiation complex TU00366	C47867H62812N16933O24180S113P3196Mg2Zn1Fe0	-3275	Transcription
transcr_ini_TU00411_cplx	transcription initiation complex TU00411	C22646H33778N7093O8774S113P612Mg2Zn1Fe0	-691	Transcription
transcr_ini_TU00414_cplx	transcription initiation complex TU00414	C42839H57046N14939O21214S113P2682Mg2Zn1Fe0	-2761	Transcription
transcr_ini_TU00415_cplx	transcription initiation complex TU00415	C38369H51874N13237O18389S113P2223Mg2Zn1Fe0	-2302	Transcription
transcr_ini_TU00417_cplx	transcription initiation complex TU00417	C47412H62255N16942O23868S113P3154Mg2Zn1Fe0	-3233	Transcription

transcr_ini_TU00418_cplx	transcription initiation complex TU00418	C47412H62255N16942O23868S113P3154Mg2Zn1Fe0	-3233	Transcription
transcr_ini_TU00426_cplx	transcription initiation complex TU00426	C25158H36700N8049O10346S113P873Mg2Zn1Fe0	-952	Transcription
transcr_ini_TU00427_cplx	transcription initiation complex TU00427	C25158H36700N8049O10346S113P873Mg2Zn1Fe0	-952	Transcription
transcr_ini_TU00429_cplx	transcription initiation complex TU00429	C31666H44210N10469O14351S113P1536Mg2Zn1Fe0	-1615	Transcription
transcr_ini_TU00430_cplx	transcription initiation complex TU00430	C27563H39480N8955O11817S113P1119Mg2Zn1Fe0	-1198	Transcription
transcr_ini_TU00434_cplx	transcription initiation complex TU00434	C56850H73177N20552O29695S113P4126Mg2Zn1Fe0	-4205	Transcription
transcr_ini_TU00435_cplx	transcription initiation complex TU00435	C56850H73177N20552O29695S113P4126Mg2Zn1Fe0	-4205	Transcription
transcr_ini_TU00436_cplx	transcription initiation complex TU00436	C34768H47743N11914O16161S113P1860Mg2Zn1Fe0	-1939	Transcription
transcr_ini_TU00437_cplx	transcription initiation complex TU00437	C34768H47743N11914O16161S113P1860Mg2Zn1Fe0	-1939	Transcription
transcr_ini_TU00438_cplx	transcription initiation complex TU00438	C34768H47743N11914O16161S113P1860Mg2Zn1Fe0	-1939	Transcription
transcr_ini_TU00440_cplx	transcription initiation complex TU00440	C38228H51728N13233O18305S113P2213Mg2Zn1Fe0	-2292	Transcription
transcr_ini_TU00442_cplx	transcription initiation complex TU00442	C26530H38260N8597O11175S113P1011Mg2Zn1Fe0	-1090	Transcription
transcr_ini_TU00460_cplx	transcription initiation complex TU00460	C31111H43607N10340O14059S113P1490Mg2Zn1Fe0	-1569	Transcription
transcr_ini_TU00471_cplx	transcription initiation complex TU00471	C38369H51874N13237O18389S113P2223Mg2Zn1Fe0	-2302	Transcription
transcr_ini_TU00472_cplx	transcription initiation complex TU00472	C18930H29535N5582O6532S113P234Mg2Zn1Fe0	-313	Transcription
transcr_ini_TU00487_cplx	transcription initiation complex TU00487	C51138H66651N18158O26263S113P3540Mg2Zn1Fe0	-3619	Transcription
transcr_ini_TU00489_cplx	transcription initiation complex TU00489	C17573H27978N5037O5721S113P95Mg2Zn1Fe0	-174	Transcription
transcr_ini_TU00490_cplx	transcription initiation complex TU00490	C17565H27969N5030O5709S113P94Mg2Zn1Fe0	-173	Transcription
transcr_ini_TU00491_cplx	transcription initiation complex TU00491	C17568H27976N5033O5716S113P95Mg2Zn1Fe0	-174	Transcription
transcr_ini_TU00492_cplx	transcription initiation complex TU00492	C20382H31210N6139O7437S113P383Mg2Zn1Fe0	-462	Transcription
transcr_ini_TU00493_cplx	transcription initiation complex TU00493	C18361H28890N5331O6212S113P176Mg2Zn1Fe0	-255	Transcription
transcr_ini_TU00494_cplx	transcription initiation complex TU00494	C17571H27978N5045O5714S113P95Mg2Zn1Fe0	-174	Transcription
transcr_ini_TU00495_cplx	transcription initiation complex TU00495	C20698H31585N6244O7645S113P416Mg2Zn1Fe0	-495	Transcription
transcr_ini_TU00496_cplx	transcription initiation complex TU00496	C19713H30437N5912O7016S113P315Mg2Zn1Fe0	-394	Transcription
transcr_ini_TU00497_cplx	transcription initiation complex TU00497	C17562H27967N5018O5717S113P94Mg2Zn1Fe0	-173	Transcription
transcr_ini_TU00498_cplx	transcription initiation complex TU00498	C17562H27967N5018O5717S113P94Mg2Zn1Fe0	-173	Transcription
transcr_ini_TU00499_cplx	transcription initiation complex TU00499	C17562H27967N5018O5717S113P94Mg2Zn1Fe0	-173	Transcription
transcr_ini_TU00500_cplx	transcription initiation complex TU00500	C17552H27955N5016O5705S113P93Mg2Zn1Fe0	-172	Transcription
transcr_ini_TU00501_cplx	transcription initiation complex TU00501	C17562H27967N5034O5709S113P94Mg2Zn1Fe0	-173	Transcription
transcr_ini_TU00502_cplx	transcription initiation complex TU00502	C17559H27966N5027O5711S113P94Mg2Zn1Fe0	-173	Transcription
transcr_ini_TU00503_cplx	transcription initiation complex TU00503	C21086H32030N6395O7888S113P455Mg2Zn1Fe0	-534	Transcription
transcr_ini_TU00504_cplx	transcription initiation complex TU00504	C21124H32076N6415O7900S113P460Mg2Zn1Fe0	-539	Transcription
transcr_ini_TU00505_cplx	transcription initiation complex TU00505	C19735H30466N5897O7038S113P317Mg2Zn1Fe0	-396	Transcription
transcr_ini_TU00506_cplx	transcription initiation complex TU00506	C17654H28067N5084O5752S113P103Mg2Zn1Fe0	-182	Transcription
transcr_ini_TU00507_cplx	transcription initiation complex TU00507	C23675H35032N7365O9490S113P722Mg2Zn1Fe0	-801	Transcription
transcr_ini_TU00508_cplx	transcription initiation complex TU00508	C17681H28098N5091O5780S113P106Mg2Zn1Fe0	-185	Transcription
transcr_ini_TU00509_cplx	transcription initiation complex TU00509	C17682H28100N5095O5776S113P106Mg2Zn1Fe0	-185	Transcription
transcr_ini_TU00510_cplx	transcription initiation complex TU00510	C17681H28098N5091O5780S113P106Mg2Zn1Fe0	-185	Transcription
transcr_ini_TU00511_cplx	transcription initiation complex TU00511	C22033H33111N6824O8428S113P554Mg2Zn1Fe0	-633	Transcription
transcr_ini_TU00512_cplx	transcription initiation complex TU00512	C19775H30517N5896O7075S113P321Mg2Zn1Fe0	-400	Transcription
transcr_ini_TU00513_cplx	transcription initiation complex TU00513	C17699H28120N5109O5782S113P108Mg2Zn1Fe0	-187	Transcription
transcr_ini_TU00514_cplx	transcription initiation complex TU00514	C18682H29256N5477O6396S113P209Mg2Zn1Fe0	-288	Transcription
transcr_ini_TU00515_cplx	transcription initiation complex TU00515	C25229H36787N8060O10395S113P880Mg2Zn1Fe0	-959	Transcription
transcr_ini_TU00516_cplx	transcription initiation complex TU00516	C17542H27945N5020O5699S113P92Mg2Zn1Fe0	-171	Transcription
transcr_ini_TU00517_cplx	transcription initiation complex TU00517	C65132H82588N24015O34675S113P4966Mg2Zn1Fe0	-5045	Transcription
transcr_ini_TU00518_cplx	transcription initiation complex TU00518	C17675H28092N5083O5777S113P105Mg2Zn1Fe0	-184	Transcription
transcr_ini_TU00519_cplx	transcription initiation complex TU00519	C17573H27978N5041O5717S113P95Mg2Zn1Fe0	-174	Transcription

transcr_ini_TU00520_cplx	transcription initiation complex TU00520	C17562H27967N5034O5709S113P94Mg2Zn1Fe0	-173	Transcription
transcr_ini_TU00521_cplx	transcription initiation complex TU00521	C19975H30740N5991O7190S113P341Mg2Zn1Fe0	-420	Transcription
transcr_ini_TU221_cplx	transcription initiation complex TU221	C25158H36700N8049O10346S113P873Mg2Zn1Fe0	-952	Transcription
transcr_ini_TU281_cplx	transcription initiation complex TU281	C43070H57367N14920O21391S113P2709Mg2Zn1Fe0	-2788	Transcription
transcr_ini_TU341_cplx	transcription initiation complex TU341	C107156H130994N40407O60493S113P9275Mg2Zn1Fe0	-9354	Transcription
transcr_ini_TU343_cplx	transcription initiation complex TU343	C17570H27976N5049O5710S113P95Mg2Zn1Fe0	-174	Transcription
transcr_ini_TU361_cplx	transcription initiation complex TU361	C40910H54814N14245O19970S113P2486Mg2Zn1Fe0	-2565	Transcription
transcr_ini_TU482_cplx	transcription initiation complex TU482	C62602H79829N22612O33429S113P4710Mg2Zn1Fe0	-4789	Transcription
transcr_ini_TU483_cplx	transcription initiation complex TU483	C50420H65784N17959O25837S113P3464Mg2Zn1Fe0	-3543	Transcription
transcr_ini_TU543_cplx	transcription initiation complex TU543	C20495H31321N6264O7479S113P395Mg2Zn1Fe0	-474	Transcription
transcr_ini_TU564_cplx	transcription initiation complex TU564	C37497H50887N12884O17872S113P2136Mg2Zn1Fe0	-2215	Transcription
transcr_ini_TU565_cplx	transcription initiation complex TU565	C37497H50887N12884O17872S113P2136Mg2Zn1Fe0	-2215	Transcription
transcr_ini_TU582_cplx	transcription initiation complex TU582	C35652H48833N12074O16824S113P1953Mg2Zn1Fe0	-2032	Transcription
transcr_ini_TU583_cplx	transcription initiation complex TU583	C35652H48833N12074O16824S113P1953Mg2Zn1Fe0	-2032	Transcription
transcr_ini_TU601_cplx	transcription initiation complex TU601	C24110H35506N7591O9709S113P765Mg2Zn1Fe0	-844	Transcription
transcr_ini_TU602_cplx	transcription initiation complex TU602	C24110H35506N7591O9709S113P765Mg2Zn1Fe0	-844	Transcription
transcr_ini_TU682_cplx	transcription initiation complex TU682	C21842H32922N6659O8362S113P535Mg2Zn1Fe0	-614	Transcription
transcr_ini_TU743_cplx	transcription initiation complex TU743	C21842H32922N6659O8362S113P535Mg2Zn1Fe0	-614	Transcription
transcr_ini_TU801_cplx	transcription initiation complex TU801	C116600H141965N44054O66490S113P10257Mg2Zn1Fe0	-10336	Transcription
transcr_ini_TU802_cplx	transcription initiation complex TU802	C116600H141965N44054O66490S113P10257Mg2Zn1Fe0	-10336	Transcription
transcr_ini_TU870_cplx	transcription initiation complex TU870	C31306H43799N10344O14160S113P1501Mg2Zn1Fe0	-1580	Transcription
transcr_ini_TU871_cplx	transcription initiation complex TU871	C27247H39139N8790O11679S113P1089Mg2Zn1Fe0	-1168	Transcription
transcr_ini_TU872_cplx	transcription initiation complex TU872	C79552H99351N29260O43746S113P6449Mg2Zn1Fe0	-6528	Transcription
transcr_ini_TU873_cplx	transcription initiation complex TU873	C79552H99351N29260O43746S113P6449Mg2Zn1Fe0	-6528	Transcription
transcr_ini_TU874_cplx	transcription initiation complex TU874	C62376H79570N22595O33168S113P4691Mg2Zn1Fe0	-4770	Transcription
transcr_ini_TU875_cplx	transcription initiation complex TU875	C48824H63986N17177O24917S113P3298Mg2Zn1Fe0	-3377	Transcription
transcr_ini_TU876_cplx	transcription initiation complex TU876	C48824H63986N17177O24917S113P3298Mg2Zn1Fe0	-3377	Transcription
transcr_ini_TU877_cplx	transcription initiation complex TU877	C40489H54372N13965O19793S113P2444Mg2Zn1Fe0	-2523	Transcription
transcr_ini_b0144_cplx	transcription initiation complex b0144	C25830H37530N8187O10830S113P945Mg2Zn1Fe0	-1024	Transcription
transcr_ini_b0206_cplx	transcription initiation complex b0206	C17573H27978N5037O5721S113P95Mg2Zn1Fe0	-174	Transcription
transcr_ini_b0503_cplx	transcription initiation complex b0503	C27504H39391N8996O11787S113P1113Mg2Zn1Fe0	-1192	Transcription
transcr_ini_b1133_cplx	transcription initiation complex b1133	C27611H39542N8965O11879S113P1125Mg2Zn1Fe0	-1204	Transcription
transcr_ini_b1211_cplx	transcription initiation complex b1211	C27376H39259N8904O11708S113P1101Mg2Zn1Fe0	-1180	Transcription
transcr_ini_b1212_cplx	transcription initiation complex b1212	C24945H36481N7898O10265S113P852Mg2Zn1Fe0	-931	Transcription
transcr_ini_b1804_cplx	transcription initiation complex b1804	C27815H39781N9026O12020S113P1146Mg2Zn1Fe0	-1225	Transcription
transcr_ini_b2745_cplx	transcription initiation complex b2745	C27086H38901N8796O11532S113P1068Mg2Zn1Fe0	-1147	Transcription
transcr_ini_b3289_cplx	transcription initiation complex b3289	C29410H41622N9621O12967S113P1308Mg2Zn1Fe0	-1387	Transcription
transcr_ini_b3760_cplx	transcription initiation complex b3760	C17573H27978N5037O5721S113P95Mg2Zn1Fe0	-174	Transcription
transcr_ini_b3761_cplx	transcription initiation complex b3761	C17564H27967N5034O5711S113P94Mg2Zn1Fe0	-173	Transcription
transcr_ini_b4143_cplx	transcription initiation complex b4143	C32902H45572N11181O15032S113P1665Mg2Zn1Fe0	-1744	Transcription
trdox	Oxidized thioredoxin	X1	0	Others
trdox[e]	Oxidized thioredoxin	X1	0	Others
trdrd	Reduced thioredoxin	X1H2	0	Others
trdrd[e]	Reduced thioredoxin	X1H2	0	Others
trp-L	L-Tryptophan	C11H12N2O2	0	Others
trp-L[e]	L-Tryptophan	C11H12N2O2	0	Others
trpT_tRNA	trpT_tRNA (b3761, uncharged tRNA)	C731H842Mg2N287O535P76S2	-73	tRNA Modification
trpT_tRNA_trp	trpT_tRNA (charged tRNA)	C742H854Mg2N289O537P76S2	-73	tRNA charging
tyr-D	D-Tyrosine	C9H11NO3	0	Others
tyr-D[e]	D-Tyrosine	C9H11NO3	0	Others

tyr-L	L-Tyrosine	C9H11NO3	0	Others
tyr-L[e]	L-Tyrosine	C9H11NO3	0	Others
tyr1_tRNA	tyr1_tRNA (uncharged tRNA)	C822H944N324O593P85S2Mg2	-82	tRNA charging
tyr1_tRNA_tyr	tyr1_tRNA (charged tRNA)	C831H955Mg2N325O596P85S2	-82	tRNA charging
tyr1_tRNA_tyr-D	tyr1_tRNA (charged tRNA)	C831H955Mg2N325O596P85S2	-82	tRNA charging
tyrT_tRNA	tyrT_tRNA (b1231, uncharged tRNA)	C822H944Mg2N323O594P85S2	-82	tRNA Modification
tyrU_tRNA	tyrU_tRNA (b3977, uncharged tRNA)	C823H945Mg2N326O592P85S2	-82	tRNA Modification
tyrV_tRNA	tyrV_tRNA (b1230, uncharged tRNA)	C822H944Mg2N323O594P85S2	-82	tRNA Modification
udp	UDP	C9H11N2O12P2	-3	Others
udp[e]	UDP	C9H11N2O12P2	-3	Others
ump	UMP	C9H11N2O9P	-2	Others
ump[e]	UMP	C9H11N2O9P	-2	Others
utp	UTP	C9H11N2O15P3	-4	Others
utp[e]	UTP	C9H11N2O15P3	-4	Others
val-L	L-Valine	C5H11NO2	0	Others
val-L[e]	L-Valine	C5H11NO2	0	Others
val1_tRNA	val1_tRNA (uncharged tRNA)	C728H832N292O534P76S1Mg2	-74	tRNA charging
val1_tRNA_val	val1_tRNA (charged tRNA)	C733H843Mg2N293O536P76S1	-74	tRNA charging
val2_tRNA	val2_tRNA (uncharged tRNA)	C738H848N291O545P77S1Mg2	-74	tRNA charging
val2_tRNA_val	val2_tRNA (charged tRNA)	C743H859Mg2N292O547P77S1	-74	tRNA charging
val3_tRNA	val3_tRNA (uncharged tRNA)	C731H837N292O537P76S1Mg2	-74	tRNA charging
val3_tRNA_val	val3_tRNA (charged tRNA)	C736H848Mg2N293O539P76S1	-74	tRNA charging
valT_tRNA	valT_tRNA (b0744, uncharged tRNA)	C728H832Mg2N292O534P76S1	-74	tRNA Modification
valU_tRNA	valU_tRNA (b2401, uncharged tRNA)	C728H832Mg2N292O534P76S1	-74	tRNA Modification
valV_tRNA	valV_tRNA (b1665, uncharged tRNA)	C738H847Mg2N290O545P77S1	-74	tRNA Modification
valW_tRNA	valW_tRNA (b1666, uncharged tRNA)	C738H850Mg2N293O545P77S1	-74	tRNA Modification
valX_tRNA	valX_tRNA (b2402, uncharged tRNA)	C728H832Mg2N292O534P76S1	-74	tRNA Modification
valY_tRNA	valY_tRNA (b2403, uncharged tRNA)	C728H832Mg2N292O534P76S1	-74	tRNA Modification
valZ_tRNA	valZ_tRNA (b0746, uncharged tRNA)	C728H832Mg2N292O534P76S1	-74	tRNA Modification
zn2	Zinc	Zn	2	Others
zn2[e]	Zinc	Zn	2	Others
