# Supplementary material for: Genome-Scale Reconstruction of Escherichia coli's Transcriptional and Translational Machinery: A Knowledge Base, Its Mathematical Formulation, and Its Functional Characterization
Source: PLoS Comput Biol. 2009 Mar 13;5(3):e1000312. doi: 10.1371/journal.pcbi.1000312 (PMC2648898; doi:10.1371/journal.pcbi.1000312)
Supplement: Table S14 — List of functional modules (0.04 MB PDF) [file pcbi.1000312.s016.pdf]

Table S14 - functional modules

Module Number	Reaction Number	Reaction Abbreviation	Subsystem	DU_23S_2449_RECYCL	KsgA_mono_RECYCL	MeST_S12_RECYCL	MeT_16S_1402_RECYCL	MeT_16S_1407_RECYCL	MeT_16S_1516_RECYCL	MeT_16S_527_RECYCL	MeT_16S_966_RECYCL	MeT_23S_1618_RECYCL	MeT_23S_1835_RECYCL	MeT_23S_1962_RECYCL	MeT_23S_2030_RECYCL	MeT_23S_2069_RECYCL	MeT_23S_2445_RECYCL	MeT_23S_2498_RECYCL	MeT_23S_2503_RECYCL	MeT_L16_RECYCL	MeT_L33_RECYCL	MeT_L7/L12_RECYCL	MeT_S11_RECYCL	PrmA_mono_RECYCL	PrmB_mono_RECYCL	RbfA_mono_RECYCL	RimI_mono_RECYCL	RimJ_mono_RECYCL	RimK_mono_RECYCL	RimL_mono_RECYCL	RimM_mono_RECYCL	RlmB_dim_RECYCL	RluB_mono_RECYCL	RluC_mono_RECYCL	RluD_mono_RECYCL	RrmA_dim_RECYCL	RrmJ_mono_RECYCL	RsmB_mono_RECYCL	RsmC_mono_RECYCL	RsuA_mono_RECYCL	RumA_mono_RECYCL	RumB_mono_RECYCL	YggJ_mono_RECYCL	YjbC_mono_RECYCL	YmfC_mono_RECYCL	YrdC_mono_RECYCL	Def_mono_RECYCL	Fmt_mono_RECYCL	IF1_RECYCL	IF3_RECYCL	RF3_mono.GDP_RECYCL	Rrf_mono_RECYCL	rib_30_ini_RECYCL	rib_50_RECYCL	IF2_RECHARG	GreA_mono_RECYCL	GreB_mono_RECYCL	Mfd_mono_RECYCL	NusA_mono_RECYCL	NusG_mono_RECYCL	RpoZ_mono_RECYCL	hRNAP_RECYCL	RNase_G_dim_RECYCL	RNase_T_dim_RECYCL	RNase_m16_RECYCL	RNase_m23_RECYCL	RNase_m5_RECYCL	TrmU_mono_RECYCL	YccK_mono_RECYCL	YheLMN_cplx_RECYCL	YhhP_mono_RECYCL	EoR_tRNA_pos34_Q_RECYCL	QueA_mono_RECYCL	QueF_dim_RECYCL	Tgt_hexa_RECYCL	RNase_E_tetra_RECYCL	RNase_Gen_RECYCL	RNase_P_cplx_RECYCL	HyL_tRNA_pos_34_ho5U_RECYCL
				rRNA Modification	rRNA Modification	Ribosomal protein modification	rRNA Modification	rRNA Modification	rRNA Modification	rRNA Modification	rRNA Modification	rRNA Modification	rRNA Modification	rRNA Modification	rRNA Modification	rRNA Modification	rRNA Modification	rRNA Modification	rRNA Modification	Ribosomal protein modification	Ribosomal protein modification	Ribosomal protein modification	Ribosomal protein modification	Ribosomal protein modification	Protein Modification	Ribosomal protein assembly	Protein Modification	Protein Modification	Protein Modification	Protein Modification	Ribosomal protein assembly	rRNA Modification	rRNA Modification	rRNA Modification	rRNA Modification	rRNA Modification	rRNA Modification	rRNA Modification	rRNA Modification	rRNA Modification	rRNA Modification	rRNA Modification	rRNA Modification	rRNA Modification	rRNA Modification	Ribosomal protein assembly	Protein Maturation	tRNA charging	Translation	Translation	Translation	Translation	Translation	Translation	Translation	Transcription	Transcription	Transcription	Transcription	Transcription	Transcription	Transcription	RNA Processing	RNA Processing	rRNA Modification	rRNA Modification	rRNA Modification	tRNA Modification	tRNA Modification	tRNA Modification	tRNA Modification	tRNA Modification	tRNA Modification	tRNA Modification	tRNA Modification	RNA Processing	RNA Processing	RNA Processing	tRNA Modification
1	1	DU_23S_2449_RECYCL	rRNA Modification	1.00	1.00	1.00	1.00	1.00	1.00	1.00	1.00	1.00	1.00	1.00	1.00	1.00	1.00	1.00	1.00	1.00	1.00	1.00	1.00	1.00	1.00	1.00	1.00	1.00	1.00	1.00	1.00	1.00	1.00	1.00	1.00	1.00	1.00	1.00	1.00	1.00	1.00	1.00	0.81	0.81	0.81	0.81	0.81	0.81	0.81	0.81	0.81	0.79	0.79	0.79	0.79	0.79	0.79	0.79	0.85	0.85	0.85	0.85	0.85	0.85	0.85	0.85	0.85	0.75	0.75	0.75	0.75	0.79	0.79	0.79	0.81
1	2	KsgA_mono_RECYCL	rRNA Modification	1.00	1.00	1.00	1.00	1.00	1.00	1.00	1.00	1.00	1.00	1.00	1.00	1.00	1.00	1.00	1.00	1.00	1.00	1.00	1.00	1.00	1.00	1.00	1.00	1.00	1.00	1.00	1.00	1.00	1.00	1.00	1.00	1.00	1.00	1.00	1.00	1.00	1.00	1.00	0.81	0.81	0.81	0.81	0.81	0.81	0.81	0.81	0.81	0.79	0.79	0.79	0.79	0.79	0.79	0.79	0.85	0.85	0.85	0.85	0.85	0.85	0.85	0.85	0.85	0.75	0.75	0.75	0.75	0.79	0.79	0.79	0.81
1	3	MeST_S12_RECYCL	Ribosomal protein modification	1.00	1.00	1.00	1.00	1.00	1.00	1.00	1.00	1.00	1.00	1.00	1.00	1.00	1.00	1.00	1.00	1.00	1.00	1.00	1.00	1.00	1.00	1.00	1.00	1.00	1.00	1.00	1.00	1.00	1.00	1.00	1.00	1.00	1.00	1.00	1.00	1.00	1.00	1.00	0.81	0.81	0.81	0.81	0.81	0.81	0.81	0.81	0.81	0.79	0.79	0.79	0.79	0.79	0.79	0.79	0.85	0.85	0.85	0.85	0.85	0.85	0.85	0.85	0.85	0.75	0.75	0.75	0.75	0.79	0.79	0.79	0.81
1	4	MeT_16S_1402_RECYCL	rRNA Modification	1.00	1.00	1.00	1.00	1.00	1.00	1.00	1.00	1.00	1.00	1.00	1.00	1.00	1.00	1.00	1.00	1.00	1.00	1.00	1.00	1.00	1.00	1.00	1.00	1.00	1.00	1.00	1.00	1.00	1.00	1.00	1.00	1.00	1.00	1.00	1.00	1.00	1.00	1.00	0.81	0.81	0.81	0.81	0.81	0.81	0.81	0.81	0.81	0.79	0.79	0.79	0.79	0.79	0.79	0.79	0.85	0.85	0.85	0.85	0.85	0.85	0.85	0.85	0.85	0.75	0.75	0.75	0.75	0.79	0.79	0.79	0.81
1	5	MeT_16S_1407_RECYCL	rRNA Modification	1.00	1.00	1.00	1.00	1.00	1.00	1.00	1.00	1.00	1.00	1.00	1.00	1.00	1.00	1.00	1.00	1.00	1.00	1.00	1.00	1.00	1.00	1.00	1.00	1.00	1.00	1.00	1.00	1.00	1.00	1.00	1.00	1.00	1.00	1.00	1.00	1.00	1.00	1.00	0.81	0.81	0.81	0.81	0.81	0.81	0.81	0.81	0.81	0.79	0.79	0.79	0.79	0.79	0.79	0.79	0.85	0.85	0.85	0.85	0.85	0.85	0.85	0.85	0.85	0.75	0.75	0.75	0.75	0.79	0.79	0.79	0.81
1	6	MeT_16S_1516_RECYCL	rRNA Modification	1.00	1.00	1.00	1.00	1.00	1.00	1.00	1.00	1.00	1.00	1.00	1.00	1.00	1.00	1.00	1.00	1.00	1.00	1.00	1.00	1.00	1.00	1.00	1.00	1.00	1.00	1.00	1.00	1.00	1.00	1.00	1.00	1.00	1.00	1.00	1.00	1.00	1.00	1.00	0.81	0.81	0.81	0.81	0.81	0.81	0.81	0.81	0.81	0.79	0.79	0.79	0.79	0.79	0.79	0.79	0.85	0.85	0.85	0.85	0.85	0.85	0.85	0.85	0.85	0.75	0.75	0.75	0.75	0.79	0.79	0.79	0.81
1	7	MeT_16S_527_RECYCL	rRNA Modification	1.00	1.00	1.00	1.00	1.00	1.00	1.00	1.00	1.00	1.00	1.00	1.00	1.00	1.00	1.00	1.00	1.00	1.00	1.00	1.00	1.00	1.00	1.00	1.00	1.00	1.00	1.00	1.00	1.00	1.00	1.00	1.00	1.00	1.00	1.00	1.00	1.00	1.00	1.00	0.81	0.81	0.81	0.81	0.81	0.81	0.81	0.81	0.81	0.79	0.79	0.79	0.79	0.79	0.79	0.79	0.85	0.85	0.85	0.85	0.85	0.85	0.85	0.85	0.85	0.75	0.75	0.75	0.75	0.79	0.79	0.79	0.81
1	8	MeT_16S_966_RECYCL	rRNA Modification	1.00	1.00	1.00	1.00	1.00	1.00	1.00	1.00	1.00	1.00	1.00	1.00	1.00	1.00	1.00	1.00	1.00	1.00	1.00	1.00	1.00	1.00	1.00	1.00	1.00	1.00	1.00	1.00	1.00	1.00	1.00	1.00	1.00	1.00	1.00	1.00	1.00	1.00	1.00	0.81	0.81	0.81	0.81	0.81	0.81	0.81	0.81	0.81	0.79	0.79	0.79	0.79	0.79	0.79	0.79	0.85	0.85	0.85	0.85	0.85	0.85	0.85	0.85	0.85	0.75	0.75	0.75	0.75	0.79	0.79	0.79	0.81
1	9	MeT_23S_1618_RECYCL	rRNA Modification	1.00	1.00	1.00	1.00	1.00	1.00	1.00	1.00	1.00	1.00	1.00	1.00	1.00	1.00	1.00	1.00	1.00	1.00	1.00	1.00	1.00	1.00	1.00	1.00	1.00	1.00	1.00	1.00	1.00	1.00	1.00	1.00	1.00	1.00	1.00	1.00	1.00	1.00	1.00	0.81	0.81	0.81	0.81	0.81	0.81	0.81	0.81	0.81	0.79	0.79	0.79	0.79	0.79	0.79	0.79	0.85	0.85	0.85	0.85	0.85	0.85	0.85	0.85	0.85	0.75	0.75	0.75	0.75	0.79	0.79	0.79	0.81
1	10	MeT_23S_1835_RECYCL	rRNA Modification	1.00	1.00	1.00	1.00	1.00	1.00	1.00	1.00	1.00	1.00	1.00	1.00	1.00	1.00	1.00	1.00	1.00	1.00	1.00	1.00	1.00	1.00	1.00	1.00	1.00	1.00	1.00	1.00	1.00	1.00	1.00	1.00	1.00	1.00	1.00	1.00	1.00	1.00	1.00	0.81	0.81	0.81	0.81	0.81	0.81	0.81	0.81	0.81	0.79	0.79	0.79	0.79	0.79	0.79	0.79	0.85	0.85	0.85	0.85	0.85	0.85	0.85	0.85	0.85	0.75	0.75	0.75	0.75	0.79	0.79	0.79	0.81
1	11	MeT_23S_1962_RECYCL	rRNA Modification	1.00	1.00	1.00	1.00	1.00	1.00	1.00	1.00	1.00	1.00	1.00	1.00	1.00	1.00	1.00	1.00	1.00	1.00	1.00	1.00	1.00	1.00	1.00	1.00	1.00	1.00	1.00	1.00	1.00	1.00	1.00	1.00	1.00	1.00	1.00	1.00	1.00	1.00	1.00	0.81	0.81	0.81	0.81	0.81	0.81	0.81	0.81	0.81	0.79	0.79	0.79	0.79	0.79	0.79	0.79	0.85	0.85	0.85	0.85	0.85	0.85	0.85	0.85	0.85	0.75	0.75	0.75	0.75	0.79	0.79	0.79	0.81
1	12	MeT_23S_2030_RECYCL	rRNA Modification	1.00	1.00	1.00	1.00	1.00	1.00	1.00	1.00	1.00	1.00	1.00	1.00	1.00	1.00	1.00	1.00	1.00	1.00	1.00	1.00	1.00	1.00	1.00	1.00	1.00	1.00	1.00	1.00	1.00	1.00	1.00	1.00	1.00	1.00	1.00	1.00	1.00	1.00	1.00	0.81	0.81	0.81	0.81	0.81	0.81	0.81	0.81	0.81	0.79	0.79	0.79	0.79	0.79	0.79	0.79	0.85	0.85	0.85	0.85	0.85	0.85	0.85	0.85	0.85	0.75	0.75	0.75	0.75	0.79	0.79	0.79	0.81
1	13	MeT_23S_2069_RECYCL	rRNA Modification	1.00	1.00	1.00	1.00	1.00	1.00	1.00	1.00	1.00	1.00	1.00	1.00	1.00	1.00	1.00	1.00	1.00	1.00	1.00	1.00	1.00	1.00	1.00	1.00	1.00	1.00	1.00	1.00	1.00	1.00	1.00	1.00	1.00	1.00	1.00	1.00	1.00	1.00	1.00	0.81	0.81	0.81	0.81	0.81	0.81	0.81	0.81	0.81	0.79	0.79	0.79	0.79	0.79	0.79	0.79	0.85	0.85	0.85	0.85	0.85	0.85	0.85	0.85	0.85	0.75	0.75	0.75	0.75	0.79	0.79	0.79	0.81
1	14	MeT_23S_2445_RECYCL	rRNA Modification	1.00	1.00	1.00	1.00	1.00	1.00	1.00	1.00	1.00	1.00	1.00	1.00	1.00	1.00	1.00	1.00	1.00	1.00	1.00	1.00	1.00	1.00	1.00	1.00	1.00	1.00	1.00	1.00	1.00	1.00	1.00	1.00	1.00	1.00	1.00	1.00	1.00	1.00	1.00	0.81	0.81	0.81	0.81	0.81	0.81	0.81	0.81	0.81	0.79	0.79	0.79	0.79	0.79	0.79	0.79	0.85	0.85	0.85	0.85	0.85	0.85	0.85	0.85	0.85	0.75	0.75	0.75	0.75	0.79	0.79	0.79	0.81
1	15	MeT_23S_2498_RECYCL	rRNA Modification	1.00	1.00	1.00	1.00	1.00	1.00	1.00	1.00	1.00	1.00	1.00	1.00	1.00	1.00	1.00	1.00	1.00	1.00	1.00	1.00	1.00	1.00	1.00	1.00	1.00	1.00	1.00	1.00	1.00	1.00	1.00	1.00	1.00	1.00	1.00	1.00	1.00	1.00	1.00	0.81	0.81	0.81	0.81	0.81	0.81	0.81	0.81	0.81	0.79	0.79	0.79	0.79	0.79	0.79	0.79	0.85	0.85	0.85	0.85	0.85	0.85	0.85	0.85	0.85	0.75	0.75	0.75	0.75	0.79	0.79	0.79	0.81
1	16	MeT_23S_2503_RECYCL	rRNA Modification	1.00	1.00	1.00	1.00	1.00	1.00	1.00	1.00	1.00	1.00	1.00	1.00	1.00	1.00	1.00	1.00	1.00	1.00	1.00	1.00	1.00	1.00	1.00	1.00	1.00	1.00	1.00	1.00	1.00	1.00	1.00	1.00	1.00	1.00	1.00	1.00	1.00	1.00	1.00	0.81	0.81	0.81	0.81	0.81	0.81	0.81	0.81	0.81	0.79	0.79	0.79	0.79	0.79	0.79	0.79	0.85	0.85	0.85	0.85	0.85	0.85	0.85	0.85	0.85	0.75	0.75	0.75	0.75	0.79	0.79	0.79	0.81
1	17	MeT_L16_RECYCL	Ribosomal protein modification	1.00	1.00	1.00	1.00	1.00	1.00	1.00	1.00	1.00	1.00	1.00	1.00	1.00	1.00	1.00	1.00	1.00	1.00	1.00	1.00	1.00	1.00	1.00	1.00	1.00	1.00	1.00	1.00	1.00	1.00	1.00	1.00	1.00	1.00	1.00	1.00	1.00	1.00	1.00	0.81	0.81	0.81	0.81	0.81	0.81	0.81	0.81	0.81	0.79	0.79	0.79	0.79	0.79	0.79	0.79	0.85	0.85	0.85	0.85	0.85	0.85	0.85	0.85	0.85	0.75	0.75	0.75	0.75	0.79	0.79	0.79	0.81
1	18	MeT_L33_RECYCL	Ribosomal protein modification	1.00	1.00	1.00	1.00	1.00	1.00	1.00	1.00	1.00	1.00	1.00	1.00	1.00	1.00	1.00	1.00	1.00	1.00	1.00	1.00	1.00	1.00	1.00	1.00	1.00	1.00	1.00	1.00	1.00	1.00	1.00	1.00	1.00	1.00	1.00	1.00	1.00	1.00	1.00	0.81	0.81	0.81	0.81	0.81	0.81	0.81	0.81	0.81	0.79	0.79	0.79	0.79	0.79	0.79	0.79	0.85	0.85	0.85	0.85	0.85	0.85	0.85	0.85	0.85	0.75	0.75	0.75	0.75	0.79	0.79	0.79	0.81
1	19	MeT_L7/L12_RECYCL	Ribosomal protein modification	1.00	1.00	1.00	1.00	1.00	1.00	1.00	1.00	1.00	1.00	1.00	1.00	1.00	1.00	1.00	1.00	1.00	1.00	1.00	1.00	1.00	1.00	1.00	1.00	1.00	1.00	1.00	1.00	1.00	1.00	1.00	1.00	1.00	1.00	1.00	1.00	1.00	1.00	1.00	0.81	0.81	0.81	0.81	0.81	0.81	0.81	0.81	0.81	0.79	0.79	0.79	0.79	0.79	0.79	0.79	0.85	0.85	0.85	0.85	0.85	0.85	0.85	0.85	0.85	0.75	0.75	0.75	0.75	0.79	0.79	0.79	0.81
1	20	MeT_S11_RECYCL	Ribosomal protein modification	1.00	1.00	1.00	1.00	1.00	1.00	1.00	1.00	1.00	1.00	1.00	1.00	1.00	1.00	1.00	1.00	1.00	1.00	1.00	1.00	1.00	1.00	1.00	1.00	1.00	1.00	1.00	1.00	1.00	1.00	1.00	1.00	1.00	1.00	1.00	1.00	1.00	1.00	1.00	0.81	0.81	0.81	0.81	0.81	0.81	0.81	0.81	0.81	0.79	0.79	0.79	0.79	0.79	0.79	0.79	0.85	0.85	0.85	0.85	0.85	0.85	0.85	0.85	0.85	0.75	0.75	0.75	0.75	0.79	0.79	0.79	0.81
1	21	PrmA_mono_RECYCL	Ribosomal protein modification	1.00	1.00	1.00	1.00	1.00	1.00	1.00	1.00	1.00	1.00	1.00	1.00	1.00	1.00	1.00	1.00	1.00	1.00	1.00	1.00	1.00	1.00	1.00	1.00	1.00	1.00	1.00	1.00	1.00	1.00	1.00	1.00	1.00	1.00	1.00	1.00	1.00	1.00	1.00	0.81	0.81	0.81	0.81	0.81	0.81	0.81	0.81	0.81	0.79	0.79	0.79	0.79	0.79	0.79	0.79	0.85	0.85	0.85	0.85	0.85	0.85	0.85	0.85	0.85	0.75	0.75	0.75	0.75	0.79	0.79	0.79	0.81
1	22	PrmB_mono_RECYCL	Protein Modification	1.00	1.00	1.00	1.00	1.00	1.00	1.00	1.00	1.00	1.00	1.00	1.00	1.00	1.00	1.00	1.00	1.00	1.00	1.00	1.00	1.00	1.00	1.00	1.00	1.00	1.00	1.00	1.00	1.00	1.00	1.00	1.00	1.00	1.00	1.00	1.00	1.00	1.00	1.00	0.81	0.81	0.81	0.81	0.81	0.81	0.81	0.81	0.81	0.79	0.79	0.79	0.79	0.79	0.79	0.79	0.85	0.85	0.85	0.85	0.85	0.85	0.85	0.85	0.85	0.75	0.75	0.75	0.75	0.79	0.79	0.79	0.81
1	23	RbfA_mono_RECYCL	Ribosomal protein assembly	1.00	1.00	1.00	1.00	1.00	1.00	1.00	1.00	1.00	1.00	1.00	1.00	1.00	1.00	1.00	1.00	1.00	1.00	1.00	1.00	1.00	1.00	1.00	1.00	1.00	1.00	1.00	1.00	1.00	1.00	1.00	1.00	1.00	1.00	1.00	1.00	1.00	1.00	1.00	0.81	0.81	0.81	0.81	0.81	0.81	0.81	0.81	0.81	0.79	0.79	0.79	0.79	0.79	0.79	0.79	0.85	0.85	0.85	0.85	0.85	0.85	0.85	0.85	0.85	0.75	0.75	0.75	0.75	0.79	0.79	0.79	0.81
1	24	RimI_mono_RECYCL	Protein Modification	1.00	1.00	1.00	1.00	1.00	1.00	1.00	1.00	1.00	1.00	1.00	1.00	1.00	1.00	1.00	1.00	1.00	1.00	1.00	1.00	1.00	1.00	1.00	1.00	1.00	1.00	1.00	1.00	1.00	1.00	1.00	1.00	1.00	1.00	1.00	1.00	1.00	1.00	1.00	0.81	0.81	0.81	0.81	0.81	0.81	0.81	0.81	0.81	0.79	0.79	0.79	0.79	0.79	0.79	0.79	0.85	0.85	0.85	0.85	0.85	0.85	0.85	0.85	0.85	0.75	0.75	0.75	0.75	0.79	0.79	0.79	0.81
1	25	RimJ_mono_RECYCL	Protein Modification	1.00	1.00	1.00	1.00	1.00	1.00	1.00	1.00	1.00	1.00	1.00	1.00	1.00	1.00	1.00	1.00	1.00	1.00	1.00	1.00	1.00	1.00	1.00	1.00	1.00	1.00	1.00	1.00	1.00	1.00	1.00	1.00	1.00	1.00	1.00	1.00	1.00	1.00	1.00	0.81	0.81	0.81	0.81	0.81	0.81	0.81	0.81	0.81	0.79	0.79	0.79	0.79	0.79	0.79	0.79	0.85	0.85	0.85	0.85	0.85	0.85	0.85	0.85	0.85	0.75	0.75	0.75	0.75	0.79	0.79	0.79	0.81
1	26	RimK_mono_RECYCL	Protein Modification	1.00	1.00	1.00	1.00	1.00	1.00	1.00	1.00	1.00	1.00	1.00	1.00	1.00	1.00	1.00	1.00	1.00	1.00	1.00	1.00	1.00	1.00	1.00	1.00	1.00	1.00	1.00	1.00	1.00	1.00	1.00	1.00	1.00	1.00	1.00	1.00	1.00	1.00	1.00	0.81	0.81	0.81	0.81	0.81	0.81	0.81	0.81	0.81	0.79	0.79	0.79	0.79	0.79	0.79	0.79	0.85	0.85	0.85	0.85	0.85	0.85	0.85	0.85	0.85	0.75	0.75	0.75	0.75	0.79	0.79	0.79	0.81
1	27	RimL_mono_RECYCL	Protein Modification	1.00	1.00	1.00	1.00	1.00	1.00	1.00	1.00	1.00	1.00	1.00	1.00	1.00	1.00	1.00	1.00	1.00	1.00	1.00	1.00	1.00	1.00	1.00	1.00	1.00	1.00	1.00	1.00	1.00	1.00	1.00	1.00	1.00	1.00	1.00	1.00	1.00	1.00	1.00	0.81	0.81	0.81	0.81	0.81	0.81	0.81	0.81	0.81	0.79	0.79	0.79	0.79	0.79	0.79	0.79	0.85	0.85	0.85	0.85	0.85	0.85	0.85	0.85	0.85	0.75	0.75	0.75	0.75	0.79	0.79	0.79	0.81
1	28	RimM_mono_RECYCL	Ribosomal protein assembly	1.00	1.00	1.00	1.00	1.00	1.00	1.00	1.00	1.00	1.00	1.00	1.00	1.00	1.00	1.00	1.00	1.00	1.00	1.00	1.00	1.00	1.00	1.00	1.00	1.00	1.00	1.00	1.00	1.00	1.00	1.00	1.00	1.00	1.00	1.00	1.00	1.00	1.00	1.00	0.81	0.81	0.81	0.81	0.81	0.81	0.81	0.81	0.81	0.79	0.79	0.79	0.79	0.79	0.79	0.79	0.85	0.85	0.85	0.85	0.85	0.85	0.85	0.85	0.85	0.75	0.75	0.75	0.75	0.79	0.79	0.79	0.81
1	29	RlmB_dim_RECYCL	rRNA Modification	1.00	1.00	1.00	1.00	1.00	1.00	1.00	1.00	1.00	1.00	1.00	1.00	1.00	1.00	1.00	1.00	1.00	1.00	1.00	1.00	1.00	1.00	1.00	1.00	1.00	1.00	1.00	1.00	1.00	1.00	1.00	1.00	1.00	1.00	1.00	1.00	1.00	1.00	1.00	0.81	0.81	0.81	0.81	0.81	0.81	0.81	0.81	0.81	0.79	0.79	0.79	0.79	0.79	0.79	0.79	0.85	0.85	0.85	0.85	0.85	0.85	0.85	0.85	0.85	0.75	0.75	0.75	0.75	0.79	0.79	0.79	0.81
1	30	RluB_mono_RECYCL	rRNA Modification	1.00	1.00	1.00	1.00	1.00	1.00	1.00	1.00	1.00	1.00	1.00	1.00	1.00	1.00	1.00	1.00	1.00	1.00	1.00	1.00	1.00	1.00	1.00	1.00	1.00	1.00	1.00	1.00	1.00	1.00	1.00	1.00	1.00	1.00	1.00	1.00	1.00	1.00	1.00	0.81	0.81	0.81	0.81	0.81	0.81	0.81	0.81	0.81	0.79	0.79	0.79	0.79	0.79	0.79	0.79	0.85	0.85	0.85	0.85	0.85	0.85	0.85	0.85	0.85	0.75	0.75	0.75	0.75	0.79	0.79	0.79	0.81
1	31	RluC_mono_RECYCL	rRNA Modification	1.00	1.00	1.00	1.00	1.00	1.00	1.00	1.00	1.00	1.00	1.00	1.00	1.00	1.00	1.00	1.00	1.00	1.00	1.00	1.00	1.00	1.00	1.00	1.00	1.00	1.00	1.00	1.00	1.00	1.00	1.00	1.00	1.00	1.00	1.00	1.00	1.00	1.00	1.00	0.81	0.81	0.81	0.81	0.81	0.81	0.81	0.81	0.81	0.79	0.79	0.79	0.79	0.79	0.79	0.79	0.85	0.85	0.85	0.85	0.85	0.85	0.85	0.85	0.85	0.75	0.75	0.75	0.75	0.79	0.79	0.79	0.81
1	32	RluD_mono_RECYCL	rRNA Modification	1.00	1.00	1.00	1.00	1.00	1.00	1.00	1.00	1.00	1.00	1.00	1.00	1.00	1.00	1.00	1.00	1.00	1.00	1.00	1.00	1.00	1.00	1.00	1.00	1.00	1.00	1.00	1.00	1.00	1.00	1.00	1.00	1.00	1.00	1.00	1.00	1.00	1.00	1.00	0.81	0.81	0.81	0.81	0.81	0.81	0.81	0.81	0.81	0.79	0.79	0.79	0.79	0.79	0.79	0.79	0.85	0.85	0.85	0.85	0.85	0.85	0.85	0.85	0.85	0.75	0.75	0.75	0.75	0.79	0.79	0.79	0.81
1	33	RrmA_dim_RECYCL	rRNA Modification	1.00	1.00	1.00	1.00	1.00	1.00	1.00	1.00	1.00	1.00	1.00	1.00	1.00	1.00	1.00	1.00	1.00	1.00	1.00	1.00	1.00	1.00	1.00	1.00	1.00	1.00	1.00	1.00	1.00	1.00	1.00	1.00	1.00	1.00	1.00	1.00	1.00	1.00	1.00	0.81	0.81	0.81	0.81	0.81	0.81	0.81	0.81	0.81	0.79	0.79	0.79	0.79	0.79	0.79	0.79	0.85	0.85	0.85	0.85	0.85	0.85	0.85	0.85	0.85	0.75	0.75	0.75	0.75	0.79	0.79	0.79	0.81
1	34	RrmJ_mono_RECYCL	rRNA Modification	1.00	1.00	1.00	1.00	1.00	1.00	1.00	1.00	1.00	1.00	1.00	1.00	1.00	1.00	1.00	1.00	1.00	1.00	1.00	1.00	1.00	1.00	1.00	1.00	1.00	1.00	1.00	1.00	1.00	1.00	1.00	1.00	1.00	1.00	1.00	1.00	1.00	1.00	1.00	0.81	0.81	0.81	0.81	0.81	0.81	0.81	0.81	0.81	0.79	0.79	0.79	0.79	0.79	0.79	0.79	0.85	0.85	0.85	0.85	0.85	0.85	0.85	0.85	0.85	0.75	0.75	0.75	0.75	0.79	0.79	0.79	0.81
1	35	RsmB_mono_RECYCL	rRNA Modification	1.00	1.00	1.00	1.00	1.00	1.00	1.00	1.00	1.00	1.00	1.00	1.00	1.00	1.00	1.00	1.00	1.00	1.00	1.00	1.00	1.00	1.00	1.00	1.00	1.00	1.00	1.00	1.00	1.00	1.00	1.00	1.00	1.00	1.00	1.00	1.00	1.00	1.00	1.00	0.81	0.81	0.81	0.81	0.81	0.81	0.81	0.81	0.81	0.79	0.79	0.79	0.79	0.79	0.79	0.79	0.85	0.85	0.85	0.85	0.85	0.85	0.85	0.85	0.85	0.75	0.75	0.75	0.75	0.79	0.79	0.79	0.81
1	36	RsmC_mono_RECYCL	rRNA Modification	1.00	1.00	1.00	1.00	1.00	1.00	1.00	1.00	1.00	1.00	1.00	1.00	1.00	1.00	1.00	1.00	1.00	1.00	1.00	1.00	1.00	1.00	1.00	1.00	1.00	1.00	1.00	1.00	1.00	1.00	1.00	1.00	1.00	1.00	1.00	1.00	1.00	1.00	1.00	0.81	0.81	0.81	0.81	0.81	0.81	0.81	0.81	0.81	0.79	0.79	0.79	0.79	0.79	0.79	0.79	0.85	0.85	0.85	0.85	0.85	0.85	0.85	0.85	0.85	0.75	0.75	0.75	0.75	0.79	0.79	0.79	0.81
1	37	RsuA_mono_RECYCL	rRNA Modification	1.00	1.00	1.00	1.00	1.00	1.00	1.00	1.00	1.00	1.00	1.00	1.00	1.00	1.00	1.00	1.00	1.00	1.00	1.00	1.00	1.00	1.00	1.00	1.00	1.00	1.00	1.00	1.00	1.00	1.00	1.00	1.00	1.00	1.00	1.00	1.00	1.00	1.00	1.00	0.81	0.81	0.81	0.81	0.81	0.81	0.81	0.81	0.81	0.79	0.79	0.79	0.79	0.79	0.79	0.79	0.85	0.85	0.85	0.85	0.85	0.85	0.85	0.85	0.85	0.75	0.75	0.75	0.75	0.79	0.79	0.79	0.81
1	38	RumA_mono_RECYCL	rRNA Modification	1.00	1.00	1.00	1.00	1.00	1.00	1.00	1.00	1.00	1.00	1.00	1.00	1.00	1.00	1.00	1.00	1.00	1.00	1.00	1.00	1.00	1.00	1.00	1.00	1.00	1.00	1.00	1.00	1.00	1.00	1.00	1.00	1.00	1.00	1.00	1.00	1.00	1.00	1.00	0.81	0.81	0.81	0.81	0.81	0.81	0.81	0.81	0.81	0.79	0.79	0.79	0.79	0.79	0.79	0.79	0.85	0.85	0.85	0.85	0.85	0.85	0.85	0.85	0.85	0.75	0.75	0.75	0.75	0.79	0.79	0.79	0.81
1	39	RumB_mono_RECYCL	rRNA Modification	1.00	1.00	1.00	1.00	1.00	1.00	1.00	1.00	1.00	1.00	1.00	1.00	1.00	1.00	1.00	1.00	1.00	1.00	1.00	1.00	1.00	1.00	1.00	1.00	1.00	1.00	1.00	1.00	1.00	1.00	1.00	1.00	1.00	1.00	1.00	1.00	1.00	1.00	1.00	0.81	0.81	0.81	0.81	0.81	0.81	0.81	0.81	0.81	0.79	0.79	0.79	0.79	0.79	0.79	0.79	0.85	0.85	0.85	0.85	0.85	0.85	0.85	0.85	0.85	0.75	0.75	0.75	0.75	0.79	0.79	0.79	0.81
1	40	YggJ_mono_RECYCL	rRNA Modification	1.00	1.00	1.00	1.00	1.00	1.00	1.00	1.00	1.00	1.00	1.00	1.00	1.00	1.00	1.00	1.00	1.00	1.00	1.00	1.00	1.00	1.00	1.00	1.00	1.00	1.00	1.00	1.00	1.00	1.00	1.00	1.00	1.00	1.00	1.00	1.00	1.00	1.00	1.00	0.81	0.81	0.81	0.81	0.81	0.81	0.81	0.81	0.81	0.79	0.79	0.79	0.79	0.79	0.79	0.79	0.85	0.85	0.85	0.85	0.85	0.85	0.85	0.85	0.85	0.75	0.75	0.75	0.75	0.79	0.79	0.79	0.81
1	41	YjbC_mono_RECYCL	rRNA Modification	1.00	1.00	1.00	1.00	1.00	1.00	1.00	1.00	1.00	1.00	1.00	1.00	1.00	1.00	1.00	1.00	1.00	1.00	1.00	1.00	1.00	1.00	1.00	1.00	1.00	1.00	1.00	1.00	1.00	1.00	1.00	1.00	1.00	1.00	1.00	1.00	1.00	1.00	1.00	0.81	0.81	0.81	0.81	0.81	0.81	0.81	0.81	0.81	0.79	0.79	0.79	0.79	0.79	0.79	0.79	0.85	0.85	0.85	0.85	0.85	0.85	0.85	0.85	0.85	0.75	0.75	0.75	0.75	0.79	0.79	0.79	0.81
1	42	YmfC_mono_RECYCL	rRNA Modification	1.00	1.00	1.00	1.00	1.00	1.00	1.00	1.00	1.00	1.00	1.00	1.00	1.00	1.00	1.00	1.00	1.00	1.00	1.00	1.00	1.00	1.00	1.00	1.00	1.00	1.00	1.00	1.00	1.00	1.00	1.00	1.00	1.00	1.00	1.00	1.00	1.00	1.00	1.00	0.81	0.81	0.81	0.81	0.81	0.81	0.81	0.81	0.81	0.79	0.79	0.79	0.79	0.79	0.79	0.79	0.85	0.85	0.85	0.85	0.85	0.85	0.85	0.85	0.85	0.75	0.75	0.75	0.75	0.79	0.79	0.79	0.81
1	43	YrdC_mono_RECYCL	Ribosomal protein assembly	1.00	1.00	1.00	1.00	1.00	1.00	1.00	1.00	1.00	1.00	1.00	1.00	1.00	1.00	1.00	1.00	1.00	1.00	1.00	1.00	1.00	1.00	1.00	1.00	1.00	1.00	1.00	1.00	1.00	1.00	1.00	1.00	1.00	1.00	1.00	1.00	1.00	1.00	1.00	1.00	1.00	1.00	1.00	0.81	0.81	0.81	0.81	0.81	0.81	0.81	0.81	0.81	0.79	0.79	0.79	0.79	0.79	0.79	0.79	0.85	0.85	0.85	0.85	0.85	0.85	0.85	0.85	0.85	0.75	0.75	0.75	0.75	0.79	0.79	0.79	0.81
2	44	Def_mono_RECYCL	Protein Maturation	0.81	0.81	0.81	0.81	0.81	0.81	0.81	0.81	0.81	0.81	0.81	0.81	0.81	0.81	0.81	0.81	0.81	0.81	0.81	0.81	0.81	0.81	0.81	0.81	0.81	0.81	0.81	0.81	0.81	0.81	0.81	0.81	0.81	0.81	0.81	0.81	0.81	0.81	0.81	0.81	0.81	0.81	0.81	1.00	1.00	1.00	1.00	1.00	1.00	1.00	1.00	1.00	0.28	0.28	0.28	0.28	0.28	0.28	0.28	0.44	0.44	0.44	0.44	0.44	0.42	0.42	0.42	0.42	0.18	0.18	0.18	0.18	0.29	0.29	0.29	0.30
2	45	Fmt_mono_RECYCL	tRNA charging	0.81	0.81	0.81	0.81	0.81	0.81	0.81	0.81	0.81	0.81	0.81	0.81	0.81	0.81	0.81	0.81	0.81	0.81	0.81	0.81	0.81	0.81	0.81	0.81	0.81	0.81	0.81	0.81	0.81	0.81	0.81	0.81	0.81	0.81	0.81	0.81	0.81	0.81	0.81	0.81	0.81	0.81	0.81	1.00	1.00	1.00	1.00	1.00	1.00	1.00	1.00	1.00	0.28	0.28	0.28	0.28	0.28	0.28	0.28	0.44	0.44	0.44	0.44	0.44	0.42	0.42	0.42	0.42	0.18	0.18	0.18	0.18	0.29	0.29	0.29	0.30
2	46	IF1_RECYCL	Translation	0.81	0.81	0.81	0.81	0.81	0.81	0.81	0.81	0.81	0.81	0.81	0.81	0.81	0.81	0.81	0.81	0.81	0.81	0.81	0.81	0.81	0.81	0.81	0.81	0.81	0.81	0.81	0.81	0.81	0.81	0.81	0.81	0.81	0.81	0.81	0.81	0.81	0.81	0.81	0.81	0.81	0.81	0.81	1.00	1.00	1.00	1.00	1.00	1.00	1.00	1.00	1.00	0.28	0.28	0.28	0.28	0.28	0.28	0.28	0.44	0.44	0.44	0.44	0.44	0.42	0.42	0.42	0.42	0.18	0.18	0.18	0.18	0.29	0.29	0.29	0.30
2	47	IF3_RECYCL	Translation	0.81	0.81	0.81	0.81	0.81	0.81	0.81	0.81	0.81	0.81	0.81	0.81	0.81	0.81	0.81	0.81	0.81	0.81	0.81	0.81	0.81	0.81	0.81	0.81	0.81	0.81	0.81	0.81	0.81	0.81	0.81	0.81	0.81	0.81	0.81	0.81	0.81	0.81	0.81	0.81	0.81	0.81	0.81	1.00	1.00	1.00	1.00	1.00	1.00	1.00	1.00	1.00	0.28	0.28	0.28	0.28	0.28	0.28	0.28	0.44	0.44	0.44	0.44	0.44	0.42	0.42	0.42	0.42	0.18	0.18	0.18	0.18	0.29	0.29	0.29	0.30
2	48	RF3_mono.GDP_RECYCL	Translation	0.81	0.81	0.81	0.81	0.81	0.81	0.81	0.81	0.81	0.81	0.81	0.81	0.81	0.81	0.81	0.81	0.81	0.81	0.81	0.81	0.81	0.81	0.81	0.81	0.81	0.81	0.81	0.81	0.81	0.81	0.81	0.81	0.81	0.81	0.81	0.81	0.81	0.81	0.81	0.81	0.81	0.81	0.81	1.00	1.00	1.00	1.00	1.00	1.00	1.00	1.00	1.00	0.28	0.28	0.28	0.28	0.28	0.28	0.28	0.44	0.44	0.44	0.44	0.44	0.42	0.42	0.42	0.42	0.18	0.18	0.18	0.18	0.29	0.29	0.29	0.30
2	49	Rrf_mono_RECYCL	Translation	0.81	0.81	0.81	0.81	0.81	0.81	0.81	0.81	0.81	0.81	0.81	0.81	0.81	0.81	0.81	0.81	0.81	0.81	0.81	0.81	0.81	0.81	0.81	0.81	0.81	0.81	0.81	0.81	0.81	0.81	0.81	0.81	0.81	0.81	0.81	0.81	0.81	0.81	0.81	0.81	0.81	0.81	0.81	1.00	1.00	1.00	1.00	1.00	1.00	1.00	1.00	1.00	0.28	0.28	0.28	0.28	0.28	0.28	0.28	0.44	0.44	0.44	0.44	0.44	0.42	0.42	0.42	0.42	0.18	0.18	0.18	0.18	0.29	0.29	0.29	0.30
2	50	rib_30_ini_RECYCL	Translation	0.81	0.81	0.81	0.81	0.81	0.81	0.81	0.81	0.81	0.81	0.81	0.81	0.81	0.81	0.81	0.81	0.81	0.81	0.81	0.81	0.81	0.81	0.81	0.81	0.81	0.81	0.81	0.81	0.81	0.81	0.81	0.81	0.81	0.81	0.81	0.81	0.81	0.81	0.81	0.81	0.81	0.81	0.81	1.00	1.00	1.00	1.00	1.00	1.00	1.00	1.00	1.00	0.28	0.28	0.28	0.28	0.28	0.28	0.28	0.44	0.44	0.44	0.44	0.44	0.43	0.43	0.43	0.43	0.18	0.18	0.18	0.18	0.29	0.29	0.29	0.31
2	51	rib_50_RECYCL	Translation	0.81	0.81	0.81	0.81	0.81	0.81	0.81	0.81	0.81	0.81	0.81	0.81	0.81	0.81	0.81	0.81	0.81	0.81	0.81	0.81	0.81	0.81	0.81	0.81	0.81	0.81	0.81	0.81	0.81	0.81	0.81	0.81	0.81	0.81	0.81	0.81	0.81	0.81	0.81	0.81	0.81	0.81	0.81	1.00	1.00	1.00	1.00	1.00	1.00	1.00	1.00	1.00	0.29	0.29	0.29	0.29	0.29	0.29	0.29	0.44	0.44	0.44	0.44	0.44	0.43	0.43	0.43	0.43	0.18	0.18	0.18	0.18	0.30	0.30	0.30	0.31
2	52	IF2_RECHARG	Translation	0.81	0.81	0.81	0.81	0.81	0.81	0.81	0.81	0.81	0.81	0.81	0.81	0.81	0.81	0.81	0.81	0.81	0.81	0.81	0.81	0.81	0.81	0.81	0.81	0.81	0.81	0.81	0.81	0.81	0.81	0.81	0.81	0.81	0.81	0.81	0.81	0.81	0.81	0.81	0.81	0.81	0.81	0.81	1.00	1.00	1.00	1.00	1.00	1.00	1.00	1.00	1.00	0.28	0.28	0.28	0.28	0.28	0.28	0.28	0.44	0.44	0.44	0.44	0.44	0.43	0.43	0.43	0.43	0.18	0.18	0.18	0.18	0.29	0.29	0.29	0.31
3	53	GreA_mono_RECYCL	Transcription	0.79	0.79	0.79	0.79	0.79	0.79	0.79	0.79	0.79	0.79	0.79	0.79	0.79	0.79	0.79	0.79	0.79	0.79	0.79	0.79	0.79	0.79	0.79	0.79	0.79	0.79	0.79	0.79	0.79	0.79	0.79	0.79	0.79	0.79	0.79	0.79	0.79	0.79	0.79	0.79	0.79	0.79	0.79	0.28	0.28	0.28	0.28	0.28	0.28	0.28	0.29	0.28	1.00	1.00	1.00	1.00	1.00	1.00	1.00	0.57	0.57	0.57	0.57	0.57	0.43	0.43	0.43	0.43	0.28	0.28	0.28	0.28	0.90	0.90	0.90	0.32
3	54	GreB_mono_RECYCL	Transcription	0.79	0.79	0.79	0.79	0.79	0.79	0.79	0.79	0.79	0.79	0.79	0.79	0.79	0.79	0.79	0.79	0.79	0.79	0.79	0.79	0.79	0.79	0.79	0.79	0.79	0.79	0.79	0.79	0.79	0.79	0.79	0.79	0.79	0.79	0.79	0.79	0.79	0.79	0.79	0.79	0.79	0.79	0.79	0.28	0.28	0.28	0.28	0.28	0.28	0.28	0.29	0.28	1.00	1.00	1.00	1.00	1.00	1.00	1.00	0.57	0.57	0.57	0.57	0.57	0.43	0.43	0.43	0.43	0.28	0.28	0.28	0.28	0.90	0.90	0.90	0.32
3	55	Mfd_mono_RECYCL	Transcription	0.79	0.79	0.79	0.79	0.79	0.79	0.79	0.79	0.79	0.79	0.79	0.79	0.79	0.79	0.79	0.79	0.79	0.79	0.79	0.79	0.79	0.79	0.79	0.79	0.79	0.79	0.79	0.79	0.79	0.79	0.79	0.79	0.79	0.79	0.79	0.79	0.79	0.79	0.79	0.79	0.79	0.79	0.79	0.28	0.28	0.28	0.28	0.28	0.28	0.28	0.29	0.28	1.00	1.00	1.00	1.00	1.00	1.00	1.00	0.57	0.57	0.57	0.57	0.57	0.43	0.43	0.43	0.43	0.28	0.28	0.28	0.28	0.90	0.90	0.90	0.32
3	56	NusA_mono_RECYCL	Transcription	0.79	0.79	0.79	0.79	0.79	0.79	0.79	0.79	0.79	0.79	0.79	0.79	0.79	0.79	0.79	0.79	0.79	0.79	0.79	0.79	0.79	0.79	0.79	0.79	0.79	0.79	0.79	0.79	0.79	0.79	0.79	0.79	0.79	0.79	0.79	0.79	0.79	0.79	0.79	0.79	0.79	0.79	0.79	0.28	0.28	0.28	0.28	0.28	0.28	0.28	0.29	0.28	1.00	1.00	1.00	1.00	1.00	1.00	1.00	0.57	0.57	0.57	0.57	0.57	0.43	0.43	0.43	0.43	0.28	0.28	0.28	0.28	0.90	0.90	0.90	0.32
3	57	NusG_mono_RECYCL	Transcription	0.79	0.79	0.79	0.79	0.79	0.79	0.79	0.79	0.79	0.79	0.79	0.79	0.79	0.79	0.79	0.79	0.79	0.79	0.79	0.79	0.79	0.79	0.79	0.79	0.79	0.79	0.79	0.79	0.79	0.79	0.79	0.79	0.79	0.79	0.79	0.79	0.79	0.79	0.79	0.79	0.79	0.79	0.79	0.28	0.28	0.28	0.28	0.28	0.28	0.28	0.29	0.28	1.00	1.00	1.00	1.00	1.00	1.00	1.00	0.57	0.57	0.57	0.57	0.57	0.43	0.43	0.43	0.43	0.28	0.28	0.28	0.28	0.90	0.90	0.90	0.32
3	58	RpoZ_mono_RECYCL	Transcription	0.79	0.79	0.79	0.79	0.79	0.79	0.79	0.79	0.79	0.79	0.79	0.79	0.79	0.79	0.79	0.79	0.79	0.79	0.79	0.79	0.79	0.79	0.79	0.79	0.79	0.79	0.79	0.79	0.79	0.79	0.79	0.79	0.79	0.79	0.79	0.79	0.79	0.79	0.79	0.79	0.79	0.79	0.79	0.28	0.28	0.28	0.28	0.28	0.28	0.28	0.29	0.28	1.00	1.00	1.00	1.00	1.00	1.00	1.00	0.57	0.57	0.57	0.57	0.57	0.43	0.43	0.43	0.43	0.28	0.28	0.28	0.28	0.90	0.90	0.90	0.32
3	59	hRNAP_RECYCL	Transcription	0.79	0.79	0.79	0.79	0.79	0.79	0.79	0.79	0.79	0.79	0.79	0.79	0.79	0.79	0.79	0.79	0.79	0.79	0.79	0.79	0.79	0.79	0.79	0.79	0.79	0.79	0.79	0.79	0.79	0.79	0.79	0.79	0.79	0.79	0.79	0.79	0.79	0.79	0.79	0.79	0.79	0.79	0.79	0.28	0.28	0.28	0.28	0.28	0.28	0.28	0.29	0.28	1.00	1.00	1.00	1.00	1.00	1.00	1.00	0.57	0.57	0.57	0.57	0.57	0.43	0.43	0.43	0.43	0.28	0.28	0.28	0.28	0.90	0.90	0.90	0.32
4	60	RNase_G_dim_RECYCL	RNA Processing	0.85	0.85	0.85	0.85	0.85	0.85	0.85	0.85	0.85	0.85	0.85	0.85	0.85	0.85	0.85	0.85	0.85	0.85	0.85	0.85	0.85	0.85	0.85	0.85	0.85	0.85	0.85	0.85	0.85	0.85	0.85	0.85	0.85	0.85	0.85	0.85	0.85	0.85	0.85	0.85	0.85	0.85	0.85	0.44	0.44	0.44	0.44	0.44	0.44	0.44	0.44	0.44	0.57	0.57	0.57	0.57	0.57	0.57	0.57	1.00	1.00	1.00	1.00	1.00	0.59	0.59	0.59	0.59	0.35	0.35	0.35	0.35	0.60	0.60	0.60	0.58
4	61	RNase_T_dim_RECYCL	RNA Processing	0.85	0.85	0.85	0.85	0.85	0.85	0.85	0.85	0.85	0.85	0.85	0.85	0.85	0.85	0.85	0.85	0.85	0.85	0.85	0.85	0.85	0.85	0.85	0.85	0.85	0.85	0.85	0.85	0.85	0.85	0.85	0.85	0.85	0.85	0.85	0.85	0.85	0.85	0.85	0.85	0.85	0.85	0.85	0.44	0.44	0.44	0.44	0.44	0.44	0.44	0.44	0.44	0.57	0.57	0.57	0.57	0.57	0.57	0.57	1.00	1.00	1.00	1.00	1.00	0.59	0.59	0.59	0.59	0.35	0.35	0.35	0.35	0.60	0.60	0.60	0.58
4	62	RNase_m16_RECYCL	rRNA Modification	0.85	0.85	0.85	0.85	0.85	0.85	0.85	0.85	0.85	0.85	0.85	0.85	0.85	0.85	0.85	0.85	0.85	0.85	0.85	0.85	0.85	0.85	0.85	0.85	0.85	0.85	0.85	0.85	0.85	0.85	0.85	0.85	0.85	0.85	0.85	0.85	0.85	0.85	0.85	0.85	0.85	0.85	0.85	0.44	0.44	0.44	0.44	0.44	0.44	0.44	0.44	0.44	0.57	0.57	0.57	0.57	0.57	0.57	0.57	1.00	1.00	1.00	1.00	1.00	0.59	0.59	0.59	0.59	0.35	0.35	0.35	0.35	0.60	0.60	0.60	0.58
4	63	RNase_m23_RECYCL	rRNA Modification	0.85	0.85	0.85	0.85	0.85	0.85	0.85	0.85	0.85	0.85	0.85	0.85	0.85	0.85	0.85	0.85	0.85	0.85	0.85	0.85	0.85	0.85	0.85	0.85	0.85	0.85	0.85	0.85	0.85	0.85	0.85	0.85	0.85	0.85	0.85	0.85	0.85	0.85	0.85	0.85	0.85	0.85	0.85	0.44	0.44	0.44	0.44	0.44	0.44	0.44	0.44	0.44	0.57	0.57	0.57	0.57	0.57	0.57	0.57	1.00	1.00	1.00	1.00	1.00	0.59	0.59	0.59	0.59	0.35	0.35	0.35	0.35	0.60	0.60	0.60	0.58
4	64	RNase_m5_RECYCL	rRNA Modification	0.85	0.85	0.85	0.85	0.85	0.85	0.85	0.85	0.85	0.85	0.85	0.85	0.85	0.85	0.85	0.85	0.85	0.85	0.85	0.85	0.85	0.85	0.85	0.85	0.85	0.85	0.85	0.85	0.85	0.85	0.85	0.85	0.85	0.85	0.85	0.85	0.85	0.85	0.85	0.85	0.85	0.85	0.85	0.44	0.44	0.44	0.44	0.44	0.44	0.44	0.44	0.44	0.57	0.57	0.57	0.57	0.57	0.57	0.57	1.00	1.00	1.00	1.00	1.00	0.59	0.59	0.59	0.59	0.35	0.35	0.35	0.35	0.60	0.60	0.60	0.58
5	65	TrmU_mono_RECYCL	tRNA Modification	0.85	0.85	0.85	0.85	0.85	0.85	0.85	0.85	0.85	0.85	0.85	0.85	0.85	0.85	0.85	0.85	0.85	0.85	0.85	0.85	0.85	0.85	0.85	0.85	0.85	0.85	0.85	0.85	0.85	0.85	0.85	0.85	0.85	0.85	0.85	0.85	0.85	0.85	0.85	0.85	0.85	0.85	0.85	0.42	0.42	0.42	0.42	0.42	0.42	0.43	0.43	0.43	0.43	0.43	0.43	0.43	0.43	0.43	0.43	0.59	0.59	0.59	0.59	0.59	1.00	1.00	1.00	1.00	0.29	0.29	0.29	0.29	0.45	0.45	0.45	0.56
5	66	YccK_mono_RECYCL	tRNA Modification	0.85	0.85	0.85	0.85	0.85	0.85	0.85	0.85	0.85	0.85	0.85	0.85	0.85	0.85	0.85	0.85	0.85	0.85	0.85	0.85	0.85	0.85	0.85	0.85	0.85	0.85	0.85	0.85	0.85	0.85	0.85	0.85	0.85	0.85	0.85	0.85	0.85	0.85	0.85	0.85	0.85	0.85	0.85	0.42	0.42	0.42	0.42	0.42	0.42	0.43	0.43	0.43	0.43	0.43	0.43	0.43	0.43	0.43	0.43	0.59	0.59	0.59	0.59	0.59	1.00	1.00	1.00	1.00	0.29	0.29	0.29	0.29	0.45	0.45	0.45	0.56
5	67	YheLMN_cplx_RECYCL	tRNA Modification	0.85	0.85	0.85	0.85	0.85	0.85	0.85	0.85	0.85	0.85	0.85	0.85	0.85	0.85	0.85	0.85	0.85	0.85	0.85	0.85	0.85	0.85	0.85	0.85	0.85	0.85	0.85	0.85	0.85	0.85	0.85	0.85	0.85	0.85	0.85	0.85	0.85	0.85	0.85	0.85	0.85	0.85	0.85	0.42	0.42	0.42	0.42	0.42	0.42	0.43	0.43	0.43	0.43	0.43	0.43	0.43	0.43	0.43	0.43	0.59	0.59	0.59	0.59	0.59	1.00	1.00	1.00	1.00	0.29	0.29	0.29	0.29	0.45	0.45	0.45	0.56
5	68	YhhP_mono_RECYCL	tRNA Modification	0.85	0.85	0.85	0.85	0.85	0.85	0.85	0.85	0.85	0.85	0.85	0.85	0.85	0.85	0.85	0.85	0.85	0.85	0.85	0.85	0.85	0.85	0.85	0.85	0.85	0.85	0.85	0.85	0.85	0.85	0.85	0.85	0.85	0.85	0.85	0.85	0.85	0.85	0.85	0.85	0.85	0.85	0.85	0.42	0.42	0.42	0.42	0.42	0.42	0.43	0.43	0.43	0.43	0.43	0.43	0.43	0.43	0.43	0.43	0.59	0.59	0.59	0.59	0.59	1.00	1.00	1.00	1.00	0.29	0.29	0.29	0.29	0.45	0.45	0.45	0.56
6	69	EoR_tRNA_pos34_Q_RECYCL	tRNA Modification	0.75	0.75	0.75	0.75	0.75	0.75	0.75	0.75	0.75	0.75	0.75	0.75	0.75	0.75	0.75	0.75	0.75	0.75	0.75	0.75	0.75	0.75	0.75	0.75	0.75	0.75	0.75	0.75	0.75	0.75	0.75	0.75	0.75	0.75	0.75	0.75	0.75	0.75	0.75	0.75	0.75	0.75	0.75	0.18	0.18	0.18	0.18	0.18	0.18	0.18	0.18	0.18	0.28	0.28	0.28	0.28	0.28	0.28	0.28	0.35	0.35	0.35	0.35	0.35	0.29	0.29	0.29	0.29	1.00	1.00	1.00	1.00	0.32	0.32	0.32	0.17
6	70	QueA_mono_RECYCL	tRNA Modification	0.75	0.75	0.75	0.75	0.75	0.75	0.75	0.75	0.75	0.75	0.75	0.75	0.75	0.75	0.75	0.75	0.75	0.75	0.75	0.75	0.75	0.75	0.75	0.75	0.75	0.75	0.75	0.75	0.75	0.75	0.75	0.75	0.75	0.75	0.75	0.75	0.75	0.75	0.75	0.75	0.75	0.75	0.75	0.18	0.18	0.18	0.18	0.18	0.18	0.18	0.18	0.18	0.28	0.28	0.28	0.28	0.28	0.28	0.28	0.35	0.35	0.35	0.35	0.35	0.29	0.29	0.29	0.29	1.00	1.00	1.00	1.00	0.32	0.32	0.32	0.17
6	71	QueF_dim_RECYCL	tRNA Modification	0.75	0.75	0.75	0.75	0.75	0.75	0.75	0.75	0.75	0.75	0.75	0.75	0.75	0.75	0.75	0.75	0.75	0.75	0.75	0.75	0.75	0.75	0.75	0.75	0.75	0.75	0.75	0.75	0.75	0.75	0.75	0.75	0.75	0.75	0.75	0.75	0.75	0.75	0.75	0.75	0.75	0.75	0.75	0.18	0.18	0.18	0.18	0.18	0.18	0.18	0.18	0.18	0.28	0.28	0.28	0.28	0.28	0.28	0.28	0.35	0.35	0.35	0.35	0.35	0.29	0.29	0.29	0.29	1.00	1.00	1.00	1.00	0.32	0.32	0.32	0.17
6	72	Tgt_hexa_RECYCL	tRNA Modification	0.75	0.75	0.75	0.75	0.75	0.75	0.75	0.75	0.75	0.75	0.75	0.75	0.75	0.75	0.75	0.75	0.75	0.75	0.75	0.75	0.75	0.75	0.75	0.75	0.75	0.75	0.75	0.75	0.75	0.75	0.75	0.75	0.75	0.75	0.75	0.75	0.75	0.75	0.75	0.75	0.75	0.75	0.75	0.18	0.18	0.18	0.18	0.18	0.18	0.18	0.18	0.18	0.28	0.28	0.28	0.28	0.28	0.28	0.28	0.35	0.35	0.35	0.35	0.35	0.29	0.29	0.29	0.29	1.00	1.00	1.00	1.00	0.32	0.32	0.32	0.17
7	73	RNase_E_tetra_RECYCL	RNA Processing	0.79	0.79	0.79	0.79	0.79	0.79	0.79	0.79	0.79	0.79	0.79	0.79	0.79	0.79	0.79	0.79	0.79	0.79	0.79	0.79	0.79	0.79	0.79	0.79	0.79	0.79	0.79	0.79	0.79	0.79	0.79	0.79	0.79	0.79	0.79	0.79	0.79	0.79	0.79	0.79	0.79	0.79	0.79	0.29	0.29	0.29	0.29	0.29	0.29	0.29	0.30	0.29	0.90	0.90	0.90	0.90	0.90	0.90	0.90	0.60	0.60	0.60	0.60	0.60	0.45	0.45	0.45	0.45	0.32	0.32	0.32	0.32	1.00	1.00	1.00	0.35
7	74	RNase_Gen_RECYCL	RNA Processing	0.79	0.79	0.79	0.79	0.79	0.79	0.79	0.79	0.79	0.79	0.79	0.79	0.79	0.79	0.79	0.79	0.79	0.79	0.79	0.79	0.79	0.79	0.79	0.79	0.79	0.79	0.79	0.79	0.79	0.79	0.79	0.79	0.79	0.79	0.79	0.79	0.79	0.79	0.79	0.79	0.79	0.79	0.79	0.29	0.29	0.29	0.29	0.29	0.29	0.29	0.30	0.29	0.90	0.90	0.90	0.90	0.90	0.90	0.90	0.60	0.60	0.60	0.60	0.60	0.45	0.45	0.45	0.45	0.32	0.32	0.32	0.32	1.00	1.00	1.00	0.35
7	75	RNase_P_cplx_RECYCL	RNA Processing	0.79	0.79	0.79	0.79	0.79	0.79	0.79	0.79	0.79	0.79	0.79	0.79	0.79	0.79	0.79	0.79	0.79	0.79	0.79	0.79	0.79	0.79	0.79	0.79	0.79	0.79	0.79	0.79	0.79	0.79	0.79	0.79	0.79	0.79	0.79	0.79	0.79	0.79	0.79	0.79	0.79	0.79	0.79	0.29	0.29	0.29	0.29	0.29	0.29	0.29	0.30	0.29	0.90	0.90	0.90	0.90	0.90	0.90	0.90	0.60	0.60	0.60	0.60	0.60	0.45	0.45	0.45	0.45	0.32	0.32	0.32	0.32	1.00	1.00	1.00	0.35
8	76	HyL_tRNA_pos_34_ho5U_RECYCL	tRNA Modification	0.81	0.81	0.81	0.81	0.81	0.81	0.81	0.81	0.81	0.81	0.81	0.81	0.81	0.81	0.81	0.81	0.81	0.81	0.81	0.81	0.81	0.81	0.81	0.81	0.81	0.81	0.81	0.81	0.81	0.81	0.81	0.81	0.81	0.81	0.81	0.81	0.81	0.81	0.81	0.81	0.81	0.81	0.81	0.30	0.30	0.30	0.30	0.30	0.30	0.31	0.31	0.31	0.32	0.32	0.32	0.32	0.32	0.32	0.32	0.58	0.58	0.58	0.58	0.58	0.56	0.56	0.56	0.56	0.17	0.17	0.17	0.17	0.35	0.35	0.35	1.00
8	77	YecO_mono_RECYCL	tRNA Modification	0.81	0.81	0.81	0.81	0.81	0.81	0.81	0.81	0.81	0.81	0.81	0.81	0.81	0.81	0.81	0.81	0.81	0.81	0.81	0.81	0.81	0.81	0.81	0.81	0.81	0.81	0.81	0.81	0.81	0.81	0.81	0.81	0.81	0.81	0.81	0.81	0.81	0.81	0.81	0.81	0.81	0.81	0.81	0.30	0.30	0.30	0.30	0.30	0.30	0.31	0.31	0.31	0.32	0.32	0.32	0.32	0.32	0.32	0.32	0.58	0.58	0.58	0.58	0.58	0.56	0.56	0.56	0.56	0.17	0.17	0.17	0.17	0.35	0.35	0.35	1.00
8	78	YecP_mono_RECYCL	tRNA Modification	0.81	0.81	0.81	0.81	0.81	0.81	0.81	0.81	0.81	0.81	0.81	0.81	0.81	0.81	0.81	0.81	0.81	0.81	0.81	0.81	0.81	0.81	0.81	0.81	0.81	0.81	0.81	0.81	0.81	0.81	0.81	0.81	0.81	0.81	0.81	0.81	0.81	0.81	0.81	0.81	0.81	0.81	0.81	0.30	0.30	0.30	0.30	0.30	0.30	0.31	0.31	0.31	0.32	0.32	0.32	0.32	0.32	0.32	0.32	0.58	0.58	0.58	0.58	0.58	0.56	0.56	0.56	0.56	0.17	0.17	0.17	0.17	0.35	0.35	0.35	1.00
9	79	GidA_mono_RECYCL	tRNA Modification	0.84	0.84	0.84	0.84	0.84	0.84	0.84	0.84	0.84	0.84	0.84	0.84	0.84	0.84	0.84	0.84	0.84	0.84	0.84	0.84	0.84	0.84	0.84	0.84	0.84	0.84	0.84	0.84	0.84	0.84	0.84	0.84	0.84	0.84	0.84	0.84	0.84	0.84	0.84	0.84	0.84	0.84	0.84	0.39	0.39	0.39	0.39	0.39	0.39	0.39	0.39	0.39	0.42	0.42	0.42	0.42	0.42	0.42	0.42	0.55	0.55	0.55	0.55	0.55	0.90	0.90	0.90	0.90	0.25	0.25	0.25	0.25	0.44	0.44	0.44	0.50
9	80	TrmC_mono_RECYCL	tRNA Modification	0.84	0.84	0.84	0.84	0.84	0.84	0.84	0.84	0.84	0.84	0.84	0.84	0.84	0.84	0.84	0.84	0.84	0.84	0.84	0.84	0.84	0.84	0.84	0.84	0.84	0.84	0.84	0.84	0.84	0.84	0.84	0.84	0.84	0.84	0.84	0.84	0.84	0.84	0.84	0.84	0.84	0.84	0.84	0.39	0.39	0.39	0.39	0.39	0.39	0.39	0.39	0.39	0.42	0.42	0.42	0.42	0.42	0.42	0.42	0.55	0.55	0.55	0.55	0.55	0.90	0.90	0.90	0.90	0.25	0.25	0.25	0.25	0.44	0.44	0.44	0.50
9	81	TrmE_dim_RECYCL	tRNA Modification	0.84	0.84	0.84	0.84	0.84	0.84	0.84	0.84	0.84	0.84	0.84	0.84	0.84	0.84	0.84	0.84	0.84	0.84	0.84	0.84	0.84	0.84	0.84	0.84	0.84	0.84	0.84	0.84	0.84	0.84	0.84	0.84	0.84	0.84	0.84	0.84	0.84	0.84	0.84	0.84	0.84	0.84	0.84	0.39	0.39	0.39	0.39	0.39	0.39	0.39	0.39	0.39	0.42	0.42	0.42	0.42	0.42	0.42	0.42	0.55	0.55	0.55	0.55	0.55	0.90	0.90	0.90	0.90	0.25	0.25	0.25	0.25	0.44	0.44	0.44	0.50
10	82	cis/transGroES_CONV	Protein Folding	0.75	0.75	0.75	0.75	0.75	0.75	0.75	0.75	0.75	0.75	0.75	0.75	0.75	0.75	0.75	0.75	0.75	0.75	0.75	0.75	0.75	0.75	0.75	0.75	0.75	0.75	0.75	0.75	0.75	0.75	0.75	0.75	0.75	0.75	0.75	0.75	0.75	0.75	0.75	0.75	0.75	0.75	0.75	0.49	0.49	0.49	0.49	0.49	0.49	0.50	0.50	0.50	0.12	0.12	0.12	0.12	0.12	0.12	0.12	0.31	0.31	0.31	0.31	0.31	0.30	0.30	0.30	0.30	0.00	0.00	0.00	0.00	0.14	0.14	0.14	0.15
10	83	GroEL.(7)ADP.cisGroES_CONV	Protein Folding	0.75	0.75	0.75	0.75	0.75	0.75	0.75	0.75	0.75	0.75	0.75	0.75	0.75	0.75	0.75	0.75	0.75	0.75	0.75	0.75	0.75	0.75	0.75	0.75	0.75	0.75	0.75	0.75	0.75	0.75	0.75	0.75	0.75	0.75	0.75	0.75	0.75	0.75	0.75	0.75	0.75	0.75	0.75	0.49	0.49	0.49	0.49	0.49	0.49	0.50	0.50	0.50	0.12	0.12	0.12	0.12	0.12	0.12	0.12	0.31	0.31	0.31	0.31	0.31	0.30	0.30	0.30	0.30	0.00	0.00	0.00	0.00	0.14	0.14	0.14	0.15
11	84	Orn_dim_RECYCL	mRNA degradation	0.75	0.75	0.75	0.75	0.75	0.75	0.75	0.75	0.75	0.75	0.75	0.75	0.75	0.75	0.75	0.75	0.75	0.75	0.75	0.75	0.75	0.75	0.75	0.75	0.75	0.75	0.75	0.75	0.75	0.75	0.75	0.75	0.75	0.75	0.75	0.75	0.75	0.75	0.75	0.75	0.75	0.75	0.75	0.18	0.18	0.18	0.18	0.18	0.18	0.18	0.19	0.18	0.21	0.21	0.21	0.21	0.21	0.21	0.21	0.31	0.31	0.31	0.31	0.31	0.29	0.29	0.29	0.29	0.00	0.00	0.00	0.00	0.13	0.13	0.13	0.15
11	85	degradosome_RECYCL	mRNA degradation	0.75	0.75	0.75	0.75	0.75	0.75	0.75	0.75	0.75	0.75	0.75	0.75	0.75	0.75	0.75	0.75	0.75	0.75	0.75	0.75	0.75	0.75	0.75	0.75	0.75	0.75	0.75	0.75	0.75	0.75	0.75	0.75	0.75	0.75	0.75	0.75	0.75	0.75	0.75	0.75	0.75	0.75	0.75	0.18	0.18	0.18	0.18	0.18	0.18	0.18	0.19	0.18	0.21	0.21	0.21	0.21	0.21	0.21	0.21	0.31	0.31	0.31	0.31	0.31	0.29	0.29	0.29	0.29	0.00	0.00	0.00	0.00	0.13	0.13	0.13	0.15
12	86	EF-Ts_RECYCL	Translation	0.79	0.79	0.79	0.79	0.79	0.79	0.79	0.79	0.79	0.79	0.79	0.79	0.79	0.79	0.79	0.79	0.79	0.79	0.79	0.79	0.79	0.79	0.79	0.79	0.79	0.79	0.79	0.79	0.79	0.79	0.79	0.79	0.79	0.79	0.79	0.79	0.79	0.79	0.79	0.79	0.79	0.79	0.79	0.84	0.84	0.84	0.84	0.84	0.84	0.84	0.84	0.84	0.25	0.25	0.25	0.25	0.25	0.25	0.25	0.41	0.41	0.41	0.41	0.41	0.39	0.39	0.39	0.39	0.14	0.14	0.14	0.14	0.26	0.26	0.26	0.27
12	87	EF-G_RECHARG	Translation	0.79	0.79	0.79	0.79	0.79	0.79	0.79	0.79	0.79	0.79	0.79	0.79	0.79	0.79	0.79	0.79	0.79	0.79	0.79	0.79	0.79	0.79	0.79	0.79	0.79	0.79	0.79	0.79	0.79	0.79	0.79	0.79	0.79	0.79	0.79	0.79	0.79	0.79	0.79	0.79	0.79	0.79	0.79	0.84	0.84	0.84	0.84	0.84	0.84	0.84	0.84	0.84	0.25	0.25	0.25	0.25	0.25	0.25	0.25	0.41	0.41	0.41	0.41	0.41	0.39	0.39	0.39	0.39	0.14	0.14	0.14	0.14	0.26	0.26	0.26	0.27
13	88	DnaJ_dim_RECYCL	Protein Folding	0.75	0.75	0.75	0.75	0.75	0.75	0.75	0.75	0.75	0.75	0.75	0.75	0.75	0.75	0.75	0.75	0.75	0.75	0.75	0.75	0.75	0.75	0.75	0.75	0.75	0.75	0.75	0.75	0.75	0.75	0.75	0.75	0.75	0.75	0.75	0.75	0.75	0.75	0.75	0.75	0.75	0.75	0.75	0.51	0.51	0.51	0.51	0.51	0.51	0.51	0.52	0.51	0.12	0.12	0.12	0.12	0.12	0.12	0.12	0.31	0.31	0.31	0.31	0.31	0.29	0.29	0.29	0.29	0.00	0.00	0.00	0.00	0.13	0.13	0.13	0.15
13	89	GrpE_dim_RECYCL	Protein Folding	0.75	0.75	0.75	0.75	0.75	0.75	0.75	0.75	0.75	0.75	0.75	0.75	0.75	0.75	0.75	0.75	0.75	0.75	0.75	0.75	0.75	0.75	0.75	0.75	0.75	0.75	0.75	0.75	0.75	0.75	0.75	0.75	0.75	0.75	0.75	0.75	0.75	0.75	0.75	0.75	0.75	0.75	0.75	0.51	0.51	0.51	0.51	0.51	0.51	0.51	0.52	0.51	0.12	0.12	0.12	0.12	0.12	0.12	0.12	0.31	0.31	0.31	0.31	0.31	0.29	0.29	0.29	0.29	0.00	0.00	0.00	0.00	0.13	0.13	0.13	0.15
14	90	AcT_EF-TU_RECYCL	Protein Modification	0.75	0.75	0.75	0.75	0.75	0.75	0.75	0.75	0.75	0.75	0.75	0.75	0.75	0.75	0.75	0.75	0.75	0.75	0.75	0.75	0.75	0.75	0.75	0.75	0.75	0.75	0.75	0.75	0.75	0.75	0.75	0.75	0.75	0.75	0.75	0.75	0.75	0.75	0.75	0.75	0.75	0.75	0.75	0.31	0.31	0.31	0.31	0.31	0.31	0.31	0.31	0.31	0.12	0.12	0.12	0.12	0.12	0.12	0.12	0.31	0.31	0.31	0.31	0.31	0.29	0.29	0.29	0.29	0.00	0.00	0.00	0.00	0.13	0.13	0.13	0.15
14	91	MeT_EF-TU_RECYCL	Protein Modification	0.75	0.75	0.75	0.75	0.75	0.75	0.75	0.75	0.75	0.75	0.75	0.75	0.75	0.75	0.75	0.75	0.75	0.75	0.75	0.75	0.75	0.75	0.75	0.75	0.75	0.75	0.75	0.75	0.75	0.75	0.75	0.75	0.75	0.75	0.75	0.75	0.75	0.75	0.75	0.75	0.75	0.75	0.75	0.31	0.31	0.31	0.31	0.31	0.31	0.31	0.31	0.31	0.12	0.12	0.12	0.12	0.12	0.12	0.12	0.31	0.31	0.31	0.31	0.31	0.29	0.29	0.29	0.29	0.00	0.00	0.00	0.00	0.13	0.13	0.13	0.15
15	92	Era_RECHARGE	Ribosomal protein assembly	0.89	0.89	0.89	0.89	0.89	0.89	0.89	0.89	0.89	0.89	0.89	0.89	0.89	0.89	0.89	0.89	0.89	0.89	0.89	0.89	0.89	0.89	0.89	0.89	0.89	0.89	0.89	0.89	0.89	0.89	0.89	0.89	0.89	0.89	0.89	0.89	0.89	0.89	0.89	0.89	0.89	0.89	0.89	0.44	0.44	0.44	0.44	0.44	0.44	0.44	0.44	0.44	0.38	0.38	0.38	0.38	0.38	0.38	0.38	0.54	0.54	0.54	0.54	0.54	0.53	0.53	0.53	0.53	0.29	0.29	0.29	0.29	0.40	0.40	0.40	0.42
16	93	YqcB_mono_RECYCL	tRNA Modification	0.82	0.82	0.82	0.82	0.82	0.82	0.82	0.82	0.82	0.82	0.82	0.82	0.82	0.82	0.82	0.82	0.82	0.82	0.82	0.82	0.82	0.82	0.82	0.82	0.82	0.82	0.82	0.82	0.82	0.82	0.82	0.82	0.82	0.82	0.82	0.82	0.82	0.82	0.82	0.82	0.82	0.82	0.82	0.32	0.32	0.32	0.32	0.32	0.32	0.32	0.33	0.32	0.34	0.34	0.34	0.34	0.34	0.34	0.34	0.47	0.47	0.47	0.47	0.47	0.44	0.44	0.44	0.44	0.49	0.49	0.49	0.49	0.37	0.37	0.37	0.45
17	94	YggH_mono_RECYCL	tRNA Modification	0.78	0.78	0.78	0.78	0.78	0.78	0.78	0.78	0.78	0.78	0.78	0.78	0.78	0.78	0.78	0.78	0.78	0.78	0.78	0.78	0.78	0.78	0.78	0.78	0.78	0.78	0.78	0.78	0.78	0.78	0.78	0.78	0.78	0.78	0.78	0.78	0.78	0.78	0.78	0.78	0.78	0.78	0.78	0.22	0.23	0.22	0.22	0.22	0.22	0.23	0.23	0.23	0.63	0.63	0.63	0.63	0.63	0.63	0.63	0.70	0.70	0.70	0.70	0.70	0.42	0.42	0.42	0.42	0.29	0.29	0.29	0.29	0.71	0.71	0.71	0.43
18	95	YdaO_mono_RECYCL	tRNA Modification	0.75	0.75	0.75	0.75	0.75	0.75	0.75	0.75	0.75	0.75	0.75	0.75	0.75	0.75	0.75	0.75	0.75	0.75	0.75	0.75	0.75	0.75	0.75	0.75	0.75	0.75	0.75	0.75	0.75	0.75	0.75	0.75	0.75	0.75	0.75	0.75	0.75	0.75	0.75	0.75	0.75	0.75	0.75	0.18	0.18	0.18	0.18	0.18	0.18	0.18	0.18	0.18	0.19	0.19	0.19	0.19	0.19	0.19	0.19	0.38	0.38	0.38	0.38	0.38	0.29	0.29	0.29	0.29	0.06	0.06	0.06	0.06	0.21	0.21	0.21	0.20
19	96	YbbB_dim_RECYCL	tRNA Modification	0.75	0.75	0.75	0.75	0.75	0.75	0.75	0.75	0.75	0.75	0.75	0.75	0.75	0.75	0.75	0.75	0.75	0.75	0.75	0.75	0.75	0.75	0.75	0.75	0.75	0.75	0.75	0.75	0.75	0.75	0.75	0.75	0.75	0.75	0.75	0.75	0.75	0.75	0.75	0.75	0.75	0.75	0.75	0.18	0.18	0.18	0.18	0.18	0.18	0.18	0.18	0.18	0.13	0.13	0.13	0.13	0.13	0.13	0.13	0.33	0.33	0.33	0.33	0.33	0.54	0.54	0.54	0.54	0.00	0.00	0.00	0.00	0.15	0.15	0.15	0.22
20	97	ValS_mono_RECYCL	tRNA charging	0.80	0.80	0.80	0.80	0.80	0.80	0.80	0.80	0.80	0.80	0.80	0.80	0.80	0.80	0.80	0.80	0.80	0.80	0.80	0.80	0.80	0.80	0.80	0.80	0.80	0.80	0.80	0.80	0.80	0.80	0.80	0.80	0.80	0.80	0.80	0.80	0.80	0.80	0.80	0.80	0.80	0.80	0.80	0.89	0.89	0.89	0.89	0.89	0.89	0.89	0.89	0.89	0.26	0.26	0.26	0.26	0.26	0.26	0.26	0.42	0.42	0.42	0.42	0.42	0.41	0.41	0.41	0.41	0.16	0.16	0.16	0.16	0.27	0.27	0.27	0.28
21	98	Up_tRNA_pos_37_t6A_RECYCL	tRNA Modification	0.79	0.79	0.79	0.79	0.79	0.79	0.79	0.79	0.79	0.79	0.79	0.79	0.79	0.79	0.79	0.79	0.79	0.79	0.79	0.79	0.79	0.79	0.79	0.79	0.79	0.79	0.79	0.79	0.79	0.79	0.79	0.79	0.79	0.79	0.79	0.79	0.79	0.79	0.79	0.79	0.79	0.79	0.79	0.24	0.24	0.24	0.24	0.24	0.24	0.24	0.24	0.24	0.41	0.41	0.41	0.41	0.41	0.41	0.41	0.50	0.50	0.50	0.50	0.50	0.46	0.46	0.46	0.46	0.17	0.17	0.17	0.17	0.46	0.46	0.46	0.37
22	99	Tyr_RS_dim_RECYCL	tRNA charging	0.76	0.76	0.76	0.76	0.76	0.76	0.76	0.76	0.76	0.76	0.76	0.76	0.76	0.76	0.76	0.76	0.76	0.76	0.76	0.76	0.76	0.76	0.76	0.76	0.76	0.76	0.76	0.76	0.76	0.76	0.76	0.76	0.76	0.76	0.76	0.76	0.76	0.76	0.76	0.76	0.76	0.76	0.76	0.44	0.44	0.44	0.44	0.44	0.44	0.44	0.44	0.44	0.14	0.14	0.14	0.14	0.14	0.14	0.14	0.32	0.32	0.32	0.32	0.32	0.29	0.29	0.29	0.29	0.00	0.00	0.00	0.00	0.15	0.15	0.15	0.15
23	100	TruD_mono_RECYCL	tRNA Modification	0.95	0.95	0.95	0.95	0.95	0.95	0.95	0.95	0.95	0.95	0.95	0.95	0.95	0.95	0.95	0.95	0.95	0.95	0.95	0.95	0.95	0.95	0.95	0.95	0.95	0.95	0.95	0.95	0.95	0.95	0.95	0.95	0.95	0.95	0.95	0.95	0.95	0.95	0.95	0.95	0.95	0.95	0.95	0.65	0.65	0.65	0.65	0.65	0.65	0.65	0.66	0.65	0.63	0.63	0.63	0.63	0.63	0.63	0.63	0.73	0.73	0.73	0.73	0.73	0.79	0.79	0.79	0.79	0.57	0.57	0.57	0.57	0.64	0.64	0.64	0.68
24	101	TruB_mono_RECYCL	tRNA Modification	0.78	0.78	0.78	0.78	0.78	0.78	0.78	0.78	0.78	0.78	0.78	0.78	0.78	0.78	0.78	0.78	0.78	0.78	0.78	0.78	0.78	0.78	0.78	0.78	0.78	0.78	0.78	0.78	0.78	0.78	0.78	0.78	0.78	0.78	0.78	0.78	0.78	0.78	0.78	0.78	0.78	0.78	0.78	0.26	0.26	0.26	0.26	0.26	0.26	0.26	0.26	0.26	0.88	0.88	0.88	0.88	0.88	0.88	0.88	0.76	0.76	0.76	0.76	0.76	0.43	0.43	0.43	0.43	0.30	0.30	0.30	0.30	0.98	0.98	0.98	0.40
25	102	TruA_dim_RECYCL	tRNA Modification	0.75	0.75	0.75	0.75	0.75	0.75	0.75	0.75	0.75	0.75	0.75	0.75	0.75	0.75	0.75	0.75	0.75	0.75	0.75	0.75	0.75	0.75	0.75	0.75	0.75	0.75	0.75	0.75	0.75	0.75	0.75	0.75	0.75	0.75	0.75	0.75	0.75	0.75	0.75	0.75	0.75	0.75	0.75	0.18	0.18	0.18	0.18	0.18	0.18	0.18	0.18	0.18	0.51	0.51	0.51	0.51	0.51	0.51	0.51	0.55	0.55	0.55	0.55	0.55	0.35	0.35	0.35	0.35	0.15	0.15	0.15	0.15	0.57	0.57	0.57	0.28
26	103	Trp_RS_dim_RECYCL	tRNA charging	0.76	0.76	0.76	0.76	0.76	0.76	0.76	0.76	0.76	0.76	0.76	0.76	0.76	0.76	0.76	0.76	0.76	0.76	0.76	0.76	0.76	0.76	0.76	0.76	0.76	0.76	0.76	0.76	0.76	0.76	0.76	0.76	0.76	0.76	0.76	0.76	0.76	0.76	0.76	0.76	0.76	0.76	0.76	0.40	0.40	0.40	0.40	0.40	0.40	0.40	0.40	0.40	0.14	0.14	0.14	0.14	0.14	0.14	0.14	0.33	0.33	0.33	0.33	0.33	0.31	0.31	0.31	0.31	0.03	0.03	0.03	0.03	0.15	0.15	0.15	0.17
27	104	TrmH_dim_RECYCL	tRNA Modification	0.75	0.75	0.75	0.75	0.75	0.75	0.75	0.75	0.75	0.75	0.75	0.75	0.75	0.75	0.75	0.75	0.75	0.75	0.75	0.75	0.75	0.75	0.75	0.75	0.75	0.75	0.75	0.75	0.75	0.75	0.75	0.75	0.75	0.75	0.75	0.75	0.75	0.75	0.75	0.75	0.75	0.75	0.75	0.18	0.18	0.18	0.18	0.18	0.18	0.18	0.18	0.18	0.34	0.34	0.34	0.34	0.34	0.34	0.34	0.47	0.47	0.47	0.47	0.47	0.29	0.29	0.29	0.29	0.05	0.05	0.05	0.05	0.39	0.39	0.39	0.17
28	105	TrmD_dim_RECYCL	tRNA Modification	0.75	0.75	0.75	0.75	0.75	0.75	0.75	0.75	0.75	0.75	0.75	0.75	0.75	0.75	0.75	0.75	0.75	0.75	0.75	0.75	0.75	0.75	0.75	0.75	0.75	0.75	0.75	0.75	0.75	0.75	0.75	0.75	0.75	0.75	0.75	0.75	0.75	0.75	0.75	0.75	0.75	0.75	0.75	0.18	0.18	0.18	0.18	0.18	0.18	0.18	0.18	0.18	0.18	0.18	0.18	0.18	0.18	0.18	0.18	0.35	0.35	0.35	0.35	0.35	0.29	0.29	0.29	0.29	0.09	0.09	0.09	0.09	0.20	0.20	0.20	0.23
29	106	TrmA_mono_RECYCL	tRNA Modification	0.79	0.79	0.79	0.79	0.79	0.79	0.79	0.79	0.79	0.79	0.79	0.79	0.79	0.79	0.79	0.79	0.79	0.79	0.79	0.79	0.79	0.79	0.79	0.79	0.79	0.79	0.79	0.79	0.79	0.79	0.79	0.79	0.79	0.79	0.79	0.79	0.79	0.79	0.79	0.79	0.79	0.79	0.79	0.26	0.26	0.26	0.26	0.26	0.26	0.26	0.26	0.26	0.86	0.86	0.86	0.86	0.86	0.86	0.86	0.76	0.76	0.76	0.76	0.76	0.44	0.44	0.44	0.44	0.30	0.30	0.30	0.30	0.96	0.96	0.96	0.41
30	107	TilS_mono_RECYCL	tRNA Modification	0.75	0.75	0.75	0.75	0.75	0.75	0.75	0.75	0.75	0.75	0.75	0.75	0.75	0.75	0.75	0.75	0.75	0.75	0.75	0.75	0.75	0.75	0.75	0.75	0.75	0.75	0.75	0.75	0.75	0.75	0.75	0.75	0.75	0.75	0.75	0.75	0.75	0.75	0.75	0.75	0.75	0.75	0.75	0.18	0.18	0.18	0.18	0.18	0.18	0.18	0.18	0.18	0.15	0.15	0.15	0.15	0.15	0.15	0.15	0.31	0.31	0.31	0.31	0.31	0.29	0.29	0.29	0.29	0.00	0.00	0.00	0.00	0.17	0.17	0.17	0.15
31	108	Thr_RS_dim_RECYCL	tRNA charging	0.77	0.77	0.77	0.77	0.77	0.77	0.77	0.77	0.77	0.77	0.77	0.77	0.77	0.77	0.77	0.77	0.77	0.77	0.77	0.77	0.77	0.77	0.77	0.77	0.77	0.77	0.77	0.77	0.77	0.77	0.77	0.77	0.77	0.77	0.77	0.77	0.77	0.77	0.77	0.77	0.77	0.77	0.77	0.65	0.65	0.65	0.65	0.65	0.65	0.65	0.66	0.65	0.17	0.17	0.17	0.17	0.17	0.17	0.17	0.35	0.35	0.35	0.35	0.35	0.34	0.34	0.34	0.34	0.06	0.06	0.06	0.06	0.19	0.19	0.19	0.20
32	109	ThiI_mono_RECYCL	tRNA Modification	0.75	0.75	0.75	0.75	0.75	0.75	0.75	0.75	0.75	0.75	0.75	0.75	0.75	0.75	0.75	0.75	0.75	0.75	0.75	0.75	0.75	0.75	0.75	0.75	0.75	0.75	0.75	0.75	0.75	0.75	0.75	0.75	0.75	0.75	0.75	0.75	0.75	0.75	0.75	0.75	0.75	0.75	0.75	0.18	0.18	0.18	0.18	0.18	0.18	0.18	0.18	0.18	0.61	0.61	0.61	0.61	0.61	0.61	0.61	0.56	0.56	0.56	0.56	0.56	0.33	0.33	0.33	0.33	0.25	0.25	0.25	0.25	0.69	0.69	0.69	0.27
33	110	TadA_dim_RECYCL	tRNA Modification	0.75	0.75	0.75	0.75	0.75	0.75	0.75	0.75	0.75	0.75	0.75	0.75	0.75	0.75	0.75	0.75	0.75	0.75	0.75	0.75	0.75	0.75	0.75	0.75	0.75	0.75	0.75	0.75	0.75	0.75	0.75	0.75	0.75	0.75	0.75	0.75	0.75	0.75	0.75	0.75	0.75	0.75	0.75	0.18	0.18	0.18	0.18	0.18	0.18	0.18	0.18	0.18	0.13	0.13	0.13	0.13	0.13	0.13	0.13	0.35	0.35	0.35	0.35	0.35	0.29	0.29	0.29	0.29	0.00	0.00	0.00	0.00	0.15	0.15	0.15	0.15
34	111	Ser_RS_dim_RECYCL	tRNA charging	0.77	0.77	0.77	0.77	0.77	0.77	0.77	0.77	0.77	0.77	0.77	0.77	0.77	0.77	0.77	0.77	0.77	0.77	0.77	0.77	0.77	0.77	0.77	0.77	0.77	0.77	0.77	0.77	0.77	0.77	0.77	0.77	0.77	0.77	0.77	0.77	0.77	0.77	0.77	0.77	0.77	0.77	0.77	0.53	0.53	0.53	0.53	0.53	0.53	0.53	0.53	0.53	0.18	0.18	0.18	0.18	0.18	0.18	0.18	0.35	0.35	0.35	0.35	0.35	0.34	0.34	0.34	0.34	0.06	0.06	0.06	0.06	0.19	0.19	0.19	0.20
35	112	SelA_deca_RECYCL	tRNA charging	0.75	0.75	0.75	0.75	0.75	0.75	0.75	0.75	0.75	0.75	0.75	0.75	0.75	0.75	0.75	0.75	0.75	0.75	0.75	0.75	0.75	0.75	0.75	0.75	0.75	0.75	0.75	0.75	0.75	0.75	0.75	0.75	0.75	0.75	0.75	0.75	0.75	0.75	0.75	0.75	0.75	0.75	0.75	0.18	0.18	0.18	0.18	0.18	0.18	0.18	0.18	0.18	0.13	0.13	0.13	0.13	0.13	0.13	0.13	0.31	0.31	0.31	0.31	0.31	0.29	0.29	0.29	0.29	0.00	0.00	0.00	0.00	0.15	0.15	0.15	0.15
36	113	RpoS_mono_RECYCL	Transcription	0.82	0.82	0.82	0.82	0.82	0.82	0.82	0.82	0.82	0.82	0.82	0.82	0.82	0.82	0.82	0.82	0.82	0.82	0.82	0.82	0.82	0.82	0.82	0.82	0.82	0.82	0.82	0.82	0.82	0.82	0.82	0.82	0.82	0.82	0.82	0.82	0.82	0.82	0.82	0.82	0.82	0.82	0.82	0.19	0.19	0.19	0.19	0.19	0.19	0.19	0.19	0.19	0.13	0.13	0.13	0.13	0.13	0.13	0.13	0.34	0.34	0.34	0.34	0.34	0.36	0.36	0.36	0.36	0.00	0.00	0.00	0.00	0.15	0.15	0.15	0.21
37	114	RpoN_mono_RECYCL	Transcription	0.75	0.75	0.75	0.75	0.75	0.75	0.75	0.75	0.75	0.75	0.75	0.75	0.75	0.75	0.75	0.75	0.75	0.75	0.75	0.75	0.75	0.75	0.75	0.75	0.75	0.75	0.75	0.75	0.75	0.75	0.75	0.75	0.75	0.75	0.75	0.75	0.75	0.75	0.75	0.75	0.75	0.75	0.75	0.18	0.18	0.18	0.18	0.18	0.18	0.18	0.18	0.18	0.12	0.12	0.12	0.12	0.12	0.12	0.12	0.31	0.31	0.31	0.31	0.31	0.29	0.29	0.29	0.29	0.00	0.00	0.00	0.00	0.13	0.13	0.13	0.15
38	115	RpoH_mono_RECYCL	Transcription	0.82	0.82	0.82	0.82	0.82	0.82	0.82	0.82	0.82	0.82	0.82	0.82	0.82	0.82	0.82	0.82	0.82	0.82	0.82	0.82	0.82	0.82	0.82	0.82	0.82	0.82	0.82	0.82	0.82	0.82	0.82	0.82	0.82	0.82	0.82	0.82	0.82	0.82	0.82	0.82	0.82	0.82	0.82	0.19	0.19	0.19	0.19	0.19	0.19	0.19	0.19	0.19	0.13	0.13	0.13	0.13	0.13	0.13	0.13	0.34	0.34	0.34	0.34	0.34	0.36	0.36	0.36	0.36	0.00	0.00	0.00	0.00	0.14	0.14	0.14	0.21
39	116	RpoE_mono_RECYCL	Transcription	0.75	0.75	0.75	0.75	0.75	0.75	0.75	0.75	0.75	0.75	0.75	0.75	0.75	0.75	0.75	0.75	0.75	0.75	0.75	0.75	0.75	0.75	0.75	0.75	0.75	0.75	0.75	0.75	0.75	0.75	0.75	0.75	0.75	0.75	0.75	0.75	0.75	0.75	0.75	0.75	0.75	0.75	0.75	0.18	0.18	0.18	0.18	0.18	0.18	0.18	0.18	0.18	0.13	0.13	0.13	0.13	0.13	0.13	0.13	0.34	0.34	0.34	0.34	0.34	0.29	0.29	0.29	0.29	0.00	0.00	0.00	0.00	0.14	0.14	0.14	0.15
40	117	RpoD_mono_RECYCL	Transcription	0.77	0.77	0.77	0.77	0.77	0.77	0.77	0.77	0.77	0.77	0.77	0.77	0.77	0.77	0.77	0.77	0.77	0.77	0.77	0.77	0.77	0.77	0.77	0.77	0.77	0.77	0.77	0.77	0.77	0.77	0.77	0.77	0.77	0.77	0.77	0.77	0.77	0.77	0.77	0.77	0.77	0.77	0.77	0.24	0.24	0.24	0.24	0.24	0.24	0.24	0.24	0.24	0.95	0.95	0.95	0.95	0.95	0.95	0.95	0.51	0.51	0.51	0.51	0.51	0.39	0.39	0.39	0.39	0.24	0.24	0.24	0.24	0.85	0.85	0.85	0.27
41	118	RluA_mono_RECYCL	rRNA Modification	0.93	0.93	0.93	0.93	0.93	0.93	0.93	0.93	0.93	0.93	0.93	0.93	0.93	0.93	0.93	0.93	0.93	0.93	0.93	0.93	0.93	0.93	0.93	0.93	0.93	0.93	0.93	0.93	0.93	0.93	0.93	0.93	0.93	0.93	0.93	0.93	0.93	0.93	0.93	0.93	0.93	0.93	0.93	0.62	0.62	0.62	0.62	0.62	0.62	0.62	0.63	0.62	0.63	0.63	0.63	0.63	0.63	0.63	0.63	0.71	0.71	0.71	0.71	0.71	0.69	0.69	0.69	0.69	0.53	0.53	0.53	0.53	0.64	0.64	0.64	0.62
42	119	Rho_hexa_RECYCL	Transcription	0.79	0.79	0.79	0.79	0.79	0.79	0.79	0.79	0.79	0.79	0.79	0.79	0.79	0.79	0.79	0.79	0.79	0.79	0.79	0.79	0.79	0.79	0.79	0.79	0.79	0.79	0.79	0.79	0.79	0.79	0.79	0.79	0.79	0.79	0.79	0.79	0.79	0.79	0.79	0.79	0.79	0.79	0.79	0.28	0.29	0.28	0.28	0.28	0.28	0.29	0.29	0.29	0.97	0.97	0.97	0.97	0.97	0.97	0.97	0.58	0.58	0.58	0.58	0.58	0.44	0.44	0.44	0.44	0.29	0.29	0.29	0.29	0.92	0.92	0.92	0.33
43	120	RNase_III_dim_RECYCL	RNA Processing	0.84	0.84	0.84	0.84	0.84	0.84	0.84	0.84	0.84	0.84	0.84	0.84	0.84	0.84	0.84	0.84	0.84	0.84	0.84	0.84	0.84	0.84	0.84	0.84	0.84	0.84	0.84	0.84	0.84	0.84	0.84	0.84	0.84	0.84	0.84	0.84	0.84	0.84	0.84	0.84	0.84	0.84	0.84	0.41	0.41	0.41	0.41	0.41	0.41	0.41	0.42	0.41	0.58	0.58	0.58	0.58	0.58	0.58	0.58	0.92	0.92	0.92	0.92	0.92	0.56	0.56	0.56	0.56	0.31	0.31	0.31	0.31	0.55	0.55	0.55	0.52
44	121	RF2_mono_RECYCL	Translation	0.75	0.75	0.75	0.75	0.75	0.75	0.75	0.75	0.75	0.75	0.75	0.75	0.75	0.75	0.75	0.75	0.75	0.75	0.75	0.75	0.75	0.75	0.75	0.75	0.75	0.75	0.75	0.75	0.75	0.75	0.75	0.75	0.75	0.75	0.75	0.75	0.75	0.75	0.75	0.75	0.75	0.75	0.75	0.46	0.46	0.46	0.46	0.46	0.46	0.46	0.46	0.46	0.12	0.12	0.12	0.12	0.12	0.12	0.12	0.31	0.31	0.31	0.31	0.31	0.30	0.30	0.30	0.30	0.00	0.00	0.00	0.00	0.13	0.13	0.13	0.15
45	122	RF1_mono_RECYCL	Translation	0.76	0.76	0.76	0.76	0.76	0.76	0.76	0.76	0.76	0.76	0.76	0.76	0.76	0.76	0.76	0.76	0.76	0.76	0.76	0.76	0.76	0.76	0.76	0.76	0.76	0.76	0.76	0.76	0.76	0.76	0.76	0.76	0.76	0.76	0.76	0.76	0.76	0.76	0.76	0.76	0.76	0.76	0.76	0.54	0.54	0.54	0.54	0.54	0.54	0.54	0.55	0.54	0.13	0.13	0.13	0.13	0.13	0.13	0.13	0.32	0.32	0.32	0.32	0.32	0.31	0.31	0.31	0.31	0.01	0.01	0.01	0.01	0.15	0.15	0.15	0.16
46	123	Pro_RS_dim_RECYCL	tRNA charging	0.77	0.77	0.77	0.77	0.77	0.77	0.77	0.77	0.77	0.77	0.77	0.77	0.77	0.77	0.77	0.77	0.77	0.77	0.77	0.77	0.77	0.77	0.77	0.77	0.77	0.77	0.77	0.77	0.77	0.77	0.77	0.77	0.77	0.77	0.77	0.77	0.77	0.77	0.77	0.77	0.77	0.77	0.77	0.60	0.60	0.60	0.60	0.60	0.60	0.60	0.60	0.60	0.16	0.16	0.16	0.16	0.16	0.16	0.16	0.34	0.34	0.34	0.34	0.34	0.32	0.32	0.32	0.32	0.04	0.04	0.04	0.04	0.17	0.17	0.17	0.19
47	124	PrmC_mono_RECYCL	Protein Modification	0.75	0.75	0.75	0.75	0.75	0.75	0.75	0.75	0.75	0.75	0.75	0.75	0.75	0.75	0.75	0.75	0.75	0.75	0.75	0.75	0.75	0.75	0.75	0.75	0.75	0.75	0.75	0.75	0.75	0.75	0.75	0.75	0.75	0.75	0.75	0.75	0.75	0.75	0.75	0.75	0.75	0.75	0.75	0.19	0.19	0.19	0.19	0.19	0.19	0.19	0.19	0.19	0.12	0.12	0.12	0.12	0.12	0.12	0.12	0.31	0.31	0.31	0.31	0.31	0.29	0.29	0.29	0.29	0.00	0.00	0.00	0.00	0.13	0.13	0.13	0.15
48	125	Phe_RS_tetra_RECYCL	tRNA charging	0.78	0.78	0.78	0.78	0.78	0.78	0.78	0.78	0.78	0.78	0.78	0.78	0.78	0.78	0.78	0.78	0.78	0.78	0.78	0.78	0.78	0.78	0.78	0.78	0.78	0.78	0.78	0.78	0.78	0.78	0.78	0.78	0.78	0.78	0.78	0.78	0.78	0.78	0.78	0.78	0.78	0.78	0.78	0.80	0.80	0.80	0.80	0.80	0.80	0.80	0.80	0.80	0.21	0.21	0.21	0.21	0.21	0.21	0.21	0.38	0.38	0.38	0.38	0.38	0.36	0.36	0.36	0.36	0.10	0.10	0.10	0.10	0.22	0.22	0.22	0.23
49	126	NusB_mono_RECYCL	Transcription	0.79	0.79	0.79	0.79	0.79	0.79	0.79	0.79	0.79	0.79	0.79	0.79	0.79	0.79	0.79	0.79	0.79	0.79	0.79	0.79	0.79	0.79	0.79	0.79	0.79	0.79	0.79	0.79	0.79	0.79	0.79	0.79	0.79	0.79	0.79	0.79	0.79	0.79	0.79	0.79	0.79	0.79	0.79	0.29	0.29	0.29	0.29	0.29	0.29	0.29	0.29	0.29	0.92	0.92	0.92	0.92	0.92	0.92	0.92	0.60	0.60	0.60	0.60	0.60	0.45	0.45	0.45	0.45	0.31	0.31	0.31	0.31	0.98	0.98	0.98	0.34
50	127	MiaB_mono_RECYCL	tRNA Modification	0.75	0.75	0.75	0.75	0.75	0.75	0.75	0.75	0.75	0.75	0.75	0.75	0.75	0.75	0.75	0.75	0.75	0.75	0.75	0.75	0.75	0.75	0.75	0.75	0.75	0.75	0.75	0.75	0.75	0.75	0.75	0.75	0.75	0.75	0.75	0.75	0.75	0.75	0.75	0.75	0.75	0.75	0.75	0.18	0.18	0.18	0.18	0.18	0.18	0.18	0.18	0.18	0.27	0.27	0.27	0.27	0.27	0.27	0.27	0.34	0.34	0.34	0.34	0.34	0.29	0.29	0.29	0.29	0.02	0.02	0.02	0.02	0.30	0.30	0.30	0.15
51	128	MiaA_dim_RECYCL	tRNA Modification	0.75	0.75	0.75	0.75	0.75	0.75	0.75	0.75	0.75	0.75	0.75	0.75	0.75	0.75	0.75	0.75	0.75	0.75	0.75	0.75	0.75	0.75	0.75	0.75	0.75	0.75	0.75	0.75	0.75	0.75	0.75	0.75	0.75	0.75	0.75	0.75	0.75	0.75	0.75	0.75	0.75	0.75	0.75	0.18	0.18	0.18	0.18	0.18	0.18	0.18	0.18	0.18	0.29	0.29	0.29	0.29	0.29	0.29	0.29	0.33	0.33	0.33	0.33	0.33	0.29	0.29	0.29	0.29	0.02	0.02	0.02	0.02	0.33	0.33	0.33	0.15
52	129	Met_RS_dim_RECYCL	tRNA charging	0.78	0.78	0.78	0.78	0.78	0.78	0.78	0.78	0.78	0.78	0.78	0.78	0.78	0.78	0.78	0.78	0.78	0.78	0.78	0.78	0.78	0.78	0.78	0.78	0.78	0.78	0.78	0.78	0.78	0.78	0.78	0.78	0.78	0.78	0.78	0.78	0.78	0.78	0.78	0.78	0.78	0.78	0.78	0.80	0.80	0.80	0.80	0.80	0.80	0.80	0.80	0.80	0.21	0.21	0.21	0.21	0.21	0.21	0.21	0.38	0.38	0.38	0.38	0.38	0.36	0.36	0.36	0.36	0.10	0.10	0.10	0.10	0.22	0.22	0.22	0.23
53	130	MeT_tRNA_pos_37_m6t6A_RECYCL	tRNA Modification	0.77	0.77	0.77	0.77	0.77	0.77	0.77	0.77	0.77	0.77	0.77	0.77	0.77	0.77	0.77	0.77	0.77	0.77	0.77	0.77	0.77	0.77	0.77	0.77	0.77	0.77	0.77	0.77	0.77	0.77	0.77	0.77	0.77	0.77	0.77	0.77	0.77	0.77	0.77	0.77	0.77	0.77	0.77	0.18	0.18	0.18	0.18	0.18	0.18	0.18	0.19	0.18	0.16	0.16	0.16	0.16	0.16	0.16	0.16	0.38	0.38	0.38	0.38	0.38	0.31	0.31	0.31	0.31	0.08	0.08	0.08	0.08	0.19	0.19	0.19	0.16
54	131	MeT_tRNA_pos_37_m6A_RECYCL	tRNA Modification	0.75	0.75	0.75	0.75	0.75	0.75	0.75	0.75	0.75	0.75	0.75	0.75	0.75	0.75	0.75	0.75	0.75	0.75	0.75	0.75	0.75	0.75	0.75	0.75	0.75	0.75	0.75	0.75	0.75	0.75	0.75	0.75	0.75	0.75	0.75	0.75	0.75	0.75	0.75	0.75	0.75	0.75	0.75	0.18	0.18	0.18	0.18	0.18	0.18	0.18	0.18	0.18	0.16	0.16	0.16	0.16	0.16	0.16	0.16	0.42	0.42	0.42	0.42	0.42	0.62	0.62	0.62	0.62	0.00	0.00	0.00	0.00	0.18	0.18	0.18	0.56
55	132	MeT_tRNA_pos_37_m2A_RECYCL	tRNA Modification	0.81	0.81	0.81	0.81	0.81	0.81	0.81	0.81	0.81	0.81	0.81	0.81	0.81	0.81	0.81	0.81	0.81	0.81	0.81	0.81	0.81	0.81	0.81	0.81	0.81	0.81	0.81	0.81	0.81	0.81	0.81	0.81	0.81	0.81	0.81	0.81	0.81	0.81	0.81	0.81	0.81	0.81	0.81	0.32	0.32	0.32	0.32	0.32	0.32	0.32	0.32	0.32	0.37	0.37	0.37	0.37	0.37	0.37	0.37	0.55	0.55	0.55	0.55	0.55	0.44	0.44	0.44	0.44	0.33	0.33	0.33	0.33	0.40	0.40	0.40	0.32
56	133	MeT_tRNA_pos_32_Um_RECYCL	tRNA Modification	0.75	0.75	0.75	0.75	0.75	0.75	0.75	0.75	0.75	0.75	0.75	0.75	0.75	0.75	0.75	0.75	0.75	0.75	0.75	0.75	0.75	0.75	0.75	0.75	0.75	0.75	0.75	0.75	0.75	0.75	0.75	0.75	0.75	0.75	0.75	0.75	0.75	0.75	0.75	0.75	0.75	0.75	0.75	0.18	0.18	0.18	0.18	0.18	0.18	0.18	0.18	0.18	0.18	0.18	0.18	0.18	0.18	0.18	0.18	0.39	0.39	0.39	0.39	0.39	0.29	0.29	0.29	0.29	0.04	0.04	0.04	0.04	0.21	0.21	0.21	0.18
57	134	MeT_tRNA_pos_32_Cm_RECYCL	tRNA Modification	0.75	0.75	0.75	0.75	0.75	0.75	0.75	0.75	0.75	0.75	0.75	0.75	0.75	0.75	0.75	0.75	0.75	0.75	0.75	0.75	0.75	0.75	0.75	0.75	0.75	0.75	0.75	0.75	0.75	0.75	0.75	0.75	0.75	0.75	0.75	0.75	0.75	0.75	0.75	0.75	0.75	0.75	0.75	0.18	0.18	0.18	0.18	0.18	0.18	0.18	0.18	0.18	0.23	0.23	0.23	0.23	0.23	0.23	0.23	0.33	0.33	0.33	0.33	0.33	0.29	0.29	0.29	0.29	0.00	0.00	0.00	0.00	0.26	0.26	0.26	0.15
58	135	Map_mono_RECYCL	Protein Maturation	0.78	0.78	0.78	0.78	0.78	0.78	0.78	0.78	0.78	0.78	0.78	0.78	0.78	0.78	0.78	0.78	0.78	0.78	0.78	0.78	0.78	0.78	0.78	0.78	0.78	0.78	0.78	0.78	0.78	0.78	0.78	0.78	0.78	0.78	0.78	0.78	0.78	0.78	0.78	0.78	0.78	0.78	0.78	0.87	0.86	0.87	0.87	0.87	0.87	0.87	0.87	0.87	0.22	0.22	0.22	0.22	0.22	0.22	0.22	0.39	0.39	0.39	0.39	0.39	0.38	0.38	0.38	0.38	0.12	0.12	0.12	0.12	0.24	0.24	0.24	0.25
59	136	LysI_RS_dim_RECYCL	tRNA charging	0.75	0.75	0.75	0.75	0.75	0.75	0.75	0.75	0.75	0.75	0.75	0.75	0.75	0.75	0.75	0.75	0.75	0.75	0.75	0.75	0.75	0.75	0.75	0.75	0.75	0.75	0.75	0.75	0.75	0.75	0.75	0.75	0.75	0.75	0.75	0.75	0.75	0.75	0.75	0.75	0.75	0.75	0.75	0.43	0.43	0.43	0.43	0.43	0.43	0.43	0.43	0.43	0.12	0.12	0.12	0.12	0.12	0.12	0.12	0.31	0.31	0.31	0.31	0.31	0.30	0.30	0.30	0.30	0.00	0.00	0.00	0.00	0.13	0.13	0.13	0.15
60	137	LysII_RS_dim_RECYCL	tRNA charging	0.75	0.75	0.75	0.75	0.75	0.75	0.75	0.75	0.75	0.75	0.75	0.75	0.75	0.75	0.75	0.75	0.75	0.75	0.75	0.75	0.75	0.75	0.75	0.75	0.75	0.75	0.75	0.75	0.75	0.75	0.75	0.75	0.75	0.75	0.75	0.75	0.75	0.75	0.75	0.75	0.75	0.75	0.75	0.43	0.43	0.43	0.43	0.43	0.43	0.43	0.43	0.43	0.12	0.12	0.12	0.12	0.12	0.12	0.12	0.31	0.31	0.31	0.31	0.31	0.30	0.30	0.30	0.30	0.00	0.00	0.00	0.00	0.13	0.13	0.13	0.15
61	138	LeuS_mono_RECYCL	tRNA charging	0.78	0.78	0.78	0.78	0.78	0.78	0.78	0.78	0.78	0.78	0.78	0.78	0.78	0.78	0.78	0.78	0.78	0.78	0.78	0.78	0.78	0.78	0.78	0.78	0.78	0.78	0.78	0.78	0.78	0.78	0.78	0.78	0.78	0.78	0.78	0.78	0.78	0.78	0.78	0.78	0.78	0.78	0.78	0.79	0.79	0.79	0.79	0.79	0.79	0.79	0.80	0.79	0.20	0.20	0.20	0.20	0.20	0.20	0.20	0.38	0.38	0.38	0.38	0.38	0.36	0.36	0.36	0.36	0.09	0.09	0.09	0.09	0.22	0.22	0.22	0.23
62	139	IleS_mono_RECYCL	tRNA charging	0.79	0.79	0.79	0.79	0.79	0.79	0.79	0.79	0.79	0.79	0.79	0.79	0.79	0.79	0.79	0.79	0.79	0.79	0.79	0.79	0.79	0.79	0.79	0.79	0.79	0.79	0.79	0.79	0.79	0.79	0.79	0.79	0.79	0.79	0.79	0.79	0.79	0.79	0.79	0.79	0.79	0.79	0.79	0.87	0.87	0.87	0.87	0.87	0.87	0.87	0.87	0.87	0.23	0.23	0.23	0.23	0.23	0.23	0.23	0.40	0.40	0.40	0.40	0.40	0.38	0.38	0.38	0.38	0.12	0.12	0.12	0.12	0.24	0.24	0.24	0.25
63	140	His_RS_dim_RECYCL	tRNA charging	0.77	0.77	0.77	0.77	0.77	0.77	0.77	0.77	0.77	0.77	0.77	0.77	0.77	0.77	0.77	0.77	0.77	0.77	0.77	0.77	0.77	0.77	0.77	0.77	0.77	0.77	0.77	0.77	0.77	0.77	0.77	0.77	0.77	0.77	0.77	0.77	0.77	0.77	0.77	0.77	0.77	0.77	0.77	0.48	0.48	0.48	0.48	0.48	0.48	0.48	0.48	0.48	0.17	0.17	0.17	0.17	0.17	0.17	0.17	0.35	0.35	0.35	0.35	0.35	0.34	0.34	0.34	0.34	0.06	0.06	0.06	0.06	0.19	0.19	0.19	0.20
64	141	Gly_RS_tetra_RECYCL	tRNA charging	0.77	0.77	0.77	0.77	0.77	0.77	0.77	0.77	0.77	0.77	0.77	0.77	0.77	0.77	0.77	0.77	0.77	0.77	0.77	0.77	0.77	0.77	0.77	0.77	0.77	0.77	0.77	0.77	0.77	0.77	0.77	0.77	0.77	0.77	0.77	0.77	0.77	0.77	0.77	0.77	0.77	0.77	0.77	0.62	0.62	0.62	0.62	0.62	0.62	0.63	0.63	0.63	0.18	0.18	0.18	0.18	0.18	0.18	0.18	0.36	0.36	0.36	0.36	0.36	0.34	0.34	0.34	0.34	0.07	0.07	0.07	0.07	0.19	0.19	0.19	0.21
65	142	GltX_mono_RECYCL	tRNA charging	0.80	0.80	0.80	0.80	0.80	0.80	0.80	0.80	0.80	0.80	0.80	0.80	0.80	0.80	0.80	0.80	0.80	0.80	0.80	0.80	0.80	0.80	0.80	0.80	0.80	0.80	0.80	0.80	0.80	0.80	0.80	0.80	0.80	0.80	0.80	0.80	0.80	0.80	0.80	0.80	0.80	0.80	0.80	0.72	0.72	0.72	0.72	0.72	0.72	0.72	0.72	0.72	0.28	0.28	0.28	0.28	0.28	0.28	0.28	0.44	0.44	0.44	0.44	0.44	0.42	0.42	0.42	0.42	0.18	0.18	0.18	0.18	0.29	0.29	0.29	0.30
66	143	GlnS_mono_RECYCL	tRNA charging	0.78	0.78	0.78	0.78	0.78	0.78	0.78	0.78	0.78	0.78	0.78	0.78	0.78	0.78	0.78	0.78	0.78	0.78	0.78	0.78	0.78	0.78	0.78	0.78	0.78	0.78	0.78	0.78	0.78	0.78	0.78	0.78	0.78	0.78	0.78	0.78	0.78	0.78	0.78	0.78	0.78	0.78	0.78	0.79	0.79	0.79	0.79	0.79	0.79	0.79	0.79	0.79	0.20	0.20	0.20	0.20	0.20	0.20	0.20	0.37	0.37	0.37	0.37	0.37	0.36	0.36	0.36	0.36	0.09	0.09	0.09	0.09	0.21	0.21	0.21	0.23
67	144	Dus_gen_RECYCL	tRNA Modification	0.77	0.77	0.77	0.77	0.77	0.77	0.77	0.77	0.77	0.77	0.77	0.77	0.77	0.77	0.77	0.77	0.77	0.77	0.77	0.77	0.77	0.77	0.77	0.77	0.77	0.77	0.77	0.77	0.77	0.77	0.77	0.77	0.77	0.77	0.77	0.77	0.77	0.77	0.77	0.77	0.77	0.77	0.77	0.21	0.21	0.21	0.21	0.21	0.21	0.21	0.21	0.21	0.77	0.77	0.77	0.77	0.77	0.77	0.77	0.68	0.68	0.68	0.68	0.68	0.38	0.38	0.38	0.38	0.27	0.27	0.27	0.27	0.87	0.87	0.87	0.39
68	145	Dtd_dim_RECYCL	tRNA charging	0.75	0.75	0.75	0.75	0.75	0.75	0.75	0.75	0.75	0.75	0.75	0.75	0.75	0.75	0.75	0.75	0.75	0.75	0.75	0.75	0.75	0.75	0.75	0.75	0.75	0.75	0.75	0.75	0.75	0.75	0.75	0.75	0.75	0.75	0.75	0.75	0.75	0.75	0.75	0.75	0.75	0.75	0.75	0.44	0.44	0.44	0.44	0.44	0.44	0.44	0.44	0.44	0.14	0.14	0.14	0.14	0.14	0.14	0.14	0.32	0.32	0.32	0.32	0.32	0.29	0.29	0.29	0.29	0.00	0.00	0.00	0.00	0.15	0.15	0.15	0.15
69	146	DnaK_mono.ATP_RECYCL	Protein Folding	0.75	0.75	0.75	0.75	0.75	0.75	0.75	0.75	0.75	0.75	0.75	0.75	0.75	0.75	0.75	0.75	0.75	0.75	0.75	0.75	0.75	0.75	0.75	0.75	0.75	0.75	0.75	0.75	0.75	0.75	0.75	0.75	0.75	0.75	0.75	0.75	0.75	0.75	0.75	0.75	0.75	0.75	0.75	0.52	0.51	0.52	0.52	0.52	0.52	0.52	0.52	0.52	0.12	0.12	0.12	0.12	0.12	0.12	0.12	0.31	0.31	0.31	0.31	0.31	0.29	0.29	0.29	0.29	0.00	0.00	0.00	0.00	0.13	0.13	0.13	0.15
70	147	CysS_mono_RECYCL	tRNA charging	0.77	0.77	0.77	0.77	0.77	0.77	0.77	0.77	0.77	0.77	0.77	0.77	0.77	0.77	0.77	0.77	0.77	0.77	0.77	0.77	0.77	0.77	0.77	0.77	0.77	0.77	0.77	0.77	0.77	0.77	0.77	0.77	0.77	0.77	0.77	0.77	0.77	0.77	0.77	0.77	0.77	0.77	0.77	0.65	0.65	0.65	0.65	0.65	0.65	0.65	0.65	0.65	0.16	0.16	0.16	0.16	0.16	0.16	0.16	0.34	0.34	0.34	0.34	0.34	0.33	0.33	0.33	0.33	0.04	0.04	0.04	0.04	0.17	0.17	0.17	0.19
71	148	Asp_RS_dim_RECYCL	tRNA charging	0.77	0.77	0.77	0.77	0.77	0.77	0.77	0.77	0.77	0.77	0.77	0.77	0.77	0.77	0.77	0.77	0.77	0.77	0.77	0.77	0.77	0.77	0.77	0.77	0.77	0.77	0.77	0.77	0.77	0.77	0.77	0.77	0.77	0.77	0.77	0.77	0.77	0.77	0.77	0.77	0.77	0.77	0.77	0.51	0.51	0.51	0.51	0.51	0.51	0.52	0.52	0.52	0.17	0.17	0.17	0.17	0.17	0.17	0.17	0.35	0.35	0.35	0.35	0.35	0.34	0.34	0.34	0.34	0.06	0.06	0.06	0.06	0.18	0.18	0.18	0.20
72	149	Asn_RS_dim_RECYCL	tRNA charging	0.79	0.79	0.79	0.79	0.79	0.79	0.79	0.79	0.79	0.79	0.79	0.79	0.79	0.79	0.79	0.79	0.79	0.79	0.79	0.79	0.79	0.79	0.79	0.79	0.79	0.79	0.79	0.79	0.79	0.79	0.79	0.79	0.79	0.79	0.79	0.79	0.79	0.79	0.79	0.79	0.79	0.79	0.79	0.84	0.84	0.84	0.84	0.84	0.84	0.84	0.84	0.84	0.23	0.23	0.23	0.23	0.23	0.23	0.23	0.39	0.39	0.39	0.39	0.39	0.38	0.38	0.38	0.38	0.12	0.12	0.12	0.12	0.24	0.24	0.24	0.25
73	150	ArgS_mono_RECYCL	tRNA charging	0.80	0.80	0.80	0.80	0.80	0.80	0.80	0.80	0.80	0.80	0.80	0.80	0.80	0.80	0.80	0.80	0.80	0.80	0.80	0.80	0.80	0.80	0.80	0.80	0.80	0.80	0.80	0.80	0.80	0.80	0.80	0.80	0.80	0.80	0.80	0.80	0.80	0.80	0.80	0.80	0.80	0.80	0.80	0.75	0.75	0.75	0.75	0.75	0.75	0.75	0.75	0.75	0.27	0.27	0.27	0.27	0.27	0.27	0.27	0.43	0.43	0.43	0.43	0.43	0.41	0.41	0.41	0.41	0.17	0.17	0.17	0.17	0.28	0.28	0.28	0.29
74	151	Ala_RS_tetra_RECYCL	tRNA charging	0.79	0.79	0.79	0.79	0.79	0.79	0.79	0.79	0.79	0.79	0.79	0.79	0.79	0.79	0.79	0.79	0.79	0.79	0.79	0.79	0.79	0.79	0.79	0.79	0.79	0.79	0.79	0.79	0.79	0.79	0.79	0.79	0.79	0.79	0.79	0.79	0.79	0.79	0.79	0.79	0.79	0.79	0.79	0.82	0.82	0.82	0.82	0.82	0.82	0.82	0.82	0.82	0.25	0.25	0.25	0.25	0.25	0.25	0.25	0.41	0.41	0.41	0.41	0.41	0.40	0.40	0.40	0.40	0.15	0.15	0.15	0.15	0.26	0.26	0.26	0.27
75	152	AcpT_tRNA_pos_47_acp3U_RECYCL	tRNA Modification	0.78	0.78	0.78	0.78	0.78	0.78	0.78	0.78	0.78	0.78	0.78	0.78	0.78	0.78	0.78	0.78	0.78	0.78	0.78	0.78	0.78	0.78	0.78	0.78	0.78	0.78	0.78	0.78	0.78	0.78	0.78	0.78	0.78	0.78	0.78	0.78	0.78	0.78	0.78	0.78	0.78	0.78	0.78	0.24	0.24	0.24	0.24	0.24	0.24	0.25	0.25	0.25	0.37	0.37	0.37	0.37	0.37	0.37	0.37	0.52	0.52	0.52	0.52	0.52	0.48	0.48	0.48	0.48	0.08	0.08	0.08	0.08	0.41	0.41	0.41	0.39
76	153	AcT_tRNA_pos_34_ac4C_RECYCL	tRNA Modification	0.75	0.75	0.75	0.75	0.75	0.75	0.75	0.75	0.75	0.75	0.75	0.75	0.75	0.75	0.75	0.75	0.75	0.75	0.75	0.75	0.75	0.75	0.75	0.75	0.75	0.75	0.75	0.75	0.75	0.75	0.75	0.75	0.75	0.75	0.75	0.75	0.75	0.75	0.75	0.75	0.75	0.75	0.75	0.18	0.18	0.18	0.18	0.18	0.18	0.18	0.18	0.18	0.13	0.13	0.13	0.13	0.13	0.13	0.13	0.35	0.35	0.35	0.35	0.35	0.29	0.29	0.29	0.29	0.00	0.00	0.00	0.00	0.15	0.15	0.15	0.15

Module Number	Reaction Number	Reaction Abbreviation	Subsystem	Values
1	1	DU_23S_2449_RECYCL	rRNA Modification	[illegible]
1	2	KsgA_mono_RECYCL	rRNA Modification	[illegible]
1	3	MeST_S12_RECYCL	Ribosomal protein modification	[illegible]
1	4	MeT_16S_1402_RECYCL	rRNA Modification	[illegible]
1	5	MeT_16S_1407_RECYCL	rRNA Modification	[illegible]
1	6	MeT_16S_1516_RECYCL	rRNA Modification	[illegible]
1	7	MeT_16S_527_RECYCL	rRNA Modification	[illegible]
1	8	MeT_16S_966_RECYCL	rRNA Modification	[illegible]
1	9	MeT_23S_1618_RECYCL	rRNA Modification	[illegible]
1	10	MeT_23S_1835_RECYCL	rRNA Modification	[illegible]
1	11	MeT_23S_1962_RECYCL	rRNA Modification	[illegible]
1	12	MeT_23S_2030_RECYCL	rRNA Modification	[illegible]
1	13	MeT_23S_2069_RECYCL	rRNA Modification	[illegible]
1	14	MeT_23S_2445_RECYCL	rRNA Modification	[illegible]
1	15	MeT_23S_2498_RECYCL	rRNA Modification	[illegible]
1	16	MeT_23S_2503_RECYCL	rRNA Modification	[illegible]
1	17	MeT_L16_RECYCL	Ribosomal protein modification	[illegible]
1	18	MeT_L33_RECYCL	Ribosomal protein modification	[illegible]
1	19	MeT_L7/L12_RECYCL	Ribosomal protein modification	[illegible]
1	20	MeT_S11_RECYCL	Ribosomal protein modification	[illegible]
1	21	PrmA_mono_RECYCL	Ribosomal protein modification	[illegible]
1	22	PrmB_mono_RECYCL	Protein Modification	[illegible]
1	23	RbfA_mono_RECYCL	Ribosomal protein assembly	[illegible]
1	24	RimI_mono_RECYCL	Protein Modification	[illegible]
1	25	RimJ_mono_RECYCL	Protein Modification	[illegible]
1	26	RimK_mono_RECYCL	Protein Modification	[illegible]
1	27	RimL_mono_RECYCL	Protein Modification	[illegible]
1	28	RimM_mono_RECYCL	Ribosomal protein assembly	[illegible]
1	29	RlmB_dim_RECYCL	rRNA Modification	[illegible]
1	30	RluB_mono_RECYCL	rRNA Modification	[illegible]
1	31	RluC_mono_RECYCL	rRNA Modification	[illegible]
1	32	RluD_mono_RECYCL	rRNA Modification	[illegible]
1	33	RrmA_dim_RECYCL	rRNA Modification	[illegible]
1	34	RrmJ_mono_RECYCL	rRNA Modification	[illegible]
1	35	RsmB_mono_RECYCL	rRNA Modification	[illegible]
1	36	RsmC_mono_RECYCL	rRNA Modification	[illegible]
1	37	RsuA_mono_RECYCL	rRNA Modification	[illegible]
1	38	RumA_mono_RECYCL	rRNA Modification	[illegible]
1	39	RumB_mono_RECYCL	rRNA Modification	[illegible]
1	40	YggJ_mono_RECYCL	rRNA Modification	[illegible]
1	41	YjbC_mono_RECYCL	rRNA Modification	[illegible]
1	42	YmfC_mono_RECYCL	rRNA Modification	[illegible]
1	43	YrdC_mono_RECYCL	Ribosomal protein assembly	[illegible]
2	44	Def_mono_RECYCL	Protein Maturation	[illegible]
2	45	Fmt_mono_RECYCL	tRNA charging	[illegible]
2	46	IF1_RECYCL	Translation	[illegible]
2	47	IF3_RECYCL	Translation	[illegible]
2	48	RF3_mono.GDP_RECYCL	Translation	[illegible]
2	49	Rrf_mono_RECYCL	Translation	[illegible]
2	50	rib_30_ini_RECYCL	Translation	[illegible]
2	51	rib_50_RECYCL	Translation	[illegible]
2	52	IF2_RECHARG	Translation	[illegible]
3	53	GreA_mono_RECYCL	Transcription	[illegible]
3	54	GreB_mono_RECYCL	Transcription	[illegible]
3	55	Mfd_mono_RECYCL	Transcription	[illegible]
3	56	NusA_mono_RECYCL	Transcription	[illegible]
3	57	NusG_mono_RECYCL	Transcription	[illegible]
3	58	RpoZ_mono_RECYCL	Transcription	[illegible]
3	59	hRNAP_RECYCL	Transcription	[illegible]
4	60	RNase_G_dim_RECYCL	RNA Processing	[illegible]
4	61	RNase_T_dim_RECYCL	RNA Processing	[illegible]
4	62	RNase_m16_RECYCL	rRNA Modification	[illegible]
4	63	RNase_m23_RECYCL	rRNA Modification	[illegible]
4	64	RNase_m5_RECYCL	rRNA Modification	[illegible]
5	65	TrmU_mono_RECYCL	tRNA Modification	[illegible]
5	66	YccK_mono_RECYCL	tRNA Modification	[illegible]
5	67	YheLMN_cplx_RECYCL	tRNA Modification	[illegible]
5	68	YhhP_mono_RECYCL	tRNA Modification	[illegible]
6	69	EoR_tRNA_pos34_Q_RECYCL	tRNA Modification	[illegible]
6	70	QueA_mono_RECYCL	tRNA Modification	[illegible]
6	71	QueF_dim_RECYCL	tRNA Modification	[illegible]
6	72	Tgt_hexa_RECYCL	tRNA Modification	[illegible]
7	73	RNase_E_tetra_RECYCL	RNA Processing	[illegible]
7	74	RNase_Gen_RECYCL	RNA Processing	[illegible]
7	75	RNase_P_cplx_RECYCL	RNA Processing	[illegible]
8	76	HyL_tRNA_pos_34_ho5U_RECYCL	tRNA Modification	[illegible]
8	77	YecO_mono_RECYCL	tRNA Modification	[illegible]
8	78	YecP_mono_RECYCL	tRNA Modification	[illegible]
9	79	GidA_mono_RECYCL	tRNA Modification	[illegible]
9	80	TrmC_mono_RECYCL	tRNA Modification	[illegible]
9	81	TrmE_dim_RECYCL	tRNA Modification	[illegible]
10	82	cis/transGroES_CONV	Protein Folding	[illegible]
10	83	GroEL.(7)ADP.cisGroES_CONV	Protein Folding	[illegible]
11	84	Orn_dim_RECYCL	mRNA degradation	[illegible]
11	85	degradosome_RECYCL	mRNA degradation	[illegible]
12	86	EF-Ts_RECYCL	Translation	[illegible]
12	87	EF-G_RECHARG	Translation	[illegible]
13	88	DnaJ_dim_RECYCL	Protein Folding	[illegible]
13	89	GrpE_dim_RECYCL	Protein Folding	[illegible]
14	90	AcT_EF-TU_RECYCL	Protein Modification	[illegible]
14	91	MeT_EF-TU_RECYCL	Protein Modification	[illegible]
15	92	Era_RECHARGE	Ribosomal protein assembly	[illegible]
16	93	YqcB_mono_RECYCL	tRNA Modification	[illegible]
17	94	YggH_mono_RECYCL	tRNA Modification	[illegible]
18	95	YdaO_mono_RECYCL	tRNA Modification	[illegible]
19	96	YbbB_dim_RECYCL	tRNA Modification	[illegible]
20	97	ValS_mono_RECYCL	tRNA charging	[illegible]
21	98	Up_tRNA_pos_37_t6A_RECYCL	tRNA Modification	[illegible]
22	99	Tyr_RS_dim_RECYCL	tRNA charging	[illegible]
23	100	TruD_mono_RECYCL	tRNA Modification	[illegible]
24	101	TruB_mono_RECYCL	tRNA Modification	[illegible]
25	102	TruA_dim_RECYCL	tRNA Modification	[illegible]
26	103	Trp_RS_dim_RECYCL	tRNA charging	[illegible]
27	104	TrmH_dim_RECYCL	tRNA Modification	[illegible]
28	105	TrmD_dim_RECYCL	tRNA Modification	[illegible]
29	106	TrmA_mono_RECYCL	tRNA Modification	[illegible]
30	107	TilS_mono_RECYCL	tRNA Modification	[illegible]
31	108	Thr_RS_dim_RECYCL	tRNA charging	[illegible]
32	109	ThiI_mono_RECYCL	tRNA Modification	[illegible]
33	110	TadA_dim_RECYCL	tRNA Modification	[illegible]
34	111	Ser_RS_dim_RECYCL	tRNA charging	[illegible]
35	112	SelA_deca_RECYCL	tRNA charging	[illegible]
36	113	RpoS_mono_RECYCL	Transcription	[illegible]
37	114	RpoN_mono_RECYCL	Transcription	[illegible]
38	115	RpoH_mono_RECYCL	Transcription	[illegible]
39	116	RpoE_mono_RECYCL	Transcription	[illegible]
40	117	RpoD_mono_RECYCL	Transcription	[illegible]
41	118	RluA_mono_RECYCL	rRNA Modification	[illegible]
42	119	Rho_hexa_RECYCL	Transcription	[illegible]
43	120	RNase_III_dim_RECYCL	RNA Processing	[illegible]
44	121	RF2_mono_RECYCL	Translation	[illegible]
45	122	RF1_mono_RECYCL	Translation	[illegible]
46	123	Pro_RS_dim_RECYCL	tRNA charging	[illegible]
47	124	PrmC_mono_RECYCL	Protein Modification	[illegible]
48	125	Phe_RS_tetra_RECYCL	tRNA charging	[illegible]
49	126	NusB_mono_RECYCL	Transcription	[illegible]
50	127	MiaB_mono_RECYCL	tRNA Modification	[illegible]
51	128	MiaA_dim_RECYCL	tRNA Modification	[illegible]
52	129	Met_RS_dim_RECYCL	tRNA charging	[illegible]
53	130	MeT_tRNA_pos_37_m6t6A_RECYCL	tRNA Modification	[illegible]
54	131	MeT_tRNA_pos_37_m6A_RECYCL	tRNA Modification	[illegible]
55	132	MeT_tRNA_pos_37_m2A_RECYCL	tRNA Modification	[illegible]
56	133	MeT_tRNA_pos_32_Um_RECYCL	tRNA Modification	[illegible]
57	134	MeT_tRNA_pos_32_Cm_RECYCL	tRNA Modification	[illegible]
58	135	Map_mono_RECYCL	Protein Maturation	[illegible]
59	136	LysI_RS_dim_RECYCL	tRNA charging	[illegible]
60	137	LysII_RS_dim_RECYCL	tRNA charging	[illegible]
61	138	LeuS_mono_RECYCL	tRNA charging	[illegible]
62	139	IleS_mono_RECYCL	tRNA charging	[illegible]
63	140	His_RS_dim_RECYCL	tRNA charging	[illegible]
64	141	Gly_RS_tetra_RECYCL	tRNA charging	[illegible]
65	142	GltX_mono_RECYCL	tRNA charging	[illegible]
66	143	GlnS_mono_RECYCL	tRNA charging	[illegible]
67	144	Dus_gen_RECYCL	tRNA Modification	[illegible]
68	145	Dtd_dim_RECYCL	tRNA charging	[illegible]
69	146	DnaK_mono.ATP_RECYCL	Protein Folding	[illegible]
70	147	CysS_mono_RECYCL	tRNA charging	[illegible]
71	148	Asp_RS_dim_RECYCL	tRNA charging	[illegible]
72	149	Asn_RS_dim_RECYCL	tRNA charging	[illegible]
73	150	ArgS_mono_RECYCL	tRNA charging	[illegible]
74	151	Ala_RS_tetra_RECYCL	tRNA charging	[illegible]
75	152	AcpT_tRNA_pos_47_acp3U_RECYCL	tRNA Modification	[illegible]
76	153	AcT_tRNA_pos_34_ac4C_RECYCL	tRNA Modification	[illegible]
