# Supplementary material for: Genome-Scale Reconstruction of Escherichia coli's Transcriptional and Translational Machinery: A Knowledge Base, Its Mathematical Formulation, and Its Functional Characterization
Source: PLoS Comput Biol. 2009 Mar 13;5(3):e1000312. doi: 10.1371/journal.pcbi.1000312 (PMC2648898; doi:10.1371/journal.pcbi.1000312)
Supplement: Table S15 — Template reactions (0.74 MB DOC) [file pcbi.1000312.s017.doc]

Thiele et al.: ‘Genome-scale reconstruction of *E. coli*'s transcriptional and translational machinery: A knowledge-base and its mathematical formulation’.

S15: Template reactions

| **Reaction Abbreviation Template** | **Reaction Name Template** | **Reaction Template** | **Directionality** | **Subsystem** | **Comments** | **References** |
| --- | --- | --- | --- | --- | --- | --- |
| TUxxx_cleavage_cplx | cleavage complex formation of mRNA | 1 TUxxx_mRNA + 1 **RNase_III_dim** --> 1 TUxxx_mRNA_cleav_cplx | reversible | Cleavage polycistronic mRNA | Besides Rnase III, Rnase P was reported to be responsible for cleavage of polycistronic mRNAs. However, in order to reduce the number of total network reactions only Rnase III was considered for cleavage in the reconstruction. | [1-3] |
| TUxxx_cleavage | cleavage of mRNA | 1 TUxxx_mRNA_cleav_cplx + h2o --> 1 xxx_mRNA + 1 RNase_III_dim_inact + amp + cmp + gmp + ump + h | irreversible | Cleavage polycistronic mRNA | Besides Rnase III, Rnase P was reported to be responsible for cleavage of polycistronic mRNAs. However, in order to reduce the number of total network reactions only Rnase III was considered for cleavage in the reconstruction. | [1-3] |
| xxx_ion_BIND | Binding of ions to | 1 xxx_m + ion --> 1 xxx_m_ions | reversible | Metallo-ion Binding | It was proposed that the binding of metallo-ions occurs prior to protein folding [4]. | [4] |
| xxx_mRNA_degr1 | Degradation of mRNA I | 1 xxx_mRNA_2 + 1 **degradosome** + 1 **Orn_dim** + atp + h2o --> 1 xxx_mRNA_2_degr + adp + pi + h | reversible | mRNA degradation | This reaction accounts for the energy necessary to unwinde double strand (ds) mRNA. Gralla and DiLisa reported that in randomly generated mRNA about 50% of the nucleotides are involved in base pairing. This the unwinding of ds mRNA has to take place prior to degradation [5]. Since no mRNA specific data for base-pairing are available we assumed that 50% of nucleotides of each mRNA are involved in base-pairing. PNPase is a pi-dependent ribonuclease (phosphorylase) [6]. However, since it is difficult to assess the amount of pi needed for the degradation action, it was not accounted for in the mRNA degradation reaction. It was assumed that the degradosome composition is the same for all mRNA transcripts. | [5-17] |
| xxx_mRNA_degr2 | Degradation of mRNA II | 1 xxx_mRNA_2_degr + h2o --> 1 **degradosome_inact** + 1 **Orn_dim_inact** + 1 atp + amp + cmp + gmp + ump + h | irreversible | mRNA degradation | This reaction accounts for the energy necessary to unwinde double strand (ds) mRNA. Gralla and DiLisa reported that in randomly generated mRNA about 50% of the nucleotides are involved in base pairing. This the unwinding of ds mRNA has to take place prior to degradation [5]. Since no mRNA specific data for base-pairing are available we assumed that 50% of nucleotides of each mRNA are involved in base-pairing. PNPase is a pi-dependent ribonuclease (phosphorylase) [6]. However, since it is difficult to assess the amount of pi needed for the degradation action, it was not accounted for in the mRNA degradation reaction. It was assumed that the degradosome composition is the same for all mRNA transcripts. | [5-17] |
| xxx_fold_spon | xxx_m folding: spontanous | 1 xxx_m(_ions) --> 1 xxx_mono | irreversible | Protein Folding | 50S ribosomal subunit is associated with tigger factor (tig, b0436) in all translational reactions. Hence, spontaneous protein folding means here that the action of the trigger factor is sufficient. In the E. coli cytosol, **nascent polypeptides interact first with trigger factor** [18-20], that **binds** to the ribosome **at proteins L23/L29** near the polypeptide exit site [21,22]. see also reaction ’Rib_50_ass3’ for more information | [18-22] |
| xxx_fold_KJE_1 | xxx_m folding: KJE mediated | 1 xxx_m(_ions) + 1 **DnaK_mono.ATP** + 1 **DnaJ_dim** --> 1 xxx_m_DnaKJ_complex | reversible | Protein Folding | DnaK/J-GrpE dependent folding reactions were based on Fig. 3 in Hartl and Hartl [23]. | [23,24] |
| xxx_fold_KJE_2 | xxx_m folding: KJE mediated | 1 xxx_m_DnaKJ_complex + 1 **GrpE_dim** + 1 h2o --> 1 xxx_DnaK_GrpE_complex + 1 pi + 1 h + 1 **DnaJ_dim_inact** + 1 adp | irreversible | Protein Folding | DnaK and GrpE have a stoichiometry of 1:2 [25,26]. DnaK/J-GrpE dependent folding reactions were based on Fig. 3 in Hartl and Hartl [23]. | [23-27] |
| xxx_fold_KJE_3 | xxx_m folding: KJE mediated | 1 xxx_DnaK_GrpE_complex + 1 atp --> 1 xxx_mono + 1 **DnaK_mono.ATP_inact** + 1 **GrpE_dim_inact** | irreversible | Protein Folding | DnaK/J-GrpE dependent folding reactions were based on Fig. 3 in Hartl and Hartl [23]. | [23,24,27] |
| xxx_fold_GroEL/ES_1 | xxx_m folding: GroEL/ES mediated; polypeptide is going in GroEL/ES complex | 1 xxx_m(_ions) + 1 **GroEL.(7)ADP.cisGroES** + 1 **transGroES_hepta** + 7 atp --> 1 xxx_m_GroEL.(7)ATP.transGroES + 1 **cisGroES_hepta** + 7 adp | irreversible | Protein Folding | In general, a polypeptide has to go through nultiple rounds of GroEL/ES folding until it reaches its final conformation. For this, it is released and recaptured b GroEL/E [23,28]. The folding requires about 100 ATP for Rhodanse and DHFR [29]. Since the amount of required ATP might differ from protein to protein we decided to account only for the cost of 1 round of folding. Hartl and Hayer-Hartl provide a very clear and comprehensive reaction mechanism in their review [23]. | [23,28-32] |
| xxx_fold_GroEL/ES_2 | xxx_m folding: GroEL/ES mediated; folding of polypeptide under ATP hydrolysis | xxx_m_GroEL.(7)ATP.transGroES + 7 h2o --> 1 xxx_GroEL.(7)ADP.transGroES + 7 h + 7 pi | irreversible | Protein Folding | In general, a polypeptide has to go through nultiple rounds of GroEL/ES folding until it reaches its final conformation. For this, it is released and recaptured b GroEL/E ([23,28]).The folding requires about 100 ATP for Rhodanse and DHFR [29]. Since the amount of required ATP might differ from protein to protein we decided to account only for the cost of 1 round of folding. Hartl and Hayer-Hartl provide a very clear and comprehensive reaction mechanism in their review [23]. | [23,28-32] |
| xxx_fold_GroEL/ES_3 | xxx_m folding: GroEL/ES mediated; release of native protein | 1 xxx_GroEL.(7)ADP.transGroES --> 1 xxx_mono + 1 **GroEL.(7)ADP.transGroES** | irreversible | Protein Folding. | In general, a polypeptide has to go through nultiple rounds of GroEL/ES folding until it reaches its final conformation. For this, it is released and recaptured b GroEL/E ([23,28]). The folding requires about 100 ATP for Rhodanse and DHFR [29]. Since the amount of required ATP might differ from protein to protein we decided to account only for the cost of 1 round of folding. Hartl and Hayer-Hartl provide a very clear and comprehensive reaction mechanism in their review [23]. | [23,28,30-32] |
| xxx_maturation1_A | polypeptide peptide deformylase complex | 1 xxx_aa + 1 **Def_mono** --> 1 xxx_def_cplx | reversible | Protein Maturation |  |  |
| xxx_maturation2_A | xxx formation | 1 xxx_def_cplx + 1 h2o --> 1 xxx_m + 1 **Def_mono_inact** + 1 for | irreversible | Protein Maturation |  |  |
| xxx_maturation1_B | polypeptide peptide deformylase and methionine aminopeptidase complex | 1 xxx_aa + 1 **Def_mono** + 1 **Map_mono** --> 1 xxx_def_map_cplx | reversible | Protein Maturation |  | [33] |
| xxx_maturation2_B | xxx formation | 1 xxx_def_map_cplx + h2o --> 1 xxx_m + 1 **Map_mono_inact** + 1 met-L + 1 **Def_mono_inact** + 1 for | irreversible | Protein Maturation |  | [33] |
| TUxxx_trimming_cplx | cutting complex formation of stable RNA | 1 TUxxx_RNA + 1 **RNase_Gen** + 1 **RNase_E_tetra** + 1 **RNase_P_cplx** --> 1 TUxxx_RNA_cut_cplx | reversible | RNA Processing | For monocistronic tRNA operons; Rnase_Gen accounts for alternatively the following Rnases: RNase II, RNase D, RNase RNase BN, RNase T, RNase PH. Based on observation of [34] and [35] one RNase is sufficient for the action | [34-46] |
| TUxxx_trimming | cutting of stable RNA $cut_tmp | 1 TUxxx_RNA_cut_cplx + h2o --> 1 xxx_RNA + amp + cmp + gmp + ump + h + 1 ppi + 1 **RNase_Gen_inact** + 1 **RNase_E_tetra_inact** + 1 **RNase_P_cplx_inact** | irreversible | RNA Processing | For monocistronic tRNA operons; Rnase_Gen accounts for alternatively the following Rnases: RNase II, RNase D, RNase RNase BN, RNase T, RNase PH. Based on observation of [34] and [35] one RNase is sufficient for the action | [34-46] |
| TUxxx_cutting_cplx | cutting complex formation of stable RNA | 1 TUxxx_RNA + 1 **RNase_III_dim** + 1 **RNase_m16** + 1 **RNase_G_dim** + 1 **RNase_P_cplx** + 1 **RNase_Gen** + 1 **RNase_m23** + 1 **Rnase_T_dim** + 1 **RNase_m5** + 1 **RNase_E_tetra -**-> 1 TUxxx_RNA_cut_cplx | reversible | RNA processing | Multicistronic stable RNA operon (which include tRNAs and rRNAs): Rnase_Gen accounts for alternatively the following Rnases: RNase II, RNase D, RNase RNase BN, RNase T, RNase PH. Based on observation of [34] and [35] that one RNase is sufficient for the action. Neidhart et al. report the existence of RNase_m23, RNase_m16, RNase_m5 necessary for the trimming of rRNA, however, the corresponding genes are unknown [47]. The *E. coli* gene for RNase PH (b3643) is a pseudogene based on the revised *E. coli* annotation [48]. Although the RNase_gen is also connected to RNase_PH no gene is associated with this protein. Furthermore, although some literature is reporting the RNase PH, no information is available for *E. coli* K12. | [34-51] |
| TUxxx_cutting | cutting of stable RNA | 1 TUxxx_RNA_cut_cplx + h2o --> 1 xxx_RNA + 1 **RNase_III_dim_inact** + 1 **RNase_m16_inact** + 1 **RNase_G_dim_inact** + 1 **RNase_P_cplx_inact** + 1 **RNase_Gen_inact** + 1 **RNase_m23_inact + 1 Rnase_T_dim_inact** + 1 **RNase_m5_inact** + 1 **RNase_E_tetra_inact** + amp + cmp + gmp + ump + h + 1 ppi | irreversible | RNA processing | Multicistronic stable RNA operon (which include tRNAs and rRNAs): Rnase_Gen accounts for alternatively the following Rnases: RNase II, RNase D, RNase RNase BN, RNase T, RNase PH. Based on observation of [34] and [35] that one RNase is sufficient for the action. Neidhart et al. report the existence of RNase_m23, RNase_m16, RNase_m5 necessary for the trimming of rRNA, however, the corresponding genes are unknown [47]. The *E. coli* gene for RNase PH (b3643) is a pseudogene based on the revised *E. coli* annotation [48]. Although the RNase_gen is also connected to RNase_PH no gene is associated with this protein. Furthermore, although some literature is reporting the RNase PH, no information is available for *E. coli* K12. | [34-51] |
| tscr_ini_TUxxx | Transcription initiation of (rho independent) | 1 TUxxx_DNA_act + 1 **RNAP_sigma_x** + atp + ctp + gtp + utp --> 1 TUxxx_transcr_ini_x_cplx + 1 **sigma_x_inact** + ppi | reversible | Transcription | The sigma factor is release when the 16th nucleotide is added to the nascent mRNA [52,53]. | [52-60] |
| tscr_elo__TUxxx _ini | formation complex for elongation of (rho independent) | 1 TUxxx_transcr_ini_x_cplx + 1 **RpoZ_mono** + 1 **NusA_mono** + 1 NusG_mono + 1 **Mfd_mono** + 1 **GreA_mono** + 1 **GreB_mono** --> 1 TUxxx_transcr_elo_x_cplx | reversible | Transcription | GreA and GreB are required for efficient transcription [61-64]. NusA increases the duration of pausing of RNAP at pausing sites, but also protects nascent mRNA from cleavage[65,66]. Mfd (transcription repair factor) reactivates or recycles stalled or arrested RNAPs during elongation. Its action is atp-dependent which was not explicitly modeled [67-69]. | [54-56,61-73] |
| tscr_elo_term_TUxxx | Transcription elongation and termination of (rho independent) | 1 TUxxx_transcr_elo_x_cplx + atp + ctp + gtp + utp --> 1 TUxxx_DNA_neu + 1 TUxxx_mRNA + 1 **hRNAP_inact** + ppi + 1 **NusA_mono_inact** + 1 **NusG_mono_inact** + 1 **Mfd_mono_inact** + 1 **GreA_mono_inact** + 1 **GreB_mono_inact** + 1 **RpoZ_mono_inact** | irreversible | Transcription | GreA and GreB are required for efficient transcription [61-64]. NusA increases the duration of pausing of RNAP at pausing sites, but also protects nascent mRNA from cleavage[65,66]. Mfd (transcription repair factor) reactivates or recycles stalled or arrested RNAPs during elongation. Its action is atp-dependent which was not explicitly modeled [67-69]. | [54-56,61-74] |
| tscr_ini_TUxxx _stab | Transcription initiation of | 1 TUxxx_DNA_act + 1 **RNAP_sigma_x** + atp + ctp + gtp + utp --> 1 TUxxx_transcr_ini_x_cplx + 1 **sigma_x_inact** + ppi | reversible | Transcription | The sigma factor is release when the 16th nucleotide is added to the nascent mRNA [52,53]. | [52-60] |
| tscr_elo_TUxxx_ini_stab | formation complex for elongation of | 1 TUxxx_transcr_ini_x_cplx + 1 **RpoZ_mono** + 1 **NusA_mono** + 1 **NusG_mono** + 1 **GreA_mono** + 1 **GreB_mono** + 1 **Mfd_mono** + 1 **RpsJ_mono** + 1 **RpsD_mono_sprm** + 1 **RplC_mono_me_sprm** + 1 **RplD_mono** + 1 **RplM_mono** + 1 **NusB_mono** + 1 **Rho_hexa** --> 1 TUxxx_transcr_elo_x_cplx | reversible | Transcription | GreA and GreB are required for efficient transcription [61-64]. Transcription of stable RNA requires NusA, NusB, NusG, S10, S4, L3,L4,L13 [75]; NusG allows Rho-dependent termination [66]. NusB is required for antitermination in rrn operon as well as NusA, NusG, RpsJ( NusE, S10) and 1 unknown factor. NusB and RpsJ (NusE, S10) bind directly to boxA. NusA increases the duration of pausing of RNAP at pausing sites, but also protects nascent mRNA from cleavage [65,66]. For rho-dependent antitermination : NusA may help to bind ribosome on nascent mRNA [66]. NusA is non-essential in rho- mutant. mfd = transcription repair factor reactivates or recycles stalled or arrested RNAPs during elongation ; action is atp-dep. (References herein: [67-69]. It was assumed that 3 ATP are consumed for the binding of RNA to Rho factor | [54-56,61-73,75-85] |
| tscr_elo_term_TUxxx_stab | Transcription elongation and termination of | 1 TUxxx_transcr_elo_x_cplx + 3 h2o + atp + ctp + gtp + utp --> 1 TUxxx_DNA_neu + 1 TUxxx_RNA + 1 **hRNAP_inact** + ppi + **1 NusA_mono_inact** + 1 **NusG_mono_inact** + 1 **GreA_mono_inact** + 1 **GreB_mono_inact** + 1 **RpoZ_mono_inact** + 1 **Mfd_mono_inact** + 1 **RpsJ_mono_inact** + 1 **RpsD_mono_sprm_inact** + 1 **RplC_mono_me_sprm_inact** + 1 **RplD_mono_inact** + 1 **RplM_mono_inact** + 1 **NusB_mono_inact** + 1 **Rho_hexa_inact** | irreversible | Transcription | GreA and GreB are required for efficient transcription [61-64]. Transcription of stable RNA requires NusA, NusB, NusG, S10, S4, L3,L4,L13 [75]. NusG allows Rho-dependent termination [66]. NusB is required for antitermination in rrn operon as well as NusA, NusG, RpsJ( NusE, S10) and 1 unknown factor. NusB and RpsJ (NusE, S10) bind directly to boxA. NusA increases the duration of pausing of RNAP at pausing sites, but also protects nascent mRNA from cleavage [65,66]. For rho-dependent antitermination : NusA may help to bind ribosome on nascent mRNA [66]. NusA is non-essential in rho- mutant. mfd = transcription repair factor reactivates or recycles stalled or arrested RNAPs during elongation ; action is atp-dep. (Refs herein: [67-69]. It was assumed that 3 ATP are consumed for the binding of RNA to Rho factor | [54-56,61-85] |
| TUxxx_DNA_act_bind | DNA binding of activator | 1 TUxxx_DNA_neu --> 1 TUxxx_DNA_act | irreversible | Transcription Regulation | This reaction was included for modeling reason. It will enable the incorporation of regulatory reactions (rules) in subsequent models. |  |
| tl_ini_xxx_x_rib | Translation initiation x ribosome(s) bound | **rib_30_ini** + 1 xxx_mRNA_1 + **fmet_tRNA_met** + **rib_50** + h2o --> 1 xxx_rib_ini_x + **IF1** + **IF2-GDP** + **IF3** +pi + h | irreversible | Translation | Beside the codon atg, ttg and gtg are also translated as fmet [86] | [54,86-92] |
| tl_elo_ xxx_x_rib1 | Translation elongation 1 x ribosome(s) | 1 xxx_rib_ini_x + mg2 + **EF-Tu.GTP.trna + EF-G.GTP** --> 1 xxx_rib_70_elo1_x_cplx | irreversible | Translation | 5 Mg2+ are required per EF-Tu.GTP.aa-tRNA for ribosome binding [93] | [54,87,89,93-99] |
| tl_elo_ xxx_x_rib2 | Translation elongation 2 x ribosome(s) | 1 xxx_rib_70_elo1_x_cplx + h2o --> 1 xxx_rib_70_elo2_x_cplx + **fmet_tRNA** + mg2 + pi + h + **EF-Tu.GDP** + EF-G.GDP + trna | irreversible | Translation |  | [54,94,95,97] |
| tl_term_ xxx_x_rib1 | Translation termination 1 x ribosome(s) | 1 xxx_rib_70_elo2_x_cplx + **RF3_mono.GDP** + **Rrf_mono** + **RF(1 or 2)** --> 1 xxx_tl_term_x_rib_cplx | reversible | Translation |  | [54,94,100-104] |
| tl_term_ xxx_x_rib2 | Translation termination 2 x ribosome(s) | 1 xxx_tl_term_x_rib_cplx2 + gtp + h2o --> **rib_70** + EF-Tu.GDP + 1 xxx_mRNA_2 + xxx_aa + gdp + h + pi + **RF(1 or 2)_inact** + **RF3_mono.GDP_inact** + **Rrf_mono_inact** + **EF-G.GDP** + trna | irreversible | Translation | For simplicity the 70S ribosome is release from this reaction (free from nascent polypeptide). The 70S ribosome reacts than in the following with IF3 and IF1 which leads to the release of the 50 S subunit. The ribosome release factor (rrf) forms a stable complex with 70S ribosome. The action of EF-G release rrf from 70S ribosome, stimulated by GTP [103]. | [54,94,100-104] |
| xxx_iron_sulfur_BIND1 | Incorporation of [4Fe-4S]2+ in xxx | 1 xxx_m + 1 IscU_dim_[4Fe-4S] --> 1 xxx_m_IscU_cplx | reversible | Iron-sulfur cluster incorporation | IscU_dim_[4Fe-4S] is used to transfer [4Fe-4S] to iron-sulfur cluster proteins | [105-110] |
| xxx_iron_sulfur_BIND2 | Incorporation of [4Fe-4S]2+ in xxx | 1 xxx_m_IscU_cplx --> 1 xxx_m_FeS + 2 IscU_mono | reversible | Iron-sulfur cluster incorporation | IscU_dim_[4Fe-4S] is used to transfer [4Fe-4S] to iron-sulfur cluster proteins | [105-110] |
| xxx_mRNA_CONV | convsersion of mRNA to mRNA_1 (synthetic rxn) | 1 xxx_mRNA --> 1 xxx_mRNA_1 | irreversible | Translation | Reaction was included for modeling reason. Many transcripts are encoded by multiple transcription units. This reaction joins the various mRNA transcripts to one mRNA specie which is than translated in subsequent reactions. |  |
| xxx_mRNA_CONV_2 | convsersion of mRNA_2 to mRNA_1 (synthetic rxn) | 1 xxx_mRNA_2 --> 1 xxx_mRNA_1 | irreversible |  | Reaction was included for modeling reason. While the synthesis rate of mRNA is dependent on the degradation rate and thus the experimentally determined mRNA concentration and mRNA half-life time (v_synth_=v_degr_=[mRNA]*ln2/T_1/2_) there is steady-states concentration of each mRNA species that can be used for transcription. The mRNA synthesis reaction only maintain this steady-state pool. These reactions are currently constraint by [mRNA]*T_d_*_relo_/(L_mRNA_/3), where T_d_ is the doubling time (s), L_mRNA_ is the mRNA length, and r_elo_ is the translation elongation rate at T_d_ |  |
| X_RECYCL | recycling of protein factor X | 1 X_inact 🡪 1 X | Irreversible | Protein Recycling | Reactions were included for modeling reasons. They represent the utilization of protein factors within the network. |  |

1. Regnier P, Grunberg-Manago M (1989) Cleavage by RNase III in the transcripts of the met Y-nus-A-infB operon of Escherichia coli releases the tRNA and initiates the decay of the downstream mRNA. J Mol Biol 210: 293-302.

2. Li Y, Altman S (2003) A specific endoribonuclease, RNase P, affects gene expression of polycistronic operon mRNAs. Proc Natl Acad Sci U S A 100: 13213-13218.

3. Perry RP (1976) Processing of RNA. Annu Rev Biochem 45: 605-629.

4. Wilson CJ, Apiyo D, Wittung-Stafshede P (2004) Role of cofactors in metalloprotein folding. Q Rev Biophys 37: 285-314.

5. Gralla J, DeLisi C (1974) mRNA is expected to form stable secondary structures. Nature 248: 330-332.

6. Symmons MF, Williams MG, Luisi BF, Jones GH, Carpousis AJ (2002) Running rings around RNA: a superfamily of phosphate-dependent RNases. Trends Biochem Sci 27: 11-18.

7. Carpousis AJ (2002) The Escherichia coli RNA degradosome: structure, function and relationship in other ribonucleolytic multienzyme complexes. Biochem Soc Trans 30: 150-155.

8. Vanzo NF, Li YS, Py B, Blum E, Higgins CF, et al. (1998) Ribonuclease E organizes the protein interactions in the Escherichia coli RNA degradosome. Genes Dev 12: 2770-2781.

9. Bernstein JA, Lin PH, Cohen SN, Lin-Chao S (2004) Global analysis of Escherichia coli RNA degradosome function using DNA microarrays. Proc Natl Acad Sci U S A 101: 2758-2763.

10. Coburn GA, Miao X, Briant DJ, Mackie GA (1999) Reconstitution of a minimal RNA degradosome demonstrates functional coordination between a 3' exonuclease and a DEAD-box RNA helicase. Genes Dev 13: 2594-2603.

11. Kushner SR (2004) mRNA decay in prokaryotes and eukaryotes: different approaches to a similar problem. IUBMB Life 56: 585-594.

12. Mackie GA (1998) Ribonuclease E is a 5'-end-dependent endonuclease. Nature 395: 720-723.

13. Py B, Higgins CF, Krisch HM, Carpousis AJ (1996) A DEAD-box RNA helicase in the Escherichia coli RNA degradosome. Nature 381: 169-172.

14. Jarrige A, Brechemier-Baey D, Mathy N, Duche O, Portier C (2002) Mutational analysis of polynucleotide phosphorylase from Escherichia coli. J Mol Biol 321: 397-409.

15. Deutscher MP (2006) Degradation of RNA in bacteria: comparison of mRNA and stable RNA. Nucleic Acids Res 34: 659-666.

16. Ghosh S, Deutscher MP (1999) Oligoribonuclease is an essential component of the mRNA decay pathway. Proc Natl Acad Sci U S A 96: 4372-4377.

17. Jain C (2002) Degradation of mRNA in Escherichia coli. IUBMB Life 54: 315-321.

18. Deuerling E, Schulze-Specking A, Tomoyasu T, Mogk A, Bukau B (1999) Trigger factor and DnaK cooperate in folding of newly synthesized proteins. Nature 400: 693-696.

19. Hesterkamp T, Hauser S, Lutcke H, Bukau B (1996) Escherichia coli trigger factor is a prolyl isomerase that associates with nascent polypeptide chains. Proc Natl Acad Sci U S A 93: 4437-4441.

20. Teter SA, Houry WA, Ang D, Tradler T, Rockabrand D, et al. (1999) Polypeptide flux through bacterial Hsp70: DnaK cooperates with trigger factor in chaperoning nascent chains. Cell 97: 755-765.

21. Kramer G, Rauch T, Rist W, Vorderwulbecke S, Patzelt H, et al. (2002) L23 protein functions as a chaperone docking site on the ribosome. Nature 419: 171-174.

22. Lill R, Crooke E, Guthrie B, Wickner W (1988) The "trigger factor cycle" includes ribosomes, presecretory proteins, and the plasma membrane. Cell 54: 1013-1018.

23. Hartl FU, Hayer-Hartl M (2002) Molecular chaperones in the cytosol: from nascent chain to folded protein. Science 295: 1852-1858.

24. Deuerling E, Patzelt H, Vorderwulbecke S, Rauch T, Kramer G, et al. (2003) Trigger Factor and DnaK possess overlapping substrate pools and binding specificities. Mol Microbiol 47: 1317-1328.

25. Schönfeld H-J, Schmidt D, Schröder H, Bukau B (1995) The DnaK Chaperone System of Escherichia coli: Quaternary Structures and Interactions of the DnaK and GrpE Components

10.1074/jbc.270.5.2183. J Biol Chem 270: 2183-2189.

26. Harrison CJ, Hayer-Hartl M, Liberto MD, Hartl F-U, Kuriyan J (1997) Crystal Structure of the Nucleotide Exchange Factor GrpE Bound to the ATPase Domain of the Molecular Chaperone DnaK

10.1126/science.276.5311.431. Science 276: 431-435.

27. Gelinas AD, Langsetmo K, Toth J, Bethoney KA, Stafford WF, et al. (2002) A Structure-based Interpretation of E. coli GrpE Thermodynamic Properties. Journal of Molecular Biology 323: 131-142.

28. Ranson NA, Clare DK, Farr GW, Houldershaw D, Horwich AL, et al. (2006) Allosteric signaling of ATP hydrolysis in GroEL-GroES complexes. 13: 147-152.

29. Martin J, Langer T, Boteva R, Schramel A, Horwich AL, et al. (1991) Chaperonin-mediated protein folding at the surface of groEL through a 'molten globule'-like intermediate. Nature 352: 36-42.

30. Kerner MJ, Naylor DJ, Ishihama Y, Maier T, Chang HC, et al. (2005) Proteome-wide analysis of chaperonin-dependent protein folding in Escherichia coli. Cell 122: 209-220.

31. Rye HS, Roseman AM, Chen S, Furtak K, Fenton WA, et al. (1999) GroEL-GroES cycling: ATP and nonnative polypeptide direct alternation of folding-active rings. Cell 97: 325-338.

32. Falke S, Tama F, Brooks CL, 3rd, Gogol EP, Fisher MT (2005) The 13 angstroms structure of a chaperonin GroEL-protein substrate complex by cryo-electron microscopy. J Mol Biol 348: 219-230.

33. Ben-Bassat A, Bauer K, Chang SY, Myambo K, Boosman A, et al. (1987) Processing of the initiation methionine from proteins: properties of the Escherichia coli methionine aminopeptidase and its gene structure. J Bacteriol 169: 751-757.

34. Reuven NB, Deutscher MP (1993) Multiple exoribonucleases are required for the 3' processing of Escherichia coli tRNA precursors in vivo. Faseb J 7: 143-148.

35. Kelly KO, Deutscher MP (1992) The presence of only one of five exoribonucleases is sufficient to support the growth of Escherichia coli. J Bacteriol 174: 6682-6684.

36. Morl M, Marchfelder A (2001) The final cut. The importance of tRNA 3'-processing. EMBO Rep 2: 17-20.

37. Li Z, Gong X, Joshi VH, Li M (2005) Co-evolution of tRNA 3' trailer sequences with 3' processing enzymes in bacteria. Rna 11: 567-577.

38. Saito H, Watanabe K, Suga H (2001) Concurrent molecular recognition of the amino acid and tRNA by a ribozyme. Rna 7: 1867-1878.

39. Pascual A, Vioque A (1999) Substrate binding and catalysis by ribonuclease P from cyanobacteria and Escherichia coli are affected differently by the 3' terminal CCA in tRNA precursors. Proc Natl Acad Sci U S A 96: 6672-6677.

40. Frank DN, Pace NR (1998) Ribonuclease P: unity and diversity in a tRNA processing ribozyme. Annu Rev Biochem 67: 153-180.

41. Lindell M, Brannvall M, Wagner EG, Kirsebom LA (2005) Lead(II) cleavage analysis of RNase P RNA in vivo. Rna 11: 1348-1354.

42. Hsieh J, Andrews AJ, Fierke CA (2004) Roles of protein subunits in RNA-protein complexes: lessons from ribonuclease P. Biopolymers 73: 79-89.

43. Meinnel T, Blanquet S (1995) Maturation of pre-tRNA(fMet) by Escherichia coli RNase P is specified by a guanosine of the 5'-flanking sequence. J Biol Chem 270: 15908-15914.

44. Li Z, Deutscher MP (1996) Maturation pathways for E. coli tRNA precursors: a random multienzyme process in vivo. Cell 86: 503-512.

45. Ow MC, Kushner SR (2002) Initiation of tRNA maturation by RNase E is essential for cell viability in E. coli. Genes Dev 16: 1102-1115.

46. Li Z, Pandit S, Deutscher MP (1998) 3' exoribonucleolytic trimming is a common feature of the maturation of small, stable RNAs in Escherichia coli. Proc Natl Acad Sci U S A 95: 2856-2861.

47. Neidhardt FC (1996) *Escherichia coli* and *Salmonella*: cellular and molecular biology. 2nd ed. Washington, D.C.: ASM Press. pp. x to y.

48. Riley M, Abe T, Arnaud MB, Berlyn MK, Blattner FR, et al. (2006) *Escherichia coli* K-12: a cooperatively developed annotation snapshot-2005. Nucleic Acids Res 34: 1-9.

49. Briant DJ, Hankins JS, Cook MA, Mackie GA (2003) The quaternary structure of RNase G from Escherichia coli. Mol Microbiol 50: 1381-1390.

50. Li Z, Pandit S, Deutscher MP (1999) RNase G (CafA protein) and RNase E are both required for the 5' maturation of 16S ribosomal RNA. Embo J 18: 2878-2885.

51. Wachi M, Umitsuki G, Shimizu M, Takada A, Nagai K (1999) Escherichia coli cafA gene encodes a novel RNase, designated as RNase G, involved in processing of the 5' end of 16S rRNA. Biochem Biophys Res Commun 259: 483-488.

52. Husnain SI, Meng W, Busby SJ, Thomas MS (2004) Escherichia coli can tolerate insertions of up to 16 amino acids in the RNA polymerase alpha subunit inter-domain linker. Biochim Biophys Acta 1678: 47-56.

53. Nickels BE, Garrity SJ, Mekler V, Minakhin L, Severinov K, et al. (2005) The interaction between sigma70 and the beta-flap of Escherichia coli RNA polymerase inhibits extension of nascent RNA during early elongation. Proc Natl Acad Sci U S A 102: 4488-4493.

54. Neidhardt FC, editor (1996) *Escherichia coli* and *Salmonella*: cellular and molecular biology. 2nd ed. Washington, D.C.: ASM Press. 2 v. (xx, 2822 , lxxvii) p.

55. Greive SJ, von Hippel PH (2005) Thinking quantitatively about transcriptional regulation. Nat Rev Mol Cell Biol 6: 221-232.

56. Toulokhonov I, Artsimovitch I, Landick R (2001) Allosteric control of RNA polymerase by a site that contacts nascent RNA hairpins. Science 292: 730-733.

57. Murakami KS, Darst SA (2003) Bacterial RNA polymerases: the wholo story. Curr Opin Struct Biol 13: 31-39.

58. Darst SA, Opalka N, Chacon P, Polyakov A, Richter C, et al. (2002) Conformational flexibility of bacterial RNA polymerase. Proc Natl Acad Sci U S A 99: 4296-4301.

59. Hsu LM (2002) Open season on RNA polymerase. Nat Struct Biol 9: 502-504.

60. Van Wynsberghe A, Li G, Cui Q (2004) Normal-mode analysis suggests protein flexibility modulation throughout RNA polymerase's functional cycle. Biochemistry 43: 13083-13096.

61. Erie DA, Hajiseyedjavadi O, Young MC, von Hippel PH (1993) Multiple RNA polymerase conformations and GreA: control of the fidelity of transcription. Science 262: 867-873.

62. Borukhov S, Polyakov A, Nikiforov V, Goldfarb A (1992) GreA protein: a transcription elongation factor from Escherichia coli. Proc Natl Acad Sci U S A 89: 8899-8902.

63. Borukhov S, Sagitov V, Goldfarb A (1993) Transcript cleavage factors from E. coli. Cell 72: 459-466.

64. Opalka N, Chlenov M, Chacon P, Rice WJ, Wriggers W, et al. (2003) Structure and function of the transcription elongation factor GreB bound to bacterial RNA polymerase. Cell 114: 335-345.

65. Mah TF, Kuznedelov K, Mushegian A, Severinov K, Greenblatt J (2000) The alpha subunit of E. coli RNA polymerase activates RNA binding by NusA. Genes Dev 14: 2664-2675.

66. Richardson JaG, J (1996) Control of RNA chain elongation and termination. In: Neidhardt FC, editor. Escherichia coli and Salmonella typhimurium. 2nd ed. Washington, DC: American Society for Microbiology. pp. 822–848.

67. Selby CP, Sancar A (1993) Molecular mechanism of transcription-repair coupling. Science 260: 53-58.

68. Park JS, Marr MT, Roberts JW (2002) E. coli Transcription repair coupling factor (Mfd protein) rescues arrested complexes by promoting forward translocation. Cell 109: 757-767.

69. Roberts J, Park JS (2004) Mfd, the bacterial transcription repair coupling factor: translocation, repair and termination. Curr Opin Microbiol 7: 120-125.

70. Borukhov S, Lee J, Laptenko O (2005) Bacterial transcription elongation factors: new insights into molecular mechanism of action. Mol Microbiol 55: 1315-1324.

71. Smith AJ, Savery NJ (2005) RNA polymerase mutants defective in the initiation of transcription-coupled DNA repair. Nucleic Acids Res 33: 755-764.

72. Korzheva N, Mustaev A, Kozlov M, Malhotra A, Nikiforov V, et al. (2000) A structural model of transcription elongation. Science 289: 619-625.

73. Carlomagno MS, Nappo A (2003) NusA modulates intragenic termination by different pathways. Gene 308: 115-128.

74. Henkin TM (1996) Control of transcription termination in prokaryotes. Annu Rev Genet 30: 35-57.

75. Torres M, Condon C, Balada JM, Squires C, Squires CL (2001) Ribosomal protein S4 is a transcription factor with properties remarkably similar to NusA, a protein involved in both non-ribosomal and ribosomal RNA antitermination. Embo J 20: 3811-3820.

76. Seoh HK, Weech M, Zhang N, Squires CL (2003) rRNA antitermination functions with heat shock promoters. J Bacteriol 185: 6486-6489.

77. Sukhodolets MV, Garges S (2003) Interaction of Escherichia coli RNA polymerase with the ribosomal protein S1 and the Sm-like ATPase Hfq. Biochemistry 42: 8022-8034.

78. Pasman Z, von Hippel PH (2000) Regulation of rho-dependent transcription termination by NusG is specific to the Escherichia coli elongation complex. Biochemistry 39: 5573-5585.

79. Burns CM, Richardson LV, Richardson JP (1998) Combinatorial effects of NusA and NusG on transcription elongation and Rho-dependent termination in Escherichia coli. J Mol Biol 278: 307-316.

80. Torres M, Balada JM, Zellars M, Squires C, Squires CL (2004) In vivo effect of NusB and NusG on rRNA transcription antitermination. J Bacteriol 186: 1304-1310.

81. Gourse RL, Gaal T, Bartlett MS, Appleman JA, Ross W (1996) rRNA transcription and growth rate-dependent regulation of ribosome synthesis in Escherichia coli. Annu Rev Microbiol 50: 645-677.

82. Greive SJ, Lins AF, von Hippel PH (2005) Assembly of an RNA-protein complex. Binding of NusB and NusE (S10) proteins to boxA RNA nucleates the formation of the antitermination complex involved in controlling rRNA transcription in Escherichia coli. J Biol Chem 280: 36397-36408.

83. Squires CL, Greenblatt J, Li J, Condon C (1993) Ribosomal RNA antitermination in vitro: requirement for Nus factors and one or more unidentified cellular components. Proc Natl Acad Sci U S A 90: 970-974.

84. Luttgen H, Robelek R, Muhlberger R, Diercks T, Schuster SC, et al. (2002) Transcriptional regulation by antitermination. Interaction of RNA with NusB protein and NusB/NusE protein complex of Escherichia coli. J Mol Biol 316: 875-885.

85. von Hippel PH, Pasman Z (2002) Reaction pathways in transcript elongation. Biophys Chem 101-102: 401-423.

86. Tong S, Porco A, Isturiz T, Conway T (1996) Cloning and molecular genetic characterization of the Escherichia coli gntR, gntK, and gntU genes of GntI, the main system for gluconate metabolism. J Bacteriol 178: 3260-3269.

87. Wu XQ, RajBhandary UL (1997) Effect of the amino acid attached to Escherichia coli initiator tRNA on its affinity for the initiation factor IF2 and on the IF2 dependence of its binding to the ribosome. J Biol Chem 272: 1891-1895.

88. Allen GS, Zavialov A, Gursky R, Ehrenberg M, Frank J (2005) The cryo-EM structure of a translation initiation complex from Escherichia coli. Cell 121: 703-712.

89. Tomsic J, Vitali LA, Daviter T, Savelsbergh A, Spurio R, et al. (2000) Late events of translation initiation in bacteria: a kinetic analysis. Embo J 19: 2127-2136.

90. Laursen BS, Sorensen HP, Mortensen KK, Sperling-Petersen HU (2005) Initiation of protein synthesis in bacteria. Microbiol Mol Biol Rev 69: 101-123.

91. Sundari RM, Pelka H, Schulman LH (1977) Structural requirements of Escherichia coli formylmethionyl transfer ribonucleic acid for ribosome binding and initiation of protein synthesis. J Biol Chem 252: 3941-3944.

92. Wakao H, Romby P, Westhof E, Laalami S, Grunberg-Manago M, et al. (1989) The solution structure of the Escherichia coli initiator tRNA and its interactions with initiation factor 2 and the ribosomal 30 S subunit. J Biol Chem 264: 20363-20371.

93. Rodnina MV, Pape T, Fricke R, Kuhn L, Wintermeyer W (1996) Initial binding of the elongation factor Tu.GTP.aminoacyl-tRNA complex preceding codon recognition on the ribosome. J Biol Chem 271: 646-652.

94. Ramakrishnan V (2002) Ribosome structure and the mechanism of translation. Cell 108: 557-572.

95. Pape T, Wintermeyer W, Rodnina MV (1998) Complete kinetic mechanism of elongation factor Tu-dependent binding of aminoacyl-tRNA to the A site of the E. coli ribosome. Embo J 17: 7490-7497.

96. Konevega AL, Soboleva NG, Makhno VI, Semenkov YP, Wintermeyer W, et al. (2004) Purine bases at position 37 of tRNA stabilize codon-anticodon interaction in the ribosomal A site by stacking and Mg2+-dependent interactions. Rna 10: 90-101.

97. Stark H, Rodnina MV, Rinke-Appel J, Brimacombe R, Wintermeyer W, et al. (1997) Visualization of elongation factor Tu on the Escherichia coli ribosome. Nature 389: 403-406.

98. Zavialov AV, Hauryliuk VV, Ehrenberg M (2005) Guanine-nucleotide exchange on ribosome-bound elongation factor G initiates the translocation of tRNAs. J Biol 4: 9.

99. Lovgren TN, Petersson A, Loftfield RB (1978) The mechanism of aminoacylation of transfer ribonucleic acid. The role of magnesium and spermine in the synthesis of isoleucyl-tRNA. J Biol Chem 253: 6702-6710.

100. Klaholz BP, Pape T, Zavialov AV, Myasnikov AG, Orlova EV, et al. (2003) Structure of the Escherichia coli ribosomal termination complex with release factor 2. Nature 421: 90-94.

101. Agrawal RK, Sharma MR, Kiel MC, Hirokawa G, Booth TM, et al. (2004) Visualization of ribosome-recycling factor on the Escherichia coli 70S ribosome: functional implications. Proc Natl Acad Sci U S A 101: 8900-8905.

102. Kisselev LL, Buckingham RH (2000) Translational termination comes of age. Trends Biochem Sci 25: 561-566.

103. Kiel MC, Raj VS, Kaji H, Kaji A (2003) Release of ribosome-bound ribosome recycling factor by elongation factor G. J Biol Chem 278: 48041-48050.

104. Tate WP, Kastner B, Edgar CD, McCaughan KK, Timms KM, et al. (1990) The ribosomal domain of the bacterial release factors. The carboxyl-terminal domain of the dimer of Escherichia coli ribosomal protein L7/L12 located in the body of the ribosome is important for release factor interaction. Eur J Biochem 187: 543-548.

105. Agar JN, Krebs C, Frazzon J, Huynh BH, Dean DR, et al. (2000) IscU as a scaffold for iron-sulfur cluster biosynthesis: sequential assembly of [2Fe-2S] and [4Fe-4S] clusters in IscU. Biochemistry 39: 7856-7862.

106. Mansy SS, Cowan JA (2004) Iron-sulfur cluster biosynthesis: toward an understanding of cellular machinery and molecular mechanism. Acc Chem Res 37: 719-725.

107. Johnson DC, Dean DR, Smith AD, Johnson MK (2005) Structure, function, and formation of biological iron-sulfur clusters. Annu Rev Biochem 74: 247-281.

108. Yang J, Bitoun JP, Ding H (2006) Interplay of IscA and IscU in biogenesis of iron-sulfur clusters. J Biol Chem.

109. Adinolfi S, Rizzo F, Masino L, Nair M, Martin SR, et al. (2004) Bacterial IscU is a well folded and functional single domain protein. Eur J Biochem 271: 2093-2100.

110. Nuth M, Yoon T, Cowan JA (2002) Iron-sulfur cluster biosynthesis: characterization of iron nucleation sites for assembly of the [2Fe-2S]2+ cluster core in IscU proteins. J Am Chem Soc 124: 8774-8775.
