# Supplementary material for: Genome-Scale Reconstruction of Escherichia coli's Transcriptional and Translational Machinery: A Knowledge Base, Its Mathematical Formulation, and Its Functional Characterization
Source: PLoS Comput Biol. 2009 Mar 13;5(3):e1000312. doi: 10.1371/journal.pcbi.1000312 (PMC2648898; doi:10.1371/journal.pcbi.1000312)
Supplement: Table S16 — Template reactions for rRNA modification (0.40 MB DOC) [file pcbi.1000312.s018.doc]

Thiele et al.: ‘Genome-scale reconstruction of *E. coli*'s transcriptional and translational machinery: A knowledge-base and its mathematical formulation’.

S16 - Template reactions for rRNA modification.

| **rRNA target** | **Position** | **Modified nucleotide** | **Protein** | **Protein Name** | **Generic Reaction** | **Comments** | **References** |
| --- | --- | --- | --- | --- | --- | --- | --- |
| 23S | 745 | G 🡪 m1G | RrmA_dim (b1822) | (23S rRNA m1G745 methyltransferase | 1 amet --> 1 ahcys + 1 h | RrmA = RlmAl. Found to be functional in dimer form [1]. | [1-4] |
| 23S | 746 | U 🡪 Y | RluA_mono (b0058) | pseudouridine synthase | --> | Pseudouridine pos 746 23S rRNA, pos 32 tRNAphe. Wrzesinski et al purified a protein of about 25kDa [5]. No information about associated ions could be found. It was reported that the enzyme has no dependence on Mg2+ [5]. | [3-6] |
| 23S | 747 | U 🡪 m5U | RumB_mono_FeS (b0859) | m(5)U747 methyltransferase | 1 amet --> 1 ahcys + 1 h | RumB is a homologe to RumA. It has a [4Fe-4S] cluster based on the discussion in [7]. No information about dimerization could be found, so the protein was assumed to be active as monomer. | [3,4,7] |
| 23S | 955 | U 🡪 Y | RluC_mono (b1086) | 23S rRNA pseudouridylate synthase | --> | RluC is a monomer [8]. No ions beside sulfate were found in the crystal structure. | [3,4,8-10] |
| 23S | 1618 | A 🡪 m6A | MeT_23S_1618 | Unknown methyltransferase | 1 amet --> 1 ahcys + 1 h | No known enzyme carries out this reaction so it is assumed to be similar to the other methylation reactions (with S-adenosyl-methionine as methyl-group donor). | [3,4] |
| 23S | 1835 | G 🡪 m2G | MeT_23S_1835 | Unknown methyltransferase | 1 amet --> 1 ahcys + 1 h | No known enzyme carries out this reaction so it is assumed to be similar to the other methylation reactions (with S-adenosyl-methionine as methyl-group donor) | [3,4,11] |
| 23S | 1911 | U 🡪 Y | RluD_mono (b2594) | 23S rRNA pseudouridine synthase | --> | RluD is active as a monomer [12]. Its crystal structure has been determined [8,13]. No ions were identified in the crystal, but the necessity of Mg2+ was reported [13]. Based on this evidence, 1 Mg2+ per monomer concluded. | [3,4,8,10,12-15] |
| 23S | 1915 | U 🡪 m3Y | RluD_mono (b2594) | 23S rRNA pseudouridine synthase | | Y formation by RluD_mono | --> | | --- | --- | | m3 formation by MeT_23S_1915 | 1 amet --> 1 ahcys + 1 h | | **Overall Reaction** | **1 amet --> 1 ahcys + 1 h** | | RluD is active as a monomer [12]. Its crystal structure has been determined [8,13]. No ions were identified in the crystal, but the necessity of Mg2+ was reported [13]. Based on this evidence, 1 Mg2+ per monomer concluded. | [3,4,8,10,12-15] |
| 23S | 1917 | U 🡪 Y | RluD_mono (b2594) | 23S rRNA pseudouridine synthase | --> | RluD is active as a monomer [12]. Its crystal structure has been determined [8,13]. No ions were identified in the crystal, but the necessity of Mg2+ was reported [13]. Based on this evidence, 1 Mg2+ per monomer concluded. | [3,4,8,10,12-15] |
| 23S | 1939 | U 🡪 m5U | RumA_mono_FeS (b2785) | 23S rRNA (uracil-5)-methyltransferase | 1 amet --> 1 ahcys + 1 h | RumA was found to be active as a monomer with [4Fe-4S]2+ cluster [7,16,17] | [3,4,7,16-18] |
| 23S | 1962 | C 🡪 m5C | MeT_23S_1962 | Unknown methyltransferase | 1 amet --> 1 ahcys + 1 h | A catalytic enzyme has not been identified for this reaction. This reaction is assumed to be similar to the other methylation reactions (with S-adenosyl-methionine as methyl-group donor) | [3,4,11] |
| 23S | 2030 | A 🡪 m6A | MeT_23S_2030 | Unknown methyltransferase | 1 amet --> 1 ahcys + 1 h | A catalytic enzyme has not been identified for this reaction. This reaction is assumed to be similar to the other methylation reactions (with S-adenosyl-methionine as methyl-group donor) | [3,4] |
| 23S | 2069 | G 🡪 m7G | MeT_23S_2069 | Unknown methyltransferase | 1 amet --> 1 ahcys + 1 h | A catalytic enzyme has not been identified for this reaction. This reaction is assumed to be analogous to the other methylation reactions (with S-adenosyl-methionine as methyl-group donor) | [3,4] |
| 23S | 2251 | G 🡪 Gm | RlmB_dim (b4180) | 23S rRNA (Gm2251)-methyltransferase | 1 amet --> 1 ahcys + 1 h | The active form is a homodimer. No ions were reported in the crystal structure [19] | [3,4,19,20] |
| 23S | 2445 | G 🡪 m2G | MeT_23S_2445 | Unknown methyltransferase | 1 amet --> 1 ahcys + 1 h | A catalytic enzyme has not been identified for this reaction. The reaction is assumed to be analogous to the other methylation reactions (with S-adenosyl-methionine as methyl-group donor) | [3,4,11] |
| 23S | 2449 | U 🡪 D | DU_23S_2449 | dihydrouridine synthetase, 23S rRNA, position 2449 | 1 nadh + 1 h --> 1 nad or 1 nadph + 1 h --> 1 nadp | The dihydrouridine modification seems to be dispensable, but a pyrimidine base at this position is essential for viability | [3,4,21,22] |
| 23S | 2457 | U 🡪 Y | YmfC_mono (b1135) | 23S rRNA pseudouridine synthase | --> | The active form is a monomer (based on a molecular weight of 24.9) [23]. No ions were identified in the crystal structure. | [23,24] [3,4] |
| 23S | 2498 | C 🡪 Cm | MeT_23S_2498 | Unknown methyltransferase | 1 amet --> 1 ahcys + 1 h | A catalytic enzyme has not been identified for this reaction. The reaction is assumed to be similar to the other methylation reactions (with S-adenosyl-methionine as methyl-group donor) | [3,4] |
| 23S | 2503 | A 🡪 m2A | MeT_23S_2503 | Unknown methyltransferase | 1 amet --> 1 ahcys + 1 h | A catalytic enzyme has not been identified for this reaction. This reaction is assumed to be analogous to the other methylation reactions (with S-adenosyl-methionine as methyl-group donor) | [3,4,11] |
| 23S | 2504 | U 🡪 Y | RluC_mono (b1086) | 23S rRNA pseudouridylate synthase | --> | The active form is a monomer [8]. Sulfate was the only ionic compound in the crystal structure. | [3,4,8,10,24] |
| 23S | 2552 | U 🡪 Um | RrmJ_mono (b3179) | 23S rRNA methyltransferase | 1 amet --> 1 ahcys + 1 h | Identification of RrmJ [25,26]. This protein exists as a monomer based on results from its purification [25]. It is strictly dependent on Mg for the methylation reaction; however, the authors suggested that this dependency might be similar to the Mg2+ requirement of RsmC which acts only on ribosomal particles but not on free 23S rRNA. Mg is more likely to be needed for structural aspects of the ribosomal particle than for the reaction [25] | [3,4,25-28] |
| 23S | 2580 | U 🡪 Y | RluC_mono (b1086) | 23S rRNA pseudouridylate synthase | --> | The active form is a monomer [8] Sulfate was the only ionic compound in the crystallized protein. | [3,4,8,10,24] |
| 23S | 2604 | U 🡪 Y | YjbC_mono (b4022) | 23S rRNA pseudouridine synthase | --> | RluF=YjbC. The protein is a monomer (based on the molecular weight of 32.5) [23]. No ions were identified in the crystallized protein. | [3,4,23] |
| 23S | 2605 | U 🡪 Y | RluB_mono (b1269) | 23S rRNA pseudouridylate synthase | --> | The active form is a monomer (based on the molecular weight of 32.7) [23]. No ions were identified in the crystallized protein. | [3,4,23,24] |
| 16S | 516 | U 🡪 Y | RsuA_mono (b2183) | 16S rRNA pseudouridylate 516 synthase | --> | The active form is a monomer [29]. No ions were identified in the crystallized protein. Deletion of protein did not affect growth rates. | [3,4,29-31] |
| 16S | 527 | G 🡪 m7G | MeT_16S_527 | Unknown methyltransferase | 1 amet --> 1 ahcys + 1 h | A catalytic enzyme has not been identified for this reaction. Reaction is assumed to be similar to the other methylation reactions (with S-adenosyl-methionine as methyl-group donor) | [3,4] |
| 16S | 966 | G 🡪 m2G | MeT_16S_966 | Unknown methyltransferase | 1 amet --> 1 ahcys + 1 h | A catalytic enzyme has not been identified for this reaction. Reaction is assumed to be similar to the other methylation reactions (with S-adenosyl-methionine as methyl-group donor) | [3,4] |
| 16S | 967 | C 🡪 m5C | RsmB_mono (b3289) | 16S rRNA m5C967 methyltransferase, S-adenosyl-L-methionine-dependent | 1 amet --> 1 ahcys + 1 h | Identification of RsmB [32,33]. It was found that Mg2+ is needed for the proper folding of substrate (16 S rRNA that is already incorporated in 30S ribsome) but NOT for the catalytic activity [32]. The protein has a specific activity of 3.1 × 10^6^ units/mg of protein at 37C, corresponding to a turnover number of 2.6 mol min-1 (mol of protein)-1 [34]. RsmB is a monomer based on molecular and crystal structure [35] | [3,4,32-36] |
| 16S | 1207 | G 🡪 m2G | RsmC_mono (b4371) | 16S RNA m2G1207 methylase | 1 amet --> 1 ahcys + 1 h | Identification of RsmC [37] It was found that Mg2+ is needed for the proper folding of substrate (16 S rRNA that is already incorporated in 30S ribsome) but NOT for the catalytic activity [37]. RsmC is a monomer according to [37]. | [3,4,37] |
| 16S | 1402 | C 🡪 m4Cm | MeT_16S_1402 | Unknown methyltransferase | 2 amet --> 2 ahcys + 2 h | A catalytic enzyme has not been identified for this reaction. The reaction is assumed to be similar to the other methylation reactions (with S-adenosyl-methionine as methyl-group donor) | [3,4,38] |
| 16S | 1407 | C 🡪 m5C | MeT_16S_1407 | Unknown methyltransferase | 1 amet --> 1 ahcys + 1 h | A catalytic enzyme has not been identified for this reaction. Reaction is assumed to be similar to the other methylation reactions (with S-adenosyl-methionine as methyl-group donor) | [3,4,38] |
| 16S | 1498 | U 🡪 m3U | YggJ_mono (b2946) | 2'O-ribose methyttansferase | 1 amet --> 1 ahcys + 1 h | yggJ = RsmE. It is not clear if it exists as a monomer, dimer, or other multimeric form. The crystal structure from homologuous gene (=RsmE family) in H. influenzae showed a dimer form. However based on these finding it was originally suggested that yggJ is a 2'O-ribose methyltransferas. Given the present evidence (or lack thereof), RsmE will be a monomer without associated ions in the model until evidence in the literature shows otherwise (function has been discovered just recently: Jan '06)[39]. | [3,4,39] |
| 16S | 1516 | G 🡪 m2G | MeT_16S_1516 | Unknown methyltransferase | 1 amet --> 1 ahcys + 1 h | A catalytic enzyme has not been identified for this reaction. Reaction is assumed to be similar to the other methylation reactions (with S-adenosyl-methionine as methyl-group donor) | [3,4] |
| 16S | 1518 | A 🡪 m6(2)A | KsgA_mono (b0051) | S-adenosylmethionine-6-N',N'-adenosyl (rRNA) dimethyltransferase | 2 amet --> 2 ahcys + 2 h | No ions were identified in the crystallized protein [40]. | [3,4,40,41] |
| 16S | 1519 | A 🡪 m6(2)A | KsgA_mono (b0051) | S-adenosylmethionine-6-N',N'-adenosyl (rRNA) dimethyltransferase | 2 amet --> 2 ahcys + 2 h | No ions were identified in the crystallized protein [40]. | [3,4,40,41] |

1. Das K, Acton T, Chiang Y, Shih L, Arnold E, et al. (2004) Crystal structure of RlmAI: implications for understanding the 23S rRNA G745/G748-methylation at the macrolide antibiotic-binding site. Proc Natl Acad Sci U S A 101: 4041-4046.

2. Gustafsson C, Persson BC (1998) Identification of the rrmA gene encoding the 23S rRNA m1G745 methyltransferase in Escherichia coli and characterization of an m1G745-deficient mutant. J Bacteriol 180: 359-365.

3. Sprinzl M, Vassilenko KS (2005) Compilation of tRNA sequences and sequences of tRNA genes. Nucleic Acids Res 33: D139-140.

4. McCloskey JA, Crain PF (1998) The RNA modification database--1998. Nucleic Acids Res 26: 196-197.

5. Wrzesinski J, Nurse K, Bakin A, Lane BG, Ofengand J (1995) A dual-specificity pseudouridine synthase: an Escherichia coli synthase purified and cloned on the basis of its specificity for psi 746 in 23S RNA is also specific for psi 32 in tRNA(phe). Rna 1: 437-448.

6. Raychaudhuri S, Niu L, Conrad J, Lane BG, Ofengand J (1999) Functional effect of deletion and mutation of the Escherichia coli ribosomal RNA and tRNA pseudouridine synthase RluA. J Biol Chem 274: 18880-18886.

7. Agarwalla S, Stroud RM, Gaffney BJ (2004) Redox reactions of the iron-sulfur cluster in a ribosomal RNA methyltransferase, RumA: optical and EPR studies. J Biol Chem 279: 34123-34129.

8. Mizutani K, Machida Y, Unzai S, Park SY, Tame JR (2004) Crystal structures of the catalytic domains of pseudouridine synthases RluC and RluD from Escherichia coli. Biochemistry 43: 4454-4463.

9. Conrad J, Sun D, Englund N, Ofengand J (1998) The rluC gene of Escherichia coli codes for a pseudouridine synthase that is solely responsible for synthesis of pseudouridine at positions 955, 2504, and 2580 in 23 S ribosomal RNA. J Biol Chem 273: 18562-18566.

10. Huang L, Ku J, Pookanjanatavip M, Gu X, Wang D, et al. (1998) Identification of two Escherichia coli pseudouridine synthases that show multisite specificity for 23S RNA. Biochemistry 37: 15951-15957.

11. Smith JE, Cooperman BS, Mitchell P (1992) Methylation sites in Escherichia coli ribosomal RNA: localization and identification of four new sites of methylation in 23S rRNA. Biochemistry 31: 10825-10834.

12. Sivaraman J, Iannuzzi P, Cygler M, Matte A (2004) Crystal structure of the RluD pseudouridine synthase catalytic module, an enzyme that modifies 23S rRNA and is essential for normal cell growth of Escherichia coli. J Mol Biol 335: 87-101.

13. Wrzesinski J, Bakin A, Ofengand J, Lane BG (2000) Isolation and properties of Escherichia coli 23S-RNA pseudouridine 1911, 1915, 1917 synthase (RluD). IUBMB Life 50: 33-37.

14. Raychaudhuri S, Conrad J, Hall BG, Ofengand J (1998) A pseudouridine synthase required for the formation of two universally conserved pseudouridines in ribosomal RNA is essential for normal growth of Escherichia coli. Rna 4: 1407-1417.

15. del Campo M, Ofengand J, Malhotra A (2004) Crystal structure of the catalytic domain of RluD, the only rRNA pseudouridine synthase required for normal growth of Escherichia coli. Rna 10: 231-239.

16. Lee TT, Agarwalla S, Stroud RM (2004) Crystal structure of RumA, an iron-sulfur cluster containing E. coli ribosomal RNA 5-methyluridine methyltransferase. Structure 12: 397-407.

17. Lee TT, Agarwalla S, Stroud RM (2005) A unique RNA Fold in the RumA-RNA-cofactor ternary complex contributes to substrate selectivity and enzymatic function. Cell 120: 599-611.

18. Agarwalla S, Kealey JT, Santi DV, Stroud RM (2002) Characterization of the 23 S ribosomal RNA m5U1939 methyltransferase from Escherichia coli. J Biol Chem 277: 8835-8840.

19. Michel G, Sauve V, Larocque R, Li Y, Matte A, et al. (2002) The structure of the RlmB 23S rRNA methyltransferase reveals a new methyltransferase fold with a unique knot. Structure 10: 1303-1315.

20. Lovgren JM, Wikstrom PM (2001) The rlmB gene is essential for formation of Gm2251 in 23S rRNA but not for ribosome maturation in Escherichia coli. J Bacteriol 183: 6957-6960.

21. Kowalak JA, Bruenger E, McCloskey JA (1995) Posttranscriptional modification of the central loop of domain V in Escherichia coli 23 S ribosomal RNA. J Biol Chem 270: 17758-17764.

22. O'Connor M, Lee WM, Mankad A, Squires CL, Dahlberg AE (2001) Mutagenesis of the peptidyltransferase center of 23S rRNA: the invariant U2449 is dispensable. Nucleic Acids Res 29: 710-715.

23. Del Campo M, Kaya Y, Ofengand J (2001) Identification and site of action of the remaining four putative pseudouridine synthases in Escherichia coli. Rna 7: 1603-1615.

24. Bakin A, Ofengand J (1993) Four newly located pseudouridylate residues in Escherichia coli 23S ribosomal RNA are all at the peptidyltransferase center: analysis by the application of a new sequencing technique. Biochemistry 32: 9754-9762.

25. Caldas T, Binet E, Bouloc P, Costa A, Desgres J, et al. (2000) The FtsJ/RrmJ heat shock protein of Escherichia coli is a 23 S ribosomal RNA methyltransferase. J Biol Chem 275: 16414-16419.

26. Hager J, Staker BL, Jakob U (2004) Substrate binding analysis of the 23S rRNA methyltransferase RrmJ. J Bacteriol 186: 6634-6642.

27. Hager J, Staker BL, Bugl H, Jakob U (2002) Active site in RrmJ, a heat shock-induced methyltransferase. J Biol Chem 277: 41978-41986.

28. Tan J, Jakob U, Bardwell JC (2002) Overexpression of two different GTPases rescues a null mutation in a heat-induced rRNA methyltransferase. J Bacteriol 184: 2692-2698.

29. Sivaraman J, Sauve V, Larocque R, Stura EA, Schrag JD, et al. (2002) Structure of the 16S rRNA pseudouridine synthase RsuA bound to uracil and UMP. Nat Struct Biol 9: 353-358.

30. Wrzesinski J, Bakin A, Nurse K, Lane BG, Ofengand J (1995) Purification, cloning, and properties of the 16S RNA pseudouridine 516 synthase from Escherichia coli. Biochemistry 34: 8904-8913.

31. Conrad J, Niu L, Rudd K, Lane BG, Ofengand J (1999) 16S ribosomal RNA pseudouridine synthase RsuA of Escherichia coli: deletion, mutation of the conserved Asp102 residue, and sequence comparison among all other pseudouridine synthases. Rna 5: 751-763.

32. Tscherne JS, Nurse K, Popienick P, Michel H, Sochacki M, et al. (1999) Purification, cloning, and characterization of the 16S RNA m5C967 methyltransferase from Escherichia coli. Biochemistry 38: 1884-1892.

33. Weitzmann C, Tumminia SJ, Boublik M, Ofengand J (1991) A paradigm for local conformational control of function in the ribosome: binding of ribosomal protein S19 to Escherichia coli 16S rRNA in the presence of S7 is required for methylation of m2G966 and blocks methylation of m5C967 by their respective methyltransferases. Nucleic Acids Res 19: 7089-7095.

34. Tscherne JS, Nurse K, Popienick P, Michel H, Sochacki M, et al. (1999) Purification, Cloning, and Characterization of the 16S RNA m<sup>5</sup>C967 Methyltransferase from <i>Escherichia coli</i>. Biochemistry 38: 1884-1892.

35. Foster PG, Nunes CR, Greene P, Moustakas D, Stroud RM (2003) The first structure of an RNA m5C methyltransferase, Fmu, provides insight into catalytic mechanism and specific binding of RNA substrate. Structure 11: 1609-1620.

36. Gu XR, Gustafsson C, Ku J, Yu M, Santi DV (1999) Identification of the 16S rRNA m5C967 methyltransferase from Escherichia coli. Biochemistry 38: 4053-4057.

37. Tscherne JS, Nurse K, Popienick P, Ofengand J (1999) Purification, cloning, and characterization of the 16 S RNA m2G1207 methyltransferase from Escherichia coli. J Biol Chem 274: 924-929.

38. Kowalak JA, Pomerantz SC, Crain PF, McCloskey JA (1993) A novel method for the determination of post-transcriptional modification in RNA by mass spectrometry. Nucleic Acids Res 21: 4577-4585.

39. Basturea GN, Rudd KE, Deutscher MP (2006) Identification and characterization of RsmE, the founding member of a new RNA base methyltransferase family. Rna 12: 426-434.

40. O'Farrell HC, Scarsdale JN, Rife JP (2004) Crystal structure of KsgA, a universally conserved rRNA adenine dimethyltransferase in Escherichia coli. J Mol Biol 339: 337-353.

41. van Buul CP, van Knippenberg PH (1985) Nucleotide sequence of the ksgA gene of Escherichia coli: comparison of methyltransferases effecting dimethylation of adenosine in ribosomal RNA. Gene 38: 65-72.
