# Supplementary material for: Genome-Scale Reconstruction of Escherichia coli's Transcriptional and Translational Machinery: A Knowledge Base, Its Mathematical Formulation, and Its Functional Characterization
Source: PLoS Comput Biol. 2009 Mar 13;5(3):e1000312. doi: 10.1371/journal.pcbi.1000312 (PMC2648898; doi:10.1371/journal.pcbi.1000312)
Supplement: Table S17 — Template reactions for tRNA modification (0.76 MB DOC) [file pcbi.1000312.s019.doc]

Thiele et al.: ‘Genome-scale reconstruction of *E. coli*'s transcriptional and translational machinery: A knowledge-base and its mathematical formulation’.

**S17 - Template reactions for tRNA modification.**

Note: The nucleotides of the tRNA that will be modified by the following reactions and their products are omitted in the reaction. (--> stand for, i.e., tRNA_U16 🡪 tRNA_D since it is only a conformational change of the base)

| **Position** | **modified nucleotide** | **Name of modified nucleotide** | **Formulae of modified nucleotide (charge)** | **Protein** | **Protein name** | **Generic reaction** | **Comments** | **References** |
| --- | --- | --- | --- | --- | --- | --- | --- | --- |
| 8 | U ( s4U | 4-thiouridine | C9H10N2O8S1P1  (-1) | ThiI_mono | sulfurtransferase required for thiamine and 4-thiouridine biosynthesis | 1 IscS_dim_S-SH + 1 atp + 1 trdrd + 1 mg2 --> 1 amp + 1 ppi + 1 h + 1 IscS_dim_S-H + 1 mg2 + 1 trdox | The reaction was based on the supplemental data in Ikeuchi et al [1]. ThiI is a monomer based on the measured molecular weight [2]. ThiI gets oxidized during reaction, thus the reaction a reduce reducing agent. Palenchar et al and Mueller et al proposed thioredoxin or glutaredoxin to reduce ThiI [3], [4]. Waterman et al found a ferrodoxin-like motif in ThiI of B. subtilis [5]. iAF1260, the most current reconstruction of the E. coli metabolism, has no ferrodoxin hence thioredoxin was choosen as reducing agent since it appears in 10 metabolic reactions in iAF1260 compared to. glutaredoxin that appears 6 times [6]. | [1-11] |
| 13 | U 🡪 Y | pseudouridine | C9H10N2O9P1  (-1) | TruD_mono | tRNA pseudouridine 13 synthase | --> | TruD seems to be a monomer. No ions were reported in the crystal structure [12,13]. | [12-14] |
| 16 | U 🡪 D | dihydrouridine | C9H12N2O9P1  (-1) | Dus_gen | tRNA dihydrouridine synthase | 1 nadph + 1 h --> 1 nadp | Dus_gen = DusA – C. These proteins are responsible for dihydrouridine formation in E.coli’s tRNA [15]. Based on blast result sequence, the dus genes have homology to genes that carry out NADPH dependent reactions. Furthermore, the reaction was reported to be NADH or NADPH dependent in yeast [16]. However, the reaction was modeled to be NADPH-dependent. The Dus protein contain probably FMN since a FMN binding site was found in sequence [15]. Also, the Dus protein might be part of a larger, yet uncharacterized complex [15]. DusA is solely responsible for tRNA fmet2 [15], meaning, that all initiator tRNAs will be solely modified by dusA. Since no information was available, a monomer conformation was assumed. No ions were reported. | [15-18] |
| 17 | U 🡪 D | dihydrouridine | C9H12N2O9P1  (-1) | Dus_gen | tRNA dihydrouridine synthase | 1 nadph + 1 h --> 1 nadp | Dus_gen = DusA – C. These proteins are responsible for dihydrouridine formation in E.coli’s tRNA [15]. Based on blast result sequence, the dus genes have homology to genes that carry out NADPH dependent reactions. Furthermore, the reaction was reported to be NADH or NADPH dependent in yeast [16]. However, the reaction was modeled to be NADPH-dependent. The Dus protein contain probably FMN since a FMN binding site was found in sequence [15]. Also, the Dus protein might be part of a larger, yet uncharacterized complex [15]. DusA is solely responsible for tRNA fmet2 [15], meaning, that all initiator tRNAs will be solely modified by dusA. Since no information was available, a monomer conformation was assumed. No ions were reported. | [15-18] |
| 18 | G 🡪 Gm | 2'-O-methylguanosine | C11H13N5O8P1  (-1) | TrmH_dim | tRNA (Gm18) 2'-O-methyltransferase | 1 amet --> 1 ahcys + 1 h | TrmH was identified by Persson et al [19]. It has been found to be a dimer in Thermus thermophilus:[20]. Based on the proposed mechanism in this paper - it should be a dimer, which acts on one tRNA per dimer. TrmH is part of SpoU protein family, beside TrmD and RlmB - 2 other E.coli proteins of this family. This protein family has been shown to be active as dimers. | [19-22] |
| 20 | U 🡪 D | dihydrouridine | C9H12N2O9P1  (-1) | Dus_gen | tRNA dihydrouridine synthase | 1 nadph + 1 h --> 1 nadp | Dus_gen = DusA – C. These proteins are responsible for dihydrouridine formation in E.coli’s tRNA [15]. Based on Blast results of the sequence, the dus genes have homology to genes that carry out NADPH dependent reactions. Furthermore, the reaction was reported to be NADH or NADPH dependent in yeast [16]. However, the reaction was modeled to be NADPH-dependent. The Dus protein contain probably FMN since a FMN binding site was found in sequence [15]. Also, the Dus protein might be part of a larger, yet uncharacterized complex [15]. DusA is solely responsible for tRNA fmet2 [15], meaning, that all initiator tRNAs will be solely modified by dusA. Since no information was available, a monomer conformation was assumed. No ions were reported. | [15-17] |
| 20A | U 🡪 D | dihydrouridine | C9H12N2O9P1  (-1) | Dus_gen | tRNA dihydrouridine synthase | 1 nadph + 1 h --> 1 nadp | Dus_gen = DusA – C. These proteins are responsible for dihydrouridine formation in E.coli’s tRNA [15]. Based on blast result sequence, the dus genes have homology to genes that carry out NADPH dependent reactions. Furthermore, the reaction was reported to be NADH or NADPH dependent in yeast [16]. However, the reaction was modeled to be NADPH-dependent. The Dus protein contain probably FMN since a FMN binding site was found in sequence [15]. Also, the Dus protein might be part of a larger, yet uncharacterized complex [15]. DusA is solely responsible for tRNA fmet2 [15], meaning, that all initiator tRNAs will be solely modified by dusA. Since no information was available, a monomer conformation was assumed. No ions were reported. | [15-17] |
| 21 | U 🡪 D | dihydrouridine | C9H12N2O9P1  (-1) | Dus_gen | tRNA dihydrouridine synthase | 1 nadph + 1 h --> 1 nadp | Dus_gen = DusA – C. These proteins are responsible for dihydrouridine formation in E.coli’s tRNA [15]. Based on blast result sequence, the dus genes have homology to genes that carry out NADPH dependent reactions. Furthermore, the reaction was reported to be NADH or NADPH dependent in yeast [16]. However, the reaction was modeled to be NADPH-dependent. The Dus protein contain probably FMN since a FMN binding site was found in sequence [15]. Also, the Dus protein might be part of a larger, yet uncharacterized complex [15]. DusA is solely responsible for tRNA fmet2 [15], meaning, that all initiator tRNAs will be solely modified by dusA. Since no information was available, a monomer conformation was assumed. No ions were reported. | [15-17] |
| 32 | C 🡪 Cm | 2'-O-methylcytidine | C10H13N3O8P1  (-1) | MeT_tRNA_pos_32_Cm | unknown Methyltransferase pos 32 (Cm) | 1 amet --> 1 ahcys + 1 h | A catalytic enzyme for this reaction has not been identified. The reaction was assumed to be similar to other methylation reactions (with S-adenosyl-methionine as methyl-group donor). | [22,23] |
| 32 | U 🡪 Um | 2'-O-methyluridine | C10H12N2O9P1  (-1) | MeT_tRNA_pos_32_Um | unknown Methyltransferase pos 32 (Um) | 1 amet --> 1 ahcys + 1 h | A catalytic enzyme for this reaction has not been identified. The reaction was assumed to be similar to other methylation reactions (with S-adenosyl-methionine as methyl-group donor). | [22,24] |
| 32 | C 🡪 s2C | 2-thiocytidine | C9H11N3O7S1P1  (-1) | YdaO_mono | C32 tRNA thiolase | 1 IscS_dim_S-SH + 1 atp + 1 trdrd + 1 mg2 --> 1 amp + 1 ppi + 1 h + 1 IscS_dim_S-H + 1 mg2 + 1 trdox | Apparently a gene called ttcA is responsible in Salmonella serovar for this transformation and it is widely distributed in different species. Authors concluded that ydaO is the ttcA gene in E. coli [25]. Reaction associated with ydaO and dependent on IscS_dim_S-SH/IscS_dim_S-H (as for s4U). No further information available about ydaO. Based on this evidence this is a monomer. | [7,11,25,26] |
| 32 | U 🡪 Y | pseudouridine | C9H10N2O9P1  (-1) | RluA_mono | 23S rRNA and tRNA pseudouridine synthase | --> | Pseudouridine pos 746 23S rRNA, pos 32 tRNAphe. No information, the paper [27] purified a protein of about 25kDa: [28]. No information about associated ions. Enzyme has no dependence on Mg2+ : [28]. | [27,28] |
| 34 | U 🡪 mnm5s2U | 5-methylaminomethyl-2-thiouridine | C11H15N3O8S1P1  (-1) | TrmU_mono,YhhP_mono,YheLMN_cplx,YccK_mono,TrmE_dim,GidA_mono,TrmC_mono | tRNA (5-methylaminomethyl-2-thiouridylate)-methyltransferase - TrmU, conserved protein required for cell growth - YhhP, YheLMN_cplx, glucose-inhibited cell-division protein - TrmE, GTPase - GidA, fused 5-methylaminomethyl-2-thiouridine-forming enzyme methyltransferase -!- FAD-dependent demodification enzyme - TrmC | | Formation of cmnm5(s2)U by at least mnmE and gidA | 1 gtp + 1 h2o --> 1 gdp + 1 pi + 1h  1 5fthf --> 1 thf + 1 gly + 1 fadh2 --> 1 fad + 1 h + 1 h2o | | --- | --- | | Formation of nm5(s2)U by mnmC | 1 fad + 1 h2o --> 1 fadh2 + 1 glx | | Formation of mnm5(s2)U by mnmC | 1 amet --> 1 ahcys + 1 h | | s2U formation by YhhP_mono,YheLMN_cplx,YccK_mono | 1 IscS_dim_S-SH + 1 atp + 1 trdrd + 1 mg2 --> 1 amp + 1 ppi + 1 h + 1 IscS_dim_S-H + 1 mg2 + 1 trdox | | **Overall reaction** | **1 IscS_dim_S-SH + 1 atp + 1 mg2 + 1 gtp + 1 h2o + 1 5fthf + 1 gly + 1 fadh2 + 1 fad + 1 amet + 1 trdrd --> 1 amp + 1 ppi + 1 IscS_dim_S-H + 1 mg2 + 1 gdp + 1 pi + 4 h + 1 thf + 1 fad + 1 fadh2 + 1 glx + 1 ahcys + 1 trdox** | | s2U formation [1]: IscS_dim interacts with tusA (tusA stimulates the desulfurase activity of iscS) tusBCD complex (2:2:2 stoichiometry) interacts with IscS/tusA; tusE mediates contact between tusBCD and mnmA, mnmA finally binds tRNA. This complicated scheme seems to be necessary for sulfur transfer. Studies until this paper showed IscS and mnmA action, however, the amount of reconstituted s2U is relatively low indicating the necessity of further proteins. tusA= yhhP=b3470. Given this information, assumed to be a monomer. (‘=’ refers to aliases). tusB=yheL=b3343, tusC=yheM=b3344, tusD=yheN=b3345, tusE=yccK=b0969 . Assumed to be a monomer. The structure of tusBCD is described by: [29]. Sulfate is the only ionic compound associated with it PDBID: 2D1P. mnmA aka TrmU: monomer based on molecular weight [30] (in the paper they carry out the characterization and purification): crystal (no structure; one crystal contained 1 mnmA-tRNA complex) [29]. The thioredoxin system had to be added to reduce trmU (oxidized by sulfur transfer reaction) [25]. Please see pos 34 (mmm5U) for mnm5 formation. | [1,7,11,22,25,29-35] |
| 34 | U ( mnm5se2U | 5-methylaminomethyl-2-selenouridine | C11H15N3O8Se1P1  (-1) | YbbB | tRNA 2-selenouridine synthase | 1 YbbB_dim + 1 selnp + 1 h2o ( 1 pi + 1 h2s + 1 h | First mnm5s2U is formed. About 5% of tRNA (Lys, Glu, Gln) contains mnm5se2U instead of mnm5s2U [36] at when selenium is present at about 1 µM concentration. It seems that the sulfur compound leaving this reaction is not known. Here, hydrogen sulfide is formed but there is no clear evidence for this formation. Formulae of selnp: H2O3PSe (-1). It seems that this reaction does not need atp hydrolysis. | [36] |
| 34 | U ( mnm5U | 5-methylaminomethyluridine | C11H15N3O9P1  (-1) | TrmE_dim, GidA_mono,TrmC_mono |  | | Formation of cmnm5(s2)U by at least mnmE and gidA | 1 gtp + 1 h2o 🡪 1 gdp + 1 pi + 1h  1 5fthf 🡪 1 thf + 1 gly + 1 fadh2 🡪 1 fad + 1 h + 1 h2o | | --- | --- | | Formation of nm5(s2)U by mnmC | 1 fad + 1 h2o 🡪 1 fadh2 + 1 glx | | Formation of mnm5(s2)U by mnmC | 1 amet 🡪 1 ahcys + 1 h | | Overall reaction | 1 gtp + 1 h2o + 1 5fthf + 1 gly + 1 fadh2 + 1 fad + 1 amet ( 1 gdp + 1 pi + 3 h + 1 thf + 1 fad + 1 fadh2 + 1 glx + 1 ahcys | | GidA has FAD bound (data were not shown but mentioned in paper that they analyzed it) [31]. Based on this evidence, this was assumed to be a monomer. From cmnm5s2U over nm5s2U to mnm5s2U is done by mnmC [32]. Described as a monomer based on the MW [33] and their comments. 78 molecules/genome equivalent in cells growing at 1.0/h. Purification, identification and function [33]. The gene is described by [32,34,35]. mnmE and trmE are aliases. MnmE is non-essential but grows slowly. MnmE of thermogata is a dimer [31]: identified with bioinformatics a THF-binding site in mnmE thermogata – and tested this by soaking the crystal in THF solution. The protein was found bound to THF, more specifically 2 THF per dimer (in this case 5-formyl-THF); they showed also 5-formyl-THF usage by E. coli (!) enzyme by ITC. They favor 5-formyl-THF for reaction but they are not sure about real odixative state of THF. They are relatively sure that 2 5fTHF were bound per dimer mnmE E. coli. They have been unable to reconstruct mnm5s2U (mnm5-moitie) in vitro with the given genes: mnmE and gidA, indicating that more gene products are needed | [11,22,25,29-35] |
| 34 | A 🡪 I | inosine | C10H10N4O8P1  (-1) | TadA_dim | tRNA-specific adenosine deaminase | 1 h2o 🡪 1 nh3 | Essential and active as a homodimer [37]. The catalytic site, carrying out the deaminase function, of this protein family (ADAR) has 3 zn2+ binding sites [37]. The crystal structure of S. Aureus of tadA contained 2 Zn2+ (per homodimer). However, the authors did not specify Zn2+ in paper [38]. Given this evidence, this will be described as a homodimer associated with 2 zn2+ | [37-39] |
| 34 | C 🡪 k2C | lysidine | C15H23N5O9P1  (-1) | TillS_mono | lysidine synthetase | 1 atp + 1 lys-L 🡪 1 ppi + 1 amp + 2 h | Existence: [40]. Structure: 1NI5. The protein seems to be a monomer without ions (isolated 48.5 kDa protein [40]). Atp-dependent ([40]) reaction. | [40,41] |
| 34 | C 🡪 ac4C | N4-acetylcytidine | C11H13N3O9P1  (-1) | AcT_tRNA_pos_34_ac4C | unknown acetyltransferase | 1 accoa 🡪 1 coa | An acetyl group is transferred. AcCoa was used, however no experimental evidence was available nor was there evidence for the catalytic enzyme or mechanism for the reaction. | [42] |
| 34 | G 🡪 Q | Queuosine | C17H21O10N5P1  (-1) | Tgt_hexa,QueA_mono,EoR_tRNA_pos34_Q | tRNA-guanine transglycosylase,  S-adenosylmethionine:tRNA ribosyltransferase-isomerase’unknown epoxide reductase | | From pre_Q1 to pre_Q1_tRNA by Tgt | 1 pre_Q1 🡪 1 gua | | --- | --- | | From pre_Q1_tRNA to oQ_tRNA by QueA | 1 amet 🡪 1 ade + 1 met-L + 2 h | | From oQ_tRNA to Q_tRNA by EoR_tRNA_pos34_Q | 1 nadh + 1 h + 1 adocbl 🡪 1 nad + 1 h2o + 1 adocbl | | **Overall reaction** | **1 pre_Q1 + 1 amet + 1 nadh + 1 h + 1 adocbl 🡪 1 gua + 1 ade + 1 met-L + 2 h + 1 h2o + 1 adocbl + 1 nad** | | A new function of S-adenosylmethionine: the ribosyl moiety of AdoMet is the precursor of the cyclopentenediol moiety of the tRNA wobble base queuine. [43]. Isolation and characterization of an Escherichia coli mutant lacking tRNA-guanine transglycosylase. Function and biosynthesis of queuosine in tRNA [44]. Tgt has a zinc binding site [45] (0.8 mol of zinc/mol of subunit). The active protein consists of a dimer of trimers [46]. E. coli TGT is normally expressed at very low levels (approximately 1 mg from 500 g cells). QueA: not metallo-ion dependence. Thermotoga structure was determined[47]. The E.coli protein was assumed to be a monomer. For the last step (oQ->Q) 2 mechanisms were proposed: 1) one mechanism would use NAD(P)H, the other one uses NAD(H). QueF is a homodimer [48]. QueC is identified by [49]. Step from GTP to pre_Q0 is unclear: apparently, 2 ribose units contribute to the formation of carbon 6,5, and cyano carbon in the case of toyocamycin (which is structurally related to pre_Q0) [50,51]. Kinetic parameters for QueA measured by Van Lanen and Iwata-Reuyl in [52] | [22,43-60] |
| 34 | U 🡪 cmo5U | uridine-5-oxyacetic-acid | C11H11N2O12P1  (-2) | YecO_mono,YecP_mono,HyL_tRNA_pos_34_ho5U | predicted methyltransferase,  predicted S-adenosyl-L-methionine-dependent methyltransferase, unknown hydroxylase | | ho5U formation | 1 chor 🡪 1 C10H8O5 | | --- | --- | | mo5U formation | 1 amet 🡪 1 ahcys + 1 h | | cmo5U formation | 1 chor 🡪 1 C9H9O4 | | Overall reaction | 2 chor + 1 amet ( 1 ahcys + 1 h + 1 C10H8O5 + 1 C9H9O4 | | It is not clear what the role of chorismate is. If chorismate is the substrate the byproducts of this reaction would have possible formula of C10H8O5 and C9H9O4. Such compounds were not found in the SimPheny or other databases nor reported in literature. If the conversion is from glutamine to glutamate and is mediated by chorismate the OH group is replaced by a NH2 group. This might be a possibility. It has at least been demonstrated hat the second chorismate is strictly necessary while the ho5U formation might slowly and with lower yield occur under chorismate absence. The ability of modified U to read all codons [61]. Nasvall et al found 2 responsible genes, yecO(b1870,comA) and yecP(b1871,comB) [24]. No further information was found, so these were assumed to be monomers. `Dummy’ compounds were created since the chorismate (or the appropriate derivative thereof) dependent reaction is still unknown (with regards to its mechanism or intermediates). | [24,61] |
| 37 | G 🡪 m1G | 1-methylguanosine | C11H13N5O8P1  (-1) | TrmD_dim | tRNA (guanine-1-)-methyltransferase | 1 amet 🡪 1 ahcys + 1 h | Identification of TrmD [62-64]. tRNA- (m1G)methyltransferase at only approximately 80 molecules/genome in a glucose minimal culture [65] The trmD gene has a codon usage typical for a protein made in low amount in accordance with the low number of tRNA-(m1G)methyltransferase molecules found in the cell. TrmD, TrmH and RlmB, are part of SpoU protein family. | [22,62-67] |
| 37 | A 🡪 m2A | 2-methyladenosine | C11H13N5O7P1  (-1) | MeT_tRNA_pos_37_m2A | unknown methyltransferase pos 37 (m2A) | 1 amet 🡪 1 ahcys + 1 h | A catalytic enzyme has not been identified yet. Reaction is assumed to be similar to the other methylation reactions (with S-adenosyl-methionine as methyl-group donor) | [22] |
| 37 | A 🡪 ms2i6A | 2-methylthio-N6-isopentenyladenosine | C16H21N5O7S1P1  (-1) | MiaA_dim,MiaB_mono | isopentenyl-adenosine A37 tRNA methylthiolase, Δ(2)-isopentenylpyrophosphate tRNA-adenosine transferase | | A to i6A by MiaA | 1 dmpp --> 1 ppi | | --- | --- | | Formation of sulfur and methyl group by MiaB (and reduction of MiaB) | 1 IscS_dim_S-SH + 2 amet + 1 fldrd --> 1 ahcys + 2 h + 1 met-L + 1 fldox + 1 dad-5 + 1 IscS_dim_S-H | | **Overall reaction** | **1 dmpp + 1 IscS_dim_S-SH + 2 amet + 1 fldrd --> 1 ppi + 1 ahcys + 2 h + 1 met-L + 1 fldox + 1 dad-5 + 1 IscS_dim_S-H** | | See pos. 37 (i6A) for comments on MiaA and i6A formation. [4Fe-4S] cluster [25,68,69]. Function of TrmD was determined [70]. The reaction schema for radical S-Adenosyl-L-methionine (amet) enzymes was described in [71,72]. Thermogota protein is monomer [70]. No further information found, so the protein was assumed to be a monomer. Pierrel et al showed that 2 S-Adenosyl-L-methionine (amet) are necessary for formation of 1 ms2i6A from i6A [70]. The sulfur source is still unknown, however, studies with labeled cysteines showed that cysteines is primary source of sulfur atom [73,74]. However, data with selenium in this study showed that the sulfur is rather incorporated in protein prior transfer (similar to IscS). Dovhoma et al proposed a mechanism for S-Adenosyl-L-methionine (amet) radical enzymes [75]. For some enzymes it has been shown that flavodoxin is used to reduce iron-sulfur protein (see [72] for examples). It was assumed that such a reaction is catalyzed by miaB. It seems that iscS is necessary for sulfur incorporation in ms2i6A [7,26], similar to all 5 thio-nucleotides in Salmonella enterica serovar typhimurium [76]. IscS_dim_S- and SH/IscS_dim_S-H are used for sulfur transfer, assuming a mechanism similar to ThiI which gets the sulfur from IscS (see [1]for details). | [1,7,11,22,25,26,68-83] |
| 37 | A 🡪 i6A | N6-isopentenyladenosine | C15H19N5O7P1  (-1) | MiaA_dim | δ(2)-isopentenylpyrophosphate tRNA-adenosine transferase | 1 dmpp --> 1 ppi | MiaA exists as a dimer [79,80]. The corresponding gene was described by [81,82] | [26,77-82] |
| 37 | A 🡪 m6A | N6-methyladenosine | C11H13N5O7P1  (-1) | MeT_tRNA_pos_37_m6A | unknown Methyltransferase | 1 amet --> 1 ahcys + 1 h | A catalytic enzyme has not been identified yet. The reaction was assumed to be similar to other methylation reactions (with S-adenosyl-methionine as methyl-group donor). | [22] |
| 37 | A 🡪 m6t6A | N6-methyl-N6-threonylcarbamoyladenosine | C16H19N6O11P1  (-2) | Up_tRNA_pos_37_t6A,MeT_tRNA_pos_37_m6t6A | Unknown protein, S-adenosylmethionine:tRNA ribosyltransferase-isomerase | | t6A by unknown protein | 1 hco3 + 1 thr-L + 1 atp + 1 mg2 --> 1 h2o + 1 ppi + 1 h + 1 mg2 + 1 amp | | --- | --- | | Methylation (m6) by unknown Methyltransferase | 1 amet --> 1 ahcys + 1 h | | **Overall reaction** | **1 hco3 + 1 thr-L + 1 atp + 1 mg2 + 1 amet --> 1 h2o + 1 ppi + 2 h + 1 mg2 + 1 ahcys + 1 amp** | | See comments to t6 formation see pos 37 (t6A). tsaA is a methyltransferase for m6t6A [84]. Apparently identical to QueA gene (ecocyc) - however, the function of QueA (ribosyl-moiety is transferred) differs from the predicted function of TsaA (methyl-group transfer). TsaA was **not** included in the reconstruction for the moment. A lack of m6 did not alter the growth rate. | [22,84-87] |
| 37 | A 🡪 t6A | N6-threonylcarbamoyladenosine | C15H17N6O11P1  (-2) | Up_tRNA_pos_37_t6A | unknown protein | 1 hco3 + 1 thr-L + 1 atp + 1 mg2 --> 1 h2o + 1 ppi + 1 h + 1 mg2 + 1 amp | Unknown protein carries out this reaction. Korner et al. purified an enzyme from E. coli that carries out this reaction, however, since then no further report of this enzyme or even its gene could be found [86]. Reaction based on Elkins et al [85]. | [85-87] |
| 38 | U 🡪 Y | pseudouridine | C9H10N2O9P1  (-1) | TruA_dim | tRNA pseudouridine synthase I | --> | TruA exists as a dimer [88]. No ion was added. A catalytic mechanism is proposed by Gu et al [89]. | [88-91] |
| 39 | U 🡪 Y | pseudouridine | C9H10N2O9P1  (-1) | TruA_dim | tRNA pseudouridine synthase I | --> | TruA exists as a dimer [88]. No ion was added. A catalytic mechanism is proposed by Gu et al [89]. | [88-91] |
| 40 | U 🡪 Y | pseudouridine | C9H10N2O9P1  (-1) | TruA_dim | tRNA pseudouridine synthase I | --> | TruA exists as a dimer [88]. No ion was added. A catalytic mechanism is proposed by Gu et al [89]. | [88-91] |
| 46 | G 🡪 m7G | 7-methylguanosine | C11H13N5O8P1  (-1) | YggH_mono | tRNA (m7G46) methyltransferase | 1 amet --> 1 ahcys + 1 h | TrmB aka yggH. Identification [92]. Monomer (based on MW via gel filtration) [92]. Kinetic data can be found in [93]. The B. Subtilis protein forms a dimer in crystal and solution [94]:”However, unlike EcTrmB, BsTrmB is shown here to be dimeric both in the crystal and in solution. “. In light of all of the evidence, this will be described as a monomer. | [22,92-94] |
| 47 | U 🡪 acp3U | 3-(3-amino-3-carboxypropyl)uridine | C13H17N3O11P1  (-1) | AcpT_tRNA_pos_47_acp3U | unknown tRNA-uridine 3-(3-amino-3-carboxypropyl)transferase | 1 amet --> 1 5mta + 1 h | Enzymatic synthesis of 3-(3-amino-3-carboxypropyl) uridine in Escherichia coli phenylalanine transfer RNA: transfer of the 3-amino-acid-3-carboxypropyl group from S-adenosylmethionine [95]. The paper could not be found, however the reaction was in KEGG: S-adenosyl-L-methionine + tRNA uridine = 5'-methylthioadenosine (5mta) + tRNA 3-(3-amino-3-carboxypropyl)-uridine | [22,95] |
| 54 | U 🡪 m5U | Ribosylthymine | C10H12N2O9P1  (-1) | TrmA_mono | tRNA (uracil-5-)-methyltransferase | 1 amet --> 1 ahcys + 1 h | TrmA is essential. The protein was assumed to be a monomer based on a molecular weight of 42kDa (purified with antibodies) [96]. No FeS cluster identified [97]. m5U54 references [98-100]; [96,101] | [22,96-104] |
| 55 | U 🡪 Y | pseudouridine | C9H10N2O9P1  (-1) | TruB_mono | tRNA pseudouridine 55 synthase | --> | Active in monomer form. No ions were reported in the crystallized form [105]. | [105-107] |
| 65 | U 🡪 Y | pseudouridine | C9H10N2O9P1  (-1) | YqcB_mono | tRNA pseudouridine 65 synthase | --> | Active in a monomer form (based on a molecular weight of 29.7) [90]. | [90] |

1. Ikeuchi Y, Shigi N, Kato J, Nishimura A, Suzuki T (2006) Mechanistic insights into sulfur relay by multiple sulfur mediators involved in thiouridine biosynthesis at tRNA wobble positions. Mol Cell 21: 97-108.

2. Kambampati R, Lauhon CT (2000) Evidence for the transfer of sulfane sulfur from IscS to ThiI during the in vitro biosynthesis of 4-thiouridine in Escherichia coli tRNA. J Biol Chem 275: 10727-10730.

3. Palenchar PM, Buck CJ, Cheng H, Larson TJ, Mueller EG (2000) Evidence that ThiI, an enzyme shared between thiamin and 4-thiouridine biosynthesis, may be a sulfurtransferase that proceeds through a persulfide intermediate. J Biol Chem 275: 8283-8286.

4. Mueller EG, Palenchar PM, Buck CJ (2001) The role of the cysteine residues of ThiI in the generation of 4-thiouridine in tRNA. J Biol Chem 276: 33588-33595.

5. Waterman DG, Ortiz-Lombardia M, Fogg MJ, Koonin EV, Antson AA (2006) Crystal structure of Bacillus anthracis ThiI, a tRNA-modifying enzyme containing the predicted RNA-binding THUMP domain. J Mol Biol 356: 97-110.

6. Feist AM, Henry CS, Reed JL, Krummenacker M, Joyce AR, et al. (2007) A genome-scale metabolic reconstruction for Escherichia coli K-12 MG1655 that accounts for 1260 ORFs and thermodynamic information. Mol Syst Biol 3.

7. Lauhon CT (2002) Requirement for IscS in biosynthesis of all thionucleosides in Escherichia coli. J Bacteriol 184: 6820-6829.

8. Lauhon CT, Erwin WM, Ton GN (2004) Substrate specificity for 4-thiouridine modification in Escherichia coli. J Biol Chem 279: 23022-23029.

9. Lauhon CT, Kambampati R (2000) The iscS gene in Escherichia coli is required for the biosynthesis of 4-thiouridine, thiamin, and NAD. J Biol Chem 275: 20096-20103.

10. Kambampati R, Lauhon CT (1999) IscS is a sulfurtransferase for the in vitro biosynthesis of 4-thiouridine in Escherichia coli tRNA. Biochemistry 38: 16561-16568.

11. Mueller EG (2006) Trafficking in persulfides: delivering sulfur in biosynthetic pathways. Nat Chem Biol 2: 185-194.

12. Hoang C, Ferre-D'Amare AR (2004) Crystal structure of the highly divergent pseudouridine synthase TruD reveals a circular permutation of a conserved fold. Rna 10: 1026-1033.

13. Ericsson UB, Nordlund P, Hallberg BM (2004) X-ray structure of tRNA pseudouridine synthase TruD reveals an inserted domain with a novel fold. FEBS Lett 565: 59-64.

14. Kaya Y, Del Campo M, Ofengand J, Malhotra A (2004) Crystal structure of TruD, a novel pseudouridine synthase with a new protein fold. J Biol Chem 279: 18107-18110.

15. Bishop AC, Xu J, Johnson RC, Schimmel P, de Crecy-Lagard V (2002) Identification of the tRNA-dihydrouridine synthase family. J Biol Chem 277: 25090-25095.

16. Xing F, Martzen MR, Phizicky EM (2002) A conserved family of Saccharomyces cerevisiae synthases effects dihydrouridine modification of tRNA. Rna 8: 370-381.

17. Green M, Cohen SS (1957) Studies on the biosynthesis of bacterial and viral pyrimidines. II. Dihydrouracil and dihydrothymine nucleosides. J Biol Chem 225: 397-407.

18. Dalluge JJ, Hashizume T, McCloskey JA (1996) Quantitative measurement of dihydrouridine in RNA using isotope dilution liquid chromatography-mass spectrometry (LC/MS). Nucleic Acids Res 24: 3242-3245.

19. Persson BC, Jager G, Gustafsson C (1997) The spoU gene of Escherichia coli, the fourth gene of the spoT operon, is essential for tRNA (Gm18) 2'-O-methyltransferase activity. Nucleic Acids Res 25: 4093-4097.

20. Watanabe K, Nureki O, Fukai S, Ishii R, Okamoto H, et al. (2005) Roles of conserved amino acid sequence motifs in the SpoU (TrmH) RNA methyltransferase family. J Biol Chem 280: 10368-10377.

21. Phelps SS, Malkiewicz A, Agris PF, Joseph S (2004) Modified nucleotides in tRNA(Lys) and tRNA(Val) are important for translocation. J Mol Biol 338: 439-444.

22. Fontecave M, Atta M, Mulliez E (2004) S-adenosylmethionine: nothing goes to waste. Trends Biochem Sci 29: 243-249.

23. Lee CH, Tinoco I, Jr. (1977) Studies of the conformation of modified dinucleoside phosphates containing 1,N6-ethenoadenosine and 2'-O-methylcytidine by 360-MHz 1H nuclear magnetic resonance spectroscopy. Investigation of the solution conformations of dinucleoside phosphates. Biochemistry 16: 5403-5414.

24. Nasvall SJ, Chen P, Bjork GR (2004) The modified wobble nucleoside uridine-5-oxyacetic acid in tRNAPro(cmo5UGG) promotes reading of all four proline codons in vivo. Rna 10: 1662-1673.

25. Jager G, Leipuviene R, Pollard MG, Qian Q, Bjork GR (2004) The conserved Cys-X1-X2-Cys motif present in the TtcA protein is required for the thiolation of cytidine in position 32 of tRNA from Salmonella enterica serovar Typhimurium. J Bacteriol 186: 750-757.

26. Lauhon CT, Skovran E, Urbina HD, Downs DM, Vickery LE (2004) Substitutions in an active site loop of Escherichia coli IscS result in specific defects in Fe-S cluster and thionucleoside biosynthesis in vivo. J Biol Chem 279: 19551-19558.

27. Raychaudhuri S, Niu L, Conrad J, Lane BG, Ofengand J (1999) Functional effect of deletion and mutation of the Escherichia coli ribosomal RNA and tRNA pseudouridine synthase RluA. J Biol Chem 274: 18880-18886.

28. Wrzesinski J, Nurse K, Bakin A, Lane BG, Ofengand J (1995) A dual-specificity pseudouridine synthase: an Escherichia coli synthase purified and cloned on the basis of its specificity for psi 746 in 23S RNA is also specific for psi 32 in tRNA(phe). Rna 1: 437-448.

29. Numata T, Fukai S, Ikeuchi Y, Suzuki T, Nureki O (2006) Structural basis for sulfur relay to RNA mediated by heterohexameric TusBCD complex. Structure 14: 357-366.

30. Kambampati R, Lauhon CT (2003) MnmA and IscS are required for in vitro 2-thiouridine biosynthesis in Escherichia coli. Biochemistry 42: 1109-1117.

31. Scrima A, Vetter IR, Armengod ME, Wittinghofer A (2005) The structure of the TrmE GTP-binding protein and its implications for tRNA modification. Embo J 24: 23-33.

32. Bujnicki JM, Oudjama Y, Roovers M, Owczarek S, Caillet J, et al. (2004) Identification of a bifunctional enzyme MnmC involved in the biosynthesis of a hypermodified uridine in the wobble position of tRNA. Rna 10: 1236-1242.

33. Hagervall TG, Edmonds CG, McCloskey JA, Bjork GR (1987) Transfer RNA(5-methylaminomethyl-2-thiouridine)-methyltransferase from Escherichia coli K-12 has two enzymatic activities. J Biol Chem 262: 8488-8495.

34. Hagervall TG, Bjork GR (1984) Genetic mapping and cloning of the gene (trmC) responsible for the synthesis of tRNA (mnm5s2U)methyltransferase in Escherichia coli K12. Mol Gen Genet 196: 201-207.

35. Bjork GR, Kjellin-Straby K (1978) Escherichia coli mutants with defects in the biosynthesis of 5-methylaminomethyl-2-thio-uridine or 1-methylguanosine in their tRNA. J Bacteriol 133: 508-517.

36. Wittwer AJ, Stadtman TC (1986) Biosynthesis of 5-methylaminomethyl-2-selenouridine, a naturally occurring nucleoside in Escherichia coli tRNA. Arch Biochem Biophys 248: 540-550.

37. Wolf J, Gerber AP, Keller W (2002) tadA, an essential tRNA-specific adenosine deaminase from Escherichia coli. Embo J 21: 3841-3851.

38. Losey HC, Ruthenburg AJ, Verdine GL (2006) Crystal structure of Staphylococcus aureus tRNA adenosine deaminase TadA in complex with RNA. Nat Struct Mol Biol 13: 153-159.

39. Elias Y, Huang RH (2005) Biochemical and structural studies of A-to-I editing by tRNA:A34 deaminases at the wobble position of transfer RNA. Biochemistry 44: 12057-12065.

40. Soma A, Ikeuchi Y, Kanemasa S, Kobayashi K, Ogasawara N, et al. (2003) An RNA-modifying enzyme that governs both the codon and amino acid specificities of isoleucine tRNA. Mol Cell 12: 689-698.

41. Ikeuchi Y, Soma A, Ote T, Kato J, Sekine Y, et al. (2005) molecular mechanism of lysidine synthesis that determines tRNA identity and codon recognition. Mol Cell 19: 235-246.

42. Stern L, Schulman LH (1978) The role of the minor base N4-acetylcytidine in the function of the Escherichia coli noninitiator methionine transfer RNA. J Biol Chem 253: 6132-6139.

43. Slany RK, Bosl M, Crain PF, Kersten H (1993) A new function of S-adenosylmethionine: the ribosyl moiety of AdoMet is the precursor of the cyclopentenediol moiety of the tRNA wobble base queuine. Biochemistry 32: 7811-7817.

44. Noguchi S, Nishimura Y, Hirota Y, Nishimura S (1982) Isolation and characterization of an Escherichia coli mutant lacking tRNA-guanine transglycosylase. Function and biosynthesis of queuosine in tRNA. J Biol Chem 257: 6544-6550.

45. Chong S, Curnow AW, Huston TJ, Garcia GA (1995) tRNA-guanine transglycosylase from Escherichia coli is a zinc metalloprotein. Site-directed mutagenesis studies to identify the zinc ligands. Biochemistry 34: 3694-3701.

46. Garcia GA, Koch KA, Chong S (1993) tRNA-guanine transglycosylase from Escherichia coli. Overexpression, purification and quaternary structure. J Mol Biol 231: 489-497.

47. Mathews I, Schwarzenbacher R, McMullan D, Abdubek P, Ambing E, et al. (2005) Crystal structure of S-adenosylmethionine:tRNA ribosyltransferase-isomerase (QueA) from Thermotoga maritima at 2.0 A resolution reveals a new fold. Proteins 59: 869-874.

48. Van Lanen SG, Reader JS, Swairjo MA, de Crecy-Lagard V, Lee B, et al. (2005) From cyclohydrolase to oxidoreductase: discovery of nitrile reductase activity in a common fold. Proc Natl Acad Sci U S A 102: 4264-4269.

49. Gaur R, Varshney U (2005) Genetic analysis identifies a function for the queC (ybaX) gene product at an initial step in the queuosine biosynthetic pathway in Escherichia coli. J Bacteriol 187: 6893-6901.

50. Smulson ME, Suhadolnik RJ (1967) The biosynthesis of the 7-deazaadenine ribonucleoside, tubercidin, by Streptomyces tubercidicus. J Biol Chem 242: 2872-2876.

51. Suhadolnik RJ, Uematsu T (1970) Biosynthesis of the pyrrolopyrimidine nucleoside antibiotic, toyocamycin. VII. Origin of the pyrrole carbons and the cyano carbon. J Biol Chem 245: 4365-4371.

52. Van Lanen SG, Iwata-Reuyl D (2003) Kinetic mechanism of the tRNA-modifying enzyme S-adenosylmethionine:tRNA ribosyltransferase-isomerase (QueA). Biochemistry 42: 5312-5320.

53. Mueller SO, Slany RK (1995) Structural analysis of the interaction of the tRNA modifying enzymes Tgt and QueA with a substrate tRNA. FEBS Lett 361: 259-264.

54. Kuchino Y, Kasai H, Nihei K, Nishimura S (1976) Biosynthesis of the modified nucleoside Q in transfer RNA. Nucleic Acids Res 3: 393-398.

55. Frey B, McCloskey J, Kersten W, Kersten H (1988) New function of vitamin B12: cobamide-dependent reduction of epoxyqueuosine to queuosine in tRNAs of Escherichia coli and Salmonella typhimurium. J Bacteriol 170: 2078-2082.

56. Iwata-Reuyl D (2003) Biosynthesis of the 7-deazaguanosine hypermodified nucleosides of transfer RNA. Bioorg Chem 31: 24-43.

57. Van Lanen SG, Kinzie SD, Matthieu S, Link T, Culp J, et al. (2003) tRNA modification by S-adenosylmethionine:tRNA ribosyltransferase-isomerase. Assay development and characterization of the recombinant enzyme. J Biol Chem 278: 10491-10499.

58. Reader JS, Metzgar D, Schimmel P, de Crecy-Lagard V (2004) Identification of four genes necessary for biosynthesis of the modified nucleoside queuosine. J Biol Chem 279: 6280-6285.

59. Brenk R, Stubbs MT, Heine A, Reuter K, Klebe G (2003) Flexible adaptations in the structure of the tRNA-modifying enzyme tRNA-guanine transglycosylase and their implications for substrate selectivity, reaction mechanism and structure-based drug design. Chembiochem 4: 1066-1077.

60. Reuter K, Slany R, Ullrich F, Kersten H (1991) Structure and organization of Escherichia coli genes involved in biosynthesis of the deazaguanine derivative queuine, a nutrient factor for eukaryotes. J Bacteriol 173: 2256-2264.

61. Yamada Y, Matsugi J, Ishikura H, Murao K (2005) Bacillus subtilis tRNA(Pro) with the anticodon mo5UGG can recognize the codon CCC. Biochim Biophys Acta 1728: 143-149.

62. Marinus MG, Morris NR, Soll D, Kwong TC (1975) Isolation and partial characterization of three Escherichia coli mutants with altered transfer ribonucleic acid methylases. J Bacteriol 122: 257-265.

63. Bystrom AS, Bjork GR (1982) The structural gene (trmD) for the tRNA(m1G)methyltransferase is part of a four polypeptide operon in Escherichia coli K-12. Mol Gen Genet 188: 447-454.

64. Bystrom AS, Bjork GR (1982) Chromosomal location and cloning of the gene (trmD) responsible for the synthesis of tRNA (m1G) methyltransferase in Escherichia coli K-12. Mol Gen Genet 188: 440-446.

65. Bystrom AS, Hjalmarsson KJ, Wikstrom PM, Bjork GR (1983) The nucleotide sequence of an Escherichia coli operon containing genes for the tRNA(m1G)methyltransferase, the ribosomal proteins S16 and L19 and a 21-K polypeptide. Embo J 2: 899-905.

66. Bystrom AS, von Gabain A, Bjork GR (1989) Differentially expressed trmD ribosomal protein operon of Escherichia coli is transcribed as a single polycistronic mRNA species. J Mol Biol 208: 575-586.

67. Elkins PA, Watts JM, Zalacain M, van Thiel A, Vitazka PR, et al. (2003) Insights into catalysis by a knotted TrmD tRNA methyltransferase. J Mol Biol 333: 931-949.

68. Pierrel F, Hernandez HL, Johnson MK, Fontecave M, Atta M (2003) MiaB protein from Thermotoga maritima. Characterization of an extremely thermophilic tRNA-methylthiotransferase. J Biol Chem 278: 29515-29524.

69. Pierrel F, Bjork GR, Fontecave M, Atta M (2002) Enzymatic modification of tRNAs: MiaB is an iron-sulfur protein. J Biol Chem 277: 13367-13370.

70. Pierrel F, Douki T, Fontecave M, Atta M (2004) MiaB protein is a bifunctional radical-S-adenosylmethionine enzyme involved in thiolation and methylation of tRNA. J Biol Chem 279: 47555-47563.

71. Layer G, Grage K, Teschner T, Schunemann V, Breckau D, et al. (2005) Radical S-adenosylmethionine enzyme coproporphyrinogen III oxidase HemN: functional features of the [4Fe-4S] cluster and the two bound S-adenosyl-L-methionines. J Biol Chem 280: 29038-29046.

72. Jarrett JT (2003) The generation of 5'-deoxyadenosyl radicals by adenosylmethionine-dependent radical enzymes. Curr Opin Chem Biol 7: 174-182.

73. Peterkofsky A, Lipsett MN (1965) The origin of the sulfur in s-RNA. Biochem Biophys Res Commun 20: 780-786.

74. Lipsett MN, Peterkofsky A (1966) Enzymatic thiolation of E. coli sRNA. Proc Natl Acad Sci U S A 55: 1169-1174.

75. Dohvoma CN (1992) Epidermoid cyst: an unusual cause of obstructive sialadenitis. Br J Oral Maxillofac Surg 30: 125-127.

76. Nilsson K, Lundgren HK, Hagervall TG, Bjork GR (2002) The cysteine desulfurase IscS is required for synthesis of all five thiolated nucleosides present in tRNA from Salmonella enterica serovar typhimurium. J Bacteriol 184: 6830-6835.

77. Moore JA, Poulter CD (1997) Escherichia coli dimethylallyl diphosphate:tRNA dimethylallyltransferase: a binding mechanism for recombinant enzyme. Biochemistry 36: 604-614.

78. Leung HC, Chen Y, Winkler ME (1997) Regulation of substrate recognition by the MiaA tRNA prenyltransferase modification enzyme of Escherichia coli K-12. J Biol Chem 272: 13073-13083.

79. Connolly DM, Winkler ME (1991) Structure of Escherichia coli K-12 miaA and characterization of the mutator phenotype caused by miaA insertion mutations. J Bacteriol 173: 1711-1721.

80. Bartz JK, Kline LK, Soll D (1970) N6-(Delta 2-isopentenyl)adenosine: biosynthesis in vitro in transfer RNA by an enzyme purified from Escherichia coli. Biochem Biophys Res Commun 40: 1481-1487.

81. Connolly DM, Winkler ME (1989) Genetic and physiological relationships among the miaA gene, 2-methylthio-N6-(delta 2-isopentenyl)-adenosine tRNA modification, and spontaneous mutagenesis in Escherichia coli K-12. J Bacteriol 171: 3233-3246.

82. Caillet J, Droogmans L (1988) Molecular cloning of the Escherichia coli miaA gene involved in the formation of delta 2-isopentenyl adenosine in tRNA. J Bacteriol 170: 4147-4152.

83. Moore JA, Mathis JR, Poulter CD (2000) Escherichia coli dimethylallyl diphosphate:tRNA dimethylallyltransferase: pre-steady-state kinetic studies. Biochim Biophys Acta 1479: 166-174.

84. Qian Q, Curran JF, Bjork GR (1998) The methyl group of the N6-methyl-N6-threonylcarbamoyladenosine in tRNA of Escherichia coli modestly improves the efficiency of the tRNA. J Bacteriol 180: 1808-1813.

85. Elkins BN, Keller EB (1974) The enzymatic synthesis of N-(purin-6-ylcarbamoyl)threonine, an anticodon-adjacent base in transfer ribonucleic acid. Biochemistry 13: 4622-4628.

86. Korner A, Soll D (1974) N-(purin-6-ylcarbamoyl)threonine: biosynthesis in vitro in transfer RNA by an enzyme purified from Escherichia coli. FEBS Lett 39: 301-306.

87. Chheda GB, Hong CI, Piskorz CF, Harmon GA (1972) Biosynthesis of N-(purin-6-ylcarbamoyl)-L-threonine riboside. Incorporation of L-threonine in vivo into modified nucleoside of transfer ribonucleic acid. Biochem J 127: 515-519.

88. Foster PG, Huang L, Santi DV, Stroud RM (2000) The structural basis for tRNA recognition and pseudouridine formation by pseudouridine synthase I. Nat Struct Biol 7: 23-27.

89. Gu X, Liu Y, Santi DV (1999) The mechanism of pseudouridine synthase I as deduced from its interaction with 5-fluorouracil-tRNA. Proc Natl Acad Sci U S A 96: 14270-14275.

90. Del Campo M, Kaya Y, Ofengand J (2001) Identification and site of action of the remaining four putative pseudouridine synthases in Escherichia coli. Rna 7: 1603-1615.

91. Bruni CB, Colantuoni V, Sbordone L, Cortese R, Blasi F (1977) Biochemical and regulatory properties of Escherichia coli K-12 hisT mutants. J Bacteriol 130: 4-10.

92. De Bie LG, Roovers M, Oudjama Y, Wattiez R, Tricot C, et al. (2003) The yggH gene of Escherichia coli encodes a tRNA (m7G46) methyltransferase. J Bacteriol 185: 3238-3243.

93. Purta E, van Vliet F, Tricot C, De Bie LG, Feder M, et al. (2005) Sequence-structure-function relationships of a tRNA (m7G46) methyltransferase studied by homology modeling and site-directed mutagenesis. Proteins 59: 482-488.

94. Zegers I, Gigot D, van Vliet F, Tricot C, Aymerich S, et al. (2006) Crystal structure of Bacillus subtilis TrmB, the tRNA (m7G46) methyltransferase. Nucleic Acids Res 34: 1925-1934.

95. Nishimura S, Taya Y, Kuchino Y, Oashi Z (1974) Enzymatic synthesis of 3-(3-amino-3-carboxypropyl)uridine in Escherichia coli phenylalanine transfer RNA: transfer of the 3-amino-acid-3-carboxypropyl group from S-adenosylmethionine. Biochem Biophys Res Commun 57: 702-708.

96. Persson BC, Gustafsson C, Berg DE, Bjork GR (1992) The gene for a tRNA modifying enzyme, m5U54-methyltransferase, is essential for viability in Escherichia coli. Proc Natl Acad Sci U S A 89: 3995-3998.

97. Agarwalla S, Stroud RM, Gaffney BJ (2004) Redox reactions of the iron-sulfur cluster in a ribosomal RNA methyltransferase, RumA: optical and EPR studies. J Biol Chem 279: 34123-34129.

98. Gustafsson C, Lindstrom PH, Hagervall TG, Esberg KB, Bjork GR (1991) The trmA promoter has regulatory features and sequence elements in common with the rRNA P1 promoter family of Escherichia coli. J Bacteriol 173: 1757-1764.

99. Bjork GR, Isaksson LA (1970) Isolation of mutants of Escherichia coli lac king 5-methyluracil in transfer ribonucleic acid or 1-methylguanine in ribosomal RNA. J Mol Biol 51: 83-100.

100. Ny T, Lindstrom HR, Hagervall TG, Bjork GR (1988) Purification of transfer RNA (m5U54)-methyltransferase from Escherichia coli. Association with RNA. Eur J Biochem 177: 467-475.

101. Ny T, Bjork GR (1980) Cloning and restriction mapping of the trmA gene coding for transfer ribonucleic acid (5-methyluridine)-methyltransferase in Escherichia coli K-12. J Bacteriol 142: 371-379.

102. Lindstrom PH, Stuber D, Bjork GR (1985) Genetic organization and transcription from the gene (trmA) responsible for synthesis of tRNA (uracil-5)-methyltransferase by Escherichia coli. J Bacteriol 164: 1117-1123.

103. Denhardt DT (1969) Formation of ribosylthymine in Escherichia coli. Studies on pulse labeling with thymine and thymidine. J Biol Chem 244: 2710-2715.

104. Gu X, Ofengand J, Santi DV (1994) In vitro methylation of Escherichia coli 16S rRNA by tRNA (m5U54)-methyltransferase. Biochemistry 33: 2255-2261.

105. Hoang C, Ferre-D'Amare AR (2001) Cocrystal structure of a tRNA Psi55 pseudouridine synthase: nucleotide flipping by an RNA-modifying enzyme. Cell 107: 929-939.

106. Pan H, Agarwalla S, Moustakas DT, Finer-Moore J, Stroud RM (2003) Structure of tRNA pseudouridine synthase TruB and its RNA complex: RNA recognition through a combination of rigid docking and induced fit. Proc Natl Acad Sci U S A 100: 12648-12653.

107. Gutgsell N, Englund N, Niu L, Kaya Y, Lane BG, et al. (2000) Deletion of the Escherichia coli pseudouridine synthase gene truB blocks formation of pseudouridine 55 in tRNA in vivo, does not affect exponential growth, but confers a strong selective disadvantage in competition with wild-type cells. Rna 6: 1870-1881.
