# Supplementary material for: Genome-Scale Reconstruction of Escherichia coli's Transcriptional and Translational Machinery: A Knowledge Base, Its Mathematical Formulation, and Its Functional Characterization
Source: PLoS Comput Biol. 2009 Mar 13;5(3):e1000312. doi: 10.1371/journal.pcbi.1000312 (PMC2648898; doi:10.1371/journal.pcbi.1000312)
Supplement: Table S18 — References for individual network reactions (3.92 MB DOC) [file pcbi.1000312.s020.doc]

Thiele et al.: ‘Genome-scale reconstruction of *E. coli*'s transcriptional and translational machinery: A knowledge-base and its mathematical formulation’.

S18 – References for individual network reactions

| **Reaction Abbreviation** | **Reaction name** | **Reaction** | **Directionality** | **Subsystem** | **Comments** | **References** |
| --- | --- | --- | --- | --- | --- | --- |
| Ala_RS_CHARG | Alanyl-tRNA synthetase charging | 1 Ala_RS_tetra + 1 ala-L + 1 atp + 1 h2o --> 1 Ala_RS_ala_amp + 1 h + 1 ppi | irreversible | Aminoacyl-tRNA synthetase charging | Class II Synthetase [1] | [1-3] |
| Arg_RS_CHARG | Arginyl-tRNA synthetase charging | 1 ArgS_mono + 1 arg-L + 1 atp + 1 h2o --> 1 Arg_RS_arg_amp + 1 h + 1 ppi | irreversible | Aminoacyl-tRNA synthetase charging | Class I Synthetase [1]. The wild-type fraction of holoenzyme of total protein is 0.81. The number of molecules per genome is 510 (E. coli NC3 grown in MOPS minimal medium with glucose, T=37°C) [4]. | [1,4-7] |
| Asn_RS_CHARG | Asparaginyl-tRNA synthetase charging | 1 Asn_RS_dim + 1 asn-L + 1 atp + 1 h2o --> 1 Asn_RS_asn_amp + 1 h + 1 ppi | irreversible | Aminoacyl-tRNA synthetase charging | Class II Synthetase [1] | [1,8,9] |
| Asp_RS_CHARGa | Aspartyl-tRNA synthetase charging | 1 Asp_RS_dim + 2 asp-L + 2 atp + 2 h2o --> 1 Asp_RS_2asp_2amp + 2 h + 2 ppi | irreversible | Aminoacyl-tRNA synthetase charging | Class II Synthetase [1] | [1,10,11] |
| Asp_RS_CHARGb | Aspartyl-tRNA synthetase charging | 1 Asp_RS_dim + 1 asp-L + 1 atp + 1 h2o --> 1 Asp_RS_asp_amp + 1 h + 1 ppi | irreversible | Aminoacyl-tRNA synthetase charging | Class II Synthetase [1] | [1,10,11] |
| Cys_RS_CHARG | Cysteinyl-tRNA synthetase charging | 1 CysS_mono + 1 atp + 1 cys-L + 1 h2o --> 1 Cys_RS_cys_amp + 1 h + 1 ppi | irreversible | Aminoacyl-tRNA synthetase charging | Class I Synthetase [1] | [1,12,13] |
| Gln_RS_CHARG | Glutaminyl-tRNA synthetase charging | 1 GlnS_mono + 1 atp + 1 gln-L + 1 h2o --> 1 Gln_RS_gln_amp + 1 h + 1 ppi | irreversible | Aminoacyl-tRNA synthetase charging | Class I Synthetase [1]. The wild-type fraction of holoenzyme of total protein is 1.06 [4]. The number of molecules per genome is 676 (E. coli NC3 grown in MOPS minimal medium with glucose, T=37°C) [4]. | [1,4,14,15] |
| Glu_RS_CHARG | Glutamyl-tRNA synthetase charging | 1 GltX_mono + 1 atp + 1 glu-L + 1 h2o --> 1 Glu_RS_glu_amp + 1 h + 1 ppi | irreversible | Aminoacyl-tRNA synthetase charging | Class I Synthetase [1]. The wild-type fraction of holoenzyme of total protein is 1.25 [4].The number of molecules per genome is 539 (E. coli NC3 grown in MOPS minimal medium with glucose, T=37°C) [4]. | [1,4] |
| Gly_RS_CHARGa | Glycyl-tRNA synthetase charging | 1 Gly_RS_tetra + 2 atp + 2 gly + 2 h2o --> 1 Gly_RS_2gly_2amp + 2 h + 2 ppi | irreversible | Aminoacyl-tRNA synthetase charging | Class II Synthetase [1]. The wild-type fraction of holoenzyme of total protein is 2.11. The number of molecules per genome is 412 (E. coli NC3 grown in MOPS minimal medium with glucose, T=37°C) [4]. | [1,4,16] |
| Gly_RS_CHARGb | Glycyl-tRNA synthetase charging | 1 Gly_RS_tetra + 1 atp + 1 gly + 1 h2o --> 1 Gly_RS_gly_amp + 1 h + 1 ppi | irreversible | Aminoacyl-tRNA synthetase charging | Class II Synthetase [1]. The wild-type fraction of holoenzyme of total protein is 2,11 [4]. The number of molecules per genomeis 412 (E. coli NC3 grown in MOPS minimal medium with glucose, T=37°C) [4]. | [1,4,16] |
| His_RS_CHARGa | Histidyl-tRNA synthetase charging | 1 His_RS_dim + 1 atp + 1 h2o + 1 his-L --> 1 His_RS_his_amp + 1 h + 1 ppi | irreversible | Aminoacyl-tRNA synthetase charging | Class II Synthetase [1] | [1,17-19] |
| His_RS_CHARGb | Histidyl-tRNA synthetase charging | 1 His_RS_dim + 2 atp + 2 h2o + 2 his-L --> 1 His_RS_2his_2amp + 2 h + 2 ppi | irreversible | Aminoacyl-tRNA synthetase charging | Class II Synthetase [1] | [1,17-19] |
| Ile_RS_CHARG | Isoleucyl-tRNA synthetase charging | 1 IleS_mono + 1 atp + 1 h2o + 1 ile-L --> 1 Ile_RS_ile_Amp + 1 h + 1 ppi | irreversible | Aminoacyl-tRNA synthetase charging | Class I Synthetase [1]. The wild-type fraction of holoenzyme of total protein is 2.29 [4]. The number of molecules per genome is 885 (E. coli NC3 grown in MOPS minimal medium with glucose, T=37°C) [4]. | [1,4,20,21] |
| Leu_RS_CHARG | Leucyl-tRNA synthetase charging | 1 LeuS_mono + 1 atp + 1 h2o + 1 leu-L --> 1 Leu_RS_leu_amp + 1 h + 1 ppi | irreversible | Aminoacyl-tRNA synthetase charging | Class I Synthetase [1]. The wild-type fraction of holoenzyme of total protein is 1.4 The number of molecules per genome is 597 (E. coli NC3 grown in MOPS minimal medium with glucose, T=37°C) [4]. | [1,4,20] |
| Lys_RSI_CHARGa | Lysyl-tRNA synthetase charging | 1 LysI_RS_dim + 1 atp + 1 h2o + 1 lys-L --> 1 LysI_RS_lys_amp + 1 h + 1 ppi | irreversible | Aminoacyl-tRNA synthetase charging | Class II Synthetase [1]. The wild-type fraction of holoenzyme of total protein is 1.05 [4]. The number of molecules per genome is 333. [4] did not distinguish between the 2 Lysyl-tRNA synthetase (E. coli NC3 grown in MOPS minimal medium with glucose, T=37°C) [4]. | [1,4,22] |
| Lys_RSI_CHARGb | Lysyl-tRNA synthetase charging | 1 LysI_RS_dim + 2 atp + 2 h2o + 2 lys-L --> 1 LysI_RS_2lys_2amp + 2 h + 2 ppi | irreversible | Aminoacyl-tRNA synthetase charging | Class II Synthetase [1]. The wild-type fraction of holoenzyme of total protein is 1.05[4]. The number of molecules per genome is 333. [4] did not distinguish between the 2 Lysyl-tRNA synthetase (E. coli NC3 grown in MOPS minimal medium with glucose, T=37°C) [4]. | [1,4,22] |
| Lys_RSII_CHARGa | Lysyl-tRNA synthetase II charging | 1 LysII_RS_dim + 1 atp + 1 h2o + 1 lys-L --> 1 LysII_RS_lys_amp + 1 h + 1 ppi | irreversible | Aminoacyl-tRNA synthetase charging | Class II Synthetase [1]. The wild-type fraction of holoenzyme of total protein is 1.05 [4]. The number of molecules per genome is 333. [4] did not distinguish between the 2 Lysyl-tRNA synthetase (E. coli NC3 grown in MOPS minimal medium with glucose, T=37°C). | [4,23-25] |
| Lys_RSII_CHARGb | Lysyl-tRNA synthetase II charging | 1 LysII_RS_dim + 2 atp + 2 h2o + 2 lys-L --> 1 LysII_RS_2lys_2amp + 2 h + 2 ppi | irreversible | Aminoacyl-tRNA synthetase charging | Class II Synthetase [1]. The wild-type fraction of holoenzyme of total protein is 1.05 [4]. The number of molecules per genome is 333. [4] did not distinguish between the 2 Lysyl-tRNA synthetase (E. coli NC3 grown in MOPS minimal medium with glucose, T=37°C) [4]. | [1,4,23-25] |
| Met_RS_CHARGa | Methionyl-tRNA synthetase charging | 1 Met_RS_dim + 2 atp + 2 h2o + 2 met-L --> 1 Met_RS_2met_2amp + 2 h + 2 ppi | irreversible | Aminoacyl-tRNA synthetase charging | Class I Synthetase [1] | [1,20,26-28] |
| Met_RS_CHARGb | Methionyl-tRNA synthetase charging | 1 Met_RS_dim + 1 atp + 1 h2o + 1 met-L --> 1 Met_RS_met_amp + 1 h + 1 ppi | irreversible | Aminoacyl-tRNA synthetase charging | Class I Synthetase [1] | [1,20,26-28] |
| Phe_RS_CHARG | Phenyl-tRNA synthetase charging | 1 Phe_RS_tetra + 1 atp + 1 h2o + 1 phe-L --> 1 Phe_RS_phe_amp + 1 h + 1 ppi | irreversible | Aminoacyl-tRNA synthetase charging | Class II Synthetase [1]. The wild-type fraction of holoenzyme of total protein is 3.94. The number of molecules per genome is 649 (E. coli NC3 grown in MOPS minimal medium with glucose, T=37°C) [4]. | [1,4,25,29] |
| Pro_RS_CHARGa | Prolyl-tRNA synthetase charging | 1 Pro_RS_dim + 2 atp + 2 h2o + 2 pro-L --> 1 Pro_RS_2pro_2amp + 2 h + 2 ppi | irreversible | Aminoacyl-tRNA synthetase charging | Class II Synthetase [1] | [1,30] |
| Pro_RS_CHARGb | Prolyl-tRNA synthetase charging | 1 Pro_RS_dim + 1 atp + 1 h2o + 1 pro-L --> 1 Pro_RS_pro_amp + 1 h + 1 ppi | irreversible | Aminoacyl-tRNA synthetase charging | Class II Synthetase [1] | [1,30] |
| Ser_RS_CHARGa | Seryl-tRNA synthetase charging | 1 Ser_RS_dim + 2 atp + 2 h2o + 2 ser-L --> 1 Ser_RS_2ser_2amp + 2 h + 2 ppi | irreversible | Aminoacyl-tRNA synthetase charging | Class II Synthetase [1] | [1,31] |
| Ser_RS_CHARGb | Seryl-tRNA synthetase charging | 1 Ser_RS_dim + 1 atp + 1 h2o + 1 ser-L --> 1 Ser_RS_ser_amp + 1 h + 1 ppi | irreversible | Aminoacyl-tRNA synthetase charging | Class II Synthetase [1] | [1,31] |
| Thr_RS_CHARGa | Threonyl-tRNA synthetase charging | 1 Thr_RS_dim + 2 atp + 2 h2o + 2 thr-L --> 1 Thr_RS_2thr_2amp + 2 h + 2 ppi | irreversible | Aminoacyl-tRNA synthetase charging | Class II Synthetase [1]. The wild-type fraction of holoenzyme of total protein is 0.92 [4]. The number of molecules per genome is 346 (E. coli NC3 grown in MOPS minimal medium with glucose, T=37°C) [4]. | [1,4,32,33] |
| Thr_RS_CHARGB | Threonyl-tRNA synthetase charging | 1 Thr_RS_dim + 1 atp + 1 h2o + 1 thr-L --> 1 Thr_RS_thr_amp + 1 h + 1 ppi | irreversible | Aminoacyl-tRNA synthetase charging | Class II Synthetase [1]. The wild-type fraction of holoenzyme of total protein is 0.92 [4]. Number of molecules per genome: 346 (E. coli NC3 grown in MOPS minimal medium with glucose, T=37°C). | [1,4,32,33] |
| Trp_RS_CHARGa | Tryptophyl-tRNA synthetase charging (tyr-D) | 1 Trp_RS_dim + 2 atp + 2 h2o + 2 trp-L --> 1 Trp_RS_2trp_2amp + 2 h + 2 ppi | irreversible | Aminoacyl-tRNA synthetase charging | Class II Synthetase [1] | [1,34-36] |
| Trp_RS_CHARGb | Tryptophyl-tRNA synthetase charging (tyr-D) | 1 Trp_RS_dim + 1 atp + 1 h2o + 1 trp-L --> 1 Trp_RS_trp_amp + 1 h + 1 ppi | irreversible | Aminoacyl-tRNA synthetase charging | Class II Synthetase [1] | [1,34-36] |
| Tyr_RS_CHARG1a | Tyrosinyl-tRNA synthetase charging | 1 Tyr_RS_dim + 1 atp + 1 h2o + 1 tyr-L --> 1 Tyr_RS_tyr_amp + 1 h + 1 ppi | irreversible | Aminoacyl-tRNA synthetase charging | Class I Synthetase [1] | [1,17,21,37,38] |
| Tyr_RS_CHARG1b | Tyrosinyl-tRNA synthetase charging | 1 Tyr_RS_dim + 2 atp + 2 h2o + 2 tyr-L --> 1 Tyr_RS_2tyr_2amp + 2 h + 2 ppi | irreversible | Aminoacyl-tRNA synthetase charging | Class I Synthetase [1] | [1,17,21,37,38] |
| Tyr_RS_CHARG2a | Tyrosinyl-tRNA synthetase charging (tyr-D) | 1 Tyr_RS_dim + 1 atp + 1 h2o + 1 tyr-D --> 1 Tyr_RS_tyr-D_amp + 1 h + 1 ppi | irreversible | Aminoacyl-tRNA synthetase charging | Class I Synthetase [1] | [1,17,37,38] |
| Tyr_RS_CHARG2b | Tyrosinyl-tRNA synthetase charging (tyr-D) | 1 Tyr_RS_dim + 2 atp + 2 h2o + 2 tyr-D --> 1 Tyr_RS_2tyr-D_2amp + 2 h + 2 ppi | irreversible | Aminoacyl-tRNA synthetase charging | Class I Synthetase [1] | [1,17,37,38] |
| Val_RS_CHARG | Valyl-tRNA synthetase charging | 1 ValS_mono + 1 atp + 1 h2o + 1 val-L --> 1 Val_RS_val_amp + 1 h + 1 ppi | irreversible | Aminoacyl-tRNA synthetase charging | Class I Synthetase [1]. The wild-type fraction of holoenzyme of total protein is 1.01 [4]. The number of molecules per genome is 425 (E. coli NC3 grown in MOPS minimal medium with glucose, T=37°C) [4]. | [1,4,20,39-41] |
| EF-Tu.EF-Ts_FORM | Formation of binary complex: EF-Tu and EF-Ts by release of GTP | 1 EF-Tu.GDP.EF-Ts --> 1 EF-Tu.EF-Ts + 1 gdp | irreversible | Charging EF-Tu | EF-Ts acts as a catalyst in the displacement of the GDP from the EF-Tu.GDP complex and allows the binding of GTP so that the ternary complex EF-Tu.GTP.aminoacyl-tRNA can be formed. The crystal structure of the complex has been solved in which no ions have been reported beside Mg2+ of EF-Tu [42]. The EF-Tu, EF-Ts complex has potentially a 1:1 stoichiometry. EF-Tu has higher affinity to GDP than to GTP. EF-Ts stimulates the dissociation of EF-Tu and GDP by formation of an tertiary complex: EF-Tu.GDP.EF-Ts (reversible). Subsequently, GDP is released. GTP binds to the binary complex EF-Tu.EF-Ts (reversible). This tertiary complex dissociates to EF-Tu.GTP and EF-Ts [43]. | [42-50] |
| EF-Tu.GDP.EF-TS_FORM | Formation of Complex of EF_Tu and EF_TS | 1 EF-Ts + 1 EF-Tu.GDP <==> 1 EF-Tu.GDP.EF-Ts | reversible | Charging EF-Tu | EF-Ts acts as a catalyst in the displacement of the GDP from the EF-Tu.GDP complex and allows the binding of GTP so that the ternary complex EF-Tu.GTP.aminoacyl-tRNA can be formed. The crystal structure of the complex has been solved in which no ions have been reported beside Mg2+ of EF-Tu [42]. The EF-Tu, EF-Ts complex has potentially a 1:1 stoichiometry. EF-Tu has higher affinity to GDP than to GTP. EF-Ts stimulates the dissociation of EF-Tu and GDP by formation of an tertiary complex: EF-Tu.GDP.EF-Ts (reversible). Subsequently, GDP is released. GTP binds to the binary complex EF-Tu.EF-Ts (reversible). This tertiary complex dissociates to EF-Tu.GTP and EF-Ts [43]. | [42-50] |
| EF-Tu.GTP.EF-Ts_FORM | formation of the ternary complex EF-Tu, GTP and EF-Ts | 1 EF-Tu.EF-Ts + 1 gtp <==> 1 EF-Tu.GTP-EF-Ts | reversible | Charging EF-Tu | EF-Ts acts as a catalyst in the displacement of the GDP from the EF-Tu.GDP complex and allows the binding of GTP so that the ternary complex EF-Tu.GTP.aminoacyl-tRNA can be formed. The crystal structure of the complex has been solved in which no ions have been reported beside Mg2+ of EF-Tu [42]. The EF-Tu, EF-Ts complex has potentially a 1:1 stoichiometry. EF-Tu has higher affinity to GDP than to GTP. EF-Ts stimulates the dissociation of EF-Tu and GDP by formation of an tertiary complex: EF-Tu.GDP.EF-Ts (reversible). Subsequently, GDP is released. GTP binds to the binary complex EF-Tu.EF-Ts (reversible). This tertiary complex dissociates to EF-Tu.GTP and EF-Ts [43]. | [42-50] |
| EF-Tu.GTP_FORM | Formation of EF-Tu.GTP by dissociation of ternary complex EF-Tu.GTP.EF-Ts | 1 EF-Tu.GTP-EF-Ts --> 1 EF-Ts_inact + 1 EF-Tu.GTP | irreversible | Charging EF-Tu | EF-Ts acts as a catalyst in the displacement of the GDP from the EF-Tu.GDP complex and allows the binding of GTP so that the ternary complex EF-Tu.GTP.aminoacyl-tRNA can be formed. The crystal structure of the complex has been solved in which no ions have been reported beside Mg2+ of EF-Tu [42]. The EF-Tu, EF-Ts complex has potentially a 1:1 stoichiometry. EF-Tu has higher affinity to GDP than to GTP. EF-Ts stimulates the dissociation of EF-Tu and GDP by formation of an tertiary complex: EF-Tu.GDP.EF-Ts (reversible). Subsequently, GDP is released. GTP binds to the binary complex EF-Tu.EF-Ts (reversible). This tertiary complex dissociates to EF-Tu.GTP and EF-Ts [43]. | [42-50] |
| [2Fe-2S]_TRANSF | formation of [2Fe-2S] cluster | 2 IscU_dim_2Fe(2)-2S --> 1 IscU_dim_[2Fe-2S]2 + 2 IscU_mono | irreversible | Iron-sulfur cluster biosynthesis | It is possible that Fe2 instead of Fe3+ is bound by IscA. However, the literature is not very clear. Regardless which iron species get bound to IscA and transferred to IscU in the iron-sulfur cluster, a reducing agent would be necessary to transfer some of the electrons from the irons in order to produce [4Fe-4S]2+ cluster. Since there is no clear information available regarding such agent (although Kato et al. suggested that glutathione might participate in one of the final cluster forming reactions [51], Fe3+ and Fe2+ sulfur clusters are combined here, to produce [4Fe-4S]2+ cluster with charge balanced reactions | [51-57] |
| [2Fe-2S]_FORM | transfer of 2 Fe2 from IscA (tetramer) to 2 IscU (monomer) | 1 IscA_tetra_Fe(2) + 2 IscU_mono_S-SH --> 1 IscA_tetra + 1 IscU_dim_2Fe(2)-2S | irreversible | Iron-sulfur cluster biosynthesis |  | [56] |
| [4Fe-4S]_FORM | formation of [4Fe-4S] cluster | 1 IscU_dim_[2Fe-2S]2 --> 1 IscU_dim_[4Fe-4S] | irreversible | Iron-sulfur cluster biosynthesis | IscU_dim_[4Fe-4S] is used to transfer [4Fe-4S] to iron-sulfur cluster proteins | [52-57] |
| IscA-Fe_FORM | 2 Fe2 bind to IscA (tetramer) | 1 IscA_tetra + 2 fe2 + 1 h + 1 nadph + 1 trdox --> 1 IscA_tetra_Fe(2) + 1 nadp + 1 trdrd | irreversible | Iron-sulfur cluster biosynthesis | **See supplemental text for details** | [58] |
| IscA_TETRA | IscA tetramer formation | 4 IscA_mono --> 1 IscA_tetra | irreversible | Iron-sulfur cluster biosynthesis | IscA binds 2 iron per tetramer: [58]. Ding et al proposed it to be ascaffold protein but it cannot replace IscU in IscU- cells (in Azotobacter vinelandii) since IscU deletion is lethal [59]. Hence, it is not clear whether IscA is a scaffold protein or a Fe-donor: [60] | [53-55,58-62] |
| IscU_dim_S-SH_FORM1 | formation of IscU and IscS complex | 1 IscS_dim_S-SH + 1 IscU_dim_SH --> 1 IscS_IscU_cplx | irreversible | Iron-sulfur cluster biosynthesis | IscS and IscU form a 1:1 complex in which IscS and IscU are linked with each other through a disulfide bond. This bond is only formed in presence of L-cys. IscS has to have IscS-(SH)2 formed for complex formation with IscU [63], [51]. To increase the turnover rate of desulfurase reaction, IscU must be dissociated from IscS immediately (after S transfer). In Kurihara et al., they achieved this with addition of DTT but propose that this might be the funciton of the two chaperones encoded in the same operon (HscA and HscB), which have been shown to interact with IscU [63]. In addition, a reducing agent is nec which has been proposed to be gluthatione [51] | [51,53-56,60,63-66] |
| IscU_dim_S-SH_FORM2 | formation of IscU S-S bound | 1 IscS_IscU_cplx --> 1 IscS_dim_S-H + 1 IscU_mono + 1 IscU_mono_S-SH | irreversible | Iron-sulfur cluster biosynthesis | IscS and IscU form a 1:1 complex in which IscS and IscU are linked with each other through a disulfide bond. This bond is only formed in presence of L-cys. IscS has to have IscS-(SH)2 formed for complex formation with IscU [63], [51]. To increase the turnover rate of desulfurase reaction, IscU must be dissociated from IscS immediately (after S transfer). In Kurihara et al., they achieved this with addition of DTT but propose that this might be the funciton of the two chaperones encoded in the same operon (HscA and HscB), which have been shown to interact with IscU [63]. In addition, a reducing agent is nec which has been proposed to be gluthatione [51] | [51,53-56,60,63-66] |
| b0661_iron_sulfur_BIND1 | Incorporation of [4Fe-4S]2+ in b0661 | 1 IscU_dim_[4Fe-4S] + 1 b0661_m <==> 1 b0661_m_IscU_cplx | reversible | Iron-sulfur cluster incorporation | MiaB contains a iron-sulfur cluster | [67-69] |
| b0661_iron_sulfur_BIND2 | Incorporation of [4Fe-4S]2+ in b0661 | 1 b0661_m_IscU_cplx <==> 2 IscU_mono + 1 b0661_m_FeS | reversible | Iron-sulfur cluster incorporation | MiaB contains a iron-sulfur cluster | [67-69] |
| b0859_iron_sulfur_BIND1 | Incorporation of [4Fe-4S]2+ in b0859 | 1 IscU_dim_[4Fe-4S] + 1 b0859_m <==> 1 b0859_m_IscU_cplx | reversible | Iron-sulfur cluster incorporation | RumB is a homologe of RumA. RumB as [4Fe-4S]cluster based on the discussion in Agarwalla et al. [70]. No information about dimerization or other homomer formation could be found, hence, a monomer structure for functional protein was assumed. | [70] |
| b0859_iron_sulfur_BIND2 | Incorporation of [4Fe-4S]2+ in b0859 | 1 b0859_m_IscU_cplx <==> 2 IscU_mono + 1 b0859_m_FeS | reversible | Iron-sulfur cluster incorporation | RumB is a homologe of RumA. RumB as [4Fe-4S]cluster based on the discussion in Agarwalla et al. [70]. No information about dimerization or other homomer formation could be found, hence, a monomer structure for functional protein was assumed. | [70] |
| b2785_iron_sulfur_BIND1 | Incorporation of [4Fe-4S]2+ in b2785 | 1 IscU_dim_[4Fe-4S] + 1 b2785_m <==> 1 b2785_m_IscU_cplx | reversible | Iron-sulfur cluster incorporation | RumA contains a iron-sulfur cluster | [71] |
| b2785_iron_sulfur_BIND2 | Incorporation of [4Fe-4S]2+ in b2785 | 1 b2785_m_IscU_cplx <==> 2 IscU_mono + 1 b2785_m_FeS | reversible | Iron-sulfur cluster incorporation | RumA contains a iron-sulfur cluster | [71] |
| b0015_ion_BIND | Binding of ions to b0015 | 1 b0015_m + 2 zn2 <==> 1 b0015_m_Zn | reversible | Metallo-ion Binding | DnaJ_mono; 2 Zn2+ ions per monomer were found | [72-74] |
| b0026_ion_BIND | Binding of ions to b0026 | 1 b0026_m + 2 zn2 <==> 1 b0026_m_Zn | reversible | Metallo-ion Binding | IleS is an iron containing protein | [75-78] |
| b0168_ion_BIND | Binding of ions to b0168 | 1 b0168_m + 2 fe2 <==> 1 b0168_m_Fe | reversible | Metallo-ion Binding | Map is an iron containing protein | [79] |
| b0406_ion_BIND | Binding of ions to b0406 | 1 b0406_m + 1 zn2 <==> 1 b0406_m_Zn | reversible | Metallo-ion Binding | Tgt. A zinc binding site was identified in protein. 0.8 mol of zinc/mol of subunit were found to be bound [80]. | [80,81] |
| b0526_ion_BIND | Binding of ions to b0526 | 1 b0526_m + 1 zn2 <==> 1 b0526_m_Zn | reversible | Metallo-ion Binding | CysS is a zinc containing protein | [12,13] |
| b0642_ion_BIND | Binding of ions to b0642 | 1 b0642_m + 1 zn2 <==> 1 b0642_m_Zn | reversible | Metallo-ion Binding | LeuS is a zinc containing protein | [82,83] |
| b1114_ion_BIND | Binding of ions to b1114 | 1 b1114_m + 1 mg2 <==> 1 b1114_m_Mg | reversible | Metallo-ion Binding | Mfd. 1 Mg2+ was found in the ATP-binding pocket [84].- Mfd was included as monomer, not bound to atp but 1 mg2+ | [84] |
| b1286_ion_BIND | Binding of ions to b1286 | 1 b1286_m + 1 mg2 <==> 1 b1286_m_Mg | reversible | Metallo-ion Binding | Rnb (aka RNase II) is a magnesium containing protein | [85] |
| b1652_ion_BIND | Binding of ions to b1652 | 1 b1652_m + 2 mg2 <==> 1 b1652_m_Mg | reversible | Metallo-ion Binding | Rnt_mono contains 2 mg2+ per monomer. | [86-91] |
| b1719_ion_BIND | Binding of ions to b1719 | 1 b1719_m + 1 zn2 <==> 1 b1719_m_Zn | reversible | Metallo-ion Binding | ThrS_mono contains 1 zn2+ per monomer. | [33,75] |
| b1804_ion_BIND | Binding of ions to b1804 | 1 b1804_m + 5 mg2 <==> 1 b1804_m_Mg | reversible | Metallo-ion Binding | Rnd (aka Rnase D) seems to be a monomer. 5 divalent cations seems to be needed [88,92] | [88,92,93] |
| b1822_ion_BIND | Binding of ions to b1822 | 1 b1822_m + 1 zn2 <==> 1 b1822_m_Zn | reversible | Metallo-ion Binding | RrmA_mono contains 1 zn2+ per monomer. | [94] |
| b1876_ion_BIND | Binding of ions to b1876 | 1 b1876_m + 1 zn2 <==> 1 b1876_m_Zn | reversible | Metallo-ion Binding | ArgS. The reaction catalyzed by ArgS needs Mg-ATP and not ATP. Since it was not accounted for Mg-ATP in other reaction it was not done here either. In any case, Mg leaves reaction as Mg-ppi and therefore is not as crucial [7]. | [5-7,75] |
| b2114_ion_BIND | Binding of ions to b2114 | 1 b2114_m + 1 zn2 <==> 1 b2114_m_Zn | reversible | Metallo-ion Binding | MetG_mono contains 1 zn2+ per monomer. | [28,78,95,96] |
| b2268_ion_BIND | Binding of ions to b2268 | 1 b2268_m + 1 zn2 <==> 1 b2268_m_Zn | reversible | Metallo-ion Binding | ElaC_mono contans 1 zn2+ per monomer | [97,98] |
| b2400_ion_BIND | Binding of ions to b2400 | 1 b2400_m + 1 zn2 <==> 1 b2400_m_Zn | reversible | Metallo-ion Binding | GltX_mono contans 1 zn2+ per monomer | [99,100] |
| b2514_ion_BIND | Binding of ions to b2514 | 1 b2514_m + 2 mg2 <==> 1 b2514_m_Mg | reversible | Metallo-ion Binding | HisS_mono contains 2 mg2+ per monomer. A metal-binding site has been reported [19], however, since the sequence has no zinc-finger motif, Mg2+ seems to be the cation. | [17-19,101] |
| b2559_ion_BIND | Binding of ions to b2559 | 1 b2559_m + 1 zn2 <==> 1 b2559_m_Zn | reversible | Metallo-ion Binding | TadA_mono. The catalytic site, carring out the deaminase function, of this protein family (ADAR) has 3 zn2+ binding sites. [102]. The crystal strucutre of S. Aureus of tadA contained 2 Zn2+ (per homodimer). However, they did not specify Zn2+ in paper [103]. 2 zn2+ per homodimer were assumed based on the above evidence. | [102,103] |
| b2567_ion_BIND | Binding of ions to b2567 | 1 b2567_m + 1 mg2 <==> 1 b2567_m_Mg | reversible | Metallo-ion Binding | Rnc_mono contains 1 mg2+ per monomer | [104,105] |
| b2594_ion_BIND | Binding of ions to b2594 | 1 b2594_m + 1 mg2 <==> 1 b2594_m_Mg | reversible | Metallo-ion Binding | RluD. No ion was reported in the crystal structure [106], but necessity of Mg2+ reported [107]. 1 Mg2+ per monomer was concluded. | [106-108] |
| b2697_ion_BIND | Binding of ions to b2697 | 1 b2697_m + 1 zn2 <==> 1 b2697_m_Zn | reversible | Metallo-ion Binding | AlaS_mono contains 1 zn2+ per monomer. | [2] |
| b2779_ion_BIND | Binding of ions to b2779 | 1 b2779_m + 2 mg2 <==> 1 b2779_m_Mg | reversible | Metallo-ion Binding | Eno_mono contains 2 mg2+ per monomer | [109-112] |
| b3287_ion_BIND | Binding of ions to b3287 | 1 b3287_m + 1 fe2 <==> 1 b3287_m_Fe | reversible | Metallo-ion Binding | Def is an iron-binding protein. | [113] |
| b3649_ion_BIND | Binding of ions to b3649 | 1 b3649_m + 1 mg2 <==> 1 b3649_m_Mg | reversible | Metallo-ion Binding | RpoZ_mono has a metallo-ion based on CCDB. | [114] |
| b3887_ion_BIND | Binding of ions to b3887 | 1 b3887_m + 6 zn2 <==> 1 b3887_m_Zn | reversible | Metallo-ion Binding | Dtd_mono contains 6 zn2+ per monomer | [115] |
| b3987_ion_BIND | Binding of ions to b3987 | 1 b3987_m + 1 zn2 <==> 1 b3987_m_Zn | reversible | Metallo-ion Binding | RpoB contains a zinc ion per monomer. 4233 Molecules/Cell In: Growth Phase, Glucose-minimal MOPS Media [44]. 2,500 Molecules/Cell In: Glucose minimal media [44] Log phase (2max): 0.67 [116]. Stationary phase (2max): 0.14 (# of RNA mol/cell) [116] | [44,116-120] |
| b3988_ion_BIND | Binding of ions to b3988 | 1 b3988_m + 2 mg2 <==> 1 b3988_m_Mg | reversible | Metallo-ion Binding | RpoC. It was decided to include only the two Mg2+ ions also some researcher reported Zn2+ binding. However, others reported that beta prime is unable to bind Zn2+. Due to the contradictory information Mg2+ was decided to be the cation in the model. | [117-121] |
| b4129_ion_BIND | Binding of ions to b4129 | 1 b4129_m + 3 mg2 <==> 1 b4129_m_Mg | reversible | Metallo-ion Binding | LysU_mono contains 3 mg2+ per monomer. | [24,101] |
| b4162_ion_BIND | Binding of ions to b4162 | 1 b4162_m + 1 mg2 <==> 1 b4162_m_Mg | reversible | Metallo-ion Binding | Orn. Oligoribonulease. | [122] |
| b4171_ion_BIND | Binding of ions to b4171 | 1 b4171_m + 1 mg2 <==> 1 b4171_m_Mg | reversible | Metallo-ion Binding | MiaA_mono. The results of Leung et al. showed that a MiaA dimer, rather than a monomer, bound to each intact tRNA molecule at saturation: [123]. Moore and Poulter concluded that it is monomeric, but it seems that most evidence is for dimeric structure for catalytic enzyme,one dimer is need to bind 1 tRNA [123,124]. Mg2+ is required [124,125]. We concluded 1 Mg2+ per monomer. The amount of MiaA corresponds to about 660 monomers per cell and a cellular MiaA concentration of about 1.0 µM, where the volume of an E. coli cell was taken as 1.0 × 10-12 ml [123]. | [123-129] |
| AlaS_RS_TETRA | formation of alanyl-tRNA synthetase (homotetramer) | 4 AlaS_mono <==> 1 Ala_RS_tetra | reversible | Protein complex formation |  | [1,2] |
| AsnS_RS_DIM | dimerization of Asparaginyl-tRNA synthetase | 2 AsnS_mono <==> 1 Asn_RS_dim | reversible | Protein complex formation |  | [1,130] |
| AspS_RS_DIM | dimerization of Aspartyl-tRNA synthetase | 2 AspS_mono <==> 1 Asp_RS_dim | reversible | Protein complex formation |  | [11,75] |
| cisGroES_FORM | formation of cis GroES | 1 GroS_hepta --> 1 cisGroES_hepta | irreversible | Protein complex formation | There is a cis and a trans version of GroES which are distinct by their binding to the GroEL complex. Both proteins are identical and interchangible (see also cis/transGroES_CONV). | Modeling reasons |
| DEGRADOSOME_FORM | Degradosome complex formation | 1 Eno_dim + 1 Pnp_trim + 1 RNase_E_tetra + 1 RhlB_dim <==> 1 degradosome | reversible | Protein complex formation | The model version of the degradosome only consists of components that seem to be necessary/ essential for its action (Eno, Pnp, RNase_E, RhlB ) but not accessory factors (such as DnaK, GroEL, PPK, PAP, S1). | [109,112,131-137] |
| DnaJ_DIM | Dimerization of DnaJ (chaperone Hsp40, co-chaperone with DnaK) | 2 DnaJ_mono <==> 1 DnaJ_dim_inact | reversible | Protein complex formation |  | [138-140] |
| DnaK_mono.ATP_Form | Binding of ATP to DnaK | 1 DnaK_mono + 1 atp --> 1 DnaK_mono.ATP_inact | irreversible | Protein complex formation | DnaK is a monomer [141]. It was found that DnaK binds to zinc in a global study of E. coli’s zinc-binding proteins [142], however, we could not find further evidence in literature. DnaJ has 2 zinc binding centers one of which is crucial for the interaction with DnaK [72]. This might be the reason why Katayama et al identified DnaK as zinc binding protein. DnaK has ATP bound; the first binding of ATP is assumed to be spontaneous because no protein for ATP transfer to DnaK has been reported. DnaK and GrpE have a stoichiometry of 1:2 [141], [143]. | [72,141-143] |
| Dtd_FORM | D-tyr-tRNA_tyr deacylase protein complex formation | 2 Dtd_mono --> 1 Dtd_dim | irreversible | Protein complex formation |  | [115] |
| Dus_gen_FORMa | formation of generic Dus | 1 DusA_mono --> 1 Dus_gen | irreversible | Protein complex formation | DusA modifies uridine in tRNA to 5,6-dihydrouridine [144]. Reaction is NADH or NADPH dependent in yeast ([145]). The protein contains probably FMN sicne a FMN binding site was found in the sequence [144]). Furthermore the protein might be part of larger protein complex [144]. DusA is solely responsible for modification in tRNA fmet (b2814 (metZ), b2815 (metW), b2816 (metV), b3171 (metY)) [144]). No information about ions could be found. A monomer was assumed due to missing information. DusA accounts for about half of the 5,6-dihydrouridine modification observed in wild-type cellular tRNA, and DusB and DusC together account for the other half [144]. | [144,145] |
| Dus_gen_FORMb | formation of generic Dus | 1 DusB_mono --> 1 Dus_gen | irreversible | Protein complex formation | DusA modifies uridine in tRNA to 5,6-dihydrouridine [144]. Reaction is NADH or NADPH dependent in yeast ([145]). The protein contains probably FMN sicne a FMN binding site was found in the sequence [144]). Furthermore the protein might be part of larger protein complex [144]. DusA is solely responsible for modification in in tRNA fmet (b2814 (metZ), b2815 (metW), b2816 (metV), b3171 (metY)) [144]). No information about ions could be found. Monomer was assumed due to missing information. DusA accounts for about half of the 5,6-dihydrouridine modification observed in wild-type cellular tRNA, and DusB and DusC together account for the other half [144]. | [144,145] |
| Dus_gen_FORMc | formation of generic Dus | 1 DusC_mono --> 1 Dus_gen | irreversible | Protein complex formation | DusA modifies uridine in tRNA to 5,6-dihydrouridine [144]. Reaction is NADH or NADPH dependent in yeast ([145]). The protein contains probably FMN sicne a FMN binding site was found in the sequence [144]). Furthermore the protein might be part of larger protein complex [144]. DusA is solely responsible for modification in in tRNA fmet (b2814 (metZ), b2815 (metW), b2816 (metV), b3171 (metY)) [144]). No information about ions could be found. Monomer was assumed due to missing information. DusA accounts for about half of the 5,6-dihydrouridine modification observed in wild-type cellular tRNA, and DusB and DusC together account for the other half [144]. | [144,145] |
| EF-G_FORM | EF-G formation (GDP binding) | 1 FusA_mono + 1 gtp + 1 h2o + 1 mg2 --> 1 EF-G.GDP + 1 h + 1 pi | irreversible | Protein complex formation | Zavialov et al. reported that the free EF-G in the cell is likely to be in the GDP-bound form [146]. | [146-148] |
| EF-TU_FORM1a | EF-TU formation 1 (Ser1 acetylation,b3339) | 1 AcT_EF-TU + 1 TufA_mono + 1 accoa --> 1 AcT_EF-TU_cplx_a | irreversible | Protein complex formation |  | [42,149-151] |
| EF-TU_FORM1b | EF-TU formation 1 (Ser1 acetylation,b3980) | 1 AcT_EF-TU + 1 TufB_mono + 1 accoa --> 1 AcT_EF-TU_cplx_b | irreversible | Protein complex formation |  | [42,149-151] |
| EF-TU_FORM2a | EF-TU formation 2 (Ser1 acetylation,b3339) | 1 AcT_EF-TU_cplx_a --> 1 AcT_EF-TU_inact + 1 coa + 1 pEF-TU_ac_a | irreversible | Protein complex formation |  | [42,149-151] |
| EF-TU_FORM2b | EF-TU formation 2 (Ser1 acetylation,b3980) | 1 AcT_EF-TU_cplx_b --> 1 AcT_EF-TU_inact + 1 coa + 1 pEF-TU_ac_b | irreversible | Protein complex formation |  | [42,149-151] |
| EF-TU_FORM3a | EF-TU formation 3 (Lys56 methylation,b3339) | 1 MeT_EF-TU + 1 amet + 1 pEF-TU_ac_a --> 1 MeT_EF-TU_cplx_a | irreversible | Protein complex formation | Noort et al. reported that the methylation of EF-Tu is correlated with E. coli’s growth rate [152]. They found monomethylation during logarithmic growth phase, while dimethyllation occurs during stationary phase. | [42,149-154] |
| EF-TU_FORM3b | EF-TU formation 3 (Lys56 methylation,b3980) | 1 MeT_EF-TU + 1 amet + 1 pEF-TU_ac_b --> 1 MeT_EF-TU_cplx_b | irreversible | Protein complex formation | Noort et al. reported that the methylation of EF-Tu is correlated with E. coli’s growth rate [152]. They found monomethylation during logarithmic growth phase, while dimethyllation occurs during stationary phase. | [42,149-154] |
| EF-TU_FORM4a | EF-TU formation 4 (Lys56 methylation,b3339) | 1 MeT_EF-TU_cplx_a --> 1 MeT_EF-TU_inact + 1 ahcys + 1 h + 1 pEF-TU_me_a | irreversible | Protein complex formation | Noort et al. reported that the methylation of EF-Tu is correlated with E. coli’s growth rate [152]. They found monomethylation during logarithmic growth phase, while dimethyllation occurs during stationary phase. | [42,149-154] |
| EF-TU_FORM4b | EF-TU formation 4 (Lys56 methylation,b3980) | 1 MeT_EF-TU_cplx_b --> 1 MeT_EF-TU_inact + 1 ahcys + 1 h + 1 pEF-TU_me_b | irreversible | Protein complex formation | Noort et al. reported that the methylation of EF-Tu is correlated with E. coli’s growth rate [152]. They found monomethylation during logarithmic growth phase, while dimethyllation occurs during stationary phase. | [42,149-154] |
| EF-TU_FORM5a | EF-TU formation 5 (GDP binding,b3339) | 1 gtp + 1 h2o + 1 mg2 + 1 pEF-TU_me_a --> 1 EF-Tu.GDP + 1 h + 1 pi | irreversible | Protein complex formation |  | [42,149-151,155] |
| EF-TU_FORM5b | EF-TU formation 5 (GDP binding,b3980) | 1 gtp + 1 h2o + 1 mg2 + 1 pEF-TU_me_b --> 1 EF-Tu.GDP + 1 h + 1 pi | irreversible | Protein complex formation | Attention, this reaction is unbalanced since the formulae of EF-TU (b3339) and EF-TU (b3980) is slightly different. Choosed EF-TU (b3339) as working EF-TU formulae (without any reason). Both species have been observed. Only difference between both is the C-terminal Ser or Gly. | [42,149-151,155] |
| Eno_DIM | Enolase dimerization | 2 Eno_mono <==> 1 Eno_dim | reversible | Protein complex formation |  | [109-112] |
| Era_DIM | dimerization of era and GTP binding | 2 Era_mono + 2 gtp --> 1 Era_dim.GTP | irreversible | Protein complex formation | It was concluded that the dimer can bind 2 GTP since each monomer subunit has its N-terminal GTPase domain. The protein is essential for growth [156]. The Km of the Era GTPase is 9.0 microM, and the maximum catalyzed rate of GTP hydrolyzed/min/mol of Era protein at 37 degrees C is 9.8 mmol. [157] | [156-159] |
| GlySQ_RS_TETRA | glycine tRNA synthetase complex formation of 2 alpha subunits and 2 beta subunits | 2 GlyQ_mono + 2 GlyS_mono <==> 1 Gly_RS_tetra | reversible | Protein complex formation |  | [16] |
| GrpE_DIM | GrpE dimerization (heat shock protein, b2614) | 2 GrpE_mono <==> 1 GrpE_dim | reversible | Protein complex formation |  | [160-162] |
| GroL_(14)_FORM | Formation of (GroL)14 | 2 GroL_hepta <==> 1 GroL_(14) | reversible | Protein complex formation | Two GroL heptamers form a GroEL 14-mer. | [163] |
| GroL_hepta_FORM | Formation of GroL heptamer | 7 GroL_mono <==> 1 GroL_hepta | reversible | Protein complex formation |  | [163] |
| GroS_hepta_FORM | Formation of GroS heptamer | 7 GroS_mono <==> 1 GroS_hepta | reversible | Protein complex formation |  | [163] |
| HisS_RS_DIM | dimerization of histidyl-tRNA synthetase | 2 HisS_mono <==> 1 His_RS_dim | reversible | Protein complex formation |  | [17-19] |
| hRNAP_FORM | holo RNA polymerase formation | 2 RpoA_mono + 1 RpoB_mono + 1 RpoC_mono <==> 1 hRNAP | reversible | Protein complex formation |  | [44,164-169] |
| IF1_RENAME | translational initiation factor IF1 formation | 1 InfA_mono --> 1 IF1 | irreversible | Protein complex formation |  | [170-173] |
| IF2_FORM | translational initiation factor IF2 formation | 1 InfB_mono + 1 gtp + 1 h2o --> 1 IF2-GDP + 1 h + 1 pi | irreversible | Protein complex formation | IF2-GDP was assumed to be the product of this reaction because it seems more likely than just the binding of GTP. Sacerdot et al. reported three different natural forms rasing from the same gene [174]. Apparently the presence of only one form is sufficient for growth but mutants are cold-sensitive [175]. For simplicity only one isoform was included. | [171,173-177] |
| IscS_DIM | Dimerization of IscS | 2 IscS_mono + 2 pydx5p <==> 1 IscS_dim_S-H | reversible | Protein complex formation | IscS needs pyridoxal-5-P. The active form is a dimer. The structure has been solved [64]. It was found that the lack of IscS leads to an overall decrease in the activity of a number of Fe-S proteins and results in a general growth defect. However, gene is not essential [64]. | [64] |
| IscU_DIM | IscU dimerization | 2 IscU_mono --> 1 IscU_dim_SH | irreversible | Protein complex formation | IscU is a scaffold protein and acts as a dimer. HscA and HscB appear to modulate IscU activity [178-180] but were not included in model due to missing detailed mechanistic information. | [53-55,178-180] |
| IF3_FORM | translational initiation factor IF3 formation | 1 InfC_mono --> 1 IF3 | irreversible | Protein complex formation |  | [171,173,181] |
| LYSII_RS_DIM | Lysyl-tRNA synthetase dimerization (inducible) | 2 LysU_mono <==> 1 LysII_RS_dim | reversible | Protein complex formation |  | [23,24] |
| LysS_RS_DIM | lysyl-tRNA synthetase dimerization (constitutive, b2890) | 2 LysS_mono <==> 1 LysI_RS_dim | reversible | Protein complex formation | There is no evidence for positive ions mediating the interaction (despite the presence of Mg2+ in the crystallization medium). | [22] |
| MiaA_DIM | Dimerization of MiaA | 2 MiaA_mono <==> 1 MiaA_dim | reversible | Protein complex formation | Experimental results showed that MiaA is a dimer rather than a monomer where each subunit is bound to each intact tRNA molecule at saturation [123]. Moore et al. concluded that protein is a monomer [124], but it seems that more evidence is for dimeric structure for catalytic active enzyme. One dimer is need to bind 1 tRNA [123]. Mg2+ is required [124,125]. It was concluded that there is 1 Mg2+ per monomer. This amount corresponds to about 660 monomers of MiaA per cell and a cellular MiaA concentration of about 1.0 µM, where the volume of an E. coli cell was taken as 1.0 × 10-12 ml [123]. | [123-125,128,129] |
| MetG_RS_DIM | dimerization of Methionyl-tRNA synthetase | 2 MetG_mono <==> 1 Met_RS_dim | reversible | Protein complex formation |  | [28,95,96] |
| PheST_RS_TETRA | formation of phenyl-tRNA synthetase (2 a;pha, 2 beta subunits) | 2 PheS_mono + 2 PheT_mono + 2 zn2 <==> 1 Phe_RS_tetra | reversible | Protein complex formation | Mayaux and Blanquet reported 8 Zn2+ per protein complex [78], while Nureki et al reported only 2 Zn2+ [75]. Furthermore, Mayaux and Blanquet found the Zn2+ ions not strongly bound to protein complex [78]. Here, 2 Zn2+ were concluded to be bound to protein complex. | [75,78,101] |
| PNPase_TRIM | PNPase trimerization | 3 Pnp_mono <==> 1 Pnp_trim | reversible | Protein complex formation | No information essential metallo ions could be found. | [109,182,183] |
| ProS_RS_DIM | dimerization of Prolyl-tRNA synthetase | 2 ProS_mono <==> 1 Pro_RS_dim | reversible | Protein complex formation |  | [30] |
| QueF_DIM | Dimerization of QueF | 2 QueF_mono <==> 1 QueF_dim | reversible | Protein complex formation |  | [184] |
| RF1_FORM1 | protein release factor RF1 formation1 (methylation) | 1 PrfA_mono + 1 PrmC_mono + 1 amet --> 1 PrmC_RF1_cplx | irreversible | Protein complex formation | The deletion of this gene leads to very poor growth on rich media and abolsihes methylation of RF1 [185]. | [185-187] |
| RF1_FORM2 | protein release factor RF1 formation2 (methylation) | 1 PrmC_RF1_cplx --> 1 PrmC_mono_inact + 1 RF1_mono_inact + 1 ahcys + 1 h | irreversible | Protein complex formation | The deletion of this gene leads to very poor growth on rich media and abolsihes methylation of RF1 [185]. | [185-187] |
| RF2_FORM1 | release factor 2 formation 1 (methylation of Gln252) | 1 PrfB_mono + 1 PrmC_mono + 1 amet <==> 1 PrmC_RF2_cplx | reversible | Protein complex formation | The deletion of this gene leads to very poor growth on rich media and abolsihes methylation of RF1 [185]. | [185,187-190] |
| RF2_FORM2 | protein release factor RF2 formation2 (methylation) | 1 PrmC_RF2_cplx --> 1 PrmC_mono_inact + 1 RF2_mono_inact + 1 ahcys + 1 h | irreversible | Protein complex formation | The deletion of this gene leads to very poor growth on rich media and abolsihes methylation of RF1 [185]. | [185,187-190] |
| RF3_FORM | protein release factor RF3 formation | 1 PrfC_mono + 1 gtp + 1 h2o --> 1 RF3_mono.GDP + 1 h + 1 pi | irreversible | Protein complex formation | The calculated molecular weight of matured protein does not fit with reported weight. However, no information could be found about post-translational modification. | [171,191] |
| RhlB_DIM | RNA helicase RhlB dimerization | 2 RhlB_mono <==> 1 RhlB_dim | reversible | Protein complex formation | The gene product catalyzea ATP consuming reaction. It is probably dimer or oligomer in the degradosome [132]. | [109,132,137] |
| RlmB_DIM | Dimerization of RlmB (b4180) | 2 RlmB_mono <==> 1 RlmB_dim | reversible | Protein complex formation | The protein is a dimer. No ions were reported in crystal structure. | [192] |
| RNAP19_FORM | RNA polymerase, sigma 19, formation | 1 FecI_mono + 1 hRNAP <==> 1 RNAP_19 | reversible | Protein complex formation |  | [44,166,193] |
| RNAP24_FORM | RNA polymerase, sigma 24, formation | 1 RpoE_mono + 1 hRNAP <==> 1 RNAP_24 | reversible | Protein complex formation | Cells with increased rpoE expression show enhanced cell lysis in early stationary phase. The rpoE induced expression was phase-specific for many genes [194]. | [44,166,193-195] |
| RNAP28_FORM | RNA polymerase, sigma 28, formation | 1 FliA_mono + 1 hRNAP <==> 1 RNAP_28 | reversible | Protein complex formation |  | [44,166,193,196,197] |
| RNAP32_FORM | RNA polymerase, sigma 32, formation | 1 RpoH_mono + 1 hRNAP <==> 1 RNAP_32 | reversible | Protein complex formation |  | [44,166,193,197] |
| RNAP38_FORM | RNA polymerase, sigma 38, formation | 1 RpoS_mono + 1 hRNAP <==> 1 RNAP_38 | reversible | Protein complex formation |  | [44,166,193,197-199] |
| RNAP54_FORM | RNA polymerase, sigma 54, formation | 1 RpoN_mono + 1 hRNAP <==> 1 RNAP_54 | reversible | Protein complex formation |  | [44,166,193,197,200,201] |
| RNAP70_FORM | RNA polymerase, sigma 70, formation | 1 RpoD_mono + 1 hRNAP <==> 1 RNAP_70 | reversible | Protein complex formation |  | [44,166,193,197] |
| RNase_BN_DIM | diemrization of ElaC_mono to form active RNase_BN | 2 ElaC_mono <==> 1 RNase_BN_dim | reversible | Protein complex formation | Rnase BN aka Rnase Z. It is responsible for 3' maturation of tRNA. | [97,98] |
| RNase_E_TETRAM | RNase E tetramerization | 4 Rne_mono + 2 zn2 <==> 1 RNase_E_tetra | reversible | Protein complex formation | RNase E together with the other components of the degradosome seems to be also responsible for the rRNA decay (which is not included in model) [202]. | [109,202-204] |
| RNase_G_DIM | dimerization of Rng_mono to form active RNase_dim | 2 Rng_mono <==> 1 RNase_G_dim | reversible | Protein complex formation | The protein is a dimer. No ions were reported. | [205,206] |
| RNase_Gen_FORMa | formation of generic RNase (consists of can be done by RNase T, PH (both show highest act.), D (Rnd_mono), II (Rnb_mono), BN(BN has poor acitivity) - presence of only one out of these 5 RNase is sufficient for growth) | 1 RNase_T_dim --> 1 RNase_Gen | irreversible | Protein complex formation | Rnase_Gen accounts for alternatively the following Rnases: RNase II, RNase D, RNase RNase BN, RNase T, RNase PH. Based on observation of [207] and [208] that one RNase is sufficient for the action. | [207,208] |
| RNase_Gen_FORMb | formation of generic RNase (consists of can be done by RNase T, PH (both show highest act.), D (Rnd_mono), II (Rnb_mono), BN(BN has poor acitivity) - presence of only one out of these 5 RNase is sufficient for growth) | 1 Rnd_mono --> 1 RNase_Gen | irreversible | Protein complex formation | Rnase_Gen accounts for alternatively the following Rnases: RNase II, RNase D, RNase RNase BN, RNase T, RNase PH. Based on observation of [207] and [208] that one RNase is sufficient for the action. | [88,92,207,208] |
| RNase_Gen_FORMc | formation of generic RNase (consists of can be done by RNase T, PH (both show highest act.), D (Rnd_mono), II (Rnb_mono), BN(BN has poor acitivity) - presence of only one out of these 5 RNase is sufficient for growth) | 1 RNase_PH --> 1 RNase_Gen | irreversible | Protein complex formation | b3643 is a pseudogen based on Riley annotation andf thus not produced in the reconstruction. Rnase_Gen accounts for alternatively the following Rnases: RNase II, RNase D, RNase RNase BN, RNase T, RNase PH. Based on observation of [207] and [208] that one RNase is sufficient for the action. | [93,207,208] |
| RNase_Gen_FORMd | formation of generic RNase (consists of can be done by RNase T, PH (both show highest act.), D (Rnd_mono), II (Rnb_mono), BN(BN has poor acitivity) - presence of only one out of these 5 RNase is sufficient for growth) | 1 Rnb_mono --> 1 RNase_Gen | irreversible | Protein complex formation | Rnase_Gen accounts for alternatively the following Rnases: RNase II, RNase D, RNase RNase BN, RNase T, RNase PH (?). Based on observation of [207] and [208] that 1 Rnase is sufficient for the action | [207-209] |
| RNase_Gen_FORMe | formation of generic RNase (consists of can be done by RNase T, PH (both show highest act.), D (Rnd_mono), II (Rnb_mono), BN(BN has poor acitivity) - presence of only one out of these 5 RNase is sufficient for growth) | 1 RNase_BN_dim --> 1 RNase_Gen | irreversible | Protein complex formation | Rnase_Gen accounts for alternatively the following Rnases: RNase II, RNase D, RNase RNase BN, RNase T, RNase PH (?) . Based on observation of 8422961 - (Reuven and Deutscher '93.143-8) and 1400219 - (Kelly and Deutscher '92.6682-4) that 1 Rnase is suffificient for the action | [97,98,207,208] |
| RNase_III_DIM | RNase III dimerization | 2 Rnc_mono <==> 1 RNase_III_dim | reversible | Protein complex formation | RNase III was found to be a dimer. It requires Mg2+. | [104,105] |
| RNase_P_FORM | formation of active RNase P | 1 RnpA_mono + 1 RnpB_RNA + 2 mg2 <==> 1 RNase_P_cplx | reversible | Protein complex formation | RNase P needs at least 2 divalent cations (preferentially Mg2+) for catalytic activity (Rnp):[210-212] | [210-215] |
| RNase_T_DIM | dimerization of Rnt_mono to form active RNase T | 2 Rnt_mono <==> 1 RNase_T_dim | reversible | Protein complex formation | Rnase T is responsible for tRNA turn-over. It has DEDD motif in sequence hence it can bind 4 Mg2+. | [86-91] |
| RP_L15_FORM | Ribosomal protein L15 formation | 1 RplO_mono + 1 spmd --> 1 RplO_mono_spmd | irreversible | Protein complex formation |  | [216-218] |
| RP_L17_FORM | Ribosomal protein L17 formation | 1 RplQ_mono + 1 spmd --> 1 RplQ_mono_spmd | irreversible | Protein complex formation |  | [216-218] |
| RP_L18_FORM | Ribosomal protein L18 formation | 1 RplR_mono + 1 spmd --> 1 RplR_mono_spmd | irreversible | Protein complex formation | Change et al report the methylation of rplR 4616944 [219] but not Arnold and Reilly 10094780 [216]. Thus the protein was not methylated in the model. | [216-219] |
| RP_L2_FORM | Ribosomal protein L2 formation | 1 RplB_mono + 1 spmd --> 1 RplB_mono_spmd | irreversible | Protein complex formation | Only those ribosomal proteins with highest label were included in complex C (ribosome, tRNA, mRNA and translation factors) [217] | [216-218] |
| RP_L3_FORM2 | Ribosomal protein L3 formation (2) | 1 PrmB_rpL3_cplx + 1 spmd --> 1 PrmB_mono_inact + 1 RplC_mono_me_spmd + 1 ahcys + 1 h | irreversible | Protein complex formation | Only those ribosomal proteins with highest label were included in complex C (ribosome, tRNA, mRNA and translation factors) [217] | [216,217,220,221] |
| RP_L6_FORM | Ribosomal protein L6 formation | 1 RplF_mono + 1 spmd --> 1 RplF_mono_spmd | irreversible | Protein complex formation | Only those ribosomal proteins with highest label were included in complex C (ribosome, tRNA, mRNA and translation factors) [217] | [216-218] |
| RP_L7/12_FORM | dimer L7/L12 formation | 1 RplL_mono_ac + 1 RplL_mono_me --> 1 rpL7/12 | irreversible | Protein complex formation | Only those ribosomal proteins with highest label were included in complex C (ribosome, tRNA, mRNA and translation factors) [217] | [44,222-227] |
| RP_S3_FORM | Ribosomal protein S3 formation | 1 RpsC_mono + 1 spmd --> 1 RpsC_mono_spmd | irreversible | Protein complex formation | Only those ribosomal proteins with highest label were included in complex C (ribosome, tRNA, mRNA and translation factors) [217] | [216,217,228] |
| RP_S4_FORM | Ribosomal protein S4 formation | 1 RpsD_mono + 1 spmd --> 1 RpsD_mono_spmd | irreversible | Protein complex formation | Only those ribosomal proteins with highest label were included in complex C (ribosome, tRNA, mRNA and translation factors) [217] | [216,217,228] |
| RrmA_DIM | Dimerization of RrmA (23S rRNA m1G745 methyltransferase (b1822)) | 2 RrmA_mono <==> 1 RrmA_dim | reversible | Protein complex formation |  | [94] |
| SelA_FORM | formation of SelA decamer (contains 1 pyridoxal-5-phosphate per monomer subunit) | 10 SelA_mono + 5 pydx5p <==> 1 SelA_deca | reversible | Protein complex formation |  | [229] |
| SerS_RS_DIM | seryl-tRNA synthetase dimerization | 2 SerS_mono <==> 1 Ser_RS_dim | reversible | Protein complex formation | No evidence could be found for the presence of metallo ions in seryl-tRNA synthetase. However, the Thermogata has a Mg2+ ion in the active site [230]. | [31,230-232] |
| TadA_DIM | Dimerization of TadA | 2 TadA_mono <==> 1 TadA_dim | reversible | Protein complex formation | The catalytic site, carring out the deaminase function, of this protein family (ADAR) has 3 zn2+ binding sites [102]. The crystal strucutre of S. Aureus of tadA contained 2 Zn2+ (per homodimer) however Losey et al. did not specify the Zn2+ presence in the paper [103]. It was conluced that TadA is a homodimer in E. coli with 2 Zn2+. | [102,103] |
| Orn_DIM | TEMP_Orn_dim | 2 Orn_mono --> 1 Orn_dim | irreversible | Protein complex formation |  | [233] |
| Rho_HEXA | TEMP_RHO | 6 Rho_mono + 3 atp + 3 mg2 --> 1 Rho_hexa | irreversible | Protein complex formation | Rho is a hexamer which had no ions in the crystal structure. It is likely that Mg-ATP rather than ATP is used for reaction. | [234-239] |
| Tgt_TRIM | Trimerization of Tgt | 3 Tgt_mono <==> 1 Tgt_trim | reversible | Protein complex formation | A zinc binding site has been identified (0.8 mol of zinc/mol of subunit) [80]. | [80,81] |
| Tgt_trim_DIM | Dimerization of 2 Tgt_trim | 2 Tgt_trim <==> 1 Tgt_hexa | reversible | Protein complex formation | Tgt is a dimer of a trimer. E. coli’s Tgt is normally expressed at very low levels (approximately 1 mg from 500 g cells) [81]. | [81] |
| ThrS_RS_DIM | dimerization of threonyl-tRNA synthetase | 2 ThrS_mono <==> 1 Thr_RS_dim | reversible | Protein complex formation |  | [33,75] |
| transGroES_FORM | formation of trans GroES | 1 GroS_hepta --> 1 transGroES_hepta | irreversible | Protein complex formation | Modeling reactions only. Since there is a cis and a trans version of GroES (just to distingish their binding to GroEL). Both proteins are identical and interchangible. (see cis/transGroES_CONV) |  |
| TrmD_DIM | Dimerization of TrmD | 2 TrmD_mono <==> 1 TrmD_dim | reversible | Protein complex formation | tRNA-(m1G)methyltransferase is presence with approximately 80 molecules/genome in a glucose minimal culture [240]. The trmD gene has a codon usage typical for a protein made in low amount in accordance with the low number of tRNA-(m1G) methyltransferase molecules found in the cell. The purified protein that correspond to a monomer rather than a dimer [241]. However, the crystal structure of the E.coli protein showed a dimer with no ions in [242] | [240-242,243 ,244,245] |
| TrmE_DIM | Dimerization of TrmE | 2 TrmE_mono <==> 1 TrmE_dim | reversible | Protein complex formation | The Thermogata [246] as well as the E. coli [247] protein has been found to be a dimer | [246,247] |
| TrmH_DIM | Dimerization of TrmH | 2 TrmH_mono <==> 1 TrmH_dim | reversible | Protein complex formation | TrmH is dimer in Thermus thermophilus [248]. Based on the proposed mechanism in this paper, the protein it should be a dimer (1 tRNA per dimer). TrmH is part of the SpoU protein family. TrmD and RlmB, 2 other E.coli prot of this family, have been shown to be dimers. Given this evidence a dimer was concluded for TrmH. | [248,249] |
| TrpS_RS_DIM | dimerization of Tryptophanyl-tRNA synthetase | 2 TrpS_mono <==> 1 Trp_RS_dim | reversible | Protein complex formation |  | [250] |
| TruA_DIM | Dimerization of TruA | 2 TruA_mono <==> 1 TruA_dim | reversible | Protein complex formation |  | [251] |
| TyrS_RS_DIM | dimerization of tyrosyl-tRNA synthetase | 2 TyrS_mono <==> 1 Tyr_RS_dim | reversible | Protein complex formation |  | [17,21,37,38] |
| YbbB_DIM | dimerization of YbbB | 2 YbbB_mono <==> 1 YbbB_dim | reversible | Protein complex formation |  | [252] |
| YheLMN_FORM | formation of YheLMN (2*YheL,2*YheM,2*YheN) | 2 YheL_mono + 2 YheM_mono + 2 YheN_mono <==> 1 YheLMN_cplx | reversible | Protein complex formation | PDBID: 2D1P. tusB=yheL=b3343; tusC=yheM=b3344; tusD=yheN=b3345 | [253,254] |
| b0049_fold_spon | b0049_m folding: spontanous | 1 b0049_m --> 1 ApaH_mono | irreversible | Protein Folding |  | [255] |
| b0050_fold_spon | b0050_m folding: spontanous | 1 b0050_m --> 1 ApaG_mono | irreversible | Protein Folding |  | [255] |
| b0051_fold_spon | b0051_m folding: spontanous | 1 b0051_m --> 1 KsgA_mono | irreversible | Protein Folding | KsgA is a monomer. No ions were reported. | [255,256] |
| b0052_fold_spon | b0052_m folding: spontanous | 1 b0052_m --> 1 PdxA_mono | irreversible | Protein Folding |  | [255] |
| b0058_fold_spon | b0058_m folding: spontanous | 1 b0058_m --> 1 RluA_mono | irreversible | Protein Folding | RluA was assumed to be a monomer. The enzyme has no dependence on Mg2+. | [257,258] |
| b0168_fold_spon | b0168_m folding: spontanous | 1 b0168_m_Fe --> 1 Map_mono | irreversible | Protein Folding |  | [79,259] |
| b0170_fold_GroEL/ES_1 | b0170_m folding: GroEL/ES mediated; polypeptide is going in GroEL/ES complex | 1 GroEL.(7)ADP.cisGroES + 7 atp + 1 b0170_m + 1 transGroES_hepta --> 7 adp + 1 b0170_m_GroEL.(7)ATP.transGroES + 1 cisGroES_hepta | irreversible | Protein Folding | EF-Ts acts as a catalyst in the displacement of the GDP from the EF-Tu.GDP complex and allows the binding of GTP so that the ternary complex EF-Tu.GTP.aminoacyl-tRNA can be formed. In the crystal structure no ions were reported beside Mg2+ of EF-Tu [42]. The EF-Tu, EF-Ts complex has potentially 1:1 stoichiometry. EF-Tu has higher affinity to GDP than to GTP [43]. EF-Ts stimulates the dissociation of EF-Tu and GDP by formation of an tertiary complex: EF-Tu.GDP.EF-Ts (reversible). Subsequently, GDP is released. GTP binds to the binary complex EF-Tu.EF-Ts (reversible). This tertiary complex dissociates to EF-Tu.GTP and EF-Ts. | [42,43,260] |
| b0170_fold_GroEL/ES_2 | b0170_m folding: GroEL/ES mediated; folding of polypeptide under ATP hydrolysis | 1 b0170_m_GroEL.(7)ATP.transGroES + 7 h2o --> 1 Tsf_GroEL.(7)ADP.transGroES + 7 h + 7 pi | irreversible | Protein Folding | EF-Ts acts as a catalyst in the displacement of the GDP from the EF-Tu.GDP complex and allows the binding of GTP so that the ternary complex EF-Tu.GTP.aminoacyl-tRNA can be formed. In the crystal structure no ions were reported beside Mg2+ of EF-Tu [42]. The EF-Tu, EF-Ts complex has potentially 1:1 stoichiometry. EF-Tu has higher affinity to GDP than to GTP [43]. EF-Ts stimulates the dissociation of EF-Tu and GDP by formation of an tertiary complex: EF-Tu.GDP.EF-Ts (reversible). Subsequently, GDP is released. GTP binds to the binary complex EF-Tu.EF-Ts (reversible). This tertiary complex dissociates to EF-Tu.GTP and EF-Ts. | [42,43,260] |
| b0170_fold_GroEL/ES_3 | b0170_m folding: GroEL/ES mediated; release of native protein | 1 Tsf_GroEL.(7)ADP.transGroES --> 1 GroEL.(7)ADP.transGroES + 1 Tsf_mono | irreversible | Protein Folding | EF-Ts acts as a catalyst in the displacement of the GDP from the EF-Tu.GDP complex and allows the binding of GTP so that the ternary complex EF-Tu.GTP.aminoacyl-tRNA can be formed. In the crystal structure no ions were reported beside Mg2+ of EF-Tu [42]. The EF-Tu, EF-Ts complex has potentially 1:1 stoichiometry. EF-Tu has higher affinity to GDP than to GTP [43]. EF-Ts stimulates the dissociation of EF-Tu and GDP by formation of an tertiary complex: EF-Tu.GDP.EF-Ts (reversible). Subsequently, GDP is released. GTP binds to the binary complex EF-Tu.EF-Ts (reversible). This tertiary complex dissociates to EF-Tu.GTP and EF-Ts. | [42,43,260] |
| b0170_fold_KJE_1 | b0170_m folding: KJE mediated | 1 DnaJ_dim + 1 DnaK_mono.ATP + 1 b0170_m <==> 1 b0170_m_DnaKJ_complex | reversible | Protein Folding | EF-Ts acts as a catalyst in the displacement of the GDP from the EF-Tu.GDP complex and allows the binding of GTP so that the ternary complex EF-Tu.GTP.aminoacyl-tRNA can be formed. In the crystal structure no ions were reported beside Mg2+ of EF-Tu [42]. The EF-Tu, EF-Ts complex has potentially 1:1 stoichiometry. EF-Tu has higher affinity to GDP than to GTP [43]. EF-Ts stimulates the dissociation of EF-Tu and GDP by formation of an tertiary complex: EF-Tu.GDP.EF-Ts (reversible). Subsequently, GDP is released. GTP binds to the binary complex EF-Tu.EF-Ts (reversible). This tertiary complex dissociates to EF-Tu.GTP and EF-Ts. | [42,43,261] |
| b0170_fold_KJE_2 | b0170_m folding: KJE mediated | 1 GrpE_dim + 1 b0170_m_DnaKJ_complex + 1 h2o --> 1 DnaJ_dim_inact + 1 Tsf_DnaK_GrpE_complex + 1 adp + 1 h + 1 pi | irreversible | Protein Folding | EF-Ts acts as a catalyst in the displacement of the GDP from the EF-Tu.GDP complex and allows the binding of GTP so that the ternary complex EF-Tu.GTP.aminoacyl-tRNA can be formed. In the crystal structure no ions were reported beside Mg2+ of EF-Tu [42]. The EF-Tu, EF-Ts complex has potentially 1:1 stoichiometry. EF-Tu has higher affinity to GDP than to GTP [43]. EF-Ts stimulates the dissociation of EF-Tu and GDP by formation of an tertiary complex: EF-Tu.GDP.EF-Ts (reversible). Subsequently, GDP is released. GTP binds to the binary complex EF-Tu.EF-Ts (reversible). This tertiary complex dissociates to EF-Tu.GTP and EF-Ts. | [42,43,261] |
| b0170_fold_KJE_3 | b0170_m folding: KJE mediated | 1 Tsf_DnaK_GrpE_complex + 1 atp --> 1 DnaK_mono.ATP_inact + 1 GrpE_dim_inact + 1 Tsf_mono | irreversible | Protein Folding | EF-Ts acts as a catalyst in the displacement of the GDP from the EF-Tu.GDP complex and allows the binding of GTP so that the ternary complex EF-Tu.GTP.aminoacyl-tRNA can be formed. In the crystal structure no ions were reported beside Mg2+ of EF-Tu [42]. The EF-Tu, EF-Ts complex has potentially 1:1 stoichiometry. EF-Tu has higher affinity to GDP than to GTP [43]. EF-Ts stimulates the dissociation of EF-Tu and GDP by formation of an tertiary complex: EF-Tu.GDP.EF-Ts (reversible). Subsequently, GDP is released. GTP binds to the binary complex EF-Tu.EF-Ts (reversible). This tertiary complex dissociates to EF-Tu.GTP and EF-Ts. | [42,43,261] |
| b0170_fold_spon | b0170_m folding: spontanous | 1 b0170_m --> 1 Tsf_mono | irreversible | Protein Folding | EF-Ts acts as a catalyst in the displacement of the GDP from the EF-Tu.GDP complex and allows the binding of GTP so that the ternary complex EF-Tu.GTP.aminoacyl-tRNA can be formed. In the crystal structure no ions were reported beside Mg2+ of EF-Tu [42]. The EF-Tu, EF-Ts complex has potentially 1:1 stoichiometry. EF-Tu has higher affinity to GDP than to GTP [43]. EF-Ts stimulates the dissociation of EF-Tu and GDP by formation of an tertiary complex: EF-Tu.GDP.EF-Ts (reversible). Subsequently, GDP is released. GTP binds to the binary complex EF-Tu.EF-Ts (reversible). This tertiary complex dissociates to EF-Tu.GTP and EF-Ts. | [42,43] |
| b0172_fold_GroEL/ES_3 | b0172_m folding: GroEL/ES mediated; release of native protein | 1 Rrf_GroEL.(7)ADP.transGroES --> 1 GroEL.(7)ADP.transGroES + 1 Rrf_mono | irreversible | Protein Folding |  | [260,262] |
| b0172_fold_KJE_3 | b0172_m folding: KJE mediated | 1 Rrf_DnaK_GrpE_complex + 1 atp --> 1 DnaK_mono.ATP_inact + 1 GrpE_dim_inact + 1 Rrf_mono | irreversible | Protein Folding |  | [261,262] |
| b0172_fold_spon | b0172_m folding: spontanous | 1 b0172_m --> 1 Rrf_mono | irreversible | Protein Folding |  | [262] |
| b0405_fold_spon | b0405_m folding: spontanous | 1 b0405_m --> 1 QueA_mono | irreversible | Protein Folding |  | [263] |
| b0406_fold_spon | b0406_m folding: spontanous | 1 b0406_m_Zn --> 1 Tgt_mono | irreversible | Protein Folding |  | [264,265] |
| b0416_fold_spon | b0416_m folding: spontanous | 1 b0416_m --> 1 NusB_mono | irreversible | Protein Folding | NusB is a monomer. No ions were reported in the crystal structure. | [266,267] |
| b0436_fold_GroEL/ES_3 | b0436_m folding: GroEL/ES mediated; release of native protein | 1 Tig_GroEL.(7)ADP.transGroES --> 1 GroEL.(7)ADP.transGroES + 1 Tig_mono | irreversible | Protein Folding | The trigger factor binds to ribosome in 1:1 stoichiometric, however, the free trigger factor is a homodimer, and seems to have additional (chaperone) function - see Liu et al. For details. The action of the dimeric trigger factor is not included in the reconstruction. In the E. coli cytosol, nascent polypeptides interact first with trigger factor (TF) [268-270], that binds to the ribosome at proteins L23/L29 near the polypeptide exit site ([271,272]. Thus, TF displayed its shielding function only in its ribosome-bound state and specifically for nascent chains still connected to the peptidyltransferase center [273]. The function of TF: TF happens to be at the exit tunnel of ribosome (near L23); it has naturally low affinity to nascent polypeptide, however, when bound to rib it can interact with polypeptide and protect it from protease digestion (degradation). Nascent polypeptide (unfolded) that leaves ribosome is not longer TF associated, and thus not protected by TF for degradation | [260,268-277] |
| b0661_fold_spon | b0661_m folding: spontanous | 1 b0661_m_FeS --> 1 MiaB_mono | irreversible | Protein Folding | MiaB is a monomer with a [4Fe-4S] cluster. b0661 is transcribed from a monocistronic operon with rho-independent termination. | [67,278] |
| b0680_fold_KJE_1 | b0680_m folding: KJE mediated | 1 DnaJ_dim + 1 DnaK_mono.ATP + 1 b0680_m <==> 1 b0680_m_DnaKJ_complex | reversible | Protein Folding | It is assumed that the GlnS assembles before tRNA binding. | [14,15,261] |
| b0680_fold_KJE_2 | b0680_m folding: KJE mediated | 1 GrpE_dim + 1 b0680_m_DnaKJ_complex + 1 h2o --> 1 DnaJ_dim_inact + 1 GlnS_DnaK_GrpE_complex + 1 adp + 1 h + 1 pi | irreversible | Protein Folding | It is assumed that the GlnS assembles before tRNA binding. | [14,15,261] |
| b0680_fold_KJE_3 | b0680_m folding: KJE mediated | 1 GlnS_DnaK_GrpE_complex + 1 atp --> 1 DnaK_mono.ATP_inact + 1 GlnS_mono + 1 GrpE_dim_inact | irreversible | Protein Folding | It is assumed that the GlnS assembles before tRNA binding. | [14,15,261] |
| b0680_fold_spon | b0680_m folding: spontanous | 1 b0680_m --> 1 GlnS_mono | irreversible | Protein Folding | It is assumed that the GlnS assembles before tRNA binding. | [15] |
| b0884_fold_spon | b0884_m folding: spontanous | 1 b0884_m --> 1 InfA_mono | irreversible | Protein Folding | InfA is a monomer. No ions were reported | [170] |
| b1066_fold_GroEL/ES_3 | b1066_m folding: GroEL/ES mediated; release of native protein | 1 RimJ_GroEL.(7)ADP.transGroES --> 1 GroEL.(7)ADP.transGroES + 1 RimJ_mono | irreversible | Protein Folding |  | [260,279] |
| b1084_fold_spon | b1084_m folding: spontanous | 1 b1084_m --> 1 Rne_mono | irreversible | Protein Folding |  | [280] |
| b1114_fold_KJE_3 | b1114_m folding: KJE mediated | 1 Mfd_DnaK_GrpE_complex + 1 atp --> 1 DnaK_mono.ATP_inact + 1 GrpE_dim_inact + 1 Mfd_mono | irreversible | Protein Folding | 1 Mg2+ in ATP-binding pocket [84] Mfd will be included as monomer, without any atp but 1 Mg2+. | [84,261] |
| b1114_fold_spon | b1114_m folding: spontanous | 1 b1114_m_Mg --> 1 Mfd_mono | irreversible | Protein Folding | 1 Mg2+ in ATP-binding pocket [84] Mfd will be included as monomer, without any atp but 1 Mg2+. | [84] |
| b1135_fold_spon | b1135_m folding: spontanous | 1 b1135_m --> 1 YmfC_mono | irreversible | Protein Folding | aka rluE | [281] |
| b1211_fold_spon | b1211_m folding: spontanous | 1 b1211_m --> 1 PrfA_mono | irreversible | Protein Folding | PrfA is a monomer based on measured molecular weight. | [282] |
| b1212_fold_spon | b1212_m folding: spontanous | 1 b1212_m --> 1 PrmC_mono | irreversible | Protein Folding | PrmC is a monomer. The deletion of this gene leads to very poor growth on rich media and abolsihes methylation of RF1 [185] | [185,186] |
| b1269_fold_GroEL/ES_3 | b1269_m folding: GroEL/ES mediated; release of native protein | 1 RluB_GroEL.(7)ADP.transGroES --> 1 GroEL.(7)ADP.transGroES + 1 RluB_mono | irreversible | Protein Folding |  | [260,281] |
| b1286_fold_spon | b1286_m folding: spontanous | 1 b1286_m_Mg --> 1 Rnb_mono | irreversible | Protein Folding | Rnb is a monomer ([85,283]). It binds Mg2+ ([85]). | [85,209,283] |
| b1427_fold_GroEL/ES_3 | b1427_m folding: GroEL/ES mediated; release of native protein | 1 RimL_GroEL.(7)ADP.transGroES --> 1 GroEL.(7)ADP.transGroES + 1 RimL_mono | irreversible | Protein Folding |  | [260,284] |
| b1427_fold_KJE_3 | b1427_m folding: KJE mediated | 1 RimL_DnaK_GrpE_complex + 1 atp --> 1 DnaK_mono.ATP_inact + 1 GrpE_dim_inact + 1 RimL_mono | irreversible | Protein Folding |  | [261,284] |
| b1652_fold_spon | b1652_m folding: spontanous | 1 b1652_m_Mg --> 1 Rnt_mono | irreversible | Protein Folding |  | [86-90] |
| b1718_fold_GroEL/ES_3 | b1718_m folding: GroEL/ES mediated; release of native protein | 1 InfC_GroEL.(7)ADP.transGroES --> 1 GroEL.(7)ADP.transGroES + 1 InfC_mono | irreversible | Protein Folding |  | [181,260] |
| b1718_fold_KJE_3 | b1718_m folding: KJE mediated | 1 InfC_DnaK_GrpE_complex + 1 atp --> 1 DnaK_mono.ATP_inact + 1 GrpE_dim_inact + 1 InfC_mono | irreversible | Protein Folding |  | [181,261] |
| b1718_fold_spon | b1718_m folding: spontanous | 1 b1718_m --> 1 InfC_mono | irreversible | Protein Folding |  | [181] |
| b1804_fold_spon | b1804_m folding: spontanous | 1 b1804_m_Mg --> 1 Rnd_mono | irreversible | Protein Folding | Rnd seems to be a monomer, 5 divalent cations seems to be needed ([88,92]. | [88,92,93] |
| b1822_fold_spon | b1822_m folding: spontanous | 1 b1822_m_Zn --> 1 RrmA_mono | irreversible | Protein Folding | RrmA is a dimer with 2 Zn2+ ions per dimer | [94] |
| b1922_fold_spon | b1922_m folding: spontanous | 1 b1922_m --> 1 FliA_mono | irreversible | Protein Folding |  | [196] |
| b2183_fold_spon | b2183_m folding: spontanous | 1 b2183_m --> 1 RsuA_mono | irreversible | Protein Folding | RsuA is a monomer. The N’terminal methionine is present in matured protein. | [285,286] |
| b2268_fold_spon | b2268_m folding: spontanous | 1 b2268_m_Zn --> 1 ElaC_mono | irreversible | Protein Folding |  | [97,98] |
| b2318_fold_spon | b2318_m folding: spontanous | 1 b2318_m --> 1 TruA_mono | irreversible | Protein Folding |  | [287] |
| b2330_fold_spon | b2330_m folding: spontanous | 1 b2330_m --> 1 PrmB_mono | irreversible | Protein Folding |  | [185] |
| b2530_fold_GroEL/ES_3 | b2530_m folding: GroEL/ES mediated; release of native protein | 1 IscS_GroEL.(7)ADP.transGroES --> 1 GroEL.(7)ADP.transGroES + 1 IscS_mono | irreversible | Protein Folding |  | [260,288] |
| b2530_fold_KJE_3 | b2530_m folding: KJE mediated | 1 IscS_DnaK_GrpE_complex + 1 atp --> 1 DnaK_mono.ATP_inact + 1 GrpE_dim_inact + 1 IscS_mono | irreversible | Protein Folding |  | [288] |
| b2559_fold_spon | b2559_m folding: spontanous | 1 b2559_m_Zn --> 1 TadA_mono | irreversible | Protein Folding | TadA is a dimer. The protein is essential. | [102] |
| b2594_fold_spon | b2594_m folding: spontanous | 1 b2594_m_Mg --> 1 RluD_mono | irreversible | Protein Folding | RluD is essential for normal growth. No ions were reported in crystal structure [106], but necessity of Mg2+ was reported in experimental study. 1 Mg2+ per monomer was concluded [107]. | [106,107] |
| b2607_fold_GroEL/ES_3 | b2607_m folding: GroEL/ES mediated; release of native protein | 1 TrmD_GroEL.(7)ADP.transGroES --> 1 GroEL.(7)ADP.transGroES + 1 TrmD_mono | irreversible | Protein Folding |  | [244,260,289,290] |
| b2608_fold_GroEL/ES_3 | b2608_m folding: GroEL/ES mediated; release of native protein | 1 RimM_GroEL.(7)ADP.transGroES --> 1 GroEL.(7)ADP.transGroES + 1 RimM_mono | irreversible | Protein Folding | RimM does not appear to be required for wild-type 16S rRNA processing [291]. Since no information was available a monomer was assumed. | [260,291,292] |
| b2608_fold_KJE_3 | b2608_m folding: KJE mediated | 1 RimM_DnaK_GrpE_complex + 1 atp --> 1 DnaK_mono.ATP_inact + 1 GrpE_dim_inact + 1 RimM_mono | irreversible | Protein Folding | RimM does not appear to be required for wild-type 16S rRNA processing [291]. Since no information was available a monomer was assumed. | [261,291,292] |
| b2745_fold_spon | b2745_m folding: spontanous | 1 b2745_m --> 1 TruD_mono | irreversible | Protein Folding | TruD is a monomer. No ions were reported | [293-295] |
| b2779_fold_GroEL/ES_3 | b2779_m folding: GroEL/ES mediated; release of native protein | 1 Eno_GroEL.(7)ADP.transGroES --> 1 Eno_mono + 1 GroEL.(7)ADP.transGroES | irreversible | Protein Folding |  | [136,260] |
| b2779_fold_KJE_3 | b2779_m folding: KJE mediated | 1 Eno_DnaK_GrpE_complex + 1 atp --> 1 DnaK_mono.ATP_inact + 1 Eno_mono + 1 GrpE_dim_inact | irreversible | Protein Folding |  | [136,261] |
| b2779_fold_spon | b2779_m folding: spontanous | 1 b2779_m_Mg --> 1 Eno_mono | irreversible | Protein Folding |  | [136] |
| b2785_fold_spon | b2785_m folding: spontanous | 1 b2785_m_FeS --> 1 RumA_mono | irreversible | Protein Folding | RumA is a monomer with a [4Fe-4S] cluster. | [71,296] |
| b2791_fold_spon | b2791_m folding: spontanous | 1 b2791_m --> 1 YqcB_mono | irreversible | Protein Folding | aka TruC | [281] |
| b2891_fold_spon | b2891_m folding: spontanous | 1 b2891_m --> 1 PrfB_mono | irreversible | Protein Folding | PrfB is a monomer. No ions were reported in crystal structure. | [188,282] |
| b2946_fold_spon | b2946_m folding: spontanous | 1 b2946_m --> 1 YggJ_mono | irreversible | Protein Folding | aka RsmE. Since no information was available a monomer was assumed. | [297] |
| b2960_fold_GroEL/ES_3 | b2960_m folding: GroEL/ES mediated; release of native protein | 1 YggH_GroEL.(7)ADP.transGroES --> 1 GroEL.(7)ADP.transGroES + 1 YggH_mono | irreversible | Protein Folding | aka TrmB. The protein is not essential. | [260,298] |
| b3164_fold_GroEL/ES_3 | b3164_m folding: GroEL/ES mediated; release of native protein | 1 Pnp_GroEL.(7)ADP.transGroES --> 1 GroEL.(7)ADP.transGroES + 1 Pnp_mono | irreversible | Protein Folding |  | [183,260] |
| b3164_fold_KJE_3 | b3164_m folding: KJE mediated | 1 Pnp_DnaK_GrpE_complex + 1 atp --> 1 DnaK_mono.ATP_inact + 1 GrpE_dim_inact + 1 Pnp_mono | irreversible | Protein Folding |  | [183,261] |
| b3164_fold_spon | b3164_m folding: spontanous | 1 b3164_m --> 1 Pnp_mono | irreversible | Protein Folding |  | [183] |
| b3166_fold_spon | b3166_m folding: spontanous | 1 b3166_m --> 1 TruB_mono | irreversible | Protein Folding | TruB is a monomer. | [299] |
| b3167_fold_spon | b3167_m folding: spontanous | 1 b3167_m --> 1 RbfA_mono | irreversible | Protein Folding | RbfA is a monomer. The ribosome-binding factor A (RbfA) from *Escherichia coli* is a cold-shock adaptation protein that is essential for bacterial growth at low (10–20 °C) temperatures [300].RbfA facilitates the ribosome assembly [292]. The E. coli RbfA appears to be essential for efficient processing of 16 S rRNA and important for the maturation/assembly of the 30 S ribosomal subunits under cold-shock conditions [292]. | [292,300] |
| b3168_fold_GroEL/ES_3 | b3168_m folding: GroEL/ES mediated; release of native protein | 1 InfB_GroEL.(7)ADP.transGroES --> 1 GroEL.(7)ADP.transGroES + 1 InfB_mono | irreversible | Protein Folding |  | [174,260] |
| b3168_fold_KJE_3 | b3168_m folding: KJE mediated | 1 InfB_DnaK_GrpE_complex + 1 atp --> 1 DnaK_mono.ATP_inact + 1 GrpE_dim_inact + 1 InfB_mono | irreversible | Protein Folding |  | [174,261] |
| b3169_fold_spon | b3169_m folding: spontanous | 1 b3169_m --> 1 NusA_mono | irreversible | Protein Folding | NusA is a monomer. No ions were reported in the Mycobacterium tuberculosis protein (1K0R) | [301] |
| b3179_fold_spon | b3179_m folding: spontanous | 1 b3179_m --> 1 RrmJ_mono | irreversible | Protein Folding |  | [302] |
| b3181_fold_GroEL/ES_3 | b3181_m folding: GroEL/ES mediated; release of native protein | 1 GreA_GroEL.(7)ADP.transGroES --> 1 GreA_mono + 1 GroEL.(7)ADP.transGroES | irreversible | Protein Folding | GreA is a monomer. | [260,303] |
| b3181_fold_KJE_3 | b3181_m folding: KJE mediated | 1 GreA_DnaK_GrpE_complex + 1 atp --> 1 DnaK_mono.ATP_inact + 1 GreA_mono + 1 GrpE_dim_inact | irreversible | Protein Folding | GreA is a monomer. | [261,303] |
| b3247_fold_spon | b3247_m folding: spontanous | 1 b3247_m --> 1 Rng_mono | irreversible | Protein Folding | Rng is a dimer. No ions were reported. | [205,206] |
| b3258_fold_spon | b3258_m folding: spontanous | 1 b3258_m --> 1 PanF_mono | irreversible | Protein Folding |  | [304] |
| b3259_fold_spon | b3259_m folding: spontanous | 1 b3259_m --> 1 PrmA_mono | irreversible | Protein Folding | The methylation seems to be dispensable,since Vanet et al. find the prmA null mutant perfectly viable [305]. | [304,305] |
| b3289_fold_spon | b3289_m folding: spontanous | 1 b3289_m --> 1 RsmB_mono | irreversible | Protein Folding | RsmB is a monomer. No ions were reported. | [306] |
| b3295_fold_GroEL/ES_3 | b3295_m folding: GroEL/ES mediated; release of native protein | 1 RpoA_GroEL.(7)ADP.transGroES --> 1 GroEL.(7)ADP.transGroES + 1 RpoA_mono | irreversible | Protein Folding |  | [165,166,260,307,308] |
| b3295_fold_KJE_3 | b3295_m folding: KJE mediated | 1 RpoA_DnaK_GrpE_complex + 1 atp --> 1 DnaK_mono.ATP_inact + 1 GrpE_dim_inact + 1 RpoA_mono | irreversible | Protein Folding |  | [261,307,308] |
| b3295_fold_spon | b3295_m folding: spontanous | 1 b3295_m --> 1 RpoA_mono | irreversible | Protein Folding |  | [307,308] |
| b3296_fold_spon | b3296_m folding: spontanous | 1 b3296_m --> 1 RpsD_mono | irreversible | Protein Folding |  | [307,308] |
| b3297_fold_spon | b3297_m folding: spontanous | 1 b3297_m --> 1 RpsK_mono | irreversible | Protein Folding |  | [307,308] |
| b3298_fold_spon | b3298_m folding: spontanous | 1 b3298_m --> 1 RpsM_mono | irreversible | Protein Folding |  | [307,308] |
| b3311_fold_spon | b3311_m folding: spontanous | 1 b3311_m --> 1 RpsQ_mono | irreversible | Protein Folding |  | [307,308] |
| b3339_fold_KJE_3 | b3339_m folding: KJE mediated | 1 TufA_DnaK_GrpE_complex + 1 atp --> 1 DnaK_mono.ATP_inact + 1 GrpE_dim_inact + 1 TufA_mono | irreversible | Protein Folding | At 2 doublings per hour: 8 molecules of EF-TU per ribosome [309]. At 0.22 doublings per hour: 14 molecules per ribosome [309]. | [150,261,309] |
| b3339_fold_spon | b3339_m folding: spontanous | 1 b3339_m --> 1 TufA_mono | irreversible | Protein Folding | At 2 doublings per hour: 8 molecules of EF-TU per ribosome [309]. At 0.22 doublings per hour: 14 molecules per ribosome [309]. | [150,309] |
| b3406_fold_spon | b3406_m folding: spontanous | 1 b3406_m --> 1 GreB_mono | irreversible | Protein Folding | GreB is a monomer. | [310] |
| b3590_fold_spon | b3590_m folding: spontanous | 1 b3590_m --> 1 SelB_mono | irreversible | Protein Folding |  | [311] |
| b3651_fold_GroEL/ES_3 | b3651_m folding: GroEL/ES mediated; release of native protein | 1 TrmH_GroEL.(7)ADP.transGroES --> 1 GroEL.(7)ADP.transGroES + 1 TrmH_mono | irreversible | Protein Folding |  | [249,260] |
| b3651_fold_KJE_3 | b3651_m folding: KJE mediated | 1 TrmH_DnaK_GrpE_complex + 1 atp --> 1 DnaK_mono.ATP_inact + 1 GrpE_dim_inact + 1 TrmH_mono | irreversible | Protein Folding |  | [249,261] |
| b3704_fold_spon | b3704_m folding: spontanous | 1 b3704_m --> 1 RnpA_mono | irreversible | Protein Folding |  | [214] |
| b3887_fold_spon | b3887_m folding: spontanous | 1 b3887_m_Zn --> 1 Dtd_mono | irreversible | Protein Folding |  | [312] |
| b3965_fold_GroEL/ES_3 | b3965_m folding: GroEL/ES mediated; release of native protein | 1 TrmA_GroEL.(7)ADP.transGroES --> 1 GroEL.(7)ADP.transGroES + 1 TrmA_mono | irreversible | Protein Folding | TrmA gene is encoded by monocistronic operon. The gene is essential. TrmA is a monomer based on molecular weigth (42kDa) [313]. TrmA has no iron-sulfur cluster : [70]. | [70,260,313-315] |
| b3980_fold_KJE_3 | b3980_m folding: KJE mediated | 1 TufB_DnaK_GrpE_complex + 1 atp --> 1 DnaK_mono.ATP_inact + 1 GrpE_dim_inact + 1 TufB_mono | irreversible | Protein Folding | At 2 doublings per hour: 8 molecules of EF-TU per ribosome [309]. At 0.22 doublings per hour: 14 molecules per ribosome [309]. | [150,261,309] |
| b3980_fold_spon | b3980_m folding: spontanous | 1 b3980_m --> 1 TufB_mono | irreversible | Protein Folding | At 2 doublings per hour: 8 molecules of EF-TU per ribosome [309]. At 0.22 doublings per hour: 14 molecules per ribosome [309]. | [150,309] |
| b4022_fold_spon | b4022_m folding: spontanous | 1 b4022_m --> 1 YjbC_mono | irreversible | Protein Folding | aka rluF | [281] |
| b4170_fold_spon | b4170_m folding: spontanous | 1 b4170_m --> 1 MutL_mono | irreversible | Protein Folding |  | [316] |
| b4171_fold_spon | b4171_m folding: spontanous | 1 b4171_m_Mg --> 1 MiaA_mono | irreversible | Protein Folding |  | [124,127,316] |
| b4180_fold_spon | b4180_m folding: spontanous | 1 b4180_m --> 1 RlmB_mono | irreversible | Protein Folding | RlmB is a dimer. No ions were reported. | [192] |
| b4373_fold_spon | b4373_m folding: spontanous | 1 b4373_m --> 1 RimI_mono | irreversible | Protein Folding |  | [279] |
| b4375_fold_KJE_3 | b4375_m folding: KJE mediated | 1 PrfC_DnaK_GrpE_complex + 1 atp --> 1 DnaK_mono.ATP_inact + 1 GrpE_dim_inact + 1 PrfC_mono | irreversible | Protein Folding |  | [191,261] |
| b4375_fold_spon | b4375_m folding: spontanous | 1 b4375_m --> 1 PrfC_mono | irreversible | Protein Folding |  | [191] |
| cis/transGroES_CONV | conversion of cisGroES to transGroES | 1 cisGroES_hepta <==> 1 transGroES_hepta | reversible | Protein Folding | This reaction is in the model since the complete cycle of chaperonine GroEL/ES cannot be modeled as it happens as follow: GroEL 14-mer binds non-native polypeptide (to the trans cave), ATP and GroES bind to GroEL-pp, ATP hydrolysis happens. This leads to conformational changes in chaperonine, allowing the "binding" of another pp in the cis cave while the mewly created natively folded protein is still present in trans cave. ATP and GroES bind to cis site of GroEL, leading to release of native protein, ADP and trans GroES from complex. Hartl and Hayer-Hartl provide a very clear and comprehensive reaction mechanism in their review [317]. | [163,317-323] |
| GroEL.(7)ADP.cisGroES_CONV | conversion of GroEL.(7)ADP.cisGroES to GroEL.(7)ADP.ctransGroES | 1 GroEL.(7)ADP.cisGroES <==> 1 GroEL.(7)ADP.transGroES | reversible | Protein Folding | This reaction is in the model since the complete cycle of chaperonine GroEL/ES cannot be modeled as it happens as follow: GroEL 14-mer binds non-native polypeptide (to the trans cave), ATP and GroES bind to GroEL-pp, ATP hydrolysis happens. This leads to conformational changes in chaperonine, allowing the "binding" of another pp in the cis cave while the mewly created natively folded protein is still present in trans cave. ATP and GroES bind to cis site of GroEL, leading to release of native protein, ADP and trans GroES from complex. Hartl and Hayer-Hartl provide a very clear and comprehensive reaction mechanism in their review [317]. | [163,317-323] |
| GroEL.(7)ADP.cisGroES_FORM | formation of ternary complex GroEL (14-mer), GroES (7-mer) and 7 ADP | 1 GroL_(14) + 7 adp + 1 cisGroES_hepta + 7 mg2 --> 1 GroEL.(7)ADP.cisGroES | irreversible | Protein Folding | This reaction is in the model since the complete cycle of chaperonine GroEL/ES cannot be modeled as it happens as follow: GroEL 14-mer binds non-native polypeptide (to the trans cave), ATP and GroES bind to GroEL-pp, ATP hydrolysis happens. This leads to conformational changes in chaperonine, allowing the "binding" of another pp in the cis cave while the mewly created natively folded protein is still present in trans cave. ATP and GroES bind to cis site of GroEL, leading to release of native protein, ADP and trans GroES from complex. Hartl and Hayer-Hartl provide a very clear and comprehensive reaction mechanism in their review [317]. | [163,317-321,323] |
| GroEL.(7)ADP.transGroES_FORM | formation of ternary complex GroEL (14-mer), GroES (7-mer) and 7 ADP | 1 GroL_(14) + 7 adp + 7 mg2 + 1 transGroES_hepta --> 1 GroEL.(7)ADP.transGroES | irreversible | Protein Folding | This reaction is in the model since the complete cycle of chaperonine GroEL/ES cannot be modeled as it happens as follow: GroEL 14-mer binds non-native polypeptide (to the trans cave), ATP and GroES bind to GroEL-pp, ATP hydrolysis happens. This leads to conformational changes in chaperonine, allowing the "binding" of another pp in the cis cave while the mewly created natively folded protein is still present in trans cave. ATP and GroES bind to cis site of GroEL, leading to release of native protein, ADP and trans GroES from complex. Hartl and Hayer-Hartl provide a very clear and comprehensive reaction mechanism in their review [317]. | [163,317-321,323] |
| Era_RECHARGE | recharging of Era | 1 Era_dim.GDP + 2 gtp --> 1 Era_dim.GTP + 2 gdp | irreversible | Ribosomal Assembly | It was concluded that both GTP are used. |  |
| Rib_30_ass1 | ribosomal subunit 30 S assembly 1 (21S) | 1 16S_rRNA + 1 Era_dim.GTP + 1 RbfA_mono + 1 RimM_mono + 1 RpsD_mono_spmd + 1 RpsE_mono_ac + 1 RpsF_mono_glu4 + 1 RpsG_mono + 1 RpsH_mono + 1 RpsI_mono + 1 RpsK_mono_me + 1 RpsL_mono_me + 1 RpsM_mono + 1 RpsO_mono + 1 RpsP_mono + 1 RpsQ_mono + 1 RpsR_mono_ac + 1 RpsS_mono + 1 RpsT_mono + 1 YrdC_mono + 2 h2o --> 1 RI_30 | irreversible | Ribosomal Assembly | Ribosome-binding factor A (RbfA) from *Escherichia coli* is a cold-shock adaptation protein that is essential for bacterial growth at low (10–20 °C) temperatures [300]. RbfA facilitates the ribosome assembly [292]. The E. coli RbfA appears to be essential for efficient processing of 16 S rRNA and important for the maturation/assembly of the 30 S ribosomal subunits under cold-shock conditions [292]. Era is essential for growth. | [44,156,291,292,300,324] |
| Rib_30_ass2a | ribosomal subunit 30 S assembly 2 (activation of RI_30, spontanous) | 1 RI_30 --> 1 Era_dim.GDP + 1 RI_30* + 1 RbfA_mono_inact + 1 RimM_mono_inact + 1 YrdC_mono_inact + 2 h + 2 pi | irreversible | Ribosomal Assembly | There are two versions of this reaction: 1. the reaction has been observed to occur spontanously; however, 2. a factor (DnaK, b0014) is normally necessary. | [156,291,292,300] |
| Rib_30_ass2b_1 | ribosomal subunit 30 S assembly 2 (activation of RI_30, DnaK mediated) | 1 DnaJ_dim + 1 DnaK_mono.ATP + 1 RI_30 --> 1 DnaKJ_RI_30_cplx + 1 Era_dim.GDP + 1 RbfA_mono_inact + 1 RimM_mono_inact + 1 YrdC_mono_inact + 2 h + 2 pi | irreversible | Ribosomal Assembly |  | [156,291,292,300] |
| Rib_30_ass2b_2 | ribosomal subunit 30 S assembly 2 (activation of RI_30, DnaK mediated) | 1 DnaKJ_RI_30_cplx + 1 GrpE_dim + 1 h2o --> 1 DnaJ_dim_inact + 1 DnaK_GrpE_RI_30_complex + 1 adp + 1 h + 1 pi | irreversible | Ribosomal Assembly |  | [156,291,292,300] |
| Rib_30_ass3 | ribosomal subunit 30 S assembly 3 | 1 RI_30* + 1 RpsA_mono + 1 RpsB_mono + 1 RpsC_mono_spmd + 1 RpsJ_mono + 1 RpsN_mono + 1 RpsU_mono + 1 Sra_mono + 60 mg2 --> 1 rib_30 | irreversible | Ribosomal Assembly | The number of Mg2+ per subunit was obtained from crystal structure [325]. A number of crystal structures are available. | [173,324-332] |
| Rib_50_ass1 | ribosomal subunit 50 S assembly 1 | 1 23S_rRNA + 1 5S_rRNA + 1 RplA_mono + 1 RplB_mono_spmd + 1 RplC_mono_me_spmd + 1 RplD_mono + 1 RplE_mono + 1 RplI_mono + 1 RplJ_mono + 1 RplK_mono_me3 + 1 RplM_mono + 1 RplQ_mono_spmd + 1 RplS_mono + 1 RplT_mono + 1 RplU_mono + 1 RplV_mono + 1 RplW_mono + 1 RplX_mono + 1 RpmC_mono + 1 RpmG_mono_me + 1 RpmH_mono + 2 rpL7/12 --> 1 RI_50 | irreversible | Ribosomal Assembly |  | [44,324] |
| Rib_50_ass2a | ribosomal subunit 50 S assembly 2 (RI_50 activation, spontanous) | 1 RI_50 --> 1 RI_50* | irreversible | Ribosomal Assembly |  |  |
| Rib_50_ass2b_1 | ribosomal subunit 50 S assembly 2 (RI_50 activation, GroEL mediated) | 1 GroEL.(7)ADP.cisGroES + 1 RI_50 + 7 atp + 1 transGroES_hepta --> 1 GroEL.(7)ATP.transGroES_RI_50_cplx + 7 adp + 1 cisGroES_hepta | irreversible | Ribosomal Assembly |  |  |
| Rib_50_ass2b_2 | ribosomal subunit 50 S assembly 2 (RI_50 activation, GroEL mediated) | 1 GroEL.(7)ATP.transGroES_RI_50_cplx + 7 h2o --> 1 GroEL.(7)ADP.transGroES_RI_50_cplx + 7 h + 7 pi | irreversible | Ribosomal Assembly |  |  |
| Rib_50_ass3 | ribosomal subunit 50 S assembly 3 | 1 RI_50* + 1 RplF_mono_spmd + 1 RplN_mono + 1 RplO_mono_spmd + 1 RplP_mono_me + 1 RplR_mono_spmd + 1 RplY_mono + 1 RpmA_mono + 1 RpmB_mono + 1 RpmD_mono + 1 RpmE_mono + 1 RpmF_mono + 1 RpmI_mono + 1 RpmJ_mono + 1 Tig_mono + 111 mg2 --> 1 rib_50_inact | irreversible | Ribosomal Assembly | In the E. coli cytosol, nascent polypeptides interact first with trigger factor (TF) [268-270], that binds to the ribosome at proteins L23/L29 near the polypeptide exit site ([271,272].Thus, TF displayed its shielding function only in its ribosome-bound state and specifically for nascent chains still connected to the peptidyltransferase center [273]. Function of TF: TF happens to be at the exit tunnel of ribosome (near L23); it has naturally low affinity to nascent polypeptide, however, when bound to rib it can interact with polypeptide and protect it from protease digestion (degradation). Nascent polypeptide (unfolded) that leaves ribosome is not longer TF associated, and thus not protected by TF for degradation. The number of Mg2+ was obtained from crystal structure [325]. | [173,268-276,324,325,327-329,331,333]: |
| RP_L7_FORM3 | Ribosomal protein L7 formation (3) - methylation (K81) | 1 MeT_L7/L12 + 1 RplL_mono + 1 amet <==> 1 MeT_L7/L12_rpL12_cplx | reversible | Ribosomal protein modification |  | [216] |
| RP_L11_FORM1 | Ribosomal protein L11 formation (1) | 1 PrmA_mono + 1 RplK_mono + 9 amet --> 1 PrmA_rpL11_cplx | irreversible | Ribosomal protein modification | The methylation seems to be dispensable,since Vanet et al. Find the prmA null mutant perfectly viable [305]. | [216,219,220,305,334,335] |
| RP_L11_FORM2 | Ribosomal protein L11 formation (2) | 1 PrmA_rpL11_cplx --> 1 PrmA_mono_inact + 1 RplK_mono_me3 + 9 ahcys + 9 h | irreversible | Ribosomal protein modification | The methylation seems to be dispensable,since Vanet et al. Find the prmA null mutant perfectly viable [305]. | [216,219,220,305,334,335] |
| RP_L16_FORM1 | Ribosomal protein L16 formation (1 )(Methyltransferase is unknown) | 1 MeT_L16 + 1 RplP_mono + 1 amet --> 1 MeT_rpL16_cplx | irreversible | Ribosomal protein modification | N-terminal methionine has been reported to be methylated [336]. Arginine at position 81 is also modified in some way, possibly hydroxylated. Arnold et al found that albeit these two modifications the calculated protein was still 14.9 Da lighter than the weight they measured.;I could not find any confirmation of hydroxylation and another modification, therefore, only methylation was included. | [216,336] |
| RP_L16_FORM2 | Ribosomal protein L16 formation (2 )(Methyltransferase is unknown) | 1 MeT_rpL16_cplx --> 1 MeT_L16_inact + 1 RplP_mono_me + 1 ahcys + 1 h | irreversible | Ribosomal protein modification | N-terminal methionine has been reported to be methylated [336]. Arginine at position 81 is also modified in some way, possibly hydroxylated. Arnold et al found that albeit these two modifications the calculated protein was still 14.9 Da lighter than the weight they measured.;I could not find any confirmation of hydroxylation and another modification, therefore, only methylation was included. | [216,336] |
| RP_L3_FORM1 | Ribosomal protein L3 formation (1) | 1 PrmB_mono + 1 RplC_mono + 1 amet --> 1 PrmB_rpL3_cplx | irreversible | Ribosomal protein modification |  | [216,220,221] |
| RP_L33_FORM1 | Ribosomal protein L33 formation (1 )(Methyltransferase is unknown) | 1 MeT_L33 + 1 RpmG_mono + 1 amet --> 1 MeT_rpL33_cplx | irreversible | Ribosomal protein modification | Unknown methyltransferase. | [216,219,334,337,338] |
| RP_L33_FORM2 | Ribosomal protein L33 formation (2 )(Methyltransferase is unknown) | 1 MeT_rpL33_cplx --> 1 MeT_L33_inact + 1 RpmG_mono_me + 1 ahcys + 1 h | irreversible | Ribosomal protein modification | Unknown methyltransferase. | [216,337,338] |
| RP_L7_FORM1 | Ribosomal protein L7 formation (1) | 1 RimL_mono + 1 RplL_mono + 1 accoa --> 1 RimL_rpL7_cplx | irreversible | Ribosomal protein modification |  | [216,226,284] |
| RP_L7_FORM2 | Ribosomal protein L7 formation (2) | 1 RimL_rpL7_cplx --> 1 RimL_mono_inact + 1 RplL_mono_ac + 1 coa | irreversible | Ribosomal protein modification |  | [216,226,284] |
| RP_L7_FORM4 | Ribosomal protein L7 formation (4) - methylation (K81) | 1 MeT_L7/L12_rpL12_cplx --> 1 MeT_L7/L12_inact + 1 RplL_mono_me + 1 ahcys + 1 h | irreversible | Ribosomal protein modification | The amount of epsilon-N-monomethyllysine in ribosomal proteins L7 and L12 in Escherichia coli is dependent upon the cell growth temperature. At 37 degrees C or above, very small amounts were detected. Dramatic increase in the content of epsilon-N-monomethyllysine in these proteins was observed when the growth temperature was lowered [339]. | [216,219,339] |
| RP_S11_FORM1 | Ribosomal protein S11 formation (1 )(Methyltransferase is unknown) | 1 MeT_S11 + 1 RpsK_mono + 1 amet --> 1 MeT_rpS11_cplx | irreversible | Ribosomal protein modification | The responsible gene for methylation could not be found. Rspk is methylated at A1. | [216,340] |
| RP_S11_FORM2 | Ribosomal protein S11 formation (2) | 1 MeT_rpS11_cplx --> 1 MeT_S11_inact + 1 RpsK_mono_me + 1 ahcys + 1 h | irreversible | Ribosomal protein modification | The responsible gene for methylation could not be found. Rspk is methylated at A1. | [216,340] |
| RP_S12_FORM1 | Ribosomal protein S12 formation (1 )(beta-methylthio-aspartic acid) | 1 MeST_S12 + 1 RpsL_mono + 1 amet + 1 cys-L + 1 fe2 --> 1 MeST_rpS12_cplx | irreversible | Ribosomal protein modification | No gene is known for beta-methylthiolated nor the mechanism. However, biosynthesis of 2-methylthio-N6-isopentyladenosine of modified tRNA was template for this reaction. There, thiolation is followed by methylation reaction where cysteine serve as sulfur donor and amet as methyl donor. | [216,341] |
| RP_S12_FORM2 | Ribosomal protein S12 formation (2 )(beta-methylthiolation) | 1 MeST_rpS12_cplx --> 1 MeST_S12_inact + 1 RpsL_mono_me + 1 ahcys + 1 ala-L + 1 fe2 + 1 h | irreversible | Ribosomal protein modification | No gene is known for beta-methylthiolated nor the mechanism. However, biosynthesis of 2-methylthio-N6-isopentyladenosine of modified tRNA was template for this reaction. There, thiolation is followed by methylation reaction where cysteine serve as sulfur donor and amet as methyl donor. | [216,341] |
| RP_S18_FORM1 | Ribosomal protein S18 formation (1) | 1 RimI_mono + 1 RpsR_mono + 1 accoa --> 1 RimI_rps18_cplx | irreversible | Ribosomal protein modification | A1 is acetylated | [216,279] |
| RP_S18_FORM2 | Ribosomal protein S18 formation (2) | 1 RimI_rps18_cplx --> 1 RimI_mono_inact + 1 RpsR_mono_ac + 1 coa | irreversible | Ribosomal protein modification | A1 is acetylated | [216,279] |
| RP_S5_FORM1 | Ribosomal protein S5 formation (1) | 1 RimJ_mono + 1 RpsE_mono + 1 accoa --> 1 RimJ_rps5_cplx | irreversible | Ribosomal protein modification | A1 is acetylated | [216,279] |
| RP_S5_FORM2 | Ribosomal protein S5 formation (2) | 1 RimJ_rps5_cplx --> 1 RimJ_mono_inact + 1 RpsE_mono_ac + 1 coa | irreversible | Ribosomal protein modification | A1 is acetylated | [216,279] |
| RP_S6_FORM1 | Ribosomal protein S6 formation (1 )(ribosomal protein S6/ rimK/ ATP/ Glu complex | 1 RimK_mono + 1 RpsF_mono + 4 atp + 4 glu-L --> 1 rps6_rimK_cplx | irreversible | Ribosomal protein modification | Galperin and Koonin proposed that RimK is a ATP-dependent ligase based on sequence similarity to other ATP-dependent ligase (ATP binding domain) [342]. No experimental verification could be found the mechanism seem to be highly probable. S6 can have up to 6 more glutamyl residues but also every other number below 6 .Based on Hitz 4 glutamyl residues were added for the model RpsF [343]. | [342-344] |
| RP_S6_FORM2 | Ribosomal protein S6 formation (2 )(ribosomal protein S6/ rimK/ ATP complex (release of phosphorylated S6 complexed with rimK) | 1 rps6_rimK_cplx --> 1 RimK_mono_inact + 1 RpsF_mono_glu4 + 4 adp + 4 h + 4 pi | irreversible | Ribosomal protein modification | Galperin and Koonin proposed that RimK is a ATP-dependent ligase based on sequence similarity to other ATP-dependent ligase (ATP binding domain) [342]. No experimental verification could be found the mechanism seem to be highly probable. S6 can have up to 6 more glutamyl residues but also every other number below 6 .Based on Hitz 4 glutamyl residues were added for the model RpsF [343]. | [342-344] |
| 5S_rRNA_FORM1 | 5S rRNA formation (generic 5S rRNA from rrfA) | 1 rrfA --> 1 5S_rRNA | irreversible | rRNA formation | 5S rRNA is not modified in E coli | Modeling reason |
| 5S_rRNA_FORM2 | 5S rRNA formation (generic 5S rRNA from rrfB) | 1 rrfB --> 1 5S_rRNA | irreversible | rRNA formation | 5S rRNA is not modified in E coli | Modeling reason |
| 5S_rRNA_FORM3 | 5S rRNA formation (generic 5S rRNA from rrfC) | 1 rrfC --> 1 5S_rRNA | irreversible | rRNA formation | 5S rRNA is not modified in E coli | Modeling reason |
| 5S_rRNA_FORM4 | 5S rRNA formation (generic 5S rRNA from rrfD) | 1 rrfD --> 1 5S_rRNA | irreversible | rRNA formation | 5S rRNA is not modified in E coli | Modeling reason |
| 5S_rRNA_FORM5 | 5S rRNA formation (generic 5S rRNA from rrfE) | 1 rrfE --> 1 5S_rRNA | irreversible | rRNA formation | 5S rRNA is not modified in E coli | Modeling reason |
| 5S_rRNA_FORM6 | 5S rRNA formation (generic 5S rRNA from rrfF) | 1 rrfF --> 1 5S_rRNA | irreversible | rRNA formation | 5S rRNA is not modified in E coli | Modeling reason |
| 5S_rRNA_FORM7 | 5S rRNA formation (generic 5S rRNA from rrfG) | 1 rrfG --> 1 5S_rRNA | irreversible | rRNA formation | 5S rRNA is not modified in E coli | Modeling reason |
| 5S_rRNA_FORM8 | 5S rRNA formation (generic 5S rRNA from rrfH) | 1 rrfH --> 1 5S_rRNA | irreversible | rRNA formation | 5S rRNA is not modified in E coli | Modeling reason |
| 16S_rRNA_FORM1 | 16S rRNA formation (generic 16S rRNA from rrsA) | 1 rrsA --> 1 16S_rRNA | irreversible | rRNA formation | A generic 16S rRNA was included in reconstruction which formula is an average of all 16S rRNA. Therefore, the reactions are unbalanced. | Modeling reason |
| 16S_rRNA_FORM2 | 16S rRNA formation (generic 16S rRNA from rrsB) | 1 rrsB --> 1 16S_rRNA | irreversible | rRNA formation | A generic 16S rRNA was included in reconstruction which formula is an average of all 16S rRNA. Therefore, the reactions are unbalanced. | Modeling reason |
| 16S_rRNA_FORM3 | 16S rRNA formation (generic 16S rRNA from rrsC) | 1 rrsC --> 1 16S_rRNA | irreversible | rRNA formation | A generic 16S rRNA was included in reconstruction which formula is an average of all 16S rRNA. Therefore, the reactions are unbalanced. | Modeling reason |
| 16S_rRNA_FORM4 | 16S rRNA formation (generic 16S rRNA from rrsD) | 1 rrsD --> 1 16S_rRNA | irreversible | rRNA formation | A generic 16S rRNA was included in reconstruction which formula is an average of all 16S rRNA. Therefore, the reactions are unbalanced. | Modeling reason |
| 16S_rRNA_FORM5 | 16S rRNA formation (generic 16S rRNA from rrsE) | 1 rrsE --> 1 16S_rRNA | irreversible | rRNA formation | A generic 16S rRNA was included in reconstruction which formula is an average of all 16S rRNA. Therefore, the reactions are unbalanced. | Modeling reason |
| 16S_rRNA_FORM6 | 16S rRNA formation (generic 16S rRNA from rrsG) | 1 rrsG --> 1 16S_rRNA | irreversible | rRNA formation | A generic 16S rRNA was included in reconstruction which formula is an average of all 16S rRNA. Therefore, the reactions are unbalanced. | Modeling reason |
| 16S_rRNA_FORM7 | 16S rRNA formation (generic 16S rRNA from rrsH) | 1 rrsH --> 1 16S_rRNA | irreversible | rRNA formation | A generic 16S rRNA was included in reconstruction which formula is an average of all 16S rRNA. Therefore, the reactions are unbalanced.s | Modeling reason |
| 23S_rRNA_FORM1 | 23S rRNA formation (generic 23S rRNA from rrlA) | 1 rrlA --> 1 23S_rRNA | irreversible | rRNA formation | A generic 23S rRNA was included in reconstruction which formula is an average of all 23S rRNA. Therefore, the reactions are unbalanced. | Modeling reason |
| 23S_rRNA_FORM2 | 23S rRNA formation (generic 23S rRNA from rrlB) | 1 rrlB --> 1 23S_rRNA | irreversible | rRNA formation | A generic 23S rRNA was included in reconstruction which formula is an average of all 23S rRNA. Therefore, the reactions are unbalanced. | Modeling reason |
| 23S_rRNA_FORM3 | 23S rRNA formation (generic 23S rRNA from rrlC) | 1 rrlC --> 1 23S_rRNA | irreversible | rRNA formation | A generic 23S rRNA was included in reconstruction which formula is an average of all 23S rRNA. Therefore, the reactions are unbalanced. | Modeling reason |
| 23S_rRNA_FORM4 | 23S rRNA formation (generic 23S rRNA from rrlD) | 1 rrlD --> 1 23S_rRNA | irreversible | rRNA formation | A generic 23S rRNA was included in reconstruction which formula is an average of all 23S rRNA. Therefore, the reactions are unbalanced. | Modeling reason |
| 23S_rRNA_FORM5 | 23S rRNA formation (generic 23S rRNA from rrlE) | 1 rrlE --> 1 23S_rRNA | irreversible | rRNA formation | A generic 23S rRNA was included in reconstruction which formula is an average of all 23S rRNA. Therefore, the reactions are unbalanced. | Modeling reason |
| 23S_rRNA_FORM6 | 23S rRNA formation (generic 23S rRNA from rrlG) | 1 rrlG --> 1 23S_rRNA | irreversible | rRNA formation | A generic 23S rRNA was included in reconstruction which formula is an average of all 23S rRNA. Therefore, the reactions are unbalanced. | Modeling reason |
| 23S_rRNA_FORM7 | 23S rRNA formation (generic 23S rRNA from rrlH) | 1 rrlH --> 1 23S_rRNA | irreversible | rRNA formation | A generic 23S rRNA was included in reconstruction which formula is an average of all 23S rRNA. Therefore, the reactions are unbalanced. | Modeling reason |
| Tsf_RENAME | TEMP_Tsf | 1 Tsf_mono --> 1 EF-Ts | irreversible | Protein complex formation | EF-Ts acts as a catalyst in the displacement of the GDP from the EF-Tu.GDP complex and allows the binding of GTP so that the ternary complex EF-Tu.GTP.aminoacyl-tRNA can be formed. The crystal structure of the complex has been solved in which no ions have been reported beside Mg2+ of EF-Tu [42]. The EF-Tu, EF-Ts complex has potentially a 1:1 stoichiometry. EF-Tu has higher affinity to GDP than to GTP. EF-Ts stimulates the dissociation of EF-Tu and GDP by formation of an tertiary complex: EF-Tu.GDP.EF-Ts (reversible). Subsequently, GDP is released. GTP binds to the binary complex EF-Tu.EF-Ts (reversible). This tertiary complex dissociates to EF-Tu.GTP and EF-Ts [43]. | [42,43] |
| EF-G_RECHARG | recharge of EF-G with GTP | 1 EF-G.GDP + 1 gtp --> 1 EF-G.GTP + 1 gdp | irreversible | Translation |  | [146-148] |
| IF2_RECHARG | recharge of IF2 with GTP | 1 IF2-GDP + 1 gtp --> 1 IF2-GTP + 1 gdp | irreversible | Translation |  | [171,173,174,176,177] |
| Rib_30_ini_FORM | formation of 30S translation initiation complex (30S subunit, IF1, IF2-GTP, IF3) | 1 IF2-GTP + 1 rib_30_IF1_IF3 --> 1 rib_30_ini_inact | irreversible | Translation |  | [173,177,325,326] |
| Rib_70_DISS | 70S ribosome dissociation | 1 IF1 + 1 IF3 + 1 rib_70 --> 1 rib_30_IF1_IF3 + 1 rib_50_inact | irreversible | Translation |  | [44,172] |
| TC_ala1 | ternary complex formation with ala1-tRNA | 1 EF-Tu.GTP + 1 ala1_tRNA_ala --> 1 EF-Tu.GTP.ala1-tRNA | irreversible | tRNA activation (EF-TU) |  | [155] |
| TC_ala2 | ternary complex formation with ala2-tRNA | 1 EF-Tu.GTP + 1 ala2_tRNA_ala --> 1 EF-Tu.GTP.ala2-tRNA | irreversible | tRNA activation (EF-TU) |  | [155] |
| TC_arg1 | ternary complex formation with arg1-tRNA | 1 EF-Tu.GTP + 1 arg1_tRNA_ala --> 1 EF-Tu.GTP.arg1-tRNA | irreversible | tRNA activation (EF-TU) |  | [155] |
| TC_argU | ternary complex formation with argU-tRNA | 1 EF-Tu.GTP + 1 argU_tRNA_ala --> 1 EF-Tu.GTP.argU-tRNA | irreversible | tRNA activation (EF-TU) |  | [155] |
| TC_argW | ternary complex formation with argW-tRNA | 1 EF-Tu.GTP + 1 argW_tRNA_arg --> 1 EF-Tu.GTP.argW-tRNA | irreversible | tRNA activation (EF-TU) |  | [155] |
| TC_argX | ternary complex formation with argX-tRNA | 1 EF-Tu.GTP + 1 argX_tRNA_arg --> 1 EF-Tu.GTP.argX-tRNA | irreversible | tRNA activation (EF-TU) |  | [155] |
| TC_asn1 | ternary complex formation with asn1-tRNA | 1 EF-Tu.GTP + 1 asn1_tRNA_asn --> 1 EF-Tu.GTP.asn1-tRNA | irreversible | tRNA activation (EF-TU) |  | [155] |
| TC_asp1 | ternary complex formation with asp1-tRNA | 1 EF-Tu.GTP + 1 asp1_tRNA_asp --> 1 EF-Tu.GTP.asp1-tRNA | irreversible | tRNA activation (EF-TU) |  | [155] |
| TC_cysT | ternary complex formation with asp1-tRNA | 1 EF-Tu.GTP + 1 cysT_tRNA_cys --> 1 EF-Tu.GTP.cysT-tRNA | irreversible | tRNA activation (EF-TU) |  | [155] |
| TC_gln1 | ternary complex formation with gln1-tRNA | 1 EF-Tu.GTP + 1 gln1_tRNA_gln --> 1 EF-Tu.GTP.gln1-tRNA | irreversible | tRNA activation (EF-TU) |  | [155] |
| TC_gln2 | ternary complex formation with gln2-tRNA | 1 EF-Tu.GTP + 1 gln2_tRNA_gln --> 1 EF-Tu.GTP.gln2-tRNA | irreversible | tRNA activation (EF-TU) |  | [155] |
| TC_glu1 | ternary complex formation with glu1-tRNA | 1 EF-Tu.GTP + 1 glu1_tRNA_glu --> 1 EF-Tu.GTP.glu1-tRNA | irreversible | tRNA activation (EF-TU) |  | [155] |
| TC_gly1 | ternary complex formation with gly1-tRNA | 1 EF-Tu.GTP + 1 gly1_tRNA_gly --> 1 EF-Tu.GTP.gly1-tRNA | irreversible | tRNA activation (EF-TU) |  | [155] |
| TC_glyT | ternary complex formation with glyT-tRNA | 1 EF-Tu.GTP + 1 glyT_tRNA_gly --> 1 EF-Tu.GTP.glyT-tRNA | irreversible | tRNA activation (EF-TU) |  | [155] |
| TC_glyU | ternary complex formation with glyU-tRNA | 1 EF-Tu.GTP + 1 glyU_tRNA_gly --> 1 EF-Tu.GTP.glyU-tRNA | irreversible | tRNA activation (EF-TU) |  | [155] |
| TC_hisR | ternary complex formation with hisR-tRNA | 1 EF-Tu.GTP + 1 hisR_tRNA_his --> 1 EF-Tu.GTP.hisR-tRNA | irreversible | tRNA activation (EF-TU) |  | [155] |
| TC_ile1 | ternary complex formation with ile1-tRNA | 1 EF-Tu.GTP + 1 ile1_tRNA_ile --> 1 EF-Tu.GTP.ile1-tRNA | irreversible | tRNA activation (EF-TU) |  | [155] |
| TC_ile2 | ternary complex formation with ile2-tRNA | 1 EF-Tu.GTP + 1 ile2_tRNA_ile --> 1 EF-Tu.GTP.ile2-tRNA | irreversible | tRNA activation (EF-TU) |  | [155] |
| TC_leu1 | ternary complex formation with leu1-tRNA | 1 EF-Tu.GTP + 1 leu1_tRNA_leu --> 1 EF-Tu.GTP.leu1-tRNA | irreversible | tRNA activation (EF-TU) |  | [155] |
| TC_leu2 | ternary complex formation with leu2-tRNA | 1 EF-Tu.GTP + 1 leu2_tRNA_leu --> 1 EF-Tu.GTP.leu2-tRNA | irreversible | tRNA activation (EF-TU) |  | [155] |
| TC_leuU | ternary complex formation with leuU-tRNA | 1 EF-Tu.GTP + 1 leuU_tRNA_leu --> 1 EF-Tu.GTP.leuU-tRNA | irreversible | tRNA activation (EF-TU) |  | [155] |
| TC_leuW | ternary complex formation with leuW-tRNA | 1 EF-Tu.GTP + 1 leuW_tRNA_leu --> 1 EF-Tu.GTP.leuW-tRNA | irreversible | tRNA activation (EF-TU) |  | [155] |
| TC_leuZ | ternary complex formation with leuZ-tRNA | 1 EF-Tu.GTP + 1 leuZ_tRNA_leu --> 1 EF-Tu.GTP.leuZ-tRNA | irreversible | tRNA activation (EF-TU) |  | [155] |
| TC_lys1 | ternary complex formation with lys1-tRNA | 1 EF-Tu.GTP + 1 lys1_tRNA_lys --> 1 EF-Tu.GTP.lys1-tRNA | irreversible | tRNA activation (EF-TU) |  | [155] |
| TC_met1 | ternary complex formation with met1-tRNA | 1 EF-Tu.GTP + 1 met1_tRNA_met --> 1 EF-Tu.GTP.met1-tRNA | irreversible | tRNA activation (EF-TU) |  | [155] |
| TC_phe1 | ternary complex formation with phe1-tRNA | 1 EF-Tu.GTP + 1 phe1_tRNA_phe --> 1 EF-Tu.GTP.phe1-tRNA | irreversible | tRNA activation (EF-TU) |  | [155] |
| TC_pro1 | ternary complex formation with pro1-tRNA | 1 EF-Tu.GTP + 1 pro1_tRNA_pro --> 1 EF-Tu.GTP.pro1-tRNA | irreversible | tRNA activation (EF-TU) |  | [155] |
| TC_pro2 | ternary complex formation with pro2-tRNA | 1 EF-Tu.GTP + 1 pro2_tRNA_pro --> 1 EF-Tu.GTP.pro2-tRNA | irreversible | tRNA activation (EF-TU) |  | [155] |
| TC_proL | ternary complex formation with proL-tRNA | 1 EF-Tu.GTP + 1 proL_tRNA_pro --> 1 EF-Tu.GTP.proL-tRNA | irreversible | tRNA activation (EF-TU) |  | [155] |
| TC_proM | ternary complex formation with proM-tRNA | 1 EF-Tu.GTP + 1 proM_tRNA_pro --> 1 EF-Tu.GTP.proM-tRNA | irreversible | tRNA activation (EF-TU) |  | [155] |
| TC_sec | ternary complex formation with sec-tRNA | 1 SelB_mono + 1 gtp + 1 sec_tRNA_sec --> 1 SelB.GTP.sec_tRNA | irreversible | tRNA activation (EF-TU) | 1100 copies of SelB per cell | [345,346] |
| TC_ser1 | ternary complex formation with ser1-tRNA | 1 EF-Tu.GTP + 1 ser1_tRNA_ser --> 1 EF-Tu.GTP.ser1-tRNA | irreversible | tRNA activation (EF-TU) |  | [155] |
| TC_ser2 | ternary complex formation with ser2-tRNA | 1 EF-Tu.GTP + 1 ser2_tRNA_ser --> 1 EF-Tu.GTP.ser2-tRNA | irreversible | tRNA activation (EF-TU) |  | [155] |
| TC_ser3 | ternary complex formation with ser3-tRNA | 1 EF-Tu.GTP + 1 ser3_tRNA_ser --> 1 EF-Tu.GTP.ser3-tRNA | irreversible | tRNA activation (EF-TU) |  | [155] |
| TC_serT | ternary complex formation with serT-tRNA | 1 EF-Tu.GTP + 1 serT_tRNA_ser --> 1 EF-Tu.GTP.serT-tRNA | irreversible | tRNA activation (EF-TU) |  | [155] |
| TC_serV | ternary complex formation with serV-tRNA | 1 EF-Tu.GTP + 1 serV_tRNA_ser --> 1 EF-Tu.GTP.serV-tRNA | irreversible | tRNA activation (EF-TU) |  | [155] |
| TC_thr1 | ternary complex formation with thr1-tRNA | 1 EF-Tu.GTP + 1 thr1_tRNA_thr --> 1 EF-Tu.GTP.thr1-tRNA | irreversible | tRNA activation (EF-TU) |  | [155] |
| TC_thr2 | ternary complex formation with thr2-tRNA | 1 EF-Tu.GTP + 1 thr2_tRNA_thr --> 1 EF-Tu.GTP.thr2-tRNA | irreversible | tRNA activation (EF-TU) |  | [155] |
| TC_thr3 | ternary complex formation with thr3-tRNA | 1 EF-Tu.GTP + 1 thr3_tRNA_thr --> 1 EF-Tu.GTP.thr3-tRNA | irreversible | tRNA activation (EF-TU) |  | [155] |
| TC_thrU | ternary complex formation with thrU-tRNA | 1 EF-Tu.GTP + 1 thrU_tRNA_thr --> 1 EF-Tu.GTP.thrU-tRNA | irreversible | tRNA activation (EF-TU) |  | [155] |
| TC_trpT | ternary complex formation with trpT-tRNA | 1 EF-Tu.GTP + 1 trpT_tRNA_trp --> 1 EF-Tu.GTP.trpT-tRNA | irreversible | tRNA activation (EF-TU) |  | [155] |
| TC_tyr1 | ternary complex formation with tyr1-tRNA | 1 EF-Tu.GTP + 1 tyr1_tRNA_tyr --> 1 EF-Tu.GTP.tyr1-tRNA | irreversible | tRNA activation (EF-TU) |  | [155] |
| TC_val1 | ternary complex formation with val1-tRNA | 1 EF-Tu.GTP + 1 val1_tRNA_val --> 1 EF-Tu.GTP.val1-tRNA | irreversible | tRNA activation (EF-TU) |  | [155] |
| TC_val2 | ternary complex formation with val2-tRNA | 1 EF-Tu.GTP + 1 val2_tRNA_val --> 1 EF-Tu.GTP.val2-tRNA | irreversible | tRNA activation (EF-TU) |  | [155] |
| TC_val3 | ternary complex formation with val3-tRNA | 1 EF-Tu.GTP + 1 val3_tRNA_val --> 1 EF-Tu.GTP.val3-tRNA | irreversible | tRNA activation (EF-TU) |  | [155] |
| ala1_tRNA_CHARG | charging ala1_tRNA | 1 Ala_RS_ala_amp + 1 ala1_tRNA --> 1 Ala_RS_tetra_inact + 1 ala1_tRNA_ala + 1 amp | irreversible | tRNA charging |  | [1-3,44] |
| ala2_tRNA_CHARG | charging ala2_tRNA | 1 Ala_RS_ala_amp + 1 ala2_tRNA --> 1 Ala_RS_tetra_inact + 1 ala2_tRNA_ala + 1 amp | irreversible | tRNA charging |  | [1-3,44] |
| alaT_to_ala1 | alaT_to_ala1 | 1 alaT_tRNA --> 1 ala1_tRNA | irreversible | tRNA charging | Reaction was included for modeling reason to represent the alternate codon reading appropriately. Assignment was done based on Solomovici et al [347]. | [347] |
| alaU_to_ala1 | alaU_to_ala1 | 1 alaU_tRNA --> 1 ala1_tRNA | irreversible | tRNA charging | Reaction was included for modeling reason to represent the alternate codon reading appropriately. Assignment was done based on Solomovici et al [347]. | [347] |
| alaV_to_ala1 | alaV_to_ala1 | 1 alaV_tRNA --> 1 ala1_tRNA | irreversible | tRNA charging | Reaction was included for modeling reason to represent the alternate codon reading appropriately. Assignment was done based on Solomovici et al [347]. | [347] |
| alaW_to_ala2 | alaW_to_ala2 | 1 alaW_tRNA --> 1 ala2_tRNA | irreversible | tRNA charging | Reaction was included for modeling reason to represent the alternate codon reading appropriately. Assignment was done based on Solomovici et al [347]. | [347] |
| alaX_to_ala2 | alaX_to_ala2 | 1 alaX_tRNA --> 1 ala2_tRNA | irreversible | tRNA charging | Reaction was included for modeling reason to represent the alternate codon reading appropriately. Assignment was done based on Solomovici et al [347]. | [347] |
| arg1_tRNA_CHARG | charging arg1_tRNA | 1 Arg_RS_arg_amp + 1 arg1_tRNA --> 1 ArgS_mono_inact + 1 amp + 1 arg1_tRNA_ala | irreversible | tRNA charging |  | [1,4-7,44] |
| argQ_to_arg1 | argQ_to_arg1 | 1 argQ_tRNA --> 1 arg1_tRNA | irreversible | tRNA charging | Reaction was included for modeling reason to represent the alternate codon reading appropriately. Assignment was done based on Solomovici et al [347]. | [347] |
| argU_tRNA_CHARG | charging argU_tRNA | 1 Arg_RS_arg_amp + 1 argU_tRNA --> 1 ArgS_mono_inact + 1 amp + 1 argU_tRNA_ala | irreversible | tRNA charging |  | [1,4-7,44] |
| argV_to_arg1 | argV_to_arg1 | 1 argV_tRNA --> 1 arg1_tRNA | irreversible | tRNA charging | Reaction was included for modeling reason to represent the alternate codon reading appropriately. Assignment was done based on Solomovici et al [347]. | [347] |
| argW_tRNA_CHARG | charging argW_tRNA | 1 Arg_RS_arg_amp + 1 argW_tRNA --> 1 ArgS_mono_inact + 1 amp + 1 argW_tRNA_arg | irreversible | tRNA charging |  | [1,4-7,44] |
| argX_tRNA_CHARG | charging argX_tRNA | 1 Arg_RS_arg_amp + 1 argX_tRNA --> 1 ArgS_mono_inact + 1 amp + 1 argX_tRNA_arg | irreversible | tRNA charging |  | [1,4-7,44] |
| argY_to_arg1 | argY_to_arg1 | 1 argY_tRNA --> 1 arg1_tRNA | irreversible | tRNA charging | Reaction was included for modeling reason to represent the alternate codon reading appropriately. Assignment was done based on Solomovici et al [347]. | [347] |
| argZ_to_arg1 | argZ_to_arg1 | 1 argZ_tRNA --> 1 arg1_tRNA | irreversible | tRNA charging | Reaction was included for modeling reason to represent the alternate codon reading appropriately. Assignment was done based on Solomovici et al [347]. | [347] |
| asn1_tRNA | charging asn1 _tRNA_CHARG | 1 Asn_RS_asn_amp + 1 asn1_tRNA --> 1 Asn_RS_dim_inact + 1 amp + 1 asn1_tRNA_asn | irreversible | tRNA charging |  | [1,8,9,44] |
| asnT_to_asn1 | asnT_to_asn1 | 1 asnT_tRNA --> 1 asn1_tRNA | irreversible | tRNA charging | Reaction was included for modeling reason to represent the alternate codon reading appropriately. Assignment was done based on Solomovici et al [347]. | [347] |
| asnU_to_asn1 | asnU_to_asn1 | 1 asnU_tRNA --> 1 asn1_tRNA | irreversible | tRNA charging | Reaction was included for modeling reason to represent the alternate codon reading appropriately. Assignment was done based on Solomovici et al [347]. | [347] |
| asnV_to_asn1 | asnV_to_asn1 | 1 asnV_tRNA --> 1 asn1_tRNA | irreversible | tRNA charging | Reaction was included for modeling reason to represent the alternate codon reading appropriately. Assignment was done based on Solomovici et al [347]. | [347] |
| asnW_to_asn1 | asnW_to_asn1 | 1 asnW_tRNA --> 1 asn1_tRNA | irreversible | tRNA charging | Reaction was included for modeling reason to represent the alternate codon reading appropriately. Assignment was done based on Solomovici et al [347]. | [347] |
| asp1_tRNA_CHARGa | charging asp1_tRNA | 1 Asp_RS_2asp_2amp + 2 asp1_tRNA --> 1 Asp_RS_dim_inact + 2 amp + 2 asp1_tRNA_asp | irreversible | tRNA charging |  | [1,10,11,44] |
| asp1_tRNA_CHARGb | charging asp1_tRNA | 1 Asp_RS_asp_amp + 1 asp1_tRNA --> 1 Asp_RS_dim_inact + 1 amp + 1 asp1_tRNA_asp | irreversible | tRNA charging |  | [1,10,11,44] |
| aspT_to_asp1 | aspT_to_asp1 | 1 aspT_tRNA --> 1 asp1_tRNA | irreversible | tRNA charging | Reaction was included for modeling reason to represent the alternate codon reading appropriately. Assignment was done based on Solomovici et al [347]. | [347] |
| aspU_to_asp1 | aspU_to_asp1 | 1 aspU_tRNA --> 1 asp1_tRNA | irreversible | tRNA charging | Reaction was included for modeling reason to represent the alternate codon reading appropriately. Assignment was done based on Solomovici et al [347]. | [347] |
| aspV_to_asp1 | aspV_to_asp1 | 1 aspV_tRNA --> 1 asp1_tRNA | irreversible | tRNA charging | Reaction was included for modeling reason to represent the alternate codon reading appropriately. Assignment was done based on Solomovici et al [347]. | [347] |
| cysT_tRNA_CHARG | charging cysT_tRNA | 1 Cys_RS_cys_amp + 1 cysT_tRNA --> 1 CysS_mono_inact + 1 amp + 1 cysT_tRNA_cys | irreversible | tRNA charging |  | [12,13,44] |
| DATYRDTRNA1 | deacylation of tyr1_tRNA_tyr-D | 1 Dtd_dim + 1 atp + 1 tyr1_tRNA_tyr-D --> 1 Dtd_dim_tyr | irreversible | tRNA charging |  | [44,115,312] |
| DATYRDTRNA2 | deacylation of tyr1_tRNA_tyr-D | 1 Dtd_dim_tyr + 1 h2o --> 1 Dtd_dim_inact + 1 adp + 1 h + 1 pi + 1 tyr-D + 1 tyr1_tRNA | irreversible | tRNA charging |  | [44,115,312] |
| fmet_tRNA_CHARGa | charging fmet_tRNA | 1 Met_RS_2met_2amp + 2 fmet_tRNA --> 1 Met_RS_dim_inact + 2 amp + 2 fmet_tRNA_met_1 | irreversible | tRNA charging |  | [44,348] |
| fmet_tRNA_CHARGb | charging fmet_tRNA | 1 Met_RS_met_amp + 1 fmet_tRNA --> 1 Met_RS_dim_inact + 1 amp + 1 fmet_tRNA_met_1 | irreversible | tRNA charging |  | [44,348] |
| fmet_tRNA_TF_1 | methionyl-tRNA formyltransferase 1 | 1 10fthf + 1 Fmt_mono + 1 fmet_tRNA_met_1 --> 1 Fmt_fmet_tRNA_met_1_cplx | irreversible | tRNA charging |  | [44,348] |
| fmet_tRNA_TF_2 | methionyl-tRNA formyltransferase 2 | 1 Fmt_fmet_tRNA_met_1_cplx --> 1 Fmt_mono_inact + 1 fmet_tRNA_met + 1 h + 1 thf | irreversible | tRNA charging |  | [44,348] |
| gln1_tRNA_CHARG | charging gln1_tRNA | 1 Gln_RS_gln_amp + 1 gln1_tRNA --> 1 GlnS_mono_inact + 1 amp + 1 gln1_tRNA_gln | irreversible | tRNA charging |  | [1,4,14,15,44] |
| gln2_tRNA_CHARG | charging gln2_tRNA | 1 Gln_RS_gln_amp + 1 gln2_tRNA --> 1 GlnS_mono_inact + 1 amp + 1 gln2_tRNA_gln | irreversible | tRNA charging |  | [1,4,14,15,44] |
| glnU_to_gln1 | glnU_to_gln1 | 1 glnU_tRNA --> 1 gln1_tRNA | irreversible | tRNA charging | Reaction was included for modeling reason to represent the alternate codon reading appropriately. Assignment was done based on Solomovici et al [347]. | [347] |
| glnV_to_gln2 | glnV_to_gln2 | 1 glnV_tRNA --> 1 gln2_tRNA | irreversible | tRNA charging | Reaction was included for modeling reason to represent the alternate codon reading appropriately. Assignment was done based on Solomovici et al [347]. | [347] |
| glnW_to_gln1 | glnW_to_gln1 | 1 glnW_tRNA --> 1 gln1_tRNA | irreversible | tRNA charging | Reaction was included for modeling reason to represent the alternate codon reading appropriately. Assignment was done based on Solomovici et al [347]. | [347] |
| glnX_to_gln2 | glnX_to_gln2 | 1 glnX_tRNA --> 1 gln2_tRNA | irreversible | tRNA charging | Reaction was included for modeling reason to represent the alternate codon reading appropriately. Assignment was done based on Solomovici et al [347]. | [347] |
| glu1_tRNA_CHARG | charging glu1_tRNA | 1 Glu_RS_glu_amp + 1 glu1_tRNA --> 1 GltX_mono_inact + 1 amp + 1 glu1_tRNA_glu | irreversible | tRNA charging |  | [1,4,44] |
| gluT_to_glu1 | gluT_to_glu1 | 1 gltT_tRNA --> 1 glu1_tRNA | irreversible | tRNA charging | Reaction was included for modeling reason to represent the alternate codon reading appropriately. Assignment was done based on Solomovici et al [347]. | [347] |
| gluU_to_glu1 | gluU_to_glu1 | 1 gltU_tRNA --> 1 glu1_tRNA | irreversible | tRNA charging | Reaction was included for modeling reason to represent the alternate codon reading appropriately. Assignment was done based on Solomovici et al [347]. | [347] |
| gluV_to_glu1 | gluV_to_glu1 | 1 gltV_tRNA --> 1 glu1_tRNA | irreversible | tRNA charging | Reaction was included for modeling reason to represent the alternate codon reading appropriately. Assignment was done based on Solomovici et al [347]. | [347] |
| gluW_to_glu1 | gluW_to_glu1 | 1 gltW_tRNA --> 1 glu1_tRNA | irreversible | tRNA charging | Reaction was included for modeling reason to represent the alternate codon reading appropriately. Assignment was done based on Solomovici et al [347]. | [347] |
| gly1_tRNA_CHARGa | charging gly1_tRNA | 1 Gly_RS_2gly_2amp + 2 gly1_tRNA --> 1 Gly_RS_tetra_inact + 2 amp + 2 gly1_tRNA_gly | irreversible | tRNA charging |  | [1,4,16,44] |
| gly1_tRNA_CHARGb | charging gly1_tRNA | 1 Gly_RS_gly_amp + 1 gly1_tRNA --> 1 Gly_RS_tetra_inact + 1 amp + 1 gly1_tRNA_gly | irreversible | tRNA charging |  | [1,4,16,44] |
| glyT_tRNA_CHARGa | charging glyT_tRNA | 1 Gly_RS_2gly_2amp + 2 glyT_tRNA --> 1 Gly_RS_tetra_inact + 2 amp + 2 glyT_tRNA_gly | irreversible | tRNA charging |  | [1,4,16,44] |
| glyT_tRNA_CHARGb | charging glyT_tRNA | 1 Gly_RS_gly_amp + 1 glyT_tRNA --> 1 Gly_RS_tetra_inact + 1 amp + 1 glyT_tRNA_gly | irreversible | tRNA charging |  | [1,4,16,44] |
| glyU_tRNA_CHARGa | charging gly2_tRNA | 1 Gly_RS_2gly_2amp + 2 glyU_tRNA --> 1 Gly_RS_tetra_inact + 2 amp + 2 glyU_tRNA_gly | irreversible | tRNA charging |  | [1,4,16,44] |
| glyU_tRNA_CHARGb | charging gly2_tRNA | 1 Gly_RS_gly_amp + 1 glyU_tRNA --> 1 Gly_RS_tetra_inact + 1 amp + 1 glyU_tRNA_gly | irreversible | tRNA charging |  | [1,4,16,44] |
| glyV_to_gly1 | glyV_to_gly1 | 1 glyV_tRNA --> 1 gly1_tRNA | irreversible | tRNA charging | Reaction was included for modeling reason to represent the alternate codon reading appropriately. Assignment was done based on Solomovici et al [347]. | [347] |
| glyW_to_gly1 | glyW_to_gly1 | 1 glyW_tRNA --> 1 gly1_tRNA | irreversible | tRNA charging | Reaction was included for modeling reason to represent the alternate codon reading appropriately. Assignment was done based on Solomovici et al [347]. | [347] |
| glyX_to_gly1 | glyX_to_gly1 | 1 glyX_tRNA --> 1 gly1_tRNA | irreversible | tRNA charging | Reaction was included for modeling reason to represent the alternate codon reading appropriately. Assignment was done based on Solomovici et al [347]. | [347] |
| glyY_to_gly1 | glyY_to_gly1 | 1 glyY_tRNA --> 1 gly1_tRNA | irreversible | tRNA charging | Reaction was included for modeling reason to represent the alternate codon reading appropriately. Assignment was done based on Solomovici et al [347]. | [347] |
| hisR_tRNA_CHARGa | charging hisR_tRNA | 1 His_RS_his_amp + 1 hisR_tRNA --> 1 His_RS_dim_inact + 1 amp + 1 hisR_tRNA_his | irreversible | tRNA charging |  | [17-19,44] |
| hisR_tRNA_CHARGb | charging hisR_tRNA | 1 His_RS_2his_2amp + 2 hisR_tRNA --> 1 His_RS_dim_inact + 2 amp + 2 hisR_tRNA_his | irreversible | tRNA charging |  | [17-19,44] |
| ile1_tRNA_CHARG | charging ile1_tRNA | 1 Ile_RS_ile_Amp + 1 ile1_tRNA --> 1 IleS_mono_inact + 1 amp + 1 ile1_tRNA_ile | irreversible | tRNA charging |  | [1,4,20,21,44] |
| ile2_tRNA_CHARG | charging ile2_tRNA | 1 Ile_RS_ile_Amp + 1 ile2_tRNA --> 1 IleS_mono_inact + 1 amp + 1 ile2_tRNA_ile | irreversible | tRNA charging |  | [1,4,20,21,44] |
| ileT_to_ile1 | ileT_to_ile1 | 1 ileT_tRNA --> 1 ile1_tRNA | irreversible | tRNA charging | Reaction was included for modeling reason to represent the alternate codon reading appropriately. Assignment was done based on Solomovici et al [347]. | [347] |
| ileU_to_ile1 | ileU_to_ile1 | 1 ileU_tRNA --> 1 ile1_tRNA | irreversible | tRNA charging | Reaction was included for modeling reason to represent the alternate codon reading appropriately. Assignment was done based on Solomovici et al [347]. | [347] |
| ileV_to_ile1 | ileV_to_ile1 | 1 ileV_tRNA --> 1 ile1_tRNA | irreversible | tRNA charging | Reaction was included for modeling reason to represent the alternate codon reading appropriately. Assignment was done based on Solomovici et al [347]. | [347] |
| ileX_to_ile2 | ileX_to_ile2 | 1 ileX_tRNA --> 1 ile2_tRNA | irreversible | tRNA charging | Reaction was included for modeling reason to represent the alternate codon reading appropriately. Assignment was done based on Solomovici et al [347]. | [347] |
| ileY_to_ile2 | ileY_to_ile2 | 1 ileY_tRNA --> 1 ile2_tRNA | irreversible | tRNA charging | Reaction was included for modeling reason to represent the alternate codon reading appropriately. Assignment was done based on Solomovici et al [347]. | [347] |
| leu1_tRNA_CHARG | charging leu1_tRNA | 1 Leu_RS_leu_amp + 1 leu1_tRNA --> 1 LeuS_mono_inact + 1 amp + 1 leu1_tRNA_leu | irreversible | tRNA charging |  | [1,4,20,44] |
| leu2_tRNA_CHARG | charging leu2_tRNA | 1 Leu_RS_leu_amp + 1 leu2_tRNA --> 1 LeuS_mono_inact + 1 amp + 1 leu2_tRNA_leu | irreversible | tRNA charging |  | [1,4,20,44] |
| leuP_to_leu1 | leuP_to_leu1 | 1 leuP_tRNA --> 1 leu1_tRNA | irreversible | tRNA charging | Reaction was included for modeling reason to represent the alternate codon reading appropriately. Assignment was done based on Solomovici et al [347]. | [347] |
| leuQ_to_leu1 | leuQ_to_leu1 | 1 leuQ_tRNA --> 1 leu1_tRNA | irreversible | tRNA charging | Reaction was included for modeling reason to represent the alternate codon reading appropriately. Assignment was done based on Solomovici et al [347]. | [347] |
| leuT_to_leu1 | leuT_to_leu1 | 1 leuT_tRNA --> 1 leu1_tRNA | irreversible | tRNA charging | Reaction was included for modeling reason to represent the alternate codon reading appropriately. Assignment was done based on Solomovici et al [347]. | [347] |
| leuU_tRNA_CHARG | charging leuU_tRNA | 1 Leu_RS_leu_amp + 1 leuU_tRNA --> 1 LeuS_mono_inact + 1 amp + 1 leuU_tRNA_leu | irreversible | tRNA charging |  | [1,4,20,44] |
| leuV_to_leu1 | leuV_to_leu1 | 1 leuV_tRNA --> 1 leu1_tRNA | irreversible | tRNA charging | Reaction was included for modeling reason to represent the alternate codon reading appropriately. Assignment was done based on Solomovici et al [347]. | [347] |
| leuW_to_leu1 | leuW_to_leu1 | 1 leuW_tRNA --> 1 leu1_tRNA | irreversible | tRNA charging | Reaction was included for modeling reason to represent the alternate codon reading appropriately. Assignment was done based on Solomovici et al [347]. | [347] |
| leuW_tRNA_CHARG | charging leuW_tRNA | 1 Leu_RS_leu_amp + 1 leuW_tRNA --> 1 LeuS_mono_inact + 1 amp + 1 leuW_tRNA_leu | irreversible | tRNA charging |  | [1,4,20,44] |
| leuX_to_leu2 | leuX_to_leu2 | 1 leuX_tRNA --> 1 leu2_tRNA | irreversible | tRNA charging | Reaction was included for modeling reason to represent the alternate codon reading appropriately. Assignment was done based on Solomovici et al [347]. | [347] |
| leuZ_to_leu2 | leuZ_to_leu2 | 1 leuZ_tRNA --> 1 leu2_tRNA | irreversible | tRNA charging | Reaction was included for modeling reason to represent the alternate codon reading appropriately. Assignment was done based on Solomovici et al [347]. | [347] |
| leuZ_tRNA_CHARG | charging leuZ_tRNA | 1 Leu_RS_leu_amp + 1 leuZ_tRNA --> 1 LeuS_mono_inact + 1 amp + 1 leuZ_tRNA_leu | irreversible | tRNA charging |  | [24,44] |
| lys1_tRNA1_CHARGa | charging lys1_tRNA (by lysI_RS) | 1 LysI_RS_lys_amp + 1 lys1_tRNA --> 1 LysI_RS_dim_inact + 1 amp + 1 lys1_tRNA_lys | irreversible | tRNA charging |  | [24,44] |
| lys1_tRNA1_CHARGb | charging lys1_tRNA (by lysI_RS) | 1 LysI_RS_2lys_2amp + 2 lys1_tRNA --> 1 LysI_RS_dim_inact + 2 amp + 2 lys1_tRNA_lys | irreversible | tRNA charging |  | [24,44] |
| lys1_tRNA2_CHARGa | charging lys1_tRNA (by lysII_RS) | 1 LysII_RS_lys_amp + 1 lys1_tRNA --> 1 LysII_RS_dim_inact + 1 amp + 1 lys1_tRNA_lys | irreversible | tRNA charging |  | [24,44] |
| lys1_tRNA2_CHARGb | charging lys1_tRNA (by lysII_RS) | 1 LysII_RS_2lys_2amp + 2 lys1_tRNA --> 1 LysII_RS_dim_inact + 2 amp + 2 lys1_tRNA_lys | irreversible | tRNA charging |  | [24,44] |
| lysQ_to_lys1 | lysQ_to_lys1 | 1 lysQ_tRNA --> 1 lys1_tRNA | irreversible | tRNA charging | Reaction was included for modeling reason to represent the alternate codon reading appropriately. Assignment was done based on Solomovici et al [347]. | [347] |
| lysT_to_lys1 | lysT_to_lys1 | 1 lysT_tRNA --> 1 lys1_tRNA | irreversible | tRNA charging | Reaction was included for modeling reason to represent the alternate codon reading appropriately. Assignment was done based on Solomovici et al [347]. | [347] |
| lysV_to_lys1 | lysV_to_lys1 | 1 lysV_tRNA --> 1 lys1_tRNA | irreversible | tRNA charging | Reaction was included for modeling reason to represent the alternate codon reading appropriately. Assignment was done based on Solomovici et al [347]. | [347] |
| lysW_to_lys1 | lysW_to_lys1 | 1 lysW_tRNA --> 1 lys1_tRNA | irreversible | tRNA charging | Reaction was included for modeling reason to represent the alternate codon reading appropriately. Assignment was done based on Solomovici et al [347]. | [347] |
| lysY_to_lys1 | lysY_to_lys1 | 1 lysY_tRNA --> 1 lys1_tRNA | irreversible | tRNA charging | Reaction was included for modeling reason to represent the alternate codon reading appropriately. Assignment was done based on Solomovici et al [347]. | [347] |
| lysZ_to_lys1 | lysZ_to_lys1 | 1 lysZ_tRNA --> 1 lys1_tRNA | irreversible | tRNA charging | Reaction was included for modeling reason to represent the alternate codon reading appropriately. Assignment was done based on Solomovici et al [347]. | [347] |
| met1_tRNA_CHARG | charging met1_tRNA | 1 Met_RS_2met_2amp + 2 met1_tRNA --> 1 Met_RS_dim_inact + 2 amp + 2 met1_tRNA_met | irreversible | tRNA charging |  | [1,20,26-28,44] |
| metT_to_met1 | metT_to_met1 | 1 metT_tRNA --> 1 met1_tRNA | irreversible | tRNA charging | Reaction was included for modeling reason to represent the alternate codon reading appropriately. Assignment was done based on Solomovici et al [347]. | [347] |
| metU_to_met1 | metU_to_met1 | 1 metU_tRNA --> 1 met1_tRNA | irreversible | tRNA charging | Reaction was included for modeling reason to represent the alternate codon reading appropriately. Assignment was done based on Solomovici et al [347]. | [347] |
| metV_to_fmet | metV_to_fmet | 1 metV_tRNA --> 1 fmet_tRNA | irreversible | tRNA charging | Reaction was included for modeling reason to represent the alternate codon reading appropriately. Assignment was done based on Solomovici et al [347]. | [347] |
| metW_to_fmet | metW_to_fmet | 1 metW_tRNA --> 1 fmet_tRNA | irreversible | tRNA charging | Reaction was included for modeling reason to represent the alternate codon reading appropriately. Assignment was done based on Solomovici et al [347]. | [347] |
| metY_to_fmet | metY_to_fmet | 1 metY_tRNA --> 1 fmet_tRNA | irreversible | tRNA charging | Reaction was included for modeling reason to represent the alternate codon reading appropriately. Assignment was done based on Solomovici et al [347]. | [347] |
| metZ_to_fmet | metZ_to_fmet | 1 metZ_tRNA --> 1 fmet_tRNA | irreversible | tRNA charging | Reaction was included for modeling reason to represent the alternate codon reading appropriately. Assignment was done based on Solomovici et al [347]. | [347] |
| phe1_tRNA_CHARG | charging phe1_tRNA | 1 Phe_RS_phe_amp + 1 phe1_tRNA --> 1 Phe_RS_tetra_inact + 1 amp + 1 phe1_tRNA_phe | irreversible | tRNA charging |  | [1,4,25,29,44] |
| pheU_to_phe1 | pheU_to_phe1 | 1 pheU_tRNA --> 1 phe1_tRNA | irreversible | tRNA charging | Reaction was included for modeling reason to represent the alternate codon reading appropriately. Assignment was done based on Solomovici et al [347]. | [347] |
| pheV_to_phe1 | pheV_to_phe1 | 1 pheV_tRNA --> 1 phe1_tRNA | irreversible | tRNA charging | Reaction was included for modeling reason to represent the alternate codon reading appropriately. Assignment was done based on Solomovici et al [347]. | [347] |
| pro1_tRNA_CHARGa | charging pro1_tRNA | 1 Pro_RS_2pro_2amp + 2 pro1_tRNA --> 1 Pro_RS_dim_inact + 2 amp + 2 pro1_tRNA_pro | irreversible | tRNA charging |  | [1,30,44] |
| pro1_tRNA_CHARGb | charging pro1_tRNA | 1 Pro_RS_pro_amp + 1 pro1_tRNA --> 1 Pro_RS_dim_inact + 1 amp + 1 pro1_tRNA_pro | irreversible | tRNA charging |  | [1,30,44] |
| pro2_tRNA_CHARGa | charging pro2_tRNA | 1 Pro_RS_2pro_2amp + 2 pro2_tRNA --> 1 Pro_RS_dim_inact + 2 amp + 2 pro2_tRNA_pro | irreversible | tRNA charging |  | [1,30,44] |
| pro2_tRNA_CHARGb | charging pro2_tRNA | 1 Pro_RS_pro_amp + 1 pro2_tRNA --> 1 Pro_RS_dim_inact + 1 amp + 1 pro2_tRNA_pro | irreversible | tRNA charging |  | [1,30,44] |
| proK_to_pro1 | proK_to_pro1 | 1 proK_tRNA --> 1 pro1_tRNA | irreversible | tRNA charging | Reaction was included for modeling reason to represent the alternate codon reading appropriately. Assignment was done based on Solomovici et al [347]. | [347] |
| proL_to_pro2 | proL_to_pro2 | 1 proL_tRNA --> 1 pro2_tRNA | irreversible | tRNA charging | Reaction was included for modeling reason to represent the alternate codon reading appropriately. Assignment was done based on Solomovici et al [347]. | [347] |
| proL_tRNA_CHARGa | charging proL_tRNA | 1 Pro_RS_2pro_2amp + 2 proL_tRNA --> 1 Pro_RS_dim_inact + 2 amp + 2 proL_tRNA_pro | irreversible | tRNA charging |  | [1,30,44] |
| proL_tRNA_CHARGb | charging proL_tRNA | 1 Pro_RS_pro_amp + 1 proL_tRNA --> 1 Pro_RS_dim_inact + 1 amp + 1 proL_tRNA_pro | irreversible | tRNA charging |  | [1,30,44] |
| proM_to_pro1 | proM_to_pro1 | 1 proM_tRNA --> 1 pro1_tRNA | irreversible | tRNA charging | Reaction was included for modeling reason to represent the alternate codon reading appropriately. Assignment was done based on Solomovici et al [347]. | [347] |
| proM_to_pro2 | proM_to_pro2 | 1 proM_tRNA --> 1 pro2_tRNA | irreversible | tRNA charging | Reaction was included for modeling reason to represent the alternate codon reading appropriately. Assignment was done based on Solomovici et al [347]. | [347] |
| proM_tRNA_CHARGa | charging proM_tRNA | 1 Pro_RS_2pro_2amp + 2 proM_tRNA --> 1 Pro_RS_dim_inact + 2 amp + 2 proM_tRNA_pro | irreversible | tRNA charging |  | [1,30,44] |
| proM_tRNA_CHARGb | charging proM_tRNA | 1 Pro_RS_pro_amp + 1 proM_tRNA --> 1 Pro_RS_dim_inact + 1 amp + 1 proM_tRNA_pro | irreversible | tRNA charging |  | [1,30,44] |
| sec_tRNA_CHARG1a | charging sec_tRNA | 1 Ser_RS_2ser_2amp + 2 selC_tRNA --> 1 Ser_RS_dim_inact + 2 amp + 2 sec_tRNA_ser | irreversible | tRNA charging |  | [44,349] |
| sec_tRNA_CHARG1b | charging sec_tRNA | 1 Ser_RS_ser_amp + 1 selC_tRNA --> 1 Ser_RS_dim_inact + 1 amp + 1 sec_tRNA_ser | irreversible | tRNA charging |  | [44,349] |
| sec_tRNA_CHARG2a | conversion of 5 sec_tRNA_ser to 5 sec_tRNA_sec | 1 SelA_deca + 5 sec_tRNA_ser --> 1 5_sec_tRNA_ser_SelA_deca_cplx + 5 h2o | irreversible | tRNA charging | The stoichiometry is up to 5 tRNA per decamer. | [229,349] |
| sec_tRNA_CHARG2b | conversion of 5 sec_tRNA_ser to 5 sec_tRNA_sec | 1 5_sec_tRNA_ser_SelA_deca_cplx + 5 h2o + 5 selnp --> 1 SelA_deca_inact + 5 h + 5 pi + 5 sec_tRNA_sec | irreversible | tRNA charging | The stoichiometry is up to 5 tRNA per decamer. | [229,349] |
| sec_tRNA_CHARG3a | conversion of 4 sec_tRNA_ser to 4 sec_tRNA_sec | 1 SelA_deca + 4 sec_tRNA_ser --> 1 4_sec_tRNA_ser_SelA_deca_cplx + 4 h2o | irreversible | tRNA charging | The stoichiometry is up to 5 tRNA per decamer. | [229,349] |
| sec_tRNA_CHARG3b | conversion of 4 sec_tRNA_ser to 4 sec_tRNA_sec | 1 4_sec_tRNA_ser_SelA_deca_cplx + 4 h2o + 4 selnp --> 1 SelA_deca_inact + 4 h + 4 pi + 4 sec_tRNA_sec | irreversible | tRNA charging | The stoichiometry is up to 5 tRNA per decamer. | [229,349] |
| sec_tRNA_CHARG4a | conversion of 3 sec_tRNA_ser to 3 sec_tRNA_sec | 1 SelA_deca + 3 sec_tRNA_ser --> 1 3_sec_tRNA_ser_SelA_deca_cplx + 3 h2o | irreversible | tRNA charging | The stoichiometry is up to 5 tRNA per decamer. | [229,349] |
| sec_tRNA_CHARG4b | conversion of 3 sec_tRNA_ser to 3 sec_tRNA_sec | 1 3_sec_tRNA_ser_SelA_deca_cplx + 3 h2o + 3 selnp --> 1 SelA_deca_inact + 3 h + 3 pi + 3 sec_tRNA_sec | irreversible | tRNA charging | The stoichiometry is up to 5 tRNA per decamer. | [229,349] |
| sec_tRNA_CHARG5a | conversion of 2 sec_tRNA_ser to 2 sec_tRNA_sec | 1 SelA_deca + 2 sec_tRNA_ser --> 1 2_sec_tRNA_ser_SelA_deca_cplx + 2 h2o | irreversible | tRNA charging | The stoichiometry is up to 5 tRNA per decamer. | [229,349] |
| sec_tRNA_CHARG5b | conversion of 2 sec_tRNA_ser to 2 sec_tRNA_sec | 1 2_sec_tRNA_ser_SelA_deca_cplx + 2 h2o + 2 selnp --> 1 SelA_deca_inact + 2 h + 2 pi + 2 sec_tRNA_sec | irreversible | tRNA charging | The stoichiometry is up to 5 tRNA per decamer. | [229,349] |
| sec_tRNA_CHARG6a | conversion of 1 sec_tRNA_ser to 1 sec_tRNA_sec | 1 SelA_deca + 1 sec_tRNA_ser --> 1 1_sec_tRNA_ser_SelA_deca_cplx + 1 h2o | irreversible | tRNA charging | The stoichiometry is up to 5 tRNA per decamer. | [229,349] |
| sec_tRNA_CHARG6b | conversion of 1 sec_tRNA_ser to 1 sec_tRNA_sec | 1 1_sec_tRNA_ser_SelA_deca_cplx + 1 h2o + 1 selnp --> 1 SelA_deca_inact + 1 h + 1 pi + 1 sec_tRNA_sec | irreversible | tRNA charging | The stoichiometry is up to 5 tRNA per decamer. | [229,349] |
| ser1_tRNA_CHARGa | charging ser1_tRNA | 1 Ser_RS_2ser_2amp + 2 ser1_tRNA --> 1 Ser_RS_dim_inact + 2 amp + 2 ser1_tRNA_ser | irreversible | tRNA charging |  | [1,31,44] |
| ser1_tRNA_CHARGb | charging ser1_tRNA | 1 Ser_RS_ser_amp + 1 ser1_tRNA --> 1 Ser_RS_dim_inact + 1 amp + 1 ser1_tRNA_ser | irreversible | tRNA charging |  | [1,31,44] |
| ser2_tRNA_CHARGa | charging ser2_tRNA | 1 Ser_RS_2ser_2amp + 2 ser2_tRNA --> 1 Ser_RS_dim_inact + 2 amp + 2 ser2_tRNA_ser | irreversible | tRNA charging |  | [1,31,44] |
| ser2_tRNA_CHARGb | charging ser2_tRNA | 1 Ser_RS_ser_amp + 1 ser2_tRNA --> 1 Ser_RS_dim_inact + 1 amp + 1 ser2_tRNA_ser | irreversible | tRNA charging |  | [1,31,44] |
| ser3_tRNA_CHARGa | charging ser3_tRNA | 1 Ser_RS_2ser_2amp + 2 ser3_tRNA --> 1 Ser_RS_dim_inact + 2 amp + 2 ser3_tRNA_ser | irreversible | tRNA charging |  | [1,31,44] |
| ser3_tRNA_CHARGb | charging ser3_tRNA | 1 Ser_RS_ser_amp + 1 ser3_tRNA --> 1 Ser_RS_dim_inact + 1 amp + 1 ser3_tRNA_ser | irreversible | tRNA charging |  | [1,31,44] |
| serT_to_ser2 | serT_to_ser2 | 1 serT_tRNA --> 1 ser2_tRNA | irreversible | tRNA charging | Reaction was included for modeling reason to represent the alternate codon reading appropriately. Assignment was done based on Solomovici et al [347]. | [347] |
| serT_to_ser3 | serT_to_ser3 | 1 serT_tRNA --> 1 ser3_tRNA | irreversible | tRNA charging | Reaction was included for modeling reason to represent the alternate codon reading appropriately. Assignment was done based on Solomovici et al [347]. | [347] |
| serT_tRNA_CHARGa | charging serT_tRNA | 1 Ser_RS_2ser_2amp + 2 serT_tRNA --> 1 Ser_RS_dim_inact + 2 amp + 2 serT_tRNA_ser | irreversible | tRNA charging |  | [1,31,44] |
| serT_tRNA_CHARGb | charging serT_tRNA | 1 Ser_RS_ser_amp + 1 serT_tRNA --> 1 Ser_RS_dim_inact + 1 amp + 1 serT_tRNA_ser | irreversible | tRNA charging |  | [1,31,44] |
| serU_to_ser3 | serU_to_ser3 | 1 serU_tRNA --> 1 ser3_tRNA | irreversible | tRNA charging | Reaction was included for modeling reason to represent the alternate codon reading appropriately. Assignment was done based on Solomovici et al [347]. | [347] |
| serV_tRNA_CHARGa | charging serV_tRNA | 1 Ser_RS_2ser_2amp + 2 serV_tRNA --> 1 Ser_RS_dim_inact + 2 amp + 2 serV_tRNA_ser | irreversible | tRNA charging |  | [1,31,44] |
| serV_tRNA_CHARGb | charging serV_tRNA | 1 Ser_RS_ser_amp + 1 serV_tRNA --> 1 Ser_RS_dim_inact + 1 amp + 1 serV_tRNA_ser | irreversible | tRNA charging |  | [1,31,44] |
| serW_to_ser1 | serW_to_ser1 | 1 serW_tRNA --> 1 ser1_tRNA | irreversible | tRNA charging | Reaction was included for modeling reason to represent the alternate codon reading appropriately. Assignment was done based on Solomovici et al [347]. | [347] |
| serW_to_ser2 | serW_to_ser2 | 1 serW_tRNA --> 1 ser2_tRNA | irreversible | tRNA charging | Reaction was included for modeling reason to represent the alternate codon reading appropriately. Assignment was done based on Solomovici et al [347]. | [347] |
| serX_to_ser1 | serX_to_ser1 | 1 serX_tRNA --> 1 ser1_tRNA | irreversible | tRNA charging | Reaction was included for modeling reason to represent the alternate codon reading appropriately. Assignment was done based on Solomovici et al [347]. | [347] |
| serX_to_ser2 | serX_to_ser2 | 1 serX_tRNA --> 1 ser2_tRNA | irreversible | tRNA charging | Reaction was included for modeling reason to represent the alternate codon reading appropriately. Assignment was done based on Solomovici et al [347]. | [347] |
| thr1_tRNA_CHARGa | charging thr1_tRNA | 1 Thr_RS_2thr_2amp + 2 thr1_tRNA --> 1 Thr_RS_dim_inact + 2 amp + 2 thr1_tRNA_thr | irreversible | tRNA charging |  | [1,4,32,33,44] |
| thr1_tRNA_CHARGb | charging thr1_tRNA | 1 Thr_RS_thr_amp + 1 thr1_tRNA --> 1 Thr_RS_dim_inact + 1 amp + 1 thr1_tRNA_thr | irreversible | tRNA charging |  | [1,4,32,33,44] |
| thr2_tRNA_CHARGa | charging thr2_tRNA | 1 Thr_RS_2thr_2amp + 2 thr2_tRNA --> 1 Thr_RS_dim_inact + 2 amp + 2 thr2_tRNA_thr | irreversible | tRNA charging |  | [1,4,32,33,44] |
| thr2_tRNA_CHARGb | charging thr2_tRNA | 1 Thr_RS_thr_amp + 1 thr2_tRNA --> 1 Thr_RS_dim_inact + 1 amp + 1 thr2_tRNA_thr | irreversible | tRNA charging |  | [1,4,32,33,44] |
| thr3_tRNA_CHARGa | charging thr3_tRNA | 1 Thr_RS_2thr_2amp + 2 thr3_tRNA --> 1 Thr_RS_dim_inact + 2 amp + 2 thr3_tRNA_thr | irreversible | tRNA charging |  | [1,4,32,33,44] |
| thr3_tRNA_CHARGB | charging thr3_tRNA | 1 Thr_RS_thr_amp + 1 thr3_tRNA --> 1 Thr_RS_dim_inact + 1 amp + 1 thr3_tRNA_thr | irreversible | tRNA charging |  | [1,4,32,33,44] |
| thrT_to_thr1 | thrT_to_thr1 | 1 thrT_tRNA --> 1 thr1_tRNA | irreversible | tRNA charging | Reaction was included for modeling reason to represent the alternate codon reading appropriately. Assignment was done based on Solomovici et al [347]. | [347] |
| thrT_to_thr2 | thrT_to_thr2 | 1 thrT_tRNA --> 1 thr2_tRNA | irreversible | tRNA charging | Reaction was included for modeling reason to represent the alternate codon reading appropriately. Assignment was done based on Solomovici et al [347]. | [347] |
| thrU_to_thr2 | thrU_to_thr2 | 1 thrU_tRNA --> 1 thr2_tRNA | irreversible | tRNA charging | Reaction was included for modeling reason to represent the alternate codon reading appropriately. Assignment was done based on Solomovici et al [347]. | [347] |
| thrU_to_thr3 | thrU_to_thr3 | 1 thrU_tRNA --> 1 thr3_tRNA | irreversible | tRNA charging | Reaction was included for modeling reason to represent the alternate codon reading appropriately. Assignment was done based on Solomovici et al [347]. | [1,4,32,33,347] |
| thrU_tRNA_CHARGa | charging thrU_tRNA | 1 Thr_RS_2thr_2amp + 2 thrU_tRNA --> 1 Thr_RS_dim_inact + 2 amp + 2 thrU_tRNA_thr | irreversible | tRNA charging |  | [1,4,32,33,44] |
| thrU_tRNA_CHARGB | charging thrU_tRNA | 1 Thr_RS_thr_amp + 1 thrU_tRNA --> 1 Thr_RS_dim_inact + 1 amp + 1 thrU_tRNA_thr | irreversible | tRNA charging |  | [1,4,32,33,44] |
| thrV_to_thr1 | thrV_to_thr1 | 1 thrV_tRNA --> 1 thr1_tRNA | irreversible | tRNA charging | Reaction was included for modeling reason to represent the alternate codon reading appropriately. Assignment was done based on Solomovici et al [347]. | [347] |
| thrV_to_thr2 | thrV_to_thr2 | 1 thrV_tRNA --> 1 thr2_tRNA | irreversible | tRNA charging | Reaction was included for modeling reason to represent the alternate codon reading appropriately. Assignment was done based on Solomovici et al [347]. | [347] |
| thrW_to_thr3 | thrW_to_thr3 | 1 thrW_tRNA --> 1 thr3_tRNA | irreversible | tRNA charging | Reaction was included for modeling reason to represent the alternate codon reading appropriately. Assignment was done based on Solomovici et al [347]. | [347] |
| trpT_tRNA_CHARGa | charging trpT_tRNA | 1 Trp_RS_2trp_2amp + 2 trpT_tRNA --> 1 Trp_RS_dim_inact + 2 amp + 2 trpT_tRNA_trp | irreversible | tRNA charging |  | [1,34-36,44] |
| trpT_tRNA_CHARGb | charging trpT_tRNA | 1 Trp_RS_trp_amp + 1 trpT_tRNA --> 1 Trp_RS_dim_inact + 1 amp + 1 trpT_tRNA_trp | irreversible | tRNA charging |  | [1,34-36,44] |
| tyr1_tRNA_CHARGa | charging tyr1_tRNA | 1 Tyr_RS_tyr_amp + 1 tyr1_tRNA --> 1 Tyr_RS_dim_inact + 1 amp + 1 tyr1_tRNA_tyr | irreversible | tRNA charging |  | [1,17,21,37,38,44] |
| tyr1_tRNA_CHARGb | charging tyr1_tRNA | 1 Tyr_RS_2tyr_2amp + 2 tyr1_tRNA --> 1 Tyr_RS_dim_inact + 2 amp + 2 tyr1_tRNA_tyr | irreversible | tRNA charging |  | [1,17,21,37,38,44] |
| tyr1_tRNA2_CHARGa | charging tyr1_tRNA (tyr-D) | 1 Tyr_RS_tyr-D_amp + 1 tyr1_tRNA --> 1 Tyr_RS_dim_inact + 1 amp + 1 tyr1_tRNA_tyr-D | irreversible | tRNA charging |  | [1,17,21,37,38,44] |
| tyr1_tRNA2_CHARGb | charging tyr1_tRNA (tyr-D) | 1 Tyr_RS_2tyr-D_2amp + 2 tyr1_tRNA --> 1 Tyr_RS_dim_inact + 2 amp + 2 tyr1_tRNA_tyr-D | irreversible | tRNA charging |  | [1,17,21,37,38,44] |
| tyrT_to_tyr1 | tyrT_to_tyr1 | 1 tyrT_tRNA --> 1 tyr1_tRNA | irreversible | tRNA charging | Reaction was included for modeling reason to represent the alternate codon reading appropriately. Assignment was done based on Solomovici et al [347]. | [347] |
| tyrU_to_tyr1 | tyrU_to_tyr1 | 1 tyrU_tRNA --> 1 tyr1_tRNA | irreversible | tRNA charging | Reaction was included for modeling reason to represent the alternate codon reading appropriately. Assignment was done based on Solomovici et al [347]. | [347] |
| tyrV_to_tyr1 | tyrV_to_tyr1 | 1 tyrV_tRNA --> 1 tyr1_tRNA | irreversible | tRNA charging | Reaction was included for modeling reason to represent the alternate codon reading appropriately. Assignment was done based on Solomovici et al [347]. | [347] |
| val1_tRNA_CHARG | charging val1_tRNA | 1 Val_RS_val_amp + 1 val1_tRNA --> 1 ValS_mono_inact + 1 amp + 1 val1_tRNA_val | irreversible | tRNA charging |  | [1,4,20,39-41,44] |
| val2_tRNA_CHARG | charging val2_tRNA | 1 Val_RS_val_amp + 1 val2_tRNA --> 1 ValS_mono_inact + 1 amp + 1 val2_tRNA_val | irreversible | tRNA charging |  | [1,4,20,39-41,44] |
| val3_tRNA_CHARG | charging val3_tRNA | 1 Val_RS_val_amp + 1 val3_tRNA --> 1 ValS_mono_inact + 1 amp + 1 val3_tRNA_val | irreversible | tRNA charging |  | [1,4,20,39-41,44] |
| valT_to_val1 | valT_to_val1 | 1 valT_tRNA --> 1 val1_tRNA | irreversible | tRNA charging | Reaction was included for modeling reason to represent the alternate codon reading appropriately. Assignment was done based on Solomovici et al [347]. | [347] |
| valT_to_val3 | valT_to_val3 | 1 valT_tRNA --> 1 val3_tRNA | irreversible | tRNA charging | Reaction was included for modeling reason to represent the alternate codon reading appropriately. Assignment was done based on Solomovici et al [347]. | [347] |
| valU_to_val1 | valU_to_val1 | 1 valU_tRNA --> 1 val1_tRNA | irreversible | tRNA charging | Reaction was included for modeling reason to represent the alternate codon reading appropriately. Assignment was done based on Solomovici et al [347]. | [347] |
| valU_to_val3 | valU_to_val3 | 1 valU_tRNA --> 1 val3_tRNA | irreversible | tRNA charging | Reaction was included for modeling reason to represent the alternate codon reading appropriately. Assignment was done based on Solomovici et al [347]. | [347] |
| valV_to_val2 | valV_to_val2 | 1 valV_tRNA --> 1 val2_tRNA | irreversible | tRNA charging | Reaction was included for modeling reason to represent the alternate codon reading appropriately. Assignment was done based on Solomovici et al [347]. | [347] |
| valV_to_val3 | valV_to_val3 | 1 valV_tRNA --> 1 val3_tRNA | irreversible | tRNA charging | Reaction was included for modeling reason to represent the alternate codon reading appropriately. Assignment was done based on Solomovici et al [347]. | [347] |
| valW_to_val2 | valW_to_val2 | 1 valW_tRNA --> 1 val2_tRNA | irreversible | tRNA charging | Reaction was included for modeling reason to represent the alternate codon reading appropriately. Assignment was done based on Solomovici et al [347]. | [347] |
| valW_to_val3 | valW_to_val3 | 1 valW_tRNA --> 1 val3_tRNA | irreversible | tRNA charging | Reaction was included for modeling reason to represent the alternate codon reading appropriately. Assignment was done based on Solomovici et al [347]. | [347] |
| valX_to_val1 | valX_to_val1 | 1 valX_tRNA --> 1 val1_tRNA | irreversible | tRNA charging | Reaction was included for modeling reason to represent the alternate codon reading appropriately. Assignment was done based on Solomovici et al [347]. | [347] |
| valX_to_val3 | valX_to_val3 | 1 valX_tRNA --> 1 val3_tRNA | irreversible | tRNA charging | Reaction was included for modeling reason to represent the alternate codon reading appropriately. Assignment was done based on Solomovici et al [347]. | [347] |
| valY_to_val1 | valY_to_val1 | 1 valY_tRNA --> 1 val1_tRNA | irreversible | tRNA charging | Reaction was included for modeling reason to represent the alternate codon reading appropriately. Assignment was done based on Solomovici et al [347]. | [347] |
| valY_to_val3 | valY_to_val3 | 1 valY_tRNA --> 1 val3_tRNA | irreversible | tRNA charging | Reaction was included for modeling reason to represent the alternate codon reading appropriately. Assignment was done based on Solomovici et al [347]. | [347] |
| valZ_to_val1 | valZ_to_val1 | 1 valZ_tRNA --> 1 val1_tRNA | irreversible | tRNA charging | Reaction was included for modeling reason to represent the alternate codon reading appropriately. Assignment was done based on Solomovici et al [347]. | [347] |
| valZ_to_val3 | valZ_to_val3 | 1 valZ_tRNA --> 1 val3_tRNA | irreversible | tRNA charging | Reaction was included for modeling reason to represent the alternate codon reading appropriately. Assignment was done based on Solomovici et al [347]. | [347] |
| IscS_dim_S-SH_FORM | formation of IscS_dim_S-SH | 1 IscS_dim_S-H + 1 cys-L --> 1 IscS_dim_S-SH + 1 ala-L | irreversible | tRNA modification | From IscS crystalization paper [64]: The active site pocket and PLP cofactor of the two monomers are ,30 A ° apart from one another in the dimer complex, suggesting that for processes involving multiple sulfur transfer reactions, e.g. iron–sulfur cluster assembly, sulfur transfer is likely to be sequential rather than a cooperative process involving both subunits. However, this might be not the case. In papers about IscS and ThiI for s4U formation, they always showed (and assumed) 1 S-S per IscS dimer. | [60,64] |
| b0014_maturation1 | Polypeptide b0014 peptide deformylase and methionine aminopeptidase complex | 1 Def_mono + 1 Map_mono + 1 b0014_aa <==> 1 b0014_def_map_cplx | reversible | Protein Maturation | The overproduced protein was purified, and Edman degradation of the protein indicated that the NH2-terminal methionine was found to be processed. | [350] |
| b0014_maturation2 | b0014 formation | 1 b0014_def_map_cplx + 2 h2o --> 1 Def_mono_inact + 1 Map_mono_inact + 1 b0014_m + 1 for + 1 met-L | irreversible | Protein Maturation | The overproduced protein was purified, and Edman degradation of the protein indicated that the NH2-terminal methionine was found to be processed. | [350] |
| b4258_fold_KJE_1 | b4258_m folding: KJE mediated | 1 DnaJ_dim + 1 DnaK_mono.ATP + 1 b4258_m <==> 1 b4258_m_DnaKJ_complex | reversible | Protein Folding |  | [39-41,261,351] |
| b4258_fold_KJE_2 | b4258_m folding: KJE mediated | 1 GrpE_dim + 1 b4258_m_DnaKJ_complex + 1 h2o --> 1 DnaJ_dim_inact + 1 ValS_DnaK_GrpE_complex + 1 adp + 1 h + 1 pi | irreversible | Protein Folding |  | [39-41,261,351] |
| b4258_fold_KJE_3 | b4258_m folding: KJE mediated | 1 ValS_DnaK_GrpE_complex + 1 atp --> 1 DnaK_mono.ATP_inact + 1 GrpE_dim_inact + 1 ValS_mono | irreversible | Protein Folding |  | [39-41,261,351] |
| b4258_fold_spon | b4258_m folding: spontanous | 1 b4258_m --> 1 ValS_mono | irreversible | Protein Folding |  | [39-41,351] |
| b0526_fold_spon | b0526_m folding: spontanous | 1 b0526_m_Zn --> 1 CysS_mono | irreversible | Protein Folding |  | [12,13,351] |
| b0026_fold_spon | b0026_m folding: spontanous | 1 b0026_m_Zn --> 1 IleS_mono | irreversible | Protein Folding |  | [351] |
| b0642_fold_KJE_1 | b0642_m folding: KJE mediated | 1 DnaJ_dim + 1 DnaK_mono.ATP + 1 b0642_m_Zn <==> 1 b0642_m_DnaKJ_complex | reversible | Protein Folding |  | [261,351] |
| b0642_fold_KJE_2 | b0642_m folding: KJE mediated | 1 GrpE_dim + 1 b0642_m_DnaKJ_complex + 1 h2o --> 1 DnaJ_dim_inact + 1 LeuS_DnaK_GrpE_complex + 1 adp + 1 h + 1 pi | irreversible | Protein Folding |  | [261,351] |
| b0642_fold_KJE_3 | b0642_m folding: KJE mediated | 1 LeuS_DnaK_GrpE_complex + 1 atp --> 1 DnaK_mono.ATP_inact + 1 GrpE_dim_inact + 1 LeuS_mono | irreversible | Protein Folding |  | [261,351] |
| b0642_fold_spon | b0642_m folding: spontanous | 1 b0642_m_Zn --> 1 LeuS_mono | irreversible | Protein Folding |  | [351] |
| b3470_fold_spon | b3470_m folding: spontanous | 1 b3470_m --> 1 YhhP_mono | irreversible | Protein Folding | aka tusA . YhhP was assumed to be monomer. | [254] |
| b0969_fold_spon | b0969_m folding: spontanous | 1 b0969_m --> 1 YccK_mono | irreversible | Protein Folding | aka tusE. YccK was assumed to be monomer. | [254] |
| b0144_fold_GroEL/ES_1 | b0144_m folding: GroEL/ES mediated; polypeptide is going in GroEL/ES complex | 1 GroEL.(7)ADP.cisGroES + 7 atp + 1 b0144_m + 1 transGroES_hepta --> 7 adp + 1 b0144_m_GroEL.(7)ATP.transGroES + 1 cisGroES_hepta | irreversible | Protein Folding | YadB is currently not connected with model | [260,352] |
| b0144_fold_GroEL/ES_2 | b0144_m folding: GroEL/ES mediated; folding of polypeptide under ATP hydrolysis | 1 b0144_m_GroEL.(7)ATP.transGroES + 7 h2o --> 1 YadB_GroEL.(7)ADP.transGroES + 7 h + 7 pi | irreversible | Protein Folding | YadB is currently not connected with model | [260,352] |
| b0144_fold_GroEL/ES_3 | b0144_m folding: GroEL/ES mediated; release of native protein | 1 YadB_GroEL.(7)ADP.transGroES --> 1 GroEL.(7)ADP.transGroES + 1 YadB_mono | irreversible | Protein Folding | YadB is currently not connected with model | [260,352] |
| b0144_fold_KJE_1 | b0144_m folding: KJE mediated | 1 DnaJ_dim + 1 DnaK_mono.ATP + 1 b0144_m <==> 1 b0144_m_DnaKJ_complex | reversible | Protein Folding | YadB is currently not connected with model | [261,352] |
| b0144_fold_KJE_2 | b0144_m folding: KJE mediated | 1 GrpE_dim + 1 b0144_m_DnaKJ_complex + 1 h2o --> 1 DnaJ_dim_inact + 1 YadB_DnaK_GrpE_complex + 1 adp + 1 h + 1 pi | irreversible | Protein Folding | YadB is currently not connected with model | [261,352] |
| b0144_fold_KJE_3 | b0144_m folding: KJE mediated | 1 YadB_DnaK_GrpE_complex + 1 atp --> 1 DnaK_mono.ATP_inact + 1 GrpE_dim_inact + 1 YadB_mono | irreversible | Protein Folding | YadB is currently not connected with model | [261,352] |
| b3282_fold_spon | b3282_m folding: spontanous | 1 b3282_m --> 1 YrdC_mono | irreversible | Protein Folding | aka RimN. YrdC is a monomer. Crystal structure has been reported [353]. | [353] |

1. Burbaum JJ, Schimmel P (1991) Structural relationships and the classification of aminoacyl-tRNA synthetases. J Biol Chem 266: 16965-16968.

2. Miller WT, Hill KA, Schimmel P (1991) Evidence for a "cysteine-histidine box" metal-binding site in an Escherichia coli aminoacyl-tRNA synthetase. Biochemistry 30: 6970-6976.

3. Putney SD, Sauer RT, Schimmel PR (1981) Purification and properties of alanine tRNA synthetase from Escherichia coli A tetramer of identical subunits. J Biol Chem 256: 198-204.

4. Neidhardt FC, Bloch PL, Pedersen S, Reeh S (1977) Chemical measurement of steady-state levels of ten aminoacyl-transfer ribonucleic acid synthetases in Escherichia coli. J Bacteriol 129: 378-387.

5. Charlier J, Gerlo E (1979) Arginyl-tRNA synthetase from Escherichia coli K12. Purification, properties, and sequence of substrate addition. Biochemistry 18: 3171-3178.

6. Lin SX, Shi JP, Cheng XD, Wang YL (1988) Arginyl-tRNA synthetase from Escherichia coli, purification by affinity chromatography, properties, and steady-state kinetics. Biochemistry 27: 6343-6348.

7. Craine JE, Peterkofsky A (1976) Studies on arginyl-tRNA synthetase from Escherichia coli B. Dual role of metals in enzyme catalysis. J Biol Chem 251: 241-246.

8. Anselme J, Hartlein M (1991) Tyr-426 of the Escherichia coli asparaginyl-tRNA synthetase, an amino acid in a C-terminal conserved motif, is involved in ATP binding. FEBS Lett 280: 163-166.

9. Madern D, Anselme J, Hartlein M (1992) Asparaginyl-tRNA synthetase from the Escherichia coli temperature-sensitive strain HO202. A proline replacement in motif 2 is responsible for a large increase in Km for asparagine and ATP. FEBS Lett 299: 85-89.

10. Eiler S, Dock-Bregeon A, Moulinier L, Thierry JC, Moras D (1999) Synthesis of aspartyl-tRNA(Asp) in Escherichia coli--a snapshot of the second step. Embo J 18: 6532-6541.

11. Moulinier L, Eiler S, Eriani G, Gangloff J, Thierry JC, et al. (2001) The structure of an AspRS-tRNA(Asp) complex reveals a tRNA-dependent control mechanism. Embo J 20: 5290-5301.

12. Newberry KJ, Hou YM, Perona JJ (2002) Structural origins of amino acid selection without editing by cysteinyl-tRNA synthetase. Embo J 21: 2778-2787.

13. Zhang CM, Christian T, Newberry KJ, Perona JJ, Hou YM (2003) Zinc-mediated amino acid discrimination in cysteinyl-tRNA synthetase. J Mol Biol 327: 911-917.

14. Sherlin LD, Perona JJ (2003) tRNA-dependent active site assembly in a class I aminoacyl-tRNA synthetase. Structure 11: 591-603.

15. Rould MA, Perona JJ, Soll D, Steitz TA (1989) Structure of E. coli glutaminyl-tRNA synthetase complexed with tRNA(Gln) and ATP at 2.8 A resolution. Science 246: 1135-1142.

16. Ostrem DL, Berg P (1974) Glycyl transfer ribonucleic acid synthetase from Escherichia coli: purification, properties, and substrate binding. Biochemistry 13: 1338-1348.

17. Kalogerakos T, Hountondji C, Berne PF, Dukta S, Blanquet S (1994) Modification of aminoacyl-tRNA synthetases with pyridoxal-5'-phosphate. Identification of the labeled amino acid residues. Biochimie 76: 33-44.

18. Gillet S, Hoang CB, Schmitter JM, Fukui T, Blanquet S, et al. (1996) Affinity labeling of Escherichia coli histidyl-tRNA synthetase with reactive ATP analogues. Identification of labeled amino acid residues by matrix assisted laser desorption-ionization mass spectrometry. Eur J Biochem 241: 133-141.

19. Arnez JG, Augustine JG, Moras D, Francklyn CS (1997) The first step of aminoacylation at the atomic level in histidyl-tRNA synthetase. Proc Natl Acad Sci U S A 94: 7144-7149.

20. Bergmann FH, Berg P, Dieckmann M (1961) The Enzymic Synthesis of Amino Acyl Derivatives of Ribonucleic Acid. II. THE PREPARATION OF LEUCYL-, VALYL-, ISOLEUCYL-, AND METHIONYL RIBONUCLEIC ACID SYNTHETASES FROM ESCHERICHIA COLI. J Biol Chem 236: 1735-1740.

21. Fersht AR, Kaethner MM (1976) Mechanism of aminoacylation of tRNA. Proof of the aminoacyl adenylate pathway for the isoleucyl- and tyrosyl-tRNA synthetases from Escherichia coli K12. Biochemistry 15: 818-823.

22. Onesti S, Desogus G, Brevet A, Chen J, Plateau P, et al. (2000) Structural studies of lysyl-tRNA synthetase: conformational changes induced by substrate binding. Biochemistry 39: 12853-12861.

23. Onesti S, Miller AD, Brick P (1995) The crystal structure of the lysyl-tRNA synthetase (LysU) from Escherichia coli. Structure 3: 163-176.

24. Hughes SJ, Tanner JA, Hindley AD, Miller AD, Gould IR (2003) Functional asymmetry in the lysyl-tRNA synthetase explored by molecular dynamics, free energy calculations and experiment. BMC Struct Biol 3: 5.

25. Kosakowski HM, Bock A (1971) Substrate complexes of phenylalanyl-tRNA synthetase from Escherichia coli. Eur J Biochem 24: 190-200.

26. Hountondji C, Schmitter JM, Fukui T, Tagaya M, Blanquet S (1990) Affinity labeling of aminoacyl-tRNA synthetases with adenosine triphosphopyridoxal: probing the Lys-Met-Ser-Lys-Ser signature sequence as the ATP-binding site in Escherichia coli methionyl-and valyl-tRNA synthetases. Biochemistry 29: 11266-11273.

27. Datta D, Vaidehi N, Zhang D, Goddard WA, 3rd (2004) Selectivity and specificity of substrate binding in methionyl-tRNA synthetase. Protein Sci 13: 2693-2705.

28. Mechulam Y, Schmitt E, Maveyraud L, Zelwer C, Nureki O, et al. (1999) Crystal structure of Escherichia coli methionyl-tRNA synthetase highlights species-specific features. J Mol Biol 294: 1287-1297.

29. Farrelly JG, Longworth JW, Stulberg MP (1971) The interaction of phenylalanyl transfer ribonucleic acid synthetase and phenylalanine transfer ribonucleic acid. Complex formation and resulting fluorescence quenching. J Biol Chem 246: 1266-1270.

30. Lee ML, Muench KH (1969) Prolyl transfer ribonucleic acid synthetase of Escherichia coli. I. Purification and evidence for subunits. J Biol Chem 244: 223-230.

31. Price S, Cusack S, Borel F, Berthet-Colominas C, Leberman R (1993) Crystallization of the seryl-tRNA synthetase:tRNAS(ser) complex of Escherichia coli. FEBS Lett 324: 167-170.

32. Torres-Larios A, Dock-Bregeon AC, Romby P, Rees B, Sankaranarayanan R, et al. (2002) Structural basis of translational control by Escherichia coli threonyl tRNA synthetase. Nat Struct Biol 9: 343-347.

33. Sankaranarayanan R, Dock-Bregeon AC, Romby P, Caillet J, Springer M, et al. (1999) The structure of threonyl-tRNA synthetase-tRNA(Thr) complex enlightens its repressor activity and reveals an essential zinc ion in the active site. Cell 97: 371-381.

34. Merle M, Trezeguet V, Graves PV, Andrews D, Muench KH, et al. (1986) Tryptophanyl adenylate formation by tryptophanyl-tRNA synthetase from Escherichia coli. Biochemistry 25: 1115-1123.

35. Muench KH (1976) Two substrate binding sites on tryptophanyl transfer ribonucleic acid synthetase of Escherichia coli. J Biol Chem 251: 5195-5199.

36. Joseph DR, Muench KH (1971) Tryptophanyl transfer ribonucleic acid synthetase of Escherichia coli. II. Molecular weight, subunit structure, sulfhydryl content, and substrate-binding properties. J Biol Chem 246: 7610-7615.

37. Kobayashi T, Takimura T, Sekine R, Kelly VP, Kamata K, et al. (2005) Structural snapshots of the KMSKS loop rearrangement for amino acid activation by bacterial tyrosyl-tRNA synthetase. J Mol Biol 346: 105-117.

38. Jakes R, Fersht AR (1975) Tyrosyl-tRNA synthetase from Escherichia coli. Stoichiometry of ligand binding and half-of-the-sites reactivity in aminoacylation. Biochemistry 14: 3344-3350.

39. Heck JD, Hatfield GW (1988) Valyl-tRNA synthetase gene of Escherichia coli K12. Molecular genetic characterization. J Biol Chem 263: 857-867.

40. Heck JD, Hatfield GW (1988) Valyl-tRNA synthetase gene of Escherichia coli K12. Primary structure and homology within a family of aminoacyl-TRNA synthetases. J Biol Chem 263: 868-877.

41. Bock A, Faiman LE, Neidhardt FC (1966) Biochemical and genetic characterization of a mutant of Escherichia coli with a temperature-sensitive valyl ribonucleic acid synthetase. J Bacteriol 92: 1076-1082.

42. Kawashima T, Berthet-Colominas C, Wulff M, Cusack S, Leberman R (1996) The structure of the Escherichia coli EF-Tu.EF-Ts complex at 2.5 A resolution. Nature 379: 511-518.

43. Gromadski KB, Wieden HJ, Rodnina MV (2002) Kinetic mechanism of elongation factor Ts-catalyzed nucleotide exchange in elongation factor Tu. Biochemistry 41: 162-169.

44. Neidhardt FC, editor (1996) *Escherichia coli* and *Salmonella*: cellular and molecular biology. 2nd ed. Washington, D.C.: ASM Press. 2 v. (xx, 2822 , lxxvii) p.

45. Arai KI, Kawakita M, Kaziro Y (1972) Studies on polypeptide elongation factors from Escherichia coli. II. Purification of factors Tu-guanosine diphosphate, Ts, and Tu-Ts, and crystallization of Tu-guanosine diphosphate and Tu-Ts. J Biol Chem 247: 7029-7037.

46. An G, Bendiak DS, Mamelak LA, Friesen JD (1981) Organization and nucleotide sequence of a new ribosomal operon in Escherichia coli containing the genes for ribosomal protein S2 and elongation factor Ts. Nucleic Acids Res 9: 4163-4172.

47. Zhang Y, Yu NJ, Spremulli LL (1998) Mutational analysis of the roles of residues in Escherichia coli elongation factor Ts in the interaction with elongation factor Tu. J Biol Chem 273: 4556-4562.

48. Manchester KL (2004) Determination of the kinetics of guanine nucleotide exchange on EF-Tu and EF-Ts: continuing uncertainties. Biochem Biophys Res Commun 314: 1-5.

49. Wieden HJ, Gromadski K, Rodnin D, Rodnina MV (2002) Mechanism of elongation factor (EF)-Ts-catalyzed nucleotide exchange in EF-Tu. Contribution of contacts at the guanine base. J Biol Chem 277: 6032-6036.

50. Alexander C, Bilgin N, Lindschau C, Mesters JR, Kraal B, et al. (1995) Phosphorylation of elongation factor Tu prevents ternary complex formation. J Biol Chem 270: 14541-14547.

51. Kato S, Mihara H, Kurihara T, Takahashi Y, Tokumoto U, et al. (2002) Cys-328 of IscS and Cys-63 of IscU are the sites of disulfide bridge formation in a covalently bound IscS/IscU complex: implications for the mechanism of iron-sulfur cluster assembly. Proc Natl Acad Sci U S A 99: 5948-5952.

52. Agar JN, Krebs C, Frazzon J, Huynh BH, Dean DR, et al. (2000) IscU as a scaffold for iron-sulfur cluster biosynthesis: sequential assembly of [2Fe-2S] and [4Fe-4S] clusters in IscU. Biochemistry 39: 7856-7862.

53. Mansy SS, Cowan JA (2004) Iron-sulfur cluster biosynthesis: toward an understanding of cellular machinery and molecular mechanism. Acc Chem Res 37: 719-725.

54. Johnson DC, Dean DR, Smith AD, Johnson MK (2005) Structure, function, and formation of biological iron-sulfur clusters. Annu Rev Biochem 74: 247-281.

55. Yang J, Bitoun JP, Ding H (2006) Interplay of IscA and IscU in biogenesis of iron-sulfur clusters. J Biol Chem.

56. Adinolfi S, Rizzo F, Masino L, Nair M, Martin SR, et al. (2004) Bacterial IscU is a well folded and functional single domain protein. Eur J Biochem 271: 2093-2100.

57. Nuth M, Yoon T, Cowan JA (2002) Iron-sulfur cluster biosynthesis: characterization of iron nucleation sites for assembly of the [2Fe-2S]2+ cluster core in IscU proteins. J Am Chem Soc 124: 8774-8775.

58. Ding H, Harrison K, Lu J (2005) Thioredoxin reductase system mediates iron binding in IscA and iron delivery for the iron-sulfur cluster assembly in IscU. J Biol Chem 280: 30432-30437.

59. Johnson DC, Dos Santos PC, Dean DR (2005) NifU and NifS are required for the maturation of nitrogenase and cannot replace the function of isc-gene products in Azotobacter vinelandii. Biochem Soc Trans 33: 90-93.

60. Ding B, Smith ES, Ding H (2005) Mobilization of the iron centre in IscA for the iron-sulphur cluster assembly in IscU. Biochem J 389: 797-802.

61. Bilder PW, Ding H, Newcomer ME (2004) Crystal structure of the ancient, Fe-S scaffold IscA reveals a novel protein fold. Biochemistry 43: 133-139.

62. Cupp-Vickery JR, Silberg JJ, Ta DT, Vickery LE (2004) Crystal structure of IscA, an iron-sulfur cluster assembly protein from Escherichia coli. J Mol Biol 338: 127-137.

63. Kurihara T, Mihara H, Kato S, Yoshimura T, Esaki N (2003) Assembly of iron-sulfur clusters mediated by cysteine desulfurases, IscS, CsdB and CSD, from Escherichia coli. Biochim Biophys Acta 1647: 303-309.

64. Cupp-Vickery JR, Urbina H, Vickery LE (2003) Crystal structure of IscS, a cysteine desulfurase from Escherichia coli. J Mol Biol 330: 1049-1059.

65. Smith AD, Agar JN, Johnson KA, Frazzon J, Amster IJ, et al. (2001) Sulfur transfer from IscS to IscU: the first step in iron-sulfur cluster biosynthesis. J Am Chem Soc 123: 11103-11104.

66. Urbina HD, Silberg JJ, Hoff KG, Vickery LE (2001) Transfer of sulfur from IscS to IscU during Fe/S cluster assembly. J Biol Chem 276: 44521-44526.

67. Pierrel F, Bjork GR, Fontecave M, Atta M (2002) Enzymatic modification of tRNAs: MiaB is an iron-sulfur protein. J Biol Chem 277: 13367-13370.

68. Jager G, Leipuviene R, Pollard MG, Qian Q, Bjork GR (2004) The conserved Cys-X1-X2-Cys motif present in the TtcA protein is required for the thiolation of cytidine in position 32 of tRNA from Salmonella enterica serovar Typhimurium. J Bacteriol 186: 750-757.

69. Pierrel F, Hernandez HL, Johnson MK, Fontecave M, Atta M (2003) MiaB protein from Thermotoga maritima. Characterization of an extremely thermophilic tRNA-methylthiotransferase. J Biol Chem 278: 29515-29524.

70. Agarwalla S, Stroud RM, Gaffney BJ (2004) Redox reactions of the iron-sulfur cluster in a ribosomal RNA methyltransferase, RumA: optical and EPR studies. J Biol Chem 279: 34123-34129.

71. Lee TT, Agarwalla S, Stroud RM (2004) Crystal structure of RumA, an iron-sulfur cluster containing E. coli ribosomal RNA 5-methyluridine methyltransferase. Structure 12: 397-407.

72. Linke K, Wolfram T, Bussemer J, Jakob U (2003) The roles of the two zinc binding sites in DnaJ. J Biol Chem 278: 44457-44466.

73. Banecki B, Liberek K, Wall D, Wawrzynow A, Georgopoulos C, et al. (1996) Structure-function analysis of the zinc finger region of the DnaJ molecular chaperone. J Biol Chem 271: 14840-14848.

74. Szabo A, Korszun R, Hartl FU, Flanagan J (1996) A zinc finger-like domain of the molecular chaperone DnaJ is involved in binding to denatured protein substrates. Embo J 15: 408-417.

75. Nureki O, Kohno T, Sakamoto K, Miyazawa T, Yokoyama S (1993) Chemical modification and mutagenesis studies on zinc binding of aminoacyl-tRNA synthetases. J Biol Chem 268: 15368-15373.

76. Schimmel P, Landro JA, Schmidt E (1993) Evidence for distinct locations for metal binding sites in two closely related class I tRNA synthetases. J Biomol Struct Dyn 11: 571-581.

77. Landro JA, Schmidt E, Schimmel P, Tierney DL, Penner-Hahn JE (1994) Thiol ligation of two zinc atoms to a class I tRNA synthetase: evidence for unshared thiols and role in amino acid binding and utilization. Biochemistry 33: 14213-14220.

78. Mayaux JF, Blanquet S (1981) Binding of zinc to Escherichia coli phenylalanyl transfer ribonucleic acid synthetase. Comparison with other aminoacyl transfer ribonucleic acid synthetases. Biochemistry 20: 4647-4654.

79. D'Souza V M, Holz RC (1999) The methionyl aminopeptidase from Escherichia coli can function as an iron(II) enzyme. Biochemistry 38: 11079-11085.

80. Chong S, Curnow AW, Huston TJ, Garcia GA (1995) tRNA-guanine transglycosylase from Escherichia coli is a zinc metalloprotein. Site-directed mutagenesis studies to identify the zinc ligands. Biochemistry 34: 3694-3701.

81. Garcia GA, Koch KA, Chong S (1993) tRNA-guanine transglycosylase from Escherichia coli. Overexpression, purification and quaternary structure. J Mol Biol 231: 489-497.

82. Cusack S, Yaremchuk A, Tukalo M (2000) The 2 A crystal structure of leucyl-tRNA synthetase and its complex with a leucyl-adenylate analogue. Embo J 19: 2351-2361.

83. Lee KW, Briggs JM (2004) Molecular modeling study of the editing active site of Escherichia coli leucyl-tRNA synthetase: two amino acid binding sites in the editing domain. Proteins 54: 693-704.

84. Deaconescu AM, Chambers AL, Smith AJ, Nickels BE, Hochschild A, et al. (2006) Structural basis for bacterial transcription-coupled DNA repair. Cell 124: 507-520.

85. Spahr PF (1964) Purification and Properties of Ribonuclease Ii from Escherichia Coli. J Biol Chem 239: 3716-3726.

86. Zuo Y, Deutscher MP (1999) The DNase activity of RNase T and its application to DNA cloning. Nucleic Acids Res 27: 4077-4082.

87. Zuo Y, Deutscher MP (2002) Mechanism of action of RNase T. II. A structural and functional model of the enzyme. J Biol Chem 277: 50160-50164.

88. Zuo Y, Wang Y, Malhotra A (2005) Crystal structure of Escherichia coli RNase D, an exoribonuclease involved in structured RNA processing. Structure 13: 973-984.

89. Li Z, Zhan L, Deutscher MP (1996) Escherichia coli RNase T functions in vivo as a dimer dependent on cysteine 168. J Biol Chem 271: 1133-1137.

90. Deutscher MP, Marlor CW (1985) Purification and characterization of Escherichia coli RNase T. J Biol Chem 260: 7067-7071.

91. Deutscher MP, Marlor CW, Zaniewski R (1985) RNase T is responsible for the end-turnover of tRNA in Escherichia coli. Proc Natl Acad Sci U S A 82: 6427-6430.

92. Cudny H, Zaniewski R, Deutscher MP (1981) Escherichia coli RNase D. Purification and structural characterization of a putative processing nuclease. J Biol Chem 256: 5627-5632.

93. Ghosh RK, Deutscher MP (1978) Identification of an Escherichia coli nuclease acting on structurally altered transfer RNA molecules. J Biol Chem 253: 997-1000.

94. Das K, Acton T, Chiang Y, Shih L, Arnold E, et al. (2004) Crystal structure of RlmAI: implications for understanding the 23S rRNA G745/G748-methylation at the macrolide antibiotic-binding site. Proc Natl Acad Sci U S A 101: 4041-4046.

95. Fourmy D, Meinnel T, Mechulam Y, Blanquet S (1993) Mapping of the zinc binding domain of Escherichia coli methionyl-tRNA synthetase. J Mol Biol 231: 1068-1077.

96. Landro JA, Schimmel P (1993) Metal-binding site in a class I tRNA synthetase localized to a cysteine cluster inserted into nucleotide-binding fold. Proc Natl Acad Sci U S A 90: 2261-2265.

97. de la Sierra-Gallay IL, Pellegrini O, Condon C (2005) Structural basis for substrate binding, cleavage and allostery in the tRNA maturase RNase Z. Nature 433: 657-661.

98. Spath B, Kirchner S, Vogel A, Schubert S, Meinlschmidt P, et al. (2005) Analysis of the functional modules of the tRNA 3' endonuclease (tRNase Z). J Biol Chem 280: 35440-35447.

99. Liu J, Gagnon Y, Gauthier J, Furenlid L, L'Heureux PJ, et al. (1995) The zinc-binding site of Escherichia coli glutamyl-tRNA synthetase is located in the acceptor-binding domain. Studies by extended x-ray absorption fine structure, molecular modeling, and site-directed mutagenesis. J Biol Chem 270: 15162-15169.

100. Liu J, Lin SX, Blochet JE, Pezolet M, Lapointe J (1993) The glutamyl-tRNA synthetase of Escherichia coli contains one atom of zinc essential for its native conformation and its catalytic activity. Biochemistry 32: 11390-11396.

101. Airas RK (1996) Differences in the magnesium dependences of the class I and class II aminoacyl-tRNA synthetases from Escherichia coli. Eur J Biochem 240: 223-231.

102. Wolf J, Gerber AP, Keller W (2002) tadA, an essential tRNA-specific adenosine deaminase from Escherichia coli. Embo J 21: 3841-3851.

103. Losey HC, Ruthenburg AJ, Verdine GL (2006) Crystal structure of Staphylococcus aureus tRNA adenosine deaminase TadA in complex with RNA. Nat Struct Mol Biol 13: 153-159.

104. Sun W, Li G, Nicholson AW (2004) Mutational analysis of the nuclease domain of Escherichia coli ribonuclease III. Identification of conserved acidic residues that are important for catalytic function in vitro. Biochemistry 43: 13054-13062.

105. Sun W, Pertzev A, Nicholson AW (2005) Catalytic mechanism of Escherichia coli ribonuclease III: kinetic and inhibitor evidence for the involvement of two magnesium ions in RNA phosphodiester hydrolysis. Nucleic Acids Res 33: 807-815.

106. Sivaraman J, Iannuzzi P, Cygler M, Matte A (2004) Crystal structure of the RluD pseudouridine synthase catalytic module, an enzyme that modifies 23S rRNA and is essential for normal cell growth of Escherichia coli. J Mol Biol 335: 87-101.

107. Wrzesinski J, Bakin A, Ofengand J, Lane BG (2000) Isolation and properties of Escherichia coli 23S-RNA pseudouridine 1911, 1915, 1917 synthase (RluD). IUBMB Life 50: 33-37.

108. Mizutani K, Machida Y, Unzai S, Park SY, Tame JR (2004) Crystal structures of the catalytic domains of pseudouridine synthases RluC and RluD from Escherichia coli. Biochemistry 43: 4454-4463.

109. Vanzo NF, Li YS, Py B, Blum E, Higgins CF, et al. (1998) Ribonuclease E organizes the protein interactions in the Escherichia coli RNA degradosome. Genes Dev 12: 2770-2781.

110. Kuhnel K, Luisi BF (2001) Crystal structure of the Escherichia coli RNA degradosome component enolase. J Mol Biol 313: 583-592.

111. Faller LD, Baroudy BM, Johnson AM, Ewall RX (1977) Magnesium ion requirements for yeast enolase activity. Biochemistry 16: 3864-3869.

112. Chandran V, Luisi BF (2006) Recognition of enolase in the Escherichia coli RNA degradosome. J Mol Biol 358: 8-15.

113. Rajagopalan PT, Grimme S, Pei D (2000) Characterization of cobalt(II)-substituted peptide deformylase: function of the metal ion and the catalytic residue Glu-133. Biochemistry 39: 779-790.

114. Sundararaj S, Guo A, Habibi-Nazhad B, Rouani M, Stothard P, et al. (2004) The CyberCell Database (CCDB): a comprehensive, self-updating, relational database to coordinate and facilitate in silico modeling of Escherichia coli. Nucleic Acids Res 32: D293-295.

115. Ferri-Fioni ML, Schmitt E, Soutourina J, Plateau P, Mechulam Y, et al. (2001) Structure of crystalline D-Tyr-tRNA(Tyr) deacylase. A representative of a new class of tRNA-dependent hydrolases. J Biol Chem 276: 47285-47290.

116. Selinger DW, Cheung KJ, Mei R, Johansson EM, Richmond CS, et al. (2000) RNA expression analysis using a 30 base pair resolution Escherichia coli genome array. Nat Biotechnol 18: 1262-1268.

117. Sujatha S, Chatterji D (1999) Detection of putative Zn(II) binding sites within Escherichia coli RNA polymerase: inconsistency between sequence-based prediction and 65Zn blotting. FEBS Lett 454: 169-171.

118. King RA, Markov D, Sen R, Severinov K, Weisberg RA (2004) A conserved zinc binding domain in the largest subunit of DNA-dependent RNA polymerase modulates intrinsic transcription termination and antitermination but does not stabilize the elongation complex. J Mol Biol 342: 1143-1154.

119. Markov D, Naryshkina T, Mustaev A, Severinov K (1999) A zinc-binding site in the largest subunit of DNA-dependent RNA polymerase is involved in enzyme assembly. Genes Dev 13: 2439-2448.

120. Loziinski T, Wierzchowski KL (2001) Mg2+ ions do not induce expansion of the melted DNA region in the open complex formed by Escherichia coli RNA polymerase at a cognate synthetic Pa promoter. A quantitative KMnO4 footprinting study. Acta Biochim Pol 48: 495-510.

121. Sosunov V, Sosunova E, Mustaev A, Bass I, Nikiforov V, et al. (2003) Unified two-metal mechanism of RNA synthesis and degradation by RNA polymerase. Embo J 22: 2234-2244.

122. Niyogi SK, Datta AK (1975) A novel oligoribonuclease of Escherichia coli. I. Isolation and properties. J Biol Chem 250: 7307-7312.

123. Leung HC, Chen Y, Winkler ME (1997) Regulation of substrate recognition by the MiaA tRNA prenyltransferase modification enzyme of Escherichia coli K-12. J Biol Chem 272: 13073-13083.

124. Moore JA, Poulter CD (1997) Escherichia coli dimethylallyl diphosphate:tRNA dimethylallyltransferase: a binding mechanism for recombinant enzyme. Biochemistry 36: 604-614.

125. Rosenbaum N, Gefter ML (1972) Delta 2 -isopentenylpyrophosphate: transfer ribonucleic acid 2 -isopentenyltransferase from Escherichia coli. Purification and properties of the enzyme. J Biol Chem 247: 5675-5680.

126. Caillet J, Droogmans L (1988) Molecular cloning of the Escherichia coli miaA gene involved in the formation of delta 2-isopentenyl adenosine in tRNA. J Bacteriol 170: 4147-4152.

127. Connolly DM, Winkler ME (1989) Genetic and physiological relationships among the miaA gene, 2-methylthio-N6-(delta 2-isopentenyl)-adenosine tRNA modification, and spontaneous mutagenesis in Escherichia coli K-12. J Bacteriol 171: 3233-3246.

128. Connolly DM, Winkler ME (1991) Structure of Escherichia coli K-12 miaA and characterization of the mutator phenotype caused by miaA insertion mutations. J Bacteriol 173: 1711-1721.

129. Bartz JK, Kline LK, Soll D (1970) N6-(Delta 2-isopentenyl)adenosine: biosynthesis in vitro in transfer RNA by an enzyme purified from Escherichia coli. Biochem Biophys Res Commun 40: 1481-1487.

130. Aoki H, Yaworsky PJ, Patel SD, Margolin-Brzezinski D, Park KS, et al. (1992) The asparaginyl-tRNA synthetase gene encodes one of the complementing factors for thermosensitive translation in the Escherichia coli mutant strain, N4316. Eur J Biochem 209: 511-521.

131. Carpousis AJ (2002) The Escherichia coli RNA degradosome: structure, function and relationship in other ribonucleolytic multienzyme complexes. Biochem Soc Trans 30: 150-155.

132. Coburn GA, Miao X, Briant DJ, Mackie GA (1999) Reconstitution of a minimal RNA degradosome demonstrates functional coordination between a 3' exonuclease and a DEAD-box RNA helicase. Genes Dev 13: 2594-2603.

133. Khemici V, Toesca I, Poljak L, Vanzo NF, Carpousis AJ (2004) The RNase E of Escherichia coli has at least two binding sites for DEAD-box RNA helicases: functional replacement of RhlB by RhlE. Mol Microbiol 54: 1422-1430.

134. Regonesi ME, Del Favero M, Basilico F, Briani F, Benazzi L, et al. (2006) Analysis of the Escherichia coli RNA degradosome composition by a proteomic approach. Biochimie 88: 151-161.

135. Khemici V, Poljak L, Toesca I, Carpousis AJ (2005) Evidence in vivo that the DEAD-box RNA helicase RhlB facilitates the degradation of ribosome-free mRNA by RNase E. Proc Natl Acad Sci U S A 102: 6913-6918.

136. Morita T, Kawamoto H, Mizota T, Inada T, Aiba H (2004) Enolase in the RNA degradosome plays a crucial role in the rapid decay of glucose transporter mRNA in the response to phosphosugar stress in Escherichia coli. Mol Microbiol 54: 1063-1075.

137. Py B, Higgins CF, Krisch HM, Carpousis AJ (1996) A DEAD-box RNA helicase in the Escherichia coli RNA degradosome. Nature 381: 169-172.

138. Zylicz M, Yamamoto T, McKittrick N, Sell S, Georgopoulos C (1985) Purification and properties of the dnaJ replication protein of Escherichia coli. J Biol Chem 260: 7591-7598.

139. Bardwell J, Tilly K, Craig E, King J, Zylicz M, et al. (1986) The nucleotide sequence of the Escherichia coli K12 dnaJ+ gene. A gene that encodes a heat shock protein. J Biol Chem 261: 1782-1785.

140. Hill RB, Flanagan JM, Prestegard JH (1995) 1H and 15N magnetic resonance assignments, secondary structure, and tertiary fold of Escherichia coli DnaJ(1-78). Biochemistry 34: 5587-5596.

141. Schonfeld HJ, Schmidt D, Schroder H, Bukau B (1995) The DnaK chaperone system of Escherichia coli: quaternary structures and interactions of the DnaK and GrpE components. J Biol Chem 270: 2183-2189.

142. Katayama A, Tsujii A, Wada A, Nishino T, Ishihama A (2002) Systematic search for zinc-binding proteins in Escherichia coli. Eur J Biochem 269: 2403-2413.

143. Harrison CJ, Hayer-Hartl M, Di Liberto M, Hartl F, Kuriyan J (1997) Crystal structure of the nucleotide exchange factor GrpE bound to the ATPase domain of the molecular chaperone DnaK. Science 276: 431-435.

144. Bishop AC, Xu J, Johnson RC, Schimmel P, de Crecy-Lagard V (2002) Identification of the tRNA-dihydrouridine synthase family. J Biol Chem 277: 25090-25095.

145. Xing F, Martzen MR, Phizicky EM (2002) A conserved family of Saccharomyces cerevisiae synthases effects dihydrouridine modification of tRNA. Rna 8: 370-381.

146. Zavialov AV, Hauryliuk VV, Ehrenberg M (2005) Guanine-nucleotide exchange on ribosome-bound elongation factor G initiates the translocation of tRNAs. J Biol 4: 9.

147. al-Karadaghi S, Aevarsson A, Garber M, Zheltonosova J, Liljas A (1996) The structure of elongation factor G in complex with GDP: conformational flexibility and nucleotide exchange. Structure 4: 555-565.

148. Rodnina MV, Savelsbergh A, Katunin VI, Wintermeyer W (1997) Hydrolysis of GTP by elongation factor G drives tRNA movement on the ribosome. Nature 385: 37-41.

149. Arai K, Clark BF, Duffy L, Jones MD, Kaziro Y, et al. (1980) Primary structure of elongation factor Tu from Escherichia coli. Proc Natl Acad Sci U S A 77: 1326-1330.

150. Laursen RA, L'Italien JJ, Nagarkatti S, Miller DL (1981) The amino acid sequence of elongation factor Tu of Escherichia coli. The complete sequence. J Biol Chem 256: 8102-8109.

151. Song H, Parsons MR, Rowsell S, Leonard G, Phillips SE (1999) Crystal structure of intact elongation factor EF-Tu from Escherichia coli in GDP conformation at 2.05 A resolution. J Mol Biol 285: 1245-1256.

152. Van Noort JM, Kraal B, Sinjorgo KM, Persoon NL, Johanns ES, et al. (1986) Methylation in vivo of elongation factor EF-Tu at lysine-56 decreases the rate of tRNA-dependent GTP hydrolysis. Eur J Biochem 160: 557-561.

153. Ames GF, Niakido K (1979) In vivo methylation of prokaryotic elongation factor Tu. J Biol Chem 254: 9947-9950.

154. Wilkins MR, Gasteiger E, Gooley AA, Herbert BR, Molloy MP, et al. (1999) High-throughput mass spectrometric discovery of protein post-translational modifications. J Mol Biol 289: 645-657.

155. Rodnina MV, Wintermeyer W (1995) GTP consumption of elongation factor Tu during translation of heteropolymeric mRNAs. Proc Natl Acad Sci U S A 92: 1945-1949.

156. March PE, Lerner CG, Ahnn J, Cui X, Inouye M (1988) The Escherichia coli Ras-like protein (Era) has GTPase activity and is essential for cell growth. Oncogene 2: 539-544.

157. Chen SM, Takiff HE, Barber AM, Dubois GC, Bardwell JC, et al. (1990) Expression and characterization of RNase III and Era proteins. Products of the rnc operon of Escherichia coli. J Biol Chem 265: 2888-2895.

158. Meier TI, Peery RB, McAllister KA, Zhao G (2000) Era GTPase of Escherichia coli: binding to 16S rRNA and modulation of GTPase activity by RNA and carbohydrates. Microbiology 146 ( Pt 5): 1071-1083.

159. Chen X, Court DL, Ji X (1999) Crystal structure of ERA: a GTPase-dependent cell cycle regulator containing an RNA binding motif. Proc Natl Acad Sci U S A 96: 8396-8401.

160. Schönfeld H-J, Schmidt D, Schröder H, Bukau B (1995) The DnaK Chaperone System of Escherichia coli: Quaternary Structures and Interactions of the DnaK and GrpE Components

10.1074/jbc.270.5.2183. J Biol Chem 270: 2183-2189.

161. Harrison CJ, Hayer-Hartl M, Liberto MD, Hartl F-U, Kuriyan J (1997) Crystal Structure of the Nucleotide Exchange Factor GrpE Bound to the ATPase Domain of the Molecular Chaperone DnaK

10.1126/science.276.5311.431. Science 276: 431-435.

162. Wu B, Wawrzynow A, Zylicz M, Georgopoulos C (1996) Structure-function analysis of the Escherichia coli GrpE heat shock protein. Embo J 15: 4806-4816.

163. Xu Z, Horwich AL, Sigler PB (1997) The crystal structure of the asymmetric GroEL-GroES-(ADP)7 chaperonin complex. Nature 388: 741-750.

164. Darst SA (2001) Bacterial RNA polymerase. Curr Opin Struct Biol 11: 155-162.

165. Blatter EE, Ross W, Tang H, Gourse RL, Ebright RH (1994) Domain organization of RNA polymerase alpha subunit: C-terminal 85 amino acids constitute a domain capable of dimerization and DNA binding. Cell 78: 889-896.

166. Negishi T, Fujita N, Ishihama A (1995) Structural map of the alpha subunit of Escherichia coli RNA polymerase: structural domains identified by proteolytic cleavage. J Mol Biol 248: 723-728.

167. Toulokhonov I, Artsimovitch I, Landick R (2001) Allosteric control of RNA polymerase by a site that contacts nascent RNA hairpins. Science 292: 730-733.

168. Murakami KS, Darst SA (2003) Bacterial RNA polymerases: the wholo story. Curr Opin Struct Biol 13: 31-39.

169. Darst SA, Opalka N, Chacon P, Polyakov A, Richter C, et al. (2002) Conformational flexibility of bacterial RNA polymerase. Proc Natl Acad Sci U S A 99: 4296-4301.

170. Sette M, van Tilborg P, Spurio R, Kaptein R, Paci M, et al. (1997) The structure of the translational initiation factor IF1 from E.coli contains an oligomer-binding motif. Embo J 16: 1436-1443.

171. Ramakrishnan V (2002) Ribosome structure and the mechanism of translation. Cell 108: 557-572.

172. Carter AP, Clemons WM, Jr., Brodersen DE, Morgan-Warren RJ, Hartsch T, et al. (2001) Crystal structure of an initiation factor bound to the 30S ribosomal subunit. Science 291: 498-501.

173. Laursen BS, Sorensen HP, Mortensen KK, Sperling-Petersen HU (2005) Initiation of protein synthesis in bacteria. Microbiol Mol Biol Rev 69: 101-123.

174. Sacerdot C, Dessen P, Hershey JW, Plumbridge JA, Grunberg-Manago M (1984) Sequence of the initiation factor IF2 gene: unusual protein features and homologies with elongation factors. Proc Natl Acad Sci U S A 81: 7787-7791.

175. Putzer HaL, S. (2003) Regulation of the expression of aminoacyl-tRNA synthetases and translation factors. In: Lapointe JB-G, L., editor. Translation Mechanisms. Georgetown, Texas, USA.: Landes Bioscience. pp. 388-415.

176. Antoun A, Pavlov MY, Andersson K, Tenson T, Ehrenberg M (2003) The roles of initiation factor 2 and guanosine triphosphate in initiation of protein synthesis. Embo J 22: 5593-5601.

177. Brock S, Szkaradkiewicz K, Sprinzl M (1998) Initiation factors of protein biosynthesis in bacteria and their structural relationship to elongation and termination factors. Mol Microbiol 29: 409-417.

178. Hoff KG, Cupp-Vickery JR, Vickery LE (2003) Contributions of the LPPVK motif of the iron-sulfur template protein IscU to interactions with the Hsc66-Hsc20 chaperone system. J Biol Chem 278: 37582-37589.

179. Silberg JJ, Tapley TL, Hoff KG, Vickery LE (2004) Regulation of the HscA ATPase reaction cycle by the co-chaperone HscB and the iron-sulfur cluster assembly protein IscU. J Biol Chem 279: 53924-53931.

180. Muhlenhoff U, Gerber J, Richhardt N, Lill R (2003) Components involved in assembly and dislocation of iron-sulfur clusters on the scaffold protein Isu1p. Embo J 22: 4815-4825.

181. Gold L, Stormo G, Saunders R (1984) Escherichia coli translational initiation factor IF3: a unique case of translational regulation. Proc Natl Acad Sci U S A 81: 7061-7065.

182. Portier C (1975) Quaternary structure of polynucleotide phosphorylase from Escherichia coli: evidence of a complex between two types of polypeptide chains. Eur J Biochem 55: 573-582.

183. Jarrige A, Brechemier-Baey D, Mathy N, Duche O, Portier C (2002) Mutational analysis of polynucleotide phosphorylase from Escherichia coli. J Mol Biol 321: 397-409.

184. Van Lanen SG, Reader JS, Swairjo MA, de Crecy-Lagard V, Lee B, et al. (2005) From cyclohydrolase to oxidoreductase: discovery of nitrile reductase activity in a common fold. Proc Natl Acad Sci U S A 102: 4264-4269.

185. Heurgue-Hamard V, Champ S, Engstrom A, Ehrenberg M, Buckingham RH (2002) The hemK gene in Escherichia coli encodes the N(5)-glutamine methyltransferase that modifies peptide release factors. Embo J 21: 769-778.

186. Yang Z, Shipman L, Zhang M, Anton BP, Roberts RJ, et al. (2004) Structural characterization and comparative phylogenetic analysis of Escherichia coli HemK, a protein (N5)-glutamine methyltransferase. J Mol Biol 340: 695-706.

187. Nakahigashi K, Kubo N, Narita S, Shimaoka T, Goto S, et al. (2002) HemK, a class of protein methyl transferase with similarity to DNA methyl transferases, methylates polypeptide chain release factors, and hemK knockout induces defects in translational termination. Proc Natl Acad Sci U S A 99: 1473-1478.

188. Vestergaard B, Van LB, Andersen GR, Nyborg J, Buckingham RH, et al. (2001) Bacterial polypeptide release factor RF2 is structurally distinct from eukaryotic eRF1. Mol Cell 8: 1375-1382.

189. Dincbas-Renqvist V, Engstrom A, Mora L, Heurgue-Hamard V, Buckingham R, et al. (2000) A post-translational modification in the GGQ motif of RF2 from Escherichia coli stimulates termination of translation. Embo J 19: 6900-6907.

190. Klaholz BP, Pape T, Zavialov AV, Myasnikov AG, Orlova EV, et al. (2003) Structure of the Escherichia coli ribosomal termination complex with release factor 2. Nature 421: 90-94.

191. Grentzmann G, Brechemier-Baey D, Heurgue V, Mora L, Buckingham RH (1994) Localization and characterization of the gene encoding release factor RF3 in Escherichia coli. Proc Natl Acad Sci U S A 91: 5848-5852.

192. Michel G, Sauve V, Larocque R, Li Y, Matte A, et al. (2002) The structure of the RlmB 23S rRNA methyltransferase reveals a new methyltransferase fold with a unique knot. Structure 10: 1303-1315.

193. Maeda H, Fujita N, Ishihama A (2000) Competition among seven Escherichia coli sigma subunits: relative binding affinities to the core RNA polymerase. Nucleic Acids Res 28: 3497-3503.

194. Kabir MS, Yamashita D, Koyama S, Oshima T, Kurokawa K, et al. (2005) Cell lysis directed by sigmaE in early stationary phase and effect of induction of the rpoE gene on global gene expression in Escherichia coli. Microbiology 151: 2721-2735.

195. Dartigalongue C, Missiakas D, Raina S (2001) Characterization of the Escherichia coli sigma E regulon. J Biol Chem 276: 20866-20875.

196. Arnosti DN, Chamberlin MJ (1989) Secondary sigma factor controls transcription of flagellar and chemotaxis genes in Escherichia coli. Proc Natl Acad Sci U S A 86: 830-834.

197. Ishihama A (2000) Functional modulation of Escherichia coli RNA polymerase. Annu Rev Microbiol 54: 499-518.

198. Muffler A, Traulsen DD, Lange R, Hengge-Aronis R (1996) Posttranscriptional osmotic regulation of the sigma(s) subunit of RNA polymerase in Escherichia coli. J Bacteriol 178: 1607-1613.

199. Weber H, Polen T, Heuveling J, Wendisch VF, Hengge R (2005) Genome-wide analysis of the general stress response network in Escherichia coli: sigmaS-dependent genes, promoters, and sigma factor selectivity. J Bacteriol 187: 1591-1603.

200. Svergun DI, Malfois M, Koch MH, Wigneshweraraj SR, Buck M (2000) Low resolution structure of the sigma54 transcription factor revealed by X-ray solution scattering. J Biol Chem 275: 4210-4214.

201. Kustu S, Santero E, Keener J, Popham D, Weiss D (1989) Expression of sigma 54 (ntrA)-dependent genes is probably united by a common mechanism. Microbiol Rev 53: 367-376.

202. Bessarab DA, Kaberdin VR, Wei CL, Liou GG, Lin-Chao S (1998) RNA components of Escherichia coli degradosome: evidence for rRNA decay. Proc Natl Acad Sci U S A 95: 3157-3161.

203. Callaghan AJ, Redko Y, Murphy LM, Grossmann JG, Yates D, et al. (2005) "Zn-link": a metal-sharing interface that organizes the quaternary structure and catalytic site of the endoribonuclease, RNase E. Biochemistry 44: 4667-4675.

204. Schubert M, Edge RE, Lario P, Cook MA, Strynadka NC, et al. (2004) Structural characterization of the RNase E S1 domain and identification of its oligonucleotide-binding and dimerization interfaces. J Mol Biol 341: 37-54.

205. Briant DJ, Hankins JS, Cook MA, Mackie GA (2003) The quaternary structure of RNase G from Escherichia coli. Mol Microbiol 50: 1381-1390.

206. Wachi M, Umitsuki G, Shimizu M, Takada A, Nagai K (1999) Escherichia coli cafA gene encodes a novel RNase, designated as RNase G, involved in processing of the 5' end of 16S rRNA. Biochem Biophys Res Commun 259: 483-488.

207. Reuven NB, Deutscher MP (1993) Multiple exoribonucleases are required for the 3' processing of Escherichia coli tRNA precursors in vivo. Faseb J 7: 143-148.

208. Kelly KO, Deutscher MP (1992) The presence of only one of five exoribonucleases is sufficient to support the growth of Escherichia coli. J Bacteriol 174: 6682-6684.

209. Cannistraro VJ, Kennell D (1994) The processive reaction mechanism of ribonuclease II. J Mol Biol 243: 930-943.

210. Kikovska E, Mikkelsen NE, Kirsebom LA (2005) The naturally trans-acting ribozyme RNase P RNA has leadzyme properties. Nucleic Acids Res 33: 6920-6930.

211. Brannvall M, Mikkelsen NE, Kirsebom LA (2001) Monitoring the structure of Escherichia coli RNase P RNA in the presence of various divalent metal ions. Nucleic Acids Res 29: 1426-1432.

212. Cuzic S, Hartmann RK (2005) Studies on Escherichia coli RNase P RNA with Zn2+ as the catalytic cofactor. Nucleic Acids Res 33: 2464-2474.

213. Wong T, Sosnick TR, Pan T (2005) Mechanistic insights on the folding of a large ribozyme during transcription. Biochemistry 44: 7535-7542.

214. Frank DN, Pace NR (1998) Ribonuclease P: unity and diversity in a tRNA processing ribozyme. Annu Rev Biochem 67: 153-180.

215. Hsieh J, Andrews AJ, Fierke CA (2004) Roles of protein subunits in RNA-protein complexes: lessons from ribonuclease P. Biopolymers 73: 79-89.

216. Arnold RJ, Reilly JP (1999) Observation of Escherichia coli ribosomal proteins and their posttranslational modifications by mass spectrometry. Anal Biochem 269: 105-112.

217. Amarantos I, Xaplanteri MA, Choli-Papadopoulou T, Kalpaxis DL (2001) Effects of two photoreactive spermine analogues on peptide bond formation and their application for labeling proteins in Escherichia coli functional ribosomal complexes. Biochemistry 40: 7641-7650.

218. Xaplanteri MA, Petropoulos AD, Dinos GP, Kalpaxis DL (2005) Localization of spermine binding sites in 23S rRNA by photoaffinity labeling: parsing the spermine contribution to ribosomal 50S subunit functions. Nucleic Acids Res 33: 2792-2805.

219. Chang FN, Chang CN, Paik WK (1974) Methylation of ribosomal proteins in Escherichia coli. J Bacteriol 120: 651-656.

220. Colson C, Lhoest J, Urlings C (1979) Genetics of ribosomal protein methylation in Escherichia coli. III. Map position of two genes, prmA and prmB, governing methylation of proteins L11 and L3. Mol Gen Genet 169: 245-250.

221. Lhoest J, Colson C (1977) Genetics of ribosomal protein methylation in Escherichia coli. II. A mutant lacking a new type of methylated amino acid, N5-methylglutamine, in protein L3. Mol Gen Genet 154: 175-180.

222. Hamman BD, Oleinikov AV, Jokhadze GG, Traut RR, Jameson DM (1996) Dimer/monomer equilibrium and domain separations of Escherichia coli ribosomal protein L7/L12. Biochemistry 35: 16680-16686.

223. Zhao Q, Nagaswamy U, Lee H, Xia Y, Huang HC, et al. (2005) NMR structure and Mg2+ binding of an RNA segment that underlies the L7/L12 stalk in the E.coli 50S ribosomal subunit. Nucleic Acids Res 33: 3145-3153.

224. Bocharov EV, Gudkov AT, Arseniev AS (1996) Topology of the secondary structure elements of ribosomal protein L7/L12 from E. coli in solution. FEBS Lett 379: 291-294.

225. Nomura T, Mochizuki R, Dabbs ER, Shimizu Y, Ueda T, et al. (2003) A point mutation in ribosomal protein L7/L12 reduces its ability to form a compact dimer structure and to assemble into the GTPase center. Biochemistry 42: 4691-4698.

226. Brot N, Weissbach H (1981) Chemistry and biology of E. coli ribosomal protein L12. Mol Cell Biochem 36: 47-63.

227. Tate WP, Kastner B, Edgar CD, McCaughan KK, Timms KM, et al. (1990) The ribosomal domain of the bacterial release factors. The carboxyl-terminal domain of the dimer of Escherichia coli ribosomal protein L7/L12 located in the body of the ribosome is important for release factor interaction. Eur J Biochem 187: 543-548.

228. Amarantos I, Zarkadis IK, Kalpaxis DL (2002) The identification of spermine binding sites in 16S rRNA allows interpretation of the spermine effect on ribosomal 30S subunit functions. Nucleic Acids Res 30: 2832-2843.

229. Forchhammer K, Bock A (1991) Selenocysteine synthase from Escherichia coli. Analysis of the reaction sequence. J Biol Chem 266: 6324-6328.

230. Vincent C, Borel F, Willison JC, Leberman R, Hartlein M (1995) Seryl-tRNA synthetase from Escherichia coli: functional evidence for cross-dimer tRNA binding during aminoacylation. Nucleic Acids Res 23: 1113-1118.

231. Cusack S, Berthet-Colominas C, Hartlein M, Nassar N, Leberman R (1990) A second class of synthetase structure revealed by X-ray analysis of Escherichia coli seryl-tRNA synthetase at 2.5 A. Nature 347: 249-255.

232. Borel F, Vincent C, Leberman R, Hartlein M (1994) Seryl-tRNA synthetase from Escherichia coli: implication of its N-terminal domain in aminoacylation activity and specificity. Nucleic Acids Res 22: 2963-2969.

233. Fiedler TJ, Vincent HA, Zuo Y, Gavrialov O, Malhotra A (2004) Purification and crystallization of Escherichia coli oligoribonuclease. Acta Crystallogr D Biol Crystallogr 60: 736-739.

234. Skordalakes E, Berger JM (2003) Structure of the Rho transcription terminator: mechanism of mRNA recognition and helicase loading. Cell 114: 135-146.

235. Stitt BL (2001) Escherichia coli transcription termination factor Rho binds and hydrolyzes ATP using a single class of three sites. Biochemistry 40: 2276-2281.

236. Walstrom KM, Dozono JM, von Hippel PH (1997) Kinetics of the RNA-DNA helicase activity of Escherichia coli transcription termination factor rho. 2. Processivity, ATP consumption, and RNA binding. Biochemistry 36: 7993-8004.

237. Adelman JL, Jeong YJ, Liao JC, Patel G, Kim DE, et al. (2006) Mechanochemistry of transcription termination factor Rho. Mol Cell 22: 611-621.

238. Weber TP, Widger WR, Kohn H (2002) The Mg2+ requirements for rho transcription termination factor: catalysis and bicyclomycin inhibition. Biochemistry 41: 12377-12383.

239. Weber TP, Widger WR, Kohn H (2003) Metal dependency for transcription factor rho activation. Biochemistry 42: 1652-1659.

240. Bystrom AS, Hjalmarsson KJ, Wikstrom PM, Bjork GR (1983) The nucleotide sequence of an Escherichia coli operon containing genes for the tRNA(m1G)methyltransferase, the ribosomal proteins S16 and L19 and a 21-K polypeptide. Embo J 2: 899-905.

241. Redlak M, Andraos-Selim C, Giege R, Florentz C, Holmes WM (1997) Interaction of tRNA with tRNA (guanosine-1)methyltransferase: binding specificity determinants involve the dinucleotide G36pG37 and tertiary structure. Biochemistry 36: 8699-8709.

242. Elkins PA, Watts JM, Zalacain M, van Thiel A, Vitazka PR, et al. (2003) Insights into catalysis by a knotted TrmD tRNA methyltransferase. J Mol Biol 333: 931-949.

243. Marinus MG, Morris NR, Soll D, Kwong TC (1975) Isolation and partial characterization of three Escherichia coli mutants with altered transfer ribonucleic acid methylases. J Bacteriol 122: 257-265.

244. Bystrom AS, Bjork GR (1982) Chromosomal location and cloning of the gene (trmD) responsible for the synthesis of tRNA (m1G) methyltransferase in Escherichia coli K-12. Mol Gen Genet 188: 440-446.

245. Bystrom AS, Bjork GR (1982) The structural gene (trmD) for the tRNA(m1G)methyltransferase is part of a four polypeptide operon in Escherichia coli K-12. Mol Gen Genet 188: 447-454.

246. Scrima A, Vetter IR, Armengod ME, Wittinghofer A (2005) The structure of the TrmE GTP-binding protein and its implications for tRNA modification. Embo J 24: 23-33.

247. Scrima A, Wittinghofer A (2006) Dimerisation-dependent GTPase reaction of MnmE: how potassium acts as GTPase-activating element. Embo J 25: 2940-2951.

248. Watanabe K, Nureki O, Fukai S, Ishii R, Okamoto H, et al. (2005) Roles of conserved amino acid sequence motifs in the SpoU (TrmH) RNA methyltransferase family. J Biol Chem 280: 10368-10377.

249. Persson BC, Jager G, Gustafsson C (1997) The spoU gene of Escherichia coli, the fourth gene of the spoT operon, is essential for tRNA (Gm18) 2'-O-methyltransferase activity. Nucleic Acids Res 25: 4093-4097.

250. Sever S, Rogers K, Rogers MJ, Carter C, Soll D (1996) Escherichia coli Tryptophanyl-tRNA Synthetase Mutants Selected for Tryptophan Auxotrophy Implicate the Dimer Interface in Optimizing Amino Acid Binding. Biochemistry 35: 32-40.

251. Foster PG, Huang L, Santi DV, Stroud RM (2000) The structural basis for tRNA recognition and pseudouridine formation by pseudouridine synthase I. Nat Struct Biol 7: 23-27.

252. Veres Z, Stadtman TC (1994) A purified selenophosphate-dependent enzyme from Salmonella typhimurium catalyzes the replacement of sulfur in 2-thiouridine residues in tRNAs with selenium. Proc Natl Acad Sci U S A 91: 8092-8096.

253. Numata T, Fukai S, Ikeuchi Y, Suzuki T, Nureki O (2006) Structural basis for sulfur relay to RNA mediated by heterohexameric TusBCD complex. Structure 14: 357-366.

254. Ikeuchi Y, Shigi N, Kato J, Nishimura A, Suzuki T (2006) Mechanistic insights into sulfur relay by multiple sulfur mediators involved in thiouridine biosynthesis at tRNA wobble positions. Mol Cell 21: 97-108.

255. Roa BB, Connolly DM, Winkler ME (1989) Overlap between pdxA and ksgA in the complex pdxA-ksgA-apaG-apaH operon of Escherichia coli K-12. J Bacteriol 171: 4767-4777.

256. O'Farrell HC, Scarsdale JN, Rife JP (2004) Crystal structure of KsgA, a universally conserved rRNA adenine dimethyltransferase in Escherichia coli. J Mol Biol 339: 337-353.

257. Wrzesinski J, Nurse K, Bakin A, Lane BG, Ofengand J (1995) A dual-specificity pseudouridine synthase: an Escherichia coli synthase purified and cloned on the basis of its specificity for psi 746 in 23S RNA is also specific for psi 32 in tRNA(phe). Rna 1: 437-448.

258. Raychaudhuri S, Niu L, Conrad J, Lane BG, Ofengand J (1999) Functional effect of deletion and mutation of the Escherichia coli ribosomal RNA and tRNA pseudouridine synthase RluA. J Biol Chem 274: 18880-18886.

259. Ben-Bassat A, Bauer K, Chang SY, Myambo K, Boosman A, et al. (1987) Processing of the initiation methionine from proteins: properties of the Escherichia coli methionine aminopeptidase and its gene structure. J Bacteriol 169: 751-757.

260. Kerner MJ, Naylor DJ, Ishihama Y, Maier T, Chang HC, et al. (2005) Proteome-wide analysis of chaperonin-dependent protein folding in Escherichia coli. Cell 122: 209-220.

261. Deuerling E, Patzelt H, Vorderwulbecke S, Rauch T, Kramer G, et al. (2003) Trigger Factor and DnaK possess overlapping substrate pools and binding specificities. Mol Microbiol 47: 1317-1328.

262. Agrawal RK, Sharma MR, Kiel MC, Hirokawa G, Booth TM, et al. (2004) Visualization of ribosome-recycling factor on the Escherichia coli 70S ribosome: functional implications. Proc Natl Acad Sci U S A 101: 8900-8905.

263. Van Lanen SG, Iwata-Reuyl D (2003) Kinetic mechanism of the tRNA-modifying enzyme S-adenosylmethionine:tRNA ribosyltransferase-isomerase (QueA). Biochemistry 42: 5312-5320.

264. Reuter K, Slany R, Ullrich F, Kersten H (1991) Structure and organization of Escherichia coli genes involved in biosynthesis of the deazaguanine derivative queuine, a nutrient factor for eukaryotes. J Bacteriol 173: 2256-2264.

265. Nonekowski ST, Garcia GA (2001) tRNA recognition by tRNA-guanine transglycosylase from Escherichia coli: the role of U33 in U-G-U sequence recognition. Rna 7: 1432-1441.

266. Altieri AS, Mazzulla MJ, Horita DA, Coats RH, Wingfield PT, et al. (2000) The structure of the transcriptional antiterminator NusB from Escherichia coli. Nat Struct Biol 7: 470-474.

267. Huenges M, Rolz C, Gschwind R, Peteranderl R, Berglechner F, et al. (1998) Solution structure of the antitermination protein NusB of Escherichia coli: a novel all-helical fold for an RNA-binding protein. Embo J 17: 4092-4100.

268. Deuerling E, Schulze-Specking A, Tomoyasu T, Mogk A, Bukau B (1999) Trigger factor and DnaK cooperate in folding of newly synthesized proteins. Nature 400: 693-696.

269. Hesterkamp T, Hauser S, Lutcke H, Bukau B (1996) Escherichia coli trigger factor is a prolyl isomerase that associates with nascent polypeptide chains. Proc Natl Acad Sci U S A 93: 4437-4441.

270. Teter SA, Houry WA, Ang D, Tradler T, Rockabrand D, et al. (1999) Polypeptide flux through bacterial Hsp70: DnaK cooperates with trigger factor in chaperoning nascent chains. Cell 97: 755-765.

271. Kramer G, Rauch T, Rist W, Vorderwulbecke S, Patzelt H, et al. (2002) L23 protein functions as a chaperone docking site on the ribosome. Nature 419: 171-174.

272. Lill R, Crooke E, Guthrie B, Wickner W (1988) The "trigger factor cycle" includes ribosomes, presecretory proteins, and the plasma membrane. Cell 54: 1013-1018.

273. Hoffmann A, Merz F, Rutkowska A, Zachmann-Brand B, Deuerling E, et al. (2006) Trigger factor forms a protective shield for nascent polypeptides at the ribosome. J Biol Chem 281: 6539-6545.

274. Ferbitz L, Maier T, Patzelt H, Bukau B, Deuerling E, et al. (2004) Trigger factor in complex with the ribosome forms a molecular cradle for nascent proteins. Nature 431: 590-596.

275. Ullers RS, Houben EN, Raine A, ten Hagen-Jongman CM, Ehrenberg M, et al. (2003) Interplay of signal recognition particle and trigger factor at L23 near the nascent chain exit site on the Escherichia coli ribosome. J Cell Biol 161: 679-684.

276. Patzelt H, Rudiger S, Brehmer D, Kramer G, Vorderwulbecke S, et al. (2001) Binding specificity of Escherichia coli trigger factor. Proc Natl Acad Sci U S A 98: 14244-14249.

277. Liu CP, Perrett S, Zhou JM (2005) Dimeric trigger factor stably binds folding-competent intermediates and cooperates with the DnaK-DnaJ-GrpE chaperone system to allow refolding. J Biol Chem 280: 13315-13320.

278. Esberg B, Leung HC, Tsui HC, Bjork GR, Winkler ME (1999) Identification of the miaB gene, involved in methylthiolation of isopentenylated A37 derivatives in the tRNA of Salmonella typhimurium and Escherichia coli. J Bacteriol 181: 7256-7265.

279. Yoshikawa A, Isono S, Sheback A, Isono K (1987) Cloning and nucleotide sequencing of the genes rimI and rimJ which encode enzymes acetylating ribosomal proteins S18 and S5 of Escherichia coli K12. Mol Gen Genet 209: 481-488.

280. Mackie GA (1998) Ribonuclease E is a 5'-end-dependent endonuclease. Nature 395: 720-723.

281. Del Campo M, Kaya Y, Ofengand J (2001) Identification and site of action of the remaining four putative pseudouridine synthases in Escherichia coli. Rna 7: 1603-1615.

282. Craigen WJ, Caskey CT (1987) The function, structure and regulation of E. coli peptide chain release factors. Biochimie 69: 1031-1041.

283. Zilhao R, Camelo L, Arraiano CM (1993) DNA sequencing and expression of the gene rnb encoding Escherichia coli ribonuclease II. Mol Microbiol 8: 43-51.

284. Tanaka S, Matsushita Y, Yoshikawa A, Isono K (1989) Cloning and molecular characterization of the gene rimL which encodes an enzyme acetylating ribosomal protein L12 of Escherichia coli K12. Mol Gen Genet 217: 289-293.

285. Sivaraman J, Sauve V, Larocque R, Stura EA, Schrag JD, et al. (2002) Structure of the 16S rRNA pseudouridine synthase RsuA bound to uracil and UMP. Nat Struct Biol 9: 353-358.

286. Conrad J, Niu L, Rudd K, Lane BG, Ofengand J (1999) 16S ribosomal RNA pseudouridine synthase RsuA of Escherichia coli: deletion, mutation of the conserved Asp102 residue, and sequence comparison among all other pseudouridine synthases. Rna 5: 751-763.

287. Kammen HO, Marvel CC, Hardy L, Penhoet EE (1988) Purification, structure, and properties of Escherichia coli tRNA pseudouridine synthase I. J Biol Chem 263: 2255-2263.

288. Flint DH (1996) Escherichia coli contains a protein that is homologous in function and N-terminal sequence to the protein encoded by the nifS gene of Azotobacter vinelandii and that can participate in the synthesis of the Fe-S cluster of dihydroxy-acid dehydratase. J Biol Chem 271: 16068-16074.

289. Bystrom AS, von Gabain A, Bjork GR (1989) Differentially expressed trmD ribosomal protein operon of Escherichia coli is transcribed as a single polycistronic mRNA species. J Mol Biol 208: 575-586.

290. Hjalmarsson KJ, Bystrom AS, Bjork GR (1983) Purification and characterization of transfer RNA (guanine-1)methyltransferase from Escherichia coli. J Biol Chem 258: 1343-1351.

291. Lovgren JM, Bylund GO, Srivastava MK, Lundberg LA, Persson OP, et al. (2004) The PRC-barrel domain of the ribosome maturation protein RimM mediates binding to ribosomal protein S19 in the 30S ribosomal subunits. Rna 10: 1798-1812.

292. Bylund GO, Wipemo LC, Lundberg LA, Wikstrom PM (1998) RimM and RbfA are essential for efficient processing of 16S rRNA in Escherichia coli. J Bacteriol 180: 73-82.

293. Kaya Y, Del Campo M, Ofengand J, Malhotra A (2004) Crystal structure of TruD, a novel pseudouridine synthase with a new protein fold. J Biol Chem 279: 18107-18110.

294. Hoang C, Ferre-D'Amare AR (2004) Crystal structure of the highly divergent pseudouridine synthase TruD reveals a circular permutation of a conserved fold. Rna 10: 1026-1033.

295. Ericsson UB, Nordlund P, Hallberg BM (2004) X-ray structure of tRNA pseudouridine synthase TruD reveals an inserted domain with a novel fold. FEBS Lett 565: 59-64.

296. Agarwalla S, Kealey JT, Santi DV, Stroud RM (2002) Characterization of the 23 S ribosomal RNA m5U1939 methyltransferase from Escherichia coli. J Biol Chem 277: 8835-8840.

297. Basturea GN, Rudd KE, Deutscher MP (2006) Identification and characterization of RsmE, the founding member of a new RNA base methyltransferase family. Rna 12: 426-434.

298. De Bie LG, Roovers M, Oudjama Y, Wattiez R, Tricot C, et al. (2003) The yggH gene of Escherichia coli encodes a tRNA (m7G46) methyltransferase. J Bacteriol 185: 3238-3243.

299. Pan H, Agarwalla S, Moustakas DT, Finer-Moore J, Stroud RM (2003) Structure of tRNA pseudouridine synthase TruB and its RNA complex: RNA recognition through a combination of rigid docking and induced fit. Proc Natl Acad Sci U S A 100: 12648-12653.

300. Huang YJ, Swapna GV, Rajan PK, Ke H, Xia B, et al. (2003) Solution NMR structure of ribosome-binding factor A (RbfA), a cold-shock adaptation protein from Escherichia coli. J Mol Biol 327: 521-536.

301. Gill SC, Yager TD, von Hippel PH (1991) Escherichia coli sigma 70 and NusA proteins. II. Physical properties and self-association states. J Mol Biol 220: 325-333.

302. Tan J, Jakob U, Bardwell JC (2002) Overexpression of two different GTPases rescues a null mutation in a heat-induced rRNA methyltransferase. J Bacteriol 184: 2692-2698.

303. Stebbins CE, Borukhov S, Orlova M, Polyakov A, Goldfarb A, et al. (1995) Crystal structure of the GreA transcript cleavage factor from Escherichia coli. Nature 373: 636-640.

304. Vanet A, Plumbridge JA, Alix JH (1993) Cotranscription of two genes necessary for ribosomal protein L11 methylation (prmA) and pantothenate transport (panF) in Escherichia coli K-12. J Bacteriol 175: 7178-7188.

305. Vanet A, Plumbridge JA, Guerin MF, Alix JH (1994) Ribosomal protein methylation in Escherichia coli: the gene prmA, encoding the ribosomal protein L11 methyltransferase, is dispensable. Mol Microbiol 14: 947-958.

306. Foster PG, Nunes CR, Greene P, Moustakas D, Stroud RM (2003) The first structure of an RNA m5C methyltransferase, Fmu, provides insight into catalytic mechanism and specific binding of RNA substrate. Structure 11: 1609-1620.

307. Post LE, Arfsten AE, Davis GR, Nomura M (1980) DNA sequence of the promoter region for the alpha ribosomal protein operon in Escherichia coli. J Biol Chem 255: 4653-4659.

308. Meek DW, Hayward RS (1984) Nucleotide sequence of the rpoA-rplQ DNA of Escherichia coli: a second regulatory binding site for protein S4? Nucleic Acids Res 12: 5813-5821.

309. Furano AV (1975) Content of elongation factor Tu in Escherichia coli. Proc Natl Acad Sci U S A 72: 4780-4784.

310. Opalka N, Chlenov M, Chacon P, Rice WJ, Wriggers W, et al. (2003) Structure and function of the transcription elongation factor GreB bound to bacterial RNA polymerase. Cell 114: 335-345.

311. Sawers G, Heider J, Zehelein E, Bock A (1991) Expression and operon structure of the sel genes of Escherichia coli and identification of a third selenium-containing formate dehydrogenase isoenzyme. J Bacteriol 173: 4983-4993.

312. Soutourina J, Plateau P, Delort F, Peirotes A, Blanquet S (1999) Functional characterization of the D-Tyr-tRNATyr deacylase from Escherichia coli. J Biol Chem 274: 19109-19114.

313. Persson BC, Gustafsson C, Berg DE, Bjork GR (1992) The gene for a tRNA modifying enzyme, m5U54-methyltransferase, is essential for viability in Escherichia coli. Proc Natl Acad Sci U S A 89: 3995-3998.

314. Lindstrom PH, Stuber D, Bjork GR (1985) Genetic organization and transcription from the gene (trmA) responsible for synthesis of tRNA (uracil-5)-methyltransferase by Escherichia coli. J Bacteriol 164: 1117-1123.

315. Gustafsson C, Lindstrom PH, Hagervall TG, Esberg KB, Bjork GR (1991) The trmA promoter has regulatory features and sequence elements in common with the rRNA P1 promoter family of Escherichia coli. J Bacteriol 173: 1757-1764.

316. Tsui HC, Winkler ME (1994) Transcriptional patterns of the mutL-miaA superoperon of Escherichia coli K-12 suggest a model for posttranscriptional regulation. Biochimie 76: 1168-1177.

317. Hartl FU, Hayer-Hartl M (2002) Molecular chaperones in the cytosol: from nascent chain to folded protein. Science 295: 1852-1858.

318. Rye HS, Burston SG, Fenton WA, Beechem JM, Xu Z, et al. (1997) Distinct actions of cis and trans ATP within the double ring of the chaperonin GroEL. Nature 388: 792-798.

319. Cliff MJ, Kad NM, Hay N, Lund PA, Webb MR, et al. (1999) A kinetic analysis of the nucleotide-induced allosteric transitions of GroEL. J Mol Biol 293: 667-684.

320. Rye HS, Roseman AM, Chen S, Furtak K, Fenton WA, et al. (1999) GroEL-GroES cycling: ATP and nonnative polypeptide direct alternation of folding-active rings. Cell 97: 325-338.

321. Ranson NA, Clare DK, Farr GW, Houldershaw D, Horwich AL, et al. (2006) Allosteric signaling of ATP hydrolysis in GroEL-GroES complexes. 13: 147-152.

322. Farr GW, Fenton WA, Chaudhuri TK, Clare DK, Saibil HR, et al. (2003) Folding with and without encapsulation by cis- and trans-only GroEL-GroES complexes. Embo J 22: 3220-3230.

323. Falke S, Tama F, Brooks CL, 3rd, Gogol EP, Fisher MT (2005) The 13 angstroms structure of a chaperonin GroEL-protein substrate complex by cryo-electron microscopy. J Mol Biol 348: 219-230.

324. Brodersen DE, Nissen P (2005) The social life of ribosomal proteins. Febs J 272: 2098-2108.

325. Schuwirth BS, Borovinskaya MA, Hau CW, Zhang W, Vila-Sanjurjo A, et al. (2005) Structures of the bacterial ribosome at 3.5 A resolution. Science 310: 827-834.

326. Tung CS, Joseph S, Sanbonmatsu KY (2002) All-atom homology model of the Escherichia coli 30S ribosomal subunit. Nat Struct Biol 9: 750-755.

327. Polacek N, Barta A (1998) Metal ion probing of rRNAs: evidence for evolutionarily conserved divalent cation binding pockets. Rna 4: 1282-1294.

328. Drygin D, Zimmermann RA (2000) Magnesium ions mediate contacts between phosphoryl oxygens at positions 2122 and 2176 of the 23S rRNA and ribosomal protein L1. Rna 6: 1714-1726.

329. Weiss RL, Kimes BW, Morris DR (1973) Cations and ribosome structure. 3. Effects on the 30S and 50S subunits of replacing bound Mg 2+ by inorganic cations. Biochemistry 12: 450-456.

330. Weiss RL, Morris DR (1973) Cations and ribosome structure. I. Effects on the 30S subunit of substituting polyamines for magnesium ion. Biochemistry 12: 435-441.

331. Hanson CL, Fucini P, Ilag LL, Nierhaus KH, Robinson CV (2003) Dissociation of intact Escherichia coli ribosomes in a mass spectrometer. Evidence for conformational change in a ribosome elongation factor G complex. J Biol Chem 278: 1259-1267.

332. Carter AP, Clemons WM, Brodersen DE, Morgan-Warren RJ, Wimberly BT, et al. (2000) Functional insights from the structure of the 30S ribosomal subunit and its interactions with antibiotics. Nature 407: 340-348.

333. Klein DJ, Moore PB, Steitz TA (2004) The contribution of metal ions to the structural stability of the large ribosomal subunit. Rna 10: 1366-1379.

334. Chang FN, Budzilowicz C (1977) Characterization of methylated neutral amino acids from Escherichia coli ribosomes. J Bacteriol 131: 105-110.

335. Colson C (1977) Genetics of ribosomal protein methylation in Escherichia coli. I. A mutant deficient in methylation of protein L11. Mol Gen Genet 154: 167-173.

336. Brosius J, Chen R (1976) The primary structure of protein L16 located at the peptidyltransferase center of Escherichia coli ribosomes. FEBS Lett 68: 105-109.

337. Maguire BA, Wild DG (1997) The effects of mutations in the rpmB,G operon of Escherichia coli on ribosome assembly and ribosomal protein synthesis. Biochim Biophys Acta 1353: 137-147.

338. Maguire BA, Wild DG (1997) The roles of proteins L28 and L33 in the assembly and function of Escherichia coli ribosomes in vivo. Mol Microbiol 23: 237-245.

339. Chang FN (1978) Temperature-dependent variation in the extent of methylation of ribosomal proteins L7 and L12 in Escherichia coli. J Bacteriol 135: 1165-1166.

340. Chen R, Chen-Schmeisser U (1977) Isopeptide linkage between N-alpha-monomethylalanine and lysine in ribosomal protein S11 from Escherichia coli. Proc Natl Acad Sci U S A 74: 4905-4908.

341. Kowalak JA, Walsh KA (1996) Beta-methylthio-aspartic acid: identification of a novel posttranslational modification in ribosomal protein S12 from Escherichia coli. Protein Sci 5: 1625-1632.

342. Galperin MY, Koonin EV (1997) A diverse superfamily of enzymes with ATP-dependent carboxylate-amine/thiol ligase activity. Protein Sci 6: 2639-2643.

343. Hitz H, Schafer D, Wittmann-Liebold B (1977) Determination of the complete amino-acid sequence of protein S6 from the wild-type and a mutant of Escherichia coli. Eur J Biochem 75: 497-512.

344. Kang WK, Icho T, Isono S, Kitakawa M, Isono K (1989) Characterization of the gene rimK responsible for the addition of glutamic acid residues to the C-terminus of ribosomal protein S6 in Escherichia coli K12. Mol Gen Genet 217: 281-288.

345. Thanbichler M, Bock A, Goody RS (2000) Kinetics of the interaction of translation factor SelB from Escherichia coli with guanosine nucleotides and selenocysteine insertion sequence RNA. J Biol Chem 275: 20458-20466.

346. Forchhammer K, Rucknagel KP, Bock A (1990) Purification and biochemical characterization of SELB, a translation factor involved in selenoprotein synthesis. J Biol Chem 265: 9346-9350.

347. Solomovici J, Lesnik T, Reiss C (1997) Does Escherichia coli optimize the economics of the translation process? J Theor Biol 185: 511-521.

348. Sundari RM, Pelka H, Schulman LH (1977) Structural requirements of Escherichia coli formylmethionyl transfer ribonucleic acid for ribosome binding and initiation of protein synthesis. J Biol Chem 252: 3941-3944.

349. Stadtman TC (1996) Selenocysteine. Annu Rev Biochem 65: 83-100.

350. Ohki M, Tamura F, Nishimura S, Uchida H (1986) Nucleotide sequence of the Escherichia coli dnaJ gene and purification of the gene product. J Biol Chem 261: 1778-1781.

351. Eriani G, Dirheimer G, Gangloff J (1991) Cysteinyl-tRNA synthetase: determination of the last E. coli aminoacyl-tRNA synthetase primary structure. Nucleic Acids Res 19: 265-269.

352. Blaise M, Becker HD, Keith G, Cambillau C, Lapointe J, et al. (2004) A minimalist glutamyl-tRNA synthetase dedicated to aminoacylation of the tRNAAsp QUC anticodon. Nucleic Acids Res 32: 2768-2775.

353. Teplova M, Tereshko V, Sanishvili R, Joachimiak A, Bushueva T, et al. (2000) The structure of the yrdC gene product from Escherichia coli reveals a new fold and suggests a role in RNA binding. Protein Sci 9: 2557-2566.
