# Supplementary material for: Genome-Scale Reconstruction of Escherichia coli's Transcriptional and Translational Machinery: A Knowledge Base, Its Mathematical Formulation, and Its Functional Characterization
Source: PLoS Comput Biol. 2009 Mar 13;5(3):e1000312. doi: 10.1371/journal.pcbi.1000312 (PMC2648898; doi:10.1371/journal.pcbi.1000312)
Supplement: Text S1 — The supplemental text describes in detail the network content, reconstruction approach, and underlying assumptions. (1.45 MB DOC) [file pcbi.1000312.s021.doc]

**Supplemental Text**

Thiele *et al.*:’ Genome-scale reconstruction of *E. coli*'s transcriptional and translational machinery: A knowledge base, its mathematical formulation, and its functional characterization’.

Content:

1. **Reconstruction approach**
2. **Basic assumptions**
3. **Reconstruction content**
4. **Properties of the ‘E-matrix’**
   1. **Protein and reaction distribution per subsystem**
   2. **Proteins without gene annotation**
   3. **rRNA operon**
   4. **Sparsity of S**
5. **Formulae used for constraints**

In this work, we present the first comprehensive, genome-scale, manually curated reconstruction of the transcriptional and translational machinery of *E. coli*, the ‘E-matrix’. The information incorporated in this reconstruction was based on almost 50 years legacy literature, and multiple databases. Hence, the reconstruction reflects the current status of knowledge of these important cellular processes in *E. coli*, since only *E. coli* specific information was incorporated into the network.

1. **Reconstruction approach**

The reconstruction process of any biological network depends heavily on the quality of the genome annotation and the amount of experimental data available for an organism [3]. The transcriptional and translational machinery of *E. coli* was selected for reconstruction, as *E. coli* is one of the most extensively studied organisms [4]. We aimed to create a high-resolution reconstruction that would accurately account for the cellular processes necessary to produce functional gene products of this machinery (Figure A-a).

The manual reconstruction of the transcriptional and translational machinery of *E. coli* was performed in an algorithmic manner (Figure A-c). First, the identification of its key components in the genome annotation resulted in an initial component list. Then, the functional roles of these key components were identified and translated into stoichiometrically accurate reactions using textbooks, reviews, and primary literature (Figure A-b). This step led to the identification of components missing in the initial component list*,* which were subsequently added to the network. In the end, this reconstruction approach led to the identification of 228 proteins and 109 RNA species*,* which were directly involved in one or more subsystems (Figure A-a, Table S1 for the Proteins, a complete list can be found in Table S10). The synthesis reactions for every network component were created using template reactions. These template reactions were carefully formulated and derived from primary and review literature. While they combine linear steps (e.g., elongation of nascent mRNA during transcription), they separate key reactions and known rate limiting steps (e.g., separation of transcription initiation and elongation). This formulation enabled the incorporation of different sets of constraints but also reduced the network size by combining linear reactions. Hence, this step-wise representation captures key event in cellular processes and can be directly used to understand their pathway/reaction mechanism at a high resolution.

**a**

b
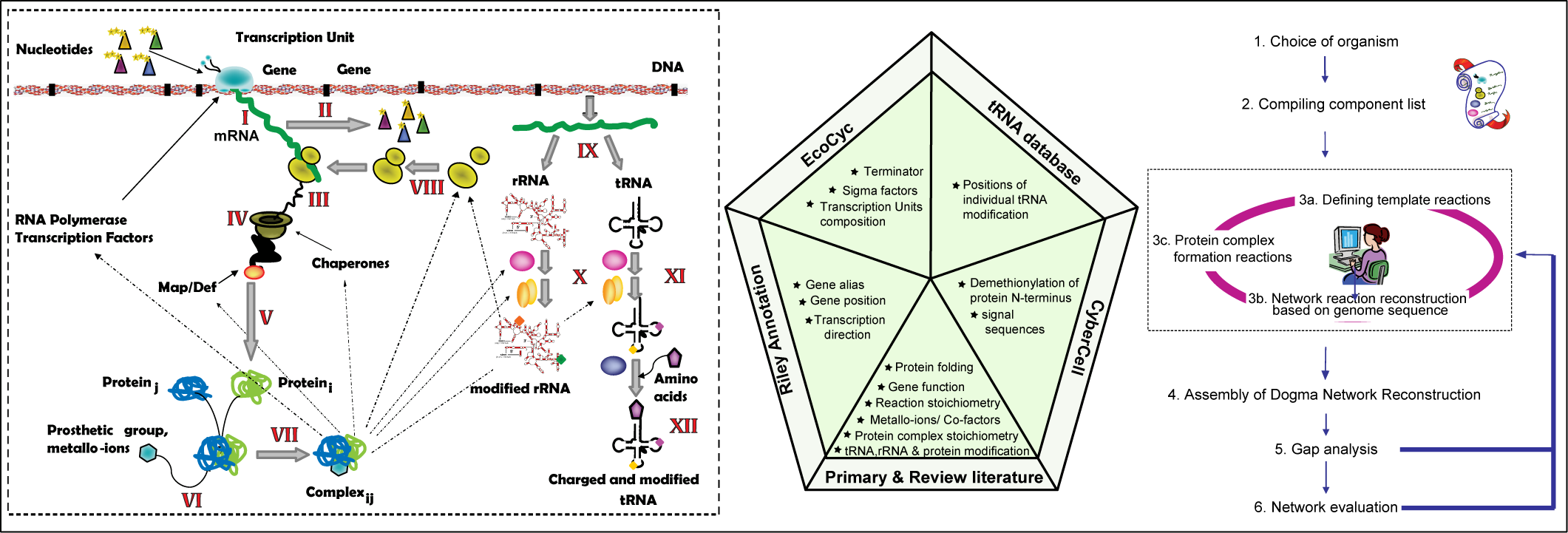


**Figure A: Reconstruction content and approach. a:** Schematic overview of the content of the ‘E-matrix’. I: Transcription; II: mRNA degradation; III: translation; IV: protein maturation; V: protein folding; VI: metallo-ion binding; VII: protein complex formation; VIII: ribosome assembly; IX: RNA processing; X: rRNA modification; XI: tRNA modification; XII: tRNA charging (see Table S2 for a complete list). **b (left panel):** The pentagram shows the main five data sources incorporated in the ‘E-matrix’ reconstruction. **b (right panel)**: The ‘E-matrix’ network was reconstructed algorithmically fashion in analogy to the process developed for metabolic network reconstruction.

***Template reactions.*** Once the main factors involved in the various processes, or subsystems, were identified the reactions carried out by one or an ensemble of these components were defined based on up-to-date literature (Table S15-S17). For a majority of the network reactions, we used the fact that the reactions were very similar for every gene or gene product. For example, transcription initiation and elongation involves RNA polymerase and transcriptional factors, such as NusA and NusB, for all genes. Subsequently, algorithmic reaction formulation could be done using template reactions for a majority of network reactions (Table S14). The generation of reactions was done using the scripting language, Perl (<http://www.perl.com/>).

The basis for the reconstruction is the genome sequence, m56 [5], the most current gene coordinates from [6], and the transcription unit (TU) definition provided by EcoCyc (version 10.6, [1]). This information, in addition to the other data resources (Figure S1b), were used to i) calculate formulae and charge for each mRNA and protein specie; ii) create gene-specific reactions from template reactions; and iii) enable the transcription of operons rather than genes. The transcriptional and translational reactions were formulated for all gene product involved in the machinery. In addition, genes that were part of such operons but not directly involved in the network functions were also included. For example, only one of four operon genes is necessary for the function of the machinery but all three other co-transcribed genes have to be transcribed and translated.

As an example of how the different information sources and the generic reactions were be used to reconstruct the network, consider TU00021:

Transcription unit TU00021 consists of the genes b3260 and b3261 (EcoCyc [1], Table S9). The genome positions and sense of b3260 (3,408,302 - 3,409,267) and of b3261 (3,409,293 - 3,409,589) can be obtained from the genome annotation [6]. Based on EcoCyc, the promoter site of TU00021 binds sigma 70. Using the genome sequence m56 [5], the necessary amount of each NTP for synthesizing the polycistronic mRNA (TU00021_mRNA) were determined. Subsequently, the transcription reactions for TU00021 can be formulated using the template reactions:

| tscr_ini_TU00021 | Transcription initiation of TU00021 (dusBp) | 1 RNAP_70 + 1 TU00021_DNA_act + 5 atp + 4 ctp + 4 gtp + 3 utp <==> 1 RpoD_mono_inact + 15 ppi + 1 transcr_ini_TU00021_cplx |
| --- | --- | --- |
| tscr_elo_TU00021_ini_rho_dep | Formation complex for elongation of TU00021 (RHO DEPENDENT TERMINATION) | 1 GreA_mono + 1 GreB_mono + 1 Mfd_mono + 1 NusA_mono + 1 NusG_mono + 1 Rho_hexa + 1 RpoZ_mono + 1 transcr_ini_TU00021_cplx <==> 1 transcr_elo_TU00021_cplx |
| tscr_elo_term_TU00021_rho_dep | Transcription elongation and RHO DEPENDENT termination of TU00021 (dusBp) | 338 atp + 309 ctp + 337 gtp + 3 h2o + 1 transcr_elo_TU00021_cplx + 291 utp --> 1 GreA_mono_inact + 1 GreB_mono_inact + 1 Mfd_mono_inact + 1 NusA_mono_inact + 1 NusG_mono_inact + 1 Rho_hexa_inact + 1 RpoZ_mono_inact + 1 TU00021_DNA_neu + 1 TU00021_mRNA + 3 adp + 3 h + 1 hRNAP_inact + 3 pi + 1272 ppi |

Similarly all other synthesis reactions for TU00021 and its gene products can be formulated.

*Network features*

The resulting network has properties distinct from metabolic networks and currently available transcriptional models:

- The ‘E-matrix’ accounts for all known gene products necessary to produce the active components (RNA, proteins) of the machinery.
- Sequence-dependent synthesis reactions were carefully formulated incorporating known reaction stoichiometry. Protein-substrate complexes were explicitly included in each network reaction. The chemical formula, charge, and molecular weight were calculated for each component assuming a pH of 7.2.
- Metallo-ions and cofactors were incorporated into proteins and modification of stable RNA and proteins were considered.
- The transcription reactions are transcription unit dependent rather than gene dependent, which accurately represents the functional genomic organization of bacterial genomes.
- The underlying formalism of template reactions can be readily extended for other gene product such as metabolic enzymes or transcription factors.

In summary, the ‘E-matrix’ reconstruction was based on *E. coli*-specific information derived from i) over 500 primary and review publications; ii) three databases: EcoCyc [1], CyberCell [7], and tRNA DB [8]; the revised genome annotation [6]; and iv) the genome sequence (m56,[5])*,* which enabled the formulation of sequence specific reactions for every network component (Figure A-c). The entire network content is available at [http://bigg.ucsd.edu/E-matrix](http://bigg.ucsd.edu/dogma).

*Iterative network reconstruction and QC/QA.*

A comprehensive, iterative quality control/ quality assurance procedure (QC/QA) ensured that the resulting network has similar properties and capabilities as *E. coli*. This QC/QA procedure included the mass- and charge balancing of most network reactions (see next section), gap analysis, and testing for the production of every network component and its intermediate form. Hence, this reconstruction follows the quality control standards developed for metabolic network reconstructions [3].

After an initial reaction list was created, as described above, a network gap analysis was performed. This procedure was an iterative process, as for metabolic network reconstructions [3], during, which further components and reactions were added (Figure A-c). Multiple iterations helped ensure completeness of the network within the pre-defined scope. Furthermore, flux balance analysis (FBA, [9,10]) was carried out to verify that every network component could be produced and consumed. Hence, a demand function for every network component was independently added to the network and maximized using linear programming. These quality control steps ensured that the final network was comprehensive and functional.

Only one network gap remained, which is the ribonuclease PH (RNase_PH) whose gene was found to be a pseudogene in *E. coli* MG1655 genome [6]. Experimental studies characterized this gene product but in different *E. coli* strains [11-13]**.**

*Mass and charge balancing*

For each network component the chemical formula, charge state at pH 7.2, and molecular weight were calculated. Almost all (99.5%) of the reactions in the ‘E-matrix’ are mass- and charge-balanced (for unbalanced reactions refer to Table S7 and S8). The remaining reactions were left unbalanced for two reasons: i) unknown electron acceptor, e.g., in the iron-sulfur-cluster biogenesis; or ii) alternate precursors with different formulae, e.g., transcription product from different overlapping transcription units*,* which were combined in subsequent reactions to reduce the overall number of network reactions.

1. **Basic model assumptions**

A number of assumptions were made to reduce the overall number of network reactions and to extrapolate the detailed knowledge for some well studied gene products to the entire network. As a consequence, some known essential genes may not be essential in the ‘E-matrix’ since they were included in all corresponding network reactions, while *in vivo* the reaction may occur even in absence of the gene products.

- 1. **Transcription**

In general there are different sets of transcription elongation factors depending on specific (pausing) sites in the DNA template, thus, the actual set of involved transcription factors might differ slightly between ORFs. We defined three different sets of transcription factors: i) stable RNA encoding ORF, ii) ORF with annotated/verified Rho-dependent transcription termination, and iii) all remaining ORF. The detailed composition of these three sets can be found in Table S15, listing the corresponding template reactions. Information about sigma factors for every gene was obtained from EcoCyc. If no information was available, sigma 70 was assumed to be needed for transcription.

In general, the model does not account for abortive products during transcription initiation, pausing sites, errors in transcription, folding, etc. It also does not account for arrested RNAP that can either resume or abort the transcription; however, both factors necessary for these actions (greA, greB) were included in the transcription reactions.

- 1. **Cleavage of RNA**

Polycistronic mRNAs were cleaved prior to translation. This modal assumption allows different translation frequencies/level for the gene products in polycistronic mRNAs [14]. The cleavage of polycistronic mRNAs was always performed by RNase III. RNase P has also been reported to be responsible for cleavage of polycistronic mRNAs [15]. However, in order to reduce the number of total network reactions only RNase III was considered for cleavage in the reconstruction [15,16]. *In vivo*, the cleavage of polycistronic mRNA may destabilize the mRNA products, although stabilization has been observed in some cases [4].

Furthermore, reactions for transcription units with overlapping codons (stop-start codon juxtaposition) were cleaved as well, whereby a full length transcript was created for the upstream (5’) gene and a shorter transcript for the downstream gene (3’). However, the translation on the shorter transcript used the full-length sequence such that the protein sequence of the gene product is complete. For more details about stop-start codon juxtaposition refer to the following studies: [14,17-20] and the review from Normark *et al*. [21]. The different frequencies of translation and half life time of cleavage products could be represented by constraints on flux rates. Decay of cleavage products occurs independent of cleavage of polycistronic mRNA. Cleavage products end with a 3' mono-phosphate group*,* which may affect the overall half-life time of cleavage products.

The processing of rRNA occurs prior to ribosome formation. Some of the posttranslational modifications as well as cleavage of rRNA require the association to ribosomal proteins. Furthermore, these factors have been shown to be involved in ribosome maturation based on 30S binding to these factors [22,23]. In the model, these factors are only involved in ribosome maturation and not in rRNA cleavage/ modification, which takes place before ribosomal assembly.

- 1. **Translation**

In many cases the transcription unit (operon) structure provides a translational coupling of the gene, where the translation of the downstream gene is dependent on the translation of the upstream gene [21]. One reason for this is that the coding region of the 1st gene may contain the Shine-Dalgarno Sequence necessary for the translation of the second gene. Such dependency was not modeled with the ‘E-matrix’.

- 1. **Protein Maturation**

CyberCell [7] lists the sequence for matured proteins, which may have the N’-terminal methionine or even signal sequences removed . Although the origin of these sequences is often not clear (experimental determination or computational prediction), the data were incorporated into the ‘E-matrix’ after sequence comparison. The removal of signal sequences is enzyme independent in the model and no action, e.g., protein export, is associated with the removal since these two functions were outside the defined scope of the ‘E-matrix’.

- 1. **mRNA degradation**

The mRNA degradation in the ‘E-matrix’ is carried out solely by the degradosome. The model degradosome consists only of components that have been found to be necessary/ essential for its action (Eno, Pnp, RNase_E, RhlB ) but not accessory factors (such as DnaK, GroEL, PPK, PAP, S1) [24-27]. No degradation of stable RNA was modeled, since tRNAs and rRNAs are known to be highly stable under normal growth conditions and are believed to be insensitive to the decay processes that lead to the fast turnover of short half-lived mRNAs [28-30].

Functions of different RNase identified in *E. coli* that were not included in the ‘E-matrix’ either because they are outside the scope of the reconstruction or their function is not well established:

- - - *RNase I* is responsible for stable RNA decay, especially 23S rRNA [31,32].
    - *RNase M* is mutated form of RNase I [33].
    - *RNase LS* was not included because little is presently known about its precise function, although it is believed to be involved in mRNA decay [34].
    - *RNase T* is responsible for tRNA turnover [35].
    - *RNase R*: Cheng *et al*. [36]showed that RNAse R is important in mRNA decay. It seems that this RNase acts on mRNAs with high secondary structures (REP elements) and that it can replace PNPase action. However, PNPase, together with other degradosome proteins, can digest mRNAs with (complex) secondary structures. It has been found that RNase R amount increase with stress conditions [37]. PNPase/RNase R both need a longer sequence before structural elements to dock on and to do degrade. Hence, it is thought that those RNA fragments get a poly(A) tail by PAP I and PAP II. It seems that RNase II has a rather protective function than degradative. Furthermore, one ribonuclease of RNase II, RNase R, PNPase can be deleted. The double deletion of RNase II and RNase R was found to be viable but double deletions of PNPase/RNaseII or PNPase/RNase R were lethal. RNase R also degraded stable RNA (whereby tRNA is poor substrate, but defectuous tRNA was a good substrate [36]). Deutscher *et al*. [38] said that the function of RNase II and RNase R will have to be re-evaluated. Hence, the reconstruction does not account for RNase R action. PNPase, together with the other degradosome proteins, seems to be sufficient under normal growth conditions.
  1. **Other assumptions**
  - All protein complex formation reactions were defined to be reversible.
  - The charge and composition of each compound in the reconstruction is based on the sequence not accounting for unknown posttranslational modification, possible water molecules associated with compound or changes in charge/composition due to protein/RNA folding.
  - Polyadenylation of mRNA was not included since its effect is not sufficiently established. While poly(A)-tail on some mRNAs lead to a prolonged half-life time (protective cap), it seems to be a degradation sign on other mRNAs [4,36,39-41]

1. **Reconstruction content**

The reconstructed network consists of 27 subsystems or pathways describing the transcriptional and translational reactions for each gene product as well as posttranslational modification reactions needed for their functionality (Table S1 and S12). A schematic representation of the network content is depicted in Figure A-a.

- 1. **Systems boundaries*.***

The scope of the reconstruction was defined to be the transcriptional and translational reactions for all components involved in these processes including the reactions needed to obtain an active form of these components (i.e., tRNA modifications). Hence, amino acids or tri-phosphate nucleotides were provided to or removed from the network by exchange reactions. A total of 76 exchange reactions were included in the reconstruction (Table S12).

- 1. **Transcription units (TU)**

The *E. coli* genome is organized in operons, or TUs. The ‘E-matrix’ accounts for this property by requiring transcription to occur in terms of TUs rather than genes. A total of 303 genes, involved in transcriptional and translational machinery, were encoded by 249 TU, while 12 further genes had no TU assignment in EcoCyc [1] (Table S9 and S10). A total of 423 gene products were synthesized, but only 303 of these gene products are directly involved in the ‘E-matrix’. Due to the TU organization of the network, 120 gene products were synthesized although they were not within the scope of the ‘E-matrix’ (i.e., metabolic or regulatory gene products). These 120 gene products were not connected to the other network components since their function was either out of scope or unknown, but corresponding demand functions were included.

- 1. **Cleavage of polycistronic mRNA and translation**

In the network, genes were transcribed as TUs under the involvement of numerous factors (see Table S1). After transcription, the polycistronic mRNA is SHAPE \* MERGEFORMAT cleaved by the action of RNase III prior to translation. *In vivo*, the translation rate depends on various factors, such as binding affinity of the ribosome to the Shine-Dalgarno sequence, tertiary structures, and mRNA degradation rates, resulting in variable transcription efficiency under different conditions, e.g., environmental stresses [4] (see section e).

In addition to the production of proteins, each tRNA and rRNA was transcribed, cleaved, and modified based on available information. In contrast to the current formulation of metabolic networks [3], proteins are part of the network reactions in the ‘E-matrix’.

As a consequence of this, the reaction substrates and proteins form a complex that is converted to the reaction products from, which the proteins are liberated in a subsequent reaction. This level of mechanistic detail explains the large number of network components*,* which are similar to the number of network reactions (Table S15 – S18).

- 1. **‘Alternate transcripts’ and Overlapping ORFs**

The intergenic regions between ORFs in an operon have varying lengths in *E. coli*. The longest intergenic region included in ‘E-matrix’ is up to 28 nucleotides long (b4167-b4168). Thus, these long intergenic regions within an operon have two energy costs associated: i) synthesis cost and ii) degradation cost. Another feature that had to be considered during the reconstruction was the presence of overlapping ORFs*,* which have been reported and studied by numerous groups [14,17-20]. In this case, the transcription occurred as polycistronic mRNA, but the second (or overlapping) gene was assumed to be cleaved after the stop codon of the upstream gene (see section 2 for more details). Again, since the translation occurs *in vivo* on the polycistronic mRNA, the overlapping does not affect the functionality of the second gene product but may affect the half-life time of its mRNA.

- 1. **Ribosomal binding to mRNA*.***

The translation initiation of an mRNA occurs relatively frequently, depending on the binding strength to the Shine-Dalgarno sequence and other factors, leading to multiple ribosomes per mRNA. It has been found that the minimum distance between two ribosomes has to be 17 amino acids [42]. This variation in mRNA occupancy is modeled by a translation reactions per mRNA with i) one ribosome per mRNA, ii) maximal possible number of ribosomes per mRNA, and iii) half of the maximal possible ribosome number per mRNA. Thus, for each mRNA there are three sets of translation reactions differing in the number of ribosomes per mRNA and polypeptide products. This simplification reduced the number of possible combinations as well as allowing adjustments of the translational rate based on translation initiation, if such data were available.

- 1. **Protein Maturation.**

Polypeptides released from the translation termination complex have a formyl-methionyl group bound to the N-terminus. While the formyl-group is removed for all polypeptides by the peptide deformylase (def, b3287), the methionyl-group is removed only from some polypeptides depending on the amino acids that follow. This latter information was obtained from CyberCell [7].

- 1. **Metallo-ions.**

Many of *E. coli*’s proteins need metallo-ions for correct folding and/or function. This information was obtained from primary crystallization literature together with the structures deposited in the Protein Database (PDB, [43]). In some cases, additional experimental studies were available, which tested the protein function with different metallo-ions. In those cases, if a favored cation was not identified, Mg^2+^ was assumed. Furthermore, some of the metallo-ions, mainly cations, are only involved in the reaction mechanism but are not directly covalently bound to the protein, and thus leave the protein after termination of the reaction. These metallo-ions were not incorporated in the proteins and hence were not part of their elementary formulae. Wilson *et al.* proposed that the metallo-ions are incorporated into the proteins prior to the protein folding [44]*,* which was implemented in the reconstruction.

- 1. **Protein Folding.**

The folding of nascent polypeptides is achieved via three distinct pathways: i) spontaneous folding; ii) through the DnaKJ-GrpE system; and iii) through GroEL/ES chaperones [45-47]. Two recently published large-scale datasets [48,49] were used for the assigning the folding pathway to the individual polypeptides. If no information was available, spontaneous protein folding was assumed. The template protein folding reactions were derived from various primary and review literature and their references can be found in Table S15 - S17.

- 1. **Iron-sulfur-cluster biogenesis.**

A number of features were included in the reconstruction such as metallo-ion binding of tRNA, rRNA, and proteins. Some of *E. coli*’s proteins have [4Fe-4S]^2+^-clusters incorporated*,* which often function as iron- or oxygen-sensor or are involved in oxidation-reduction reactions [50]. These iron-sulfur clusters are formed outside of the target protein and the [4Fe-4S]^2+^ cluster is transferred to the corresponding proteins by an IscU dimer (b2529) [51-57]. Following extensive perusal of the primary literature regarding iron-sulfur-cluster biogenesis, a mechanism was used for this reconstruction*,* which summarized the current consensus rather than copying a specific proposed mechanism (see below and Table S18). There does not seem to be a general consensus on the biogenesis of the iron-sulfur-cluster in the scientific community.

In the current reconstruction, two of the biogenesis reactions are not balanced because the formation of [2Fe-2S]^2+^ and [4Fe-4S]^2+^ requires an electron acceptor. Kato *et al*. [57] proposed glutathione as electron acceptor, however, glutathione may involve a proton transfer*,* which would require an additional acceptor. In short, since an appropriate electron transfer mechanism could not be found, the two reactions had to remain unbalanced. The two chaperones, HscA (b2526) and HscB (b2527), were not included in the reconstruction since their functions in the iron-sulfur-cluster biogenesis has not been completely elucidated [58-62].

Other players:

It seems to be clear that cysteine provides the sulfur for the cluster via IscS; however, the Fe2+ source is not clear since iron is toxic to the cells, so that its cytoplasmic, soluble concentration is low [63]. In addition, *E. coli* has a number of iron-binding proteins*,* which remove free iron. YggX (b2962) is another protein that has been shown to be able to bind iron [64,65]; however, a recent report did not manifest the ability of YggX to bind iron [66]. Another candidate is CyaY (b3807), a frataxin homolog and tetramer in solution*,* which can bind 6 to 26 Fe3+ ions when there is an excess of intracellular iron [67,68]. The deletion of cyaY does not have any apparent effect on biogenesis of iron-sulfur cluster in *E. coli* [69] and in yeast [70], but *Salmonella enterica* strains lacking the cyaY show defects in Fe-S cluster metabolism *in vivo* [71]. Since the function of cyaY has not been completely elucidated, we did not include this gene product into the reconstruction.

Three mechanism for iron-sulfur cluster formation have been proposed [72].

1. Iron binds to IscU, then the sulfur is transfered from IscS to IscU (based on observation of a stable iron-IscU complex in the case of *T. maritima* IscU [56]). However, there is no experimental evidence that the addition of sulfur atoms to an iron-loaded IscU gives rise to a cluster. For reasons probably related to structural differences between IscU proteins [53], IscU from *E. coli* and *A. vinelandii* do not bind iron [55,73,74].
2. Sulfur binding occurs first, and is followed by iron binding. This is supported by the finding that sulfur transferred from IscS to IscU through transpersulfuration reactions is effective [74,75]. However, there is no evidence for iron-sulfur cluster formation upon the addition of iron to sulfur-containing forms of IscU either [56]. Further work is needed to show that this mechanism is possible.
3. The frataxin homolog CyaY binds Fe3+ and forms a complex with IscS, which is bound to IscU. A cysteine molecule persulfates IscS with release of alanine. Using a second cysteine as electron donor for Fe3+ reduction, Fe2+ is transferred to IscS. And IscU eventually contains the [2Fe-2S] cluster [72].

More details can be found in the reviews in Layer *et al* [72] and Mansy *et al*. [52].

Young *et al* [54] studied the iron-sulfur cluster formation under physiologically relevant conditions. They found that IscU is the preferred scaffold protein when iron, L-cysteine, and IscS are present. When L-cysteine is not present in incubation solution, IscA acts as an iron chaperon*,* which binds ‘free’ iron. The iron binding in IscA appears to prevent the formation of inaccessible ferric hydroxide under aerobic conditions, subsequent addition of L-cysteine mobilizes the iron center in IscA and transfers the iron for the iron-sulfur cluster assembly in IscU even under aerobic conditions.

Reasons against the CyaY model (3): Li *et al*. found that deletion of CyaY did not affect cellular iron content and growth behavior [69]. This is in agreement with an IscU deletion being lethal since IscA is delivering iron but does not function as cluster assembly scaffold protein. However, CyaY may act au lieu of IscA. The iron binding capacity/affinity differs between the references. While [72] listed a number of references showing a high iron binding capacity for CyaY, [54] cited references*,* which claim poor binding affinities for CyaY. However, this model does not contradict the models rejected by [72].

- 1. **Template reactions for tRNA and rRNA processing***.*

The tRNA and rRNA modification reactions were created sequentially, allowing the representation of each modification reaction within the network (Table S16 and S17). The tRNA modification positions were obtained from a tRNA database [8]. Each tRNA sequence obtained from the genome sequence and its genome coordinates was aligned to the tRNAs listed in the tRNA database, since different nomenclature was used in the databases. For three *E. coli* tRNA, namely ProK (b3545), ProL (b2189), and ProM (b3799), no entries were available in the tRNA database and no reports of identified modifications could be found. *S. typhi* modifications were used for these three tRNAs (tRNA database entries RP1700, RP1701, and RP1702). The positions for rRNA modifications were obtained from the RNA modification database [76-78].

The formula for each modified nucleotide (tRNA and rRNA) was calculated based on its structure found in [4], RNA modification databases [76-78], and primary literature (see Table S16 and S17 for details). The modification reactions were obtained based on primary literature. In some cases, a consensus mechanism could not be derived from the literature, thus, the most popular mechanism was chosen for the reconstruction.

- 1. **Codon usage and tRNA assignment.**

The anticodon and codons for each of the 86 tRNA species was obtained from EcoCyc [1] and the Riley annotation [6]. Since some of the tRNAs can read more than one codon or have overlapping functions with other tRNAs due to the wobble position, template tRNA were created for those cases. For example, tRNA^gltT^, tRNA^gltU^, tRNA^gltV^, and tRNA^gltW^ have an UUC anticodon and can translate the codons GAA and GAG. Instead of having this variation on level of the translation reactions, which would lead to a combinatorial explosion of the number of network reactions, a generic tRNA^glt1^ specie was used for the translation reactions. Additionally, four reactions were created by converting each tRNA^glt^ species into tRNA^glt1^ (irreversibly). This simplification enabled a dramatic reduction in the number of network reactions, while conserving the intrinsic property of redundant codon reading (Table S11).

- 1. **Sink reactions.**

Sink reactions were created for proteins with unknown genes and some metabolic compounds whose biosynthetic pathways are unknown, i.e., pre-Q0 needed for the tRNA modification queonosine (Table S12). The reconstruction contains a total of 35 sink reactions.

- 1. **Demand functions.**

For every network component that is needed for the transcription and translation of other genes, i.e., metabolic genes, a demand function was created. These 302 network components represent the transcription and translation machinery of *E. coli* (Table S12).

1. **Properties of the ‘E-matrix’**
   1. **Protein and reaction distribution per subsystem**

There are a total of 303 components (RNA and proteins) involved in various processes of the ‘E-matrix’ machinery (Table S1 and S10). 228 of these components are proteins, consisting of one or more subunits. The pie chart in Figure B shows the percentage of these protein components involved in 13 of the 27 subsystems. As expected, the translational reactions occupy a third of these protein components. Another third is involved in the modification of stable RNA (33%). Furthermore, 14 protein components (6%) are involved in the posttranslational modification of network proteins, i.e., translation elongation factor Tu (EF-Tu), and ribosomal proteins (Table S1).

**Figure B:** Protein composition of network subsystems. A total of 228 proteins are involved in various processes included in the ‘E-matrix’ matrix. The pie chart displays the distributions of proteins in the different subsystems.

- 1. **rRNA operons**

Application of the steady state mass conservation assumption requires that input fluxes of every component are balanced by output fluxes. Consequently, only those reactions whose substrates and products can be balanced within the network can carry a non-zero flux. In the ‘E-matrix’ there is one reaction, the transcription of the rRNA operon rrnD (b3272-b3278)*,* which is stoichiometrically unbalanced (Figure C). In contrast to the other rRNA operons, the transcription of this operon produces two 5S rRNA (rrfD,b3272 and rrfF, b3274), while only one 23S rRNA and one 16S rRNA is produced. However, ribosomes contain only one copy of each rRNA type. In terms of mass conservation, the transcription of this operon is infeasible since both of the 5S rRNAs cannot be incorporated into one ribosome, and accumulation of rrfD is not allowed by the steady-state assumption. Since this operon also encodes for three tRNA molecules, namely ileU (b3277), alaU (b3276), and thrV (b3273), we created a sink reaction for rrfD 5S rRNA. The operon structure for the rrfD 5S rRNA has been experimentally determined and sequenced by Duester and Holmes [79].

SHAPE \* MERGEFORMAT


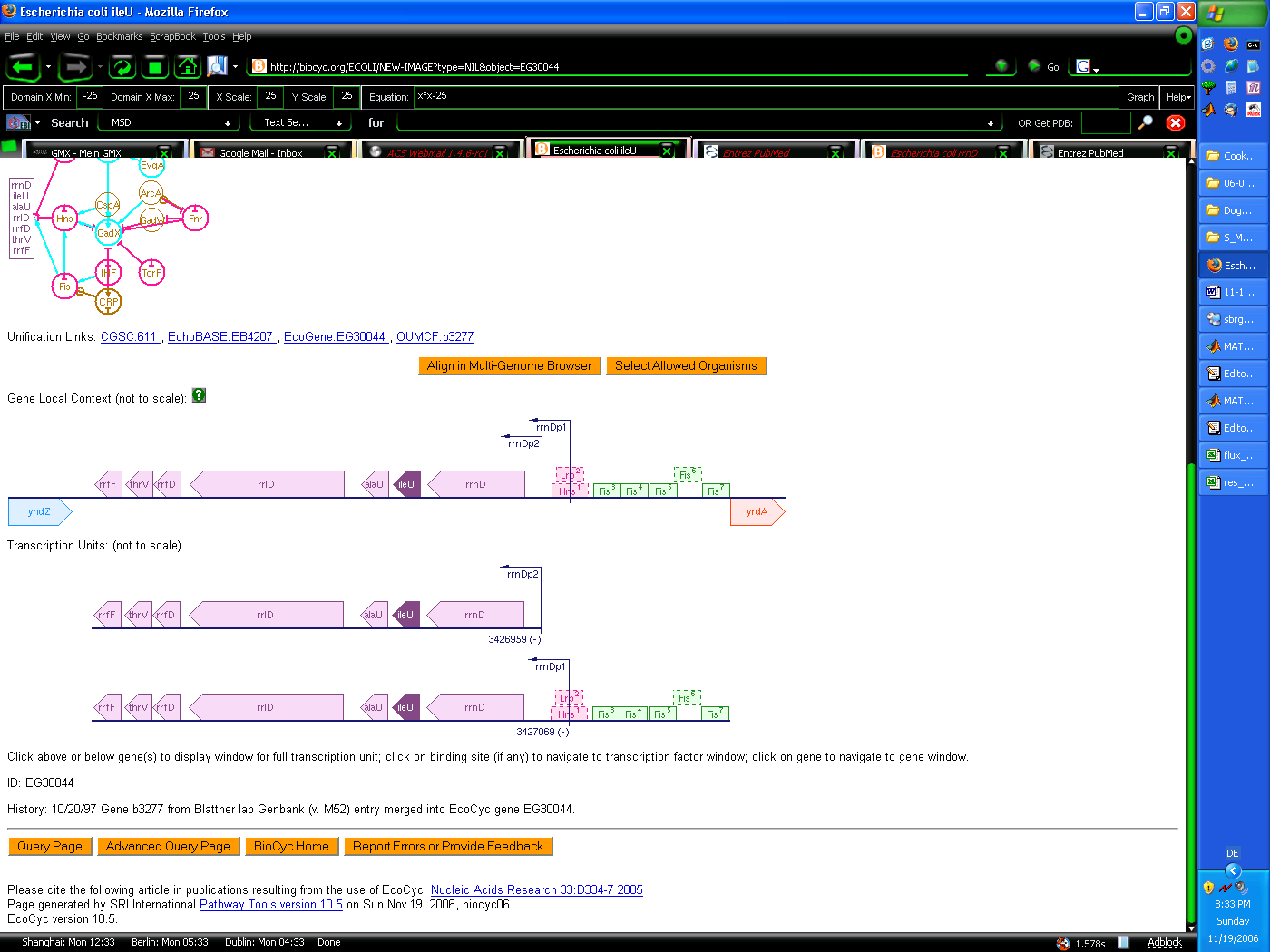


**Figure C:** Schematic representation of the stoichiometrically unbalanced rrnD operon (b3272-b3278). Taken from EcoCyc [1]

- 1. **Sparsity of S**

Stoichiometric matrices of metabolic reconstructions are known to be very sparse since there are typically only two to four compounds participating in a network reaction out of hundreds of network compounds [80]. The metabolic reconstruction of *E. coli* [81], with 761 metabolites and 1075 reactions, has only 0.55% non-zero elements. The ‘E-matrix’ is different since a component appeared on average in 16 reactions (median=2, range= 1-6886) and each network reaction contained on average 14 components (median=9, range=1-53). This higher number of participating components per reaction was due to the fact that most network reactions are lumped reactions (summing up a linear chain of reactions into a single, overall reaction). The resulting number of nonzero elements in the stoichiometric matrix of ‘E-matrix’ was 0.11%. Additionally, the matrix has a ‘linear structure’ (Figure D-a) with only few components participating in multiple reactions or even subsystems (also, compare to Figure D-b). This means that most of the components were only used ‘locally’ being passed from one reaction to the next. The by far the largest subsystem was translation due to the alternate ribosome occupation per mRNA. This subsystem also has the components with highest connectivity, such as the elongation factor G (EF-G) and the ribosome release factor (RRF).

**a
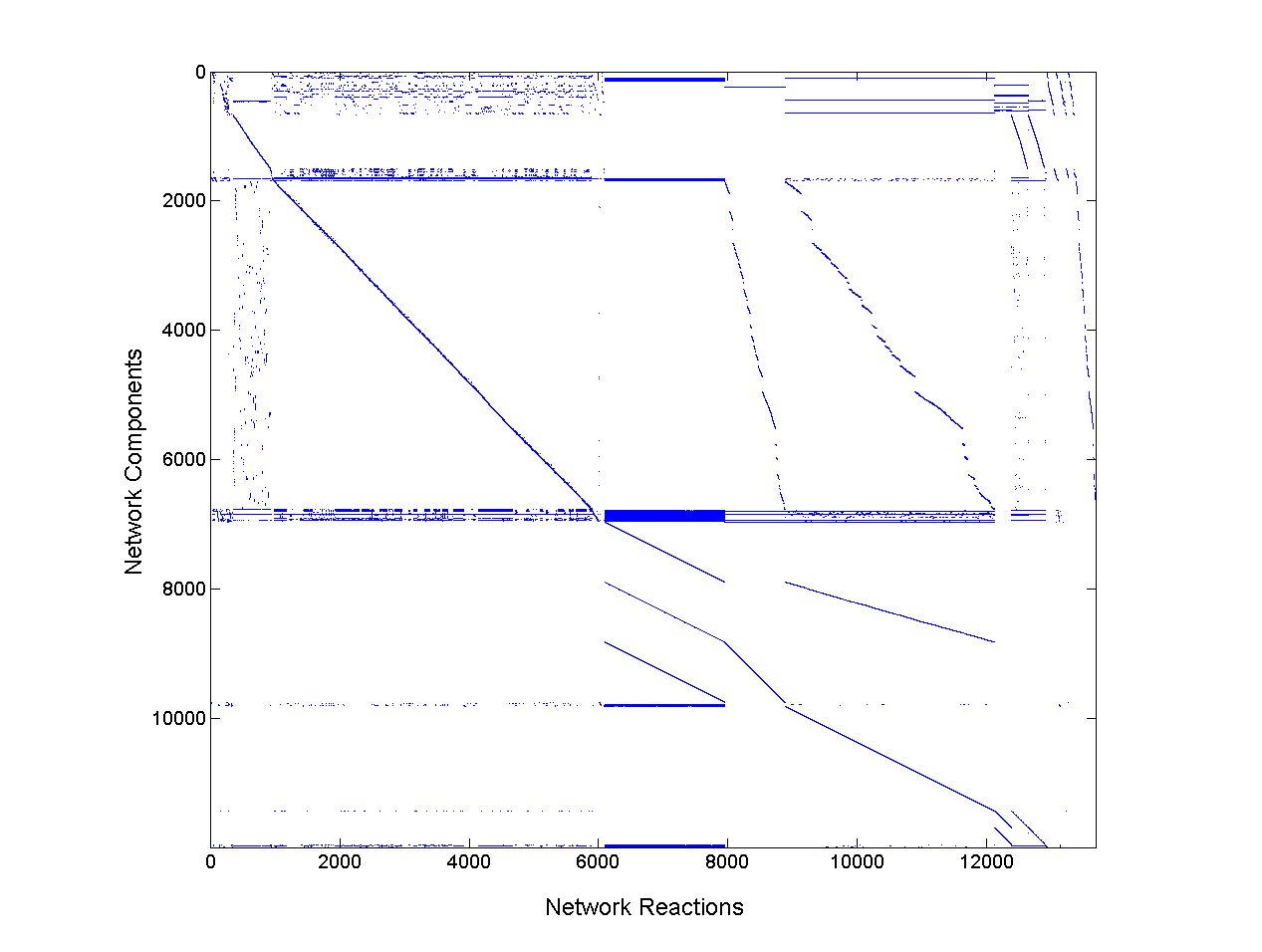
**

**b
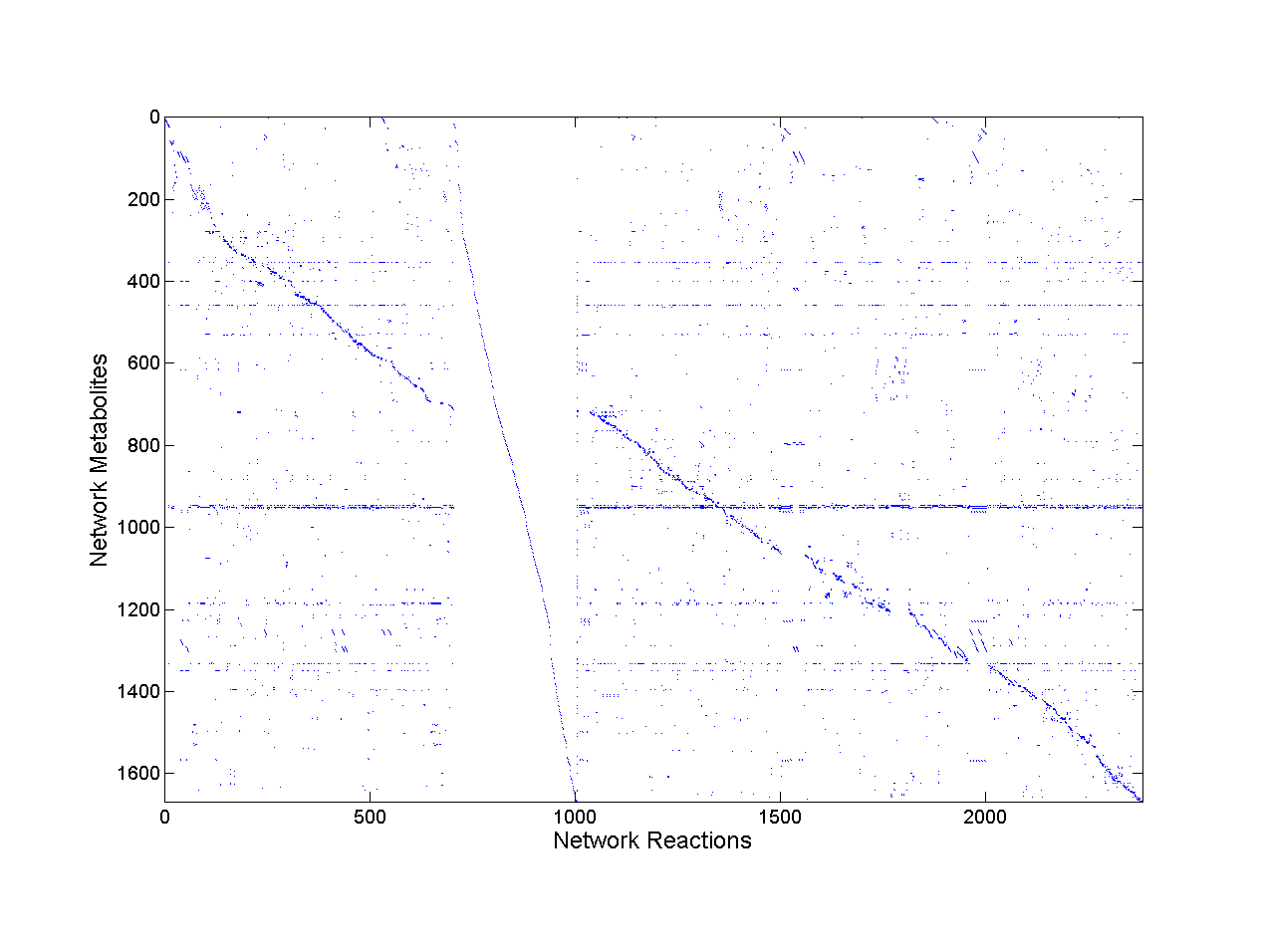
**

**Figure D-a:** Representation of the ‘E-matrix’. The blue dots represent non-zero entries in the ‘E-matrix’*, which* consists of 11991 components (y axis) and 13694 reactions (x axis). **Figure D-b:** Representation of the *i*AF1260, the most recent reconstruction of *E. coli*’s metabolism [2]. The blue dots represent non-zero entries in the ‘E-matrix’*, which* consists of 1668 components (y axis) and 2382 reactions (x axis).

1. **Formulae used to calculate reaction constraints**

**General information**

Avogadro number, N_A_:

Conversion from doubling time, $T_{D}$, to growth rate, *µ*:

Cell mass, *m_c_*, was taken from Table 2 in Neidhardt [82] (see also below), which lists: .

We used the data for the listed doubling times to calculate the cell mass at $T_{D}$=90 min. We obtained best fit of data with power law: (R^2^ = 0.9976). The cell mass values used in this study are listed in the following table:

| T_D_ (min) | µ (1/h) | *m_c_* |
| --- | --- | --- |
| 100 | 0.42 | 148 |
| 60 | 0.69 | 258 |
| 40 | 1.04 | 433 |
| 30 | 1.39 | 641 |
| 24 | 1.73 | 865 |
| 90 | 0.46 | 162 |

**Conversion from to :**

, where z is the cell mass .

**Adjusting the *in vivo* ribosome number per cell per** doubling time

| **Doubling time (min)** | **30** | **100** | **60** | **40** | **24** |
| --- | --- | --- | --- | --- | --- |
| Ribosome number (per cell) [82] | 45100 | 6800 | 13500 | 26300 | 72000 |
| Produced ribosome number | 31985.82 | 4822.695 | 9574.468 | 18652.48 | 51063.83 |

The produced ribosome number accounts for the fact that an average cell population has an cell age of 0.41 (defined so that 50% of the cells in the population are younger and 50% are older [82]). Hence, the number of produced ribosomes was scaled by 1/1.41. The resulting number of ribosomes for a given growth rate were in agreement with the maximal rRNA elongation rate observed in *E. coli* cells [82].

**Stable RNA transcription rates:**

The stable transcription initiation rate, *i_rrn_*, is given in Neidhardt (Table 3 in [82]) in initiations/min/gene. We have to account for the number of each stable RNA gene due to gene dosage effect (see below). Furthermore, the initiation rate has to be extended to the number of initiations per doubling time. It thus follows:

, where *y* is the doubling time in minutes, *i_rrn_* is the initiation rate at rrn genes in initiations/min/gene (from Table 3 in [82]):

| Doubling time (min) | **30** | **90** | **100** | **60** | **40** | **24** |
| --- | --- | --- | --- | --- | --- | --- |
| i_rrn_ (initiations/min/gene) | 39 | 5 | 4 | 10 | 23 | 58 |

Information for the different rRNA operons used for the calculation of the transcription rates (The basic gene information was obtained from Riley *et al*. [6]):

| **Transcription unit (Promoter)** | **Gene names** | **Gene alias** | **Strand** | **Coordinates (in base pairs)** | **Genes/ cell** | | | | | |
| --- | --- | --- | --- | --- | --- | --- | --- | --- | --- | --- |
|  |  |  |  |  | T_D_ = 30 min | T_D_ = 90 min | T_D_ = 100 min | T_D_ = 60 min | T_D_ = 40 min | T_D_ = 24 min |
| TU0-1181 (P1) | b3851 - b3855 | rrsA-ileT-alaT-rrlA-rrF | forward | 4,033,554 - 4,038,659 | 4.49 | 2.07 | 1.92 | 2.37 | 3.24 | 6.17 |
| TU0-1182 (P1) | b3968 - b3971 | rrsB-gltT-rrlB-rrfB | forward | 4,164,682 - 4,169,779 | 4.24 | 2.01 | 1.87 | 2.29 | 3.10 | 5.77 |
| TU0-1186 (P1) | b4007 - b4010 | rrsE-gltV-rrlE-rrfE | forward | 4,206,170 - 4,211,182 | 4.17 | 1.99 | 1.85 | 2.27 | 3.06 | 5.64 |
| TU0-1189 (P1); TU0-1190 (P2) | b0201 - b0205 | rrsH-ileV-alaV-rrlH-rrfH | forward | 223,771 - 228,875 | 3.15 | 1.72 | 1.62 | 1.93 | 2.45 | 4.00 |
| TU0-1187 (P1); TU0-1188 (P2) | b2588 - b2591 | rrsG-gltW-rrlG-rrfG | complement | 2,727,638 - 2,724,210 | 2.81 | 1.62 | 1.54 | 1.80 | 2.25 | 3.49 |
| TU0-1191 (P1); TU0-1192 (P2) | b3272 - b3278 | rrsD-ileU-alaU-rrlD-rrfD-thrV-rrfF | complement | 3,425,243 - 3,421,564 | 3.79 | 1.90 | 1.77 | 2.15 | 2.84 | 5.02 |
| TU0-1183 (P1); TU0-1184 (P2) | b3756 - b3759 | rrsC-gltU-rrlC-rrfC | forward | 3,939,831 – 3,944,842 | 4.67 | 2.12 | 1.95 | 2.42 | 3.35 | 6.48 |

**Conversion of microarray data and mRNA half life times into constraints of mRNA degradation reactions**

The microarray data were preprocessed to convert the log_2_ value for ever gene into molecules per cell. Therefore, the relative signal, s_i_, was calculated and multiplied by the total number of mRNA per cell at the corresponding doubling time. In the case of 30 minutes doubling time, the total number was mRNA molecules was reported to be 4600 [83].

where y is the doubling time in minutes, z is the cell mass.

Information for half-life time ($T_{1/2,i}$) and microarray data (s_i_) were taken from supplemental data from Bon *et al*. [83].

**Exchange constraints**

Neidhardt *et al* reported the amino acid and nucleotide triphosphate composition for an *E. coli* cell at 40 minutes doubling time [84]. Furthermore, these values are generally incorporated into the biomass function in the metabolic network of *E. coli* [2,81]. These values were taken to calculate the fractional distribution of the metabolites and then scaled by the protein and RNA concentration at a given doubling time:

| **Metabolite** | **mmol/g_DW_ (from** [84]**)** | **sum** | **Fraction of each metabolite** | **Uptake rate per doubling time (µmol/gDW T_D_)** | | | | | |
| --- | --- | --- | --- | --- | --- | --- | --- | --- | --- |
| T_D_ (min) |  |  |  | 90 | 30 | 100 | 60 | 40 | 24 |
| atp | 0.165 | 0.63 | -26.19 | -10.86 | -16.55 | -10.87 | -12.31 | -14.36 | -19.61 |
| ctp | 0.126 |  | -0.20 | -0.08 | -0.13 | -0.08 | -0.09 | -0.11 | -0.15 |
| gtp | 0.203 |  | -32.22 | -13.36 | -20.37 | -13.38 | -15.14 | -17.67 | -24.12 |
| utp | 0.136 |  | -0.22 | -0.09 | -0.14 | -0.09 | -0.10 | -0.12 | -0.16 |
| ala-L | 0.488 | 5.081 | -0.10 | -0.58 | -0.47 | -0.60 | -0.54 | -0.48 | -0.46 |
| arg-L | 0.281 |  | -0.06 | -0.34 | -0.27 | -0.35 | -0.31 | -0.28 | -0.27 |
| asn-L | 0.229 |  | -0.05 | -0.27 | -0.22 | -0.28 | -0.25 | -0.22 | -0.22 |
| asp-L | 0.229 |  | -0.05 | -0.27 | -0.22 | -0.28 | -0.25 | -0.22 | -0.22 |
| cys-L | 0.087 |  | -0.02 | -0.10 | -0.08 | -0.11 | -0.10 | -0.09 | -0.08 |
| gln-L | 0.25 |  | -0.05 | -0.30 | -0.24 | -0.31 | -0.28 | -0.25 | -0.24 |
| glu-L | 0.25 |  | -0.05 | -0.30 | -0.24 | -0.31 | -0.28 | -0.25 | -0.24 |
| gly | 0.582 |  | -0.11 | -0.69 | -0.56 | -0.72 | -0.64 | -0.57 | -0.55 |
| his-L | 0.09 |  | -0.02 | -0.11 | -0.09 | -0.11 | -0.10 | -0.09 | -0.09 |
| ile-L | 0.276 |  | -0.05 | -0.33 | -0.27 | -0.34 | -0.30 | -0.27 | -0.26 |
| leu-L | 0.428 |  | -0.08 | -0.51 | -0.41 | -0.53 | -0.47 | -0.42 | -0.40 |
| lys-L | 0.326 |  | -0.06 | -0.39 | -0.31 | -0.40 | -0.36 | -0.32 | -0.31 |
| met-L | 0.146 |  | -0.03 | -0.17 | -0.14 | -0.18 | -0.16 | -0.14 | -0.14 |
| phe-L | 0.176 |  | -0.03 | -0.21 | -0.17 | -0.22 | -0.19 | -0.17 | -0.17 |
| pro-L | 0.21 |  | -0.04 | -0.25 | -0.20 | -0.26 | -0.23 | -0.21 | -0.20 |
| ser-L | 0.205 |  | -0.04 | -0.24 | -0.20 | -0.25 | -0.23 | -0.20 | -0.19 |
| thr-L | 0.241 |  | -0.05 | -0.29 | -0.23 | -0.30 | -0.27 | -0.24 | -0.23 |
| trp-L | 0.054 |  | -0.01 | -0.06 | -0.05 | -0.07 | -0.06 | -0.05 | -0.05 |
| tyr-L | 0.131 |  | -0.03 | -0.16 | -0.13 | -0.16 | -0.14 | -0.13 | -0.12 |
| val-L | 0.402 |  | -0.08 | -0.48 | -0.39 | -0.50 | -0.44 | -0.39 | -0.38 |

The following equations were used:

The number of was reported in (Table 2 in [82], see also below) and was given in at a specific doubling time.

The number of was reported in (Table 2 in [82], see also below) and was given in at a specific doubling time.

Conversion from to (the amino acids conversion can be done similarly):

Where *z* is the cell mass and *x* is the number of nucleotide residues per cell.

For every nucleotide triphosphate species the corresponding fraction was multiplied by the RNA factor for each doubling time:

| doubling time (min) | protein (aa residues per cell) | protein (µmol aa /gDW) | RNA (nt per cell) | RNA (µmol nt /gDW) | µg DW mass/10^9 cell |
| --- | --- | --- | --- | --- | --- |
| 100 | 5.60E+08 | 6.28E+03 | 37000000 | 415.13 | 148 |
| 60 | 8.70E+08 | 5.60E+03 | 73000000 | 469.84 | 258 |
| 40 | 1.30E+09 | 4.99E+03 | 143000000 | 548.40 | 433 |
| 30 | 1.89E+09 | 4.90E+03 | 244000000 | 632.09 | 641 |
| 24 | 2.50E+09 | 4.80E+03 | 390000000 | 748.68 | 865 |
| 90 | 5.94E+08 | 6.07E+03 | 40609460 | 414.58 | 162.65 |

**References**

1. Karp PD, Arnaud M, Collado-Vides J, Ingraham J, Paulsen IT, et al. (2004) The *E. coli* EcoCyc Database: No Longer Just a Metabolic Pathway Database. ASM News 70: 25-30.

2. Feist AM, Henry CS, Reed JL, Krummenacker M, Joyce AR, et al. (2007) A genome-scale metabolic reconstruction for *Escherichia coli* K-12 MG1655 that accounts for 1260 ORFs and thermodynamic information. Mol Syst Biol 3.

3. Reed JL, Famili I, Thiele I, Palsson BO (2006) Towards multidimensional genome annotation. Nat Rev Genet 7: 130-141.

4. Neidhardt FC, editor (1996) *Escherichia coli* and *Salmonella*: cellular and molecular biology. 2nd ed. Washington, D.C.: ASM Press. 2 v. (xx, 2822 , lxxvii) p.

5. Blattner FR, Plunkett G, 3rd, Bloch CA, Perna NT, Burland V, et al. (1997) The complete genome sequence of *Escherichia coli* K-12. Science 277: 1453-1474.

6. Riley M, Abe T, Arnaud MB, Berlyn MK, Blattner FR, et al. (2006) *Escherichia coli* K-12: a cooperatively developed annotation snapshot-2005. Nucleic Acids Res 34: 1-9.

7. Sundararaj S, Guo A, Habibi-Nazhad B, Rouani M, Stothard P, et al. (2004) The CyberCell Database (CCDB): a comprehensive, self-updating, relational database to coordinate and facilitate in silico modeling of Escherichia coli. Nucleic Acids Res 32: D293-295.

8. Sprinzl M, Vassilenko KS (2005) Compilation of tRNA sequences and sequences of tRNA genes. Nucleic Acids Res 33: D139-140.

9. Edwards JS, Covert M, Palsson B (2002) Metabolic modeling of microbes: the flux-balance approach. Environmental Microbiology 4: 133-140.

10. Varma A, Palsson BO (1994) Metabolic Flux Balancing: Basic concepts, Scientific and Practical Use. Nat Biotechnol 12: 994-998.

11. Li Z, Deutscher MP (1994) The role of individual exoribonucleases in processing at the 3' end of Escherichia coli tRNA precursors. J Biol Chem 269: 6064-6071.

12. Symmons MF, Williams MG, Luisi BF, Jones GH, Carpousis AJ (2002) Running rings around RNA: a superfamily of phosphate-dependent RNases. Trends Biochem Sci 27: 11-18.

13. Ost KA, Deutscher MP (1990) RNase PH catalyzes a synthetic reaction, the addition of nucleotides to the 3' end of RNA. Biochimie 72: 813-818.

14. Patel AM, Dunn SD (1992) RNase E-dependent cleavages in the 5' and 3' regions of the Escherichia coli unc mRNA. J Bacteriol 174: 3541-3548.

15. Li Y, Altman S (2003) A specific endoribonuclease, RNase P, affects gene expression of polycistronic operon mRNAs. Proc Natl Acad Sci U S A 100: 13213-13218.

16. Regnier P, Grunberg-Manago M (1989) Cleavage by RNase III in the transcripts of the met Y-nus-A-infB operon of Escherichia coli releases the tRNA and initiates the decay of the downstream mRNA. J Mol Biol 210: 293-302.

17. Das A, Yanofsky C (1989) Restoration of a translational stop-start overlap reinstates translational coupling in a mutant trpB'-trpA gene pair of the Escherichia coli tryptophan operon. Nucleic Acids Res 17: 9333-9340.

18. Roa BB, Connolly DM, Winkler ME (1989) Overlap between pdxA and ksgA in the complex pdxA-ksgA-apaG-apaH operon of Escherichia coli K-12. J Bacteriol 171: 4767-4777.

19. Aksoy S, Squires CL, Squires C (1984) Translational coupling of the trpB and trpA genes in the Escherichia coli tryptophan operon. J Bacteriol 157: 363-367.

20. Oppenheim DS, Yanofsky C (1980) Translational coupling during expression of the tryptophan operon of Escherichia coli. Genetics 95: 785-795.

21. Normark S, Bergstrom S, Edlund T, Grundstrom T, Jaurin B, et al. (1983) Overlapping genes. Annu Rev Genet 17: 499-525.

22. Bylund GO, Wipemo LC, Lundberg LA, Wikstrom PM (1998) RimM and RbfA are essential for efficient processing of 16S rRNA in Escherichia coli. J Bacteriol 180: 73-82.

23. Li Z, Pandit S, Deutscher MP (1998) 3' exoribonucleolytic trimming is a common feature of the maturation of small, stable RNAs in Escherichia coli. Proc Natl Acad Sci U S A 95: 2856-2861.

24. Carpousis AJ (2002) The Escherichia coli RNA degradosome: structure, function and relationship in other ribonucleolytic multienzyme complexes. Biochem Soc Trans 30: 150-155.

25. Coburn GA, Miao X, Briant DJ, Mackie GA (1999) Reconstitution of a minimal RNA degradosome demonstrates functional coordination between a 3' exonuclease and a DEAD-box RNA helicase. Genes Dev 13: 2594-2603.

26. Vanzo NF, Li YS, Py B, Blum E, Higgins CF, et al. (1998) Ribonuclease E organizes the protein interactions in the Escherichia coli RNA degradosome. Genes Dev 12: 2770-2781.

27. Khemici V, Toesca I, Poljak L, Vanzo NF, Carpousis AJ (2004) The RNase E of Escherichia coli has at least two binding sites for DEAD-box RNA helicases: functional replacement of RhlB by RhlE. Mol Microbiol 54: 1422-1430.

28. Neidhardt FC (1964) The regulation RNA synthesis in bacteria. Prog Nucleic Acid Res Mol Biol 3: 145-181.

29. Ben-Hamida F, Schlessinger D (1966) Synthesis and breakdown of ribonucleic acid in Escherichia coli starving for nitrogen. Biochim Biophys Acta 119: 183-191.

30. Bessarab DA, Kaberdin VR, Wei CL, Liou GG, Lin-Chao S (1998) RNA components of Escherichia coli degradosome: evidence for rRNA decay. Proc Natl Acad Sci U S A 95: 3157-3161.

31. Ghosh S, Deutscher MP (1999) Oligoribonuclease is an essential component of the mRNA decay pathway. Proc Natl Acad Sci U S A 96: 4372-4377.

32. Fiedler TJ, Vincent HA, Zuo Y, Gavrialov O, Malhotra A (2004) Purification and crystallization of Escherichia coli oligoribonuclease. Acta Crystallogr D Biol Crystallogr 60: 736-739.

33. Subbarayan PR, Deutscher MP (2001) Escherichia coli RNase M is a multiply altered form of RNase I. Rna 7: 1702-1707.

34. Otsuka Y, Yonesaki T (2005) A novel endoribonuclease, RNase LS, in Escherichia coli. Genetics 169: 13-20.

35. Zuo Y, Deutscher MP (1999) The DNase activity of RNase T and its application to DNA cloning. Nucleic Acids Res 27: 4077-4082.

36. Cheng ZF, Deutscher MP (2005) An important role for RNase R in mRNA decay. Mol Cell 17: 313-318.

37. Chen C, Deutscher MP (2005) Elevation of RNase R in response to multiple stress conditions. J Biol Chem 280: 34393-34396.

38. Deutscher MP (2006) Degradation of RNA in bacteria: comparison of mRNA and stable RNA. Nucleic Acids Res 34: 659-666.

39. Hajnsdorf E, Regnier P (1999) E. coli RpsO mRNA decay: RNase E processing at the beginning of the coding sequence stimulates poly(A)-dependent degradation of the mRNA. J Mol Biol 286: 1033-1043.

40. Jain C (2002) Degradation of mRNA in Escherichia coli. IUBMB Life 54: 315-321.

41. Sarkar N (1997) Polyadenylation of mRNA in prokaryotes. Annu Rev Biochem 66: 173-197.

42. Kang CW, Cantor CR (1985) Structure of ribosome-bound messenger RNA as revealed by enzymatic accessibility studies. J Mol Biol 181: 241-251.

43. Berman HM, Westbrook J, Feng Z, Gilliland G, Bhat TN, et al. (2000) The Protein Data Bank. Nucleic Acids Res 28: 235-242.

44. Wilson CJ, Apiyo D, Wittung-Stafshede P (2004) Role of cofactors in metalloprotein folding. Q Rev Biophys 37: 285-314.

45. Hartl FU, Hayer-Hartl M (2002) Molecular chaperones in the cytosol: from nascent chain to folded protein. Science 295: 1852-1858.

46. Ranson NA, Clare DK, Farr GW, Houldershaw D, Horwich AL, et al. (2006) Allosteric signaling of ATP hydrolysis in GroEL-GroES complexes. Nat Struct Mol Biol 13: 147-152.

47. Martin J, Langer T, Boteva R, Schramel A, Horwich AL, et al. (1991) Chaperonin-mediated protein folding at the surface of groEL through a 'molten globule'-like intermediate. Nature 352: 36-42.

48. Deuerling E, Patzelt H, Vorderwulbecke S, Rauch T, Kramer G, et al. (2003) Trigger Factor and DnaK possess overlapping substrate pools and binding specificities. Mol Microbiol 47: 1317-1328.

49. Kerner MJ, Naylor DJ, Ishihama Y, Maier T, Chang HC, et al. (2005) Proteome-wide analysis of chaperonin-dependent protein folding in Escherichia coli. Cell 122: 209-220.

50. Beinert H, Holm RH, Munck E (1997) Iron-sulfur clusters: nature's modular, multipurpose structures. Science 277: 653-659.

51. Agar JN, Krebs C, Frazzon J, Huynh BH, Dean DR, et al. (2000) IscU as a scaffold for iron-sulfur cluster biosynthesis: sequential assembly of [2Fe-2S] and [4Fe-4S] clusters in IscU. Biochemistry 39: 7856-7862.

52. Mansy SS, Cowan JA (2004) Iron-sulfur cluster biosynthesis: toward an understanding of cellular machinery and molecular mechanism. Acc Chem Res 37: 719-725.

53. Johnson DC, Dean DR, Smith AD, Johnson MK (2005) Structure, function, and formation of biological iron-sulfur clusters. Annu Rev Biochem 74: 247-281.

54. Yang J, Bitoun JP, Ding H (2006) Interplay of IscA and IscU in biogenesis of iron-sulfur clusters. J Biol Chem.

55. Adinolfi S, Rizzo F, Masino L, Nair M, Martin SR, et al. (2004) Bacterial IscU is a well folded and functional single domain protein. Eur J Biochem 271: 2093-2100.

56. Nuth M, Yoon T, Cowan JA (2002) Iron-sulfur cluster biosynthesis: characterization of iron nucleation sites for assembly of the [2Fe-2S]2+ cluster core in IscU proteins. J Am Chem Soc 124: 8774-8775.

57. Kato S, Mihara H, Kurihara T, Takahashi Y, Tokumoto U, et al. (2002) Cys-328 of IscS and Cys-63 of IscU are the sites of disulfide bridge formation in a covalently bound IscS/IscU complex: implications for the mechanism of iron-sulfur cluster assembly. Proc Natl Acad Sci U S A 99: 5948-5952.

58. Kurihara T, Mihara H, Kato S, Yoshimura T, Esaki N (2003) Assembly of iron-sulfur clusters mediated by cysteine desulfurases, IscS, CsdB and CSD, from Escherichia coli. Biochim Biophys Acta 1647: 303-309.

59. Hoff KG, Cupp-Vickery JR, Vickery LE (2003) Contributions of the LPPVK motif of the iron-sulfur template protein IscU to interactions with the Hsc66-Hsc20 chaperone system. J Biol Chem 278: 37582-37589.

60. Silberg JJ, Tapley TL, Hoff KG, Vickery LE (2004) Regulation of the HscA ATPase reaction cycle by the co-chaperone HscB and the iron-sulfur cluster assembly protein IscU. J Biol Chem 279: 53924-53931.

61. Takahashi Y, Nakamura M (1999) Functional assignment of the ORF2-iscS-iscU-iscA-hscB-hscA-fdx-ORF3 gene cluster involved in the assembly of Fe-S clusters in Escherichia coli. J Biochem (Tokyo) 126: 917-926.

62. Zheng L, Cash VL, Flint DH, Dean DR (1998) Assembly of iron-sulfur clusters. Identification of an iscSUA-hscBA-fdx gene cluster from Azotobacter vinelandii. J Biol Chem 273: 13264-13272.

63. Aslund F, Beckwith J (1999) The thioredoxin superfamily: redundancy, specificity, and gray-area genomics. J Bacteriol 181: 1375-1379.

64. Gralnick JA, Downs DM (2003) The YggX protein of Salmonella enterica is involved in Fe(II) trafficking and minimizes the DNA damage caused by hydroxyl radicals: residue CYS-7 is essential for YggX function. J Biol Chem 278: 20708-20715.

65. Gralnick J, Downs D (2001) Protection from superoxide damage associated with an increased level of the YggX protein in Salmonella enterica. Proc Natl Acad Sci U S A 98: 8030-8035.

66. Osborne MJ, Siddiqui N, Landgraf D, Pomposiello PJ, Gehring K (2005) The solution structure of the oxidative stress-related protein YggX from Escherichia coli. Protein Sci 14: 1673-1678.

67. Adinolfi S, Trifuoggi M, Politou AS, Martin S, Pastore A (2002) A structural approach to understanding the iron-binding properties of phylogenetically different frataxins. Hum Mol Genet 11: 1865-1877.

68. Bou-Abdallah F, Lewin AC, Le Brun NE, Moore GR, Chasteen ND (2002) Iron detoxification properties of Escherichia coli bacterioferritin. Attenuation of oxyradical chemistry. J Biol Chem 277: 37064-37069.

69. Li DS, Ohshima K, Jiralerspong S, Bojanowski MW, Pandolfo M (1999) Knock-out of the cyaY gene in Escherichia coli does not affect cellular iron content and sensitivity to oxidants. FEBS Lett 456: 13-16.

70. Duby G, Foury F, Ramazzotti A, Herrmann J, Lutz T (2002) A non-essential function for yeast frataxin in iron-sulfur cluster assembly. Hum Mol Genet 11: 2635-2643.

71. Vivas E, Skovran E, Downs DM (2006) Salmonella enterica strains lacking the frataxin homolog CyaY show defects in Fe-S cluster metabolism in vivo. J Bacteriol 188: 1175-1179.

72. Layer G, Ollagnier-de Choudens S, Sanakis Y, Fontecave M (2006) Iron-sulfur cluster biosynthesis: characterization of Escherichia coli CYaY as an iron donor for the assembly of [2Fe-2S] clusters in the scaffold IscU. J Biol Chem 281: 16256-16263.

73. Ding B, Smith ES, Ding H (2005) Mobilization of the iron centre in IscA for the iron-sulphur cluster assembly in IscU. Biochem J 389: 797-802.

74. Smith AD, Agar JN, Johnson KA, Frazzon J, Amster IJ, et al. (2001) Sulfur transfer from IscS to IscU: the first step in iron-sulfur cluster biosynthesis. J Am Chem Soc 123: 11103-11104.

75. Urbina HD, Silberg JJ, Hoff KG, Vickery LE (2001) Transfer of sulfur from IscS to IscU during Fe/S cluster assembly. J Biol Chem 276: 44521-44526.

76. Limbach PA, Crain PF, McCloskey JA (1994) Summary: the modified nucleosides of RNA. Nucleic Acids Res 22: 2183-2196.

77. Rozenski J, Crain PF, McCloskey JA (1999) The RNA Modification Database: 1999 update. Nucleic Acids Res 27: 196-197.

78. McCloskey JA, Rozenski J (2005) The Small Subunit rRNA Modification Database. Nucleic Acids Res 33: D135-138.

79. Duester GL, Holmes WM (1980) The distal end of the ribosomal RNA operon rrnD of Escherichia coli contains a tRNA1thr gene, two 5s rRNA genes and a transcription terminator. Nucleic Acids Res 8: 3793-3807.

80. Palsson BO (2006) Systems biology: properties of reconstructed networks. New York: Cambridge University Press.

81. Reed JL, Vo TD, Schilling CH, Palsson BO (2003) An expanded genome-scale model of *Escherichia coli* K-12 (*i*JR904 GSM/GPR). Genome Biology 4: R54.51-R54.12.

82. Neidhardt FC (1996) *Escherichia coli* and *Salmonella*: cellular and molecular biology. 2nd ed. Washington, D.C.: ASM Press. pp. x to y.

83. Bon M, McGowan SJ, Cook PR (2006) Many expressed genes in bacteria and yeast are transcribed only once per cell cycle. Faseb J 20: 1721-1723.

84. Neidhardt FC, Ingraham JL, Schaechter M (1990) Physiology of the bacterial cell: a molecular approach. Sunderland, Mass.: Sinauer Associates. xii, 506 p.
