# Supplementary material for: Genome-Scale Reconstruction of Escherichia coli's Transcriptional and Translational Machinery: A Knowledge Base, Its Mathematical Formulation, and Its Functional Characterization
Source: PLoS Comput Biol. 2009 Mar 13;5(3):e1000312. doi: 10.1371/journal.pcbi.1000312 (PMC2648898; doi:10.1371/journal.pcbi.1000312)
Supplement: Table S12 — Complete model reaction list and flux variability (FVA) results (1.75 MB PDF) [file pcbi.1000312.s014.pdf]

Table S12 Reaction List+FVA results

Unit for all flux values: **nmol/g dry weight per 30 min doubling time**

					LB medium constraints (all) (30 min doubling time)		Flux Variability Results -Only Exchange Reactions were constraint			Flux Variability Results -Exchange Reactions + stable RNA transcription were constraint			Flux Variability Results -Exchange Reactions + stable RNA transcription + mRNA degradation were constraint		
Reaction Abbreviation	Reaction Name	Reaction	Direction	Subsystem	Lower Bound	Upper Bound	Minimum Flux Value	Maximum Flux Value	Flux Span	Minimum Flux Value	Maximum Flux Value	Flux Span	Minimum Flux Value	Maximum Flux Value	Flux Span
10FTHFt	10-Formyltetrahydrofolate transport	1 10fthf[e] <==> 1 10fthf	reversible	Transport	-1.00E+09	1.00E+09	5.23E+03	2.30E+04	1.78E+04	3.85E+03	2.37E+04	19852.92	3.85E+03	2.15E+04	17644.16
16S_rRNA_FORM1	16S rRNA formation (generic 16S rRNA from rrsA)	1 rrsA --> 1 16S_rRNA	irreversible	rRNA formation	0.00E+00	1.00E+09	0	1.12E+02	1.12E+02	0	1.36E+01	13.6	0	1.36E+01	13.6
16S_rRNA_FORM2	16S rRNA formation (generic 16S rRNA from rrsB)	1 rrsB --> 1 16S_rRNA	irreversible	rRNA formation	0.00E+00	1.00E+09	0	1.11E+02	1.11E+02	0	1.29E+01	12.9	0	1.29E+01	12.9
16S_rRNA_FORM3	16S rRNA formation (generic 16S rRNA from rrsC)	1 rrsC --> 1 16S_rRNA	irreversible	rRNA formation	0.00E+00	1.00E+09	0	1.12E+02	1.12E+02	0	1.42E+01	14.2	0	1.42E+01	14.2
16S_rRNA_FORM4	16S rRNA formation (generic 16S rRNA from rrsD)	1 rrsD --> 1 16S_rRNA	irreversible	rRNA formation	0.00E+00	1.00E+09	0	1.06E+02	1.06E+02	0	1.15E+01	11.5	0	1.15E+01	11.5
16S_rRNA_FORM5	16S rRNA formation (generic 16S rRNA from rrsE)	1 rrsE --> 1 16S_rRNA	irreversible	rRNA formation	0.00E+00	1.00E+09	0	1.13E+02	1.13E+02	0	1.26E+01	12.6	0	1.26E+01	12.6
16S_rRNA_FORM6	16S rRNA formation (generic 16S rRNA from rrsG)	1 rrsG --> 1 16S_rRNA	irreversible	rRNA formation	0.00E+00	1.00E+09	0	1.11E+02	1.11E+02	0	8.54E+00	8.54	0	8.54E+00	8.54
16S_rRNA_FORM7	16S rRNA formation (generic 16S rRNA from rrsH)	1 rrsH --> 1 16S_rRNA	irreversible	rRNA formation	0.00E+00	1.00E+09	0	1.11E+02	1.11E+02	0	9.54E+00	9.54	0	9.54E+00	9.54
23S_rRNA_FORM1	23S rRNA formation (generic 23S rRNA from rrlA)	1 rrlA --> 1 23S_rRNA	irreversible	rRNA formation	0.00E+00	1.00E+09	0	1.12E+02	1.12E+02	0	1.36E+01	13.6	0	1.36E+01	13.6
23S_rRNA_FORM2	23S rRNA formation (generic 23S rRNA from rrlB)	1 rrlB --> 1 23S_rRNA	irreversible	rRNA formation	0.00E+00	1.00E+09	0	1.11E+02	1.11E+02	0	1.29E+01	12.9	0	1.29E+01	12.9
23S_rRNA_FORM3	23S rRNA formation (generic 23S rRNA from rrlC)	1 rrlC --> 1 23S_rRNA	irreversible	rRNA formation	0.00E+00	1.00E+09	0	1.12E+02	1.12E+02	0	1.42E+01	14.2	0	1.42E+01	14.2
23S_rRNA_FORM4	23S rRNA formation (generic 23S rRNA from rrlD)	1 rrlD --> 1 23S_rRNA	irreversible	rRNA formation	0.00E+00	1.00E+09	0	1.06E+02	1.06E+02	0	1.15E+01	11.5	0	1.15E+01	11.5
23S_rRNA_FORM5	23S rRNA formation (generic 23S rRNA from rrlE)	1 rrlE --> 1 23S_rRNA	irreversible	rRNA formation	0.00E+00	1.00E+09	0	1.13E+02	1.13E+02	0	1.26E+01	12.6	0	1.26E+01	12.6
23S_rRNA_FORM6	23S rRNA formation (generic 23S rRNA from rrlG)	1 rrlG --> 1 23S_rRNA	irreversible	rRNA formation	0.00E+00	1.00E+09	0	1.11E+02	1.11E+02	0	8.54E+00	8.54	0	8.54E+00	8.54
23S_rRNA_FORM7	23S rRNA formation (generic 23S rRNA from rrlH)	1 rrlH --> 1 23S_rRNA	irreversible	rRNA formation	0.00E+00	1.00E+09	0	1.11E+02	1.11E+02	0	9.54E+00	9.54	0	9.54E+00	9.54
5FTHFt	transport of 5fthf	1 5fthf <==> 1 5fthf[e]	reversible	Transport	-1.00E+09	1.00E+09	-1.86E+03	0	1.86E+03	-1.09E+02	-2.75E+01	81.96	-1.09E+02	-2.75E+01	81.96
5MTAt	transport of 5mta	1 5mta <==> 1 5mta[e]	reversible	Transport	-1.00E+09	1.00E+09	0	1.81E+03	1.81E+03	1.39E+01	1.83E+02	169.4	1.39E+01	1.83E+02	169.4
5S_rRNA_FORM1	5S rRNA formation (generic 5S rRNA from rrfA)	1 rrfA --> 1 5S_rRNA	irreversible	rRNA formation	0.00E+00	1.00E+09	0	1.12E+02	1.12E+02	0	1.36E+01	13.6	0	1.36E+01	13.6
5S_rRNA_FORM2	5S rRNA formation (generic 5S rRNA from rrfB)	1 rrfB --> 1 5S_rRNA	irreversible	rRNA formation	0.00E+00	1.00E+09	0	1.11E+02	1.11E+02	0	1.29E+01	12.9	0	1.29E+01	12.9
5S_rRNA_FORM3	5S rRNA formation (generic 5S rRNA from rrfC)	1 rrfC --> 1 5S_rRNA	irreversible	rRNA formation	0.00E+00	1.00E+09	0	1.12E+02	1.12E+02	0	1.42E+01	14.2	0	1.42E+01	14.2
5S_rRNA_FORM4	5S rRNA formation (generic 5S rRNA from rrfD)	1 rrfD --> 1 5S_rRNA	irreversible	rRNA formation	0.00E+00	1.00E+09	0	0	0.00E+00	0	0	0	0	0	0
5S_rRNA_FORM5	5S rRNA formation (generic 5S rRNA from rrfE)	1 rrfE --> 1 5S_rRNA	irreversible	rRNA formation	0.00E+00	1.00E+09	0	1.13E+02	1.13E+02	0	1.26E+01	12.6	0	1.26E+01	12.6
5S_rRNA_FORM6	5S rRNA formation (generic 5S rRNA from rrfF)	1 rrfF --> 1 5S_rRNA	irreversible	rRNA formation	0.00E+00	1.00E+09	0	1.06E+02	1.06E+02	0	1.15E+01	11.5	0	1.15E+01	11.5
5S_rRNA_FORM7	5S rRNA formation (generic 5S rRNA from rrfG)	1 rrfG --> 1 5S_rRNA	irreversible	rRNA formation	0.00E+00	1.00E+09	0	1.11E+02	1.11E+02	0	8.54E+00	8.54	0	8.54E+00	8.54
5S_rRNA_FORM8	5S rRNA formation (generic 5S rRNA from rrfH)	1 rrfH --> 1 5S_rRNA	irreversible	rRNA formation	0.00E+00	1.00E+09	0	1.11E+02	1.11E+02	0	9.54E+00	9.54	0	9.54E+00	9.54
ACCOAt	transport of accoa	1 accoa <==> 1 accoa[e]	reversible	Transport	-1.00E+09	1.00E+09	-5.24E+03	-3.38E+02	4.90E+03	-5.59E+03	-2.49E+02	5343.775	-2.91E+03	-2.49E+02	2664.524
AHCYSt	S-adenosyl-L-homocysteine exchange	1 ahcys <==> 1 ahcys[e]	reversible	Transport	-1.00E+09	1.00E+09	3.88E+03	1.32E+04	9.34E+03	2.89E+03	1.03E+04	7399.09	2.89E+03	8.54E+03	5652.935
ALAt5r	L-alanine transport via diffusion	1 ala-L[e] <==> 1 ala-L	reversible	Transport	-1.00E+09	1.00E+09	8.26E+04	4.70E+05	3.88E+05	6.22E+04	4.70E+05	408040.84	6.25E+04	4.29E+05	366966.42
AMETt	S-adenosyl-L-methionine exchange	1 amet <==> 1 amet[e]	reversible	Transport	-1.00E+09	1.00E+09	-1.46E+04	-3.88E+03	1.07E+04	-1.07E+04	-2.90E+03	7840.78	-8.90E+03	-2.90E+03	5993.993
ARGt5r	L-arginine transport via diffusion	1 arg-L[e] <==> 1 arg-L	reversible	Transport	-1.00E+09	1.00E+09	6.29E+04	2.71E+05	2.08E+05	4.63E+04	2.71E+05	224466.7	4.63E+04	2.71E+05	224466.7
ASNt5r	L-asparagine transport via diffusion	1 asn-L[e] <==> 1 asn-L	reversible	Transport	-1.00E+09	1.00E+09	2.64E+04	1.77E+05	1.50E+05	1.95E+04	1.88E+05	168543.42	1.95E+04	1.62E+05	142880.92
ASPt5r	L-aspartate transport via diffusion	1 asp-L[e] <==> 1 asp-L	reversible	Transport	-1.00E+09	1.00E+09	3.55E+04	2.21E+05	1.85E+05	2.62E+04	2.21E+05	194498.54	2.62E+04	2.21E+05	194498.54
AlaS_RS_TETRA	formation of alanyl-tRNA synthetase (homotetramer)	4 AlaS_mono <==> 1 Ala_RS_tetra	reversible	Protein complex formation	-1.00E+09	1.00E+09	0	6.64E+02	6.64E+02	0	7.70E+02	770.0907	0	3.14E+01	31.35225
Ala_RS_CHARG	Alanyl-tRNA synthetase charging	1 Ala_RS_tetra + 1 ala-L + 1 atp + 1 h2o --> 1 Ala_RS_ala_amp + 1 h + 1 ppi	irreversible	Aminoacyl-tRNA synthetase charging	0.00E+00	1.00E+09	8.58E+04	4.74E+05	3.88E+05	6.32E+04	4.79E+05	415453.04	6.32E+04	4.33E+05	369977.64
Arg_RS_CHARG	Arginyl-tRNA synthetase charging	1 ArgS_mono + 1 arg-L + 1 atp + 1 h2o --> 1 Arg_RS_arg_amp + 1 h + 1 ppi	irreversible	Aminoacyl-tRNA synthetase charging	0.00E+00	1.00E+09	6.29E+04	2.75E+05	2.12E+05	4.63E+04	2.77E+05	230699.9	4.63E+04	2.73E+05	226306.7
AsnS_RS_DIM	dimerization of Asparaginyl-tRNA synthetase	2 AsnS_mono <==> 1 Asn_RS_dim	reversible	Protein complex formation	-1.00E+09	1.00E+09	0	1.29E+03	1.29E+03	0	2.23E+03	2233.246	0	7.34E+01	73.359
Asn_RS_CHARG	Asparaginyl-tRNA synthetase charging	1 Asn_RS_dim + 1 asn-L + 1 atp + 1 h2o --> 1 Asn_RS_asn_amp + 1 h + 1 ppi	irreversible	Aminoacyl-tRNA synthetase charging	0.00E+00	1.00E+09	2.64E+04	1.77E+05	1.51E+05	1.95E+04	1.89E+05	169232.42	1.95E+04	1.63E+05	143663.82
AspS_RS_DIM	dimerization of Aspartyl-tRNA synthetase	2 AspS_mono <==> 1 Asp_RS_dim	reversible	Protein complex formation	-1.00E+09	1.00E+09	0	1.24E+03	1.24E+03	0	2.11E+03	2114.115	0	3.93E+01	39.338
Asp_RS_CHARGa	Aspartyl-tRNA synthetase charging	1 Asp_RS_dim + 2 asp-L + 2 atp + 2 h2o --> 1 Asp_RS_2asp_2amp + 2 h + 2 ppi	irreversible	Aminoacyl-tRNA synthetase charging	0.00E+00	1.00E+09	0	1.10E+05	1.10E+05	0	1.11E+05	110520.4	0	1.10E+05	110377.2
Asp_RS_CHARGb	Aspartyl-tRNA synthetase charging	1 Asp_RS_dim + 1 asp-L + 1 atp + 1 h2o --> 1 Asp_RS_asp_amp + 1 h + 1 ppi	irreversible	Aminoacyl-tRNA synthetase charging	0.00E+00	1.00E+09	0	2.21E+05	2.21E+05	0	2.21E+05	221040.8	0	2.21E+05	220754.4
C10H8O5t	transport of C10H8O5	1 C10H8O5 <==> 1 C10H8O5[e]	reversible	Transport	-1.00E+09	1.00E+09	0	1.38E+03	1.38E+03	1.39E+01	9.38E+01	79.83	1.39E+01	9.38E+01	79.83
C9H9O4t	transport of C9H9O4	1 C9H9O4 <==> 1 C9H9O4[e]	reversible	Transport	-1.00E+09	1.00E+09	0	1.38E+03	1.38E+03	1.39E+01	9.38E+01	79.83	1.39E+01	9.38E+01	79.83
CYStec	L-cysteine transport via diffusion (extracellular to cytosol)	1 cys-L[e] <==> 1 cys-L	reversible	Transport	-1.00E+09	1.00E+09	3.46E+03	7.34E+04	6.99E+04	2.58E+03	8.38E+04	81258.46	2.58E+03	5.79E+04	55298.11
Cys_RS_CHARG	Cysteinyl-tRNA synthetase charging	1 CysS_mono + 1 atp + 1 cys-L + 1 h2o --> 1 Cys_RS_cys_amp + 1 h + 1 ppi	irreversible	Aminoacyl-tRNA synthetase charging	0.00E+00	1.00E+09	3.38E+03	6.85E+04	6.51E+04	2.49E+03	7.80E+04	75550.35	2.49E+03	5.61E+04	53616.26
DATYRDTRNA1	deacylation of tyr1_tRNA_tyr-D	1 Dtd_dim + 1 atp + 1 tyr1_tRNA_tyr-D --> 1 Dtd_dim_tyr	irreversible	tRNA charging	0.00E+00	1.00E+09	0	7.10E+06	7.10E+06	0	7.41E+06	7412071	0	7.41E+06	7412071
DATYRDTRNA2	deacylation of tyr1_tRNA_tyr-D	1 Dtd_dim_tyr + 1 h2o --> 1 Dtd_dim_inact + 1 adp + 1 h + 1 pi + 1 tyr-D + 1 tyr1_tRNA	irreversible	tRNA charging	0.00E+00	1.00E+09	0	7.10E+06	7.10E+06	0	7.41E+06	7412071	0	7.41E+06	7412071
DEGRADOSOME_FORM	Degradosome complex formation	1 Eno_dim + 1 Pnp_trim + 1 RNase_E_tetra + 1 RhlB_dim <==> 1 degradosome	reversible	Protein complex formation	-1.00E+09	1.00E+09	0	2.20E+02	2.20E+02	0	2.39E+02	238.7058	0	2.88E+01	28.8145
DnaJ_DIM	Dimerization of DnaJ (chaperone Hsp40, co-chaperone with DnaK)	2 DnaJ_mono <==> 1 DnaJ_dim_inact	reversible	Protein complex formation	-1.00E+09	1.00E+09	0	4.98E+02	4.98E+02	0	8.45E+02	844.9304	0	4.84E+01	48.411
DnaK_mono.ATP_Form	Binding of ATP to DnaK	1 DnaK_mono + 1 atp --> 1 DnaK_mono.ATP_inact	irreversible	Protein complex formation	0.00E+00	1.00E+09	0	1.76E+03	1.76E+03	0	2.93E+03	2933.059	0	2.54E+02	253.857

Dtd_FORM	D-tyr-tRNA_tyr deacylase protein complex formation	2 Dtd_mono --> 1 Dtd_dim	irreversible	Protein complex formation	0.00E+00	1.00E+09	0	2.16E+02	2.16E+02	0	3.51E+02	351.3855	0	3.90E+01	39.016
Dus_gen_FORMa	formation of generic Dus	1 DusA_mono --> 1 Dus_gen	irreversible	Protein complex formation	0.00E+00	1.00E+09	0	2.73E+03	2.73E+03	0	4.75E+03	4751.471	0	9.56E+01	95.6
Dus_gen_FORMb	formation of generic Dus	1 DusB_mono --> 1 Dus_gen	irreversible	Protein complex formation	0.00E+00	1.00E+09	0	1.90E+03	1.90E+03	0	3.31E+03	3305.097	0	6.51E+01	65.124
Dus_gen_FORMc	formation of generic Dus	1 DusC_mono --> 1 Dus_gen	irreversible	Protein complex formation	0.00E+00	1.00E+09	0	2.78E+03	2.78E+03	0	4.83E+03	4832.749	0	9.38E+01	93.834
EF-G_FORM	EF-G formation (GDP binding)	1 FusA_mono + 1 gtp + 1 h2o + 1 mg2 --> 1 EF-G.GDP + 1 h + 1 pi	irreversible	Protein complex formation	0.00E+00	1.00E+09	0	2.04E+03	2.04E+03	0	3.14E+03	3140.993	0	5.11E+02	510.778
EF-G_RECHARG	recharge of EF-G with GTP	1 EF-G.GDP + 1 gtp --> 1 EF-G.GTP + 1 gdp	irreversible	Translation	0.00E+00	1.00E+09	7.64E+05	4.24E+06	3.48E+06	5.62E+05	4.31E+06	3743538.3	5.62E+05	4.01E+06	3444862.3
EF-TU_FORM1a	EF-TU formation 1 (Ser1 acetylation,b3339)	1 AcT_EF-TU + 1 TufA_mono + 1 accoa --> 1 AcT_EF-TU_cplx_a	irreversible	Protein Modification	0.00E+00	1.00E+09	0	2.13E+03	2.13E+03	0	2.80E+03	2800.46	0	1.27E+03	1268.634
EF-TU_FORM1b	EF-TU formation 1 (Ser1 acetylation,b3980)	1 AcT_EF-TU + 1 TufB_mono + 1 accoa --> 1 AcT_EF-TU_cplx_b	irreversible	Protein Modification	0.00E+00	1.00E+09	0	3.66E+03	3.66E+03	0	5.33E+03	5328.195	0	1.30E+03	1297.43
EF-TU_FORM2a	EF-TU formation 2 (Ser1 acetylation,b3339)	1 AcT_EF-TU_cplx_a --> 1 AcT_EF-TU_inact + 1 coa + 1 pEF-TU_ac_a	irreversible	Protein Modification	0.00E+00	1.00E+09	0	2.13E+03	2.13E+03	0	2.80E+03	2800.46	0	1.27E+03	1268.634
EF-TU_FORM2b	EF-TU formation 2 (Ser1 acetylation,b3980)	1 AcT_EF-TU_cplx_b --> 1 AcT_EF-TU_inact + 1 coa + 1 pEF-TU_ac_b	irreversible	Protein Modification	0.00E+00	1.00E+09	0	3.66E+03	3.66E+03	0	5.33E+03	5328.195	0	1.30E+03	1297.43
EF-TU_FORM3a	EF-TU formation 3 (Lys56 methylation,b3339)	1 MeT_EF-TU + 1 amet + 1 pEF-TU_ac_a --> 1 MeT_EF-TU_cplx_a	irreversible	Protein Modification	0.00E+00	1.00E+09	0	2.13E+03	2.13E+03	0	2.80E+03	2800.46	0	1.27E+03	1268.634
EF-TU_FORM3b	EF-TU formation 3 (Lys56 methylation,b3980)	1 MeT_EF-TU + 1 amet + 1 pEF-TU_ac_b --> 1 MeT_EF-TU_cplx_b	irreversible	Protein Modification	0.00E+00	1.00E+09	0	3.66E+03	3.66E+03	0	5.33E+03	5328.195	0	1.30E+03	1297.43
EF-TU_FORM4a	EF-TU formation 4 (Lys56 methylation,b3339)	1 MeT_EF-TU_cplx_a --> 1 MeT_EF-TU_inact + 1 ahcys + 1 h + 1 pEF-TU_me_a	irreversible	Protein Modification	0.00E+00	1.00E+09	0	2.13E+03	2.13E+03	0	2.80E+03	2800.46	0	1.27E+03	1268.634
EF-TU_FORM4b	EF-TU formation 4 (Lys56 methylation,b3980)	1 MeT_EF-TU_cplx_b --> 1 MeT_EF-TU_inact + 1 ahcys + 1 h + 1 pEF-TU_me_b	irreversible	Protein Modification	0.00E+00	1.00E+09	0	3.66E+03	3.66E+03	0	5.33E+03	5328.195	0	1.30E+03	1297.43
EF-TU_FORM5a	EF-TU formation 5 (GDP binding,b3339)	1 gtp + 1 h2o + 1 mg2 + 1 pEF-TU_me_a --> 1 EF-Tu.GDP + 1 h + 1 pi	irreversible	Protein complex formation	0.00E+00	1.00E+09	0	2.13E+03	2.13E+03	0	2.80E+03	2800.46	0	1.27E+03	1268.634
EF-TU_FORM5b	EF-TU formation 5 (GDP binding,b3980)	1 gtp + 1 h2o + 1 mg2 + 1 pEF-TU_me_b --> 1 EF-Tu.GDP + 1 h + 1 pi	irreversible	Protein complex formation	0.00E+00	1.00E+09	0	3.66E+03	3.66E+03	0	5.33E+03	5328.195	0	1.30E+03	1297.43
EF-Tu.EF-Ts_FORM	Formation of binary complex: EF-Tu and EF-Ts by release of GTP	1 EF-Tu.GDP.EF-Ts --> 1 EF-Tu.EF-Ts + 1 gdp	irreversible	Charging EF-Tu	0.00E+00	1.00E+09	7.64E+05	4.24E+06	3.48E+06	5.62E+05	4.31E+06	3743156.3	5.62E+05	4.01E+06	3444862.3
EF-Tu.GDP.EF-TS_FORM	Formation of Complex of EF_Tu and EF_TS	1 EF-Ts + 1 EF-Tu.GDP <==> 1 EF-Tu.GDP.EF-Ts	reversible	Charging EF-Tu	-1.00E+09	1.00E+09	7.64E+05	4.24E+06	3.48E+06	5.62E+05	4.31E+06	3743156.3	5.62E+05	4.01E+06	3444862.3
EF-Tu.GTP.EF-Ts_FORM	formation of the ternary complex EF-Tu, GTP and EF-Ts	1 EF-Tu.EF-Ts + 1 gtp <==> 1 EF-Tu.GTP-EF-Ts	reversible	Charging EF-Tu	-1.00E+09	1.00E+09	7.64E+05	4.24E+06	3.48E+06	5.62E+05	4.31E+06	3743156.3	5.62E+05	4.01E+06	3444862.3
EF-Tu.GTP_FORM	Formation of EF-Tu.GTP by dissociation of ternary complex EF-Tu.GTP.EF-Ts	1 EF-Tu.GTP-EF-Ts --> 1 EF-Ts_inact + 1 EF-Tu.GTP	irreversible	Charging EF-Tu	0.00E+00	1.00E+09	7.64E+05	4.24E+06	3.48E+06	5.62E+05	4.31E+06	3743156.3	5.62E+05	4.01E+06	3444862.3
Eno_DIM	Enolase dimerization	2 Eno_mono <==> 1 Eno_dim	reversible	Protein complex formation	-1.00E+09	1.00E+09	0	2.20E+02	2.20E+02	0	2.39E+02	238.7058	0	2.88E+01	28.8145
Era_DIM	dimerization of era and GTP binding	2 Era_mono + 2 gtp --> 1 Era_dim.GTP	irreversible	Protein Complex formation	0.00E+00	1.00E+09	0	7.33E+02	7.33E+02	0	1.27E+03	1265.676	0	3.80E+01	38.0375
Era_RECHARGE	recharging of Era	1 Era_dim.GDP + 2 gtp --> 1 Era_dim.GTP + 2 gdp	irreversible	Protein complex formation	0.00E+00	1.00E+09	0	1.13E+02	1.13E+02	0	8.29E+01	82.88	2.41E+01	8.29E+01	58.7575
Exch_10fthf[e]	Exchange of 10fthf[e]	1 10fthf[e] <==>	reversible	Exchange reactions	-1.00E+09	1.00E+09	-2.30E+04	-5.23E+03	1.78E+04	-2.37E+04	-3.85E+03	19852.92	-2.15E+04	-3.85E+03	17644.16
Exch_5fthf[e]	Exchange of 5fthf	1 5fthf[e] <==>	reversible	Exchange reactions	-1.00E+09	1.00E+09	-1.86E+03	0	1.86E+03	-1.09E+02	-2.75E+01	81.96	-1.09E+02	-2.75E+01	81.96
Exch_5mta[e]	Exchange of 5mta	1 5mta[e] <==>	reversible	Exchange reactions	-1.00E+09	1.00E+09	0	1.81E+03	1.81E+03	1.39E+01	1.83E+02	169.4	1.39E+01	1.83E+02	169.4
Exch_C10H8O5[e]	Exchange of C10H8O5	1 C10H8O5[e] <==>	reversible	Exchange reactions	-1.00E+09	1.00E+09	0	1.38E+03	1.38E+03	1.39E+01	9.38E+01	79.83	1.39E+01	9.38E+01	79.83
Exch_C9H9O4[e]	Exchange of C9H9O4	1 C9H9O4[e] <==>	reversible	Exchange reactions	-1.00E+09	1.00E+09	0	1.38E+03	1.38E+03	1.39E+01	9.38E+01	79.83	1.39E+01	9.38E+01	79.83
Exch_Fe(2)[e]	Exchange of Fe2+	1 fe2[e] <==>	reversible	Exchange reactions	-1.00E+09	1.00E+09	-1.15E+04	0	1.15E+04	-1.91E+04	0	19065.9	-1.55E+03	0	1552.616
Exch_Fe(3)[e]	Exchange of Fe3+	1 fe3[e] <==>	reversible	Exchange reactions	-1.00E+09	1.00E+09	0	0	0.00E+00	0	0	0	0	0	0
Exch_accoa[e]	Exchange of accoa[e]	1 accoa[e] <==>	reversible	Exchange reactions	-1.00E+09	1.00E+09	-5.24E+03	-3.38E+02	4.90E+03	-5.59E+03	-2.49E+02	5343.775	-2.91E+03	-2.49E+02	2664.524
Exch_ade[e]	Exchange of ade	1 ade[e] <==>	reversible	Exchange reactions	-1.00E+09	1.00E+09	0	1.77E+03	1.77E+03	0	7.90E+02	789.686	0	8.58E+01	85.794
Exch_adocbl[e]	Exchange of adocbl[e]	1 adocbl[e] <==>	reversible	Exchange reactions	-1.00E+09	1.00E+09	0	0	0.00E+00	0	0	0	0	0	0
Exch_adp[e]	Exchange of adp[e]	1 adp[e] <==>	reversible	Exchange reactions	-1.00E+09	1.00E+12	5.64E+02	7.11E+06	7.11E+06	3.86E+02	7.41E+06	7413166.4	3.86E+02	7.41E+06	7413166.4
Exch_ahcys[e]	Exchange of ahcys[e]	1 ahcys[e] <==>	reversible	Exchange reactions	-1.00E+09	1.00E+09	3.88E+03	1.32E+04	9.34E+03	2.89E+03	1.03E+04	7399.09	2.89E+03	8.54E+03	5652.935
Exch_ala-L[e]	Exchange of ala-L[e]	1 ala-L[e] <==>	reversible	Exchange reactions	-4.70E+05	0.00E+00	-4.70E+05	-8.26E+04	3.88E+05	-4.70E+05	-6.22E+04	408040.84	-4.29E+05	-6.25E+04	366966.42
Exch_amet[e]	Exchange of amet[e]	1 amet[e] <==>	reversible	Exchange reactions	-1.00E+09	1.00E+09	-1.46E+04	-3.88E+03	1.07E+04	-1.07E+04	-2.90E+03	7840.78	-8.90E+03	-2.90E+03	5993.993
Exch_amp[e]	Exchange of amp[e]	1 amp[e] <==>	reversible	Exchange reactions	-1.00E+09	1.00E+12	7.79E+05	7.88E+06	7.10E+06	5.74E+05	7.99E+06	7412102.1	5.74E+05	7.99E+06	7412102.1
Exch_arg-L[e]	Exchange of arg-L[e]	1 arg-L[e] <==>	reversible	Exchange reactions	-2.71E+05	1.00E+12	-2.71E+05	-6.29E+04	2.08E+05	-2.71E+05	-4.63E+04	224466.7	-2.71E+05	-4.63E+04	224466.7
Exch_asn-L[e]	Exchange of asn-L[e]	1 asn-L[e] <==>	reversible	Exchange reactions	-2.21E+05	1.00E+12	-1.77E+05	-2.64E+04	1.50E+05	-1.88E+05	-1.95E+04	168543.42	-1.62E+05	-1.95E+04	142880.92
Exch_asp-L[e]	Exchange of asp-L[e]	1 asp-L[e] <==>	reversible	Exchange reactions	-2.21E+05	1.00E+12	-2.21E+05	-3.55E+04	1.85E+05	-2.21E+05	-2.62E+04	194498.54	-2.21E+05	-2.62E+04	194498.54
Exch_atp[e]	Exchange of atp[e]	1 atp[e] <==>	reversible	Exchange reactions	-1.66E+07	1.00E+12	-1.51E+07	-8.80E+05	1.42E+07	-1.55E+07	-6.49E+05	14828678.9	-1.55E+07	-6.49E+05	14828679
Exch_cdp[e]	Exchange of cdp[e]	1 cdp[e] <==>	reversible	Exchange reactions	-1.00E+09	1.00E+09	0	0	0.00E+00	0	0	0	0	0	0
Exch_chor[e]	Exchange of chor	1 chor[e] <==>	reversible	Exchange reactions	-1.00E+09	1.00E+09	-2.76E+03	0	2.76E+03	-1.88E+02	-2.78E+01	159.66	-1.88E+02	-2.78E+01	159.66
Exch_cmp[e]	Exchange of cmp[e]	1 cmp[e] <==>	reversible	Exchange reactions	-1.00E+09	1.00E+09	5.49E+03	3.75E+04	3.20E+04	4.17E+03	6.08E+04	56611.87	4.17E+03	2.62E+04	22046.26
Exch_coa[e]	Exchange of coa[e]	1 coa[e] <==>	reversible	Exchange reactions	-1.00E+09	1.00E+09	3.38E+02	5.24E+03	4.90E+03	2.49E+02	5.59E+03	5343.775	2.49E+02	2.91E+03	2664.524
Exch_ctp[e]	Exchange of ctp[e]	1 ctp[e] <==>	reversible	Exchange reactions	-1.26E+05	0.00E+00	-1.26E+05	-9.45E+04	3.19E+04	-1.26E+05	-7.00E+04	56379.6	-1.26E+05	-7.00E+04	56379.6
Exch_cys-L[e]	Exchange of cys-L[e]	1 cys-L[e] <==>	reversible	Exchange reactions	-8.38E+04	1.00E+12	-7.34E+04	-3.46E+03	6.99E+04	-8.38E+04	-2.58E+03	81258.46	-5.79E+04	-2.58E+03	55298.11
Exch_dad-5[e]	Exchange of dad-5	1 dad-5[e] <==>	reversible	Exchange reactions	-1.00E+09	1.00E+09	0	1.52E+03	1.52E+03	0	7.95E+02	795.0555	0	8.59E+01	85.864
Exch_dmpp[e]	Exchange of dmpp	1 dmpp[e] <==>	reversible	Exchange reactions	-1.00E+09	1.00E+09	-1.52E+03	0	1.52E+03	-8.03E+02	0	803.4932	-9.96E+01	0	99.564
Exch_fad[e]	Exchange of fad[e]	1 fad[e] <==>	reversible	Exchange reactions	-1.00E+09	1.00E+09	0	0	0.00E+00	0	0	0	0	0	0
Exch_fadh2[e]	Exchange of fadh2[e]	1 fadh2[e] <==>	reversible	Exchange reactions	-1.00E+09	1.00E+09	0	0	0.00E+00	0	0	0	0	0	0
Exch_fldox[e]	Exchange of fldox	1 fldox[e] <==>	reversible	Exchange reactions	-1.00E+09	1.00E+09	0	1.52E+03	1.52E+03	0	7.95E+02	795.0555	0	8.59E+01	85.864
Exch_fldrd[e]	Exchange of fldrd	1 fldrd[e] <==>	reversible	Exchange reactions	-1.00E+09	1.00E+09	-1.52E+03	0	1.52E+03	-7.95E+02	0	795.0555	-8.59E+01	0	85.864
Exch_for[e]	Exchange of for[e]	1 for[e] <==>	reversible	Exchange reactions	-1.00E+09	1.00E+09	5.23E+03	2.30E+04	1.78E+04	3.85E+03	2.37E+04	19852.92	3.85E+03	2.14E+04	17593.9
Exch_gdp[e]	Exchange of gdp[e]	1 gdp[e] <==>	reversible	Exchange reactions	-1.00E+09	1.00E+09	1.54E+06	8.51E+06	6.97E+06	1.13E+06	8.64E+06	7509213	1.13E+06	8.04E+06	6911881

Exch_gln-L[e]	Exchange of gln-L[e]	1 gln-L[e] <==>	reversible	Exchange reactions	-2.41E+05	1.00E+12	-2.41E+05	-2.64E+04	2.14E+05	-2.41E+05	-1.95E+04	221447.82	-2.16E+05	-1.95E+04	196657.22
Exch_glu-L[e]	Exchange of glu-L[e]	1 glu-L[e] <==>	reversible	Exchange reactions	-2.41E+05	1.00E+12	-2.41E+05	-5.94E+04	1.81E+05	-2.41E+05	-4.38E+04	197143.26	-2.41E+05	-4.38E+04	197143.26
Exch_glx[e]	Exchange of glx	1 glx[e] <==>	reversible	Exchange reactions	-1.00E+09	1.00E+09	0	1.86E+03	1.86E+03	2.75E+01	1.09E+02	81.96	2.75E+01	1.09E+02	81.96
Exch_gly[e]	Exchange of gly[e]	1 gly[e] <==>	reversible	Exchange reactions	-5.61E+05	1.00E+12	-3.73E+05	-6.08E+04	3.12E+05	-3.94E+05	-4.48E+04	348719.18	-3.32E+05	-4.48E+04	286829.28
Exch_gmp[e]	Exchange of gmp[e]	1 gmp[e] <==>	reversible	Exchange reactions	-1.00E+09	1.00E+09	6.84E+03	5.42E+04	4.73E+04	5.26E+03	8.85E+04	83260.94	5.26E+03	2.99E+04	24655.18
Exch_gtp[e]	Exchange of gtp[e]	1 gtp[e] <==>	reversible	Exchange reactions	-2.04E+07	1.00E+12	-8.68E+06	-1.67E+06	7.01E+06	-8.80E+06	-1.23E+06	7573145	-8.20E+06	-1.23E+06	6970628
Exch_gua[e]	Exchange of gua	1 gua[e] <==>	reversible	Exchange reactions	-1.00E+09	1.00E+09	0	1.77E+03	1.77E+03	0	7.90E+02	789.686	0	8.58E+01	85.794
Exch_h2o[e]	Exchange of h2o[e]	1 h2o[e] <==>	reversible	Exchange reactions	-1.66E+07	1.00E+12	-1.58E+07	-1.58E+06	1.42E+07	-1.60E+07	-1.17E+06	14824244	-1.60E+07	-1.17E+06	14824244
Exch_h2s[e]	Exchange of h[e]	1 h2s[e] <==>	reversible	Exchange reactions	-1.00E+09	1.00E+09	0	1.86E+03	1.86E+03	0	9.36E+01	93.62	0	9.36E+01	93.62
Exch_h[e]	Exchange of h[e]	1 h[e] <==>	reversible	Exchange reactions	-1.00E+03	1.66E+07	2.35E+06	16554815	1.42E+07	1.73E+06	16554815	14824142	1.73E+06	16554815	14824142
Exch_hco3[e]	Exchange of hco3	1 hco3[e] <==>	reversible	Exchange reactions	-1.00E+09	1.00E+09	-1.81E+03	0	1.81E+03	-2.12E+02	-1.39E+01	197.71	-2.12E+02	-1.39E+01	197.71
Exch_his-L[e]	Exchange of his-L[e]	1 his-L[e] <==>	reversible	Exchange reactions	-8.67E+04	1.00E+12	-8.67E+04	-1.44E+04	7.23E+04	-8.67E+04	-1.06E+04	76096.02	-8.67E+04	-1.06E+04	76096.02
Exch_ile-L[e]	Exchange of ile-L[e]	1 ile-L[e] <==>	reversible	Exchange reactions	-2.66E+05	1.00E+12	-2.66E+05	-4.25E+04	2.23E+05	-2.66E+05	-3.13E+04	234691.42	-2.50E+05	-3.13E+04	218779.62
Exch_leu-L[e]	Exchange of leu-L[e]	1 leu-L[e] <==>	reversible	Exchange reactions	-4.12E+05	1.00E+12	-4.12E+05	-5.32E+04	3.59E+05	-4.12E+05	-3.92E+04	373266.6	-4.12E+05	-3.92E+04	373266.6
Exch_lys-L[e]	Exchange of lys-L[e]	1 lys-L[e] <==>	reversible	Exchange reactions	-3.14E+05	1.00E+12	-2.80E+05	-7.19E+04	2.08E+05	-2.99E+05	-5.30E+04	245920.18	-2.46E+05	-5.30E+04	192575.98
Exch_met-L[e]	Exchange of met-L[e]	1 met-L[e] <==>	reversible	Exchange reactions	-1.41E+05	0.00E+00	-1.33E+05	-1.47E+04	1.18E+05	-1.41E+05	-1.09E+04	129758.02	-1.15E+05	-1.19E+04	103535.85
Exch_mg2[e]	Exchange of mg2[e]	1 mg2[e] <==>	reversible	Exchange reactions	-1.00E+09	1.00E+09	-3.07E+04	-1.46E+04	1.61E+04	-3.38E+04	-1.08E+04	23040	-2.09E+04	-1.08E+04	10161.68
Exch_nad[e]	Exchange of nad	1 nad[e] <==>	reversible	Exchange reactions	-1.00E+09	1.00E+12	0	1.86E+03	1.86E+03	0	8.52E+02	851.846	0	1.69E+02	168.674
Exch_nadh[e]	Exchange of nadh	1 nadh[e] <==>	reversible	Exchange reactions	-1.00E+09	1.00E+12	-1.86E+03	0	1.86E+03	-8.52E+02	0	851.846	-1.69E+02	0	168.674
Exch_nadp[e]	Exchange of nadp	1 nadp[e] <==>	reversible	Exchange reactions	-1.00E+09	1.00E+12	0	8.00E+03	8.00E+03	4.18E+01	9.61E+03	9564.167	4.18E+01	2.37E+03	2326.953
Exch_nadph[e]	Exchange of nadph	1 nadph[e] <==>	reversible	Exchange reactions	-1.00E+09	1.00E+12	-8.00E+03	0	8.00E+03	-9.61E+03	-4.18E+01	9564.167	-2.37E+03	-4.18E+01	2326.953
Exch_nh3[e]	Exchange of nh3	1 nh3[e] <==>	reversible	Exchange reactions	-1.00E+09	1.00E+09	0	5.86E+02	5.86E+02	0	3.55E+01	35.48	0	3.55E+01	35.48
Exch_phe-L[e]	Exchange of phe-L[e]	1 phe-L[e] <==>	reversible	Exchange reactions	-1.70E+05	1.00E+12	-1.70E+05	-2.17E+04	1.48E+05	-1.70E+05	-1.60E+04	153621.18	-1.62E+05	-1.60E+04	146446.98
Exch_pi[e]	Exchange of pi[e]	1 pi[e] <==>	reversible	Exchange reactions	-1.00E+09	1.00E+12	1.54E+06	1.03E+07	8.80E+06	1.13E+06	1.03E+07	9209854	1.13E+06	1.03E+07	9135134
Exch_ppi[e]	Exchange of ppi[e]	1 ppi[e] <==>	reversible	Exchange reactions	-1.00E+09	1.00E+12	1.19E+06	8.43E+06	7.24E+06	8.81E+05	8.44E+06	7556292.8	8.81E+05	8.38E+06	7498028.8
Exch_pro-L[e]	Exchange of pro-L[e]	1 pro-L[e] <==>	reversible	Exchange reactions	-2.02E+05	1.00E+12	-2.02E+05	-2.35E+04	1.79E+05	-2.02E+05	-1.73E+04	185078.72	-2.00E+05	-1.73E+04	182852.72
Exch_pydx5p	Exchange of pydx5p	1 pydx5p[e] <==>	reversible	Exchange reactions	-1.00E+09	1.00E+09	-1.30E+03	0	1.30E+03	-2.19E+03	0	2188.119	-1.43E+02	0	142.705
Exch_selnp[e]	Exchange of selnp[e]	1 selnp[e] <==>	reversible	Exchange reactions	-1.00E+09	1.00E+09	-1.86E+03	0	1.86E+03	-1.07E+02	0	107.32	-1.07E+02	0	107.32
Exch_ser-L[e]	Exchange of ser-L[e]	1 ser-L[e] <==>	reversible	Exchange reactions	-1.98E+05	1.00E+12	-1.98E+05	-3.45E+04	1.63E+05	-1.98E+05	-2.54E+04	172117.76	-1.98E+05	-2.54E+04	172117.76
Exch_so4[e]	Exchange of so4[e]	1 so4[e] <==>	reversible	Exchange reactions	-1.00E+09	1.00E+09	0	0	0.00E+00	0	0	0	0	0	0
Exch_spmd[e]	Exchange of spmd[e]	1 spmd[e] <==>	reversible	Exchange reactions	-1.00E+09	1.00E+12	-9.01E+02	-6.75E+02	2.25E+02	-6.63E+02	-4.97E+02	165.76	-6.63E+02	-4.97E+02	165.76
Exch_thf[e]	Exchange of thf[e]	1 thf[e] <==>	reversible	Exchange reactions	-1.00E+09	1.00E+09	5.23E+03	2.33E+04	1.80E+04	3.88E+03	2.38E+04	19872.77	3.88E+03	2.16E+04	17726.12
Exch_thr-L[e]	Exchange of thr-L[e]	1 thr-L[e] <==>	reversible	Exchange reactions	-2.32E+05	1.00E+12	-2.32E+05	-3.78E+04	1.94E+05	-2.32E+05	-2.79E+04	204369.7	-2.32E+05	-2.79E+04	204369.7
Exch_trdox[e]	Exchange of trdox	1 trdox[e] <==>	reversible	Exchange reactions	-1.00E+09	1.00E+09	-5.49E+03	1.86E+03	7.34E+03	-9.34E+03	1.09E+03	10432.265	-5.89E+02	5.27E+02	1116.229
Exch_trdrd[e]	Exchange of trdrd	1 trdrd[e] <==>	reversible	Exchange reactions	-1.00E+09	1.00E+09	-1.86E+03	5.49E+03	7.34E+03	-1.09E+03	9.34E+03	10432.265	-5.27E+02	5.89E+02	1116.229
Exch_trp-L[e]	Exchange of trp-L[e]	1 trp-L[e] <==>	reversible	Exchange reactions	-5.20E+04	1.00E+12	-5.20E+04	-3.63E+03	4.84E+04	-5.20E+04	-2.67E+03	49362.35	-5.20E+04	-2.67E+03	49362.35
Exch_tyr-D[e]	Exchange of tyr-D[e]	1 tyr-D[e] <==>	reversible	Exchange reactions	-1.00E+09	1.00E+09	0	0	0.00E+00	0	0	0	0	0	0
Exch_tyr-L[e]	Exchange of tyr-L[e]	1 tyr-L[e] <==>	reversible	Exchange reactions	-1.26E+05	1.00E+12	-1.26E+05	-1.27E+04	1.13E+05	-1.26E+05	-9.39E+03	116847.44	-1.17E+05	-9.39E+03	107184.64
Exch_udp[e]	Exchange of udp[e]	1 udp[e] <==>	reversible	Exchange reactions	-1.00E+09	1.00E+09	0	0	0.00E+00	0	0	0	0	0	0
Exch_ump[e]	Exchange of ump[e]	1 ump[e] <==>	reversible	Exchange reactions	-1.00E+09	1.00E+09	8.44E+03	5.05E+04	4.21E+04	6.67E+03	7.78E+04	71153.83	6.67E+03	2.86E+04	21935.62
Exch_utp[e]	Exchange of utp[e]	1 utp[e] <==>	reversible	Exchange reactions	-1.36E+05	0.00E+00	-1.31E+05	-8.76E+04	4.35E+04	-1.36E+05	-6.54E+04	71006.98	-1.17E+05	-6.54E+04	51524.48
Exch_val-L[e]	Exchange of val-L[e]	1 val-L[e] <==>	reversible	Exchange reactions	-3.87E+05	1.00E+12	-3.76E+05	-7.33E+04	3.03E+05	-3.87E+05	-5.40E+04	333418.52	-3.38E+05	-5.40E+04	283575.22
Exch_zn2[e]	Exchange of zn2[e]	1 zn2[e] <==>	reversible	Exchange reactions	-1.00E+09	1.00E+09	-4.85E+03	0	4.85E+03	-6.60E+03	0	6599.788	-2.55E+03	0	2553.129
FORt	formate transport via diffusion	1 for[e] <==> 1 for	reversible	Transport	-1.00E+09	1.00E+09	-2.30E+04	-5.23E+03	1.78E+04	-2.37E+04	-3.85E+03	19852.92	-2.14E+04	-3.85E+03	17593.9
Fe(2)xt	transport of Fe2+	1 fe2 <==> 1 fe2[e]	reversible	Transport	-1.00E+09	1.00E+09	-1.15E+04	0	1.15E+04	-1.91E+04	0	19065.9	-1.55E+03	0	1552.616
Fe(3)xt	transport of Fe3+	1 fe3 <==> 1 fe3[e]	reversible	Transport	-1.00E+09	1.00E+09	0	0	0.00E+00	0	0	0	0	0	0
GLNt5r	L-glutamine transport via diffusion	1 gln-L[e] <==> 1 gln-L	reversible	Transport	-1.00E+09	1.00E+09	2.64E+04	2.41E+05	2.14E+05	1.95E+04	2.41E+05	221447.82	1.95E+04	2.16E+05	196657.22
GLUt5r	L-glutamate transport via diffusion	1 glu-L[e] <==> 1 glu-L	reversible	Transport	-1.00E+09	1.00E+09	5.94E+04	2.41E+05	1.81E+05	4.38E+04	2.41E+05	197143.26	4.38E+04	2.41E+05	197143.26
GLYt	Glycine transport via facilitate diffusion	1 gly[e] <==> 1 gly	reversible	Transport	-1.00E+09	1.00E+09	6.08E+04	3.73E+05	3.12E+05	4.48E+04	3.94E+05	348719.18	4.48E+04	3.32E+05	286829.28
Gln_RS_CHARG	Glutaminyl-tRNA synthetase charging	1 GlnS_mono + 1 atp + 1 gln-L + 1 h2o --> 1 Gln_RS_gln_amp + 1 h + 1 ppi	irreversible	Aminoacyl-tRNA synthetase charging	0.00E+00	1.00E+09	2.64E+04	2.42E+05	2.16E+05	1.95E+04	2.45E+05	225156.12	1.95E+04	2.16E+05	196962.32
Glu_RS_CHARG	Glutamyl-tRNA synthetase charging	1 GltX_mono + 1 atp + 1 glu-L + 1 h2o --> 1 Glu_RS_glu_amp + 1 h 1 ppi	irreversible	Aminoacyl-tRNA synthetase charging	0.00E+00	1.00E+09	5.91E+04	2.44E+05	1.85E+05	4.35E+04	2.46E+05	202239.9	4.35E+04	2.41E+05	197591.3
GlySQ_RS_TETRA	glycine tRNA synthetase complex formation of 2 alpha subunits and 2 beta subunits	2 GlyQ_mono + 2 GlyS_mono <==> 1 Gly_RS_tetra	reversible	Protein complex formation	-1.00E+09	1.00E+09	0	3.76E+02	3.76E+02	0	6.38E+02	637.5599	0	3.58E+01	35.7595
Gly_RS_CHARGa	Glycyl-tRNA synthetase charging	1 Gly_RS_tetra + 2 atp + 2 gly + 2 h2o --> 1 Gly_RS_2gly_2amp + 2 + 2 ppi	irreversible	Aminoacyl-tRNA synthetase charging	0.00E+00	1.00E+09	0	1.87E+05	1.87E+05	0	1.98E+05	198330.9	0	1.67E+05	166532.3
Gly_RS_CHARGb	Glycyl-tRNA synthetase charging	1 Gly_RS_tetra + 1 atp + 1 gly + 1 h2o --> 1 Gly_RS_gly_amp + 1 h + 1 ppi	irreversible	Aminoacyl-tRNA synthetase charging	0.00E+00	1.00E+09	0	3.74E+05	3.74E+05	0	3.97E+05	396661.7	0	3.33E+05	333064.7
GroEL.(7)ADP.cisGroES_CONV	conversion of GroEL.(7)ADP.cisGroES to GroEL.(7)ADP.ctransGroES	1 GroEL.(7)ADP.cisGroES <==> 1 GroEL.(7)ADP.transGroES	reversible	Protein Folding	-1.00E+09	1.00E+09	-7.94E+03	1.49E+02	8.09E+03	-9.80E+03	2.45E+02	10048.1279	-5.82E+03	1.67E+01	5837.6464

GroEL.(7)ADP.cisGroES_FORM	formation of ternary complex GroEL (14-mer), GroES (7-mer) and 7 ADP	1 GroL_(14) + 7 adp + 1 cisGroES_hepta + 7 mg2 --> 1 GroEL.(7)ADP.cisGroES	irreversible	Protein Folding	0.00E+00	1.00E+09	0	1.49E+02	1.49E+02	0	2.45E+02	244.8199	0	1.67E+01	16.66743
GroEL.(7)ADP.transGroES_FORM	formation of ternary complex GroEL (14-mer), GroES (7-mer) and 7 ADP	1 GroL_(14) + 7 adp + 7 mg2 + 1 transGroES_hepta --> 1 GroEL.(7)ADP.transGroES	irreversible	Protein Folding	0.00E+00	1.00E+09	0	1.49E+02	1.49E+02	0	2.45E+02	244.8199	0	1.67E+01	16.66743
GroL_(14)_FORM	Formation of (GroL)14	2 GroL_hepta <==> 1 GroL_(14)	reversible	Protein complex formation	-1.00E+09	1.00E+09	0	1.49E+02	1.49E+02	0	2.45E+02	244.8199	0	1.67E+01	16.66743
GroL_hepta_FORM	Formation of GroL heptamer	7 GroL_mono <==> 1 GroL_hepta	reversible	Protein complex formation	-1.00E+09	1.00E+09	0	2.99E+02	2.99E+02	0	4.90E+02	489.6399	0	3.33E+01	33.33486
GroS_hepta_FORM	Formation of GroS heptamer	7 GroS_mono <==> 1 GroS_hepta	reversible	Protein complex formation	-1.00E+09	1.00E+09	0	4.23E+02	4.23E+02	0	7.14E+02	714.4714	0	4.37E+01	43.73
GrpE_DIM	GrpE dimerization (heat shock protein, b2614)	2 GrpE_mono <==> 1 GrpE_dim	reversible	Protein complex formation	-1.00E+09	1.00E+09	0	1.55E+03	1.55E+03	0	2.66E+03	2664.459	0	1.03E+02	103.3285
H2Ot	H2O transport via diffusion	1 h2o[e] <==> 1 h2o	reversible	Transport	-1.00E+09	1.00E+09	1.58E+06	1.58E+07	1.42E+07	1.17E+06	1.60E+07	14824244	1.17E+06	1.60E+07	14824244
HISt5r	L-histidine transport via diffusion	1 his-L[e] <==> 1 his-L	reversible	Transport	-1.00E+09	1.00E+09	1.44E+04	8.67E+04	7.23E+04	1.06E+04	8.67E+04	76096.02	1.06E+04	8.67E+04	76096.02
HisS_RS_DIM	dimerization of histidyl-tRNA synthetase	2 HisS_mono <==> 1 His_RS_dim	reversible	Protein complex formation	-1.00E+09	1.00E+09	0	1.28E+03	1.28E+03	0	2.22E+03	2216.958	0	6.26E+01	62.628
His_RS_CHARGa	Histidyl-tRNA synthetase charging	1 His_RS_dim + 1 atp + 1 h2o + 1 his-L --> 1 His_RS_his_amp + 1 h + 1 ppi	irreversible	Aminoacyl-tRNA synthetase charging	0.00E+00	1.00E+09	0	8.97E+04	8.97E+04	0	9.17E+04	91685.86	0	8.70E+04	87033.61
His_RS_CHARGb	Histidyl-tRNA synthetase charging	1 His_RS_dim + 2 atp + 2 h2o + 2 his-L --> 1 His_RS_2his_2amp + 2 h + 2 ppi	irreversible	Aminoacyl-tRNA synthetase charging	0.00E+00	1.00E+09	0	4.48E+04	4.48E+04	0	4.58E+04	45842.93	0	4.35E+04	43516.8
Ht	proton diffusion	1 h <==> 1 h[e]	reversible	Transport	-1.00E+09	1.00E+09	2.35E+06	16554815	1.42E+07	1.73E+06	16554815	14824142	1.73E+06	16554815	14824142
IF1_RENAME	translational initiation factor IF1 formation	1 InfA_mono --> 1 IF1	irreversible	Protein complex formation	0.00E+00	1.00E+09	8.44E+01	4.56E+03	4.48E+03	6.22E+01	6.34E+03	6276.458	6.22E+01	2.27E+03	2203.364
IF2_FORM	translational initiation factor IF2 formation	1 InfB_mono + 1 gtp + 1 h2o --> 1 IF2-GDP + 1 h + 1 pi	irreversible	Protein complex formation	0.00E+00	1.00E+09	8.44E+01	1.60E+03	1.52E+03	6.22E+01	2.03E+03	1965.924	6.22E+01	1.11E+02	49.12
IF2_RECHARG	recharge of IF2 with GTP	1 IF2-GDP + 1 gtp --> 1 IF2-GTP + 1 gdp	irreversible	Translation	0.00E+00	1.00E+09	5.32E+03	2.31E+04	1.78E+04	3.92E+03	2.38E+04	19852.92	3.92E+03	2.15E+04	17614.62
IF3_FORM	translational initiation factor IF3 formation	1 InfC_mono --> 1 IF3	irreversible	Protein complex formation	0.00E+00	1.00E+09	8.44E+01	3.02E+03	2.94E+03	6.22E+01	5.11E+03	5046.609	6.22E+01	2.96E+02	234.06
ILEtec	L-isoleucine transport via diffusion (extracellular to cytosol)	1 ile-L[e] <==> 1 ile-L	reversible	Transport	-1.00E+09	1.00E+09	4.25E+04	2.66E+05	2.23E+05	3.13E+04	2.66E+05	234691.42	3.13E+04	2.50E+05	218779.62
Ile_RS_CHARG	Isoleucyl-tRNA synthetase charging	1 IleS_mono + 1 atp + 1 h2o + 1 ile-L --> 1 Ile_RS_ile_Amp + 1 h + 1 ppi	irreversible	Aminoacyl-tRNA synthetase charging	0.00E+00	1.00E+09	4.25E+04	2.68E+05	2.26E+05	3.13E+04	2.70E+05	238579.52	3.13E+04	2.51E+05	219614.72
IscA-Fe_FORM	2 Fe2 bind to IscA (tetramer)	1 IscA_tetra + 2 fe2 + 1 h + 1 nadph + 1 trdox --> 1 IscA_tetra_Fe(2) 1 nadp + 1 trdrd	irreversible	Iron-sulfur cluster biosynthesis	0.00E+00	1.00E+09	0	5.57E+03	5.57E+03	0	9.38E+03	9377.848	0	6.17E+02	616.825
IscA_TETRA	IscA tetramer formation	4 IscA_mono --> 1 IscA_tetra	irreversible	Protein complex formation	0.00E+00	1.00E+09	0	9.93E+01	9.93E+01	0	1.57E+02	157.3623	0	2.38E+01	23.802
IscS_DIM	Dimerization of IscS	2 IscS_mono + 2 pydx5p <==> 1 IscS_dim_S-H	reversible	Protein complex formation	-1.00E+09	1.00E+09	0	6.26E+02	6.26E+02	0	1.07E+03	1070.975	0	4.70E+01	47.012
IscS_dim_S-SH_FORM	formation of IscS_dim_S-SH	1 IscS_dim_S-H + 1 cys-L --> 1 IscS_dim_S-SH + 1 ala-L	irreversible	tRNA modification	0.00E+00	1.00E+09	0	1.13E+04	1.13E+04	2.75E+01	1.88E+04	18812.28	2.75E+01	1.89E+03	1865.93
IscU_DIM	IscU dimerization	2 IscU_mono --> 1 IscU_dim_SH	irreversible	Protein complex formation	0.00E+00	1.00E+09	0	1.11E+04	1.11E+04	0	1.88E+04	18755.7	0	1.23E+03	1233.65
IscU_dim_S-SH_FORM1	formation of IscU and IscS complex	1 IscS_dim_S-SH + 1 IscU_dim_SH --> 1 IscS_IscU_cplx	irreversible	Iron-sulfur cluster biosynthesis	0.00E+00	1.00E+09	0	1.11E+04	1.11E+04	0	1.88E+04	18755.7	0	1.23E+03	1233.65
IscU_dim_S-SH_FORM2	formation of IscU S-S bound	1 IscS_IscU_cplx --> 1 IscS_dim_S-H + 1 IscU_mono + 1 IscU_mono_S-SH	irreversible	Iron-sulfur cluster biosynthesis	0.00E+00	1.00E+09	0	1.11E+04	1.11E+04	0	1.88E+04	18755.7	0	1.23E+03	1233.65
LEUtec	L-leucine transport via diffusion (extracellular to cytosol)	1 leu-L[e] <==> 1 leu-L	reversible	Transport	-1.00E+09	1.00E+09	5.32E+04	4.12E+05	3.59E+05	3.92E+04	4.12E+05	373266.6	3.92E+04	4.12E+05	373266.6
LYSII_RS_DIM	Lysyl-tRNA synthetase dimerization (inducible)	2 LysU_mono <==> 1 LysII_RS_dim	reversible	Protein complex formation	-1.00E+09	1.00E+09	0	1.42E+03	1.42E+03	0	2.14E+03	2142.861	0	5.93E+01	59.2615
LYSt5r	L-lysine transport via diffusion	1 lys-L[e] <==> 1 lys-L	reversible	Transport	-1.00E+09	1.00E+09	7.19E+04	2.80E+05	2.08E+05	5.30E+04	2.99E+05	245920.18	5.30E+04	2.46E+05	192575.98
Leu_RS_CHARG	Leucyl-tRNA synthetase charging	1 LeuS_mono + 1 atp + 1 h2o + 1 leu-L --> 1 Leu_RS_leu_amp + 1 h + 1 ppi	irreversible	Aminoacyl-tRNA synthetase charging	0.00E+00	1.00E+09	5.32E+04	4.21E+05	3.68E+05	3.92E+04	4.25E+05	385360.1	3.92E+04	4.17E+05	377769.6
LysS_RS_DIM	lysyl-tRNA synthetase dimerization (constitutive, b2890)	2 LysS_mono <==> 1 LysI_RS_dim	reversible	Protein complex formation	-1.00E+09	1.00E+09	0	7.36E+02	7.36E+02	0	1.26E+03	1257.488	0	5.77E+01	57.7245
Lys_RSII_CHARGa	Lysyl-tRNA synthetase II charging	1 LysII_RS_dim + 1 atp + 1 h2o + 1 lys-L --> 1 LysII_RS_lys_amp + 1 h + 1 ppi	irreversible	Aminoacyl-tRNA synthetase charging	0.00E+00	1.00E+09	0	2.81E+05	2.81E+05	0	3.00E+05	299732.5	0	2.46E+05	246432.4
Lys_RSII_CHARGb	Lysyl-tRNA synthetase II charging	1 LysII_RS_dim + 2 atp + 2 h2o + 2 lys-L --> 1 LysII_RS_2lys_2amp 2 h + 2 ppi	irreversible	tRNA charging	0.00E+00	1.00E+09	0	1.40E+05	1.40E+05	0	1.50E+05	149866.2	0	1.23E+05	123216.2
Lys_RSI_CHARGa	Lysyl-tRNA synthetase charging	1 LysI_RS_dim + 1 atp + 1 h2o + 1 lys-L --> 1 LysI_RS_lys_amp + 1 + 1 ppi	irreversible	Aminoacyl-tRNA synthetase charging	0.00E+00	1.00E+09	0	2.81E+05	2.81E+05	0	3.00E+05	299732.5	0	2.46E+05	246432.4
Lys_RSI_CHARGb	Lysyl-tRNA synthetase charging	1 LysI_RS_dim + 2 atp + 2 h2o + 2 lys-L --> 1 LysI_RS_2lys_2amp + 2 h + 2 ppi	irreversible	Aminoacyl-tRNA synthetase charging	0.00E+00	1.00E+09	0	1.40E+05	1.40E+05	0	1.50E+05	149866.2	0	1.23E+05	123216.2
METtec	L-methionine transport via diffusion (extracellular to cytosol)	1 met-L[e] <==> 1 met-L	reversible	Transport	-1.00E+09	1.00E+09	1.47E+04	1.33E+05	1.18E+05	1.09E+04	1.41E+05	129758.02	1.19E+04	1.15E+05	103535.85
MGt5	magnesium transport in/out via permease (no H+)	1 mg2 <==> 1 mg2[e]	reversible	Transport	-1.00E+09	1.00E+09	-3.07E+04	-1.46E+04	1.61E+04	-3.38E+04	-1.08E+04	23040	-2.09E+04	-1.08E+04	10161.68
MetG_RS_DIM	dimerization of Methionyl-tRNA synthetase	2 MetG_mono <==> 1 Met_RS_dim	reversible	Protein complex formation	-1.00E+09	1.00E+09	0	1.21E+03	1.21E+03	0	2.08E+03	2075.962	0	5.56E+01	55.6335
Met_RS_CHARGa	Methionyl-tRNA synthetase charging	1 Met_RS_dim + 2 atp + 2 h2o + 2 met-L --> 1 Met_RS_2met_2amp 2 h + 2 ppi	irreversible	Aminoacyl-tRNA synthetase charging	0.00E+00	1.00E+09	7.18E+03	7.32E+04	6.61E+04	5.28E+03	7.89E+04	73616.67	5.28E+03	6.44E+04	59069.45
Met_RS_CHARGb	Methionyl-tRNA synthetase charging	1 Met_RS_dim + 1 atp + 1 h2o + 1 met-L --> 1 Met_RS_met_amp + 1 h + 1 ppi	irreversible	Aminoacyl-tRNA synthetase charging	0.00E+00	1.00E+09	0	2.30E+04	2.30E+04	0	2.37E+04	23706.84	0	2.15E+04	21498.08
MiaA_DIM	Dimerization of MiaA	2 MiaA_mono <==> 1 MiaA_dim	reversible	Protein complex formation	-1.00E+09	1.00E+09	0	1.90E+02	1.90E+02	0	2.74E+02	273.6907	0	8.06E+01	80.586
Orn_DIM	TEMP_Orn_dim	2 Orn_mono --> 1 Orn_dim	irreversible	Protein complex formation	0.00E+00	1.00E+09	0	1.24E+03	1.24E+03	0	2.16E+03	2155.584	0	3.68E+01	36.76
PHEtec	L-phenylalanine transport via diffusion (extracellular to cytosol)	1 phe-L[e] <==> 1 phe-L	reversible	Transport	-1.00E+09	1.00E+09	2.17E+04	1.70E+05	1.48E+05	1.60E+04	1.70E+05	153621.18	1.60E+04	1.62E+05	146446.98
PIt	Inorganic phosphate exchange, diffusion	1 pi <==> 1 pi[e]	reversible	Transport	-1.00E+09	1.00E+09	1.54E+06	1.03E+07	8.80E+06	1.13E+06	1.03E+07	9209854	1.13E+06	1.03E+07	9135134
PNPase_TRIM	PNPase trimerization	3 Pnp_mono <==> 1 Pnp_trim	reversible	Protein complex formation	-1.00E+09	1.00E+09	0	2.20E+02	2.20E+02	0	2.39E+02	238.7058	0	2.88E+01	28.8145
PROt5r	L-proline transport via diffusion	1 pro-L[e] <==> 1 pro-L	reversible	Transport	-1.00E+09	1.00E+09	2.35E+04	2.02E+05	1.79E+05	1.73E+04	2.02E+05	185078.72	1.73E+04	2.00E+05	182852.72
PheST_RS_TETRA	formation of phenyl-tRNA synthetase (2 a;pha, 2 beta subunits)	2 PheS_mono + 2 PheT_mono + 2 zn2 <==> 1 Phe_RS_tetra	reversible	Protein complex formation	-1.00E+09	1.00E+09	0	3.64E+02	3.64E+02	0	5.97E+02	596.7828	0	5.36E+01	53.5624
Phe_RS_CHARG	Phenyl-tRNA synthetase charging	1 Phe_RS_tetra + 1 atp + 1 h2o + 1 phe-L --> 1 Phe_RS_phe_amp + 1 h + 1 ppi	irreversible	Aminoacyl-tRNA synthetase charging	0.00E+00	1.00E+09	2.17E+04	1.73E+05	1.52E+05	1.60E+04	1.76E+05	159664.48	1.60E+04	1.63E+05	147228.38
ProS_RS_DIM	dimerization of Prolyl-tRNA synthetase	2 ProS_mono <==> 1 Pro_RS_dim	reversible	Protein complex formation	-1.00E+09	1.00E+09	0	1.24E+03	1.24E+03	0	2.05E+03	2053.575	0	5.72E+01	57.2055

Pro_RS_CHARGa	Prolyl-tRNA synthetase charging	1 Pro_RS_dim + 2 atp + 2 h2o + 2 pro-L --> 1 Pro_RS_2pro_2amp + 2 h + 2 ppi	irreversible	Aminoacyl-tRNA synthetase charging	0.00E+00	1.00E+09	0	1.03E+05	1.03E+05	0	1.05E+05	104716.7	0	1.00E+05	100346.3
Pro_RS_CHARGb	Prolyl-tRNA synthetase charging	1 Pro_RS_dim + 1 atp + 1 h2o + 1 pro-L --> 1 Pro_RS_pro_amp + 1 + 1 ppi	irreversible	Aminoacyl-tRNA synthetase charging	0.00E+00	1.00E+09	0	2.07E+05	2.07E+05	0	2.09E+05	209433.4	0	2.01E+05	200692.5
QueF_DIM	Dimerization of QueF	2 QueF_mono <==> 1 QueF_dim	reversible	Protein complex formation	-1.00E+09	1.00E+09	0	1.26E+03	1.26E+03	0	2.20E+03	2200.206	0	3.26E+01	32.592
RF1_FORM1	protein release factor RF1 formation1 (methylation)	1 PrfA_mono + 1 PrmC_mono + 1 amet --> 1 PrmC_RF1_cplx	irreversible	Protein Modification	0.00E+00	1.00E+09	0	2.52E+03	2.52E+03	0	4.38E+03	4382.152	0	9.89E+01	98.868
RF1_FORM2	protein release factor RF1 formation2 (methylation)	1 PrmC_RF1_cplx --> 1 PrmC_mono_inact + 1 RF1_mono_inact + 1 ahcys + 1 h	irreversible	Protein Modification	0.00E+00	1.00E+09	0	2.52E+03	2.52E+03	0	4.38E+03	4382.152	0	9.89E+01	98.868
RF2_FORM1	release factor 2 formation 1 (methylation of Gln252)	1 PrfB_mono + 1 PrmC_mono + 1 amet <==> 1 PrmC_RF2_cplx	reversible	Protein Modification	-1.00E+09	1.00E+09	0	1.07E+03	1.07E+03	0	1.82E+03	1824.129	0	8.65E+01	86.541
RF2_FORM2	protein release factor RF2 formation2 (methylation)	1 PrmC_RF2_cplx --> 1 PrmC_mono_inact + 1 RF2_mono_inact + 1 ahcys + 1 h	irreversible	Protein Modification	0.00E+00	1.00E+09	0	1.07E+03	1.07E+03	0	1.82E+03	1824.129	0	8.65E+01	86.541
RF3_FORM	protein release factor RF3 formation	1 PrfC_mono + 1 gtp + 1 h2o --> 1 RF3_mono.GDP + 1 h + 1 pi	irreversible	Protein complex formation	0.00E+00	1.00E+09	0	2.49E+03	2.49E+03	0	4.33E+03	4334.315	0	9.14E+01	91.388
RNAP19_FORM	RNA polymerase, sigma 19, formation	1 FecI_mono + 1 hRNAP <==> 1 RNAP_19	reversible	Protein complex formation	-1.00E+09	1.00E+09	0	0	0.00E+00	0	0	0	0	0	0
RNAP24_FORM	RNA polymerase, sigma 24, formation	1 RpoE_mono + 1 hRNAP <==> 1 RNAP_24	reversible	Protein complex formation	-1.00E+09	1.00E+09	0	3.99E+02	3.99E+02	0	5.44E+02	543.5469	0	6.81E+00	6.814
RNAP28_FORM	RNA polymerase, sigma 28, formation	1 FliA_mono + 1 hRNAP <==> 1 RNAP_28	reversible	Protein complex formation	-1.00E+09	1.00E+09	0	0	0.00E+00	0	0	0	0	0	0
RNAP32_FORM	RNA polymerase, sigma 32, formation	1 RpoH_mono + 1 hRNAP <==> 1 RNAP_32	reversible	Protein complex formation	-1.00E+09	1.00E+09	0	3.37E+02	3.37E+02	0	4.79E+02	478.8246	0	1.43E+01	14.262
RNAP38_FORM	RNA polymerase, sigma 38, formation	1 RpoS_mono + 1 hRNAP <==> 1 RNAP_38	reversible	Protein complex formation	-1.00E+09	1.00E+09	0	2.28E+02	2.28E+02	0	2.73E+02	273.4138	0	1.52E+01	15.16
RNAP54_FORM	RNA polymerase, sigma 54, formation	1 RpoN_mono + 1 hRNAP <==> 1 RNAP_54	reversible	Protein complex formation	-1.00E+09	1.00E+09	0	5.93E+02	5.93E+02	0	9.40E+02	939.6599	0	6.22E-01	0.622
RNAP70_FORM	RNA polymerase, sigma 70, formation	1 RpoD_mono + 1 hRNAP <==> 1 RNAP_70	reversible	Protein complex formation	-1.00E+09	1.00E+09	0	1.86E+03	1.86E+03	3.57E+01	1.26E+03	1222.624	3.57E+01	4.97E+02	460.9116
RNase_BN_DIM	diemrization of ElaC_mono to form active RNase_BN	2 ElaC_mono <==> 1 RNase_BN_dim	reversible	Protein complex formation	-1.00E+09	1.00E+09	0	1.21E+03	1.21E+03	0	2.10E+03	2100.966	0	4.57E+01	45.6535
RNase_E_TETRAM	RNase E tetramerization	4 Rne_mono + 2 zn2 <==> 1 RNase_E_tetra	reversible	Protein complex formation	-1.00E+09	1.00E+09	0	3.49E+02	3.49E+02	0	3.79E+02	379.1216	0	2.88E+01	28.8145
RNase_G_DIM	dimerization of Rng_mono to form active RNase_dim	2 Rng_mono <==> 1 RNase_G_dim	reversible	Protein complex formation	-1.00E+09	1.00E+09	0	4.23E+02	4.23E+02	0	7.19E+02	718.9986	0	3.88E+01	38.836
RNase_Gen_FORMa	formation of generic RNase (consists of can be done by RNase T, PH (both show highest act.), D (Rnd_mono), II (Rnb_mono), BN(BN has poor acitivity - presence of only one out of these 5 RNase is sufficient for growth)	1 RNase_T_dim --> 1 RNase_Gen	irreversible	Protein Complex formation	0.00E+00	1.00E+09	0	1.73E+02	1.73E+02	0	2.71E+02	271.4044	0	4.52E+01	45.246
RNase_Gen_FORMb	formation of generic RNase (consists of can be done by RNase T, PH (both show highest act.), D (Rnd_mono), II (Rnb_mono), BN(BN has poor acitivity - presence of only one out of these 5 RNase is sufficient for growth)	1 Rnd_mono --> 1 RNase_Gen	irreversible	Protein Complex formation	0.00E+00	1.00E+09	0	2.50E+03	2.50E+03	0	4.11E+03	4113.529	0	7.31E+01	73.139
RNase_Gen_FORMc	formation of generic RNase (consists of can be done by RNase T, PH (both show highest act.), D (Rnd_mono), II (Rnb_mono), BN(BN has poor acitivity - presence of only one out of these 5 RNase is sufficient for growth)	1 RNase_PH --> 1 RNase_Gen	irreversible	Protein Complex formation	0.00E+00	1.00E+09	0	0	0.00E+00	0	0	0	0	0	0
RNase_Gen_FORMd	formation of generic RNase (consists of can be done by RNase T, PH (both show highest act.), D (Rnd_mono), II (Rnb_mono), BN(BN has poor acitivity - presence of only one out of these 5 RNase is sufficient for growth)	1 Rnb_mono --> 1 RNase_Gen	irreversible	Protein Complex formation	0.00E+00	1.00E+09	0	2.38E+03	2.38E+03	0	3.79E+03	3791.216	0	1.25E+02	125.43
RNase_Gen_FORMe	formation of generic RNase (consists of can be done by RNase T, PH (both show highest act.), D (Rnd_mono), II (Rnb_mono), BN(BN has poor acitivity - presence of only one out of these 5 RNase is sufficient for growth)	1 RNase_BN_dim --> 1 RNase_Gen	irreversible	Protein Complex formation	0.00E+00	1.00E+09	0	1.21E+03	1.21E+03	0	2.10E+03	2100.966	0	4.57E+01	45.6535
RNase_III_DIM	RNase III dimerization	2 Rnc_mono <==> 1 RNase_III_dim	reversible	Protein complex formation	-1.00E+09	1.00E+09	0	5.64E+02	5.64E+02	0	9.72E+02	971.8349	0	3.31E+01	33.0525
RNase_P_FORM	formation of active RNase P	1 RnpA_mono + 1 RnpB_RNA + 2 mg2 <==> 1 RNase_P_cplx	reversible	Protein complex formation	-1.00E+09	1.00E+09	0	2.81E+02	2.81E+02	0	5.93E+01	59.25644	0	1.08E+01	10.8499
RNase_T_DIM	dimerization of Rnt_mono to form active RNase T	2 Rnt_mono <==> 1 RNase_T_dim	reversible	Protein complex formation	-1.00E+09	1.00E+09	0	1.73E+02	1.73E+02	0	2.71E+02	271.4044	0	4.52E+01	45.246
RP_L11_FORM1	Ribosomal protein L11 formation (1)	1 PrmA_mono + 1 RplK_mono + 9 amet --> 1 PrmA_rpL11_cplx	irreversible	Ribosomal protein modification	0.00E+00	1.00E+09	8.44E+01	1.13E+02	2.81E+01	6.22E+01	8.29E+01	20.72	6.22E+01	8.29E+01	20.72
RP_L11_FORM2	Ribosomal protein L11 formation (2)	1 PrmA_rpL11_cplx --> 1 PrmA_mono_inact + 1 RplK_mono_me3 + ahcys + 9 h	irreversible	Ribosomal protein modification	0.00E+00	1.00E+09	8.44E+01	1.13E+02	2.81E+01	6.22E+01	8.29E+01	20.72	6.22E+01	8.29E+01	20.72
RP_L15_FORM	Ribosomal protein L15 formation	1 RplO_mono + 1 spmd --> 1 RplO_mono_spmd	irreversible	Protein complex formation	0.00E+00	1.00E+09	8.44E+01	1.13E+02	2.81E+01	6.22E+01	8.29E+01	20.72	6.22E+01	8.29E+01	20.72
RP_L16_FORM1	Ribosomal protein L16 formation (1) (Methyltransferase is unknown)	1 MeT_L16 + 1 RplP_mono + 1 amet --> 1 MeT_rpL16_cplx	irreversible	Ribosomal protein modification	0.00E+00	1.00E+09	8.44E+01	1.13E+02	2.81E+01	6.22E+01	8.29E+01	20.72	6.22E+01	8.29E+01	20.72
RP_L16_FORM2	Ribosomal protein L16 formation (2) (Methyltransferase is unknown)	1 MeT_rpL16_cplx --> 1 MeT_L16_inact + 1 RplP_mono_me + 1 ahcys + 1 h	irreversible	Ribosomal protein modification	0.00E+00	1.00E+09	8.44E+01	1.13E+02	2.81E+01	6.22E+01	8.29E+01	20.72	6.22E+01	8.29E+01	20.72
RP_L17_FORM	Ribosomal protein L17 formation	1 RplQ_mono + 1 spmd --> 1 RplQ_mono_spmd	irreversible	Protein complex formation	0.00E+00	1.00E+09	8.44E+01	1.13E+02	2.81E+01	6.22E+01	8.29E+01	20.72	6.22E+01	8.29E+01	20.72
RP_L18_FORM	Ribosomal protein L18 formation	1 RplR_mono + 1 spmd --> 1 RplR_mono_spmd	irreversible	Protein complex formation	0.00E+00	1.00E+09	8.44E+01	1.13E+02	2.81E+01	6.22E+01	8.29E+01	20.72	6.22E+01	8.29E+01	20.72
RP_L2_FORM	Ribosomal protein L2 formation	1 RplB_mono + 1 spmd --> 1 RplB_mono_spmd	irreversible	Protein complex formation	0.00E+00	1.00E+09	8.44E+01	1.13E+02	2.81E+01	6.22E+01	8.29E+01	20.72	6.22E+01	8.29E+01	20.72
RP_L33_FORM1	Ribosomal protein L33 formation (1) (Methyltransferase is unknown)	1 MeT_L33 + 1 RpmG_mono + 1 amet --> 1 MeT_rpL33_cplx	irreversible	Ribosomal protein modification	0.00E+00	1.00E+09	8.44E+01	1.13E+02	2.81E+01	6.22E+01	8.29E+01	20.72	6.22E+01	8.29E+01	20.72
RP_L33_FORM2	Ribosomal protein L33 formation (2) (Methyltransferase is unknown)	1 MeT_rpL33_cplx --> 1 MeT_L33_inact + 1 RpmG_mono_me + 1 ahcys + 1 h	irreversible	Ribosomal protein modification	0.00E+00	1.00E+09	8.44E+01	1.13E+02	2.81E+01	6.22E+01	8.29E+01	20.72	6.22E+01	8.29E+01	20.72

RP_L3_FORM1	Ribosomal protein L3 formation (1)	1 PrmB_mono + 1 RplC_mono + 1 amet --> 1 PrmB_rpL3_cplx	irreversible	Ribosomal protein modification	0.00E+00	1.00E+09	8.44E+01	1.13E+02	2.81E+01	6.22E+01	8.29E+01	20.72	6.22E+01	8.29E+01	20.72
RP_L3_FORM2	Ribosomal protein L3 formation (2)	1 PrmB_rpL3_cplx + 1 spmd --> 1 PrmB_mono_inact + 1 RplC_mono_me_spmd + 1 ahcys + 1 h	irreversible	Protein complex formation	0.00E+00	1.00E+09	8.44E+01	1.13E+02	2.81E+01	6.22E+01	8.29E+01	20.72	6.22E+01	8.29E+01	20.72
RP_L6_FORM	Ribosomal protein L6 formation	1 RplF_mono + 1 spmd --> 1 RplF_mono_spmd	irreversible	Protein complex formation	0.00E+00	1.00E+09	8.44E+01	1.13E+02	2.81E+01	6.22E+01	8.29E+01	20.72	6.22E+01	8.29E+01	20.72
RP_L7/12_FORM	dimer L7/L12 formation	1 RplL_mono_ac + 1 RplL_mono_me --> 1 rpL7/12	irreversible	Protein complex formation	0.00E+00	1.00E+09	1.69E+02	2.25E+02	5.63E+01	1.24E+02	1.66E+02	41.44	1.24E+02	1.66E+02	41.44
RP_L7_FORM1	Ribosomal protein L7 formation (1)	1 RimL_mono + 1 RplL_mono + 1 accoa --> 1 RimL_rpL7_cplx	irreversible	Ribosomal protein modification	0.00E+00	1.00E+09	1.69E+02	2.25E+02	5.63E+01	1.24E+02	1.66E+02	41.44	1.24E+02	1.66E+02	41.44
RP_L7_FORM2	Ribosomal protein L7 formation (2)	1 RimL_rpL7_cplx --> 1 RimL_mono_inact + 1 RplL_mono_ac + 1 coa	irreversible	Ribosomal protein modification	0.00E+00	1.00E+09	1.69E+02	2.25E+02	5.63E+01	1.24E+02	1.66E+02	41.44	1.24E+02	1.66E+02	41.44
RP_L7_FORM3	Ribosomal protein L7 formation (3) - methylation (K81)	1 MeT_L7/L12 + 1 RplL_mono + 1 amet <==> 1 MeT_L7/L12_rpL12_cplx	reversible	Ribosomal protein modification	-1.00E+09	1.00E+09	1.69E+02	2.25E+02	5.63E+01	1.24E+02	1.66E+02	41.44	1.24E+02	1.66E+02	41.44
RP_L7_FORM4	Ribosomal protein L7 formation (4) - methylation (K81)	1 MeT_L7/L12_rpL12_cplx --> 1 MeT_L7/L12_inact + 1 RplL_mono_me + 1 ahcys + 1 h	irreversible	Ribosomal protein modification	0.00E+00	1.00E+09	1.69E+02	2.25E+02	5.63E+01	1.24E+02	1.66E+02	41.44	1.24E+02	1.66E+02	41.44
RP_S11_FORM1	Ribosomal protein S11 formation (1) (Methyltransferase is unknown)	1 MeT_S11 + 1 RpsK_mono + 1 amet --> 1 MeT_rpS11_cplx	irreversible	Ribosomal protein modification	0.00E+00	1.00E+09	8.44E+01	1.13E+02	2.81E+01	6.22E+01	8.29E+01	20.72	6.22E+01	8.29E+01	20.72
RP_S11_FORM2	Ribosomal protein S11 formation (2)	1 MeT_rpS11_cplx --> 1 MeT_S11_inact + 1 RpsK_mono_me + 1 ahcys + 1 h	irreversible	Ribosomal protein modification	0.00E+00	1.00E+09	8.44E+01	1.13E+02	2.81E+01	6.22E+01	8.29E+01	20.72	6.22E+01	8.29E+01	20.72
RP_S12_FORM1	Ribosomal protein S12 formation (1) (beta-methylthio-aspartic acid)	1 MeST_S12 + 1 RpsL_mono + 1 amet + 1 cys-L + 1 fe2 --> 1 MeST_rpS12_cplx	irreversible	Ribosomal protein modification	0.00E+00	1.00E+09	8.44E+01	1.13E+02	2.81E+01	6.22E+01	8.29E+01	20.72	6.22E+01	8.29E+01	20.72
RP_S12_FORM2	Ribosomal protein S12 formation (2) (beta-methylthiolation)	1 MeST_rpS12_cplx --> 1 MeST_S12_inact + 1 RpsL_mono_me + 1 ahcys + 1 ala-L + 1 fe2 + 1 h	irreversible	Ribosomal protein modification	0.00E+00	1.00E+09	8.44E+01	1.13E+02	2.81E+01	6.22E+01	8.29E+01	20.72	6.22E+01	8.29E+01	20.72
RP_S18_FORM1	Ribosomal protein S18 formation (1)	1 RimI_mono + 1 RpsR_mono + 1 accoa --> 1 RimI_rps18_cplx	irreversible	Ribosomal protein modification	0.00E+00	1.00E+09	8.44E+01	1.13E+02	2.81E+01	6.22E+01	8.29E+01	20.72	6.22E+01	8.29E+01	20.72
RP_S18_FORM2	Ribosomal protein S18 formation (2)	1 RimI_rps18_cplx --> 1 RimI_mono_inact + 1 RpsR_mono_ac + 1 coa	irreversible	Ribosomal protein modification	0.00E+00	1.00E+09	8.44E+01	1.13E+02	2.81E+01	6.22E+01	8.29E+01	20.72	6.22E+01	8.29E+01	20.72
RP_S3_FORM	Ribosomal protein S3 formation	1 RpsC_mono + 1 spmd --> 1 RpsC_mono_spmd	irreversible	Protein complex formation	0.00E+00	1.00E+09	8.44E+01	1.13E+02	2.81E+01	6.22E+01	8.29E+01	20.72	6.22E+01	8.29E+01	20.72
RP_S4_FORM	Ribosomal protein S4 formation	1 RpsD_mono + 1 spmd --> 1 RpsD_mono_spmd	irreversible	Protein complex formation	0.00E+00	1.00E+09	8.44E+01	1.13E+02	2.81E+01	6.22E+01	8.29E+01	20.72	6.22E+01	8.29E+01	20.72
RP_S5_FORM1	Ribosomal protein S5 formation (1)	1 RimJ_mono + 1 RpsE_mono + 1 accoa --> 1 RimJ_rps5_cplx	irreversible	Ribosomal protein modification	0.00E+00	1.00E+09	8.44E+01	1.13E+02	2.81E+01	6.22E+01	8.29E+01	20.72	6.22E+01	8.29E+01	20.72
RP_S5_FORM2	Ribosomal protein S5 formation (2)	1 RimJ_rps5_cplx --> 1 RimJ_mono_inact + 1 RpsE_mono_ac + 1 coa	irreversible	Ribosomal protein modification	0.00E+00	1.00E+09	8.44E+01	1.13E+02	2.81E+01	6.22E+01	8.29E+01	20.72	6.22E+01	8.29E+01	20.72
RP_S6_FORM1	Ribosomal protein S6 formation (1) (ribosomal protein S6/ rimK/ ATP/ Glu complex	1 RimK_mono + 1 RpsF_mono + 4 atp + 4 glu-L --> 1 rps6_rimK_cplx	irreversible	Ribosomal protein modification	0.00E+00	1.00E+09	8.44E+01	1.13E+02	2.81E+01	6.22E+01	8.29E+01	20.72	6.22E+01	8.29E+01	20.72
RP_S6_FORM2	Ribosomal protein S6 formation (2) (ribosomal protein S6/ rimK/ ATP complex (release of phosphorylated S complexed with rimK)	1 rps6_rimK_cplx --> 1 RimK_mono_inact + 1 RpsF_mono_glu4 + 4 adp + 4 h + 4 pi	irreversible	Ribosomal protein modification	0.00E+00	1.00E+09	8.44E+01	1.13E+02	2.81E+01	6.22E+01	8.29E+01	20.72	6.22E+01	8.29E+01	20.72
RhlB_DIM	RNA helicase RhlB dimerization	2 RhlB_mono <==> 1 RhlB_dim	reversible	Protein complex formation	-1.00E+09	1.00E+09	0	2.20E+02	2.20E+02	0	2.39E+02	238.7058	0	2.88E+01	28.8145
Rho_HEXA	formation of Rho hexamer	6 Rho_mono + 3 atp + 3 mg2 --> 1 Rho_hexa	irreversible	Protein complex formation	0.00E+00	1.00E+09	0	3.67E+02	3.67E+02	0	6.32E+02	632.4217	0	2.04E+01	20.428
Rib_30_ass1	ribosomal subunit 30 S assembly 1 (21S)	1 16S_rRNA + 1 Era_dim.GTP + 1 RbfA_mono + 1 RimM_mono + 1 RpsD_mono_spmd + 1 RpsE_mono_ac + 1 RpsF_mono_glu4 + 1 RpsG_mono + 1 RpsH_mono + 1 RpsI_mono + 1 RpsK_mono_me + 1 RpsL_mono_me + 1 RpsM_mono + 1 RpsO_mono + 1 RpsP_mono + 1 RpsQ_mono + 1 RpsR_mono_ac + 1 RpsS_mono + 1 RpsT_mono + 1 YrdC_mono + 2 h2o --> 1 RI_30	irreversible	Ribosomal Assembly	0.00E+00	1.00E+09	8.44E+01	1.13E+02	2.81E+01	6.22E+01	8.29E+01	20.72	6.22E+01	8.29E+01	20.72
Rib_30_ass2a	ribosomal subunit 30 S assembly 2 (activation of RI_30, spontanous)	1 RI_30 --> 1 Era_dim.GDP + 1 RI_30* + 1 RbfA_mono_inact + 1 RimM_mono_inact + 1 YrdC_mono_inact + 2 h + 2 pi	irreversible	Ribosomal Assembly	0.00E+00	1.00E+09	0	1.13E+02	1.13E+02	0	8.29E+01	82.88	0	8.29E+01	82.88
Rib_30_ass2b_1	ribosomal subunit 30 S assembly 2 (activation of RI_30, DnaK mediated)	1 DnaJ_dim + 1 DnaK_mono.ATP + 1 RI_30 --> 1 DnaKJ_RI_30_cpl + 1 Era_dim.GDP + 1 RbfA_mono_inact + 1 RimM_mono_inact + 1 YrdC_mono_inact + 2 h + 2 pi	irreversible	Ribosomal Assembly	0.00E+00	1.00E+09	0	1.13E+02	1.13E+02	0	8.29E+01	82.88	0	8.29E+01	82.88
Rib_30_ass2b_2	ribosomal subunit 30 S assembly 2 (activation of RI_30, DnaK mediated)	1 DnaKJ_RI_30_cplx + 1 GrpE_dim + 1 h2o --> 1 DnaJ_dim_inact + DnaK_GrpE_RI_30_complex + 1 adp + 1 h + 1 pi	irreversible	Ribosomal Assembly	0.00E+00	1.00E+09	0	1.13E+02	1.13E+02	0	8.29E+01	82.88	0	8.29E+01	82.88
Rib_30_ass2b_3	ribosomal subunit 30 S assembly 2 (activation of RI_30, DnaK mediated)	1 DnaK_GrpE_RI_30_complex + 1 atp --> 1 DnaK_mono.ATP_inact 1 GrpE_dim_inact + 1 RI_30*	irreversible	Ribosomal Assembly	0.00E+00	1.00E+09	0	1.13E+02	1.13E+02	0	8.29E+01	82.88	0	8.29E+01	82.88
Rib_30_ass3	ribosomal subunit 30 S assembly 3	1 RI_30* + 1 RpsA_mono + 1 RpsB_mono + 1 RpsC_mono_spmd + RpsJ_mono + 1 RpsN_mono + 1 RpsU_mono + 1 Sra_mono + 60 mg2 --> 1 rib_30	irreversible	Ribosomal Assembly	0.00E+00	1.00E+09	8.44E+01	1.13E+02	2.81E+01	6.22E+01	8.29E+01	20.72	6.22E+01	8.29E+01	20.72
Rib_30_ini_FORM	formation of 30S translation initiation complex (30S subunit, IF1, IF2-GTP, IF3)	1 IF2-GTP + 1 rib_30_IF1_IF3 --> 1 rib_30_ini_inact	irreversible	Translation	0.00E+00	1.00E+09	5.32E+03	2.31E+04	1.78E+04	3.92E+03	2.38E+04	19852.92	3.92E+03	2.15E+04	17614.62
Rib_50_ass1	ribosomal subunit 50 S assembly 1	1 23S_rRNA + 1 5S_rRNA + 1 RplA_mono + 1 RplB_mono_spmd + RplC_mono_me_spmd + 1 RplD_mono + 1 RplE_mono + 1 RplI_mono + 1 RplJ_mono + 1 RplK_mono_me3 + 1 RplM_mono + 1 RplQ_mono_spmd + 1 RplS_mono + 1 RplT_mono + 1 RplU_mono + 1 RplV_mono + 1 RplW_mono + 1 RplX_mono + 1 RpmC_mono + 1 RpmG_mono_me + 1 RpmH_mono + 2 rpL7/12 --> 1 RI_50	irreversible	Ribosomal Assembly	0.00E+00	1.00E+09	8.44E+01	1.13E+02	2.81E+01	6.22E+01	8.29E+01	20.72	6.22E+01	8.29E+01	20.72
Rib_50_ass2a	ribosomal subunit 50 S assembly 2 (RI_50 activation, spontanous)	1 RI_50 --> 1 RI_50*	irreversible	Ribosomal Assembly	0.00E+00	1.00E+09	0	1.13E+02	1.13E+02	0	8.29E+01	82.88	0	8.29E+01	82.88
Rib_50_ass2b_1	ribosomal subunit 50 S assembly 2 (RI_50 activation, GroEL mediated)	1 GroEL.(7)ADP.cisGroES + 1 RI_50 + 7 atp + 1 transGroES_hepta -> 1 GroEL.(7)ATP.transGroES_RI_50_cplx + 7 adp + 1 cisGroES_hepta	irreversible	Ribosomal Assembly	0.00E+00	1.00E+09	0	1.13E+02	1.13E+02	0	8.29E+01	82.88	0	8.29E+01	82.88
Rib_50_ass2b_2	ribosomal subunit 50 S assembly 2 (RI_50 activation, GroEL mediated)	1 GroEL.(7)ATP.transGroES_RI_50_cplx + 7 h2o --> 1 GroEL.(7)ADP.transGroES_RI_50_cplx + 7 h + 7 pi	irreversible	Ribosomal Assembly	0.00E+00	1.00E+09	0	1.13E+02	1.13E+02	0	8.29E+01	82.88	0	8.29E+01	82.88
Rib_50_ass2b_3	ribosomal subunit 50 S assembly 2 (RI_50 activation, GroEL mediated)	1 GroEL.(7)ADP.transGroES_RI_50_cplx --> 1 GroEL.(7)ADP.transGroES + 1 RI_50*	irreversible	Ribosomal Assembly	0.00E+00	1.00E+09	0	1.13E+02	1.13E+02	0	8.29E+01	82.88	0	8.29E+01	82.88
Rib_50_ass3	ribosomal subunit 50 S assembly 3	1 RI_50* + 1 RplF_mono_spmd + 1 RplN_mono + 1 RplO_mono_spmd + 1 RplP_mono_me + 1 RplR_mono_spmd + 1 RplY_mono + 1 RpmA_mono + 1 RpmB_mono + 1 RpmD_mono + 1 RpmE_mono + 1 RpmF_mono + 1 RpmI_mono + 1 RpmJ_mono + 1 Tig_mono + 111 mg2 --> 1 rib_50_inact	irreversible	Ribosomal Assembly	0.00E+00	1.00E+09	8.44E+01	1.13E+02	2.81E+01	6.22E+01	8.29E+01	20.72	6.22E+01	8.29E+01	20.72
Rib_70_DISS	70S ribosome dissociation	1 IF1 + 1 IF3 + 1 rib_70 --> 1 rib_30_IF1_IF3 + 1 rib_50_inact	irreversible	Translation	0.00E+00	1.00E+09	5.32E+03	2.31E+04	1.78E+04	3.92E+03	2.38E+04	19852.92	3.92E+03	2.15E+04	17614.62
Rib_70_ass	70S ribosome formation	1 rib_30 + 1 rib_50 --> 1 rib_70	irreversible	Ribosomal Assembly	0.00E+00	1.00E+09	8.44E+01	1.13E+02	2.81E+01	6.22E+01	8.29E+01	20.72	6.22E+01	8.29E+01	20.72

RlmB_DIM	Dimerization of RlmB (b4180)	2 RlmB_mono <==> 1 RlmB_dim	reversible	Protein complex formation	-1.00E+09	1.00E+09	0	2.81E+02	2.81E+02	0	4.61E+02	460.6318	0	4.65E+01	46.466
RnpBa_RENAME	TEMP_RnpBa	1 b3123_RNA --> 1 RnpB_RNA	irreversible	rRNA formation	0.00E+00	1.00E+09	0	2.81E+02	2.81E+02	0	1.08E+01	10.8	0	1.08E+01	10.8
RnpBb_RENAME	TEMP_RnpBb	1 b3123_v1_RNA --> 1 RnpB_RNA	irreversible	rRNA formation	0.00E+00	1.00E+09	0	2.80E+01	2.80E+01	0	4.94E+01	49.41244	0	4.99E-02	0.0499
RrmA_DIM	Dimerization of RrmA (23S rRNA m1G745 methyltransferase (b1822))	2 RrmA_mono <==> 1 RrmA_dim	reversible	Protein complex formation	-1.00E+09	1.00E+09	0	1.26E+03	1.26E+03	0	2.19E+03	2190.606	0	4.48E+01	44.7735
SELNPt	transport of selnp	1 selnp <==> 1 selnp[e]	reversible	Transport	-1.00E+09	1.00E+09	-1.86E+03	0	1.86E+03	-1.07E+02	0	107.32	-1.07E+02	0	107.32
SERt5r	L-serine transport via diffusion	1 ser-L[e] <==> 1 ser-L	reversible	Transport	-1.00E+09	1.00E+09	3.45E+04	1.98E+05	1.63E+05	2.54E+04	1.98E+05	172117.76	2.54E+04	1.98E+05	172117.76
SO4tr	sulfate reversible uniport	1 so4[e] <==> 1 so4	reversible	Transport	-1.00E+09	1.00E+09	0	0	0.00E+00	0	0	0	0	0	0
SPMDti	spermidine irreversible uniport	1 spmd[e] --> 1 spmd	irreversible	Transport	0.00E+00	1.00E+09	6.75E+02	9.01E+02	2.25E+02	4.97E+02	6.63E+02	165.76	4.97E+02	6.63E+02	165.76
SelA_FORM	formation of SelA decamer (contains 1 pyridoxal-5-phosphate per monomer subunit)	10 SelA_mono + 5 pydx5p <==> 1 SelA_deca	reversible	Protein complex formation	-1.00E+09	1.00E+09	0	1.09E+02	1.09E+02	0	4.62E+01	46.224	0	9.74E+00	9.7362
SerS_RS_DIM	seryl-tRNA synthetase dimerization	2 SerS_mono <==> 1 Ser_RS_dim	reversible	Protein complex formation	-1.00E+09	1.00E+09	0	1.34E+03	1.34E+03	0	2.30E+03	2297.203	0	1.05E+02	105.2375
Ser_RS_CHARGa	Seryl-tRNA synthetase charging	1 Ser_RS_dim + 2 atp + 2 h2o + 2 ser-L --> 1 Ser_RS_2ser_2amp + h + 2 ppi	irreversible	Aminoacyl-tRNA synthetase charging	0.00E+00	1.00E+09	0	1.03E+05	1.03E+05	0	1.05E+05	104805.4	0	1.00E+05	100188.8
Ser_RS_CHARGb	Seryl-tRNA synthetase charging	1 Ser_RS_dim + 1 atp + 1 h2o + 1 ser-L --> 1 Ser_RS_ser_amp + 1 h + 1 ppi	irreversible	Aminoacyl-tRNA synthetase charging	0.00E+00	1.00E+09	0	2.06E+05	2.06E+05	0	2.10E+05	209610.8	0	2.00E+05	200377.6
TC_ala1	ternary complex formation with ala1-tRNA	1 EF-Tu.GTP + 1 ala1_tRNA_ala --> 1 EF-Tu.GTP.ala1-tRNA	irreversible	tRNA activation (EF-TU)	0.00E+00	1.00E+09	7.83E+04	3.88E+05	3.09E+05	5.76E+04	4.04E+05	345895.78	5.76E+04	3.52E+05	294635.08
TC_ala2	ternary complex formation with ala2-tRNA	1 EF-Tu.GTP + 1 ala2_tRNA_ala --> 1 EF-Tu.GTP.ala2-tRNA	irreversible	tRNA activation (EF-TU)	0.00E+00	1.00E+09	7.51E+03	1.23E+05	1.16E+05	5.53E+03	1.42E+05	136727.26	5.53E+03	1.05E+05	99582.36
TC_arg1	ternary complex formation with arg1-tRNA	1 EF-Tu.GTP + 1 arg1_tRNA_arg --> 1 EF-Tu.GTP.arg1-tRNA	irreversible	tRNA activation (EF-TU)	0.00E+00	1.00E+09	6.21E+04	2.70E+05	2.08E+05	4.57E+04	2.72E+05	225864.1	4.57E+04	2.60E+05	214349.6
TC_argU	ternary complex formation with argU-tRNA	1 EF-Tu.GTP + 1 argU_tRNA_ala --> 1 EF-Tu.GTP.argU-tRNA	irreversible	tRNA activation (EF-TU)	0.00E+00	1.00E+09	5.07E+02	1.59E+04	1.54E+04	3.73E+02	2.10E+04	20599.27	3.73E+02	9.06E+03	8686.903
TC_argW	ternary complex formation with argW-tRNA	1 EF-Tu.GTP + 1 argW_tRNA_arg --> 1 EF-Tu.GTP.argW-tRNA	irreversible	tRNA activation (EF-TU)	0.00E+00	1.00E+09	0	1.14E+04	1.14E+04	0	1.33E+04	13314.72	0	7.04E+03	7036.922
TC_argX	ternary complex formation with argX-tRNA	1 EF-Tu.GTP + 1 argX_tRNA_arg --> 1 EF-Tu.GTP.argX-tRNA	irreversible	tRNA activation (EF-TU)	0.00E+00	1.00E+09	3.38E+02	4.51E+04	4.48E+04	2.49E+02	5.26E+04	52370.69	2.49E+02	3.08E+04	30511.28
TC_asn1	ternary complex formation with asn1-tRNA	1 EF-Tu.GTP + 1 asn1_tRNA_asn --> 1 EF-Tu.GTP.asn1-tRNA	irreversible	tRNA activation (EF-TU)	0.00E+00	1.00E+09	2.64E+04	1.77E+05	1.51E+05	1.95E+04	1.89E+05	169232.42	1.95E+04	1.63E+05	143663.82
TC_asp1	ternary complex formation with asp1-tRNA	1 EF-Tu.GTP + 1 asp1_tRNA_asp --> 1 EF-Tu.GTP.asp1-tRNA	irreversible	tRNA activation (EF-TU)	0.00E+00	1.00E+09	3.55E+04	2.21E+05	1.85E+05	2.62E+04	2.21E+05	194871.44	2.62E+04	2.21E+05	194585.04
TC_cysT	ternary complex formation with asp1-tRNA	1 EF-Tu.GTP + 1 cysT_tRNA_cys --> 1 EF-Tu.GTP.cysT-tRNA	irreversible	tRNA activation (EF-TU)	0.00E+00	1.00E+09	3.38E+03	6.85E+04	6.51E+04	2.49E+03	7.80E+04	75550.35	2.49E+03	5.61E+04	53616.26
TC_gln1	ternary complex formation with gln1-tRNA	1 EF-Tu.GTP + 1 gln1_tRNA_gln --> 1 EF-Tu.GTP.gln1-tRNA	irreversible	tRNA activation (EF-TU)	0.00E+00	1.00E+09	1.95E+04	1.61E+05	1.42E+05	1.44E+04	1.74E+05	159569.24	1.44E+04	1.48E+05	133738.94
TC_gln2	ternary complex formation with gln2-tRNA	1 EF-Tu.GTP + 1 gln2_tRNA_gln --> 1 EF-Tu.GTP.gln2-tRNA	irreversible	tRNA activation (EF-TU)	0.00E+00	1.00E+09	6.92E+03	1.10E+05	1.03E+05	5.10E+03	1.25E+05	119982.98	5.10E+03	8.11E+04	76038.87
TC_glu1	ternary complex formation with glu1-tRNA	1 EF-Tu.GTP + 1 glu1_tRNA_glu --> 1 EF-Tu.GTP.glu1-tRNA	irreversible	tRNA activation (EF-TU)	0.00E+00	1.00E+09	5.91E+04	2.44E+05	1.85E+05	4.35E+04	2.46E+05	202239.9	4.35E+04	2.41E+05	197591.3
TC_gly1	ternary complex formation with gly1-tRNA	1 EF-Tu.GTP + 1 gly1_tRNA_gly --> 1 EF-Tu.GTP.gly1-tRNA	irreversible	tRNA activation (EF-TU)	0.00E+00	1.00E+09	5.94E+04	3.22E+05	2.63E+05	4.37E+04	3.41E+05	297767.62	4.37E+04	2.89E+05	245349.42
TC_glyT	ternary complex formation with glyT-tRNA	1 EF-Tu.GTP + 1 glyT_tRNA_gly --> 1 EF-Tu.GTP.glyT-tRNA	irreversible	tRNA activation (EF-TU)	0.00E+00	1.00E+09	7.60E+02	4.14E+04	4.07E+04	5.59E+02	4.71E+04	46499.04	5.59E+02	3.61E+04	35512.63
TC_glyU	ternary complex formation with glyU-tRNA	1 EF-Tu.GTP + 1 glyU_tRNA_gly --> 1 EF-Tu.GTP.glyU-tRNA	irreversible	tRNA activation (EF-TU)	0.00E+00	1.00E+09	6.75E+02	5.39E+04	5.32E+04	4.97E+02	6.25E+04	62041.16	4.97E+02	4.60E+04	45491.6
TC_hisR	ternary complex formation with hisR-tRNA	1 EF-Tu.GTP + 1 hisR_tRNA_his --> 1 EF-Tu.GTP.hisR-tRNA	irreversible	tRNA activation (EF-TU)	0.00E+00	1.00E+09	1.44E+04	8.97E+04	7.52E+04	1.06E+04	9.17E+04	81056.5	1.06E+04	8.70E+04	76404.25
TC_ile1	ternary complex formation with ile1-tRNA	1 EF-Tu.GTP + 1 ile1_tRNA_ile --> 1 EF-Tu.GTP.ile1-tRNA	irreversible	tRNA activation (EF-TU)	0.00E+00	1.00E+09	4.22E+04	2.66E+05	2.24E+05	3.11E+04	2.68E+05	237268.6	3.11E+04	2.44E+05	212773.4
TC_ile2	ternary complex formation with ile2-tRNA	1 EF-Tu.GTP + 1 ile2_tRNA_ile --> 1 EF-Tu.GTP.ile2-tRNA	irreversible	tRNA activation (EF-TU)	0.00E+00	1.00E+09	2.53E+02	2.35E+04	2.32E+04	1.86E+02	2.88E+04	28643.74	1.86E+02	1.69E+04	16706.63
TC_leu1	ternary complex formation with leu1-tRNA	1 EF-Tu.GTP + 1 leu1_tRNA_leu --> 1 EF-Tu.GTP.leu1-tRNA	irreversible	tRNA activation (EF-TU)	0.00E+00	1.00E+09	4.42E+04	2.86E+05	2.42E+05	3.26E+04	2.97E+05	264787.16	3.26E+04	2.51E+05	218188.56
TC_leu2	ternary complex formation with leu2-tRNA	1 EF-Tu.GTP + 1 leu2_tRNA_leu --> 1 EF-Tu.GTP.leu2-tRNA	irreversible	tRNA activation (EF-TU)	0.00E+00	1.00E+09	2.11E+03	7.59E+04	7.38E+04	1.55E+03	8.47E+04	83125.76	1.55E+03	6.29E+04	61390.68
TC_leuU	ternary complex formation with leuU-tRNA	1 EF-Tu.GTP + 1 leuU_tRNA_leu --> 1 EF-Tu.GTP.leuU-tRNA	irreversible	tRNA activation (EF-TU)	0.00E+00	1.00E+09	5.23E+03	1.07E+05	1.02E+05	3.85E+03	1.18E+05	113903.98	3.85E+03	9.56E+04	91727.67
TC_leuW	ternary complex formation with leuW-tRNA	1 EF-Tu.GTP + 1 leuW_tRNA_leu --> 1 EF-Tu.GTP.leuW-tRNA	irreversible	tRNA activation (EF-TU)	0.00E+00	1.00E+09	8.44E+01	2.16E+04	2.15E+04	6.22E+01	2.54E+04	25300.64	6.22E+01	1.72E+04	17178.58
TC_leuZ	ternary complex formation with leuZ-tRNA	1 EF-Tu.GTP + 1 leuZ_tRNA_leu --> 1 EF-Tu.GTP.leuZ-tRNA	irreversible	tRNA activation (EF-TU)	0.00E+00	1.00E+09	1.52E+03	8.27E+04	8.12E+04	1.12E+03	9.15E+04	90351.38	1.12E+03	5.88E+04	57667.91
TC_lys1	ternary complex formation with lys1-tRNA	1 EF-Tu.GTP + 1 lys1_tRNA_lys --> 1 EF-Tu.GTP.lys1-tRNA	irreversible	tRNA activation (EF-TU)	0.00E+00	1.00E+09	7.19E+04	2.81E+05	2.09E+05	5.30E+04	3.00E+05	246772.18	5.30E+04	2.46E+05	193472.08
TC_met1	ternary complex formation with met1-tRNA	1 EF-Tu.GTP + 1 met1_tRNA_met --> 1 EF-Tu.GTP.met1-tRNA	irreversible	tRNA activation (EF-TU)	0.00E+00	1.00E+09	1.44E+04	1.30E+05	1.15E+05	1.06E+04	1.41E+05	130816.8	1.06E+04	1.13E+05	102388.4
TC_phe1	ternary complex formation with phe1-tRNA	1 EF-Tu.GTP + 1 phe1_tRNA_phe --> 1 EF-Tu.GTP.phe1-tRNA	irreversible	tRNA activation (EF-TU)	0.00E+00	1.00E+09	2.17E+04	1.73E+05	1.52E+05	1.60E+04	1.76E+05	159664.48	1.60E+04	1.63E+05	147228.38
TC_pro1	ternary complex formation with pro1-tRNA	1 EF-Tu.GTP + 1 pro1_tRNA_pro --> 1 EF-Tu.GTP.pro1-tRNA	irreversible	tRNA activation (EF-TU)	0.00E+00	1.00E+09	1.67E+04	1.38E+05	1.21E+05	1.23E+04	1.51E+05	138552.52	1.23E+04	1.28E+05	115401.02
TC_pro2	ternary complex formation with pro2-tRNA	1 EF-Tu.GTP + 1 pro2_tRNA_pro --> 1 EF-Tu.GTP.pro2-tRNA	irreversible	tRNA activation (EF-TU)	0.00E+00	1.00E+09	3.55E+03	5.39E+04	5.03E+04	2.61E+03	6.18E+04	59223.94	2.61E+03	4.19E+04	39334.93
TC_proL	ternary complex formation with proL-tRNA	1 EF-Tu.GTP + 1 proL_tRNA_pro --> 1 EF-Tu.GTP.proL-tRNA	irreversible	tRNA activation (EF-TU)	0.00E+00	1.00E+09	2.53E+02	3.86E+04	3.83E+04	1.86E+02	4.57E+04	45486.69	1.86E+02	2.81E+04	27870.28
TC_proM	ternary complex formation with proM-tRNA	1 EF-Tu.GTP + 1 proM_tRNA_pro --> 1 EF-Tu.GTP.proM-tRNA	irreversible	tRNA activation (EF-TU)	0.00E+00	1.00E+09	2.96E+03	5.23E+04	4.94E+04	2.18E+03	6.13E+04	59156.69	2.18E+03	4.45E+04	42283.27
TC_sec	ternary complex formation with sec-tRNA	1 SelB_mono + 1 gtp + 1 sec_tRNA_sec --> 1 SelB.GTP.sec_tRNA	irreversible	tRNA activation (EF-TU)	0.00E+00	1.00E+09	0	6.39E+02	6.39E+02	0	1.37E+01	13.7	0	1.37E+01	13.7
TC_ser1	ternary complex formation with ser1-tRNA	1 EF-Tu.GTP + 1 ser1_tRNA_ser --> 1 EF-Tu.GTP.ser1-tRNA	irreversible	tRNA activation (EF-TU)	0.00E+00	1.00E+09	8.70E+03	5.45E+04	4.58E+04	6.40E+03	5.88E+04	52383.65	6.40E+03	4.83E+04	41871.29
TC_ser2	ternary complex formation with ser2-tRNA	1 EF-Tu.GTP + 1 ser2_tRNA_ser --> 1 EF-Tu.GTP.ser2-tRNA	irreversible	tRNA activation (EF-TU)	0.00E+00	1.00E+09	1.35E+04	7.19E+04	5.84E+04	9.95E+03	7.49E+04	64973.44	9.95E+03	6.51E+04	55196.43
TC_ser3	ternary complex formation with ser3-tRNA	1 EF-Tu.GTP + 1 ser3_tRNA_ser --> 1 EF-Tu.GTP.ser3-tRNA	irreversible	tRNA activation (EF-TU)	0.00E+00	1.00E+09	7.60E+02	4.39E+04	4.31E+04	5.59E+02	5.09E+04	50383.93	5.59E+02	3.60E+04	35446.53
TC_serT	ternary complex formation with serT-tRNA	1 EF-Tu.GTP + 1 serT_tRNA_ser --> 1 EF-Tu.GTP.serT-tRNA	irreversible	tRNA activation (EF-TU)	0.00E+00	1.00E+09	1.10E+03	3.41E+04	3.30E+04	8.08E+02	3.94E+04	38627.56	8.08E+02	2.82E+04	27377.49
TC_serV	ternary complex formation with serV-tRNA	1 EF-Tu.GTP + 1 serV_tRNA_ser --> 1 EF-Tu.GTP.serV-tRNA	irreversible	tRNA activation (EF-TU)	0.00E+00	1.00E+09	1.05E+04	1.14E+05	1.04E+05	7.71E+03	1.24E+05	116319.36	7.71E+03	1.04E+05	96377.16
TC_thr1	ternary complex formation with thr1-tRNA	1 EF-Tu.GTP + 1 thr1_tRNA_thr --> 1 EF-Tu.GTP.thr1-tRNA	irreversible	tRNA activation (EF-TU)	0.00E+00	1.00E+09	1.64E+04	1.27E+05	1.11E+05	1.21E+04	1.37E+05	125299.76	1.21E+04	1.15E+05	103317.86
TC_thr2	ternary complex formation with thr2-tRNA	1 EF-Tu.GTP + 1 thr2_tRNA_thr --> 1 EF-Tu.GTP.thr2-tRNA	irreversible	tRNA activation (EF-TU)	0.00E+00	1.00E+09	1.74E+04	8.45E+04	6.71E+04	1.28E+04	8.67E+04	73881.9	1.28E+04	7.80E+04	65237.21
TC_thr3	ternary complex formation with thr3-tRNA	1 EF-Tu.GTP + 1 thr3_tRNA_thr --> 1 EF-Tu.GTP.thr3-tRNA	irreversible	tRNA activation (EF-TU)	0.00E+00	1.00E+09	2.53E+03	7.89E+04	7.64E+04	1.86E+03	8.73E+04	85415.28	1.86E+03	6.83E+04	66480.6
TC_thrU	ternary complex formation with thrU-tRNA	1 EF-Tu.GTP + 1 thrU_tRNA_thr --> 1 EF-Tu.GTP.thrU-tRNA	irreversible	tRNA activation (EF-TU)	0.00E+00	1.00E+09	1.52E+03	3.87E+04	3.71E+04	1.12E+03	4.61E+04	44982.49	1.12E+03	2.97E+04	28541.32
TC_trpT	ternary complex formation with trpT-tRNA	1 EF-Tu.GTP + 1 trpT_tRNA_trp --> 1 EF-Tu.GTP.trpT-tRNA	irreversible	tRNA activation (EF-TU)	0.00E+00	1.00E+09	3.63E+03	5.38E+04	5.02E+04	2.67E+03	5.48E+04	52095.4	2.67E+03	5.25E+04	49845.16
TC_tyr1	ternary complex formation with tyr1-tRNA	1 EF-Tu.GTP + 1 tyr1_tRNA_tyr --> 1 EF-Tu.GTP.tyr1-tRNA	irreversible	tRNA activation (EF-TU)	0.00E+00	1.00E+09	1.27E+04	1.27E+05	1.14E+05	9.39E+03	1.29E+05	119488.84	9.39E+03	1.17E+05	107468.44
TC_val1	ternary complex formation with val1-tRNA	1 EF-Tu.GTP + 1 val1_tRNA_val --> 1 EF-Tu.GTP.val1-tRNA	irreversible	tRNA activation (EF-TU)	0.00E+00	1.00E+09	2.99E+04	2.02E+05	1.72E+05	2.20E+04	2.18E+05	195531.36	2.20E+04	1.73E+05	150984.96
TC_val2	ternary complex formation with val2-tRNA	1 EF-Tu.GTP + 1 val2_tRNA_val --> 1 EF-Tu.GTP.val2-tRNA	irreversible	tRNA activation (EF-TU)	0.00E+00	1.00E+09	6.50E+03	9.11E+04	8.46E+04	4.79E+03	1.06E+05	101600.58	4.79E+03	7.33E+04	68522.62

TC_val3	ternary complex formation with val3-tRNA	1 EF-Tu.GTP + 1 val3_tRNA_val --> 1 EF-Tu.GTP.val3-tRNA	irreversible	tRNA activation (EF-TU)	0.00E+00	1.00E+09	3.69E+04	1.59E+05	1.22E+05	2.72E+04	1.63E+05	135578.28	2.72E+04	1.54E+05	126576.38
TEMP_gltT_tRNA	TEMP_gltT_tRNA	1 gltT_tRNA_Se --> 1 gltT_tRNA	irreversible	tRNA modification	0.00E+00	1.00E+09	0	1.11E+02	1.11E+02	0	1.29E+01	12.9	0	1.29E+01	12.9
TEMP_gltU_tRNA	TEMP_gltU_tRNA	1 gltU_tRNA_Se --> 1 gltU_tRNA	irreversible	tRNA modification	0.00E+00	1.00E+09	0	1.12E+02	1.12E+02	0	1.42E+01	14.2	0	1.42E+01	14.2
TEMP_gltV_tRNA	TEMP_gltV_tRNA	1 gltV_tRNA_Se --> 1 gltV_tRNA	irreversible	tRNA modification	0.00E+00	1.00E+09	0	1.13E+02	1.13E+02	0	1.26E+01	12.6	0	1.26E+01	12.6
TEMP_gltW_tRNA	TEMP_gltW_tRNA	1 gltW_tRNA_Se --> 1 gltW_tRNA	irreversible	tRNA modification	0.00E+00	1.00E+09	0	1.11E+02	1.11E+02	0	8.54E+00	8.54	0	8.54E+00	8.54
TEMP_lysQ_tRNA	TEMP_lysQ_tRNA	1 lysQ_tRNA_Se --> 1 lysQ_tRNA	irreversible	tRNA modification	0.00E+00	1.00E+09	0	1.77E+03	1.77E+03	0	7.51E+00	7.51	0	7.51E+00	7.51
TEMP_lysT_tRNA	TEMP_lysT_tRNA	1 lysT_tRNA_Se --> 1 lysT_tRNA	irreversible	tRNA modification	0.00E+00	1.00E+09	0	3.51E+02	3.51E+02	0	7.52E+00	7.52	0	7.52E+00	7.52
TEMP_lysV_tRNA	TEMP_lysV_tRNA	1 lysV_tRNA_Se --> 1 lysV_tRNA	irreversible	tRNA modification	0.00E+00	1.00E+09	0	3.01E+02	3.01E+02	0	7.81E+00	7.81	0	7.81E+00	7.81
TEMP_lysW_tRNA	TEMP_lysW_tRNA	1 lysW_tRNA_Se --> 1 lysW_tRNA	irreversible	tRNA modification	0.00E+00	1.00E+09	0	3.51E+02	3.51E+02	0	7.52E+00	7.52	0	7.52E+00	7.52
TEMP_lysY_tRNA	TEMP_lysY_tRNA	1 lysY_tRNA_Se --> 1 lysY_tRNA	irreversible	tRNA modification	0.00E+00	1.00E+09	0	7.43E+02	7.43E+02	0	7.51E+00	7.51	0	7.51E+00	7.51
TEMP_lysZ_tRNA	TEMP_lysZ_tRNA	1 lysZ_tRNA_Se --> 1 lysZ_tRNA	irreversible	tRNA modification	0.00E+00	1.00E+09	0	1.77E+03	1.77E+03	0	7.51E+00	7.51	0	7.51E+00	7.51
THFt	5,6,7,8-Tetrahydrofolate transport	1 thf <==> 1 thf[e]	reversible	Transport	-1.00E+09	1.00E+09	5.23E+03	2.33E+04	1.80E+04	3.88E+03	2.38E+04	19872.77	3.88E+03	2.16E+04	17726.12
THRt5r	L-threonine transport via diffusion	1 thr-L[e] <==> 1 thr-L	reversible	Transport	-1.00E+09	1.00E+09	3.78E+04	2.32E+05	1.94E+05	2.79E+04	2.32E+05	204369.7	2.79E+04	2.32E+05	204369.7
TRPt	L-tryptophan transport	1 trp-L[e] <==> 1 trp-L	reversible	Transport	-1.00E+09	1.00E+09	3.63E+03	5.20E+04	4.84E+04	2.67E+03	5.20E+04	49362.35	2.67E+03	5.20E+04	49362.35
TU-8389_DNA_act_bind	TU-8389_DNA binding of activator	1 TU-8389_DNA_neu --> 1 TU-8389_DNA_act	irreversible	Transcription Regulation	0.00E+00	1.00E+09	0	5.81E+02	5.81E+02	0	1.03E+03	1025.084	0	6.81E+00	6.81
TU-8390_DNA_act_bind	TU-8390_DNA binding of activator	1 TU-8390_DNA_neu --> 1 TU-8390_DNA_act	irreversible	Transcription Regulation	0.00E+00	1.00E+09	0	5.81E+02	5.81E+02	0	1.03E+03	1025.084	0	6.81E+00	6.81
TU-8392_DNA_act_bind	TU-8392_DNA binding of activator	1 TU-8392_DNA_neu --> 1 TU-8392_DNA_act	irreversible	Transcription Regulation	0.00E+00	1.00E+09	0	3.54E+01	3.54E+01	0	6.24E+01	62.43587	0	1.42E-01	0.142
TU-8392_cleavage	Cleavage of TU-8392_mRNA	1 TU-8392_mRNA_cleav_cplx + 97 h2o --> 1 RNase_III_dim_inact + 29 amp + 1 b2891_v1_mRNA + 1 b2892_mRNA + 1 b2893_v1_mRNA + 24 cmp + 16 gmp + 97 h + 26 ump	irreversible	Cleavage polycistronic mRNA	0.00E+00	1.00E+09	0	3.54E+01	3.54E+01	0	6.24E+01	62.43587	0	1.42E-01	0.142
TU-8392_cleavage_cplx	Cleavage complex formation of TU-8392_mRNA (dsbCp2, no_common_name_3124)	1 RNase_III_dim + 1 TU-8392_mRNA <==> 1 TU-8392_mRNA_cleav_cplx	reversible	Cleavage polycistronic mRNA	-1.00E+09	1.00E+09	0	3.54E+01	3.54E+01	0	6.24E+01	62.43587	0	1.42E-01	0.142
TU-8397_DNA_act_bind	TU-8397_DNA binding of activator	1 TU-8397_DNA_neu --> 1 TU-8397_DNA_act	irreversible	Transcription Regulation	0.00E+00	1.00E+09	0	3.07E+02	3.07E+02	0	5.42E+02	542.1115	0	2.45E-01	0.245
TU-8398_DNA_act_bind	TU-8398_DNA binding of activator	1 TU-8398_DNA_neu --> 1 TU-8398_DNA_act	irreversible	Transcription Regulation	0.00E+00	1.00E+09	0	3.04E+01	3.04E+01	0	5.37E+01	53.74604	0	2.07E-01	0.207
TU-8398_cleavage	Cleavage of TU-8398_mRNA	1 TU-8398_mRNA_cleav_cplx + 235 h2o --> 1 RNase_III_dim_inact + 54 amp + 1 b3201_mRNA + 1 b3202_v2_mRNA + 1 b3203_v1_mRNA + 1 b3204_v1_mRNA + 1 b3205_v1_mRNA + 43 cmp + 67 gmp + 235 h + 67 ump	irreversible	Cleavage polycistronic mRNA	0.00E+00	1.00E+09	0	3.04E+01	3.04E+01	0	5.37E+01	53.74604	0	2.07E-01	0.207
TU-8398_cleavage_cplx	Cleavage complex formation of TU-8398_mRNA (lptBp, no_common_name_3119)	1 RNase_III_dim + 1 TU-8398_mRNA <==> 1 TU-8398_mRNA_cleav_cplx	reversible	Cleavage polycistronic mRNA	-1.00E+09	1.00E+09	0	3.04E+01	3.04E+01	0	5.37E+01	53.74604	0	2.07E-01	0.207
TU-8407_DNA_act_bind	TU-8407_DNA binding of activator	1 TU-8407_DNA_neu --> 1 TU-8407_DNA_act	irreversible	Transcription Regulation	0.00E+00	1.00E+09	0	3.60E+01	3.60E+01	0	6.36E+01	63.56211	0	1.89E-01	0.189
TU0-1181_DNA_act_bind	TU0-1181_DNA binding of activator	1 TU0-1181_DNA_neu --> 1 TU0-1181_DNA_act	irreversible	Transcription Regulation	0.00E+00	1.00E+09	0	1.12E+02	1.12E+02	0	1.36E+01	13.6	0	1.36E+01	13.6
TU0-1181_cutting	Cutting of stable TU0-1181_RNA	1 TU0-1181_RNA_cut_cplx + 391 h2o --> 1 RNase_E_tetra_inact + 1 RNase_G_dim_inact + 1 RNase_Gen_inact + 1 RNase_III_dim_inact + 1 RNase_P_cplx_inact + 1 RNase_T_dim_inact + 1 RNase_m16_inact + 1 RNase_m23_inact + 1 RNase_m5_inact + 13[illegible] amp + 1 b3851_RNA + 1 b3852_RNA + 1 b3853_RNA + 1 b3854_RNA + 1 b3855_RNA + 65 cmp + 81 gmp + 391 h + 1 ppi + 107 ump	irreversible	RNA processing	0.00E+00	1.00E+09	0	1.12E+02	1.12E+02	0	1.36E+01	13.6	0	1.36E+01	13.6
TU0-1181_cutting_cplx	Cutting complex formation of stable TU0-1181_RNA (rrnAp1, no_common_name_3075)	1 RNase_E_tetra + 1 RNase_G_dim + 1 RNase_Gen + 1 RNase_III_dim + 1 RNase_P_cplx + 1 RNase_T_dim + 1 RNase_m1[illegible] + 1 RNase_m23 + 1 RNase_m5 + 1 TU0-1181_RNA <==> 1 TU0-1181_RNA_cut_cplx	reversible	RNA processing	-1.00E+09	1.00E+09	0	1.12E+02	1.12E+02	0	1.36E+01	13.6	0	1.36E+01	13.6
TU0-1182_DNA_act_bind	TU0-1182_DNA binding of activator	1 TU0-1182_DNA_neu --> 1 TU0-1182_DNA_act	irreversible	Transcription Regulation	0.00E+00	1.00E+09	0	1.11E+02	1.11E+02	0	1.29E+01	12.9	0	1.29E+01	12.9
TU0-1182_cutting	Cutting of stable TU0-1182_RNA	1 TU0-1182_RNA_cut_cplx + 460 h2o --> 1 RNase_E_tetra_inact + 1 RNase_G_dim_inact + 1 RNase_Gen_inact + 1 RNase_III_dim_inact + 1 RNase_P_cplx_inact + 1 RNase_T_dim_inact + 1 RNase_m16_inact + 1 RNase_m23_inact + 1 RNase_m5_inact + 14[illegible] amp + 1 b3968_v1_RNA + 1 b3969_v1_RNA + 1 b3970_v1_RNA + 1 b3971_v1_RNA + 83 cmp + 106 gmp + 460 h + 1 ppi + 122 ump	irreversible	RNA processing	0.00E+00	1.00E+09	0	1.11E+02	1.11E+02	0	1.29E+01	12.9	0	1.29E+01	12.9
TU0-1182_cutting_cplx	Cutting complex formation of stable TU0-1182_RNA (rrsBp1, no_common_name_3074)	1 RNase_E_tetra + 1 RNase_G_dim + 1 RNase_Gen + 1 RNase_III_dim + 1 RNase_P_cplx + 1 RNase_T_dim + 1 RNase_m1[illegible] + 1 RNase_m23 + 1 RNase_m5 + 1 TU0-1182_RNA <==> 1 TU0-1182_RNA_cut_cplx	reversible	RNA processing	-1.00E+09	1.00E+09	0	1.11E+02	1.11E+02	0	1.29E+01	12.9	0	1.29E+01	12.9
TU0-1183_DNA_act_bind	TU0-1183_DNA binding of activator	1 TU0-1183_DNA_neu --> 1 TU0-1183_DNA_act	irreversible	Transcription Regulation	0.00E+00	1.00E+09	0	1.12E+02	1.12E+02	0	1.42E+01	14.2	0	1.42E+01	14.2
TU0-1183_cutting	Cutting of stable TU0-1183_RNA	1 TU0-1183_RNA_cut_cplx + 374 h2o --> 1 RNase_E_tetra_inact + 1 RNase_G_dim_inact + 1 RNase_Gen_inact + 1 RNase_III_dim_inact + 1 RNase_P_cplx_inact + 1 RNase_T_dim_inact + 1 RNase_m16_inact + 1 RNase_m23_inact + 1 RNase_m5_inact + 11[illegible] amp + 1 b3756_v1_RNA + 1 b3757_v1_RNA + 1 b3758_v1_RNA + 1 b3759_v1_RNA + 66 cmp + 86 gmp + 374 h + 1 ppi + 100 ump	irreversible	RNA processing	0.00E+00	1.00E+09	0	1.12E+02	1.12E+02	0	1.42E+01	14.2	0	1.42E+01	14.2
TU0-1183_cutting_cplx	Cutting complex formation of stable TU0-1183_RNA (rrnCp1, no_common_name_3073)	1 RNase_E_tetra + 1 RNase_G_dim + 1 RNase_Gen + 1 RNase_III_dim + 1 RNase_P_cplx + 1 RNase_T_dim + 1 RNase_m1[illegible] + 1 RNase_m23 + 1 RNase_m5 + 1 TU0-1183_RNA <==> 1 TU0-1183_RNA_cut_cplx	reversible	RNA processing	-1.00E+09	1.00E+09	0	1.12E+02	1.12E+02	0	1.42E+01	14.2	0	1.42E+01	14.2
TU0-1184_DNA_act_bind	TU0-1184_DNA binding of activator	1 TU0-1184_DNA_neu --> 1 TU0-1184_DNA_act	irreversible	Transcription Regulation	0.00E+00	1.00E+09	0	1.12E+02	1.12E+02	0	0	0	0	0	0
TU0-1184_cutting	Cutting of stable TU0-1184_RNA	1 TU0-1184_RNA_cut_cplx + 374 h2o --> 1 RNase_E_tetra_inact + 1 RNase_G_dim_inact + 1 RNase_Gen_inact + 1 RNase_III_dim_inact + 1 RNase_P_cplx_inact + 1 RNase_T_dim_inact + 1 RNase_m16_inact + 1 RNase_m23_inact + 1 RNase_m5_inact + 11[illegible] amp + 1 b3756_RNA + 1 b3757_RNA + 1 b3758_RNA + 1 b3759_RNA + 66 cmp + 86 gmp + 374 h + 1 ppi + 100 ump	irreversible	RNA processing	0.00E+00	1.00E+09	0	1.12E+02	1.12E+02	0	0	0	0	0	0
TU0-1184_cutting_cplx	Cutting complex formation of stable TU0-1184_RNA (rrnCp2, no_common_name_3072)	1 RNase_E_tetra + 1 RNase_G_dim + 1 RNase_Gen + 1 RNase_III_dim + 1 RNase_P_cplx + 1 RNase_T_dim + 1 RNase_m1[illegible] + 1 RNase_m23 + 1 RNase_m5 + 1 TU0-1184_RNA <==> 1 TU0-1184_RNA_cut_cplx	reversible	RNA processing	-1.00E+09	1.00E+09	0	1.12E+02	1.12E+02	0	0	0	0	0	0
TU0-1186_DNA_act_bind	TU0-1186_DNA binding of activator	1 TU0-1186_DNA_neu --> 1 TU0-1186_DNA_act	irreversible	Transcription Regulation	0.00E+00	1.00E+09	0	1.13E+02	1.13E+02	0	1.26E+01	12.6	0	1.26E+01	12.6

TU0-1186_cutting	Cutting of stable TU0-1186_RNA	1 TU0-1186_RNA_cut_cplx + 375 h2o --> 1 RNase_E_tetra_inact + 1 RNase_G_dim_inact + 1 RNase_Gen_inact + 1 RNase_III_dim_inact + 1 RNase_P_cplx_inact + 1 RNase_T_dim_inact + 1 RNase_m16_inact + 1 RNase_m23_inact + 1 RNase_m5_inact + 118 amp + 1 b4007_RNA + 1 b4008_RNA + 1 b4009_RNA + 1 b4010_RNA + 66 cmp + 85 gmp + 375 h + 1 ppi + 102 ump	irreversible	RNA processing	0.00E+00	1.00E+09	0	1.13E+02	1.13E+02	0	1.26E+01	12.6	0	1.26E+01	12.6
TU0-1186_cutting_cplx	Cutting complex formation of stable TU0-1186_RNA (rrnEp, no_common_name_3071)	1 RNase_E_tetra + 1 RNase_G_dim + 1 RNase_Gen + 1 RNase_III_dim + 1 RNase_P_cplx + 1 RNase_T_dim + 1 RNase_m1 + 1 RNase_m23 + 1 RNase_m5 + 1 TU0-1186_RNA <==> 1 TU0-1186_RNA_cut_cplx	reversible	RNA processing	-1.00E+09	1.00E+09	0	1.13E+02	1.13E+02	0	1.26E+01	12.6	0	1.26E+01	12.6
TU0-1187_DNA_act_bind	TU0-1187_DNA binding of activator	1 TU0-1187_DNA_neu --> 1 TU0-1187_DNA_act	irreversible	Transcription Regulation	0.00E+00	1.00E+09	0	1.11E+02	1.11E+02	0	8.54E+00	8.54	0	8.54E+00	8.54
TU0-1187_cutting	Cutting of stable TU0-1187_RNA	1 TU0-1187_RNA_cut_cplx + 451 h2o --> 1 RNase_E_tetra_inact + 1 RNase_G_dim_inact + 1 RNase_Gen_inact + 1 RNase_III_dim_inact + 1 RNase_P_cplx_inact + 1 RNase_T_dim_inact + 1 RNase_m16_inact + 1 RNase_m23_inact + 1 RNase_m5_inact + 141 amp + 1 b2588_v1_RNA + 1 b2589_v1_RNA + 1 b2590_v1_RNA + 1 b2591_v1_RNA + 82 cmp + 101 gmp + 451 h + 1 ppi + 123 ump	irreversible	RNA processing	0.00E+00	1.00E+09	0	1.11E+02	1.11E+02	0	8.54E+00	8.54	0	8.54E+00	8.54
TU0-1187_cutting_cplx	Cutting complex formation of stable TU0-1187_RNA (rrsGp1, no_common_name_3070)	1 RNase_E_tetra + 1 RNase_G_dim + 1 RNase_Gen + 1 RNase_III_dim + 1 RNase_P_cplx + 1 RNase_T_dim + 1 RNase_m1 + 1 RNase_m23 + 1 RNase_m5 + 1 TU0-1187_RNA <==> 1 TU0-1187_RNA_cut_cplx	reversible	RNA processing	-1.00E+09	1.00E+09	0	1.11E+02	1.11E+02	0	8.54E+00	8.54	0	8.54E+00	8.54
TU0-1188_DNA_act_bind	TU0-1188_DNA binding of activator	1 TU0-1188_DNA_neu --> 1 TU0-1188_DNA_act	irreversible	Transcription Regulation	0.00E+00	1.00E+09	0	1.11E+02	1.11E+02	0	0	0	0	0	0
TU0-1188_cutting	Cutting of stable TU0-1188_RNA	1 TU0-1188_RNA_cut_cplx + 451 h2o --> 1 RNase_E_tetra_inact + 1 RNase_G_dim_inact + 1 RNase_Gen_inact + 1 RNase_III_dim_inact + 1 RNase_P_cplx_inact + 1 RNase_T_dim_inact + 1 RNase_m16_inact + 1 RNase_m23_inact + 1 RNase_m5_inact + 141 amp + 1 b2588_RNA + 1 b2589_RNA + 1 b2590_RNA + 1 b2591_RNA + 82 cmp + 101 gmp + 451 h + 1 ppi + 123 ump	irreversible	RNA processing	0.00E+00	1.00E+09	0	1.11E+02	1.11E+02	0	0	0	0	0	0
TU0-1188_cutting_cplx	Cutting complex formation of stable TU0-1188_RNA (rrsGp2, no_common_name_3069)	1 RNase_E_tetra + 1 RNase_G_dim + 1 RNase_Gen + 1 RNase_III_dim + 1 RNase_P_cplx + 1 RNase_T_dim + 1 RNase_m1 + 1 RNase_m23 + 1 RNase_m5 + 1 TU0-1188_RNA <==> 1 TU0-1188_RNA_cut_cplx	reversible	RNA processing	-1.00E+09	1.00E+09	0	1.11E+02	1.11E+02	0	0	0	0	0	0
TU0-1189_DNA_act_bind	TU0-1189_DNA binding of activator	1 TU0-1189_DNA_neu --> 1 TU0-1189_DNA_act	irreversible	Transcription Regulation	0.00E+00	1.00E+09	0	1.11E+02	1.11E+02	0	9.54E+00	9.54	0	9.54E+00	9.54
TU0-1189_cutting	Cutting of stable TU0-1189_RNA	1 TU0-1189_RNA_cut_cplx + 391 h2o --> 1 RNase_E_tetra_inact + 1 RNase_G_dim_inact + 1 RNase_Gen_inact + 1 RNase_III_dim_inact + 1 RNase_P_cplx_inact + 1 RNase_T_dim_inact + 1 RNase_m16_inact + 1 RNase_m23_inact + 1 RNase_m5_inact + 130 amp + 1 b0201_v1_RNA + 1 b0202_v1_RNA + 1 b0203_v1_RNA + 1 b0204_v1_RNA + 1 b0205_v1_RNA + 67 cmp + 82 gmp + 391 h + 1 ppi + 107 ump	irreversible	RNA processing	0.00E+00	1.00E+09	0	1.11E+02	1.11E+02	0	9.54E+00	9.54	0	9.54E+00	9.54
TU0-1189_cutting_cplx	Cutting complex formation of stable TU0-1189_RNA (rrsHp1, no_common_name_3068)	1 RNase_E_tetra + 1 RNase_G_dim + 1 RNase_Gen + 1 RNase_III_dim + 1 RNase_P_cplx + 1 RNase_T_dim + 1 RNase_m1 + 1 RNase_m23 + 1 RNase_m5 + 1 TU0-1189_RNA <==> 1 TU0-1189_RNA_cut_cplx	reversible	RNA processing	-1.00E+09	1.00E+09	0	1.11E+02	1.11E+02	0	9.54E+00	9.54	0	9.54E+00	9.54
TU0-1190_DNA_act_bind	TU0-1190_DNA binding of activator	1 TU0-1190_DNA_neu --> 1 TU0-1190_DNA_act	irreversible	Transcription Regulation	0.00E+00	1.00E+09	0	1.11E+02	1.11E+02	0	0	0	0	0	0
TU0-1190_cutting	Cutting of stable TU0-1190_RNA	1 TU0-1190_RNA_cut_cplx + 391 h2o --> 1 RNase_E_tetra_inact + 1 RNase_G_dim_inact + 1 RNase_Gen_inact + 1 RNase_III_dim_inact + 1 RNase_P_cplx_inact + 1 RNase_T_dim_inact + 1 RNase_m16_inact + 1 RNase_m23_inact + 1 RNase_m5_inact + 130 amp + 1 b0201_RNA + 1 b0202_RNA + 1 b0203_RNA + 1 b0204_RNA + 1 b0205_RNA + 67 cmp + 82 gmp + 391 h + 1 ppi + 107 ump	irreversible	RNA processing	0.00E+00	1.00E+09	0	1.11E+02	1.11E+02	0	0	0	0	0	0
TU0-1190_cutting_cplx	Cutting complex formation of stable TU0-1190_RNA (rrsHp2, no_common_name_3067)	1 RNase_E_tetra + 1 RNase_G_dim + 1 RNase_Gen + 1 RNase_III_dim + 1 RNase_P_cplx + 1 RNase_T_dim + 1 RNase_m1 + 1 RNase_m23 + 1 RNase_m5 + 1 TU0-1190_RNA <==> 1 TU0-1190_RNA_cut_cplx	reversible	RNA processing	-1.00E+09	1.00E+09	0	1.11E+02	1.11E+02	0	0	0	0	0	0
TU0-1191_DNA_act_bind	TU0-1191_DNA binding of activator	1 TU0-1191_DNA_neu --> 1 TU0-1191_DNA_act	irreversible	Transcription Regulation	0.00E+00	1.00E+09	0	1.06E+02	1.06E+02	0	1.15E+01	11.5	0	1.15E+01	11.5
TU0-1191_cutting	Cutting of stable TU0-1191_RNA	1 TU0-1191_RNA_cut_cplx + 432 h2o --> 1 RNase_E_tetra_inact + 1 RNase_G_dim_inact + 1 RNase_Gen_inact + 1 RNase_III_dim_inact + 1 RNase_P_cplx_inact + 1 RNase_T_dim_inact + 1 RNase_m16_inact + 1 RNase_m23_inact + 1 RNase_m5_inact + 144 amp + 1 b3272_v1_RNA + 1 b3273_v1_RNA + 1 b3274_v1_RNA + 1 b3275_v1_RNA + 1 b3276_v1_RNA + 1 b3277_v1_RNA + 1 b3278_v1_RNA + 71 cmp + 88 gmp + 432 h + 1 ppi + 122 ump	irreversible	RNA processing	0.00E+00	1.00E+09	0	1.06E+02	1.06E+02	0	1.15E+01	11.5	0	1.15E+01	11.5
TU0-1191_cutting_cplx	Cutting complex formation of stable TU0-1191_RNA (rrnDp1, no_common_name_3066)	1 RNase_E_tetra + 1 RNase_G_dim + 1 RNase_Gen + 1 RNase_III_dim + 1 RNase_P_cplx + 1 RNase_T_dim + 1 RNase_m1 + 1 RNase_m23 + 1 RNase_m5 + 1 TU0-1191_RNA <==> 1 TU0-1191_RNA_cut_cplx	reversible	RNA processing	-1.00E+09	1.00E+09	0	1.06E+02	1.06E+02	0	1.15E+01	11.5	0	1.15E+01	11.5
TU0-1192_DNA_act_bind	TU0-1192_DNA binding of activator	1 TU0-1192_DNA_neu --> 1 TU0-1192_DNA_act	irreversible	Transcription Regulation	0.00E+00	1.00E+09	0	1.06E+02	1.06E+02	0	0	0	0	0	0
TU0-1192_cutting	Cutting of stable TU0-1192_RNA	1 TU0-1192_RNA_cut_cplx + 432 h2o --> 1 RNase_E_tetra_inact + 1 RNase_G_dim_inact + 1 RNase_Gen_inact + 1 RNase_III_dim_inact + 1 RNase_P_cplx_inact + 1 RNase_T_dim_inact + 1 RNase_m16_inact + 1 RNase_m23_inact + 1 RNase_m5_inact + 144 amp + 1 b3272_RNA + 1 b3273_RNA + 1 b3274_RNA + 1 b3275_RNA + 1 b3276_RNA + 1 b3277_RNA + 1 b3278_RNA + 71 cmp + 88 gmp + 432 h + 1 ppi + 122 ump	irreversible	RNA processing	0.00E+00	1.00E+09	0	1.06E+02	1.06E+02	0	0	0	0	0	0
TU0-1192_cutting_cplx	Cutting complex formation of stable TU0-1192_RNA (rrnDp2, no_common_name_3065)	1 RNase_E_tetra + 1 RNase_G_dim + 1 RNase_Gen + 1 RNase_III_dim + 1 RNase_P_cplx + 1 RNase_T_dim + 1 RNase_m1 + 1 RNase_m23 + 1 RNase_m5 + 1 TU0-1192_RNA <==> 1 TU0-1192_RNA_cut_cplx	reversible	RNA processing	-1.00E+09	1.00E+09	0	1.06E+02	1.06E+02	0	0	0	0	0	0
TU0-12803_DNA_act_bind	TU0-12803_DNA binding of activator	1 TU0-12803_DNA_neu --> 1 TU0-12803_DNA_act	irreversible	Transcription Regulation	0.00E+00	1.00E+09	0	3.42E+01	3.42E+01	0	6.04E+01	60.36359	0	1.86E-01	0.186
TU0-12803_cleavage	Cleavage of TU0-12803_mRNA	1 TU0-12803_mRNA_cleav_cplx + 12 h2o --> 1 RNase_III_dim_inact + 4 amp + 1 b0058_mRNA + 1 b0059_v1_mRNA + 3 cmp + 4 gmp + 12 h	irreversible	Cleavage polycistronic mRNA	0.00E+00	1.00E+09	0	3.42E+01	3.42E+01	0	6.04E+01	60.36359	0	1.86E-01	0.186
TU0-12803_cleavage_cpl	Cleavage complex formation of TU0-12803_mRNA (, no_common_name_3037)	1 RNase_III_dim + 1 TU0-12803_mRNA <==> 1 TU0-12803_mRNA_cleav_cplx	reversible	Cleavage polycistronic mRNA	-1.00E+09	1.00E+09	0	3.42E+01	3.42E+01	0	6.04E+01	60.36359	0	1.86E-01	0.186
TU0-12827_DNA_act_bind	TU0-12827_DNA binding of activator	1 TU0-12827_DNA_neu --> 1 TU0-12827_DNA_act	irreversible	Transcription Regulation	0.00E+00	1.00E+09	0	2.75E+01	2.75E+01	0	4.86E+01	48.6031	0	2.07E-01	0.207
TU0-12827_cleavage	Cleavage of TU0-12827_mRNA	1 TU0-12827_mRNA_cleav_cplx + 93 h2o --> 1 RNase_III_dim_inact + 26 amp + 1 b0166_v1_mRNA + 1 b0167_mRNA + 1 b0168_mRNA + 13 cmp + 23 gmp + 93 h + 29 ump	irreversible	Cleavage polycistronic mRNA	0.00E+00	1.00E+09	0	2.75E+01	2.75E+01	0	4.86E+01	48.6031	0	2.07E-01	0.207
TU0-12827_cleavage_cpl	Cleavage complex formation of TU0-12827_mRNA (, no_common_name_3012)	1 RNase_III_dim + 1 TU0-12827_mRNA <==> 1 TU0-12827_mRNA_cleav_cplx	reversible	Cleavage polycistronic mRNA	-1.00E+09	1.00E+09	0	2.75E+01	2.75E+01	0	4.86E+01	48.6031	0	2.07E-01	0.207

TU0-12830_DNA_act_bind	TU0-12830_DNA binding of activator	1 TU0-12830_DNA_neu --> 1 TU0-12830_DNA_act	irreversible	Transcription Regulation	0.00E+00	1.00E+09	0	2.46E+02	2.46E+02	0	4.34E+02	433.6892	0	3.99E-01	0.399
TU0-12833_DNA_act_bind	TU0-12833_DNA binding of activator	1 TU0-12833_DNA_neu --> 1 TU0-12833_DNA_act	irreversible	Transcription Regulation	0.00E+00	1.00E+09	0	9.48E+01	9.48E+01	0	1.67E+02	167.2985	0	1.66E-01	0.166
TU0-12921_DNA_act_bind	TU0-12921_DNA binding of activator	1 TU0-12921_DNA_neu --> 1 TU0-12921_DNA_act	irreversible	Transcription Regulation	0.00E+00	1.00E+09	0	4.96E+01	4.96E+01	0	8.75E+01	87.54595	0	9.13E-02	0.0913
TU0-12921_cleavage	Cleavage of TU0-12921_mRNA	1 TU0-12921_mRNA_cleav_cplx + 113 h2o --> 1 RNase_III_dim_inact + 35 amp + 1 b0413_mRNA + 1 b0414_mRNA + 1 b0415_mRNA + 1 b0416_mRNA + 31 cmp + 28 gmp + 113 h + 16 ump	irreversible	Cleavage polycistronic mRNA	0.00E+00	1.00E+09	0	4.96E+01	4.96E+01	0	8.75E+01	87.54595	0	9.13E-02	0.0913
TU0-12921_cleavage_cpl	Cleavage complex formation of TU0-12921_mRNA (, no_common_name_2917)	1 RNase_III_dim + 1 TU0-12921_mRNA <==> 1 TU0-12921_mRNA_cleav_cplx	reversible	Cleavage polycistronic mRNA	-1.00E+09	1.00E+09	0	4.96E+01	4.96E+01	0	8.75E+01	87.54595	0	9.13E-02	0.0913
TU0-12928_DNA_act_bind	TU0-12928_DNA binding of activator	1 TU0-12928_DNA_neu --> 1 TU0-12928_DNA_act	irreversible	Transcription Regulation	0.00E+00	1.00E+09	0	8.92E+01	8.92E+01	0	8.29E+01	82.88	0	3.08E-01	0.308
TU0-13010_DNA_act_bind	TU0-13010_DNA binding of activator	1 TU0-13010_DNA_neu --> 1 TU0-13010_DNA_act	irreversible	Transcription Regulation	0.00E+00	1.00E+09	0	2.29E+01	2.29E+01	0	4.04E+01	40.38653	0	1.46E-01	0.146
TU0-13010_cleavage	Cleavage of TU0-13010_mRNA , 1 nt short (b0640)	1 TU0-13010_mRNA_cleav_cplx + 42 h2o --> 1 RNase_III_dim_inact + 7 amp + 1 b0638_mRNA + 1 b0639_mRNA + 1 b0640_mRNA + 1 b0641_mRNA + 1 b0642_mRNA + 8 cmp + 16 gmp + 42 h + 7 ump	irreversible	Cleavage polycistronic mRNA	0.00E+00	1.00E+09	0	2.29E+01	2.29E+01	0	4.04E+01	40.38653	0	1.46E-01	0.146
TU0-13010_cleavage_cpl	Cleavage complex formation of TU0-13010_mRNA (, no_common_name_2829) , 1 nt short (b0640)	1 RNase_III_dim + 1 TU0-13010_mRNA <==> 1 TU0-13010_mRNA_cleav_cplx	reversible	Cleavage polycistronic mRNA	-1.00E+09	1.00E+09	0	2.29E+01	2.29E+01	0	4.04E+01	40.38653	0	1.46E-01	0.146
TU0-13018_DNA_act_bind	TU0-13018_DNA binding of activator	1 TU0-13018_DNA_neu --> 1 TU0-13018_DNA_act	irreversible	Transcription Regulation	0.00E+00	1.00E+09	0	8.54E+01	8.54E+01	0	1.51E+02	150.7476	0	1.92E-01	0.192
TU0-13020_DNA_act_bind	TU0-13020_DNA binding of activator	1 TU0-13020_DNA_neu --> 1 TU0-13020_DNA_act	irreversible	Transcription Regulation	0.00E+00	1.00E+09	0	7.55E+01	7.55E+01	0	1.33E+02	133.2851	0	4.14E-01	0.414
TU0-13034_DNA_act_bind	TU0-13034_DNA binding of activator	1 TU0-13034_DNA_neu --> 1 TU0-13034_DNA_act	irreversible	Transcription Regulation	0.00E+00	1.00E+09	0	7.43E+02	7.43E+02	0	7.51E+00	7.51	0	7.51E+00	7.51
TU0-13034_cutting	Cutting of stable TU0-13034_RNA	1 TU0-13034_RNA_cut_cplx + 5 h2o --> 1 RNase_E_tetra_inact + 1 RNase_G_dim_inact + 1 RNase_Gen_inact + 1 RNase_III_dim_inact + 1 RNase_P_cplx_inact + 1 RNase_T_dim_inact + 1 RNase_m16_inact + 1 RNase_m23_inact + 1 RNase_m5_inact + 1 b0746_RNA + 1 b0747_RNA + 2 cmp + 5 h + 1 ppi + 1 ump	irreversible	RNA processing	0.00E+00	1.00E+09	0	7.43E+02	7.43E+02	0	7.51E+00	7.51	0	7.51E+00	7.51
TU0-13034_cutting_cplx	Cutting complex formation of stable TU0-13034_RNA (, no_common_name_2805)	1 RNase_E_tetra + 1 RNase_G_dim + 1 RNase_Gen + 1 RNase_III_dim + 1 RNase_P_cplx + 1 RNase_T_dim + 1 RNase_m1 + 1 RNase_m23 + 1 RNase_m5 + 1 TU0-13034_RNA <==> 1 TU0-13034_RNA_cut_cplx	reversible	RNA processing	-1.00E+09	1.00E+09	0	7.43E+02	7.43E+02	0	7.51E+00	7.51	0	7.51E+00	7.51
TU0-13035_DNA_act_bind	TU0-13035_DNA binding of activator	1 TU0-13035_DNA_neu --> 1 TU0-13035_DNA_act	irreversible	Transcription Regulation	0.00E+00	1.00E+09	0	1.77E+03	1.77E+03	0	7.51E+00	7.51	0	7.51E+00	7.51
TU0-13035_trimming	Cutting of stable TU0-13035_RNA	1 TU0-13035_RNA_cut_cplx + 1 h2o --> 1 RNase_E_tetra_inact + 1 RNase_Gen_inact + 1 RNase_P_cplx_inact + 1 b0748_RNA + 1 h + ppi	irreversible	RNA Processing	0.00E+00	1.00E+09	0	1.77E+03	1.77E+03	0	7.51E+00	7.51	0	7.51E+00	7.51
TU0-13035_trimming_cplx	Cutting complex formation of stable TU0-13035_RNA (, no_common_name_2804)	1 RNase_E_tetra + 1 RNase_Gen + 1 RNase_P_cplx + 1 TU0-13035_RNA <==> 1 TU0-13035_RNA_cut_cplx	reversible	RNA Processing	-1.00E+09	1.00E+09	0	1.77E+03	1.77E+03	0	7.51E+00	7.51	0	7.51E+00	7.51
TU0-13036_DNA_act_bind	TU0-13036_DNA binding of activator	1 TU0-13036_DNA_neu --> 1 TU0-13036_DNA_act	irreversible	Transcription Regulation	0.00E+00	1.00E+09	0	1.77E+03	1.77E+03	0	7.51E+00	7.51	0	7.51E+00	7.51
TU0-13036_trimming	Cutting of stable TU0-13036_RNA	1 TU0-13036_RNA_cut_cplx + 1 h2o --> 1 RNase_E_tetra_inact + 1 RNase_Gen_inact + 1 RNase_P_cplx_inact + 1 b0749_RNA + 1 h + ppi	irreversible	RNA Processing	0.00E+00	1.00E+09	0	1.77E+03	1.77E+03	0	7.51E+00	7.51	0	7.51E+00	7.51
TU0-13036_trimming_cplx	Cutting complex formation of stable TU0-13036_RNA (, no_common_name_2803)	1 RNase_E_tetra + 1 RNase_Gen + 1 RNase_P_cplx + 1 TU0-13036_RNA <==> 1 TU0-13036_RNA_cut_cplx	reversible	RNA Processing	-1.00E+09	1.00E+09	0	1.77E+03	1.77E+03	0	7.51E+00	7.51	0	7.51E+00	7.51
TU0-13072_DNA_act_bind	TU0-13072_DNA binding of activator	1 TU0-13072_DNA_neu --> 1 TU0-13072_DNA_act	irreversible	Transcription Regulation	0.00E+00	1.00E+09	0	7.59E+01	7.59E+01	0	1.34E+02	133.9183	0	1.04E-01	0.104
TU0-13072_cleavage	Cleavage of TU0-13072_mRNA	1 TU0-13072_mRNA_cleav_cplx + 41 h2o --> 1 RNase_III_dim_inact + 9 amp + 1 b0858_mRNA + 1 b0859_mRNA + 7 cmp + 11 gmp + 41 h + 13 ump	irreversible	Cleavage polycistronic mRNA	0.00E+00	1.00E+09	0	7.59E+01	7.59E+01	0	1.34E+02	133.9183	0	1.04E-01	0.104
TU0-13072_cleavage_cpl	Cleavage complex formation of TU0-13072_mRNA (, no_common_name_2767)	1 RNase_III_dim + 1 TU0-13072_mRNA <==> 1 TU0-13072_mRNA_cleav_cplx	reversible	Cleavage polycistronic mRNA	-1.00E+09	1.00E+09	0	7.59E+01	7.59E+01	0	1.34E+02	133.9183	0	1.04E-01	0.104
TU0-13080_DNA_act_bind	TU0-13080_DNA binding of activator	1 TU0-13080_DNA_neu --> 1 TU0-13080_DNA_act	irreversible	Transcription Regulation	0.00E+00	1.00E+09	0	9.98E+01	9.98E+01	0	1.76E+02	176.1862	0	8.29E-01	0.829
TU0-13093_DNA_act_bind	TU0-13093_DNA binding of activator	1 TU0-13093_DNA_neu --> 1 TU0-13093_DNA_act	irreversible	Transcription Regulation	0.00E+00	1.00E+09	0	9.10E+01	9.10E+01	0	1.61E+02	160.6256	0	6.34E-01	0.634
TU0-13104_DNA_act_bind	TU0-13104_DNA binding of activator	1 TU0-13104_DNA_neu --> 1 TU0-13104_DNA_act	irreversible	Transcription Regulation	0.00E+00	1.00E+09	0	4.99E+02	4.99E+02	0	8.81E+02	880.9312	0	1.54E-01	0.154
TU0-13134_DNA_act_bind	TU0-13134_DNA binding of activator	1 TU0-13134_DNA_neu --> 1 TU0-13134_DNA_act	irreversible	Transcription Regulation	0.00E+00	1.00E+09	0	1.51E+02	1.51E+02	0	2.66E+02	265.9415	0	2.07E-01	0.207
TU0-13139_DNA_act_bind	TU0-13139_DNA binding of activator	1 TU0-13139_DNA_neu --> 1 TU0-13139_DNA_act	irreversible	Transcription Regulation	0.00E+00	1.00E+09	0	3.53E+01	3.53E+01	0	6.23E+01	62.29789	0	2.07E-01	0.207
TU0-13145_DNA_act_bind	TU0-13145_DNA binding of activator	1 TU0-13145_DNA_neu --> 1 TU0-13145_DNA_act	irreversible	Transcription Regulation	0.00E+00	1.00E+09	0	1.13E+02	1.13E+02	0	2.00E+02	199.9276	0	1.28E-01	0.128
TU0-13145_cleavage	Cleavage of TU0-13145_mRNA	1 TU0-13145_mRNA_cleav_cplx + 10 h2o --> 1 RNase_III_dim_inact + 4 amp + 1 b1134_mRNA + 1 b1135_mRNA + 1 cmp + 3 gmp + 10 + 1 ump	irreversible	Cleavage polycistronic mRNA	0.00E+00	1.00E+09	0	1.13E+02	1.13E+02	0	2.00E+02	199.9276	0	1.28E-01	0.128
TU0-13145_cleavage_cpl	Cleavage complex formation of TU0-13145_mRNA (, no_common_name_2694)	1 RNase_III_dim + 1 TU0-13145_mRNA <==> 1 TU0-13145_mRNA_cleav_cplx	reversible	Cleavage polycistronic mRNA	-1.00E+09	1.00E+09	0	1.13E+02	1.13E+02	0	2.00E+02	199.9276	0	1.28E-01	0.128
TU0-13195_DNA_act_bind	TU0-13195_DNA binding of activator	1 TU0-13195_DNA_neu --> 1 TU0-13195_DNA_act	irreversible	Transcription Regulation	0.00E+00	1.00E+09	0	1.56E+02	1.56E+02	0	2.75E+02	275.0224	0	2.45E-01	0.245
TU0-13225_DNA_act_bind	TU0-13225_DNA binding of activator	1 TU0-13225_DNA_neu --> 1 TU0-13225_DNA_act	irreversible	Transcription Regulation	0.00E+00	1.00E+09	0	1.53E+02	1.53E+02	0	2.70E+02	269.7588	0	1.89E-01	0.189
TU0-13421_DNA_act_bind	TU0-13421_DNA binding of activator	1 TU0-13421_DNA_neu --> 1 TU0-13421_DNA_act	irreversible	Transcription Regulation	0.00E+00	1.00E+09	0	1.62E+02	1.62E+02	0	2.86E+02	286.1908	0	7.98E-02	0.0798
TU0-13443_DNA_act_bind	TU0-13443_DNA binding of activator	1 TU0-13443_DNA_neu --> 1 TU0-13443_DNA_act	irreversible	Transcription Regulation	0.00E+00	1.00E+09	0	6.13E+01	6.13E+01	0	1.08E+02	108.2142	0	1.58E-01	0.158
TU0-13443_cleavage	Cleavage of TU0-13443_mRNA , 4 nt short (b1871)	1 TU0-13443_mRNA_cleav_cplx + 42 h2o --> 1 RNase_III_dim_inact + 7 amp + 1 b1869_mRNA + 1 b1870_mRNA + 1 b1871_mRNA + 12 cmp + 5 gmp + 42 h + 16 ump	irreversible	Cleavage polycistronic mRNA	0.00E+00	1.00E+09	0	6.13E+01	6.13E+01	0	1.08E+02	108.2142	0	1.58E-01	0.158
TU0-13443_cleavage_cpl	Cleavage complex formation of TU0-13443_mRNA (, no_common_name_2396) , 4 nt short (b1871)	1 RNase_III_dim + 1 TU0-13443_mRNA <==> 1 TU0-13443_mRNA_cleav_cplx	reversible	Cleavage polycistronic mRNA	-1.00E+09	1.00E+09	0	6.13E+01	6.13E+01	0	1.08E+02	108.2142	0	1.58E-01	0.158
TU0-13444_DNA_act_bind	TU0-13444_DNA binding of activator	1 TU0-13444_DNA_neu --> 1 TU0-13444_DNA_act	irreversible	Transcription Regulation	0.00E+00	1.00E+09	0	7.32E+01	7.32E+01	0	1.29E+02	129.311	0	3.26E-01	0.326
TU0-13519_DNA_act_bind	TU0-13519_DNA binding of activator	1 TU0-13519_DNA_neu --> 1 TU0-13519_DNA_act	irreversible	Transcription Regulation	0.00E+00	1.00E+09	0	5.98E+01	5.98E+01	0	1.06E+02	105.5798	0	5.13E-01	0.513
TU0-13534_DNA_act_bind	TU0-13534_DNA binding of activator	1 TU0-13534_DNA_neu --> 1 TU0-13534_DNA_act	irreversible	Transcription Regulation	0.00E+00	1.00E+09	0	1.49E+02	1.49E+02	0	2.63E+02	263.456	0	1.83E-01	0.183
TU0-13555_DNA_act_bind	TU0-13555_DNA binding of activator	1 TU0-13555_DNA_neu --> 1 TU0-13555_DNA_act	irreversible	Transcription Regulation	0.00E+00	1.00E+09	0	1.78E+02	1.78E+02	0	3.15E+02	314.9698	0	1.86E-01	0.186
TU0-13557_DNA_act_bind	TU0-13557_DNA binding of activator	1 TU0-13557_DNA_neu --> 1 TU0-13557_DNA_act	irreversible	Transcription Regulation	0.00E+00	1.00E+09	0	1.06E+02	1.06E+02	0	8.29E+01	82.88	0	3.50E-01	0.35
TU0-13573_DNA_act_bind	TU0-13573_DNA binding of activator	1 TU0-13573_DNA_neu --> 1 TU0-13573_DNA_act	irreversible	Transcription Regulation	0.00E+00	1.00E+09	0	1.37E+02	1.37E+02	0	2.42E+02	241.9725	0	1.71E-01	0.171
TU0-13593_DNA_act_bind	TU0-13593_DNA binding of activator	1 TU0-13593_DNA_neu --> 1 TU0-13593_DNA_act	irreversible	Transcription Regulation	0.00E+00	1.00E+09	0	2.70E+01	2.70E+01	0	4.77E+01	47.69847	0	8.76E-02	0.0876

TU0-13593_cleavage	Cleavage of TU0-13593_mRNA , 1 nt short (b2326), 1 nt short (b2327)	1 TU0-13593_mRNA_cleav_cplx + 75 h2o --> 1 RNase_III_dim_inact + 30 amp + 1 b2325_mRNA + 1 b2326_mRNA + 1 b2327_mRNA + 1 b2328_mRNA + 1 b2329_mRNA + 1 b2330_mRNA + 16 cmp + 16 gmp + 75 h + 8 ump	irreversible	Cleavage polycistronic mRNA	0.00E+00	1.00E+09	0	2.70E+01	2.70E+01	0	4.77E+01	47.69847	0	8.76E-02	0.0876
TU0-13593_cleavage_cpl	Cleavage complex formation of TU0-13593_mRNA (, no_common_name_2246) , 1 nt short (b2326), 1 nt short (b2327)	1 RNase_III_dim + 1 TU0-13593_mRNA <==> 1 TU0-13593_mRNA_cleav_cplx	reversible	Cleavage polycistronic mRNA	-1.00E+09	1.00E+09	0	2.70E+01	2.70E+01	0	4.77E+01	47.69847	0	8.76E-02	0.0876
TU0-13667_DNA_act_bind	TU0-13667_DNA binding of activator	1 TU0-13667_DNA_neu --> 1 TU0-13667_DNA_act	irreversible	Transcription Regulation	0.00E+00	1.00E+09	0	1.12E+02	1.12E+02	0	1.98E+02	197.8231	0	1.31E-01	0.131
TU0-13667_cleavage	Cleavage of TU0-13667_mRNA	1 TU0-13667_mRNA_cleav_cplx + 25 h2o --> 1 RNase_III_dim_inact + 8 amp + 1 b2559_mRNA + 1 b2560_mRNA + 4 cmp + 6 gmp + 25 + 6 ump	irreversible	Cleavage polycistronic mRNA	0.00E+00	1.00E+09	0	1.12E+02	1.12E+02	0	1.98E+02	197.8231	0	1.31E-01	0.131
TU0-13667_cleavage_cpl	Cleavage complex formation of TU0-13667_mRNA (, no_common_name_2172)	1 RNase_III_dim + 1 TU0-13667_mRNA <==> 1 TU0-13667_mRNA_cleav_cplx	reversible	Cleavage polycistronic mRNA	-1.00E+09	1.00E+09	0	1.12E+02	1.12E+02	0	1.98E+02	197.8231	0	1.31E-01	0.131
TU0-13675_DNA_act_bind	TU0-13675_DNA binding of activator	1 TU0-13675_DNA_neu --> 1 TU0-13675_DNA_act	irreversible	Transcription Regulation	0.00E+00	1.00E+09	0	7.46E+01	7.46E+01	0	1.32E+02	131.728	0	1.96E-01	0.196
TU0-13675_cleavage	Cleavage of TU0-13675_mRNA , 4 nt short (b2593)	1 TU0-13675_mRNA_cleav_cplx + 1 h2o --> 1 RNase_III_dim_inact 1 b2593_mRNA + 1 b2594_mRNA + 1 h	irreversible	Cleavage polycistronic mRNA	0.00E+00	1.00E+09	0	7.46E+01	7.46E+01	0	1.32E+02	131.728	0	1.96E-01	0.196
TU0-13675_cleavage_cpl	Cleavage complex formation of TU0-13675_mRNA (, no_common_name_2164) , 4 nt short (b2593)	1 RNase_III_dim + 1 TU0-13675_mRNA <==> 1 TU0-13675_mRNA_cleav_cplx	reversible	Cleavage polycistronic mRNA	-1.00E+09	1.00E+09	0	7.46E+01	7.46E+01	0	1.32E+02	131.728	0	1.96E-01	0.196
TU0-13704_DNA_act_bind	TU0-13704_DNA binding of activator	1 TU0-13704_DNA_neu --> 1 TU0-13704_DNA_act	irreversible	Transcription Regulation	0.00E+00	1.00E+09	0	1.52E+03	1.52E+03	0	8.74E+00	8.740001	0	8.74E+00	8.740001
TU0-13704_trimming	Cutting of stable TU0-13704_RNA	1 TU0-13704_RNA_cut_cplx + 1 h2o --> 1 RNase_E_tetra_inact + 1 RNase_Gen_inact + 1 RNase_P_cplx_inact + 1 b2652_RNA + 1 h + ppi	irreversible	RNA Processing	0.00E+00	1.00E+09	0	1.52E+03	1.52E+03	0	8.74E+00	8.740001	0	8.74E+00	8.740001
TU0-13704_trimming_cplx	Cutting complex formation of stable TU0-13704_RNA (, no_common_name_2135)	1 RNase_E_tetra + 1 RNase_Gen + 1 RNase_P_cplx + 1 TU0-13704_RNA <==> 1 TU0-13704_RNA_cut_cplx	reversible	RNA Processing	-1.00E+09	1.00E+09	0	1.52E+03	1.52E+03	0	8.74E+00	8.740001	0	8.74E+00	8.740001
TU0-13717_DNA_act_bind	TU0-13717_DNA binding of activator	1 TU0-13717_DNA_neu --> 1 TU0-13717_DNA_act	irreversible	Transcription Regulation	0.00E+00	1.00E+09	0	5.01E+01	5.01E+01	0	8.85E+01	88.508	0	5.39E-01	0.539
TU0-13734_DNA_act_bind	TU0-13734_DNA binding of activator	1 TU0-13734_DNA_neu --> 1 TU0-13734_DNA_act	irreversible	Transcription Regulation	0.00E+00	1.00E+09	0	4.31E+01	4.31E+01	0	7.52E+01	75.1818	0	4.14E-01	0.414
TU0-13734_cleavage	Cleavage of TU0-13734_mRNA	1 TU0-13734_mRNA_cleav_cplx + 88 h2o --> 1 RNase_III_dim_inact + 24 amp + 1 b2779_mRNA + 1 b2780_v2_mRNA + 15 cmp + 24 gmp + 88 h + 24 ump	irreversible	Cleavage polycistronic mRNA	0.00E+00	1.00E+09	0	4.31E+01	4.31E+01	0	7.52E+01	75.1818	0	4.14E-01	0.414
TU0-13734_cleavage_cpl	Cleavage complex formation of TU0-13734_mRNA (, no_common_name_2105)	1 RNase_III_dim + 1 TU0-13734_mRNA <==> 1 TU0-13734_mRNA_cleav_cplx	reversible	Cleavage polycistronic mRNA	-1.00E+09	1.00E+09	0	4.31E+01	4.31E+01	0	7.52E+01	75.1818	0	4.14E-01	0.414
TU0-13735_DNA_act_bind	TU0-13735_DNA binding of activator	1 TU0-13735_DNA_neu --> 1 TU0-13735_DNA_act	irreversible	Transcription Regulation	0.00E+00	1.00E+09	0	9.95E+01	9.95E+01	0	1.76E+02	175.6374	0	9.45E-02	0.0945
TU0-13736_DNA_act_bind	TU0-13736_DNA binding of activator	1 TU0-13736_DNA_neu --> 1 TU0-13736_DNA_act	irreversible	Transcription Regulation	0.00E+00	1.00E+09	0	8.27E+01	8.27E+01	0	1.46E+02	146.0611	0	1.89E-01	0.189
TU0-13736_cleavage	Cleavage of TU0-13736_mRNA , 1 nt short (b2791)	1 TU0-13736_mRNA_cleav_cplx + 19 h2o --> 1 RNase_III_dim_inact + 6 amp + 1 b2790_mRNA + 1 b2791_mRNA + 1 b2792_mRNA + 3 cmp + 6 gmp + 19 h + 2 ump	irreversible	Cleavage polycistronic mRNA	0.00E+00	1.00E+09	0	8.27E+01	8.27E+01	0	1.46E+02	146.0611	0	1.89E-01	0.189
TU0-13736_cleavage_cpl	Cleavage complex formation of TU0-13736_mRNA (, no_common_name_2103) , 1 nt short (b2791)	1 RNase_III_dim + 1 TU0-13736_mRNA <==> 1 TU0-13736_mRNA_cleav_cplx	reversible	Cleavage polycistronic mRNA	-1.00E+09	1.00E+09	0	8.27E+01	8.27E+01	0	1.46E+02	146.0611	0	1.89E-01	0.189
TU0-13738_DNA_act_bind	TU0-13738_DNA binding of activator	1 TU0-13738_DNA_neu --> 1 TU0-13738_DNA_act	irreversible	Transcription Regulation	0.00E+00	1.00E+09	0	1.54E+02	1.54E+02	0	2.71E+02	271.0557	0	1.04E-01	0.104
TU0-13805_DNA_act_bind	TU0-13805_DNA binding of activator	1 TU0-13805_DNA_neu --> 1 TU0-13805_DNA_act	irreversible	Transcription Regulation	0.00E+00	1.00E+09	0	7.46E+01	7.46E+01	0	1.32E+02	131.728	0	1.96E-01	0.196
TU0-13805_cleavage	Cleavage of TU0-13805_mRNA	1 TU0-13805_mRNA_cleav_cplx + 13 h2o --> 1 RNase_III_dim_inact + 6 amp + 1 b2946_mRNA + 1 b2947_mRNA + 1 cmp + 4 gmp + 13 + 1 ump	irreversible	Cleavage polycistronic mRNA	0.00E+00	1.00E+09	0	7.46E+01	7.46E+01	0	1.32E+02	131.728	0	1.96E-01	0.196
TU0-13805_cleavage_cpl	Cleavage complex formation of TU0-13805_mRNA (, no_common_name_2034)	1 RNase_III_dim + 1 TU0-13805_mRNA <==> 1 TU0-13805_mRNA_cleav_cplx	reversible	Cleavage polycistronic mRNA	-1.00E+09	1.00E+09	0	7.46E+01	7.46E+01	0	1.32E+02	131.728	0	1.96E-01	0.196
TU0-13809_DNA_act_bind	TU0-13809_DNA binding of activator	1 TU0-13809_DNA_neu --> 1 TU0-13809_DNA_act	irreversible	Transcription Regulation	0.00E+00	1.00E+09	0	1.38E+02	1.38E+02	0	2.44E+02	244.0675	0	1.52E-01	0.152
TU0-13809_cleavage	Cleavage of TU0-13809_mRNA , 1 nt short (b2959)	1 TU0-13809_mRNA_cleav_cplx + 1 h2o --> 1 RNase_III_dim_inact 1 b2959_mRNA + 1 b2960_mRNA + 1 h	irreversible	Cleavage polycistronic mRNA	0.00E+00	1.00E+09	0	1.38E+02	1.38E+02	0	2.44E+02	244.0675	0	1.52E-01	0.152
TU0-13809_cleavage_cpl	Cleavage complex formation of TU0-13809_mRNA (, no_common_name_2030) , 1 nt short (b2959)	1 RNase_III_dim + 1 TU0-13809_mRNA <==> 1 TU0-13809_mRNA_cleav_cplx	reversible	Cleavage polycistronic mRNA	-1.00E+09	1.00E+09	0	1.38E+02	1.38E+02	0	2.44E+02	244.0675	0	1.52E-01	0.152
TU0-13915_DNA_act_bind	TU0-13915_DNA binding of activator	1 TU0-13915_DNA_neu --> 1 TU0-13915_DNA_act	irreversible	Transcription Regulation	0.00E+00	1.00E+09	0	7.43E+01	7.43E+01	0	1.31E+02	131.1153	0	1.12E-01	0.112
TU0-13915_cleavage	Cleavage of TU0-13915_mRNA , 1 nt short (b3344), 1 nt short (b3345)	1 TU0-13915_mRNA_cleav_cplx + 10 h2o --> 1 RNase_III_dim_inact + 1 b3343_mRNA + 1 b3344_mRNA + 1 b3345_mRNA + 1 b3346_mRNA + 1 cmp + 3 gmp + 10 h + 3 ump	irreversible	Cleavage polycistronic mRNA	0.00E+00	1.00E+09	0	7.43E+01	7.43E+01	0	1.31E+02	131.1153	0	1.12E-01	0.112
TU0-13915_cleavage_cpl	Cleavage complex formation of TU0-13915_mRNA (, no_common_name_1924) , 1 nt short (b3344), 1 nt short (b3345)	1 RNase_III_dim + 1 TU0-13915_mRNA <==> 1 TU0-13915_mRNA_cleav_cplx	reversible	Cleavage polycistronic mRNA	-1.00E+09	1.00E+09	0	7.43E+01	7.43E+01	0	1.31E+02	131.1153	0	1.12E-01	0.112
TU0-13931_DNA_act_bind	TU0-13931_DNA binding of activator	1 TU0-13931_DNA_neu --> 1 TU0-13931_DNA_act	irreversible	Transcription Regulation	0.00E+00	1.00E+09	0	2.90E+02	2.90E+02	0	5.13E+02	512.5418	0	9.45E-02	0.0945
TU0-14007_DNA_act_bind	TU0-14007_DNA binding of activator	1 TU0-14007_DNA_neu --> 1 TU0-14007_DNA_act	irreversible	Transcription Regulation	0.00E+00	1.00E+09	0	6.39E+02	6.39E+02	0	1.37E+01	13.7	0	1.37E+01	13.7
TU0-14007_trimming	Cutting of stable TU0-14007_RNA	1 TU0-14007_RNA_cut_cplx + 1 h2o --> 1 RNase_E_tetra_inact + 1 RNase_Gen_inact + 1 RNase_P_cplx_inact + 1 b3658_RNA + 1 h + ppi	irreversible	RNA Processing	0.00E+00	1.00E+09	0	6.39E+02	6.39E+02	0	1.37E+01	13.7	0	1.37E+01	13.7
TU0-14007_trimming_cplx	Cutting complex formation of stable TU0-14007_RNA (, no_common_name_1831)	1 RNase_E_tetra + 1 RNase_Gen + 1 RNase_P_cplx + 1 TU0-14007_RNA <==> 1 TU0-14007_RNA_cut_cplx	reversible	RNA Processing	-1.00E+09	1.00E+09	0	6.39E+02	6.39E+02	0	1.37E+01	13.7	0	1.37E+01	13.7
TU0-14021_DNA_act_bind	TU0-14021_DNA binding of activator	1 TU0-14021_DNA_neu --> 1 TU0-14021_DNA_act	irreversible	Transcription Regulation	0.00E+00	1.00E+09	0	8.73E+01	8.73E+01	0	1.54E+02	154.0426	0	2.63E-01	0.263
TU0-14029_DNA_act_bind	TU0-14029_DNA binding of activator	1 TU0-14029_DNA_neu --> 1 TU0-14029_DNA_act	irreversible	Transcription Regulation	0.00E+00	1.00E+09	0	4.80E+01	4.80E+01	0	8.48E+01	84.78135	0	2.07E-01	0.207
TU0-14029_cleavage	Cleavage of TU0-14029_mRNA	1 TU0-14029_mRNA_cleav_cplx + 64 h2o --> 1 RNase_III_dim_inact + 22 amp + 1 b3740_mRNA + 1 b3741_mRNA + 19 cmp + 12 gmp + 64 h + 10 ump	irreversible	Cleavage polycistronic mRNA	0.00E+00	1.00E+09	0	4.80E+01	4.80E+01	0	8.48E+01	84.78135	0	2.07E-01	0.207
TU0-14029_cleavage_cpl	Cleavage complex formation of TU0-14029_mRNA (, no_common_name_1808)	1 RNase_III_dim + 1 TU0-14029_mRNA <==> 1 TU0-14029_mRNA_cleav_cplx	reversible	Cleavage polycistronic mRNA	-1.00E+09	1.00E+09	0	4.80E+01	4.80E+01	0	8.48E+01	84.78135	0	2.07E-01	0.207
TU0-14037_DNA_act_bind	TU0-14037_DNA binding of activator	1 TU0-14037_DNA_neu --> 1 TU0-14037_DNA_act	irreversible	Transcription Regulation	0.00E+00	1.00E+09	0	9.34E+01	9.34E+01	0	1.59E+02	159.0788	0	3.26E-01	0.326
TU0-14038_DNA_act_bind	TU0-14038_DNA binding of activator	1 TU0-14038_DNA_neu --> 1 TU0-14038_DNA_act	irreversible	Transcription Regulation	0.00E+00	1.00E+09	0	8.68E+01	8.68E+01	0	1.53E+02	153.2054	0	2.07E-01	0.207
TU0-14038_cleavage	Cleavage of TU0-14038_mRNA	1 TU0-14038_mRNA_cleav_cplx + 85 h2o --> 1 RNase_III_dim_inact + 28 amp + 1 b3782_v1_mRNA + 1 b3783_v1_mRNA + 24 cmp + 12 gmp + 85 h + 20 ump	irreversible	Cleavage polycistronic mRNA	0.00E+00	1.00E+09	0	8.68E+01	8.68E+01	0	1.53E+02	153.2054	0	2.07E-01	0.207
TU0-14038_cleavage_cpl	Cleavage complex formation of TU0-14038_mRNA (, no_common_name_1799)	1 RNase_III_dim + 1 TU0-14038_mRNA <==> 1 TU0-14038_mRNA_cleav_cplx	reversible	Cleavage polycistronic mRNA	-1.00E+09	1.00E+09	0	8.68E+01	8.68E+01	0	1.53E+02	153.2054	0	2.07E-01	0.207
TU0-14070_DNA_act_bind	TU0-14070_DNA binding of activator	1 TU0-14070_DNA_neu --> 1 TU0-14070_DNA_act	irreversible	Transcription Regulation	0.00E+00	1.00E+09	0	4.43E+01	4.43E+01	0	7.82E+01	78.19639	0	1.04E-01	0.104

TU0-14070_cleavage	Cleavage of TU0-14070_mRNA , 7 nt short (b3886), 4 nt short (b3887), 4 nt short (b3888)	1 TU0-14070_mRNA_cleav_cplx + 3 h2o --> 1 RNase_III_dim_inact 1 b3885_mRNA + 1 b3886_mRNA + 1 b3887_mRNA + 1 b3888_mRNA + 3 h	irreversible	Cleavage polycistronic mRNA	0.00E+00	1.00E+09	0	4.43E+01	4.43E+01	0	7.82E+01	78.19639	0	1.04E-01	0.104
TU0-14070_cleavage_cpl	Cleavage complex formation of TU0-14070_mRNA (, no_common_name_1767) , 7 nt short (b3886), 4 nt short (b3887), 4 nt short (b3888)	1 RNase_III_dim + 1 TU0-14070_mRNA <==> 1 TU0-14070_mRNA_cleav_cplx	reversible	Cleavage polycistronic mRNA	-1.00E+09	1.00E+09	0	4.43E+01	4.43E+01	0	7.82E+01	78.19639	0	1.04E-01	0.104
TU0-14100_DNA_act_bind	TU0-14100_DNA binding of activator	1 TU0-14100_DNA_neu --> 1 TU0-14100_DNA_act	irreversible	Transcription Regulation	0.00E+00	1.00E+09	0	1.04E+02	1.04E+02	0	1.84E+02	183.6469	0	1.51E+00	1.51
TU0-14118_DNA_act_bind	TU0-14118_DNA binding of activator	1 TU0-14118_DNA_neu --> 1 TU0-14118_DNA_act	irreversible	Transcription Regulation	0.00E+00	1.00E+09	0	1.32E+02	1.32E+02	0	2.33E+02	232.9735	0	1.80E-01	0.18
TU0-14151_DNA_act_bind	TU0-14151_DNA binding of activator	1 TU0-14151_DNA_neu --> 1 TU0-14151_DNA_act	irreversible	Transcription Regulation	0.00E+00	1.00E+09	0	2.40E+02	2.40E+02	0	4.24E+02	423.9067	0	1.42E-01	0.142
TU0-14215_DNA_act_bind	TU0-14215_DNA binding of activator	1 TU0-14215_DNA_neu --> 1 TU0-14215_DNA_act	irreversible	Transcription Regulation	0.00E+00	1.00E+09	0	1.17E+02	1.17E+02	0	2.07E+02	207.2779	0	3.37E-01	0.337
TU0-14216_DNA_act_bind	TU0-14216_DNA binding of activator	1 TU0-14216_DNA_neu --> 1 TU0-14216_DNA_act	irreversible	Transcription Regulation	0.00E+00	1.00E+09	0	7.75E+01	7.75E+01	0	1.37E+02	136.8437	0	9.29E-02	0.0929
TU0-14216_cleavage	Cleavage of TU0-14216_mRNA , 32 n short (b4373)	1 TU0-14216_mRNA_cleav_cplx + 16 h2o --> 1 RNase_III_dim_inact + 6 amp + 1 b4372_mRNA + 1 b4373_mRNA + 1 b4374_mRNA + 1 cmp + 4 gmp + 16 h + 3 ump	irreversible	Cleavage polycistronic mRNA	0.00E+00	1.00E+09	0	7.75E+01	7.75E+01	0	1.37E+02	136.8437	0	9.29E-02	0.0929
TU0-14216_cleavage_cpl	Cleavage complex formation of TU0-14216_mRNA (, no_common_name_1620) , 32 nt shor (b4373)	1 RNase_III_dim + 1 TU0-14216_mRNA <==> 1 TU0-14216_mRNA_cleav_cplx	reversible	Cleavage polycistronic mRNA	-1.00E+09	1.00E+09	0	7.75E+01	7.75E+01	0	1.37E+02	136.8437	0	9.29E-02	0.0929
TU0-14217_DNA_act_bind	TU0-14217_DNA binding of activator	1 TU0-14217_DNA_neu --> 1 TU0-14217_DNA_act	irreversible	Transcription Regulation	0.00E+00	1.00E+09	0	7.77E+01	7.77E+01	0	1.37E+02	137.1766	0	3.08E-01	0.308
TU0-14252_DNA_act_bind	TU0-14252_DNA binding of activator	1 TU0-14252_DNA_neu --> 1 TU0-14252_DNA_act	irreversible	Transcription Regulation	0.00E+00	1.00E+09	0	8.92E+01	8.92E+01	0	8.29E+01	82.88	0	3.08E-01	0.308
TU0-1881_DNA_act_bind	TU0-1881_DNA binding of activator	1 TU0-1881_DNA_neu --> 1 TU0-1881_DNA_act	irreversible	Transcription Regulation	0.00E+00	1.00E+09	0	5.05E+01	5.05E+01	0	8.92E+01	89.20822	0	1.68E-01	0.168
TU0-1881_cleavage	Cleavage of TU0-1881_mRNA	1 TU0-1881_mRNA_cleav_cplx + 157 h2o --> 1 RNase_III_dim_inact + 45 amp + 1 b2528_mRNA + 1 b2529_mRNA + 1 b2530_mRNA + 1 b2531_mRNA + 26 cmp + 41 gmp + 157 h + 42 ump	irreversible	Cleavage polycistronic mRNA	0.00E+00	1.00E+09	0	5.05E+01	5.05E+01	0	8.92E+01	89.20822	0	1.68E-01	0.168
TU0-1881_cleavage_cplx	Cleavage complex formation of TU0-1881_mRNA (iscRp, no_common_name_1548)	1 RNase_III_dim + 1 TU0-1881_mRNA <==> 1 TU0-1881_mRNA_cleav_cplx	reversible	Cleavage polycistronic mRNA	-1.00E+09	1.00E+09	0	5.05E+01	5.05E+01	0	8.92E+01	89.20822	0	1.68E-01	0.168
TU0-1_DNA_act_bind	TU0-1_DNA binding of activator	1 TU0-1_DNA_neu --> 1 TU0-1_DNA_act	irreversible	Transcription Regulation	0.00E+00	1.00E+09	0	3.27E+01	3.27E+01	0	5.77E+01	57.70685	0	2.07E-01	0.207
TU0-1_cleavage	Cleavage of TU0-1_mRNA	1 TU0-1_mRNA_cleav_cplx + 178 h2o --> 1 RNase_III_dim_inact + 56 amp + 1 b1089_v2_mRNA + 1 b1090_v1_mRNA + 1 b1091_v2_mRNA + 1 b1092_v3_mRNA + 1 b1093_v4_mRNA + 39 cmp + 49 gmp + 178 h + 30 ump	irreversible	Cleavage polycistronic mRNA	0.00E+00	1.00E+09	0	3.27E+01	3.27E+01	0	5.77E+01	57.70685	0	2.07E-01	0.207
TU0-1_cleavage_cplx	Cleavage complex formation of TU0-1_mRNA (rpmFp, no_common_name_3093)	1 RNase_III_dim + 1 TU0-1_mRNA <==> 1 TU0-1_mRNA_cleav_cplx	reversible	Cleavage polycistronic mRNA	-1.00E+09	1.00E+09	0	3.27E+01	3.27E+01	0	5.77E+01	57.70685	0	2.07E-01	0.207
TU0-2081_DNA_act_bind	TU0-2081_DNA binding of activator	1 TU0-2081_DNA_neu --> 1 TU0-2081_DNA_act	irreversible	Transcription Regulation	0.00E+00	1.00E+09	0	3.53E+01	3.53E+01	0	6.23E+01	62.29789	0	2.07E-01	0.207
TU0-2082_DNA_act_bind	TU0-2082_DNA binding of activator	1 TU0-2082_DNA_neu --> 1 TU0-2082_DNA_act	irreversible	Transcription Regulation	0.00E+00	1.00E+09	0	3.53E+01	3.53E+01	0	6.23E+01	62.29789	0	2.07E-01	0.207
TU0-2101_DNA_act_bind	TU0-2101_DNA binding of activator	1 TU0-2101_DNA_neu --> 1 TU0-2101_DNA_act	irreversible	Transcription Regulation	0.00E+00	1.00E+09	0	5.24E+01	5.24E+01	0	9.26E+01	92.57733	0	2.07E-01	0.207
TU0-2101_cleavage	Cleavage of TU0-2101_mRNA , 17 nt short (b0851)	1 TU0-2101_mRNA_cleav_cplx + 150 h2o --> 1 RNase_III_dim_inact + 33 amp + 1 b0850_mRNA + 1 b0851_mRNA + 1 b0852_v1_mRNA + 1 b0853_v1_mRNA + 31 cmp + 39 gmp + 150 h + 44 ump	irreversible	Cleavage polycistronic mRNA	0.00E+00	1.00E+09	0	5.24E+01	5.24E+01	0	9.26E+01	92.57733	0	2.07E-01	0.207
TU0-2101_cleavage_cplx	Cleavage complex formation of TU0-2101_mRNA (ybjCp, no_common_name_1535) , 17 nt shor (b0851)	1 RNase_III_dim + 1 TU0-2101_mRNA <==> 1 TU0-2101_mRNA_cleav_cplx	reversible	Cleavage polycistronic mRNA	-1.00E+09	1.00E+09	0	5.24E+01	5.24E+01	0	9.26E+01	92.57733	0	2.07E-01	0.207
TU0-2121_DNA_act_bind	TU0-2121_DNA binding of activator	1 TU0-2121_DNA_neu --> 1 TU0-2121_DNA_act	irreversible	Transcription Regulation	0.00E+00	1.00E+09	0	9.18E+01	9.18E+01	0	1.62E+02	162.0103	0	2.07E-01	0.207
TU0-2121_cleavage	Cleavage of TU0-2121_mRNA	1 TU0-2121_mRNA_cleav_cplx + 88 h2o --> 1 RNase_III_dim_inact 19 amp + 1 b0852_mRNA + 1 b0853_mRNA + 17 cmp + 19 gmp + 8 h + 32 ump	irreversible	Cleavage polycistronic mRNA	0.00E+00	1.00E+09	0	9.18E+01	9.18E+01	0	1.62E+02	162.0103	0	2.07E-01	0.207
TU0-2121_cleavage_cplx	Cleavage complex formation of TU0-2121_mRNA (rimKp, no_common_name_1534)	1 RNase_III_dim + 1 TU0-2121_mRNA <==> 1 TU0-2121_mRNA_cleav_cplx	reversible	Cleavage polycistronic mRNA	-1.00E+09	1.00E+09	0	9.18E+01	9.18E+01	0	1.62E+02	162.0103	0	2.07E-01	0.207
TU0-3304_DNA_act_bind	TU0-3304_DNA binding of activator	1 TU0-3304_DNA_neu --> 1 TU0-3304_DNA_act	irreversible	Transcription Regulation	0.00E+00	1.00E+09	0	2.73E+02	2.73E+02	0	4.82E+02	481.8769	0	6.22E-01	0.622
TU0-3305_DNA_act_bind	TU0-3305_DNA binding of activator	1 TU0-3305_DNA_neu --> 1 TU0-3305_DNA_act	irreversible	Transcription Regulation	0.00E+00	1.00E+09	0	2.73E+02	2.73E+02	0	4.82E+02	481.8769	0	6.22E-01	0.622
TU0-3364_DNA_act_bind	TU0-3364_DNA binding of activator	1 TU0-3364_DNA_neu --> 1 TU0-3364_DNA_act	irreversible	Transcription Regulation	0.00E+00	1.00E+09	0	1.11E+02	1.11E+02	0	0	0	0	0	0
TU0-3364_cutting	Cutting of stable TU0-3364_RNA	1 TU0-3364_RNA_cut_cplx + 460 h2o --> 1 RNase_E_tetra_inact + 1 RNase_G_dim_inact + 1 RNase_Gen_inact + 1 RNase_III_dim_inact + 1 RNase_P_cplx_inact + 1 RNase_T_dim_inact + 1 RNase_m16_inact + 1 RNase_m23_inact + 1 RNase_m5_inact + 14 amp + 1 b3968_RNA + 1 b3969_RNA + 1 b3970_RNA + 1 b3971_RNA + 83 cmp + 106 gmp + 460 h + 1 ppi + 122 ump	irreversible	RNA processing	0.00E+00	1.00E+09	0	1.11E+02	1.11E+02	0	0	0	0	0	0
TU0-3364_cutting_cplx	Cutting complex formation of stable TU0-3364_RNA (rrsBp2, no_common_name_1483)	1 RNase_E_tetra + 1 RNase_G_dim + 1 RNase_Gen + 1 RNase_III_dim + 1 RNase_P_cplx + 1 RNase_T_dim + 1 RNase_m1 + 1 RNase_m23 + 1 RNase_m5 + 1 TU0-3364_RNA <==> 1 TU0-3364_RNA_cut_cplx	reversible	RNA processing	-1.00E+09	1.00E+09	0	1.11E+02	1.11E+02	0	0	0	0	0	0
TU0-3561_DNA_act_bind	TU0-3561_DNA binding of activator	1 TU0-3561_DNA_neu --> 1 TU0-3561_DNA_act	irreversible	Transcription Regulation	0.00E+00	1.00E+09	0	1.77E+03	1.77E+03	0	6.40E+00	6.4	0	6.40E+00	6.4
TU0-3561_trimming	Cutting of stable TU0-3561_RNA	1 TU0-3561_RNA_cut_cplx + 1 h2o --> 1 RNase_E_tetra_inact + 1 RNase_Gen_inact + 1 RNase_P_cplx_inact + 1 b1984_RNA + 1 h + ppi	irreversible	RNA Processing	0.00E+00	1.00E+09	0	1.77E+03	1.77E+03	0	6.40E+00	6.4	0	6.40E+00	6.4
TU0-3561_trimming_cplx	Cutting complex formation of stable TU0-3561_RNA (asnWp, no_common_name_1467)	1 RNase_E_tetra + 1 RNase_Gen + 1 RNase_P_cplx + 1 TU0-3561_RNA <==> 1 TU0-3561_RNA_cut_cplx	reversible	RNA Processing	-1.00E+09	1.00E+09	0	1.77E+03	1.77E+03	0	6.40E+00	6.4	0	6.40E+00	6.4
TU0-3901_DNA_act_bind	TU0-3901_DNA binding of activator	1 TU0-3901_DNA_neu --> 1 TU0-3901_DNA_act	irreversible	Transcription Regulation	0.00E+00	1.00E+09	0	1.09E+02	1.09E+02	0	8.29E+01	82.88	0	3.10E+00	3.1
TU0-4925_DNA_act_bind	TU0-4925_DNA binding of activator	1 TU0-4925_DNA_neu --> 1 TU0-4925_DNA_act	irreversible	Transcription Regulation	0.00E+00	1.00E+09	0	1.04E+02	1.04E+02	0	1.84E+02	183.6469	0	1.51E+00	1.51
TU0-5003_DNA_act_bind	TU0-5003_DNA binding of activator	1 TU0-5003_DNA_neu --> 1 TU0-5003_DNA_act	irreversible	Transcription Regulation	0.00E+00	1.00E+09	0	5.32E+02	5.32E+02	0	9.40E+02	939.6599	0	6.22E-01	0.622
TU0-5121_DNA_act_bind	TU0-5121_DNA binding of activator	1 TU0-5121_DNA_neu --> 1 TU0-5121_DNA_act	irreversible	Transcription Regulation	0.00E+00	1.00E+09	0	3.82E+01	3.82E+01	0	6.74E+01	67.35913	0	7.58E-01	0.758
TU0-5121_cleavage	Cleavage of TU0-5121_mRNA	1 TU0-5121_mRNA_cleav_cplx + 71 h2o --> 1 RNase_III_dim_inact 21 amp + 1 b3339_v1_mRNA + 1 b3340_v1_mRNA + 16 cmp + 18 gmp + 71 h + 15 ump	irreversible	Cleavage polycistronic mRNA	0.00E+00	1.00E+09	0	3.82E+01	3.82E+01	0	6.74E+01	67.35913	0	7.58E-01	0.758
TU0-5121_cleavage_cplx	Cleavage complex formation of TU0-5121_mRNA (fusAp, no_common_name_1357)	1 RNase_III_dim + 1 TU0-5121_mRNA <==> 1 TU0-5121_mRNA_cleav_cplx	reversible	Cleavage polycistronic mRNA	-1.00E+09	1.00E+09	0	3.82E+01	3.82E+01	0	6.74E+01	67.35913	0	7.58E-01	0.758
TU0-5182_DNA_act_bind	TU0-5182_DNA binding of activator	1 TU0-5182_DNA_neu --> 1 TU0-5182_DNA_act	irreversible	Transcription Regulation	0.00E+00	1.00E+09	0	3.50E+01	3.50E+01	0	6.18E+01	61.81973	0	9.45E-02	0.0945
TU0-5182_cleavage	Cleavage of TU0-5182_mRNA , 1 nt short (b2318_v1)	1 TU0-5182_mRNA_cleav_cplx + 150 h2o --> 1 RNase_III_dim_inact + 41 amp + 1 b2317_v1_mRNA + 1 b2318_v1_mRNA + 1 b2319_v1_mRNA + 1 b2320_mRNA + 33 cmp + 34 gmp + 150 h + 3 ump	irreversible	Cleavage polycistronic mRNA	0.00E+00	1.00E+09	0	3.50E+01	3.50E+01	0	6.18E+01	61.81973	0	9.45E-02	0.0945

TU0-5182_cleavage_cplx	Cleavage complex formation of TU0-5182_mRNA (pdxBp, no_common_name_1354) , 1 nt short (b2318_v1)	1 RNase_III_dim + 1 TU0-5182_mRNA <==> 1 TU0-5182_mRNA_cleav_cplx	reversible	Cleavage polycistronic mRNA	-1.00E+09	1.00E+09	0	3.50E+01	3.50E+01	0	6.18E+01	61.81973	0	9.45E-02	0.0945
TU0-5183_DNA_act_bind	TU0-5183_DNA binding of activator	1 TU0-5183_DNA_neu --> 1 TU0-5183_DNA_act	irreversible	Transcription Regulation	0.00E+00	1.00E+09	0	5.09E+01	5.09E+01	0	8.99E+01	89.91961	0	9.45E-02	0.0945
TU0-5183_cleavage	Cleavage of TU0-5183_mRNA , 1 nt short (b2318)	1 TU0-5183_mRNA_cleav_cplx + 84 h2o --> 1 RNase_III_dim_inact + 29 amp + 1 b2317_mRNA + 1 b2318_mRNA + 1 b2319_mRNA + 16 cmp + 21 gmp + 84 h + 16 ump	irreversible	Cleavage polycistronic mRNA	0.00E+00	1.00E+09	0	5.09E+01	5.09E+01	0	8.99E+01	89.91961	0	9.45E-02	0.0945
TU0-5183_cleavage_cplx	Cleavage complex formation of TU0-5183_mRNA (usgp, no_common_name_1353) , 1 nt short (b2318)	1 RNase_III_dim + 1 TU0-5183_mRNA <==> 1 TU0-5183_mRNA_cleav_cplx	reversible	Cleavage polycistronic mRNA	-1.00E+09	1.00E+09	0	5.09E+01	5.09E+01	0	8.99E+01	89.91961	0	9.45E-02	0.0945
TU0-5186_DNA_act_bind	TU0-5186_DNA binding of activator	1 TU0-5186_DNA_neu --> 1 TU0-5186_DNA_act	irreversible	Transcription Regulation	0.00E+00	1.00E+09	0	5.90E+01	5.90E+01	0	1.04E+02	104.2137	0	1.68E-01	0.168
TU0-5186_cleavage	Cleavage of TU0-5186_mRNA	1 TU0-5186_mRNA_cleav_cplx + 10 h2o --> 1 RNase_III_dim_inact + 4 amp + 1 b0049_v3_mRNA + 1 b0050_v3_mRNA + 1 b0051_v3_mRNA + 1 cmp + 2 gmp + 10 h + 1 ump	irreversible	Cleavage polycistronic mRNA	0.00E+00	1.00E+09	0	5.90E+01	5.90E+01	0	1.04E+02	104.2137	0	1.68E-01	0.168
TU0-5186_cleavage_cplx	Cleavage complex formation of TU0-5186_mRNA (rsmAp, no_common_name_1351)	1 RNase_III_dim + 1 TU0-5186_mRNA <==> 1 TU0-5186_mRNA_cleav_cplx	reversible	Cleavage polycistronic mRNA	-1.00E+09	1.00E+09	0	5.90E+01	5.90E+01	0	1.04E+02	104.2137	0	1.68E-01	0.168
TU0-5201_DNA_act_bind	TU0-5201_DNA binding of activator	1 TU0-5201_DNA_neu --> 1 TU0-5201_DNA_act	irreversible	Transcription Regulation	0.00E+00	1.00E+09	0	3.90E+01	3.90E+01	0	6.89E+01	68.92371	0	1.68E-01	0.168
TU0-5201_cleavage	Cleavage of TU0-5201_mRNA , 4 nt short (b0051_v2)	1 TU0-5201_mRNA_cleav_cplx + 11 h2o --> 1 RNase_III_dim_inact + 4 amp + 1 b0049_v2_mRNA + 1 b0050_v2_mRNA + 1 b0051_v2_mRNA + 1 b0052_v2_mRNA + 1 cmp + 2 gmp + 11 h + 1 ump	irreversible	Cleavage polycistronic mRNA	0.00E+00	1.00E+09	0	3.90E+01	3.90E+01	0	6.89E+01	68.92371	0	1.68E-01	0.168
TU0-5201_cleavage_cplx	Cleavage complex formation of TU0-5201_mRNA (pdxAp, no_common_name_1350) , 4 nt short (b0051_v2)	1 RNase_III_dim + 1 TU0-5201_mRNA <==> 1 TU0-5201_mRNA_cleav_cplx	reversible	Cleavage polycistronic mRNA	-1.00E+09	1.00E+09	0	3.90E+01	3.90E+01	0	6.89E+01	68.92371	0	1.68E-01	0.168
TU0-5221_DNA_act_bind	TU0-5221_DNA binding of activator	1 TU0-5221_DNA_neu --> 1 TU0-5221_DNA_act	irreversible	Transcription Regulation	0.00E+00	1.00E+09	0	6.43E+01	6.43E+01	0	1.13E+02	113.4398	0	1.68E-01	0.168
TU0-5221_cleavage	Cleavage of TU0-5221_mRNA , 4 nt short (b0051_v1)	1 TU0-5221_mRNA_cleav_cplx + 1 h2o --> 1 RNase_III_dim_inact + 1 b0051_v1_mRNA + 1 b0052_v1_mRNA + 1 h	irreversible	Cleavage polycistronic mRNA	0.00E+00	1.00E+09	0	6.43E+01	6.43E+01	0	1.13E+02	113.4398	0	1.68E-01	0.168
TU0-5221_cleavage_cplx	Cleavage complex formation of TU0-5221_mRNA (pdxAp, no_common_name_1348) , 4 nt short (b0051_v1)	1 RNase_III_dim + 1 TU0-5221_mRNA <==> 1 TU0-5221_mRNA_cleav_cplx	reversible	Cleavage polycistronic mRNA	-1.00E+09	1.00E+09	0	6.43E+01	6.43E+01	0	1.13E+02	113.4398	0	1.68E-01	0.168
TU0-5522_DNA_act_bind	TU0-5522_DNA binding of activator	1 TU0-5522_DNA_neu --> 1 TU0-5522_DNA_act	irreversible	Transcription Regulation	0.00E+00	1.00E+09	0	6.30E+01	6.30E+01	0	7.71E+01	77.08692	0	1.04E-01	0.104
TU0-5522_cleavage	Cleavage of TU0-5522_mRNA	1 TU0-5522_mRNA_cleav_cplx + 336 h2o --> 1 RNase_III_dim_inact + 75 amp + 1 b3635_v3_mRNA + 1 b3636_v3_mRNA + 1 b3637_v3_mRNA + 1 b3638_v1_mRNA + 81 cmp + 82 gmp + 336 h + 95 ump	irreversible	Cleavage polycistronic mRNA	0.00E+00	1.00E+09	0	6.30E+01	6.30E+01	0	7.71E+01	77.08692	0	1.04E-01	0.104
TU0-5522_cleavage_cplx	Cleavage complex formation of TU0-5522_mRNA (yicRp, no_common_name_1339)	1 RNase_III_dim + 1 TU0-5522_mRNA <==> 1 TU0-5522_mRNA_cleav_cplx	reversible	Cleavage polycistronic mRNA	-1.00E+09	1.00E+09	0	6.30E+01	6.30E+01	0	7.71E+01	77.08692	0	1.04E-01	0.104
TU0-5543_DNA_act_bind	TU0-5543_DNA binding of activator	1 TU0-5543_DNA_neu --> 1 TU0-5543_DNA_act	irreversible	Transcription Regulation	0.00E+00	1.00E+09	0	8.85E+01	8.85E+01	0	8.29E+01	82.88	0	1.92E-01	0.192
TU0-5543_cleavage	Cleavage of TU0-5543_mRNA	1 TU0-5543_mRNA_cleav_cplx + 119 h2o --> 1 RNase_III_dim_inact + 26 amp + 1 b3635_v1_mRNA + 1 b3636_v2_mRNA + 1 b3637_v2_mRNA + 27 cmp + 24 gmp + 119 h + 40 ump	irreversible	Cleavage polycistronic mRNA	0.00E+00	1.00E+09	0	8.85E+01	8.85E+01	0	8.29E+01	82.88	0	1.92E-01	0.192
TU0-5543_cleavage_cplx	Cleavage complex formation of TU0-5543_mRNA (rpmBp, no_common_name_1337)	1 RNase_III_dim + 1 TU0-5543_mRNA <==> 1 TU0-5543_mRNA_cleav_cplx	reversible	Cleavage polycistronic mRNA	-1.00E+09	1.00E+09	0	8.85E+01	8.85E+01	0	8.29E+01	82.88	0	1.92E-01	0.192
TU0-5601_DNA_act_bind	TU0-5601_DNA binding of activator	1 TU0-5601_DNA_neu --> 1 TU0-5601_DNA_act	irreversible	Transcription Regulation	0.00E+00	1.00E+09	0	8.92E+01	8.92E+01	0	8.29E+01	82.88	0	1.04E-01	0.104
TU0-5601_cleavage	Cleavage of TU0-5601_mRNA	1 TU0-5601_mRNA_cleav_cplx + 238 h2o --> 1 RNase_III_dim_inact + 58 amp + 1 b3636_v1_mRNA + 1 b3637_v1_mRNA + 1 b3638_mRNA + 56 cmp + 62 gmp + 238 h + 60 ump	irreversible	Cleavage polycistronic mRNA	0.00E+00	1.00E+09	0	8.92E+01	8.92E+01	0	8.29E+01	82.88	0	1.04E-01	0.104
TU0-5601_cleavage_cplx	Cleavage complex formation of TU0-5601_mRNA (yicRp, no_common_name_1329)	1 RNase_III_dim + 1 TU0-5601_mRNA <==> 1 TU0-5601_mRNA_cleav_cplx	reversible	Cleavage polycistronic mRNA	-1.00E+09	1.00E+09	0	8.92E+01	8.92E+01	0	8.29E+01	82.88	0	1.04E-01	0.104
TU0-6223_DNA_act_bind	TU0-6223_DNA binding of activator	1 TU0-6223_DNA_neu --> 1 TU0-6223_DNA_act	irreversible	Transcription Regulation	0.00E+00	1.00E+09	0	1.43E+01	1.43E+01	0	2.52E+01	25.16946	0	1.46E-01	0.146
TU0-6223_cleavage	Cleavage of TU0-6223_mRNA , 1 nt short (b3166_v1)	1 TU0-6223_mRNA_cleav_cplx + 752 h2o --> 1 RNase_III_dim_inact + 175 amp + 1 b3164_v3_mRNA + 1 b3165_v3_mRNA + 1 b3166_v1_mRNA + 1 b3167_v1_mRNA + 1 b3168_v3_mRNA + 1 b3169_v3_mRNA + 1 b3170_v3_mRNA + 1 b3171_v4_RNA_pre + 148 cmp + 193 gmp + 752 h + 229 ump	irreversible	Cleavage polycistronic mRNA	0.00E+00	1.00E+09	0	1.43E+01	1.43E+01	0	2.52E+01	25.16946	0	1.46E-01	0.146
TU0-6223_cleavage_cplx	Cleavage complex formation of TU0-6223_mRNA (metYp1, no_common_name_1307) , 1 nt short (b3166_v1)	1 RNase_III_dim + 1 TU0-6223_mRNA <==> 1 TU0-6223_mRNA_cleav_cplx	reversible	Cleavage polycistronic mRNA	-1.00E+09	1.00E+09	0	1.43E+01	1.43E+01	0	2.52E+01	25.16946	0	1.46E-01	0.146
TU0-6405_DNA_act_bind	TU0-6405_DNA binding of activator	1 TU0-6405_DNA_neu --> 1 TU0-6405_DNA_act	irreversible	Transcription Regulation	0.00E+00	1.00E+09	0	4.21E+01	4.21E+01	0	7.43E+01	74.28142	0	4.90E-01	0.49
TU0-6409_DNA_act_bind	TU0-6409_DNA binding of activator	1 TU0-6409_DNA_neu --> 1 TU0-6409_DNA_act	irreversible	Transcription Regulation	0.00E+00	1.00E+09	0	9.02E+01	9.02E+01	0	1.59E+02	159.2644	0	5.77E-01	0.577
TU0-6423_DNA_act_bind	TU0-6423_DNA binding of activator	1 TU0-6423_DNA_neu --> 1 TU0-6423_DNA_act	irreversible	Transcription Regulation	0.00E+00	1.00E+09	0	2.93E+02	2.93E+02	0	4.97E+02	496.5524	0	1.15E-01	0.115
TU0-6441_DNA_act_bind	TU0-6441_DNA binding of activator	1 TU0-6441_DNA_neu --> 1 TU0-6441_DNA_act	irreversible	Transcription Regulation	0.00E+00	1.00E+09	0	5.01E+01	5.01E+01	0	8.85E+01	88.508	0	5.39E-01	0.539
TU0-6504_DNA_act_bind	TU0-6504_DNA binding of activator	1 TU0-6504_DNA_neu --> 1 TU0-6504_DNA_act	irreversible	Transcription Regulation	0.00E+00	1.00E+09	0	7.55E+01	7.55E+01	0	1.33E+02	133.2851	0	4.14E-01	0.414
TU0-6506_DNA_act_bind	TU0-6506_DNA binding of activator	1 TU0-6506_DNA_neu --> 1 TU0-6506_DNA_act	irreversible	Transcription Regulation	0.00E+00	1.00E+09	0	1.02E+02	1.02E+02	0	1.80E+02	180.1265	0	5.99E-01	0.599
TU0-6512_DNA_act_bind	TU0-6512_DNA binding of activator	1 TU0-6512_DNA_neu --> 1 TU0-6512_DNA_act	irreversible	Transcription Regulation	0.00E+00	1.00E+09	0	1.14E+01	1.14E+01	0	2.02E+01	20.15717	0	1.89E-01	0.189
TU0-6512_cleavage	Cleavage of TU0-6512_mRNA	1 TU0-6512_mRNA_cleav_cplx + 881 h2o --> 1 RNase_III_dim_inact + 221 amp + 1 b3983_mRNA + 1 b3984_mRNA + 1 b3985_v1_mRNA + 1 b3986_v2_mRNA + 1 b3987_v2_mRNA + 1 b3988_v2_mRNA + 201 cmp + 215 gmp + 881 h + 239 ump	irreversible	Cleavage polycistronic mRNA	0.00E+00	1.00E+09	0	1.14E+01	1.14E+01	0	2.02E+01	20.15717	0	1.89E-01	0.189
TU0-6512_cleavage_cplx	Cleavage complex formation of TU0-6512_mRNA (rplKp, no_common_name_1269)	1 RNase_III_dim + 1 TU0-6512_mRNA <==> 1 TU0-6512_mRNA_cleav_cplx	reversible	Cleavage polycistronic mRNA	-1.00E+09	1.00E+09	0	1.14E+01	1.14E+01	0	2.02E+01	20.15717	0	1.89E-01	0.189
TU0-6550_DNA_act_bind	TU0-6550_DNA binding of activator	1 TU0-6550_DNA_neu --> 1 TU0-6550_DNA_act	irreversible	Transcription Regulation	0.00E+00	1.00E+09	0	9.02E+01	9.02E+01	0	1.59E+02	159.2644	0	5.77E-01	0.577
TU0-6551_DNA_act_bind	TU0-6551_DNA binding of activator	1 TU0-6551_DNA_neu --> 1 TU0-6551_DNA_act	irreversible	Transcription Regulation	0.00E+00	1.00E+09	0	9.02E+01	9.02E+01	0	1.59E+02	159.2644	0	5.77E-01	0.577
TU0-6562_DNA_act_bind	TU0-6562_DNA binding of activator	1 TU0-6562_DNA_neu --> 1 TU0-6562_DNA_act	irreversible	Transcription Regulation	0.00E+00	1.00E+09	0	7.21E+01	7.21E+01	0	1.27E+02	127.2677	0	2.07E-01	0.207
TU0-6626_DNA_act_bind	TU0-6626_DNA binding of activator	1 TU0-6626_DNA_neu --> 1 TU0-6626_DNA_act	irreversible	Transcription Regulation	0.00E+00	1.00E+09	0	3.20E+01	3.20E+01	0	5.65E+01	56.54924	0	9.88E-02	0.0988
TU0-6626_cleavage	Cleavage of TU0-6626_mRNA	1 TU0-6626_mRNA_cleav_cplx + 304 h2o --> 1 RNase_III_dim_inact + 96 amp + 1 b1712_v1_mRNA + 1 b1713_mRNA + 1 b1714_mRNA + 1 b1715_v2_mRNA + 67 cmp + 82 gmp + 304 h + 56 ump	irreversible	Cleavage polycistronic mRNA	0.00E+00	1.00E+09	0	3.20E+01	3.20E+01	0	5.65E+01	56.54924	0	9.88E-02	0.0988

TU0-6626_cleavage_cplx	Cleavage complex formation of TU0-6626_mRNA (pheMp, no_common_name_1244)	1 RNase_III_dim + 1 TU0-6626_mRNA <==> 1 TU0-6626_mRNA_cleav_cplx	reversible	Cleavage polycistronic mRNA	-1.00E+09	1.00E+09	0	3.20E+01	3.20E+01	0	5.65E+01	56.54924	0	9.88E-02	0.0988
TU0-6654_DNA_act_bind	TU0-6654_DNA binding of activator	1 TU0-6654_DNA_neu --> 1 TU0-6654_DNA_act	irreversible	Transcription Regulation	0.00E+00	1.00E+09	0	5.71E+01	5.71E+01	0	1.00E+02	100.0923	0	6.46E-01	0.646
TU0-6657_DNA_act_bind	TU0-6657_DNA binding of activator	1 TU0-6657_DNA_neu --> 1 TU0-6657_DNA_act	irreversible	Transcription Regulation	0.00E+00	1.00E+09	0	1.05E+02	1.05E+02	0	8.29E+01	82.88	0	1.65E-01	0.165
TU0-6658_DNA_act_bind	TU0-6658_DNA binding of activator	1 TU0-6658_DNA_neu --> 1 TU0-6658_DNA_act	irreversible	Transcription Regulation	0.00E+00	1.00E+09	0	1.47E+02	1.47E+02	0	2.60E+02	259.8138	0	2.99E-01	0.299
TU0-6660_DNA_act_bind	TU0-6660_DNA binding of activator	1 TU0-6660_DNA_neu --> 1 TU0-6660_DNA_act	irreversible	Transcription Regulation	0.00E+00	1.00E+09	0	4.77E+01	4.77E+01	0	7.03E+01	70.31333	0	4.81E-01	0.481
TU0-6660_cleavage	Cleavage of TU0-6660_mRNA	1 TU0-6660_mRNA_cleav_cplx + 178 h2o --> 1 RNase_III_dim_inact + 39 amp + 1 b3164_v1_mRNA + 1 b3165_v2_mRNA + 35 cmp + 52 gmp + 178 h + 51 ump	irreversible	Cleavage polycistronic mRNA	0.00E+00	1.00E+09	0	4.77E+01	4.77E+01	0	7.03E+01	70.31333	0	4.81E-01	0.481
TU0-6660_cleavage_cplx	Cleavage complex formation of TU0-6660_mRNA (rpsOp, no_common_name_1226)	1 RNase_III_dim + 1 TU0-6660_mRNA <==> 1 TU0-6660_mRNA_cleav_cplx	reversible	Cleavage polycistronic mRNA	-1.00E+09	1.00E+09	0	4.77E+01	4.77E+01	0	7.03E+01	70.31333	0	4.81E-01	0.481
TU0-6687_DNA_act_bind	TU0-6687_DNA binding of activator	1 TU0-6687_DNA_neu --> 1 TU0-6687_DNA_act	irreversible	Transcription Regulation	0.00E+00	1.00E+09	0	4.21E+01	4.21E+01	0	7.43E+01	74.28142	0	4.90E-01	0.49
TU0-6941_DNA_act_bind	TU0-6941_DNA binding of activator	1 TU0-6941_DNA_neu --> 1 TU0-6941_DNA_act	irreversible	Transcription Regulation	0.00E+00	1.00E+09	0	8.68E+01	8.68E+01	0	1.53E+02	153.2054	0	2.07E-01	0.207
TU0-6941_cleavage	Cleavage of TU0-6941_mRNA	1 TU0-6941_mRNA_cleav_cplx + 85 h2o --> 1 RNase_III_dim_inact + 28 amp + 1 b3782_mRNA + 1 b3783_mRNA + 24 cmp + 12 gmp + 85 h + 20 ump	irreversible	Cleavage polycistronic mRNA	0.00E+00	1.00E+09	0	8.68E+01	8.68E+01	0	1.53E+02	153.2054	0	2.07E-01	0.207
TU0-6941_cleavage_cplx	Cleavage complex formation of TU0-6941_mRNA (rhoLp, no_common_name_1208)	1 RNase_III_dim + 1 TU0-6941_mRNA <==> 1 TU0-6941_mRNA_cleav_cplx	reversible	Cleavage polycistronic mRNA	-1.00E+09	1.00E+09	0	8.68E+01	8.68E+01	0	1.53E+02	153.2054	0	2.07E-01	0.207
TU0-7002_DNA_act_bind	TU0-7002_DNA binding of activator	1 TU0-7002_DNA_neu --> 1 TU0-7002_DNA_act	irreversible	Transcription Regulation	0.00E+00	1.00E+09	0	1.49E+01	1.49E+01	0	2.63E+01	26.30872	0	1.89E-01	0.189
TU0-7002_cleavage	Cleavage of TU0-7002_mRNA	1 TU0-7002_mRNA_cleav_cplx + 77 h2o --> 1 RNase_III_dim_inact + 18 amp + 1 b3987_v1_mRNA + 1 b3988_v1_mRNA + 22 cmp + 20 gmp + 77 h + 16 ump	irreversible	Cleavage polycistronic mRNA	0.00E+00	1.00E+09	0	1.49E+01	1.49E+01	0	2.63E+01	26.30872	0	1.89E-01	0.189
TU0-7002_cleavage_cplx	Cleavage complex formation of TU0-7002_mRNA (rpoBp, no_common_name_1201)	1 RNase_III_dim + 1 TU0-7002_mRNA <==> 1 TU0-7002_mRNA_cleav_cplx	reversible	Cleavage polycistronic mRNA	-1.00E+09	1.00E+09	0	1.49E+01	1.49E+01	0	2.63E+01	26.30872	0	1.89E-01	0.189
TU0-7141_DNA_act_bind	TU0-7141_DNA binding of activator	1 TU0-7141_DNA_neu --> 1 TU0-7141_DNA_act	irreversible	Transcription Regulation	0.00E+00	1.00E+09	0	1.03E+02	1.03E+02	0	8.29E+01	82.88	0	1.89E-01	0.189
TU0-7141_cleavage	Cleavage of TU0-7141_mRNA	1 TU0-7141_mRNA_cleav_cplx + 102 h2o --> 1 RNase_III_dim_inact + 24 amp + 1 b1480_mRNA + 1 b1481_mRNA + 18 cmp + 27 gmp + 102 h + 32 ump	irreversible	Cleavage polycistronic mRNA	0.00E+00	1.00E+09	0	1.03E+02	1.03E+02	0	8.29E+01	82.88	0	1.89E-01	0.189
TU0-7141_cleavage_cplx	Cleavage complex formation of TU0-7141_mRNA (bdmp, no_common_name_1194)	1 RNase_III_dim + 1 TU0-7141_mRNA <==> 1 TU0-7141_mRNA_cleav_cplx	reversible	Cleavage polycistronic mRNA	-1.00E+09	1.00E+09	0	1.03E+02	1.03E+02	0	8.29E+01	82.88	0	1.89E-01	0.189
TU0-7281_DNA_act_bind	TU0-7281_DNA binding of activator	1 TU0-7281_DNA_neu --> 1 TU0-7281_DNA_act	irreversible	Transcription Regulation	0.00E+00	1.00E+09	0	6.27E+01	6.27E+01	0	1.11E+02	110.7654	0	1.22E-01	0.122
TU0-7281_cleavage	Cleavage of TU0-7281_mRNA , 4 nt short (b3280)	1 TU0-7281_mRNA_cleav_cplx + 11 h2o --> 1 RNase_III_dim_inact + 4 amp + 1 b3280_mRNA + 1 b3281_mRNA + 1 b3282_mRNA + 1 b3283_mRNA + 2 cmp + 11 h + 2 ump	irreversible	Cleavage polycistronic mRNA	0.00E+00	1.00E+09	0	6.27E+01	6.27E+01	0	1.11E+02	110.7654	0	1.22E-01	0.122
TU0-7281_cleavage_cplx	Cleavage complex formation of TU0-7281_mRNA (yrdDp, no_common_name_1183) , 4 nt short (b3280)	1 RNase_III_dim + 1 TU0-7281_mRNA <==> 1 TU0-7281_mRNA_cleav_cplx	reversible	Cleavage polycistronic mRNA	-1.00E+09	1.00E+09	0	6.27E+01	6.27E+01	0	1.11E+02	110.7654	0	1.22E-01	0.122
TU0-7842_DNA_act_bind	TU0-7842_DNA binding of activator	1 TU0-7842_DNA_neu --> 1 TU0-7842_DNA_act	irreversible	Transcription Regulation	0.00E+00	1.00E+09	0	1.06E+02	1.06E+02	0	8.29E+01	82.88	0	2.92E+00	2.92
TU0-7844_DNA_act_bind	TU0-7844_DNA binding of activator	1 TU0-7844_DNA_neu --> 1 TU0-7844_DNA_act	irreversible	Transcription Regulation	0.00E+00	1.00E+09	0	1.06E+02	1.06E+02	0	8.29E+01	82.88	0	2.92E+00	2.92
TU0-8084_DNA_act_bind	TU0-8084_DNA binding of activator	1 TU0-8084_DNA_neu --> 1 TU0-8084_DNA_act	irreversible	Transcription Regulation	0.00E+00	1.00E+09	0	7.08E+01	7.08E+01	0	1.25E+02	125.0102	0	1.04E-01	0.104
TU0-8281_DNA_act_bind	TU0-8281_DNA binding of activator	1 TU0-8281_DNA_neu --> 1 TU0-8281_DNA_act	irreversible	Transcription Regulation	0.00E+00	1.00E+09	0	2.28E+02	2.28E+02	0	4.03E+02	402.7114	0	3.11E-01	0.311
TU0-8464_DNA_act_bind	TU0-8464_DNA binding of activator	1 TU0-8464_DNA_neu --> 1 TU0-8464_DNA_act	irreversible	Transcription Regulation	0.00E+00	1.00E+09	0	3.61E+02	3.61E+02	0	3.32E+02	331.52	0	1.70E+00	1.7
TU0-8474_DNA_act_bind	TU0-8474_DNA binding of activator	1 TU0-8474_DNA_neu --> 1 TU0-8474_DNA_act	irreversible	Transcription Regulation	0.00E+00	1.00E+09	0	1.06E+02	1.06E+02	0	8.29E+01	82.88	0	4.81E-01	0.481
TU0-8476_DNA_act_bind	TU0-8476_DNA binding of activator	1 TU0-8476_DNA_neu --> 1 TU0-8476_DNA_act	irreversible	Transcription Regulation	0.00E+00	1.00E+09	0	1.23E+03	1.23E+03	0	11	11	0	11	11
TU0-8476_trimming	Cutting of stable TU0-8476_RNA	1 TU0-8476_RNA_cut_cplx + 1 h2o --> 1 RNase_E_tetra_inact + 1 RNase_Gen_inact + 1 RNase_P_cplx_inact + 1 b3171_v3_RNA + 1 + 1 ppi	irreversible	RNA Processing	0.00E+00	1.00E+09	0	1.23E+03	1.23E+03	0	11	11	0	11	11
TU0-8476_trimming_cplx	Cutting complex formation of stable TU0-8476_RNA (metYp2, no_common_name_1038)	1 RNase_E_tetra + 1 RNase_Gen + 1 RNase_P_cplx + 1 TU0-8476_RNA <==> 1 TU0-8476_RNA_cut_cplx	reversible	RNA Processing	-1.00E+09	1.00E+09	0	1.23E+03	1.23E+03	0	11	11	0	11	11
TU0-8477_DNA_act_bind	TU0-8477_DNA binding of activator	1 TU0-8477_DNA_neu --> 1 TU0-8477_DNA_act	irreversible	Transcription Regulation	0.00E+00	1.00E+09	0	9.18E+01	9.18E+01	0	1.62E+02	162.0103	0	2.76E-01	0.276
TU0-8502_DNA_act_bind	TU0-8502_DNA binding of activator	1 TU0-8502_DNA_neu --> 1 TU0-8502_DNA_act	irreversible	Transcription Regulation	0.00E+00	1.00E+09	0	3.36E+01	3.36E+01	0	5.93E+01	59.28454	0	1.18E-01	0.118
TU0-8502_cleavage	Cleavage of TU0-8502_mRNA	1 TU0-8502_mRNA_cleav_cplx + 219 h2o --> 1 RNase_III_dim_inact + 74 amp + 1 b4178_mRNA + 1 b4179_mRNA + 1 b4180_mRNA + 46 cmp + 49 gmp + 219 h + 48 ump	irreversible	Cleavage polycistronic mRNA	0.00E+00	1.00E+09	0	3.36E+01	3.36E+01	0	5.93E+01	59.28454	0	1.18E-01	0.118
TU0-8502_cleavage_cplx	Cleavage complex formation of TU0-8502_mRNA (nsrRp, no_common_name_1013)	1 RNase_III_dim + 1 TU0-8502_mRNA <==> 1 TU0-8502_mRNA_cleav_cplx	reversible	Cleavage polycistronic mRNA	-1.00E+09	1.00E+09	0	3.36E+01	3.36E+01	0	5.93E+01	59.28454	0	1.18E-01	0.118
TU0-8505_DNA_act_bind	TU0-8505_DNA binding of activator	1 TU0-8505_DNA_neu --> 1 TU0-8505_DNA_act	irreversible	Transcription Regulation	0.00E+00	1.00E+09	0	1.54E+02	1.54E+02	0	2.71E+02	271.0557	0	1.71E-01	0.171
TU0-8506_DNA_act_bind	TU0-8506_DNA binding of activator	1 TU0-8506_DNA_neu --> 1 TU0-8506_DNA_act	irreversible	Transcription Regulation	0.00E+00	1.00E+09	0	1.07E+02	1.07E+02	0	8.29E+01	82.88	0	3.72E-01	0.372
TU0-8510_DNA_act_bind	TU0-8510_DNA binding of activator	1 TU0-8510_DNA_neu --> 1 TU0-8510_DNA_act	irreversible	Transcription Regulation	0.00E+00	1.00E+09	0	4.77E+02	4.77E+02	0	8.41E+02	841.4865	0	2.00E-01	0.2
TU0-8529_DNA_act_bind	TU0-8529_DNA binding of activator	1 TU0-8529_DNA_neu --> 1 TU0-8529_DNA_act	irreversible	Transcription Regulation	0.00E+00	1.00E+09	0	8.92E+01	8.92E+01	0	1.57E+02	157.4849	0	1.86E-01	0.186
TU0-8823_DNA_act_bind	TU0-8823_DNA binding of activator	1 TU0-8823_DNA_neu --> 1 TU0-8823_DNA_act	irreversible	Transcription Regulation	0.00E+00	1.00E+09	0	6.65E+01	6.65E+01	0	1.17E+02	117.4575	0	1.17E-01	0.117
TU0-8855_DNA_act_bind	TU0-8855_DNA binding of activator	1 TU0-8855_DNA_neu --> 1 TU0-8855_DNA_act	irreversible	Transcription Regulation	0.00E+00	1.00E+09	0	1.02E+02	1.02E+02	0	8.29E+01	82.88	0	9.88E-02	0.0988
TU0-8855_cleavage	Cleavage of TU0-8855_mRNA	1 TU0-8855_mRNA_cleav_cplx + 123 h2o --> 1 RNase_III_dim_inact + 36 amp + 1 b1715_v1_mRNA + 1 b1716_v1_mRNA + 28 cmp + 24 gmp + 123 h + 34 ump	irreversible	Cleavage polycistronic mRNA	0.00E+00	1.00E+09	0	1.02E+02	1.02E+02	0	8.29E+01	82.88	0	9.88E-02	0.0988
TU0-8855_cleavage_cplx	Cleavage complex formation of TU0-8855_mRNA (rplTp, no_common_name_685)	1 RNase_III_dim + 1 TU0-8855_mRNA <==> 1 TU0-8855_mRNA_cleav_cplx	reversible	Cleavage polycistronic mRNA	-1.00E+09	1.00E+09	0	1.02E+02	1.02E+02	0	8.29E+01	82.88	0	9.88E-02	0.0988
TU0-8862_DNA_act_bind	TU0-8862_DNA binding of activator	1 TU0-8862_DNA_neu --> 1 TU0-8862_DNA_act	irreversible	Transcription Regulation	0.00E+00	1.00E+09	0	1.30E+02	1.30E+02	0	2.30E+02	230.1208	0	1.89E-01	0.189
TU0-8864_DNA_act_bind	TU0-8864_DNA binding of activator	1 TU0-8864_DNA_neu --> 1 TU0-8864_DNA_act	irreversible	Transcription Regulation	0.00E+00	1.00E+09	0	4.46E+01	4.46E+01	0	7.87E+01	78.74245	0	1.04E-01	0.104
TU0-8864_cleavage	Cleavage of TU0-8864_mRNA	1 TU0-8864_mRNA_cleav_cplx + 188 h2o --> 1 RNase_III_dim_inact + 49 amp + 1 b1636_v1_mRNA + 1 b1637_v1_mRNA + 1 b1638_mRNA + 35 cmp + 45 gmp + 188 h + 57 ump	irreversible	Cleavage polycistronic mRNA	0.00E+00	1.00E+09	0	4.46E+01	4.46E+01	0	7.87E+01	78.74245	0	1.04E-01	0.104
TU0-8864_cleavage_cplx	Cleavage complex formation of TU0-8864_mRNA (pdxHp, no_common_name_681)	1 RNase_III_dim + 1 TU0-8864_mRNA <==> 1 TU0-8864_mRNA_cleav_cplx	reversible	Cleavage polycistronic mRNA	-1.00E+09	1.00E+09	0	4.46E+01	4.46E+01	0	7.87E+01	78.74245	0	1.04E-01	0.104
TU0-8865_DNA_act_bind	TU0-8865_DNA binding of activator	1 TU0-8865_DNA_neu --> 1 TU0-8865_DNA_act	irreversible	Transcription Regulation	0.00E+00	1.00E+09	0	6.09E+01	6.09E+01	0	1.08E+02	107.5946	0	2.24E-01	0.224

TU0-8865_cleavage	Cleavage of TU0-8865_mRNA	1 TU0-8865_mRNA_cleav_cplx + 59 h2o --> 1 RNase_III_dim_inact · 17 amp + 1 b1636_mRNA + 1 b1637_mRNA + 10 cmp + 17 gmp + 5 h + 14 ump	irreversible	Cleavage polycistronic mRNA	0.00E+00	1.00E+09	0	6.09E+01	6.09E+01	0	1.08E+02	107.5946	0	2.24E-01	0.224
TU0-8865_cleavage_cplx	Cleavage complex formation of TU0-8865_mRNA (tyrSp, no_common_name_680)	1 RNase_III_dim + 1 TU0-8865_mRNA <==> 1 TU0-8865_mRNA_cleav_cplx	reversible	Cleavage polycistronic mRNA	-1.00E+09	1.00E+09	0	6.09E+01	6.09E+01	0	1.08E+02	107.5946	0	2.24E-01	0.224
TU00021_DNA_act_bind	TU00021_DNA binding of activator	1 TU00021_DNA_neu --> 1 TU00021_DNA_act	irreversible	Transcription Regulation	0.00E+00	1.00E+09	0	1.02E+02	1.02E+02	0	1.80E+02	180.1265	0	1.28E-01	0.128
TU00021_cleavage	Cleavage of TU00021_mRNA	1 TU00021_mRNA_cleav_cplx + 26 h2o --> 1 RNase_III_dim_inact + 13 amp + 1 b3260_mRNA + 1 b3261_mRNA + 4 cmp + 5 gmp + 26 h + 3 ump	irreversible	Cleavage polycistronic mRNA	0.00E+00	1.00E+09	0	1.02E+02	1.02E+02	0	1.80E+02	180.1265	0	1.28E-01	0.128
TU00021_cleavage_cplx	Cleavage complex formation of TU00021_mRNA (dusBp, no_common_name_644)	1 RNase_III_dim + 1 TU00021_mRNA <==> 1 TU00021_mRNA_cleav_cplx	reversible	Cleavage polycistronic mRNA	-1.00E+09	1.00E+09	0	1.02E+02	1.02E+02	0	1.80E+02	180.1265	0	1.28E-01	0.128
TU00083_DNA_act_bind	TU00083_DNA binding of activator	1 TU00083_DNA_neu --> 1 TU00083_DNA_act	irreversible	Transcription Regulation	0.00E+00	1.00E+09	0	1.47E+02	1.47E+02	0	2.60E+02	259.8138	0	2.99E-01	0.299
TU00084_DNA_act_bind	TU00084_DNA binding of activator	1 TU00084_DNA_neu --> 1 TU00084_DNA_act	irreversible	Transcription Regulation	0.00E+00	1.00E+09	0	1.47E+02	1.47E+02	0	2.60E+02	259.8138	0	2.99E-01	0.299
TU00221_DNA_act_bind	TU00221_DNA binding of activator	1 TU00221_DNA_neu --> 1 TU00221_DNA_act	irreversible	Transcription Regulation	0.00E+00	1.00E+09	0	9.39E+01	9.39E+01	0	1.66E+02	165.8223	0	2.07E-01	0.207
TU00236_DNA_act_bind	TU00236_DNA binding of activator	1 TU00236_DNA_neu --> 1 TU00236_DNA_act	irreversible	Transcription Regulation	0.00E+00	1.00E+09	0	1.22E+01	1.22E+01	0	2.16E+01	21.56007	0	1.04E-01	0.104
TU00236_cleavage	Cleavage of TU00236_mRNA , 29 nt short (b4168_v2), 8 nt short (b4171_v2)	1 TU00236_mRNA_cleav_cplx + 282 h2o --> 1 RNase_III_dim_inact + 78 amp + 1 b4167_v2_mRNA + 1 b4168_v2_mRNA + 1 b4169_v2_mRNA + 1 b4170_v2_mRNA + 1 b4171_v2_mRNA + 1 b4172_v2_mRNA + 1 b4173_v2_mRNA + 1 b4174_v2_mRNA + 1 b4175_v2_mRNA + 54 cmp + 77 gmp + 282 h + 65 ump	irreversible	Cleavage polycistronic mRNA	0.00E+00	1.00E+09	0	1.22E+01	1.22E+01	0	2.16E+01	21.56007	0	1.04E-01	0.104
TU00236_cleavage_cplx	Cleavage complex formation of TU00236_mRNA (yjeFp1, no_common_name_432) , 29 nt short (b4168_v2), 8 nt short (b4171_v2)	1 RNase_III_dim + 1 TU00236_mRNA <==> 1 TU00236_mRNA_cleav_cplx	reversible	Cleavage polycistronic mRNA	-1.00E+09	1.00E+09	0	1.22E+01	1.22E+01	0	2.16E+01	21.56007	0	1.04E-01	0.104
TU00258_DNA_act_bind	TU00258_DNA binding of activator	1 TU00258_DNA_neu --> 1 TU00258_DNA_act	irreversible	Transcription Regulation	0.00E+00	1.00E+09	0	9.07E+01	9.07E+01	0	1.60E+02	160.1693	0	2.84E-01	0.284
TU00258_cleavage	Cleavage of TU00258_mRNA	1 TU00258_mRNA_cleav_cplx + 15 h2o --> 1 RNase_III_dim_inact + 7 amp + 1 b3287_mRNA + 1 b3288_mRNA + 2 cmp + 3 gmp + 15 h 2 ump	irreversible	Cleavage polycistronic mRNA	0.00E+00	1.00E+09	0	9.07E+01	9.07E+01	0	1.60E+02	160.1693	0	2.84E-01	0.284
TU00258_cleavage_cplx	Cleavage complex formation of TU00258_mRNA (defp, no_common_name_410)	1 RNase_III_dim + 1 TU00258_mRNA <==> 1 TU00258_mRNA_cleav_cplx	reversible	Cleavage polycistronic mRNA	-1.00E+09	1.00E+09	0	9.07E+01	9.07E+01	0	1.60E+02	160.1693	0	2.84E-01	0.284
TU00260_DNA_act_bind	TU00260_DNA binding of activator	1 TU00260_DNA_neu --> 1 TU00260_DNA_act	irreversible	Transcription Regulation	0.00E+00	1.00E+09	0	4.13E+01	4.13E+01	0	7.28E+01	72.84185	0	4.31E-01	0.431
TU00260_cleavage	Cleavage of TU00260_mRNA	1 TU00260_mRNA_cleav_cplx + 89 h2o --> 1 RNase_III_dim_inact + 25 amp + 1 b0014_v2_mRNA + 1 b0015_v2_mRNA + 21 cmp + 21 gmp + 89 h + 21 ump	irreversible	Cleavage polycistronic mRNA	0.00E+00	1.00E+09	0	4.13E+01	4.13E+01	0	7.28E+01	72.84185	0	4.31E-01	0.431
TU00260_cleavage_cplx	Cleavage complex formation of TU00260_mRNA (dnaKp1, no_common_name_408)	1 RNase_III_dim + 1 TU00260_mRNA <==> 1 TU00260_mRNA_cleav_cplx	reversible	Cleavage polycistronic mRNA	-1.00E+09	1.00E+09	0	4.13E+01	4.13E+01	0	7.28E+01	72.84185	0	4.31E-01	0.431
TU00277_DNA_act_bind	TU00277_DNA binding of activator	1 TU00277_DNA_neu --> 1 TU00277_DNA_act	irreversible	Transcription Regulation	0.00E+00	1.00E+09	0	5.09E+01	5.09E+01	0	8.98E+01	89.77642	0	2.45E-01	0.245
TU00277_cleavage	Cleavage of TU00277_mRNA	1 TU00277_mRNA_cleav_cplx + 100 h2o --> 1 RNase_III_dim_inact + 30 amp + 1 b3178_v1_mRNA + 1 b3179_v1_mRNA + 15 cmp + 25 gmp + 100 h + 29 ump	irreversible	Cleavage polycistronic mRNA	0.00E+00	1.00E+09	0	5.09E+01	5.09E+01	0	8.98E+01	89.77642	0	2.45E-01	0.245
TU00277_cleavage_cplx	Cleavage complex formation of TU00277_mRNA (rrmJp1, no_common_name_392)	1 RNase_III_dim + 1 TU00277_mRNA <==> 1 TU00277_mRNA_cleav_cplx	reversible	Cleavage polycistronic mRNA	-1.00E+09	1.00E+09	0	5.09E+01	5.09E+01	0	8.98E+01	89.77642	0	2.45E-01	0.245
TU00281_DNA_act_bind	TU00281_DNA binding of activator	1 TU00281_DNA_neu --> 1 TU00281_DNA_act	irreversible	Transcription Regulation	0.00E+00	1.00E+09	0	4.02E+01	4.02E+01	0	7.10E+01	71.00705	0	2.07E-01	0.207
TU00281_cleavage	Cleavage of TU00281_mRNA	1 TU00281_mRNA_cleav_cplx + 10 h2o --> 1 RNase_III_dim_inact + 1 amp + 1 b3559_mRNA + 1 b3560_mRNA + 2 cmp + 5 gmp + 10 h 1 ump	irreversible	Cleavage polycistronic mRNA	0.00E+00	1.00E+09	0	4.02E+01	4.02E+01	0	7.10E+01	71.00705	0	2.07E-01	0.207
TU00281_cleavage_cplx	Cleavage complex formation of TU00281_mRNA (glyQp, no_common_name_388)	1 RNase_III_dim + 1 TU00281_mRNA <==> 1 TU00281_mRNA_cleav_cplx	reversible	Cleavage polycistronic mRNA	-1.00E+09	1.00E+09	0	4.02E+01	4.02E+01	0	7.10E+01	71.00705	0	2.07E-01	0.207
TU00291_DNA_act_bind	TU00291_DNA binding of activator	1 TU00291_DNA_neu --> 1 TU00291_DNA_act	irreversible	Transcription Regulation	0.00E+00	1.00E+09	0	2.14E+01	2.14E+01	0	3.78E+01	37.83865	0	9.45E-02	0.0945
TU00291_cleavage	Cleavage of TU00291_mRNA , 1 nt short (b0027)	1 TU00291_mRNA_cleav_cplx + 171 h2o --> 1 RNase_III_dim_inact + 50 amp + 1 b0025_mRNA + 1 b0026_mRNA + 1 b0027_mRNA + 1 b0028_mRNA + 1 b0029_mRNA + 44 cmp + 38 gmp + 171 h + 35 ump	irreversible	Cleavage polycistronic mRNA	0.00E+00	1.00E+09	0	2.14E+01	2.14E+01	0	3.78E+01	37.83865	0	9.45E-02	0.0945
TU00291_cleavage_cplx	Cleavage complex formation of TU00291_mRNA (ribFp, no_common_name_379) , 1 nt short (b0027)	1 RNase_III_dim + 1 TU00291_mRNA <==> 1 TU00291_mRNA_cleav_cplx	reversible	Cleavage polycistronic mRNA	-1.00E+09	1.00E+09	0	2.14E+01	2.14E+01	0	3.78E+01	37.83865	0	9.45E-02	0.0945
TU00304_DNA_act_bind	TU00304_DNA binding of activator	1 TU00304_DNA_neu --> 1 TU00304_DNA_act	irreversible	Transcription Regulation	0.00E+00	1.00E+09	0	2.76E+01	2.76E+01	0	4.87E+01	48.68704	0	1.04E-01	0.104
TU00304_cleavage	Cleavage of TU00304_mRNA , 11 nt short (b3247), 1 nt short (b3249)	1 TU00304_mRNA_cleav_cplx + 77 h2o --> 1 RNase_III_dim_inact + 16 amp + 1 b3247_mRNA + 1 b3248_mRNA + 1 b3249_mRNA + 1 b3250_mRNA + 1 b3251_mRNA + 15 cmp + 21 gmp + 77 h + 21 um	irreversible	Cleavage polycistronic mRNA	0.00E+00	1.00E+09	0	2.76E+01	2.76E+01	0	4.87E+01	48.68704	0	1.04E-01	0.104
TU00304_cleavage_cplx	Cleavage complex formation of TU00304_mRNA (mreBp, no_common_name_367) , 11 nt short (b3247), 1 nt short (b3249)	1 RNase_III_dim + 1 TU00304_mRNA <==> 1 TU00304_mRNA_cleav_cplx	reversible	Cleavage polycistronic mRNA	-1.00E+09	1.00E+09	0	2.76E+01	2.76E+01	0	4.87E+01	48.68704	0	1.04E-01	0.104
TU00309_DNA_act_bind	TU00309_DNA binding of activator	1 TU00309_DNA_neu --> 1 TU00309_DNA_act	irreversible	Transcription Regulation	0.00E+00	1.00E+09	0	5.83E+01	5.83E+01	0	1.03E+02	102.8825	0	2.31E-01	0.231
TU00309_cleavage	Cleavage of TU00309_mRNA	1 TU00309_mRNA_cleav_cplx + 63 h2o --> 1 RNase_III_dim_inact + 13 amp + 1 b2741_v2_mRNA + 1 b2742_v1_mRNA + 15 cmp + 19 gmp + 63 h + 15 ump	irreversible	Cleavage polycistronic mRNA	0.00E+00	1.00E+09	0	5.83E+01	5.83E+01	0	1.03E+02	102.8825	0	2.31E-01	0.231
TU00309_cleavage_cplx	Cleavage complex formation of TU00309_mRNA (nlpDp1, no_common_name_362)	1 RNase_III_dim + 1 TU00309_mRNA <==> 1 TU00309_mRNA_cleav_cplx	reversible	Cleavage polycistronic mRNA	-1.00E+09	1.00E+09	0	5.83E+01	5.83E+01	0	1.03E+02	102.8825	0	2.31E-01	0.231
TU00311_DNA_act_bind	TU00311_DNA binding of activator	1 TU00311_DNA_neu --> 1 TU00311_DNA_act	irreversible	Transcription Regulation	0.00E+00	1.00E+09	0	2.88E+01	2.88E+01	0	5.08E+01	50.83823	0	1.50E-01	0.15
TU00311_cleavage	Cleavage of TU00311_mRNA	1 TU00311_mRNA_cleav_cplx + 53 h2o --> 1 RNase_III_dim_inact + 17 amp + 1 b3168_v2_mRNA + 1 b3169_v2_mRNA + 1 b3170_v2_mRNA + 9 cmp + 17 gmp + 53 h + 8 ump	irreversible	Cleavage polycistronic mRNA	0.00E+00	1.00E+09	0	2.88E+01	2.88E+01	0	5.08E+01	50.83823	0	1.50E-01	0.15
TU00311_cleavage_cplx	Cleavage complex formation of TU00311_mRNA (yhbCp, no_common_name_360)	1 RNase_III_dim + 1 TU00311_mRNA <==> 1 TU00311_mRNA_cleav_cplx	reversible	Cleavage polycistronic mRNA	-1.00E+09	1.00E+09	0	2.88E+01	2.88E+01	0	5.08E+01	50.83823	0	1.50E-01	0.15
TU00314_DNA_act_bind	TU00314_DNA binding of activator	1 TU00314_DNA_neu --> 1 TU00314_DNA_act	irreversible	Transcription Regulation	0.00E+00	1.00E+09	0	4.88E+01	4.88E+01	0	8.61E+01	86.07572	0	1.96E-01	0.196
TU00314_cleavage	Cleavage of TU00314_mRNA , 11 nt short (b3258)	1 TU00314_mRNA_cleav_cplx + 13 h2o --> 1 RNase_III_dim_inact + 2 amp + 1 b3257_mRNA + 1 b3258_mRNA + 1 b3259_mRNA + 2 cmp + 3 gmp + 13 h + 4 ump	irreversible	Cleavage polycistronic mRNA	0.00E+00	1.00E+09	0	4.88E+01	4.88E+01	0	8.61E+01	86.07572	0	1.96E-01	0.196
TU00314_cleavage_cplx	Cleavage complex formation of TU00314_mRNA (yhdTp, no_common_name_357) , 11 nt short (b3258)	1 RNase_III_dim + 1 TU00314_mRNA <==> 1 TU00314_mRNA_cleav_cplx	reversible	Cleavage polycistronic mRNA	-1.00E+09	1.00E+09	0	4.88E+01	4.88E+01	0	8.61E+01	86.07572	0	1.96E-01	0.196
TU00324_DNA_act_bind	TU00324_DNA binding of activator	1 TU00324_DNA_neu --> 1 TU00324_DNA_act	irreversible	Transcription Regulation	0.00E+00	1.00E+09	0	4.70E+01	4.70E+01	0	8.30E+01	83.03328	0	2.91E-01	0.291

TU00324_cleavage	Cleavage of TU00324_mRNA	1 TU00324_mRNA_cleav_cplx + 10 h2o --> 1 RNase_III_dim_inact + 4 amp + 1 b2890_mRNA + 1 b2891_mRNA + 3 cmp + 2 gmp + 10 h	irreversible	Cleavage polycistronic mRNA	0.00E+00	1.00E+09	0	4.70E+01	4.70E+01	0	8.30E+01	83.03328	0	2.91E-01	0.291
TU00324_cleavage_cplx	Cleavage complex formation of TU00324_mRNA (prfBp, no_common_name_349)	1 RNase_III_dim + 1 TU00324_mRNA <==> 1 TU00324_mRNA_cleav_cplx	reversible	Cleavage polycistronic mRNA	-1.00E+09	1.00E+09	0	4.70E+01	4.70E+01	0	8.30E+01	83.03328	0	2.91E-01	0.291
TU00332_DNA_act_bind	TU00332_DNA binding of activator	1 TU00332_DNA_neu --> 1 TU00332_DNA_act	irreversible	Transcription Regulation	0.00E+00	1.00E+09	0	8.19E+01	8.19E+01	0	1.45E+02	144.5631	0	1.35E-01	0.135
TU00332_cleavage	Cleavage of TU00332_mRNA , 4 nt short (b2566_v1)	1 TU00332_mRNA_cleav_cplx + 1 h2o --> 1 RNase_III_dim_inact + b2566_v1_mRNA + 1 b2567_v1_mRNA + 1 h	irreversible	Cleavage polycistronic mRNA	0.00E+00	1.00E+09	0	8.19E+01	8.19E+01	0	1.45E+02	144.5631	0	1.35E-01	0.135
TU00332_cleavage_cplx	Cleavage complex formation of TU00332_mRNA (mcp, no_common_name_342) , 4 nt short (b2566_v1)	1 RNase_III_dim + 1 TU00332_mRNA <==> 1 TU00332_mRNA_cleav_cplx	reversible	Cleavage polycistronic mRNA	-1.00E+09	1.00E+09	0	8.19E+01	8.19E+01	0	1.45E+02	144.5631	0	1.35E-01	0.135
TU00333_DNA_act_bind	TU00333_DNA binding of activator	1 TU00333_DNA_neu --> 1 TU00333_DNA_act	irreversible	Transcription Regulation	0.00E+00	1.00E+09	0	2.14E+01	2.14E+01	0	3.79E+01	37.86407	0	1.71E-01	0.171
TU00333_cleavage	Cleavage of TU00333_mRNA	1 TU00333_mRNA_cleav_cplx + 93 h2o --> 1 RNase_III_dim_inact + 24 amp + 1 b1652_mRNA + 1 b1653_mRNA + 20 cmp + 23 gmp + 9 h + 25 ump	irreversible	Cleavage polycistronic mRNA	0.00E+00	1.00E+09	0	2.14E+01	2.14E+01	0	3.79E+01	37.86407	0	1.71E-01	0.171
TU00333_cleavage_cplx	Cleavage complex formation of TU00333_mRNA (rntp, no_common_name_341)	1 RNase_III_dim + 1 TU00333_mRNA <==> 1 TU00333_mRNA_cleav_cplx	reversible	Cleavage polycistronic mRNA	-1.00E+09	1.00E+09	0	2.14E+01	2.14E+01	0	3.79E+01	37.86407	0	1.71E-01	0.171
TU00335_DNA_act_bind	TU00335_DNA binding of activator	1 TU00335_DNA_neu --> 1 TU00335_DNA_act	irreversible	Transcription Regulation	0.00E+00	1.00E+09	0	1.32E+01	1.32E+01	0	2.33E+01	23.27811	0	1.89E-01	0.189
TU00335_cleavage	Cleavage of TU00335_mRNA	1 TU00335_mRNA_cleav_cplx + 464 h2o --> 1 RNase_III_dim_inact + 113 amp + 1 b3985_mRNA + 1 b3986_mRNA + 1 b3987_mRNA + 1 b3988_mRNA + 109 cmp + 107 gmp + 464 h + 132 ump	irreversible	Cleavage polycistronic mRNA	0.00E+00	1.00E+09	0	1.32E+01	1.32E+01	0	2.33E+01	23.27811	0	1.89E-01	0.189
TU00335_cleavage_cplx	Cleavage complex formation of TU00335_mRNA (rplJp, no_common_name_339)	1 RNase_III_dim + 1 TU00335_mRNA <==> 1 TU00335_mRNA_cleav_cplx	reversible	Cleavage polycistronic mRNA	-1.00E+09	1.00E+09	0	1.32E+01	1.32E+01	0	2.33E+01	23.27811	0	1.89E-01	0.189
TU00336_DNA_act_bind	TU00336_DNA binding of activator	1 TU00336_DNA_neu --> 1 TU00336_DNA_act	irreversible	Transcription Regulation	0.00E+00	1.00E+09	0	9.48E+01	9.48E+01	0	8.29E+01	82.88	0	4.68E-01	0.468
TU00336_cleavage	Cleavage of TU00336_mRNA	1 TU00336_mRNA_cleav_cplx + 16 h2o --> 1 RNase_III_dim_inact + 4 amp + 1 b3230_mRNA + 1 b3231_mRNA + 2 cmp + 5 gmp + 16 h 4 ump	irreversible	Cleavage polycistronic mRNA	0.00E+00	1.00E+09	0	9.48E+01	9.48E+01	0	8.29E+01	82.88	0	4.68E-01	0.468
TU00336_cleavage_cplx	Cleavage complex formation of TU00336_mRNA (rplMp, no_common_name_338)	1 RNase_III_dim + 1 TU00336_mRNA <==> 1 TU00336_mRNA_cleav_cplx	reversible	Cleavage polycistronic mRNA	-1.00E+09	1.00E+09	0	9.48E+01	9.48E+01	0	8.29E+01	82.88	0	4.68E-01	0.468
TU00337_DNA_act_bind	TU00337_DNA binding of activator	1 TU00337_DNA_neu --> 1 TU00337_DNA_act	irreversible	Transcription Regulation	0.00E+00	1.00E+09	0	2.46E+01	2.46E+01	0	4.34E+01	43.43574	0	1.46E-01	0.146
TU00337_cleavage	Cleavage of TU00337_mRNA	1 TU00337_mRNA_cleav_cplx + 161 h2o --> 1 RNase_III_dim_inact + 49 amp + 1 b3299_mRNA + 1 b3300_mRNA + 1 b3301_mRNA + 1 b3302_mRNA + 1 b3303_mRNA + 1 b3304_mRNA + 1 b3305_mRNA + 1 b3306_mRNA + 1 b3307_mRNA + 1 b3308_mRNA + 1 b3309_mRNA + 1 b3310_mRNA + 21 cmp + 50 gmp + 161 h + 30 ump	irreversible	Cleavage polycistronic mRNA	0.00E+00	1.00E+09	0	2.46E+01	2.46E+01	0	4.34E+01	43.43574	0	1.46E-01	0.146
TU00337_cleavage_cplx	Cleavage complex formation of TU00337_mRNA (rplNp, no_common_name_337)	1 RNase_III_dim + 1 TU00337_mRNA <==> 1 TU00337_mRNA_cleav_cplx	reversible	Cleavage polycistronic mRNA	-1.00E+09	1.00E+09	0	2.46E+01	2.46E+01	0	4.34E+01	43.43574	0	1.46E-01	0.146
TU00338_DNA_act_bind	TU00338_DNA binding of activator	1 TU00338_DNA_neu --> 1 TU00338_DNA_act	irreversible	Transcription Regulation	0.00E+00	1.00E+09	0	1.01E+02	1.01E+02	0	8.29E+01	82.88	0	5.39E-01	0.539
TU00338_cleavage	Cleavage of TU00338_mRNA	1 TU00338_mRNA_cleav_cplx + 21 h2o --> 1 RNase_III_dim_inact + 7 amp + 1 b3185_mRNA + 1 b3186_mRNA + 3 cmp + 6 gmp + 21 h 4 ump	irreversible	Cleavage polycistronic mRNA	0.00E+00	1.00E+09	0	1.01E+02	1.01E+02	0	8.29E+01	82.88	0	5.39E-01	0.539
TU00338_cleavage_cplx	Cleavage complex formation of TU00338_mRNA (rplUp, no_common_name_336)	1 RNase_III_dim + 1 TU00338_mRNA <==> 1 TU00338_mRNA_cleav_cplx	reversible	Cleavage polycistronic mRNA	-1.00E+09	1.00E+09	0	1.01E+02	1.01E+02	0	8.29E+01	82.88	0	5.39E-01	0.539
TU00339_DNA_act_bind	TU00339_DNA binding of activator	1 TU00339_DNA_neu --> 1 TU00339_DNA_act	irreversible	Transcription Regulation	0.00E+00	1.00E+09	0	1.04E+02	1.04E+02	0	8.29E+01	82.88	0	6.41E-01	0.641
TU00339_cleavage	Cleavage of TU00339_mRNA	1 TU00339_mRNA_cleav_cplx + 21 h2o --> 1 RNase_III_dim_inact + 9 amp + 1 b3636_mRNA + 1 b3637_mRNA + 2 cmp + 4 gmp + 21 h 5 ump	irreversible	Cleavage polycistronic mRNA	0.00E+00	1.00E+09	0	1.04E+02	1.04E+02	0	8.29E+01	82.88	0	6.41E-01	0.641
TU00339_cleavage_cplx	Cleavage complex formation of TU00339_mRNA (rpmBp, no_common_name_335)	1 RNase_III_dim + 1 TU00339_mRNA <==> 1 TU00339_mRNA_cleav_cplx	reversible	Cleavage polycistronic mRNA	-1.00E+09	1.00E+09	0	1.04E+02	1.04E+02	0	8.29E+01	82.88	0	6.41E-01	0.641
TU00340_DNA_act_bind	TU00340_DNA binding of activator	1 TU00340_DNA_neu --> 1 TU00340_DNA_act	irreversible	Transcription Regulation	0.00E+00	1.00E+09	0	9.23E+01	9.23E+01	0	5.24E+01	52.37149	0	3.26E-01	0.326
TU00340_cleavage	Cleavage of TU00340_mRNA	1 TU00340_mRNA_cleav_cplx + 17 h2o --> 1 RNase_III_dim_inact + 6 amp + 1 b3703_v2_mRNA + 1 b3704_v2_mRNA + 5 cmp + 2 gmp + 17 h + 3 ump	irreversible	Cleavage polycistronic mRNA	0.00E+00	1.00E+09	0	9.23E+01	9.23E+01	0	5.24E+01	52.37149	0	3.26E-01	0.326
TU00340_cleavage_cplx	Cleavage complex formation of TU00340_mRNA (rpmHp1, no_common_name_334)	1 RNase_III_dim + 1 TU00340_mRNA <==> 1 TU00340_mRNA_cleav_cplx	reversible	Cleavage polycistronic mRNA	-1.00E+09	1.00E+09	0	9.23E+01	9.23E+01	0	5.24E+01	52.37149	0	3.26E-01	0.326
TU00342_DNA_act_bind	TU00342_DNA binding of activator	1 TU00342_DNA_neu --> 1 TU00342_DNA_act	irreversible	Transcription Regulation	0.00E+00	1.00E+09	0	4.80E+01	4.80E+01	0	8.47E+01	84.65405	0	2.99E-01	0.299
TU00342_cleavage	Cleavage of TU00342_mRNA , 4 nt short (b2570_v2), 1 nt short (b2571_v2)	1 TU00342_mRNA_cleav_cplx + 35 h2o --> 1 RNase_III_dim_inact + 9 amp + 1 b2570_v2_mRNA + 1 b2571_v2_mRNA + 1 b2572_v2_mRNA + 1 b2573_v1_mRNA + 4 cmp + 12 gmp + 35 h + [illegible] ump	irreversible	Cleavage polycistronic mRNA	0.00E+00	1.00E+09	0	4.80E+01	4.80E+01	0	8.47E+01	84.65405	0	2.99E-01	0.299
TU00342_cleavage_cplx	Cleavage complex formation of TU00342_mRNA (rpoEp2, no_common_name_333) , 4 nt short (b2570_v2), 1 nt short (b2571_v2)	1 RNase_III_dim + 1 TU00342_mRNA <==> 1 TU00342_mRNA_cleav_cplx	reversible	Cleavage polycistronic mRNA	-1.00E+09	1.00E+09	0	4.80E+01	4.80E+01	0	8.47E+01	84.65405	0	2.99E-01	0.299
TU00343_DNA_act_bind	TU00343_DNA binding of activator	1 TU00343_DNA_neu --> 1 TU00343_DNA_act	irreversible	Transcription Regulation	0.00E+00	1.00E+09	0	8.36E+01	8.36E+01	0	1.48E+02	147.5906	0	2.07E-01	0.207
TU00344_DNA_act_bind	TU00344_DNA binding of activator	1 TU00344_DNA_neu --> 1 TU00344_DNA_act	irreversible	Transcription Regulation	0.00E+00	1.00E+09	0	2.25E+01	2.25E+01	0	3.96E+01	39.6481	0	1.92E-01	0.192
TU00344_cleavage	Cleavage of TU00344_mRNA	1 TU00344_mRNA_cleav_cplx + 32 h2o --> 1 RNase_III_dim_inact + 4 amp + 1 b3649_mRNA + 1 b3650_mRNA + 1 b3651_mRNA + 1 b3652_mRNA + 9 cmp + 8 gmp + 32 h + 8 ump	irreversible	Cleavage polycistronic mRNA	0.00E+00	1.00E+09	0	2.25E+01	2.25E+01	0	3.96E+01	39.6481	0	1.92E-01	0.192
TU00344_cleavage_cplx	Cleavage complex formation of TU00344_mRNA (rpoZp, no_common_name_331)	1 RNase_III_dim + 1 TU00344_mRNA <==> 1 TU00344_mRNA_cleav_cplx	reversible	Cleavage polycistronic mRNA	-1.00E+09	1.00E+09	0	2.25E+01	2.25E+01	0	3.96E+01	39.6481	0	1.92E-01	0.192
TU00345_DNA_act_bind	TU00345_DNA binding of activator	1 TU00345_DNA_neu --> 1 TU00345_DNA_act	irreversible	Transcription Regulation	0.00E+00	1.00E+09	0	7.50E+01	7.50E+01	0	8.09E+01	80.94127	0	5.18E-01	0.518
TU00345_cleavage	Cleavage of TU00345_mRNA	1 TU00345_mRNA_cleav_cplx + 258 h2o --> 1 RNase_III_dim_inact + 67 amp + 1 b0169_mRNA + 1 b0170_mRNA + 56 cmp + 73 gmp + 258 h + 61 ump	irreversible	Cleavage polycistronic mRNA	0.00E+00	1.00E+09	0	7.50E+01	7.50E+01	0	8.09E+01	80.94127	0	5.18E-01	0.518
TU00345_cleavage_cplx	Cleavage complex formation of TU00345_mRNA (rpsBp, no_common_name_330)	1 RNase_III_dim + 1 TU00345_mRNA <==> 1 TU00345_mRNA_cleav_cplx	reversible	Cleavage polycistronic mRNA	-1.00E+09	1.00E+09	0	7.50E+01	7.50E+01	0	8.09E+01	80.94127	0	5.18E-01	0.518
TU00346_DNA_act_bind	TU00346_DNA binding of activator	1 TU00346_DNA_neu --> 1 TU00346_DNA_act	irreversible	Transcription Regulation	0.00E+00	1.00E+09	0	8.54E+01	8.54E+01	0	8.29E+01	82.88	0	2.89E-01	0.289
TU00346_cleavage	Cleavage of TU00346_mRNA	1 TU00346_mRNA_cleav_cplx + 54 h2o --> 1 RNase_III_dim_inact + 13 amp + 1 b4200_mRNA + 1 b4201_mRNA + 1 b4202_mRNA + 1 b4203_mRNA + 10 cmp + 12 gmp + 54 h + 16 ump	irreversible	Cleavage polycistronic mRNA	0.00E+00	1.00E+09	0	8.54E+01	8.54E+01	0	8.29E+01	82.88	0	2.89E-01	0.289
TU00346_cleavage_cplx	Cleavage complex formation of TU00346_mRNA (rpsFp, no_common_name_329)	1 RNase_III_dim + 1 TU00346_mRNA <==> 1 TU00346_mRNA_cleav_cplx	reversible	Cleavage polycistronic mRNA	-1.00E+09	1.00E+09	0	8.54E+01	8.54E+01	0	8.29E+01	82.88	0	2.89E-01	0.289

TU00347_DNA_act_bind	TU00347_DNA binding of activator	1 TU00347_DNA_neu --> 1 TU00347_DNA_act	irreversible	Transcription Regulation	0.00E+00	1.00E+09	0	2.69E+01	2.69E+01	0	4.75E+01	47.45757	0	1.72E-01	0.172
TU00347_cleavage	Cleavage of TU00347_mRNA , 1 nt short (b3311), 1 nt short (b3312), 4 nt short (b3318)	1 TU00347_mRNA_cleav_cplx + 128 h2o --> 1 RNase_III_dim_inact + 36 amp + 1 b3311_mRNA + 1 b3312_mRNA + 1 b3313_mRNA + 1 b3314_mRNA + 1 b3315_mRNA + 1 b3316_mRNA + 1 b3317_mRNA + 1 b3318_mRNA + 1 b3319_mRNA + 1 b3320_mRNA + 1 b3321_mRNA + 16 cmp + 43 gmp + 128 h + 23 ump	irreversible	Cleavage polycistronic mRNA	0.00E+00	1.00E+09	0	2.69E+01	2.69E+01	0	4.75E+01	47.45757	0	1.72E-01	0.172
TU00347_cleavage_cplx	Cleavage complex formation of TU00347_mRNA (rpsJp, no_common_name_328) , 1 nt short (b3311), 1 nt short (b3312), 4 nt short (b3318)	1 RNase_III_dim + 1 TU00347_mRNA <==> 1 TU00347_mRNA_cleav_cplx	reversible	Cleavage polycistronic mRNA	-1.00E+09	1.00E+09	0	2.69E+01	2.69E+01	0	4.75E+01	47.45757	0	1.72E-01	0.172
TU00348_DNA_act_bind	TU00348_DNA binding of activator	1 TU00348_DNA_neu --> 1 TU00348_DNA_act	irreversible	Transcription Regulation	0.00E+00	1.00E+09	0	2.93E+01	2.93E+01	0	5.17E+01	51.7244	0	5.67E-01	0.567
TU00348_cleavage	Cleavage of TU00348_mRNA	1 TU00348_mRNA_cleav_cplx + 196 h2o --> 1 RNase_III_dim_inact + 67 amp + 1 b3339_mRNA + 1 b3340_mRNA + 1 b3341_mRNA + 1 b3342_mRNA + 39 cmp + 40 gmp + 196 h + 47 ump	irreversible	Cleavage polycistronic mRNA	0.00E+00	1.00E+09	0	2.93E+01	2.93E+01	0	5.17E+01	51.7244	0	5.67E-01	0.567
TU00348_cleavage_cplx	Cleavage complex formation of TU00348_mRNA (rpsLp, no_common_name_327)	1 RNase_III_dim + 1 TU00348_mRNA <==> 1 TU00348_mRNA_cleav_cplx	reversible	Cleavage polycistronic mRNA	-1.00E+09	1.00E+09	0	2.93E+01	2.93E+01	0	5.17E+01	51.7244	0	5.67E-01	0.567
TU00349_DNA_act_bind	TU00349_DNA binding of activator	1 TU00349_DNA_neu --> 1 TU00349_DNA_act	irreversible	Transcription Regulation	0.00E+00	1.00E+09	0	4.61E+01	4.61E+01	0	6.94E+01	69.42961	0	4.02E-01	0.402
TU00349_cleavage	Cleavage of TU00349_mRNA	1 TU00349_mRNA_cleav_cplx + 118 h2o --> 1 RNase_III_dim_inact + 35 amp + 1 b3294_mRNA + 1 b3295_mRNA + 1 b3296_mRNA + 1 b3297_mRNA + 1 b3298_mRNA + 18 cmp + 33 gmp + 118 h + 28 ump	irreversible	Cleavage polycistronic mRNA	0.00E+00	1.00E+09	0	4.61E+01	4.61E+01	0	6.94E+01	69.42961	0	4.02E-01	0.402
TU00349_cleavage_cplx	Cleavage complex formation of TU00349_mRNA (rpsMp, no_common_name_326)	1 RNase_III_dim + 1 TU00349_mRNA <==> 1 TU00349_mRNA_cleav_cplx	reversible	Cleavage polycistronic mRNA	-1.00E+09	1.00E+09	0	4.61E+01	4.61E+01	0	6.94E+01	69.42961	0	4.02E-01	0.402
TU00351_DNA_act_bind	TU00351_DNA binding of activator	1 TU00351_DNA_neu --> 1 TU00351_DNA_act	irreversible	Transcription Regulation	0.00E+00	1.00E+09	0	6.90E+01	6.90E+01	0	7.91E+01	79.13389	0	2.79E-01	0.279
TU00351_cleavage	Cleavage of TU00351_mRNA	1 TU00351_mRNA_cleav_cplx + 92 h2o --> 1 RNase_III_dim_inact + 27 amp + 1 b2606_mRNA + 1 b2607_mRNA + 1 b2608_mRNA + 1 b2609_mRNA + 21 cmp + 22 gmp + 92 h + 19 ump	irreversible	Cleavage polycistronic mRNA	0.00E+00	1.00E+09	0	6.90E+01	6.90E+01	0	7.91E+01	79.13389	0	2.79E-01	0.279
TU00351_cleavage_cplx	Cleavage complex formation of TU00351_mRNA (rpsPp, no_common_name_325)	1 RNase_III_dim + 1 TU00351_mRNA <==> 1 TU00351_mRNA_cleav_cplx	reversible	Cleavage polycistronic mRNA	-1.00E+09	1.00E+09	0	6.90E+01	6.90E+01	0	7.91E+01	79.13389	0	2.79E-01	0.279
TU00352_DNA_act_bind	TU00352_DNA binding of activator	1 TU00352_DNA_neu --> 1 TU00352_DNA_act	irreversible	Transcription Regulation	0.00E+00	1.00E+09	0	3.03E+01	3.03E+01	0	5.34E+01	53.44037	0	2.45E-01	0.245
TU00352_cleavage	Cleavage of TU00352_mRNA	1 TU00352_mRNA_cleav_cplx + 306 h2o --> 1 RNase_III_dim_inact + 78 amp + 1 b3065_v3_mRNA + 1 b3066_v2_mRNA + 1 b3067_v5_mRNA + 74 cmp + 80 gmp + 306 h + 72 ump	irreversible	Cleavage polycistronic mRNA	0.00E+00	1.00E+09	0	3.03E+01	3.03E+01	0	5.34E+01	53.44037	0	2.45E-01	0.245
TU00352_cleavage_cplx	Cleavage complex formation of TU00352_mRNA (rpsUp1, no_common_name_324)	1 RNase_III_dim + 1 TU00352_mRNA <==> 1 TU00352_mRNA_cleav_cplx	reversible	Cleavage polycistronic mRNA	-1.00E+09	1.00E+09	0	3.03E+01	3.03E+01	0	5.34E+01	53.44037	0	2.45E-01	0.245
TU00354_DNA_act_bind	TU00354_DNA binding of activator	1 TU00354_DNA_neu --> 1 TU00354_DNA_act	irreversible	Transcription Regulation	0.00E+00	1.00E+09	0	1.49E+02	1.49E+02	0	2.63E+02	263.456	0	2.39E-01	0.239
TU00354_cleavage	Cleavage of TU00354_mRNA	1 TU00354_mRNA_cleav_cplx + 2 h2o --> 1 RNase_III_dim_inact + b3981_mRNA + 1 b3982_mRNA + 1 gmp + 2 h	irreversible	Cleavage polycistronic mRNA	0.00E+00	1.00E+09	0	1.49E+02	1.49E+02	0	2.63E+02	263.456	0	2.39E-01	0.239
TU00354_cleavage_cplx	Cleavage complex formation of TU00354_mRNA (secEp, no_common_name_322)	1 RNase_III_dim + 1 TU00354_mRNA <==> 1 TU00354_mRNA_cleav_cplx	reversible	Cleavage polycistronic mRNA	-1.00E+09	1.00E+09	0	1.49E+02	1.49E+02	0	2.63E+02	263.456	0	2.39E-01	0.239
TU00355_DNA_act_bind	TU00355_DNA binding of activator	1 TU00355_DNA_neu --> 1 TU00355_DNA_act	irreversible	Transcription Regulation	0.00E+00	1.00E+09	0	3.70E+01	3.70E+01	0	1.37E+01	13.7	0	1.86E-01	0.186
TU00355_cleavage	Cleavage of TU00355_mRNA , 4 nt short (b3590)	1 TU00355_mRNA_cleav_cplx + 1 h2o --> 1 RNase_III_dim_inact + b3590_mRNA + 1 b3591_mRNA + 1 h	irreversible	Cleavage polycistronic mRNA	0.00E+00	1.00E+09	0	3.70E+01	3.70E+01	0	1.37E+01	13.7	0	1.86E-01	0.186
TU00355_cleavage_cplx	Cleavage complex formation of TU00355_mRNA (selAp, no_common_name_321) , 4 nt short (b3590)	1 RNase_III_dim + 1 TU00355_mRNA <==> 1 TU00355_mRNA_cleav_cplx	reversible	Cleavage polycistronic mRNA	-1.00E+09	1.00E+09	0	3.70E+01	3.70E+01	0	1.37E+01	13.7	0	1.86E-01	0.186
TU00362_DNA_act_bind	TU00362_DNA binding of activator	1 TU00362_DNA_neu --> 1 TU00362_DNA_act	irreversible	Transcription Regulation	0.00E+00	1.00E+09	0	2.75E+01	2.75E+01	0	4.85E+01	48.51944	0	1.68E-01	0.168
TU00362_cleavage	Cleavage of TU00362_mRNA , 4 nt short (b0051), 1 nt short (b0052)	1 TU00362_mRNA_cleav_cplx + 12 h2o --> 1 RNase_III_dim_inact + 4 amp + 1 b0049_mRNA + 1 b0050_mRNA + 1 b0051_mRNA + 1 b0052_mRNA + 1 b0053_mRNA + 1 cmp + 2 gmp + 12 h + 1 ump	irreversible	Cleavage polycistronic mRNA	0.00E+00	1.00E+09	0	2.75E+01	2.75E+01	0	4.85E+01	48.51944	0	1.68E-01	0.168
TU00362_cleavage_cplx	Cleavage complex formation of TU00362_mRNA (surAp, no_common_name_314) , 4 nt short (b0051), 1 nt short (b0052)	1 RNase_III_dim + 1 TU00362_mRNA <==> 1 TU00362_mRNA_cleav_cplx	reversible	Cleavage polycistronic mRNA	-1.00E+09	1.00E+09	0	2.75E+01	2.75E+01	0	4.85E+01	48.51944	0	1.68E-01	0.168
TU00366_DNA_act_bind	TU00366_DNA binding of activator	1 TU00366_DNA_neu --> 1 TU00366_DNA_act	irreversible	Transcription Regulation	0.00E+00	1.00E+09	0	4.35E+01	4.35E+01	0	6.79E+01	67.90817	0	1.65E-01	0.165
TU00366_cleavage	Cleavage of TU00366_mRNA	1 TU00366_mRNA_cleav_cplx + 154 h2o --> 1 RNase_III_dim_inact + 38 amp + 1 b1716_mRNA + 1 b1717_mRNA + 1 b1718_mRNA + 1 b1719_mRNA + 26 cmp + 35 gmp + 154 h + 52 ump	irreversible	Cleavage polycistronic mRNA	0.00E+00	1.00E+09	0	4.35E+01	4.35E+01	0	6.79E+01	67.90817	0	1.65E-01	0.165
TU00366_cleavage_cplx	Cleavage complex formation of TU00366_mRNA (thrSp, no_common_name_310)	1 RNase_III_dim + 1 TU00366_mRNA <==> 1 TU00366_mRNA_cleav_cplx	reversible	Cleavage polycistronic mRNA	-1.00E+09	1.00E+09	0	4.35E+01	4.35E+01	0	6.79E+01	67.90817	0	1.65E-01	0.165
TU00411_DNA_act_bind	TU00411_DNA binding of activator	1 TU00411_DNA_neu --> 1 TU00411_DNA_act	irreversible	Transcription Regulation	0.00E+00	1.00E+09	0	2.64E+02	2.64E+02	0	4.66E+02	465.9471	0	2.87E-01	0.287
TU00414_DNA_act_bind	TU00414_DNA binding of activator	1 TU00414_DNA_neu --> 1 TU00414_DNA_act	irreversible	Transcription Regulation	0.00E+00	1.00E+09	0	5.09E+01	5.09E+01	0	8.98E+01	89.77642	0	2.45E-01	0.245
TU00414_cleavage	Cleavage of TU00414_mRNA	1 TU00414_mRNA_cleav_cplx + 100 h2o --> 1 RNase_III_dim_inact + 30 amp + 1 b3178_mRNA + 1 b3179_mRNA + 15 cmp + 25 gmp + 100 h + 29 ump	irreversible	Cleavage polycistronic mRNA	0.00E+00	1.00E+09	0	5.09E+01	5.09E+01	0	8.98E+01	89.77642	0	2.45E-01	0.245
TU00414_cleavage_cplx	Cleavage complex formation of TU00414_mRNA (rrmJp2, no_common_name_264)	1 RNase_III_dim + 1 TU00414_mRNA <==> 1 TU00414_mRNA_cleav_cplx	reversible	Cleavage polycistronic mRNA	-1.00E+09	1.00E+09	0	5.09E+01	5.09E+01	0	8.98E+01	89.77642	0	2.45E-01	0.245
TU00415_DNA_act_bind	TU00415_DNA binding of activator	1 TU00415_DNA_neu --> 1 TU00415_DNA_act	irreversible	Transcription Regulation	0.00E+00	1.00E+09	0	6.35E+01	6.35E+01	0	1.12E+02	112.0867	0	1.61E-01	0.161
TU00415_cleavage	Cleavage of TU00415_mRNA	1 TU00415_mRNA_cleav_cplx + 134 h2o --> 1 RNase_III_dim_inact + 43 amp + 1 b1920_v1_mRNA + 1 b1921_v1_mRNA + 1 b1922_v1_mRNA + 24 cmp + 27 gmp + 134 h + 38 ump	irreversible	Cleavage polycistronic mRNA	0.00E+00	1.00E+09	0	6.35E+01	6.35E+01	0	1.12E+02	112.0867	0	1.61E-01	0.161
TU00415_cleavage_cplx	Cleavage complex formation of TU00415_mRNA (fliAp1, no_common_name_263)	1 RNase_III_dim + 1 TU00415_mRNA <==> 1 TU00415_mRNA_cleav_cplx	reversible	Cleavage polycistronic mRNA	-1.00E+09	1.00E+09	0	6.35E+01	6.35E+01	0	1.12E+02	112.0867	0	1.61E-01	0.161
TU00417_DNA_act_bind	TU00417_DNA binding of activator	1 TU00417_DNA_neu --> 1 TU00417_DNA_act	irreversible	Transcription Regulation	0.00E+00	1.00E+09	0	4.13E+01	4.13E+01	0	7.28E+01	72.84185	0	4.31E-01	0.431
TU00417_cleavage	Cleavage of TU00417_mRNA	1 TU00417_mRNA_cleav_cplx + 89 h2o --> 1 RNase_III_dim_inact + 25 amp + 1 b0014_v1_mRNA + 1 b0015_v1_mRNA + 21 cmp + 21 gmp + 89 h + 21 ump	irreversible	Cleavage polycistronic mRNA	0.00E+00	1.00E+09	0	4.13E+01	4.13E+01	0	7.28E+01	72.84185	0	4.31E-01	0.431
TU00417_cleavage_cplx	Cleavage complex formation of TU00417_mRNA (dnaKp2, no_common_name_261)	1 RNase_III_dim + 1 TU00417_mRNA <==> 1 TU00417_mRNA_cleav_cplx	reversible	Cleavage polycistronic mRNA	-1.00E+09	1.00E+09	0	4.13E+01	4.13E+01	0	7.28E+01	72.84185	0	4.31E-01	0.431
TU00418_DNA_act_bind	TU00418_DNA binding of activator	1 TU00418_DNA_neu --> 1 TU00418_DNA_act	irreversible	Transcription Regulation	0.00E+00	1.00E+09	0	4.13E+01	4.13E+01	0	7.28E+01	72.84185	0	4.31E-01	0.431

TU00418_cleavage	Cleavage of TU00418_mRNA	1 TU00418_mRNA_cleav_cplx + 89 h2o --> 1 RNase_III_dim_inact + 25 amp + 1 b0014_mRNA + 1 b0015_mRNA + 21 cmp + 21 gmp + 89 h + 21 ump	irreversible	Cleavage polycistronic mRNA	0.00E+00	1.00E+09	0	4.13E+01	4.13E+01	0	7.28E+01	72.84185	0	4.31E-01	0.431
TU00418_cleavage_cplx	Cleavage complex formation of TU00418_mRNA (dnaKp3, no_common_name_260)	1 RNase_III_dim + 1 TU00418_mRNA <==> 1 TU00418_mRNA_cleav_cplx	reversible	Cleavage polycistronic mRNA	-1.00E+09	1.00E+09	0	4.13E+01	4.13E+01	0	7.28E+01	72.84185	0	4.31E-01	0.431
TU00426_DNA_act_bind	TU00426_DNA binding of activator	1 TU00426_DNA_neu --> 1 TU00426_DNA_act	irreversible	Transcription Regulation	0.00E+00	1.00E+09	0	1.47E+02	1.47E+02	0	2.60E+02	259.8138	0	2.99E-01	0.299
TU00427_DNA_act_bind	TU00427_DNA binding of activator	1 TU00427_DNA_neu --> 1 TU00427_DNA_act	irreversible	Transcription Regulation	0.00E+00	1.00E+09	0	1.47E+02	1.47E+02	0	2.60E+02	259.8138	0	2.99E-01	0.299
TU00429_DNA_act_bind	TU00429_DNA binding of activator	1 TU00429_DNA_neu --> 1 TU00429_DNA_act	irreversible	Transcription Regulation	0.00E+00	1.00E+09	0	9.39E+01	9.39E+01	0	1.66E+02	165.8223	0	2.07E-01	0.207
TU00430_DNA_act_bind	TU00430_DNA binding of activator	1 TU00430_DNA_neu --> 1 TU00430_DNA_act	irreversible	Transcription Regulation	0.00E+00	1.00E+09	0	1.19E+02	1.19E+02	0	2.10E+02	209.5896	0	9.79E-02	0.0979
TU00434_DNA_act_bind	TU00434_DNA binding of activator	1 TU00434_DNA_neu --> 1 TU00434_DNA_act	irreversible	Transcription Regulation	0.00E+00	1.00E+09	0	3.03E+01	3.03E+01	0	5.34E+01	53.44037	0	2.45E-01	0.245
TU00434_cleavage	Cleavage of TU00434_mRNA	1 TU00434_mRNA_cleav_cplx + 306 h2o --> 1 RNase_III_dim_inact + 78 amp + 1 b3065_v2_mRNA + 1 b3066_v1_mRNA + 1 b3067_v4_mRNA + 74 cmp + 80 gmp + 306 h + 72 ump	irreversible	Cleavage polycistronic mRNA	0.00E+00	1.00E+09	0	3.03E+01	3.03E+01	0	5.34E+01	53.44037	0	2.45E-01	0.245
TU00434_cleavage_cplx	Cleavage complex formation of TU00434_mRNA (rpsUp2, no_common_name_244)	1 RNase_III_dim + 1 TU00434_mRNA <==> 1 TU00434_mRNA_cleav_cplx	reversible	Cleavage polycistronic mRNA	-1.00E+09	1.00E+09	0	3.03E+01	3.03E+01	0	5.34E+01	53.44037	0	2.45E-01	0.245
TU00435_DNA_act_bind	TU00435_DNA binding of activator	1 TU00435_DNA_neu --> 1 TU00435_DNA_act	irreversible	Transcription Regulation	0.00E+00	1.00E+09	0	3.03E+01	3.03E+01	0	5.34E+01	53.44037	0	2.45E-01	0.245
TU00435_cleavage	Cleavage of TU00435_mRNA	1 TU00435_mRNA_cleav_cplx + 306 h2o --> 1 RNase_III_dim_inact + 78 amp + 1 b3065_v1_mRNA + 1 b3066_mRNA + 1 b3067_v3_mRNA + 74 cmp + 80 gmp + 306 h + 72 ump	irreversible	Cleavage polycistronic mRNA	0.00E+00	1.00E+09	0	3.03E+01	3.03E+01	0	5.34E+01	53.44037	0	2.45E-01	0.245
TU00435_cleavage_cplx	Cleavage complex formation of TU00435_mRNA (rpsUp3, no_common_name_243)	1 RNase_III_dim + 1 TU00435_mRNA <==> 1 TU00435_mRNA_cleav_cplx	reversible	Cleavage polycistronic mRNA	-1.00E+09	1.00E+09	0	3.03E+01	3.03E+01	0	5.34E+01	53.44037	0	2.45E-01	0.245
TU00436_DNA_act_bind	TU00436_DNA binding of activator	1 TU00436_DNA_neu --> 1 TU00436_DNA_act	irreversible	Transcription Regulation	0.00E+00	1.00E+09	0	6.70E+01	6.70E+01	0	1.18E+02	118.1962	0	3.99E-01	0.399
TU00437_DNA_act_bind	TU00437_DNA binding of activator	1 TU00437_DNA_neu --> 1 TU00437_DNA_act	irreversible	Transcription Regulation	0.00E+00	1.00E+09	0	6.70E+01	6.70E+01	0	1.18E+02	118.1962	0	3.99E-01	0.399
TU00438_DNA_act_bind	TU00438_DNA binding of activator	1 TU00438_DNA_neu --> 1 TU00438_DNA_act	irreversible	Transcription Regulation	0.00E+00	1.00E+09	0	6.70E+01	6.70E+01	0	1.18E+02	118.1962	0	3.99E-01	0.399
TU00440_DNA_act_bind	TU00440_DNA binding of activator	1 TU00440_DNA_neu --> 1 TU00440_DNA_act	irreversible	Transcription Regulation	0.00E+00	1.00E+09	0	5.83E+01	5.83E+01	0	1.03E+02	102.8825	0	2.31E-01	0.231
TU00440_cleavage	Cleavage of TU00440_mRNA	1 TU00440_mRNA_cleav_cplx + 63 h2o --> 1 RNase_III_dim_inact + 13 amp + 1 b2741_v1_mRNA + 1 b2742_mRNA + 15 cmp + 19 gmp + 63 h + 15 ump	irreversible	Cleavage polycistronic mRNA	0.00E+00	1.00E+09	0	5.83E+01	5.83E+01	0	1.03E+02	102.8825	0	2.31E-01	0.231
TU00440_cleavage_cplx	Cleavage complex formation of TU00440_mRNA (nlpDp2, no_common_name_238)	1 RNase_III_dim + 1 TU00440_mRNA <==> 1 TU00440_mRNA_cleav_cplx	reversible	Cleavage polycistronic mRNA	-1.00E+09	1.00E+09	0	5.83E+01	5.83E+01	0	1.03E+02	102.8825	0	2.31E-01	0.231
TU00442_DNA_act_bind	TU00442_DNA binding of activator	1 TU00442_DNA_neu --> 1 TU00442_DNA_act	irreversible	Transcription Regulation	0.00E+00	1.00E+09	0	1.42E+02	1.42E+02	0	2.51E+02	250.576	0	2.31E-01	0.231
TU00460_DNA_act_bind	TU00460_DNA binding of activator	1 TU00460_DNA_neu --> 1 TU00460_DNA_act	irreversible	Transcription Regulation	0.00E+00	1.00E+09	0	0	0.00E+00	0	0	0	0	0	0
TU00460_cleavage	Cleavage of TU00460_mRNA , 4 nt short (b4292)	1 TU00460_mRNA_cleav_cplx + 1 h2o --> 1 RNase_III_dim_inact + b4292_mRNA + 1 b4293_mRNA + 1 h	irreversible	Cleavage polycistronic mRNA	0.00E+00	1.00E+09	0	0	0.00E+00	0	0	0	0	0	0
TU00460_cleavage_cplx	Cleavage complex formation of TU00460_mRNA (feclp, no_common_name_220) , 4 nt short (b4292)	1 RNase_III_dim + 1 TU00460_mRNA <==> 1 TU00460_mRNA_cleav_cplx	reversible	Cleavage polycistronic mRNA	-1.00E+09	1.00E+09	0	0	0.00E+00	0	0	0	0	0	0
TU00471_DNA_act_bind	TU00471_DNA binding of activator	1 TU00471_DNA_neu --> 1 TU00471_DNA_act	irreversible	Transcription Regulation	0.00E+00	1.00E+09	0	6.35E+01	6.35E+01	0	1.12E+02	112.0867	0	1.61E-01	0.161
TU00471_cleavage	Cleavage of TU00471_mRNA	1 TU00471_mRNA_cleav_cplx + 134 h2o --> 1 RNase_III_dim_inact + 43 amp + 1 b1920_mRNA + 1 b1921_mRNA + 1 b1922_mRNA + 24 cmp + 27 gmp + 134 h + 38 ump	irreversible	Cleavage polycistronic mRNA	0.00E+00	1.00E+09	0	6.35E+01	6.35E+01	0	1.12E+02	112.0867	0	1.61E-01	0.161
TU00471_cleavage_cplx	Cleavage complex formation of TU00471_mRNA (fliAp2, no_common_name_210)	1 RNase_III_dim + 1 TU00471_mRNA <==> 1 TU00471_mRNA_cleav_cplx	reversible	Cleavage polycistronic mRNA	-1.00E+09	1.00E+09	0	6.35E+01	6.35E+01	0	1.12E+02	112.0867	0	1.61E-01	0.161
TU00472_DNA_act_bind	TU00472_DNA binding of activator	1 TU00472_DNA_neu --> 1 TU00472_DNA_act	irreversible	Transcription Regulation	0.00E+00	1.00E+09	0	1.07E+02	1.07E+02	0	8.29E+01	82.88	0	2.19E+00	2.19
TU00487_DNA_act_bind	TU00487_DNA binding of activator	1 TU00487_DNA_neu --> 1 TU00487_DNA_act	irreversible	Transcription Regulation	0.00E+00	1.00E+09	0	3.52E+01	3.52E+01	0	6.22E+01	62.16052	0	2.07E-01	0.207
TU00487_cleavage	Cleavage of TU00487_mRNA , 4 nt short (b3206)	1 TU00487_mRNA_cleav_cplx + 188 h2o --> 1 RNase_III_dim_inact + 41 amp + 1 b3202_mRNA + 1 b3203_mRNA + 1 b3204_mRNA + 1 b3205_mRNA + 1 b3206_mRNA + 37 cmp + 52 gmp + 188 h + 54 ump	irreversible	Cleavage polycistronic mRNA	0.00E+00	1.00E+09	0	3.52E+01	3.52E+01	0	6.22E+01	62.16052	0	2.07E-01	0.207
TU00487_cleavage_cplx	Cleavage complex formation of TU00487_mRNA (rpoNp, no_common_name_194) , 4 nt short (b3206)	1 RNase_III_dim + 1 TU00487_mRNA <==> 1 TU00487_mRNA_cleav_cplx	reversible	Cleavage polycistronic mRNA	-1.00E+09	1.00E+09	0	3.52E+01	3.52E+01	0	6.22E+01	62.16052	0	2.07E-01	0.207
TU00489_DNA_act_bind	TU00489_DNA binding of activator	1 TU00489_DNA_neu --> 1 TU00489_DNA_act	irreversible	Transcription Regulation	0.00E+00	1.00E+09	0	1.45E+03	1.45E+03	0	9.48E+00	9.48	0	9.48E+00	9.48
TU00489_trimming	Cutting of stable TU00489_RNA	1 TU00489_RNA_cut_cplx + 1 h2o --> 1 RNase_E_tetra_inact + 1 RNase_Gen_inact + 1 RNase_P_cplx_inact + 1 b0216_RNA + 1 h + ppi	irreversible	RNA Processing	0.00E+00	1.00E+09	0	1.45E+03	1.45E+03	0	9.48E+00	9.48	0	9.48E+00	9.48
TU00489_trimming_cplx	Cutting complex formation of stable TU00489_RNA (aspV, no_common_name_192)	1 RNase_E_tetra + 1 RNase_Gen + 1 RNase_P_cplx + 1 TU00489_RNA <==> 1 TU00489_RNA_cut_cplx	reversible	RNA Processing	-1.00E+09	1.00E+09	0	1.45E+03	1.45E+03	0	9.48E+00	9.48	0	9.48E+00	9.48
TU00490_DNA_act_bind	TU00490_DNA binding of activator	1 TU00490_DNA_neu --> 1 TU00490_DNA_act	irreversible	Transcription Regulation	0.00E+00	1.00E+09	0	1.77E+03	1.77E+03	0	9.38E+00	9.38	0	9.38E+00	9.38
TU00490_trimming	Cutting of stable TU00490_RNA	1 TU00490_RNA_cut_cplx + 1 h2o --> 1 RNase_E_tetra_inact + 1 RNase_Gen_inact + 1 RNase_P_cplx_inact + 1 b0244_RNA + 1 h + ppi	irreversible	RNA Processing	0.00E+00	1.00E+09	0	1.77E+03	1.77E+03	0	9.38E+00	9.38	0	9.38E+00	9.38
TU00490_trimming_cplx	Cutting complex formation of stable TU00490_RNA (thrWp, no_common_name_191)	1 RNase_E_tetra + 1 RNase_Gen + 1 RNase_P_cplx + 1 TU00490_RNA <==> 1 TU00490_RNA_cut_cplx	reversible	RNA Processing	-1.00E+09	1.00E+09	0	1.77E+03	1.77E+03	0	9.38E+00	9.38	0	9.38E+00	9.38
TU00491_DNA_act_bind	TU00491_DNA binding of activator	1 TU00491_DNA_neu --> 1 TU00491_DNA_act	irreversible	Transcription Regulation	0.00E+00	1.00E+09	0	1.39E+03	1.39E+03	0	8.24E+00	8.24	0	8.24E+00	8.24
TU00491_trimming	Cutting of stable TU00491_RNA	1 TU00491_RNA_cut_cplx + 1 h2o --> 1 RNase_E_tetra_inact + 1 RNase_Gen_inact + 1 RNase_P_cplx_inact + 1 b0536_RNA + 1 h + ppi	irreversible	RNA Processing	0.00E+00	1.00E+09	0	1.39E+03	1.39E+03	0	8.24E+00	8.24	0	8.24E+00	8.24
TU00491_trimming_cplx	Cutting complex formation of stable TU00491_RNA (argUp, no_common_name_190)	1 RNase_E_tetra + 1 RNase_Gen + 1 RNase_P_cplx + 1 TU00491_RNA <==> 1 TU00491_RNA_cut_cplx	reversible	RNA Processing	-1.00E+09	1.00E+09	0	1.39E+03	1.39E+03	0	8.24E+00	8.24	0	8.24E+00	8.24
TU00492_DNA_act_bind	TU00492_DNA binding of activator	1 TU00492_DNA_neu --> 1 TU00492_DNA_act	irreversible	Transcription Regulation	0.00E+00	1.00E+09	0	3.51E+02	3.51E+02	0	7.52E+00	7.52	0	7.52E+00	7.52
TU00492_cutting	Cutting of stable TU00492_RNA	1 TU00492_RNA_cut_cplx + 140 h2o --> 1 RNase_E_tetra_inact + 1 RNase_G_dim_inact + 1 RNase_Gen_inact + 1 RNase_III_dim_inact + 1 RNase_P_cplx_inact + 1 RNase_T_dim_inact + 1 RNase_m16_inact + 1 RNase_m23_inact + 1 RNase_m5_inact + 41 amp + 1 b0743_RNA + 1 b0744_RNA + 1 b0745_RNA + 32 cmp + 38 gmp + 140 h + 1 ppi + 26 ump	irreversible	RNA processing	0.00E+00	1.00E+09	0	3.51E+02	3.51E+02	0	7.52E+00	7.52	0	7.52E+00	7.52
TU00492_cutting_cplx	Cutting complex formation of stable TU00492_RNA (lysTp, no_common_name_189)	1 RNase_E_tetra + 1 RNase_G_dim + 1 RNase_Gen + 1 RNase_III_dim + 1 RNase_P_cplx + 1 RNase_T_dim + 1 RNase_m1 + 1 RNase_m23 + 1 RNase_m5 + 1 TU00492_RNA <==> 1 TU00492_RNA_cut_cplx	reversible	RNA processing	-1.00E+09	1.00E+09	0	3.51E+02	3.51E+02	0	7.52E+00	7.52	0	7.52E+00	7.52

TU00493_DNA_act_bind	TU00493_DNA binding of activator	1 TU00493_DNA_neu --> 1 TU00493_DNA_act	irreversible	Transcription Regulation	0.00E+00	1.00E+09	0	7.79E+02	7.79E+02	0	5.60E+00	5.6	0	5.60E+00	5.6
TU00493_cutting	Cutting of stable TU00493_RNA	1 TU00493_RNA_cut_cplx + 6 h2o --> 1 RNase_E_tetra_inact + 1 RNase_G_dim_inact + 1 RNase_Gen_inact + 1 RNase_III_dim_inact + 1 RNase_P_cplx_inact + 1 RNase_T_dim_inact + 1 RNase_m16_inact + 1 RNase_m23_inact + 1 RNase_m5_inact + 1 b1665_RNA + 1 b1666_RNA + 2 cmp + 6 h + 1 ppi + 2 ump	irreversible	RNA processing	0.00E+00	1.00E+09	0	7.79E+02	7.79E+02	0	5.60E+00	5.6	0	5.60E+00	5.6
TU00493_cutting_cplx	Cutting complex formation of stable TU00493_RNA (valVp, no_common_name_188)	1 RNase_E_tetra + 1 RNase_G_dim + 1 RNase_Gen + 1 RNase_III_dim + 1 RNase_P_cplx + 1 RNase_T_dim + 1 RNase_m16 + 1 RNase_m23 + 1 RNase_m5 + 1 TU00493_RNA <==> 1 TU00493_RNA_cut_cplx	reversible	RNA processing	-1.00E+09	1.00E+09	0	7.79E+02	7.79E+02	0	5.60E+00	5.6	0	5.60E+00	5.6
TU00494_DNA_act_bind	TU00494_DNA binding of activator	1 TU00494_DNA_neu --> 1 TU00494_DNA_act	irreversible	Transcription Regulation	0.00E+00	1.00E+09	0	1.39E+03	1.39E+03	0	7.06E+00	7.06	0	7.06E+00	7.06
TU00494_trimming	Cutting of stable TU00494_RNA	1 TU00494_RNA_cut_cplx + 1 h2o --> 1 RNase_E_tetra_inact + 1 RNase_Gen_inact + 1 RNase_P_cplx_inact + 1 b2189_RNA + 1 h + ppi	irreversible	RNA Processing	0.00E+00	1.00E+09	0	1.39E+03	1.39E+03	0	7.06E+00	7.06	0	7.06E+00	7.06
TU00494_trimming_cplx	Cutting complex formation of stable TU00494_RNA (proLp, no_common_name_187)	1 RNase_E_tetra + 1 RNase_Gen + 1 RNase_P_cplx + 1 TU00494_RNA <==> 1 TU00494_RNA_cut_cplx	reversible	RNA Processing	-1.00E+09	1.00E+09	0	1.39E+03	1.39E+03	0	7.06E+00	7.06	0	7.06E+00	7.06
TU00495_DNA_act_bind	TU00495_DNA binding of activator	1 TU00495_DNA_neu --> 1 TU00495_DNA_act	irreversible	Transcription Regulation	0.00E+00	1.00E+09	0	3.01E+02	3.01E+02	0	7.81E+00	7.81	0	7.81E+00	7.81
TU00495_cutting	Cutting of stable TU00495_RNA	1 TU00495_RNA_cut_cplx + 98 h2o --> 1 RNase_E_tetra_inact + 1 RNase_G_dim_inact + 1 RNase_Gen_inact + 1 RNase_III_dim_inact + 1 RNase_P_cplx_inact + 1 RNase_T_dim_inact + 1 RNase_m16_inact + 1 RNase_m23_inact + 1 RNase_m5_inact + 27 amp + 1 b2401_RNA + 1 b2402_RNA + 1 b2403_RNA + 1 b2404_RNA + 19 cmp + 18 gmp + 98 h + 1 ppi + 30 ump	irreversible	RNA processing	0.00E+00	1.00E+09	0	3.01E+02	3.01E+02	0	7.81E+00	7.81	0	7.81E+00	7.81
TU00495_cutting_cplx	Cutting complex formation of stable TU00495_RNA (valUp, no_common_name_186)	1 RNase_E_tetra + 1 RNase_G_dim + 1 RNase_Gen + 1 RNase_III_dim + 1 RNase_P_cplx + 1 RNase_T_dim + 1 RNase_m16 + 1 RNase_m23 + 1 RNase_m5 + 1 TU00495_RNA <==> 1 TU00495_RNA_cut_cplx	reversible	RNA processing	-1.00E+09	1.00E+09	0	3.01E+02	3.01E+02	0	7.81E+00	7.81	0	7.81E+00	7.81
TU00496_DNA_act_bind	TU00496_DNA binding of activator	1 TU00496_DNA_neu --> 1 TU00496_DNA_act	irreversible	Transcription Regulation	0.00E+00	1.00E+09	0	3.85E+02	3.85E+02	0	9.37E+00	9.37	0	9.37E+00	9.37
TU00496_cutting	Cutting of stable TU00496_RNA	1 TU00496_RNA_cut_cplx + 69 h2o --> 1 RNase_E_tetra_inact + 1 RNase_G_dim_inact + 1 RNase_Gen_inact + 1 RNase_III_dim_inact + 1 RNase_P_cplx_inact + 1 RNase_T_dim_inact + 1 RNase_m16_inact + 1 RNase_m23_inact + 1 RNase_m5_inact + 28 amp + 1 b2814_RNA + 1 b2815_RNA + 1 b2816_RNA + 5 cmp + 16 gmp + 69 h + 1 ppi + 17 ump	irreversible	RNA processing	0.00E+00	1.00E+09	0	3.85E+02	3.85E+02	0	9.37E+00	9.37	0	9.37E+00	9.37
TU00496_cutting_cplx	Cutting complex formation of stable TU00496_RNA (metZp, no_common_name_185)	1 RNase_E_tetra + 1 RNase_G_dim + 1 RNase_Gen + 1 RNase_III_dim + 1 RNase_P_cplx + 1 RNase_T_dim + 1 RNase_m16 + 1 RNase_m23 + 1 RNase_m5 + 1 TU00496_RNA <==> 1 TU00496_RNA_cut_cplx	reversible	RNA processing	-1.00E+09	1.00E+09	0	3.85E+02	3.85E+02	0	9.37E+00	9.37	0	9.37E+00	9.37
TU00497_DNA_act_bind	TU00497_DNA binding of activator	1 TU00497_DNA_neu --> 1 TU00497_DNA_act	irreversible	Transcription Regulation	0.00E+00	1.00E+09	0	1.77E+03	1.77E+03	0	6.36E+00	6.36	0	6.36E+00	6.36
TU00497_trimming	Cutting of stable TU00497_RNA	1 TU00497_RNA_cut_cplx + 1 h2o --> 1 RNase_E_tetra_inact + 1 RNase_Gen_inact + 1 RNase_P_cplx_inact + 1 b1977_RNA + 1 h + ppi	irreversible	RNA Processing	0.00E+00	1.00E+09	0	1.77E+03	1.77E+03	0	6.36E+00	6.36	0	6.36E+00	6.36
TU00497_trimming_cplx	Cutting complex formation of stable TU00497_RNA (asnTp, no_common_name_184)	1 RNase_E_tetra + 1 RNase_Gen + 1 RNase_P_cplx + 1 TU00497_RNA <==> 1 TU00497_RNA_cut_cplx	reversible	RNA Processing	-1.00E+09	1.00E+09	0	1.77E+03	1.77E+03	0	6.36E+00	6.36	0	6.36E+00	6.36
TU00498_DNA_act_bind	TU00498_DNA binding of activator	1 TU00498_DNA_neu --> 1 TU00498_DNA_act	irreversible	Transcription Regulation	0.00E+00	1.00E+09	0	1.77E+03	1.77E+03	0	6.41E+00	6.41	0	6.41E+00	6.41
TU00498_trimming	Cutting of stable TU00498_RNA	1 TU00498_RNA_cut_cplx + 1 h2o --> 1 RNase_E_tetra_inact + 1 RNase_Gen_inact + 1 RNase_P_cplx_inact + 1 b1986_RNA + 1 h + ppi	irreversible	RNA Processing	0.00E+00	1.00E+09	0	1.77E+03	1.77E+03	0	6.41E+00	6.41	0	6.41E+00	6.41
TU00498_trimming_cplx	Cutting complex formation of stable TU00498_RNA (asnUp, no_common_name_183)	1 RNase_E_tetra + 1 RNase_Gen + 1 RNase_P_cplx + 1 TU00498_RNA <==> 1 TU00498_RNA_cut_cplx	reversible	RNA Processing	-1.00E+09	1.00E+09	0	1.77E+03	1.77E+03	0	6.41E+00	6.41	0	6.41E+00	6.41
TU00499_DNA_act_bind	TU00499_DNA binding of activator	1 TU00499_DNA_neu --> 1 TU00499_DNA_act	irreversible	Transcription Regulation	0.00E+00	1.00E+09	0	1.77E+03	1.77E+03	0	6.41E+00	6.41	0	6.41E+00	6.41
TU00499_trimming	Cutting of stable TU00499_RNA	1 TU00499_RNA_cut_cplx + 1 h2o --> 1 RNase_E_tetra_inact + 1 RNase_Gen_inact + 1 RNase_P_cplx_inact + 1 b1989_RNA + 1 h + ppi	irreversible	RNA Processing	0.00E+00	1.00E+09	0	1.77E+03	1.77E+03	0	6.41E+00	6.41	0	6.41E+00	6.41
TU00499_trimming_cplx	Cutting complex formation of stable TU00499_RNA (asnVp, no_common_name_182)	1 RNase_E_tetra + 1 RNase_Gen + 1 RNase_P_cplx + 1 TU00499_RNA <==> 1 TU00499_RNA_cut_cplx	reversible	RNA Processing	-1.00E+09	1.00E+09	0	1.77E+03	1.77E+03	0	6.41E+00	6.41	0	6.41E+00	6.41
TU00500_DNA_act_bind	TU00500_DNA binding of activator	1 TU00500_DNA_neu --> 1 TU00500_DNA_act	irreversible	Transcription Regulation	0.00E+00	1.00E+09	0	1.68E+03	1.68E+03	0	7.62E+00	7.62	0	7.62E+00	7.62
TU00500_trimming	Cutting of stable TU00500_RNA	1 TU00500_RNA_cut_cplx + 1 h2o --> 1 RNase_E_tetra_inact + 1 RNase_Gen_inact + 1 RNase_P_cplx_inact + 1 b2348_RNA + 1 h + ppi	irreversible	RNA Processing	0.00E+00	1.00E+09	0	1.68E+03	1.68E+03	0	7.62E+00	7.62	0	7.62E+00	7.62
TU00500_trimming_cplx	Cutting complex formation of stable TU00500_RNA (argWp, no_common_name_181)	1 RNase_E_tetra + 1 RNase_Gen + 1 RNase_P_cplx + 1 TU00500_RNA <==> 1 TU00500_RNA_cut_cplx	reversible	RNA Processing	-1.00E+09	1.00E+09	0	1.68E+03	1.68E+03	0	7.62E+00	7.62	0	7.62E+00	7.62
TU00501_DNA_act_bind	TU00501_DNA binding of activator	1 TU00501_DNA_neu --> 1 TU00501_DNA_act	irreversible	Transcription Regulation	0.00E+00	1.00E+09	0	1.52E+03	1.52E+03	0	10	10	0	10	10
TU00501_trimming	Cutting of stable TU00501_RNA	1 TU00501_RNA_cut_cplx + 1 h2o --> 1 RNase_E_tetra_inact + 1 RNase_Gen_inact + 1 RNase_P_cplx_inact + 1 b2967_RNA + 1 h + ppi	irreversible	RNA Processing	0.00E+00	1.00E+09	0	1.52E+03	1.52E+03	0	10	10	0	10	10
TU00501_trimming_cplx	Cutting complex formation of stable TU00501_RNA (pheVp, no_common_name_180)	1 RNase_E_tetra + 1 RNase_Gen + 1 RNase_P_cplx + 1 TU00501_RNA <==> 1 TU00501_RNA_cut_cplx	reversible	RNA Processing	-1.00E+09	1.00E+09	0	1.52E+03	1.52E+03	0	10	10	0	10	10
TU00502_DNA_act_bind	TU00502_DNA binding of activator	1 TU00502_DNA_neu --> 1 TU00502_DNA_act	irreversible	Transcription Regulation	0.00E+00	1.00E+09	0	1.45E+03	1.45E+03	0	1.05E+01	10.5	0	1.05E+01	10.5
TU00502_trimming	Cutting of stable TU00502_RNA	1 TU00502_RNA_cut_cplx + 1 h2o --> 1 RNase_E_tetra_inact + 1 RNase_Gen_inact + 1 RNase_P_cplx_inact + 1 b3069_RNA + 1 h + ppi	irreversible	RNA Processing	0.00E+00	1.00E+09	0	1.45E+03	1.45E+03	0	1.05E+01	10.5	0	1.05E+01	10.5
TU00502_trimming_cplx	Cutting complex formation of stable TU00502_RNA (ileXp, no_common_name_179)	1 RNase_E_tetra + 1 RNase_Gen + 1 RNase_P_cplx + 1 TU00502_RNA <==> 1 TU00502_RNA_cut_cplx	reversible	RNA Processing	-1.00E+09	1.00E+09	0	1.45E+03	1.45E+03	0	1.05E+01	10.5	0	1.05E+01	10.5
TU00503_DNA_act_bind	TU00503_DNA binding of activator	1 TU00503_DNA_neu --> 1 TU00503_DNA_act	irreversible	Transcription Regulation	0.00E+00	1.00E+09	0	3.01E+02	3.01E+02	0	1.39E+01	13.9	0	1.39E+01	13.9
TU00503_cutting	Cutting of stable TU00503_RNA	1 TU00503_RNA_cut_cplx + 123 h2o --> 1 RNase_E_tetra_inact + 1 RNase_G_dim_inact + 1 RNase_Gen_inact + 1 RNase_III_dim_inact + 1 RNase_P_cplx_inact + 1 RNase_T_dim_inact + 1 RNase_m16_inact + 1 RNase_m23_inact + 1 RNase_m5_inact + 39 amp + 1 b3796_RNA + 1 b3797_RNA + 1 b3798_RNA + 1 b3799_RNA + 17 cmp + 26 gmp + 123 h + 1 ppi + 37 ump	irreversible	RNA processing	0.00E+00	1.00E+09	0	3.01E+02	3.01E+02	0	1.39E+01	13.9	0	1.39E+01	13.9
TU00503_cutting_cplx	Cutting complex formation of stable TU00503_RNA (argXp, no_common_name_178)	1 RNase_E_tetra + 1 RNase_G_dim + 1 RNase_Gen + 1 RNase_III_dim + 1 RNase_P_cplx + 1 RNase_T_dim + 1 RNase_m16 + 1 RNase_m23 + 1 RNase_m5 + 1 TU00503_RNA <==> 1 TU00503_RNA_cut_cplx	reversible	RNA processing	-1.00E+09	1.00E+09	0	3.01E+02	3.01E+02	0	1.39E+01	13.9	0	1.39E+01	13.9
TU00504_DNA_act_bind	TU00504_DNA binding of activator	1 TU00504_DNA_neu --> 1 TU00504_DNA_act	irreversible	Transcription Regulation	0.00E+00	1.00E+09	0	2.71E+02	2.71E+02	0	1.28E+01	12.8	0	1.28E+01	12.8

TU00504_cutting	Cutting of stable TU00504_RNA	1 TU00504_RNA_cut_cplx + 134 h2o --> 1 RNase_E_tetra_inact + 1 RNase_G_dim_inact + 1 RNase_Gen_inact + 1 RNase_III_dim_inact + 1 RNase_P_cplx_inact + 1 RNase_T_dim_inact + 1 RNase_m16_inact + 1 RNase_m23_inact + 1 RNase_m5_inact + 36 amp + 1 b3976_RNA + 1 b3977_RNA + 1 b3978_RNA + 1 b3979_RNA + 30 cmp + 34 gmp + 134 h + 1 ppi + 30 ump	irreversible	RNA processing	0.00E+00	1.00E+09	0	2.71E+02	2.71E+02	0	1.28E+01	12.8	0	1.28E+01	12.8
TU00504_cutting_cplx	Cutting complex formation of stable TU00504_RNA (thrUp, no_common_name_177)	1 RNase_E_tetra + 1 RNase_G_dim + 1 RNase_Gen + 1 RNase_III_dim + 1 RNase_P_cplx + 1 RNase_T_dim + 1 RNase_m1 + 1 RNase_m23 + 1 RNase_m5 + 1 TU00504_RNA <==> 1 TU00504_RNA_cut_cplx	reversible	RNA processing	-1.00E+09	1.00E+09	0	2.71E+02	2.71E+02	0	1.28E+01	12.8	0	1.28E+01	12.8
TU00505_DNA_act_bind	TU00505_DNA binding of activator	1 TU00505_DNA_neu --> 1 TU00505_DNA_act	irreversible	Transcription Regulation	0.00E+00	1.00E+09	0	3.99E+02	3.99E+02	0	1.17E+01	11.7	0	1.17E+01	11.7
TU00505_cutting	Cutting of stable TU00505_RNA	1 TU00505_RNA_cut_cplx + 74 h2o --> 1 RNase_E_tetra_inact + 1 RNase_G_dim_inact + 1 RNase_Gen_inact + 1 RNase_III_dim_inact + 1 RNase_P_cplx_inact + 1 RNase_T_dim_inact + 1 RNase_m16_inact + 1 RNase_m23_inact + 1 RNase_m5_inact + 30 amp + 1 b4163_RNA + 1 b4164_RNA + 1 b4165_RNA + 17 cmp + 1[illegible] gmp + 74 h + 1 ppi + 11 ump	irreversible	RNA processing	0.00E+00	1.00E+09	0	3.99E+02	3.99E+02	0	1.17E+01	11.7	0	1.17E+01	11.7
TU00505_cutting_cplx	Cutting complex formation of stable TU00505_RNA (glyVp, no_common_name_176)	1 RNase_E_tetra + 1 RNase_G_dim + 1 RNase_Gen + 1 RNase_III_dim + 1 RNase_P_cplx + 1 RNase_T_dim + 1 RNase_m1 + 1 RNase_m23 + 1 RNase_m5 + 1 TU00505_RNA <==> 1 TU00505_RNA_cut_cplx	reversible	RNA processing	-1.00E+09	1.00E+09	0	3.99E+02	3.99E+02	0	1.17E+01	11.7	0	1.17E+01	11.7
TU00506_DNA_act_bind	TU00506_DNA binding of activator	1 TU00506_DNA_neu --> 1 TU00506_DNA_act	irreversible	Transcription Regulation	0.00E+00	1.00E+09	0	1.45E+03	1.45E+03	0	1.12E+01	11.2	0	1.12E+01	11.2
TU00506_trimming	Cutting of stable TU00506_RNA	1 TU00506_RNA_cut_cplx + 1 h2o --> 1 RNase_E_tetra_inact + 1 RNase_Gen_inact + 1 RNase_P_cplx_inact + 1 b4270_RNA + 1 h + ppi	irreversible	RNA Processing	0.00E+00	1.00E+09	0	1.45E+03	1.45E+03	0	1.12E+01	11.2	0	1.12E+01	11.2
TU00506_trimming_cplx	Cutting complex formation of stable TU00506_RNA (leuXp, no_common_name_175)	1 RNase_E_tetra + 1 RNase_Gen + 1 RNase_P_cplx + 1 TU00506_RNA <==> 1 TU00506_RNA_cut_cplx	reversible	RNA Processing	-1.00E+09	1.00E+09	0	1.45E+03	1.45E+03	0	1.12E+01	11.2	0	1.12E+01	11.2
TU00507_DNA_act_bind	TU00507_DNA binding of activator	1 TU00507_DNA_neu --> 1 TU00507_DNA_act	irreversible	Transcription Regulation	0.00E+00	1.00E+09	0	1.70E+02	1.70E+02	0	7.79E+00	7.79	0	7.79E+00	7.79
TU00507_cutting	Cutting of stable TU00507_RNA	1 TU00507_RNA_cut_cplx + 172 h2o --> 1 RNase_E_tetra_inact + 1 RNase_G_dim_inact + 1 RNase_Gen_inact + 1 RNase_III_dim_inact + 1 RNase_P_cplx_inact + 1 RNase_T_dim_inact + 1 RNase_m16_inact + 1 RNase_m23_inact + 1 RNase_m5_inact + 46 amp + 1 b0664_RNA + 1 b0665_RNA + 1 b0666_RNA + 1 b0668_RNA + 1 b0670_RNA + 1 b0672_RNA + 1 b0673_RNA + 35 cmp + 29 gmp + 172 h + 1 ppi + 55 ump	irreversible	RNA processing	0.00E+00	1.00E+09	0	1.70E+02	1.70E+02	0	7.79E+00	7.79	0	7.79E+00	7.79
TU00507_cutting_cplx	Cutting complex formation of stable TU00507_RNA (metTp, no_common_name_174)	1 RNase_E_tetra + 1 RNase_G_dim + 1 RNase_Gen + 1 RNase_III_dim + 1 RNase_P_cplx + 1 RNase_T_dim + 1 RNase_m1 + 1 RNase_m23 + 1 RNase_m5 + 1 TU00507_RNA <==> 1 TU00507_RNA_cut_cplx	reversible	RNA processing	-1.00E+09	1.00E+09	0	1.70E+02	1.70E+02	0	7.79E+00	7.79	0	7.79E+00	7.79
TU00508_DNA_act_bind	TU00508_DNA binding of activator	1 TU00508_DNA_neu --> 1 TU00508_DNA_act	irreversible	Transcription Regulation	0.00E+00	1.00E+09	0	1.33E+03	1.33E+03	0	7.06E+00	7.06	0	7.06E+00	7.06
TU00508_trimming	Cutting of stable TU00508_RNA	1 TU00508_RNA_cut_cplx + 1 h2o --> 1 RNase_E_tetra_inact + 1 RNase_Gen_inact + 1 RNase_P_cplx_inact + 1 b0883_RNA + 1 h + ppi	irreversible	RNA Processing	0.00E+00	1.00E+09	0	1.33E+03	1.33E+03	0	7.06E+00	7.06	0	7.06E+00	7.06
TU00508_trimming_cplx	Cutting complex formation of stable TU00508_RNA (serWp, no_common_name_173)	1 RNase_E_tetra + 1 RNase_Gen + 1 RNase_P_cplx + 1 TU00508_RNA <==> 1 TU00508_RNA_cut_cplx	reversible	RNA Processing	-1.00E+09	1.00E+09	0	1.33E+03	1.33E+03	0	7.06E+00	7.06	0	7.06E+00	7.06
TU00509_DNA_act_bind	TU00509_DNA binding of activator	1 TU00509_DNA_neu --> 1 TU00509_DNA_act	irreversible	Transcription Regulation	0.00E+00	1.00E+09	0	1.33E+03	1.33E+03	0	6.75E+00	6.75	0	6.75E+00	6.75
TU00509_trimming	Cutting of stable TU00509_RNA	1 TU00509_RNA_cut_cplx + 1 h2o --> 1 RNase_E_tetra_inact + 1 RNase_Gen_inact + 1 RNase_P_cplx_inact + 1 b0971_RNA + 1 h + ppi	irreversible	RNA Processing	0.00E+00	1.00E+09	0	1.33E+03	1.33E+03	0	6.75E+00	6.75	0	6.75E+00	6.75
TU00509_trimming_cplx	Cutting complex formation of stable TU00509_RNA (serTp, no_common_name_172)	1 RNase_E_tetra + 1 RNase_Gen + 1 RNase_P_cplx + 1 TU00509_RNA <==> 1 TU00509_RNA_cut_cplx	reversible	RNA Processing	-1.00E+09	1.00E+09	0	1.33E+03	1.33E+03	0	6.75E+00	6.75	0	6.75E+00	6.75
TU00510_DNA_act_bind	TU00510_DNA binding of activator	1 TU00510_DNA_neu --> 1 TU00510_DNA_act	irreversible	Transcription Regulation	0.00E+00	1.00E+09	0	1.33E+03	1.33E+03	0	6.56E+00	6.56	0	6.56E+00	6.56
TU00510_trimming	Cutting of stable TU00510_RNA	1 TU00510_RNA_cut_cplx + 1 h2o --> 1 RNase_E_tetra_inact + 1 RNase_Gen_inact + 1 RNase_P_cplx_inact + 1 b1032_RNA + 1 h + ppi	irreversible	RNA Processing	0.00E+00	1.00E+09	0	1.33E+03	1.33E+03	0	6.56E+00	6.56	0	6.56E+00	6.56
TU00510_trimming_cplx	Cutting complex formation of stable TU00510_RNA (serXp, no_common_name_171)	1 RNase_E_tetra + 1 RNase_Gen + 1 RNase_P_cplx + 1 TU00510_RNA <==> 1 TU00510_RNA_cut_cplx	reversible	RNA Processing	-1.00E+09	1.00E+09	0	1.33E+03	1.33E+03	0	6.56E+00	6.56	0	6.56E+00	6.56
TU00511_DNA_act_bind	TU00511_DNA binding of activator	1 TU00511_DNA_neu --> 1 TU00511_DNA_act	irreversible	Transcription Regulation	0.00E+00	1.00E+09	0	2.11E+02	2.11E+02	0	3.73E+02	373.3748	0	2.07E-01	0.207
TU00511_cleavage	Cleavage of TU00511_mRNA	1 TU00511_mRNA_cleav_cplx + 266 h2o --> 1 RNase_III_dim_inact + 78 amp + 1 b1229_mRNA + 1 b1230_RNA_pre + 1 b1231_RNA_pre + 70 cmp + 58 gmp + 266 h + 58 ump	irreversible	Cleavage polycistronic mRNA	0.00E+00	1.00E+09	0	2.11E+02	2.11E+02	0	3.73E+02	373.3748	0	2.07E-01	0.207
TU00511_cleavage_cplx	Cleavage complex formation of TU00511_mRNA (tyrTp, no_common_name_170)	1 RNase_III_dim + 1 TU00511_mRNA <==> 1 TU00511_mRNA_cleav_cplx	reversible	Cleavage polycistronic mRNA	-1.00E+09	1.00E+09	0	2.11E+02	2.11E+02	0	3.73E+02	373.3748	0	2.07E-01	0.207
TU00512_DNA_act_bind	TU00512_DNA binding of activator	1 TU00512_DNA_neu --> 1 TU00512_DNA_act	irreversible	Transcription Regulation	0.00E+00	1.00E+09	0	3.99E+02	3.99E+02	0	6.22E+00	6.22	0	6.22E+00	6.22
TU00512_cutting	Cutting of stable TU00512_RNA	1 TU00512_RNA_cut_cplx + 69 h2o --> 1 RNase_E_tetra_inact + 1 RNase_G_dim_inact + 1 RNase_Gen_inact + 1 RNase_III_dim_inact + 1 RNase_P_cplx_inact + 1 RNase_T_dim_inact + 1 RNase_m16_inact + 1 RNase_m23_inact + 1 RNase_m5_inact + 17 amp + 1 b1909_RNA + 1 b1910_RNA + 1 b1911_RNA + 14 cmp + 1[illegible] gmp + 69 h + 1 ppi + 18 ump	irreversible	RNA processing	0.00E+00	1.00E+09	0	3.99E+02	3.99E+02	0	6.22E+00	6.22	0	6.22E+00	6.22
TU00512_cutting_cplx	Cutting complex formation of stable TU00512_RNA (glyWp, no_common_name_169)	1 RNase_E_tetra + 1 RNase_G_dim + 1 RNase_Gen + 1 RNase_III_dim + 1 RNase_P_cplx + 1 RNase_T_dim + 1 RNase_m1 + 1 RNase_m23 + 1 RNase_m5 + 1 TU00512_RNA <==> 1 TU00512_RNA_cut_cplx	reversible	RNA processing	-1.00E+09	1.00E+09	0	3.99E+02	3.99E+02	0	6.22E+00	6.22	0	6.22E+00	6.22
TU00513_DNA_act_bind	TU00513_DNA binding of activator	1 TU00513_DNA_neu --> 1 TU00513_DNA_act	irreversible	Transcription Regulation	0.00E+00	1.00E+09	0	1.14E+03	1.14E+03	0	6.36E+00	6.36	0	6.36E+00	6.36
TU00513_trimming	Cutting of stable TU00513_RNA	1 TU00513_RNA_cut_cplx + 1 h2o --> 1 RNase_E_tetra_inact + 1 RNase_Gen_inact + 1 RNase_P_cplx_inact + 1 b1975_RNA + 1 h + ppi	irreversible	RNA Processing	0.00E+00	1.00E+09	0	1.14E+03	1.14E+03	0	6.36E+00	6.36	0	6.36E+00	6.36
TU00513_trimming_cplx	Cutting complex formation of stable TU00513_RNA (serUp, no_common_name_168)	1 RNase_E_tetra + 1 RNase_Gen + 1 RNase_P_cplx + 1 TU00513_RNA <==> 1 TU00513_RNA_cut_cplx	reversible	RNA Processing	-1.00E+09	1.00E+09	0	1.14E+03	1.14E+03	0	6.36E+00	6.36	0	6.36E+00	6.36
TU00514_DNA_act_bind	TU00514_DNA binding of activator	1 TU00514_DNA_neu --> 1 TU00514_DNA_act	irreversible	Transcription Regulation	0.00E+00	1.00E+09	0	6.14E+02	6.14E+02	0	7.80E+00	7.8	0	7.80E+00	7.8
TU00514_cutting	Cutting of stable TU00514_RNA	1 TU00514_RNA_cut_cplx + 41 h2o --> 1 RNase_E_tetra_inact + 1 RNase_G_dim_inact + 1 RNase_Gen_inact + 1 RNase_III_dim_inact + 1 RNase_P_cplx_inact + 1 RNase_T_dim_inact + 1 RNase_m16_inact + 1 RNase_m23_inact + 1 RNase_m5_inact + 12 amp + 1 b2396_RNA + 1 b2397_RNA + 10 cmp + 7 gmp + 41 h + 1 ppi + 10 ump	irreversible	RNA processing	0.00E+00	1.00E+09	0	6.14E+02	6.14E+02	0	7.80E+00	7.8	0	7.80E+00	7.8
TU00514_cutting_cplx	Cutting complex formation of stable TU00514_RNA (alaWp, no_common_name_167)	1 RNase_E_tetra + 1 RNase_G_dim + 1 RNase_Gen + 1 RNase_III_dim + 1 RNase_P_cplx + 1 RNase_T_dim + 1 RNase_m1 + 1 RNase_m23 + 1 RNase_m5 + 1 TU00514_RNA <==> 1 TU00514_RNA_cut_cplx	reversible	RNA processing	-1.00E+09	1.00E+09	0	6.14E+02	6.14E+02	0	7.80E+00	7.8	0	7.80E+00	7.8

TU00515_DNA_act_bind	TU00515_DNA binding of activator	1 TU00515_DNA_neu --> 1 TU00515_DNA_act	irreversible	Transcription Regulation	0.00E+00	1.00E+09	0	1.46E+02	1.46E+02	0	8.87E+00	8.87	0	8.87E+00	8.87
TU00515_cutting	Cutting of stable TU00515_RNA	1 TU00515_RNA_cut_cplx + 466 h2o --> 1 RNase_E_tetra_inact + 1 RNase_G_dim_inact + 1 RNase_Gen_inact + 1 RNase_III_dim_inact + 1 RNase_P_cplx_inact + 1 RNase_T_dim_inact + 1 RNase_m16_inact + 1 RNase_m23_inact + 1 RNase_m5_inact + 13 amp + 1 b2691_RNA + 1 b2692_RNA + 1 b2693_RNA + 1 b2694_RNA + 1 b2695_RNA + 98 cmp + 114 gmp + 466 h + 1 ppi + 118 ump	irreversible	RNA processing	0.00E+00	1.00E+09	0	1.46E+02	1.46E+02	0	8.87E+00	8.87	0	8.87E+00	8.87
TU00515_cutting_cplx	Cutting complex formation of stable TU00515_RNA (serVp, no_common_name_166)	1 RNase_E_tetra + 1 RNase_G_dim + 1 RNase_Gen + 1 RNase_III_dim + 1 RNase_P_cplx + 1 RNase_T_dim + 1 RNase_m1 + 1 RNase_m23 + 1 RNase_m5 + 1 TU00515_RNA <==> 1 TU00515_RNA_cut_cplx	reversible	RNA processing	-1.00E+09	1.00E+09	0	1.46E+02	1.46E+02	0	8.87E+00	8.87	0	8.87E+00	8.87
TU00516_DNA_act_bind	TU00516_DNA binding of activator	1 TU00516_DNA_neu --> 1 TU00516_DNA_act	irreversible	Transcription Regulation	0.00E+00	1.00E+09	0	1.45E+03	1.45E+03	0	9.58E+00	9.58	0	9.58E+00	9.58
TU00516_trimming	Cutting of stable TU00516_RNA	1 TU00516_RNA_cut_cplx + 1 h2o --> 1 RNase_E_tetra_inact + 1 RNase_Gen_inact + 1 RNase_P_cplx_inact + 1 b2864_RNA + 1 h + ppi	irreversible	RNA Processing	0.00E+00	1.00E+09	0	1.45E+03	1.45E+03	0	9.58E+00	9.58	0	9.58E+00	9.58
TU00516_trimming_cplx	Cutting complex formation of stable TU00516_RNA (glyUp, no_common_name_165)	1 RNase_E_tetra + 1 RNase_Gen + 1 RNase_P_cplx + 1 TU00516_RNA <==> 1 TU00516_RNA_cut_cplx	reversible	RNA Processing	-1.00E+09	1.00E+09	0	1.45E+03	1.45E+03	0	9.58E+00	9.58	0	9.58E+00	9.58
TU00517_DNA_act_bind	TU00517_DNA binding of activator	1 TU00517_DNA_neu --> 1 TU00517_DNA_act	irreversible	Transcription Regulation	0.00E+00	1.00E+09	0	2.72E+01	2.72E+01	0	4.80E+01	48.0235	0	1.50E-01	0.15
TU00517_cleavage	Cleavage of TU00517_mRNA	1 TU00517_mRNA_cleav_cplx + 260 h2o --> 1 RNase_III_dim_inact + 61 amp + 1 b3168_v1_mRNA + 1 b3169_v1_mRNA + 1 b3170_v1_mRNA + 1 b3171_v2_RNA_pre + 48 cmp + 67 gmp + 260 h + 81 ump	irreversible	Cleavage polycistronic mRNA	0.00E+00	1.00E+09	0	2.72E+01	2.72E+01	0	4.80E+01	48.0235	0	1.50E-01	0.15
TU00517_cleavage_cplx	Cleavage complex formation of TU00517_mRNA (metYp1, no_common_name_164)	1 RNase_III_dim + 1 TU00517_mRNA <==> 1 TU00517_mRNA_cleav_cplx	reversible	Cleavage polycistronic mRNA	-1.00E+09	1.00E+09	0	2.72E+01	2.72E+01	0	4.80E+01	48.0235	0	1.50E-01	0.15
TU00518_DNA_act_bind	TU00518_DNA binding of activator	1 TU00518_DNA_neu --> 1 TU00518_DNA_act	irreversible	Transcription Regulation	0.00E+00	1.00E+09	0	1.52E+03	1.52E+03	0	11	11	0	11	11
TU00518_trimming	Cutting of stable TU00518_RNA	1 TU00518_RNA_cut_cplx + 1 h2o --> 1 RNase_E_tetra_inact + 1 RNase_Gen_inact + 1 RNase_P_cplx_inact + 1 b3174_RNA + 1 h + ppi	irreversible	RNA Processing	0.00E+00	1.00E+09	0	1.52E+03	1.52E+03	0	11	11	0	11	11
TU00518_trimming_cplx	Cutting complex formation of stable TU00518_RNA (leuUp, no_common_name_163)	1 RNase_E_tetra + 1 RNase_Gen + 1 RNase_P_cplx + 1 TU00518_RNA <==> 1 TU00518_RNA_cut_cplx	reversible	RNA Processing	-1.00E+09	1.00E+09	0	1.52E+03	1.52E+03	0	11	11	0	11	11
TU00519_DNA_act_bind	TU00519_DNA binding of activator	1 TU00519_DNA_neu --> 1 TU00519_DNA_act	irreversible	Transcription Regulation	0.00E+00	1.00E+09	0	1.52E+03	1.52E+03	0	13	13	0	13	13
TU00519_trimming	Cutting of stable TU00519_RNA	1 TU00519_RNA_cut_cplx + 1 h2o --> 1 RNase_E_tetra_inact + 1 RNase_Gen_inact + 1 RNase_P_cplx_inact + 1 b3545_RNA + 1 h + ppi	irreversible	RNA Processing	0.00E+00	1.00E+09	0	1.52E+03	1.52E+03	0	13	13	0	13	13
TU00519_trimming_cplx	Cutting complex formation of stable TU00519_RNA (proKp, no_common_name_162)	1 RNase_E_tetra + 1 RNase_Gen + 1 RNase_P_cplx + 1 TU00519_RNA <==> 1 TU00519_RNA_cut_cplx	reversible	RNA Processing	-1.00E+09	1.00E+09	0	1.52E+03	1.52E+03	0	13	13	0	13	13
TU00520_DNA_act_bind	TU00520_DNA binding of activator	1 TU00520_DNA_neu --> 1 TU00520_DNA_act	irreversible	Transcription Regulation	0.00E+00	1.00E+09	0	1.52E+03	1.52E+03	0	1.18E+01	11.8	0	1.18E+01	11.8
TU00520_trimming	Cutting of stable TU00520_RNA	1 TU00520_RNA_cut_cplx + 1 h2o --> 1 RNase_E_tetra_inact + 1 RNase_Gen_inact + 1 RNase_P_cplx_inact + 1 b4134_RNA + 1 h + ppi	irreversible	RNA Processing	0.00E+00	1.00E+09	0	1.52E+03	1.52E+03	0	1.18E+01	11.8	0	1.18E+01	11.8
TU00520_trimming_cplx	Cutting complex formation of stable TU00520_RNA (pheUp, no_common_name_161)	1 RNase_E_tetra + 1 RNase_Gen + 1 RNase_P_cplx + 1 TU00520_RNA <==> 1 TU00520_RNA_cut_cplx	reversible	RNA Processing	-1.00E+09	1.00E+09	0	1.52E+03	1.52E+03	0	1.18E+01	11.8	0	1.18E+01	11.8
TU00521_DNA_act_bind	TU00521_DNA binding of activator	1 TU00521_DNA_neu --> 1 TU00521_DNA_act	irreversible	Transcription Regulation	0.00E+00	1.00E+09	0	3.89E+02	3.89E+02	0	1.07E+01	10.7	0	1.07E+01	10.7
TU00521_cutting	Cutting of stable TU00521_RNA	1 TU00521_RNA_cut_cplx + 65 h2o --> 1 RNase_E_tetra_inact + 1 RNase_G_dim_inact + 1 RNase_Gen_inact + 1 RNase_III_dim_inact + 1 RNase_P_cplx_inact + 1 RNase_T_dim_inact + 1 RNase_m16_inact + 1 RNase_m23_inact + 1 RNase_m5_inact + 24 amp + 1 b4368_RNA + 1 b4369_RNA + 1 b4370_RNA + 11 cmp + 14 gmp + 65 h + 1 ppi + 13 ump	irreversible	RNA processing	0.00E+00	1.00E+09	0	3.89E+02	3.89E+02	0	1.07E+01	10.7	0	1.07E+01	10.7
TU00521_cutting_cplx	Cutting complex formation of stable TU00521_RNA (leuQp, no_common_name_160)	1 RNase_E_tetra + 1 RNase_G_dim + 1 RNase_Gen + 1 RNase_III_dim + 1 RNase_P_cplx + 1 RNase_T_dim + 1 RNase_m1 + 1 RNase_m23 + 1 RNase_m5 + 1 TU00521_RNA <==> 1 TU00521_RNA_cut_cplx	reversible	RNA processing	-1.00E+09	1.00E+09	0	3.89E+02	3.89E+02	0	1.07E+01	10.7	0	1.07E+01	10.7
TU221_DNA_act_bind	TU221_DNA binding of activator	1 TU221_DNA_neu --> 1 TU221_DNA_act	irreversible	Transcription Regulation	0.00E+00	1.00E+09	0	1.47E+02	1.47E+02	0	2.60E+02	259.8138	0	2.99E-01	0.299
TU281_DNA_act_bind	TU281_DNA binding of activator	1 TU281_DNA_neu --> 1 TU281_DNA_act	irreversible	Transcription Regulation	0.00E+00	1.00E+09	0	4.80E+01	4.80E+01	0	8.47E+01	84.65405	0	2.99E-01	0.299
TU281_cleavage	Cleavage of TU281_mRNA , 4 nt short (b2570), 1 nt short (b2571)	1 TU281_mRNA_cleav_cplx + 35 h2o --> 1 RNase_III_dim_inact + 9 amp + 1 b2570_mRNA + 1 b2571_mRNA + 1 b2572_mRNA + 1 b2573_mRNA + 4 cmp + 12 gmp + 35 h + 7 ump	irreversible	Cleavage polycistronic mRNA	0.00E+00	1.00E+09	0	4.80E+01	4.80E+01	0	8.47E+01	84.65405	0	2.99E-01	0.299
TU281_cleavage_cplx	Cleavage complex formation of TU281_mRNA (rpoEp1, no_common_name_129) , 4 nt short (b2570), 1 nt short (b2571)	1 RNase_III_dim + 1 TU281_mRNA <==> 1 TU281_mRNA_cleav_cplx	reversible	Cleavage polycistronic mRNA	-1.00E+09	1.00E+09	0	4.80E+01	4.80E+01	0	8.47E+01	84.65405	0	2.99E-01	0.299
TU341_DNA_act_bind	TU341_DNA binding of activator	1 TU341_DNA_neu --> 1 TU341_DNA_act	irreversible	Transcription Regulation	0.00E+00	1.00E+09	0	1.43E+01	1.43E+01	0	2.52E+01	25.16946	0	1.46E-01	0.146
TU341_cleavage	Cleavage of TU341_mRNA , 1 nt short (b3166)	1 TU341_mRNA_cleav_cplx + 752 h2o --> 1 RNase_III_dim_inact + 175 amp + 1 b3164_mRNA + 1 b3165_mRNA + 1 b3166_mRNA + 1 b3167_mRNA + 1 b3168_mRNA + 1 b3169_mRNA + 1 b3170_mRNA + 1 b3171_v1_RNA_pre + 148 cmp + 193 gmp + 752 + 229 ump	irreversible	Cleavage polycistronic mRNA	0.00E+00	1.00E+09	0	1.43E+01	1.43E+01	0	2.52E+01	25.16946	0	1.46E-01	0.146
TU341_cleavage_cplx	Cleavage complex formation of TU341_mRNA (metYp2, no_common_name_123) , 1 nt short (b3166)	1 RNase_III_dim + 1 TU341_mRNA <==> 1 TU341_mRNA_cleav_cplx	reversible	Cleavage polycistronic mRNA	-1.00E+09	1.00E+09	0	1.43E+01	1.43E+01	0	2.52E+01	25.16946	0	1.46E-01	0.146
TU343_DNA_act_bind	TU343_DNA binding of activator	1 TU343_DNA_neu --> 1 TU343_DNA_act	irreversible	Transcription Regulation	0.00E+00	1.00E+09	0	1.23E+03	1.23E+03	0	11	11	0	11	11
TU343_trimming	Cutting of stable TU343_RNA	1 TU343_RNA_cut_cplx + 1 h2o --> 1 RNase_E_tetra_inact + 1 RNase_Gen_inact + 1 RNase_P_cplx_inact + 1 b3171_RNA + 1 h + ppi	irreversible	RNA Processing	0.00E+00	1.00E+09	0	1.23E+03	1.23E+03	0	11	11	0	11	11
TU343_trimming_cplx	Cutting complex formation of stable TU343_RNA (metYp1, no_common_name_122)	1 RNase_E_tetra + 1 RNase_Gen + 1 RNase_P_cplx + 1 TU343_RNA <==> 1 TU343_RNA_cut_cplx	reversible	RNA Processing	-1.00E+09	1.00E+09	0	1.23E+03	1.23E+03	0	11	11	0	11	11
TU361_DNA_act_bind	TU361_DNA binding of activator	1 TU361_DNA_neu --> 1 TU361_DNA_act	irreversible	Transcription Regulation	0.00E+00	1.00E+09	0	5.39E+01	5.39E+01	0	7.34E+01	73.37709	0	3.44E-01	0.344
TU361_cleavage	Cleavage of TU361_mRNA	1 TU361_mRNA_cleav_cplx + 111 h2o --> 1 RNase_III_dim_inact + 35 amp + 1 b0910_mRNA + 1 b0911_v2_mRNA + 26 cmp + 27 gmp + 111 h + 22 ump	irreversible	Cleavage polycistronic mRNA	0.00E+00	1.00E+09	0	5.39E+01	5.39E+01	0	7.34E+01	73.37709	0	3.44E-01	0.344
TU361_cleavage_cplx	Cleavage complex formation of TU361_mRNA (cmkp, no_common_name_110)	1 RNase_III_dim + 1 TU361_mRNA <==> 1 TU361_mRNA_cleav_cplx	reversible	Cleavage polycistronic mRNA	-1.00E+09	1.00E+09	0	5.39E+01	5.39E+01	0	7.34E+01	73.37709	0	3.44E-01	0.344
TU482_DNA_act_bind	TU482_DNA binding of activator	1 TU482_DNA_neu --> 1 TU482_DNA_act	irreversible	Transcription Regulation	0.00E+00	1.00E+09	0	2.80E+01	2.80E+01	0	4.94E+01	49.41244	0	4.99E-02	0.0499
TU482_cleavage	Cleavage of TU482_mRNA	1 TU482_mRNA_cleav_cplx + 95 h2o --> 1 RNase_III_dim_inact + 2 amp + 1 b3123_v1_RNA_pre + 1 b3124_mRNA + 1 b3125_mRNA + 1 b3126_mRNA + 1 b3127_mRNA + 28 cmp + 25 gmp + 95 h + 13 ump	irreversible	Cleavage polycistronic mRNA	0.00E+00	1.00E+09	0	2.80E+01	2.80E+01	0	4.94E+01	49.41244	0	4.99E-02	0.0499

TU482_cleavage_cplx	Cleavage complex formation of TU482_mRNA (garPp, no_common_name_92)	1 RNase_III_dim + 1 TU482_mRNA <==> 1 TU482_mRNA_cleav_cplx	reversible	Cleavage polycistronic mRNA	-1.00E+09	1.00E+09	0	2.80E+01	2.80E+01	0	4.94E+01	49.41244	0	4.99E-02	0.0499
TU483_DNA_act_bind	TU483_DNA binding of activator	1 TU483_DNA_neu --> 1 TU483_DNA_act	irreversible	Transcription Regulation	0.00E+00	1.00E+09	0	3.70E+01	3.70E+01	0	6.53E+01	65.32977	0	1.06E-01	0.106
TU483_cleavage	Cleavage of TU483_mRNA , 1 nt short (b2563_v1), 4 nt short (b2566)	1 TU483_mRNA_cleav_cplx + 26 h2o --> 1 RNase_III_dim_inact + 5 amp + 1 b2563_v1_mRNA + 1 b2564_v1_mRNA + 1 b2565_mRNA + 1 b2566_mRNA + 1 b2567_mRNA + 4 cmp + 7 gmp + 26 h + 6 ump	irreversible	Cleavage polycistronic mRNA	0.00E+00	1.00E+09	0	3.70E+01	3.70E+01	0	6.53E+01	65.32977	0	1.06E-01	0.106
TU483_cleavage_cplx	Cleavage complex formation of TU483_mRNA (rncp, no_common_name_91) , 1 nt short (b2563_v1), 4 nt short (b2566)	1 RNase_III_dim + 1 TU483_mRNA <==> 1 TU483_mRNA_cleav_cplx	reversible	Cleavage polycistronic mRNA	-1.00E+09	1.00E+09	0	3.70E+01	3.70E+01	0	6.53E+01	65.32977	0	1.06E-01	0.106
TU543_DNA_act_bind	TU543_DNA binding of activator	1 TU543_DNA_neu --> 1 TU543_DNA_act	irreversible	Transcription Regulation	0.00E+00	1.00E+09	0	2.81E+02	2.81E+02	0	1.08E+01	10.8	0	1.08E+01	10.8
TU543_cleav	Cutting of stable TU543_RNA	1 TU543_RNA_cleav_cplx + 1 h2o --> 1 RNase_III_dim_inact + 1 b3123_RNA + 1 h + 1 ppi	irreversible	RNA Processing	0.00E+00	1.00E+09	0	2.81E+02	2.81E+02	0	1.08E+01	10.8	0	1.08E+01	10.8
TU543_cleav_cplx	Cutting complex formation of stable TU543_RNA (rnpBp, no_common_name_71)	1 RNase_III_dim + 1 TU543_RNA <==> 1 TU543_RNA_cleav_cplx	reversible	RNA Processing	-1.00E+09	1.00E+09	0	2.81E+02	2.81E+02	0	1.08E+01	10.8	0	1.08E+01	10.8
TU564_DNA_act_bind	TU564_DNA binding of activator	1 TU564_DNA_neu --> 1 TU564_DNA_act	irreversible	Transcription Regulation	0.00E+00	1.00E+09	0	6.32E+01	6.32E+01	0	7.72E+01	77.17767	0	4.73E-01	0.473
TU564_cleavage	Cleavage of TU564_mRNA	1 TU564_mRNA_cleav_cplx + 160 h2o --> 1 RNase_III_dim_inact + 37 amp + 1 b0911_v1_mRNA + 1 b0912_v1_mRNA + 37 cmp + 37 gmp + 160 h + 48 ump	irreversible	Cleavage polycistronic mRNA	0.00E+00	1.00E+09	0	6.32E+01	6.32E+01	0	7.72E+01	77.17767	0	4.73E-01	0.473
TU564_cleavage_cplx	Cleavage complex formation of TU564_mRNA (rpsAp1, no_common_name_66)	1 RNase_III_dim + 1 TU564_mRNA <==> 1 TU564_mRNA_cleav_cplx	reversible	Cleavage polycistronic mRNA	-1.00E+09	1.00E+09	0	6.32E+01	6.32E+01	0	7.72E+01	77.17767	0	4.73E-01	0.473
TU565_DNA_act_bind	TU565_DNA binding of activator	1 TU565_DNA_neu --> 1 TU565_DNA_act	irreversible	Transcription Regulation	0.00E+00	1.00E+09	0	6.32E+01	6.32E+01	0	7.72E+01	77.17767	0	4.73E-01	0.473
TU565_cleavage	Cleavage of TU565_mRNA	1 TU565_mRNA_cleav_cplx + 160 h2o --> 1 RNase_III_dim_inact + 37 amp + 1 b0911_mRNA + 1 b0912_mRNA + 37 cmp + 37 gmp + 160 h + 48 ump	irreversible	Cleavage polycistronic mRNA	0.00E+00	1.00E+09	0	6.32E+01	6.32E+01	0	7.72E+01	77.17767	0	4.73E-01	0.473
TU565_cleavage_cplx	Cleavage complex formation of TU565_mRNA (rpsAp3, no_common_name_65)	1 RNase_III_dim + 1 TU565_mRNA <==> 1 TU565_mRNA_cleav_cplx	reversible	Cleavage polycistronic mRNA	-1.00E+09	1.00E+09	0	6.32E+01	6.32E+01	0	7.72E+01	77.17767	0	4.73E-01	0.473
TU582_DNA_act_bind	TU582_DNA binding of activator	1 TU582_DNA_neu --> 1 TU582_DNA_act	irreversible	Transcription Regulation	0.00E+00	1.00E+09	0	6.13E+01	6.13E+01	0	1.08E+02	108.2142	0	4.90E-01	0.49
TU583_DNA_act_bind	TU583_DNA binding of activator	1 TU583_DNA_neu --> 1 TU583_DNA_act	irreversible	Transcription Regulation	0.00E+00	1.00E+09	0	6.13E+01	6.13E+01	0	1.08E+02	108.2142	0	4.90E-01	0.49
TU601_DNA_act_bind	TU601_DNA binding of activator	1 TU601_DNA_neu --> 1 TU601_DNA_act	irreversible	Transcription Regulation	0.00E+00	1.00E+09	0	9.68E+01	9.68E+01	0	8.29E+01	82.88	0	4.55E-01	0.455
TU601_cleavage	Cleavage of TU601_mRNA	1 TU601_mRNA_cleav_cplx + 52 h2o --> 1 RNase_III_dim_inact + 1 amp + 1 b1088_v1_mRNA + 1 b1089_v1_mRNA + 10 cmp + 17 gmp + 52 h + 13 ump	irreversible	Cleavage polycistronic mRNA	0.00E+00	1.00E+09	0	9.68E+01	9.68E+01	0	8.29E+01	82.88	0	4.55E-01	0.455
TU601_cleavage_cplx	Cleavage complex formation of TU601_mRNA (yceDp1, no_common_name_56)	1 RNase_III_dim + 1 TU601_mRNA <==> 1 TU601_mRNA_cleav_cplx	reversible	Cleavage polycistronic mRNA	-1.00E+09	1.00E+09	0	9.68E+01	9.68E+01	0	8.29E+01	82.88	0	4.55E-01	0.455
TU602_DNA_act_bind	TU602_DNA binding of activator	1 TU602_DNA_neu --> 1 TU602_DNA_act	irreversible	Transcription Regulation	0.00E+00	1.00E+09	0	9.68E+01	9.68E+01	0	8.29E+01	82.88	0	4.55E-01	0.455
TU602_cleavage	Cleavage of TU602_mRNA	1 TU602_mRNA_cleav_cplx + 52 h2o --> 1 RNase_III_dim_inact + 1 amp + 1 b1088_mRNA + 1 b1089_mRNA + 10 cmp + 17 gmp + 52 h + 13 ump	irreversible	Cleavage polycistronic mRNA	0.00E+00	1.00E+09	0	9.68E+01	9.68E+01	0	8.29E+01	82.88	0	4.55E-01	0.455
TU602_cleavage_cplx	Cleavage complex formation of TU602_mRNA (yceDp2, no_common_name_55)	1 RNase_III_dim + 1 TU602_mRNA <==> 1 TU602_mRNA_cleav_cplx	reversible	Cleavage polycistronic mRNA	-1.00E+09	1.00E+09	0	9.68E+01	9.68E+01	0	8.29E+01	82.88	0	4.55E-01	0.455
TU682_DNA_act_bind	TU682_DNA binding of activator	1 TU682_DNA_neu --> 1 TU682_DNA_act	irreversible	Transcription Regulation	0.00E+00	1.00E+09	0	9.23E+01	9.23E+01	0	5.24E+01	52.37149	0	3.26E-01	0.326
TU682_cleavage	Cleavage of TU682_mRNA	1 TU682_mRNA_cleav_cplx + 17 h2o --> 1 RNase_III_dim_inact + 6 amp + 1 b3703_v1_mRNA + 1 b3704_v1_mRNA + 5 cmp + 2 gmp + 17 h + 3 ump	irreversible	Cleavage polycistronic mRNA	0.00E+00	1.00E+09	0	9.23E+01	9.23E+01	0	5.24E+01	52.37149	0	3.26E-01	0.326
TU682_cleavage_cplx	Cleavage complex formation of TU682_mRNA (rpmHp2, no_common_name_45)	1 RNase_III_dim + 1 TU682_mRNA <==> 1 TU682_mRNA_cleav_cplx	reversible	Cleavage polycistronic mRNA	-1.00E+09	1.00E+09	0	9.23E+01	9.23E+01	0	5.24E+01	52.37149	0	3.26E-01	0.326
TU743_DNA_act_bind	TU743_DNA binding of activator	1 TU743_DNA_neu --> 1 TU743_DNA_act	irreversible	Transcription Regulation	0.00E+00	1.00E+09	0	9.23E+01	9.23E+01	0	5.24E+01	52.37149	0	3.26E-01	0.326
TU743_cleavage	Cleavage of TU743_mRNA	1 TU743_mRNA_cleav_cplx + 17 h2o --> 1 RNase_III_dim_inact + 6 amp + 1 b3703_mRNA + 1 b3704_mRNA + 5 cmp + 2 gmp + 17 h + ump	irreversible	Cleavage polycistronic mRNA	0.00E+00	1.00E+09	0	9.23E+01	9.23E+01	0	5.24E+01	52.37149	0	3.26E-01	0.326
TU743_cleavage_cplx	Cleavage complex formation of TU743_mRNA (rpmHp3, no_common_name_35)	1 RNase_III_dim + 1 TU743_mRNA <==> 1 TU743_mRNA_cleav_cplx	reversible	Cleavage polycistronic mRNA	-1.00E+09	1.00E+09	0	9.23E+01	9.23E+01	0	5.24E+01	52.37149	0	3.26E-01	0.326
TU801_DNA_act_bind	TU801_DNA binding of activator	1 TU801_DNA_neu --> 1 TU801_DNA_act	irreversible	Transcription Regulation	0.00E+00	1.00E+09	0	1.22E+01	1.22E+01	0	2.16E+01	21.56007	0	1.04E-01	0.104
TU801_cleavage	Cleavage of TU801_mRNA , 29 nt short (b4168_v1), 8 nt short (b4171_v1)	1 TU801_mRNA_cleav_cplx + 282 h2o --> 1 RNase_III_dim_inact + 78 amp + 1 b4167_v1_mRNA + 1 b4168_v1_mRNA + 1 b4169_v1_mRNA + 1 b4170_v1_mRNA + 1 b4171_v1_mRNA + 1 b4172_v1_mRNA + 1 b4173_v1_mRNA + 1 b4174_v1_mRNA + 1 b4175_v1_mRNA + 54 cmp + 77 gmp + 282 h + 65 ump	irreversible	Cleavage polycistronic mRNA	0.00E+00	1.00E+09	0	1.22E+01	1.22E+01	0	2.16E+01	21.56007	0	1.04E-01	0.104
TU801_cleavage_cplx	Cleavage complex formation of TU801_mRNA (yjeFp2, no_common_name_19) , 29 nt short (b4168_v1), 8 nt short (b4171_v1)	1 RNase_III_dim + 1 TU801_mRNA <==> 1 TU801_mRNA_cleav_cplx	reversible	Cleavage polycistronic mRNA	-1.00E+09	1.00E+09	0	1.22E+01	1.22E+01	0	2.16E+01	21.56007	0	1.04E-01	0.104
TU802_DNA_act_bind	TU802_DNA binding of activator	1 TU802_DNA_neu --> 1 TU802_DNA_act	irreversible	Transcription Regulation	0.00E+00	1.00E+09	0	1.22E+01	1.22E+01	0	2.16E+01	21.56007	0	1.04E-01	0.104
TU802_cleavage	Cleavage of TU802_mRNA , 29 nt short (b4168), 8 nt short (b4171)	1 TU802_mRNA_cleav_cplx + 282 h2o --> 1 RNase_III_dim_inact + 78 amp + 1 b4167_mRNA + 1 b4168_mRNA + 1 b4169_mRNA + 1 b4170_mRNA + 1 b4171_mRNA + 1 b4172_mRNA + 1 b4173_mRNA + 1 b4174_mRNA + 1 b4175_mRNA + 54 cmp + 77 gmp + 282 h + 65 ump	irreversible	Cleavage polycistronic mRNA	0.00E+00	1.00E+09	0	1.22E+01	1.22E+01	0	2.16E+01	21.56007	0	1.04E-01	0.104
TU802_cleavage_cplx	Cleavage complex formation of TU802_mRNA (yjeFp3, no_common_name_18) , 29 nt short (b4168), 8 nt short (b4171)	1 RNase_III_dim + 1 TU802_mRNA <==> 1 TU802_mRNA_cleav_cplx	reversible	Cleavage polycistronic mRNA	-1.00E+09	1.00E+09	0	1.22E+01	1.22E+01	0	2.16E+01	21.56007	0	1.04E-01	0.104
TU870_DNA_act_bind	TU870_DNA binding of activator	1 TU870_DNA_neu --> 1 TU870_DNA_act	irreversible	Transcription Regulation	0.00E+00	1.00E+09	0	9.15E+01	9.15E+01	0	1.62E+02	161.5461	0	1.96E-01	0.196
TU870_cleavage	Cleavage of TU870_mRNA	1 TU870_mRNA_cleav_cplx + 23 h2o --> 1 RNase_III_dim_inact + 1[illegible] amp + 1 b0406_mRNA + 1 b0407_mRNA + 4 gmp + 23 h + 8 ump	irreversible	Cleavage polycistronic mRNA	0.00E+00	1.00E+09	0	9.15E+01	9.15E+01	0	1.62E+02	161.5461	0	1.96E-01	0.196
TU870_cleavage_cplx	Cleavage complex formation of TU870_mRNA (tgtp, no_common_name_9)	1 RNase_III_dim + 1 TU870_mRNA <==> 1 TU870_mRNA_cleav_cplx	reversible	Cleavage polycistronic mRNA	-1.00E+09	1.00E+09	0	9.15E+01	9.15E+01	0	1.62E+02	161.5461	0	1.96E-01	0.196
TU871_DNA_act_bind	TU871_DNA binding of activator	1 TU871_DNA_neu --> 1 TU871_DNA_act	irreversible	Transcription Regulation	0.00E+00	1.00E+09	0	1.12E+02	1.12E+02	0	1.98E+02	197.8231	0	3.39E-02	0.0339
TU872_DNA_act_bind	TU872_DNA binding of activator	1 TU872_DNA_neu --> 1 TU872_DNA_act	irreversible	Transcription Regulation	0.00E+00	1.00E+09	0	2.01E+01	2.01E+01	0	3.56E+01	35.57072	0	1.04E-01	0.104
TU872_cleavage	Cleavage of TU872_mRNA , 8 nt short (b3384_v5), 8 nt short (b3385_v5)	1 TU872_mRNA_cleav_cplx + 276 h2o --> 1 RNase_III_dim_inact + 63 amp + 1 b3384_v5_mRNA + 1 b3385_v5_mRNA + 1 b3386_v5_mRNA + 1 b3387_v4_mRNA + 1 b3388_v2_mRNA + 1 b3389_v1_mRNA + 1 b3390_v1_mRNA + 52 cmp + 76 gmp + 276 h + 79 ump	irreversible	Cleavage polycistronic mRNA	0.00E+00	1.00E+09	0	2.01E+01	2.01E+01	0	3.56E+01	35.57072	0	1.04E-01	0.104

TU872_cleavage_cplx	Cleavage complex formation of TU872_mRNA (aroKp1, no_common_name_7) , 8 nt short (b3384_v5), 8 nt short (b3385_v5)	1 RNase_III_dim + 1 TU872_mRNA <==> 1 TU872_mRNA_cleav_cplx	reversible	Cleavage polycistronic mRNA	-1.00E+09	1.00E+09	0	2.01E+01	2.01E+01	0	3.56E+01	35.57072	0	1.04E-01	0.104
TU873_DNA_act_bind	TU873_DNA binding of activator	1 TU873_DNA_neu --> 1 TU873_DNA_act	irreversible	Transcription Regulation	0.00E+00	1.00E+09	0	2.01E+01	2.01E+01	0	3.56E+01	35.57072	0	1.04E-01	0.104
TU873_cleavage	Cleavage of TU873_mRNA , 8 nt short (b3384_v4), 8 nt short (b3385_v4)	1 TU873_mRNA_cleav_cplx + 276 h2o --> 1 RNase_III_dim_inact + 63 amp + 1 b3384_v4_mRNA + 1 b3385_v4_mRNA + 1 b3386_v4_mRNA + 1 b3387_v3_mRNA + 1 b3388_v1_mRNA + 1 b3389_mRNA + 1 b3390_mRNA + 52 cmp + 76 gmp + 276 h + 79 ump	irreversible	Cleavage polycistronic mRNA	0.00E+00	1.00E+09	0	2.01E+01	2.01E+01	0	3.56E+01	35.57072	0	1.04E-01	0.104
TU873_cleavage_cplx	Cleavage complex formation of TU873_mRNA (aroKp2, no_common_name_6) , 8 nt short (b3384_v4), 8 nt short (b3385_v4)	1 RNase_III_dim + 1 TU873_mRNA <==> 1 TU873_mRNA_cleav_cplx	reversible	Cleavage polycistronic mRNA	-1.00E+09	1.00E+09	0	2.01E+01	2.01E+01	0	3.56E+01	35.57072	0	1.04E-01	0.104
TU874_DNA_act_bind	TU874_DNA binding of activator	1 TU874_DNA_neu --> 1 TU874_DNA_act	irreversible	Transcription Regulation	0.00E+00	1.00E+09	0	2.71E+01	2.71E+01	0	4.78E+01	47.81984	0	1.74E-01	0.174
TU874_cleavage	Cleavage of TU874_mRNA , 8 nt short (b3384_v3), 8 nt short (b3385_v3)	1 TU874_mRNA_cleav_cplx + 127 h2o --> 1 RNase_III_dim_inact + 26 amp + 1 b3384_v3_mRNA + 1 b3385_v3_mRNA + 1 b3386_v3_mRNA + 1 b3387_v2_mRNA + 1 b3388_mRNA + 26 cmp + 38 gmp + 127 h + 33 ump	irreversible	Cleavage polycistronic mRNA	0.00E+00	1.00E+09	0	2.71E+01	2.71E+01	0	4.78E+01	47.81984	0	1.74E-01	0.174
TU874_cleavage_cplx	Cleavage complex formation of TU874_mRNA (damXp, no_common_name_5) , 8 nt short (b3384_v3), 8 nt short (b3385_v3)	1 RNase_III_dim + 1 TU874_mRNA <==> 1 TU874_mRNA_cleav_cplx	reversible	Cleavage polycistronic mRNA	-1.00E+09	1.00E+09	0	2.71E+01	2.71E+01	0	4.78E+01	47.81984	0	1.74E-01	0.174
TU875_DNA_act_bind	TU875_DNA binding of activator	1 TU875_DNA_neu --> 1 TU875_DNA_act	irreversible	Transcription Regulation	0.00E+00	1.00E+09	0	3.98E+01	3.98E+01	0	7.02E+01	70.2112	0	1.74E-01	0.174
TU875_cleavage	Cleavage of TU875_mRNA , 8 nt short (b3384_v2), 8 nt short (b3385_v2)	1 TU875_mRNA_cleav_cplx + 20 h2o --> 1 RNase_III_dim_inact + 5 amp + 1 b3384_v2_mRNA + 1 b3385_v2_mRNA + 1 b3386_v2_mRNA + 1 b3387_v1_mRNA + 3 cmp + 6 gmp + 20 h + 3 ump	irreversible	Cleavage polycistronic mRNA	0.00E+00	1.00E+09	0	3.98E+01	3.98E+01	0	7.02E+01	70.2112	0	1.74E-01	0.174
TU875_cleavage_cplx	Cleavage complex formation of TU875_mRNA (damp1, no_common_name_4) , 8 nt short (b3384_v2), 8 nt short (b3385_v2)	1 RNase_III_dim + 1 TU875_mRNA <==> 1 TU875_mRNA_cleav_cplx	reversible	Cleavage polycistronic mRNA	-1.00E+09	1.00E+09	0	3.98E+01	3.98E+01	0	7.02E+01	70.2112	0	1.74E-01	0.174
TU876_DNA_act_bind	TU876_DNA binding of activator	1 TU876_DNA_neu --> 1 TU876_DNA_act	irreversible	Transcription Regulation	0.00E+00	1.00E+09	0	3.98E+01	3.98E+01	0	7.02E+01	70.2112	0	1.74E-01	0.174
TU876_cleavage	Cleavage of TU876_mRNA , 8 nt short (b3384_v1), 8 nt short (b3385_v1)	1 TU876_mRNA_cleav_cplx + 20 h2o --> 1 RNase_III_dim_inact + 5 amp + 1 b3384_v1_mRNA + 1 b3385_v1_mRNA + 1 b3386_v1_mRNA + 1 b3387_mRNA + 3 cmp + 6 gmp + 20 h + 3 ump	irreversible	Cleavage polycistronic mRNA	0.00E+00	1.00E+09	0	3.98E+01	3.98E+01	0	7.02E+01	70.2112	0	1.74E-01	0.174
TU876_cleavage_cplx	Cleavage complex formation of TU876_mRNA (damp2, no_common_name_3) , 8 nt short (b3384_v1), 8 nt short (b3385_v1)	1 RNase_III_dim + 1 TU876_mRNA <==> 1 TU876_mRNA_cleav_cplx	reversible	Cleavage polycistronic mRNA	-1.00E+09	1.00E+09	0	3.98E+01	3.98E+01	0	7.02E+01	70.2112	0	1.74E-01	0.174
TU877_DNA_act_bind	TU877_DNA binding of activator	1 TU877_DNA_neu --> 1 TU877_DNA_act	irreversible	Transcription Regulation	0.00E+00	1.00E+09	0	5.36E+01	5.36E+01	0	9.46E+01	94.59664	0	1.89E-01	0.189
TU877_cleavage	Cleavage of TU877_mRNA , 8 nt short (b3384), 8 nt short (b3385)	1 TU877_mRNA_cleav_cplx + 2 h2o --> 1 RNase_III_dim_inact + 1 b3384_mRNA + 1 b3385_mRNA + 1 b3386_mRNA + 2 h	irreversible	Cleavage polycistronic mRNA	0.00E+00	1.00E+09	0	5.36E+01	5.36E+01	0	9.46E+01	94.59664	0	1.89E-01	0.189
TU877_cleavage_cplx	Cleavage complex formation of TU877_mRNA (rpep, no_common_name_2) , 8 nt short (b3384), 8 nt short (b3385)	1 RNase_III_dim + 1 TU877_mRNA <==> 1 TU877_mRNA_cleav_cplx	reversible	Cleavage polycistronic mRNA	-1.00E+09	1.00E+09	0	5.36E+01	5.36E+01	0	9.46E+01	94.59664	0	1.89E-01	0.189
TYRt	L-tyrosine transport	1 tyr-L[e] <==> 1 tyr-L	reversible	Transport	-1.00E+09	1.00E+09	1.27E+04	1.26E+05	1.13E+05	9.39E+03	1.26E+05	116847.44	9.39E+03	1.17E+05	107184.64
TadA_DIM	Dimerization of TadA	2 TadA_mono <==> 1 TadA_dim	reversible	Protein complex formation	-1.00E+09	1.00E+09	0	6.05E+02	6.05E+02	0	1.03E+03	1033.666	0	4.52E+01	45.205
Tgt_TRIM	Trimerization of Tgt	3 Tgt_mono <==> 1 Tgt_trim	reversible	Protein complex formation	-1.00E+09	1.00E+09	0	7.00E+02	7.00E+02	0	1.21E+03	1213.858	0	3.06E+01	30.624
Tgt_trim_DIM	Dimerization of 2 Tgt_trim	2 Tgt_trim <==> 1 Tgt_hexa	reversible	Protein complex formation	-1.00E+09	1.00E+09	0	3.50E+02	3.50E+02	0	6.07E+02	606.9291	0	1.53E+01	15.312
ThrS_RS_DIM	dimerization of threonyl-tRNA synthetase	2 ThrS_mono <==> 1 Thr_RS_dim	reversible	Protein complex formation	-1.00E+09	1.00E+09	0	9.61E+02	9.61E+02	0	1.41E+03	1412.256	0	1.59E+02	159.0075
Thr_RS_CHARGB	Threonyl-tRNA synthetase charging	1 Thr_RS_dim + 1 atp + 1 h2o + 1 thr-L --> 1 Thr_RS_thr_amp + 1 h + 1 ppi	irreversible	Aminoacyl-tRNA synthetase charging	0.00E+00	1.00E+09	0	2.36E+05	2.36E+05	0	2.38E+05	237541.8	0	2.34E+05	233580.6
Thr_RS_CHARGa	Threonyl-tRNA synthetase charging	1 Thr_RS_dim + 2 atp + 2 h2o + 2 thr-L --> 1 Thr_RS_2thr_2amp + 2 h + 2 ppi	irreversible	Aminoacyl-tRNA synthetase charging	0.00E+00	1.00E+09	0	1.18E+05	1.18E+05	0	1.19E+05	118770.9	0	1.17E+05	116790.3
TrmD_DIM	Dimerization of TrmD	2 TrmD_mono <==> 1 TrmD_dim	reversible	Protein complex formation	-1.00E+09	1.00E+09	0	7.06E+02	7.06E+02	0	7.83E+02	782.5042	0	1.91E+02	191.0925
TrmE_DIM	Dimerization of TrmE	2 TrmE_mono <==> 1 TrmE_dim	reversible	Protein complex formation	-1.00E+09	1.00E+09	0	1.17E+03	1.17E+03	0	2.04E+03	2042.594	0	4.35E+01	43.459
TrmH_DIM	Dimerization of TrmH	2 TrmH_mono <==> 1 TrmH_dim	reversible	Protein complex formation	-1.00E+09	1.00E+09	0	1.83E+02	1.83E+02	0	2.95E+02	294.7626	0	3.83E+01	38.298
TrpS_RS_DIM	dimerization of Tryptophanyl-tRNA synthetase	2 TrpS_mono <==> 1 Trp_RS_dim	reversible	Protein complex formation	-1.00E+09	1.00E+09	0	1.30E+03	1.30E+03	0	2.24E+03	2244.478	0	6.01E+01	60.1255
Trp_RS_CHARGa	Tryptophyl-tRNA synthetase charging (tyr-D)	1 Trp_RS_dim + 2 atp + 2 h2o + 2 trp-L --> 1 Trp_RS_2trp_2amp + 2 h + 2 ppi	irreversible	Aminoacyl-tRNA synthetase charging	0.00E+00	1.00E+09	0	2.69E+04	2.69E+04	0	2.74E+04	27384.14	0	2.63E+04	26259.02
Trp_RS_CHARGb	Tryptophyl-tRNA synthetase charging (tyr-D)	1 Trp_RS_dim + 1 atp + 1 h2o + 1 trp-L --> 1 Trp_RS_trp_amp + 1 h + 1 ppi	irreversible	Aminoacyl-tRNA synthetase charging	0.00E+00	1.00E+09	0	5.38E+04	5.38E+04	0	5.48E+04	54768.28	0	5.25E+04	52518.04
TruA_DIM	Dimerization of TruA	2 TruA_mono <==> 1 TruA_dim	reversible	Protein complex formation	-1.00E+09	1.00E+09	0	4.31E+02	4.31E+02	0	7.24E+02	723.5971	0	4.99E+01	49.90875
Tsf_RENAME	TEMP_Tsf	1 Tsf_mono --> 1 EF-Ts	irreversible	Protein complex formation	0.00E+00	1.00E+09	0	1.45E+03	1.45E+03	0	1.54E+03	1543.06	0	2.56E+02	256.288
TyrS_RS_DIM	dimerization of tyrosyl-tRNA synthetas	2 TyrS_mono <==> 1 Tyr_RS_dim	reversible	Protein complex formation	-1.00E+09	1.00E+09	0	7.76E+02	7.76E+02	0	1.34E+03	1336.016	0	4.76E+01	47.568
Tyr_RS_CHARG1a	Tyrosinyl-tRNA synthetase charging	1 Tyr_RS_dim + 1 atp + 1 h2o + 1 tyr-L --> 1 Tyr_RS_tyr_amp + 1 h + 1 ppi	irreversible	Aminoacyl-tRNA synthetase charging	0.00E+00	1.00E+09	0	1.27E+05	1.27E+05	0	1.29E+05	128875	0	1.17E+05	116854.6
Tyr_RS_CHARG1b	Tyrosinyl-tRNA synthetase charging	1 Tyr_RS_dim + 2 atp + 2 h2o + 2 tyr-L --> 1 Tyr_RS_2tyr_2amp + 2 h + 2 ppi	irreversible	Aminoacyl-tRNA synthetase charging	0.00E+00	1.00E+09	0	6.35E+04	6.35E+04	0	6.44E+04	64437.48	0	5.84E+04	58427.28
Tyr_RS_CHARG2a	Tyrosinyl-tRNA synthetase charging (tyr-D)	1 Tyr_RS_dim + 1 atp + 1 h2o + 1 tyr-D --> 1 Tyr_RS_tyr-D_amp + 1 h + 1 ppi	irreversible	Aminoacyl-tRNA synthetase charging	0.00E+00	1.00E+09	0	7.10E+06	7.10E+06	0	7.41E+06	7412071	0	7.41E+06	7412071
Tyr_RS_CHARG2b	Tyrosinyl-tRNA synthetase charging (tyr-D)	1 Tyr_RS_dim + 2 atp + 2 h2o + 2 tyr-D --> 1 Tyr_RS_2tyr-D_2amp + 2 h + 2 ppi	irreversible	Aminoacyl-tRNA synthetase charging	0.00E+00	1.00E+09	0	3.55E+06	3.55E+06	0	3.71E+06	3706036	0	3.71E+06	3706036
VALtec	L-valine transport via diffusion (extracellular to cytosol)	1 val-L[e] <==> 1 val-L	reversible	Transport	-1.00E+09	1.00E+09	7.33E+04	3.76E+05	3.03E+05	5.40E+04	3.87E+05	333418.52	5.40E+04	3.38E+05	283575.22
Val_RS_CHARG	Valyl-tRNA synthetase charging	1 ValS_mono + 1 atp + 1 h2o + 1 val-L --> 1 Val_RS_val_amp + 1 h + 1 ppi	irreversible	Aminoacyl-tRNA synthetase charging	0.00E+00	1.00E+09	7.33E+04	3.77E+05	3.04E+05	5.40E+04	3.90E+05	335576.92	5.40E+04	3.39E+05	285093.62
YbbB_DIM	dimerization of YbbB	2 YbbB_mono <==> 1 YbbB_dim	reversible	Protein complex formation	-1.00E+09	1.00E+09	0	1.27E+03	1.27E+03	0	2.21E+03	2214.226	0	4.72E+01	47.208
YheLMN_FORM	formation of YheLMN (2*YheL,2*YheM,2*YheN)	2 YheL_mono + 2 YheM_mono + 2 YheN_mono <==> 1 YheLMN_cplx	reversible	Protein complex formation	-1.00E+09	1.00E+09	0	2.22E+02	2.22E+02	0	3.65E+02	364.5383	0	3.35E+01	33.537

ZN2t	Zn2+ tranport	1 zn2[e] <==> 1 zn2	reversible	Transport	-1.00E+09	1.00E+09	0	4.85E+03	4.85E+03	0	6.60E+03	6599.788	0	2.55E+03	2553.129
[2Fe-2S]_FORM	transfer of 2 Fe2 from IscA (tetramer) to 2 IscU (monomer)	1 IscA_tetra_Fe(2) + 2 IscU_mono_S-SH --> 1 IscA_tetra + 1 IscU_dim_2Fe(2)-2S	irreversible	Iron-sulfur cluster biosynthesis	0.00E+00	1.00E+09	0	5.57E+03	5.57E+03	0	9.38E+03	9377.848	0	6.17E+02	616.825
[2Fe-2S]_TRANSF	formation of [2Fe-2S] cluster	2 IscU_dim_2Fe(2)-2S --> 1 IscU_dim_[2Fe-2S]2 + 2 IscU_mono	irreversible	Iron-sulfur cluster biosynthesis	0.00E+00	1.00E+09	0	2.79E+03	2.79E+03	0	4.69E+03	4688.924	0	3.08E+02	308.4125
[4Fe-4S]_FORM	formation of [4Fe-4S] cluster	1 IscU_dim_[2Fe-2S]2 --> 1 IscU_dim_[4Fe-4S]	irreversible	Iron-sulfur cluster biosynthesis	0.00E+00	1.00E+09	0	2.79E+03	2.79E+03	0	4.69E+03	4688.924	0	3.08E+02	308.4125
adet	transport of ade	1 ade <==> 1 ade[e]	reversible	Transport	-1.00E+09	1.00E+09	0	1.77E+03	1.77E+03	0	7.90E+02	789.686	0	8.58E+01	85.794
adocblt	transport of adocbl	1 adocbl <==> 1 adocbl[e]	reversible	Transport	-1.00E+09	1.00E+09	0	0	0.00E+00	0	0	0	0	0	0
adp_exch	adp_exch	1 adp <==> 1 adp[e]	reversible	Transport	-1.00E+09	1.00E+09	5.64E+02	7.11E+06	7.11E+06	3.86E+02	7.41E+06	7413166.4	3.86E+02	7.41E+06	7413166.4
ala1_tRNA_CHARG	charging ala1_tRNA	1 Ala_RS_ala_amp + 1 ala1_tRNA --> 1 Ala_RS_tetra_inact + 1 ala1_tRNA_ala + 1 amp	irreversible	tRNA charging	0.00E+00	1.00E+09	7.83E+04	3.88E+05	3.09E+05	5.76E+04	4.04E+05	345895.78	5.76E+04	3.52E+05	294635.08
ala2_tRNA_CHARG	charging ala2_tRNA	1 Ala_RS_ala_amp + 1 ala2_tRNA --> 1 Ala_RS_tetra_inact + 1 ala2_tRNA_ala + 1 amp	irreversible	tRNA charging	0.00E+00	1.00E+09	7.51E+03	1.23E+05	1.16E+05	5.53E+03	1.42E+05	136727.26	5.53E+03	1.05E+05	99582.36
alaT_to_ala1	alaT_to_ala1	1 alaT_tRNA --> 1 ala1_tRNA	irreversible	tRNA charging	0.00E+00	1.00E+09	0	1.12E+02	1.12E+02	0	1.36E+01	13.6	0	1.36E+01	13.6
alaU_to_ala1	alaU_to_ala1	1 alaU_tRNA --> 1 ala1_tRNA	irreversible	tRNA charging	0.00E+00	1.00E+09	0	1.06E+02	1.06E+02	0	1.15E+01	11.5	0	1.15E+01	11.5
alaV_to_ala1	alaV_to_ala1	1 alaV_tRNA --> 1 ala1_tRNA	irreversible	tRNA charging	0.00E+00	1.00E+09	0	1.11E+02	1.11E+02	0	9.54E+00	9.54	0	9.54E+00	9.54
alaW_to_ala2	alaW_to_ala2	1 alaW_tRNA --> 1 ala2_tRNA	irreversible	tRNA charging	0.00E+00	1.00E+09	0	6.14E+02	6.14E+02	0	7.80E+00	7.8	0	7.80E+00	7.8
alaX_to_ala2	alaX_to_ala2	1 alaX_tRNA --> 1 ala2_tRNA	irreversible	tRNA charging	0.00E+00	1.00E+09	0	6.14E+02	6.14E+02	0	7.80E+00	7.8	0	7.80E+00	7.8
amp_exch	amp transport	1 amp[e] <==> 1 amp	reversible	Transport	-1.00E+09	1.00E+09	-7.88E+06	-7.79E+05	7.10E+06	-7.99E+06	-5.74E+05	7412102.1	-7.99E+06	-5.74E+05	7412102.1
arg1_tRNA_CHARG	charging arg1_tRNA	1 Arg_RS_arg_amp + 1 arg1_tRNA --> 1 ArgS_mono_inact + 1 amp + 1 arg1_tRNA_ala	irreversible	tRNA charging	0.00E+00	1.00E+09	6.21E+04	2.70E+05	2.08E+05	4.57E+04	2.72E+05	225864.1	4.57E+04	2.60E+05	214349.6
argQ_to_arg1	argQ_to_arg1	1 argQ_tRNA --> 1 arg1_tRNA	irreversible	tRNA charging	0.00E+00	1.00E+09	0	1.46E+02	1.46E+02	0	8.87E+00	8.87	0	8.87E+00	8.87
argU_tRNA_CHARG	charging argU_tRNA	1 Arg_RS_arg_amp + 1 argU_tRNA --> 1 ArgS_mono_inact + 1 amp + 1 argU_tRNA_ala	irreversible	tRNA charging	0.00E+00	1.00E+09	5.07E+02	1.59E+04	1.54E+04	3.73E+02	2.10E+04	20599.27	3.73E+02	9.06E+03	8686.903
argV_to_arg1	argV_to_arg1	1 argV_tRNA --> 1 arg1_tRNA	irreversible	tRNA charging	0.00E+00	1.00E+09	0	1.46E+02	1.46E+02	0	8.87E+00	8.87	0	8.87E+00	8.87
argW_tRNA_CHARG	charging argW_tRNA	1 Arg_RS_arg_amp + 1 argW_tRNA --> 1 ArgS_mono_inact + 1 amp + 1 argW_tRNA_arg	irreversible	tRNA charging	0.00E+00	1.00E+09	0	1.14E+04	1.14E+04	0	1.33E+04	13314.72	0	7.04E+03	7036.922
argX_tRNA_CHARG	charging argX_tRNA	1 Arg_RS_arg_amp + 1 argX_tRNA --> 1 ArgS_mono_inact + 1 amp + 1 argX_tRNA_arg	irreversible	tRNA charging	0.00E+00	1.00E+09	3.38E+02	4.51E+04	4.48E+04	2.49E+02	5.26E+04	52370.69	2.49E+02	3.08E+04	30511.28
argY_to_arg1	argY_to_arg1	1 argY_tRNA --> 1 arg1_tRNA	irreversible	tRNA charging	0.00E+00	1.00E+09	0	1.46E+02	1.46E+02	0	8.87E+00	8.87	0	8.87E+00	8.87
argZ_to_arg1	argZ_to_arg1	1 argZ_tRNA --> 1 arg1_tRNA	irreversible	tRNA charging	0.00E+00	1.00E+09	0	1.46E+02	1.46E+02	0	8.87E+00	8.87	0	8.87E+00	8.87
asn1_tRNA	charging asn1 _tRNA_CHARG	1 Asn_RS_asn_amp + 1 asn1_tRNA --> 1 Asn_RS_dim_inact + 1 amp + 1 asn1_tRNA_asn	irreversible	tRNA charging	0.00E+00	1.00E+09	2.64E+04	1.77E+05	1.51E+05	1.95E+04	1.89E+05	169232.42	1.95E+04	1.63E+05	143663.82
asnT_to_asn1	asnT_to_asn1	1 asnT_tRNA --> 1 asn1_tRNA	irreversible	tRNA charging	0.00E+00	1.00E+09	0	1.77E+03	1.77E+03	0	6.36E+00	6.36	0	6.36E+00	6.36
asnU_to_asn1	asnU_to_asn1	1 asnU_tRNA --> 1 asn1_tRNA	irreversible	tRNA charging	0.00E+00	1.00E+09	0	1.77E+03	1.77E+03	0	6.41E+00	6.41	0	6.41E+00	6.41
asnV_to_asn1	asnV_to_asn1	1 asnV_tRNA --> 1 asn1_tRNA	irreversible	tRNA charging	0.00E+00	1.00E+09	0	1.77E+03	1.77E+03	0	6.41E+00	6.41	0	6.41E+00	6.41
asnW_to_asn1	asnW_to_asn1	1 asnW_tRNA --> 1 asn1_tRNA	irreversible	tRNA charging	0.00E+00	1.00E+09	0	1.77E+03	1.77E+03	0	6.40E+00	6.4	0	6.40E+00	6.4
asp1_tRNA_CHARGa	charging asp1_tRNA	1 Asp_RS_2asp_2amp + 2 asp1_tRNA --> 1 Asp_RS_dim_inact + 2 amp + 2 asp1_tRNA_asp	irreversible	tRNA charging	0.00E+00	1.00E+09	0	1.10E+05	1.10E+05	0	1.11E+05	110520.4	0	1.10E+05	110377.2
asp1_tRNA_CHARGb	charging asp1_tRNA	1 Asp_RS_asp_amp + 1 asp1_tRNA --> 1 Asp_RS_dim_inact + 1 amp + 1 asp1_tRNA_asp	irreversible	tRNA charging	0.00E+00	1.00E+09	0	2.21E+05	2.21E+05	0	2.21E+05	221040.8	0	2.21E+05	220754.4
aspT_to_asp1	aspT_to_asp1	1 aspT_tRNA --> 1 asp1_tRNA	irreversible	tRNA charging	0.00E+00	1.00E+09	0	1.45E+03	1.45E+03	0	1.41E+01	14.1	0	1.41E+01	14.1
aspU_to_asp1	aspU_to_asp1	1 aspU_tRNA --> 1 asp1_tRNA	irreversible	tRNA charging	0.00E+00	1.00E+09	0	1.45E+03	1.45E+03	0	9.52E+00	9.52	0	9.52E+00	9.52
aspV_to_asp1	aspV_to_asp1	1 aspV_tRNA --> 1 asp1_tRNA	irreversible	tRNA charging	0.00E+00	1.00E+09	0	1.45E+03	1.45E+03	0	9.48E+00	9.48	0	9.48E+00	9.48
atp_exch	atp_exch	1 atp <==> 1 atp[e]	reversible	Transport	-1.00E+09	1.00E+09	-1.51E+07	-8.80E+05	1.42E+07	-1.55E+07	-6.49E+05	14828678.9	-1.55E+07	-6.49E+05	14828679
b0014_fold_GroEL/ES_1	b0014_m folding: GroEL/ES mediated: polypeptide is going in GroEL/ES complex	1 GroEL.(7)ADP.cisGroES + 7 atp + 1 b0014_m + 1 transGroES_hepta --> 7 adp + 1 b0014_m_GroEL.(7)ATP.transGroES + 1 cisGroES_hepta	irreversible	Protein Folding	0.00E+00	1.00E+09	0	1.76E+03	1.76E+03	0	2.93E+03	2933.059	0	2.54E+02	253.857
b0014_fold_GroEL/ES_2	b0014_m folding: GroEL/ES mediated: folding of polypeptide under ATP hydrolysis	1 b0014_m_GroEL.(7)ATP.transGroES + 7 h2o --> 1 DnaK_GroEL.(7)ADP.transGroES + 7 h + 7 pi	irreversible	Protein Folding	0.00E+00	1.00E+09	0	1.76E+03	1.76E+03	0	2.93E+03	2933.059	0	2.54E+02	253.857
b0014_fold_GroEL/ES_3	b0014_m folding: GroEL/ES mediated: release of native protein	1 DnaK_GroEL.(7)ADP.transGroES --> 1 DnaK_mono + 1 GroEL.(7)ADP.transGroES	irreversible	Protein Folding	0.00E+00	1.00E+09	0	1.76E+03	1.76E+03	0	2.93E+03	2933.059	0	2.54E+02	253.857
b0014_fold_KJE_1	b0014_m folding: KJE mediated	1 DnaJ_dim + 1 DnaK_mono.ATP + 1 b0014_m <==> 1 b0014_m_DnaKJ_complex	reversible	Protein Folding	-1.00E+09	1.00E+09	0	1.76E+03	1.76E+03	0	2.93E+03	2933.059	0	2.54E+02	253.857
b0014_fold_KJE_2	b0014_m folding: KJE mediated	1 GrpE_dim + 1 b0014_m_DnaKJ_complex + 1 h2o --> 1 DnaJ_dim_inact + 1 DnaK_DnaK_GrpE_complex + 1 adp + 1 h + 1 pi	irreversible	Protein Folding	0.00E+00	1.00E+09	0	1.76E+03	1.76E+03	0	2.93E+03	2933.059	0	2.54E+02	253.857
b0014_fold_KJE_3	b0014_m folding: KJE mediated	1 DnaK_DnaK_GrpE_complex + 1 atp --> 1 DnaK_mono + 1 DnaK_mono.ATP_inact + 1 GrpE_dim_inact	irreversible	Protein Folding	0.00E+00	1.00E+09	0	1.76E+03	1.76E+03	0	2.93E+03	2933.059	0	2.54E+02	253.857
b0014_mRNA_CONV	convsersion of mRNA to mRNA_1 (synthetic rxn)	1 b0014_mRNA --> 1 b0014_mRNA_1	irreversible	Translation	0.00E+00	1.00E+09	0	4.13E+01	4.13E+01	0	7.28E+01	72.84185	0	4.31E-01	0.431
b0014_mRNA_degr1	Degradation of b0014_mRNA I	1 Orn_dim + 479 atp + 1 b0014_mRNA_2 + 1 degradosome + 479 h2o <==> 479 adp + 1 b0014_mRNA_2_degr + 479 h + 479 pi	reversible	mRNA degradation	-1.00E+09	1.00E+09	0	4.13E+01	4.13E+01	0	7.28E+01	72.84185	0	4.31E-01	0.431
b0014_mRNA_degr2	Degradation of b0014_mRNA II	1 b0014_mRNA_2_degr + 1916 h2o --> 1 Orn_dim_inact + 542 amp + 1 atp + 475 cmp + 1 degradosome_inact + 505 gmp + 1916 h + 394 ump	irreversible	mRNA degradation	0.00E+00	7.70E-01	0	4.13E+01	4.13E+01	0	7.28E+01	72.84185	0	4.31E-01	0.431
b0014_maturation1	Polypeptide b0014 peptide deformylase and methionine aminopeptidase complex	1 Def_mono + 1 Map_mono + 1 b0014_aa <==> 1 b0014_def_map_cplx	reversible	Protein Maturation	-1.00E+09	1.00E+09	0	1.76E+03	1.76E+03	0	2.93E+03	2933.059	0	2.54E+02	253.857
b0014_maturation2	b0014 formation	1 b0014_def_map_cplx + 2 h2o --> 1 Def_mono_inact + 1 Map_mono_inact + 1 b0014_m + 1 for + 1 met-L	irreversible	Protein Maturation	0.00E+00	1.00E+09	0	1.76E+03	1.76E+03	0	2.93E+03	2933.059	0	2.54E+02	253.857
b0014_v1_mRNA_CONV	convsersion of mRNA to mRNA_1 (synthetic rxn)	1 b0014_v1_mRNA --> 1 b0014_mRNA_1	irreversible	Translation	0.00E+00	1.00E+09	0	4.13E+01	4.13E+01	0	7.28E+01	72.84185	0	4.31E-01	0.431
b0014_v2_mRNA_CONV	convsersion of mRNA to mRNA_1 (synthetic rxn)	1 b0014_v2_mRNA --> 1 b0014_mRNA_1	irreversible	Translation	0.00E+00	1.00E+09	0	4.13E+01	4.13E+01	0	7.28E+01	72.84185	0	4.31E-01	0.431
b0015_fold_GroEL/ES_1	b0015_m folding: GroEL/ES mediated: polypeptide is going in GroEL/ES complex	1 GroEL.(7)ADP.cisGroES + 7 atp + 1 b0015_m_Zn + 1 transGroES_hepta --> 7 adp + 1 b0015_m_GroEL.(7)ATP.transGroES + 1 cisGroES_hepta	irreversible	Protein Folding	0.00E+00	1.00E+09	0	9.95E+02	9.95E+02	0	1.69E+03	1689.861	0	9.68E+01	96.822
b0015_fold_GroEL/ES_2	b0015_m folding: GroEL/ES mediated: folding of polypeptide under ATP hydrolysis	1 b0015_m_GroEL.(7)ATP.transGroES + 7 h2o --> 1 DnaJ_GroEL.(7)ADP.transGroES + 7 h + 7 pi	irreversible	Protein Folding	0.00E+00	1.00E+09	0	9.95E+02	9.95E+02	0	1.69E+03	1689.861	0	9.68E+01	96.822
b0015_fold_GroEL/ES_3	b0015_m folding: GroEL/ES mediated: release of native protein	1 DnaJ_GroEL.(7)ADP.transGroES --> 1 DnaJ_mono + 1 GroEL.(7)ADP.transGroES	irreversible	Protein Folding	0.00E+00	1.00E+09	0	9.95E+02	9.95E+02	0	1.69E+03	1689.861	0	9.68E+01	96.822
b0015_ion_BIND	Binding of ions to b0015	1 b0015_m + 2 zn2 <==> 1 b0015_m_Zn	reversible	Metallo-ion Binding	-1.00E+09	1.00E+09	0	9.95E+02	9.95E+02	0	1.69E+03	1689.861	0	9.68E+01	96.822
b0015_mRNA_CONV	convsersion of mRNA to mRNA_1 (synthetic rxn)	1 b0015_mRNA --> 1 b0015_mRNA_1	irreversible	Translation	0.00E+00	1.00E+09	0	4.13E+01	4.13E+01	0	7.28E+01	72.84185	0	4.31E-01	0.431

b0015_mRNA_degr1	Degradation of b0015_mRNA I	1 Orn_dim + 282 atp + 1 b0015_mRNA_2 + 1 degradosome + 282 h2o <==> 282 adp + 1 b0015_mRNA_2_degr + 282 h + 282 pi	reversible	mRNA degradation	-1.00E+09	1.00E+09	0	4.13E+01	4.13E+01	0	7.28E+01	72.84185	0	4.31E-01	0.431
b0015_mRNA_degr2	Degradation of b0015_mRNA II	1 b0015_mRNA_2_degr + 1130 h2o --> 1 Orn_dim_inact + 276 amp 278 cmp + 1 degradosome_inact + 346 gmp + 1130 h + 231 ump	irreversible	mRNA degradation	0.00E+00	4.31E-01	0	4.13E+01	4.13E+01	0	7.28E+01	72.84185	0	4.31E-01	0.431
b0015_maturation1	Polypeptide b0015 peptide deformylas and methionine aminopeptidase complex	1 Def_mono + 1 Map_mono + 1 b0015_aa <==> 1 b0015_def_map_cplx	reversible	Protein Maturation	-1.00E+09	1.00E+09	0	9.95E+02	9.95E+02	0	1.69E+03	1689.861	0	9.68E+01	96.822
b0015_maturation2	b0015 formation	1 b0015_def_map_cplx + 2 h2o --> 1 Def_mono_inact + 1 Map_mono_inact + 1 b0015_m + 1 for + 1 met-L	irreversible	Protein Maturation	0.00E+00	1.00E+09	0	9.95E+02	9.95E+02	0	1.69E+03	1689.861	0	9.68E+01	96.822
b0015_v1_mRNA_CONV	convsersion of mRNA to mRNA_1 (synthetic rxn)	1 b0015_v1_mRNA --> 1 b0015_mRNA_1	irreversible	Translation	0.00E+00	1.00E+09	0	4.13E+01	4.13E+01	0	7.28E+01	72.84185	0	4.31E-01	0.431
b0015_v2_mRNA_CONV	convsersion of mRNA to mRNA_1 (synthetic rxn)	1 b0015_v2_mRNA --> 1 b0015_mRNA_1	irreversible	Translation	0.00E+00	1.00E+09	0	4.13E+01	4.13E+01	0	7.28E+01	72.84185	0	4.31E-01	0.431
b0023_fold_spon	b0023_m folding: spontanous	1 b0023_m --> 1 RpsT_mono	irreversible	Protein Folding	0.00E+00	1.00E+09	8.44E+01	1.13E+02	2.81E+01	6.22E+01	8.29E+01	20.72	6.22E+01	8.29E+01	20.72
b0023_mRNA_CONV	convsersion of mRNA to mRNA_1 (synthetic rxn)	1 b0023_mRNA --> 1 b0023_mRNA_1	irreversible	Translation	0.00E+00	1.00E+09	0	1.06E+02	1.06E+02	0	8.29E+01	82.88	0	2.92E+00	2.92
b0023_mRNA_degr1	Degradation of b0023_mRNA I	1 Orn_dim + 66 atp + 1 b0023_mRNA_2 + 1 degradosome + 66 h2o <==> 66 adp + 1 b0023_mRNA_2_degr + 66 h + 66 pi	reversible	mRNA degradation	-1.00E+09	1.00E+09	0	1.06E+02	1.06E+02	0	8.29E+01	82.88	0	2.92E+00	2.92
b0023_mRNA_degr2	Degradation of b0023_mRNA II	1 b0023_mRNA_2_degr + 263 h2o --> 1 Orn_dim_inact + 89 amp + 66 cmp + 1 degradosome_inact + 57 gmp + 263 h + 51 ump + 1 utp	irreversible	mRNA degradation	0.00E+00	2.92E+00	0	1.06E+02	1.06E+02	0	8.29E+01	82.88	0	2.92E+00	2.92
b0023_maturation1	Polypeptide b0023 peptide deformylas and methionine aminopeptidase complex	1 Def_mono + 1 Map_mono + 1 b0023_aa <==> 1 b0023_def_map_cplx	reversible	Protein Maturation	-1.00E+09	1.00E+09	8.44E+01	1.13E+02	2.81E+01	6.22E+01	8.29E+01	20.72	6.22E+01	8.29E+01	20.72
b0023_maturation2	b0023 formation	1 b0023_def_map_cplx + 2 h2o --> 1 Def_mono_inact + 1 Map_mono_inact + 1 b0023_m + 1 for + 1 met-L	irreversible	Protein Maturation	0.00E+00	1.00E+09	8.44E+01	1.13E+02	2.81E+01	6.22E+01	8.29E+01	20.72	6.22E+01	8.29E+01	20.72
b0023_v1_mRNA_CONV	convsersion of mRNA to mRNA_1 (synthetic rxn)	1 b0023_v1_mRNA --> 1 b0023_mRNA_1	irreversible	Translation	0.00E+00	1.00E+09	0	1.06E+02	1.06E+02	0	8.29E+01	82.88	0	2.92E+00	2.92
b0025_fold_spon	b0025_m folding: spontanous	1 b0025_m --> 1 RibF_mono	irreversible	Protein Folding	0.00E+00	1.00E+09	0	5.15E+02	5.15E+02	0	8.10E+02	809.7958	0	1.30E+02	130.401
b0025_mRNA_CONV	convsersion of mRNA to mRNA_1 (synthetic rxn)	1 b0025_mRNA --> 1 b0025_mRNA_1	irreversible	Translation	0.00E+00	1.00E+09	0	2.14E+01	2.14E+01	0	3.78E+01	37.83865	0	9.45E-02	0.0945
b0025_mRNA_degr1	Degradation of b0025_mRNA I	1 Orn_dim + 235 atp + 1 b0025_mRNA_2 + 1 degradosome + 235 h2o <==> 235 adp + 1 b0025_mRNA_2_degr + 235 h + 235 pi	reversible	mRNA degradation	-1.00E+09	1.00E+09	0	2.14E+01	2.14E+01	0	3.78E+01	37.83865	0	9.45E-02	0.0945
b0025_mRNA_degr2	Degradation of b0025_mRNA II	1 b0025_mRNA_2_degr + 941 h2o --> 1 Orn_dim_inact + 210 amp + 1 atp + 227 cmp + 1 degradosome_inact + 278 gmp + 941 h + 226 ump	irreversible	mRNA degradation	0.00E+00	1.89E-01	0	2.14E+01	2.14E+01	0	3.78E+01	37.83865	0	9.45E-02	0.0945
b0025_maturation1	Polypeptide b0025 peptide deformylas and methionine aminopeptidase complex	1 Def_mono + 1 Map_mono + 1 b0025_aa <==> 1 b0025_def_map_cplx	reversible	Protein Maturation	-1.00E+09	1.00E+09	0	5.15E+02	5.15E+02	0	8.10E+02	809.7958	0	1.30E+02	130.401
b0025_maturation2	b0025 formation	1 b0025_def_map_cplx + 2 h2o --> 1 Def_mono_inact + 1 Map_mono_inact + 1 b0025_m + 1 for + 1 met-L	irreversible	Protein Maturation	0.00E+00	1.00E+09	0	5.15E+02	5.15E+02	0	8.10E+02	809.7958	0	1.30E+02	130.401
b0026_fold_spon	b0026_m folding: spontanous	1 b0026_m_Zn --> 1 IleS_mono	irreversible	Protein Folding	0.00E+00	1.00E+09	0	1.28E+03	1.28E+03	0	2.13E+03	2133.32	0	1.04E+02	103.6475
b0026_ion_BIND	Binding of ions to b0026	1 b0026_m + 2 zn2 <==> 1 b0026_m_Zn	reversible	Metallo-ion Binding	-1.00E+09	1.00E+09	0	1.28E+03	1.28E+03	0	2.13E+03	2133.32	0	1.04E+02	103.6475
b0026_mRNA_CONV	convsersion of mRNA to mRNA_1 (synthetic rxn)	1 b0026_mRNA --> 1 b0026_mRNA_1	irreversible	Translation	0.00E+00	1.00E+09	0	2.14E+01	2.14E+01	0	3.78E+01	37.83865	0	9.45E-02	0.0945
b0026_mRNA_degr1	Degradation of b0026_mRNA I	1 Orn_dim + 704 atp + 1 b0026_mRNA_2 + 1 degradosome + 704 h2o <==> 704 adp + 1 b0026_mRNA_2_degr + 704 h + 704 pi	reversible	mRNA degradation	-1.00E+09	1.00E+09	0	2.14E+01	2.14E+01	0	3.78E+01	37.83865	0	9.45E-02	0.0945
b0026_mRNA_degr2	Degradation of b0026_mRNA II	1 b0026_mRNA_2_degr + 2816 h2o --> 1 Orn_dim_inact + 655 amp 747 cmp + 1 degradosome_inact + 824 gmp + 2816 h + 591 ump	irreversible	mRNA degradation	0.00E+00	5.39E-01	0	2.14E+01	2.14E+01	0	3.78E+01	37.83865	0	9.45E-02	0.0945
b0026_maturation1	Polypeptide b0026 peptide deformylas and methionine aminopeptidase complex	1 Def_mono + 1 Map_mono + 1 b0026_aa <==> 1 b0026_def_map_cplx	reversible	Protein Maturation	-1.00E+09	1.00E+09	0	1.28E+03	1.28E+03	0	2.13E+03	2133.32	0	1.04E+02	103.6475
b0026_maturation2	b0026 formation	1 b0026_def_map_cplx + 2 h2o --> 1 Def_mono_inact + 1 Map_mono_inact + 1 b0026_m + 1 for + 1 met-L	irreversible	Protein Maturation	0.00E+00	1.00E+09	0	1.28E+03	1.28E+03	0	2.13E+03	2133.32	0	1.04E+02	103.6475
b0027_fold_spon	b0027_m folding: spontanous	1 b0027_m --> 1 LspA_mono	irreversible	Protein Folding	0.00E+00	1.00E+09	0	2.80E+02	2.80E+02	0	4.28E+02	427.5779	0	8.79E+01	87.8805
b0027_mRNA_CONV	convsersion of mRNA to mRNA_1 (synthetic rxn)	1 b0027_mRNA --> 1 b0027_mRNA_1	irreversible	Translation	0.00E+00	1.00E+09	0	2.14E+01	2.14E+01	0	3.78E+01	37.83865	0	9.45E-02	0.0945
b0027_mRNA_degr1	Degradation of b0027_mRNA I	1 Orn_dim + 123 atp + 1 b0027_mRNA_2 + 1 degradosome + 123 h2o <==> 123 adp + 1 b0027_mRNA_2_degr + 123 h + 123 pi	reversible	mRNA degradation	-1.00E+09	1.00E+09	0	2.14E+01	2.14E+01	0	3.78E+01	37.83865	0	9.45E-02	0.0945
b0027_mRNA_degr2	Degradation of b0027_mRNA II	1 b0027_mRNA_2_degr + 493 h2o --> 1 Orn_dim_inact + 91 amp + 124 cmp + 1 degradosome_inact + 141 gmp + 493 h + 138 ump	irreversible	mRNA degradation	0.00E+00	2.51E-01	0	2.14E+01	2.14E+01	0	3.78E+01	37.83865	0	9.45E-02	0.0945
b0027_maturation1	Polypeptide b0027 peptide deformylas and methionine aminopeptidase complex	1 Def_mono + 1 Map_mono + 1 b0027_aa <==> 1 b0027_def_map_cplx	reversible	Protein Maturation	-1.00E+09	1.00E+09	0	2.80E+02	2.80E+02	0	4.28E+02	427.5779	0	8.79E+01	87.8805
b0027_maturation2	b0027 formation	1 b0027_def_map_cplx + 2 h2o --> 1 Def_mono_inact + 1 Map_mono_inact + 1 b0027_m + 1 for + 1 met-L	irreversible	Protein Maturation	0.00E+00	1.00E+09	0	2.80E+02	2.80E+02	0	4.28E+02	427.5779	0	8.79E+01	87.8805
b0028_fold_spon	b0028_m folding: spontanous	1 b0028_m --> 1 FkpB_mono	irreversible	Protein Folding	0.00E+00	1.00E+09	0	2.42E+02	2.42E+02	0	3.73E+02	372.7892	0	7.08E+01	70.836
b0028_mRNA_CONV	convsersion of mRNA to mRNA_1 (synthetic rxn)	1 b0028_mRNA --> 1 b0028_mRNA_1	irreversible	Translation	0.00E+00	1.00E+09	0	2.14E+01	2.14E+01	0	3.78E+01	37.83865	0	9.45E-02	0.0945
b0028_mRNA_degr1	Degradation of b0028_mRNA I	1 Orn_dim + 112 atp + 1 b0028_mRNA_2 + 1 degradosome + 112 h2o <==> 112 adp + 1 b0028_mRNA_2_degr + 112 h + 112 pi	reversible	mRNA degradation	-1.00E+09	1.00E+09	0	2.14E+01	2.14E+01	0	3.78E+01	37.83865	0	9.45E-02	0.0945
b0028_mRNA_degr2	Degradation of b0028_mRNA II	1 b0028_mRNA_2_degr + 449 h2o --> 1 Orn_dim_inact + 100 amp + 120 cmp + 1 degradosome_inact + 125 gmp + 449 h + 105 ump	irreversible	mRNA degradation	0.00E+00	9.45E-02	0	2.14E+01	2.14E+01	0	3.78E+01	37.83865	0	9.45E-02	0.0945
b0028_maturation1	Polypeptide b0028 peptide deformylas and methionine aminopeptidase complex	1 Def_mono + 1 Map_mono + 1 b0028_aa <==> 1 b0028_def_map_cplx	reversible	Protein Maturation	-1.00E+09	1.00E+09	0	2.42E+02	2.42E+02	0	3.73E+02	372.7892	0	7.08E+01	70.836
b0028_maturation2	b0028 formation	1 b0028_def_map_cplx + 2 h2o --> 1 Def_mono_inact + 1 Map_mono_inact + 1 b0028_m + 1 for + 1 met-L	irreversible	Protein Maturation	0.00E+00	1.00E+09	0	2.42E+02	2.42E+02	0	3.73E+02	372.7892	0	7.08E+01	70.836
b0029_fold_spon	b0029_m folding: spontanous	1 b0029_m --> 1 IspH_mono	irreversible	Protein Folding	0.00E+00	1.00E+09	0	4.76E+02	4.76E+02	0	7.71E+02	771.4558	0	9.21E+01	92.061
b0029_mRNA_CONV	convsersion of mRNA to mRNA_1 (synthetic rxn)	1 b0029_mRNA --> 1 b0029_mRNA_1	irreversible	Translation	0.00E+00	1.00E+09	0	2.14E+01	2.14E+01	0	3.78E+01	37.83865	0	9.45E-02	0.0945
b0029_mRNA_degr1	Degradation of b0029_mRNA I	1 Orn_dim + 237 atp + 1 b0029_mRNA_2 + 1 degradosome + 237 h2o <==> 237 adp + 1 b0029_mRNA_2_degr + 237 h + 237 pi	reversible	mRNA degradation	-1.00E+09	1.00E+09	0	2.14E+01	2.14E+01	0	3.78E+01	37.83865	0	9.45E-02	0.0945
b0029_mRNA_degr2	Degradation of b0029_mRNA II	1 b0029_mRNA_2_degr + 950 h2o --> 1 Orn_dim_inact + 220 amp + 228 cmp + 1 degradosome_inact + 296 gmp + 950 h + 207 ump	irreversible	mRNA degradation	0.00E+00	1.89E-01	0	2.14E+01	2.14E+01	0	3.78E+01	37.83865	0	9.45E-02	0.0945
b0029_maturation1	Polypeptide b0029 peptide deformylas and methionine aminopeptidase complex	1 Def_mono + 1 Map_mono + 1 b0029_aa <==> 1 b0029_def_map_cplx	reversible	Protein Maturation	-1.00E+09	1.00E+09	0	4.76E+02	4.76E+02	0	7.71E+02	771.4558	0	9.21E+01	92.061
b0029_maturation2	b0029 formation	1 b0029_def_map_cplx + 2 h2o --> 1 Def_mono_inact + 1 Map_mono_inact + 1 b0029_m + 1 for + 1 met-L	irreversible	Protein Maturation	0.00E+00	1.00E+09	0	4.76E+02	4.76E+02	0	7.71E+02	771.4558	0	9.21E+01	92.061
b0049_fold_spon	b0049_m folding: spontanous	1 b0049_m --> 1 ApaH_mono	irreversible	Protein Folding	0.00E+00	1.00E+09	0	1.03E+03	1.03E+03	0	1.75E+03	1752.699	0	8.80E+01	87.968
b0049_mRNA_CONV	convsersion of mRNA to mRNA_1 (synthetic rxn)	1 b0049_mRNA --> 1 b0049_mRNA_1	irreversible	Translation	0.00E+00	1.00E+09	0	2.75E+01	2.75E+01	0	4.85E+01	48.51944	0	1.68E-01	0.168
b0049_mRNA_degr1	Degradation of b0049_mRNA I	1 Orn_dim + 210 atp + 1 b0049_mRNA_2 + 1 degradosome + 210 h2o <==> 210 adp + 1 b0049_mRNA_2_degr + 210 h + 210 pi	reversible	mRNA degradation	-1.00E+09	1.00E+09	0	5.90E+01	5.90E+01	0	1.04E+02	104.2137	0	1.68E-01	0.168

b0049_mRNA_degr2	Degradation of b0049_mRNA II	1 b0049_mRNA_2_degr + 842 h2o --> 1 Orn_dim_inact + 185 amp + 218 cmp + 1 degradosome_inact + 252 gmp + 842 h + 188 ump	irreversible	mRNA degradation	0.00E+00	2.99E-01	0	5.90E+01	5.90E+01	0	1.04E+02	104.2137	0	1.68E-01	0.168
b0049_maturation1	Polypeptide b0049 peptide deformylas and methionine aminopeptidase complex	1 Def_mono + 1 Map_mono + 1 b0049_aa <==> 1 b0049_def_map_cplx	reversible	Protein Maturation	-1.00E+09	1.00E+09	0	1.03E+03	1.03E+03	0	1.75E+03	1752.699	0	8.80E+01	87.968
b0049_maturation2	b0049 formation	1 b0049_def_map_cplx + 2 h2o --> 1 Def_mono_inact + 1 Map_mono_inact + 1 b0049_m + 1 for + 1 met-L	irreversible	Protein Maturation	0.00E+00	1.00E+09	0	1.03E+03	1.03E+03	0	1.75E+03	1752.699	0	8.80E+01	87.968
b0049_v2_mRNA_CONV	convsersion of mRNA to mRNA_1 (synthetic rxn)	1 b0049_v2_mRNA --> 1 b0049_mRNA_1	irreversible	Translation	0.00E+00	1.00E+09	0	3.90E+01	3.90E+01	0	6.89E+01	68.92371	0	1.68E-01	0.168
b0049_v3_mRNA_CONV	convsersion of mRNA to mRNA_1 (synthetic rxn)	1 b0049_v3_mRNA --> 1 b0049_mRNA_1	irreversible	Translation	0.00E+00	1.00E+09	0	5.90E+01	5.90E+01	0	1.04E+02	104.2137	0	1.68E-01	0.168
b0050_fold_spon	b0050_m folding: spontanous	1 b0050_m --> 1 ApaG_mono	irreversible	Protein Folding	0.00E+00	1.00E+09	0	5.02E+02	5.02E+02	0	8.18E+02	818.3957	0	9.01E+01	90.076
b0050_mRNA_CONV	convsersion of mRNA to mRNA_1 (synthetic rxn)	1 b0050_mRNA --> 1 b0050_mRNA_1	irreversible	Translation	0.00E+00	1.00E+09	0	2.75E+01	2.75E+01	0	4.85E+01	48.51944	0	1.68E-01	0.168
b0050_mRNA_degr1	Degradation of b0050_mRNA I	1 Orn_dim + 94 atp + 1 b0050_mRNA_2 + 1 degradosome + 94 h2o <==> 94 adp + 1 b0050_mRNA_2_degr + 94 h + 94 pi	reversible	mRNA degradation	-1.00E+09	1.00E+09	0	5.90E+01	5.90E+01	0	1.04E+02	104.2137	0	1.68E-01	0.168
b0050_mRNA_degr2	Degradation of b0050_mRNA II	1 b0050_mRNA_2_degr + 377 h2o --> 1 Orn_dim_inact + 92 amp + 102 cmp + 1 degradosome_inact + 94 gmp + 377 h + 90 ump	irreversible	mRNA degradation	0.00E+00	2.34E-01	0	5.90E+01	5.90E+01	0	1.04E+02	104.2137	0	1.68E-01	0.168
b0050_maturation1	Polypeptide b0050 peptide deformylas and methionine aminopeptidase complex	1 Def_mono + 1 Map_mono + 1 b0050_aa <==> 1 b0050_def_map_cplx	reversible	Protein Maturation	-1.00E+09	1.00E+09	0	5.02E+02	5.02E+02	0	8.18E+02	818.3957	0	9.01E+01	90.076
b0050_maturation2	b0050 formation	1 b0050_def_map_cplx + 2 h2o --> 1 Def_mono_inact + 1 Map_mono_inact + 1 b0050_m + 1 for + 1 met-L	irreversible	Protein Maturation	0.00E+00	1.00E+09	0	5.02E+02	5.02E+02	0	8.18E+02	818.3957	0	9.01E+01	90.076
b0050_v2_mRNA_CONV	convsersion of mRNA to mRNA_1 (synthetic rxn)	1 b0050_v2_mRNA --> 1 b0050_mRNA_1	irreversible	Translation	0.00E+00	1.00E+09	0	3.90E+01	3.90E+01	0	6.89E+01	68.92371	0	1.68E-01	0.168
b0050_v3_mRNA_CONV	convsersion of mRNA to mRNA_1 (synthetic rxn)	1 b0050_v3_mRNA --> 1 b0050_mRNA_1	irreversible	Translation	0.00E+00	1.00E+09	0	5.90E+01	5.90E+01	0	1.04E+02	104.2137	0	1.68E-01	0.168
b0051_fold_spon	b0051_m folding: spontanous	1 b0051_m --> 1 KsgA_mono	irreversible	Protein Folding	0.00E+00	1.00E+09	0	1.10E+03	1.10E+03	0	1.89E+03	1891.517	0	7.92E+01	79.168
b0051_mRNA_CONV	convsersion of mRNA to mRNA_1 (synthetic rxn)	1 b0051_mRNA --> 1 b0051_mRNA_1	irreversible	Translation	0.00E+00	1.00E+09	0	2.75E+01	2.75E+01	0	4.85E+01	48.51944	0	1.68E-01	0.168
b0051_mRNA_degr1	Degradation of b0051_mRNA I	1 Orn_dim + 204 atp + 1 b0051_mRNA_2 + 1 degradosome + 204 h2o <==> 204 adp + 1 b0051_mRNA_2_degr + 204 h + 204 pi	reversible	mRNA degradation	-1.00E+09	1.00E+09	0	6.43E+01	6.43E+01	0	1.13E+02	113.4398	0	1.68E-01	0.168
b0051_mRNA_degr2	Degradation of b0051_mRNA II	1 b0051_mRNA_2_degr + 817 h2o --> 1 Orn_dim_inact + 195 amp + 220 cmp + 1 degradosome_inact + 215 gmp + 817 h + 188 ump	irreversible	mRNA degradation	0.00E+00	1.68E-01	0	6.43E+01	6.43E+01	0	1.13E+02	113.4398	0	1.68E-01	0.168
b0051_maturation1	Polypeptide b0051 peptide deformylas and methionine aminopeptidase complex	1 Def_mono + 1 Map_mono + 1 b0051_aa <==> 1 b0051_def_map_cplx	reversible	Protein Maturation	-1.00E+09	1.00E+09	0	1.10E+03	1.10E+03	0	1.89E+03	1891.517	0	7.92E+01	79.168
b0051_maturation2	b0051 formation	1 b0051_def_map_cplx + 2 h2o --> 1 Def_mono_inact + 1 Map_mono_inact + 1 b0051_m + 1 for + 1 met-L	irreversible	Protein Maturation	0.00E+00	1.00E+09	0	1.10E+03	1.10E+03	0	1.89E+03	1891.517	0	7.92E+01	79.168
b0051_v1_mRNA_CONV	convsersion of mRNA to mRNA_1 (synthetic rxn)	1 b0051_v1_mRNA --> 1 b0051_mRNA_1	irreversible	Translation	0.00E+00	1.00E+09	0	6.43E+01	6.43E+01	0	1.13E+02	113.4398	0	1.68E-01	0.168
b0051_v2_mRNA_CONV	convsersion of mRNA to mRNA_1 (synthetic rxn)	1 b0051_v2_mRNA --> 1 b0051_mRNA_1	irreversible	Translation	0.00E+00	1.00E+09	0	3.90E+01	3.90E+01	0	6.89E+01	68.92371	0	1.68E-01	0.168
b0051_v3_mRNA_CONV	convsersion of mRNA to mRNA_1 (synthetic rxn)	1 b0051_v3_mRNA --> 1 b0051_mRNA_1	irreversible	Translation	0.00E+00	1.00E+09	0	5.90E+01	5.90E+01	0	1.04E+02	104.2137	0	1.68E-01	0.168
b0052_fold_spon	b0052_m folding: spontanous	1 b0052_m --> 1 PdxA_mono	irreversible	Protein Folding	0.00E+00	1.00E+09	0	1.31E+03	1.31E+03	0	2.24E+03	2241.617	0	8.95E+01	89.452
b0052_mRNA_CONV	convsersion of mRNA to mRNA_1 (synthetic rxn)	1 b0052_mRNA --> 1 b0052_mRNA_1	irreversible	Translation	0.00E+00	1.00E+09	0	2.75E+01	2.75E+01	0	4.85E+01	48.51944	0	1.68E-01	0.168
b0052_mRNA_degr1	Degradation of b0052_mRNA I	1 Orn_dim + 247 atp + 1 b0052_mRNA_2 + 1 degradosome + 247 h2o <==> 247 adp + 1 b0052_mRNA_2_degr + 247 h + 247 pi	reversible	mRNA degradation	-1.00E+09	1.00E+09	0	6.43E+01	6.43E+01	0	1.13E+02	113.4398	0	1.68E-01	0.168
b0052_mRNA_degr2	Degradation of b0052_mRNA II	1 b0052_mRNA_2_degr + 988 h2o --> 1 Orn_dim_inact + 204 amp + 277 cmp + 1 degradosome_inact + 281 gmp + 988 h + 227 ump	irreversible	mRNA degradation	0.00E+00	2.07E-01	0	6.43E+01	6.43E+01	0	1.13E+02	113.4398	0	1.68E-01	0.168
b0052_maturation1	Polypeptide b0052 peptide deformylas and methionine aminopeptidase complex	1 Def_mono + 1 Map_mono + 1 b0052_aa <==> 1 b0052_def_map_cplx	reversible	Protein Maturation	-1.00E+09	1.00E+09	0	1.31E+03	1.31E+03	0	2.24E+03	2241.617	0	8.95E+01	89.452
b0052_maturation2	b0052 formation	1 b0052_def_map_cplx + 2 h2o --> 1 Def_mono_inact + 1 Map_mono_inact + 1 b0052_m + 1 for + 1 met-L	irreversible	Protein Maturation	0.00E+00	1.00E+09	0	1.31E+03	1.31E+03	0	2.24E+03	2241.617	0	8.95E+01	89.452
b0052_v1_mRNA_CONV	convsersion of mRNA to mRNA_1 (synthetic rxn)	1 b0052_v1_mRNA --> 1 b0052_mRNA_1	irreversible	Translation	0.00E+00	1.00E+09	0	6.43E+01	6.43E+01	0	1.13E+02	113.4398	0	1.68E-01	0.168
b0052_v2_mRNA_CONV	convsersion of mRNA to mRNA_1 (synthetic rxn)	1 b0052_v2_mRNA --> 1 b0052_mRNA_1	irreversible	Translation	0.00E+00	1.00E+09	0	3.90E+01	3.90E+01	0	6.89E+01	68.92371	0	1.68E-01	0.168
b0053_fold_spon	b0053_m folding: spontanous	1 b0053_m --> 1 SurA_mono	irreversible	Protein Folding	0.00E+00	1.00E+09	0	7.96E+02	7.96E+02	0	1.32E+03	1321.986	0	1.13E+02	113.2
b0053_mRNA_CONV	convsersion of mRNA to mRNA_1 (synthetic rxn)	1 b0053_mRNA --> 1 b0053_mRNA_1	irreversible	Translation	0.00E+00	1.00E+09	0	2.75E+01	2.75E+01	0	4.85E+01	48.51944	0	1.68E-01	0.168
b0053_mRNA_degr1	Degradation of b0053_mRNA I	1 Orn_dim + 321 atp + 1 b0053_mRNA_2 + 1 degradosome + 321 h2o <==> 321 adp + 1 b0053_mRNA_2_degr + 321 h + 321 pi	reversible	mRNA degradation	-1.00E+09	1.00E+09	0	2.75E+01	2.75E+01	0	4.85E+01	48.51944	0	1.68E-01	0.168
b0053_mRNA_degr2	Degradation of b0053_mRNA II	1 b0053_mRNA_2_degr + 1286 h2o --> 1 Orn_dim_inact + 336 amp 1 atp + 344 cmp + 1 degradosome_inact + 349 gmp + 1286 h + 257 ump	irreversible	mRNA degradation	0.00E+00	2.07E-01	0	2.75E+01	2.75E+01	0	4.85E+01	48.51944	0	1.68E-01	0.168
b0053_maturation1	Polypeptide b0053 peptide deformylas complex	1 Def_mono + 1 b0053_aa <==> 1 b0053_def_cplx	reversible	Protein Maturation	-1.00E+09	1.00E+09	0	7.96E+02	7.96E+02	0	1.32E+03	1321.986	0	1.13E+02	113.2
b0053_maturation2	b0053 formation	1 b0053_def_cplx + 9 h2o --> 1 Def_mono_inact + 1 ala-L + 1 asn-L 1 b0053_m + 1 for + 1 ile-L + 1 leu-L + 1 lys-L + 1 ser-L + 1 tyr-L + 1 val-L	irreversible	Protein Maturation	0.00E+00	1.00E+09	0	7.96E+02	7.96E+02	0	1.32E+03	1321.986	0	1.13E+02	113.2
b0058_fold_spon	b0058_m folding: spontanous	1 b0058_m --> 1 RluA_mono	irreversible	Protein Folding	0.00E+00	1.00E+09	0	4.97E+02	4.97E+02	0	8.11E+02	811.2431	0	8.91E+01	89.112
b0058_mRNA_CONV	convsersion of mRNA to mRNA_1 (synthetic rxn)	1 b0058_mRNA --> 1 b0058_mRNA_1	irreversible	Translation	0.00E+00	1.00E+09	0	3.42E+01	3.42E+01	0	6.04E+01	60.36359	0	1.86E-01	0.186
b0058_mRNA_degr1	Degradation of b0058_mRNA I	1 Orn_dim + 165 atp + 1 b0058_mRNA_2 + 1 degradosome + 165 h2o <==> 165 adp + 1 b0058_mRNA_2_degr + 165 h + 165 pi	reversible	mRNA degradation	-1.00E+09	1.00E+09	0	3.42E+01	3.42E+01	0	6.04E+01	60.36359	0	1.86E-01	0.186
b0058_mRNA_degr2	Degradation of b0058_mRNA II	1 b0058_mRNA_2_degr + 659 h2o --> 1 Orn_dim_inact + 161 amp + 154 cmp + 1 degradosome_inact + 202 gmp + 659 h + 143 ump	irreversible	mRNA degradation	0.00E+00	1.86E-01	0	3.42E+01	3.42E+01	0	6.04E+01	60.36359	0	1.86E-01	0.186
b0058_maturation1	Polypeptide b0058 peptide deformylas and methionine aminopeptidase complex	1 Def_mono + 1 Map_mono + 1 b0058_aa <==> 1 b0058_def_map_cplx	reversible	Protein Maturation	-1.00E+09	1.00E+09	0	4.97E+02	4.97E+02	0	8.11E+02	811.2431	0	8.91E+01	89.112
b0058_maturation2	b0058 formation	1 b0058_def_map_cplx + 2 h2o --> 1 Def_mono_inact + 1 Map_mono_inact + 1 b0058_m + 1 for + 1 met-L	irreversible	Protein Maturation	0.00E+00	1.00E+09	0	4.97E+02	4.97E+02	0	8.11E+02	811.2431	0	8.91E+01	89.112
b0059_fold_KJE_1	b0059_m folding: KJE mediated	1 DnaJ_dim + 1 DnaK_mono.ATP + 1 b0059_m <==> 1 b0059_m_DnaKJ_complex	reversible	Protein Folding	-1.00E+09	1.00E+09	0	2.01E+03	2.01E+03	0	2.40E+03	2396.157	0	1.02E+02	102.256
b0059_fold_KJE_2	b0059_m folding: KJE mediated	1 GrpE_dim + 1 b0059_m_DnaKJ_complex + 1 h2o --> 1 DnaJ_dim_inact + 1 HepA_DnaK_GrpE_complex + 1 adp + 1 h + 1 pi	irreversible	Protein Folding	0.00E+00	1.00E+09	0	2.01E+03	2.01E+03	0	2.40E+03	2396.157	0	1.02E+02	102.256
b0059_fold_KJE_3	b0059_m folding: KJE mediated	1 HepA_DnaK_GrpE_complex + 1 atp --> 1 DnaK_mono.ATP_inact + 1 GrpE_dim_inact + 1 HepA_mono	irreversible	Protein Folding	0.00E+00	1.00E+09	0	2.01E+03	2.01E+03	0	2.40E+03	2396.157	0	1.02E+02	102.256
b0059_fold_spon	b0059_m folding: spontanous	1 b0059_m --> 1 HepA_mono	irreversible	Protein Folding	0.00E+00	1.00E+09	0	2.01E+03	2.01E+03	0	2.40E+03	2396.157	0	1.02E+02	102.256
b0059_mRNA_degr1	Degradation of b0059_mRNA I	1 Orn_dim + 726 atp + 1 b0059_mRNA_2 + 1 degradosome + 726 h2o <==> 726 adp + 1 b0059_mRNA_2_degr + 726 h + 726 pi	reversible	mRNA degradation	-1.00E+09	1.00E+09	0	3.42E+01	3.42E+01	0	6.04E+01	60.36359	0	1.86E-01	0.186
b0059_mRNA_degr2	Degradation of b0059_mRNA II	1 b0059_mRNA_2_degr + 2906 h2o --> 1 Orn_dim_inact + 676 amp 1 atp + 777 cmp + 1 degradosome_inact + 819 gmp + 2906 h + 634 ump	irreversible	mRNA degradation	0.00E+00	1.89E-01	0	3.42E+01	3.42E+01	0	6.04E+01	60.36359	0	1.86E-01	0.186

b0059_maturation1	Polypeptide b0059 peptide deformylas and methionine aminopeptidase complex	1 Def_mono + 1 Map_mono + 1 b0059_aa <==> 1 b0059_def_map_cplx	reversible	Protein Maturation	-1.00E+09	1.00E+09	0	2.01E+03	2.01E+03	0	2.40E+03	2396.157	0	1.02E+02	102.256
b0059_maturation2	b0059 formation	1 b0059_def_map_cplx + 2 h2o --> 1 Def_mono_inact + 1 Map_mono_inact + 1 b0059_m + 1 for + 1 met-L	irreversible	Protein Maturation	0.00E+00	1.00E+09	0	2.01E+03	2.01E+03	0	2.40E+03	2396.157	0	1.02E+02	102.256
b0059_v1_mRNA_CONV	convsersion of mRNA to mRNA_1 (synthetic rxn)	1 b0059_v1_mRNA --> 1 b0059_mRNA_1	irreversible	Translation	0.00E+00	1.00E+09	0	3.42E+01	3.42E+01	0	6.04E+01	60.36359	0	1.86E-01	0.186
b0144_DNA_act_bind	b0144_DNA binding of activator	1 b0144_DNA_neu --> 1 b0144_DNA_act	irreversible	Transcription Regulation	0.00E+00	1.00E+09	0	1.23E+02	1.23E+02	0	2.18E+02	217.6818	0	2.03E-01	0.203
b0144_fold_GroEL/ES_1	b0144_m folding: GroEL/ES mediated: polypeptide is going in GroEL/ES complex	1 GroEL.(7)ADP.cisGroES + 7 atp + 1 b0144_m + 1 transGroES_hepta --> 7 adp + 1 b0144_m_GroEL.(7)ATP.transGroES + 1 cisGroES_hepta	irreversible	Protein Folding	0.00E+00	1.00E+09	0	2.31E+03	2.31E+03	0	4.01E+03	4010.973	0	9.64E+01	96.354
b0144_fold_GroEL/ES_2	b0144_m folding: GroEL/ES mediated: folding of polypeptide under ATP hydrolysis	1 b0144_m_GroEL.(7)ATP.transGroES + 7 h2o --> 1 YadB_GroEL.(7)ADP.transGroES + 7 h + 7 pi	irreversible	Protein Folding	0.00E+00	1.00E+09	0	2.31E+03	2.31E+03	0	4.01E+03	4010.973	0	9.64E+01	96.354
b0144_fold_GroEL/ES_3	b0144_m folding: GroEL/ES mediated: release of native protein	1 YadB_GroEL.(7)ADP.transGroES --> 1 GroEL.(7)ADP.transGroES + 1 YadB_mono	irreversible	Protein Folding	0.00E+00	1.00E+09	0	2.31E+03	2.31E+03	0	4.01E+03	4010.973	0	9.64E+01	96.354
b0144_fold_KJE_1	b0144_m folding: KJE mediated	1 DnaJ_dim + 1 DnaK_mono.ATP + 1 b0144_m <==> 1 b0144_m_DnaKJ_complex	reversible	Protein Folding	-1.00E+09	1.00E+09	0	2.31E+03	2.31E+03	0	4.01E+03	4010.973	0	9.64E+01	96.354
b0144_fold_KJE_2	b0144_m folding: KJE mediated	1 GrpE_dim + 1 b0144_m_DnaKJ_complex + 1 h2o --> 1 DnaJ_dim_inact + 1 YadB_DnaK_GrpE_complex + 1 adp + 1 h + 1 pi	irreversible	Protein Folding	0.00E+00	1.00E+09	0	2.31E+03	2.31E+03	0	4.01E+03	4010.973	0	9.64E+01	96.354
b0144_fold_KJE_3	b0144_m folding: KJE mediated	1 YadB_DnaK_GrpE_complex + 1 atp --> 1 DnaK_mono.ATP_inact + 1 GrpE_dim_inact + 1 YadB_mono	irreversible	Protein Folding	0.00E+00	1.00E+09	0	2.31E+03	2.31E+03	0	4.01E+03	4010.973	0	9.64E+01	96.354
b0144_mRNA_CONV	convsersion of mRNA to mRNA_1 (synthetic rxn)	1 b0144_mRNA --> 1 b0144_mRNA_1	irreversible	Translation	0.00E+00	1.00E+09	0	1.23E+02	1.23E+02	0	2.18E+02	217.6818	0	2.03E-01	0.203
b0144_mRNA_degr1	Degradation of b0144_mRNA I	1 Orn_dim + 231 atp + 1 b0144_mRNA_2 + 1 degradosome + 231 h2o <==> 231 adp + 1 b0144_mRNA_2_degr + 231 h + 231 pi	reversible	mRNA degradation	-1.00E+09	1.00E+09	0	1.23E+02	1.23E+02	0	2.18E+02	217.6818	0	2.03E-01	0.203
b0144_mRNA_degr2	Degradation of b0144_mRNA II	1 b0144_mRNA_2_degr + 926 h2o --> 1 Orn_dim_inact + 208 amp + 1 atp + 259 cmp + 1 degradosome_inact + 238 gmp + 926 h + 221 ump	irreversible	mRNA degradation	0.00E+00	2.03E-01	0	1.23E+02	1.23E+02	0	2.18E+02	217.6818	0	2.03E-01	0.203
b0144_maturation1	Polypeptide b0144 peptide deformylas and methionine aminopeptidase complex	1 Def_mono + 1 Map_mono + 1 b0144_aa <==> 1 b0144_def_map_cplx	reversible	Protein Maturation	-1.00E+09	1.00E+09	0	2.31E+03	2.31E+03	0	4.01E+03	4010.973	0	9.64E+01	96.354
b0144_maturation2	b0144 formation	1 b0144_def_map_cplx + 12 h2o --> 1 Def_mono_inact + 1 Map_mono_inact + 1 b0144_m + 1 for + 1 glu-L + 2 leu-L + 1 lys-L + 1 met-L + 2 phe-L + 2 pro-L + 1 tyr-L	irreversible	Protein Maturation	0.00E+00	1.00E+09	0	2.31E+03	2.31E+03	0	4.01E+03	4010.973	0	9.64E+01	96.354
b0166_fold_spon	b0166_m folding: spontanous	1 b0166_m --> 1 DapD_mono	irreversible	Protein Folding	0.00E+00	1.00E+09	0	5.33E+02	5.33E+02	0	8.70E+02	870.2896	0	9.60E+01	95.952
b0166_mRNA_degr1	Degradation of b0166_mRNA I	1 Orn_dim + 206 atp + 1 b0166_mRNA_2 + 1 degradosome + 206 h2o <==> 206 adp + 1 b0166_mRNA_2_degr + 206 h + 206 pi	reversible	mRNA degradation	-1.00E+09	1.00E+09	0	2.75E+01	2.75E+01	0	4.86E+01	48.6031	0	2.07E-01	0.207
b0166_mRNA_degr2	Degradation of b0166_mRNA II	1 b0166_mRNA_2_degr + 824 h2o --> 1 Orn_dim_inact + 198 amp + 1 atp + 204 cmp + 1 degradosome_inact + 234 gmp + 824 h + 188 ump	irreversible	mRNA degradation	0.00E+00	2.91E-01	0	2.75E+01	2.75E+01	0	4.86E+01	48.6031	0	2.07E-01	0.207
b0166_maturation1	Polypeptide b0166 peptide deformylas complex	1 Def_mono + 1 b0166_aa <==> 1 b0166_def_cplx	reversible	Protein Maturation	-1.00E+09	1.00E+09	0	5.33E+02	5.33E+02	0	8.70E+02	870.2896	0	9.60E+01	95.952
b0166_maturation2	b0166 formation	1 b0166_def_cplx + 1 h2o --> 1 Def_mono_inact + 1 b0166_m + 1 for	irreversible	Protein Maturation	0.00E+00	1.00E+09	0	5.33E+02	5.33E+02	0	8.70E+02	870.2896	0	9.60E+01	95.952
b0166_v1_mRNA_CONV	convsersion of mRNA to mRNA_1 (synthetic rxn)	1 b0166_v1_mRNA --> 1 b0166_mRNA_1	irreversible	Translation	0.00E+00	1.00E+09	0	2.75E+01	2.75E+01	0	4.86E+01	48.6031	0	2.07E-01	0.207
b0167_fold_GroEL/ES_1	b0167_m folding: GroEL/ES mediated: polypeptide is going in GroEL/ES complex	1 GroEL.(7)ADP.cisGroES + 7 atp + 1 b0167_m + 1 transGroES_hepta --> 7 adp + 1 b0167_m_GroEL.(7)ATP.transGroES + 1 cisGroES_hepta	irreversible	Protein Folding	0.00E+00	1.00E+09	0	1.54E+03	1.54E+03	0	1.99E+03	1991.064	0	1.14E+02	114.244
b0167_fold_GroEL/ES_2	b0167_m folding: GroEL/ES mediated: folding of polypeptide under ATP hydrolysis	1 b0167_m_GroEL.(7)ATP.transGroES + 7 h2o --> 1 GlnD_GroEL.(7)ADP.transGroES + 7 h + 7 pi	irreversible	Protein Folding	0.00E+00	1.00E+09	0	1.54E+03	1.54E+03	0	1.99E+03	1991.064	0	1.14E+02	114.244
b0167_fold_GroEL/ES_3	b0167_m folding: GroEL/ES mediated: release of native protein	1 GlnD_GroEL.(7)ADP.transGroES --> 1 GlnD_mono + 1 GroEL.(7)ADP.transGroES	irreversible	Protein Folding	0.00E+00	1.00E+09	0	1.54E+03	1.54E+03	0	1.99E+03	1991.064	0	1.14E+02	114.244
b0167_fold_KJE_1	b0167_m folding: KJE mediated	1 DnaJ_dim + 1 DnaK_mono.ATP + 1 b0167_m <==> 1 b0167_m_DnaKJ_complex	reversible	Protein Folding	-1.00E+09	1.00E+09	0	1.54E+03	1.54E+03	0	1.99E+03	1991.064	0	1.14E+02	114.244
b0167_fold_KJE_2	b0167_m folding: KJE mediated	1 GrpE_dim + 1 b0167_m_DnaKJ_complex + 1 h2o --> 1 DnaJ_dim_inact + 1 GlnD_DnaK_GrpE_complex + 1 adp + 1 h + 1 pi	irreversible	Protein Folding	0.00E+00	1.00E+09	0	1.54E+03	1.54E+03	0	1.99E+03	1991.064	0	1.14E+02	114.244
b0167_fold_KJE_3	b0167_m folding: KJE mediated	1 GlnD_DnaK_GrpE_complex + 1 atp --> 1 DnaK_mono.ATP_inact + 1 GlnD_mono + 1 GrpE_dim_inact	irreversible	Protein Folding	0.00E+00	1.00E+09	0	1.54E+03	1.54E+03	0	1.99E+03	1991.064	0	1.14E+02	114.244
b0167_mRNA_CONV	convsersion of mRNA to mRNA_1 (synthetic rxn)	1 b0167_mRNA --> 1 b0167_mRNA_1	irreversible	Translation	0.00E+00	1.00E+09	0	2.75E+01	2.75E+01	0	4.86E+01	48.6031	0	2.07E-01	0.207
b0167_mRNA_degr1	Degradation of b0167_mRNA I	1 Orn_dim + 668 atp + 1 b0167_mRNA_2 + 1 degradosome + 668 h2o <==> 668 adp + 1 b0167_mRNA_2_degr + 668 h + 668 pi	reversible	mRNA degradation	-1.00E+09	1.00E+09	0	2.75E+01	2.75E+01	0	4.86E+01	48.6031	0	2.07E-01	0.207
b0167_mRNA_degr2	Degradation of b0167_mRNA II	1 b0167_mRNA_2_degr + 2672 h2o --> 1 Orn_dim_inact + 623 amp 747 cmp + 1 degradosome_inact + 695 gmp + 2672 h + 608 ump	irreversible	mRNA degradation	0.00E+00	2.34E-01	0	2.75E+01	2.75E+01	0	4.86E+01	48.6031	0	2.07E-01	0.207
b0167_maturation1	Polypeptide b0167 peptide deformylas and methionine aminopeptidase complex	1 Def_mono + 1 Map_mono + 1 b0167_aa <==> 1 b0167_def_map_cplx	reversible	Protein Maturation	-1.00E+09	1.00E+09	0	1.54E+03	1.54E+03	0	1.99E+03	1991.064	0	1.14E+02	114.244
b0167_maturation2	b0167 formation	1 b0167_def_map_cplx + 2 h2o --> 1 Def_mono_inact + 1 Map_mono_inact + 1 b0167_m + 1 for + 1 met-L	irreversible	Protein Maturation	0.00E+00	1.00E+09	0	1.54E+03	1.54E+03	0	1.99E+03	1991.064	0	1.14E+02	114.244
b0168_fold_spon	b0168_m folding: spontanous	1 b0168_m_Fe --> 1 Map_mono	irreversible	Protein Folding	0.00E+00	1.00E+09	0	5.14E+02	5.14E+02	0	8.30E+02	829.6965	0	1.04E+02	103.755
b0168_ion_BIND	Binding of ions to b0168	1 b0168_m + 2 fe2 <==> 1 b0168_m_Fe	reversible	Metallo-ion Binding	-1.00E+09	1.00E+09	0	5.14E+02	5.14E+02	0	8.30E+02	829.6965	0	1.04E+02	103.755
b0168_mRNA_CONV	convsersion of mRNA to mRNA_1 (synthetic rxn)	1 b0168_mRNA --> 1 b0168_mRNA_1	irreversible	Translation	0.00E+00	1.00E+09	0	2.75E+01	2.75E+01	0	4.86E+01	48.6031	0	2.07E-01	0.207
b0168_mRNA_degr1	Degradation of b0168_mRNA I	1 Orn_dim + 198 atp + 1 b0168_mRNA_2 + 1 degradosome + 198 h2o <==> 198 adp + 1 b0168_mRNA_2_degr + 198 h + 198 pi	reversible	mRNA degradation	-1.00E+09	1.00E+09	0	2.75E+01	2.75E+01	0	4.86E+01	48.6031	0	2.07E-01	0.207
b0168_mRNA_degr2	Degradation of b0168_mRNA II	1 b0168_mRNA_2_degr + 794 h2o --> 1 Orn_dim_inact + 217 amp + 1 atp + 196 cmp + 1 degradosome_inact + 205 gmp + 794 h + 176 ump	irreversible	mRNA degradation	0.00E+00	2.07E-01	0	2.75E+01	2.75E+01	0	4.86E+01	48.6031	0	2.07E-01	0.207
b0168_maturation1	Polypeptide b0168 peptide deformylas and methionine aminopeptidase complex	1 Def_mono + 1 Map_mono + 1 b0168_aa <==> 1 b0168_def_map_cplx	reversible	Protein Maturation	-1.00E+09	1.00E+09	0	5.14E+02	5.14E+02	0	8.30E+02	829.6965	0	1.04E+02	103.755
b0168_maturation2	b0168 formation	1 b0168_def_map_cplx + 2 h2o --> 1 Def_mono_inact + 1 Map_mono_inact + 1 b0168_m + 1 for + 1 met-L	irreversible	Protein Maturation	0.00E+00	1.00E+09	0	5.14E+02	5.14E+02	0	8.30E+02	829.6965	0	1.04E+02	103.755
b0169_fold_spon	b0169_m folding: spontanous	1 b0169_m --> 1 RpsB_mono	irreversible	Protein Folding	0.00E+00	1.00E+09	8.44E+01	1.13E+02	2.81E+01	6.22E+01	8.29E+01	20.72	6.22E+01	8.29E+01	20.72
b0169_mRNA_CONV	convsersion of mRNA to mRNA_1 (synthetic rxn)	1 b0169_mRNA --> 1 b0169_mRNA_1	irreversible	Translation	0.00E+00	1.00E+09	0	7.50E+01	7.50E+01	0	8.09E+01	80.94127	0	5.18E-01	0.518
b0169_mRNA_degr1	Degradation of b0169_mRNA I	1 Orn_dim + 181 atp + 1 b0169_mRNA_2 + 1 degradosome + 181 h2o <==> 181 adp + 1 b0169_mRNA_2_degr + 181 h + 181 pi	reversible	mRNA degradation	-1.00E+09	1.00E+09	0	7.50E+01	7.50E+01	0	8.09E+01	80.94127	0	5.18E-01	0.518
b0169_mRNA_degr2	Degradation of b0169_mRNA II	1 b0169_mRNA_2_degr + 725 h2o --> 1 Orn_dim_inact + 186 amp + 1 atp + 184 cmp + 1 degradosome_inact + 189 gmp + 725 h + 166 ump	irreversible	mRNA degradation	0.00E+00	5.18E-01	0	7.50E+01	7.50E+01	0	8.09E+01	80.94127	0	5.18E-01	0.518
b0169_maturation1	Polypeptide b0169 peptide deformylas complex	1 Def_mono + 1 b0169_aa <==> 1 b0169_def_cplx	reversible	Protein Maturation	-1.00E+09	1.00E+09	8.44E+01	1.13E+02	2.81E+01	6.22E+01	8.29E+01	20.72	6.22E+01	8.29E+01	20.72
b0169_maturation2	b0169 formation	1 b0169_def_cplx + 2 h2o --> 1 Def_mono_inact + 1 b0169_m + 1 for + 1 met-L	irreversible	Protein Maturation	0.00E+00	1.00E+09	8.44E+01	1.13E+02	2.81E+01	6.22E+01	8.29E+01	20.72	6.22E+01	8.29E+01	20.72

b0170_fold_GroEL/ES_1	b0170_m folding: GroEL/ES mediated: polypeptide is going in GroEL/ES complex	1 GroEL.(7)ADP.cisGroES + 7 atp + 1 b0170_m + 1 transGroES_hepta --> 7 adp + 1 b0170_m_GroEL.(7)ATP.transGroES + 1 cisGroES_hepta	irreversible	Protein Folding	0.00E+00	1.00E+09	0	1.45E+03	1.45E+03	0	1.54E+03	1543.06	0	2.56E+02	256.288
b0170_fold_GroEL/ES_2	b0170_m folding: GroEL/ES mediated: folding of polypeptide under ATP hydrolysis	1 b0170_m_GroEL.(7)ATP.transGroES + 7 h2o --> 1 Tsf_GroEL.(7)ADP.transGroES + 7 h + 7 pi	irreversible	Protein Folding	0.00E+00	1.00E+09	0	1.45E+03	1.45E+03	0	1.54E+03	1543.06	0	2.56E+02	256.288
b0170_fold_GroEL/ES_3	b0170_m folding: GroEL/ES mediated: release of native protein	1 Tsf_GroEL.(7)ADP.transGroES --> 1 GroEL.(7)ADP.transGroES + 1 Tsf_mono	irreversible	Protein Folding	0.00E+00	1.00E+09	0	1.45E+03	1.45E+03	0	1.54E+03	1543.06	0	2.56E+02	256.288
b0170_fold_KJE_1	b0170_m folding: KJE mediated	1 DnaJ_dim + 1 DnaK_mono.ATP + 1 b0170_m <==> 1 b0170_m_DnaKJ_complex	reversible	Protein Folding	-1.00E+09	1.00E+09	0	1.45E+03	1.45E+03	0	1.54E+03	1543.06	0	2.56E+02	256.288
b0170_fold_KJE_2	b0170_m folding: KJE mediated	1 GrpE_dim + 1 b0170_m_DnaKJ_complex + 1 h2o --> 1 DnaJ_dim_inact + 1 Tsf_DnaK_GrpE_complex + 1 adp + 1 h + 1 pi	irreversible	Protein Folding	0.00E+00	1.00E+09	0	1.45E+03	1.45E+03	0	1.54E+03	1543.06	0	2.56E+02	256.288
b0170_fold_KJE_3	b0170_m folding: KJE mediated	1 Tsf_DnaK_GrpE_complex + 1 atp --> 1 DnaK_mono.ATP_inact + 1 GrpE_dim_inact + 1 Tsf_mono	irreversible	Protein Folding	0.00E+00	1.00E+09	0	1.45E+03	1.45E+03	0	1.54E+03	1543.06	0	2.56E+02	256.288
b0170_fold_spon	b0170_m folding: spontanous	1 b0170_m --> 1 Tsf_mono	irreversible	Protein Folding	0.00E+00	1.00E+09	0	1.45E+03	1.45E+03	0	1.54E+03	1543.06	0	2.56E+02	256.288
b0170_mRNA_CONV	convsersion of mRNA to mRNA_1 (synthetic rxn)	1 b0170_mRNA --> 1 b0170_mRNA_1	irreversible	Translation	0.00E+00	1.00E+09	0	7.50E+01	7.50E+01	0	8.09E+01	80.94127	0	5.18E-01	0.518
b0170_mRNA_degr1	Degradation of b0170_mRNA I	1 Orn_dim + 213 atp + 1 b0170_mRNA_2 + 1 degradosome + 213 h2o <==> 213 adp + 1 b0170_mRNA_2_degr + 213 h + 213 pi	reversible	mRNA degradation	-1.00E+09	1.00E+09	0	7.50E+01	7.50E+01	0	8.09E+01	80.94127	0	5.18E-01	0.518
b0170_mRNA_degr2	Degradation of b0170_mRNA II	1 b0170_mRNA_2_degr + 851 h2o --> 1 Orn_dim_inact + 247 amp + 186 cmp + 1 degradosome_inact + 239 gmp + 851 h + 180 ump	irreversible	mRNA degradation	0.00E+00	5.18E-01	0	7.50E+01	7.50E+01	0	8.09E+01	80.94127	0	5.18E-01	0.518
b0170_maturation1	Polypeptide b0170 peptide deformylas and methionine aminopeptidase complex	1 Def_mono + 1 Map_mono + 1 b0170_aa <==> 1 b0170_def_map_cplx	reversible	Protein Maturation	-1.00E+09	1.00E+09	0	1.45E+03	1.45E+03	0	1.54E+03	1543.06	0	2.56E+02	256.288
b0170_maturation2	b0170 formation	1 b0170_def_map_cplx + 2 h2o --> 1 Def_mono_inact + 1 Map_mono_inact + 1 b0170_m + 1 for + 1 met-L	irreversible	Protein Maturation	0.00E+00	1.00E+09	0	1.45E+03	1.45E+03	0	1.54E+03	1543.06	0	2.56E+02	256.288
b0172_fold_GroEL/ES_1	b0172_m folding: GroEL/ES mediated: polypeptide is going in GroEL/ES complex	1 GroEL.(7)ADP.cisGroES + 7 atp + 1 b0172_m + 1 transGroES_hepta --> 7 adp + 1 b0172_m_GroEL.(7)ATP.transGroES + 1 cisGroES_hepta	irreversible	Protein Folding	0.00E+00	1.00E+09	0	2.65E+03	2.65E+03	0	4.53E+03	4528.892	0	1.96E+02	195.99
b0172_fold_GroEL/ES_2	b0172_m folding: GroEL/ES mediated: folding of polypeptide under ATP hydrolysis	1 b0172_m_GroEL.(7)ATP.transGroES + 7 h2o --> 1 Rrf_GroEL.(7)ADP.transGroES + 7 h + 7 pi	irreversible	Protein Folding	0.00E+00	1.00E+09	0	2.65E+03	2.65E+03	0	4.53E+03	4528.892	0	1.96E+02	195.99
b0172_fold_GroEL/ES_3	b0172_m folding: GroEL/ES mediated: release of native protein	1 Rrf_GroEL.(7)ADP.transGroES --> 1 GroEL.(7)ADP.transGroES + 1 Rrf_mono	irreversible	Protein Folding	0.00E+00	1.00E+09	0	2.65E+03	2.65E+03	0	4.53E+03	4528.892	0	1.96E+02	195.99
b0172_fold_KJE_1	b0172_m folding: KJE mediated	1 DnaJ_dim + 1 DnaK_mono.ATP + 1 b0172_m <==> 1 b0172_m_DnaKJ_complex	reversible	Protein Folding	-1.00E+09	1.00E+09	0	2.65E+03	2.65E+03	0	4.53E+03	4528.892	0	1.96E+02	195.99
b0172_fold_KJE_2	b0172_m folding: KJE mediated	1 GrpE_dim + 1 b0172_m_DnaKJ_complex + 1 h2o --> 1 DnaJ_dim_inact + 1 Rrf_DnaK_GrpE_complex + 1 adp + 1 h + 1 pi	irreversible	Protein Folding	0.00E+00	1.00E+09	0	2.65E+03	2.65E+03	0	4.53E+03	4528.892	0	1.96E+02	195.99
b0172_fold_KJE_3	b0172_m folding: KJE mediated	1 Rrf_DnaK_GrpE_complex + 1 atp --> 1 DnaK_mono.ATP_inact + 1 GrpE_dim_inact + 1 Rrf_mono	irreversible	Protein Folding	0.00E+00	1.00E+09	0	2.65E+03	2.65E+03	0	4.53E+03	4528.892	0	1.96E+02	195.99
b0172_fold_spon	b0172_m folding: spontanous	1 b0172_m --> 1 Rrf_mono	irreversible	Protein Folding	0.00E+00	1.00E+09	0	2.65E+03	2.65E+03	0	4.53E+03	4528.892	0	1.96E+02	195.99
b0172_mRNA_CONV	convsersion of mRNA to mRNA_1 (synthetic rxn)	1 b0172_mRNA --> 1 b0172_mRNA_1	irreversible	Translation	0.00E+00	1.00E+09	0	2.46E+02	2.46E+02	0	4.34E+02	433.6892	0	3.99E-01	0.399
b0172_mRNA_degr1	Degradation of b0172_mRNA I	1 Orn_dim + 139 atp + 1 b0172_mRNA_2 + 1 degradosome + 139 h2o <==> 139 adp + 1 b0172_mRNA_2_degr + 139 h + 139 pi	reversible	mRNA degradation	-1.00E+09	1.00E+09	0	2.46E+02	2.46E+02	0	4.34E+02	433.6892	0	3.99E-01	0.399
b0172_mRNA_degr2	Degradation of b0172_mRNA II	1 b0172_mRNA_2_degr + 557 h2o --> 1 Orn_dim_inact + 163 amp + 130 cmp + 1 degradosome_inact + 153 gmp + 1 gtp + 557 h + 111 ump	irreversible	mRNA degradation	0.00E+00	3.99E-01	0	2.46E+02	2.46E+02	0	4.34E+02	433.6892	0	3.99E-01	0.399
b0172_maturation1	Polypeptide b0172 peptide deformylas complex	1 Def_mono + 1 b0172_aa <==> 1 b0172_def_cplx	reversible	Protein Maturation	-1.00E+09	1.00E+09	0	2.65E+03	2.65E+03	0	4.53E+03	4528.892	0	1.96E+02	195.99
b0172_maturation2	b0172 formation	1 b0172_def_cplx + 1 h2o --> 1 Def_mono_inact + 1 b0172_m + 1 for	irreversible	Protein Maturation	0.00E+00	1.00E+09	0	2.65E+03	2.65E+03	0	4.53E+03	4528.892	0	1.96E+02	195.99
b0188_fold_spon	b0188_m folding: spontanous	1 b0188_m --> 1 TilS_mono	irreversible	Protein Folding	0.00E+00	1.00E+09	0	2.46E+03	2.46E+03	0	3.29E+03	3290.823	0	9.59E+01	95.9
b0188_mRNA_CONV	convsersion of mRNA to mRNA_1 (synthetic rxn)	1 b0188_mRNA --> 1 b0188_mRNA_1	irreversible	Translation	0.00E+00	1.00E+09	0	9.48E+01	9.48E+01	0	1.67E+02	167.2985	0	1.66E-01	0.166
b0188_mRNA_degr1	Degradation of b0188_mRNA I	1 Orn_dim + 324 atp + 1 b0188_mRNA_2 + 1 degradosome + 324 h2o <==> 324 adp + 1 b0188_mRNA_2_degr + 324 h + 324 pi	reversible	mRNA degradation	-1.00E+09	1.00E+09	0	9.48E+01	9.48E+01	0	1.67E+02	167.2985	0	1.66E-01	0.166
b0188_mRNA_degr2	Degradation of b0188_mRNA II	1 b0188_mRNA_2_degr + 1298 h2o --> 1 Orn_dim_inact + 264 amp 1 atp + 337 cmp + 1 degradosome_inact + 417 gmp + 1298 h + 280 ump	irreversible	mRNA degradation	0.00E+00	1.66E-01	0	9.48E+01	9.48E+01	0	1.67E+02	167.2985	0	1.66E-01	0.166
b0188_maturation1	Polypeptide b0188 peptide deformylas and methionine aminopeptidase complex	1 Def_mono + 1 Map_mono + 1 b0188_aa <==> 1 b0188_def_map_cplx	reversible	Protein Maturation	-1.00E+09	1.00E+09	0	2.46E+03	2.46E+03	0	3.29E+03	3290.823	0	9.59E+01	95.9
b0188_maturation2	b0188 formation	1 b0188_def_map_cplx + 2 h2o --> 1 Def_mono_inact + 1 Map_mono_inact + 1 b0188_m + 1 for + 1 met-L	irreversible	Protein Maturation	0.00E+00	1.00E+09	0	2.46E+03	2.46E+03	0	3.29E+03	3290.823	0	9.59E+01	95.9
b0194_fold_KJE_1	b0194_m folding: KJE mediated	1 DnaJ_dim + 1 DnaK_mono.ATP + 1 b0194_m <==> 1 b0194_m_DnaKJ_complex	reversible	Protein Folding	-1.00E+09	1.00E+09	0	2.49E+03	2.49E+03	0	4.11E+03	4107.15	0	1.14E+02	114.411
b0194_fold_KJE_2	b0194_m folding: KJE mediated	1 GrpE_dim + 1 b0194_m_DnaKJ_complex + 1 h2o --> 1 DnaJ_dim_inact + 1 ProS_DnaK_GrpE_complex + 1 adp + 1 h + 1 pi	irreversible	Protein Folding	0.00E+00	1.00E+09	0	2.49E+03	2.49E+03	0	4.11E+03	4107.15	0	1.14E+02	114.411
b0194_fold_KJE_3	b0194_m folding: KJE mediated	1 ProS_DnaK_GrpE_complex + 1 atp --> 1 DnaK_mono.ATP_inact + 1 GrpE_dim_inact + 1 ProS_mono	irreversible	Protein Folding	0.00E+00	1.00E+09	0	2.49E+03	2.49E+03	0	4.11E+03	4107.15	0	1.14E+02	114.411
b0194_fold_spon	b0194_m folding: spontanous	1 b0194_m --> 1 ProS_mono	irreversible	Protein Folding	0.00E+00	1.00E+09	0	2.49E+03	2.49E+03	0	4.11E+03	4107.15	0	1.14E+02	114.411
b0194_mRNA_CONV	convsersion of mRNA to mRNA_1 (synthetic rxn)	1 b0194_mRNA --> 1 b0194_mRNA_1	irreversible	Translation	0.00E+00	1.00E+09	0	7.21E+01	7.21E+01	0	1.27E+02	127.2677	0	2.07E-01	0.207
b0194_mRNA_degr1	Degradation of b0194_mRNA I	1 Orn_dim + 429 atp + 1 b0194_mRNA_2 + 1 degradosome + 429 h2o <==> 429 adp + 1 b0194_mRNA_2_degr + 429 h + 429 pi	reversible	mRNA degradation	-1.00E+09	1.00E+09	0	7.21E+01	7.21E+01	0	1.27E+02	127.2677	0	2.07E-01	0.207
b0194_mRNA_degr2	Degradation of b0194_mRNA II	1 b0194_mRNA_2_degr + 1718 h2o --> 1 Orn_dim_inact + 411 amp 1 atp + 443 cmp + 1 degradosome_inact + 494 gmp + 1718 h + 370 ump	irreversible	mRNA degradation	0.00E+00	2.07E-01	0	7.21E+01	7.21E+01	0	1.27E+02	127.2677	0	2.07E-01	0.207
b0194_maturation1	Polypeptide b0194 peptide deformylas complex	1 Def_mono + 1 b0194_aa <==> 1 b0194_def_cplx	reversible	Protein Maturation	-1.00E+09	1.00E+09	0	2.49E+03	2.49E+03	0	4.11E+03	4107.15	0	1.14E+02	114.411
b0194_maturation2	b0194 formation	1 b0194_def_cplx + 1 h2o --> 1 Def_mono_inact + 1 b0194_m + 1 for	irreversible	Protein Maturation	0.00E+00	1.00E+09	0	2.49E+03	2.49E+03	0	4.11E+03	4107.15	0	1.14E+02	114.411
b0201_RNA_10_modif_1	b0201_RNA modification (16S rRNA): position: 967, m5C by RsmB_mono	1 RsmB_mono + 1 amet + 1 b0201_RNA_10 <==> 1 b0201_RNA_10_RsmB_mono	reversible	rRNA modification	-1.00E+09	1.00E+09	0	1.11E+02	1.11E+02	0	0	0	0	0	0
b0201_RNA_10_modif_2	b0201_RNA modification (16S rRNA): position: 967, m5C by RsmB_mono	1 b0201_RNA_10_RsmB_mono --> 1 RsmB_mono_inact + 1 ahcys + 1 h + 1 rrsH	irreversible	rRNA modification	0.00E+00	1.00E+09	0	1.11E+02	1.11E+02	0	0	0	0	0	0
b0201_RNA_1_modif_1	b0201_RNA modification (16S rRNA): position: 1402, m4Cm by MeT_16S_1402	1 MeT_16S_1402 + 2 amet + 1 b0201_RNA_1 <==> 1 b0201_RNA_1_MeT_16S_1402	reversible	rRNA modification	-1.00E+09	1.00E+09	0	1.11E+02	1.11E+02	0	0	0	0	0	0
b0201_RNA_1_modif_2	b0201_RNA modification (16S rRNA): position: 1402, m4Cm by MeT_16S_1402	1 b0201_RNA_1_MeT_16S_1402 --> 1 MeT_16S_1402_inact + 2 ahcys + 1 b0201_RNA_2 + 2 h	irreversible	rRNA modification	0.00E+00	1.00E+09	0	1.11E+02	1.11E+02	0	0	0	0	0	0
b0201_RNA_2_modif_1	b0201_RNA modification (16S rRNA): position: 1407, m5C by MeT_16S_1407	1 MeT_16S_1407 + 1 amet + 1 b0201_RNA_2 <==> 1 b0201_RNA_2_MeT_16S_1407	reversible	rRNA modification	-1.00E+09	1.00E+09	0	1.11E+02	1.11E+02	0	0	0	0	0	0

b0201_RNA_2_modif_2	b0201_RNA modification (16S rRNA): position: 1407, m5C by MeT_16S_1407	1 b0201_RNA_2_MeT_16S_1407 --> 1 MeT_16S_1407_inact + 1 ahcys + 1 b0201_RNA_3 + 1 h	irreversible	rRNA modification	0.00E+00	1.00E+09	0	1.11E+02	1.11E+02	0	0	0	0	0	0
b0201_RNA_3_modif_1	b0201_RNA modification (16S rRNA): position: 1498, m3U by YggJ_mono	1 YggJ_mono + 1 amet + 1 b0201_RNA_3 <==> 1 b0201_RNA_3_YggJ_mono	reversible	rRNA modification	-1.00E+09	1.00E+09	0	1.11E+02	1.11E+02	0	0	0	0	0	0
b0201_RNA_3_modif_2	b0201_RNA modification (16S rRNA): position: 1498, m3U by YggJ_mono	1 b0201_RNA_3_YggJ_mono --> 1 YggJ_mono_inact + 1 ahcys + 1 b0201_RNA_4 + 1 h	irreversible	rRNA modification	0.00E+00	1.00E+09	0	1.11E+02	1.11E+02	0	0	0	0	0	0
b0201_RNA_4_modif_1	b0201_RNA modification (16S rRNA): position: 1516, m2G by MeT_16S_1516	1 MeT_16S_1516 + 1 amet + 1 b0201_RNA_4 <==> 1 b0201_RNA_4_MeT_16S_1516	reversible	rRNA modification	-1.00E+09	1.00E+09	0	1.11E+02	1.11E+02	0	0	0	0	0	0
b0201_RNA_4_modif_2	b0201_RNA modification (16S rRNA): position: 1516, m2G by MeT_16S_1516	1 b0201_RNA_4_MeT_16S_1516 --> 1 MeT_16S_1516_inact + 1 ahcys + 1 b0201_RNA_5 + 1 h	irreversible	rRNA modification	0.00E+00	1.00E+09	0	1.11E+02	1.11E+02	0	0	0	0	0	0
b0201_RNA_5_modif_1	b0201_RNA modification (16S rRNA): position: 1518, m6(2)A by KsgA_mono	1 KsgA_mono + 2 amet + 1 b0201_RNA_5 <==> 1 b0201_RNA_5_KsgA_mono	reversible	rRNA modification	-1.00E+09	1.00E+09	0	1.11E+02	1.11E+02	0	0	0	0	0	0
b0201_RNA_5_modif_2	b0201_RNA modification (16S rRNA): position: 1518, m6(2)A by KsgA_mono	1 b0201_RNA_5_KsgA_mono --> 1 KsgA_mono_inact + 2 ahcys + 1 b0201_RNA_6 + 2 h	irreversible	rRNA modification	0.00E+00	1.00E+09	0	1.11E+02	1.11E+02	0	0	0	0	0	0
b0201_RNA_6_modif_1	b0201_RNA modification (16S rRNA): position: 1519, m6(2)A by KsgA_mono	1 KsgA_mono + 2 amet + 1 b0201_RNA_6 <==> 1 b0201_RNA_6_KsgA_mono	reversible	rRNA modification	-1.00E+09	1.00E+09	0	1.11E+02	1.11E+02	0	0	0	0	0	0
b0201_RNA_6_modif_2	b0201_RNA modification (16S rRNA): position: 1519, m6(2)A by KsgA_mono	1 b0201_RNA_6_KsgA_mono --> 1 KsgA_mono_inact + 2 ahcys + 1 b0201_RNA_7 + 2 h	irreversible	rRNA modification	0.00E+00	1.00E+09	0	1.11E+02	1.11E+02	0	0	0	0	0	0
b0201_RNA_7_modif_1	b0201_RNA modification (16S rRNA): position: 516, Y by RsuA_mono	1 RsuA_mono + 1 b0201_RNA_7 <==> 1 b0201_RNA_7_RsuA_mono	reversible	rRNA modification	-1.00E+09	1.00E+09	0	1.11E+02	1.11E+02	0	0	0	0	0	0
b0201_RNA_7_modif_2	b0201_RNA modification (16S rRNA): position: 516, Y by RsuA_mono	1 b0201_RNA_7_RsuA_mono --> 1 RsuA_mono_inact + 1 b0201_RNA_8	irreversible	rRNA modification	0.00E+00	1.00E+09	0	1.11E+02	1.11E+02	0	0	0	0	0	0
b0201_RNA_8_modif_1	b0201_RNA modification (16S rRNA): position: 527, m7G by MeT_16S_527	1 MeT_16S_527 + 1 amet + 1 b0201_RNA_8 <==> 1 b0201_RNA_8_MeT_16S_527	reversible	rRNA modification	-1.00E+09	1.00E+09	0	1.11E+02	1.11E+02	0	0	0	0	0	0
b0201_RNA_8_modif_2	b0201_RNA modification (16S rRNA): position: 527, m7G by MeT_16S_527	1 b0201_RNA_8_MeT_16S_527 --> 1 MeT_16S_527_inact + 1 ahcy + 1 b0201_RNA_9 + 1 h	irreversible	rRNA modification	0.00E+00	1.00E+09	0	1.11E+02	1.11E+02	0	0	0	0	0	0
b0201_RNA_9_modif_1	b0201_RNA modification (16S rRNA): position: 966, m2G by MeT_16S_966	1 MeT_16S_966 + 1 amet + 1 b0201_RNA_9 <==> 1 b0201_RNA_9_MeT_16S_966	reversible	rRNA modification	-1.00E+09	1.00E+09	0	1.11E+02	1.11E+02	0	0	0	0	0	0
b0201_RNA_9_modif_2	b0201_RNA modification (16S rRNA): position: 966, m2G by MeT_16S_966	1 b0201_RNA_9_MeT_16S_966 --> 1 MeT_16S_966_inact + 1 ahcy + 1 b0201_RNA_10 + 1 h	irreversible	rRNA modification	0.00E+00	1.00E+09	0	1.11E+02	1.11E+02	0	0	0	0	0	0
b0201_RNA_modif_1	b0201_RNA modification (16S rRNA): position: 1207, m2G by RsmC_mono	1 RsmC_mono + 1 amet + 1 b0201_RNA <==> 1 b0201_RNA_RsmC_mono	reversible	rRNA modification	-1.00E+09	1.00E+09	0	1.11E+02	1.11E+02	0	0	0	0	0	0
b0201_RNA_modif_2	b0201_RNA modification (16S rRNA): position: 1207, m2G by RsmC_mono	1 b0201_RNA_RsmC_mono --> 1 RsmC_mono_inact + 1 ahcys + 1 b0201_RNA_1 + 1 h	irreversible	rRNA modification	0.00E+00	1.00E+09	0	1.11E+02	1.11E+02	0	0	0	0	0	0
b0201_v1_RNA_10_modif _1	b0201_v1_RNA modification (16S rRNA): position: 967, m5C by RsmB_mono	1 RsmB_mono + 1 amet + 1 b0201_v1_RNA_10 <==> 1 b0201_v1_RNA_10_RsmB_mono	reversible	rRNA modification	-1.00E+09	1.00E+09	0	1.11E+02	1.11E+02	0	9.54E+00	9.54	0	9.54E+00	9.54
b0201_v1_RNA_10_modif _2	b0201_v1_RNA modification (16S rRNA): position: 967, m5C by RsmB_mono	1 b0201_v1_RNA_10_RsmB_mono --> 1 RsmB_mono_inact + 1 ahcys + 1 h + 1 rrsH	irreversible	rRNA modification	0.00E+00	1.00E+09	0	1.11E+02	1.11E+02	0	9.54E+00	9.54	0	9.54E+00	9.54
b0201_v1_RNA_1_modif_ 1	b0201_v1_RNA modification (16S rRNA): position: 1402, m4Cm by MeT_16S_1402	1 MeT_16S_1402 + 2 amet + 1 b0201_v1_RNA_1 <==> 1 b0201_v1_RNA_1_MeT_16S_1402	reversible	rRNA modification	-1.00E+09	1.00E+09	0	1.11E+02	1.11E+02	0	9.54E+00	9.54	0	9.54E+00	9.54
b0201_v1_RNA_1_modif_ 2	b0201_v1_RNA modification (16S rRNA): position: 1402, m4Cm by MeT_16S_1402	1 b0201_v1_RNA_1_MeT_16S_1402 --> 1 MeT_16S_1402_inact + 2 ahcys + 1 b0201_v1_RNA_2 + 2 h	irreversible	rRNA modification	0.00E+00	1.00E+09	0	1.11E+02	1.11E+02	0	9.54E+00	9.54	0	9.54E+00	9.54
b0201_v1_RNA_2_modif_ 1	b0201_v1_RNA modification (16S rRNA): position: 1407, m5C by MeT_16S_1407	1 MeT_16S_1407 + 1 amet + 1 b0201_v1_RNA_2 <==> 1 b0201_v1_RNA_2_MeT_16S_1407	reversible	rRNA modification	-1.00E+09	1.00E+09	0	1.11E+02	1.11E+02	0	9.54E+00	9.54	0	9.54E+00	9.54
b0201_v1_RNA_2_modif_ 2	b0201_v1_RNA modification (16S rRNA): position: 1407, m5C by MeT_16S_1407	1 b0201_v1_RNA_2_MeT_16S_1407 --> 1 MeT_16S_1407_inact + 1 ahcys + 1 b0201_v1_RNA_3 + 1 h	irreversible	rRNA modification	0.00E+00	1.00E+09	0	1.11E+02	1.11E+02	0	9.54E+00	9.54	0	9.54E+00	9.54
b0201_v1_RNA_3_modif_ 1	b0201_v1_RNA modification (16S rRNA): position: 1498, m3U by YggJ_mono	1 YggJ_mono + 1 amet + 1 b0201_v1_RNA_3 <==> 1 b0201_v1_RNA_3_YggJ_mono	reversible	rRNA modification	-1.00E+09	1.00E+09	0	1.11E+02	1.11E+02	0	9.54E+00	9.54	0	9.54E+00	9.54
b0201_v1_RNA_3_modif_ 2	b0201_v1_RNA modification (16S rRNA): position: 1498, m3U by YggJ_mono	1 b0201_v1_RNA_3_YggJ_mono --> 1 YggJ_mono_inact + 1 ahcys 1 b0201_v1_RNA_4 + 1 h	irreversible	rRNA modification	0.00E+00	1.00E+09	0	1.11E+02	1.11E+02	0	9.54E+00	9.54	0	9.54E+00	9.54
b0201_v1_RNA_4_modif_ 1	b0201_v1_RNA modification (16S rRNA): position: 1516, m2G by MeT_16S_1516	1 MeT_16S_1516 + 1 amet + 1 b0201_v1_RNA_4 <==> 1 b0201_v1_RNA_4_MeT_16S_1516	reversible	rRNA modification	-1.00E+09	1.00E+09	0	1.11E+02	1.11E+02	0	9.54E+00	9.54	0	9.54E+00	9.54
b0201_v1_RNA_4_modif_ 2	b0201_v1_RNA modification (16S rRNA): position: 1516, m2G by MeT_16S_1516	1 b0201_v1_RNA_4_MeT_16S_1516 --> 1 MeT_16S_1516_inact + 1 ahcys + 1 b0201_v1_RNA_5 + 1 h	irreversible	rRNA modification	0.00E+00	1.00E+09	0	1.11E+02	1.11E+02	0	9.54E+00	9.54	0	9.54E+00	9.54
b0201_v1_RNA_5_modif_ 1	b0201_v1_RNA modification (16S rRNA): position: 1518, m6(2)A by KsgA_mono	1 KsgA_mono + 2 amet + 1 b0201_v1_RNA_5 <==> 1 b0201_v1_RNA_5_KsgA_mono	reversible	rRNA modification	-1.00E+09	1.00E+09	0	1.11E+02	1.11E+02	0	9.54E+00	9.54	0	9.54E+00	9.54
b0201_v1_RNA_5_modif_ 2	b0201_v1_RNA modification (16S rRNA): position: 1518, m6(2)A by KsgA_mono	1 b0201_v1_RNA_5_KsgA_mono --> 1 KsgA_mono_inact + 2 ahcys + 1 b0201_v1_RNA_6 + 2 h	irreversible	rRNA modification	0.00E+00	1.00E+09	0	1.11E+02	1.11E+02	0	9.54E+00	9.54	0	9.54E+00	9.54
b0201_v1_RNA_6_modif_ 1	b0201_v1_RNA modification (16S rRNA): position: 1519, m6(2)A by KsgA_mono	1 KsgA_mono + 2 amet + 1 b0201_v1_RNA_6 <==> 1 b0201_v1_RNA_6_KsgA_mono	reversible	rRNA modification	-1.00E+09	1.00E+09	0	1.11E+02	1.11E+02	0	9.54E+00	9.54	0	9.54E+00	9.54
b0201_v1_RNA_6_modif_ 2	b0201_v1_RNA modification (16S rRNA): position: 1519, m6(2)A by KsgA_mono	1 b0201_v1_RNA_6_KsgA_mono --> 1 KsgA_mono_inact + 2 ahcys + 1 b0201_v1_RNA_7 + 2 h	irreversible	rRNA modification	0.00E+00	1.00E+09	0	1.11E+02	1.11E+02	0	9.54E+00	9.54	0	9.54E+00	9.54
b0201_v1_RNA_7_modif_ 1	b0201_v1_RNA modification (16S rRNA): position: 516, Y by RsuA_mono	1 RsuA_mono + 1 b0201_v1_RNA_7 <==> 1 b0201_v1_RNA_7_RsuA_mono	reversible	rRNA modification	-1.00E+09	1.00E+09	0	1.11E+02	1.11E+02	0	9.54E+00	9.54	0	9.54E+00	9.54
b0201_v1_RNA_7_modif_ 2	b0201_v1_RNA modification (16S rRNA): position: 516, Y by RsuA_mono	1 b0201_v1_RNA_7_RsuA_mono --> 1 RsuA_mono_inact + 1 b0201_v1_RNA_8	irreversible	rRNA modification	0.00E+00	1.00E+09	0	1.11E+02	1.11E+02	0	9.54E+00	9.54	0	9.54E+00	9.54
b0201_v1_RNA_8_modif_ 1	b0201_v1_RNA modification (16S rRNA): position: 527, m7G by MeT_16S_527	1 MeT_16S_527 + 1 amet + 1 b0201_v1_RNA_8 <==> 1 b0201_v1_RNA_8_MeT_16S_527	reversible	rRNA modification	-1.00E+09	1.00E+09	0	1.11E+02	1.11E+02	0	9.54E+00	9.54	0	9.54E+00	9.54
b0201_v1_RNA_8_modif_ 2	b0201_v1_RNA modification (16S rRNA): position: 527, m7G by MeT_16S_527	1 b0201_v1_RNA_8_MeT_16S_527 --> 1 MeT_16S_527_inact + 1 ahcys + 1 b0201_v1_RNA_9 + 1 h	irreversible	rRNA modification	0.00E+00	1.00E+09	0	1.11E+02	1.11E+02	0	9.54E+00	9.54	0	9.54E+00	9.54
b0201_v1_RNA_9_modif_ 1	b0201_v1_RNA modification (16S rRNA): position: 966, m2G by MeT_16S_966	1 MeT_16S_966 + 1 amet + 1 b0201_v1_RNA_9 <==> 1 b0201_v1_RNA_9_MeT_16S_966	reversible	rRNA modification	-1.00E+09	1.00E+09	0	1.11E+02	1.11E+02	0	9.54E+00	9.54	0	9.54E+00	9.54
b0201_v1_RNA_9_modif_ 2	b0201_v1_RNA modification (16S rRNA): position: 966, m2G by MeT_16S_966	1 b0201_v1_RNA_9_MeT_16S_966 --> 1 MeT_16S_966_inact + 1 ahcys + 1 b0201_v1_RNA_10 + 1 h	irreversible	rRNA modification	0.00E+00	1.00E+09	0	1.11E+02	1.11E+02	0	9.54E+00	9.54	0	9.54E+00	9.54
b0201_v1_RNA_modif_1	b0201_v1_RNA modification (16S rRNA): position: 1207, m2G by RsmC_mono	1 RsmC_mono + 1 amet + 1 b0201_v1_RNA <==> 1 b0201_v1_RNA_RsmC_mono	reversible	rRNA modification	-1.00E+09	1.00E+09	0	1.11E+02	1.11E+02	0	9.54E+00	9.54	0	9.54E+00	9.54

b0201_v1_RNA_modif_2	b0201_v1_RNA modification (16S rRNA): position: 1207, m2G by RsmC_mono	1 b0201_v1_RNA_RsmC_mono --> 1 RsmC_mono_inact + 1 ahcys + 1 b0201_v1_RNA_1 + 1 h	irreversible	rRNA modification	0.00E+00	1.00E+09	0	1.11E+02	1.11E+02	0	9.54E+00	9.54	0	9.54E+00	9.54
b0202_tRNA_20A_MODIF_1	modification of position 20A nt (D) in ileV by Dus_gen	1 Dus_gen + 1 b0202_tRNA_1 + 1 h + 1 nadph <==> 1 b0202_tRNA_1_Dus_gen_cplx	reversible	tRNA modification	-1.00E+09	1.00E+09	0	1.11E+02	1.11E+02	0	0	0	0	0	0
b0202_tRNA_20A_MODIF_2	modification of position 20A nt (D) in ileV by Dus_gen	1 b0202_tRNA_1_Dus_gen_cplx --> 1 Dus_gen_inact + 1 b0202_tRNA_2 + 1 nadp	irreversible	tRNA modification	0.00E+00	1.00E+09	0	1.11E+02	1.11E+02	0	0	0	0	0	0
b0202_tRNA_20_MODIF_1	modification of position 20 nt (D) in ileV by Dus_gen	1 Dus_gen + 1 b0202_tRNA_Mg2 + 1 h + 1 nadph <==> 1 b0202_tRNA_Mg2_Dus_gen_cplx	reversible	tRNA modification	-1.00E+09	1.00E+09	0	1.11E+02	1.11E+02	0	0	0	0	0	0
b0202_tRNA_20_MODIF_2	modification of position 20 nt (D) in ileV by Dus_gen	1 b0202_tRNA_Mg2_Dus_gen_cplx --> 1 Dus_gen_inact + 1 b0202_tRNA_1 + 1 nadp	irreversible	tRNA modification	0.00E+00	1.00E+09	0	1.11E+02	1.11E+02	0	0	0	0	0	0
b0202_tRNA_37_MODIF_1	modification of position 37 nt (t6A) in ileV by Up_tRNA_pos_37_t6A	1 Up_tRNA_pos_37_t6A + 1 atp + 1 b0202_tRNA_2 + 1 hco3 + 1 mg2 + 1 thr-L <==> 1 b0202_tRNA_2_Up_tRNA_pos_37_t6A_cplx	reversible	tRNA modification	-1.00E+09	1.00E+09	0	1.11E+02	1.11E+02	0	0	0	0	0	0
b0202_tRNA_37_MODIF_2	modification of position 37 nt (t6A) in ileV by Up_tRNA_pos_37_t6A	1 b0202_tRNA_2_Up_tRNA_pos_37_t6A_cplx --> 1 Up_tRNA_pos_37_t6A_inact + 1 amp + 1 b0202_tRNA_3 + 1 h + 1 h2o + 1 mg2 + 1 ppi	irreversible	tRNA modification	0.00E+00	1.00E+09	0	1.11E+02	1.11E+02	0	0	0	0	0	0
b0202_tRNA_46_MODIF_1	modification of position 46 nt (m7G) in ileV by YggH_mono	1 YggH_mono + 1 amet + 1 b0202_tRNA_3 <==> 1 b0202_tRNA_3_YggH_mono_cplx	reversible	tRNA modification	-1.00E+09	1.00E+09	0	1.11E+02	1.11E+02	0	0	0	0	0	0
b0202_tRNA_46_MODIF_2	modification of position 46 nt (m7G) in ileV by YggH_mono	1 b0202_tRNA_3_YggH_mono_cplx --> 1 YggH_mono_inact + 1 ahcys + 1 b0202_tRNA_4 + 1 h	irreversible	tRNA modification	0.00E+00	1.00E+09	0	1.11E+02	1.11E+02	0	0	0	0	0	0
b0202_tRNA_47_MODIF_1	modification of position 47 nt (acp3U) in ileV by AcpT_tRNA_pos_47_acp3U	1 AcpT_tRNA_pos_47_acp3U + 1 amet + 1 b0202_tRNA_4 <==> 1 b0202_tRNA_4_AcpT_tRNA_pos_47_acp3U_cplx	reversible	tRNA modification	-1.00E+09	1.00E+09	0	1.11E+02	1.11E+02	0	0	0	0	0	0
b0202_tRNA_47_MODIF_2	modification of position 47 nt (acp3U) in ileV by AcpT_tRNA_pos_47_acp3U	1 b0202_tRNA_4_AcpT_tRNA_pos_47_acp3U_cplx --> 1 5mta + 1 AcpT_tRNA_pos_47_acp3U_inact + 1 b0202_tRNA_5 + 1 h	irreversible	tRNA modification	0.00E+00	1.00E+09	0	1.11E+02	1.11E+02	0	0	0	0	0	0
b0202_tRNA_54_MODIF_1	modification of position 54 nt (m5U) in ileV by TrmA_mono	1 TrmA_mono + 1 amet + 1 b0202_tRNA_5 <==> 1 b0202_tRNA_5_TrmA_mono_cplx	reversible	tRNA modification	-1.00E+09	1.00E+09	0	1.11E+02	1.11E+02	0	0	0	0	0	0
b0202_tRNA_54_MODIF_2	modification of position 54 nt (m5U) in ileV by TrmA_mono	1 b0202_tRNA_5_TrmA_mono_cplx --> 1 TrmA_mono_inact + 1 ahcys + 1 b0202_tRNA_6 + 1 h	irreversible	tRNA modification	0.00E+00	1.00E+09	0	1.11E+02	1.11E+02	0	0	0	0	0	0
b0202_tRNA_55_MODIF_1	modification of position 55 nt (Y) in ileV by TruB_mono	1 TruB_mono + 1 b0202_tRNA_6 <==> 1 b0202_tRNA_6_TruB_mono_cplx	reversible	tRNA modification	-1.00E+09	1.00E+09	0	1.11E+02	1.11E+02	0	0	0	0	0	0
b0202_tRNA_55_MODIF_2	modification of position 55 nt (Y) in ileV by TruB_mono	1 b0202_tRNA_6_TruB_mono_cplx --> 1 TruB_mono_inact + 1 b0202_tRNA_7	irreversible	tRNA modification	0.00E+00	1.00E+09	0	1.11E+02	1.11E+02	0	0	0	0	0	0
b0202_tRNA_65_MODIF_1	modification of position 65 nt (Y) in ileV by YqcB_mono	1 YqcB_mono + 1 b0202_tRNA_7 <==> 1 b0202_tRNA_7_YqcB_mono_cplx	reversible	tRNA modification	-1.00E+09	1.00E+09	0	1.11E+02	1.11E+02	0	0	0	0	0	0
b0202_tRNA_65_MODIF_2	modification of position 65 nt (Y) in ileV by YqcB_mono	1 b0202_tRNA_7_YqcB_mono_cplx --> 1 YqcB_mono_inact + 1 ileV_tRNA	irreversible	tRNA modification	0.00E+00	1.00E+09	0	1.11E+02	1.11E+02	0	0	0	0	0	0
b0202_tRNA_Mg_BIND	Binding of two Mg2 to b0202_tRNA	1 b0202_RNA + 2 mg2 <==> 1 b0202_tRNA_Mg2	reversible	Metallo-ion binding	-1.00E+09	1.00E+09	0	1.11E+02	1.11E+02	0	0	0	0	0	0
b0202_v1_tRNA_20A_MODIF_1	modification of position 20A nt (D) in ileV by Dus_gen	1 Dus_gen + 1 b0202_v1_tRNA_1 + 1 h + 1 nadph <==> 1 b0202_v1_tRNA_1_Dus_gen_cplx	reversible	tRNA modification	-1.00E+09	1.00E+09	0	1.11E+02	1.11E+02	0	9.54E+00	9.54	0	9.54E+00	9.54
b0202_v1_tRNA_20A_MODIF_2	modification of position 20A nt (D) in ileV by Dus_gen	1 b0202_v1_tRNA_1_Dus_gen_cplx --> 1 Dus_gen_inact + 1 b0202_v1_tRNA_2 + 1 nadp	irreversible	tRNA modification	0.00E+00	1.00E+09	0	1.11E+02	1.11E+02	0	9.54E+00	9.54	0	9.54E+00	9.54
b0202_v1_tRNA_20_MODIF_1	modification of position 20 nt (D) in ileV by Dus_gen	1 Dus_gen + 1 b0202_v1_tRNA_Mg2 + 1 h + 1 nadph <==> 1 b0202_v1_tRNA_Mg2_Dus_gen_cplx	reversible	tRNA modification	-1.00E+09	1.00E+09	0	1.11E+02	1.11E+02	0	9.54E+00	9.54	0	9.54E+00	9.54
b0202_v1_tRNA_20_MODIF_2	modification of position 20 nt (D) in ileV by Dus_gen	1 b0202_v1_tRNA_Mg2_Dus_gen_cplx --> 1 Dus_gen_inact + 1 b0202_v1_tRNA_1 + 1 nadp	irreversible	tRNA modification	0.00E+00	1.00E+09	0	1.11E+02	1.11E+02	0	9.54E+00	9.54	0	9.54E+00	9.54
b0202_v1_tRNA_37_MODIF_1	modification of position 37 nt (t6A) in ileV by Up_tRNA_pos_37_t6A	1 Up_tRNA_pos_37_t6A + 1 atp + 1 b0202_v1_tRNA_2 + 1 hco3 + 1 mg2 + 1 thr-L <==> 1 b0202_v1_tRNA_2_Up_tRNA_pos_37_t6A_cplx	reversible	tRNA modification	-1.00E+09	1.00E+09	0	1.11E+02	1.11E+02	0	9.54E+00	9.54	0	9.54E+00	9.54
b0202_v1_tRNA_37_MODIF_2	modification of position 37 nt (t6A) in ileV by Up_tRNA_pos_37_t6A	1 b0202_v1_tRNA_2_Up_tRNA_pos_37_t6A_cplx --> 1 Up_tRNA_pos_37_t6A_inact + 1 amp + 1 b0202_v1_tRNA_3 + 1 h + 1 h2o + 1 mg2 + 1 ppi	irreversible	tRNA modification	0.00E+00	1.00E+09	0	1.11E+02	1.11E+02	0	9.54E+00	9.54	0	9.54E+00	9.54
b0202_v1_tRNA_46_MODIF_1	modification of position 46 nt (m7G) in ileV by YggH_mono	1 YggH_mono + 1 amet + 1 b0202_v1_tRNA_3 <==> 1 b0202_v1_tRNA_3_YggH_mono_cplx	reversible	tRNA modification	-1.00E+09	1.00E+09	0	1.11E+02	1.11E+02	0	9.54E+00	9.54	0	9.54E+00	9.54
b0202_v1_tRNA_46_MODIF_2	modification of position 46 nt (m7G) in ileV by YggH_mono	1 b0202_v1_tRNA_3_YggH_mono_cplx --> 1 YggH_mono_inact + 1 ahcys + 1 b0202_v1_tRNA_4 + 1 h	irreversible	tRNA modification	0.00E+00	1.00E+09	0	1.11E+02	1.11E+02	0	9.54E+00	9.54	0	9.54E+00	9.54
b0202_v1_tRNA_47_MODIF_1	modification of position 47 nt (acp3U) in ileV by AcpT_tRNA_pos_47_acp3U	1 AcpT_tRNA_pos_47_acp3U + 1 amet + 1 b0202_v1_tRNA_4 <==> 1 b0202_v1_tRNA_4_AcpT_tRNA_pos_47_acp3U_cplx	reversible	tRNA modification	-1.00E+09	1.00E+09	0	1.11E+02	1.11E+02	0	9.54E+00	9.54	0	9.54E+00	9.54
b0202_v1_tRNA_47_MODIF_2	modification of position 47 nt (acp3U) in ileV by AcpT_tRNA_pos_47_acp3U	1 b0202_v1_tRNA_4_AcpT_tRNA_pos_47_acp3U_cplx --> 1 5mta + 1 AcpT_tRNA_pos_47_acp3U_inact + 1 b0202_v1_tRNA_5 + 1 h	irreversible	tRNA modification	0.00E+00	1.00E+09	0	1.11E+02	1.11E+02	0	9.54E+00	9.54	0	9.54E+00	9.54
b0202_v1_tRNA_54_MODIF_1	modification of position 54 nt (m5U) in ileV by TrmA_mono	1 TrmA_mono + 1 amet + 1 b0202_v1_tRNA_5 <==> 1 b0202_v1_tRNA_5_TrmA_mono_cplx	reversible	tRNA modification	-1.00E+09	1.00E+09	0	1.11E+02	1.11E+02	0	9.54E+00	9.54	0	9.54E+00	9.54
b0202_v1_tRNA_54_MODIF_2	modification of position 54 nt (m5U) in ileV by TrmA_mono	1 b0202_v1_tRNA_5_TrmA_mono_cplx --> 1 TrmA_mono_inact + 1 ahcys + 1 b0202_v1_tRNA_6 + 1 h	irreversible	tRNA modification	0.00E+00	1.00E+09	0	1.11E+02	1.11E+02	0	9.54E+00	9.54	0	9.54E+00	9.54
b0202_v1_tRNA_55_MODIF_1	modification of position 55 nt (Y) in ileV by TruB_mono	1 TruB_mono + 1 b0202_v1_tRNA_6 <==> 1 b0202_v1_tRNA_6_TruB_mono_cplx	reversible	tRNA modification	-1.00E+09	1.00E+09	0	1.11E+02	1.11E+02	0	9.54E+00	9.54	0	9.54E+00	9.54
b0202_v1_tRNA_55_MODIF_2	modification of position 55 nt (Y) in ileV by TruB_mono	1 b0202_v1_tRNA_6_TruB_mono_cplx --> 1 TruB_mono_inact + 1 b0202_v1_tRNA_7	irreversible	tRNA modification	0.00E+00	1.00E+09	0	1.11E+02	1.11E+02	0	9.54E+00	9.54	0	9.54E+00	9.54
b0202_v1_tRNA_65_MODIF_1	modification of position 65 nt (Y) in ileV by YqcB_mono	1 YqcB_mono + 1 b0202_v1_tRNA_7 <==> 1 b0202_v1_tRNA_7_YqcB_mono_cplx	reversible	tRNA modification	-1.00E+09	1.00E+09	0	1.11E+02	1.11E+02	0	9.54E+00	9.54	0	9.54E+00	9.54
b0202_v1_tRNA_65_MODIF_2	modification of position 65 nt (Y) in ileV by YqcB_mono	1 b0202_v1_tRNA_7_YqcB_mono_cplx --> 1 YqcB_mono_inact + 1 ileV_tRNA	irreversible	tRNA modification	0.00E+00	1.00E+09	0	1.11E+02	1.11E+02	0	9.54E+00	9.54	0	9.54E+00	9.54
b0202_v1_tRNA_Mg_BIND	Binding of two Mg2 to b0202_v1_tRNA	1 b0202_v1_RNA + 2 mg2 <==> 1 b0202_v1_tRNA_Mg2	reversible	Metallo-ion binding	-1.00E+09	1.00E+09	0	1.11E+02	1.11E+02	0	9.54E+00	9.54	0	9.54E+00	9.54
b0203_tRNA_17_MODIF_1	modification of position 17 nt (D) in alaV by Dus_gen	1 Dus_gen + 1 b0203_tRNA_Mg2 + 1 h + 1 nadph <==> 1 b0203_tRNA_Mg2_Dus_gen_cplx	reversible	tRNA modification	-1.00E+09	1.00E+09	0	1.11E+02	1.11E+02	0	0	0	0	0	0
b0203_tRNA_17_MODIF_2	modification of position 17 nt (D) in alaV by Dus_gen	1 b0203_tRNA_Mg2_Dus_gen_cplx --> 1 Dus_gen_inact + 1 b0203_tRNA_1 + 1 nadp	irreversible	tRNA modification	0.00E+00	1.00E+09	0	1.11E+02	1.11E+02	0	0	0	0	0	0
b0203_tRNA_34_MODIF_1	modification of position 34 nt (cmo5U) in alaV by YecO_mono-YecP_mono-HyL_tRNA_pos_34_ho5U	1 HyL_tRNA_pos_34_ho5U + 1 YecO_mono + 1 YecP_mono + 1 amet + 1 b0203_tRNA_1 + 2 chor <==> 1 b0203_tRNA_1_YecO_mono-YecP_mono-HyL_tRNA_pos_34_ho5U_cplx	reversible	tRNA modification	-1.00E+09	1.00E+09	0	1.11E+02	1.11E+02	0	0	0	0	0	0
b0203_tRNA_34_MODIF_2	modification of position 34 nt (cmo5U) in alaV by YecO_mono-YecP_mono-HyL_tRNA_pos_34_ho5U	1 b0203_tRNA_1_YecO_mono-YecP_mono-HyL_tRNA_pos_34_ho5U_cplx --> 1 C10H8O5 + 1 C9H9O4 + 1 HyL_tRNA_pos_34_ho5U_inact + 1 YecO_mono_inact + 1 YecP_mono_inact + 1 ahcys + 1 b0203_tRNA_2 + 1 h	irreversible	tRNA modification	0.00E+00	1.00E+09	0	1.11E+02	1.11E+02	0	0	0	0	0	0
b0203_tRNA_46_MODIF_1	modification of position 46 nt (m7G) in alaV by YggH_mono	1 YggH_mono + 1 amet + 1 b0203_tRNA_2 <==> 1 b0203_tRNA_2_YggH_mono_cplx	reversible	tRNA modification	-1.00E+09	1.00E+09	0	1.11E+02	1.11E+02	0	0	0	0	0	0
b0203_tRNA_46_MODIF_2	modification of position 46 nt (m7G) in alaV by YggH_mono	1 b0203_tRNA_2_YggH_mono_cplx --> 1 YggH_mono_inact + 1 ahcys + 1 b0203_tRNA_3 + 1 h	irreversible	tRNA modification	0.00E+00	1.00E+09	0	1.11E+02	1.11E+02	0	0	0	0	0	0
b0203_tRNA_54_MODIF_1	modification of position 54 nt (m5U) in alaV by TrmA_mono	1 TrmA_mono + 1 amet + 1 b0203_tRNA_3 <==> 1 b0203_tRNA_3_TrmA_mono_cplx	reversible	tRNA modification	-1.00E+09	1.00E+09	0	1.11E+02	1.11E+02	0	0	0	0	0	0
b0203_tRNA_54_MODIF_2	modification of position 54 nt (m5U) in alaV by TrmA_mono	1 b0203_tRNA_3_TrmA_mono_cplx --> 1 TrmA_mono_inact + 1 ahcys + 1 b0203_tRNA_4 + 1 h	irreversible	tRNA modification	0.00E+00	1.00E+09	0	1.11E+02	1.11E+02	0	0	0	0	0	0
b0203_tRNA_55_MODIF_1	modification of position 55 nt (Y) in alaV by TruB_mono	1 TruB_mono + 1 b0203_tRNA_4 <==> 1 b0203_tRNA_4_TruB_mono_cplx	reversible	tRNA modification	-1.00E+09	1.00E+09	0	1.11E+02	1.11E+02	0	0	0	0	0	0
b0203_tRNA_55_MODIF_2	modification of position 55 nt (Y) in alaV by TruB_mono	1 b0203_tRNA_4_TruB_mono_cplx --> 1 TruB_mono_inact + 1 alaV_tRNA	irreversible	tRNA modification	0.00E+00	1.00E+09	0	1.11E+02	1.11E+02	0	0	0	0	0	0
b0203_tRNA_Mg_BIND	Binding of two Mg2 to b0203_tRNA	1 b0203_RNA + 2 mg2 <==> 1 b0203_tRNA_Mg2	reversible	Metallo-ion binding	-1.00E+09	1.00E+09	0	1.11E+02	1.11E+02	0	0	0	0	0	0
b0203_v1_tRNA_17_MODIF_1	modification of position 17 nt (D) in alaV by Dus_gen	1 Dus_gen + 1 b0203_v1_tRNA_Mg2 + 1 h + 1 nadph <==> 1 b0203_v1_tRNA_Mg2_Dus_gen_cplx	reversible	tRNA modification	-1.00E+09	1.00E+09	0	1.11E+02	1.11E+02	0	9.54E+00	9.54	0	9.54E+00	9.54
b0203_v1_tRNA_17_MODIF_2	modification of position 17 nt (D) in alaV by Dus_gen	1 b0203_v1_tRNA_Mg2_Dus_gen_cplx --> 1 Dus_gen_inact + 1 b0203_v1_tRNA_1 + 1 nadp	irreversible	tRNA modification	0.00E+00	1.00E+09	0	1.11E+02	1.11E+02	0	9.54E+00	9.54	0	9.54E+00	9.54
b0203_v1_tRNA_34_MODIF_1	modification of position 34 nt (cmo5U) in alaV by YecO_mono-YecP_mono-HyL_tRNA_pos_34_ho5U	1 HyL_tRNA_pos_34_ho5U + 1 YecO_mono + 1 YecP_mono + 1 amet + 1 b0203_v1_tRNA_1 + 2 chor <==> 1 b0203_v1_tRNA_1_YecO_mono-YecP_mono-HyL_tRNA_pos_34_ho5U_cplx	reversible	tRNA modification	-1.00E+09	1.00E+09	0	1.11E+02	1.11E+02	0	9.54E+00	9.54	0	9.54E+00	9.54

b0203_v1_tRNA_34_MODIF_2	modification of position 34 nt (cmo5U) in alaV by YecO_mono-YecP_mono-HyL_tRNA_pos_34_ho5U	1 b0203_v1_tRNA_1_YecO_mono-YecP_mono-HyL_tRNA_pos_34_ho5U_cplx --> 1 C10H8O5 + 1 C9H9O4 + 1 HyL_tRNA_pos_34_ho5U_inact + 1 YecO_mono_inact + 1 YecP_mono_inact + 1 ahcys + 1 b0203_v1_tRNA_2 + 1 h	irreversible	tRNA modification	0.00E+00	1.00E+09	0	1.11E+02	1.11E+02	0	9.54E+00	9.54	0	9.54E+00	9.54
b0203_v1_tRNA_46_MODIF_1	modification of position 46 nt (m7G) in alaV by YggH_mono	1 YggH_mono + 1 amet + 1 b0203_v1_tRNA_2 <==> 1 b0203_v1_tRNA_2_YggH_mono_cplx	reversible	tRNA modification	-1.00E+09	1.00E+09	0	1.11E+02	1.11E+02	0	9.54E+00	9.54	0	9.54E+00	9.54
b0203_v1_tRNA_46_MODIF_2	modification of position 46 nt (m7G) in alaV by YggH_mono	1 b0203_v1_tRNA_2_YggH_mono_cplx --> 1 YggH_mono_inact + 1 ahcys + 1 b0203_v1_tRNA_3 + 1 h	irreversible	tRNA modification	0.00E+00	1.00E+09	0	1.11E+02	1.11E+02	0	9.54E+00	9.54	0	9.54E+00	9.54
b0203_v1_tRNA_54_MODIF_1	modification of position 54 nt (m5U) in alaV by TrmA_mono	1 TrmA_mono + 1 amet + 1 b0203_v1_tRNA_3 <==> 1 b0203_v1_tRNA_3_TrmA_mono_cplx	reversible	tRNA modification	-1.00E+09	1.00E+09	0	1.11E+02	1.11E+02	0	9.54E+00	9.54	0	9.54E+00	9.54
b0203_v1_tRNA_54_MODIF_2	modification of position 54 nt (m5U) in alaV by TrmA_mono	1 b0203_v1_tRNA_3_TrmA_mono_cplx --> 1 TrmA_mono_inact + 1 ahcys + 1 b0203_v1_tRNA_4 + 1 h	irreversible	tRNA modification	0.00E+00	1.00E+09	0	1.11E+02	1.11E+02	0	9.54E+00	9.54	0	9.54E+00	9.54
b0203_v1_tRNA_55_MODIF_1	modification of position 55 nt (Y) in alaV by TruB_mono	1 TruB_mono + 1 b0203_v1_tRNA_4 <==> 1 b0203_v1_tRNA_4_TruB_mono_cplx	reversible	tRNA modification	-1.00E+09	1.00E+09	0	1.11E+02	1.11E+02	0	9.54E+00	9.54	0	9.54E+00	9.54
b0203_v1_tRNA_55_MODIF_2	modification of position 55 nt (Y) in alaV by TruB_mono	1 b0203_v1_tRNA_4_TruB_mono_cplx --> 1 TruB_mono_inact + 1 alaV_tRNA	irreversible	tRNA modification	0.00E+00	1.00E+09	0	1.11E+02	1.11E+02	0	9.54E+00	9.54	0	9.54E+00	9.54
b0203_v1_tRNA_Mg_BIND	Binding of two Mg2 to b0203_v1_tRNA	1 b0203_v1_RNA + 2 mg2 <==> 1 b0203_v1_tRNA_Mg2	reversible	Metallo-ion binding	-1.00E+09	1.00E+09	0	1.11E+02	1.11E+02	0	9.54E+00	9.54	0	9.54E+00	9.54
b0204_RNA_10_modif_1	b0204_RNA modification (23S rRNA): position: 2445, m2G by MeT_23S_2445	1 MeT_23S_2445 + 1 amet + 1 b0204_RNA_10 <==> 1 b0204_RNA_10_MeT_23S_2445	reversible	rRNA modification	-1.00E+09	1.00E+09	0	1.11E+02	1.11E+02	0	0	0	0	0	0
b0204_RNA_10_modif_2	b0204_RNA modification (23S rRNA): position: 2445, m2G by MeT_23S_2445	1 b0204_RNA_10_MeT_23S_2445 --> 1 MeT_23S_2445_inact + 1 ahcys + 1 b0204_RNA_11 + 1 h	irreversible	rRNA modification	0.00E+00	1.00E+09	0	1.11E+02	1.11E+02	0	0	0	0	0	0
b0204_RNA_11_a_modif_1	b0204_RNA modification (23S rRNA): position: 2449, D by DU_23S_2449 (NADH)	1 DU_23S_2449 + 1 b0204_RNA_11 + 1 h + 1 nadh <==> 1 b0204_RNA_11_DU_23S_2449_a	reversible	rRNA modification	-1.00E+09	1.00E+09	0	1.11E+02	1.11E+02	0	0	0	0	0	0
b0204_RNA_11_a_modif_2	b0204_RNA modification (23S rRNA): position: 2449, D by DU_23S_2449 (NADH)	1 b0204_RNA_11_DU_23S_2449_a --> 1 DU_23S_2449_inact + 1 b0204_RNA_12 + 1 nad	irreversible	rRNA modification	0.00E+00	1.00E+09	0	1.11E+02	1.11E+02	0	0	0	0	0	0
b0204_RNA_11_b_modif_1	b0204_RNA modification (23S rRNA): position: 2449, D by DU_23S_2449 (NADPH)	1 DU_23S_2449 + 1 b0204_RNA_11 + 1 h + 1 nadph <==> 1 b0204_RNA_11_DU_23S_2449_b	reversible	rRNA modification	-1.00E+09	1.00E+09	0	1.11E+02	1.11E+02	0	0	0	0	0	0
b0204_RNA_11_b_modif_2	b0204_RNA modification (23S rRNA): position: 2449, D by DU_23S_2449 (NADPH)	1 b0204_RNA_11_DU_23S_2449_b --> 1 DU_23S_2449_inact + 1 b0204_RNA_12 + 1 nadp	irreversible	rRNA modification	0.00E+00	1.00E+09	0	1.11E+02	1.11E+02	0	0	0	0	0	0
b0204_RNA_12_modif_1	b0204_RNA modification (23S rRNA): position: 2457, Y by YmfC_mono	1 YmfC_mono + 1 b0204_RNA_12 <==> 1 b0204_RNA_12_YmfC_mono	reversible	rRNA modification	-1.00E+09	1.00E+09	0	1.11E+02	1.11E+02	0	0	0	0	0	0
b0204_RNA_12_modif_2	b0204_RNA modification (23S rRNA): position: 2457, Y by YmfC_mono	1 b0204_RNA_12_YmfC_mono --> 1 YmfC_mono_inact + 1 b0204_RNA_13	irreversible	rRNA modification	0.00E+00	1.00E+09	0	1.11E+02	1.11E+02	0	0	0	0	0	0
b0204_RNA_13_modif_1	b0204_RNA modification (23S rRNA): position: 2498, Cm by MeT_23S_2498	1 MeT_23S_2498 + 1 amet + 1 b0204_RNA_13 <==> 1 b0204_RNA_13_MeT_23S_2498	reversible	rRNA modification	-1.00E+09	1.00E+09	0	1.11E+02	1.11E+02	0	0	0	0	0	0
b0204_RNA_13_modif_2	b0204_RNA modification (23S rRNA): position: 2498, Cm by MeT_23S_2498	1 b0204_RNA_13_MeT_23S_2498 --> 1 MeT_23S_2498_inact + 1 ahcys + 1 b0204_RNA_14 + 1 h	irreversible	rRNA modification	0.00E+00	1.00E+09	0	1.11E+02	1.11E+02	0	0	0	0	0	0
b0204_RNA_14_modif_1	b0204_RNA modification (23S rRNA): position: 2503, m2A by MeT_23S_2503	1 MeT_23S_2503 + 1 amet + 1 b0204_RNA_14 <==> 1 b0204_RNA_14_MeT_23S_2503	reversible	rRNA modification	-1.00E+09	1.00E+09	0	1.11E+02	1.11E+02	0	0	0	0	0	0
b0204_RNA_14_modif_2	b0204_RNA modification (23S rRNA): position: 2503, m2A by MeT_23S_2503	1 b0204_RNA_14_MeT_23S_2503 --> 1 MeT_23S_2503_inact + 1 ahcys + 1 b0204_RNA_15 + 1 h	irreversible	rRNA modification	0.00E+00	1.00E+09	0	1.11E+02	1.11E+02	0	0	0	0	0	0
b0204_RNA_15_modif_1	b0204_RNA modification (23S rRNA): position: 2504, Y by RluC_mono	1 RluC_mono + 1 b0204_RNA_15 <==> 1 b0204_RNA_15_RluC_mono	reversible	rRNA modification	-1.00E+09	1.00E+09	0	1.11E+02	1.11E+02	0	0	0	0	0	0
b0204_RNA_15_modif_2	b0204_RNA modification (23S rRNA): position: 2504, Y by RluC_mono	1 b0204_RNA_15_RluC_mono --> 1 RluC_mono_inact + 1 b0204_RNA_16	irreversible	rRNA modification	0.00E+00	1.00E+09	0	1.11E+02	1.11E+02	0	0	0	0	0	0
b0204_RNA_16_modif_1	b0204_RNA modification (23S rRNA): position: 2552, Um by RrmJ_mono	1 RrmJ_mono + 1 amet + 1 b0204_RNA_16 <==> 1 b0204_RNA_16_RrmJ_mono	reversible	rRNA modification	-1.00E+09	1.00E+09	0	1.11E+02	1.11E+02	0	0	0	0	0	0
b0204_RNA_16_modif_2	b0204_RNA modification (23S rRNA): position: 2552, Um by RrmJ_mono	1 b0204_RNA_16_RrmJ_mono --> 1 RrmJ_mono_inact + 1 ahcys + b0204_RNA_17 + 1 h	irreversible	rRNA modification	0.00E+00	1.00E+09	0	1.11E+02	1.11E+02	0	0	0	0	0	0
b0204_RNA_17_modif_1	b0204_RNA modification (23S rRNA): position: 2580, Y by RluC_mono	1 RluC_mono + 1 b0204_RNA_17 <==> 1 b0204_RNA_17_RluC_mono	reversible	rRNA modification	-1.00E+09	1.00E+09	0	1.11E+02	1.11E+02	0	0	0	0	0	0
b0204_RNA_17_modif_2	b0204_RNA modification (23S rRNA): position: 2580, Y by RluC_mono	1 b0204_RNA_17_RluC_mono --> 1 RluC_mono_inact + 1 b0204_RNA_18	irreversible	rRNA modification	0.00E+00	1.00E+09	0	1.11E+02	1.11E+02	0	0	0	0	0	0
b0204_RNA_18_modif_1	b0204_RNA modification (23S rRNA): position: 2604, Y by YjbC_mono	1 YjbC_mono + 1 b0204_RNA_18 <==> 1 b0204_RNA_18_YjbC_mono	reversible	rRNA modification	-1.00E+09	1.00E+09	0	1.11E+02	1.11E+02	0	0	0	0	0	0
b0204_RNA_18_modif_2	b0204_RNA modification (23S rRNA): position: 2604, Y by YjbC_mono	1 b0204_RNA_18_YjbC_mono --> 1 YjbC_mono_inact + 1 b0204_RNA_19	irreversible	rRNA modification	0.00E+00	1.00E+09	0	1.11E+02	1.11E+02	0	0	0	0	0	0
b0204_RNA_19_modif_1	b0204_RNA modification (23S rRNA): position: 2605, Y by RluB_mono	1 RluB_mono + 1 b0204_RNA_19 <==> 1 b0204_RNA_19_RluB_mono	reversible	rRNA modification	-1.00E+09	1.00E+09	0	1.11E+02	1.11E+02	0	0	0	0	0	0
b0204_RNA_19_modif_2	b0204_RNA modification (23S rRNA): position: 2605, Y by RluB_mono	1 b0204_RNA_19_RluB_mono --> 1 RluB_mono_inact + 1 b0204_RNA_20	irreversible	rRNA modification	0.00E+00	1.00E+09	0	1.11E+02	1.11E+02	0	0	0	0	0	0
b0204_RNA_1_modif_1	b0204_RNA modification (23S rRNA): position: 1835, m2G by MeT_23S_1835	1 MeT_23S_1835 + 1 amet + 1 b0204_RNA_1 <==> 1 b0204_RNA_1_MeT_23S_1835	reversible	rRNA modification	-1.00E+09	1.00E+09	0	1.11E+02	1.11E+02	0	0	0	0	0	0
b0204_RNA_1_modif_2	b0204_RNA modification (23S rRNA): position: 1835, m2G by MeT_23S_1835	1 b0204_RNA_1_MeT_23S_1835 --> 1 MeT_23S_1835_inact + 1 ahcys + 1 b0204_RNA_2 + 1 h	irreversible	rRNA modification	0.00E+00	1.00E+09	0	1.11E+02	1.11E+02	0	0	0	0	0	0
b0204_RNA_20_modif_1	b0204_RNA modification (23S rRNA): position: 745, m1G by RrmA_dim	1 RrmA_dim + 1 amet + 1 b0204_RNA_20 <==> 1 b0204_RNA_20_RrmA_dim	reversible	rRNA modification	-1.00E+09	1.00E+09	0	1.11E+02	1.11E+02	0	0	0	0	0	0
b0204_RNA_20_modif_2	b0204_RNA modification (23S rRNA): position: 745, m1G by RrmA_dim	1 b0204_RNA_20_RrmA_dim --> 1 RrmA_dim_inact + 1 ahcys + 1 b0204_RNA_21 + 1 h	irreversible	rRNA modification	0.00E+00	1.00E+09	0	1.11E+02	1.11E+02	0	0	0	0	0	0
b0204_RNA_21_modif_1	b0204_RNA modification (23S rRNA): position: 746, Y by RluA_mono	1 RluA_mono + 1 b0204_RNA_21 <==> 1 b0204_RNA_21_RluA_mono	reversible	rRNA modification	-1.00E+09	1.00E+09	0	1.11E+02	1.11E+02	0	0	0	0	0	0
b0204_RNA_21_modif_2	b0204_RNA modification (23S rRNA): position: 746, Y by RluA_mono	1 b0204_RNA_21_RluA_mono --> 1 RluA_mono_inact + 1 b0204_RNA_22	irreversible	rRNA modification	0.00E+00	1.00E+09	0	1.11E+02	1.11E+02	0	0	0	0	0	0
b0204_RNA_22_modif_1	b0204_RNA modification (23S rRNA): position: 747, m5U by RumB_mono	1 RumB_mono + 1 amet + 1 b0204_RNA_22 <==> 1 b0204_RNA_22_RumB_mono	reversible	rRNA modification	-1.00E+09	1.00E+09	0	1.11E+02	1.11E+02	0	0	0	0	0	0
b0204_RNA_22_modif_2	b0204_RNA modification (23S rRNA): position: 747, m5U by RumB_mono	1 b0204_RNA_22_RumB_mono --> 1 RumB_mono_inact + 1 ahcys + 1 b0204_RNA_23 + 1 h	irreversible	rRNA modification	0.00E+00	1.00E+09	0	1.11E+02	1.11E+02	0	0	0	0	0	0
b0204_RNA_23_modif_1	b0204_RNA modification (23S rRNA): position: 955, Y by RluC_mono	1 RluC_mono + 1 b0204_RNA_23 <==> 1 b0204_RNA_23_RluC_mono	reversible	rRNA modification	-1.00E+09	1.00E+09	0	1.11E+02	1.11E+02	0	0	0	0	0	0
b0204_RNA_23_modif_2	b0204_RNA modification (23S rRNA): position: 955, Y by RluC_mono	1 b0204_RNA_23_RluC_mono --> 1 RluC_mono_inact + 1 rrlH	irreversible	rRNA modification	0.00E+00	1.00E+09	0	1.11E+02	1.11E+02	0	0	0	0	0	0

b0204_RNA_2_modif_1	b0204_RNA modification (23S rRNA): position: 1911, Y by RluD_mono	1 RluD_mono + 1 b0204_RNA_2 <==> 1 b0204_RNA_2_RluD_mono	reversible	rRNA modification	-1.00E+09	1.00E+09	0	1.11E+02	1.11E+02	0	0	0	0	0	0
b0204_RNA_2_modif_2	b0204_RNA modification (23S rRNA): position: 1911, Y by RluD_mono	1 b0204_RNA_2_RluD_mono --> 1 RluD_mono_inact + 1 b0204_RNA_3	irreversible	rRNA modification	0.00E+00	1.00E+09	0	1.11E+02	1.11E+02	0	0	0	0	0	0
b0204_RNA_3_modif_1	b0204_RNA modification (23S rRNA): position: 1915, m3Y by RluD_mono	1 RluD_mono + 1 amet + 1 b0204_RNA_3 <==> 1 b0204_RNA_3_RluD_mono	reversible	rRNA modification	-1.00E+09	1.00E+09	0	1.11E+02	1.11E+02	0	0	0	0	0	0
b0204_RNA_3_modif_2	b0204_RNA modification (23S rRNA): position: 1915, m3Y by RluD_mono	1 b0204_RNA_3_RluD_mono --> 1 RluD_mono_inact + 1 ahcys + 1 b0204_RNA_4 + 1 h	irreversible	rRNA modification	0.00E+00	1.00E+09	0	1.11E+02	1.11E+02	0	0	0	0	0	0
b0204_RNA_4_modif_1	b0204_RNA modification (23S rRNA): position: 1917, Y by RluD_mono	1 RluD_mono + 1 b0204_RNA_4 <==> 1 b0204_RNA_4_RluD_mono	reversible	rRNA modification	-1.00E+09	1.00E+09	0	1.11E+02	1.11E+02	0	0	0	0	0	0
b0204_RNA_4_modif_2	b0204_RNA modification (23S rRNA): position: 1917, Y by RluD_mono	1 b0204_RNA_4_RluD_mono --> 1 RluD_mono_inact + 1 b0204_RNA_5	irreversible	rRNA modification	0.00E+00	1.00E+09	0	1.11E+02	1.11E+02	0	0	0	0	0	0
b0204_RNA_5_modif_1	b0204_RNA modification (23S rRNA): position: 1939, m5U by RumA_mono	1 RumA_mono + 1 amet + 1 b0204_RNA_5 <==> 1 b0204_RNA_5_RumA_mono	reversible	rRNA modification	-1.00E+09	1.00E+09	0	1.11E+02	1.11E+02	0	0	0	0	0	0
b0204_RNA_5_modif_2	b0204_RNA modification (23S rRNA): position: 1939, m5U by RumA_mono	1 b0204_RNA_5_RumA_mono --> 1 RumA_mono_inact + 1 ahcys + 1 b0204_RNA_6 + 1 h	irreversible	rRNA modification	0.00E+00	1.00E+09	0	1.11E+02	1.11E+02	0	0	0	0	0	0
b0204_RNA_6_modif_1	b0204_RNA modification (23S rRNA): position: 1962, m5C by MeT_23S_1962	1 MeT_23S_1962 + 1 amet + 1 b0204_RNA_6 <==> 1 b0204_RNA_6_MeT_23S_1962	reversible	rRNA modification	-1.00E+09	1.00E+09	0	1.11E+02	1.11E+02	0	0	0	0	0	0
b0204_RNA_6_modif_2	b0204_RNA modification (23S rRNA): position: 1962, m5C by MeT_23S_1962	1 b0204_RNA_6_MeT_23S_1962 --> 1 MeT_23S_1962_inact + 1 ahcys + 1 b0204_RNA_7 + 1 h	irreversible	rRNA modification	0.00E+00	1.00E+09	0	1.11E+02	1.11E+02	0	0	0	0	0	0
b0204_RNA_7_modif_1	b0204_RNA modification (23S rRNA): position: 2030, m6A by MeT_23S_2030	1 MeT_23S_2030 + 1 amet + 1 b0204_RNA_7 <==> 1 b0204_RNA_7_MeT_23S_2030	reversible	rRNA modification	-1.00E+09	1.00E+09	0	1.11E+02	1.11E+02	0	0	0	0	0	0
b0204_RNA_7_modif_2	b0204_RNA modification (23S rRNA): position: 2030, m6A by MeT_23S_2030	1 b0204_RNA_7_MeT_23S_2030 --> 1 MeT_23S_2030_inact + 1 ahcys + 1 b0204_RNA_8 + 1 h	irreversible	rRNA modification	0.00E+00	1.00E+09	0	1.11E+02	1.11E+02	0	0	0	0	0	0
b0204_RNA_8_modif_1	b0204_RNA modification (23S rRNA): position: 2069, m7G by MeT_23S_2069	1 MeT_23S_2069 + 1 amet + 1 b0204_RNA_8 <==> 1 b0204_RNA_8_MeT_23S_2069	reversible	rRNA modification	-1.00E+09	1.00E+09	0	1.11E+02	1.11E+02	0	0	0	0	0	0
b0204_RNA_8_modif_2	b0204_RNA modification (23S rRNA): position: 2069, m7G by MeT_23S_2069	1 b0204_RNA_8_MeT_23S_2069 --> 1 MeT_23S_2069_inact + 1 ahcys + 1 b0204_RNA_9 + 1 h	irreversible	rRNA modification	0.00E+00	1.00E+09	0	1.11E+02	1.11E+02	0	0	0	0	0	0
b0204_RNA_9_modif_1	b0204_RNA modification (23S rRNA): position: 2251, Gm by RlmB_dim	1 RlmB_dim + 1 amet + 1 b0204_RNA_9 <==> 1 b0204_RNA_9_RlmB_dim	reversible	rRNA modification	-1.00E+09	1.00E+09	0	1.11E+02	1.11E+02	0	0	0	0	0	0
b0204_RNA_9_modif_2	b0204_RNA modification (23S rRNA): position: 2251, Gm by RlmB_dim	1 b0204_RNA_9_RlmB_dim --> 1 RlmB_dim_inact + 1 ahcys + 1 b0204_RNA_10 + 1 h	irreversible	rRNA modification	0.00E+00	1.00E+09	0	1.11E+02	1.11E+02	0	0	0	0	0	0
b0204_RNA_modif_1	b0204_RNA modification (23S rRNA): position: 1618, m6A by MeT_23S_1618	1 MeT_23S_1618 + 1 amet + 1 b0204_RNA <==> 1 b0204_RNA_MeT_23S_1618	reversible	rRNA modification	-1.00E+09	1.00E+09	0	1.11E+02	1.11E+02	0	0	0	0	0	0
b0204_RNA_modif_2	b0204_RNA modification (23S rRNA): position: 1618, m6A by MeT_23S_1618	1 b0204_RNA_MeT_23S_1618 --> 1 MeT_23S_1618_inact + 1 ahcy + 1 b0204_RNA_1 + 1 h	irreversible	rRNA modification	0.00E+00	1.00E+09	0	1.11E+02	1.11E+02	0	0	0	0	0	0
b0204_v1_RNA_10_modif _1	b0204_v1_RNA modification (23S rRNA): position: 2445, m2G by MeT_23S_2445	1 MeT_23S_2445 + 1 amet + 1 b0204_v1_RNA_10 <==> 1 b0204_v1_RNA_10_MeT_23S_2445	reversible	rRNA modification	-1.00E+09	1.00E+09	0	1.11E+02	1.11E+02	0	9.54E+00	9.54	0	9.54E+00	9.54
b0204_v1_RNA_10_modif _2	b0204_v1_RNA modification (23S rRNA): position: 2445, m2G by MeT_23S_2445	1 b0204_v1_RNA_10_MeT_23S_2445 --> 1 MeT_23S_2445_inact + 1 ahcys + 1 b0204_v1_RNA_11 + 1 h	irreversible	rRNA modification	0.00E+00	1.00E+09	0	1.11E+02	1.11E+02	0	9.54E+00	9.54	0	9.54E+00	9.54
b0204_v1_RNA_11_a_mo dif_1	b0204_v1_RNA modification (23S rRNA): position: 2449, D by DU_23S_2449 (NADH)	1 DU_23S_2449 + 1 b0204_v1_RNA_11 + 1 h + 1 nadh <==> 1 b0204_v1_RNA_11_DU_23S_2449_a	reversible	rRNA modification	-1.00E+09	1.00E+09	0	1.11E+02	1.11E+02	0	9.54E+00	9.54	0	9.54E+00	9.54
b0204_v1_RNA_11_a_mo dif_2	b0204_v1_RNA modification (23S rRNA): position: 2449, D by DU_23S_2449 (NADH)	1 b0204_v1_RNA_11_DU_23S_2449_a --> 1 DU_23S_2449_inact + 1 b0204_v1_RNA_12 + 1 nad	irreversible	rRNA modification	0.00E+00	1.00E+09	0	1.11E+02	1.11E+02	0	9.54E+00	9.54	0	9.54E+00	9.54
b0204_v1_RNA_11_b_mo dif_1	b0204_v1_RNA modification (23S rRNA): position: 2449, D by DU_23S_2449 (NADPH)	1 DU_23S_2449 + 1 b0204_v1_RNA_11 + 1 h + 1 nadph <==> 1 b0204_v1_RNA_11_DU_23S_2449_b	reversible	rRNA modification	-1.00E+09	1.00E+09	0	1.11E+02	1.11E+02	0	9.54E+00	9.54	0	9.54E+00	9.54
b0204_v1_RNA_11_b_mo dif_2	b0204_v1_RNA modification (23S rRNA): position: 2449, D by DU_23S_2449 (NADPH)	1 b0204_v1_RNA_11_DU_23S_2449_b --> 1 DU_23S_2449_inact + 1 b0204_v1_RNA_12 + 1 nadp	irreversible	rRNA modification	0.00E+00	1.00E+09	0	1.11E+02	1.11E+02	0	9.54E+00	9.54	0	9.54E+00	9.54
b0204_v1_RNA_12_modif _1	b0204_v1_RNA modification (23S rRNA): position: 2457, Y by YmfC_mono	1 YmfC_mono + 1 b0204_v1_RNA_12 <==> 1 b0204_v1_RNA_12_YmfC_mono	reversible	rRNA modification	-1.00E+09	1.00E+09	0	1.11E+02	1.11E+02	0	9.54E+00	9.54	0	9.54E+00	9.54
b0204_v1_RNA_12_modif _2	b0204_v1_RNA modification (23S rRNA): position: 2457, Y by YmfC_mono	1 b0204_v1_RNA_12_YmfC_mono --> 1 YmfC_mono_inact + 1 b0204_v1_RNA_13	irreversible	rRNA modification	0.00E+00	1.00E+09	0	1.11E+02	1.11E+02	0	9.54E+00	9.54	0	9.54E+00	9.54
b0204_v1_RNA_13_modif _1	b0204_v1_RNA modification (23S rRNA): position: 2498, Cm by MeT_23S_2498	1 MeT_23S_2498 + 1 amet + 1 b0204_v1_RNA_13 <==> 1 b0204_v1_RNA_13_MeT_23S_2498	reversible	rRNA modification	-1.00E+09	1.00E+09	0	1.11E+02	1.11E+02	0	9.54E+00	9.54	0	9.54E+00	9.54
b0204_v1_RNA_13_modif _2	b0204_v1_RNA modification (23S rRNA): position: 2498, Cm by MeT_23S_2498	1 b0204_v1_RNA_13_MeT_23S_2498 --> 1 MeT_23S_2498_inact + 1 ahcys + 1 b0204_v1_RNA_14 + 1 h	irreversible	rRNA modification	0.00E+00	1.00E+09	0	1.11E+02	1.11E+02	0	9.54E+00	9.54	0	9.54E+00	9.54
b0204_v1_RNA_14_modif _1	b0204_v1_RNA modification (23S rRNA): position: 2503, m2A by MeT_23S_2503	1 MeT_23S_2503 + 1 amet + 1 b0204_v1_RNA_14 <==> 1 b0204_v1_RNA_14_MeT_23S_2503	reversible	rRNA modification	-1.00E+09	1.00E+09	0	1.11E+02	1.11E+02	0	9.54E+00	9.54	0	9.54E+00	9.54
b0204_v1_RNA_14_modif _2	b0204_v1_RNA modification (23S rRNA): position: 2503, m2A by MeT_23S_2503	1 b0204_v1_RNA_14_MeT_23S_2503 --> 1 MeT_23S_2503_inact + 1 ahcys + 1 b0204_v1_RNA_15 + 1 h	irreversible	rRNA modification	0.00E+00	1.00E+09	0	1.11E+02	1.11E+02	0	9.54E+00	9.54	0	9.54E+00	9.54
b0204_v1_RNA_15_modif _1	b0204_v1_RNA modification (23S rRNA): position: 2504, Y by RluC_mono	1 RluC_mono + 1 b0204_v1_RNA_15 <==> 1 b0204_v1_RNA_15_RluC_mono	reversible	rRNA modification	-1.00E+09	1.00E+09	0	1.11E+02	1.11E+02	0	9.54E+00	9.54	0	9.54E+00	9.54
b0204_v1_RNA_15_modif _2	b0204_v1_RNA modification (23S rRNA): position: 2504, Y by RluC_mono	1 b0204_v1_RNA_15_RluC_mono --> 1 RluC_mono_inact + 1 b0204_v1_RNA_16	irreversible	rRNA modification	0.00E+00	1.00E+09	0	1.11E+02	1.11E+02	0	9.54E+00	9.54	0	9.54E+00	9.54
b0204_v1_RNA_16_modif _1	b0204_v1_RNA modification (23S rRNA): position: 2552, Um by RrmJ_mono	1 RrmJ_mono + 1 amet + 1 b0204_v1_RNA_16 <==> 1 b0204_v1_RNA_16_RrmJ_mono	reversible	rRNA modification	-1.00E+09	1.00E+09	0	1.11E+02	1.11E+02	0	9.54E+00	9.54	0	9.54E+00	9.54
b0204_v1_RNA_16_modif _2	b0204_v1_RNA modification (23S rRNA): position: 2552, Um by RrmJ_mono	1 b0204_v1_RNA_16_RrmJ_mono --> 1 RrmJ_mono_inact + 1 ahcy + 1 b0204_v1_RNA_17 + 1 h	irreversible	rRNA modification	0.00E+00	1.00E+09	0	1.11E+02	1.11E+02	0	9.54E+00	9.54	0	9.54E+00	9.54
b0204_v1_RNA_17_modif _1	b0204_v1_RNA modification (23S rRNA): position: 2580, Y by RluC_mono	1 RluC_mono + 1 b0204_v1_RNA_17 <==> 1 b0204_v1_RNA_17_RluC_mono	reversible	rRNA modification	-1.00E+09	1.00E+09	0	1.11E+02	1.11E+02	0	9.54E+00	9.54	0	9.54E+00	9.54
b0204_v1_RNA_17_modif _2	b0204_v1_RNA modification (23S rRNA): position: 2580, Y by RluC_mono	1 b0204_v1_RNA_17_RluC_mono --> 1 RluC_mono_inact + 1 b0204_v1_RNA_18	irreversible	rRNA modification	0.00E+00	1.00E+09	0	1.11E+02	1.11E+02	0	9.54E+00	9.54	0	9.54E+00	9.54
b0204_v1_RNA_18_modif _1	b0204_v1_RNA modification (23S rRNA): position: 2604, Y by YjbC_mono	1 YjbC_mono + 1 b0204_v1_RNA_18 <==> 1 b0204_v1_RNA_18_YjbC_mono	reversible	rRNA modification	-1.00E+09	1.00E+09	0	1.11E+02	1.11E+02	0	9.54E+00	9.54	0	9.54E+00	9.54
b0204_v1_RNA_18_modif _2	b0204_v1_RNA modification (23S rRNA): position: 2604, Y by YjbC_mono	1 b0204_v1_RNA_18_YjbC_mono --> 1 YjbC_mono_inact + 1 b0204_v1_RNA_19	irreversible	rRNA modification	0.00E+00	1.00E+09	0	1.11E+02	1.11E+02	0	9.54E+00	9.54	0	9.54E+00	9.54

b0204_v1_RNA_19_modif_1	b0204_v1_RNA modification (23S rRNA): position: 2605, Y by RluB_mono	1 RluB_mono + 1 b0204_v1_RNA_19 <==> 1 b0204_v1_RNA_19_RluB_mono	reversible	rRNA modification	-1.00E+09	1.00E+09	0	1.11E+02	1.11E+02	0	9.54E+00	9.54	0	9.54E+00	9.54
b0204_v1_RNA_19_modif_2	b0204_v1_RNA modification (23S rRNA): position: 2605, Y by RluB_mono	1 b0204_v1_RNA_19_RluB_mono --> 1 RluB_mono_inact + 1 b0204_v1_RNA_20	irreversible	rRNA modification	0.00E+00	1.00E+09	0	1.11E+02	1.11E+02	0	9.54E+00	9.54	0	9.54E+00	9.54
b0204_v1_RNA_1_modif_1	b0204_v1_RNA modification (23S rRNA): position: 1835, m2G by MeT_23S_1835	1 MeT_23S_1835 + 1 amet + 1 b0204_v1_RNA_1 <==> 1 b0204_v1_RNA_1_MeT_23S_1835	reversible	rRNA modification	-1.00E+09	1.00E+09	0	1.11E+02	1.11E+02	0	9.54E+00	9.54	0	9.54E+00	9.54
b0204_v1_RNA_1_modif_2	b0204_v1_RNA modification (23S rRNA): position: 1835, m2G by MeT_23S_1835	1 b0204_v1_RNA_1_MeT_23S_1835 --> 1 MeT_23S_1835_inact + 1 ahcys + 1 b0204_v1_RNA_2 + 1 h	irreversible	rRNA modification	0.00E+00	1.00E+09	0	1.11E+02	1.11E+02	0	9.54E+00	9.54	0	9.54E+00	9.54
b0204_v1_RNA_20_modif_1	b0204_v1_RNA modification (23S rRNA): position: 745, m1G by RrmA_dim	1 RrmA_dim + 1 amet + 1 b0204_v1_RNA_20 <==> 1 b0204_v1_RNA_20_RrmA_dim	reversible	rRNA modification	-1.00E+09	1.00E+09	0	1.11E+02	1.11E+02	0	9.54E+00	9.54	0	9.54E+00	9.54
b0204_v1_RNA_20_modif_2	b0204_v1_RNA modification (23S rRNA): position: 745, m1G by RrmA_dim	1 b0204_v1_RNA_20_RrmA_dim --> 1 RrmA_dim_inact + 1 ahcys + 1 b0204_v1_RNA_21 + 1 h	irreversible	rRNA modification	0.00E+00	1.00E+09	0	1.11E+02	1.11E+02	0	9.54E+00	9.54	0	9.54E+00	9.54
b0204_v1_RNA_21_modif_1	b0204_v1_RNA modification (23S rRNA): position: 746, Y by RluA_mono	1 RluA_mono + 1 b0204_v1_RNA_21 <==> 1 b0204_v1_RNA_21_RluA_mono	reversible	rRNA modification	-1.00E+09	1.00E+09	0	1.11E+02	1.11E+02	0	9.54E+00	9.54	0	9.54E+00	9.54
b0204_v1_RNA_21_modif_2	b0204_v1_RNA modification (23S rRNA): position: 746, Y by RluA_mono	1 b0204_v1_RNA_21_RluA_mono --> 1 RluA_mono_inact + 1 b0204_v1_RNA_22	irreversible	rRNA modification	0.00E+00	1.00E+09	0	1.11E+02	1.11E+02	0	9.54E+00	9.54	0	9.54E+00	9.54
b0204_v1_RNA_22_modif_1	b0204_v1_RNA modification (23S rRNA): position: 747, m5U by RumB_mono	1 RumB_mono + 1 amet + 1 b0204_v1_RNA_22 <==> 1 b0204_v1_RNA_22_RumB_mono	reversible	rRNA modification	-1.00E+09	1.00E+09	0	1.11E+02	1.11E+02	0	9.54E+00	9.54	0	9.54E+00	9.54
b0204_v1_RNA_22_modif_2	b0204_v1_RNA modification (23S rRNA): position: 747, m5U by RumB_mono	1 b0204_v1_RNA_22_RumB_mono --> 1 RumB_mono_inact + 1 ahcys + 1 b0204_v1_RNA_23 + 1 h	irreversible	rRNA modification	0.00E+00	1.00E+09	0	1.11E+02	1.11E+02	0	9.54E+00	9.54	0	9.54E+00	9.54
b0204_v1_RNA_23_modif_1	b0204_v1_RNA modification (23S rRNA): position: 955, Y by RluC_mono	1 RluC_mono + 1 b0204_v1_RNA_23 <==> 1 b0204_v1_RNA_23_RluC_mono	reversible	rRNA modification	-1.00E+09	1.00E+09	0	1.11E+02	1.11E+02	0	9.54E+00	9.54	0	9.54E+00	9.54
b0204_v1_RNA_23_modif_2	b0204_v1_RNA modification (23S rRNA): position: 955, Y by RluC_mono	1 b0204_v1_RNA_23_RluC_mono --> 1 RluC_mono_inact + 1 rrlH	irreversible	rRNA modification	0.00E+00	1.00E+09	0	1.11E+02	1.11E+02	0	9.54E+00	9.54	0	9.54E+00	9.54
b0204_v1_RNA_2_modif_1	b0204_v1_RNA modification (23S rRNA): position: 1911, Y by RluD_mono	1 RluD_mono + 1 b0204_v1_RNA_2 <==> 1 b0204_v1_RNA_2_RluD_mono	reversible	rRNA modification	-1.00E+09	1.00E+09	0	1.11E+02	1.11E+02	0	9.54E+00	9.54	0	9.54E+00	9.54
b0204_v1_RNA_2_modif_2	b0204_v1_RNA modification (23S rRNA): position: 1911, Y by RluD_mono	1 b0204_v1_RNA_2_RluD_mono --> 1 RluD_mono_inact + 1 b0204_v1_RNA_3	irreversible	rRNA modification	0.00E+00	1.00E+09	0	1.11E+02	1.11E+02	0	9.54E+00	9.54	0	9.54E+00	9.54
b0204_v1_RNA_3_modif_1	b0204_v1_RNA modification (23S rRNA): position: 1915, m3Y by RluD_mono	1 RluD_mono + 1 amet + 1 b0204_v1_RNA_3 <==> 1 b0204_v1_RNA_3_RluD_mono	reversible	rRNA modification	-1.00E+09	1.00E+09	0	1.11E+02	1.11E+02	0	9.54E+00	9.54	0	9.54E+00	9.54
b0204_v1_RNA_3_modif_2	b0204_v1_RNA modification (23S rRNA): position: 1915, m3Y by RluD_mono	1 b0204_v1_RNA_3_RluD_mono --> 1 RluD_mono_inact + 1 ahcys + 1 b0204_v1_RNA_4 + 1 h	irreversible	rRNA modification	0.00E+00	1.00E+09	0	1.11E+02	1.11E+02	0	9.54E+00	9.54	0	9.54E+00	9.54
b0204_v1_RNA_4_modif_1	b0204_v1_RNA modification (23S rRNA): position: 1917, Y by RluD_mono	1 RluD_mono + 1 b0204_v1_RNA_4 <==> 1 b0204_v1_RNA_4_RluD_mono	reversible	rRNA modification	-1.00E+09	1.00E+09	0	1.11E+02	1.11E+02	0	9.54E+00	9.54	0	9.54E+00	9.54
b0204_v1_RNA_4_modif_2	b0204_v1_RNA modification (23S rRNA): position: 1917, Y by RluD_mono	1 b0204_v1_RNA_4_RluD_mono --> 1 RluD_mono_inact + 1 b0204_v1_RNA_5	irreversible	rRNA modification	0.00E+00	1.00E+09	0	1.11E+02	1.11E+02	0	9.54E+00	9.54	0	9.54E+00	9.54
b0204_v1_RNA_5_modif_1	b0204_v1_RNA modification (23S rRNA): position: 1939, m5U by RumA_mono	1 RumA_mono + 1 amet + 1 b0204_v1_RNA_5 <==> 1 b0204_v1_RNA_5_RumA_mono	reversible	rRNA modification	-1.00E+09	1.00E+09	0	1.11E+02	1.11E+02	0	9.54E+00	9.54	0	9.54E+00	9.54
b0204_v1_RNA_5_modif_2	b0204_v1_RNA modification (23S rRNA): position: 1939, m5U by RumA_mono	1 b0204_v1_RNA_5_RumA_mono --> 1 RumA_mono_inact + 1 ahcy + 1 b0204_v1_RNA_6 + 1 h	irreversible	rRNA modification	0.00E+00	1.00E+09	0	1.11E+02	1.11E+02	0	9.54E+00	9.54	0	9.54E+00	9.54
b0204_v1_RNA_6_modif_1	b0204_v1_RNA modification (23S rRNA): position: 1962, m5C by MeT_23S_1962	1 MeT_23S_1962 + 1 amet + 1 b0204_v1_RNA_6 <==> 1 b0204_v1_RNA_6_MeT_23S_1962	reversible	rRNA modification	-1.00E+09	1.00E+09	0	1.11E+02	1.11E+02	0	9.54E+00	9.54	0	9.54E+00	9.54
b0204_v1_RNA_6_modif_2	b0204_v1_RNA modification (23S rRNA): position: 1962, m5C by MeT_23S_1962	1 b0204_v1_RNA_6_MeT_23S_1962 --> 1 MeT_23S_1962_inact + 1 ahcys + 1 b0204_v1_RNA_7 + 1 h	irreversible	rRNA modification	0.00E+00	1.00E+09	0	1.11E+02	1.11E+02	0	9.54E+00	9.54	0	9.54E+00	9.54
b0204_v1_RNA_7_modif_1	b0204_v1_RNA modification (23S rRNA): position: 2030, m6A by MeT_23S_2030	1 MeT_23S_2030 + 1 amet + 1 b0204_v1_RNA_7 <==> 1 b0204_v1_RNA_7_MeT_23S_2030	reversible	rRNA modification	-1.00E+09	1.00E+09	0	1.11E+02	1.11E+02	0	9.54E+00	9.54	0	9.54E+00	9.54
b0204_v1_RNA_7_modif_2	b0204_v1_RNA modification (23S rRNA): position: 2030, m6A by MeT_23S_2030	1 b0204_v1_RNA_7_MeT_23S_2030 --> 1 MeT_23S_2030_inact + 1 ahcys + 1 b0204_v1_RNA_8 + 1 h	irreversible	rRNA modification	0.00E+00	1.00E+09	0	1.11E+02	1.11E+02	0	9.54E+00	9.54	0	9.54E+00	9.54
b0204_v1_RNA_8_modif_1	b0204_v1_RNA modification (23S rRNA): position: 2069, m7G by MeT_23S_2069	1 MeT_23S_2069 + 1 amet + 1 b0204_v1_RNA_8 <==> 1 b0204_v1_RNA_8_MeT_23S_2069	reversible	rRNA modification	-1.00E+09	1.00E+09	0	1.11E+02	1.11E+02	0	9.54E+00	9.54	0	9.54E+00	9.54
b0204_v1_RNA_8_modif_2	b0204_v1_RNA modification (23S rRNA): position: 2069, m7G by MeT_23S_2069	1 b0204_v1_RNA_8_MeT_23S_2069 --> 1 MeT_23S_2069_inact + 1 ahcys + 1 b0204_v1_RNA_9 + 1 h	irreversible	rRNA modification	0.00E+00	1.00E+09	0	1.11E+02	1.11E+02	0	9.54E+00	9.54	0	9.54E+00	9.54
b0204_v1_RNA_9_modif_1	b0204_v1_RNA modification (23S rRNA): position: 2251, Gm by RlmB_dim	1 RlmB_dim + 1 amet + 1 b0204_v1_RNA_9 <==> 1 b0204_v1_RNA_9_RlmB_dim	reversible	rRNA modification	-1.00E+09	1.00E+09	0	1.11E+02	1.11E+02	0	9.54E+00	9.54	0	9.54E+00	9.54
b0204_v1_RNA_9_modif_2	b0204_v1_RNA modification (23S rRNA): position: 2251, Gm by RlmB_dim	1 b0204_v1_RNA_9_RlmB_dim --> 1 RlmB_dim_inact + 1 ahcys + 1 b0204_v1_RNA_10 + 1 h	irreversible	rRNA modification	0.00E+00	1.00E+09	0	1.11E+02	1.11E+02	0	9.54E+00	9.54	0	9.54E+00	9.54
b0204_v1_RNA_modif_1	b0204_v1_RNA modification (23S rRNA): position: 1618, m6A by MeT_23S_1618	1 MeT_23S_1618 + 1 amet + 1 b0204_v1_RNA <==> 1 b0204_v1_RNA_MeT_23S_1618	reversible	rRNA modification	-1.00E+09	1.00E+09	0	1.11E+02	1.11E+02	0	9.54E+00	9.54	0	9.54E+00	9.54
b0204_v1_RNA_modif_2	b0204_v1_RNA modification (23S rRNA): position: 1618, m6A by MeT_23S_1618	1 b0204_v1_RNA_MeT_23S_1618 --> 1 MeT_23S_1618_inact + 1 ahcys + 1 b0204_v1_RNA_1 + 1 h	irreversible	rRNA modification	0.00E+00	1.00E+09	0	1.11E+02	1.11E+02	0	9.54E+00	9.54	0	9.54E+00	9.54
b0206_DNA_act_bind	b0206_DNA binding of activator	1 b0206_DNA_neu --> 1 b0206_DNA_act	irreversible	Transcription Regulation	0.00E+00	1.00E+09	0	1.45E+03	1.45E+03	0	9.52E+00	9.52	0	9.52E+00	9.52
b0206_tRNA_16_MODIF_1	modification of position 16 nt (D) in aspU by Dus_gen	1 Dus_gen + 1 b0206_tRNA_Mg2 + 1 h + 1 nadph <==> 1 b0206_tRNA_Mg2_Dus_gen_cplx	reversible	tRNA modification	-1.00E+09	1.00E+09	0	1.45E+03	1.45E+03	0	9.52E+00	9.52	0	9.52E+00	9.52
b0206_tRNA_16_MODIF_2	modification of position 16 nt (D) in aspU by Dus_gen	1 b0206_tRNA_Mg2_Dus_gen_cplx --> 1 Dus_gen_inact + 1 b0206_tRNA_1 + 1 nadp	irreversible	tRNA modification	0.00E+00	1.00E+09	0	1.45E+03	1.45E+03	0	9.52E+00	9.52	0	9.52E+00	9.52
b0206_tRNA_20A_MODIF_1	modification of position 20A nt (D) in aspU by Dus_gen	1 Dus_gen + 1 b0206_tRNA_2 + 1 h + 1 nadph <==> 1 b0206_tRNA_2_Dus_gen_cplx	reversible	tRNA modification	-1.00E+09	1.00E+09	0	1.45E+03	1.45E+03	0	9.52E+00	9.52	0	9.52E+00	9.52
b0206_tRNA_20A_MODIF_2	modification of position 20A nt (D) in aspU by Dus_gen	1 b0206_tRNA_2_Dus_gen_cplx --> 1 Dus_gen_inact + 1 b0206_tRNA_3 + 1 nadp	irreversible	tRNA modification	0.00E+00	1.00E+09	0	1.45E+03	1.45E+03	0	9.52E+00	9.52	0	9.52E+00	9.52
b0206_tRNA_20_MODIF_1	modification of position 20 nt (D) in aspU by Dus_gen	1 Dus_gen + 1 b0206_tRNA_1 + 1 h + 1 nadph <==> 1 b0206_tRNA_1_Dus_gen_cplx	reversible	tRNA modification	-1.00E+09	1.00E+09	0	1.45E+03	1.45E+03	0	9.52E+00	9.52	0	9.52E+00	9.52
b0206_tRNA_20_MODIF_2	modification of position 20 nt (D) in aspU by Dus_gen	1 b0206_tRNA_1_Dus_gen_cplx --> 1 Dus_gen_inact + 1 b0206_tRNA_2 + 1 nadp	irreversible	tRNA modification	0.00E+00	1.00E+09	0	1.45E+03	1.45E+03	0	9.52E+00	9.52	0	9.52E+00	9.52
b0206_tRNA_34_MODIF_1	modification of position 34 nt (Q) in aspU by Tgt_hexa-QueA_mono-EoR_tRNA_pos34_Q	1 EoR_tRNA_pos34_Q + 1 QueA_mono + 1 Tgt_hexa + 1 adocbl + 1 amet + 1 b0206_tRNA_3 + 1 h + 1 nadh + 1 pre_Q1 <==> 1 b0206_tRNA_3_Tgt_hexa-QueA_mono-EoR_tRNA_pos34_Q_cplx	reversible	tRNA modification	-1.00E+09	1.00E+09	0	1.45E+03	1.45E+03	0	9.52E+00	9.52	0	9.52E+00	9.52
b0206_tRNA_34_MODIF_2	modification of position 34 nt (Q) in aspU by Tgt_hexa-QueA_mono-EoR_tRNA_pos34_Q	1 b0206_tRNA_3_Tgt_hexa-QueA_mono-EoR_tRNA_pos34_Q_cplx > 1 EoR_tRNA_pos34_Q_inact + 1 QueA_mono_inact + 1 Tgt_hexa_inact + 1 ade + 1 adocbl + 1 b0206_tRNA_4 + 1 gua + 2 h 1 h2o + 1 met-L + 1 nad	irreversible	tRNA modification	0.00E+00	1.00E+09	0	1.45E+03	1.45E+03	0	9.52E+00	9.52	0	9.52E+00	9.52
b0206_tRNA_37_MODIF_1	modification of position 37 nt (m2A) in aspU by MeT_tRNA_pos_37_m2A	1 MeT_tRNA_pos_37_m2A + 1 amet + 1 b0206_tRNA_4 <==> 1 b0206_tRNA_4_MeT_tRNA_pos_37_m2A_cplx	reversible	tRNA modification	-1.00E+09	1.00E+09	0	1.45E+03	1.45E+03	0	9.52E+00	9.52	0	9.52E+00	9.52

b0206_tRNA_37_MODIF_2	modification of position 37 nt (m2A) in aspU by MeT_tRNA_pos_37_m2A	1 b0206_tRNA_4_MeT_tRNA_pos_37_m2A_cplx --> 1 MeT_tRNA_pos_37_m2A_inact + 1 ahcys + 1 b0206_tRNA_5 + 1 h	irreversible	tRNA modification	0.00E+00	1.00E+09	0	1.45E+03	1.45E+03	0	9.52E+00	9.52	0	9.52E+00	9.52
b0206_tRNA_46_MODIF_1	modification of position 46 nt (m7G) in aspU by YggH_mono	1 YggH_mono + 1 amet + 1 b0206_tRNA_5 <==> 1 b0206_tRNA_5_YggH_mono_cplx	reversible	tRNA modification	-1.00E+09	1.00E+09	0	1.45E+03	1.45E+03	0	9.52E+00	9.52	0	9.52E+00	9.52
b0206_tRNA_46_MODIF_2	modification of position 46 nt (m7G) in aspU by YggH_mono	1 b0206_tRNA_5_YggH_mono_cplx --> 1 YggH_mono_inact + 1 ahcys + 1 b0206_tRNA_6 + 1 h	irreversible	tRNA modification	0.00E+00	1.00E+09	0	1.45E+03	1.45E+03	0	9.52E+00	9.52	0	9.52E+00	9.52
b0206_tRNA_54_MODIF_1	modification of position 54 nt (m5U) in aspU by TrmA_mono	1 TrmA_mono + 1 amet + 1 b0206_tRNA_6 <==> 1 b0206_tRNA_6_TrmA_mono_cplx	reversible	tRNA modification	-1.00E+09	1.00E+09	0	1.45E+03	1.45E+03	0	9.52E+00	9.52	0	9.52E+00	9.52
b0206_tRNA_54_MODIF_2	modification of position 54 nt (m5U) in aspU by TrmA_mono	1 b0206_tRNA_6_TrmA_mono_cplx --> 1 TrmA_mono_inact + 1 ahcys + 1 b0206_tRNA_7 + 1 h	irreversible	tRNA modification	0.00E+00	1.00E+09	0	1.45E+03	1.45E+03	0	9.52E+00	9.52	0	9.52E+00	9.52
b0206_tRNA_55_MODIF_1	modification of position 55 nt (Y) in aspU by TruB_mono	1 TruB_mono + 1 b0206_tRNA_7 <==> 1 b0206_tRNA_7_TruB_mono_cplx	reversible	tRNA modification	-1.00E+09	1.00E+09	0	1.45E+03	1.45E+03	0	9.52E+00	9.52	0	9.52E+00	9.52
b0206_tRNA_55_MODIF_2	modification of position 55 nt (Y) in aspU by TruB_mono	1 b0206_tRNA_7_TruB_mono_cplx --> 1 TruB_mono_inact + 1 b0206_tRNA_8	irreversible	tRNA modification	0.00E+00	1.00E+09	0	1.45E+03	1.45E+03	0	9.52E+00	9.52	0	9.52E+00	9.52
b0206_tRNA_65_MODIF_1	modification of position 65 nt (Y) in aspU by YqcB_mono	1 YqcB_mono + 1 b0206_tRNA_8 <==> 1 b0206_tRNA_8_YqcB_mono_cplx	reversible	tRNA modification	-1.00E+09	1.00E+09	0	1.45E+03	1.45E+03	0	9.52E+00	9.52	0	9.52E+00	9.52
b0206_tRNA_65_MODIF_2	modification of position 65 nt (Y) in aspU by YqcB_mono	1 b0206_tRNA_8_YqcB_mono_cplx --> 1 YqcB_mono_inact + 1 b0206_tRNA_9	irreversible	tRNA modification	0.00E+00	1.00E+09	0	1.45E+03	1.45E+03	0	9.52E+00	9.52	0	9.52E+00	9.52
b0206_tRNA_8_MODIF_1	modification of position 8 nt (s4U) in aspU by ThiI_mono	1 IscS_dim_S-SH + 1 ThiI_mono + 1 atp + 1 b0206_tRNA_9 + 1 mg2 + 1 trdrd <==> 1 b0206_tRNA_9_ThiI_mono_cplx	reversible	tRNA modification	-1.00E+09	1.00E+09	0	1.45E+03	1.45E+03	0	9.52E+00	9.52	0	9.52E+00	9.52
b0206_tRNA_8_MODIF_2	modification of position 8 nt (s4U) in aspU by ThiI_mono	1 b0206_tRNA_9_ThiI_mono_cplx --> 1 IscS_dim_S-H + 1 ThiI_mono_inact + 1 amp + 1 aspU_tRNA + 1 h + 1 mg2 + 1 ppi + 1 trdox	irreversible	tRNA modification	0.00E+00	1.00E+09	0	1.45E+03	1.45E+03	0	9.52E+00	9.52	0	9.52E+00	9.52
b0206_tRNA_Mg_BIND	Binding of two Mg2 to b0206_tRNA	1 b0206_RNA + 2 mg2 <==> 1 b0206_tRNA_Mg2	reversible	Metallo-ion binding	-1.00E+09	1.00E+09	0	1.45E+03	1.45E+03	0	9.52E+00	9.52	0	9.52E+00	9.52
b0206_trimming	Cutting of stable b0206_RNA	1 b0206_RNA_cut_cplx + 1 h2o --> 1 RNase_E_tetra_inact + 1 RNase_Gen_inact + 1 RNase_P_cplx_inact + 1 b0206_RNA + 1 h + ppi	irreversible	RNA Processing	0.00E+00	1.00E+09	0	1.45E+03	1.45E+03	0	9.52E+00	9.52	0	9.52E+00	9.52
b0206_trimming_cplx	Cutting complex formation of stable b0206_RNA (, b0206)	1 RNase_E_tetra + 1 RNase_Gen + 1 RNase_P_cplx + 1 b0206_RNA_pre <==> 1 b0206_RNA_cut_cplx	reversible	RNA Processing	-1.00E+09	1.00E+09	0	1.45E+03	1.45E+03	0	9.52E+00	9.52	0	9.52E+00	9.52
b0216_tRNA_16_MODIF_1	modification of position 16 nt (D) in aspV by Dus_gen	1 Dus_gen + 1 b0216_tRNA_Mg2 + 1 h + 1 nadph <==> 1 b0216_tRNA_Mg2_Dus_gen_cplx	reversible	tRNA modification	-1.00E+09	1.00E+09	0	1.45E+03	1.45E+03	0	9.48E+00	9.48	0	9.48E+00	9.48
b0216_tRNA_16_MODIF_2	modification of position 16 nt (D) in aspV by Dus_gen	1 b0216_tRNA_Mg2_Dus_gen_cplx --> 1 Dus_gen_inact + 1 b0216_tRNA_1 + 1 nadp	irreversible	tRNA modification	0.00E+00	1.00E+09	0	1.45E+03	1.45E+03	0	9.48E+00	9.48	0	9.48E+00	9.48
b0216_tRNA_20A_MODIF_1	modification of position 20A nt (D) in aspV by Dus_gen	1 Dus_gen + 1 b0216_tRNA_2 + 1 h + 1 nadph <==> 1 b0216_tRNA_2_Dus_gen_cplx	reversible	tRNA modification	-1.00E+09	1.00E+09	0	1.45E+03	1.45E+03	0	9.48E+00	9.48	0	9.48E+00	9.48
b0216_tRNA_20A_MODIF_2	modification of position 20A nt (D) in aspV by Dus_gen	1 b0216_tRNA_2_Dus_gen_cplx --> 1 Dus_gen_inact + 1 b0216_tRNA_3 + 1 nadp	irreversible	tRNA modification	0.00E+00	1.00E+09	0	1.45E+03	1.45E+03	0	9.48E+00	9.48	0	9.48E+00	9.48
b0216_tRNA_20_MODIF_1	modification of position 20 nt (D) in aspV by Dus_gen	1 Dus_gen + 1 b0216_tRNA_1 + 1 h + 1 nadph <==> 1 b0216_tRNA_1_Dus_gen_cplx	reversible	tRNA modification	-1.00E+09	1.00E+09	0	1.45E+03	1.45E+03	0	9.48E+00	9.48	0	9.48E+00	9.48
b0216_tRNA_20_MODIF_2	modification of position 20 nt (D) in aspV by Dus_gen	1 b0216_tRNA_1_Dus_gen_cplx --> 1 Dus_gen_inact + 1 b0216_tRNA_2 + 1 nadp	irreversible	tRNA modification	0.00E+00	1.00E+09	0	1.45E+03	1.45E+03	0	9.48E+00	9.48	0	9.48E+00	9.48
b0216_tRNA_34_MODIF_1	modification of position 34 nt (Q) in aspV by Tgt_hexa-QueA_mono-EoR_tRNA_pos34_Q	1 EoR_tRNA_pos34_Q + 1 QueA_mono + 1 Tgt_hexa + 1 adocbl + 1 amet + 1 b0216_tRNA_3 + 1 h + 1 nadh + 1 pre_Q1 <==> 1 b0216_tRNA_3_Tgt_hexa-QueA_mono-EoR_tRNA_pos34_Q_cplx	reversible	tRNA modification	-1.00E+09	1.00E+09	0	1.45E+03	1.45E+03	0	9.48E+00	9.48	0	9.48E+00	9.48
b0216_tRNA_34_MODIF_2	modification of position 34 nt (Q) in aspV by Tgt_hexa-QueA_mono-EoR_tRNA_pos34_Q	1 b0216_tRNA_3_Tgt_hexa-QueA_mono-EoR_tRNA_pos34_Q_cplx > 1 EoR_tRNA_pos34_Q_inact + 1 QueA_mono_inact + 1 Tgt_hexa_inact + 1 ade + 1 adocbl + 1 b0216_tRNA_4 + 1 gua + 2 h 1 h2o + 1 met-L + 1 nad	irreversible	tRNA modification	0.00E+00	1.00E+09	0	1.45E+03	1.45E+03	0	9.48E+00	9.48	0	9.48E+00	9.48
b0216_tRNA_37_MODIF_1	modification of position 37 nt (m2A) in aspV by MeT_tRNA_pos_37_m2A	1 MeT_tRNA_pos_37_m2A + 1 amet + 1 b0216_tRNA_4 <==> 1 b0216_tRNA_4_MeT_tRNA_pos_37_m2A_cplx	reversible	tRNA modification	-1.00E+09	1.00E+09	0	1.45E+03	1.45E+03	0	9.48E+00	9.48	0	9.48E+00	9.48
b0216_tRNA_37_MODIF_2	modification of position 37 nt (m2A) in aspV by MeT_tRNA_pos_37_m2A	1 b0216_tRNA_4_MeT_tRNA_pos_37_m2A_cplx --> 1 MeT_tRNA_pos_37_m2A_inact + 1 ahcys + 1 b0216_tRNA_5 + 1 h	irreversible	tRNA modification	0.00E+00	1.00E+09	0	1.45E+03	1.45E+03	0	9.48E+00	9.48	0	9.48E+00	9.48
b0216_tRNA_46_MODIF_1	modification of position 46 nt (m7G) in aspV by YggH_mono	1 YggH_mono + 1 amet + 1 b0216_tRNA_5 <==> 1 b0216_tRNA_5_YggH_mono_cplx	reversible	tRNA modification	-1.00E+09	1.00E+09	0	1.45E+03	1.45E+03	0	9.48E+00	9.48	0	9.48E+00	9.48
b0216_tRNA_46_MODIF_2	modification of position 46 nt (m7G) in aspV by YggH_mono	1 b0216_tRNA_5_YggH_mono_cplx --> 1 YggH_mono_inact + 1 ahcys + 1 b0216_tRNA_6 + 1 h	irreversible	tRNA modification	0.00E+00	1.00E+09	0	1.45E+03	1.45E+03	0	9.48E+00	9.48	0	9.48E+00	9.48
b0216_tRNA_54_MODIF_1	modification of position 54 nt (m5U) in aspV by TrmA_mono	1 TrmA_mono + 1 amet + 1 b0216_tRNA_6 <==> 1 b0216_tRNA_6_TrmA_mono_cplx	reversible	tRNA modification	-1.00E+09	1.00E+09	0	1.45E+03	1.45E+03	0	9.48E+00	9.48	0	9.48E+00	9.48
b0216_tRNA_54_MODIF_2	modification of position 54 nt (m5U) in aspV by TrmA_mono	1 b0216_tRNA_6_TrmA_mono_cplx --> 1 TrmA_mono_inact + 1 ahcys + 1 b0216_tRNA_7 + 1 h	irreversible	tRNA modification	0.00E+00	1.00E+09	0	1.45E+03	1.45E+03	0	9.48E+00	9.48	0	9.48E+00	9.48
b0216_tRNA_55_MODIF_1	modification of position 55 nt (Y) in aspV by TruB_mono	1 TruB_mono + 1 b0216_tRNA_7 <==> 1 b0216_tRNA_7_TruB_mono_cplx	reversible	tRNA modification	-1.00E+09	1.00E+09	0	1.45E+03	1.45E+03	0	9.48E+00	9.48	0	9.48E+00	9.48
b0216_tRNA_55_MODIF_2	modification of position 55 nt (Y) in aspV by TruB_mono	1 b0216_tRNA_7_TruB_mono_cplx --> 1 TruB_mono_inact + 1 b0216_tRNA_8	irreversible	tRNA modification	0.00E+00	1.00E+09	0	1.45E+03	1.45E+03	0	9.48E+00	9.48	0	9.48E+00	9.48
b0216_tRNA_65_MODIF_1	modification of position 65 nt (Y) in aspV by YqcB_mono	1 YqcB_mono + 1 b0216_tRNA_8 <==> 1 b0216_tRNA_8_YqcB_mono_cplx	reversible	tRNA modification	-1.00E+09	1.00E+09	0	1.45E+03	1.45E+03	0	9.48E+00	9.48	0	9.48E+00	9.48
b0216_tRNA_65_MODIF_2	modification of position 65 nt (Y) in aspV by YqcB_mono	1 b0216_tRNA_8_YqcB_mono_cplx --> 1 YqcB_mono_inact + 1 b0216_tRNA_9	irreversible	tRNA modification	0.00E+00	1.00E+09	0	1.45E+03	1.45E+03	0	9.48E+00	9.48	0	9.48E+00	9.48
b0216_tRNA_8_MODIF_1	modification of position 8 nt (s4U) in aspV by ThiI_mono	1 IscS_dim_S-SH + 1 ThiI_mono + 1 atp + 1 b0216_tRNA_9 + 1 mg2 + 1 trdrd <==> 1 b0216_tRNA_9_ThiI_mono_cplx	reversible	tRNA modification	-1.00E+09	1.00E+09	0	1.45E+03	1.45E+03	0	9.48E+00	9.48	0	9.48E+00	9.48
b0216_tRNA_8_MODIF_2	modification of position 8 nt (s4U) in aspV by ThiI_mono	1 b0216_tRNA_9_ThiI_mono_cplx --> 1 IscS_dim_S-H + 1 ThiI_mono_inact + 1 amp + 1 aspV_tRNA + 1 h + 1 mg2 + 1 ppi + 1 trdox	irreversible	tRNA modification	0.00E+00	1.00E+09	0	1.45E+03	1.45E+03	0	9.48E+00	9.48	0	9.48E+00	9.48
b0216_tRNA_Mg_BIND	Binding of two Mg2 to b0216_tRNA	1 b0216_RNA + 2 mg2 <==> 1 b0216_tRNA_Mg2	reversible	Metallo-ion binding	-1.00E+09	1.00E+09	0	1.45E+03	1.45E+03	0	9.48E+00	9.48	0	9.48E+00	9.48
b0244_tRNA_16_MODIF_1	modification of position 16 nt (D) in thrW by Dus_gen	1 Dus_gen + 1 b0244_tRNA_Mg2 + 1 h + 1 nadph <==> 1 b0244_tRNA_Mg2_Dus_gen_cplx	reversible	tRNA modification	-1.00E+09	1.00E+09	0	1.77E+03	1.77E+03	0	9.38E+00	9.38	0	9.38E+00	9.38
b0244_tRNA_16_MODIF_2	modification of position 16 nt (D) in thrW by Dus_gen	1 b0244_tRNA_Mg2_Dus_gen_cplx --> 1 Dus_gen_inact + 1 b0244_tRNA_1 + 1 nadp	irreversible	tRNA modification	0.00E+00	1.00E+09	0	1.77E+03	1.77E+03	0	9.38E+00	9.38	0	9.38E+00	9.38
b0244_tRNA_17_MODIF_1	modification of position 17 nt (D) in thrW by Dus_gen	1 Dus_gen + 1 b0244_tRNA_1 + 1 h + 1 nadph <==> 1 b0244_tRNA_1_Dus_gen_cplx	reversible	tRNA modification	-1.00E+09	1.00E+09	0	1.77E+03	1.77E+03	0	9.38E+00	9.38	0	9.38E+00	9.38
b0244_tRNA_17_MODIF_2	modification of position 17 nt (D) in thrW by Dus_gen	1 b0244_tRNA_1_Dus_gen_cplx --> 1 Dus_gen_inact + 1 b0244_tRNA_2 + 1 nadp	irreversible	tRNA modification	0.00E+00	1.00E+09	0	1.77E+03	1.77E+03	0	9.38E+00	9.38	0	9.38E+00	9.38
b0244_tRNA_20_MODIF_1	modification of position 20 nt (D) in thrW by Dus_gen	1 Dus_gen + 1 b0244_tRNA_2 + 1 h + 1 nadph <==> 1 b0244_tRNA_2_Dus_gen_cplx	reversible	tRNA modification	-1.00E+09	1.00E+09	0	1.77E+03	1.77E+03	0	9.38E+00	9.38	0	9.38E+00	9.38
b0244_tRNA_20_MODIF_2	modification of position 20 nt (D) in thrW by Dus_gen	1 b0244_tRNA_2_Dus_gen_cplx --> 1 Dus_gen_inact + 1 b0244_tRNA_3 + 1 nadp	irreversible	tRNA modification	0.00E+00	1.00E+09	0	1.77E+03	1.77E+03	0	9.38E+00	9.38	0	9.38E+00	9.38
b0244_tRNA_37_MODIF_1	modification of position 37 nt (m6t6A) in thrW by Up_tRNA_pos_37_t6A-MeT_tRNA_pos_37_m6t6A	1 MeT_tRNA_pos_37_m6t6A + 1 Up_tRNA_pos_37_t6A + 1 amet + atp + 1 b0244_tRNA_3 + 1 hco3 + 1 mg2 + 1 thr-L <==> 1 b0244_tRNA_3_Up_tRNA_pos_37_t6A-MeT_tRNA_pos_37_m6t6A_cplx	reversible	tRNA modification	-1.00E+09	1.00E+09	0	1.77E+03	1.77E+03	0	9.38E+00	9.38	0	9.38E+00	9.38
b0244_tRNA_37_MODIF_2	modification of position 37 nt (m6t6A) in thrW by Up_tRNA_pos_37_t6A-MeT_tRNA_pos_37_m6t6A	1 b0244_tRNA_3_Up_tRNA_pos_37_t6A-MeT_tRNA_pos_37_m6t6A_cplx --> 1 MeT_tRNA_pos_37_m6t6A_inact + 1 Up_tRNA_pos_37_t6A_inact + 1 ahcys + 1 amp + 1 b0244_tRNA_4 + 2 h + 1 h2o + 1 mg2 + 1 ppi	irreversible	tRNA modification	0.00E+00	1.00E+09	0	1.77E+03	1.77E+03	0	9.38E+00	9.38	0	9.38E+00	9.38
b0244_tRNA_46_MODIF_1	modification of position 46 nt (m7G) in thrW by YggH_mono	1 YggH_mono + 1 amet + 1 b0244_tRNA_4 <==> 1 b0244_tRNA_4_YggH_mono_cplx	reversible	tRNA modification	-1.00E+09	1.00E+09	0	1.77E+03	1.77E+03	0	9.38E+00	9.38	0	9.38E+00	9.38
b0244_tRNA_46_MODIF_2	modification of position 46 nt (m7G) in thrW by YggH_mono	1 b0244_tRNA_4_YggH_mono_cplx --> 1 YggH_mono_inact + 1 ahcys + 1 b0244_tRNA_5 + 1 h	irreversible	tRNA modification	0.00E+00	1.00E+09	0	1.77E+03	1.77E+03	0	9.38E+00	9.38	0	9.38E+00	9.38
b0244_tRNA_54_MODIF_1	modification of position 54 nt (m5U) in thrW by TrmA_mono	1 TrmA_mono + 1 amet + 1 b0244_tRNA_5 <==> 1 b0244_tRNA_5_TrmA_mono_cplx	reversible	tRNA modification	-1.00E+09	1.00E+09	0	1.77E+03	1.77E+03	0	9.38E+00	9.38	0	9.38E+00	9.38
b0244_tRNA_54_MODIF_2	modification of position 54 nt (m5U) in thrW by TrmA_mono	1 b0244_tRNA_5_TrmA_mono_cplx --> 1 TrmA_mono_inact + 1 ahcys + 1 b0244_tRNA_6 + 1 h	irreversible	tRNA modification	0.00E+00	1.00E+09	0	1.77E+03	1.77E+03	0	9.38E+00	9.38	0	9.38E+00	9.38
b0244_tRNA_55_MODIF_1	modification of position 55 nt (Y) in thrW by TruB_mono	1 TruB_mono + 1 b0244_tRNA_6 <==> 1 b0244_tRNA_6_TruB_mono_cplx	reversible	tRNA modification	-1.00E+09	1.00E+09	0	1.77E+03	1.77E+03	0	9.38E+00	9.38	0	9.38E+00	9.38
b0244_tRNA_55_MODIF_2	modification of position 55 nt (Y) in thrW by TruB_mono	1 b0244_tRNA_6_TruB_mono_cplx --> 1 TruB_mono_inact + 1 thrW_tRNA	irreversible	tRNA modification	0.00E+00	1.00E+09	0	1.77E+03	1.77E+03	0	9.38E+00	9.38	0	9.38E+00	9.38
b0244_tRNA_Mg_BIND	Binding of two Mg2 to b0244_tRNA	1 b0244_RNA + 2 mg2 <==> 1 b0244_tRNA_Mg2	reversible	Metallo-ion binding	-1.00E+09	1.00E+09	0	1.77E+03	1.77E+03	0	9.38E+00	9.38	0	9.38E+00	9.38

b0405_fold_spon	b0405_m folding: spontanous	1 b0405_m --> 1 QueA_mono	irreversible	Protein Folding	0.00E+00	1.00E+09	0	2.30E+03	2.30E+03	0	4.02E+03	4019.263	0	6.35E+01	63.478
b0405_mRNA_CONV	convsersion of mRNA to mRNA_1 (synthetic rxn)	1 b0405_mRNA --> 1 b0405_mRNA_1	irreversible	Translation	0.00E+00	1.00E+09	0	1.12E+02	1.12E+02	0	1.98E+02	197.8231	0	3.39E-02	0.0339
b0405_mRNA_degr1	Degradation of b0405_mRNA I	1 Orn_dim + 267 atp + 1 b0405_mRNA_2 + 1 degradosome + 267 h2o <==> 267 adp + 1 b0405_mRNA_2_degr + 267 h + 267 pi	reversible	mRNA degradation	-1.00E+09	1.00E+09	0	1.12E+02	1.12E+02	0	1.98E+02	197.8231	0	3.39E-02	0.0339
b0405_mRNA_degr2	Degradation of b0405_mRNA II	1 b0405_mRNA_2_degr + 1070 h2o --> 1 Orn_dim_inact + 245 amp 1 atp + 285 cmp + 1 degradosome_inact + 295 gmp + 1070 h + 245 ump	irreversible	mRNA degradation	0.00E+00	3.39E-02	0	1.12E+02	1.12E+02	0	1.98E+02	197.8231	0	3.39E-02	0.0339
b0405_maturation1	Polypeptide b0405 peptide deformylas and methionine aminopeptidase complex	1 Def_mono + 1 Map_mono + 1 b0405_aa <==> 1 b0405_def_map_cplx	reversible	Protein Maturation	-1.00E+09	1.00E+09	0	2.30E+03	2.30E+03	0	4.02E+03	4019.263	0	6.35E+01	63.478
b0405_maturation2	b0405 formation	1 b0405_def_map_cplx + 2 h2o --> 1 Def_mono_inact + 1 Map_mono_inact + 1 b0405_m + 1 for + 1 met-L	irreversible	Protein Maturation	0.00E+00	1.00E+09	0	2.30E+03	2.30E+03	0	4.02E+03	4019.263	0	6.35E+01	63.478
b0406_fold_spon	b0406_m folding: spontanous	1 b0406_m_Zn --> 1 Tgt_mono	irreversible	Protein Folding	0.00E+00	1.00E+09	0	2.10E+03	2.10E+03	0	3.64E+03	3641.575	0	9.19E+01	91.872
b0406_ion_BIND	Binding of ions to b0406	1 b0406_m + 1 zn2 <==> 1 b0406_m_Zn	reversible	Metallo-ion Binding	-1.00E+09	1.00E+09	0	2.10E+03	2.10E+03	0	3.64E+03	3641.575	0	9.19E+01	91.872
b0406_mRNA_CONV	convsersion of mRNA to mRNA_1 (synthetic rxn)	1 b0406_mRNA --> 1 b0406_mRNA_1	irreversible	Translation	0.00E+00	1.00E+09	0	9.15E+01	9.15E+01	0	1.62E+02	161.5461	0	1.96E-01	0.196
b0406_mRNA_degr1	Degradation of b0406_mRNA I	1 Orn_dim + 282 atp + 1 b0406_mRNA_2 + 1 degradosome + 282 h2o <==> 282 adp + 1 b0406_mRNA_2_degr + 282 h + 282 pi	reversible	mRNA degradation	-1.00E+09	1.00E+09	0	9.15E+01	9.15E+01	0	1.62E+02	161.5461	0	1.96E-01	0.196
b0406_mRNA_degr2	Degradation of b0406_mRNA II	1 b0406_mRNA_2_degr + 1127 h2o --> 1 Orn_dim_inact + 267 amp 1 atp + 274 cmp + 1 degradosome_inact + 305 gmp + 1127 h + 281 ump	irreversible	mRNA degradation	0.00E+00	2.57E-01	0	9.15E+01	9.15E+01	0	1.62E+02	161.5461	0	1.96E-01	0.196
b0406_maturation1	Polypeptide b0406 peptide deformylas complex	1 Def_mono + 1 b0406_aa <==> 1 b0406_def_cplx	reversible	Protein Maturation	-1.00E+09	1.00E+09	0	2.10E+03	2.10E+03	0	3.64E+03	3641.575	0	9.19E+01	91.872
b0406_maturation2	b0406 formation	1 b0406_def_cplx + 1 h2o --> 1 Def_mono_inact + 1 b0406_m + 1 for	irreversible	Protein Maturation	0.00E+00	1.00E+09	0	2.10E+03	2.10E+03	0	3.64E+03	3641.575	0	9.19E+01	91.872
b0407_fold_spon	b0407_m folding: spontanous	1 b0407_m --> 1 YajC_mono	irreversible	Protein Folding	0.00E+00	1.00E+09	0	6.56E+02	6.56E+02	0	1.08E+03	1076.077	0	1.08E+02	107.976
b0407_mRNA_CONV	convsersion of mRNA to mRNA_1 (synthetic rxn)	1 b0407_mRNA --> 1 b0407_mRNA_1	irreversible	Translation	0.00E+00	1.00E+09	0	9.15E+01	9.15E+01	0	1.62E+02	161.5461	0	1.96E-01	0.196
b0407_mRNA_degr1	Degradation of b0407_mRNA I	1 Orn_dim + 83 atp + 1 b0407_mRNA_2 + 1 degradosome + 83 h2o <==> 83 adp + 1 b0407_mRNA_2_degr + 83 h + 83 pi	reversible	mRNA degradation	-1.00E+09	1.00E+09	0	9.15E+01	9.15E+01	0	1.62E+02	161.5461	0	1.96E-01	0.196
b0407_mRNA_degr2	Degradation of b0407_mRNA II	1 b0407_mRNA_2_degr + 332 h2o --> 1 Orn_dim_inact + 84 amp + 75 cmp + 1 degradosome_inact + 90 gmp + 332 h + 84 ump	irreversible	mRNA degradation	0.00E+00	1.96E-01	0	9.15E+01	9.15E+01	0	1.62E+02	161.5461	0	1.96E-01	0.196
b0407_maturation1	Polypeptide b0407 peptide deformylas and methionine aminopeptidase complex	1 Def_mono + 1 Map_mono + 1 b0407_aa <==> 1 b0407_def_map_cplx	reversible	Protein Maturation	-1.00E+09	1.00E+09	0	6.56E+02	6.56E+02	0	1.08E+03	1076.077	0	1.08E+02	107.976
b0407_maturation2	b0407 formation	1 b0407_def_map_cplx + 2 h2o --> 1 Def_mono_inact + 1 Map_mono_inact + 1 b0407_m + 1 for + 1 met-L	irreversible	Protein Maturation	0.00E+00	1.00E+09	0	6.56E+02	6.56E+02	0	1.08E+03	1076.077	0	1.08E+02	107.976
b0413_fold_spon	b0413_m folding: spontanous	1 b0413_m --> 1 YbaD_mono	irreversible	Protein Folding	0.00E+00	1.00E+09	0	4.62E+02	4.62E+02	0	7.65E+02	765.4876	0	6.59E+01	65.8504
b0413_mRNA_CONV	convsersion of mRNA to mRNA_1 (synthetic rxn)	1 b0413_mRNA --> 1 b0413_mRNA_1	irreversible	Translation	0.00E+00	1.00E+09	0	4.96E+01	4.96E+01	0	8.75E+01	87.54595	0	9.13E-02	0.0913
b0413_mRNA_degr1	Degradation of b0413_mRNA I	1 Orn_dim + 112 atp + 1 b0413_mRNA_2 + 1 degradosome + 112 h2o <==> 112 adp + 1 b0413_mRNA_2_degr + 112 h + 112 pi	reversible	mRNA degradation	-1.00E+09	1.00E+09	0	4.96E+01	4.96E+01	0	8.75E+01	87.54595	0	9.13E-02	0.0913
b0413_mRNA_degr2	Degradation of b0413_mRNA II	1 b0413_mRNA_2_degr + 449 h2o --> 1 Orn_dim_inact + 110 amp + 1 atp + 100 cmp + 1 degradosome_inact + 131 gmp + 449 h + 108 ump	irreversible	mRNA degradation	0.00E+00	9.13E-02	0	4.96E+01	4.96E+01	0	8.75E+01	87.54595	0	9.13E-02	0.0913
b0413_maturation1	Polypeptide b0413 peptide deformylas and methionine aminopeptidase complex	1 Def_mono + 1 Map_mono + 1 b0413_aa <==> 1 b0413_def_map_cplx	reversible	Protein Maturation	-1.00E+09	1.00E+09	0	4.62E+02	4.62E+02	0	7.65E+02	765.4876	0	6.59E+01	65.8504
b0413_maturation2	b0413 formation	1 b0413_def_map_cplx + 2 h2o --> 1 Def_mono_inact + 1 Map_mono_inact + 1 b0413_m + 1 for + 1 met-L	irreversible	Protein Maturation	0.00E+00	1.00E+09	0	4.62E+02	4.62E+02	0	7.65E+02	765.4876	0	6.59E+01	65.8504
b0414_fold_spon	b0414_m folding: spontanous	1 b0414_m --> 1 RibD_mono	irreversible	Protein Folding	0.00E+00	1.00E+09	0	1.13E+03	1.13E+03	0	1.93E+03	1929.185	0	9.26E+01	92.6373
b0414_mRNA_CONV	convsersion of mRNA to mRNA_1 (synthetic rxn)	1 b0414_mRNA --> 1 b0414_mRNA_1	irreversible	Translation	0.00E+00	1.00E+09	0	4.96E+01	4.96E+01	0	8.75E+01	87.54595	0	9.13E-02	0.0913
b0414_mRNA_degr1	Degradation of b0414_mRNA I	1 Orn_dim + 276 atp + 1 b0414_mRNA_2 + 1 degradosome + 276 h2o <==> 276 adp + 1 b0414_mRNA_2_degr + 276 h + 276 pi	reversible	mRNA degradation	-1.00E+09	1.00E+09	0	4.96E+01	4.96E+01	0	8.75E+01	87.54595	0	9.13E-02	0.0913
b0414_mRNA_degr2	Degradation of b0414_mRNA II	1 b0414_mRNA_2_degr + 1103 h2o --> 1 Orn_dim_inact + 256 amp 289 cmp + 1 degradosome_inact + 322 gmp + 1103 h + 237 ump	irreversible	mRNA degradation	0.00E+00	3.17E-01	0	4.96E+01	4.96E+01	0	8.75E+01	87.54595	0	9.13E-02	0.0913
b0414_maturation1	Polypeptide b0414 peptide deformylas and methionine aminopeptidase complex	1 Def_mono + 1 Map_mono + 1 b0414_aa <==> 1 b0414_def_map_cplx	reversible	Protein Maturation	-1.00E+09	1.00E+09	0	1.13E+03	1.13E+03	0	1.93E+03	1929.185	0	9.26E+01	92.6373
b0414_maturation2	b0414 formation	1 b0414_def_map_cplx + 2 h2o --> 1 Def_mono_inact + 1 Map_mono_inact + 1 b0414_m + 1 for + 1 met-L	irreversible	Protein Maturation	0.00E+00	1.00E+09	0	1.13E+03	1.13E+03	0	1.93E+03	1929.185	0	9.26E+01	92.6373
b0415_fold_spon	b0415_m folding: spontanous	1 b0415_m --> 1 RibE_mono	irreversible	Protein Folding	0.00E+00	1.00E+09	0	5.49E+02	5.49E+02	0	8.91E+02	890.5136	0	1.03E+02	103.4217
b0415_mRNA_CONV	convsersion of mRNA to mRNA_1 (synthetic rxn)	1 b0415_mRNA --> 1 b0415_mRNA_1	irreversible	Translation	0.00E+00	1.00E+09	0	4.96E+01	4.96E+01	0	8.75E+01	87.54595	0	9.13E-02	0.0913
b0415_mRNA_degr1	Degradation of b0415_mRNA I	1 Orn_dim + 117 atp + 1 b0415_mRNA_2 + 1 degradosome + 117 h2o <==> 117 adp + 1 b0415_mRNA_2_degr + 117 h + 117 pi	reversible	mRNA degradation	-1.00E+09	1.00E+09	0	4.96E+01	4.96E+01	0	8.75E+01	87.54595	0	9.13E-02	0.0913
b0415_mRNA_degr2	Degradation of b0415_mRNA II	1 b0415_mRNA_2_degr + 470 h2o --> 1 Orn_dim_inact + 117 amp + 113 cmp + 1 degradosome_inact + 133 gmp + 470 h + 108 ump	irreversible	mRNA degradation	0.00E+00	3.08E-01	0	4.96E+01	4.96E+01	0	8.75E+01	87.54595	0	9.13E-02	0.0913
b0415_maturation1	Polypeptide b0415 peptide deformylas and methionine aminopeptidase complex	1 Def_mono + 1 Map_mono + 1 b0415_aa <==> 1 b0415_def_map_cplx	reversible	Protein Maturation	-1.00E+09	1.00E+09	0	5.49E+02	5.49E+02	0	8.91E+02	890.5136	0	1.03E+02	103.4217
b0415_maturation2	b0415 formation	1 b0415_def_map_cplx + 2 h2o --> 1 Def_mono_inact + 1 Map_mono_inact + 1 b0415_m + 1 for + 1 met-L	irreversible	Protein Maturation	0.00E+00	1.00E+09	0	5.49E+02	5.49E+02	0	8.91E+02	890.5136	0	1.03E+02	103.4217
b0416_fold_spon	b0416_m folding: spontanous	1 b0416_m --> 1 NusB_mono	irreversible	Protein Folding	0.00E+00	1.00E+09	0	4.98E+02	4.98E+02	0	8.02E+02	801.9676	0	1.02E+02	102.3304
b0416_mRNA_CONV	convsersion of mRNA to mRNA_1 (synthetic rxn)	1 b0416_mRNA --> 1 b0416_mRNA_1	irreversible	Translation	0.00E+00	1.00E+09	0	4.96E+01	4.96E+01	0	8.75E+01	87.54595	0	9.13E-02	0.0913
b0416_mRNA_degr1	Degradation of b0416_mRNA I	1 Orn_dim + 105 atp + 1 b0416_mRNA_2 + 1 degradosome + 105 h2o <==> 105 adp + 1 b0416_mRNA_2_degr + 105 h + 105 pi	reversible	mRNA degradation	-1.00E+09	1.00E+09	0	4.96E+01	4.96E+01	0	8.75E+01	87.54595	0	9.13E-02	0.0913
b0416_mRNA_degr2	Degradation of b0416_mRNA II	1 b0416_mRNA_2_degr + 419 h2o --> 1 Orn_dim_inact + 107 amp + 111 cmp + 1 degradosome_inact + 115 gmp + 419 h + 87 ump	irreversible	mRNA degradation	0.00E+00	3.17E-01	0	4.96E+01	4.96E+01	0	8.75E+01	87.54595	0	9.13E-02	0.0913
b0416_maturation1	Polypeptide b0416 peptide deformylas and methionine aminopeptidase complex	1 Def_mono + 1 Map_mono + 1 b0416_aa <==> 1 b0416_def_map_cplx	reversible	Protein Maturation	-1.00E+09	1.00E+09	0	4.98E+02	4.98E+02	0	8.02E+02	801.9676	0	1.02E+02	102.3304
b0416_maturation2	b0416 formation	1 b0416_def_map_cplx + 2 h2o --> 1 Def_mono_inact + 1 Map_mono_inact + 1 b0416_m + 1 for + 1 met-L	irreversible	Protein Maturation	0.00E+00	1.00E+09	0	4.98E+02	4.98E+02	0	8.02E+02	801.9676	0	1.02E+02	102.3304
b0423_fold_spon	b0423_m folding: spontanous	1 b0423_m --> 1 ThiI_mono	irreversible	Protein Folding	0.00E+00	1.00E+09	0	2.59E+03	2.59E+03	0	4.50E+03	4501.697	0	9.73E+01	97.328
b0423_mRNA_CONV	convsersion of mRNA to mRNA_1 (synthetic rxn)	1 b0423_mRNA --> 1 b0423_mRNA_1	irreversible	Translation	0.00E+00	1.00E+09	0	8.92E+01	8.92E+01	0	1.57E+02	157.4849	0	1.86E-01	0.186
b0423_mRNA_degr1	Degradation of b0423_mRNA I	1 Orn_dim + 362 atp + 1 b0423_mRNA_2 + 1 degradosome + 362 h2o <==> 362 adp + 1 b0423_mRNA_2_degr + 362 h + 362 pi	reversible	mRNA degradation	-1.00E+09	1.00E+09	0	8.92E+01	8.92E+01	0	1.57E+02	157.4849	0	1.86E-01	0.186
b0423_mRNA_degr2	Degradation of b0423_mRNA II	1 b0423_mRNA_2_degr + 1448 h2o --> 1 Orn_dim_inact + 348 amp 1 atp + 358 cmp + 1 degradosome_inact + 398 gmp + 1448 h + 344 ump	irreversible	mRNA degradation	0.00E+00	1.86E-01	0	8.92E+01	8.92E+01	0	1.57E+02	157.4849	0	1.86E-01	0.186

b0423_maturation1	Polypeptide b0423 peptide deformylase and methionine aminopeptidase complex	1 Def_mono + 1 Map_mono + 1 b0423_aa <==> 1 b0423_def_map_cplx	reversible	Protein Maturation	-1.00E+09	1.00E+09	0	2.59E+03	2.59E+03	0	4.50E+03	4501.697	0	9.73E+01	97.328
b0423_maturation2	b0423 formation	1 b0423_def_map_cplx + 2 h2o --> 1 Def_mono_inact + 1 Map_mono_inact + 1 b0423_m + 1 for + 1 met-L	irreversible	Protein Maturation	0.00E+00	1.00E+09	0	2.59E+03	2.59E+03	0	4.50E+03	4501.697	0	9.73E+01	97.328
b0436_fold_GroEL/ES_1	b0436_m folding: GroEL/ES mediated: polypeptide is going in GroEL/ES complex	1 GroEL.(7)ADP.cisGroES + 7 atp + 1 b0436_m + 1 transGroES_hepta --> 7 adp + 1 b0436_m_GroEL.(7)ATP.transGroES + 1 cisGroES_hepta	irreversible	Protein Folding	0.00E+00	1.00E+09	0	1.13E+02	1.13E+02	0	8.29E+01	82.88	0	8.29E+01	82.88
b0436_fold_GroEL/ES_2	b0436_m folding: GroEL/ES mediated: folding of polypeptide under ATP hydrolysis	1 b0436_m_GroEL.(7)ATP.transGroES + 7 h2o --> 1 Tig_GroEL.(7)ADP.transGroES + 7 h + 7 pi	irreversible	Protein Folding	0.00E+00	1.00E+09	0	1.13E+02	1.13E+02	0	8.29E+01	82.88	0	8.29E+01	82.88
b0436_fold_GroEL/ES_3	b0436_m folding: GroEL/ES mediated: release of native protein	1 Tig_GroEL.(7)ADP.transGroES --> 1 GroEL.(7)ADP.transGroES + 1 Tig_mono	irreversible	Protein Folding	0.00E+00	1.00E+09	0	1.13E+02	1.13E+02	0	8.29E+01	82.88	0	8.29E+01	82.88
b0436_fold_KJE_1	b0436_m folding: KJE mediated	1 DnaJ_dim + 1 DnaK_mono.ATP + 1 b0436_m <==> 1 b0436_m_DnaKJ_complex	reversible	Protein Folding	-1.00E+09	1.00E+09	0	1.13E+02	1.13E+02	0	8.29E+01	82.88	0	8.29E+01	82.88
b0436_fold_KJE_2	b0436_m folding: KJE mediated	1 GrpE_dim + 1 b0436_m_DnaKJ_complex + 1 h2o --> 1 DnaJ_dim_inact + 1 Tig_DnaK_GrpE_complex + 1 adp + 1 h + 1 pi	irreversible	Protein Folding	0.00E+00	1.00E+09	0	1.13E+02	1.13E+02	0	8.29E+01	82.88	0	8.29E+01	82.88
b0436_fold_KJE_3	b0436_m folding: KJE mediated	1 Tig_DnaK_GrpE_complex + 1 atp --> 1 DnaK_mono.ATP_inact + 1 GrpE_dim_inact + 1 Tig_mono	irreversible	Protein Folding	0.00E+00	1.00E+09	0	1.13E+02	1.13E+02	0	8.29E+01	82.88	0	8.29E+01	82.88
b0436_fold_spon	b0436_m folding: spontanous	1 b0436_m --> 1 Tig_mono	irreversible	Protein Folding	0.00E+00	1.00E+09	0	1.13E+02	1.13E+02	0	8.29E+01	82.88	0	8.29E+01	82.88
b0436_mRNA_CONV	convsersion of mRNA to mRNA_1 (synthetic rxn)	1 b0436_mRNA --> 1 b0436_mRNA_1	irreversible	Translation	0.00E+00	1.00E+09	0	8.92E+01	8.92E+01	0	8.29E+01	82.88	0	3.08E-01	0.308
b0436_mRNA_degr1	Degradation of b0436_mRNA I	1 Orn_dim + 324 atp + 1 b0436_mRNA_2 + 1 degradosome + 324 h2o <==> 324 adp + 1 b0436_mRNA_2_degr + 324 h + 324 pi	reversible	mRNA degradation	-1.00E+09	1.00E+09	0	8.92E+01	8.92E+01	0	8.29E+01	82.88	0	3.08E-01	0.308
b0436_mRNA_degr2	Degradation of b0436_mRNA II	1 b0436_mRNA_2_degr + 1298 h2o --> 1 Orn_dim_inact + 374 amp 1 atp + 298 cmp + 1 degradosome_inact + 367 gmp + 1298 h + 259 ump	irreversible	mRNA degradation	0.00E+00	3.08E-01	0	8.92E+01	8.92E+01	0	8.29E+01	82.88	0	3.08E-01	0.308
b0436_maturation1	Polypeptide b0436 peptide deformylase complex	1 Def_mono + 1 b0436_aa <==> 1 b0436_def_cplx	reversible	Protein Maturation	-1.00E+09	1.00E+09	8.44E+01	1.13E+02	2.81E+01	6.22E+01	8.29E+01	20.72	6.22E+01	8.29E+01	20.72
b0436_maturation2	b0436 formation	1 b0436_def_cplx + 1 h2o --> 1 Def_mono_inact + 1 b0436_m + 1 for	irreversible	Protein Maturation	0.00E+00	1.00E+09	8.44E+01	1.13E+02	2.81E+01	6.22E+01	8.29E+01	20.72	6.22E+01	8.29E+01	20.72
b0436_v1_mRNA_CONV	convsersion of mRNA to mRNA_1 (synthetic rxn)	1 b0436_v1_mRNA --> 1 b0436_mRNA_1	irreversible	Translation	0.00E+00	1.00E+09	0	8.92E+01	8.92E+01	0	8.29E+01	82.88	0	3.08E-01	0.308
b0503_DNA_act_bind	b0503_DNA binding of activator	1 b0503_DNA_neu --> 1 b0503_DNA_act	irreversible	Transcription Regulation	0.00E+00	1.00E+09	0	1.17E+02	1.17E+02	0	2.07E+02	206.5187	0	1.36E-01	0.136
b0503_fold_GroEL/ES_1	b0503_m folding: GroEL/ES mediated: polypeptide is going in GroEL/ES complex	1 GroEL.(7)ADP.cisGroES + 7 atp + 1 b0503_m + 1 transGroES_hepta --> 7 adp + 1 b0503_m_GroEL.(7)ATP.transGroES + 1 cisGroES_hepta	irreversible	Protein Folding	0.00E+00	1.00E+09	0	2.55E+03	2.55E+03	0	4.43E+03	4428.452	0	9.44E+01	94.416
b0503_fold_GroEL/ES_2	b0503_m folding: GroEL/ES mediated: folding of polypeptide under ATP hydrolysis	1 b0503_m_GroEL.(7)ATP.transGroES + 7 h2o --> 1 YbbB_GroEL.(7)ADP.transGroES + 7 h + 7 pi	irreversible	Protein Folding	0.00E+00	1.00E+09	0	2.55E+03	2.55E+03	0	4.43E+03	4428.452	0	9.44E+01	94.416
b0503_fold_GroEL/ES_3	b0503_m folding: GroEL/ES mediated: release of native protein	1 YbbB_GroEL.(7)ADP.transGroES --> 1 GroEL.(7)ADP.transGroES + 1 YbbB_mono	irreversible	Protein Folding	0.00E+00	1.00E+09	0	2.55E+03	2.55E+03	0	4.43E+03	4428.452	0	9.44E+01	94.416
b0503_fold_KJE_1	b0503_m folding: KJE mediated	1 DnaJ_dim + 1 DnaK_mono.ATP + 1 b0503_m <==> 1 b0503_m_DnaKJ_complex	reversible	Protein Folding	-1.00E+09	1.00E+09	0	2.55E+03	2.55E+03	0	4.43E+03	4428.452	0	9.44E+01	94.416
b0503_fold_KJE_2	b0503_m folding: KJE mediated	1 GrpE_dim + 1 b0503_m_DnaKJ_complex + 1 h2o --> 1 DnaJ_dim_inact + 1 YbbB_DnaK_GrpE_complex + 1 adp + 1 h + 1 pi	irreversible	Protein Folding	0.00E+00	1.00E+09	0	2.55E+03	2.55E+03	0	4.43E+03	4428.452	0	9.44E+01	94.416
b0503_fold_KJE_3	b0503_m folding: KJE mediated	1 YbbB_DnaK_GrpE_complex + 1 atp --> 1 DnaK_mono.ATP_inact + 1 GrpE_dim_inact + 1 YbbB_mono	irreversible	Protein Folding	0.00E+00	1.00E+09	0	2.55E+03	2.55E+03	0	4.43E+03	4428.452	0	9.44E+01	94.416
b0503_mRNA_CONV	convsersion of mRNA to mRNA_1 (synthetic rxn)	1 b0503_mRNA --> 1 b0503_mRNA_1	irreversible	Translation	0.00E+00	1.00E+09	0	1.17E+02	1.17E+02	0	2.07E+02	206.5187	0	1.36E-01	0.136
b0503_mRNA_degr1	Degradation of b0503_mRNA I	1 Orn_dim + 273 atp + 1 b0503_mRNA_2 + 1 degradosome + 273 h2o <==> 273 adp + 1 b0503_mRNA_2_degr + 273 h + 273 pi	reversible	mRNA degradation	-1.00E+09	1.00E+09	0	1.17E+02	1.17E+02	0	2.07E+02	206.5187	0	1.36E-01	0.136
b0503_mRNA_degr2	Degradation of b0503_mRNA II	1 b0503_mRNA_2_degr + 1094 h2o --> 1 Orn_dim_inact + 256 amp 1 atp + 273 cmp + 1 degradosome_inact + 336 gmp + 1094 h + 229 ump	irreversible	mRNA degradation	0.00E+00	1.36E-01	0	1.17E+02	1.17E+02	0	2.07E+02	206.5187	0	1.36E-01	0.136
b0503_maturation1	Polypeptide b0503 peptide deformylase and methionine aminopeptidase complex	1 Def_mono + 1 Map_mono + 1 b0503_aa <==> 1 b0503_def_map_cplx	reversible	Protein Maturation	-1.00E+09	1.00E+09	0	2.55E+03	2.55E+03	0	4.43E+03	4428.452	0	9.44E+01	94.416
b0503_maturation2	b0503 formation	1 b0503_def_map_cplx + 2 h2o --> 1 Def_mono_inact + 1 Map_mono_inact + 1 b0503_m + 1 for + 1 met-L	irreversible	Protein Maturation	0.00E+00	1.00E+09	0	2.55E+03	2.55E+03	0	4.43E+03	4428.452	0	9.44E+01	94.416
b0526_fold_spon	b0526_m folding: spontanous	1 b0526_m_Zn --> 1 CysS_mono	irreversible	Protein Folding	0.00E+00	1.00E+09	0	2.57E+03	2.57E+03	0	4.46E+03	4461.759	0	9.49E+01	94.932
b0526_ion_BIND	Binding of ions to b0526	1 b0526_m + 1 zn2 <==> 1 b0526_m_Zn	reversible	Metallo-ion Binding	-1.00E+09	1.00E+09	0	2.57E+03	2.57E+03	0	4.46E+03	4461.759	0	9.49E+01	94.932
b0526_mRNA_CONV	convsersion of mRNA to mRNA_1 (synthetic rxn)	1 b0526_mRNA --> 1 b0526_mRNA_1	irreversible	Translation	0.00E+00	1.00E+09	0	9.18E+01	9.18E+01	0	1.62E+02	162.0103	0	2.76E-01	0.276
b0526_mRNA_degr1	Degradation of b0526_mRNA I	1 Orn_dim + 346 atp + 1 b0526_mRNA_2 + 1 degradosome + 346 h2o <==> 346 adp + 1 b0526_mRNA_2_degr + 346 h + 346 pi	reversible	mRNA degradation	-1.00E+09	1.00E+09	0	9.18E+01	9.18E+01	0	1.62E+02	162.0103	0	2.76E-01	0.276
b0526_mRNA_degr2	Degradation of b0526_mRNA II	1 b0526_mRNA_2_degr + 1385 h2o --> 1 Orn_dim_inact + 335 amp 1 atp + 348 cmp + 1 degradosome_inact + 407 gmp + 1385 h + 295 ump	irreversible	mRNA degradation	0.00E+00	2.76E-01	0	9.18E+01	9.18E+01	0	1.62E+02	162.0103	0	2.76E-01	0.276
b0526_maturation1	Polypeptide b0526 peptide deformylase complex	1 Def_mono + 1 b0526_aa <==> 1 b0526_def_cplx	reversible	Protein Maturation	-1.00E+09	1.00E+09	0	2.57E+03	2.57E+03	0	4.46E+03	4461.759	0	9.49E+01	94.932
b0526_maturation2	b0526 formation	1 b0526_def_cplx + 1 h2o --> 1 Def_mono_inact + 1 b0526_m + 1 for	irreversible	Protein Maturation	0.00E+00	1.00E+09	0	2.57E+03	2.57E+03	0	4.46E+03	4461.759	0	9.49E+01	94.932
b0536_tRNA_32_MODIF_1	modification of position 32 nt (s2C) in argU by YdaO_mono	1 IscS_dim_S-SH + 1 YdaO_mono + 1 atp + 1 b0536_tRNA_Mg2 + 1 mg2 + 1 trdrd <==> 1 b0536_tRNA_Mg2_YdaO_mono_cplx	reversible	tRNA modification	-1.00E+09	1.00E+09	0	1.39E+03	1.39E+03	0	8.24E+00	8.24	0	8.24E+00	8.24
b0536_tRNA_32_MODIF_2	modification of position 32 nt (s2C) in argU by YdaO_mono	1 b0536_tRNA_Mg2_YdaO_mono_cplx --> 1 IscS_dim_S-H + 1 YdaO_mono_inact + 1 amp + 1 b0536_tRNA_1 + 1 h + 1 mg2 + 1 ppi + 1 trdox	irreversible	tRNA modification	0.00E+00	1.00E+09	0	1.39E+03	1.39E+03	0	8.24E+00	8.24	0	8.24E+00	8.24
b0536_tRNA_34_MODIF_1	modification of position 34 nt (mnm5U) in argU by TrmE_dim-GidA_mono-TrmC_mono	1 5fthf + 1 GidA_mono + 1 TrmC_mono + 1 TrmE_dim + 1 amet + 1 b0536_tRNA_1 + 1 fad + 1 fadh2 + 1 gly + 1 gtp + 1 h2o <==> 1 b0536_tRNA_1_TrmE_dim-GidA_mono-TrmC_mono_cplx	reversible	tRNA modification	-1.00E+09	1.00E+09	0	1.39E+03	1.39E+03	0	8.24E+00	8.24	0	8.24E+00	8.24
b0536_tRNA_34_MODIF_2	modification of position 34 nt (mnm5U) in argU by TrmE_dim-GidA_mono-TrmC_mono	1 b0536_tRNA_1_TrmE_dim-GidA_mono-TrmC_mono_cplx --> 1 GidA_mono_inact + 1 TrmC_mono_inact + 1 TrmE_dim_inact + 1 ahcys + 1 b0536_tRNA_2 + 1 fad + 1 fadh2 + 1 gdp + 1 glx + 3 h + 1 pi + 1 thf	irreversible	tRNA modification	0.00E+00	1.00E+09	0	1.39E+03	1.39E+03	0	8.24E+00	8.24	0	8.24E+00	8.24
b0536_tRNA_37_MODIF_1	modification of position 37 nt (t6A) in argU by Up_tRNA_pos_37_t6A	1 Up_tRNA_pos_37_t6A + 1 atp + 1 b0536_tRNA_2 + 1 hco3 + 1 mg2 + 1 thr-L <==> 1 b0536_tRNA_2_Up_tRNA_pos_37_t6A_cplx	reversible	tRNA modification	-1.00E+09	1.00E+09	0	1.39E+03	1.39E+03	0	8.24E+00	8.24	0	8.24E+00	8.24
b0536_tRNA_37_MODIF_2	modification of position 37 nt (t6A) in argU by Up_tRNA_pos_37_t6A	1 b0536_tRNA_2_Up_tRNA_pos_37_t6A_cplx --> 1 Up_tRNA_pos_37_t6A_inact + 1 amp + 1 b0536_tRNA_3 + 1 h + 1 h2o + 1 mg2 + 1 ppi	irreversible	tRNA modification	0.00E+00	1.00E+09	0	1.39E+03	1.39E+03	0	8.24E+00	8.24	0	8.24E+00	8.24
b0536_tRNA_40_MODIF_1	modification of position 40 nt (Y) in argU by TruA_dim	1 TruA_dim + 1 b0536_tRNA_3 <==> 1 b0536_tRNA_3_TruA_dim_cplx	reversible	tRNA modification	-1.00E+09	1.00E+09	0	1.39E+03	1.39E+03	0	8.24E+00	8.24	0	8.24E+00	8.24
b0536_tRNA_40_MODIF_2	modification of position 40 nt (Y) in argU by TruA_dim	1 b0536_tRNA_3_TruA_dim_cplx --> 1 TruA_dim_inact + 1 b0536_tRNA_4	irreversible	tRNA modification	0.00E+00	1.00E+09	0	1.39E+03	1.39E+03	0	8.24E+00	8.24	0	8.24E+00	8.24
b0536_tRNA_54_MODIF_1	modification of position 54 nt (m5U) in argU by TrmA_mono	1 TrmA_mono + 1 amet + 1 b0536_tRNA_4 <==> 1 b0536_tRNA_4_TrmA_mono_cplx	reversible	tRNA modification	-1.00E+09	1.00E+09	0	1.39E+03	1.39E+03	0	8.24E+00	8.24	0	8.24E+00	8.24
b0536_tRNA_54_MODIF_2	modification of position 54 nt (m5U) in argU by TrmA_mono	1 b0536_tRNA_4_TrmA_mono_cplx --> 1 TrmA_mono_inact + 1 ahcys + 1 b0536_tRNA_5 + 1 h	irreversible	tRNA modification	0.00E+00	1.00E+09	0	1.39E+03	1.39E+03	0	8.24E+00	8.24	0	8.24E+00	8.24
b0536_tRNA_55_MODIF_1	modification of position 55 nt (Y) in argU by TruB_mono	1 TruB_mono + 1 b0536_tRNA_5 <==> 1 b0536_tRNA_5_TruB_mono_cplx	reversible	tRNA modification	-1.00E+09	1.00E+09	0	1.39E+03	1.39E+03	0	8.24E+00	8.24	0	8.24E+00	8.24

b0536_tRNA_55_MODIF_2	modification of position 55 nt (Y) in argU by TruB_mono	1 b0536_tRNA_5_TruB_mono_cplx --> 1 TruB_mono_inact + 1 argU_tRNA	irreversible	tRNA modification	0.00E+00	1.00E+09	0	1.39E+03	1.39E+03	0	8.24E+00	8.24	0	8.24E+00	8.24
b0536_tRNA_Mg_BIND	Binding of two Mg2 to b0536_tRNA	1 b0536_RNA + 2 mg2 <==> 1 b0536_tRNA_Mg2	reversible	Metallo-ion binding	-1.00E+09	1.00E+09	0	1.39E+03	1.39E+03	0	8.24E+00	8.24	0	8.24E+00	8.24
b0638_fold_spon	b0638_m folding: spontanous	1 b0638_m --> 1 CobC_mono	irreversible	Protein Folding	0.00E+00	1.00E+09	0	3.12E+02	3.12E+02	0	5.05E+02	504.8618	0	6.22E+01	62.216
b0638_mRNA_CONV	convsersion of mRNA to mRNA_1 (synthetic rxn)	1 b0638_mRNA --> 1 b0638_mRNA_1	irreversible	Translation	0.00E+00	1.00E+09	0	2.29E+01	2.29E+01	0	4.04E+01	40.38653	0	1.46E-01	0.146
b0638_mRNA_degr1	Degradation of b0638_mRNA I	1 Orn_dim + 153 atp + 1 b0638_mRNA_2 + 1 degradosome + 153 h2o <==> 153 adp + 1 b0638_mRNA_2_degr + 153 h + 153 pi	reversible	mRNA degradation	-1.00E+09	1.00E+09	0	2.29E+01	2.29E+01	0	4.04E+01	40.38653	0	1.46E-01	0.146
b0638_mRNA_degr2	Degradation of b0638_mRNA II	1 b0638_mRNA_2_degr + 611 h2o --> 1 Orn_dim_inact + 148 amp + 153 cmp + 1 degradosome_inact + 160 gmp + 611 h + 151 ump	irreversible	mRNA degradation	0.00E+00	1.46E-01	0	2.29E+01	2.29E+01	0	4.04E+01	40.38653	0	1.46E-01	0.146
b0638_maturation1	Polypeptide b0638 peptide deformylas and methionine aminopeptidase complex	1 Def_mono + 1 Map_mono + 1 b0638_aa <==> 1 b0638_def_map_cplx	reversible	Protein Maturation	-1.00E+09	1.00E+09	0	3.12E+02	3.12E+02	0	5.05E+02	504.8618	0	6.22E+01	62.216
b0638_maturation2	b0638 formation	1 b0638_def_map_cplx + 2 h2o --> 1 Def_mono_inact + 1 Map_mono_inact + 1 b0638_m + 1 for + 1 met-L	irreversible	Protein Maturation	0.00E+00	1.00E+09	0	3.12E+02	3.12E+02	0	5.05E+02	504.8618	0	6.22E+01	62.216
b0639_fold_spon	b0639_m folding: spontanous	1 b0639_m --> 1 NadD_mono	irreversible	Protein Folding	0.00E+00	1.00E+09	0	3.48E+02	3.48E+02	0	5.58E+02	558.1984	0	7.53E+01	75.312
b0639_mRNA_CONV	convsersion of mRNA to mRNA_1 (synthetic rxn)	1 b0639_mRNA --> 1 b0639_mRNA_1	irreversible	Translation	0.00E+00	1.00E+09	0	2.29E+01	2.29E+01	0	4.04E+01	40.38653	0	1.46E-01	0.146
b0639_mRNA_degr1	Degradation of b0639_mRNA I	1 Orn_dim + 160 atp + 1 b0639_mRNA_2 + 1 degradosome + 160 h2o <==> 160 adp + 1 b0639_mRNA_2_degr + 160 h + 160 pi	reversible	mRNA degradation	-1.00E+09	1.00E+09	0	2.29E+01	2.29E+01	0	4.04E+01	40.38653	0	1.46E-01	0.146
b0639_mRNA_degr2	Degradation of b0639_mRNA II	1 b0639_mRNA_2_degr + 641 h2o --> 1 Orn_dim_inact + 168 amp + 169 cmp + 1 degradosome_inact + 152 gmp + 641 h + 153 ump	irreversible	mRNA degradation	0.00E+00	1.63E-01	0	2.29E+01	2.29E+01	0	4.04E+01	40.38653	0	1.46E-01	0.146
b0639_maturation1	Polypeptide b0639 peptide deformylas and methionine aminopeptidase complex	1 Def_mono + 1 Map_mono + 1 b0639_aa <==> 1 b0639_def_map_cplx	reversible	Protein Maturation	-1.00E+09	1.00E+09	0	3.48E+02	3.48E+02	0	5.58E+02	558.1984	0	7.53E+01	75.312
b0639_maturation2	b0639 formation	1 b0639_def_map_cplx + 2 h2o --> 1 Def_mono_inact + 1 Map_mono_inact + 1 b0639_m + 1 for + 1 met-L	irreversible	Protein Maturation	0.00E+00	1.00E+09	0	3.48E+02	3.48E+02	0	5.58E+02	558.1984	0	7.53E+01	75.312
b0640_fold_spon	b0640_m folding: spontanous	1 b0640_m --> 1 HolA_mono	irreversible	Protein Folding	0.00E+00	1.00E+09	0	5.39E+02	5.39E+02	0	8.89E+02	889.3306	0	8.45E+01	84.52
b0640_mRNA_CONV	convsersion of mRNA to mRNA_1 (synthetic rxn)	1 b0640_mRNA --> 1 b0640_mRNA_1	irreversible	Translation	0.00E+00	1.00E+09	0	2.29E+01	2.29E+01	0	4.04E+01	40.38653	0	1.46E-01	0.146
b0640_mRNA_degr1	Degradation of b0640_mRNA I	1 Orn_dim + 257 atp + 1 b0640_mRNA_2 + 1 degradosome + 257 h2o <==> 257 adp + 1 b0640_mRNA_2_degr + 257 h + 257 pi	reversible	mRNA degradation	-1.00E+09	1.00E+09	0	2.29E+01	2.29E+01	0	4.04E+01	40.38653	0	1.46E-01	0.146
b0640_mRNA_degr2	Degradation of b0640_mRNA II	1 b0640_mRNA_2_degr + 1030 h2o --> 1 Orn_dim_inact + 240 amp 262 cmp + 1 degradosome_inact + 278 gmp + 1030 h + 251 ump	irreversible	mRNA degradation	0.00E+00	3.37E-01	0	2.29E+01	2.29E+01	0	4.04E+01	40.38653	0	1.46E-01	0.146
b0640_maturation1	Polypeptide b0640 peptide deformylas complex	1 Def_mono + 1 b0640_aa <==> 1 b0640_def_cplx	reversible	Protein Maturation	-1.00E+09	1.00E+09	0	5.39E+02	5.39E+02	0	8.89E+02	889.3306	0	8.45E+01	84.52
b0640_maturation2	b0640 formation	1 b0640_def_cplx + 1 h2o --> 1 Def_mono_inact + 1 b0640_m + 1 for	irreversible	Protein Maturation	0.00E+00	1.00E+09	0	5.39E+02	5.39E+02	0	8.89E+02	889.3306	0	8.45E+01	84.52
b0641_fold_spon	b0641_m folding: spontanous	1 b0641_m --> 1 RlpB_mono	irreversible	Protein Folding	0.00E+00	1.00E+09	0	3.42E+02	3.42E+02	0	5.35E+02	534.5618	0	9.19E+01	91.916
b0641_mRNA_CONV	convsersion of mRNA to mRNA_1 (synthetic rxn)	1 b0641_mRNA --> 1 b0641_mRNA_1	irreversible	Translation	0.00E+00	1.00E+09	0	2.29E+01	2.29E+01	0	4.04E+01	40.38653	0	1.46E-01	0.146
b0641_mRNA_degr1	Degradation of b0641_mRNA I	1 Orn_dim + 145 atp + 1 b0641_mRNA_2 + 1 degradosome + 145 h2o <==> 145 adp + 1 b0641_mRNA_2_degr + 145 h + 145 pi	reversible	mRNA degradation	-1.00E+09	1.00E+09	0	2.29E+01	2.29E+01	0	4.04E+01	40.38653	0	1.46E-01	0.146
b0641_mRNA_degr2	Degradation of b0641_mRNA II	1 b0641_mRNA_2_degr + 581 h2o --> 1 Orn_dim_inact + 145 amp + 150 cmp + 1 degradosome_inact + 154 gmp + 581 h + 133 ump	irreversible	mRNA degradation	0.00E+00	4.14E-01	0	2.29E+01	2.29E+01	0	4.04E+01	40.38653	0	1.46E-01	0.146
b0641_maturation1	Polypeptide b0641 peptide deformylas and methionine aminopeptidase complex	1 Def_mono + 1 Map_mono + 1 b0641_aa <==> 1 b0641_def_map_cplx	reversible	Protein Maturation	-1.00E+09	1.00E+09	0	3.42E+02	3.42E+02	0	5.35E+02	534.5618	0	9.19E+01	91.916
b0641_maturation2	b0641 formation	1 b0641_def_map_cplx + 19 h2o --> 1 Def_mono_inact + 1 Map_mono_inact + 3 ala-L + 1 arg-L + 1 b0641_m + 1 for + 1 gly + 1 ile-L + 6 leu-L + 1 met-L + 1 ser-L + 2 thr-L + 1 tyr-L + 1 val-L	irreversible	Protein Maturation	0.00E+00	1.00E+09	0	3.42E+02	3.42E+02	0	5.35E+02	534.5618	0	9.19E+01	91.916
b0642_fold_KJE_1	b0642_m folding: KJE mediated	1 DnaJ_dim + 1 DnaK_mono.ATP + 1 b0642_m_Zn <==> 1 b0642_m_DnaKJ_complex	reversible	Protein Folding	-1.00E+09	1.00E+09	0	1.25E+03	1.25E+03	0	2.13E+03	2127.826	0	1.16E+02	115.8
b0642_fold_KJE_2	b0642_m folding: KJE mediated	1 GrpE_dim + 1 b0642_m_DnaKJ_complex + 1 h2o --> 1 DnaJ_dim_inact + 1 LeuS_DnaK_GrpE_complex + 1 adp + 1 h + 1 pi	irreversible	Protein Folding	0.00E+00	1.00E+09	0	1.25E+03	1.25E+03	0	2.13E+03	2127.826	0	1.16E+02	115.8
b0642_fold_KJE_3	b0642_m folding: KJE mediated	1 LeuS_DnaK_GrpE_complex + 1 atp --> 1 DnaK_mono.ATP_inact + 1 GrpE_dim_inact + 1 LeuS_mono	irreversible	Protein Folding	0.00E+00	1.00E+09	0	1.25E+03	1.25E+03	0	2.13E+03	2127.826	0	1.16E+02	115.8
b0642_fold_spon	b0642_m folding: spontanous	1 b0642_m_Zn --> 1 LeuS_mono	irreversible	Protein Folding	0.00E+00	1.00E+09	0	1.25E+03	1.25E+03	0	2.13E+03	2127.826	0	1.16E+02	115.8
b0642_ion_BIND	Binding of ions to b0642	1 b0642_m + 1 zn2 <==> 1 b0642_m_Zn	reversible	Metallo-ion Binding	-1.00E+09	1.00E+09	0	1.25E+03	1.25E+03	0	2.13E+03	2127.826	0	1.16E+02	115.8
b0642_mRNA_CONV	convsersion of mRNA to mRNA_1 (synthetic rxn)	1 b0642_mRNA --> 1 b0642_mRNA_1	irreversible	Translation	0.00E+00	1.00E+09	0	2.29E+01	2.29E+01	0	4.04E+01	40.38653	0	1.46E-01	0.146
b0642_mRNA_degr1	Degradation of b0642_mRNA I	1 Orn_dim + 645 atp + 1 b0642_mRNA_2 + 1 degradosome + 645 h2o <==> 645 adp + 1 b0642_mRNA_2_degr + 645 h + 645 pi	reversible	mRNA degradation	-1.00E+09	1.00E+09	0	2.29E+01	2.29E+01	0	4.04E+01	40.38653	0	1.46E-01	0.146
b0642_mRNA_degr2	Degradation of b0642_mRNA II	1 b0642_mRNA_2_degr + 2582 h2o --> 1 Orn_dim_inact + 660 amp 1 atp + 654 cmp + 1 degradosome_inact + 724 gmp + 2582 h + 544 ump	irreversible	mRNA degradation	0.00E+00	4.90E-01	0	2.29E+01	2.29E+01	0	4.04E+01	40.38653	0	1.46E-01	0.146
b0642_maturation1	Polypeptide b0642 peptide deformylas and methionine aminopeptidase complex	1 Def_mono + 1 Map_mono + 1 b0642_aa <==> 1 b0642_def_map_cplx	reversible	Protein Maturation	-1.00E+09	1.00E+09	0	1.25E+03	1.25E+03	0	2.13E+03	2127.826	0	1.16E+02	115.8
b0642_maturation2	b0642 formation	1 b0642_def_map_cplx + 2 h2o --> 1 Def_mono_inact + 1 Map_mono_inact + 1 b0642_m + 1 for + 1 met-L	irreversible	Protein Maturation	0.00E+00	1.00E+09	0	1.25E+03	1.25E+03	0	2.13E+03	2127.826	0	1.16E+02	115.8
b0661_fold_spon	b0661_m folding: spontanous	1 b0661_m_FeS --> 1 MiaB_mono	irreversible	Protein Folding	0.00E+00	1.00E+09	0	2.40E+03	2.40E+03	0	4.16E+03	4160.365	0	9.54E+01	95.364
b0661_iron_sulfur_BIND1	Incorporation of [4Fe-4S]2+ in b0661	1 IscU_dim_[4Fe-4S] + 1 b0661_m <==> 1 b0661_m_IscU_cplx	reversible	Iron-sulfur cluster incorporation	-1.00E+09	1.00E+09	0	2.40E+03	2.40E+03	0	4.16E+03	4160.365	0	9.54E+01	95.364
b0661_iron_sulfur_BIND2	Incorporation of [4Fe-4S]2+ in b0661	1 b0661_m_IscU_cplx <==> 2 IscU_mono + 1 b0661_m_FeS	reversible	Iron-sulfur cluster incorporation	-1.00E+09	1.00E+09	0	2.40E+03	2.40E+03	0	4.16E+03	4160.365	0	9.54E+01	95.364
b0661_mRNA_CONV	convsersion of mRNA to mRNA_1 (synthetic rxn)	1 b0661_mRNA --> 1 b0661_mRNA_1	irreversible	Translation	0.00E+00	1.00E+09	0	8.54E+01	8.54E+01	0	1.51E+02	150.7476	0	1.92E-01	0.192
b0661_mRNA_degr1	Degradation of b0661_mRNA I	1 Orn_dim + 356 atp + 1 b0661_mRNA_2 + 1 degradosome + 356 h2o <==> 356 adp + 1 b0661_mRNA_2_degr + 356 h + 356 pi	reversible	mRNA degradation	-1.00E+09	1.00E+09	0	8.54E+01	8.54E+01	0	1.51E+02	150.7476	0	1.92E-01	0.192
b0661_mRNA_degr2	Degradation of b0661_mRNA II	1 b0661_mRNA_2_degr + 1424 h2o --> 1 Orn_dim_inact + 344 amp 1 atp + 374 cmp + 1 degradosome_inact + 398 gmp + 1424 h + 308 ump	irreversible	mRNA degradation	0.00E+00	1.92E-01	0	8.54E+01	8.54E+01	0	1.51E+02	150.7476	0	1.92E-01	0.192
b0661_maturation1	Polypeptide b0661 peptide deformylas and methionine aminopeptidase complex	1 Def_mono + 1 Map_mono + 1 b0661_aa <==> 1 b0661_def_map_cplx	reversible	Protein Maturation	-1.00E+09	1.00E+09	0	2.40E+03	2.40E+03	0	4.16E+03	4160.365	0	9.54E+01	95.364
b0661_maturation2	b0661 formation	1 b0661_def_map_cplx + 2 h2o --> 1 Def_mono_inact + 1 Map_mono_inact + 1 b0661_m + 1 for + 1 met-L	irreversible	Protein Maturation	0.00E+00	1.00E+09	0	2.40E+03	2.40E+03	0	4.16E+03	4160.365	0	9.54E+01	95.364
b0664_tRNA_18_MODIF_1	modification of position 18 nt (Gm) in glnX by TrmH_dim	1 TrmH_dim + 1 amet + 1 b0664_tRNA_Mg2 <==> 1 b0664_tRNA_Mg2_TrmH_dim_cplx	reversible	tRNA modification	-1.00E+09	1.00E+09	0	1.70E+02	1.70E+02	0	7.79E+00	7.79	0	7.79E+00	7.79
b0664_tRNA_18_MODIF_2	modification of position 18 nt (Gm) in glnX by TrmH_dim	1 b0664_tRNA_Mg2_TrmH_dim_cplx --> 1 TrmH_dim_inact + 1 ahcys + 1 b0664_tRNA_1 + 1 h	irreversible	tRNA modification	0.00E+00	1.00E+09	0	1.70E+02	1.70E+02	0	7.79E+00	7.79	0	7.79E+00	7.79
b0664_tRNA_20_MODIF_1	modification of position 20 nt (D) in glnX by Dus_gen	1 Dus_gen + 1 b0664_tRNA_1 + 1 h + 1 nadph <==> 1 b0664_tRNA_1_Dus_gen_cplx	reversible	tRNA modification	-1.00E+09	1.00E+09	0	1.70E+02	1.70E+02	0	7.79E+00	7.79	0	7.79E+00	7.79

b0664_tRNA_20_MODIF_2	modification of position 20 nt (D) in glnX by Dus_gen	1 b0664_tRNA_1_Dus_gen_cplx --> 1 Dus_gen_inact + 1 b0664_tRNA_2 + 1 nadp	irreversible	tRNA modification	0.00E+00	1.00E+09	0	1.70E+02	1.70E+02	0	7.79E+00	7.79	0	7.79E+00	7.79
b0664_tRNA_32_MODIF_1	modification of position 32 nt (Um) in glnX by MeT_tRNA_pos_32_Um	1 MeT_tRNA_pos_32_Um + 1 amet + 1 b0664_tRNA_2 <==> 1 b0664_tRNA_2_MeT_tRNA_pos_32_Um_cplx	reversible	tRNA modification	-1.00E+09	1.00E+09	0	1.70E+02	1.70E+02	0	7.79E+00	7.79	0	7.79E+00	7.79
b0664_tRNA_32_MODIF_2	modification of position 32 nt (Um) in glnX by MeT_tRNA_pos_32_Um	1 b0664_tRNA_2_MeT_tRNA_pos_32_Um_cplx --> 1 MeT_tRNA_pos_32_Um_inact + 1 ahcys + 1 b0664_tRNA_3 + 1 h	irreversible	tRNA modification	0.00E+00	1.00E+09	0	1.70E+02	1.70E+02	0	7.79E+00	7.79	0	7.79E+00	7.79
b0664_tRNA_37_MODIF_1	modification of position 37 nt (m2A) in glnX by MeT_tRNA_pos_37_m2A	1 MeT_tRNA_pos_37_m2A + 1 amet + 1 b0664_tRNA_3 <==> 1 b0664_tRNA_3_MeT_tRNA_pos_37_m2A_cplx	reversible	tRNA modification	-1.00E+09	1.00E+09	0	1.70E+02	1.70E+02	0	7.79E+00	7.79	0	7.79E+00	7.79
b0664_tRNA_37_MODIF_2	modification of position 37 nt (m2A) in glnX by MeT_tRNA_pos_37_m2A	1 b0664_tRNA_3_MeT_tRNA_pos_37_m2A_cplx --> 1 MeT_tRNA_pos_37_m2A_inact + 1 ahcys + 1 b0664_tRNA_4 + 1 h	irreversible	tRNA modification	0.00E+00	1.00E+09	0	1.70E+02	1.70E+02	0	7.79E+00	7.79	0	7.79E+00	7.79
b0664_tRNA_38_MODIF_1	modification of position 38 nt (Y) in glnX by TruA_dim	1 TruA_dim + 1 b0664_tRNA_4 <==> 1 b0664_tRNA_4_TruA_dim_cplx	reversible	tRNA modification	-1.00E+09	1.00E+09	0	1.70E+02	1.70E+02	0	7.79E+00	7.79	0	7.79E+00	7.79
b0664_tRNA_38_MODIF_2	modification of position 38 nt (Y) in glnX by TruA_dim	1 b0664_tRNA_4_TruA_dim_cplx --> 1 TruA_dim_inact + 1 b0664_tRNA_5	irreversible	tRNA modification	0.00E+00	1.00E+09	0	1.70E+02	1.70E+02	0	7.79E+00	7.79	0	7.79E+00	7.79
b0664_tRNA_54_MODIF_1	modification of position 54 nt (m5U) in glnX by TrmA_mono	1 TrmA_mono + 1 amet + 1 b0664_tRNA_5 <==> 1 b0664_tRNA_5_TrmA_mono_cplx	reversible	tRNA modification	-1.00E+09	1.00E+09	0	1.70E+02	1.70E+02	0	7.79E+00	7.79	0	7.79E+00	7.79
b0664_tRNA_54_MODIF_2	modification of position 54 nt (m5U) in glnX by TrmA_mono	1 b0664_tRNA_5_TrmA_mono_cplx --> 1 TrmA_mono_inact + 1 ahcys + 1 b0664_tRNA_6 + 1 h	irreversible	tRNA modification	0.00E+00	1.00E+09	0	1.70E+02	1.70E+02	0	7.79E+00	7.79	0	7.79E+00	7.79
b0664_tRNA_55_MODIF_1	modification of position 55 nt (Y) in glnX by TruB_mono	1 TruB_mono + 1 b0664_tRNA_6 <==> 1 b0664_tRNA_6_TruB_mono_cplx	reversible	tRNA modification	-1.00E+09	1.00E+09	0	1.70E+02	1.70E+02	0	7.79E+00	7.79	0	7.79E+00	7.79
b0664_tRNA_55_MODIF_2	modification of position 55 nt (Y) in glnX by TruB_mono	1 b0664_tRNA_6_TruB_mono_cplx --> 1 TruB_mono_inact + 1 b0664_tRNA_7	irreversible	tRNA modification	0.00E+00	1.00E+09	0	1.70E+02	1.70E+02	0	7.79E+00	7.79	0	7.79E+00	7.79
b0664_tRNA_8_MODIF_1	modification of position 8 nt (s4U) in glnX by ThiI_mono	1 IscS_dim_S-SH + 1 ThiI_mono + 1 atp + 1 b0664_tRNA_7 + 1 mg2 + 1 trdrd <==> 1 b0664_tRNA_7_ThiI_mono_cplx	reversible	tRNA modification	-1.00E+09	1.00E+09	0	1.70E+02	1.70E+02	0	7.79E+00	7.79	0	7.79E+00	7.79
b0664_tRNA_8_MODIF_2	modification of position 8 nt (s4U) in glnX by ThiI_mono	1 b0664_tRNA_7_ThiI_mono_cplx --> 1 IscS_dim_S-H + 1 ThiI_mono_inact + 1 amp + 1 glnX_tRNA + 1 h + 1 mg2 + 1 ppi + 1 trdox	irreversible	tRNA modification	0.00E+00	1.00E+09	0	1.70E+02	1.70E+02	0	7.79E+00	7.79	0	7.79E+00	7.79
b0664_tRNA_Mg_BIND	Binding of two Mg2 to b0664_tRNA	1 b0664_RNA + 2 mg2 <==> 1 b0664_tRNA_Mg2	reversible	Metallo-ion binding	-1.00E+09	1.00E+09	0	1.70E+02	1.70E+02	0	7.79E+00	7.79	0	7.79E+00	7.79
b0665_tRNA_18_MODIF_1	modification of position 18 nt (Gm) in glnV by TrmH_dim	1 TrmH_dim + 1 amet + 1 b0665_tRNA_Mg2 <==> 1 b0665_tRNA_Mg2_TrmH_dim_cplx	reversible	tRNA modification	-1.00E+09	1.00E+09	0	1.70E+02	1.70E+02	0	7.79E+00	7.79	0	7.79E+00	7.79
b0665_tRNA_18_MODIF_2	modification of position 18 nt (Gm) in glnV by TrmH_dim	1 b0665_tRNA_Mg2_TrmH_dim_cplx --> 1 TrmH_dim_inact + 1 ahcys + 1 b0665_tRNA_1 + 1 h	irreversible	tRNA modification	0.00E+00	1.00E+09	0	1.70E+02	1.70E+02	0	7.79E+00	7.79	0	7.79E+00	7.79
b0665_tRNA_20_MODIF_1	modification of position 20 nt (D) in glnV by Dus_gen	1 Dus_gen + 1 b0665_tRNA_1 + 1 h + 1 nadph <==> 1 b0665_tRNA_1_Dus_gen_cplx	reversible	tRNA modification	-1.00E+09	1.00E+09	0	1.70E+02	1.70E+02	0	7.79E+00	7.79	0	7.79E+00	7.79
b0665_tRNA_20_MODIF_2	modification of position 20 nt (D) in glnV by Dus_gen	1 b0665_tRNA_1_Dus_gen_cplx --> 1 Dus_gen_inact + 1 b0665_tRNA_2 + 1 nadp	irreversible	tRNA modification	0.00E+00	1.00E+09	0	1.70E+02	1.70E+02	0	7.79E+00	7.79	0	7.79E+00	7.79
b0665_tRNA_32_MODIF_1	modification of position 32 nt (Um) in glnV by MeT_tRNA_pos_32_Um	1 MeT_tRNA_pos_32_Um + 1 amet + 1 b0665_tRNA_2 <==> 1 b0665_tRNA_2_MeT_tRNA_pos_32_Um_cplx	reversible	tRNA modification	-1.00E+09	1.00E+09	0	1.70E+02	1.70E+02	0	7.79E+00	7.79	0	7.79E+00	7.79
b0665_tRNA_32_MODIF_2	modification of position 32 nt (Um) in glnV by MeT_tRNA_pos_32_Um	1 b0665_tRNA_2_MeT_tRNA_pos_32_Um_cplx --> 1 MeT_tRNA_pos_32_Um_inact + 1 ahcys + 1 b0665_tRNA_3 + 1 h	irreversible	tRNA modification	0.00E+00	1.00E+09	0	1.70E+02	1.70E+02	0	7.79E+00	7.79	0	7.79E+00	7.79
b0665_tRNA_37_MODIF_1	modification of position 37 nt (m2A) in glnV by MeT_tRNA_pos_37_m2A	1 MeT_tRNA_pos_37_m2A + 1 amet + 1 b0665_tRNA_3 <==> 1 b0665_tRNA_3_MeT_tRNA_pos_37_m2A_cplx	reversible	tRNA modification	-1.00E+09	1.00E+09	0	1.70E+02	1.70E+02	0	7.79E+00	7.79	0	7.79E+00	7.79
b0665_tRNA_37_MODIF_2	modification of position 37 nt (m2A) in glnV by MeT_tRNA_pos_37_m2A	1 b0665_tRNA_3_MeT_tRNA_pos_37_m2A_cplx --> 1 MeT_tRNA_pos_37_m2A_inact + 1 ahcys + 1 b0665_tRNA_4 + 1 h	irreversible	tRNA modification	0.00E+00	1.00E+09	0	1.70E+02	1.70E+02	0	7.79E+00	7.79	0	7.79E+00	7.79
b0665_tRNA_38_MODIF_1	modification of position 38 nt (Y) in glnV by TruA_dim	1 TruA_dim + 1 b0665_tRNA_4 <==> 1 b0665_tRNA_4_TruA_dim_cplx	reversible	tRNA modification	-1.00E+09	1.00E+09	0	1.70E+02	1.70E+02	0	7.79E+00	7.79	0	7.79E+00	7.79
b0665_tRNA_38_MODIF_2	modification of position 38 nt (Y) in glnV by TruA_dim	1 b0665_tRNA_4_TruA_dim_cplx --> 1 TruA_dim_inact + 1 b0665_tRNA_5	irreversible	tRNA modification	0.00E+00	1.00E+09	0	1.70E+02	1.70E+02	0	7.79E+00	7.79	0	7.79E+00	7.79
b0665_tRNA_54_MODIF_1	modification of position 54 nt (m5U) in glnV by TrmA_mono	1 TrmA_mono + 1 amet + 1 b0665_tRNA_5 <==> 1 b0665_tRNA_5_TrmA_mono_cplx	reversible	tRNA modification	-1.00E+09	1.00E+09	0	1.70E+02	1.70E+02	0	7.79E+00	7.79	0	7.79E+00	7.79
b0665_tRNA_54_MODIF_2	modification of position 54 nt (m5U) in glnV by TrmA_mono	1 b0665_tRNA_5_TrmA_mono_cplx --> 1 TrmA_mono_inact + 1 ahcys + 1 b0665_tRNA_6 + 1 h	irreversible	tRNA modification	0.00E+00	1.00E+09	0	1.70E+02	1.70E+02	0	7.79E+00	7.79	0	7.79E+00	7.79
b0665_tRNA_55_MODIF_1	modification of position 55 nt (Y) in glnV by TruB_mono	1 TruB_mono + 1 b0665_tRNA_6 <==> 1 b0665_tRNA_6_TruB_mono_cplx	reversible	tRNA modification	-1.00E+09	1.00E+09	0	1.70E+02	1.70E+02	0	7.79E+00	7.79	0	7.79E+00	7.79
b0665_tRNA_55_MODIF_2	modification of position 55 nt (Y) in glnV by TruB_mono	1 b0665_tRNA_6_TruB_mono_cplx --> 1 TruB_mono_inact + 1 b0665_tRNA_7	irreversible	tRNA modification	0.00E+00	1.00E+09	0	1.70E+02	1.70E+02	0	7.79E+00	7.79	0	7.79E+00	7.79
b0665_tRNA_8_MODIF_1	modification of position 8 nt (s4U) in glnV by ThiI_mono	1 IscS_dim_S-SH + 1 ThiI_mono + 1 atp + 1 b0665_tRNA_7 + 1 mg2 + 1 trdrd <==> 1 b0665_tRNA_7_ThiI_mono_cplx	reversible	tRNA modification	-1.00E+09	1.00E+09	0	1.70E+02	1.70E+02	0	7.79E+00	7.79	0	7.79E+00	7.79
b0665_tRNA_8_MODIF_2	modification of position 8 nt (s4U) in glnV by ThiI_mono	1 b0665_tRNA_7_ThiI_mono_cplx --> 1 IscS_dim_S-H + 1 ThiI_mono_inact + 1 amp + 1 glnV_tRNA + 1 h + 1 mg2 + 1 ppi + 1 trdox	irreversible	tRNA modification	0.00E+00	1.00E+09	0	1.70E+02	1.70E+02	0	7.79E+00	7.79	0	7.79E+00	7.79
b0665_tRNA_Mg_BIND	Binding of two Mg2 to b0665_tRNA	1 b0665_RNA + 2 mg2 <==> 1 b0665_tRNA_Mg2	reversible	Metallo-ion binding	-1.00E+09	1.00E+09	0	1.70E+02	1.70E+02	0	7.79E+00	7.79	0	7.79E+00	7.79
b0666_tRNA_16_MODIF_1	modification of position 16 nt (D) in metU by DusA_mono	1 DusA_mono + 1 b0666_tRNA_Mg2 + 1 h + 1 nadph <==> 1 b0666_tRNA_Mg2_DusA_mono_cplx	reversible	tRNA modification	-1.00E+09	1.00E+09	0	1.70E+02	1.70E+02	0	7.79E+00	7.79	0	7.79E+00	7.79
b0666_tRNA_16_MODIF_2	modification of position 16 nt (D) in metU by DusA_mono	1 b0666_tRNA_Mg2_DusA_mono_cplx --> 1 DusA_mono + 1 b0666_tRNA_1 + 1 nadp	irreversible	tRNA modification	0.00E+00	1.00E+09	0	1.70E+02	1.70E+02	0	7.79E+00	7.79	0	7.79E+00	7.79
b0666_tRNA_17_MODIF_1	modification of position 17 nt (D) in metU by DusA_mono	1 DusA_mono + 1 b0666_tRNA_1 + 1 h + 1 nadph <==> 1 b0666_tRNA_1_DusA_mono_cplx	reversible	tRNA modification	-1.00E+09	1.00E+09	0	1.70E+02	1.70E+02	0	7.79E+00	7.79	0	7.79E+00	7.79
b0666_tRNA_17_MODIF_2	modification of position 17 nt (D) in metU by DusA_mono	1 b0666_tRNA_1_DusA_mono_cplx --> 1 DusA_mono + 1 b0666_tRNA_2 + 1 nadp	irreversible	tRNA modification	0.00E+00	1.00E+09	0	1.70E+02	1.70E+02	0	7.79E+00	7.79	0	7.79E+00	7.79
b0666_tRNA_18_MODIF_1	modification of position 18 nt (Gm) in metU by TrmH_dim	1 TrmH_dim + 1 amet + 1 b0666_tRNA_2 <==> 1 b0666_tRNA_2_TrmH_dim_cplx	reversible	tRNA modification	-1.00E+09	1.00E+09	0	1.70E+02	1.70E+02	0	7.79E+00	7.79	0	7.79E+00	7.79
b0666_tRNA_18_MODIF_2	modification of position 18 nt (Gm) in metU by TrmH_dim	1 b0666_tRNA_2_TrmH_dim_cplx --> 1 TrmH_dim_inact + 1 ahcys + 1 b0666_tRNA_3 + 1 h	irreversible	tRNA modification	0.00E+00	1.00E+09	0	1.70E+02	1.70E+02	0	7.79E+00	7.79	0	7.79E+00	7.79
b0666_tRNA_20A_MODIF_1	modification of position 20A nt (D) in metU by DusA_mono	1 DusA_mono + 1 b0666_tRNA_4 + 1 h + 1 nadph <==> 1 b0666_tRNA_4_DusA_mono_cplx	reversible	tRNA modification	-1.00E+09	1.00E+09	0	1.70E+02	1.70E+02	0	7.79E+00	7.79	0	7.79E+00	7.79
b0666_tRNA_20A_MODIF_2	modification of position 20A nt (D) in metU by DusA_mono	1 b0666_tRNA_4_DusA_mono_cplx --> 1 DusA_mono + 1 b0666_tRNA_5 + 1 nadp	irreversible	tRNA modification	0.00E+00	1.00E+09	0	1.70E+02	1.70E+02	0	7.79E+00	7.79	0	7.79E+00	7.79
b0666_tRNA_20_MODIF_1	modification of position 20 nt (D) in metU by DusA_mono	1 DusA_mono + 1 b0666_tRNA_3 + 1 h + 1 nadph <==> 1 b0666_tRNA_3_DusA_mono_cplx	reversible	tRNA modification	-1.00E+09	1.00E+09	0	1.70E+02	1.70E+02	0	7.79E+00	7.79	0	7.79E+00	7.79
b0666_tRNA_20_MODIF_2	modification of position 20 nt (D) in metU by DusA_mono	1 b0666_tRNA_3_DusA_mono_cplx --> 1 DusA_mono + 1 b0666_tRNA_4 + 1 nadp	irreversible	tRNA modification	0.00E+00	1.00E+09	0	1.70E+02	1.70E+02	0	7.79E+00	7.79	0	7.79E+00	7.79
b0666_tRNA_34_MODIF_1	modification of position 34 nt (ac4C) in metU by AcT_tRNA_pos_34_ac4C	1 AcT_tRNA_pos_34_ac4C + 1 accoa + 1 b0666_tRNA_5 <==> 1 b0666_tRNA_5_AcT_tRNA_pos_34_ac4C_cplx	reversible	tRNA modification	-1.00E+09	1.00E+09	0	1.70E+02	1.70E+02	0	7.79E+00	7.79	0	7.79E+00	7.79
b0666_tRNA_34_MODIF_2	modification of position 34 nt (ac4C) in metU by AcT_tRNA_pos_34_ac4C	1 b0666_tRNA_5_AcT_tRNA_pos_34_ac4C_cplx --> 1 AcT_tRNA_pos_34_ac4C_inact + 1 b0666_tRNA_6 + 1 coa	irreversible	tRNA modification	0.00E+00	1.00E+09	0	1.70E+02	1.70E+02	0	7.79E+00	7.79	0	7.79E+00	7.79
b0666_tRNA_37_MODIF_1	modification of position 37 nt (t6A) in metU by Up_tRNA_pos_37_t6A	1 Up_tRNA_pos_37_t6A + 1 atp + 1 b0666_tRNA_6 + 1 hco3 + 1 mg2 + 1 thr-L <==> 1 b0666_tRNA_6_Up_tRNA_pos_37_t6A_cplx	reversible	tRNA modification	-1.00E+09	1.00E+09	0	1.70E+02	1.70E+02	0	7.79E+00	7.79	0	7.79E+00	7.79
b0666_tRNA_37_MODIF_2	modification of position 37 nt (t6A) in metU by Up_tRNA_pos_37_t6A	1 b0666_tRNA_6_Up_tRNA_pos_37_t6A_cplx --> 1 Up_tRNA_pos_37_t6A_inact + 1 amp + 1 b0666_tRNA_7 + 1 h + 1 h2o + 1 mg2 + 1 ppi	irreversible	tRNA modification	0.00E+00	1.00E+09	0	1.70E+02	1.70E+02	0	7.79E+00	7.79	0	7.79E+00	7.79
b0666_tRNA_39_MODIF_1	modification of position 39 nt (Y) in metU by TruA_dim	1 TruA_dim + 1 b0666_tRNA_7 <==> 1 b0666_tRNA_7_TruA_dim_cplx	reversible	tRNA modification	-1.00E+09	1.00E+09	0	1.70E+02	1.70E+02	0	7.79E+00	7.79	0	7.79E+00	7.79
b0666_tRNA_39_MODIF_2	modification of position 39 nt (Y) in metU by TruA_dim	1 b0666_tRNA_7_TruA_dim_cplx --> 1 TruA_dim_inact + 1 b0666_tRNA_8	irreversible	tRNA modification	0.00E+00	1.00E+09	0	1.70E+02	1.70E+02	0	7.79E+00	7.79	0	7.79E+00	7.79
b0666_tRNA_46_MODIF_1	modification of position 46 nt (m7G) in metU by YggH_mono	1 YggH_mono + 1 amet + 1 b0666_tRNA_8 <==> 1 b0666_tRNA_8_YggH_mono_cplx	reversible	tRNA modification	-1.00E+09	1.00E+09	0	1.70E+02	1.70E+02	0	7.79E+00	7.79	0	7.79E+00	7.79
b0666_tRNA_46_MODIF_2	modification of position 46 nt (m7G) in metU by YggH_mono	1 b0666_tRNA_8_YggH_mono_cplx --> 1 YggH_mono_inact + 1 ahcys + 1 b0666_tRNA_9 + 1 h	irreversible	tRNA modification	0.00E+00	1.00E+09	0	1.70E+02	1.70E+02	0	7.79E+00	7.79	0	7.79E+00	7.79
b0666_tRNA_47_MODIF_1	modification of position 47 nt (acp3U) in metU by AcpT_tRNA_pos_47_acp3U	1 AcpT_tRNA_pos_47_acp3U + 1 amet + 1 b0666_tRNA_9 <==> 1 b0666_tRNA_9_AcpT_tRNA_pos_47_acp3U_cplx	reversible	tRNA modification	-1.00E+09	1.00E+09	0	1.70E+02	1.70E+02	0	7.79E+00	7.79	0	7.79E+00	7.79

b0666_tRNA_47_MODIF_2	modification of position 47 nt (acp3U) in metU by AcpT_tRNA_pos_47_acp3U	1 b0666_tRNA_9_AcpT_tRNA_pos_47_acp3U_cplx --> 1 5mta + 1 AcpT_tRNA_pos_47_acp3U_inact + 1 b0666_tRNA_10 + 1 h	irreversible	tRNA modification	0.00E+00	1.00E+09	0	1.70E+02	1.70E+02	0	7.79E+00	7.79	0	7.79E+00	7.79
b0666_tRNA_54_MODIF_1	modification of position 54 nt (m5U) in metU by TrmA_mono	1 TrmA_mono + 1 amet + 1 b0666_tRNA_10 <==> 1 b0666_tRNA_10_TrmA_mono_cplx	reversible	tRNA modification	-1.00E+09	1.00E+09	0	1.70E+02	1.70E+02	0	7.79E+00	7.79	0	7.79E+00	7.79
b0666_tRNA_54_MODIF_2	modification of position 54 nt (m5U) in metU by TrmA_mono	1 b0666_tRNA_10_TrmA_mono_cplx --> 1 TrmA_mono_inact + 1 ahcys + 1 b0666_tRNA_11 + 1 h	irreversible	tRNA modification	0.00E+00	1.00E+09	0	1.70E+02	1.70E+02	0	7.79E+00	7.79	0	7.79E+00	7.79
b0666_tRNA_55_MODIF_1	modification of position 55 nt (Y) in metU by TruB_mono	1 TruB_mono + 1 b0666_tRNA_11 <==> 1 b0666_tRNA_11_TruB_mono_cplx	reversible	tRNA modification	-1.00E+09	1.00E+09	0	1.70E+02	1.70E+02	0	7.79E+00	7.79	0	7.79E+00	7.79
b0666_tRNA_55_MODIF_2	modification of position 55 nt (Y) in metU by TruB_mono	1 b0666_tRNA_11_TruB_mono_cplx --> 1 TruB_mono_inact + 1 b0666_tRNA_12	irreversible	tRNA modification	0.00E+00	1.00E+09	0	1.70E+02	1.70E+02	0	7.79E+00	7.79	0	7.79E+00	7.79
b0666_tRNA_8_MODIF_1	modification of position 8 nt (s4U) in metU by ThiI_mono	1 IscS_dim_S-SH + 1 ThiI_mono + 1 atp + 1 b0666_tRNA_12 + 1 mg2 + 1 trdrd <==> 1 b0666_tRNA_12_ThiI_mono_cplx	reversible	tRNA modification	-1.00E+09	1.00E+09	0	1.70E+02	1.70E+02	0	7.79E+00	7.79	0	7.79E+00	7.79
b0666_tRNA_8_MODIF_2	modification of position 8 nt (s4U) in metU by ThiI_mono	1 b0666_tRNA_12_ThiI_mono_cplx --> 1 IscS_dim_S-H + 1 ThiI_mono_inact + 1 amp + 1 h + 1 metU_tRNA + 1 mg2 + 1 ppi + 1 trdox	irreversible	tRNA modification	0.00E+00	1.00E+09	0	1.70E+02	1.70E+02	0	7.79E+00	7.79	0	7.79E+00	7.79
b0666_tRNA_Mg_BIND	Binding of two Mg2 to b0666_tRNA	1 b0666_RNA + 2 mg2 <==> 1 b0666_tRNA_Mg2	reversible	Metallo-ion binding	-1.00E+09	1.00E+09	0	1.70E+02	1.70E+02	0	7.79E+00	7.79	0	7.79E+00	7.79
b0668_tRNA_18_MODIF_1	modification of position 18 nt (Gm) in glnW by TrmH_dim	1 TrmH_dim + 1 amet + 1 b0668_tRNA_Mg2 <==> 1 b0668_tRNA_Mg2_TrmH_dim_cplx	reversible	tRNA modification	-1.00E+09	1.00E+09	0	1.70E+02	1.70E+02	0	7.79E+00	7.79	0	7.79E+00	7.79
b0668_tRNA_18_MODIF_2	modification of position 18 nt (Gm) in glnW by TrmH_dim	1 b0668_tRNA_Mg2_TrmH_dim_cplx --> 1 TrmH_dim_inact + 1 ahcys + 1 b0668_tRNA_1 + 1 h	irreversible	tRNA modification	0.00E+00	1.00E+09	0	1.70E+02	1.70E+02	0	7.79E+00	7.79	0	7.79E+00	7.79
b0668_tRNA_20_MODIF_1	modification of position 20 nt (D) in glnW by Dus_gen	1 Dus_gen + 1 b0668_tRNA_1 + 1 h + 1 nadph <==> 1 b0668_tRNA_1_Dus_gen_cplx	reversible	tRNA modification	-1.00E+09	1.00E+09	0	1.70E+02	1.70E+02	0	7.79E+00	7.79	0	7.79E+00	7.79
b0668_tRNA_20_MODIF_2	modification of position 20 nt (D) in glnW by Dus_gen	1 b0668_tRNA_1_Dus_gen_cplx --> 1 Dus_gen_inact + 1 b0668_tRNA_2 + 1 nadp	irreversible	tRNA modification	0.00E+00	1.00E+09	0	1.70E+02	1.70E+02	0	7.79E+00	7.79	0	7.79E+00	7.79
b0668_tRNA_32_MODIF_1	modification of position 32 nt (Um) in glnW by MeT_tRNA_pos_32_Um	1 MeT_tRNA_pos_32_Um + 1 amet + 1 b0668_tRNA_2 <==> 1 b0668_tRNA_2_MeT_tRNA_pos_32_Um_cplx	reversible	tRNA modification	-1.00E+09	1.00E+09	0	1.70E+02	1.70E+02	0	7.79E+00	7.79	0	7.79E+00	7.79
b0668_tRNA_32_MODIF_2	modification of position 32 nt (Um) in glnW by MeT_tRNA_pos_32_Um	1 b0668_tRNA_2_MeT_tRNA_pos_32_Um_cplx --> 1 MeT_tRNA_pos_32_Um_inact + 1 ahcys + 1 b0668_tRNA_3 + 1 h	irreversible	tRNA modification	0.00E+00	1.00E+09	0	1.70E+02	1.70E+02	0	7.79E+00	7.79	0	7.79E+00	7.79
b0668_tRNA_37_MODIF_1	modification of position 37 nt (m2A) in glnW by MeT_tRNA_pos_37_m2A	1 MeT_tRNA_pos_37_m2A + 1 amet + 1 b0668_tRNA_3 <==> 1 b0668_tRNA_3_MeT_tRNA_pos_37_m2A_cplx	reversible	tRNA modification	-1.00E+09	1.00E+09	0	1.70E+02	1.70E+02	0	7.79E+00	7.79	0	7.79E+00	7.79
b0668_tRNA_37_MODIF_2	modification of position 37 nt (m2A) in glnW by MeT_tRNA_pos_37_m2A	1 b0668_tRNA_3_MeT_tRNA_pos_37_m2A_cplx --> 1 MeT_tRNA_pos_37_m2A_inact + 1 ahcys + 1 b0668_tRNA_4 + 1 h	irreversible	tRNA modification	0.00E+00	1.00E+09	0	1.70E+02	1.70E+02	0	7.79E+00	7.79	0	7.79E+00	7.79
b0668_tRNA_38_MODIF_1	modification of position 38 nt (Y) in glnW by TruA_dim	1 TruA_dim + 1 b0668_tRNA_4 <==> 1 b0668_tRNA_4_TruA_dim_cplx	reversible	tRNA modification	-1.00E+09	1.00E+09	0	1.70E+02	1.70E+02	0	7.79E+00	7.79	0	7.79E+00	7.79
b0668_tRNA_38_MODIF_2	modification of position 38 nt (Y) in glnW by TruA_dim	1 b0668_tRNA_4_TruA_dim_cplx --> 1 TruA_dim_inact + 1 b0668_tRNA_5	irreversible	tRNA modification	0.00E+00	1.00E+09	0	1.70E+02	1.70E+02	0	7.79E+00	7.79	0	7.79E+00	7.79
b0668_tRNA_39_MODIF_1	modification of position 39 nt (Y) in glnW by TruA_dim	1 TruA_dim + 1 b0668_tRNA_5 <==> 1 b0668_tRNA_5_TruA_dim_cplx	reversible	tRNA modification	-1.00E+09	1.00E+09	0	1.70E+02	1.70E+02	0	7.79E+00	7.79	0	7.79E+00	7.79
b0668_tRNA_39_MODIF_2	modification of position 39 nt (Y) in glnW by TruA_dim	1 b0668_tRNA_5_TruA_dim_cplx --> 1 TruA_dim_inact + 1 b0668_tRNA_6	irreversible	tRNA modification	0.00E+00	1.00E+09	0	1.70E+02	1.70E+02	0	7.79E+00	7.79	0	7.79E+00	7.79
b0668_tRNA_54_MODIF_1	modification of position 54 nt (m5U) in glnW by TrmA_mono	1 TrmA_mono + 1 amet + 1 b0668_tRNA_6 <==> 1 b0668_tRNA_6_TrmA_mono_cplx	reversible	tRNA modification	-1.00E+09	1.00E+09	0	1.70E+02	1.70E+02	0	7.79E+00	7.79	0	7.79E+00	7.79
b0668_tRNA_54_MODIF_2	modification of position 54 nt (m5U) in glnW by TrmA_mono	1 b0668_tRNA_6_TrmA_mono_cplx --> 1 TrmA_mono_inact + 1 ahcys + 1 b0668_tRNA_7 + 1 h	irreversible	tRNA modification	0.00E+00	1.00E+09	0	1.70E+02	1.70E+02	0	7.79E+00	7.79	0	7.79E+00	7.79
b0668_tRNA_55_MODIF_1	modification of position 55 nt (Y) in glnW by TruB_mono	1 TruB_mono + 1 b0668_tRNA_7 <==> 1 b0668_tRNA_7_TruB_mono_cplx	reversible	tRNA modification	-1.00E+09	1.00E+09	0	1.70E+02	1.70E+02	0	7.79E+00	7.79	0	7.79E+00	7.79
b0668_tRNA_55_MODIF_2	modification of position 55 nt (Y) in glnW by TruB_mono	1 b0668_tRNA_7_TruB_mono_cplx --> 1 TruB_mono_inact + 1 b0668_tRNA_8	irreversible	tRNA modification	0.00E+00	1.00E+09	0	1.70E+02	1.70E+02	0	7.79E+00	7.79	0	7.79E+00	7.79
b0668_tRNA_8_MODIF_1	modification of position 8 nt (s4U) in glnW by ThiI_mono	1 IscS_dim_S-SH + 1 ThiI_mono + 1 atp + 1 b0668_tRNA_8 + 1 mg2 + 1 trdrd <==> 1 b0668_tRNA_8_ThiI_mono_cplx	reversible	tRNA modification	-1.00E+09	1.00E+09	0	1.70E+02	1.70E+02	0	7.79E+00	7.79	0	7.79E+00	7.79
b0668_tRNA_8_MODIF_2	modification of position 8 nt (s4U) in glnW by ThiI_mono	1 b0668_tRNA_8_ThiI_mono_cplx --> 1 IscS_dim_S-H + 1 ThiI_mono_inact + 1 amp + 1 glnW_tRNA + 1 h + 1 mg2 + 1 ppi + 1 trdox	irreversible	tRNA modification	0.00E+00	1.00E+09	0	1.70E+02	1.70E+02	0	7.79E+00	7.79	0	7.79E+00	7.79
b0668_tRNA_Mg_BIND	Binding of two Mg2 to b0668_tRNA	1 b0668_RNA + 2 mg2 <==> 1 b0668_tRNA_Mg2	reversible	Metallo-ion binding	-1.00E+09	1.00E+09	0	1.70E+02	1.70E+02	0	7.79E+00	7.79	0	7.79E+00	7.79
b0670_tRNA_18_MODIF_1	modification of position 18 nt (Gm) in glnU by TrmH_dim	1 TrmH_dim + 1 amet + 1 b0670_tRNA_Mg2 <==> 1 b0670_tRNA_Mg2_TrmH_dim_cplx	reversible	tRNA modification	-1.00E+09	1.00E+09	0	1.70E+02	1.70E+02	0	7.79E+00	7.79	0	7.79E+00	7.79
b0670_tRNA_18_MODIF_2	modification of position 18 nt (Gm) in glnU by TrmH_dim	1 b0670_tRNA_Mg2_TrmH_dim_cplx --> 1 TrmH_dim_inact + 1 ahcys + 1 b0670_tRNA_1 + 1 h	irreversible	tRNA modification	0.00E+00	1.00E+09	0	1.70E+02	1.70E+02	0	7.79E+00	7.79	0	7.79E+00	7.79
b0670_tRNA_20_MODIF_1	modification of position 20 nt (D) in glnU by Dus_gen	1 Dus_gen + 1 b0670_tRNA_1 + 1 h + 1 nadph <==> 1 b0670_tRNA_1_Dus_gen_cplx	reversible	tRNA modification	-1.00E+09	1.00E+09	0	1.70E+02	1.70E+02	0	7.79E+00	7.79	0	7.79E+00	7.79
b0670_tRNA_20_MODIF_2	modification of position 20 nt (D) in glnU by Dus_gen	1 b0670_tRNA_1_Dus_gen_cplx --> 1 Dus_gen_inact + 1 b0670_tRNA_2 + 1 nadp	irreversible	tRNA modification	0.00E+00	1.00E+09	0	1.70E+02	1.70E+02	0	7.79E+00	7.79	0	7.79E+00	7.79
b0670_tRNA_32_MODIF_1	modification of position 32 nt (Um) in glnU by MeT_tRNA_pos_32_Um	1 MeT_tRNA_pos_32_Um + 1 amet + 1 b0670_tRNA_2 <==> 1 b0670_tRNA_2_MeT_tRNA_pos_32_Um_cplx	reversible	tRNA modification	-1.00E+09	1.00E+09	0	1.70E+02	1.70E+02	0	7.79E+00	7.79	0	7.79E+00	7.79
b0670_tRNA_32_MODIF_2	modification of position 32 nt (Um) in glnU by MeT_tRNA_pos_32_Um	1 b0670_tRNA_2_MeT_tRNA_pos_32_Um_cplx --> 1 MeT_tRNA_pos_32_Um_inact + 1 ahcys + 1 b0670_tRNA_3 + 1 h	irreversible	tRNA modification	0.00E+00	1.00E+09	0	1.70E+02	1.70E+02	0	7.79E+00	7.79	0	7.79E+00	7.79
b0670_tRNA_37_MODIF_1	modification of position 37 nt (m2A) in glnU by MeT_tRNA_pos_37_m2A	1 MeT_tRNA_pos_37_m2A + 1 amet + 1 b0670_tRNA_3 <==> 1 b0670_tRNA_3_MeT_tRNA_pos_37_m2A_cplx	reversible	tRNA modification	-1.00E+09	1.00E+09	0	1.70E+02	1.70E+02	0	7.79E+00	7.79	0	7.79E+00	7.79
b0670_tRNA_37_MODIF_2	modification of position 37 nt (m2A) in glnU by MeT_tRNA_pos_37_m2A	1 b0670_tRNA_3_MeT_tRNA_pos_37_m2A_cplx --> 1 MeT_tRNA_pos_37_m2A_inact + 1 ahcys + 1 b0670_tRNA_4 + 1 h	irreversible	tRNA modification	0.00E+00	1.00E+09	0	1.70E+02	1.70E+02	0	7.79E+00	7.79	0	7.79E+00	7.79
b0670_tRNA_38_MODIF_1	modification of position 38 nt (Y) in glnU by TruA_dim	1 TruA_dim + 1 b0670_tRNA_4 <==> 1 b0670_tRNA_4_TruA_dim_cplx	reversible	tRNA modification	-1.00E+09	1.00E+09	0	1.70E+02	1.70E+02	0	7.79E+00	7.79	0	7.79E+00	7.79
b0670_tRNA_38_MODIF_2	modification of position 38 nt (Y) in glnU by TruA_dim	1 b0670_tRNA_4_TruA_dim_cplx --> 1 TruA_dim_inact + 1 b0670_tRNA_5	irreversible	tRNA modification	0.00E+00	1.00E+09	0	1.70E+02	1.70E+02	0	7.79E+00	7.79	0	7.79E+00	7.79
b0670_tRNA_39_MODIF_1	modification of position 39 nt (Y) in glnU by TruA_dim	1 TruA_dim + 1 b0670_tRNA_5 <==> 1 b0670_tRNA_5_TruA_dim_cplx	reversible	tRNA modification	-1.00E+09	1.00E+09	0	1.70E+02	1.70E+02	0	7.79E+00	7.79	0	7.79E+00	7.79
b0670_tRNA_39_MODIF_2	modification of position 39 nt (Y) in glnU by TruA_dim	1 b0670_tRNA_5_TruA_dim_cplx --> 1 TruA_dim_inact + 1 b0670_tRNA_6	irreversible	tRNA modification	0.00E+00	1.00E+09	0	1.70E+02	1.70E+02	0	7.79E+00	7.79	0	7.79E+00	7.79
b0670_tRNA_54_MODIF_1	modification of position 54 nt (m5U) in glnU by TrmA_mono	1 TrmA_mono + 1 amet + 1 b0670_tRNA_6 <==> 1 b0670_tRNA_6_TrmA_mono_cplx	reversible	tRNA modification	-1.00E+09	1.00E+09	0	1.70E+02	1.70E+02	0	7.79E+00	7.79	0	7.79E+00	7.79
b0670_tRNA_54_MODIF_2	modification of position 54 nt (m5U) in glnU by TrmA_mono	1 b0670_tRNA_6_TrmA_mono_cplx --> 1 TrmA_mono_inact + 1 ahcys + 1 b0670_tRNA_7 + 1 h	irreversible	tRNA modification	0.00E+00	1.00E+09	0	1.70E+02	1.70E+02	0	7.79E+00	7.79	0	7.79E+00	7.79
b0670_tRNA_55_MODIF_1	modification of position 55 nt (Y) in glnU by TruB_mono	1 TruB_mono + 1 b0670_tRNA_7 <==> 1 b0670_tRNA_7_TruB_mono_cplx	reversible	tRNA modification	-1.00E+09	1.00E+09	0	1.70E+02	1.70E+02	0	7.79E+00	7.79	0	7.79E+00	7.79
b0670_tRNA_55_MODIF_2	modification of position 55 nt (Y) in glnU by TruB_mono	1 b0670_tRNA_7_TruB_mono_cplx --> 1 TruB_mono_inact + 1 b0670_tRNA_8	irreversible	tRNA modification	0.00E+00	1.00E+09	0	1.70E+02	1.70E+02	0	7.79E+00	7.79	0	7.79E+00	7.79
b0670_tRNA_8_MODIF_1	modification of position 8 nt (s4U) in glnU by ThiI_mono	1 IscS_dim_S-SH + 1 ThiI_mono + 1 atp + 1 b0670_tRNA_8 + 1 mg2 + 1 trdrd <==> 1 b0670_tRNA_8_ThiI_mono_cplx	reversible	tRNA modification	-1.00E+09	1.00E+09	0	1.70E+02	1.70E+02	0	7.79E+00	7.79	0	7.79E+00	7.79
b0670_tRNA_8_MODIF_2	modification of position 8 nt (s4U) in glnU by ThiI_mono	1 b0670_tRNA_8_ThiI_mono_cplx --> 1 IscS_dim_S-H + 1 ThiI_mono_inact + 1 amp + 1 glnU_tRNA + 1 h + 1 mg2 + 1 ppi + 1 trdox	irreversible	tRNA modification	0.00E+00	1.00E+09	0	1.70E+02	1.70E+02	0	7.79E+00	7.79	0	7.79E+00	7.79
b0670_tRNA_Mg_BIND	Binding of two Mg2 to b0670_tRNA	1 b0670_RNA + 2 mg2 <==> 1 b0670_tRNA_Mg2	reversible	Metallo-ion binding	-1.00E+09	1.00E+09	0	1.70E+02	1.70E+02	0	7.79E+00	7.79	0	7.79E+00	7.79
b0672_tRNA_16_MODIF_1	modification of position 16 nt (D) in leuW by Dus_gen	1 Dus_gen + 1 b0672_tRNA_Mg2 + 1 h + 1 nadph <==> 1 b0672_tRNA_Mg2_Dus_gen_cplx	reversible	tRNA modification	-1.00E+09	1.00E+09	0	1.70E+02	1.70E+02	0	7.79E+00	7.79	0	7.79E+00	7.79
b0672_tRNA_16_MODIF_2	modification of position 16 nt (D) in leuW by Dus_gen	1 b0672_tRNA_Mg2_Dus_gen_cplx --> 1 Dus_gen_inact + 1 b0672_tRNA_1 + 1 nadp	irreversible	tRNA modification	0.00E+00	1.00E+09	0	1.70E+02	1.70E+02	0	7.79E+00	7.79	0	7.79E+00	7.79
b0672_tRNA_17_MODIF_1	modification of position 17 nt (D) in leuW by Dus_gen	1 Dus_gen + 1 b0672_tRNA_1 + 1 h + 1 nadph <==> 1 b0672_tRNA_1_Dus_gen_cplx	reversible	tRNA modification	-1.00E+09	1.00E+09	0	1.70E+02	1.70E+02	0	7.79E+00	7.79	0	7.79E+00	7.79
b0672_tRNA_17_MODIF_2	modification of position 17 nt (D) in leuW by Dus_gen	1 b0672_tRNA_1_Dus_gen_cplx --> 1 Dus_gen_inact + 1 b0672_tRNA_2 + 1 nadp	irreversible	tRNA modification	0.00E+00	1.00E+09	0	1.70E+02	1.70E+02	0	7.79E+00	7.79	0	7.79E+00	7.79
b0672_tRNA_18_MODIF_1	modification of position 18 nt (Gm) in leuW by TrmH_dim	1 TrmH_dim + 1 amet + 1 b0672_tRNA_2 <==> 1 b0672_tRNA_2_TrmH_dim_cplx	reversible	tRNA modification	-1.00E+09	1.00E+09	0	1.70E+02	1.70E+02	0	7.79E+00	7.79	0	7.79E+00	7.79

b0672_tRNA_18_MODIF_2	modification of position 18 nt (Gm) in leuW by TrmH_dim	1 b0672_tRNA_2_TrmH_dim_cplx --> 1 TrmH_dim_inact + 1 ahcys + 1 b0672_tRNA_3 + 1 h	irreversible	tRNA modification	0.00E+00	1.00E+09	0	1.70E+02	1.70E+02	0	7.79E+00	7.79	0	7.79E+00	7.79
b0672_tRNA_20_MODIF_1	modification of position 20 nt (D) in leuW by Dus_gen	1 Dus_gen + 1 b0672_tRNA_3 + 1 h + 1 nadph <==> 1 b0672_tRNA_3_Dus_gen_cplx	reversible	tRNA modification	-1.00E+09	1.00E+09	0	1.70E+02	1.70E+02	0	7.79E+00	7.79	0	7.79E+00	7.79
b0672_tRNA_20_MODIF_2	modification of position 20 nt (D) in leuW by Dus_gen	1 b0672_tRNA_3_Dus_gen_cplx --> 1 Dus_gen_inact + 1 b0672_tRNA_4 + 1 nadp	irreversible	tRNA modification	0.00E+00	1.00E+09	0	1.70E+02	1.70E+02	0	7.79E+00	7.79	0	7.79E+00	7.79
b0672_tRNA_38_MODIF_1	modification of position 38 nt (Y) in leuW by TruA_dim	1 TruA_dim + 1 b0672_tRNA_4 <==> 1 b0672_tRNA_4_TruA_dim_cplx	reversible	tRNA modification	-1.00E+09	1.00E+09	0	1.70E+02	1.70E+02	0	7.79E+00	7.79	0	7.79E+00	7.79
b0672_tRNA_38_MODIF_2	modification of position 38 nt (Y) in leuW by TruA_dim	1 b0672_tRNA_4_TruA_dim_cplx --> 1 TruA_dim_inact + 1 b0672_tRNA_5	irreversible	tRNA modification	0.00E+00	1.00E+09	0	1.70E+02	1.70E+02	0	7.79E+00	7.79	0	7.79E+00	7.79
b0672_tRNA_55_MODIF_1	modification of position 55 nt (Y) in leuW by TruB_mono	1 TruB_mono + 1 b0672_tRNA_5 <==> 1 b0672_tRNA_5_TruB_mono_cplx	reversible	tRNA modification	-1.00E+09	1.00E+09	0	1.70E+02	1.70E+02	0	7.79E+00	7.79	0	7.79E+00	7.79
b0672_tRNA_55_MODIF_2	modification of position 55 nt (Y) in leuW by TruB_mono	1 b0672_tRNA_5_TruB_mono_cplx --> 1 TruB_mono_inact + 1 leuW_tRNA	irreversible	tRNA modification	0.00E+00	1.00E+09	0	1.70E+02	1.70E+02	0	7.79E+00	7.79	0	7.79E+00	7.79
b0672_tRNA_Mg_BIND	Binding of two Mg2 to b0672_tRNA	1 b0672_RNA + 2 mg2 <==> 1 b0672_tRNA_Mg2	reversible	Metallo-ion binding	-1.00E+09	1.00E+09	0	1.70E+02	1.70E+02	0	7.79E+00	7.79	0	7.79E+00	7.79
b0673_tRNA_16_MODIF_1	modification of position 16 nt (D) in metT by DusA_mono	1 DusA_mono + 1 b0673_tRNA_Mg2 + 1 h + 1 nadph <==> 1 b0673_tRNA_Mg2_DusA_mono_cplx	reversible	tRNA modification	-1.00E+09	1.00E+09	0	1.70E+02	1.70E+02	0	7.79E+00	7.79	0	7.79E+00	7.79
b0673_tRNA_16_MODIF_2	modification of position 16 nt (D) in metT by DusA_mono	1 b0673_tRNA_Mg2_DusA_mono_cplx --> 1 DusA_mono + 1 b0673_tRNA_1 + 1 nadp	irreversible	tRNA modification	0.00E+00	1.00E+09	0	1.70E+02	1.70E+02	0	7.79E+00	7.79	0	7.79E+00	7.79
b0673_tRNA_17_MODIF_1	modification of position 17 nt (D) in metT by DusA_mono	1 DusA_mono + 1 b0673_tRNA_1 + 1 h + 1 nadph <==> 1 b0673_tRNA_1_DusA_mono_cplx	reversible	tRNA modification	-1.00E+09	1.00E+09	0	1.70E+02	1.70E+02	0	7.79E+00	7.79	0	7.79E+00	7.79
b0673_tRNA_17_MODIF_2	modification of position 17 nt (D) in metT by DusA_mono	1 b0673_tRNA_1_DusA_mono_cplx --> 1 DusA_mono + 1 b0673_tRNA_2 + 1 nadp	irreversible	tRNA modification	0.00E+00	1.00E+09	0	1.70E+02	1.70E+02	0	7.79E+00	7.79	0	7.79E+00	7.79
b0673_tRNA_18_MODIF_1	modification of position 18 nt (Gm) in metT by TrmH_dim	1 TrmH_dim + 1 amet + 1 b0673_tRNA_2 <==> 1 b0673_tRNA_2_TrmH_dim_cplx	reversible	tRNA modification	-1.00E+09	1.00E+09	0	1.70E+02	1.70E+02	0	7.79E+00	7.79	0	7.79E+00	7.79
b0673_tRNA_18_MODIF_2	modification of position 18 nt (Gm) in metT by TrmH_dim	1 b0673_tRNA_2_TrmH_dim_cplx --> 1 TrmH_dim_inact + 1 ahcys + 1 b0673_tRNA_3 + 1 h	irreversible	tRNA modification	0.00E+00	1.00E+09	0	1.70E+02	1.70E+02	0	7.79E+00	7.79	0	7.79E+00	7.79
b0673_tRNA_20A_MODIF_1	modification of position 20A nt (D) in metT by DusA_mono	1 DusA_mono + 1 b0673_tRNA_4 + 1 h + 1 nadph <==> 1 b0673_tRNA_4_DusA_mono_cplx	reversible	tRNA modification	-1.00E+09	1.00E+09	0	1.70E+02	1.70E+02	0	7.79E+00	7.79	0	7.79E+00	7.79
b0673_tRNA_20A_MODIF_2	modification of position 20A nt (D) in metT by DusA_mono	1 b0673_tRNA_4_DusA_mono_cplx --> 1 DusA_mono + 1 b0673_tRNA_5 + 1 nadp	irreversible	tRNA modification	0.00E+00	1.00E+09	0	1.70E+02	1.70E+02	0	7.79E+00	7.79	0	7.79E+00	7.79
b0673_tRNA_20_MODIF_1	modification of position 20 nt (D) in metT by DusA_mono	1 DusA_mono + 1 b0673_tRNA_3 + 1 h + 1 nadph <==> 1 b0673_tRNA_3_DusA_mono_cplx	reversible	tRNA modification	-1.00E+09	1.00E+09	0	1.70E+02	1.70E+02	0	7.79E+00	7.79	0	7.79E+00	7.79
b0673_tRNA_20_MODIF_2	modification of position 20 nt (D) in metT by DusA_mono	1 b0673_tRNA_3_DusA_mono_cplx --> 1 DusA_mono + 1 b0673_tRNA_4 + 1 nadp	irreversible	tRNA modification	0.00E+00	1.00E+09	0	1.70E+02	1.70E+02	0	7.79E+00	7.79	0	7.79E+00	7.79
b0673_tRNA_34_MODIF_1	modification of position 34 nt (ac4C) in metT by AcT_tRNA_pos_34_ac4C	1 AcT_tRNA_pos_34_ac4C + 1 accoa + 1 b0673_tRNA_5 <==> 1 b0673_tRNA_5_AcT_tRNA_pos_34_ac4C_cplx	reversible	tRNA modification	-1.00E+09	1.00E+09	0	1.70E+02	1.70E+02	0	7.79E+00	7.79	0	7.79E+00	7.79
b0673_tRNA_34_MODIF_2	modification of position 34 nt (ac4C) in metT by AcT_tRNA_pos_34_ac4C	1 b0673_tRNA_5_AcT_tRNA_pos_34_ac4C_cplx --> 1 AcT_tRNA_pos_34_ac4C_inact + 1 b0673_tRNA_6 + 1 coa	irreversible	tRNA modification	0.00E+00	1.00E+09	0	1.70E+02	1.70E+02	0	7.79E+00	7.79	0	7.79E+00	7.79
b0673_tRNA_37_MODIF_1	modification of position 37 nt (t6A) in metT by Up_tRNA_pos_37_t6A	1 Up_tRNA_pos_37_t6A + 1 atp + 1 b0673_tRNA_6 + 1 hco3 + 1 mg2 + 1 thr-L <==> 1 b0673_tRNA_6_Up_tRNA_pos_37_t6A_cplx	reversible	tRNA modification	-1.00E+09	1.00E+09	0	1.70E+02	1.70E+02	0	7.79E+00	7.79	0	7.79E+00	7.79
b0673_tRNA_37_MODIF_2	modification of position 37 nt (t6A) in metT by Up_tRNA_pos_37_t6A	1 b0673_tRNA_6_Up_tRNA_pos_37_t6A_cplx --> 1 Up_tRNA_pos_37_t6A_inact + 1 amp + 1 b0673_tRNA_7 + 1 h + 1 h2o + 1 mg2 + 1 ppi	irreversible	tRNA modification	0.00E+00	1.00E+09	0	1.70E+02	1.70E+02	0	7.79E+00	7.79	0	7.79E+00	7.79
b0673_tRNA_39_MODIF_1	modification of position 39 nt (Y) in metT by TruA_dim	1 TruA_dim + 1 b0673_tRNA_7 <==> 1 b0673_tRNA_7_TruA_dim_cplx	reversible	tRNA modification	-1.00E+09	1.00E+09	0	1.70E+02	1.70E+02	0	7.79E+00	7.79	0	7.79E+00	7.79
b0673_tRNA_39_MODIF_2	modification of position 39 nt (Y) in metT by TruA_dim	1 b0673_tRNA_7_TruA_dim_cplx --> 1 TruA_dim_inact + 1 b0673_tRNA_8	irreversible	tRNA modification	0.00E+00	1.00E+09	0	1.70E+02	1.70E+02	0	7.79E+00	7.79	0	7.79E+00	7.79
b0673_tRNA_46_MODIF_1	modification of position 46 nt (m7G) in metT by YggH_mono	1 YggH_mono + 1 amet + 1 b0673_tRNA_8 <==> 1 b0673_tRNA_8_YggH_mono_cplx	reversible	tRNA modification	-1.00E+09	1.00E+09	0	1.70E+02	1.70E+02	0	7.79E+00	7.79	0	7.79E+00	7.79
b0673_tRNA_46_MODIF_2	modification of position 46 nt (m7G) in metT by YggH_mono	1 b0673_tRNA_8_YggH_mono_cplx --> 1 YggH_mono_inact + 1 ahcys + 1 b0673_tRNA_9 + 1 h	irreversible	tRNA modification	0.00E+00	1.00E+09	0	1.70E+02	1.70E+02	0	7.79E+00	7.79	0	7.79E+00	7.79
b0673_tRNA_47_MODIF_1	modification of position 47 nt (acp3U) in metT by AcpT_tRNA_pos_47_acp3U	1 AcpT_tRNA_pos_47_acp3U + 1 amet + 1 b0673_tRNA_9 <==> 1 b0673_tRNA_9_AcpT_tRNA_pos_47_acp3U_cplx	reversible	tRNA modification	-1.00E+09	1.00E+09	0	1.70E+02	1.70E+02	0	7.79E+00	7.79	0	7.79E+00	7.79
b0673_tRNA_47_MODIF_2	modification of position 47 nt (acp3U) in metT by AcpT_tRNA_pos_47_acp3U	1 b0673_tRNA_9_AcpT_tRNA_pos_47_acp3U_cplx --> 1 5mta + 1 AcpT_tRNA_pos_47_acp3U_inact + 1 b0673_tRNA_10 + 1 h	irreversible	tRNA modification	0.00E+00	1.00E+09	0	1.70E+02	1.70E+02	0	7.79E+00	7.79	0	7.79E+00	7.79
b0673_tRNA_54_MODIF_1	modification of position 54 nt (m5U) in metT by TrmA_mono	1 TrmA_mono + 1 amet + 1 b0673_tRNA_10 <==> 1 b0673_tRNA_10_TrmA_mono_cplx	reversible	tRNA modification	-1.00E+09	1.00E+09	0	1.70E+02	1.70E+02	0	7.79E+00	7.79	0	7.79E+00	7.79
b0673_tRNA_54_MODIF_2	modification of position 54 nt (m5U) in metT by TrmA_mono	1 b0673_tRNA_10_TrmA_mono_cplx --> 1 TrmA_mono_inact + 1 ahcys + 1 b0673_tRNA_11 + 1 h	irreversible	tRNA modification	0.00E+00	1.00E+09	0	1.70E+02	1.70E+02	0	7.79E+00	7.79	0	7.79E+00	7.79
b0673_tRNA_55_MODIF_1	modification of position 55 nt (Y) in metT by TruB_mono	1 TruB_mono + 1 b0673_tRNA_11 <==> 1 b0673_tRNA_11_TruB_mono_cplx	reversible	tRNA modification	-1.00E+09	1.00E+09	0	1.70E+02	1.70E+02	0	7.79E+00	7.79	0	7.79E+00	7.79
b0673_tRNA_55_MODIF_2	modification of position 55 nt (Y) in metT by TruB_mono	1 b0673_tRNA_11_TruB_mono_cplx --> 1 TruB_mono_inact + 1 b0673_tRNA_12	irreversible	tRNA modification	0.00E+00	1.00E+09	0	1.70E+02	1.70E+02	0	7.79E+00	7.79	0	7.79E+00	7.79
b0673_tRNA_8_MODIF_1	modification of position 8 nt (s4U) in metT by ThiI_mono	1 IscS_dim_S-SH + 1 ThiI_mono + 1 atp + 1 b0673_tRNA_12 + 1 mg2 + 1 trdrd <==> 1 b0673_tRNA_12_ThiI_mono_cplx	reversible	tRNA modification	-1.00E+09	1.00E+09	0	1.70E+02	1.70E+02	0	7.79E+00	7.79	0	7.79E+00	7.79
b0673_tRNA_8_MODIF_2	modification of position 8 nt (s4U) in metT by ThiI_mono	1 b0673_tRNA_12_ThiI_mono_cplx --> 1 IscS_dim_S-H + 1 ThiI_mono_inact + 1 amp + 1 h + 1 metT_tRNA + 1 mg2 + 1 ppi + 1 trdox	irreversible	tRNA modification	0.00E+00	1.00E+09	0	1.70E+02	1.70E+02	0	7.79E+00	7.79	0	7.79E+00	7.79
b0673_tRNA_Mg_BIND	Binding of two Mg2 to b0673_tRNA	1 b0673_RNA + 2 mg2 <==> 1 b0673_tRNA_Mg2	reversible	Metallo-ion binding	-1.00E+09	1.00E+09	0	1.70E+02	1.70E+02	0	7.79E+00	7.79	0	7.79E+00	7.79
b0680_fold_KJE_1	b0680_m folding: KJE mediated	1 DnaJ_dim + 1 DnaK_mono.ATP + 1 b0680_m <==> 1 b0680_m_DnaKJ_complex	reversible	Protein Folding	-1.00E+09	1.00E+09	0	2.60E+03	2.60E+03	0	4.29E+03	4285.722	0	2.02E+02	201.728
b0680_fold_KJE_2	b0680_m folding: KJE mediated	1 GrpE_dim + 1 b0680_m_DnaKJ_complex + 1 h2o --> 1 DnaJ_dim_inact + 1 GlnS_DnaK_GrpE_complex + 1 adp + 1 h + 1 pi	irreversible	Protein Folding	0.00E+00	1.00E+09	0	2.60E+03	2.60E+03	0	4.29E+03	4285.722	0	2.02E+02	201.728
b0680_fold_KJE_3	b0680_m folding: KJE mediated	1 GlnS_DnaK_GrpE_complex + 1 atp --> 1 DnaK_mono.ATP_inact + 1 GlnS_mono + 1 GrpE_dim_inact	irreversible	Protein Folding	0.00E+00	1.00E+09	0	2.60E+03	2.60E+03	0	4.29E+03	4285.722	0	2.02E+02	201.728
b0680_fold_spon	b0680_m folding: spontanous	1 b0680_m --> 1 GlnS_mono	irreversible	Protein Folding	0.00E+00	1.00E+09	0	2.60E+03	2.60E+03	0	4.29E+03	4285.722	0	2.02E+02	201.728
b0680_mRNA_CONV	convsersion of mRNA to mRNA_1 (synthetic rxn)	1 b0680_mRNA --> 1 b0680_mRNA_1	irreversible	Translation	0.00E+00	1.00E+09	0	7.55E+01	7.55E+01	0	1.33E+02	133.2851	0	4.14E-01	0.414
b0680_mRNA_degr1	Degradation of b0680_mRNA I	1 Orn_dim + 416 atp + 1 b0680_mRNA_2 + 1 degradosome + 416 h2o <==> 416 adp + 1 b0680_mRNA_2_degr + 416 h + 416 pi	reversible	mRNA degradation	-1.00E+09	1.00E+09	0	7.55E+01	7.55E+01	0	1.33E+02	133.2851	0	4.14E-01	0.414
b0680_mRNA_degr2	Degradation of b0680_mRNA II	1 b0680_mRNA_2_degr + 1664 h2o --> 1 Orn_dim_inact + 414 amp 1 atp + 423 cmp + 1 degradosome_inact + 451 gmp + 1664 h + 376 ump	irreversible	mRNA degradation	0.00E+00	4.14E-01	0	7.55E+01	7.55E+01	0	1.33E+02	133.2851	0	4.14E-01	0.414
b0680_maturation1	Polypeptide b0680 peptide deformylas and methionine aminopeptidase complex	1 Def_mono + 1 Map_mono + 1 b0680_aa <==> 1 b0680_def_map_cplx	reversible	Protein Maturation	-1.00E+09	1.00E+09	0	2.60E+03	2.60E+03	0	4.29E+03	4285.722	0	2.02E+02	201.728
b0680_maturation2	b0680 formation	1 b0680_def_map_cplx + 2 h2o --> 1 Def_mono_inact + 1 Map_mono_inact + 1 b0680_m + 1 for + 1 met-L	irreversible	Protein Maturation	0.00E+00	1.00E+09	0	2.60E+03	2.60E+03	0	4.29E+03	4285.722	0	2.02E+02	201.728
b0680_v1_mRNA_CONV	convsersion of mRNA to mRNA_1 (synthetic rxn)	1 b0680_v1_mRNA --> 1 b0680_mRNA_1	irreversible	Translation	0.00E+00	1.00E+09	0	7.55E+01	7.55E+01	0	1.33E+02	133.2851	0	4.14E-01	0.414
b0743_tRNA_16_MODIF_1	modification of position 16 nt (D) in lysT by Dus_gen	1 Dus_gen + 1 b0743_tRNA_Mg2 + 1 h + 1 nadph <==> 1 b0743_tRNA_Mg2_Dus_gen_cplx	reversible	tRNA modification	-1.00E+09	1.00E+09	0	3.51E+02	3.51E+02	0	7.52E+00	7.52	0	7.52E+00	7.52
b0743_tRNA_16_MODIF_2	modification of position 16 nt (D) in lysT by Dus_gen	1 b0743_tRNA_Mg2_Dus_gen_cplx --> 1 Dus_gen_inact + 1 b0743_tRNA_1 + 1 nadp	irreversible	tRNA modification	0.00E+00	1.00E+09	0	3.51E+02	3.51E+02	0	7.52E+00	7.52	0	7.52E+00	7.52
b0743_tRNA_17_MODIF_1	modification of position 17 nt (D) in lysT by Dus_gen	1 Dus_gen + 1 b0743_tRNA_1 + 1 h + 1 nadph <==> 1 b0743_tRNA_1_Dus_gen_cplx	reversible	tRNA modification	-1.00E+09	1.00E+09	0	3.51E+02	3.51E+02	0	7.52E+00	7.52	0	7.52E+00	7.52
b0743_tRNA_17_MODIF_2	modification of position 17 nt (D) in lysT by Dus_gen	1 b0743_tRNA_1_Dus_gen_cplx --> 1 Dus_gen_inact + 1 b0743_tRNA_2 + 1 nadp	irreversible	tRNA modification	0.00E+00	1.00E+09	0	3.51E+02	3.51E+02	0	7.52E+00	7.52	0	7.52E+00	7.52
b0743_tRNA_20_MODIF_1	modification of position 20 nt (D) in lysT by Dus_gen	1 Dus_gen + 1 b0743_tRNA_2 + 1 h + 1 nadph <==> 1 b0743_tRNA_2_Dus_gen_cplx	reversible	tRNA modification	-1.00E+09	1.00E+09	0	3.51E+02	3.51E+02	0	7.52E+00	7.52	0	7.52E+00	7.52
b0743_tRNA_20_MODIF_2	modification of position 20 nt (D) in lysT by Dus_gen	1 b0743_tRNA_2_Dus_gen_cplx --> 1 Dus_gen_inact + 1 b0743_tRNA_3 + 1 nadp	irreversible	tRNA modification	0.00E+00	1.00E+09	0	3.51E+02	3.51E+02	0	7.52E+00	7.52	0	7.52E+00	7.52

b0743_tRNA_34_MODIF_1	modification of position 34 nt (mnm5s2U) in lysT by TrmU_mono-YhhP_mono-YheLMN_cplx-YccK_mono-TrmE_dim-GidA_mono-TrmC_mono	1 5fthf + 1 GidA_mono + 1 IscS_dim_S-SH + 1 TrmC_mono + 1 TrmE_dim + 1 TrmU_mono + 1 YccK_mono + 1 YheLMN_cplx + 1 YhhP_mono + 1 amet + 1 atp + 1 b0743_tRNA_3 + 1 fad + 1 fadh2 + 1 gly + 1 gtp + 1 h2o + 1 mg2 + 1 trdrd <==> 1 b0743_tRNA_3_TrmU_mono-YhhP_mono-YheLMN_cplx-YccK_mon TrmE_dim-GidA_mono-TrmC_mono_cplx	reversible	tRNA modification	-1.00E+09	1.00E+09	0	3.51E+02	3.51E+02	0	7.52E+00	7.52	0	7.52E+00	7.52
b0743_tRNA_34_MODIF_2	modification of position 34 nt (mnm5s2U) in lysT by TrmU_mono-YhhP_mono-YheLMN_cplx-YccK_mono-TrmE_dim-GidA_mono-TrmC_mono	1 b0743_tRNA_3_TrmU_mono-YhhP_mono-YheLMN_cplx-YccK_mono-TrmE_dim-GidA_mono-TrmC_mono_cplx --> 1 GidA_mono_inact + 1 IscS_dim_S-H + 1 TrmC_mono_inact + 1 TrmE_dim_inact + 1 TrmU_mono_inact + 1 YccK_mono_inact + 1 YheLMN_cplx_inact + 1 YhhP_mono_inact + 1 ahcys + 1 amp + 1 b0743_tRNA_4 + 1 fad + 1 fadh2 + 1 gdp + 1 glx + 4 h + 1 mg2 + 1 pi + 1 ppi + 1 thf + 1 trdox	irreversible	tRNA modification	0.00E+00	1.00E+09	0	3.51E+02	3.51E+02	0	7.52E+00	7.52	0	7.52E+00	7.52
b0743_tRNA_34_MODIF_3	replacement of sulfur with selenium: b0743_tRNA_4	1 YbbB_dim + 1 b0743_tRNA_4 + 1 h2o + 1 selnp <==> 1 b0743_tRNA_4_YbbB_dim_cplx	reversible	tRNA modification	-1.00E+09	1.00E+09	0	3.51E+02	3.51E+02	0	7.52E+00	7.52	0	7.52E+00	7.52
b0743_tRNA_34_MODIF_4	replacement of sulfur with selenium: b0743_tRNA_4	1 b0743_tRNA_4_YbbB_dim_cplx --> 1 YbbB_dim_inact + 1 b0743_tRNA_4_Se + 1 h + 1 h2s + 1 pi	irreversible	tRNA modification	0.00E+00	1.00E+09	0	3.51E+02	3.51E+02	0	7.52E+00	7.52	0	7.52E+00	7.52
b0743_tRNA_37_MODIF_1	modification of position 37 nt (t6A) in lysT by Up_tRNA_pos_37_t6A	1 Up_tRNA_pos_37_t6A + 1 atp + 1 b0743_tRNA_4 + 1 hco3 + 1 mg2 + 1 thr-L <==> 1 b0743_tRNA_4_Up_tRNA_pos_37_t6A_cplx	reversible	tRNA modification	-1.00E+09	1.00E+09	0	3.51E+02	3.51E+02	0	7.52E+00	7.52	0	7.52E+00	7.52
b0743_tRNA_37_MODIF_1_Se	modification of position 37 nt (t6A) in lysT by Up_tRNA_pos_37_t6A	1 Up_tRNA_pos_37_t6A + 1 atp + 1 b0743_tRNA_4_Se + 1 hco3 + 1 mg2 + 1 thr-L <==> 1 b0743_tRNA_4_Se_Up_tRNA_pos_37_t6A_cplx	reversible	tRNA modification	-1.00E+09	1.00E+09	0	3.51E+02	3.51E+02	0	7.52E+00	7.52	0	7.52E+00	7.52
b0743_tRNA_37_MODIF_2	modification of position 37 nt (t6A) in lysT by Up_tRNA_pos_37_t6A	1 b0743_tRNA_4_Up_tRNA_pos_37_t6A_cplx --> 1 Up_tRNA_pos_37_t6A_inact + 1 amp + 1 b0743_tRNA_5 + 1 h + 1 h2o + 1 mg2 + 1 ppi	irreversible	tRNA modification	0.00E+00	1.00E+09	0	3.51E+02	3.51E+02	0	7.52E+00	7.52	0	7.52E+00	7.52
b0743_tRNA_37_MODIF_2_Se	modification of position 37 nt (t6A) in lysT by Up_tRNA_pos_37_t6A	1 b0743_tRNA_4_Se_Up_tRNA_pos_37_t6A_cplx --> 1 Up_tRNA_pos_37_t6A_inact + 1 amp + 1 b0743_tRNA_5_Se + 1 h + 1 h2o + 1 mg2 + 1 ppi	irreversible	tRNA modification	0.00E+00	1.00E+09	0	3.51E+02	3.51E+02	0	7.52E+00	7.52	0	7.52E+00	7.52
b0743_tRNA_39_MODIF_1	modification of position 39 nt (Y) in lysT by TruA_dim	1 TruA_dim + 1 b0743_tRNA_5 <==> 1 b0743_tRNA_5_TruA_dim_cplx	reversible	tRNA modification	-1.00E+09	1.00E+09	0	3.51E+02	3.51E+02	0	7.52E+00	7.52	0	7.52E+00	7.52
b0743_tRNA_39_MODIF_1_Se	modification of position 39 nt (Y) in lysT by TruA_dim	1 TruA_dim + 1 b0743_tRNA_5_Se <==> 1 b0743_tRNA_5_Se_TruA_dim_cplx	reversible	tRNA modification	-1.00E+09	1.00E+09	0	3.51E+02	3.51E+02	0	7.52E+00	7.52	0	7.52E+00	7.52
b0743_tRNA_39_MODIF_2	modification of position 39 nt (Y) in lysT by TruA_dim	1 b0743_tRNA_5_TruA_dim_cplx --> 1 TruA_dim_inact + 1 b0743_tRNA_6	irreversible	tRNA modification	0.00E+00	1.00E+09	0	3.51E+02	3.51E+02	0	7.52E+00	7.52	0	7.52E+00	7.52
b0743_tRNA_39_MODIF_2_Se	modification of position 39 nt (Y) in lysT by TruA_dim	1 b0743_tRNA_5_Se_TruA_dim_cplx --> 1 TruA_dim_inact + 1 b0743_tRNA_6_Se	irreversible	tRNA modification	0.00E+00	1.00E+09	0	3.51E+02	3.51E+02	0	7.52E+00	7.52	0	7.52E+00	7.52
b0743_tRNA_46_MODIF_1	modification of position 46 nt (m7G) in lysT by YggH_mono	1 YggH_mono + 1 amet + 1 b0743_tRNA_6 <==> 1 b0743_tRNA_6_YggH_mono_cplx	reversible	tRNA modification	-1.00E+09	1.00E+09	0	3.51E+02	3.51E+02	0	7.52E+00	7.52	0	7.52E+00	7.52
b0743_tRNA_46_MODIF_1_Se	modification of position 46 nt (m7G) in lysT by YggH_mono	1 YggH_mono + 1 amet + 1 b0743_tRNA_6_Se <==> 1 b0743_tRNA_6_Se_YggH_mono_cplx	reversible	tRNA modification	-1.00E+09	1.00E+09	0	3.51E+02	3.51E+02	0	7.52E+00	7.52	0	7.52E+00	7.52
b0743_tRNA_46_MODIF_2	modification of position 46 nt (m7G) in lysT by YggH_mono	1 b0743_tRNA_6_YggH_mono_cplx --> 1 YggH_mono_inact + 1 ahcys + 1 b0743_tRNA_7 + 1 h	irreversible	tRNA modification	0.00E+00	1.00E+09	0	3.51E+02	3.51E+02	0	7.52E+00	7.52	0	7.52E+00	7.52
b0743_tRNA_46_MODIF_2_Se	modification of position 46 nt (m7G) in lysT by YggH_mono	1 b0743_tRNA_6_Se_YggH_mono_cplx --> 1 YggH_mono_inact + 1 ahcys + 1 b0743_tRNA_7_Se + 1 h	irreversible	tRNA modification	0.00E+00	1.00E+09	0	3.51E+02	3.51E+02	0	7.52E+00	7.52	0	7.52E+00	7.52
b0743_tRNA_47_MODIF_1	modification of position 47 nt (acp3U) in lysT by AcpT_tRNA_pos_47_acp3U	1 AcpT_tRNA_pos_47_acp3U + 1 amet + 1 b0743_tRNA_7 <==> 1 b0743_tRNA_7_AcpT_tRNA_pos_47_acp3U_cplx	reversible	tRNA modification	-1.00E+09	1.00E+09	0	3.51E+02	3.51E+02	0	7.52E+00	7.52	0	7.52E+00	7.52
b0743_tRNA_47_MODIF_1_Se	modification of position 47 nt (acp3U) in lysT by AcpT_tRNA_pos_47_acp3U	1 AcpT_tRNA_pos_47_acp3U + 1 amet + 1 b0743_tRNA_7_Se <==: 1 b0743_tRNA_7_Se_AcpT_tRNA_pos_47_acp3U_cplx	reversible	tRNA modification	-1.00E+09	1.00E+09	0	3.51E+02	3.51E+02	0	7.52E+00	7.52	0	7.52E+00	7.52
b0743_tRNA_47_MODIF_2	modification of position 47 nt (acp3U) in lysT by AcpT_tRNA_pos_47_acp3U	1 b0743_tRNA_7_AcpT_tRNA_pos_47_acp3U_cplx --> 1 5mta + 1 AcpT_tRNA_pos_47_acp3U_inact + 1 b0743_tRNA_8 + 1 h	irreversible	tRNA modification	0.00E+00	1.00E+09	0	3.51E+02	3.51E+02	0	7.52E+00	7.52	0	7.52E+00	7.52
b0743_tRNA_47_MODIF_2_Se	modification of position 47 nt (acp3U) in lysT by AcpT_tRNA_pos_47_acp3U	1 b0743_tRNA_7_Se_AcpT_tRNA_pos_47_acp3U_cplx --> 1 5mta + 1 AcpT_tRNA_pos_47_acp3U_inact + 1 b0743_tRNA_8_Se + 1 h	irreversible	tRNA modification	0.00E+00	1.00E+09	0	3.51E+02	3.51E+02	0	7.52E+00	7.52	0	7.52E+00	7.52
b0743_tRNA_54_MODIF_1	modification of position 54 nt (m5U) in lysT by TrmA_mono	1 TrmA_mono + 1 amet + 1 b0743_tRNA_8 <==> 1 b0743_tRNA_8_TrmA_mono_cplx	reversible	tRNA modification	-1.00E+09	1.00E+09	0	3.51E+02	3.51E+02	0	7.52E+00	7.52	0	7.52E+00	7.52
b0743_tRNA_54_MODIF_1_Se	modification of position 54 nt (m5U) in lysT by TrmA_mono	1 TrmA_mono + 1 amet + 1 b0743_tRNA_8_Se <==> 1 b0743_tRNA_8_Se_TrmA_mono_cplx	reversible	tRNA modification	-1.00E+09	1.00E+09	0	3.51E+02	3.51E+02	0	7.52E+00	7.52	0	7.52E+00	7.52
b0743_tRNA_54_MODIF_2	modification of position 54 nt (m5U) in lysT by TrmA_mono	1 b0743_tRNA_8_TrmA_mono_cplx --> 1 TrmA_mono_inact + 1 ahcys + 1 b0743_tRNA_9 + 1 h	irreversible	tRNA modification	0.00E+00	1.00E+09	0	3.51E+02	3.51E+02	0	7.52E+00	7.52	0	7.52E+00	7.52
b0743_tRNA_54_MODIF_2_Se	modification of position 54 nt (m5U) in lysT by TrmA_mono	1 b0743_tRNA_8_Se_TrmA_mono_cplx --> 1 TrmA_mono_inact + 1 ahcys + 1 b0743_tRNA_9_Se + 1 h	irreversible	tRNA modification	0.00E+00	1.00E+09	0	3.51E+02	3.51E+02	0	7.52E+00	7.52	0	7.52E+00	7.52
b0743_tRNA_55_MODIF_1	modification of position 55 nt (Y) in lysT by TruB_mono	1 TruB_mono + 1 b0743_tRNA_9 <==> 1 b0743_tRNA_9_TruB_mono_cplx	reversible	tRNA modification	-1.00E+09	1.00E+09	0	3.51E+02	3.51E+02	0	7.52E+00	7.52	0	7.52E+00	7.52
b0743_tRNA_55_MODIF_1_Se	modification of position 55 nt (Y) in lysT by TruB_mono	1 TruB_mono + 1 b0743_tRNA_9_Se <==> 1 b0743_tRNA_9_Se_TruB_mono_cplx	reversible	tRNA modification	-1.00E+09	1.00E+09	0	3.51E+02	3.51E+02	0	7.52E+00	7.52	0	7.52E+00	7.52
b0743_tRNA_55_MODIF_2	modification of position 55 nt (Y) in lysT by TruB_mono	1 b0743_tRNA_9_TruB_mono_cplx --> 1 TruB_mono_inact + 1 lysT_tRNA	irreversible	tRNA modification	0.00E+00	1.00E+09	0	3.51E+02	3.51E+02	0	7.52E+00	7.52	0	7.52E+00	7.52
b0743_tRNA_55_MODIF_2_Se	modification of position 55 nt (Y) in lysT by TruB_mono	1 b0743_tRNA_9_Se_TruB_mono_cplx --> 1 TruB_mono_inact + 1 lysT_tRNA_Se	irreversible	tRNA modification	0.00E+00	1.00E+09	0	3.51E+02	3.51E+02	0	7.52E+00	7.52	0	7.52E+00	7.52
b0743_tRNA_Mg_BIND	Binding of two Mg2 to b0743_tRNA	1 b0743_RNA + 2 mg2 <==> 1 b0743_tRNA_Mg2	reversible	Metallo-ion binding	-1.00E+09	1.00E+09	0	3.51E+02	3.51E+02	0	7.52E+00	7.52	0	7.52E+00	7.52
b0744_tRNA_17_MODIF_1	modification of position 17 nt (D) in valT by Dus_gen	1 Dus_gen + 1 b0744_tRNA_Mg2 + 1 h + 1 nadph <==> 1 b0744_tRNA_Mg2_Dus_gen_cplx	reversible	tRNA modification	-1.00E+09	1.00E+09	0	3.51E+02	3.51E+02	0	7.52E+00	7.52	0	7.52E+00	7.52
b0744_tRNA_17_MODIF_2	modification of position 17 nt (D) in valT by Dus_gen	1 b0744_tRNA_Mg2_Dus_gen_cplx --> 1 Dus_gen_inact + 1 b0744_tRNA_1 + 1 nadp	irreversible	tRNA modification	0.00E+00	1.00E+09	0	3.51E+02	3.51E+02	0	7.52E+00	7.52	0	7.52E+00	7.52
b0744_tRNA_34_MODIF_1	modification of position 34 nt (cmo5U) in valT by YecO_mono-YecP_mono-HyL_tRNA_pos_34_ho5U	1 HyL_tRNA_pos_34_ho5U + 1 YecO_mono + 1 YecP_mono + 1 amet + 1 b0744_tRNA_1 + 2 chor <==> 1 b0744_tRNA_1_YecO_mono-YecP_mono-HyL_tRNA_pos_34_ho5U_cplx	reversible	tRNA modification	-1.00E+09	1.00E+09	0	3.51E+02	3.51E+02	0	7.52E+00	7.52	0	7.52E+00	7.52
b0744_tRNA_34_MODIF_2	modification of position 34 nt (cmo5U) in valT by YecO_mono-YecP_mono-HyL_tRNA_pos_34_ho5U	1 b0744_tRNA_1_YecO_mono-YecP_mono-HyL_tRNA_pos_34_ho5U_cplx --> 1 C10H8O5 + 1 C9H9O4 + 1 HyL_tRNA_pos_34_ho5U_inact + 1 YecO_mono_inact + 1 YecP_mono_inact + 1 ahcys + 1 b0744_tRNA_2 + 1 h	irreversible	tRNA modification	0.00E+00	1.00E+09	0	3.51E+02	3.51E+02	0	7.52E+00	7.52	0	7.52E+00	7.52
b0744_tRNA_37_MODIF_1	modification of position 37 nt (m6A) in valT by MeT_tRNA_pos_37_m6A	1 MeT_tRNA_pos_37_m6A + 1 amet + 1 b0744_tRNA_2 <==> 1 b0744_tRNA_2_MeT_tRNA_pos_37_m6A_cplx	reversible	tRNA modification	-1.00E+09	1.00E+09	0	3.51E+02	3.51E+02	0	7.52E+00	7.52	0	7.52E+00	7.52
b0744_tRNA_37_MODIF_2	modification of position 37 nt (m6A) in valT by MeT_tRNA_pos_37_m6A	1 b0744_tRNA_2_MeT_tRNA_pos_37_m6A_cplx --> 1 MeT_tRNA_pos_37_m6A_inact + 1 ahcys + 1 b0744_tRNA_3 + 1 h	irreversible	tRNA modification	0.00E+00	1.00E+09	0	3.51E+02	3.51E+02	0	7.52E+00	7.52	0	7.52E+00	7.52
b0744_tRNA_46_MODIF_1	modification of position 46 nt (m7G) in valT by YggH_mono	1 YggH_mono + 1 amet + 1 b0744_tRNA_3 <==> 1 b0744_tRNA_3_YggH_mono_cplx	reversible	tRNA modification	-1.00E+09	1.00E+09	0	3.51E+02	3.51E+02	0	7.52E+00	7.52	0	7.52E+00	7.52
b0744_tRNA_46_MODIF_2	modification of position 46 nt (m7G) in valT by YggH_mono	1 b0744_tRNA_3_YggH_mono_cplx --> 1 YggH_mono_inact + 1 ahcys + 1 b0744_tRNA_4 + 1 h	irreversible	tRNA modification	0.00E+00	1.00E+09	0	3.51E+02	3.51E+02	0	7.52E+00	7.52	0	7.52E+00	7.52
b0744_tRNA_54_MODIF_1	modification of position 54 nt (m5U) in valT by TrmA_mono	1 TrmA_mono + 1 amet + 1 b0744_tRNA_4 <==> 1 b0744_tRNA_4_TrmA_mono_cplx	reversible	tRNA modification	-1.00E+09	1.00E+09	0	3.51E+02	3.51E+02	0	7.52E+00	7.52	0	7.52E+00	7.52
b0744_tRNA_54_MODIF_2	modification of position 54 nt (m5U) in valT by TrmA_mono	1 b0744_tRNA_4_TrmA_mono_cplx --> 1 TrmA_mono_inact + 1 ahcys + 1 b0744_tRNA_5 + 1 h	irreversible	tRNA modification	0.00E+00	1.00E+09	0	3.51E+02	3.51E+02	0	7.52E+00	7.52	0	7.52E+00	7.52
b0744_tRNA_55_MODIF_1	modification of position 55 nt (Y) in valT by TruB_mono	1 TruB_mono + 1 b0744_tRNA_5 <==> 1 b0744_tRNA_5_TruB_mono_cplx	reversible	tRNA modification	-1.00E+09	1.00E+09	0	3.51E+02	3.51E+02	0	7.52E+00	7.52	0	7.52E+00	7.52
b0744_tRNA_55_MODIF_2	modification of position 55 nt (Y) in valT by TruB_mono	1 b0744_tRNA_5_TruB_mono_cplx --> 1 TruB_mono_inact + 1 b0744_tRNA_6	irreversible	tRNA modification	0.00E+00	1.00E+09	0	3.51E+02	3.51E+02	0	7.52E+00	7.52	0	7.52E+00	7.52
b0744_tRNA_8_MODIF_1	modification of position 8 nt (s4U) in valT by ThiI_mono	1 IscS_dim_S-SH + 1 ThiI_mono + 1 atp + 1 b0744_tRNA_6 + 1 mg2 + 1 trdrd <==> 1 b0744_tRNA_6_ThiI_mono_cplx	reversible	tRNA modification	-1.00E+09	1.00E+09	0	3.51E+02	3.51E+02	0	7.52E+00	7.52	0	7.52E+00	7.52
b0744_tRNA_8_MODIF_2	modification of position 8 nt (s4U) in valT by ThiI_mono	1 b0744_tRNA_6_ThiI_mono_cplx --> 1 IscS_dim_S-H + 1 ThiI_mono_inact + 1 amp + 1 h + 1 mg2 + 1 ppi + 1 trdox + 1 valT_tRNA	irreversible	tRNA modification	0.00E+00	1.00E+09	0	3.51E+02	3.51E+02	0	7.52E+00	7.52	0	7.52E+00	7.52
b0744_tRNA_Mg_BIND	Binding of two Mg2 to b0744_tRNA	1 b0744_RNA + 2 mg2 <==> 1 b0744_tRNA_Mg2	reversible	Metallo-ion binding	-1.00E+09	1.00E+09	0	3.51E+02	3.51E+02	0	7.52E+00	7.52	0	7.52E+00	7.52
b0745_tRNA_16_MODIF_1	modification of position 16 nt (D) in lysW by Dus_gen	1 Dus_gen + 1 b0745_tRNA_Mg2 + 1 h + 1 nadph <==> 1 b0745_tRNA_Mg2_Dus_gen_cplx	reversible	tRNA modification	-1.00E+09	1.00E+09	0	3.51E+02	3.51E+02	0	7.52E+00	7.52	0	7.52E+00	7.52

b0745_tRNA_16_MODIF_2	modification of position 16 nt (D) in lysW by Dus_gen	1 b0745_tRNA_Mg2_Dus_gen_cplx --> 1 Dus_gen_inact + 1 b0745_tRNA_1 + 1 nadp	irreversible	tRNA modification	0.00E+00	1.00E+09	0	3.51E+02	3.51E+02	0	7.52E+00	7.52	0	7.52E+00	7.52
b0745_tRNA_17_MODIF_1	modification of position 17 nt (D) in lysW by Dus_gen	1 Dus_gen + 1 b0745_tRNA_1 + 1 h + 1 nadph <==> 1 b0745_tRNA_1_Dus_gen_cplx	reversible	tRNA modification	-1.00E+09	1.00E+09	0	3.51E+02	3.51E+02	0	7.52E+00	7.52	0	7.52E+00	7.52
b0745_tRNA_17_MODIF_2	modification of position 17 nt (D) in lysW by Dus_gen	1 b0745_tRNA_1_Dus_gen_cplx --> 1 Dus_gen_inact + 1 b0745_tRNA_2 + 1 nadp	irreversible	tRNA modification	0.00E+00	1.00E+09	0	3.51E+02	3.51E+02	0	7.52E+00	7.52	0	7.52E+00	7.52
b0745_tRNA_20_MODIF_1	modification of position 20 nt (D) in lysW by Dus_gen	1 Dus_gen + 1 b0745_tRNA_2 + 1 h + 1 nadph <==> 1 b0745_tRNA_2_Dus_gen_cplx	reversible	tRNA modification	-1.00E+09	1.00E+09	0	3.51E+02	3.51E+02	0	7.52E+00	7.52	0	7.52E+00	7.52
b0745_tRNA_20_MODIF_2	modification of position 20 nt (D) in lysW by Dus_gen	1 b0745_tRNA_2_Dus_gen_cplx --> 1 Dus_gen_inact + 1 b0745_tRNA_3 + 1 nadp	irreversible	tRNA modification	0.00E+00	1.00E+09	0	3.51E+02	3.51E+02	0	7.52E+00	7.52	0	7.52E+00	7.52
b0745_tRNA_34_MODIF_1	modification of position 34 nt (mnm5s2U) in lysW by TrmU_mono-YhhP_mono-YheLMN_cplx-YccK_mono-TrmE_dim-GidA_mono-TrmC_mono	1 5fthf + 1 GidA_mono + 1 IscS_dim_S-SH + 1 TrmC_mono + 1 TrmE_dim + 1 TrmU_mono + 1 YccK_mono + 1 YheLMN_cplx + 1 YhhP_mono + 1 amet + 1 atp + 1 b0745_tRNA_3 + 1 fad + 1 fadh2 + 1 gly + 1 gtp + 1 h2o + 1 mg2 + 1 trdrd <==> 1 b0745_tRNA_3_TrmU_mono-YhhP_mono-YheLMN_cplx-YccK_mon TrmE_dim-GidA_mono-TrmC_mono_cplx	reversible	tRNA modification	-1.00E+09	1.00E+09	0	3.51E+02	3.51E+02	0	7.52E+00	7.52	0	7.52E+00	7.52
b0745_tRNA_34_MODIF_2	modification of position 34 nt (mnm5s2U) in lysW by TrmU_mono-YhhP_mono-YheLMN_cplx-YccK_mono-TrmE_dim-GidA_mono-TrmC_mono	1 b0745_tRNA_3_TrmU_mono-YhhP_mono-YheLMN_cplx-YccK_mono-TrmE_dim-GidA_mono-TrmC_mono_cplx --> 1 GidA_mono_inact + 1 IscS_dim_S-H + 1 TrmC_mono_inact + 1 TrmE_dim_inact + 1 TrmU_mono_inact + 1 YccK_mono_inact + 1 YheLMN_cplx_inact + 1 YhhP_mono_inact + 1 ahcys + 1 amp + 1 b0745_tRNA_4 + 1 fad + 1 fadh2 + 1 gdp + 1 glx + 4 h + 1 mg2 + 1 pi + 1 ppi + 1 thf + 1 trdox	irreversible	tRNA modification	0.00E+00	1.00E+09	0	3.51E+02	3.51E+02	0	7.52E+00	7.52	0	7.52E+00	7.52
b0745_tRNA_34_MODIF_3	replacement of sulfur with selenium: b0745_tRNA_4	1 YbbB_dim + 1 b0745_tRNA_4 + 1 h2o + 1 selnp <==> 1 b0745_tRNA_4_YbbB_dim_cplx	reversible	tRNA modification	-1.00E+09	1.00E+09	0	3.51E+02	3.51E+02	0	7.52E+00	7.52	0	7.52E+00	7.52
b0745_tRNA_34_MODIF_4	replacement of sulfur with selenium: b0745_tRNA_4	1 b0745_tRNA_4_YbbB_dim_cplx --> 1 YbbB_dim_inact + 1 b0745_tRNA_4_Se + 1 h + 1 h2s + 1 pi	irreversible	tRNA modification	0.00E+00	1.00E+09	0	3.51E+02	3.51E+02	0	7.52E+00	7.52	0	7.52E+00	7.52
b0745_tRNA_37_MODIF_1	modification of position 37 nt (t6A) in lysW by Up_tRNA_pos_37_t6A	1 Up_tRNA_pos_37_t6A + 1 atp + 1 b0745_tRNA_4 + 1 hco3 + 1 mg2 + 1 thr-L <==> 1 b0745_tRNA_4_Up_tRNA_pos_37_t6A_cplx	reversible	tRNA modification	-1.00E+09	1.00E+09	0	3.51E+02	3.51E+02	0	7.52E+00	7.52	0	7.52E+00	7.52
b0745_tRNA_37_MODIF_1_Se	modification of position 37 nt (t6A) in lysW by Up_tRNA_pos_37_t6A	1 Up_tRNA_pos_37_t6A + 1 atp + 1 b0745_tRNA_4_Se + 1 hco3 + 1 mg2 + 1 thr-L <==> 1 b0745_tRNA_4_Se_Up_tRNA_pos_37_t6A_cplx	reversible	tRNA modification	-1.00E+09	1.00E+09	0	3.51E+02	3.51E+02	0	7.52E+00	7.52	0	7.52E+00	7.52
b0745_tRNA_37_MODIF_2	modification of position 37 nt (t6A) in lysW by Up_tRNA_pos_37_t6A	1 b0745_tRNA_4_Up_tRNA_pos_37_t6A_cplx --> 1 Up_tRNA_pos_37_t6A_inact + 1 amp + 1 b0745_tRNA_5 + 1 h + 1 h2o + 1 mg2 + 1 ppi	irreversible	tRNA modification	0.00E+00	1.00E+09	0	3.51E+02	3.51E+02	0	7.52E+00	7.52	0	7.52E+00	7.52
b0745_tRNA_37_MODIF_2_Se	modification of position 37 nt (t6A) in lysW by Up_tRNA_pos_37_t6A	1 b0745_tRNA_4_Se_Up_tRNA_pos_37_t6A_cplx --> 1 Up_tRNA_pos_37_t6A_inact + 1 amp + 1 b0745_tRNA_5_Se + 1 h + 1 h2o + 1 mg2 + 1 ppi	irreversible	tRNA modification	0.00E+00	1.00E+09	0	3.51E+02	3.51E+02	0	7.52E+00	7.52	0	7.52E+00	7.52
b0745_tRNA_39_MODIF_1	modification of position 39 nt (Y) in lysW by TruA_dim	1 TruA_dim + 1 b0745_tRNA_5 <==> 1 b0745_tRNA_5_TruA_dim_cplx	reversible	tRNA modification	-1.00E+09	1.00E+09	0	3.51E+02	3.51E+02	0	7.52E+00	7.52	0	7.52E+00	7.52
b0745_tRNA_39_MODIF_1_Se	modification of position 39 nt (Y) in lysW by TruA_dim	1 TruA_dim + 1 b0745_tRNA_5_Se <==> 1 b0745_tRNA_5_Se_TruA_dim_cplx	reversible	tRNA modification	-1.00E+09	1.00E+09	0	3.51E+02	3.51E+02	0	7.52E+00	7.52	0	7.52E+00	7.52
b0745_tRNA_39_MODIF_2	modification of position 39 nt (Y) in lysW by TruA_dim	1 b0745_tRNA_5_TruA_dim_cplx --> 1 TruA_dim_inact + 1 b0745_tRNA_6	irreversible	tRNA modification	0.00E+00	1.00E+09	0	3.51E+02	3.51E+02	0	7.52E+00	7.52	0	7.52E+00	7.52
b0745_tRNA_39_MODIF_2_Se	modification of position 39 nt (Y) in lysW by TruA_dim	1 b0745_tRNA_5_Se_TruA_dim_cplx --> 1 TruA_dim_inact + 1 b0745_tRNA_6_Se	irreversible	tRNA modification	0.00E+00	1.00E+09	0	3.51E+02	3.51E+02	0	7.52E+00	7.52	0	7.52E+00	7.52
b0745_tRNA_46_MODIF_1	modification of position 46 nt (m7G) in lysW by YggH_mono	1 YggH_mono + 1 amet + 1 b0745_tRNA_6 <==> 1 b0745_tRNA_6_YggH_mono_cplx	reversible	tRNA modification	-1.00E+09	1.00E+09	0	3.51E+02	3.51E+02	0	7.52E+00	7.52	0	7.52E+00	7.52
b0745_tRNA_46_MODIF_1_Se	modification of position 46 nt (m7G) in lysW by YggH_mono	1 YggH_mono + 1 amet + 1 b0745_tRNA_6_Se <==> 1 b0745_tRNA_6_Se_YggH_mono_cplx	reversible	tRNA modification	-1.00E+09	1.00E+09	0	3.51E+02	3.51E+02	0	7.52E+00	7.52	0	7.52E+00	7.52
b0745_tRNA_46_MODIF_2	modification of position 46 nt (m7G) in lysW by YggH_mono	1 b0745_tRNA_6_YggH_mono_cplx --> 1 YggH_mono_inact + 1 ahcys + 1 b0745_tRNA_7 + 1 h	irreversible	tRNA modification	0.00E+00	1.00E+09	0	3.51E+02	3.51E+02	0	7.52E+00	7.52	0	7.52E+00	7.52
b0745_tRNA_46_MODIF_2_Se	modification of position 46 nt (m7G) in lysW by YggH_mono	1 b0745_tRNA_6_Se_YggH_mono_cplx --> 1 YggH_mono_inact + 1 ahcys + 1 b0745_tRNA_7_Se + 1 h	irreversible	tRNA modification	0.00E+00	1.00E+09	0	3.51E+02	3.51E+02	0	7.52E+00	7.52	0	7.52E+00	7.52
b0745_tRNA_47_MODIF_1	modification of position 47 nt (acp3U) in lysW by AcpT_tRNA_pos_47_acp3U	1 AcpT_tRNA_pos_47_acp3U + 1 amet + 1 b0745_tRNA_7 <==> 1 b0745_tRNA_7_AcpT_tRNA_pos_47_acp3U_cplx	reversible	tRNA modification	-1.00E+09	1.00E+09	0	3.51E+02	3.51E+02	0	7.52E+00	7.52	0	7.52E+00	7.52
b0745_tRNA_47_MODIF_1_Se	modification of position 47 nt (acp3U) in lysW by AcpT_tRNA_pos_47_acp3U	1 AcpT_tRNA_pos_47_acp3U + 1 amet + 1 b0745_tRNA_7_Se <==> 1 b0745_tRNA_7_Se_AcpT_tRNA_pos_47_acp3U_cplx	reversible	tRNA modification	-1.00E+09	1.00E+09	0	3.51E+02	3.51E+02	0	7.52E+00	7.52	0	7.52E+00	7.52
b0745_tRNA_47_MODIF_2	modification of position 47 nt (acp3U) in lysW by AcpT_tRNA_pos_47_acp3U	1 b0745_tRNA_7_AcpT_tRNA_pos_47_acp3U_cplx --> 1 5mta + 1 AcpT_tRNA_pos_47_acp3U_inact + 1 b0745_tRNA_8 + 1 h	irreversible	tRNA modification	0.00E+00	1.00E+09	0	3.51E+02	3.51E+02	0	7.52E+00	7.52	0	7.52E+00	7.52
b0745_tRNA_47_MODIF_2_Se	modification of position 47 nt (acp3U) in lysW by AcpT_tRNA_pos_47_acp3U	1 b0745_tRNA_7_Se_AcpT_tRNA_pos_47_acp3U_cplx --> 1 5mta + 1 AcpT_tRNA_pos_47_acp3U_inact + 1 b0745_tRNA_8_Se + 1 h	irreversible	tRNA modification	0.00E+00	1.00E+09	0	3.51E+02	3.51E+02	0	7.52E+00	7.52	0	7.52E+00	7.52
b0745_tRNA_54_MODIF_1	modification of position 54 nt (m5U) in lysW by TrmA_mono	1 TrmA_mono + 1 amet + 1 b0745_tRNA_8 <==> 1 b0745_tRNA_8_TrmA_mono_cplx	reversible	tRNA modification	-1.00E+09	1.00E+09	0	3.51E+02	3.51E+02	0	7.52E+00	7.52	0	7.52E+00	7.52
b0745_tRNA_54_MODIF_1_Se	modification of position 54 nt (m5U) in lysW by TrmA_mono	1 TrmA_mono + 1 amet + 1 b0745_tRNA_8_Se <==> 1 b0745_tRNA_8_Se_TrmA_mono_cplx	reversible	tRNA modification	-1.00E+09	1.00E+09	0	3.51E+02	3.51E+02	0	7.52E+00	7.52	0	7.52E+00	7.52
b0745_tRNA_54_MODIF_2	modification of position 54 nt (m5U) in lysW by TrmA_mono	1 b0745_tRNA_8_TrmA_mono_cplx --> 1 TrmA_mono_inact + 1 ahcys + 1 b0745_tRNA_9 + 1 h	irreversible	tRNA modification	0.00E+00	1.00E+09	0	3.51E+02	3.51E+02	0	7.52E+00	7.52	0	7.52E+00	7.52
b0745_tRNA_54_MODIF_2_Se	modification of position 54 nt (m5U) in lysW by TrmA_mono	1 b0745_tRNA_8_Se_TrmA_mono_cplx --> 1 TrmA_mono_inact + 1 ahcys + 1 b0745_tRNA_9_Se + 1 h	irreversible	tRNA modification	0.00E+00	1.00E+09	0	3.51E+02	3.51E+02	0	7.52E+00	7.52	0	7.52E+00	7.52
b0745_tRNA_55_MODIF_1	modification of position 55 nt (Y) in lysW by TruB_mono	1 TruB_mono + 1 b0745_tRNA_9 <==> 1 b0745_tRNA_9_TruB_mono_cplx	reversible	tRNA modification	-1.00E+09	1.00E+09	0	3.51E+02	3.51E+02	0	7.52E+00	7.52	0	7.52E+00	7.52
b0745_tRNA_55_MODIF_1_Se	modification of position 55 nt (Y) in lysW by TruB_mono	1 TruB_mono + 1 b0745_tRNA_9_Se <==> 1 b0745_tRNA_9_Se_TruB_mono_cplx	reversible	tRNA modification	-1.00E+09	1.00E+09	0	3.51E+02	3.51E+02	0	7.52E+00	7.52	0	7.52E+00	7.52
b0745_tRNA_55_MODIF_2	modification of position 55 nt (Y) in lysW by TruB_mono	1 b0745_tRNA_9_TruB_mono_cplx --> 1 TruB_mono_inact + 1 lysW_tRNA	irreversible	tRNA modification	0.00E+00	1.00E+09	0	3.51E+02	3.51E+02	0	7.52E+00	7.52	0	7.52E+00	7.52
b0745_tRNA_55_MODIF_2_Se	modification of position 55 nt (Y) in lysW by TruB_mono	1 b0745_tRNA_9_Se_TruB_mono_cplx --> 1 TruB_mono_inact + 1 lysW_tRNA_Se	irreversible	tRNA modification	0.00E+00	1.00E+09	0	3.51E+02	3.51E+02	0	7.52E+00	7.52	0	7.52E+00	7.52
b0745_tRNA_Mg_BIND	Binding of two Mg2 to b0745_tRNA	1 b0745_RNA + 2 mg2 <==> 1 b0745_tRNA_Mg2	reversible	Metallo-ion binding	-1.00E+09	1.00E+09	0	3.51E+02	3.51E+02	0	7.52E+00	7.52	0	7.52E+00	7.52
b0746_tRNA_17_MODIF_1	modification of position 17 nt (D) in valZ by Dus_gen	1 Dus_gen + 1 b0746_tRNA_Mg2 + 1 h + 1 nadph <==> 1 b0746_tRNA_Mg2_Dus_gen_cplx	reversible	tRNA modification	-1.00E+09	1.00E+09	0	7.43E+02	7.43E+02	0	7.51E+00	7.51	0	7.51E+00	7.51
b0746_tRNA_17_MODIF_2	modification of position 17 nt (D) in valZ by Dus_gen	1 b0746_tRNA_Mg2_Dus_gen_cplx --> 1 Dus_gen_inact + 1 b0746_tRNA_1 + 1 nadp	irreversible	tRNA modification	0.00E+00	1.00E+09	0	7.43E+02	7.43E+02	0	7.51E+00	7.51	0	7.51E+00	7.51
b0746_tRNA_34_MODIF_1	modification of position 34 nt (cmo5U) in valZ by YecO_mono-YecP_mono-HyL_tRNA_pos_34_ho5U	1 HyL_tRNA_pos_34_ho5U + 1 YecO_mono + 1 YecP_mono + 1 amet + 1 b0746_tRNA_1 + 2 chor <==> 1 b0746_tRNA_1_YecO_mono-YecP_mono-HyL_tRNA_pos_34_ho5U_cplx	reversible	tRNA modification	-1.00E+09	1.00E+09	0	7.43E+02	7.43E+02	0	7.51E+00	7.51	0	7.51E+00	7.51
b0746_tRNA_34_MODIF_2	modification of position 34 nt (cmo5U) in valZ by YecO_mono-YecP_mono-HyL_tRNA_pos_34_ho5U	1 b0746_tRNA_1_YecO_mono-YecP_mono-HyL_tRNA_pos_34_ho5U_cplx --> 1 C10H8O5 + 1 C9H9O4 + 1 HyL_tRNA_pos_34_ho5U_inact + 1 YecO_mono_inact + 1 YecP_mono_inact + 1 ahcys + 1 b0746_tRNA_2 + 1 h	irreversible	tRNA modification	0.00E+00	1.00E+09	0	7.43E+02	7.43E+02	0	7.51E+00	7.51	0	7.51E+00	7.51
b0746_tRNA_37_MODIF_1	modification of position 37 nt (m6A) in valZ by MeT_tRNA_pos_37_m6A	1 MeT_tRNA_pos_37_m6A + 1 amet + 1 b0746_tRNA_2 <==> 1 b0746_tRNA_2_MeT_tRNA_pos_37_m6A_cplx	reversible	tRNA modification	-1.00E+09	1.00E+09	0	7.43E+02	7.43E+02	0	7.51E+00	7.51	0	7.51E+00	7.51
b0746_tRNA_37_MODIF_2	modification of position 37 nt (m6A) in valZ by MeT_tRNA_pos_37_m6A	1 b0746_tRNA_2_MeT_tRNA_pos_37_m6A_cplx --> 1 MeT_tRNA_pos_37_m6A_inact + 1 ahcys + 1 b0746_tRNA_3 + 1 h	irreversible	tRNA modification	0.00E+00	1.00E+09	0	7.43E+02	7.43E+02	0	7.51E+00	7.51	0	7.51E+00	7.51
b0746_tRNA_46_MODIF_1	modification of position 46 nt (m7G) in valZ by YggH_mono	1 YggH_mono + 1 amet + 1 b0746_tRNA_3 <==> 1 b0746_tRNA_3_YggH_mono_cplx	reversible	tRNA modification	-1.00E+09	1.00E+09	0	7.43E+02	7.43E+02	0	7.51E+00	7.51	0	7.51E+00	7.51
b0746_tRNA_46_MODIF_2	modification of position 46 nt (m7G) in valZ by YggH_mono	1 b0746_tRNA_3_YggH_mono_cplx --> 1 YggH_mono_inact + 1 ahcys + 1 b0746_tRNA_4 + 1 h	irreversible	tRNA modification	0.00E+00	1.00E+09	0	7.43E+02	7.43E+02	0	7.51E+00	7.51	0	7.51E+00	7.51
b0746_tRNA_54_MODIF_1	modification of position 54 nt (m5U) in valZ by TrmA_mono	1 TrmA_mono + 1 amet + 1 b0746_tRNA_4 <==> 1 b0746_tRNA_4_TrmA_mono_cplx	reversible	tRNA modification	-1.00E+09	1.00E+09	0	7.43E+02	7.43E+02	0	7.51E+00	7.51	0	7.51E+00	7.51
b0746_tRNA_54_MODIF_2	modification of position 54 nt (m5U) in valZ by TrmA_mono	1 b0746_tRNA_4_TrmA_mono_cplx --> 1 TrmA_mono_inact + 1 ahcys + 1 b0746_tRNA_5 + 1 h	irreversible	tRNA modification	0.00E+00	1.00E+09	0	7.43E+02	7.43E+02	0	7.51E+00	7.51	0	7.51E+00	7.51
b0746_tRNA_55_MODIF_1	modification of position 55 nt (Y) in valZ by TruB_mono	1 TruB_mono + 1 b0746_tRNA_5 <==> 1 b0746_tRNA_5_TruB_mono_cplx	reversible	tRNA modification	-1.00E+09	1.00E+09	0	7.43E+02	7.43E+02	0	7.51E+00	7.51	0	7.51E+00	7.51

b0746_tRNA_55_MODIF_2	modification of position 55 nt (Y) in valZ by TruB_mono	1 b0746_tRNA_5_TruB_mono_cplx --> 1 TruB_mono_inact + 1 b0746_tRNA_6	irreversible	tRNA modification	0.00E+00	1.00E+09	0	7.43E+02	7.43E+02	0	7.51E+00	7.51	0	7.51E+00	7.51
b0746_tRNA_8_MODIF_1	modification of position 8 nt (s4U) in valZ by ThiI_mono	1 IscS_dim_S-SH + 1 ThiI_mono + 1 atp + 1 b0746_tRNA_6 + 1 mg2 + 1 trdrd <==> 1 b0746_tRNA_6_ThiI_mono_cplx	reversible	tRNA modification	-1.00E+09	1.00E+09	0	7.43E+02	7.43E+02	0	7.51E+00	7.51	0	7.51E+00	7.51
b0746_tRNA_8_MODIF_2	modification of position 8 nt (s4U) in valZ by ThiI_mono	1 b0746_tRNA_6_ThiI_mono_cplx --> 1 IscS_dim_S-H + 1 ThiI_mono_inact + 1 amp + 1 h + 1 mg2 + 1 ppi + 1 trdox + 1 valZ_tRNA	irreversible	tRNA modification	0.00E+00	1.00E+09	0	7.43E+02	7.43E+02	0	7.51E+00	7.51	0	7.51E+00	7.51
b0746_tRNA_Mg_BIND	Binding of two Mg2 to b0746_tRNA	1 b0746_RNA + 2 mg2 <==> 1 b0746_tRNA_Mg2	reversible	Metallo-ion binding	-1.00E+09	1.00E+09	0	7.43E+02	7.43E+02	0	7.51E+00	7.51	0	7.51E+00	7.51
b0747_tRNA_16_MODIF_1	modification of position 16 nt (D) in lysY by Dus_gen	1 Dus_gen + 1 b0747_tRNA_Mg2 + 1 h + 1 nadph <==> 1 b0747_tRNA_Mg2_Dus_gen_cplx	reversible	tRNA modification	-1.00E+09	1.00E+09	0	7.43E+02	7.43E+02	0	7.51E+00	7.51	0	7.51E+00	7.51
b0747_tRNA_16_MODIF_2	modification of position 16 nt (D) in lysY by Dus_gen	1 b0747_tRNA_Mg2_Dus_gen_cplx --> 1 Dus_gen_inact + 1 b0747_tRNA_1 + 1 nadp	irreversible	tRNA modification	0.00E+00	1.00E+09	0	7.43E+02	7.43E+02	0	7.51E+00	7.51	0	7.51E+00	7.51
b0747_tRNA_17_MODIF_1	modification of position 17 nt (D) in lysY by Dus_gen	1 Dus_gen + 1 b0747_tRNA_1 + 1 h + 1 nadph <==> 1 b0747_tRNA_1_Dus_gen_cplx	reversible	tRNA modification	-1.00E+09	1.00E+09	0	7.43E+02	7.43E+02	0	7.51E+00	7.51	0	7.51E+00	7.51
b0747_tRNA_17_MODIF_2	modification of position 17 nt (D) in lysY by Dus_gen	1 b0747_tRNA_1_Dus_gen_cplx --> 1 Dus_gen_inact + 1 b0747_tRNA_2 + 1 nadp	irreversible	tRNA modification	0.00E+00	1.00E+09	0	7.43E+02	7.43E+02	0	7.51E+00	7.51	0	7.51E+00	7.51
b0747_tRNA_20_MODIF_1	modification of position 20 nt (D) in lysY by Dus_gen	1 Dus_gen + 1 b0747_tRNA_2 + 1 h + 1 nadph <==> 1 b0747_tRNA_2_Dus_gen_cplx	reversible	tRNA modification	-1.00E+09	1.00E+09	0	7.43E+02	7.43E+02	0	7.51E+00	7.51	0	7.51E+00	7.51
b0747_tRNA_20_MODIF_2	modification of position 20 nt (D) in lysY by Dus_gen	1 b0747_tRNA_2_Dus_gen_cplx --> 1 Dus_gen_inact + 1 b0747_tRNA_3 + 1 nadp	irreversible	tRNA modification	0.00E+00	1.00E+09	0	7.43E+02	7.43E+02	0	7.51E+00	7.51	0	7.51E+00	7.51
b0747_tRNA_34_MODIF_1	modification of position 34 nt (mnm5s2U) in lysY by TrmU_mono-YhhP_mono-YheLMN_cplx-YccK_mono-TrmE_dim-GidA_mono-TrmC_mono	1 5fthf + 1 GidA_mono + 1 IscS_dim_S-SH + 1 TrmC_mono + 1 TrmE_dim + 1 TrmU_mono + 1 YccK_mono + 1 YheLMN_cplx + 1 YhhP_mono + 1 amet + 1 atp + 1 b0747_tRNA_3 + 1 fad + 1 fadh2 + 1 gly + 1 gtp + 1 h2o + 1 mg2 + 1 trdrd <==> 1 b0747_tRNA_3_TrmU_mono-YhhP_mono-YheLMN_cplx-YccK_mon TrmE_dim-GidA_mono-TrmC_mono_cplx	reversible	tRNA modification	-1.00E+09	1.00E+09	0	7.43E+02	7.43E+02	0	7.51E+00	7.51	0	7.51E+00	7.51
b0747_tRNA_34_MODIF_2	modification of position 34 nt (mnm5s2U) in lysY by TrmU_mono-YhhP_mono-YheLMN_cplx-YccK_mono-TrmE_dim-GidA_mono-TrmC_mono	1 b0747_tRNA_3_TrmU_mono-YhhP_mono-YheLMN_cplx-YccK_mono-TrmE_dim-GidA_mono-TrmC_mono_cplx --> 1 GidA_mono_inact + 1 IscS_dim_S-H + 1 TrmC_mono_inact + 1 TrmE_dim_inact + 1 TrmU_mono_inact + 1 YccK_mono_inact + 1 YheLMN_cplx_inact + 1 YhhP_mono_inact + 1 ahcys + 1 amp + 1 b0747_tRNA_4 + 1 fad + 1 fadh2 + 1 gdp + 1 glx + 4 h + 1 mg2 + 1 pi + 1 ppi + 1 thf + 1 trdox	irreversible	tRNA modification	0.00E+00	1.00E+09	0	7.43E+02	7.43E+02	0	7.51E+00	7.51	0	7.51E+00	7.51
b0747_tRNA_34_MODIF_3	replacement of sulfur with selenium: b0747_tRNA_4	1 YbbB_dim + 1 b0747_tRNA_4 + 1 h2o + 1 selnp <==> 1 b0747_tRNA_4_YbbB_dim_cplx	reversible	tRNA modification	-1.00E+09	1.00E+09	0	7.43E+02	7.43E+02	0	7.51E+00	7.51	0	7.51E+00	7.51
b0747_tRNA_34_MODIF_4	replacement of sulfur with selenium: b0747_tRNA_4	1 b0747_tRNA_4_YbbB_dim_cplx --> 1 YbbB_dim_inact + 1 b0747_tRNA_4_Se + 1 h + 1 h2s + 1 pi	irreversible	tRNA modification	0.00E+00	1.00E+09	0	7.43E+02	7.43E+02	0	7.51E+00	7.51	0	7.51E+00	7.51
b0747_tRNA_37_MODIF_1	modification of position 37 nt (t6A) in lysY by Up_tRNA_pos_37_t6A	1 Up_tRNA_pos_37_t6A + 1 atp + 1 b0747_tRNA_4 + 1 hco3 + 1 mg2 + 1 thr-L <==> 1 b0747_tRNA_4_Up_tRNA_pos_37_t6A_cplx	reversible	tRNA modification	-1.00E+09	1.00E+09	0	7.43E+02	7.43E+02	0	7.51E+00	7.51	0	7.51E+00	7.51
b0747_tRNA_37_MODIF_1_Se	modification of position 37 nt (t6A) in lysY by Up_tRNA_pos_37_t6A	1 Up_tRNA_pos_37_t6A + 1 atp + 1 b0747_tRNA_4_Se + 1 hco3 + 1 mg2 + 1 thr-L <==> 1 b0747_tRNA_4_Se_Up_tRNA_pos_37_t6A_cplx	reversible	tRNA modification	-1.00E+09	1.00E+09	0	7.43E+02	7.43E+02	0	7.51E+00	7.51	0	7.51E+00	7.51
b0747_tRNA_37_MODIF_2	modification of position 37 nt (t6A) in lysY by Up_tRNA_pos_37_t6A	1 b0747_tRNA_4_Up_tRNA_pos_37_t6A_cplx --> 1 Up_tRNA_pos_37_t6A_inact + 1 amp + 1 b0747_tRNA_5 + 1 h + 1 h2o + 1 mg2 + 1 ppi	irreversible	tRNA modification	0.00E+00	1.00E+09	0	7.43E+02	7.43E+02	0	7.51E+00	7.51	0	7.51E+00	7.51
b0747_tRNA_37_MODIF_2_Se	modification of position 37 nt (t6A) in lysY by Up_tRNA_pos_37_t6A	1 b0747_tRNA_4_Se_Up_tRNA_pos_37_t6A_cplx --> 1 Up_tRNA_pos_37_t6A_inact + 1 amp + 1 b0747_tRNA_5_Se + 1 h + 1 h2o + 1 mg2 + 1 ppi	irreversible	tRNA modification	0.00E+00	1.00E+09	0	7.43E+02	7.43E+02	0	7.51E+00	7.51	0	7.51E+00	7.51
b0747_tRNA_39_MODIF_1	modification of position 39 nt (Y) in lysY by TruA_dim	1 TruA_dim + 1 b0747_tRNA_5 <==> 1 b0747_tRNA_5_TruA_dim_cplx	reversible	tRNA modification	-1.00E+09	1.00E+09	0	7.43E+02	7.43E+02	0	7.51E+00	7.51	0	7.51E+00	7.51
b0747_tRNA_39_MODIF_1_Se	modification of position 39 nt (Y) in lysY by TruA_dim	1 TruA_dim + 1 b0747_tRNA_5_Se <==> 1 b0747_tRNA_5_Se_TruA_dim_cplx	reversible	tRNA modification	-1.00E+09	1.00E+09	0	7.43E+02	7.43E+02	0	7.51E+00	7.51	0	7.51E+00	7.51
b0747_tRNA_39_MODIF_2	modification of position 39 nt (Y) in lysY by TruA_dim	1 b0747_tRNA_5_TruA_dim_cplx --> 1 TruA_dim_inact + 1 b0747_tRNA_6	irreversible	tRNA modification	0.00E+00	1.00E+09	0	7.43E+02	7.43E+02	0	7.51E+00	7.51	0	7.51E+00	7.51
b0747_tRNA_39_MODIF_2_Se	modification of position 39 nt (Y) in lysY by TruA_dim	1 b0747_tRNA_5_Se_TruA_dim_cplx --> 1 TruA_dim_inact + 1 b0747_tRNA_6_Se	irreversible	tRNA modification	0.00E+00	1.00E+09	0	7.43E+02	7.43E+02	0	7.51E+00	7.51	0	7.51E+00	7.51
b0747_tRNA_46_MODIF_1	modification of position 46 nt (m7G) in lysY by YggH_mono	1 YggH_mono + 1 amet + 1 b0747_tRNA_6 <==> 1 b0747_tRNA_6_YggH_mono_cplx	reversible	tRNA modification	-1.00E+09	1.00E+09	0	7.43E+02	7.43E+02	0	7.51E+00	7.51	0	7.51E+00	7.51
b0747_tRNA_46_MODIF_1_Se	modification of position 46 nt (m7G) in lysY by YggH_mono	1 YggH_mono + 1 amet + 1 b0747_tRNA_6_Se <==> 1 b0747_tRNA_6_Se_YggH_mono_cplx	reversible	tRNA modification	-1.00E+09	1.00E+09	0	7.43E+02	7.43E+02	0	7.51E+00	7.51	0	7.51E+00	7.51
b0747_tRNA_46_MODIF_2	modification of position 46 nt (m7G) in lysY by YggH_mono	1 b0747_tRNA_6_YggH_mono_cplx --> 1 YggH_mono_inact + 1 ahcys + 1 b0747_tRNA_7 + 1 h	irreversible	tRNA modification	0.00E+00	1.00E+09	0	7.43E+02	7.43E+02	0	7.51E+00	7.51	0	7.51E+00	7.51
b0747_tRNA_46_MODIF_2_Se	modification of position 46 nt (m7G) in lysY by YggH_mono	1 b0747_tRNA_6_Se_YggH_mono_cplx --> 1 YggH_mono_inact + 1 ahcys + 1 b0747_tRNA_7_Se + 1 h	irreversible	tRNA modification	0.00E+00	1.00E+09	0	7.43E+02	7.43E+02	0	7.51E+00	7.51	0	7.51E+00	7.51
b0747_tRNA_47_MODIF_1	modification of position 47 nt (acp3U) in lysY by AcpT_tRNA_pos_47_acp3U	1 AcpT_tRNA_pos_47_acp3U + 1 amet + 1 b0747_tRNA_7 <==> 1 b0747_tRNA_7_AcpT_tRNA_pos_47_acp3U_cplx	reversible	tRNA modification	-1.00E+09	1.00E+09	0	7.43E+02	7.43E+02	0	7.51E+00	7.51	0	7.51E+00	7.51
b0747_tRNA_47_MODIF_1_Se	modification of position 47 nt (acp3U) in lysY by AcpT_tRNA_pos_47_acp3U	1 AcpT_tRNA_pos_47_acp3U + 1 amet + 1 b0747_tRNA_7_Se <==> 1 b0747_tRNA_7_Se_AcpT_tRNA_pos_47_acp3U_cplx	reversible	tRNA modification	-1.00E+09	1.00E+09	0	7.43E+02	7.43E+02	0	7.51E+00	7.51	0	7.51E+00	7.51
b0747_tRNA_47_MODIF_2	modification of position 47 nt (acp3U) in lysY by AcpT_tRNA_pos_47_acp3U	1 b0747_tRNA_7_AcpT_tRNA_pos_47_acp3U_cplx --> 1 5mta + 1 AcpT_tRNA_pos_47_acp3U_inact + 1 b0747_tRNA_8 + 1 h	irreversible	tRNA modification	0.00E+00	1.00E+09	0	7.43E+02	7.43E+02	0	7.51E+00	7.51	0	7.51E+00	7.51
b0747_tRNA_47_MODIF_2_Se	modification of position 47 nt (acp3U) in lysY by AcpT_tRNA_pos_47_acp3U	1 b0747_tRNA_7_Se_AcpT_tRNA_pos_47_acp3U_cplx --> 1 5mta + 1 AcpT_tRNA_pos_47_acp3U_inact + 1 b0747_tRNA_8_Se + 1 h	irreversible	tRNA modification	0.00E+00	1.00E+09	0	7.43E+02	7.43E+02	0	7.51E+00	7.51	0	7.51E+00	7.51
b0747_tRNA_54_MODIF_1	modification of position 54 nt (m5U) in lysY by TrmA_mono	1 TrmA_mono + 1 amet + 1 b0747_tRNA_8 <==> 1 b0747_tRNA_8_TrmA_mono_cplx	reversible	tRNA modification	-1.00E+09	1.00E+09	0	7.43E+02	7.43E+02	0	7.51E+00	7.51	0	7.51E+00	7.51
b0747_tRNA_54_MODIF_1_Se	modification of position 54 nt (m5U) in lysY by TrmA_mono	1 TrmA_mono + 1 amet + 1 b0747_tRNA_8_Se <==> 1 b0747_tRNA_8_Se_TrmA_mono_cplx	reversible	tRNA modification	-1.00E+09	1.00E+09	0	7.43E+02	7.43E+02	0	7.51E+00	7.51	0	7.51E+00	7.51
b0747_tRNA_54_MODIF_2	modification of position 54 nt (m5U) in lysY by TrmA_mono	1 b0747_tRNA_8_TrmA_mono_cplx --> 1 TrmA_mono_inact + 1 ahcys + 1 b0747_tRNA_9 + 1 h	irreversible	tRNA modification	0.00E+00	1.00E+09	0	7.43E+02	7.43E+02	0	7.51E+00	7.51	0	7.51E+00	7.51
b0747_tRNA_54_MODIF_2_Se	modification of position 54 nt (m5U) in lysY by TrmA_mono	1 b0747_tRNA_8_Se_TrmA_mono_cplx --> 1 TrmA_mono_inact + 1 ahcys + 1 b0747_tRNA_9_Se + 1 h	irreversible	tRNA modification	0.00E+00	1.00E+09	0	7.43E+02	7.43E+02	0	7.51E+00	7.51	0	7.51E+00	7.51
b0747_tRNA_55_MODIF_1	modification of position 55 nt (Y) in lysY by TruB_mono	1 TruB_mono + 1 b0747_tRNA_9 <==> 1 b0747_tRNA_9_TruB_mono_cplx	reversible	tRNA modification	-1.00E+09	1.00E+09	0	7.43E+02	7.43E+02	0	7.51E+00	7.51	0	7.51E+00	7.51
b0747_tRNA_55_MODIF_1_Se	modification of position 55 nt (Y) in lysY by TruB_mono	1 TruB_mono + 1 b0747_tRNA_9_Se <==> 1 b0747_tRNA_9_Se_TruB_mono_cplx	reversible	tRNA modification	-1.00E+09	1.00E+09	0	7.43E+02	7.43E+02	0	7.51E+00	7.51	0	7.51E+00	7.51
b0747_tRNA_55_MODIF_2	modification of position 55 nt (Y) in lysY by TruB_mono	1 b0747_tRNA_9_TruB_mono_cplx --> 1 TruB_mono_inact + 1 lysY_tRNA	irreversible	tRNA modification	0.00E+00	1.00E+09	0	7.43E+02	7.43E+02	0	7.51E+00	7.51	0	7.51E+00	7.51
b0747_tRNA_55_MODIF_2_Se	modification of position 55 nt (Y) in lysY by TruB_mono	1 b0747_tRNA_9_Se_TruB_mono_cplx --> 1 TruB_mono_inact + 1 lysY_tRNA_Se	irreversible	tRNA modification	0.00E+00	1.00E+09	0	7.43E+02	7.43E+02	0	7.51E+00	7.51	0	7.51E+00	7.51
b0747_tRNA_Mg_BIND	Binding of two Mg2 to b0747_tRNA	1 b0747_RNA + 2 mg2 <==> 1 b0747_tRNA_Mg2	reversible	Metallo-ion binding	-1.00E+09	1.00E+09	0	7.43E+02	7.43E+02	0	7.51E+00	7.51	0	7.51E+00	7.51
b0748_tRNA_16_MODIF_1	modification of position 16 nt (D) in lysZ by Dus_gen	1 Dus_gen + 1 b0748_tRNA_Mg2 + 1 h + 1 nadph <==> 1 b0748_tRNA_Mg2_Dus_gen_cplx	reversible	tRNA modification	-1.00E+09	1.00E+09	0	1.77E+03	1.77E+03	0	7.51E+00	7.51	0	7.51E+00	7.51
b0748_tRNA_16_MODIF_2	modification of position 16 nt (D) in lysZ by Dus_gen	1 b0748_tRNA_Mg2_Dus_gen_cplx --> 1 Dus_gen_inact + 1 b0748_tRNA_1 + 1 nadp	irreversible	tRNA modification	0.00E+00	1.00E+09	0	1.77E+03	1.77E+03	0	7.51E+00	7.51	0	7.51E+00	7.51
b0748_tRNA_17_MODIF_1	modification of position 17 nt (D) in lysZ by Dus_gen	1 Dus_gen + 1 b0748_tRNA_1 + 1 h + 1 nadph <==> 1 b0748_tRNA_1_Dus_gen_cplx	reversible	tRNA modification	-1.00E+09	1.00E+09	0	1.77E+03	1.77E+03	0	7.51E+00	7.51	0	7.51E+00	7.51
b0748_tRNA_17_MODIF_2	modification of position 17 nt (D) in lysZ by Dus_gen	1 b0748_tRNA_1_Dus_gen_cplx --> 1 Dus_gen_inact + 1 b0748_tRNA_2 + 1 nadp	irreversible	tRNA modification	0.00E+00	1.00E+09	0	1.77E+03	1.77E+03	0	7.51E+00	7.51	0	7.51E+00	7.51
b0748_tRNA_20_MODIF_1	modification of position 20 nt (D) in lysZ by Dus_gen	1 Dus_gen + 1 b0748_tRNA_2 + 1 h + 1 nadph <==> 1 b0748_tRNA_2_Dus_gen_cplx	reversible	tRNA modification	-1.00E+09	1.00E+09	0	1.77E+03	1.77E+03	0	7.51E+00	7.51	0	7.51E+00	7.51
b0748_tRNA_20_MODIF_2	modification of position 20 nt (D) in lysZ by Dus_gen	1 b0748_tRNA_2_Dus_gen_cplx --> 1 Dus_gen_inact + 1 b0748_tRNA_3 + 1 nadp	irreversible	tRNA modification	0.00E+00	1.00E+09	0	1.77E+03	1.77E+03	0	7.51E+00	7.51	0	7.51E+00	7.51
b0748_tRNA_34_MODIF_1	modification of position 34 nt (mnm5s2U) in lysZ by TrmU_mono-YhhP_mono-YheLMN_cplx-YccK_mono-TrmE_dim-GidA_mono-TrmC_mono	1 5fthf + 1 GidA_mono + 1 IscS_dim_S-SH + 1 TrmC_mono + 1 TrmE_dim + 1 TrmU_mono + 1 YccK_mono + 1 YheLMN_cplx + 1 YhhP_mono + 1 amet + 1 atp + 1 b0748_tRNA_3 + 1 fad + 1 fadh2 + 1 gly + 1 gtp + 1 h2o + 1 mg2 + 1 trdrd <==> 1 b0748_tRNA_3_TrmU_mono-YhhP_mono-YheLMN_cplx-YccK_mon TrmE_dim-GidA_mono-TrmC_mono_cplx	reversible	tRNA modification	-1.00E+09	1.00E+09	0	1.77E+03	1.77E+03	0	7.51E+00	7.51	0	7.51E+00	7.51

b0748_tRNA_34_MODIF_2	modification of position 34 nt (mnm5s2U) in lysZ by TrmU_mono-YhhP_mono-YheLMN_cplx-YccK_mono-TrmE_dim-GidA_mono-TrmC_mono	1 b0748_tRNA_3_TrmU_mono-YhhP_mono-YheLMN_cplx-YccK_mono-TrmE_dim-GidA_mono-TrmC_mono_cplx --> 1 GidA_mono_inact + 1 IscS_dim_S-H + 1 TrmC_mono_inact + 1 TrmE_dim_inact + 1 TrmU_mono_inact + 1 YccK_mono_inact + 1 YheLMN_cplx_inact + 1 YhhP_mono_inact + 1 ahcys + 1 amp + 1 b0748_tRNA_4 + 1 fad + 1 fadh2 + 1 gdp + 1 glx + 4 h + 1 mg2 + 1 pi + 1 ppi + 1 thf + 1 trdox	irreversible	tRNA modification	0.00E+00	1.00E+09	0	1.77E+03	1.77E+03	0	7.51E+00	7.51	0	7.51E+00	7.51
b0748_tRNA_34_MODIF_3	replacement of sulfur with selenium: b0748_tRNA_4	1 YbbB_dim + 1 b0748_tRNA_4 + 1 h2o + 1 selnp <==> 1 b0748_tRNA_4_YbbB_dim_cplx	reversible	tRNA modification	-1.00E+09	1.00E+09	0	1.77E+03	1.77E+03	0	7.51E+00	7.51	0	7.51E+00	7.51
b0748_tRNA_34_MODIF_4	replacement of sulfur with selenium: b0748_tRNA_4	1 b0748_tRNA_4_YbbB_dim_cplx --> 1 YbbB_dim_inact + 1 b0748_tRNA_4_Se + 1 h + 1 h2s + 1 pi	irreversible	tRNA modification	0.00E+00	1.00E+09	0	1.77E+03	1.77E+03	0	7.51E+00	7.51	0	7.51E+00	7.51
b0748_tRNA_37_MODIF_1	modification of position 37 nt (t6A) in lysZ by Up_tRNA_pos_37_t6A	1 Up_tRNA_pos_37_t6A + 1 atp + 1 b0748_tRNA_4 + 1 hco3 + 1 mg2 + 1 thr-L <==> 1 b0748_tRNA_4_Up_tRNA_pos_37_t6A_cplx	reversible	tRNA modification	-1.00E+09	1.00E+09	0	1.77E+03	1.77E+03	0	7.51E+00	7.51	0	7.51E+00	7.51
b0748_tRNA_37_MODIF_1_Se	modification of position 37 nt (t6A) in lysZ by Up_tRNA_pos_37_t6A	1 Up_tRNA_pos_37_t6A + 1 atp + 1 b0748_tRNA_4_Se + 1 hco3 + 1 mg2 + 1 thr-L <==> 1 b0748_tRNA_4_Se_Up_tRNA_pos_37_t6A_cplx	reversible	tRNA modification	-1.00E+09	1.00E+09	0	1.77E+03	1.77E+03	0	7.51E+00	7.51	0	7.51E+00	7.51
b0748_tRNA_37_MODIF_2	modification of position 37 nt (t6A) in lysZ by Up_tRNA_pos_37_t6A	1 b0748_tRNA_4_Up_tRNA_pos_37_t6A_cplx --> 1 Up_tRNA_pos_37_t6A_inact + 1 amp + 1 b0748_tRNA_5 + 1 h + 1 h2o + 1 mg2 + 1 ppi	irreversible	tRNA modification	0.00E+00	1.00E+09	0	1.77E+03	1.77E+03	0	7.51E+00	7.51	0	7.51E+00	7.51
b0748_tRNA_37_MODIF_2_Se	modification of position 37 nt (t6A) in lysZ by Up_tRNA_pos_37_t6A	1 b0748_tRNA_4_Se_Up_tRNA_pos_37_t6A_cplx --> 1 Up_tRNA_pos_37_t6A_inact + 1 amp + 1 b0748_tRNA_5_Se + 1 h + 1 h2o + 1 mg2 + 1 ppi	irreversible	tRNA modification	0.00E+00	1.00E+09	0	1.77E+03	1.77E+03	0	7.51E+00	7.51	0	7.51E+00	7.51
b0748_tRNA_39_MODIF_1	modification of position 39 nt (Y) in lysZ by TruA_dim	1 TruA_dim + 1 b0748_tRNA_5 <==> 1 b0748_tRNA_5_TruA_dim_cplx	reversible	tRNA modification	-1.00E+09	1.00E+09	0	1.77E+03	1.77E+03	0	7.51E+00	7.51	0	7.51E+00	7.51
b0748_tRNA_39_MODIF_1_Se	modification of position 39 nt (Y) in lysZ by TruA_dim	1 TruA_dim + 1 b0748_tRNA_5_Se <==> 1 b0748_tRNA_5_Se_TruA_dim_cplx	reversible	tRNA modification	-1.00E+09	1.00E+09	0	1.77E+03	1.77E+03	0	7.51E+00	7.51	0	7.51E+00	7.51
b0748_tRNA_39_MODIF_2	modification of position 39 nt (Y) in lysZ by TruA_dim	1 b0748_tRNA_5_TruA_dim_cplx --> 1 TruA_dim_inact + 1 b0748_tRNA_6	irreversible	tRNA modification	0.00E+00	1.00E+09	0	1.77E+03	1.77E+03	0	7.51E+00	7.51	0	7.51E+00	7.51
b0748_tRNA_39_MODIF_2_Se	modification of position 39 nt (Y) in lysZ by TruA_dim	1 b0748_tRNA_5_Se_TruA_dim_cplx --> 1 TruA_dim_inact + 1 b0748_tRNA_6_Se	irreversible	tRNA modification	0.00E+00	1.00E+09	0	1.77E+03	1.77E+03	0	7.51E+00	7.51	0	7.51E+00	7.51
b0748_tRNA_46_MODIF_1	modification of position 46 nt (m7G) in lysZ by YggH_mono	1 YggH_mono + 1 amet + 1 b0748_tRNA_6 <==> 1 b0748_tRNA_6_YggH_mono_cplx	reversible	tRNA modification	-1.00E+09	1.00E+09	0	1.77E+03	1.77E+03	0	7.51E+00	7.51	0	7.51E+00	7.51
b0748_tRNA_46_MODIF_1_Se	modification of position 46 nt (m7G) in lysZ by YggH_mono	1 YggH_mono + 1 amet + 1 b0748_tRNA_6_Se <==> 1 b0748_tRNA_6_Se_YggH_mono_cplx	reversible	tRNA modification	-1.00E+09	1.00E+09	0	1.77E+03	1.77E+03	0	7.51E+00	7.51	0	7.51E+00	7.51
b0748_tRNA_46_MODIF_2	modification of position 46 nt (m7G) in lysZ by YggH_mono	1 b0748_tRNA_6_YggH_mono_cplx --> 1 YggH_mono_inact + 1 ahcys + 1 b0748_tRNA_7 + 1 h	irreversible	tRNA modification	0.00E+00	1.00E+09	0	1.77E+03	1.77E+03	0	7.51E+00	7.51	0	7.51E+00	7.51
b0748_tRNA_46_MODIF_2_Se	modification of position 46 nt (m7G) in lysZ by YggH_mono	1 b0748_tRNA_6_Se_YggH_mono_cplx --> 1 YggH_mono_inact + 1 ahcys + 1 b0748_tRNA_7_Se + 1 h	irreversible	tRNA modification	0.00E+00	1.00E+09	0	1.77E+03	1.77E+03	0	7.51E+00	7.51	0	7.51E+00	7.51
b0748_tRNA_47_MODIF_1	modification of position 47 nt (acp3U) in lysZ by AcpT_tRNA_pos_47_acp3U	1 AcpT_tRNA_pos_47_acp3U + 1 amet + 1 b0748_tRNA_7 <==> 1 b0748_tRNA_7_AcpT_tRNA_pos_47_acp3U_cplx	reversible	tRNA modification	-1.00E+09	1.00E+09	0	1.77E+03	1.77E+03	0	7.51E+00	7.51	0	7.51E+00	7.51
b0748_tRNA_47_MODIF_1_Se	modification of position 47 nt (acp3U) in lysZ by AcpT_tRNA_pos_47_acp3U	1 AcpT_tRNA_pos_47_acp3U + 1 amet + 1 b0748_tRNA_7_Se <==> 1 b0748_tRNA_7_Se_AcpT_tRNA_pos_47_acp3U_cplx	reversible	tRNA modification	-1.00E+09	1.00E+09	0	1.77E+03	1.77E+03	0	7.51E+00	7.51	0	7.51E+00	7.51
b0748_tRNA_47_MODIF_2	modification of position 47 nt (acp3U) in lysZ by AcpT_tRNA_pos_47_acp3U	1 b0748_tRNA_7_AcpT_tRNA_pos_47_acp3U_cplx --> 1 5mta + 1 AcpT_tRNA_pos_47_acp3U_inact + 1 b0748_tRNA_8 + 1 h	irreversible	tRNA modification	0.00E+00	1.00E+09	0	1.77E+03	1.77E+03	0	7.51E+00	7.51	0	7.51E+00	7.51
b0748_tRNA_47_MODIF_2_Se	modification of position 47 nt (acp3U) in lysZ by AcpT_tRNA_pos_47_acp3U	1 b0748_tRNA_7_Se_AcpT_tRNA_pos_47_acp3U_cplx --> 1 5mta + 1 AcpT_tRNA_pos_47_acp3U_inact + 1 b0748_tRNA_8_Se + 1 h	irreversible	tRNA modification	0.00E+00	1.00E+09	0	1.77E+03	1.77E+03	0	7.51E+00	7.51	0	7.51E+00	7.51
b0748_tRNA_54_MODIF_1	modification of position 54 nt (m5U) in lysZ by TrmA_mono	1 TrmA_mono + 1 amet + 1 b0748_tRNA_8 <==> 1 b0748_tRNA_8_TrmA_mono_cplx	reversible	tRNA modification	-1.00E+09	1.00E+09	0	1.77E+03	1.77E+03	0	7.51E+00	7.51	0	7.51E+00	7.51
b0748_tRNA_54_MODIF_1_Se	modification of position 54 nt (m5U) in lysZ by TrmA_mono	1 TrmA_mono + 1 amet + 1 b0748_tRNA_8_Se <==> 1 b0748_tRNA_8_Se_TrmA_mono_cplx	reversible	tRNA modification	-1.00E+09	1.00E+09	0	1.77E+03	1.77E+03	0	7.51E+00	7.51	0	7.51E+00	7.51
b0748_tRNA_54_MODIF_2	modification of position 54 nt (m5U) in lysZ by TrmA_mono	1 b0748_tRNA_8_TrmA_mono_cplx --> 1 TrmA_mono_inact + 1 ahcys + 1 b0748_tRNA_9 + 1 h	irreversible	tRNA modification	0.00E+00	1.00E+09	0	1.77E+03	1.77E+03	0	7.51E+00	7.51	0	7.51E+00	7.51
b0748_tRNA_54_MODIF_2_Se	modification of position 54 nt (m5U) in lysZ by TrmA_mono	1 b0748_tRNA_8_Se_TrmA_mono_cplx --> 1 TrmA_mono_inact + 1 ahcys + 1 b0748_tRNA_9_Se + 1 h	irreversible	tRNA modification	0.00E+00	1.00E+09	0	1.77E+03	1.77E+03	0	7.51E+00	7.51	0	7.51E+00	7.51
b0748_tRNA_55_MODIF_1	modification of position 55 nt (Y) in lysZ by TruB_mono	1 TruB_mono + 1 b0748_tRNA_9 <==> 1 b0748_tRNA_9_TruB_mono_cplx	reversible	tRNA modification	-1.00E+09	1.00E+09	0	1.77E+03	1.77E+03	0	7.51E+00	7.51	0	7.51E+00	7.51
b0748_tRNA_55_MODIF_1_Se	modification of position 55 nt (Y) in lysZ by TruB_mono	1 TruB_mono + 1 b0748_tRNA_9_Se <==> 1 b0748_tRNA_9_Se_TruB_mono_cplx	reversible	tRNA modification	-1.00E+09	1.00E+09	0	1.77E+03	1.77E+03	0	7.51E+00	7.51	0	7.51E+00	7.51
b0748_tRNA_55_MODIF_2	modification of position 55 nt (Y) in lysZ by TruB_mono	1 b0748_tRNA_9_TruB_mono_cplx --> 1 TruB_mono_inact + 1 lysZ_tRNA	irreversible	tRNA modification	0.00E+00	1.00E+09	0	1.77E+03	1.77E+03	0	7.51E+00	7.51	0	7.51E+00	7.51
b0748_tRNA_55_MODIF_2_Se	modification of position 55 nt (Y) in lysZ by TruB_mono	1 b0748_tRNA_9_Se_TruB_mono_cplx --> 1 TruB_mono_inact + 1 lysZ_tRNA_Se	irreversible	tRNA modification	0.00E+00	1.00E+09	0	1.77E+03	1.77E+03	0	7.51E+00	7.51	0	7.51E+00	7.51
b0748_tRNA_Mg_BIND	Binding of two Mg2 to b0748_tRNA	1 b0748_RNA + 2 mg2 <==> 1 b0748_tRNA_Mg2	reversible	Metallo-ion binding	-1.00E+09	1.00E+09	0	1.77E+03	1.77E+03	0	7.51E+00	7.51	0	7.51E+00	7.51
b0749_tRNA_16_MODIF_1	modification of position 16 nt (D) in lysQ by Dus_gen	1 Dus_gen + 1 b0749_tRNA_Mg2 + 1 h + 1 nadph <==> 1 b0749_tRNA_Mg2_Dus_gen_cplx	reversible	tRNA modification	-1.00E+09	1.00E+09	0	1.77E+03	1.77E+03	0	7.51E+00	7.51	0	7.51E+00	7.51
b0749_tRNA_16_MODIF_2	modification of position 16 nt (D) in lysQ by Dus_gen	1 b0749_tRNA_Mg2_Dus_gen_cplx --> 1 Dus_gen_inact + 1 b0749_tRNA_1 + 1 nadp	irreversible	tRNA modification	0.00E+00	1.00E+09	0	1.77E+03	1.77E+03	0	7.51E+00	7.51	0	7.51E+00	7.51
b0749_tRNA_17_MODIF_1	modification of position 17 nt (D) in lysQ by Dus_gen	1 Dus_gen + 1 b0749_tRNA_1 + 1 h + 1 nadph <==> 1 b0749_tRNA_1_Dus_gen_cplx	reversible	tRNA modification	-1.00E+09	1.00E+09	0	1.77E+03	1.77E+03	0	7.51E+00	7.51	0	7.51E+00	7.51
b0749_tRNA_17_MODIF_2	modification of position 17 nt (D) in lysQ by Dus_gen	1 b0749_tRNA_1_Dus_gen_cplx --> 1 Dus_gen_inact + 1 b0749_tRNA_2 + 1 nadp	irreversible	tRNA modification	0.00E+00	1.00E+09	0	1.77E+03	1.77E+03	0	7.51E+00	7.51	0	7.51E+00	7.51
b0749_tRNA_20_MODIF_1	modification of position 20 nt (D) in lysQ by Dus_gen	1 Dus_gen + 1 b0749_tRNA_2 + 1 h + 1 nadph <==> 1 b0749_tRNA_2_Dus_gen_cplx	reversible	tRNA modification	-1.00E+09	1.00E+09	0	1.77E+03	1.77E+03	0	7.51E+00	7.51	0	7.51E+00	7.51
b0749_tRNA_20_MODIF_2	modification of position 20 nt (D) in lysQ by Dus_gen	1 b0749_tRNA_2_Dus_gen_cplx --> 1 Dus_gen_inact + 1 b0749_tRNA_3 + 1 nadp	irreversible	tRNA modification	0.00E+00	1.00E+09	0	1.77E+03	1.77E+03	0	7.51E+00	7.51	0	7.51E+00	7.51
b0749_tRNA_34_MODIF_1	modification of position 34 nt (mnm5s2U) in lysQ by TrmU_mono-YhhP_mono-YheLMN_cplx-YccK_mono-TrmE_dim-GidA_mono-TrmC_mono	1 5fthf + 1 GidA_mono + 1 IscS_dim_S-SH + 1 TrmC_mono + 1 TrmE_dim + 1 TrmU_mono + 1 YccK_mono + 1 YheLMN_cplx + 1 YhhP_mono + 1 amet + 1 atp + 1 b0749_tRNA_3 + 1 fad + 1 fadh2 + 1 gly + 1 gtp + 1 h2o + 1 mg2 + 1 trdrd <==> 1 b0749_tRNA_3_TrmU_mono-YhhP_mono-YheLMN_cplx-YccK_mono-TrmE_dim-GidA_mono-TrmC_mono_cplx	reversible	tRNA modification	-1.00E+09	1.00E+09	0	1.77E+03	1.77E+03	0	7.51E+00	7.51	0	7.51E+00	7.51
b0749_tRNA_34_MODIF_2	modification of position 34 nt (mnm5s2U) in lysQ by TrmU_mono-YhhP_mono-YheLMN_cplx-YccK_mono-TrmE_dim-GidA_mono-TrmC_mono	1 b0749_tRNA_3_TrmU_mono-YhhP_mono-YheLMN_cplx-YccK_mono-TrmE_dim-GidA_mono-TrmC_mono_cplx --> 1 GidA_mono_inact + 1 IscS_dim_S-H + 1 TrmC_mono_inact + 1 TrmE_dim_inact + 1 TrmU_mono_inact + 1 YccK_mono_inact + 1 YheLMN_cplx_inact + 1 YhhP_mono_inact + 1 ahcys + 1 amp + 1 b0749_tRNA_4 + 1 fad + 1 fadh2 + 1 gdp + 1 glx + 4 h + 1 mg2 + 1 pi + 1 ppi + 1 thf + 1 trdox	irreversible	tRNA modification	0.00E+00	1.00E+09	0	1.77E+03	1.77E+03	0	7.51E+00	7.51	0	7.51E+00	7.51
b0749_tRNA_34_MODIF_3	replacement of sulfur with selenium: b0749_tRNA_4	1 YbbB_dim + 1 b0749_tRNA_4 + 1 h2o + 1 selnp <==> 1 b0749_tRNA_4_YbbB_dim_cplx	reversible	tRNA modification	-1.00E+09	1.00E+09	0	1.77E+03	1.77E+03	0	7.51E+00	7.51	0	7.51E+00	7.51
b0749_tRNA_34_MODIF_4	replacement of sulfur with selenium: b0749_tRNA_4	1 b0749_tRNA_4_YbbB_dim_cplx --> 1 YbbB_dim_inact + 1 b0749_tRNA_4_Se + 1 h + 1 h2s + 1 pi	irreversible	tRNA modification	0.00E+00	1.00E+09	0	1.77E+03	1.77E+03	0	7.51E+00	7.51	0	7.51E+00	7.51
b0749_tRNA_37_MODIF_1	modification of position 37 nt (t6A) in lysQ by Up_tRNA_pos_37_t6A	1 Up_tRNA_pos_37_t6A + 1 atp + 1 b0749_tRNA_4 + 1 hco3 + 1 mg2 + 1 thr-L <==> 1 b0749_tRNA_4_Up_tRNA_pos_37_t6A_cplx	reversible	tRNA modification	-1.00E+09	1.00E+09	0	1.77E+03	1.77E+03	0	7.51E+00	7.51	0	7.51E+00	7.51
b0749_tRNA_37_MODIF_1_Se	modification of position 37 nt (t6A) in lysQ by Up_tRNA_pos_37_t6A	1 Up_tRNA_pos_37_t6A + 1 atp + 1 b0749_tRNA_4_Se + 1 hco3 + 1 mg2 + 1 thr-L <==> 1 b0749_tRNA_4_Se_Up_tRNA_pos_37_t6A_cplx	reversible	tRNA modification	-1.00E+09	1.00E+09	0	1.77E+03	1.77E+03	0	7.51E+00	7.51	0	7.51E+00	7.51
b0749_tRNA_37_MODIF_2	modification of position 37 nt (t6A) in lysQ by Up_tRNA_pos_37_t6A	1 b0749_tRNA_4_Up_tRNA_pos_37_t6A_cplx --> 1 Up_tRNA_pos_37_t6A_inact + 1 amp + 1 b0749_tRNA_5 + 1 h + 1 h2o + 1 mg2 + 1 ppi	irreversible	tRNA modification	0.00E+00	1.00E+09	0	1.77E+03	1.77E+03	0	7.51E+00	7.51	0	7.51E+00	7.51
b0749_tRNA_37_MODIF_2_Se	modification of position 37 nt (t6A) in lysQ by Up_tRNA_pos_37_t6A	1 b0749_tRNA_4_Se_Up_tRNA_pos_37_t6A_cplx --> 1 Up_tRNA_pos_37_t6A_inact + 1 amp + 1 b0749_tRNA_5_Se + 1 h + 1 h2o + 1 mg2 + 1 ppi	irreversible	tRNA modification	0.00E+00	1.00E+09	0	1.77E+03	1.77E+03	0	7.51E+00	7.51	0	7.51E+00	7.51
b0749_tRNA_39_MODIF_1	modification of position 39 nt (Y) in lysQ by TruA_dim	1 TruA_dim + 1 b0749_tRNA_5 <==> 1 b0749_tRNA_5_TruA_dim_cplx	reversible	tRNA modification	-1.00E+09	1.00E+09	0	1.77E+03	1.77E+03	0	7.51E+00	7.51	0	7.51E+00	7.51

b0749_tRNA_39_MODIF_1_Se	modification of position 39 nt (Y) in lysQ by TruA_dim	1 TruA_dim + 1 b0749_tRNA_5_Se <==> 1 b0749_tRNA_5_Se_TruA_dim_cplx	reversible	tRNA modification	-1.00E+09	1.00E+09	0	1.77E+03	1.77E+03	0	7.51E+00	7.51	0	7.51E+00	7.51
b0749_tRNA_39_MODIF_2	modification of position 39 nt (Y) in lysQ by TruA_dim	1 b0749_tRNA_5_TruA_dim_cplx --> 1 TruA_dim_inact + 1 b0749_tRNA_6	irreversible	tRNA modification	0.00E+00	1.00E+09	0	1.77E+03	1.77E+03	0	7.51E+00	7.51	0	7.51E+00	7.51
b0749_tRNA_39_MODIF_2_Se	modification of position 39 nt (Y) in lysQ by TruA_dim	1 b0749_tRNA_5_Se_TruA_dim_cplx --> 1 TruA_dim_inact + 1 b0749_tRNA_6_Se	irreversible	tRNA modification	0.00E+00	1.00E+09	0	1.77E+03	1.77E+03	0	7.51E+00	7.51	0	7.51E+00	7.51
b0749_tRNA_46_MODIF_1	modification of position 46 nt (m7G) in lysQ by YggH_mono	1 YggH_mono + 1 amet + 1 b0749_tRNA_6 <==> 1 b0749_tRNA_6_YggH_mono_cplx	reversible	tRNA modification	-1.00E+09	1.00E+09	0	1.77E+03	1.77E+03	0	7.51E+00	7.51	0	7.51E+00	7.51
b0749_tRNA_46_MODIF_1_Se	modification of position 46 nt (m7G) in lysQ by YggH_mono	1 YggH_mono + 1 amet + 1 b0749_tRNA_6_Se <==> 1 b0749_tRNA_6_Se_YggH_mono_cplx	reversible	tRNA modification	-1.00E+09	1.00E+09	0	1.77E+03	1.77E+03	0	7.51E+00	7.51	0	7.51E+00	7.51
b0749_tRNA_46_MODIF_2	modification of position 46 nt (m7G) in lysQ by YggH_mono	1 b0749_tRNA_6_YggH_mono_cplx --> 1 YggH_mono_inact + 1 ahcys + 1 b0749_tRNA_7 + 1 h	irreversible	tRNA modification	0.00E+00	1.00E+09	0	1.77E+03	1.77E+03	0	7.51E+00	7.51	0	7.51E+00	7.51
b0749_tRNA_46_MODIF_2_Se	modification of position 46 nt (m7G) in lysQ by YggH_mono	1 b0749_tRNA_6_Se_YggH_mono_cplx --> 1 YggH_mono_inact + 1 ahcys + 1 b0749_tRNA_7_Se + 1 h	irreversible	tRNA modification	0.00E+00	1.00E+09	0	1.77E+03	1.77E+03	0	7.51E+00	7.51	0	7.51E+00	7.51
b0749_tRNA_47_MODIF_1	modification of position 47 nt (acp3U) in lysQ by AcpT_tRNA_pos_47_acp3U	1 AcpT_tRNA_pos_47_acp3U + 1 amet + 1 b0749_tRNA_7 <==> 1 b0749_tRNA_7_AcpT_tRNA_pos_47_acp3U_cplx	reversible	tRNA modification	-1.00E+09	1.00E+09	0	1.77E+03	1.77E+03	0	7.51E+00	7.51	0	7.51E+00	7.51
b0749_tRNA_47_MODIF_1_Se	modification of position 47 nt (acp3U) in lysQ by AcpT_tRNA_pos_47_acp3U	1 AcpT_tRNA_pos_47_acp3U + 1 amet + 1 b0749_tRNA_7_Se <==> 1 b0749_tRNA_7_Se_AcpT_tRNA_pos_47_acp3U_cplx	reversible	tRNA modification	-1.00E+09	1.00E+09	0	1.77E+03	1.77E+03	0	7.51E+00	7.51	0	7.51E+00	7.51
b0749_tRNA_47_MODIF_2	modification of position 47 nt (acp3U) in lysQ by AcpT_tRNA_pos_47_acp3U	1 b0749_tRNA_7_AcpT_tRNA_pos_47_acp3U_cplx --> 1 5mta + 1 AcpT_tRNA_pos_47_acp3U_inact + 1 b0749_tRNA_8 + 1 h	irreversible	tRNA modification	0.00E+00	1.00E+09	0	1.77E+03	1.77E+03	0	7.51E+00	7.51	0	7.51E+00	7.51
b0749_tRNA_47_MODIF_2_Se	modification of position 47 nt (acp3U) in lysQ by AcpT_tRNA_pos_47_acp3U	1 b0749_tRNA_7_Se_AcpT_tRNA_pos_47_acp3U_cplx --> 1 5mta + 1 AcpT_tRNA_pos_47_acp3U_inact + 1 b0749_tRNA_8_Se + 1 h	irreversible	tRNA modification	0.00E+00	1.00E+09	0	1.77E+03	1.77E+03	0	7.51E+00	7.51	0	7.51E+00	7.51
b0749_tRNA_54_MODIF_1	modification of position 54 nt (m5U) in lysQ by TrmA_mono	1 TrmA_mono + 1 amet + 1 b0749_tRNA_8 <==> 1 b0749_tRNA_8_TrmA_mono_cplx	reversible	tRNA modification	-1.00E+09	1.00E+09	0	1.77E+03	1.77E+03	0	7.51E+00	7.51	0	7.51E+00	7.51
b0749_tRNA_54_MODIF_1_Se	modification of position 54 nt (m5U) in lysQ by TrmA_mono	1 TrmA_mono + 1 amet + 1 b0749_tRNA_8_Se <==> 1 b0749_tRNA_8_Se_TrmA_mono_cplx	reversible	tRNA modification	-1.00E+09	1.00E+09	0	1.77E+03	1.77E+03	0	7.51E+00	7.51	0	7.51E+00	7.51
b0749_tRNA_54_MODIF_2	modification of position 54 nt (m5U) in lysQ by TrmA_mono	1 b0749_tRNA_8_TrmA_mono_cplx --> 1 TrmA_mono_inact + 1 ahcys + 1 b0749_tRNA_9 + 1 h	irreversible	tRNA modification	0.00E+00	1.00E+09	0	1.77E+03	1.77E+03	0	7.51E+00	7.51	0	7.51E+00	7.51
b0749_tRNA_54_MODIF_2_Se	modification of position 54 nt (m5U) in lysQ by TrmA_mono	1 b0749_tRNA_8_Se_TrmA_mono_cplx --> 1 TrmA_mono_inact + 1 ahcys + 1 b0749_tRNA_9_Se + 1 h	irreversible	tRNA modification	0.00E+00	1.00E+09	0	1.77E+03	1.77E+03	0	7.51E+00	7.51	0	7.51E+00	7.51
b0749_tRNA_55_MODIF_1	modification of position 55 nt (Y) in lysQ by TruB_mono	1 TruB_mono + 1 b0749_tRNA_9 <==> 1 b0749_tRNA_9_TruB_mono_cplx	reversible	tRNA modification	-1.00E+09	1.00E+09	0	1.77E+03	1.77E+03	0	7.51E+00	7.51	0	7.51E+00	7.51
b0749_tRNA_55_MODIF_1_Se	modification of position 55 nt (Y) in lysQ by TruB_mono	1 TruB_mono + 1 b0749_tRNA_9_Se <==> 1 b0749_tRNA_9_Se_TruB_mono_cplx	reversible	tRNA modification	-1.00E+09	1.00E+09	0	1.77E+03	1.77E+03	0	7.51E+00	7.51	0	7.51E+00	7.51
b0749_tRNA_55_MODIF_2	modification of position 55 nt (Y) in lysQ by TruB_mono	1 b0749_tRNA_9_TruB_mono_cplx --> 1 TruB_mono_inact + 1 lysQ_tRNA	irreversible	tRNA modification	0.00E+00	1.00E+09	0	1.77E+03	1.77E+03	0	7.51E+00	7.51	0	7.51E+00	7.51
b0749_tRNA_55_MODIF_2_Se	modification of position 55 nt (Y) in lysQ by TruB_mono	1 b0749_tRNA_9_Se_TruB_mono_cplx --> 1 TruB_mono_inact + 1 lysQ_tRNA_Se	irreversible	tRNA modification	0.00E+00	1.00E+09	0	1.77E+03	1.77E+03	0	7.51E+00	7.51	0	7.51E+00	7.51
b0749_tRNA_Mg_BIND	Binding of two Mg2 to b0749_tRNA	1 b0749_RNA + 2 mg2 <==> 1 b0749_tRNA_Mg2	reversible	Metallo-ion binding	-1.00E+09	1.00E+09	0	1.77E+03	1.77E+03	0	7.51E+00	7.51	0	7.51E+00	7.51
b0850_fold_spon	b0850_m folding: spontanous	1 b0850_m --> 1 YbjC_mono	irreversible	Protein Folding	0.00E+00	1.00E+09	0	3.46E+02	3.46E+02	0	5.46E+02	546.3867	0	8.45E+01	84.535
b0850_mRNA_CONV	convsersion of mRNA to mRNA_1 (synthetic rxn)	1 b0850_mRNA --> 1 b0850_mRNA_1	irreversible	Translation	0.00E+00	1.00E+09	0	5.24E+01	5.24E+01	0	9.26E+01	92.57733	0	2.07E-01	0.207
b0850_mRNA_degr1	Degradation of b0850_mRNA I	1 Orn_dim + 72 atp + 1 b0850_mRNA_2 + 1 degradosome + 72 h2o <==> 72 adp + 1 b0850_mRNA_2_degr + 72 h + 72 pi	reversible	mRNA degradation	-1.00E+09	1.00E+09	0	5.24E+01	5.24E+01	0	9.26E+01	92.57733	0	2.07E-01	0.207
b0850_mRNA_degr2	Degradation of b0850_mRNA II	1 b0850_mRNA_2_degr + 287 h2o --> 1 Orn_dim_inact + 63 amp + 1 atp + 69 cmp + 1 degradosome_inact + 76 gmp + 287 h + 79 ump	irreversible	mRNA degradation	0.00E+00	2.20E-01	0	5.24E+01	5.24E+01	0	9.26E+01	92.57733	0	2.07E-01	0.207
b0850_maturation1	Polypeptide b0850 peptide deformylas and methionine aminopeptidase complex	1 Def_mono + 1 Map_mono + 1 b0850_aa <==> 1 b0850_def_map_cplx	reversible	Protein Maturation	-1.00E+09	1.00E+09	0	3.46E+02	3.46E+02	0	5.46E+02	546.3867	0	8.45E+01	84.535
b0850_maturation2	b0850 formation	1 b0850_def_map_cplx + 2 h2o --> 1 Def_mono_inact + 1 Map_mono_inact + 1 b0850_m + 1 for + 1 met-L	irreversible	Protein Maturation	0.00E+00	1.00E+09	0	3.46E+02	3.46E+02	0	5.46E+02	546.3867	0	8.45E+01	84.535
b0851_fold_spon	b0851_m folding: spontanous	1 b0851_m --> 1 NfsA_mono	irreversible	Protein Folding	0.00E+00	1.00E+09	0	8.65E+02	8.65E+02	0	1.43E+03	1426.703	0	1.34E+02	133.518
b0851_mRNA_CONV	convsersion of mRNA to mRNA_1 (synthetic rxn)	1 b0851_mRNA --> 1 b0851_mRNA_1	irreversible	Translation	0.00E+00	1.00E+09	0	5.24E+01	5.24E+01	0	9.26E+01	92.57733	0	2.07E-01	0.207
b0851_mRNA_degr1	Degradation of b0851_mRNA I	1 Orn_dim + 176 atp + 1 b0851_mRNA_2 + 1 degradosome + 176 h2o <==> 176 adp + 1 b0851_mRNA_2_degr + 176 h + 176 pi	reversible	mRNA degradation	-1.00E+09	1.00E+09	0	5.24E+01	5.24E+01	0	9.26E+01	92.57733	0	2.07E-01	0.207
b0851_mRNA_degr2	Degradation of b0851_mRNA II	1 b0851_mRNA_2_degr + 705 h2o --> 1 Orn_dim_inact + 165 amp + 178 cmp + 1 degradosome_inact + 194 gmp + 705 h + 169 ump	irreversible	mRNA degradation	0.00E+00	2.07E-01	0	5.24E+01	5.24E+01	0	9.26E+01	92.57733	0	2.07E-01	0.207
b0851_maturation1	Polypeptide b0851 peptide deformylas and methionine aminopeptidase complex	1 Def_mono + 1 Map_mono + 1 b0851_aa <==> 1 b0851_def_map_cplx	reversible	Protein Maturation	-1.00E+09	1.00E+09	0	8.65E+02	8.65E+02	0	1.43E+03	1426.703	0	1.34E+02	133.518
b0851_maturation2	b0851 formation	1 b0851_def_map_cplx + 2 h2o --> 1 Def_mono_inact + 1 Map_mono_inact + 1 b0851_m + 1 for + 1 met-L	irreversible	Protein Maturation	0.00E+00	1.00E+09	0	8.65E+02	8.65E+02	0	1.43E+03	1426.703	0	1.34E+02	133.518
b0852_fold_spon	b0852_m folding: spontanous	1 b0852_m --> 1 RimK_mono	irreversible	Protein Folding	0.00E+00	1.00E+09	0	1.64E+03	1.64E+03	0	2.83E+03	2833.396	0	8.27E+01	82.739
b0852_mRNA_CONV	convsersion of mRNA to mRNA_1 (synthetic rxn)	1 b0852_mRNA --> 1 b0852_mRNA_1	irreversible	Translation	0.00E+00	1.00E+09	0	9.18E+01	9.18E+01	0	1.62E+02	162.0103	0	2.07E-01	0.207
b0852_mRNA_degr1	Degradation of b0852_mRNA I	1 Orn_dim + 225 atp + 1 b0852_mRNA_2 + 1 degradosome + 225 h2o <==> 225 adp + 1 b0852_mRNA_2_degr + 225 h + 225 pi	reversible	mRNA degradation	-1.00E+09	1.00E+09	0	9.18E+01	9.18E+01	0	1.62E+02	162.0103	0	2.07E-01	0.207
b0852_mRNA_degr2	Degradation of b0852_mRNA II	1 b0852_mRNA_2_degr + 902 h2o --> 1 Orn_dim_inact + 192 amp + 220 cmp + 1 degradosome_inact + 287 gmp + 1 gtp + 902 h + 203 ump	irreversible	mRNA degradation	0.00E+00	2.07E-01	0	9.18E+01	9.18E+01	0	1.62E+02	162.0103	0	2.07E-01	0.207
b0852_maturation1	Polypeptide b0852 peptide deformylas and methionine aminopeptidase complex	1 Def_mono + 1 Map_mono + 1 b0852_aa <==> 1 b0852_def_map_cplx	reversible	Protein Maturation	-1.00E+09	1.00E+09	0	1.64E+03	1.64E+03	0	2.83E+03	2833.396	0	8.27E+01	82.739
b0852_maturation2	b0852 formation	1 b0852_def_map_cplx + 2 h2o --> 1 Def_mono_inact + 1 Map_mono_inact + 1 b0852_m + 1 for + 1 met-L	irreversible	Protein Maturation	0.00E+00	1.00E+09	0	1.64E+03	1.64E+03	0	2.83E+03	2833.396	0	8.27E+01	82.739
b0852_v1_mRNA_CONV	convsersion of mRNA to mRNA_1 (synthetic rxn)	1 b0852_v1_mRNA --> 1 b0852_mRNA_1	irreversible	Translation	0.00E+00	1.00E+09	0	5.24E+01	5.24E+01	0	9.26E+01	92.57733	0	2.07E-01	0.207
b0853_fold_spon	b0853_m folding: spontanous	1 b0853_m --> 1 YbjN_mono	irreversible	Protein Folding	0.00E+00	1.00E+09	0	9.69E+02	9.69E+02	0	1.60E+03	1601.193	0	1.45E+02	144.963
b0853_mRNA_CONV	convsersion of mRNA to mRNA_1 (synthetic rxn)	1 b0853_mRNA --> 1 b0853_mRNA_1	irreversible	Translation	0.00E+00	1.00E+09	0	9.18E+01	9.18E+01	0	1.62E+02	162.0103	0	2.07E-01	0.207
b0853_mRNA_degr1	Degradation of b0853_mRNA I	1 Orn_dim + 119 atp + 1 b0853_mRNA_2 + 1 degradosome + 119 h2o <==> 119 adp + 1 b0853_mRNA_2_degr + 119 h + 119 pi	reversible	mRNA degradation	-1.00E+09	1.00E+09	0	9.18E+01	9.18E+01	0	1.62E+02	162.0103	0	2.07E-01	0.207
b0853_mRNA_degr2	Degradation of b0853_mRNA II	1 b0853_mRNA_2_degr + 476 h2o --> 1 Orn_dim_inact + 109 amp + 111 cmp + 1 degradosome_inact + 120 gmp + 476 h + 137 ump	irreversible	mRNA degradation	0.00E+00	2.99E-01	0	9.18E+01	9.18E+01	0	1.62E+02	162.0103	0	2.07E-01	0.207
b0853_maturation1	Polypeptide b0853 peptide deformylas and methionine aminopeptidase complex	1 Def_mono + 1 Map_mono + 1 b0853_aa <==> 1 b0853_def_map_cplx	reversible	Protein Maturation	-1.00E+09	1.00E+09	0	9.69E+02	9.69E+02	0	1.60E+03	1601.193	0	1.45E+02	144.963
b0853_maturation2	b0853 formation	1 b0853_def_map_cplx + 2 h2o --> 1 Def_mono_inact + 1 Map_mono_inact + 1 b0853_m + 1 for + 1 met-L	irreversible	Protein Maturation	0.00E+00	1.00E+09	0	9.69E+02	9.69E+02	0	1.60E+03	1601.193	0	1.45E+02	144.963
b0853_v1_mRNA_CONV	convsersion of mRNA to mRNA_1 (synthetic rxn)	1 b0853_v1_mRNA --> 1 b0853_mRNA_1	irreversible	Translation	0.00E+00	1.00E+09	0	5.24E+01	5.24E+01	0	9.26E+01	92.57733	0	2.07E-01	0.207
b0858_fold_spon	b0858_m folding: spontanous	1 b0858_m --> 1 YbjO_mono	irreversible	Protein Folding	0.00E+00	1.00E+09	0	7.71E+02	7.71E+02	0	1.29E+03	1293.374	0	8.90E+01	89.046
b0858_mRNA_CONV	convsersion of mRNA to mRNA_1 (synthetic rxn)	1 b0858_mRNA --> 1 b0858_mRNA_1	irreversible	Translation	0.00E+00	1.00E+09	0	7.59E+01	7.59E+01	0	1.34E+02	133.9183	0	1.04E-01	0.104
b0858_mRNA_degr1	Degradation of b0858_mRNA I	1 Orn_dim + 122 atp + 1 b0858_mRNA_2 + 1 degradosome + 122 h2o <==> 122 adp + 1 b0858_mRNA_2_degr + 122 h + 122 pi	reversible	mRNA degradation	-1.00E+09	1.00E+09	0	7.59E+01	7.59E+01	0	1.34E+02	133.9183	0	1.04E-01	0.104

b0858_mRNA_degr2	Degradation of b0858_mRNA II	1 b0858_mRNA_2_degr + 488 h2o --> 1 Orn_dim_inact + 93 amp + 1 atp + 117 cmp + 1 degradosome_inact + 138 gmp + 488 h + 140 ump	irreversible	mRNA degradation	0.00E+00	2.07E-01	0	7.59E+01	7.59E+01	0	1.34E+02	133.9183	0	1.04E-01	0.104
b0858_maturation1	Polypeptide b0858 peptide deformylas and methionine aminopeptidase complex	1 Def_mono + 1 Map_mono + 1 b0858_aa <==> 1 b0858_def_map_cplx	reversible	Protein Maturation	-1.00E+09	1.00E+09	0	7.71E+02	7.71E+02	0	1.29E+03	1293.374	0	8.90E+01	89.046
b0858_maturation2	b0858 formation	1 b0858_def_map_cplx + 2 h2o --> 1 Def_mono_inact + 1 Map_mono_inact + 1 b0858_m + 1 for + 1 met-L	irreversible	Protein Maturation	0.00E+00	1.00E+09	0	7.71E+02	7.71E+02	0	1.29E+03	1293.374	0	8.90E+01	89.046
b0859_fold_spon	b0859_m folding: spontanous	1 b0859_m_FeS --> 1 RumB_mono	irreversible	Protein Folding	0.00E+00	1.00E+09	0	1.74E+03	1.74E+03	0	3.02E+03	3017.262	0	7.33E+01	73.348
b0859_iron_sulfur_BIND1	Incorporation of [4Fe-4S]2+ in b0859	1 IscU_dim_[4Fe-4S] + 1 b0859_m <==> 1 b0859_m_IscU_cplx	reversible	Iron-sulfur cluster incorporation	-1.00E+09	1.00E+09	0	1.74E+03	1.74E+03	0	3.02E+03	3017.262	0	7.33E+01	73.348
b0859_iron_sulfur_BIND2	Incorporation of [4Fe-4S]2+ in b0859	1 b0859_m_IscU_cplx <==> 2 IscU_mono + 1 b0859_m_FeS	reversible	Iron-sulfur cluster incorporation	-1.00E+09	1.00E+09	0	1.74E+03	1.74E+03	0	3.02E+03	3017.262	0	7.33E+01	73.348
b0859_mRNA_CONV	convsersion of mRNA to mRNA_1 (synthetic rxn)	1 b0859_mRNA --> 1 b0859_mRNA_1	irreversible	Translation	0.00E+00	1.00E+09	0	7.59E+01	7.59E+01	0	1.34E+02	133.9183	0	1.04E-01	0.104
b0859_mRNA_degr1	Degradation of b0859_mRNA I	1 Orn_dim + 282 atp + 1 b0859_mRNA_2 + 1 degradosome + 282 h2o <==> 282 adp + 1 b0859_mRNA_2_degr + 282 h + 282 pi	reversible	mRNA degradation	-1.00E+09	1.00E+09	0	7.59E+01	7.59E+01	0	1.34E+02	133.9183	0	1.04E-01	0.104
b0859_mRNA_degr2	Degradation of b0859_mRNA II	1 b0859_mRNA_2_degr + 1127 h2o --> 1 Orn_dim_inact + 248 amp 297 cmp + 1 degradosome_inact + 319 gmp + 1127 h + 264 ump	irreversible	mRNA degradation	0.00E+00	1.04E-01	0	7.59E+01	7.59E+01	0	1.34E+02	133.9183	0	1.04E-01	0.104
b0859_maturation1	Polypeptide b0859 peptide deformylas and methionine aminopeptidase complex	1 Def_mono + 1 Map_mono + 1 b0859_aa <==> 1 b0859_def_map_cplx	reversible	Protein Maturation	-1.00E+09	1.00E+09	0	1.74E+03	1.74E+03	0	3.02E+03	3017.262	0	7.33E+01	73.348
b0859_maturation2	b0859 formation	1 b0859_def_map_cplx + 2 h2o --> 1 Def_mono_inact + 1 Map_mono_inact + 1 b0859_m + 1 for + 1 met-L	irreversible	Protein Maturation	0.00E+00	1.00E+09	0	1.74E+03	1.74E+03	0	3.02E+03	3017.262	0	7.33E+01	73.348
b0883_tRNA_18_MODIF_1	modification of position 18 nt (Gm) in serW by TrmH_dim	1 TrmH_dim + 1 amet + 1 b0883_tRNA_Mg2 <==> 1 b0883_tRNA_Mg2_TrmH_dim_cplx	reversible	tRNA modification	-1.00E+09	1.00E+09	0	1.33E+03	1.33E+03	0	7.06E+00	7.06	0	7.06E+00	7.06
b0883_tRNA_18_MODIF_2	modification of position 18 nt (Gm) in serW by TrmH_dim	1 b0883_tRNA_Mg2_TrmH_dim_cplx --> 1 TrmH_dim_inact + 1 ahcys + 1 b0883_tRNA_1 + 1 h	irreversible	tRNA modification	0.00E+00	1.00E+09	0	1.33E+03	1.33E+03	0	7.06E+00	7.06	0	7.06E+00	7.06
b0883_tRNA_20A_MODIF_1	modification of position 20A nt (D) in serW by Dus_gen	1 Dus_gen + 1 b0883_tRNA_1 + 1 h + 1 nadph <==> 1 b0883_tRNA_1_Dus_gen_cplx	reversible	tRNA modification	-1.00E+09	1.00E+09	0	1.33E+03	1.33E+03	0	7.06E+00	7.06	0	7.06E+00	7.06
b0883_tRNA_20A_MODIF_2	modification of position 20A nt (D) in serW by Dus_gen	1 b0883_tRNA_1_Dus_gen_cplx --> 1 Dus_gen_inact + 1 b0883_tRNA_2 + 1 nadp	irreversible	tRNA modification	0.00E+00	1.00E+09	0	1.33E+03	1.33E+03	0	7.06E+00	7.06	0	7.06E+00	7.06
b0883_tRNA_40_MODIF_1	modification of position 40 nt (Y) in serW by TruA_dim	1 TruA_dim + 1 b0883_tRNA_2 <==> 1 b0883_tRNA_2_TruA_dim_cplx	reversible	tRNA modification	-1.00E+09	1.00E+09	0	1.33E+03	1.33E+03	0	7.06E+00	7.06	0	7.06E+00	7.06
b0883_tRNA_40_MODIF_2	modification of position 40 nt (Y) in serW by TruA_dim	1 b0883_tRNA_2_TruA_dim_cplx --> 1 TruA_dim_inact + 1 b0883_tRNA_3	irreversible	tRNA modification	0.00E+00	1.00E+09	0	1.33E+03	1.33E+03	0	7.06E+00	7.06	0	7.06E+00	7.06
b0883_tRNA_54_MODIF_1	modification of position 54 nt (m5U) in serW by TrmA_mono	1 TrmA_mono + 1 amet + 1 b0883_tRNA_3 <==> 1 b0883_tRNA_3_TrmA_mono_cplx	reversible	tRNA modification	-1.00E+09	1.00E+09	0	1.33E+03	1.33E+03	0	7.06E+00	7.06	0	7.06E+00	7.06
b0883_tRNA_54_MODIF_2	modification of position 54 nt (m5U) in serW by TrmA_mono	1 b0883_tRNA_3_TrmA_mono_cplx --> 1 TrmA_mono_inact + 1 ahcys + 1 b0883_tRNA_4 + 1 h	irreversible	tRNA modification	0.00E+00	1.00E+09	0	1.33E+03	1.33E+03	0	7.06E+00	7.06	0	7.06E+00	7.06
b0883_tRNA_55_MODIF_1	modification of position 55 nt (Y) in serW by TruB_mono	1 TruB_mono + 1 b0883_tRNA_4 <==> 1 b0883_tRNA_4_TruB_mono_cplx	reversible	tRNA modification	-1.00E+09	1.00E+09	0	1.33E+03	1.33E+03	0	7.06E+00	7.06	0	7.06E+00	7.06
b0883_tRNA_55_MODIF_2	modification of position 55 nt (Y) in serW by TruB_mono	1 b0883_tRNA_4_TruB_mono_cplx --> 1 TruB_mono_inact + 1 serW_tRNA	irreversible	tRNA modification	0.00E+00	1.00E+09	0	1.33E+03	1.33E+03	0	7.06E+00	7.06	0	7.06E+00	7.06
b0883_tRNA_Mg_BIND	Binding of two Mg2 to b0883_tRNA	1 b0883_RNA + 2 mg2 <==> 1 b0883_tRNA_Mg2	reversible	Metallo-ion binding	-1.00E+09	1.00E+09	0	1.33E+03	1.33E+03	0	7.06E+00	7.06	0	7.06E+00	7.06
b0884_fold_spon	b0884_m folding: spontanous	1 b0884_m --> 1 InfA_mono	irreversible	Protein Folding	0.00E+00	1.00E+09	8.44E+01	4.56E+03	4.48E+03	6.22E+01	6.34E+03	6276.458	6.22E+01	2.27E+03	2203.364
b0884_mRNA_CONV	convsersion of mRNA to mRNA_1 (synthetic rxn)	1 b0884_mRNA --> 1 b0884_mRNA_1	irreversible	Translation	0.00E+00	1.00E+09	0	5.81E+02	5.81E+02	0	1.03E+03	1025.084	0	6.81E+00	6.81
b0884_mRNA_degr1	Degradation of b0884_mRNA I	1 Orn_dim + 54 atp + 1 b0884_mRNA_2 + 1 degradosome + 54 h2o <==> 54 adp + 1 b0884_mRNA_2_degr + 54 h + 54 pi	reversible	mRNA degradation	-1.00E+09	1.00E+09	0	5.81E+02	5.81E+02	0	1.03E+03	1025.084	0	6.81E+00	6.81
b0884_mRNA_degr2	Degradation of b0884_mRNA II	1 b0884_mRNA_2_degr + 218 h2o --> 1 Orn_dim_inact + 64 amp + 1 atp + 55 cmp + 1 degradosome_inact + 51 gmp + 218 h + 48 ump	irreversible	mRNA degradation	0.00E+00	6.81E+00	0	5.81E+02	5.81E+02	0	1.03E+03	1025.084	0	6.81E+00	6.81
b0884_maturation1	Polypeptide b0884 peptide deformylas and methionine aminopeptidase complex	1 Def_mono + 1 Map_mono + 1 b0884_aa <==> 1 b0884_def_map_cplx	reversible	Protein Maturation	-1.00E+09	1.00E+09	8.44E+01	4.56E+03	4.48E+03	6.22E+01	6.34E+03	6276.458	6.22E+01	2.27E+03	2203.364
b0884_maturation2	b0884 formation	1 b0884_def_map_cplx + 2 h2o --> 1 Def_mono_inact + 1 Map_mono_inact + 1 b0884_m + 1 for + 1 met-L	irreversible	Protein Maturation	0.00E+00	1.00E+09	8.44E+01	4.56E+03	4.48E+03	6.22E+01	6.34E+03	6276.458	6.22E+01	2.27E+03	2203.364
b0884_v1_mRNA_CONV	convsersion of mRNA to mRNA_1 (synthetic rxn)	1 b0884_v1_mRNA --> 1 b0884_mRNA_1	irreversible	Translation	0.00E+00	1.00E+09	0	5.81E+02	5.81E+02	0	1.03E+03	1025.084	0	6.81E+00	6.81
b0893_fold_KJE_1	b0893_m folding: KJE mediated	1 DnaJ_dim + 1 DnaK_mono.ATP + 1 b0893_m <==> 1 b0893_m_DnaKJ_complex	reversible	Protein Folding	-1.00E+09	1.00E+09	0	2.68E+03	2.68E+03	0	4.59E+03	4594.406	0	2.10E+02	210.475
b0893_fold_KJE_2	b0893_m folding: KJE mediated	1 GrpE_dim + 1 b0893_m_DnaKJ_complex + 1 h2o --> 1 DnaJ_dim_inact + 1 SerS_DnaK_GrpE_complex + 1 adp + 1 h + 1 pi	irreversible	Protein Folding	0.00E+00	1.00E+09	0	2.68E+03	2.68E+03	0	4.59E+03	4594.406	0	2.10E+02	210.475
b0893_fold_KJE_3	b0893_m folding: KJE mediated	1 SerS_DnaK_GrpE_complex + 1 atp --> 1 DnaK_mono.ATP_inact + 1 GrpE_dim_inact + 1 SerS_mono	irreversible	Protein Folding	0.00E+00	1.00E+09	0	2.68E+03	2.68E+03	0	4.59E+03	4594.406	0	2.10E+02	210.475
b0893_fold_spon	b0893_m folding: spontanous	1 b0893_m --> 1 SerS_mono	irreversible	Protein Folding	0.00E+00	1.00E+09	0	2.68E+03	2.68E+03	0	4.59E+03	4594.406	0	2.10E+02	210.475
b0893_mRNA_CONV	convsersion of mRNA to mRNA_1 (synthetic rxn)	1 b0893_mRNA --> 1 b0893_mRNA_1	irreversible	Translation	0.00E+00	1.00E+09	0	9.98E+01	9.98E+01	0	1.76E+02	176.1862	0	8.29E-01	0.829
b0893_mRNA_degr1	Degradation of b0893_mRNA I	1 Orn_dim + 323 atp + 1 b0893_mRNA_2 + 1 degradosome + 323 h2o <==> 323 adp + 1 b0893_mRNA_2_degr + 323 h + 323 pi	reversible	mRNA degradation	-1.00E+09	1.00E+09	0	9.98E+01	9.98E+01	0	1.76E+02	176.1862	0	8.29E-01	0.829
b0893_mRNA_degr2	Degradation of b0893_mRNA II	1 b0893_mRNA_2_degr + 1292 h2o --> 1 Orn_dim_inact + 322 amp 1 atp + 320 cmp + 1 degradosome_inact + 361 gmp + 1292 h + 289 ump	irreversible	mRNA degradation	0.00E+00	8.29E-01	0	9.98E+01	9.98E+01	0	1.76E+02	176.1862	0	8.29E-01	0.829
b0893_maturation1	Polypeptide b0893 peptide deformylas and methionine aminopeptidase complex	1 Def_mono + 1 Map_mono + 1 b0893_aa <==> 1 b0893_def_map_cplx	reversible	Protein Maturation	-1.00E+09	1.00E+09	0	2.68E+03	2.68E+03	0	4.59E+03	4594.406	0	2.10E+02	210.475
b0893_maturation2	b0893 formation	1 b0893_def_map_cplx + 2 h2o --> 1 Def_mono_inact + 1 Map_mono_inact + 1 b0893_m + 1 for + 1 met-L	irreversible	Protein Maturation	0.00E+00	1.00E+09	0	2.68E+03	2.68E+03	0	4.59E+03	4594.406	0	2.10E+02	210.475
b0910_fold_spon	b0910_m folding: spontanous	1 b0910_m --> 1 Cmk_mono	irreversible	Protein Folding	0.00E+00	1.00E+09	0	8.34E+02	8.34E+02	0	1.09E+03	1087.802	0	1.38E+02	138.372
b0910_mRNA_CONV	convsersion of mRNA to mRNA_1 (synthetic rxn)	1 b0910_mRNA --> 1 b0910_mRNA_1	irreversible	Translation	0.00E+00	1.00E+09	0	5.39E+01	5.39E+01	0	7.34E+01	73.37709	0	3.44E-01	0.344
b0910_mRNA_degr1	Degradation of b0910_mRNA I	1 Orn_dim + 171 atp + 1 b0910_mRNA_2 + 1 degradosome + 171 h2o <==> 171 adp + 1 b0910_mRNA_2_degr + 171 h + 171 pi	reversible	mRNA degradation	-1.00E+09	1.00E+09	0	5.39E+01	5.39E+01	0	7.34E+01	73.37709	0	3.44E-01	0.344
b0910_mRNA_degr2	Degradation of b0910_mRNA II	1 b0910_mRNA_2_degr + 683 h2o --> 1 Orn_dim_inact + 156 amp + 1 atp + 169 cmp + 1 degradosome_inact + 198 gmp + 683 h + 160 ump	irreversible	mRNA degradation	0.00E+00	3.44E-01	0	5.39E+01	5.39E+01	0	7.34E+01	73.37709	0	3.44E-01	0.344
b0910_maturation1	Polypeptide b0910 peptide deformylas and methionine aminopeptidase complex	1 Def_mono + 1 Map_mono + 1 b0910_aa <==> 1 b0910_def_map_cplx	reversible	Protein Maturation	-1.00E+09	1.00E+09	0	8.34E+02	8.34E+02	0	1.09E+03	1087.802	0	1.38E+02	138.372
b0910_maturation2	b0910 formation	1 b0910_def_map_cplx + 2 h2o --> 1 Def_mono_inact + 1 Map_mono_inact + 1 b0910_m + 1 for + 1 met-L	irreversible	Protein Maturation	0.00E+00	1.00E+09	0	8.34E+02	8.34E+02	0	1.09E+03	1087.802	0	1.38E+02	138.372
b0911_fold_spon	b0911_m folding: spontanous	1 b0911_m --> 1 RpsA_mono	irreversible	Protein Folding	0.00E+00	1.00E+09	8.44E+01	1.13E+02	2.81E+01	6.22E+01	8.29E+01	20.72	6.22E+01	8.29E+01	20.72
b0911_mRNA_CONV	convsersion of mRNA to mRNA_1 (synthetic rxn)	1 b0911_mRNA --> 1 b0911_mRNA_1	irreversible	Translation	0.00E+00	1.00E+09	0	6.32E+01	6.32E+01	0	7.72E+01	77.17767	0	4.73E-01	0.473
b0911_mRNA_degr1	Degradation of b0911_mRNA I	1 Orn_dim + 418 atp + 1 b0911_mRNA_2 + 1 degradosome + 418 h2o <==> 418 adp + 1 b0911_mRNA_2_degr + 418 h + 418 pi	reversible	mRNA degradation	-1.00E+09	1.00E+09	0	6.32E+01	6.32E+01	0	7.72E+01	77.17767	0	7.39E-01	0.739
b0911_mRNA_degr2	Degradation of b0911_mRNA II	1 b0911_mRNA_2_degr + 1673 h2o --> 1 Orn_dim_inact + 447 amp 1 atp + 398 cmp + 1 degradosome_inact + 459 gmp + 1673 h + 369 ump	irreversible	mRNA degradation	0.00E+00	7.39E-01	0	6.32E+01	6.32E+01	0	7.72E+01	77.17767	0	7.39E-01	0.739

b0911_maturation1	Polypeptide b0911 peptide deformylas complex	1 Def_mono + 1 b0911_aa <==> 1 b0911_def_cplx	reversible	Protein Maturation	-1.00E+09	1.00E+09	8.44E+01	1.13E+02	2.81E+01	6.22E+01	8.29E+01	20.72	6.22E+01	8.29E+01	20.72
b0911_maturation2	b0911 formation	1 b0911_def_cplx + 1 h2o --> 1 Def_mono_inact + 1 b0911_m + 1 for	irreversible	Protein Maturation	0.00E+00	1.00E+09	8.44E+01	1.13E+02	2.81E+01	6.22E+01	8.29E+01	20.72	6.22E+01	8.29E+01	20.72
b0911_v1_mRNA_CONV	convsersion of mRNA to mRNA_1 (synthetic rxn)	1 b0911_v1_mRNA --> 1 b0911_mRNA_1	irreversible	Translation	0.00E+00	1.00E+09	0	6.32E+01	6.32E+01	0	7.72E+01	77.17767	0	4.73E-01	0.473
b0911_v2_mRNA_CONV	convsersion of mRNA to mRNA_1 (synthetic rxn)	1 b0911_v2_mRNA --> 1 b0911_mRNA_1	irreversible	Translation	0.00E+00	1.00E+09	0	5.39E+01	5.39E+01	0	7.34E+01	73.37709	0	3.44E-01	0.344
b0912_fold_spon	b0912_m folding: spontanous	1 b0912_m --> 1 IhfB_mono	irreversible	Protein Folding	0.00E+00	1.00E+09	0	5.44E+02	5.44E+02	0	6.14E+02	613.8883	0	2.30E+02	230.365
b0912_mRNA_CONV	convsersion of mRNA to mRNA_1 (synthetic rxn)	1 b0912_mRNA --> 1 b0912_mRNA_1	irreversible	Translation	0.00E+00	1.00E+09	0	6.32E+01	6.32E+01	0	7.72E+01	77.17767	0	4.73E-01	0.473
b0912_mRNA_degr1	Degradation of b0912_mRNA I	1 Orn_dim + 71 atp + 1 b0912_mRNA_2 + 1 degradosome + 71 h2o <==> 71 adp + 1 b0912_mRNA_2_degr + 71 h + 71 pi	reversible	mRNA degradation	-1.00E+09	1.00E+09	0	6.32E+01	6.32E+01	0	7.72E+01	77.17767	0	4.73E-01	0.473
b0912_mRNA_degr2	Degradation of b0912_mRNA II	1 b0912_mRNA_2_degr + 284 h2o --> 1 Orn_dim_inact + 84 amp + 70 cmp + 1 degradosome_inact + 70 gmp + 284 h + 61 ump	irreversible	mRNA degradation	0.00E+00	4.73E-01	0	6.32E+01	6.32E+01	0	7.72E+01	77.17767	0	4.73E-01	0.473
b0912_maturation1	Polypeptide b0912 peptide deformylas complex	1 Def_mono + 1 b0912_aa <==> 1 b0912_def_cplx	reversible	Protein Maturation	-1.00E+09	1.00E+09	0	5.44E+02	5.44E+02	0	6.14E+02	613.8883	0	2.30E+02	230.365
b0912_maturation2	b0912 formation	1 b0912_def_cplx + 1 h2o --> 1 Def_mono_inact + 1 b0912_m + 1 for	irreversible	Protein Maturation	0.00E+00	1.00E+09	0	5.44E+02	5.44E+02	0	6.14E+02	613.8883	0	2.30E+02	230.365
b0912_v1_mRNA_CONV	convsersion of mRNA to mRNA_1 (synthetic rxn)	1 b0912_v1_mRNA --> 1 b0912_mRNA_1	irreversible	Translation	0.00E+00	1.00E+09	0	6.32E+01	6.32E+01	0	7.72E+01	77.17767	0	4.73E-01	0.473
b0930_fold_GroEL/ES_1	b0930_m folding: GroEL/ES mediated; polypeptide is going in GroEL/ES complex	1 GroEL.(7)ADP.cisGroES + 7 atp + 1 b0930_m + 1 transGroES_hepta --> 7 adp + 1 b0930_m_GroEL.(7)ATP.transGroES + 1 cisGroES_hepta	irreversible	Protein Folding	0.00E+00	1.00E+09	0	2.59E+03	2.59E+03	0	4.47E+03	4466.492	0	1.47E+02	146.718
b0930_fold_GroEL/ES_2	b0930_m folding: GroEL/ES mediated; folding of polypeptide under ATP hydrolysis	1 b0930_m_GroEL.(7)ATP.transGroES + 7 h2o --> 1 AsnS_GroEL.(7)ADP.transGroES + 7 h + 7 pi	irreversible	Protein Folding	0.00E+00	1.00E+09	0	2.59E+03	2.59E+03	0	4.47E+03	4466.492	0	1.47E+02	146.718
b0930_fold_GroEL/ES_3	b0930_m folding: GroEL/ES mediated; release of native protein	1 AsnS_GroEL.(7)ADP.transGroES --> 1 AsnS_mono + 1 GroEL.(7)ADP.transGroES	irreversible	Protein Folding	0.00E+00	1.00E+09	0	2.59E+03	2.59E+03	0	4.47E+03	4466.492	0	1.47E+02	146.718
b0930_fold_KJE_1	b0930_m folding: KJE mediated	1 DnaJ_dim + 1 DnaK_mono.ATP + 1 b0930_m <==> 1 b0930_m_DnaKJ_complex	reversible	Protein Folding	-1.00E+09	1.00E+09	0	2.59E+03	2.59E+03	0	4.47E+03	4466.492	0	1.47E+02	146.718
b0930_fold_KJE_2	b0930_m folding: KJE mediated	1 GrpE_dim + 1 b0930_m_DnaKJ_complex + 1 h2o --> 1 AsnS_DnaK_GrpE_complex + 1 DnaJ_dim_inact + 1 adp + 1 h + 1 pi	irreversible	Protein Folding	0.00E+00	1.00E+09	0	2.59E+03	2.59E+03	0	4.47E+03	4466.492	0	1.47E+02	146.718
b0930_fold_KJE_3	b0930_m folding: KJE mediated	1 AsnS_DnaK_GrpE_complex + 1 atp --> 1 AsnS_mono + 1 DnaK_mono.ATP_inact + 1 GrpE_dim_inact	irreversible	Protein Folding	0.00E+00	1.00E+09	0	2.59E+03	2.59E+03	0	4.47E+03	4466.492	0	1.47E+02	146.718
b0930_fold_spon	b0930_m folding: spontanous	1 b0930_m --> 1 AsnS_mono	irreversible	Protein Folding	0.00E+00	1.00E+09	0	2.59E+03	2.59E+03	0	4.47E+03	4466.492	0	1.47E+02	146.718
b0930_mRNA_CONV	convsersion of mRNA to mRNA_1 (synthetic rxn)	1 b0930_mRNA --> 1 b0930_mRNA_1	irreversible	Translation	0.00E+00	1.00E+09	0	9.10E+01	9.10E+01	0	1.61E+02	160.6256	0	6.34E-01	0.634
b0930_mRNA_degr1	Degradation of b0930_mRNA I	1 Orn_dim + 350 atp + 1 b0930_mRNA_2 + 1 degradosome + 350 h2o <==> 350 adp + 1 b0930_mRNA_2_degr + 350 h + 350 pi	reversible	mRNA degradation	-1.00E+09	1.00E+09	0	9.10E+01	9.10E+01	0	1.61E+02	160.6256	0	6.34E-01	0.634
b0930_mRNA_degr2	Degradation of b0930_mRNA II	1 b0930_mRNA_2_degr + 1400 h2o --> 1 Orn_dim_inact + 318 amp 1 atp + 351 cmp + 1 degradosome_inact + 383 gmp + 1400 h + 348 ump	irreversible	mRNA degradation	0.00E+00	6.34E-01	0	9.10E+01	9.10E+01	0	1.61E+02	160.6256	0	6.34E-01	0.634
b0930_maturation1	Polypeptide b0930 peptide deformylas and methionine aminopeptidase complex	1 Def_mono + 1 Map_mono + 1 b0930_aa <==> 1 b0930_def_map_cplx	reversible	Protein Maturation	-1.00E+09	1.00E+09	0	2.59E+03	2.59E+03	0	4.47E+03	4466.492	0	1.47E+02	146.718
b0930_maturation2	b0930 formation	1 b0930_def_map_cplx + 2 h2o --> 1 Def_mono_inact + 1 Map_mono_inact + 1 b0930_m + 1 for + 1 met-L	irreversible	Protein Maturation	0.00E+00	1.00E+09	0	2.59E+03	2.59E+03	0	4.47E+03	4466.492	0	1.47E+02	146.718
b0969_fold_spon	b0969_m folding: spontanous	1 b0969_m --> 1 YccK_mono	irreversible	Protein Folding	0.00E+00	1.00E+09	0	3.07E+03	3.07E+03	0	5.36E+03	5362.387	0	7.77E+01	77.724
b0969_mRNA_CONV	convsersion of mRNA to mRNA_1 (synthetic rxn)	1 b0969_mRNA --> 1 b0969_mRNA_1	irreversible	Translation	0.00E+00	1.00E+09	0	4.99E+02	4.99E+02	0	8.81E+02	880.9312	0	1.54E-01	0.154
b0969_mRNA_degr1	Degradation of b0969_mRNA I	1 Orn_dim + 82 atp + 1 b0969_mRNA_2 + 1 degradosome + 82 h2o <==> 82 adp + 1 b0969_mRNA_2_degr + 82 h + 82 pi	reversible	mRNA degradation	-1.00E+09	1.00E+09	0	4.99E+02	4.99E+02	0	8.81E+02	880.9312	0	1.54E-01	0.154
b0969_mRNA_degr2	Degradation of b0969_mRNA II	1 b0969_mRNA_2_degr + 329 h2o --> 1 Orn_dim_inact + 97 amp + 1 atp + 64 cmp + 1 degradosome_inact + 90 gmp + 329 h + 78 ump	irreversible	mRNA degradation	0.00E+00	1.54E-01	0	4.99E+02	4.99E+02	0	8.81E+02	880.9312	0	1.54E-01	0.154
b0969_maturation1	Polypeptide b0969 peptide deformylas and methionine aminopeptidase complex	1 Def_mono + 1 Map_mono + 1 b0969_aa <==> 1 b0969_def_map_cplx	reversible	Protein Maturation	-1.00E+09	1.00E+09	0	3.07E+03	3.07E+03	0	5.36E+03	5362.387	0	7.77E+01	77.724
b0969_maturation2	b0969 formation	1 b0969_def_map_cplx + 2 h2o --> 1 Def_mono_inact + 1 Map_mono_inact + 1 b0969_m + 1 for + 1 met-L	irreversible	Protein Maturation	0.00E+00	1.00E+09	0	3.07E+03	3.07E+03	0	5.36E+03	5362.387	0	7.77E+01	77.724
b0971_tRNA_18_MODIF_1	modification of position 18 nt (Gm) in serT by TrmH_dim	1 TrmH_dim + 1 amet + 1 b0971_tRNA_Mg2 <==> 1 b0971_tRNA_Mg2_TrmH_dim_cplx	reversible	tRNA modification	-1.00E+09	1.00E+09	0	1.33E+03	1.33E+03	0	6.75E+00	6.75	0	6.75E+00	6.75
b0971_tRNA_18_MODIF_2	modification of position 18 nt (Gm) in serT by TrmH_dim	1 b0971_tRNA_Mg2_TrmH_dim_cplx --> 1 TrmH_dim_inact + 1 ahcys + 1 b0971_tRNA_1 + 1 h	irreversible	tRNA modification	0.00E+00	1.00E+09	0	1.33E+03	1.33E+03	0	6.75E+00	6.75	0	6.75E+00	6.75
b0971_tRNA_20A_MODIF_1	modification of position 20A nt (D) in serT by Dus_gen	1 Dus_gen + 1 b0971_tRNA_2 + 1 h + 1 nadph <==> 1 b0971_tRNA_2_Dus_gen_cplx	reversible	tRNA modification	-1.00E+09	1.00E+09	0	1.33E+03	1.33E+03	0	6.75E+00	6.75	0	6.75E+00	6.75
b0971_tRNA_20A_MODIF_2	modification of position 20A nt (D) in serT by Dus_gen	1 b0971_tRNA_2_Dus_gen_cplx --> 1 Dus_gen_inact + 1 b0971_tRNA_3 + 1 nadp	irreversible	tRNA modification	0.00E+00	1.00E+09	0	1.33E+03	1.33E+03	0	6.75E+00	6.75	0	6.75E+00	6.75
b0971_tRNA_20_MODIF_1	modification of position 20 nt (D) in serT by Dus_gen	1 Dus_gen + 1 b0971_tRNA_1 + 1 h + 1 nadph <==> 1 b0971_tRNA_1_Dus_gen_cplx	reversible	tRNA modification	-1.00E+09	1.00E+09	0	1.33E+03	1.33E+03	0	6.75E+00	6.75	0	6.75E+00	6.75
b0971_tRNA_20_MODIF_2	modification of position 20 nt (D) in serT by Dus_gen	1 b0971_tRNA_1_Dus_gen_cplx --> 1 Dus_gen_inact + 1 b0971_tRNA_2 + 1 nadp	irreversible	tRNA modification	0.00E+00	1.00E+09	0	1.33E+03	1.33E+03	0	6.75E+00	6.75	0	6.75E+00	6.75
b0971_tRNA_32_MODIF_1	modification of position 32 nt (Cm) in serT by MeT_tRNA_pos_32_Cm	1 MeT_tRNA_pos_32_Cm + 1 amet + 1 b0971_tRNA_3 <==> 1 b0971_tRNA_3_MeT_tRNA_pos_32_Cm_cplx	reversible	tRNA modification	-1.00E+09	1.00E+09	0	1.33E+03	1.33E+03	0	6.75E+00	6.75	0	6.75E+00	6.75
b0971_tRNA_32_MODIF_2	modification of position 32 nt (Cm) in serT by MeT_tRNA_pos_32_Cm	1 b0971_tRNA_3_MeT_tRNA_pos_32_Cm_cplx --> 1 MeT_tRNA_pos_32_Cm_inact + 1 ahcys + 1 b0971_tRNA_4 + 1 h	irreversible	tRNA modification	0.00E+00	1.00E+09	0	1.33E+03	1.33E+03	0	6.75E+00	6.75	0	6.75E+00	6.75
b0971_tRNA_34_MODIF_1	modification of position 34 nt (cmo5U) in serT by YecO_mono-YecP_mono-HyL_tRNA_pos_34_ho5U	1 HyL_tRNA_pos_34_ho5U + 1 YecO_mono + 1 YecP_mono + 1 amet + 1 b0971_tRNA_4 + 2 chor <==> 1 b0971_tRNA_4_YecO_mono-YecP_mono-HyL_tRNA_pos_34_ho5U_cplx	reversible	tRNA modification	-1.00E+09	1.00E+09	0	1.33E+03	1.33E+03	0	6.75E+00	6.75	0	6.75E+00	6.75
b0971_tRNA_34_MODIF_2	modification of position 34 nt (cmo5U) in serT by YecO_mono-YecP_mono-HyL_tRNA_pos_34_ho5U	1 b0971_tRNA_4_YecO_mono-YecP_mono-HyL_tRNA_pos_34_ho5U_cplx --> 1 C10H8O5 + 1 C9H9O4 + 1 HyL_tRNA_pos_34_ho5U_inact + 1 YecO_mono_inact + 1 YecP_mono_inact + 1 ahcys + 1 b0971_tRNA_5 + 1 h	irreversible	tRNA modification	0.00E+00	1.00E+09	0	1.33E+03	1.33E+03	0	6.75E+00	6.75	0	6.75E+00	6.75
b0971_tRNA_37_MODIF_1	modification of position 37 nt (ms2i6A) in serT by MiaA_dim-MiaB_mono	1 IscS_dim_S-SH + 1 MiaA_dim + 1 MiaB_mono + 2 amet + 1 b0971_tRNA_5 + 1 dmpp + 1 fldrd <==> 1 b0971_tRNA_5_MiaA_dim MiaB_mono_cplx	reversible	tRNA modification	-1.00E+09	1.00E+09	0	1.33E+03	1.33E+03	0	6.75E+00	6.75	0	6.75E+00	6.75
b0971_tRNA_37_MODIF_2	modification of position 37 nt (ms2i6A) in serT by MiaA_dim-MiaB_mono	1 b0971_tRNA_5_MiaA_dim-MiaB_mono_cplx --> 1 IscS_dim_S-H + 1 MiaA_dim_inact + 1 MiaB_mono_inact + 1 ahcys + 1 b0971_tRNA_6 + 1 dad-5 + 1 fldox + 2 h + 1 met-L + 1 ppi	irreversible	tRNA modification	0.00E+00	1.00E+09	0	1.33E+03	1.33E+03	0	6.75E+00	6.75	0	6.75E+00	6.75
b0971_tRNA_54_MODIF_1	modification of position 54 nt (m5U) in serT by TrmA_mono	1 TrmA_mono + 1 amet + 1 b0971_tRNA_6 <==> 1 b0971_tRNA_6_TrmA_mono_cplx	reversible	tRNA modification	-1.00E+09	1.00E+09	0	1.33E+03	1.33E+03	0	6.75E+00	6.75	0	6.75E+00	6.75
b0971_tRNA_54_MODIF_2	modification of position 54 nt (m5U) in serT by TrmA_mono	1 b0971_tRNA_6_TrmA_mono_cplx --> 1 TrmA_mono_inact + 1 ahcys + 1 b0971_tRNA_7 + 1 h	irreversible	tRNA modification	0.00E+00	1.00E+09	0	1.33E+03	1.33E+03	0	6.75E+00	6.75	0	6.75E+00	6.75
b0971_tRNA_55_MODIF_1	modification of position 55 nt (Y) in serT by TruB_mono	1 TruB_mono + 1 b0971_tRNA_7 <==> 1 b0971_tRNA_7_TruB_mono_cplx	reversible	tRNA modification	-1.00E+09	1.00E+09	0	1.33E+03	1.33E+03	0	6.75E+00	6.75	0	6.75E+00	6.75
b0971_tRNA_55_MODIF_2	modification of position 55 nt (Y) in serT by TruB_mono	1 b0971_tRNA_7_TruB_mono_cplx --> 1 TruB_mono_inact + 1 b0971_tRNA_8	irreversible	tRNA modification	0.00E+00	1.00E+09	0	1.33E+03	1.33E+03	0	6.75E+00	6.75	0	6.75E+00	6.75
b0971_tRNA_8_MODIF_1	modification of position 8 nt (s4U) in serT by ThiI_mono	1 IscS_dim_S-SH + 1 ThiI_mono + 1 atp + 1 b0971_tRNA_8 + 1 mg2 + 1 trdrd <==> 1 b0971_tRNA_8_ThiI_mono_cplx	reversible	tRNA modification	-1.00E+09	1.00E+09	0	1.33E+03	1.33E+03	0	6.75E+00	6.75	0	6.75E+00	6.75
b0971_tRNA_8_MODIF_2	modification of position 8 nt (s4U) in serT by ThiI_mono	1 b0971_tRNA_8_ThiI_mono_cplx --> 1 IscS_dim_S-H + 1 ThiI_mono_inact + 1 amp + 1 h + 1 mg2 + 1 ppi + 1 serT_tRNA + 1 trdox	irreversible	tRNA modification	0.00E+00	1.00E+09	0	1.33E+03	1.33E+03	0	6.75E+00	6.75	0	6.75E+00	6.75

b0971_tRNA_Mg_BIND	Binding of two Mg2 to b0971_tRNA	1 b0971_RNA + 2 mg2 <==> 1 b0971_tRNA_Mg2	reversible	Metallo-ion binding	-1.00E+09	1.00E+09	0	1.33E+03	1.33E+03	0	6.75E+00	6.75	0	6.75E+00	6.75
b1032_tRNA_18_MODIF_1	modification of position 18 nt (Gm) in serX by TrmH_dim	1 TrmH_dim + 1 amet + 1 b1032_tRNA_Mg2 <==> 1 b1032_tRNA_Mg2_TrmH_dim_cplx	reversible	tRNA modification	-1.00E+09	1.00E+09	0	1.33E+03	1.33E+03	0	6.56E+00	6.56	0	6.56E+00	6.56
b1032_tRNA_18_MODIF_2	modification of position 18 nt (Gm) in serX by TrmH_dim	1 b1032_tRNA_Mg2_TrmH_dim_cplx --> 1 TrmH_dim_inact + 1 ahcys + 1 b1032_tRNA_1 + 1 h	irreversible	tRNA modification	0.00E+00	1.00E+09	0	1.33E+03	1.33E+03	0	6.56E+00	6.56	0	6.56E+00	6.56
b1032_tRNA_20A_MODIF_1	modification of position 20A nt (D) in serX by Dus_gen	1 Dus_gen + 1 b1032_tRNA_1 + 1 h + 1 nadph <==> 1 b1032_tRNA_1_Dus_gen_cplx	reversible	tRNA modification	-1.00E+09	1.00E+09	0	1.33E+03	1.33E+03	0	6.56E+00	6.56	0	6.56E+00	6.56
b1032_tRNA_20A_MODIF_2	modification of position 20A nt (D) in serX by Dus_gen	1 b1032_tRNA_1_Dus_gen_cplx --> 1 Dus_gen_inact + 1 b1032_tRNA_2 + 1 nadp	irreversible	tRNA modification	0.00E+00	1.00E+09	0	1.33E+03	1.33E+03	0	6.56E+00	6.56	0	6.56E+00	6.56
b1032_tRNA_40_MODIF_1	modification of position 40 nt (Y) in serX by TruA_dim	1 TruA_dim + 1 b1032_tRNA_2 <==> 1 b1032_tRNA_2_TruA_dim_cplx	reversible	tRNA modification	-1.00E+09	1.00E+09	0	1.33E+03	1.33E+03	0	6.56E+00	6.56	0	6.56E+00	6.56
b1032_tRNA_40_MODIF_2	modification of position 40 nt (Y) in serX by TruA_dim	1 b1032_tRNA_2_TruA_dim_cplx --> 1 TruA_dim_inact + 1 b1032_tRNA_3	irreversible	tRNA modification	0.00E+00	1.00E+09	0	1.33E+03	1.33E+03	0	6.56E+00	6.56	0	6.56E+00	6.56
b1032_tRNA_54_MODIF_1	modification of position 54 nt (m5U) in serX by TrmA_mono	1 TrmA_mono + 1 amet + 1 b1032_tRNA_3 <==> 1 b1032_tRNA_3_TrmA_mono_cplx	reversible	tRNA modification	-1.00E+09	1.00E+09	0	1.33E+03	1.33E+03	0	6.56E+00	6.56	0	6.56E+00	6.56
b1032_tRNA_54_MODIF_2	modification of position 54 nt (m5U) in serX by TrmA_mono	1 b1032_tRNA_3_TrmA_mono_cplx --> 1 TrmA_mono_inact + 1 ahcys + 1 b1032_tRNA_4 + 1 h	irreversible	tRNA modification	0.00E+00	1.00E+09	0	1.33E+03	1.33E+03	0	6.56E+00	6.56	0	6.56E+00	6.56
b1032_tRNA_55_MODIF_1	modification of position 55 nt (Y) in serX by TruB_mono	1 TruB_mono + 1 b1032_tRNA_4 <==> 1 b1032_tRNA_4_TruB_mono_cplx	reversible	tRNA modification	-1.00E+09	1.00E+09	0	1.33E+03	1.33E+03	0	6.56E+00	6.56	0	6.56E+00	6.56
b1032_tRNA_55_MODIF_2	modification of position 55 nt (Y) in serX by TruB_mono	1 b1032_tRNA_4_TruB_mono_cplx --> 1 TruB_mono_inact + 1 serX_tRNA	irreversible	tRNA modification	0.00E+00	1.00E+09	0	1.33E+03	1.33E+03	0	6.56E+00	6.56	0	6.56E+00	6.56
b1032_tRNA_Mg_BIND	Binding of two Mg2 to b1032_tRNA	1 b1032_RNA + 2 mg2 <==> 1 b1032_tRNA_Mg2	reversible	Metallo-ion binding	-1.00E+09	1.00E+09	0	1.33E+03	1.33E+03	0	6.56E+00	6.56	0	6.56E+00	6.56
b1066_fold_GroEL/ES_1	b1066_m folding: GroEL/ES mediated: polypeptide is going in GroEL/ES complex	1 GroEL.(7)ADP.cisGroES + 7 atp + 1 b1066_m + 1 transGroES_hepta --> 7 adp + 1 b1066_m_GroEL.(7)ATP.transGroES + 1 cisGroES_hepta	irreversible	Protein Folding	0.00E+00	1.00E+09	0	2.66E+03	2.66E+03	0	4.58E+03	4578.325	0	1.52E+02	151.921
b1066_fold_GroEL/ES_2	b1066_m folding: GroEL/ES mediated: folding of polypeptide under ATP hydrolysis	1 b1066_m_GroEL.(7)ATP.transGroES + 7 h2o --> 1 RimJ_GroEL.(7)ADP.transGroES + 7 h + 7 pi	irreversible	Protein Folding	0.00E+00	1.00E+09	0	2.66E+03	2.66E+03	0	4.58E+03	4578.325	0	1.52E+02	151.921
b1066_fold_GroEL/ES_3	b1066_m folding: GroEL/ES mediated: release of native protein	1 RimJ_GroEL.(7)ADP.transGroES --> 1 GroEL.(7)ADP.transGroES + 1 RimJ_mono	irreversible	Protein Folding	0.00E+00	1.00E+09	0	2.66E+03	2.66E+03	0	4.58E+03	4578.325	0	1.52E+02	151.921
b1066_mRNA_CONV	convsersion of mRNA to mRNA_1 (synthetic rxn)	1 b1066_mRNA --> 1 b1066_mRNA_1	irreversible	Translation	0.00E+00	1.00E+09	0	2.28E+02	2.28E+02	0	4.03E+02	402.7114	0	3.11E-01	0.311
b1066_mRNA_degr1	Degradation of b1066_mRNA I	1 Orn_dim + 146 atp + 1 b1066_mRNA_2 + 1 degradosome + 146 h2o <==> 146 adp + 1 b1066_mRNA_2_degr + 146 h + 146 pi	reversible	mRNA degradation	-1.00E+09	1.00E+09	0	2.28E+02	2.28E+02	0	4.03E+02	402.7114	0	3.11E-01	0.311
b1066_mRNA_degr2	Degradation of b1066_mRNA II	1 b1066_mRNA_2_degr + 584 h2o --> 1 Orn_dim_inact + 144 amp + 1 atp + 140 cmp + 1 degradosome_inact + 149 gmp + 584 h + 151 ump	irreversible	mRNA degradation	0.00E+00	3.11E-01	0	2.28E+02	2.28E+02	0	4.03E+02	402.7114	0	3.11E-01	0.311
b1066_maturation1	Polypeptide b1066 peptide deformylas and methionine aminopeptidase complex	1 Def_mono + 1 Map_mono + 1 b1066_aa <==> 1 b1066_def_map_cplx	reversible	Protein Maturation	-1.00E+09	1.00E+09	0	2.66E+03	2.66E+03	0	4.58E+03	4578.325	0	1.52E+02	151.921
b1066_maturation2	b1066 formation	1 b1066_def_map_cplx + 2 h2o --> 1 Def_mono_inact + 1 Map_mono_inact + 1 b1066_m + 1 for + 1 met-L	irreversible	Protein Maturation	0.00E+00	1.00E+09	0	2.66E+03	2.66E+03	0	4.58E+03	4578.325	0	1.52E+02	151.921
b1084_fold_spon	b1084_m folding: spontanous	1 b1084_m --> 1 Rne_mono	irreversible	Protein Folding	0.00E+00	1.00E+09	0	1.40E+03	1.40E+03	0	1.52E+03	1516.486	0	1.15E+02	115.258
b1084_mRNA_CONV	convsersion of mRNA to mRNA_1 (synthetic rxn)	1 b1084_mRNA --> 1 b1084_mRNA_1	irreversible	Translation	0.00E+00	1.00E+09	0	3.60E+01	3.60E+01	0	6.36E+01	63.56211	0	1.89E-01	0.189
b1084_mRNA_degr1	Degradation of b1084_mRNA I	1 Orn_dim + 796 atp + 1 b1084_mRNA_2 + 1 degradosome + 796 h2o <==> 796 adp + 1 b1084_mRNA_2_degr + 796 h + 796 pi	reversible	mRNA degradation	-1.00E+09	1.00E+09	0	3.60E+01	3.60E+01	0	6.36E+01	63.56211	0	1.89E-01	0.189
b1084_mRNA_degr2	Degradation of b1084_mRNA II	1 b1084_mRNA_2_degr + 3185 h2o --> 1 Orn_dim_inact + 797 amp 1 atp + 887 cmp + 1 degradosome_inact + 886 gmp + 3185 h + 615 ump	irreversible	mRNA degradation	0.00E+00	1.89E-01	0	3.60E+01	3.60E+01	0	6.36E+01	63.56211	0	1.89E-01	0.189
b1084_maturation1	Polypeptide b1084 peptide deformylas complex	1 Def_mono + 1 b1084_aa <==> 1 b1084_def_cplx	reversible	Protein Maturation	-1.00E+09	1.00E+09	0	1.40E+03	1.40E+03	0	1.52E+03	1516.486	0	1.15E+02	115.258
b1084_maturation2	b1084 formation	1 b1084_def_cplx + 1 h2o --> 1 Def_mono_inact + 1 b1084_m + 1 for	irreversible	Protein Maturation	0.00E+00	1.00E+09	0	1.40E+03	1.40E+03	0	1.52E+03	1516.486	0	1.15E+02	115.258
b1086_fold_GroEL/ES_1	b1086_m folding: GroEL/ES mediated: polypeptide is going in GroEL/ES complex	1 GroEL.(7)ADP.cisGroES + 7 atp + 1 b1086_m + 1 transGroES_hepta --> 7 adp + 1 b1086_m_GroEL.(7)ATP.transGroES + 1 cisGroES_hepta	irreversible	Protein Folding	0.00E+00	1.00E+09	0	2.79E+03	2.79E+03	0	4.87E+03	4865.967	0	8.27E+01	82.746
b1086_fold_GroEL/ES_2	b1086_m folding: GroEL/ES mediated: folding of polypeptide under ATP hydrolysis	1 b1086_m_GroEL.(7)ATP.transGroES + 7 h2o --> 1 RluC_GroEL.(7)ADP.transGroES + 7 h + 7 pi	irreversible	Protein Folding	0.00E+00	1.00E+09	0	2.79E+03	2.79E+03	0	4.87E+03	4865.967	0	8.27E+01	82.746
b1086_fold_GroEL/ES_3	b1086_m folding: GroEL/ES mediated: release of native protein	1 RluC_GroEL.(7)ADP.transGroES --> 1 GroEL.(7)ADP.transGroES + 1 RluC_mono	irreversible	Protein Folding	0.00E+00	1.00E+09	0	2.79E+03	2.79E+03	0	4.87E+03	4865.967	0	8.27E+01	82.746
b1086_mRNA_CONV	convsersion of mRNA to mRNA_1 (synthetic rxn)	1 b1086_mRNA --> 1 b1086_mRNA_1	irreversible	Translation	0.00E+00	1.00E+09	0	1.51E+02	1.51E+02	0	2.66E+02	265.9415	0	2.07E-01	0.207
b1086_mRNA_degr1	Degradation of b1086_mRNA I	1 Orn_dim + 240 atp + 1 b1086_mRNA_2 + 1 degradosome + 240 h2o <==> 240 adp + 1 b1086_mRNA_2_degr + 240 h + 240 pi	reversible	mRNA degradation	-1.00E+09	1.00E+09	0	1.51E+02	1.51E+02	0	2.66E+02	265.9415	0	2.07E-01	0.207
b1086_mRNA_degr2	Degradation of b1086_mRNA II	1 b1086_mRNA_2_degr + 959 h2o --> 1 Orn_dim_inact + 241 amp + 1 atp + 212 cmp + 1 degradosome_inact + 282 gmp + 959 h + 224 ump	irreversible	mRNA degradation	0.00E+00	2.07E-01	0	1.51E+02	1.51E+02	0	2.66E+02	265.9415	0	2.07E-01	0.207
b1086_maturation1	Polypeptide b1086 peptide deformylas and methionine aminopeptidase complex	1 Def_mono + 1 Map_mono + 1 b1086_aa <==> 1 b1086_def_map_cplx	reversible	Protein Maturation	-1.00E+09	1.00E+09	0	2.79E+03	2.79E+03	0	4.87E+03	4865.967	0	8.27E+01	82.746
b1086_maturation2	b1086 formation	1 b1086_def_map_cplx + 2 h2o --> 1 Def_mono_inact + 1 Map_mono_inact + 1 b1086_m + 1 for + 1 met-L	irreversible	Protein Maturation	0.00E+00	1.00E+09	0	2.79E+03	2.79E+03	0	4.87E+03	4865.967	0	8.27E+01	82.746
b1088_fold_spon	b1088_m folding: spontanous	1 b1088_m --> 1 YceD_mono	irreversible	Protein Folding	0.00E+00	1.00E+09	0	1.30E+03	1.30E+03	0	1.16E+03	1162.8	0	3.39E+02	338.55
b1088_mRNA_CONV	convsersion of mRNA to mRNA_1 (synthetic rxn)	1 b1088_mRNA --> 1 b1088_mRNA_1	irreversible	Translation	0.00E+00	1.00E+09	0	9.68E+01	9.68E+01	0	8.29E+01	82.88	0	4.55E-01	0.455
b1088_mRNA_degr1	Degradation of b1088_mRNA I	1 Orn_dim + 130 atp + 1 b1088_mRNA_2 + 1 degradosome + 130 h2o <==> 130 adp + 1 b1088_mRNA_2_degr + 130 h + 130 pi	reversible	mRNA degradation	-1.00E+09	1.00E+09	0	9.68E+01	9.68E+01	0	8.29E+01	82.88	0	4.55E-01	0.455
b1088_mRNA_degr2	Degradation of b1088_mRNA II	1 b1088_mRNA_2_degr + 521 h2o --> 1 Orn_dim_inact + 133 amp + 1 atp + 121 cmp + 1 degradosome_inact + 138 gmp + 521 h + 129 ump	irreversible	mRNA degradation	0.00E+00	4.55E-01	0	9.68E+01	9.68E+01	0	8.29E+01	82.88	0	4.55E-01	0.455
b1088_maturation1	Polypeptide b1088 peptide deformylas and methionine aminopeptidase complex	1 Def_mono + 1 Map_mono + 1 b1088_aa <==> 1 b1088_def_map_cplx	reversible	Protein Maturation	-1.00E+09	1.00E+09	0	1.30E+03	1.30E+03	0	1.16E+03	1162.8	0	3.39E+02	338.55
b1088_maturation2	b1088 formation	1 b1088_def_map_cplx + 2 h2o --> 1 Def_mono_inact + 1 Map_mono_inact + 1 b1088_m + 1 for + 1 met-L	irreversible	Protein Maturation	0.00E+00	1.00E+09	0	1.30E+03	1.30E+03	0	1.16E+03	1162.8	0	3.39E+02	338.55
b1088_v1_mRNA_CONV	convsersion of mRNA to mRNA_1 (synthetic rxn)	1 b1088_v1_mRNA --> 1 b1088_mRNA_1	irreversible	Translation	0.00E+00	1.00E+09	0	9.68E+01	9.68E+01	0	8.29E+01	82.88	0	4.55E-01	0.455
b1089_fold_spon	b1089_m folding: spontanous	1 b1089_m --> 1 RpmF_mono	irreversible	Protein Folding	0.00E+00	1.00E+09	8.44E+01	1.13E+02	2.81E+01	6.22E+01	8.29E+01	20.72	6.22E+01	8.29E+01	20.72
b1089_mRNA_CONV	convsersion of mRNA to mRNA_1 (synthetic rxn)	1 b1089_mRNA --> 1 b1089_mRNA_1	irreversible	Translation	0.00E+00	1.00E+09	0	9.68E+01	9.68E+01	0	8.29E+01	82.88	0	4.55E-01	0.455
b1089_mRNA_degr1	Degradation of b1089_mRNA I	1 Orn_dim + 43 atp + 1 b1089_mRNA_2 + 1 degradosome + 43 h2o <==> 43 adp + 1 b1089_mRNA_2_degr + 43 h + 43 pi	reversible	mRNA degradation	-1.00E+09	1.00E+09	0	9.68E+01	9.68E+01	0	8.29E+01	82.88	0	6.62E-01	0.662
b1089_mRNA_degr2	Degradation of b1089_mRNA II	1 b1089_mRNA_2_degr + 173 h2o --> 1 Orn_dim_inact + 48 amp + 56 cmp + 1 degradosome_inact + 38 gmp + 173 h + 32 ump	irreversible	mRNA degradation	0.00E+00	8.82E-01	0	9.68E+01	9.68E+01	0	8.29E+01	82.88	0	6.62E-01	0.662
b1089_maturation1	Polypeptide b1089 peptide deformylas and methionine aminopeptidase complex	1 Def_mono + 1 Map_mono + 1 b1089_aa <==> 1 b1089_def_map_cplx	reversible	Protein Maturation	-1.00E+09	1.00E+09	8.44E+01	1.13E+02	2.81E+01	6.22E+01	8.29E+01	20.72	6.22E+01	8.29E+01	20.72
b1089_maturation2	b1089 formation	1 b1089_def_map_cplx + 2 h2o --> 1 Def_mono_inact + 1 Map_mono_inact + 1 b1089_m + 1 for + 1 met-L	irreversible	Protein Maturation	0.00E+00	1.00E+09	8.44E+01	1.13E+02	2.81E+01	6.22E+01	8.29E+01	20.72	6.22E+01	8.29E+01	20.72

b1089_v1_mRNA_CONV	convsersion of mRNA to mRNA_1 (synthetic rxn)	1 b1089_v1_mRNA --> 1 b1089_mRNA_1	irreversible	Translation	0.00E+00	1.00E+09	0	9.68E+01	9.68E+01	0	8.29E+01	82.88	0	4.55E-01	0.455
b1089_v2_mRNA_CONV	convsersion of mRNA to mRNA_1 (synthetic rxn)	1 b1089_v2_mRNA --> 1 b1089_mRNA_1	irreversible	Translation	0.00E+00	1.00E+09	0	3.27E+01	3.27E+01	0	5.77E+01	57.70685	0	2.07E-01	0.207
b1090_fold_spon	b1090_m folding: spontanous	1 b1090_m --> 1 PlsX_mono	irreversible	Protein Folding	0.00E+00	1.00E+09	0	7.53E+02	7.53E+02	0	1.25E+03	1253.737	0	1.04E+02	103.74
b1090_mRNA_degr1	Degradation of b1090_mRNA I	1 Orn_dim + 267 atp + 1 b1090_mRNA_2 + 1 degradosome + 267 h2o <==> 267 adp + 1 b1090_mRNA_2_degr + 267 h + 267 pi	reversible	mRNA degradation	-1.00E+09	1.00E+09	0	3.27E+01	3.27E+01	0	5.77E+01	57.70685	0	2.07E-01	0.207
b1090_mRNA_degr2	Degradation of b1090_mRNA II	1 b1090_mRNA_2_degr + 1070 h2o --> 1 Orn_dim_inact + 250 amp 249 cmp + 1 degradosome_inact + 309 gmp + 1070 h + 262 ump + 1 utp	irreversible	mRNA degradation	0.00E+00	2.07E-01	0	3.27E+01	3.27E+01	0	5.77E+01	57.70685	0	2.07E-01	0.207
b1090_maturation1	Polypeptide b1090 peptide deformylas and methionine aminopeptidase complex	1 Def_mono + 1 Map_mono + 1 b1090_aa <==> 1 b1090_def_map_cplx	reversible	Protein Maturation	-1.00E+09	1.00E+09	0	7.53E+02	7.53E+02	0	1.25E+03	1253.737	0	1.04E+02	103.74
b1090_maturation2	b1090 formation	1 b1090_def_map_cplx + 2 h2o --> 1 Def_mono_inact + 1 Map_mono_inact + 1 b1090_m + 1 for + 1 met-L	irreversible	Protein Maturation	0.00E+00	1.00E+09	0	7.53E+02	7.53E+02	0	1.25E+03	1253.737	0	1.04E+02	103.74
b1090_v1_mRNA_CONV	convsersion of mRNA to mRNA_1 (synthetic rxn)	1 b1090_v1_mRNA --> 1 b1090_mRNA_1	irreversible	Translation	0.00E+00	1.00E+09	0	3.27E+01	3.27E+01	0	5.77E+01	57.70685	0	2.07E-01	0.207
b1091_fold_spon	b1091_m folding: spontanous	1 b1091_m --> 1 FabH_mono	irreversible	Protein Folding	0.00E+00	1.00E+09	0	7.05E+02	7.05E+02	0	1.16E+03	1155.183	0	1.20E+02	120.186
b1091_mRNA_degr1	Degradation of b1091_mRNA I	1 Orn_dim + 238 atp + 1 b1091_mRNA_2 + 1 degradosome + 238 h2o <==> 238 adp + 1 b1091_mRNA_2_degr + 238 h + 238 pi	reversible	mRNA degradation	-1.00E+09	1.00E+09	0	3.27E+01	3.27E+01	0	5.77E+01	57.70685	0	2.07E-01	0.207
b1091_mRNA_degr2	Degradation of b1091_mRNA II	1 b1091_mRNA_2_degr + 953 h2o --> 1 Orn_dim_inact + 205 amp + 1 atp + 240 cmp + 1 degradosome_inact + 282 gmp + 953 h + 226 ump	irreversible	mRNA degradation	0.00E+00	4.49E-01	0	3.27E+01	3.27E+01	0	5.77E+01	57.70685	0	2.07E-01	0.207
b1091_maturation1	Polypeptide b1091 peptide deformylas complex	1 Def_mono + 1 b1091_aa <==> 1 b1091_def_cplx	reversible	Protein Maturation	-1.00E+09	1.00E+09	0	7.05E+02	7.05E+02	0	1.16E+03	1155.183	0	1.20E+02	120.186
b1091_maturation2	b1091 formation	1 b1091_def_cplx + 1 h2o --> 1 Def_mono_inact + 1 b1091_m + 1 for	irreversible	Protein Maturation	0.00E+00	1.00E+09	0	7.05E+02	7.05E+02	0	1.16E+03	1155.183	0	1.20E+02	120.186
b1091_v2_mRNA_CONV	convsersion of mRNA to mRNA_1 (synthetic rxn)	1 b1091_v2_mRNA --> 1 b1091_mRNA_1	irreversible	Translation	0.00E+00	1.00E+09	0	3.27E+01	3.27E+01	0	5.77E+01	57.70685	0	2.07E-01	0.207
b1092_fold_spon	b1092_m folding: spontanous	1 b1092_m --> 1 FabD_mono	irreversible	Protein Folding	0.00E+00	1.00E+09	0	7.79E+02	7.79E+02	0	1.23E+03	1229.523	0	1.95E+02	194.526
b1092_mRNA_degr1	Degradation of b1092_mRNA I	1 Orn_dim + 232 atp + 1 b1092_mRNA_2 + 1 degradosome + 232 h2o <==> 232 adp + 1 b1092_mRNA_2_degr + 232 h + 232 pi	reversible	mRNA degradation	-1.00E+09	1.00E+09	0	3.27E+01	3.27E+01	0	5.77E+01	57.70685	0	2.07E-01	0.207
b1092_mRNA_degr2	Degradation of b1092_mRNA II	1 b1092_mRNA_2_degr + 929 h2o --> 1 Orn_dim_inact + 211 amp + 1 atp + 228 cmp + 1 degradosome_inact + 285 gmp + 929 h + 205 ump	irreversible	mRNA degradation	0.00E+00	7.43E-01	0	3.27E+01	3.27E+01	0	5.77E+01	57.70685	0	2.07E-01	0.207
b1092_maturation1	Polypeptide b1092 peptide deformylas and methionine aminopeptidase complex	1 Def_mono + 1 Map_mono + 1 b1092_aa <==> 1 b1092_def_map_cplx	reversible	Protein Maturation	-1.00E+09	1.00E+09	0	7.79E+02	7.79E+02	0	1.23E+03	1229.523	0	1.95E+02	194.526
b1092_maturation2	b1092 formation	1 b1092_def_map_cplx + 2 h2o --> 1 Def_mono_inact + 1 Map_mono_inact + 1 b1092_m + 1 for + 1 met-L	irreversible	Protein Maturation	0.00E+00	1.00E+09	0	7.79E+02	7.79E+02	0	1.23E+03	1229.523	0	1.95E+02	194.526
b1092_v3_mRNA_CONV	convsersion of mRNA to mRNA_1 (synthetic rxn)	1 b1092_v3_mRNA --> 1 b1092_mRNA_1	irreversible	Translation	0.00E+00	1.00E+09	0	3.27E+01	3.27E+01	0	5.77E+01	57.70685	0	2.07E-01	0.207
b1093_fold_GroEL/ES_1	b1093_m folding: GroEL/ES mediated: polypeptide is going in GroEL/ES complex	1 GroEL.(7)ADP.cisGroES + 7 atp + 1 b1093_m + 1 transGroES_hepta --> 7 adp + 1 b1093_m_GroEL.(7)ATP.transGroES + 1 cisGroES_hepta	irreversible	Protein Folding	0.00E+00	1.00E+09	0	6.66E+02	6.66E+02	0	1.02E+03	1016.496	0	2.11E+02	211.498
b1093_fold_GroEL/ES_2	b1093_m folding: GroEL/ES mediated: folding of polypeptide under ATP hydrolysis	1 b1093_m_GroEL.(7)ATP.transGroES + 7 h2o --> 1 FabG_GroEL.(7)ADP.transGroES + 7 h + 7 pi	irreversible	Protein Folding	0.00E+00	1.00E+09	0	6.66E+02	6.66E+02	0	1.02E+03	1016.496	0	2.11E+02	211.498
b1093_fold_GroEL/ES_3	b1093_m folding: GroEL/ES mediated: release of native protein	1 FabG_GroEL.(7)ADP.transGroES --> 1 FabG_mono + 1 GroEL.(7)ADP.transGroES	irreversible	Protein Folding	0.00E+00	1.00E+09	0	6.66E+02	6.66E+02	0	1.02E+03	1016.496	0	2.11E+02	211.498
b1093_fold_KJE_1	b1093_m folding: KJE mediated	1 DnaJ_dim + 1 DnaK_mono.ATP + 1 b1093_m <==> 1 b1093_m_DnaKJ_complex	reversible	Protein Folding	-1.00E+09	1.00E+09	0	6.66E+02	6.66E+02	0	1.02E+03	1016.496	0	2.11E+02	211.498
b1093_fold_KJE_2	b1093_m folding: KJE mediated	1 GrpE_dim + 1 b1093_m_DnaKJ_complex + 1 h2o --> 1 DnaJ_dim_inact + 1 FabG_DnaK_GrpE_complex + 1 adp + 1 h + 1 pi	irreversible	Protein Folding	0.00E+00	1.00E+09	0	6.66E+02	6.66E+02	0	1.02E+03	1016.496	0	2.11E+02	211.498
b1093_fold_KJE_3	b1093_m folding: KJE mediated	1 FabG_DnaK_GrpE_complex + 1 atp --> 1 DnaK_mono.ATP_inact + 1 FabG_mono + 1 GrpE_dim_inact	irreversible	Protein Folding	0.00E+00	1.00E+09	0	6.66E+02	6.66E+02	0	1.02E+03	1016.496	0	2.11E+02	211.498
b1093_mRNA_degr1	Degradation of b1093_mRNA I	1 Orn_dim + 183 atp + 1 b1093_mRNA_2 + 1 degradosome + 183 h2o <==> 183 adp + 1 b1093_mRNA_2_degr + 183 h + 183 pi	reversible	mRNA degradation	-1.00E+09	1.00E+09	0	3.27E+01	3.27E+01	0	5.77E+01	57.70685	0	2.07E-01	0.207
b1093_mRNA_degr2	Degradation of b1093_mRNA II	1 b1093_mRNA_2_degr + 734 h2o --> 1 Orn_dim_inact + 181 amp + 1 atp + 165 cmp + 1 degradosome_inact + 214 gmp + 734 h + 174 ump	irreversible	mRNA degradation	0.00E+00	3.78E-01	0	3.27E+01	3.27E+01	0	5.77E+01	57.70685	0	2.07E-01	0.207
b1093_maturation1	Polypeptide b1093 peptide deformylas and methionine aminopeptidase complex	1 Def_mono + 1 Map_mono + 1 b1093_aa <==> 1 b1093_def_map_cplx	reversible	Protein Maturation	-1.00E+09	1.00E+09	0	6.66E+02	6.66E+02	0	1.02E+03	1016.496	0	2.11E+02	211.498
b1093_maturation2	b1093 formation	1 b1093_def_map_cplx + 2 h2o --> 1 Def_mono_inact + 1 Map_mono_inact + 1 b1093_m + 1 for + 1 met-L	irreversible	Protein Maturation	0.00E+00	1.00E+09	0	6.66E+02	6.66E+02	0	1.02E+03	1016.496	0	2.11E+02	211.498
b1093_v4_mRNA_CONV	convsersion of mRNA to mRNA_1 (synthetic rxn)	1 b1093_v4_mRNA --> 1 b1093_mRNA_1	irreversible	Translation	0.00E+00	1.00E+09	0	3.27E+01	3.27E+01	0	5.77E+01	57.70685	0	2.07E-01	0.207
b1114_fold_KJE_1	b1114_m folding: KJE mediated	1 DnaJ_dim + 1 DnaK_mono.ATP + 1 b1114_m_Mg <==> 1 b1114_m_DnaKJ_complex	reversible	Protein Folding	-1.00E+09	1.00E+09	0	1.95E+03	1.95E+03	0	2.12E+03	2119.82	0	7.92E+01	79.194
b1114_fold_KJE_2	b1114_m folding: KJE mediated	1 GrpE_dim + 1 b1114_m_DnaKJ_complex + 1 h2o --> 1 DnaJ_dim_inact + 1 Mfd_DnaK_GrpE_complex + 1 adp + 1 h + 1 pi	irreversible	Protein Folding	0.00E+00	1.00E+09	0	1.95E+03	1.95E+03	0	2.12E+03	2119.82	0	7.92E+01	79.194
b1114_fold_KJE_3	b1114_m folding: KJE mediated	1 Mfd_DnaK_GrpE_complex + 1 atp --> 1 DnaK_mono.ATP_inact + 1 GrpE_dim_inact + 1 Mfd_mono	irreversible	Protein Folding	0.00E+00	1.00E+09	0	1.95E+03	1.95E+03	0	2.12E+03	2119.82	0	7.92E+01	79.194
b1114_fold_spon	b1114_m folding: spontanous	1 b1114_m_Mg --> 1 Mfd_mono	irreversible	Protein Folding	0.00E+00	1.00E+09	0	1.95E+03	1.95E+03	0	2.12E+03	2119.82	0	7.92E+01	79.194
b1114_ion_BIND	Binding of ions to b1114	1 b1114_m + 1 mg2 <==> 1 b1114_m_Mg	reversible	Metallo-ion Binding	-1.00E+09	1.00E+09	0	1.95E+03	1.95E+03	0	2.12E+03	2119.82	0	7.92E+01	79.194
b1114_mRNA_CONV	convsersion of mRNA to mRNA_1 (synthetic rxn)	1 b1114_mRNA --> 1 b1114_mRNA_1	irreversible	Translation	0.00E+00	1.00E+09	0	3.53E+01	3.53E+01	0	6.23E+01	62.29789	0	2.07E-01	0.207
b1114_mRNA_degr1	Degradation of b1114_mRNA I	1 Orn_dim + 861 atp + 1 b1114_mRNA_2 + 1 degradosome + 861 h2o <==> 861 adp + 1 b1114_mRNA_2_degr + 861 h + 861 pi	reversible	mRNA degradation	-1.00E+09	1.00E+09	0	3.53E+01	3.53E+01	0	6.23E+01	62.29789	0	2.07E-01	0.207
b1114_mRNA_degr2	Degradation of b1114_mRNA II	1 b1114_mRNA_2_degr + 3446 h2o --> 1 Orn_dim_inact + 806 amp 1 atp + 905 cmp + 1 degradosome_inact + 983 gmp + 3446 h + 752 ump	irreversible	mRNA degradation	0.00E+00	2.07E-01	0	3.53E+01	3.53E+01	0	6.23E+01	62.29789	0	2.07E-01	0.207
b1114_maturation1	Polypeptide b1114 peptide deformylas and methionine aminopeptidase complex	1 Def_mono + 1 Map_mono + 1 b1114_aa <==> 1 b1114_def_map_cplx	reversible	Protein Maturation	-1.00E+09	1.00E+09	0	1.95E+03	1.95E+03	0	2.12E+03	2119.82	0	7.92E+01	79.194
b1114_maturation2	b1114 formation	1 b1114_def_map_cplx + 2 h2o --> 1 Def_mono_inact + 1 Map_mono_inact + 1 b1114_m + 1 for + 1 met-L	irreversible	Protein Maturation	0.00E+00	1.00E+09	0	1.95E+03	1.95E+03	0	2.12E+03	2119.82	0	7.92E+01	79.194
b1114_v1_mRNA_CONV	convsersion of mRNA to mRNA_1 (synthetic rxn)	1 b1114_v1_mRNA --> 1 b1114_mRNA_1	irreversible	Translation	0.00E+00	1.00E+09	0	3.53E+01	3.53E+01	0	6.23E+01	62.29789	0	2.07E-01	0.207
b1114_v2_mRNA_CONV	convsersion of mRNA to mRNA_1 (synthetic rxn)	1 b1114_v2_mRNA --> 1 b1114_mRNA_1	irreversible	Translation	0.00E+00	1.00E+09	0	3.53E+01	3.53E+01	0	6.23E+01	62.29789	0	2.07E-01	0.207
b1133_DNA_act_bind	b1133_DNA binding of activator	1 b1133_DNA_neu --> 1 b1133_DNA_act	irreversible	Transcription Regulation	0.00E+00	1.00E+09	0	1.13E+02	1.13E+02	0	1.99E+02	199.2212	0	2.07E-01	0.207
b1133_fold_spon	b1133_m folding: spontanous	1 b1133_m --> 1 TrmU_mono	irreversible	Protein Folding	0.00E+00	1.00E+09	0	2.47E+03	2.47E+03	0	4.28E+03	4284.865	0	1.06E+02	105.567
b1133_mRNA_CONV	convsersion of mRNA to mRNA_1 (synthetic rxn)	1 b1133_mRNA --> 1 b1133_mRNA_1	irreversible	Translation	0.00E+00	1.00E+09	0	1.13E+02	1.13E+02	0	1.99E+02	199.2212	0	2.07E-01	0.207
b1133_mRNA_degr1	Degradation of b1133_mRNA I	1 Orn_dim + 276 atp + 1 b1133_mRNA_2 + 1 degradosome + 276 h2o <==> 276 adp + 1 b1133_mRNA_2_degr + 276 h + 276 pi	reversible	mRNA degradation	-1.00E+09	1.00E+09	0	1.13E+02	1.13E+02	0	1.99E+02	199.2212	0	2.07E-01	0.207

b1133_mRNA_degr2	Degradation of b1133_mRNA II	1 b1133_mRNA_2_degr + 1106 h2o --> 1 Orn_dim_inact + 261 amp + 1 atp + 283 cmp + 1 degradosome_inact + 312 gmp + 1106 h + 250 ump	irreversible	mRNA degradation	0.00E+00	2.07E-01	0	1.13E+02	1.13E+02	0	1.99E+02	199.2212	0	2.07E-01	0.207
b1133_maturation1	Polypeptide b1133 peptide deformylas and methionine aminopeptidase complex	1 Def_mono + 1 Map_mono + 1 b1133_aa <==> 1 b1133_def_map_cplx	reversible	Protein Maturation	-1.00E+09	1.00E+09	0	2.47E+03	2.47E+03	0	4.28E+03	4284.865	0	1.06E+02	105.567
b1133_maturation2	b1133 formation	1 b1133_def_map_cplx + 2 h2o --> 1 Def_mono_inact + 1 Map_mono_inact + 1 b1133_m + 1 for + 1 met-L	irreversible	Protein Maturation	0.00E+00	1.00E+09	0	2.47E+03	2.47E+03	0	4.28E+03	4284.865	0	1.06E+02	105.567
b1134_fold_spon	b1134_m folding: spontanous	1 b1134_m --> 1 YmfB_mono	irreversible	Protein Folding	0.00E+00	1.00E+09	0	1.11E+03	1.11E+03	0	1.89E+03	1892.949	0	9.48E+01	94.752
b1134_mRNA_CONV	convsersion of mRNA to mRNA_1 (synthetic rxn)	1 b1134_mRNA --> 1 b1134_mRNA_1	irreversible	Translation	0.00E+00	1.00E+09	0	1.13E+02	1.13E+02	0	2.00E+02	199.9276	0	1.28E-01	0.128
b1134_mRNA_degr1	Degradation of b1134_mRNA I	1 Orn_dim + 115 atp + 1 b1134_mRNA_2 + 1 degradosome + 115 h2o <==> 115 adp + 1 b1134_mRNA_2_degr + 115 h + 115 pi	reversible	mRNA degradation	-1.00E+09	1.00E+09	0	1.13E+02	1.13E+02	0	2.00E+02	199.9276	0	1.28E-01	0.128
b1134_mRNA_degr2	Degradation of b1134_mRNA II	1 b1134_mRNA_2_degr + 461 h2o --> 1 Orn_dim_inact + 113 amp + 109 cmp + 1 degradosome_inact + 123 gmp + 461 h + 117 ump	irreversible	mRNA degradation	0.00E+00	2.07E-01	0	1.13E+02	1.13E+02	0	2.00E+02	199.9276	0	1.28E-01	0.128
b1134_maturation1	Polypeptide b1134 peptide deformylas and methionine aminopeptidase complex	1 Def_mono + 1 Map_mono + 1 b1134_aa <==> 1 b1134_def_map_cplx	reversible	Protein Maturation	-1.00E+09	1.00E+09	0	1.11E+03	1.11E+03	0	1.89E+03	1892.949	0	9.48E+01	94.752
b1134_maturation2	b1134 formation	1 b1134_def_map_cplx + 2 h2o --> 1 Def_mono_inact + 1 Map_mono_inact + 1 b1134_m + 1 for + 1 met-L	irreversible	Protein Maturation	0.00E+00	1.00E+09	0	1.11E+03	1.11E+03	0	1.89E+03	1892.949	0	9.48E+01	94.752
b1135_fold_spon	b1135_m folding: spontanous	1 b1135_m --> 1 YmfC_mono	irreversible	Protein Folding	0.00E+00	1.00E+09	0	1.44E+03	1.44E+03	0	2.48E+03	2481.692	0	8.41E+01	84.096
b1135_mRNA_CONV	convsersion of mRNA to mRNA_1 (synthetic rxn)	1 b1135_mRNA --> 1 b1135_mRNA_1	irreversible	Translation	0.00E+00	1.00E+09	0	1.13E+02	1.13E+02	0	2.00E+02	199.9276	0	1.28E-01	0.128
b1135_mRNA_degr1	Degradation of b1135_mRNA I	1 Orn_dim + 163 atp + 1 b1135_mRNA_2 + 1 degradosome + 163 h2o <==> 163 adp + 1 b1135_mRNA_2_degr + 163 h + 163 pi	reversible	mRNA degradation	-1.00E+09	1.00E+09	0	1.13E+02	1.13E+02	0	2.00E+02	199.9276	0	1.28E-01	0.128
b1135_mRNA_degr2	Degradation of b1135_mRNA II	1 b1135_mRNA_2_degr + 653 h2o --> 1 Orn_dim_inact + 172 amp + 1 atp + 172 cmp + 1 degradosome_inact + 163 gmp + 653 h + 146 ump	irreversible	mRNA degradation	0.00E+00	1.28E-01	0	1.13E+02	1.13E+02	0	2.00E+02	199.9276	0	1.28E-01	0.128
b1135_maturation1	Polypeptide b1135 peptide deformylas and methionine aminopeptidase complex	1 Def_mono + 1 Map_mono + 1 b1135_aa <==> 1 b1135_def_map_cplx	reversible	Protein Maturation	-1.00E+09	1.00E+09	0	1.44E+03	1.44E+03	0	2.48E+03	2481.692	0	8.41E+01	84.096
b1135_maturation2	b1135 formation	1 b1135_def_map_cplx + 2 h2o --> 1 Def_mono_inact + 1 Map_mono_inact + 1 b1135_m + 1 for + 1 met-L	irreversible	Protein Maturation	0.00E+00	1.00E+09	0	1.44E+03	1.44E+03	0	2.48E+03	2481.692	0	8.41E+01	84.096
b1211_DNA_act_bind	b1211_DNA binding of activator	1 b1211_DNA_neu --> 1 b1211_DNA_act	irreversible	Transcription Regulation	0.00E+00	1.00E+09	0	1.16E+02	1.16E+02	0	2.04E+02	204.2739	0	3.08E-01	0.308
b1211_fold_spon	b1211_m folding: spontanous	1 b1211_m --> 1 PrfA_mono	irreversible	Protein Folding	0.00E+00	1.00E+09	0	2.52E+03	2.52E+03	0	4.38E+03	4382.152	0	9.89E+01	98.868
b1211_mRNA_CONV	convsersion of mRNA to mRNA_1 (synthetic rxn)	1 b1211_mRNA --> 1 b1211_mRNA_1	irreversible	Translation	0.00E+00	1.00E+09	0	1.16E+02	1.16E+02	0	2.04E+02	204.2739	0	3.08E-01	0.308
b1211_mRNA_degr1	Degradation of b1211_mRNA I	1 Orn_dim + 270 atp + 1 b1211_mRNA_2 + 1 degradosome + 270 h2o <==> 270 adp + 1 b1211_mRNA_2_degr + 270 h + 270 pi	reversible	mRNA degradation	-1.00E+09	1.00E+09	0	1.16E+02	1.16E+02	0	2.04E+02	204.2739	0	3.08E-01	0.308
b1211_mRNA_degr2	Degradation of b1211_mRNA II	1 b1211_mRNA_2_degr + 1082 h2o --> 1 Orn_dim_inact + 275 amp + 1 atp + 276 cmp + 1 degradosome_inact + 298 gmp + 1082 h + 233 ump	irreversible	mRNA degradation	0.00E+00	3.08E-01	0	1.16E+02	1.16E+02	0	2.04E+02	204.2739	0	3.08E-01	0.308
b1211_maturation1	Polypeptide b1211 peptide deformylas and methionine aminopeptidase complex	1 Def_mono + 1 Map_mono + 1 b1211_aa <==> 1 b1211_def_map_cplx	reversible	Protein Maturation	-1.00E+09	1.00E+09	0	2.52E+03	2.52E+03	0	4.38E+03	4382.152	0	9.89E+01	98.868
b1211_maturation2	b1211 formation	1 b1211_def_map_cplx + 2 h2o --> 1 Def_mono_inact + 1 Map_mono_inact + 1 b1211_m + 1 for + 1 met-L	irreversible	Protein Maturation	0.00E+00	1.00E+09	0	2.52E+03	2.52E+03	0	4.38E+03	4382.152	0	9.89E+01	98.868
b1212_DNA_act_bind	b1212_DNA binding of activator	1 b1212_DNA_neu --> 1 b1212_DNA_act	irreversible	Transcription Regulation	0.00E+00	1.00E+09	0	1.46E+02	1.46E+02	0	2.57E+02	257.4411	0	2.07E-01	0.207
b1212_fold_spon	b1212_m folding: spontanous	1 b1212_m --> 1 PrmC_mono	irreversible	Protein Folding	0.00E+00	1.00E+09	0	2.42E+03	2.42E+03	0	4.21E+03	4210.577	0	9.48E+01	94.832
b1212_mRNA_CONV	convsersion of mRNA to mRNA_1 (synthetic rxn)	1 b1212_mRNA --> 1 b1212_mRNA_1	irreversible	Translation	0.00E+00	1.00E+09	0	1.46E+02	1.46E+02	0	2.57E+02	257.4411	0	2.07E-01	0.207
b1212_mRNA_degr1	Degradation of b1212_mRNA I	1 Orn_dim + 208 atp + 1 b1212_mRNA_2 + 1 degradosome + 208 h2o <==> 208 adp + 1 b1212_mRNA_2_degr + 208 h + 208 pi	reversible	mRNA degradation	-1.00E+09	1.00E+09	0	1.46E+02	1.46E+02	0	2.57E+02	257.4411	0	2.07E-01	0.207
b1212_mRNA_degr2	Degradation of b1212_mRNA II	1 b1212_mRNA_2_degr + 833 h2o --> 1 Orn_dim_inact + 180 amp + 1 atp + 219 cmp + 1 degradosome_inact + 241 gmp + 833 h + 193 ump	irreversible	mRNA degradation	0.00E+00	2.07E-01	0	1.46E+02	1.46E+02	0	2.57E+02	257.4411	0	2.07E-01	0.207
b1212_maturation1	Polypeptide b1212 peptide deformylas and methionine aminopeptidase complex	1 Def_mono + 1 Map_mono + 1 b1212_aa <==> 1 b1212_def_map_cplx	reversible	Protein Maturation	-1.00E+09	1.00E+09	0	2.42E+03	2.42E+03	0	4.21E+03	4210.577	0	9.48E+01	94.832
b1212_maturation2	b1212 formation	1 b1212_def_map_cplx + 2 h2o --> 1 Def_mono_inact + 1 Map_mono_inact + 1 b1212_m + 1 for + 1 met-L	irreversible	Protein Maturation	0.00E+00	1.00E+09	0	2.42E+03	2.42E+03	0	4.21E+03	4210.577	0	9.48E+01	94.832
b1229_fold_spon	b1229_m folding: spontanous	1 b1229_m --> 1 Tpr_mono	irreversible	Protein Folding	0.00E+00	1.00E+09	0	2.59E+02	2.59E+02	0	4.21E+02	421.3748	0	4.82E+01	48.207
b1229_mRNA_CONV	convsersion of mRNA to mRNA_1 (synthetic rxn)	1 b1229_mRNA --> 1 b1229_mRNA_1	irreversible	Translation	0.00E+00	1.00E+09	0	2.11E+02	2.11E+02	0	3.73E+02	373.3748	0	2.07E-01	0.207
b1229_mRNA_degr1	Degradation of b1229_mRNA I	1 Orn_dim + 25 atp + 1 b1229_mRNA_2 + 1 degradosome + 25 h2o <==> 25 adp + 1 b1229_mRNA_2_degr + 25 h + 25 pi	reversible	mRNA degradation	-1.00E+09	1.00E+09	0	2.11E+02	2.11E+02	0	3.73E+02	373.3748	0	2.07E-01	0.207
b1229_mRNA_degr2	Degradation of b1229_mRNA II	1 b1229_mRNA_2_degr + 101 h2o --> 1 Orn_dim_inact + 26 amp + 27 cmp + 1 degradosome_inact + 39 gmp + 101 h + 10 ump	irreversible	mRNA degradation	0.00E+00	2.07E-01	0	2.11E+02	2.11E+02	0	3.73E+02	373.3748	0	2.07E-01	0.207
b1229_maturation1	Polypeptide b1229 peptide deformylas and methionine aminopeptidase complex	1 Def_mono + 1 Map_mono + 1 b1229_aa <==> 1 b1229_def_map_cplx	reversible	Protein Maturation	-1.00E+09	1.00E+09	0	2.59E+02	2.59E+02	0	4.21E+02	421.3748	0	4.82E+01	48.207
b1229_maturation2	b1229 formation	1 b1229_def_map_cplx + 2 h2o --> 1 Def_mono_inact + 1 Map_mono_inact + 1 b1229_m + 1 for + 1 met-L	irreversible	Protein Maturation	0.00E+00	1.00E+09	0	2.59E+02	2.59E+02	0	4.21E+02	421.3748	0	4.82E+01	48.207
b1230_tRNA_18_MODIF_1	modification of position 18 nt (Gm) in tyrV by TrmH_dim	1 TrmH_dim + 1 amet + 1 b1230_tRNA_Mg2 <==> 1 b1230_tRNA_Mg2_TrmH_dim_cplx	reversible	tRNA modification	-1.00E+09	1.00E+09	0	2.11E+02	2.11E+02	0	3.73E+02	373.3748	0	2.07E-01	0.207
b1230_tRNA_18_MODIF_2	modification of position 18 nt (Gm) in tyrV by TrmH_dim	1 b1230_tRNA_Mg2_TrmH_dim_cplx --> 1 TrmH_dim_inact + 1 ahcys + 1 b1230_tRNA_1 + 1 h	irreversible	tRNA modification	0.00E+00	1.00E+09	0	2.11E+02	2.11E+02	0	3.73E+02	373.3748	0	2.07E-01	0.207
b1230_tRNA_34_MODIF_1	modification of position 34 nt (Q) in tyrV by Tgt_hexa-QueA_mono-EoR_tRNA_pos34_Q	1 EoR_tRNA_pos34_Q + 1 QueA_mono + 1 Tgt_hexa + 1 adocbl + 1 amet + 1 b1230_tRNA_1 + 1 h + 1 nadh + 1 pre_Q1 <==> 1 b1230_tRNA_1_Tgt_hexa-QueA_mono-EoR_tRNA_pos34_Q_cplx	reversible	tRNA modification	-1.00E+09	1.00E+09	0	2.11E+02	2.11E+02	0	3.73E+02	373.3748	0	2.07E-01	0.207
b1230_tRNA_34_MODIF_2	modification of position 34 nt (Q) in tyrV by Tgt_hexa-QueA_mono-EoR_tRNA_pos34_Q	1 b1230_tRNA_1_Tgt_hexa-QueA_mono-EoR_tRNA_pos34_Q_cplx > 1 EoR_tRNA_pos34_Q_inact + 1 QueA_mono_inact + 1 Tgt_hexa_inact + 1 ade + 1 adocbl + 1 b1230_tRNA_2 + 1 gua + 2 h 1 h2o + 1 met-L + 1 nad	irreversible	tRNA modification	0.00E+00	1.00E+09	0	2.11E+02	2.11E+02	0	3.73E+02	373.3748	0	2.07E-01	0.207
b1230_tRNA_37_MODIF_1	modification of position 37 nt (ms2i6A) in tyrV by MiaA_dim-MiaB_mono	1 IscS_dim_S-SH + 1 MiaA_dim + 1 MiaB_mono + 2 amet + 1 b1230_tRNA_2 + 1 dmpp + 1 fldrd <==> 1 b1230_tRNA_2_MiaA_dim MiaB_mono_cplx	reversible	tRNA modification	-1.00E+09	1.00E+09	0	2.11E+02	2.11E+02	0	3.73E+02	373.3748	0	2.07E-01	0.207
b1230_tRNA_37_MODIF_2	modification of position 37 nt (ms2i6A) in tyrV by MiaA_dim-MiaB_mono	1 b1230_tRNA_2_MiaA_dim-MiaB_mono_cplx --> 1 IscS_dim_S-H + 1 MiaA_dim_inact + 1 MiaB_mono_inact + 1 ahcys + 1 b1230_tRNA_3 + 1 dad-5 + 1 fldox + 2 h + 1 met-L + 1 ppi	irreversible	tRNA modification	0.00E+00	1.00E+09	0	2.11E+02	2.11E+02	0	3.73E+02	373.3748	0	2.07E-01	0.207
b1230_tRNA_39_MODIF_1	modification of position 39 nt (Y) in tyrV by TruA_dim	1 TruA_dim + 1 b1230_tRNA_3 <==> 1 b1230_tRNA_3_TruA_dim_cplx	reversible	tRNA modification	-1.00E+09	1.00E+09	0	2.11E+02	2.11E+02	0	3.73E+02	373.3748	0	2.07E-01	0.207
b1230_tRNA_39_MODIF_2	modification of position 39 nt (Y) in tyrV by TruA_dim	1 b1230_tRNA_3_TruA_dim_cplx --> 1 TruA_dim_inact + 1 b1230_tRNA_4	irreversible	tRNA modification	0.00E+00	1.00E+09	0	2.11E+02	2.11E+02	0	3.73E+02	373.3748	0	2.07E-01	0.207
b1230_tRNA_54_MODIF_1	modification of position 54 nt (m5U) in tyrV by TrmA_mono	1 TrmA_mono + 1 amet + 1 b1230_tRNA_4 <==> 1 b1230_tRNA_4_TrmA_mono_cplx	reversible	tRNA modification	-1.00E+09	1.00E+09	0	2.11E+02	2.11E+02	0	3.73E+02	373.3748	0	2.07E-01	0.207
b1230_tRNA_54_MODIF_2	modification of position 54 nt (m5U) in tyrV by TrmA_mono	1 b1230_tRNA_4_TrmA_mono_cplx --> 1 TrmA_mono_inact + 1 ahcys + 1 b1230_tRNA_5 + 1 h	irreversible	tRNA modification	0.00E+00	1.00E+09	0	2.11E+02	2.11E+02	0	3.73E+02	373.3748	0	2.07E-01	0.207
b1230_tRNA_55_MODIF_1	modification of position 55 nt (Y) in tyrV by TruB_mono	1 TruB_mono + 1 b1230_tRNA_5 <==> 1 b1230_tRNA_5_TruB_mono_cplx	reversible	tRNA modification	-1.00E+09	1.00E+09	0	2.11E+02	2.11E+02	0	3.73E+02	373.3748	0	2.07E-01	0.207

b1230_tRNA_55_MODIF_2	modification of position 55 nt (Y) in tyrV by TruB_mono	1 b1230_tRNA_5_TruB_mono_cplx --> 1 TruB_mono_inact + 1 b1230_tRNA_6	irreversible	tRNA modification	0.00E+00	1.00E+09	0	2.11E+02	2.11E+02	0	3.73E+02	373.3748	0	2.07E-01	0.207
b1230_tRNA_8_MODIF_1	modification of position 8 nt (s4U) in tyrV by ThiI_mono	1 IscS_dim_S-SH + 1 ThiI_mono + 1 atp + 1 b1230_tRNA_6 + 1 mg2 + 1 trdrd <==> 1 b1230_tRNA_6_ThiI_mono_cplx	reversible	tRNA modification	-1.00E+09	1.00E+09	0	2.11E+02	2.11E+02	0	3.73E+02	373.3748	0	2.07E-01	0.207
b1230_tRNA_8_MODIF_2	modification of position 8 nt (s4U) in tyrV by ThiI_mono	1 b1230_tRNA_6_ThiI_mono_cplx --> 1 IscS_dim_S-H + 1 ThiI_mono_inact + 1 amp + 1 h + 1 mg2 + 1 ppi + 1 trdox + 1 tyrV_tRNA	irreversible	tRNA modification	0.00E+00	1.00E+09	0	2.11E+02	2.11E+02	0	3.73E+02	373.3748	0	2.07E-01	0.207
b1230_tRNA_Mg_BIND	Binding of two Mg2 to b1230_tRNA	1 b1230_RNA + 2 mg2 <==> 1 b1230_tRNA_Mg2	reversible	Metallo-ion binding	-1.00E+09	1.00E+09	0	2.11E+02	2.11E+02	0	3.73E+02	373.3748	0	2.07E-01	0.207
b1230_trimming	Cutting of stable b1230_RNA	1 b1230_RNA_cut_cplx + 1 h2o --> 1 RNase_E_tetra_inact + 1 RNase_Gen_inact + 1 RNase_P_cplx_inact + 1 b1230_RNA + 1 h + ppi	irreversible	RNA Processing	0.00E+00	1.00E+09	0	2.11E+02	2.11E+02	0	3.73E+02	373.3748	0	2.07E-01	0.207
b1230_trimming_cplx	Cutting complex formation of stable b1230_RNA (,)	1 RNase_E_tetra + 1 RNase_Gen + 1 RNase_P_cplx + 1 b1230_RNA_pre <==> 1 b1230_RNA_cut_cplx	reversible	RNA Processing	-1.00E+09	1.00E+09	0	2.11E+02	2.11E+02	0	3.73E+02	373.3748	0	2.07E-01	0.207
b1231_tRNA_18_MODIF_1	modification of position 18 nt (Gm) in tyrT by TrmH_dim	1 TrmH_dim + 1 amet + 1 b1231_tRNA_Mg2 <==> 1 b1231_tRNA_Mg2_TrmH_dim_cplx	reversible	tRNA modification	-1.00E+09	1.00E+09	0	2.11E+02	2.11E+02	0	3.73E+02	373.3748	0	2.07E-01	0.207
b1231_tRNA_18_MODIF_2	modification of position 18 nt (Gm) in tyrT by TrmH_dim	1 b1231_tRNA_Mg2_TrmH_dim_cplx --> 1 TrmH_dim_inact + 1 ahcys + 1 b1231_tRNA_1 + 1 h	irreversible	tRNA modification	0.00E+00	1.00E+09	0	2.11E+02	2.11E+02	0	3.73E+02	373.3748	0	2.07E-01	0.207
b1231_tRNA_34_MODIF_1	modification of position 34 nt (Q) in tyrT by Tgt_hexa-QueA_mono-EoR_tRNA_pos34_Q	1 EoR_tRNA_pos34_Q + 1 QueA_mono + 1 Tgt_hexa + 1 adocbl + 1 amet + 1 b1231_tRNA_1 + 1 h + 1 nadh + 1 pre_Q1 <==> 1 b1231_tRNA_1_Tgt_hexa-QueA_mono-EoR_tRNA_pos34_Q_cplx	reversible	tRNA modification	-1.00E+09	1.00E+09	0	2.11E+02	2.11E+02	0	3.73E+02	373.3748	0	2.07E-01	0.207
b1231_tRNA_34_MODIF_2	modification of position 34 nt (Q) in tyrT by Tgt_hexa-QueA_mono-EoR_tRNA_pos34_Q	1 b1231_tRNA_1_Tgt_hexa-QueA_mono-EoR_tRNA_pos34_Q_cplx > 1 EoR_tRNA_pos34_Q_inact + 1 QueA_mono_inact + 1 Tgt_hexa_inact + 1 ade + 1 adocbl + 1 b1231_tRNA_2 + 1 gua + 2 h 1 h2o + 1 met-L + 1 nad	irreversible	tRNA modification	0.00E+00	1.00E+09	0	2.11E+02	2.11E+02	0	3.73E+02	373.3748	0	2.07E-01	0.207
b1231_tRNA_37_MODIF_1	modification of position 37 nt (ms2i6A) in tyrT by MiaA_dim-MiaB_mono	1 IscS_dim_S-SH + 1 MiaA_dim + 1 MiaB_mono + 2 amet + 1 b1231_tRNA_2 + 1 dmpp + 1 fldrd <==> 1 b1231_tRNA_2_MiaA_dim MiaB_mono_cplx	reversible	tRNA modification	-1.00E+09	1.00E+09	0	2.11E+02	2.11E+02	0	3.73E+02	373.3748	0	2.07E-01	0.207
b1231_tRNA_37_MODIF_2	modification of position 37 nt (ms2i6A) in tyrT by MiaA_dim-MiaB_mono	1 b1231_tRNA_2_MiaA_dim-MiaB_mono_cplx --> 1 IscS_dim_S-H + 1 MiaA_dim_inact + 1 MiaB_mono_inact + 1 ahcys + 1 b1231_tRNA_3 + 1 dad-5 + 1 fldox + 2 h + 1 met-L + 1 ppi	irreversible	tRNA modification	0.00E+00	1.00E+09	0	2.11E+02	2.11E+02	0	3.73E+02	373.3748	0	2.07E-01	0.207
b1231_tRNA_39_MODIF_1	modification of position 39 nt (Y) in tyrT by TruA_dim	1 TruA_dim + 1 b1231_tRNA_3 <==> 1 b1231_tRNA_3_TruA_dim_cplx	reversible	tRNA modification	-1.00E+09	1.00E+09	0	2.11E+02	2.11E+02	0	3.73E+02	373.3748	0	2.07E-01	0.207
b1231_tRNA_39_MODIF_2	modification of position 39 nt (Y) in tyrT by TruA_dim	1 b1231_tRNA_3_TruA_dim_cplx --> 1 TruA_dim_inact + 1 b1231_tRNA_4	irreversible	tRNA modification	0.00E+00	1.00E+09	0	2.11E+02	2.11E+02	0	3.73E+02	373.3748	0	2.07E-01	0.207
b1231_tRNA_54_MODIF_1	modification of position 54 nt (m5U) in tyrT by TrmA_mono	1 TrmA_mono + 1 amet + 1 b1231_tRNA_4 <==> 1 b1231_tRNA_4_TrmA_mono_cplx	reversible	tRNA modification	-1.00E+09	1.00E+09	0	2.11E+02	2.11E+02	0	3.73E+02	373.3748	0	2.07E-01	0.207
b1231_tRNA_54_MODIF_2	modification of position 54 nt (m5U) in tyrT by TrmA_mono	1 b1231_tRNA_4_TrmA_mono_cplx --> 1 TrmA_mono_inact + 1 ahcys + 1 b1231_tRNA_5 + 1 h	irreversible	tRNA modification	0.00E+00	1.00E+09	0	2.11E+02	2.11E+02	0	3.73E+02	373.3748	0	2.07E-01	0.207
b1231_tRNA_55_MODIF_1	modification of position 55 nt (Y) in tyrT by TruB_mono	1 TruB_mono + 1 b1231_tRNA_5 <==> 1 b1231_tRNA_5_TruB_mono_cplx	reversible	tRNA modification	-1.00E+09	1.00E+09	0	2.11E+02	2.11E+02	0	3.73E+02	373.3748	0	2.07E-01	0.207
b1231_tRNA_55_MODIF_2	modification of position 55 nt (Y) in tyrT by TruB_mono	1 b1231_tRNA_5_TruB_mono_cplx --> 1 TruB_mono_inact + 1 b1231_tRNA_6	irreversible	tRNA modification	0.00E+00	1.00E+09	0	2.11E+02	2.11E+02	0	3.73E+02	373.3748	0	2.07E-01	0.207
b1231_tRNA_8_MODIF_1	modification of position 8 nt (s4U) in tyrT by ThiI_mono	1 IscS_dim_S-SH + 1 ThiI_mono + 1 atp + 1 b1231_tRNA_6 + 1 mg2 + 1 trdrd <==> 1 b1231_tRNA_6_ThiI_mono_cplx	reversible	tRNA modification	-1.00E+09	1.00E+09	0	2.11E+02	2.11E+02	0	3.73E+02	373.3748	0	2.07E-01	0.207
b1231_tRNA_8_MODIF_2	modification of position 8 nt (s4U) in tyrT by ThiI_mono	1 b1231_tRNA_6_ThiI_mono_cplx --> 1 IscS_dim_S-H + 1 ThiI_mono_inact + 1 amp + 1 h + 1 mg2 + 1 ppi + 1 trdox + 1 tyrT_tRNA	irreversible	tRNA modification	0.00E+00	1.00E+09	0	2.11E+02	2.11E+02	0	3.73E+02	373.3748	0	2.07E-01	0.207
b1231_tRNA_Mg_BIND	Binding of two Mg2 to b1231_tRNA	1 b1231_RNA + 2 mg2 <==> 1 b1231_tRNA_Mg2	reversible	Metallo-ion binding	-1.00E+09	1.00E+09	0	2.11E+02	2.11E+02	0	3.73E+02	373.3748	0	2.07E-01	0.207
b1231_trimming	Cutting of stable b1231_RNA	1 b1231_RNA_cut_cplx + 1 h2o --> 1 RNase_E_tetra_inact + 1 RNase_Gen_inact + 1 RNase_P_cplx_inact + 1 b1231_RNA + 1 h + ppi	irreversible	RNA Processing	0.00E+00	1.00E+09	0	2.11E+02	2.11E+02	0	3.73E+02	373.3748	0	2.07E-01	0.207
b1231_trimming_cplx	Cutting complex formation of stable b1231_RNA (,)	1 RNase_E_tetra + 1 RNase_Gen + 1 RNase_P_cplx + 1 b1231_RNA_pre <==> 1 b1231_RNA_cut_cplx	reversible	RNA Processing	-1.00E+09	1.00E+09	0	2.11E+02	2.11E+02	0	3.73E+02	373.3748	0	2.07E-01	0.207
b1269_fold_GroEL/ES_1	b1269_m folding: GroEL/ES mediated: polypeptide is going in GroEL/ES complex	1 GroEL.(7)ADP.cisGroES + 7 atp + 1 b1269_m + 1 transGroES_hepta --> 7 adp + 1 b1269_m_GroEL.(7)ATP.transGroES + 1 cisGroES_hepta	irreversible	Protein Folding	0.00E+00	1.00E+09	0	2.74E+03	2.74E+03	0	4.77E+03	4768.031	0	9.68E+01	96.815
b1269_fold_GroEL/ES_2	b1269_m folding: GroEL/ES mediated: folding of polypeptide under ATP hydrolysis	1 b1269_m_GroEL.(7)ATP.transGroES + 7 h2o --> 1 RluB_GroEL.(7)ADP.transGroES + 7 h + 7 pi	irreversible	Protein Folding	0.00E+00	1.00E+09	0	2.74E+03	2.74E+03	0	4.77E+03	4768.031	0	9.68E+01	96.815
b1269_fold_GroEL/ES_3	b1269_m folding: GroEL/ES mediated: release of native protein	1 RluB_GroEL.(7)ADP.transGroES --> 1 GroEL.(7)ADP.transGroES + 1 RluB_mono	irreversible	Protein Folding	0.00E+00	1.00E+09	0	2.74E+03	2.74E+03	0	4.77E+03	4768.031	0	9.68E+01	96.815
b1269_fold_KJE_1	b1269_m folding: KJE mediated	1 DnaJ_dim + 1 DnaK_mono.ATP + 1 b1269_m <==> 1 b1269_m_DnaKJ_complex	reversible	Protein Folding	-1.00E+09	1.00E+09	0	2.74E+03	2.74E+03	0	4.77E+03	4768.031	0	9.68E+01	96.815
b1269_fold_KJE_2	b1269_m folding: KJE mediated	1 GrpE_dim + 1 b1269_m_DnaKJ_complex + 1 h2o --> 1 DnaJ_dim_inact + 1 RluB_DnaK_GrpE_complex + 1 adp + 1 h + 1 pi	irreversible	Protein Folding	0.00E+00	1.00E+09	0	2.74E+03	2.74E+03	0	4.77E+03	4768.031	0	9.68E+01	96.815
b1269_fold_KJE_3	b1269_m folding: KJE mediated	1 RluB_DnaK_GrpE_complex + 1 atp --> 1 DnaK_mono.ATP_inact + 1 GrpE_dim_inact + 1 RluB_mono	irreversible	Protein Folding	0.00E+00	1.00E+09	0	2.74E+03	2.74E+03	0	4.77E+03	4768.031	0	9.68E+01	96.815
b1269_mRNA_CONV	convsersion of mRNA to mRNA_1 (synthetic rxn)	1 b1269_mRNA --> 1 b1269_mRNA_1	irreversible	Translation	0.00E+00	1.00E+09	0	1.56E+02	1.56E+02	0	2.75E+02	275.0224	0	2.45E-01	0.245
b1269_mRNA_degr1	Degradation of b1269_mRNA I	1 Orn_dim + 219 atp + 1 b1269_mRNA_2 + 1 degradosome + 219 h2o <==> 219 adp + 1 b1269_mRNA_2_degr + 219 h + 219 pi	reversible	mRNA degradation	-1.00E+09	1.00E+09	0	1.56E+02	1.56E+02	0	2.75E+02	275.0224	0	2.45E-01	0.245
b1269_mRNA_degr2	Degradation of b1269_mRNA II	1 b1269_mRNA_2_degr + 875 h2o --> 1 Orn_dim_inact + 200 amp + 1 atp + 205 cmp + 1 degradosome_inact + 266 gmp + 875 h + 204 ump	irreversible	mRNA degradation	0.00E+00	2.45E-01	0	1.56E+02	1.56E+02	0	2.75E+02	275.0224	0	2.45E-01	0.245
b1269_maturation1	Polypeptide b1269 peptide deformylase and methionine aminopeptidase complex	1 Def_mono + 1 Map_mono + 1 b1269_aa <==> 1 b1269_def_map_cplx	reversible	Protein Maturation	-1.00E+09	1.00E+09	0	2.74E+03	2.74E+03	0	4.77E+03	4768.031	0	9.68E+01	96.815
b1269_maturation2	b1269 formation	1 b1269_def_map_cplx + 2 h2o --> 1 Def_mono_inact + 1 Map_mono_inact + 1 b1269_m + 1 for + 1 met-L	irreversible	Protein Maturation	0.00E+00	1.00E+09	0	2.74E+03	2.74E+03	0	4.77E+03	4768.031	0	9.68E+01	96.815
b1286_fold_spon	b1286_m folding: spontanous	1 b1286_m_Mg --> 1 Rnb_mono	irreversible	Protein Folding	0.00E+00	1.00E+09	0	2.38E+03	2.38E+03	0	3.79E+03	3791.216	0	1.25E+02	125.43
b1286_ion_BIND	Binding of ions to b1286	1 b1286_m + 1 mg2 <==> 1 b1286_m_Mg	reversible	Metallo-ion Binding	-1.00E+09	1.00E+09	0	2.38E+03	2.38E+03	0	3.79E+03	3791.216	0	1.25E+02	125.43
b1286_mRNA_CONV	convsersion of mRNA to mRNA_1 (synthetic rxn)	1 b1286_mRNA --> 1 b1286_mRNA_1	irreversible	Translation	0.00E+00	1.00E+09	0	6.13E+01	6.13E+01	0	1.08E+02	108.2142	0	4.90E-01	0.49
b1286_mRNA_degr1	Degradation of b1286_mRNA I	1 Orn_dim + 483 atp + 1 b1286_mRNA_2 + 1 degradosome + 483 h2o <==> 483 adp + 1 b1286_mRNA_2_degr + 483 h + 483 pi	reversible	mRNA degradation	-1.00E+09	1.00E+09	0	6.13E+01	6.13E+01	0	1.08E+02	108.2142	0	4.90E-01	0.49
b1286_mRNA_degr2	Degradation of b1286_mRNA II	1 b1286_mRNA_2_degr + 1934 h2o --> 1 Orn_dim_inact + 470 amp 1 atp + 521 cmp + 1 degradosome_inact + 509 gmp + 1934 h + 434 ump	irreversible	mRNA degradation	0.00E+00	4.90E-01	0	6.13E+01	6.13E+01	0	1.08E+02	108.2142	0	4.90E-01	0.49
b1286_maturation1	Polypeptide b1286 peptide deformylase and methionine aminopeptidase complex	1 Def_mono + 1 Map_mono + 1 b1286_aa <==> 1 b1286_def_map_cplx	reversible	Protein Maturation	-1.00E+09	1.00E+09	0	2.38E+03	2.38E+03	0	3.79E+03	3791.216	0	1.25E+02	125.43
b1286_maturation2	b1286 formation	1 b1286_def_map_cplx + 2 h2o --> 1 Def_mono_inact + 1 Map_mono_inact + 1 b1286_m + 1 for + 1 met-L	irreversible	Protein Maturation	0.00E+00	1.00E+09	0	2.38E+03	2.38E+03	0	3.79E+03	3791.216	0	1.25E+02	125.43
b1286_v1_mRNA_CONV	convsersion of mRNA to mRNA_1 (synthetic rxn)	1 b1286_v1_mRNA --> 1 b1286_mRNA_1	irreversible	Translation	0.00E+00	1.00E+09	0	6.13E+01	6.13E+01	0	1.08E+02	108.2142	0	4.90E-01	0.49
b1344_fold_spon	b1344_m folding: spontanous	1 b1344_m --> 1 YdaO_mono	irreversible	Protein Folding	0.00E+00	1.00E+09	0	2.84E+03	2.84E+03	0	4.95E+03	4947.459	0	9.52E+01	95.202
b1344_mRNA_CONV	convsersion of mRNA to mRNA_1 (synthetic rxn)	1 b1344_mRNA --> 1 b1344_mRNA_1	irreversible	Translation	0.00E+00	1.00E+09	0	1.53E+02	1.53E+02	0	2.70E+02	269.7588	0	1.89E-01	0.189
b1344_mRNA_degr1	Degradation of b1344_mRNA I	1 Orn_dim + 234 atp + 1 b1344_mRNA_2 + 1 degradosome + 234 h2o <==> 234 adp + 1 b1344_mRNA_2_degr + 234 h + 234 pi	reversible	mRNA degradation	-1.00E+09	1.00E+09	0	1.53E+02	1.53E+02	0	2.70E+02	269.7588	0	1.89E-01	0.189

b1344_mRNA_degr2	Degradation of b1344_mRNA II	1 b1344_mRNA_2_degr + 935 h2o --> 1 Orn_dim_inact + 257 amp + 1 atp + 209 cmp + 1 degradosome_inact + 246 gmp + 935 h + 223 ump	irreversible	mRNA degradation	0.00E+00	1.89E-01	0	1.53E+02	1.53E+02	0	2.70E+02	269.7588	0	1.89E-01	0.189
b1344_maturation1	Polypeptide b1344 peptide deformylas and methionine aminopeptidase complex	1 Def_mono + 1 Map_mono + 1 b1344_aa <==> 1 b1344_def_map_cplx	reversible	Protein Maturation	-1.00E+09	1.00E+09	0	2.84E+03	2.84E+03	0	4.95E+03	4947.459	0	9.52E+01	95.202
b1344_maturation2	b1344 formation	1 b1344_def_map_cplx + 2 h2o --> 1 Def_mono_inact + 1 Map_mono_inact + 1 b1344_m + 1 for + 1 met-L	irreversible	Protein Maturation	0.00E+00	1.00E+09	0	2.84E+03	2.84E+03	0	4.95E+03	4947.459	0	9.52E+01	95.202
b1427_fold_GroEL/ES_1	b1427_m folding: GroEL/ES mediated polypeptide is going in GroEL/ES complex	1 GroEL.(7)ADP.cisGroES + 7 atp + 1 b1427_m + 1 transGroES_hepta --> 7 adp + 1 b1427_m_GroEL.(7)ATP.transGroES + 1 cisGroES_hepta	irreversible	Protein Folding	0.00E+00	1.00E+09	0	3.02E+03	3.02E+03	0	5.05E+03	5054.124	0	8.98E+01	89.75
b1427_fold_GroEL/ES_2	b1427_m folding: GroEL/ES mediated folding of polypeptide under ATP hydrolysis	1 b1427_m_GroEL.(7)ATP.transGroES + 7 h2o --> 1 RimL_GroEL.(7)ADP.transGroES + 7 h + 7 pi	irreversible	Protein Folding	0.00E+00	1.00E+09	0	3.02E+03	3.02E+03	0	5.05E+03	5054.124	0	8.98E+01	89.75
b1427_fold_GroEL/ES_3	b1427_m folding: GroEL/ES mediated release of native protein	1 RimL_GroEL.(7)ADP.transGroES --> 1 GroEL.(7)ADP.transGroES + 1 RimL_mono	irreversible	Protein Folding	0.00E+00	1.00E+09	0	3.02E+03	3.02E+03	0	5.05E+03	5054.124	0	8.98E+01	89.75
b1427_fold_KJE_1	b1427_m folding: KJE mediated	1 DnaJ_dim + 1 DnaK_mono.ATP + 1 b1427_m <==> 1 b1427_m_DnaKJ_complex	reversible	Protein Folding	-1.00E+09	1.00E+09	0	3.02E+03	3.02E+03	0	5.05E+03	5054.124	0	8.98E+01	89.75
b1427_fold_KJE_2	b1427_m folding: KJE mediated	1 GrpE_dim + 1 b1427_m_DnaKJ_complex + 1 h2o --> 1 DnaJ_dim_inact + 1 RimL_DnaK_GrpE_complex + 1 adp + 1 h + 1 pi	irreversible	Protein Folding	0.00E+00	1.00E+09	0	3.02E+03	3.02E+03	0	5.05E+03	5054.124	0	8.98E+01	89.75
b1427_fold_KJE_3	b1427_m folding: KJE mediated	1 RimL_DnaK_GrpE_complex + 1 atp --> 1 DnaK_mono.ATP_inact + 1 GrpE_dim_inact + 1 RimL_mono	irreversible	Protein Folding	0.00E+00	1.00E+09	0	3.02E+03	3.02E+03	0	5.05E+03	5054.124	0	8.98E+01	89.75
b1427_mRNA_CONV	convsersion of mRNA to mRNA_1 (synthetic rxn)	1 b1427_mRNA --> 1 b1427_mRNA_1	irreversible	Translation	0.00E+00	1.00E+09	0	2.93E+02	2.93E+02	0	4.97E+02	496.5524	0	1.15E-01	0.115
b1427_mRNA_degr1	Degradation of b1427_mRNA I	1 Orn_dim + 135 atp + 1 b1427_mRNA_2 + 1 degradosome + 135 h2o <==> 135 adp + 1 b1427_mRNA_2_degr + 135 h + 135 pi	reversible	mRNA degradation	-1.00E+09	1.00E+09	0	2.93E+02	2.93E+02	0	4.97E+02	496.5524	0	1.15E-01	0.115
b1427_mRNA_degr2	Degradation of b1427_mRNA II	1 b1427_mRNA_2_degr + 539 h2o --> 1 Orn_dim_inact + 162 amp + 1 atp + 109 cmp + 1 degradosome_inact + 125 gmp + 539 h + 143 ump	irreversible	mRNA degradation	0.00E+00	1.15E-01	0	2.93E+02	2.93E+02	0	4.97E+02	496.5524	0	1.15E-01	0.115
b1427_maturation1	Polypeptide b1427 peptide deformylas and methionine aminopeptidase complex	1 Def_mono + 1 Map_mono + 1 b1427_aa <==> 1 b1427_def_map_cplx	reversible	Protein Maturation	-1.00E+09	1.00E+09	0	3.02E+03	3.02E+03	0	5.05E+03	5054.124	0	8.98E+01	89.75
b1427_maturation2	b1427 formation	1 b1427_def_map_cplx + 2 h2o --> 1 Def_mono_inact + 1 Map_mono_inact + 1 b1427_m + 1 for + 1 met-L	irreversible	Protein Maturation	0.00E+00	1.00E+09	0	3.02E+03	3.02E+03	0	5.05E+03	5054.124	0	8.98E+01	89.75
b1480_fold_spon	b1480_m folding: spontanous	1 b1480_m --> 1 Sra_mono	irreversible	Protein Folding	0.00E+00	1.00E+09	8.44E+01	1.13E+02	2.81E+01	6.22E+01	8.29E+01	20.72	6.22E+01	8.29E+01	20.72
b1480_mRNA_CONV	convsersion of mRNA to mRNA_1 (synthetic rxn)	1 b1480_mRNA --> 1 b1480_mRNA_1	irreversible	Translation	0.00E+00	1.00E+09	0	1.03E+02	1.03E+02	0	8.29E+01	82.88	0	1.89E-01	0.189
b1480_mRNA_degr1	Degradation of b1480_mRNA I	1 Orn_dim + 34 atp + 1 b1480_mRNA_2 + 1 degradosome + 34 h2o <==> 34 adp + 1 b1480_mRNA_2_degr + 34 h + 34 pi	reversible	mRNA degradation	-1.00E+09	1.00E+09	0	1.09E+02	1.09E+02	0	8.29E+01	82.88	0	3.10E+00	3.1
b1480_mRNA_degr2	Degradation of b1480_mRNA II	1 b1480_mRNA_2_degr + 137 h2o --> 1 Orn_dim_inact + 52 amp + 34 cmp + 1 degradosome_inact + 27 gmp + 137 h + 25 ump	irreversible	mRNA degradation	0.00E+00	3.10E+00	0	1.09E+02	1.09E+02	0	8.29E+01	82.88	0	3.10E+00	3.1
b1480_maturation1	Polypeptide b1480 peptide deformylas complex	1 Def_mono + 1 b1480_aa <==> 1 b1480_def_cplx	reversible	Protein Maturation	-1.00E+09	1.00E+09	8.44E+01	1.13E+02	2.81E+01	6.22E+01	8.29E+01	20.72	6.22E+01	8.29E+01	20.72
b1480_maturation2	b1480 formation	1 b1480_def_cplx + 1 h2o --> 1 Def_mono_inact + 1 b1480_m + 1 for	irreversible	Protein Maturation	0.00E+00	1.00E+09	8.44E+01	1.13E+02	2.81E+01	6.22E+01	8.29E+01	20.72	6.22E+01	8.29E+01	20.72
b1480_v1_mRNA_CONV	convsersion of mRNA to mRNA_1 (synthetic rxn)	1 b1480_v1_mRNA --> 1 b1480_mRNA_1	irreversible	Translation	0.00E+00	1.00E+09	0	1.09E+02	1.09E+02	0	8.29E+01	82.88	0	3.10E+00	3.1
b1481_fold_spon	b1481_m folding: spontanous	1 b1481_m --> 1 Bdm_mono	irreversible	Protein Folding	0.00E+00	1.00E+09	0	5.03E+02	5.03E+02	0	4.21E+02	420.72	0	9.00E+01	89.956
b1481_mRNA_CONV	convsersion of mRNA to mRNA_1 (synthetic rxn)	1 b1481_mRNA --> 1 b1481_mRNA_1	irreversible	Translation	0.00E+00	1.00E+09	0	1.03E+02	1.03E+02	0	8.29E+01	82.88	0	1.89E-01	0.189
b1481_mRNA_degr1	Degradation of b1481_mRNA I	1 Orn_dim + 54 atp + 1 b1481_mRNA_2 + 1 degradosome + 54 h2o <==> 54 adp + 1 b1481_mRNA_2_degr + 54 h + 54 pi	reversible	mRNA degradation	-1.00E+09	1.00E+09	0	1.03E+02	1.03E+02	0	8.29E+01	82.88	0	1.89E-01	0.189
b1481_mRNA_degr2	Degradation of b1481_mRNA II	1 b1481_mRNA_2_degr + 215 h2o --> 1 Orn_dim_inact + 61 amp + 1 atp + 50 cmp + 1 degradosome_inact + 48 gmp + 215 h + 56 ump	irreversible	mRNA degradation	0.00E+00	1.89E-01	0	1.03E+02	1.03E+02	0	8.29E+01	82.88	0	1.89E-01	0.189
b1481_maturation1	Polypeptide b1481 peptide deformylas and methionine aminopeptidase complex	1 Def_mono + 1 Map_mono + 1 b1481_aa <==> 1 b1481_def_map_cplx	reversible	Protein Maturation	-1.00E+09	1.00E+09	0	5.03E+02	5.03E+02	0	4.21E+02	420.72	0	9.00E+01	89.956
b1481_maturation2	b1481 formation	1 b1481_def_map_cplx + 2 h2o --> 1 Def_mono_inact + 1 Map_mono_inact + 1 b1481_m + 1 for + 1 met-L	irreversible	Protein Maturation	0.00E+00	1.00E+09	0	5.03E+02	5.03E+02	0	4.21E+02	420.72	0	9.00E+01	89.956
b1636_fold_spon	b1636_m folding: spontanous	1 b1636_m --> 1 PdxY_mono	irreversible	Protein Folding	0.00E+00	1.00E+09	0	1.06E+03	1.06E+03	0	1.81E+03	1809.834	0	9.19E+01	91.904
b1636_mRNA_CONV	convsersion of mRNA to mRNA_1 (synthetic rxn)	1 b1636_mRNA --> 1 b1636_mRNA_1	irreversible	Translation	0.00E+00	1.00E+09	0	6.09E+01	6.09E+01	0	1.08E+02	107.5946	0	2.24E-01	0.224
b1636_mRNA_degr1	Degradation of b1636_mRNA I	1 Orn_dim + 216 atp + 1 b1636_mRNA_2 + 1 degradosome + 216 h2o <==> 216 adp + 1 b1636_mRNA_2_degr + 216 h + 216 pi	reversible	mRNA degradation	-1.00E+09	1.00E+09	0	6.09E+01	6.09E+01	0	1.08E+02	107.5946	0	2.24E-01	0.224
b1636_mRNA_degr2	Degradation of b1636_mRNA II	1 b1636_mRNA_2_degr + 863 h2o --> 1 Orn_dim_inact + 204 amp + 208 cmp + 1 degradosome_inact + 250 gmp + 863 h + 202 ump	irreversible	mRNA degradation	0.00E+00	2.24E-01	0	6.09E+01	6.09E+01	0	1.08E+02	107.5946	0	2.24E-01	0.224
b1636_maturation1	Polypeptide b1636 peptide deformylas and methionine aminopeptidase complex	1 Def_mono + 1 Map_mono + 1 b1636_aa <==> 1 b1636_def_map_cplx	reversible	Protein Maturation	-1.00E+09	1.00E+09	0	1.06E+03	1.06E+03	0	1.81E+03	1809.834	0	9.19E+01	91.904
b1636_maturation2	b1636 formation	1 b1636_def_map_cplx + 2 h2o --> 1 Def_mono_inact + 1 Map_mono_inact + 1 b1636_m + 1 for + 1 met-L	irreversible	Protein Maturation	0.00E+00	1.00E+09	0	1.06E+03	1.06E+03	0	1.81E+03	1809.834	0	9.19E+01	91.904
b1636_v1_mRNA_CONV	convsersion of mRNA to mRNA_1 (synthetic rxn)	1 b1636_v1_mRNA --> 1 b1636_mRNA_1	irreversible	Translation	0.00E+00	1.00E+09	0	4.46E+01	4.46E+01	0	7.87E+01	78.74245	0	1.04E-01	0.104
b1637_fold_spon	b1637_m folding: spontanous	1 b1637_m --> 1 TyrS_mono	irreversible	Protein Folding	0.00E+00	1.00E+09	0	1.55E+03	1.55E+03	0	2.67E+03	2672.032	0	9.51E+01	95.136
b1637_mRNA_CONV	convsersion of mRNA to mRNA_1 (synthetic rxn)	1 b1637_mRNA --> 1 b1637_mRNA_1	irreversible	Translation	0.00E+00	1.00E+09	0	6.09E+01	6.09E+01	0	1.08E+02	107.5946	0	2.24E-01	0.224
b1637_mRNA_degr1	Degradation of b1637_mRNA I	1 Orn_dim + 318 atp + 1 b1637_mRNA_2 + 1 degradosome + 318 h2o <==> 318 adp + 1 b1637_mRNA_2_degr + 318 h + 318 pi	reversible	mRNA degradation	-1.00E+09	1.00E+09	0	6.09E+01	6.09E+01	0	1.08E+02	107.5946	0	2.24E-01	0.224
b1637_mRNA_degr2	Degradation of b1637_mRNA II	1 b1637_mRNA_2_degr + 1274 h2o --> 1 Orn_dim_inact + 321 amp 1 atp + 306 cmp + 1 degradosome_inact + 351 gmp + 1274 h + 296 ump	irreversible	mRNA degradation	0.00E+00	4.49E-01	0	6.09E+01	6.09E+01	0	1.08E+02	107.5946	0	2.24E-01	0.224
b1637_maturation1	Polypeptide b1637 peptide deformylas and methionine aminopeptidase complex	1 Def_mono + 1 Map_mono + 1 b1637_aa <==> 1 b1637_def_map_cplx	reversible	Protein Maturation	-1.00E+09	1.00E+09	0	1.55E+03	1.55E+03	0	2.67E+03	2672.032	0	9.51E+01	95.136
b1637_maturation2	b1637 formation	1 b1637_def_map_cplx + 2 h2o --> 1 Def_mono_inact + 1 Map_mono_inact + 1 b1637_m + 1 for + 1 met-L	irreversible	Protein Maturation	0.00E+00	1.00E+09	0	1.55E+03	1.55E+03	0	2.67E+03	2672.032	0	9.51E+01	95.136
b1637_v1_mRNA_CONV	convsersion of mRNA to mRNA_1 (synthetic rxn)	1 b1637_v1_mRNA --> 1 b1637_mRNA_1	irreversible	Translation	0.00E+00	1.00E+09	0	4.46E+01	4.46E+01	0	7.87E+01	78.74245	0	1.04E-01	0.104
b1638_fold_spon	b1638_m folding: spontanous	1 b1638_m --> 1 PdxH_mono	irreversible	Protein Folding	0.00E+00	1.00E+09	0	6.07E+02	6.07E+02	0	1.02E+03	1016.789	0	7.31E+01	73.128
b1638_mRNA_CONV	convsersion of mRNA to mRNA_1 (synthetic rxn)	1 b1638_mRNA --> 1 b1638_mRNA_1	irreversible	Translation	0.00E+00	1.00E+09	0	4.46E+01	4.46E+01	0	7.87E+01	78.74245	0	1.04E-01	0.104
b1638_mRNA_degr1	Degradation of b1638_mRNA I	1 Orn_dim + 164 atp + 1 b1638_mRNA_2 + 1 degradosome + 164 h2o <==> 164 adp + 1 b1638_mRNA_2_degr + 164 h + 164 pi	reversible	mRNA degradation	-1.00E+09	1.00E+09	0	4.46E+01	4.46E+01	0	7.87E+01	78.74245	0	1.04E-01	0.104
b1638_mRNA_degr2	Degradation of b1638_mRNA II	1 b1638_mRNA_2_degr + 656 h2o --> 1 Orn_dim_inact + 154 amp + 1 atp + 167 cmp + 1 degradosome_inact + 174 gmp + 656 h + 161 ump	irreversible	mRNA degradation	0.00E+00	1.04E-01	0	4.46E+01	4.46E+01	0	7.87E+01	78.74245	0	1.04E-01	0.104
b1638_maturation1	Polypeptide b1638 peptide deformylas and methionine aminopeptidase complex	1 Def_mono + 1 Map_mono + 1 b1638_aa <==> 1 b1638_def_map_cplx	reversible	Protein Maturation	-1.00E+09	1.00E+09	0	6.07E+02	6.07E+02	0	1.02E+03	1016.789	0	7.31E+01	73.128
b1638_maturation2	b1638 formation	1 b1638_def_map_cplx + 2 h2o --> 1 Def_mono_inact + 1 Map_mono_inact + 1 b1638_m + 1 for + 1 met-L	irreversible	Protein Maturation	0.00E+00	1.00E+09	0	6.07E+02	6.07E+02	0	1.02E+03	1016.789	0	7.31E+01	73.128

b1652_fold_spon	b1652_m folding: spontanous	1 b1652_m_Mg --> 1 Rnt_mono	irreversible	Protein Folding	0.00E+00	1.00E+09	0	3.46E+02	3.46E+02	0	5.43E+02	542.8088	0	9.05E+01	90.492
b1652_ion_BIND	Binding of ions to b1652	1 b1652_m + 2 mg2 <==> 1 b1652_m_Mg	reversible	Metallo-ion Binding	-1.00E+09	1.00E+09	0	3.46E+02	3.46E+02	0	5.43E+02	542.8088	0	9.05E+01	90.492
b1652_mRNA_CONV	convsersion of mRNA to mRNA_1 (synthetic rxn)	1 b1652_mRNA --> 1 b1652_mRNA_1	irreversible	Translation	0.00E+00	1.00E+09	0	2.14E+01	2.14E+01	0	3.79E+01	37.86407	0	1.71E-01	0.171
b1652_mRNA_degr1	Degradation of b1652_mRNA I	1 Orn_dim + 162 atp + 1 b1652_mRNA_2 + 1 degradosome + 162 h2o <==> 162 adp + 1 b1652_mRNA_2_degr + 162 h + 162 pi	reversible	mRNA degradation	-1.00E+09	1.00E+09	0	2.14E+01	2.14E+01	0	3.79E+01	37.86407	0	1.71E-01	0.171
b1652_mRNA_degr2	Degradation of b1652_mRNA II	1 b1652_mRNA_2_degr + 647 h2o --> 1 Orn_dim_inact + 150 amp + 1 atp + 186 cmp + 1 degradosome_inact + 165 gmp + 647 h + 146 ump	irreversible	mRNA degradation	0.00E+00	1.71E-01	0	2.14E+01	2.14E+01	0	3.79E+01	37.86407	0	1.71E-01	0.171
b1652_maturation1	Polypeptide b1652 peptide deformylas and methionine aminopeptidase complex	1 Def_mono + 1 Map_mono + 1 b1652_aa <==> 1 b1652_def_map_cplx	reversible	Protein Maturation	-1.00E+09	1.00E+09	0	3.46E+02	3.46E+02	0	5.43E+02	542.8088	0	9.05E+01	90.492
b1652_maturation2	b1652 formation	1 b1652_def_map_cplx + 2 h2o --> 1 Def_mono_inact + 1 Map_mono_inact + 1 b1652_m + 1 for + 1 met-L	irreversible	Protein Maturation	0.00E+00	1.00E+09	0	3.46E+02	3.46E+02	0	5.43E+02	542.8088	0	9.05E+01	90.492
b1653_fold_spon	b1653_m folding: spontanous	1 b1653_m --> 1 Lhr_mono	irreversible	Protein Folding	0.00E+00	1.00E+09	0	1.41E+03	1.41E+03	0	1.53E+03	1525.901	0	1.08E+02	108.09
b1653_mRNA_CONV	convsersion of mRNA to mRNA_1 (synthetic rxn)	1 b1653_mRNA --> 1 b1653_mRNA_1	irreversible	Translation	0.00E+00	1.00E+09	0	2.14E+01	2.14E+01	0	3.79E+01	37.86407	0	1.71E-01	0.171
b1653_mRNA_degr1	Degradation of b1653_mRNA I	1 Orn_dim + 1154 atp + 1 b1653_mRNA_2 + 1 degradosome + 1154 h2o <==> 1154 adp + 1 b1653_mRNA_2_degr + 1154 h + 1154 pi	reversible	mRNA degradation	-1.00E+09	1.00E+09	0	2.14E+01	2.14E+01	0	3.79E+01	37.86407	0	1.71E-01	0.171
b1653_mRNA_degr2	Degradation of b1653_mRNA II	1 b1653_mRNA_2_degr + 4616 h2o --> 1 Orn_dim_inact + 969 amp 1283 cmp + 1 degradosome_inact + 1344 gmp + 4616 h + 1021 ump	irreversible	mRNA degradation	0.00E+00	1.92E-01	0	2.14E+01	2.14E+01	0	3.79E+01	37.86407	0	1.71E-01	0.171
b1653_maturation1	Polypeptide b1653 peptide deformylas and methionine aminopeptidase complex	1 Def_mono + 1 Map_mono + 1 b1653_aa <==> 1 b1653_def_map_cplx	reversible	Protein Maturation	-1.00E+09	1.00E+09	0	1.41E+03	1.41E+03	0	1.53E+03	1525.901	0	1.08E+02	108.09
b1653_maturation2	b1653 formation	1 b1653_def_map_cplx + 2 h2o --> 1 Def_mono_inact + 1 Map_mono_inact + 1 b1653_m + 1 for + 1 met-L	irreversible	Protein Maturation	0.00E+00	1.00E+09	0	1.41E+03	1.41E+03	0	1.53E+03	1525.901	0	1.08E+02	108.09
b1665_tRNA_16_MODIF_1	modification of position 16 nt (D) in valV by Dus_gen	1 Dus_gen + 1 b1665_tRNA_Mg2 + 1 h + 1 nadph <==> 1 b1665_tRNA_Mg2_Dus_gen_cplx	reversible	tRNA modification	-1.00E+09	1.00E+09	0	7.79E+02	7.79E+02	0	5.60E+00	5.6	0	5.60E+00	5.6
b1665_tRNA_16_MODIF_2	modification of position 16 nt (D) in valV by Dus_gen	1 b1665_tRNA_Mg2_Dus_gen_cplx --> 1 Dus_gen_inact + 1 b1665_tRNA_1 + 1 nadp	irreversible	tRNA modification	0.00E+00	1.00E+09	0	7.79E+02	7.79E+02	0	5.60E+00	5.6	0	5.60E+00	5.6
b1665_tRNA_17_MODIF_1	modification of position 17 nt (D) in valV by Dus_gen	1 Dus_gen + 1 b1665_tRNA_1 + 1 h + 1 nadph <==> 1 b1665_tRNA_1_Dus_gen_cplx	reversible	tRNA modification	-1.00E+09	1.00E+09	0	7.79E+02	7.79E+02	0	5.60E+00	5.6	0	5.60E+00	5.6
b1665_tRNA_17_MODIF_2	modification of position 17 nt (D) in valV by Dus_gen	1 b1665_tRNA_1_Dus_gen_cplx --> 1 Dus_gen_inact + 1 b1665_tRNA_2 + 1 nadp	irreversible	tRNA modification	0.00E+00	1.00E+09	0	7.79E+02	7.79E+02	0	5.60E+00	5.6	0	5.60E+00	5.6
b1665_tRNA_20A_MODIF_1	modification of position 20A nt (D) in valV by Dus_gen	1 Dus_gen + 1 b1665_tRNA_3 + 1 h + 1 nadph <==> 1 b1665_tRNA_3_Dus_gen_cplx	reversible	tRNA modification	-1.00E+09	1.00E+09	0	7.79E+02	7.79E+02	0	5.60E+00	5.6	0	5.60E+00	5.6
b1665_tRNA_20A_MODIF_2	modification of position 20A nt (D) in valV by Dus_gen	1 b1665_tRNA_3_Dus_gen_cplx --> 1 Dus_gen_inact + 1 b1665_tRNA_4 + 1 nadp	irreversible	tRNA modification	0.00E+00	1.00E+09	0	7.79E+02	7.79E+02	0	5.60E+00	5.6	0	5.60E+00	5.6
b1665_tRNA_20_MODIF_1	modification of position 20 nt (D) in valV by Dus_gen	1 Dus_gen + 1 b1665_tRNA_2 + 1 h + 1 nadph <==> 1 b1665_tRNA_2_Dus_gen_cplx	reversible	tRNA modification	-1.00E+09	1.00E+09	0	7.79E+02	7.79E+02	0	5.60E+00	5.6	0	5.60E+00	5.6
b1665_tRNA_20_MODIF_2	modification of position 20 nt (D) in valV by Dus_gen	1 b1665_tRNA_2_Dus_gen_cplx --> 1 Dus_gen_inact + 1 b1665_tRNA_3 + 1 nadp	irreversible	tRNA modification	0.00E+00	1.00E+09	0	7.79E+02	7.79E+02	0	5.60E+00	5.6	0	5.60E+00	5.6
b1665_tRNA_46_MODIF_1	modification of position 46 nt (m7G) in valV by YggH_mono	1 YggH_mono + 1 amet + 1 b1665_tRNA_4 <==> 1 b1665_tRNA_4_YggH_mono_cplx	reversible	tRNA modification	-1.00E+09	1.00E+09	0	7.79E+02	7.79E+02	0	5.60E+00	5.6	0	5.60E+00	5.6
b1665_tRNA_46_MODIF_2	modification of position 46 nt (m7G) in valV by YggH_mono	1 b1665_tRNA_4_YggH_mono_cplx --> 1 YggH_mono_inact + 1 ahcys + 1 b1665_tRNA_5 + 1 h	irreversible	tRNA modification	0.00E+00	1.00E+09	0	7.79E+02	7.79E+02	0	5.60E+00	5.6	0	5.60E+00	5.6
b1665_tRNA_47_MODIF_1	modification of position 47 nt (acp3U) in valV by AcpT_tRNA_pos_47_acp3U	1 AcpT_tRNA_pos_47_acp3U + 1 amet + 1 b1665_tRNA_5 <==> 1 b1665_tRNA_5_AcpT_tRNA_pos_47_acp3U_cplx	reversible	tRNA modification	-1.00E+09	1.00E+09	0	7.79E+02	7.79E+02	0	5.60E+00	5.6	0	5.60E+00	5.6
b1665_tRNA_47_MODIF_2	modification of position 47 nt (acp3U) in valV by AcpT_tRNA_pos_47_acp3U	1 b1665_tRNA_5_AcpT_tRNA_pos_47_acp3U_cplx --> 1 5mta + 1 AcpT_tRNA_pos_47_acp3U_inact + 1 b1665_tRNA_6 + 1 h	irreversible	tRNA modification	0.00E+00	1.00E+09	0	7.79E+02	7.79E+02	0	5.60E+00	5.6	0	5.60E+00	5.6
b1665_tRNA_54_MODIF_1	modification of position 54 nt (m5U) in valV by TrmA_mono	1 TrmA_mono + 1 amet + 1 b1665_tRNA_6 <==> 1 b1665_tRNA_6_TrmA_mono_cplx	reversible	tRNA modification	-1.00E+09	1.00E+09	0	7.79E+02	7.79E+02	0	5.60E+00	5.6	0	5.60E+00	5.6
b1665_tRNA_54_MODIF_2	modification of position 54 nt (m5U) in valV by TrmA_mono	1 b1665_tRNA_6_TrmA_mono_cplx --> 1 TrmA_mono_inact + 1 ahcys + 1 b1665_tRNA_7 + 1 h	irreversible	tRNA modification	0.00E+00	1.00E+09	0	7.79E+02	7.79E+02	0	5.60E+00	5.6	0	5.60E+00	5.6
b1665_tRNA_55_MODIF_1	modification of position 55 nt (Y) in valV by TruB_mono	1 TruB_mono + 1 b1665_tRNA_7 <==> 1 b1665_tRNA_7_TruB_mono_cplx	reversible	tRNA modification	-1.00E+09	1.00E+09	0	7.79E+02	7.79E+02	0	5.60E+00	5.6	0	5.60E+00	5.6
b1665_tRNA_55_MODIF_2	modification of position 55 nt (Y) in valV by TruB_mono	1 b1665_tRNA_7_TruB_mono_cplx --> 1 TruB_mono_inact + 1 b1665_tRNA_8	irreversible	tRNA modification	0.00E+00	1.00E+09	0	7.79E+02	7.79E+02	0	5.60E+00	5.6	0	5.60E+00	5.6
b1665_tRNA_8_MODIF_1	modification of position 8 nt (s4U) in valV by ThiI_mono	1 IscS_dim_S-SH + 1 ThiI_mono + 1 atp + 1 b1665_tRNA_8 + 1 mg2 + 1 trdrd <==> 1 b1665_tRNA_8_ThiI_mono_cplx	reversible	tRNA modification	-1.00E+09	1.00E+09	0	7.79E+02	7.79E+02	0	5.60E+00	5.6	0	5.60E+00	5.6
b1665_tRNA_8_MODIF_2	modification of position 8 nt (s4U) in valV by ThiI_mono	1 b1665_tRNA_8_ThiI_mono_cplx --> 1 IscS_dim_S-H + 1 ThiI_mono_inact + 1 amp + 1 h + 1 mg2 + 1 ppi + 1 trdox + 1 valV_tRNA	irreversible	tRNA modification	0.00E+00	1.00E+09	0	7.79E+02	7.79E+02	0	5.60E+00	5.6	0	5.60E+00	5.6
b1665_tRNA_Mg_BIND	Binding of two Mg2 to b1665_tRNA	1 b1665_RNA + 2 mg2 <==> 1 b1665_tRNA_Mg2	reversible	Metallo-ion binding	-1.00E+09	1.00E+09	0	7.79E+02	7.79E+02	0	5.60E+00	5.6	0	5.60E+00	5.6
b1666_tRNA_16_MODIF_1	modification of position 16 nt (D) in valW by Dus_gen	1 Dus_gen + 1 b1666_tRNA_Mg2 + 1 h + 1 nadph <==> 1 b1666_tRNA_Mg2_Dus_gen_cplx	reversible	tRNA modification	-1.00E+09	1.00E+09	0	7.79E+02	7.79E+02	0	5.60E+00	5.6	0	5.60E+00	5.6
b1666_tRNA_16_MODIF_2	modification of position 16 nt (D) in valW by Dus_gen	1 b1666_tRNA_Mg2_Dus_gen_cplx --> 1 Dus_gen_inact + 1 b1666_tRNA_1 + 1 nadp	irreversible	tRNA modification	0.00E+00	1.00E+09	0	7.79E+02	7.79E+02	0	5.60E+00	5.6	0	5.60E+00	5.6
b1666_tRNA_17_MODIF_1	modification of position 17 nt (D) in valW by Dus_gen	1 Dus_gen + 1 b1666_tRNA_1 + 1 h + 1 nadph <==> 1 b1666_tRNA_1_Dus_gen_cplx	reversible	tRNA modification	-1.00E+09	1.00E+09	0	7.79E+02	7.79E+02	0	5.60E+00	5.6	0	5.60E+00	5.6
b1666_tRNA_17_MODIF_2	modification of position 17 nt (D) in valW by Dus_gen	1 b1666_tRNA_1_Dus_gen_cplx --> 1 Dus_gen_inact + 1 b1666_tRNA_2 + 1 nadp	irreversible	tRNA modification	0.00E+00	1.00E+09	0	7.79E+02	7.79E+02	0	5.60E+00	5.6	0	5.60E+00	5.6
b1666_tRNA_20A_MODIF_1	modification of position 20A nt (D) in valW by Dus_gen	1 Dus_gen + 1 b1666_tRNA_3 + 1 h + 1 nadph <==> 1 b1666_tRNA_3_Dus_gen_cplx	reversible	tRNA modification	-1.00E+09	1.00E+09	0	7.79E+02	7.79E+02	0	5.60E+00	5.6	0	5.60E+00	5.6
b1666_tRNA_20A_MODIF_2	modification of position 20A nt (D) in valW by Dus_gen	1 b1666_tRNA_3_Dus_gen_cplx --> 1 Dus_gen_inact + 1 b1666_tRNA_4 + 1 nadp	irreversible	tRNA modification	0.00E+00	1.00E+09	0	7.79E+02	7.79E+02	0	5.60E+00	5.6	0	5.60E+00	5.6
b1666_tRNA_20_MODIF_1	modification of position 20 nt (D) in valW by Dus_gen	1 Dus_gen + 1 b1666_tRNA_2 + 1 h + 1 nadph <==> 1 b1666_tRNA_2_Dus_gen_cplx	reversible	tRNA modification	-1.00E+09	1.00E+09	0	7.79E+02	7.79E+02	0	5.60E+00	5.6	0	5.60E+00	5.6
b1666_tRNA_20_MODIF_2	modification of position 20 nt (D) in valW by Dus_gen	1 b1666_tRNA_2_Dus_gen_cplx --> 1 Dus_gen_inact + 1 b1666_tRNA_3 + 1 nadp	irreversible	tRNA modification	0.00E+00	1.00E+09	0	7.79E+02	7.79E+02	0	5.60E+00	5.6	0	5.60E+00	5.6
b1666_tRNA_46_MODIF_1	modification of position 46 nt (m7G) in valW by YggH_mono	1 YggH_mono + 1 amet + 1 b1666_tRNA_4 <==> 1 b1666_tRNA_4_YggH_mono_cplx	reversible	tRNA modification	-1.00E+09	1.00E+09	0	7.79E+02	7.79E+02	0	5.60E+00	5.6	0	5.60E+00	5.6
b1666_tRNA_46_MODIF_2	modification of position 46 nt (m7G) in valW by YggH_mono	1 b1666_tRNA_4_YggH_mono_cplx --> 1 YggH_mono_inact + 1 ahcys + 1 b1666_tRNA_5 + 1 h	irreversible	tRNA modification	0.00E+00	1.00E+09	0	7.79E+02	7.79E+02	0	5.60E+00	5.6	0	5.60E+00	5.6
b1666_tRNA_47_MODIF_1	modification of position 47 nt (acp3U) in valW by AcpT_tRNA_pos_47_acp3U	1 AcpT_tRNA_pos_47_acp3U + 1 amet + 1 b1666_tRNA_5 <==> 1 b1666_tRNA_5_AcpT_tRNA_pos_47_acp3U_cplx	reversible	tRNA modification	-1.00E+09	1.00E+09	0	7.79E+02	7.79E+02	0	5.60E+00	5.6	0	5.60E+00	5.6
b1666_tRNA_47_MODIF_2	modification of position 47 nt (acp3U) in valW by AcpT_tRNA_pos_47_acp3U	1 b1666_tRNA_5_AcpT_tRNA_pos_47_acp3U_cplx --> 1 5mta + 1 AcpT_tRNA_pos_47_acp3U_inact + 1 b1666_tRNA_6 + 1 h	irreversible	tRNA modification	0.00E+00	1.00E+09	0	7.79E+02	7.79E+02	0	5.60E+00	5.6	0	5.60E+00	5.6
b1666_tRNA_54_MODIF_1	modification of position 54 nt (m5U) in valW by TrmA_mono	1 TrmA_mono + 1 amet + 1 b1666_tRNA_6 <==> 1 b1666_tRNA_6_TrmA_mono_cplx	reversible	tRNA modification	-1.00E+09	1.00E+09	0	7.79E+02	7.79E+02	0	5.60E+00	5.6	0	5.60E+00	5.6
b1666_tRNA_54_MODIF_2	modification of position 54 nt (m5U) in valW by TrmA_mono	1 b1666_tRNA_6_TrmA_mono_cplx --> 1 TrmA_mono_inact + 1 ahcys + 1 b1666_tRNA_7 + 1 h	irreversible	tRNA modification	0.00E+00	1.00E+09	0	7.79E+02	7.79E+02	0	5.60E+00	5.6	0	5.60E+00	5.6
b1666_tRNA_55_MODIF_1	modification of position 55 nt (Y) in valW by TruB_mono	1 TruB_mono + 1 b1666_tRNA_7 <==> 1 b1666_tRNA_7_TruB_mono_cplx	reversible	tRNA modification	-1.00E+09	1.00E+09	0	7.79E+02	7.79E+02	0	5.60E+00	5.6	0	5.60E+00	5.6
b1666_tRNA_55_MODIF_2	modification of position 55 nt (Y) in valW by TruB_mono	1 b1666_tRNA_7_TruB_mono_cplx --> 1 TruB_mono_inact + 1 b1666_tRNA_8	irreversible	tRNA modification	0.00E+00	1.00E+09	0	7.79E+02	7.79E+02	0	5.60E+00	5.6	0	5.60E+00	5.6
b1666_tRNA_8_MODIF_1	modification of position 8 nt (s4U) in valW by ThiI_mono	1 IscS_dim_S-SH + 1 ThiI_mono + 1 atp + 1 b1666_tRNA_8 + 1 mg2 + 1 trdrd <==> 1 b1666_tRNA_8_ThiI_mono_cplx	reversible	tRNA modification	-1.00E+09	1.00E+09	0	7.79E+02	7.79E+02	0	5.60E+00	5.6	0	5.60E+00	5.6
b1666_tRNA_8_MODIF_2	modification of position 8 nt (s4U) in valW by ThiI_mono	1 b1666_tRNA_8_ThiI_mono_cplx --> 1 IscS_dim_S-H + 1 ThiI_mono_inact + 1 amp + 1 h + 1 mg2 + 1 ppi + 1 trdox + 1 valW_tRNA	irreversible	tRNA modification	0.00E+00	1.00E+09	0	7.79E+02	7.79E+02	0	5.60E+00	5.6	0	5.60E+00	5.6
b1666_tRNA_Mg_BIND	Binding of two Mg2 to b1666_tRNA	1 b1666_RNA + 2 mg2 <==> 1 b1666_tRNA_Mg2	reversible	Metallo-ion binding	-1.00E+09	1.00E+09	0	7.79E+02	7.79E+02	0	5.60E+00	5.6	0	5.60E+00	5.6

b1712_fold_spon	b1712_m folding: spontanous	1 b1712_m --> 1 IhfA_mono	irreversible	Protein Folding	0.00E+00	1.00E+09	0	2.83E+02	2.83E+02	0	4.05E+02	405.2462	0	1.23E+02	122.994
b1712_mRNA_degr1	Degradation of b1712_mRNA I	1 Orn_dim + 75 atp + 1 b1712_mRNA_2 + 1 degradosome + 75 h2o <==> 75 adp + 1 b1712_mRNA_2_degr + 75 h + 75 pi	reversible	mRNA degradation	-1.00E+09	1.00E+09	0	3.20E+01	3.20E+01	0	5.65E+01	56.54924	0	9.88E-02	0.0988
b1712_mRNA_degr2	Degradation of b1712_mRNA II	1 b1712_mRNA_2_degr + 299 h2o --> 1 Orn_dim_inact + 86 amp + atp + 67 cmp + 1 degradosome_inact + 83 gmp + 299 h + 63 ump	irreversible	mRNA degradation	0.00E+00	2.45E-01	0	3.20E+01	3.20E+01	0	5.65E+01	56.54924	0	9.88E-02	0.0988
b1712_maturation1	Polypeptide b1712 peptide deformylas and methionine aminopeptidase complex	1 Def_mono + 1 Map_mono + 1 b1712_aa <==> 1 b1712_def_map_cplx	reversible	Protein Maturation	-1.00E+09	1.00E+09	0	2.83E+02	2.83E+02	0	4.05E+02	405.2462	0	1.23E+02	122.994
b1712_maturation2	b1712 formation	1 b1712_def_map_cplx + 2 h2o --> 1 Def_mono_inact + 1 Map_mono_inact + 1 b1712_m + 1 for + 1 met-L	irreversible	Protein Maturation	0.00E+00	1.00E+09	0	2.83E+02	2.83E+02	0	4.05E+02	405.2462	0	1.23E+02	122.994
b1712_v1_mRNA_CONV	convsersion of mRNA to mRNA_1 (synthetic rxn)	1 b1712_v1_mRNA --> 1 b1712_mRNA_1	irreversible	Translation	0.00E+00	1.00E+09	0	3.20E+01	3.20E+01	0	5.65E+01	56.54924	0	9.88E-02	0.0988
b1713_fold_KJE_1	b1713_m folding: KJE mediated	1 DnaJ_dim + 1 DnaK_mono.ATP + 1 b1713_m <==> 1 b1713_m_DnaKJ_complex	reversible	Protein Folding	-1.00E+09	1.00E+09	0	7.28E+02	7.28E+02	0	1.19E+03	1193.566	0	1.07E+02	107.1248
b1713_fold_KJE_2	b1713_m folding: KJE mediated	1 GrpE_dim + 1 b1713_m_DnaKJ_complex + 1 h2o --> 1 DnaJ_dim_inact + 1 PheT_DnaK_GrpE_complex + 1 adp + 1 h + 1 pi	irreversible	Protein Folding	0.00E+00	1.00E+09	0	7.28E+02	7.28E+02	0	1.19E+03	1193.566	0	1.07E+02	107.1248
b1713_fold_KJE_3	b1713_m folding: KJE mediated	1 PheT_DnaK_GrpE_complex + 1 atp --> 1 DnaK_mono.ATP_inact + 1 GrpE_dim_inact + 1 PheT_mono	irreversible	Protein Folding	0.00E+00	1.00E+09	0	7.28E+02	7.28E+02	0	1.19E+03	1193.566	0	1.07E+02	107.1248
b1713_fold_spon	b1713_m folding: spontanous	1 b1713_m --> 1 PheT_mono	irreversible	Protein Folding	0.00E+00	1.00E+09	0	7.28E+02	7.28E+02	0	1.19E+03	1193.566	0	1.07E+02	107.1248
b1713_mRNA_CONV	convsersion of mRNA to mRNA_1 (synthetic rxn)	1 b1713_mRNA --> 1 b1713_mRNA_1	irreversible	Translation	0.00E+00	1.00E+09	0	3.20E+01	3.20E+01	0	5.65E+01	56.54924	0	9.88E-02	0.0988
b1713_mRNA_degr1	Degradation of b1713_mRNA I	1 Orn_dim + 597 atp + 1 b1713_mRNA_2 + 1 degradosome + 597 h2o <==> 597 adp + 1 b1713_mRNA_2_degr + 597 h + 597 pi	reversible	mRNA degradation	-1.00E+09	1.00E+09	0	3.20E+01	3.20E+01	0	5.65E+01	56.54924	0	9.88E-02	0.0988
b1713_mRNA_degr2	Degradation of b1713_mRNA II	1 b1713_mRNA_2_degr + 2387 h2o --> 1 Orn_dim_inact + 553 amp 588 cmp + 1 degradosome_inact + 710 gmp + 2387 h + 537 ump	irreversible	mRNA degradation	0.00E+00	2.84E-01	0	3.20E+01	3.20E+01	0	5.65E+01	56.54924	0	9.88E-02	0.0988
b1713_maturation1	Polypeptide b1713 peptide deformylas and methionine aminopeptidase complex	1 Def_mono + 1 Map_mono + 1 b1713_aa <==> 1 b1713_def_map_cplx	reversible	Protein Maturation	-1.00E+09	1.00E+09	0	7.28E+02	7.28E+02	0	1.19E+03	1193.566	0	1.07E+02	107.1248
b1713_maturation2	b1713 formation	1 b1713_def_map_cplx + 2 h2o --> 1 Def_mono_inact + 1 Map_mono_inact + 1 b1713_m + 1 for + 1 met-L	irreversible	Protein Maturation	0.00E+00	1.00E+09	0	7.28E+02	7.28E+02	0	1.19E+03	1193.566	0	1.07E+02	107.1248
b1714_fold_spon	b1714_m folding: spontanous	1 b1714_m --> 1 PheS_mono	irreversible	Protein Folding	0.00E+00	1.00E+09	0	7.28E+02	7.28E+02	0	1.19E+03	1193.566	0	1.07E+02	107.1248
b1714_mRNA_CONV	convsersion of mRNA to mRNA_1 (synthetic rxn)	1 b1714_mRNA --> 1 b1714_mRNA_1	irreversible	Translation	0.00E+00	1.00E+09	0	3.20E+01	3.20E+01	0	5.65E+01	56.54924	0	9.88E-02	0.0988
b1714_mRNA_degr1	Degradation of b1714_mRNA I	1 Orn_dim + 246 atp + 1 b1714_mRNA_2 + 1 degradosome + 246 h2o <==> 246 adp + 1 b1714_mRNA_2_degr + 246 h + 246 pi	reversible	mRNA degradation	-1.00E+09	1.00E+09	0	3.20E+01	3.20E+01	0	5.65E+01	56.54924	0	9.88E-02	0.0988
b1714_mRNA_degr2	Degradation of b1714_mRNA II	1 b1714_mRNA_2_degr + 983 h2o --> 1 Orn_dim_inact + 223 amp + 265 cmp + 1 degradosome_inact + 265 gmp + 983 h + 231 ump	irreversible	mRNA degradation	0.00E+00	3.72E-01	0	3.20E+01	3.20E+01	0	5.65E+01	56.54924	0	9.88E-02	0.0988
b1714_maturation1	Polypeptide b1714 peptide deformylas and methionine aminopeptidase complex	1 Def_mono + 1 Map_mono + 1 b1714_aa <==> 1 b1714_def_map_cplx	reversible	Protein Maturation	-1.00E+09	1.00E+09	0	7.28E+02	7.28E+02	0	1.19E+03	1193.566	0	1.07E+02	107.1248
b1714_maturation2	b1714 formation	1 b1714_def_map_cplx + 2 h2o --> 1 Def_mono_inact + 1 Map_mono_inact + 1 b1714_m + 1 for + 1 met-L	irreversible	Protein Maturation	0.00E+00	1.00E+09	0	7.28E+02	7.28E+02	0	1.19E+03	1193.566	0	1.07E+02	107.1248
b1715_fold_spon	b1715_m folding: spontanous	1 b1715_m --> 1 PheM_mono	irreversible	Protein Folding	0.00E+00	1.00E+09	0	2.19E+02	2.19E+02	0	2.24E+02	224.2242	0	1.13E+02	113.3448
b1715_mRNA_degr1	Degradation of b1715_mRNA I	1 Orn_dim + 11 atp + 1 b1715_mRNA_2 + 1 degradosome + 11 h2o <==> 11 adp + 1 b1715_mRNA_2_degr + 11 h + 11 pi	reversible	mRNA degradation	-1.00E+09	1.00E+09	0	1.06E+02	1.06E+02	0	1.11E+02	110.9782	0	9.88E-02	0.0988
b1715_mRNA_degr2	Degradation of b1715_mRNA II	1 b1715_mRNA_2_degr + 44 h2o --> 1 Orn_dim_inact + 7 amp + 1 atp + 10 cmp + 1 degradosome_inact + 6 gmp + 44 h + 21 ump	irreversible	mRNA degradation	0.00E+00	9.88E-02	0	1.06E+02	1.06E+02	0	1.11E+02	110.9782	0	9.88E-02	0.0988
b1715_maturation1	Polypeptide b1715 peptide deformylas and methionine aminopeptidase complex	1 Def_mono + 1 Map_mono + 1 b1715_aa <==> 1 b1715_def_map_cplx	reversible	Protein Maturation	-1.00E+09	1.00E+09	0	2.19E+02	2.19E+02	0	2.24E+02	224.2242	0	1.13E+02	113.3448
b1715_maturation2	b1715 formation	1 b1715_def_map_cplx + 2 h2o --> 1 Def_mono_inact + 1 Map_mono_inact + 1 b1715_m + 1 for + 1 met-L	irreversible	Protein Maturation	0.00E+00	1.00E+09	0	2.19E+02	2.19E+02	0	2.24E+02	224.2242	0	1.13E+02	113.3448
b1715_v1_mRNA_CONV	convsersion of mRNA to mRNA_1 (synthetic rxn)	1 b1715_v1_mRNA --> 1 b1715_mRNA_1	irreversible	Translation	0.00E+00	1.00E+09	0	1.02E+02	1.02E+02	0	8.29E+01	82.88	0	9.88E-02	0.0988
b1715_v2_mRNA_CONV	convsersion of mRNA to mRNA_1 (synthetic rxn)	1 b1715_v2_mRNA --> 1 b1715_mRNA_1	irreversible	Translation	0.00E+00	1.00E+09	0	3.20E+01	3.20E+01	0	5.65E+01	56.54924	0	9.88E-02	0.0988
b1716_fold_spon	b1716_m folding: spontanous	1 b1716_m --> 1 RplT_mono	irreversible	Protein Folding	0.00E+00	1.00E+09	8.44E+01	1.13E+02	2.81E+01	6.22E+01	8.29E+01	20.72	6.22E+01	8.29E+01	20.72
b1716_mRNA_CONV	convsersion of mRNA to mRNA_1 (synthetic rxn)	1 b1716_mRNA --> 1 b1716_mRNA_1	irreversible	Translation	0.00E+00	1.00E+09	0	4.35E+01	4.35E+01	0	6.79E+01	67.90817	0	1.65E-01	0.165
b1716_mRNA_degr1	Degradation of b1716_mRNA I	1 Orn_dim + 89 atp + 1 b1716_mRNA_2 + 1 degradosome + 89 h2o <==> 89 adp + 1 b1716_mRNA_2_degr + 89 h + 89 pi	reversible	mRNA degradation	-1.00E+09	1.00E+09	0	1.05E+02	1.05E+02	0	8.29E+01	82.88	0	1.65E-01	0.165
b1716_mRNA_degr2	Degradation of b1716_mRNA II	1 b1716_mRNA_2_degr + 356 h2o --> 1 Orn_dim_inact + 97 amp + 86 cmp + 1 degradosome_inact + 86 gmp + 356 h + 88 ump	irreversible	mRNA degradation	0.00E+00	1.65E-01	0	1.05E+02	1.05E+02	0	8.29E+01	82.88	0	1.65E-01	0.165
b1716_maturation1	Polypeptide b1716 peptide deformylas and methionine aminopeptidase complex	1 Def_mono + 1 Map_mono + 1 b1716_aa <==> 1 b1716_def_map_cplx	reversible	Protein Maturation	-1.00E+09	1.00E+09	8.44E+01	1.13E+02	2.81E+01	6.22E+01	8.29E+01	20.72	6.22E+01	8.29E+01	20.72
b1716_maturation2	b1716 formation	1 b1716_def_map_cplx + 2 h2o --> 1 Def_mono_inact + 1 Map_mono_inact + 1 b1716_m + 1 for + 1 met-L	irreversible	Protein Maturation	0.00E+00	1.00E+09	8.44E+01	1.13E+02	2.81E+01	6.22E+01	8.29E+01	20.72	6.22E+01	8.29E+01	20.72
b1716_v1_mRNA_CONV	convsersion of mRNA to mRNA_1 (synthetic rxn)	1 b1716_v1_mRNA --> 1 b1716_mRNA_1	irreversible	Translation	0.00E+00	1.00E+09	0	1.02E+02	1.02E+02	0	8.29E+01	82.88	0	9.88E-02	0.0988
b1716_v2_mRNA_CONV	convsersion of mRNA to mRNA_1 (synthetic rxn)	1 b1716_v2_mRNA --> 1 b1716_mRNA_1	irreversible	Translation	0.00E+00	1.00E+09	0	1.05E+02	1.05E+02	0	8.29E+01	82.88	0	1.65E-01	0.165
b1717_fold_spon	b1717_m folding: spontanous	1 b1717_m --> 1 RpmI_mono	irreversible	Protein Folding	0.00E+00	1.00E+09	8.44E+01	1.13E+02	2.81E+01	6.22E+01	8.29E+01	20.72	6.22E+01	8.29E+01	20.72
b1717_mRNA_CONV	convsersion of mRNA to mRNA_1 (synthetic rxn)	1 b1717_mRNA --> 1 b1717_mRNA_1	irreversible	Translation	0.00E+00	1.00E+09	0	4.35E+01	4.35E+01	0	6.79E+01	67.90817	0	1.65E-01	0.165
b1717_mRNA_degr1	Degradation of b1717_mRNA I	1 Orn_dim + 49 atp + 1 b1717_mRNA_2 + 1 degradosome + 49 h2o <==> 49 adp + 1 b1717_mRNA_2_degr + 49 h + 49 pi	reversible	mRNA degradation	-1.00E+09	1.00E+09	0	4.35E+01	4.35E+01	0	6.79E+01	67.90817	0	1.65E-01	0.165
b1717_mRNA_degr2	Degradation of b1717_mRNA II	1 b1717_mRNA_2_degr + 197 h2o --> 1 Orn_dim_inact + 61 amp + 53 cmp + 1 degradosome_inact + 46 gmp + 197 h + 38 ump	irreversible	mRNA degradation	0.00E+00	2.62E-01	0	4.35E+01	4.35E+01	0	6.79E+01	67.90817	0	1.65E-01	0.165
b1717_maturation1	Polypeptide b1717 peptide deformylas and methionine aminopeptidase complex	1 Def_mono + 1 Map_mono + 1 b1717_aa <==> 1 b1717_def_map_cplx	reversible	Protein Maturation	-1.00E+09	1.00E+09	8.44E+01	1.13E+02	2.81E+01	6.22E+01	8.29E+01	20.72	6.22E+01	8.29E+01	20.72
b1717_maturation2	b1717 formation	1 b1717_def_map_cplx + 2 h2o --> 1 Def_mono_inact + 1 Map_mono_inact + 1 b1717_m + 1 for + 1 met-L	irreversible	Protein Maturation	0.00E+00	1.00E+09	8.44E+01	1.13E+02	2.81E+01	6.22E+01	8.29E+01	20.72	6.22E+01	8.29E+01	20.72
b1718_fold_GroEL/ES_1	b1718_m folding: GroEL/ES mediated: polypeptide is going in GroEL/ES complex	1 GroEL.(7)ADP.cisGroES + 7 atp + 1 b1718_m + 1 transGroES_hepta --> 7 adp + 1 b1718_m_GroEL.(7)ATP.transGroES + 1 cisGroES_hepta	irreversible	Protein Folding	0.00E+00	1.00E+09	0	3.02E+03	3.02E+03	0	5.11E+03	5108.769	0	2.96E+02	296.22
b1718_fold_GroEL/ES_2	b1718_m folding: GroEL/ES mediated: folding of polypeptide under ATP hydrolysis	1 b1718_m_GroEL.(7)ATP.transGroES + 7 h2o --> 1 InfC_GroEL.(7)ADP.transGroES + 7 h + 7 pi	irreversible	Protein Folding	0.00E+00	1.00E+09	0	3.02E+03	3.02E+03	0	5.11E+03	5108.769	0	2.96E+02	296.22
b1718_fold_GroEL/ES_3	b1718_m folding: GroEL/ES mediated: release of native protein	1 InfC_GroEL.(7)ADP.transGroES --> 1 GroEL.(7)ADP.transGroES + 1 InfC_mono	irreversible	Protein Folding	0.00E+00	1.00E+09	0	3.02E+03	3.02E+03	0	5.11E+03	5108.769	0	2.96E+02	296.22
b1718_fold_KJE_1	b1718_m folding: KJE mediated	1 DnaJ_dim + 1 DnaK_mono.ATP + 1 b1718_m <==> 1 b1718_m_DnaKJ_complex	reversible	Protein Folding	-1.00E+09	1.00E+09	0	3.02E+03	3.02E+03	0	5.11E+03	5108.769	0	2.96E+02	296.22
b1718_fold_KJE_2	b1718_m folding: KJE mediated	1 GrpE_dim + 1 b1718_m_DnaKJ_complex + 1 h2o --> 1 DnaJ_dim_inact + 1 InfC_DnaK_GrpE_complex + 1 adp + 1 h + 1 pi	irreversible	Protein Folding	0.00E+00	1.00E+09	0	3.02E+03	3.02E+03	0	5.11E+03	5108.769	0	2.96E+02	296.22
b1718_fold_KJE_3	b1718_m folding: KJE mediated	1 InfC_DnaK_GrpE_complex + 1 atp --> 1 DnaK_mono.ATP_inact + GrpE_dim_inact + 1 InfC_mono	irreversible	Protein Folding	0.00E+00	1.00E+09	0	3.02E+03	3.02E+03	0	5.11E+03	5108.769	0	2.96E+02	296.22
b1718_fold_spon	b1718_m folding: spontanous	1 b1718_m --> 1 InfC_mono	irreversible	Protein Folding	0.00E+00	1.00E+09	0	3.02E+03	3.02E+03	0	5.11E+03	5108.769	0	2.96E+02	296.22

b1718_mRNA_CONV	convsersion of mRNA to mRNA_1 (synthetic rxn)	1 b1718_mRNA --> 1 b1718_mRNA_1	irreversible	Translation	0.00E+00	1.00E+09	0	4.35E+01	4.35E+01	0	6.79E+01	67.90817	0	1.65E-01	0.165
b1718_mRNA_degr1	Degradation of b1718_mRNA I	1 Orn_dim + 135 atp + 1 b1718_mRNA_2 + 1 degradosome + 135 h2o <==> 135 adp + 1 b1718_mRNA_2_degr + 135 h + 135 pi	reversible	mRNA degradation	-1.00E+09	1.00E+09	0	2.73E+02	2.73E+02	0	4.82E+02	481.8769	0	6.22E-01	0.622
b1718_mRNA_degr2	Degradation of b1718_mRNA II	1 b1718_mRNA_2_degr + 542 h2o --> 1 Orn_dim_inact + 169 amp + 117 cmp + 1 degradosome_inact + 147 gmp + 542 h + 110 ump	irreversible	mRNA degradation	0.00E+00	6.22E-01	0	2.73E+02	2.73E+02	0	4.82E+02	481.8769	0	6.22E-01	0.622
b1718_maturation1	Polypeptide b1718 peptide deformylas complex	1 Def_mono + 1 b1718_aa <==> 1 b1718_def_cplx	reversible	Protein Maturation	-1.00E+09	1.00E+09	8.44E+01	3.02E+03	2.94E+03	6.22E+01	5.11E+03	5046.609	6.22E+01	2.96E+02	234.06
b1718_maturation2	b1718 formation	1 b1718_def_cplx + 1 h2o --> 1 Def_mono_inact + 1 b1718_m + 1 for	irreversible	Protein Maturation	0.00E+00	1.00E+09	8.44E+01	3.02E+03	2.94E+03	6.22E+01	5.11E+03	5046.609	6.22E+01	2.96E+02	234.06
b1718_v1_mRNA_CONV	convsersion of mRNA to mRNA_1 (synthetic rxn)	1 b1718_v1_mRNA --> 1 b1718_mRNA_1	irreversible	Translation	0.00E+00	1.00E+09	0	2.73E+02	2.73E+02	0	4.82E+02	481.8769	0	6.22E-01	0.622
b1718_v2_mRNA_CONV	convsersion of mRNA to mRNA_1 (synthetic rxn)	1 b1718_v2_mRNA --> 1 b1718_mRNA_1	irreversible	Translation	0.00E+00	1.00E+09	0	2.73E+02	2.73E+02	0	4.82E+02	481.8769	0	6.22E-01	0.622
b1719_fold_GroEL/ES_1	b1719_m folding: GroEL/ES mediated: polypeptide is going in GroEL/ES complex	1 GroEL.(7)ADP.cisGroES + 7 atp + 1 b1719_m_Zn + 1 transGroES_hepta --> 7 adp + 1 b1719_m_GroEL.(7)ATP.transGroES + 1 cisGroES_hepta	irreversible	Protein Folding	0.00E+00	1.00E+09	0	1.92E+03	1.92E+03	0	2.82E+03	2824.512	0	3.18E+02	318.015
b1719_fold_GroEL/ES_2	b1719_m folding: GroEL/ES mediated: folding of polypeptide under ATP hydrolysis	1 b1719_m_GroEL.(7)ATP.transGroES + 7 h2o --> 1 ThrS_GroEL.(7)ADP.transGroES + 7 h + 7 pi	irreversible	Protein Folding	0.00E+00	1.00E+09	0	1.92E+03	1.92E+03	0	2.82E+03	2824.512	0	3.18E+02	318.015
b1719_fold_GroEL/ES_3	b1719_m folding: GroEL/ES mediated: release of native protein	1 ThrS_GroEL.(7)ADP.transGroES --> 1 GroEL.(7)ADP.transGroES + 1 ThrS_mono	irreversible	Protein Folding	0.00E+00	1.00E+09	0	1.92E+03	1.92E+03	0	2.82E+03	2824.512	0	3.18E+02	318.015
b1719_fold_KJE_1	b1719_m folding: KJE mediated	1 DnaJ_dim + 1 DnaK_mono.ATP + 1 b1719_m_Zn <==> 1 b1719_m_DnaKJ_complex	reversible	Protein Folding	-1.00E+09	1.00E+09	0	1.92E+03	1.92E+03	0	2.82E+03	2824.512	0	3.18E+02	318.015
b1719_fold_KJE_2	b1719_m folding: KJE mediated	1 GrpE_dim + 1 b1719_m_DnaKJ_complex + 1 h2o --> 1 DnaJ_dim_inact + 1 ThrS_DnaK_GrpE_complex + 1 adp + 1 h + 1 pi	irreversible	Protein Folding	0.00E+00	1.00E+09	0	1.92E+03	1.92E+03	0	2.82E+03	2824.512	0	3.18E+02	318.015
b1719_fold_KJE_3	b1719_m folding: KJE mediated	1 ThrS_DnaK_GrpE_complex + 1 atp --> 1 DnaK_mono.ATP_inact + 1 GrpE_dim_inact + 1 ThrS_mono	irreversible	Protein Folding	0.00E+00	1.00E+09	0	1.92E+03	1.92E+03	0	2.82E+03	2824.512	0	3.18E+02	318.015
b1719_ion_BIND	Binding of ions to b1719	1 b1719_m + 1 zn2 <==> 1 b1719_m_Zn	reversible	Metallo-ion Binding	-1.00E+09	1.00E+09	0	1.92E+03	1.92E+03	0	2.82E+03	2824.512	0	3.18E+02	318.015
b1719_mRNA_CONV	convsersion of mRNA to mRNA_1 (synthetic rxn)	1 b1719_mRNA --> 1 b1719_mRNA_1	irreversible	Translation	0.00E+00	1.00E+09	0	4.35E+01	4.35E+01	0	6.79E+01	67.90817	0	1.65E-01	0.165
b1719_mRNA_degr1	Degradation of b1719_mRNA I	1 Orn_dim + 482 atp + 1 b1719_mRNA_2 + 1 degradosome + 482 h2o <==> 482 adp + 1 b1719_mRNA_2_degr + 482 h + 482 pi	reversible	mRNA degradation	-1.00E+09	1.00E+09	0	4.35E+01	4.35E+01	0	6.79E+01	67.90817	0	1.65E-01	0.165
b1719_mRNA_degr2	Degradation of b1719_mRNA II	1 b1719_mRNA_2_degr + 1928 h2o --> 1 Orn_dim_inact + 518 amp 1 atp + 452 cmp + 1 degradosome_inact + 504 gmp + 1928 h + 454 ump	irreversible	mRNA degradation	0.00E+00	5.67E-01	0	4.35E+01	4.35E+01	0	6.79E+01	67.90817	0	1.65E-01	0.165
b1719_maturation1	Polypeptide b1719 peptide deformylas and methionine aminopeptidase complex	1 Def_mono + 1 Map_mono + 1 b1719_aa <==> 1 b1719_def_map_cplx	reversible	Protein Maturation	-1.00E+09	1.00E+09	0	1.92E+03	1.92E+03	0	2.82E+03	2824.512	0	3.18E+02	318.015
b1719_maturation2	b1719 formation	1 b1719_def_map_cplx + 2 h2o --> 1 Def_mono_inact + 1 Map_mono_inact + 1 b1719_m + 1 for + 1 met-L	irreversible	Protein Maturation	0.00E+00	1.00E+09	0	1.92E+03	1.92E+03	0	2.82E+03	2824.512	0	3.18E+02	318.015
b1804_DNA_act_bind	b1804_DNA binding of activator	1 b1804_DNA_neu --> 1 b1804_DNA_act	irreversible	Transcription Regulation	0.00E+00	1.00E+09	0	1.11E+02	1.11E+02	0	1.95E+02	195.0851	0	9.45E-02	0.0945
b1804_fold_spon	b1804_m folding: spontanous	1 b1804_m_Mg --> 1 Rnd_mono	irreversible	Protein Folding	0.00E+00	1.00E+09	0	2.50E+03	2.50E+03	0	4.11E+03	4113.529	0	7.31E+01	73.139
b1804_ion_BIND	Binding of ions to b1804	1 b1804_m + 5 mg2 <==> 1 b1804_m_Mg	reversible	Metallo-ion Binding	-1.00E+09	1.00E+09	0	2.50E+03	2.50E+03	0	4.11E+03	4113.529	0	7.31E+01	73.139
b1804_mRNA_CONV	convsersion of mRNA to mRNA_1 (synthetic rxn)	1 b1804_mRNA --> 1 b1804_mRNA_1	irreversible	Translation	0.00E+00	1.00E+09	0	1.11E+02	1.11E+02	0	1.95E+02	195.0851	0	9.45E-02	0.0945
b1804_mRNA_degr1	Degradation of b1804_mRNA I	1 Orn_dim + 282 atp + 1 b1804_mRNA_2 + 1 degradosome + 282 h2o <==> 282 adp + 1 b1804_mRNA_2_degr + 282 h + 282 pi	reversible	mRNA degradation	-1.00E+09	1.00E+09	0	1.11E+02	1.11E+02	0	1.95E+02	195.0851	0	9.45E-02	0.0945
b1804_mRNA_degr2	Degradation of b1804_mRNA II	1 b1804_mRNA_2_degr + 1127 h2o --> 1 Orn_dim_inact + 258 amp 289 cmp + 1 degradosome_inact + 321 gmp + 1127 h + 259 ump + 1 utp	irreversible	mRNA degradation	0.00E+00	9.45E-02	0	1.11E+02	1.11E+02	0	1.95E+02	195.0851	0	9.45E-02	0.0945
b1804_maturation1	Polypeptide b1804 peptide deformylas complex	1 Def_mono + 1 b1804_aa <==> 1 b1804_def_cplx	reversible	Protein Maturation	-1.00E+09	1.00E+09	0	2.50E+03	2.50E+03	0	4.11E+03	4113.529	0	7.31E+01	73.139
b1804_maturation2	b1804 formation	1 b1804_def_cplx + 1 h2o --> 1 Def_mono_inact + 1 b1804_m + 1 for	irreversible	Protein Maturation	0.00E+00	1.00E+09	0	2.50E+03	2.50E+03	0	4.11E+03	4113.529	0	7.31E+01	73.139
b1822_fold_spon	b1822_m folding: spontanous	1 b1822_m_Zn --> 1 RrmA_mono	irreversible	Protein Folding	0.00E+00	1.00E+09	0	2.52E+03	2.52E+03	0	4.38E+03	4381.213	0	8.95E+01	89.547
b1822_ion_BIND	Binding of ions to b1822	1 b1822_m + 1 zn2 <==> 1 b1822_m_Zn	reversible	Metallo-ion Binding	-1.00E+09	1.00E+09	0	2.52E+03	2.52E+03	0	4.38E+03	4381.213	0	8.95E+01	89.547
b1822_mRNA_CONV	convsersion of mRNA to mRNA_1 (synthetic rxn)	1 b1822_mRNA --> 1 b1822_mRNA_1	irreversible	Translation	0.00E+00	1.00E+09	0	1.62E+02	1.62E+02	0	2.86E+02	286.1908	0	7.98E-02	0.0798
b1822_mRNA_degr1	Degradation of b1822_mRNA I	1 Orn_dim + 202 atp + 1 b1822_mRNA_2 + 1 degradosome + 202 h2o <==> 202 adp + 1 b1822_mRNA_2_degr + 202 h + 202 pi	reversible	mRNA degradation	-1.00E+09	1.00E+09	0	1.62E+02	1.62E+02	0	2.86E+02	286.1908	0	7.98E-02	0.0798
b1822_mRNA_degr2	Degradation of b1822_mRNA II	1 b1822_mRNA_2_degr + 809 h2o --> 1 Orn_dim_inact + 193 amp + 1 atp + 197 cmp + 1 degradosome_inact + 219 gmp + 809 h + 200 ump	irreversible	mRNA degradation	0.00E+00	7.98E-02	0	1.62E+02	1.62E+02	0	2.86E+02	286.1908	0	7.98E-02	0.0798
b1822_maturation1	Polypeptide b1822 peptide deformylas and methionine aminopeptidase complex	1 Def_mono + 1 Map_mono + 1 b1822_aa <==> 1 b1822_def_map_cplx	reversible	Protein Maturation	-1.00E+09	1.00E+09	0	2.52E+03	2.52E+03	0	4.38E+03	4381.213	0	8.95E+01	89.547
b1822_maturation2	b1822 formation	1 b1822_def_map_cplx + 2 h2o --> 1 Def_mono_inact + 1 Map_mono_inact + 1 b1822_m + 1 for + 1 met-L	irreversible	Protein Maturation	0.00E+00	1.00E+09	0	2.52E+03	2.52E+03	0	4.38E+03	4381.213	0	8.95E+01	89.547
b1866_fold_spon	b1866_m folding: spontanous	1 b1866_m --> 1 AspS_mono	irreversible	Protein Folding	0.00E+00	1.00E+09	0	2.48E+03	2.48E+03	0	4.23E+03	4228.23	0	7.87E+01	78.676
b1866_mRNA_CONV	convsersion of mRNA to mRNA_1 (synthetic rxn)	1 b1866_mRNA --> 1 b1866_mRNA_1	irreversible	Translation	0.00E+00	1.00E+09	0	7.08E+01	7.08E+01	0	1.25E+02	125.0102	0	1.04E-01	0.104
b1866_mRNA_degr1	Degradation of b1866_mRNA I	1 Orn_dim + 443 atp + 1 b1866_mRNA_2 + 1 degradosome + 443 h2o <==> 443 adp + 1 b1866_mRNA_2_degr + 443 h + 443 pi	reversible	mRNA degradation	-1.00E+09	1.00E+09	0	7.08E+01	7.08E+01	0	1.25E+02	125.0102	0	1.04E-01	0.104
b1866_mRNA_degr2	Degradation of b1866_mRNA II	1 b1866_mRNA_2_degr + 1772 h2o --> 1 Orn_dim_inact + 416 amp 1 atp + 451 cmp + 1 degradosome_inact + 502 gmp + 1772 h + 403 ump	irreversible	mRNA degradation	0.00E+00	1.04E-01	0	7.08E+01	7.08E+01	0	1.25E+02	125.0102	0	1.04E-01	0.104
b1866_maturation1	Polypeptide b1866 peptide deformylas complex	1 Def_mono + 1 b1866_aa <==> 1 b1866_def_cplx	reversible	Protein Maturation	-1.00E+09	1.00E+09	0	2.48E+03	2.48E+03	0	4.23E+03	4228.23	0	7.87E+01	78.676
b1866_maturation2	b1866 formation	1 b1866_def_cplx + 1 h2o --> 1 Def_mono_inact + 1 b1866_m + 1 for	irreversible	Protein Maturation	0.00E+00	1.00E+09	0	2.48E+03	2.48E+03	0	4.23E+03	4228.23	0	7.87E+01	78.676
b1869_fold_spon	b1869_m folding: spontanous	1 b1869_m --> 1 YecN_mono	irreversible	Protein Folding	0.00E+00	1.00E+09	0	5.14E+02	5.14E+02	0	8.42E+02	842.1994	0	8.58E+01	85.806
b1869_mRNA_CONV	convsersion of mRNA to mRNA_1 (synthetic rxn)	1 b1869_mRNA --> 1 b1869_mRNA_1	irreversible	Translation	0.00E+00	1.00E+09	0	6.13E+01	6.13E+01	0	1.08E+02	108.2142	0	1.58E-01	0.158
b1869_mRNA_degr1	Degradation of b1869_mRNA I	1 Orn_dim + 99 atp + 1 b1869_mRNA_2 + 1 degradosome + 99 h2o <==> 99 adp + 1 b1869_mRNA_2_degr + 99 h + 99 pi	reversible	mRNA degradation	-1.00E+09	1.00E+09	0	6.13E+01	6.13E+01	0	1.08E+02	108.2142	0	1.58E-01	0.158
b1869_mRNA_degr2	Degradation of b1869_mRNA II	1 b1869_mRNA_2_degr + 395 h2o --> 1 Orn_dim_inact + 66 amp + [illegible] atp + 86 cmp + 1 degradosome_inact + 115 gmp + 395 h + 128 ump	irreversible	mRNA degradation	0.00E+00	1.77E-01	0	6.13E+01	6.13E+01	0	1.08E+02	108.2142	0	1.58E-01	0.158
b1869_maturation1	Polypeptide b1869 peptide deformylas and methionine aminopeptidase complex	1 Def_mono + 1 Map_mono + 1 b1869_aa <==> 1 b1869_def_map_cplx	reversible	Protein Maturation	-1.00E+09	1.00E+09	0	5.14E+02	5.14E+02	0	8.42E+02	842.1994	0	8.58E+01	85.806
b1869_maturation2	b1869 formation	1 b1869_def_map_cplx + 2 h2o --> 1 Def_mono_inact + 1 Map_mono_inact + 1 b1869_m + 1 for + 1 met-L	irreversible	Protein Maturation	0.00E+00	1.00E+09	0	5.14E+02	5.14E+02	0	8.42E+02	842.1994	0	8.58E+01	85.806
b1870_fold_spon	b1870_m folding: spontanous	1 b1870_m --> 1 YecO_mono	irreversible	Protein Folding	0.00E+00	1.00E+09	0	9.48E+02	9.48E+02	0	1.60E+03	1604.879	0	9.21E+01	92.092
b1870_mRNA_CONV	convsersion of mRNA to mRNA_1 (synthetic rxn)	1 b1870_mRNA --> 1 b1870_mRNA_1	irreversible	Translation	0.00E+00	1.00E+09	0	6.13E+01	6.13E+01	0	1.08E+02	108.2142	0	1.58E-01	0.158

b1870_mRNA_degr1	Degradation of b1870_mRNA I	1 Orn_dim + 186 atp + 1 b1870_mRNA_2 + 1 degradosome + 186 h2o <==> 186 adp + 1 b1870_mRNA_2_degr + 186 h + 186 pi	reversible	mRNA degradation	-1.00E+09	1.00E+09	0	6.13E+01	6.13E+01	0	1.08E+02	108.2142	0	1.58E-01	0.158
b1870_mRNA_degr2	Degradation of b1870_mRNA II	1 b1870_mRNA_2_degr + 743 h2o --> 1 Orn_dim_inact + 177 amp + 188 cmp + 1 degradosome_inact + 189 gmp + 743 h + 190 ump	irreversible	mRNA degradation	0.00E+00	2.84E-01	0	6.13E+01	6.13E+01	0	1.08E+02	108.2142	0	1.58E-01	0.158
b1870_maturation1	Polypeptide b1870 peptide deformylas and methionine aminopeptidase complex	1 Def_mono + 1 Map_mono + 1 b1870_aa <==> 1 b1870_def_map_cplx	reversible	Protein Maturation	-1.00E+09	1.00E+09	0	9.48E+02	9.48E+02	0	1.60E+03	1604.879	0	9.21E+01	92.092
b1870_maturation2	b1870 formation	1 b1870_def_map_cplx + 2 h2o --> 1 Def_mono_inact + 1 Map_mono_inact + 1 b1870_m + 1 for + 1 met-L	irreversible	Protein Maturation	0.00E+00	1.00E+09	0	9.48E+02	9.48E+02	0	1.60E+03	1604.879	0	9.21E+01	92.092
b1871_fold_spon	b1871_m folding: spontanous	1 b1871_m --> 1 YecP_mono	irreversible	Protein Folding	0.00E+00	1.00E+09	0	1.26E+03	1.26E+03	0	2.15E+03	2149.36	0	9.63E+01	96.292
b1871_mRNA_CONV	convsersion of mRNA to mRNA_1 (synthetic rxn)	1 b1871_mRNA --> 1 b1871_mRNA_1	irreversible	Translation	0.00E+00	1.00E+09	0	6.13E+01	6.13E+01	0	1.08E+02	108.2142	0	1.58E-01	0.158
b1871_mRNA_degr1	Degradation of b1871_mRNA I	1 Orn_dim + 242 atp + 1 b1871_mRNA_2 + 1 degradosome + 242 h2o <==> 242 adp + 1 b1871_mRNA_2_degr + 242 h + 242 pi	reversible	mRNA degradation	-1.00E+09	1.00E+09	0	6.13E+01	6.13E+01	0	1.08E+02	108.2142	0	1.58E-01	0.158
b1871_mRNA_degr2	Degradation of b1871_mRNA II	1 b1871_mRNA_2_degr + 967 h2o --> 1 Orn_dim_inact + 225 amp + 235 cmp + 1 degradosome_inact + 270 gmp + 967 h + 238 ump	irreversible	mRNA degradation	0.00E+00	1.58E-01	0	6.13E+01	6.13E+01	0	1.08E+02	108.2142	0	1.58E-01	0.158
b1871_maturation1	Polypeptide b1871 peptide deformylas and methionine aminopeptidase complex	1 Def_mono + 1 Map_mono + 1 b1871_aa <==> 1 b1871_def_map_cplx	reversible	Protein Maturation	-1.00E+09	1.00E+09	0	1.26E+03	1.26E+03	0	2.15E+03	2149.36	0	9.63E+01	96.292
b1871_maturation2	b1871 formation	1 b1871_def_map_cplx + 2 h2o --> 1 Def_mono_inact + 1 Map_mono_inact + 1 b1871_m + 1 for + 1 met-L	irreversible	Protein Maturation	0.00E+00	1.00E+09	0	1.26E+03	1.26E+03	0	2.15E+03	2149.36	0	9.63E+01	96.292
b1876_fold_spon	b1876_m folding: spontanous	1 b1876_m --> 1 ArgS_mono	irreversible	Protein Folding	0.00E+00	1.00E+09	0	2.51E+03	2.51E+03	0	4.11E+03	4107.15	0	1.02E+02	101.508
b1876_mRNA_CONV	convsersion of mRNA to mRNA_1 (synthetic rxn)	1 b1876_mRNA --> 1 b1876_mRNA_1	irreversible	Translation	0.00E+00	1.00E+09	0	7.32E+01	7.32E+01	0	1.29E+02	129.311	0	3.26E-01	0.326
b1876_mRNA_degr1	Degradation of b1876_mRNA I	1 Orn_dim + 433 atp + 1 b1876_mRNA_2 + 1 degradosome + 433 h2o <==> 433 adp + 1 b1876_mRNA_2_degr + 433 h + 433 pi	reversible	mRNA degradation	-1.00E+09	1.00E+09	0	7.32E+01	7.32E+01	0	1.29E+02	129.311	0	3.26E-01	0.326
b1876_mRNA_degr2	Degradation of b1876_mRNA II	1 b1876_mRNA_2_degr + 1733 h2o --> 1 Orn_dim_inact + 445 amp 436 cmp + 1 degradosome_inact + 487 gmp + 1 gtp + 1733 h + 365 ump	irreversible	mRNA degradation	0.00E+00	3.26E-01	0	7.32E+01	7.32E+01	0	1.29E+02	129.311	0	3.26E-01	0.326
b1876_maturation1	Polypeptide b1876 peptide deformylas complex	1 Def_mono + 1 b1876_aa <==> 1 b1876_def_cplx	reversible	Protein Maturation	-1.00E+09	1.00E+09	0	2.51E+03	2.51E+03	0	4.11E+03	4107.15	0	1.02E+02	101.508
b1876_maturation2	b1876 formation	1 b1876_def_cplx + 1 h2o --> 1 Def_mono_inact + 1 b1876_m + 1 for	irreversible	Protein Maturation	0.00E+00	1.00E+09	0	2.51E+03	2.51E+03	0	4.11E+03	4107.15	0	1.02E+02	101.508
b1909_tRNA_16_MODIF_1	modification of position 16 nt (D) in leuZ by Dus_gen	1 Dus_gen + 1 b1909_tRNA_Mg2 + 1 h + 1 nadph <==> 1 b1909_tRNA_Mg2_Dus_gen_cplx	reversible	tRNA modification	-1.00E+09	1.00E+09	0	3.99E+02	3.99E+02	0	6.22E+00	6.22	0	6.22E+00	6.22
b1909_tRNA_16_MODIF_2	modification of position 16 nt (D) in leuZ by Dus_gen	1 b1909_tRNA_Mg2_Dus_gen_cplx --> 1 Dus_gen_inact + 1 b1909_tRNA_1 + 1 nadp	irreversible	tRNA modification	0.00E+00	1.00E+09	0	3.99E+02	3.99E+02	0	6.22E+00	6.22	0	6.22E+00	6.22
b1909_tRNA_18_MODIF_1	modification of position 18 nt (Gm) in leuZ by TrmH_dim	1 TrmH_dim + 1 amet + 1 b1909_tRNA_1 <==> 1 b1909_tRNA_1_TrmH_dim_cplx	reversible	tRNA modification	-1.00E+09	1.00E+09	0	3.99E+02	3.99E+02	0	6.22E+00	6.22	0	6.22E+00	6.22
b1909_tRNA_18_MODIF_2	modification of position 18 nt (Gm) in leuZ by TrmH_dim	1 b1909_tRNA_1_TrmH_dim_cplx --> 1 TrmH_dim_inact + 1 ahcys + 1 b1909_tRNA_2 + 1 h	irreversible	tRNA modification	0.00E+00	1.00E+09	0	3.99E+02	3.99E+02	0	6.22E+00	6.22	0	6.22E+00	6.22
b1909_tRNA_20_MODIF_1	modification of position 20 nt (D) in leuZ by Dus_gen	1 Dus_gen + 1 b1909_tRNA_2 + 1 h + 1 nadph <==> 1 b1909_tRNA_2_Dus_gen_cplx	reversible	tRNA modification	-1.00E+09	1.00E+09	0	3.99E+02	3.99E+02	0	6.22E+00	6.22	0	6.22E+00	6.22
b1909_tRNA_20_MODIF_2	modification of position 20 nt (D) in leuZ by Dus_gen	1 b1909_tRNA_2_Dus_gen_cplx --> 1 Dus_gen_inact + 1 b1909_tRNA_3 + 1 nadp	irreversible	tRNA modification	0.00E+00	1.00E+09	0	3.99E+02	3.99E+02	0	6.22E+00	6.22	0	6.22E+00	6.22
b1909_tRNA_32_MODIF_1	modification of position 32 nt (Y) in leuZ by RluA_mono	1 RluA_mono + 1 b1909_tRNA_3 <==> 1 b1909_tRNA_3_RluA_mono_cplx	reversible	tRNA modification	-1.00E+09	1.00E+09	0	3.99E+02	3.99E+02	0	6.22E+00	6.22	0	6.22E+00	6.22
b1909_tRNA_32_MODIF_2	modification of position 32 nt (Y) in leuZ by RluA_mono	1 b1909_tRNA_3_RluA_mono_cplx --> 1 RluA_mono_inact + 1 b1909_tRNA_4	irreversible	tRNA modification	0.00E+00	1.00E+09	0	3.99E+02	3.99E+02	0	6.22E+00	6.22	0	6.22E+00	6.22
b1909_tRNA_37_MODIF_1	modification of position 37 nt (ms2i6A) in leuZ by MiaA_dim-MiaB_mono	1 IscS_dim_S-SH + 1 MiaA_dim + 1 MiaB_mono + 2 amet + 1 b1909_tRNA_4 + 1 dmpp + 1 fldrd <==> 1 b1909_tRNA_4_MiaA_dim MiaB_mono_cplx	reversible	tRNA modification	-1.00E+09	1.00E+09	0	3.99E+02	3.99E+02	0	6.22E+00	6.22	0	6.22E+00	6.22
b1909_tRNA_37_MODIF_2	modification of position 37 nt (ms2i6A) in leuZ by MiaA_dim-MiaB_mono	1 b1909_tRNA_4_MiaA_dim-MiaB_mono_cplx --> 1 IscS_dim_S-H + 1 MiaA_dim_inact + 1 MiaB_mono_inact + 1 ahcys + 1 b1909_tRNA_5 + 1 dad-5 + 1 fldox + 2 h + 1 met-L + 1 ppi	irreversible	tRNA modification	0.00E+00	1.00E+09	0	3.99E+02	3.99E+02	0	6.22E+00	6.22	0	6.22E+00	6.22
b1909_tRNA_39_MODIF_1	modification of position 39 nt (Y) in leuZ by TruA_dim	1 TruA_dim + 1 b1909_tRNA_5 <==> 1 b1909_tRNA_5_TruA_dim_cplx	reversible	tRNA modification	-1.00E+09	1.00E+09	0	3.99E+02	3.99E+02	0	6.22E+00	6.22	0	6.22E+00	6.22
b1909_tRNA_39_MODIF_2	modification of position 39 nt (Y) in leuZ by TruA_dim	1 b1909_tRNA_5_TruA_dim_cplx --> 1 TruA_dim_inact + 1 b1909_tRNA_6	irreversible	tRNA modification	0.00E+00	1.00E+09	0	3.99E+02	3.99E+02	0	6.22E+00	6.22	0	6.22E+00	6.22
b1909_tRNA_54_MODIF_1	modification of position 54 nt (m5U) in leuZ by TrmA_mono	1 TrmA_mono + 1 amet + 1 b1909_tRNA_6 <==> 1 b1909_tRNA_6_TrmA_mono_cplx	reversible	tRNA modification	-1.00E+09	1.00E+09	0	3.99E+02	3.99E+02	0	6.22E+00	6.22	0	6.22E+00	6.22
b1909_tRNA_54_MODIF_2	modification of position 54 nt (m5U) in leuZ by TrmA_mono	1 b1909_tRNA_6_TrmA_mono_cplx --> 1 TrmA_mono_inact + 1 ahcys + 1 b1909_tRNA_7 + 1 h	irreversible	tRNA modification	0.00E+00	1.00E+09	0	3.99E+02	3.99E+02	0	6.22E+00	6.22	0	6.22E+00	6.22
b1909_tRNA_55_MODIF_1	modification of position 55 nt (Y) in leuZ by TruB_mono	1 TruB_mono + 1 b1909_tRNA_7 <==> 1 b1909_tRNA_7_TruB_mono_cplx	reversible	tRNA modification	-1.00E+09	1.00E+09	0	3.99E+02	3.99E+02	0	6.22E+00	6.22	0	6.22E+00	6.22
b1909_tRNA_55_MODIF_2	modification of position 55 nt (Y) in leuZ by TruB_mono	1 b1909_tRNA_7_TruB_mono_cplx --> 1 TruB_mono_inact + 1 b1909_tRNA_8	irreversible	tRNA modification	0.00E+00	1.00E+09	0	3.99E+02	3.99E+02	0	6.22E+00	6.22	0	6.22E+00	6.22
b1909_tRNA_8_MODIF_1	modification of position 8 nt (s4U) in leuZ by ThiI_mono	1 IscS_dim_S-SH + 1 ThiI_mono + 1 atp + 1 b1909_tRNA_8 + 1 mg2 + 1 trdrd <==> 1 b1909_tRNA_8_ThiI_mono_cplx	reversible	tRNA modification	-1.00E+09	1.00E+09	0	3.99E+02	3.99E+02	0	6.22E+00	6.22	0	6.22E+00	6.22
b1909_tRNA_8_MODIF_2	modification of position 8 nt (s4U) in leuZ by ThiI_mono	1 b1909_tRNA_8_ThiI_mono_cplx --> 1 IscS_dim_S-H + 1 ThiI_mono_inact + 1 amp + 1 h + 1 leuZ_tRNA + 1 mg2 + 1 ppi + 1 trdox	irreversible	tRNA modification	0.00E+00	1.00E+09	0	3.99E+02	3.99E+02	0	6.22E+00	6.22	0	6.22E+00	6.22
b1909_tRNA_Mg_BIND	Binding of two Mg2 to b1909_tRNA	1 b1909_RNA + 2 mg2 <==> 1 b1909_tRNA_Mg2	reversible	Metallo-ion binding	-1.00E+09	1.00E+09	0	3.99E+02	3.99E+02	0	6.22E+00	6.22	0	6.22E+00	6.22
b1910_tRNA_20_MODIF_1	modification of position 20 nt (D) in cysT by Dus_gen	1 Dus_gen + 1 b1910_tRNA_Mg2 + 1 h + 1 nadph <==> 1 b1910_tRNA_Mg2_Dus_gen_cplx	reversible	tRNA modification	-1.00E+09	1.00E+09	0	3.99E+02	3.99E+02	0	6.22E+00	6.22	0	6.22E+00	6.22
b1910_tRNA_20_MODIF_2	modification of position 20 nt (D) in cysT by Dus_gen	1 b1910_tRNA_Mg2_Dus_gen_cplx --> 1 Dus_gen_inact + 1 b1910_tRNA_1 + 1 nadp	irreversible	tRNA modification	0.00E+00	1.00E+09	0	3.99E+02	3.99E+02	0	6.22E+00	6.22	0	6.22E+00	6.22
b1910_tRNA_21_MODIF_1	modification of position 21 nt (D) in cysT by Dus_gen	1 Dus_gen + 1 b1910_tRNA_1 + 1 h + 1 nadph <==> 1 b1910_tRNA_1_Dus_gen_cplx	reversible	tRNA modification	-1.00E+09	1.00E+09	0	3.99E+02	3.99E+02	0	6.22E+00	6.22	0	6.22E+00	6.22
b1910_tRNA_21_MODIF_2	modification of position 21 nt (D) in cysT by Dus_gen	1 b1910_tRNA_1_Dus_gen_cplx --> 1 Dus_gen_inact + 1 b1910_tRNA_2 + 1 nadp	irreversible	tRNA modification	0.00E+00	1.00E+09	0	3.99E+02	3.99E+02	0	6.22E+00	6.22	0	6.22E+00	6.22
b1910_tRNA_32_MODIF_1	modification of position 32 nt (Y) in cysT by RluA_mono	1 RluA_mono + 1 b1910_tRNA_2 <==> 1 b1910_tRNA_2_RluA_mono_cplx	reversible	tRNA modification	-1.00E+09	1.00E+09	0	3.99E+02	3.99E+02	0	6.22E+00	6.22	0	6.22E+00	6.22
b1910_tRNA_32_MODIF_2	modification of position 32 nt (Y) in cysT by RluA_mono	1 b1910_tRNA_2_RluA_mono_cplx --> 1 RluA_mono_inact + 1 b1910_tRNA_3	irreversible	tRNA modification	0.00E+00	1.00E+09	0	3.99E+02	3.99E+02	0	6.22E+00	6.22	0	6.22E+00	6.22
b1910_tRNA_37_MODIF_1	modification of position 37 nt (ms2i6A) in cysT by MiaA_dim-MiaB_mono	1 IscS_dim_S-SH + 1 MiaA_dim + 1 MiaB_mono + 2 amet + 1 b1910_tRNA_3 + 1 dmpp + 1 fldrd <==> 1 b1910_tRNA_3_MiaA_dim MiaB_mono_cplx	reversible	tRNA modification	-1.00E+09	1.00E+09	0	3.99E+02	3.99E+02	0	6.22E+00	6.22	0	6.22E+00	6.22
b1910_tRNA_37_MODIF_2	modification of position 37 nt (ms2i6A) in cysT by MiaA_dim-MiaB_mono	1 b1910_tRNA_3_MiaA_dim-MiaB_mono_cplx --> 1 IscS_dim_S-H + 1 MiaA_dim_inact + 1 MiaB_mono_inact + 1 ahcys + 1 b1910_tRNA_4 + 1 dad-5 + 1 fldox + 2 h + 1 met-L + 1 ppi	irreversible	tRNA modification	0.00E+00	1.00E+09	0	3.99E+02	3.99E+02	0	6.22E+00	6.22	0	6.22E+00	6.22
b1910_tRNA_39_MODIF_1	modification of position 39 nt (Y) in cysT by TruA_dim	1 TruA_dim + 1 b1910_tRNA_4 <==> 1 b1910_tRNA_4_TruA_dim_cplx	reversible	tRNA modification	-1.00E+09	1.00E+09	0	3.99E+02	3.99E+02	0	6.22E+00	6.22	0	6.22E+00	6.22
b1910_tRNA_39_MODIF_2	modification of position 39 nt (Y) in cysT by TruA_dim	1 b1910_tRNA_4_TruA_dim_cplx --> 1 TruA_dim_inact + 1 b1910_tRNA_5	irreversible	tRNA modification	0.00E+00	1.00E+09	0	3.99E+02	3.99E+02	0	6.22E+00	6.22	0	6.22E+00	6.22
b1910_tRNA_54_MODIF_1	modification of position 54 nt (m5U) in cysT by TrmA_mono	1 TrmA_mono + 1 amet + 1 b1910_tRNA_5 <==> 1 b1910_tRNA_5_TrmA_mono_cplx	reversible	tRNA modification	-1.00E+09	1.00E+09	0	3.99E+02	3.99E+02	0	6.22E+00	6.22	0	6.22E+00	6.22
b1910_tRNA_54_MODIF_2	modification of position 54 nt (m5U) in cysT by TrmA_mono	1 b1910_tRNA_5_TrmA_mono_cplx --> 1 TrmA_mono_inact + 1 ahcys + 1 b1910_tRNA_6 + 1 h	irreversible	tRNA modification	0.00E+00	1.00E+09	0	3.99E+02	3.99E+02	0	6.22E+00	6.22	0	6.22E+00	6.22
b1910_tRNA_55_MODIF_1	modification of position 55 nt (Y) in cysT by TruB_mono	1 TruB_mono + 1 b1910_tRNA_6 <==> 1 b1910_tRNA_6_TruB_mono_cplx	reversible	tRNA modification	-1.00E+09	1.00E+09	0	3.99E+02	3.99E+02	0	6.22E+00	6.22	0	6.22E+00	6.22
b1910_tRNA_55_MODIF_2	modification of position 55 nt (Y) in cysT by TruB_mono	1 b1910_tRNA_6_TruB_mono_cplx --> 1 TruB_mono_inact + 1 b1910_tRNA_7	irreversible	tRNA modification	0.00E+00	1.00E+09	0	3.99E+02	3.99E+02	0	6.22E+00	6.22	0	6.22E+00	6.22
b1910_tRNA_8_MODIF_1	modification of position 8 nt (s4U) in cysT by ThiI_mono	1 IscS_dim_S-SH + 1 ThiI_mono + 1 atp + 1 b1910_tRNA_7 + 1 mg2 + 1 trdrd <==> 1 b1910_tRNA_7_ThiI_mono_cplx	reversible	tRNA modification	-1.00E+09	1.00E+09	0	3.99E+02	3.99E+02	0	6.22E+00	6.22	0	6.22E+00	6.22

b1910_tRNA_8_MODIF_2	modification of position 8 nt (s4U) in cysT by ThiI_mono	1 b1910_tRNA_7_ThiI_mono_cplx --> 1 IscS_dim_S-H + 1 ThiI_mono_inact + 1 amp + 1 cysT_tRNA + 1 h + 1 mg2 + 1 ppi + 1 trdox	irreversible	tRNA modification	0.00E+00	1.00E+09	0	3.99E+02	3.99E+02	0	6.22E+00	6.22	0	6.22E+00	6.22
b1910_tRNA_Mg_BIND	Binding of two Mg2 to b1910_tRNA	1 b1910_RNA + 2 mg2 <==> 1 b1910_tRNA_Mg2	reversible	Metallo-ion binding	-1.00E+09	1.00E+09	0	3.99E+02	3.99E+02	0	6.22E+00	6.22	0	6.22E+00	6.22
b1911_tRNA_16_MODIF_1	modification of position 16 nt (D) in glyW by Dus_gen	1 Dus_gen + 1 b1911_tRNA_Mg2 + 1 h + 1 nadph <==> 1 b1911_tRNA_Mg2_Dus_gen_cplx	reversible	tRNA modification	-1.00E+09	1.00E+09	0	3.99E+02	3.99E+02	0	6.22E+00	6.22	0	6.22E+00	6.22
b1911_tRNA_16_MODIF_2	modification of position 16 nt (D) in glyW by Dus_gen	1 b1911_tRNA_Mg2_Dus_gen_cplx --> 1 Dus_gen_inact + 1 b1911_tRNA_1 + 1 nadp	irreversible	tRNA modification	0.00E+00	1.00E+09	0	3.99E+02	3.99E+02	0	6.22E+00	6.22	0	6.22E+00	6.22
b1911_tRNA_17_MODIF_1	modification of position 17 nt (D) in glyW by Dus_gen	1 Dus_gen + 1 b1911_tRNA_1 + 1 h + 1 nadph <==> 1 b1911_tRNA_1_Dus_gen_cplx	reversible	tRNA modification	-1.00E+09	1.00E+09	0	3.99E+02	3.99E+02	0	6.22E+00	6.22	0	6.22E+00	6.22
b1911_tRNA_17_MODIF_2	modification of position 17 nt (D) in glyW by Dus_gen	1 b1911_tRNA_1_Dus_gen_cplx --> 1 Dus_gen_inact + 1 b1911_tRNA_2 + 1 nadp	irreversible	tRNA modification	0.00E+00	1.00E+09	0	3.99E+02	3.99E+02	0	6.22E+00	6.22	0	6.22E+00	6.22
b1911_tRNA_20_MODIF_1	modification of position 20 nt (D) in glyW by Dus_gen	1 Dus_gen + 1 b1911_tRNA_2 + 1 h + 1 nadph <==> 1 b1911_tRNA_2_Dus_gen_cplx	reversible	tRNA modification	-1.00E+09	1.00E+09	0	3.99E+02	3.99E+02	0	6.22E+00	6.22	0	6.22E+00	6.22
b1911_tRNA_20_MODIF_2	modification of position 20 nt (D) in glyW by Dus_gen	1 b1911_tRNA_2_Dus_gen_cplx --> 1 Dus_gen_inact + 1 b1911_tRNA_3 + 1 nadp	irreversible	tRNA modification	0.00E+00	1.00E+09	0	3.99E+02	3.99E+02	0	6.22E+00	6.22	0	6.22E+00	6.22
b1911_tRNA_46_MODIF_1	modification of position 46 nt (m7G) in glyW by YggH_mono	1 YggH_mono + 1 amet + 1 b1911_tRNA_3 <==> 1 b1911_tRNA_3_YggH_mono_cplx	reversible	tRNA modification	-1.00E+09	1.00E+09	0	3.99E+02	3.99E+02	0	6.22E+00	6.22	0	6.22E+00	6.22
b1911_tRNA_46_MODIF_2	modification of position 46 nt (m7G) in glyW by YggH_mono	1 b1911_tRNA_3_YggH_mono_cplx --> 1 YggH_mono_inact + 1 ahcys + 1 b1911_tRNA_4 + 1 h	irreversible	tRNA modification	0.00E+00	1.00E+09	0	3.99E+02	3.99E+02	0	6.22E+00	6.22	0	6.22E+00	6.22
b1911_tRNA_54_MODIF_1	modification of position 54 nt (m5U) in glyW by TrmA_mono	1 TrmA_mono + 1 amet + 1 b1911_tRNA_4 <==> 1 b1911_tRNA_4_TrmA_mono_cplx	reversible	tRNA modification	-1.00E+09	1.00E+09	0	3.99E+02	3.99E+02	0	6.22E+00	6.22	0	6.22E+00	6.22
b1911_tRNA_54_MODIF_2	modification of position 54 nt (m5U) in glyW by TrmA_mono	1 b1911_tRNA_4_TrmA_mono_cplx --> 1 TrmA_mono_inact + 1 ahcys + 1 b1911_tRNA_5 + 1 h	irreversible	tRNA modification	0.00E+00	1.00E+09	0	3.99E+02	3.99E+02	0	6.22E+00	6.22	0	6.22E+00	6.22
b1911_tRNA_55_MODIF_1	modification of position 55 nt (Y) in glyW by TruB_mono	1 TruB_mono + 1 b1911_tRNA_5 <==> 1 b1911_tRNA_5_TruB_mono_cplx	reversible	tRNA modification	-1.00E+09	1.00E+09	0	3.99E+02	3.99E+02	0	6.22E+00	6.22	0	6.22E+00	6.22
b1911_tRNA_55_MODIF_2	modification of position 55 nt (Y) in glyW by TruB_mono	1 b1911_tRNA_5_TruB_mono_cplx --> 1 TruB_mono_inact + 1 glyW_tRNA	irreversible	tRNA modification	0.00E+00	1.00E+09	0	3.99E+02	3.99E+02	0	6.22E+00	6.22	0	6.22E+00	6.22
b1911_tRNA_Mg_BIND	Binding of two Mg2 to b1911_tRNA	1 b1911_RNA + 2 mg2 <==> 1 b1911_tRNA_Mg2	reversible	Metallo-ion binding	-1.00E+09	1.00E+09	0	3.99E+02	3.99E+02	0	6.22E+00	6.22	0	6.22E+00	6.22
b1920_fold_spon	b1920_m folding: spontanous	1 b1920_m --> 1 FliY_mono	irreversible	Protein Folding	0.00E+00	1.00E+09	0	1.07E+03	1.07E+03	0	1.80E+03	1796.95	0	1.18E+02	118.065
b1920_mRNA_CONV	convsersion of mRNA to mRNA_1 (synthetic rxn)	1 b1920_mRNA --> 1 b1920_mRNA_1	irreversible	Translation	0.00E+00	1.00E+09	0	6.35E+01	6.35E+01	0	1.12E+02	112.0867	0	1.61E-01	0.161
b1920_mRNA_degr1	Degradation of b1920_mRNA I	1 Orn_dim + 200 atp + 1 b1920_mRNA_2 + 1 degradosome + 200 h2o <==> 200 adp + 1 b1920_mRNA_2_degr + 200 h + 200 pi	reversible	mRNA degradation	-1.00E+09	1.00E+09	0	6.35E+01	6.35E+01	0	1.12E+02	112.0867	0	1.61E-01	0.161
b1920_mRNA_degr2	Degradation of b1920_mRNA II	1 b1920_mRNA_2_degr + 800 h2o --> 1 Orn_dim_inact + 220 amp + 162 cmp + 1 degradosome_inact + 237 gmp + 800 h + 182 ump	irreversible	mRNA degradation	0.00E+00	1.96E-01	0	6.35E+01	6.35E+01	0	1.12E+02	112.0867	0	1.61E-01	0.161
b1920_maturation1	Polypeptide b1920 peptide deformylas and methionine aminopeptidase complex	1 Def_mono + 1 Map_mono + 1 b1920_aa <==> 1 b1920_def_map_cplx	reversible	Protein Maturation	-1.00E+09	1.00E+09	0	1.07E+03	1.07E+03	0	1.80E+03	1796.95	0	1.18E+02	118.065
b1920_maturation2	b1920 formation	1 b1920_def_map_cplx + 30 h2o --> 1 Def_mono_inact + 1 Map_mono_inact + 6 ala-L + 1 arg-L + 1 b1920_m + 1 for + 1 gln-L + 3 gly + 1 his-L + 4 leu-L + 2 lys-L + 4 met-L + 1 phe-L + 2 ser-L + 4 va L	irreversible	Protein Maturation	0.00E+00	1.00E+09	0	1.07E+03	1.07E+03	0	1.80E+03	1796.95	0	1.18E+02	118.065
b1920_v1_mRNA_CONV	convsersion of mRNA to mRNA_1 (synthetic rxn)	1 b1920_v1_mRNA --> 1 b1920_mRNA_1	irreversible	Translation	0.00E+00	1.00E+09	0	6.35E+01	6.35E+01	0	1.12E+02	112.0867	0	1.61E-01	0.161
b1921_fold_spon	b1921_m folding: spontanous	1 b1921_m --> 1 FliZ_mono	irreversible	Protein Folding	0.00E+00	1.00E+09	0	7.47E+02	7.47E+02	0	1.23E+03	1232.867	0	1.14E+02	113.61
b1921_mRNA_CONV	convsersion of mRNA to mRNA_1 (synthetic rxn)	1 b1921_mRNA --> 1 b1921_mRNA_1	irreversible	Translation	0.00E+00	1.00E+09	0	6.35E+01	6.35E+01	0	1.12E+02	112.0867	0	1.61E-01	0.161
b1921_mRNA_degr1	Degradation of b1921_mRNA I	1 Orn_dim + 138 atp + 1 b1921_mRNA_2 + 1 degradosome + 138 h2o <==> 138 adp + 1 b1921_mRNA_2_degr + 138 h + 138 pi	reversible	mRNA degradation	-1.00E+09	1.00E+09	0	6.35E+01	6.35E+01	0	1.12E+02	112.0867	0	1.61E-01	0.161
b1921_mRNA_degr2	Degradation of b1921_mRNA II	1 b1921_mRNA_2_degr + 551 h2o --> 1 Orn_dim_inact + 153 amp + 138 cmp + 1 degradosome_inact + 119 gmp + 551 h + 142 ump	irreversible	mRNA degradation	0.00E+00	1.77E-01	0	6.35E+01	6.35E+01	0	1.12E+02	112.0867	0	1.61E-01	0.161
b1921_maturation1	Polypeptide b1921 peptide deformylas and methionine aminopeptidase complex	1 Def_mono + 1 Map_mono + 1 b1921_aa <==> 1 b1921_def_map_cplx	reversible	Protein Maturation	-1.00E+09	1.00E+09	0	7.47E+02	7.47E+02	0	1.23E+03	1232.867	0	1.14E+02	113.61
b1921_maturation2	b1921 formation	1 b1921_def_map_cplx + 2 h2o --> 1 Def_mono_inact + 1 Map_mono_inact + 1 b1921_m + 1 for + 1 met-L	irreversible	Protein Maturation	0.00E+00	1.00E+09	0	7.47E+02	7.47E+02	0	1.23E+03	1232.867	0	1.14E+02	113.61
b1921_v1_mRNA_CONV	convsersion of mRNA to mRNA_1 (synthetic rxn)	1 b1921_v1_mRNA --> 1 b1921_mRNA_1	irreversible	Translation	0.00E+00	1.00E+09	0	6.35E+01	6.35E+01	0	1.12E+02	112.0867	0	1.61E-01	0.161
b1922_fold_spon	b1922_m folding: spontanous	1 b1922_m --> 1 FliA_mono	irreversible	Protein Folding	0.00E+00	1.00E+09	0	9.82E+02	9.82E+02	0	1.66E+03	1662.033	0	9.51E+01	95.074
b1922_mRNA_CONV	convsersion of mRNA to mRNA_1 (synthetic rxn)	1 b1922_mRNA --> 1 b1922_mRNA_1	irreversible	Translation	0.00E+00	1.00E+09	0	6.35E+01	6.35E+01	0	1.12E+02	112.0867	0	1.61E-01	0.161
b1922_mRNA_degr1	Degradation of b1922_mRNA I	1 Orn_dim + 180 atp + 1 b1922_mRNA_2 + 1 degradosome + 180 h2o <==> 180 adp + 1 b1922_mRNA_2_degr + 180 h + 180 pi	reversible	mRNA degradation	-1.00E+09	1.00E+09	0	6.35E+01	6.35E+01	0	1.12E+02	112.0867	0	1.61E-01	0.161
b1922_mRNA_degr2	Degradation of b1922_mRNA II	1 b1922_mRNA_2_degr + 719 h2o --> 1 Orn_dim_inact + 185 amp + 179 cmp + 1 degradosome_inact + 210 gmp + 1 gtp + 719 h + 145 ump	irreversible	mRNA degradation	0.00E+00	1.61E-01	0	6.35E+01	6.35E+01	0	1.12E+02	112.0867	0	1.61E-01	0.161
b1922_maturation1	Polypeptide b1922 peptide deformylas and methionine aminopeptidase complex	1 Def_mono + 1 Map_mono + 1 b1922_aa <==> 1 b1922_def_map_cplx	reversible	Protein Maturation	-1.00E+09	1.00E+09	0	9.82E+02	9.82E+02	0	1.66E+03	1662.033	0	9.51E+01	95.074
b1922_maturation2	b1922 formation	1 b1922_def_map_cplx + 2 h2o --> 1 Def_mono_inact + 1 Map_mono_inact + 1 b1922_m + 1 for + 1 met-L	irreversible	Protein Maturation	0.00E+00	1.00E+09	0	9.82E+02	9.82E+02	0	1.66E+03	1662.033	0	9.51E+01	95.074
b1922_v1_mRNA_CONV	convsersion of mRNA to mRNA_1 (synthetic rxn)	1 b1922_v1_mRNA --> 1 b1922_mRNA_1	irreversible	Translation	0.00E+00	1.00E+09	0	6.35E+01	6.35E+01	0	1.12E+02	112.0867	0	1.61E-01	0.161
b1975_tRNA_18_MODIF_1	modification of position 18 nt (Gm) in serU by TrmH_dim	1 TrmH_dim + 1 amet + 1 b1975_tRNA_Mg2 <==> 1 b1975_tRNA_Mg2_TrmH_dim_cplx	reversible	tRNA modification	-1.00E+09	1.00E+09	0	1.14E+03	1.14E+03	0	6.36E+00	6.36	0	6.36E+00	6.36
b1975_tRNA_18_MODIF_2	modification of position 18 nt (Gm) in serU by TrmH_dim	1 b1975_tRNA_Mg2_TrmH_dim_cplx --> 1 TrmH_dim_inact + 1 ahcys + 1 b1975_tRNA_1 + 1 h	irreversible	tRNA modification	0.00E+00	1.00E+09	0	1.14E+03	1.14E+03	0	6.36E+00	6.36	0	6.36E+00	6.36
b1975_tRNA_20A_MODIF_1	modification of position 20A nt (D) in serU by Dus_gen	1 Dus_gen + 1 b1975_tRNA_1 + 1 h + 1 nadph <==> 1 b1975_tRNA_1_Dus_gen_cplx	reversible	tRNA modification	-1.00E+09	1.00E+09	0	1.14E+03	1.14E+03	0	6.36E+00	6.36	0	6.36E+00	6.36
b1975_tRNA_20A_MODIF_2	modification of position 20A nt (D) in serU by Dus_gen	1 b1975_tRNA_1_Dus_gen_cplx --> 1 Dus_gen_inact + 1 b1975_tRNA_2 + 1 nadp	irreversible	tRNA modification	0.00E+00	1.00E+09	0	1.14E+03	1.14E+03	0	6.36E+00	6.36	0	6.36E+00	6.36
b1975_tRNA_37_MODIF_1	modification of position 37 nt (ms2i6A) in serU by MiaA_dim-MiaB_mono	1 IscS_dim_S-SH + 1 MiaA_dim + 1 MiaB_mono + 2 amet + 1 b1975_tRNA_2 + 1 dmpp + 1 fldrd <==> 1 b1975_tRNA_2_MiaA_dim MiaB_mono_cplx	reversible	tRNA modification	-1.00E+09	1.00E+09	0	1.14E+03	1.14E+03	0	6.36E+00	6.36	0	6.36E+00	6.36
b1975_tRNA_37_MODIF_2	modification of position 37 nt (ms2i6A) in serU by MiaA_dim-MiaB_mono	1 b1975_tRNA_2_MiaA_dim-MiaB_mono_cplx --> 1 IscS_dim_S-H + 1 MiaA_dim_inact + 1 MiaB_mono_inact + 1 ahcys + 1 b1975_tRNA_3 + 1 dad-5 + 1 fldox + 2 h + 1 met-L + 1 ppi	irreversible	tRNA modification	0.00E+00	1.00E+09	0	1.14E+03	1.14E+03	0	6.36E+00	6.36	0	6.36E+00	6.36
b1975_tRNA_54_MODIF_1	modification of position 54 nt (m5U) in serU by TrmA_mono	1 TrmA_mono + 1 amet + 1 b1975_tRNA_3 <==> 1 b1975_tRNA_3_TrmA_mono_cplx	reversible	tRNA modification	-1.00E+09	1.00E+09	0	1.14E+03	1.14E+03	0	6.36E+00	6.36	0	6.36E+00	6.36
b1975_tRNA_54_MODIF_2	modification of position 54 nt (m5U) in serU by TrmA_mono	1 b1975_tRNA_3_TrmA_mono_cplx --> 1 TrmA_mono_inact + 1 ahcys + 1 b1975_tRNA_4 + 1 h	irreversible	tRNA modification	0.00E+00	1.00E+09	0	1.14E+03	1.14E+03	0	6.36E+00	6.36	0	6.36E+00	6.36
b1975_tRNA_55_MODIF_1	modification of position 55 nt (Y) in serU by TruB_mono	1 TruB_mono + 1 b1975_tRNA_4 <==> 1 b1975_tRNA_4_TruB_mono_cplx	reversible	tRNA modification	-1.00E+09	1.00E+09	0	1.14E+03	1.14E+03	0	6.36E+00	6.36	0	6.36E+00	6.36
b1975_tRNA_55_MODIF_2	modification of position 55 nt (Y) in serU by TruB_mono	1 b1975_tRNA_4_TruB_mono_cplx --> 1 TruB_mono_inact + 1 serU_tRNA	irreversible	tRNA modification	0.00E+00	1.00E+09	0	1.14E+03	1.14E+03	0	6.36E+00	6.36	0	6.36E+00	6.36
b1975_tRNA_Mg_BIND	Binding of two Mg2 to b1975_tRNA	1 b1975_RNA + 2 mg2 <==> 1 b1975_tRNA_Mg2	reversible	Metallo-ion binding	-1.00E+09	1.00E+09	0	1.14E+03	1.14E+03	0	6.36E+00	6.36	0	6.36E+00	6.36
b1977_tRNA_16_MODIF_1	modification of position 16 nt (D) in asnT by Dus_gen	1 Dus_gen + 1 b1977_tRNA_Mg2 + 1 h + 1 nadph <==> 1 b1977_tRNA_Mg2_Dus_gen_cplx	reversible	tRNA modification	-1.00E+09	1.00E+09	0	1.77E+03	1.77E+03	0	6.36E+00	6.36	0	6.36E+00	6.36
b1977_tRNA_16_MODIF_2	modification of position 16 nt (D) in asnT by Dus_gen	1 b1977_tRNA_Mg2_Dus_gen_cplx --> 1 Dus_gen_inact + 1 b1977_tRNA_1 + 1 nadp	irreversible	tRNA modification	0.00E+00	1.00E+09	0	1.77E+03	1.77E+03	0	6.36E+00	6.36	0	6.36E+00	6.36
b1977_tRNA_20_MODIF_1	modification of position 20 nt (D) in asnT by Dus_gen	1 Dus_gen + 1 b1977_tRNA_1 + 1 h + 1 nadph <==> 1 b1977_tRNA_1_Dus_gen_cplx	reversible	tRNA modification	-1.00E+09	1.00E+09	0	1.77E+03	1.77E+03	0	6.36E+00	6.36	0	6.36E+00	6.36

b1977_tRNA_20_MODIF_2	modification of position 20 nt (D) in asnT by Dus_gen	1 b1977_tRNA_1_Dus_gen_cplx --> 1 Dus_gen_inact + 1 b1977_tRNA_2 + 1 nadp	irreversible	tRNA modification	0.00E+00	1.00E+09	0	1.77E+03	1.77E+03	0	6.36E+00	6.36	0	6.36E+00	6.36
b1977_tRNA_34_MODIF_1	modification of position 34 nt (Q) in asnT by Tgt_hexa-QueA_mono-EoR_tRNA_pos34_Q	1 EoR_tRNA_pos34_Q + 1 QueA_mono + 1 Tgt_hexa + 1 adocbl + 1 amet + 1 b1977_tRNA_2 + 1 h + 1 nadh + 1 pre_Q1 <==> 1 b1977_tRNA_2_Tgt_hexa-QueA_mono-EoR_tRNA_pos34_Q_cplx	reversible	tRNA modification	-1.00E+09	1.00E+09	0	1.77E+03	1.77E+03	0	6.36E+00	6.36	0	6.36E+00	6.36
b1977_tRNA_34_MODIF_2	modification of position 34 nt (Q) in asnT by Tgt_hexa-QueA_mono-EoR_tRNA_pos34_Q	1 b1977_tRNA_2_Tgt_hexa-QueA_mono-EoR_tRNA_pos34_Q_cplx > 1 EoR_tRNA_pos34_Q_inact + 1 QueA_mono_inact + 1 Tgt_hexa_inact + 1 ade + 1 adocbl + 1 b1977_tRNA_3 + 1 gua + 2 h 1 h2o + 1 met-L + 1 nad	irreversible	tRNA modification	0.00E+00	1.00E+09	0	1.77E+03	1.77E+03	0	6.36E+00	6.36	0	6.36E+00	6.36
b1977_tRNA_37_MODIF_1	modification of position 37 nt (t6A) in asnT by Up_tRNA_pos_37_t6A	1 Up_tRNA_pos_37_t6A + 1 atp + 1 b1977_tRNA_3 + 1 hco3 + 1 mg2 + 1 thr-L <==> 1 b1977_tRNA_3_Up_tRNA_pos_37_t6A_cplx	reversible	tRNA modification	-1.00E+09	1.00E+09	0	1.77E+03	1.77E+03	0	6.36E+00	6.36	0	6.36E+00	6.36
b1977_tRNA_37_MODIF_2	modification of position 37 nt (t6A) in asnT by Up_tRNA_pos_37_t6A	1 b1977_tRNA_3_Up_tRNA_pos_37_t6A_cplx --> 1 Up_tRNA_pos_37_t6A_inact + 1 amp + 1 b1977_tRNA_4 + 1 h + 1 h2o + 1 mg2 + 1 ppi	irreversible	tRNA modification	0.00E+00	1.00E+09	0	1.77E+03	1.77E+03	0	6.36E+00	6.36	0	6.36E+00	6.36
b1977_tRNA_39_MODIF_1	modification of position 39 nt (Y) in asnT by TruA_dim	1 TruA_dim + 1 b1977_tRNA_4 <==> 1 b1977_tRNA_4_TruA_dim_cplx	reversible	tRNA modification	-1.00E+09	1.00E+09	0	1.77E+03	1.77E+03	0	6.36E+00	6.36	0	6.36E+00	6.36
b1977_tRNA_39_MODIF_2	modification of position 39 nt (Y) in asnT by TruA_dim	1 b1977_tRNA_4_TruA_dim_cplx --> 1 TruA_dim_inact + 1 b1977_tRNA_5	irreversible	tRNA modification	0.00E+00	1.00E+09	0	1.77E+03	1.77E+03	0	6.36E+00	6.36	0	6.36E+00	6.36
b1977_tRNA_46_MODIF_1	modification of position 46 nt (m7G) in asnT by YggH_mono	1 YggH_mono + 1 amet + 1 b1977_tRNA_5 <==> 1 b1977_tRNA_5_YggH_mono_cplx	reversible	tRNA modification	-1.00E+09	1.00E+09	0	1.77E+03	1.77E+03	0	6.36E+00	6.36	0	6.36E+00	6.36
b1977_tRNA_46_MODIF_2	modification of position 46 nt (m7G) in asnT by YggH_mono	1 b1977_tRNA_5_YggH_mono_cplx --> 1 YggH_mono_inact + 1 ahcys + 1 b1977_tRNA_6 + 1 h	irreversible	tRNA modification	0.00E+00	1.00E+09	0	1.77E+03	1.77E+03	0	6.36E+00	6.36	0	6.36E+00	6.36
b1977_tRNA_54_MODIF_1	modification of position 54 nt (m5U) in asnT by TrmA_mono	1 TrmA_mono + 1 amet + 1 b1977_tRNA_6 <==> 1 b1977_tRNA_6_TrmA_mono_cplx	reversible	tRNA modification	-1.00E+09	1.00E+09	0	1.77E+03	1.77E+03	0	6.36E+00	6.36	0	6.36E+00	6.36
b1977_tRNA_54_MODIF_2	modification of position 54 nt (m5U) in asnT by TrmA_mono	1 b1977_tRNA_6_TrmA_mono_cplx --> 1 TrmA_mono_inact + 1 ahcys + 1 b1977_tRNA_7 + 1 h	irreversible	tRNA modification	0.00E+00	1.00E+09	0	1.77E+03	1.77E+03	0	6.36E+00	6.36	0	6.36E+00	6.36
b1977_tRNA_55_MODIF_1	modification of position 55 nt (Y) in asnT by TruB_mono	1 TruB_mono + 1 b1977_tRNA_7 <==> 1 b1977_tRNA_7_TruB_mono_cplx	reversible	tRNA modification	-1.00E+09	1.00E+09	0	1.77E+03	1.77E+03	0	6.36E+00	6.36	0	6.36E+00	6.36
b1977_tRNA_55_MODIF_2	modification of position 55 nt (Y) in asnT by TruB_mono	1 b1977_tRNA_7_TruB_mono_cplx --> 1 TruB_mono_inact + 1 b1977_tRNA_8	irreversible	tRNA modification	0.00E+00	1.00E+09	0	1.77E+03	1.77E+03	0	6.36E+00	6.36	0	6.36E+00	6.36
b1977_tRNA_8_MODIF_1	modification of position 8 nt (s4U) in asnT by ThiI_mono	1 IscS_dim_S-SH + 1 ThiI_mono + 1 atp + 1 b1977_tRNA_8 + 1 mg2 + 1 trdrd <==> 1 b1977_tRNA_8_ThiI_mono_cplx	reversible	tRNA modification	-1.00E+09	1.00E+09	0	1.77E+03	1.77E+03	0	6.36E+00	6.36	0	6.36E+00	6.36
b1977_tRNA_8_MODIF_2	modification of position 8 nt (s4U) in asnT by ThiI_mono	1 b1977_tRNA_8_ThiI_mono_cplx --> 1 IscS_dim_S-H + 1 ThiI_mono_inact + 1 amp + 1 asnT_tRNA + 1 h + 1 mg2 + 1 ppi + 1 trdox	irreversible	tRNA modification	0.00E+00	1.00E+09	0	1.77E+03	1.77E+03	0	6.36E+00	6.36	0	6.36E+00	6.36
b1977_tRNA_Mg_BIND	Binding of two Mg2 to b1977_tRNA	1 b1977_RNA + 2 mg2 <==> 1 b1977_tRNA_Mg2	reversible	Metallo-ion binding	-1.00E+09	1.00E+09	0	1.77E+03	1.77E+03	0	6.36E+00	6.36	0	6.36E+00	6.36
b1984_tRNA_16_MODIF_1	modification of position 16 nt (D) in asnW by Dus_gen	1 Dus_gen + 1 b1984_tRNA_Mg2 + 1 h + 1 nadph <==> 1 b1984_tRNA_Mg2_Dus_gen_cplx	reversible	tRNA modification	-1.00E+09	1.00E+09	0	1.77E+03	1.77E+03	0	6.40E+00	6.4	0	6.40E+00	6.4
b1984_tRNA_16_MODIF_2	modification of position 16 nt (D) in asnW by Dus_gen	1 b1984_tRNA_Mg2_Dus_gen_cplx --> 1 Dus_gen_inact + 1 b1984_tRNA_1 + 1 nadp	irreversible	tRNA modification	0.00E+00	1.00E+09	0	1.77E+03	1.77E+03	0	6.40E+00	6.4	0	6.40E+00	6.4
b1984_tRNA_20_MODIF_1	modification of position 20 nt (D) in asnW by Dus_gen	1 Dus_gen + 1 b1984_tRNA_1 + 1 h + 1 nadph <==> 1 b1984_tRNA_1_Dus_gen_cplx	reversible	tRNA modification	-1.00E+09	1.00E+09	0	1.77E+03	1.77E+03	0	6.40E+00	6.4	0	6.40E+00	6.4
b1984_tRNA_20_MODIF_2	modification of position 20 nt (D) in asnW by Dus_gen	1 b1984_tRNA_1_Dus_gen_cplx --> 1 Dus_gen_inact + 1 b1984_tRNA_2 + 1 nadp	irreversible	tRNA modification	0.00E+00	1.00E+09	0	1.77E+03	1.77E+03	0	6.40E+00	6.4	0	6.40E+00	6.4
b1984_tRNA_34_MODIF_1	modification of position 34 nt (Q) in asnW by Tgt_hexa-QueA_mono-EoR_tRNA_pos34_Q	1 EoR_tRNA_pos34_Q + 1 QueA_mono + 1 Tgt_hexa + 1 adocbl + 1 amet + 1 b1984_tRNA_2 + 1 h + 1 nadh + 1 pre_Q1 <==> 1 b1984_tRNA_2_Tgt_hexa-QueA_mono-EoR_tRNA_pos34_Q_cplx	reversible	tRNA modification	-1.00E+09	1.00E+09	0	1.77E+03	1.77E+03	0	6.40E+00	6.4	0	6.40E+00	6.4
b1984_tRNA_34_MODIF_2	modification of position 34 nt (Q) in asnW by Tgt_hexa-QueA_mono-EoR_tRNA_pos34_Q	1 b1984_tRNA_2_Tgt_hexa-QueA_mono-EoR_tRNA_pos34_Q_cplx > 1 EoR_tRNA_pos34_Q_inact + 1 QueA_mono_inact + 1 Tgt_hexa_inact + 1 ade + 1 adocbl + 1 b1984_tRNA_3 + 1 gua + 2 h 1 h2o + 1 met-L + 1 nad	irreversible	tRNA modification	0.00E+00	1.00E+09	0	1.77E+03	1.77E+03	0	6.40E+00	6.4	0	6.40E+00	6.4
b1984_tRNA_37_MODIF_1	modification of position 37 nt (t6A) in asnW by Up_tRNA_pos_37_t6A	1 Up_tRNA_pos_37_t6A + 1 atp + 1 b1984_tRNA_3 + 1 hco3 + 1 mg2 + 1 thr-L <==> 1 b1984_tRNA_3_Up_tRNA_pos_37_t6A_cplx	reversible	tRNA modification	-1.00E+09	1.00E+09	0	1.77E+03	1.77E+03	0	6.40E+00	6.4	0	6.40E+00	6.4
b1984_tRNA_37_MODIF_2	modification of position 37 nt (t6A) in asnW by Up_tRNA_pos_37_t6A	1 b1984_tRNA_3_Up_tRNA_pos_37_t6A_cplx --> 1 Up_tRNA_pos_37_t6A_inact + 1 amp + 1 b1984_tRNA_4 + 1 h + 1 h2o + 1 mg2 + 1 ppi	irreversible	tRNA modification	0.00E+00	1.00E+09	0	1.77E+03	1.77E+03	0	6.40E+00	6.4	0	6.40E+00	6.4
b1984_tRNA_39_MODIF_1	modification of position 39 nt (Y) in asnW by TruA_dim	1 TruA_dim + 1 b1984_tRNA_4 <==> 1 b1984_tRNA_4_TruA_dim_cplx	reversible	tRNA modification	-1.00E+09	1.00E+09	0	1.77E+03	1.77E+03	0	6.40E+00	6.4	0	6.40E+00	6.4
b1984_tRNA_39_MODIF_2	modification of position 39 nt (Y) in asnW by TruA_dim	1 b1984_tRNA_4_TruA_dim_cplx --> 1 TruA_dim_inact + 1 b1984_tRNA_5	irreversible	tRNA modification	0.00E+00	1.00E+09	0	1.77E+03	1.77E+03	0	6.40E+00	6.4	0	6.40E+00	6.4
b1984_tRNA_46_MODIF_1	modification of position 46 nt (m7G) in asnW by YggH_mono	1 YggH_mono + 1 amet + 1 b1984_tRNA_5 <==> 1 b1984_tRNA_5_YggH_mono_cplx	reversible	tRNA modification	-1.00E+09	1.00E+09	0	1.77E+03	1.77E+03	0	6.40E+00	6.4	0	6.40E+00	6.4
b1984_tRNA_46_MODIF_2	modification of position 46 nt (m7G) in asnW by YggH_mono	1 b1984_tRNA_5_YggH_mono_cplx --> 1 YggH_mono_inact + 1 ahcys + 1 b1984_tRNA_6 + 1 h	irreversible	tRNA modification	0.00E+00	1.00E+09	0	1.77E+03	1.77E+03	0	6.40E+00	6.4	0	6.40E+00	6.4
b1984_tRNA_54_MODIF_1	modification of position 54 nt (m5U) in asnW by TrmA_mono	1 TrmA_mono + 1 amet + 1 b1984_tRNA_6 <==> 1 b1984_tRNA_6_TrmA_mono_cplx	reversible	tRNA modification	-1.00E+09	1.00E+09	0	1.77E+03	1.77E+03	0	6.40E+00	6.4	0	6.40E+00	6.4
b1984_tRNA_54_MODIF_2	modification of position 54 nt (m5U) in asnW by TrmA_mono	1 b1984_tRNA_6_TrmA_mono_cplx --> 1 TrmA_mono_inact + 1 ahcys + 1 b1984_tRNA_7 + 1 h	irreversible	tRNA modification	0.00E+00	1.00E+09	0	1.77E+03	1.77E+03	0	6.40E+00	6.4	0	6.40E+00	6.4
b1984_tRNA_55_MODIF_1	modification of position 55 nt (Y) in asnW by TruB_mono	1 TruB_mono + 1 b1984_tRNA_7 <==> 1 b1984_tRNA_7_TruB_mono_cplx	reversible	tRNA modification	-1.00E+09	1.00E+09	0	1.77E+03	1.77E+03	0	6.40E+00	6.4	0	6.40E+00	6.4
b1984_tRNA_55_MODIF_2	modification of position 55 nt (Y) in asnW by TruB_mono	1 b1984_tRNA_7_TruB_mono_cplx --> 1 TruB_mono_inact + 1 b1984_tRNA_8	irreversible	tRNA modification	0.00E+00	1.00E+09	0	1.77E+03	1.77E+03	0	6.40E+00	6.4	0	6.40E+00	6.4
b1984_tRNA_8_MODIF_1	modification of position 8 nt (s4U) in asnW by ThiI_mono	1 IscS_dim_S-SH + 1 ThiI_mono + 1 atp + 1 b1984_tRNA_8 + 1 mg2 + 1 trdrd <==> 1 b1984_tRNA_8_ThiI_mono_cplx	reversible	tRNA modification	-1.00E+09	1.00E+09	0	1.77E+03	1.77E+03	0	6.40E+00	6.4	0	6.40E+00	6.4
b1984_tRNA_8_MODIF_2	modification of position 8 nt (s4U) in asnW by ThiI_mono	1 b1984_tRNA_8_ThiI_mono_cplx --> 1 IscS_dim_S-H + 1 ThiI_mono_inact + 1 amp + 1 asnW_tRNA + 1 h + 1 mg2 + 1 ppi + 1 trdox	irreversible	tRNA modification	0.00E+00	1.00E+09	0	1.77E+03	1.77E+03	0	6.40E+00	6.4	0	6.40E+00	6.4
b1984_tRNA_Mg_BIND	Binding of two Mg2 to b1984_tRNA	1 b1984_RNA + 2 mg2 <==> 1 b1984_tRNA_Mg2	reversible	Metallo-ion binding	-1.00E+09	1.00E+09	0	1.77E+03	1.77E+03	0	6.40E+00	6.4	0	6.40E+00	6.4
b1986_tRNA_16_MODIF_1	modification of position 16 nt (D) in asnU by Dus_gen	1 Dus_gen + 1 b1986_tRNA_Mg2 + 1 h + 1 nadph <==> 1 b1986_tRNA_Mg2_Dus_gen_cplx	reversible	tRNA modification	-1.00E+09	1.00E+09	0	1.77E+03	1.77E+03	0	6.41E+00	6.41	0	6.41E+00	6.41
b1986_tRNA_16_MODIF_2	modification of position 16 nt (D) in asnU by Dus_gen	1 b1986_tRNA_Mg2_Dus_gen_cplx --> 1 Dus_gen_inact + 1 b1986_tRNA_1 + 1 nadp	irreversible	tRNA modification	0.00E+00	1.00E+09	0	1.77E+03	1.77E+03	0	6.41E+00	6.41	0	6.41E+00	6.41
b1986_tRNA_20_MODIF_1	modification of position 20 nt (D) in asnU by Dus_gen	1 Dus_gen + 1 b1986_tRNA_1 + 1 h + 1 nadph <==> 1 b1986_tRNA_1_Dus_gen_cplx	reversible	tRNA modification	-1.00E+09	1.00E+09	0	1.77E+03	1.77E+03	0	6.41E+00	6.41	0	6.41E+00	6.41
b1986_tRNA_20_MODIF_2	modification of position 20 nt (D) in asnU by Dus_gen	1 b1986_tRNA_1_Dus_gen_cplx --> 1 Dus_gen_inact + 1 b1986_tRNA_2 + 1 nadp	irreversible	tRNA modification	0.00E+00	1.00E+09	0	1.77E+03	1.77E+03	0	6.41E+00	6.41	0	6.41E+00	6.41
b1986_tRNA_34_MODIF_1	modification of position 34 nt (Q) in asnU by Tgt_hexa-QueA_mono-EoR_tRNA_pos34_Q	1 EoR_tRNA_pos34_Q + 1 QueA_mono + 1 Tgt_hexa + 1 adocbl + 1 amet + 1 b1986_tRNA_2 + 1 h + 1 nadh + 1 pre_Q1 <==> 1 b1986_tRNA_2_Tgt_hexa-QueA_mono-EoR_tRNA_pos34_Q_cplx	reversible	tRNA modification	-1.00E+09	1.00E+09	0	1.77E+03	1.77E+03	0	6.41E+00	6.41	0	6.41E+00	6.41
b1986_tRNA_34_MODIF_2	modification of position 34 nt (Q) in asnU by Tgt_hexa-QueA_mono-EoR_tRNA_pos34_Q	1 b1986_tRNA_2_Tgt_hexa-QueA_mono-EoR_tRNA_pos34_Q_cplx > 1 EoR_tRNA_pos34_Q_inact + 1 QueA_mono_inact + 1 Tgt_hexa_inact + 1 ade + 1 adocbl + 1 b1986_tRNA_3 + 1 gua + 2 h 1 h2o + 1 met-L + 1 nad	irreversible	tRNA modification	0.00E+00	1.00E+09	0	1.77E+03	1.77E+03	0	6.41E+00	6.41	0	6.41E+00	6.41
b1986_tRNA_37_MODIF_1	modification of position 37 nt (t6A) in asnU by Up_tRNA_pos_37_t6A	1 Up_tRNA_pos_37_t6A + 1 atp + 1 b1986_tRNA_3 + 1 hco3 + 1 mg2 + 1 thr-L <==> 1 b1986_tRNA_3_Up_tRNA_pos_37_t6A_cplx	reversible	tRNA modification	-1.00E+09	1.00E+09	0	1.77E+03	1.77E+03	0	6.41E+00	6.41	0	6.41E+00	6.41
b1986_tRNA_37_MODIF_2	modification of position 37 nt (t6A) in asnU by Up_tRNA_pos_37_t6A	1 b1986_tRNA_3_Up_tRNA_pos_37_t6A_cplx --> 1 Up_tRNA_pos_37_t6A_inact + 1 amp + 1 b1986_tRNA_4 + 1 h + 1 h2o + 1 mg2 + 1 ppi	irreversible	tRNA modification	0.00E+00	1.00E+09	0	1.77E+03	1.77E+03	0	6.41E+00	6.41	0	6.41E+00	6.41
b1986_tRNA_39_MODIF_1	modification of position 39 nt (Y) in asnU by TruA_dim	1 TruA_dim + 1 b1986_tRNA_4 <==> 1 b1986_tRNA_4_TruA_dim_cplx	reversible	tRNA modification	-1.00E+09	1.00E+09	0	1.77E+03	1.77E+03	0	6.41E+00	6.41	0	6.41E+00	6.41
b1986_tRNA_39_MODIF_2	modification of position 39 nt (Y) in asnU by TruA_dim	1 b1986_tRNA_4_TruA_dim_cplx --> 1 TruA_dim_inact + 1 b1986_tRNA_5	irreversible	tRNA modification	0.00E+00	1.00E+09	0	1.77E+03	1.77E+03	0	6.41E+00	6.41	0	6.41E+00	6.41
b1986_tRNA_46_MODIF_1	modification of position 46 nt (m7G) in asnU by YggH_mono	1 YggH_mono + 1 amet + 1 b1986_tRNA_5 <==> 1 b1986_tRNA_5_YggH_mono_cplx	reversible	tRNA modification	-1.00E+09	1.00E+09	0	1.77E+03	1.77E+03	0	6.41E+00	6.41	0	6.41E+00	6.41
b1986_tRNA_46_MODIF_2	modification of position 46 nt (m7G) in asnU by YggH_mono	1 b1986_tRNA_5_YggH_mono_cplx --> 1 YggH_mono_inact + 1 ahcys + 1 b1986_tRNA_6 + 1 h	irreversible	tRNA modification	0.00E+00	1.00E+09	0	1.77E+03	1.77E+03	0	6.41E+00	6.41	0	6.41E+00	6.41

b1986_tRNA_54_MODIF_1	modification of position 54 nt (m5U) in asnU by TrmA_mono	1 TrmA_mono + 1 amet + 1 b1986_tRNA_6 <==> 1 b1986_tRNA_6_TrmA_mono_cplx	reversible	tRNA modification	-1.00E+09	1.00E+09	0	1.77E+03	1.77E+03	0	6.41E+00	6.41	0	6.41E+00	6.41
b1986_tRNA_54_MODIF_2	modification of position 54 nt (m5U) in asnU by TrmA_mono	1 b1986_tRNA_6_TrmA_mono_cplx --> 1 TrmA_mono_inact + 1 ahcys + 1 b1986_tRNA_7 + 1 h	irreversible	tRNA modification	0.00E+00	1.00E+09	0	1.77E+03	1.77E+03	0	6.41E+00	6.41	0	6.41E+00	6.41
b1986_tRNA_55_MODIF_1	modification of position 55 nt (Y) in asnU by TruB_mono	1 TruB_mono + 1 b1986_tRNA_7 <==> 1 b1986_tRNA_7_TruB_mono_cplx	reversible	tRNA modification	-1.00E+09	1.00E+09	0	1.77E+03	1.77E+03	0	6.41E+00	6.41	0	6.41E+00	6.41
b1986_tRNA_55_MODIF_2	modification of position 55 nt (Y) in asnU by TruB_mono	1 b1986_tRNA_7_TruB_mono_cplx --> 1 TruB_mono_inact + 1 b1986_tRNA_8	irreversible	tRNA modification	0.00E+00	1.00E+09	0	1.77E+03	1.77E+03	0	6.41E+00	6.41	0	6.41E+00	6.41
b1986_tRNA_8_MODIF_1	modification of position 8 nt (s4U) in asnU by ThiI_mono	1 IscS_dim_S-SH + 1 ThiI_mono + 1 atp + 1 b1986_tRNA_8 + 1 mg2 + 1 trdrd <==> 1 b1986_tRNA_8_ThiI_mono_cplx	reversible	tRNA modification	-1.00E+09	1.00E+09	0	1.77E+03	1.77E+03	0	6.41E+00	6.41	0	6.41E+00	6.41
b1986_tRNA_8_MODIF_2	modification of position 8 nt (s4U) in asnU by ThiI_mono	1 b1986_tRNA_8_ThiI_mono_cplx --> 1 IscS_dim_S-H + 1 ThiI_mono_inact + 1 amp + 1 asnU_tRNA + 1 h + 1 mg2 + 1 ppi + 1 trdox	irreversible	tRNA modification	0.00E+00	1.00E+09	0	1.77E+03	1.77E+03	0	6.41E+00	6.41	0	6.41E+00	6.41
b1986_tRNA_Mg_BIND	Binding of two Mg2 to b1986_tRNA	1 b1986_RNA + 2 mg2 <==> 1 b1986_tRNA_Mg2	reversible	Metallo-ion binding	-1.00E+09	1.00E+09	0	1.77E+03	1.77E+03	0	6.41E+00	6.41	0	6.41E+00	6.41
b1989_tRNA_16_MODIF_1	modification of position 16 nt (D) in asnV by Dus_gen	1 Dus_gen + 1 b1989_tRNA_Mg2 + 1 h + 1 nadph <==> 1 b1989_tRNA_Mg2_Dus_gen_cplx	reversible	tRNA modification	-1.00E+09	1.00E+09	0	1.77E+03	1.77E+03	0	6.41E+00	6.41	0	6.41E+00	6.41
b1989_tRNA_16_MODIF_2	modification of position 16 nt (D) in asnV by Dus_gen	1 b1989_tRNA_Mg2_Dus_gen_cplx --> 1 Dus_gen_inact + 1 b1989_tRNA_1 + 1 nadp	irreversible	tRNA modification	0.00E+00	1.00E+09	0	1.77E+03	1.77E+03	0	6.41E+00	6.41	0	6.41E+00	6.41
b1989_tRNA_20_MODIF_1	modification of position 20 nt (D) in asnV by Dus_gen	1 Dus_gen + 1 b1989_tRNA_1 + 1 h + 1 nadph <==> 1 b1989_tRNA_1_Dus_gen_cplx	reversible	tRNA modification	-1.00E+09	1.00E+09	0	1.77E+03	1.77E+03	0	6.41E+00	6.41	0	6.41E+00	6.41
b1989_tRNA_20_MODIF_2	modification of position 20 nt (D) in asnV by Dus_gen	1 b1989_tRNA_1_Dus_gen_cplx --> 1 Dus_gen_inact + 1 b1989_tRNA_2 + 1 nadp	irreversible	tRNA modification	0.00E+00	1.00E+09	0	1.77E+03	1.77E+03	0	6.41E+00	6.41	0	6.41E+00	6.41
b1989_tRNA_34_MODIF_1	modification of position 34 nt (Q) in asnV by Tgt_hexa-QueA_mono-EoR_tRNA_pos34_Q	1 EoR_tRNA_pos34_Q + 1 QueA_mono + 1 Tgt_hexa + 1 adocbl + 1 amet + 1 b1989_tRNA_2 + 1 h + 1 nadh + 1 pre_Q1 <==> 1 b1989_tRNA_2_Tgt_hexa-QueA_mono-EoR_tRNA_pos34_Q_cplx	reversible	tRNA modification	-1.00E+09	1.00E+09	0	1.77E+03	1.77E+03	0	6.41E+00	6.41	0	6.41E+00	6.41
b1989_tRNA_34_MODIF_2	modification of position 34 nt (Q) in asnV by Tgt_hexa-QueA_mono-EoR_tRNA_pos34_Q	1 b1989_tRNA_2_Tgt_hexa-QueA_mono-EoR_tRNA_pos34_Q_cplx > 1 EoR_tRNA_pos34_Q_inact + 1 QueA_mono_inact + 1 Tgt_hexa_inact + 1 ade + 1 adocbl + 1 b1989_tRNA_3 + 1 gua + 2 h 1 h2o + 1 met-L + 1 nad	irreversible	tRNA modification	0.00E+00	1.00E+09	0	1.77E+03	1.77E+03	0	6.41E+00	6.41	0	6.41E+00	6.41
b1989_tRNA_37_MODIF_1	modification of position 37 nt (t6A) in asnV by Up_tRNA_pos_37_t6A	1 Up_tRNA_pos_37_t6A + 1 atp + 1 b1989_tRNA_3 + 1 hco3 + 1 mg2 + 1 thr-L <==> 1 b1989_tRNA_3_Up_tRNA_pos_37_t6A_cplx	reversible	tRNA modification	-1.00E+09	1.00E+09	0	1.77E+03	1.77E+03	0	6.41E+00	6.41	0	6.41E+00	6.41
b1989_tRNA_37_MODIF_2	modification of position 37 nt (t6A) in asnV by Up_tRNA_pos_37_t6A	1 b1989_tRNA_3_Up_tRNA_pos_37_t6A_cplx --> 1 Up_tRNA_pos_37_t6A_inact + 1 amp + 1 b1989_tRNA_4 + 1 h + 1 h2o + 1 mg2 + 1 ppi	irreversible	tRNA modification	0.00E+00	1.00E+09	0	1.77E+03	1.77E+03	0	6.41E+00	6.41	0	6.41E+00	6.41
b1989_tRNA_39_MODIF_1	modification of position 39 nt (Y) in asnV by TruA_dim	1 TruA_dim + 1 b1989_tRNA_4 <==> 1 b1989_tRNA_4_TruA_dim_cplx	reversible	tRNA modification	-1.00E+09	1.00E+09	0	1.77E+03	1.77E+03	0	6.41E+00	6.41	0	6.41E+00	6.41
b1989_tRNA_39_MODIF_2	modification of position 39 nt (Y) in asnV by TruA_dim	1 b1989_tRNA_4_TruA_dim_cplx --> 1 TruA_dim_inact + 1 b1989_tRNA_5	irreversible	tRNA modification	0.00E+00	1.00E+09	0	1.77E+03	1.77E+03	0	6.41E+00	6.41	0	6.41E+00	6.41
b1989_tRNA_46_MODIF_1	modification of position 46 nt (m7G) in asnV by YggH_mono	1 YggH_mono + 1 amet + 1 b1989_tRNA_5 <==> 1 b1989_tRNA_5_YggH_mono_cplx	reversible	tRNA modification	-1.00E+09	1.00E+09	0	1.77E+03	1.77E+03	0	6.41E+00	6.41	0	6.41E+00	6.41
b1989_tRNA_46_MODIF_2	modification of position 46 nt (m7G) in asnV by YggH_mono	1 b1989_tRNA_5_YggH_mono_cplx --> 1 YggH_mono_inact + 1 ahcys + 1 b1989_tRNA_6 + 1 h	irreversible	tRNA modification	0.00E+00	1.00E+09	0	1.77E+03	1.77E+03	0	6.41E+00	6.41	0	6.41E+00	6.41
b1989_tRNA_54_MODIF_1	modification of position 54 nt (m5U) in asnV by TrmA_mono	1 TrmA_mono + 1 amet + 1 b1989_tRNA_6 <==> 1 b1989_tRNA_6_TrmA_mono_cplx	reversible	tRNA modification	-1.00E+09	1.00E+09	0	1.77E+03	1.77E+03	0	6.41E+00	6.41	0	6.41E+00	6.41
b1989_tRNA_54_MODIF_2	modification of position 54 nt (m5U) in asnV by TrmA_mono	1 b1989_tRNA_6_TrmA_mono_cplx --> 1 TrmA_mono_inact + 1 ahcys + 1 b1989_tRNA_7 + 1 h	irreversible	tRNA modification	0.00E+00	1.00E+09	0	1.77E+03	1.77E+03	0	6.41E+00	6.41	0	6.41E+00	6.41
b1989_tRNA_55_MODIF_1	modification of position 55 nt (Y) in asnV by TruB_mono	1 TruB_mono + 1 b1989_tRNA_7 <==> 1 b1989_tRNA_7_TruB_mono_cplx	reversible	tRNA modification	-1.00E+09	1.00E+09	0	1.77E+03	1.77E+03	0	6.41E+00	6.41	0	6.41E+00	6.41
b1989_tRNA_55_MODIF_2	modification of position 55 nt (Y) in asnV by TruB_mono	1 b1989_tRNA_7_TruB_mono_cplx --> 1 TruB_mono_inact + 1 b1989_tRNA_8	irreversible	tRNA modification	0.00E+00	1.00E+09	0	1.77E+03	1.77E+03	0	6.41E+00	6.41	0	6.41E+00	6.41
b1989_tRNA_8_MODIF_1	modification of position 8 nt (s4U) in asnV by ThiI_mono	1 IscS_dim_S-SH + 1 ThiI_mono + 1 atp + 1 b1989_tRNA_8 + 1 mg2 + 1 trdrd <==> 1 b1989_tRNA_8_ThiI_mono_cplx	reversible	tRNA modification	-1.00E+09	1.00E+09	0	1.77E+03	1.77E+03	0	6.41E+00	6.41	0	6.41E+00	6.41
b1989_tRNA_8_MODIF_2	modification of position 8 nt (s4U) in asnV by ThiI_mono	1 b1989_tRNA_8_ThiI_mono_cplx --> 1 IscS_dim_S-H + 1 ThiI_mono_inact + 1 amp + 1 asnV_tRNA + 1 h + 1 mg2 + 1 ppi + 1 trdox	irreversible	tRNA modification	0.00E+00	1.00E+09	0	1.77E+03	1.77E+03	0	6.41E+00	6.41	0	6.41E+00	6.41
b1989_tRNA_Mg_BIND	Binding of two Mg2 to b1989_tRNA	1 b1989_RNA + 2 mg2 <==> 1 b1989_tRNA_Mg2	reversible	Metallo-ion binding	-1.00E+09	1.00E+09	0	1.77E+03	1.77E+03	0	6.41E+00	6.41	0	6.41E+00	6.41
b2114_fold_KJE_1	b2114_m folding: KJE mediated	1 DnaJ_dim + 1 DnaK_mono.ATP + 1 b2114_m_Zn <==> 1 b2114_m_DnaKJ_complex	reversible	Protein Folding	-1.00E+09	1.00E+09	0	2.42E+03	2.42E+03	0	4.15E+03	4151.924	0	1.11E+02	111.267
b2114_fold_KJE_2	b2114_m folding: KJE mediated	1 GrpE_dim + 1 b2114_m_DnaKJ_complex + 1 h2o --> 1 DnaJ_dim_inact + 1 MetG_DnaK_GrpE_complex + 1 adp + 1 h + 1 pi	irreversible	Protein Folding	0.00E+00	1.00E+09	0	2.42E+03	2.42E+03	0	4.15E+03	4151.924	0	1.11E+02	111.267
b2114_fold_KJE_3	b2114_m folding: KJE mediated	1 MetG_DnaK_GrpE_complex + 1 atp --> 1 DnaK_mono.ATP + 1 GrpE_dim_inact + 1 MetG_mono	irreversible	Protein Folding	0.00E+00	1.00E+09	0	2.42E+03	2.42E+03	0	4.15E+03	4151.924	0	1.11E+02	111.267
b2114_fold_spon	b2114_m folding: spontanous	1 b2114_m_Zn --> 1 MetG_mono	irreversible	Protein Folding	0.00E+00	1.00E+09	0	2.42E+03	2.42E+03	0	4.15E+03	4151.924	0	1.11E+02	111.267
b2114_ion_BIND	Binding of ions to b2114	1 b2114_m + 1 zn2 <==> 1 b2114_m_Zn	reversible	Metallo-ion Binding	-1.00E+09	1.00E+09	0	2.42E+03	2.42E+03	0	4.15E+03	4151.924	0	1.11E+02	111.267
b2114_mRNA_CONV	convsersion of mRNA to mRNA_1 (synthetic rxn)	1 b2114_mRNA --> 1 b2114_mRNA_1	irreversible	Translation	0.00E+00	1.00E+09	0	5.98E+01	5.98E+01	0	1.06E+02	105.5798	0	5.13E-01	0.513
b2114_mRNA_degr1	Degradation of b2114_mRNA I	1 Orn_dim + 508 atp + 1 b2114_mRNA_2 + 1 degradosome + 508 h2o <==> 508 adp + 1 b2114_mRNA_2_degr + 508 h + 508 pi	reversible	mRNA degradation	-1.00E+09	1.00E+09	0	5.98E+01	5.98E+01	0	1.06E+02	105.5798	0	5.13E-01	0.513
b2114_mRNA_degr2	Degradation of b2114_mRNA II	1 b2114_mRNA_2_degr + 2033 h2o --> 1 Orn_dim_inact + 492 amp 1 atp + 534 cmp + 1 degradosome_inact + 545 gmp + 2033 h + 462 ump	irreversible	mRNA degradation	0.00E+00	5.13E-01	0	5.98E+01	5.98E+01	0	1.06E+02	105.5798	0	5.13E-01	0.513
b2114_maturation1	Polypeptide b2114 peptide deformylas and methionine aminopeptidase complex	1 Def_mono + 1 Map_mono + 1 b2114_aa <==> 1 b2114_def_map_cplx	reversible	Protein Maturation	-1.00E+09	1.00E+09	0	2.42E+03	2.42E+03	0	4.15E+03	4151.924	0	1.11E+02	111.267
b2114_maturation2	b2114 formation	1 b2114_def_map_cplx + 2 h2o --> 1 Def_mono_inact + 1 Map_mono_inact + 1 b2114_m + 1 for + 1 met-L	irreversible	Protein Maturation	0.00E+00	1.00E+09	0	2.42E+03	2.42E+03	0	4.15E+03	4151.924	0	1.11E+02	111.267
b2140_fold_GroEL/ES_1	b2140_m folding: GroEL/ES mediated: polypeptide is going in GroEL/ES complex	1 GroEL.(7)ADP.cisGroES + 7 atp + 1 b2140_m + 1 transGroES_hepta --> 7 adp + 1 b2140_m_GroEL.(7)ATP.transGroES + 1 cisGroES_hepta	irreversible	Protein Folding	0.00E+00	1.00E+09	0	2.78E+03	2.78E+03	0	4.83E+03	4832.749	0	9.38E+01	93.834
b2140_fold_GroEL/ES_2	b2140_m folding: GroEL/ES mediated folding of polypeptide under ATP hydrolysis	1 b2140_m_GroEL.(7)ATP.transGroES + 7 h2o --> 1 DusC_GroEL.(7)ADP.transGroES + 7 h + 7 pi	irreversible	Protein Folding	0.00E+00	1.00E+09	0	2.78E+03	2.78E+03	0	4.83E+03	4832.749	0	9.38E+01	93.834
b2140_fold_GroEL/ES_3	b2140_m folding: GroEL/ES mediated release of native protein	1 DusC_GroEL.(7)ADP.transGroES --> 1 DusC_mono + 1 GroEL.(7)ADP.transGroES	irreversible	Protein Folding	0.00E+00	1.00E+09	0	2.78E+03	2.78E+03	0	4.83E+03	4832.749	0	9.38E+01	93.834
b2140_mRNA_CONV	convsersion of mRNA to mRNA_1 (synthetic rxn)	1 b2140_mRNA --> 1 b2140_mRNA_1	irreversible	Translation	0.00E+00	1.00E+09	0	1.49E+02	1.49E+02	0	2.63E+02	263.456	0	1.83E-01	0.183
b2140_mRNA_degr1	Degradation of b2140_mRNA I	1 Orn_dim + 237 atp + 1 b2140_mRNA_2 + 1 degradosome + 237 h2o <==> 237 adp + 1 b2140_mRNA_2_degr + 237 h + 237 pi	reversible	mRNA degradation	-1.00E+09	1.00E+09	0	1.49E+02	1.49E+02	0	2.63E+02	263.456	0	1.83E-01	0.183
b2140_mRNA_degr2	Degradation of b2140_mRNA II	1 b2140_mRNA_2_degr + 947 h2o --> 1 Orn_dim_inact + 220 amp + 1 atp + 214 cmp + 1 degradosome_inact + 300 gmp + 947 h + 213 ump	irreversible	mRNA degradation	0.00E+00	1.83E-01	0	1.49E+02	1.49E+02	0	2.63E+02	263.456	0	1.83E-01	0.183
b2140_maturation1	Polypeptide b2140 peptide deformylas and methionine aminopeptidase complex	1 Def_mono + 1 Map_mono + 1 b2140_aa <==> 1 b2140_def_map_cplx	reversible	Protein Maturation	-1.00E+09	1.00E+09	0	2.78E+03	2.78E+03	0	4.83E+03	4832.749	0	9.38E+01	93.834
b2140_maturation2	b2140 formation	1 b2140_def_map_cplx + 2 h2o --> 1 Def_mono_inact + 1 Map_mono_inact + 1 b2140_m + 1 for + 1 met-L	irreversible	Protein Maturation	0.00E+00	1.00E+09	0	2.78E+03	2.78E+03	0	4.83E+03	4832.749	0	9.38E+01	93.834
b2183_fold_spon	b2183_m folding: spontanous	1 b2183_m --> 1 RsuA_mono	irreversible	Protein Folding	0.00E+00	1.00E+09	0	2.41E+03	2.41E+03	0	4.18E+03	4183.787	0	9.16E+01	91.598
b2183_mRNA_CONV	convsersion of mRNA to mRNA_1 (synthetic rxn)	1 b2183_mRNA --> 1 b2183_mRNA_1	irreversible	Translation	0.00E+00	1.00E+09	0	1.78E+02	1.78E+02	0	3.15E+02	314.9698	0	1.86E-01	0.186
b2183_mRNA_degr1	Degradation of b2183_mRNA I	1 Orn_dim + 174 atp + 1 b2183_mRNA_2 + 1 degradosome + 174 h2o <==> 174 adp + 1 b2183_mRNA_2_degr + 174 h + 174 pi	reversible	mRNA degradation	-1.00E+09	1.00E+09	0	1.78E+02	1.78E+02	0	3.15E+02	314.9698	0	1.86E-01	0.186

b2183_mRNA_degr2	Degradation of b2183_mRNA II	1 b2183_mRNA_2_degr + 695 h2o --> 1 Orn_dim_inact + 161 amp + 1 atp + 179 cmp + 1 degradosome_inact + 189 gmp + 695 h + 166 ump	irreversible	mRNA degradation	0.00E+00	1.86E-01	0	1.78E+02	1.78E+02	0	3.15E+02	314.9698	0	1.86E-01	0.186
b2183_maturation1	Polypeptide b2183 peptide deformylas complex	1 Def_mono + 1 b2183_aa <==> 1 b2183_def_cplx	reversible	Protein Maturation	-1.00E+09	1.00E+09	0	2.41E+03	2.41E+03	0	4.18E+03	4183.787	0	9.16E+01	91.598
b2183_maturation2	b2183 formation	1 b2183_def_cplx + 1 h2o --> 1 Def_mono_inact + 1 b2183_m + 1 for	irreversible	Protein Maturation	0.00E+00	1.00E+09	0	2.41E+03	2.41E+03	0	4.18E+03	4183.787	0	9.16E+01	91.598
b2185_fold_spon	b2185_m folding: spontanous	1 b2185_m --> 1 RplY_mono	irreversible	Protein Folding	0.00E+00	1.00E+09	8.44E+01	1.13E+02	2.81E+01	6.22E+01	8.29E+01	20.72	6.22E+01	8.29E+01	20.72
b2185_mRNA_CONV	convsersion of mRNA to mRNA_1 (synthetic rxn)	1 b2185_mRNA --> 1 b2185_mRNA_1	irreversible	Translation	0.00E+00	1.00E+09	0	1.06E+02	1.06E+02	0	8.29E+01	82.88	0	3.50E-01	0.35
b2185_mRNA_degr1	Degradation of b2185_mRNA I	1 Orn_dim + 71 atp + 1 b2185_mRNA_2 + 1 degradosome + 71 h2o <==> 71 adp + 1 b2185_mRNA_2_degr + 71 h + 71 pi	reversible	mRNA degradation	-1.00E+09	1.00E+09	0	1.06E+02	1.06E+02	0	8.29E+01	82.88	0	3.50E-01	0.35
b2185_mRNA_degr2	Degradation of b2185_mRNA II	1 b2185_mRNA_2_degr + 284 h2o --> 1 Orn_dim_inact + 84 amp + 1 atp + 72 cmp + 1 degradosome_inact + 72 gmp + 284 h + 56 ump	irreversible	mRNA degradation	0.00E+00	3.50E-01	0	1.06E+02	1.06E+02	0	8.29E+01	82.88	0	3.50E-01	0.35
b2185_maturation1	Polypeptide b2185 peptide deformylas complex	1 Def_mono + 1 b2185_aa <==> 1 b2185_def_cplx	reversible	Protein Maturation	-1.00E+09	1.00E+09	8.44E+01	1.13E+02	2.81E+01	6.22E+01	8.29E+01	20.72	6.22E+01	8.29E+01	20.72
b2185_maturation2	b2185 formation	1 b2185_def_cplx + 1 h2o --> 1 Def_mono_inact + 1 b2185_m + 1 for	irreversible	Protein Maturation	0.00E+00	1.00E+09	8.44E+01	1.13E+02	2.81E+01	6.22E+01	8.29E+01	20.72	6.22E+01	8.29E+01	20.72
b2189_tRNA_20_MODIF_1	modification of position 20 nt (D) in proL by Dus_gen	1 Dus_gen + 1 b2189_tRNA_Mg2 + 1 h + 1 nadph <==> 1 b2189_tRNA_Mg2_Dus_gen_cplx	reversible	tRNA modification	-1.00E+09	1.00E+09	0	1.39E+03	1.39E+03	0	7.06E+00	7.06	0	7.06E+00	7.06
b2189_tRNA_20_MODIF_2	modification of position 20 nt (D) in proL by Dus_gen	1 b2189_tRNA_Mg2_Dus_gen_cplx --> 1 Dus_gen_inact + 1 b2189_tRNA_1 + 1 nadp	irreversible	tRNA modification	0.00E+00	1.00E+09	0	1.39E+03	1.39E+03	0	7.06E+00	7.06	0	7.06E+00	7.06
b2189_tRNA_32_MODIF_1	modification of position 32 nt (Cm) in proL by MeT_tRNA_pos_32_Cm	1 MeT_tRNA_pos_32_Cm + 1 amet + 1 b2189_tRNA_1 <==> 1 b2189_tRNA_1_MeT_tRNA_pos_32_Cm_cplx	reversible	tRNA modification	-1.00E+09	1.00E+09	0	1.39E+03	1.39E+03	0	7.06E+00	7.06	0	7.06E+00	7.06
b2189_tRNA_32_MODIF_2	modification of position 32 nt (Cm) in proL by MeT_tRNA_pos_32_Cm	1 b2189_tRNA_1_MeT_tRNA_pos_32_Cm_cplx --> 1 MeT_tRNA_pos_32_Cm_inact + 1 ahcys + 1 b2189_tRNA_2 + 1 h	irreversible	tRNA modification	0.00E+00	1.00E+09	0	1.39E+03	1.39E+03	0	7.06E+00	7.06	0	7.06E+00	7.06
b2189_tRNA_37_MODIF_1	modification of position 37 nt (m1G) in proL by TrmD_dim	1 TrmD_dim + 1 amet + 1 b2189_tRNA_2 <==> 1 b2189_tRNA_2_TrmD_dim_cplx	reversible	tRNA modification	-1.00E+09	1.00E+09	0	1.39E+03	1.39E+03	0	7.06E+00	7.06	0	7.06E+00	7.06
b2189_tRNA_37_MODIF_2	modification of position 37 nt (m1G) in proL by TrmD_dim	1 b2189_tRNA_2_TrmD_dim_cplx --> 1 TrmD_dim_inact + 1 ahcys + 1 b2189_tRNA_3 + 1 h	irreversible	tRNA modification	0.00E+00	1.00E+09	0	1.39E+03	1.39E+03	0	7.06E+00	7.06	0	7.06E+00	7.06
b2189_tRNA_39_MODIF_1	modification of position 39 nt (Y) in proL by TruA_dim	1 TruA_dim + 1 b2189_tRNA_3 <==> 1 b2189_tRNA_3_TruA_dim_cplx	reversible	tRNA modification	-1.00E+09	1.00E+09	0	1.39E+03	1.39E+03	0	7.06E+00	7.06	0	7.06E+00	7.06
b2189_tRNA_39_MODIF_2	modification of position 39 nt (Y) in proL by TruA_dim	1 b2189_tRNA_3_TruA_dim_cplx --> 1 TruA_dim_inact + 1 b2189_tRNA_4	irreversible	tRNA modification	0.00E+00	1.00E+09	0	1.39E+03	1.39E+03	0	7.06E+00	7.06	0	7.06E+00	7.06
b2189_tRNA_46_MODIF_1	modification of position 46 nt (m7G) in proL by YggH_mono	1 YggH_mono + 1 amet + 1 b2189_tRNA_4 <==> 1 b2189_tRNA_4_YggH_mono_cplx	reversible	tRNA modification	-1.00E+09	1.00E+09	0	1.39E+03	1.39E+03	0	7.06E+00	7.06	0	7.06E+00	7.06
b2189_tRNA_46_MODIF_2	modification of position 46 nt (m7G) in proL by YggH_mono	1 b2189_tRNA_4_YggH_mono_cplx --> 1 YggH_mono_inact + 1 ahcys + 1 b2189_tRNA_5 + 1 h	irreversible	tRNA modification	0.00E+00	1.00E+09	0	1.39E+03	1.39E+03	0	7.06E+00	7.06	0	7.06E+00	7.06
b2189_tRNA_54_MODIF_1	modification of position 54 nt (m5U) in proL by TrmA_mono	1 TrmA_mono + 1 amet + 1 b2189_tRNA_5 <==> 1 b2189_tRNA_5_TrmA_mono_cplx	reversible	tRNA modification	-1.00E+09	1.00E+09	0	1.39E+03	1.39E+03	0	7.06E+00	7.06	0	7.06E+00	7.06
b2189_tRNA_54_MODIF_2	modification of position 54 nt (m5U) in proL by TrmA_mono	1 b2189_tRNA_5_TrmA_mono_cplx --> 1 TrmA_mono_inact + 1 ahcys + 1 b2189_tRNA_6 + 1 h	irreversible	tRNA modification	0.00E+00	1.00E+09	0	1.39E+03	1.39E+03	0	7.06E+00	7.06	0	7.06E+00	7.06
b2189_tRNA_55_MODIF_1	modification of position 55 nt (Y) in proL by TruB_mono	1 TruB_mono + 1 b2189_tRNA_6 <==> 1 b2189_tRNA_6_TruB_mono_cplx	reversible	tRNA modification	-1.00E+09	1.00E+09	0	1.39E+03	1.39E+03	0	7.06E+00	7.06	0	7.06E+00	7.06
b2189_tRNA_55_MODIF_2	modification of position 55 nt (Y) in proL by TruB_mono	1 b2189_tRNA_6_TruB_mono_cplx --> 1 TruB_mono_inact + 1 b2189_tRNA_7	irreversible	tRNA modification	0.00E+00	1.00E+09	0	1.39E+03	1.39E+03	0	7.06E+00	7.06	0	7.06E+00	7.06
b2189_tRNA_8_MODIF_1	modification of position 8 nt (s4U) in proL by ThiI_mono	1 IscS_dim_S-SH + 1 ThiI_mono + 1 atp + 1 b2189_tRNA_7 + 1 mg2 + 1 trdrd <==> 1 b2189_tRNA_7_ThiI_mono_cplx	reversible	tRNA modification	-1.00E+09	1.00E+09	0	1.39E+03	1.39E+03	0	7.06E+00	7.06	0	7.06E+00	7.06
b2189_tRNA_8_MODIF_2	modification of position 8 nt (s4U) in proL by ThiI_mono	1 b2189_tRNA_7_ThiI_mono_cplx --> 1 IscS_dim_S-H + 1 ThiI_mono_inact + 1 amp + 1 h + 1 mg2 + 1 ppi + 1 proL_tRNA + 1 trdox	irreversible	tRNA modification	0.00E+00	1.00E+09	0	1.39E+03	1.39E+03	0	7.06E+00	7.06	0	7.06E+00	7.06
b2189_tRNA_Mg_BIND	Binding of two Mg2 to b2189_tRNA	1 b2189_RNA + 2 mg2 <==> 1 b2189_tRNA_Mg2	reversible	Metallo-ion binding	-1.00E+09	1.00E+09	0	1.39E+03	1.39E+03	0	7.06E+00	7.06	0	7.06E+00	7.06
b2268_fold_spon	b2268_m folding: spontanous	1 b2268_m_Zn --> 1 ElaC_mono	irreversible	Protein Folding	0.00E+00	1.00E+09	0	2.42E+03	2.42E+03	0	4.20E+03	4201.933	0	9.13E+01	91.307
b2268_ion_BIND	Binding of ions to b2268	1 b2268_m + 1 zn2 <==> 1 b2268_m_Zn	reversible	Metallo-ion Binding	-1.00E+09	1.00E+09	0	2.42E+03	2.42E+03	0	4.20E+03	4201.933	0	9.13E+01	91.307
b2268_mRNA_CONV	convsersion of mRNA to mRNA_1 (synthetic rxn)	1 b2268_mRNA --> 1 b2268_mRNA_1	irreversible	Translation	0.00E+00	1.00E+09	0	1.37E+02	1.37E+02	0	2.42E+02	241.9725	0	1.71E-01	0.171
b2268_mRNA_degr1	Degradation of b2268_mRNA I	1 Orn_dim + 229 atp + 1 b2268_mRNA_2 + 1 degradosome + 229 h2o <==> 229 adp + 1 b2268_mRNA_2_degr + 229 h + 229 pi	reversible	mRNA degradation	-1.00E+09	1.00E+09	0	1.37E+02	1.37E+02	0	2.42E+02	241.9725	0	1.71E-01	0.171
b2268_mRNA_degr2	Degradation of b2268_mRNA II	1 b2268_mRNA_2_degr + 917 h2o --> 1 Orn_dim_inact + 226 amp + 1 atp + 233 cmp + 1 degradosome_inact + 233 gmp + 917 h + 225 ump	irreversible	mRNA degradation	0.00E+00	1.71E-01	0	1.37E+02	1.37E+02	0	2.42E+02	241.9725	0	1.71E-01	0.171
b2268_maturation1	Polypeptide b2268 peptide deformylas and methionine aminopeptidase complex	1 Def_mono + 1 Map_mono + 1 b2268_aa <==> 1 b2268_def_map_cplx	reversible	Protein Maturation	-1.00E+09	1.00E+09	0	2.42E+03	2.42E+03	0	4.20E+03	4201.933	0	9.13E+01	91.307
b2268_maturation2	b2268 formation	1 b2268_def_map_cplx + 2 h2o --> 1 Def_mono_inact + 1 Map_mono_inact + 1 b2268_m + 1 for + 1 met-L	irreversible	Protein Maturation	0.00E+00	1.00E+09	0	2.42E+03	2.42E+03	0	4.20E+03	4201.933	0	9.13E+01	91.307
b2317_fold_spon	b2317_m folding: spontanous	1 b2317_m --> 1 DedA_mono	irreversible	Protein Folding	0.00E+00	1.00E+09	0	6.98E+02	6.98E+02	0	1.17E+03	1165.915	0	8.80E+01	88.014
b2317_mRNA_CONV	convsersion of mRNA to mRNA_1 (synthetic rxn)	1 b2317_mRNA --> 1 b2317_mRNA_1	irreversible	Translation	0.00E+00	1.00E+09	0	5.09E+01	5.09E+01	0	8.99E+01	89.91961	0	9.45E-02	0.0945
b2317_mRNA_degr1	Degradation of b2317_mRNA I	1 Orn_dim + 165 atp + 1 b2317_mRNA_2 + 1 degradosome + 165 h2o <==> 165 adp + 1 b2317_mRNA_2_degr + 165 h + 165 pi	reversible	mRNA degradation	-1.00E+09	1.00E+09	0	5.09E+01	5.09E+01	0	8.99E+01	89.91961	0	9.45E-02	0.0945
b2317_mRNA_degr2	Degradation of b2317_mRNA II	1 b2317_mRNA_2_degr + 659 h2o --> 1 Orn_dim_inact + 135 amp + 154 cmp + 1 degradosome_inact + 169 gmp + 659 h + 202 ump	irreversible	mRNA degradation	0.00E+00	1.89E-01	0	5.09E+01	5.09E+01	0	8.99E+01	89.91961	0	9.45E-02	0.0945
b2317_maturation1	Polypeptide b2317 peptide deformylas and methionine aminopeptidase complex	1 Def_mono + 1 Map_mono + 1 b2317_aa <==> 1 b2317_def_map_cplx	reversible	Protein Maturation	-1.00E+09	1.00E+09	0	6.98E+02	6.98E+02	0	1.17E+03	1165.915	0	8.80E+01	88.014
b2317_maturation2	b2317 formation	1 b2317_def_map_cplx + 2 h2o --> 1 Def_mono_inact + 1 Map_mono_inact + 1 b2317_m + 1 for + 1 met-L	irreversible	Protein Maturation	0.00E+00	1.00E+09	0	6.98E+02	6.98E+02	0	1.17E+03	1165.915	0	8.80E+01	88.014
b2317_v1_mRNA_CONV	convsersion of mRNA to mRNA_1 (synthetic rxn)	1 b2317_v1_mRNA --> 1 b2317_mRNA_1	irreversible	Translation	0.00E+00	1.00E+09	0	3.50E+01	3.50E+01	0	6.18E+01	61.81973	0	9.45E-02	0.0945
b2318_fold_spon	b2318_m folding: spontanous	1 b2318_m --> 1 TruA_mono	irreversible	Protein Folding	0.00E+00	1.00E+09	0	8.62E+02	8.62E+02	0	1.45E+03	1447.194	0	9.98E+01	99.8175
b2318_mRNA_CONV	convsersion of mRNA to mRNA_1 (synthetic rxn)	1 b2318_mRNA --> 1 b2318_mRNA_1	irreversible	Translation	0.00E+00	1.00E+09	0	5.09E+01	5.09E+01	0	8.99E+01	89.91961	0	9.45E-02	0.0945
b2318_mRNA_degr1	Degradation of b2318_mRNA I	1 Orn_dim + 203 atp + 1 b2318_mRNA_2 + 1 degradosome + 203 h2o <==> 203 adp + 1 b2318_mRNA_2_degr + 203 h + 203 pi	reversible	mRNA degradation	-1.00E+09	1.00E+09	0	5.09E+01	5.09E+01	0	8.99E+01	89.91961	0	9.45E-02	0.0945
b2318_mRNA_degr2	Degradation of b2318_mRNA II	1 b2318_mRNA_2_degr + 811 h2o --> 1 Orn_dim_inact + 188 amp + 208 cmp + 1 degradosome_inact + 242 gmp + 811 h + 174 ump	irreversible	mRNA degradation	0.00E+00	1.89E-01	0	5.09E+01	5.09E+01	0	8.99E+01	89.91961	0	9.45E-02	0.0945
b2318_maturation1	Polypeptide b2318 peptide deformylas and methionine aminopeptidase complex	1 Def_mono + 1 Map_mono + 1 b2318_aa <==> 1 b2318_def_map_cplx	reversible	Protein Maturation	-1.00E+09	1.00E+09	0	8.62E+02	8.62E+02	0	1.45E+03	1447.194	0	9.98E+01	99.8175
b2318_maturation2	b2318 formation	1 b2318_def_map_cplx + 2 h2o --> 1 Def_mono_inact + 1 Map_mono_inact + 1 b2318_m + 1 for + 1 met-L	irreversible	Protein Maturation	0.00E+00	1.00E+09	0	8.62E+02	8.62E+02	0	1.45E+03	1447.194	0	9.98E+01	99.8175
b2318_v1_mRNA_CONV	convsersion of mRNA to mRNA_1 (synthetic rxn)	1 b2318_v1_mRNA --> 1 b2318_mRNA_1	irreversible	Translation	0.00E+00	1.00E+09	0	3.50E+01	3.50E+01	0	6.18E+01	61.81973	0	9.45E-02	0.0945
b2319_fold_spon	b2319_m folding: spontanous	1 b2319_m --> 1 Usg_mono	irreversible	Protein Folding	0.00E+00	1.00E+09	0	1.03E+03	1.03E+03	0	1.77E+03	1771.553	0	6.49E+01	64.8755
b2319_mRNA_CONV	convsersion of mRNA to mRNA_1 (synthetic rxn)	1 b2319_mRNA --> 1 b2319_mRNA_1	irreversible	Translation	0.00E+00	1.00E+09	0	5.09E+01	5.09E+01	0	8.99E+01	89.91961	0	9.45E-02	0.0945
b2319_mRNA_degr1	Degradation of b2319_mRNA I	1 Orn_dim + 253 atp + 1 b2319_mRNA_2 + 1 degradosome + 253 h2o <==> 253 adp + 1 b2319_mRNA_2_degr + 253 h + 253 pi	reversible	mRNA degradation	-1.00E+09	1.00E+09	0	5.09E+01	5.09E+01	0	8.99E+01	89.91961	0	9.45E-02	0.0945

b2319_mRNA_degr2	Degradation of b2319_mRNA II	1 b2319_mRNA_2_degr + 1013 h2o --> 1 Orn_dim_inact + 230 amp 1 atp + 249 cmp + 1 degradosome_inact + 308 gmp + 1013 h + 226 ump	irreversible	mRNA degradation	0.00E+00	9.45E-02	0	5.09E+01	5.09E+01	0	8.99E+01	89.91961	0	9.45E-02	0.0945
b2319_maturation1	Polypeptide b2319 peptide deformylas complex	1 Def_mono + 1 b2319_aa <==> 1 b2319_def_cplx	reversible	Protein Maturation	-1.00E+09	1.00E+09	0	1.03E+03	1.03E+03	0	1.77E+03	1771.553	0	6.49E+01	64.8755
b2319_maturation2	b2319 formation	1 b2319_def_cplx + 1 h2o --> 1 Def_mono_inact + 1 b2319_m + 1 for	irreversible	Protein Maturation	0.00E+00	1.00E+09	0	1.03E+03	1.03E+03	0	1.77E+03	1771.553	0	6.49E+01	64.8755
b2319_v1_mRNA_CONV	convsersion of mRNA to mRNA_1 (synthetic rxn)	1 b2319_v1_mRNA --> 1 b2319_mRNA_1	irreversible	Translation	0.00E+00	1.00E+09	0	3.50E+01	3.50E+01	0	6.18E+01	61.81973	0	9.45E-02	0.0945
b2320_fold_spon	b2320_m folding: spontanous	1 b2320_m --> 1 PdxB_mono	irreversible	Protein Folding	0.00E+00	1.00E+09	0	8.46E+02	8.46E+02	0	1.44E+03	1435.934	0	7.80E+01	77.979
b2320_mRNA_CONV	convsersion of mRNA to mRNA_1 (synthetic rxn)	1 b2320_mRNA --> 1 b2320_mRNA_1	irreversible	Translation	0.00E+00	1.00E+09	0	3.50E+01	3.50E+01	0	6.18E+01	61.81973	0	9.45E-02	0.0945
b2320_mRNA_degr1	Degradation of b2320_mRNA I	1 Orn_dim + 284 atp + 1 b2320_mRNA_2 + 1 degradosome + 284 h2o <==> 284 adp + 1 b2320_mRNA_2_degr + 284 h + 284 pi	reversible	mRNA degradation	-1.00E+09	1.00E+09	0	3.50E+01	3.50E+01	0	6.18E+01	61.81973	0	9.45E-02	0.0945
b2320_mRNA_degr2	Degradation of b2320_mRNA II	1 b2320_mRNA_2_degr + 1136 h2o --> 1 Orn_dim_inact + 251 amp 268 cmp + 1 degradosome_inact + 334 gmp + 1 gtp + 1136 h + 283 ump	irreversible	mRNA degradation	0.00E+00	1.35E-01	0	3.50E+01	3.50E+01	0	6.18E+01	61.81973	0	9.45E-02	0.0945
b2320_maturation1	Polypeptide b2320 peptide deformylas and methionine aminopeptidase complex	1 Def_mono + 1 Map_mono + 1 b2320_aa <==> 1 b2320_def_map_cplx	reversible	Protein Maturation	-1.00E+09	1.00E+09	0	8.46E+02	8.46E+02	0	1.44E+03	1435.934	0	7.80E+01	77.979
b2320_maturation2	b2320 formation	1 b2320_def_map_cplx + 2 h2o --> 1 Def_mono_inact + 1 Map_mono_inact + 1 b2320_m + 1 for + 1 met-L	irreversible	Protein Maturation	0.00E+00	1.00E+09	0	8.46E+02	8.46E+02	0	1.44E+03	1435.934	0	7.80E+01	77.979
b2324_fold_spon	b2324_m folding: spontanous	1 b2324_m --> 1 TrmC_mono	irreversible	Protein Folding	0.00E+00	1.00E+09	0	2.66E+03	2.66E+03	0	3.29E+03	3290.823	0	7.01E+01	70.083
b2324_mRNA_CONV	convsersion of mRNA to mRNA_1 (synthetic rxn)	1 b2324_mRNA --> 1 b2324_mRNA_1	irreversible	Translation	0.00E+00	1.00E+09	0	6.65E+01	6.65E+01	0	1.17E+02	117.4575	0	1.17E-01	0.117
b2324_mRNA_degr1	Degradation of b2324_mRNA I	1 Orn_dim + 501 atp + 1 b2324_mRNA_2 + 1 degradosome + 501 h2o <==> 501 adp + 1 b2324_mRNA_2_degr + 501 h + 501 pi	reversible	mRNA degradation	-1.00E+09	1.00E+09	0	6.65E+01	6.65E+01	0	1.17E+02	117.4575	0	1.17E-01	0.117
b2324_mRNA_degr2	Degradation of b2324_mRNA II	1 b2324_mRNA_2_degr + 2006 h2o --> 1 Orn_dim_inact + 460 amp 480 cmp + 1 degradosome_inact + 580 gmp + 1 gtp + 2006 h + 486 ump	irreversible	mRNA degradation	0.00E+00	1.17E-01	0	6.65E+01	6.65E+01	0	1.17E+02	117.4575	0	1.17E-01	0.117
b2324_maturation1	Polypeptide b2324 peptide deformylas and methionine aminopeptidase complex	1 Def_mono + 1 Map_mono + 1 b2324_aa <==> 1 b2324_def_map_cplx	reversible	Protein Maturation	-1.00E+09	1.00E+09	0	2.66E+03	2.66E+03	0	3.29E+03	3290.823	0	7.01E+01	70.083
b2324_maturation2	b2324 formation	1 b2324_def_map_cplx + 2 h2o --> 1 Def_mono_inact + 1 Map_mono_inact + 1 b2324_m + 1 for + 1 met-L	irreversible	Protein Maturation	0.00E+00	1.00E+09	0	2.66E+03	2.66E+03	0	3.29E+03	3290.823	0	7.01E+01	70.083
b2325_fold_spon	b2325_m folding: spontanous	1 b2325_m --> 1 YfcL_mono	irreversible	Protein Folding	0.00E+00	1.00E+09	0	2.21E+02	2.21E+02	0	3.24E+02	324.4924	0	8.64E+01	86.438
b2325_mRNA_CONV	convsersion of mRNA to mRNA_1 (synthetic rxn)	1 b2325_mRNA --> 1 b2325_mRNA_1	irreversible	Translation	0.00E+00	1.00E+09	0	2.70E+01	2.70E+01	0	4.77E+01	47.69847	0	8.76E-02	0.0876
b2325_mRNA_degr1	Degradation of b2325_mRNA I	1 Orn_dim + 69 atp + 1 b2325_mRNA_2 + 1 degradosome + 69 h2o <==> 69 adp + 1 b2325_mRNA_2_degr + 69 h + 69 pi	reversible	mRNA degradation	-1.00E+09	1.00E+09	0	2.70E+01	2.70E+01	0	4.77E+01	47.69847	0	8.76E-02	0.0876
b2325_mRNA_degr2	Degradation of b2325_mRNA II	1 b2325_mRNA_2_degr + 278 h2o --> 1 Orn_dim_inact + 65 amp + 62 cmp + 1 degradosome_inact + 85 gmp + 278 h + 67 ump	irreversible	mRNA degradation	0.00E+00	2.07E-01	0	2.70E+01	2.70E+01	0	4.77E+01	47.69847	0	8.76E-02	0.0876
b2325_maturation1	Polypeptide b2325 peptide deformylas and methionine aminopeptidase complex	1 Def_mono + 1 Map_mono + 1 b2325_aa <==> 1 b2325_def_map_cplx	reversible	Protein Maturation	-1.00E+09	1.00E+09	0	2.21E+02	2.21E+02	0	3.24E+02	324.4924	0	8.64E+01	86.438
b2325_maturation2	b2325 formation	1 b2325_def_map_cplx + 2 h2o --> 1 Def_mono_inact + 1 Map_mono_inact + 1 b2325_m + 1 for + 1 met-L	irreversible	Protein Maturation	0.00E+00	1.00E+09	0	2.21E+02	2.21E+02	0	3.24E+02	324.4924	0	8.64E+01	86.438
b2326_fold_spon	b2326_m folding: spontanous	1 b2326_m --> 1 YfcM_mono	irreversible	Protein Folding	0.00E+00	1.00E+09	0	3.27E+02	3.27E+02	0	5.33E+02	533.3847	0	5.73E+01	57.276
b2326_mRNA_CONV	convsersion of mRNA to mRNA_1 (synthetic rxn)	1 b2326_mRNA --> 1 b2326_mRNA_1	irreversible	Translation	0.00E+00	1.00E+09	0	2.70E+01	2.70E+01	0	4.77E+01	47.69847	0	8.76E-02	0.0876
b2326_mRNA_degr1	Degradation of b2326_mRNA I	1 Orn_dim + 137 atp + 1 b2326_mRNA_2 + 1 degradosome + 137 h2o <==> 137 adp + 1 b2326_mRNA_2_degr + 137 h + 137 pi	reversible	mRNA degradation	-1.00E+09	1.00E+09	0	2.70E+01	2.70E+01	0	4.77E+01	47.69847	0	8.76E-02	0.0876
b2326_mRNA_degr2	Degradation of b2326_mRNA II	1 b2326_mRNA_2_degr + 547 h2o --> 1 Orn_dim_inact + 133 amp + 127 cmp + 1 degradosome_inact + 149 gmp + 547 h + 139 ump	irreversible	mRNA degradation	0.00E+00	1.04E-01	0	2.70E+01	2.70E+01	0	4.77E+01	47.69847	0	8.76E-02	0.0876
b2326_maturation1	Polypeptide b2326 peptide deformylas and methionine aminopeptidase complex	1 Def_mono + 1 Map_mono + 1 b2326_aa <==> 1 b2326_def_map_cplx	reversible	Protein Maturation	-1.00E+09	1.00E+09	0	3.27E+02	3.27E+02	0	5.33E+02	533.3847	0	5.73E+01	57.276
b2326_maturation2	b2326 formation	1 b2326_def_map_cplx + 2 h2o --> 1 Def_mono_inact + 1 Map_mono_inact + 1 b2326_m + 1 for + 1 met-L	irreversible	Protein Maturation	0.00E+00	1.00E+09	0	3.27E+02	3.27E+02	0	5.33E+02	533.3847	0	5.73E+01	57.276
b2327_fold_spon	b2327_m folding: spontanous	1 b2327_m --> 1 YfcA_mono	irreversible	Protein Folding	0.00E+00	1.00E+09	0	4.94E+02	4.94E+02	0	8.04E+02	803.8271	0	8.97E+01	89.664
b2327_mRNA_CONV	convsersion of mRNA to mRNA_1 (synthetic rxn)	1 b2327_mRNA --> 1 b2327_mRNA_1	irreversible	Translation	0.00E+00	1.00E+09	0	2.70E+01	2.70E+01	0	4.77E+01	47.69847	0	8.76E-02	0.0876
b2327_mRNA_degr1	Degradation of b2327_mRNA I	1 Orn_dim + 202 atp + 1 b2327_mRNA_2 + 1 degradosome + 202 h2o <==> 202 adp + 1 b2327_mRNA_2_degr + 202 h + 202 pi	reversible	mRNA degradation	-1.00E+09	1.00E+09	0	2.70E+01	2.70E+01	0	4.77E+01	47.69847	0	8.76E-02	0.0876
b2327_mRNA_degr2	Degradation of b2327_mRNA II	1 b2327_mRNA_2_degr + 808 h2o --> 1 Orn_dim_inact + 156 amp + 202 cmp + 1 degradosome_inact + 225 gmp + 808 h + 226 ump	irreversible	mRNA degradation	0.00E+00	8.76E-02	0	2.70E+01	2.70E+01	0	4.77E+01	47.69847	0	8.76E-02	0.0876
b2327_maturation1	Polypeptide b2327 peptide deformylas and methionine aminopeptidase complex	1 Def_mono + 1 Map_mono + 1 b2327_aa <==> 1 b2327_def_map_cplx	reversible	Protein Maturation	-1.00E+09	1.00E+09	0	4.94E+02	4.94E+02	0	8.04E+02	803.8271	0	8.97E+01	89.664
b2327_maturation2	b2327 formation	1 b2327_def_map_cplx + 2 h2o --> 1 Def_mono_inact + 1 Map_mono_inact + 1 b2327_m + 1 for + 1 met-L	irreversible	Protein Maturation	0.00E+00	1.00E+09	0	4.94E+02	4.94E+02	0	8.04E+02	803.8271	0	8.97E+01	89.664
b2328_fold_spon	b2328_m folding: spontanous	1 b2328_m --> 1 MepA_mono	irreversible	Protein Folding	0.00E+00	1.00E+09	0	5.03E+02	5.03E+02	0	8.34E+02	833.8956	0	7.21E+01	72.1216
b2328_mRNA_CONV	convsersion of mRNA to mRNA_1 (synthetic rxn)	1 b2328_mRNA --> 1 b2328_mRNA_1	irreversible	Translation	0.00E+00	1.00E+09	0	2.70E+01	2.70E+01	0	4.77E+01	47.69847	0	8.76E-02	0.0876
b2328_mRNA_degr1	Degradation of b2328_mRNA I	1 Orn_dim + 206 atp + 1 b2328_mRNA_2 + 1 degradosome + 206 h2o <==> 206 adp + 1 b2328_mRNA_2_degr + 206 h + 206 pi	reversible	mRNA degradation	-1.00E+09	1.00E+09	0	2.70E+01	2.70E+01	0	4.77E+01	47.69847	0	8.76E-02	0.0876
b2328_mRNA_degr2	Degradation of b2328_mRNA II	1 b2328_mRNA_2_degr + 824 h2o --> 1 Orn_dim_inact + 186 amp + 231 cmp + 1 degradosome_inact + 226 gmp + 824 h + 182 ump	irreversible	mRNA degradation	0.00E+00	1.74E-01	0	2.70E+01	2.70E+01	0	4.77E+01	47.69847	0	8.76E-02	0.0876
b2328_maturation1	Polypeptide b2328 peptide deformylas and methionine aminopeptidase complex	1 Def_mono + 1 Map_mono + 1 b2328_aa <==> 1 b2328_def_map_cplx	reversible	Protein Maturation	-1.00E+09	1.00E+09	0	5.03E+02	5.03E+02	0	8.34E+02	833.8956	0	7.21E+01	72.1216
b2328_maturation2	b2328 formation	1 b2328_def_map_cplx + 20 h2o --> 1 Def_mono_inact + 1 Map_mono_inact + 6 ala-L + 1 asn-L + 1 b2328_m + 1 for + 1 ile-L + leu-L + 1 lys-L + 1 met-L + 3 ser-L + 1 thr-L	irreversible	Protein Maturation	0.00E+00	1.00E+09	0	5.03E+02	5.03E+02	0	8.34E+02	833.8956	0	7.21E+01	72.1216
b2329_fold_spon	b2329_m folding: spontanous	1 b2329_m --> 1 AroC_mono	irreversible	Protein Folding	0.00E+00	1.00E+09	0	6.71E+02	6.71E+02	0	1.10E+03	1104.778	0	1.05E+02	104.9496
b2329_mRNA_CONV	convsersion of mRNA to mRNA_1 (synthetic rxn)	1 b2329_mRNA --> 1 b2329_mRNA_1	irreversible	Translation	0.00E+00	1.00E+09	0	2.70E+01	2.70E+01	0	4.77E+01	47.69847	0	8.76E-02	0.0876
b2329_mRNA_degr1	Degradation of b2329_mRNA I	1 Orn_dim + 271 atp + 1 b2329_mRNA_2 + 1 degradosome + 271 h2o <==> 271 adp + 1 b2329_mRNA_2_degr + 271 h + 271 pi	reversible	mRNA degradation	-1.00E+09	1.00E+09	0	2.70E+01	2.70E+01	0	4.77E+01	47.69847	0	8.76E-02	0.0876
b2329_mRNA_degr2	Degradation of b2329_mRNA II	1 b2329_mRNA_2_degr + 1085 h2o --> 1 Orn_dim_inact + 247 amp 305 cmp + 1 degradosome_inact + 311 gmp + 1085 h + 223 ump	irreversible	mRNA degradation	0.00E+00	2.57E-01	0	2.70E+01	2.70E+01	0	4.77E+01	47.69847	0	8.76E-02	0.0876
b2329_maturation1	Polypeptide b2329 peptide deformylas and methionine aminopeptidase complex	1 Def_mono + 1 Map_mono + 1 b2329_aa <==> 1 b2329_def_map_cplx	reversible	Protein Maturation	-1.00E+09	1.00E+09	0	6.71E+02	6.71E+02	0	1.10E+03	1104.778	0	1.05E+02	104.9496
b2329_maturation2	b2329 formation	1 b2329_def_map_cplx + 2 h2o --> 1 Def_mono_inact + 1 Map_mono_inact + 1 b2329_m + 1 for + 1 met-L	irreversible	Protein Maturation	0.00E+00	1.00E+09	0	6.71E+02	6.71E+02	0	1.10E+03	1104.778	0	1.05E+02	104.9496
b2330_fold_spon	b2330_m folding: spontanous	1 b2330_m --> 1 PrmB_mono	irreversible	Protein Folding	0.00E+00	1.00E+09	0	5.78E+02	5.78E+02	0	9.51E+02	950.5525	0	9.36E+01	93.5568

b2330_mRNA_CONV	conversion of mRNA to mRNA_1 (synthetic rxn)	1 b2330_mRNA --> 1 b2330_mRNA_1	irreversible	Translation	0.00E+00	1.00E+09	0	2.70E+01	2.70E+01	0	4.77E+01	47.69847	0	8.76E-02	0.0876
b2330_mRNA_degr1	Degradation of b2330_mRNA I	1 Orn_dim + 233 atp + 1 b2330_mRNA_2 + 1 degradosome + 233 h2o <==> 233 adp + 1 b2330_mRNA_2_degr + 233 h + 233 pi	reversible	mRNA degradation	-1.00E+09	1.00E+09	0	2.70E+01	2.70E+01	0	4.77E+01	47.69847	0	8.76E-02	0.0876
b2330_mRNA_degr2	Degradation of b2330_mRNA II	1 b2330_mRNA_2_degr + 932 h2o --> 1 Orn_dim_inact + 218 amp + 239 cmp + 1 degradosome_inact + 250 gmp + 1 gtp + 932 h + 225 ump	irreversible	mRNA degradation	0.00E+00	1.77E-01	0	2.70E+01	2.70E+01	0	4.77E+01	47.69847	0	8.76E-02	0.0876
b2330_maturation1	Polypeptide b2330 peptide deformylas and methionine aminopeptidase complex	1 Def_mono + 1 Map_mono + 1 b2330_aa <==> 1 b2330_def_map_cplx	reversible	Protein Maturation	-1.00E+09	1.00E+09	0	5.78E+02	5.78E+02	0	9.51E+02	950.5525	0	9.36E+01	93.5568
b2330_maturation2	b2330 formation	1 b2330_def_map_cplx + 2 h2o --> 1 Def_mono_inact + 1 Map_mono_inact + 1 b2330_m + 1 for + 1 met-L	irreversible	Protein Maturation	0.00E+00	1.00E+09	0	5.78E+02	5.78E+02	0	9.51E+02	950.5525	0	9.36E+01	93.5568
b2348_tRNA_20A_MODIF_1	modification of position 20A nt (D) in argW by Dus_gen	1 Dus_gen + 1 b2348_tRNA_Mg2 + 1 h + 1 nadph <==> 1 b2348_tRNA_Mg2_Dus_gen_cplx	reversible	tRNA modification	-1.00E+09	1.00E+09	0	1.68E+03	1.68E+03	0	7.62E+00	7.62	0	7.62E+00	7.62
b2348_tRNA_20A_MODIF_2	modification of position 20A nt (D) in argW by Dus_gen	1 b2348_tRNA_Mg2_Dus_gen_cplx --> 1 Dus_gen_inact + 1 b2348_tRNA_1 + 1 nadp	irreversible	tRNA modification	0.00E+00	1.00E+09	0	1.68E+03	1.68E+03	0	7.62E+00	7.62	0	7.62E+00	7.62
b2348_tRNA_32_MODIF_1	modification of position 32 nt (s2C) in argW by YdaO_mono	1 IscS_dim_S-SH + 1 YdaO_mono + 1 atp + 1 b2348_tRNA_1 + 1 mg2 + 1 trdrd <==> 1 b2348_tRNA_1_YdaO_mono_cplx	reversible	tRNA modification	-1.00E+09	1.00E+09	0	1.68E+03	1.68E+03	0	7.62E+00	7.62	0	7.62E+00	7.62
b2348_tRNA_32_MODIF_2	modification of position 32 nt (s2C) in argW by YdaO_mono	1 b2348_tRNA_1_YdaO_mono_cplx --> 1 IscS_dim_S-H + 1 YdaO_mono_inact + 1 amp + 1 b2348_tRNA_2 + 1 h + 1 mg2 + 1 ppi + 1 trdox	irreversible	tRNA modification	0.00E+00	1.00E+09	0	1.68E+03	1.68E+03	0	7.62E+00	7.62	0	7.62E+00	7.62
b2348_tRNA_34_MODIF_1	modification of position 34 nt (mnm5U) in argW by TrmE_dim-GidA_mono-TrmC_mono	1 5fthf + 1 GidA_mono + 1 TrmC_mono + 1 TrmE_dim + 1 amet + 1 b2348_tRNA_2 + 1 fad + 1 fadh2 + 1 gly + 1 gtp + 1 h2o <==> 1 b2348_tRNA_2_TrmE_dim-GidA_mono-TrmC_mono_cplx	reversible	tRNA modification	-1.00E+09	1.00E+09	0	1.68E+03	1.68E+03	0	7.62E+00	7.62	0	7.62E+00	7.62
b2348_tRNA_34_MODIF_2	modification of position 34 nt (mnm5U) in argW by TrmE_dim-GidA_mono-TrmC_mono	1 b2348_tRNA_2_TrmE_dim-GidA_mono-TrmC_mono_cplx --> 1 GidA_mono_inact + 1 TrmC_mono_inact + 1 TrmE_dim_inact + 1 ahcys + 1 b2348_tRNA_3 + 1 fad + 1 fadh2 + 1 gdp + 1 glx + 3 h + 1 pi + 1 thf	irreversible	tRNA modification	0.00E+00	1.00E+09	0	1.68E+03	1.68E+03	0	7.62E+00	7.62	0	7.62E+00	7.62
b2348_tRNA_37_MODIF_1	modification of position 37 nt (t6A) in argW by Up_tRNA_pos_37_t6A	1 Up_tRNA_pos_37_t6A + 1 atp + 1 b2348_tRNA_3 + 1 hco3 + 1 mg2 + 1 thr-L <==> 1 b2348_tRNA_3_Up_tRNA_pos_37_t6A_cplx	reversible	tRNA modification	-1.00E+09	1.00E+09	0	1.68E+03	1.68E+03	0	7.62E+00	7.62	0	7.62E+00	7.62
b2348_tRNA_37_MODIF_2	modification of position 37 nt (t6A) in argW by Up_tRNA_pos_37_t6A	1 b2348_tRNA_3_Up_tRNA_pos_37_t6A_cplx --> 1 Up_tRNA_pos_37_t6A_inact + 1 amp + 1 b2348_tRNA_4 + 1 h + 1 h2o + 1 mg2 + 1 ppi	irreversible	tRNA modification	0.00E+00	1.00E+09	0	1.68E+03	1.68E+03	0	7.62E+00	7.62	0	7.62E+00	7.62
b2348_tRNA_54_MODIF_1	modification of position 54 nt (m5U) in argW by TrmA_mono	1 TrmA_mono + 1 amet + 1 b2348_tRNA_4 <==> 1 b2348_tRNA_4_TrmA_mono_cplx	reversible	tRNA modification	-1.00E+09	1.00E+09	0	1.68E+03	1.68E+03	0	7.62E+00	7.62	0	7.62E+00	7.62
b2348_tRNA_54_MODIF_2	modification of position 54 nt (m5U) in argW by TrmA_mono	1 b2348_tRNA_4_TrmA_mono_cplx --> 1 TrmA_mono_inact + 1 ahcys + 1 b2348_tRNA_5 + 1 h	irreversible	tRNA modification	0.00E+00	1.00E+09	0	1.68E+03	1.68E+03	0	7.62E+00	7.62	0	7.62E+00	7.62
b2348_tRNA_55_MODIF_1	modification of position 55 nt (Y) in argW by TruB_mono	1 TruB_mono + 1 b2348_tRNA_5 <==> 1 b2348_tRNA_5_TruB_mono_cplx	reversible	tRNA modification	-1.00E+09	1.00E+09	0	1.68E+03	1.68E+03	0	7.62E+00	7.62	0	7.62E+00	7.62
b2348_tRNA_55_MODIF_2	modification of position 55 nt (Y) in argW by TruB_mono	1 b2348_tRNA_5_TruB_mono_cplx --> 1 TruB_mono_inact + 1 argW_tRNA	irreversible	tRNA modification	0.00E+00	1.00E+09	0	1.68E+03	1.68E+03	0	7.62E+00	7.62	0	7.62E+00	7.62
b2348_tRNA_Mg_BIND	Binding of two Mg2 to b2348_tRNA	1 b2348_RNA + 2 mg2 <==> 1 b2348_tRNA_Mg2	reversible	Metallo-ion binding	-1.00E+09	1.00E+09	0	1.68E+03	1.68E+03	0	7.62E+00	7.62	0	7.62E+00	7.62
b2396_tRNA_17_MODIF_1	modification of position 17 nt (D) in alaX by Dus_gen	1 Dus_gen + 1 b2396_tRNA_Mg2 + 1 h + 1 nadph <==> 1 b2396_tRNA_Mg2_Dus_gen_cplx	reversible	tRNA modification	-1.00E+09	1.00E+09	0	6.14E+02	6.14E+02	0	7.80E+00	7.8	0	7.80E+00	7.8
b2396_tRNA_17_MODIF_2	modification of position 17 nt (D) in alaX by Dus_gen	1 b2396_tRNA_Mg2_Dus_gen_cplx --> 1 Dus_gen_inact + 1 b2396_tRNA_1 + 1 nadp	irreversible	tRNA modification	0.00E+00	1.00E+09	0	6.14E+02	6.14E+02	0	7.80E+00	7.8	0	7.80E+00	7.8
b2396_tRNA_46_MODIF_1	modification of position 46 nt (m7G) in alaX by YggH_mono	1 YggH_mono + 1 amet + 1 b2396_tRNA_1 <==> 1 b2396_tRNA_1_YggH_mono_cplx	reversible	tRNA modification	-1.00E+09	1.00E+09	0	6.14E+02	6.14E+02	0	7.80E+00	7.8	0	7.80E+00	7.8
b2396_tRNA_46_MODIF_2	modification of position 46 nt (m7G) in alaX by YggH_mono	1 b2396_tRNA_1_YggH_mono_cplx --> 1 YggH_mono_inact + 1 ahcys + 1 b2396_tRNA_2 + 1 h	irreversible	tRNA modification	0.00E+00	1.00E+09	0	6.14E+02	6.14E+02	0	7.80E+00	7.8	0	7.80E+00	7.8
b2396_tRNA_54_MODIF_1	modification of position 54 nt (m5U) in alaX by TrmA_mono	1 TrmA_mono + 1 amet + 1 b2396_tRNA_2 <==> 1 b2396_tRNA_2_TrmA_mono_cplx	reversible	tRNA modification	-1.00E+09	1.00E+09	0	6.14E+02	6.14E+02	0	7.80E+00	7.8	0	7.80E+00	7.8
b2396_tRNA_54_MODIF_2	modification of position 54 nt (m5U) in alaX by TrmA_mono	1 b2396_tRNA_2_TrmA_mono_cplx --> 1 TrmA_mono_inact + 1 ahcys + 1 b2396_tRNA_3 + 1 h	irreversible	tRNA modification	0.00E+00	1.00E+09	0	6.14E+02	6.14E+02	0	7.80E+00	7.8	0	7.80E+00	7.8
b2396_tRNA_55_MODIF_1	modification of position 55 nt (Y) in alaX by TruB_mono	1 TruB_mono + 1 b2396_tRNA_3 <==> 1 b2396_tRNA_3_TruB_mono_cplx	reversible	tRNA modification	-1.00E+09	1.00E+09	0	6.14E+02	6.14E+02	0	7.80E+00	7.8	0	7.80E+00	7.8
b2396_tRNA_55_MODIF_2	modification of position 55 nt (Y) in alaX by TruB_mono	1 b2396_tRNA_3_TruB_mono_cplx --> 1 TruB_mono_inact + 1 alaX_tRNA	irreversible	tRNA modification	0.00E+00	1.00E+09	0	6.14E+02	6.14E+02	0	7.80E+00	7.8	0	7.80E+00	7.8
b2396_tRNA_Mg_BIND	Binding of two Mg2 to b2396_tRNA	1 b2396_RNA + 2 mg2 <==> 1 b2396_tRNA_Mg2	reversible	Metallo-ion binding	-1.00E+09	1.00E+09	0	6.14E+02	6.14E+02	0	7.80E+00	7.8	0	7.80E+00	7.8
b2397_tRNA_17_MODIF_1	modification of position 17 nt (D) in alaW by Dus_gen	1 Dus_gen + 1 b2397_tRNA_Mg2 + 1 h + 1 nadph <==> 1 b2397_tRNA_Mg2_Dus_gen_cplx	reversible	tRNA modification	-1.00E+09	1.00E+09	0	6.14E+02	6.14E+02	0	7.80E+00	7.8	0	7.80E+00	7.8
b2397_tRNA_17_MODIF_2	modification of position 17 nt (D) in alaW by Dus_gen	1 b2397_tRNA_Mg2_Dus_gen_cplx --> 1 Dus_gen_inact + 1 b2397_tRNA_1 + 1 nadp	irreversible	tRNA modification	0.00E+00	1.00E+09	0	6.14E+02	6.14E+02	0	7.80E+00	7.8	0	7.80E+00	7.8
b2397_tRNA_46_MODIF_1	modification of position 46 nt (m7G) in alaW by YggH_mono	1 YggH_mono + 1 amet + 1 b2397_tRNA_1 <==> 1 b2397_tRNA_1_YggH_mono_cplx	reversible	tRNA modification	-1.00E+09	1.00E+09	0	6.14E+02	6.14E+02	0	7.80E+00	7.8	0	7.80E+00	7.8
b2397_tRNA_46_MODIF_2	modification of position 46 nt (m7G) in alaW by YggH_mono	1 b2397_tRNA_1_YggH_mono_cplx --> 1 YggH_mono_inact + 1 ahcys + 1 b2397_tRNA_2 + 1 h	irreversible	tRNA modification	0.00E+00	1.00E+09	0	6.14E+02	6.14E+02	0	7.80E+00	7.8	0	7.80E+00	7.8
b2397_tRNA_54_MODIF_1	modification of position 54 nt (m5U) in alaW by TrmA_mono	1 TrmA_mono + 1 amet + 1 b2397_tRNA_2 <==> 1 b2397_tRNA_2_TrmA_mono_cplx	reversible	tRNA modification	-1.00E+09	1.00E+09	0	6.14E+02	6.14E+02	0	7.80E+00	7.8	0	7.80E+00	7.8
b2397_tRNA_54_MODIF_2	modification of position 54 nt (m5U) in alaW by TrmA_mono	1 b2397_tRNA_2_TrmA_mono_cplx --> 1 TrmA_mono_inact + 1 ahcys + 1 b2397_tRNA_3 + 1 h	irreversible	tRNA modification	0.00E+00	1.00E+09	0	6.14E+02	6.14E+02	0	7.80E+00	7.8	0	7.80E+00	7.8
b2397_tRNA_55_MODIF_1	modification of position 55 nt (Y) in alaW by TruB_mono	1 TruB_mono + 1 b2397_tRNA_3 <==> 1 b2397_tRNA_3_TruB_mono_cplx	reversible	tRNA modification	-1.00E+09	1.00E+09	0	6.14E+02	6.14E+02	0	7.80E+00	7.8	0	7.80E+00	7.8
b2397_tRNA_55_MODIF_2	modification of position 55 nt (Y) in alaW by TruB_mono	1 b2397_tRNA_3_TruB_mono_cplx --> 1 TruB_mono_inact + 1 alaW_tRNA	irreversible	tRNA modification	0.00E+00	1.00E+09	0	6.14E+02	6.14E+02	0	7.80E+00	7.8	0	7.80E+00	7.8
b2397_tRNA_Mg_BIND	Binding of two Mg2 to b2397_tRNA	1 b2397_RNA + 2 mg2 <==> 1 b2397_tRNA_Mg2	reversible	Metallo-ion binding	-1.00E+09	1.00E+09	0	6.14E+02	6.14E+02	0	7.80E+00	7.8	0	7.80E+00	7.8
b2400_fold_spon	b2400_m folding: spontanous	1 b2400_m_Zn --> 1 GltX_mono	irreversible	Protein Folding	0.00E+00	1.00E+09	0	2.61E+03	2.61E+03	0	4.01E+03	4005.054	0	1.87E+02	186.759
b2400_ion_BIND	Binding of ions to b2400	1 b2400_m + 1 zn2 <==> 1 b2400_m_Zn	reversible	Metallo-ion Binding	-1.00E+09	1.00E+09	0	2.61E+03	2.61E+03	0	4.01E+03	4005.054	0	1.87E+02	186.759
b2400_mRNA_CONV	conversion of mRNA to mRNA_1 (synthetic rxn)	1 b2400_mRNA --> 1 b2400_mRNA_1	irreversible	Translation	0.00E+00	1.00E+09	0	9.02E+01	9.02E+01	0	1.59E+02	159.2644	0	5.77E-01	0.577
b2400_mRNA_degr1	Degradation of b2400_mRNA I	1 Orn_dim + 354 atp + 1 b2400_mRNA_2 + 1 degradosome + 354 h2o <==> 354 adp + 1 b2400_mRNA_2_degr + 354 h + 354 pi	reversible	mRNA degradation	-1.00E+09	1.00E+09	0	9.02E+01	9.02E+01	0	1.59E+02	159.2644	0	5.77E-01	0.577
b2400_mRNA_degr2	Degradation of b2400_mRNA II	1 b2400_mRNA_2_degr + 1415 h2o --> 1 Orn_dim_inact + 348 amp + 1 atp + 354 cmp + 1 degradosome_inact + 390 gmp + 1415 h + 323 ump	irreversible	mRNA degradation	0.00E+00	5.77E-01	0	9.02E+01	9.02E+01	0	1.59E+02	159.2644	0	5.77E-01	0.577
b2400_maturation1	Polypeptide b2400 peptide deformylas and methionine aminopeptidase complex	1 Def_mono + 1 Map_mono + 1 b2400_aa <==> 1 b2400_def_map_cplx	reversible	Protein Maturation	-1.00E+09	1.00E+09	0	2.61E+03	2.61E+03	0	4.01E+03	4005.054	0	1.87E+02	186.759
b2400_maturation2	b2400 formation	1 b2400_def_map_cplx + 2 h2o --> 1 Def_mono_inact + 1 Map_mono_inact + 1 b2400_m + 1 for + 1 met-L	irreversible	Protein Maturation	0.00E+00	1.00E+09	0	2.61E+03	2.61E+03	0	4.01E+03	4005.054	0	1.87E+02	186.759
b2400_v1_mRNA_CONV	conversion of mRNA to mRNA_1 (synthetic rxn)	1 b2400_v1_mRNA --> 1 b2400_mRNA_1	irreversible	Translation	0.00E+00	1.00E+09	0	9.02E+01	9.02E+01	0	1.59E+02	159.2644	0	5.77E-01	0.577
b2400_v2_mRNA_CONV	conversion of mRNA to mRNA_1 (synthetic rxn)	1 b2400_v2_mRNA --> 1 b2400_mRNA_1	irreversible	Translation	0.00E+00	1.00E+09	0	9.02E+01	9.02E+01	0	1.59E+02	159.2644	0	5.77E-01	0.577
b2401_tRNA_17_MODIF_1	modification of position 17 nt (D) in valU by Dus_gen	1 Dus_gen + 1 b2401_tRNA_Mg2 + 1 h + 1 nadph <==> 1 b2401_tRNA_Mg2_Dus_gen_cplx	reversible	tRNA modification	-1.00E+09	1.00E+09	0	3.01E+02	3.01E+02	0	7.81E+00	7.81	0	7.81E+00	7.81
b2401_tRNA_17_MODIF_2	modification of position 17 nt (D) in valU by Dus_gen	1 b2401_tRNA_Mg2_Dus_gen_cplx --> 1 Dus_gen_inact + 1 b2401_tRNA_1 + 1 nadp	irreversible	tRNA modification	0.00E+00	1.00E+09	0	3.01E+02	3.01E+02	0	7.81E+00	7.81	0	7.81E+00	7.81
b2401_tRNA_34_MODIF_1	modification of position 34 nt (cmo5U) in valU by YecO_mono-YecP_mono-HyL_tRNA_pos_34_ho5U	1 HyL_tRNA_pos_34_ho5U + 1 YecO_mono + 1 YecP_mono + 1 amet + 1 b2401_tRNA_1 + 2 chor <==> 1 b2401_tRNA_1_YecO_mono-YecP_mono-HyL_tRNA_pos_34_ho5U_cplx	reversible	tRNA modification	-1.00E+09	1.00E+09	0	3.01E+02	3.01E+02	0	7.81E+00	7.81	0	7.81E+00	7.81
b2401_tRNA_34_MODIF_2	modification of position 34 nt (cmo5U) in valU by YecO_mono-YecP_mono-HyL_tRNA_pos_34_ho5U	1 b2401_tRNA_1_YecO_mono-YecP_mono-HyL_tRNA_pos_34_ho5U_cplx --> 1 C10H8O5 + 1 C9H9O4 + 1 HyL_tRNA_pos_34_ho5U_inact + 1 YecO_mono_inact + 1 YecP_mono_inact + 1 ahcys + 1 b2401_tRNA_2 + 1 h	irreversible	tRNA modification	0.00E+00	1.00E+09	0	3.01E+02	3.01E+02	0	7.81E+00	7.81	0	7.81E+00	7.81

b2401_tRNA_37_MODIF_1	modification of position 37 nt (m6A) in valU by MeT_tRNA_pos_37_m6A	1 MeT_tRNA_pos_37_m6A + 1 amet + 1 b2401_tRNA_2 <==> 1 b2401_tRNA_2_MeT_tRNA_pos_37_m6A_cplx	reversible	tRNA modification	-1.00E+09	1.00E+09	0	3.01E+02	3.01E+02	0	7.81E+00	7.81	0	7.81E+00	7.81
b2401_tRNA_37_MODIF_2	modification of position 37 nt (m6A) in valU by MeT_tRNA_pos_37_m6A	1 b2401_tRNA_2_MeT_tRNA_pos_37_m6A_cplx --> 1 MeT_tRNA_pos_37_m6A_inact + 1 ahcys + 1 b2401_tRNA_3 + 1 h	irreversible	tRNA modification	0.00E+00	1.00E+09	0	3.01E+02	3.01E+02	0	7.81E+00	7.81	0	7.81E+00	7.81
b2401_tRNA_46_MODIF_1	modification of position 46 nt (m7G) in valU by YggH_mono	1 YggH_mono + 1 amet + 1 b2401_tRNA_3 <==> 1 b2401_tRNA_3_YggH_mono_cplx	reversible	tRNA modification	-1.00E+09	1.00E+09	0	3.01E+02	3.01E+02	0	7.81E+00	7.81	0	7.81E+00	7.81
b2401_tRNA_46_MODIF_2	modification of position 46 nt (m7G) in valU by YggH_mono	1 b2401_tRNA_3_YggH_mono_cplx --> 1 YggH_mono_inact + 1 ahcys + 1 b2401_tRNA_4 + 1 h	irreversible	tRNA modification	0.00E+00	1.00E+09	0	3.01E+02	3.01E+02	0	7.81E+00	7.81	0	7.81E+00	7.81
b2401_tRNA_54_MODIF_1	modification of position 54 nt (m5U) in valU by TrmA_mono	1 TrmA_mono + 1 amet + 1 b2401_tRNA_4 <==> 1 b2401_tRNA_4_TrmA_mono_cplx	reversible	tRNA modification	-1.00E+09	1.00E+09	0	3.01E+02	3.01E+02	0	7.81E+00	7.81	0	7.81E+00	7.81
b2401_tRNA_54_MODIF_2	modification of position 54 nt (m5U) in valU by TrmA_mono	1 b2401_tRNA_4_TrmA_mono_cplx --> 1 TrmA_mono_inact + 1 ahcys + 1 b2401_tRNA_5 + 1 h	irreversible	tRNA modification	0.00E+00	1.00E+09	0	3.01E+02	3.01E+02	0	7.81E+00	7.81	0	7.81E+00	7.81
b2401_tRNA_55_MODIF_1	modification of position 55 nt (Y) in valU by TruB_mono	1 TruB_mono + 1 b2401_tRNA_5 <==> 1 b2401_tRNA_5_TruB_mono_cplx	reversible	tRNA modification	-1.00E+09	1.00E+09	0	3.01E+02	3.01E+02	0	7.81E+00	7.81	0	7.81E+00	7.81
b2401_tRNA_55_MODIF_2	modification of position 55 nt (Y) in valU by TruB_mono	1 b2401_tRNA_5_TruB_mono_cplx --> 1 TruB_mono_inact + 1 b2401_tRNA_6	irreversible	tRNA modification	0.00E+00	1.00E+09	0	3.01E+02	3.01E+02	0	7.81E+00	7.81	0	7.81E+00	7.81
b2401_tRNA_8_MODIF_1	modification of position 8 nt (s4U) in valU by ThiI_mono	1 IscS_dim_S-SH + 1 ThiI_mono + 1 atp + 1 b2401_tRNA_6 + 1 mg2 + 1 trdrd <==> 1 b2401_tRNA_6_ThiI_mono_cplx	reversible	tRNA modification	-1.00E+09	1.00E+09	0	3.01E+02	3.01E+02	0	7.81E+00	7.81	0	7.81E+00	7.81
b2401_tRNA_8_MODIF_2	modification of position 8 nt (s4U) in valU by ThiI_mono	1 b2401_tRNA_6_ThiI_mono_cplx --> 1 IscS_dim_S-H + 1 ThiI_mono_inact + 1 amp + 1 h + 1 mg2 + 1 ppi + 1 trdox + 1 valU_tRNA	irreversible	tRNA modification	0.00E+00	1.00E+09	0	3.01E+02	3.01E+02	0	7.81E+00	7.81	0	7.81E+00	7.81
b2401_tRNA_Mg_BIND	Binding of two Mg2 to b2401_tRNA	1 b2401_RNA + 2 mg2 <==> 1 b2401_tRNA_Mg2	reversible	Metallo-ion binding	-1.00E+09	1.00E+09	0	3.01E+02	3.01E+02	0	7.81E+00	7.81	0	7.81E+00	7.81
b2402_tRNA_17_MODIF_1	modification of position 17 nt (D) in valX by Dus_gen	1 Dus_gen + 1 b2402_tRNA_Mg2 + 1 h + 1 nadph <==> 1 b2402_tRNA_Mg2_Dus_gen_cplx	reversible	tRNA modification	-1.00E+09	1.00E+09	0	3.01E+02	3.01E+02	0	7.81E+00	7.81	0	7.81E+00	7.81
b2402_tRNA_17_MODIF_2	modification of position 17 nt (D) in valX by Dus_gen	1 b2402_tRNA_Mg2_Dus_gen_cplx --> 1 Dus_gen_inact + 1 b2402_tRNA_1 + 1 nadp	irreversible	tRNA modification	0.00E+00	1.00E+09	0	3.01E+02	3.01E+02	0	7.81E+00	7.81	0	7.81E+00	7.81
b2402_tRNA_34_MODIF_1	modification of position 34 nt (cmo5U) in valX by YecO_mono-YecP_mono-HyL_tRNA_pos_34_ho5U	1 HyL_tRNA_pos_34_ho5U + 1 YecO_mono + 1 YecP_mono + 1 amet + 1 b2402_tRNA_1 + 2 chor <==> 1 b2402_tRNA_1_YecO_mono-YecP_mono-HyL_tRNA_pos_34_ho5U_cplx	reversible	tRNA modification	-1.00E+09	1.00E+09	0	3.01E+02	3.01E+02	0	7.81E+00	7.81	0	7.81E+00	7.81
b2402_tRNA_34_MODIF_2	modification of position 34 nt (cmo5U) in valX by YecO_mono-YecP_mono-HyL_tRNA_pos_34_ho5U	1 b2402_tRNA_1_YecO_mono-YecP_mono-HyL_tRNA_pos_34_ho5U_cplx --> 1 C10H8O5 + 1 C9H9O4 + 1 HyL_tRNA_pos_34_ho5U_inact + 1 YecO_mono_inact + 1 YecP_mono_inact + 1 ahcys + 1 b2402_tRNA_2 + 1 h	irreversible	tRNA modification	0.00E+00	1.00E+09	0	3.01E+02	3.01E+02	0	7.81E+00	7.81	0	7.81E+00	7.81
b2402_tRNA_37_MODIF_1	modification of position 37 nt (m6A) in valX by MeT_tRNA_pos_37_m6A	1 MeT_tRNA_pos_37_m6A + 1 amet + 1 b2402_tRNA_2 <==> 1 b2402_tRNA_2_MeT_tRNA_pos_37_m6A_cplx	reversible	tRNA modification	-1.00E+09	1.00E+09	0	3.01E+02	3.01E+02	0	7.81E+00	7.81	0	7.81E+00	7.81
b2402_tRNA_37_MODIF_2	modification of position 37 nt (m6A) in valX by MeT_tRNA_pos_37_m6A	1 b2402_tRNA_2_MeT_tRNA_pos_37_m6A_cplx --> 1 MeT_tRNA_pos_37_m6A_inact + 1 ahcys + 1 b2402_tRNA_3 + 1 h	irreversible	tRNA modification	0.00E+00	1.00E+09	0	3.01E+02	3.01E+02	0	7.81E+00	7.81	0	7.81E+00	7.81
b2402_tRNA_46_MODIF_1	modification of position 46 nt (m7G) in valX by YggH_mono	1 YggH_mono + 1 amet + 1 b2402_tRNA_3 <==> 1 b2402_tRNA_3_YggH_mono_cplx	reversible	tRNA modification	-1.00E+09	1.00E+09	0	3.01E+02	3.01E+02	0	7.81E+00	7.81	0	7.81E+00	7.81
b2402_tRNA_46_MODIF_2	modification of position 46 nt (m7G) in valX by YggH_mono	1 b2402_tRNA_3_YggH_mono_cplx --> 1 YggH_mono_inact + 1 ahcys + 1 b2402_tRNA_4 + 1 h	irreversible	tRNA modification	0.00E+00	1.00E+09	0	3.01E+02	3.01E+02	0	7.81E+00	7.81	0	7.81E+00	7.81
b2402_tRNA_54_MODIF_1	modification of position 54 nt (m5U) in valX by TrmA_mono	1 TrmA_mono + 1 amet + 1 b2402_tRNA_4 <==> 1 b2402_tRNA_4_TrmA_mono_cplx	reversible	tRNA modification	-1.00E+09	1.00E+09	0	3.01E+02	3.01E+02	0	7.81E+00	7.81	0	7.81E+00	7.81
b2402_tRNA_54_MODIF_2	modification of position 54 nt (m5U) in valX by TrmA_mono	1 b2402_tRNA_4_TrmA_mono_cplx --> 1 TrmA_mono_inact + 1 ahcys + 1 b2402_tRNA_5 + 1 h	irreversible	tRNA modification	0.00E+00	1.00E+09	0	3.01E+02	3.01E+02	0	7.81E+00	7.81	0	7.81E+00	7.81
b2402_tRNA_55_MODIF_1	modification of position 55 nt (Y) in valX by TruB_mono	1 TruB_mono + 1 b2402_tRNA_5 <==> 1 b2402_tRNA_5_TruB_mono_cplx	reversible	tRNA modification	-1.00E+09	1.00E+09	0	3.01E+02	3.01E+02	0	7.81E+00	7.81	0	7.81E+00	7.81
b2402_tRNA_55_MODIF_2	modification of position 55 nt (Y) in valX by TruB_mono	1 b2402_tRNA_5_TruB_mono_cplx --> 1 TruB_mono_inact + 1 b2402_tRNA_6	irreversible	tRNA modification	0.00E+00	1.00E+09	0	3.01E+02	3.01E+02	0	7.81E+00	7.81	0	7.81E+00	7.81
b2402_tRNA_8_MODIF_1	modification of position 8 nt (s4U) in valX by ThiI_mono	1 IscS_dim_S-SH + 1 ThiI_mono + 1 atp + 1 b2402_tRNA_6 + 1 mg2 + 1 trdrd <==> 1 b2402_tRNA_6_ThiI_mono_cplx	reversible	tRNA modification	-1.00E+09	1.00E+09	0	3.01E+02	3.01E+02	0	7.81E+00	7.81	0	7.81E+00	7.81
b2402_tRNA_8_MODIF_2	modification of position 8 nt (s4U) in valX by ThiI_mono	1 b2402_tRNA_6_ThiI_mono_cplx --> 1 IscS_dim_S-H + 1 ThiI_mono_inact + 1 amp + 1 h + 1 mg2 + 1 ppi + 1 trdox + 1 valX_tRNA	irreversible	tRNA modification	0.00E+00	1.00E+09	0	3.01E+02	3.01E+02	0	7.81E+00	7.81	0	7.81E+00	7.81
b2402_tRNA_Mg_BIND	Binding of two Mg2 to b2402_tRNA	1 b2402_RNA + 2 mg2 <==> 1 b2402_tRNA_Mg2	reversible	Metallo-ion binding	-1.00E+09	1.00E+09	0	3.01E+02	3.01E+02	0	7.81E+00	7.81	0	7.81E+00	7.81
b2403_tRNA_17_MODIF_1	modification of position 17 nt (D) in valY by Dus_gen	1 Dus_gen + 1 b2403_tRNA_Mg2 + 1 h + 1 nadph <==> 1 b2403_tRNA_Mg2_Dus_gen_cplx	reversible	tRNA modification	-1.00E+09	1.00E+09	0	3.01E+02	3.01E+02	0	7.81E+00	7.81	0	7.81E+00	7.81
b2403_tRNA_17_MODIF_2	modification of position 17 nt (D) in valY by Dus_gen	1 b2403_tRNA_Mg2_Dus_gen_cplx --> 1 Dus_gen_inact + 1 b2403_tRNA_1 + 1 nadp	irreversible	tRNA modification	0.00E+00	1.00E+09	0	3.01E+02	3.01E+02	0	7.81E+00	7.81	0	7.81E+00	7.81
b2403_tRNA_34_MODIF_1	modification of position 34 nt (cmo5U) in valY by YecO_mono-YecP_mono-HyL_tRNA_pos_34_ho5U	1 HyL_tRNA_pos_34_ho5U + 1 YecO_mono + 1 YecP_mono + 1 amet + 1 b2403_tRNA_1 + 2 chor <==> 1 b2403_tRNA_1_YecO_mono-YecP_mono-HyL_tRNA_pos_34_ho5U_cplx	reversible	tRNA modification	-1.00E+09	1.00E+09	0	3.01E+02	3.01E+02	0	7.81E+00	7.81	0	7.81E+00	7.81
b2403_tRNA_34_MODIF_2	modification of position 34 nt (cmo5U) in valY by YecO_mono-YecP_mono-HyL_tRNA_pos_34_ho5U	1 b2403_tRNA_1_YecO_mono-YecP_mono-HyL_tRNA_pos_34_ho5U_cplx --> 1 C10H8O5 + 1 C9H9O4 + 1 HyL_tRNA_pos_34_ho5U_inact + 1 YecO_mono_inact + 1 YecP_mono_inact + 1 ahcys + 1 b2403_tRNA_2 + 1 h	irreversible	tRNA modification	0.00E+00	1.00E+09	0	3.01E+02	3.01E+02	0	7.81E+00	7.81	0	7.81E+00	7.81
b2403_tRNA_37_MODIF_1	modification of position 37 nt (m6A) in valY by MeT_tRNA_pos_37_m6A	1 MeT_tRNA_pos_37_m6A + 1 amet + 1 b2403_tRNA_2 <==> 1 b2403_tRNA_2_MeT_tRNA_pos_37_m6A_cplx	reversible	tRNA modification	-1.00E+09	1.00E+09	0	3.01E+02	3.01E+02	0	7.81E+00	7.81	0	7.81E+00	7.81
b2403_tRNA_37_MODIF_2	modification of position 37 nt (m6A) in valY by MeT_tRNA_pos_37_m6A	1 b2403_tRNA_2_MeT_tRNA_pos_37_m6A_cplx --> 1 MeT_tRNA_pos_37_m6A_inact + 1 ahcys + 1 b2403_tRNA_3 + 1 h	irreversible	tRNA modification	0.00E+00	1.00E+09	0	3.01E+02	3.01E+02	0	7.81E+00	7.81	0	7.81E+00	7.81
b2403_tRNA_46_MODIF_1	modification of position 46 nt (m7G) in valY by YggH_mono	1 YggH_mono + 1 amet + 1 b2403_tRNA_3 <==> 1 b2403_tRNA_3_YggH_mono_cplx	reversible	tRNA modification	-1.00E+09	1.00E+09	0	3.01E+02	3.01E+02	0	7.81E+00	7.81	0	7.81E+00	7.81
b2403_tRNA_46_MODIF_2	modification of position 46 nt (m7G) in valY by YggH_mono	1 b2403_tRNA_3_YggH_mono_cplx --> 1 YggH_mono_inact + 1 ahcys + 1 b2403_tRNA_4 + 1 h	irreversible	tRNA modification	0.00E+00	1.00E+09	0	3.01E+02	3.01E+02	0	7.81E+00	7.81	0	7.81E+00	7.81
b2403_tRNA_54_MODIF_1	modification of position 54 nt (m5U) in valY by TrmA_mono	1 TrmA_mono + 1 amet + 1 b2403_tRNA_4 <==> 1 b2403_tRNA_4_TrmA_mono_cplx	reversible	tRNA modification	-1.00E+09	1.00E+09	0	3.01E+02	3.01E+02	0	7.81E+00	7.81	0	7.81E+00	7.81
b2403_tRNA_54_MODIF_2	modification of position 54 nt (m5U) in valY by TrmA_mono	1 b2403_tRNA_4_TrmA_mono_cplx --> 1 TrmA_mono_inact + 1 ahcys + 1 b2403_tRNA_5 + 1 h	irreversible	tRNA modification	0.00E+00	1.00E+09	0	3.01E+02	3.01E+02	0	7.81E+00	7.81	0	7.81E+00	7.81
b2403_tRNA_55_MODIF_1	modification of position 55 nt (Y) in valY by TruB_mono	1 TruB_mono + 1 b2403_tRNA_5 <==> 1 b2403_tRNA_5_TruB_mono_cplx	reversible	tRNA modification	-1.00E+09	1.00E+09	0	3.01E+02	3.01E+02	0	7.81E+00	7.81	0	7.81E+00	7.81
b2403_tRNA_55_MODIF_2	modification of position 55 nt (Y) in valY by TruB_mono	1 b2403_tRNA_5_TruB_mono_cplx --> 1 TruB_mono_inact + 1 b2403_tRNA_6	irreversible	tRNA modification	0.00E+00	1.00E+09	0	3.01E+02	3.01E+02	0	7.81E+00	7.81	0	7.81E+00	7.81
b2403_tRNA_8_MODIF_1	modification of position 8 nt (s4U) in valY by ThiI_mono	1 IscS_dim_S-SH + 1 ThiI_mono + 1 atp + 1 b2403_tRNA_6 + 1 mg2 + 1 trdrd <==> 1 b2403_tRNA_6_ThiI_mono_cplx	reversible	tRNA modification	-1.00E+09	1.00E+09	0	3.01E+02	3.01E+02	0	7.81E+00	7.81	0	7.81E+00	7.81
b2403_tRNA_8_MODIF_2	modification of position 8 nt (s4U) in valY by ThiI_mono	1 b2403_tRNA_6_ThiI_mono_cplx --> 1 IscS_dim_S-H + 1 ThiI_mono_inact + 1 amp + 1 h + 1 mg2 + 1 ppi + 1 trdox + 1 valY_tRNA	irreversible	tRNA modification	0.00E+00	1.00E+09	0	3.01E+02	3.01E+02	0	7.81E+00	7.81	0	7.81E+00	7.81
b2403_tRNA_Mg_BIND	Binding of two Mg2 to b2403_tRNA	1 b2403_RNA + 2 mg2 <==> 1 b2403_tRNA_Mg2	reversible	Metallo-ion binding	-1.00E+09	1.00E+09	0	3.01E+02	3.01E+02	0	7.81E+00	7.81	0	7.81E+00	7.81
b2404_tRNA_16_MODIF_1	modification of position 16 nt (D) in lysV by Dus_gen	1 Dus_gen + 1 b2404_tRNA_Mg2 + 1 h + 1 nadph <==> 1 b2404_tRNA_Mg2_Dus_gen_cplx	reversible	tRNA modification	-1.00E+09	1.00E+09	0	3.01E+02	3.01E+02	0	7.81E+00	7.81	0	7.81E+00	7.81
b2404_tRNA_16_MODIF_2	modification of position 16 nt (D) in lysV by Dus_gen	1 b2404_tRNA_Mg2_Dus_gen_cplx --> 1 Dus_gen_inact + 1 b2404_tRNA_1 + 1 nadp	irreversible	tRNA modification	0.00E+00	1.00E+09	0	3.01E+02	3.01E+02	0	7.81E+00	7.81	0	7.81E+00	7.81
b2404_tRNA_17_MODIF_1	modification of position 17 nt (D) in lysV by Dus_gen	1 Dus_gen + 1 b2404_tRNA_1 + 1 h + 1 nadph <==> 1 b2404_tRNA_1_Dus_gen_cplx	reversible	tRNA modification	-1.00E+09	1.00E+09	0	3.01E+02	3.01E+02	0	7.81E+00	7.81	0	7.81E+00	7.81
b2404_tRNA_17_MODIF_2	modification of position 17 nt (D) in lysV by Dus_gen	1 b2404_tRNA_1_Dus_gen_cplx --> 1 Dus_gen_inact + 1 b2404_tRNA_2 + 1 nadp	irreversible	tRNA modification	0.00E+00	1.00E+09	0	3.01E+02	3.01E+02	0	7.81E+00	7.81	0	7.81E+00	7.81
b2404_tRNA_20_MODIF_1	modification of position 20 nt (D) in lysV by Dus_gen	1 Dus_gen + 1 b2404_tRNA_2 + 1 h + 1 nadph <==> 1 b2404_tRNA_2_Dus_gen_cplx	reversible	tRNA modification	-1.00E+09	1.00E+09	0	3.01E+02	3.01E+02	0	7.81E+00	7.81	0	7.81E+00	7.81
b2404_tRNA_20_MODIF_2	modification of position 20 nt (D) in lysV by Dus_gen	1 b2404_tRNA_2_Dus_gen_cplx --> 1 Dus_gen_inact + 1 b2404_tRNA_3 + 1 nadp	irreversible	tRNA modification	0.00E+00	1.00E+09	0	3.01E+02	3.01E+02	0	7.81E+00	7.81	0	7.81E+00	7.81

b2404_tRNA_34_MODIF_ 1	modification of position 34 nt (mnm5s2U) in lysV by TrmU_mono-YhhP_mono-YheLMN_cplx-YccK_mono-TrmE_dim-GidA_mono-TrmC_mono	1 5fthf + 1 GidA_mono + 1 IscS_dim_S-SH + 1 TrmC_mono + 1 TrmE_dim + 1 TrmU_mono + 1 YccK_mono + 1 YheLMN_cplx + 1 YhhP_mono + 1 amet + 1 atp + 1 b2404_tRNA_3 + 1 fad + 1 fadh2 + 1 gly + 1 gtp + 1 h2o + 1 mg2 + 1 trdrd <==> 1 b2404_tRNA_3_TrmU_mono-YhhP_mono-YheLMN_cplx-YccK_mon TrmE_dim-GidA_mono-TrmC_mono_cplx	reversible	tRNA modification	-1.00E+09	1.00E+09	0	3.01E+02	3.01E+02	0	7.81E+00	7.81	0	7.81E+00	7.81
b2404_tRNA_34_MODIF_ 2	modification of position 34 nt (mnm5s2U) in lysV by TrmU_mono-YhhP_mono-YheLMN_cplx-YccK_mono-TrmE_dim-GidA_mono-TrmC_mono	1 b2404_tRNA_3_TrmU_mono-YhhP_mono-YheLMN_cplx-YccK_mono-TrmE_dim-GidA_mono-TrmC_mono_cplx --> 1 GidA_mono_inact + 1 IscS_dim_S-H + 1 TrmC_mono_inact + 1 TrmE_dim_inact + 1 TrmU_mono_inact + 1 YccK_mono_inact + 1 YheLMN_cplx_inact + 1 YhhP_mono_inact + 1 ahcys + 1 amp + 1 b2404_tRNA_4 + 1 fad + 1 fadh2 + 1 gdp + 1 glx + 4 h + 1 mg2 + 1 pi + 1 ppi + 1 thf + 1 trdox	irreversible	tRNA modification	0.00E+00	1.00E+09	0	3.01E+02	3.01E+02	0	7.81E+00	7.81	0	7.81E+00	7.81
b2404_tRNA_34_MODIF_ 3	replacement of sulfur with selenium: b2404_tRNA_4	1 YbbB_dim + 1 b2404_tRNA_4 + 1 h2o + 1 selnp <==> 1 b2404_tRNA_4_YbbB_dim_cplx	reversible	tRNA modification	-1.00E+09	1.00E+09	0	3.01E+02	3.01E+02	0	7.81E+00	7.81	0	7.81E+00	7.81
b2404_tRNA_34_MODIF_ 4	replacement of sulfur with selenium: b2404_tRNA_4	1 b2404_tRNA_4_YbbB_dim_cplx --> 1 YbbB_dim_inact + 1 b2404_tRNA_4_Se + 1 h + 1 h2s + 1 pi	irreversible	tRNA modification	0.00E+00	1.00E+09	0	3.01E+02	3.01E+02	0	7.81E+00	7.81	0	7.81E+00	7.81
b2404_tRNA_37_MODIF_ 1	modification of position 37 nt (t6A) in lysV by Up_tRNA_pos_37_t6A	1 Up_tRNA_pos_37_t6A + 1 atp + 1 b2404_tRNA_4 + 1 hco3 + 1 mg2 + 1 thr-L <==> 1 b2404_tRNA_4_Up_tRNA_pos_37_t6A_cplx	reversible	tRNA modification	-1.00E+09	1.00E+09	0	3.01E+02	3.01E+02	0	7.81E+00	7.81	0	7.81E+00	7.81
b2404_tRNA_37_MODIF_ 1_Se	modification of position 37 nt (t6A) in lysV by Up_tRNA_pos_37_t6A	1 Up_tRNA_pos_37_t6A + 1 atp + 1 b2404_tRNA_4_Se + 1 hco3 + 1 mg2 + 1 thr-L <==> 1 b2404_tRNA_4_Se_Up_tRNA_pos_37_t6A_cplx	reversible	tRNA modification	-1.00E+09	1.00E+09	0	3.01E+02	3.01E+02	0	7.81E+00	7.81	0	7.81E+00	7.81
b2404_tRNA_37_MODIF_ 2	modification of position 37 nt (t6A) in lysV by Up_tRNA_pos_37_t6A	1 b2404_tRNA_4_Up_tRNA_pos_37_t6A_cplx --> 1 Up_tRNA_pos_37_t6A_inact + 1 amp + 1 b2404_tRNA_5 + 1 h + 1 h2o + 1 mg2 + 1 ppi	irreversible	tRNA modification	0.00E+00	1.00E+09	0	3.01E+02	3.01E+02	0	7.81E+00	7.81	0	7.81E+00	7.81
b2404_tRNA_37_MODIF_ 2_Se	modification of position 37 nt (t6A) in lysV by Up_tRNA_pos_37_t6A	1 b2404_tRNA_4_Se_Up_tRNA_pos_37_t6A_cplx --> 1 Up_tRNA_pos_37_t6A_inact + 1 amp + 1 b2404_tRNA_5_Se + 1 h + 1 h2o + 1 mg2 + 1 ppi	irreversible	tRNA modification	0.00E+00	1.00E+09	0	3.01E+02	3.01E+02	0	7.81E+00	7.81	0	7.81E+00	7.81
b2404_tRNA_39_MODIF_ 1	modification of position 39 nt (Y) in lysV by TruA_dim	1 TruA_dim + 1 b2404_tRNA_5 <==> 1 b2404_tRNA_5_TruA_dim_cplx	reversible	tRNA modification	-1.00E+09	1.00E+09	0	3.01E+02	3.01E+02	0	7.81E+00	7.81	0	7.81E+00	7.81
b2404_tRNA_39_MODIF_ 1_Se	modification of position 39 nt (Y) in lysV by TruA_dim	1 TruA_dim + 1 b2404_tRNA_5_Se <==> 1 b2404_tRNA_5_Se_TruA_dim_cplx	reversible	tRNA modification	-1.00E+09	1.00E+09	0	3.01E+02	3.01E+02	0	7.81E+00	7.81	0	7.81E+00	7.81
b2404_tRNA_39_MODIF_ 2	modification of position 39 nt (Y) in lysV by TruA_dim	1 b2404_tRNA_5_TruA_dim_cplx --> 1 TruA_dim_inact + 1 b2404_tRNA_6	irreversible	tRNA modification	0.00E+00	1.00E+09	0	3.01E+02	3.01E+02	0	7.81E+00	7.81	0	7.81E+00	7.81
b2404_tRNA_39_MODIF_ 2_Se	modification of position 39 nt (Y) in lysV by TruA_dim	1 b2404_tRNA_5_Se_TruA_dim_cplx --> 1 TruA_dim_inact + 1 b2404_tRNA_6_Se	irreversible	tRNA modification	0.00E+00	1.00E+09	0	3.01E+02	3.01E+02	0	7.81E+00	7.81	0	7.81E+00	7.81
b2404_tRNA_46_MODIF_ 1	modification of position 46 nt (m7G) in lysV by YggH_mono	1 YggH_mono + 1 amet + 1 b2404_tRNA_6 <==> 1 b2404_tRNA_6_YggH_mono_cplx	reversible	tRNA modification	-1.00E+09	1.00E+09	0	3.01E+02	3.01E+02	0	7.81E+00	7.81	0	7.81E+00	7.81
b2404_tRNA_46_MODIF_ 1_Se	modification of position 46 nt (m7G) in lysV by YggH_mono	1 YggH_mono + 1 amet + 1 b2404_tRNA_6_Se <==> 1 b2404_tRNA_6_Se_YggH_mono_cplx	reversible	tRNA modification	-1.00E+09	1.00E+09	0	3.01E+02	3.01E+02	0	7.81E+00	7.81	0	7.81E+00	7.81
b2404_tRNA_46_MODIF_ 2	modification of position 46 nt (m7G) in lysV by YggH_mono	1 b2404_tRNA_6_YggH_mono_cplx --> 1 YggH_mono_inact + 1 ahcys + 1 b2404_tRNA_7 + 1 h	irreversible	tRNA modification	0.00E+00	1.00E+09	0	3.01E+02	3.01E+02	0	7.81E+00	7.81	0	7.81E+00	7.81
b2404_tRNA_46_MODIF_ 2_Se	modification of position 46 nt (m7G) in lysV by YggH_mono	1 b2404_tRNA_6_Se_YggH_mono_cplx --> 1 YggH_mono_inact + 1 ahcys + 1 b2404_tRNA_7_Se + 1 h	irreversible	tRNA modification	0.00E+00	1.00E+09	0	3.01E+02	3.01E+02	0	7.81E+00	7.81	0	7.81E+00	7.81
b2404_tRNA_47_MODIF_ 1	modification of position 47 nt (acp3U) in lysV by AcpT_tRNA_pos_47_acp3U	1 AcpT_tRNA_pos_47_acp3U + 1 amet + 1 b2404_tRNA_7 <==> 1 b2404_tRNA_7_AcpT_tRNA_pos_47_acp3U_cplx	reversible	tRNA modification	-1.00E+09	1.00E+09	0	3.01E+02	3.01E+02	0	7.81E+00	7.81	0	7.81E+00	7.81
b2404_tRNA_47_MODIF_ 1_Se	modification of position 47 nt (acp3U) in lysV by AcpT_tRNA_pos_47_acp3U	1 AcpT_tRNA_pos_47_acp3U + 1 amet + 1 b2404_tRNA_7_Se <==> 1 b2404_tRNA_7_Se_AcpT_tRNA_pos_47_acp3U_cplx	reversible	tRNA modification	-1.00E+09	1.00E+09	0	3.01E+02	3.01E+02	0	7.81E+00	7.81	0	7.81E+00	7.81
b2404_tRNA_47_MODIF_ 2	modification of position 47 nt (acp3U) in lysV by AcpT_tRNA_pos_47_acp3U	1 b2404_tRNA_7_AcpT_tRNA_pos_47_acp3U_cplx --> 1 5mta + 1 AcpT_tRNA_pos_47_acp3U_inact + 1 b2404_tRNA_8 + 1 h	irreversible	tRNA modification	0.00E+00	1.00E+09	0	3.01E+02	3.01E+02	0	7.81E+00	7.81	0	7.81E+00	7.81
b2404_tRNA_47_MODIF_ 2_Se	modification of position 47 nt (acp3U) in lysV by AcpT_tRNA_pos_47_acp3U	1 b2404_tRNA_7_Se_AcpT_tRNA_pos_47_acp3U_cplx --> 1 5mta + 1 AcpT_tRNA_pos_47_acp3U_inact + 1 b2404_tRNA_8_Se + 1 h	irreversible	tRNA modification	0.00E+00	1.00E+09	0	3.01E+02	3.01E+02	0	7.81E+00	7.81	0	7.81E+00	7.81
b2404_tRNA_54_MODIF_ 1	modification of position 54 nt (m5U) in lysV by TrmA_mono	1 TrmA_mono + 1 amet + 1 b2404_tRNA_8 <==> 1 b2404_tRNA_8_TrmA_mono_cplx	reversible	tRNA modification	-1.00E+09	1.00E+09	0	3.01E+02	3.01E+02	0	7.81E+00	7.81	0	7.81E+00	7.81
b2404_tRNA_54_MODIF_ 1_Se	modification of position 54 nt (m5U) in lysV by TrmA_mono	1 TrmA_mono + 1 amet + 1 b2404_tRNA_8_Se <==> 1 b2404_tRNA_8_Se_TrmA_mono_cplx	reversible	tRNA modification	-1.00E+09	1.00E+09	0	3.01E+02	3.01E+02	0	7.81E+00	7.81	0	7.81E+00	7.81
b2404_tRNA_54_MODIF_ 2	modification of position 54 nt (m5U) in lysV by TrmA_mono	1 b2404_tRNA_8_TrmA_mono_cplx --> 1 TrmA_mono_inact + 1 ahcys + 1 b2404_tRNA_9 + 1 h	irreversible	tRNA modification	0.00E+00	1.00E+09	0	3.01E+02	3.01E+02	0	7.81E+00	7.81	0	7.81E+00	7.81
b2404_tRNA_54_MODIF_ 2_Se	modification of position 54 nt (m5U) in lysV by TrmA_mono	1 b2404_tRNA_8_Se_TrmA_mono_cplx --> 1 TrmA_mono_inact + 1 ahcys + 1 b2404_tRNA_9_Se + 1 h	irreversible	tRNA modification	0.00E+00	1.00E+09	0	3.01E+02	3.01E+02	0	7.81E+00	7.81	0	7.81E+00	7.81
b2404_tRNA_55_MODIF_ 1	modification of position 55 nt (Y) in lysV by TruB_mono	1 TruB_mono + 1 b2404_tRNA_9 <==> 1 b2404_tRNA_9_TruB_mono_cplx	reversible	tRNA modification	-1.00E+09	1.00E+09	0	3.01E+02	3.01E+02	0	7.81E+00	7.81	0	7.81E+00	7.81
b2404_tRNA_55_MODIF_ 1_Se	modification of position 55 nt (Y) in lysV by TruB_mono	1 TruB_mono + 1 b2404_tRNA_9_Se <==> 1 b2404_tRNA_9_Se_TruB_mono_cplx	reversible	tRNA modification	-1.00E+09	1.00E+09	0	3.01E+02	3.01E+02	0	7.81E+00	7.81	0	7.81E+00	7.81
b2404_tRNA_55_MODIF_ 2	modification of position 55 nt (Y) in lysV by TruB_mono	1 b2404_tRNA_9_TruB_mono_cplx --> 1 TruB_mono_inact + 1 lysV_tRNA	irreversible	tRNA modification	0.00E+00	1.00E+09	0	3.01E+02	3.01E+02	0	7.81E+00	7.81	0	7.81E+00	7.81
b2404_tRNA_55_MODIF_ 2_Se	modification of position 55 nt (Y) in lysV by TruB_mono	1 b2404_tRNA_9_Se_TruB_mono_cplx --> 1 TruB_mono_inact + 1 lysV_tRNA_Se	irreversible	tRNA modification	0.00E+00	1.00E+09	0	3.01E+02	3.01E+02	0	7.81E+00	7.81	0	7.81E+00	7.81
b2404_tRNA_Mg_BIND	Binding of two Mg2 to b2404_tRNA	1 b2404_RNA + 2 mg2 <==> 1 b2404_tRNA_Mg2	reversible	Metallo-ion binding	-1.00E+09	1.00E+09	0	3.01E+02	3.01E+02	0	7.81E+00	7.81	0	7.81E+00	7.81
b2514_fold_spon	b2514_m folding: spontanous	1 b2514_m_Mg --> 1 HisS_mono	irreversible	Protein Folding	0.00E+00	1.00E+09	0	2.56E+03	2.56E+03	0	4.43E+03	4433.916	0	1.25E+02	125.256
b2514_ion_BIND	Binding of ions to b2514	1 b2514_m + 2 mg2 <==> 1 b2514_m_Mg	reversible	Metallo-ion Binding	-1.00E+09	1.00E+09	0	2.56E+03	2.56E+03	0	4.43E+03	4433.916	0	1.25E+02	125.256
b2514_mRNA_CONV	convsersion of mRNA to mRNA_1 (synthetic rxn)	1 b2514_mRNA --> 1 b2514_mRNA_1	irreversible	Translation	0.00E+00	1.00E+09	0	1.02E+02	1.02E+02	0	1.80E+02	180.1265	0	5.99E-01	0.599
b2514_mRNA_degr1	Degradation of b2514_mRNA I	1 Orn_dim + 318 atp + 1 b2514_mRNA_2 + 1 degradosome + 318 h2o <==> 318 adp + 1 b2514_mRNA_2_degr + 318 h + 318 pi	reversible	mRNA degradation	-1.00E+09	1.00E+09	0	1.02E+02	1.02E+02	0	1.80E+02	180.1265	0	5.99E-01	0.599
b2514_mRNA_degr2	Degradation of b2514_mRNA II	1 b2514_mRNA_2_degr + 1274 h2o --> 1 Orn_dim_inact + 282 amp 313 cmp + 1 degradosome_inact + 384 gmp + 1 gtp + 1274 h + 295 ump	irreversible	mRNA degradation	0.00E+00	5.99E-01	0	1.02E+02	1.02E+02	0	1.80E+02	180.1265	0	5.99E-01	0.599
b2514_maturation1	Polypeptide b2514 peptide deformylas and methionine aminopeptidase complex	1 Def_mono + 1 Map_mono + 1 b2514_aa <==> 1 b2514_def_map_cplx	reversible	Protein Maturation	-1.00E+09	1.00E+09	0	2.56E+03	2.56E+03	0	4.43E+03	4433.916	0	1.25E+02	125.256
b2514_maturation2	b2514 formation	1 b2514_def_map_cplx + 2 h2o --> 1 Def_mono_inact + 1 Map_mono_inact + 1 b2514_m + 1 for + 1 met-L	irreversible	Protein Maturation	0.00E+00	1.00E+09	0	2.56E+03	2.56E+03	0	4.43E+03	4433.916	0	1.25E+02	125.256
b2528_fold_spon	b2528_m folding: spontanous	1 b2528_m --> 1 IscA_mono	irreversible	Protein Folding	0.00E+00	1.00E+09	0	3.97E+02	3.97E+02	0	6.29E+02	629.4493	0	9.52E+01	95.208
b2528_mRNA_CONV	convsersion of mRNA to mRNA_1 (synthetic rxn)	1 b2528_mRNA --> 1 b2528_mRNA_1	irreversible	Translation	0.00E+00	1.00E+09	0	5.05E+01	5.05E+01	0	8.92E+01	89.20822	0	1.68E-01	0.168
b2528_mRNA_degr1	Degradation of b2528_mRNA I	1 Orn_dim + 81 atp + 1 b2528_mRNA_2 + 1 degradosome + 81 h2o <==> 81 adp + 1 b2528_mRNA_2_degr + 81 h + 81 pi	reversible	mRNA degradation	-1.00E+09	1.00E+09	0	5.05E+01	5.05E+01	0	8.92E+01	89.20822	0	1.68E-01	0.168
b2528_mRNA_degr2	Degradation of b2528_mRNA II	1 b2528_mRNA_2_degr + 323 h2o --> 1 Orn_dim_inact + 83 amp + 68 cmp + 1 degradosome_inact + 99 gmp + 323 h + 74 ump	irreversible	mRNA degradation	0.00E+00	1.89E-01	0	5.05E+01	5.05E+01	0	8.92E+01	89.20822	0	1.68E-01	0.168
b2528_maturation1	Polypeptide b2528 peptide deformylas and methionine aminopeptidase complex	1 Def_mono + 1 Map_mono + 1 b2528_aa <==> 1 b2528_def_map_cplx	reversible	Protein Maturation	-1.00E+09	1.00E+09	0	3.97E+02	3.97E+02	0	6.29E+02	629.4493	0	9.52E+01	95.208
b2528_maturation2	b2528 formation	1 b2528_def_map_cplx + 2 h2o --> 1 Def_mono_inact + 1 Map_mono_inact + 1 b2528_m + 1 for + 1 met-L	irreversible	Protein Maturation	0.00E+00	1.00E+09	0	3.97E+02	3.97E+02	0	6.29E+02	629.4493	0	9.52E+01	95.208
b2529_fold_spon	b2529_m folding: spontanous	1 b2529_m --> 1 IscU_mono	irreversible	Protein Folding	0.00E+00	1.00E+09	0	4.76E+02	4.76E+02	0	7.47E+02	746.9575	0	1.24E+02	123.676
b2529_mRNA_CONV	convsersion of mRNA to mRNA_1 (synthetic rxn)	1 b2529_mRNA --> 1 b2529_mRNA_1	irreversible	Translation	0.00E+00	1.00E+09	0	5.05E+01	5.05E+01	0	8.92E+01	89.20822	0	1.68E-01	0.168
b2529_mRNA_degr1	Degradation of b2529_mRNA I	1 Orn_dim + 96 atp + 1 b2529_mRNA_2 + 1 degradosome + 96 h2o <==> 96 adp + 1 b2529_mRNA_2_degr + 96 h + 96 pi	reversible	mRNA degradation	-1.00E+09	1.00E+09	0	5.05E+01	5.05E+01	0	8.92E+01	89.20822	0	1.68E-01	0.168
b2529_mRNA_degr2	Degradation of b2529_mRNA II	1 b2529_mRNA_2_degr + 386 h2o --> 1 Orn_dim_inact + 114 amp + 89 cmp + 1 degradosome_inact + 105 gmp + 386 h + 79 ump	irreversible	mRNA degradation	0.00E+00	2.07E-01	0	5.05E+01	5.05E+01	0	8.92E+01	89.20822	0	1.68E-01	0.168

b2529_maturation1	Polypeptide b2529 peptide deformylas and methionine aminopeptidase complex	1 Def_mono + 1 Map_mono + 1 b2529_aa <==> 1 b2529_def_map_cplx	reversible	Protein Maturation	-1.00E+09	1.00E+09	0	4.76E+02	4.76E+02	0	7.47E+02	746.9575	0	1.24E+02	123.676
b2529_maturation2	b2529 formation	1 b2529_def_map_cplx + 2 h2o --> 1 Def_mono_inact + 1 Map_mono_inact + 1 b2529_m + 1 for + 1 met-L	irreversible	Protein Maturation	0.00E+00	1.00E+09	0	4.76E+02	4.76E+02	0	7.47E+02	746.9575	0	1.24E+02	123.676
b2530_fold_GroEL/ES_1	b2530_m folding: GroEL/ES mediated: polypeptide is going in GroEL/ES complex	1 GroEL.(7)ADP.cisGroES + 7 atp + 1 b2530_m + 1 transGroES_hepta --> 7 adp + 1 b2530_m_GroEL.(7)ATP.transGroES + 1 cisGroES_hepta	irreversible	Protein Folding	0.00E+00	1.00E+09	0	1.25E+03	1.25E+03	0	2.14E+03	2141.949	0	9.40E+01	94.024
b2530_fold_GroEL/ES_2	b2530_m folding: GroEL/ES mediated: folding of polypeptide under ATP hydrolysis	1 b2530_m_GroEL.(7)ATP.transGroES + 7 h2o --> 1 IscS_GroEL.(7)ADP.transGroES + 7 h + 7 pi	irreversible	Protein Folding	0.00E+00	1.00E+09	0	1.25E+03	1.25E+03	0	2.14E+03	2141.949	0	9.40E+01	94.024
b2530_fold_GroEL/ES_3	b2530_m folding: GroEL/ES mediated: release of native protein	1 IscS_GroEL.(7)ADP.transGroES --> 1 GroEL.(7)ADP.transGroES + 1 IscS_mono	irreversible	Protein Folding	0.00E+00	1.00E+09	0	1.25E+03	1.25E+03	0	2.14E+03	2141.949	0	9.40E+01	94.024
b2530_fold_KJE_1	b2530_m folding: KJE mediated	1 DnaJ_dim + 1 DnaK_mono.ATP + 1 b2530_m <==> 1 b2530_m_DnaKJ_complex	reversible	Protein Folding	-1.00E+09	1.00E+09	0	1.25E+03	1.25E+03	0	2.14E+03	2141.949	0	9.40E+01	94.024
b2530_fold_KJE_2	b2530_m folding: KJE mediated	1 GrpE_dim + 1 b2530_m_DnaKJ_complex + 1 h2o --> 1 DnaJ_dim_inact + 1 IscS_DnaK_GrpE_complex + 1 adp + 1 h + 1 pi	irreversible	Protein Folding	0.00E+00	1.00E+09	0	1.25E+03	1.25E+03	0	2.14E+03	2141.949	0	9.40E+01	94.024
b2530_fold_KJE_3	b2530_m folding: KJE mediated	1 IscS_DnaK_GrpE_complex + 1 atp --> 1 DnaK_mono.ATP_inact + 1 GrpE_dim_inact + 1 IscS_mono	irreversible	Protein Folding	0.00E+00	1.00E+09	0	1.25E+03	1.25E+03	0	2.14E+03	2141.949	0	9.40E+01	94.024
b2530_mRNA_CONV	convsersion of mRNA to mRNA_1 (synthetic rxn)	1 b2530_mRNA --> 1 b2530_mRNA_1	irreversible	Translation	0.00E+00	1.00E+09	0	5.05E+01	5.05E+01	0	8.92E+01	89.20822	0	1.68E-01	0.168
b2530_mRNA_degr1	Degradation of b2530_mRNA I	1 Orn_dim + 303 atp + 1 b2530_mRNA_2 + 1 degradosome + 303 h2o <==> 303 adp + 1 b2530_mRNA_2_degr + 303 h + 303 pi	reversible	mRNA degradation	-1.00E+09	1.00E+09	0	5.05E+01	5.05E+01	0	8.92E+01	89.20822	0	1.68E-01	0.168
b2530_mRNA_degr2	Degradation of b2530_mRNA II	1 b2530_mRNA_2_degr + 1214 h2o --> 1 Orn_dim_inact + 298 amp + 322 cmp + 1 degradosome_inact + 326 gmp + 1214 h + 269 ump	irreversible	mRNA degradation	0.00E+00	3.72E-01	0	5.05E+01	5.05E+01	0	8.92E+01	89.20822	0	1.68E-01	0.168
b2530_maturation1	Polypeptide b2530 peptide deformylas and methionine aminopeptidase complex	1 Def_mono + 1 Map_mono + 1 b2530_aa <==> 1 b2530_def_map_cplx	reversible	Protein Maturation	-1.00E+09	1.00E+09	0	1.25E+03	1.25E+03	0	2.14E+03	2141.949	0	9.40E+01	94.024
b2530_maturation2	b2530 formation	1 b2530_def_map_cplx + 2 h2o --> 1 Def_mono_inact + 1 Map_mono_inact + 1 b2530_m + 1 for + 1 met-L	irreversible	Protein Maturation	0.00E+00	1.00E+09	0	1.25E+03	1.25E+03	0	2.14E+03	2141.949	0	9.40E+01	94.024
b2531_fold_spon	b2531_m folding: spontanous	1 b2531_m --> 1 IscR_mono	irreversible	Protein Folding	0.00E+00	1.00E+09	0	5.43E+02	5.43E+02	0	8.91E+02	890.984	0	8.96E+01	89.622
b2531_mRNA_CONV	convsersion of mRNA to mRNA_1 (synthetic rxn)	1 b2531_mRNA --> 1 b2531_mRNA_1	irreversible	Translation	0.00E+00	1.00E+09	0	5.05E+01	5.05E+01	0	8.92E+01	89.20822	0	1.68E-01	0.168
b2531_mRNA_degr1	Degradation of b2531_mRNA I	1 Orn_dim + 122 atp + 1 b2531_mRNA_2 + 1 degradosome + 122 h2o <==> 122 adp + 1 b2531_mRNA_2_degr + 122 h + 122 pi	reversible	mRNA degradation	-1.00E+09	1.00E+09	0	5.05E+01	5.05E+01	0	8.92E+01	89.20822	0	1.68E-01	0.168
b2531_mRNA_degr2	Degradation of b2531_mRNA II	1 b2531_mRNA_2_degr + 488 h2o --> 1 Orn_dim_inact + 109 amp + 1 atp + 127 cmp + 1 degradosome_inact + 135 gmp + 488 h + 117 ump	irreversible	mRNA degradation	0.00E+00	1.68E-01	0	5.05E+01	5.05E+01	0	8.92E+01	89.20822	0	1.68E-01	0.168
b2531_maturation1	Polypeptide b2531 peptide deformylas and methionine aminopeptidase complex	1 Def_mono + 1 Map_mono + 1 b2531_aa <==> 1 b2531_def_map_cplx	reversible	Protein Maturation	-1.00E+09	1.00E+09	0	5.43E+02	5.43E+02	0	8.91E+02	890.984	0	8.96E+01	89.622
b2531_maturation2	b2531 formation	1 b2531_def_map_cplx + 2 h2o --> 1 Def_mono_inact + 1 Map_mono_inact + 1 b2531_m + 1 for + 1 met-L	irreversible	Protein Maturation	0.00E+00	1.00E+09	0	5.43E+02	5.43E+02	0	8.91E+02	890.984	0	8.96E+01	89.622
b2559_fold_spon	b2559_m folding: spontanous	1 b2559_m_Zn --> 1 TadA_mono	irreversible	Protein Folding	0.00E+00	1.00E+09	0	1.21E+03	1.21E+03	0	2.07E+03	2067.331	0	9.04E+01	90.41
b2559_ion_BIND	Binding of ions to b2559	1 b2559_m + 1 zn2 <==> 1 b2559_m_Zn	reversible	Metallo-ion Binding	-1.00E+09	1.00E+09	0	1.21E+03	1.21E+03	0	2.07E+03	2067.331	0	9.04E+01	90.41
b2559_mRNA_CONV	convsersion of mRNA to mRNA_1 (synthetic rxn)	1 b2559_mRNA --> 1 b2559_mRNA_1	irreversible	Translation	0.00E+00	1.00E+09	0	1.12E+02	1.12E+02	0	1.98E+02	197.8231	0	1.31E-01	0.131
b2559_mRNA_degr1	Degradation of b2559_mRNA I	1 Orn_dim + 134 atp + 1 b2559_mRNA_2 + 1 degradosome + 134 h2o <==> 134 adp + 1 b2559_mRNA_2_degr + 134 h + 134 pi	reversible	mRNA degradation	-1.00E+09	1.00E+09	0	1.12E+02	1.12E+02	0	1.98E+02	197.8231	0	1.31E-01	0.131
b2559_mRNA_degr2	Degradation of b2559_mRNA II	1 b2559_mRNA_2_degr + 536 h2o --> 1 Orn_dim_inact + 121 amp + 126 cmp + 1 degradosome_inact + 165 gmp + 536 h + 125 ump	irreversible	mRNA degradation	0.00E+00	1.31E-01	0	1.12E+02	1.12E+02	0	1.98E+02	197.8231	0	1.31E-01	0.131
b2559_maturation1	Polypeptide b2559 peptide deformylas and methionine aminopeptidase complex	1 Def_mono + 1 Map_mono + 1 b2559_aa <==> 1 b2559_def_map_cplx	reversible	Protein Maturation	-1.00E+09	1.00E+09	0	1.21E+03	1.21E+03	0	2.07E+03	2067.331	0	9.04E+01	90.41
b2559_maturation2	b2559 formation	1 b2559_def_map_cplx + 2 h2o --> 1 Def_mono_inact + 1 Map_mono_inact + 1 b2559_m + 1 for + 1 met-L	irreversible	Protein Maturation	0.00E+00	1.00E+09	0	1.21E+03	1.21E+03	0	2.07E+03	2067.331	0	9.04E+01	90.41
b2560_fold_spon	b2560_m folding: spontanous	1 b2560_m --> 1 YfhB_mono	irreversible	Protein Folding	0.00E+00	1.00E+09	0	1.43E+03	1.43E+03	0	2.46E+03	2463.998	0	9.17E+01	91.692
b2560_mRNA_CONV	convsersion of mRNA to mRNA_1 (synthetic rxn)	1 b2560_mRNA --> 1 b2560_mRNA_1	irreversible	Translation	0.00E+00	1.00E+09	0	1.12E+02	1.12E+02	0	1.98E+02	197.8231	0	1.31E-01	0.131
b2560_mRNA_degr1	Degradation of b2560_mRNA I	1 Orn_dim + 159 atp + 1 b2560_mRNA_2 + 1 degradosome + 159 h2o <==> 159 adp + 1 b2560_mRNA_2_degr + 159 h + 159 pi	reversible	mRNA degradation	-1.00E+09	1.00E+09	0	1.12E+02	1.12E+02	0	1.98E+02	197.8231	0	1.31E-01	0.131
b2560_mRNA_degr2	Degradation of b2560_mRNA II	1 b2560_mRNA_2_degr + 635 h2o --> 1 Orn_dim_inact + 126 amp + 155 cmp + 1 degradosome_inact + 177 gmp + 635 h + 177 ump + 1 utp	irreversible	mRNA degradation	0.00E+00	2.00E-01	0	1.12E+02	1.12E+02	0	1.98E+02	197.8231	0	1.31E-01	0.131
b2560_maturation1	Polypeptide b2560 peptide deformylas and methionine aminopeptidase complex	1 Def_mono + 1 Map_mono + 1 b2560_aa <==> 1 b2560_def_map_cplx	reversible	Protein Maturation	-1.00E+09	1.00E+09	0	1.43E+03	1.43E+03	0	2.46E+03	2463.998	0	9.17E+01	91.692
b2560_maturation2	b2560 formation	1 b2560_def_map_cplx + 2 h2o --> 1 Def_mono_inact + 1 Map_mono_inact + 1 b2560_m + 1 for + 1 met-L	irreversible	Protein Maturation	0.00E+00	1.00E+09	0	1.43E+03	1.43E+03	0	2.46E+03	2463.998	0	9.17E+01	91.692
b2563_fold_spon	b2563_m folding: spontanous	1 b2563_m --> 1 AcpS_mono	irreversible	Protein Folding	0.00E+00	1.00E+09	0	3.32E+02	3.32E+02	0	5.30E+02	530.1084	0	7.35E+01	73.542
b2563_mRNA_degr1	Degradation of b2563_mRNA I	1 Orn_dim + 95 atp + 1 b2563_mRNA_2 + 1 degradosome + 95 h2o <==> 95 adp + 1 b2563_mRNA_2_degr + 95 h + 95 pi	reversible	mRNA degradation	-1.00E+09	1.00E+09	0	3.70E+01	3.70E+01	0	6.53E+01	65.32977	0	1.06E-01	0.106
b2563_mRNA_degr2	Degradation of b2563_mRNA II	1 b2563_mRNA_2_degr + 379 h2o --> 1 Orn_dim_inact + 97 amp + 82 cmp + 1 degradosome_inact + 114 gmp + 379 h + 87 ump	irreversible	mRNA degradation	0.00E+00	1.25E-01	0	3.70E+01	3.70E+01	0	6.53E+01	65.32977	0	1.06E-01	0.106
b2563_maturation1	Polypeptide b2563 peptide deformylas and methionine aminopeptidase complex	1 Def_mono + 1 Map_mono + 1 b2563_aa <==> 1 b2563_def_map_cplx	reversible	Protein Maturation	-1.00E+09	1.00E+09	0	3.32E+02	3.32E+02	0	5.30E+02	530.1084	0	7.35E+01	73.542
b2563_maturation2	b2563 formation	1 b2563_def_map_cplx + 2 h2o --> 1 Def_mono_inact + 1 Map_mono_inact + 1 b2563_m + 1 for + 1 met-L	irreversible	Protein Maturation	0.00E+00	1.00E+09	0	3.32E+02	3.32E+02	0	5.30E+02	530.1084	0	7.35E+01	73.542
b2563_v1_mRNA_CONV	convsersion of mRNA to mRNA_1 (synthetic rxn)	1 b2563_v1_mRNA --> 1 b2563_mRNA_1	irreversible	Translation	0.00E+00	1.00E+09	0	3.70E+01	3.70E+01	0	6.53E+01	65.32977	0	1.06E-01	0.106
b2564_fold_spon	b2564_m folding: spontanous	1 b2564_m --> 1 PdxJ_mono	irreversible	Protein Folding	0.00E+00	1.00E+09	0	6.09E+02	6.09E+02	0	1.01E+03	1005.897	0	9.28E+01	92.764
b2564_mRNA_degr1	Degradation of b2564_mRNA I	1 Orn_dim + 183 atp + 1 b2564_mRNA_2 + 1 degradosome + 183 h2o <==> 183 adp + 1 b2564_mRNA_2_degr + 183 h + 183 pi	reversible	mRNA degradation	-1.00E+09	1.00E+09	0	3.70E+01	3.70E+01	0	6.53E+01	65.32977	0	1.06E-01	0.106
b2564_mRNA_degr2	Degradation of b2564_mRNA II	1 b2564_mRNA_2_degr + 731 h2o --> 1 Orn_dim_inact + 172 amp + 1 atp + 192 cmp + 1 degradosome_inact + 221 gmp + 731 h + 146 ump	irreversible	mRNA degradation	0.00E+00	2.69E-01	0	3.70E+01	3.70E+01	0	6.53E+01	65.32977	0	1.06E-01	0.106
b2564_maturation1	Polypeptide b2564 peptide deformylas and methionine aminopeptidase complex	1 Def_mono + 1 Map_mono + 1 b2564_aa <==> 1 b2564_def_map_cplx	reversible	Protein Maturation	-1.00E+09	1.00E+09	0	6.09E+02	6.09E+02	0	1.01E+03	1005.897	0	9.28E+01	92.764
b2564_maturation2	b2564 formation	1 b2564_def_map_cplx + 2 h2o --> 1 Def_mono_inact + 1 Map_mono_inact + 1 b2564_m + 1 for + 1 met-L	irreversible	Protein Maturation	0.00E+00	1.00E+09	0	6.09E+02	6.09E+02	0	1.01E+03	1005.897	0	9.28E+01	92.764
b2564_v1_mRNA_CONV	convsersion of mRNA to mRNA_1 (synthetic rxn)	1 b2564_v1_mRNA --> 1 b2564_mRNA_1	irreversible	Translation	0.00E+00	1.00E+09	0	3.70E+01	3.70E+01	0	6.53E+01	65.32977	0	1.06E-01	0.106
b2565_fold_spon	b2565_m folding: spontanous	1 b2565_m --> 1 RecO_mono	irreversible	Protein Folding	0.00E+00	1.00E+09	0	5.83E+02	5.83E+02	0	9.79E+02	979.2968	0	6.62E+01	66.164
b2565_mRNA_CONV	convsersion of mRNA to mRNA_1 (synthetic rxn)	1 b2565_mRNA --> 1 b2565_mRNA_1	irreversible	Translation	0.00E+00	1.00E+09	0	3.70E+01	3.70E+01	0	6.53E+01	65.32977	0	1.06E-01	0.106

b2565_mRNA_degr1	Degradation of b2565_mRNA I	1 Orn_dim + 182 atp + 1 b2565_mRNA_2 + 1 degradosome + 182 h2o <==> 182 adp + 1 b2565_mRNA_2_degr + 182 h + 182 pi	reversible	mRNA degradation	-1.00E+09	1.00E+09	0	3.70E+01	3.70E+01	0	6.53E+01	65.32977	0	1.06E-01	0.106
b2565_mRNA_degr2	Degradation of b2565_mRNA II	1 b2565_mRNA_2_degr + 728 h2o --> 1 Orn_dim_inact + 165 amp + 195 cmp + 1 degradosome_inact + 199 gmp + 728 h + 170 ump	irreversible	mRNA degradation	0.00E+00	1.06E-01	0	3.70E+01	3.70E+01	0	6.53E+01	65.32977	0	1.06E-01	0.106
b2565_maturation1	Polypeptide b2565 peptide deformylas and methionine aminopeptidase complex	1 Def_mono + 1 Map_mono + 1 b2565_aa <==> 1 b2565_def_map_cplx	reversible	Protein Maturation	-1.00E+09	1.00E+09	0	5.83E+02	5.83E+02	0	9.79E+02	979.2968	0	6.62E+01	66.164
b2565_maturation2	b2565 formation	1 b2565_def_map_cplx + 2 h2o --> 1 Def_mono_inact + 1 Map_mono_inact + 1 b2565_m + 1 for + 1 met-L	irreversible	Protein Maturation	0.00E+00	1.00E+09	0	5.83E+02	5.83E+02	0	9.79E+02	979.2968	0	6.62E+01	66.164
b2566_fold_spon	b2566_m folding: spontanous	1 b2566_m --> 1 Era_mono	irreversible	Protein Folding	0.00E+00	1.00E+09	0	1.47E+03	1.47E+03	0	2.53E+03	2531.352	0	7.61E+01	76.075
b2566_mRNA_CONV	convsersion of mRNA to mRNA_1 (synthetic rxn)	1 b2566_mRNA --> 1 b2566_mRNA_1	irreversible	Translation	0.00E+00	1.00E+09	0	3.70E+01	3.70E+01	0	6.53E+01	65.32977	0	1.06E-01	0.106
b2566_mRNA_degr1	Degradation of b2566_mRNA I	1 Orn_dim + 225 atp + 1 b2566_mRNA_2 + 1 degradosome + 225 h2o <==> 225 adp + 1 b2566_mRNA_2_degr + 225 h + 225 pi	reversible	mRNA degradation	-1.00E+09	1.00E+09	0	8.19E+01	8.19E+01	0	1.45E+02	144.5631	0	1.35E-01	0.135
b2566_mRNA_degr2	Degradation of b2566_mRNA II	1 b2566_mRNA_2_degr + 901 h2o --> 1 Orn_dim_inact + 236 amp + 229 cmp + 1 degradosome_inact + 244 gmp + 901 h + 193 ump	irreversible	mRNA degradation	0.00E+00	1.86E-01	0	8.19E+01	8.19E+01	0	1.45E+02	144.5631	0	1.35E-01	0.135
b2566_maturation1	Polypeptide b2566 peptide deformylas and methionine aminopeptidase complex	1 Def_mono + 1 Map_mono + 1 b2566_aa <==> 1 b2566_def_map_cplx	reversible	Protein Maturation	-1.00E+09	1.00E+09	0	1.47E+03	1.47E+03	0	2.53E+03	2531.352	0	7.61E+01	76.075
b2566_maturation2	b2566 formation	1 b2566_def_map_cplx + 2 h2o --> 1 Def_mono_inact + 1 Map_mono_inact + 1 b2566_m + 1 for + 1 met-L	irreversible	Protein Maturation	0.00E+00	1.00E+09	0	1.47E+03	1.47E+03	0	2.53E+03	2531.352	0	7.61E+01	76.075
b2566_v1_mRNA_CONV	convsersion of mRNA to mRNA_1 (synthetic rxn)	1 b2566_v1_mRNA --> 1 b2566_mRNA_1	irreversible	Translation	0.00E+00	1.00E+09	0	8.19E+01	8.19E+01	0	1.45E+02	144.5631	0	1.35E-01	0.135
b2567_fold_spon	b2567_m folding: spontanous	1 b2567_m_Mg --> 1 Rnc_mono	irreversible	Protein Folding	0.00E+00	1.00E+09	0	1.13E+03	1.13E+03	0	1.94E+03	1943.67	0	6.61E+01	66.105
b2567_ion_BIND	Binding of ions to b2567	1 b2567_m + 1 mg2 <==> 1 b2567_m_Mg	reversible	Metallo-ion Binding	-1.00E+09	1.00E+09	0	1.13E+03	1.13E+03	0	1.94E+03	1943.67	0	6.61E+01	66.105
b2567_mRNA_CONV	convsersion of mRNA to mRNA_1 (synthetic rxn)	1 b2567_mRNA --> 1 b2567_mRNA_1	irreversible	Translation	0.00E+00	1.00E+09	0	3.70E+01	3.70E+01	0	6.53E+01	65.32977	0	1.06E-01	0.106
b2567_mRNA_degr1	Degradation of b2567_mRNA I	1 Orn_dim + 170 atp + 1 b2567_mRNA_2 + 1 degradosome + 170 h2o <==> 170 adp + 1 b2567_mRNA_2_degr + 170 h + 170 pi	reversible	mRNA degradation	-1.00E+09	1.00E+09	0	8.19E+01	8.19E+01	0	1.45E+02	144.5631	0	1.35E-01	0.135
b2567_mRNA_degr2	Degradation of b2567_mRNA II	1 b2567_mRNA_2_degr + 680 h2o --> 1 Orn_dim_inact + 174 amp + 1 atp + 161 cmp + 1 degradosome_inact + 184 gmp + 680 h + 161 ump	irreversible	mRNA degradation	0.00E+00	1.35E-01	0	8.19E+01	8.19E+01	0	1.45E+02	144.5631	0	1.35E-01	0.135
b2567_maturation1	Polypeptide b2567 peptide deformylas and methionine aminopeptidase complex	1 Def_mono + 1 Map_mono + 1 b2567_aa <==> 1 b2567_def_map_cplx	reversible	Protein Maturation	-1.00E+09	1.00E+09	0	1.13E+03	1.13E+03	0	1.94E+03	1943.67	0	6.61E+01	66.105
b2567_maturation2	b2567 formation	1 b2567_def_map_cplx + 2 h2o --> 1 Def_mono_inact + 1 Map_mono_inact + 1 b2567_m + 1 for + 1 met-L	irreversible	Protein Maturation	0.00E+00	1.00E+09	0	1.13E+03	1.13E+03	0	1.94E+03	1943.67	0	6.61E+01	66.105
b2567_v1_mRNA_CONV	convsersion of mRNA to mRNA_1 (synthetic rxn)	1 b2567_v1_mRNA --> 1 b2567_mRNA_1	irreversible	Translation	0.00E+00	1.00E+09	0	8.19E+01	8.19E+01	0	1.45E+02	144.5631	0	1.35E-01	0.135
b2570_fold_spon	b2570_m folding: spontanous	1 b2570_m --> 1 RseC_mono	irreversible	Protein Folding	0.00E+00	1.00E+09	0	5.21E+02	5.21E+02	0	8.52E+02	851.6164	0	9.24E+01	92.421
b2570_mRNA_CONV	convsersion of mRNA to mRNA_1 (synthetic rxn)	1 b2570_mRNA --> 1 b2570_mRNA_1	irreversible	Translation	0.00E+00	1.00E+09	0	4.80E+01	4.80E+01	0	8.47E+01	84.65405	0	2.99E-01	0.299
b2570_mRNA_degr1	Degradation of b2570_mRNA I	1 Orn_dim + 119 atp + 1 b2570_mRNA_2 + 1 degradosome + 119 h2o <==> 119 adp + 1 b2570_mRNA_2_degr + 119 h + 119 pi	reversible	mRNA degradation	-1.00E+09	1.00E+09	0	4.80E+01	4.80E+01	0	8.47E+01	84.65405	0	2.99E-01	0.299
b2570_mRNA_degr2	Degradation of b2570_mRNA II	1 b2570_mRNA_2_degr + 475 h2o --> 1 Orn_dim_inact + 90 amp + 122 cmp + 1 degradosome_inact + 140 gmp + 475 h + 124 ump	irreversible	mRNA degradation	0.00E+00	2.99E-01	0	4.80E+01	4.80E+01	0	8.47E+01	84.65405	0	2.99E-01	0.299
b2570_maturation1	Polypeptide b2570 peptide deformylas and methionine aminopeptidase complex	1 Def_mono + 1 Map_mono + 1 b2570_aa <==> 1 b2570_def_map_cplx	reversible	Protein Maturation	-1.00E+09	1.00E+09	0	5.21E+02	5.21E+02	0	8.52E+02	851.6164	0	9.24E+01	92.421
b2570_maturation2	b2570 formation	1 b2570_def_map_cplx + 2 h2o --> 1 Def_mono_inact + 1 Map_mono_inact + 1 b2570_m + 1 for + 1 met-L	irreversible	Protein Maturation	0.00E+00	1.00E+09	0	5.21E+02	5.21E+02	0	8.52E+02	851.6164	0	9.24E+01	92.421
b2570_v2_mRNA_CONV	convsersion of mRNA to mRNA_1 (synthetic rxn)	1 b2570_v2_mRNA --> 1 b2570_mRNA_1	irreversible	Translation	0.00E+00	1.00E+09	0	4.80E+01	4.80E+01	0	8.47E+01	84.65405	0	2.99E-01	0.299
b2571_fold_spon	b2571_m folding: spontanous	1 b2571_m --> 1 RseB_mono	irreversible	Protein Folding	0.00E+00	1.00E+09	0	9.74E+02	9.74E+02	0	1.63E+03	1634.653	0	1.16E+02	116.262
b2571_mRNA_CONV	convsersion of mRNA to mRNA_1 (synthetic rxn)	1 b2571_mRNA --> 1 b2571_mRNA_1	irreversible	Translation	0.00E+00	1.00E+09	0	4.80E+01	4.80E+01	0	8.47E+01	84.65405	0	2.99E-01	0.299
b2571_mRNA_degr1	Degradation of b2571_mRNA I	1 Orn_dim + 239 atp + 1 b2571_mRNA_2 + 1 degradosome + 239 h2o <==> 239 adp + 1 b2571_mRNA_2_degr + 239 h + 239 pi	reversible	mRNA degradation	-1.00E+09	1.00E+09	0	4.80E+01	4.80E+01	0	8.47E+01	84.65405	0	2.99E-01	0.299
b2571_mRNA_degr2	Degradation of b2571_mRNA II	1 b2571_mRNA_2_degr + 955 h2o --> 1 Orn_dim_inact + 227 amp + 249 cmp + 1 degradosome_inact + 237 gmp + 955 h + 243 ump	irreversible	mRNA degradation	0.00E+00	3.48E-01	0	4.80E+01	4.80E+01	0	8.47E+01	84.65405	0	2.99E-01	0.299
b2571_maturation1	Polypeptide b2571 peptide deformylas and methionine aminopeptidase complex	1 Def_mono + 1 Map_mono + 1 b2571_aa <==> 1 b2571_def_map_cplx	reversible	Protein Maturation	-1.00E+09	1.00E+09	0	9.74E+02	9.74E+02	0	1.63E+03	1634.653	0	1.16E+02	116.262
b2571_maturation2	b2571 formation	1 b2571_def_map_cplx + 24 h2o --> 1 Def_mono_inact + 1 Map_mono_inact + 4 ala-L + 1 asn-L + 1 b2571_m + 1 for + 1 gln-L + 1 gly + 4 leu-L + 1 lys-L + 2 met-L + 2 phe-L + 4 ser-L + 1 thr-L + 1 trp L + 1 val-L	irreversible	Protein Maturation	0.00E+00	1.00E+09	0	9.74E+02	9.74E+02	0	1.63E+03	1634.653	0	1.16E+02	116.262
b2571_v2_mRNA_CONV	convsersion of mRNA to mRNA_1 (synthetic rxn)	1 b2571_v2_mRNA --> 1 b2571_mRNA_1	irreversible	Translation	0.00E+00	1.00E+09	0	4.80E+01	4.80E+01	0	8.47E+01	84.65405	0	2.99E-01	0.299
b2572_fold_spon	b2572_m folding: spontanous	1 b2572_m --> 1 RseA_mono	irreversible	Protein Folding	0.00E+00	1.00E+09	0	7.16E+02	7.16E+02	0	1.16E+03	1156.249	0	1.44E+02	143.988
b2572_mRNA_CONV	convsersion of mRNA to mRNA_1 (synthetic rxn)	1 b2572_mRNA --> 1 b2572_mRNA_1	irreversible	Translation	0.00E+00	1.00E+09	0	4.80E+01	4.80E+01	0	8.47E+01	84.65405	0	2.99E-01	0.299
b2572_mRNA_degr1	Degradation of b2572_mRNA I	1 Orn_dim + 162 atp + 1 b2572_mRNA_2 + 1 degradosome + 162 h2o <==> 162 adp + 1 b2572_mRNA_2_degr + 162 h + 162 pi	reversible	mRNA degradation	-1.00E+09	1.00E+09	0	4.80E+01	4.80E+01	0	8.47E+01	84.65405	0	2.99E-01	0.299
b2572_mRNA_degr2	Degradation of b2572_mRNA II	1 b2572_mRNA_2_degr + 650 h2o --> 1 Orn_dim_inact + 183 amp + 173 cmp + 1 degradosome_inact + 167 gmp + 650 h + 128 ump	irreversible	mRNA degradation	0.00E+00	5.39E-01	0	4.80E+01	4.80E+01	0	8.47E+01	84.65405	0	2.99E-01	0.299
b2572_maturation1	Polypeptide b2572 peptide deformylas and methionine aminopeptidase complex	1 Def_mono + 1 Map_mono + 1 b2572_aa <==> 1 b2572_def_map_cplx	reversible	Protein Maturation	-1.00E+09	1.00E+09	0	7.16E+02	7.16E+02	0	1.16E+03	1156.249	0	1.44E+02	143.988
b2572_maturation2	b2572 formation	1 b2572_def_map_cplx + 2 h2o --> 1 Def_mono_inact + 1 Map_mono_inact + 1 b2572_m + 1 for + 1 met-L	irreversible	Protein Maturation	0.00E+00	1.00E+09	0	7.16E+02	7.16E+02	0	1.16E+03	1156.249	0	1.44E+02	143.988
b2572_v2_mRNA_CONV	convsersion of mRNA to mRNA_1 (synthetic rxn)	1 b2572_v2_mRNA --> 1 b2572_mRNA_1	irreversible	Translation	0.00E+00	1.00E+09	0	4.80E+01	4.80E+01	0	8.47E+01	84.65405	0	2.99E-01	0.299
b2573_fold_spon	b2573_m folding: spontanous	1 b2573_m --> 1 RpoE_mono	irreversible	Protein Folding	0.00E+00	1.00E+09	0	6.30E+02	6.30E+02	0	1.03E+03	1033.275	0	1.05E+02	105.369
b2573_mRNA_CONV	convsersion of mRNA to mRNA_1 (synthetic rxn)	1 b2573_mRNA --> 1 b2573_mRNA_1	irreversible	Translation	0.00E+00	1.00E+09	0	4.80E+01	4.80E+01	0	8.47E+01	84.65405	0	2.99E-01	0.299
b2573_mRNA_degr1	Degradation of b2573_mRNA I	1 Orn_dim + 144 atp + 1 b2573_mRNA_2 + 1 degradosome + 144 h2o <==> 144 adp + 1 b2573_mRNA_2_degr + 144 h + 144 pi	reversible	mRNA degradation	-1.00E+09	1.00E+09	0	4.80E+01	4.80E+01	0	8.47E+01	84.65405	0	2.99E-01	0.299
b2573_mRNA_degr2	Degradation of b2573_mRNA II	1 b2573_mRNA_2_degr + 575 h2o --> 1 Orn_dim_inact + 145 amp + 1 atp + 118 cmp + 1 degradosome_inact + 163 gmp + 575 h + 149 ump	irreversible	mRNA degradation	0.00E+00	3.08E-01	0	4.80E+01	4.80E+01	0	8.47E+01	84.65405	0	2.99E-01	0.299
b2573_maturation1	Polypeptide b2573 peptide deformylas and methionine aminopeptidase complex	1 Def_mono + 1 Map_mono + 1 b2573_aa <==> 1 b2573_def_map_cplx	reversible	Protein Maturation	-1.00E+09	1.00E+09	0	6.30E+02	6.30E+02	0	1.03E+03	1033.275	0	1.05E+02	105.369
b2573_maturation2	b2573 formation	1 b2573_def_map_cplx + 2 h2o --> 1 Def_mono_inact + 1 Map_mono_inact + 1 b2573_m + 1 for + 1 met-L	irreversible	Protein Maturation	0.00E+00	1.00E+09	0	6.30E+02	6.30E+02	0	1.03E+03	1033.275	0	1.05E+02	105.369

b2573_v1_mRNA_CONV	convsersion of mRNA to mRNA_1 (synthetic rxn)	1 b2573_v1_mRNA --> 1 b2573_mRNA_1	irreversible	Translation	0.00E+00	1.00E+09	0	4.80E+01	4.80E+01	0	8.47E+01	84.65405	0	2.99E-01	0.299
b2589_RNA_10_modif_1	b2589_RNA modification (23S rRNA): position: 2445, m2G by MeT_23S_2445	1 MeT_23S_2445 + 1 amet + 1 b2589_RNA_10 <==> 1 b2589_RNA_10_MeT_23S_2445	reversible	rRNA modification	-1.00E+09	1.00E+09	0	1.11E+02	1.11E+02	0	0	0	0	0	0
b2589_RNA_10_modif_2	b2589_RNA modification (23S rRNA): position: 2445, m2G by MeT_23S_2445	1 b2589_RNA_10_MeT_23S_2445 --> 1 MeT_23S_2445_inact + 1 ahcys + 1 b2589_RNA_11 + 1 h	irreversible	rRNA modification	0.00E+00	1.00E+09	0	1.11E+02	1.11E+02	0	0	0	0	0	0
b2589_RNA_11_a_modif_1	b2589_RNA modification (23S rRNA): position: 2449, D by DU_23S_2449 (NADH)	1 DU_23S_2449 + 1 b2589_RNA_11 + 1 h + 1 nadh <==> 1 b2589_RNA_11_DU_23S_2449_a	reversible	rRNA modification	-1.00E+09	1.00E+09	0	1.11E+02	1.11E+02	0	0	0	0	0	0
b2589_RNA_11_a_modif_2	b2589_RNA modification (23S rRNA): position: 2449, D by DU_23S_2449 (NADH)	1 b2589_RNA_11_DU_23S_2449_a --> 1 DU_23S_2449_inact + 1 b2589_RNA_12 + 1 nad	irreversible	rRNA modification	0.00E+00	1.00E+09	0	1.11E+02	1.11E+02	0	0	0	0	0	0
b2589_RNA_11_b_modif_1	b2589_RNA modification (23S rRNA): position: 2449, D by DU_23S_2449 (NADPH)	1 DU_23S_2449 + 1 b2589_RNA_11 + 1 h + 1 nadph <==> 1 b2589_RNA_11_DU_23S_2449_b	reversible	rRNA modification	-1.00E+09	1.00E+09	0	1.11E+02	1.11E+02	0	0	0	0	0	0
b2589_RNA_11_b_modif_2	b2589_RNA modification (23S rRNA): position: 2449, D by DU_23S_2449 (NADPH)	1 b2589_RNA_11_DU_23S_2449_b --> 1 DU_23S_2449_inact + 1 b2589_RNA_12 + 1 nadp	irreversible	rRNA modification	0.00E+00	1.00E+09	0	1.11E+02	1.11E+02	0	0	0	0	0	0
b2589_RNA_12_modif_1	b2589_RNA modification (23S rRNA): position: 2457, Y by YmfC_mono	1 YmfC_mono + 1 b2589_RNA_12 <==> 1 b2589_RNA_12_YmfC_mono	reversible	rRNA modification	-1.00E+09	1.00E+09	0	1.11E+02	1.11E+02	0	0	0	0	0	0
b2589_RNA_12_modif_2	b2589_RNA modification (23S rRNA): position: 2457, Y by YmfC_mono	1 b2589_RNA_12_YmfC_mono --> 1 YmfC_mono_inact + 1 b2589_RNA_13	irreversible	rRNA modification	0.00E+00	1.00E+09	0	1.11E+02	1.11E+02	0	0	0	0	0	0
b2589_RNA_13_modif_1	b2589_RNA modification (23S rRNA): position: 2498, Cm by MeT_23S_2498	1 MeT_23S_2498 + 1 amet + 1 b2589_RNA_13 <==> 1 b2589_RNA_13_MeT_23S_2498	reversible	rRNA modification	-1.00E+09	1.00E+09	0	1.11E+02	1.11E+02	0	0	0	0	0	0
b2589_RNA_13_modif_2	b2589_RNA modification (23S rRNA): position: 2498, Cm by MeT_23S_2498	1 b2589_RNA_13_MeT_23S_2498 --> 1 MeT_23S_2498_inact + 1 ahcys + 1 b2589_RNA_14 + 1 h	irreversible	rRNA modification	0.00E+00	1.00E+09	0	1.11E+02	1.11E+02	0	0	0	0	0	0
b2589_RNA_14_modif_1	b2589_RNA modification (23S rRNA): position: 2503, m2A by MeT_23S_2503	1 MeT_23S_2503 + 1 amet + 1 b2589_RNA_14 <==> 1 b2589_RNA_14_MeT_23S_2503	reversible	rRNA modification	-1.00E+09	1.00E+09	0	1.11E+02	1.11E+02	0	0	0	0	0	0
b2589_RNA_14_modif_2	b2589_RNA modification (23S rRNA): position: 2503, m2A by MeT_23S_2503	1 b2589_RNA_14_MeT_23S_2503 --> 1 MeT_23S_2503_inact + 1 ahcys + 1 b2589_RNA_15 + 1 h	irreversible	rRNA modification	0.00E+00	1.00E+09	0	1.11E+02	1.11E+02	0	0	0	0	0	0
b2589_RNA_15_modif_1	b2589_RNA modification (23S rRNA): position: 2504, Y by RluC_mono	1 RluC_mono + 1 b2589_RNA_15 <==> 1 b2589_RNA_15_RluC_mono	reversible	rRNA modification	-1.00E+09	1.00E+09	0	1.11E+02	1.11E+02	0	0	0	0	0	0
b2589_RNA_15_modif_2	b2589_RNA modification (23S rRNA): position: 2504, Y by RluC_mono	1 b2589_RNA_15_RluC_mono --> 1 RluC_mono_inact + 1 b2589_RNA_16	irreversible	rRNA modification	0.00E+00	1.00E+09	0	1.11E+02	1.11E+02	0	0	0	0	0	0
b2589_RNA_16_modif_1	b2589_RNA modification (23S rRNA): position: 2552, Um by RrmJ_mono	1 RrmJ_mono + 1 amet + 1 b2589_RNA_16 <==> 1 b2589_RNA_16_RrmJ_mono	reversible	rRNA modification	-1.00E+09	1.00E+09	0	1.11E+02	1.11E+02	0	0	0	0	0	0
b2589_RNA_16_modif_2	b2589_RNA modification (23S rRNA): position: 2552, Um by RrmJ_mono	1 b2589_RNA_16_RrmJ_mono --> 1 RrmJ_mono_inact + 1 ahcys + b2589_RNA_17 + 1 h	irreversible	rRNA modification	0.00E+00	1.00E+09	0	1.11E+02	1.11E+02	0	0	0	0	0	0
b2589_RNA_17_modif_1	b2589_RNA modification (23S rRNA): position: 2580, Y by RluC_mono	1 RluC_mono + 1 b2589_RNA_17 <==> 1 b2589_RNA_17_RluC_mono	reversible	rRNA modification	-1.00E+09	1.00E+09	0	1.11E+02	1.11E+02	0	0	0	0	0	0
b2589_RNA_17_modif_2	b2589_RNA modification (23S rRNA): position: 2580, Y by RluC_mono	1 b2589_RNA_17_RluC_mono --> 1 RluC_mono_inact + 1 b2589_RNA_18	irreversible	rRNA modification	0.00E+00	1.00E+09	0	1.11E+02	1.11E+02	0	0	0	0	0	0
b2589_RNA_18_modif_1	b2589_RNA modification (23S rRNA): position: 2604, Y by YjbC_mono	1 YjbC_mono + 1 b2589_RNA_18 <==> 1 b2589_RNA_18_YjbC_mono	reversible	rRNA modification	-1.00E+09	1.00E+09	0	1.11E+02	1.11E+02	0	0	0	0	0	0
b2589_RNA_18_modif_2	b2589_RNA modification (23S rRNA): position: 2604, Y by YjbC_mono	1 b2589_RNA_18_YjbC_mono --> 1 YjbC_mono_inact + 1 b2589_RNA_19	irreversible	rRNA modification	0.00E+00	1.00E+09	0	1.11E+02	1.11E+02	0	0	0	0	0	0
b2589_RNA_19_modif_1	b2589_RNA modification (23S rRNA): position: 2605, Y by RluB_mono	1 RluB_mono + 1 b2589_RNA_19 <==> 1 b2589_RNA_19_RluB_mono	reversible	rRNA modification	-1.00E+09	1.00E+09	0	1.11E+02	1.11E+02	0	0	0	0	0	0
b2589_RNA_19_modif_2	b2589_RNA modification (23S rRNA): position: 2605, Y by RluB_mono	1 b2589_RNA_19_RluB_mono --> 1 RluB_mono_inact + 1 b2589_RNA_20	irreversible	rRNA modification	0.00E+00	1.00E+09	0	1.11E+02	1.11E+02	0	0	0	0	0	0
b2589_RNA_1_modif_1	b2589_RNA modification (23S rRNA): position: 1835, m2G by MeT_23S_1835	1 MeT_23S_1835 + 1 amet + 1 b2589_RNA_1 <==> 1 b2589_RNA_1_MeT_23S_1835	reversible	rRNA modification	-1.00E+09	1.00E+09	0	1.11E+02	1.11E+02	0	0	0	0	0	0
b2589_RNA_1_modif_2	b2589_RNA modification (23S rRNA): position: 1835, m2G by MeT_23S_1835	1 b2589_RNA_1_MeT_23S_1835 --> 1 MeT_23S_1835_inact + 1 ahcys + 1 b2589_RNA_2 + 1 h	irreversible	rRNA modification	0.00E+00	1.00E+09	0	1.11E+02	1.11E+02	0	0	0	0	0	0
b2589_RNA_20_modif_1	b2589_RNA modification (23S rRNA): position: 745, m1G by RrmA_dim	1 RrmA_dim + 1 amet + 1 b2589_RNA_20 <==> 1 b2589_RNA_20_RrmA_dim	reversible	rRNA modification	-1.00E+09	1.00E+09	0	1.11E+02	1.11E+02	0	0	0	0	0	0
b2589_RNA_20_modif_2	b2589_RNA modification (23S rRNA): position: 745, m1G by RrmA_dim	1 b2589_RNA_20_RrmA_dim --> 1 RrmA_dim_inact + 1 ahcys + 1 b2589_RNA_21 + 1 h	irreversible	rRNA modification	0.00E+00	1.00E+09	0	1.11E+02	1.11E+02	0	0	0	0	0	0
b2589_RNA_21_modif_1	b2589_RNA modification (23S rRNA): position: 746, Y by RluA_mono	1 RluA_mono + 1 b2589_RNA_21 <==> 1 b2589_RNA_21_RluA_mono	reversible	rRNA modification	-1.00E+09	1.00E+09	0	1.11E+02	1.11E+02	0	0	0	0	0	0
b2589_RNA_21_modif_2	b2589_RNA modification (23S rRNA): position: 746, Y by RluA_mono	1 b2589_RNA_21_RluA_mono --> 1 RluA_mono_inact + 1 b2589_RNA_22	irreversible	rRNA modification	0.00E+00	1.00E+09	0	1.11E+02	1.11E+02	0	0	0	0	0	0
b2589_RNA_22_modif_1	b2589_RNA modification (23S rRNA): position: 747, m5U by RumB_mono	1 RumB_mono + 1 amet + 1 b2589_RNA_22 <==> 1 b2589_RNA_22_RumB_mono	reversible	rRNA modification	-1.00E+09	1.00E+09	0	1.11E+02	1.11E+02	0	0	0	0	0	0
b2589_RNA_22_modif_2	b2589_RNA modification (23S rRNA): position: 747, m5U by RumB_mono	1 b2589_RNA_22_RumB_mono --> 1 RumB_mono_inact + 1 ahcys + 1 b2589_RNA_23 + 1 h	irreversible	rRNA modification	0.00E+00	1.00E+09	0	1.11E+02	1.11E+02	0	0	0	0	0	0
b2589_RNA_23_modif_1	b2589_RNA modification (23S rRNA): position: 955, Y by RluC_mono	1 RluC_mono + 1 b2589_RNA_23 <==> 1 b2589_RNA_23_RluC_mono	reversible	rRNA modification	-1.00E+09	1.00E+09	0	1.11E+02	1.11E+02	0	0	0	0	0	0
b2589_RNA_23_modif_2	b2589_RNA modification (23S rRNA): position: 955, Y by RluC_mono	1 b2589_RNA_23_RluC_mono --> 1 RluC_mono_inact + 1 rrlG	irreversible	rRNA modification	0.00E+00	1.00E+09	0	1.11E+02	1.11E+02	0	0	0	0	0	0
b2589_RNA_2_modif_1	b2589_RNA modification (23S rRNA): position: 1911, Y by RluD_mono	1 RluD_mono + 1 b2589_RNA_2 <==> 1 b2589_RNA_2_RluD_mono	reversible	rRNA modification	-1.00E+09	1.00E+09	0	1.11E+02	1.11E+02	0	0	0	0	0	0
b2589_RNA_2_modif_2	b2589_RNA modification (23S rRNA): position: 1911, Y by RluD_mono	1 b2589_RNA_2_RluD_mono --> 1 RluD_mono_inact + 1 b2589_RNA_3	irreversible	rRNA modification	0.00E+00	1.00E+09	0	1.11E+02	1.11E+02	0	0	0	0	0	0
b2589_RNA_3_modif_1	b2589_RNA modification (23S rRNA): position: 1915, m3Y by RluD_mono	1 RluD_mono + 1 amet + 1 b2589_RNA_3 <==> 1 b2589_RNA_3_RluD_mono	reversible	rRNA modification	-1.00E+09	1.00E+09	0	1.11E+02	1.11E+02	0	0	0	0	0	0
b2589_RNA_3_modif_2	b2589_RNA modification (23S rRNA): position: 1915, m3Y by RluD_mono	1 b2589_RNA_3_RluD_mono --> 1 RluD_mono_inact + 1 ahcys + 1 b2589_RNA_4 + 1 h	irreversible	rRNA modification	0.00E+00	1.00E+09	0	1.11E+02	1.11E+02	0	0	0	0	0	0
b2589_RNA_4_modif_1	b2589_RNA modification (23S rRNA): position: 1917, Y by RluD_mono	1 RluD_mono + 1 b2589_RNA_4 <==> 1 b2589_RNA_4_RluD_mono	reversible	rRNA modification	-1.00E+09	1.00E+09	0	1.11E+02	1.11E+02	0	0	0	0	0	0

b2589_RNA_4_modif_2	b2589_RNA modification (23S rRNA): position: 1917, Y by RluD_mono	1 b2589_RNA_4_RluD_mono --> 1 RluD_mono_inact + 1 b2589_RNA_5	irreversible	rRNA modification	0.00E+00	1.00E+09	0	1.11E+02	1.11E+02	0	0	0	0	0	0
b2589_RNA_5_modif_1	b2589_RNA modification (23S rRNA): position: 1939, m5U by RumA_mono	1 RumA_mono + 1 amet + 1 b2589_RNA_5 <==> 1 b2589_RNA_5_RumA_mono	reversible	rRNA modification	-1.00E+09	1.00E+09	0	1.11E+02	1.11E+02	0	0	0	0	0	0
b2589_RNA_5_modif_2	b2589_RNA modification (23S rRNA): position: 1939, m5U by RumA_mono	1 b2589_RNA_5_RumA_mono --> 1 RumA_mono_inact + 1 ahcys + 1 b2589_RNA_6 + 1 h	irreversible	rRNA modification	0.00E+00	1.00E+09	0	1.11E+02	1.11E+02	0	0	0	0	0	0
b2589_RNA_6_modif_1	b2589_RNA modification (23S rRNA): position: 1962, m5C by MeT_23S_1962	1 MeT_23S_1962 + 1 amet + 1 b2589_RNA_6 <==> 1 b2589_RNA_6_MeT_23S_1962	reversible	rRNA modification	-1.00E+09	1.00E+09	0	1.11E+02	1.11E+02	0	0	0	0	0	0
b2589_RNA_6_modif_2	b2589_RNA modification (23S rRNA): position: 1962, m5C by MeT_23S_1962	1 b2589_RNA_6_MeT_23S_1962 --> 1 MeT_23S_1962_inact + 1 ahcys + 1 b2589_RNA_7 + 1 h	irreversible	rRNA modification	0.00E+00	1.00E+09	0	1.11E+02	1.11E+02	0	0	0	0	0	0
b2589_RNA_7_modif_1	b2589_RNA modification (23S rRNA): position: 2030, m6A by MeT_23S_2030	1 MeT_23S_2030 + 1 amet + 1 b2589_RNA_7 <==> 1 b2589_RNA_7_MeT_23S_2030	reversible	rRNA modification	-1.00E+09	1.00E+09	0	1.11E+02	1.11E+02	0	0	0	0	0	0
b2589_RNA_7_modif_2	b2589_RNA modification (23S rRNA): position: 2030, m6A by MeT_23S_2030	1 b2589_RNA_7_MeT_23S_2030 --> 1 MeT_23S_2030_inact + 1 ahcys + 1 b2589_RNA_8 + 1 h	irreversible	rRNA modification	0.00E+00	1.00E+09	0	1.11E+02	1.11E+02	0	0	0	0	0	0
b2589_RNA_8_modif_1	b2589_RNA modification (23S rRNA): position: 2069, m7G by MeT_23S_2069	1 MeT_23S_2069 + 1 amet + 1 b2589_RNA_8 <==> 1 b2589_RNA_8_MeT_23S_2069	reversible	rRNA modification	-1.00E+09	1.00E+09	0	1.11E+02	1.11E+02	0	0	0	0	0	0
b2589_RNA_8_modif_2	b2589_RNA modification (23S rRNA): position: 2069, m7G by MeT_23S_2069	1 b2589_RNA_8_MeT_23S_2069 --> 1 MeT_23S_2069_inact + 1 ahcys + 1 b2589_RNA_9 + 1 h	irreversible	rRNA modification	0.00E+00	1.00E+09	0	1.11E+02	1.11E+02	0	0	0	0	0	0
b2589_RNA_9_modif_1	b2589_RNA modification (23S rRNA): position: 2251, Gm by RlmB_dim	1 RlmB_dim + 1 amet + 1 b2589_RNA_9 <==> 1 b2589_RNA_9_RlmB_dim	reversible	rRNA modification	-1.00E+09	1.00E+09	0	1.11E+02	1.11E+02	0	0	0	0	0	0
b2589_RNA_9_modif_2	b2589_RNA modification (23S rRNA): position: 2251, Gm by RlmB_dim	1 b2589_RNA_9_RlmB_dim --> 1 RlmB_dim_inact + 1 ahcys + 1 b2589_RNA_10 + 1 h	irreversible	rRNA modification	0.00E+00	1.00E+09	0	1.11E+02	1.11E+02	0	0	0	0	0	0
b2589_RNA_modif_1	b2589_RNA modification (23S rRNA): position: 1618, m6A by MeT_23S_1618	1 MeT_23S_1618 + 1 amet + 1 b2589_RNA <==> 1 b2589_RNA_MeT_23S_1618	reversible	rRNA modification	-1.00E+09	1.00E+09	0	1.11E+02	1.11E+02	0	0	0	0	0	0
b2589_RNA_modif_2	b2589_RNA modification (23S rRNA): position: 1618, m6A by MeT_23S_1618	1 b2589_RNA_MeT_23S_1618 --> 1 MeT_23S_1618_inact + 1 ahcy + 1 b2589_RNA_1 + 1 h	irreversible	rRNA modification	0.00E+00	1.00E+09	0	1.11E+02	1.11E+02	0	0	0	0	0	0
b2589_v1_RNA_10_modif _1	b2589_v1_RNA modification (23S rRNA): position: 2445, m2G by MeT_23S_2445	1 MeT_23S_2445 + 1 amet + 1 b2589_v1_RNA_10 <==> 1 b2589_v1_RNA_10_MeT_23S_2445	reversible	rRNA modification	-1.00E+09	1.00E+09	0	1.11E+02	1.11E+02	0	8.54E+00	8.54	0	8.54E+00	8.54
b2589_v1_RNA_10_modif _2	b2589_v1_RNA modification (23S rRNA): position: 2445, m2G by MeT_23S_2445	1 b2589_v1_RNA_10_MeT_23S_2445 --> 1 MeT_23S_2445_inact + 1 ahcys + 1 b2589_v1_RNA_11 + 1 h	irreversible	rRNA modification	0.00E+00	1.00E+09	0	1.11E+02	1.11E+02	0	8.54E+00	8.54	0	8.54E+00	8.54
b2589_v1_RNA_11_a_mo dif_1	b2589_v1_RNA modification (23S rRNA): position: 2449, D by DU_23S_2449 (NADH)	1 DU_23S_2449 + 1 b2589_v1_RNA_11 + 1 h + 1 nadh <==> 1 b2589_v1_RNA_11_DU_23S_2449_a	reversible	rRNA modification	-1.00E+09	1.00E+09	0	1.11E+02	1.11E+02	0	8.54E+00	8.54	0	8.54E+00	8.54
b2589_v1_RNA_11_a_mo dif_2	b2589_v1_RNA modification (23S rRNA): position: 2449, D by DU_23S_2449 (NADH)	1 b2589_v1_RNA_11_DU_23S_2449_a --> 1 DU_23S_2449_inact + 1 b2589_v1_RNA_12 + 1 nad	irreversible	rRNA modification	0.00E+00	1.00E+09	0	1.11E+02	1.11E+02	0	8.54E+00	8.54	0	8.54E+00	8.54
b2589_v1_RNA_11_b_mo dif_1	b2589_v1_RNA modification (23S rRNA): position: 2449, D by DU_23S_2449 (NADPH)	1 DU_23S_2449 + 1 b2589_v1_RNA_11 + 1 h + 1 nadph <==> 1 b2589_v1_RNA_11_DU_23S_2449_b	reversible	rRNA modification	-1.00E+09	1.00E+09	0	1.11E+02	1.11E+02	0	8.54E+00	8.54	0	8.54E+00	8.54
b2589_v1_RNA_11_b_mo dif_2	b2589_v1_RNA modification (23S rRNA): position: 2449, D by DU_23S_2449 (NADPH)	1 b2589_v1_RNA_11_DU_23S_2449_b --> 1 DU_23S_2449_inact + 1 b2589_v1_RNA_12 + 1 nadp	irreversible	rRNA modification	0.00E+00	1.00E+09	0	1.11E+02	1.11E+02	0	8.54E+00	8.54	0	8.54E+00	8.54
b2589_v1_RNA_12_modif _1	b2589_v1_RNA modification (23S rRNA): position: 2457, Y by YmfC_mono	1 YmfC_mono + 1 b2589_v1_RNA_12 <==> 1 b2589_v1_RNA_12_YmfC_mono	reversible	rRNA modification	-1.00E+09	1.00E+09	0	1.11E+02	1.11E+02	0	8.54E+00	8.54	0	8.54E+00	8.54
b2589_v1_RNA_12_modif _2	b2589_v1_RNA modification (23S rRNA): position: 2457, Y by YmfC_mono	1 b2589_v1_RNA_12_YmfC_mono --> 1 YmfC_mono_inact + 1 b2589_v1_RNA_13	irreversible	rRNA modification	0.00E+00	1.00E+09	0	1.11E+02	1.11E+02	0	8.54E+00	8.54	0	8.54E+00	8.54
b2589_v1_RNA_13_modif _1	b2589_v1_RNA modification (23S rRNA): position: 2498, Cm by MeT_23S_2498	1 MeT_23S_2498 + 1 amet + 1 b2589_v1_RNA_13 <==> 1 b2589_v1_RNA_13_MeT_23S_2498	reversible	rRNA modification	-1.00E+09	1.00E+09	0	1.11E+02	1.11E+02	0	8.54E+00	8.54	0	8.54E+00	8.54
b2589_v1_RNA_13_modif _2	b2589_v1_RNA modification (23S rRNA): position: 2498, Cm by MeT_23S_2498	1 b2589_v1_RNA_13_MeT_23S_2498 --> 1 MeT_23S_2498_inact + 1 ahcys + 1 b2589_v1_RNA_14 + 1 h	irreversible	rRNA modification	0.00E+00	1.00E+09	0	1.11E+02	1.11E+02	0	8.54E+00	8.54	0	8.54E+00	8.54
b2589_v1_RNA_14_modif _1	b2589_v1_RNA modification (23S rRNA): position: 2503, m2A by MeT_23S_2503	1 MeT_23S_2503 + 1 amet + 1 b2589_v1_RNA_14 <==> 1 b2589_v1_RNA_14_MeT_23S_2503	reversible	rRNA modification	-1.00E+09	1.00E+09	0	1.11E+02	1.11E+02	0	8.54E+00	8.54	0	8.54E+00	8.54
b2589_v1_RNA_14_modif _2	b2589_v1_RNA modification (23S rRNA): position: 2503, m2A by MeT_23S_2503	1 b2589_v1_RNA_14_MeT_23S_2503 --> 1 MeT_23S_2503_inact + 1 ahcys + 1 b2589_v1_RNA_15 + 1 h	irreversible	rRNA modification	0.00E+00	1.00E+09	0	1.11E+02	1.11E+02	0	8.54E+00	8.54	0	8.54E+00	8.54
b2589_v1_RNA_15_modif _1	b2589_v1_RNA modification (23S rRNA): position: 2504, Y by RluC_mono	1 RluC_mono + 1 b2589_v1_RNA_15 <==> 1 b2589_v1_RNA_15_RluC_mono	reversible	rRNA modification	-1.00E+09	1.00E+09	0	1.11E+02	1.11E+02	0	8.54E+00	8.54	0	8.54E+00	8.54
b2589_v1_RNA_15_modif _2	b2589_v1_RNA modification (23S rRNA): position: 2504, Y by RluC_mono	1 b2589_v1_RNA_15_RluC_mono --> 1 RluC_mono_inact + 1 b2589_v1_RNA_16	irreversible	rRNA modification	0.00E+00	1.00E+09	0	1.11E+02	1.11E+02	0	8.54E+00	8.54	0	8.54E+00	8.54
b2589_v1_RNA_16_modif _1	b2589_v1_RNA modification (23S rRNA): position: 2552, Um by RrmJ_mono	1 RrmJ_mono + 1 amet + 1 b2589_v1_RNA_16 <==> 1 b2589_v1_RNA_16_RrmJ_mono	reversible	rRNA modification	-1.00E+09	1.00E+09	0	1.11E+02	1.11E+02	0	8.54E+00	8.54	0	8.54E+00	8.54
b2589_v1_RNA_16_modif _2	b2589_v1_RNA modification (23S rRNA): position: 2552, Um by RrmJ_mono	1 b2589_v1_RNA_16_RrmJ_mono --> 1 RrmJ_mono_inact + 1 ahcy + 1 b2589_v1_RNA_17 + 1 h	irreversible	rRNA modification	0.00E+00	1.00E+09	0	1.11E+02	1.11E+02	0	8.54E+00	8.54	0	8.54E+00	8.54
b2589_v1_RNA_17_modif _1	b2589_v1_RNA modification (23S rRNA): position: 2580, Y by RluC_mono	1 RluC_mono + 1 b2589_v1_RNA_17 <==> 1 b2589_v1_RNA_17_RluC_mono	reversible	rRNA modification	-1.00E+09	1.00E+09	0	1.11E+02	1.11E+02	0	8.54E+00	8.54	0	8.54E+00	8.54
b2589_v1_RNA_17_modif _2	b2589_v1_RNA modification (23S rRNA): position: 2580, Y by RluC_mono	1 b2589_v1_RNA_17_RluC_mono --> 1 RluC_mono_inact + 1 b2589_v1_RNA_18	irreversible	rRNA modification	0.00E+00	1.00E+09	0	1.11E+02	1.11E+02	0	8.54E+00	8.54	0	8.54E+00	8.54
b2589_v1_RNA_18_modif _1	b2589_v1_RNA modification (23S rRNA): position: 2604, Y by YjbC_mono	1 YjbC_mono + 1 b2589_v1_RNA_18 <==> 1 b2589_v1_RNA_18_YjbC_mono	reversible	rRNA modification	-1.00E+09	1.00E+09	0	1.11E+02	1.11E+02	0	8.54E+00	8.54	0	8.54E+00	8.54
b2589_v1_RNA_18_modif _2	b2589_v1_RNA modification (23S rRNA): position: 2604, Y by YjbC_mono	1 b2589_v1_RNA_18_YjbC_mono --> 1 YjbC_mono_inact + 1 b2589_v1_RNA_19	irreversible	rRNA modification	0.00E+00	1.00E+09	0	1.11E+02	1.11E+02	0	8.54E+00	8.54	0	8.54E+00	8.54
b2589_v1_RNA_19_modif _1	b2589_v1_RNA modification (23S rRNA): position: 2605, Y by RluB_mono	1 RluB_mono + 1 b2589_v1_RNA_19 <==> 1 b2589_v1_RNA_19_RluB_mono	reversible	rRNA modification	-1.00E+09	1.00E+09	0	1.11E+02	1.11E+02	0	8.54E+00	8.54	0	8.54E+00	8.54
b2589_v1_RNA_19_modif _2	b2589_v1_RNA modification (23S rRNA): position: 2605, Y by RluB_mono	1 b2589_v1_RNA_19_RluB_mono --> 1 RluB_mono_inact + 1 b2589_v1_RNA_20	irreversible	rRNA modification	0.00E+00	1.00E+09	0	1.11E+02	1.11E+02	0	8.54E+00	8.54	0	8.54E+00	8.54
b2589_v1_RNA_1_modif_ 1	b2589_v1_RNA modification (23S rRNA): position: 1835, m2G by MeT_23S_1835	1 MeT_23S_1835 + 1 amet + 1 b2589_v1_RNA_1 <==> 1 b2589_v1_RNA_1_MeT_23S_1835	reversible	rRNA modification	-1.00E+09	1.00E+09	0	1.11E+02	1.11E+02	0	8.54E+00	8.54	0	8.54E+00	8.54
b2589_v1_RNA_1_modif_ 2	b2589_v1_RNA modification (23S rRNA): position: 1835, m2G by MeT_23S_1835	1 b2589_v1_RNA_1_MeT_23S_1835 --> 1 MeT_23S_1835_inact + 1 ahcys + 1 b2589_v1_RNA_2 + 1 h	irreversible	rRNA modification	0.00E+00	1.00E+09	0	1.11E+02	1.11E+02	0	8.54E+00	8.54	0	8.54E+00	8.54
b2589_v1_RNA_20_modif _1	b2589_v1_RNA modification (23S rRNA): position: 745, m1G by RrmA_dim	1 RrmA_dim + 1 amet + 1 b2589_v1_RNA_20 <==> 1 b2589_v1_RNA_20_RrmA_dim	reversible	rRNA modification	-1.00E+09	1.00E+09	0	1.11E+02	1.11E+02	0	8.54E+00	8.54	0	8.54E+00	8.54

b2589_v1_RNA_20_modif_2	b2589_v1_RNA modification (23S rRNA): position: 745, m1G by RrmA_dim	1 b2589_v1_RNA_20_RrmA_dim --> 1 RrmA_dim_inact + 1 ahcys + 1 b2589_v1_RNA_21 + 1 h	irreversible	rRNA modification	0.00E+00	1.00E+09	0	1.11E+02	1.11E+02	0	8.54E+00	8.54	0	8.54E+00	8.54
b2589_v1_RNA_21_modif_1	b2589_v1_RNA modification (23S rRNA): position: 746, Y by RluA_mono	1 RluA_mono + 1 b2589_v1_RNA_21 <==> 1 b2589_v1_RNA_21_RluA_mono	reversible	rRNA modification	-1.00E+09	1.00E+09	0	1.11E+02	1.11E+02	0	8.54E+00	8.54	0	8.54E+00	8.54
b2589_v1_RNA_21_modif_2	b2589_v1_RNA modification (23S rRNA): position: 746, Y by RluA_mono	1 b2589_v1_RNA_21_RluA_mono --> 1 RluA_mono_inact + 1 b2589_v1_RNA_22	irreversible	rRNA modification	0.00E+00	1.00E+09	0	1.11E+02	1.11E+02	0	8.54E+00	8.54	0	8.54E+00	8.54
b2589_v1_RNA_22_modif_1	b2589_v1_RNA modification (23S rRNA): position: 747, m5U by RumB_mono	1 RumB_mono + 1 amet + 1 b2589_v1_RNA_22 <==> 1 b2589_v1_RNA_22_RumB_mono	reversible	rRNA modification	-1.00E+09	1.00E+09	0	1.11E+02	1.11E+02	0	8.54E+00	8.54	0	8.54E+00	8.54
b2589_v1_RNA_22_modif_2	b2589_v1_RNA modification (23S rRNA): position: 747, m5U by RumB_mono	1 b2589_v1_RNA_22_RumB_mono --> 1 RumB_mono_inact + 1 ahcys + 1 b2589_v1_RNA_23 + 1 h	irreversible	rRNA modification	0.00E+00	1.00E+09	0	1.11E+02	1.11E+02	0	8.54E+00	8.54	0	8.54E+00	8.54
b2589_v1_RNA_23_modif_1	b2589_v1_RNA modification (23S rRNA): position: 955, Y by RluC_mono	1 RluC_mono + 1 b2589_v1_RNA_23 <==> 1 b2589_v1_RNA_23_RluC_mono	reversible	rRNA modification	-1.00E+09	1.00E+09	0	1.11E+02	1.11E+02	0	8.54E+00	8.54	0	8.54E+00	8.54
b2589_v1_RNA_23_modif_2	b2589_v1_RNA modification (23S rRNA): position: 955, Y by RluC_mono	1 b2589_v1_RNA_23_RluC_mono --> 1 RluC_mono_inact + 1 rrlG	irreversible	rRNA modification	0.00E+00	1.00E+09	0	1.11E+02	1.11E+02	0	8.54E+00	8.54	0	8.54E+00	8.54
b2589_v1_RNA_2_modif_1	b2589_v1_RNA modification (23S rRNA): position: 1911, Y by RluD_mono	1 RluD_mono + 1 b2589_v1_RNA_2 <==> 1 b2589_v1_RNA_2_RluD_mono	reversible	rRNA modification	-1.00E+09	1.00E+09	0	1.11E+02	1.11E+02	0	8.54E+00	8.54	0	8.54E+00	8.54
b2589_v1_RNA_2_modif_2	b2589_v1_RNA modification (23S rRNA): position: 1911, Y by RluD_mono	1 b2589_v1_RNA_2_RluD_mono --> 1 RluD_mono_inact + 1 b2589_v1_RNA_3	irreversible	rRNA modification	0.00E+00	1.00E+09	0	1.11E+02	1.11E+02	0	8.54E+00	8.54	0	8.54E+00	8.54
b2589_v1_RNA_3_modif_1	b2589_v1_RNA modification (23S rRNA): position: 1915, m3Y by RluD_mono	1 RluD_mono + 1 amet + 1 b2589_v1_RNA_3 <==> 1 b2589_v1_RNA_3_RluD_mono	reversible	rRNA modification	-1.00E+09	1.00E+09	0	1.11E+02	1.11E+02	0	8.54E+00	8.54	0	8.54E+00	8.54
b2589_v1_RNA_3_modif_2	b2589_v1_RNA modification (23S rRNA): position: 1915, m3Y by RluD_mono	1 b2589_v1_RNA_3_RluD_mono --> 1 RluD_mono_inact + 1 ahcys + 1 b2589_v1_RNA_4 + 1 h	irreversible	rRNA modification	0.00E+00	1.00E+09	0	1.11E+02	1.11E+02	0	8.54E+00	8.54	0	8.54E+00	8.54
b2589_v1_RNA_4_modif_1	b2589_v1_RNA modification (23S rRNA): position: 1917, Y by RluD_mono	1 RluD_mono + 1 b2589_v1_RNA_4 <==> 1 b2589_v1_RNA_4_RluD_mono	reversible	rRNA modification	-1.00E+09	1.00E+09	0	1.11E+02	1.11E+02	0	8.54E+00	8.54	0	8.54E+00	8.54
b2589_v1_RNA_4_modif_2	b2589_v1_RNA modification (23S rRNA): position: 1917, Y by RluD_mono	1 b2589_v1_RNA_4_RluD_mono --> 1 RluD_mono_inact + 1 b2589_v1_RNA_5	irreversible	rRNA modification	0.00E+00	1.00E+09	0	1.11E+02	1.11E+02	0	8.54E+00	8.54	0	8.54E+00	8.54
b2589_v1_RNA_5_modif_1	b2589_v1_RNA modification (23S rRNA): position: 1939, m5U by RumA_mono	1 RumA_mono + 1 amet + 1 b2589_v1_RNA_5 <==> 1 b2589_v1_RNA_5_RumA_mono	reversible	rRNA modification	-1.00E+09	1.00E+09	0	1.11E+02	1.11E+02	0	8.54E+00	8.54	0	8.54E+00	8.54
b2589_v1_RNA_5_modif_2	b2589_v1_RNA modification (23S rRNA): position: 1939, m5U by RumA_mono	1 b2589_v1_RNA_5_RumA_mono --> 1 RumA_mono_inact + 1 ahcy + 1 b2589_v1_RNA_6 + 1 h	irreversible	rRNA modification	0.00E+00	1.00E+09	0	1.11E+02	1.11E+02	0	8.54E+00	8.54	0	8.54E+00	8.54
b2589_v1_RNA_6_modif_1	b2589_v1_RNA modification (23S rRNA): position: 1962, m5C by MeT_23S_1962	1 MeT_23S_1962 + 1 amet + 1 b2589_v1_RNA_6 <==> 1 b2589_v1_RNA_6_MeT_23S_1962	reversible	rRNA modification	-1.00E+09	1.00E+09	0	1.11E+02	1.11E+02	0	8.54E+00	8.54	0	8.54E+00	8.54
b2589_v1_RNA_6_modif_2	b2589_v1_RNA modification (23S rRNA): position: 1962, m5C by MeT_23S_1962	1 b2589_v1_RNA_6_MeT_23S_1962 --> 1 MeT_23S_1962_inact + 1 ahcys + 1 b2589_v1_RNA_7 + 1 h	irreversible	rRNA modification	0.00E+00	1.00E+09	0	1.11E+02	1.11E+02	0	8.54E+00	8.54	0	8.54E+00	8.54
b2589_v1_RNA_7_modif_1	b2589_v1_RNA modification (23S rRNA): position: 2030, m6A by MeT_23S_2030	1 MeT_23S_2030 + 1 amet + 1 b2589_v1_RNA_7 <==> 1 b2589_v1_RNA_7_MeT_23S_2030	reversible	rRNA modification	-1.00E+09	1.00E+09	0	1.11E+02	1.11E+02	0	8.54E+00	8.54	0	8.54E+00	8.54
b2589_v1_RNA_7_modif_2	b2589_v1_RNA modification (23S rRNA): position: 2030, m6A by MeT_23S_2030	1 b2589_v1_RNA_7_MeT_23S_2030 --> 1 MeT_23S_2030_inact + 1 ahcys + 1 b2589_v1_RNA_8 + 1 h	irreversible	rRNA modification	0.00E+00	1.00E+09	0	1.11E+02	1.11E+02	0	8.54E+00	8.54	0	8.54E+00	8.54
b2589_v1_RNA_8_modif_1	b2589_v1_RNA modification (23S rRNA): position: 2069, m7G by MeT_23S_2069	1 MeT_23S_2069 + 1 amet + 1 b2589_v1_RNA_8 <==> 1 b2589_v1_RNA_8_MeT_23S_2069	reversible	rRNA modification	-1.00E+09	1.00E+09	0	1.11E+02	1.11E+02	0	8.54E+00	8.54	0	8.54E+00	8.54
b2589_v1_RNA_8_modif_2	b2589_v1_RNA modification (23S rRNA): position: 2069, m7G by MeT_23S_2069	1 b2589_v1_RNA_8_MeT_23S_2069 --> 1 MeT_23S_2069_inact + 1 ahcys + 1 b2589_v1_RNA_9 + 1 h	irreversible	rRNA modification	0.00E+00	1.00E+09	0	1.11E+02	1.11E+02	0	8.54E+00	8.54	0	8.54E+00	8.54
b2589_v1_RNA_9_modif_1	b2589_v1_RNA modification (23S rRNA): position: 2251, Gm by RlmB_dim	1 RlmB_dim + 1 amet + 1 b2589_v1_RNA_9 <==> 1 b2589_v1_RNA_9_RlmB_dim	reversible	rRNA modification	-1.00E+09	1.00E+09	0	1.11E+02	1.11E+02	0	8.54E+00	8.54	0	8.54E+00	8.54
b2589_v1_RNA_9_modif_2	b2589_v1_RNA modification (23S rRNA): position: 2251, Gm by RlmB_dim	1 b2589_v1_RNA_9_RlmB_dim --> 1 RlmB_dim_inact + 1 ahcys + 1 b2589_v1_RNA_10 + 1 h	irreversible	rRNA modification	0.00E+00	1.00E+09	0	1.11E+02	1.11E+02	0	8.54E+00	8.54	0	8.54E+00	8.54
b2589_v1_RNA_modif_1	b2589_v1_RNA modification (23S rRNA): position: 1618, m6A by MeT_23S_1618	1 MeT_23S_1618 + 1 amet + 1 b2589_v1_RNA <==> 1 b2589_v1_RNA_MeT_23S_1618	reversible	rRNA modification	-1.00E+09	1.00E+09	0	1.11E+02	1.11E+02	0	8.54E+00	8.54	0	8.54E+00	8.54
b2589_v1_RNA_modif_2	b2589_v1_RNA modification (23S rRNA): position: 1618, m6A by MeT_23S_1618	1 b2589_v1_RNA_MeT_23S_1618 --> 1 MeT_23S_1618_inact + 1 ahcys + 1 b2589_v1_RNA_1 + 1 h	irreversible	rRNA modification	0.00E+00	1.00E+09	0	1.11E+02	1.11E+02	0	8.54E+00	8.54	0	8.54E+00	8.54
b2590_tRNA_13_MODIF_1	modification of position 13 nt (Y) in gltW by TruD_mono	1 TruD_mono + 1 b2590_tRNA_Mg2 <==> 1 b2590_tRNA_Mg2_TruD_mono_cplx	reversible	tRNA modification	-1.00E+09	1.00E+09	0	1.11E+02	1.11E+02	0	0	0	0	0	0
b2590_tRNA_13_MODIF_2	modification of position 13 nt (Y) in gltW by TruD_mono	1 b2590_tRNA_Mg2_TruD_mono_cplx --> 1 TruD_mono_inact + 1 b2590_tRNA_1	irreversible	tRNA modification	0.00E+00	1.00E+09	0	1.11E+02	1.11E+02	0	0	0	0	0	0
b2590_tRNA_34_MODIF_1	modification of position 34 nt (mnm5s2U) in gltW by TrmU_mono-YhhP_mono-YheLMN_cplx-YccK_mono-TrmE_dim-GidA_mono-TrmC_mono	1 5fthf + 1 GidA_mono + 1 IscS_dim_S-SH + 1 TrmC_mono + 1 TrmE_dim + 1 TrmU_mono + 1 YccK_mono + 1 YheLMN_cplx + 1 YhhP_mono + 1 amet + 1 atp + 1 b2590_tRNA_1 + 1 fad + 1 fadh2 + 1 gly + 1 gtp + 1 h2o + 1 mg2 + 1 trdrd <==> 1 b2590_tRNA_1_TrmU_mono-YhhP_mono-YheLMN_cplx-YccK_mono-TrmE_dim-GidA_mono-TrmC_mono_cplx	reversible	tRNA modification	-1.00E+09	1.00E+09	0	1.11E+02	1.11E+02	0	0	0	0	0	0
b2590_tRNA_34_MODIF_2	modification of position 34 nt (mnm5s2U) in gltW by TrmU_mono-YhhP_mono-YheLMN_cplx-YccK_mono-TrmE_dim-GidA_mono-TrmC_mono	1 b2590_tRNA_1_TrmU_mono-YhhP_mono-YheLMN_cplx-YccK_mono-TrmE_dim-GidA_mono-TrmC_mono_cplx --> 1 GidA_mono_inact + 1 IscS_dim_S-H + 1 TrmC_mono_inact + 1 TrmE_dim_inact + 1 TrmU_mono_inact + 1 YccK_mono_inact + 1 YheLMN_cplx_inact + 1 YhhP_mono_inact + 1 ahcys + 1 amp + 1 b2590_tRNA_2 + 1 fad + 1 fadh2 + 1 gdp + 1 glx + 4 h + 1 mg2 + 1 pi + 1 ppi + 1 thf + 1 trdox	irreversible	tRNA modification	0.00E+00	1.00E+09	0	1.11E+02	1.11E+02	0	0	0	0	0	0
b2590_tRNA_34_MODIF_3	replacement of sulfur with selenium: b2590_tRNA_2	1 YbbB_dim + 1 b2590_tRNA_2 + 1 h2o + 1 selnp <==> 1 b2590_tRNA_2_YbbB_dim_cplx	reversible	tRNA modification	-1.00E+09	1.00E+09	0	1.11E+02	1.11E+02	0	0	0	0	0	0
b2590_tRNA_34_MODIF_4	replacement of sulfur with selenium: b2590_tRNA_2	1 b2590_tRNA_2_YbbB_dim_cplx --> 1 YbbB_dim_inact + 1 b2590_tRNA_2_Se + 1 h + 1 h2s + 1 pi	irreversible	tRNA modification	0.00E+00	1.00E+09	0	1.11E+02	1.11E+02	0	0	0	0	0	0
b2590_tRNA_37_MODIF_1	modification of position 37 nt (m2A) in gltW by MeT_tRNA_pos_37_m2A	1 MeT_tRNA_pos_37_m2A + 1 amet + 1 b2590_tRNA_2 <==> 1 b2590_tRNA_2_MeT_tRNA_pos_37_m2A_cplx	reversible	tRNA modification	-1.00E+09	1.00E+09	0	1.11E+02	1.11E+02	0	0	0	0	0	0
b2590_tRNA_37_MODIF_1_Se	modification of position 37 nt (m2A) in gltW by MeT_tRNA_pos_37_m2A	1 MeT_tRNA_pos_37_m2A + 1 amet + 1 b2590_tRNA_2_Se <==> 1 b2590_tRNA_2_Se_MeT_tRNA_pos_37_m2A_cplx	reversible	tRNA modification	-1.00E+09	1.00E+09	0	1.11E+02	1.11E+02	0	0	0	0	0	0
b2590_tRNA_37_MODIF_2	modification of position 37 nt (m2A) in gltW by MeT_tRNA_pos_37_m2A	1 b2590_tRNA_2_MeT_tRNA_pos_37_m2A_cplx --> 1 MeT_tRNA_pos_37_m2A_inact + 1 ahcys + 1 b2590_tRNA_3 + 1 h	irreversible	tRNA modification	0.00E+00	1.00E+09	0	1.11E+02	1.11E+02	0	0	0	0	0	0
b2590_tRNA_37_MODIF_2_Se	modification of position 37 nt (m2A) in gltW by MeT_tRNA_pos_37_m2A	1 b2590_tRNA_2_Se_MeT_tRNA_pos_37_m2A_cplx --> 1 MeT_tRNA_pos_37_m2A_inact + 1 ahcys + 1 b2590_tRNA_3_Se + h	irreversible	tRNA modification	0.00E+00	1.00E+09	0	1.11E+02	1.11E+02	0	0	0	0	0	0
b2590_tRNA_54_MODIF_1	modification of position 54 nt (m5U) in gltW by TrmA_mono	1 TrmA_mono + 1 amet + 1 b2590_tRNA_3 <==> 1 b2590_tRNA_3_TrmA_mono_cplx	reversible	tRNA modification	-1.00E+09	1.00E+09	0	1.11E+02	1.11E+02	0	0	0	0	0	0
b2590_tRNA_54_MODIF_1_Se	modification of position 54 nt (m5U) in gltW by TrmA_mono	1 TrmA_mono + 1 amet + 1 b2590_tRNA_3_Se <==> 1 b2590_tRNA_3_Se_TrmA_mono_cplx	reversible	tRNA modification	-1.00E+09	1.00E+09	0	1.11E+02	1.11E+02	0	0	0	0	0	0
b2590_tRNA_54_MODIF_2	modification of position 54 nt (m5U) in gltW by TrmA_mono	1 b2590_tRNA_3_TrmA_mono_cplx --> 1 TrmA_mono_inact + 1 ahcys + 1 b2590_tRNA_4 + 1 h	irreversible	tRNA modification	0.00E+00	1.00E+09	0	1.11E+02	1.11E+02	0	0	0	0	0	0

b2590_tRNA_54_MODIF_2_Se	modification of position 54 nt (m5U) in gltW by TrmA_mono	1 b2590_tRNA_3_Se_TrmA_mono_cplx --> 1 TrmA_mono_inact + 1 ahcys + 1 b2590_tRNA_4_Se + 1 h	irreversible	tRNA modification	0.00E+00	1.00E+09	0	1.11E+02	1.11E+02	0	0	0	0	0	0
b2590_tRNA_55_MODIF_1	modification of position 55 nt (Y) in gltW by TruB_mono	1 TruB_mono + 1 b2590_tRNA_4 <==> 1 b2590_tRNA_4_TruB_mono_cplx	reversible	tRNA modification	-1.00E+09	1.00E+09	0	1.11E+02	1.11E+02	0	0	0	0	0	0
b2590_tRNA_55_MODIF_1_Se	modification of position 55 nt (Y) in gltW by TruB_mono	1 TruB_mono + 1 b2590_tRNA_4_Se <==> 1 b2590_tRNA_4_Se_TruB_mono_cplx	reversible	tRNA modification	-1.00E+09	1.00E+09	0	1.11E+02	1.11E+02	0	0	0	0	0	0
b2590_tRNA_55_MODIF_2	modification of position 55 nt (Y) in gltW by TruB_mono	1 b2590_tRNA_4_TruB_mono_cplx --> 1 TruB_mono_inact + 1 gltW_tRNA	irreversible	tRNA modification	0.00E+00	1.00E+09	0	1.11E+02	1.11E+02	0	0	0	0	0	0
b2590_tRNA_55_MODIF_2_Se	modification of position 55 nt (Y) in gltW by TruB_mono	1 b2590_tRNA_4_Se_TruB_mono_cplx --> 1 TruB_mono_inact + 1 gltW_tRNA_Se	irreversible	tRNA modification	0.00E+00	1.00E+09	0	1.11E+02	1.11E+02	0	0	0	0	0	0
b2590_tRNA_Mg_BIND	Binding of two Mg2 to b2590_tRNA	1 b2590_RNA + 2 mg2 <==> 1 b2590_tRNA_Mg2	reversible	Metallo-ion binding	-1.00E+09	1.00E+09	0	1.11E+02	1.11E+02	0	0	0	0	0	0
b2590_v1_tRNA_13_MODIF_1	modification of position 13 nt (Y) in gltW by TruD_mono	1 TruD_mono + 1 b2590_v1_tRNA_Mg2 <==> 1 b2590_v1_tRNA_Mg2_TruD_mono_cplx	reversible	tRNA modification	-1.00E+09	1.00E+09	0	1.11E+02	1.11E+02	0	8.54E+00	8.54	0	8.54E+00	8.54
b2590_v1_tRNA_13_MODIF_2	modification of position 13 nt (Y) in gltW by TruD_mono	1 b2590_v1_tRNA_Mg2_TruD_mono_cplx --> 1 TruD_mono_inact + b2590_v1_tRNA_1	irreversible	tRNA modification	0.00E+00	1.00E+09	0	1.11E+02	1.11E+02	0	8.54E+00	8.54	0	8.54E+00	8.54
b2590_v1_tRNA_34_MODIF_1	modification of position 34 nt (mnm5s2U) in gltW by TrmU_mono-YhhP_mono-YheLMN_cplx-YccK_mono-TrmE_dim-GidA_mono-TrmC_mono	1 5fthf + 1 GidA_mono + 1 IscS_dim_S-SH + 1 TrmC_mono + 1 TrmE_dim + 1 TrmU_mono + 1 YccK_mono + 1 YheLMN_cplx + 1 YhhP_mono + 1 amet + 1 atp + 1 b2590_v1_tRNA_1 + 1 fad + 1 fadh2 + 1 gly + 1 gtp + 1 h2o + 1 mg2 + 1 trdrd <==> 1 b2590_v1_tRNA_1_TrmU_mono-YhhP_mono-YheLMN_cplx-YccK_mono-TrmE_dim-GidA_mono-TrmC_mono_cplx	reversible	tRNA modification	-1.00E+09	1.00E+09	0	1.11E+02	1.11E+02	0	8.54E+00	8.54	0	8.54E+00	8.54
b2590_v1_tRNA_34_MODIF_2	modification of position 34 nt (mnm5s2U) in gltW by TrmU_mono-YhhP_mono-YheLMN_cplx-YccK_mono-TrmE_dim-GidA_mono-TrmC_mono	1 b2590_v1_tRNA_1_TrmU_mono-YhhP_mono-YheLMN_cplx-YccK_mono-TrmE_dim-GidA_mono-TrmC_mono_cplx --> 1 GidA_mono_inact + 1 IscS_dim_S-H + 1 TrmC_mono_inact + 1 TrmE_dim_inact + 1 TrmU_mono_inact + 1 YccK_mono_inact + 1 YheLMN_cplx_inact + 1 YhhP_mono_inact + 1 ahcys + 1 amp + 1 b2590_v1_tRNA_2 + 1 fad + 1 fadh2 + 1 gdp + 1 glx + 4 h + 1 mg2 + 1 pi + 1 ppi + 1 thf + 1 trdox	irreversible	tRNA modification	0.00E+00	1.00E+09	0	1.11E+02	1.11E+02	0	8.54E+00	8.54	0	8.54E+00	8.54
b2590_v1_tRNA_34_MODIF_3	replacement of sulfur with selenium: b2590_tRNA_2	1 YbbB_dim + 1 b2590_v1_tRNA_2 + 1 h2o + 1 selnp <==> 1 b2590_v1_tRNA_2_YbbB_dim_cplx	reversible	tRNA modification	-1.00E+09	1.00E+09	0	1.11E+02	1.11E+02	0	8.54E+00	8.54	0	8.54E+00	8.54
b2590_v1_tRNA_34_MODIF_4	replacement of sulfur with selenium: b2590_tRNA_2	1 b2590_v1_tRNA_2_YbbB_dim_cplx --> 1 YbbB_dim_inact + 1 b2590_v1_tRNA_2_Se + 1 h + 1 h2s + 1 pi	irreversible	tRNA modification	0.00E+00	1.00E+09	0	1.11E+02	1.11E+02	0	8.54E+00	8.54	0	8.54E+00	8.54
b2590_v1_tRNA_37_MODIF_1	modification of position 37 nt (m2A) in gltW by MeT_tRNA_pos_37_m2A	1 MeT_tRNA_pos_37_m2A + 1 amet + 1 b2590_v1_tRNA_2 <==> 1 b2590_v1_tRNA_2_MeT_tRNA_pos_37_m2A_cplx	reversible	tRNA modification	-1.00E+09	1.00E+09	0	1.11E+02	1.11E+02	0	8.54E+00	8.54	0	8.54E+00	8.54
b2590_v1_tRNA_37_MODIF_1_Se	modification of position 37 nt (m2A) in gltW by MeT_tRNA_pos_37_m2A	1 MeT_tRNA_pos_37_m2A + 1 amet + 1 b2590_v1_tRNA_2_Se <==> 1 b2590_v1_tRNA_2_Se_MeT_tRNA_pos_37_m2A_cplx	reversible	tRNA modification	-1.00E+09	1.00E+09	0	1.11E+02	1.11E+02	0	8.54E+00	8.54	0	8.54E+00	8.54
b2590_v1_tRNA_37_MODIF_2	modification of position 37 nt (m2A) in gltW by MeT_tRNA_pos_37_m2A	1 b2590_v1_tRNA_2_MeT_tRNA_pos_37_m2A_cplx --> 1 MeT_tRNA_pos_37_m2A_inact + 1 ahcys + 1 b2590_v1_tRNA_3 + 1 h	irreversible	tRNA modification	0.00E+00	1.00E+09	0	1.11E+02	1.11E+02	0	8.54E+00	8.54	0	8.54E+00	8.54
b2590_v1_tRNA_37_MODIF_2_Se	modification of position 37 nt (m2A) in gltW by MeT_tRNA_pos_37_m2A	1 b2590_v1_tRNA_2_Se_MeT_tRNA_pos_37_m2A_cplx --> 1 MeT_tRNA_pos_37_m2A_inact + 1 ahcys + 1 b2590_v1_tRNA_3_Se + 1 h	irreversible	tRNA modification	0.00E+00	1.00E+09	0	1.11E+02	1.11E+02	0	8.54E+00	8.54	0	8.54E+00	8.54
b2590_v1_tRNA_54_MODIF_1	modification of position 54 nt (m5U) in gltW by TrmA_mono	1 TrmA_mono + 1 amet + 1 b2590_v1_tRNA_3 <==> 1 b2590_v1_tRNA_3_TrmA_mono_cplx	reversible	tRNA modification	-1.00E+09	1.00E+09	0	1.11E+02	1.11E+02	0	8.54E+00	8.54	0	8.54E+00	8.54
b2590_v1_tRNA_54_MODIF_1_Se	modification of position 54 nt (m5U) in gltW by TrmA_mono	1 TrmA_mono + 1 amet + 1 b2590_v1_tRNA_3_Se <==> 1 b2590_v1_tRNA_3_Se_TrmA_mono_cplx	reversible	tRNA modification	-1.00E+09	1.00E+09	0	1.11E+02	1.11E+02	0	8.54E+00	8.54	0	8.54E+00	8.54
b2590_v1_tRNA_54_MODIF_2	modification of position 54 nt (m5U) in gltW by TrmA_mono	1 b2590_v1_tRNA_3_TrmA_mono_cplx --> 1 TrmA_mono_inact + 1 ahcys + 1 b2590_v1_tRNA_4 + 1 h	irreversible	tRNA modification	0.00E+00	1.00E+09	0	1.11E+02	1.11E+02	0	8.54E+00	8.54	0	8.54E+00	8.54
b2590_v1_tRNA_54_MODIF_2_Se	modification of position 54 nt (m5U) in gltW by TrmA_mono	1 b2590_v1_tRNA_3_Se_TrmA_mono_cplx --> 1 TrmA_mono_inact 1 ahcys + 1 b2590_v1_tRNA_4_Se + 1 h	irreversible	tRNA modification	0.00E+00	1.00E+09	0	1.11E+02	1.11E+02	0	8.54E+00	8.54	0	8.54E+00	8.54
b2590_v1_tRNA_55_MODIF_1	modification of position 55 nt (Y) in gltW by TruB_mono	1 TruB_mono + 1 b2590_v1_tRNA_4 <==> 1 b2590_v1_tRNA_4_TruB_mono_cplx	reversible	tRNA modification	-1.00E+09	1.00E+09	0	1.11E+02	1.11E+02	0	8.54E+00	8.54	0	8.54E+00	8.54
b2590_v1_tRNA_55_MODIF_1_Se	modification of position 55 nt (Y) in gltW by TruB_mono	1 TruB_mono + 1 b2590_v1_tRNA_4_Se <==> 1 b2590_v1_tRNA_4_Se_TruB_mono_cplx	reversible	tRNA modification	-1.00E+09	1.00E+09	0	1.11E+02	1.11E+02	0	8.54E+00	8.54	0	8.54E+00	8.54
b2590_v1_tRNA_55_MODIF_2	modification of position 55 nt (Y) in gltW by TruB_mono	1 b2590_v1_tRNA_4_TruB_mono_cplx --> 1 TruB_mono_inact + 1 gltW_tRNA	irreversible	tRNA modification	0.00E+00	1.00E+09	0	1.11E+02	1.11E+02	0	8.54E+00	8.54	0	8.54E+00	8.54
b2590_v1_tRNA_55_MODIF_2_Se	modification of position 55 nt (Y) in gltW by TruB_mono	1 b2590_v1_tRNA_4_Se_TruB_mono_cplx --> 1 TruB_mono_inact + 1 gltW_tRNA_Se	irreversible	tRNA modification	0.00E+00	1.00E+09	0	1.11E+02	1.11E+02	0	8.54E+00	8.54	0	8.54E+00	8.54
b2590_v1_tRNA_Mg_BIND	Binding of two Mg2 to b2590_v1_tRNA	1 b2590_v1_RNA + 2 mg2 <==> 1 b2590_v1_tRNA_Mg2	reversible	Metallo-ion binding	-1.00E+09	1.00E+09	0	1.11E+02	1.11E+02	0	8.54E+00	8.54	0	8.54E+00	8.54
b2591_RNA_10_modif_1	b2591_RNA modification (16S rRNA): position: 967, m5C by RsmB_mono	1 RsmB_mono + 1 amet + 1 b2591_RNA_10 <==> 1 b2591_RNA_10_RsmB_mono	reversible	rRNA modification	-1.00E+09	1.00E+09	0	1.11E+02	1.11E+02	0	0	0	0	0	0
b2591_RNA_10_modif_2	b2591_RNA modification (16S rRNA): position: 967, m5C by RsmB_mono	1 b2591_RNA_10_RsmB_mono --> 1 RsmB_mono_inact + 1 ahcys + 1 h + 1 rrsG	irreversible	rRNA modification	0.00E+00	1.00E+09	0	1.11E+02	1.11E+02	0	0	0	0	0	0
b2591_RNA_1_modif_1	b2591_RNA modification (16S rRNA): position: 1402, m4Cm by MeT_16S_1402	1 MeT_16S_1402 + 2 amet + 1 b2591_RNA_1 <==> 1 b2591_RNA_1_MeT_16S_1402	reversible	rRNA modification	-1.00E+09	1.00E+09	0	1.11E+02	1.11E+02	0	0	0	0	0	0
b2591_RNA_1_modif_2	b2591_RNA modification (16S rRNA): position: 1402, m4Cm by MeT_16S_1402	1 b2591_RNA_1_MeT_16S_1402 --> 1 MeT_16S_1402_inact + 2 ahcys + 1 b2591_RNA_2 + 2 h	irreversible	rRNA modification	0.00E+00	1.00E+09	0	1.11E+02	1.11E+02	0	0	0	0	0	0
b2591_RNA_2_modif_1	b2591_RNA modification (16S rRNA): position: 1407, m5C by MeT_16S_1407	1 MeT_16S_1407 + 1 amet + 1 b2591_RNA_2 <==> 1 b2591_RNA_2_MeT_16S_1407	reversible	rRNA modification	-1.00E+09	1.00E+09	0	1.11E+02	1.11E+02	0	0	0	0	0	0
b2591_RNA_2_modif_2	b2591_RNA modification (16S rRNA): position: 1407, m5C by MeT_16S_1407	1 b2591_RNA_2_MeT_16S_1407 --> 1 MeT_16S_1407_inact + 1 ahcys + 1 b2591_RNA_3 + 1 h	irreversible	rRNA modification	0.00E+00	1.00E+09	0	1.11E+02	1.11E+02	0	0	0	0	0	0
b2591_RNA_3_modif_1	b2591_RNA modification (16S rRNA): position: 1498, m3U by YggJ_mono	1 YggJ_mono + 1 amet + 1 b2591_RNA_3 <==> 1 b2591_RNA_3_YggJ_mono	reversible	rRNA modification	-1.00E+09	1.00E+09	0	1.11E+02	1.11E+02	0	0	0	0	0	0
b2591_RNA_3_modif_2	b2591_RNA modification (16S rRNA): position: 1498, m3U by YggJ_mono	1 b2591_RNA_3_YggJ_mono --> 1 YggJ_mono_inact + 1 ahcys + 1 b2591_RNA_4 + 1 h	irreversible	rRNA modification	0.00E+00	1.00E+09	0	1.11E+02	1.11E+02	0	0	0	0	0	0
b2591_RNA_4_modif_1	b2591_RNA modification (16S rRNA): position: 1516, m2G by MeT_16S_1516	1 MeT_16S_1516 + 1 amet + 1 b2591_RNA_4 <==> 1 b2591_RNA_4_MeT_16S_1516	reversible	rRNA modification	-1.00E+09	1.00E+09	0	1.11E+02	1.11E+02	0	0	0	0	0	0
b2591_RNA_4_modif_2	b2591_RNA modification (16S rRNA): position: 1516, m2G by MeT_16S_1516	1 b2591_RNA_4_MeT_16S_1516 --> 1 MeT_16S_1516_inact + 1 ahcys + 1 b2591_RNA_5 + 1 h	irreversible	rRNA modification	0.00E+00	1.00E+09	0	1.11E+02	1.11E+02	0	0	0	0	0	0
b2591_RNA_5_modif_1	b2591_RNA modification (16S rRNA): position: 1518, m6(2)A by KsgA_mono	1 KsgA_mono + 2 amet + 1 b2591_RNA_5 <==> 1 b2591_RNA_5_KsgA_mono	reversible	rRNA modification	-1.00E+09	1.00E+09	0	1.11E+02	1.11E+02	0	0	0	0	0	0
b2591_RNA_5_modif_2	b2591_RNA modification (16S rRNA): position: 1518, m6(2)A by KsgA_mono	1 b2591_RNA_5_KsgA_mono --> 1 KsgA_mono_inact + 2 ahcys + 1 b2591_RNA_6 + 2 h	irreversible	rRNA modification	0.00E+00	1.00E+09	0	1.11E+02	1.11E+02	0	0	0	0	0	0
b2591_RNA_6_modif_1	b2591_RNA modification (16S rRNA): position: 1519, m6(2)A by KsgA_mono	1 KsgA_mono + 2 amet + 1 b2591_RNA_6 <==> 1 b2591_RNA_6_KsgA_mono	reversible	rRNA modification	-1.00E+09	1.00E+09	0	1.11E+02	1.11E+02	0	0	0	0	0	0
b2591_RNA_6_modif_2	b2591_RNA modification (16S rRNA): position: 1519, m6(2)A by KsgA_mono	1 b2591_RNA_6_KsgA_mono --> 1 KsgA_mono_inact + 2 ahcys + 1 b2591_RNA_7 + 2 h	irreversible	rRNA modification	0.00E+00	1.00E+09	0	1.11E+02	1.11E+02	0	0	0	0	0	0
b2591_RNA_7_modif_1	b2591_RNA modification (16S rRNA): position: 516, Y by RsuA_mono	1 RsuA_mono + 1 b2591_RNA_7 <==> 1 b2591_RNA_7_RsuA_mono	reversible	rRNA modification	-1.00E+09	1.00E+09	0	1.11E+02	1.11E+02	0	0	0	0	0	0
b2591_RNA_7_modif_2	b2591_RNA modification (16S rRNA): position: 516, Y by RsuA_mono	1 b2591_RNA_7_RsuA_mono --> 1 RsuA_mono_inact + 1 b2591_RNA_8	irreversible	rRNA modification	0.00E+00	1.00E+09	0	1.11E+02	1.11E+02	0	0	0	0	0	0

b2591_RNA_8_modif_1	b2591_RNA modification (16S rRNA): position: 527, m7G by MeT_16S_527	1 MeT_16S_527 + 1 amet + 1 b2591_RNA_8 <==> 1 b2591_RNA_8_MeT_16S_527	reversible	rRNA modification	-1.00E+09	1.00E+09	0	1.11E+02	1.11E+02	0	0	0	0	0	0
b2591_RNA_8_modif_2	b2591_RNA modification (16S rRNA): position: 527, m7G by MeT_16S_527	1 b2591_RNA_8_MeT_16S_527 --> 1 MeT_16S_527_inact + 1 ahcy + 1 b2591_RNA_9 + 1 h	irreversible	rRNA modification	0.00E+00	1.00E+09	0	1.11E+02	1.11E+02	0	0	0	0	0	0
b2591_RNA_9_modif_1	b2591_RNA modification (16S rRNA): position: 966, m2G by MeT_16S_966	1 MeT_16S_966 + 1 amet + 1 b2591_RNA_9 <==> 1 b2591_RNA_9_MeT_16S_966	reversible	rRNA modification	-1.00E+09	1.00E+09	0	1.11E+02	1.11E+02	0	0	0	0	0	0
b2591_RNA_9_modif_2	b2591_RNA modification (16S rRNA): position: 966, m2G by MeT_16S_966	1 b2591_RNA_9_MeT_16S_966 --> 1 MeT_16S_966_inact + 1 ahcy + 1 b2591_RNA_10 + 1 h	irreversible	rRNA modification	0.00E+00	1.00E+09	0	1.11E+02	1.11E+02	0	0	0	0	0	0
b2591_RNA_modif_1	b2591_RNA modification (16S rRNA): position: 1207, m2G by RsmC_mono	1 RsmC_mono + 1 amet + 1 b2591_RNA <==> 1 b2591_RNA_RsmC_mono	reversible	rRNA modification	-1.00E+09	1.00E+09	0	1.11E+02	1.11E+02	0	0	0	0	0	0
b2591_RNA_modif_2	b2591_RNA modification (16S rRNA): position: 1207, m2G by RsmC_mono	1 b2591_RNA_RsmC_mono --> 1 RsmC_mono_inact + 1 ahcys + 1 b2591_RNA_1 + 1 h	irreversible	rRNA modification	0.00E+00	1.00E+09	0	1.11E+02	1.11E+02	0	0	0	0	0	0
b2591_v1_RNA_10_modif_1	b2591_v1_RNA modification (16S rRNA): position: 967, m5C by RsmB_mono	1 RsmB_mono + 1 amet + 1 b2591_v1_RNA_10 <==> 1 b2591_v1_RNA_10_RsmB_mono	reversible	rRNA modification	-1.00E+09	1.00E+09	0	1.11E+02	1.11E+02	0	8.54E+00	8.54	0	8.54E+00	8.54
b2591_v1_RNA_10_modif_2	b2591_v1_RNA modification (16S rRNA): position: 967, m5C by RsmB_mono	1 b2591_v1_RNA_10_RsmB_mono --> 1 RsmB_mono_inact + 1 ahcys + 1 h + 1 rrsG	irreversible	rRNA modification	0.00E+00	1.00E+09	0	1.11E+02	1.11E+02	0	8.54E+00	8.54	0	8.54E+00	8.54
b2591_v1_RNA_1_modif_1	b2591_v1_RNA modification (16S rRNA): position: 1402, m4Cm by MeT_16S_1402	1 MeT_16S_1402 + 2 amet + 1 b2591_v1_RNA_1 <==> 1 b2591_v1_RNA_1_MeT_16S_1402	reversible	rRNA modification	-1.00E+09	1.00E+09	0	1.11E+02	1.11E+02	0	8.54E+00	8.54	0	8.54E+00	8.54
b2591_v1_RNA_1_modif_2	b2591_v1_RNA modification (16S rRNA): position: 1402, m4Cm by MeT_16S_1402	1 b2591_v1_RNA_1_MeT_16S_1402 --> 1 MeT_16S_1402_inact + 2 ahcys + 1 b2591_v1_RNA_2 + 2 h	irreversible	rRNA modification	0.00E+00	1.00E+09	0	1.11E+02	1.11E+02	0	8.54E+00	8.54	0	8.54E+00	8.54
b2591_v1_RNA_2_modif_1	b2591_v1_RNA modification (16S rRNA): position: 1407, m5C by MeT_16S_1407	1 MeT_16S_1407 + 1 amet + 1 b2591_v1_RNA_2 <==> 1 b2591_v1_RNA_2_MeT_16S_1407	reversible	rRNA modification	-1.00E+09	1.00E+09	0	1.11E+02	1.11E+02	0	8.54E+00	8.54	0	8.54E+00	8.54
b2591_v1_RNA_2_modif_2	b2591_v1_RNA modification (16S rRNA): position: 1407, m5C by MeT_16S_1407	1 b2591_v1_RNA_2_MeT_16S_1407 --> 1 MeT_16S_1407_inact + 1 ahcys + 1 b2591_v1_RNA_3 + 1 h	irreversible	rRNA modification	0.00E+00	1.00E+09	0	1.11E+02	1.11E+02	0	8.54E+00	8.54	0	8.54E+00	8.54
b2591_v1_RNA_3_modif_1	b2591_v1_RNA modification (16S rRNA): position: 1498, m3U by YggJ_mono	1 YggJ_mono + 1 amet + 1 b2591_v1_RNA_3 <==> 1 b2591_v1_RNA_3_YggJ_mono	reversible	rRNA modification	-1.00E+09	1.00E+09	0	1.11E+02	1.11E+02	0	8.54E+00	8.54	0	8.54E+00	8.54
b2591_v1_RNA_3_modif_2	b2591_v1_RNA modification (16S rRNA): position: 1498, m3U by YggJ_mono	1 b2591_v1_RNA_3_YggJ_mono --> 1 YggJ_mono_inact + 1 ahcys 1 b2591_v1_RNA_4 + 1 h	irreversible	rRNA modification	0.00E+00	1.00E+09	0	1.11E+02	1.11E+02	0	8.54E+00	8.54	0	8.54E+00	8.54
b2591_v1_RNA_4_modif_1	b2591_v1_RNA modification (16S rRNA): position: 1516, m2G by MeT_16S_1516	1 MeT_16S_1516 + 1 amet + 1 b2591_v1_RNA_4 <==> 1 b2591_v1_RNA_4_MeT_16S_1516	reversible	rRNA modification	-1.00E+09	1.00E+09	0	1.11E+02	1.11E+02	0	8.54E+00	8.54	0	8.54E+00	8.54
b2591_v1_RNA_4_modif_2	b2591_v1_RNA modification (16S rRNA): position: 1516, m2G by MeT_16S_1516	1 b2591_v1_RNA_4_MeT_16S_1516 --> 1 MeT_16S_1516_inact + 1 ahcys + 1 b2591_v1_RNA_5 + 1 h	irreversible	rRNA modification	0.00E+00	1.00E+09	0	1.11E+02	1.11E+02	0	8.54E+00	8.54	0	8.54E+00	8.54
b2591_v1_RNA_5_modif_1	b2591_v1_RNA modification (16S rRNA): position: 1518, m6(2)A by KsgA_mono	1 KsgA_mono + 2 amet + 1 b2591_v1_RNA_5 <==> 1 b2591_v1_RNA_5_KsgA_mono	reversible	rRNA modification	-1.00E+09	1.00E+09	0	1.11E+02	1.11E+02	0	8.54E+00	8.54	0	8.54E+00	8.54
b2591_v1_RNA_5_modif_2	b2591_v1_RNA modification (16S rRNA): position: 1518, m6(2)A by KsgA_mono	1 b2591_v1_RNA_5_KsgA_mono --> 1 KsgA_mono_inact + 2 ahcys + 1 b2591_v1_RNA_6 + 2 h	irreversible	rRNA modification	0.00E+00	1.00E+09	0	1.11E+02	1.11E+02	0	8.54E+00	8.54	0	8.54E+00	8.54
b2591_v1_RNA_6_modif_1	b2591_v1_RNA modification (16S rRNA): position: 1519, m6(2)A by KsgA_mono	1 KsgA_mono + 2 amet + 1 b2591_v1_RNA_6 <==> 1 b2591_v1_RNA_6_KsgA_mono	reversible	rRNA modification	-1.00E+09	1.00E+09	0	1.11E+02	1.11E+02	0	8.54E+00	8.54	0	8.54E+00	8.54
b2591_v1_RNA_6_modif_2	b2591_v1_RNA modification (16S rRNA): position: 1519, m6(2)A by KsgA_mono	1 b2591_v1_RNA_6_KsgA_mono --> 1 KsgA_mono_inact + 2 ahcys + 1 b2591_v1_RNA_7 + 2 h	irreversible	rRNA modification	0.00E+00	1.00E+09	0	1.11E+02	1.11E+02	0	8.54E+00	8.54	0	8.54E+00	8.54
b2591_v1_RNA_7_modif_1	b2591_v1_RNA modification (16S rRNA): position: 516, Y by RsuA_mono	1 RsuA_mono + 1 b2591_v1_RNA_7 <==> 1 b2591_v1_RNA_7_RsuA_mono	reversible	rRNA modification	-1.00E+09	1.00E+09	0	1.11E+02	1.11E+02	0	8.54E+00	8.54	0	8.54E+00	8.54
b2591_v1_RNA_7_modif_2	b2591_v1_RNA modification (16S rRNA): position: 516, Y by RsuA_mono	1 b2591_v1_RNA_7_RsuA_mono --> 1 RsuA_mono_inact + 1 b2591_v1_RNA_8	irreversible	rRNA modification	0.00E+00	1.00E+09	0	1.11E+02	1.11E+02	0	8.54E+00	8.54	0	8.54E+00	8.54
b2591_v1_RNA_8_modif_1	b2591_v1_RNA modification (16S rRNA): position: 527, m7G by MeT_16S_527	1 MeT_16S_527 + 1 amet + 1 b2591_v1_RNA_8 <==> 1 b2591_v1_RNA_8_MeT_16S_527	reversible	rRNA modification	-1.00E+09	1.00E+09	0	1.11E+02	1.11E+02	0	8.54E+00	8.54	0	8.54E+00	8.54
b2591_v1_RNA_8_modif_2	b2591_v1_RNA modification (16S rRNA): position: 527, m7G by MeT_16S_527	1 b2591_v1_RNA_8_MeT_16S_527 --> 1 MeT_16S_527_inact + 1 ahcys + 1 b2591_v1_RNA_9 + 1 h	irreversible	rRNA modification	0.00E+00	1.00E+09	0	1.11E+02	1.11E+02	0	8.54E+00	8.54	0	8.54E+00	8.54
b2591_v1_RNA_9_modif_1	b2591_v1_RNA modification (16S rRNA): position: 966, m2G by MeT_16S_966	1 MeT_16S_966 + 1 amet + 1 b2591_v1_RNA_9 <==> 1 b2591_v1_RNA_9_MeT_16S_966	reversible	rRNA modification	-1.00E+09	1.00E+09	0	1.11E+02	1.11E+02	0	8.54E+00	8.54	0	8.54E+00	8.54
b2591_v1_RNA_9_modif_2	b2591_v1_RNA modification (16S rRNA): position: 966, m2G by MeT_16S_966	1 b2591_v1_RNA_9_MeT_16S_966 --> 1 MeT_16S_966_inact + 1 ahcys + 1 b2591_v1_RNA_10 + 1 h	irreversible	rRNA modification	0.00E+00	1.00E+09	0	1.11E+02	1.11E+02	0	8.54E+00	8.54	0	8.54E+00	8.54
b2591_v1_RNA_modif_1	b2591_v1_RNA modification (16S rRNA): position: 1207, m2G by RsmC_mono	1 RsmC_mono + 1 amet + 1 b2591_v1_RNA <==> 1 b2591_v1_RNA_RsmC_mono	reversible	rRNA modification	-1.00E+09	1.00E+09	0	1.11E+02	1.11E+02	0	8.54E+00	8.54	0	8.54E+00	8.54
b2591_v1_RNA_modif_2	b2591_v1_RNA modification (16S rRNA): position: 1207, m2G by RsmC_mono	1 b2591_v1_RNA_RsmC_mono --> 1 RsmC_mono_inact + 1 ahcys + 1 b2591_v1_RNA_1 + 1 h	irreversible	rRNA modification	0.00E+00	1.00E+09	0	1.11E+02	1.11E+02	0	8.54E+00	8.54	0	8.54E+00	8.54
b2593_fold_spon	b2593_m folding: spontanous	1 b2593_m --> 1 YfiH_mono	irreversible	Protein Folding	0.00E+00	1.00E+09	0	1.14E+03	1.14E+03	0	1.94E+03	1935.472	0	9.40E+01	94.024
b2593_mRNA_CONV	convsersion of mRNA to mRNA_1 (synthetic rxn)	1 b2593_mRNA --> 1 b2593_mRNA_1	irreversible	Translation	0.00E+00	1.00E+09	0	7.46E+01	7.46E+01	0	1.32E+02	131.728	0	1.96E-01	0.196
b2593_mRNA_degr1	Degradation of b2593_mRNA I	1 Orn_dim + 182 atp + 1 b2593_mRNA_2 + 1 degradosome + 182 h2o <==> 182 adp + 1 b2593_mRNA_2_degr + 182 h + 182 pi	reversible	mRNA degradation	-1.00E+09	1.00E+09	0	7.46E+01	7.46E+01	0	1.32E+02	131.728	0	1.96E-01	0.196
b2593_mRNA_degr2	Degradation of b2593_mRNA II	1 b2593_mRNA_2_degr + 727 h2o --> 1 Orn_dim_inact + 148 amp + 181 cmp + 1 degradosome_inact + 219 gmp + 727 h + 180 ump	irreversible	mRNA degradation	0.00E+00	1.96E-01	0	7.46E+01	7.46E+01	0	1.32E+02	131.728	0	1.96E-01	0.196
b2593_maturation1	Polypeptide b2593 peptide deformylase and methionine aminopeptidase complex	1 Def_mono + 1 Map_mono + 1 b2593_aa <==> 1 b2593_def_map_cplx	reversible	Protein Maturation	-1.00E+09	1.00E+09	0	1.14E+03	1.14E+03	0	1.94E+03	1935.472	0	9.40E+01	94.024
b2593_maturation2	b2593 formation	1 b2593_def_map_cplx + 2 h2o --> 1 Def_mono_inact + 1 Map_mono_inact + 1 b2593_m + 1 for + 1 met-L	irreversible	Protein Maturation	0.00E+00	1.00E+09	0	1.14E+03	1.14E+03	0	1.94E+03	1935.472	0	9.40E+01	94.024
b2594_fold_spon	b2594_m folding: spontanous	1 b2594_m_Mg --> 1 RluD_mono	irreversible	Protein Folding	0.00E+00	1.00E+09	0	1.53E+03	1.53E+03	0	2.61E+03	2611.512	0	1.12E+02	112.404
b2594_ion_BIND	Binding of ions to b2594	1 b2594_m + 1 mg2 <==> 1 b2594_m_Mg	reversible	Metallo-ion Binding	-1.00E+09	1.00E+09	0	1.53E+03	1.53E+03	0	2.61E+03	2611.512	0	1.12E+02	112.404
b2594_mRNA_CONV	convsersion of mRNA to mRNA_1 (synthetic rxn)	1 b2594_mRNA --> 1 b2594_mRNA_1	irreversible	Translation	0.00E+00	1.00E+09	0	7.46E+01	7.46E+01	0	1.32E+02	131.728	0	1.96E-01	0.196
b2594_mRNA_degr1	Degradation of b2594_mRNA I	1 Orn_dim + 245 atp + 1 b2594_mRNA_2 + 1 degradosome + 245 h2o <==> 245 adp + 1 b2594_mRNA_2_degr + 245 h + 245 pi	reversible	mRNA degradation	-1.00E+09	1.00E+09	0	7.46E+01	7.46E+01	0	1.32E+02	131.728	0	1.96E-01	0.196
b2594_mRNA_degr2	Degradation of b2594_mRNA II	1 b2594_mRNA_2_degr + 980 h2o --> 1 Orn_dim_inact + 241 amp + 1 atp + 247 cmp + 1 degradosome_inact + 271 gmp + 980 h + 221 ump	irreversible	mRNA degradation	0.00E+00	3.72E-01	0	7.46E+01	7.46E+01	0	1.32E+02	131.728	0	1.96E-01	0.196
b2594_maturation1	Polypeptide b2594 peptide deformylase and methionine aminopeptidase complex	1 Def_mono + 1 Map_mono + 1 b2594_aa <==> 1 b2594_def_map_cplx	reversible	Protein Maturation	-1.00E+09	1.00E+09	0	1.53E+03	1.53E+03	0	2.61E+03	2611.512	0	1.12E+02	112.404

b2594_maturation2	b2594 formation	1 b2594_def_map_cplx + 2 h2o --> 1 Def_mono_inact + 1 Map_mono_inact + 1 b2594_m + 1 for + 1 met-L	irreversible	Protein Maturation	0.00E+00	1.00E+09	0	1.53E+03	1.53E+03	0	2.61E+03	2611.512	0	1.12E+02	112.404
b2606_fold_spon	b2606_m folding: spontanous	1 b2606_m --> 1 RplS_mono	irreversible	Protein Folding	0.00E+00	1.00E+09	8.44E+01	1.13E+02	2.81E+01	6.22E+01	8.29E+01	20.72	6.22E+01	8.29E+01	20.72
b2606_mRNA_CONV	convsersion of mRNA to mRNA_1 (synthetic rxn)	1 b2606_mRNA --> 1 b2606_mRNA_1	irreversible	Translation	0.00E+00	1.00E+09	0	6.90E+01	6.90E+01	0	7.91E+01	79.13389	0	2.79E-01	0.279
b2606_mRNA_degr1	Degradation of b2606_mRNA I	1 Orn_dim + 87 atp + 1 b2606_mRNA_2 + 1 degradosome + 87 h2o <==> 87 adp + 1 b2606_mRNA_2_degr + 87 h + 87 pi	reversible	mRNA degradation	-1.00E+09	1.00E+09	0	6.90E+01	6.90E+01	0	7.91E+01	79.13389	0	2.79E-01	0.279
b2606_mRNA_degr2	Degradation of b2606_mRNA II	1 b2606_mRNA_2_degr + 347 h2o --> 1 Orn_dim_inact + 88 amp + 78 cmp + 1 degradosome_inact + 90 gmp + 347 h + 92 ump	irreversible	mRNA degradation	0.00E+00	3.37E-01	0	6.90E+01	6.90E+01	0	7.91E+01	79.13389	0	2.79E-01	0.279
b2606_maturation1	Polypeptide b2606 peptide deformylas and methionine aminopeptidase complex	1 Def_mono + 1 Map_mono + 1 b2606_aa <==> 1 b2606_def_map_cplx	reversible	Protein Maturation	-1.00E+09	1.00E+09	8.44E+01	1.13E+02	2.81E+01	6.22E+01	8.29E+01	20.72	6.22E+01	8.29E+01	20.72
b2606_maturation2	b2606 formation	1 b2606_def_map_cplx + 2 h2o --> 1 Def_mono_inact + 1 Map_mono_inact + 1 b2606_m + 1 for + 1 met-L	irreversible	Protein Maturation	0.00E+00	1.00E+09	8.44E+01	1.13E+02	2.81E+01	6.22E+01	8.29E+01	20.72	6.22E+01	8.29E+01	20.72
b2607_fold_GroEL/ES_1	b2607_m folding: GroEL/ES mediated; polypeptide is going in GroEL/ES complex	1 GroEL.(7)ADP.cisGroES + 7 atp + 1 b2607_m + 1 transGroES_hepta --> 7 adp + 1 b2607_m_GroEL.(7)ATP.transGroES + 1 cisGroES_hepta	irreversible	Protein Folding	0.00E+00	1.00E+09	0	1.41E+03	1.41E+03	0	1.57E+03	1565.008	0	3.82E+02	382.185
b2607_fold_GroEL/ES_2	b2607_m folding: GroEL/ES mediated; folding of polypeptide under ATP hydrolysis	1 b2607_m_GroEL.(7)ATP.transGroES + 7 h2o --> 1 TrmD_GroEL.(7)ADP.transGroES + 7 h + 7 pi	irreversible	Protein Folding	0.00E+00	1.00E+09	0	1.41E+03	1.41E+03	0	1.57E+03	1565.008	0	3.82E+02	382.185
b2607_fold_GroEL/ES_3	b2607_m folding: GroEL/ES mediated; release of native protein	1 TrmD_GroEL.(7)ADP.transGroES --> 1 GroEL.(7)ADP.transGroES + 1 TrmD_mono	irreversible	Protein Folding	0.00E+00	1.00E+09	0	1.41E+03	1.41E+03	0	1.57E+03	1565.008	0	3.82E+02	382.185
b2607_mRNA_CONV	convsersion of mRNA to mRNA_1 (synthetic rxn)	1 b2607_mRNA --> 1 b2607_mRNA_1	irreversible	Translation	0.00E+00	1.00E+09	0	6.90E+01	6.90E+01	0	7.91E+01	79.13389	0	2.79E-01	0.279
b2607_mRNA_degr1	Degradation of b2607_mRNA I	1 Orn_dim + 192 atp + 1 b2607_mRNA_2 + 1 degradosome + 192 h2o <==> 192 adp + 1 b2607_mRNA_2_degr + 192 h + 192 pi	reversible	mRNA degradation	-1.00E+09	1.00E+09	0	6.90E+01	6.90E+01	0	7.91E+01	79.13389	0	2.79E-01	0.279
b2607_mRNA_degr2	Degradation of b2607_mRNA II	1 b2607_mRNA_2_degr + 767 h2o --> 1 Orn_dim_inact + 192 amp + 179 cmp + 1 degradosome_inact + 231 gmp + 767 h + 166 ump	irreversible	mRNA degradation	0.00E+00	6.18E-01	0	6.90E+01	6.90E+01	0	7.91E+01	79.13389	0	2.79E-01	0.279
b2607_maturation1	Polypeptide b2607 peptide deformylas complex	1 Def_mono + 1 b2607_aa <==> 1 b2607_def_cplx	reversible	Protein Maturation	-1.00E+09	1.00E+09	0	1.41E+03	1.41E+03	0	1.57E+03	1565.008	0	3.82E+02	382.185
b2607_maturation2	b2607 formation	1 b2607_def_cplx + 1 h2o --> 1 Def_mono_inact + 1 b2607_m + 1 for	irreversible	Protein Maturation	0.00E+00	1.00E+09	0	1.41E+03	1.41E+03	0	1.57E+03	1565.008	0	3.82E+02	382.185
b2608_fold_GroEL/ES_1	b2608_m folding: GroEL/ES mediated; polypeptide is going in GroEL/ES complex	1 GroEL.(7)ADP.cisGroES + 7 atp + 1 b2608_m + 1 transGroES_hepta --> 7 adp + 1 b2608_m_GroEL.(7)ATP.transGroES + 1 cisGroES_hepta	irreversible	Protein Folding	0.00E+00	1.00E+09	0	8.59E+02	8.59E+02	0	9.60E+02	960.3389	0	1.72E+02	171.79
b2608_fold_GroEL/ES_2	b2608_m folding: GroEL/ES mediated; folding of polypeptide under ATP hydrolysis	1 b2608_m_GroEL.(7)ATP.transGroES + 7 h2o --> 1 RimM_GroEL.(7)ADP.transGroES + 7 h + 7 pi	irreversible	Protein Folding	0.00E+00	1.00E+09	0	8.59E+02	8.59E+02	0	9.60E+02	960.3389	0	1.72E+02	171.79
b2608_fold_GroEL/ES_3	b2608_m folding: GroEL/ES mediated; release of native protein	1 RimM_GroEL.(7)ADP.transGroES --> 1 GroEL.(7)ADP.transGroES + 1 RimM_mono	irreversible	Protein Folding	0.00E+00	1.00E+09	0	8.59E+02	8.59E+02	0	9.60E+02	960.3389	0	1.72E+02	171.79
b2608_fold_KJE_1	b2608_m folding: KJE mediated	1 DnaJ_dim + 1 DnaK_mono.ATP + 1 b2608_m <==> 1 b2608_m_DnaKJ_complex	reversible	Protein Folding	-1.00E+09	1.00E+09	0	8.59E+02	8.59E+02	0	9.60E+02	960.3389	0	1.72E+02	171.79
b2608_fold_KJE_2	b2608_m folding: KJE mediated	1 GrpE_dim + 1 b2608_m_DnaKJ_complex + 1 h2o --> 1 DnaJ_dim_inact + 1 RimM_DnaK_GrpE_complex + 1 adp + 1 h + 1 pi	irreversible	Protein Folding	0.00E+00	1.00E+09	0	8.59E+02	8.59E+02	0	9.60E+02	960.3389	0	1.72E+02	171.79
b2608_fold_KJE_3	b2608_m folding: KJE mediated	1 RimM_DnaK_GrpE_complex + 1 atp --> 1 DnaK_mono.ATP_inact + 1 GrpE_dim_inact + 1 RimM_mono	irreversible	Protein Folding	0.00E+00	1.00E+09	0	8.59E+02	8.59E+02	0	9.60E+02	960.3389	0	1.72E+02	171.79
b2608_mRNA_CONV	convsersion of mRNA to mRNA_1 (synthetic rxn)	1 b2608_mRNA --> 1 b2608_mRNA_1	irreversible	Translation	0.00E+00	1.00E+09	0	6.90E+01	6.90E+01	0	7.91E+01	79.13389	0	2.79E-01	0.279
b2608_mRNA_degr1	Degradation of b2608_mRNA I	1 Orn_dim + 137 atp + 1 b2608_mRNA_2 + 1 degradosome + 137 h2o <==> 137 adp + 1 b2608_mRNA_2_degr + 137 h + 137 pi	reversible	mRNA degradation	-1.00E+09	1.00E+09	0	6.90E+01	6.90E+01	0	7.91E+01	79.13389	0	2.79E-01	0.279
b2608_mRNA_degr2	Degradation of b2608_mRNA II	1 b2608_mRNA_2_degr + 548 h2o --> 1 Orn_dim_inact + 145 amp + 121 cmp + 1 degradosome_inact + 148 gmp + 548 h + 135 ump	irreversible	mRNA degradation	0.00E+00	3.11E-01	0	6.90E+01	6.90E+01	0	7.91E+01	79.13389	0	2.79E-01	0.279
b2608_maturation1	Polypeptide b2608 peptide deformylas and methionine aminopeptidase complex	1 Def_mono + 1 Map_mono + 1 b2608_aa <==> 1 b2608_def_map_cplx	reversible	Protein Maturation	-1.00E+09	1.00E+09	0	8.59E+02	8.59E+02	0	9.60E+02	960.3389	0	1.72E+02	171.79
b2608_maturation2	b2608 formation	1 b2608_def_map_cplx + 2 h2o --> 1 Def_mono_inact + 1 Map_mono_inact + 1 b2608_m + 1 for + 1 met-L	irreversible	Protein Maturation	0.00E+00	1.00E+09	0	8.59E+02	8.59E+02	0	9.60E+02	960.3389	0	1.72E+02	171.79
b2609_fold_spon	b2609_m folding: spontanous	1 b2609_m --> 1 RpsP_mono	irreversible	Protein Folding	0.00E+00	1.00E+09	8.44E+01	1.13E+02	2.81E+01	6.22E+01	8.29E+01	20.72	6.22E+01	8.29E+01	20.72
b2609_mRNA_CONV	convsersion of mRNA to mRNA_1 (synthetic rxn)	1 b2609_mRNA --> 1 b2609_mRNA_1	irreversible	Translation	0.00E+00	1.00E+09	0	6.90E+01	6.90E+01	0	7.91E+01	79.13389	0	2.79E-01	0.279
b2609_mRNA_degr1	Degradation of b2609_mRNA I	1 Orn_dim + 62 atp + 1 b2609_mRNA_2 + 1 degradosome + 62 h2o <==> 62 adp + 1 b2609_mRNA_2_degr + 62 h + 62 pi	reversible	mRNA degradation	-1.00E+09	1.00E+09	0	6.90E+01	6.90E+01	0	7.91E+01	79.13389	0	2.79E-01	0.279
b2609_mRNA_degr2	Degradation of b2609_mRNA II	1 b2609_mRNA_2_degr + 248 h2o --> 1 Orn_dim_inact + 61 amp + 1 atp + 64 cmp + 1 degradosome_inact + 64 gmp + 248 h + 59 ump	irreversible	mRNA degradation	0.00E+00	2.79E-01	0	6.90E+01	6.90E+01	0	7.91E+01	79.13389	0	2.79E-01	0.279
b2609_maturation1	Polypeptide b2609 peptide deformylas complex	1 Def_mono + 1 b2609_aa <==> 1 b2609_def_cplx	reversible	Protein Maturation	-1.00E+09	1.00E+09	8.44E+01	1.13E+02	2.81E+01	6.22E+01	8.29E+01	20.72	6.22E+01	8.29E+01	20.72
b2609_maturation2	b2609 formation	1 b2609_def_cplx + 1 h2o --> 1 Def_mono_inact + 1 b2609_m + 1 for	irreversible	Protein Maturation	0.00E+00	1.00E+09	8.44E+01	1.13E+02	2.81E+01	6.22E+01	8.29E+01	20.72	6.22E+01	8.29E+01	20.72
b2614_fold_GroEL/ES_1	b2614_m folding: GroEL/ES mediated; polypeptide is going in GroEL/ES complex	1 GroEL.(7)ADP.cisGroES + 7 atp + 1 b2614_m + 1 transGroES_hepta --> 7 adp + 1 b2614_m_GroEL.(7)ATP.transGroES + 1 cisGroES_hepta	irreversible	Protein Folding	0.00E+00	1.00E+09	0	3.11E+03	3.11E+03	0	5.33E+03	5328.918	0	2.07E+02	206.657
b2614_fold_GroEL/ES_2	b2614_m folding: GroEL/ES mediated; folding of polypeptide under ATP hydrolysis	1 b2614_m_GroEL.(7)ATP.transGroES + 7 h2o --> 1 GrpE_GroEL.(7)ADP.transGroES + 7 h + 7 pi	irreversible	Protein Folding	0.00E+00	1.00E+09	0	3.11E+03	3.11E+03	0	5.33E+03	5328.918	0	2.07E+02	206.657
b2614_fold_GroEL/ES_3	b2614_m folding: GroEL/ES mediated; release of native protein	1 GrpE_GroEL.(7)ADP.transGroES --> 1 GroEL.(7)ADP.transGroES + 1 GrpE_mono	irreversible	Protein Folding	0.00E+00	1.00E+09	0	3.11E+03	3.11E+03	0	5.33E+03	5328.918	0	2.07E+02	206.657
b2614_fold_KJE_1	b2614_m folding: KJE mediated	1 DnaJ_dim + 1 DnaK_mono.ATP + 1 b2614_m <==> 1 b2614_m_DnaKJ_complex	reversible	Protein Folding	-1.00E+09	1.00E+09	0	3.11E+03	3.11E+03	0	5.33E+03	5328.918	0	2.07E+02	206.657
b2614_fold_KJE_2	b2614_m folding: KJE mediated	1 GrpE_dim + 1 b2614_m_DnaKJ_complex + 1 h2o --> 1 DnaJ_dim_inact + 1 GrpE_DnaK_GrpE_complex + 1 adp + 1 h + 1 pi	irreversible	Protein Folding	0.00E+00	1.00E+09	0	3.11E+03	3.11E+03	0	5.33E+03	5328.918	0	2.07E+02	206.657
b2614_fold_KJE_3	b2614_m folding: KJE mediated	1 GrpE_DnaK_GrpE_complex + 1 atp --> 1 DnaK_mono.ATP_inact + 1 GrpE_dim_inact + 1 GrpE_mono	irreversible	Protein Folding	0.00E+00	1.00E+09	0	3.11E+03	3.11E+03	0	5.33E+03	5328.918	0	2.07E+02	206.657
b2614_fold_spon	b2614_m folding: spontanous	1 b2614_m --> 1 GrpE_mono	irreversible	Protein Folding	0.00E+00	1.00E+09	0	3.11E+03	3.11E+03	0	5.33E+03	5328.918	0	2.07E+02	206.657
b2614_mRNA_CONV	convsersion of mRNA to mRNA_1 (synthetic rxn)	1 b2614_mRNA --> 1 b2614_mRNA_1	irreversible	Translation	0.00E+00	1.00E+09	0	2.64E+02	2.64E+02	0	4.66E+02	465.9471	0	2.87E-01	0.287
b2614_mRNA_degr1	Degradation of b2614_mRNA I	1 Orn_dim + 148 atp + 1 b2614_mRNA_2 + 1 degradosome + 148 h2o <==> 148 adp + 1 b2614_mRNA_2_degr + 148 h + 148 pi	reversible	mRNA degradation	-1.00E+09	1.00E+09	0	2.64E+02	2.64E+02	0	4.66E+02	465.9471	0	2.87E-01	0.287
b2614_mRNA_degr2	Degradation of b2614_mRNA II	1 b2614_mRNA_2_degr + 593 h2o --> 1 Orn_dim_inact + 165 amp + 1 atp + 121 cmp + 1 degradosome_inact + 178 gmp + 593 h + 129 ump	irreversible	mRNA degradation	0.00E+00	2.87E-01	0	2.64E+02	2.64E+02	0	4.66E+02	465.9471	0	2.87E-01	0.287
b2614_maturation1	Polypeptide b2614 peptide deformylas and methionine aminopeptidase complex	1 Def_mono + 1 Map_mono + 1 b2614_aa <==> 1 b2614_def_map_cplx	reversible	Protein Maturation	-1.00E+09	1.00E+09	0	3.11E+03	3.11E+03	0	5.33E+03	5328.918	0	2.07E+02	206.657
b2614_maturation2	b2614 formation	1 b2614_def_map_cplx + 2 h2o --> 1 Def_mono_inact + 1 Map_mono_inact + 1 b2614_m + 1 for + 1 met-L	irreversible	Protein Maturation	0.00E+00	1.00E+09	0	3.11E+03	3.11E+03	0	5.33E+03	5328.918	0	2.07E+02	206.657
b2652_tRNA_18_MODIF_1	modification of position 18 nt (Gm) in ileY by TrmH_dim	1 TrmH_dim + 1 amet + 1 b2652_tRNA_Mg2 <==> 1 b2652_tRNA_Mg2_TrmH_dim_cplx	reversible	tRNA modification	-1.00E+09	1.00E+09	0	1.52E+03	1.52E+03	0	8.74E+00	8.740001	0	8.74E+00	8.740001

b2652_tRNA_18_MODIF_2	modification of position 18 nt (Gm) in ileY by TrmH_dim	1 b2652_tRNA_Mg2_TrmH_dim_cplx --> 1 TrmH_dim_inact + 1 ahcys + 1 b2652_tRNA_1 + 1 h	irreversible	tRNA modification	0.00E+00	1.00E+09	0	1.52E+03	1.52E+03	0	8.74E+00	8.740001	0	8.74E+00	8.740001
b2652_tRNA_20A_MODIF_1	modification of position 20A nt (D) in ileY by Dus_gen	1 Dus_gen + 1 b2652_tRNA_2 + 1 h + 1 nadph <==> 1 b2652_tRNA_2_Dus_gen_cplx	reversible	tRNA modification	-1.00E+09	1.00E+09	0	1.52E+03	1.52E+03	0	8.74E+00	8.740001	0	8.74E+00	8.740001
b2652_tRNA_20A_MODIF_2	modification of position 20A nt (D) in ileY by Dus_gen	1 b2652_tRNA_2_Dus_gen_cplx --> 1 Dus_gen_inact + 1 b2652_tRNA_3 + 1 nadp	irreversible	tRNA modification	0.00E+00	1.00E+09	0	1.52E+03	1.52E+03	0	8.74E+00	8.740001	0	8.74E+00	8.740001
b2652_tRNA_20_MODIF_1	modification of position 20 nt (D) in ileY by Dus_gen	1 Dus_gen + 1 b2652_tRNA_1 + 1 h + 1 nadph <==> 1 b2652_tRNA_1_Dus_gen_cplx	reversible	tRNA modification	-1.00E+09	1.00E+09	0	1.52E+03	1.52E+03	0	8.74E+00	8.740001	0	8.74E+00	8.740001
b2652_tRNA_20_MODIF_2	modification of position 20 nt (D) in ileY by Dus_gen	1 b2652_tRNA_1_Dus_gen_cplx --> 1 Dus_gen_inact + 1 b2652_tRNA_2 + 1 nadp	irreversible	tRNA modification	0.00E+00	1.00E+09	0	1.52E+03	1.52E+03	0	8.74E+00	8.740001	0	8.74E+00	8.740001
b2652_tRNA_34_MODIF_1	modification of position 34 nt (k2C) in ileY by TilS_mono	1 TilS_mono + 1 atp + 1 b2652_tRNA_3 + 1 lys-L <==> 1 b2652_tRNA_3_TilS_mono_cplx	reversible	tRNA modification	-1.00E+09	1.00E+09	0	1.52E+03	1.52E+03	0	8.74E+00	8.740001	0	8.74E+00	8.740001
b2652_tRNA_34_MODIF_2	modification of position 34 nt (k2C) in ileY by TilS_mono	1 b2652_tRNA_3_TilS_mono_cplx --> 1 TilS_mono_inact + 1 amp + b2652_tRNA_4 + 2 h + 1 ppi	irreversible	tRNA modification	0.00E+00	1.00E+09	0	1.52E+03	1.52E+03	0	8.74E+00	8.740001	0	8.74E+00	8.740001
b2652_tRNA_37_MODIF_1	modification of position 37 nt (t6A) in ileY by Up_tRNA_pos_37_t6A	1 Up_tRNA_pos_37_t6A + 1 atp + 1 b2652_tRNA_4 + 1 hco3 + 1 mg2 + 1 thr-L <==> 1 b2652_tRNA_4_Up_tRNA_pos_37_t6A_cplx	reversible	tRNA modification	-1.00E+09	1.00E+09	0	1.52E+03	1.52E+03	0	8.74E+00	8.740001	0	8.74E+00	8.740001
b2652_tRNA_37_MODIF_2	modification of position 37 nt (t6A) in ileY by Up_tRNA_pos_37_t6A	1 b2652_tRNA_4_Up_tRNA_pos_37_t6A_cplx --> 1 Up_tRNA_pos_37_t6A_inact + 1 amp + 1 b2652_tRNA_5 + 1 h + 1 h2o + 1 mg2 + 1 ppi	irreversible	tRNA modification	0.00E+00	1.00E+09	0	1.52E+03	1.52E+03	0	8.74E+00	8.740001	0	8.74E+00	8.740001
b2652_tRNA_39_MODIF_1	modification of position 39 nt (Y) in ileY by TruA_dim	1 TruA_dim + 1 b2652_tRNA_5 <==> 1 b2652_tRNA_5_TruA_dim_cplx	reversible	tRNA modification	-1.00E+09	1.00E+09	0	1.52E+03	1.52E+03	0	8.74E+00	8.740001	0	8.74E+00	8.740001
b2652_tRNA_39_MODIF_2	modification of position 39 nt (Y) in ileY by TruA_dim	1 b2652_tRNA_5_TruA_dim_cplx --> 1 TruA_dim_inact + 1 b2652_tRNA_6	irreversible	tRNA modification	0.00E+00	1.00E+09	0	1.52E+03	1.52E+03	0	8.74E+00	8.740001	0	8.74E+00	8.740001
b2652_tRNA_46_MODIF_1	modification of position 46 nt (m7G) in ileY by YggH_mono	1 YggH_mono + 1 amet + 1 b2652_tRNA_6 <==> 1 b2652_tRNA_6_YggH_mono_cplx	reversible	tRNA modification	-1.00E+09	1.00E+09	0	1.52E+03	1.52E+03	0	8.74E+00	8.740001	0	8.74E+00	8.740001
b2652_tRNA_46_MODIF_2	modification of position 46 nt (m7G) in ileY by YggH_mono	1 b2652_tRNA_6_YggH_mono_cplx --> 1 YggH_mono_inact + 1 ahcys + 1 b2652_tRNA_7 + 1 h	irreversible	tRNA modification	0.00E+00	1.00E+09	0	1.52E+03	1.52E+03	0	8.74E+00	8.740001	0	8.74E+00	8.740001
b2652_tRNA_47_MODIF_1	modification of position 47 nt (acp3U) in ileY by AcpT_tRNA_pos_47_acp3U	1 AcpT_tRNA_pos_47_acp3U + 1 amet + 1 b2652_tRNA_7 <==> 1 b2652_tRNA_7_AcpT_tRNA_pos_47_acp3U_cplx	reversible	tRNA modification	-1.00E+09	1.00E+09	0	1.52E+03	1.52E+03	0	8.74E+00	8.740001	0	8.74E+00	8.740001
b2652_tRNA_47_MODIF_2	modification of position 47 nt (acp3U) in ileY by AcpT_tRNA_pos_47_acp3U	1 b2652_tRNA_7_AcpT_tRNA_pos_47_acp3U_cplx --> 1 5mta + 1 AcpT_tRNA_pos_47_acp3U_inact + 1 b2652_tRNA_8 + 1 h	irreversible	tRNA modification	0.00E+00	1.00E+09	0	1.52E+03	1.52E+03	0	8.74E+00	8.740001	0	8.74E+00	8.740001
b2652_tRNA_54_MODIF_1	modification of position 54 nt (m5U) in ileY by TrmA_mono	1 TrmA_mono + 1 amet + 1 b2652_tRNA_8 <==> 1 b2652_tRNA_8_TrmA_mono_cplx	reversible	tRNA modification	-1.00E+09	1.00E+09	0	1.52E+03	1.52E+03	0	8.74E+00	8.740001	0	8.74E+00	8.740001
b2652_tRNA_54_MODIF_2	modification of position 54 nt (m5U) in ileY by TrmA_mono	1 b2652_tRNA_8_TrmA_mono_cplx --> 1 TrmA_mono_inact + 1 ahcys + 1 b2652_tRNA_9 + 1 h	irreversible	tRNA modification	0.00E+00	1.00E+09	0	1.52E+03	1.52E+03	0	8.74E+00	8.740001	0	8.74E+00	8.740001
b2652_tRNA_55_MODIF_1	modification of position 55 nt (Y) in ileY by TruB_mono	1 TruB_mono + 1 b2652_tRNA_9 <==> 1 b2652_tRNA_9_TruB_mono_cplx	reversible	tRNA modification	-1.00E+09	1.00E+09	0	1.52E+03	1.52E+03	0	8.74E+00	8.740001	0	8.74E+00	8.740001
b2652_tRNA_55_MODIF_2	modification of position 55 nt (Y) in ileY by TruB_mono	1 b2652_tRNA_9_TruB_mono_cplx --> 1 TruB_mono_inact + 1 b2652_tRNA_10	irreversible	tRNA modification	0.00E+00	1.00E+09	0	1.52E+03	1.52E+03	0	8.74E+00	8.740001	0	8.74E+00	8.740001
b2652_tRNA_8_MODIF_1	modification of position 8 nt (s4U) in ileY by ThiI_mono	1 IscS_dim_S-SH + 1 ThiI_mono + 1 atp + 1 b2652_tRNA_10 + 1 mg2 + 1 trdrd <==> 1 b2652_tRNA_10_ThiI_mono_cplx	reversible	tRNA modification	-1.00E+09	1.00E+09	0	1.52E+03	1.52E+03	0	8.74E+00	8.740001	0	8.74E+00	8.740001
b2652_tRNA_8_MODIF_2	modification of position 8 nt (s4U) in ileY by ThiI_mono	1 b2652_tRNA_10_ThiI_mono_cplx --> 1 IscS_dim_S-H + 1 ThiI_mono_inact + 1 amp + 1 h + 1 ileY_tRNA + 1 mg2 + 1 ppi + 1 trdox	irreversible	tRNA modification	0.00E+00	1.00E+09	0	1.52E+03	1.52E+03	0	8.74E+00	8.740001	0	8.74E+00	8.740001
b2652_tRNA_Mg_BIND	Binding of two Mg2 to b2652_tRNA	1 b2652_RNA + 2 mg2 <==> 1 b2652_tRNA_Mg2	reversible	Metallo-ion binding	-1.00E+09	1.00E+09	0	1.52E+03	1.52E+03	0	8.74E+00	8.740001	0	8.74E+00	8.740001
b2691_tRNA_17_MODIF_1	modification of position 17 nt (D) in argQ by Dus_gen	1 Dus_gen + 1 b2691_tRNA_Mg2 + 1 h + 1 nadph <==> 1 b2691_tRNA_Mg2_Dus_gen_cplx	reversible	tRNA modification	-1.00E+09	1.00E+09	0	1.46E+02	1.46E+02	0	8.87E+00	8.87	0	8.87E+00	8.87
b2691_tRNA_17_MODIF_2	modification of position 17 nt (D) in argQ by Dus_gen	1 b2691_tRNA_Mg2_Dus_gen_cplx --> 1 Dus_gen_inact + 1 b2691_tRNA_1 + 1 nadp	irreversible	tRNA modification	0.00E+00	1.00E+09	0	1.46E+02	1.46E+02	0	8.87E+00	8.87	0	8.87E+00	8.87
b2691_tRNA_20A_MODIF_1	modification of position 20A nt (D) in argQ by Dus_gen	1 Dus_gen + 1 b2691_tRNA_1 + 1 h + 1 nadph <==> 1 b2691_tRNA_1_Dus_gen_cplx	reversible	tRNA modification	-1.00E+09	1.00E+09	0	1.46E+02	1.46E+02	0	8.87E+00	8.87	0	8.87E+00	8.87
b2691_tRNA_20A_MODIF_2	modification of position 20A nt (D) in argQ by Dus_gen	1 b2691_tRNA_1_Dus_gen_cplx --> 1 Dus_gen_inact + 1 b2691_tRNA_2 + 1 nadp	irreversible	tRNA modification	0.00E+00	1.00E+09	0	1.46E+02	1.46E+02	0	8.87E+00	8.87	0	8.87E+00	8.87
b2691_tRNA_34_MODIF_1	modification of position 34 nt (I) in argQ by TadA_dim	1 TadA_dim + 1 b2691_tRNA_2 + 1 h2o <==> 1 b2691_tRNA_2_TadA_dim_cplx	reversible	tRNA modification	-1.00E+09	1.00E+09	0	1.46E+02	1.46E+02	0	8.87E+00	8.87	0	8.87E+00	8.87
b2691_tRNA_34_MODIF_2	modification of position 34 nt (I) in argQ by TadA_dim	1 b2691_tRNA_2_TadA_dim_cplx --> 1 TadA_dim_inact + 1 b2691_tRNA_3 + 1 nh3	irreversible	tRNA modification	0.00E+00	1.00E+09	0	1.46E+02	1.46E+02	0	8.87E+00	8.87	0	8.87E+00	8.87
b2691_tRNA_37_MODIF_1	modification of position 37 nt (m2A) in argQ by MeT_tRNA_pos_37_m2A	1 MeT_tRNA_pos_37_m2A + 1 amet + 1 b2691_tRNA_3 <==> 1 b2691_tRNA_3_MeT_tRNA_pos_37_m2A_cplx	reversible	tRNA modification	-1.00E+09	1.00E+09	0	1.46E+02	1.46E+02	0	8.87E+00	8.87	0	8.87E+00	8.87
b2691_tRNA_37_MODIF_2	modification of position 37 nt (m2A) in argQ by MeT_tRNA_pos_37_m2A	1 b2691_tRNA_3_MeT_tRNA_pos_37_m2A_cplx --> 1 MeT_tRNA_pos_37_m2A_inact + 1 ahcys + 1 b2691_tRNA_4 + 1 h	irreversible	tRNA modification	0.00E+00	1.00E+09	0	1.46E+02	1.46E+02	0	8.87E+00	8.87	0	8.87E+00	8.87
b2691_tRNA_46_MODIF_1	modification of position 46 nt (m7G) in argQ by YggH_mono	1 YggH_mono + 1 amet + 1 b2691_tRNA_4 <==> 1 b2691_tRNA_4_YggH_mono_cplx	reversible	tRNA modification	-1.00E+09	1.00E+09	0	1.46E+02	1.46E+02	0	8.87E+00	8.87	0	8.87E+00	8.87
b2691_tRNA_46_MODIF_2	modification of position 46 nt (m7G) in argQ by YggH_mono	1 b2691_tRNA_4_YggH_mono_cplx --> 1 YggH_mono_inact + 1 ahcys + 1 b2691_tRNA_5 + 1 h	irreversible	tRNA modification	0.00E+00	1.00E+09	0	1.46E+02	1.46E+02	0	8.87E+00	8.87	0	8.87E+00	8.87
b2691_tRNA_47_MODIF_1	modification of position 47 nt (acp3U) in argQ by AcpT_tRNA_pos_47_acp3U	1 AcpT_tRNA_pos_47_acp3U + 1 amet + 1 b2691_tRNA_5 <==> 1 b2691_tRNA_5_AcpT_tRNA_pos_47_acp3U_cplx	reversible	tRNA modification	-1.00E+09	1.00E+09	0	1.46E+02	1.46E+02	0	8.87E+00	8.87	0	8.87E+00	8.87
b2691_tRNA_47_MODIF_2	modification of position 47 nt (acp3U) in argQ by AcpT_tRNA_pos_47_acp3U	1 b2691_tRNA_5_AcpT_tRNA_pos_47_acp3U_cplx --> 1 5mta + 1 AcpT_tRNA_pos_47_acp3U_inact + 1 b2691_tRNA_6 + 1 h	irreversible	tRNA modification	0.00E+00	1.00E+09	0	1.46E+02	1.46E+02	0	8.87E+00	8.87	0	8.87E+00	8.87
b2691_tRNA_54_MODIF_1	modification of position 54 nt (m5U) in argQ by TrmA_mono	1 TrmA_mono + 1 amet + 1 b2691_tRNA_6 <==> 1 b2691_tRNA_6_TrmA_mono_cplx	reversible	tRNA modification	-1.00E+09	1.00E+09	0	1.46E+02	1.46E+02	0	8.87E+00	8.87	0	8.87E+00	8.87
b2691_tRNA_54_MODIF_2	modification of position 54 nt (m5U) in argQ by TrmA_mono	1 b2691_tRNA_6_TrmA_mono_cplx --> 1 TrmA_mono_inact + 1 ahcys + 1 b2691_tRNA_7 + 1 h	irreversible	tRNA modification	0.00E+00	1.00E+09	0	1.46E+02	1.46E+02	0	8.87E+00	8.87	0	8.87E+00	8.87
b2691_tRNA_55_MODIF_1	modification of position 55 nt (Y) in argQ by TruB_mono	1 TruB_mono + 1 b2691_tRNA_7 <==> 1 b2691_tRNA_7_TruB_mono_cplx	reversible	tRNA modification	-1.00E+09	1.00E+09	0	1.46E+02	1.46E+02	0	8.87E+00	8.87	0	8.87E+00	8.87
b2691_tRNA_55_MODIF_2	modification of position 55 nt (Y) in argQ by TruB_mono	1 b2691_tRNA_7_TruB_mono_cplx --> 1 TruB_mono_inact + 1 b2691_tRNA_8	irreversible	tRNA modification	0.00E+00	1.00E+09	0	1.46E+02	1.46E+02	0	8.87E+00	8.87	0	8.87E+00	8.87
b2691_tRNA_8_MODIF_1	modification of position 8 nt (s4U) in argQ by ThiI_mono	1 IscS_dim_S-SH + 1 ThiI_mono + 1 atp + 1 b2691_tRNA_8 + 1 mg2 + 1 trdrd <==> 1 b2691_tRNA_8_ThiI_mono_cplx	reversible	tRNA modification	-1.00E+09	1.00E+09	0	1.46E+02	1.46E+02	0	8.87E+00	8.87	0	8.87E+00	8.87
b2691_tRNA_8_MODIF_2	modification of position 8 nt (s4U) in argQ by ThiI_mono	1 b2691_tRNA_8_ThiI_mono_cplx --> 1 IscS_dim_S-H + 1 ThiI_mono_inact + 1 amp + 1 argQ_tRNA + 1 h + 1 mg2 + 1 ppi + 1 trdox	irreversible	tRNA modification	0.00E+00	1.00E+09	0	1.46E+02	1.46E+02	0	8.87E+00	8.87	0	8.87E+00	8.87
b2691_tRNA_Mg_BIND	Binding of two Mg2 to b2691_tRNA	1 b2691_RNA + 2 mg2 <==> 1 b2691_tRNA_Mg2	reversible	Metallo-ion binding	-1.00E+09	1.00E+09	0	1.46E+02	1.46E+02	0	8.87E+00	8.87	0	8.87E+00	8.87
b2692_tRNA_17_MODIF_1	modification of position 17 nt (D) in argZ by Dus_gen	1 Dus_gen + 1 b2692_tRNA_Mg2 + 1 h + 1 nadph <==> 1 b2692_tRNA_Mg2_Dus_gen_cplx	reversible	tRNA modification	-1.00E+09	1.00E+09	0	1.46E+02	1.46E+02	0	8.87E+00	8.87	0	8.87E+00	8.87
b2692_tRNA_17_MODIF_2	modification of position 17 nt (D) in argZ by Dus_gen	1 b2692_tRNA_Mg2_Dus_gen_cplx --> 1 Dus_gen_inact + 1 b2692_tRNA_1 + 1 nadp	irreversible	tRNA modification	0.00E+00	1.00E+09	0	1.46E+02	1.46E+02	0	8.87E+00	8.87	0	8.87E+00	8.87
b2692_tRNA_20A_MODIF_1	modification of position 20A nt (D) in argZ by Dus_gen	1 Dus_gen + 1 b2692_tRNA_1 + 1 h + 1 nadph <==> 1 b2692_tRNA_1_Dus_gen_cplx	reversible	tRNA modification	-1.00E+09	1.00E+09	0	1.46E+02	1.46E+02	0	8.87E+00	8.87	0	8.87E+00	8.87
b2692_tRNA_20A_MODIF_2	modification of position 20A nt (D) in argZ by Dus_gen	1 b2692_tRNA_1_Dus_gen_cplx --> 1 Dus_gen_inact + 1 b2692_tRNA_2 + 1 nadp	irreversible	tRNA modification	0.00E+00	1.00E+09	0	1.46E+02	1.46E+02	0	8.87E+00	8.87	0	8.87E+00	8.87
b2692_tRNA_34_MODIF_1	modification of position 34 nt (I) in argZ by TadA_dim	1 TadA_dim + 1 b2692_tRNA_2 + 1 h2o <==> 1 b2692_tRNA_2_TadA_dim_cplx	reversible	tRNA modification	-1.00E+09	1.00E+09	0	1.46E+02	1.46E+02	0	8.87E+00	8.87	0	8.87E+00	8.87
b2692_tRNA_34_MODIF_2	modification of position 34 nt (I) in argZ by TadA_dim	1 b2692_tRNA_2_TadA_dim_cplx --> 1 TadA_dim_inact + 1 b2692_tRNA_3 + 1 nh3	irreversible	tRNA modification	0.00E+00	1.00E+09	0	1.46E+02	1.46E+02	0	8.87E+00	8.87	0	8.87E+00	8.87
b2692_tRNA_37_MODIF_1	modification of position 37 nt (m2A) in argZ by MeT_tRNA_pos_37_m2A	1 MeT_tRNA_pos_37_m2A + 1 amet + 1 b2692_tRNA_3 <==> 1 b2692_tRNA_3_MeT_tRNA_pos_37_m2A_cplx	reversible	tRNA modification	-1.00E+09	1.00E+09	0	1.46E+02	1.46E+02	0	8.87E+00	8.87	0	8.87E+00	8.87
b2692_tRNA_37_MODIF_2	modification of position 37 nt (m2A) in argZ by MeT_tRNA_pos_37_m2A	1 b2692_tRNA_3_MeT_tRNA_pos_37_m2A_cplx --> 1 MeT_tRNA_pos_37_m2A_inact + 1 ahcys + 1 b2692_tRNA_4 + 1 h	irreversible	tRNA modification	0.00E+00	1.00E+09	0	1.46E+02	1.46E+02	0	8.87E+00	8.87	0	8.87E+00	8.87
b2692_tRNA_46_MODIF_1	modification of position 46 nt (m7G) in argZ by YggH_mono	1 YggH_mono + 1 amet + 1 b2692_tRNA_4 <==> 1 b2692_tRNA_4_YggH_mono_cplx	reversible	tRNA modification	-1.00E+09	1.00E+09	0	1.46E+02	1.46E+02	0	8.87E+00	8.87	0	8.87E+00	8.87
b2692_tRNA_46_MODIF_2	modification of position 46 nt (m7G) in argZ by YggH_mono	1 b2692_tRNA_4_YggH_mono_cplx --> 1 YggH_mono_inact + 1 ahcys + 1 b2692_tRNA_5 + 1 h	irreversible	tRNA modification	0.00E+00	1.00E+09	0	1.46E+02	1.46E+02	0	8.87E+00	8.87	0	8.87E+00	8.87

b2692_tRNA_47_MODIF_1	modification of position 47 nt (acp3U) in argZ by AcpT_tRNA_pos_47_acp3U	1 AcpT_tRNA_pos_47_acp3U + 1 amet + 1 b2692_tRNA_5 <==> 1 b2692_tRNA_5_AcpT_tRNA_pos_47_acp3U_cplx	reversible	tRNA modification	-1.00E+09	1.00E+09	0	1.46E+02	1.46E+02	0	8.87E+00	8.87	0	8.87E+00	8.87
b2692_tRNA_47_MODIF_2	modification of position 47 nt (acp3U) in argZ by AcpT_tRNA_pos_47_acp3U	1 b2692_tRNA_5_AcpT_tRNA_pos_47_acp3U_cplx --> 1 5mta + 1 AcpT_tRNA_pos_47_acp3U_inact + 1 b2692_tRNA_6 + 1 h	irreversible	tRNA modification	0.00E+00	1.00E+09	0	1.46E+02	1.46E+02	0	8.87E+00	8.87	0	8.87E+00	8.87
b2692_tRNA_54_MODIF_1	modification of position 54 nt (m5U) in argZ by TrmA_mono	1 TrmA_mono + 1 amet + 1 b2692_tRNA_6 <==> 1 b2692_tRNA_6_TrmA_mono_cplx	reversible	tRNA modification	-1.00E+09	1.00E+09	0	1.46E+02	1.46E+02	0	8.87E+00	8.87	0	8.87E+00	8.87
b2692_tRNA_54_MODIF_2	modification of position 54 nt (m5U) in argZ by TrmA_mono	1 b2692_tRNA_6_TrmA_mono_cplx --> 1 TrmA_mono_inact + 1 ahcys + 1 b2692_tRNA_7 + 1 h	irreversible	tRNA modification	0.00E+00	1.00E+09	0	1.46E+02	1.46E+02	0	8.87E+00	8.87	0	8.87E+00	8.87
b2692_tRNA_55_MODIF_1	modification of position 55 nt (Y) in argZ by TruB_mono	1 TruB_mono + 1 b2692_tRNA_7 <==> 1 b2692_tRNA_7_TruB_mono_cplx	reversible	tRNA modification	-1.00E+09	1.00E+09	0	1.46E+02	1.46E+02	0	8.87E+00	8.87	0	8.87E+00	8.87
b2692_tRNA_55_MODIF_2	modification of position 55 nt (Y) in argZ by TruB_mono	1 b2692_tRNA_7_TruB_mono_cplx --> 1 TruB_mono_inact + 1 b2692_tRNA_8	irreversible	tRNA modification	0.00E+00	1.00E+09	0	1.46E+02	1.46E+02	0	8.87E+00	8.87	0	8.87E+00	8.87
b2692_tRNA_8_MODIF_1	modification of position 8 nt (s4U) in argZ by ThiI_mono	1 IscS_dim_S-SH + 1 ThiI_mono + 1 atp + 1 b2692_tRNA_8 + 1 mg2 + 1 trdrd <==> 1 b2692_tRNA_8_ThiI_mono_cplx	reversible	tRNA modification	-1.00E+09	1.00E+09	0	1.46E+02	1.46E+02	0	8.87E+00	8.87	0	8.87E+00	8.87
b2692_tRNA_8_MODIF_2	modification of position 8 nt (s4U) in argZ by ThiI_mono	1 b2692_tRNA_8_ThiI_mono_cplx --> 1 IscS_dim_S-H + 1 ThiI_mono_inact + 1 amp + 1 argZ_tRNA + 1 h + 1 mg2 + 1 ppi + 1 trdox	irreversible	tRNA modification	0.00E+00	1.00E+09	0	1.46E+02	1.46E+02	0	8.87E+00	8.87	0	8.87E+00	8.87
b2692_tRNA_Mg_BIND	Binding of two Mg2 to b2692_tRNA	1 b2692_RNA + 2 mg2 <==> 1 b2692_tRNA_Mg2	reversible	Metallo-ion binding	-1.00E+09	1.00E+09	0	1.46E+02	1.46E+02	0	8.87E+00	8.87	0	8.87E+00	8.87
b2693_tRNA_17_MODIF_1	modification of position 17 nt (D) in argY by Dus_gen	1 Dus_gen + 1 b2693_tRNA_Mg2 + 1 h + 1 nadph <==> 1 b2693_tRNA_Mg2_Dus_gen_cplx	reversible	tRNA modification	-1.00E+09	1.00E+09	0	1.46E+02	1.46E+02	0	8.87E+00	8.87	0	8.87E+00	8.87
b2693_tRNA_17_MODIF_2	modification of position 17 nt (D) in argY by Dus_gen	1 b2693_tRNA_Mg2_Dus_gen_cplx --> 1 Dus_gen_inact + 1 b2693_tRNA_1 + 1 nadp	irreversible	tRNA modification	0.00E+00	1.00E+09	0	1.46E+02	1.46E+02	0	8.87E+00	8.87	0	8.87E+00	8.87
b2693_tRNA_20A_MODIF_1	modification of position 20A nt (D) in argY by Dus_gen	1 Dus_gen + 1 b2693_tRNA_1 + 1 h + 1 nadph <==> 1 b2693_tRNA_1_Dus_gen_cplx	reversible	tRNA modification	-1.00E+09	1.00E+09	0	1.46E+02	1.46E+02	0	8.87E+00	8.87	0	8.87E+00	8.87
b2693_tRNA_20A_MODIF_2	modification of position 20A nt (D) in argY by Dus_gen	1 b2693_tRNA_1_Dus_gen_cplx --> 1 Dus_gen_inact + 1 b2693_tRNA_2 + 1 nadp	irreversible	tRNA modification	0.00E+00	1.00E+09	0	1.46E+02	1.46E+02	0	8.87E+00	8.87	0	8.87E+00	8.87
b2693_tRNA_34_MODIF_1	modification of position 34 nt (I) in argY by TadA_dim	1 TadA_dim + 1 b2693_tRNA_2 + 1 h2o <==> 1 b2693_tRNA_2_TadA_dim_cplx	reversible	tRNA modification	-1.00E+09	1.00E+09	0	1.46E+02	1.46E+02	0	8.87E+00	8.87	0	8.87E+00	8.87
b2693_tRNA_34_MODIF_2	modification of position 34 nt (I) in argY by TadA_dim	1 b2693_tRNA_2_TadA_dim_cplx --> 1 TadA_dim_inact + 1 b2693_tRNA_3 + 1 nh3	irreversible	tRNA modification	0.00E+00	1.00E+09	0	1.46E+02	1.46E+02	0	8.87E+00	8.87	0	8.87E+00	8.87
b2693_tRNA_37_MODIF_1	modification of position 37 nt (m2A) in argY by MeT_tRNA_pos_37_m2A	1 MeT_tRNA_pos_37_m2A + 1 amet + 1 b2693_tRNA_3 <==> 1 b2693_tRNA_3_MeT_tRNA_pos_37_m2A_cplx	reversible	tRNA modification	-1.00E+09	1.00E+09	0	1.46E+02	1.46E+02	0	8.87E+00	8.87	0	8.87E+00	8.87
b2693_tRNA_37_MODIF_2	modification of position 37 nt (m2A) in argY by MeT_tRNA_pos_37_m2A	1 b2693_tRNA_3_MeT_tRNA_pos_37_m2A_cplx --> 1 MeT_tRNA_pos_37_m2A_inact + 1 ahcys + 1 b2693_tRNA_4 + 1 h	irreversible	tRNA modification	0.00E+00	1.00E+09	0	1.46E+02	1.46E+02	0	8.87E+00	8.87	0	8.87E+00	8.87
b2693_tRNA_46_MODIF_1	modification of position 46 nt (m7G) in argY by YggH_mono	1 YggH_mono + 1 amet + 1 b2693_tRNA_4 <==> 1 b2693_tRNA_4_YggH_mono_cplx	reversible	tRNA modification	-1.00E+09	1.00E+09	0	1.46E+02	1.46E+02	0	8.87E+00	8.87	0	8.87E+00	8.87
b2693_tRNA_46_MODIF_2	modification of position 46 nt (m7G) in argY by YggH_mono	1 b2693_tRNA_4_YggH_mono_cplx --> 1 YggH_mono_inact + 1 ahcys + 1 b2693_tRNA_5 + 1 h	irreversible	tRNA modification	0.00E+00	1.00E+09	0	1.46E+02	1.46E+02	0	8.87E+00	8.87	0	8.87E+00	8.87
b2693_tRNA_47_MODIF_1	modification of position 47 nt (acp3U) in argY by AcpT_tRNA_pos_47_acp3U	1 AcpT_tRNA_pos_47_acp3U + 1 amet + 1 b2693_tRNA_5 <==> 1 b2693_tRNA_5_AcpT_tRNA_pos_47_acp3U_cplx	reversible	tRNA modification	-1.00E+09	1.00E+09	0	1.46E+02	1.46E+02	0	8.87E+00	8.87	0	8.87E+00	8.87
b2693_tRNA_47_MODIF_2	modification of position 47 nt (acp3U) in argY by AcpT_tRNA_pos_47_acp3U	1 b2693_tRNA_5_AcpT_tRNA_pos_47_acp3U_cplx --> 1 5mta + 1 AcpT_tRNA_pos_47_acp3U_inact + 1 b2693_tRNA_6 + 1 h	irreversible	tRNA modification	0.00E+00	1.00E+09	0	1.46E+02	1.46E+02	0	8.87E+00	8.87	0	8.87E+00	8.87
b2693_tRNA_54_MODIF_1	modification of position 54 nt (m5U) in argY by TrmA_mono	1 TrmA_mono + 1 amet + 1 b2693_tRNA_6 <==> 1 b2693_tRNA_6_TrmA_mono_cplx	reversible	tRNA modification	-1.00E+09	1.00E+09	0	1.46E+02	1.46E+02	0	8.87E+00	8.87	0	8.87E+00	8.87
b2693_tRNA_54_MODIF_2	modification of position 54 nt (m5U) in argY by TrmA_mono	1 b2693_tRNA_6_TrmA_mono_cplx --> 1 TrmA_mono_inact + 1 ahcys + 1 b2693_tRNA_7 + 1 h	irreversible	tRNA modification	0.00E+00	1.00E+09	0	1.46E+02	1.46E+02	0	8.87E+00	8.87	0	8.87E+00	8.87
b2693_tRNA_55_MODIF_1	modification of position 55 nt (Y) in argY by TruB_mono	1 TruB_mono + 1 b2693_tRNA_7 <==> 1 b2693_tRNA_7_TruB_mono_cplx	reversible	tRNA modification	-1.00E+09	1.00E+09	0	1.46E+02	1.46E+02	0	8.87E+00	8.87	0	8.87E+00	8.87
b2693_tRNA_55_MODIF_2	modification of position 55 nt (Y) in argY by TruB_mono	1 b2693_tRNA_7_TruB_mono_cplx --> 1 TruB_mono_inact + 1 b2693_tRNA_8	irreversible	tRNA modification	0.00E+00	1.00E+09	0	1.46E+02	1.46E+02	0	8.87E+00	8.87	0	8.87E+00	8.87
b2693_tRNA_8_MODIF_1	modification of position 8 nt (s4U) in argY by ThiI_mono	1 IscS_dim_S-SH + 1 ThiI_mono + 1 atp + 1 b2693_tRNA_8 + 1 mg2 + 1 trdrd <==> 1 b2693_tRNA_8_ThiI_mono_cplx	reversible	tRNA modification	-1.00E+09	1.00E+09	0	1.46E+02	1.46E+02	0	8.87E+00	8.87	0	8.87E+00	8.87
b2693_tRNA_8_MODIF_2	modification of position 8 nt (s4U) in argY by ThiI_mono	1 b2693_tRNA_8_ThiI_mono_cplx --> 1 IscS_dim_S-H + 1 ThiI_mono_inact + 1 amp + 1 argY_tRNA + 1 h + 1 mg2 + 1 ppi + 1 trdox	irreversible	tRNA modification	0.00E+00	1.00E+09	0	1.46E+02	1.46E+02	0	8.87E+00	8.87	0	8.87E+00	8.87
b2693_tRNA_Mg_BIND	Binding of two Mg2 to b2693_tRNA	1 b2693_RNA + 2 mg2 <==> 1 b2693_tRNA_Mg2	reversible	Metallo-ion binding	-1.00E+09	1.00E+09	0	1.46E+02	1.46E+02	0	8.87E+00	8.87	0	8.87E+00	8.87
b2694_tRNA_17_MODIF_1	modification of position 17 nt (D) in argV by Dus_gen	1 Dus_gen + 1 b2694_tRNA_Mg2 + 1 h + 1 nadph <==> 1 b2694_tRNA_Mg2_Dus_gen_cplx	reversible	tRNA modification	-1.00E+09	1.00E+09	0	1.46E+02	1.46E+02	0	8.87E+00	8.87	0	8.87E+00	8.87
b2694_tRNA_17_MODIF_2	modification of position 17 nt (D) in argV by Dus_gen	1 b2694_tRNA_Mg2_Dus_gen_cplx --> 1 Dus_gen_inact + 1 b2694_tRNA_1 + 1 nadp	irreversible	tRNA modification	0.00E+00	1.00E+09	0	1.46E+02	1.46E+02	0	8.87E+00	8.87	0	8.87E+00	8.87
b2694_tRNA_20A_MODIF_1	modification of position 20A nt (D) in argV by Dus_gen	1 Dus_gen + 1 b2694_tRNA_1 + 1 h + 1 nadph <==> 1 b2694_tRNA_1_Dus_gen_cplx	reversible	tRNA modification	-1.00E+09	1.00E+09	0	1.46E+02	1.46E+02	0	8.87E+00	8.87	0	8.87E+00	8.87
b2694_tRNA_20A_MODIF_2	modification of position 20A nt (D) in argV by Dus_gen	1 b2694_tRNA_1_Dus_gen_cplx --> 1 Dus_gen_inact + 1 b2694_tRNA_2 + 1 nadp	irreversible	tRNA modification	0.00E+00	1.00E+09	0	1.46E+02	1.46E+02	0	8.87E+00	8.87	0	8.87E+00	8.87
b2694_tRNA_34_MODIF_1	modification of position 34 nt (I) in argV by TadA_dim	1 TadA_dim + 1 b2694_tRNA_2 + 1 h2o <==> 1 b2694_tRNA_2_TadA_dim_cplx	reversible	tRNA modification	-1.00E+09	1.00E+09	0	1.46E+02	1.46E+02	0	8.87E+00	8.87	0	8.87E+00	8.87
b2694_tRNA_34_MODIF_2	modification of position 34 nt (I) in argV by TadA_dim	1 b2694_tRNA_2_TadA_dim_cplx --> 1 TadA_dim_inact + 1 b2694_tRNA_3 + 1 nh3	irreversible	tRNA modification	0.00E+00	1.00E+09	0	1.46E+02	1.46E+02	0	8.87E+00	8.87	0	8.87E+00	8.87
b2694_tRNA_37_MODIF_1	modification of position 37 nt (m2A) in argV by MeT_tRNA_pos_37_m2A	1 MeT_tRNA_pos_37_m2A + 1 amet + 1 b2694_tRNA_3 <==> 1 b2694_tRNA_3_MeT_tRNA_pos_37_m2A_cplx	reversible	tRNA modification	-1.00E+09	1.00E+09	0	1.46E+02	1.46E+02	0	8.87E+00	8.87	0	8.87E+00	8.87
b2694_tRNA_37_MODIF_2	modification of position 37 nt (m2A) in argV by MeT_tRNA_pos_37_m2A	1 b2694_tRNA_3_MeT_tRNA_pos_37_m2A_cplx --> 1 MeT_tRNA_pos_37_m2A_inact + 1 ahcys + 1 b2694_tRNA_4 + 1 h	irreversible	tRNA modification	0.00E+00	1.00E+09	0	1.46E+02	1.46E+02	0	8.87E+00	8.87	0	8.87E+00	8.87
b2694_tRNA_46_MODIF_1	modification of position 46 nt (m7G) in argV by YggH_mono	1 YggH_mono + 1 amet + 1 b2694_tRNA_4 <==> 1 b2694_tRNA_4_YggH_mono_cplx	reversible	tRNA modification	-1.00E+09	1.00E+09	0	1.46E+02	1.46E+02	0	8.87E+00	8.87	0	8.87E+00	8.87
b2694_tRNA_46_MODIF_2	modification of position 46 nt (m7G) in argV by YggH_mono	1 b2694_tRNA_4_YggH_mono_cplx --> 1 YggH_mono_inact + 1 ahcys + 1 b2694_tRNA_5 + 1 h	irreversible	tRNA modification	0.00E+00	1.00E+09	0	1.46E+02	1.46E+02	0	8.87E+00	8.87	0	8.87E+00	8.87
b2694_tRNA_47_MODIF_1	modification of position 47 nt (acp3U) in argV by AcpT_tRNA_pos_47_acp3U	1 AcpT_tRNA_pos_47_acp3U + 1 amet + 1 b2694_tRNA_5 <==> 1 b2694_tRNA_5_AcpT_tRNA_pos_47_acp3U_cplx	reversible	tRNA modification	-1.00E+09	1.00E+09	0	1.46E+02	1.46E+02	0	8.87E+00	8.87	0	8.87E+00	8.87
b2694_tRNA_47_MODIF_2	modification of position 47 nt (acp3U) in argV by AcpT_tRNA_pos_47_acp3U	1 b2694_tRNA_5_AcpT_tRNA_pos_47_acp3U_cplx --> 1 5mta + 1 AcpT_tRNA_pos_47_acp3U_inact + 1 b2694_tRNA_6 + 1 h	irreversible	tRNA modification	0.00E+00	1.00E+09	0	1.46E+02	1.46E+02	0	8.87E+00	8.87	0	8.87E+00	8.87
b2694_tRNA_54_MODIF_1	modification of position 54 nt (m5U) in argV by TrmA_mono	1 TrmA_mono + 1 amet + 1 b2694_tRNA_6 <==> 1 b2694_tRNA_6_TrmA_mono_cplx	reversible	tRNA modification	-1.00E+09	1.00E+09	0	1.46E+02	1.46E+02	0	8.87E+00	8.87	0	8.87E+00	8.87
b2694_tRNA_54_MODIF_2	modification of position 54 nt (m5U) in argV by TrmA_mono	1 b2694_tRNA_6_TrmA_mono_cplx --> 1 TrmA_mono_inact + 1 ahcys + 1 b2694_tRNA_7 + 1 h	irreversible	tRNA modification	0.00E+00	1.00E+09	0	1.46E+02	1.46E+02	0	8.87E+00	8.87	0	8.87E+00	8.87
b2694_tRNA_55_MODIF_1	modification of position 55 nt (Y) in argV by TruB_mono	1 TruB_mono + 1 b2694_tRNA_7 <==> 1 b2694_tRNA_7_TruB_mono_cplx	reversible	tRNA modification	-1.00E+09	1.00E+09	0	1.46E+02	1.46E+02	0	8.87E+00	8.87	0	8.87E+00	8.87
b2694_tRNA_55_MODIF_2	modification of position 55 nt (Y) in argV by TruB_mono	1 b2694_tRNA_7_TruB_mono_cplx --> 1 TruB_mono_inact + 1 b2694_tRNA_8	irreversible	tRNA modification	0.00E+00	1.00E+09	0	1.46E+02	1.46E+02	0	8.87E+00	8.87	0	8.87E+00	8.87
b2694_tRNA_8_MODIF_1	modification of position 8 nt (s4U) in argV by ThiI_mono	1 IscS_dim_S-SH + 1 ThiI_mono + 1 atp + 1 b2694_tRNA_8 + 1 mg2 + 1 trdrd <==> 1 b2694_tRNA_8_ThiI_mono_cplx	reversible	tRNA modification	-1.00E+09	1.00E+09	0	1.46E+02	1.46E+02	0	8.87E+00	8.87	0	8.87E+00	8.87
b2694_tRNA_8_MODIF_2	modification of position 8 nt (s4U) in argV by ThiI_mono	1 b2694_tRNA_8_ThiI_mono_cplx --> 1 IscS_dim_S-H + 1 ThiI_mono_inact + 1 amp + 1 argV_tRNA + 1 h + 1 mg2 + 1 ppi + 1 trdox	irreversible	tRNA modification	0.00E+00	1.00E+09	0	1.46E+02	1.46E+02	0	8.87E+00	8.87	0	8.87E+00	8.87
b2694_tRNA_Mg_BIND	Binding of two Mg2 to b2694_tRNA	1 b2694_RNA + 2 mg2 <==> 1 b2694_tRNA_Mg2	reversible	Metallo-ion binding	-1.00E+09	1.00E+09	0	1.46E+02	1.46E+02	0	8.87E+00	8.87	0	8.87E+00	8.87
b2695_tRNA_20A_MODIF_1	modification of position 20A nt (D) in serV by Dus_gen	1 Dus_gen + 1 b2695_tRNA_Mg2 + 1 h + 1 nadph <==> 1 b2695_tRNA_Mg2_Dus_gen_cplx	reversible	tRNA modification	-1.00E+09	1.00E+09	0	1.46E+02	1.46E+02	0	8.87E+00	8.87	0	8.87E+00	8.87
b2695_tRNA_20A_MODIF_2	modification of position 20A nt (D) in serV by Dus_gen	1 b2695_tRNA_Mg2_Dus_gen_cplx --> 1 Dus_gen_inact + 1 b2695_tRNA_1 + 1 nadp	irreversible	tRNA modification	0.00E+00	1.00E+09	0	1.46E+02	1.46E+02	0	8.87E+00	8.87	0	8.87E+00	8.87
b2695_tRNA_32_MODIF_1	modification of position 32 nt (s2C) in serV by YdaO_mono	1 IscS_dim_S-SH + 1 YdaO_mono + 1 atp + 1 b2695_tRNA_1 + 1 mg2 + 1 trdrd <==> 1 b2695_tRNA_1_YdaO_mono_cplx	reversible	tRNA modification	-1.00E+09	1.00E+09	0	1.46E+02	1.46E+02	0	8.87E+00	8.87	0	8.87E+00	8.87

b2695_tRNA_32_MODIF_2	modification of position 32 nt (s2C) in serV by YdaO_mono	1 b2695_tRNA_1_YdaO_mono_cplx --> 1 IscS_dim_S-H + 1 YdaO_mono_inact + 1 amp + 1 b2695_tRNA_2 + 1 h + 1 mg2 + 1 ppi + 1 trdox	irreversible	tRNA modification	0.00E+00	1.00E+09	0	1.46E+02	1.46E+02	0	8.87E+00	8.87	0	8.87E+00	8.87
b2695_tRNA_37_MODIF_1	modification of position 37 nt (t6A) in serV by Up_tRNA_pos_37_t6A	1 Up_tRNA_pos_37_t6A + 1 atp + 1 b2695_tRNA_2 + 1 hco3 + 1 mg2 + 1 thr-L <==> 1 b2695_tRNA_2_Up_tRNA_pos_37_t6A_cplx	reversible	tRNA modification	-1.00E+09	1.00E+09	0	1.46E+02	1.46E+02	0	8.87E+00	8.87	0	8.87E+00	8.87
b2695_tRNA_37_MODIF_2	modification of position 37 nt (t6A) in serV by Up_tRNA_pos_37_t6A	1 b2695_tRNA_2_Up_tRNA_pos_37_t6A_cplx --> 1 Up_tRNA_pos_37_t6A_inact + 1 amp + 1 b2695_tRNA_3 + 1 h + 1 h2o + 1 mg2 + 1 ppi	irreversible	tRNA modification	0.00E+00	1.00E+09	0	1.46E+02	1.46E+02	0	8.87E+00	8.87	0	8.87E+00	8.87
b2695_tRNA_54_MODIF_1	modification of position 54 nt (m5U) in serV by TrmA_mono	1 TrmA_mono + 1 amet + 1 b2695_tRNA_3 <==> 1 b2695_tRNA_3_TrmA_mono_cplx	reversible	tRNA modification	-1.00E+09	1.00E+09	0	1.46E+02	1.46E+02	0	8.87E+00	8.87	0	8.87E+00	8.87
b2695_tRNA_54_MODIF_2	modification of position 54 nt (m5U) in serV by TrmA_mono	1 b2695_tRNA_3_TrmA_mono_cplx --> 1 TrmA_mono_inact + 1 ahcys + 1 b2695_tRNA_4 + 1 h	irreversible	tRNA modification	0.00E+00	1.00E+09	0	1.46E+02	1.46E+02	0	8.87E+00	8.87	0	8.87E+00	8.87
b2695_tRNA_55_MODIF_1	modification of position 55 nt (Y) in serV by TruB_mono	1 TruB_mono + 1 b2695_tRNA_4 <==> 1 b2695_tRNA_4_TruB_mono_cplx	reversible	tRNA modification	-1.00E+09	1.00E+09	0	1.46E+02	1.46E+02	0	8.87E+00	8.87	0	8.87E+00	8.87
b2695_tRNA_55_MODIF_2	modification of position 55 nt (Y) in serV by TruB_mono	1 b2695_tRNA_4_TruB_mono_cplx --> 1 TruB_mono_inact + 1 b2695_tRNA_5	irreversible	tRNA modification	0.00E+00	1.00E+09	0	1.46E+02	1.46E+02	0	8.87E+00	8.87	0	8.87E+00	8.87
b2695_tRNA_8_MODIF_1	modification of position 8 nt (s4U) in serV by ThiI_mono	1 IscS_dim_S-SH + 1 ThiI_mono + 1 atp + 1 b2695_tRNA_5 + 1 mg2 + 1 trdrd <==> 1 b2695_tRNA_5_ThiI_mono_cplx	reversible	tRNA modification	-1.00E+09	1.00E+09	0	1.46E+02	1.46E+02	0	8.87E+00	8.87	0	8.87E+00	8.87
b2695_tRNA_8_MODIF_2	modification of position 8 nt (s4U) in serV by ThiI_mono	1 b2695_tRNA_5_ThiI_mono_cplx --> 1 IscS_dim_S-H + 1 ThiI_mono_inact + 1 amp + 1 h + 1 mg2 + 1 ppi + 1 serV_tRNA + 1 trdox	irreversible	tRNA modification	0.00E+00	1.00E+09	0	1.46E+02	1.46E+02	0	8.87E+00	8.87	0	8.87E+00	8.87
b2695_tRNA_Mg_BIND	Binding of two Mg2 to b2695_tRNA	1 b2695_RNA + 2 mg2 <==> 1 b2695_tRNA_Mg2	reversible	Metallo-ion binding	-1.00E+09	1.00E+09	0	1.46E+02	1.46E+02	0	8.87E+00	8.87	0	8.87E+00	8.87
b2697_fold_KJE_1	b2697_m folding: KJE mediated	1 DnaJ_dim + 1 DnaK_mono.ATP + 1 b2697_m_Zn <==> 1 b2697_m_DnaKJ_complex	reversible	Protein Folding	-1.00E+09	1.00E+09	0	2.65E+03	2.65E+03	0	3.08E+03	3080.363	0	1.25E+02	125.409
b2697_fold_KJE_2	b2697_m folding: KJE mediated	1 GrpE_dim + 1 b2697_m_DnaKJ_complex + 1 h2o --> 1 AlaS_DnaK_GrpE_complex + 1 DnaJ_dim_inact + 1 adp + 1 h + 1 pi	irreversible	Protein Folding	0.00E+00	1.00E+09	0	2.65E+03	2.65E+03	0	3.08E+03	3080.363	0	1.25E+02	125.409
b2697_fold_KJE_3	b2697_m folding: KJE mediated	1 AlaS_DnaK_GrpE_complex + 1 atp --> 1 AlaS_mono + 1 DnaK_mono.ATP_inact + 1 GrpE_dim_inact	irreversible	Protein Folding	0.00E+00	1.00E+09	0	2.65E+03	2.65E+03	0	3.08E+03	3080.363	0	1.25E+02	125.409
b2697_fold_spon	b2697_m folding: spontanous	1 b2697_m_Zn --> 1 AlaS_mono	irreversible	Protein Folding	0.00E+00	1.00E+09	0	2.65E+03	2.65E+03	0	3.08E+03	3080.363	0	1.25E+02	125.409
b2697_ion_BIND	Binding of ions to b2697	1 b2697_m + 1 zn2 <==> 1 b2697_m_Zn	reversible	Metallo-ion Binding	-1.00E+09	1.00E+09	0	2.65E+03	2.65E+03	0	3.08E+03	3080.363	0	1.25E+02	125.409
b2697_mRNA_CONV	convsersion of mRNA to mRNA_1 (synthetic rxn)	1 b2697_mRNA --> 1 b2697_mRNA_1	irreversible	Translation	0.00E+00	1.00E+09	0	5.01E+01	5.01E+01	0	8.85E+01	88.508	0	5.39E-01	0.539
b2697_mRNA_degr1	Degradation of b2697_mRNA I	1 Orn_dim + 657 atp + 1 b2697_mRNA_2 + 1 degradosome + 657 h2o <==> 657 adp + 1 b2697_mRNA_2_degr + 657 h + 657 pi	reversible	mRNA degradation	-1.00E+09	1.00E+09	0	5.01E+01	5.01E+01	0	8.85E+01	88.508	0	5.39E-01	0.539
b2697_mRNA_degr2	Degradation of b2697_mRNA II	1 b2697_mRNA_2_degr + 2630 h2o --> 1 Orn_dim_inact + 626 amp 1 atp + 637 cmp + 1 degradosome_inact + 774 gmp + 2630 h + 593 ump	irreversible	mRNA degradation	0.00E+00	5.39E-01	0	5.01E+01	5.01E+01	0	8.85E+01	88.508	0	5.39E-01	0.539
b2697_maturation1	Polypeptide b2697 peptide deformylas and methionine aminopeptidase complex	1 Def_mono + 1 Map_mono + 1 b2697_aa <==> 1 b2697_def_map_cplx	reversible	Protein Maturation	-1.00E+09	1.00E+09	0	2.65E+03	2.65E+03	0	3.08E+03	3080.363	0	1.25E+02	125.409
b2697_maturation2	b2697 formation	1 b2697_def_map_cplx + 2 h2o --> 1 Def_mono_inact + 1 Map_mono_inact + 1 b2697_m + 1 for + 1 met-L	irreversible	Protein Maturation	0.00E+00	1.00E+09	0	2.65E+03	2.65E+03	0	3.08E+03	3080.363	0	1.25E+02	125.409
b2697_v1_mRNA_CONV	convsersion of mRNA to mRNA_1 (synthetic rxn)	1 b2697_v1_mRNA --> 1 b2697_mRNA_1	irreversible	Translation	0.00E+00	1.00E+09	0	5.01E+01	5.01E+01	0	8.85E+01	88.508	0	5.39E-01	0.539
b2741_fold_spon	b2741_m folding: spontanous	1 b2741_m --> 1 RpoS_mono	irreversible	Protein Folding	0.00E+00	1.00E+09	0	2.83E+03	2.83E+03	0	4.81E+03	4808.371	0	1.39E+02	138.719
b2741_mRNA_CONV	convsersion of mRNA to mRNA_1 (synthetic rxn)	1 b2741_mRNA --> 1 b2741_mRNA_1	irreversible	Translation	0.00E+00	1.00E+09	0	1.42E+02	1.42E+02	0	2.51E+02	250.576	0	2.31E-01	0.231
b2741_mRNA_degr1	Degradation of b2741_mRNA I	1 Orn_dim + 248 atp + 1 b2741_mRNA_2 + 1 degradosome + 248 h2o <==> 248 adp + 1 b2741_mRNA_2_degr + 248 h + 248 pi	reversible	mRNA degradation	-1.00E+09	1.00E+09	0	1.42E+02	1.42E+02	0	2.51E+02	250.576	0	2.31E-01	0.231
b2741_mRNA_degr2	Degradation of b2741_mRNA II	1 b2741_mRNA_2_degr + 992 h2o --> 1 Orn_dim_inact + 253 amp + 1 atp + 225 cmp + 1 degradosome_inact + 292 gmp + 992 h + 222 ump	irreversible	mRNA degradation	0.00E+00	2.31E-01	0	1.42E+02	1.42E+02	0	2.51E+02	250.576	0	2.31E-01	0.231
b2741_maturation1	Polypeptide b2741 peptide deformylas and methionine aminopeptidase complex	1 Def_mono + 1 Map_mono + 1 b2741_aa <==> 1 b2741_def_map_cplx	reversible	Protein Maturation	-1.00E+09	1.00E+09	0	2.83E+03	2.83E+03	0	4.81E+03	4808.371	0	1.39E+02	138.719
b2741_maturation2	b2741 formation	1 b2741_def_map_cplx + 2 h2o --> 1 Def_mono_inact + 1 Map_mono_inact + 1 b2741_m + 1 for + 1 met-L	irreversible	Protein Maturation	0.00E+00	1.00E+09	0	2.83E+03	2.83E+03	0	4.81E+03	4808.371	0	1.39E+02	138.719
b2741_v1_mRNA_CONV	convsersion of mRNA to mRNA_1 (synthetic rxn)	1 b2741_v1_mRNA --> 1 b2741_mRNA_1	irreversible	Translation	0.00E+00	1.00E+09	0	5.83E+01	5.83E+01	0	1.03E+02	102.8825	0	2.31E-01	0.231
b2741_v2_mRNA_CONV	convsersion of mRNA to mRNA_1 (synthetic rxn)	1 b2741_v2_mRNA --> 1 b2741_mRNA_1	irreversible	Translation	0.00E+00	1.00E+09	0	5.83E+01	5.83E+01	0	1.03E+02	102.8825	0	2.31E-01	0.231
b2742_fold_spon	b2742_m folding: spontanous	1 b2742_m --> 1 NlpD_mono	irreversible	Protein Folding	0.00E+00	1.00E+09	0	1.55E+03	1.55E+03	0	2.53E+03	2529.614	0	2.71E+02	271.282
b2742_mRNA_CONV	convsersion of mRNA to mRNA_1 (synthetic rxn)	1 b2742_mRNA --> 1 b2742_mRNA_1	irreversible	Translation	0.00E+00	1.00E+09	0	5.83E+01	5.83E+01	0	1.03E+02	102.8825	0	2.31E-01	0.231
b2742_mRNA_degr1	Degradation of b2742_mRNA I	1 Orn_dim + 285 atp + 1 b2742_mRNA_2 + 1 degradosome + 285 h2o <==> 285 adp + 1 b2742_mRNA_2_degr + 285 h + 285 pi	reversible	mRNA degradation	-1.00E+09	1.00E+09	0	5.83E+01	5.83E+01	0	1.03E+02	102.8825	0	2.31E-01	0.231
b2742_mRNA_degr2	Degradation of b2742_mRNA II	1 b2742_mRNA_2_degr + 1139 h2o --> 1 Orn_dim_inact + 312 amp 1 atp + 308 cmp + 1 degradosome_inact + 290 gmp + 1139 h + 229 ump	irreversible	mRNA degradation	0.00E+00	4.02E-01	0	5.83E+01	5.83E+01	0	1.03E+02	102.8825	0	2.31E-01	0.231
b2742_maturation1	Polypeptide b2742 peptide deformylas and methionine aminopeptidase complex	1 Def_mono + 1 Map_mono + 1 b2742_aa <==> 1 b2742_def_map_cplx	reversible	Protein Maturation	-1.00E+09	1.00E+09	0	1.55E+03	1.55E+03	0	2.53E+03	2529.614	0	2.71E+02	271.282
b2742_maturation2	b2742 formation	1 b2742_def_map_cplx + 26 h2o --> 1 Def_mono_inact + 1 Map_mono_inact + 4 ala-L + 2 arg-L + 1 b2742_m + 1 for + 2 gly + 1 ile-L + 4 leu-L + 1 lys-L + 1 met-L + 1 phe-L + 1 pro-L + 4 ser-L + 1 th L + 1 trp-L + 2 val-L	irreversible	Protein Maturation	0.00E+00	1.00E+09	0	1.55E+03	1.55E+03	0	2.53E+03	2529.614	0	2.71E+02	271.282
b2742_v1_mRNA_CONV	convsersion of mRNA to mRNA_1 (synthetic rxn)	1 b2742_v1_mRNA --> 1 b2742_mRNA_1	irreversible	Translation	0.00E+00	1.00E+09	0	5.83E+01	5.83E+01	0	1.03E+02	102.8825	0	2.31E-01	0.231
b2745_DNA_act_bind	b2745_DNA binding of activator	1 b2745_DNA_neu --> 1 b2745_DNA_act	irreversible	Transcription Regulation	0.00E+00	1.00E+09	0	1.34E+02	1.34E+02	0	2.37E+02	236.8891	0	2.29E-01	0.229
b2745_fold_spon	b2745_m folding: spontanous	1 b2745_m --> 1 TruD_mono	irreversible	Protein Folding	0.00E+00	1.00E+09	0	2.77E+03	2.77E+03	0	4.82E+03	4823.381	0	9.02E+01	90.18
b2745_mRNA_CONV	convsersion of mRNA to mRNA_1 (synthetic rxn)	1 b2745_mRNA --> 1 b2745_mRNA_1	irreversible	Translation	0.00E+00	1.00E+09	0	1.34E+02	1.34E+02	0	2.37E+02	236.8891	0	2.29E-01	0.229
b2745_mRNA_degr1	Degradation of b2745_mRNA I	1 Orn_dim + 262 atp + 1 b2745_mRNA_2 + 1 degradosome + 262 h2o <==> 262 adp + 1 b2745_mRNA_2_degr + 262 h + 262 pi	reversible	mRNA degradation	-1.00E+09	1.00E+09	0	1.34E+02	1.34E+02	0	2.37E+02	236.8891	0	2.29E-01	0.229
b2745_mRNA_degr2	Degradation of b2745_mRNA II	1 b2745_mRNA_2_degr + 1049 h2o --> 1 Orn_dim_inact + 261 amp 1 atp + 238 cmp + 1 degradosome_inact + 310 gmp + 1049 h + 240 ump	irreversible	mRNA degradation	0.00E+00	2.29E-01	0	1.34E+02	1.34E+02	0	2.37E+02	236.8891	0	2.29E-01	0.229
b2745_maturation1	Polypeptide b2745 peptide deformylas complex	1 Def_mono + 1 b2745_aa <==> 1 b2745_def_cplx	reversible	Protein Maturation	-1.00E+09	1.00E+09	0	2.77E+03	2.77E+03	0	4.82E+03	4823.381	0	9.02E+01	90.18
b2745_maturation2	b2745 formation	1 b2745_def_cplx + 1 h2o --> 1 Def_mono_inact + 1 b2745_m + 1 for	irreversible	Protein Maturation	0.00E+00	1.00E+09	0	2.77E+03	2.77E+03	0	4.82E+03	4823.381	0	9.02E+01	90.18
b2779_fold_GroEL/ES_1	b2779_m folding: GroEL/ES mediated: polypeptide is going in GroEL/ES complex	1 GroEL.(7)ADP.cisGroES + 7 atp + 1 b2779_m_Mg + 1 transGroES_hepta --> 7 adp + 1 b2779_m_GroEL.(7)ATP.transGroES + 1 cisGroES_hepta	irreversible	Protein Folding	0.00E+00	1.00E+09	0	4.40E+02	4.40E+02	0	4.77E+02	477.4116	0	5.76E+01	57.629
b2779_fold_GroEL/ES_2	b2779_m folding: GroEL/ES mediated: folding of polypeptide under ATP hydrolysis	1 b2779_m_GroEL.(7)ATP.transGroES + 7 h2o --> 1 Eno_GroEL.(7)ADP.transGroES + 7 h + 7 pi	irreversible	Protein Folding	0.00E+00	1.00E+09	0	4.40E+02	4.40E+02	0	4.77E+02	477.4116	0	5.76E+01	57.629
b2779_fold_GroEL/ES_3	b2779_m folding: GroEL/ES mediated: release of native protein	1 Eno_GroEL.(7)ADP.transGroES --> 1 Eno_mono + 1 GroEL.(7)ADP.transGroES	irreversible	Protein Folding	0.00E+00	1.00E+09	0	4.40E+02	4.40E+02	0	4.77E+02	477.4116	0	5.76E+01	57.629

b2779_fold_KJE_1	b2779_m folding: KJE mediated	1 DnaJ_dim + 1 DnaK_mono.ATP + 1 b2779_m_Mg <==> 1 b2779_m_DnaKJ_complex	reversible	Protein Folding	-1.00E+09	1.00E+09	0	4.40E+02	4.40E+02	0	4.77E+02	477.4116	0	5.76E+01	57.629
b2779_fold_KJE_2	b2779_m folding: KJE mediated	1 GrpE_dim + 1 b2779_m_DnaKJ_complex + 1 h2o --> 1 DnaJ_dim_inact + 1 Eno_DnaK_GrpE_complex + 1 adp + 1 h + 1 pi	irreversible	Protein Folding	0.00E+00	1.00E+09	0	4.40E+02	4.40E+02	0	4.77E+02	477.4116	0	5.76E+01	57.629
b2779_fold_KJE_3	b2779_m folding: KJE mediated	1 Eno_DnaK_GrpE_complex + 1 atp --> 1 DnaK_mono.ATP_inact + Eno_mono + 1 GrpE_dim_inact	irreversible	Protein Folding	0.00E+00	1.00E+09	0	4.40E+02	4.40E+02	0	4.77E+02	477.4116	0	5.76E+01	57.629
b2779_fold_spon	b2779_m folding: spontanous	1 b2779_m_Mg --> 1 Eno_mono	irreversible	Protein Folding	0.00E+00	1.00E+09	0	4.40E+02	4.40E+02	0	4.77E+02	477.4116	0	5.76E+01	57.629
b2779_ion_BIND	Binding of ions to b2779	1 b2779_m + 2 mg2 <==> 1 b2779_m_Mg	reversible	Metallo-ion Binding	-1.00E+09	1.00E+09	0	4.40E+02	4.40E+02	0	4.77E+02	477.4116	0	5.76E+01	57.629
b2779_mRNA_CONV	convsersion of mRNA to mRNA_1 (synthetic rxn)	1 b2779_mRNA --> 1 b2779_mRNA_1	irreversible	Translation	0.00E+00	1.00E+09	0	4.31E+01	4.31E+01	0	7.52E+01	75.1818	0	4.14E-01	0.414
b2779_mRNA_degr1	Degradation of b2779_mRNA I	1 Orn_dim + 324 atp + 1 b2779_mRNA_2 + 1 degradosome + 324 h2o <==> 324 adp + 1 b2779_mRNA_2_degr + 324 h + 324 pi	reversible	mRNA degradation	-1.00E+09	1.00E+09	0	4.31E+01	4.31E+01	0	7.52E+01	75.1818	0	4.14E-01	0.414
b2779_mRNA_degr2	Degradation of b2779_mRNA II	1 b2779_mRNA_2_degr + 1298 h2o --> 1 Orn_dim_inact + 354 amp 320 cmp + 1 degradosome_inact + 334 gmp + 1298 h + 291 ump	irreversible	mRNA degradation	0.00E+00	4.14E-01	0	4.31E+01	4.31E+01	0	7.52E+01	75.1818	0	4.14E-01	0.414
b2779_maturation1	Polypeptide b2779 peptide deformylas and methionine aminopeptidase complex	1 Def_mono + 1 Map_mono + 1 b2779_aa <==> 1 b2779_def_map_cplx	reversible	Protein Maturation	-1.00E+09	1.00E+09	0	4.40E+02	4.40E+02	0	4.77E+02	477.4116	0	5.76E+01	57.629
b2779_maturation2	b2779 formation	1 b2779_def_map_cplx + 2 h2o --> 1 Def_mono_inact + 1 Map_mono_inact + 1 b2779_m + 1 for + 1 met-L	irreversible	Protein Maturation	0.00E+00	1.00E+09	0	4.40E+02	4.40E+02	0	4.77E+02	477.4116	0	5.76E+01	57.629
b2780_fold_KJE_1	b2780_m folding: KJE mediated	1 DnaJ_dim + 1 DnaK_mono.ATP + 1 b2780_m <==> 1 b2780_m_DnaKJ_complex	reversible	Protein Folding	-1.00E+09	1.00E+09	0	1.57E+03	1.57E+03	0	2.60E+03	2597.498	0	2.05E+02	204.928
b2780_fold_KJE_2	b2780_m folding: KJE mediated	1 GrpE_dim + 1 b2780_m_DnaKJ_complex + 1 h2o --> 1 DnaJ_dim_inact + 1 PyrG_DnaK_GrpE_complex + 1 adp + 1 h + 1 pi	irreversible	Protein Folding	0.00E+00	1.00E+09	0	1.57E+03	1.57E+03	0	2.60E+03	2597.498	0	2.05E+02	204.928
b2780_fold_KJE_3	b2780_m folding: KJE mediated	1 PyrG_DnaK_GrpE_complex + 1 atp --> 1 DnaK_mono.ATP_inact + 1 GrpE_dim_inact + 1 PyrG_mono	irreversible	Protein Folding	0.00E+00	1.00E+09	0	1.57E+03	1.57E+03	0	2.60E+03	2597.498	0	2.05E+02	204.928
b2780_fold_spon	b2780_m folding: spontanous	1 b2780_m --> 1 PyrG_mono	irreversible	Protein Folding	0.00E+00	1.00E+09	0	1.57E+03	1.57E+03	0	2.60E+03	2597.498	0	2.05E+02	204.928
b2780_mRNA_degr1	Degradation of b2780_mRNA I	1 Orn_dim + 409 atp + 1 b2780_mRNA_2 + 1 degradosome + 409 h2o <==> 409 adp + 1 b2780_mRNA_2_degr + 409 h + 409 pi	reversible	mRNA degradation	-1.00E+09	1.00E+09	0	4.31E+01	4.31E+01	0	7.52E+01	75.1818	0	4.14E-01	0.414
b2780_mRNA_degr2	Degradation of b2780_mRNA II	1 b2780_mRNA_2_degr + 1637 h2o --> 1 Orn_dim_inact + 397 amp 1 atp + 406 cmp + 1 degradosome_inact + 444 gmp + 1637 h + 390 ump	irreversible	mRNA degradation	0.00E+00	5.82E-01	0	4.31E+01	4.31E+01	0	7.52E+01	75.1818	0	4.14E-01	0.414
b2780_maturation1	Polypeptide b2780 peptide deformylas and methionine aminopeptidase complex	1 Def_mono + 1 Map_mono + 1 b2780_aa <==> 1 b2780_def_map_cplx	reversible	Protein Maturation	-1.00E+09	1.00E+09	0	1.57E+03	1.57E+03	0	2.60E+03	2597.498	0	2.05E+02	204.928
b2780_maturation2	b2780 formation	1 b2780_def_map_cplx + 2 h2o --> 1 Def_mono_inact + 1 Map_mono_inact + 1 b2780_m + 1 for + 1 met-L	irreversible	Protein Maturation	0.00E+00	1.00E+09	0	1.57E+03	1.57E+03	0	2.60E+03	2597.498	0	2.05E+02	204.928
b2780_v2_mRNA_CONV	convsersion of mRNA to mRNA_1 (synthetic rxn)	1 b2780_v2_mRNA --> 1 b2780_mRNA_1	irreversible	Translation	0.00E+00	1.00E+09	0	4.31E+01	4.31E+01	0	7.52E+01	75.1818	0	4.14E-01	0.414
b2785_fold_spon	b2785_m folding: spontanous	1 b2785_m_FeS --> 1 RumA_mono	irreversible	Protein Folding	0.00E+00	1.00E+09	0	2.56E+03	2.56E+03	0	4.47E+03	4466.434	0	7.79E+01	77.8625
b2785_iron_sulfur_BIND1	Incorporation of [4Fe-4S]2+ in b2785	1 IscU_dim_[4Fe-4S] + 1 b2785_m <==> 1 b2785_m_IscU_cplx	reversible	Iron-sulfur cluster incorporation	-1.00E+09	1.00E+09	0	2.56E+03	2.56E+03	0	4.47E+03	4466.434	0	7.79E+01	77.8625
b2785_iron_sulfur_BIND2	Incorporation of [4Fe-4S]2+ in b2785	1 b2785_m_IscU_cplx <==> 2 IscU_mono + 1 b2785_m_FeS	reversible	Iron-sulfur cluster incorporation	-1.00E+09	1.00E+09	0	2.56E+03	2.56E+03	0	4.47E+03	4466.434	0	7.79E+01	77.8625
b2785_mRNA_CONV	convsersion of mRNA to mRNA_1 (synthetic rxn)	1 b2785_mRNA --> 1 b2785_mRNA_1	irreversible	Translation	0.00E+00	1.00E+09	0	9.95E+01	9.95E+01	0	1.76E+02	175.6374	0	9.45E-02	0.0945
b2785_mRNA_degr1	Degradation of b2785_mRNA I	1 Orn_dim + 325 atp + 1 b2785_mRNA_2 + 1 degradosome + 325 h2o <==> 325 adp + 1 b2785_mRNA_2_degr + 325 h + 325 pi	reversible	mRNA degradation	-1.00E+09	1.00E+09	0	9.95E+01	9.95E+01	0	1.76E+02	175.6374	0	9.45E-02	0.0945
b2785_mRNA_degr2	Degradation of b2785_mRNA II	1 b2785_mRNA_2_degr + 1301 h2o --> 1 Orn_dim_inact + 330 amp 1 atp + 321 cmp + 1 degradosome_inact + 349 gmp + 1301 h + 301 ump	irreversible	mRNA degradation	0.00E+00	9.45E-02	0	9.95E+01	9.95E+01	0	1.76E+02	175.6374	0	9.45E-02	0.0945
b2785_maturation1	Polypeptide b2785 peptide deformylas and methionine aminopeptidase complex	1 Def_mono + 1 Map_mono + 1 b2785_aa <==> 1 b2785_def_map_cplx	reversible	Protein Maturation	-1.00E+09	1.00E+09	0	2.56E+03	2.56E+03	0	4.47E+03	4466.434	0	7.79E+01	77.8625
b2785_maturation2	b2785 formation	1 b2785_def_map_cplx + 2 h2o --> 1 Def_mono_inact + 1 Map_mono_inact + 1 b2785_m + 1 for + 1 met-L	irreversible	Protein Maturation	0.00E+00	1.00E+09	0	2.56E+03	2.56E+03	0	4.47E+03	4466.434	0	7.79E+01	77.8625
b2790_fold_spon	b2790_m folding: spontanous	1 b2790_m --> 1 YqcA_mono	irreversible	Protein Folding	0.00E+00	1.00E+09	0	7.47E+02	7.47E+02	0	1.25E+03	1253.289	0	8.63E+01	86.312
b2790_mRNA_CONV	convsersion of mRNA to mRNA_1 (synthetic rxn)	1 b2790_mRNA --> 1 b2790_mRNA_1	irreversible	Translation	0.00E+00	1.00E+09	0	8.27E+01	8.27E+01	0	1.46E+02	146.0611	0	1.89E-01	0.189
b2790_mRNA_degr1	Degradation of b2790_mRNA I	1 Orn_dim + 112 atp + 1 b2790_mRNA_2 + 1 degradosome + 112 h2o <==> 112 adp + 1 b2790_mRNA_2_degr + 112 h + 112 pi	reversible	mRNA degradation	-1.00E+09	1.00E+09	0	8.27E+01	8.27E+01	0	1.46E+02	146.0611	0	1.89E-01	0.189
b2790_mRNA_degr2	Degradation of b2790_mRNA II	1 b2790_mRNA_2_degr + 449 h2o --> 1 Orn_dim_inact + 107 amp + 100 cmp + 1 degradosome_inact + 137 gmp + 449 h + 106 ump	irreversible	mRNA degradation	0.00E+00	2.69E-01	0	8.27E+01	8.27E+01	0	1.46E+02	146.0611	0	1.89E-01	0.189
b2790_maturation1	Polypeptide b2790 peptide deformylas and methionine aminopeptidase complex	1 Def_mono + 1 Map_mono + 1 b2790_aa <==> 1 b2790_def_map_cplx	reversible	Protein Maturation	-1.00E+09	1.00E+09	0	7.47E+02	7.47E+02	0	1.25E+03	1253.289	0	8.63E+01	86.312
b2790_maturation2	b2790 formation	1 b2790_def_map_cplx + 2 h2o --> 1 Def_mono_inact + 1 Map_mono_inact + 1 b2790_m + 1 for + 1 met-L	irreversible	Protein Maturation	0.00E+00	1.00E+09	0	7.47E+02	7.47E+02	0	1.25E+03	1253.289	0	8.63E+01	86.312
b2791_fold_spon	b2791_m folding: spontanous	1 b2791_m --> 1 YqcB_mono	irreversible	Protein Folding	0.00E+00	1.00E+09	0	1.33E+03	1.33E+03	0	2.28E+03	2282.417	0	9.43E+01	94.335
b2791_mRNA_CONV	convsersion of mRNA to mRNA_1 (synthetic rxn)	1 b2791_mRNA --> 1 b2791_mRNA_1	irreversible	Translation	0.00E+00	1.00E+09	0	8.27E+01	8.27E+01	0	1.46E+02	146.0611	0	1.89E-01	0.189
b2791_mRNA_degr1	Degradation of b2791_mRNA I	1 Orn_dim + 195 atp + 1 b2791_mRNA_2 + 1 degradosome + 195 h2o <==> 195 adp + 1 b2791_mRNA_2_degr + 195 h + 195 pi	reversible	mRNA degradation	-1.00E+09	1.00E+09	0	8.27E+01	8.27E+01	0	1.46E+02	146.0611	0	1.89E-01	0.189
b2791_mRNA_degr2	Degradation of b2791_mRNA II	1 b2791_mRNA_2_degr + 781 h2o --> 1 Orn_dim_inact + 184 amp + 196 cmp + 1 degradosome_inact + 227 gmp + 781 h + 175 ump	irreversible	mRNA degradation	0.00E+00	1.89E-01	0	8.27E+01	8.27E+01	0	1.46E+02	146.0611	0	1.89E-01	0.189
b2791_maturation1	Polypeptide b2791 peptide deformylas and methionine aminopeptidase complex	1 Def_mono + 1 Map_mono + 1 b2791_aa <==> 1 b2791_def_map_cplx	reversible	Protein Maturation	-1.00E+09	1.00E+09	0	1.33E+03	1.33E+03	0	2.28E+03	2282.417	0	9.43E+01	94.335
b2791_maturation2	b2791 formation	1 b2791_def_map_cplx + 2 h2o --> 1 Def_mono_inact + 1 Map_mono_inact + 1 b2791_m + 1 for + 1 met-L	irreversible	Protein Maturation	0.00E+00	1.00E+09	0	1.33E+03	1.33E+03	0	2.28E+03	2282.417	0	9.43E+01	94.335
b2792_fold_spon	b2792_m folding: spontanous	1 b2792_m --> 1 YqcC_mono	irreversible	Protein Folding	0.00E+00	1.00E+09	0	5.73E+02	5.73E+02	0	9.53E+02	953.1668	0	7.79E+01	77.934
b2792_mRNA_CONV	convsersion of mRNA to mRNA_1 (synthetic rxn)	1 b2792_mRNA --> 1 b2792_mRNA_1	irreversible	Translation	0.00E+00	1.00E+09	0	8.27E+01	8.27E+01	0	1.46E+02	146.0611	0	1.89E-01	0.189
b2792_mRNA_degr1	Degradation of b2792_mRNA I	1 Orn_dim + 82 atp + 1 b2792_mRNA_2 + 1 degradosome + 82 h2o <==> 82 adp + 1 b2792_mRNA_2_degr + 82 h + 82 pi	reversible	mRNA degradation	-1.00E+09	1.00E+09	0	8.27E+01	8.27E+01	0	1.46E+02	146.0611	0	1.89E-01	0.189
b2792_mRNA_degr2	Degradation of b2792_mRNA II	1 b2792_mRNA_2_degr + 329 h2o --> 1 Orn_dim_inact + 77 amp + 1 atp + 87 cmp + 1 degradosome_inact + 86 gmp + 329 h + 79 ump	irreversible	mRNA degradation	0.00E+00	2.03E-01	0	8.27E+01	8.27E+01	0	1.46E+02	146.0611	0	1.89E-01	0.189
b2792_maturation1	Polypeptide b2792 peptide deformylas and methionine aminopeptidase complex	1 Def_mono + 1 Map_mono + 1 b2792_aa <==> 1 b2792_def_map_cplx	reversible	Protein Maturation	-1.00E+09	1.00E+09	0	5.73E+02	5.73E+02	0	9.53E+02	953.1668	0	7.79E+01	77.934
b2792_maturation2	b2792 formation	1 b2792_def_map_cplx + 2 h2o --> 1 Def_mono_inact + 1 Map_mono_inact + 1 b2792_m + 1 for + 1 met-L	irreversible	Protein Maturation	0.00E+00	1.00E+09	0	5.73E+02	5.73E+02	0	9.53E+02	953.1668	0	7.79E+01	77.934
b2794_fold_spon	b2794_m folding: spontanous	1 b2794_m --> 1 QueF_mono	irreversible	Protein Folding	0.00E+00	1.00E+09	0	2.52E+03	2.52E+03	0	4.40E+03	4400.412	0	6.52E+01	65.184
b2794_mRNA_CONV	convsersion of mRNA to mRNA_1 (synthetic rxn)	1 b2794_mRNA --> 1 b2794_mRNA_1	irreversible	Translation	0.00E+00	1.00E+09	0	1.54E+02	1.54E+02	0	2.71E+02	271.0557	0	1.04E-01	0.104

b2794_mRNA_degr1	Degradation of b2794_mRNA I	1 Orn_dim + 212 atp + 1 b2794_mRNA_2 + 1 degradosome + 212 h2o <==> 212 adp + 1 b2794_mRNA_2_degr + 212 h + 212 pi	reversible	mRNA degradation	-1.00E+09	1.00E+09	0	1.54E+02	1.54E+02	0	2.71E+02	271.0557	0	1.04E-01	0.104
b2794_mRNA_degr2	Degradation of b2794_mRNA II	1 b2794_mRNA_2_degr + 848 h2o --> 1 Orn_dim_inact + 219 amp + 1 atp + 208 cmp + 1 degradosome_inact + 207 gmp + 848 h + 214 ump	irreversible	mRNA degradation	0.00E+00	1.04E-01	0	1.54E+02	1.54E+02	0	2.71E+02	271.0557	0	1.04E-01	0.104
b2794_maturation1	Polypeptide b2794 peptide deformylas and methionine aminopeptidase complex	1 Def_mono + 1 Map_mono + 1 b2794_aa <==> 1 b2794_def_map_cplx	reversible	Protein Maturation	-1.00E+09	1.00E+09	0	2.52E+03	2.52E+03	0	4.40E+03	4400.412	0	6.52E+01	65.184
b2794_maturation2	b2794 formation	1 b2794_def_map_cplx + 2 h2o --> 1 Def_mono_inact + 1 Map_mono_inact + 1 b2794_m + 1 for + 1 met-L	irreversible	Protein Maturation	0.00E+00	1.00E+09	0	2.52E+03	2.52E+03	0	4.40E+03	4400.412	0	6.52E+01	65.184
b2814_tRNA_20_MODIF_1	modification of position 20 nt (D) in metZ by DusA_mono	1 DusA_mono + 1 b2814_tRNA_Mg2 + 1 h + 1 nadph <==> 1 b2814_tRNA_Mg2_DusA_mono_cplx	reversible	tRNA modification	-1.00E+09	1.00E+09	0	3.85E+02	3.85E+02	0	9.37E+00	9.37	0	9.37E+00	9.37
b2814_tRNA_20_MODIF_2	modification of position 20 nt (D) in metZ by DusA_mono	1 b2814_tRNA_Mg2_DusA_mono_cplx --> 1 DusA_mono + 1 b2814_tRNA_1 + 1 nadp	irreversible	tRNA modification	0.00E+00	1.00E+09	0	3.85E+02	3.85E+02	0	9.37E+00	9.37	0	9.37E+00	9.37
b2814_tRNA_32_MODIF_1	modification of position 32 nt (Cm) in metZ by MeT_tRNA_pos_32_Cm	1 MeT_tRNA_pos_32_Cm + 1 amet + 1 b2814_tRNA_1 <==> 1 b2814_tRNA_1_MeT_tRNA_pos_32_Cm_cplx	reversible	tRNA modification	-1.00E+09	1.00E+09	0	3.85E+02	3.85E+02	0	9.37E+00	9.37	0	9.37E+00	9.37
b2814_tRNA_32_MODIF_2	modification of position 32 nt (Cm) in metZ by MeT_tRNA_pos_32_Cm	1 b2814_tRNA_1_MeT_tRNA_pos_32_Cm_cplx --> 1 MeT_tRNA_pos_32_Cm_inact + 1 ahcys + 1 b2814_tRNA_2 + 1 h	irreversible	tRNA modification	0.00E+00	1.00E+09	0	3.85E+02	3.85E+02	0	9.37E+00	9.37	0	9.37E+00	9.37
b2814_tRNA_46_MODIF_1	modification of position 46 nt (m7G) in metZ by YggH_mono	1 YggH_mono + 1 amet + 1 b2814_tRNA_2 <==> 1 b2814_tRNA_2_YggH_mono_cplx	reversible	tRNA modification	-1.00E+09	1.00E+09	0	3.85E+02	3.85E+02	0	9.37E+00	9.37	0	9.37E+00	9.37
b2814_tRNA_46_MODIF_2	modification of position 46 nt (m7G) in metZ by YggH_mono	1 b2814_tRNA_2_YggH_mono_cplx --> 1 YggH_mono_inact + 1 ahcys + 1 b2814_tRNA_3 + 1 h	irreversible	tRNA modification	0.00E+00	1.00E+09	0	3.85E+02	3.85E+02	0	9.37E+00	9.37	0	9.37E+00	9.37
b2814_tRNA_54_MODIF_1	modification of position 54 nt (m5U) in metZ by TrmA_mono	1 TrmA_mono + 1 amet + 1 b2814_tRNA_3 <==> 1 b2814_tRNA_3_TrmA_mono_cplx	reversible	tRNA modification	-1.00E+09	1.00E+09	0	3.85E+02	3.85E+02	0	9.37E+00	9.37	0	9.37E+00	9.37
b2814_tRNA_54_MODIF_2	modification of position 54 nt (m5U) in metZ by TrmA_mono	1 b2814_tRNA_3_TrmA_mono_cplx --> 1 TrmA_mono_inact + 1 ahcys + 1 b2814_tRNA_4 + 1 h	irreversible	tRNA modification	0.00E+00	1.00E+09	0	3.85E+02	3.85E+02	0	9.37E+00	9.37	0	9.37E+00	9.37
b2814_tRNA_55_MODIF_1	modification of position 55 nt (Y) in metZ by TruB_mono	1 TruB_mono + 1 b2814_tRNA_4 <==> 1 b2814_tRNA_4_TruB_mono_cplx	reversible	tRNA modification	-1.00E+09	1.00E+09	0	3.85E+02	3.85E+02	0	9.37E+00	9.37	0	9.37E+00	9.37
b2814_tRNA_55_MODIF_2	modification of position 55 nt (Y) in metZ by TruB_mono	1 b2814_tRNA_4_TruB_mono_cplx --> 1 TruB_mono_inact + 1 b2814_tRNA_5	irreversible	tRNA modification	0.00E+00	1.00E+09	0	3.85E+02	3.85E+02	0	9.37E+00	9.37	0	9.37E+00	9.37
b2814_tRNA_8_MODIF_1	modification of position 8 nt (s4U) in metZ by ThiI_mono	1 IscS_dim_S-SH + 1 ThiI_mono + 1 atp + 1 b2814_tRNA_5 + 1 mg2 + 1 trdrd <==> 1 b2814_tRNA_5_ThiI_mono_cplx	reversible	tRNA modification	-1.00E+09	1.00E+09	0	3.85E+02	3.85E+02	0	9.37E+00	9.37	0	9.37E+00	9.37
b2814_tRNA_8_MODIF_2	modification of position 8 nt (s4U) in metZ by ThiI_mono	1 b2814_tRNA_5_ThiI_mono_cplx --> 1 IscS_dim_S-H + 1 ThiI_mono_inact + 1 amp + 1 h + 1 metZ_tRNA + 1 mg2 + 1 ppi + 1 trdox	irreversible	tRNA modification	0.00E+00	1.00E+09	0	3.85E+02	3.85E+02	0	9.37E+00	9.37	0	9.37E+00	9.37
b2814_tRNA_Mg_BIND	Binding of two Mg2 to b2814_tRNA	1 b2814_RNA + 2 mg2 <==> 1 b2814_tRNA_Mg2	reversible	Metallo-ion binding	-1.00E+09	1.00E+09	0	3.85E+02	3.85E+02	0	9.37E+00	9.37	0	9.37E+00	9.37
b2815_tRNA_20_MODIF_1	modification of position 20 nt (D) in metW by DusA_mono	1 DusA_mono + 1 b2815_tRNA_Mg2 + 1 h + 1 nadph <==> 1 b2815_tRNA_Mg2_DusA_mono_cplx	reversible	tRNA modification	-1.00E+09	1.00E+09	0	3.85E+02	3.85E+02	0	9.37E+00	9.37	0	9.37E+00	9.37
b2815_tRNA_20_MODIF_2	modification of position 20 nt (D) in metW by DusA_mono	1 b2815_tRNA_Mg2_DusA_mono_cplx --> 1 DusA_mono + 1 b2815_tRNA_1 + 1 nadp	irreversible	tRNA modification	0.00E+00	1.00E+09	0	3.85E+02	3.85E+02	0	9.37E+00	9.37	0	9.37E+00	9.37
b2815_tRNA_32_MODIF_1	modification of position 32 nt (Cm) in metW by MeT_tRNA_pos_32_Cm	1 MeT_tRNA_pos_32_Cm + 1 amet + 1 b2815_tRNA_1 <==> 1 b2815_tRNA_1_MeT_tRNA_pos_32_Cm_cplx	reversible	tRNA modification	-1.00E+09	1.00E+09	0	3.85E+02	3.85E+02	0	9.37E+00	9.37	0	9.37E+00	9.37
b2815_tRNA_32_MODIF_2	modification of position 32 nt (Cm) in metW by MeT_tRNA_pos_32_Cm	1 b2815_tRNA_1_MeT_tRNA_pos_32_Cm_cplx --> 1 MeT_tRNA_pos_32_Cm_inact + 1 ahcys + 1 b2815_tRNA_2 + 1 h	irreversible	tRNA modification	0.00E+00	1.00E+09	0	3.85E+02	3.85E+02	0	9.37E+00	9.37	0	9.37E+00	9.37
b2815_tRNA_46_MODIF_1	modification of position 46 nt (m7G) in metW by YggH_mono	1 YggH_mono + 1 amet + 1 b2815_tRNA_2 <==> 1 b2815_tRNA_2_YggH_mono_cplx	reversible	tRNA modification	-1.00E+09	1.00E+09	0	3.85E+02	3.85E+02	0	9.37E+00	9.37	0	9.37E+00	9.37
b2815_tRNA_46_MODIF_2	modification of position 46 nt (m7G) in metW by YggH_mono	1 b2815_tRNA_2_YggH_mono_cplx --> 1 YggH_mono_inact + 1 ahcys + 1 b2815_tRNA_3 + 1 h	irreversible	tRNA modification	0.00E+00	1.00E+09	0	3.85E+02	3.85E+02	0	9.37E+00	9.37	0	9.37E+00	9.37
b2815_tRNA_54_MODIF_1	modification of position 54 nt (m5U) in metW by TrmA_mono	1 TrmA_mono + 1 amet + 1 b2815_tRNA_3 <==> 1 b2815_tRNA_3_TrmA_mono_cplx	reversible	tRNA modification	-1.00E+09	1.00E+09	0	3.85E+02	3.85E+02	0	9.37E+00	9.37	0	9.37E+00	9.37
b2815_tRNA_54_MODIF_2	modification of position 54 nt (m5U) in metW by TrmA_mono	1 b2815_tRNA_3_TrmA_mono_cplx --> 1 TrmA_mono_inact + 1 ahcys + 1 b2815_tRNA_4 + 1 h	irreversible	tRNA modification	0.00E+00	1.00E+09	0	3.85E+02	3.85E+02	0	9.37E+00	9.37	0	9.37E+00	9.37
b2815_tRNA_55_MODIF_1	modification of position 55 nt (Y) in metW by TruB_mono	1 TruB_mono + 1 b2815_tRNA_4 <==> 1 b2815_tRNA_4_TruB_mono_cplx	reversible	tRNA modification	-1.00E+09	1.00E+09	0	3.85E+02	3.85E+02	0	9.37E+00	9.37	0	9.37E+00	9.37
b2815_tRNA_55_MODIF_2	modification of position 55 nt (Y) in metW by TruB_mono	1 b2815_tRNA_4_TruB_mono_cplx --> 1 TruB_mono_inact + 1 b2815_tRNA_5	irreversible	tRNA modification	0.00E+00	1.00E+09	0	3.85E+02	3.85E+02	0	9.37E+00	9.37	0	9.37E+00	9.37
b2815_tRNA_8_MODIF_1	modification of position 8 nt (s4U) in metW by ThiI_mono	1 IscS_dim_S-SH + 1 ThiI_mono + 1 atp + 1 b2815_tRNA_5 + 1 mg2 + 1 trdrd <==> 1 b2815_tRNA_5_ThiI_mono_cplx	reversible	tRNA modification	-1.00E+09	1.00E+09	0	3.85E+02	3.85E+02	0	9.37E+00	9.37	0	9.37E+00	9.37
b2815_tRNA_8_MODIF_2	modification of position 8 nt (s4U) in metW by ThiI_mono	1 b2815_tRNA_5_ThiI_mono_cplx --> 1 IscS_dim_S-H + 1 ThiI_mono_inact + 1 amp + 1 h + 1 metW_tRNA + 1 mg2 + 1 ppi + 1 trdox	irreversible	tRNA modification	0.00E+00	1.00E+09	0	3.85E+02	3.85E+02	0	9.37E+00	9.37	0	9.37E+00	9.37
b2815_tRNA_Mg_BIND	Binding of two Mg2 to b2815_tRNA	1 b2815_RNA + 2 mg2 <==> 1 b2815_tRNA_Mg2	reversible	Metallo-ion binding	-1.00E+09	1.00E+09	0	3.85E+02	3.85E+02	0	9.37E+00	9.37	0	9.37E+00	9.37
b2816_tRNA_20_MODIF_1	modification of position 20 nt (D) in metV by DusA_mono	1 DusA_mono + 1 b2816_tRNA_Mg2 + 1 h + 1 nadph <==> 1 b2816_tRNA_Mg2_DusA_mono_cplx	reversible	tRNA modification	-1.00E+09	1.00E+09	0	3.85E+02	3.85E+02	0	9.37E+00	9.37	0	9.37E+00	9.37
b2816_tRNA_20_MODIF_2	modification of position 20 nt (D) in metV by DusA_mono	1 b2816_tRNA_Mg2_DusA_mono_cplx --> 1 DusA_mono + 1 b2816_tRNA_1 + 1 nadp	irreversible	tRNA modification	0.00E+00	1.00E+09	0	3.85E+02	3.85E+02	0	9.37E+00	9.37	0	9.37E+00	9.37
b2816_tRNA_32_MODIF_1	modification of position 32 nt (Cm) in metV by MeT_tRNA_pos_32_Cm	1 MeT_tRNA_pos_32_Cm + 1 amet + 1 b2816_tRNA_1 <==> 1 b2816_tRNA_1_MeT_tRNA_pos_32_Cm_cplx	reversible	tRNA modification	-1.00E+09	1.00E+09	0	3.85E+02	3.85E+02	0	9.37E+00	9.37	0	9.37E+00	9.37
b2816_tRNA_32_MODIF_2	modification of position 32 nt (Cm) in metV by MeT_tRNA_pos_32_Cm	1 b2816_tRNA_1_MeT_tRNA_pos_32_Cm_cplx --> 1 MeT_tRNA_pos_32_Cm_inact + 1 ahcys + 1 b2816_tRNA_2 + 1 h	irreversible	tRNA modification	0.00E+00	1.00E+09	0	3.85E+02	3.85E+02	0	9.37E+00	9.37	0	9.37E+00	9.37
b2816_tRNA_46_MODIF_1	modification of position 46 nt (m7G) in metV by YggH_mono	1 YggH_mono + 1 amet + 1 b2816_tRNA_2 <==> 1 b2816_tRNA_2_YggH_mono_cplx	reversible	tRNA modification	-1.00E+09	1.00E+09	0	3.85E+02	3.85E+02	0	9.37E+00	9.37	0	9.37E+00	9.37
b2816_tRNA_46_MODIF_2	modification of position 46 nt (m7G) in metV by YggH_mono	1 b2816_tRNA_2_YggH_mono_cplx --> 1 YggH_mono_inact + 1 ahcys + 1 b2816_tRNA_3 + 1 h	irreversible	tRNA modification	0.00E+00	1.00E+09	0	3.85E+02	3.85E+02	0	9.37E+00	9.37	0	9.37E+00	9.37
b2816_tRNA_54_MODIF_1	modification of position 54 nt (m5U) in metV by TrmA_mono	1 TrmA_mono + 1 amet + 1 b2816_tRNA_3 <==> 1 b2816_tRNA_3_TrmA_mono_cplx	reversible	tRNA modification	-1.00E+09	1.00E+09	0	3.85E+02	3.85E+02	0	9.37E+00	9.37	0	9.37E+00	9.37
b2816_tRNA_54_MODIF_2	modification of position 54 nt (m5U) in metV by TrmA_mono	1 b2816_tRNA_3_TrmA_mono_cplx --> 1 TrmA_mono_inact + 1 ahcys + 1 b2816_tRNA_4 + 1 h	irreversible	tRNA modification	0.00E+00	1.00E+09	0	3.85E+02	3.85E+02	0	9.37E+00	9.37	0	9.37E+00	9.37
b2816_tRNA_55_MODIF_1	modification of position 55 nt (Y) in metV by TruB_mono	1 TruB_mono + 1 b2816_tRNA_4 <==> 1 b2816_tRNA_4_TruB_mono_cplx	reversible	tRNA modification	-1.00E+09	1.00E+09	0	3.85E+02	3.85E+02	0	9.37E+00	9.37	0	9.37E+00	9.37
b2816_tRNA_55_MODIF_2	modification of position 55 nt (Y) in metV by TruB_mono	1 b2816_tRNA_4_TruB_mono_cplx --> 1 TruB_mono_inact + 1 b2816_tRNA_5	irreversible	tRNA modification	0.00E+00	1.00E+09	0	3.85E+02	3.85E+02	0	9.37E+00	9.37	0	9.37E+00	9.37
b2816_tRNA_8_MODIF_1	modification of position 8 nt (s4U) in metV by ThiI_mono	1 IscS_dim_S-SH + 1 ThiI_mono + 1 atp + 1 b2816_tRNA_5 + 1 mg2 + 1 trdrd <==> 1 b2816_tRNA_5_ThiI_mono_cplx	reversible	tRNA modification	-1.00E+09	1.00E+09	0	3.85E+02	3.85E+02	0	9.37E+00	9.37	0	9.37E+00	9.37
b2816_tRNA_8_MODIF_2	modification of position 8 nt (s4U) in metV by ThiI_mono	1 b2816_tRNA_5_ThiI_mono_cplx --> 1 IscS_dim_S-H + 1 ThiI_mono_inact + 1 amp + 1 h + 1 metV_tRNA + 1 mg2 + 1 ppi + 1 trdox	irreversible	tRNA modification	0.00E+00	1.00E+09	0	3.85E+02	3.85E+02	0	9.37E+00	9.37	0	9.37E+00	9.37
b2816_tRNA_Mg_BIND	Binding of two Mg2 to b2816_tRNA	1 b2816_RNA + 2 mg2 <==> 1 b2816_tRNA_Mg2	reversible	Metallo-ion binding	-1.00E+09	1.00E+09	0	3.85E+02	3.85E+02	0	9.37E+00	9.37	0	9.37E+00	9.37
b2864_tRNA_20_MODIF_1	modification of position 20 nt (D) in glyU by Dus_gen	1 Dus_gen + 1 b2864_tRNA_Mg2 + 1 h + 1 nadph <==> 1 b2864_tRNA_Mg2_Dus_gen_cplx	reversible	tRNA modification	-1.00E+09	1.00E+09	0	1.45E+03	1.45E+03	0	9.58E+00	9.58	0	9.58E+00	9.58
b2864_tRNA_20_MODIF_2	modification of position 20 nt (D) in glyU by Dus_gen	1 b2864_tRNA_Mg2_Dus_gen_cplx --> 1 Dus_gen_inact + 1 b2864_tRNA_1 + 1 nadp	irreversible	tRNA modification	0.00E+00	1.00E+09	0	1.45E+03	1.45E+03	0	9.58E+00	9.58	0	9.58E+00	9.58
b2864_tRNA_54_MODIF_1	modification of position 54 nt (m5U) in glyU by TrmA_mono	1 TrmA_mono + 1 amet + 1 b2864_tRNA_1 <==> 1 b2864_tRNA_1_TrmA_mono_cplx	reversible	tRNA modification	-1.00E+09	1.00E+09	0	1.45E+03	1.45E+03	0	9.58E+00	9.58	0	9.58E+00	9.58
b2864_tRNA_54_MODIF_2	modification of position 54 nt (m5U) in glyU by TrmA_mono	1 b2864_tRNA_1_TrmA_mono_cplx --> 1 TrmA_mono_inact + 1 ahcys + 1 b2864_tRNA_2 + 1 h	irreversible	tRNA modification	0.00E+00	1.00E+09	0	1.45E+03	1.45E+03	0	9.58E+00	9.58	0	9.58E+00	9.58
b2864_tRNA_55_MODIF_1	modification of position 55 nt (Y) in glyU by TruB_mono	1 TruB_mono + 1 b2864_tRNA_2 <==> 1 b2864_tRNA_2_TruB_mono_cplx	reversible	tRNA modification	-1.00E+09	1.00E+09	0	1.45E+03	1.45E+03	0	9.58E+00	9.58	0	9.58E+00	9.58
b2864_tRNA_55_MODIF_2	modification of position 55 nt (Y) in glyU by TruB_mono	1 b2864_tRNA_2_TruB_mono_cplx --> 1 TruB_mono_inact + 1 b2864_tRNA_3	irreversible	tRNA modification	0.00E+00	1.00E+09	0	1.45E+03	1.45E+03	0	9.58E+00	9.58	0	9.58E+00	9.58
b2864_tRNA_8_MODIF_1	modification of position 8 nt (s4U) in glyU by ThiI_mono	1 IscS_dim_S-SH + 1 ThiI_mono + 1 atp + 1 b2864_tRNA_3 + 1 mg2 + 1 trdrd <==> 1 b2864_tRNA_3_ThiI_mono_cplx	reversible	tRNA modification	-1.00E+09	1.00E+09	0	1.45E+03	1.45E+03	0	9.58E+00	9.58	0	9.58E+00	9.58
b2864_tRNA_8_MODIF_2	modification of position 8 nt (s4U) in glyU by ThiI_mono	1 b2864_tRNA_3_ThiI_mono_cplx --> 1 IscS_dim_S-H + 1 ThiI_mono_inact + 1 amp + 1 glyU_tRNA + 1 h + 1 mg2 + 1 ppi + 1 trdox	irreversible	tRNA modification	0.00E+00	1.00E+09	0	1.45E+03	1.45E+03	0	9.58E+00	9.58	0	9.58E+00	9.58

b2864_tRNA_Mg_BIND	Binding of two Mg2 to b2864_tRNA	1 b2864_RNA + 2 mg2 <==> 1 b2864_tRNA_Mg2	reversible	Metallo-ion binding	-1.00E+09	1.00E+09	0	1.45E+03	1.45E+03	0	9.58E+00	9.58	0	9.58E+00	9.58
b2864_v1_tRNA_20_MODIF_1	modification of position 20 nt (D) in glyU by Dus_gen	1 Dus_gen + 1 b2864_v1_tRNA_Mg2 + 1 h + 1 nadph <==> 1 b2864_v1_tRNA_Mg2_Dus_gen_cplx	reversible	tRNA modification	-1.00E+09	1.00E+09	0	0	0.00E+00	0	0	0	0	0	0
b2864_v1_tRNA_20_MODIF_2	modification of position 20 nt (D) in glyU by Dus_gen	1 b2864_v1_tRNA_Mg2_Dus_gen_cplx --> 1 Dus_gen_inact + 1 b2864_v1_tRNA_1 + 1 nadp	irreversible	tRNA modification	0.00E+00	1.00E+09	0	0	0.00E+00	0	0	0	0	0	0
b2864_v1_tRNA_54_MODIF_1	modification of position 54 nt (m5U) in glyU by TrmA_mono	1 TrmA_mono + 1 amet + 1 b2864_v1_tRNA_1 <==> 1 b2864_v1_tRNA_1_TrmA_mono_cplx	reversible	tRNA modification	-1.00E+09	1.00E+09	0	0	0.00E+00	0	0	0	0	0	0
b2864_v1_tRNA_54_MODIF_2	modification of position 54 nt (m5U) in glyU by TrmA_mono	1 b2864_v1_tRNA_1_TrmA_mono_cplx --> 1 TrmA_mono_inact + 1 ahcys + 1 b2864_v1_tRNA_2 + 1 h	irreversible	tRNA modification	0.00E+00	1.00E+09	0	0	0.00E+00	0	0	0	0	0	0
b2864_v1_tRNA_55_MODIF_1	modification of position 55 nt (Y) in glyU by TruB_mono	1 TruB_mono + 1 b2864_v1_tRNA_2 <==> 1 b2864_v1_tRNA_2_TruB_mono_cplx	reversible	tRNA modification	-1.00E+09	1.00E+09	0	0	0.00E+00	0	0	0	0	0	0
b2864_v1_tRNA_55_MODIF_2	modification of position 55 nt (Y) in glyU by TruB_mono	1 b2864_v1_tRNA_2_TruB_mono_cplx --> 1 TruB_mono_inact + 1 b2864_v1_tRNA_3	irreversible	tRNA modification	0.00E+00	1.00E+09	0	0	0.00E+00	0	0	0	0	0	0
b2864_v1_tRNA_8_MODIF_1	modification of position 8 nt (s4U) in glyU by ThiI_mono	1 IscS_dim_S-SH + 1 ThiI_mono + 1 atp + 1 b2864_v1_tRNA_3 + 1 mg2 + 1 trdrd <==> 1 b2864_v1_tRNA_3_ThiI_mono_cplx	reversible	tRNA modification	-1.00E+09	1.00E+09	0	0	0.00E+00	0	0	0	0	0	0
b2864_v1_tRNA_8_MODIF_2	modification of position 8 nt (s4U) in glyU by ThiI_mono	1 b2864_v1_tRNA_3_ThiI_mono_cplx --> 1 IscS_dim_S-H + 1 ThiI_mono_inact + 1 amp + 1 glyU_tRNA + 1 h + 1 mg2 + 1 ppi + 1 trdox	irreversible	tRNA modification	0.00E+00	1.00E+09	0	0	0.00E+00	0	0	0	0	0	0
b2864_v1_tRNA_Mg_BIND	Binding of two Mg2 to b2864_v1_tRNA	1 b2864_v1_RNA + 2 mg2 <==> 1 b2864_v1_tRNA_Mg2	reversible	Metallo-ion binding	-1.00E+09	1.00E+09	0	0	0.00E+00	0	0	0	0	0	0
b2890_fold_spon	b2890_m folding: spontanous	1 b2890_m --> 1 LysS_mono	irreversible	Protein Folding	0.00E+00	1.00E+09	0	1.47E+03	1.47E+03	0	2.51E+03	2514.975	0	1.15E+02	115.449
b2890_mRNA_CONV	convsersion of mRNA to mRNA_1 (synthetic rxn)	1 b2890_mRNA --> 1 b2890_mRNA_1	irreversible	Translation	0.00E+00	1.00E+09	0	4.70E+01	4.70E+01	0	8.30E+01	83.03328	0	2.91E-01	0.291
b2890_mRNA_degr1	Degradation of b2890_mRNA I	1 Orn_dim + 379 atp + 1 b2890_mRNA_2 + 1 degradosome + 379 h2o <==> 379 adp + 1 b2890_mRNA_2_degr + 379 h + 379 pi	reversible	mRNA degradation	-1.00E+09	1.00E+09	0	4.70E+01	4.70E+01	0	8.30E+01	83.03328	0	2.91E-01	0.291
b2890_mRNA_degr2	Degradation of b2890_mRNA II	1 b2890_mRNA_2_degr + 1517 h2o --> 1 Orn_dim_inact + 376 amp 402 cmp + 1 degradosome_inact + 404 gmp + 1517 h + 336 ump	irreversible	mRNA degradation	0.00E+00	4.90E-01	0	4.70E+01	4.70E+01	0	8.30E+01	83.03328	0	2.91E-01	0.291
b2890_maturation1	Polypeptide b2890 peptide deformylase and methionine aminopeptidase complex	1 Def_mono + 1 Map_mono + 1 b2890_aa <==> 1 b2890_def_map_cplx	reversible	Protein Maturation	-1.00E+09	1.00E+09	0	1.47E+03	1.47E+03	0	2.51E+03	2514.975	0	1.15E+02	115.449
b2890_maturation2	b2890 formation	1 b2890_def_map_cplx + 2 h2o --> 1 Def_mono_inact + 1 Map_mono_inact + 1 b2890_m + 1 for + 1 met-L	irreversible	Protein Maturation	0.00E+00	1.00E+09	0	1.47E+03	1.47E+03	0	2.51E+03	2514.975	0	1.15E+02	115.449
b2891_fold_spon	b2891_m folding: spontanous	1 b2891_m --> 1 PrfB_mono	irreversible	Protein Folding	0.00E+00	1.00E+09	0	1.07E+03	1.07E+03	0	1.82E+03	1824.129	0	8.65E+01	86.541
b2891_mRNA_CONV	convsersion of mRNA to mRNA_1 (synthetic rxn)	1 b2891_mRNA --> 1 b2891_mRNA_1	irreversible	Translation	0.00E+00	1.00E+09	0	4.70E+01	4.70E+01	0	8.30E+01	83.03328	0	2.91E-01	0.291
b2891_mRNA_degr1	Degradation of b2891_mRNA I	1 Orn_dim + 274 atp + 1 b2891_mRNA_2 + 1 degradosome + 274 h2o <==> 274 adp + 1 b2891_mRNA_2_degr + 274 h + 274 pi	reversible	mRNA degradation	-1.00E+09	1.00E+09	0	4.70E+01	4.70E+01	0	8.30E+01	83.03328	0	2.91E-01	0.291
b2891_mRNA_degr2	Degradation of b2891_mRNA II	1 b2891_mRNA_2_degr + 1098 h2o --> 1 Orn_dim_inact + 286 amp 1 atp + 274 cmp + 1 degradosome_inact + 314 gmp + 1098 h + 224 ump	irreversible	mRNA degradation	0.00E+00	2.91E-01	0	4.70E+01	4.70E+01	0	8.30E+01	83.03328	0	2.91E-01	0.291
b2891_maturation1	Polypeptide b2891 peptide deformylase complex	1 Def_mono + 1 b2891_aa <==> 1 b2891_def_cplx	reversible	Protein Maturation	-1.00E+09	1.00E+09	0	1.07E+03	1.07E+03	0	1.82E+03	1824.129	0	8.65E+01	86.541
b2891_maturation2	b2891 formation	1 b2891_def_cplx + 1 h2o --> 1 Def_mono_inact + 1 b2891_m + 1 for	irreversible	Protein Maturation	0.00E+00	1.00E+09	0	1.07E+03	1.07E+03	0	1.82E+03	1824.129	0	8.65E+01	86.541
b2891_v1_mRNA_CONV	convsersion of mRNA to mRNA_1 (synthetic rxn)	1 b2891_v1_mRNA --> 1 b2891_mRNA_1	irreversible	Translation	0.00E+00	1.00E+09	0	3.54E+01	3.54E+01	0	6.24E+01	62.43587	0	1.42E-01	0.142
b2892_fold_spon	b2892_m folding: spontanous	1 b2892_m --> 1 RecJ_mono	irreversible	Protein Folding	0.00E+00	1.00E+09	0	1.23E+03	1.23E+03	0	2.12E+03	2119.124	0	6.34E+01	63.426
b2892_mRNA_CONV	convsersion of mRNA to mRNA_1 (synthetic rxn)	1 b2892_mRNA --> 1 b2892_mRNA_1	irreversible	Translation	0.00E+00	1.00E+09	0	3.54E+01	3.54E+01	0	6.24E+01	62.43587	0	1.42E-01	0.142
b2892_mRNA_degr1	Degradation of b2892_mRNA I	1 Orn_dim + 433 atp + 1 b2892_mRNA_2 + 1 degradosome + 433 h2o <==> 433 adp + 1 b2892_mRNA_2_degr + 433 h + 433 pi	reversible	mRNA degradation	-1.00E+09	1.00E+09	0	3.54E+01	3.54E+01	0	6.24E+01	62.43587	0	1.42E-01	0.142
b2892_mRNA_degr2	Degradation of b2892_mRNA II	1 b2892_mRNA_2_degr + 1733 h2o --> 1 Orn_dim_inact + 364 amp 449 cmp + 1 degradosome_inact + 533 gmp + 1733 h + 388 ump	irreversible	mRNA degradation	0.00E+00	1.42E-01	0	3.54E+01	3.54E+01	0	6.24E+01	62.43587	0	1.42E-01	0.142
b2892_maturation1	Polypeptide b2892 peptide deformylase and methionine aminopeptidase complex	1 Def_mono + 1 Map_mono + 1 b2892_aa <==> 1 b2892_def_map_cplx	reversible	Protein Maturation	-1.00E+09	1.00E+09	0	1.23E+03	1.23E+03	0	2.12E+03	2119.124	0	6.34E+01	63.426
b2892_maturation2	b2892 formation	1 b2892_def_map_cplx + 2 h2o --> 1 Def_mono_inact + 1 Map_mono_inact + 1 b2892_m + 1 for + 1 met-L	irreversible	Protein Maturation	0.00E+00	1.00E+09	0	1.23E+03	1.23E+03	0	2.12E+03	2119.124	0	6.34E+01	63.426
b2893_fold_spon	b2893_m folding: spontanous	1 b2893_m --> 1 DsbC_mono	irreversible	Protein Folding	0.00E+00	1.00E+09	0	5.47E+02	5.47E+02	0	8.99E+02	899.0264	0	8.92E+01	89.206
b2893_mRNA_degr1	Degradation of b2893_mRNA I	1 Orn_dim + 177 atp + 1 b2893_mRNA_2 + 1 degradosome + 177 h2o <==> 177 adp + 1 b2893_mRNA_2_degr + 177 h + 177 pi	reversible	mRNA degradation	-1.00E+09	1.00E+09	0	3.54E+01	3.54E+01	0	6.24E+01	62.43587	0	1.42E-01	0.142
b2893_mRNA_degr2	Degradation of b2893_mRNA II	1 b2893_mRNA_2_degr + 710 h2o --> 1 Orn_dim_inact + 202 amp + 1 atp + 156 cmp + 1 degradosome_inact + 188 gmp + 710 h + 164 ump	irreversible	mRNA degradation	0.00E+00	3.48E-01	0	3.54E+01	3.54E+01	0	6.24E+01	62.43587	0	1.42E-01	0.142
b2893_maturation1	Polypeptide b2893 peptide deformylase and methionine aminopeptidase complex	1 Def_mono + 1 Map_mono + 1 b2893_aa <==> 1 b2893_def_map_cplx	reversible	Protein Maturation	-1.00E+09	1.00E+09	0	5.47E+02	5.47E+02	0	8.99E+02	899.0264	0	8.92E+01	89.206
b2893_maturation2	b2893 formation	1 b2893_def_map_cplx + 21 h2o --> 1 Def_mono_inact + 1 Map_mono_inact + 4 ala-L + 1 b2893_m + 1 for + 1 gln-L + 2 gly + 3 leu-L + 2 lys-L + 2 met-L + 4 phe-L + 1 ser-L + 1 thr-L	irreversible	Protein Maturation	0.00E+00	1.00E+09	0	5.47E+02	5.47E+02	0	8.99E+02	899.0264	0	8.92E+01	89.206
b2893_v1_mRNA_CONV	convsersion of mRNA to mRNA_1 (synthetic rxn)	1 b2893_v1_mRNA --> 1 b2893_mRNA_1	irreversible	Translation	0.00E+00	1.00E+09	0	3.54E+01	3.54E+01	0	6.24E+01	62.43587	0	1.42E-01	0.142
b2946_fold_spon	b2946_m folding: spontanous	1 b2946_m --> 1 YggJ_mono	irreversible	Protein Folding	0.00E+00	1.00E+09	0	1.13E+03	1.13E+03	0	1.92E+03	1924.832	0	8.34E+01	83.384
b2946_mRNA_CONV	convsersion of mRNA to mRNA_1 (synthetic rxn)	1 b2946_mRNA --> 1 b2946_mRNA_1	irreversible	Translation	0.00E+00	1.00E+09	0	7.46E+01	7.46E+01	0	1.32E+02	131.728	0	1.96E-01	0.196
b2946_mRNA_degr1	Degradation of b2946_mRNA I	1 Orn_dim + 183 atp + 1 b2946_mRNA_2 + 1 degradosome + 183 h2o <==> 183 adp + 1 b2946_mRNA_2_degr + 183 h + 183 pi	reversible	mRNA degradation	-1.00E+09	1.00E+09	0	7.46E+01	7.46E+01	0	1.32E+02	131.728	0	1.96E-01	0.196
b2946_mRNA_degr2	Degradation of b2946_mRNA II	1 b2946_mRNA_2_degr + 731 h2o --> 1 Orn_dim_inact + 175 amp + 1 atp + 188 cmp + 1 degradosome_inact + 201 gmp + 731 h + 167 ump	irreversible	mRNA degradation	0.00E+00	1.96E-01	0	7.46E+01	7.46E+01	0	1.32E+02	131.728	0	1.96E-01	0.196
b2946_maturation1	Polypeptide b2946 peptide deformylase and methionine aminopeptidase complex	1 Def_mono + 1 Map_mono + 1 b2946_aa <==> 1 b2946_def_map_cplx	reversible	Protein Maturation	-1.00E+09	1.00E+09	0	1.13E+03	1.13E+03	0	1.92E+03	1924.832	0	8.34E+01	83.384
b2946_maturation2	b2946 formation	1 b2946_def_map_cplx + 2 h2o --> 1 Def_mono_inact + 1 Map_mono_inact + 1 b2946_m + 1 for + 1 met-L	irreversible	Protein Maturation	0.00E+00	1.00E+09	0	1.13E+03	1.13E+03	0	1.92E+03	1924.832	0	8.34E+01	83.384
b2947_fold_spon	b2947_m folding: spontanous	1 b2947_m --> 1 GshB_mono	irreversible	Protein Folding	0.00E+00	1.00E+09	0	1.47E+03	1.47E+03	0	2.50E+03	2498.544	0	1.31E+02	130.968
b2947_mRNA_CONV	convsersion of mRNA to mRNA_1 (synthetic rxn)	1 b2947_mRNA --> 1 b2947_mRNA_1	irreversible	Translation	0.00E+00	1.00E+09	0	7.46E+01	7.46E+01	0	1.32E+02	131.728	0	1.96E-01	0.196
b2947_mRNA_degr1	Degradation of b2947_mRNA I	1 Orn_dim + 237 atp + 1 b2947_mRNA_2 + 1 degradosome + 237 h2o <==> 237 adp + 1 b2947_mRNA_2_degr + 237 h + 237 pi	reversible	mRNA degradation	-1.00E+09	1.00E+09	0	7.46E+01	7.46E+01	0	1.32E+02	131.728	0	1.96E-01	0.196
b2947_mRNA_degr2	Degradation of b2947_mRNA II	1 b2947_mRNA_2_degr + 950 h2o --> 1 Orn_dim_inact + 238 amp + 239 cmp + 1 degradosome_inact + 269 gmp + 950 h + 205 ump	irreversible	mRNA degradation	0.00E+00	4.14E-01	0	7.46E+01	7.46E+01	0	1.32E+02	131.728	0	1.96E-01	0.196
b2947_maturation1	Polypeptide b2947 peptide deformylase complex	1 Def_mono + 1 b2947_aa <==> 1 b2947_def_cplx	reversible	Protein Maturation	-1.00E+09	1.00E+09	0	1.47E+03	1.47E+03	0	2.50E+03	2498.544	0	1.31E+02	130.968
b2947_maturation2	b2947 formation	1 b2947_def_cplx + 1 h2o --> 1 Def_mono_inact + 1 b2947_m + 1 for	irreversible	Protein Maturation	0.00E+00	1.00E+09	0	1.47E+03	1.47E+03	0	2.50E+03	2498.544	0	1.31E+02	130.968
b2959_fold_spon	b2959_m folding: spontanous	1 b2959_m --> 1 YggL_mono	irreversible	Protein Folding	0.00E+00	1.00E+09	0	9.54E+02	9.54E+02	0	1.59E+03	1588.605	0	1.25E+02	125.112

b2959_mRNA_CONV	convsersion of mRNA to mRNA_1 (synthetic rxn)	1 b2959_mRNA --> 1 b2959_mRNA_1	irreversible	Translation	0.00E+00	1.00E+09	0	1.38E+02	1.38E+02	0	2.44E+02	244.0675	0	1.52E-01	0.152
b2959_mRNA_degr1	Degradation of b2959_mRNA I	1 Orn_dim + 81 atp + 1 b2959_mRNA_2 + 1 degradosome + 81 h2o <==> 81 adp + 1 b2959_mRNA_2_degr + 81 h + 81 pi	reversible	mRNA degradation	-1.00E+09	1.00E+09	0	1.38E+02	1.38E+02	0	2.44E+02	244.0675	0	1.52E-01	0.152
b2959_mRNA_degr2	Degradation of b2959_mRNA II	1 b2959_mRNA_2_degr + 325 h2o --> 1 Orn_dim_inact + 95 amp + 65 cmp + 1 degradosome_inact + 94 gmp + 325 h + 72 ump	irreversible	mRNA degradation	0.00E+00	1.52E-01	0	1.38E+02	1.38E+02	0	2.44E+02	244.0675	0	1.52E-01	0.152
b2959_maturation1	Polypeptide b2959 peptide deformylas and methionine aminopeptidase complex	1 Def_mono + 1 Map_mono + 1 b2959_aa <==> 1 b2959_def_map_cplx	reversible	Protein Maturation	-1.00E+09	1.00E+09	0	9.54E+02	9.54E+02	0	1.59E+03	1588.605	0	1.25E+02	125.112
b2959_maturation2	b2959 formation	1 b2959_def_map_cplx + 2 h2o --> 1 Def_mono_inact + 1 Map_mono_inact + 1 b2959_m + 1 for + 1 met-L	irreversible	Protein Maturation	0.00E+00	1.00E+09	0	9.54E+02	9.54E+02	0	1.59E+03	1588.605	0	1.25E+02	125.112
b2960_fold_GroEL/ES_1	b2960_m folding: GroEL/ES mediated: polypeptide is going in GroEL/ES complex	1 GroEL.(7)ADP.cisGroES + 7 atp + 1 b2960_m + 1 transGroES_hepta --> 7 adp + 1 b2960_m_GroEL.(7)ATP.transGroES + 1 cisGroES_hepta	irreversible	Protein Folding	0.00E+00	1.00E+09	0	2.03E+03	2.03E+03	0	3.51E+03	3509.765	0	9.49E+01	94.948
b2960_fold_GroEL/ES_2	b2960_m folding: GroEL/ES mediated: folding of polypeptide under ATP hydrolysis	1 b2960_m_GroEL.(7)ATP.transGroES + 7 h2o --> 1 YggH_GroEL.(7)ADP.transGroES + 7 h + 7 pi	irreversible	Protein Folding	0.00E+00	1.00E+09	0	2.03E+03	2.03E+03	0	3.51E+03	3509.765	0	9.49E+01	94.948
b2960_fold_GroEL/ES_3	b2960_m folding: GroEL/ES mediated: release of native protein	1 YggH_GroEL.(7)ADP.transGroES --> 1 GroEL.(7)ADP.transGroES + 1 YggH_mono	irreversible	Protein Folding	0.00E+00	1.00E+09	0	2.03E+03	2.03E+03	0	3.51E+03	3509.765	0	9.49E+01	94.948
b2960_mRNA_CONV	convsersion of mRNA to mRNA_1 (synthetic rxn)	1 b2960_mRNA --> 1 b2960_mRNA_1	irreversible	Translation	0.00E+00	1.00E+09	0	1.38E+02	1.38E+02	0	2.44E+02	244.0675	0	1.52E-01	0.152
b2960_mRNA_degr1	Degradation of b2960_mRNA I	1 Orn_dim + 180 atp + 1 b2960_mRNA_2 + 1 degradosome + 180 h2o <==> 180 adp + 1 b2960_mRNA_2_degr + 180 h + 180 pi	reversible	mRNA degradation	-1.00E+09	1.00E+09	0	1.38E+02	1.38E+02	0	2.44E+02	244.0675	0	1.52E-01	0.152
b2960_mRNA_degr2	Degradation of b2960_mRNA II	1 b2960_mRNA_2_degr + 719 h2o --> 1 Orn_dim_inact + 166 amp + 1 atp + 166 cmp + 1 degradosome_inact + 213 gmp + 719 h + 174 ump	irreversible	mRNA degradation	0.00E+00	2.84E-01	0	1.38E+02	1.38E+02	0	2.44E+02	244.0675	0	1.52E-01	0.152
b2960_maturation1	Polypeptide b2960 peptide deformylas and methionine aminopeptidase complex	1 Def_mono + 1 Map_mono + 1 b2960_aa <==> 1 b2960_def_map_cplx	reversible	Protein Maturation	-1.00E+09	1.00E+09	0	2.03E+03	2.03E+03	0	3.51E+03	3509.765	0	9.49E+01	94.948
b2960_maturation2	b2960 formation	1 b2960_def_map_cplx + 2 h2o --> 1 Def_mono_inact + 1 Map_mono_inact + 1 b2960_m + 1 for + 1 met-L	irreversible	Protein Maturation	0.00E+00	1.00E+09	0	2.03E+03	2.03E+03	0	3.51E+03	3509.765	0	9.49E+01	94.948
b2967_tRNA_16_MODIF_1	modification of position 16 nt (D) in pheV by Dus_gen	1 Dus_gen + 1 b2967_tRNA_Mg2 + 1 h + 1 nadph <==> 1 b2967_tRNA_Mg2_Dus_gen_cplx	reversible	tRNA modification	-1.00E+09	1.00E+09	0	1.52E+03	1.52E+03	0	10	10	0	10	10
b2967_tRNA_16_MODIF_2	modification of position 16 nt (D) in pheV by Dus_gen	1 b2967_tRNA_Mg2_Dus_gen_cplx --> 1 Dus_gen_inact + 1 b2967_tRNA_1 + 1 nadp	irreversible	tRNA modification	0.00E+00	1.00E+09	0	1.52E+03	1.52E+03	0	10	10	0	10	10
b2967_tRNA_20_MODIF_1	modification of position 20 nt (D) in pheV by Dus_gen	1 Dus_gen + 1 b2967_tRNA_1 + 1 h + 1 nadph <==> 1 b2967_tRNA_1_Dus_gen_cplx	reversible	tRNA modification	-1.00E+09	1.00E+09	0	1.52E+03	1.52E+03	0	10	10	0	10	10
b2967_tRNA_20_MODIF_2	modification of position 20 nt (D) in pheV by Dus_gen	1 b2967_tRNA_1_Dus_gen_cplx --> 1 Dus_gen_inact + 1 b2967_tRNA_2 + 1 nadp	irreversible	tRNA modification	0.00E+00	1.00E+09	0	1.52E+03	1.52E+03	0	10	10	0	10	10
b2967_tRNA_32_MODIF_1	modification of position 32 nt (Y) in pheV by RluA_mono	1 RluA_mono + 1 b2967_tRNA_2 <==> 1 b2967_tRNA_2_RluA_mono_cplx	reversible	tRNA modification	-1.00E+09	1.00E+09	0	1.52E+03	1.52E+03	0	10	10	0	10	10
b2967_tRNA_32_MODIF_2	modification of position 32 nt (Y) in pheV by RluA_mono	1 b2967_tRNA_2_RluA_mono_cplx --> 1 RluA_mono_inact + 1 b2967_tRNA_3	irreversible	tRNA modification	0.00E+00	1.00E+09	0	1.52E+03	1.52E+03	0	10	10	0	10	10
b2967_tRNA_37_MODIF_1	modification of position 37 nt (ms2i6A) in pheV by MiaA_dim-MiaB_mono	1 IscS_dim_S-SH + 1 MiaA_dim + 1 MiaB_mono + 2 amet + 1 b2967_tRNA_3 + 1 dmpp + 1 fldrd <==> 1 b2967_tRNA_3_MiaA_dim MiaB_mono_cplx	reversible	tRNA modification	-1.00E+09	1.00E+09	0	1.52E+03	1.52E+03	0	10	10	0	10	10
b2967_tRNA_37_MODIF_2	modification of position 37 nt (ms2i6A) in pheV by MiaA_dim-MiaB_mono	1 b2967_tRNA_3_MiaA_dim-MiaB_mono_cplx --> 1 IscS_dim_S-SH + 1 MiaA_dim_inact + 1 MiaB_mono_inact + 1 ahcys + 1 b2967_tRNA_4 + 1 dad-5 + 1 fldox + 2 h + 1 met-L + 1 ppi	irreversible	tRNA modification	0.00E+00	1.00E+09	0	1.52E+03	1.52E+03	0	10	10	0	10	10
b2967_tRNA_39_MODIF_1	modification of position 39 nt (Y) in pheV by TruA_dim	1 TruA_dim + 1 b2967_tRNA_4 <==> 1 b2967_tRNA_4_TruA_dim_cplx	reversible	tRNA modification	-1.00E+09	1.00E+09	0	1.52E+03	1.52E+03	0	10	10	0	10	10
b2967_tRNA_39_MODIF_2	modification of position 39 nt (Y) in pheV by TruA_dim	1 b2967_tRNA_4_TruA_dim_cplx --> 1 TruA_dim_inact + 1 b2967_tRNA_5	irreversible	tRNA modification	0.00E+00	1.00E+09	0	1.52E+03	1.52E+03	0	10	10	0	10	10
b2967_tRNA_46_MODIF_1	modification of position 46 nt (m7G) in pheV by YggH_mono	1 YggH_mono + 1 amet + 1 b2967_tRNA_5 <==> 1 b2967_tRNA_5_YggH_mono_cplx	reversible	tRNA modification	-1.00E+09	1.00E+09	0	1.52E+03	1.52E+03	0	10	10	0	10	10
b2967_tRNA_46_MODIF_2	modification of position 46 nt (m7G) in pheV by YggH_mono	1 b2967_tRNA_5_YggH_mono_cplx --> 1 YggH_mono_inact + 1 ahcys + 1 b2967_tRNA_6 + 1 h	irreversible	tRNA modification	0.00E+00	1.00E+09	0	1.52E+03	1.52E+03	0	10	10	0	10	10
b2967_tRNA_47_MODIF_1	modification of position 47 nt (acp3U) in pheV by AcpT_tRNA_pos_47_acp3U	1 AcpT_tRNA_pos_47_acp3U + 1 amet + 1 b2967_tRNA_6 <==> 1 b2967_tRNA_6_AcpT_tRNA_pos_47_acp3U_cplx	reversible	tRNA modification	-1.00E+09	1.00E+09	0	1.52E+03	1.52E+03	0	10	10	0	10	10
b2967_tRNA_47_MODIF_2	modification of position 47 nt (acp3U) in pheV by AcpT_tRNA_pos_47_acp3U	1 b2967_tRNA_6_AcpT_tRNA_pos_47_acp3U_cplx --> 1 5mta + 1 AcpT_tRNA_pos_47_acp3U_inact + 1 b2967_tRNA_7 + 1 h	irreversible	tRNA modification	0.00E+00	1.00E+09	0	1.52E+03	1.52E+03	0	10	10	0	10	10
b2967_tRNA_54_MODIF_1	modification of position 54 nt (m5U) in pheV by TrmA_mono	1 TrmA_mono + 1 amet + 1 b2967_tRNA_7 <==> 1 b2967_tRNA_7_TrmA_mono_cplx	reversible	tRNA modification	-1.00E+09	1.00E+09	0	1.52E+03	1.52E+03	0	10	10	0	10	10
b2967_tRNA_54_MODIF_2	modification of position 54 nt (m5U) in pheV by TrmA_mono	1 b2967_tRNA_7_TrmA_mono_cplx --> 1 TrmA_mono_inact + 1 ahcys + 1 b2967_tRNA_8 + 1 h	irreversible	tRNA modification	0.00E+00	1.00E+09	0	1.52E+03	1.52E+03	0	10	10	0	10	10
b2967_tRNA_55_MODIF_1	modification of position 55 nt (Y) in pheV by TruB_mono	1 TruB_mono + 1 b2967_tRNA_8 <==> 1 b2967_tRNA_8_TruB_mono_cplx	reversible	tRNA modification	-1.00E+09	1.00E+09	0	1.52E+03	1.52E+03	0	10	10	0	10	10
b2967_tRNA_55_MODIF_2	modification of position 55 nt (Y) in pheV by TruB_mono	1 b2967_tRNA_8_TruB_mono_cplx --> 1 TruB_mono_inact + 1 b2967_tRNA_9	irreversible	tRNA modification	0.00E+00	1.00E+09	0	1.52E+03	1.52E+03	0	10	10	0	10	10
b2967_tRNA_8_MODIF_1	modification of position 8 nt (s4U) in pheV by ThiI_mono	1 IscS_dim_S-SH + 1 ThiI_mono + 1 atp + 1 b2967_tRNA_9 + 1 mg2 + 1 trdrd <==> 1 b2967_tRNA_9_ThiI_mono_cplx	reversible	tRNA modification	-1.00E+09	1.00E+09	0	1.52E+03	1.52E+03	0	10	10	0	10	10
b2967_tRNA_8_MODIF_2	modification of position 8 nt (s4U) in pheV by ThiI_mono	1 b2967_tRNA_9_ThiI_mono_cplx --> 1 IscS_dim_S-H + 1 ThiI_mono_inact + 1 amp + 1 h + 1 mg2 + 1 pheV_tRNA + 1 ppi + 1 trdox	irreversible	tRNA modification	0.00E+00	1.00E+09	0	1.52E+03	1.52E+03	0	10	10	0	10	10
b2967_tRNA_Mg_BIND	Binding of two Mg2 to b2967_tRNA	1 b2967_RNA + 2 mg2 <==> 1 b2967_tRNA_Mg2	reversible	Metallo-ion binding	-1.00E+09	1.00E+09	0	1.52E+03	1.52E+03	0	10	10	0	10	10
b3065_fold_spon	b3065_m folding: spontanous	1 b3065_m --> 1 RpsU_mono	irreversible	Protein Folding	0.00E+00	1.00E+09	8.44E+01	1.13E+02	2.81E+01	6.22E+01	8.29E+01	20.72	6.22E+01	8.29E+01	20.72
b3065_mRNA_CONV	convsersion of mRNA to mRNA_1 (synthetic rxn)	1 b3065_mRNA --> 1 b3065_mRNA_1	irreversible	Translation	0.00E+00	1.00E+09	0	1.07E+02	1.07E+02	0	8.29E+01	82.88	0	2.19E+00	2.19
b3065_mRNA_degr1	Degradation of b3065_mRNA I	1 Orn_dim + 54 atp + 1 b3065_mRNA_2 + 1 degradosome + 54 h2o <==> 54 adp + 1 b3065_mRNA_2_degr + 54 h + 54 pi	reversible	mRNA degradation	-1.00E+09	1.00E+09	0	1.07E+02	1.07E+02	0	8.29E+01	82.88	0	2.19E+00	2.19
b3065_mRNA_degr2	Degradation of b3065_mRNA II	1 b3065_mRNA_2_degr + 215 h2o --> 1 Orn_dim_inact + 58 amp + 1 atp + 55 cmp + 1 degradosome_inact + 57 gmp + 215 h + 45 ump	irreversible	mRNA degradation	0.00E+00	2.19E+00	0	1.07E+02	1.07E+02	0	8.29E+01	82.88	0	2.19E+00	2.19
b3065_maturation1	Polypeptide b3065 peptide deformylas and methionine aminopeptidase complex	1 Def_mono + 1 Map_mono + 1 b3065_aa <==> 1 b3065_def_map_cplx	reversible	Protein Maturation	-1.00E+09	1.00E+09	8.44E+01	1.13E+02	2.81E+01	6.22E+01	8.29E+01	20.72	6.22E+01	8.29E+01	20.72
b3065_maturation2	b3065 formation	1 b3065_def_map_cplx + 2 h2o --> 1 Def_mono_inact + 1 Map_mono_inact + 1 b3065_m + 1 for + 1 met-L	irreversible	Protein Maturation	0.00E+00	1.00E+09	8.44E+01	1.13E+02	2.81E+01	6.22E+01	8.29E+01	20.72	6.22E+01	8.29E+01	20.72
b3065_v1_mRNA_CONV	convsersion of mRNA to mRNA_1 (synthetic rxn)	1 b3065_v1_mRNA --> 1 b3065_mRNA_1	irreversible	Translation	0.00E+00	1.00E+09	0	3.03E+01	3.03E+01	0	5.34E+01	53.44037	0	2.45E-01	0.245
b3065_v2_mRNA_CONV	convsersion of mRNA to mRNA_1 (synthetic rxn)	1 b3065_v2_mRNA --> 1 b3065_mRNA_1	irreversible	Translation	0.00E+00	1.00E+09	0	3.03E+01	3.03E+01	0	5.34E+01	53.44037	0	2.45E-01	0.245
b3065_v3_mRNA_CONV	convsersion of mRNA to mRNA_1 (synthetic rxn)	1 b3065_v3_mRNA --> 1 b3065_mRNA_1	irreversible	Translation	0.00E+00	1.00E+09	0	3.03E+01	3.03E+01	0	5.34E+01	53.44037	0	2.45E-01	0.245
b3066_fold_spon	b3066_m folding: spontanous	1 b3066_m --> 1 DnaG_mono	irreversible	Protein Folding	0.00E+00	1.00E+09	0	1.09E+03	1.09E+03	0	1.88E+03	1877.153	0	6.85E+01	68.51
b3066_mRNA_CONV	convsersion of mRNA to mRNA_1 (synthetic rxn)	1 b3066_mRNA --> 1 b3066_mRNA_1	irreversible	Translation	0.00E+00	1.00E+09	0	3.03E+01	3.03E+01	0	5.34E+01	53.44037	0	2.45E-01	0.245
b3066_mRNA_degr1	Degradation of b3066_mRNA I	1 Orn_dim + 436 atp + 1 b3066_mRNA_2 + 1 degradosome + 436 h2o <==> 436 adp + 1 b3066_mRNA_2_degr + 436 h + 436 pi	reversible	mRNA degradation	-1.00E+09	1.00E+09	0	3.03E+01	3.03E+01	0	5.34E+01	53.44037	0	2.45E-01	0.245
b3066_mRNA_degr2	Degradation of b3066_mRNA II	1 b3066_mRNA_2_degr + 1745 h2o --> 1 Orn_dim_inact + 439 amp 449 cmp + 1 degradosome_inact + 464 gmp + 1745 h + 394 ump	irreversible	mRNA degradation	0.00E+00	2.45E-01	0	3.03E+01	3.03E+01	0	5.34E+01	53.44037	0	2.45E-01	0.245
b3066_maturation1	Polypeptide b3066 peptide deformylas and methionine aminopeptidase complex	1 Def_mono + 1 Map_mono + 1 b3066_aa <==> 1 b3066_def_map_cplx	reversible	Protein Maturation	-1.00E+09	1.00E+09	0	1.09E+03	1.09E+03	0	1.88E+03	1877.153	0	6.85E+01	68.51

b3066_maturation2	b3066 formation	1 b3066_def_map_cplx + 2 h2o --> 1 Def_mono_inact + 1 Map_mono_inact + 1 b3066_m + 1 for + 1 met-L	irreversible	Protein Maturation	0.00E+00	1.00E+09	0	1.09E+03	1.09E+03	0	1.88E+03	1877.153	0	6.85E+01	68.51
b3066_v1_mRNA_CONV	convsersion of mRNA to mRNA_1 (synthetic rxn)	1 b3066_v1_mRNA --> 1 b3066_mRNA_1	irreversible	Translation	0.00E+00	1.00E+09	0	3.03E+01	3.03E+01	0	5.34E+01	53.44037	0	2.45E-01	0.245
b3066_v2_mRNA_CONV	convsersion of mRNA to mRNA_1 (synthetic rxn)	1 b3066_v2_mRNA --> 1 b3066_mRNA_1	irreversible	Translation	0.00E+00	1.00E+09	0	3.03E+01	3.03E+01	0	5.34E+01	53.44037	0	2.45E-01	0.245
b3067_fold_KJE_1	b3067_m folding: KJE mediated	1 DnaJ_dim + 1 DnaK_mono.ATP + 1 b3067_m <==> 1 b3067_m_DnaKJ_complex	reversible	Protein Folding	-1.00E+09	1.00E+09	0	2.50E+03	2.50E+03	0	2.78E+03	2776.665	0	1.02E+02	101.844
b3067_fold_KJE_2	b3067_m folding: KJE mediated	1 GrpE_dim + 1 b3067_m_DnaKJ_complex + 1 h2o --> 1 DnaJ_dim_inact + 1 RpoD_DnaK_GrpE_complex + 1 adp + 1 h + 1 pi	irreversible	Protein Folding	0.00E+00	1.00E+09	0	2.50E+03	2.50E+03	0	2.78E+03	2776.665	0	1.02E+02	101.844
b3067_fold_KJE_3	b3067_m folding: KJE mediated	1 RpoD_DnaK_GrpE_complex + 1 atp --> 1 DnaK_mono.ATP_inact + 1 GrpE_dim_inact + 1 RpoD_mono	irreversible	Protein Folding	0.00E+00	1.00E+09	0	2.50E+03	2.50E+03	0	2.78E+03	2776.665	0	1.02E+02	101.844
b3067_fold_spon	b3067_m folding: spontanous	1 b3067_m --> 1 RpoD_mono	irreversible	Protein Folding	0.00E+00	1.00E+09	0	2.50E+03	2.50E+03	0	2.78E+03	2776.665	0	1.02E+02	101.844
b3067_mRNA_CONV	convsersion of mRNA to mRNA_1 (synthetic rxn)	1 b3067_mRNA --> 1 b3067_mRNA_1	irreversible	Translation	0.00E+00	1.00E+09	0	6.70E+01	6.70E+01	0	1.18E+02	118.1962	0	3.99E-01	0.399
b3067_mRNA_degr1	Degradation of b3067_mRNA I	1 Orn_dim + 460 atp + 1 b3067_mRNA_2 + 1 degradosome + 460 h2o <==> 460 adp + 1 b3067_mRNA_2_degr + 460 h + 460 pi	reversible	mRNA degradation	-1.00E+09	1.00E+09	0	6.70E+01	6.70E+01	0	1.18E+02	118.1962	0	3.99E-01	0.399
b3067_mRNA_degr2	Degradation of b3067_mRNA II	1 b3067_mRNA_2_degr + 1841 h2o --> 1 Orn_dim_inact + 501 amp + 1 atp + 477 cmp + 1 degradosome_inact + 499 gmp + 1841 h + 364 ump	irreversible	mRNA degradation	0.00E+00	3.99E-01	0	6.70E+01	6.70E+01	0	1.18E+02	118.1962	0	3.99E-01	0.399
b3067_maturation1	Polypeptide b3067 peptide deformylase complex	1 Def_mono + 1 b3067_aa <==> 1 b3067_def_cplx	reversible	Protein Maturation	-1.00E+09	1.00E+09	0	2.50E+03	2.50E+03	0	2.78E+03	2776.665	0	1.02E+02	101.844
b3067_maturation2	b3067 formation	1 b3067_def_cplx + 1 h2o --> 1 Def_mono_inact + 1 b3067_m + 1 for	irreversible	Protein Maturation	0.00E+00	1.00E+09	0	2.50E+03	2.50E+03	0	2.78E+03	2776.665	0	1.02E+02	101.844
b3067_v1_mRNA_CONV	convsersion of mRNA to mRNA_1 (synthetic rxn)	1 b3067_v1_mRNA --> 1 b3067_mRNA_1	irreversible	Translation	0.00E+00	1.00E+09	0	6.70E+01	6.70E+01	0	1.18E+02	118.1962	0	3.99E-01	0.399
b3067_v2_mRNA_CONV	convsersion of mRNA to mRNA_1 (synthetic rxn)	1 b3067_v2_mRNA --> 1 b3067_mRNA_1	irreversible	Translation	0.00E+00	1.00E+09	0	6.70E+01	6.70E+01	0	1.18E+02	118.1962	0	3.99E-01	0.399
b3067_v3_mRNA_CONV	convsersion of mRNA to mRNA_1 (synthetic rxn)	1 b3067_v3_mRNA --> 1 b3067_mRNA_1	irreversible	Translation	0.00E+00	1.00E+09	0	3.03E+01	3.03E+01	0	5.34E+01	53.44037	0	2.45E-01	0.245
b3067_v4_mRNA_CONV	convsersion of mRNA to mRNA_1 (synthetic rxn)	1 b3067_v4_mRNA --> 1 b3067_mRNA_1	irreversible	Translation	0.00E+00	1.00E+09	0	3.03E+01	3.03E+01	0	5.34E+01	53.44037	0	2.45E-01	0.245
b3067_v5_mRNA_CONV	convsersion of mRNA to mRNA_1 (synthetic rxn)	1 b3067_v5_mRNA --> 1 b3067_mRNA_1	irreversible	Translation	0.00E+00	1.00E+09	0	3.03E+01	3.03E+01	0	5.34E+01	53.44037	0	2.45E-01	0.245
b3069_tRNA_18_MODIF_1	modification of position 18 nt (Gm) in ileX by TrmH_dim	1 TrmH_dim + 1 amet + 1 b3069_tRNA_Mg2 <==> 1 b3069_tRNA_Mg2_TrmH_dim_cplx	reversible	tRNA modification	-1.00E+09	1.00E+09	0	1.45E+03	1.45E+03	0	1.05E+01	10.5	0	1.05E+01	10.5
b3069_tRNA_18_MODIF_2	modification of position 18 nt (Gm) in ileX by TrmH_dim	1 b3069_tRNA_Mg2_TrmH_dim_cplx --> 1 TrmH_dim_inact + 1 ahcys + 1 b3069_tRNA_1 + 1 h	irreversible	tRNA modification	0.00E+00	1.00E+09	0	1.45E+03	1.45E+03	0	1.05E+01	10.5	0	1.05E+01	10.5
b3069_tRNA_20A_MODIF_1	modification of position 20A nt (D) in ileX by Dus_gen	1 Dus_gen + 1 b3069_tRNA_2 + 1 h + 1 nadph <==> 1 b3069_tRNA_2_Dus_gen_cplx	reversible	tRNA modification	-1.00E+09	1.00E+09	0	1.45E+03	1.45E+03	0	1.05E+01	10.5	0	1.05E+01	10.5
b3069_tRNA_20A_MODIF_2	modification of position 20A nt (D) in ileX by Dus_gen	1 b3069_tRNA_2_Dus_gen_cplx --> 1 Dus_gen_inact + 1 b3069_tRNA_3 + 1 nadp	irreversible	tRNA modification	0.00E+00	1.00E+09	0	1.45E+03	1.45E+03	0	1.05E+01	10.5	0	1.05E+01	10.5
b3069_tRNA_20_MODIF_1	modification of position 20 nt (D) in ileX by Dus_gen	1 Dus_gen + 1 b3069_tRNA_1 + 1 h + 1 nadph <==> 1 b3069_tRNA_1_Dus_gen_cplx	reversible	tRNA modification	-1.00E+09	1.00E+09	0	1.45E+03	1.45E+03	0	1.05E+01	10.5	0	1.05E+01	10.5
b3069_tRNA_20_MODIF_2	modification of position 20 nt (D) in ileX by Dus_gen	1 b3069_tRNA_1_Dus_gen_cplx --> 1 Dus_gen_inact + 1 b3069_tRNA_2 + 1 nadp	irreversible	tRNA modification	0.00E+00	1.00E+09	0	1.45E+03	1.45E+03	0	1.05E+01	10.5	0	1.05E+01	10.5
b3069_tRNA_34_MODIF_1	modification of position 34 nt (k2C) in ileX by TilS_mono	1 TilS_mono + 1 atp + 1 b3069_tRNA_3 + 1 lys-L <==> 1 b3069_tRNA_3_TilS_mono_cplx	reversible	tRNA modification	-1.00E+09	1.00E+09	0	1.45E+03	1.45E+03	0	1.05E+01	10.5	0	1.05E+01	10.5
b3069_tRNA_34_MODIF_2	modification of position 34 nt (k2C) in ileX by TilS_mono	1 b3069_tRNA_3_TilS_mono_cplx --> 1 TilS_mono_inact + 1 amp + 1 b3069_tRNA_4 + 2 h + 1 ppi	irreversible	tRNA modification	0.00E+00	1.00E+09	0	1.45E+03	1.45E+03	0	1.05E+01	10.5	0	1.05E+01	10.5
b3069_tRNA_37_MODIF_1	modification of position 37 nt (t6A) in ileX by Up_tRNA_pos_37_t6A	1 Up_tRNA_pos_37_t6A + 1 atp + 1 b3069_tRNA_4 + 1 hco3 + 1 mg2 + 1 thr-L <==> 1 b3069_tRNA_4_Up_tRNA_pos_37_t6A_cplx	reversible	tRNA modification	-1.00E+09	1.00E+09	0	1.45E+03	1.45E+03	0	1.05E+01	10.5	0	1.05E+01	10.5
b3069_tRNA_37_MODIF_2	modification of position 37 nt (t6A) in ileX by Up_tRNA_pos_37_t6A	1 b3069_tRNA_4_Up_tRNA_pos_37_t6A_cplx --> 1 Up_tRNA_pos_37_t6A_inact + 1 amp + 1 b3069_tRNA_5 + 1 h + 1 h2o + 1 mg2 + 1 ppi	irreversible	tRNA modification	0.00E+00	1.00E+09	0	1.45E+03	1.45E+03	0	1.05E+01	10.5	0	1.05E+01	10.5
b3069_tRNA_39_MODIF_1	modification of position 39 nt (Y) in ileX by TruA_dim	1 TruA_dim + 1 b3069_tRNA_5 <==> 1 b3069_tRNA_5_TruA_dim_cplx	reversible	tRNA modification	-1.00E+09	1.00E+09	0	1.45E+03	1.45E+03	0	1.05E+01	10.5	0	1.05E+01	10.5
b3069_tRNA_39_MODIF_2	modification of position 39 nt (Y) in ileX by TruA_dim	1 b3069_tRNA_5_TruA_dim_cplx --> 1 TruA_dim_inact + 1 b3069_tRNA_6	irreversible	tRNA modification	0.00E+00	1.00E+09	0	1.45E+03	1.45E+03	0	1.05E+01	10.5	0	1.05E+01	10.5
b3069_tRNA_46_MODIF_1	modification of position 46 nt (m7G) in ileX by YggH_mono	1 YggH_mono + 1 amet + 1 b3069_tRNA_6 <==> 1 b3069_tRNA_6_YggH_mono_cplx	reversible	tRNA modification	-1.00E+09	1.00E+09	0	1.45E+03	1.45E+03	0	1.05E+01	10.5	0	1.05E+01	10.5
b3069_tRNA_46_MODIF_2	modification of position 46 nt (m7G) in ileX by YggH_mono	1 b3069_tRNA_6_YggH_mono_cplx --> 1 YggH_mono_inact + 1 ahcys + 1 b3069_tRNA_7 + 1 h	irreversible	tRNA modification	0.00E+00	1.00E+09	0	1.45E+03	1.45E+03	0	1.05E+01	10.5	0	1.05E+01	10.5
b3069_tRNA_47_MODIF_1	modification of position 47 nt (acp3U) in ileX by AcpT_tRNA_pos_47_acp3U	1 AcpT_tRNA_pos_47_acp3U + 1 amet + 1 b3069_tRNA_7 <==> 1 b3069_tRNA_7_AcpT_tRNA_pos_47_acp3U_cplx	reversible	tRNA modification	-1.00E+09	1.00E+09	0	1.45E+03	1.45E+03	0	1.05E+01	10.5	0	1.05E+01	10.5
b3069_tRNA_47_MODIF_2	modification of position 47 nt (acp3U) in ileX by AcpT_tRNA_pos_47_acp3U	1 b3069_tRNA_7_AcpT_tRNA_pos_47_acp3U_cplx --> 1 5mta + 1 AcpT_tRNA_pos_47_acp3U_inact + 1 b3069_tRNA_8 + 1 h	irreversible	tRNA modification	0.00E+00	1.00E+09	0	1.45E+03	1.45E+03	0	1.05E+01	10.5	0	1.05E+01	10.5
b3069_tRNA_54_MODIF_1	modification of position 54 nt (m5U) in ileX by TrmA_mono	1 TrmA_mono + 1 amet + 1 b3069_tRNA_8 <==> 1 b3069_tRNA_8_TrmA_mono_cplx	reversible	tRNA modification	-1.00E+09	1.00E+09	0	1.45E+03	1.45E+03	0	1.05E+01	10.5	0	1.05E+01	10.5
b3069_tRNA_54_MODIF_2	modification of position 54 nt (m5U) in ileX by TrmA_mono	1 b3069_tRNA_8_TrmA_mono_cplx --> 1 TrmA_mono_inact + 1 ahcys + 1 b3069_tRNA_9 + 1 h	irreversible	tRNA modification	0.00E+00	1.00E+09	0	1.45E+03	1.45E+03	0	1.05E+01	10.5	0	1.05E+01	10.5
b3069_tRNA_55_MODIF_1	modification of position 55 nt (Y) in ileX by TruB_mono	1 TruB_mono + 1 b3069_tRNA_9 <==> 1 b3069_tRNA_9_TruB_mono_cplx	reversible	tRNA modification	-1.00E+09	1.00E+09	0	1.45E+03	1.45E+03	0	1.05E+01	10.5	0	1.05E+01	10.5
b3069_tRNA_55_MODIF_2	modification of position 55 nt (Y) in ileX by TruB_mono	1 b3069_tRNA_9_TruB_mono_cplx --> 1 TruB_mono_inact + 1 b3069_tRNA_10	irreversible	tRNA modification	0.00E+00	1.00E+09	0	1.45E+03	1.45E+03	0	1.05E+01	10.5	0	1.05E+01	10.5
b3069_tRNA_8_MODIF_1	modification of position 8 nt (s4U) in ileX by ThiI_mono	1 IscS_dim_S-SH + 1 ThiI_mono + 1 atp + 1 b3069_tRNA_10 + 1 mg2 + 1 trdrd <==> 1 b3069_tRNA_10_ThiI_mono_cplx	reversible	tRNA modification	-1.00E+09	1.00E+09	0	1.45E+03	1.45E+03	0	1.05E+01	10.5	0	1.05E+01	10.5
b3069_tRNA_8_MODIF_2	modification of position 8 nt (s4U) in ileX by ThiI_mono	1 b3069_tRNA_10_ThiI_mono_cplx --> 1 IscS_dim_S-H + 1 ThiI_mono_inact + 1 amp + 1 h + 1 ileX_tRNA + 1 mg2 + 1 ppi + 1 trdox	irreversible	tRNA modification	0.00E+00	1.00E+09	0	1.45E+03	1.45E+03	0	1.05E+01	10.5	0	1.05E+01	10.5
b3069_tRNA_Mg_BIND	Binding of two Mg2 to b3069_tRNA	1 b3069_RNA + 2 mg2 <==> 1 b3069_tRNA_Mg2	reversible	Metallo-ion binding	-1.00E+09	1.00E+09	0	1.45E+03	1.45E+03	0	1.05E+01	10.5	0	1.05E+01	10.5
b3123_v1_trimming	Cutting of stable b3123_v1_RNA	1 b3123_v1_RNA_cut_cplx + 1 h2o --> 1 RNase_E_tetra_inact + 1 RNase_Gen_inact + 1 RNase_P_cplx_inact + 1 b3123_v1_RNA + 1 + 1 ppi	irreversible	RNA Processing	0.00E+00	1.00E+09	0	2.80E+01	2.80E+01	0	4.94E+01	49.41244	0	4.99E-02	0.0499
b3123_v1_trimming_cplx	Cutting complex formation of stable b3123_v1_RNA (,)	1 RNase_E_tetra + 1 RNase_Gen + 1 RNase_P_cplx + 1 b3123_v1_RNA_pre <==> 1 b3123_v1_RNA_cut_cplx	reversible	RNA Processing	-1.00E+09	1.00E+09	0	2.80E+01	2.80E+01	0	4.94E+01	49.41244	0	4.99E-02	0.0499
b3124_fold_spon	b3124_m folding: spontanous	1 b3124_m --> 1 GarK_mono	irreversible	Protein Folding	0.00E+00	1.00E+09	0	7.65E+02	7.65E+02	0	1.28E+03	1279.259	0	9.46E+01	94.55759
b3124_mRNA_CONV	convsersion of mRNA to mRNA_1 (synthetic rxn)	1 b3124_mRNA --> 1 b3124_mRNA_1	irreversible	Translation	0.00E+00	1.00E+09	0	2.80E+01	2.80E+01	0	4.94E+01	49.41244	0	4.99E-02	0.0499
b3124_mRNA_degr1	Degradation of b3124_mRNA I	1 Orn_dim + 306 atp + 1 b3124_mRNA_2 + 1 degradosome + 306 h2o <==> 306 adp + 1 b3124_mRNA_2_degr + 306 h + 306 pi	reversible	mRNA degradation	-1.00E+09	1.00E+09	0	2.80E+01	2.80E+01	0	4.94E+01	49.41244	0	4.99E-02	0.0499
b3124_mRNA_degr2	Degradation of b3124_mRNA II	1 b3124_mRNA_2_degr + 1226 h2o --> 1 Orn_dim_inact + 294 amp + 275 cmp + 1 degradosome_inact + 386 gmp + 1226 h + 272 ump	irreversible	mRNA degradation	0.00E+00	2.07E-01	0	2.80E+01	2.80E+01	0	4.94E+01	49.41244	0	4.99E-02	0.0499
b3124_maturation1	Polypeptide b3124 peptide deformylase and methionine aminopeptidase complex	1 Def_mono + 1 Map_mono + 1 b3124_aa <==> 1 b3124_def_map_cplx	reversible	Protein Maturation	-1.00E+09	1.00E+09	0	7.65E+02	7.65E+02	0	1.28E+03	1279.259	0	9.46E+01	94.55759
b3124_maturation2	b3124 formation	1 b3124_def_map_cplx + 29 h2o --> 1 Def_mono_inact + 1 Map_mono_inact + 2 ala-L + 4 arg-L + 3 asn-L + 1 b3124_m + 1 cys-L + 1 for + 1 glu-L + 3 gly + 1 his-L + 1 ile-L + 2 leu-L + 1 lys-L + 2 met-L + 2 pro-L + 1 ser-L + 1 thr-L + 1 tyr-L + 2 val-L	irreversible	Protein Maturation	0.00E+00	1.00E+09	0	7.65E+02	7.65E+02	0	1.28E+03	1279.259	0	9.46E+01	94.55759
b3125_fold_spon	b3125_m folding: spontanous	1 b3125_m --> 1 GarR_mono	irreversible	Protein Folding	0.00E+00	1.00E+09	0	5.40E+02	5.40E+02	0	9.04E+02	904.2715	0	6.51E+01	65.1083
b3125_mRNA_CONV	convsersion of mRNA to mRNA_1 (synthetic rxn)	1 b3125_mRNA --> 1 b3125_mRNA_1	irreversible	Translation	0.00E+00	1.00E+09	0	2.80E+01	2.80E+01	0	4.94E+01	49.41244	0	4.99E-02	0.0499

b3125_mRNA_degr1	Degradation of b3125_mRNA I	1 Orn_dim + 222 atp + 1 b3125_mRNA_2 + 1 degradosome + 222 h2o <==> 222 adp + 1 b3125_mRNA_2_degr + 222 h + 222 pi	reversible	mRNA degradation	-1.00E+09	1.00E+09	0	2.80E+01	2.80E+01	0	4.94E+01	49.41244	0	4.99E-02	0.0499
b3125_mRNA_degr2	Degradation of b3125_mRNA II	1 b3125_mRNA_2_degr + 890 h2o --> 1 Orn_dim_inact + 224 amp + 198 cmp + 1 degradosome_inact + 273 gmp + 890 h + 196 ump	irreversible	mRNA degradation	0.00E+00	9.62E-02	0	2.80E+01	2.80E+01	0	4.94E+01	49.41244	0	4.99E-02	0.0499
b3125_maturation1	Polypeptide b3125 peptide deformylas and methionine aminopeptidase complex	1 Def_mono + 1 Map_mono + 1 b3125_aa <==> 1 b3125_def_map_cplx	reversible	Protein Maturation	-1.00E+09	1.00E+09	0	5.40E+02	5.40E+02	0	9.04E+02	904.2715	0	6.51E+01	65.1083
b3125_maturation2	b3125 formation	1 b3125_def_map_cplx + 4 h2o --> 1 Def_mono_inact + 1 Map_mono_inact + 1 b3125_m + 1 for + 2 met-L + 1 thr-L	irreversible	Protein Maturation	0.00E+00	1.00E+09	0	5.40E+02	5.40E+02	0	9.04E+02	904.2715	0	6.51E+01	65.1083
b3126_fold_spon	b3126_m folding: spontanous	1 b3126_m --> 1 GarL_mono	irreversible	Protein Folding	0.00E+00	1.00E+09	0	5.13E+02	5.13E+02	0	8.34E+02	834.0366	0	9.36E+01	93.5985
b3126_mRNA_CONV	convsersion of mRNA to mRNA_1 (synthetic rxn)	1 b3126_mRNA --> 1 b3126_mRNA_1	irreversible	Translation	0.00E+00	1.00E+09	0	2.80E+01	2.80E+01	0	4.94E+01	49.41244	0	4.99E-02	0.0499
b3126_mRNA_degr1	Degradation of b3126_mRNA I	1 Orn_dim + 192 atp + 1 b3126_mRNA_2 + 1 degradosome + 192 h2o <==> 192 adp + 1 b3126_mRNA_2_degr + 192 h + 192 pi	reversible	mRNA degradation	-1.00E+09	1.00E+09	0	2.80E+01	2.80E+01	0	4.94E+01	49.41244	0	4.99E-02	0.0499
b3126_mRNA_degr2	Degradation of b3126_mRNA II	1 b3126_mRNA_2_degr + 770 h2o --> 1 Orn_dim_inact + 183 amp + 206 cmp + 1 degradosome_inact + 202 gmp + 770 h + 180 ump	irreversible	mRNA degradation	0.00E+00	1.71E-01	0	2.80E+01	2.80E+01	0	4.94E+01	49.41244	0	4.99E-02	0.0499
b3126_maturation1	Polypeptide b3126 peptide deformylas complex	1 Def_mono + 1 b3126_aa <==> 1 b3126_def_cplx	reversible	Protein Maturation	-1.00E+09	1.00E+09	0	5.13E+02	5.13E+02	0	8.34E+02	834.0366	0	9.36E+01	93.5985
b3126_maturation2	b3126 formation	1 b3126_def_cplx + 1 h2o --> 1 Def_mono_inact + 1 b3126_m + 1 for	irreversible	Protein Maturation	0.00E+00	1.00E+09	0	5.13E+02	5.13E+02	0	8.34E+02	834.0366	0	9.36E+01	93.5985
b3127_fold_spon	b3127_m folding: spontanous	1 b3127_m --> 1 GarP_mono	irreversible	Protein Folding	0.00E+00	1.00E+09	0	7.88E+02	7.88E+02	0	1.34E+03	1344.783	0	6.14E+01	61.3574
b3127_mRNA_CONV	convsersion of mRNA to mRNA_1 (synthetic rxn)	1 b3127_mRNA --> 1 b3127_mRNA_1	irreversible	Translation	0.00E+00	1.00E+09	0	2.80E+01	2.80E+01	0	4.94E+01	49.41244	0	4.99E-02	0.0499
b3127_mRNA_degr1	Degradation of b3127_mRNA I	1 Orn_dim + 333 atp + 1 b3127_mRNA_2 + 1 degradosome + 333 h2o <==> 333 adp + 1 b3127_mRNA_2_degr + 333 h + 333 pi	reversible	mRNA degradation	-1.00E+09	1.00E+09	0	2.80E+01	2.80E+01	0	4.94E+01	49.41244	0	4.99E-02	0.0499
b3127_mRNA_degr2	Degradation of b3127_mRNA II	1 b3127_mRNA_2_degr + 1334 h2o --> 1 Orn_dim_inact + 264 amp 1 atp + 333 cmp + 1 degradosome_inact + 352 gmp + 1334 h + 385 ump	irreversible	mRNA degradation	0.00E+00	4.99E-02	0	2.80E+01	2.80E+01	0	4.94E+01	49.41244	0	4.99E-02	0.0499
b3127_maturation1	Polypeptide b3127 peptide deformylas and methionine aminopeptidase complex	1 Def_mono + 1 Map_mono + 1 b3127_aa <==> 1 b3127_def_map_cplx	reversible	Protein Maturation	-1.00E+09	1.00E+09	0	7.88E+02	7.88E+02	0	1.34E+03	1344.783	0	6.14E+01	61.3574
b3127_maturation2	b3127 formation	1 b3127_def_map_cplx + 2 h2o --> 1 Def_mono_inact + 1 Map_mono_inact + 1 b3127_m + 1 for + 1 met-L	irreversible	Protein Maturation	0.00E+00	1.00E+09	0	7.88E+02	7.88E+02	0	1.34E+03	1344.783	0	6.14E+01	61.3574
b3164_fold_GroEL/ES_1	b3164_m folding: GroEL/ES mediated: polypeptide is going in GroEL/ES complex	1 GroEL.(7)ADP.cisGroES + 7 atp + 1 b3164_m + 1 transGroES_hepta --> 7 adp + 1 b3164_m_GroEL.(7)ATP.transGroES + 1 cisGroES_hepta	irreversible	Protein Folding	0.00E+00	1.00E+09	0	6.59E+02	6.59E+02	0	7.16E+02	716.1174	0	8.64E+01	86.4435
b3164_fold_GroEL/ES_2	b3164_m folding: GroEL/ES mediated: folding of polypeptide under ATP hydrolysis	1 b3164_m_GroEL.(7)ATP.transGroES + 7 h2o --> 1 Pnp_GroEL.(7)ADP.transGroES + 7 h + 7 pi	irreversible	Protein Folding	0.00E+00	1.00E+09	0	6.59E+02	6.59E+02	0	7.16E+02	716.1174	0	8.64E+01	86.4435
b3164_fold_GroEL/ES_3	b3164_m folding: GroEL/ES mediated: release of native protein	1 Pnp_GroEL.(7)ADP.transGroES --> 1 GroEL.(7)ADP.transGroES + 1 Pnp_mono	irreversible	Protein Folding	0.00E+00	1.00E+09	0	6.59E+02	6.59E+02	0	7.16E+02	716.1174	0	8.64E+01	86.4435
b3164_fold_KJE_1	b3164_m folding: KJE mediated	1 DnaJ_dim + 1 DnaK_mono.ATP + 1 b3164_m <==> 1 b3164_m_DnaKJ_complex	reversible	Protein Folding	-1.00E+09	1.00E+09	0	6.59E+02	6.59E+02	0	7.16E+02	716.1174	0	8.64E+01	86.4435
b3164_fold_KJE_2	b3164_m folding: KJE mediated	1 GrpE_dim + 1 b3164_m_DnaKJ_complex + 1 h2o --> 1 DnaJ_dim_inact + 1 Pnp_DnaK_GrpE_complex + 1 adp + 1 h + 1 pi	irreversible	Protein Folding	0.00E+00	1.00E+09	0	6.59E+02	6.59E+02	0	7.16E+02	716.1174	0	8.64E+01	86.4435
b3164_fold_KJE_3	b3164_m folding: KJE mediated	1 Pnp_DnaK_GrpE_complex + 1 atp --> 1 DnaK_mono.ATP_inact + GrpE_dim_inact + 1 Pnp_mono	irreversible	Protein Folding	0.00E+00	1.00E+09	0	6.59E+02	6.59E+02	0	7.16E+02	716.1174	0	8.64E+01	86.4435
b3164_fold_spon	b3164_m folding: spontanous	1 b3164_m --> 1 Pnp_mono	irreversible	Protein Folding	0.00E+00	1.00E+09	0	6.59E+02	6.59E+02	0	7.16E+02	716.1174	0	8.64E+01	86.4435
b3164_mRNA_CONV	convsersion of mRNA to mRNA_1 (synthetic rxn)	1 b3164_mRNA --> 1 b3164_mRNA_1	irreversible	Translation	0.00E+00	1.00E+09	0	1.43E+01	1.43E+01	0	2.52E+01	25.16946	0	1.46E-01	0.146
b3164_mRNA_degr1	Degradation of b3164_mRNA I	1 Orn_dim + 551 atp + 1 b3164_mRNA_2 + 1 degradosome + 551 h2o <==> 551 adp + 1 b3164_mRNA_2_degr + 551 h + 551 pi	reversible	mRNA degradation	-1.00E+09	1.00E+09	0	5.71E+01	5.71E+01	0	1.00E+02	100.0923	0	6.46E-01	0.646
b3164_mRNA_degr2	Degradation of b3164_mRNA II	1 b3164_mRNA_2_degr + 2204 h2o --> 1 Orn_dim_inact + 531 amp 559 cmp + 1 degradosome_inact + 625 gmp + 2204 h + 490 ump	irreversible	mRNA degradation	0.00E+00	6.46E-01	0	5.71E+01	5.71E+01	0	1.00E+02	100.0923	0	6.46E-01	0.646
b3164_maturation1	Polypeptide b3164 peptide deformylas complex	1 Def_mono + 1 b3164_aa <==> 1 b3164_def_cplx	reversible	Protein Maturation	-1.00E+09	1.00E+09	0	6.59E+02	6.59E+02	0	7.16E+02	716.1174	0	8.64E+01	86.4435
b3164_maturation2	b3164 formation	1 b3164_def_cplx + 24 h2o --> 1 Def_mono_inact + 1 ala-L + 6 arg-L + 1 asn-L + 1 asp-L + 1 b3164_m + 1 cys-L + 1 for + 1 gly + 2 ile-L + leu-L + 2 lys-L + 2 ser-L + 2 thr-L + 1 tyr-L + 1 val-L	irreversible	Protein Maturation	0.00E+00	1.00E+09	0	6.59E+02	6.59E+02	0	7.16E+02	716.1174	0	8.64E+01	86.4435
b3164_v1_mRNA_CONV	convsersion of mRNA to mRNA_1 (synthetic rxn)	1 b3164_v1_mRNA --> 1 b3164_mRNA_1	irreversible	Translation	0.00E+00	1.00E+09	0	4.77E+01	4.77E+01	0	7.03E+01	70.31333	0	4.81E-01	0.481
b3164_v2_mRNA_CONV	conversion of mRNA to mRNA_1 (synthetic rxn)	1 b3164_v2_mRNA --> 1 b3164_mRNA_1	irreversible	Translation	0.00E+00	1.00E+09	0	5.71E+01	5.71E+01	0	1.00E+02	100.0923	0	6.46E-01	0.646
b3164_v3_mRNA_CONV	conversion of mRNA to mRNA_1 (synthetic rxn)	1 b3164_v3_mRNA --> 1 b3164_mRNA_1	irreversible	Translation	0.00E+00	1.00E+09	0	1.43E+01	1.43E+01	0	2.52E+01	25.16946	0	1.46E-01	0.146
b3165_fold_spon	b3165_m folding: spontanous	1 b3165_m --> 1 RpsO_mono	irreversible	Protein Folding	0.00E+00	1.00E+09	8.44E+01	1.13E+02	2.81E+01	6.22E+01	8.29E+01	20.72	6.22E+01	8.29E+01	20.72
b3165_mRNA_CONV	convsersion of mRNA to mRNA_1 (synthetic rxn)	1 b3165_mRNA --> 1 b3165_mRNA_1	irreversible	Translation	0.00E+00	1.00E+09	0	1.43E+01	1.43E+01	0	2.52E+01	25.16946	0	1.46E-01	0.146
b3165_mRNA_degr1	Degradation of b3165_mRNA I	1 Orn_dim + 67 atp + 1 b3165_mRNA_2 + 1 degradosome + 67 h2o <==> 67 adp + 1 b3165_mRNA_2_degr + 67 h + 67 pi	reversible	mRNA degradation	-1.00E+09	1.00E+09	0	1.06E+02	1.06E+02	0	8.29E+01	82.88	0	4.81E-01	0.481
b3165_mRNA_degr2	Degradation of b3165_mRNA II	1 b3165_mRNA_2_degr + 269 h2o --> 1 Orn_dim_inact + 71 amp + 76 cmp + 1 degradosome_inact + 64 gmp + 269 h + 59 ump	irreversible	mRNA degradation	0.00E+00	4.81E-01	0	1.06E+02	1.06E+02	0	8.29E+01	82.88	0	4.81E-01	0.481
b3165_maturation1	Polypeptide b3165 peptide deformylas and methionine aminopeptidase complex	1 Def_mono + 1 Map_mono + 1 b3165_aa <==> 1 b3165_def_map_cplx	reversible	Protein Maturation	-1.00E+09	1.00E+09	8.44E+01	1.13E+02	2.81E+01	6.22E+01	8.29E+01	20.72	6.22E+01	8.29E+01	20.72
b3165_maturation2	b3165 formation	1 b3165_def_map_cplx + 2 h2o --> 1 Def_mono_inact + 1 Map_mono_inact + 1 b3165_m + 1 for + 1 met-L	irreversible	Protein Maturation	0.00E+00	1.00E+09	8.44E+01	1.13E+02	2.81E+01	6.22E+01	8.29E+01	20.72	6.22E+01	8.29E+01	20.72
b3165_v1_mRNA_CONV	convsersion of mRNA to mRNA_1 (synthetic rxn)	1 b3165_v1_mRNA --> 1 b3165_mRNA_1	irreversible	Translation	0.00E+00	1.00E+09	0	1.06E+02	1.06E+02	0	8.29E+01	82.88	0	4.81E-01	0.481
b3165_v2_mRNA_CONV	convsersion of mRNA to mRNA_1 (synthetic rxn)	1 b3165_v2_mRNA --> 1 b3165_mRNA_1	irreversible	Translation	0.00E+00	1.00E+09	0	4.77E+01	4.77E+01	0	7.03E+01	70.31333	0	4.81E-01	0.481
b3165_v3_mRNA_CONV	conversion of mRNA to mRNA_1 (synthetic rxn)	1 b3165_v3_mRNA --> 1 b3165_mRNA_1	irreversible	Translation	0.00E+00	1.00E+09	0	1.43E+01	1.43E+01	0	2.52E+01	25.16946	0	1.46E-01	0.146
b3166_fold_spon	b3166_m folding: spontanous	1 b3166_m --> 1 TruB_mono	irreversible	Protein Folding	0.00E+00	1.00E+09	0	3.26E+02	3.26E+02	0	5.23E+02	522.5303	0	7.21E+01	72.108
b3166_mRNA_CONV	convsersion of mRNA to mRNA_1 (synthetic rxn)	1 b3166_mRNA --> 1 b3166_mRNA_1	irreversible	Translation	0.00E+00	1.00E+09	0	1.43E+01	1.43E+01	0	2.52E+01	25.16946	0	1.46E-01	0.146
b3166_mRNA_degr1	Degradation of b3166_mRNA I	1 Orn_dim + 236 atp + 1 b3166_mRNA_2 + 1 degradosome + 236 h2o <==> 236 adp + 1 b3166_mRNA_2_degr + 236 h + 236 pi	reversible	mRNA degradation	-1.00E+09	1.00E+09	0	1.43E+01	1.43E+01	0	2.52E+01	25.16946	0	1.46E-01	0.146
b3166_mRNA_degr2	Degradation of b3166_mRNA II	1 b3166_mRNA_2_degr + 943 h2o --> 1 Orn_dim_inact + 220 amp + 231 cmp + 1 degradosome_inact + 265 gmp + 943 h + 228 ump	irreversible	mRNA degradation	0.00E+00	1.58E-01	0	1.43E+01	1.43E+01	0	2.52E+01	25.16946	0	1.46E-01	0.146
b3166_maturation1	Polypeptide b3166 peptide deformylas and methionine aminopeptidase complex	1 Def_mono + 1 Map_mono + 1 b3166_aa <==> 1 b3166_def_map_cplx	reversible	Protein Maturation	-1.00E+09	1.00E+09	0	3.26E+02	3.26E+02	0	5.23E+02	522.5303	0	7.21E+01	72.108
b3166_maturation2	b3166 formation	1 b3166_def_map_cplx + 2 h2o --> 1 Def_mono_inact + 1 Map_mono_inact + 1 b3166_m + 1 for + 1 met-L	irreversible	Protein Maturation	0.00E+00	1.00E+09	0	3.26E+02	3.26E+02	0	5.23E+02	522.5303	0	7.21E+01	72.108
b3166_v1_mRNA_CONV	conversion of mRNA to mRNA_1 (synthetic rxn)	1 b3166_v1_mRNA --> 1 b3166_mRNA_1	irreversible	Translation	0.00E+00	1.00E+09	0	1.43E+01	1.43E+01	0	2.52E+01	25.16946	0	1.46E-01	0.146

b3167_fold_spon	b3167_m folding: spontanous	1 b3167_m --> 1 RbfA_mono	irreversible	Protein Folding	0.00E+00	1.00E+09	0	1.69E+02	1.69E+02	0	2.45E+02	244.9262	0	6.98E+01	69.762
b3167_mRNA_CONV	convsersion of mRNA to mRNA_1 (synthetic rxn)	1 b3167_mRNA --> 1 b3167_mRNA_1	irreversible	Translation	0.00E+00	1.00E+09	0	1.43E+01	1.43E+01	0	2.52E+01	25.16946	0	1.46E-01	0.146
b3167_mRNA_degr1	Degradation of b3167_mRNA I	1 Orn_dim + 100 atp + 1 b3167_mRNA_2 + 1 degradosome + 100 h2o <==> 100 adp + 1 b3167_mRNA_2_degr + 100 h + 100 pi	reversible	mRNA degradation	-1.00E+09	1.00E+09	0	1.43E+01	1.43E+01	0	2.52E+01	25.16946	0	1.46E-01	0.146
b3167_mRNA_degr2	Degradation of b3167_mRNA II	1 b3167_mRNA_2_degr + 401 h2o --> 1 Orn_dim_inact + 107 amp + 100 cmp + 1 degradosome_inact + 113 gmp + 401 h + 82 ump	irreversible	mRNA degradation	0.00E+00	1.46E-01	0	1.43E+01	1.43E+01	0	2.52E+01	25.16946	0	1.46E-01	0.146
b3167_maturation1	Polypeptide b3167 peptide deformylas and methionine aminopeptidase complex	1 Def_mono + 1 Map_mono + 1 b3167_aa <==> 1 b3167_def_map_cplx	reversible	Protein Maturation	-1.00E+09	1.00E+09	0	1.69E+02	1.69E+02	0	2.45E+02	244.9262	0	6.98E+01	69.762
b3167_maturation2	b3167 formation	1 b3167_def_map_cplx + 2 h2o --> 1 Def_mono_inact + 1 Map_mono_inact + 1 b3167_m + 1 for + 1 met-L	irreversible	Protein Maturation	0.00E+00	1.00E+09	0	1.69E+02	1.69E+02	0	2.45E+02	244.9262	0	6.98E+01	69.762
b3167_v1_mRNA_CONV	convsersion of mRNA to mRNA_1 (synthetic rxn)	1 b3167_v1_mRNA --> 1 b3167_mRNA_1	irreversible	Translation	0.00E+00	1.00E+09	0	1.43E+01	1.43E+01	0	2.52E+01	25.16946	0	1.46E-01	0.146
b3168_fold_GroEL/ES_1	b3168_m folding: GroEL/ES mediated: polypeptide is going in GroEL/ES complex	1 GroEL.(7)ADP.cisGroES + 7 atp + 1 b3168_m + 1 transGroES_hepta --> 7 adp + 1 b3168_m_GroEL.(7)ATP.transGroES + 1 cisGroES_hepta	irreversible	Protein Folding	0.00E+00	1.00E+09	0	1.60E+03	1.60E+03	0	2.03E+03	2028.084	0	1.11E+02	111.28
b3168_fold_GroEL/ES_2	b3168_m folding: GroEL/ES mediated: folding of polypeptide under ATP hydrolysis	1 b3168_m_GroEL.(7)ATP.transGroES + 7 h2o --> 1 InfB_GroEL.(7)ADP.transGroES + 7 h + 7 pi	irreversible	Protein Folding	0.00E+00	1.00E+09	0	1.60E+03	1.60E+03	0	2.03E+03	2028.084	0	1.11E+02	111.28
b3168_fold_GroEL/ES_3	b3168_m folding: GroEL/ES mediated: release of native protein	1 InfB_GroEL.(7)ADP.transGroES --> 1 GroEL.(7)ADP.transGroES + 1 InfB_mono	irreversible	Protein Folding	0.00E+00	1.00E+09	0	1.60E+03	1.60E+03	0	2.03E+03	2028.084	0	1.11E+02	111.28
b3168_fold_KJE_1	b3168_m folding: KJE mediated	1 DnaJ_dim + 1 DnaK_mono.ATP + 1 b3168_m <==> 1 b3168_m_DnaKJ_complex	reversible	Protein Folding	-1.00E+09	1.00E+09	0	1.60E+03	1.60E+03	0	2.03E+03	2028.084	0	1.11E+02	111.28
b3168_fold_KJE_2	b3168_m folding: KJE mediated	1 GrpE_dim + 1 b3168_m_DnaKJ_complex + 1 h2o --> 1 DnaJ_dim_inact + 1 InfB_DnaK_GrpE_complex + 1 adp + 1 h + 1 pi	irreversible	Protein Folding	0.00E+00	1.00E+09	0	1.60E+03	1.60E+03	0	2.03E+03	2028.084	0	1.11E+02	111.28
b3168_fold_KJE_3	b3168_m folding: KJE mediated	1 InfB_DnaK_GrpE_complex + 1 atp --> 1 DnaK_mono.ATP_inact + GrpE_dim_inact + 1 InfB_mono	irreversible	Protein Folding	0.00E+00	1.00E+09	0	1.60E+03	1.60E+03	0	2.03E+03	2028.084	0	1.11E+02	111.28
b3168_mRNA_CONV	convsersion of mRNA to mRNA_1 (synthetic rxn)	1 b3168_mRNA --> 1 b3168_mRNA_1	irreversible	Translation	0.00E+00	1.00E+09	0	1.43E+01	1.43E+01	0	2.52E+01	25.16946	0	1.46E-01	0.146
b3168_mRNA_degr1	Degradation of b3168_mRNA I	1 Orn_dim + 668 atp + 1 b3168_mRNA_2 + 1 degradosome + 668 h2o <==> 668 adp + 1 b3168_mRNA_2_degr + 668 h + 668 pi	reversible	mRNA degradation	-1.00E+09	1.00E+09	0	2.88E+01	2.88E+01	0	5.08E+01	50.83823	0	1.50E-01	0.15
b3168_mRNA_degr2	Degradation of b3168_mRNA II	1 b3168_mRNA_2_degr + 2672 h2o --> 1 Orn_dim_inact + 730 amp 647 cmp + 1 degradosome_inact + 781 gmp + 2672 h + 515 ump	irreversible	mRNA degradation	0.00E+00	4.31E-01	0	2.88E+01	2.88E+01	0	5.08E+01	50.83823	0	1.50E-01	0.15
b3168_maturation1	Polypeptide b3168 peptide deformylas complex	1 Def_mono + 1 b3168_aa <==> 1 b3168_def_cplx	reversible	Protein Maturation	-1.00E+09	1.00E+09	8.44E+01	1.60E+03	1.52E+03	6.22E+01	2.03E+03	1965.924	6.22E+01	1.11E+02	49.12
b3168_maturation2	b3168 formation	1 b3168_def_cplx + 1 h2o --> 1 Def_mono_inact + 1 b3168_m + 1 for	irreversible	Protein Maturation	0.00E+00	1.00E+09	8.44E+01	1.60E+03	1.52E+03	6.22E+01	2.03E+03	1965.924	6.22E+01	1.11E+02	49.12
b3168_v1_mRNA_CONV	convsersion of mRNA to mRNA_1 (synthetic rxn)	1 b3168_v1_mRNA --> 1 b3168_mRNA_1	irreversible	Translation	0.00E+00	1.00E+09	0	2.72E+01	2.72E+01	0	4.80E+01	48.0235	0	1.50E-01	0.15
b3168_v2_mRNA_CONV	convsersion of mRNA to mRNA_1 (synthetic rxn)	1 b3168_v2_mRNA --> 1 b3168_mRNA_1	irreversible	Translation	0.00E+00	1.00E+09	0	2.88E+01	2.88E+01	0	5.08E+01	50.83823	0	1.50E-01	0.15
b3168_v3_mRNA_CONV	convsersion of mRNA to mRNA_1 (synthetic rxn)	1 b3168_v3_mRNA --> 1 b3168_mRNA_1	irreversible	Translation	0.00E+00	1.00E+09	0	1.43E+01	1.43E+01	0	2.52E+01	25.16946	0	1.46E-01	0.146
b3169_fold_spon	b3169_m folding: spontanous	1 b3169_m --> 1 NusA_mono	irreversible	Protein Folding	0.00E+00	1.00E+09	0	9.12E+02	9.12E+02	0	1.55E+03	1550.869	0	8.09E+01	80.91
b3169_mRNA_CONV	convsersion of mRNA to mRNA_1 (synthetic rxn)	1 b3169_mRNA --> 1 b3169_mRNA_1	irreversible	Translation	0.00E+00	1.00E+09	0	1.43E+01	1.43E+01	0	2.52E+01	25.16946	0	1.46E-01	0.146
b3169_mRNA_degr1	Degradation of b3169_mRNA I	1 Orn_dim + 372 atp + 1 b3169_mRNA_2 + 1 degradosome + 372 h2o <==> 372 adp + 1 b3169_mRNA_2_degr + 372 h + 372 pi	reversible	mRNA degradation	-1.00E+09	1.00E+09	0	2.88E+01	2.88E+01	0	5.08E+01	50.83823	0	1.50E-01	0.15
b3169_mRNA_degr2	Degradation of b3169_mRNA II	1 b3169_mRNA_2_degr + 1487 h2o --> 1 Orn_dim_inact + 387 amp 352 cmp + 1 degradosome_inact + 433 gmp + 1487 h + 316 ump	irreversible	mRNA degradation	0.00E+00	1.50E-01	0	2.88E+01	2.88E+01	0	5.08E+01	50.83823	0	1.50E-01	0.15
b3169_maturation1	Polypeptide b3169 peptide deformylas complex	1 Def_mono + 1 b3169_aa <==> 1 b3169_def_cplx	reversible	Protein Maturation	-1.00E+09	1.00E+09	0	9.12E+02	9.12E+02	0	1.55E+03	1550.869	0	8.09E+01	80.91
b3169_maturation2	b3169 formation	1 b3169_def_cplx + 1 h2o --> 1 Def_mono_inact + 1 b3169_m + 1 for	irreversible	Protein Maturation	0.00E+00	1.00E+09	0	9.12E+02	9.12E+02	0	1.55E+03	1550.869	0	8.09E+01	80.91
b3169_v1_mRNA_CONV	convsersion of mRNA to mRNA_1 (synthetic rxn)	1 b3169_v1_mRNA --> 1 b3169_mRNA_1	irreversible	Translation	0.00E+00	1.00E+09	0	2.72E+01	2.72E+01	0	4.80E+01	48.0235	0	1.50E-01	0.15
b3169_v2_mRNA_CONV	convsersion of mRNA to mRNA_1 (synthetic rxn)	1 b3169_v2_mRNA --> 1 b3169_mRNA_1	irreversible	Translation	0.00E+00	1.00E+09	0	2.88E+01	2.88E+01	0	5.08E+01	50.83823	0	1.50E-01	0.15
b3169_v3_mRNA_CONV	convsersion of mRNA to mRNA_1 (synthetic rxn)	1 b3169_v3_mRNA --> 1 b3169_mRNA_1	irreversible	Translation	0.00E+00	1.00E+09	0	1.43E+01	1.43E+01	0	2.52E+01	25.16946	0	1.46E-01	0.146
b3170_fold_spon	b3170_m folding: spontanous	1 b3170_m --> 1 YhbC_mono	irreversible	Protein Folding	0.00E+00	1.00E+09	0	3.10E+02	3.10E+02	0	4.86E+02	486.3058	0	8.08E+01	80.8
b3170_mRNA_CONV	convsersion of mRNA to mRNA_1 (synthetic rxn)	1 b3170_mRNA --> 1 b3170_mRNA_1	irreversible	Translation	0.00E+00	1.00E+09	0	1.43E+01	1.43E+01	0	2.52E+01	25.16946	0	1.46E-01	0.146
b3170_mRNA_degr1	Degradation of b3170_mRNA I	1 Orn_dim + 113 atp + 1 b3170_mRNA_2 + 1 degradosome + 113 h2o <==> 113 adp + 1 b3170_mRNA_2_degr + 113 h + 113 pi	reversible	mRNA degradation	-1.00E+09	1.00E+09	0	2.88E+01	2.88E+01	0	5.08E+01	50.83823	0	1.50E-01	0.15
b3170_mRNA_degr2	Degradation of b3170_mRNA II	1 b3170_mRNA_2_degr + 452 h2o --> 1 Orn_dim_inact + 114 amp + 101 cmp + 1 degradosome_inact + 119 gmp + 452 h + 119 ump	irreversible	mRNA degradation	0.00E+00	2.07E-01	0	2.88E+01	2.88E+01	0	5.08E+01	50.83823	0	1.50E-01	0.15
b3170_maturation1	Polypeptide b3170 peptide deformylas and methionine aminopeptidase complex	1 Def_mono + 1 Map_mono + 1 b3170_aa <==> 1 b3170_def_map_cplx	reversible	Protein Maturation	-1.00E+09	1.00E+09	0	3.10E+02	3.10E+02	0	4.86E+02	486.3058	0	8.08E+01	80.8
b3170_maturation2	b3170 formation	1 b3170_def_map_cplx + 12 h2o --> 1 Def_mono_inact + 1 Map_mono_inact + 1 b3170_m + 1 for + 1 gln-L + 2 glu-L + 2 leu-L + lys-L + 2 met-L + 1 ser-L + 2 thr-L	irreversible	Protein Maturation	0.00E+00	1.00E+09	0	3.10E+02	3.10E+02	0	4.86E+02	486.3058	0	8.08E+01	80.8
b3170_v1_mRNA_CONV	convsersion of mRNA to mRNA_1 (synthetic rxn)	1 b3170_v1_mRNA --> 1 b3170_mRNA_1	irreversible	Translation	0.00E+00	1.00E+09	0	2.72E+01	2.72E+01	0	4.80E+01	48.0235	0	1.50E-01	0.15
b3170_v2_mRNA_CONV	convsersion of mRNA to mRNA_1 (synthetic rxn)	1 b3170_v2_mRNA --> 1 b3170_mRNA_1	irreversible	Translation	0.00E+00	1.00E+09	0	2.88E+01	2.88E+01	0	5.08E+01	50.83823	0	1.50E-01	0.15
b3170_v3_mRNA_CONV	convsersion of mRNA to mRNA_1 (synthetic rxn)	1 b3170_v3_mRNA --> 1 b3170_mRNA_1	irreversible	Translation	0.00E+00	1.00E+09	0	1.43E+01	1.43E+01	0	2.52E+01	25.16946	0	1.46E-01	0.146
b3171_tRNA_20_MODIF_1	modification of position 20 nt (D) in metY by DusA_mono	1 DusA_mono + 1 b3171_tRNA_Mg2 + 1 h + 1 nadph <==> 1 b3171_tRNA_Mg2_DusA_mono_cplx	reversible	tRNA modification	-1.00E+09	1.00E+09	0	1.23E+03	1.23E+03	0	11	11	0	11	11
b3171_tRNA_20_MODIF_2	modification of position 20 nt (D) in metY by DusA_mono	1 b3171_tRNA_Mg2_DusA_mono_cplx --> 1 DusA_mono + 1 b3171_tRNA_1 + 1 nadp	irreversible	tRNA modification	0.00E+00	1.00E+09	0	1.23E+03	1.23E+03	0	11	11	0	11	11
b3171_tRNA_32_MODIF_1	modification of position 32 nt (Cm) in metY by MeT_tRNA_pos_32_Cm	1 MeT_tRNA_pos_32_Cm + 1 amet + 1 b3171_tRNA_1 <==> 1 b3171_tRNA_1_MeT_tRNA_pos_32_Cm_cplx	reversible	tRNA modification	-1.00E+09	1.00E+09	0	1.23E+03	1.23E+03	0	11	11	0	11	11
b3171_tRNA_32_MODIF_2	modification of position 32 nt (Cm) in metY by MeT_tRNA_pos_32_Cm	1 b3171_tRNA_1_MeT_tRNA_pos_32_Cm_cplx --> 1 MeT_tRNA_pos_32_Cm_inact + 1 ahcys + 1 b3171_tRNA_2 + 1 h	irreversible	tRNA modification	0.00E+00	1.00E+09	0	1.23E+03	1.23E+03	0	11	11	0	11	11
b3171_tRNA_54_MODIF_1	modification of position 54 nt (m5U) in metY by TrmA_mono	1 TrmA_mono + 1 amet + 1 b3171_tRNA_2 <==> 1 b3171_tRNA_2_TrmA_mono_cplx	reversible	tRNA modification	-1.00E+09	1.00E+09	0	1.23E+03	1.23E+03	0	11	11	0	11	11
b3171_tRNA_54_MODIF_2	modification of position 54 nt (m5U) in metY by TrmA_mono	1 b3171_tRNA_2_TrmA_mono_cplx --> 1 TrmA_mono_inact + 1 ahcys + 1 b3171_tRNA_3 + 1 h	irreversible	tRNA modification	0.00E+00	1.00E+09	0	1.23E+03	1.23E+03	0	11	11	0	11	11
b3171_tRNA_55_MODIF_1	modification of position 55 nt (Y) in metY by TruB_mono	1 TruB_mono + 1 b3171_tRNA_3 <==> 1 b3171_tRNA_3_TruB_mono_cplx	reversible	tRNA modification	-1.00E+09	1.00E+09	0	1.23E+03	1.23E+03	0	11	11	0	11	11
b3171_tRNA_55_MODIF_2	modification of position 55 nt (Y) in metY by TruB_mono	1 b3171_tRNA_3_TruB_mono_cplx --> 1 TruB_mono_inact + 1 b3171_tRNA_4	irreversible	tRNA modification	0.00E+00	1.00E+09	0	1.23E+03	1.23E+03	0	11	11	0	11	11
b3171_tRNA_8_MODIF_1	modification of position 8 nt (s4U) in metY by ThiI_mono	1 IscS_dim_S-SH + 1 ThiI_mono + 1 atp + 1 b3171_tRNA_4 + 1 mg2 + 1 trdrd <==> 1 b3171_tRNA_4_ThiI_mono_cplx	reversible	tRNA modification	-1.00E+09	1.00E+09	0	1.23E+03	1.23E+03	0	11	11	0	11	11

b3171_tRNA_8_MODIF_2	modification of position 8 nt (s4U) in metY by ThiI_mono	1 b3171_tRNA_4_ThiI_mono_cplx --> 1 IscS_dim_S-H + 1 ThiI_mono_inact + 1 amp + 1 h + 1 metY_tRNA + 1 mg2 + 1 ppi + 1 trdox	irreversible	tRNA modification	0.00E+00	1.00E+09	0	1.23E+03	1.23E+03	0	11	11	0	11	11
b3171_tRNA_Mg_BIND	Binding of two Mg2 to b3171_tRNA	1 b3171_RNA + 2 mg2 <==> 1 b3171_tRNA_Mg2	reversible	Metallo-ion binding	-1.00E+09	1.00E+09	0	1.23E+03	1.23E+03	0	11	11	0	11	11
b3171_v1_tRNA_20_MODIF_1	modification of position 20 nt (D) in metY by DusA_mono	1 DusA_mono + 1 b3171_v1_tRNA_Mg2 + 1 h + 1 nadph <==> 1 b3171_v1_tRNA_Mg2_DusA_mono_cplx	reversible	tRNA modification	-1.00E+09	1.00E+09	0	1.43E+01	1.43E+01	0	2.52E+01	25.16946	0	1.46E-01	0.146
b3171_v1_tRNA_20_MODIF_2	modification of position 20 nt (D) in metY by DusA_mono	1 b3171_v1_tRNA_Mg2_DusA_mono_cplx --> 1 DusA_mono + 1 b3171_v1_tRNA_1 + 1 nadp	irreversible	tRNA modification	0.00E+00	1.00E+09	0	1.43E+01	1.43E+01	0	2.52E+01	25.16946	0	1.46E-01	0.146
b3171_v1_tRNA_32_MODIF_1	modification of position 32 nt (Cm) in metY by MeT_tRNA_pos_32_Cm	1 MeT_tRNA_pos_32_Cm + 1 amet + 1 b3171_v1_tRNA_1 <==> 1 b3171_v1_tRNA_1_MeT_tRNA_pos_32_Cm_cplx	reversible	tRNA modification	-1.00E+09	1.00E+09	0	1.43E+01	1.43E+01	0	2.52E+01	25.16946	0	1.46E-01	0.146
b3171_v1_tRNA_32_MODIF_2	modification of position 32 nt (Cm) in metY by MeT_tRNA_pos_32_Cm	1 b3171_v1_tRNA_1_MeT_tRNA_pos_32_Cm_cplx --> 1 MeT_tRNA_pos_32_Cm_inact + 1 ahcys + 1 b3171_v1_tRNA_2 + 1 h	irreversible	tRNA modification	0.00E+00	1.00E+09	0	1.43E+01	1.43E+01	0	2.52E+01	25.16946	0	1.46E-01	0.146
b3171_v1_tRNA_54_MODIF_1	modification of position 54 nt (m5U) in metY by TrmA_mono	1 TrmA_mono + 1 amet + 1 b3171_v1_tRNA_2 <==> 1 b3171_v1_tRNA_2_TrmA_mono_cplx	reversible	tRNA modification	-1.00E+09	1.00E+09	0	1.43E+01	1.43E+01	0	2.52E+01	25.16946	0	1.46E-01	0.146
b3171_v1_tRNA_54_MODIF_2	modification of position 54 nt (m5U) in metY by TrmA_mono	1 b3171_v1_tRNA_2_TrmA_mono_cplx --> 1 TrmA_mono_inact + 1 ahcys + 1 b3171_v1_tRNA_3 + 1 h	irreversible	tRNA modification	0.00E+00	1.00E+09	0	1.43E+01	1.43E+01	0	2.52E+01	25.16946	0	1.46E-01	0.146
b3171_v1_tRNA_55_MODIF_1	modification of position 55 nt (Y) in metY by TruB_mono	1 TruB_mono + 1 b3171_v1_tRNA_3 <==> 1 b3171_v1_tRNA_3_TruB_mono_cplx	reversible	tRNA modification	-1.00E+09	1.00E+09	0	1.43E+01	1.43E+01	0	2.52E+01	25.16946	0	1.46E-01	0.146
b3171_v1_tRNA_55_MODIF_2	modification of position 55 nt (Y) in metY by TruB_mono	1 b3171_v1_tRNA_3_TruB_mono_cplx --> 1 TruB_mono_inact + 1 b3171_v1_tRNA_4	irreversible	tRNA modification	0.00E+00	1.00E+09	0	1.43E+01	1.43E+01	0	2.52E+01	25.16946	0	1.46E-01	0.146
b3171_v1_tRNA_8_MODIF_1	modification of position 8 nt (s4U) in metY by ThiI_mono	1 IscS_dim_S-SH + 1 ThiI_mono + 1 atp + 1 b3171_v1_tRNA_4 + 1 mg2 + 1 trdrd <==> 1 b3171_v1_tRNA_4_ThiI_mono_cplx	reversible	tRNA modification	-1.00E+09	1.00E+09	0	1.43E+01	1.43E+01	0	2.52E+01	25.16946	0	1.46E-01	0.146
b3171_v1_tRNA_8_MODIF_2	modification of position 8 nt (s4U) in metY by ThiI_mono	1 b3171_v1_tRNA_4_ThiI_mono_cplx --> 1 IscS_dim_S-H + 1 ThiI_mono_inact + 1 amp + 1 h + 1 metY_tRNA + 1 mg2 + 1 ppi + 1 trdox	irreversible	tRNA modification	0.00E+00	1.00E+09	0	1.43E+01	1.43E+01	0	2.52E+01	25.16946	0	1.46E-01	0.146
b3171_v1_tRNA_Mg_BIND	Binding of two Mg2 to b3171_v1_tRNA	1 b3171_v1_RNA + 2 mg2 <==> 1 b3171_v1_tRNA_Mg2	reversible	Metallo-ion binding	-1.00E+09	1.00E+09	0	1.43E+01	1.43E+01	0	2.52E+01	25.16946	0	1.46E-01	0.146
b3171_v1_trimming	Cutting of stable b3171_v1_RNA	1 b3171_v1_RNA_cut_cplx + 1 h2o --> 1 RNase_E_tetra_inact + 1 RNase_Gen_inact + 1 RNase_P_cplx_inact + 1 b3171_v1_RNA + 1 ppi	irreversible	RNA Processing	0.00E+00	1.00E+09	0	1.43E+01	1.43E+01	0	2.52E+01	25.16946	0	1.46E-01	0.146
b3171_v1_trimming_cplx	Cutting complex formation of stable b3171_v1_RNA (,)	1 RNase_E_tetra + 1 RNase_Gen + 1 RNase_P_cplx + 1 b3171_v1_RNA_pre <==> 1 b3171_v1_RNA_cut_cplx	reversible	RNA Processing	-1.00E+09	1.00E+09	0	1.43E+01	1.43E+01	0	2.52E+01	25.16946	0	1.46E-01	0.146
b3171_v2_tRNA_20_MODIF_1	modification of position 20 nt (D) in metY by DusA_mono	1 DusA_mono + 1 b3171_v2_tRNA_Mg2 + 1 h + 1 nadph <==> 1 b3171_v2_tRNA_Mg2_DusA_mono_cplx	reversible	tRNA modification	-1.00E+09	1.00E+09	0	2.72E+01	2.72E+01	0	4.80E+01	48.0235	0	1.50E-01	0.15
b3171_v2_tRNA_20_MODIF_2	modification of position 20 nt (D) in metY by DusA_mono	1 b3171_v2_tRNA_Mg2_DusA_mono_cplx --> 1 DusA_mono + 1 b3171_v2_tRNA_1 + 1 nadp	irreversible	tRNA modification	0.00E+00	1.00E+09	0	2.72E+01	2.72E+01	0	4.80E+01	48.0235	0	1.50E-01	0.15
b3171_v2_tRNA_32_MODIF_1	modification of position 32 nt (Cm) in metY by MeT_tRNA_pos_32_Cm	1 MeT_tRNA_pos_32_Cm + 1 amet + 1 b3171_v2_tRNA_1 <==> 1 b3171_v2_tRNA_1_MeT_tRNA_pos_32_Cm_cplx	reversible	tRNA modification	-1.00E+09	1.00E+09	0	2.72E+01	2.72E+01	0	4.80E+01	48.0235	0	1.50E-01	0.15
b3171_v2_tRNA_32_MODIF_2	modification of position 32 nt (Cm) in metY by MeT_tRNA_pos_32_Cm	1 b3171_v2_tRNA_1_MeT_tRNA_pos_32_Cm_cplx --> 1 MeT_tRNA_pos_32_Cm_inact + 1 ahcys + 1 b3171_v2_tRNA_2 + 1 h	irreversible	tRNA modification	0.00E+00	1.00E+09	0	2.72E+01	2.72E+01	0	4.80E+01	48.0235	0	1.50E-01	0.15
b3171_v2_tRNA_54_MODIF_1	modification of position 54 nt (m5U) in metY by TrmA_mono	1 TrmA_mono + 1 amet + 1 b3171_v2_tRNA_2 <==> 1 b3171_v2_tRNA_2_TrmA_mono_cplx	reversible	tRNA modification	-1.00E+09	1.00E+09	0	2.72E+01	2.72E+01	0	4.80E+01	48.0235	0	1.50E-01	0.15
b3171_v2_tRNA_54_MODIF_2	modification of position 54 nt (m5U) in metY by TrmA_mono	1 b3171_v2_tRNA_2_TrmA_mono_cplx --> 1 TrmA_mono_inact + 1 ahcys + 1 b3171_v2_tRNA_3 + 1 h	irreversible	tRNA modification	0.00E+00	1.00E+09	0	2.72E+01	2.72E+01	0	4.80E+01	48.0235	0	1.50E-01	0.15
b3171_v2_tRNA_55_MODIF_1	modification of position 55 nt (Y) in metY by TruB_mono	1 TruB_mono + 1 b3171_v2_tRNA_3 <==> 1 b3171_v2_tRNA_3_TruB_mono_cplx	reversible	tRNA modification	-1.00E+09	1.00E+09	0	2.72E+01	2.72E+01	0	4.80E+01	48.0235	0	1.50E-01	0.15
b3171_v2_tRNA_55_MODIF_2	modification of position 55 nt (Y) in metY by TruB_mono	1 b3171_v2_tRNA_3_TruB_mono_cplx --> 1 TruB_mono_inact + 1 b3171_v2_tRNA_4	irreversible	tRNA modification	0.00E+00	1.00E+09	0	2.72E+01	2.72E+01	0	4.80E+01	48.0235	0	1.50E-01	0.15
b3171_v2_tRNA_8_MODIF_1	modification of position 8 nt (s4U) in metY by ThiI_mono	1 IscS_dim_S-SH + 1 ThiI_mono + 1 atp + 1 b3171_v2_tRNA_4 + 1 mg2 + 1 trdrd <==> 1 b3171_v2_tRNA_4_ThiI_mono_cplx	reversible	tRNA modification	-1.00E+09	1.00E+09	0	2.72E+01	2.72E+01	0	4.80E+01	48.0235	0	1.50E-01	0.15
b3171_v2_tRNA_8_MODIF_2	modification of position 8 nt (s4U) in metY by ThiI_mono	1 b3171_v2_tRNA_4_ThiI_mono_cplx --> 1 IscS_dim_S-H + 1 ThiI_mono_inact + 1 amp + 1 h + 1 metY_tRNA + 1 mg2 + 1 ppi + 1 trdox	irreversible	tRNA modification	0.00E+00	1.00E+09	0	2.72E+01	2.72E+01	0	4.80E+01	48.0235	0	1.50E-01	0.15
b3171_v2_tRNA_Mg_BIND	Binding of two Mg2 to b3171_v2_tRNA	1 b3171_v2_RNA + 2 mg2 <==> 1 b3171_v2_tRNA_Mg2	reversible	Metallo-ion binding	-1.00E+09	1.00E+09	0	2.72E+01	2.72E+01	0	4.80E+01	48.0235	0	1.50E-01	0.15
b3171_v2_trimming	Cutting of stable b3171_v2_RNA	1 b3171_v2_RNA_cut_cplx + 1 h2o --> 1 RNase_E_tetra_inact + 1 RNase_Gen_inact + 1 RNase_P_cplx_inact + 1 b3171_v2_RNA + 1 ppi	irreversible	RNA Processing	0.00E+00	1.00E+09	0	2.72E+01	2.72E+01	0	4.80E+01	48.0235	0	1.50E-01	0.15
b3171_v2_trimming_cplx	Cutting complex formation of stable b3171_v2_RNA (,)	1 RNase_E_tetra + 1 RNase_Gen + 1 RNase_P_cplx + 1 b3171_v2_RNA_pre <==> 1 b3171_v2_RNA_cut_cplx	reversible	RNA Processing	-1.00E+09	1.00E+09	0	2.72E+01	2.72E+01	0	4.80E+01	48.0235	0	1.50E-01	0.15
b3171_v3_tRNA_20_MODIF_1	modification of position 20 nt (D) in metY by DusA_mono	1 DusA_mono + 1 b3171_v3_tRNA_Mg2 + 1 h + 1 nadph <==> 1 b3171_v3_tRNA_Mg2_DusA_mono_cplx	reversible	tRNA modification	-1.00E+09	1.00E+09	0	1.23E+03	1.23E+03	0	11	11	0	11	11
b3171_v3_tRNA_20_MODIF_2	modification of position 20 nt (D) in metY by DusA_mono	1 b3171_v3_tRNA_Mg2_DusA_mono_cplx --> 1 DusA_mono + 1 b3171_v3_tRNA_1 + 1 nadp	irreversible	tRNA modification	0.00E+00	1.00E+09	0	1.23E+03	1.23E+03	0	11	11	0	11	11
b3171_v3_tRNA_32_MODIF_1	modification of position 32 nt (Cm) in metY by MeT_tRNA_pos_32_Cm	1 MeT_tRNA_pos_32_Cm + 1 amet + 1 b3171_v3_tRNA_1 <==> 1 b3171_v3_tRNA_1_MeT_tRNA_pos_32_Cm_cplx	reversible	tRNA modification	-1.00E+09	1.00E+09	0	1.23E+03	1.23E+03	0	11	11	0	11	11
b3171_v3_tRNA_32_MODIF_2	modification of position 32 nt (Cm) in metY by MeT_tRNA_pos_32_Cm	1 b3171_v3_tRNA_1_MeT_tRNA_pos_32_Cm_cplx --> 1 MeT_tRNA_pos_32_Cm_inact + 1 ahcys + 1 b3171_v3_tRNA_2 + 1 h	irreversible	tRNA modification	0.00E+00	1.00E+09	0	1.23E+03	1.23E+03	0	11	11	0	11	11
b3171_v3_tRNA_54_MODIF_1	modification of position 54 nt (m5U) in metY by TrmA_mono	1 TrmA_mono + 1 amet + 1 b3171_v3_tRNA_2 <==> 1 b3171_v3_tRNA_2_TrmA_mono_cplx	reversible	tRNA modification	-1.00E+09	1.00E+09	0	1.23E+03	1.23E+03	0	11	11	0	11	11
b3171_v3_tRNA_54_MODIF_2	modification of position 54 nt (m5U) in metY by TrmA_mono	1 b3171_v3_tRNA_2_TrmA_mono_cplx --> 1 TrmA_mono_inact + 1 ahcys + 1 b3171_v3_tRNA_3 + 1 h	irreversible	tRNA modification	0.00E+00	1.00E+09	0	1.23E+03	1.23E+03	0	11	11	0	11	11
b3171_v3_tRNA_55_MODIF_1	modification of position 55 nt (Y) in metY by TruB_mono	1 TruB_mono + 1 b3171_v3_tRNA_3 <==> 1 b3171_v3_tRNA_3_TruB_mono_cplx	reversible	tRNA modification	-1.00E+09	1.00E+09	0	1.23E+03	1.23E+03	0	11	11	0	11	11
b3171_v3_tRNA_55_MODIF_2	modification of position 55 nt (Y) in metY by TruB_mono	1 b3171_v3_tRNA_3_TruB_mono_cplx --> 1 TruB_mono_inact + 1 b3171_v3_tRNA_4	irreversible	tRNA modification	0.00E+00	1.00E+09	0	1.23E+03	1.23E+03	0	11	11	0	11	11
b3171_v3_tRNA_8_MODIF_1	modification of position 8 nt (s4U) in metY by ThiI_mono	1 IscS_dim_S-SH + 1 ThiI_mono + 1 atp + 1 b3171_v3_tRNA_4 + 1 mg2 + 1 trdrd <==> 1 b3171_v3_tRNA_4_ThiI_mono_cplx	reversible	tRNA modification	-1.00E+09	1.00E+09	0	1.23E+03	1.23E+03	0	11	11	0	11	11
b3171_v3_tRNA_8_MODIF_2	modification of position 8 nt (s4U) in metY by ThiI_mono	1 b3171_v3_tRNA_4_ThiI_mono_cplx --> 1 IscS_dim_S-H + 1 ThiI_mono_inact + 1 amp + 1 h + 1 metY_tRNA + 1 mg2 + 1 ppi + 1 trdox	irreversible	tRNA modification	0.00E+00	1.00E+09	0	1.23E+03	1.23E+03	0	11	11	0	11	11
b3171_v3_tRNA_Mg_BIND	Binding of two Mg2 to b3171_v3_tRNA	1 b3171_v3_RNA + 2 mg2 <==> 1 b3171_v3_tRNA_Mg2	reversible	Metallo-ion binding	-1.00E+09	1.00E+09	0	1.23E+03	1.23E+03	0	11	11	0	11	11
b3171_v4_tRNA_20_MODIF_1	modification of position 20 nt (D) in metY by DusA_mono	1 DusA_mono + 1 b3171_v4_tRNA_Mg2 + 1 h + 1 nadph <==> 1 b3171_v4_tRNA_Mg2_DusA_mono_cplx	reversible	tRNA modification	-1.00E+09	1.00E+09	0	1.43E+01	1.43E+01	0	2.52E+01	25.16946	0	1.46E-01	0.146
b3171_v4_tRNA_20_MODIF_2	modification of position 20 nt (D) in metY by DusA_mono	1 b3171_v4_tRNA_Mg2_DusA_mono_cplx --> 1 DusA_mono + 1 b3171_v4_tRNA_1 + 1 nadp	irreversible	tRNA modification	0.00E+00	1.00E+09	0	1.43E+01	1.43E+01	0	2.52E+01	25.16946	0	1.46E-01	0.146
b3171_v4_tRNA_32_MODIF_1	modification of position 32 nt (Cm) in metY by MeT_tRNA_pos_32_Cm	1 MeT_tRNA_pos_32_Cm + 1 amet + 1 b3171_v4_tRNA_1 <==> 1 b3171_v4_tRNA_1_MeT_tRNA_pos_32_Cm_cplx	reversible	tRNA modification	-1.00E+09	1.00E+09	0	1.43E+01	1.43E+01	0	2.52E+01	25.16946	0	1.46E-01	0.146
b3171_v4_tRNA_32_MODIF_2	modification of position 32 nt (Cm) in metY by MeT_tRNA_pos_32_Cm	1 b3171_v4_tRNA_1_MeT_tRNA_pos_32_Cm_cplx --> 1 MeT_tRNA_pos_32_Cm_inact + 1 ahcys + 1 b3171_v4_tRNA_2 + 1 h	irreversible	tRNA modification	0.00E+00	1.00E+09	0	1.43E+01	1.43E+01	0	2.52E+01	25.16946	0	1.46E-01	0.146
b3171_v4_tRNA_54_MODIF_1	modification of position 54 nt (m5U) in metY by TrmA_mono	1 TrmA_mono + 1 amet + 1 b3171_v4_tRNA_2 <==> 1 b3171_v4_tRNA_2_TrmA_mono_cplx	reversible	tRNA modification	-1.00E+09	1.00E+09	0	1.43E+01	1.43E+01	0	2.52E+01	25.16946	0	1.46E-01	0.146
b3171_v4_tRNA_54_MODIF_2	modification of position 54 nt (m5U) in metY by TrmA_mono	1 b3171_v4_tRNA_2_TrmA_mono_cplx --> 1 TrmA_mono_inact + 1 ahcys + 1 b3171_v4_tRNA_3 + 1 h	irreversible	tRNA modification	0.00E+00	1.00E+09	0	1.43E+01	1.43E+01	0	2.52E+01	25.16946	0	1.46E-01	0.146
b3171_v4_tRNA_55_MODIF_1	modification of position 55 nt (Y) in metY by TruB_mono	1 TruB_mono + 1 b3171_v4_tRNA_3 <==> 1 b3171_v4_tRNA_3_TruB_mono_cplx	reversible	tRNA modification	-1.00E+09	1.00E+09	0	1.43E+01	1.43E+01	0	2.52E+01	25.16946	0	1.46E-01	0.146
b3171_v4_tRNA_55_MODIF_2	modification of position 55 nt (Y) in metY by TruB_mono	1 b3171_v4_tRNA_3_TruB_mono_cplx --> 1 TruB_mono_inact + 1 b3171_v4_tRNA_4	irreversible	tRNA modification	0.00E+00	1.00E+09	0	1.43E+01	1.43E+01	0	2.52E+01	25.16946	0	1.46E-01	0.146
b3171_v4_tRNA_8_MODIF_1	modification of position 8 nt (s4U) in metY by ThiI_mono	1 IscS_dim_S-SH + 1 ThiI_mono + 1 atp + 1 b3171_v4_tRNA_4 + 1 mg2 + 1 trdrd <==> 1 b3171_v4_tRNA_4_ThiI_mono_cplx	reversible	tRNA modification	-1.00E+09	1.00E+09	0	1.43E+01	1.43E+01	0	2.52E+01	25.16946	0	1.46E-01	0.146
b3171_v4_tRNA_8_MODIF_2	modification of position 8 nt (s4U) in metY by ThiI_mono	1 b3171_v4_tRNA_4_ThiI_mono_cplx --> 1 IscS_dim_S-H + 1 ThiI_mono_inact + 1 amp + 1 h + 1 metY_tRNA + 1 mg2 + 1 ppi + 1 trdox	irreversible	tRNA modification	0.00E+00	1.00E+09	0	1.43E+01	1.43E+01	0	2.52E+01	25.16946	0	1.46E-01	0.146
b3171_v4_tRNA_Mg_BIND	Binding of two Mg2 to b3171_v4_tRNA	1 b3171_v4_RNA + 2 mg2 <==> 1 b3171_v4_tRNA_Mg2	reversible	Metallo-ion binding	-1.00E+09	1.00E+09	0	1.43E+01	1.43E+01	0	2.52E+01	25.16946	0	1.46E-01	0.146

b3171_v4_trimming	Cutting of stable b3171_v4_RNA	1 b3171_v4_RNA_cut_cplx + 1 h2o --> 1 RNase_E_tetra_inact + 1 RNase_Gen_inact + 1 RNase_P_cplx_inact + 1 b3171_v4_RNA + 1 + 1 ppi	irreversible	RNA Processing	0.00E+00	1.00E+09	0	1.43E+01	1.43E+01	0	2.52E+01	25.16946	0	1.46E-01	0.146
b3171_v4_trimming_cplx	Cutting complex formation of stable b3171_v4_RNA (,)	1 RNase_E_tetra + 1 RNase_Gen + 1 RNase_P_cplx + 1 b3171_v4_RNA_pre <==> 1 b3171_v4_RNA_cut_cplx	reversible	RNA Processing	-1.00E+09	1.00E+09	0	1.43E+01	1.43E+01	0	2.52E+01	25.16946	0	1.46E-01	0.146
b3174_tRNA_16_MODIF_1	modification of position 16 nt (D) in leuU by Dus_gen	1 Dus_gen + 1 b3174_tRNA_Mg2 + 1 h + 1 nadph <==> 1 b3174_tRNA_Mg2_Dus_gen_cplx	reversible	tRNA modification	-1.00E+09	1.00E+09	0	1.52E+03	1.52E+03	0	11	11	0	11	11
b3174_tRNA_16_MODIF_2	modification of position 16 nt (D) in leuU by Dus_gen	1 b3174_tRNA_Mg2_Dus_gen_cplx --> 1 Dus_gen_inact + 1 b3174_tRNA_1 + 1 nadp	irreversible	tRNA modification	0.00E+00	1.00E+09	0	1.52E+03	1.52E+03	0	11	11	0	11	11
b3174_tRNA_17_MODIF_1	modification of position 17 nt (D) in leuU by Dus_gen	1 Dus_gen + 1 b3174_tRNA_1 + 1 h + 1 nadph <==> 1 b3174_tRNA_1_Dus_gen_cplx	reversible	tRNA modification	-1.00E+09	1.00E+09	0	1.52E+03	1.52E+03	0	11	11	0	11	11
b3174_tRNA_17_MODIF_2	modification of position 17 nt (D) in leuU by Dus_gen	1 b3174_tRNA_1_Dus_gen_cplx --> 1 Dus_gen_inact + 1 b3174_tRNA_2 + 1 nadp	irreversible	tRNA modification	0.00E+00	1.00E+09	0	1.52E+03	1.52E+03	0	11	11	0	11	11
b3174_tRNA_18_MODIF_1	modification of position 18 nt (Gm) in leuU by TrmH_dim	1 TrmH_dim + 1 amet + 1 b3174_tRNA_2 <==> 1 b3174_tRNA_2_TrmH_dim_cplx	reversible	tRNA modification	-1.00E+09	1.00E+09	0	1.52E+03	1.52E+03	0	11	11	0	11	11
b3174_tRNA_18_MODIF_2	modification of position 18 nt (Gm) in leuU by TrmH_dim	1 b3174_tRNA_2_TrmH_dim_cplx --> 1 TrmH_dim_inact + 1 ahcys + 1 b3174_tRNA_3 + 1 h	irreversible	tRNA modification	0.00E+00	1.00E+09	0	1.52E+03	1.52E+03	0	11	11	0	11	11
b3174_tRNA_20_MODIF_1	modification of position 20 nt (D) in leuU by Dus_gen	1 Dus_gen + 1 b3174_tRNA_3 + 1 h + 1 nadph <==> 1 b3174_tRNA_3_Dus_gen_cplx	reversible	tRNA modification	-1.00E+09	1.00E+09	0	1.52E+03	1.52E+03	0	11	11	0	11	11
b3174_tRNA_20_MODIF_2	modification of position 20 nt (D) in leuU by Dus_gen	1 b3174_tRNA_3_Dus_gen_cplx --> 1 Dus_gen_inact + 1 b3174_tRNA_4 + 1 nadp	irreversible	tRNA modification	0.00E+00	1.00E+09	0	1.52E+03	1.52E+03	0	11	11	0	11	11
b3174_tRNA_38_MODIF_1	modification of position 38 nt (Y) in leuU by TruA_dim	1 TruA_dim + 1 b3174_tRNA_4 <==> 1 b3174_tRNA_4_TruA_dim_cplx	reversible	tRNA modification	-1.00E+09	1.00E+09	0	1.52E+03	1.52E+03	0	11	11	0	11	11
b3174_tRNA_38_MODIF_2	modification of position 38 nt (Y) in leuU by TruA_dim	1 b3174_tRNA_4_TruA_dim_cplx --> 1 TruA_dim_inact + 1 b3174_tRNA_5	irreversible	tRNA modification	0.00E+00	1.00E+09	0	1.52E+03	1.52E+03	0	11	11	0	11	11
b3174_tRNA_54_MODIF_1	modification of position 54 nt (m5U) in leuU by TrmA_mono	1 TrmA_mono + 1 amet + 1 b3174_tRNA_5 <==> 1 b3174_tRNA_5_TrmA_mono_cplx	reversible	tRNA modification	-1.00E+09	1.00E+09	0	1.52E+03	1.52E+03	0	11	11	0	11	11
b3174_tRNA_54_MODIF_2	modification of position 54 nt (m5U) in leuU by TrmA_mono	1 b3174_tRNA_5_TrmA_mono_cplx --> 1 TrmA_mono_inact + 1 ahcys + 1 b3174_tRNA_6 + 1 h	irreversible	tRNA modification	0.00E+00	1.00E+09	0	1.52E+03	1.52E+03	0	11	11	0	11	11
b3174_tRNA_55_MODIF_1	modification of position 55 nt (Y) in leuU by TruB_mono	1 TruB_mono + 1 b3174_tRNA_6 <==> 1 b3174_tRNA_6_TruB_mono_cplx	reversible	tRNA modification	-1.00E+09	1.00E+09	0	1.52E+03	1.52E+03	0	11	11	0	11	11
b3174_tRNA_55_MODIF_2	modification of position 55 nt (Y) in leuU by TruB_mono	1 b3174_tRNA_6_TruB_mono_cplx --> 1 TruB_mono_inact + 1 leuU_tRNA	irreversible	tRNA modification	0.00E+00	1.00E+09	0	1.52E+03	1.52E+03	0	11	11	0	11	11
b3174_tRNA_Mg_BIND	Binding of two Mg2 to b3174_tRNA	1 b3174_RNA + 2 mg2 <==> 1 b3174_tRNA_Mg2	reversible	Metallo-ion binding	-1.00E+09	1.00E+09	0	1.52E+03	1.52E+03	0	11	11	0	11	11
b3178_fold_spon	b3178_m folding: spontanous	1 b3178_m --> 1 FtsH_mono	irreversible	Protein Folding	0.00E+00	1.00E+09	0	1.97E+03	1.97E+03	0	3.41E+03	3407.568	0	9.49E+01	94.905
b3178_mRNA_CONV	convsersion of mRNA to mRNA_1 (synthetic rxn)	1 b3178_mRNA --> 1 b3178_mRNA_1	irreversible	Translation	0.00E+00	1.00E+09	0	5.09E+01	5.09E+01	0	8.98E+01	89.77642	0	2.45E-01	0.245
b3178_mRNA_degr1	Degradation of b3178_mRNA I	1 Orn_dim + 483 atp + 1 b3178_mRNA_2 + 1 degradosome + 483 h2o <==> 483 adp + 1 b3178_mRNA_2_degr + 483 h + 483 pi	reversible	mRNA degradation	-1.00E+09	1.00E+09	0	5.09E+01	5.09E+01	0	8.98E+01	89.77642	0	2.45E-01	0.245
b3178_mRNA_degr2	Degradation of b3178_mRNA II	1 b3178_mRNA_2_degr + 1934 h2o --> 1 Orn_dim_inact + 462 amp 476 cmp + 1 degradosome_inact + 561 gmp + 1934 h + 436 ump	irreversible	mRNA degradation	0.00E+00	4.90E-01	0	5.09E+01	5.09E+01	0	8.98E+01	89.77642	0	2.45E-01	0.245
b3178_maturation1	Polypeptide b3178 peptide deformylas and methionine aminopeptidase complex	1 Def_mono + 1 Map_mono + 1 b3178_aa <==> 1 b3178_def_map_cplx	reversible	Protein Maturation	-1.00E+09	1.00E+09	0	1.97E+03	1.97E+03	0	3.41E+03	3407.568	0	9.49E+01	94.905
b3178_maturation2	b3178 formation	1 b3178_def_map_cplx + 2 h2o --> 1 Def_mono_inact + 1 Map_mono_inact + 1 b3178_m + 1 for + 1 met-L	irreversible	Protein Maturation	0.00E+00	1.00E+09	0	1.97E+03	1.97E+03	0	3.41E+03	3407.568	0	9.49E+01	94.905
b3178_v1_mRNA_CONV	convsersion of mRNA to mRNA_1 (synthetic rxn)	1 b3178_v1_mRNA --> 1 b3178_mRNA_1	irreversible	Translation	0.00E+00	1.00E+09	0	5.09E+01	5.09E+01	0	8.98E+01	89.77642	0	2.45E-01	0.245
b3179_fold_spon	b3179_m folding: spontanous	1 b3179_m --> 1 RrmJ_mono	irreversible	Protein Folding	0.00E+00	1.00E+09	0	7.23E+02	7.23E+02	0	1.19E+03	1189.757	0	1.15E+02	115.38
b3179_mRNA_CONV	convsersion of mRNA to mRNA_1 (synthetic rxn)	1 b3179_mRNA --> 1 b3179_mRNA_1	irreversible	Translation	0.00E+00	1.00E+09	0	5.09E+01	5.09E+01	0	8.98E+01	89.77642	0	2.45E-01	0.245
b3179_mRNA_degr1	Degradation of b3179_mRNA I	1 Orn_dim + 157 atp + 1 b3179_mRNA_2 + 1 degradosome + 157 h2o <==> 157 adp + 1 b3179_mRNA_2_degr + 157 h + 157 pi	reversible	mRNA degradation	-1.00E+09	1.00E+09	0	5.09E+01	5.09E+01	0	8.98E+01	89.77642	0	2.45E-01	0.245
b3179_mRNA_degr2	Degradation of b3179_mRNA II	1 b3179_mRNA_2_degr + 629 h2o --> 1 Orn_dim_inact + 150 amp + 1 atp + 137 cmp + 1 degradosome_inact + 181 gmp + 629 h + 161 ump	irreversible	mRNA degradation	0.00E+00	2.45E-01	0	5.09E+01	5.09E+01	0	8.98E+01	89.77642	0	2.45E-01	0.245
b3179_maturation1	Polypeptide b3179 peptide deformylas and methionine aminopeptidase complex	1 Def_mono + 1 Map_mono + 1 b3179_aa <==> 1 b3179_def_map_cplx	reversible	Protein Maturation	-1.00E+09	1.00E+09	0	7.23E+02	7.23E+02	0	1.19E+03	1189.757	0	1.15E+02	115.38
b3179_maturation2	b3179 formation	1 b3179_def_map_cplx + 2 h2o --> 1 Def_mono_inact + 1 Map_mono_inact + 1 b3179_m + 1 for + 1 met-L	irreversible	Protein Maturation	0.00E+00	1.00E+09	0	7.23E+02	7.23E+02	0	1.19E+03	1189.757	0	1.15E+02	115.38
b3179_v1_mRNA_CONV	convsersion of mRNA to mRNA_1 (synthetic rxn)	1 b3179_v1_mRNA --> 1 b3179_mRNA_1	irreversible	Translation	0.00E+00	1.00E+09	0	5.09E+01	5.09E+01	0	8.98E+01	89.77642	0	2.45E-01	0.245
b3181_fold_GroEL/ES_1	b3181_m folding: GroEL/ES mediated: polypeptide is going in GroEL/ES complex	1 GroEL.(7)ADP.cisGroES + 7 atp + 1 b3181_m + 1 transGroES_hepta --> 7 adp + 1 b3181_m_GroEL.(7)ATP.transGroES + 1 cisGroES_hepta	irreversible	Protein Folding	0.00E+00	1.00E+09	0	2.85E+03	2.85E+03	0	4.97E+03	4969.003	0	9.22E+01	92.205
b3181_fold_GroEL/ES_2	b3181_m folding: GroEL/ES mediated: folding of polypeptide under ATP hydrolysis	1 b3181_m_GroEL.(7)ATP.transGroES + 7 h2o --> 1 GreA_GroEL.(7)ADP.transGroES + 7 h + 7 pi	irreversible	Protein Folding	0.00E+00	1.00E+09	0	2.85E+03	2.85E+03	0	4.97E+03	4969.003	0	9.22E+01	92.205
b3181_fold_GroEL/ES_3	b3181_m folding: GroEL/ES mediated: release of native protein	1 GreA_GroEL.(7)ADP.transGroES --> 1 GreA_mono + 1 GroEL.(7)ADP.transGroES	irreversible	Protein Folding	0.00E+00	1.00E+09	0	2.85E+03	2.85E+03	0	4.97E+03	4969.003	0	9.22E+01	92.205
b3181_fold_KJE_1	b3181_m folding: KJE mediated	1 DnaJ_dim + 1 DnaK_mono.ATP + 1 b3181_m <==> 1 b3181_m_DnaKJ_complex	reversible	Protein Folding	-1.00E+09	1.00E+09	0	2.85E+03	2.85E+03	0	4.97E+03	4969.003	0	9.22E+01	92.205
b3181_fold_KJE_2	b3181_m folding: KJE mediated	1 GrpE_dim + 1 b3181_m_DnaKJ_complex + 1 h2o --> 1 DnaJ_dim_inact + 1 GreA_DnaK_GrpE_complex + 1 adp + 1 h + 1 pi	irreversible	Protein Folding	0.00E+00	1.00E+09	0	2.85E+03	2.85E+03	0	4.97E+03	4969.003	0	9.22E+01	92.205
b3181_fold_KJE_3	b3181_m folding: KJE mediated	1 GreA_DnaK_GrpE_complex + 1 atp --> 1 DnaK_mono.ATP_inact + 1 GreA_mono + 1 GrpE_dim_inact	irreversible	Protein Folding	0.00E+00	1.00E+09	0	2.85E+03	2.85E+03	0	4.97E+03	4969.003	0	9.22E+01	92.205
b3181_mRNA_CONV	convsersion of mRNA to mRNA_1 (synthetic rxn)	1 b3181_mRNA --> 1 b3181_mRNA_1	irreversible	Translation	0.00E+00	1.00E+09	0	3.07E+02	3.07E+02	0	5.42E+02	542.1115	0	2.45E-01	0.245
b3181_mRNA_degr1	Degradation of b3181_mRNA I	1 Orn_dim + 119 atp + 1 b3181_mRNA_2 + 1 degradosome + 119 h2o <==> 119 adp + 1 b3181_mRNA_2_degr + 119 h + 119 pi	reversible	mRNA degradation	-1.00E+09	1.00E+09	0	3.07E+02	3.07E+02	0	5.42E+02	542.1115	0	2.45E-01	0.245
b3181_mRNA_degr2	Degradation of b3181_mRNA II	1 b3181_mRNA_2_degr + 476 h2o --> 1 Orn_dim_inact + 135 amp + 1 atp + 104 cmp + 1 degradosome_inact + 127 gmp + 476 h + 110 ump	irreversible	mRNA degradation	0.00E+00	2.45E-01	0	3.07E+02	3.07E+02	0	5.42E+02	542.1115	0	2.45E-01	0.245
b3181_maturation1	Polypeptide b3181 peptide deformylas complex	1 Def_mono + 1 b3181_aa <==> 1 b3181_def_cplx	reversible	Protein Maturation	-1.00E+09	1.00E+09	0	2.85E+03	2.85E+03	0	4.97E+03	4969.003	0	9.22E+01	92.205
b3181_maturation2	b3181 formation	1 b3181_def_cplx + 1 h2o --> 1 Def_mono_inact + 1 b3181_m + 1 for	irreversible	Protein Maturation	0.00E+00	1.00E+09	0	2.85E+03	2.85E+03	0	4.97E+03	4969.003	0	9.22E+01	92.205
b3185_fold_spon	b3185_m folding: spontanous	1 b3185_m --> 1 RpmA_mono	irreversible	Protein Folding	0.00E+00	1.00E+09	8.44E+01	1.13E+02	2.81E+01	6.22E+01	8.29E+01	20.72	6.22E+01	8.29E+01	20.72
b3185_mRNA_CONV	convsersion of mRNA to mRNA_1 (synthetic rxn)	1 b3185_mRNA --> 1 b3185_mRNA_1	irreversible	Translation	0.00E+00	1.00E+09	0	1.01E+02	1.01E+02	0	8.29E+01	82.88	0	5.39E-01	0.539
b3185_mRNA_degr1	Degradation of b3185_mRNA I	1 Orn_dim + 64 atp + 1 b3185_mRNA_2 + 1 degradosome + 64 h2o <==> 64 adp + 1 b3185_mRNA_2_degr + 64 h + 64 pi	reversible	mRNA degradation	-1.00E+09	1.00E+09	0	1.01E+02	1.01E+02	0	8.29E+01	82.88	0	5.39E-01	0.539
b3185_mRNA_degr2	Degradation of b3185_mRNA II	1 b3185_mRNA_2_degr + 257 h2o --> 1 Orn_dim_inact + 72 amp + 60 cmp + 1 degradosome_inact + 69 gmp + 257 h + 57 ump	irreversible	mRNA degradation	0.00E+00	5.39E-01	0	1.01E+02	1.01E+02	0	8.29E+01	82.88	0	5.39E-01	0.539
b3185_maturation1	Polypeptide b3185 peptide deformylas and methionine aminopeptidase complex	1 Def_mono + 1 Map_mono + 1 b3185_aa <==> 1 b3185_def_map_cplx	reversible	Protein Maturation	-1.00E+09	1.00E+09	8.44E+01	1.13E+02	2.81E+01	6.22E+01	8.29E+01	20.72	6.22E+01	8.29E+01	20.72
b3185_maturation2	b3185 formation	1 b3185_def_map_cplx + 2 h2o --> 1 Def_mono_inact + 1 Map_mono_inact + 1 b3185_m + 1 for + 1 met-L	irreversible	Protein Maturation	0.00E+00	1.00E+09	8.44E+01	1.13E+02	2.81E+01	6.22E+01	8.29E+01	20.72	6.22E+01	8.29E+01	20.72
b3186_fold_spon	b3186_m folding: spontanous	1 b3186_m --> 1 RplU_mono	irreversible	Protein Folding	0.00E+00	1.00E+09	8.44E+01	1.13E+02	2.81E+01	6.22E+01	8.29E+01	20.72	6.22E+01	8.29E+01	20.72
b3186_mRNA_CONV	convsersion of mRNA to mRNA_1 (synthetic rxn)	1 b3186_mRNA --> 1 b3186_mRNA_1	irreversible	Translation	0.00E+00	1.00E+09	0	1.01E+02	1.01E+02	0	8.29E+01	82.88	0	5.39E-01	0.539

b3186_mRNA_degr1	Degradation of b3186_mRNA I	1 Orn_dim + 78 atp + 1 b3186_mRNA_2 + 1 degradosome + 78 h2o <==> 78 adp + 1 b3186_mRNA_2_degr + 78 h + 78 pi	reversible	mRNA degradation	-1.00E+09	1.00E+09	0	1.01E+02	1.01E+02	0	8.29E+01	82.88	0	5.39E-01	0.539
b3186_mRNA_degr2	Degradation of b3186_mRNA II	1 b3186_mRNA_2_degr + 311 h2o --> 1 Orn_dim_inact + 85 amp + 1 atp + 64 cmp + 1 degradosome_inact + 87 gmp + 311 h + 75 ump	irreversible	mRNA degradation	0.00E+00	9.29E-01	0	1.01E+02	1.01E+02	0	8.29E+01	82.88	0	5.39E-01	0.539
b3186_maturation1	Polypeptide b3186 peptide deformylas complex	1 Def_mono + 1 b3186_aa <==> 1 b3186_def_cplx	reversible	Protein Maturation	-1.00E+09	1.00E+09	8.44E+01	1.13E+02	2.81E+01	6.22E+01	8.29E+01	20.72	6.22E+01	8.29E+01	20.72
b3186_maturation2	b3186 formation	1 b3186_def_cplx + 1 h2o --> 1 Def_mono_inact + 1 b3186_m + 1 for	irreversible	Protein Maturation	0.00E+00	1.00E+09	8.44E+01	1.13E+02	2.81E+01	6.22E+01	8.29E+01	20.72	6.22E+01	8.29E+01	20.72
b3201_fold_spon	b3201_m folding: spontanous	1 b3201_m --> 1 YhbG_mono	irreversible	Protein Folding	0.00E+00	1.00E+09	0	5.07E+02	5.07E+02	0	8.34E+02	833.6445	0	8.41E+01	84.098
b3201_mRNA_CONV	convsersion of mRNA to mRNA_1 (synthetic rxn)	1 b3201_mRNA --> 1 b3201_mRNA_1	irreversible	Translation	0.00E+00	1.00E+09	0	3.04E+01	3.04E+01	0	5.37E+01	53.74604	0	2.07E-01	0.207
b3201_mRNA_degr1	Degradation of b3201_mRNA I	1 Orn_dim + 181 atp + 1 b3201_mRNA_2 + 1 degradosome + 181 h2o <==> 181 adp + 1 b3201_mRNA_2_degr + 181 h + 181 pi	reversible	mRNA degradation	-1.00E+09	1.00E+09	0	3.04E+01	3.04E+01	0	5.37E+01	53.74604	0	2.07E-01	0.207
b3201_mRNA_degr2	Degradation of b3201_mRNA II	1 b3201_mRNA_2_degr + 725 h2o --> 1 Orn_dim_inact + 169 amp + 1 atp + 195 cmp + 1 degradosome_inact + 200 gmp + 725 h + 161 ump	irreversible	mRNA degradation	0.00E+00	5.13E-01	0	3.04E+01	3.04E+01	0	5.37E+01	53.74604	0	2.07E-01	0.207
b3201_maturation1	Polypeptide b3201 peptide deformylas and methionine aminopeptidase complex	1 Def_mono + 1 Map_mono + 1 b3201_aa <==> 1 b3201_def_map_cplx	reversible	Protein Maturation	-1.00E+09	1.00E+09	0	5.07E+02	5.07E+02	0	8.34E+02	833.6445	0	8.41E+01	84.098
b3201_maturation2	b3201 formation	1 b3201_def_map_cplx + 2 h2o --> 1 Def_mono_inact + 1 Map_mono_inact + 1 b3201_m + 1 for + 1 met-L	irreversible	Protein Maturation	0.00E+00	1.00E+09	0	5.07E+02	5.07E+02	0	8.34E+02	833.6445	0	8.41E+01	84.098
b3202_fold_spon	b3202_m folding: spontanous	1 b3202_m --> 1 RpoN_mono	irreversible	Protein Folding	0.00E+00	1.00E+09	0	2.44E+03	2.44E+03	0	4.24E+03	4236.416	0	1.10E+02	109.676
b3202_mRNA_CONV	convsersion of mRNA to mRNA_1 (synthetic rxn)	1 b3202_mRNA --> 1 b3202_mRNA_1	irreversible	Translation	0.00E+00	1.00E+09	0	3.52E+01	3.52E+01	0	6.22E+01	62.16052	0	2.07E-01	0.207
b3202_mRNA_degr1	Degradation of b3202_mRNA I	1 Orn_dim + 358 atp + 1 b3202_mRNA_2 + 1 degradosome + 358 h2o <==> 358 adp + 1 b3202_mRNA_2_degr + 358 h + 358 pi	reversible	mRNA degradation	-1.00E+09	1.00E+09	0	8.36E+01	8.36E+01	0	1.48E+02	147.5906	0	2.07E-01	0.207
b3202_mRNA_degr2	Degradation of b3202_mRNA II	1 b3202_mRNA_2_degr + 1433 h2o --> 1 Orn_dim_inact + 376 amp 1 atp + 382 cmp + 1 degradosome_inact + 376 gmp + 1433 h + 299 ump	irreversible	mRNA degradation	0.00E+00	2.07E-01	0	8.36E+01	8.36E+01	0	1.48E+02	147.5906	0	2.07E-01	0.207
b3202_maturation1	Polypeptide b3202 peptide deformylas and methionine aminopeptidase complex	1 Def_mono + 1 Map_mono + 1 b3202_aa <==> 1 b3202_def_map_cplx	reversible	Protein Maturation	-1.00E+09	1.00E+09	0	2.44E+03	2.44E+03	0	4.24E+03	4236.416	0	1.10E+02	109.676
b3202_maturation2	b3202 formation	1 b3202_def_map_cplx + 2 h2o --> 1 Def_mono_inact + 1 Map_mono_inact + 1 b3202_m + 1 for + 1 met-L	irreversible	Protein Maturation	0.00E+00	1.00E+09	0	2.44E+03	2.44E+03	0	4.24E+03	4236.416	0	1.10E+02	109.676
b3202_v1_mRNA_CONV	convsersion of mRNA to mRNA_1 (synthetic rxn)	1 b3202_v1_mRNA --> 1 b3202_mRNA_1	irreversible	Translation	0.00E+00	1.00E+09	0	8.36E+01	8.36E+01	0	1.48E+02	147.5906	0	2.07E-01	0.207
b3202_v2_mRNA_CONV	convsersion of mRNA to mRNA_1 (synthetic rxn)	1 b3202_v2_mRNA --> 1 b3202_mRNA_1	irreversible	Translation	0.00E+00	1.00E+09	0	3.04E+01	3.04E+01	0	5.37E+01	53.74604	0	2.07E-01	0.207
b3203_fold_spon	b3203_m folding: spontanous	1 b3203_m --> 1 YhbH_mono	irreversible	Protein Folding	0.00E+00	1.00E+09	0	3.43E+02	3.43E+02	0	4.78E+02	477.8026	0	1.68E+02	168.035
b3203_mRNA_CONV	convsersion of mRNA to mRNA_1 (synthetic rxn)	1 b3203_mRNA --> 1 b3203_mRNA_1	irreversible	Translation	0.00E+00	1.00E+09	0	3.52E+01	3.52E+01	0	6.22E+01	62.16052	0	2.07E-01	0.207
b3203_mRNA_degr1	Degradation of b3203_mRNA I	1 Orn_dim + 72 atp + 1 b3203_mRNA_2 + 1 degradosome + 72 h2o <==> 72 adp + 1 b3203_mRNA_2_degr + 72 h + 72 pi	reversible	mRNA degradation	-1.00E+09	1.00E+09	0	3.52E+01	3.52E+01	0	6.22E+01	62.16052	0	2.07E-01	0.207
b3203_mRNA_degr2	Degradation of b3203_mRNA II	1 b3203_mRNA_2_degr + 287 h2o --> 1 Orn_dim_inact + 96 amp + 66 cmp + 1 degradosome_inact + 63 gmp + 287 h + 63 ump	irreversible	mRNA degradation	0.00E+00	2.84E-01	0	3.52E+01	3.52E+01	0	6.22E+01	62.16052	0	2.07E-01	0.207
b3203_maturation1	Polypeptide b3203 peptide deformylas complex	1 Def_mono + 1 b3203_aa <==> 1 b3203_def_cplx	reversible	Protein Maturation	-1.00E+09	1.00E+09	0	3.43E+02	3.43E+02	0	4.78E+02	477.8026	0	1.68E+02	168.035
b3203_maturation2	b3203 formation	1 b3203_def_cplx + 1 h2o --> 1 Def_mono_inact + 1 b3203_m + 1 for	irreversible	Protein Maturation	0.00E+00	1.00E+09	0	3.43E+02	3.43E+02	0	4.78E+02	477.8026	0	1.68E+02	168.035
b3203_v1_mRNA_CONV	convsersion of mRNA to mRNA_1 (synthetic rxn)	1 b3203_v1_mRNA --> 1 b3203_mRNA_1	irreversible	Translation	0.00E+00	1.00E+09	0	3.04E+01	3.04E+01	0	5.37E+01	53.74604	0	2.07E-01	0.207
b3204_fold_spon	b3204_m folding: spontanous	1 b3204_m --> 1 PtsN_mono	irreversible	Protein Folding	0.00E+00	1.00E+09	0	4.55E+02	4.55E+02	0	6.98E+02	698.0447	0	1.40E+02	140.463
b3204_mRNA_CONV	convsersion of mRNA to mRNA_1 (synthetic rxn)	1 b3204_mRNA --> 1 b3204_mRNA_1	irreversible	Translation	0.00E+00	1.00E+09	0	3.52E+01	3.52E+01	0	6.22E+01	62.16052	0	2.07E-01	0.207
b3204_mRNA_degr1	Degradation of b3204_mRNA I	1 Orn_dim + 123 atp + 1 b3204_mRNA_2 + 1 degradosome + 123 h2o <==> 123 adp + 1 b3204_mRNA_2_degr + 123 h + 123 pi	reversible	mRNA degradation	-1.00E+09	1.00E+09	0	3.52E+01	3.52E+01	0	6.22E+01	62.16052	0	2.07E-01	0.207
b3204_mRNA_degr2	Degradation of b3204_mRNA II	1 b3204_mRNA_2_degr + 491 h2o --> 1 Orn_dim_inact + 131 amp + 130 cmp + 1 degradosome_inact + 127 gmp + 491 h + 104 ump	irreversible	mRNA degradation	0.00E+00	4.14E-01	0	3.52E+01	3.52E+01	0	6.22E+01	62.16052	0	2.07E-01	0.207
b3204_maturation1	Polypeptide b3204 peptide deformylas and methionine aminopeptidase complex	1 Def_mono + 1 Map_mono + 1 b3204_aa <==> 1 b3204_def_map_cplx	reversible	Protein Maturation	-1.00E+09	1.00E+09	0	4.55E+02	4.55E+02	0	6.98E+02	698.0447	0	1.40E+02	140.463
b3204_maturation2	b3204 formation	1 b3204_def_map_cplx + 2 h2o --> 1 Def_mono_inact + 1 Map_mono_inact + 1 b3204_m + 1 for + 1 met-L	irreversible	Protein Maturation	0.00E+00	1.00E+09	0	4.55E+02	4.55E+02	0	6.98E+02	698.0447	0	1.40E+02	140.463
b3204_v1_mRNA_CONV	convsersion of mRNA to mRNA_1 (synthetic rxn)	1 b3204_v1_mRNA --> 1 b3204_mRNA_1	irreversible	Translation	0.00E+00	1.00E+09	0	3.04E+01	3.04E+01	0	5.37E+01	53.74604	0	2.07E-01	0.207
b3205_fold_spon	b3205_m folding: spontanous	1 b3205_m --> 1 YhbJ_mono	irreversible	Protein Folding	0.00E+00	1.00E+09	0	6.89E+02	6.89E+02	0	1.12E+03	1120.648	0	1.29E+02	129.392
b3205_mRNA_CONV	convsersion of mRNA to mRNA_1 (synthetic rxn)	1 b3205_mRNA --> 1 b3205_mRNA_1	irreversible	Translation	0.00E+00	1.00E+09	0	3.52E+01	3.52E+01	0	6.22E+01	62.16052	0	2.07E-01	0.207
b3205_mRNA_degr1	Degradation of b3205_mRNA I	1 Orn_dim + 213 atp + 1 b3205_mRNA_2 + 1 degradosome + 213 h2o <==> 213 adp + 1 b3205_mRNA_2_degr + 213 h + 213 pi	reversible	mRNA degradation	-1.00E+09	1.00E+09	0	3.52E+01	3.52E+01	0	6.22E+01	62.16052	0	2.07E-01	0.207
b3205_mRNA_degr2	Degradation of b3205_mRNA II	1 b3205_mRNA_2_degr + 854 h2o --> 1 Orn_dim_inact + 198 amp + 233 cmp + 1 degradosome_inact + 213 gmp + 854 h + 211 ump	irreversible	mRNA degradation	0.00E+00	2.07E-01	0	3.52E+01	3.52E+01	0	6.22E+01	62.16052	0	2.07E-01	0.207
b3205_maturation1	Polypeptide b3205 peptide deformylas and methionine aminopeptidase complex	1 Def_mono + 1 Map_mono + 1 b3205_aa <==> 1 b3205_def_map_cplx	reversible	Protein Maturation	-1.00E+09	1.00E+09	0	6.89E+02	6.89E+02	0	1.12E+03	1120.648	0	1.29E+02	129.392
b3205_maturation2	b3205 formation	1 b3205_def_map_cplx + 2 h2o --> 1 Def_mono_inact + 1 Map_mono_inact + 1 b3205_m + 1 for + 1 met-L	irreversible	Protein Maturation	0.00E+00	1.00E+09	0	6.89E+02	6.89E+02	0	1.12E+03	1120.648	0	1.29E+02	129.392
b3205_v1_mRNA_CONV	convsersion of mRNA to mRNA_1 (synthetic rxn)	1 b3205_v1_mRNA --> 1 b3205_mRNA_1	irreversible	Translation	0.00E+00	1.00E+09	0	3.04E+01	3.04E+01	0	5.37E+01	53.74604	0	2.07E-01	0.207
b3206_fold_spon	b3206_m folding: spontanous	1 b3206_m --> 1 Npr_mono	irreversible	Protein Folding	0.00E+00	1.00E+09	0	2.85E+02	2.85E+02	0	4.20E+02	419.8026	0	1.10E+02	110.035
b3206_mRNA_CONV	convsersion of mRNA to mRNA_1 (synthetic rxn)	1 b3206_mRNA --> 1 b3206_mRNA_1	irreversible	Translation	0.00E+00	1.00E+09	0	3.52E+01	3.52E+01	0	6.22E+01	62.16052	0	2.07E-01	0.207
b3206_mRNA_degr1	Degradation of b3206_mRNA I	1 Orn_dim + 67 atp + 1 b3206_mRNA_2 + 1 degradosome + 67 h2o <==> 67 adp + 1 b3206_mRNA_2_degr + 67 h + 67 pi	reversible	mRNA degradation	-1.00E+09	1.00E+09	0	3.52E+01	3.52E+01	0	6.22E+01	62.16052	0	2.07E-01	0.207
b3206_mRNA_degr2	Degradation of b3206_mRNA II	1 b3206_mRNA_2_degr + 268 h2o --> 1 Orn_dim_inact + 72 amp + 59 cmp + 1 degradosome_inact + 74 gmp + 268 h + 64 ump	irreversible	mRNA degradation	0.00E+00	2.07E-01	0	3.52E+01	3.52E+01	0	6.22E+01	62.16052	0	2.07E-01	0.207
b3206_maturation1	Polypeptide b3206 peptide deformylas and methionine aminopeptidase complex	1 Def_mono + 1 Map_mono + 1 b3206_aa <==> 1 b3206_def_map_cplx	reversible	Protein Maturation	-1.00E+09	1.00E+09	0	2.85E+02	2.85E+02	0	4.20E+02	419.8026	0	1.10E+02	110.035
b3206_maturation2	b3206 formation	1 b3206_def_map_cplx + 2 h2o --> 1 Def_mono_inact + 1 Map_mono_inact + 1 b3206_m + 1 for + 1 met-L	irreversible	Protein Maturation	0.00E+00	1.00E+09	0	2.85E+02	2.85E+02	0	4.20E+02	419.8026	0	1.10E+02	110.035
b3230_fold_spon	b3230_m folding: spontanous	1 b3230_m --> 1 RpsI_mono	irreversible	Protein Folding	0.00E+00	1.00E+09	8.44E+01	1.13E+02	2.81E+01	6.22E+01	8.29E+01	20.72	6.22E+01	8.29E+01	20.72
b3230_mRNA_CONV	conversion of mRNA to mRNA_1 (synthetic rxn)	1 b3230_mRNA --> 1 b3230_mRNA_1	irreversible	Translation	0.00E+00	1.00E+09	0	9.48E+01	9.48E+01	0	8.29E+01	82.88	0	4.68E-01	0.468
b3230_mRNA_degr1	Degradation of b3230_mRNA I	1 Orn_dim + 98 atp + 1 b3230_mRNA_2 + 1 degradosome + 98 h2o <==> 98 adp + 1 b3230_mRNA_2_degr + 98 h + 98 pi	reversible	mRNA degradation	-1.00E+09	1.00E+09	0	9.48E+01	9.48E+01	0	8.29E+01	82.88	0	4.68E-01	0.468
b3230_mRNA_degr2	Degradation of b3230_mRNA II	1 b3230_mRNA_2_degr + 392 h2o --> 1 Orn_dim_inact + 93 amp + 102 cmp + 1 degradosome_inact + 104 gmp + 392 h + 94 ump	irreversible	mRNA degradation	0.00E+00	4.68E-01	0	9.48E+01	9.48E+01	0	8.29E+01	82.88	0	4.68E-01	0.468

b3230_maturation1	Polypeptide b3230 peptide deformylas and methionine aminopeptidase complex	1 Def_mono + 1 Map_mono + 1 b3230_aa <==> 1 b3230_def_map_cplx	reversible	Protein Maturation	-1.00E+09	1.00E+09	8.44E+01	1.13E+02	2.81E+01	6.22E+01	8.29E+01	20.72	6.22E+01	8.29E+01	20.72
b3230_maturation2	b3230 formation	1 b3230_def_map_cplx + 2 h2o --> 1 Def_mono_inact + 1 Map_mono_inact + 1 b3230_m + 1 for + 1 met-L	irreversible	Protein Maturation	0.00E+00	1.00E+09	8.44E+01	1.13E+02	2.81E+01	6.22E+01	8.29E+01	20.72	6.22E+01	8.29E+01	20.72
b3231_fold_spon	b3231_m folding: spontanous	1 b3231_m --> 1 RplM_mono	irreversible	Protein Folding	0.00E+00	1.00E+09	8.44E+01	1.13E+02	2.81E+01	6.22E+01	8.29E+01	20.72	6.22E+01	8.29E+01	20.72
b3231_mRNA_CONV	convsersion of mRNA to mRNA_1 (synthetic rxn)	1 b3231_mRNA --> 1 b3231_mRNA_1	irreversible	Translation	0.00E+00	1.00E+09	0	9.48E+01	9.48E+01	0	8.29E+01	82.88	0	4.68E-01	0.468
b3231_mRNA_degr1	Degradation of b3231_mRNA I	1 Orn_dim + 107 atp + 1 b3231_mRNA_2 + 1 degradosome + 107 h2o <==> 107 adp + 1 b3231_mRNA_2_degr + 107 h + 107 pi	reversible	mRNA degradation	-1.00E+09	1.00E+09	0	9.48E+01	9.48E+01	0	8.29E+01	82.88	0	4.68E-01	0.468
b3231_mRNA_degr2	Degradation of b3231_mRNA II	1 b3231_mRNA_2_degr + 428 h2o --> 1 Orn_dim_inact + 120 amp + 1 atp + 110 cmp + 1 degradosome_inact + 107 gmp + 428 h + 91 ump	irreversible	mRNA degradation	0.00E+00	1.08E+00	0	9.48E+01	9.48E+01	0	8.29E+01	82.88	0	4.68E-01	0.468
b3231_maturation1	Polypeptide b3231 peptide deformylas complex	1 Def_mono + 1 b3231_aa <==> 1 b3231_def_cplx	reversible	Protein Maturation	-1.00E+09	1.00E+09	8.44E+01	1.13E+02	2.81E+01	6.22E+01	8.29E+01	20.72	6.22E+01	8.29E+01	20.72
b3231_maturation2	b3231 formation	1 b3231_def_cplx + 1 h2o --> 1 Def_mono_inact + 1 b3231_m + 1 for	irreversible	Protein Maturation	0.00E+00	1.00E+09	8.44E+01	1.13E+02	2.81E+01	6.22E+01	8.29E+01	20.72	6.22E+01	8.29E+01	20.72
b3247_fold_spon	b3247_m folding: spontanous	1 b3247_m --> 1 Rng_mono	irreversible	Protein Folding	0.00E+00	1.00E+09	0	8.47E+02	8.47E+02	0	1.44E+03	1437.997	0	7.77E+01	77.672
b3247_mRNA_CONV	convsersion of mRNA to mRNA_1 (synthetic rxn)	1 b3247_mRNA --> 1 b3247_mRNA_1	irreversible	Translation	0.00E+00	1.00E+09	0	2.76E+01	2.76E+01	0	4.87E+01	48.68704	0	1.04E-01	0.104
b3247_mRNA_degr1	Degradation of b3247_mRNA I	1 Orn_dim + 364 atp + 1 b3247_mRNA_2 + 1 degradosome + 364 h2o <==> 364 adp + 1 b3247_mRNA_2_degr + 364 h + 364 pi	reversible	mRNA degradation	-1.00E+09	1.00E+09	0	2.76E+01	2.76E+01	0	4.87E+01	48.68704	0	1.04E-01	0.104
b3247_mRNA_degr2	Degradation of b3247_mRNA II	1 b3247_mRNA_2_degr + 1458 h2o --> 1 Orn_dim_inact + 365 amp 347 cmp + 1 degradosome_inact + 413 gmp + 1458 h + 334 ump	irreversible	mRNA degradation	0.00E+00	2.07E-01	0	2.76E+01	2.76E+01	0	4.87E+01	48.68704	0	1.04E-01	0.104
b3247_maturation1	Polypeptide b3247 peptide deformylas and methionine aminopeptidase complex	1 Def_mono + 1 Map_mono + 1 b3247_aa <==> 1 b3247_def_map_cplx	reversible	Protein Maturation	-1.00E+09	1.00E+09	0	8.47E+02	8.47E+02	0	1.44E+03	1437.997	0	7.77E+01	77.672
b3247_maturation2	b3247 formation	1 b3247_def_map_cplx + 2 h2o --> 1 Def_mono_inact + 1 Map_mono_inact + 1 b3247_m + 1 for + 1 met-L	irreversible	Protein Maturation	0.00E+00	1.00E+09	0	8.47E+02	8.47E+02	0	1.44E+03	1437.997	0	7.77E+01	77.672
b3248_fold_spon	b3248_m folding: spontanous	1 b3248_m --> 1 YhdE_mono	irreversible	Protein Folding	0.00E+00	1.00E+09	0	3.76E+02	3.76E+02	0	6.08E+02	608.4875	0	7.41E+01	74.074
b3248_mRNA_CONV	convsersion of mRNA to mRNA_1 (synthetic rxn)	1 b3248_mRNA --> 1 b3248_mRNA_1	irreversible	Translation	0.00E+00	1.00E+09	0	2.76E+01	2.76E+01	0	4.87E+01	48.68704	0	1.04E-01	0.104
b3248_mRNA_degr1	Degradation of b3248_mRNA I	1 Orn_dim + 148 atp + 1 b3248_mRNA_2 + 1 degradosome + 148 h2o <==> 148 adp + 1 b3248_mRNA_2_degr + 148 h + 148 pi	reversible	mRNA degradation	-1.00E+09	1.00E+09	0	2.76E+01	2.76E+01	0	4.87E+01	48.68704	0	1.04E-01	0.104
b3248_mRNA_degr2	Degradation of b3248_mRNA II	1 b3248_mRNA_2_degr + 593 h2o --> 1 Orn_dim_inact + 141 amp + 133 cmp + 1 degradosome_inact + 186 gmp + 593 h + 134 ump	irreversible	mRNA degradation	0.00E+00	1.86E-01	0	2.76E+01	2.76E+01	0	4.87E+01	48.68704	0	1.04E-01	0.104
b3248_maturation1	Polypeptide b3248 peptide deformylas and methionine aminopeptidase complex	1 Def_mono + 1 Map_mono + 1 b3248_aa <==> 1 b3248_def_map_cplx	reversible	Protein Maturation	-1.00E+09	1.00E+09	0	3.76E+02	3.76E+02	0	6.08E+02	608.4875	0	7.41E+01	74.074
b3248_maturation2	b3248 formation	1 b3248_def_map_cplx + 2 h2o --> 1 Def_mono_inact + 1 Map_mono_inact + 1 b3248_m + 1 for + 1 met-L	irreversible	Protein Maturation	0.00E+00	1.00E+09	0	3.76E+02	3.76E+02	0	6.08E+02	608.4875	0	7.41E+01	74.074
b3249_fold_spon	b3249_m folding: spontanous	1 b3249_m --> 1 MreD_mono	irreversible	Protein Folding	0.00E+00	1.00E+09	0	3.36E+02	3.36E+02	0	5.26E+02	526.2934	0	8.90E+01	89.046
b3249_mRNA_CONV	convsersion of mRNA to mRNA_1 (synthetic rxn)	1 b3249_mRNA --> 1 b3249_mRNA_1	irreversible	Translation	0.00E+00	1.00E+09	0	2.76E+01	2.76E+01	0	4.87E+01	48.68704	0	1.04E-01	0.104
b3249_mRNA_degr1	Degradation of b3249_mRNA I	1 Orn_dim + 122 atp + 1 b3249_mRNA_2 + 1 degradosome + 122 h2o <==> 122 adp + 1 b3249_mRNA_2_degr + 122 h + 122 pi	reversible	mRNA degradation	-1.00E+09	1.00E+09	0	2.76E+01	2.76E+01	0	4.87E+01	48.68704	0	1.04E-01	0.104
b3249_mRNA_degr2	Degradation of b3249_mRNA II	1 b3249_mRNA_2_degr + 487 h2o --> 1 Orn_dim_inact + 85 amp + 109 cmp + 1 degradosome_inact + 141 gmp + 487 h + 153 ump	irreversible	mRNA degradation	0.00E+00	2.15E-01	0	2.76E+01	2.76E+01	0	4.87E+01	48.68704	0	1.04E-01	0.104
b3249_maturation1	Polypeptide b3249 peptide deformylas and methionine aminopeptidase complex	1 Def_mono + 1 Map_mono + 1 b3249_aa <==> 1 b3249_def_map_cplx	reversible	Protein Maturation	-1.00E+09	1.00E+09	0	3.36E+02	3.36E+02	0	5.26E+02	526.2934	0	8.90E+01	89.046
b3249_maturation2	b3249 formation	1 b3249_def_map_cplx + 2 h2o --> 1 Def_mono_inact + 1 Map_mono_inact + 1 b3249_m + 1 for + 1 met-L	irreversible	Protein Maturation	0.00E+00	1.00E+09	0	3.36E+02	3.36E+02	0	5.26E+02	526.2934	0	8.90E+01	89.046
b3250_fold_spon	b3250_m folding: spontanous	1 b3250_m --> 1 MreC_mono	irreversible	Protein Folding	0.00E+00	1.00E+09	0	6.48E+02	6.48E+02	0	1.09E+03	1091.728	0	7.15E+01	71.484
b3250_mRNA_CONV	convsersion of mRNA to mRNA_1 (synthetic rxn)	1 b3250_mRNA --> 1 b3250_mRNA_1	irreversible	Translation	0.00E+00	1.00E+09	0	2.76E+01	2.76E+01	0	4.87E+01	48.68704	0	1.04E-01	0.104
b3250_mRNA_degr1	Degradation of b3250_mRNA I	1 Orn_dim + 276 atp + 1 b3250_mRNA_2 + 1 degradosome + 276 h2o <==> 276 adp + 1 b3250_mRNA_2_degr + 276 h + 276 pi	reversible	mRNA degradation	-1.00E+09	1.00E+09	0	2.76E+01	2.76E+01	0	4.87E+01	48.68704	0	1.04E-01	0.104
b3250_mRNA_degr2	Degradation of b3250_mRNA II	1 b3250_mRNA_2_degr + 1103 h2o --> 1 Orn_dim_inact + 227 amp 290 cmp + 1 degradosome_inact + 328 gmp + 1103 h + 259 ump	irreversible	mRNA degradation	0.00E+00	1.04E-01	0	2.76E+01	2.76E+01	0	4.87E+01	48.68704	0	1.04E-01	0.104
b3250_maturation1	Polypeptide b3250 peptide deformylas and methionine aminopeptidase complex	1 Def_mono + 1 Map_mono + 1 b3250_aa <==> 1 b3250_def_map_cplx	reversible	Protein Maturation	-1.00E+09	1.00E+09	0	6.48E+02	6.48E+02	0	1.09E+03	1091.728	0	7.15E+01	71.484
b3250_maturation2	b3250 formation	1 b3250_def_map_cplx + 2 h2o --> 1 Def_mono_inact + 1 Map_mono_inact + 1 b3250_m + 1 for + 1 met-L	irreversible	Protein Maturation	0.00E+00	1.00E+09	0	6.48E+02	6.48E+02	0	1.09E+03	1091.728	0	7.15E+01	71.484
b3251_fold_GroEL/ES_1	b3251_m folding: GroEL/ES mediated: polypeptide is going in GroEL/ES complex	1 GroEL.(7)ADP.cisGroES + 7 atp + 1 b3251_m + 1 transGroES_hepta --> 7 adp + 1 b3251_m_GroEL.(7)ATP.transGroES + 1 cisGroES_hepta	irreversible	Protein Folding	0.00E+00	1.00E+09	0	6.64E+02	6.64E+02	0	1.09E+03	1086.541	0	1.15E+02	114.88
b3251_fold_GroEL/ES_2	b3251_m folding: GroEL/ES mediated: folding of polypeptide under ATP hydrolysis	1 b3251_m_GroEL.(7)ATP.transGroES + 7 h2o --> 1 MreB_GroEL.(7)ADP.transGroES + 7 h + 7 pi	irreversible	Protein Folding	0.00E+00	1.00E+09	0	6.64E+02	6.64E+02	0	1.09E+03	1086.541	0	1.15E+02	114.88
b3251_fold_GroEL/ES_3	b3251_m folding: GroEL/ES mediated: release of native protein	1 MreB_GroEL.(7)ADP.transGroES --> 1 GroEL.(7)ADP.transGroES + 1 MreB_mono	irreversible	Protein Folding	0.00E+00	1.00E+09	0	6.64E+02	6.64E+02	0	1.09E+03	1086.541	0	1.15E+02	114.88
b3251_fold_KJE_1	b3251_m folding: KJE mediated	1 DnaJ_dim + 1 DnaK_mono.ATP + 1 b3251_m <==> 1 b3251_m_DnaKJ_complex	reversible	Protein Folding	-1.00E+09	1.00E+09	0	6.64E+02	6.64E+02	0	1.09E+03	1086.541	0	1.15E+02	114.88
b3251_fold_KJE_2	b3251_m folding: KJE mediated	1 GrpE_dim + 1 b3251_m_DnaKJ_complex + 1 h2o --> 1 DnaJ_dim_inact + 1 MreB_DnaK_GrpE_complex + 1 adp + 1 h + 1 pi	irreversible	Protein Folding	0.00E+00	1.00E+09	0	6.64E+02	6.64E+02	0	1.09E+03	1086.541	0	1.15E+02	114.88
b3251_fold_KJE_3	b3251_m folding: KJE mediated	1 MreB_DnaK_GrpE_complex + 1 atp --> 1 DnaK_mono.ATP_inact + 1 GrpE_dim_inact + 1 MreB_mono	irreversible	Protein Folding	0.00E+00	1.00E+09	0	6.64E+02	6.64E+02	0	1.09E+03	1086.541	0	1.15E+02	114.88
b3251_mRNA_CONV	convsersion of mRNA to mRNA_1 (synthetic rxn)	1 b3251_mRNA --> 1 b3251_mRNA_1	irreversible	Translation	0.00E+00	1.00E+09	0	2.76E+01	2.76E+01	0	4.87E+01	48.68704	0	1.04E-01	0.104
b3251_mRNA_degr1	Degradation of b3251_mRNA I	1 Orn_dim + 261 atp + 1 b3251_mRNA_2 + 1 degradosome + 261 h2o <==> 261 adp + 1 b3251_mRNA_2_degr + 261 h + 261 pi	reversible	mRNA degradation	-1.00E+09	1.00E+09	0	2.76E+01	2.76E+01	0	4.87E+01	48.68704	0	1.04E-01	0.104
b3251_mRNA_degr2	Degradation of b3251_mRNA II	1 b3251_mRNA_2_degr + 1043 h2o --> 1 Orn_dim_inact + 222 amp 1 atp + 264 cmp + 1 degradosome_inact + 303 gmp + 1043 h + 254 ump	irreversible	mRNA degradation	0.00E+00	3.08E-01	0	2.76E+01	2.76E+01	0	4.87E+01	48.68704	0	1.04E-01	0.104
b3251_maturation1	Polypeptide b3251 peptide deformylas complex	1 Def_mono + 1 b3251_aa <==> 1 b3251_def_cplx	reversible	Protein Maturation	-1.00E+09	1.00E+09	0	6.64E+02	6.64E+02	0	1.09E+03	1086.541	0	1.15E+02	114.88
b3251_maturation2	b3251 formation	1 b3251_def_cplx + 1 h2o --> 1 Def_mono_inact + 1 b3251_m + 1 for	irreversible	Protein Maturation	0.00E+00	1.00E+09	0	6.64E+02	6.64E+02	0	1.09E+03	1086.541	0	1.15E+02	114.88
b3257_fold_spon	b3257_m folding: spontanous	1 b3257_m --> 1 YhdT_mono	irreversible	Protein Folding	0.00E+00	1.00E+09	0	2.74E+02	2.74E+02	0	4.24E+02	423.5029	0	8.00E+01	79.984
b3257_mRNA_CONV	convsersion of mRNA to mRNA_1 (synthetic rxn)	1 b3257_mRNA --> 1 b3257_mRNA_1	irreversible	Translation	0.00E+00	1.00E+09	0	4.88E+01	4.88E+01	0	8.61E+01	86.07572	0	1.96E-01	0.196
b3257_mRNA_degr1	Degradation of b3257_mRNA I	1 Orn_dim + 60 atp + 1 b3257_mRNA_2 + 1 degradosome + 60 h2o <==> 60 adp + 1 b3257_mRNA_2_degr + 60 h + 60 pi	reversible	mRNA degradation	-1.00E+09	1.00E+09	0	4.88E+01	4.88E+01	0	8.61E+01	86.07572	0	1.96E-01	0.196

b3257_mRNA_degr2	Degradation of b3257_mRNA II	1 b3257_mRNA_2_degr + 242 h2o --> 1 Orn_dim_inact + 37 amp + 1 atp + 59 cmp + 1 degradosome_inact + 73 gmp + 242 h + 73 ump	irreversible	mRNA degradation	0.00E+00	1.96E-01	0	4.88E+01	4.88E+01	0	8.61E+01	86.07572	0	1.96E-01	0.196
b3257_maturation1	Polypeptide b3257 peptide deformylas and methionine aminopeptidase complex	1 Def_mono + 1 Map_mono + 1 b3257_aa <==> 1 b3257_def_map_cplx	reversible	Protein Maturation	-1.00E+09	1.00E+09	0	2.74E+02	2.74E+02	0	4.24E+02	423.5029	0	8.00E+01	79.984
b3257_maturation2	b3257 formation	1 b3257_def_map_cplx + 2 h2o --> 1 Def_mono_inact + 1 Map_mono_inact + 1 b3257_m + 1 for + 1 met-L	irreversible	Protein Maturation	0.00E+00	1.00E+09	0	2.74E+02	2.74E+02	0	4.24E+02	423.5029	0	8.00E+01	79.984
b3258_fold_spon	b3258_m folding: spontanous	1 b3258_m --> 1 PanF_mono	irreversible	Protein Folding	0.00E+00	1.00E+09	0	1.46E+03	1.46E+03	0	2.50E+03	2501.96	0	9.73E+01	97.328
b3258_mRNA_CONV	convsersion of mRNA to mRNA_1 (synthetic rxn)	1 b3258_mRNA --> 1 b3258_mRNA_1	irreversible	Translation	0.00E+00	1.00E+09	0	4.88E+01	4.88E+01	0	8.61E+01	86.07572	0	1.96E-01	0.196
b3258_mRNA_degr1	Degradation of b3258_mRNA I	1 Orn_dim + 360 atp + 1 b3258_mRNA_2 + 1 degradosome + 360 h2o <==> 360 adp + 1 b3258_mRNA_2_degr + 360 h + 360 pi	reversible	mRNA degradation	-1.00E+09	1.00E+09	0	4.88E+01	4.88E+01	0	8.61E+01	86.07572	0	1.96E-01	0.196
b3258_mRNA_degr2	Degradation of b3258_mRNA II	1 b3258_mRNA_2_degr + 1440 h2o --> 1 Orn_dim_inact + 261 amp 369 cmp + 1 degradosome_inact + 415 gmp + 1440 h + 396 ump	irreversible	mRNA degradation	0.00E+00	2.29E-01	0	4.88E+01	4.88E+01	0	8.61E+01	86.07572	0	1.96E-01	0.196
b3258_maturation1	Polypeptide b3258 peptide deformylas and methionine aminopeptidase complex	1 Def_mono + 1 Map_mono + 1 b3258_aa <==> 1 b3258_def_map_cplx	reversible	Protein Maturation	-1.00E+09	1.00E+09	0	1.46E+03	1.46E+03	0	2.50E+03	2501.96	0	9.73E+01	97.328
b3258_maturation2	b3258 formation	1 b3258_def_map_cplx + 2 h2o --> 1 Def_mono_inact + 1 Map_mono_inact + 1 b3258_m + 1 for + 1 met-L	irreversible	Protein Maturation	0.00E+00	1.00E+09	0	1.46E+03	1.46E+03	0	2.50E+03	2501.96	0	9.73E+01	97.328
b3259_fold_spon	b3259_m folding: spontanous	1 b3259_m --> 1 PrmA_mono	irreversible	Protein Folding	0.00E+00	1.00E+09	0	9.21E+02	9.21E+02	0	1.56E+03	1555.257	0	9.53E+01	95.302
b3259_mRNA_CONV	convsersion of mRNA to mRNA_1 (synthetic rxn)	1 b3259_mRNA --> 1 b3259_mRNA_1	irreversible	Translation	0.00E+00	1.00E+09	0	4.88E+01	4.88E+01	0	8.61E+01	86.07572	0	1.96E-01	0.196
b3259_mRNA_degr1	Degradation of b3259_mRNA I	1 Orn_dim + 220 atp + 1 b3259_mRNA_2 + 1 degradosome + 220 h2o <==> 220 adp + 1 b3259_mRNA_2_degr + 220 h + 220 pi	reversible	mRNA degradation	-1.00E+09	1.00E+09	0	4.88E+01	4.88E+01	0	8.61E+01	86.07572	0	1.96E-01	0.196
b3259_mRNA_degr2	Degradation of b3259_mRNA II	1 b3259_mRNA_2_degr + 881 h2o --> 1 Orn_dim_inact + 208 amp + 225 cmp + 1 degradosome_inact + 254 gmp + 881 h + 195 ump	irreversible	mRNA degradation	0.00E+00	2.07E-01	0	4.88E+01	4.88E+01	0	8.61E+01	86.07572	0	1.96E-01	0.196
b3259_maturation1	Polypeptide b3259 peptide deformylas and methionine aminopeptidase complex	1 Def_mono + 1 Map_mono + 1 b3259_aa <==> 1 b3259_def_map_cplx	reversible	Protein Maturation	-1.00E+09	1.00E+09	0	9.21E+02	9.21E+02	0	1.56E+03	1555.257	0	9.53E+01	95.302
b3259_maturation2	b3259 formation	1 b3259_def_map_cplx + 2 h2o --> 1 Def_mono_inact + 1 Map_mono_inact + 1 b3259_m + 1 for + 1 met-L	irreversible	Protein Maturation	0.00E+00	1.00E+09	0	9.21E+02	9.21E+02	0	1.56E+03	1555.257	0	9.53E+01	95.302
b3260_fold_GroEL/ES_1	b3260_m folding: GroEL/ES mediated: polypeptide is going in GroEL/ES complex	1 GroEL.(7)ADP.cisGroES + 7 atp + 1 b3260_m + 1 transGroES_hepta --> 7 adp + 1 b3260_m_GroEL.(7)ATP.transGroES + 1 cisGroES_hepta	irreversible	Protein Folding	0.00E+00	1.00E+09	0	1.90E+03	1.90E+03	0	3.31E+03	3305.097	0	6.51E+01	65.124
b3260_fold_GroEL/ES_2	b3260_m folding: GroEL/ES mediated: folding of polypeptide under ATP hydrolysis	1 b3260_m_GroEL.(7)ATP.transGroES + 7 h2o --> 1 DusB_GroEL.(7)ADP.transGroES + 7 h + 7 pi	irreversible	Protein Folding	0.00E+00	1.00E+09	0	1.90E+03	1.90E+03	0	3.31E+03	3305.097	0	6.51E+01	65.124
b3260_fold_GroEL/ES_3	b3260_m folding: GroEL/ES mediated: release of native protein	1 DusB_GroEL.(7)ADP.transGroES --> 1 DusB_mono + 1 GroEL.(7)ADP.transGroES	irreversible	Protein Folding	0.00E+00	1.00E+09	0	1.90E+03	1.90E+03	0	3.31E+03	3305.097	0	6.51E+01	65.124
b3260_mRNA_CONV	convsersion of mRNA to mRNA_1 (synthetic rxn)	1 b3260_mRNA --> 1 b3260_mRNA_1	irreversible	Translation	0.00E+00	1.00E+09	0	1.02E+02	1.02E+02	0	1.80E+02	180.1265	0	1.28E-01	0.128
b3260_mRNA_degr1	Degradation of b3260_mRNA I	1 Orn_dim + 241 atp + 1 b3260_mRNA_2 + 1 degradosome + 241 h2o <==> 241 adp + 1 b3260_mRNA_2_degr + 241 h + 241 pi	reversible	mRNA degradation	-1.00E+09	1.00E+09	0	1.02E+02	1.02E+02	0	1.80E+02	180.1265	0	1.28E-01	0.128
b3260_mRNA_degr2	Degradation of b3260_mRNA II	1 b3260_mRNA_2_degr + 965 h2o --> 1 Orn_dim_inact + 238 amp + 1 atp + 240 cmp + 1 degradosome_inact + 265 gmp + 965 h + 222 ump	irreversible	mRNA degradation	0.00E+00	1.28E-01	0	1.02E+02	1.02E+02	0	1.80E+02	180.1265	0	1.28E-01	0.128
b3260_maturation1	Polypeptide b3260 peptide deformylas and methionine aminopeptidase complex	1 Def_mono + 1 Map_mono + 1 b3260_aa <==> 1 b3260_def_map_cplx	reversible	Protein Maturation	-1.00E+09	1.00E+09	0	1.90E+03	1.90E+03	0	3.31E+03	3305.097	0	6.51E+01	65.124
b3260_maturation2	b3260 formation	1 b3260_def_map_cplx + 2 h2o --> 1 Def_mono_inact + 1 Map_mono_inact + 1 b3260_m + 1 for + 1 met-L	irreversible	Protein Maturation	0.00E+00	1.00E+09	0	1.90E+03	1.90E+03	0	3.31E+03	3305.097	0	6.51E+01	65.124
b3261_fold_spon	b3261_m folding: spontanous	1 b3261_m --> 1 Fis_mono	irreversible	Protein Folding	0.00E+00	1.00E+09	0	6.34E+02	6.34E+02	0	1.02E+03	1024.133	0	1.24E+02	124.14
b3261_mRNA_CONV	convsersion of mRNA to mRNA_1 (synthetic rxn)	1 b3261_mRNA --> 1 b3261_mRNA_1	irreversible	Translation	0.00E+00	1.00E+09	0	1.02E+02	1.02E+02	0	1.80E+02	180.1265	0	1.28E-01	0.128
b3261_mRNA_degr1	Degradation of b3261_mRNA I	1 Orn_dim + 74 atp + 1 b3261_mRNA_2 + 1 degradosome + 74 h2o <==> 74 adp + 1 b3261_mRNA_2_degr + 74 h + 74 pi	reversible	mRNA degradation	-1.00E+09	1.00E+09	0	1.02E+02	1.02E+02	0	1.80E+02	180.1265	0	1.28E-01	0.128
b3261_mRNA_degr2	Degradation of b3261_mRNA II	1 b3261_mRNA_2_degr + 296 h2o --> 1 Orn_dim_inact + 88 amp + 69 cmp + 1 degradosome_inact + 71 gmp + 296 h + 69 ump	irreversible	mRNA degradation	0.00E+00	4.90E-01	0	1.02E+02	1.02E+02	0	1.80E+02	180.1265	0	1.28E-01	0.128
b3261_maturation1	Polypeptide b3261 peptide deformylas complex	1 Def_mono + 1 b3261_aa <==> 1 b3261_def_cplx	reversible	Protein Maturation	-1.00E+09	1.00E+09	0	6.34E+02	6.34E+02	0	1.02E+03	1024.133	0	1.24E+02	124.14
b3261_maturation2	b3261 formation	1 b3261_def_cplx + 1 h2o --> 1 Def_mono_inact + 1 b3261_m + 1 for	irreversible	Protein Maturation	0.00E+00	1.00E+09	0	6.34E+02	6.34E+02	0	1.02E+03	1024.133	0	1.24E+02	124.14
b3273_tRNA_16_MODIF_1	modification of position 16 nt (D) in thrV by Dus_gen	1 Dus_gen + 1 b3273_tRNA_Mg2 + 1 h + 1 nadph <==> 1 b3273_tRNA_Mg2_Dus_gen_cplx	reversible	tRNA modification	-1.00E+09	1.00E+09	0	1.06E+02	1.06E+02	0	0	0	0	0	0
b3273_tRNA_16_MODIF_2	modification of position 16 nt (D) in thrV by Dus_gen	1 b3273_tRNA_Mg2_Dus_gen_cplx --> 1 Dus_gen_inact + 1 b3273_tRNA_1 + 1 nadp	irreversible	tRNA modification	0.00E+00	1.00E+09	0	1.06E+02	1.06E+02	0	0	0	0	0	0
b3273_tRNA_17_MODIF_1	modification of position 17 nt (D) in thrV by Dus_gen	1 Dus_gen + 1 b3273_tRNA_1 + 1 h + 1 nadph <==> 1 b3273_tRNA_1_Dus_gen_cplx	reversible	tRNA modification	-1.00E+09	1.00E+09	0	1.06E+02	1.06E+02	0	0	0	0	0	0
b3273_tRNA_17_MODIF_2	modification of position 17 nt (D) in thrV by Dus_gen	1 b3273_tRNA_1_Dus_gen_cplx --> 1 Dus_gen_inact + 1 b3273_tRNA_2 + 1 nadp	irreversible	tRNA modification	0.00E+00	1.00E+09	0	1.06E+02	1.06E+02	0	0	0	0	0	0
b3273_tRNA_20_MODIF_1	modification of position 20 nt (D) in thrV by Dus_gen	1 Dus_gen + 1 b3273_tRNA_2 + 1 h + 1 nadph <==> 1 b3273_tRNA_2_Dus_gen_cplx	reversible	tRNA modification	-1.00E+09	1.00E+09	0	1.06E+02	1.06E+02	0	0	0	0	0	0
b3273_tRNA_20_MODIF_2	modification of position 20 nt (D) in thrV by Dus_gen	1 b3273_tRNA_2_Dus_gen_cplx --> 1 Dus_gen_inact + 1 b3273_tRNA_3 + 1 nadp	irreversible	tRNA modification	0.00E+00	1.00E+09	0	1.06E+02	1.06E+02	0	0	0	0	0	0
b3273_tRNA_37_MODIF_1	modification of position 37 nt (m6t6A) in thrV by Up_tRNA_pos_37_t6A-MeT_tRNA_pos_37_m6t6A	1 MeT_tRNA_pos_37_m6t6A + 1 Up_tRNA_pos_37_t6A + 1 amet + atp + 1 b3273_tRNA_3 + 1 hco3 + 1 mg2 + 1 thr-L <==> 1 b3273_tRNA_3_Up_tRNA_pos_37_t6A-MeT_tRNA_pos_37_m6t6A_cplx	reversible	tRNA modification	-1.00E+09	1.00E+09	0	1.06E+02	1.06E+02	0	0	0	0	0	0
b3273_tRNA_37_MODIF_2	modification of position 37 nt (m6t6A) in thrV by Up_tRNA_pos_37_t6A-MeT_tRNA_pos_37_m6t6A	1 b3273_tRNA_3_Up_tRNA_pos_37_t6A-MeT_tRNA_pos_37_m6t6A_cplx --> 1 MeT_tRNA_pos_37_m6t6A_inact + 1 Up_tRNA_pos_37_t6A_inact + 1 ahcys + 1 amp + 1 b3273_tRNA_4 + 2 h + 1 h2o + 1 mg2 + 1 ppi	irreversible	tRNA modification	0.00E+00	1.00E+09	0	1.06E+02	1.06E+02	0	0	0	0	0	0
b3273_tRNA_46_MODIF_1	modification of position 46 nt (m7G) in thrV by YggH_mono	1 YggH_mono + 1 amet + 1 b3273_tRNA_4 <==> 1 b3273_tRNA_4_YggH_mono_cplx	reversible	tRNA modification	-1.00E+09	1.00E+09	0	1.06E+02	1.06E+02	0	0	0	0	0	0
b3273_tRNA_46_MODIF_2	modification of position 46 nt (m7G) in thrV by YggH_mono	1 b3273_tRNA_4_YggH_mono_cplx --> 1 YggH_mono_inact + 1 ahcys + 1 b3273_tRNA_5 + 1 h	irreversible	tRNA modification	0.00E+00	1.00E+09	0	1.06E+02	1.06E+02	0	0	0	0	0	0
b3273_tRNA_54_MODIF_1	modification of position 54 nt (m5U) in thrV by TrmA_mono	1 TrmA_mono + 1 amet + 1 b3273_tRNA_5 <==> 1 b3273_tRNA_5_TrmA_mono_cplx	reversible	tRNA modification	-1.00E+09	1.00E+09	0	1.06E+02	1.06E+02	0	0	0	0	0	0
b3273_tRNA_54_MODIF_2	modification of position 54 nt (m5U) in thrV by TrmA_mono	1 b3273_tRNA_5_TrmA_mono_cplx --> 1 TrmA_mono_inact + 1 ahcys + 1 b3273_tRNA_6 + 1 h	irreversible	tRNA modification	0.00E+00	1.00E+09	0	1.06E+02	1.06E+02	0	0	0	0	0	0
b3273_tRNA_55_MODIF_1	modification of position 55 nt (Y) in thrV by TruB_mono	1 TruB_mono + 1 b3273_tRNA_6 <==> 1 b3273_tRNA_6_TruB_mono_cplx	reversible	tRNA modification	-1.00E+09	1.00E+09	0	1.06E+02	1.06E+02	0	0	0	0	0	0
b3273_tRNA_55_MODIF_2	modification of position 55 nt (Y) in thrV by TruB_mono	1 b3273_tRNA_6_TruB_mono_cplx --> 1 TruB_mono_inact + 1 thrV_tRNA	irreversible	tRNA modification	0.00E+00	1.00E+09	0	1.06E+02	1.06E+02	0	0	0	0	0	0
b3273_tRNA_Mg_BIND	Binding of two Mg2 to b3273_tRNA	1 b3273_RNA + 2 mg2 <==> 1 b3273_tRNA_Mg2	reversible	Metallo-ion binding	-1.00E+09	1.00E+09	0	1.06E+02	1.06E+02	0	0	0	0	0	0
b3273_v1_tRNA_16_MODIF_1	modification of position 16 nt (D) in thrV by Dus_gen	1 Dus_gen + 1 b3273_v1_tRNA_Mg2 + 1 h + 1 nadph <==> 1 b3273_v1_tRNA_Mg2_Dus_gen_cplx	reversible	tRNA modification	-1.00E+09	1.00E+09	0	1.06E+02	1.06E+02	0	1.15E+01	11.5	0	1.15E+01	11.5
b3273_v1_tRNA_16_MODIF_2	modification of position 16 nt (D) in thrV by Dus_gen	1 b3273_v1_tRNA_Mg2_Dus_gen_cplx --> 1 Dus_gen_inact + 1 b3273_v1_tRNA_1 + 1 nadp	irreversible	tRNA modification	0.00E+00	1.00E+09	0	1.06E+02	1.06E+02	0	1.15E+01	11.5	0	1.15E+01	11.5
b3273_v1_tRNA_17_MODIF_1	modification of position 17 nt (D) in thrV by Dus_gen	1 Dus_gen + 1 b3273_v1_tRNA_1 + 1 h + 1 nadph <==> 1 b3273_v1_tRNA_1_Dus_gen_cplx	reversible	tRNA modification	-1.00E+09	1.00E+09	0	1.06E+02	1.06E+02	0	1.15E+01	11.5	0	1.15E+01	11.5

b3273_v1_tRNA_17_MODIF_2	modification of position 17 nt (D) in thrV by Dus_gen	1 b3273_v1_tRNA_1_Dus_gen_cplx --> 1 Dus_gen_inact + 1 b3273_v1_tRNA_2 + 1 nadp	irreversible	tRNA modification	0.00E+00	1.00E+09	0	1.06E+02	1.06E+02	0	1.15E+01	11.5	0	1.15E+01	11.5
b3273_v1_tRNA_20_MODIF_1	modification of position 20 nt (D) in thrV by Dus_gen	1 Dus_gen + 1 b3273_v1_tRNA_2 + 1 h + 1 nadph <==> 1 b3273_v1_tRNA_2_Dus_gen_cplx	reversible	tRNA modification	-1.00E+09	1.00E+09	0	1.06E+02	1.06E+02	0	1.15E+01	11.5	0	1.15E+01	11.5
b3273_v1_tRNA_20_MODIF_2	modification of position 20 nt (D) in thrV by Dus_gen	1 b3273_v1_tRNA_2_Dus_gen_cplx --> 1 Dus_gen_inact + 1 b3273_v1_tRNA_3 + 1 nadp	irreversible	tRNA modification	0.00E+00	1.00E+09	0	1.06E+02	1.06E+02	0	1.15E+01	11.5	0	1.15E+01	11.5
b3273_v1_tRNA_37_MODIF_1	modification of position 37 nt (m6t6A) in thrV by Up_tRNA_pos_37_t6A-MeT_tRNA_pos_37_m6t6A	1 MeT_tRNA_pos_37_m6t6A + 1 Up_tRNA_pos_37_t6A + 1 amet + atp + 1 b3273_v1_tRNA_3 + 1 hco3 + 1 mg2 + 1 thr-L <==> 1 b3273_v1_tRNA_3_Up_tRNA_pos_37_t6A-MeT_tRNA_pos_37_m6t6A_cplx	reversible	tRNA modification	-1.00E+09	1.00E+09	0	1.06E+02	1.06E+02	0	1.15E+01	11.5	0	1.15E+01	11.5
b3273_v1_tRNA_37_MODIF_2	modification of position 37 nt (m6t6A) in thrV by Up_tRNA_pos_37_t6A-MeT_tRNA_pos_37_m6t6A	1 b3273_v1_tRNA_3_Up_tRNA_pos_37_t6A-MeT_tRNA_pos_37_m6t6A_cplx --> 1 MeT_tRNA_pos_37_m6t6A_inact + 1 Up_tRNA_pos_37_t6A_inact + 1 ahcys + 1 amp + 1 b3273_v1_tRNA_4 + 2 h + 1 h2o + 1 mg2 + 1 p	irreversible	tRNA modification	0.00E+00	1.00E+09	0	1.06E+02	1.06E+02	0	1.15E+01	11.5	0	1.15E+01	11.5
b3273_v1_tRNA_46_MODIF_1	modification of position 46 nt (m7G) in thrV by YggH_mono	1 YggH_mono + 1 amet + 1 b3273_v1_tRNA_4 <==> 1 b3273_v1_tRNA_4_YggH_mono_cplx	reversible	tRNA modification	-1.00E+09	1.00E+09	0	1.06E+02	1.06E+02	0	1.15E+01	11.5	0	1.15E+01	11.5
b3273_v1_tRNA_46_MODIF_2	modification of position 46 nt (m7G) in thrV by YggH_mono	1 b3273_v1_tRNA_4_YggH_mono_cplx --> 1 YggH_mono_inact + 1 ahcys + 1 b3273_v1_tRNA_5 + 1 h	irreversible	tRNA modification	0.00E+00	1.00E+09	0	1.06E+02	1.06E+02	0	1.15E+01	11.5	0	1.15E+01	11.5
b3273_v1_tRNA_54_MODIF_1	modification of position 54 nt (m5U) in thrV by TrmA_mono	1 TrmA_mono + 1 amet + 1 b3273_v1_tRNA_5 <==> 1 b3273_v1_tRNA_5_TrmA_mono_cplx	reversible	tRNA modification	-1.00E+09	1.00E+09	0	1.06E+02	1.06E+02	0	1.15E+01	11.5	0	1.15E+01	11.5
b3273_v1_tRNA_54_MODIF_2	modification of position 54 nt (m5U) in thrV by TrmA_mono	1 b3273_v1_tRNA_5_TrmA_mono_cplx --> 1 TrmA_mono_inact + 1 ahcys + 1 b3273_v1_tRNA_6 + 1 h	irreversible	tRNA modification	0.00E+00	1.00E+09	0	1.06E+02	1.06E+02	0	1.15E+01	11.5	0	1.15E+01	11.5
b3273_v1_tRNA_55_MODIF_1	modification of position 55 nt (Y) in thrV by TruB_mono	1 TruB_mono + 1 b3273_v1_tRNA_6 <==> 1 b3273_v1_tRNA_6_TruB_mono_cplx	reversible	tRNA modification	-1.00E+09	1.00E+09	0	1.06E+02	1.06E+02	0	1.15E+01	11.5	0	1.15E+01	11.5
b3273_v1_tRNA_55_MODIF_2	modification of position 55 nt (Y) in thrV by TruB_mono	1 b3273_v1_tRNA_6_TruB_mono_cplx --> 1 TruB_mono_inact + 1 thrV_tRNA	irreversible	tRNA modification	0.00E+00	1.00E+09	0	1.06E+02	1.06E+02	0	1.15E+01	11.5	0	1.15E+01	11.5
b3273_v1_tRNA_Mg_BIND	Binding of two Mg2 to b3273_v1_tRNA	1 b3273_v1_RNA + 2 mg2 <==> 1 b3273_v1_tRNA_Mg2	reversible	Metallo-ion binding	-1.00E+09	1.00E+09	0	1.06E+02	1.06E+02	0	1.15E+01	11.5	0	1.15E+01	11.5
b3275_RNA_10_modif_1	b3275_RNA modification (23S rRNA): position: 2445, m2G by MeT_23S_2445	1 MeT_23S_2445 + 1 amet + 1 b3275_RNA_10 <==> 1 b3275_RNA_10_MeT_23S_2445	reversible	rRNA modification	-1.00E+09	1.00E+09	0	1.06E+02	1.06E+02	0	0	0	0	0	0
b3275_RNA_10_modif_2	b3275_RNA modification (23S rRNA): position: 2445, m2G by MeT_23S_2445	1 b3275_RNA_10_MeT_23S_2445 --> 1 MeT_23S_2445_inact + 1 ahcys + 1 b3275_RNA_11 + 1 h	irreversible	rRNA modification	0.00E+00	1.00E+09	0	1.06E+02	1.06E+02	0	0	0	0	0	0
b3275_RNA_11_a_modif_1	b3275_RNA modification (23S rRNA): position: 2449, D by DU_23S_2449 (NADH)	1 DU_23S_2449 + 1 b3275_RNA_11 + 1 h + 1 nadh <==> 1 b3275_RNA_11_DU_23S_2449_a	reversible	rRNA modification	-1.00E+09	1.00E+09	0	1.06E+02	1.06E+02	0	0	0	0	0	0
b3275_RNA_11_a_modif_2	b3275_RNA modification (23S rRNA): position: 2449, D by DU_23S_2449 (NADH)	1 b3275_RNA_11_DU_23S_2449_a --> 1 DU_23S_2449_inact + 1 b3275_RNA_12 + 1 nad	irreversible	rRNA modification	0.00E+00	1.00E+09	0	1.06E+02	1.06E+02	0	0	0	0	0	0
b3275_RNA_11_b_modif_1	b3275_RNA modification (23S rRNA): position: 2449, D by DU_23S_2449 (NADPH)	1 DU_23S_2449 + 1 b3275_RNA_11 + 1 h + 1 nadph <==> 1 b3275_RNA_11_DU_23S_2449_b	reversible	rRNA modification	-1.00E+09	1.00E+09	0	1.06E+02	1.06E+02	0	0	0	0	0	0
b3275_RNA_11_b_modif_2	b3275_RNA modification (23S rRNA): position: 2449, D by DU_23S_2449 (NADPH)	1 b3275_RNA_11_DU_23S_2449_b --> 1 DU_23S_2449_inact + 1 b3275_RNA_12 + 1 nadp	irreversible	rRNA modification	0.00E+00	1.00E+09	0	1.06E+02	1.06E+02	0	0	0	0	0	0
b3275_RNA_12_modif_1	b3275_RNA modification (23S rRNA): position: 2457, Y by YmfC_mono	1 YmfC_mono + 1 b3275_RNA_12 <==> 1 b3275_RNA_12_YmfC_mono	reversible	rRNA modification	-1.00E+09	1.00E+09	0	1.06E+02	1.06E+02	0	0	0	0	0	0
b3275_RNA_12_modif_2	b3275_RNA modification (23S rRNA): position: 2457, Y by YmfC_mono	1 b3275_RNA_12_YmfC_mono --> 1 YmfC_mono_inact + 1 b3275_RNA_13	irreversible	rRNA modification	0.00E+00	1.00E+09	0	1.06E+02	1.06E+02	0	0	0	0	0	0
b3275_RNA_13_modif_1	b3275_RNA modification (23S rRNA): position: 2498, Cm by MeT_23S_2498	1 MeT_23S_2498 + 1 amet + 1 b3275_RNA_13 <==> 1 b3275_RNA_13_MeT_23S_2498	reversible	rRNA modification	-1.00E+09	1.00E+09	0	1.06E+02	1.06E+02	0	0	0	0	0	0
b3275_RNA_13_modif_2	b3275_RNA modification (23S rRNA): position: 2498, Cm by MeT_23S_2498	1 b3275_RNA_13_MeT_23S_2498 --> 1 MeT_23S_2498_inact + 1 ahcys + 1 b3275_RNA_14 + 1 h	irreversible	rRNA modification	0.00E+00	1.00E+09	0	1.06E+02	1.06E+02	0	0	0	0	0	0
b3275_RNA_14_modif_1	b3275_RNA modification (23S rRNA): position: 2503, m2A by MeT_23S_2503	1 MeT_23S_2503 + 1 amet + 1 b3275_RNA_14 <==> 1 b3275_RNA_14_MeT_23S_2503	reversible	rRNA modification	-1.00E+09	1.00E+09	0	1.06E+02	1.06E+02	0	0	0	0	0	0
b3275_RNA_14_modif_2	b3275_RNA modification (23S rRNA): position: 2503, m2A by MeT_23S_2503	1 b3275_RNA_14_MeT_23S_2503 --> 1 MeT_23S_2503_inact + 1 ahcys + 1 b3275_RNA_15 + 1 h	irreversible	rRNA modification	0.00E+00	1.00E+09	0	1.06E+02	1.06E+02	0	0	0	0	0	0
b3275_RNA_15_modif_1	b3275_RNA modification (23S rRNA): position: 2504, Y by RluC_mono	1 RluC_mono + 1 b3275_RNA_15 <==> 1 b3275_RNA_15_RluC_mono	reversible	rRNA modification	-1.00E+09	1.00E+09	0	1.06E+02	1.06E+02	0	0	0	0	0	0
b3275_RNA_15_modif_2	b3275_RNA modification (23S rRNA): position: 2504, Y by RluC_mono	1 b3275_RNA_15_RluC_mono --> 1 RluC_mono_inact + 1 b3275_RNA_16	irreversible	rRNA modification	0.00E+00	1.00E+09	0	1.06E+02	1.06E+02	0	0	0	0	0	0
b3275_RNA_16_modif_1	b3275_RNA modification (23S rRNA): position: 2552, Um by RrmJ_mono	1 RrmJ_mono + 1 amet + 1 b3275_RNA_16 <==> 1 b3275_RNA_16_RrmJ_mono	reversible	rRNA modification	-1.00E+09	1.00E+09	0	1.06E+02	1.06E+02	0	0	0	0	0	0
b3275_RNA_16_modif_2	b3275_RNA modification (23S rRNA): position: 2552, Um by RrmJ_mono	1 b3275_RNA_16_RrmJ_mono --> 1 RrmJ_mono_inact + 1 ahcys + b3275_RNA_17 + 1 h	irreversible	rRNA modification	0.00E+00	1.00E+09	0	1.06E+02	1.06E+02	0	0	0	0	0	0
b3275_RNA_17_modif_1	b3275_RNA modification (23S rRNA): position: 2580, Y by RluC_mono	1 RluC_mono + 1 b3275_RNA_17 <==> 1 b3275_RNA_17_RluC_mono	reversible	rRNA modification	-1.00E+09	1.00E+09	0	1.06E+02	1.06E+02	0	0	0	0	0	0
b3275_RNA_17_modif_2	b3275_RNA modification (23S rRNA): position: 2580, Y by RluC_mono	1 b3275_RNA_17_RluC_mono --> 1 RluC_mono_inact + 1 b3275_RNA_18	irreversible	rRNA modification	0.00E+00	1.00E+09	0	1.06E+02	1.06E+02	0	0	0	0	0	0
b3275_RNA_18_modif_1	b3275_RNA modification (23S rRNA): position: 2604, Y by YjbC_mono	1 YjbC_mono + 1 b3275_RNA_18 <==> 1 b3275_RNA_18_YjbC_mono	reversible	rRNA modification	-1.00E+09	1.00E+09	0	1.06E+02	1.06E+02	0	0	0	0	0	0
b3275_RNA_18_modif_2	b3275_RNA modification (23S rRNA): position: 2604, Y by YjbC_mono	1 b3275_RNA_18_YjbC_mono --> 1 YjbC_mono_inact + 1 b3275_RNA_19	irreversible	rRNA modification	0.00E+00	1.00E+09	0	1.06E+02	1.06E+02	0	0	0	0	0	0
b3275_RNA_19_modif_1	b3275_RNA modification (23S rRNA): position: 2605, Y by RluB_mono	1 RluB_mono + 1 b3275_RNA_19 <==> 1 b3275_RNA_19_RluB_mono	reversible	rRNA modification	-1.00E+09	1.00E+09	0	1.06E+02	1.06E+02	0	0	0	0	0	0
b3275_RNA_19_modif_2	b3275_RNA modification (23S rRNA): position: 2605, Y by RluB_mono	1 b3275_RNA_19_RluB_mono --> 1 RluB_mono_inact + 1 b3275_RNA_20	irreversible	rRNA modification	0.00E+00	1.00E+09	0	1.06E+02	1.06E+02	0	0	0	0	0	0
b3275_RNA_1_modif_1	b3275_RNA modification (23S rRNA): position: 1835, m2G by MeT_23S_1835	1 MeT_23S_1835 + 1 amet + 1 b3275_RNA_1 <==> 1 b3275_RNA_1_MeT_23S_1835	reversible	rRNA modification	-1.00E+09	1.00E+09	0	1.06E+02	1.06E+02	0	0	0	0	0	0
b3275_RNA_1_modif_2	b3275_RNA modification (23S rRNA): position: 1835, m2G by MeT_23S_1835	1 b3275_RNA_1_MeT_23S_1835 --> 1 MeT_23S_1835_inact + 1 ahcys + 1 b3275_RNA_2 + 1 h	irreversible	rRNA modification	0.00E+00	1.00E+09	0	1.06E+02	1.06E+02	0	0	0	0	0	0
b3275_RNA_20_modif_1	b3275_RNA modification (23S rRNA): position: 745, m1G by RrmA_dim	1 RrmA_dim + 1 amet + 1 b3275_RNA_20 <==> 1 b3275_RNA_20_RrmA_dim	reversible	rRNA modification	-1.00E+09	1.00E+09	0	1.06E+02	1.06E+02	0	0	0	0	0	0
b3275_RNA_20_modif_2	b3275_RNA modification (23S rRNA): position: 745, m1G by RrmA_dim	1 b3275_RNA_20_RrmA_dim --> 1 RrmA_dim_inact + 1 ahcys + 1 b3275_RNA_21 + 1 h	irreversible	rRNA modification	0.00E+00	1.00E+09	0	1.06E+02	1.06E+02	0	0	0	0	0	0
b3275_RNA_21_modif_1	b3275_RNA modification (23S rRNA): position: 746, Y by RluA_mono	1 RluA_mono + 1 b3275_RNA_21 <==> 1 b3275_RNA_21_RluA_mono	reversible	rRNA modification	-1.00E+09	1.00E+09	0	1.06E+02	1.06E+02	0	0	0	0	0	0
b3275_RNA_21_modif_2	b3275_RNA modification (23S rRNA): position: 746, Y by RluA_mono	1 b3275_RNA_21_RluA_mono --> 1 RluA_mono_inact + 1 b3275_RNA_22	irreversible	rRNA modification	0.00E+00	1.00E+09	0	1.06E+02	1.06E+02	0	0	0	0	0	0

b3275_RNA_22_modif_1	b3275_RNA modification (23S rRNA): position: 747, m5U by RumB_mono	1 RumB_mono + 1 amet + 1 b3275_RNA_22 <==> 1 b3275_RNA_22_RumB_mono	reversible	rRNA modification	-1.00E+09	1.00E+09	0	1.06E+02	1.06E+02	0	0	0	0	0	0
b3275_RNA_22_modif_2	b3275_RNA modification (23S rRNA): position: 747, m5U by RumB_mono	1 b3275_RNA_22_RumB_mono --> 1 RumB_mono_inact + 1 ahcys + 1 b3275_RNA_23 + 1 h	irreversible	rRNA modification	0.00E+00	1.00E+09	0	1.06E+02	1.06E+02	0	0	0	0	0	0
b3275_RNA_23_modif_1	b3275_RNA modification (23S rRNA): position: 955, Y by RluC_mono	1 RluC_mono + 1 b3275_RNA_23 <==> 1 b3275_RNA_23_RluC_mono	reversible	rRNA modification	-1.00E+09	1.00E+09	0	1.06E+02	1.06E+02	0	0	0	0	0	0
b3275_RNA_23_modif_2	b3275_RNA modification (23S rRNA): position: 955, Y by RluC_mono	1 b3275_RNA_23_RluC_mono --> 1 RluC_mono_inact + 1 rrlD	irreversible	rRNA modification	0.00E+00	1.00E+09	0	1.06E+02	1.06E+02	0	0	0	0	0	0
b3275_RNA_2_modif_1	b3275_RNA modification (23S rRNA): position: 1911, Y by RluD_mono	1 RluD_mono + 1 b3275_RNA_2 <==> 1 b3275_RNA_2_RluD_mono	reversible	rRNA modification	-1.00E+09	1.00E+09	0	1.06E+02	1.06E+02	0	0	0	0	0	0
b3275_RNA_2_modif_2	b3275_RNA modification (23S rRNA): position: 1911, Y by RluD_mono	1 b3275_RNA_2_RluD_mono --> 1 RluD_mono_inact + 1 b3275_RNA_3	irreversible	rRNA modification	0.00E+00	1.00E+09	0	1.06E+02	1.06E+02	0	0	0	0	0	0
b3275_RNA_3_modif_1	b3275_RNA modification (23S rRNA): position: 1915, m3Y by RluD_mono	1 RluD_mono + 1 amet + 1 b3275_RNA_3 <==> 1 b3275_RNA_3_RluD_mono	reversible	rRNA modification	-1.00E+09	1.00E+09	0	1.06E+02	1.06E+02	0	0	0	0	0	0
b3275_RNA_3_modif_2	b3275_RNA modification (23S rRNA): position: 1915, m3Y by RluD_mono	1 b3275_RNA_3_RluD_mono --> 1 RluD_mono_inact + 1 ahcys + 1 b3275_RNA_4 + 1 h	irreversible	rRNA modification	0.00E+00	1.00E+09	0	1.06E+02	1.06E+02	0	0	0	0	0	0
b3275_RNA_4_modif_1	b3275_RNA modification (23S rRNA): position: 1917, Y by RluD_mono	1 RluD_mono + 1 b3275_RNA_4 <==> 1 b3275_RNA_4_RluD_mono	reversible	rRNA modification	-1.00E+09	1.00E+09	0	1.06E+02	1.06E+02	0	0	0	0	0	0
b3275_RNA_4_modif_2	b3275_RNA modification (23S rRNA): position: 1917, Y by RluD_mono	1 b3275_RNA_4_RluD_mono --> 1 RluD_mono_inact + 1 b3275_RNA_5	irreversible	rRNA modification	0.00E+00	1.00E+09	0	1.06E+02	1.06E+02	0	0	0	0	0	0
b3275_RNA_5_modif_1	b3275_RNA modification (23S rRNA): position: 1939, m5U by RumA_mono	1 RumA_mono + 1 amet + 1 b3275_RNA_5 <==> 1 b3275_RNA_5_RumA_mono	reversible	rRNA modification	-1.00E+09	1.00E+09	0	1.06E+02	1.06E+02	0	0	0	0	0	0
b3275_RNA_5_modif_2	b3275_RNA modification (23S rRNA): position: 1939, m5U by RumA_mono	1 b3275_RNA_5_RumA_mono --> 1 RumA_mono_inact + 1 ahcys + 1 b3275_RNA_6 + 1 h	irreversible	rRNA modification	0.00E+00	1.00E+09	0	1.06E+02	1.06E+02	0	0	0	0	0	0
b3275_RNA_6_modif_1	b3275_RNA modification (23S rRNA): position: 1962, m5C by MeT_23S_1962	1 MeT_23S_1962 + 1 amet + 1 b3275_RNA_6 <==> 1 b3275_RNA_6_MeT_23S_1962	reversible	rRNA modification	-1.00E+09	1.00E+09	0	1.06E+02	1.06E+02	0	0	0	0	0	0
b3275_RNA_6_modif_2	b3275_RNA modification (23S rRNA): position: 1962, m5C by MeT_23S_1962	1 b3275_RNA_6_MeT_23S_1962 --> 1 MeT_23S_1962_inact + 1 ahcys + 1 b3275_RNA_7 + 1 h	irreversible	rRNA modification	0.00E+00	1.00E+09	0	1.06E+02	1.06E+02	0	0	0	0	0	0
b3275_RNA_7_modif_1	b3275_RNA modification (23S rRNA): position: 2030, m6A by MeT_23S_2030	1 MeT_23S_2030 + 1 amet + 1 b3275_RNA_7 <==> 1 b3275_RNA_7_MeT_23S_2030	reversible	rRNA modification	-1.00E+09	1.00E+09	0	1.06E+02	1.06E+02	0	0	0	0	0	0
b3275_RNA_7_modif_2	b3275_RNA modification (23S rRNA): position: 2030, m6A by MeT_23S_2030	1 b3275_RNA_7_MeT_23S_2030 --> 1 MeT_23S_2030_inact + 1 ahcys + 1 b3275_RNA_8 + 1 h	irreversible	rRNA modification	0.00E+00	1.00E+09	0	1.06E+02	1.06E+02	0	0	0	0	0	0
b3275_RNA_8_modif_1	b3275_RNA modification (23S rRNA): position: 2069, m7G by MeT_23S_2069	1 MeT_23S_2069 + 1 amet + 1 b3275_RNA_8 <==> 1 b3275_RNA_8_MeT_23S_2069	reversible	rRNA modification	-1.00E+09	1.00E+09	0	1.06E+02	1.06E+02	0	0	0	0	0	0
b3275_RNA_8_modif_2	b3275_RNA modification (23S rRNA): position: 2069, m7G by MeT_23S_2069	1 b3275_RNA_8_MeT_23S_2069 --> 1 MeT_23S_2069_inact + 1 ahcys + 1 b3275_RNA_9 + 1 h	irreversible	rRNA modification	0.00E+00	1.00E+09	0	1.06E+02	1.06E+02	0	0	0	0	0	0
b3275_RNA_9_modif_1	b3275_RNA modification (23S rRNA): position: 2251, Gm by RlmB_dim	1 RlmB_dim + 1 amet + 1 b3275_RNA_9 <==> 1 b3275_RNA_9_RlmB_dim	reversible	rRNA modification	-1.00E+09	1.00E+09	0	1.06E+02	1.06E+02	0	0	0	0	0	0
b3275_RNA_9_modif_2	b3275_RNA modification (23S rRNA): position: 2251, Gm by RlmB_dim	1 b3275_RNA_9_RlmB_dim --> 1 RlmB_dim_inact + 1 ahcys + 1 b3275_RNA_10 + 1 h	irreversible	rRNA modification	0.00E+00	1.00E+09	0	1.06E+02	1.06E+02	0	0	0	0	0	0
b3275_RNA_modif_1	b3275_RNA modification (23S rRNA): position: 1618, m6A by MeT_23S_1618	1 MeT_23S_1618 + 1 amet + 1 b3275_RNA <==> 1 b3275_RNA_MeT_23S_1618	reversible	rRNA modification	-1.00E+09	1.00E+09	0	1.06E+02	1.06E+02	0	0	0	0	0	0
b3275_RNA_modif_2	b3275_RNA modification (23S rRNA): position: 1618, m6A by MeT_23S_1618	1 b3275_RNA_MeT_23S_1618 --> 1 MeT_23S_1618_inact + 1 ahcy + 1 b3275_RNA_1 + 1 h	irreversible	rRNA modification	0.00E+00	1.00E+09	0	1.06E+02	1.06E+02	0	0	0	0	0	0
b3275_v1_RNA_10_modif_1	b3275_v1_RNA modification (23S rRNA): position: 2445, m2G by MeT_23S_2445	1 MeT_23S_2445 + 1 amet + 1 b3275_v1_RNA_10 <==> 1 b3275_v1_RNA_10_MeT_23S_2445	reversible	rRNA modification	-1.00E+09	1.00E+09	0	1.06E+02	1.06E+02	0	1.15E+01	11.5	0	1.15E+01	11.5
b3275_v1_RNA_10_modif_2	b3275_v1_RNA modification (23S rRNA): position: 2445, m2G by MeT_23S_2445	1 b3275_v1_RNA_10_MeT_23S_2445 --> 1 MeT_23S_2445_inact + 1 ahcys + 1 b3275_v1_RNA_11 + 1 h	irreversible	rRNA modification	0.00E+00	1.00E+09	0	1.06E+02	1.06E+02	0	1.15E+01	11.5	0	1.15E+01	11.5
b3275_v1_RNA_11_a_modif_1	b3275_v1_RNA modification (23S rRNA): position: 2449, D by DU_23S_2449 (NADH)	1 DU_23S_2449 + 1 b3275_v1_RNA_11 + 1 h + 1 nadh <==> 1 b3275_v1_RNA_11_DU_23S_2449_a	reversible	rRNA modification	-1.00E+09	1.00E+09	0	1.06E+02	1.06E+02	0	1.15E+01	11.5	0	1.15E+01	11.5
b3275_v1_RNA_11_a_modif_2	b3275_v1_RNA modification (23S rRNA): position: 2449, D by DU_23S_2449 (NADH)	1 b3275_v1_RNA_11_DU_23S_2449_a --> 1 DU_23S_2449_inact + 1 b3275_v1_RNA_12 + 1 nad	irreversible	rRNA modification	0.00E+00	1.00E+09	0	1.06E+02	1.06E+02	0	1.15E+01	11.5	0	1.15E+01	11.5
b3275_v1_RNA_11_b_modif_1	b3275_v1_RNA modification (23S rRNA): position: 2449, D by DU_23S_2449 (NADPH)	1 DU_23S_2449 + 1 b3275_v1_RNA_11 + 1 h + 1 nadph <==> 1 b3275_v1_RNA_11_DU_23S_2449_b	reversible	rRNA modification	-1.00E+09	1.00E+09	0	1.06E+02	1.06E+02	0	1.15E+01	11.5	0	1.15E+01	11.5
b3275_v1_RNA_11_b_modif_2	b3275_v1_RNA modification (23S rRNA): position: 2449, D by DU_23S_2449 (NADPH)	1 b3275_v1_RNA_11_DU_23S_2449_b --> 1 DU_23S_2449_inact + 1 b3275_v1_RNA_12 + 1 nadp	irreversible	rRNA modification	0.00E+00	1.00E+09	0	1.06E+02	1.06E+02	0	1.15E+01	11.5	0	1.15E+01	11.5
b3275_v1_RNA_12_modif_1	b3275_v1_RNA modification (23S rRNA): position: 2457, Y by YmfC_mono	1 YmfC_mono + 1 b3275_v1_RNA_12 <==> 1 b3275_v1_RNA_12_YmfC_mono	reversible	rRNA modification	-1.00E+09	1.00E+09	0	1.06E+02	1.06E+02	0	1.15E+01	11.5	0	1.15E+01	11.5
b3275_v1_RNA_12_modif_2	b3275_v1_RNA modification (23S rRNA): position: 2457, Y by YmfC_mono	1 b3275_v1_RNA_12_YmfC_mono --> 1 YmfC_mono_inact + 1 b3275_v1_RNA_13	irreversible	rRNA modification	0.00E+00	1.00E+09	0	1.06E+02	1.06E+02	0	1.15E+01	11.5	0	1.15E+01	11.5
b3275_v1_RNA_13_modif_1	b3275_v1_RNA modification (23S rRNA): position: 2498, Cm by MeT_23S_2498	1 MeT_23S_2498 + 1 amet + 1 b3275_v1_RNA_13 <==> 1 b3275_v1_RNA_13_MeT_23S_2498	reversible	rRNA modification	-1.00E+09	1.00E+09	0	1.06E+02	1.06E+02	0	1.15E+01	11.5	0	1.15E+01	11.5
b3275_v1_RNA_13_modif_2	b3275_v1_RNA modification (23S rRNA): position: 2498, Cm by MeT_23S_2498	1 b3275_v1_RNA_13_MeT_23S_2498 --> 1 MeT_23S_2498_inact + 1 ahcys + 1 b3275_v1_RNA_14 + 1 h	irreversible	rRNA modification	0.00E+00	1.00E+09	0	1.06E+02	1.06E+02	0	1.15E+01	11.5	0	1.15E+01	11.5
b3275_v1_RNA_14_modif_1	b3275_v1_RNA modification (23S rRNA): position: 2503, m2A by MeT_23S_2503	1 MeT_23S_2503 + 1 amet + 1 b3275_v1_RNA_14 <==> 1 b3275_v1_RNA_14_MeT_23S_2503	reversible	rRNA modification	-1.00E+09	1.00E+09	0	1.06E+02	1.06E+02	0	1.15E+01	11.5	0	1.15E+01	11.5
b3275_v1_RNA_14_modif_2	b3275_v1_RNA modification (23S rRNA): position: 2503, m2A by MeT_23S_2503	1 b3275_v1_RNA_14_MeT_23S_2503 --> 1 MeT_23S_2503_inact + 1 ahcys + 1 b3275_v1_RNA_15 + 1 h	irreversible	rRNA modification	0.00E+00	1.00E+09	0	1.06E+02	1.06E+02	0	1.15E+01	11.5	0	1.15E+01	11.5
b3275_v1_RNA_15_modif_1	b3275_v1_RNA modification (23S rRNA): position: 2504, Y by RluC_mono	1 RluC_mono + 1 b3275_v1_RNA_15 <==> 1 b3275_v1_RNA_15_RluC_mono	reversible	rRNA modification	-1.00E+09	1.00E+09	0	1.06E+02	1.06E+02	0	1.15E+01	11.5	0	1.15E+01	11.5
b3275_v1_RNA_15_modif_2	b3275_v1_RNA modification (23S rRNA): position: 2504, Y by RluC_mono	1 b3275_v1_RNA_15_RluC_mono --> 1 RluC_mono_inact + 1 b3275_v1_RNA_16	irreversible	rRNA modification	0.00E+00	1.00E+09	0	1.06E+02	1.06E+02	0	1.15E+01	11.5	0	1.15E+01	11.5
b3275_v1_RNA_16_modif_1	b3275_v1_RNA modification (23S rRNA): position: 2552, Um by RrmJ_mono	1 RrmJ_mono + 1 amet + 1 b3275_v1_RNA_16 <==> 1 b3275_v1_RNA_16_RrmJ_mono	reversible	rRNA modification	-1.00E+09	1.00E+09	0	1.06E+02	1.06E+02	0	1.15E+01	11.5	0	1.15E+01	11.5
b3275_v1_RNA_16_modif_2	b3275_v1_RNA modification (23S rRNA): position: 2552, Um by RrmJ_mono	1 b3275_v1_RNA_16_RrmJ_mono --> 1 RrmJ_mono_inact + 1 ahcys + 1 b3275_v1_RNA_17 + 1 h	irreversible	rRNA modification	0.00E+00	1.00E+09	0	1.06E+02	1.06E+02	0	1.15E+01	11.5	0	1.15E+01	11.5

b3275_v1_RNA_17_modif_1	b3275_v1_RNA modification (23S rRNA): position: 2580, Y by RluC_mono	1 RluC_mono + 1 b3275_v1_RNA_17 <==> 1 b3275_v1_RNA_17_RluC_mono	reversible	rRNA modification	-1.00E+09	1.00E+09	0	1.06E+02	1.06E+02	0	1.15E+01	11.5	0	1.15E+01	11.5
b3275_v1_RNA_17_modif_2	b3275_v1_RNA modification (23S rRNA): position: 2580, Y by RluC_mono	1 b3275_v1_RNA_17_RluC_mono --> 1 RluC_mono_inact + 1 b3275_v1_RNA_18	irreversible	rRNA modification	0.00E+00	1.00E+09	0	1.06E+02	1.06E+02	0	1.15E+01	11.5	0	1.15E+01	11.5
b3275_v1_RNA_18_modif_1	b3275_v1_RNA modification (23S rRNA): position: 2604, Y by YjbC_mono	1 YjbC_mono + 1 b3275_v1_RNA_18 <==> 1 b3275_v1_RNA_18_YjbC_mono	reversible	rRNA modification	-1.00E+09	1.00E+09	0	1.06E+02	1.06E+02	0	1.15E+01	11.5	0	1.15E+01	11.5
b3275_v1_RNA_18_modif_2	b3275_v1_RNA modification (23S rRNA): position: 2604, Y by YjbC_mono	1 b3275_v1_RNA_18_YjbC_mono --> 1 YjbC_mono_inact + 1 b3275_v1_RNA_19	irreversible	rRNA modification	0.00E+00	1.00E+09	0	1.06E+02	1.06E+02	0	1.15E+01	11.5	0	1.15E+01	11.5
b3275_v1_RNA_19_modif_1	b3275_v1_RNA modification (23S rRNA): position: 2605, Y by RluB_mono	1 RluB_mono + 1 b3275_v1_RNA_19 <==> 1 b3275_v1_RNA_19_RluB_mono	reversible	rRNA modification	-1.00E+09	1.00E+09	0	1.06E+02	1.06E+02	0	1.15E+01	11.5	0	1.15E+01	11.5
b3275_v1_RNA_19_modif_2	b3275_v1_RNA modification (23S rRNA): position: 2605, Y by RluB_mono	1 b3275_v1_RNA_19_RluB_mono --> 1 RluB_mono_inact + 1 b3275_v1_RNA_20	irreversible	rRNA modification	0.00E+00	1.00E+09	0	1.06E+02	1.06E+02	0	1.15E+01	11.5	0	1.15E+01	11.5
b3275_v1_RNA_1_modif_1	b3275_v1_RNA modification (23S rRNA): position: 1835, m2G by MeT_23S_1835	1 MeT_23S_1835 + 1 amet + 1 b3275_v1_RNA_1 <==> 1 b3275_v1_RNA_1_MeT_23S_1835	reversible	rRNA modification	-1.00E+09	1.00E+09	0	1.06E+02	1.06E+02	0	1.15E+01	11.5	0	1.15E+01	11.5
b3275_v1_RNA_1_modif_2	b3275_v1_RNA modification (23S rRNA): position: 1835, m2G by MeT_23S_1835	1 b3275_v1_RNA_1_MeT_23S_1835 --> 1 MeT_23S_1835_inact + 1 ahcys + 1 b3275_v1_RNA_2 + 1 h	irreversible	rRNA modification	0.00E+00	1.00E+09	0	1.06E+02	1.06E+02	0	1.15E+01	11.5	0	1.15E+01	11.5
b3275_v1_RNA_20_modif_1	b3275_v1_RNA modification (23S rRNA): position: 745, m1G by RrmA_dim	1 RrmA_dim + 1 amet + 1 b3275_v1_RNA_20 <==> 1 b3275_v1_RNA_20_RrmA_dim	reversible	rRNA modification	-1.00E+09	1.00E+09	0	1.06E+02	1.06E+02	0	1.15E+01	11.5	0	1.15E+01	11.5
b3275_v1_RNA_20_modif_2	b3275_v1_RNA modification (23S rRNA): position: 745, m1G by RrmA_dim	1 b3275_v1_RNA_20_RrmA_dim --> 1 RrmA_dim_inact + 1 ahcys + 1 b3275_v1_RNA_21 + 1 h	irreversible	rRNA modification	0.00E+00	1.00E+09	0	1.06E+02	1.06E+02	0	1.15E+01	11.5	0	1.15E+01	11.5
b3275_v1_RNA_21_modif_1	b3275_v1_RNA modification (23S rRNA): position: 746, Y by RluA_mono	1 RluA_mono + 1 b3275_v1_RNA_21 <==> 1 b3275_v1_RNA_21_RluA_mono	reversible	rRNA modification	-1.00E+09	1.00E+09	0	1.06E+02	1.06E+02	0	1.15E+01	11.5	0	1.15E+01	11.5
b3275_v1_RNA_21_modif_2	b3275_v1_RNA modification (23S rRNA): position: 746, Y by RluA_mono	1 b3275_v1_RNA_21_RluA_mono --> 1 RluA_mono_inact + 1 b3275_v1_RNA_22	irreversible	rRNA modification	0.00E+00	1.00E+09	0	1.06E+02	1.06E+02	0	1.15E+01	11.5	0	1.15E+01	11.5
b3275_v1_RNA_22_modif_1	b3275_v1_RNA modification (23S rRNA): position: 747, m5U by RumB_mono	1 RumB_mono + 1 amet + 1 b3275_v1_RNA_22 <==> 1 b3275_v1_RNA_22_RumB_mono	reversible	rRNA modification	-1.00E+09	1.00E+09	0	1.06E+02	1.06E+02	0	1.15E+01	11.5	0	1.15E+01	11.5
b3275_v1_RNA_22_modif_2	b3275_v1_RNA modification (23S rRNA): position: 747, m5U by RumB_mono	1 b3275_v1_RNA_22_RumB_mono --> 1 RumB_mono_inact + 1 ahcys + 1 b3275_v1_RNA_23 + 1 h	irreversible	rRNA modification	0.00E+00	1.00E+09	0	1.06E+02	1.06E+02	0	1.15E+01	11.5	0	1.15E+01	11.5
b3275_v1_RNA_23_modif_1	b3275_v1_RNA modification (23S rRNA): position: 955, Y by RluC_mono	1 RluC_mono + 1 b3275_v1_RNA_23 <==> 1 b3275_v1_RNA_23_RluC_mono	reversible	rRNA modification	-1.00E+09	1.00E+09	0	1.06E+02	1.06E+02	0	1.15E+01	11.5	0	1.15E+01	11.5
b3275_v1_RNA_23_modif_2	b3275_v1_RNA modification (23S rRNA): position: 955, Y by RluC_mono	1 b3275_v1_RNA_23_RluC_mono --> 1 RluC_mono_inact + 1 rrlD	irreversible	rRNA modification	0.00E+00	1.00E+09	0	1.06E+02	1.06E+02	0	1.15E+01	11.5	0	1.15E+01	11.5
b3275_v1_RNA_2_modif_1	b3275_v1_RNA modification (23S rRNA): position: 1911, Y by RluD_mono	1 RluD_mono + 1 b3275_v1_RNA_2 <==> 1 b3275_v1_RNA_2_RluD_mono	reversible	rRNA modification	-1.00E+09	1.00E+09	0	1.06E+02	1.06E+02	0	1.15E+01	11.5	0	1.15E+01	11.5
b3275_v1_RNA_2_modif_2	b3275_v1_RNA modification (23S rRNA): position: 1911, Y by RluD_mono	1 b3275_v1_RNA_2_RluD_mono --> 1 RluD_mono_inact + 1 b3275_v1_RNA_3	irreversible	rRNA modification	0.00E+00	1.00E+09	0	1.06E+02	1.06E+02	0	1.15E+01	11.5	0	1.15E+01	11.5
b3275_v1_RNA_3_modif_1	b3275_v1_RNA modification (23S rRNA): position: 1915, m3Y by RluD_mono	1 RluD_mono + 1 amet + 1 b3275_v1_RNA_3 <==> 1 b3275_v1_RNA_3_RluD_mono	reversible	rRNA modification	-1.00E+09	1.00E+09	0	1.06E+02	1.06E+02	0	1.15E+01	11.5	0	1.15E+01	11.5
b3275_v1_RNA_3_modif_2	b3275_v1_RNA modification (23S rRNA): position: 1915, m3Y by RluD_mono	1 b3275_v1_RNA_3_RluD_mono --> 1 RluD_mono_inact + 1 ahcys + 1 b3275_v1_RNA_4 + 1 h	irreversible	rRNA modification	0.00E+00	1.00E+09	0	1.06E+02	1.06E+02	0	1.15E+01	11.5	0	1.15E+01	11.5
b3275_v1_RNA_4_modif_1	b3275_v1_RNA modification (23S rRNA): position: 1917, Y by RluD_mono	1 RluD_mono + 1 b3275_v1_RNA_4 <==> 1 b3275_v1_RNA_4_RluD_mono	reversible	rRNA modification	-1.00E+09	1.00E+09	0	1.06E+02	1.06E+02	0	1.15E+01	11.5	0	1.15E+01	11.5
b3275_v1_RNA_4_modif_2	b3275_v1_RNA modification (23S rRNA): position: 1917, Y by RluD_mono	1 b3275_v1_RNA_4_RluD_mono --> 1 RluD_mono_inact + 1 b3275_v1_RNA_5	irreversible	rRNA modification	0.00E+00	1.00E+09	0	1.06E+02	1.06E+02	0	1.15E+01	11.5	0	1.15E+01	11.5
b3275_v1_RNA_5_modif_1	b3275_v1_RNA modification (23S rRNA): position: 1939, m5U by RumA_mono	1 RumA_mono + 1 amet + 1 b3275_v1_RNA_5 <==> 1 b3275_v1_RNA_5_RumA_mono	reversible	rRNA modification	-1.00E+09	1.00E+09	0	1.06E+02	1.06E+02	0	1.15E+01	11.5	0	1.15E+01	11.5
b3275_v1_RNA_5_modif_2	b3275_v1_RNA modification (23S rRNA): position: 1939, m5U by RumA_mono	1 b3275_v1_RNA_5_RumA_mono --> 1 RumA_mono_inact + 1 ahcy + 1 b3275_v1_RNA_6 + 1 h	irreversible	rRNA modification	0.00E+00	1.00E+09	0	1.06E+02	1.06E+02	0	1.15E+01	11.5	0	1.15E+01	11.5
b3275_v1_RNA_6_modif_1	b3275_v1_RNA modification (23S rRNA): position: 1962, m5C by MeT_23S_1962	1 MeT_23S_1962 + 1 amet + 1 b3275_v1_RNA_6 <==> 1 b3275_v1_RNA_6_MeT_23S_1962	reversible	rRNA modification	-1.00E+09	1.00E+09	0	1.06E+02	1.06E+02	0	1.15E+01	11.5	0	1.15E+01	11.5
b3275_v1_RNA_6_modif_2	b3275_v1_RNA modification (23S rRNA): position: 1962, m5C by MeT_23S_1962	1 b3275_v1_RNA_6_MeT_23S_1962 --> 1 MeT_23S_1962_inact + 1 ahcys + 1 b3275_v1_RNA_7 + 1 h	irreversible	rRNA modification	0.00E+00	1.00E+09	0	1.06E+02	1.06E+02	0	1.15E+01	11.5	0	1.15E+01	11.5
b3275_v1_RNA_7_modif_1	b3275_v1_RNA modification (23S rRNA): position: 2030, m6A by MeT_23S_2030	1 MeT_23S_2030 + 1 amet + 1 b3275_v1_RNA_7 <==> 1 b3275_v1_RNA_7_MeT_23S_2030	reversible	rRNA modification	-1.00E+09	1.00E+09	0	1.06E+02	1.06E+02	0	1.15E+01	11.5	0	1.15E+01	11.5
b3275_v1_RNA_7_modif_2	b3275_v1_RNA modification (23S rRNA): position: 2030, m6A by MeT_23S_2030	1 b3275_v1_RNA_7_MeT_23S_2030 --> 1 MeT_23S_2030_inact + 1 ahcys + 1 b3275_v1_RNA_8 + 1 h	irreversible	rRNA modification	0.00E+00	1.00E+09	0	1.06E+02	1.06E+02	0	1.15E+01	11.5	0	1.15E+01	11.5
b3275_v1_RNA_8_modif_1	b3275_v1_RNA modification (23S rRNA): position: 2069, m7G by MeT_23S_2069	1 MeT_23S_2069 + 1 amet + 1 b3275_v1_RNA_8 <==> 1 b3275_v1_RNA_8_MeT_23S_2069	reversible	rRNA modification	-1.00E+09	1.00E+09	0	1.06E+02	1.06E+02	0	1.15E+01	11.5	0	1.15E+01	11.5
b3275_v1_RNA_8_modif_2	b3275_v1_RNA modification (23S rRNA): position: 2069, m7G by MeT_23S_2069	1 b3275_v1_RNA_8_MeT_23S_2069 --> 1 MeT_23S_2069_inact + 1 ahcys + 1 b3275_v1_RNA_9 + 1 h	irreversible	rRNA modification	0.00E+00	1.00E+09	0	1.06E+02	1.06E+02	0	1.15E+01	11.5	0	1.15E+01	11.5
b3275_v1_RNA_9_modif_1	b3275_v1_RNA modification (23S rRNA): position: 2251, Gm by RlmB_dim	1 RlmB_dim + 1 amet + 1 b3275_v1_RNA_9 <==> 1 b3275_v1_RNA_9_RlmB_dim	reversible	rRNA modification	-1.00E+09	1.00E+09	0	1.06E+02	1.06E+02	0	1.15E+01	11.5	0	1.15E+01	11.5
b3275_v1_RNA_9_modif_2	b3275_v1_RNA modification (23S rRNA): position: 2251, Gm by RlmB_dim	1 b3275_v1_RNA_9_RlmB_dim --> 1 RlmB_dim_inact + 1 ahcys + 1 b3275_v1_RNA_10 + 1 h	irreversible	rRNA modification	0.00E+00	1.00E+09	0	1.06E+02	1.06E+02	0	1.15E+01	11.5	0	1.15E+01	11.5
b3275_v1_RNA_modif_1	b3275_v1_RNA modification (23S rRNA): position: 1618, m6A by MeT_23S_1618	1 MeT_23S_1618 + 1 amet + 1 b3275_v1_RNA <==> 1 b3275_v1_RNA_MeT_23S_1618	reversible	rRNA modification	-1.00E+09	1.00E+09	0	1.06E+02	1.06E+02	0	1.15E+01	11.5	0	1.15E+01	11.5
b3275_v1_RNA_modif_2	b3275_v1_RNA modification (23S rRNA): position: 1618, m6A by MeT_23S_1618	1 b3275_v1_RNA_MeT_23S_1618 --> 1 MeT_23S_1618_inact + 1 ahcys + 1 b3275_v1_RNA_1 + 1 h	irreversible	rRNA modification	0.00E+00	1.00E+09	0	1.06E+02	1.06E+02	0	1.15E+01	11.5	0	1.15E+01	11.5
b3276_tRNA_17_MODIF_1	modification of position 17 nt (D) in alaU by Dus_gen	1 Dus_gen + 1 b3276_tRNA_Mg2 + 1 h + 1 nadph <==> 1 b3276_tRNA_Mg2_Dus_gen_cplx	reversible	tRNA modification	-1.00E+09	1.00E+09	0	1.06E+02	1.06E+02	0	0	0	0	0	0
b3276_tRNA_17_MODIF_2	modification of position 17 nt (D) in alaU by Dus_gen	1 b3276_tRNA_Mg2_Dus_gen_cplx --> 1 Dus_gen_inact + 1 b3276_tRNA_1 + 1 nadp	irreversible	tRNA modification	0.00E+00	1.00E+09	0	1.06E+02	1.06E+02	0	0	0	0	0	0
b3276_tRNA_34_MODIF_1	modification of position 34 nt (cmo5U) in alaU by YecO_mono-YecP_mono-HyL_tRNA_pos_34_ho5U	1 HyL_tRNA_pos_34_ho5U + 1 YecO_mono + 1 YecP_mono + 1 amet + 1 b3276_tRNA_1 + 2 chor <==> 1 b3276_tRNA_1_YecO_mono-YecP_mono-HyL_tRNA_pos_34_ho5U_cplx	reversible	tRNA modification	-1.00E+09	1.00E+09	0	1.06E+02	1.06E+02	0	0	0	0	0	0
b3276_tRNA_34_MODIF_2	modification of position 34 nt (cmo5U) in alaU by YecO_mono-YecP_mono-HyL_tRNA_pos_34_ho5U	1 b3276_tRNA_1_YecO_mono-YecP_mono-HyL_tRNA_pos_34_ho5U_cplx --> 1 C10H8O5 + 1 C9H9O4 + 1 HyL_tRNA_pos_34_ho5U_inact + 1 YecO_mono_inact + 1 YecP_mono_inact + 1 ahcys + 1 b3276_tRNA_2 + 1 h	irreversible	tRNA modification	0.00E+00	1.00E+09	0	1.06E+02	1.06E+02	0	0	0	0	0	0

b3276_tRNA_46_MODIF_1	modification of position 46 nt (m7G) in alaU by YggH_mono	1 YggH_mono + 1 amet + 1 b3276_tRNA_2 <==> 1 b3276_tRNA_2_YggH_mono_cplx	reversible	tRNA modification	-1.00E+09	1.00E+09	0	1.06E+02	1.06E+02	0	0	0	0	0	0
b3276_tRNA_46_MODIF_2	modification of position 46 nt (m7G) in alaU by YggH_mono	1 b3276_tRNA_2_YggH_mono_cplx --> 1 YggH_mono_inact + 1 ahcys + 1 b3276_tRNA_3 + 1 h	irreversible	tRNA modification	0.00E+00	1.00E+09	0	1.06E+02	1.06E+02	0	0	0	0	0	0
b3276_tRNA_54_MODIF_1	modification of position 54 nt (m5U) in alaU by TrmA_mono	1 TrmA_mono + 1 amet + 1 b3276_tRNA_3 <==> 1 b3276_tRNA_3_TrmA_mono_cplx	reversible	tRNA modification	-1.00E+09	1.00E+09	0	1.06E+02	1.06E+02	0	0	0	0	0	0
b3276_tRNA_54_MODIF_2	modification of position 54 nt (m5U) in alaU by TrmA_mono	1 b3276_tRNA_3_TrmA_mono_cplx --> 1 TrmA_mono_inact + 1 ahcys + 1 b3276_tRNA_4 + 1 h	irreversible	tRNA modification	0.00E+00	1.00E+09	0	1.06E+02	1.06E+02	0	0	0	0	0	0
b3276_tRNA_55_MODIF_1	modification of position 55 nt (Y) in alaU by TruB_mono	1 TruB_mono + 1 b3276_tRNA_4 <==> 1 b3276_tRNA_4_TruB_mono_cplx	reversible	tRNA modification	-1.00E+09	1.00E+09	0	1.06E+02	1.06E+02	0	0	0	0	0	0
b3276_tRNA_55_MODIF_2	modification of position 55 nt (Y) in alaU by TruB_mono	1 b3276_tRNA_4_TruB_mono_cplx --> 1 TruB_mono_inact + 1 alaU_tRNA	irreversible	tRNA modification	0.00E+00	1.00E+09	0	1.06E+02	1.06E+02	0	0	0	0	0	0
b3276_tRNA_Mg_BIND	Binding of two Mg2 to b3276_tRNA	1 b3276_RNA + 2 mg2 <==> 1 b3276_tRNA_Mg2	reversible	Metallo-ion binding	-1.00E+09	1.00E+09	0	1.06E+02	1.06E+02	0	0	0	0	0	0
b3276_v1_tRNA_17_MODIF_1	modification of position 17 nt (D) in alaU by Dus_gen	1 Dus_gen + 1 b3276_v1_tRNA_Mg2 + 1 h + 1 nadph <==> 1 b3276_v1_tRNA_Mg2_Dus_gen_cplx	reversible	tRNA modification	-1.00E+09	1.00E+09	0	1.06E+02	1.06E+02	0	1.15E+01	11.5	0	1.15E+01	11.5
b3276_v1_tRNA_17_MODIF_2	modification of position 17 nt (D) in alaU by Dus_gen	1 b3276_v1_tRNA_Mg2_Dus_gen_cplx --> 1 Dus_gen_inact + 1 b3276_v1_tRNA_1 + 1 nadp	irreversible	tRNA modification	0.00E+00	1.00E+09	0	1.06E+02	1.06E+02	0	1.15E+01	11.5	0	1.15E+01	11.5
b3276_v1_tRNA_34_MODIF_1	modification of position 34 nt (cmo5U) in alaU by YecO_mono-YecP_mono-HyL_tRNA_pos_34_ho5U	1 HyL_tRNA_pos_34_ho5U + 1 YecO_mono + 1 YecP_mono + 1 amet + 1 b3276_v1_tRNA_1 + 2 chor <==> 1 b3276_v1_tRNA_1_YecO_mono-YecP_mono-HyL_tRNA_pos_34_ho5U_cplx	reversible	tRNA modification	-1.00E+09	1.00E+09	0	1.06E+02	1.06E+02	0	1.15E+01	11.5	0	1.15E+01	11.5
b3276_v1_tRNA_34_MODIF_2	modification of position 34 nt (cmo5U) in alaU by YecO_mono-YecP_mono-HyL_tRNA_pos_34_ho5U	1 b3276_v1_tRNA_1_YecO_mono-YecP_mono-HyL_tRNA_pos_34_ho5U_cplx --> 1 C10H8O5 + 1 C9H9O4 + 1 HyL_tRNA_pos_34_ho5U_inact + 1 YecO_mono_inact + 1 YecP_mono_inact + 1 ahcys + 1 b3276_v1_tRNA_2 + 1 h	irreversible	tRNA modification	0.00E+00	1.00E+09	0	1.06E+02	1.06E+02	0	1.15E+01	11.5	0	1.15E+01	11.5
b3276_v1_tRNA_46_MODIF_1	modification of position 46 nt (m7G) in alaU by YggH_mono	1 YggH_mono + 1 amet + 1 b3276_v1_tRNA_2 <==> 1 b3276_v1_tRNA_2_YggH_mono_cplx	reversible	tRNA modification	-1.00E+09	1.00E+09	0	1.06E+02	1.06E+02	0	1.15E+01	11.5	0	1.15E+01	11.5
b3276_v1_tRNA_46_MODIF_2	modification of position 46 nt (m7G) in alaU by YggH_mono	1 b3276_v1_tRNA_2_YggH_mono_cplx --> 1 YggH_mono_inact + 1 ahcys + 1 b3276_v1_tRNA_3 + 1 h	irreversible	tRNA modification	0.00E+00	1.00E+09	0	1.06E+02	1.06E+02	0	1.15E+01	11.5	0	1.15E+01	11.5
b3276_v1_tRNA_54_MODIF_1	modification of position 54 nt (m5U) in alaU by TrmA_mono	1 TrmA_mono + 1 amet + 1 b3276_v1_tRNA_3 <==> 1 b3276_v1_tRNA_3_TrmA_mono_cplx	reversible	tRNA modification	-1.00E+09	1.00E+09	0	1.06E+02	1.06E+02	0	1.15E+01	11.5	0	1.15E+01	11.5
b3276_v1_tRNA_54_MODIF_2	modification of position 54 nt (m5U) in alaU by TrmA_mono	1 b3276_v1_tRNA_3_TrmA_mono_cplx --> 1 TrmA_mono_inact + 1 ahcys + 1 b3276_v1_tRNA_4 + 1 h	irreversible	tRNA modification	0.00E+00	1.00E+09	0	1.06E+02	1.06E+02	0	1.15E+01	11.5	0	1.15E+01	11.5
b3276_v1_tRNA_55_MODIF_1	modification of position 55 nt (Y) in alaU by TruB_mono	1 TruB_mono + 1 b3276_v1_tRNA_4 <==> 1 b3276_v1_tRNA_4_TruB_mono_cplx	reversible	tRNA modification	-1.00E+09	1.00E+09	0	1.06E+02	1.06E+02	0	1.15E+01	11.5	0	1.15E+01	11.5
b3276_v1_tRNA_55_MODIF_2	modification of position 55 nt (Y) in alaU by TruB_mono	1 b3276_v1_tRNA_4_TruB_mono_cplx --> 1 TruB_mono_inact + 1 alaU_tRNA	irreversible	tRNA modification	0.00E+00	1.00E+09	0	1.06E+02	1.06E+02	0	1.15E+01	11.5	0	1.15E+01	11.5
b3276_v1_tRNA_Mg_BIND	Binding of two Mg2 to b3276_v1_tRNA	1 b3276_v1_RNA + 2 mg2 <==> 1 b3276_v1_tRNA_Mg2	reversible	Metallo-ion binding	-1.00E+09	1.00E+09	0	1.06E+02	1.06E+02	0	1.15E+01	11.5	0	1.15E+01	11.5
b3277_tRNA_20A_MODIF_1	modification of position 20A nt (D) in ileU by Dus_gen	1 Dus_gen + 1 b3277_tRNA_1 + 1 h + 1 nadph <==> 1 b3277_tRNA_1_Dus_gen_cplx	reversible	tRNA modification	-1.00E+09	1.00E+09	0	1.06E+02	1.06E+02	0	0	0	0	0	0
b3277_tRNA_20A_MODIF_2	modification of position 20A nt (D) in ileU by Dus_gen	1 b3277_tRNA_1_Dus_gen_cplx --> 1 Dus_gen_inact + 1 b3277_tRNA_2 + 1 nadp	irreversible	tRNA modification	0.00E+00	1.00E+09	0	1.06E+02	1.06E+02	0	0	0	0	0	0
b3277_tRNA_20_MODIF_1	modification of position 20 nt (D) in ileU by Dus_gen	1 Dus_gen + 1 b3277_tRNA_Mg2 + 1 h + 1 nadph <==> 1 b3277_tRNA_Mg2_Dus_gen_cplx	reversible	tRNA modification	-1.00E+09	1.00E+09	0	1.06E+02	1.06E+02	0	0	0	0	0	0
b3277_tRNA_20_MODIF_2	modification of position 20 nt (D) in ileU by Dus_gen	1 b3277_tRNA_Mg2_Dus_gen_cplx --> 1 Dus_gen_inact + 1 b3277_tRNA_1 + 1 nadp	irreversible	tRNA modification	0.00E+00	1.00E+09	0	1.06E+02	1.06E+02	0	0	0	0	0	0
b3277_tRNA_37_MODIF_1	modification of position 37 nt (t6A) in ileU by Up_tRNA_pos_37_t6A	1 Up_tRNA_pos_37_t6A + 1 atp + 1 b3277_tRNA_2 + 1 hco3 + 1 mg2 + 1 thr-L <==> 1 b3277_tRNA_2_Up_tRNA_pos_37_t6A_cplx	reversible	tRNA modification	-1.00E+09	1.00E+09	0	1.06E+02	1.06E+02	0	0	0	0	0	0
b3277_tRNA_37_MODIF_2	modification of position 37 nt (t6A) in ileU by Up_tRNA_pos_37_t6A	1 b3277_tRNA_2_Up_tRNA_pos_37_t6A_cplx --> 1 Up_tRNA_pos_37_t6A_inact + 1 amp + 1 b3277_tRNA_3 + 1 h + 1 h2o + 1 mg2 + 1 ppi	irreversible	tRNA modification	0.00E+00	1.00E+09	0	1.06E+02	1.06E+02	0	0	0	0	0	0
b3277_tRNA_46_MODIF_1	modification of position 46 nt (m7G) in ileU by YggH_mono	1 YggH_mono + 1 amet + 1 b3277_tRNA_3 <==> 1 b3277_tRNA_3_YggH_mono_cplx	reversible	tRNA modification	-1.00E+09	1.00E+09	0	1.06E+02	1.06E+02	0	0	0	0	0	0
b3277_tRNA_46_MODIF_2	modification of position 46 nt (m7G) in ileU by YggH_mono	1 b3277_tRNA_3_YggH_mono_cplx --> 1 YggH_mono_inact + 1 ahcys + 1 b3277_tRNA_4 + 1 h	irreversible	tRNA modification	0.00E+00	1.00E+09	0	1.06E+02	1.06E+02	0	0	0	0	0	0
b3277_tRNA_47_MODIF_1	modification of position 47 nt (acp3U) in ileU by AcpT_tRNA_pos_47_acp3U	1 AcpT_tRNA_pos_47_acp3U + 1 amet + 1 b3277_tRNA_4 <==> 1 b3277_tRNA_4_AcpT_tRNA_pos_47_acp3U_cplx	reversible	tRNA modification	-1.00E+09	1.00E+09	0	1.06E+02	1.06E+02	0	0	0	0	0	0
b3277_tRNA_47_MODIF_2	modification of position 47 nt (acp3U) in ileU by AcpT_tRNA_pos_47_acp3U	1 b3277_tRNA_4_AcpT_tRNA_pos_47_acp3U_cplx --> 1 5mta + 1 AcpT_tRNA_pos_47_acp3U_inact + 1 b3277_tRNA_5 + 1 h	irreversible	tRNA modification	0.00E+00	1.00E+09	0	1.06E+02	1.06E+02	0	0	0	0	0	0
b3277_tRNA_54_MODIF_1	modification of position 54 nt (m5U) in ileU by TrmA_mono	1 TrmA_mono + 1 amet + 1 b3277_tRNA_5 <==> 1 b3277_tRNA_5_TrmA_mono_cplx	reversible	tRNA modification	-1.00E+09	1.00E+09	0	1.06E+02	1.06E+02	0	0	0	0	0	0
b3277_tRNA_54_MODIF_2	modification of position 54 nt (m5U) in ileU by TrmA_mono	1 b3277_tRNA_5_TrmA_mono_cplx --> 1 TrmA_mono_inact + 1 ahcys + 1 b3277_tRNA_6 + 1 h	irreversible	tRNA modification	0.00E+00	1.00E+09	0	1.06E+02	1.06E+02	0	0	0	0	0	0
b3277_tRNA_55_MODIF_1	modification of position 55 nt (Y) in ileU by TruB_mono	1 TruB_mono + 1 b3277_tRNA_6 <==> 1 b3277_tRNA_6_TruB_mono_cplx	reversible	tRNA modification	-1.00E+09	1.00E+09	0	1.06E+02	1.06E+02	0	0	0	0	0	0
b3277_tRNA_55_MODIF_2	modification of position 55 nt (Y) in ileU by TruB_mono	1 b3277_tRNA_6_TruB_mono_cplx --> 1 TruB_mono_inact + 1 b3277_tRNA_7	irreversible	tRNA modification	0.00E+00	1.00E+09	0	1.06E+02	1.06E+02	0	0	0	0	0	0
b3277_tRNA_65_MODIF_1	modification of position 65 nt (Y) in ileU by YqcB_mono	1 YqcB_mono + 1 b3277_tRNA_7 <==> 1 b3277_tRNA_7_YqcB_mono_cplx	reversible	tRNA modification	-1.00E+09	1.00E+09	0	1.06E+02	1.06E+02	0	0	0	0	0	0
b3277_tRNA_65_MODIF_2	modification of position 65 nt (Y) in ileU by YqcB_mono	1 b3277_tRNA_7_YqcB_mono_cplx --> 1 YqcB_mono_inact + 1 ileU_tRNA	irreversible	tRNA modification	0.00E+00	1.00E+09	0	1.06E+02	1.06E+02	0	0	0	0	0	0
b3277_tRNA_Mg_BIND	Binding of two Mg2 to b3277_tRNA	1 b3277_RNA + 2 mg2 <==> 1 b3277_tRNA_Mg2	reversible	Metallo-ion binding	-1.00E+09	1.00E+09	0	1.06E+02	1.06E+02	0	0	0	0	0	0
b3277_v1_tRNA_20A_MODIF_1	modification of position 20A nt (D) in ileU by Dus_gen	1 Dus_gen + 1 b3277_v1_tRNA_1 + 1 h + 1 nadph <==> 1 b3277_v1_tRNA_1_Dus_gen_cplx	reversible	tRNA modification	-1.00E+09	1.00E+09	0	1.06E+02	1.06E+02	0	1.15E+01	11.5	0	1.15E+01	11.5
b3277_v1_tRNA_20A_MODIF_2	modification of position 20A nt (D) in ileU by Dus_gen	1 b3277_v1_tRNA_1_Dus_gen_cplx --> 1 Dus_gen_inact + 1 b3277_v1_tRNA_2 + 1 nadp	irreversible	tRNA modification	0.00E+00	1.00E+09	0	1.06E+02	1.06E+02	0	1.15E+01	11.5	0	1.15E+01	11.5
b3277_v1_tRNA_20_MODIF_1	modification of position 20 nt (D) in ileU by Dus_gen	1 Dus_gen + 1 b3277_v1_tRNA_Mg2 + 1 h + 1 nadph <==> 1 b3277_v1_tRNA_Mg2_Dus_gen_cplx	reversible	tRNA modification	-1.00E+09	1.00E+09	0	1.06E+02	1.06E+02	0	1.15E+01	11.5	0	1.15E+01	11.5
b3277_v1_tRNA_20_MODIF_2	modification of position 20 nt (D) in ileU by Dus_gen	1 b3277_v1_tRNA_Mg2_Dus_gen_cplx --> 1 Dus_gen_inact + 1 b3277_v1_tRNA_1 + 1 nadp	irreversible	tRNA modification	0.00E+00	1.00E+09	0	1.06E+02	1.06E+02	0	1.15E+01	11.5	0	1.15E+01	11.5
b3277_v1_tRNA_37_MODIF_1	modification of position 37 nt (t6A) in ileU by Up_tRNA_pos_37_t6A	1 Up_tRNA_pos_37_t6A + 1 atp + 1 b3277_v1_tRNA_2 + 1 hco3 + 1 mg2 + 1 thr-L <==> 1 b3277_v1_tRNA_2_Up_tRNA_pos_37_t6A_cplx	reversible	tRNA modification	-1.00E+09	1.00E+09	0	1.06E+02	1.06E+02	0	1.15E+01	11.5	0	1.15E+01	11.5
b3277_v1_tRNA_37_MODIF_2	modification of position 37 nt (t6A) in ileU by Up_tRNA_pos_37_t6A	1 b3277_v1_tRNA_2_Up_tRNA_pos_37_t6A_cplx --> 1 Up_tRNA_pos_37_t6A_inact + 1 amp + 1 b3277_v1_tRNA_3 + 1 h + 1 h2o + 1 mg2 + 1 ppi	irreversible	tRNA modification	0.00E+00	1.00E+09	0	1.06E+02	1.06E+02	0	1.15E+01	11.5	0	1.15E+01	11.5
b3277_v1_tRNA_46_MODIF_1	modification of position 46 nt (m7G) in ileU by YggH_mono	1 YggH_mono + 1 amet + 1 b3277_v1_tRNA_3 <==> 1 b3277_v1_tRNA_3_YggH_mono_cplx	reversible	tRNA modification	-1.00E+09	1.00E+09	0	1.06E+02	1.06E+02	0	1.15E+01	11.5	0	1.15E+01	11.5
b3277_v1_tRNA_46_MODIF_2	modification of position 46 nt (m7G) in ileU by YggH_mono	1 b3277_v1_tRNA_3_YggH_mono_cplx --> 1 YggH_mono_inact + 1 ahcys + 1 b3277_v1_tRNA_4 + 1 h	irreversible	tRNA modification	0.00E+00	1.00E+09	0	1.06E+02	1.06E+02	0	1.15E+01	11.5	0	1.15E+01	11.5
b3277_v1_tRNA_47_MODIF_1	modification of position 47 nt (acp3U) in ileU by AcpT_tRNA_pos_47_acp3U	1 AcpT_tRNA_pos_47_acp3U + 1 amet + 1 b3277_v1_tRNA_4 <==> 1 b3277_v1_tRNA_4_AcpT_tRNA_pos_47_acp3U_cplx	reversible	tRNA modification	-1.00E+09	1.00E+09	0	1.06E+02	1.06E+02	0	1.15E+01	11.5	0	1.15E+01	11.5
b3277_v1_tRNA_47_MODIF_2	modification of position 47 nt (acp3U) in ileU by AcpT_tRNA_pos_47_acp3U	1 b3277_v1_tRNA_4_AcpT_tRNA_pos_47_acp3U_cplx --> 1 5mta + 1 AcpT_tRNA_pos_47_acp3U_inact + 1 b3277_v1_tRNA_5 + 1 h	irreversible	tRNA modification	0.00E+00	1.00E+09	0	1.06E+02	1.06E+02	0	1.15E+01	11.5	0	1.15E+01	11.5
b3277_v1_tRNA_54_MODIF_1	modification of position 54 nt (m5U) in ileU by TrmA_mono	1 TrmA_mono + 1 amet + 1 b3277_v1_tRNA_5 <==> 1 b3277_v1_tRNA_5_TrmA_mono_cplx	reversible	tRNA modification	-1.00E+09	1.00E+09	0	1.06E+02	1.06E+02	0	1.15E+01	11.5	0	1.15E+01	11.5
b3277_v1_tRNA_54_MODIF_2	modification of position 54 nt (m5U) in ileU by TrmA_mono	1 b3277_v1_tRNA_5_TrmA_mono_cplx --> 1 TrmA_mono_inact + 1 ahcys + 1 b3277_v1_tRNA_6 + 1 h	irreversible	tRNA modification	0.00E+00	1.00E+09	0	1.06E+02	1.06E+02	0	1.15E+01	11.5	0	1.15E+01	11.5
b3277_v1_tRNA_55_MODIF_1	modification of position 55 nt (Y) in ileU by TruB_mono	1 TruB_mono + 1 b3277_v1_tRNA_6 <==> 1 b3277_v1_tRNA_6_TruB_mono_cplx	reversible	tRNA modification	-1.00E+09	1.00E+09	0	1.06E+02	1.06E+02	0	1.15E+01	11.5	0	1.15E+01	11.5
b3277_v1_tRNA_55_MODIF_2	modification of position 55 nt (Y) in ileU by TruB_mono	1 b3277_v1_tRNA_6_TruB_mono_cplx --> 1 TruB_mono_inact + 1 b3277_v1_tRNA_7	irreversible	tRNA modification	0.00E+00	1.00E+09	0	1.06E+02	1.06E+02	0	1.15E+01	11.5	0	1.15E+01	11.5
b3277_v1_tRNA_65_MODIF_1	modification of position 65 nt (Y) in ileU by YqcB_mono	1 YqcB_mono + 1 b3277_v1_tRNA_7 <==> 1 b3277_v1_tRNA_7_YqcB_mono_cplx	reversible	tRNA modification	-1.00E+09	1.00E+09	0	1.06E+02	1.06E+02	0	1.15E+01	11.5	0	1.15E+01	11.5
b3277_v1_tRNA_65_MODIF_2	modification of position 65 nt (Y) in ileU by YqcB_mono	1 b3277_v1_tRNA_7_YqcB_mono_cplx --> 1 YqcB_mono_inact + 1 ileU_tRNA	irreversible	tRNA modification	0.00E+00	1.00E+09	0	1.06E+02	1.06E+02	0	1.15E+01	11.5	0	1.15E+01	11.5

b3277_v1_tRNA_Mg_BIND	Binding of two Mg2 to b3277_v1_tRNA	1 b3277_v1_RNA + 2 mg2 <==> 1 b3277_v1_tRNA_Mg2	reversible	Metallo-ion binding	-1.00E+09	1.00E+09	0	1.06E+02	1.06E+02	0	1.15E+01	11.5	0	1.15E+01	11.5
b3278_RNA_10_modif_1	b3278_RNA modification (16S rRNA): position: 967, m5C by RsmB_mono	1 RsmB_mono + 1 amet + 1 b3278_RNA_10 <==> 1 b3278_RNA_10_RsmB_mono	reversible	rRNA modification	-1.00E+09	1.00E+09	0	1.06E+02	1.06E+02	0	0	0	0	0	0
b3278_RNA_10_modif_2	b3278_RNA modification (16S rRNA): position: 967, m5C by RsmB_mono	1 b3278_RNA_10_RsmB_mono --> 1 RsmB_mono_inact + 1 ahcys + 1 h + 1 rrsD	irreversible	rRNA modification	0.00E+00	1.00E+09	0	1.06E+02	1.06E+02	0	0	0	0	0	0
b3278_RNA_1_modif_1	b3278_RNA modification (16S rRNA): position: 1402, m4Cm by MeT_16S_1402	1 MeT_16S_1402 + 2 amet + 1 b3278_RNA_1 <==> 1 b3278_RNA_1_MeT_16S_1402	reversible	rRNA modification	-1.00E+09	1.00E+09	0	1.06E+02	1.06E+02	0	0	0	0	0	0
b3278_RNA_1_modif_2	b3278_RNA modification (16S rRNA): position: 1402, m4Cm by MeT_16S_1402	1 b3278_RNA_1_MeT_16S_1402 --> 1 MeT_16S_1402_inact + 2 ahcys + 1 b3278_RNA_2 + 2 h	irreversible	rRNA modification	0.00E+00	1.00E+09	0	1.06E+02	1.06E+02	0	0	0	0	0	0
b3278_RNA_2_modif_1	b3278_RNA modification (16S rRNA): position: 1407, m5C by MeT_16S_1407	1 MeT_16S_1407 + 1 amet + 1 b3278_RNA_2 <==> 1 b3278_RNA_2_MeT_16S_1407	reversible	rRNA modification	-1.00E+09	1.00E+09	0	1.06E+02	1.06E+02	0	0	0	0	0	0
b3278_RNA_2_modif_2	b3278_RNA modification (16S rRNA): position: 1407, m5C by MeT_16S_1407	1 b3278_RNA_2_MeT_16S_1407 --> 1 MeT_16S_1407_inact + 1 ahcys + 1 b3278_RNA_3 + 1 h	irreversible	rRNA modification	0.00E+00	1.00E+09	0	1.06E+02	1.06E+02	0	0	0	0	0	0
b3278_RNA_3_modif_1	b3278_RNA modification (16S rRNA): position: 1498, m3U by YggJ_mono	1 YggJ_mono + 1 amet + 1 b3278_RNA_3 <==> 1 b3278_RNA_3_YggJ_mono	reversible	rRNA modification	-1.00E+09	1.00E+09	0	1.06E+02	1.06E+02	0	0	0	0	0	0
b3278_RNA_3_modif_2	b3278_RNA modification (16S rRNA): position: 1498, m3U by YggJ_mono	1 b3278_RNA_3_YggJ_mono --> 1 YggJ_mono_inact + 1 ahcys + 1 b3278_RNA_4 + 1 h	irreversible	rRNA modification	0.00E+00	1.00E+09	0	1.06E+02	1.06E+02	0	0	0	0	0	0
b3278_RNA_4_modif_1	b3278_RNA modification (16S rRNA): position: 1516, m2G by MeT_16S_1516	1 MeT_16S_1516 + 1 amet + 1 b3278_RNA_4 <==> 1 b3278_RNA_4_MeT_16S_1516	reversible	rRNA modification	-1.00E+09	1.00E+09	0	1.06E+02	1.06E+02	0	0	0	0	0	0
b3278_RNA_4_modif_2	b3278_RNA modification (16S rRNA): position: 1516, m2G by MeT_16S_1516	1 b3278_RNA_4_MeT_16S_1516 --> 1 MeT_16S_1516_inact + 1 ahcys + 1 b3278_RNA_5 + 1 h	irreversible	rRNA modification	0.00E+00	1.00E+09	0	1.06E+02	1.06E+02	0	0	0	0	0	0
b3278_RNA_5_modif_1	b3278_RNA modification (16S rRNA): position: 1518, m6(2)A by KsgA_mono	1 KsgA_mono + 2 amet + 1 b3278_RNA_5 <==> 1 b3278_RNA_5_KsgA_mono	reversible	rRNA modification	-1.00E+09	1.00E+09	0	1.06E+02	1.06E+02	0	0	0	0	0	0
b3278_RNA_5_modif_2	b3278_RNA modification (16S rRNA): position: 1518, m6(2)A by KsgA_mono	1 b3278_RNA_5_KsgA_mono --> 1 KsgA_mono_inact + 2 ahcys + 1 b3278_RNA_6 + 2 h	irreversible	rRNA modification	0.00E+00	1.00E+09	0	1.06E+02	1.06E+02	0	0	0	0	0	0
b3278_RNA_6_modif_1	b3278_RNA modification (16S rRNA): position: 1519, m6(2)A by KsgA_mono	1 KsgA_mono + 2 amet + 1 b3278_RNA_6 <==> 1 b3278_RNA_6_KsgA_mono	reversible	rRNA modification	-1.00E+09	1.00E+09	0	1.06E+02	1.06E+02	0	0	0	0	0	0
b3278_RNA_6_modif_2	b3278_RNA modification (16S rRNA): position: 1519, m6(2)A by KsgA_mono	1 b3278_RNA_6_KsgA_mono --> 1 KsgA_mono_inact + 2 ahcys + 1 b3278_RNA_7 + 2 h	irreversible	rRNA modification	0.00E+00	1.00E+09	0	1.06E+02	1.06E+02	0	0	0	0	0	0
b3278_RNA_7_modif_1	b3278_RNA modification (16S rRNA): position: 516, Y by RsuA_mono	1 RsuA_mono + 1 b3278_RNA_7 <==> 1 b3278_RNA_7_RsuA_mono	reversible	rRNA modification	-1.00E+09	1.00E+09	0	1.06E+02	1.06E+02	0	0	0	0	0	0
b3278_RNA_7_modif_2	b3278_RNA modification (16S rRNA): position: 516, Y by RsuA_mono	1 b3278_RNA_7_RsuA_mono --> 1 RsuA_mono_inact + 1 b3278_RNA_8	irreversible	rRNA modification	0.00E+00	1.00E+09	0	1.06E+02	1.06E+02	0	0	0	0	0	0
b3278_RNA_8_modif_1	b3278_RNA modification (16S rRNA): position: 527, m7G by MeT_16S_527	1 MeT_16S_527 + 1 amet + 1 b3278_RNA_8 <==> 1 b3278_RNA_8_MeT_16S_527	reversible	rRNA modification	-1.00E+09	1.00E+09	0	1.06E+02	1.06E+02	0	0	0	0	0	0
b3278_RNA_8_modif_2	b3278_RNA modification (16S rRNA): position: 527, m7G by MeT_16S_527	1 b3278_RNA_8_MeT_16S_527 --> 1 MeT_16S_527_inact + 1 ahcy + 1 b3278_RNA_9 + 1 h	irreversible	rRNA modification	0.00E+00	1.00E+09	0	1.06E+02	1.06E+02	0	0	0	0	0	0
b3278_RNA_9_modif_1	b3278_RNA modification (16S rRNA): position: 966, m2G by MeT_16S_966	1 MeT_16S_966 + 1 amet + 1 b3278_RNA_9 <==> 1 b3278_RNA_9_MeT_16S_966	reversible	rRNA modification	-1.00E+09	1.00E+09	0	1.06E+02	1.06E+02	0	0	0	0	0	0
b3278_RNA_9_modif_2	b3278_RNA modification (16S rRNA): position: 966, m2G by MeT_16S_966	1 b3278_RNA_9_MeT_16S_966 --> 1 MeT_16S_966_inact + 1 ahcy + 1 b3278_RNA_10 + 1 h	irreversible	rRNA modification	0.00E+00	1.00E+09	0	1.06E+02	1.06E+02	0	0	0	0	0	0
b3278_RNA_modif_1	b3278_RNA modification (16S rRNA): position: 1207, m2G by RsmC_mono	1 RsmC_mono + 1 amet + 1 b3278_RNA <==> 1 b3278_RNA_RsmC_mono	reversible	rRNA modification	-1.00E+09	1.00E+09	0	1.06E+02	1.06E+02	0	0	0	0	0	0
b3278_RNA_modif_2	b3278_RNA modification (16S rRNA): position: 1207, m2G by RsmC_mono	1 b3278_RNA_RsmC_mono --> 1 RsmC_mono_inact + 1 ahcys + 1 b3278_RNA_1 + 1 h	irreversible	rRNA modification	0.00E+00	1.00E+09	0	1.06E+02	1.06E+02	0	0	0	0	0	0
b3278_v1_RNA_10_modif_1	b3278_v1_RNA modification (16S rRNA): position: 967, m5C by RsmB_mono	1 RsmB_mono + 1 amet + 1 b3278_v1_RNA_10 <==> 1 b3278_v1_RNA_10_RsmB_mono	reversible	rRNA modification	-1.00E+09	1.00E+09	0	1.06E+02	1.06E+02	0	1.15E+01	11.5	0	1.15E+01	11.5
b3278_v1_RNA_10_modif_2	b3278_v1_RNA modification (16S rRNA): position: 967, m5C by RsmB_mono	1 b3278_v1_RNA_10_RsmB_mono --> 1 RsmB_mono_inact + 1 ahcys + 1 h + 1 rrsD	irreversible	rRNA modification	0.00E+00	1.00E+09	0	1.06E+02	1.06E+02	0	1.15E+01	11.5	0	1.15E+01	11.5
b3278_v1_RNA_1_modif_1	b3278_v1_RNA modification (16S rRNA): position: 1402, m4Cm by MeT_16S_1402	1 MeT_16S_1402 + 2 amet + 1 b3278_v1_RNA_1 <==> 1 b3278_v1_RNA_1_MeT_16S_1402	reversible	rRNA modification	-1.00E+09	1.00E+09	0	1.06E+02	1.06E+02	0	1.15E+01	11.5	0	1.15E+01	11.5
b3278_v1_RNA_1_modif_2	b3278_v1_RNA modification (16S rRNA): position: 1402, m4Cm by MeT_16S_1402	1 b3278_v1_RNA_1_MeT_16S_1402 --> 1 MeT_16S_1402_inact + 2 ahcys + 1 b3278_v1_RNA_2 + 2 h	irreversible	rRNA modification	0.00E+00	1.00E+09	0	1.06E+02	1.06E+02	0	1.15E+01	11.5	0	1.15E+01	11.5
b3278_v1_RNA_2_modif_1	b3278_v1_RNA modification (16S rRNA): position: 1407, m5C by MeT_16S_1407	1 MeT_16S_1407 + 1 amet + 1 b3278_v1_RNA_2 <==> 1 b3278_v1_RNA_2_MeT_16S_1407	reversible	rRNA modification	-1.00E+09	1.00E+09	0	1.06E+02	1.06E+02	0	1.15E+01	11.5	0	1.15E+01	11.5
b3278_v1_RNA_2_modif_2	b3278_v1_RNA modification (16S rRNA): position: 1407, m5C by MeT_16S_1407	1 b3278_v1_RNA_2_MeT_16S_1407 --> 1 MeT_16S_1407_inact + 1 ahcys + 1 b3278_v1_RNA_3 + 1 h	irreversible	rRNA modification	0.00E+00	1.00E+09	0	1.06E+02	1.06E+02	0	1.15E+01	11.5	0	1.15E+01	11.5
b3278_v1_RNA_3_modif_1	b3278_v1_RNA modification (16S rRNA): position: 1498, m3U by YggJ_mono	1 YggJ_mono + 1 amet + 1 b3278_v1_RNA_3 <==> 1 b3278_v1_RNA_3_YggJ_mono	reversible	rRNA modification	-1.00E+09	1.00E+09	0	1.06E+02	1.06E+02	0	1.15E+01	11.5	0	1.15E+01	11.5
b3278_v1_RNA_3_modif_2	b3278_v1_RNA modification (16S rRNA): position: 1498, m3U by YggJ_mono	1 b3278_v1_RNA_3_YggJ_mono --> 1 YggJ_mono_inact + 1 ahcys + 1 b3278_v1_RNA_4 + 1 h	irreversible	rRNA modification	0.00E+00	1.00E+09	0	1.06E+02	1.06E+02	0	1.15E+01	11.5	0	1.15E+01	11.5
b3278_v1_RNA_4_modif_1	b3278_v1_RNA modification (16S rRNA): position: 1516, m2G by MeT_16S_1516	1 MeT_16S_1516 + 1 amet + 1 b3278_v1_RNA_4 <==> 1 b3278_v1_RNA_4_MeT_16S_1516	reversible	rRNA modification	-1.00E+09	1.00E+09	0	1.06E+02	1.06E+02	0	1.15E+01	11.5	0	1.15E+01	11.5
b3278_v1_RNA_4_modif_2	b3278_v1_RNA modification (16S rRNA): position: 1516, m2G by MeT_16S_1516	1 b3278_v1_RNA_4_MeT_16S_1516 --> 1 MeT_16S_1516_inact + 1 ahcys + 1 b3278_v1_RNA_5 + 1 h	irreversible	rRNA modification	0.00E+00	1.00E+09	0	1.06E+02	1.06E+02	0	1.15E+01	11.5	0	1.15E+01	11.5
b3278_v1_RNA_5_modif_1	b3278_v1_RNA modification (16S rRNA): position: 1518, m6(2)A by KsgA_mono	1 KsgA_mono + 2 amet + 1 b3278_v1_RNA_5 <==> 1 b3278_v1_RNA_5_KsgA_mono	reversible	rRNA modification	-1.00E+09	1.00E+09	0	1.06E+02	1.06E+02	0	1.15E+01	11.5	0	1.15E+01	11.5
b3278_v1_RNA_5_modif_2	b3278_v1_RNA modification (16S rRNA): position: 1518, m6(2)A by KsgA_mono	1 b3278_v1_RNA_5_KsgA_mono --> 1 KsgA_mono_inact + 2 ahcys + 1 b3278_v1_RNA_6 + 2 h	irreversible	rRNA modification	0.00E+00	1.00E+09	0	1.06E+02	1.06E+02	0	1.15E+01	11.5	0	1.15E+01	11.5
b3278_v1_RNA_6_modif_1	b3278_v1_RNA modification (16S rRNA): position: 1519, m6(2)A by KsgA_mono	1 KsgA_mono + 2 amet + 1 b3278_v1_RNA_6 <==> 1 b3278_v1_RNA_6_KsgA_mono	reversible	rRNA modification	-1.00E+09	1.00E+09	0	1.06E+02	1.06E+02	0	1.15E+01	11.5	0	1.15E+01	11.5
b3278_v1_RNA_6_modif_2	b3278_v1_RNA modification (16S rRNA): position: 1519, m6(2)A by KsgA_mono	1 b3278_v1_RNA_6_KsgA_mono --> 1 KsgA_mono_inact + 2 ahcys + 1 b3278_v1_RNA_7 + 2 h	irreversible	rRNA modification	0.00E+00	1.00E+09	0	1.06E+02	1.06E+02	0	1.15E+01	11.5	0	1.15E+01	11.5
b3278_v1_RNA_7_modif_1	b3278_v1_RNA modification (16S rRNA): position: 516, Y by RsuA_mono	1 RsuA_mono + 1 b3278_v1_RNA_7 <==> 1 b3278_v1_RNA_7_RsuA_mono	reversible	rRNA modification	-1.00E+09	1.00E+09	0	1.06E+02	1.06E+02	0	1.15E+01	11.5	0	1.15E+01	11.5

b3278_v1_RNA_7_modif_2	b3278_v1_RNA modification (16S rRNA): position: 516, Y by RsuA_mon	1 b3278_v1_RNA_7_RsuA_mono --> 1 RsuA_mono_inact + 1 b3278_v1_RNA_8	irreversible	rRNA modification	0.00E+00	1.00E+09	0	1.06E+02	1.06E+02	0	1.15E+01	11.5	0	1.15E+01	11.5
b3278_v1_RNA_8_modif_1	b3278_v1_RNA modification (16S rRNA): position: 527, m7G by MeT_16S_527	1 MeT_16S_527 + 1 amet + 1 b3278_v1_RNA_8 <==> 1 b3278_v1_RNA_8_MeT_16S_527	reversible	rRNA modification	-1.00E+09	1.00E+09	0	1.06E+02	1.06E+02	0	1.15E+01	11.5	0	1.15E+01	11.5
b3278_v1_RNA_8_modif_2	b3278_v1_RNA modification (16S rRNA): position: 527, m7G by MeT_16S_527	1 b3278_v1_RNA_8_MeT_16S_527 --> 1 MeT_16S_527_inact + 1 ahcys + 1 b3278_v1_RNA_9 + 1 h	irreversible	rRNA modification	0.00E+00	1.00E+09	0	1.06E+02	1.06E+02	0	1.15E+01	11.5	0	1.15E+01	11.5
b3278_v1_RNA_9_modif_1	b3278_v1_RNA modification (16S rRNA): position: 966, m2G by MeT_16S_966	1 MeT_16S_966 + 1 amet + 1 b3278_v1_RNA_9 <==> 1 b3278_v1_RNA_9_MeT_16S_966	reversible	rRNA modification	-1.00E+09	1.00E+09	0	1.06E+02	1.06E+02	0	1.15E+01	11.5	0	1.15E+01	11.5
b3278_v1_RNA_9_modif_2	b3278_v1_RNA modification (16S rRNA): position: 966, m2G by MeT_16S_966	1 b3278_v1_RNA_9_MeT_16S_966 --> 1 MeT_16S_966_inact + 1 ahcys + 1 b3278_v1_RNA_10 + 1 h	irreversible	rRNA modification	0.00E+00	1.00E+09	0	1.06E+02	1.06E+02	0	1.15E+01	11.5	0	1.15E+01	11.5
b3278_v1_RNA_modif_1	b3278_v1_RNA modification (16S rRNA): position: 1207, m2G by RsmC_mono	1 RsmC_mono + 1 amet + 1 b3278_v1_RNA <==> 1 b3278_v1_RNA_RsmC_mono	reversible	rRNA modification	-1.00E+09	1.00E+09	0	1.06E+02	1.06E+02	0	1.15E+01	11.5	0	1.15E+01	11.5
b3278_v1_RNA_modif_2	b3278_v1_RNA modification (16S rRNA): position: 1207, m2G by RsmC_mono	1 b3278_v1_RNA_RsmC_mono --> 1 RsmC_mono_inact + 1 ahcys + 1 b3278_v1_RNA_1 + 1 h	irreversible	rRNA modification	0.00E+00	1.00E+09	0	1.06E+02	1.06E+02	0	1.15E+01	11.5	0	1.15E+01	11.5
b3280_fold_spon	b3280_m folding: spontanous	1 b3280_m --> 1 YrdB_mono	irreversible	Protein Folding	0.00E+00	1.00E+09	0	4.07E+02	4.07E+02	0	6.47E+02	647.3271	0	9.41E+01	94.11
b3280_mRNA_CONV	convsersion of mRNA to mRNA_1 (synthetic rxn)	1 b3280_mRNA --> 1 b3280_mRNA_1	irreversible	Translation	0.00E+00	1.00E+09	0	6.27E+01	6.27E+01	0	1.11E+02	110.7654	0	1.22E-01	0.122
b3280_mRNA_degr1	Degradation of b3280_mRNA I	1 Orn_dim + 63 atp + 1 b3280_mRNA_2 + 1 degradosome + 63 h2o <==> 63 adp + 1 b3280_mRNA_2_degr + 63 h + 63 pi	reversible	mRNA degradation	-1.00E+09	1.00E+09	0	6.27E+01	6.27E+01	0	1.11E+02	110.7654	0	1.22E-01	0.122
b3280_mRNA_degr2	Degradation of b3280_mRNA II	1 b3280_mRNA_2_degr + 253 h2o --> 1 Orn_dim_inact + 70 amp + 50 cmp + 1 degradosome_inact + 75 gmp + 253 h + 59 ump	irreversible	mRNA degradation	0.00E+00	3.08E-01	0	6.27E+01	6.27E+01	0	1.11E+02	110.7654	0	1.22E-01	0.122
b3280_maturation1	Polypeptide b3280 peptide deformylas and methionine aminopeptidase complex	1 Def_mono + 1 Map_mono + 1 b3280_aa <==> 1 b3280_def_map_cplx	reversible	Protein Maturation	-1.00E+09	1.00E+09	0	4.07E+02	4.07E+02	0	6.47E+02	647.3271	0	9.41E+01	94.11
b3280_maturation2	b3280 formation	1 b3280_def_map_cplx + 2 h2o --> 1 Def_mono_inact + 1 Map_mono_inact + 1 b3280_m + 1 for + 1 met-L	irreversible	Protein Maturation	0.00E+00	1.00E+09	0	4.07E+02	4.07E+02	0	6.47E+02	647.3271	0	9.41E+01	94.11
b3281_fold_spon	b3281_m folding: spontanous	1 b3281_m --> 1 AroE_mono	irreversible	Protein Folding	0.00E+00	1.00E+09	0	1.08E+03	1.08E+03	0	1.84E+03	1843.607	0	7.33E+01	73.312
b3281_mRNA_CONV	convsersion of mRNA to mRNA_1 (synthetic rxn)	1 b3281_mRNA --> 1 b3281_mRNA_1	irreversible	Translation	0.00E+00	1.00E+09	0	6.27E+01	6.27E+01	0	1.11E+02	110.7654	0	1.22E-01	0.122
b3281_mRNA_degr1	Degradation of b3281_mRNA I	1 Orn_dim + 204 atp + 1 b3281_mRNA_2 + 1 degradosome + 204 h2o <==> 204 adp + 1 b3281_mRNA_2_degr + 204 h + 204 pi	reversible	mRNA degradation	-1.00E+09	1.00E+09	0	6.27E+01	6.27E+01	0	1.11E+02	110.7654	0	1.22E-01	0.122
b3281_mRNA_degr2	Degradation of b3281_mRNA II	1 b3281_mRNA_2_degr + 818 h2o --> 1 Orn_dim_inact + 188 amp + 183 cmp + 1 degradosome_inact + 218 gmp + 818 h + 230 ump	irreversible	mRNA degradation	0.00E+00	1.22E-01	0	6.27E+01	6.27E+01	0	1.11E+02	110.7654	0	1.22E-01	0.122
b3281_maturation1	Polypeptide b3281 peptide deformylas complex	1 Def_mono + 1 b3281_aa <==> 1 b3281_def_cplx	reversible	Protein Maturation	-1.00E+09	1.00E+09	0	1.08E+03	1.08E+03	0	1.84E+03	1843.607	0	7.33E+01	73.312
b3281_maturation2	b3281 formation	1 b3281_def_cplx + 1 h2o --> 1 Def_mono_inact + 1 b3281_m + 1 for	irreversible	Protein Maturation	0.00E+00	1.00E+09	0	1.08E+03	1.08E+03	0	1.84E+03	1843.607	0	7.33E+01	73.312
b3282_fold_spon	b3282_m folding: spontanous	1 b3282_m --> 1 YrdC_mono	irreversible	Protein Folding	0.00E+00	1.00E+09	0	7.82E+02	7.82E+02	0	1.31E+03	1310.16	0	9.31E+01	93.082
b3282_mRNA_CONV	convsersion of mRNA to mRNA_1 (synthetic rxn)	1 b3282_mRNA --> 1 b3282_mRNA_1	irreversible	Translation	0.00E+00	1.00E+09	0	6.27E+01	6.27E+01	0	1.11E+02	110.7654	0	1.22E-01	0.122
b3282_mRNA_degr1	Degradation of b3282_mRNA I	1 Orn_dim + 143 atp + 1 b3282_mRNA_2 + 1 degradosome + 143 h2o <==> 143 adp + 1 b3282_mRNA_2_degr + 143 h + 143 pi	reversible	mRNA degradation	-1.00E+09	1.00E+09	0	6.27E+01	6.27E+01	0	1.11E+02	110.7654	0	1.22E-01	0.122
b3282_mRNA_degr2	Degradation of b3282_mRNA II	1 b3282_mRNA_2_degr + 572 h2o --> 1 Orn_dim_inact + 126 amp + 140 cmp + 1 degradosome_inact + 159 gmp + 572 h + 148 ump	irreversible	mRNA degradation	0.00E+00	3.99E-01	0	6.27E+01	6.27E+01	0	1.11E+02	110.7654	0	1.22E-01	0.122
b3282_maturation1	Polypeptide b3282 peptide deformylas and methionine aminopeptidase complex	1 Def_mono + 1 Map_mono + 1 b3282_aa <==> 1 b3282_def_map_cplx	reversible	Protein Maturation	-1.00E+09	1.00E+09	0	7.82E+02	7.82E+02	0	1.31E+03	1310.16	0	9.31E+01	93.082
b3282_maturation2	b3282 formation	1 b3282_def_map_cplx + 2 h2o --> 1 Def_mono_inact + 1 Map_mono_inact + 1 b3282_m + 1 for + 1 met-L	irreversible	Protein Maturation	0.00E+00	1.00E+09	0	7.82E+02	7.82E+02	0	1.31E+03	1310.16	0	9.31E+01	93.082
b3283_fold_spon	b3283_m folding: spontanous	1 b3283_m --> 1 YrdD_mono	irreversible	Protein Folding	0.00E+00	1.00E+09	0	7.10E+02	7.10E+02	0	1.19E+03	1190.554	0	8.41E+01	84.12
b3283_mRNA_CONV	convsersion of mRNA to mRNA_1 (synthetic rxn)	1 b3283_mRNA --> 1 b3283_mRNA_1	irreversible	Translation	0.00E+00	1.00E+09	0	6.27E+01	6.27E+01	0	1.11E+02	110.7654	0	1.22E-01	0.122
b3283_mRNA_degr1	Degradation of b3283_mRNA I	1 Orn_dim + 135 atp + 1 b3283_mRNA_2 + 1 degradosome + 135 h2o <==> 135 adp + 1 b3283_mRNA_2_degr + 135 h + 135 pi	reversible	mRNA degradation	-1.00E+09	1.00E+09	0	6.27E+01	6.27E+01	0	1.11E+02	110.7654	0	1.22E-01	0.122
b3283_mRNA_degr2	Degradation of b3283_mRNA II	1 b3283_mRNA_2_degr + 542 h2o --> 1 Orn_dim_inact + 137 amp + 1 atp + 134 cmp + 1 degradosome_inact + 136 gmp + 542 h + 135 ump	irreversible	mRNA degradation	0.00E+00	3.26E-01	0	6.27E+01	6.27E+01	0	1.11E+02	110.7654	0	1.22E-01	0.122
b3283_maturation1	Polypeptide b3283 peptide deformylas and methionine aminopeptidase complex	1 Def_mono + 1 Map_mono + 1 b3283_aa <==> 1 b3283_def_map_cplx	reversible	Protein Maturation	-1.00E+09	1.00E+09	0	7.10E+02	7.10E+02	0	1.19E+03	1190.554	0	8.41E+01	84.12
b3283_maturation2	b3283 formation	1 b3283_def_map_cplx + 2 h2o --> 1 Def_mono_inact + 1 Map_mono_inact + 1 b3283_m + 1 for + 1 met-L	irreversible	Protein Maturation	0.00E+00	1.00E+09	0	7.10E+02	7.10E+02	0	1.19E+03	1190.554	0	8.41E+01	84.12
b3287_fold_spon	b3287_m folding: spontanous	1 b3287_m_Fe --> 1 Def_mono	irreversible	Protein Folding	0.00E+00	1.00E+09	0	9.25E+02	9.25E+02	0	1.55E+03	1550.424	0	1.11E+02	111.456
b3287_ion_BIND	Binding of ions to b3287	1 b3287_m + 1 fe2 <==> 1 b3287_m_Fe	reversible	Metallo-ion Binding	-1.00E+09	1.00E+09	0	9.25E+02	9.25E+02	0	1.55E+03	1550.424	0	1.11E+02	111.456
b3287_mRNA_CONV	convsersion of mRNA to mRNA_1 (synthetic rxn)	1 b3287_mRNA --> 1 b3287_mRNA_1	irreversible	Translation	0.00E+00	1.00E+09	0	9.07E+01	9.07E+01	0	1.60E+02	160.1693	0	2.84E-01	0.284
b3287_mRNA_degr1	Degradation of b3287_mRNA I	1 Orn_dim + 127 atp + 1 b3287_mRNA_2 + 1 degradosome + 127 h2o <==> 127 adp + 1 b3287_mRNA_2_degr + 127 h + 127 pi	reversible	mRNA degradation	-1.00E+09	1.00E+09	0	9.07E+01	9.07E+01	0	1.60E+02	160.1693	0	2.84E-01	0.284
b3287_mRNA_degr2	Degradation of b3287_mRNA II	1 b3287_mRNA_2_degr + 509 h2o --> 1 Orn_dim_inact + 148 amp + 1 atp + 105 cmp + 1 degradosome_inact + 131 gmp + 509 h + 125 ump	irreversible	mRNA degradation	0.00E+00	2.84E-01	0	9.07E+01	9.07E+01	0	1.60E+02	160.1693	0	2.84E-01	0.284
b3287_maturation1	Polypeptide b3287 peptide deformylas and methionine aminopeptidase complex	1 Def_mono + 1 Map_mono + 1 b3287_aa <==> 1 b3287_def_map_cplx	reversible	Protein Maturation	-1.00E+09	1.00E+09	0	9.25E+02	9.25E+02	0	1.55E+03	1550.424	0	1.11E+02	111.456
b3287_maturation2	b3287 formation	1 b3287_def_map_cplx + 2 h2o --> 1 Def_mono_inact + 1 Map_mono_inact + 1 b3287_m + 1 for + 1 met-L	irreversible	Protein Maturation	0.00E+00	1.00E+09	0	9.25E+02	9.25E+02	0	1.55E+03	1550.424	0	1.11E+02	111.456
b3288_fold_spon	b3288_m folding: spontanous	1 b3288_m --> 1 Fmt_mono	irreversible	Protein Folding	0.00E+00	1.00E+09	0	1.71E+03	1.71E+03	0	2.96E+03	2962.967	0	8.50E+01	85.032
b3288_mRNA_CONV	convsersion of mRNA to mRNA_1 (synthetic rxn)	1 b3288_mRNA --> 1 b3288_mRNA_1	irreversible	Translation	0.00E+00	1.00E+09	0	9.07E+01	9.07E+01	0	1.60E+02	160.1693	0	2.84E-01	0.284
b3288_mRNA_degr1	Degradation of b3288_mRNA I	1 Orn_dim + 237 atp + 1 b3288_mRNA_2 + 1 degradosome + 237 h2o <==> 237 adp + 1 b3288_mRNA_2_degr + 237 h + 237 pi	reversible	mRNA degradation	-1.00E+09	1.00E+09	0	9.07E+01	9.07E+01	0	1.60E+02	160.1693	0	2.84E-01	0.284
b3288_mRNA_degr2	Degradation of b3288_mRNA II	1 b3288_mRNA_2_degr + 947 h2o --> 1 Orn_dim_inact + 229 amp + 245 cmp + 1 degradosome_inact + 256 gmp + 947 h + 218 ump	irreversible	mRNA degradation	0.00E+00	3.37E-01	0	9.07E+01	9.07E+01	0	1.60E+02	160.1693	0	2.84E-01	0.284
b3288_maturation1	Polypeptide b3288 peptide deformylas and methionine aminopeptidase complex	1 Def_mono + 1 Map_mono + 1 b3288_aa <==> 1 b3288_def_map_cplx	reversible	Protein Maturation	-1.00E+09	1.00E+09	0	1.71E+03	1.71E+03	0	2.96E+03	2962.967	0	8.50E+01	85.032
b3288_maturation2	b3288 formation	1 b3288_def_map_cplx + 2 h2o --> 1 Def_mono_inact + 1 Map_mono_inact + 1 b3288_m + 1 for + 1 met-L	irreversible	Protein Maturation	0.00E+00	1.00E+09	0	1.71E+03	1.71E+03	0	2.96E+03	2962.967	0	8.50E+01	85.032
b3289_DNA_act_bind	b3289_DNA binding of activator	1 b3289_DNA_neu --> 1 b3289_DNA_act	irreversible	Transcription Regulation	0.00E+00	1.00E+09	0	1.01E+02	1.01E+02	0	1.78E+02	177.8536	0	3.17E-01	0.317
b3289_fold_spon	b3289_m folding: spontanous	1 b3289_m --> 1 RsmB_mono	irreversible	Protein Folding	0.00E+00	1.00E+09	0	2.59E+03	2.59E+03	0	4.52E+03	4517.09	0	7.87E+01	78.675

b3289_mRNA_CONV	convsersion of mRNA to mRNA_1 (synthetic rxn)	1 b3289_mRNA --> 1 b3289_mRNA_1	irreversible	Translation	0.00E+00	1.00E+09	0	1.01E+02	1.01E+02	0	1.78E+02	177.8536	0	3.17E-01	0.317
b3289_mRNA_degr1	Degradation of b3289_mRNA I	1 Orn_dim + 322 atp + 1 b3289_mRNA_2 + 1 degradosome + 322 h2o <==> 322 adp + 1 b3289_mRNA_2_degr + 322 h + 322 pi	reversible	mRNA degradation	-1.00E+09	1.00E+09	0	1.01E+02	1.01E+02	0	1.78E+02	177.8536	0	3.17E-01	0.317
b3289_mRNA_degr2	Degradation of b3289_mRNA II	1 b3289_mRNA_2_degr + 1289 h2o --> 1 Orn_dim_inact + 332 amp 1 atp + 317 cmp + 1 degradosome_inact + 324 gmp + 1289 h + 316 ump	irreversible	mRNA degradation	0.00E+00	3.17E-01	0	1.01E+02	1.01E+02	0	1.78E+02	177.8536	0	3.17E-01	0.317
b3289_maturation1	Polypeptide b3289 peptide deformylas complex	1 Def_mono + 1 b3289_aa <==> 1 b3289_def_cplx	reversible	Protein Maturation	-1.00E+09	1.00E+09	0	2.59E+03	2.59E+03	0	4.52E+03	4517.09	0	7.87E+01	78.675
b3289_maturation2	b3289 formation	1 b3289_def_cplx + 1 h2o --> 1 Def_mono_inact + 1 b3289_m + 1 for	irreversible	Protein Maturation	0.00E+00	1.00E+09	0	2.59E+03	2.59E+03	0	4.52E+03	4517.09	0	7.87E+01	78.675
b3294_fold_spon	b3294_m folding: spontanous	1 b3294_m --> 1 RplQ_mono	irreversible	Protein Folding	0.00E+00	1.00E+09	8.44E+01	1.13E+02	2.81E+01	6.22E+01	8.29E+01	20.72	6.22E+01	8.29E+01	20.72
b3294_mRNA_CONV	convsersion of mRNA to mRNA_1 (synthetic rxn)	1 b3294_mRNA --> 1 b3294_mRNA_1	irreversible	Translation	0.00E+00	1.00E+09	0	4.61E+01	4.61E+01	0	6.94E+01	69.42961	0	4.02E-01	0.402
b3294_mRNA_degr1	Degradation of b3294_mRNA I	1 Orn_dim + 96 atp + 1 b3294_mRNA_2 + 1 degradosome + 96 h2o <==> 96 adp + 1 b3294_mRNA_2_degr + 96 h + 96 pi	reversible	mRNA degradation	-1.00E+09	1.00E+09	0	4.61E+01	4.61E+01	0	6.94E+01	69.42961	0	4.02E-01	0.402
b3294_mRNA_degr2	Degradation of b3294_mRNA II	1 b3294_mRNA_2_degr + 383 h2o --> 1 Orn_dim_inact + 90 amp + 99 cmp + 1 degradosome_inact + 107 gmp + 383 h + 88 ump	irreversible	mRNA degradation	0.00E+00	4.73E-01	0	4.61E+01	4.61E+01	0	6.94E+01	69.42961	0	4.02E-01	0.402
b3294_maturation1	Polypeptide b3294 peptide deformylas complex	1 Def_mono + 1 b3294_aa <==> 1 b3294_def_cplx	reversible	Protein Maturation	-1.00E+09	1.00E+09	8.44E+01	1.13E+02	2.81E+01	6.22E+01	8.29E+01	20.72	6.22E+01	8.29E+01	20.72
b3294_maturation2	b3294 formation	1 b3294_def_cplx + 1 h2o --> 1 Def_mono_inact + 1 b3294_m + 1 for	irreversible	Protein Maturation	0.00E+00	1.00E+09	8.44E+01	1.13E+02	2.81E+01	6.22E+01	8.29E+01	20.72	6.22E+01	8.29E+01	20.72
b3295_fold_GroEL/ES_1	b3295_m folding: GroEL/ES mediated: polypeptide is going in GroEL/ES complex	1 GroEL.(7)ADP.cisGroES + 7 atp + 1 b3295_m + 1 transGroES_hepta --> 7 adp + 1 b3295_m_GroEL.(7)ATP.transGroES + 1 cisGroES_hepta	irreversible	Protein Folding	0.00E+00	1.00E+09	0	9.65E+02	9.65E+02	0	1.30E+03	1301.275	0	2.05E+02	204.836
b3295_fold_GroEL/ES_2	b3295_m folding: GroEL/ES mediated folding of polypeptide under ATP hydrolysis	1 b3295_m_GroEL.(7)ATP.transGroES + 7 h2o --> 1 RpoA_GroEL.(7)ADP.transGroES + 7 h + 7 pi	irreversible	Protein Folding	0.00E+00	1.00E+09	0	9.65E+02	9.65E+02	0	1.30E+03	1301.275	0	2.05E+02	204.836
b3295_fold_GroEL/ES_3	b3295_m folding: GroEL/ES mediated release of native protein	1 RpoA_GroEL.(7)ADP.transGroES --> 1 GroEL.(7)ADP.transGroES + 1 RpoA_mono	irreversible	Protein Folding	0.00E+00	1.00E+09	0	9.65E+02	9.65E+02	0	1.30E+03	1301.275	0	2.05E+02	204.836
b3295_fold_KJE_1	b3295_m folding: KJE mediated	1 DnaJ_dim + 1 DnaK_mono.ATP + 1 b3295_m <==> 1 b3295_m_DnaKJ_complex	reversible	Protein Folding	-1.00E+09	1.00E+09	0	9.65E+02	9.65E+02	0	1.30E+03	1301.275	0	2.05E+02	204.836
b3295_fold_KJE_2	b3295_m folding: KJE mediated	1 GrpE_dim + 1 b3295_m_DnaKJ_complex + 1 h2o --> 1 DnaJ_dim_inact + 1 RpoA_DnaK_GrpE_complex + 1 adp + 1 h + 1 pi	irreversible	Protein Folding	0.00E+00	1.00E+09	0	9.65E+02	9.65E+02	0	1.30E+03	1301.275	0	2.05E+02	204.836
b3295_fold_KJE_3	b3295_m folding: KJE mediated	1 RpoA_DnaK_GrpE_complex + 1 atp --> 1 DnaK_mono.ATP_inact + 1 GrpE_dim_inact + 1 RpoA_mono	irreversible	Protein Folding	0.00E+00	1.00E+09	0	9.65E+02	9.65E+02	0	1.30E+03	1301.275	0	2.05E+02	204.836
b3295_fold_spon	b3295_m folding: spontanous	1 b3295_m --> 1 RpoA_mono	irreversible	Protein Folding	0.00E+00	1.00E+09	0	9.65E+02	9.65E+02	0	1.30E+03	1301.275	0	2.05E+02	204.836
b3295_mRNA_CONV	convsersion of mRNA to mRNA_1 (synthetic rxn)	1 b3295_mRNA --> 1 b3295_mRNA_1	irreversible	Translation	0.00E+00	1.00E+09	0	4.61E+01	4.61E+01	0	6.94E+01	69.42961	0	4.02E-01	0.402
b3295_mRNA_degr1	Degradation of b3295_mRNA I	1 Orn_dim + 247 atp + 1 b3295_mRNA_2 + 1 degradosome + 247 h2o <==> 247 adp + 1 b3295_mRNA_2_degr + 247 h + 247 pi	reversible	mRNA degradation	-1.00E+09	1.00E+09	0	4.61E+01	4.61E+01	0	6.94E+01	69.42961	0	4.02E-01	0.402
b3295_mRNA_degr2	Degradation of b3295_mRNA II	1 b3295_mRNA_2_degr + 989 h2o --> 1 Orn_dim_inact + 244 amp + 246 cmp + 1 degradosome_inact + 271 gmp + 989 h + 229 ump	irreversible	mRNA degradation	0.00E+00	6.62E-01	0	4.61E+01	4.61E+01	0	6.94E+01	69.42961	0	4.02E-01	0.402
b3295_maturation1	Polypeptide b3295 peptide deformylas complex	1 Def_mono + 1 b3295_aa <==> 1 b3295_def_cplx	reversible	Protein Maturation	-1.00E+09	1.00E+09	0	9.65E+02	9.65E+02	0	1.30E+03	1301.275	0	2.05E+02	204.836
b3295_maturation2	b3295 formation	1 b3295_def_cplx + 1 h2o --> 1 Def_mono_inact + 1 b3295_m + 1 for	irreversible	Protein Maturation	0.00E+00	1.00E+09	0	9.65E+02	9.65E+02	0	1.30E+03	1301.275	0	2.05E+02	204.836
b3296_fold_spon	b3296_m folding: spontanous	1 b3296_m --> 1 RpsD_mono	irreversible	Protein Folding	0.00E+00	1.00E+09	8.44E+01	1.13E+02	2.81E+01	6.22E+01	8.29E+01	20.72	6.22E+01	8.29E+01	20.72
b3296_mRNA_CONV	convsersion of mRNA to mRNA_1 (synthetic rxn)	1 b3296_mRNA --> 1 b3296_mRNA_1	irreversible	Translation	0.00E+00	1.00E+09	0	4.61E+01	4.61E+01	0	6.94E+01	69.42961	0	4.02E-01	0.402
b3296_mRNA_degr1	Degradation of b3296_mRNA I	1 Orn_dim + 155 atp + 1 b3296_mRNA_2 + 1 degradosome + 155 h2o <==> 155 adp + 1 b3296_mRNA_2_degr + 155 h + 155 pi	reversible	mRNA degradation	-1.00E+09	1.00E+09	0	4.61E+01	4.61E+01	0	6.94E+01	69.42961	0	4.02E-01	0.402
b3296_mRNA_degr2	Degradation of b3296_mRNA II	1 b3296_mRNA_2_degr + 620 h2o --> 1 Orn_dim_inact + 158 amp + 139 cmp + 1 degradosome_inact + 176 gmp + 620 h + 148 ump	irreversible	mRNA degradation	0.00E+00	6.62E-01	0	4.61E+01	4.61E+01	0	6.94E+01	69.42961	0	4.02E-01	0.402
b3296_maturation1	Polypeptide b3296 peptide deformylas and methionine aminopeptidase complex	1 Def_mono + 1 Map_mono + 1 b3296_aa <==> 1 b3296_def_map_cplx	reversible	Protein Maturation	-1.00E+09	1.00E+09	8.44E+01	1.13E+02	2.81E+01	6.22E+01	8.29E+01	20.72	6.22E+01	8.29E+01	20.72
b3296_maturation2	b3296 formation	1 b3296_def_map_cplx + 2 h2o --> 1 Def_mono_inact + 1 Map_mono_inact + 1 b3296_m + 1 for + 1 met-L	irreversible	Protein Maturation	0.00E+00	1.00E+09	8.44E+01	1.13E+02	2.81E+01	6.22E+01	8.29E+01	20.72	6.22E+01	8.29E+01	20.72
b3297_fold_spon	b3297_m folding: spontanous	1 b3297_m --> 1 RpsK_mono	irreversible	Protein Folding	0.00E+00	1.00E+09	8.44E+01	1.13E+02	2.81E+01	6.22E+01	8.29E+01	20.72	6.22E+01	8.29E+01	20.72
b3297_mRNA_CONV	convsersion of mRNA to mRNA_1 (synthetic rxn)	1 b3297_mRNA --> 1 b3297_mRNA_1	irreversible	Translation	0.00E+00	1.00E+09	0	4.61E+01	4.61E+01	0	6.94E+01	69.42961	0	4.02E-01	0.402
b3297_mRNA_degr1	Degradation of b3297_mRNA I	1 Orn_dim + 97 atp + 1 b3297_mRNA_2 + 1 degradosome + 97 h2o <==> 97 adp + 1 b3297_mRNA_2_degr + 97 h + 97 pi	reversible	mRNA degradation	-1.00E+09	1.00E+09	0	4.61E+01	4.61E+01	0	6.94E+01	69.42961	0	4.02E-01	0.402
b3297_mRNA_degr2	Degradation of b3297_mRNA II	1 b3297_mRNA_2_degr + 389 h2o --> 1 Orn_dim_inact + 95 amp + 102 cmp + 1 degradosome_inact + 99 gmp + 389 h + 94 ump	irreversible	mRNA degradation	0.00E+00	4.02E-01	0	4.61E+01	4.61E+01	0	6.94E+01	69.42961	0	4.02E-01	0.402
b3297_maturation1	Polypeptide b3297 peptide deformylas and methionine aminopeptidase complex	1 Def_mono + 1 Map_mono + 1 b3297_aa <==> 1 b3297_def_map_cplx	reversible	Protein Maturation	-1.00E+09	1.00E+09	8.44E+01	1.13E+02	2.81E+01	6.22E+01	8.29E+01	20.72	6.22E+01	8.29E+01	20.72
b3297_maturation2	b3297 formation	1 b3297_def_map_cplx + 2 h2o --> 1 Def_mono_inact + 1 Map_mono_inact + 1 b3297_m + 1 for + 1 met-L	irreversible	Protein Maturation	0.00E+00	1.00E+09	8.44E+01	1.13E+02	2.81E+01	6.22E+01	8.29E+01	20.72	6.22E+01	8.29E+01	20.72
b3298_fold_spon	b3298_m folding: spontanous	1 b3298_m --> 1 RpsM_mono	irreversible	Protein Folding	0.00E+00	1.00E+09	8.44E+01	1.13E+02	2.81E+01	6.22E+01	8.29E+01	20.72	6.22E+01	8.29E+01	20.72
b3298_mRNA_CONV	convsersion of mRNA to mRNA_1 (synthetic rxn)	1 b3298_mRNA --> 1 b3298_mRNA_1	irreversible	Translation	0.00E+00	1.00E+09	0	4.61E+01	4.61E+01	0	6.94E+01	69.42961	0	4.02E-01	0.402
b3298_mRNA_degr1	Degradation of b3298_mRNA I	1 Orn_dim + 89 atp + 1 b3298_mRNA_2 + 1 degradosome + 89 h2o <==> 89 adp + 1 b3298_mRNA_2_degr + 89 h + 89 pi	reversible	mRNA degradation	-1.00E+09	1.00E+09	0	4.61E+01	4.61E+01	0	6.94E+01	69.42961	0	4.02E-01	0.402
b3298_mRNA_degr2	Degradation of b3298_mRNA II	1 b3298_mRNA_2_degr + 356 h2o --> 1 Orn_dim_inact + 88 amp + 89 cmp + 1 degradosome_inact + 95 gmp + 1 gtp + 356 h + 84 ump	irreversible	mRNA degradation	0.00E+00	1.11E+00	0	4.61E+01	4.61E+01	0	6.94E+01	69.42961	0	4.02E-01	0.402
b3298_maturation1	Polypeptide b3298 peptide deformylas and methionine aminopeptidase complex	1 Def_mono + 1 Map_mono + 1 b3298_aa <==> 1 b3298_def_map_cplx	reversible	Protein Maturation	-1.00E+09	1.00E+09	8.44E+01	1.13E+02	2.81E+01	6.22E+01	8.29E+01	20.72	6.22E+01	8.29E+01	20.72
b3298_maturation2	b3298 formation	1 b3298_def_map_cplx + 2 h2o --> 1 Def_mono_inact + 1 Map_mono_inact + 1 b3298_m + 1 for + 1 met-L	irreversible	Protein Maturation	0.00E+00	1.00E+09	8.44E+01	1.13E+02	2.81E+01	6.22E+01	8.29E+01	20.72	6.22E+01	8.29E+01	20.72
b3299_fold_spon	b3299_m folding: spontanous	1 b3299_m --> 1 RpmJ_mono	irreversible	Protein Folding	0.00E+00	1.00E+09	8.44E+01	1.13E+02	2.81E+01	6.22E+01	8.29E+01	20.72	6.22E+01	8.29E+01	20.72
b3299_mRNA_CONV	convsersion of mRNA to mRNA_1 (synthetic rxn)	1 b3299_mRNA --> 1 b3299_mRNA_1	irreversible	Translation	0.00E+00	1.00E+09	0	2.46E+01	2.46E+01	0	4.34E+01	43.43574	0	1.46E-01	0.146
b3299_mRNA_degr1	Degradation of b3299_mRNA I	1 Orn_dim + 29 atp + 1 b3299_mRNA_2 + 1 degradosome + 29 h2o <==> 29 adp + 1 b3299_mRNA_2_degr + 29 h + 29 pi	reversible	mRNA degradation	-1.00E+09	1.00E+09	0	2.46E+01	2.46E+01	0	4.34E+01	43.43574	0	1.46E-01	0.146
b3299_mRNA_degr2	Degradation of b3299_mRNA II	1 b3299_mRNA_2_degr + 116 h2o --> 1 Orn_dim_inact + 33 amp + 25 cmp + 1 degradosome_inact + 31 gmp + 116 h + 28 ump	irreversible	mRNA degradation	0.00E+00	5.99E-01	0	2.46E+01	2.46E+01	0	4.34E+01	43.43574	0	1.46E-01	0.146
b3299_maturation1	Polypeptide b3299 peptide deformylas complex	1 Def_mono + 1 b3299_aa <==> 1 b3299_def_cplx	reversible	Protein Maturation	-1.00E+09	1.00E+09	8.44E+01	1.13E+02	2.81E+01	6.22E+01	8.29E+01	20.72	6.22E+01	8.29E+01	20.72
b3299_maturation2	b3299 formation	1 b3299_def_cplx + 1 h2o --> 1 Def_mono_inact + 1 b3299_m + 1 for	irreversible	Protein Maturation	0.00E+00	1.00E+09	8.44E+01	1.13E+02	2.81E+01	6.22E+01	8.29E+01	20.72	6.22E+01	8.29E+01	20.72
b3300_fold_spon	b3300_m folding: spontanous	1 b3300_m --> 1 SecY_mono	irreversible	Protein Folding	0.00E+00	1.00E+09	0	8.26E+02	8.26E+02	0	1.32E+03	1315.489	0	1.90E+02	189.956
b3300_mRNA_CONV	convsersion of mRNA to mRNA_1 (synthetic rxn)	1 b3300_mRNA --> 1 b3300_mRNA_1	irreversible	Translation	0.00E+00	1.00E+09	0	2.46E+01	2.46E+01	0	4.34E+01	43.43574	0	1.46E-01	0.146

b3300_mRNA_degr1	Degradation of b3300_mRNA I	1 Orn_dim + 333 atp + 1 b3300_mRNA_2 + 1 degradosome + 333 h2o <==> 333 adp + 1 b3300_mRNA_2_degr + 333 h + 333 pi	reversible	mRNA degradation	-1.00E+09	1.00E+09	0	2.46E+01	2.46E+01	0	4.34E+01	43.43574	0	1.46E-01	0.146
b3300_mRNA_degr2	Degradation of b3300_mRNA II	1 b3300_mRNA_2_degr + 1331 h2o --> 1 Orn_dim_inact + 286 amp + 312 cmp + 1 degradosome_inact + 350 gmp + 1331 h + 384 ump	irreversible	mRNA degradation	0.00E+00	1.46E-01	0	2.46E+01	2.46E+01	0	4.34E+01	43.43574	0	1.46E-01	0.146
b3300_maturation1	Polypeptide b3300 peptide deformylas and methionine aminopeptidase complex	1 Def_mono + 1 Map_mono + 1 b3300_aa <==> 1 b3300_def_map_cplx	reversible	Protein Maturation	-1.00E+09	1.00E+09	0	8.26E+02	8.26E+02	0	1.32E+03	1315.489	0	1.90E+02	189.956
b3300_maturation2	b3300 formation	1 b3300_def_map_cplx + 2 h2o --> 1 Def_mono_inact + 1 Map_mono_inact + 1 b3300_m + 1 for + 1 met-L	irreversible	Protein Maturation	0.00E+00	1.00E+09	0	8.26E+02	8.26E+02	0	1.32E+03	1315.489	0	1.90E+02	189.956
b3301_fold_spon	b3301_m folding: spontanous	1 b3301_m --> 1 RplO_mono	irreversible	Protein Folding	0.00E+00	1.00E+09	8.44E+01	1.13E+02	2.81E+01	6.22E+01	8.29E+01	20.72	6.22E+01	8.29E+01	20.72
b3301_mRNA_CONV	convsersion of mRNA to mRNA_1 (synthetic rxn)	1 b3301_mRNA --> 1 b3301_mRNA_1	irreversible	Translation	0.00E+00	1.00E+09	0	2.46E+01	2.46E+01	0	4.34E+01	43.43574	0	1.46E-01	0.146
b3301_mRNA_degr1	Degradation of b3301_mRNA I	1 Orn_dim + 108 atp + 1 b3301_mRNA_2 + 1 degradosome + 108 h2o <==> 108 adp + 1 b3301_mRNA_2_degr + 108 h + 108 pi	reversible	mRNA degradation	-1.00E+09	1.00E+09	0	2.46E+01	2.46E+01	0	4.34E+01	43.43574	0	1.46E-01	0.146
b3301_mRNA_degr2	Degradation of b3301_mRNA II	1 b3301_mRNA_2_degr + 434 h2o --> 1 Orn_dim_inact + 97 amp + 102 cmp + 1 degradosome_inact + 131 gmp + 434 h + 105 ump	irreversible	mRNA degradation	0.00E+00	3.37E-01	0	2.46E+01	2.46E+01	0	4.34E+01	43.43574	0	1.46E-01	0.146
b3301_maturation1	Polypeptide b3301 peptide deformylas complex	1 Def_mono + 1 b3301_aa <==> 1 b3301_def_cplx	reversible	Protein Maturation	-1.00E+09	1.00E+09	8.44E+01	1.13E+02	2.81E+01	6.22E+01	8.29E+01	20.72	6.22E+01	8.29E+01	20.72
b3301_maturation2	b3301 formation	1 b3301_def_cplx + 1 h2o --> 1 Def_mono_inact + 1 b3301_m + 1 for	irreversible	Protein Maturation	0.00E+00	1.00E+09	8.44E+01	1.13E+02	2.81E+01	6.22E+01	8.29E+01	20.72	6.22E+01	8.29E+01	20.72
b3302_fold_spon	b3302_m folding: spontanous	1 b3302_m --> 1 RpmD_mono	irreversible	Protein Folding	0.00E+00	1.00E+09	8.44E+01	1.13E+02	2.81E+01	6.22E+01	8.29E+01	20.72	6.22E+01	8.29E+01	20.72
b3302_mRNA_CONV	convsersion of mRNA to mRNA_1 (synthetic rxn)	1 b3302_mRNA --> 1 b3302_mRNA_1	irreversible	Translation	0.00E+00	1.00E+09	0	2.46E+01	2.46E+01	0	4.34E+01	43.43574	0	1.46E-01	0.146
b3302_mRNA_degr1	Degradation of b3302_mRNA I	1 Orn_dim + 45 atp + 1 b3302_mRNA_2 + 1 degradosome + 45 h2o <==> 45 adp + 1 b3302_mRNA_2_degr + 45 h + 45 pi	reversible	mRNA degradation	-1.00E+09	1.00E+09	0	2.46E+01	2.46E+01	0	4.34E+01	43.43574	0	1.46E-01	0.146
b3302_mRNA_degr2	Degradation of b3302_mRNA II	1 b3302_mRNA_2_degr + 179 h2o --> 1 Orn_dim_inact + 46 amp + 43 cmp + 1 degradosome_inact + 47 gmp + 179 h + 44 ump	irreversible	mRNA degradation	0.00E+00	2.90E-01	0	2.46E+01	2.46E+01	0	4.34E+01	43.43574	0	1.46E-01	0.146
b3302_maturation1	Polypeptide b3302 peptide deformylas and methionine aminopeptidase complex	1 Def_mono + 1 Map_mono + 1 b3302_aa <==> 1 b3302_def_map_cplx	reversible	Protein Maturation	-1.00E+09	1.00E+09	8.44E+01	1.13E+02	2.81E+01	6.22E+01	8.29E+01	20.72	6.22E+01	8.29E+01	20.72
b3302_maturation2	b3302 formation	1 b3302_def_map_cplx + 2 h2o --> 1 Def_mono_inact + 1 Map_mono_inact + 1 b3302_m + 1 for + 1 met-L	irreversible	Protein Maturation	0.00E+00	1.00E+09	8.44E+01	1.13E+02	2.81E+01	6.22E+01	8.29E+01	20.72	6.22E+01	8.29E+01	20.72
b3303_fold_spon	b3303_m folding: spontanous	1 b3303_m --> 1 RpsE_mono	irreversible	Protein Folding	0.00E+00	1.00E+09	8.44E+01	1.13E+02	2.81E+01	6.22E+01	8.29E+01	20.72	6.22E+01	8.29E+01	20.72
b3303_mRNA_CONV	convsersion of mRNA to mRNA_1 (synthetic rxn)	1 b3303_mRNA --> 1 b3303_mRNA_1	irreversible	Translation	0.00E+00	1.00E+09	0	2.46E+01	2.46E+01	0	4.34E+01	43.43574	0	1.46E-01	0.146
b3303_mRNA_degr1	Degradation of b3303_mRNA I	1 Orn_dim + 126 atp + 1 b3303_mRNA_2 + 1 degradosome + 126 h2o <==> 126 adp + 1 b3303_mRNA_2_degr + 126 h + 126 pi	reversible	mRNA degradation	-1.00E+09	1.00E+09	0	2.46E+01	2.46E+01	0	4.34E+01	43.43574	0	1.46E-01	0.146
b3303_mRNA_degr2	Degradation of b3303_mRNA II	1 b3303_mRNA_2_degr + 503 h2o --> 1 Orn_dim_inact + 130 amp + 117 cmp + 1 degradosome_inact + 136 gmp + 503 h + 121 ump	irreversible	mRNA degradation	0.00E+00	4.49E-01	0	2.46E+01	2.46E+01	0	4.34E+01	43.43574	0	1.46E-01	0.146
b3303_maturation1	Polypeptide b3303 peptide deformylas and methionine aminopeptidase complex	1 Def_mono + 1 Map_mono + 1 b3303_aa <==> 1 b3303_def_map_cplx	reversible	Protein Maturation	-1.00E+09	1.00E+09	8.44E+01	1.13E+02	2.81E+01	6.22E+01	8.29E+01	20.72	6.22E+01	8.29E+01	20.72
b3303_maturation2	b3303 formation	1 b3303_def_map_cplx + 2 h2o --> 1 Def_mono_inact + 1 Map_mono_inact + 1 b3303_m + 1 for + 1 met-L	irreversible	Protein Maturation	0.00E+00	1.00E+09	8.44E+01	1.13E+02	2.81E+01	6.22E+01	8.29E+01	20.72	6.22E+01	8.29E+01	20.72
b3304_fold_spon	b3304_m folding: spontanous	1 b3304_m --> 1 RplR_mono	irreversible	Protein Folding	0.00E+00	1.00E+09	8.44E+01	1.13E+02	2.81E+01	6.22E+01	8.29E+01	20.72	6.22E+01	8.29E+01	20.72
b3304_mRNA_CONV	convsersion of mRNA to mRNA_1 (synthetic rxn)	1 b3304_mRNA --> 1 b3304_mRNA_1	irreversible	Translation	0.00E+00	1.00E+09	0	2.46E+01	2.46E+01	0	4.34E+01	43.43574	0	1.46E-01	0.146
b3304_mRNA_degr1	Degradation of b3304_mRNA I	1 Orn_dim + 88 atp + 1 b3304_mRNA_2 + 1 degradosome + 88 h2o <==> 88 adp + 1 b3304_mRNA_2_degr + 88 h + 88 pi	reversible	mRNA degradation	-1.00E+09	1.00E+09	0	2.46E+01	2.46E+01	0	4.34E+01	43.43574	0	1.46E-01	0.146
b3304_mRNA_degr2	Degradation of b3304_mRNA II	1 b3304_mRNA_2_degr + 353 h2o --> 1 Orn_dim_inact + 85 amp + 95 cmp + 1 degradosome_inact + 95 gmp + 353 h + 79 ump	irreversible	mRNA degradation	0.00E+00	1.20E+00	0	2.46E+01	2.46E+01	0	4.34E+01	43.43574	0	1.46E-01	0.146
b3304_maturation1	Polypeptide b3304 peptide deformylas complex	1 Def_mono + 1 b3304_aa <==> 1 b3304_def_cplx	reversible	Protein Maturation	-1.00E+09	1.00E+09	8.44E+01	1.13E+02	2.81E+01	6.22E+01	8.29E+01	20.72	6.22E+01	8.29E+01	20.72
b3304_maturation2	b3304 formation	1 b3304_def_cplx + 1 h2o --> 1 Def_mono_inact + 1 b3304_m + 1 for	irreversible	Protein Maturation	0.00E+00	1.00E+09	8.44E+01	1.13E+02	2.81E+01	6.22E+01	8.29E+01	20.72	6.22E+01	8.29E+01	20.72
b3305_fold_spon	b3305_m folding: spontanous	1 b3305_m --> 1 RplF_mono	irreversible	Protein Folding	0.00E+00	1.00E+09	8.44E+01	1.13E+02	2.81E+01	6.22E+01	8.29E+01	20.72	6.22E+01	8.29E+01	20.72
b3305_mRNA_CONV	convsersion of mRNA to mRNA_1 (synthetic rxn)	1 b3305_mRNA --> 1 b3305_mRNA_1	irreversible	Translation	0.00E+00	1.00E+09	0	2.46E+01	2.46E+01	0	4.34E+01	43.43574	0	1.46E-01	0.146
b3305_mRNA_degr1	Degradation of b3305_mRNA I	1 Orn_dim + 133 atp + 1 b3305_mRNA_2 + 1 degradosome + 133 h2o <==> 133 adp + 1 b3305_mRNA_2_degr + 133 h + 133 pi	reversible	mRNA degradation	-1.00E+09	1.00E+09	0	2.46E+01	2.46E+01	0	4.34E+01	43.43574	0	1.46E-01	0.146
b3305_mRNA_degr2	Degradation of b3305_mRNA II	1 b3305_mRNA_2_degr + 533 h2o --> 1 Orn_dim_inact + 125 amp + 126 cmp + 1 degradosome_inact + 152 gmp + 533 h + 131 ump	irreversible	mRNA degradation	0.00E+00	7.92E-01	0	2.46E+01	2.46E+01	0	4.34E+01	43.43574	0	1.46E-01	0.146
b3305_maturation1	Polypeptide b3305 peptide deformylas and methionine aminopeptidase complex	1 Def_mono + 1 Map_mono + 1 b3305_aa <==> 1 b3305_def_map_cplx	reversible	Protein Maturation	-1.00E+09	1.00E+09	8.44E+01	1.13E+02	2.81E+01	6.22E+01	8.29E+01	20.72	6.22E+01	8.29E+01	20.72
b3305_maturation2	b3305 formation	1 b3305_def_map_cplx + 2 h2o --> 1 Def_mono_inact + 1 Map_mono_inact + 1 b3305_m + 1 for + 1 met-L	irreversible	Protein Maturation	0.00E+00	1.00E+09	8.44E+01	1.13E+02	2.81E+01	6.22E+01	8.29E+01	20.72	6.22E+01	8.29E+01	20.72
b3306_fold_spon	b3306_m folding: spontanous	1 b3306_m --> 1 RpsH_mono	irreversible	Protein Folding	0.00E+00	1.00E+09	8.44E+01	1.13E+02	2.81E+01	6.22E+01	8.29E+01	20.72	6.22E+01	8.29E+01	20.72
b3306_mRNA_CONV	convsersion of mRNA to mRNA_1 (synthetic rxn)	1 b3306_mRNA --> 1 b3306_mRNA_1	irreversible	Translation	0.00E+00	1.00E+09	0	2.46E+01	2.46E+01	0	4.34E+01	43.43574	0	1.46E-01	0.146
b3306_mRNA_degr1	Degradation of b3306_mRNA I	1 Orn_dim + 98 atp + 1 b3306_mRNA_2 + 1 degradosome + 98 h2o <==> 98 adp + 1 b3306_mRNA_2_degr + 98 h + 98 pi	reversible	mRNA degradation	-1.00E+09	1.00E+09	0	2.46E+01	2.46E+01	0	4.34E+01	43.43574	0	1.46E-01	0.146
b3306_mRNA_degr2	Degradation of b3306_mRNA II	1 b3306_mRNA_2_degr + 392 h2o --> 1 Orn_dim_inact + 102 amp + 90 cmp + 1 degradosome_inact + 107 gmp + 392 h + 94 ump	irreversible	mRNA degradation	0.00E+00	3.95E+00	0	2.46E+01	2.46E+01	0	4.34E+01	43.43574	0	1.46E-01	0.146
b3306_maturation1	Polypeptide b3306 peptide deformylas and methionine aminopeptidase complex	1 Def_mono + 1 Map_mono + 1 b3306_aa <==> 1 b3306_def_map_cplx	reversible	Protein Maturation	-1.00E+09	1.00E+09	8.44E+01	1.13E+02	2.81E+01	6.22E+01	8.29E+01	20.72	6.22E+01	8.29E+01	20.72
b3306_maturation2	b3306 formation	1 b3306_def_map_cplx + 2 h2o --> 1 Def_mono_inact + 1 Map_mono_inact + 1 b3306_m + 1 for + 1 met-L	irreversible	Protein Maturation	0.00E+00	1.00E+09	8.44E+01	1.13E+02	2.81E+01	6.22E+01	8.29E+01	20.72	6.22E+01	8.29E+01	20.72
b3307_fold_spon	b3307_m folding: spontanous	1 b3307_m --> 1 RpsN_mono	irreversible	Protein Folding	0.00E+00	1.00E+09	8.44E+01	1.13E+02	2.81E+01	6.22E+01	8.29E+01	20.72	6.22E+01	8.29E+01	20.72
b3307_mRNA_CONV	convsersion of mRNA to mRNA_1 (synthetic rxn)	1 b3307_mRNA --> 1 b3307_mRNA_1	irreversible	Translation	0.00E+00	1.00E+09	0	2.46E+01	2.46E+01	0	4.34E+01	43.43574	0	1.46E-01	0.146
b3307_mRNA_degr1	Degradation of b3307_mRNA I	1 Orn_dim + 76 atp + 1 b3307_mRNA_2 + 1 degradosome + 76 h2o <==> 76 adp + 1 b3307_mRNA_2_degr + 76 h + 76 pi	reversible	mRNA degradation	-1.00E+09	1.00E+09	0	2.46E+01	2.46E+01	0	4.34E+01	43.43574	0	1.46E-01	0.146
b3307_mRNA_degr2	Degradation of b3307_mRNA II	1 b3307_mRNA_2_degr + 305 h2o --> 1 Orn_dim_inact + 77 amp + 77 cmp + 1 degradosome_inact + 84 gmp + 305 h + 68 ump	irreversible	mRNA degradation	0.00E+00	2.15E+00	0	2.46E+01	2.46E+01	0	4.34E+01	43.43574	0	1.46E-01	0.146
b3307_maturation1	Polypeptide b3307 peptide deformylas complex	1 Def_mono + 1 b3307_aa <==> 1 b3307_def_cplx	reversible	Protein Maturation	-1.00E+09	1.00E+09	8.44E+01	1.13E+02	2.81E+01	6.22E+01	8.29E+01	20.72	6.22E+01	8.29E+01	20.72
b3307_maturation2	b3307 formation	1 b3307_def_cplx + 2 h2o --> 1 Def_mono_inact + 1 b3307_m + 1 for + 1 met-L	irreversible	Protein Maturation	0.00E+00	1.00E+09	8.44E+01	1.13E+02	2.81E+01	6.22E+01	8.29E+01	20.72	6.22E+01	8.29E+01	20.72
b3308_fold_spon	b3308_m folding: spontanous	1 b3308_m --> 1 RplE_mono	irreversible	Protein Folding	0.00E+00	1.00E+09	8.44E+01	1.13E+02	2.81E+01	6.22E+01	8.29E+01	20.72	6.22E+01	8.29E+01	20.72
b3308_mRNA_CONV	convsersion of mRNA to mRNA_1 (synthetic rxn)	1 b3308_mRNA --> 1 b3308_mRNA_1	irreversible	Translation	0.00E+00	1.00E+09	0	2.46E+01	2.46E+01	0	4.34E+01	43.43574	0	1.46E-01	0.146
b3308_mRNA_degr1	Degradation of b3308_mRNA I	1 Orn_dim + 135 atp + 1 b3308_mRNA_2 + 1 degradosome + 135 h2o <==> 135 adp + 1 b3308_mRNA_2_degr + 135 h + 135 pi	reversible	mRNA degradation	-1.00E+09	1.00E+09	0	2.46E+01	2.46E+01	0	4.34E+01	43.43574	0	1.46E-01	0.146

b3308_mRNA_degr2	Degradation of b3308_mRNA II	1 b3308_mRNA_2_degr + 539 h2o --> 1 Orn_dim_inact + 141 amp + 137 cmp + 1 degradosome_inact + 132 gmp + 539 h + 130 ump	irreversible	mRNA degradation	0.00E+00	1.35E+00	0	2.46E+01	2.46E+01	0	4.34E+01	43.43574	0	1.46E-01	0.146
b3308_maturation1	Polypeptide b3308 peptide deformylas and methionine aminopeptidase complex	1 Def_mono + 1 Map_mono + 1 b3308_aa <==> 1 b3308_def_map_cplx	reversible	Protein Maturation	-1.00E+09	1.00E+09	8.44E+01	1.13E+02	2.81E+01	6.22E+01	8.29E+01	20.72	6.22E+01	8.29E+01	20.72
b3308_maturation2	b3308 formation	1 b3308_def_map_cplx + 2 h2o --> 1 Def_mono_inact + 1 Map_mono_inact + 1 b3308_m + 1 for + 1 met-L	irreversible	Protein Maturation	0.00E+00	1.00E+09	8.44E+01	1.13E+02	2.81E+01	6.22E+01	8.29E+01	20.72	6.22E+01	8.29E+01	20.72
b3309_fold_spon	b3309_m folding: spontanous	1 b3309_m --> 1 RplX_mono	irreversible	Protein Folding	0.00E+00	1.00E+09	8.44E+01	1.13E+02	2.81E+01	6.22E+01	8.29E+01	20.72	6.22E+01	8.29E+01	20.72
b3309_mRNA_CONV	convsersion of mRNA to mRNA_1 (synthetic rxn)	1 b3309_mRNA --> 1 b3309_mRNA_1	irreversible	Translation	0.00E+00	1.00E+09	0	2.46E+01	2.46E+01	0	4.34E+01	43.43574	0	1.46E-01	0.146
b3309_mRNA_degr1	Degradation of b3309_mRNA I	1 Orn_dim + 78 atp + 1 b3309_mRNA_2 + 1 degradosome + 78 h2o <==> 78 adp + 1 b3309_mRNA_2_degr + 78 h + 78 pi	reversible	mRNA degradation	-1.00E+09	1.00E+09	0	2.46E+01	2.46E+01	0	4.34E+01	43.43574	0	1.46E-01	0.146
b3309_mRNA_degr2	Degradation of b3309_mRNA II	1 b3309_mRNA_2_degr + 314 h2o --> 1 Orn_dim_inact + 98 amp + 66 cmp + 1 degradosome_inact + 79 gmp + 314 h + 72 ump	irreversible	mRNA degradation	0.00E+00	1.00E+09	0	2.46E+01	2.46E+01	0	4.34E+01	43.43574	0	1.46E-01	0.146
b3309_maturation1	Polypeptide b3309 peptide deformylas and methionine aminopeptidase complex	1 Def_mono + 1 Map_mono + 1 b3309_aa <==> 1 b3309_def_map_cplx	reversible	Protein Maturation	-1.00E+09	1.00E+09	8.44E+01	1.13E+02	2.81E+01	6.22E+01	8.29E+01	20.72	6.22E+01	8.29E+01	20.72
b3309_maturation2	b3309 formation	1 b3309_def_map_cplx + 2 h2o --> 1 Def_mono_inact + 1 Map_mono_inact + 1 b3309_m + 1 for + 1 met-L	irreversible	Protein Maturation	0.00E+00	1.00E+09	8.44E+01	1.13E+02	2.81E+01	6.22E+01	8.29E+01	20.72	6.22E+01	8.29E+01	20.72
b3310_fold_spon	b3310_m folding: spontanous	1 b3310_m --> 1 RplN_mono	irreversible	Protein Folding	0.00E+00	1.00E+09	8.44E+01	1.13E+02	2.81E+01	6.22E+01	8.29E+01	20.72	6.22E+01	8.29E+01	20.72
b3310_mRNA_CONV	convsersion of mRNA to mRNA_1 (synthetic rxn)	1 b3310_mRNA --> 1 b3310_mRNA_1	irreversible	Translation	0.00E+00	1.00E+09	0	2.46E+01	2.46E+01	0	4.34E+01	43.43574	0	1.46E-01	0.146
b3310_mRNA_degr1	Degradation of b3310_mRNA I	1 Orn_dim + 93 atp + 1 b3310_mRNA_2 + 1 degradosome + 93 h2o <==> 93 adp + 1 b3310_mRNA_2_degr + 93 h + 93 pi	reversible	mRNA degradation	-1.00E+09	1.00E+09	0	2.46E+01	2.46E+01	0	4.34E+01	43.43574	0	1.46E-01	0.146
b3310_mRNA_degr2	Degradation of b3310_mRNA II	1 b3310_mRNA_2_degr + 371 h2o --> 1 Orn_dim_inact + 92 amp + 1 atp + 87 cmp + 1 degradosome_inact + 103 gmp + 371 h + 89 ump	irreversible	mRNA degradation	0.00E+00	7.56E-01	0	2.46E+01	2.46E+01	0	4.34E+01	43.43574	0	1.46E-01	0.146
b3310_maturation1	Polypeptide b3310 peptide deformylas complex	1 Def_mono + 1 b3310_aa <==> 1 b3310_def_cplx	reversible	Protein Maturation	-1.00E+09	1.00E+09	8.44E+01	1.13E+02	2.81E+01	6.22E+01	8.29E+01	20.72	6.22E+01	8.29E+01	20.72
b3310_maturation2	b3310 formation	1 b3310_def_cplx + 1 h2o --> 1 Def_mono_inact + 1 b3310_m + 1 for	irreversible	Protein Maturation	0.00E+00	1.00E+09	8.44E+01	1.13E+02	2.81E+01	6.22E+01	8.29E+01	20.72	6.22E+01	8.29E+01	20.72
b3311_fold_spon	b3311_m folding: spontanous	1 b3311_m --> 1 RpsQ_mono	irreversible	Protein Folding	0.00E+00	1.00E+09	8.44E+01	1.13E+02	2.81E+01	6.22E+01	8.29E+01	20.72	6.22E+01	8.29E+01	20.72
b3311_mRNA_CONV	convsersion of mRNA to mRNA_1 (synthetic rxn)	1 b3311_mRNA --> 1 b3311_mRNA_1	irreversible	Translation	0.00E+00	1.00E+09	0	2.69E+01	2.69E+01	0	4.75E+01	47.45757	0	1.72E-01	0.172
b3311_mRNA_degr1	Degradation of b3311_mRNA I	1 Orn_dim + 63 atp + 1 b3311_mRNA_2 + 1 degradosome + 63 h2o <==> 63 adp + 1 b3311_mRNA_2_degr + 63 h + 63 pi	reversible	mRNA degradation	-1.00E+09	1.00E+09	0	2.69E+01	2.69E+01	0	4.75E+01	47.45757	0	1.72E-01	0.172
b3311_mRNA_degr2	Degradation of b3311_mRNA II	1 b3311_mRNA_2_degr + 253 h2o --> 1 Orn_dim_inact + 72 amp + 57 cmp + 1 degradosome_inact + 64 gmp + 253 h + 61 ump	irreversible	mRNA degradation	0.00E+00	5.67E-01	0	2.69E+01	2.69E+01	0	4.75E+01	47.45757	0	1.72E-01	0.172
b3311_maturation1	Polypeptide b3311 peptide deformylas and methionine aminopeptidase complex	1 Def_mono + 1 Map_mono + 1 b3311_aa <==> 1 b3311_def_map_cplx	reversible	Protein Maturation	-1.00E+09	1.00E+09	8.44E+01	1.13E+02	2.81E+01	6.22E+01	8.29E+01	20.72	6.22E+01	8.29E+01	20.72
b3311_maturation2	b3311 formation	1 b3311_def_map_cplx + 2 h2o --> 1 Def_mono_inact + 1 Map_mono_inact + 1 b3311_m + 1 for + 1 met-L	irreversible	Protein Maturation	0.00E+00	1.00E+09	8.44E+01	1.13E+02	2.81E+01	6.22E+01	8.29E+01	20.72	6.22E+01	8.29E+01	20.72
b3312_fold_spon	b3312_m folding: spontanous	1 b3312_m --> 1 RpmC_mono	irreversible	Protein Folding	0.00E+00	1.00E+09	8.44E+01	1.13E+02	2.81E+01	6.22E+01	8.29E+01	20.72	6.22E+01	8.29E+01	20.72
b3312_mRNA_CONV	convsersion of mRNA to mRNA_1 (synthetic rxn)	1 b3312_mRNA --> 1 b3312_mRNA_1	irreversible	Translation	0.00E+00	1.00E+09	0	2.69E+01	2.69E+01	0	4.75E+01	47.45757	0	1.72E-01	0.172
b3312_mRNA_degr1	Degradation of b3312_mRNA I	1 Orn_dim + 47 atp + 1 b3312_mRNA_2 + 1 degradosome + 47 h2o <==> 47 adp + 1 b3312_mRNA_2_degr + 47 h + 47 pi	reversible	mRNA degradation	-1.00E+09	1.00E+09	0	2.69E+01	2.69E+01	0	4.75E+01	47.45757	0	1.72E-01	0.172
b3312_mRNA_degr2	Degradation of b3312_mRNA II	1 b3312_mRNA_2_degr + 190 h2o --> 1 Orn_dim_inact + 51 amp + 44 cmp + 1 degradosome_inact + 60 gmp + 190 h + 36 ump	irreversible	mRNA degradation	0.00E+00	1.61E+00	0	2.69E+01	2.69E+01	0	4.75E+01	47.45757	0	1.72E-01	0.172
b3312_maturation1	Polypeptide b3312 peptide deformylas complex	1 Def_mono + 1 b3312_aa <==> 1 b3312_def_cplx	reversible	Protein Maturation	-1.00E+09	1.00E+09	8.44E+01	1.13E+02	2.81E+01	6.22E+01	8.29E+01	20.72	6.22E+01	8.29E+01	20.72
b3312_maturation2	b3312 formation	1 b3312_def_cplx + 1 h2o --> 1 Def_mono_inact + 1 b3312_m + 1 for	irreversible	Protein Maturation	0.00E+00	1.00E+09	8.44E+01	1.13E+02	2.81E+01	6.22E+01	8.29E+01	20.72	6.22E+01	8.29E+01	20.72
b3313_fold_spon	b3313_m folding: spontanous	1 b3313_m --> 1 RplP_mono	irreversible	Protein Folding	0.00E+00	1.00E+09	8.44E+01	1.13E+02	2.81E+01	6.22E+01	8.29E+01	20.72	6.22E+01	8.29E+01	20.72
b3313_mRNA_CONV	convsersion of mRNA to mRNA_1 (synthetic rxn)	1 b3313_mRNA --> 1 b3313_mRNA_1	irreversible	Translation	0.00E+00	1.00E+09	0	2.69E+01	2.69E+01	0	4.75E+01	47.45757	0	1.72E-01	0.172
b3313_mRNA_degr1	Degradation of b3313_mRNA I	1 Orn_dim + 102 atp + 1 b3313_mRNA_2 + 1 degradosome + 102 h2o <==> 102 adp + 1 b3313_mRNA_2_degr + 102 h + 102 pi	reversible	mRNA degradation	-1.00E+09	1.00E+09	0	2.69E+01	2.69E+01	0	4.75E+01	47.45757	0	1.72E-01	0.172
b3313_mRNA_degr2	Degradation of b3313_mRNA II	1 b3313_mRNA_2_degr + 410 h2o --> 1 Orn_dim_inact + 107 amp + 92 cmp + 1 degradosome_inact + 121 gmp + 410 h + 91 ump	irreversible	mRNA degradation	0.00E+00	2.84E-01	0	2.69E+01	2.69E+01	0	4.75E+01	47.45757	0	1.72E-01	0.172
b3313_maturation1	Polypeptide b3313 peptide deformylas complex	1 Def_mono + 1 b3313_aa <==> 1 b3313_def_cplx	reversible	Protein Maturation	-1.00E+09	1.00E+09	8.44E+01	1.13E+02	2.81E+01	6.22E+01	8.29E+01	20.72	6.22E+01	8.29E+01	20.72
b3313_maturation2	b3313 formation	1 b3313_def_cplx + 1 h2o --> 1 Def_mono_inact + 1 b3313_m + 1 for	irreversible	Protein Maturation	0.00E+00	1.00E+09	8.44E+01	1.13E+02	2.81E+01	6.22E+01	8.29E+01	20.72	6.22E+01	8.29E+01	20.72
b3314_fold_spon	b3314_m folding: spontanous	1 b3314_m --> 1 RpsC_mono	irreversible	Protein Folding	0.00E+00	1.00E+09	8.44E+01	1.13E+02	2.81E+01	6.22E+01	8.29E+01	20.72	6.22E+01	8.29E+01	20.72
b3314_mRNA_CONV	convsersion of mRNA to mRNA_1 (synthetic rxn)	1 b3314_mRNA --> 1 b3314_mRNA_1	irreversible	Translation	0.00E+00	1.00E+09	0	2.69E+01	2.69E+01	0	4.75E+01	47.45757	0	1.72E-01	0.172
b3314_mRNA_degr1	Degradation of b3314_mRNA I	1 Orn_dim + 175 atp + 1 b3314_mRNA_2 + 1 degradosome + 175 h2o <==> 175 adp + 1 b3314_mRNA_2_degr + 175 h + 175 pi	reversible	mRNA degradation	-1.00E+09	1.00E+09	0	2.69E+01	2.69E+01	0	4.75E+01	47.45757	0	1.72E-01	0.172
b3314_mRNA_degr2	Degradation of b3314_mRNA II	1 b3314_mRNA_2_degr + 701 h2o --> 1 Orn_dim_inact + 189 amp + 171 cmp + 1 degradosome_inact + 187 gmp + 701 h + 155 ump	irreversible	mRNA degradation	0.00E+00	1.72E-01	0	2.69E+01	2.69E+01	0	4.75E+01	47.45757	0	1.72E-01	0.172
b3314_maturation1	Polypeptide b3314 peptide deformylas and methionine aminopeptidase complex	1 Def_mono + 1 Map_mono + 1 b3314_aa <==> 1 b3314_def_map_cplx	reversible	Protein Maturation	-1.00E+09	1.00E+09	8.44E+01	1.13E+02	2.81E+01	6.22E+01	8.29E+01	20.72	6.22E+01	8.29E+01	20.72
b3314_maturation2	b3314 formation	1 b3314_def_map_cplx + 2 h2o --> 1 Def_mono_inact + 1 Map_mono_inact + 1 b3314_m + 1 for + 1 met-L	irreversible	Protein Maturation	0.00E+00	1.00E+09	8.44E+01	1.13E+02	2.81E+01	6.22E+01	8.29E+01	20.72	6.22E+01	8.29E+01	20.72
b3315_fold_spon	b3315_m folding: spontanous	1 b3315_m --> 1 RplV_mono	irreversible	Protein Folding	0.00E+00	1.00E+09	8.44E+01	1.13E+02	2.81E+01	6.22E+01	8.29E+01	20.72	6.22E+01	8.29E+01	20.72
b3315_mRNA_CONV	convsersion of mRNA to mRNA_1 (synthetic rxn)	1 b3315_mRNA --> 1 b3315_mRNA_1	irreversible	Translation	0.00E+00	1.00E+09	0	2.69E+01	2.69E+01	0	4.75E+01	47.45757	0	1.72E-01	0.172
b3315_mRNA_degr1	Degradation of b3315_mRNA I	1 Orn_dim + 83 atp + 1 b3315_mRNA_2 + 1 degradosome + 83 h2o <==> 83 adp + 1 b3315_mRNA_2_degr + 83 h + 83 pi	reversible	mRNA degradation	-1.00E+09	1.00E+09	0	2.69E+01	2.69E+01	0	4.75E+01	47.45757	0	1.72E-01	0.172
b3315_mRNA_degr2	Degradation of b3315_mRNA II	1 b3315_mRNA_2_degr + 332 h2o --> 1 Orn_dim_inact + 88 amp + 82 cmp + 1 degradosome_inact + 84 gmp + 332 h + 79 ump	irreversible	mRNA degradation	0.00E+00	2.29E-01	0	2.69E+01	2.69E+01	0	4.75E+01	47.45757	0	1.72E-01	0.172
b3315_maturation1	Polypeptide b3315 peptide deformylas complex	1 Def_mono + 1 b3315_aa <==> 1 b3315_def_cplx	reversible	Protein Maturation	-1.00E+09	1.00E+09	8.44E+01	1.13E+02	2.81E+01	6.22E+01	8.29E+01	20.72	6.22E+01	8.29E+01	20.72
b3315_maturation2	b3315 formation	1 b3315_def_cplx + 1 h2o --> 1 Def_mono_inact + 1 b3315_m + 1 for	irreversible	Protein Maturation	0.00E+00	1.00E+09	8.44E+01	1.13E+02	2.81E+01	6.22E+01	8.29E+01	20.72	6.22E+01	8.29E+01	20.72
b3316_fold_spon	b3316_m folding: spontanous	1 b3316_m --> 1 RpsS_mono	irreversible	Protein Folding	0.00E+00	1.00E+09	8.44E+01	1.13E+02	2.81E+01	6.22E+01	8.29E+01	20.72	6.22E+01	8.29E+01	20.72
b3316_mRNA_CONV	convsersion of mRNA to mRNA_1 (synthetic rxn)	1 b3316_mRNA --> 1 b3316_mRNA_1	irreversible	Translation	0.00E+00	1.00E+09	0	2.69E+01	2.69E+01	0	4.75E+01	47.45757	0	1.72E-01	0.172
b3316_mRNA_degr1	Degradation of b3316_mRNA I	1 Orn_dim + 69 atp + 1 b3316_mRNA_2 + 1 degradosome + 69 h2o <==> 69 adp + 1 b3316_mRNA_2_degr + 69 h + 69 pi	reversible	mRNA degradation	-1.00E+09	1.00E+09	0	2.69E+01	2.69E+01	0	4.75E+01	47.45757	0	1.72E-01	0.172
b3316_mRNA_degr2	Degradation of b3316_mRNA II	1 b3316_mRNA_2_degr + 278 h2o --> 1 Orn_dim_inact + 77 amp + 67 cmp + 1 degradosome_inact + 69 gmp + 278 h + 66 ump	irreversible	mRNA degradation	0.00E+00	1.39E+00	0	2.69E+01	2.69E+01	0	4.75E+01	47.45757	0	1.72E-01	0.172
b3316_maturation1	Polypeptide b3316 peptide deformylas and methionine aminopeptidase complex	1 Def_mono + 1 Map_mono + 1 b3316_aa <==> 1 b3316_def_map_cplx	reversible	Protein Maturation	-1.00E+09	1.00E+09	8.44E+01	1.13E+02	2.81E+01	6.22E+01	8.29E+01	20.72	6.22E+01	8.29E+01	20.72
b3316_maturation2	b3316 formation	1 b3316_def_map_cplx + 2 h2o --> 1 Def_mono_inact + 1 Map_mono_inact + 1 b3316_m + 1 for + 1 met-L	irreversible	Protein Maturation	0.00E+00	1.00E+09	8.44E+01	1.13E+02	2.81E+01	6.22E+01	8.29E+01	20.72	6.22E+01	8.29E+01	20.72
b3317_fold_spon	b3317_m folding: spontanous	1 b3317_m --> 1 RplB_mono	irreversible	Protein Folding	0.00E+00	1.00E+09	8.44E+01	1.13E+02	2.81E+01	6.22E+01	8.29E+01	20.72	6.22E+01	8.29E+01	20.72

b3317_mRNA_CONV	convsersion of mRNA to mRNA_1 (synthetic rxn)	1 b3317_mRNA --> 1 b3317_mRNA_1	irreversible	Translation	0.00E+00	1.00E+09	0	2.69E+01	2.69E+01	0	4.75E+01	47.45757	0	1.72E-01	0.172
b3317_mRNA_degr1	Degradation of b3317_mRNA I	1 Orn_dim + 205 atp + 1 b3317_mRNA_2 + 1 degradosome + 205 h2o <==> 205 adp + 1 b3317_mRNA_2_degr + 205 h + 205 pi	reversible	mRNA degradation	-1.00E+09	1.00E+09	0	2.69E+01	2.69E+01	0	4.75E+01	47.45757	0	1.72E-01	0.172
b3317_mRNA_degr2	Degradation of b3317_mRNA II	1 b3317_mRNA_2_degr + 821 h2o --> 1 Orn_dim_inact + 203 amp + 209 cmp + 1 degradosome_inact + 224 gmp + 821 h + 186 ump	irreversible	mRNA degradation	0.00E+00	5.53E-01	0	2.69E+01	2.69E+01	0	4.75E+01	47.45757	0	1.72E-01	0.172
b3317_maturation1	Polypeptide b3317 peptide deformylas and methionine aminopeptidase complex	1 Def_mono + 1 Map_mono + 1 b3317_aa <==> 1 b3317_def_map_cplx	reversible	Protein Maturation	-1.00E+09	1.00E+09	8.44E+01	1.13E+02	2.81E+01	6.22E+01	8.29E+01	20.72	6.22E+01	8.29E+01	20.72
b3317_maturation2	b3317 formation	1 b3317_def_map_cplx + 2 h2o --> 1 Def_mono_inact + 1 Map_mono_inact + 1 b3317_m + 1 for + 1 met-L	irreversible	Protein Maturation	0.00E+00	1.00E+09	8.44E+01	1.13E+02	2.81E+01	6.22E+01	8.29E+01	20.72	6.22E+01	8.29E+01	20.72
b3318_fold_spon	b3318_m folding: spontanous	1 b3318_m --> 1 RplW_mono	irreversible	Protein Folding	0.00E+00	1.00E+09	8.44E+01	1.13E+02	2.81E+01	6.22E+01	8.29E+01	20.72	6.22E+01	8.29E+01	20.72
b3318_mRNA_CONV	convsersion of mRNA to mRNA_1 (synthetic rxn)	1 b3318_mRNA --> 1 b3318_mRNA_1	irreversible	Translation	0.00E+00	1.00E+09	0	2.69E+01	2.69E+01	0	4.75E+01	47.45757	0	1.72E-01	0.172
b3318_mRNA_degr1	Degradation of b3318_mRNA I	1 Orn_dim + 74 atp + 1 b3318_mRNA_2 + 1 degradosome + 74 h2o <==> 74 adp + 1 b3318_mRNA_2_degr + 74 h + 74 pi	reversible	mRNA degradation	-1.00E+09	1.00E+09	0	2.69E+01	2.69E+01	0	4.75E+01	47.45757	0	1.72E-01	0.172
b3318_mRNA_degr2	Degradation of b3318_mRNA II	1 b3318_mRNA_2_degr + 298 h2o --> 1 Orn_dim_inact + 92 amp + 66 cmp + 1 degradosome_inact + 79 gmp + 298 h + 62 ump	irreversible	mRNA degradation	0.00E+00	1.09E+00	0	2.69E+01	2.69E+01	0	4.75E+01	47.45757	0	1.72E-01	0.172
b3318_maturation1	Polypeptide b3318 peptide deformylas complex	1 Def_mono + 1 b3318_aa <==> 1 b3318_def_cplx	reversible	Protein Maturation	-1.00E+09	1.00E+09	8.44E+01	1.13E+02	2.81E+01	6.22E+01	8.29E+01	20.72	6.22E+01	8.29E+01	20.72
b3318_maturation2	b3318 formation	1 b3318_def_cplx + 1 h2o --> 1 Def_mono_inact + 1 b3318_m + 1 for	irreversible	Protein Maturation	0.00E+00	1.00E+09	8.44E+01	1.13E+02	2.81E+01	6.22E+01	8.29E+01	20.72	6.22E+01	8.29E+01	20.72
b3319_fold_spon	b3319_m folding: spontanous	1 b3319_m --> 1 RplD_mono	irreversible	Protein Folding	0.00E+00	1.00E+09	8.44E+01	1.13E+02	2.81E+01	6.22E+01	8.29E+01	20.72	6.22E+01	8.29E+01	20.72
b3319_mRNA_CONV	convsersion of mRNA to mRNA_1 (synthetic rxn)	1 b3319_mRNA --> 1 b3319_mRNA_1	irreversible	Translation	0.00E+00	1.00E+09	0	2.69E+01	2.69E+01	0	4.75E+01	47.45757	0	1.72E-01	0.172
b3319_mRNA_degr1	Degradation of b3319_mRNA I	1 Orn_dim + 151 atp + 1 b3319_mRNA_2 + 1 degradosome + 151 h2o <==> 151 adp + 1 b3319_mRNA_2_degr + 151 h + 151 pi	reversible	mRNA degradation	-1.00E+09	1.00E+09	0	2.69E+01	2.69E+01	0	4.75E+01	47.45757	0	1.72E-01	0.172
b3319_mRNA_degr2	Degradation of b3319_mRNA II	1 b3319_mRNA_2_degr + 605 h2o --> 1 Orn_dim_inact + 145 amp + 146 cmp + 1 degradosome_inact + 175 gmp + 605 h + 140 ump	irreversible	mRNA degradation	0.00E+00	2.69E-01	0	2.69E+01	2.69E+01	0	4.75E+01	47.45757	0	1.72E-01	0.172
b3319_maturation1	Polypeptide b3319 peptide deformylas complex	1 Def_mono + 1 b3319_aa <==> 1 b3319_def_cplx	reversible	Protein Maturation	-1.00E+09	1.00E+09	8.44E+01	1.13E+02	2.81E+01	6.22E+01	8.29E+01	20.72	6.22E+01	8.29E+01	20.72
b3319_maturation2	b3319 formation	1 b3319_def_cplx + 1 h2o --> 1 Def_mono_inact + 1 b3319_m + 1 for	irreversible	Protein Maturation	0.00E+00	1.00E+09	8.44E+01	1.13E+02	2.81E+01	6.22E+01	8.29E+01	20.72	6.22E+01	8.29E+01	20.72
b3320_fold_spon	b3320_m folding: spontanous	1 b3320_m --> 1 RplC_mono	irreversible	Protein Folding	0.00E+00	1.00E+09	8.44E+01	1.13E+02	2.81E+01	6.22E+01	8.29E+01	20.72	6.22E+01	8.29E+01	20.72
b3320_mRNA_CONV	convsersion of mRNA to mRNA_1 (synthetic rxn)	1 b3320_mRNA --> 1 b3320_mRNA_1	irreversible	Translation	0.00E+00	1.00E+09	0	2.69E+01	2.69E+01	0	4.75E+01	47.45757	0	1.72E-01	0.172
b3320_mRNA_degr1	Degradation of b3320_mRNA I	1 Orn_dim + 157 atp + 1 b3320_mRNA_2 + 1 degradosome + 157 h2o <==> 157 adp + 1 b3320_mRNA_2_degr + 157 h + 157 pi	reversible	mRNA degradation	-1.00E+09	1.00E+09	0	2.69E+01	2.69E+01	0	4.75E+01	47.45757	0	1.72E-01	0.172
b3320_mRNA_degr2	Degradation of b3320_mRNA II	1 b3320_mRNA_2_degr + 629 h2o --> 1 Orn_dim_inact + 162 amp + 148 cmp + 1 degradosome_inact + 176 gmp + 629 h + 144 ump	irreversible	mRNA degradation	0.00E+00	6.22E-01	0	2.69E+01	2.69E+01	0	4.75E+01	47.45757	0	1.72E-01	0.172
b3320_maturation1	Polypeptide b3320 peptide deformylas complex	1 Def_mono + 1 b3320_aa <==> 1 b3320_def_cplx	reversible	Protein Maturation	-1.00E+09	1.00E+09	8.44E+01	1.13E+02	2.81E+01	6.22E+01	8.29E+01	20.72	6.22E+01	8.29E+01	20.72
b3320_maturation2	b3320 formation	1 b3320_def_cplx + 1 h2o --> 1 Def_mono_inact + 1 b3320_m + 1 for	irreversible	Protein Maturation	0.00E+00	1.00E+09	8.44E+01	1.13E+02	2.81E+01	6.22E+01	8.29E+01	20.72	6.22E+01	8.29E+01	20.72
b3321_fold_spon	b3321_m folding: spontanous	1 b3321_m --> 1 RpsJ_mono	irreversible	Protein Folding	0.00E+00	1.00E+09	8.44E+01	1.13E+02	2.81E+01	6.22E+01	8.29E+01	20.72	6.22E+01	8.29E+01	20.72
b3321_mRNA_CONV	convsersion of mRNA to mRNA_1 (synthetic rxn)	1 b3321_mRNA --> 1 b3321_mRNA_1	irreversible	Translation	0.00E+00	1.00E+09	0	2.69E+01	2.69E+01	0	4.75E+01	47.45757	0	1.72E-01	0.172
b3321_mRNA_degr1	Degradation of b3321_mRNA I	1 Orn_dim + 78 atp + 1 b3321_mRNA_2 + 1 degradosome + 78 h2o <==> 78 adp + 1 b3321_mRNA_2_degr + 78 h + 78 pi	reversible	mRNA degradation	-1.00E+09	1.00E+09	0	2.69E+01	2.69E+01	0	4.75E+01	47.45757	0	1.72E-01	0.172
b3321_mRNA_degr2	Degradation of b3321_mRNA II	1 b3321_mRNA_2_degr + 311 h2o --> 1 Orn_dim_inact + 73 amp + 1 atp + 90 cmp + 1 degradosome_inact + 83 gmp + 311 h + 65 ump	irreversible	mRNA degradation	0.00E+00	1.21E+00	0	2.69E+01	2.69E+01	0	4.75E+01	47.45757	0	1.72E-01	0.172
b3321_maturation1	Polypeptide b3321 peptide deformylas complex	1 Def_mono + 1 b3321_aa <==> 1 b3321_def_cplx	reversible	Protein Maturation	-1.00E+09	1.00E+09	8.44E+01	1.13E+02	2.81E+01	6.22E+01	8.29E+01	20.72	6.22E+01	8.29E+01	20.72
b3321_maturation2	b3321 formation	1 b3321_def_cplx + 1 h2o --> 1 Def_mono_inact + 1 b3321_m + 1 for	irreversible	Protein Maturation	0.00E+00	1.00E+09	8.44E+01	1.13E+02	2.81E+01	6.22E+01	8.29E+01	20.72	6.22E+01	8.29E+01	20.72
b3339_fold_KJE_1	b3339_m folding: KJE mediated	1 DnaJ_dim + 1 DnaK_mono.ATP + 1 b3339_m <==> 1 b3339_m_DnaKJ_complex	reversible	Protein Folding	-1.00E+09	1.00E+09	0	2.13E+03	2.13E+03	0	2.80E+03	2800.46	0	1.27E+03	1268.634
b3339_fold_KJE_2	b3339_m folding: KJE mediated	1 GrpE_dim + 1 b3339_m_DnaKJ_complex + 1 h2o --> 1 DnaJ_dim_inact + 1 TufA_DnaK_GrpE_complex + 1 adp + 1 h + 1 pi	irreversible	Protein Folding	0.00E+00	1.00E+09	0	2.13E+03	2.13E+03	0	2.80E+03	2800.46	0	1.27E+03	1268.634
b3339_fold_KJE_3	b3339_m folding: KJE mediated	1 TufA_DnaK_GrpE_complex + 1 atp --> 1 DnaK_mono.ATP_inact + 1 GrpE_dim_inact + 1 TufA_mono	irreversible	Protein Folding	0.00E+00	1.00E+09	0	2.13E+03	2.13E+03	0	2.80E+03	2800.46	0	1.27E+03	1268.634
b3339_fold_spon	b3339_m folding: spontanous	1 b3339_m --> 1 TufA_mono	irreversible	Protein Folding	0.00E+00	1.00E+09	0	2.13E+03	2.13E+03	0	2.80E+03	2800.46	0	1.27E+03	1268.634
b3339_mRNA_CONV	convsersion of mRNA to mRNA_1 (synthetic rxn)	1 b3339_mRNA --> 1 b3339_mRNA_1	irreversible	Translation	0.00E+00	1.00E+09	0	2.93E+01	2.93E+01	0	5.17E+01	51.7244	0	5.67E-01	0.567
b3339_mRNA_degr1	Degradation of b3339_mRNA I	1 Orn_dim + 296 atp + 1 b3339_mRNA_2 + 1 degradosome + 296 h2o <==> 296 adp + 1 b3339_mRNA_2_degr + 296 h + 296 pi	reversible	mRNA degradation	-1.00E+09	1.00E+09	0	3.82E+01	3.82E+01	0	6.74E+01	67.35913	0	7.58E-01	0.758
b3339_mRNA_degr2	Degradation of b3339_mRNA II	1 b3339_mRNA_2_degr + 1184 h2o --> 1 Orn_dim_inact + 286 amp 306 cmp + 1 degradosome_inact + 325 gmp + 1184 h + 268 ump	irreversible	mRNA degradation	0.00E+00	1.77E+00	0	3.82E+01	3.82E+01	0	6.74E+01	67.35913	0	7.58E-01	0.758
b3339_maturation1	Polypeptide b3339 peptide deformylas and methionine aminopeptidase complex	1 Def_mono + 1 Map_mono + 1 b3339_aa <==> 1 b3339_def_map_cplx	reversible	Protein Maturation	-1.00E+09	1.00E+09	0	2.13E+03	2.13E+03	0	2.80E+03	2800.46	0	1.27E+03	1268.634
b3339_maturation2	b3339 formation	1 b3339_def_map_cplx + 2 h2o --> 1 Def_mono_inact + 1 Map_mono_inact + 1 b3339_m + 1 for + 1 met-L	irreversible	Protein Maturation	0.00E+00	1.00E+09	0	2.13E+03	2.13E+03	0	2.80E+03	2800.46	0	1.27E+03	1268.634
b3339_v1_mRNA_CONV	convsersion of mRNA to mRNA_1 (synthetic rxn)	1 b3339_v1_mRNA --> 1 b3339_mRNA_1	irreversible	Translation	0.00E+00	1.00E+09	0	3.82E+01	3.82E+01	0	6.74E+01	67.35913	0	7.58E-01	0.758
b3340_fold_GroEL/ES_1	b3340_m folding: GroEL/ES mediated: polypeptide is going in GroEL/ES complex	1 GroEL.(7)ADP.cisGroES + 7 atp + 1 b3340_m + 1 transGroES_hepta --> 7 adp + 1 b3340_m_GroEL.(7)ATP.transGroES + 1 cisGroES_hepta	irreversible	Protein Folding	0.00E+00	1.00E+09	0	2.04E+03	2.04E+03	0	3.14E+03	3140.993	0	5.11E+02	510.778
b3340_fold_GroEL/ES_2	b3340_m folding: GroEL/ES mediated: folding of polypeptide under ATP hydrolysis	1 b3340_m_GroEL.(7)ATP.transGroES + 7 h2o --> 1 FusA_GroEL.(7)ADP.transGroES + 7 h + 7 pi	irreversible	Protein Folding	0.00E+00	1.00E+09	0	2.04E+03	2.04E+03	0	3.14E+03	3140.993	0	5.11E+02	510.778
b3340_fold_GroEL/ES_3	b3340_m folding: GroEL/ES mediated: release of native protein	1 FusA_GroEL.(7)ADP.transGroES --> 1 FusA_mono + 1 GroEL.(7)ADP.transGroES	irreversible	Protein Folding	0.00E+00	1.00E+09	0	2.04E+03	2.04E+03	0	3.14E+03	3140.993	0	5.11E+02	510.778
b3340_fold_KJE_1	b3340_m folding: KJE mediated	1 DnaJ_dim + 1 DnaK_mono.ATP + 1 b3340_m <==> 1 b3340_m_DnaKJ_complex	reversible	Protein Folding	-1.00E+09	1.00E+09	0	2.04E+03	2.04E+03	0	3.14E+03	3140.993	0	5.11E+02	510.778
b3340_fold_KJE_2	b3340_m folding: KJE mediated	1 GrpE_dim + 1 b3340_m_DnaKJ_complex + 1 h2o --> 1 DnaJ_dim_inact + 1 FusA_DnaK_GrpE_complex + 1 adp + 1 h + 1 pi	irreversible	Protein Folding	0.00E+00	1.00E+09	0	2.04E+03	2.04E+03	0	3.14E+03	3140.993	0	5.11E+02	510.778
b3340_fold_KJE_3	b3340_m folding: KJE mediated	1 FusA_DnaK_GrpE_complex + 1 atp --> 1 DnaK_mono.ATP_inact + 1 FusA_mono + 1 GrpE_dim_inact	irreversible	Protein Folding	0.00E+00	1.00E+09	0	2.04E+03	2.04E+03	0	3.14E+03	3140.993	0	5.11E+02	510.778
b3340_fold_spon	b3340_m folding: spontanous	1 b3340_m --> 1 FusA_mono	irreversible	Protein Folding	0.00E+00	1.00E+09	0	2.04E+03	2.04E+03	0	3.14E+03	3140.993	0	5.11E+02	510.778
b3340_mRNA_CONV	conversion of mRNA to mRNA_1 (synthetic rxn)	1 b3340_mRNA --> 1 b3340_mRNA_1	irreversible	Translation	0.00E+00	1.00E+09	0	2.93E+01	2.93E+01	0	5.17E+01	51.7244	0	5.67E-01	0.567
b3340_mRNA_degr1	Degradation of b3340_mRNA I	1 Orn_dim + 528 atp + 1 b3340_mRNA_2 + 1 degradosome + 528 h2o <==> 528 adp + 1 b3340_mRNA_2_degr + 528 h + 528 pi	reversible	mRNA degradation	-1.00E+09	1.00E+09	0	3.82E+01	3.82E+01	0	6.74E+01	67.35913	0	7.58E-01	0.758

b3340_mRNA_degr2	Degradation of b3340_mRNA II	1 b3340_mRNA_2_degr + 2114 h2o --> 1 Orn_dim_inact + 551 amp 515 cmp + 1 degradosome_inact + 560 gmp + 2114 h + 489 ump	irreversible	mRNA degradation	0.00E+00	7.58E-01	0	3.82E+01	3.82E+01	0	6.74E+01	67.35913	0	7.58E-01	0.758
b3340_maturation1	Polypeptide b3340 peptide deformylas and methionine aminopeptidase complex	1 Def_mono + 1 Map_mono + 1 b3340_aa <==> 1 b3340_def_map_cplx	reversible	Protein Maturation	-1.00E+09	1.00E+09	0	2.04E+03	2.04E+03	0	3.14E+03	3140.993	0	5.11E+02	510.778
b3340_maturation2	b3340 formation	1 b3340_def_map_cplx + 2 h2o --> 1 Def_mono_inact + 1 Map_mono_inact + 1 b3340_m + 1 for + 1 met-L	irreversible	Protein Maturation	0.00E+00	1.00E+09	0	2.04E+03	2.04E+03	0	3.14E+03	3140.993	0	5.11E+02	510.778
b3340_v1_mRNA_CONV	convsersion of mRNA to mRNA_1 (synthetic rxn)	1 b3340_v1_mRNA --> 1 b3340_mRNA_1	irreversible	Translation	0.00E+00	1.00E+09	0	3.82E+01	3.82E+01	0	6.74E+01	67.35913	0	7.58E-01	0.758
b3341_fold_spon	b3341_m folding: spontanous	1 b3341_m --> 1 RpsG_mono	irreversible	Protein Folding	0.00E+00	1.00E+09	8.44E+01	1.13E+02	2.81E+01	6.22E+01	8.29E+01	20.72	6.22E+01	8.29E+01	20.72
b3341_mRNA_CONV	convsersion of mRNA to mRNA_1 (synthetic rxn)	1 b3341_mRNA --> 1 b3341_mRNA_1	irreversible	Translation	0.00E+00	1.00E+09	0	2.93E+01	2.93E+01	0	5.17E+01	51.7244	0	5.67E-01	0.567
b3341_mRNA_degr1	Degradation of b3341_mRNA I	1 Orn_dim + 135 atp + 1 b3341_mRNA_2 + 1 degradosome + 135 h2o <==> 135 adp + 1 b3341_mRNA_2_degr + 135 h + 135 pi	reversible	mRNA degradation	-1.00E+09	1.00E+09	0	2.93E+01	2.93E+01	0	5.17E+01	51.7244	0	5.67E-01	0.567
b3341_mRNA_degr2	Degradation of b3341_mRNA II	1 b3341_mRNA_2_degr + 539 h2o --> 1 Orn_dim_inact + 132 amp + 129 cmp + 1 degradosome_inact + 142 gmp + 539 h + 137 ump	irreversible	mRNA degradation	0.00E+00	5.67E-01	0	2.93E+01	2.93E+01	0	5.17E+01	51.7244	0	5.67E-01	0.567
b3341_maturation1	Polypeptide b3341 peptide deformylas and methionine aminopeptidase complex	1 Def_mono + 1 Map_mono + 1 b3341_aa <==> 1 b3341_def_map_cplx	reversible	Protein Maturation	-1.00E+09	1.00E+09	8.44E+01	1.13E+02	2.81E+01	6.22E+01	8.29E+01	20.72	6.22E+01	8.29E+01	20.72
b3341_maturation2	b3341 formation	1 b3341_def_map_cplx + 2 h2o --> 1 Def_mono_inact + 1 Map_mono_inact + 1 b3341_m + 1 for + 1 met-L	irreversible	Protein Maturation	0.00E+00	1.00E+09	8.44E+01	1.13E+02	2.81E+01	6.22E+01	8.29E+01	20.72	6.22E+01	8.29E+01	20.72
b3342_fold_spon	b3342_m folding: spontanous	1 b3342_m --> 1 RpsL_mono	irreversible	Protein Folding	0.00E+00	1.00E+09	8.44E+01	1.13E+02	2.81E+01	6.22E+01	8.29E+01	20.72	6.22E+01	8.29E+01	20.72
b3342_mRNA_CONV	convsersion of mRNA to mRNA_1 (synthetic rxn)	1 b3342_mRNA --> 1 b3342_mRNA_1	irreversible	Translation	0.00E+00	1.00E+09	0	2.93E+01	2.93E+01	0	5.17E+01	51.7244	0	5.67E-01	0.567
b3342_mRNA_degr1	Degradation of b3342_mRNA I	1 Orn_dim + 93 atp + 1 b3342_mRNA_2 + 1 degradosome + 93 h2o <==> 93 adp + 1 b3342_mRNA_2_degr + 93 h + 93 pi	reversible	mRNA degradation	-1.00E+09	1.00E+09	0	2.93E+01	2.93E+01	0	5.17E+01	51.7244	0	5.67E-01	0.567
b3342_mRNA_degr2	Degradation of b3342_mRNA II	1 b3342_mRNA_2_degr + 374 h2o --> 1 Orn_dim_inact + 90 amp + 1 atp + 101 cmp + 1 degradosome_inact + 99 gmp + 374 h + 84 ump	irreversible	mRNA degradation	0.00E+00	6.73E-01	0	2.93E+01	2.93E+01	0	5.17E+01	51.7244	0	5.67E-01	0.567
b3342_maturation1	Polypeptide b3342 peptide deformylas and methionine aminopeptidase complex	1 Def_mono + 1 Map_mono + 1 b3342_aa <==> 1 b3342_def_map_cplx	reversible	Protein Maturation	-1.00E+09	1.00E+09	8.44E+01	1.13E+02	2.81E+01	6.22E+01	8.29E+01	20.72	6.22E+01	8.29E+01	20.72
b3342_maturation2	b3342 formation	1 b3342_def_map_cplx + 2 h2o --> 1 Def_mono_inact + 1 Map_mono_inact + 1 b3342_m + 1 for + 1 met-L	irreversible	Protein Maturation	0.00E+00	1.00E+09	8.44E+01	1.13E+02	2.81E+01	6.22E+01	8.29E+01	20.72	6.22E+01	8.29E+01	20.72
b3343_fold_spon	b3343_m folding: spontanous	1 b3343_m --> 1 YheL_mono	irreversible	Protein Folding	0.00E+00	1.00E+09	0	4.45E+02	4.45E+02	0	7.29E+02	729.0767	0	6.71E+01	67.074
b3343_mRNA_CONV	convsersion of mRNA to mRNA_1 (synthetic rxn)	1 b3343_mRNA --> 1 b3343_mRNA_1	irreversible	Translation	0.00E+00	1.00E+09	0	7.43E+01	7.43E+01	0	1.31E+02	131.1153	0	1.12E-01	0.112
b3343_mRNA_degr1	Degradation of b3343_mRNA I	1 Orn_dim + 72 atp + 1 b3343_mRNA_2 + 1 degradosome + 72 h2o <==> 72 adp + 1 b3343_mRNA_2_degr + 72 h + 72 pi	reversible	mRNA degradation	-1.00E+09	1.00E+09	0	7.43E+01	7.43E+01	0	1.31E+02	131.1153	0	1.12E-01	0.112
b3343_mRNA_degr2	Degradation of b3343_mRNA II	1 b3343_mRNA_2_degr + 287 h2o --> 1 Orn_dim_inact + 67 amp + 77 cmp + 1 degradosome_inact + 68 gmp + 287 h + 76 ump	irreversible	mRNA degradation	0.00E+00	2.99E-01	0	7.43E+01	7.43E+01	0	1.31E+02	131.1153	0	1.12E-01	0.112
b3343_maturation1	Polypeptide b3343 peptide deformylas and methionine aminopeptidase complex	1 Def_mono + 1 Map_mono + 1 b3343_aa <==> 1 b3343_def_map_cplx	reversible	Protein Maturation	-1.00E+09	1.00E+09	0	4.45E+02	4.45E+02	0	7.29E+02	729.0767	0	6.71E+01	67.074
b3343_maturation2	b3343 formation	1 b3343_def_map_cplx + 2 h2o --> 1 Def_mono_inact + 1 Map_mono_inact + 1 b3343_m + 1 for + 1 met-L	irreversible	Protein Maturation	0.00E+00	1.00E+09	0	4.45E+02	4.45E+02	0	7.29E+02	729.0767	0	6.71E+01	67.074
b3344_fold_spon	b3344_m folding: spontanous	1 b3344_m --> 1 YheM_mono	irreversible	Protein Folding	0.00E+00	1.00E+09	0	4.45E+02	4.45E+02	0	7.29E+02	729.0767	0	6.71E+01	67.074
b3344_mRNA_CONV	convsersion of mRNA to mRNA_1 (synthetic rxn)	1 b3344_mRNA --> 1 b3344_mRNA_1	irreversible	Translation	0.00E+00	1.00E+09	0	7.43E+01	7.43E+01	0	1.31E+02	131.1153	0	1.12E-01	0.112
b3344_mRNA_degr1	Degradation of b3344_mRNA I	1 Orn_dim + 89 atp + 1 b3344_mRNA_2 + 1 degradosome + 89 h2o <==> 89 adp + 1 b3344_mRNA_2_degr + 89 h + 89 pi	reversible	mRNA degradation	-1.00E+09	1.00E+09	0	7.43E+01	7.43E+01	0	1.31E+02	131.1153	0	1.12E-01	0.112
b3344_mRNA_degr2	Degradation of b3344_mRNA II	1 b3344_mRNA_2_degr + 358 h2o --> 1 Orn_dim_inact + 73 amp + 87 cmp + 1 degradosome_inact + 94 gmp + 358 h + 105 ump	irreversible	mRNA degradation	0.00E+00	1.40E-01	0	7.43E+01	7.43E+01	0	1.31E+02	131.1153	0	1.12E-01	0.112
b3344_maturation1	Polypeptide b3344 peptide deformylas and methionine aminopeptidase complex	1 Def_mono + 1 Map_mono + 1 b3344_aa <==> 1 b3344_def_map_cplx	reversible	Protein Maturation	-1.00E+09	1.00E+09	0	4.45E+02	4.45E+02	0	7.29E+02	729.0767	0	6.71E+01	67.074
b3344_maturation2	b3344 formation	1 b3344_def_map_cplx + 2 h2o --> 1 Def_mono_inact + 1 Map_mono_inact + 1 b3344_m + 1 for + 1 met-L	irreversible	Protein Maturation	0.00E+00	1.00E+09	0	4.45E+02	4.45E+02	0	7.29E+02	729.0767	0	6.71E+01	67.074
b3345_fold_spon	b3345_m folding: spontanous	1 b3345_m --> 1 YheN_mono	irreversible	Protein Folding	0.00E+00	1.00E+09	0	4.45E+02	4.45E+02	0	7.29E+02	729.0767	0	6.71E+01	67.074
b3345_mRNA_CONV	convsersion of mRNA to mRNA_1 (synthetic rxn)	1 b3345_mRNA --> 1 b3345_mRNA_1	irreversible	Translation	0.00E+00	1.00E+09	0	7.43E+01	7.43E+01	0	1.31E+02	131.1153	0	1.12E-01	0.112
b3345_mRNA_degr1	Degradation of b3345_mRNA I	1 Orn_dim + 96 atp + 1 b3345_mRNA_2 + 1 degradosome + 96 h2o <==> 96 adp + 1 b3345_mRNA_2_degr + 96 h + 96 pi	reversible	mRNA degradation	-1.00E+09	1.00E+09	0	7.43E+01	7.43E+01	0	1.31E+02	131.1153	0	1.12E-01	0.112
b3345_mRNA_degr2	Degradation of b3345_mRNA II	1 b3345_mRNA_2_degr + 385 h2o --> 1 Orn_dim_inact + 82 amp + 91 cmp + 1 degradosome_inact + 123 gmp + 385 h + 90 ump	irreversible	mRNA degradation	0.00E+00	1.12E-01	0	7.43E+01	7.43E+01	0	1.31E+02	131.1153	0	1.12E-01	0.112
b3345_maturation1	Polypeptide b3345 peptide deformylas and methionine aminopeptidase complex	1 Def_mono + 1 Map_mono + 1 b3345_aa <==> 1 b3345_def_map_cplx	reversible	Protein Maturation	-1.00E+09	1.00E+09	0	4.45E+02	4.45E+02	0	7.29E+02	729.0767	0	6.71E+01	67.074
b3345_maturation2	b3345 formation	1 b3345_def_map_cplx + 2 h2o --> 1 Def_mono_inact + 1 Map_mono_inact + 1 b3345_m + 1 for + 1 met-L	irreversible	Protein Maturation	0.00E+00	1.00E+09	0	4.45E+02	4.45E+02	0	7.29E+02	729.0767	0	6.71E+01	67.074
b3346_fold_spon	b3346_m folding: spontanous	1 b3346_m --> 1 YheO_mono	irreversible	Protein Folding	0.00E+00	1.00E+09	0	1.12E+03	1.12E+03	0	1.92E+03	1917.235	0	8.32E+01	83.188
b3346_mRNA_CONV	convsersion of mRNA to mRNA_1 (synthetic rxn)	1 b3346_mRNA --> 1 b3346_mRNA_1	irreversible	Translation	0.00E+00	1.00E+09	0	7.43E+01	7.43E+01	0	1.31E+02	131.1153	0	1.12E-01	0.112
b3346_mRNA_degr1	Degradation of b3346_mRNA I	1 Orn_dim + 180 atp + 1 b3346_mRNA_2 + 1 degradosome + 180 h2o <==> 180 adp + 1 b3346_mRNA_2_degr + 180 h + 180 pi	reversible	mRNA degradation	-1.00E+09	1.00E+09	0	7.43E+01	7.43E+01	0	1.31E+02	131.1153	0	1.12E-01	0.112
b3346_mRNA_degr2	Degradation of b3346_mRNA II	1 b3346_mRNA_2_degr + 722 h2o --> 1 Orn_dim_inact + 187 amp + 1 atp + 174 cmp + 1 degradosome_inact + 168 gmp + 722 h + 193 ump	irreversible	mRNA degradation	0.00E+00	3.17E-01	0	7.43E+01	7.43E+01	0	1.31E+02	131.1153	0	1.12E-01	0.112
b3346_maturation1	Polypeptide b3346 peptide deformylas and methionine aminopeptidase complex	1 Def_mono + 1 Map_mono + 1 b3346_aa <==> 1 b3346_def_map_cplx	reversible	Protein Maturation	-1.00E+09	1.00E+09	0	1.12E+03	1.12E+03	0	1.92E+03	1917.235	0	8.32E+01	83.188
b3346_maturation2	b3346 formation	1 b3346_def_map_cplx + 2 h2o --> 1 Def_mono_inact + 1 Map_mono_inact + 1 b3346_m + 1 for + 1 met-L	irreversible	Protein Maturation	0.00E+00	1.00E+09	0	1.12E+03	1.12E+03	0	1.92E+03	1917.235	0	8.32E+01	83.188
b3384_fold_KJE_1	b3384_m folding: KJE mediated	1 DnaJ_dim + 1 DnaK_mono.ATP + 1 b3384_m <==> 1 b3384_m_DnaKJ_complex	reversible	Protein Folding	-1.00E+09	1.00E+09	0	2.59E+03	2.59E+03	0	4.49E+03	4488.955	0	1.20E+02	120.251
b3384_fold_KJE_2	b3384_m folding: KJE mediated	1 GrpE_dim + 1 b3384_m_DnaKJ_complex + 1 h2o --> 1 DnaJ_dim_inact + 1 TrpS_DnaK_GrpE_complex + 1 adp + 1 h + 1 pi	irreversible	Protein Folding	0.00E+00	1.00E+09	0	2.59E+03	2.59E+03	0	4.49E+03	4488.955	0	1.20E+02	120.251
b3384_fold_KJE_3	b3384_m folding: KJE mediated	1 TrpS_DnaK_GrpE_complex + 1 atp --> 1 DnaK_mono.ATP_inact + 1 GrpE_dim_inact + 1 TrpS_mono	irreversible	Protein Folding	0.00E+00	1.00E+09	0	2.59E+03	2.59E+03	0	4.49E+03	4488.955	0	1.20E+02	120.251
b3384_fold_spon	b3384_m folding: spontanous	1 b3384_m --> 1 TrpS_mono	irreversible	Protein Folding	0.00E+00	1.00E+09	0	2.59E+03	2.59E+03	0	4.49E+03	4488.955	0	1.20E+02	120.251
b3384_mRNA_CONV	convsersion of mRNA to mRNA_1 (synthetic rxn)	1 b3384_mRNA --> 1 b3384_mRNA_1	irreversible	Translation	0.00E+00	1.00E+09	0	5.36E+01	5.36E+01	0	9.46E+01	94.59664	0	1.89E-01	0.189
b3384_mRNA_degr1	Degradation of b3384_mRNA I	1 Orn_dim + 249 atp + 1 b3384_mRNA_2 + 1 degradosome + 249 h2o <==> 249 adp + 1 b3384_mRNA_2_degr + 249 h + 249 pi	reversible	mRNA degradation	-1.00E+09	1.00E+09	0	1.30E+02	1.30E+02	0	2.30E+02	230.1208	0	1.89E-01	0.189
b3384_mRNA_degr2	Degradation of b3384_mRNA II	1 b3384_mRNA_2_degr + 996 h2o --> 1 Orn_dim_inact + 251 amp + 244 cmp + 1 degradosome_inact + 277 gmp + 996 h + 225 ump	irreversible	mRNA degradation	0.00E+00	1.89E-01	0	1.30E+02	1.30E+02	0	2.30E+02	230.1208	0	1.89E-01	0.189
b3384_maturation1	Polypeptide b3384 peptide deformylas and methionine aminopeptidase complex	1 Def_mono + 1 Map_mono + 1 b3384_aa <==> 1 b3384_def_map_cplx	reversible	Protein Maturation	-1.00E+09	1.00E+09	0	2.59E+03	2.59E+03	0	4.49E+03	4488.955	0	1.20E+02	120.251

b3384_maturation2	b3384 formation	1 b3384_def_map_cplx + 2 h2o --> 1 Def_mono_inact + 1 Map_mono_inact + 1 b3384_m + 1 for + 1 met-L	irreversible	Protein Maturation	0.00E+00	1.00E+09	0	2.59E+03	2.59E+03	0	4.49E+03	4488.955	0	1.20E+02	120.251
b3384_v1_mRNA_CONV	convsersion of mRNA to mRNA_1 (synthetic rxn)	1 b3384_v1_mRNA --> 1 b3384_mRNA_1	irreversible	Translation	0.00E+00	1.00E+09	0	3.98E+01	3.98E+01	0	7.02E+01	70.2112	0	1.74E-01	0.174
b3384_v2_mRNA_CONV	convsersion of mRNA to mRNA_1 (synthetic rxn)	1 b3384_v2_mRNA --> 1 b3384_mRNA_1	irreversible	Translation	0.00E+00	1.00E+09	0	3.98E+01	3.98E+01	0	7.02E+01	70.2112	0	1.74E-01	0.174
b3384_v3_mRNA_CONV	convsersion of mRNA to mRNA_1 (synthetic rxn)	1 b3384_v3_mRNA --> 1 b3384_mRNA_1	irreversible	Translation	0.00E+00	1.00E+09	0	2.71E+01	2.71E+01	0	4.78E+01	47.81984	0	1.74E-01	0.174
b3384_v4_mRNA_CONV	convsersion of mRNA to mRNA_1 (synthetic rxn)	1 b3384_v4_mRNA --> 1 b3384_mRNA_1	irreversible	Translation	0.00E+00	1.00E+09	0	2.01E+01	2.01E+01	0	3.56E+01	35.57072	0	1.04E-01	0.104
b3384_v5_mRNA_CONV	convsersion of mRNA to mRNA_1 (synthetic rxn)	1 b3384_v5_mRNA --> 1 b3384_mRNA_1	irreversible	Translation	0.00E+00	1.00E+09	0	2.01E+01	2.01E+01	0	3.56E+01	35.57072	0	1.04E-01	0.104
b3384_v6_mRNA_CONV	convsersion of mRNA to mRNA_1 (synthetic rxn)	1 b3384_v6_mRNA --> 1 b3384_mRNA_1	irreversible	Translation	0.00E+00	1.00E+09	0	1.30E+02	1.30E+02	0	2.30E+02	230.1208	0	1.89E-01	0.189
b3385_fold_spon	b3385_m folding: spontanous	1 b3385_m --> 1 Gph_mono	irreversible	Protein Folding	0.00E+00	1.00E+09	0	8.33E+02	8.33E+02	0	1.41E+03	1407.233	0	8.55E+01	85.526
b3385_mRNA_CONV	convsersion of mRNA to mRNA_1 (synthetic rxn)	1 b3385_mRNA --> 1 b3385_mRNA_1	irreversible	Translation	0.00E+00	1.00E+09	0	5.36E+01	5.36E+01	0	9.46E+01	94.59664	0	1.89E-01	0.189
b3385_mRNA_degr1	Degradation of b3385_mRNA I	1 Orn_dim + 187 atp + 1 b3385_mRNA_2 + 1 degradosome + 187 h2o <==> 187 adp + 1 b3385_mRNA_2_degr + 187 h + 187 pi	reversible	mRNA degradation	-1.00E+09	1.00E+09	0	5.36E+01	5.36E+01	0	9.46E+01	94.59664	0	1.89E-01	0.189
b3385_mRNA_degr2	Degradation of b3385_mRNA II	1 b3385_mRNA_2_degr + 750 h2o --> 1 Orn_dim_inact + 173 amp + 183 cmp + 1 degradosome_inact + 217 gmp + 750 h + 178 ump	irreversible	mRNA degradation	0.00E+00	1.89E-01	0	5.36E+01	5.36E+01	0	9.46E+01	94.59664	0	1.89E-01	0.189
b3385_maturation1	Polypeptide b3385 peptide deformylas and methionine aminopeptidase complex	1 Def_mono + 1 Map_mono + 1 b3385_aa <==> 1 b3385_def_map_cplx	reversible	Protein Maturation	-1.00E+09	1.00E+09	0	8.33E+02	8.33E+02	0	1.41E+03	1407.233	0	8.55E+01	85.526
b3385_maturation2	b3385 formation	1 b3385_def_map_cplx + 2 h2o --> 1 Def_mono_inact + 1 Map_mono_inact + 1 b3385_m + 1 for + 1 met-L	irreversible	Protein Maturation	0.00E+00	1.00E+09	0	8.33E+02	8.33E+02	0	1.41E+03	1407.233	0	8.55E+01	85.526
b3385_v1_mRNA_CONV	convsersion of mRNA to mRNA_1 (synthetic rxn)	1 b3385_v1_mRNA --> 1 b3385_mRNA_1	irreversible	Translation	0.00E+00	1.00E+09	0	3.98E+01	3.98E+01	0	7.02E+01	70.2112	0	1.74E-01	0.174
b3385_v2_mRNA_CONV	convsersion of mRNA to mRNA_1 (synthetic rxn)	1 b3385_v2_mRNA --> 1 b3385_mRNA_1	irreversible	Translation	0.00E+00	1.00E+09	0	3.98E+01	3.98E+01	0	7.02E+01	70.2112	0	1.74E-01	0.174
b3385_v3_mRNA_CONV	convsersion of mRNA to mRNA_1 (synthetic rxn)	1 b3385_v3_mRNA --> 1 b3385_mRNA_1	irreversible	Translation	0.00E+00	1.00E+09	0	2.71E+01	2.71E+01	0	4.78E+01	47.81984	0	1.74E-01	0.174
b3385_v4_mRNA_CONV	convsersion of mRNA to mRNA_1 (synthetic rxn)	1 b3385_v4_mRNA --> 1 b3385_mRNA_1	irreversible	Translation	0.00E+00	1.00E+09	0	2.01E+01	2.01E+01	0	3.56E+01	35.57072	0	1.04E-01	0.104
b3385_v5_mRNA_CONV	convsersion of mRNA to mRNA_1 (synthetic rxn)	1 b3385_v5_mRNA --> 1 b3385_mRNA_1	irreversible	Translation	0.00E+00	1.00E+09	0	2.01E+01	2.01E+01	0	3.56E+01	35.57072	0	1.04E-01	0.104
b3386_fold_spon	b3386_m folding: spontanous	1 b3386_m --> 1 Rpe_mono	irreversible	Protein Folding	0.00E+00	1.00E+09	0	8.20E+02	8.20E+02	0	1.35E+03	1353.646	0	1.26E+02	126.347
b3386_mRNA_CONV	convsersion of mRNA to mRNA_1 (synthetic rxn)	1 b3386_mRNA --> 1 b3386_mRNA_1	irreversible	Translation	0.00E+00	1.00E+09	0	5.36E+01	5.36E+01	0	9.46E+01	94.59664	0	1.89E-01	0.189
b3386_mRNA_degr1	Degradation of b3386_mRNA I	1 Orn_dim + 169 atp + 1 b3386_mRNA_2 + 1 degradosome + 169 h2o <==> 169 adp + 1 b3386_mRNA_2_degr + 169 h + 169 pi	reversible	mRNA degradation	-1.00E+09	1.00E+09	0	5.36E+01	5.36E+01	0	9.46E+01	94.59664	0	1.89E-01	0.189
b3386_mRNA_degr2	Degradation of b3386_mRNA II	1 b3386_mRNA_2_degr + 677 h2o --> 1 Orn_dim_inact + 161 amp + 1 atp + 169 cmp + 1 degradosome_inact + 177 gmp + 677 h + 170 ump	irreversible	mRNA degradation	0.00E+00	1.89E-01	0	5.36E+01	5.36E+01	0	9.46E+01	94.59664	0	1.89E-01	0.189
b3386_maturation1	Polypeptide b3386 peptide deformylas and methionine aminopeptidase complex	1 Def_mono + 1 Map_mono + 1 b3386_aa <==> 1 b3386_def_map_cplx	reversible	Protein Maturation	-1.00E+09	1.00E+09	0	8.20E+02	8.20E+02	0	1.35E+03	1353.646	0	1.26E+02	126.347
b3386_maturation2	b3386 formation	1 b3386_def_map_cplx + 2 h2o --> 1 Def_mono_inact + 1 Map_mono_inact + 1 b3386_m + 1 for + 1 met-L	irreversible	Protein Maturation	0.00E+00	1.00E+09	0	8.20E+02	8.20E+02	0	1.35E+03	1353.646	0	1.26E+02	126.347
b3386_v1_mRNA_CONV	convsersion of mRNA to mRNA_1 (synthetic rxn)	1 b3386_v1_mRNA --> 1 b3386_mRNA_1	irreversible	Translation	0.00E+00	1.00E+09	0	3.98E+01	3.98E+01	0	7.02E+01	70.2112	0	1.74E-01	0.174
b3386_v2_mRNA_CONV	convsersion of mRNA to mRNA_1 (synthetic rxn)	1 b3386_v2_mRNA --> 1 b3386_mRNA_1	irreversible	Translation	0.00E+00	1.00E+09	0	3.98E+01	3.98E+01	0	7.02E+01	70.2112	0	1.74E-01	0.174
b3386_v3_mRNA_CONV	convsersion of mRNA to mRNA_1 (synthetic rxn)	1 b3386_v3_mRNA --> 1 b3386_mRNA_1	irreversible	Translation	0.00E+00	1.00E+09	0	2.71E+01	2.71E+01	0	4.78E+01	47.81984	0	1.74E-01	0.174
b3386_v4_mRNA_CONV	convsersion of mRNA to mRNA_1 (synthetic rxn)	1 b3386_v4_mRNA --> 1 b3386_mRNA_1	irreversible	Translation	0.00E+00	1.00E+09	0	2.01E+01	2.01E+01	0	3.56E+01	35.57072	0	1.04E-01	0.104
b3386_v5_mRNA_CONV	convsersion of mRNA to mRNA_1 (synthetic rxn)	1 b3386_v5_mRNA --> 1 b3386_mRNA_1	irreversible	Translation	0.00E+00	1.00E+09	0	2.01E+01	2.01E+01	0	3.56E+01	35.57072	0	1.04E-01	0.104
b3387_fold_spon	b3387_m folding: spontanous	1 b3387_m --> 1 Dam_mono	irreversible	Protein Folding	0.00E+00	1.00E+09	0	7.06E+02	7.06E+02	0	1.19E+03	1193.139	0	7.25E+01	72.544
b3387_mRNA_CONV	convsersion of mRNA to mRNA_1 (synthetic rxn)	1 b3387_mRNA --> 1 b3387_mRNA_1	irreversible	Translation	0.00E+00	1.00E+09	0	3.98E+01	3.98E+01	0	7.02E+01	70.2112	0	1.74E-01	0.174
b3387_mRNA_degr1	Degradation of b3387_mRNA I	1 Orn_dim + 209 atp + 1 b3387_mRNA_2 + 1 degradosome + 209 h2o <==> 209 adp + 1 b3387_mRNA_2_degr + 209 h + 209 pi	reversible	mRNA degradation	-1.00E+09	1.00E+09	0	3.98E+01	3.98E+01	0	7.02E+01	70.2112	0	1.74E-01	0.174
b3387_mRNA_degr2	Degradation of b3387_mRNA II	1 b3387_mRNA_2_degr + 836 h2o --> 1 Orn_dim_inact + 207 amp + 1 atp + 204 cmp + 1 degradosome_inact + 211 gmp + 836 h + 214 ump	irreversible	mRNA degradation	0.00E+00	1.74E-01	0	3.98E+01	3.98E+01	0	7.02E+01	70.2112	0	1.74E-01	0.174
b3387_maturation1	Polypeptide b3387 peptide deformylas and methionine aminopeptidase complex	1 Def_mono + 1 Map_mono + 1 b3387_aa <==> 1 b3387_def_map_cplx	reversible	Protein Maturation	-1.00E+09	1.00E+09	0	7.06E+02	7.06E+02	0	1.19E+03	1193.139	0	7.25E+01	72.544
b3387_maturation2	b3387 formation	1 b3387_def_map_cplx + 2 h2o --> 1 Def_mono_inact + 1 Map_mono_inact + 1 b3387_m + 1 for + 1 met-L	irreversible	Protein Maturation	0.00E+00	1.00E+09	0	7.06E+02	7.06E+02	0	1.19E+03	1193.139	0	7.25E+01	72.544
b3387_v1_mRNA_CONV	convsersion of mRNA to mRNA_1 (synthetic rxn)	1 b3387_v1_mRNA --> 1 b3387_mRNA_1	irreversible	Translation	0.00E+00	1.00E+09	0	3.98E+01	3.98E+01	0	7.02E+01	70.2112	0	1.74E-01	0.174
b3387_v2_mRNA_CONV	convsersion of mRNA to mRNA_1 (synthetic rxn)	1 b3387_v2_mRNA --> 1 b3387_mRNA_1	irreversible	Translation	0.00E+00	1.00E+09	0	2.71E+01	2.71E+01	0	4.78E+01	47.81984	0	1.74E-01	0.174
b3387_v3_mRNA_CONV	convsersion of mRNA to mRNA_1 (synthetic rxn)	1 b3387_v3_mRNA --> 1 b3387_mRNA_1	irreversible	Translation	0.00E+00	1.00E+09	0	2.01E+01	2.01E+01	0	3.56E+01	35.57072	0	1.04E-01	0.104
b3387_v4_mRNA_CONV	convsersion of mRNA to mRNA_1 (synthetic rxn)	1 b3387_v4_mRNA --> 1 b3387_mRNA_1	irreversible	Translation	0.00E+00	1.00E+09	0	2.01E+01	2.01E+01	0	3.56E+01	35.57072	0	1.04E-01	0.104
b3388_fold_spon	b3388_m folding: spontanous	1 b3388_m --> 1 DamX_mono	irreversible	Protein Folding	0.00E+00	1.00E+09	0	7.48E+02	7.48E+02	0	1.27E+03	1266.246	0	7.51E+01	75.1
b3388_mRNA_CONV	convsersion of mRNA to mRNA_1 (synthetic rxn)	1 b3388_mRNA --> 1 b3388_mRNA_1	irreversible	Translation	0.00E+00	1.00E+09	0	2.71E+01	2.71E+01	0	4.78E+01	47.81984	0	1.74E-01	0.174
b3388_mRNA_degr1	Degradation of b3388_mRNA I	1 Orn_dim + 321 atp + 1 b3388_mRNA_2 + 1 degradosome + 321 h2o <==> 321 adp + 1 b3388_mRNA_2_degr + 321 h + 321 pi	reversible	mRNA degradation	-1.00E+09	1.00E+09	0	2.71E+01	2.71E+01	0	4.78E+01	47.81984	0	1.74E-01	0.174
b3388_mRNA_degr2	Degradation of b3388_mRNA II	1 b3388_mRNA_2_degr + 1286 h2o --> 1 Orn_dim_inact + 351 amp + 1 atp + 353 cmp + 1 degradosome_inact + 349 gmp + 1286 h + 233 ump	irreversible	mRNA degradation	0.00E+00	2.00E-01	0	2.71E+01	2.71E+01	0	4.78E+01	47.81984	0	1.74E-01	0.174
b3388_maturation1	Polypeptide b3388 peptide deformylas and methionine aminopeptidase complex	1 Def_mono + 1 Map_mono + 1 b3388_aa <==> 1 b3388_def_map_cplx	reversible	Protein Maturation	-1.00E+09	1.00E+09	0	7.48E+02	7.48E+02	0	1.27E+03	1266.246	0	7.51E+01	75.1
b3388_maturation2	b3388 formation	1 b3388_def_map_cplx + 2 h2o --> 1 Def_mono_inact + 1 Map_mono_inact + 1 b3388_m + 1 for + 1 met-L	irreversible	Protein Maturation	0.00E+00	1.00E+09	0	7.48E+02	7.48E+02	0	1.27E+03	1266.246	0	7.51E+01	75.1
b3388_v1_mRNA_CONV	convsersion of mRNA to mRNA_1 (synthetic rxn)	1 b3388_v1_mRNA --> 1 b3388_mRNA_1	irreversible	Translation	0.00E+00	1.00E+09	0	2.01E+01	2.01E+01	0	3.56E+01	35.57072	0	1.04E-01	0.104
b3388_v2_mRNA_CONV	convsersion of mRNA to mRNA_1 (synthetic rxn)	1 b3388_v2_mRNA --> 1 b3388_mRNA_1	irreversible	Translation	0.00E+00	1.00E+09	0	2.01E+01	2.01E+01	0	3.56E+01	35.57072	0	1.04E-01	0.104
b3389_fold_spon	b3389_m folding: spontanous	1 b3389_m --> 1 AroB_mono	irreversible	Protein Folding	0.00E+00	1.00E+09	0	4.99E+02	4.99E+02	0	8.23E+02	822.7952	0	7.80E+01	77.994
b3389_mRNA_CONV	convsersion of mRNA to mRNA_1 (synthetic rxn)	1 b3389_mRNA --> 1 b3389_mRNA_1	irreversible	Translation	0.00E+00	1.00E+09	0	2.01E+01	2.01E+01	0	3.56E+01	35.57072	0	1.04E-01	0.104
b3389_mRNA_degr1	Degradation of b3389_mRNA I	1 Orn_dim + 272 atp + 1 b3389_mRNA_2 + 1 degradosome + 272 h2o <==> 272 adp + 1 b3389_mRNA_2_degr + 272 h + 272 pi	reversible	mRNA degradation	-1.00E+09	1.00E+09	0	2.01E+01	2.01E+01	0	3.56E+01	35.57072	0	1.04E-01	0.104

b3389_mRNA_degr2	Degradation of b3389_mRNA II	1 b3389_mRNA_2_degr + 1088 h2o --> 1 Orn_dim_inact + 223 amp 265 cmp + 1 degradosome_inact + 325 gmp + 1088 h + 276 ump	irreversible	mRNA degradation	0.00E+00	3.17E-01	0	2.01E+01	2.01E+01	0	3.56E+01	35.57072	0	1.04E-01	0.104
b3389_maturation1	Polypeptide b3389 peptide deformylas and methionine aminopeptidase complex	1 Def_mono + 1 Map_mono + 1 b3389_aa <==> 1 b3389_def_map_cplx	reversible	Protein Maturation	-1.00E+09	1.00E+09	0	4.99E+02	4.99E+02	0	8.23E+02	822.7952	0	7.80E+01	77.994
b3389_maturation2	b3389 formation	1 b3389_def_map_cplx + 2 h2o --> 1 Def_mono_inact + 1 Map_mono_inact + 1 b3389_m + 1 for + 1 met-L	irreversible	Protein Maturation	0.00E+00	1.00E+09	0	4.99E+02	4.99E+02	0	8.23E+02	822.7952	0	7.80E+01	77.994
b3389_v1_mRNA_CONV	convsersion of mRNA to mRNA_1 (synthetic rxn)	1 b3389_v1_mRNA --> 1 b3389_mRNA_1	irreversible	Translation	0.00E+00	1.00E+09	0	2.01E+01	2.01E+01	0	3.56E+01	35.57072	0	1.04E-01	0.104
b3390_fold_GroEL/ES_1	b3390_m folding: GroEL/ES mediated polypeptide is going in GroEL/ES complex	1 GroEL.(7)ADP.cisGroES + 7 atp + 1 b3390_m + 1 transGroES_hepta --> 7 adp + 1 b3390_m_GroEL.(7)ATP.transGroES + 1 cisGroES_hepta	irreversible	Protein Folding	0.00E+00	1.00E+09	0	2.77E+02	2.77E+02	0	4.31E+02	431.2072	0	7.65E+01	76.54
b3390_fold_GroEL/ES_2	b3390_m folding: GroEL/ES mediated: folding of polypeptide under ATP hydrolysis	1 b3390_m_GroEL.(7)ATP.transGroES + 7 h2o --> 1 AroK_GroEL.(7)ADP.transGroES + 7 h + 7 pi	irreversible	Protein Folding	0.00E+00	1.00E+09	0	2.77E+02	2.77E+02	0	4.31E+02	431.2072	0	7.65E+01	76.54
b3390_fold_GroEL/ES_3	b3390_m folding: GroEL/ES mediated: release of native protein	1 AroK_GroEL.(7)ADP.transGroES --> 1 AroK_mono + 1 GroEL.(7)ADP.transGroES	irreversible	Protein Folding	0.00E+00	1.00E+09	0	2.77E+02	2.77E+02	0	4.31E+02	431.2072	0	7.65E+01	76.54
b3390_fold_KJE_1	b3390_m folding: KJE mediated	1 DnaJ_dim + 1 DnaK_mono.ATP + 1 b3390_m <==> 1 b3390_m_DnaKJ_complex	reversible	Protein Folding	-1.00E+09	1.00E+09	0	2.77E+02	2.77E+02	0	4.31E+02	431.2072	0	7.65E+01	76.54
b3390_fold_KJE_2	b3390_m folding: KJE mediated	1 GrpE_dim + 1 b3390_m_DnaKJ_complex + 1 h2o --> 1 AroK_DnaK_GrpE_complex + 1 DnaJ_dim_inact + 1 adp + 1 h + 1 pi	irreversible	Protein Folding	0.00E+00	1.00E+09	0	2.77E+02	2.77E+02	0	4.31E+02	431.2072	0	7.65E+01	76.54
b3390_fold_KJE_3	b3390_m folding: KJE mediated	1 AroK_DnaK_GrpE_complex + 1 atp --> 1 AroK_mono + 1 DnaK_mono.ATP_inact + 1 GrpE_dim_inact	irreversible	Protein Folding	0.00E+00	1.00E+09	0	2.77E+02	2.77E+02	0	4.31E+02	431.2072	0	7.65E+01	76.54
b3390_mRNA_CONV	convsersion of mRNA to mRNA_1 (synthetic rxn)	1 b3390_mRNA --> 1 b3390_mRNA_1	irreversible	Translation	0.00E+00	1.00E+09	0	2.01E+01	2.01E+01	0	3.56E+01	35.57072	0	1.04E-01	0.104
b3390_mRNA_degr1	Degradation of b3390_mRNA I	1 Orn_dim + 130 atp + 1 b3390_mRNA_2 + 1 degradosome + 130 h2o <==> 130 adp + 1 b3390_mRNA_2_degr + 130 h + 130 pi	reversible	mRNA degradation	-1.00E+09	1.00E+09	0	2.01E+01	2.01E+01	0	3.56E+01	35.57072	0	1.04E-01	0.104
b3390_mRNA_degr2	Degradation of b3390_mRNA II	1 b3390_mRNA_2_degr + 521 h2o --> 1 Orn_dim_inact + 146 amp + 1 atp + 115 cmp + 1 degradosome_inact + 142 gmp + 521 h + 118 ump	irreversible	mRNA degradation	0.00E+00	1.04E-01	0	2.01E+01	2.01E+01	0	3.56E+01	35.57072	0	1.04E-01	0.104
b3390_maturation1	Polypeptide b3390 peptide deformylas and methionine aminopeptidase complex	1 Def_mono + 1 Map_mono + 1 b3390_aa <==> 1 b3390_def_map_cplx	reversible	Protein Maturation	-1.00E+09	1.00E+09	0	2.77E+02	2.77E+02	0	4.31E+02	431.2072	0	7.65E+01	76.54
b3390_maturation2	b3390 formation	1 b3390_def_map_cplx + 2 h2o --> 1 Def_mono_inact + 1 Map_mono_inact + 1 b3390_m + 1 for + 1 met-L	irreversible	Protein Maturation	0.00E+00	1.00E+09	0	2.77E+02	2.77E+02	0	4.31E+02	431.2072	0	7.65E+01	76.54
b3390_v1_mRNA_CONV	convsersion of mRNA to mRNA_1 (synthetic rxn)	1 b3390_v1_mRNA --> 1 b3390_mRNA_1	irreversible	Translation	0.00E+00	1.00E+09	0	2.01E+01	2.01E+01	0	3.56E+01	35.57072	0	1.04E-01	0.104
b3406_fold_spon	b3406_m folding: spontanous	1 b3406_m --> 1 GreB_mono	irreversible	Protein Folding	0.00E+00	1.00E+09	0	2.69E+03	2.69E+03	0	4.69E+03	4687.216	0	7.52E+01	75.1905
b3406_mRNA_CONV	convsersion of mRNA to mRNA_1 (synthetic rxn)	1 b3406_mRNA --> 1 b3406_mRNA_1	irreversible	Translation	0.00E+00	1.00E+09	0	2.90E+02	2.90E+02	0	5.13E+02	512.5418	0	9.45E-02	0.0945
b3406_mRNA_degr1	Degradation of b3406_mRNA I	1 Orn_dim + 119 atp + 1 b3406_mRNA_2 + 1 degradosome + 119 h2o <==> 119 adp + 1 b3406_mRNA_2_degr + 119 h + 119 pi	reversible	mRNA degradation	-1.00E+09	1.00E+09	0	2.90E+02	2.90E+02	0	5.13E+02	512.5418	0	9.45E-02	0.0945
b3406_mRNA_degr2	Degradation of b3406_mRNA II	1 b3406_mRNA_2_degr + 476 h2o --> 1 Orn_dim_inact + 132 amp + 1 atp + 110 cmp + 1 degradosome_inact + 128 gmp + 476 h + 106 ump	irreversible	mRNA degradation	0.00E+00	9.45E-02	0	2.90E+02	2.90E+02	0	5.13E+02	512.5418	0	9.45E-02	0.0945
b3406_maturation1	Polypeptide b3406 peptide deformylas complex	1 Def_mono + 1 b3406_aa <==> 1 b3406_def_cplx	reversible	Protein Maturation	-1.00E+09	1.00E+09	0	2.69E+03	2.69E+03	0	4.69E+03	4687.216	0	7.52E+01	75.1905
b3406_maturation2	b3406 formation	1 b3406_def_cplx + 1 h2o --> 1 Def_mono_inact + 1 b3406_m + 1 for	irreversible	Protein Maturation	0.00E+00	1.00E+09	0	2.69E+03	2.69E+03	0	4.69E+03	4687.216	0	7.52E+01	75.1905
b3461_fold_spon	b3461_m folding: spontanous	1 b3461_m --> 1 RpoH_mono	irreversible	Protein Folding	0.00E+00	1.00E+09	0	2.45E+03	2.45E+03	0	4.25E+03	4251.581	0	9.93E+01	99.344
b3461_mRNA_CONV	convsersion of mRNA to mRNA_1 (synthetic rxn)	1 b3461_mRNA --> 1 b3461_mRNA_1	irreversible	Translation	0.00E+00	1.00E+09	0	1.47E+02	1.47E+02	0	2.60E+02	259.8138	0	2.99E-01	0.299
b3461_mRNA_degr1	Degradation of b3461_mRNA I	1 Orn_dim + 213 atp + 1 b3461_mRNA_2 + 1 degradosome + 213 h2o <==> 213 adp + 1 b3461_mRNA_2_degr + 213 h + 213 pi	reversible	mRNA degradation	-1.00E+09	1.00E+09	0	1.47E+02	1.47E+02	0	2.60E+02	259.8138	0	2.99E-01	0.299
b3461_mRNA_degr2	Degradation of b3461_mRNA II	1 b3461_mRNA_2_degr + 854 h2o --> 1 Orn_dim_inact + 211 amp + 1 atp + 217 cmp + 1 degradosome_inact + 246 gmp + 854 h + 180 ump	irreversible	mRNA degradation	0.00E+00	2.99E-01	0	1.47E+02	1.47E+02	0	2.60E+02	259.8138	0	2.99E-01	0.299
b3461_maturation1	Polypeptide b3461 peptide deformylas and methionine aminopeptidase complex	1 Def_mono + 1 Map_mono + 1 b3461_aa <==> 1 b3461_def_map_cplx	reversible	Protein Maturation	-1.00E+09	1.00E+09	0	2.45E+03	2.45E+03	0	4.25E+03	4251.581	0	9.93E+01	99.344
b3461_maturation2	b3461 formation	1 b3461_def_map_cplx + 2 h2o --> 1 Def_mono_inact + 1 Map_mono_inact + 1 b3461_m + 1 for + 1 met-L	irreversible	Protein Maturation	0.00E+00	1.00E+09	0	2.45E+03	2.45E+03	0	4.25E+03	4251.581	0	9.93E+01	99.344
b3461_v1_mRNA_CONV	convsersion of mRNA to mRNA_1 (synthetic rxn)	1 b3461_v1_mRNA --> 1 b3461_mRNA_1	irreversible	Translation	0.00E+00	1.00E+09	0	1.47E+02	1.47E+02	0	2.60E+02	259.8138	0	2.99E-01	0.299
b3461_v2_mRNA_CONV	convsersion of mRNA to mRNA_1 (synthetic rxn)	1 b3461_v2_mRNA --> 1 b3461_mRNA_1	irreversible	Translation	0.00E+00	1.00E+09	0	1.47E+02	1.47E+02	0	2.60E+02	259.8138	0	2.99E-01	0.299
b3461_v3_mRNA_CONV	convsersion of mRNA to mRNA_1 (synthetic rxn)	1 b3461_v3_mRNA --> 1 b3461_mRNA_1	irreversible	Translation	0.00E+00	1.00E+09	0	1.47E+02	1.47E+02	0	2.60E+02	259.8138	0	2.99E-01	0.299
b3461_v4_mRNA_CONV	convsersion of mRNA to mRNA_1 (synthetic rxn)	1 b3461_v4_mRNA --> 1 b3461_mRNA_1	irreversible	Translation	0.00E+00	1.00E+09	0	1.47E+02	1.47E+02	0	2.60E+02	259.8138	0	2.99E-01	0.299
b3461_v5_mRNA_CONV	convsersion of mRNA to mRNA_1 (synthetic rxn)	1 b3461_v5_mRNA --> 1 b3461_mRNA_1	irreversible	Translation	0.00E+00	1.00E+09	0	1.47E+02	1.47E+02	0	2.60E+02	259.8138	0	2.99E-01	0.299
b3470_fold_spon	b3470_m folding: spontanous	1 b3470_m --> 1 YhhP_mono	irreversible	Protein Folding	0.00E+00	1.00E+09	0	1.99E+03	1.99E+03	0	3.45E+03	3448.746	0	8.36E+01	83.6
b3470_mRNA_CONV	convsersion of mRNA to mRNA_1 (synthetic rxn)	1 b3470_mRNA --> 1 b3470_mRNA_1	irreversible	Translation	0.00E+00	1.00E+09	0	4.77E+02	4.77E+02	0	8.41E+02	841.4865	0	2.00E-01	0.2
b3470_mRNA_degr1	Degradation of b3470_mRNA I	1 Orn_dim + 61 atp + 1 b3470_mRNA_2 + 1 degradosome + 61 h2o <==> 61 adp + 1 b3470_mRNA_2_degr + 61 h + 61 pi	reversible	mRNA degradation	-1.00E+09	1.00E+09	0	4.77E+02	4.77E+02	0	8.41E+02	841.4865	0	2.00E-01	0.2
b3470_mRNA_degr2	Degradation of b3470_mRNA II	1 b3470_mRNA_2_degr + 245 h2o --> 1 Orn_dim_inact + 52 amp + 1 atp + 67 cmp + 1 degradosome_inact + 65 gmp + 245 h + 61 ump	irreversible	mRNA degradation	0.00E+00	2.00E-01	0	4.77E+02	4.77E+02	0	8.41E+02	841.4865	0	2.00E-01	0.2
b3470_maturation1	Polypeptide b3470 peptide deformylas and methionine aminopeptidase complex	1 Def_mono + 1 Map_mono + 1 b3470_aa <==> 1 b3470_def_map_cplx	reversible	Protein Maturation	-1.00E+09	1.00E+09	0	1.99E+03	1.99E+03	0	3.45E+03	3448.746	0	8.36E+01	83.6
b3470_maturation2	b3470 formation	1 b3470_def_map_cplx + 2 h2o --> 1 Def_mono_inact + 1 Map_mono_inact + 1 b3470_m + 1 for + 1 met-L	irreversible	Protein Maturation	0.00E+00	1.00E+09	0	1.99E+03	1.99E+03	0	3.45E+03	3448.746	0	8.36E+01	83.6
b3545_tRNA_20_MODIF_1	modification of position 20 nt (D) in proK by Dus_gen	1 Dus_gen + 1 b3545_tRNA_Mg2 + 1 h + 1 nadph <==> 1 b3545_tRNA_Mg2_Dus_gen_cplx	reversible	tRNA modification	-1.00E+09	1.00E+09	0	1.52E+03	1.52E+03	0	13	13	0	13	13
b3545_tRNA_20_MODIF_2	modification of position 20 nt (D) in proK by Dus_gen	1 b3545_tRNA_Mg2_Dus_gen_cplx --> 1 Dus_gen_inact + 1 b3545_tRNA_1 + 1 nadp	irreversible	tRNA modification	0.00E+00	1.00E+09	0	1.52E+03	1.52E+03	0	13	13	0	13	13
b3545_tRNA_32_MODIF_1	modification of position 32 nt (Um) in proK by MeT_tRNA_pos_32_Um	1 MeT_tRNA_pos_32_Um + 1 amet + 1 b3545_tRNA_1 <==> 1 b3545_tRNA_1_MeT_tRNA_pos_32_Um_cplx	reversible	tRNA modification	-1.00E+09	1.00E+09	0	1.52E+03	1.52E+03	0	13	13	0	13	13
b3545_tRNA_32_MODIF_2	modification of position 32 nt (Um) in proK by MeT_tRNA_pos_32_Um	1 b3545_tRNA_1_MeT_tRNA_pos_32_Um_cplx --> 1 MeT_tRNA_pos_32_Um_inact + 1 ahcys + 1 b3545_tRNA_2 + 1 h	irreversible	tRNA modification	0.00E+00	1.00E+09	0	1.52E+03	1.52E+03	0	13	13	0	13	13
b3545_tRNA_37_MODIF_1	modification of position 37 nt (m1G) in proK by TrmD_dim	1 TrmD_dim + 1 amet + 1 b3545_tRNA_2 <==> 1 b3545_tRNA_2_TrmD_dim_cplx	reversible	tRNA modification	-1.00E+09	1.00E+09	0	1.52E+03	1.52E+03	0	13	13	0	13	13
b3545_tRNA_37_MODIF_2	modification of position 37 nt (m1G) in proK by TrmD_dim	1 b3545_tRNA_2_TrmD_dim_cplx --> 1 TrmD_dim_inact + 1 ahcys + 1 b3545_tRNA_3 + 1 h	irreversible	tRNA modification	0.00E+00	1.00E+09	0	1.52E+03	1.52E+03	0	13	13	0	13	13
b3545_tRNA_46_MODIF_1	modification of position 46 nt (m7G) in proK by YggH_mono	1 YggH_mono + 1 amet + 1 b3545_tRNA_3 <==> 1 b3545_tRNA_3_YggH_mono_cplx	reversible	tRNA modification	-1.00E+09	1.00E+09	0	1.52E+03	1.52E+03	0	13	13	0	13	13
b3545_tRNA_46_MODIF_2	modification of position 46 nt (m7G) in proK by YggH_mono	1 b3545_tRNA_3_YggH_mono_cplx --> 1 YggH_mono_inact + 1 ahcys + 1 b3545_tRNA_4 + 1 h	irreversible	tRNA modification	0.00E+00	1.00E+09	0	1.52E+03	1.52E+03	0	13	13	0	13	13
b3545_tRNA_54_MODIF_1	modification of position 54 nt (m5U) in proK by TrmA_mono	1 TrmA_mono + 1 amet + 1 b3545_tRNA_4 <==> 1 b3545_tRNA_4_TrmA_mono_cplx	reversible	tRNA modification	-1.00E+09	1.00E+09	0	1.52E+03	1.52E+03	0	13	13	0	13	13

b3545_tRNA_54_MODIF_2	modification of position 54 nt (m5U) in proK by TrmA_mono	1 b3545_tRNA_4_TrmA_mono_cplx --> 1 TrmA_mono_inact + 1 ahcys + 1 b3545_tRNA_5 + 1 h	irreversible	tRNA modification	0.00E+00	1.00E+09	0	1.52E+03	1.52E+03	0	13	13	0	13	13
b3545_tRNA_55_MODIF_1	modification of position 55 nt (Y) in proK by TruB_mono	1 TruB_mono + 1 b3545_tRNA_5 <==> 1 b3545_tRNA_5_TruB_mono_cplx	reversible	tRNA modification	-1.00E+09	1.00E+09	0	1.52E+03	1.52E+03	0	13	13	0	13	13
b3545_tRNA_55_MODIF_2	modification of position 55 nt (Y) in proK by TruB_mono	1 b3545_tRNA_5_TruB_mono_cplx --> 1 TruB_mono_inact + 1 b3545_tRNA_6	irreversible	tRNA modification	0.00E+00	1.00E+09	0	1.52E+03	1.52E+03	0	13	13	0	13	13
b3545_tRNA_8_MODIF_1	modification of position 8 nt (s4U) in proK by ThiI_mono	1 IscS_dim_S-SH + 1 ThiI_mono + 1 atp + 1 b3545_tRNA_6 + 1 mg2 + 1 trdrd <==> 1 b3545_tRNA_6_ThiI_mono_cplx	reversible	tRNA modification	-1.00E+09	1.00E+09	0	1.52E+03	1.52E+03	0	13	13	0	13	13
b3545_tRNA_8_MODIF_2	modification of position 8 nt (s4U) in proK by ThiI_mono	1 b3545_tRNA_6_ThiI_mono_cplx --> 1 IscS_dim_S-H + 1 ThiI_mono_inact + 1 amp + 1 h + 1 mg2 + 1 ppi + 1 proK_tRNA + 1 trdox	irreversible	tRNA modification	0.00E+00	1.00E+09	0	1.52E+03	1.52E+03	0	13	13	0	13	13
b3545_tRNA_Mg_BIND	Binding of two Mg2 to b3545_tRNA	1 b3545_RNA + 2 mg2 <==> 1 b3545_tRNA_Mg2	reversible	Metallo-ion binding	-1.00E+09	1.00E+09	0	1.52E+03	1.52E+03	0	13	13	0	13	13
b3559_fold_spon	b3559_m folding: spontanous	1 b3559_m --> 1 GlyS_mono	irreversible	Protein Folding	0.00E+00	1.00E+09	0	7.52E+02	7.52E+02	0	1.28E+03	1275.12	0	7.15E+01	71.519
b3559_mRNA_CONV	convsersion of mRNA to mRNA_1 (synthetic rxn)	1 b3559_mRNA --> 1 b3559_mRNA_1	irreversible	Translation	0.00E+00	1.00E+09	0	4.02E+01	4.02E+01	0	7.10E+01	71.00705	0	2.07E-01	0.207
b3559_mRNA_degr1	Degradation of b3559_mRNA I	1 Orn_dim + 517 atp + 1 b3559_mRNA_2 + 1 degradosome + 517 h2o <==> 517 adp + 1 b3559_mRNA_2_degr + 517 h + 517 pi	reversible	mRNA degradation	-1.00E+09	1.00E+09	0	4.02E+01	4.02E+01	0	7.10E+01	71.00705	0	2.07E-01	0.207
b3559_mRNA_degr2	Degradation of b3559_mRNA II	1 b3559_mRNA_2_degr + 2069 h2o --> 1 Orn_dim_inact + 472 amp 533 cmp + 1 degradosome_inact + 614 gmp + 2069 h + 451 ump	irreversible	mRNA degradation	0.00E+00	2.07E-01	0	4.02E+01	4.02E+01	0	7.10E+01	71.00705	0	2.07E-01	0.207
b3559_maturation1	Polypeptide b3559 peptide deformylas and methionine aminopeptidase complex	1 Def_mono + 1 Map_mono + 1 b3559_aa <==> 1 b3559_def_map_cplx	reversible	Protein Maturation	-1.00E+09	1.00E+09	0	7.52E+02	7.52E+02	0	1.28E+03	1275.12	0	7.15E+01	71.519
b3559_maturation2	b3559 formation	1 b3559_def_map_cplx + 2 h2o --> 1 Def_mono_inact + 1 Map_mono_inact + 1 b3559_m + 1 for + 1 met-L	irreversible	Protein Maturation	0.00E+00	1.00E+09	0	7.52E+02	7.52E+02	0	1.28E+03	1275.12	0	7.15E+01	71.519
b3560_fold_spon	b3560_m folding: spontanous	1 b3560_m --> 1 GlyQ_mono	irreversible	Protein Folding	0.00E+00	1.00E+09	0	7.52E+02	7.52E+02	0	1.28E+03	1275.12	0	7.15E+01	71.519
b3560_mRNA_CONV	convsersion of mRNA to mRNA_1 (synthetic rxn)	1 b3560_mRNA --> 1 b3560_mRNA_1	irreversible	Translation	0.00E+00	1.00E+09	0	4.02E+01	4.02E+01	0	7.10E+01	71.00705	0	2.07E-01	0.207
b3560_mRNA_degr1	Degradation of b3560_mRNA I	1 Orn_dim + 228 atp + 1 b3560_mRNA_2 + 1 degradosome + 228 h2o <==> 228 adp + 1 b3560_mRNA_2_degr + 228 h + 228 pi	reversible	mRNA degradation	-1.00E+09	1.00E+09	0	4.02E+01	4.02E+01	0	7.10E+01	71.00705	0	2.07E-01	0.207
b3560_mRNA_degr2	Degradation of b3560_mRNA II	1 b3560_mRNA_2_degr + 911 h2o --> 1 Orn_dim_inact + 209 amp + 1 atp + 259 cmp + 1 degradosome_inact + 253 gmp + 911 h + 190 ump	irreversible	mRNA degradation	0.00E+00	2.34E-01	0	4.02E+01	4.02E+01	0	7.10E+01	71.00705	0	2.07E-01	0.207
b3560_maturation1	Polypeptide b3560 peptide deformylas and methionine aminopeptidase complex	1 Def_mono + 1 Map_mono + 1 b3560_aa <==> 1 b3560_def_map_cplx	reversible	Protein Maturation	-1.00E+09	1.00E+09	0	7.52E+02	7.52E+02	0	1.28E+03	1275.12	0	7.15E+01	71.519
b3560_maturation2	b3560 formation	1 b3560_def_map_cplx + 2 h2o --> 1 Def_mono_inact + 1 Map_mono_inact + 1 b3560_m + 1 for + 1 met-L	irreversible	Protein Maturation	0.00E+00	1.00E+09	0	7.52E+02	7.52E+02	0	1.28E+03	1275.12	0	7.15E+01	71.519
b3590_fold_spon	b3590_m folding: spontanous	1 b3590_m --> 1 SelB_mono	irreversible	Protein Folding	0.00E+00	1.00E+09	0	6.39E+02	6.39E+02	0	1.37E+01	13.7	0	1.37E+01	13.7
b3590_mRNA_CONV	convsersion of mRNA to mRNA_1 (synthetic rxn)	1 b3590_mRNA --> 1 b3590_mRNA_1	irreversible	Translation	0.00E+00	1.00E+09	0	3.70E+01	3.70E+01	0	1.37E+01	13.7	0	1.86E-01	0.186
b3590_mRNA_degr1	Degradation of b3590_mRNA I	1 Orn_dim + 460 atp + 1 b3590_mRNA_2 + 1 degradosome + 460 h2o <==> 460 adp + 1 b3590_mRNA_2_degr + 460 h + 460 pi	reversible	mRNA degradation	-1.00E+09	1.00E+09	0	3.70E+01	3.70E+01	0	1.37E+01	13.7	0	1.86E-01	0.186
b3590_mRNA_degr2	Degradation of b3590_mRNA II	1 b3590_mRNA_2_degr + 1840 h2o --> 1 Orn_dim_inact + 414 amp 484 cmp + 1 degradosome_inact + 555 gmp + 1840 h + 388 ump	irreversible	mRNA degradation	0.00E+00	1.86E-01	0	3.70E+01	3.70E+01	0	1.37E+01	13.7	0	1.86E-01	0.186
b3590_maturation1	Polypeptide b3590 peptide deformylas complex	1 Def_mono + 1 b3590_aa <==> 1 b3590_def_cplx	reversible	Protein Maturation	-1.00E+09	1.00E+09	0	6.39E+02	6.39E+02	0	1.37E+01	13.7	0	1.37E+01	13.7
b3590_maturation2	b3590 formation	1 b3590_def_cplx + 1 h2o --> 1 Def_mono_inact + 1 b3590_m + 1 for	irreversible	Protein Maturation	0.00E+00	1.00E+09	0	6.39E+02	6.39E+02	0	1.37E+01	13.7	0	1.37E+01	13.7
b3591_fold_spon	b3591_m folding: spontanous	1 b3591_m --> 1 SelA_mono	irreversible	Protein Folding	0.00E+00	1.00E+09	0	1.09E+03	1.09E+03	0	4.62E+02	462.24	0	9.74E+01	97.362
b3591_mRNA_CONV	convsersion of mRNA to mRNA_1 (synthetic rxn)	1 b3591_mRNA --> 1 b3591_mRNA_1	irreversible	Translation	0.00E+00	1.00E+09	0	3.70E+01	3.70E+01	0	1.37E+01	13.7	0	1.86E-01	0.186
b3591_mRNA_degr1	Degradation of b3591_mRNA I	1 Orn_dim + 348 atp + 1 b3591_mRNA_2 + 1 degradosome + 348 h2o <==> 348 adp + 1 b3591_mRNA_2_degr + 348 h + 348 pi	reversible	mRNA degradation	-1.00E+09	1.00E+09	0	3.70E+01	3.70E+01	0	1.37E+01	13.7	0	1.86E-01	0.186
b3591_mRNA_degr2	Degradation of b3591_mRNA II	1 b3591_mRNA_2_degr + 1391 h2o --> 1 Orn_dim_inact + 299 amp 1 atp + 351 cmp + 1 degradosome_inact + 429 gmp + 1391 h + 312 ump	irreversible	mRNA degradation	0.00E+00	2.99E-01	0	3.70E+01	3.70E+01	0	1.37E+01	13.7	0	1.86E-01	0.186
b3591_maturation1	Polypeptide b3591 peptide deformylas and methionine aminopeptidase complex	1 Def_mono + 1 Map_mono + 1 b3591_aa <==> 1 b3591_def_map_cplx	reversible	Protein Maturation	-1.00E+09	1.00E+09	0	1.09E+03	1.09E+03	0	4.62E+02	462.24	0	9.74E+01	97.362
b3591_maturation2	b3591 formation	1 b3591_def_map_cplx + 2 h2o --> 1 Def_mono_inact + 1 Map_mono_inact + 1 b3591_m + 1 for + 1 met-L	irreversible	Protein Maturation	0.00E+00	1.00E+09	0	1.09E+03	1.09E+03	0	4.62E+02	462.24	0	9.74E+01	97.362
b3635_fold_spon	b3635_m folding: spontanous	1 b3635_m --> 1 MutM_mono	irreversible	Protein Folding	0.00E+00	1.00E+09	0	1.42E+03	1.42E+03	0	1.33E+03	1331.55	0	9.12E+01	91.23
b3635_mRNA_degr1	Degradation of b3635_mRNA I	1 Orn_dim + 202 atp + 1 b3635_mRNA_2 + 1 degradosome + 202 h2o <==> 202 adp + 1 b3635_mRNA_2_degr + 202 h + 202 pi	reversible	mRNA degradation	-1.00E+09	1.00E+09	0	8.85E+01	8.85E+01	0	8.29E+01	82.88	0	1.92E-01	0.192
b3635_mRNA_degr2	Degradation of b3635_mRNA II	1 b3635_mRNA_2_degr + 809 h2o --> 1 Orn_dim_inact + 186 amp + 1 atp + 184 cmp + 1 degradosome_inact + 252 gmp + 809 h + 187 ump	irreversible	mRNA degradation	0.00E+00	1.92E-01	0	8.85E+01	8.85E+01	0	8.29E+01	82.88	0	1.92E-01	0.192
b3635_maturation1	Polypeptide b3635 peptide deformylas and methionine aminopeptidase complex	1 Def_mono + 1 Map_mono + 1 b3635_aa <==> 1 b3635_def_map_cplx	reversible	Protein Maturation	-1.00E+09	1.00E+09	0	1.42E+03	1.42E+03	0	1.33E+03	1331.55	0	9.12E+01	91.23
b3635_maturation2	b3635 formation	1 b3635_def_map_cplx + 2 h2o --> 1 Def_mono_inact + 1 Map_mono_inact + 1 b3635_m + 1 for + 1 met-L	irreversible	Protein Maturation	0.00E+00	1.00E+09	0	1.42E+03	1.42E+03	0	1.33E+03	1331.55	0	9.12E+01	91.23
b3635_v1_mRNA_CONV	conversion of mRNA to mRNA_1 (synthetic rxn)	1 b3635_v1_mRNA --> 1 b3635_mRNA_1	irreversible	Translation	0.00E+00	1.00E+09	0	8.85E+01	8.85E+01	0	8.29E+01	82.88	0	1.92E-01	0.192
b3635_v3_mRNA_CONV	conversion of mRNA to mRNA_1 (synthetic rxn)	1 b3635_v3_mRNA --> 1 b3635_mRNA_1	irreversible	Translation	0.00E+00	1.00E+09	0	6.30E+01	6.30E+01	0	7.71E+01	77.08692	0	1.04E-01	0.104
b3636_fold_spon	b3636_m folding: spontanous	1 b3636_m --> 1 RpmG_mono	irreversible	Protein Folding	0.00E+00	1.00E+09	8.44E+01	1.13E+02	2.81E+01	6.22E+01	8.29E+01	20.72	6.22E+01	8.29E+01	20.72
b3636_mRNA_CONV	convsersion of mRNA to mRNA_1 (synthetic rxn)	1 b3636_mRNA --> 1 b3636_mRNA_1	irreversible	Translation	0.00E+00	1.00E+09	0	1.04E+02	1.04E+02	0	8.29E+01	82.88	0	6.41E-01	0.641
b3636_mRNA_degr1	Degradation of b3636_mRNA I	1 Orn_dim + 42 atp + 1 b3636_mRNA_2 + 1 degradosome + 42 h2o <==> 42 adp + 1 b3636_mRNA_2_degr + 42 h + 42 pi	reversible	mRNA degradation	-1.00E+09	1.00E+09	0	1.04E+02	1.04E+02	0	8.29E+01	82.88	0	6.41E-01	0.641
b3636_mRNA_degr2	Degradation of b3636_mRNA II	1 b3636_mRNA_2_degr + 167 h2o --> 1 Orn_dim_inact + 61 amp + 34 cmp + 1 degradosome_inact + 34 gmp + 167 h + 39 ump	irreversible	mRNA degradation	0.00E+00	6.41E-01	0	1.04E+02	1.04E+02	0	8.29E+01	82.88	0	6.41E-01	0.641
b3636_maturation1	Polypeptide b3636 peptide deformylas and methionine aminopeptidase complex	1 Def_mono + 1 Map_mono + 1 b3636_aa <==> 1 b3636_def_map_cplx	reversible	Protein Maturation	-1.00E+09	1.00E+09	8.44E+01	1.13E+02	2.81E+01	6.22E+01	8.29E+01	20.72	6.22E+01	8.29E+01	20.72
b3636_maturation2	b3636 formation	1 b3636_def_map_cplx + 2 h2o --> 1 Def_mono_inact + 1 Map_mono_inact + 1 b3636_m + 1 for + 1 met-L	irreversible	Protein Maturation	0.00E+00	1.00E+09	8.44E+01	1.13E+02	2.81E+01	6.22E+01	8.29E+01	20.72	6.22E+01	8.29E+01	20.72
b3636_v1_mRNA_CONV	conversion of mRNA to mRNA_1 (synthetic rxn)	1 b3636_v1_mRNA --> 1 b3636_mRNA_1	irreversible	Translation	0.00E+00	1.00E+09	0	8.92E+01	8.92E+01	0	8.29E+01	82.88	0	1.04E-01	0.104
b3636_v2_mRNA_CONV	conversion of mRNA to mRNA_1 (synthetic rxn)	1 b3636_v2_mRNA --> 1 b3636_mRNA_1	irreversible	Translation	0.00E+00	1.00E+09	0	8.85E+01	8.85E+01	0	8.29E+01	82.88	0	1.92E-01	0.192
b3636_v3_mRNA_CONV	conversion of mRNA to mRNA_1 (synthetic rxn)	1 b3636_v3_mRNA --> 1 b3636_mRNA_1	irreversible	Translation	0.00E+00	1.00E+09	0	6.30E+01	6.30E+01	0	7.71E+01	77.08692	0	1.04E-01	0.104
b3637_fold_spon	b3637_m folding: spontanous	1 b3637_m --> 1 RpmB_mono	irreversible	Protein Folding	0.00E+00	1.00E+09	8.44E+01	1.13E+02	2.81E+01	6.22E+01	8.29E+01	20.72	6.22E+01	8.29E+01	20.72
b3637_mRNA_CONV	convsersion of mRNA to mRNA_1 (synthetic rxn)	1 b3637_mRNA --> 1 b3637_mRNA_1	irreversible	Translation	0.00E+00	1.00E+09	0	1.04E+02	1.04E+02	0	8.29E+01	82.88	0	6.41E-01	0.641
b3637_mRNA_degr1	Degradation of b3637_mRNA I	1 Orn_dim + 59 atp + 1 b3637_mRNA_2 + 1 degradosome + 59 h2o <==> 59 adp + 1 b3637_mRNA_2_degr + 59 h + 59 pi	reversible	mRNA degradation	-1.00E+09	1.00E+09	0	1.04E+02	1.04E+02	0	8.29E+01	82.88	0	6.41E-01	0.641

b3637_mRNA_degr2	Degradation of b3637_mRNA II	1 b3637_mRNA_2_degr + 236 h2o --> 1 Orn_dim_inact + 56 amp + 1 atp + 65 cmp + 1 degradosome_inact + 61 gmp + 236 h + 54 ump	irreversible	mRNA degradation	0.00E+00	1.18E+00	0	1.04E+02	1.04E+02	0	8.29E+01	82.88	0	6.41E-01	0.641
b3637_maturation1	Polypeptide b3637 peptide deformylas and methionine aminopeptidase complex	1 Def_mono + 1 Map_mono + 1 b3637_aa <==> 1 b3637_def_map_cplx	reversible	Protein Maturation	-1.00E+09	1.00E+09	8.44E+01	1.13E+02	2.81E+01	6.22E+01	8.29E+01	20.72	6.22E+01	8.29E+01	20.72
b3637_maturation2	b3637 formation	1 b3637_def_map_cplx + 2 h2o --> 1 Def_mono_inact + 1 Map_mono_inact + 1 b3637_m + 1 for + 1 met-L	irreversible	Protein Maturation	0.00E+00	1.00E+09	8.44E+01	1.13E+02	2.81E+01	6.22E+01	8.29E+01	20.72	6.22E+01	8.29E+01	20.72
b3637_v1_mRNA_CONV	convsersion of mRNA to mRNA_1 (synthetic rxn)	1 b3637_v1_mRNA --> 1 b3637_mRNA_1	irreversible	Translation	0.00E+00	1.00E+09	0	8.92E+01	8.92E+01	0	8.29E+01	82.88	0	1.04E-01	0.104
b3637_v2_mRNA_CONV	convsersion of mRNA to mRNA_1 (synthetic rxn)	1 b3637_v2_mRNA --> 1 b3637_mRNA_1	irreversible	Translation	0.00E+00	1.00E+09	0	8.85E+01	8.85E+01	0	8.29E+01	82.88	0	1.92E-01	0.192
b3637_v3_mRNA_CONV	convsersion of mRNA to mRNA_1 (synthetic rxn)	1 b3637_v3_mRNA --> 1 b3637_mRNA_1	irreversible	Translation	0.00E+00	1.00E+09	0	6.30E+01	6.30E+01	0	7.71E+01	77.08692	0	1.04E-01	0.104
b3638_fold_spon	b3638_m folding: spontanous	1 b3638_m --> 1 YicR_mono	irreversible	Protein Folding	0.00E+00	1.00E+09	0	1.23E+03	1.23E+03	0	1.14E+03	1142.96	0	6.69E+01	66.872
b3638_mRNA_CONV	convsersion of mRNA to mRNA_1 (synthetic rxn)	1 b3638_mRNA --> 1 b3638_mRNA_1	irreversible	Translation	0.00E+00	1.00E+09	0	8.92E+01	8.92E+01	0	8.29E+01	82.88	0	1.04E-01	0.104
b3638_mRNA_degr1	Degradation of b3638_mRNA I	1 Orn_dim + 167 atp + 1 b3638_mRNA_2 + 1 degradosome + 167 h2o <==> 167 adp + 1 b3638_mRNA_2_degr + 167 h + 167 pi	reversible	mRNA degradation	-1.00E+09	1.00E+09	0	8.92E+01	8.92E+01	0	8.29E+01	82.88	0	1.04E-01	0.104
b3638_mRNA_degr2	Degradation of b3638_mRNA II	1 b3638_mRNA_2_degr + 668 h2o --> 1 Orn_dim_inact + 173 amp + 143 cmp + 1 degradosome_inact + 177 gmp + 1 gtp + 668 h + 175 ump	irreversible	mRNA degradation	0.00E+00	1.04E-01	0	8.92E+01	8.92E+01	0	8.29E+01	82.88	0	1.04E-01	0.104
b3638_maturation1	Polypeptide b3638 peptide deformylas and methionine aminopeptidase complex	1 Def_mono + 1 Map_mono + 1 b3638_aa <==> 1 b3638_def_map_cplx	reversible	Protein Maturation	-1.00E+09	1.00E+09	0	1.23E+03	1.23E+03	0	1.14E+03	1142.96	0	6.69E+01	66.872
b3638_maturation2	b3638 formation	1 b3638_def_map_cplx + 2 h2o --> 1 Def_mono_inact + 1 Map_mono_inact + 1 b3638_m + 1 for + 1 met-L	irreversible	Protein Maturation	0.00E+00	1.00E+09	0	1.23E+03	1.23E+03	0	1.14E+03	1142.96	0	6.69E+01	66.872
b3638_v1_mRNA_CONV	convsersion of mRNA to mRNA_1 (synthetic rxn)	1 b3638_v1_mRNA --> 1 b3638_mRNA_1	irreversible	Translation	0.00E+00	1.00E+09	0	6.30E+01	6.30E+01	0	7.71E+01	77.08692	0	1.04E-01	0.104
b3649_fold_spon	b3649_m folding: spontanous	1 b3649_m_Mg --> 1 RpoZ_mono	irreversible	Protein Folding	0.00E+00	1.00E+09	0	1.99E+02	1.99E+02	0	2.85E+02	285.2405	0	8.80E+01	87.96
b3649_ion_BIND	Binding of ions to b3649	1 b3649_m + 1 mg2 <==> 1 b3649_m_Mg	reversible	Metallo-ion Binding	-1.00E+09	1.00E+09	0	1.99E+02	1.99E+02	0	2.85E+02	285.2405	0	8.80E+01	87.96
b3649_mRNA_CONV	convsersion of mRNA to mRNA_1 (synthetic rxn)	1 b3649_mRNA --> 1 b3649_mRNA_1	irreversible	Translation	0.00E+00	1.00E+09	0	2.25E+01	2.25E+01	0	3.96E+01	39.6481	0	1.92E-01	0.192
b3649_mRNA_degr1	Degradation of b3649_mRNA I	1 Orn_dim + 69 atp + 1 b3649_mRNA_2 + 1 degradosome + 69 h2o <==> 69 adp + 1 b3649_mRNA_2_degr + 69 h + 69 pi	reversible	mRNA degradation	-1.00E+09	1.00E+09	0	2.25E+01	2.25E+01	0	3.96E+01	39.6481	0	1.92E-01	0.192
b3649_mRNA_degr2	Degradation of b3649_mRNA II	1 b3649_mRNA_2_degr + 275 h2o --> 1 Orn_dim_inact + 76 amp + 1 atp + 68 cmp + 1 degradosome_inact + 79 gmp + 275 h + 52 ump	irreversible	mRNA degradation	0.00E+00	2.63E-01	0	2.25E+01	2.25E+01	0	3.96E+01	39.6481	0	1.92E-01	0.192
b3649_maturation1	Polypeptide b3649 peptide deformylas and methionine aminopeptidase complex	1 Def_mono + 1 Map_mono + 1 b3649_aa <==> 1 b3649_def_map_cplx	reversible	Protein Maturation	-1.00E+09	1.00E+09	0	1.99E+02	1.99E+02	0	2.85E+02	285.2405	0	8.80E+01	87.96
b3649_maturation2	b3649 formation	1 b3649_def_map_cplx + 2 h2o --> 1 Def_mono_inact + 1 Map_mono_inact + 1 b3649_m + 1 for + 1 met-L	irreversible	Protein Maturation	0.00E+00	1.00E+09	0	1.99E+02	1.99E+02	0	2.85E+02	285.2405	0	8.80E+01	87.96
b3650_fold_GroEL/ES_1	b3650_m folding: GroEL/ES mediated: polypeptide is going in GroEL/ES complex	1 GroEL.(7)ADP.cisGroES + 7 atp + 1 b3650_m + 1 transGroES_hepta --> 7 adp + 1 b3650_m_GroEL.(7)ATP.transGroES + 1 cisGroES_hepta	irreversible	Protein Folding	0.00E+00	1.00E+09	0	1.02E+03	1.02E+03	0	1.73E+03	1728.892	0	1.11E+02	111.192
b3650_fold_GroEL/ES_2	b3650_m folding: GroEL/ES mediated: folding of polypeptide under ATP hydrolysis	1 b3650_m_GroEL.(7)ATP.transGroES + 7 h2o --> 1 SpoT_GroEL.(7)ADP.transGroES + 7 h + 7 pi	irreversible	Protein Folding	0.00E+00	1.00E+09	0	1.02E+03	1.02E+03	0	1.73E+03	1728.892	0	1.11E+02	111.192
b3650_fold_GroEL/ES_3	b3650_m folding: GroEL/ES mediated: release of native protein	1 SpoT_GroEL.(7)ADP.transGroES --> 1 GroEL.(7)ADP.transGroES + 1 SpoT_mono	irreversible	Protein Folding	0.00E+00	1.00E+09	0	1.02E+03	1.02E+03	0	1.73E+03	1728.892	0	1.11E+02	111.192
b3650_fold_KJE_1	b3650_m folding: KJE mediated	1 DnaJ_dim + 1 DnaK_mono.ATP + 1 b3650_m <==> 1 b3650_m_DnaKJ_complex	reversible	Protein Folding	-1.00E+09	1.00E+09	0	1.02E+03	1.02E+03	0	1.73E+03	1728.892	0	1.11E+02	111.192
b3650_fold_KJE_2	b3650_m folding: KJE mediated	1 GrpE_dim + 1 b3650_m_DnaKJ_complex + 1 h2o --> 1 DnaJ_dim_inact + 1 SpoT_DnaK_GrpE_complex + 1 adp + 1 h + 1 pi	irreversible	Protein Folding	0.00E+00	1.00E+09	0	1.02E+03	1.02E+03	0	1.73E+03	1728.892	0	1.11E+02	111.192
b3650_fold_KJE_3	b3650_m folding: KJE mediated	1 SpoT_DnaK_GrpE_complex + 1 atp --> 1 DnaK_mono.ATP_inact + 1 GrpE_dim_inact + 1 SpoT_mono	irreversible	Protein Folding	0.00E+00	1.00E+09	0	1.02E+03	1.02E+03	0	1.73E+03	1728.892	0	1.11E+02	111.192
b3650_mRNA_CONV	convsersion of mRNA to mRNA_1 (synthetic rxn)	1 b3650_mRNA --> 1 b3650_mRNA_1	irreversible	Translation	0.00E+00	1.00E+09	0	2.25E+01	2.25E+01	0	3.96E+01	39.6481	0	1.92E-01	0.192
b3650_mRNA_degr1	Degradation of b3650_mRNA I	1 Orn_dim + 527 atp + 1 b3650_mRNA_2 + 1 degradosome + 527 h2o <==> 527 adp + 1 b3650_mRNA_2_degr + 527 h + 527 pi	reversible	mRNA degradation	-1.00E+09	1.00E+09	0	2.25E+01	2.25E+01	0	3.96E+01	39.6481	0	1.92E-01	0.192
b3650_mRNA_degr2	Degradation of b3650_mRNA II	1 b3650_mRNA_2_degr + 2108 h2o --> 1 Orn_dim_inact + 523 amp 566 cmp + 1 degradosome_inact + 567 gmp + 2108 h + 453 ump	irreversible	mRNA degradation	0.00E+00	3.17E-01	0	2.25E+01	2.25E+01	0	3.96E+01	39.6481	0	1.92E-01	0.192
b3650_maturation1	Polypeptide b3650 peptide deformylas and methionine aminopeptidase complex	1 Def_mono + 1 Map_mono + 1 b3650_aa <==> 1 b3650_def_map_cplx	reversible	Protein Maturation	-1.00E+09	1.00E+09	0	1.02E+03	1.02E+03	0	1.73E+03	1728.892	0	1.11E+02	111.192
b3650_maturation2	b3650 formation	1 b3650_def_map_cplx + 2 h2o --> 1 Def_mono_inact + 1 Map_mono_inact + 1 b3650_m + 1 for + 1 met-L	irreversible	Protein Maturation	0.00E+00	1.00E+09	0	1.02E+03	1.02E+03	0	1.73E+03	1728.892	0	1.11E+02	111.192
b3651_fold_GroEL/ES_1	b3651_m folding: GroEL/ES mediated: polypeptide is going in GroEL/ES complex	1 GroEL.(7)ADP.cisGroES + 7 atp + 1 b3651_m + 1 transGroES_hepta --> 7 adp + 1 b3651_m_GroEL.(7)ATP.transGroES + 1 cisGroES_hepta	irreversible	Protein Folding	0.00E+00	1.00E+09	0	3.66E+02	3.66E+02	0	5.90E+02	589.5253	0	7.66E+01	76.596
b3651_fold_GroEL/ES_2	b3651_m folding: GroEL/ES mediated: folding of polypeptide under ATP hydrolysis	1 b3651_m_GroEL.(7)ATP.transGroES + 7 h2o --> 1 TrmH_GroEL.(7)ADP.transGroES + 7 h + 7 pi	irreversible	Protein Folding	0.00E+00	1.00E+09	0	3.66E+02	3.66E+02	0	5.90E+02	589.5253	0	7.66E+01	76.596
b3651_fold_GroEL/ES_3	b3651_m folding: GroEL/ES mediated: release of native protein	1 TrmH_GroEL.(7)ADP.transGroES --> 1 GroEL.(7)ADP.transGroES + 1 TrmH_mono	irreversible	Protein Folding	0.00E+00	1.00E+09	0	3.66E+02	3.66E+02	0	5.90E+02	589.5253	0	7.66E+01	76.596
b3651_fold_KJE_1	b3651_m folding: KJE mediated	1 DnaJ_dim + 1 DnaK_mono.ATP + 1 b3651_m <==> 1 b3651_m_DnaKJ_complex	reversible	Protein Folding	-1.00E+09	1.00E+09	0	3.66E+02	3.66E+02	0	5.90E+02	589.5253	0	7.66E+01	76.596
b3651_fold_KJE_2	b3651_m folding: KJE mediated	1 GrpE_dim + 1 b3651_m_DnaKJ_complex + 1 h2o --> 1 DnaJ_dim_inact + 1 TrmH_DnaK_GrpE_complex + 1 adp + 1 h + 1 pi	irreversible	Protein Folding	0.00E+00	1.00E+09	0	3.66E+02	3.66E+02	0	5.90E+02	589.5253	0	7.66E+01	76.596
b3651_fold_KJE_3	b3651_m folding: KJE mediated	1 TrmH_DnaK_GrpE_complex + 1 atp --> 1 DnaK_mono.ATP_inact + 1 GrpE_dim_inact + 1 TrmH_mono	irreversible	Protein Folding	0.00E+00	1.00E+09	0	3.66E+02	3.66E+02	0	5.90E+02	589.5253	0	7.66E+01	76.596
b3651_mRNA_CONV	convsersion of mRNA to mRNA_1 (synthetic rxn)	1 b3651_mRNA --> 1 b3651_mRNA_1	irreversible	Translation	0.00E+00	1.00E+09	0	2.25E+01	2.25E+01	0	3.96E+01	39.6481	0	1.92E-01	0.192
b3651_mRNA_degr1	Degradation of b3651_mRNA I	1 Orn_dim + 172 atp + 1 b3651_mRNA_2 + 1 degradosome + 172 h2o <==> 172 adp + 1 b3651_mRNA_2_degr + 172 h + 172 pi	reversible	mRNA degradation	-1.00E+09	1.00E+09	0	2.25E+01	2.25E+01	0	3.96E+01	39.6481	0	1.92E-01	0.192
b3651_mRNA_degr2	Degradation of b3651_mRNA II	1 b3651_mRNA_2_degr + 689 h2o --> 1 Orn_dim_inact + 165 amp + 192 cmp + 1 degradosome_inact + 190 gmp + 689 h + 143 ump	irreversible	mRNA degradation	0.00E+00	2.45E-01	0	2.25E+01	2.25E+01	0	3.96E+01	39.6481	0	1.92E-01	0.192
b3651_maturation1	Polypeptide b3651 peptide deformylas and methionine aminopeptidase complex	1 Def_mono + 1 Map_mono + 1 b3651_aa <==> 1 b3651_def_map_cplx	reversible	Protein Maturation	-1.00E+09	1.00E+09	0	3.66E+02	3.66E+02	0	5.90E+02	589.5253	0	7.66E+01	76.596
b3651_maturation2	b3651 formation	1 b3651_def_map_cplx + 2 h2o --> 1 Def_mono_inact + 1 Map_mono_inact + 1 b3651_m + 1 for + 1 met-L	irreversible	Protein Maturation	0.00E+00	1.00E+09	0	3.66E+02	3.66E+02	0	5.90E+02	589.5253	0	7.66E+01	76.596
b3652_fold_spon	b3652_m folding: spontanous	1 b3652_m --> 1 RecG_mono	irreversible	Protein Folding	0.00E+00	1.00E+09	0	9.63E+02	9.63E+02	0	1.65E+03	1650.324	0	7.21E+01	72.08
b3652_mRNA_CONV	convsersion of mRNA to mRNA_1 (synthetic rxn)	1 b3652_mRNA --> 1 b3652_mRNA_1	irreversible	Translation	0.00E+00	1.00E+09	0	2.25E+01	2.25E+01	0	3.96E+01	39.6481	0	1.92E-01	0.192
b3652_mRNA_degr1	Degradation of b3652_mRNA I	1 Orn_dim + 520 atp + 1 b3652_mRNA_2 + 1 degradosome + 520 h2o <==> 520 adp + 1 b3652_mRNA_2_degr + 520 h + 520 pi	reversible	mRNA degradation	-1.00E+09	1.00E+09	0	2.25E+01	2.25E+01	0	3.96E+01	39.6481	0	1.92E-01	0.192

b3652_mRNA_degr2	Degradation of b3652_mRNA II	1 b3652_mRNA_2_degr + 2081 h2o --> 1 Orn_dim_inact + 481 amp 587 cmp + 1 degradosome_inact + 604 gmp + 2081 h + 410 ump	irreversible	mRNA degradation	0.00E+00	1.92E-01	0	2.25E+01	2.25E+01	0	3.96E+01	39.6481	0	1.92E-01	0.192
b3652_maturation1	Polypeptide b3652 peptide deformylas and methionine aminopeptidase complex	1 Def_mono + 1 Map_mono + 1 b3652_aa <==> 1 b3652_def_map_cplx	reversible	Protein Maturation	-1.00E+09	1.00E+09	0	9.63E+02	9.63E+02	0	1.65E+03	1650.324	0	7.21E+01	72.08
b3652_maturation2	b3652 formation	1 b3652_def_map_cplx + 2 h2o --> 1 Def_mono_inact + 1 Map_mono_inact + 1 b3652_m + 1 for + 1 met-L	irreversible	Protein Maturation	0.00E+00	1.00E+09	0	9.63E+02	9.63E+02	0	1.65E+03	1650.324	0	7.21E+01	72.08
b3658_tRNA_20_MODIF_ 1	modification of position 20 nt (D) in selC by Dus_gen	1 Dus_gen + 1 b3658_tRNA_Mg2 + 1 h + 1 nadph <==> 1 b3658_tRNA_Mg2_Dus_gen_cplx	reversible	tRNA modification	-1.00E+09	1.00E+09	0	6.39E+02	6.39E+02	0	1.37E+01	13.7	0	1.37E+01	13.7
b3658_tRNA_20_MODIF_ 2	modification of position 20 nt (D) in selC by Dus_gen	1 b3658_tRNA_Mg2_Dus_gen_cplx --> 1 Dus_gen_inact + 1 b3658_tRNA_1 + 1 nadp	irreversible	tRNA modification	0.00E+00	1.00E+09	0	6.39E+02	6.39E+02	0	1.37E+01	13.7	0	1.37E+01	13.7
b3658_tRNA_37_MODIF_ 1	modification of position 37 nt (i6A) in selC by MiaA_dim	1 MiaA_dim + 1 b3658_tRNA_1 + 1 dmpp <==> 1 b3658_tRNA_1_MiaA_dim_cplx	reversible	tRNA modification	-1.00E+09	1.00E+09	0	6.39E+02	6.39E+02	0	1.37E+01	13.7	0	1.37E+01	13.7
b3658_tRNA_37_MODIF_ 2	modification of position 37 nt (i6A) in selC by MiaA_dim	1 b3658_tRNA_1_MiaA_dim_cplx --> 1 MiaA_dim_inact + 1 b3658_tRNA_2 + 1 ppi	irreversible	tRNA modification	0.00E+00	1.00E+09	0	6.39E+02	6.39E+02	0	1.37E+01	13.7	0	1.37E+01	13.7
b3658_tRNA_54_MODIF_ 1	modification of position 54 nt (m5U) in selC by TrmA_mono	1 TrmA_mono + 1 amet + 1 b3658_tRNA_2 <==> 1 b3658_tRNA_2_TrmA_mono_cplx	reversible	tRNA modification	-1.00E+09	1.00E+09	0	6.39E+02	6.39E+02	0	1.37E+01	13.7	0	1.37E+01	13.7
b3658_tRNA_54_MODIF_ 2	modification of position 54 nt (m5U) in selC by TrmA_mono	1 b3658_tRNA_2_TrmA_mono_cplx --> 1 TrmA_mono_inact + 1 ahcys + 1 b3658_tRNA_3 + 1 h	irreversible	tRNA modification	0.00E+00	1.00E+09	0	6.39E+02	6.39E+02	0	1.37E+01	13.7	0	1.37E+01	13.7
b3658_tRNA_55_MODIF_ 1	modification of position 55 nt (Y) in selC by TruB_mono	1 TruB_mono + 1 b3658_tRNA_3 <==> 1 b3658_tRNA_3_TruB_mono_cplx	reversible	tRNA modification	-1.00E+09	1.00E+09	0	6.39E+02	6.39E+02	0	1.37E+01	13.7	0	1.37E+01	13.7
b3658_tRNA_55_MODIF_ 2	modification of position 55 nt (Y) in selC by TruB_mono	1 b3658_tRNA_3_TruB_mono_cplx --> 1 TruB_mono_inact + 1 selC_tRNA	irreversible	tRNA modification	0.00E+00	1.00E+09	0	6.39E+02	6.39E+02	0	1.37E+01	13.7	0	1.37E+01	13.7
b3658_tRNA_Mg_BIND	Binding of two Mg2 to b3658_tRNA	1 b3658_RNA + 2 mg2 <==> 1 b3658_tRNA_Mg2	reversible	Metallo-ion binding	-1.00E+09	1.00E+09	0	6.39E+02	6.39E+02	0	1.37E+01	13.7	0	1.37E+01	13.7
b3703_fold_spon	b3703_m folding: spontanous	1 b3703_m --> 1 RpmH_mono	irreversible	Protein Folding	0.00E+00	1.00E+09	8.44E+01	1.13E+02	2.81E+01	6.22E+01	8.29E+01	20.72	6.22E+01	8.29E+01	20.72
b3703_mRNA_CONV	convsersion of mRNA to mRNA_1 (synthetic rxn)	1 b3703_mRNA --> 1 b3703_mRNA_1	irreversible	Translation	0.00E+00	1.00E+09	0	9.23E+01	9.23E+01	0	5.24E+01	52.37149	0	3.26E-01	0.326
b3703_mRNA_degr1	Degradation of b3703_mRNA I	1 Orn_dim + 35 atp + 1 b3703_mRNA_2 + 1 degradosome + 35 h2o <==> 35 adp + 1 b3703_mRNA_2_degr + 35 h + 35 pi	reversible	mRNA degradation	-1.00E+09	1.00E+09	0	9.23E+01	9.23E+01	0	5.24E+01	52.37149	0	3.26E-01	0.326
b3703_mRNA_degr2	Degradation of b3703_mRNA II	1 b3703_mRNA_2_degr + 140 h2o --> 1 Orn_dim_inact + 29 amp + 1 atp + 39 cmp + 1 degradosome_inact + 34 gmp + 140 h + 38 ump	irreversible	mRNA degradation	0.00E+00	5.99E-01	0	9.23E+01	9.23E+01	0	5.24E+01	52.37149	0	3.26E-01	0.326
b3703_maturation1	Polypeptide b3703 peptide deformylas complex	1 Def_mono + 1 b3703_aa <==> 1 b3703_def_cplx	reversible	Protein Maturation	-1.00E+09	1.00E+09	8.44E+01	1.13E+02	2.81E+01	6.22E+01	8.29E+01	20.72	6.22E+01	8.29E+01	20.72
b3703_maturation2	b3703 formation	1 b3703_def_cplx + 1 h2o --> 1 Def_mono_inact + 1 b3703_m + 1 for	irreversible	Protein Maturation	0.00E+00	1.00E+09	8.44E+01	1.13E+02	2.81E+01	6.22E+01	8.29E+01	20.72	6.22E+01	8.29E+01	20.72
b3703_v1_mRNA_CONV	convsersion of mRNA to mRNA_1 (synthetic rxn)	1 b3703_v1_mRNA --> 1 b3703_mRNA_1	irreversible	Translation	0.00E+00	1.00E+09	0	9.23E+01	9.23E+01	0	5.24E+01	52.37149	0	3.26E-01	0.326
b3703_v2_mRNA_CONV	convsersion of mRNA to mRNA_1 (synthetic rxn)	1 b3703_v2_mRNA --> 1 b3703_mRNA_1	irreversible	Translation	0.00E+00	1.00E+09	0	9.23E+01	9.23E+01	0	5.24E+01	52.37149	0	3.26E-01	0.326
b3704_fold_spon	b3704_m folding: spontanous	1 b3704_m --> 1 RnpA_mono	irreversible	Protein Folding	0.00E+00	1.00E+09	0	2.81E+02	2.81E+02	0	5.93E+01	59.25644	0	1.08E+01	10.8499
b3704_mRNA_CONV	convsersion of mRNA to mRNA_1 (synthetic rxn)	1 b3704_mRNA --> 1 b3704_mRNA_1	irreversible	Translation	0.00E+00	1.00E+09	0	9.23E+01	9.23E+01	0	5.24E+01	52.37149	0	3.26E-01	0.326
b3704_mRNA_degr1	Degradation of b3704_mRNA I	1 Orn_dim + 90 atp + 1 b3704_mRNA_2 + 1 degradosome + 90 h2o <==> 90 adp + 1 b3704_mRNA_2_degr + 90 h + 90 pi	reversible	mRNA degradation	-1.00E+09	1.00E+09	0	9.23E+01	9.23E+01	0	5.24E+01	52.37149	0	3.26E-01	0.326
b3704_mRNA_degr2	Degradation of b3704_mRNA II	1 b3704_mRNA_2_degr + 359 h2o --> 1 Orn_dim_inact + 78 amp + 106 cmp + 1 degradosome_inact + 95 gmp + 359 h + 81 ump	irreversible	mRNA degradation	0.00E+00	3.26E-01	0	9.23E+01	9.23E+01	0	5.24E+01	52.37149	0	3.26E-01	0.326
b3704_maturation1	Polypeptide b3704 peptide deformylas and methionine aminopeptidase complex	1 Def_mono + 1 Map_mono + 1 b3704_aa <==> 1 b3704_def_map_cplx	reversible	Protein Maturation	-1.00E+09	1.00E+09	0	2.81E+02	2.81E+02	0	5.93E+01	59.25644	0	1.08E+01	10.8499
b3704_maturation2	b3704 formation	1 b3704_def_map_cplx + 2 h2o --> 1 Def_mono_inact + 1 Map_mono_inact + 1 b3704_m + 1 for + 1 met-L	irreversible	Protein Maturation	0.00E+00	1.00E+09	0	2.81E+02	2.81E+02	0	5.93E+01	59.25644	0	1.08E+01	10.8499
b3704_v1_mRNA_CONV	convsersion of mRNA to mRNA_1 (synthetic rxn)	1 b3704_v1_mRNA --> 1 b3704_mRNA_1	irreversible	Translation	0.00E+00	1.00E+09	0	9.23E+01	9.23E+01	0	5.24E+01	52.37149	0	3.26E-01	0.326
b3704_v2_mRNA_CONV	convsersion of mRNA to mRNA_1 (synthetic rxn)	1 b3704_v2_mRNA --> 1 b3704_mRNA_1	irreversible	Translation	0.00E+00	1.00E+09	0	9.23E+01	9.23E+01	0	5.24E+01	52.37149	0	3.26E-01	0.326
b3706_fold_spon	b3706_m folding: spontanous	1 b3706_m --> 1 TrmE_mono	irreversible	Protein Folding	0.00E+00	1.00E+09	0	2.35E+03	2.35E+03	0	4.09E+03	4085.188	0	8.69E+01	86.918
b3706_mRNA_CONV	convsersion of mRNA to mRNA_1 (synthetic rxn)	1 b3706_mRNA --> 1 b3706_mRNA_1	irreversible	Translation	0.00E+00	1.00E+09	0	8.73E+01	8.73E+01	0	1.54E+02	154.0426	0	2.63E-01	0.263
b3706_mRNA_degr1	Degradation of b3706_mRNA I	1 Orn_dim + 341 atp + 1 b3706_mRNA_2 + 1 degradosome + 341 h2o <==> 341 adp + 1 b3706_mRNA_2_degr + 341 h + 341 pi	reversible	mRNA degradation	-1.00E+09	1.00E+09	0	8.73E+01	8.73E+01	0	1.54E+02	154.0426	0	2.63E-01	0.263
b3706_mRNA_degr2	Degradation of b3706_mRNA II	1 b3706_mRNA_2_degr + 1364 h2o --> 1 Orn_dim_inact + 307 amp 1 atp + 366 cmp + 1 degradosome_inact + 403 gmp + 1364 h + 288 ump	irreversible	mRNA degradation	0.00E+00	2.63E-01	0	8.73E+01	8.73E+01	0	1.54E+02	154.0426	0	2.63E-01	0.263
b3706_maturation1	Polypeptide b3706 peptide deformylas and methionine aminopeptidase complex	1 Def_mono + 1 Map_mono + 1 b3706_aa <==> 1 b3706_def_map_cplx	reversible	Protein Maturation	-1.00E+09	1.00E+09	0	2.35E+03	2.35E+03	0	4.09E+03	4085.188	0	8.69E+01	86.918
b3706_maturation2	b3706 formation	1 b3706_def_map_cplx + 2 h2o --> 1 Def_mono_inact + 1 Map_mono_inact + 1 b3706_m + 1 for + 1 met-L	irreversible	Protein Maturation	0.00E+00	1.00E+09	0	2.35E+03	2.35E+03	0	4.09E+03	4085.188	0	8.69E+01	86.918
b3740_fold_spon	b3740_m folding: spontanous	1 b3740_m --> 1 GidB_mono	irreversible	Protein Folding	0.00E+00	1.00E+09	0	6.68E+02	6.68E+02	0	1.11E+03	1109.296	0	9.44E+01	94.404
b3740_mRNA_CONV	convsersion of mRNA to mRNA_1 (synthetic rxn)	1 b3740_mRNA --> 1 b3740_mRNA_1	irreversible	Translation	0.00E+00	1.00E+09	0	4.80E+01	4.80E+01	0	8.48E+01	84.78135	0	2.07E-01	0.207
b3740_mRNA_degr1	Degradation of b3740_mRNA I	1 Orn_dim + 156 atp + 1 b3740_mRNA_2 + 1 degradosome + 156 h2o <==> 156 adp + 1 b3740_mRNA_2_degr + 156 h + 156 pi	reversible	mRNA degradation	-1.00E+09	1.00E+09	0	4.80E+01	4.80E+01	0	8.48E+01	84.78135	0	2.07E-01	0.207
b3740_mRNA_degr2	Degradation of b3740_mRNA II	1 b3740_mRNA_2_degr + 623 h2o --> 1 Orn_dim_inact + 160 amp + 149 cmp + 1 degradosome_inact + 155 gmp + 623 h + 160 ump	irreversible	mRNA degradation	0.00E+00	2.07E-01	0	4.80E+01	4.80E+01	0	8.48E+01	84.78135	0	2.07E-01	0.207
b3740_maturation1	Polypeptide b3740 peptide deformylas and methionine aminopeptidase complex	1 Def_mono + 1 Map_mono + 1 b3740_aa <==> 1 b3740_def_map_cplx	reversible	Protein Maturation	-1.00E+09	1.00E+09	0	6.68E+02	6.68E+02	0	1.11E+03	1109.296	0	9.44E+01	94.404
b3740_maturation2	b3740 formation	1 b3740_def_map_cplx + 2 h2o --> 1 Def_mono_inact + 1 Map_mono_inact + 1 b3740_m + 1 for + 1 met-L	irreversible	Protein Maturation	0.00E+00	1.00E+09	0	6.68E+02	6.68E+02	0	1.11E+03	1109.296	0	9.44E+01	94.404
b3741_fold_GroEL/ES_1	b3741_m folding: GroEL/ES mediated: polypeptide is going in GroEL/ES complex	1 GroEL.(7)ADP.cisGroES + 7 atp + 1 b3741_m + 1 transGroES_hepta --> 7 adp + 1 b3741_m_GroEL.(7)ATP.transGroES + 1 cisGroES_hepta	irreversible	Protein Folding	0.00E+00	1.00E+09	0	1.84E+03	1.84E+03	0	3.20E+03	3202.77	0	7.35E+01	73.519
b3741_fold_GroEL/ES_2	b3741_m folding: GroEL/ES mediated folding of polypeptide under ATP hydrolysis	1 b3741_m_GroEL.(7)ATP.transGroES + 7 h2o --> 1 GidA_GroEL.(7)ADP.transGroES + 7 h + 7 pi	irreversible	Protein Folding	0.00E+00	1.00E+09	0	1.84E+03	1.84E+03	0	3.20E+03	3202.77	0	7.35E+01	73.519
b3741_fold_GroEL/ES_3	b3741_m folding: GroEL/ES mediated: release of native protein	1 GidA_GroEL.(7)ADP.transGroES --> 1 GidA_mono + 1 GroEL.(7)ADP.transGroES	irreversible	Protein Folding	0.00E+00	1.00E+09	0	1.84E+03	1.84E+03	0	3.20E+03	3202.77	0	7.35E+01	73.519
b3741_fold_KJE_1	b3741_m folding: KJE mediated	1 DnaJ_dim + 1 DnaK_mono.ATP + 1 b3741_m <==> 1 b3741_m_DnaKJ_complex	reversible	Protein Folding	-1.00E+09	1.00E+09	0	1.84E+03	1.84E+03	0	3.20E+03	3202.77	0	7.35E+01	73.519
b3741_fold_KJE_2	b3741_m folding: KJE mediated	1 GrpE_dim + 1 b3741_m_DnaKJ_complex + 1 h2o --> 1 DnaJ_dim_inact + 1 GidA_DnaK_GrpE_complex + 1 adp + 1 h + 1 pi	irreversible	Protein Folding	0.00E+00	1.00E+09	0	1.84E+03	1.84E+03	0	3.20E+03	3202.77	0	7.35E+01	73.519
b3741_fold_KJE_3	b3741_m folding: KJE mediated	1 GidA_DnaK_GrpE_complex + 1 atp --> 1 DnaK_mono.ATP_inact + 1 GidA_mono + 1 GrpE_dim_inact	irreversible	Protein Folding	0.00E+00	1.00E+09	0	1.84E+03	1.84E+03	0	3.20E+03	3202.77	0	7.35E+01	73.519
b3741_mRNA_CONV	convsersion of mRNA to mRNA_1 (synthetic rxn)	1 b3741_mRNA --> 1 b3741_mRNA_1	irreversible	Translation	0.00E+00	1.00E+09	0	4.80E+01	4.80E+01	0	8.48E+01	84.78135	0	2.07E-01	0.207
b3741_mRNA_degr1	Degradation of b3741_mRNA I	1 Orn_dim + 472 atp + 1 b3741_mRNA_2 + 1 degradosome + 472 h2o <==> 472 adp + 1 b3741_mRNA_2_degr + 472 h + 472 pi	reversible	mRNA degradation	-1.00E+09	1.00E+09	0	4.80E+01	4.80E+01	0	8.48E+01	84.78135	0	2.07E-01	0.207

b3741_mRNA_degr2	Degradation of b3741_mRNA II	1 b3741_mRNA_2_degr + 1889 h2o --> 1 Orn_dim_inact + 437 amp 1 atp + 497 cmp + 1 degradosome_inact + 531 gmp + 1889 h + 424 ump	irreversible	mRNA degradation	0.00E+00	2.15E-01	0	4.80E+01	4.80E+01	0	8.48E+01	84.78135	0	2.07E-01	0.207
b3741_maturation1	Polypeptide b3741 peptide deformylas and methionine aminopeptidase complex	1 Def_mono + 1 Map_mono + 1 b3741_aa <==> 1 b3741_def_map_cplx	reversible	Protein Maturation	-1.00E+09	1.00E+09	0	1.84E+03	1.84E+03	0	3.20E+03	3202.77	0	7.35E+01	73.519
b3741_maturation2	b3741 formation	1 b3741_def_map_cplx + 2 h2o --> 1 Def_mono_inact + 1 Map_mono_inact + 1 b3741_m + 1 for + 1 met-L	irreversible	Protein Maturation	0.00E+00	1.00E+09	0	1.84E+03	1.84E+03	0	3.20E+03	3202.77	0	7.35E+01	73.519
b3756_RNA_10_modif_1	b3756_RNA modification (16S rRNA): position: 967, m5C by RsmB_mono	1 RsmB_mono + 1 amet + 1 b3756_RNA_10 <==> 1 b3756_RNA_10_RsmB_mono	reversible	rRNA modification	-1.00E+09	1.00E+09	0	1.12E+02	1.12E+02	0	0	0	0	0	0
b3756_RNA_10_modif_2	b3756_RNA modification (16S rRNA): position: 967, m5C by RsmB_mono	1 b3756_RNA_10_RsmB_mono --> 1 RsmB_mono_inact + 1 ahcys + 1 h + 1 rrsC	irreversible	rRNA modification	0.00E+00	1.00E+09	0	1.12E+02	1.12E+02	0	0	0	0	0	0
b3756_RNA_1_modif_1	b3756_RNA modification (16S rRNA): position: 1402, m4Cm by MeT_16S_1402	1 MeT_16S_1402 + 2 amet + 1 b3756_RNA_1 <==> 1 b3756_RNA_1_MeT_16S_1402	reversible	rRNA modification	-1.00E+09	1.00E+09	0	1.12E+02	1.12E+02	0	0	0	0	0	0
b3756_RNA_1_modif_2	b3756_RNA modification (16S rRNA): position: 1402, m4Cm by MeT_16S_1402	1 b3756_RNA_1_MeT_16S_1402 --> 1 MeT_16S_1402_inact + 2 ahcys + 1 b3756_RNA_2 + 2 h	irreversible	rRNA modification	0.00E+00	1.00E+09	0	1.12E+02	1.12E+02	0	0	0	0	0	0
b3756_RNA_2_modif_1	b3756_RNA modification (16S rRNA): position: 1407, m5C by MeT_16S_1407	1 MeT_16S_1407 + 1 amet + 1 b3756_RNA_2 <==> 1 b3756_RNA_2_MeT_16S_1407	reversible	rRNA modification	-1.00E+09	1.00E+09	0	1.12E+02	1.12E+02	0	0	0	0	0	0
b3756_RNA_2_modif_2	b3756_RNA modification (16S rRNA): position: 1407, m5C by MeT_16S_1407	1 b3756_RNA_2_MeT_16S_1407 --> 1 MeT_16S_1407_inact + 1 ahcys + 1 b3756_RNA_3 + 1 h	irreversible	rRNA modification	0.00E+00	1.00E+09	0	1.12E+02	1.12E+02	0	0	0	0	0	0
b3756_RNA_3_modif_1	b3756_RNA modification (16S rRNA): position: 1498, m3U by YggJ_mono	1 YggJ_mono + 1 amet + 1 b3756_RNA_3 <==> 1 b3756_RNA_3_YggJ_mono	reversible	rRNA modification	-1.00E+09	1.00E+09	0	1.12E+02	1.12E+02	0	0	0	0	0	0
b3756_RNA_3_modif_2	b3756_RNA modification (16S rRNA): position: 1498, m3U by YggJ_mono	1 b3756_RNA_3_YggJ_mono --> 1 YggJ_mono_inact + 1 ahcys + 1 b3756_RNA_4 + 1 h	irreversible	rRNA modification	0.00E+00	1.00E+09	0	1.12E+02	1.12E+02	0	0	0	0	0	0
b3756_RNA_4_modif_1	b3756_RNA modification (16S rRNA): position: 1516, m2G by MeT_16S_1516	1 MeT_16S_1516 + 1 amet + 1 b3756_RNA_4 <==> 1 b3756_RNA_4_MeT_16S_1516	reversible	rRNA modification	-1.00E+09	1.00E+09	0	1.12E+02	1.12E+02	0	0	0	0	0	0
b3756_RNA_4_modif_2	b3756_RNA modification (16S rRNA): position: 1516, m2G by MeT_16S_1516	1 b3756_RNA_4_MeT_16S_1516 --> 1 MeT_16S_1516_inact + 1 ahcys + 1 b3756_RNA_5 + 1 h	irreversible	rRNA modification	0.00E+00	1.00E+09	0	1.12E+02	1.12E+02	0	0	0	0	0	0
b3756_RNA_5_modif_1	b3756_RNA modification (16S rRNA): position: 1518, m6(2)A by KsgA_mono	1 KsgA_mono + 2 amet + 1 b3756_RNA_5 <==> 1 b3756_RNA_5_KsgA_mono	reversible	rRNA modification	-1.00E+09	1.00E+09	0	1.12E+02	1.12E+02	0	0	0	0	0	0
b3756_RNA_5_modif_2	b3756_RNA modification (16S rRNA): position: 1518, m6(2)A by KsgA_mono	1 b3756_RNA_5_KsgA_mono --> 1 KsgA_mono_inact + 2 ahcys + 1 b3756_RNA_6 + 2 h	irreversible	rRNA modification	0.00E+00	1.00E+09	0	1.12E+02	1.12E+02	0	0	0	0	0	0
b3756_RNA_6_modif_1	b3756_RNA modification (16S rRNA): position: 1519, m6(2)A by KsgA_mono	1 KsgA_mono + 2 amet + 1 b3756_RNA_6 <==> 1 b3756_RNA_6_KsgA_mono	reversible	rRNA modification	-1.00E+09	1.00E+09	0	1.12E+02	1.12E+02	0	0	0	0	0	0
b3756_RNA_6_modif_2	b3756_RNA modification (16S rRNA): position: 1519, m6(2)A by KsgA_mono	1 b3756_RNA_6_KsgA_mono --> 1 KsgA_mono_inact + 2 ahcys + 1 b3756_RNA_7 + 2 h	irreversible	rRNA modification	0.00E+00	1.00E+09	0	1.12E+02	1.12E+02	0	0	0	0	0	0
b3756_RNA_7_modif_1	b3756_RNA modification (16S rRNA): position: 516, Y by RsuA_mono	1 RsuA_mono + 1 b3756_RNA_7 <==> 1 b3756_RNA_7_RsuA_mono	reversible	rRNA modification	-1.00E+09	1.00E+09	0	1.12E+02	1.12E+02	0	0	0	0	0	0
b3756_RNA_7_modif_2	b3756_RNA modification (16S rRNA): position: 516, Y by RsuA_mono	1 b3756_RNA_7_RsuA_mono --> 1 RsuA_mono_inact + 1 b3756_RNA_8	irreversible	rRNA modification	0.00E+00	1.00E+09	0	1.12E+02	1.12E+02	0	0	0	0	0	0
b3756_RNA_8_modif_1	b3756_RNA modification (16S rRNA): position: 527, m7G by MeT_16S_527	1 MeT_16S_527 + 1 amet + 1 b3756_RNA_8 <==> 1 b3756_RNA_8_MeT_16S_527	reversible	rRNA modification	-1.00E+09	1.00E+09	0	1.12E+02	1.12E+02	0	0	0	0	0	0
b3756_RNA_8_modif_2	b3756_RNA modification (16S rRNA): position: 527, m7G by MeT_16S_527	1 b3756_RNA_8_MeT_16S_527 --> 1 MeT_16S_527_inact + 1 ahcy + 1 b3756_RNA_9 + 1 h	irreversible	rRNA modification	0.00E+00	1.00E+09	0	1.12E+02	1.12E+02	0	0	0	0	0	0
b3756_RNA_9_modif_1	b3756_RNA modification (16S rRNA): position: 966, m2G by MeT_16S_966	1 MeT_16S_966 + 1 amet + 1 b3756_RNA_9 <==> 1 b3756_RNA_9_MeT_16S_966	reversible	rRNA modification	-1.00E+09	1.00E+09	0	1.12E+02	1.12E+02	0	0	0	0	0	0
b3756_RNA_9_modif_2	b3756_RNA modification (16S rRNA): position: 966, m2G by MeT_16S_966	1 b3756_RNA_9_MeT_16S_966 --> 1 MeT_16S_966_inact + 1 ahcy + 1 b3756_RNA_10 + 1 h	irreversible	rRNA modification	0.00E+00	1.00E+09	0	1.12E+02	1.12E+02	0	0	0	0	0	0
b3756_RNA_modif_1	b3756_RNA modification (16S rRNA): position: 1207, m2G by RsmC_mono	1 RsmC_mono + 1 amet + 1 b3756_RNA <==> 1 b3756_RNA_RsmC_mono	reversible	rRNA modification	-1.00E+09	1.00E+09	0	1.12E+02	1.12E+02	0	0	0	0	0	0
b3756_RNA_modif_2	b3756_RNA modification (16S rRNA): position: 1207, m2G by RsmC_mono	1 b3756_RNA_RsmC_mono --> 1 RsmC_mono_inact + 1 ahcys + 1 b3756_RNA_1 + 1 h	irreversible	rRNA modification	0.00E+00	1.00E+09	0	1.12E+02	1.12E+02	0	0	0	0	0	0
b3756_v1_RNA_10_modif_1	b3756_v1_RNA modification (16S rRNA): position: 967, m5C by RsmB_mono	1 RsmB_mono + 1 amet + 1 b3756_v1_RNA_10 <==> 1 b3756_v1_RNA_10_RsmB_mono	reversible	rRNA modification	-1.00E+09	1.00E+09	0	1.12E+02	1.12E+02	0	1.42E+01	14.2	0	1.42E+01	14.2
b3756_v1_RNA_10_modif_2	b3756_v1_RNA modification (16S rRNA): position: 967, m5C by RsmB_mono	1 b3756_v1_RNA_10_RsmB_mono --> 1 RsmB_mono_inact + 1 ahcys + 1 h + 1 rrsC	irreversible	rRNA modification	0.00E+00	1.00E+09	0	1.12E+02	1.12E+02	0	1.42E+01	14.2	0	1.42E+01	14.2
b3756_v1_RNA_1_modif_1	b3756_v1_RNA modification (16S rRNA): position: 1402, m4Cm by MeT_16S_1402	1 MeT_16S_1402 + 2 amet + 1 b3756_v1_RNA_1 <==> 1 b3756_v1_RNA_1_MeT_16S_1402	reversible	rRNA modification	-1.00E+09	1.00E+09	0	1.12E+02	1.12E+02	0	1.42E+01	14.2	0	1.42E+01	14.2
b3756_v1_RNA_1_modif_2	b3756_v1_RNA modification (16S rRNA): position: 1402, m4Cm by MeT_16S_1402	1 b3756_v1_RNA_1_MeT_16S_1402 --> 1 MeT_16S_1402_inact + 2 ahcys + 1 b3756_v1_RNA_2 + 2 h	irreversible	rRNA modification	0.00E+00	1.00E+09	0	1.12E+02	1.12E+02	0	1.42E+01	14.2	0	1.42E+01	14.2
b3756_v1_RNA_2_modif_1	b3756_v1_RNA modification (16S rRNA): position: 1407, m5C by MeT_16S_1407	1 MeT_16S_1407 + 1 amet + 1 b3756_v1_RNA_2 <==> 1 b3756_v1_RNA_2_MeT_16S_1407	reversible	rRNA modification	-1.00E+09	1.00E+09	0	1.12E+02	1.12E+02	0	1.42E+01	14.2	0	1.42E+01	14.2
b3756_v1_RNA_2_modif_2	b3756_v1_RNA modification (16S rRNA): position: 1407, m5C by MeT_16S_1407	1 b3756_v1_RNA_2_MeT_16S_1407 --> 1 MeT_16S_1407_inact + 1 ahcys + 1 b3756_v1_RNA_3 + 1 h	irreversible	rRNA modification	0.00E+00	1.00E+09	0	1.12E+02	1.12E+02	0	1.42E+01	14.2	0	1.42E+01	14.2
b3756_v1_RNA_3_modif_1	b3756_v1_RNA modification (16S rRNA): position: 1498, m3U by YggJ_mono	1 YggJ_mono + 1 amet + 1 b3756_v1_RNA_3 <==> 1 b3756_v1_RNA_3_YggJ_mono	reversible	rRNA modification	-1.00E+09	1.00E+09	0	1.12E+02	1.12E+02	0	1.42E+01	14.2	0	1.42E+01	14.2
b3756_v1_RNA_3_modif_2	b3756_v1_RNA modification (16S rRNA): position: 1498, m3U by YggJ_mono	1 b3756_v1_RNA_3_YggJ_mono --> 1 YggJ_mono_inact + 1 ahcys 1 b3756_v1_RNA_4 + 1 h	irreversible	rRNA modification	0.00E+00	1.00E+09	0	1.12E+02	1.12E+02	0	1.42E+01	14.2	0	1.42E+01	14.2
b3756_v1_RNA_4_modif_1	b3756_v1_RNA modification (16S rRNA): position: 1516, m2G by MeT_16S_1516	1 MeT_16S_1516 + 1 amet + 1 b3756_v1_RNA_4 <==> 1 b3756_v1_RNA_4_MeT_16S_1516	reversible	rRNA modification	-1.00E+09	1.00E+09	0	1.12E+02	1.12E+02	0	1.42E+01	14.2	0	1.42E+01	14.2
b3756_v1_RNA_4_modif_2	b3756_v1_RNA modification (16S rRNA): position: 1516, m2G by MeT_16S_1516	1 b3756_v1_RNA_4_MeT_16S_1516 --> 1 MeT_16S_1516_inact + 1 ahcys + 1 b3756_v1_RNA_5 + 1 h	irreversible	rRNA modification	0.00E+00	1.00E+09	0	1.12E+02	1.12E+02	0	1.42E+01	14.2	0	1.42E+01	14.2
b3756_v1_RNA_5_modif_1	b3756_v1_RNA modification (16S rRNA): position: 1518, m6(2)A by KsgA_mono	1 KsgA_mono + 2 amet + 1 b3756_v1_RNA_5 <==> 1 b3756_v1_RNA_5_KsgA_mono	reversible	rRNA modification	-1.00E+09	1.00E+09	0	1.12E+02	1.12E+02	0	1.42E+01	14.2	0	1.42E+01	14.2
b3756_v1_RNA_5_modif_2	b3756_v1_RNA modification (16S rRNA): position: 1518, m6(2)A by KsgA_mono	1 b3756_v1_RNA_5_KsgA_mono --> 1 KsgA_mono_inact + 2 ahcys + 1 b3756_v1_RNA_6 + 2 h	irreversible	rRNA modification	0.00E+00	1.00E+09	0	1.12E+02	1.12E+02	0	1.42E+01	14.2	0	1.42E+01	14.2
b3756_v1_RNA_6_modif_1	b3756_v1_RNA modification (16S rRNA): position: 1519, m6(2)A by KsgA_mono	1 KsgA_mono + 2 amet + 1 b3756_v1_RNA_6 <==> 1 b3756_v1_RNA_6_KsgA_mono	reversible	rRNA modification	-1.00E+09	1.00E+09	0	1.12E+02	1.12E+02	0	1.42E+01	14.2	0	1.42E+01	14.2

b3756_v1_RNA_6_modif_2	b3756_v1_RNA modification (16S rRNA): position: 1519, m6(2)A by KsgA_mono	1 b3756_v1_RNA_6_KsgA_mono --> 1 KsgA_mono_inact + 2 ahcys + 1 b3756_v1_RNA_7 + 2 h	irreversible	rRNA modification	0.00E+00	1.00E+09	0	1.12E+02	1.12E+02	0	1.42E+01	14.2	0	1.42E+01	14.2
b3756_v1_RNA_7_modif_1	b3756_v1_RNA modification (16S rRNA): position: 516, Y by RsuA_mon	1 RsuA_mono + 1 b3756_v1_RNA_7 <==> 1 b3756_v1_RNA_7_RsuA_mono	reversible	rRNA modification	-1.00E+09	1.00E+09	0	1.12E+02	1.12E+02	0	1.42E+01	14.2	0	1.42E+01	14.2
b3756_v1_RNA_7_modif_2	b3756_v1_RNA modification (16S rRNA): position: 516, Y by RsuA_mon	1 b3756_v1_RNA_7_RsuA_mono --> 1 RsuA_mono_inact + 1 b3756_v1_RNA_8	irreversible	rRNA modification	0.00E+00	1.00E+09	0	1.12E+02	1.12E+02	0	1.42E+01	14.2	0	1.42E+01	14.2
b3756_v1_RNA_8_modif_1	b3756_v1_RNA modification (16S rRNA): position: 527, m7G by MeT_16S_527	1 MeT_16S_527 + 1 amet + 1 b3756_v1_RNA_8 <==> 1 b3756_v1_RNA_8_MeT_16S_527	reversible	rRNA modification	-1.00E+09	1.00E+09	0	1.12E+02	1.12E+02	0	1.42E+01	14.2	0	1.42E+01	14.2
b3756_v1_RNA_8_modif_2	b3756_v1_RNA modification (16S rRNA): position: 527, m7G by MeT_16S_527	1 b3756_v1_RNA_8_MeT_16S_527 --> 1 MeT_16S_527_inact + 1 ahcys + 1 b3756_v1_RNA_9 + 1 h	irreversible	rRNA modification	0.00E+00	1.00E+09	0	1.12E+02	1.12E+02	0	1.42E+01	14.2	0	1.42E+01	14.2
b3756_v1_RNA_9_modif_1	b3756_v1_RNA modification (16S rRNA): position: 966, m2G by MeT_16S_966	1 MeT_16S_966 + 1 amet + 1 b3756_v1_RNA_9 <==> 1 b3756_v1_RNA_9_MeT_16S_966	reversible	rRNA modification	-1.00E+09	1.00E+09	0	1.12E+02	1.12E+02	0	1.42E+01	14.2	0	1.42E+01	14.2
b3756_v1_RNA_9_modif_2	b3756_v1_RNA modification (16S rRNA): position: 966, m2G by MeT_16S_966	1 b3756_v1_RNA_9_MeT_16S_966 --> 1 MeT_16S_966_inact + 1 ahcys + 1 b3756_v1_RNA_10 + 1 h	irreversible	rRNA modification	0.00E+00	1.00E+09	0	1.12E+02	1.12E+02	0	1.42E+01	14.2	0	1.42E+01	14.2
b3756_v1_RNA_modif_1	b3756_v1_RNA modification (16S rRNA): position: 1207, m2G by RsmC_mono	1 RsmC_mono + 1 amet + 1 b3756_v1_RNA <==> 1 b3756_v1_RNA_RsmC_mono	reversible	rRNA modification	-1.00E+09	1.00E+09	0	1.12E+02	1.12E+02	0	1.42E+01	14.2	0	1.42E+01	14.2
b3756_v1_RNA_modif_2	b3756_v1_RNA modification (16S rRNA): position: 1207, m2G by RsmC_mono	1 b3756_v1_RNA_RsmC_mono --> 1 RsmC_mono_inact + 1 ahcys + 1 b3756_v1_RNA_1 + 1 h	irreversible	rRNA modification	0.00E+00	1.00E+09	0	1.12E+02	1.12E+02	0	1.42E+01	14.2	0	1.42E+01	14.2
b3757_tRNA_13_MODIF_1	modification of position 13 nt (Y) in gltU by TruD_mono	1 TruD_mono + 1 b3757_tRNA_Mg2 <==> 1 b3757_tRNA_Mg2_TruD_mono_cplx	reversible	tRNA modification	-1.00E+09	1.00E+09	0	1.12E+02	1.12E+02	0	0	0	0	0	0
b3757_tRNA_13_MODIF_2	modification of position 13 nt (Y) in gltU by TruD_mono	1 b3757_tRNA_Mg2_TruD_mono_cplx --> 1 TruD_mono_inact + 1 b3757_tRNA_1	irreversible	tRNA modification	0.00E+00	1.00E+09	0	1.12E+02	1.12E+02	0	0	0	0	0	0
b3757_tRNA_34_MODIF_1	modification of position 34 nt (mnm5s2U) in gltU by TrmU_mono-YhhP_mono-YheLMN_cplx-YccK_mono-TrmE_dim-GidA_mono-TrmC_mono	1 5fthf + 1 GidA_mono + 1 IscS_dim_S-SH + 1 TrmC_mono + 1 TrmE_dim + 1 TrmU_mono + 1 YccK_mono + 1 YheLMN_cplx + 1 YhhP_mono + 1 amet + 1 atp + 1 b3757_tRNA_1 + 1 fad + 1 fadh2 + 1 gly + 1 gtp + 1 h2o + 1 mg2 + 1 trdrd <==> 1 b3757_tRNA_1_TrmU_mono-YhhP_mono-YheLMN_cplx-YccK_mon TrmE_dim-GidA_mono-TrmC_mono_cplx	reversible	tRNA modification	-1.00E+09	1.00E+09	0	1.12E+02	1.12E+02	0	0	0	0	0	0
b3757_tRNA_34_MODIF_2	modification of position 34 nt (mnm5s2U) in gltU by TrmU_mono-YhhP_mono-YheLMN_cplx-YccK_mono-TrmE_dim-GidA_mono-TrmC_mono	1 b3757_tRNA_1_TrmU_mono-YhhP_mono-YheLMN_cplx-YccK_mono-TrmE_dim-GidA_mono-TrmC_mono_cplx --> 1 GidA_mono_inact + 1 IscS_dim_S-H + 1 TrmC_mono_inact + 1 TrmE_dim_inact + 1 TrmU_mono_inact + 1 YccK_mono_inact + 1 YheLMN_cplx_inact + 1 YhhP_mono_inact + 1 ahcys + 1 amp + 1 b3757_tRNA_2 + 1 fad + 1 fadh2 + 1 gdp + 1 glx + 4 h + 1 mg2 + 1 pi + 1 ppi + 1 thf + 1 trdox	irreversible	tRNA modification	0.00E+00	1.00E+09	0	1.12E+02	1.12E+02	0	0	0	0	0	0
b3757_tRNA_34_MODIF_3	replacement of sulfur with selenium: b3757_tRNA_2	1 YbbB_dim + 1 b3757_tRNA_2 + 1 h2o + 1 selnp <==> 1 b3757_tRNA_2_YbbB_dim_cplx	reversible	tRNA modification	-1.00E+09	1.00E+09	0	1.12E+02	1.12E+02	0	0	0	0	0	0
b3757_tRNA_34_MODIF_4	replacement of sulfur with selenium: b3757_tRNA_2	1 b3757_tRNA_2_YbbB_dim_cplx --> 1 YbbB_dim_inact + 1 b3757_tRNA_2_Se + 1 h + 1 h2s + 1 pi	irreversible	tRNA modification	0.00E+00	1.00E+09	0	1.12E+02	1.12E+02	0	0	0	0	0	0
b3757_tRNA_37_MODIF_1	modification of position 37 nt (m2A) in gltU by MeT_tRNA_pos_37_m2A	1 MeT_tRNA_pos_37_m2A + 1 amet + 1 b3757_tRNA_2 <==> 1 b3757_tRNA_2_MeT_tRNA_pos_37_m2A_cplx	reversible	tRNA modification	-1.00E+09	1.00E+09	0	1.12E+02	1.12E+02	0	0	0	0	0	0
b3757_tRNA_37_MODIF_1_Se	modification of position 37 nt (m2A) in gltU by MeT_tRNA_pos_37_m2A	1 MeT_tRNA_pos_37_m2A + 1 amet + 1 b3757_tRNA_2_Se <==> 1 b3757_tRNA_2_Se_MeT_tRNA_pos_37_m2A_cplx	reversible	tRNA modification	-1.00E+09	1.00E+09	0	1.12E+02	1.12E+02	0	0	0	0	0	0
b3757_tRNA_37_MODIF_2	modification of position 37 nt (m2A) in gltU by MeT_tRNA_pos_37_m2A	1 b3757_tRNA_2_MeT_tRNA_pos_37_m2A_cplx --> 1 MeT_tRNA_pos_37_m2A_inact + 1 ahcys + 1 b3757_tRNA_3 + 1 h	irreversible	tRNA modification	0.00E+00	1.00E+09	0	1.12E+02	1.12E+02	0	0	0	0	0	0
b3757_tRNA_37_MODIF_2_Se	modification of position 37 nt (m2A) in gltU by MeT_tRNA_pos_37_m2A	1 b3757_tRNA_2_Se_MeT_tRNA_pos_37_m2A_cplx --> 1 MeT_tRNA_pos_37_m2A_inact + 1 ahcys + 1 b3757_tRNA_3_Se + h	irreversible	tRNA modification	0.00E+00	1.00E+09	0	1.12E+02	1.12E+02	0	0	0	0	0	0
b3757_tRNA_54_MODIF_1	modification of position 54 nt (m5U) in gltU by TrmA_mono	1 TrmA_mono + 1 amet + 1 b3757_tRNA_3 <==> 1 b3757_tRNA_3_TrmA_mono_cplx	reversible	tRNA modification	-1.00E+09	1.00E+09	0	1.12E+02	1.12E+02	0	0	0	0	0	0
b3757_tRNA_54_MODIF_1_Se	modification of position 54 nt (m5U) in gltU by TrmA_mono	1 TrmA_mono + 1 amet + 1 b3757_tRNA_3_Se <==> 1 b3757_tRNA_3_Se_TrmA_mono_cplx	reversible	tRNA modification	-1.00E+09	1.00E+09	0	1.12E+02	1.12E+02	0	0	0	0	0	0
b3757_tRNA_54_MODIF_2	modification of position 54 nt (m5U) in gltU by TrmA_mono	1 b3757_tRNA_3_TrmA_mono_cplx --> 1 TrmA_mono_inact + 1 ahcys + 1 b3757_tRNA_4 + 1 h	irreversible	tRNA modification	0.00E+00	1.00E+09	0	1.12E+02	1.12E+02	0	0	0	0	0	0
b3757_tRNA_54_MODIF_2_Se	modification of position 54 nt (m5U) in gltU by TrmA_mono	1 b3757_tRNA_3_Se_TrmA_mono_cplx --> 1 TrmA_mono_inact + 1 ahcys + 1 b3757_tRNA_4_Se + 1 h	irreversible	tRNA modification	0.00E+00	1.00E+09	0	1.12E+02	1.12E+02	0	0	0	0	0	0
b3757_tRNA_55_MODIF_1	modification of position 55 nt (Y) in gltU by TruB_mono	1 TruB_mono + 1 b3757_tRNA_4 <==> 1 b3757_tRNA_4_TruB_mono_cplx	reversible	tRNA modification	-1.00E+09	1.00E+09	0	1.12E+02	1.12E+02	0	0	0	0	0	0
b3757_tRNA_55_MODIF_1_Se	modification of position 55 nt (Y) in gltU by TruB_mono	1 TruB_mono + 1 b3757_tRNA_4_Se <==> 1 b3757_tRNA_4_Se_TruB_mono_cplx	reversible	tRNA modification	-1.00E+09	1.00E+09	0	1.12E+02	1.12E+02	0	0	0	0	0	0
b3757_tRNA_55_MODIF_2	modification of position 55 nt (Y) in gltU by TruB_mono	1 b3757_tRNA_4_TruB_mono_cplx --> 1 TruB_mono_inact + 1 gltU_tRNA	irreversible	tRNA modification	0.00E+00	1.00E+09	0	1.12E+02	1.12E+02	0	0	0	0	0	0
b3757_tRNA_55_MODIF_2_Se	modification of position 55 nt (Y) in gltU by TruB_mono	1 b3757_tRNA_4_Se_TruB_mono_cplx --> 1 TruB_mono_inact + 1 gltU_tRNA_Se	irreversible	tRNA modification	0.00E+00	1.00E+09	0	1.12E+02	1.12E+02	0	0	0	0	0	0
b3757_tRNA_Mg_BIND	Binding of two Mg2 to b3757_tRNA	1 b3757_RNA + 2 mg2 <==> 1 b3757_tRNA_Mg2	reversible	Metallo-ion binding	-1.00E+09	1.00E+09	0	1.12E+02	1.12E+02	0	0	0	0	0	0
b3757_v1_tRNA_13_MODIF_1	modification of position 13 nt (Y) in gltU by TruD_mono	1 TruD_mono + 1 b3757_v1_tRNA_Mg2 <==> 1 b3757_v1_tRNA_Mg2_TruD_mono_cplx	reversible	tRNA modification	-1.00E+09	1.00E+09	0	1.12E+02	1.12E+02	0	1.42E+01	14.2	0	1.42E+01	14.2
b3757_v1_tRNA_13_MODIF_2	modification of position 13 nt (Y) in gltU by TruD_mono	1 b3757_v1_tRNA_Mg2_TruD_mono_cplx --> 1 TruD_mono_inact + 1 b3757_v1_tRNA_1	irreversible	tRNA modification	0.00E+00	1.00E+09	0	1.12E+02	1.12E+02	0	1.42E+01	14.2	0	1.42E+01	14.2
b3757_v1_tRNA_34_MODIF_1	modification of position 34 nt (mnm5s2U) in gltU by TrmU_mono-YhhP_mono-YheLMN_cplx-YccK_mono-TrmE_dim-GidA_mono-TrmC_mono	1 5fthf + 1 GidA_mono + 1 IscS_dim_S-SH + 1 TrmC_mono + 1 TrmE_dim + 1 TrmU_mono + 1 YccK_mono + 1 YheLMN_cplx + 1 YhhP_mono + 1 amet + 1 atp + 1 b3757_v1_tRNA_1 + 1 fad + 1 fadh2 + 1 gly + 1 gtp + 1 h2o + 1 mg2 + 1 trdrd <==> 1 b3757_v1_tRNA_1_TrmU_mono-YhhP_mono-YheLMN_cplx-YccK_mono-TrmE_dim-GidA_mono-TrmC_mono_cplx	reversible	tRNA modification	-1.00E+09	1.00E+09	0	1.12E+02	1.12E+02	0	1.42E+01	14.2	0	1.42E+01	14.2
b3757_v1_tRNA_34_MODIF_2	modification of position 34 nt (mnm5s2U) in gltU by TrmU_mono-YhhP_mono-YheLMN_cplx-YccK_mono-TrmE_dim-GidA_mono-TrmC_mono	1 b3757_v1_tRNA_1_TrmU_mono-YhhP_mono-YheLMN_cplx-YccK_mono-TrmE_dim-GidA_mono-TrmC_mono_cplx --> 1 GidA_mono_inact + 1 IscS_dim_S-H + 1 TrmC_mono_inact + 1 TrmE_dim_inact + 1 TrmU_mono_inact + 1 YccK_mono_inact + 1 YheLMN_cplx_inact + 1 YhhP_mono_inact + 1 ahcys + 1 amp + 1 b3757_v1_tRNA_2 + 1 fad + 1 fadh2 + 1 gdp + 1 glx + 4 h + 1 mg2 + 1 pi + 1 ppi + 1 thf + 1 trdox	irreversible	tRNA modification	0.00E+00	1.00E+09	0	1.12E+02	1.12E+02	0	1.42E+01	14.2	0	1.42E+01	14.2
b3757_v1_tRNA_34_MODIF_3	replacement of sulfur with selenium: b3757_tRNA_2	1 YbbB_dim + 1 b3757_v1_tRNA_2 + 1 h2o + 1 selnp <==> 1 b3757_v1_tRNA_2_YbbB_dim_cplx	reversible	tRNA modification	-1.00E+09	1.00E+09	0	1.12E+02	1.12E+02	0	1.42E+01	14.2	0	1.42E+01	14.2
b3757_v1_tRNA_34_MODIF_4	replacement of sulfur with selenium: b3757_tRNA_2	1 b3757_v1_tRNA_2_YbbB_dim_cplx --> 1 YbbB_dim_inact + 1 b3757_v1_tRNA_2_Se + 1 h + 1 h2s + 1 pi	irreversible	tRNA modification	0.00E+00	1.00E+09	0	1.12E+02	1.12E+02	0	1.42E+01	14.2	0	1.42E+01	14.2
b3757_v1_tRNA_37_MODIF_1	modification of position 37 nt (m2A) in gltU by MeT_tRNA_pos_37_m2A	1 MeT_tRNA_pos_37_m2A + 1 amet + 1 b3757_v1_tRNA_2 <==> 1 b3757_v1_tRNA_2_MeT_tRNA_pos_37_m2A_cplx	reversible	tRNA modification	-1.00E+09	1.00E+09	0	1.12E+02	1.12E+02	0	1.42E+01	14.2	0	1.42E+01	14.2
b3757_v1_tRNA_37_MODIF_1_Se	modification of position 37 nt (m2A) in gltU by MeT_tRNA_pos_37_m2A	1 MeT_tRNA_pos_37_m2A + 1 amet + 1 b3757_v1_tRNA_2_Se <==> 1 b3757_v1_tRNA_2_Se_MeT_tRNA_pos_37_m2A_cplx	reversible	tRNA modification	-1.00E+09	1.00E+09	0	1.12E+02	1.12E+02	0	1.42E+01	14.2	0	1.42E+01	14.2
b3757_v1_tRNA_37_MODIF_2	modification of position 37 nt (m2A) in gltU by MeT_tRNA_pos_37_m2A	1 b3757_v1_tRNA_2_MeT_tRNA_pos_37_m2A_cplx --> 1 MeT_tRNA_pos_37_m2A_inact + 1 ahcys + 1 b3757_v1_tRNA_3 + 1 h	irreversible	tRNA modification	0.00E+00	1.00E+09	0	1.12E+02	1.12E+02	0	1.42E+01	14.2	0	1.42E+01	14.2
b3757_v1_tRNA_37_MODIF_2_Se	modification of position 37 nt (m2A) in gltU by MeT_tRNA_pos_37_m2A	1 b3757_v1_tRNA_2_Se_MeT_tRNA_pos_37_m2A_cplx --> 1 MeT_tRNA_pos_37_m2A_inact + 1 ahcys + 1 b3757_v1_tRNA_3_Se + 1 h	irreversible	tRNA modification	0.00E+00	1.00E+09	0	1.12E+02	1.12E+02	0	1.42E+01	14.2	0	1.42E+01	14.2

b3757_v1_tRNA_54_MODIF_1	modification of position 54 nt (m5U) in gltU by TrmA_mono	1 TrmA_mono + 1 amet + 1 b3757_v1_tRNA_3 <==> 1 b3757_v1_tRNA_3_TrmA_mono_cplx	reversible	tRNA modification	-1.00E+09	1.00E+09	0	1.12E+02	1.12E+02	0	1.42E+01	14.2	0	1.42E+01	14.2
b3757_v1_tRNA_54_MODIF_1_Se	modification of position 54 nt (m5U) in gltU by TrmA_mono	1 TrmA_mono + 1 amet + 1 b3757_v1_tRNA_3_Se <==> 1 b3757_v1_tRNA_3_Se_TrmA_mono_cplx	reversible	tRNA modification	-1.00E+09	1.00E+09	0	1.12E+02	1.12E+02	0	1.42E+01	14.2	0	1.42E+01	14.2
b3757_v1_tRNA_54_MODIF_2	modification of position 54 nt (m5U) in gltU by TrmA_mono	1 b3757_v1_tRNA_3_TrmA_mono_cplx --> 1 TrmA_mono_inact + 1 ahcys + 1 b3757_v1_tRNA_4 + 1 h	irreversible	tRNA modification	0.00E+00	1.00E+09	0	1.12E+02	1.12E+02	0	1.42E+01	14.2	0	1.42E+01	14.2
b3757_v1_tRNA_54_MODIF_2_Se	modification of position 54 nt (m5U) in gltU by TrmA_mono	1 b3757_v1_tRNA_3_Se_TrmA_mono_cplx --> 1 TrmA_mono_inact 1 ahcys + 1 b3757_v1_tRNA_4_Se + 1 h	irreversible	tRNA modification	0.00E+00	1.00E+09	0	1.12E+02	1.12E+02	0	1.42E+01	14.2	0	1.42E+01	14.2
b3757_v1_tRNA_55_MODIF_1	modification of position 55 nt (Y) in gltU by TruB_mono	1 TruB_mono + 1 b3757_v1_tRNA_4 <==> 1 b3757_v1_tRNA_4_TruB_mono_cplx	reversible	tRNA modification	-1.00E+09	1.00E+09	0	1.12E+02	1.12E+02	0	1.42E+01	14.2	0	1.42E+01	14.2
b3757_v1_tRNA_55_MODIF_1_Se	modification of position 55 nt (Y) in gltU by TruB_mono	1 TruB_mono + 1 b3757_v1_tRNA_4_Se <==> 1 b3757_v1_tRNA_4_Se_TruB_mono_cplx	reversible	tRNA modification	-1.00E+09	1.00E+09	0	1.12E+02	1.12E+02	0	1.42E+01	14.2	0	1.42E+01	14.2
b3757_v1_tRNA_55_MODIF_2	modification of position 55 nt (Y) in gltU by TruB_mono	1 b3757_v1_tRNA_4_TruB_mono_cplx --> 1 TruB_mono_inact + 1 gltU_tRNA	irreversible	tRNA modification	0.00E+00	1.00E+09	0	1.12E+02	1.12E+02	0	1.42E+01	14.2	0	1.42E+01	14.2
b3757_v1_tRNA_55_MODIF_2_Se	modification of position 55 nt (Y) in gltU by TruB_mono	1 b3757_v1_tRNA_4_Se_TruB_mono_cplx --> 1 TruB_mono_inact + 1 gltU_tRNA_Se	irreversible	tRNA modification	0.00E+00	1.00E+09	0	1.12E+02	1.12E+02	0	1.42E+01	14.2	0	1.42E+01	14.2
b3757_v1_tRNA_Mg_BIND	Binding of two Mg2 to b3757_v1_tRNA	1 b3757_v1_RNA + 2 mg2 <==> 1 b3757_v1_tRNA_Mg2	reversible	Metallo-ion binding	-1.00E+09	1.00E+09	0	1.12E+02	1.12E+02	0	1.42E+01	14.2	0	1.42E+01	14.2
b3758_RNA_10_modif_1	b3758_RNA modification (23S rRNA): position: 2445, m2G by MeT_23S_2445	1 MeT_23S_2445 + 1 amet + 1 b3758_RNA_10 <==> 1 b3758_RNA_10_MeT_23S_2445	reversible	rRNA modification	-1.00E+09	1.00E+09	0	1.12E+02	1.12E+02	0	0	0	0	0	0
b3758_RNA_10_modif_2	b3758_RNA modification (23S rRNA): position: 2445, m2G by MeT_23S_2445	1 b3758_RNA_10_MeT_23S_2445 --> 1 MeT_23S_2445_inact + 1 ahcys + 1 b3758_RNA_11 + 1 h	irreversible	rRNA modification	0.00E+00	1.00E+09	0	1.12E+02	1.12E+02	0	0	0	0	0	0
b3758_RNA_11_a_modif_1	b3758_RNA modification (23S rRNA): position: 2449, D by DU_23S_2449 (NADH)	1 DU_23S_2449 + 1 b3758_RNA_11 + 1 h + 1 nadh <==> 1 b3758_RNA_11_DU_23S_2449_a	reversible	rRNA modification	-1.00E+09	1.00E+09	0	1.12E+02	1.12E+02	0	0	0	0	0	0
b3758_RNA_11_a_modif_2	b3758_RNA modification (23S rRNA): position: 2449, D by DU_23S_2449 (NADH)	1 b3758_RNA_11_DU_23S_2449_a --> 1 DU_23S_2449_inact + 1 b3758_RNA_12 + 1 nad	irreversible	rRNA modification	0.00E+00	1.00E+09	0	1.12E+02	1.12E+02	0	0	0	0	0	0
b3758_RNA_11_b_modif_1	b3758_RNA modification (23S rRNA): position: 2449, D by DU_23S_2449 (NADPH)	1 DU_23S_2449 + 1 b3758_RNA_11 + 1 h + 1 nadph <==> 1 b3758_RNA_11_DU_23S_2449_b	reversible	rRNA modification	-1.00E+09	1.00E+09	0	1.12E+02	1.12E+02	0	0	0	0	0	0
b3758_RNA_11_b_modif_2	b3758_RNA modification (23S rRNA): position: 2449, D by DU_23S_2449 (NADPH)	1 b3758_RNA_11_DU_23S_2449_b --> 1 DU_23S_2449_inact + 1 b3758_RNA_12 + 1 nadp	irreversible	rRNA modification	0.00E+00	1.00E+09	0	1.12E+02	1.12E+02	0	0	0	0	0	0
b3758_RNA_12_modif_1	b3758_RNA modification (23S rRNA): position: 2457, Y by YmfC_mono	1 YmfC_mono + 1 b3758_RNA_12 <==> 1 b3758_RNA_12_YmfC_mono	reversible	rRNA modification	-1.00E+09	1.00E+09	0	1.12E+02	1.12E+02	0	0	0	0	0	0
b3758_RNA_12_modif_2	b3758_RNA modification (23S rRNA): position: 2457, Y by YmfC_mono	1 b3758_RNA_12_YmfC_mono --> 1 YmfC_mono_inact + 1 b3758_RNA_13	irreversible	rRNA modification	0.00E+00	1.00E+09	0	1.12E+02	1.12E+02	0	0	0	0	0	0
b3758_RNA_13_modif_1	b3758_RNA modification (23S rRNA): position: 2498, Cm by MeT_23S_2498	1 MeT_23S_2498 + 1 amet + 1 b3758_RNA_13 <==> 1 b3758_RNA_13_MeT_23S_2498	reversible	rRNA modification	-1.00E+09	1.00E+09	0	1.12E+02	1.12E+02	0	0	0	0	0	0
b3758_RNA_13_modif_2	b3758_RNA modification (23S rRNA): position: 2498, Cm by MeT_23S_2498	1 b3758_RNA_13_MeT_23S_2498 --> 1 MeT_23S_2498_inact + 1 ahcys + 1 b3758_RNA_14 + 1 h	irreversible	rRNA modification	0.00E+00	1.00E+09	0	1.12E+02	1.12E+02	0	0	0	0	0	0
b3758_RNA_14_modif_1	b3758_RNA modification (23S rRNA): position: 2503, m2A by MeT_23S_2503	1 MeT_23S_2503 + 1 amet + 1 b3758_RNA_14 <==> 1 b3758_RNA_14_MeT_23S_2503	reversible	rRNA modification	-1.00E+09	1.00E+09	0	1.12E+02	1.12E+02	0	0	0	0	0	0
b3758_RNA_14_modif_2	b3758_RNA modification (23S rRNA): position: 2503, m2A by MeT_23S_2503	1 b3758_RNA_14_MeT_23S_2503 --> 1 MeT_23S_2503_inact + 1 ahcys + 1 b3758_RNA_15 + 1 h	irreversible	rRNA modification	0.00E+00	1.00E+09	0	1.12E+02	1.12E+02	0	0	0	0	0	0
b3758_RNA_15_modif_1	b3758_RNA modification (23S rRNA): position: 2504, Y by RluC_mono	1 RluC_mono + 1 b3758_RNA_15 <==> 1 b3758_RNA_15_RluC_mono	reversible	rRNA modification	-1.00E+09	1.00E+09	0	1.12E+02	1.12E+02	0	0	0	0	0	0
b3758_RNA_15_modif_2	b3758_RNA modification (23S rRNA): position: 2504, Y by RluC_mono	1 b3758_RNA_15_RluC_mono --> 1 RluC_mono_inact + 1 b3758_RNA_16	irreversible	rRNA modification	0.00E+00	1.00E+09	0	1.12E+02	1.12E+02	0	0	0	0	0	0
b3758_RNA_16_modif_1	b3758_RNA modification (23S rRNA): position: 2552, Um by RrmJ_mono	1 RrmJ_mono + 1 amet + 1 b3758_RNA_16 <==> 1 b3758_RNA_16_RrmJ_mono	reversible	rRNA modification	-1.00E+09	1.00E+09	0	1.12E+02	1.12E+02	0	0	0	0	0	0
b3758_RNA_16_modif_2	b3758_RNA modification (23S rRNA): position: 2552, Um by RrmJ_mono	1 b3758_RNA_16_RrmJ_mono --> 1 RrmJ_mono_inact + 1 ahcys + b3758_RNA_17 + 1 h	irreversible	rRNA modification	0.00E+00	1.00E+09	0	1.12E+02	1.12E+02	0	0	0	0	0	0
b3758_RNA_17_modif_1	b3758_RNA modification (23S rRNA): position: 2580, Y by RluC_mono	1 RluC_mono + 1 b3758_RNA_17 <==> 1 b3758_RNA_17_RluC_mono	reversible	rRNA modification	-1.00E+09	1.00E+09	0	1.12E+02	1.12E+02	0	0	0	0	0	0
b3758_RNA_17_modif_2	b3758_RNA modification (23S rRNA): position: 2580, Y by RluC_mono	1 b3758_RNA_17_RluC_mono --> 1 RluC_mono_inact + 1 b3758_RNA_18	irreversible	rRNA modification	0.00E+00	1.00E+09	0	1.12E+02	1.12E+02	0	0	0	0	0	0
b3758_RNA_18_modif_1	b3758_RNA modification (23S rRNA): position: 2604, Y by YjbC_mono	1 YjbC_mono + 1 b3758_RNA_18 <==> 1 b3758_RNA_18_YjbC_mono	reversible	rRNA modification	-1.00E+09	1.00E+09	0	1.12E+02	1.12E+02	0	0	0	0	0	0
b3758_RNA_18_modif_2	b3758_RNA modification (23S rRNA): position: 2604, Y by YjbC_mono	1 b3758_RNA_18_YjbC_mono --> 1 YjbC_mono_inact + 1 b3758_RNA_19	irreversible	rRNA modification	0.00E+00	1.00E+09	0	1.12E+02	1.12E+02	0	0	0	0	0	0
b3758_RNA_19_modif_1	b3758_RNA modification (23S rRNA): position: 2605, Y by RluB_mono	1 RluB_mono + 1 b3758_RNA_19 <==> 1 b3758_RNA_19_RluB_mono	reversible	rRNA modification	-1.00E+09	1.00E+09	0	1.12E+02	1.12E+02	0	0	0	0	0	0
b3758_RNA_19_modif_2	b3758_RNA modification (23S rRNA): position: 2605, Y by RluB_mono	1 b3758_RNA_19_RluB_mono --> 1 RluB_mono_inact + 1 b3758_RNA_20	irreversible	rRNA modification	0.00E+00	1.00E+09	0	1.12E+02	1.12E+02	0	0	0	0	0	0
b3758_RNA_1_modif_1	b3758_RNA modification (23S rRNA): position: 1835, m2G by MeT_23S_1835	1 MeT_23S_1835 + 1 amet + 1 b3758_RNA_1 <==> 1 b3758_RNA_1_MeT_23S_1835	reversible	rRNA modification	-1.00E+09	1.00E+09	0	1.12E+02	1.12E+02	0	0	0	0	0	0
b3758_RNA_1_modif_2	b3758_RNA modification (23S rRNA): position: 1835, m2G by MeT_23S_1835	1 b3758_RNA_1_MeT_23S_1835 --> 1 MeT_23S_1835_inact + 1 ahcys + 1 b3758_RNA_2 + 1 h	irreversible	rRNA modification	0.00E+00	1.00E+09	0	1.12E+02	1.12E+02	0	0	0	0	0	0
b3758_RNA_20_modif_1	b3758_RNA modification (23S rRNA): position: 745, m1G by RrmA_dim	1 RrmA_dim + 1 amet + 1 b3758_RNA_20 <==> 1 b3758_RNA_20_RrmA_dim	reversible	rRNA modification	-1.00E+09	1.00E+09	0	1.12E+02	1.12E+02	0	0	0	0	0	0
b3758_RNA_20_modif_2	b3758_RNA modification (23S rRNA): position: 745, m1G by RrmA_dim	1 b3758_RNA_20_RrmA_dim --> 1 RrmA_dim_inact + 1 ahcys + 1 b3758_RNA_21 + 1 h	irreversible	rRNA modification	0.00E+00	1.00E+09	0	1.12E+02	1.12E+02	0	0	0	0	0	0
b3758_RNA_21_modif_1	b3758_RNA modification (23S rRNA): position: 746, Y by RluA_mono	1 RluA_mono + 1 b3758_RNA_21 <==> 1 b3758_RNA_21_RluA_mono	reversible	rRNA modification	-1.00E+09	1.00E+09	0	1.12E+02	1.12E+02	0	0	0	0	0	0
b3758_RNA_21_modif_2	b3758_RNA modification (23S rRNA): position: 746, Y by RluA_mono	1 b3758_RNA_21_RluA_mono --> 1 RluA_mono_inact + 1 b3758_RNA_22	irreversible	rRNA modification	0.00E+00	1.00E+09	0	1.12E+02	1.12E+02	0	0	0	0	0	0
b3758_RNA_22_modif_1	b3758_RNA modification (23S rRNA): position: 747, m5U by RumB_mono	1 RumB_mono + 1 amet + 1 b3758_RNA_22 <==> 1 b3758_RNA_22_RumB_mono	reversible	rRNA modification	-1.00E+09	1.00E+09	0	1.12E+02	1.12E+02	0	0	0	0	0	0
b3758_RNA_22_modif_2	b3758_RNA modification (23S rRNA): position: 747, m5U by RumB_mono	1 b3758_RNA_22_RumB_mono --> 1 RumB_mono_inact + 1 ahcys 1 b3758_RNA_23 + 1 h	irreversible	rRNA modification	0.00E+00	1.00E+09	0	1.12E+02	1.12E+02	0	0	0	0	0	0
b3758_RNA_23_modif_1	b3758_RNA modification (23S rRNA): position: 955, Y by RluC_mono	1 RluC_mono + 1 b3758_RNA_23 <==> 1 b3758_RNA_23_RluC_mono	reversible	rRNA modification	-1.00E+09	1.00E+09	0	1.12E+02	1.12E+02	0	0	0	0	0	0
b3758_RNA_23_modif_2	b3758_RNA modification (23S rRNA): position: 955, Y by RluC_mono	1 b3758_RNA_23_RluC_mono --> 1 RluC_mono_inact + 1 rrlC	irreversible	rRNA modification	0.00E+00	1.00E+09	0	1.12E+02	1.12E+02	0	0	0	0	0	0

b3758_RNA_2_modif_1	b3758_RNA modification (23S rRNA): position: 1911, Y by RluD_mono	1 RluD_mono + 1 b3758_RNA_2 <==> 1 b3758_RNA_2_RluD_mono	reversible	rRNA modification	-1.00E+09	1.00E+09	0	1.12E+02	1.12E+02	0	0	0	0	0	0
b3758_RNA_2_modif_2	b3758_RNA modification (23S rRNA): position: 1911, Y by RluD_mono	1 b3758_RNA_2_RluD_mono --> 1 RluD_mono_inact + 1 b3758_RNA_3	irreversible	rRNA modification	0.00E+00	1.00E+09	0	1.12E+02	1.12E+02	0	0	0	0	0	0
b3758_RNA_3_modif_1	b3758_RNA modification (23S rRNA): position: 1915, m3Y by RluD_mono	1 RluD_mono + 1 amet + 1 b3758_RNA_3 <==> 1 b3758_RNA_3_RluD_mono	reversible	rRNA modification	-1.00E+09	1.00E+09	0	1.12E+02	1.12E+02	0	0	0	0	0	0
b3758_RNA_3_modif_2	b3758_RNA modification (23S rRNA): position: 1915, m3Y by RluD_mono	1 b3758_RNA_3_RluD_mono --> 1 RluD_mono_inact + 1 ahcys + 1 b3758_RNA_4 + 1 h	irreversible	rRNA modification	0.00E+00	1.00E+09	0	1.12E+02	1.12E+02	0	0	0	0	0	0
b3758_RNA_4_modif_1	b3758_RNA modification (23S rRNA): position: 1917, Y by RluD_mono	1 RluD_mono + 1 b3758_RNA_4 <==> 1 b3758_RNA_4_RluD_mono	reversible	rRNA modification	-1.00E+09	1.00E+09	0	1.12E+02	1.12E+02	0	0	0	0	0	0
b3758_RNA_4_modif_2	b3758_RNA modification (23S rRNA): position: 1917, Y by RluD_mono	1 b3758_RNA_4_RluD_mono --> 1 RluD_mono_inact + 1 b3758_RNA_5	irreversible	rRNA modification	0.00E+00	1.00E+09	0	1.12E+02	1.12E+02	0	0	0	0	0	0
b3758_RNA_5_modif_1	b3758_RNA modification (23S rRNA): position: 1939, m5U by RumA_mono	1 RumA_mono + 1 amet + 1 b3758_RNA_5 <==> 1 b3758_RNA_5_RumA_mono	reversible	rRNA modification	-1.00E+09	1.00E+09	0	1.12E+02	1.12E+02	0	0	0	0	0	0
b3758_RNA_5_modif_2	b3758_RNA modification (23S rRNA): position: 1939, m5U by RumA_mono	1 b3758_RNA_5_RumA_mono --> 1 RumA_mono_inact + 1 ahcys + 1 b3758_RNA_6 + 1 h	irreversible	rRNA modification	0.00E+00	1.00E+09	0	1.12E+02	1.12E+02	0	0	0	0	0	0
b3758_RNA_6_modif_1	b3758_RNA modification (23S rRNA): position: 1962, m5C by MeT_23S_1962	1 MeT_23S_1962 + 1 amet + 1 b3758_RNA_6 <==> 1 b3758_RNA_6_MeT_23S_1962	reversible	rRNA modification	-1.00E+09	1.00E+09	0	1.12E+02	1.12E+02	0	0	0	0	0	0
b3758_RNA_6_modif_2	b3758_RNA modification (23S rRNA): position: 1962, m5C by MeT_23S_1962	1 b3758_RNA_6_MeT_23S_1962 --> 1 MeT_23S_1962_inact + 1 ahcys + 1 b3758_RNA_7 + 1 h	irreversible	rRNA modification	0.00E+00	1.00E+09	0	1.12E+02	1.12E+02	0	0	0	0	0	0
b3758_RNA_7_modif_1	b3758_RNA modification (23S rRNA): position: 2030, m6A by MeT_23S_2030	1 MeT_23S_2030 + 1 amet + 1 b3758_RNA_7 <==> 1 b3758_RNA_7_MeT_23S_2030	reversible	rRNA modification	-1.00E+09	1.00E+09	0	1.12E+02	1.12E+02	0	0	0	0	0	0
b3758_RNA_7_modif_2	b3758_RNA modification (23S rRNA): position: 2030, m6A by MeT_23S_2030	1 b3758_RNA_7_MeT_23S_2030 --> 1 MeT_23S_2030_inact + 1 ahcys + 1 b3758_RNA_8 + 1 h	irreversible	rRNA modification	0.00E+00	1.00E+09	0	1.12E+02	1.12E+02	0	0	0	0	0	0
b3758_RNA_8_modif_1	b3758_RNA modification (23S rRNA): position: 2069, m7G by MeT_23S_2069	1 MeT_23S_2069 + 1 amet + 1 b3758_RNA_8 <==> 1 b3758_RNA_8_MeT_23S_2069	reversible	rRNA modification	-1.00E+09	1.00E+09	0	1.12E+02	1.12E+02	0	0	0	0	0	0
b3758_RNA_8_modif_2	b3758_RNA modification (23S rRNA): position: 2069, m7G by MeT_23S_2069	1 b3758_RNA_8_MeT_23S_2069 --> 1 MeT_23S_2069_inact + 1 ahcys + 1 b3758_RNA_9 + 1 h	irreversible	rRNA modification	0.00E+00	1.00E+09	0	1.12E+02	1.12E+02	0	0	0	0	0	0
b3758_RNA_9_modif_1	b3758_RNA modification (23S rRNA): position: 2251, Gm by RlmB_dim	1 RlmB_dim + 1 amet + 1 b3758_RNA_9 <==> 1 b3758_RNA_9_RlmB_dim	reversible	rRNA modification	-1.00E+09	1.00E+09	0	1.12E+02	1.12E+02	0	0	0	0	0	0
b3758_RNA_9_modif_2	b3758_RNA modification (23S rRNA): position: 2251, Gm by RlmB_dim	1 b3758_RNA_9_RlmB_dim --> 1 RlmB_dim_inact + 1 ahcys + 1 b3758_RNA_10 + 1 h	irreversible	rRNA modification	0.00E+00	1.00E+09	0	1.12E+02	1.12E+02	0	0	0	0	0	0
b3758_RNA_modif_1	b3758_RNA modification (23S rRNA): position: 1618, m6A by MeT_23S_1618	1 MeT_23S_1618 + 1 amet + 1 b3758_RNA <==> 1 b3758_RNA_MeT_23S_1618	reversible	rRNA modification	-1.00E+09	1.00E+09	0	1.12E+02	1.12E+02	0	0	0	0	0	0
b3758_RNA_modif_2	b3758_RNA modification (23S rRNA): position: 1618, m6A by MeT_23S_1618	1 b3758_RNA_MeT_23S_1618 --> 1 MeT_23S_1618_inact + 1 ahcy + 1 b3758_RNA_1 + 1 h	irreversible	rRNA modification	0.00E+00	1.00E+09	0	1.12E+02	1.12E+02	0	0	0	0	0	0
b3758_v1_RNA_10_modif_1	b3758_v1_RNA modification (23S rRNA): position: 2445, m2G by MeT_23S_2445	1 MeT_23S_2445 + 1 amet + 1 b3758_v1_RNA_10 <==> 1 b3758_v1_RNA_10_MeT_23S_2445	reversible	rRNA modification	-1.00E+09	1.00E+09	0	1.12E+02	1.12E+02	0	1.42E+01	14.2	0	1.42E+01	14.2
b3758_v1_RNA_10_modif_2	b3758_v1_RNA modification (23S rRNA): position: 2445, m2G by MeT_23S_2445	1 b3758_v1_RNA_10_MeT_23S_2445 --> 1 MeT_23S_2445_inact + 1 ahcys + 1 b3758_v1_RNA_11 + 1 h	irreversible	rRNA modification	0.00E+00	1.00E+09	0	1.12E+02	1.12E+02	0	1.42E+01	14.2	0	1.42E+01	14.2
b3758_v1_RNA_11_a_modif_1	b3758_v1_RNA modification (23S rRNA): position: 2449, D by DU_23S_2449 (NADH)	1 DU_23S_2449 + 1 b3758_v1_RNA_11 + 1 h + 1 nadh <==> 1 b3758_v1_RNA_11_DU_23S_2449_a	reversible	rRNA modification	-1.00E+09	1.00E+09	0	1.12E+02	1.12E+02	0	1.42E+01	14.2	0	1.42E+01	14.2
b3758_v1_RNA_11_a_modif_2	b3758_v1_RNA modification (23S rRNA): position: 2449, D by DU_23S_2449 (NADH)	1 b3758_v1_RNA_11_DU_23S_2449_a --> 1 DU_23S_2449_inact + 1 b3758_v1_RNA_12 + 1 nad	irreversible	rRNA modification	0.00E+00	1.00E+09	0	1.12E+02	1.12E+02	0	1.42E+01	14.2	0	1.42E+01	14.2
b3758_v1_RNA_11_b_modif_1	b3758_v1_RNA modification (23S rRNA): position: 2449, D by DU_23S_2449 (NADPH)	1 DU_23S_2449 + 1 b3758_v1_RNA_11 + 1 h + 1 nadph <==> 1 b3758_v1_RNA_11_DU_23S_2449_b	reversible	rRNA modification	-1.00E+09	1.00E+09	0	1.12E+02	1.12E+02	0	1.42E+01	14.2	0	1.42E+01	14.2
b3758_v1_RNA_11_b_modif_2	b3758_v1_RNA modification (23S rRNA): position: 2449, D by DU_23S_2449 (NADPH)	1 b3758_v1_RNA_11_DU_23S_2449_b --> 1 DU_23S_2449_inact + 1 b3758_v1_RNA_12 + 1 nadp	irreversible	rRNA modification	0.00E+00	1.00E+09	0	1.12E+02	1.12E+02	0	1.42E+01	14.2	0	1.42E+01	14.2
b3758_v1_RNA_12_modif_1	b3758_v1_RNA modification (23S rRNA): position: 2457, Y by YmfC_mono	1 YmfC_mono + 1 b3758_v1_RNA_12 <==> 1 b3758_v1_RNA_12_YmfC_mono	reversible	rRNA modification	-1.00E+09	1.00E+09	0	1.12E+02	1.12E+02	0	1.42E+01	14.2	0	1.42E+01	14.2
b3758_v1_RNA_12_modif_2	b3758_v1_RNA modification (23S rRNA): position: 2457, Y by YmfC_mono	1 b3758_v1_RNA_12_YmfC_mono --> 1 YmfC_mono_inact + 1 b3758_v1_RNA_13	irreversible	rRNA modification	0.00E+00	1.00E+09	0	1.12E+02	1.12E+02	0	1.42E+01	14.2	0	1.42E+01	14.2
b3758_v1_RNA_13_modif_1	b3758_v1_RNA modification (23S rRNA): position: 2498, Cm by MeT_23S_2498	1 MeT_23S_2498 + 1 amet + 1 b3758_v1_RNA_13 <==> 1 b3758_v1_RNA_13_MeT_23S_2498	reversible	rRNA modification	-1.00E+09	1.00E+09	0	1.12E+02	1.12E+02	0	1.42E+01	14.2	0	1.42E+01	14.2
b3758_v1_RNA_13_modif_2	b3758_v1_RNA modification (23S rRNA): position: 2498, Cm by MeT_23S_2498	1 b3758_v1_RNA_13_MeT_23S_2498 --> 1 MeT_23S_2498_inact + 1 ahcys + 1 b3758_v1_RNA_14 + 1 h	irreversible	rRNA modification	0.00E+00	1.00E+09	0	1.12E+02	1.12E+02	0	1.42E+01	14.2	0	1.42E+01	14.2
b3758_v1_RNA_14_modif_1	b3758_v1_RNA modification (23S rRNA): position: 2503, m2A by MeT_23S_2503	1 MeT_23S_2503 + 1 amet + 1 b3758_v1_RNA_14 <==> 1 b3758_v1_RNA_14_MeT_23S_2503	reversible	rRNA modification	-1.00E+09	1.00E+09	0	1.12E+02	1.12E+02	0	1.42E+01	14.2	0	1.42E+01	14.2
b3758_v1_RNA_14_modif_2	b3758_v1_RNA modification (23S rRNA): position: 2503, m2A by MeT_23S_2503	1 b3758_v1_RNA_14_MeT_23S_2503 --> 1 MeT_23S_2503_inact + 1 ahcys + 1 b3758_v1_RNA_15 + 1 h	irreversible	rRNA modification	0.00E+00	1.00E+09	0	1.12E+02	1.12E+02	0	1.42E+01	14.2	0	1.42E+01	14.2
b3758_v1_RNA_15_modif_1	b3758_v1_RNA modification (23S rRNA): position: 2504, Y by RluC_mono	1 RluC_mono + 1 b3758_v1_RNA_15 <==> 1 b3758_v1_RNA_15_RluC_mono	reversible	rRNA modification	-1.00E+09	1.00E+09	0	1.12E+02	1.12E+02	0	1.42E+01	14.2	0	1.42E+01	14.2
b3758_v1_RNA_15_modif_2	b3758_v1_RNA modification (23S rRNA): position: 2504, Y by RluC_mono	1 b3758_v1_RNA_15_RluC_mono --> 1 RluC_mono_inact + 1 b3758_v1_RNA_16	irreversible	rRNA modification	0.00E+00	1.00E+09	0	1.12E+02	1.12E+02	0	1.42E+01	14.2	0	1.42E+01	14.2
b3758_v1_RNA_16_modif_1	b3758_v1_RNA modification (23S rRNA): position: 2552, Um by RrmJ_mono	1 RrmJ_mono + 1 amet + 1 b3758_v1_RNA_16 <==> 1 b3758_v1_RNA_16_RrmJ_mono	reversible	rRNA modification	-1.00E+09	1.00E+09	0	1.12E+02	1.12E+02	0	1.42E+01	14.2	0	1.42E+01	14.2
b3758_v1_RNA_16_modif_2	b3758_v1_RNA modification (23S rRNA): position: 2552, Um by RrmJ_mono	1 b3758_v1_RNA_16_RrmJ_mono --> 1 RrmJ_mono_inact + 1 ahcy + 1 b3758_v1_RNA_17 + 1 h	irreversible	rRNA modification	0.00E+00	1.00E+09	0	1.12E+02	1.12E+02	0	1.42E+01	14.2	0	1.42E+01	14.2
b3758_v1_RNA_17_modif_1	b3758_v1_RNA modification (23S rRNA): position: 2580, Y by RluC_mono	1 RluC_mono + 1 b3758_v1_RNA_17 <==> 1 b3758_v1_RNA_17_RluC_mono	reversible	rRNA modification	-1.00E+09	1.00E+09	0	1.12E+02	1.12E+02	0	1.42E+01	14.2	0	1.42E+01	14.2
b3758_v1_RNA_17_modif_2	b3758_v1_RNA modification (23S rRNA): position: 2580, Y by RluC_mono	1 b3758_v1_RNA_17_RluC_mono --> 1 RluC_mono_inact + 1 b3758_v1_RNA_18	irreversible	rRNA modification	0.00E+00	1.00E+09	0	1.12E+02	1.12E+02	0	1.42E+01	14.2	0	1.42E+01	14.2
b3758_v1_RNA_18_modif_1	b3758_v1_RNA modification (23S rRNA): position: 2604, Y by YjbC_mono	1 YjbC_mono + 1 b3758_v1_RNA_18 <==> 1 b3758_v1_RNA_18_YjbC_mono	reversible	rRNA modification	-1.00E+09	1.00E+09	0	1.12E+02	1.12E+02	0	1.42E+01	14.2	0	1.42E+01	14.2
b3758_v1_RNA_18_modif_2	b3758_v1_RNA modification (23S rRNA): position: 2604, Y by YjbC_mono	1 b3758_v1_RNA_18_YjbC_mono --> 1 YjbC_mono_inact + 1 b3758_v1_RNA_19	irreversible	rRNA modification	0.00E+00	1.00E+09	0	1.12E+02	1.12E+02	0	1.42E+01	14.2	0	1.42E+01	14.2

b3758_v1_RNA_19_modif_1	b3758_v1_RNA modification (23S rRNA): position: 2605, Y by RluB_mono	1 RluB_mono + 1 b3758_v1_RNA_19 <==> 1 b3758_v1_RNA_19_RluB_mono	reversible	rRNA modification	-1.00E+09	1.00E+09	0	1.12E+02	1.12E+02	0	1.42E+01	14.2	0	1.42E+01	14.2
b3758_v1_RNA_19_modif_2	b3758_v1_RNA modification (23S rRNA): position: 2605, Y by RluB_mono	1 b3758_v1_RNA_19_RluB_mono --> 1 RluB_mono_inact + 1 b3758_v1_RNA_20	irreversible	rRNA modification	0.00E+00	1.00E+09	0	1.12E+02	1.12E+02	0	1.42E+01	14.2	0	1.42E+01	14.2
b3758_v1_RNA_1_modif_1	b3758_v1_RNA modification (23S rRNA): position: 1835, m2G by MeT_23S_1835	1 MeT_23S_1835 + 1 amet + 1 b3758_v1_RNA_1 <==> 1 b3758_v1_RNA_1_MeT_23S_1835	reversible	rRNA modification	-1.00E+09	1.00E+09	0	1.12E+02	1.12E+02	0	1.42E+01	14.2	0	1.42E+01	14.2
b3758_v1_RNA_1_modif_2	b3758_v1_RNA modification (23S rRNA): position: 1835, m2G by MeT_23S_1835	1 b3758_v1_RNA_1_MeT_23S_1835 --> 1 MeT_23S_1835_inact + 1 ahcys + 1 b3758_v1_RNA_2 + 1 h	irreversible	rRNA modification	0.00E+00	1.00E+09	0	1.12E+02	1.12E+02	0	1.42E+01	14.2	0	1.42E+01	14.2
b3758_v1_RNA_20_modif_1	b3758_v1_RNA modification (23S rRNA): position: 745, m1G by RrmA_dim	1 RrmA_dim + 1 amet + 1 b3758_v1_RNA_20 <==> 1 b3758_v1_RNA_20_RrmA_dim	reversible	rRNA modification	-1.00E+09	1.00E+09	0	1.12E+02	1.12E+02	0	1.42E+01	14.2	0	1.42E+01	14.2
b3758_v1_RNA_20_modif_2	b3758_v1_RNA modification (23S rRNA): position: 745, m1G by RrmA_dim	1 b3758_v1_RNA_20_RrmA_dim --> 1 RrmA_dim_inact + 1 ahcys + 1 b3758_v1_RNA_21 + 1 h	irreversible	rRNA modification	0.00E+00	1.00E+09	0	1.12E+02	1.12E+02	0	1.42E+01	14.2	0	1.42E+01	14.2
b3758_v1_RNA_21_modif_1	b3758_v1_RNA modification (23S rRNA): position: 746, Y by RluA_mono	1 RluA_mono + 1 b3758_v1_RNA_21 <==> 1 b3758_v1_RNA_21_RluA_mono	reversible	rRNA modification	-1.00E+09	1.00E+09	0	1.12E+02	1.12E+02	0	1.42E+01	14.2	0	1.42E+01	14.2
b3758_v1_RNA_21_modif_2	b3758_v1_RNA modification (23S rRNA): position: 746, Y by RluA_mono	1 b3758_v1_RNA_21_RluA_mono --> 1 RluA_mono_inact + 1 b3758_v1_RNA_22	irreversible	rRNA modification	0.00E+00	1.00E+09	0	1.12E+02	1.12E+02	0	1.42E+01	14.2	0	1.42E+01	14.2
b3758_v1_RNA_22_modif_1	b3758_v1_RNA modification (23S rRNA): position: 747, m5U by RumB_mono	1 RumB_mono + 1 amet + 1 b3758_v1_RNA_22 <==> 1 b3758_v1_RNA_22_RumB_mono	reversible	rRNA modification	-1.00E+09	1.00E+09	0	1.12E+02	1.12E+02	0	1.42E+01	14.2	0	1.42E+01	14.2
b3758_v1_RNA_22_modif_2	b3758_v1_RNA modification (23S rRNA): position: 747, m5U by RumB_mono	1 b3758_v1_RNA_22_RumB_mono --> 1 RumB_mono_inact + 1 ahcys + 1 b3758_v1_RNA_23 + 1 h	irreversible	rRNA modification	0.00E+00	1.00E+09	0	1.12E+02	1.12E+02	0	1.42E+01	14.2	0	1.42E+01	14.2
b3758_v1_RNA_23_modif_1	b3758_v1_RNA modification (23S rRNA): position: 955, Y by RluC_mono	1 RluC_mono + 1 b3758_v1_RNA_23 <==> 1 b3758_v1_RNA_23_RluC_mono	reversible	rRNA modification	-1.00E+09	1.00E+09	0	1.12E+02	1.12E+02	0	1.42E+01	14.2	0	1.42E+01	14.2
b3758_v1_RNA_23_modif_2	b3758_v1_RNA modification (23S rRNA): position: 955, Y by RluC_mono	1 b3758_v1_RNA_23_RluC_mono --> 1 RluC_mono_inact + 1 rrlC	irreversible	rRNA modification	0.00E+00	1.00E+09	0	1.12E+02	1.12E+02	0	1.42E+01	14.2	0	1.42E+01	14.2
b3758_v1_RNA_2_modif_1	b3758_v1_RNA modification (23S rRNA): position: 1911, Y by RluD_mono	1 RluD_mono + 1 b3758_v1_RNA_2 <==> 1 b3758_v1_RNA_2_RluD_mono	reversible	rRNA modification	-1.00E+09	1.00E+09	0	1.12E+02	1.12E+02	0	1.42E+01	14.2	0	1.42E+01	14.2
b3758_v1_RNA_2_modif_2	b3758_v1_RNA modification (23S rRNA): position: 1911, Y by RluD_mono	1 b3758_v1_RNA_2_RluD_mono --> 1 RluD_mono_inact + 1 b3758_v1_RNA_3	irreversible	rRNA modification	0.00E+00	1.00E+09	0	1.12E+02	1.12E+02	0	1.42E+01	14.2	0	1.42E+01	14.2
b3758_v1_RNA_3_modif_1	b3758_v1_RNA modification (23S rRNA): position: 1915, m3Y by RluD_mono	1 RluD_mono + 1 amet + 1 b3758_v1_RNA_3 <==> 1 b3758_v1_RNA_3_RluD_mono	reversible	rRNA modification	-1.00E+09	1.00E+09	0	1.12E+02	1.12E+02	0	1.42E+01	14.2	0	1.42E+01	14.2
b3758_v1_RNA_3_modif_2	b3758_v1_RNA modification (23S rRNA): position: 1915, m3Y by RluD_mono	1 b3758_v1_RNA_3_RluD_mono --> 1 RluD_mono_inact + 1 ahcys + 1 b3758_v1_RNA_4 + 1 h	irreversible	rRNA modification	0.00E+00	1.00E+09	0	1.12E+02	1.12E+02	0	1.42E+01	14.2	0	1.42E+01	14.2
b3758_v1_RNA_4_modif_1	b3758_v1_RNA modification (23S rRNA): position: 1917, Y by RluD_mono	1 RluD_mono + 1 b3758_v1_RNA_4 <==> 1 b3758_v1_RNA_4_RluD_mono	reversible	rRNA modification	-1.00E+09	1.00E+09	0	1.12E+02	1.12E+02	0	1.42E+01	14.2	0	1.42E+01	14.2
b3758_v1_RNA_4_modif_2	b3758_v1_RNA modification (23S rRNA): position: 1917, Y by RluD_mono	1 b3758_v1_RNA_4_RluD_mono --> 1 RluD_mono_inact + 1 b3758_v1_RNA_5	irreversible	rRNA modification	0.00E+00	1.00E+09	0	1.12E+02	1.12E+02	0	1.42E+01	14.2	0	1.42E+01	14.2
b3758_v1_RNA_5_modif_1	b3758_v1_RNA modification (23S rRNA): position: 1939, m5U by RumA_mono	1 RumA_mono + 1 amet + 1 b3758_v1_RNA_5 <==> 1 b3758_v1_RNA_5_RumA_mono	reversible	rRNA modification	-1.00E+09	1.00E+09	0	1.12E+02	1.12E+02	0	1.42E+01	14.2	0	1.42E+01	14.2
b3758_v1_RNA_5_modif_2	b3758_v1_RNA modification (23S rRNA): position: 1939, m5U by RumA_mono	1 b3758_v1_RNA_5_RumA_mono --> 1 RumA_mono_inact + 1 ahcy + 1 b3758_v1_RNA_6 + 1 h	irreversible	rRNA modification	0.00E+00	1.00E+09	0	1.12E+02	1.12E+02	0	1.42E+01	14.2	0	1.42E+01	14.2
b3758_v1_RNA_6_modif_1	b3758_v1_RNA modification (23S rRNA): position: 1962, m5C by MeT_23S_1962	1 MeT_23S_1962 + 1 amet + 1 b3758_v1_RNA_6 <==> 1 b3758_v1_RNA_6_MeT_23S_1962	reversible	rRNA modification	-1.00E+09	1.00E+09	0	1.12E+02	1.12E+02	0	1.42E+01	14.2	0	1.42E+01	14.2
b3758_v1_RNA_6_modif_2	b3758_v1_RNA modification (23S rRNA): position: 1962, m5C by MeT_23S_1962	1 b3758_v1_RNA_6_MeT_23S_1962 --> 1 MeT_23S_1962_inact + 1 ahcys + 1 b3758_v1_RNA_7 + 1 h	irreversible	rRNA modification	0.00E+00	1.00E+09	0	1.12E+02	1.12E+02	0	1.42E+01	14.2	0	1.42E+01	14.2
b3758_v1_RNA_7_modif_1	b3758_v1_RNA modification (23S rRNA): position: 2030, m6A by MeT_23S_2030	1 MeT_23S_2030 + 1 amet + 1 b3758_v1_RNA_7 <==> 1 b3758_v1_RNA_7_MeT_23S_2030	reversible	rRNA modification	-1.00E+09	1.00E+09	0	1.12E+02	1.12E+02	0	1.42E+01	14.2	0	1.42E+01	14.2
b3758_v1_RNA_7_modif_2	b3758_v1_RNA modification (23S rRNA): position: 2030, m6A by MeT_23S_2030	1 b3758_v1_RNA_7_MeT_23S_2030 --> 1 MeT_23S_2030_inact + 1 ahcys + 1 b3758_v1_RNA_8 + 1 h	irreversible	rRNA modification	0.00E+00	1.00E+09	0	1.12E+02	1.12E+02	0	1.42E+01	14.2	0	1.42E+01	14.2
b3758_v1_RNA_8_modif_1	b3758_v1_RNA modification (23S rRNA): position: 2069, m7G by MeT_23S_2069	1 MeT_23S_2069 + 1 amet + 1 b3758_v1_RNA_8 <==> 1 b3758_v1_RNA_8_MeT_23S_2069	reversible	rRNA modification	-1.00E+09	1.00E+09	0	1.12E+02	1.12E+02	0	1.42E+01	14.2	0	1.42E+01	14.2
b3758_v1_RNA_8_modif_2	b3758_v1_RNA modification (23S rRNA): position: 2069, m7G by MeT_23S_2069	1 b3758_v1_RNA_8_MeT_23S_2069 --> 1 MeT_23S_2069_inact + 1 ahcys + 1 b3758_v1_RNA_9 + 1 h	irreversible	rRNA modification	0.00E+00	1.00E+09	0	1.12E+02	1.12E+02	0	1.42E+01	14.2	0	1.42E+01	14.2
b3758_v1_RNA_9_modif_1	b3758_v1_RNA modification (23S rRNA): position: 2251, Gm by RlmB_dim	1 RlmB_dim + 1 amet + 1 b3758_v1_RNA_9 <==> 1 b3758_v1_RNA_9_RlmB_dim	reversible	rRNA modification	-1.00E+09	1.00E+09	0	1.12E+02	1.12E+02	0	1.42E+01	14.2	0	1.42E+01	14.2
b3758_v1_RNA_9_modif_2	b3758_v1_RNA modification (23S rRNA): position: 2251, Gm by RlmB_dim	1 b3758_v1_RNA_9_RlmB_dim --> 1 RlmB_dim_inact + 1 ahcys + 1 b3758_v1_RNA_10 + 1 h	irreversible	rRNA modification	0.00E+00	1.00E+09	0	1.12E+02	1.12E+02	0	1.42E+01	14.2	0	1.42E+01	14.2
b3758_v1_RNA_modif_1	b3758_v1_RNA modification (23S rRNA): position: 1618, m6A by MeT_23S_1618	1 MeT_23S_1618 + 1 amet + 1 b3758_v1_RNA <==> 1 b3758_v1_RNA_MeT_23S_1618	reversible	rRNA modification	-1.00E+09	1.00E+09	0	1.12E+02	1.12E+02	0	1.42E+01	14.2	0	1.42E+01	14.2
b3758_v1_RNA_modif_2	b3758_v1_RNA modification (23S rRNA): position: 1618, m6A by MeT_23S_1618	1 b3758_v1_RNA_MeT_23S_1618 --> 1 MeT_23S_1618_inact + 1 ahcys + 1 b3758_v1_RNA_1 + 1 h	irreversible	rRNA modification	0.00E+00	1.00E+09	0	1.12E+02	1.12E+02	0	1.42E+01	14.2	0	1.42E+01	14.2
b3760_DNA_act_bind	b3760_DNA binding of activator	1 b3760_DNA_neu --> 1 b3760_DNA_act	irreversible	Transcription Regulation	0.00E+00	1.00E+09	0	1.45E+03	1.45E+03	0	1.41E+01	14.1	0	1.41E+01	14.1
b3760_tRNA_16_MODIF_1	modification of position 16 nt (D) in aspT by Dus_gen	1 Dus_gen + 1 b3760_tRNA_Mg2 + 1 h + 1 nadph <==> 1 b3760_tRNA_Mg2_Dus_gen_cplx	reversible	tRNA modification	-1.00E+09	1.00E+09	0	1.45E+03	1.45E+03	0	1.41E+01	14.1	0	1.41E+01	14.1
b3760_tRNA_16_MODIF_2	modification of position 16 nt (D) in aspT by Dus_gen	1 b3760_tRNA_Mg2_Dus_gen_cplx --> 1 Dus_gen_inact + 1 b3760_tRNA_1 + 1 nadp	irreversible	tRNA modification	0.00E+00	1.00E+09	0	1.45E+03	1.45E+03	0	1.41E+01	14.1	0	1.41E+01	14.1
b3760_tRNA_20A_MODIF_1	modification of position 20A nt (D) in aspT by Dus_gen	1 Dus_gen + 1 b3760_tRNA_2 + 1 h + 1 nadph <==> 1 b3760_tRNA_2_Dus_gen_cplx	reversible	tRNA modification	-1.00E+09	1.00E+09	0	1.45E+03	1.45E+03	0	1.41E+01	14.1	0	1.41E+01	14.1
b3760_tRNA_20A_MODIF_2	modification of position 20A nt (D) in aspT by Dus_gen	1 b3760_tRNA_2_Dus_gen_cplx --> 1 Dus_gen_inact + 1 b3760_tRNA_3 + 1 nadp	irreversible	tRNA modification	0.00E+00	1.00E+09	0	1.45E+03	1.45E+03	0	1.41E+01	14.1	0	1.41E+01	14.1
b3760_tRNA_20_MODIF_1	modification of position 20 nt (D) in aspT by Dus_gen	1 Dus_gen + 1 b3760_tRNA_1 + 1 h + 1 nadph <==> 1 b3760_tRNA_1_Dus_gen_cplx	reversible	tRNA modification	-1.00E+09	1.00E+09	0	1.45E+03	1.45E+03	0	1.41E+01	14.1	0	1.41E+01	14.1
b3760_tRNA_20_MODIF_2	modification of position 20 nt (D) in aspT by Dus_gen	1 b3760_tRNA_1_Dus_gen_cplx --> 1 Dus_gen_inact + 1 b3760_tRNA_2 + 1 nadp	irreversible	tRNA modification	0.00E+00	1.00E+09	0	1.45E+03	1.45E+03	0	1.41E+01	14.1	0	1.41E+01	14.1
b3760_tRNA_34_MODIF_1	modification of position 34 nt (Q) in aspT by Tgt_hexa-QueA_mono-EoR_tRNA_pos34_Q	1 EoR_tRNA_pos34_Q + 1 QueA_mono + 1 Tgt_hexa + 1 adocbl + 1 amet + 1 b3760_tRNA_3 + 1 h + 1 nadh + 1 pre_Q1 <==> 1 b3760_tRNA_3_Tgt_hexa-QueA_mono-EoR_tRNA_pos34_Q_cplx	reversible	tRNA modification	-1.00E+09	1.00E+09	0	1.45E+03	1.45E+03	0	1.41E+01	14.1	0	1.41E+01	14.1
b3760_tRNA_34_MODIF_2	modification of position 34 nt (Q) in aspT by Tgt_hexa-QueA_mono-EoR_tRNA_pos34_Q	1 b3760_tRNA_3_Tgt_hexa-QueA_mono-EoR_tRNA_pos34_Q_cplx > 1 EoR_tRNA_pos34_Q_inact + 1 QueA_mono_inact + 1 Tgt_hexa_inact + 1 ade + 1 adocbl + 1 b3760_tRNA_4 + 1 gua + 2 h 1 h2o + 1 met-L + 1 nad	irreversible	tRNA modification	0.00E+00	1.00E+09	0	1.45E+03	1.45E+03	0	1.41E+01	14.1	0	1.41E+01	14.1
b3760_tRNA_37_MODIF_1	modification of position 37 nt (m2A) in aspT by MeT_tRNA_pos_37_m2A	1 MeT_tRNA_pos_37_m2A + 1 amet + 1 b3760_tRNA_4 <==> 1 b3760_tRNA_4_MeT_tRNA_pos_37_m2A_cplx	reversible	tRNA modification	-1.00E+09	1.00E+09	0	1.45E+03	1.45E+03	0	1.41E+01	14.1	0	1.41E+01	14.1

b3760_tRNA_37_MODIF_2	modification of position 37 nt (m2A) in aspT by MeT_tRNA_pos_37_m2A	1 b3760_tRNA_4_MeT_tRNA_pos_37_m2A_cplx --> 1 MeT_tRNA_pos_37_m2A_inact + 1 ahcys + 1 b3760_tRNA_5 + 1 h	irreversible	tRNA modification	0.00E+00	1.00E+09	0	1.45E+03	1.45E+03	0	1.41E+01	14.1	0	1.41E+01	14.1
b3760_tRNA_46_MODIF_1	modification of position 46 nt (m7G) in aspT by YggH_mono	1 YggH_mono + 1 amet + 1 b3760_tRNA_5 <==> 1 b3760_tRNA_5_YggH_mono_cplx	reversible	tRNA modification	-1.00E+09	1.00E+09	0	1.45E+03	1.45E+03	0	1.41E+01	14.1	0	1.41E+01	14.1
b3760_tRNA_46_MODIF_2	modification of position 46 nt (m7G) in aspT by YggH_mono	1 b3760_tRNA_5_YggH_mono_cplx --> 1 YggH_mono_inact + 1 ahcys + 1 b3760_tRNA_6 + 1 h	irreversible	tRNA modification	0.00E+00	1.00E+09	0	1.45E+03	1.45E+03	0	1.41E+01	14.1	0	1.41E+01	14.1
b3760_tRNA_54_MODIF_1	modification of position 54 nt (m5U) in aspT by TrmA_mono	1 TrmA_mono + 1 amet + 1 b3760_tRNA_6 <==> 1 b3760_tRNA_6_TrmA_mono_cplx	reversible	tRNA modification	-1.00E+09	1.00E+09	0	1.45E+03	1.45E+03	0	1.41E+01	14.1	0	1.41E+01	14.1
b3760_tRNA_54_MODIF_2	modification of position 54 nt (m5U) in aspT by TrmA_mono	1 b3760_tRNA_6_TrmA_mono_cplx --> 1 TrmA_mono_inact + 1 ahcys + 1 b3760_tRNA_7 + 1 h	irreversible	tRNA modification	0.00E+00	1.00E+09	0	1.45E+03	1.45E+03	0	1.41E+01	14.1	0	1.41E+01	14.1
b3760_tRNA_55_MODIF_1	modification of position 55 nt (Y) in aspT by TruB_mono	1 TruB_mono + 1 b3760_tRNA_7 <==> 1 b3760_tRNA_7_TruB_mono_cplx	reversible	tRNA modification	-1.00E+09	1.00E+09	0	1.45E+03	1.45E+03	0	1.41E+01	14.1	0	1.41E+01	14.1
b3760_tRNA_55_MODIF_2	modification of position 55 nt (Y) in aspT by TruB_mono	1 b3760_tRNA_7_TruB_mono_cplx --> 1 TruB_mono_inact + 1 b3760_tRNA_8	irreversible	tRNA modification	0.00E+00	1.00E+09	0	1.45E+03	1.45E+03	0	1.41E+01	14.1	0	1.41E+01	14.1
b3760_tRNA_65_MODIF_1	modification of position 65 nt (Y) in aspT by YqcB_mono	1 YqcB_mono + 1 b3760_tRNA_8 <==> 1 b3760_tRNA_8_YqcB_mono_cplx	reversible	tRNA modification	-1.00E+09	1.00E+09	0	1.45E+03	1.45E+03	0	1.41E+01	14.1	0	1.41E+01	14.1
b3760_tRNA_65_MODIF_2	modification of position 65 nt (Y) in aspT by YqcB_mono	1 b3760_tRNA_8_YqcB_mono_cplx --> 1 YqcB_mono_inact + 1 b3760_tRNA_9	irreversible	tRNA modification	0.00E+00	1.00E+09	0	1.45E+03	1.45E+03	0	1.41E+01	14.1	0	1.41E+01	14.1
b3760_tRNA_8_MODIF_1	modification of position 8 nt (s4U) in aspT by ThiI_mono	1 IscS_dim_S-SH + 1 ThiI_mono + 1 atp + 1 b3760_tRNA_9 + 1 mg2 + 1 trdrd <==> 1 b3760_tRNA_9_ThiI_mono_cplx	reversible	tRNA modification	-1.00E+09	1.00E+09	0	1.45E+03	1.45E+03	0	1.41E+01	14.1	0	1.41E+01	14.1
b3760_tRNA_8_MODIF_2	modification of position 8 nt (s4U) in aspT by ThiI_mono	1 b3760_tRNA_9_ThiI_mono_cplx --> 1 IscS_dim_S-H + 1 ThiI_mono_inact + 1 amp + 1 aspT_tRNA + 1 h + 1 mg2 + 1 ppi + 1 trdox	irreversible	tRNA modification	0.00E+00	1.00E+09	0	1.45E+03	1.45E+03	0	1.41E+01	14.1	0	1.41E+01	14.1
b3760_tRNA_Mg_BIND	Binding of two Mg2 to b3760_tRNA	1 b3760_RNA + 2 mg2 <==> 1 b3760_tRNA_Mg2	reversible	Metallo-ion binding	-1.00E+09	1.00E+09	0	1.45E+03	1.45E+03	0	1.41E+01	14.1	0	1.41E+01	14.1
b3760_trimming	Cutting of stable b3760_RNA	1 b3760_RNA_cut_cplx + 1 h2o --> 1 RNase_E_tetra_inact + 1 RNase_Gen_inact + 1 RNase_P_cplx_inact + 1 b3760_RNA + 1 h + ppi	irreversible	RNA Processing	0.00E+00	1.00E+09	0	1.45E+03	1.45E+03	0	1.41E+01	14.1	0	1.41E+01	14.1
b3760_trimming_cplx	Cutting complex formation of stable b3760_RNA (, b3760)	1 RNase_E_tetra + 1 RNase_Gen + 1 RNase_P_cplx + 1 b3760_RNA_pre <==> 1 b3760_RNA_cut_cplx	reversible	RNA Processing	-1.00E+09	1.00E+09	0	1.45E+03	1.45E+03	0	1.41E+01	14.1	0	1.41E+01	14.1
b3761_DNA_act_bind	b3761_DNA binding of activator	1 b3761_DNA_neu --> 1 b3761_DNA_act	irreversible	Transcription Regulation	0.00E+00	1.00E+09	0	1.52E+03	1.52E+03	0	1.41E+01	14.1	0	1.41E+01	14.1
b3761_tRNA_16_MODIF_1	modification of position 16 nt (D) in trpT by Dus_gen	1 Dus_gen + 1 b3761_tRNA_Mg2 + 1 h + 1 nadph <==> 1 b3761_tRNA_Mg2_Dus_gen_cplx	reversible	tRNA modification	-1.00E+09	1.00E+09	0	1.52E+03	1.52E+03	0	1.41E+01	14.1	0	1.41E+01	14.1
b3761_tRNA_16_MODIF_2	modification of position 16 nt (D) in trpT by Dus_gen	1 b3761_tRNA_Mg2_Dus_gen_cplx --> 1 Dus_gen_inact + 1 b3761_tRNA_1 + 1 nadp	irreversible	tRNA modification	0.00E+00	1.00E+09	0	1.52E+03	1.52E+03	0	1.41E+01	14.1	0	1.41E+01	14.1
b3761_tRNA_17_MODIF_1	modification of position 17 nt (D) in trpT by Dus_gen	1 Dus_gen + 1 b3761_tRNA_1 + 1 h + 1 nadph <==> 1 b3761_tRNA_1_Dus_gen_cplx	reversible	tRNA modification	-1.00E+09	1.00E+09	0	1.52E+03	1.52E+03	0	1.41E+01	14.1	0	1.41E+01	14.1
b3761_tRNA_17_MODIF_2	modification of position 17 nt (D) in trpT by Dus_gen	1 b3761_tRNA_1_Dus_gen_cplx --> 1 Dus_gen_inact + 1 b3761_tRNA_2 + 1 nadp	irreversible	tRNA modification	0.00E+00	1.00E+09	0	1.52E+03	1.52E+03	0	1.41E+01	14.1	0	1.41E+01	14.1
b3761_tRNA_20_MODIF_1	modification of position 20 nt (D) in trpT by Dus_gen	1 Dus_gen + 1 b3761_tRNA_2 + 1 h + 1 nadph <==> 1 b3761_tRNA_2_Dus_gen_cplx	reversible	tRNA modification	-1.00E+09	1.00E+09	0	1.52E+03	1.52E+03	0	1.41E+01	14.1	0	1.41E+01	14.1
b3761_tRNA_20_MODIF_2	modification of position 20 nt (D) in trpT by Dus_gen	1 b3761_tRNA_2_Dus_gen_cplx --> 1 Dus_gen_inact + 1 b3761_tRNA_3 + 1 nadp	irreversible	tRNA modification	0.00E+00	1.00E+09	0	1.52E+03	1.52E+03	0	1.41E+01	14.1	0	1.41E+01	14.1
b3761_tRNA_32_MODIF_1	modification of position 32 nt (Cm) in trpT by MeT_tRNA_pos_32_Cm	1 MeT_tRNA_pos_32_Cm + 1 amet + 1 b3761_tRNA_3 <==> 1 b3761_tRNA_3_MeT_tRNA_pos_32_Cm_cplx	reversible	tRNA modification	-1.00E+09	1.00E+09	0	1.52E+03	1.52E+03	0	1.41E+01	14.1	0	1.41E+01	14.1
b3761_tRNA_32_MODIF_2	modification of position 32 nt (Cm) in trpT by MeT_tRNA_pos_32_Cm	1 b3761_tRNA_3_MeT_tRNA_pos_32_Cm_cplx --> 1 MeT_tRNA_pos_32_Cm_inact + 1 ahcys + 1 b3761_tRNA_4 + 1 h	irreversible	tRNA modification	0.00E+00	1.00E+09	0	1.52E+03	1.52E+03	0	1.41E+01	14.1	0	1.41E+01	14.1
b3761_tRNA_37_MODIF_1	modification of position 37 nt (ms2i6A) in trpT by MiaA_dim-MiaB_mono	1 IscS_dim_S-SH + 1 MiaA_dim + 1 MiaB_mono + 2 amet + 1 b3761_tRNA_4 + 1 dmpp + 1 fldrd <==> 1 b3761_tRNA_4_MiaA_dim MiaB_mono_cplx	reversible	tRNA modification	-1.00E+09	1.00E+09	0	1.52E+03	1.52E+03	0	1.41E+01	14.1	0	1.41E+01	14.1
b3761_tRNA_37_MODIF_2	modification of position 37 nt (ms2i6A) in trpT by MiaA_dim-MiaB_mono	1 b3761_tRNA_4_MiaA_dim-MiaB_mono_cplx --> 1 IscS_dim_S-H + 1 MiaA_dim_inact + 1 MiaB_mono_inact + 1 ahcys + 1 b3761_tRNA_5 + 1 dad-5 + 1 fldox + 2 h + 1 met-L + 1 ppi	irreversible	tRNA modification	0.00E+00	1.00E+09	0	1.52E+03	1.52E+03	0	1.41E+01	14.1	0	1.41E+01	14.1
b3761_tRNA_46_MODIF_1	modification of position 46 nt (m7G) in trpT by YggH_mono	1 YggH_mono + 1 amet + 1 b3761_tRNA_5 <==> 1 b3761_tRNA_5_YggH_mono_cplx	reversible	tRNA modification	-1.00E+09	1.00E+09	0	1.52E+03	1.52E+03	0	1.41E+01	14.1	0	1.41E+01	14.1
b3761_tRNA_46_MODIF_2	modification of position 46 nt (m7G) in trpT by YggH_mono	1 b3761_tRNA_5_YggH_mono_cplx --> 1 YggH_mono_inact + 1 ahcys + 1 b3761_tRNA_6 + 1 h	irreversible	tRNA modification	0.00E+00	1.00E+09	0	1.52E+03	1.52E+03	0	1.41E+01	14.1	0	1.41E+01	14.1
b3761_tRNA_54_MODIF_1	modification of position 54 nt (m5U) in trpT by TrmA_mono	1 TrmA_mono + 1 amet + 1 b3761_tRNA_6 <==> 1 b3761_tRNA_6_TrmA_mono_cplx	reversible	tRNA modification	-1.00E+09	1.00E+09	0	1.52E+03	1.52E+03	0	1.41E+01	14.1	0	1.41E+01	14.1
b3761_tRNA_54_MODIF_2	modification of position 54 nt (m5U) in trpT by TrmA_mono	1 b3761_tRNA_6_TrmA_mono_cplx --> 1 TrmA_mono_inact + 1 ahcys + 1 b3761_tRNA_7 + 1 h	irreversible	tRNA modification	0.00E+00	1.00E+09	0	1.52E+03	1.52E+03	0	1.41E+01	14.1	0	1.41E+01	14.1
b3761_tRNA_55_MODIF_1	modification of position 55 nt (Y) in trpT by TruB_mono	1 TruB_mono + 1 b3761_tRNA_7 <==> 1 b3761_tRNA_7_TruB_mono_cplx	reversible	tRNA modification	-1.00E+09	1.00E+09	0	1.52E+03	1.52E+03	0	1.41E+01	14.1	0	1.41E+01	14.1
b3761_tRNA_55_MODIF_2	modification of position 55 nt (Y) in trpT by TruB_mono	1 b3761_tRNA_7_TruB_mono_cplx --> 1 TruB_mono_inact + 1 b3761_tRNA_8	irreversible	tRNA modification	0.00E+00	1.00E+09	0	1.52E+03	1.52E+03	0	1.41E+01	14.1	0	1.41E+01	14.1
b3761_tRNA_8_MODIF_1	modification of position 8 nt (s4U) in trpT by ThiI_mono	1 IscS_dim_S-SH + 1 ThiI_mono + 1 atp + 1 b3761_tRNA_8 + 1 mg2 + 1 trdrd <==> 1 b3761_tRNA_8_ThiI_mono_cplx	reversible	tRNA modification	-1.00E+09	1.00E+09	0	1.52E+03	1.52E+03	0	1.41E+01	14.1	0	1.41E+01	14.1
b3761_tRNA_8_MODIF_2	modification of position 8 nt (s4U) in trpT by ThiI_mono	1 b3761_tRNA_8_ThiI_mono_cplx --> 1 IscS_dim_S-H + 1 ThiI_mono_inact + 1 amp + 1 h + 1 mg2 + 1 ppi + 1 trdox + 1 trpT_tRNA	irreversible	tRNA modification	0.00E+00	1.00E+09	0	1.52E+03	1.52E+03	0	1.41E+01	14.1	0	1.41E+01	14.1
b3761_tRNA_Mg_BIND	Binding of two Mg2 to b3761_tRNA	1 b3761_RNA + 2 mg2 <==> 1 b3761_tRNA_Mg2	reversible	Metallo-ion binding	-1.00E+09	1.00E+09	0	1.52E+03	1.52E+03	0	1.41E+01	14.1	0	1.41E+01	14.1
b3761_trimming	Cutting of stable b3761_RNA	1 b3761_RNA_cut_cplx + 1 h2o --> 1 RNase_E_tetra_inact + 1 RNase_Gen_inact + 1 RNase_P_cplx_inact + 1 b3761_RNA + 1 h + ppi	irreversible	RNA Processing	0.00E+00	1.00E+09	0	1.52E+03	1.52E+03	0	1.41E+01	14.1	0	1.41E+01	14.1
b3761_trimming_cplx	Cutting complex formation of stable b3761_RNA (, b3761)	1 RNase_E_tetra + 1 RNase_Gen + 1 RNase_P_cplx + 1 b3761_RNA_pre <==> 1 b3761_RNA_cut_cplx	reversible	RNA Processing	-1.00E+09	1.00E+09	0	1.52E+03	1.52E+03	0	1.41E+01	14.1	0	1.41E+01	14.1
b3780_fold_GroEL/ES_1	b3780_m folding: GroEL/ES mediated: polypeptide is going in GroEL/ES complex	1 GroEL.(7)ADP.cisGroES + 7 atp + 1 b3780_m + 1 transGroES_hepta --> 7 adp + 1 b3780_m_GroEL.(7)ATP.transGroES + 1 cisGroES_hepta	irreversible	Protein Folding	0.00E+00	1.00E+09	0	4.40E+02	4.40E+02	0	4.77E+02	477.4116	0	5.76E+01	57.629
b3780_fold_GroEL/ES_2	b3780_m folding: GroEL/ES mediated: folding of polypeptide under ATP hydrolysis	1 b3780_m_GroEL.(7)ATP.transGroES + 7 h2o --> 1 RhlB_GroEL.(7)ADP.transGroES + 7 h + 7 pi	irreversible	Protein Folding	0.00E+00	1.00E+09	0	4.40E+02	4.40E+02	0	4.77E+02	477.4116	0	5.76E+01	57.629
b3780_fold_GroEL/ES_3	b3780_m folding: GroEL/ES mediated: release of native protein	1 RhlB_GroEL.(7)ADP.transGroES --> 1 GroEL.(7)ADP.transGroES + 1 RhlB_mono	irreversible	Protein Folding	0.00E+00	1.00E+09	0	4.40E+02	4.40E+02	0	4.77E+02	477.4116	0	5.76E+01	57.629
b3780_fold_KJE_1	b3780_m folding: KJE mediated	1 DnaJ_dim + 1 DnaK_mono.ATP + 1 b3780_m <==> 1 b3780_m_DnaKJ_complex	reversible	Protein Folding	-1.00E+09	1.00E+09	0	4.40E+02	4.40E+02	0	4.77E+02	477.4116	0	5.76E+01	57.629
b3780_fold_KJE_2	b3780_m folding: KJE mediated	1 GrpE_dim + 1 b3780_m_DnaKJ_complex + 1 h2o --> 1 DnaJ_dim_inact + 1 RhlB_DnaK_GrpE_complex + 1 adp + 1 h + 1 pi	irreversible	Protein Folding	0.00E+00	1.00E+09	0	4.40E+02	4.40E+02	0	4.77E+02	477.4116	0	5.76E+01	57.629
b3780_fold_KJE_3	b3780_m folding: KJE mediated	1 RhlB_DnaK_GrpE_complex + 1 atp --> 1 DnaK_mono.ATP_inact + 1 GrpE_dim_inact + 1 RhlB_mono	irreversible	Protein Folding	0.00E+00	1.00E+09	0	4.40E+02	4.40E+02	0	4.77E+02	477.4116	0	5.76E+01	57.629
b3780_mRNA_CONV	convsersion of mRNA to mRNA_1 (synthetic rxn)	1 b3780_mRNA --> 1 b3780_mRNA_1	irreversible	Translation	0.00E+00	1.00E+09	0	9.34E+01	9.34E+01	0	1.59E+02	159.0788	0	3.26E-01	0.326
b3780_mRNA_degr1	Degradation of b3780_mRNA I	1 Orn_dim + 316 atp + 1 b3780_mRNA_2 + 1 degradosome + 316 h2o <==> 316 adp + 1 b3780_mRNA_2_degr + 316 h + 316 pi	reversible	mRNA degradation	-1.00E+09	1.00E+09	0	9.34E+01	9.34E+01	0	1.59E+02	159.0788	0	3.26E-01	0.326
b3780_mRNA_degr2	Degradation of b3780_mRNA II	1 b3780_mRNA_2_degr + 1265 h2o --> 1 Orn_dim_inact + 305 amp 1 atp + 329 cmp + 1 degradosome_inact + 336 gmp + 1265 h + 295 ump	irreversible	mRNA degradation	0.00E+00	3.26E-01	0	9.34E+01	9.34E+01	0	1.59E+02	159.0788	0	3.26E-01	0.326
b3780_maturation1	Polypeptide b3780 peptide deformylase and methionine aminopeptidase complex	1 Def_mono + 1 Map_mono + 1 b3780_aa <==> 1 b3780_def_map_cplx	reversible	Protein Maturation	-1.00E+09	1.00E+09	0	4.40E+02	4.40E+02	0	4.77E+02	477.4116	0	5.76E+01	57.629
b3780_maturation2	b3780 formation	1 b3780_def_map_cplx + 2 h2o --> 1 Def_mono_inact + 1 Map_mono_inact + 1 b3780_m + 1 for + 1 met-L	irreversible	Protein Maturation	0.00E+00	1.00E+09	0	4.40E+02	4.40E+02	0	4.77E+02	477.4116	0	5.76E+01	57.629
b3782_fold_spon	b3782_m folding: spontanous	1 b3782_m --> 1 RhoL_mono	irreversible	Protein Folding	0.00E+00	1.00E+09	0	1.72E+02	1.72E+02	0	2.38E+02	238.0054	0	8.50E+01	85.007

b3782_mRNA_CONV	convsersion of mRNA to mRNA_1 (synthetic rxn)	1 b3782_mRNA --> 1 b3782_mRNA_1	irreversible	Translation	0.00E+00	1.00E+09	0	8.68E+01	8.68E+01	0	1.53E+02	153.2054	0	2.07E-01	0.207
b3782_mRNA_degr1	Degradation of b3782_mRNA I	1 Orn_dim + 25 atp + 1 b3782_mRNA_2 + 1 degradosome + 25 h2o <==> 25 adp + 1 b3782_mRNA_2_degr + 25 h + 25 pi	reversible	mRNA degradation	-1.00E+09	1.00E+09	0	8.68E+01	8.68E+01	0	1.53E+02	153.2054	0	2.07E-01	0.207
b3782_mRNA_degr2	Degradation of b3782_mRNA II	1 b3782_mRNA_2_degr + 101 h2o --> 1 Orn_dim_inact + 23 amp + 1 atp + 25 cmp + 1 degradosome_inact + 25 gmp + 101 h + 28 ump	irreversible	mRNA degradation	0.00E+00	2.38E-01	0	8.68E+01	8.68E+01	0	1.53E+02	153.2054	0	2.07E-01	0.207
b3782_maturation1	Polypeptide b3782 peptide deformylas and methionine aminopeptidase complex	1 Def_mono + 1 Map_mono + 1 b3782_aa <==> 1 b3782_def_map_cplx	reversible	Protein Maturation	-1.00E+09	1.00E+09	0	1.72E+02	1.72E+02	0	2.38E+02	238.0054	0	8.50E+01	85.007
b3782_maturation2	b3782 formation	1 b3782_def_map_cplx + 2 h2o --> 1 Def_mono_inact + 1 Map_mono_inact + 1 b3782_m + 1 for + 1 met-L	irreversible	Protein Maturation	0.00E+00	1.00E+09	0	1.72E+02	1.72E+02	0	2.38E+02	238.0054	0	8.50E+01	85.007
b3782_v1_mRNA_CONV	convsersion of mRNA to mRNA_1 (synthetic rxn)	1 b3782_v1_mRNA --> 1 b3782_mRNA_1	irreversible	Translation	0.00E+00	1.00E+09	0	8.68E+01	8.68E+01	0	1.53E+02	153.2054	0	2.07E-01	0.207
b3783_fold_GroEL/ES_1	b3783_m folding: GroEL/ES mediated; polypeptide is going in GroEL/ES complex	1 GroEL.(7)ADP.cisGroES + 7 atp + 1 b3783_m + 1 transGroES_hepta --> 7 adp + 1 b3783_m_GroEL.(7)ATP.transGroES + 1 cisGroES_hepta	irreversible	Protein Folding	0.00E+00	1.00E+09	0	2.20E+03	2.20E+03	0	3.79E+03	3794.53	0	1.23E+02	122.568
b3783_fold_GroEL/ES_2	b3783_m folding: GroEL/ES mediated; folding of polypeptide under ATP hydrolysis	1 b3783_m_GroEL.(7)ATP.transGroES + 7 h2o --> 1 Rho_GroEL.(7)ADP.transGroES + 7 h + 7 pi	irreversible	Protein Folding	0.00E+00	1.00E+09	0	2.20E+03	2.20E+03	0	3.79E+03	3794.53	0	1.23E+02	122.568
b3783_fold_GroEL/ES_3	b3783_m folding: GroEL/ES mediated; release of native protein	1 Rho_GroEL.(7)ADP.transGroES --> 1 GroEL.(7)ADP.transGroES + 1 Rho_mono	irreversible	Protein Folding	0.00E+00	1.00E+09	0	2.20E+03	2.20E+03	0	3.79E+03	3794.53	0	1.23E+02	122.568
b3783_fold_KJE_1	b3783_m folding: KJE mediated	1 DnaJ_dim + 1 DnaK_mono.ATP + 1 b3783_m <==> 1 b3783_m_DnaKJ_complex	reversible	Protein Folding	-1.00E+09	1.00E+09	0	2.20E+03	2.20E+03	0	3.79E+03	3794.53	0	1.23E+02	122.568
b3783_fold_KJE_2	b3783_m folding: KJE mediated	1 GrpE_dim + 1 b3783_m_DnaKJ_complex + 1 h2o --> 1 DnaJ_dim_inact + 1 Rho_DnaK_GrpE_complex + 1 adp + 1 h + 1 pi	irreversible	Protein Folding	0.00E+00	1.00E+09	0	2.20E+03	2.20E+03	0	3.79E+03	3794.53	0	1.23E+02	122.568
b3783_fold_KJE_3	b3783_m folding: KJE mediated	1 Rho_DnaK_GrpE_complex + 1 atp --> 1 DnaK_mono.ATP_inact + GrpE_dim_inact + 1 Rho_mono	irreversible	Protein Folding	0.00E+00	1.00E+09	0	2.20E+03	2.20E+03	0	3.79E+03	3794.53	0	1.23E+02	122.568
b3783_mRNA_CONV	convsersion of mRNA to mRNA_1 (synthetic rxn)	1 b3783_mRNA --> 1 b3783_mRNA_1	irreversible	Translation	0.00E+00	1.00E+09	0	8.68E+01	8.68E+01	0	1.53E+02	153.2054	0	2.07E-01	0.207
b3783_mRNA_degr1	Degradation of b3783_mRNA I	1 Orn_dim + 315 atp + 1 b3783_mRNA_2 + 1 degradosome + 315 h2o <==> 315 adp + 1 b3783_mRNA_2_degr + 315 h + 315 pi	reversible	mRNA degradation	-1.00E+09	1.00E+09	0	8.68E+01	8.68E+01	0	1.53E+02	153.2054	0	2.07E-01	0.207
b3783_mRNA_degr2	Degradation of b3783_mRNA II	1 b3783_mRNA_2_degr + 1259 h2o --> 1 Orn_dim_inact + 328 amp 319 cmp + 1 degradosome_inact + 315 gmp + 1259 h + 298 ump	irreversible	mRNA degradation	0.00E+00	2.07E-01	0	8.68E+01	8.68E+01	0	1.53E+02	153.2054	0	2.07E-01	0.207
b3783_maturation1	Polypeptide b3783 peptide deformylas complex	1 Def_mono + 1 b3783_aa <==> 1 b3783_def_cplx	reversible	Protein Maturation	-1.00E+09	1.00E+09	0	2.20E+03	2.20E+03	0	3.79E+03	3794.53	0	1.23E+02	122.568
b3783_maturation2	b3783 formation	1 b3783_def_cplx + 1 h2o --> 1 Def_mono_inact + 1 b3783_m + 1 for	irreversible	Protein Maturation	0.00E+00	1.00E+09	0	2.20E+03	2.20E+03	0	3.79E+03	3794.53	0	1.23E+02	122.568
b3783_v1_mRNA_CONV	convsersion of mRNA to mRNA_1 (synthetic rxn)	1 b3783_v1_mRNA --> 1 b3783_mRNA_1	irreversible	Translation	0.00E+00	1.00E+09	0	8.68E+01	8.68E+01	0	1.53E+02	153.2054	0	2.07E-01	0.207
b3796_tRNA_17_MODIF_1	modification of position 17 nt (D) in argX by Dus_gen	1 Dus_gen + 1 b3796_tRNA_Mg2 + 1 h + 1 nadph <==> 1 b3796_tRNA_Mg2_Dus_gen_cplx	reversible	tRNA modification	-1.00E+09	1.00E+09	0	3.01E+02	3.01E+02	0	1.39E+01	13.9	0	1.39E+01	13.9
b3796_tRNA_17_MODIF_2	modification of position 17 nt (D) in argX by Dus_gen	1 b3796_tRNA_Mg2_Dus_gen_cplx --> 1 Dus_gen_inact + 1 b3796_tRNA_1 + 1 nadp	irreversible	tRNA modification	0.00E+00	1.00E+09	0	3.01E+02	3.01E+02	0	1.39E+01	13.9	0	1.39E+01	13.9
b3796_tRNA_20A_MODIF_1	modification of position 20A nt (D) in argX by Dus_gen	1 Dus_gen + 1 b3796_tRNA_1 + 1 h + 1 nadph <==> 1 b3796_tRNA_1_Dus_gen_cplx	reversible	tRNA modification	-1.00E+09	1.00E+09	0	3.01E+02	3.01E+02	0	1.39E+01	13.9	0	1.39E+01	13.9
b3796_tRNA_20A_MODIF_2	modification of position 20A nt (D) in argX by Dus_gen	1 b3796_tRNA_1_Dus_gen_cplx --> 1 Dus_gen_inact + 1 b3796_tRNA_2 + 1 nadp	irreversible	tRNA modification	0.00E+00	1.00E+09	0	3.01E+02	3.01E+02	0	1.39E+01	13.9	0	1.39E+01	13.9
b3796_tRNA_32_MODIF_1	modification of position 32 nt (s2C) in argX by YdaO_mono	1 IscS_dim_S-SH + 1 YdaO_mono + 1 atp + 1 b3796_tRNA_2 + 1 mg2 + 1 trdrd <==> 1 b3796_tRNA_2_YdaO_mono_cplx	reversible	tRNA modification	-1.00E+09	1.00E+09	0	3.01E+02	3.01E+02	0	1.39E+01	13.9	0	1.39E+01	13.9
b3796_tRNA_32_MODIF_2	modification of position 32 nt (s2C) in argX by YdaO_mono	1 b3796_tRNA_2_YdaO_mono_cplx --> 1 IscS_dim_S-H + 1 YdaO_mono_inact + 1 amp + 1 b3796_tRNA_3 + 1 h + 1 mg2 + 1 ppi + 1 trdox	irreversible	tRNA modification	0.00E+00	1.00E+09	0	3.01E+02	3.01E+02	0	1.39E+01	13.9	0	1.39E+01	13.9
b3796_tRNA_37_MODIF_1	modification of position 37 nt (m1G) in argX by TrmD_dim	1 TrmD_dim + 1 amet + 1 b3796_tRNA_3 <==> 1 b3796_tRNA_3_TrmD_dim_cplx	reversible	tRNA modification	-1.00E+09	1.00E+09	0	3.01E+02	3.01E+02	0	1.39E+01	13.9	0	1.39E+01	13.9
b3796_tRNA_37_MODIF_2	modification of position 37 nt (m1G) in argX by TrmD_dim	1 b3796_tRNA_3_TrmD_dim_cplx --> 1 TrmD_dim_inact + 1 ahcys + 1 b3796_tRNA_4 + 1 h	irreversible	tRNA modification	0.00E+00	1.00E+09	0	3.01E+02	3.01E+02	0	1.39E+01	13.9	0	1.39E+01	13.9
b3796_tRNA_46_MODIF_1	modification of position 46 nt (m7G) in argX by YggH_mono	1 YggH_mono + 1 amet + 1 b3796_tRNA_4 <==> 1 b3796_tRNA_4_YggH_mono_cplx	reversible	tRNA modification	-1.00E+09	1.00E+09	0	3.01E+02	3.01E+02	0	1.39E+01	13.9	0	1.39E+01	13.9
b3796_tRNA_46_MODIF_2	modification of position 46 nt (m7G) in argX by YggH_mono	1 b3796_tRNA_4_YggH_mono_cplx --> 1 YggH_mono_inact + 1 ahcys + 1 b3796_tRNA_5 + 1 h	irreversible	tRNA modification	0.00E+00	1.00E+09	0	3.01E+02	3.01E+02	0	1.39E+01	13.9	0	1.39E+01	13.9
b3796_tRNA_54_MODIF_1	modification of position 54 nt (m5U) in argX by TrmA_mono	1 TrmA_mono + 1 amet + 1 b3796_tRNA_5 <==> 1 b3796_tRNA_5_TrmA_mono_cplx	reversible	tRNA modification	-1.00E+09	1.00E+09	0	3.01E+02	3.01E+02	0	1.39E+01	13.9	0	1.39E+01	13.9
b3796_tRNA_54_MODIF_2	modification of position 54 nt (m5U) in argX by TrmA_mono	1 b3796_tRNA_5_TrmA_mono_cplx --> 1 TrmA_mono_inact + 1 ahcys + 1 b3796_tRNA_6 + 1 h	irreversible	tRNA modification	0.00E+00	1.00E+09	0	3.01E+02	3.01E+02	0	1.39E+01	13.9	0	1.39E+01	13.9
b3796_tRNA_55_MODIF_1	modification of position 55 nt (Y) in argX by TruB_mono	1 TruB_mono + 1 b3796_tRNA_6 <==> 1 b3796_tRNA_6_TruB_mono_cplx	reversible	tRNA modification	-1.00E+09	1.00E+09	0	3.01E+02	3.01E+02	0	1.39E+01	13.9	0	1.39E+01	13.9
b3796_tRNA_55_MODIF_2	modification of position 55 nt (Y) in argX by TruB_mono	1 b3796_tRNA_6_TruB_mono_cplx --> 1 TruB_mono_inact + 1 argX_tRNA	irreversible	tRNA modification	0.00E+00	1.00E+09	0	3.01E+02	3.01E+02	0	1.39E+01	13.9	0	1.39E+01	13.9
b3796_tRNA_Mg_BIND	Binding of two Mg2 to b3796_tRNA	1 b3796_RNA + 2 mg2 <==> 1 b3796_tRNA_Mg2	reversible	Metallo-ion binding	-1.00E+09	1.00E+09	0	3.01E+02	3.01E+02	0	1.39E+01	13.9	0	1.39E+01	13.9
b3797_tRNA_16_MODIF_1	modification of position 16 nt (D) in hisR by Dus_gen	1 Dus_gen + 1 b3797_tRNA_Mg2 + 1 h + 1 nadph <==> 1 b3797_tRNA_Mg2_Dus_gen_cplx	reversible	tRNA modification	-1.00E+09	1.00E+09	0	3.01E+02	3.01E+02	0	1.39E+01	13.9	0	1.39E+01	13.9
b3797_tRNA_16_MODIF_2	modification of position 16 nt (D) in hisR by Dus_gen	1 b3797_tRNA_Mg2_Dus_gen_cplx --> 1 Dus_gen_inact + 1 b3797_tRNA_1 + 1 nadp	irreversible	tRNA modification	0.00E+00	1.00E+09	0	3.01E+02	3.01E+02	0	1.39E+01	13.9	0	1.39E+01	13.9
b3797_tRNA_17_MODIF_1	modification of position 17 nt (D) in hisR by Dus_gen	1 Dus_gen + 1 b3797_tRNA_1 + 1 h + 1 nadph <==> 1 b3797_tRNA_1_Dus_gen_cplx	reversible	tRNA modification	-1.00E+09	1.00E+09	0	3.01E+02	3.01E+02	0	1.39E+01	13.9	0	1.39E+01	13.9
b3797_tRNA_17_MODIF_2	modification of position 17 nt (D) in hisR by Dus_gen	1 b3797_tRNA_1_Dus_gen_cplx --> 1 Dus_gen_inact + 1 b3797_tRNA_2 + 1 nadp	irreversible	tRNA modification	0.00E+00	1.00E+09	0	3.01E+02	3.01E+02	0	1.39E+01	13.9	0	1.39E+01	13.9
b3797_tRNA_20_MODIF_1	modification of position 20 nt (D) in hisR by Dus_gen	1 Dus_gen + 1 b3797_tRNA_2 + 1 h + 1 nadph <==> 1 b3797_tRNA_2_Dus_gen_cplx	reversible	tRNA modification	-1.00E+09	1.00E+09	0	3.01E+02	3.01E+02	0	1.39E+01	13.9	0	1.39E+01	13.9
b3797_tRNA_20_MODIF_2	modification of position 20 nt (D) in hisR by Dus_gen	1 b3797_tRNA_2_Dus_gen_cplx --> 1 Dus_gen_inact + 1 b3797_tRNA_3 + 1 nadp	irreversible	tRNA modification	0.00E+00	1.00E+09	0	3.01E+02	3.01E+02	0	1.39E+01	13.9	0	1.39E+01	13.9
b3797_tRNA_34_MODIF_1	modification of position 34 nt (Q) in hisR by Tgt_hexa-QueA_mono-EoR_tRNA_pos34_Q	1 EoR_tRNA_pos34_Q + 1 QueA_mono + 1 Tgt_hexa + 1 adocbl + 1 amet + 1 b3797_tRNA_3 + 1 h + 1 nadh + 1 pre_Q1 <==> 1 b3797_tRNA_3_Tgt_hexa-QueA_mono-EoR_tRNA_pos34_Q_cplx	reversible	tRNA modification	-1.00E+09	1.00E+09	0	3.01E+02	3.01E+02	0	1.39E+01	13.9	0	1.39E+01	13.9
b3797_tRNA_34_MODIF_2	modification of position 34 nt (Q) in hisR by Tgt_hexa-QueA_mono-EoR_tRNA_pos34_Q	1 b3797_tRNA_3_Tgt_hexa-QueA_mono-EoR_tRNA_pos34_Q_cplx > 1 EoR_tRNA_pos34_Q_inact + 1 QueA_mono_inact + 1 Tgt_hexa_inact + 1 ade + 1 adocbl + 1 b3797_tRNA_4 + 1 gua + 2 h 1 h2o + 1 met-L + 1 nad	irreversible	tRNA modification	0.00E+00	1.00E+09	0	3.01E+02	3.01E+02	0	1.39E+01	13.9	0	1.39E+01	13.9
b3797_tRNA_37_MODIF_1	modification of position 37 nt (m2A) in hisR by MeT_tRNA_pos_37_m2A	1 MeT_tRNA_pos_37_m2A + 1 amet + 1 b3797_tRNA_4 <==> 1 b3797_tRNA_4_MeT_tRNA_pos_37_m2A_cplx	reversible	tRNA modification	-1.00E+09	1.00E+09	0	3.01E+02	3.01E+02	0	1.39E+01	13.9	0	1.39E+01	13.9
b3797_tRNA_37_MODIF_2	modification of position 37 nt (m2A) in hisR by MeT_tRNA_pos_37_m2A	1 b3797_tRNA_4_MeT_tRNA_pos_37_m2A_cplx --> 1 MeT_tRNA_pos_37_m2A_inact + 1 ahcys + 1 b3797_tRNA_5 + 1 h	irreversible	tRNA modification	0.00E+00	1.00E+09	0	3.01E+02	3.01E+02	0	1.39E+01	13.9	0	1.39E+01	13.9
b3797_tRNA_38_MODIF_1	modification of position 38 nt (Y) in hisR by TruA_dim	1 TruA_dim + 1 b3797_tRNA_5 <==> 1 b3797_tRNA_5_TruA_dim_cplx	reversible	tRNA modification	-1.00E+09	1.00E+09	0	3.01E+02	3.01E+02	0	1.39E+01	13.9	0	1.39E+01	13.9
b3797_tRNA_38_MODIF_2	modification of position 38 nt (Y) in hisR by TruA_dim	1 b3797_tRNA_5_TruA_dim_cplx --> 1 TruA_dim_inact + 1 b3797_tRNA_6	irreversible	tRNA modification	0.00E+00	1.00E+09	0	3.01E+02	3.01E+02	0	1.39E+01	13.9	0	1.39E+01	13.9
b3797_tRNA_39_MODIF_1	modification of position 39 nt (Y) in hisR by TruA_dim	1 TruA_dim + 1 b3797_tRNA_6 <==> 1 b3797_tRNA_6_TruA_dim_cplx	reversible	tRNA modification	-1.00E+09	1.00E+09	0	3.01E+02	3.01E+02	0	1.39E+01	13.9	0	1.39E+01	13.9
b3797_tRNA_39_MODIF_2	modification of position 39 nt (Y) in hisR by TruA_dim	1 b3797_tRNA_6_TruA_dim_cplx --> 1 TruA_dim_inact + 1 b3797_tRNA_7	irreversible	tRNA modification	0.00E+00	1.00E+09	0	3.01E+02	3.01E+02	0	1.39E+01	13.9	0	1.39E+01	13.9
b3797_tRNA_46_MODIF_1	modification of position 46 nt (m7G) in hisR by YggH_mono	1 YggH_mono + 1 amet + 1 b3797_tRNA_7 <==> 1 b3797_tRNA_7_YggH_mono_cplx	reversible	tRNA modification	-1.00E+09	1.00E+09	0	3.01E+02	3.01E+02	0	1.39E+01	13.9	0	1.39E+01	13.9
b3797_tRNA_46_MODIF_2	modification of position 46 nt (m7G) in hisR by YggH_mono	1 b3797_tRNA_7_YggH_mono_cplx --> 1 YggH_mono_inact + 1 ahcys + 1 b3797_tRNA_8 + 1 h	irreversible	tRNA modification	0.00E+00	1.00E+09	0	3.01E+02	3.01E+02	0	1.39E+01	13.9	0	1.39E+01	13.9

b3797_tRNA_54_MODIF_1	modification of position 54 nt (m5U) in hisR by TrmA_mono	1 TrmA_mono + 1 amet + 1 b3797_tRNA_8 <==> 1 b3797_tRNA_8_TrmA_mono_cplx	reversible	tRNA modification	-1.00E+09	1.00E+09	0	3.01E+02	3.01E+02	0	1.39E+01	13.9	0	1.39E+01	13.9
b3797_tRNA_54_MODIF_2	modification of position 54 nt (m5U) in hisR by TrmA_mono	1 b3797_tRNA_8_TrmA_mono_cplx --> 1 TrmA_mono_inact + 1 ahcys + 1 b3797_tRNA_9 + 1 h	irreversible	tRNA modification	0.00E+00	1.00E+09	0	3.01E+02	3.01E+02	0	1.39E+01	13.9	0	1.39E+01	13.9
b3797_tRNA_55_MODIF_1	modification of position 55 nt (Y) in hisR by TruB_mono	1 TruB_mono + 1 b3797_tRNA_9 <==> 1 b3797_tRNA_9_TruB_mono_cplx	reversible	tRNA modification	-1.00E+09	1.00E+09	0	3.01E+02	3.01E+02	0	1.39E+01	13.9	0	1.39E+01	13.9
b3797_tRNA_55_MODIF_2	modification of position 55 nt (Y) in hisR by TruB_mono	1 b3797_tRNA_9_TruB_mono_cplx --> 1 TruB_mono_inact + 1 b3797_tRNA_10	irreversible	tRNA modification	0.00E+00	1.00E+09	0	3.01E+02	3.01E+02	0	1.39E+01	13.9	0	1.39E+01	13.9
b3797_tRNA_8_MODIF_1	modification of position 8 nt (s4U) in hisR by ThiI_mono	1 IscS_dim_S-SH + 1 ThiI_mono + 1 atp + 1 b3797_tRNA_10 + 1 mg2 + 1 trdrd <==> 1 b3797_tRNA_10_ThiI_mono_cplx	reversible	tRNA modification	-1.00E+09	1.00E+09	0	3.01E+02	3.01E+02	0	1.39E+01	13.9	0	1.39E+01	13.9
b3797_tRNA_8_MODIF_2	modification of position 8 nt (s4U) in hisR by ThiI_mono	1 b3797_tRNA_10_ThiI_mono_cplx --> 1 IscS_dim_S-H + 1 ThiI_mono_inact + 1 amp + 1 h + 1 hisR_tRNA + 1 mg2 + 1 ppi + 1 trdox	irreversible	tRNA modification	0.00E+00	1.00E+09	0	3.01E+02	3.01E+02	0	1.39E+01	13.9	0	1.39E+01	13.9
b3797_tRNA_Mg_BIND	Binding of two Mg2 to b3797_tRNA	1 b3797_RNA + 2 mg2 <==> 1 b3797_tRNA_Mg2	reversible	Metallo-ion binding	-1.00E+09	1.00E+09	0	3.01E+02	3.01E+02	0	1.39E+01	13.9	0	1.39E+01	13.9
b3798_tRNA_16_MODIF_1	modification of position 16 nt (D) in leuT by Dus_gen	1 Dus_gen + 1 b3798_tRNA_Mg2 + 1 h + 1 nadph <==> 1 b3798_tRNA_Mg2_Dus_gen_cplx	reversible	tRNA modification	-1.00E+09	1.00E+09	0	3.01E+02	3.01E+02	0	1.39E+01	13.9	0	1.39E+01	13.9
b3798_tRNA_16_MODIF_2	modification of position 16 nt (D) in leuT by Dus_gen	1 b3798_tRNA_Mg2_Dus_gen_cplx --> 1 Dus_gen_inact + 1 b3798_tRNA_1 + 1 nadp	irreversible	tRNA modification	0.00E+00	1.00E+09	0	3.01E+02	3.01E+02	0	1.39E+01	13.9	0	1.39E+01	13.9
b3798_tRNA_17_MODIF_1	modification of position 17 nt (D) in leuT by Dus_gen	1 Dus_gen + 1 b3798_tRNA_1 + 1 h + 1 nadph <==> 1 b3798_tRNA_1_Dus_gen_cplx	reversible	tRNA modification	-1.00E+09	1.00E+09	0	3.01E+02	3.01E+02	0	1.39E+01	13.9	0	1.39E+01	13.9
b3798_tRNA_17_MODIF_2	modification of position 17 nt (D) in leuT by Dus_gen	1 b3798_tRNA_1_Dus_gen_cplx --> 1 Dus_gen_inact + 1 b3798_tRNA_2 + 1 nadp	irreversible	tRNA modification	0.00E+00	1.00E+09	0	3.01E+02	3.01E+02	0	1.39E+01	13.9	0	1.39E+01	13.9
b3798_tRNA_18_MODIF_1	modification of position 18 nt (Gm) in leuT by TrmH_dim	1 TrmH_dim + 1 amet + 1 b3798_tRNA_2 <==> 1 b3798_tRNA_2_TrmH_dim_cplx	reversible	tRNA modification	-1.00E+09	1.00E+09	0	3.01E+02	3.01E+02	0	1.39E+01	13.9	0	1.39E+01	13.9
b3798_tRNA_18_MODIF_2	modification of position 18 nt (Gm) in leuT by TrmH_dim	1 b3798_tRNA_2_TrmH_dim_cplx --> 1 TrmH_dim_inact + 1 ahcys + 1 b3798_tRNA_3 + 1 h	irreversible	tRNA modification	0.00E+00	1.00E+09	0	3.01E+02	3.01E+02	0	1.39E+01	13.9	0	1.39E+01	13.9
b3798_tRNA_20_MODIF_1	modification of position 20 nt (D) in leuT by Dus_gen	1 Dus_gen + 1 b3798_tRNA_3 + 1 h + 1 nadph <==> 1 b3798_tRNA_3_Dus_gen_cplx	reversible	tRNA modification	-1.00E+09	1.00E+09	0	3.01E+02	3.01E+02	0	1.39E+01	13.9	0	1.39E+01	13.9
b3798_tRNA_20_MODIF_2	modification of position 20 nt (D) in leuT by Dus_gen	1 b3798_tRNA_3_Dus_gen_cplx --> 1 Dus_gen_inact + 1 b3798_tRNA_4 + 1 nadp	irreversible	tRNA modification	0.00E+00	1.00E+09	0	3.01E+02	3.01E+02	0	1.39E+01	13.9	0	1.39E+01	13.9
b3798_tRNA_38_MODIF_1	modification of position 38 nt (Y) in leuT by TruA_dim	1 TruA_dim + 1 b3798_tRNA_4 <==> 1 b3798_tRNA_4_TruA_dim_cplx	reversible	tRNA modification	-1.00E+09	1.00E+09	0	3.01E+02	3.01E+02	0	1.39E+01	13.9	0	1.39E+01	13.9
b3798_tRNA_38_MODIF_2	modification of position 38 nt (Y) in leuT by TruA_dim	1 b3798_tRNA_4_TruA_dim_cplx --> 1 TruA_dim_inact + 1 b3798_tRNA_5	irreversible	tRNA modification	0.00E+00	1.00E+09	0	3.01E+02	3.01E+02	0	1.39E+01	13.9	0	1.39E+01	13.9
b3798_tRNA_40_MODIF_1	modification of position 40 nt (Y) in leuT by TruA_dim	1 TruA_dim + 1 b3798_tRNA_5 <==> 1 b3798_tRNA_5_TruA_dim_cplx	reversible	tRNA modification	-1.00E+09	1.00E+09	0	3.01E+02	3.01E+02	0	1.39E+01	13.9	0	1.39E+01	13.9
b3798_tRNA_40_MODIF_2	modification of position 40 nt (Y) in leuT by TruA_dim	1 b3798_tRNA_5_TruA_dim_cplx --> 1 TruA_dim_inact + 1 b3798_tRNA_6	irreversible	tRNA modification	0.00E+00	1.00E+09	0	3.01E+02	3.01E+02	0	1.39E+01	13.9	0	1.39E+01	13.9
b3798_tRNA_54_MODIF_1	modification of position 54 nt (m5U) in leuT by TrmA_mono	1 TrmA_mono + 1 amet + 1 b3798_tRNA_6 <==> 1 b3798_tRNA_6_TrmA_mono_cplx	reversible	tRNA modification	-1.00E+09	1.00E+09	0	3.01E+02	3.01E+02	0	1.39E+01	13.9	0	1.39E+01	13.9
b3798_tRNA_54_MODIF_2	modification of position 54 nt (m5U) in leuT by TrmA_mono	1 b3798_tRNA_6_TrmA_mono_cplx --> 1 TrmA_mono_inact + 1 ahcys + 1 b3798_tRNA_7 + 1 h	irreversible	tRNA modification	0.00E+00	1.00E+09	0	3.01E+02	3.01E+02	0	1.39E+01	13.9	0	1.39E+01	13.9
b3798_tRNA_55_MODIF_1	modification of position 55 nt (Y) in leuT by TruB_mono	1 TruB_mono + 1 b3798_tRNA_7 <==> 1 b3798_tRNA_7_TruB_mono_cplx	reversible	tRNA modification	-1.00E+09	1.00E+09	0	3.01E+02	3.01E+02	0	1.39E+01	13.9	0	1.39E+01	13.9
b3798_tRNA_55_MODIF_2	modification of position 55 nt (Y) in leuT by TruB_mono	1 b3798_tRNA_7_TruB_mono_cplx --> 1 TruB_mono_inact + 1 leuT_tRNA	irreversible	tRNA modification	0.00E+00	1.00E+09	0	3.01E+02	3.01E+02	0	1.39E+01	13.9	0	1.39E+01	13.9
b3798_tRNA_Mg_BIND	Binding of two Mg2 to b3798_tRNA	1 b3798_RNA + 2 mg2 <==> 1 b3798_tRNA_Mg2	reversible	Metallo-ion binding	-1.00E+09	1.00E+09	0	3.01E+02	3.01E+02	0	1.39E+01	13.9	0	1.39E+01	13.9
b3799_tRNA_20_MODIF_1	modification of position 20 nt (D) in proM by Dus_gen	1 Dus_gen + 1 b3799_tRNA_Mg2 + 1 h + 1 nadph <==> 1 b3799_tRNA_Mg2_Dus_gen_cplx	reversible	tRNA modification	-1.00E+09	1.00E+09	0	3.01E+02	3.01E+02	0	1.39E+01	13.9	0	1.39E+01	13.9
b3799_tRNA_20_MODIF_2	modification of position 20 nt (D) in proM by Dus_gen	1 b3799_tRNA_Mg2_Dus_gen_cplx --> 1 Dus_gen_inact + 1 b3799_tRNA_1 + 1 nadp	irreversible	tRNA modification	0.00E+00	1.00E+09	0	3.01E+02	3.01E+02	0	1.39E+01	13.9	0	1.39E+01	13.9
b3799_tRNA_32_MODIF_1	modification of position 32 nt (Um) in proM by MeT_tRNA_pos_32_Um	1 MeT_tRNA_pos_32_Um + 1 amet + 1 b3799_tRNA_1 <==> 1 b3799_tRNA_1_MeT_tRNA_pos_32_Um_cplx	reversible	tRNA modification	-1.00E+09	1.00E+09	0	3.01E+02	3.01E+02	0	1.39E+01	13.9	0	1.39E+01	13.9
b3799_tRNA_32_MODIF_2	modification of position 32 nt (Um) in proM by MeT_tRNA_pos_32_Um	1 b3799_tRNA_1_MeT_tRNA_pos_32_Um_cplx --> 1 MeT_tRNA_pos_32_Um_inact + 1 ahcys + 1 b3799_tRNA_2 + 1 h	irreversible	tRNA modification	0.00E+00	1.00E+09	0	3.01E+02	3.01E+02	0	1.39E+01	13.9	0	1.39E+01	13.9
b3799_tRNA_34_MODIF_1	modification of position 34 nt (cmo5U) in proM by YecO_mono-YecP_mono-HyL_tRNA_pos_34_ho5U	1 HyL_tRNA_pos_34_ho5U + 1 YecO_mono + 1 YecP_mono + 1 amet + 1 b3799_tRNA_2 + 2 chor <==> 1 b3799_tRNA_2_YecO_mono-YecP_mono-HyL_tRNA_pos_34_ho5U_cplx	reversible	tRNA modification	-1.00E+09	1.00E+09	0	3.01E+02	3.01E+02	0	1.39E+01	13.9	0	1.39E+01	13.9
b3799_tRNA_34_MODIF_2	modification of position 34 nt (cmo5U) in proM by YecO_mono-YecP_mono-HyL_tRNA_pos_34_ho5U	1 b3799_tRNA_2_YecO_mono-YecP_mono-HyL_tRNA_pos_34_ho5U_cplx --> 1 C10H8O5 + 1 C9H9O4 + 1 HyL_tRNA_pos_34_ho5U_inact + 1 YecO_mono_inact + 1 YecP_mono_inact + 1 ahcys + 1 b3799_tRNA_3 + 1 h	irreversible	tRNA modification	0.00E+00	1.00E+09	0	3.01E+02	3.01E+02	0	1.39E+01	13.9	0	1.39E+01	13.9
b3799_tRNA_37_MODIF_1	modification of position 37 nt (m1G) in proM by TrmD_dim	1 TrmD_dim + 1 amet + 1 b3799_tRNA_3 <==> 1 b3799_tRNA_3_TrmD_dim_cplx	reversible	tRNA modification	-1.00E+09	1.00E+09	0	3.01E+02	3.01E+02	0	1.39E+01	13.9	0	1.39E+01	13.9
b3799_tRNA_37_MODIF_2	modification of position 37 nt (m1G) in proM by TrmD_dim	1 b3799_tRNA_3_TrmD_dim_cplx --> 1 TrmD_dim_inact + 1 ahcys + 1 b3799_tRNA_4 + 1 h	irreversible	tRNA modification	0.00E+00	1.00E+09	0	3.01E+02	3.01E+02	0	1.39E+01	13.9	0	1.39E+01	13.9
b3799_tRNA_46_MODIF_1	modification of position 46 nt (m7G) in proM by YggH_mono	1 YggH_mono + 1 amet + 1 b3799_tRNA_4 <==> 1 b3799_tRNA_4_YggH_mono_cplx	reversible	tRNA modification	-1.00E+09	1.00E+09	0	3.01E+02	3.01E+02	0	1.39E+01	13.9	0	1.39E+01	13.9
b3799_tRNA_46_MODIF_2	modification of position 46 nt (m7G) in proM by YggH_mono	1 b3799_tRNA_4_YggH_mono_cplx --> 1 YggH_mono_inact + 1 ahcys + 1 b3799_tRNA_5 + 1 h	irreversible	tRNA modification	0.00E+00	1.00E+09	0	3.01E+02	3.01E+02	0	1.39E+01	13.9	0	1.39E+01	13.9
b3799_tRNA_54_MODIF_1	modification of position 54 nt (m5U) in proM by TrmA_mono	1 TrmA_mono + 1 amet + 1 b3799_tRNA_5 <==> 1 b3799_tRNA_5_TrmA_mono_cplx	reversible	tRNA modification	-1.00E+09	1.00E+09	0	3.01E+02	3.01E+02	0	1.39E+01	13.9	0	1.39E+01	13.9
b3799_tRNA_54_MODIF_2	modification of position 54 nt (m5U) in proM by TrmA_mono	1 b3799_tRNA_5_TrmA_mono_cplx --> 1 TrmA_mono_inact + 1 ahcys + 1 b3799_tRNA_6 + 1 h	irreversible	tRNA modification	0.00E+00	1.00E+09	0	3.01E+02	3.01E+02	0	1.39E+01	13.9	0	1.39E+01	13.9
b3799_tRNA_55_MODIF_1	modification of position 55 nt (Y) in proM by TruB_mono	1 TruB_mono + 1 b3799_tRNA_6 <==> 1 b3799_tRNA_6_TruB_mono_cplx	reversible	tRNA modification	-1.00E+09	1.00E+09	0	3.01E+02	3.01E+02	0	1.39E+01	13.9	0	1.39E+01	13.9
b3799_tRNA_55_MODIF_2	modification of position 55 nt (Y) in proM by TruB_mono	1 b3799_tRNA_6_TruB_mono_cplx --> 1 TruB_mono_inact + 1 b3799_tRNA_7	irreversible	tRNA modification	0.00E+00	1.00E+09	0	3.01E+02	3.01E+02	0	1.39E+01	13.9	0	1.39E+01	13.9
b3799_tRNA_65_MODIF_1	modification of position 65 nt (Y) in proM by YqcB_mono	1 YqcB_mono + 1 b3799_tRNA_7 <==> 1 b3799_tRNA_7_YqcB_mono_cplx	reversible	tRNA modification	-1.00E+09	1.00E+09	0	3.01E+02	3.01E+02	0	1.39E+01	13.9	0	1.39E+01	13.9
b3799_tRNA_65_MODIF_2	modification of position 65 nt (Y) in proM by YqcB_mono	1 b3799_tRNA_7_YqcB_mono_cplx --> 1 YqcB_mono_inact + 1 b3799_tRNA_8	irreversible	tRNA modification	0.00E+00	1.00E+09	0	3.01E+02	3.01E+02	0	1.39E+01	13.9	0	1.39E+01	13.9
b3799_tRNA_8_MODIF_1	modification of position 8 nt (s4U) in proM by ThiI_mono	1 IscS_dim_S-SH + 1 ThiI_mono + 1 atp + 1 b3799_tRNA_8 + 1 mg2 + 1 trdrd <==> 1 b3799_tRNA_8_ThiI_mono_cplx	reversible	tRNA modification	-1.00E+09	1.00E+09	0	3.01E+02	3.01E+02	0	1.39E+01	13.9	0	1.39E+01	13.9
b3799_tRNA_8_MODIF_2	modification of position 8 nt (s4U) in proM by ThiI_mono	1 b3799_tRNA_8_ThiI_mono_cplx --> 1 IscS_dim_S-H + 1 ThiI_mono_inact + 1 amp + 1 h + 1 mg2 + 1 ppi + 1 proM_tRNA + 1 trdox	irreversible	tRNA modification	0.00E+00	1.00E+09	0	3.01E+02	3.01E+02	0	1.39E+01	13.9	0	1.39E+01	13.9
b3799_tRNA_Mg_BIND	Binding of two Mg2 to b3799_tRNA	1 b3799_RNA + 2 mg2 <==> 1 b3799_tRNA_Mg2	reversible	Metallo-ion binding	-1.00E+09	1.00E+09	0	3.01E+02	3.01E+02	0	1.39E+01	13.9	0	1.39E+01	13.9
b3851_RNA_10_modif_1	b3851_RNA modification (16S rRNA): position: 967, m5C by RsmB_mono	1 RsmB_mono + 1 amet + 1 b3851_RNA_10 <==> 1 b3851_RNA_10_RsmB_mono	reversible	rRNA modification	-1.00E+09	1.00E+09	0	1.12E+02	1.12E+02	0	1.36E+01	13.6	0	1.36E+01	13.6
b3851_RNA_10_modif_2	b3851_RNA modification (16S rRNA): position: 967, m5C by RsmB_mono	1 b3851_RNA_10_RsmB_mono --> 1 RsmB_mono_inact + 1 ahcys + 1 h + 1 rrsA	irreversible	rRNA modification	0.00E+00	1.00E+09	0	1.12E+02	1.12E+02	0	1.36E+01	13.6	0	1.36E+01	13.6
b3851_RNA_1_modif_1	b3851_RNA modification (16S rRNA): position: 1402, m4Cm by MeT_16S_1402	1 MeT_16S_1402 + 2 amet + 1 b3851_RNA_1 <==> 1 b3851_RNA_1_MeT_16S_1402	reversible	rRNA modification	-1.00E+09	1.00E+09	0	1.12E+02	1.12E+02	0	1.36E+01	13.6	0	1.36E+01	13.6
b3851_RNA_1_modif_2	b3851_RNA modification (16S rRNA): position: 1402, m4Cm by MeT_16S_1402	1 b3851_RNA_1_MeT_16S_1402 --> 1 MeT_16S_1402_inact + 2 ahcys + 1 b3851_RNA_2 + 2 h	irreversible	rRNA modification	0.00E+00	1.00E+09	0	1.12E+02	1.12E+02	0	1.36E+01	13.6	0	1.36E+01	13.6
b3851_RNA_2_modif_1	b3851_RNA modification (16S rRNA): position: 1407, m5C by MeT_16S_1407	1 MeT_16S_1407 + 1 amet + 1 b3851_RNA_2 <==> 1 b3851_RNA_2_MeT_16S_1407	reversible	rRNA modification	-1.00E+09	1.00E+09	0	1.12E+02	1.12E+02	0	1.36E+01	13.6	0	1.36E+01	13.6
b3851_RNA_2_modif_2	b3851_RNA modification (16S rRNA): position: 1407, m5C by MeT_16S_1407	1 b3851_RNA_2_MeT_16S_1407 --> 1 MeT_16S_1407_inact + 1 ahcys + 1 b3851_RNA_3 + 1 h	irreversible	rRNA modification	0.00E+00	1.00E+09	0	1.12E+02	1.12E+02	0	1.36E+01	13.6	0	1.36E+01	13.6
b3851_RNA_3_modif_1	b3851_RNA modification (16S rRNA): position: 1498, m3U by YggJ_mono	1 YggJ_mono + 1 amet + 1 b3851_RNA_3 <==> 1 b3851_RNA_3_YggJ_mono	reversible	rRNA modification	-1.00E+09	1.00E+09	0	1.12E+02	1.12E+02	0	1.36E+01	13.6	0	1.36E+01	13.6

b3851_RNA_3_modif_2	b3851_RNA modification (16S rRNA): position: 1498, m3U by YggJ_mono	1 b3851_RNA_3_YggJ_mono --> 1 YggJ_mono_inact + 1 ahcys + 1 b3851_RNA_4 + 1 h	irreversible	rRNA modification	0.00E+00	1.00E+09	0	1.12E+02	1.12E+02	0	1.36E+01	13.6	0	1.36E+01	13.6
b3851_RNA_4_modif_1	b3851_RNA modification (16S rRNA): position: 1516, m2G by MeT_16S_1516	1 MeT_16S_1516 + 1 amet + 1 b3851_RNA_4 <==> 1 b3851_RNA_4_MeT_16S_1516	reversible	rRNA modification	-1.00E+09	1.00E+09	0	1.12E+02	1.12E+02	0	1.36E+01	13.6	0	1.36E+01	13.6
b3851_RNA_4_modif_2	b3851_RNA modification (16S rRNA): position: 1516, m2G by MeT_16S_1516	1 b3851_RNA_4_MeT_16S_1516 --> 1 MeT_16S_1516_inact + 1 ahcys + 1 b3851_RNA_5 + 1 h	irreversible	rRNA modification	0.00E+00	1.00E+09	0	1.12E+02	1.12E+02	0	1.36E+01	13.6	0	1.36E+01	13.6
b3851_RNA_5_modif_1	b3851_RNA modification (16S rRNA): position: 1518, m6(2)A by KsgA_mono	1 KsgA_mono + 2 amet + 1 b3851_RNA_5 <==> 1 b3851_RNA_5_KsgA_mono	reversible	rRNA modification	-1.00E+09	1.00E+09	0	1.12E+02	1.12E+02	0	1.36E+01	13.6	0	1.36E+01	13.6
b3851_RNA_5_modif_2	b3851_RNA modification (16S rRNA): position: 1518, m6(2)A by KsgA_mono	1 b3851_RNA_5_KsgA_mono --> 1 KsgA_mono_inact + 2 ahcys + 1 b3851_RNA_6 + 2 h	irreversible	rRNA modification	0.00E+00	1.00E+09	0	1.12E+02	1.12E+02	0	1.36E+01	13.6	0	1.36E+01	13.6
b3851_RNA_6_modif_1	b3851_RNA modification (16S rRNA): position: 1519, m6(2)A by KsgA_mono	1 KsgA_mono + 2 amet + 1 b3851_RNA_6 <==> 1 b3851_RNA_6_KsgA_mono	reversible	rRNA modification	-1.00E+09	1.00E+09	0	1.12E+02	1.12E+02	0	1.36E+01	13.6	0	1.36E+01	13.6
b3851_RNA_6_modif_2	b3851_RNA modification (16S rRNA): position: 1519, m6(2)A by KsgA_mono	1 b3851_RNA_6_KsgA_mono --> 1 KsgA_mono_inact + 2 ahcys + 1 b3851_RNA_7 + 2 h	irreversible	rRNA modification	0.00E+00	1.00E+09	0	1.12E+02	1.12E+02	0	1.36E+01	13.6	0	1.36E+01	13.6
b3851_RNA_7_modif_1	b3851_RNA modification (16S rRNA): position: 516, Y by RsuA_mono	1 RsuA_mono + 1 b3851_RNA_7 <==> 1 b3851_RNA_7_RsuA_mono	reversible	rRNA modification	-1.00E+09	1.00E+09	0	1.12E+02	1.12E+02	0	1.36E+01	13.6	0	1.36E+01	13.6
b3851_RNA_7_modif_2	b3851_RNA modification (16S rRNA): position: 516, Y by RsuA_mono	1 b3851_RNA_7_RsuA_mono --> 1 RsuA_mono_inact + 1 b3851_RNA_8	irreversible	rRNA modification	0.00E+00	1.00E+09	0	1.12E+02	1.12E+02	0	1.36E+01	13.6	0	1.36E+01	13.6
b3851_RNA_8_modif_1	b3851_RNA modification (16S rRNA): position: 527, m7G by MeT_16S_527	1 MeT_16S_527 + 1 amet + 1 b3851_RNA_8 <==> 1 b3851_RNA_8_MeT_16S_527	reversible	rRNA modification	-1.00E+09	1.00E+09	0	1.12E+02	1.12E+02	0	1.36E+01	13.6	0	1.36E+01	13.6
b3851_RNA_8_modif_2	b3851_RNA modification (16S rRNA): position: 527, m7G by MeT_16S_527	1 b3851_RNA_8_MeT_16S_527 --> 1 MeT_16S_527_inact + 1 ahcy + 1 b3851_RNA_9 + 1 h	irreversible	rRNA modification	0.00E+00	1.00E+09	0	1.12E+02	1.12E+02	0	1.36E+01	13.6	0	1.36E+01	13.6
b3851_RNA_9_modif_1	b3851_RNA modification (16S rRNA): position: 966, m2G by MeT_16S_966	1 MeT_16S_966 + 1 amet + 1 b3851_RNA_9 <==> 1 b3851_RNA_9_MeT_16S_966	reversible	rRNA modification	-1.00E+09	1.00E+09	0	1.12E+02	1.12E+02	0	1.36E+01	13.6	0	1.36E+01	13.6
b3851_RNA_9_modif_2	b3851_RNA modification (16S rRNA): position: 966, m2G by MeT_16S_966	1 b3851_RNA_9_MeT_16S_966 --> 1 MeT_16S_966_inact + 1 ahcy + 1 b3851_RNA_10 + 1 h	irreversible	rRNA modification	0.00E+00	1.00E+09	0	1.12E+02	1.12E+02	0	1.36E+01	13.6	0	1.36E+01	13.6
b3851_RNA_modif_1	b3851_RNA modification (16S rRNA): position: 1207, m2G by RsmC_mono	1 RsmC_mono + 1 amet + 1 b3851_RNA <==> 1 b3851_RNA_RsmC_mono	reversible	rRNA modification	-1.00E+09	1.00E+09	0	1.12E+02	1.12E+02	0	1.36E+01	13.6	0	1.36E+01	13.6
b3851_RNA_modif_2	b3851_RNA modification (16S rRNA): position: 1207, m2G by RsmC_mono	1 b3851_RNA_RsmC_mono --> 1 RsmC_mono_inact + 1 ahcys + 1 b3851_RNA_1 + 1 h	irreversible	rRNA modification	0.00E+00	1.00E+09	0	1.12E+02	1.12E+02	0	1.36E+01	13.6	0	1.36E+01	13.6
b3852_tRNA_20A_MODIF_1	modification of position 20A nt (D) in ileT by Dus_gen	1 Dus_gen + 1 b3852_tRNA_1 + 1 h + 1 nadph <==> 1 b3852_tRNA_1_Dus_gen_cplx	reversible	tRNA modification	-1.00E+09	1.00E+09	0	1.12E+02	1.12E+02	0	1.36E+01	13.6	0	1.36E+01	13.6
b3852_tRNA_20A_MODIF_2	modification of position 20A nt (D) in ileT by Dus_gen	1 b3852_tRNA_1_Dus_gen_cplx --> 1 Dus_gen_inact + 1 b3852_tRNA_2 + 1 nadp	irreversible	tRNA modification	0.00E+00	1.00E+09	0	1.12E+02	1.12E+02	0	1.36E+01	13.6	0	1.36E+01	13.6
b3852_tRNA_20_MODIF_1	modification of position 20 nt (D) in ileT by Dus_gen	1 Dus_gen + 1 b3852_tRNA_Mg2 + 1 h + 1 nadph <==> 1 b3852_tRNA_Mg2_Dus_gen_cplx	reversible	tRNA modification	-1.00E+09	1.00E+09	0	1.12E+02	1.12E+02	0	1.36E+01	13.6	0	1.36E+01	13.6
b3852_tRNA_20_MODIF_2	modification of position 20 nt (D) in ileT by Dus_gen	1 b3852_tRNA_Mg2_Dus_gen_cplx --> 1 Dus_gen_inact + 1 b3852_tRNA_1 + 1 nadp	irreversible	tRNA modification	0.00E+00	1.00E+09	0	1.12E+02	1.12E+02	0	1.36E+01	13.6	0	1.36E+01	13.6
b3852_tRNA_37_MODIF_1	modification of position 37 nt (t6A) in ileT by Up_tRNA_pos_37_t6A	1 Up_tRNA_pos_37_t6A + 1 atp + 1 b3852_tRNA_2 + 1 hco3 + 1 mg2 + 1 thr-L <==> 1 b3852_tRNA_2_Up_tRNA_pos_37_t6A_cplx	reversible	tRNA modification	-1.00E+09	1.00E+09	0	1.12E+02	1.12E+02	0	1.36E+01	13.6	0	1.36E+01	13.6
b3852_tRNA_37_MODIF_2	modification of position 37 nt (t6A) in ileT by Up_tRNA_pos_37_t6A	1 b3852_tRNA_2_Up_tRNA_pos_37_t6A_cplx --> 1 Up_tRNA_pos_37_t6A_inact + 1 amp + 1 b3852_tRNA_3 + 1 h + 1 h2o + 1 mg2 + 1 ppi	irreversible	tRNA modification	0.00E+00	1.00E+09	0	1.12E+02	1.12E+02	0	1.36E+01	13.6	0	1.36E+01	13.6
b3852_tRNA_46_MODIF_1	modification of position 46 nt (m7G) in ileT by YggH_mono	1 YggH_mono + 1 amet + 1 b3852_tRNA_3 <==> 1 b3852_tRNA_3_YggH_mono_cplx	reversible	tRNA modification	-1.00E+09	1.00E+09	0	1.12E+02	1.12E+02	0	1.36E+01	13.6	0	1.36E+01	13.6
b3852_tRNA_46_MODIF_2	modification of position 46 nt (m7G) in ileT by YggH_mono	1 b3852_tRNA_3_YggH_mono_cplx --> 1 YggH_mono_inact + 1 ahcys + 1 b3852_tRNA_4 + 1 h	irreversible	tRNA modification	0.00E+00	1.00E+09	0	1.12E+02	1.12E+02	0	1.36E+01	13.6	0	1.36E+01	13.6
b3852_tRNA_47_MODIF_1	modification of position 47 nt (acp3U) in ileT by AcpT_tRNA_pos_47_acp3U	1 AcpT_tRNA_pos_47_acp3U + 1 amet + 1 b3852_tRNA_4 <==> 1 b3852_tRNA_4_AcpT_tRNA_pos_47_acp3U_cplx	reversible	tRNA modification	-1.00E+09	1.00E+09	0	1.12E+02	1.12E+02	0	1.36E+01	13.6	0	1.36E+01	13.6
b3852_tRNA_47_MODIF_2	modification of position 47 nt (acp3U) in ileT by AcpT_tRNA_pos_47_acp3U	1 b3852_tRNA_4_AcpT_tRNA_pos_47_acp3U_cplx --> 1 5mta + 1 AcpT_tRNA_pos_47_acp3U_inact + 1 b3852_tRNA_5 + 1 h	irreversible	tRNA modification	0.00E+00	1.00E+09	0	1.12E+02	1.12E+02	0	1.36E+01	13.6	0	1.36E+01	13.6
b3852_tRNA_54_MODIF_1	modification of position 54 nt (m5U) in ileT by TrmA_mono	1 TrmA_mono + 1 amet + 1 b3852_tRNA_5 <==> 1 b3852_tRNA_5_TrmA_mono_cplx	reversible	tRNA modification	-1.00E+09	1.00E+09	0	1.12E+02	1.12E+02	0	1.36E+01	13.6	0	1.36E+01	13.6
b3852_tRNA_54_MODIF_2	modification of position 54 nt (m5U) in ileT by TrmA_mono	1 b3852_tRNA_5_TrmA_mono_cplx --> 1 TrmA_mono_inact + 1 ahcys + 1 b3852_tRNA_6 + 1 h	irreversible	tRNA modification	0.00E+00	1.00E+09	0	1.12E+02	1.12E+02	0	1.36E+01	13.6	0	1.36E+01	13.6
b3852_tRNA_55_MODIF_1	modification of position 55 nt (Y) in ileT by TruB_mono	1 TruB_mono + 1 b3852_tRNA_6 <==> 1 b3852_tRNA_6_TruB_mono_cplx	reversible	tRNA modification	-1.00E+09	1.00E+09	0	1.12E+02	1.12E+02	0	1.36E+01	13.6	0	1.36E+01	13.6
b3852_tRNA_55_MODIF_2	modification of position 55 nt (Y) in ileT by TruB_mono	1 b3852_tRNA_6_TruB_mono_cplx --> 1 TruB_mono_inact + 1 b3852_tRNA_7	irreversible	tRNA modification	0.00E+00	1.00E+09	0	1.12E+02	1.12E+02	0	1.36E+01	13.6	0	1.36E+01	13.6
b3852_tRNA_65_MODIF_1	modification of position 65 nt (Y) in ileT by YqcB_mono	1 YqcB_mono + 1 b3852_tRNA_7 <==> 1 b3852_tRNA_7_YqcB_mono_cplx	reversible	tRNA modification	-1.00E+09	1.00E+09	0	1.12E+02	1.12E+02	0	1.36E+01	13.6	0	1.36E+01	13.6
b3852_tRNA_65_MODIF_2	modification of position 65 nt (Y) in ileT by YqcB_mono	1 b3852_tRNA_7_YqcB_mono_cplx --> 1 YqcB_mono_inact + 1 ileT_tRNA	irreversible	tRNA modification	0.00E+00	1.00E+09	0	1.12E+02	1.12E+02	0	1.36E+01	13.6	0	1.36E+01	13.6
b3852_tRNA_Mg_BIND	Binding of two Mg2 to b3852_tRNA	1 b3852_RNA + 2 mg2 <==> 1 b3852_tRNA_Mg2	reversible	Metallo-ion binding	-1.00E+09	1.00E+09	0	1.12E+02	1.12E+02	0	1.36E+01	13.6	0	1.36E+01	13.6
b3853_tRNA_17_MODIF_1	modification of position 17 nt (D) in alaT by Dus_gen	1 Dus_gen + 1 b3853_tRNA_Mg2 + 1 h + 1 nadph <==> 1 b3853_tRNA_Mg2_Dus_gen_cplx	reversible	tRNA modification	-1.00E+09	1.00E+09	0	1.12E+02	1.12E+02	0	1.36E+01	13.6	0	1.36E+01	13.6
b3853_tRNA_17_MODIF_2	modification of position 17 nt (D) in alaT by Dus_gen	1 b3853_tRNA_Mg2_Dus_gen_cplx --> 1 Dus_gen_inact + 1 b3853_tRNA_1 + 1 nadp	irreversible	tRNA modification	0.00E+00	1.00E+09	0	1.12E+02	1.12E+02	0	1.36E+01	13.6	0	1.36E+01	13.6
b3853_tRNA_34_MODIF_1	modification of position 34 nt (cmo5U) in alaT by YecO_mono-YecP_mono-HyL_tRNA_pos_34_ho5U	1 HyL_tRNA_pos_34_ho5U + 1 YecO_mono + 1 YecP_mono + 1 amet + 1 b3853_tRNA_1 + 2 chor <==> 1 b3853_tRNA_1_YecO_mono-YecP_mono-HyL_tRNA_pos_34_ho5U_cplx	reversible	tRNA modification	-1.00E+09	1.00E+09	0	1.12E+02	1.12E+02	0	1.36E+01	13.6	0	1.36E+01	13.6
b3853_tRNA_34_MODIF_2	modification of position 34 nt (cmo5U) in alaT by YecO_mono-YecP_mono-HyL_tRNA_pos_34_ho5U	1 b3853_tRNA_1_YecO_mono-YecP_mono-HyL_tRNA_pos_34_ho5U_cplx --> 1 C10H8O5 + 1 C9H9O4 + 1 HyL_tRNA_pos_34_ho5U_inact + 1 YecO_mono_inact + 1 YecP_mono_inact + 1 ahcys + 1 b3853_tRNA_2 + 1 h	irreversible	tRNA modification	0.00E+00	1.00E+09	0	1.12E+02	1.12E+02	0	1.36E+01	13.6	0	1.36E+01	13.6
b3853_tRNA_46_MODIF_1	modification of position 46 nt (m7G) in alaT by YggH_mono	1 YggH_mono + 1 amet + 1 b3853_tRNA_2 <==> 1 b3853_tRNA_2_YggH_mono_cplx	reversible	tRNA modification	-1.00E+09	1.00E+09	0	1.12E+02	1.12E+02	0	1.36E+01	13.6	0	1.36E+01	13.6
b3853_tRNA_46_MODIF_2	modification of position 46 nt (m7G) in alaT by YggH_mono	1 b3853_tRNA_2_YggH_mono_cplx --> 1 YggH_mono_inact + 1 ahcys + 1 b3853_tRNA_3 + 1 h	irreversible	tRNA modification	0.00E+00	1.00E+09	0	1.12E+02	1.12E+02	0	1.36E+01	13.6	0	1.36E+01	13.6
b3853_tRNA_54_MODIF_1	modification of position 54 nt (m5U) in alaT by TrmA_mono	1 TrmA_mono + 1 amet + 1 b3853_tRNA_3 <==> 1 b3853_tRNA_3_TrmA_mono_cplx	reversible	tRNA modification	-1.00E+09	1.00E+09	0	1.12E+02	1.12E+02	0	1.36E+01	13.6	0	1.36E+01	13.6
b3853_tRNA_54_MODIF_2	modification of position 54 nt (m5U) in alaT by TrmA_mono	1 b3853_tRNA_3_TrmA_mono_cplx --> 1 TrmA_mono_inact + 1 ahcys + 1 b3853_tRNA_4 + 1 h	irreversible	tRNA modification	0.00E+00	1.00E+09	0	1.12E+02	1.12E+02	0	1.36E+01	13.6	0	1.36E+01	13.6
b3853_tRNA_55_MODIF_1	modification of position 55 nt (Y) in alaT by TruB_mono	1 TruB_mono + 1 b3853_tRNA_4 <==> 1 b3853_tRNA_4_TruB_mono_cplx	reversible	tRNA modification	-1.00E+09	1.00E+09	0	1.12E+02	1.12E+02	0	1.36E+01	13.6	0	1.36E+01	13.6
b3853_tRNA_55_MODIF_2	modification of position 55 nt (Y) in alaT by TruB_mono	1 b3853_tRNA_4_TruB_mono_cplx --> 1 TruB_mono_inact + 1 alaT_tRNA	irreversible	tRNA modification	0.00E+00	1.00E+09	0	1.12E+02	1.12E+02	0	1.36E+01	13.6	0	1.36E+01	13.6
b3853_tRNA_Mg_BIND	Binding of two Mg2 to b3853_tRNA	1 b3853_RNA + 2 mg2 <==> 1 b3853_tRNA_Mg2	reversible	Metallo-ion binding	-1.00E+09	1.00E+09	0	1.12E+02	1.12E+02	0	1.36E+01	13.6	0	1.36E+01	13.6
b3854_RNA_10_modif_1	b3854_RNA modification (23S rRNA): position: 2445, m2G by MeT_23S_2445	1 MeT_23S_2445 + 1 amet + 1 b3854_RNA_10 <==> 1 b3854_RNA_10_MeT_23S_2445	reversible	rRNA modification	-1.00E+09	1.00E+09	0	1.12E+02	1.12E+02	0	1.36E+01	13.6	0	1.36E+01	13.6
b3854_RNA_10_modif_2	b3854_RNA modification (23S rRNA): position: 2445, m2G by MeT_23S_2445	1 b3854_RNA_10_MeT_23S_2445 --> 1 MeT_23S_2445_inact + 1 ahcys + 1 b3854_RNA_11 + 1 h	irreversible	rRNA modification	0.00E+00	1.00E+09	0	1.12E+02	1.12E+02	0	1.36E+01	13.6	0	1.36E+01	13.6

b3854_RNA_11_a_modif_1	b3854_RNA modification (23S rRNA): position: 2449, D by DU_23S_2449 (NADH)	1 DU_23S_2449 + 1 b3854_RNA_11 + 1 h + 1 nadh <==> 1 b3854_RNA_11_DU_23S_2449_a	reversible	rRNA modification	-1.00E+09	1.00E+09	0	1.12E+02	1.12E+02	0	1.36E+01	13.6	0	1.36E+01	13.6
b3854_RNA_11_a_modif_2	b3854_RNA modification (23S rRNA): position: 2449, D by DU_23S_2449 (NADH)	1 b3854_RNA_11_DU_23S_2449_a --> 1 DU_23S_2449_inact + 1 b3854_RNA_12 + 1 nad	irreversible	rRNA modification	0.00E+00	1.00E+09	0	1.12E+02	1.12E+02	0	1.36E+01	13.6	0	1.36E+01	13.6
b3854_RNA_11_b_modif_1	b3854_RNA modification (23S rRNA): position: 2449, D by DU_23S_2449 (NADPH)	1 DU_23S_2449 + 1 b3854_RNA_11 + 1 h + 1 nadph <==> 1 b3854_RNA_11_DU_23S_2449_b	reversible	rRNA modification	-1.00E+09	1.00E+09	0	1.12E+02	1.12E+02	0	1.36E+01	13.6	0	1.36E+01	13.6
b3854_RNA_11_b_modif_2	b3854_RNA modification (23S rRNA): position: 2449, D by DU_23S_2449 (NADPH)	1 b3854_RNA_11_DU_23S_2449_b --> 1 DU_23S_2449_inact + 1 b3854_RNA_12 + 1 nadp	irreversible	rRNA modification	0.00E+00	1.00E+09	0	1.12E+02	1.12E+02	0	1.36E+01	13.6	0	1.36E+01	13.6
b3854_RNA_12_modif_1	b3854_RNA modification (23S rRNA): position: 2457, Y by YmfC_mono	1 YmfC_mono + 1 b3854_RNA_12 <==> 1 b3854_RNA_12_YmfC_mono	reversible	rRNA modification	-1.00E+09	1.00E+09	0	1.12E+02	1.12E+02	0	1.36E+01	13.6	0	1.36E+01	13.6
b3854_RNA_12_modif_2	b3854_RNA modification (23S rRNA): position: 2457, Y by YmfC_mono	1 b3854_RNA_12_YmfC_mono --> 1 YmfC_mono_inact + 1 b3854_RNA_13	irreversible	rRNA modification	0.00E+00	1.00E+09	0	1.12E+02	1.12E+02	0	1.36E+01	13.6	0	1.36E+01	13.6
b3854_RNA_13_modif_1	b3854_RNA modification (23S rRNA): position: 2498, Cm by MeT_23S_2498	1 MeT_23S_2498 + 1 amet + 1 b3854_RNA_13 <==> 1 b3854_RNA_13_MeT_23S_2498	reversible	rRNA modification	-1.00E+09	1.00E+09	0	1.12E+02	1.12E+02	0	1.36E+01	13.6	0	1.36E+01	13.6
b3854_RNA_13_modif_2	b3854_RNA modification (23S rRNA): position: 2498, Cm by MeT_23S_2498	1 b3854_RNA_13_MeT_23S_2498 --> 1 MeT_23S_2498_inact + 1 ahcys + 1 b3854_RNA_14 + 1 h	irreversible	rRNA modification	0.00E+00	1.00E+09	0	1.12E+02	1.12E+02	0	1.36E+01	13.6	0	1.36E+01	13.6
b3854_RNA_14_modif_1	b3854_RNA modification (23S rRNA): position: 2503, m2A by MeT_23S_2503	1 MeT_23S_2503 + 1 amet + 1 b3854_RNA_14 <==> 1 b3854_RNA_14_MeT_23S_2503	reversible	rRNA modification	-1.00E+09	1.00E+09	0	1.12E+02	1.12E+02	0	1.36E+01	13.6	0	1.36E+01	13.6
b3854_RNA_14_modif_2	b3854_RNA modification (23S rRNA): position: 2503, m2A by MeT_23S_2503	1 b3854_RNA_14_MeT_23S_2503 --> 1 MeT_23S_2503_inact + 1 ahcys + 1 b3854_RNA_15 + 1 h	irreversible	rRNA modification	0.00E+00	1.00E+09	0	1.12E+02	1.12E+02	0	1.36E+01	13.6	0	1.36E+01	13.6
b3854_RNA_15_modif_1	b3854_RNA modification (23S rRNA): position: 2504, Y by RluC_mono	1 RluC_mono + 1 b3854_RNA_15 <==> 1 b3854_RNA_15_RluC_mono	reversible	rRNA modification	-1.00E+09	1.00E+09	0	1.12E+02	1.12E+02	0	1.36E+01	13.6	0	1.36E+01	13.6
b3854_RNA_15_modif_2	b3854_RNA modification (23S rRNA): position: 2504, Y by RluC_mono	1 b3854_RNA_15_RluC_mono --> 1 RluC_mono_inact + 1 b3854_RNA_16	irreversible	rRNA modification	0.00E+00	1.00E+09	0	1.12E+02	1.12E+02	0	1.36E+01	13.6	0	1.36E+01	13.6
b3854_RNA_16_modif_1	b3854_RNA modification (23S rRNA): position: 2552, Um by RrmJ_mono	1 RrmJ_mono + 1 amet + 1 b3854_RNA_16 <==> 1 b3854_RNA_16_RrmJ_mono	reversible	rRNA modification	-1.00E+09	1.00E+09	0	1.12E+02	1.12E+02	0	1.36E+01	13.6	0	1.36E+01	13.6
b3854_RNA_16_modif_2	b3854_RNA modification (23S rRNA): position: 2552, Um by RrmJ_mono	1 b3854_RNA_16_RrmJ_mono --> 1 RrmJ_mono_inact + 1 ahcys + b3854_RNA_17 + 1 h	irreversible	rRNA modification	0.00E+00	1.00E+09	0	1.12E+02	1.12E+02	0	1.36E+01	13.6	0	1.36E+01	13.6
b3854_RNA_17_modif_1	b3854_RNA modification (23S rRNA): position: 2580, Y by RluC_mono	1 RluC_mono + 1 b3854_RNA_17 <==> 1 b3854_RNA_17_RluC_mono	reversible	rRNA modification	-1.00E+09	1.00E+09	0	1.12E+02	1.12E+02	0	1.36E+01	13.6	0	1.36E+01	13.6
b3854_RNA_17_modif_2	b3854_RNA modification (23S rRNA): position: 2580, Y by RluC_mono	1 b3854_RNA_17_RluC_mono --> 1 RluC_mono_inact + 1 b3854_RNA_18	irreversible	rRNA modification	0.00E+00	1.00E+09	0	1.12E+02	1.12E+02	0	1.36E+01	13.6	0	1.36E+01	13.6
b3854_RNA_18_modif_1	b3854_RNA modification (23S rRNA): position: 2604, Y by YjbC_mono	1 YjbC_mono + 1 b3854_RNA_18 <==> 1 b3854_RNA_18_YjbC_mono	reversible	rRNA modification	-1.00E+09	1.00E+09	0	1.12E+02	1.12E+02	0	1.36E+01	13.6	0	1.36E+01	13.6
b3854_RNA_18_modif_2	b3854_RNA modification (23S rRNA): position: 2604, Y by YjbC_mono	1 b3854_RNA_18_YjbC_mono --> 1 YjbC_mono_inact + 1 b3854_RNA_19	irreversible	rRNA modification	0.00E+00	1.00E+09	0	1.12E+02	1.12E+02	0	1.36E+01	13.6	0	1.36E+01	13.6
b3854_RNA_19_modif_1	b3854_RNA modification (23S rRNA): position: 2605, Y by RluB_mono	1 RluB_mono + 1 b3854_RNA_19 <==> 1 b3854_RNA_19_RluB_mono	reversible	rRNA modification	-1.00E+09	1.00E+09	0	1.12E+02	1.12E+02	0	1.36E+01	13.6	0	1.36E+01	13.6
b3854_RNA_19_modif_2	b3854_RNA modification (23S rRNA): position: 2605, Y by RluB_mono	1 b3854_RNA_19_RluB_mono --> 1 RluB_mono_inact + 1 b3854_RNA_20	irreversible	rRNA modification	0.00E+00	1.00E+09	0	1.12E+02	1.12E+02	0	1.36E+01	13.6	0	1.36E+01	13.6
b3854_RNA_1_modif_1	b3854_RNA modification (23S rRNA): position: 1835, m2G by MeT_23S_1835	1 MeT_23S_1835 + 1 amet + 1 b3854_RNA_1 <==> 1 b3854_RNA_1_MeT_23S_1835	reversible	rRNA modification	-1.00E+09	1.00E+09	0	1.12E+02	1.12E+02	0	1.36E+01	13.6	0	1.36E+01	13.6
b3854_RNA_1_modif_2	b3854_RNA modification (23S rRNA): position: 1835, m2G by MeT_23S_1835	1 b3854_RNA_1_MeT_23S_1835 --> 1 MeT_23S_1835_inact + 1 ahcys + 1 b3854_RNA_2 + 1 h	irreversible	rRNA modification	0.00E+00	1.00E+09	0	1.12E+02	1.12E+02	0	1.36E+01	13.6	0	1.36E+01	13.6
b3854_RNA_20_modif_1	b3854_RNA modification (23S rRNA): position: 745, m1G by RrmA_dim	1 RrmA_dim + 1 amet + 1 b3854_RNA_20 <==> 1 b3854_RNA_20_RrmA_dim	reversible	rRNA modification	-1.00E+09	1.00E+09	0	1.12E+02	1.12E+02	0	1.36E+01	13.6	0	1.36E+01	13.6
b3854_RNA_20_modif_2	b3854_RNA modification (23S rRNA): position: 745, m1G by RrmA_dim	1 b3854_RNA_20_RrmA_dim --> 1 RrmA_dim_inact + 1 ahcys + 1 b3854_RNA_21 + 1 h	irreversible	rRNA modification	0.00E+00	1.00E+09	0	1.12E+02	1.12E+02	0	1.36E+01	13.6	0	1.36E+01	13.6
b3854_RNA_21_modif_1	b3854_RNA modification (23S rRNA): position: 746, Y by RluA_mono	1 RluA_mono + 1 b3854_RNA_21 <==> 1 b3854_RNA_21_RluA_mono	reversible	rRNA modification	-1.00E+09	1.00E+09	0	1.12E+02	1.12E+02	0	1.36E+01	13.6	0	1.36E+01	13.6
b3854_RNA_21_modif_2	b3854_RNA modification (23S rRNA): position: 746, Y by RluA_mono	1 b3854_RNA_21_RluA_mono --> 1 RluA_mono_inact + 1 b3854_RNA_22	irreversible	rRNA modification	0.00E+00	1.00E+09	0	1.12E+02	1.12E+02	0	1.36E+01	13.6	0	1.36E+01	13.6
b3854_RNA_22_modif_1	b3854_RNA modification (23S rRNA): position: 747, m5U by RumB_mono	1 RumB_mono + 1 amet + 1 b3854_RNA_22 <==> 1 b3854_RNA_22_RumB_mono	reversible	rRNA modification	-1.00E+09	1.00E+09	0	1.12E+02	1.12E+02	0	1.36E+01	13.6	0	1.36E+01	13.6
b3854_RNA_22_modif_2	b3854_RNA modification (23S rRNA): position: 747, m5U by RumB_mono	1 b3854_RNA_22_RumB_mono --> 1 RumB_mono_inact + 1 ahcys + 1 b3854_RNA_23 + 1 h	irreversible	rRNA modification	0.00E+00	1.00E+09	0	1.12E+02	1.12E+02	0	1.36E+01	13.6	0	1.36E+01	13.6
b3854_RNA_23_modif_1	b3854_RNA modification (23S rRNA): position: 955, Y by RluC_mono	1 RluC_mono + 1 b3854_RNA_23 <==> 1 b3854_RNA_23_RluC_mono	reversible	rRNA modification	-1.00E+09	1.00E+09	0	1.12E+02	1.12E+02	0	1.36E+01	13.6	0	1.36E+01	13.6
b3854_RNA_23_modif_2	b3854_RNA modification (23S rRNA): position: 955, Y by RluC_mono	1 b3854_RNA_23_RluC_mono --> 1 RluC_mono_inact + 1 rrlA	irreversible	rRNA modification	0.00E+00	1.00E+09	0	1.12E+02	1.12E+02	0	1.36E+01	13.6	0	1.36E+01	13.6
b3854_RNA_2_modif_1	b3854_RNA modification (23S rRNA): position: 1911, Y by RluD_mono	1 RluD_mono + 1 b3854_RNA_2 <==> 1 b3854_RNA_2_RluD_mono	reversible	rRNA modification	-1.00E+09	1.00E+09	0	1.12E+02	1.12E+02	0	1.36E+01	13.6	0	1.36E+01	13.6
b3854_RNA_2_modif_2	b3854_RNA modification (23S rRNA): position: 1911, Y by RluD_mono	1 b3854_RNA_2_RluD_mono --> 1 RluD_mono_inact + 1 b3854_RNA_3	irreversible	rRNA modification	0.00E+00	1.00E+09	0	1.12E+02	1.12E+02	0	1.36E+01	13.6	0	1.36E+01	13.6
b3854_RNA_3_modif_1	b3854_RNA modification (23S rRNA): position: 1915, m3Y by RluD_mono	1 RluD_mono + 1 amet + 1 b3854_RNA_3 <==> 1 b3854_RNA_3_RluD_mono	reversible	rRNA modification	-1.00E+09	1.00E+09	0	1.12E+02	1.12E+02	0	1.36E+01	13.6	0	1.36E+01	13.6
b3854_RNA_3_modif_2	b3854_RNA modification (23S rRNA): position: 1915, m3Y by RluD_mono	1 b3854_RNA_3_RluD_mono --> 1 RluD_mono_inact + 1 ahcys + 1 b3854_RNA_4 + 1 h	irreversible	rRNA modification	0.00E+00	1.00E+09	0	1.12E+02	1.12E+02	0	1.36E+01	13.6	0	1.36E+01	13.6
b3854_RNA_4_modif_1	b3854_RNA modification (23S rRNA): position: 1917, Y by RluD_mono	1 RluD_mono + 1 b3854_RNA_4 <==> 1 b3854_RNA_4_RluD_mono	reversible	rRNA modification	-1.00E+09	1.00E+09	0	1.12E+02	1.12E+02	0	1.36E+01	13.6	0	1.36E+01	13.6
b3854_RNA_4_modif_2	b3854_RNA modification (23S rRNA): position: 1917, Y by RluD_mono	1 b3854_RNA_4_RluD_mono --> 1 RluD_mono_inact + 1 b3854_RNA_5	irreversible	rRNA modification	0.00E+00	1.00E+09	0	1.12E+02	1.12E+02	0	1.36E+01	13.6	0	1.36E+01	13.6
b3854_RNA_5_modif_1	b3854_RNA modification (23S rRNA): position: 1939, m5U by RumA_mono	1 RumA_mono + 1 amet + 1 b3854_RNA_5 <==> 1 b3854_RNA_5_RumA_mono	reversible	rRNA modification	-1.00E+09	1.00E+09	0	1.12E+02	1.12E+02	0	1.36E+01	13.6	0	1.36E+01	13.6
b3854_RNA_5_modif_2	b3854_RNA modification (23S rRNA): position: 1939, m5U by RumA_mono	1 b3854_RNA_5_RumA_mono --> 1 RumA_mono_inact + 1 ahcys + 1 b3854_RNA_6 + 1 h	irreversible	rRNA modification	0.00E+00	1.00E+09	0	1.12E+02	1.12E+02	0	1.36E+01	13.6	0	1.36E+01	13.6

b3854_RNA_6_modif_1	b3854_RNA modification (23S rRNA): position: 1962, m5C by MeT_23S_1962	1 MeT_23S_1962 + 1 amet + 1 b3854_RNA_6 <==> 1 b3854_RNA_6_MeT_23S_1962	reversible	rRNA modification	-1.00E+09	1.00E+09	0	1.12E+02	1.12E+02	0	1.36E+01	13.6	0	1.36E+01	13.6
b3854_RNA_6_modif_2	b3854_RNA modification (23S rRNA): position: 1962, m5C by MeT_23S_1962	1 b3854_RNA_6_MeT_23S_1962 --> 1 MeT_23S_1962_inact + 1 ahcys + 1 b3854_RNA_7 + 1 h	irreversible	rRNA modification	0.00E+00	1.00E+09	0	1.12E+02	1.12E+02	0	1.36E+01	13.6	0	1.36E+01	13.6
b3854_RNA_7_modif_1	b3854_RNA modification (23S rRNA): position: 2030, m6A by MeT_23S_2030	1 MeT_23S_2030 + 1 amet + 1 b3854_RNA_7 <==> 1 b3854_RNA_7_MeT_23S_2030	reversible	rRNA modification	-1.00E+09	1.00E+09	0	1.12E+02	1.12E+02	0	1.36E+01	13.6	0	1.36E+01	13.6
b3854_RNA_7_modif_2	b3854_RNA modification (23S rRNA): position: 2030, m6A by MeT_23S_2030	1 b3854_RNA_7_MeT_23S_2030 --> 1 MeT_23S_2030_inact + 1 ahcys + 1 b3854_RNA_8 + 1 h	irreversible	rRNA modification	0.00E+00	1.00E+09	0	1.12E+02	1.12E+02	0	1.36E+01	13.6	0	1.36E+01	13.6
b3854_RNA_8_modif_1	b3854_RNA modification (23S rRNA): position: 2069, m7G by MeT_23S_2069	1 MeT_23S_2069 + 1 amet + 1 b3854_RNA_8 <==> 1 b3854_RNA_8_MeT_23S_2069	reversible	rRNA modification	-1.00E+09	1.00E+09	0	1.12E+02	1.12E+02	0	1.36E+01	13.6	0	1.36E+01	13.6
b3854_RNA_8_modif_2	b3854_RNA modification (23S rRNA): position: 2069, m7G by MeT_23S_2069	1 b3854_RNA_8_MeT_23S_2069 --> 1 MeT_23S_2069_inact + 1 ahcys + 1 b3854_RNA_9 + 1 h	irreversible	rRNA modification	0.00E+00	1.00E+09	0	1.12E+02	1.12E+02	0	1.36E+01	13.6	0	1.36E+01	13.6
b3854_RNA_9_modif_1	b3854_RNA modification (23S rRNA): position: 2251, Gm by RlmB_dim	1 RlmB_dim + 1 amet + 1 b3854_RNA_9 <==> 1 b3854_RNA_9_RlmB_dim	reversible	rRNA modification	-1.00E+09	1.00E+09	0	1.12E+02	1.12E+02	0	1.36E+01	13.6	0	1.36E+01	13.6
b3854_RNA_9_modif_2	b3854_RNA modification (23S rRNA): position: 2251, Gm by RlmB_dim	1 b3854_RNA_9_RlmB_dim --> 1 RlmB_dim_inact + 1 ahcys + 1 b3854_RNA_10 + 1 h	irreversible	rRNA modification	0.00E+00	1.00E+09	0	1.12E+02	1.12E+02	0	1.36E+01	13.6	0	1.36E+01	13.6
b3854_RNA_modif_1	b3854_RNA modification (23S rRNA): position: 1618, m6A by MeT_23S_1618	1 MeT_23S_1618 + 1 amet + 1 b3854_RNA <==> 1 b3854_RNA_MeT_23S_1618	reversible	rRNA modification	-1.00E+09	1.00E+09	0	1.12E+02	1.12E+02	0	1.36E+01	13.6	0	1.36E+01	13.6
b3854_RNA_modif_2	b3854_RNA modification (23S rRNA): position: 1618, m6A by MeT_23S_1618	1 b3854_RNA_MeT_23S_1618 --> 1 MeT_23S_1618_inact + 1 ahcy + 1 b3854_RNA_1 + 1 h	irreversible	rRNA modification	0.00E+00	1.00E+09	0	1.12E+02	1.12E+02	0	1.36E+01	13.6	0	1.36E+01	13.6
b3885_fold_spon	b3885_m folding: spontanous	1 b3885_m --> 1 YihX_mono	irreversible	Protein Folding	0.00E+00	1.00E+09	0	5.90E+02	5.90E+02	0	9.63E+02	963.2302	0	1.04E+02	104.214
b3885_mRNA_CONV	convsersion of mRNA to mRNA_1 (synthetic rxn)	1 b3885_mRNA --> 1 b3885_mRNA_1	irreversible	Translation	0.00E+00	1.00E+09	0	4.43E+01	4.43E+01	0	7.82E+01	78.19639	0	1.04E-01	0.104
b3885_mRNA_degr1	Degradation of b3885_mRNA I	1 Orn_dim + 150 atp + 1 b3885_mRNA_2 + 1 degradosome + 150 h2o <==> 150 adp + 1 b3885_mRNA_2_degr + 150 h + 150 pi	reversible	mRNA degradation	-1.00E+09	1.00E+09	0	4.43E+01	4.43E+01	0	7.82E+01	78.19639	0	1.04E-01	0.104
b3885_mRNA_degr2	Degradation of b3885_mRNA II	1 b3885_mRNA_2_degr + 599 h2o --> 1 Orn_dim_inact + 142 amp + 1 atp + 138 cmp + 1 degradosome_inact + 165 gmp + 599 h + 154 ump	irreversible	mRNA degradation	0.00E+00	1.89E-01	0	4.43E+01	4.43E+01	0	7.82E+01	78.19639	0	1.04E-01	0.104
b3885_maturation1	Polypeptide b3885 peptide deformylas and methionine aminopeptidase complex	1 Def_mono + 1 Map_mono + 1 b3885_aa <==> 1 b3885_def_map_cplx	reversible	Protein Maturation	-1.00E+09	1.00E+09	0	5.90E+02	5.90E+02	0	9.63E+02	963.2302	0	1.04E+02	104.214
b3885_maturation2	b3885 formation	1 b3885_def_map_cplx + 2 h2o --> 1 Def_mono_inact + 1 Map_mono_inact + 1 b3885_m + 1 for + 1 met-L	irreversible	Protein Maturation	0.00E+00	1.00E+09	0	5.90E+02	5.90E+02	0	9.63E+02	963.2302	0	1.04E+02	104.214
b3886_fold_spon	b3886_m folding: spontanous	1 b3886_m --> 1 Rbn_mono	irreversible	Protein Folding	0.00E+00	1.00E+09	0	8.24E+02	8.24E+02	0	1.40E+03	1400.399	0	7.28E+01	72.828
b3886_mRNA_CONV	convsersion of mRNA to mRNA_1 (synthetic rxn)	1 b3886_mRNA --> 1 b3886_mRNA_1	irreversible	Translation	0.00E+00	1.00E+09	0	4.43E+01	4.43E+01	0	7.82E+01	78.19639	0	1.04E-01	0.104
b3886_mRNA_degr1	Degradation of b3886_mRNA I	1 Orn_dim + 216 atp + 1 b3886_mRNA_2 + 1 degradosome + 216 h2o <==> 216 adp + 1 b3886_mRNA_2_degr + 216 h + 216 pi	reversible	mRNA degradation	-1.00E+09	1.00E+09	0	4.43E+01	4.43E+01	0	7.82E+01	78.19639	0	1.04E-01	0.104
b3886_mRNA_degr2	Degradation of b3886_mRNA II	1 b3886_mRNA_2_degr + 865 h2o --> 1 Orn_dim_inact + 177 amp + 229 cmp + 1 degradosome_inact + 213 gmp + 865 h + 247 ump	irreversible	mRNA degradation	0.00E+00	1.04E-01	0	4.43E+01	4.43E+01	0	7.82E+01	78.19639	0	1.04E-01	0.104
b3886_maturation1	Polypeptide b3886 peptide deformylas and methionine aminopeptidase complex	1 Def_mono + 1 Map_mono + 1 b3886_aa <==> 1 b3886_def_map_cplx	reversible	Protein Maturation	-1.00E+09	1.00E+09	0	8.24E+02	8.24E+02	0	1.40E+03	1400.399	0	7.28E+01	72.828
b3886_maturation2	b3886 formation	1 b3886_def_map_cplx + 2 h2o --> 1 Def_mono_inact + 1 Map_mono_inact + 1 b3886_m + 1 for + 1 met-L	irreversible	Protein Maturation	0.00E+00	1.00E+09	0	8.24E+02	8.24E+02	0	1.40E+03	1400.399	0	7.28E+01	72.828
b3887_fold_spon	b3887_m folding: spontanous	1 b3887_m_Zn --> 1 Dtd_mono	irreversible	Protein Folding	0.00E+00	1.00E+09	0	4.32E+02	4.32E+02	0	7.03E+02	702.7711	0	7.80E+01	78.032
b3887_ion_BIND	Binding of ions to b3887	1 b3887_m + 6 zn2 <==> 1 b3887_m_Zn	reversible	Metallo-ion Binding	-1.00E+09	1.00E+09	0	4.32E+02	4.32E+02	0	7.03E+02	702.7711	0	7.80E+01	78.032
b3887_mRNA_CONV	convsersion of mRNA to mRNA_1 (synthetic rxn)	1 b3887_mRNA --> 1 b3887_mRNA_1	irreversible	Translation	0.00E+00	1.00E+09	0	4.43E+01	4.43E+01	0	7.82E+01	78.19639	0	1.04E-01	0.104
b3887_mRNA_degr1	Degradation of b3887_mRNA I	1 Orn_dim + 108 atp + 1 b3887_mRNA_2 + 1 degradosome + 108 h2o <==> 108 adp + 1 b3887_mRNA_2_degr + 108 h + 108 pi	reversible	mRNA degradation	-1.00E+09	1.00E+09	0	4.43E+01	4.43E+01	0	7.82E+01	78.19639	0	1.04E-01	0.104
b3887_mRNA_degr2	Degradation of b3887_mRNA II	1 b3887_mRNA_2_degr + 433 h2o --> 1 Orn_dim_inact + 102 amp + 105 cmp + 1 degradosome_inact + 135 gmp + 433 h + 92 ump	irreversible	mRNA degradation	0.00E+00	2.07E-01	0	4.43E+01	4.43E+01	0	7.82E+01	78.19639	0	1.04E-01	0.104
b3887_maturation1	Polypeptide b3887 peptide deformylas complex	1 Def_mono + 1 b3887_aa <==> 1 b3887_def_cplx	reversible	Protein Maturation	-1.00E+09	1.00E+09	0	4.32E+02	4.32E+02	0	7.03E+02	702.7711	0	7.80E+01	78.032
b3887_maturation2	b3887 formation	1 b3887_def_cplx + 1 h2o --> 1 Def_mono_inact + 1 b3887_m + 1 for	irreversible	Protein Maturation	0.00E+00	1.00E+09	0	4.32E+02	4.32E+02	0	7.03E+02	702.7711	0	7.80E+01	78.032
b3888_fold_spon	b3888_m folding: spontanous	1 b3888_m --> 1 YiiD_mono	irreversible	Protein Folding	0.00E+00	1.00E+09	0	9.22E+02	9.22E+02	0	1.57E+03	1566.481	0	8.27E+01	82.726
b3888_mRNA_CONV	convsersion of mRNA to mRNA_1 (synthetic rxn)	1 b3888_mRNA --> 1 b3888_mRNA_1	irreversible	Translation	0.00E+00	1.00E+09	0	4.43E+01	4.43E+01	0	7.82E+01	78.19639	0	1.04E-01	0.104
b3888_mRNA_degr1	Degradation of b3888_mRNA I	1 Orn_dim + 246 atp + 1 b3888_mRNA_2 + 1 degradosome + 246 h2o <==> 246 adp + 1 b3888_mRNA_2_degr + 246 h + 246 pi	reversible	mRNA degradation	-1.00E+09	1.00E+09	0	4.43E+01	4.43E+01	0	7.82E+01	78.19639	0	1.04E-01	0.104
b3888_mRNA_degr2	Degradation of b3888_mRNA II	1 b3888_mRNA_2_degr + 985 h2o --> 1 Orn_dim_inact + 234 amp + 249 cmp + 1 degradosome_inact + 295 gmp + 985 h + 208 ump	irreversible	mRNA degradation	0.00E+00	1.17E-01	0	4.43E+01	4.43E+01	0	7.82E+01	78.19639	0	1.04E-01	0.104
b3888_maturation1	Polypeptide b3888 peptide deformylas and methionine aminopeptidase complex	1 Def_mono + 1 Map_mono + 1 b3888_aa <==> 1 b3888_def_map_cplx	reversible	Protein Maturation	-1.00E+09	1.00E+09	0	9.22E+02	9.22E+02	0	1.57E+03	1566.481	0	8.27E+01	82.726
b3888_maturation2	b3888 formation	1 b3888_def_map_cplx + 2 h2o --> 1 Def_mono_inact + 1 Map_mono_inact + 1 b3888_m + 1 for + 1 met-L	irreversible	Protein Maturation	0.00E+00	1.00E+09	0	9.22E+02	9.22E+02	0	1.57E+03	1566.481	0	8.27E+01	82.726
b3936_fold_spon	b3936_m folding: spontanous	1 b3936_m --> 1 RpmE_mono	irreversible	Protein Folding	0.00E+00	1.00E+09	8.44E+01	1.13E+02	2.81E+01	6.22E+01	8.29E+01	20.72	6.22E+01	8.29E+01	20.72
b3936_mRNA_CONV	convsersion of mRNA to mRNA_1 (synthetic rxn)	1 b3936_mRNA --> 1 b3936_mRNA_1	irreversible	Translation	0.00E+00	1.00E+09	0	1.07E+02	1.07E+02	0	8.29E+01	82.88	0	3.72E-01	0.372
b3936_mRNA_degr1	Degradation of b3936_mRNA I	1 Orn_dim + 53 atp + 1 b3936_mRNA_2 + 1 degradosome + 53 h2o <==> 53 adp + 1 b3936_mRNA_2_degr + 53 h + 53 pi	reversible	mRNA degradation	-1.00E+09	1.00E+09	0	1.07E+02	1.07E+02	0	8.29E+01	82.88	0	3.72E-01	0.372
b3936_mRNA_degr2	Degradation of b3936_mRNA II	1 b3936_mRNA_2_degr + 212 h2o --> 1 Orn_dim_inact + 59 amp + 1 atp + 57 cmp + 1 degradosome_inact + 48 gmp + 212 h + 48 ump	irreversible	mRNA degradation	0.00E+00	3.72E-01	0	1.07E+02	1.07E+02	0	8.29E+01	82.88	0	3.72E-01	0.372
b3936_maturation1	Polypeptide b3936 peptide deformylas complex	1 Def_mono + 1 b3936_aa <==> 1 b3936_def_cplx	reversible	Protein Maturation	-1.00E+09	1.00E+09	8.44E+01	1.13E+02	2.81E+01	6.22E+01	8.29E+01	20.72	6.22E+01	8.29E+01	20.72
b3936_maturation2	b3936 formation	1 b3936_def_cplx + 1 h2o --> 1 Def_mono_inact + 1 b3936_m + 1 for	irreversible	Protein Maturation	0.00E+00	1.00E+09	8.44E+01	1.13E+02	2.81E+01	6.22E+01	8.29E+01	20.72	6.22E+01	8.29E+01	20.72
b3965_fold_GroEL/ES_1	b3965_m folding: GroEL/ES mediated: polypeptide is going in GroEL/ES complex	1 GroEL.(7)ADP.cisGroES + 7 atp + 1 b3965_m + 1 transGroES_hepta --> 7 adp + 1 b3965_m_GroEL.(7)ATP.transGroES + 1 cisGroES_hepta	irreversible	Protein Folding	0.00E+00	1.00E+09	0	2.56E+03	2.56E+03	0	4.47E+03	4470.891	0	7.16E+01	71.5659
b3965_fold_GroEL/ES_2	b3965_m folding: GroEL/ES mediated: folding of polypeptide under ATP hydrolysis	1 b3965_m_GroEL.(7)ATP.transGroES + 7 h2o --> 1 TrmA_GroEL.(7)ADP.transGroES + 7 h + 7 pi	irreversible	Protein Folding	0.00E+00	1.00E+09	0	2.56E+03	2.56E+03	0	4.47E+03	4470.891	0	7.16E+01	71.5659
b3965_fold_GroEL/ES_3	b3965_m folding: GroEL/ES mediated: release of native protein	1 TrmA_GroEL.(7)ADP.transGroES --> 1 GroEL.(7)ADP.transGroES + 1 TrmA_mono	irreversible	Protein Folding	0.00E+00	1.00E+09	0	2.56E+03	2.56E+03	0	4.47E+03	4470.891	0	7.16E+01	71.5659

b3965_mRNA_CONV	convsersion of mRNA to mRNA_1 (synthetic rxn)	1 b3965_mRNA --> 1 b3965_mRNA_1	irreversible	Translation	0.00E+00	1.00E+09	0	1.19E+02	1.19E+02	0	2.10E+02	209.5896	0	9.79E-02	0.0979
b3965_mRNA_degr1	Degradation of b3965_mRNA I	1 Orn_dim + 275 atp + 1 b3965_mRNA_2 + 1 degradosome + 275 h2o <==> 275 adp + 1 b3965_mRNA_2_degr + 275 h + 275 pi	reversible	mRNA degradation	-1.00E+09	1.00E+09	0	1.19E+02	1.19E+02	0	2.10E+02	209.5896	0	9.79E-02	0.0979
b3965_mRNA_degr2	Degradation of b3965_mRNA II	1 b3965_mRNA_2_degr + 1100 h2o --> 1 Orn_dim_inact + 288 amp 1 atp + 269 cmp + 1 degradosome_inact + 291 gmp + 1100 h + 252 ump	irreversible	mRNA degradation	0.00E+00	9.79E-02	0	1.19E+02	1.19E+02	0	2.10E+02	209.5896	0	9.79E-02	0.0979
b3965_maturation1	Polypeptide b3965 peptide deformylas complex	1 Def_mono + 1 b3965_aa <==> 1 b3965_def_cplx	reversible	Protein Maturation	-1.00E+09	1.00E+09	0	2.56E+03	2.56E+03	0	4.47E+03	4470.891	0	7.16E+01	71.5659
b3965_maturation2	b3965 formation	1 b3965_def_cplx + 1 h2o --> 1 Def_mono_inact + 1 b3965_m + 1 for	irreversible	Protein Maturation	0.00E+00	1.00E+09	0	2.56E+03	2.56E+03	0	4.47E+03	4470.891	0	7.16E+01	71.5659
b3968_RNA_10_modif_1	b3968_RNA modification (16S rRNA): position: 967, m5C by RsmB_mono	1 RsmB_mono + 1 amet + 1 b3968_RNA_10 <==> 1 b3968_RNA_10_RsmB_mono	reversible	rRNA modification	-1.00E+09	1.00E+09	0	1.11E+02	1.11E+02	0	0	0	0	0	0
b3968_RNA_10_modif_2	b3968_RNA modification (16S rRNA): position: 967, m5C by RsmB_mono	1 b3968_RNA_10_RsmB_mono --> 1 RsmB_mono_inact + 1 ahcys + 1 h + 1 rrsB	irreversible	rRNA modification	0.00E+00	1.00E+09	0	1.11E+02	1.11E+02	0	0	0	0	0	0
b3968_RNA_1_modif_1	b3968_RNA modification (16S rRNA): position: 1402, m4Cm by MeT_16S_1402	1 MeT_16S_1402 + 2 amet + 1 b3968_RNA_1 <==> 1 b3968_RNA_1_MeT_16S_1402	reversible	rRNA modification	-1.00E+09	1.00E+09	0	1.11E+02	1.11E+02	0	0	0	0	0	0
b3968_RNA_1_modif_2	b3968_RNA modification (16S rRNA): position: 1402, m4Cm by MeT_16S_1402	1 b3968_RNA_1_MeT_16S_1402 --> 1 MeT_16S_1402_inact + 2 ahcys + 1 b3968_RNA_2 + 2 h	irreversible	rRNA modification	0.00E+00	1.00E+09	0	1.11E+02	1.11E+02	0	0	0	0	0	0
b3968_RNA_2_modif_1	b3968_RNA modification (16S rRNA): position: 1407, m5C by MeT_16S_1407	1 MeT_16S_1407 + 1 amet + 1 b3968_RNA_2 <==> 1 b3968_RNA_2_MeT_16S_1407	reversible	rRNA modification	-1.00E+09	1.00E+09	0	1.11E+02	1.11E+02	0	0	0	0	0	0
b3968_RNA_2_modif_2	b3968_RNA modification (16S rRNA): position: 1407, m5C by MeT_16S_1407	1 b3968_RNA_2_MeT_16S_1407 --> 1 MeT_16S_1407_inact + 1 ahcys + 1 b3968_RNA_3 + 1 h	irreversible	rRNA modification	0.00E+00	1.00E+09	0	1.11E+02	1.11E+02	0	0	0	0	0	0
b3968_RNA_3_modif_1	b3968_RNA modification (16S rRNA): position: 1498, m3U by YggJ_mono	1 YggJ_mono + 1 amet + 1 b3968_RNA_3 <==> 1 b3968_RNA_3_YggJ_mono	reversible	rRNA modification	-1.00E+09	1.00E+09	0	1.11E+02	1.11E+02	0	0	0	0	0	0
b3968_RNA_3_modif_2	b3968_RNA modification (16S rRNA): position: 1498, m3U by YggJ_mono	1 b3968_RNA_3_YggJ_mono --> 1 YggJ_mono_inact + 1 ahcys + 1 b3968_RNA_4 + 1 h	irreversible	rRNA modification	0.00E+00	1.00E+09	0	1.11E+02	1.11E+02	0	0	0	0	0	0
b3968_RNA_4_modif_1	b3968_RNA modification (16S rRNA): position: 1516, m2G by MeT_16S_1516	1 MeT_16S_1516 + 1 amet + 1 b3968_RNA_4 <==> 1 b3968_RNA_4_MeT_16S_1516	reversible	rRNA modification	-1.00E+09	1.00E+09	0	1.11E+02	1.11E+02	0	0	0	0	0	0
b3968_RNA_4_modif_2	b3968_RNA modification (16S rRNA): position: 1516, m2G by MeT_16S_1516	1 b3968_RNA_4_MeT_16S_1516 --> 1 MeT_16S_1516_inact + 1 ahcys + 1 b3968_RNA_5 + 1 h	irreversible	rRNA modification	0.00E+00	1.00E+09	0	1.11E+02	1.11E+02	0	0	0	0	0	0
b3968_RNA_5_modif_1	b3968_RNA modification (16S rRNA): position: 1518, m6(2)A by KsgA_mono	1 KsgA_mono + 2 amet + 1 b3968_RNA_5 <==> 1 b3968_RNA_5_KsgA_mono	reversible	rRNA modification	-1.00E+09	1.00E+09	0	1.11E+02	1.11E+02	0	0	0	0	0	0
b3968_RNA_5_modif_2	b3968_RNA modification (16S rRNA): position: 1518, m6(2)A by KsgA_mono	1 b3968_RNA_5_KsgA_mono --> 1 KsgA_mono_inact + 2 ahcys + 1 b3968_RNA_6 + 2 h	irreversible	rRNA modification	0.00E+00	1.00E+09	0	1.11E+02	1.11E+02	0	0	0	0	0	0
b3968_RNA_6_modif_1	b3968_RNA modification (16S rRNA): position: 1519, m6(2)A by KsgA_mono	1 KsgA_mono + 2 amet + 1 b3968_RNA_6 <==> 1 b3968_RNA_6_KsgA_mono	reversible	rRNA modification	-1.00E+09	1.00E+09	0	1.11E+02	1.11E+02	0	0	0	0	0	0
b3968_RNA_6_modif_2	b3968_RNA modification (16S rRNA): position: 1519, m6(2)A by KsgA_mono	1 b3968_RNA_6_KsgA_mono --> 1 KsgA_mono_inact + 2 ahcys + 1 b3968_RNA_7 + 2 h	irreversible	rRNA modification	0.00E+00	1.00E+09	0	1.11E+02	1.11E+02	0	0	0	0	0	0
b3968_RNA_7_modif_1	b3968_RNA modification (16S rRNA): position: 516, Y by RsuA_mono	1 RsuA_mono + 1 b3968_RNA_7 <==> 1 b3968_RNA_7_RsuA_mono	reversible	rRNA modification	-1.00E+09	1.00E+09	0	1.11E+02	1.11E+02	0	0	0	0	0	0
b3968_RNA_7_modif_2	b3968_RNA modification (16S rRNA): position: 516, Y by RsuA_mono	1 b3968_RNA_7_RsuA_mono --> 1 RsuA_mono_inact + 1 b3968_RNA_8	irreversible	rRNA modification	0.00E+00	1.00E+09	0	1.11E+02	1.11E+02	0	0	0	0	0	0
b3968_RNA_8_modif_1	b3968_RNA modification (16S rRNA): position: 527, m7G by MeT_16S_527	1 MeT_16S_527 + 1 amet + 1 b3968_RNA_8 <==> 1 b3968_RNA_8_MeT_16S_527	reversible	rRNA modification	-1.00E+09	1.00E+09	0	1.11E+02	1.11E+02	0	0	0	0	0	0
b3968_RNA_8_modif_2	b3968_RNA modification (16S rRNA): position: 527, m7G by MeT_16S_527	1 b3968_RNA_8_MeT_16S_527 --> 1 MeT_16S_527_inact + 1 ahcy + 1 b3968_RNA_9 + 1 h	irreversible	rRNA modification	0.00E+00	1.00E+09	0	1.11E+02	1.11E+02	0	0	0	0	0	0
b3968_RNA_9_modif_1	b3968_RNA modification (16S rRNA): position: 966, m2G by MeT_16S_966	1 MeT_16S_966 + 1 amet + 1 b3968_RNA_9 <==> 1 b3968_RNA_9_MeT_16S_966	reversible	rRNA modification	-1.00E+09	1.00E+09	0	1.11E+02	1.11E+02	0	0	0	0	0	0
b3968_RNA_9_modif_2	b3968_RNA modification (16S rRNA): position: 966, m2G by MeT_16S_966	1 b3968_RNA_9_MeT_16S_966 --> 1 MeT_16S_966_inact + 1 ahcy + 1 b3968_RNA_10 + 1 h	irreversible	rRNA modification	0.00E+00	1.00E+09	0	1.11E+02	1.11E+02	0	0	0	0	0	0
b3968_RNA_modif_1	b3968_RNA modification (16S rRNA): position: 1207, m2G by RsmC_mono	1 RsmC_mono + 1 amet + 1 b3968_RNA <==> 1 b3968_RNA_RsmC_mono	reversible	rRNA modification	-1.00E+09	1.00E+09	0	1.11E+02	1.11E+02	0	0	0	0	0	0
b3968_RNA_modif_2	b3968_RNA modification (16S rRNA): position: 1207, m2G by RsmC_mono	1 b3968_RNA_RsmC_mono --> 1 RsmC_mono_inact + 1 ahcys + 1 b3968_RNA_1 + 1 h	irreversible	rRNA modification	0.00E+00	1.00E+09	0	1.11E+02	1.11E+02	0	0	0	0	0	0
b3968_v1_RNA_10_modif _1	b3968_v1_RNA modification (16S rRNA): position: 967, m5C by RsmB_mono	1 RsmB_mono + 1 amet + 1 b3968_v1_RNA_10 <==> 1 b3968_v1_RNA_10_RsmB_mono	reversible	rRNA modification	-1.00E+09	1.00E+09	0	1.11E+02	1.11E+02	0	1.29E+01	12.9	0	1.29E+01	12.9
b3968_v1_RNA_10_modif _2	b3968_v1_RNA modification (16S rRNA): position: 967, m5C by RsmB_mono	1 b3968_v1_RNA_10_RsmB_mono --> 1 RsmB_mono_inact + 1 ahcys + 1 h + 1 rrsB	irreversible	rRNA modification	0.00E+00	1.00E+09	0	1.11E+02	1.11E+02	0	1.29E+01	12.9	0	1.29E+01	12.9
b3968_v1_RNA_1_modif_ 1	b3968_v1_RNA modification (16S rRNA): position: 1402, m4Cm by MeT_16S_1402	1 MeT_16S_1402 + 2 amet + 1 b3968_v1_RNA_1 <==> 1 b3968_v1_RNA_1_MeT_16S_1402	reversible	rRNA modification	-1.00E+09	1.00E+09	0	1.11E+02	1.11E+02	0	1.29E+01	12.9	0	1.29E+01	12.9
b3968_v1_RNA_1_modif_ 2	b3968_v1_RNA modification (16S rRNA): position: 1402, m4Cm by MeT_16S_1402	1 b3968_v1_RNA_1_MeT_16S_1402 --> 1 MeT_16S_1402_inact + 2 ahcys + 1 b3968_v1_RNA_2 + 2 h	irreversible	rRNA modification	0.00E+00	1.00E+09	0	1.11E+02	1.11E+02	0	1.29E+01	12.9	0	1.29E+01	12.9
b3968_v1_RNA_2_modif_ 1	b3968_v1_RNA modification (16S rRNA): position: 1407, m5C by MeT_16S_1407	1 MeT_16S_1407 + 1 amet + 1 b3968_v1_RNA_2 <==> 1 b3968_v1_RNA_2_MeT_16S_1407	reversible	rRNA modification	-1.00E+09	1.00E+09	0	1.11E+02	1.11E+02	0	1.29E+01	12.9	0	1.29E+01	12.9
b3968_v1_RNA_2_modif_ 2	b3968_v1_RNA modification (16S rRNA): position: 1407, m5C by MeT_16S_1407	1 b3968_v1_RNA_2_MeT_16S_1407 --> 1 MeT_16S_1407_inact + 1 ahcys + 1 b3968_v1_RNA_3 + 1 h	irreversible	rRNA modification	0.00E+00	1.00E+09	0	1.11E+02	1.11E+02	0	1.29E+01	12.9	0	1.29E+01	12.9
b3968_v1_RNA_3_modif_ 1	b3968_v1_RNA modification (16S rRNA): position: 1498, m3U by YggJ_mono	1 YggJ_mono + 1 amet + 1 b3968_v1_RNA_3 <==> 1 b3968_v1_RNA_3_YggJ_mono	reversible	rRNA modification	-1.00E+09	1.00E+09	0	1.11E+02	1.11E+02	0	1.29E+01	12.9	0	1.29E+01	12.9
b3968_v1_RNA_3_modif_ 2	b3968_v1_RNA modification (16S rRNA): position: 1498, m3U by YggJ_mono	1 b3968_v1_RNA_3_YggJ_mono --> 1 YggJ_mono_inact + 1 ahcys + 1 b3968_v1_RNA_4 + 1 h	irreversible	rRNA modification	0.00E+00	1.00E+09	0	1.11E+02	1.11E+02	0	1.29E+01	12.9	0	1.29E+01	12.9
b3968_v1_RNA_4_modif_ 1	b3968_v1_RNA modification (16S rRNA): position: 1516, m2G by MeT_16S_1516	1 MeT_16S_1516 + 1 amet + 1 b3968_v1_RNA_4 <==> 1 b3968_v1_RNA_4_MeT_16S_1516	reversible	rRNA modification	-1.00E+09	1.00E+09	0	1.11E+02	1.11E+02	0	1.29E+01	12.9	0	1.29E+01	12.9
b3968_v1_RNA_4_modif_ 2	b3968_v1_RNA modification (16S rRNA): position: 1516, m2G by MeT_16S_1516	1 b3968_v1_RNA_4_MeT_16S_1516 --> 1 MeT_16S_1516_inact + 1 ahcys + 1 b3968_v1_RNA_5 + 1 h	irreversible	rRNA modification	0.00E+00	1.00E+09	0	1.11E+02	1.11E+02	0	1.29E+01	12.9	0	1.29E+01	12.9
b3968_v1_RNA_5_modif_ 1	b3968_v1_RNA modification (16S rRNA): position: 1518, m6(2)A by KsgA_mono	1 KsgA_mono + 2 amet + 1 b3968_v1_RNA_5 <==> 1 b3968_v1_RNA_5_KsgA_mono	reversible	rRNA modification	-1.00E+09	1.00E+09	0	1.11E+02	1.11E+02	0	1.29E+01	12.9	0	1.29E+01	12.9
b3968_v1_RNA_5_modif_ 2	b3968_v1_RNA modification (16S rRNA): position: 1518, m6(2)A by KsgA_mono	1 b3968_v1_RNA_5_KsgA_mono --> 1 KsgA_mono_inact + 2 ahcys + 1 b3968_v1_RNA_6 + 2 h	irreversible	rRNA modification	0.00E+00	1.00E+09	0	1.11E+02	1.11E+02	0	1.29E+01	12.9	0	1.29E+01	12.9

b3968_v1_RNA_6_modif_1	b3968_v1_RNA modification (16S rRNA): position: 1519, m6(2)A by KsgA_mono	1 KsgA_mono + 2 amet + 1 b3968_v1_RNA_6 <==> 1 b3968_v1_RNA_6_KsgA_mono	reversible	rRNA modification	-1.00E+09	1.00E+09	0	1.11E+02	1.11E+02	0	1.29E+01	12.9	0	1.29E+01	12.9
b3968_v1_RNA_6_modif_2	b3968_v1_RNA modification (16S rRNA): position: 1519, m6(2)A by KsgA_mono	1 b3968_v1_RNA_6_KsgA_mono --> 1 KsgA_mono_inact + 2 ahcys + 1 b3968_v1_RNA_7 + 2 h	irreversible	rRNA modification	0.00E+00	1.00E+09	0	1.11E+02	1.11E+02	0	1.29E+01	12.9	0	1.29E+01	12.9
b3968_v1_RNA_7_modif_1	b3968_v1_RNA modification (16S rRNA): position: 516, Y by RsuA_mono	1 RsuA_mono + 1 b3968_v1_RNA_7 <==> 1 b3968_v1_RNA_7_RsuA_mono	reversible	rRNA modification	-1.00E+09	1.00E+09	0	1.11E+02	1.11E+02	0	1.29E+01	12.9	0	1.29E+01	12.9
b3968_v1_RNA_7_modif_2	b3968_v1_RNA modification (16S rRNA): position: 516, Y by RsuA_mono	1 b3968_v1_RNA_7_RsuA_mono --> 1 RsuA_mono_inact + 1 b3968_v1_RNA_8	irreversible	rRNA modification	0.00E+00	1.00E+09	0	1.11E+02	1.11E+02	0	1.29E+01	12.9	0	1.29E+01	12.9
b3968_v1_RNA_8_modif_1	b3968_v1_RNA modification (16S rRNA): position: 527, m7G by MeT_16S_527	1 MeT_16S_527 + 1 amet + 1 b3968_v1_RNA_8 <==> 1 b3968_v1_RNA_8_MeT_16S_527	reversible	rRNA modification	-1.00E+09	1.00E+09	0	1.11E+02	1.11E+02	0	1.29E+01	12.9	0	1.29E+01	12.9
b3968_v1_RNA_8_modif_2	b3968_v1_RNA modification (16S rRNA): position: 527, m7G by MeT_16S_527	1 b3968_v1_RNA_8_MeT_16S_527 --> 1 MeT_16S_527_inact + 1 ahcys + 1 b3968_v1_RNA_9 + 1 h	irreversible	rRNA modification	0.00E+00	1.00E+09	0	1.11E+02	1.11E+02	0	1.29E+01	12.9	0	1.29E+01	12.9
b3968_v1_RNA_9_modif_1	b3968_v1_RNA modification (16S rRNA): position: 966, m2G by MeT_16S_966	1 MeT_16S_966 + 1 amet + 1 b3968_v1_RNA_9 <==> 1 b3968_v1_RNA_9_MeT_16S_966	reversible	rRNA modification	-1.00E+09	1.00E+09	0	1.11E+02	1.11E+02	0	1.29E+01	12.9	0	1.29E+01	12.9
b3968_v1_RNA_9_modif_2	b3968_v1_RNA modification (16S rRNA): position: 966, m2G by MeT_16S_966	1 b3968_v1_RNA_9_MeT_16S_966 --> 1 MeT_16S_966_inact + 1 ahcys + 1 b3968_v1_RNA_10 + 1 h	irreversible	rRNA modification	0.00E+00	1.00E+09	0	1.11E+02	1.11E+02	0	1.29E+01	12.9	0	1.29E+01	12.9
b3968_v1_RNA_modif_1	b3968_v1_RNA modification (16S rRNA): position: 1207, m2G by RsmC_mono	1 RsmC_mono + 1 amet + 1 b3968_v1_RNA <==> 1 b3968_v1_RNA_RsmC_mono	reversible	rRNA modification	-1.00E+09	1.00E+09	0	1.11E+02	1.11E+02	0	1.29E+01	12.9	0	1.29E+01	12.9
b3968_v1_RNA_modif_2	b3968_v1_RNA modification (16S rRNA): position: 1207, m2G by RsmC_mono	1 b3968_v1_RNA_RsmC_mono --> 1 RsmC_mono_inact + 1 ahcys + 1 b3968_v1_RNA_1 + 1 h	irreversible	rRNA modification	0.00E+00	1.00E+09	0	1.11E+02	1.11E+02	0	1.29E+01	12.9	0	1.29E+01	12.9
b3969_tRNA_13_MODIF_1	modification of position 13 nt (Y) in gltT by TruD_mono	1 TruD_mono + 1 b3969_tRNA_Mg2 <==> 1 b3969_tRNA_Mg2_TruD_mono_cplx	reversible	tRNA modification	-1.00E+09	1.00E+09	0	1.11E+02	1.11E+02	0	0	0	0	0	0
b3969_tRNA_13_MODIF_2	modification of position 13 nt (Y) in gltT by TruD_mono	1 b3969_tRNA_Mg2_TruD_mono_cplx --> 1 TruD_mono_inact + 1 b3969_tRNA_1	irreversible	tRNA modification	0.00E+00	1.00E+09	0	1.11E+02	1.11E+02	0	0	0	0	0	0
b3969_tRNA_34_MODIF_1	modification of position 34 nt (mnm5s2U) in gltT by TrmU_mono-YhhP_mono-YheLMN_cplx-YccK_mono-TrmE_dim-GidA_mono-TrmC_mono	1 5fthf + 1 GidA_mono + 1 IscS_dim_S-SH + 1 TrmC_mono + 1 TrmE_dim + 1 TrmU_mono + 1 YccK_mono + 1 YheLMN_cplx + 1 YhhP_mono + 1 amet + 1 atp + 1 b3969_tRNA_1 + 1 fad + 1 fadh2 + 1 gly + 1 gtp + 1 h2o + 1 mg2 + 1 trdrd <==> 1 b3969_tRNA_1_TrmU_mono-YhhP_mono-YheLMN_cplx-YccK_mono-TrmE_dim-GidA_mono-TrmC_mono_cplx	reversible	tRNA modification	-1.00E+09	1.00E+09	0	1.11E+02	1.11E+02	0	0	0	0	0	0
b3969_tRNA_34_MODIF_2	modification of position 34 nt (mnm5s2U) in gltT by TrmU_mono-YhhP_mono-YheLMN_cplx-YccK_mono-TrmE_dim-GidA_mono-TrmC_mono	1 b3969_tRNA_1_TrmU_mono-YhhP_mono-YheLMN_cplx-YccK_mono-TrmE_dim-GidA_mono-TrmC_mono_cplx --> 1 GidA_mono_inact + 1 IscS_dim_S-H + 1 TrmC_mono_inact + 1 TrmE_dim_inact + 1 TrmU_mono_inact + 1 YccK_mono_inact + 1 YheLMN_cplx_inact + 1 YhhP_mono_inact + 1 ahcys + 1 amp + 1 b3969_tRNA_2 + 1 fad + 1 fadh2 + 1 gdp + 1 glx + 4 h + 1 mg2 + 1 pi + 1 ppi + 1 thf + 1 trdox	irreversible	tRNA modification	0.00E+00	1.00E+09	0	1.11E+02	1.11E+02	0	0	0	0	0	0
b3969_tRNA_34_MODIF_3	replacement of sulfur with selenium: b3969_tRNA_2	1 YbbB_dim + 1 b3969_tRNA_2 + 1 h2o + 1 selnp <==> 1 b3969_tRNA_2_YbbB_dim_cplx	reversible	tRNA modification	-1.00E+09	1.00E+09	0	1.11E+02	1.11E+02	0	0	0	0	0	0
b3969_tRNA_34_MODIF_4	replacement of sulfur with selenium: b3969_tRNA_2	1 b3969_tRNA_2_YbbB_dim_cplx --> 1 YbbB_dim_inact + 1 b3969_tRNA_2_Se + 1 h + 1 h2s + 1 pi	irreversible	tRNA modification	0.00E+00	1.00E+09	0	1.11E+02	1.11E+02	0	0	0	0	0	0
b3969_tRNA_37_MODIF_1	modification of position 37 nt (m2A) in gltT by MeT_tRNA_pos_37_m2A	1 MeT_tRNA_pos_37_m2A + 1 amet + 1 b3969_tRNA_2 <==> 1 b3969_tRNA_2_MeT_tRNA_pos_37_m2A_cplx	reversible	tRNA modification	-1.00E+09	1.00E+09	0	1.11E+02	1.11E+02	0	0	0	0	0	0
b3969_tRNA_37_MODIF_1_Se	modification of position 37 nt (m2A) in gltT by MeT_tRNA_pos_37_m2A	1 MeT_tRNA_pos_37_m2A + 1 amet + 1 b3969_tRNA_2_Se <==> 1 b3969_tRNA_2_Se_MeT_tRNA_pos_37_m2A_cplx	reversible	tRNA modification	-1.00E+09	1.00E+09	0	1.11E+02	1.11E+02	0	0	0	0	0	0
b3969_tRNA_37_MODIF_2	modification of position 37 nt (m2A) in gltT by MeT_tRNA_pos_37_m2A	1 b3969_tRNA_2_MeT_tRNA_pos_37_m2A_cplx --> 1 MeT_tRNA_pos_37_m2A_inact + 1 ahcys + 1 b3969_tRNA_3 + 1 h	irreversible	tRNA modification	0.00E+00	1.00E+09	0	1.11E+02	1.11E+02	0	0	0	0	0	0
b3969_tRNA_37_MODIF_2_Se	modification of position 37 nt (m2A) in gltT by MeT_tRNA_pos_37_m2A	1 b3969_tRNA_2_Se_MeT_tRNA_pos_37_m2A_cplx --> 1 MeT_tRNA_pos_37_m2A_inact + 1 ahcys + 1 b3969_tRNA_3_Se + h	irreversible	tRNA modification	0.00E+00	1.00E+09	0	1.11E+02	1.11E+02	0	0	0	0	0	0
b3969_tRNA_54_MODIF_1	modification of position 54 nt (m5U) in gltT by TrmA_mono	1 TrmA_mono + 1 amet + 1 b3969_tRNA_3 <==> 1 b3969_tRNA_3_TrmA_mono_cplx	reversible	tRNA modification	-1.00E+09	1.00E+09	0	1.11E+02	1.11E+02	0	0	0	0	0	0
b3969_tRNA_54_MODIF_1_Se	modification of position 54 nt (m5U) in gltT by TrmA_mono	1 TrmA_mono + 1 amet + 1 b3969_tRNA_3_Se <==> 1 b3969_tRNA_3_Se_TrmA_mono_cplx	reversible	tRNA modification	-1.00E+09	1.00E+09	0	1.11E+02	1.11E+02	0	0	0	0	0	0
b3969_tRNA_54_MODIF_2	modification of position 54 nt (m5U) in gltT by TrmA_mono	1 b3969_tRNA_3_TrmA_mono_cplx --> 1 TrmA_mono_inact + 1 ahcys + 1 b3969_tRNA_4 + 1 h	irreversible	tRNA modification	0.00E+00	1.00E+09	0	1.11E+02	1.11E+02	0	0	0	0	0	0
b3969_tRNA_54_MODIF_2_Se	modification of position 54 nt (m5U) in gltT by TrmA_mono	1 b3969_tRNA_3_Se_TrmA_mono_cplx --> 1 TrmA_mono_inact + 1 ahcys + 1 b3969_tRNA_4_Se + 1 h	irreversible	tRNA modification	0.00E+00	1.00E+09	0	1.11E+02	1.11E+02	0	0	0	0	0	0
b3969_tRNA_55_MODIF_1	modification of position 55 nt (Y) in gltT by TruB_mono	1 TruB_mono + 1 b3969_tRNA_4 <==> 1 b3969_tRNA_4_TruB_mono_cplx	reversible	tRNA modification	-1.00E+09	1.00E+09	0	1.11E+02	1.11E+02	0	0	0	0	0	0
b3969_tRNA_55_MODIF_1_Se	modification of position 55 nt (Y) in gltT by TruB_mono	1 TruB_mono + 1 b3969_tRNA_4_Se <==> 1 b3969_tRNA_4_Se_TruB_mono_cplx	reversible	tRNA modification	-1.00E+09	1.00E+09	0	1.11E+02	1.11E+02	0	0	0	0	0	0
b3969_tRNA_55_MODIF_2	modification of position 55 nt (Y) in gltT by TruB_mono	1 b3969_tRNA_4_TruB_mono_cplx --> 1 TruB_mono_inact + 1 gltT_tRNA	irreversible	tRNA modification	0.00E+00	1.00E+09	0	1.11E+02	1.11E+02	0	0	0	0	0	0
b3969_tRNA_55_MODIF_2_Se	modification of position 55 nt (Y) in gltT by TruB_mono	1 b3969_tRNA_4_Se_TruB_mono_cplx --> 1 TruB_mono_inact + 1 gltT_tRNA_Se	irreversible	tRNA modification	0.00E+00	1.00E+09	0	1.11E+02	1.11E+02	0	0	0	0	0	0
b3969_tRNA_Mg_BIND	Binding of two Mg2 to b3969_tRNA	1 b3969_RNA + 2 mg2 <==> 1 b3969_tRNA_Mg2	reversible	Metallo-ion binding	-1.00E+09	1.00E+09	0	1.11E+02	1.11E+02	0	0	0	0	0	0
b3969_v1_tRNA_13_MODIF_1	modification of position 13 nt (Y) in gltT by TruD_mono	1 TruD_mono + 1 b3969_v1_tRNA_Mg2 <==> 1 b3969_v1_tRNA_Mg2_TruD_mono_cplx	reversible	tRNA modification	-1.00E+09	1.00E+09	0	1.11E+02	1.11E+02	0	1.29E+01	12.9	0	1.29E+01	12.9
b3969_v1_tRNA_13_MODIF_2	modification of position 13 nt (Y) in gltT by TruD_mono	1 b3969_v1_tRNA_Mg2_TruD_mono_cplx --> 1 TruD_mono_inact + b3969_v1_tRNA_1	irreversible	tRNA modification	0.00E+00	1.00E+09	0	1.11E+02	1.11E+02	0	1.29E+01	12.9	0	1.29E+01	12.9
b3969_v1_tRNA_34_MODIF_1	modification of position 34 nt (mnm5s2U) in gltT by TrmU_mono-YhhP_mono-YheLMN_cplx-YccK_mono-TrmE_dim-GidA_mono-TrmC_mono	1 5fthf + 1 GidA_mono + 1 IscS_dim_S-SH + 1 TrmC_mono + 1 TrmE_dim + 1 TrmU_mono + 1 YccK_mono + 1 YheLMN_cplx + 1 YhhP_mono + 1 amet + 1 atp + 1 b3969_v1_tRNA_1 + 1 fad + 1 fadh2 + 1 gly + 1 gtp + 1 h2o + 1 mg2 + 1 trdrd <==> 1 b3969_v1_tRNA_1_TrmU_mono-YhhP_mono-YheLMN_cplx-YccK_mono-TrmE_dim-GidA_mono-TrmC_mono_cplx	reversible	tRNA modification	-1.00E+09	1.00E+09	0	1.11E+02	1.11E+02	0	1.29E+01	12.9	0	1.29E+01	12.9
b3969_v1_tRNA_34_MODIF_2	modification of position 34 nt (mnm5s2U) in gltT by TrmU_mono-YhhP_mono-YheLMN_cplx-YccK_mono-TrmE_dim-GidA_mono-TrmC_mono	1 b3969_v1_tRNA_1_TrmU_mono-YhhP_mono-YheLMN_cplx-YccK_mono-TrmE_dim-GidA_mono-TrmC_mono_cplx --> 1 GidA_mono_inact + 1 IscS_dim_S-H + 1 TrmC_mono_inact + 1 TrmE_dim_inact + 1 TrmU_mono_inact + 1 YccK_mono_inact + 1 YheLMN_cplx_inact + 1 YhhP_mono_inact + 1 ahcys + 1 amp + 1 b3969_v1_tRNA_2 + 1 fad + 1 fadh2 + 1 gdp + 1 glx + 4 h + 1 mg2 + 1 pi + 1 ppi + 1 thf + 1 trdox	irreversible	tRNA modification	0.00E+00	1.00E+09	0	1.11E+02	1.11E+02	0	1.29E+01	12.9	0	1.29E+01	12.9
b3969_v1_tRNA_34_MODIF_3	replacement of sulfur with selenium: b3969_tRNA_2	1 YbbB_dim + 1 b3969_v1_tRNA_2 + 1 h2o + 1 selnp <==> 1 b3969_v1_tRNA_2_YbbB_dim_cplx	reversible	tRNA modification	-1.00E+09	1.00E+09	0	1.11E+02	1.11E+02	0	1.29E+01	12.9	0	1.29E+01	12.9
b3969_v1_tRNA_34_MODIF_4	replacement of sulfur with selenium: b3969_tRNA_2	1 b3969_v1_tRNA_2_YbbB_dim_cplx --> 1 YbbB_dim_inact + 1 b3969_v1_tRNA_2_Se + 1 h + 1 h2s + 1 pi	irreversible	tRNA modification	0.00E+00	1.00E+09	0	1.11E+02	1.11E+02	0	1.29E+01	12.9	0	1.29E+01	12.9
b3969_v1_tRNA_37_MODIF_1	modification of position 37 nt (m2A) in gltT by MeT_tRNA_pos_37_m2A	1 MeT_tRNA_pos_37_m2A + 1 amet + 1 b3969_v1_tRNA_2 <==> 1 b3969_v1_tRNA_2_MeT_tRNA_pos_37_m2A_cplx	reversible	tRNA modification	-1.00E+09	1.00E+09	0	1.11E+02	1.11E+02	0	1.29E+01	12.9	0	1.29E+01	12.9
b3969_v1_tRNA_37_MODIF_1_Se	modification of position 37 nt (m2A) in gltT by MeT_tRNA_pos_37_m2A	1 MeT_tRNA_pos_37_m2A + 1 amet + 1 b3969_v1_tRNA_2_Se <==> 1 b3969_v1_tRNA_2_Se_MeT_tRNA_pos_37_m2A_cplx	reversible	tRNA modification	-1.00E+09	1.00E+09	0	1.11E+02	1.11E+02	0	1.29E+01	12.9	0	1.29E+01	12.9
b3969_v1_tRNA_37_MODIF_2	modification of position 37 nt (m2A) in gltT by MeT_tRNA_pos_37_m2A	1 b3969_v1_tRNA_2_MeT_tRNA_pos_37_m2A_cplx --> 1 MeT_tRNA_pos_37_m2A_inact + 1 ahcys + 1 b3969_v1_tRNA_3 + 1 h	irreversible	tRNA modification	0.00E+00	1.00E+09	0	1.11E+02	1.11E+02	0	1.29E+01	12.9	0	1.29E+01	12.9

b3969_v1_tRNA_37_MODIF_2_Se	modification of position 37 nt (m2A) in gltT by MeT_tRNA_pos_37_m2A	1 b3969_v1_tRNA_2_Se_MeT_tRNA_pos_37_m2A_cplx --> 1 MeT_tRNA_pos_37_m2A_inact + 1 ahcys + 1 b3969_v1_tRNA_3_Se + 1 h	irreversible	tRNA modification	0.00E+00	1.00E+09	0	1.11E+02	1.11E+02	0	1.29E+01	12.9	0	1.29E+01	12.9
b3969_v1_tRNA_54_MODIF_1	modification of position 54 nt (m5U) in gltT by TrmA_mono	1 TrmA_mono + 1 amet + 1 b3969_v1_tRNA_3 <==> 1 b3969_v1_tRNA_3_TrmA_mono_cplx	reversible	tRNA modification	-1.00E+09	1.00E+09	0	1.11E+02	1.11E+02	0	1.29E+01	12.9	0	1.29E+01	12.9
b3969_v1_tRNA_54_MODIF_1_Se	modification of position 54 nt (m5U) in gltT by TrmA_mono	1 TrmA_mono + 1 amet + 1 b3969_v1_tRNA_3_Se <==> 1 b3969_v1_tRNA_3_Se_TrmA_mono_cplx	reversible	tRNA modification	-1.00E+09	1.00E+09	0	1.11E+02	1.11E+02	0	1.29E+01	12.9	0	1.29E+01	12.9
b3969_v1_tRNA_54_MODIF_2	modification of position 54 nt (m5U) in gltT by TrmA_mono	1 b3969_v1_tRNA_3_TrmA_mono_cplx --> 1 TrmA_mono_inact + 1 ahcys + 1 b3969_v1_tRNA_4 + 1 h	irreversible	tRNA modification	0.00E+00	1.00E+09	0	1.11E+02	1.11E+02	0	1.29E+01	12.9	0	1.29E+01	12.9
b3969_v1_tRNA_54_MODIF_2_Se	modification of position 54 nt (m5U) in gltT by TrmA_mono	1 b3969_v1_tRNA_3_Se_TrmA_mono_cplx --> 1 TrmA_mono_inact + 1 ahcys + 1 b3969_v1_tRNA_4_Se + 1 h	irreversible	tRNA modification	0.00E+00	1.00E+09	0	1.11E+02	1.11E+02	0	1.29E+01	12.9	0	1.29E+01	12.9
b3969_v1_tRNA_55_MODIF_1	modification of position 55 nt (Y) in gltT by TruB_mono	1 TruB_mono + 1 b3969_v1_tRNA_4 <==> 1 b3969_v1_tRNA_4_TruB_mono_cplx	reversible	tRNA modification	-1.00E+09	1.00E+09	0	1.11E+02	1.11E+02	0	1.29E+01	12.9	0	1.29E+01	12.9
b3969_v1_tRNA_55_MODIF_1_Se	modification of position 55 nt (Y) in gltT by TruB_mono	1 TruB_mono + 1 b3969_v1_tRNA_4_Se <==> 1 b3969_v1_tRNA_4_Se_TruB_mono_cplx	reversible	tRNA modification	-1.00E+09	1.00E+09	0	1.11E+02	1.11E+02	0	1.29E+01	12.9	0	1.29E+01	12.9
b3969_v1_tRNA_55_MODIF_2	modification of position 55 nt (Y) in gltT by TruB_mono	1 b3969_v1_tRNA_4_TruB_mono_cplx --> 1 TruB_mono_inact + 1 gltT_tRNA	irreversible	tRNA modification	0.00E+00	1.00E+09	0	1.11E+02	1.11E+02	0	1.29E+01	12.9	0	1.29E+01	12.9
b3969_v1_tRNA_55_MODIF_2_Se	modification of position 55 nt (Y) in gltT by TruB_mono	1 b3969_v1_tRNA_4_Se_TruB_mono_cplx --> 1 TruB_mono_inact + 1 gltT_tRNA_Se	irreversible	tRNA modification	0.00E+00	1.00E+09	0	1.11E+02	1.11E+02	0	1.29E+01	12.9	0	1.29E+01	12.9
b3969_v1_tRNA_Mg_BIND	Binding of two Mg2 to b3969_v1_tRNA	1 b3969_v1_RNA + 2 mg2 <==> 1 b3969_v1_tRNA_Mg2	reversible	Metallo-ion binding	-1.00E+09	1.00E+09	0	1.11E+02	1.11E+02	0	1.29E+01	12.9	0	1.29E+01	12.9
b3970_RNA_10_modif_1	b3970_RNA modification (23S rRNA): position: 2445, m2G by MeT_23S_2445	1 MeT_23S_2445 + 1 amet + 1 b3970_RNA_10 <==> 1 b3970_RNA_10_MeT_23S_2445	reversible	rRNA modification	-1.00E+09	1.00E+09	0	1.11E+02	1.11E+02	0	0	0	0	0	0
b3970_RNA_10_modif_2	b3970_RNA modification (23S rRNA): position: 2445, m2G by MeT_23S_2445	1 b3970_RNA_10_MeT_23S_2445 --> 1 MeT_23S_2445_inact + 1 ahcys + 1 b3970_RNA_11 + 1 h	irreversible	rRNA modification	0.00E+00	1.00E+09	0	1.11E+02	1.11E+02	0	0	0	0	0	0
b3970_RNA_11_a_modif_1	b3970_RNA modification (23S rRNA): position: 2449, D by DU_23S_2449 (NADH)	1 DU_23S_2449 + 1 b3970_RNA_11 + 1 h + 1 nadh <==> 1 b3970_RNA_11_DU_23S_2449_a	reversible	rRNA modification	-1.00E+09	1.00E+09	0	1.11E+02	1.11E+02	0	0	0	0	0	0
b3970_RNA_11_a_modif_2	b3970_RNA modification (23S rRNA): position: 2449, D by DU_23S_2449 (NADH)	1 b3970_RNA_11_DU_23S_2449_a --> 1 DU_23S_2449_inact + 1 b3970_RNA_12 + 1 nad	irreversible	rRNA modification	0.00E+00	1.00E+09	0	1.11E+02	1.11E+02	0	0	0	0	0	0
b3970_RNA_11_b_modif_1	b3970_RNA modification (23S rRNA): position: 2449, D by DU_23S_2449 (NADPH)	1 DU_23S_2449 + 1 b3970_RNA_11 + 1 h + 1 nadph <==> 1 b3970_RNA_11_DU_23S_2449_b	reversible	rRNA modification	-1.00E+09	1.00E+09	0	1.11E+02	1.11E+02	0	0	0	0	0	0
b3970_RNA_11_b_modif_2	b3970_RNA modification (23S rRNA): position: 2449, D by DU_23S_2449 (NADPH)	1 b3970_RNA_11_DU_23S_2449_b --> 1 DU_23S_2449_inact + 1 b3970_RNA_12 + 1 nadp	irreversible	rRNA modification	0.00E+00	1.00E+09	0	1.11E+02	1.11E+02	0	0	0	0	0	0
b3970_RNA_12_modif_1	b3970_RNA modification (23S rRNA): position: 2457, Y by YmfC_mono	1 YmfC_mono + 1 b3970_RNA_12 <==> 1 b3970_RNA_12_YmfC_mono	reversible	rRNA modification	-1.00E+09	1.00E+09	0	1.11E+02	1.11E+02	0	0	0	0	0	0
b3970_RNA_12_modif_2	b3970_RNA modification (23S rRNA): position: 2457, Y by YmfC_mono	1 b3970_RNA_12_YmfC_mono --> 1 YmfC_mono_inact + 1 b3970_RNA_13	irreversible	rRNA modification	0.00E+00	1.00E+09	0	1.11E+02	1.11E+02	0	0	0	0	0	0
b3970_RNA_13_modif_1	b3970_RNA modification (23S rRNA): position: 2498, Cm by MeT_23S_2498	1 MeT_23S_2498 + 1 amet + 1 b3970_RNA_13 <==> 1 b3970_RNA_13_MeT_23S_2498	reversible	rRNA modification	-1.00E+09	1.00E+09	0	1.11E+02	1.11E+02	0	0	0	0	0	0
b3970_RNA_13_modif_2	b3970_RNA modification (23S rRNA): position: 2498, Cm by MeT_23S_2498	1 b3970_RNA_13_MeT_23S_2498 --> 1 MeT_23S_2498_inact + 1 ahcys + 1 b3970_RNA_14 + 1 h	irreversible	rRNA modification	0.00E+00	1.00E+09	0	1.11E+02	1.11E+02	0	0	0	0	0	0
b3970_RNA_14_modif_1	b3970_RNA modification (23S rRNA): position: 2503, m2A by MeT_23S_2503	1 MeT_23S_2503 + 1 amet + 1 b3970_RNA_14 <==> 1 b3970_RNA_14_MeT_23S_2503	reversible	rRNA modification	-1.00E+09	1.00E+09	0	1.11E+02	1.11E+02	0	0	0	0	0	0
b3970_RNA_14_modif_2	b3970_RNA modification (23S rRNA): position: 2503, m2A by MeT_23S_2503	1 b3970_RNA_14_MeT_23S_2503 --> 1 MeT_23S_2503_inact + 1 ahcys + 1 b3970_RNA_15 + 1 h	irreversible	rRNA modification	0.00E+00	1.00E+09	0	1.11E+02	1.11E+02	0	0	0	0	0	0
b3970_RNA_15_modif_1	b3970_RNA modification (23S rRNA): position: 2504, Y by RluC_mono	1 RluC_mono + 1 b3970_RNA_15 <==> 1 b3970_RNA_15_RluC_mono	reversible	rRNA modification	-1.00E+09	1.00E+09	0	1.11E+02	1.11E+02	0	0	0	0	0	0
b3970_RNA_15_modif_2	b3970_RNA modification (23S rRNA): position: 2504, Y by RluC_mono	1 b3970_RNA_15_RluC_mono --> 1 RluC_mono_inact + 1 b3970_RNA_16	irreversible	rRNA modification	0.00E+00	1.00E+09	0	1.11E+02	1.11E+02	0	0	0	0	0	0
b3970_RNA_16_modif_1	b3970_RNA modification (23S rRNA): position: 2552, Um by RrmJ_mono	1 RrmJ_mono + 1 amet + 1 b3970_RNA_16 <==> 1 b3970_RNA_16_RrmJ_mono	reversible	rRNA modification	-1.00E+09	1.00E+09	0	1.11E+02	1.11E+02	0	0	0	0	0	0
b3970_RNA_16_modif_2	b3970_RNA modification (23S rRNA): position: 2552, Um by RrmJ_mono	1 b3970_RNA_16_RrmJ_mono --> 1 RrmJ_mono_inact + 1 ahcys + 1 b3970_RNA_17 + 1 h	irreversible	rRNA modification	0.00E+00	1.00E+09	0	1.11E+02	1.11E+02	0	0	0	0	0	0
b3970_RNA_17_modif_1	b3970_RNA modification (23S rRNA): position: 2580, Y by RluC_mono	1 RluC_mono + 1 b3970_RNA_17 <==> 1 b3970_RNA_17_RluC_mono	reversible	rRNA modification	-1.00E+09	1.00E+09	0	1.11E+02	1.11E+02	0	0	0	0	0	0
b3970_RNA_17_modif_2	b3970_RNA modification (23S rRNA): position: 2580, Y by RluC_mono	1 b3970_RNA_17_RluC_mono --> 1 RluC_mono_inact + 1 b3970_RNA_18	irreversible	rRNA modification	0.00E+00	1.00E+09	0	1.11E+02	1.11E+02	0	0	0	0	0	0
b3970_RNA_18_modif_1	b3970_RNA modification (23S rRNA): position: 2604, Y by YjbC_mono	1 YjbC_mono + 1 b3970_RNA_18 <==> 1 b3970_RNA_18_YjbC_mono	reversible	rRNA modification	-1.00E+09	1.00E+09	0	1.11E+02	1.11E+02	0	0	0	0	0	0
b3970_RNA_18_modif_2	b3970_RNA modification (23S rRNA): position: 2604, Y by YjbC_mono	1 b3970_RNA_18_YjbC_mono --> 1 YjbC_mono_inact + 1 b3970_RNA_19	irreversible	rRNA modification	0.00E+00	1.00E+09	0	1.11E+02	1.11E+02	0	0	0	0	0	0
b3970_RNA_19_modif_1	b3970_RNA modification (23S rRNA): position: 2605, Y by RluB_mono	1 RluB_mono + 1 b3970_RNA_19 <==> 1 b3970_RNA_19_RluB_mono	reversible	rRNA modification	-1.00E+09	1.00E+09	0	1.11E+02	1.11E+02	0	0	0	0	0	0
b3970_RNA_19_modif_2	b3970_RNA modification (23S rRNA): position: 2605, Y by RluB_mono	1 b3970_RNA_19_RluB_mono --> 1 RluB_mono_inact + 1 b3970_RNA_20	irreversible	rRNA modification	0.00E+00	1.00E+09	0	1.11E+02	1.11E+02	0	0	0	0	0	0
b3970_RNA_1_modif_1	b3970_RNA modification (23S rRNA): position: 1835, m2G by MeT_23S_1835	1 MeT_23S_1835 + 1 amet + 1 b3970_RNA_1 <==> 1 b3970_RNA_1_MeT_23S_1835	reversible	rRNA modification	-1.00E+09	1.00E+09	0	1.11E+02	1.11E+02	0	0	0	0	0	0
b3970_RNA_1_modif_2	b3970_RNA modification (23S rRNA): position: 1835, m2G by MeT_23S_1835	1 b3970_RNA_1_MeT_23S_1835 --> 1 MeT_23S_1835_inact + 1 ahcys + 1 b3970_RNA_2 + 1 h	irreversible	rRNA modification	0.00E+00	1.00E+09	0	1.11E+02	1.11E+02	0	0	0	0	0	0
b3970_RNA_20_modif_1	b3970_RNA modification (23S rRNA): position: 745, m1G by RrmA_dim	1 RrmA_dim + 1 amet + 1 b3970_RNA_20 <==> 1 b3970_RNA_20_RrmA_dim	reversible	rRNA modification	-1.00E+09	1.00E+09	0	1.11E+02	1.11E+02	0	0	0	0	0	0
b3970_RNA_20_modif_2	b3970_RNA modification (23S rRNA): position: 745, m1G by RrmA_dim	1 b3970_RNA_20_RrmA_dim --> 1 RrmA_dim_inact + 1 ahcys + 1 b3970_RNA_21 + 1 h	irreversible	rRNA modification	0.00E+00	1.00E+09	0	1.11E+02	1.11E+02	0	0	0	0	0	0
b3970_RNA_21_modif_1	b3970_RNA modification (23S rRNA): position: 746, Y by RluA_mono	1 RluA_mono + 1 b3970_RNA_21 <==> 1 b3970_RNA_21_RluA_mono	reversible	rRNA modification	-1.00E+09	1.00E+09	0	1.11E+02	1.11E+02	0	0	0	0	0	0
b3970_RNA_21_modif_2	b3970_RNA modification (23S rRNA): position: 746, Y by RluA_mono	1 b3970_RNA_21_RluA_mono --> 1 RluA_mono_inact + 1 b3970_RNA_22	irreversible	rRNA modification	0.00E+00	1.00E+09	0	1.11E+02	1.11E+02	0	0	0	0	0	0
b3970_RNA_22_modif_1	b3970_RNA modification (23S rRNA): position: 747, m5U by RumB_mono	1 RumB_mono + 1 amet + 1 b3970_RNA_22 <==> 1 b3970_RNA_22_RumB_mono	reversible	rRNA modification	-1.00E+09	1.00E+09	0	1.11E+02	1.11E+02	0	0	0	0	0	0
b3970_RNA_22_modif_2	b3970_RNA modification (23S rRNA): position: 747, m5U by RumB_mono	1 b3970_RNA_22_RumB_mono --> 1 RumB_mono_inact + 1 ahcys + 1 b3970_RNA_23 + 1 h	irreversible	rRNA modification	0.00E+00	1.00E+09	0	1.11E+02	1.11E+02	0	0	0	0	0	0
b3970_RNA_23_modif_1	b3970_RNA modification (23S rRNA): position: 955, Y by RluC_mono	1 RluC_mono + 1 b3970_RNA_23 <==> 1 b3970_RNA_23_RluC_mono	reversible	rRNA modification	-1.00E+09	1.00E+09	0	1.11E+02	1.11E+02	0	0	0	0	0	0

b3970_RNA_23_modif_2	b3970_RNA modification (23S rRNA): position: 955, Y by RluC_mono	1 b3970_RNA_23_RluC_mono --> 1 RluC_mono_inact + 1 rrlB	irreversible	rRNA modification	0.00E+00	1.00E+09	0	1.11E+02	1.11E+02	0	0	0	0	0	0
b3970_RNA_2_modif_1	b3970_RNA modification (23S rRNA): position: 1911, Y by RluD_mono	1 RluD_mono + 1 b3970_RNA_2 <==> 1 b3970_RNA_2_RluD_mono	reversible	rRNA modification	-1.00E+09	1.00E+09	0	1.11E+02	1.11E+02	0	0	0	0	0	0
b3970_RNA_2_modif_2	b3970_RNA modification (23S rRNA): position: 1911, Y by RluD_mono	1 b3970_RNA_2_RluD_mono --> 1 RluD_mono_inact + 1 b3970_RNA_3	irreversible	rRNA modification	0.00E+00	1.00E+09	0	1.11E+02	1.11E+02	0	0	0	0	0	0
b3970_RNA_3_modif_1	b3970_RNA modification (23S rRNA): position: 1915, m3Y by RluD_mono	1 RluD_mono + 1 amet + 1 b3970_RNA_3 <==> 1 b3970_RNA_3_RluD_mono	reversible	rRNA modification	-1.00E+09	1.00E+09	0	1.11E+02	1.11E+02	0	0	0	0	0	0
b3970_RNA_3_modif_2	b3970_RNA modification (23S rRNA): position: 1915, m3Y by RluD_mono	1 b3970_RNA_3_RluD_mono --> 1 RluD_mono_inact + 1 ahcys + 1 b3970_RNA_4 + 1 h	irreversible	rRNA modification	0.00E+00	1.00E+09	0	1.11E+02	1.11E+02	0	0	0	0	0	0
b3970_RNA_4_modif_1	b3970_RNA modification (23S rRNA): position: 1917, Y by RluD_mono	1 RluD_mono + 1 b3970_RNA_4 <==> 1 b3970_RNA_4_RluD_mono	reversible	rRNA modification	-1.00E+09	1.00E+09	0	1.11E+02	1.11E+02	0	0	0	0	0	0
b3970_RNA_4_modif_2	b3970_RNA modification (23S rRNA): position: 1917, Y by RluD_mono	1 b3970_RNA_4_RluD_mono --> 1 RluD_mono_inact + 1 b3970_RNA_5	irreversible	rRNA modification	0.00E+00	1.00E+09	0	1.11E+02	1.11E+02	0	0	0	0	0	0
b3970_RNA_5_modif_1	b3970_RNA modification (23S rRNA): position: 1939, m5U by RumA_mono	1 RumA_mono + 1 amet + 1 b3970_RNA_5 <==> 1 b3970_RNA_5_RumA_mono	reversible	rRNA modification	-1.00E+09	1.00E+09	0	1.11E+02	1.11E+02	0	0	0	0	0	0
b3970_RNA_5_modif_2	b3970_RNA modification (23S rRNA): position: 1939, m5U by RumA_mono	1 b3970_RNA_5_RumA_mono --> 1 RumA_mono_inact + 1 ahcys + 1 b3970_RNA_6 + 1 h	irreversible	rRNA modification	0.00E+00	1.00E+09	0	1.11E+02	1.11E+02	0	0	0	0	0	0
b3970_RNA_6_modif_1	b3970_RNA modification (23S rRNA): position: 1962, m5C by MeT_23S_1962	1 MeT_23S_1962 + 1 amet + 1 b3970_RNA_6 <==> 1 b3970_RNA_6_MeT_23S_1962	reversible	rRNA modification	-1.00E+09	1.00E+09	0	1.11E+02	1.11E+02	0	0	0	0	0	0
b3970_RNA_6_modif_2	b3970_RNA modification (23S rRNA): position: 1962, m5C by MeT_23S_1962	1 b3970_RNA_6_MeT_23S_1962 --> 1 MeT_23S_1962_inact + 1 ahcys + 1 b3970_RNA_7 + 1 h	irreversible	rRNA modification	0.00E+00	1.00E+09	0	1.11E+02	1.11E+02	0	0	0	0	0	0
b3970_RNA_7_modif_1	b3970_RNA modification (23S rRNA): position: 2030, m6A by MeT_23S_2030	1 MeT_23S_2030 + 1 amet + 1 b3970_RNA_7 <==> 1 b3970_RNA_7_MeT_23S_2030	reversible	rRNA modification	-1.00E+09	1.00E+09	0	1.11E+02	1.11E+02	0	0	0	0	0	0
b3970_RNA_7_modif_2	b3970_RNA modification (23S rRNA): position: 2030, m6A by MeT_23S_2030	1 b3970_RNA_7_MeT_23S_2030 --> 1 MeT_23S_2030_inact + 1 ahcys + 1 b3970_RNA_8 + 1 h	irreversible	rRNA modification	0.00E+00	1.00E+09	0	1.11E+02	1.11E+02	0	0	0	0	0	0
b3970_RNA_8_modif_1	b3970_RNA modification (23S rRNA): position: 2069, m7G by MeT_23S_2069	1 MeT_23S_2069 + 1 amet + 1 b3970_RNA_8 <==> 1 b3970_RNA_8_MeT_23S_2069	reversible	rRNA modification	-1.00E+09	1.00E+09	0	1.11E+02	1.11E+02	0	0	0	0	0	0
b3970_RNA_8_modif_2	b3970_RNA modification (23S rRNA): position: 2069, m7G by MeT_23S_2069	1 b3970_RNA_8_MeT_23S_2069 --> 1 MeT_23S_2069_inact + 1 ahcys + 1 b3970_RNA_9 + 1 h	irreversible	rRNA modification	0.00E+00	1.00E+09	0	1.11E+02	1.11E+02	0	0	0	0	0	0
b3970_RNA_9_modif_1	b3970_RNA modification (23S rRNA): position: 2251, Gm by RlmB_dim	1 RlmB_dim + 1 amet + 1 b3970_RNA_9 <==> 1 b3970_RNA_9_RlmB_dim	reversible	rRNA modification	-1.00E+09	1.00E+09	0	1.11E+02	1.11E+02	0	0	0	0	0	0
b3970_RNA_9_modif_2	b3970_RNA modification (23S rRNA): position: 2251, Gm by RlmB_dim	1 b3970_RNA_9_RlmB_dim --> 1 RlmB_dim_inact + 1 ahcys + 1 b3970_RNA_10 + 1 h	irreversible	rRNA modification	0.00E+00	1.00E+09	0	1.11E+02	1.11E+02	0	0	0	0	0	0
b3970_RNA_modif_1	b3970_RNA modification (23S rRNA): position: 1618, m6A by MeT_23S_1618	1 MeT_23S_1618 + 1 amet + 1 b3970_RNA <==> 1 b3970_RNA_MeT_23S_1618	reversible	rRNA modification	-1.00E+09	1.00E+09	0	1.11E+02	1.11E+02	0	0	0	0	0	0
b3970_RNA_modif_2	b3970_RNA modification (23S rRNA): position: 1618, m6A by MeT_23S_1618	1 b3970_RNA_MeT_23S_1618 --> 1 MeT_23S_1618_inact + 1 ahcy + 1 b3970_RNA_1 + 1 h	irreversible	rRNA modification	0.00E+00	1.00E+09	0	1.11E+02	1.11E+02	0	0	0	0	0	0
b3970_v1_RNA_10_modif_1	b3970_v1_RNA modification (23S rRNA): position: 2445, m2G by MeT_23S_2445	1 MeT_23S_2445 + 1 amet + 1 b3970_v1_RNA_10 <==> 1 b3970_v1_RNA_10_MeT_23S_2445	reversible	rRNA modification	-1.00E+09	1.00E+09	0	1.11E+02	1.11E+02	0	1.29E+01	12.9	0	1.29E+01	12.9
b3970_v1_RNA_10_modif_2	b3970_v1_RNA modification (23S rRNA): position: 2445, m2G by MeT_23S_2445	1 b3970_v1_RNA_10_MeT_23S_2445 --> 1 MeT_23S_2445_inact + 1 ahcys + 1 b3970_v1_RNA_11 + 1 h	irreversible	rRNA modification	0.00E+00	1.00E+09	0	1.11E+02	1.11E+02	0	1.29E+01	12.9	0	1.29E+01	12.9
b3970_v1_RNA_11_a_modif_1	b3970_v1_RNA modification (23S rRNA): position: 2449, D by DU_23S_2449 (NADH)	1 DU_23S_2449 + 1 b3970_v1_RNA_11 + 1 h + 1 nadh <==> 1 b3970_v1_RNA_11_DU_23S_2449_a	reversible	rRNA modification	-1.00E+09	1.00E+09	0	1.11E+02	1.11E+02	0	1.29E+01	12.9	0	1.29E+01	12.9
b3970_v1_RNA_11_a_modif_2	b3970_v1_RNA modification (23S rRNA): position: 2449, D by DU_23S_2449 (NADH)	1 b3970_v1_RNA_11_DU_23S_2449_a --> 1 DU_23S_2449_inact + 1 b3970_v1_RNA_12 + 1 nad	irreversible	rRNA modification	0.00E+00	1.00E+09	0	1.11E+02	1.11E+02	0	1.29E+01	12.9	0	1.29E+01	12.9
b3970_v1_RNA_11_b_modif_1	b3970_v1_RNA modification (23S rRNA): position: 2449, D by DU_23S_2449 (NADPH)	1 DU_23S_2449 + 1 b3970_v1_RNA_11 + 1 h + 1 nadph <==> 1 b3970_v1_RNA_11_DU_23S_2449_b	reversible	rRNA modification	-1.00E+09	1.00E+09	0	1.11E+02	1.11E+02	0	1.29E+01	12.9	0	1.29E+01	12.9
b3970_v1_RNA_11_b_modif_2	b3970_v1_RNA modification (23S rRNA): position: 2449, D by DU_23S_2449 (NADPH)	1 b3970_v1_RNA_11_DU_23S_2449_b --> 1 DU_23S_2449_inact + 1 b3970_v1_RNA_12 + 1 nadp	irreversible	rRNA modification	0.00E+00	1.00E+09	0	1.11E+02	1.11E+02	0	1.29E+01	12.9	0	1.29E+01	12.9
b3970_v1_RNA_12_modif_1	b3970_v1_RNA modification (23S rRNA): position: 2457, Y by YmfC_mono	1 YmfC_mono + 1 b3970_v1_RNA_12 <==> 1 b3970_v1_RNA_12_YmfC_mono	reversible	rRNA modification	-1.00E+09	1.00E+09	0	1.11E+02	1.11E+02	0	1.29E+01	12.9	0	1.29E+01	12.9
b3970_v1_RNA_12_modif_2	b3970_v1_RNA modification (23S rRNA): position: 2457, Y by YmfC_mono	1 b3970_v1_RNA_12_YmfC_mono --> 1 YmfC_mono_inact + 1 b3970_v1_RNA_13	irreversible	rRNA modification	0.00E+00	1.00E+09	0	1.11E+02	1.11E+02	0	1.29E+01	12.9	0	1.29E+01	12.9
b3970_v1_RNA_13_modif_1	b3970_v1_RNA modification (23S rRNA): position: 2498, Cm by MeT_23S_2498	1 MeT_23S_2498 + 1 amet + 1 b3970_v1_RNA_13 <==> 1 b3970_v1_RNA_13_MeT_23S_2498	reversible	rRNA modification	-1.00E+09	1.00E+09	0	1.11E+02	1.11E+02	0	1.29E+01	12.9	0	1.29E+01	12.9
b3970_v1_RNA_13_modif_2	b3970_v1_RNA modification (23S rRNA): position: 2498, Cm by MeT_23S_2498	1 b3970_v1_RNA_13_MeT_23S_2498 --> 1 MeT_23S_2498_inact + 1 ahcys + 1 b3970_v1_RNA_14 + 1 h	irreversible	rRNA modification	0.00E+00	1.00E+09	0	1.11E+02	1.11E+02	0	1.29E+01	12.9	0	1.29E+01	12.9
b3970_v1_RNA_14_modif_1	b3970_v1_RNA modification (23S rRNA): position: 2503, m2A by MeT_23S_2503	1 MeT_23S_2503 + 1 amet + 1 b3970_v1_RNA_14 <==> 1 b3970_v1_RNA_14_MeT_23S_2503	reversible	rRNA modification	-1.00E+09	1.00E+09	0	1.11E+02	1.11E+02	0	1.29E+01	12.9	0	1.29E+01	12.9
b3970_v1_RNA_14_modif_2	b3970_v1_RNA modification (23S rRNA): position: 2503, m2A by MeT_23S_2503	1 b3970_v1_RNA_14_MeT_23S_2503 --> 1 MeT_23S_2503_inact + 1 ahcys + 1 b3970_v1_RNA_15 + 1 h	irreversible	rRNA modification	0.00E+00	1.00E+09	0	1.11E+02	1.11E+02	0	1.29E+01	12.9	0	1.29E+01	12.9
b3970_v1_RNA_15_modif_1	b3970_v1_RNA modification (23S rRNA): position: 2504, Y by RluC_mono	1 RluC_mono + 1 b3970_v1_RNA_15 <==> 1 b3970_v1_RNA_15_RluC_mono	reversible	rRNA modification	-1.00E+09	1.00E+09	0	1.11E+02	1.11E+02	0	1.29E+01	12.9	0	1.29E+01	12.9
b3970_v1_RNA_15_modif_2	b3970_v1_RNA modification (23S rRNA): position: 2504, Y by RluC_mono	1 b3970_v1_RNA_15_RluC_mono --> 1 RluC_mono_inact + 1 b3970_v1_RNA_16	irreversible	rRNA modification	0.00E+00	1.00E+09	0	1.11E+02	1.11E+02	0	1.29E+01	12.9	0	1.29E+01	12.9
b3970_v1_RNA_16_modif_1	b3970_v1_RNA modification (23S rRNA): position: 2552, Um by RrmJ_mono	1 RrmJ_mono + 1 amet + 1 b3970_v1_RNA_16 <==> 1 b3970_v1_RNA_16_RrmJ_mono	reversible	rRNA modification	-1.00E+09	1.00E+09	0	1.11E+02	1.11E+02	0	1.29E+01	12.9	0	1.29E+01	12.9
b3970_v1_RNA_16_modif_2	b3970_v1_RNA modification (23S rRNA): position: 2552, Um by RrmJ_mono	1 b3970_v1_RNA_16_RrmJ_mono --> 1 RrmJ_mono_inact + 1 ahcy + 1 b3970_v1_RNA_17 + 1 h	irreversible	rRNA modification	0.00E+00	1.00E+09	0	1.11E+02	1.11E+02	0	1.29E+01	12.9	0	1.29E+01	12.9
b3970_v1_RNA_17_modif_1	b3970_v1_RNA modification (23S rRNA): position: 2580, Y by RluC_mono	1 RluC_mono + 1 b3970_v1_RNA_17 <==> 1 b3970_v1_RNA_17_RluC_mono	reversible	rRNA modification	-1.00E+09	1.00E+09	0	1.11E+02	1.11E+02	0	1.29E+01	12.9	0	1.29E+01	12.9
b3970_v1_RNA_17_modif_2	b3970_v1_RNA modification (23S rRNA): position: 2580, Y by RluC_mono	1 b3970_v1_RNA_17_RluC_mono --> 1 RluC_mono_inact + 1 b3970_v1_RNA_18	irreversible	rRNA modification	0.00E+00	1.00E+09	0	1.11E+02	1.11E+02	0	1.29E+01	12.9	0	1.29E+01	12.9
b3970_v1_RNA_18_modif_1	b3970_v1_RNA modification (23S rRNA): position: 2604, Y by YjbC_mono	1 YjbC_mono + 1 b3970_v1_RNA_18 <==> 1 b3970_v1_RNA_18_YjbC_mono	reversible	rRNA modification	-1.00E+09	1.00E+09	0	1.11E+02	1.11E+02	0	1.29E+01	12.9	0	1.29E+01	12.9

b3970_v1_RNA_18_modif_2	b3970_v1_RNA modification (23S rRNA): position: 2604, Y by YjbC_mono	1 b3970_v1_RNA_18_YjbC_mono --> 1 YjbC_mono_inact + 1 b3970_v1_RNA_19	irreversible	rRNA modification	0.00E+00	1.00E+09	0	1.11E+02	1.11E+02	0	1.29E+01	12.9	0	1.29E+01	12.9
b3970_v1_RNA_19_modif_1	b3970_v1_RNA modification (23S rRNA): position: 2605, Y by RluB_mono	1 RluB_mono + 1 b3970_v1_RNA_19 <==> 1 b3970_v1_RNA_19_RluB_mono	reversible	rRNA modification	-1.00E+09	1.00E+09	0	1.11E+02	1.11E+02	0	1.29E+01	12.9	0	1.29E+01	12.9
b3970_v1_RNA_19_modif_2	b3970_v1_RNA modification (23S rRNA): position: 2605, Y by RluB_mono	1 b3970_v1_RNA_19_RluB_mono --> 1 RluB_mono_inact + 1 b3970_v1_RNA_20	irreversible	rRNA modification	0.00E+00	1.00E+09	0	1.11E+02	1.11E+02	0	1.29E+01	12.9	0	1.29E+01	12.9
b3970_v1_RNA_1_modif_1	b3970_v1_RNA modification (23S rRNA): position: 1835, m2G by MeT_23S_1835	1 MeT_23S_1835 + 1 amet + 1 b3970_v1_RNA_1 <==> 1 b3970_v1_RNA_1_MeT_23S_1835	reversible	rRNA modification	-1.00E+09	1.00E+09	0	1.11E+02	1.11E+02	0	1.29E+01	12.9	0	1.29E+01	12.9
b3970_v1_RNA_1_modif_2	b3970_v1_RNA modification (23S rRNA): position: 1835, m2G by MeT_23S_1835	1 b3970_v1_RNA_1_MeT_23S_1835 --> 1 MeT_23S_1835_inact + 1 ahcys + 1 b3970_v1_RNA_2 + 1 h	irreversible	rRNA modification	0.00E+00	1.00E+09	0	1.11E+02	1.11E+02	0	1.29E+01	12.9	0	1.29E+01	12.9
b3970_v1_RNA_20_modif_1	b3970_v1_RNA modification (23S rRNA): position: 745, m1G by RrmA_dim	1 RrmA_dim + 1 amet + 1 b3970_v1_RNA_20 <==> 1 b3970_v1_RNA_20_RrmA_dim	reversible	rRNA modification	-1.00E+09	1.00E+09	0	1.11E+02	1.11E+02	0	1.29E+01	12.9	0	1.29E+01	12.9
b3970_v1_RNA_20_modif_2	b3970_v1_RNA modification (23S rRNA): position: 745, m1G by RrmA_dim	1 b3970_v1_RNA_20_RrmA_dim --> 1 RrmA_dim_inact + 1 ahcys + 1 b3970_v1_RNA_21 + 1 h	irreversible	rRNA modification	0.00E+00	1.00E+09	0	1.11E+02	1.11E+02	0	1.29E+01	12.9	0	1.29E+01	12.9
b3970_v1_RNA_21_modif_1	b3970_v1_RNA modification (23S rRNA): position: 746, Y by RluA_mono	1 RluA_mono + 1 b3970_v1_RNA_21 <==> 1 b3970_v1_RNA_21_RluA_mono	reversible	rRNA modification	-1.00E+09	1.00E+09	0	1.11E+02	1.11E+02	0	1.29E+01	12.9	0	1.29E+01	12.9
b3970_v1_RNA_21_modif_2	b3970_v1_RNA modification (23S rRNA): position: 746, Y by RluA_mono	1 b3970_v1_RNA_21_RluA_mono --> 1 RluA_mono_inact + 1 b3970_v1_RNA_22	irreversible	rRNA modification	0.00E+00	1.00E+09	0	1.11E+02	1.11E+02	0	1.29E+01	12.9	0	1.29E+01	12.9
b3970_v1_RNA_22_modif_1	b3970_v1_RNA modification (23S rRNA): position: 747, m5U by RumB_mono	1 RumB_mono + 1 amet + 1 b3970_v1_RNA_22 <==> 1 b3970_v1_RNA_22_RumB_mono	reversible	rRNA modification	-1.00E+09	1.00E+09	0	1.11E+02	1.11E+02	0	1.29E+01	12.9	0	1.29E+01	12.9
b3970_v1_RNA_22_modif_2	b3970_v1_RNA modification (23S rRNA): position: 747, m5U by RumB_mono	1 b3970_v1_RNA_22_RumB_mono --> 1 RumB_mono_inact + 1 ahcys + 1 b3970_v1_RNA_23 + 1 h	irreversible	rRNA modification	0.00E+00	1.00E+09	0	1.11E+02	1.11E+02	0	1.29E+01	12.9	0	1.29E+01	12.9
b3970_v1_RNA_23_modif_1	b3970_v1_RNA modification (23S rRNA): position: 955, Y by RluC_mono	1 RluC_mono + 1 b3970_v1_RNA_23 <==> 1 b3970_v1_RNA_23_RluC_mono	reversible	rRNA modification	-1.00E+09	1.00E+09	0	1.11E+02	1.11E+02	0	1.29E+01	12.9	0	1.29E+01	12.9
b3970_v1_RNA_23_modif_2	b3970_v1_RNA modification (23S rRNA): position: 955, Y by RluC_mono	1 b3970_v1_RNA_23_RluC_mono --> 1 RluC_mono_inact + 1 rrlB	irreversible	rRNA modification	0.00E+00	1.00E+09	0	1.11E+02	1.11E+02	0	1.29E+01	12.9	0	1.29E+01	12.9
b3970_v1_RNA_2_modif_1	b3970_v1_RNA modification (23S rRNA): position: 1911, Y by RluD_mono	1 RluD_mono + 1 b3970_v1_RNA_2 <==> 1 b3970_v1_RNA_2_RluD_mono	reversible	rRNA modification	-1.00E+09	1.00E+09	0	1.11E+02	1.11E+02	0	1.29E+01	12.9	0	1.29E+01	12.9
b3970_v1_RNA_2_modif_2	b3970_v1_RNA modification (23S rRNA): position: 1911, Y by RluD_mono	1 b3970_v1_RNA_2_RluD_mono --> 1 RluD_mono_inact + 1 b3970_v1_RNA_3	irreversible	rRNA modification	0.00E+00	1.00E+09	0	1.11E+02	1.11E+02	0	1.29E+01	12.9	0	1.29E+01	12.9
b3970_v1_RNA_3_modif_1	b3970_v1_RNA modification (23S rRNA): position: 1915, m3Y by RluD_mono	1 RluD_mono + 1 amet + 1 b3970_v1_RNA_3 <==> 1 b3970_v1_RNA_3_RluD_mono	reversible	rRNA modification	-1.00E+09	1.00E+09	0	1.11E+02	1.11E+02	0	1.29E+01	12.9	0	1.29E+01	12.9
b3970_v1_RNA_3_modif_2	b3970_v1_RNA modification (23S rRNA): position: 1915, m3Y by RluD_mono	1 b3970_v1_RNA_3_RluD_mono --> 1 RluD_mono_inact + 1 ahcys + 1 b3970_v1_RNA_4 + 1 h	irreversible	rRNA modification	0.00E+00	1.00E+09	0	1.11E+02	1.11E+02	0	1.29E+01	12.9	0	1.29E+01	12.9
b3970_v1_RNA_4_modif_1	b3970_v1_RNA modification (23S rRNA): position: 1917, Y by RluD_mono	1 RluD_mono + 1 b3970_v1_RNA_4 <==> 1 b3970_v1_RNA_4_RluD_mono	reversible	rRNA modification	-1.00E+09	1.00E+09	0	1.11E+02	1.11E+02	0	1.29E+01	12.9	0	1.29E+01	12.9
b3970_v1_RNA_4_modif_2	b3970_v1_RNA modification (23S rRNA): position: 1917, Y by RluD_mono	1 b3970_v1_RNA_4_RluD_mono --> 1 RluD_mono_inact + 1 b3970_v1_RNA_5	irreversible	rRNA modification	0.00E+00	1.00E+09	0	1.11E+02	1.11E+02	0	1.29E+01	12.9	0	1.29E+01	12.9
b3970_v1_RNA_5_modif_1	b3970_v1_RNA modification (23S rRNA): position: 1939, m5U by RumA_mono	1 RumA_mono + 1 amet + 1 b3970_v1_RNA_5 <==> 1 b3970_v1_RNA_5_RumA_mono	reversible	rRNA modification	-1.00E+09	1.00E+09	0	1.11E+02	1.11E+02	0	1.29E+01	12.9	0	1.29E+01	12.9
b3970_v1_RNA_5_modif_2	b3970_v1_RNA modification (23S rRNA): position: 1939, m5U by RumA_mono	1 b3970_v1_RNA_5_RumA_mono --> 1 RumA_mono_inact + 1 ahcy + 1 b3970_v1_RNA_6 + 1 h	irreversible	rRNA modification	0.00E+00	1.00E+09	0	1.11E+02	1.11E+02	0	1.29E+01	12.9	0	1.29E+01	12.9
b3970_v1_RNA_6_modif_1	b3970_v1_RNA modification (23S rRNA): position: 1962, m5C by MeT_23S_1962	1 MeT_23S_1962 + 1 amet + 1 b3970_v1_RNA_6 <==> 1 b3970_v1_RNA_6_MeT_23S_1962	reversible	rRNA modification	-1.00E+09	1.00E+09	0	1.11E+02	1.11E+02	0	1.29E+01	12.9	0	1.29E+01	12.9
b3970_v1_RNA_6_modif_2	b3970_v1_RNA modification (23S rRNA): position: 1962, m5C by MeT_23S_1962	1 b3970_v1_RNA_6_MeT_23S_1962 --> 1 MeT_23S_1962_inact + 1 ahcys + 1 b3970_v1_RNA_7 + 1 h	irreversible	rRNA modification	0.00E+00	1.00E+09	0	1.11E+02	1.11E+02	0	1.29E+01	12.9	0	1.29E+01	12.9
b3970_v1_RNA_7_modif_1	b3970_v1_RNA modification (23S rRNA): position: 2030, m6A by MeT_23S_2030	1 MeT_23S_2030 + 1 amet + 1 b3970_v1_RNA_7 <==> 1 b3970_v1_RNA_7_MeT_23S_2030	reversible	rRNA modification	-1.00E+09	1.00E+09	0	1.11E+02	1.11E+02	0	1.29E+01	12.9	0	1.29E+01	12.9
b3970_v1_RNA_7_modif_2	b3970_v1_RNA modification (23S rRNA): position: 2030, m6A by MeT_23S_2030	1 b3970_v1_RNA_7_MeT_23S_2030 --> 1 MeT_23S_2030_inact + 1 ahcys + 1 b3970_v1_RNA_8 + 1 h	irreversible	rRNA modification	0.00E+00	1.00E+09	0	1.11E+02	1.11E+02	0	1.29E+01	12.9	0	1.29E+01	12.9
b3970_v1_RNA_8_modif_1	b3970_v1_RNA modification (23S rRNA): position: 2069, m7G by MeT_23S_2069	1 MeT_23S_2069 + 1 amet + 1 b3970_v1_RNA_8 <==> 1 b3970_v1_RNA_8_MeT_23S_2069	reversible	rRNA modification	-1.00E+09	1.00E+09	0	1.11E+02	1.11E+02	0	1.29E+01	12.9	0	1.29E+01	12.9
b3970_v1_RNA_8_modif_2	b3970_v1_RNA modification (23S rRNA): position: 2069, m7G by MeT_23S_2069	1 b3970_v1_RNA_8_MeT_23S_2069 --> 1 MeT_23S_2069_inact + 1 ahcys + 1 b3970_v1_RNA_9 + 1 h	irreversible	rRNA modification	0.00E+00	1.00E+09	0	1.11E+02	1.11E+02	0	1.29E+01	12.9	0	1.29E+01	12.9
b3970_v1_RNA_9_modif_1	b3970_v1_RNA modification (23S rRNA): position: 2251, Gm by RlmB_dim	1 RlmB_dim + 1 amet + 1 b3970_v1_RNA_9 <==> 1 b3970_v1_RNA_9_RlmB_dim	reversible	rRNA modification	-1.00E+09	1.00E+09	0	1.11E+02	1.11E+02	0	1.29E+01	12.9	0	1.29E+01	12.9
b3970_v1_RNA_9_modif_2	b3970_v1_RNA modification (23S rRNA): position: 2251, Gm by RlmB_dim	1 b3970_v1_RNA_9_RlmB_dim --> 1 RlmB_dim_inact + 1 ahcys + 1 b3970_v1_RNA_10 + 1 h	irreversible	rRNA modification	0.00E+00	1.00E+09	0	1.11E+02	1.11E+02	0	1.29E+01	12.9	0	1.29E+01	12.9
b3970_v1_RNA_modif_1	b3970_v1_RNA modification (23S rRNA): position: 1618, m6A by MeT_23S_1618	1 MeT_23S_1618 + 1 amet + 1 b3970_v1_RNA <==> 1 b3970_v1_RNA_MeT_23S_1618	reversible	rRNA modification	-1.00E+09	1.00E+09	0	1.11E+02	1.11E+02	0	1.29E+01	12.9	0	1.29E+01	12.9
b3970_v1_RNA_modif_2	b3970_v1_RNA modification (23S rRNA): position: 1618, m6A by MeT_23S_1618	1 b3970_v1_RNA_MeT_23S_1618 --> 1 MeT_23S_1618_inact + 1 ahcys + 1 b3970_v1_RNA_1 + 1 h	irreversible	rRNA modification	0.00E+00	1.00E+09	0	1.11E+02	1.11E+02	0	1.29E+01	12.9	0	1.29E+01	12.9
b3976_tRNA_16_MODIF_1	modification of position 16 nt (D) in thrU by Dus_gen	1 Dus_gen + 1 b3976_tRNA_Mg2 + 1 h + 1 nadph <==> 1 b3976_tRNA_Mg2_Dus_gen_cplx	reversible	tRNA modification	-1.00E+09	1.00E+09	0	2.71E+02	2.71E+02	0	1.28E+01	12.8	0	1.28E+01	12.8
b3976_tRNA_16_MODIF_2	modification of position 16 nt (D) in thrU by Dus_gen	1 b3976_tRNA_Mg2_Dus_gen_cplx --> 1 Dus_gen_inact + 1 b3976_tRNA_1 + 1 nadp	irreversible	tRNA modification	0.00E+00	1.00E+09	0	2.71E+02	2.71E+02	0	1.28E+01	12.8	0	1.28E+01	12.8
b3976_tRNA_20_MODIF_1	modification of position 20 nt (D) in thrU by Dus_gen	1 Dus_gen + 1 b3976_tRNA_1 + 1 h + 1 nadph <==> 1 b3976_tRNA_1_Dus_gen_cplx	reversible	tRNA modification	-1.00E+09	1.00E+09	0	2.71E+02	2.71E+02	0	1.28E+01	12.8	0	1.28E+01	12.8
b3976_tRNA_20_MODIF_2	modification of position 20 nt (D) in thrU by Dus_gen	1 b3976_tRNA_1_Dus_gen_cplx --> 1 Dus_gen_inact + 1 b3976_tRNA_2 + 1 nadp	irreversible	tRNA modification	0.00E+00	1.00E+09	0	2.71E+02	2.71E+02	0	1.28E+01	12.8	0	1.28E+01	12.8
b3976_tRNA_37_MODIF_1	modification of position 37 nt (m6t6A) in thrU by Up_tRNA_pos_37_t6A-MeT_tRNA_pos_37_m6t6A	1 MeT_tRNA_pos_37_m6t6A + 1 Up_tRNA_pos_37_t6A + 1 amet + atp + 1 b3976_tRNA_2 + 1 hco3 + 1 mg2 + 1 thr-L <==> 1 b3976_tRNA_2_Up_tRNA_pos_37_t6A-MeT_tRNA_pos_37_m6t6A_cplx	reversible	tRNA modification	-1.00E+09	1.00E+09	0	2.71E+02	2.71E+02	0	1.28E+01	12.8	0	1.28E+01	12.8
b3976_tRNA_37_MODIF_2	modification of position 37 nt (m6t6A) in thrU by Up_tRNA_pos_37_t6A-MeT_tRNA_pos_37_m6t6A	1 b3976_tRNA_2_Up_tRNA_pos_37_t6A-MeT_tRNA_pos_37_m6t6A_cplx --> 1 MeT_tRNA_pos_37_m6t6A_inact + 1 Up_tRNA_pos_37_t6A_inact + 1 ahcys + 1 amp + 1 b3976_tRNA_3 + 2 h + 1 h2o + 1 mg2 + 1 ppi	irreversible	tRNA modification	0.00E+00	1.00E+09	0	2.71E+02	2.71E+02	0	1.28E+01	12.8	0	1.28E+01	12.8
b3976_tRNA_46_MODIF_1	modification of position 46 nt (m7G) in thrU by YggH_mono	1 YggH_mono + 1 amet + 1 b3976_tRNA_3 <==> 1 b3976_tRNA_3_YggH_mono_cplx	reversible	tRNA modification	-1.00E+09	1.00E+09	0	2.71E+02	2.71E+02	0	1.28E+01	12.8	0	1.28E+01	12.8
b3976_tRNA_46_MODIF_2	modification of position 46 nt (m7G) in thrU by YggH_mono	1 b3976_tRNA_3_YggH_mono_cplx --> 1 YggH_mono_inact + 1 ahcys + 1 b3976_tRNA_4 + 1 h	irreversible	tRNA modification	0.00E+00	1.00E+09	0	2.71E+02	2.71E+02	0	1.28E+01	12.8	0	1.28E+01	12.8

b3976_tRNA_54_MODIF_1	modification of position 54 nt (m5U) in thrU by TrmA_mono	1 TrmA_mono + 1 amet + 1 b3976_tRNA_4 <==> 1 b3976_tRNA_4_TrmA_mono_cplx	reversible	tRNA modification	-1.00E+09	1.00E+09	0	2.71E+02	2.71E+02	0	1.28E+01	12.8	0	1.28E+01	12.8
b3976_tRNA_54_MODIF_2	modification of position 54 nt (m5U) in thrU by TrmA_mono	1 b3976_tRNA_4_TrmA_mono_cplx --> 1 TrmA_mono_inact + 1 ahcys + 1 b3976_tRNA_5 + 1 h	irreversible	tRNA modification	0.00E+00	1.00E+09	0	2.71E+02	2.71E+02	0	1.28E+01	12.8	0	1.28E+01	12.8
b3976_tRNA_55_MODIF_1	modification of position 55 nt (Y) in thrU by TruB_mono	1 TruB_mono + 1 b3976_tRNA_5 <==> 1 b3976_tRNA_5_TruB_mono_cplx	reversible	tRNA modification	-1.00E+09	1.00E+09	0	2.71E+02	2.71E+02	0	1.28E+01	12.8	0	1.28E+01	12.8
b3976_tRNA_55_MODIF_2	modification of position 55 nt (Y) in thrU by TruB_mono	1 b3976_tRNA_5_TruB_mono_cplx --> 1 TruB_mono_inact + 1 thrU_tRNA	irreversible	tRNA modification	0.00E+00	1.00E+09	0	2.71E+02	2.71E+02	0	1.28E+01	12.8	0	1.28E+01	12.8
b3976_tRNA_Mg_BIND	Binding of two Mg2 to b3976_tRNA	1 b3976_RNA + 2 mg2 <==> 1 b3976_tRNA_Mg2	reversible	Metallo-ion binding	-1.00E+09	1.00E+09	0	2.71E+02	2.71E+02	0	1.28E+01	12.8	0	1.28E+01	12.8
b3977_tRNA_18_MODIF_1	modification of position 18 nt (Gm) in tyrU by TrmH_dim	1 TrmH_dim + 1 amet + 1 b3977_tRNA_Mg2 <==> 1 b3977_tRNA_Mg2_TrmH_dim_cplx	reversible	tRNA modification	-1.00E+09	1.00E+09	0	2.71E+02	2.71E+02	0	1.28E+01	12.8	0	1.28E+01	12.8
b3977_tRNA_18_MODIF_2	modification of position 18 nt (Gm) in tyrU by TrmH_dim	1 b3977_tRNA_Mg2_TrmH_dim_cplx --> 1 TrmH_dim_inact + 1 ahcys + 1 b3977_tRNA_1 + 1 h	irreversible	tRNA modification	0.00E+00	1.00E+09	0	2.71E+02	2.71E+02	0	1.28E+01	12.8	0	1.28E+01	12.8
b3977_tRNA_34_MODIF_1	modification of position 34 nt (Q) in tyrU by Tgt_hexa-QueA_mono-EoR_tRNA_pos34_Q	1 EoR_tRNA_pos34_Q + 1 QueA_mono + 1 Tgt_hexa + 1 adocbl + 1 amet + 1 b3977_tRNA_1 + 1 h + 1 nadh + 1 pre_Q1 <==> 1 b3977_tRNA_1_Tgt_hexa-QueA_mono-EoR_tRNA_pos34_Q_cplx	reversible	tRNA modification	-1.00E+09	1.00E+09	0	2.71E+02	2.71E+02	0	1.28E+01	12.8	0	1.28E+01	12.8
b3977_tRNA_34_MODIF_2	modification of position 34 nt (Q) in tyrU by Tgt_hexa-QueA_mono-EoR_tRNA_pos34_Q	1 b3977_tRNA_1_Tgt_hexa-QueA_mono-EoR_tRNA_pos34_Q_cplx > 1 EoR_tRNA_pos34_Q_inact + 1 QueA_mono_inact + 1 Tgt_hexa_inact + 1 ade + 1 adocbl + 1 b3977_tRNA_2 + 1 gua + 2 h 1 h2o + 1 met-L + 1 nad	irreversible	tRNA modification	0.00E+00	1.00E+09	0	2.71E+02	2.71E+02	0	1.28E+01	12.8	0	1.28E+01	12.8
b3977_tRNA_37_MODIF_1	modification of position 37 nt (ms2i6A) in tyrU by MiaA_dim-MiaB_mono	1 IscS_dim_S-SH + 1 MiaA_dim + 1 MiaB_mono + 2 amet + 1 b3977_tRNA_2 + 1 dmpp + 1 fldrd <==> 1 b3977_tRNA_2_MiaA_dim MiaB_mono_cplx	reversible	tRNA modification	-1.00E+09	1.00E+09	0	2.71E+02	2.71E+02	0	1.28E+01	12.8	0	1.28E+01	12.8
b3977_tRNA_37_MODIF_2	modification of position 37 nt (ms2i6A) in tyrU by MiaA_dim-MiaB_mono	1 b3977_tRNA_2_MiaA_dim-MiaB_mono_cplx --> 1 IscS_dim_S-H + 1 MiaA_dim_inact + 1 MiaB_mono_inact + 1 ahcys + 1 b3977_tRNA_3 + 1 dad-5 + 1 fldox + 2 h + 1 met-L + 1 ppi	irreversible	tRNA modification	0.00E+00	1.00E+09	0	2.71E+02	2.71E+02	0	1.28E+01	12.8	0	1.28E+01	12.8
b3977_tRNA_39_MODIF_1	modification of position 39 nt (Y) in tyrU by TruA_dim	1 TruA_dim + 1 b3977_tRNA_3 <==> 1 b3977_tRNA_3_TruA_dim_cplx	reversible	tRNA modification	-1.00E+09	1.00E+09	0	2.71E+02	2.71E+02	0	1.28E+01	12.8	0	1.28E+01	12.8
b3977_tRNA_39_MODIF_2	modification of position 39 nt (Y) in tyrU by TruA_dim	1 b3977_tRNA_3_TruA_dim_cplx --> 1 TruA_dim_inact + 1 b3977_tRNA_4	irreversible	tRNA modification	0.00E+00	1.00E+09	0	2.71E+02	2.71E+02	0	1.28E+01	12.8	0	1.28E+01	12.8
b3977_tRNA_54_MODIF_1	modification of position 54 nt (m5U) in tyrU by TrmA_mono	1 TrmA_mono + 1 amet + 1 b3977_tRNA_4 <==> 1 b3977_tRNA_4_TrmA_mono_cplx	reversible	tRNA modification	-1.00E+09	1.00E+09	0	2.71E+02	2.71E+02	0	1.28E+01	12.8	0	1.28E+01	12.8
b3977_tRNA_54_MODIF_2	modification of position 54 nt (m5U) in tyrU by TrmA_mono	1 b3977_tRNA_4_TrmA_mono_cplx --> 1 TrmA_mono_inact + 1 ahcys + 1 b3977_tRNA_5 + 1 h	irreversible	tRNA modification	0.00E+00	1.00E+09	0	2.71E+02	2.71E+02	0	1.28E+01	12.8	0	1.28E+01	12.8
b3977_tRNA_55_MODIF_1	modification of position 55 nt (Y) in tyrU by TruB_mono	1 TruB_mono + 1 b3977_tRNA_5 <==> 1 b3977_tRNA_5_TruB_mono_cplx	reversible	tRNA modification	-1.00E+09	1.00E+09	0	2.71E+02	2.71E+02	0	1.28E+01	12.8	0	1.28E+01	12.8
b3977_tRNA_55_MODIF_2	modification of position 55 nt (Y) in tyrU by TruB_mono	1 b3977_tRNA_5_TruB_mono_cplx --> 1 TruB_mono_inact + 1 b3977_tRNA_6	irreversible	tRNA modification	0.00E+00	1.00E+09	0	2.71E+02	2.71E+02	0	1.28E+01	12.8	0	1.28E+01	12.8
b3977_tRNA_8_MODIF_1	modification of position 8 nt (s4U) in tyrU by ThiI_mono	1 IscS_dim_S-SH + 1 ThiI_mono + 1 atp + 1 b3977_tRNA_6 + 1 mg2 + 1 trdrd <==> 1 b3977_tRNA_6_ThiI_mono_cplx	reversible	tRNA modification	-1.00E+09	1.00E+09	0	2.71E+02	2.71E+02	0	1.28E+01	12.8	0	1.28E+01	12.8
b3977_tRNA_8_MODIF_2	modification of position 8 nt (s4U) in tyrU by ThiI_mono	1 b3977_tRNA_6_ThiI_mono_cplx --> 1 IscS_dim_S-H + 1 ThiI_mono_inact + 1 amp + 1 h + 1 mg2 + 1 ppi + 1 trdox + 1 tyrU_tRNA	irreversible	tRNA modification	0.00E+00	1.00E+09	0	2.71E+02	2.71E+02	0	1.28E+01	12.8	0	1.28E+01	12.8
b3977_tRNA_Mg_BIND	Binding of two Mg2 to b3977_tRNA	1 b3977_RNA + 2 mg2 <==> 1 b3977_tRNA_Mg2	reversible	Metallo-ion binding	-1.00E+09	1.00E+09	0	2.71E+02	2.71E+02	0	1.28E+01	12.8	0	1.28E+01	12.8
b3978_tRNA_54_MODIF_1	modification of position 54 nt (m5U) in glyT by TrmA_mono	1 TrmA_mono + 1 amet + 1 b3978_tRNA_Mg2 <==> 1 b3978_tRNA_Mg2_TrmA_mono_cplx	reversible	tRNA modification	-1.00E+09	1.00E+09	0	2.71E+02	2.71E+02	0	1.28E+01	12.8	0	1.28E+01	12.8
b3978_tRNA_54_MODIF_2	modification of position 54 nt (m5U) in glyT by TrmA_mono	1 b3978_tRNA_Mg2_TrmA_mono_cplx --> 1 TrmA_mono_inact + 1 ahcys + 1 b3978_tRNA_1 + 1 h	irreversible	tRNA modification	0.00E+00	1.00E+09	0	2.71E+02	2.71E+02	0	1.28E+01	12.8	0	1.28E+01	12.8
b3978_tRNA_55_MODIF_1	modification of position 55 nt (Y) in glyT by TruB_mono	1 TruB_mono + 1 b3978_tRNA_1 <==> 1 b3978_tRNA_1_TruB_mono_cplx	reversible	tRNA modification	-1.00E+09	1.00E+09	0	2.71E+02	2.71E+02	0	1.28E+01	12.8	0	1.28E+01	12.8
b3978_tRNA_55_MODIF_2	modification of position 55 nt (Y) in glyT by TruB_mono	1 b3978_tRNA_1_TruB_mono_cplx --> 1 TruB_mono_inact + 1 glyT_tRNA	irreversible	tRNA modification	0.00E+00	1.00E+09	0	2.71E+02	2.71E+02	0	1.28E+01	12.8	0	1.28E+01	12.8
b3978_tRNA_Mg_BIND	Binding of two Mg2 to b3978_tRNA	1 b3978_RNA + 2 mg2 <==> 1 b3978_tRNA_Mg2	reversible	Metallo-ion binding	-1.00E+09	1.00E+09	0	2.71E+02	2.71E+02	0	1.28E+01	12.8	0	1.28E+01	12.8
b3979_tRNA_16_MODIF_1	modification of position 16 nt (D) in thrT by Dus_gen	1 Dus_gen + 1 b3979_tRNA_Mg2 + 1 h + 1 nadph <==> 1 b3979_tRNA_Mg2_Dus_gen_cplx	reversible	tRNA modification	-1.00E+09	1.00E+09	0	2.71E+02	2.71E+02	0	1.28E+01	12.8	0	1.28E+01	12.8
b3979_tRNA_16_MODIF_2	modification of position 16 nt (D) in thrT by Dus_gen	1 b3979_tRNA_Mg2_Dus_gen_cplx --> 1 Dus_gen_inact + 1 b3979_tRNA_1 + 1 nadp	irreversible	tRNA modification	0.00E+00	1.00E+09	0	2.71E+02	2.71E+02	0	1.28E+01	12.8	0	1.28E+01	12.8
b3979_tRNA_17_MODIF_1	modification of position 17 nt (D) in thrT by Dus_gen	1 Dus_gen + 1 b3979_tRNA_1 + 1 h + 1 nadph <==> 1 b3979_tRNA_1_Dus_gen_cplx	reversible	tRNA modification	-1.00E+09	1.00E+09	0	2.71E+02	2.71E+02	0	1.28E+01	12.8	0	1.28E+01	12.8
b3979_tRNA_17_MODIF_2	modification of position 17 nt (D) in thrT by Dus_gen	1 b3979_tRNA_1_Dus_gen_cplx --> 1 Dus_gen_inact + 1 b3979_tRNA_2 + 1 nadp	irreversible	tRNA modification	0.00E+00	1.00E+09	0	2.71E+02	2.71E+02	0	1.28E+01	12.8	0	1.28E+01	12.8
b3979_tRNA_20_MODIF_1	modification of position 20 nt (D) in thrT by Dus_gen	1 Dus_gen + 1 b3979_tRNA_2 + 1 h + 1 nadph <==> 1 b3979_tRNA_2_Dus_gen_cplx	reversible	tRNA modification	-1.00E+09	1.00E+09	0	2.71E+02	2.71E+02	0	1.28E+01	12.8	0	1.28E+01	12.8
b3979_tRNA_20_MODIF_2	modification of position 20 nt (D) in thrT by Dus_gen	1 b3979_tRNA_2_Dus_gen_cplx --> 1 Dus_gen_inact + 1 b3979_tRNA_3 + 1 nadp	irreversible	tRNA modification	0.00E+00	1.00E+09	0	2.71E+02	2.71E+02	0	1.28E+01	12.8	0	1.28E+01	12.8
b3979_tRNA_37_MODIF_1	modification of position 37 nt (m6t6A) in thrT by Up_tRNA_pos_37_t6A-MeT_tRNA_pos_37_m6t6A	1 MeT_tRNA_pos_37_m6t6A + 1 Up_tRNA_pos_37_t6A + 1 amet + atp + 1 b3979_tRNA_3 + 1 hco3 + 1 mg2 + 1 thr-L <==> 1 b3979_tRNA_3_Up_tRNA_pos_37_t6A-MeT_tRNA_pos_37_m6t6A_cplx	reversible	tRNA modification	-1.00E+09	1.00E+09	0	2.71E+02	2.71E+02	0	1.28E+01	12.8	0	1.28E+01	12.8
b3979_tRNA_37_MODIF_2	modification of position 37 nt (m6t6A) in thrT by Up_tRNA_pos_37_t6A-MeT_tRNA_pos_37_m6t6A	1 b3979_tRNA_3_Up_tRNA_pos_37_t6A-MeT_tRNA_pos_37_m6t6A_cplx --> 1 MeT_tRNA_pos_37_m6t6A_inact + 1 Up_tRNA_pos_37_t6A_inact + 1 ahcys + 1 amp + 1 b3979_tRNA_4 + 2 h + 1 h2o + 1 mg2 + 1 ppi	irreversible	tRNA modification	0.00E+00	1.00E+09	0	2.71E+02	2.71E+02	0	1.28E+01	12.8	0	1.28E+01	12.8
b3979_tRNA_46_MODIF_1	modification of position 46 nt (m7G) in thrT by YggH_mono	1 YggH_mono + 1 amet + 1 b3979_tRNA_4 <==> 1 b3979_tRNA_4_YggH_mono_cplx	reversible	tRNA modification	-1.00E+09	1.00E+09	0	2.71E+02	2.71E+02	0	1.28E+01	12.8	0	1.28E+01	12.8
b3979_tRNA_46_MODIF_2	modification of position 46 nt (m7G) in thrT by YggH_mono	1 b3979_tRNA_4_YggH_mono_cplx --> 1 YggH_mono_inact + 1 ahcys + 1 b3979_tRNA_5 + 1 h	irreversible	tRNA modification	0.00E+00	1.00E+09	0	2.71E+02	2.71E+02	0	1.28E+01	12.8	0	1.28E+01	12.8
b3979_tRNA_54_MODIF_1	modification of position 54 nt (m5U) in thrT by TrmA_mono	1 TrmA_mono + 1 amet + 1 b3979_tRNA_5 <==> 1 b3979_tRNA_5_TrmA_mono_cplx	reversible	tRNA modification	-1.00E+09	1.00E+09	0	2.71E+02	2.71E+02	0	1.28E+01	12.8	0	1.28E+01	12.8
b3979_tRNA_54_MODIF_2	modification of position 54 nt (m5U) in thrT by TrmA_mono	1 b3979_tRNA_5_TrmA_mono_cplx --> 1 TrmA_mono_inact + 1 ahcys + 1 b3979_tRNA_6 + 1 h	irreversible	tRNA modification	0.00E+00	1.00E+09	0	2.71E+02	2.71E+02	0	1.28E+01	12.8	0	1.28E+01	12.8
b3979_tRNA_55_MODIF_1	modification of position 55 nt (Y) in thrT by TruB_mono	1 TruB_mono + 1 b3979_tRNA_6 <==> 1 b3979_tRNA_6_TruB_mono_cplx	reversible	tRNA modification	-1.00E+09	1.00E+09	0	2.71E+02	2.71E+02	0	1.28E+01	12.8	0	1.28E+01	12.8
b3979_tRNA_55_MODIF_2	modification of position 55 nt (Y) in thrT by TruB_mono	1 b3979_tRNA_6_TruB_mono_cplx --> 1 TruB_mono_inact + 1 thrT_tRNA	irreversible	tRNA modification	0.00E+00	1.00E+09	0	2.71E+02	2.71E+02	0	1.28E+01	12.8	0	1.28E+01	12.8
b3979_tRNA_Mg_BIND	Binding of two Mg2 to b3979_tRNA	1 b3979_RNA + 2 mg2 <==> 1 b3979_tRNA_Mg2	reversible	Metallo-ion binding	-1.00E+09	1.00E+09	0	2.71E+02	2.71E+02	0	1.28E+01	12.8	0	1.28E+01	12.8
b3980_fold_KJE_1	b3980_m folding: KJE mediated	1 DnaJ_dim + 1 DnaK_mono.ATP + 1 b3980_m <==> 1 b3980_m_DnaKJ_complex	reversible	Protein Folding	-1.00E+09	1.00E+09	0	3.66E+03	3.66E+03	0	5.33E+03	5328.195	0	1.30E+03	1297.43
b3980_fold_KJE_2	b3980_m folding: KJE mediated	1 GrpE_dim + 1 b3980_m_DnaKJ_complex + 1 h2o --> 1 DnaJ_dim_inact + 1 TufB_DnaK_GrpE_complex + 1 adp + 1 h + 1 pi	irreversible	Protein Folding	0.00E+00	1.00E+09	0	3.66E+03	3.66E+03	0	5.33E+03	5328.195	0	1.30E+03	1297.43
b3980_fold_KJE_3	b3980_m folding: KJE mediated	1 TufB_DnaK_GrpE_complex + 1 atp --> 1 DnaK_mono.ATP_inact + 1 GrpE_dim_inact + 1 TufB_mono	irreversible	Protein Folding	0.00E+00	1.00E+09	0	3.66E+03	3.66E+03	0	5.33E+03	5328.195	0	1.30E+03	1297.43
b3980_fold_spon	b3980_m folding: spontanous	1 b3980_m --> 1 TufB_mono	irreversible	Protein Folding	0.00E+00	1.00E+09	0	3.66E+03	3.66E+03	0	5.33E+03	5328.195	0	1.30E+03	1297.43
b3980_mRNA_CONV	convsersion of mRNA to mRNA_1 (synthetic rxn)	1 b3980_mRNA --> 1 b3980_mRNA_1	irreversible	Translation	0.00E+00	1.00E+09	0	1.04E+02	1.04E+02	0	1.84E+02	183.6469	0	1.51E+00	1.51
b3980_mRNA_degr1	Degradation of b3980_mRNA I	1 Orn_dim + 296 atp + 1 b3980_mRNA_2 + 1 degradosome + 296 h2o <==> 296 adp + 1 b3980_mRNA_2_degr + 296 h + 296 pi	reversible	mRNA degradation	-1.00E+09	1.00E+09	0	1.04E+02	1.04E+02	0	1.84E+02	183.6469	0	1.51E+00	1.51
b3980_mRNA_degr2	Degradation of b3980_mRNA II	1 b3980_mRNA_2_degr + 1184 h2o --> 1 Orn_dim_inact + 289 amp 1 atp + 307 cmp + 1 degradosome_inact + 326 gmp + 1184 h + 262 ump	irreversible	mRNA degradation	0.00E+00	1.51E+00	0	1.04E+02	1.04E+02	0	1.84E+02	183.6469	0	1.51E+00	1.51
b3980_maturation1	Polypeptide b3980 peptide deformylas and methionine aminopeptidase complex	1 Def_mono + 1 Map_mono + 1 b3980_aa <==> 1 b3980_def_map_cplx	reversible	Protein Maturation	-1.00E+09	1.00E+09	0	3.66E+03	3.66E+03	0	5.33E+03	5328.195	0	1.30E+03	1297.43
b3980_maturation2	b3980 formation	1 b3980_def_map_cplx + 2 h2o --> 1 Def_mono_inact + 1 Map_mono_inact + 1 b3980_m + 1 for + 1 met-L	irreversible	Protein Maturation	0.00E+00	1.00E+09	0	3.66E+03	3.66E+03	0	5.33E+03	5328.195	0	1.30E+03	1297.43

b3980_v1_mRNA_CONV	convsersion of mRNA to mRNA_1 (synthetic rxn)	1 b3980_v1_mRNA --> 1 b3980_mRNA_1	irreversible	Translation	0.00E+00	1.00E+09	0	1.04E+02	1.04E+02	0	1.84E+02	183.6469	0	1.51E+00	1.51
b3981_fold_spon	b3981_m folding: spontanous	1 b3981_m --> 1 SecE_mono	irreversible	Protein Folding	0.00E+00	1.00E+09	0	1.13E+03	1.13E+03	0	1.93E+03	1931.692	0	8.92E+01	89.173
b3981_mRNA_CONV	convsersion of mRNA to mRNA_1 (synthetic rxn)	1 b3981_mRNA --> 1 b3981_mRNA_1	irreversible	Translation	0.00E+00	1.00E+09	0	1.49E+02	1.49E+02	0	2.63E+02	263.456	0	2.39E-01	0.239
b3981_mRNA_degr1	Degradation of b3981_mRNA I	1 Orn_dim + 96 atp + 1 b3981_mRNA_2 + 1 degradosome + 96 h2o <==> 96 adp + 1 b3981_mRNA_2_degr + 96 h + 96 pi	reversible	mRNA degradation	-1.00E+09	1.00E+09	0	1.49E+02	1.49E+02	0	2.63E+02	263.456	0	2.39E-01	0.239
b3981_mRNA_degr2	Degradation of b3981_mRNA II	1 b3981_mRNA_2_degr + 383 h2o --> 1 Orn_dim_inact + 72 amp + 1 atp + 90 cmp + 1 degradosome_inact + 121 gmp + 383 h + 100 ump	irreversible	mRNA degradation	0.00E+00	2.39E-01	0	1.49E+02	1.49E+02	0	2.63E+02	263.456	0	2.39E-01	0.239
b3981_maturation1	Polypeptide b3981 peptide deformylas and methionine aminopeptidase complex	1 Def_mono + 1 Map_mono + 1 b3981_aa <==> 1 b3981_def_map_cplx	reversible	Protein Maturation	-1.00E+09	1.00E+09	0	1.13E+03	1.13E+03	0	1.93E+03	1931.692	0	8.92E+01	89.173
b3981_maturation2	b3981 formation	1 b3981_def_map_cplx + 2 h2o --> 1 Def_mono_inact + 1 Map_mono_inact + 1 b3981_m + 1 for + 1 met-L	irreversible	Protein Maturation	0.00E+00	1.00E+09	0	1.13E+03	1.13E+03	0	1.93E+03	1931.692	0	8.92E+01	89.173
b3982_fold_GroEL/ES_1	b3982_m folding: GroEL/ES mediated; polypeptide is going in GroEL/ES complex	1 GroEL.(7)ADP.cisGroES + 7 atp + 1 b3982_m + 1 transGroES_hepta --> 7 adp + 1 b3982_m_GroEL.(7)ATP.transGroES + 1 cisGroES_hepta	irreversible	Protein Folding	0.00E+00	1.00E+09	0	1.58E+03	1.58E+03	0	2.72E+03	2722.16	0	9.00E+01	89.99
b3982_fold_GroEL/ES_2	b3982_m folding: GroEL/ES mediated; folding of polypeptide under ATP hydrolysis	1 b3982_m_GroEL.(7)ATP.transGroES + 7 h2o --> 1 NusG_GroEL.(7)ADP.transGroES + 7 h + 7 pi	irreversible	Protein Folding	0.00E+00	1.00E+09	0	1.58E+03	1.58E+03	0	2.72E+03	2722.16	0	9.00E+01	89.99
b3982_fold_GroEL/ES_3	b3982_m folding: GroEL/ES mediated; release of native protein	1 NusG_GroEL.(7)ADP.transGroES --> 1 GroEL.(7)ADP.transGroES + 1 NusG_mono	irreversible	Protein Folding	0.00E+00	1.00E+09	0	1.58E+03	1.58E+03	0	2.72E+03	2722.16	0	9.00E+01	89.99
b3982_fold_KJE_1	b3982_m folding: KJE mediated	1 DnaJ_dim + 1 DnaK_mono.ATP + 1 b3982_m <==> 1 b3982_m_DnaKJ_complex	reversible	Protein Folding	-1.00E+09	1.00E+09	0	1.58E+03	1.58E+03	0	2.72E+03	2722.16	0	9.00E+01	89.99
b3982_fold_KJE_2	b3982_m folding: KJE mediated	1 GrpE_dim + 1 b3982_m_DnaKJ_complex + 1 h2o --> 1 DnaJ_dim_inact + 1 NusG_DnaK_GrpE_complex + 1 adp + 1 h + 1 pi	irreversible	Protein Folding	0.00E+00	1.00E+09	0	1.58E+03	1.58E+03	0	2.72E+03	2722.16	0	9.00E+01	89.99
b3982_fold_KJE_3	b3982_m folding: KJE mediated	1 NusG_DnaK_GrpE_complex + 1 atp --> 1 DnaK_mono.ATP_inact + 1 GrpE_dim_inact + 1 NusG_mono	irreversible	Protein Folding	0.00E+00	1.00E+09	0	1.58E+03	1.58E+03	0	2.72E+03	2722.16	0	9.00E+01	89.99
b3982_mRNA_CONV	convsersion of mRNA to mRNA_1 (synthetic rxn)	1 b3982_mRNA --> 1 b3982_mRNA_1	irreversible	Translation	0.00E+00	1.00E+09	0	1.49E+02	1.49E+02	0	2.63E+02	263.456	0	2.39E-01	0.239
b3982_mRNA_degr1	Degradation of b3982_mRNA I	1 Orn_dim + 136 atp + 1 b3982_mRNA_2 + 1 degradosome + 136 h2o <==> 136 adp + 1 b3982_mRNA_2_degr + 136 h + 136 pi	reversible	mRNA degradation	-1.00E+09	1.00E+09	0	1.49E+02	1.49E+02	0	2.63E+02	263.456	0	2.39E-01	0.239
b3982_mRNA_degr2	Degradation of b3982_mRNA II	1 b3982_mRNA_2_degr + 545 h2o --> 1 Orn_dim_inact + 128 amp + 124 cmp + 1 degradosome_inact + 158 gmp + 545 h + 136 ump	irreversible	mRNA degradation	0.00E+00	3.26E-01	0	1.49E+02	1.49E+02	0	2.63E+02	263.456	0	2.39E-01	0.239
b3982_maturation1	Polypeptide b3982 peptide deformylas and methionine aminopeptidase complex	1 Def_mono + 1 Map_mono + 1 b3982_aa <==> 1 b3982_def_map_cplx	reversible	Protein Maturation	-1.00E+09	1.00E+09	0	1.58E+03	1.58E+03	0	2.72E+03	2722.16	0	9.00E+01	89.99
b3982_maturation2	b3982 formation	1 b3982_def_map_cplx + 2 h2o --> 1 Def_mono_inact + 1 Map_mono_inact + 1 b3982_m + 1 for + 1 met-L	irreversible	Protein Maturation	0.00E+00	1.00E+09	0	1.58E+03	1.58E+03	0	2.72E+03	2722.16	0	9.00E+01	89.99
b3983_fold_spon	b3983_m folding: spontanous	1 b3983_m --> 1 RplK_mono	irreversible	Protein Folding	0.00E+00	1.00E+09	8.44E+01	1.13E+02	2.81E+01	6.22E+01	8.29E+01	20.72	6.22E+01	8.29E+01	20.72
b3983_mRNA_CONV	convsersion of mRNA to mRNA_1 (synthetic rxn)	1 b3983_mRNA --> 1 b3983_mRNA_1	irreversible	Translation	0.00E+00	1.00E+09	0	1.14E+01	1.14E+01	0	2.02E+01	20.15717	0	1.89E-01	0.189
b3983_mRNA_degr1	Degradation of b3983_mRNA I	1 Orn_dim + 107 atp + 1 b3983_mRNA_2 + 1 degradosome + 107 h2o <==> 107 adp + 1 b3983_mRNA_2_degr + 107 h + 107 pi	reversible	mRNA degradation	-1.00E+09	1.00E+09	0	1.14E+01	1.14E+01	0	2.02E+01	20.15717	0	1.89E-01	0.189
b3983_mRNA_degr2	Degradation of b3983_mRNA II	1 b3983_mRNA_2_degr + 428 h2o --> 1 Orn_dim_inact + 113 amp + 1 atp + 110 cmp + 1 degradosome_inact + 114 gmp + 428 h + 91 ump	irreversible	mRNA degradation	0.00E+00	1.08E+00	0	1.14E+01	1.14E+01	0	2.02E+01	20.15717	0	1.89E-01	0.189
b3983_maturation1	Polypeptide b3983 peptide deformylas and methionine aminopeptidase complex	1 Def_mono + 1 Map_mono + 1 b3983_aa <==> 1 b3983_def_map_cplx	reversible	Protein Maturation	-1.00E+09	1.00E+09	8.44E+01	1.13E+02	2.81E+01	6.22E+01	8.29E+01	20.72	6.22E+01	8.29E+01	20.72
b3983_maturation2	b3983 formation	1 b3983_def_map_cplx + 2 h2o --> 1 Def_mono_inact + 1 Map_mono_inact + 1 b3983_m + 1 for + 1 met-L	irreversible	Protein Maturation	0.00E+00	1.00E+09	8.44E+01	1.13E+02	2.81E+01	6.22E+01	8.29E+01	20.72	6.22E+01	8.29E+01	20.72
b3984_fold_spon	b3984_m folding: spontanous	1 b3984_m --> 1 RplA_mono	irreversible	Protein Folding	0.00E+00	1.00E+09	8.44E+01	1.13E+02	2.81E+01	6.22E+01	8.29E+01	20.72	6.22E+01	8.29E+01	20.72
b3984_mRNA_CONV	convsersion of mRNA to mRNA_1 (synthetic rxn)	1 b3984_mRNA --> 1 b3984_mRNA_1	irreversible	Translation	0.00E+00	1.00E+09	0	1.14E+01	1.14E+01	0	2.02E+01	20.15717	0	1.89E-01	0.189
b3984_mRNA_degr1	Degradation of b3984_mRNA I	1 Orn_dim + 176 atp + 1 b3984_mRNA_2 + 1 degradosome + 176 h2o <==> 176 adp + 1 b3984_mRNA_2_degr + 176 h + 176 pi	reversible	mRNA degradation	-1.00E+09	1.00E+09	0	1.14E+01	1.14E+01	0	2.02E+01	20.15717	0	1.89E-01	0.189
b3984_mRNA_degr2	Degradation of b3984_mRNA II	1 b3984_mRNA_2_degr + 704 h2o --> 1 Orn_dim_inact + 196 amp + 173 cmp + 1 degradosome_inact + 191 gmp + 704 h + 145 ump	irreversible	mRNA degradation	0.00E+00	4.62E-01	0	1.14E+01	1.14E+01	0	2.02E+01	20.15717	0	1.89E-01	0.189
b3984_maturation1	Polypeptide b3984 peptide deformylas and methionine aminopeptidase complex	1 Def_mono + 1 Map_mono + 1 b3984_aa <==> 1 b3984_def_map_cplx	reversible	Protein Maturation	-1.00E+09	1.00E+09	8.44E+01	1.13E+02	2.81E+01	6.22E+01	8.29E+01	20.72	6.22E+01	8.29E+01	20.72
b3984_maturation2	b3984 formation	1 b3984_def_map_cplx + 2 h2o --> 1 Def_mono_inact + 1 Map_mono_inact + 1 b3984_m + 1 for + 1 met-L	irreversible	Protein Maturation	0.00E+00	1.00E+09	8.44E+01	1.13E+02	2.81E+01	6.22E+01	8.29E+01	20.72	6.22E+01	8.29E+01	20.72
b3985_fold_spon	b3985_m folding: spontanous	1 b3985_m --> 1 RplJ_mono	irreversible	Protein Folding	0.00E+00	1.00E+09	8.44E+01	1.13E+02	2.81E+01	6.22E+01	8.29E+01	20.72	6.22E+01	8.29E+01	20.72
b3985_mRNA_CONV	convsersion of mRNA to mRNA_1 (synthetic rxn)	1 b3985_mRNA --> 1 b3985_mRNA_1	irreversible	Translation	0.00E+00	1.00E+09	0	1.32E+01	1.32E+01	0	2.33E+01	23.27811	0	1.89E-01	0.189
b3985_mRNA_degr1	Degradation of b3985_mRNA I	1 Orn_dim + 124 atp + 1 b3985_mRNA_2 + 1 degradosome + 124 h2o <==> 124 adp + 1 b3985_mRNA_2_degr + 124 h + 124 pi	reversible	mRNA degradation	-1.00E+09	1.00E+09	0	1.32E+01	1.32E+01	0	2.33E+01	23.27811	0	1.89E-01	0.189
b3985_mRNA_degr2	Degradation of b3985_mRNA II	1 b3985_mRNA_2_degr + 497 h2o --> 1 Orn_dim_inact + 120 amp + 1 atp + 122 cmp + 1 degradosome_inact + 139 gmp + 497 h + 116 ump	irreversible	mRNA degradation	0.00E+00	4.04E-01	0	1.32E+01	1.32E+01	0	2.33E+01	23.27811	0	1.89E-01	0.189
b3985_maturation1	Polypeptide b3985 peptide deformylas and methionine aminopeptidase complex	1 Def_mono + 1 Map_mono + 1 b3985_aa <==> 1 b3985_def_map_cplx	reversible	Protein Maturation	-1.00E+09	1.00E+09	8.44E+01	1.13E+02	2.81E+01	6.22E+01	8.29E+01	20.72	6.22E+01	8.29E+01	20.72
b3985_maturation2	b3985 formation	1 b3985_def_map_cplx + 2 h2o --> 1 Def_mono_inact + 1 Map_mono_inact + 1 b3985_m + 1 for + 1 met-L	irreversible	Protein Maturation	0.00E+00	1.00E+09	8.44E+01	1.13E+02	2.81E+01	6.22E+01	8.29E+01	20.72	6.22E+01	8.29E+01	20.72
b3985_v1_mRNA_CONV	convsersion of mRNA to mRNA_1 (synthetic rxn)	1 b3985_v1_mRNA --> 1 b3985_mRNA_1	irreversible	Translation	0.00E+00	1.00E+09	0	1.14E+01	1.14E+01	0	2.02E+01	20.15717	0	1.89E-01	0.189
b3986_fold_spon	b3986_m folding: spontanous	1 b3986_m --> 1 RplL_mono	irreversible	Protein Folding	0.00E+00	1.00E+09	3.38E+02	4.50E+02	1.13E+02	2.49E+02	3.32E+02	82.88	2.49E+02	3.32E+02	82.88
b3986_mRNA_CONV	convsersion of mRNA to mRNA_1 (synthetic rxn)	1 b3986_mRNA --> 1 b3986_mRNA_1	irreversible	Translation	0.00E+00	1.00E+09	0	1.32E+01	1.32E+01	0	2.33E+01	23.27811	0	1.89E-01	0.189
b3986_mRNA_degr1	Degradation of b3986_mRNA I	1 Orn_dim + 91 atp + 1 b3986_mRNA_2 + 1 degradosome + 91 h2o <==> 91 adp + 1 b3986_mRNA_2_degr + 91 h + 91 pi	reversible	mRNA degradation	-1.00E+09	1.00E+09	0	3.61E+02	3.61E+02	0	3.32E+02	331.52	0	1.70E+00	1.7
b3986_mRNA_degr2	Degradation of b3986_mRNA II	1 b3986_mRNA_2_degr + 365 h2o --> 1 Orn_dim_inact + 112 amp + 70 cmp + 1 degradosome_inact + 99 gmp + 365 h + 85 ump	irreversible	mRNA degradation	0.00E+00	1.70E+00	0	3.61E+02	3.61E+02	0	3.32E+02	331.52	0	1.70E+00	1.7
b3986_maturation1	Polypeptide b3986 peptide deformylas and methionine aminopeptidase complex	1 Def_mono + 1 Map_mono + 1 b3986_aa <==> 1 b3986_def_map_cplx	reversible	Protein Maturation	-1.00E+09	1.00E+09	3.38E+02	4.50E+02	1.13E+02	2.49E+02	3.32E+02	82.88	2.49E+02	3.32E+02	82.88
b3986_maturation2	b3986 formation	1 b3986_def_map_cplx + 2 h2o --> 1 Def_mono_inact + 1 Map_mono_inact + 1 b3986_m + 1 for + 1 met-L	irreversible	Protein Maturation	0.00E+00	1.00E+09	3.38E+02	4.50E+02	1.13E+02	2.49E+02	3.32E+02	82.88	2.49E+02	3.32E+02	82.88
b3986_v1_mRNA_CONV	convsersion of mRNA to mRNA_1 (synthetic rxn)	1 b3986_v1_mRNA --> 1 b3986_mRNA_1	irreversible	Translation	0.00E+00	1.00E+09	0	3.61E+02	3.61E+02	0	3.32E+02	331.52	0	1.70E+00	1.7
b3986_v2_mRNA_CONV	convsersion of mRNA to mRNA_1 (synthetic rxn)	1 b3986_v2_mRNA --> 1 b3986_mRNA_1	irreversible	Translation	0.00E+00	1.00E+09	0	1.14E+01	1.14E+01	0	2.02E+01	20.15717	0	1.89E-01	0.189
b3987_fold_GroEL/ES_1	b3987_m folding: GroEL/ES mediated; polypeptide is going in GroEL/ES complex	1 GroEL.(7)ADP.cisGroES + 7 atp + 1 b3987_m_Zn + 1 transGroES_hepta --> 7 adp + 1 b3987_m_GroEL.(7)ATP.transGroES + 1 cisGroES_hepta	irreversible	Protein Folding	0.00E+00	1.00E+09	0	4.82E+02	4.82E+02	0	6.51E+02	650.6377	0	1.02E+02	102.418

b3987_fold_GroEL/ES_2	b3987_m folding: GroEL/ES mediated: folding of polypeptide under ATP hydrolysis	1 b3987_m_GroEL.(7)ATP.transGroES + 7 h2o --> 1 RpoB_GroEL.(7)ADP.transGroES + 7 h + 7 pi	irreversible	Protein Folding	0.00E+00	1.00E+09	0	4.82E+02	4.82E+02	0	6.51E+02	650.6377	0	1.02E+02	102.418
b3987_fold_GroEL/ES_3	b3987_m folding: GroEL/ES mediated: release of native protein	1 RpoB_GroEL.(7)ADP.transGroES --> 1 GroEL.(7)ADP.transGroES + 1 RpoB_mono	irreversible	Protein Folding	0.00E+00	1.00E+09	0	4.82E+02	4.82E+02	0	6.51E+02	650.6377	0	1.02E+02	102.418
b3987_fold_KJE_1	b3987_m folding: KJE mediated	1 DnaJ_dim + 1 DnaK_mono.ATP + 1 b3987_m_Zn <==> 1 b3987_m_DnaKJ_complex	reversible	Protein Folding	-1.00E+09	1.00E+09	0	4.82E+02	4.82E+02	0	6.51E+02	650.6377	0	1.02E+02	102.418
b3987_fold_KJE_2	b3987_m folding: KJE mediated	1 GrpE_dim + 1 b3987_m_DnaKJ_complex + 1 h2o --> 1 DnaJ_dim_inact + 1 RpoB_DnaK_GrpE_complex + 1 adp + 1 h + 1 pi	irreversible	Protein Folding	0.00E+00	1.00E+09	0	4.82E+02	4.82E+02	0	6.51E+02	650.6377	0	1.02E+02	102.418
b3987_fold_KJE_3	b3987_m folding: KJE mediated	1 RpoB_DnaK_GrpE_complex + 1 atp --> 1 DnaK_mono.ATP_inact + 1 GrpE_dim_inact + 1 RpoB_mono	irreversible	Protein Folding	0.00E+00	1.00E+09	0	4.82E+02	4.82E+02	0	6.51E+02	650.6377	0	1.02E+02	102.418
b3987_ion_BIND	Binding of ions to b3987	1 b3987_m + 1 zn2 <==> 1 b3987_m_Zn	reversible	Metallo-ion Binding	-1.00E+09	1.00E+09	0	4.82E+02	4.82E+02	0	6.51E+02	650.6377	0	1.02E+02	102.418
b3987_mRNA_CONV	convsersion of mRNA to mRNA_1 (synthetic rxn)	1 b3987_mRNA --> 1 b3987_mRNA_1	irreversible	Translation	0.00E+00	1.00E+09	0	1.32E+01	1.32E+01	0	2.33E+01	23.27811	0	1.89E-01	0.189
b3987_mRNA_degr1	Degradation of b3987_mRNA I	1 Orn_dim + 1007 atp + 1 b3987_mRNA_2 + 1 degradosome + 1007 h2o <==> 1007 adp + 1 b3987_mRNA_2_degr + 1007 h + 1007 pi	reversible	mRNA degradation	-1.00E+09	1.00E+09	0	1.49E+01	1.49E+01	0	2.63E+01	26.30872	0	1.89E-01	0.189
b3987_mRNA_degr2	Degradation of b3987_mRNA II	1 b3987_mRNA_2_degr + 4028 h2o --> 1 Orn_dim_inact + 1017 amp + 1033 cmp + 1 degradosome_inact + 1087 gmp + 4028 h + 892 ump	irreversible	mRNA degradation	0.00E+00	2.84E-01	0	1.49E+01	1.49E+01	0	2.63E+01	26.30872	0	1.89E-01	0.189
b3987_maturation1	Polypeptide b3987 peptide deformylase complex	1 Def_mono + 1 b3987_aa <==> 1 b3987_def_cplx	reversible	Protein Maturation	-1.00E+09	1.00E+09	0	4.82E+02	4.82E+02	0	6.51E+02	650.6377	0	1.02E+02	102.418
b3987_maturation2	b3987 formation	1 b3987_def_cplx + 1 h2o --> 1 Def_mono_inact + 1 b3987_m + 1 for	irreversible	Protein Maturation	0.00E+00	1.00E+09	0	4.82E+02	4.82E+02	0	6.51E+02	650.6377	0	1.02E+02	102.418
b3987_v1_mRNA_CONV	convsersion of mRNA to mRNA_1 (synthetic rxn)	1 b3987_v1_mRNA --> 1 b3987_mRNA_1	irreversible	Translation	0.00E+00	1.00E+09	0	1.49E+01	1.49E+01	0	2.63E+01	26.30872	0	1.89E-01	0.189
b3987_v2_mRNA_CONV	convsersion of mRNA to mRNA_1 (synthetic rxn)	1 b3987_v2_mRNA --> 1 b3987_mRNA_1	irreversible	Translation	0.00E+00	1.00E+09	0	1.14E+01	1.14E+01	0	2.02E+01	20.15717	0	1.89E-01	0.189
b3988_fold_GroEL/ES_1	b3988_m folding: GroEL/ES mediated: polypeptide is going in GroEL/ES complex	1 GroEL.(7)ADP.cisGroES + 7 atp + 1 b3988_m_Mg + 1 transGroES_hepta --> 7 adp + 1 b3988_m_GroEL.(7)ATP.transGroES + 1 cisGroES_hepta	irreversible	Protein Folding	0.00E+00	1.00E+09	0	4.82E+02	4.82E+02	0	6.51E+02	650.6377	0	1.02E+02	102.418
b3988_fold_GroEL/ES_2	b3988_m folding: GroEL/ES mediated: folding of polypeptide under ATP hydrolysis	1 b3988_m_GroEL.(7)ATP.transGroES + 7 h2o --> 1 RpoC_GroEL.(7)ADP.transGroES + 7 h + 7 pi	irreversible	Protein Folding	0.00E+00	1.00E+09	0	4.82E+02	4.82E+02	0	6.51E+02	650.6377	0	1.02E+02	102.418
b3988_fold_GroEL/ES_3	b3988_m folding: GroEL/ES mediated: release of native protein	1 RpoC_GroEL.(7)ADP.transGroES --> 1 GroEL.(7)ADP.transGroES + 1 RpoC_mono	irreversible	Protein Folding	0.00E+00	1.00E+09	0	4.82E+02	4.82E+02	0	6.51E+02	650.6377	0	1.02E+02	102.418
b3988_fold_KJE_1	b3988_m folding: KJE mediated	1 DnaJ_dim + 1 DnaK_mono.ATP + 1 b3988_m_Mg <==> 1 b3988_m_DnaKJ_complex	reversible	Protein Folding	-1.00E+09	1.00E+09	0	4.82E+02	4.82E+02	0	6.51E+02	650.6377	0	1.02E+02	102.418
b3988_fold_KJE_2	b3988_m folding: KJE mediated	1 GrpE_dim + 1 b3988_m_DnaKJ_complex + 1 h2o --> 1 DnaJ_dim_inact + 1 RpoC_DnaK_GrpE_complex + 1 adp + 1 h + 1 pi	irreversible	Protein Folding	0.00E+00	1.00E+09	0	4.82E+02	4.82E+02	0	6.51E+02	650.6377	0	1.02E+02	102.418
b3988_fold_KJE_3	b3988_m folding: KJE mediated	1 RpoC_DnaK_GrpE_complex + 1 atp --> 1 DnaK_mono.ATP_inact + 1 GrpE_dim_inact + 1 RpoC_mono	irreversible	Protein Folding	0.00E+00	1.00E+09	0	4.82E+02	4.82E+02	0	6.51E+02	650.6377	0	1.02E+02	102.418
b3988_ion_BIND	Binding of ions to b3988	1 b3988_m + 2 mg2 <==> 1 b3988_m_Mg	reversible	Metallo-ion Binding	-1.00E+09	1.00E+09	0	4.82E+02	4.82E+02	0	6.51E+02	650.6377	0	1.02E+02	102.418
b3988_mRNA_CONV	convsersion of mRNA to mRNA_1 (synthetic rxn)	1 b3988_mRNA --> 1 b3988_mRNA_1	irreversible	Translation	0.00E+00	1.00E+09	0	1.32E+01	1.32E+01	0	2.33E+01	23.27811	0	1.89E-01	0.189
b3988_mRNA_degr1	Degradation of b3988_mRNA I	1 Orn_dim + 1056 atp + 1 b3988_mRNA_2 + 1 degradosome + 1056 h2o <==> 1056 adp + 1 b3988_mRNA_2_degr + 1056 h + 1056 pi	reversible	mRNA degradation	-1.00E+09	1.00E+09	0	1.49E+01	1.49E+01	0	2.63E+01	26.30872	0	1.89E-01	0.189
b3988_mRNA_degr2	Degradation of b3988_mRNA II	1 b3988_mRNA_2_degr + 4223 h2o --> 1 Orn_dim_inact + 1012 amp + 1088 cmp + 1 degradosome_inact + 1186 gmp + 4223 h + 938 ump	irreversible	mRNA degradation	0.00E+00	1.89E-01	0	1.49E+01	1.49E+01	0	2.63E+01	26.30872	0	1.89E-01	0.189
b3988_maturation1	Polypeptide b3988 peptide deformylase complex	1 Def_mono + 1 b3988_aa <==> 1 b3988_def_cplx	reversible	Protein Maturation	-1.00E+09	1.00E+09	0	4.82E+02	4.82E+02	0	6.51E+02	650.6377	0	1.02E+02	102.418
b3988_maturation2	b3988 formation	1 b3988_def_cplx + 1 h2o --> 1 Def_mono_inact + 1 b3988_m + 1 for	irreversible	Protein Maturation	0.00E+00	1.00E+09	0	4.82E+02	4.82E+02	0	6.51E+02	650.6377	0	1.02E+02	102.418
b3988_v1_mRNA_CONV	convsersion of mRNA to mRNA_1 (synthetic rxn)	1 b3988_v1_mRNA --> 1 b3988_mRNA_1	irreversible	Translation	0.00E+00	1.00E+09	0	1.49E+01	1.49E+01	0	2.63E+01	26.30872	0	1.89E-01	0.189
b3988_v2_mRNA_CONV	convsersion of mRNA to mRNA_1 (synthetic rxn)	1 b3988_v2_mRNA --> 1 b3988_mRNA_1	irreversible	Translation	0.00E+00	1.00E+09	0	1.14E+01	1.14E+01	0	2.02E+01	20.15717	0	1.89E-01	0.189
b4007_RNA_10_modif_1	b4007_RNA modification (16S rRNA): position: 967, m5C by RsmB_mono	1 RsmB_mono + 1 amet + 1 b4007_RNA_10 <==> 1 b4007_RNA_10_RsmB_mono	reversible	rRNA modification	-1.00E+09	1.00E+09	0	1.13E+02	1.13E+02	0	1.26E+01	12.6	0	1.26E+01	12.6
b4007_RNA_10_modif_2	b4007_RNA modification (16S rRNA): position: 967, m5C by RsmB_mono	1 b4007_RNA_10_RsmB_mono --> 1 RsmB_mono_inact + 1 ahcys + 1 h + 1 rrsE	irreversible	rRNA modification	0.00E+00	1.00E+09	0	1.13E+02	1.13E+02	0	1.26E+01	12.6	0	1.26E+01	12.6
b4007_RNA_1_modif_1	b4007_RNA modification (16S rRNA): position: 1402, m4Cm by MeT_16S_1402	1 MeT_16S_1402 + 2 amet + 1 b4007_RNA_1 <==> 1 b4007_RNA_1_MeT_16S_1402	reversible	rRNA modification	-1.00E+09	1.00E+09	0	1.13E+02	1.13E+02	0	1.26E+01	12.6	0	1.26E+01	12.6
b4007_RNA_1_modif_2	b4007_RNA modification (16S rRNA): position: 1402, m4Cm by MeT_16S_1402	1 b4007_RNA_1_MeT_16S_1402 --> 1 MeT_16S_1402_inact + 2 ahcys + 1 b4007_RNA_2 + 2 h	irreversible	rRNA modification	0.00E+00	1.00E+09	0	1.13E+02	1.13E+02	0	1.26E+01	12.6	0	1.26E+01	12.6
b4007_RNA_2_modif_1	b4007_RNA modification (16S rRNA): position: 1407, m5C by MeT_16S_1407	1 MeT_16S_1407 + 1 amet + 1 b4007_RNA_2 <==> 1 b4007_RNA_2_MeT_16S_1407	reversible	rRNA modification	-1.00E+09	1.00E+09	0	1.13E+02	1.13E+02	0	1.26E+01	12.6	0	1.26E+01	12.6
b4007_RNA_2_modif_2	b4007_RNA modification (16S rRNA): position: 1407, m5C by MeT_16S_1407	1 b4007_RNA_2_MeT_16S_1407 --> 1 MeT_16S_1407_inact + 1 ahcys + 1 b4007_RNA_3 + 1 h	irreversible	rRNA modification	0.00E+00	1.00E+09	0	1.13E+02	1.13E+02	0	1.26E+01	12.6	0	1.26E+01	12.6
b4007_RNA_3_modif_1	b4007_RNA modification (16S rRNA): position: 1498, m3U by YggJ_mono	1 YggJ_mono + 1 amet + 1 b4007_RNA_3 <==> 1 b4007_RNA_3_YggJ_mono	reversible	rRNA modification	-1.00E+09	1.00E+09	0	1.13E+02	1.13E+02	0	1.26E+01	12.6	0	1.26E+01	12.6
b4007_RNA_3_modif_2	b4007_RNA modification (16S rRNA): position: 1498, m3U by YggJ_mono	1 b4007_RNA_3_YggJ_mono --> 1 YggJ_mono_inact + 1 ahcys + 1 b4007_RNA_4 + 1 h	irreversible	rRNA modification	0.00E+00	1.00E+09	0	1.13E+02	1.13E+02	0	1.26E+01	12.6	0	1.26E+01	12.6
b4007_RNA_4_modif_1	b4007_RNA modification (16S rRNA): position: 1516, m2G by MeT_16S_1516	1 MeT_16S_1516 + 1 amet + 1 b4007_RNA_4 <==> 1 b4007_RNA_4_MeT_16S_1516	reversible	rRNA modification	-1.00E+09	1.00E+09	0	1.13E+02	1.13E+02	0	1.26E+01	12.6	0	1.26E+01	12.6
b4007_RNA_4_modif_2	b4007_RNA modification (16S rRNA): position: 1516, m2G by MeT_16S_1516	1 b4007_RNA_4_MeT_16S_1516 --> 1 MeT_16S_1516_inact + 1 ahcys + 1 b4007_RNA_5 + 1 h	irreversible	rRNA modification	0.00E+00	1.00E+09	0	1.13E+02	1.13E+02	0	1.26E+01	12.6	0	1.26E+01	12.6
b4007_RNA_5_modif_1	b4007_RNA modification (16S rRNA): position: 1518, m6(2)A by KsgA_mono	1 KsgA_mono + 2 amet + 1 b4007_RNA_5 <==> 1 b4007_RNA_5_KsgA_mono	reversible	rRNA modification	-1.00E+09	1.00E+09	0	1.13E+02	1.13E+02	0	1.26E+01	12.6	0	1.26E+01	12.6
b4007_RNA_5_modif_2	b4007_RNA modification (16S rRNA): position: 1518, m6(2)A by KsgA_mono	1 b4007_RNA_5_KsgA_mono --> 1 KsgA_mono_inact + 2 ahcys + 1 b4007_RNA_6 + 2 h	irreversible	rRNA modification	0.00E+00	1.00E+09	0	1.13E+02	1.13E+02	0	1.26E+01	12.6	0	1.26E+01	12.6
b4007_RNA_6_modif_1	b4007_RNA modification (16S rRNA): position: 1519, m6(2)A by KsgA_mono	1 KsgA_mono + 2 amet + 1 b4007_RNA_6 <==> 1 b4007_RNA_6_KsgA_mono	reversible	rRNA modification	-1.00E+09	1.00E+09	0	1.13E+02	1.13E+02	0	1.26E+01	12.6	0	1.26E+01	12.6
b4007_RNA_6_modif_2	b4007_RNA modification (16S rRNA): position: 1519, m6(2)A by KsgA_mono	1 b4007_RNA_6_KsgA_mono --> 1 KsgA_mono_inact + 2 ahcys + 1 b4007_RNA_7 + 2 h	irreversible	rRNA modification	0.00E+00	1.00E+09	0	1.13E+02	1.13E+02	0	1.26E+01	12.6	0	1.26E+01	12.6
b4007_RNA_7_modif_1	b4007_RNA modification (16S rRNA): position: 516, Y by RsuA_mono	1 RsuA_mono + 1 b4007_RNA_7 <==> 1 b4007_RNA_7_RsuA_mono	reversible	rRNA modification	-1.00E+09	1.00E+09	0	1.13E+02	1.13E+02	0	1.26E+01	12.6	0	1.26E+01	12.6
b4007_RNA_7_modif_2	b4007_RNA modification (16S rRNA): position: 516, Y by RsuA_mono	1 b4007_RNA_7_RsuA_mono --> 1 RsuA_mono_inact + 1 b4007_RNA_8	irreversible	rRNA modification	0.00E+00	1.00E+09	0	1.13E+02	1.13E+02	0	1.26E+01	12.6	0	1.26E+01	12.6

b4007_RNA_8_modif_1	b4007_RNA modification (16S rRNA): position: 527, m7G by MeT_16S_527	1 MeT_16S_527 + 1 amet + 1 b4007_RNA_8 <==> 1 b4007_RNA_8_MeT_16S_527	reversible	rRNA modification	-1.00E+09	1.00E+09	0	1.13E+02	1.13E+02	0	1.26E+01	12.6	0	1.26E+01	12.6
b4007_RNA_8_modif_2	b4007_RNA modification (16S rRNA): position: 527, m7G by MeT_16S_527	1 b4007_RNA_8_MeT_16S_527 --> 1 MeT_16S_527_inact + 1 ahcy + 1 b4007_RNA_9 + 1 h	irreversible	rRNA modification	0.00E+00	1.00E+09	0	1.13E+02	1.13E+02	0	1.26E+01	12.6	0	1.26E+01	12.6
b4007_RNA_9_modif_1	b4007_RNA modification (16S rRNA): position: 966, m2G by MeT_16S_966	1 MeT_16S_966 + 1 amet + 1 b4007_RNA_9 <==> 1 b4007_RNA_9_MeT_16S_966	reversible	rRNA modification	-1.00E+09	1.00E+09	0	1.13E+02	1.13E+02	0	1.26E+01	12.6	0	1.26E+01	12.6
b4007_RNA_9_modif_2	b4007_RNA modification (16S rRNA): position: 966, m2G by MeT_16S_966	1 b4007_RNA_9_MeT_16S_966 --> 1 MeT_16S_966_inact + 1 ahcy + 1 b4007_RNA_10 + 1 h	irreversible	rRNA modification	0.00E+00	1.00E+09	0	1.13E+02	1.13E+02	0	1.26E+01	12.6	0	1.26E+01	12.6
b4007_RNA_modif_1	b4007_RNA modification (16S rRNA): position: 1207, m2G by RsmC_mono	1 RsmC_mono + 1 amet + 1 b4007_RNA <==> 1 b4007_RNA_RsmC_mono	reversible	rRNA modification	-1.00E+09	1.00E+09	0	1.13E+02	1.13E+02	0	1.26E+01	12.6	0	1.26E+01	12.6
b4007_RNA_modif_2	b4007_RNA modification (16S rRNA): position: 1207, m2G by RsmC_mono	1 b4007_RNA_RsmC_mono --> 1 RsmC_mono_inact + 1 ahcys + 1 b4007_RNA_1 + 1 h	irreversible	rRNA modification	0.00E+00	1.00E+09	0	1.13E+02	1.13E+02	0	1.26E+01	12.6	0	1.26E+01	12.6
b4008_tRNA_13_MODIF_1	modification of position 13 nt (Y) in gltV by TruD_mono	1 TruD_mono + 1 b4008_tRNA_Mg2 <==> 1 b4008_tRNA_Mg2_TruD_mono_cplx	reversible	tRNA modification	-1.00E+09	1.00E+09	0	1.13E+02	1.13E+02	0	1.26E+01	12.6	0	1.26E+01	12.6
b4008_tRNA_13_MODIF_2	modification of position 13 nt (Y) in gltV by TruD_mono	1 b4008_tRNA_Mg2_TruD_mono_cplx --> 1 TruD_mono_inact + 1 b4008_tRNA_1	irreversible	tRNA modification	0.00E+00	1.00E+09	0	1.13E+02	1.13E+02	0	1.26E+01	12.6	0	1.26E+01	12.6
b4008_tRNA_34_MODIF_1	modification of position 34 nt (mnm5s2U) in gltV by TrmU_mono-YhhP_mono-YheLMN_cplx-YccK_mono-TrmE_dim-GidA_mono-TrmC_mono	1 5fthf + 1 GidA_mono + 1 IscS_dim_S-SH + 1 TrmC_mono + 1 TrmE_dim + 1 TrmU_mono + 1 YccK_mono + 1 YheLMN_cplx + 1 YhhP_mono + 1 amet + 1 atp + 1 b4008_tRNA_1 + 1 fad + 1 fadh2 + 1 gly + 1 gtp + 1 h2o + 1 mg2 + 1 trdrd <==> 1 b4008_tRNA_1_TrmU_mono-YhhP_mono-YheLMN_cplx-YccK_mon TrmE_dim-GidA_mono-TrmC_mono_cplx	reversible	tRNA modification	-1.00E+09	1.00E+09	0	1.13E+02	1.13E+02	0	1.26E+01	12.6	0	1.26E+01	12.6
b4008_tRNA_34_MODIF_2	modification of position 34 nt (mnm5s2U) in gltV by TrmU_mono-YhhP_mono-YheLMN_cplx-YccK_mono-TrmE_dim-GidA_mono-TrmC_mono	1 b4008_tRNA_1_TrmU_mono-YhhP_mono-YheLMN_cplx-YccK_mono-TrmE_dim-GidA_mono-TrmC_mono_cplx --> 1 GidA_mono_inact + 1 IscS_dim_S-H + 1 TrmC_mono_inact + 1 TrmE_dim_inact + 1 TrmU_mono_inact + 1 YccK_mono_inact + 1 YheLMN_cplx_inact + 1 YhhP_mono_inact + 1 ahcys + 1 amp + 1 b4008_tRNA_2 + 1 fad + 1 fadh2 + 1 gdp + 1 glx + 4 h + 1 mg2 + 1 pi + 1 ppi + 1 thf + 1 trdox	irreversible	tRNA modification	0.00E+00	1.00E+09	0	1.13E+02	1.13E+02	0	1.26E+01	12.6	0	1.26E+01	12.6
b4008_tRNA_34_MODIF_3	replacement of sulfur with selenium: b4008_tRNA_2	1 YbbB_dim + 1 b4008_tRNA_2 + 1 h2o + 1 selnp <==> 1 b4008_tRNA_2_YbbB_dim_cplx	reversible	tRNA modification	-1.00E+09	1.00E+09	0	1.13E+02	1.13E+02	0	1.26E+01	12.6	0	1.26E+01	12.6
b4008_tRNA_34_MODIF_4	replacement of sulfur with selenium: b4008_tRNA_2	1 b4008_tRNA_2_YbbB_dim_cplx --> 1 YbbB_dim_inact + 1 b4008_tRNA_2_Se + 1 h + 1 h2s + 1 pi	irreversible	tRNA modification	0.00E+00	1.00E+09	0	1.13E+02	1.13E+02	0	1.26E+01	12.6	0	1.26E+01	12.6
b4008_tRNA_37_MODIF_1	modification of position 37 nt (m2A) in gltV by MeT_tRNA_pos_37_m2A	1 MeT_tRNA_pos_37_m2A + 1 amet + 1 b4008_tRNA_2 <==> 1 b4008_tRNA_2_MeT_tRNA_pos_37_m2A_cplx	reversible	tRNA modification	-1.00E+09	1.00E+09	0	1.13E+02	1.13E+02	0	1.26E+01	12.6	0	1.26E+01	12.6
b4008_tRNA_37_MODIF_1_Se	modification of position 37 nt (m2A) in gltV by MeT_tRNA_pos_37_m2A	1 MeT_tRNA_pos_37_m2A + 1 amet + 1 b4008_tRNA_2_Se <==> 1 b4008_tRNA_2_Se_MeT_tRNA_pos_37_m2A_cplx	reversible	tRNA modification	-1.00E+09	1.00E+09	0	1.13E+02	1.13E+02	0	1.26E+01	12.6	0	1.26E+01	12.6
b4008_tRNA_37_MODIF_2	modification of position 37 nt (m2A) in gltV by MeT_tRNA_pos_37_m2A	1 b4008_tRNA_2_MeT_tRNA_pos_37_m2A_cplx --> 1 MeT_tRNA_pos_37_m2A_inact + 1 ahcys + 1 b4008_tRNA_3 + 1 h	irreversible	tRNA modification	0.00E+00	1.00E+09	0	1.13E+02	1.13E+02	0	1.26E+01	12.6	0	1.26E+01	12.6
b4008_tRNA_37_MODIF_2_Se	modification of position 37 nt (m2A) in gltV by MeT_tRNA_pos_37_m2A	1 b4008_tRNA_2_Se_MeT_tRNA_pos_37_m2A_cplx --> 1 MeT_tRNA_pos_37_m2A_inact + 1 ahcys + 1 b4008_tRNA_3_Se + h	irreversible	tRNA modification	0.00E+00	1.00E+09	0	1.13E+02	1.13E+02	0	1.26E+01	12.6	0	1.26E+01	12.6
b4008_tRNA_54_MODIF_1	modification of position 54 nt (m5U) in gltV by TrmA_mono	1 TrmA_mono + 1 amet + 1 b4008_tRNA_3 <==> 1 b4008_tRNA_3_TrmA_mono_cplx	reversible	tRNA modification	-1.00E+09	1.00E+09	0	1.13E+02	1.13E+02	0	1.26E+01	12.6	0	1.26E+01	12.6
b4008_tRNA_54_MODIF_1_Se	modification of position 54 nt (m5U) in gltV by TrmA_mono	1 TrmA_mono + 1 amet + 1 b4008_tRNA_3_Se <==> 1 b4008_tRNA_3_Se_TrmA_mono_cplx	reversible	tRNA modification	-1.00E+09	1.00E+09	0	1.13E+02	1.13E+02	0	1.26E+01	12.6	0	1.26E+01	12.6
b4008_tRNA_54_MODIF_2	modification of position 54 nt (m5U) in gltV by TrmA_mono	1 b4008_tRNA_3_TrmA_mono_cplx --> 1 TrmA_mono_inact + 1 ahcys + 1 b4008_tRNA_4 + 1 h	irreversible	tRNA modification	0.00E+00	1.00E+09	0	1.13E+02	1.13E+02	0	1.26E+01	12.6	0	1.26E+01	12.6
b4008_tRNA_54_MODIF_2_Se	modification of position 54 nt (m5U) in gltV by TrmA_mono	1 b4008_tRNA_3_Se_TrmA_mono_cplx --> 1 TrmA_mono_inact + 1 ahcys + 1 b4008_tRNA_4_Se + 1 h	irreversible	tRNA modification	0.00E+00	1.00E+09	0	1.13E+02	1.13E+02	0	1.26E+01	12.6	0	1.26E+01	12.6
b4008_tRNA_55_MODIF_1	modification of position 55 nt (Y) in gltV by TruB_mono	1 TruB_mono + 1 b4008_tRNA_4 <==> 1 b4008_tRNA_4_TruB_mono_cplx	reversible	tRNA modification	-1.00E+09	1.00E+09	0	1.13E+02	1.13E+02	0	1.26E+01	12.6	0	1.26E+01	12.6
b4008_tRNA_55_MODIF_1_Se	modification of position 55 nt (Y) in gltV by TruB_mono	1 TruB_mono + 1 b4008_tRNA_4_Se <==> 1 b4008_tRNA_4_Se_TruB_mono_cplx	reversible	tRNA modification	-1.00E+09	1.00E+09	0	1.13E+02	1.13E+02	0	1.26E+01	12.6	0	1.26E+01	12.6
b4008_tRNA_55_MODIF_2	modification of position 55 nt (Y) in gltV by TruB_mono	1 b4008_tRNA_4_TruB_mono_cplx --> 1 TruB_mono_inact + 1 gltV_tRNA	irreversible	tRNA modification	0.00E+00	1.00E+09	0	1.13E+02	1.13E+02	0	1.26E+01	12.6	0	1.26E+01	12.6
b4008_tRNA_55_MODIF_2_Se	modification of position 55 nt (Y) in gltV by TruB_mono	1 b4008_tRNA_4_Se_TruB_mono_cplx --> 1 TruB_mono_inact + 1 gltV_tRNA_Se	irreversible	tRNA modification	0.00E+00	1.00E+09	0	1.13E+02	1.13E+02	0	1.26E+01	12.6	0	1.26E+01	12.6
b4008_tRNA_Mg_BIND	Binding of two Mg2 to b4008_tRNA	1 b4008_RNA + 2 mg2 <==> 1 b4008_tRNA_Mg2	reversible	Metallo-ion binding	-1.00E+09	1.00E+09	0	1.13E+02	1.13E+02	0	1.26E+01	12.6	0	1.26E+01	12.6
b4009_RNA_10_modif_1	b4009_RNA modification (23S rRNA): position: 2445, m2G by MeT_23S_2445	1 MeT_23S_2445 + 1 amet + 1 b4009_RNA_10 <==> 1 b4009_RNA_10_MeT_23S_2445	reversible	rRNA modification	-1.00E+09	1.00E+09	0	1.13E+02	1.13E+02	0	1.26E+01	12.6	0	1.26E+01	12.6
b4009_RNA_10_modif_2	b4009_RNA modification (23S rRNA): position: 2445, m2G by MeT_23S_2445	1 b4009_RNA_10_MeT_23S_2445 --> 1 MeT_23S_2445_inact + 1 ahcys + 1 b4009_RNA_11 + 1 h	irreversible	rRNA modification	0.00E+00	1.00E+09	0	1.13E+02	1.13E+02	0	1.26E+01	12.6	0	1.26E+01	12.6
b4009_RNA_11_a_modif_1	b4009_RNA modification (23S rRNA): position: 2449, D by DU_23S_2449 (NADH)	1 DU_23S_2449 + 1 b4009_RNA_11 + 1 h + 1 nadh <==> 1 b4009_RNA_11_DU_23S_2449_a	reversible	rRNA modification	-1.00E+09	1.00E+09	0	1.13E+02	1.13E+02	0	1.26E+01	12.6	0	1.26E+01	12.6
b4009_RNA_11_a_modif_2	b4009_RNA modification (23S rRNA): position: 2449, D by DU_23S_2449 (NADH)	1 b4009_RNA_11_DU_23S_2449_a --> 1 DU_23S_2449_inact + 1 b4009_RNA_12 + 1 nad	irreversible	rRNA modification	0.00E+00	1.00E+09	0	1.13E+02	1.13E+02	0	1.26E+01	12.6	0	1.26E+01	12.6
b4009_RNA_11_b_modif_1	b4009_RNA modification (23S rRNA): position: 2449, D by DU_23S_2449 (NADPH)	1 DU_23S_2449 + 1 b4009_RNA_11 + 1 h + 1 nadph <==> 1 b4009_RNA_11_DU_23S_2449_b	reversible	rRNA modification	-1.00E+09	1.00E+09	0	1.13E+02	1.13E+02	0	1.26E+01	12.6	0	1.26E+01	12.6
b4009_RNA_11_b_modif_2	b4009_RNA modification (23S rRNA): position: 2449, D by DU_23S_2449 (NADPH)	1 b4009_RNA_11_DU_23S_2449_b --> 1 DU_23S_2449_inact + 1 b4009_RNA_12 + 1 nadp	irreversible	rRNA modification	0.00E+00	1.00E+09	0	1.13E+02	1.13E+02	0	1.26E+01	12.6	0	1.26E+01	12.6
b4009_RNA_12_modif_1	b4009_RNA modification (23S rRNA): position: 2457, Y by YmfC_mono	1 YmfC_mono + 1 b4009_RNA_12 <==> 1 b4009_RNA_12_YmfC_mono	reversible	rRNA modification	-1.00E+09	1.00E+09	0	1.13E+02	1.13E+02	0	1.26E+01	12.6	0	1.26E+01	12.6
b4009_RNA_12_modif_2	b4009_RNA modification (23S rRNA): position: 2457, Y by YmfC_mono	1 b4009_RNA_12_YmfC_mono --> 1 YmfC_mono_inact + 1 b4009_RNA_13	irreversible	rRNA modification	0.00E+00	1.00E+09	0	1.13E+02	1.13E+02	0	1.26E+01	12.6	0	1.26E+01	12.6
b4009_RNA_13_modif_1	b4009_RNA modification (23S rRNA): position: 2498, Cm by MeT_23S_2498	1 MeT_23S_2498 + 1 amet + 1 b4009_RNA_13 <==> 1 b4009_RNA_13_MeT_23S_2498	reversible	rRNA modification	-1.00E+09	1.00E+09	0	1.13E+02	1.13E+02	0	1.26E+01	12.6	0	1.26E+01	12.6
b4009_RNA_13_modif_2	b4009_RNA modification (23S rRNA): position: 2498, Cm by MeT_23S_2498	1 b4009_RNA_13_MeT_23S_2498 --> 1 MeT_23S_2498_inact + 1 ahcys + 1 b4009_RNA_14 + 1 h	irreversible	rRNA modification	0.00E+00	1.00E+09	0	1.13E+02	1.13E+02	0	1.26E+01	12.6	0	1.26E+01	12.6
b4009_RNA_14_modif_1	b4009_RNA modification (23S rRNA): position: 2503, m2A by MeT_23S_2503	1 MeT_23S_2503 + 1 amet + 1 b4009_RNA_14 <==> 1 b4009_RNA_14_MeT_23S_2503	reversible	rRNA modification	-1.00E+09	1.00E+09	0	1.13E+02	1.13E+02	0	1.26E+01	12.6	0	1.26E+01	12.6
b4009_RNA_14_modif_2	b4009_RNA modification (23S rRNA): position: 2503, m2A by MeT_23S_2503	1 b4009_RNA_14_MeT_23S_2503 --> 1 MeT_23S_2503_inact + 1 ahcys + 1 b4009_RNA_15 + 1 h	irreversible	rRNA modification	0.00E+00	1.00E+09	0	1.13E+02	1.13E+02	0	1.26E+01	12.6	0	1.26E+01	12.6
b4009_RNA_15_modif_1	b4009_RNA modification (23S rRNA): position: 2504, Y by RluC_mono	1 RluC_mono + 1 b4009_RNA_15 <==> 1 b4009_RNA_15_RluC_mono	reversible	rRNA modification	-1.00E+09	1.00E+09	0	1.13E+02	1.13E+02	0	1.26E+01	12.6	0	1.26E+01	12.6
b4009_RNA_15_modif_2	b4009_RNA modification (23S rRNA): position: 2504, Y by RluC_mono	1 b4009_RNA_15_RluC_mono --> 1 RluC_mono_inact + 1 b4009_RNA_16	irreversible	rRNA modification	0.00E+00	1.00E+09	0	1.13E+02	1.13E+02	0	1.26E+01	12.6	0	1.26E+01	12.6
b4009_RNA_16_modif_1	b4009_RNA modification (23S rRNA): position: 2552, Um by RrmJ_mono	1 RrmJ_mono + 1 amet + 1 b4009_RNA_16 <==> 1 b4009_RNA_16_RrmJ_mono	reversible	rRNA modification	-1.00E+09	1.00E+09	0	1.13E+02	1.13E+02	0	1.26E+01	12.6	0	1.26E+01	12.6

b4009_RNA_16_modif_2	b4009_RNA modification (23S rRNA): position: 2552, Um by RrmJ_mono	1 b4009_RNA_16_RrmJ_mono --> 1 RrmJ_mono_inact + 1 ahcys + b4009_RNA_17 + 1 h	irreversible	rRNA modification	0.00E+00	1.00E+09	0	1.13E+02	1.13E+02	0	1.26E+01	12.6	0	1.26E+01	12.6
b4009_RNA_17_modif_1	b4009_RNA modification (23S rRNA): position: 2580, Y by RluC_mono	1 RluC_mono + 1 b4009_RNA_17 <==> 1 b4009_RNA_17_RluC_mono	reversible	rRNA modification	-1.00E+09	1.00E+09	0	1.13E+02	1.13E+02	0	1.26E+01	12.6	0	1.26E+01	12.6
b4009_RNA_17_modif_2	b4009_RNA modification (23S rRNA): position: 2580, Y by RluC_mono	1 b4009_RNA_17_RluC_mono --> 1 RluC_mono_inact + 1 b4009_RNA_18	irreversible	rRNA modification	0.00E+00	1.00E+09	0	1.13E+02	1.13E+02	0	1.26E+01	12.6	0	1.26E+01	12.6
b4009_RNA_18_modif_1	b4009_RNA modification (23S rRNA): position: 2604, Y by YjbC_mono	1 YjbC_mono + 1 b4009_RNA_18 <==> 1 b4009_RNA_18_YjbC_mono	reversible	rRNA modification	-1.00E+09	1.00E+09	0	1.13E+02	1.13E+02	0	1.26E+01	12.6	0	1.26E+01	12.6
b4009_RNA_18_modif_2	b4009_RNA modification (23S rRNA): position: 2604, Y by YjbC_mono	1 b4009_RNA_18_YjbC_mono --> 1 YjbC_mono_inact + 1 b4009_RNA_19	irreversible	rRNA modification	0.00E+00	1.00E+09	0	1.13E+02	1.13E+02	0	1.26E+01	12.6	0	1.26E+01	12.6
b4009_RNA_19_modif_1	b4009_RNA modification (23S rRNA): position: 2605, Y by RluB_mono	1 RluB_mono + 1 b4009_RNA_19 <==> 1 b4009_RNA_19_RluB_mono	reversible	rRNA modification	-1.00E+09	1.00E+09	0	1.13E+02	1.13E+02	0	1.26E+01	12.6	0	1.26E+01	12.6
b4009_RNA_19_modif_2	b4009_RNA modification (23S rRNA): position: 2605, Y by RluB_mono	1 b4009_RNA_19_RluB_mono --> 1 RluB_mono_inact + 1 b4009_RNA_20	irreversible	rRNA modification	0.00E+00	1.00E+09	0	1.13E+02	1.13E+02	0	1.26E+01	12.6	0	1.26E+01	12.6
b4009_RNA_1_modif_1	b4009_RNA modification (23S rRNA): position: 1835, m2G by MeT_23S_1835	1 MeT_23S_1835 + 1 amet + 1 b4009_RNA_1 <==> 1 b4009_RNA_1_MeT_23S_1835	reversible	rRNA modification	-1.00E+09	1.00E+09	0	1.13E+02	1.13E+02	0	1.26E+01	12.6	0	1.26E+01	12.6
b4009_RNA_1_modif_2	b4009_RNA modification (23S rRNA): position: 1835, m2G by MeT_23S_1835	1 b4009_RNA_1_MeT_23S_1835 --> 1 MeT_23S_1835_inact + 1 ahcys + 1 b4009_RNA_2 + 1 h	irreversible	rRNA modification	0.00E+00	1.00E+09	0	1.13E+02	1.13E+02	0	1.26E+01	12.6	0	1.26E+01	12.6
b4009_RNA_20_modif_1	b4009_RNA modification (23S rRNA): position: 745, m1G by RrmA_dim	1 RrmA_dim + 1 amet + 1 b4009_RNA_20 <==> 1 b4009_RNA_20_RrmA_dim	reversible	rRNA modification	-1.00E+09	1.00E+09	0	1.13E+02	1.13E+02	0	1.26E+01	12.6	0	1.26E+01	12.6
b4009_RNA_20_modif_2	b4009_RNA modification (23S rRNA): position: 745, m1G by RrmA_dim	1 b4009_RNA_20_RrmA_dim --> 1 RrmA_dim_inact + 1 ahcys + 1 b4009_RNA_21 + 1 h	irreversible	rRNA modification	0.00E+00	1.00E+09	0	1.13E+02	1.13E+02	0	1.26E+01	12.6	0	1.26E+01	12.6
b4009_RNA_21_modif_1	b4009_RNA modification (23S rRNA): position: 746, Y by RluA_mono	1 RluA_mono + 1 b4009_RNA_21 <==> 1 b4009_RNA_21_RluA_mono	reversible	rRNA modification	-1.00E+09	1.00E+09	0	1.13E+02	1.13E+02	0	1.26E+01	12.6	0	1.26E+01	12.6
b4009_RNA_21_modif_2	b4009_RNA modification (23S rRNA): position: 746, Y by RluA_mono	1 b4009_RNA_21_RluA_mono --> 1 RluA_mono_inact + 1 b4009_RNA_22	irreversible	rRNA modification	0.00E+00	1.00E+09	0	1.13E+02	1.13E+02	0	1.26E+01	12.6	0	1.26E+01	12.6
b4009_RNA_22_modif_1	b4009_RNA modification (23S rRNA): position: 747, m5U by RumB_mono	1 RumB_mono + 1 amet + 1 b4009_RNA_22 <==> 1 b4009_RNA_22_RumB_mono	reversible	rRNA modification	-1.00E+09	1.00E+09	0	1.13E+02	1.13E+02	0	1.26E+01	12.6	0	1.26E+01	12.6
b4009_RNA_22_modif_2	b4009_RNA modification (23S rRNA): position: 747, m5U by RumB_mono	1 b4009_RNA_22_RumB_mono --> 1 RumB_mono_inact + 1 ahcys + 1 b4009_RNA_23 + 1 h	irreversible	rRNA modification	0.00E+00	1.00E+09	0	1.13E+02	1.13E+02	0	1.26E+01	12.6	0	1.26E+01	12.6
b4009_RNA_23_modif_1	b4009_RNA modification (23S rRNA): position: 955, Y by RluC_mono	1 RluC_mono + 1 b4009_RNA_23 <==> 1 b4009_RNA_23_RluC_mono	reversible	rRNA modification	-1.00E+09	1.00E+09	0	1.13E+02	1.13E+02	0	1.26E+01	12.6	0	1.26E+01	12.6
b4009_RNA_23_modif_2	b4009_RNA modification (23S rRNA): position: 955, Y by RluC_mono	1 b4009_RNA_23_RluC_mono --> 1 RluC_mono_inact + 1 rrlE	irreversible	rRNA modification	0.00E+00	1.00E+09	0	1.13E+02	1.13E+02	0	1.26E+01	12.6	0	1.26E+01	12.6
b4009_RNA_2_modif_1	b4009_RNA modification (23S rRNA): position: 1911, Y by RluD_mono	1 RluD_mono + 1 b4009_RNA_2 <==> 1 b4009_RNA_2_RluD_mono	reversible	rRNA modification	-1.00E+09	1.00E+09	0	1.13E+02	1.13E+02	0	1.26E+01	12.6	0	1.26E+01	12.6
b4009_RNA_2_modif_2	b4009_RNA modification (23S rRNA): position: 1911, Y by RluD_mono	1 b4009_RNA_2_RluD_mono --> 1 RluD_mono_inact + 1 b4009_RNA_3	irreversible	rRNA modification	0.00E+00	1.00E+09	0	1.13E+02	1.13E+02	0	1.26E+01	12.6	0	1.26E+01	12.6
b4009_RNA_3_modif_1	b4009_RNA modification (23S rRNA): position: 1915, m3Y by RluD_mono	1 RluD_mono + 1 amet + 1 b4009_RNA_3 <==> 1 b4009_RNA_3_RluD_mono	reversible	rRNA modification	-1.00E+09	1.00E+09	0	1.13E+02	1.13E+02	0	1.26E+01	12.6	0	1.26E+01	12.6
b4009_RNA_3_modif_2	b4009_RNA modification (23S rRNA): position: 1915, m3Y by RluD_mono	1 b4009_RNA_3_RluD_mono --> 1 RluD_mono_inact + 1 ahcys + 1 b4009_RNA_4 + 1 h	irreversible	rRNA modification	0.00E+00	1.00E+09	0	1.13E+02	1.13E+02	0	1.26E+01	12.6	0	1.26E+01	12.6
b4009_RNA_4_modif_1	b4009_RNA modification (23S rRNA): position: 1917, Y by RluD_mono	1 RluD_mono + 1 b4009_RNA_4 <==> 1 b4009_RNA_4_RluD_mono	reversible	rRNA modification	-1.00E+09	1.00E+09	0	1.13E+02	1.13E+02	0	1.26E+01	12.6	0	1.26E+01	12.6
b4009_RNA_4_modif_2	b4009_RNA modification (23S rRNA): position: 1917, Y by RluD_mono	1 b4009_RNA_4_RluD_mono --> 1 RluD_mono_inact + 1 b4009_RNA_5	irreversible	rRNA modification	0.00E+00	1.00E+09	0	1.13E+02	1.13E+02	0	1.26E+01	12.6	0	1.26E+01	12.6
b4009_RNA_5_modif_1	b4009_RNA modification (23S rRNA): position: 1939, m5U by RumA_mono	1 RumA_mono + 1 amet + 1 b4009_RNA_5 <==> 1 b4009_RNA_5_RumA_mono	reversible	rRNA modification	-1.00E+09	1.00E+09	0	1.13E+02	1.13E+02	0	1.26E+01	12.6	0	1.26E+01	12.6
b4009_RNA_5_modif_2	b4009_RNA modification (23S rRNA): position: 1939, m5U by RumA_mono	1 b4009_RNA_5_RumA_mono --> 1 RumA_mono_inact + 1 ahcys + 1 b4009_RNA_6 + 1 h	irreversible	rRNA modification	0.00E+00	1.00E+09	0	1.13E+02	1.13E+02	0	1.26E+01	12.6	0	1.26E+01	12.6
b4009_RNA_6_modif_1	b4009_RNA modification (23S rRNA): position: 1962, m5C by MeT_23S_1962	1 MeT_23S_1962 + 1 amet + 1 b4009_RNA_6 <==> 1 b4009_RNA_6_MeT_23S_1962	reversible	rRNA modification	-1.00E+09	1.00E+09	0	1.13E+02	1.13E+02	0	1.26E+01	12.6	0	1.26E+01	12.6
b4009_RNA_6_modif_2	b4009_RNA modification (23S rRNA): position: 1962, m5C by MeT_23S_1962	1 b4009_RNA_6_MeT_23S_1962 --> 1 MeT_23S_1962_inact + 1 ahcys + 1 b4009_RNA_7 + 1 h	irreversible	rRNA modification	0.00E+00	1.00E+09	0	1.13E+02	1.13E+02	0	1.26E+01	12.6	0	1.26E+01	12.6
b4009_RNA_7_modif_1	b4009_RNA modification (23S rRNA): position: 2030, m6A by MeT_23S_2030	1 MeT_23S_2030 + 1 amet + 1 b4009_RNA_7 <==> 1 b4009_RNA_7_MeT_23S_2030	reversible	rRNA modification	-1.00E+09	1.00E+09	0	1.13E+02	1.13E+02	0	1.26E+01	12.6	0	1.26E+01	12.6
b4009_RNA_7_modif_2	b4009_RNA modification (23S rRNA): position: 2030, m6A by MeT_23S_2030	1 b4009_RNA_7_MeT_23S_2030 --> 1 MeT_23S_2030_inact + 1 ahcys + 1 b4009_RNA_8 + 1 h	irreversible	rRNA modification	0.00E+00	1.00E+09	0	1.13E+02	1.13E+02	0	1.26E+01	12.6	0	1.26E+01	12.6
b4009_RNA_8_modif_1	b4009_RNA modification (23S rRNA): position: 2069, m7G by MeT_23S_2069	1 MeT_23S_2069 + 1 amet + 1 b4009_RNA_8 <==> 1 b4009_RNA_8_MeT_23S_2069	reversible	rRNA modification	-1.00E+09	1.00E+09	0	1.13E+02	1.13E+02	0	1.26E+01	12.6	0	1.26E+01	12.6
b4009_RNA_8_modif_2	b4009_RNA modification (23S rRNA): position: 2069, m7G by MeT_23S_2069	1 b4009_RNA_8_MeT_23S_2069 --> 1 MeT_23S_2069_inact + 1 ahcys + 1 b4009_RNA_9 + 1 h	irreversible	rRNA modification	0.00E+00	1.00E+09	0	1.13E+02	1.13E+02	0	1.26E+01	12.6	0	1.26E+01	12.6
b4009_RNA_9_modif_1	b4009_RNA modification (23S rRNA): position: 2251, Gm by RlmB_dim	1 RlmB_dim + 1 amet + 1 b4009_RNA_9 <==> 1 b4009_RNA_9_RlmB_dim	reversible	rRNA modification	-1.00E+09	1.00E+09	0	1.13E+02	1.13E+02	0	1.26E+01	12.6	0	1.26E+01	12.6
b4009_RNA_9_modif_2	b4009_RNA modification (23S rRNA): position: 2251, Gm by RlmB_dim	1 b4009_RNA_9_RlmB_dim --> 1 RlmB_dim_inact + 1 ahcys + 1 b4009_RNA_10 + 1 h	irreversible	rRNA modification	0.00E+00	1.00E+09	0	1.13E+02	1.13E+02	0	1.26E+01	12.6	0	1.26E+01	12.6
b4009_RNA_modif_1	b4009_RNA modification (23S rRNA): position: 1618, m6A by MeT_23S_1618	1 MeT_23S_1618 + 1 amet + 1 b4009_RNA <==> 1 b4009_RNA_MeT_23S_1618	reversible	rRNA modification	-1.00E+09	1.00E+09	0	1.13E+02	1.13E+02	0	1.26E+01	12.6	0	1.26E+01	12.6
b4009_RNA_modif_2	b4009_RNA modification (23S rRNA): position: 1618, m6A by MeT_23S_1618	1 b4009_RNA_MeT_23S_1618 --> 1 MeT_23S_1618_inact + 1 ahcy + 1 b4009_RNA_1 + 1 h	irreversible	rRNA modification	0.00E+00	1.00E+09	0	1.13E+02	1.13E+02	0	1.26E+01	12.6	0	1.26E+01	12.6
b4022_fold_spon	b4022_m folding: spontanous	1 b4022_m --> 1 YjbC_mono	irreversible	Protein Folding	0.00E+00	1.00E+09	0	2.70E+03	2.70E+03	0	4.70E+03	4700.938	0	9.59E+01	95.897
b4022_mRNA_CONV	convsersion of mRNA to mRNA_1 (synthetic rxn)	1 b4022_mRNA --> 1 b4022_mRNA_1	irreversible	Translation	0.00E+00	1.00E+09	0	1.54E+02	1.54E+02	0	2.71E+02	271.0557	0	1.71E-01	0.171
b4022_mRNA_degr1	Degradation of b4022_mRNA I	1 Orn_dim + 218 atp + 1 b4022_mRNA_2 + 1 degradosome + 218 h2o <==> 218 adp + 1 b4022_mRNA_2_degr + 218 h + 218 pi	reversible	mRNA degradation	-1.00E+09	1.00E+09	0	1.54E+02	1.54E+02	0	2.71E+02	271.0557	0	1.71E-01	0.171
b4022_mRNA_degr2	Degradation of b4022_mRNA II	1 b4022_mRNA_2_degr + 872 h2o --> 1 Orn_dim_inact + 241 amp + 1 atp + 208 cmp + 1 degradosome_inact + 242 gmp + 872 h + 181 ump	irreversible	mRNA degradation	0.00E+00	1.71E-01	0	1.54E+02	1.54E+02	0	2.71E+02	271.0557	0	1.71E-01	0.171

b4022_maturation1	Polypeptide b4022 peptide deformylas and methionine aminopeptidase complex	1 Def_mono + 1 Map_mono + 1 b4022_aa <==> 1 b4022_def_map_cplx	reversible	Protein Maturation	-1.00E+09	1.00E+09	0	2.70E+03	2.70E+03	0	4.70E+03	4700.938	0	9.59E+01	95.897
b4022_maturation2	b4022 formation	1 b4022_def_map_cplx + 2 h2o --> 1 Def_mono_inact + 1 Map_mono_inact + 1 b4022_m + 1 for + 1 met-L	irreversible	Protein Maturation	0.00E+00	1.00E+09	0	2.70E+03	2.70E+03	0	4.70E+03	4700.938	0	9.59E+01	95.897
b4049_fold_GroEL/ES_1	b4049_m folding: GroEL/ES mediated: polypeptide is going in GroEL/ES complex	1 GroEL.(7)ADP.cisGroES + 7 atp + 1 b4049_m + 1 transGroES_hepta --> 7 adp + 1 b4049_m_GroEL.(7)ATP.transGroES + 1 cisGroES_hepta	irreversible	Protein Folding	0.00E+00	1.00E+09	0	2.73E+03	2.73E+03	0	4.75E+03	4751.471	0	9.56E+01	95.6
b4049_fold_GroEL/ES_2	b4049_m folding: GroEL/ES mediated: folding of polypeptide under ATP hydrolysis	1 b4049_m_GroEL.(7)ATP.transGroES + 7 h2o --> 1 DusA_GroEL.(7)ADP.transGroES + 7 h + 7 pi	irreversible	Protein Folding	0.00E+00	1.00E+09	0	2.73E+03	2.73E+03	0	4.75E+03	4751.471	0	9.56E+01	95.6
b4049_fold_GroEL/ES_3	b4049_m folding: GroEL/ES mediated: release of native protein	1 DusA_GroEL.(7)ADP.transGroES --> 1 DusA_mono + 1 GroEL.(7)ADP.transGroES	irreversible	Protein Folding	0.00E+00	1.00E+09	0	2.73E+03	2.73E+03	0	4.75E+03	4751.471	0	9.56E+01	95.6
b4049_fold_KJE_1	b4049_m folding: KJE mediated	1 DnaJ_dim + 1 DnaK_mono.ATP + 1 b4049_m <==> 1 b4049_m_DnaKJ_complex	reversible	Protein Folding	-1.00E+09	1.00E+09	0	2.73E+03	2.73E+03	0	4.75E+03	4751.471	0	9.56E+01	95.6
b4049_fold_KJE_2	b4049_m folding: KJE mediated	1 GrpE_dim + 1 b4049_m_DnaKJ_complex + 1 h2o --> 1 DnaJ_dim_inact + 1 DusA_DnaK_GrpE_complex + 1 adp + 1 h + 1 pi	irreversible	Protein Folding	0.00E+00	1.00E+09	0	2.73E+03	2.73E+03	0	4.75E+03	4751.471	0	9.56E+01	95.6
b4049_fold_KJE_3	b4049_m folding: KJE mediated	1 DusA_DnaK_GrpE_complex + 1 atp --> 1 DnaK_mono.ATP_inact + 1 DusA_mono + 1 GrpE_dim_inact	irreversible	Protein Folding	0.00E+00	1.00E+09	0	2.73E+03	2.73E+03	0	4.75E+03	4751.471	0	9.56E+01	95.6
b4049_mRNA_CONV	convsersion of mRNA to mRNA_1 (synthetic rxn)	1 b4049_mRNA --> 1 b4049_mRNA_1	irreversible	Translation	0.00E+00	1.00E+09	0	1.32E+02	1.32E+02	0	2.33E+02	232.9735	0	1.80E-01	0.18
b4049_mRNA_degr1	Degradation of b4049_mRNA I	1 Orn_dim + 259 atp + 1 b4049_mRNA_2 + 1 degradosome + 259 h2o <==> 259 adp + 1 b4049_mRNA_2_degr + 259 h + 259 pi	reversible	mRNA degradation	-1.00E+09	1.00E+09	0	1.32E+02	1.32E+02	0	2.33E+02	232.9735	0	1.80E-01	0.18
b4049_mRNA_degr2	Degradation of b4049_mRNA II	1 b4049_mRNA_2_degr + 1037 h2o --> 1 Orn_dim_inact + 243 amp + 1 atp + 242 cmp + 1 degradosome_inact + 315 gmp + 1037 h + 237 ump	irreversible	mRNA degradation	0.00E+00	1.80E-01	0	1.32E+02	1.32E+02	0	2.33E+02	232.9735	0	1.80E-01	0.18
b4049_maturation1	Polypeptide b4049 peptide deformylas and methionine aminopeptidase complex	1 Def_mono + 1 Map_mono + 1 b4049_aa <==> 1 b4049_def_map_cplx	reversible	Protein Maturation	-1.00E+09	1.00E+09	0	2.73E+03	2.73E+03	0	4.75E+03	4751.471	0	9.56E+01	95.6
b4049_maturation2	b4049 formation	1 b4049_def_map_cplx + 17 h2o --> 1 Def_mono_inact + 1 Map_mono_inact + 1 ala-L + 2 asn-L + 1 b4049_m + 1 for + 2 gln-L + 1 glu-L + 1 gly + 1 his-L + 1 ile-L + 1 lys-L + 3 met-L + 2 ser-L + 1 thr-L	irreversible	Protein Maturation	0.00E+00	1.00E+09	0	2.73E+03	2.73E+03	0	4.75E+03	4751.471	0	9.56E+01	95.6
b4129_fold_KJE_1	b4129_m folding: KJE mediated	1 DnaJ_dim + 1 DnaK_mono.ATP + 1 b4129_m_Mg <==> 1 b4129_m_DnaKJ_complex	reversible	Protein Folding	-1.00E+09	1.00E+09	0	2.84E+03	2.84E+03	0	4.29E+03	4285.722	0	1.19E+02	118.523
b4129_fold_KJE_2	b4129_m folding: KJE mediated	1 GrpE_dim + 1 b4129_m_DnaKJ_complex + 1 h2o --> 1 DnaJ_dim_inact + 1 LysU_DnaK_GrpE_complex + 1 adp + 1 h + 1 pi	irreversible	Protein Folding	0.00E+00	1.00E+09	0	2.84E+03	2.84E+03	0	4.29E+03	4285.722	0	1.19E+02	118.523
b4129_fold_KJE_3	b4129_m folding: KJE mediated	1 LysU_DnaK_GrpE_complex + 1 atp --> 1 DnaK_mono.ATP_inact + 1 GrpE_dim_inact + 1 LysU_mono	irreversible	Protein Folding	0.00E+00	1.00E+09	0	2.84E+03	2.84E+03	0	4.29E+03	4285.722	0	1.19E+02	118.523
b4129_fold_spon	b4129_m folding: spontanous	1 b4129_m_Mg --> 1 LysU_mono	irreversible	Protein Folding	0.00E+00	1.00E+09	0	2.84E+03	2.84E+03	0	4.29E+03	4285.722	0	1.19E+02	118.523
b4129_ion_BIND	Binding of ions to b4129	1 b4129_m + 3 mg2 <==> 1 b4129_m_Mg	reversible	Metallo-ion Binding	-1.00E+09	1.00E+09	0	2.84E+03	2.84E+03	0	4.29E+03	4285.722	0	1.19E+02	118.523
b4129_mRNA_CONV	convsersion of mRNA to mRNA_1 (synthetic rxn)	1 b4129_mRNA --> 1 b4129_mRNA_1	irreversible	Translation	0.00E+00	1.00E+09	0	9.39E+01	9.39E+01	0	1.66E+02	165.8223	0	2.07E-01	0.207
b4129_mRNA_degr1	Degradation of b4129_mRNA I	1 Orn_dim + 379 atp + 1 b4129_mRNA_2 + 1 degradosome + 379 h2o <==> 379 adp + 1 b4129_mRNA_2_degr + 379 h + 379 pi	reversible	mRNA degradation	-1.00E+09	1.00E+09	0	9.39E+01	9.39E+01	0	1.66E+02	165.8223	0	2.07E-01	0.207
b4129_mRNA_degr2	Degradation of b4129_mRNA II	1 b4129_mRNA_2_degr + 1517 h2o --> 1 Orn_dim_inact + 400 amp + 1 atp + 340 cmp + 1 degradosome_inact + 380 gmp + 1517 h + 397 ump	irreversible	mRNA degradation	0.00E+00	2.07E-01	0	9.39E+01	9.39E+01	0	1.66E+02	165.8223	0	2.07E-01	0.207
b4129_maturation1	Polypeptide b4129 peptide deformylas and methionine aminopeptidase complex	1 Def_mono + 1 Map_mono + 1 b4129_aa <==> 1 b4129_def_map_cplx	reversible	Protein Maturation	-1.00E+09	1.00E+09	0	2.84E+03	2.84E+03	0	4.29E+03	4285.722	0	1.19E+02	118.523
b4129_maturation2	b4129 formation	1 b4129_def_map_cplx + 2 h2o --> 1 Def_mono_inact + 1 Map_mono_inact + 1 b4129_m + 1 for + 1 met-L	irreversible	Protein Maturation	0.00E+00	1.00E+09	0	2.84E+03	2.84E+03	0	4.29E+03	4285.722	0	1.19E+02	118.523
b4129_v1_mRNA_CONV	convsersion of mRNA to mRNA_1 (synthetic rxn)	1 b4129_v1_mRNA --> 1 b4129_mRNA_1	irreversible	Translation	0.00E+00	1.00E+09	0	9.39E+01	9.39E+01	0	1.66E+02	165.8223	0	2.07E-01	0.207
b4134_tRNA_16_MODIF_1	modification of position 16 nt (D) in pheU by Dus_gen	1 Dus_gen + 1 b4134_tRNA_Mg2 + 1 h + 1 nadph <==> 1 b4134_tRNA_Mg2_Dus_gen_cplx	reversible	tRNA modification	-1.00E+09	1.00E+09	0	1.52E+03	1.52E+03	0	1.18E+01	11.8	0	1.18E+01	11.8
b4134_tRNA_16_MODIF_2	modification of position 16 nt (D) in pheU by Dus_gen	1 b4134_tRNA_Mg2_Dus_gen_cplx --> 1 Dus_gen_inact + 1 b4134_tRNA_1 + 1 nadp	irreversible	tRNA modification	0.00E+00	1.00E+09	0	1.52E+03	1.52E+03	0	1.18E+01	11.8	0	1.18E+01	11.8
b4134_tRNA_20_MODIF_1	modification of position 20 nt (D) in pheU by Dus_gen	1 Dus_gen + 1 b4134_tRNA_1 + 1 h + 1 nadph <==> 1 b4134_tRNA_1_Dus_gen_cplx	reversible	tRNA modification	-1.00E+09	1.00E+09	0	1.52E+03	1.52E+03	0	1.18E+01	11.8	0	1.18E+01	11.8
b4134_tRNA_20_MODIF_2	modification of position 20 nt (D) in pheU by Dus_gen	1 b4134_tRNA_1_Dus_gen_cplx --> 1 Dus_gen_inact + 1 b4134_tRNA_2 + 1 nadp	irreversible	tRNA modification	0.00E+00	1.00E+09	0	1.52E+03	1.52E+03	0	1.18E+01	11.8	0	1.18E+01	11.8
b4134_tRNA_32_MODIF_1	modification of position 32 nt (Y) in pheU by RluA_mono	1 RluA_mono + 1 b4134_tRNA_2 <==> 1 b4134_tRNA_2_RluA_mono_cplx	reversible	tRNA modification	-1.00E+09	1.00E+09	0	1.52E+03	1.52E+03	0	1.18E+01	11.8	0	1.18E+01	11.8
b4134_tRNA_32_MODIF_2	modification of position 32 nt (Y) in pheU by RluA_mono	1 b4134_tRNA_2_RluA_mono_cplx --> 1 RluA_mono_inact + 1 b4134_tRNA_3	irreversible	tRNA modification	0.00E+00	1.00E+09	0	1.52E+03	1.52E+03	0	1.18E+01	11.8	0	1.18E+01	11.8
b4134_tRNA_37_MODIF_1	modification of position 37 nt (ms2i6A) in pheU by MiaA_dim-MiaB_mono	1 IscS_dim_S-SH + 1 MiaA_dim + 1 MiaB_mono + 2 amet + 1 b4134_tRNA_3 + 1 dmpp + 1 fldrd <==> 1 b4134_tRNA_3_MiaA_dim MiaB_mono_cplx	reversible	tRNA modification	-1.00E+09	1.00E+09	0	1.52E+03	1.52E+03	0	1.18E+01	11.8	0	1.18E+01	11.8
b4134_tRNA_37_MODIF_2	modification of position 37 nt (ms2i6A) in pheU by MiaA_dim-MiaB_mono	1 b4134_tRNA_3_MiaA_dim-MiaB_mono_cplx --> 1 IscS_dim_S-H + 1 MiaA_dim_inact + 1 MiaB_mono_inact + 1 ahcys + 1 b4134_tRNA_4 + 1 dad-5 + 1 fldox + 2 h + 1 met-L + 1 ppi	irreversible	tRNA modification	0.00E+00	1.00E+09	0	1.52E+03	1.52E+03	0	1.18E+01	11.8	0	1.18E+01	11.8
b4134_tRNA_39_MODIF_1	modification of position 39 nt (Y) in pheU by TruA_dim	1 TruA_dim + 1 b4134_tRNA_4 <==> 1 b4134_tRNA_4_TruA_dim_cplx	reversible	tRNA modification	-1.00E+09	1.00E+09	0	1.52E+03	1.52E+03	0	1.18E+01	11.8	0	1.18E+01	11.8
b4134_tRNA_39_MODIF_2	modification of position 39 nt (Y) in pheU by TruA_dim	1 b4134_tRNA_4_TruA_dim_cplx --> 1 TruA_dim_inact + 1 b4134_tRNA_5	irreversible	tRNA modification	0.00E+00	1.00E+09	0	1.52E+03	1.52E+03	0	1.18E+01	11.8	0	1.18E+01	11.8
b4134_tRNA_46_MODIF_1	modification of position 46 nt (m7G) in pheU by YggH_mono	1 YggH_mono + 1 amet + 1 b4134_tRNA_5 <==> 1 b4134_tRNA_5_YggH_mono_cplx	reversible	tRNA modification	-1.00E+09	1.00E+09	0	1.52E+03	1.52E+03	0	1.18E+01	11.8	0	1.18E+01	11.8
b4134_tRNA_46_MODIF_2	modification of position 46 nt (m7G) in pheU by YggH_mono	1 b4134_tRNA_5_YggH_mono_cplx --> 1 YggH_mono_inact + 1 ahcys + 1 b4134_tRNA_6 + 1 h	irreversible	tRNA modification	0.00E+00	1.00E+09	0	1.52E+03	1.52E+03	0	1.18E+01	11.8	0	1.18E+01	11.8
b4134_tRNA_47_MODIF_1	modification of position 47 nt (acp3U) in pheU by AcpT_tRNA_pos_47_acp3U	1 AcpT_tRNA_pos_47_acp3U + 1 amet + 1 b4134_tRNA_6 <==> 1 b4134_tRNA_6_AcpT_tRNA_pos_47_acp3U_cplx	reversible	tRNA modification	-1.00E+09	1.00E+09	0	1.52E+03	1.52E+03	0	1.18E+01	11.8	0	1.18E+01	11.8
b4134_tRNA_47_MODIF_2	modification of position 47 nt (acp3U) in pheU by AcpT_tRNA_pos_47_acp3U	1 b4134_tRNA_6_AcpT_tRNA_pos_47_acp3U_cplx --> 1 5mta + 1 AcpT_tRNA_pos_47_acp3U_inact + 1 b4134_tRNA_7 + 1 h	irreversible	tRNA modification	0.00E+00	1.00E+09	0	1.52E+03	1.52E+03	0	1.18E+01	11.8	0	1.18E+01	11.8
b4134_tRNA_54_MODIF_1	modification of position 54 nt (m5U) in pheU by TrmA_mono	1 TrmA_mono + 1 amet + 1 b4134_tRNA_7 <==> 1 b4134_tRNA_7_TrmA_mono_cplx	reversible	tRNA modification	-1.00E+09	1.00E+09	0	1.52E+03	1.52E+03	0	1.18E+01	11.8	0	1.18E+01	11.8
b4134_tRNA_54_MODIF_2	modification of position 54 nt (m5U) in pheU by TrmA_mono	1 b4134_tRNA_7_TrmA_mono_cplx --> 1 TrmA_mono_inact + 1 ahcys + 1 b4134_tRNA_8 + 1 h	irreversible	tRNA modification	0.00E+00	1.00E+09	0	1.52E+03	1.52E+03	0	1.18E+01	11.8	0	1.18E+01	11.8
b4134_tRNA_55_MODIF_1	modification of position 55 nt (Y) in pheU by TruB_mono	1 TruB_mono + 1 b4134_tRNA_8 <==> 1 b4134_tRNA_8_TruB_mono_cplx	reversible	tRNA modification	-1.00E+09	1.00E+09	0	1.52E+03	1.52E+03	0	1.18E+01	11.8	0	1.18E+01	11.8
b4134_tRNA_55_MODIF_2	modification of position 55 nt (Y) in pheU by TruB_mono	1 b4134_tRNA_8_TruB_mono_cplx --> 1 TruB_mono_inact + 1 b4134_tRNA_9	irreversible	tRNA modification	0.00E+00	1.00E+09	0	1.52E+03	1.52E+03	0	1.18E+01	11.8	0	1.18E+01	11.8
b4134_tRNA_8_MODIF_1	modification of position 8 nt (s4U) in pheU by ThiI_mono	1 IscS_dim_S-SH + 1 ThiI_mono + 1 atp + 1 b4134_tRNA_9 + 1 mg2 + 1 trdrd <==> 1 b4134_tRNA_9_ThiI_mono_cplx	reversible	tRNA modification	-1.00E+09	1.00E+09	0	1.52E+03	1.52E+03	0	1.18E+01	11.8	0	1.18E+01	11.8
b4134_tRNA_8_MODIF_2	modification of position 8 nt (s4U) in pheU by ThiI_mono	1 b4134_tRNA_9_ThiI_mono_cplx --> 1 IscS_dim_S-H + 1 ThiI_mono_inact + 1 amp + 1 h + 1 mg2 + 1 pheU_tRNA + 1 ppi + 1 trdox	irreversible	tRNA modification	0.00E+00	1.00E+09	0	1.52E+03	1.52E+03	0	1.18E+01	11.8	0	1.18E+01	11.8
b4134_tRNA_Mg_BIND	Binding of two Mg2 to b4134_tRNA	1 b4134_RNA + 2 mg2 <==> 1 b4134_tRNA_Mg2	reversible	Metallo-ion binding	-1.00E+09	1.00E+09	0	1.52E+03	1.52E+03	0	1.18E+01	11.8	0	1.18E+01	11.8
b4142_fold_spon	b4142_m folding: spontanous	1 b4142_m --> 1 GroS_mono	irreversible	Protein Folding	0.00E+00	1.00E+09	0	2.96E+03	2.96E+03	0	5.00E+03	5001.3	0	3.06E+02	306.11
b4142_mRNA_CONV	convsersion of mRNA to mRNA_1 (synthetic rxn)	1 b4142_mRNA --> 1 b4142_mRNA_1	irreversible	Translation	0.00E+00	1.00E+09	0	5.32E+02	5.32E+02	0	9.40E+02	939.6599	0	6.22E-01	0.622

b4142_mRNA_degr1	Degradation of b4142_mRNA I	1 Orn_dim + 73 atp + 1 b4142_mRNA_2 + 1 degradosome + 73 h2o <==> 73 adp + 1 b4142_mRNA_2_degr + 73 h + 73 pi	reversible	mRNA degradation	-1.00E+09	1.00E+09	0	5.32E+02	5.32E+02	0	9.40E+02	939.6599	0	6.22E-01	0.622
b4142_mRNA_degr2	Degradation of b4142_mRNA II	1 b4142_mRNA_2_degr + 293 h2o --> 1 Orn_dim_inact + 78 amp + 1 atp + 60 cmp + 1 degradosome_inact + 83 gmp + 293 h + 72 ump	irreversible	mRNA degradation	0.00E+00	6.22E-01	0	5.32E+02	5.32E+02	0	9.40E+02	939.6599	0	6.22E-01	0.622
b4142_maturation1	Polypeptide b4142 peptide deformylas complex	1 Def_mono + 1 b4142_aa <==> 1 b4142_def_cplx	reversible	Protein Maturation	-1.00E+09	1.00E+09	0	2.96E+03	2.96E+03	0	5.00E+03	5001.3	0	3.06E+02	306.11
b4142_maturation2	b4142 formation	1 b4142_def_cplx + 1 h2o --> 1 Def_mono_inact + 1 b4142_m + 1 for	irreversible	Protein Maturation	0.00E+00	1.00E+09	0	2.96E+03	2.96E+03	0	5.00E+03	5001.3	0	3.06E+02	306.11
b4143_DNA_act_bind	b4143_DNA binding of activator	1 b4143_DNA_neu --> 1 b4143_DNA_act	irreversible	Transcription Regulation	0.00E+00	1.00E+09	0	8.11E+01	8.11E+01	0	1.43E+02	143.0954	0	3.12E-01	0.312
b4143_fold_spon	b4143_m folding: spontanous	1 b4143_m --> 1 GroL_mono	irreversible	Protein Folding	0.00E+00	1.00E+09	0	2.09E+03	2.09E+03	0	3.43E+03	3427.479	0	2.33E+02	233.344
b4143_mRNA_CONV	convsersion of mRNA to mRNA_1 (synthetic rxn)	1 b4143_mRNA --> 1 b4143_mRNA_1	irreversible	Translation	0.00E+00	1.00E+09	0	8.11E+01	8.11E+01	0	1.43E+02	143.0954	0	3.12E-01	0.312
b4143_mRNA_degr1	Degradation of b4143_mRNA I	1 Orn_dim + 411 atp + 1 b4143_mRNA_2 + 1 degradosome + 411 h2o <==> 411 adp + 1 b4143_mRNA_2_degr + 411 h + 411 pi	reversible	mRNA degradation	-1.00E+09	1.00E+09	0	8.11E+01	8.11E+01	0	1.43E+02	143.0954	0	3.12E-01	0.312
b4143_mRNA_degr2	Degradation of b4143_mRNA II	1 b4143_mRNA_2_degr + 1646 h2o --> 1 Orn_dim_inact + 436 amp + 1 atp + 394 cmp + 1 degradosome_inact + 477 gmp + 1646 h + 339 ump	irreversible	mRNA degradation	0.00E+00	3.12E-01	0	8.11E+01	8.11E+01	0	1.43E+02	143.0954	0	3.12E-01	0.312
b4143_maturation1	Polypeptide b4143 peptide deformylas and methionine aminopeptidase complex	1 Def_mono + 1 Map_mono + 1 b4143_aa <==> 1 b4143_def_map_cplx	reversible	Protein Maturation	-1.00E+09	1.00E+09	0	2.09E+03	2.09E+03	0	3.43E+03	3427.479	0	2.33E+02	233.344
b4143_maturation2	b4143 formation	1 b4143_def_map_cplx + 2 h2o --> 1 Def_mono_inact + 1 Map_mono_inact + 1 b4143_m + 1 for + 1 met-L	irreversible	Protein Maturation	0.00E+00	1.00E+09	0	2.09E+03	2.09E+03	0	3.43E+03	3427.479	0	2.33E+02	233.344
b4162_fold_spon	b4162_m folding: spontanous	1 b4162_m_Mg --> 1 Orn_mono	irreversible	Protein Folding	0.00E+00	1.00E+09	0	2.47E+03	2.47E+03	0	4.31E+03	4311.167	0	7.35E+01	73.52
b4162_ion_BIND	Binding of ions to b4162	1 b4162_m + 1 mg2 <==> 1 b4162_m_Mg	reversible	Metallo-ion Binding	-1.00E+09	1.00E+09	0	2.47E+03	2.47E+03	0	4.31E+03	4311.167	0	7.35E+01	73.52
b4162_mRNA_CONV	convsersion of mRNA to mRNA_1 (synthetic rxn)	1 b4162_mRNA --> 1 b4162_mRNA_1	irreversible	Translation	0.00E+00	1.00E+09	0	2.40E+02	2.40E+02	0	4.24E+02	423.9067	0	1.42E-01	0.142
b4162_mRNA_degr1	Degradation of b4162_mRNA I	1 Orn_dim + 136 atp + 1 b4162_mRNA_2 + 1 degradosome + 136 h2o <==> 136 adp + 1 b4162_mRNA_2_degr + 136 h + 136 pi	reversible	mRNA degradation	-1.00E+09	1.00E+09	0	2.40E+02	2.40E+02	0	4.24E+02	423.9067	0	1.42E-01	0.142
b4162_mRNA_degr2	Degradation of b4162_mRNA II	1 b4162_mRNA_2_degr + 545 h2o --> 1 Orn_dim_inact + 133 amp + 1 atp + 133 cmp + 1 degradosome_inact + 162 gmp + 545 h + 117 ump	irreversible	mRNA degradation	0.00E+00	1.42E-01	0	2.40E+02	2.40E+02	0	4.24E+02	423.9067	0	1.42E-01	0.142
b4162_maturation1	Polypeptide b4162 peptide deformylas and methionine aminopeptidase complex	1 Def_mono + 1 Map_mono + 1 b4162_aa <==> 1 b4162_def_map_cplx	reversible	Protein Maturation	-1.00E+09	1.00E+09	0	2.47E+03	2.47E+03	0	4.31E+03	4311.167	0	7.35E+01	73.52
b4162_maturation2	b4162 formation	1 b4162_def_map_cplx + 2 h2o --> 1 Def_mono_inact + 1 Map_mono_inact + 1 b4162_m + 1 for + 1 met-L	irreversible	Protein Maturation	0.00E+00	1.00E+09	0	2.47E+03	2.47E+03	0	4.31E+03	4311.167	0	7.35E+01	73.52
b4163_tRNA_16_MODIF_1	modification of position 16 nt (D) in glyV by Dus_gen	1 Dus_gen + 1 b4163_tRNA_Mg2 + 1 h + 1 nadph <==> 1 b4163_tRNA_Mg2_Dus_gen_cplx	reversible	tRNA modification	-1.00E+09	1.00E+09	0	3.99E+02	3.99E+02	0	1.17E+01	11.7	0	1.17E+01	11.7
b4163_tRNA_16_MODIF_2	modification of position 16 nt (D) in glyV by Dus_gen	1 b4163_tRNA_Mg2_Dus_gen_cplx --> 1 Dus_gen_inact + 1 b4163_tRNA_1 + 1 nadp	irreversible	tRNA modification	0.00E+00	1.00E+09	0	3.99E+02	3.99E+02	0	1.17E+01	11.7	0	1.17E+01	11.7
b4163_tRNA_17_MODIF_1	modification of position 17 nt (D) in glyV by Dus_gen	1 Dus_gen + 1 b4163_tRNA_1 + 1 h + 1 nadph <==> 1 b4163_tRNA_1_Dus_gen_cplx	reversible	tRNA modification	-1.00E+09	1.00E+09	0	3.99E+02	3.99E+02	0	1.17E+01	11.7	0	1.17E+01	11.7
b4163_tRNA_17_MODIF_2	modification of position 17 nt (D) in glyV by Dus_gen	1 b4163_tRNA_1_Dus_gen_cplx --> 1 Dus_gen_inact + 1 b4163_tRNA_2 + 1 nadp	irreversible	tRNA modification	0.00E+00	1.00E+09	0	3.99E+02	3.99E+02	0	1.17E+01	11.7	0	1.17E+01	11.7
b4163_tRNA_20_MODIF_1	modification of position 20 nt (D) in glyV by Dus_gen	1 Dus_gen + 1 b4163_tRNA_2 + 1 h + 1 nadph <==> 1 b4163_tRNA_2_Dus_gen_cplx	reversible	tRNA modification	-1.00E+09	1.00E+09	0	3.99E+02	3.99E+02	0	1.17E+01	11.7	0	1.17E+01	11.7
b4163_tRNA_20_MODIF_2	modification of position 20 nt (D) in glyV by Dus_gen	1 b4163_tRNA_2_Dus_gen_cplx --> 1 Dus_gen_inact + 1 b4163_tRNA_3 + 1 nadp	irreversible	tRNA modification	0.00E+00	1.00E+09	0	3.99E+02	3.99E+02	0	1.17E+01	11.7	0	1.17E+01	11.7
b4163_tRNA_46_MODIF_1	modification of position 46 nt (m7G) in glyV by YggH_mono	1 YggH_mono + 1 amet + 1 b4163_tRNA_3 <==> 1 b4163_tRNA_3_YggH_mono_cplx	reversible	tRNA modification	-1.00E+09	1.00E+09	0	3.99E+02	3.99E+02	0	1.17E+01	11.7	0	1.17E+01	11.7
b4163_tRNA_46_MODIF_2	modification of position 46 nt (m7G) in glyV by YggH_mono	1 b4163_tRNA_3_YggH_mono_cplx --> 1 YggH_mono_inact + 1 ahcys + 1 b4163_tRNA_4 + 1 h	irreversible	tRNA modification	0.00E+00	1.00E+09	0	3.99E+02	3.99E+02	0	1.17E+01	11.7	0	1.17E+01	11.7
b4163_tRNA_54_MODIF_1	modification of position 54 nt (m5U) in glyV by TrmA_mono	1 TrmA_mono + 1 amet + 1 b4163_tRNA_4 <==> 1 b4163_tRNA_4_TrmA_mono_cplx	reversible	tRNA modification	-1.00E+09	1.00E+09	0	3.99E+02	3.99E+02	0	1.17E+01	11.7	0	1.17E+01	11.7
b4163_tRNA_54_MODIF_2	modification of position 54 nt (m5U) in glyV by TrmA_mono	1 b4163_tRNA_4_TrmA_mono_cplx --> 1 TrmA_mono_inact + 1 ahcys + 1 b4163_tRNA_5 + 1 h	irreversible	tRNA modification	0.00E+00	1.00E+09	0	3.99E+02	3.99E+02	0	1.17E+01	11.7	0	1.17E+01	11.7
b4163_tRNA_55_MODIF_1	modification of position 55 nt (Y) in glyV by TruB_mono	1 TruB_mono + 1 b4163_tRNA_5 <==> 1 b4163_tRNA_5_TruB_mono_cplx	reversible	tRNA modification	-1.00E+09	1.00E+09	0	3.99E+02	3.99E+02	0	1.17E+01	11.7	0	1.17E+01	11.7
b4163_tRNA_55_MODIF_2	modification of position 55 nt (Y) in glyV by TruB_mono	1 b4163_tRNA_5_TruB_mono_cplx --> 1 TruB_mono_inact + 1 glyV_tRNA	irreversible	tRNA modification	0.00E+00	1.00E+09	0	3.99E+02	3.99E+02	0	1.17E+01	11.7	0	1.17E+01	11.7
b4163_tRNA_Mg_BIND	Binding of two Mg2 to b4163_tRNA	1 b4163_RNA + 2 mg2 <==> 1 b4163_tRNA_Mg2	reversible	Metallo-ion binding	-1.00E+09	1.00E+09	0	3.99E+02	3.99E+02	0	1.17E+01	11.7	0	1.17E+01	11.7
b4164_tRNA_16_MODIF_1	modification of position 16 nt (D) in glyX by Dus_gen	1 Dus_gen + 1 b4164_tRNA_Mg2 + 1 h + 1 nadph <==> 1 b4164_tRNA_Mg2_Dus_gen_cplx	reversible	tRNA modification	-1.00E+09	1.00E+09	0	3.99E+02	3.99E+02	0	1.17E+01	11.7	0	1.17E+01	11.7
b4164_tRNA_16_MODIF_2	modification of position 16 nt (D) in glyX by Dus_gen	1 b4164_tRNA_Mg2_Dus_gen_cplx --> 1 Dus_gen_inact + 1 b4164_tRNA_1 + 1 nadp	irreversible	tRNA modification	0.00E+00	1.00E+09	0	3.99E+02	3.99E+02	0	1.17E+01	11.7	0	1.17E+01	11.7
b4164_tRNA_17_MODIF_1	modification of position 17 nt (D) in glyX by Dus_gen	1 Dus_gen + 1 b4164_tRNA_1 + 1 h + 1 nadph <==> 1 b4164_tRNA_1_Dus_gen_cplx	reversible	tRNA modification	-1.00E+09	1.00E+09	0	3.99E+02	3.99E+02	0	1.17E+01	11.7	0	1.17E+01	11.7
b4164_tRNA_17_MODIF_2	modification of position 17 nt (D) in glyX by Dus_gen	1 b4164_tRNA_1_Dus_gen_cplx --> 1 Dus_gen_inact + 1 b4164_tRNA_2 + 1 nadp	irreversible	tRNA modification	0.00E+00	1.00E+09	0	3.99E+02	3.99E+02	0	1.17E+01	11.7	0	1.17E+01	11.7
b4164_tRNA_20_MODIF_1	modification of position 20 nt (D) in glyX by Dus_gen	1 Dus_gen + 1 b4164_tRNA_2 + 1 h + 1 nadph <==> 1 b4164_tRNA_2_Dus_gen_cplx	reversible	tRNA modification	-1.00E+09	1.00E+09	0	3.99E+02	3.99E+02	0	1.17E+01	11.7	0	1.17E+01	11.7
b4164_tRNA_20_MODIF_2	modification of position 20 nt (D) in glyX by Dus_gen	1 b4164_tRNA_2_Dus_gen_cplx --> 1 Dus_gen_inact + 1 b4164_tRNA_3 + 1 nadp	irreversible	tRNA modification	0.00E+00	1.00E+09	0	3.99E+02	3.99E+02	0	1.17E+01	11.7	0	1.17E+01	11.7
b4164_tRNA_46_MODIF_1	modification of position 46 nt (m7G) in glyX by YggH_mono	1 YggH_mono + 1 amet + 1 b4164_tRNA_3 <==> 1 b4164_tRNA_3_YggH_mono_cplx	reversible	tRNA modification	-1.00E+09	1.00E+09	0	3.99E+02	3.99E+02	0	1.17E+01	11.7	0	1.17E+01	11.7
b4164_tRNA_46_MODIF_2	modification of position 46 nt (m7G) in glyX by YggH_mono	1 b4164_tRNA_3_YggH_mono_cplx --> 1 YggH_mono_inact + 1 ahcys + 1 b4164_tRNA_4 + 1 h	irreversible	tRNA modification	0.00E+00	1.00E+09	0	3.99E+02	3.99E+02	0	1.17E+01	11.7	0	1.17E+01	11.7
b4164_tRNA_54_MODIF_1	modification of position 54 nt (m5U) in glyX by TrmA_mono	1 TrmA_mono + 1 amet + 1 b4164_tRNA_4 <==> 1 b4164_tRNA_4_TrmA_mono_cplx	reversible	tRNA modification	-1.00E+09	1.00E+09	0	3.99E+02	3.99E+02	0	1.17E+01	11.7	0	1.17E+01	11.7
b4164_tRNA_54_MODIF_2	modification of position 54 nt (m5U) in glyX by TrmA_mono	1 b4164_tRNA_4_TrmA_mono_cplx --> 1 TrmA_mono_inact + 1 ahcys + 1 b4164_tRNA_5 + 1 h	irreversible	tRNA modification	0.00E+00	1.00E+09	0	3.99E+02	3.99E+02	0	1.17E+01	11.7	0	1.17E+01	11.7
b4164_tRNA_55_MODIF_1	modification of position 55 nt (Y) in glyX by TruB_mono	1 TruB_mono + 1 b4164_tRNA_5 <==> 1 b4164_tRNA_5_TruB_mono_cplx	reversible	tRNA modification	-1.00E+09	1.00E+09	0	3.99E+02	3.99E+02	0	1.17E+01	11.7	0	1.17E+01	11.7
b4164_tRNA_55_MODIF_2	modification of position 55 nt (Y) in glyX by TruB_mono	1 b4164_tRNA_5_TruB_mono_cplx --> 1 TruB_mono_inact + 1 glyX_tRNA	irreversible	tRNA modification	0.00E+00	1.00E+09	0	3.99E+02	3.99E+02	0	1.17E+01	11.7	0	1.17E+01	11.7
b4164_tRNA_Mg_BIND	Binding of two Mg2 to b4164_tRNA	1 b4164_RNA + 2 mg2 <==> 1 b4164_tRNA_Mg2	reversible	Metallo-ion binding	-1.00E+09	1.00E+09	0	3.99E+02	3.99E+02	0	1.17E+01	11.7	0	1.17E+01	11.7
b4165_tRNA_16_MODIF_1	modification of position 16 nt (D) in glyY by Dus_gen	1 Dus_gen + 1 b4165_tRNA_Mg2 + 1 h + 1 nadph <==> 1 b4165_tRNA_Mg2_Dus_gen_cplx	reversible	tRNA modification	-1.00E+09	1.00E+09	0	3.99E+02	3.99E+02	0	1.17E+01	11.7	0	1.17E+01	11.7
b4165_tRNA_16_MODIF_2	modification of position 16 nt (D) in glyY by Dus_gen	1 b4165_tRNA_Mg2_Dus_gen_cplx --> 1 Dus_gen_inact + 1 b4165_tRNA_1 + 1 nadp	irreversible	tRNA modification	0.00E+00	1.00E+09	0	3.99E+02	3.99E+02	0	1.17E+01	11.7	0	1.17E+01	11.7
b4165_tRNA_17_MODIF_1	modification of position 17 nt (D) in glyY by Dus_gen	1 Dus_gen + 1 b4165_tRNA_1 + 1 h + 1 nadph <==> 1 b4165_tRNA_1_Dus_gen_cplx	reversible	tRNA modification	-1.00E+09	1.00E+09	0	3.99E+02	3.99E+02	0	1.17E+01	11.7	0	1.17E+01	11.7
b4165_tRNA_17_MODIF_2	modification of position 17 nt (D) in glyY by Dus_gen	1 b4165_tRNA_1_Dus_gen_cplx --> 1 Dus_gen_inact + 1 b4165_tRNA_2 + 1 nadp	irreversible	tRNA modification	0.00E+00	1.00E+09	0	3.99E+02	3.99E+02	0	1.17E+01	11.7	0	1.17E+01	11.7
b4165_tRNA_20_MODIF_1	modification of position 20 nt (D) in glyY by Dus_gen	1 Dus_gen + 1 b4165_tRNA_2 + 1 h + 1 nadph <==> 1 b4165_tRNA_2_Dus_gen_cplx	reversible	tRNA modification	-1.00E+09	1.00E+09	0	3.99E+02	3.99E+02	0	1.17E+01	11.7	0	1.17E+01	11.7
b4165_tRNA_20_MODIF_2	modification of position 20 nt (D) in glyY by Dus_gen	1 b4165_tRNA_2_Dus_gen_cplx --> 1 Dus_gen_inact + 1 b4165_tRNA_3 + 1 nadp	irreversible	tRNA modification	0.00E+00	1.00E+09	0	3.99E+02	3.99E+02	0	1.17E+01	11.7	0	1.17E+01	11.7
b4165_tRNA_46_MODIF_1	modification of position 46 nt (m7G) in glyY by YggH_mono	1 YggH_mono + 1 amet + 1 b4165_tRNA_3 <==> 1 b4165_tRNA_3_YggH_mono_cplx	reversible	tRNA modification	-1.00E+09	1.00E+09	0	3.99E+02	3.99E+02	0	1.17E+01	11.7	0	1.17E+01	11.7
b4165_tRNA_46_MODIF_2	modification of position 46 nt (m7G) in glyY by YggH_mono	1 b4165_tRNA_3_YggH_mono_cplx --> 1 YggH_mono_inact + 1 ahcys + 1 b4165_tRNA_4 + 1 h	irreversible	tRNA modification	0.00E+00	1.00E+09	0	3.99E+02	3.99E+02	0	1.17E+01	11.7	0	1.17E+01	11.7
b4165_tRNA_54_MODIF_1	modification of position 54 nt (m5U) in glyY by TrmA_mono	1 TrmA_mono + 1 amet + 1 b4165_tRNA_4 <==> 1 b4165_tRNA_4_TrmA_mono_cplx	reversible	tRNA modification	-1.00E+09	1.00E+09	0	3.99E+02	3.99E+02	0	1.17E+01	11.7	0	1.17E+01	11.7
b4165_tRNA_54_MODIF_2	modification of position 54 nt (m5U) in glyY by TrmA_mono	1 b4165_tRNA_4_TrmA_mono_cplx --> 1 TrmA_mono_inact + 1 ahcys + 1 b4165_tRNA_5 + 1 h	irreversible	tRNA modification	0.00E+00	1.00E+09	0	3.99E+02	3.99E+02	0	1.17E+01	11.7	0	1.17E+01	11.7

b4165_tRNA_55_MODIF_1	modification of position 55 nt (Y) in glyY by TruB_mono	1 TruB_mono + 1 b4165_tRNA_5 <==> 1 b4165_tRNA_5_TruB_mono_cplx	reversible	tRNA modification	-1.00E+09	1.00E+09	0	3.99E+02	3.99E+02	0	1.17E+01	11.7	0	1.17E+01	11.7
b4165_tRNA_55_MODIF_2	modification of position 55 nt (Y) in glyY by TruB_mono	1 b4165_tRNA_5_TruB_mono_cplx --> 1 TruB_mono_inact + 1 glyY_tRNA	irreversible	tRNA modification	0.00E+00	1.00E+09	0	3.99E+02	3.99E+02	0	1.17E+01	11.7	0	1.17E+01	11.7
b4165_tRNA_Mg_BIND	Binding of two Mg2 to b4165_tRNA	1 b4165_RNA + 2 mg2 <==> 1 b4165_tRNA_Mg2	reversible	Metallo-ion binding	-1.00E+09	1.00E+09	0	3.99E+02	3.99E+02	0	1.17E+01	11.7	0	1.17E+01	11.7
b4167_fold_spon	b4167_m folding: spontanous	1 b4167_m --> 1 YjeF_mono	irreversible	Protein Folding	0.00E+00	1.00E+09	0	4.59E+02	4.59E+02	0	7.39E+02	739.2022	0	9.55E+01	95.52
b4167_mRNA_CONV	convsersion of mRNA to mRNA_1 (synthetic rxn)	1 b4167_mRNA --> 1 b4167_mRNA_1	irreversible	Translation	0.00E+00	1.00E+09	0	1.22E+01	1.22E+01	0	2.16E+01	21.56007	0	1.04E-01	0.104
b4167_mRNA_degr1	Degradation of b4167_mRNA I	1 Orn_dim + 387 atp + 1 b4167_mRNA_2 + 1 degradosome + 387 h2o <==> 387 adp + 1 b4167_mRNA_2_degr + 387 h + 387 pi	reversible	mRNA degradation	-1.00E+09	1.00E+09	0	1.22E+01	1.22E+01	0	2.16E+01	21.56007	0	1.04E-01	0.104
b4167_mRNA_degr2	Degradation of b4167_mRNA II	1 b4167_mRNA_2_degr + 1547 h2o --> 1 Orn_dim_inact + 328 amp 1 atp + 402 cmp + 1 degradosome_inact + 482 gmp + 1547 h + 335 ump	irreversible	mRNA degradation	0.00E+00	2.07E-01	0	1.22E+01	1.22E+01	0	2.16E+01	21.56007	0	1.04E-01	0.104
b4167_maturation1	Polypeptide b4167 peptide deformylas complex	1 Def_mono + 1 b4167_aa <==> 1 b4167_def_cplx	reversible	Protein Maturation	-1.00E+09	1.00E+09	0	4.59E+02	4.59E+02	0	7.39E+02	739.2022	0	9.55E+01	95.52
b4167_maturation2	b4167 formation	1 b4167_def_cplx + 2 h2o --> 1 Def_mono_inact + 1 b4167_m + 1 for + 1 met-L	irreversible	Protein Maturation	0.00E+00	1.00E+09	0	4.59E+02	4.59E+02	0	7.39E+02	739.2022	0	9.55E+01	95.52
b4167_v1_mRNA_CONV	convsersion of mRNA to mRNA_1 (synthetic rxn)	1 b4167_v1_mRNA --> 1 b4167_mRNA_1	irreversible	Translation	0.00E+00	1.00E+09	0	1.22E+01	1.22E+01	0	2.16E+01	21.56007	0	1.04E-01	0.104
b4167_v2_mRNA_CONV	convsersion of mRNA to mRNA_1 (synthetic rxn)	1 b4167_v2_mRNA --> 1 b4167_mRNA_1	irreversible	Translation	0.00E+00	1.00E+09	0	1.22E+01	1.22E+01	0	2.16E+01	21.56007	0	1.04E-01	0.104
b4168_fold_spon	b4168_m folding: spontanous	1 b4168_m --> 1 YjeE_mono	irreversible	Protein Folding	0.00E+00	1.00E+09	0	1.70E+02	1.70E+02	0	2.54E+02	254.4307	0	6.13E+01	61.326
b4168_mRNA_CONV	convsersion of mRNA to mRNA_1 (synthetic rxn)	1 b4168_mRNA --> 1 b4168_mRNA_1	irreversible	Translation	0.00E+00	1.00E+09	0	1.22E+01	1.22E+01	0	2.16E+01	21.56007	0	1.04E-01	0.104
b4168_mRNA_degr1	Degradation of b4168_mRNA I	1 Orn_dim + 108 atp + 1 b4168_mRNA_2 + 1 degradosome + 108 h2o <==> 108 adp + 1 b4168_mRNA_2_degr + 108 h + 108 pi	reversible	mRNA degradation	-1.00E+09	1.00E+09	0	1.22E+01	1.22E+01	0	2.16E+01	21.56007	0	1.04E-01	0.104
b4168_mRNA_degr2	Degradation of b4168_mRNA II	1 b4168_mRNA_2_degr + 432 h2o --> 1 Orn_dim_inact + 95 amp + 105 cmp + 1 degradosome_inact + 124 gmp + 432 h + 109 ump	irreversible	mRNA degradation	0.00E+00	1.04E-01	0	1.22E+01	1.22E+01	0	2.16E+01	21.56007	0	1.04E-01	0.104
b4168_maturation1	Polypeptide b4168 peptide deformylas and methionine aminopeptidase complex	1 Def_mono + 1 Map_mono + 1 b4168_aa <==> 1 b4168_def_map_cplx	reversible	Protein Maturation	-1.00E+09	1.00E+09	0	1.70E+02	1.70E+02	0	2.54E+02	254.4307	0	6.13E+01	61.326
b4168_maturation2	b4168 formation	1 b4168_def_map_cplx + 2 h2o --> 1 Def_mono_inact + 1 Map_mono_inact + 1 b4168_m + 1 for + 1 met-L	irreversible	Protein Maturation	0.00E+00	1.00E+09	0	1.70E+02	1.70E+02	0	2.54E+02	254.4307	0	6.13E+01	61.326
b4168_v1_mRNA_CONV	convsersion of mRNA to mRNA_1 (synthetic rxn)	1 b4168_v1_mRNA --> 1 b4168_mRNA_1	irreversible	Translation	0.00E+00	1.00E+09	0	1.22E+01	1.22E+01	0	2.16E+01	21.56007	0	1.04E-01	0.104
b4168_v2_mRNA_CONV	convsersion of mRNA to mRNA_1 (synthetic rxn)	1 b4168_v2_mRNA --> 1 b4168_mRNA_1	irreversible	Translation	0.00E+00	1.00E+09	0	1.22E+01	1.22E+01	0	2.16E+01	21.56007	0	1.04E-01	0.104
b4169_fold_spon	b4169_m folding: spontanous	1 b4169_m --> 1 AmiB_mono	irreversible	Protein Folding	0.00E+00	1.00E+09	0	4.10E+02	4.10E+02	0	6.53E+02	653.1219	0	9.53E+01	95.264
b4169_mRNA_CONV	convsersion of mRNA to mRNA_1 (synthetic rxn)	1 b4169_mRNA --> 1 b4169_mRNA_1	irreversible	Translation	0.00E+00	1.00E+09	0	1.22E+01	1.22E+01	0	2.16E+01	21.56007	0	1.04E-01	0.104
b4169_mRNA_degr1	Degradation of b4169_mRNA I	1 Orn_dim + 334 atp + 1 b4169_mRNA_2 + 1 degradosome + 334 h2o <==> 334 adp + 1 b4169_mRNA_2_degr + 334 h + 334 pi	reversible	mRNA degradation	-1.00E+09	1.00E+09	0	1.22E+01	1.22E+01	0	2.16E+01	21.56007	0	1.04E-01	0.104
b4169_mRNA_degr2	Degradation of b4169_mRNA II	1 b4169_mRNA_2_degr + 1337 h2o --> 1 Orn_dim_inact + 318 amp 356 cmp + 1 degradosome_inact + 381 gmp + 1337 h + 283 ump	irreversible	mRNA degradation	0.00E+00	2.91E-01	0	1.22E+01	1.22E+01	0	2.16E+01	21.56007	0	1.04E-01	0.104
b4169_maturation1	Polypeptide b4169 peptide deformylas complex	1 Def_mono + 1 b4169_aa <==> 1 b4169_def_cplx	reversible	Protein Maturation	-1.00E+09	1.00E+09	0	4.10E+02	4.10E+02	0	6.53E+02	653.1219	0	9.53E+01	95.264
b4169_maturation2	b4169 formation	1 b4169_def_cplx + 23 h2o --> 1 Def_mono_inact + 2 ala-L + 2 arg-L + 1 asn-L + 1 b4169_m + 1 cys-L + 1 for + 1 gly + 1 ile-L + 5 leu-L + 2 met-L + 1 pro-L + 2 thr-L + 1 trp-L + 1 tyr-L + 2 val-L	irreversible	Protein Maturation	0.00E+00	1.00E+09	0	4.10E+02	4.10E+02	0	6.53E+02	653.1219	0	9.53E+01	95.264
b4169_v1_mRNA_CONV	convsersion of mRNA to mRNA_1 (synthetic rxn)	1 b4169_v1_mRNA --> 1 b4169_mRNA_1	irreversible	Translation	0.00E+00	1.00E+09	0	1.22E+01	1.22E+01	0	2.16E+01	21.56007	0	1.04E-01	0.104
b4169_v2_mRNA_CONV	convsersion of mRNA to mRNA_1 (synthetic rxn)	1 b4169_v2_mRNA --> 1 b4169_mRNA_1	irreversible	Translation	0.00E+00	1.00E+09	0	1.22E+01	1.22E+01	0	2.16E+01	21.56007	0	1.04E-01	0.104
b4170_fold_spon	b4170_m folding: spontanous	1 b4170_m --> 1 MutL_mono	irreversible	Protein Folding	0.00E+00	1.00E+09	0	5.05E+02	5.05E+02	0	8.42E+02	841.6827	0	6.93E+01	69.264
b4170_mRNA_CONV	convsersion of mRNA to mRNA_1 (synthetic rxn)	1 b4170_mRNA --> 1 b4170_mRNA_1	irreversible	Translation	0.00E+00	1.00E+09	0	1.22E+01	1.22E+01	0	2.16E+01	21.56007	0	1.04E-01	0.104
b4170_mRNA_degr1	Degradation of b4170_mRNA I	1 Orn_dim + 462 atp + 1 b4170_mRNA_2 + 1 degradosome + 462 h2o <==> 462 adp + 1 b4170_mRNA_2_degr + 462 h + 462 pi	reversible	mRNA degradation	-1.00E+09	1.00E+09	0	1.22E+01	1.22E+01	0	2.16E+01	21.56007	0	1.04E-01	0.104
b4170_mRNA_degr2	Degradation of b4170_mRNA II	1 b4170_mRNA_2_degr + 1847 h2o --> 1 Orn_dim_inact + 436 amp 516 cmp + 1 degradosome_inact + 524 gmp + 1847 h + 372 ump	irreversible	mRNA degradation	0.00E+00	2.00E-01	0	1.22E+01	1.22E+01	0	2.16E+01	21.56007	0	1.04E-01	0.104
b4170_maturation1	Polypeptide b4170 peptide deformylas and methionine aminopeptidase complex	1 Def_mono + 1 Map_mono + 1 b4170_aa <==> 1 b4170_def_map_cplx	reversible	Protein Maturation	-1.00E+09	1.00E+09	0	5.05E+02	5.05E+02	0	8.42E+02	841.6827	0	6.93E+01	69.264
b4170_maturation2	b4170 formation	1 b4170_def_map_cplx + 2 h2o --> 1 Def_mono_inact + 1 Map_mono_inact + 1 b4170_m + 1 for + 1 met-L	irreversible	Protein Maturation	0.00E+00	1.00E+09	0	5.05E+02	5.05E+02	0	8.42E+02	841.6827	0	6.93E+01	69.264
b4170_v1_mRNA_CONV	convsersion of mRNA to mRNA_1 (synthetic rxn)	1 b4170_v1_mRNA --> 1 b4170_mRNA_1	irreversible	Translation	0.00E+00	1.00E+09	0	1.22E+01	1.22E+01	0	2.16E+01	21.56007	0	1.04E-01	0.104
b4170_v2_mRNA_CONV	convsersion of mRNA to mRNA_1 (synthetic rxn)	1 b4170_v2_mRNA --> 1 b4170_mRNA_1	irreversible	Translation	0.00E+00	1.00E+09	0	1.22E+01	1.22E+01	0	2.16E+01	21.56007	0	1.04E-01	0.104
b4171_fold_spon	b4171_m folding: spontanous	1 b4171_m_Mg --> 1 MiaA_mono	irreversible	Protein Folding	0.00E+00	1.00E+09	0	3.79E+02	3.79E+02	0	5.47E+02	547.3813	0	1.61E+02	161.172
b4171_ion_BIND	Binding of ions to b4171	1 b4171_m + 1 mg2 <==> 1 b4171_m_Mg	reversible	Metallo-ion Binding	-1.00E+09	1.00E+09	0	3.79E+02	3.79E+02	0	5.47E+02	547.3813	0	1.61E+02	161.172
b4171_mRNA_CONV	convsersion of mRNA to mRNA_1 (synthetic rxn)	1 b4171_mRNA --> 1 b4171_mRNA_1	irreversible	Translation	0.00E+00	1.00E+09	0	1.22E+01	1.22E+01	0	2.16E+01	21.56007	0	1.04E-01	0.104
b4171_mRNA_degr1	Degradation of b4171_mRNA I	1 Orn_dim + 235 atp + 1 b4171_mRNA_2 + 1 degradosome + 235 h2o <==> 235 adp + 1 b4171_mRNA_2_degr + 235 h + 235 pi	reversible	mRNA degradation	-1.00E+09	1.00E+09	0	1.22E+01	1.22E+01	0	2.16E+01	21.56007	0	1.04E-01	0.104
b4171_mRNA_degr2	Degradation of b4171_mRNA II	1 b4171_mRNA_2_degr + 942 h2o --> 1 Orn_dim_inact + 211 amp + 219 cmp + 1 degradosome_inact + 286 gmp + 942 h + 227 ump	irreversible	mRNA degradation	0.00E+00	2.61E-01	0	1.22E+01	1.22E+01	0	2.16E+01	21.56007	0	1.04E-01	0.104
b4171_maturation1	Polypeptide b4171 peptide deformylas and methionine aminopeptidase complex	1 Def_mono + 1 Map_mono + 1 b4171_aa <==> 1 b4171_def_map_cplx	reversible	Protein Maturation	-1.00E+09	1.00E+09	0	3.79E+02	3.79E+02	0	5.47E+02	547.3813	0	1.61E+02	161.172
b4171_maturation2	b4171 formation	1 b4171_def_map_cplx + 2 h2o --> 1 Def_mono_inact + 1 Map_mono_inact + 1 b4171_m + 1 for + 1 met-L	irreversible	Protein Maturation	0.00E+00	1.00E+09	0	3.79E+02	3.79E+02	0	5.47E+02	547.3813	0	1.61E+02	161.172
b4171_v1_mRNA_CONV	convsersion of mRNA to mRNA_1 (synthetic rxn)	1 b4171_v1_mRNA --> 1 b4171_mRNA_1	irreversible	Translation	0.00E+00	1.00E+09	0	1.22E+01	1.22E+01	0	2.16E+01	21.56007	0	1.04E-01	0.104
b4171_v2_mRNA_CONV	convsersion of mRNA to mRNA_1 (synthetic rxn)	1 b4171_v2_mRNA --> 1 b4171_mRNA_1	irreversible	Translation	0.00E+00	1.00E+09	0	1.22E+01	1.22E+01	0	2.16E+01	21.56007	0	1.04E-01	0.104
b4172_fold_spon	b4172_m folding: spontanous	1 b4172_m --> 1 Hfq_mono	irreversible	Protein Folding	0.00E+00	1.00E+09	0	6.82E+02	6.82E+02	0	7.38E+02	738.3065	0	6.10E+02	609.57
b4172_mRNA_CONV	convsersion of mRNA to mRNA_1 (synthetic rxn)	1 b4172_mRNA --> 1 b4172_mRNA_1	irreversible	Translation	0.00E+00	1.00E+09	0	1.22E+01	1.22E+01	0	2.16E+01	21.56007	0	1.04E-01	0.104
b4172_mRNA_degr1	Degradation of b4172_mRNA I	1 Orn_dim + 77 atp + 1 b4172_mRNA_2 + 1 degradosome + 77 h2o <==> 77 adp + 1 b4172_mRNA_2_degr + 77 h + 77 pi	reversible	mRNA degradation	-1.00E+09	1.00E+09	0	1.22E+01	1.22E+01	0	2.16E+01	21.56007	0	1.04E-01	0.104
b4172_mRNA_degr2	Degradation of b4172_mRNA II	1 b4172_mRNA_2_degr + 308 h2o --> 1 Orn_dim_inact + 81 amp + 75 cmp + 1 degradosome_inact + 76 gmp + 308 h + 77 ump	irreversible	mRNA degradation	0.00E+00	8.23E-01	0	1.22E+01	1.22E+01	0	2.16E+01	21.56007	0	1.04E-01	0.104
b4172_maturation1	Polypeptide b4172 peptide deformylas and methionine aminopeptidase complex	1 Def_mono + 1 Map_mono + 1 b4172_aa <==> 1 b4172_def_map_cplx	reversible	Protein Maturation	-1.00E+09	1.00E+09	0	6.82E+02	6.82E+02	0	7.38E+02	738.3065	0	6.10E+02	609.57
b4172_maturation2	b4172 formation	1 b4172_def_map_cplx + 2 h2o --> 1 Def_mono_inact + 1 Map_mono_inact + 1 b4172_m + 1 for + 1 met-L	irreversible	Protein Maturation	0.00E+00	1.00E+09	0	6.82E+02	6.82E+02	0	7.38E+02	738.3065	0	6.10E+02	609.57

b4172_v1_mRNA_CONV	convsersion of mRNA to mRNA_1 (synthetic rxn)	1 b4172_v1_mRNA --> 1 b4172_mRNA_1	irreversible	Translation	0.00E+00	1.00E+09	0	1.22E+01	1.22E+01	0	2.16E+01	21.56007	0	1.04E-01	0.104
b4172_v2_mRNA_CONV	convsersion of mRNA to mRNA_1 (synthetic rxn)	1 b4172_v2_mRNA --> 1 b4172_mRNA_1	irreversible	Translation	0.00E+00	1.00E+09	0	1.22E+01	1.22E+01	0	2.16E+01	21.56007	0	1.04E-01	0.104
b4173_fold_spon	b4173_m folding: spontanous	1 b4173_m --> 1 HflX_mono	irreversible	Protein Folding	0.00E+00	1.00E+09	0	3.93E+02	3.93E+02	0	6.27E+02	626.5019	0	9.01E+01	90.1
b4173_mRNA_CONV	convsersion of mRNA to mRNA_1 (synthetic rxn)	1 b4173_mRNA --> 1 b4173_mRNA_1	irreversible	Translation	0.00E+00	1.00E+09	0	1.22E+01	1.22E+01	0	2.16E+01	21.56007	0	1.04E-01	0.104
b4173_mRNA_degr1	Degradation of b4173_mRNA I	1 Orn_dim + 320 atp + 1 b4173_mRNA_2 + 1 degradosome + 320 h2o <==> 320 adp + 1 b4173_mRNA_2_degr + 320 h + 320 pi	reversible	mRNA degradation	-1.00E+09	1.00E+09	0	1.22E+01	1.22E+01	0	2.16E+01	21.56007	0	1.04E-01	0.104
b4173_mRNA_degr2	Degradation of b4173_mRNA II	1 b4173_mRNA_2_degr + 1280 h2o --> 1 Orn_dim_inact + 298 amp 312 cmp + 1 degradosome_inact + 378 gmp + 1280 h + 293 ump	irreversible	mRNA degradation	0.00E+00	2.84E-01	0	1.22E+01	1.22E+01	0	2.16E+01	21.56007	0	1.04E-01	0.104
b4173_maturation1	Polypeptide b4173 peptide deformylas and methionine aminopeptidase complex	1 Def_mono + 1 Map_mono + 1 b4173_aa <==> 1 b4173_def_map_cplx	reversible	Protein Maturation	-1.00E+09	1.00E+09	0	3.93E+02	3.93E+02	0	6.27E+02	626.5019	0	9.01E+01	90.1
b4173_maturation2	b4173 formation	1 b4173_def_map_cplx + 2 h2o --> 1 Def_mono_inact + 1 Map_mono_inact + 1 b4173_m + 1 for + 1 met-L	irreversible	Protein Maturation	0.00E+00	1.00E+09	0	3.93E+02	3.93E+02	0	6.27E+02	626.5019	0	9.01E+01	90.1
b4173_v1_mRNA_CONV	convsersion of mRNA to mRNA_1 (synthetic rxn)	1 b4173_v1_mRNA --> 1 b4173_mRNA_1	irreversible	Translation	0.00E+00	1.00E+09	0	1.22E+01	1.22E+01	0	2.16E+01	21.56007	0	1.04E-01	0.104
b4173_v2_mRNA_CONV	convsersion of mRNA to mRNA_1 (synthetic rxn)	1 b4173_v2_mRNA --> 1 b4173_mRNA_1	irreversible	Translation	0.00E+00	1.00E+09	0	1.22E+01	1.22E+01	0	2.16E+01	21.56007	0	1.04E-01	0.104
b4174_fold_spon	b4174_m folding: spontanous	1 b4174_m --> 1 HflK_mono	irreversible	Protein Folding	0.00E+00	1.00E+09	0	3.95E+02	3.95E+02	0	6.19E+02	618.9618	0	1.04E+02	104.016
b4174_mRNA_CONV	convsersion of mRNA to mRNA_1 (synthetic rxn)	1 b4174_mRNA --> 1 b4174_mRNA_1	irreversible	Translation	0.00E+00	1.00E+09	0	1.22E+01	1.22E+01	0	2.16E+01	21.56007	0	1.04E-01	0.104
b4174_mRNA_degr1	Degradation of b4174_mRNA I	1 Orn_dim + 315 atp + 1 b4174_mRNA_2 + 1 degradosome + 315 h2o <==> 315 adp + 1 b4174_mRNA_2_degr + 315 h + 315 pi	reversible	mRNA degradation	-1.00E+09	1.00E+09	0	1.22E+01	1.22E+01	0	2.16E+01	21.56007	0	1.04E-01	0.104
b4174_mRNA_degr2	Degradation of b4174_mRNA II	1 b4174_mRNA_2_degr + 1259 h2o --> 1 Orn_dim_inact + 317 amp 327 cmp + 1 degradosome_inact + 381 gmp + 1259 h + 235 ump	irreversible	mRNA degradation	0.00E+00	4.90E-01	0	1.22E+01	1.22E+01	0	2.16E+01	21.56007	0	1.04E-01	0.104
b4174_maturation1	Polypeptide b4174 peptide deformylas and methionine aminopeptidase complex	1 Def_mono + 1 Map_mono + 1 b4174_aa <==> 1 b4174_def_map_cplx	reversible	Protein Maturation	-1.00E+09	1.00E+09	0	3.95E+02	3.95E+02	0	6.19E+02	618.9618	0	1.04E+02	104.016
b4174_maturation2	b4174 formation	1 b4174_def_map_cplx + 2 h2o --> 1 Def_mono_inact + 1 Map_mono_inact + 1 b4174_m + 1 for + 1 met-L	irreversible	Protein Maturation	0.00E+00	1.00E+09	0	3.95E+02	3.95E+02	0	6.19E+02	618.9618	0	1.04E+02	104.016
b4174_v1_mRNA_CONV	convsersion of mRNA to mRNA_1 (synthetic rxn)	1 b4174_v1_mRNA --> 1 b4174_mRNA_1	irreversible	Translation	0.00E+00	1.00E+09	0	1.22E+01	1.22E+01	0	2.16E+01	21.56007	0	1.04E-01	0.104
b4174_v2_mRNA_CONV	convsersion of mRNA to mRNA_1 (synthetic rxn)	1 b4174_v2_mRNA --> 1 b4174_mRNA_1	irreversible	Translation	0.00E+00	1.00E+09	0	1.22E+01	1.22E+01	0	2.16E+01	21.56007	0	1.04E-01	0.104
b4175_fold_spon	b4175_m folding: spontanous	1 b4175_m --> 1 HflC_mono	irreversible	Protein Folding	0.00E+00	1.00E+09	0	3.28E+02	3.28E+02	0	5.05E+02	505.2114	0	9.75E+01	97.546
b4175_mRNA_CONV	convsersion of mRNA to mRNA_1 (synthetic rxn)	1 b4175_mRNA --> 1 b4175_mRNA_1	irreversible	Translation	0.00E+00	1.00E+09	0	1.22E+01	1.22E+01	0	2.16E+01	21.56007	0	1.04E-01	0.104
b4175_mRNA_degr1	Degradation of b4175_mRNA I	1 Orn_dim + 251 atp + 1 b4175_mRNA_2 + 1 degradosome + 251 h2o <==> 251 adp + 1 b4175_mRNA_2_degr + 251 h + 251 pi	reversible	mRNA degradation	-1.00E+09	1.00E+09	0	1.22E+01	1.22E+01	0	2.16E+01	21.56007	0	1.04E-01	0.104
b4175_mRNA_degr2	Degradation of b4175_mRNA II	1 b4175_mRNA_2_degr + 1004 h2o --> 1 Orn_dim_inact + 248 amp 249 cmp + 1 degradosome_inact + 284 gmp + 1004 h + 224 ump	irreversible	mRNA degradation	0.00E+00	3.37E-01	0	1.22E+01	1.22E+01	0	2.16E+01	21.56007	0	1.04E-01	0.104
b4175_maturation1	Polypeptide b4175 peptide deformylas and methionine aminopeptidase complex	1 Def_mono + 1 Map_mono + 1 b4175_aa <==> 1 b4175_def_map_cplx	reversible	Protein Maturation	-1.00E+09	1.00E+09	0	3.28E+02	3.28E+02	0	5.05E+02	505.2114	0	9.75E+01	97.546
b4175_maturation2	b4175 formation	1 b4175_def_map_cplx + 2 h2o --> 1 Def_mono_inact + 1 Map_mono_inact + 1 b4175_m + 1 for + 1 met-L	irreversible	Protein Maturation	0.00E+00	1.00E+09	0	3.28E+02	3.28E+02	0	5.05E+02	505.2114	0	9.75E+01	97.546
b4175_v1_mRNA_CONV	conversion of mRNA to mRNA_1 (synthetic rxn)	1 b4175_v1_mRNA --> 1 b4175_mRNA_1	irreversible	Translation	0.00E+00	1.00E+09	0	1.22E+01	1.22E+01	0	2.16E+01	21.56007	0	1.04E-01	0.104
b4175_v2_mRNA_CONV	conversion of mRNA to mRNA_1 (synthetic rxn)	1 b4175_v2_mRNA --> 1 b4175_mRNA_1	irreversible	Translation	0.00E+00	1.00E+09	0	1.22E+01	1.22E+01	0	2.16E+01	21.56007	0	1.04E-01	0.104
b4178_fold_spon	b4178_m folding: spontanous	1 b4178_m --> 1 YjeB_mono	irreversible	Protein Folding	0.00E+00	1.00E+09	0	3.53E+02	3.53E+02	0	5.59E+02	559.0763	0	8.57E+01	85.744
b4178_mRNA_CONV	conversion of mRNA to mRNA_1 (synthetic rxn)	1 b4178_mRNA --> 1 b4178_mRNA_1	irreversible	Translation	0.00E+00	1.00E+09	0	3.36E+01	3.36E+01	0	5.93E+01	59.28454	0	1.18E-01	0.118
b4178_mRNA_degr1	Degradation of b4178_mRNA I	1 Orn_dim + 106 atp + 1 b4178_mRNA_2 + 1 degradosome + 106 h2o <==> 106 adp + 1 b4178_mRNA_2_degr + 106 h + 106 pi	reversible	mRNA degradation	-1.00E+09	1.00E+09	0	3.36E+01	3.36E+01	0	5.93E+01	59.28454	0	1.18E-01	0.118
b4178_mRNA_degr2	Degradation of b4178_mRNA II	1 b4178_mRNA_2_degr + 425 h2o --> 1 Orn_dim_inact + 103 amp + 89 cmp + 1 degradosome_inact + 116 gmp + 1 gtp + 425 h + 117 ump	irreversible	mRNA degradation	0.00E+00	1.18E-01	0	3.36E+01	3.36E+01	0	5.93E+01	59.28454	0	1.18E-01	0.118
b4178_maturation1	Polypeptide b4178 peptide deformylas and methionine aminopeptidase complex	1 Def_mono + 1 Map_mono + 1 b4178_aa <==> 1 b4178_def_map_cplx	reversible	Protein Maturation	-1.00E+09	1.00E+09	0	3.53E+02	3.53E+02	0	5.59E+02	559.0763	0	8.57E+01	85.744
b4178_maturation2	b4178 formation	1 b4178_def_map_cplx + 2 h2o --> 1 Def_mono_inact + 1 Map_mono_inact + 1 b4178_m + 1 for + 1 met-L	irreversible	Protein Maturation	0.00E+00	1.00E+09	0	3.53E+02	3.53E+02	0	5.59E+02	559.0763	0	8.57E+01	85.744
b4179_fold_GroEL/ES_1	b4179_m folding: GroEL/ES mediated: polypeptide is going in GroEL/ES complex	1 GroEL.(7)ADP.cisGroES + 7 atp + 1 b4179_m + 1 transGroES_hepta --> 7 adp + 1 b4179_m_GroEL.(7)ATP.transGroES + 1 cisGroES_hepta	irreversible	Protein Folding	0.00E+00	1.00E+09	0	1.65E+03	1.65E+03	0	2.79E+03	2794.799	0	7.56E+01	75.576
b4179_fold_GroEL/ES_2	b4179_m folding: GroEL/ES mediated: folding of polypeptide under ATP hydrolysis	1 b4179_m_GroEL.(7)ATP.transGroES + 7 h2o --> 1 Rnr_GroEL.(7)ADP.transGroES + 7 h + 7 pi	irreversible	Protein Folding	0.00E+00	1.00E+09	0	1.65E+03	1.65E+03	0	2.79E+03	2794.799	0	7.56E+01	75.576
b4179_fold_GroEL/ES_3	b4179_m folding: GroEL/ES mediated: release of native protein	1 Rnr_GroEL.(7)ADP.transGroES --> 1 GroEL.(7)ADP.transGroES + 1 Rnr_mono	irreversible	Protein Folding	0.00E+00	1.00E+09	0	1.65E+03	1.65E+03	0	2.79E+03	2794.799	0	7.56E+01	75.576
b4179_fold_KJE_1	b4179_m folding: KJE mediated	1 DnaJ_dim + 1 DnaK_mono.ATP + 1 b4179_m <==> 1 b4179_m_DnaKJ_complex	reversible	Protein Folding	-1.00E+09	1.00E+09	0	1.65E+03	1.65E+03	0	2.79E+03	2794.799	0	7.56E+01	75.576
b4179_fold_KJE_2	b4179_m folding: KJE mediated	1 GrpE_dim + 1 b4179_m_DnaKJ_complex + 1 h2o --> 1 DnaJ_dim_inact + 1 Rnr_DnaK_GrpE_complex + 1 adp + 1 h + 1 pi	irreversible	Protein Folding	0.00E+00	1.00E+09	0	1.65E+03	1.65E+03	0	2.79E+03	2794.799	0	7.56E+01	75.576
b4179_fold_KJE_3	b4179_m folding: KJE mediated	1 Rnr_DnaK_GrpE_complex + 1 atp --> 1 DnaK_mono.ATP_inact + 1 GrpE_dim_inact + 1 Rnr_mono	irreversible	Protein Folding	0.00E+00	1.00E+09	0	1.65E+03	1.65E+03	0	2.79E+03	2794.799	0	7.56E+01	75.576
b4179_mRNA_CONV	convsersion of mRNA to mRNA_1 (synthetic rxn)	1 b4179_mRNA --> 1 b4179_mRNA_1	irreversible	Translation	0.00E+00	1.00E+09	0	3.36E+01	3.36E+01	0	5.93E+01	59.28454	0	1.18E-01	0.118
b4179_mRNA_degr1	Degradation of b4179_mRNA I	1 Orn_dim + 610 atp + 1 b4179_mRNA_2 + 1 degradosome + 610 h2o <==> 610 adp + 1 b4179_mRNA_2_degr + 610 h + 610 pi	reversible	mRNA degradation	-1.00E+09	1.00E+09	0	3.36E+01	3.36E+01	0	5.93E+01	59.28454	0	1.18E-01	0.118
b4179_mRNA_degr2	Degradation of b4179_mRNA II	1 b4179_mRNA_2_degr + 2441 h2o --> 1 Orn_dim_inact + 589 amp 622 cmp + 1 degradosome_inact + 728 gmp + 2441 h + 503 ump	irreversible	mRNA degradation	0.00E+00	1.35E-01	0	3.36E+01	3.36E+01	0	5.93E+01	59.28454	0	1.18E-01	0.118
b4179_maturation1	Polypeptide b4179 peptide deformylas and methionine aminopeptidase complex	1 Def_mono + 1 Map_mono + 1 b4179_aa <==> 1 b4179_def_map_cplx	reversible	Protein Maturation	-1.00E+09	1.00E+09	0	1.65E+03	1.65E+03	0	2.79E+03	2794.799	0	7.56E+01	75.576
b4179_maturation2	b4179 formation	1 b4179_def_map_cplx + 2 h2o --> 1 Def_mono_inact + 1 Map_mono_inact + 1 b4179_m + 1 for + 1 met-L	irreversible	Protein Maturation	0.00E+00	1.00E+09	0	1.65E+03	1.65E+03	0	2.79E+03	2794.799	0	7.56E+01	75.576
b4180_fold_spon	b4180_m folding: spontanous	1 b4180_m --> 1 RlmB_mono	irreversible	Protein Folding	0.00E+00	1.00E+09	0	5.61E+02	5.61E+02	0	9.21E+02	921.2635	0	9.29E+01	92.932
b4180_mRNA_CONV	conversion of mRNA to mRNA_1 (synthetic rxn)	1 b4180_mRNA --> 1 b4180_mRNA_1	irreversible	Translation	0.00E+00	1.00E+09	0	3.36E+01	3.36E+01	0	5.93E+01	59.28454	0	1.18E-01	0.118
b4180_mRNA_degr1	Degradation of b4180_mRNA I	1 Orn_dim + 183 atp + 1 b4180_mRNA_2 + 1 degradosome + 183 h2o <==> 183 adp + 1 b4180_mRNA_2_degr + 183 h + 183 pi	reversible	mRNA degradation	-1.00E+09	1.00E+09	0	3.36E+01	3.36E+01	0	5.93E+01	59.28454	0	1.18E-01	0.118

b4180_mRNA_degr2	Degradation of b4180_mRNA II	1 b4180_mRNA_2_degr + 731 h2o --> 1 Orn_dim_inact + 163 amp + 194 cmp + 1 degradosome_inact + 218 gmp + 731 h + 157 ump	irreversible	mRNA degradation	0.00E+00	3.26E-01	0	3.36E+01	3.36E+01	0	5.93E+01	59.28454	0	1.18E-01	0.118
b4180_maturation1	Polypeptide b4180 peptide deformylas and methionine aminopeptidase complex	1 Def_mono + 1 Map_mono + 1 b4180_aa <==> 1 b4180_def_map_cplx	reversible	Protein Maturation	-1.00E+09	1.00E+09	0	5.61E+02	5.61E+02	0	9.21E+02	921.2635	0	9.29E+01	92.932
b4180_maturation2	b4180 formation	1 b4180_def_map_cplx + 2 h2o --> 1 Def_mono_inact + 1 Map_mono_inact + 1 b4180_m + 1 for + 1 met-L	irreversible	Protein Maturation	0.00E+00	1.00E+09	0	5.61E+02	5.61E+02	0	9.21E+02	921.2635	0	9.29E+01	92.932
b4200_fold_spon	b4200_m folding: spontanous	1 b4200_m --> 1 RpsF_mono	irreversible	Protein Folding	0.00E+00	1.00E+09	8.44E+01	1.13E+02	2.81E+01	6.22E+01	8.29E+01	20.72	6.22E+01	8.29E+01	20.72
b4200_mRNA_CONV	convsersion of mRNA to mRNA_1 (synthetic rxn)	1 b4200_mRNA --> 1 b4200_mRNA_1	irreversible	Translation	0.00E+00	1.00E+09	0	8.54E+01	8.54E+01	0	8.29E+01	82.88	0	2.89E-01	0.289
b4200_mRNA_degr1	Degradation of b4200_mRNA I	1 Orn_dim + 99 atp + 1 b4200_mRNA_2 + 1 degradosome + 99 h2o <==> 99 adp + 1 b4200_mRNA_2_degr + 99 h + 99 pi	reversible	mRNA degradation	-1.00E+09	1.00E+09	0	8.54E+01	8.54E+01	0	8.29E+01	82.88	0	2.89E-01	0.289
b4200_mRNA_degr2	Degradation of b4200_mRNA II	1 b4200_mRNA_2_degr + 395 h2o --> 1 Orn_dim_inact + 103 amp + 1 atp + 102 cmp + 1 degradosome_inact + 106 gmp + 395 h + 84 ump	irreversible	mRNA degradation	0.00E+00	7.70E-01	0	8.54E+01	8.54E+01	0	8.29E+01	82.88	0	2.89E-01	0.289
b4200_maturation1	Polypeptide b4200 peptide deformylas complex	1 Def_mono + 1 b4200_aa <==> 1 b4200_def_cplx	reversible	Protein Maturation	-1.00E+09	1.00E+09	8.44E+01	1.13E+02	2.81E+01	6.22E+01	8.29E+01	20.72	6.22E+01	8.29E+01	20.72
b4200_maturation2	b4200 formation	1 b4200_def_cplx + 1 h2o --> 1 Def_mono_inact + 1 b4200_m + 1 for	irreversible	Protein Maturation	0.00E+00	1.00E+09	8.44E+01	1.13E+02	2.81E+01	6.22E+01	8.29E+01	20.72	6.22E+01	8.29E+01	20.72
b4201_fold_spon	b4201_m folding: spontanous	1 b4201_m --> 1 PriB_mono	irreversible	Protein Folding	0.00E+00	1.00E+09	0	8.89E+02	8.89E+02	0	8.74E+02	874.08	0	3.79E+02	378.534
b4201_mRNA_CONV	convsersion of mRNA to mRNA_1 (synthetic rxn)	1 b4201_mRNA --> 1 b4201_mRNA_1	irreversible	Translation	0.00E+00	1.00E+09	0	8.54E+01	8.54E+01	0	8.29E+01	82.88	0	2.89E-01	0.289
b4201_mRNA_degr1	Degradation of b4201_mRNA I	1 Orn_dim + 78 atp + 1 b4201_mRNA_2 + 1 degradosome + 78 h2o <==> 78 adp + 1 b4201_mRNA_2_degr + 78 h + 78 pi	reversible	mRNA degradation	-1.00E+09	1.00E+09	0	8.54E+01	8.54E+01	0	8.29E+01	82.88	0	2.89E-01	0.289
b4201_mRNA_degr2	Degradation of b4201_mRNA II	1 b4201_mRNA_2_degr + 314 h2o --> 1 Orn_dim_inact + 76 amp + 78 cmp + 1 degradosome_inact + 87 gmp + 314 h + 74 ump	irreversible	mRNA degradation	0.00E+00	5.55E-01	0	8.54E+01	8.54E+01	0	8.29E+01	82.88	0	2.89E-01	0.289
b4201_maturation1	Polypeptide b4201 peptide deformylas and methionine aminopeptidase complex	1 Def_mono + 1 Map_mono + 1 b4201_aa <==> 1 b4201_def_map_cplx	reversible	Protein Maturation	-1.00E+09	1.00E+09	0	8.89E+02	8.89E+02	0	8.74E+02	874.08	0	3.79E+02	378.534
b4201_maturation2	b4201 formation	1 b4201_def_map_cplx + 2 h2o --> 1 Def_mono_inact + 1 Map_mono_inact + 1 b4201_m + 1 for + 1 met-L	irreversible	Protein Maturation	0.00E+00	1.00E+09	0	8.89E+02	8.89E+02	0	8.74E+02	874.08	0	3.79E+02	378.534
b4202_fold_spon	b4202_m folding: spontanous	1 b4202_m --> 1 RpsR_mono	irreversible	Protein Folding	0.00E+00	1.00E+09	8.44E+01	1.13E+02	2.81E+01	6.22E+01	8.29E+01	20.72	6.22E+01	8.29E+01	20.72
b4202_mRNA_CONV	convsersion of mRNA to mRNA_1 (synthetic rxn)	1 b4202_mRNA --> 1 b4202_mRNA_1	irreversible	Translation	0.00E+00	1.00E+09	0	8.54E+01	8.54E+01	0	8.29E+01	82.88	0	2.89E-01	0.289
b4202_mRNA_degr1	Degradation of b4202_mRNA I	1 Orn_dim + 57 atp + 1 b4202_mRNA_2 + 1 degradosome + 57 h2o <==> 57 adp + 1 b4202_mRNA_2_degr + 57 h + 57 pi	reversible	mRNA degradation	-1.00E+09	1.00E+09	0	8.54E+01	8.54E+01	0	8.29E+01	82.88	0	2.89E-01	0.289
b4202_mRNA_degr2	Degradation of b4202_mRNA II	1 b4202_mRNA_2_degr + 227 h2o --> 1 Orn_dim_inact + 58 amp + 69 cmp + 1 degradosome_inact + 51 gmp + 227 h + 50 ump	irreversible	mRNA degradation	0.00E+00	1.04E+00	0	8.54E+01	8.54E+01	0	8.29E+01	82.88	0	2.89E-01	0.289
b4202_maturation1	Polypeptide b4202 peptide deformylas and methionine aminopeptidase complex	1 Def_mono + 1 Map_mono + 1 b4202_aa <==> 1 b4202_def_map_cplx	reversible	Protein Maturation	-1.00E+09	1.00E+09	8.44E+01	1.13E+02	2.81E+01	6.22E+01	8.29E+01	20.72	6.22E+01	8.29E+01	20.72
b4202_maturation2	b4202 formation	1 b4202_def_map_cplx + 2 h2o --> 1 Def_mono_inact + 1 Map_mono_inact + 1 b4202_m + 1 for + 1 met-L	irreversible	Protein Maturation	0.00E+00	1.00E+09	8.44E+01	1.13E+02	2.81E+01	6.22E+01	8.29E+01	20.72	6.22E+01	8.29E+01	20.72
b4203_fold_spon	b4203_m folding: spontanous	1 b4203_m --> 1 RplI_mono	irreversible	Protein Folding	0.00E+00	1.00E+09	8.44E+01	1.13E+02	2.81E+01	6.22E+01	8.29E+01	20.72	6.22E+01	8.29E+01	20.72
b4203_mRNA_CONV	convsersion of mRNA to mRNA_1 (synthetic rxn)	1 b4203_mRNA --> 1 b4203_mRNA_1	irreversible	Translation	0.00E+00	1.00E+09	0	8.54E+01	8.54E+01	0	8.29E+01	82.88	0	2.89E-01	0.289
b4203_mRNA_degr1	Degradation of b4203_mRNA I	1 Orn_dim + 112 atp + 1 b4203_mRNA_2 + 1 degradosome + 112 h2o <==> 112 adp + 1 b4203_mRNA_2_degr + 112 h + 112 pi	reversible	mRNA degradation	-1.00E+09	1.00E+09	0	8.54E+01	8.54E+01	0	8.29E+01	82.88	0	2.89E-01	0.289
b4203_mRNA_degr2	Degradation of b4203_mRNA II	1 b4203_mRNA_2_degr + 449 h2o --> 1 Orn_dim_inact + 121 amp + 102 cmp + 1 degradosome_inact + 122 gmp + 449 h + 105 ump	irreversible	mRNA degradation	0.00E+00	2.89E-01	0	8.54E+01	8.54E+01	0	8.29E+01	82.88	0	2.89E-01	0.289
b4203_maturation1	Polypeptide b4203 peptide deformylas complex	1 Def_mono + 1 b4203_aa <==> 1 b4203_def_cplx	reversible	Protein Maturation	-1.00E+09	1.00E+09	8.44E+01	1.13E+02	2.81E+01	6.22E+01	8.29E+01	20.72	6.22E+01	8.29E+01	20.72
b4203_maturation2	b4203 formation	1 b4203_def_cplx + 1 h2o --> 1 Def_mono_inact + 1 b4203_m + 1 for	irreversible	Protein Maturation	0.00E+00	1.00E+09	8.44E+01	1.13E+02	2.81E+01	6.22E+01	8.29E+01	20.72	6.22E+01	8.29E+01	20.72
b4258_fold_KJE_1	b4258_m folding: KJE mediated	1 DnaJ_dim + 1 DnaK_mono.ATP + 1 b4258_m <==> 1 b4258_m_DnaKJ_complex	reversible	Protein Folding	-1.00E+09	1.00E+09	0	2.02E+03	2.02E+03	0	2.06E+03	2056.765	0	1.19E+02	118.8
b4258_fold_KJE_2	b4258_m folding: KJE mediated	1 GrpE_dim + 1 b4258_m_DnaKJ_complex + 1 h2o --> 1 DnaJ_dim_inact + 1 ValS_DnaK_GrpE_complex + 1 adp + 1 h + 1 pi	irreversible	Protein Folding	0.00E+00	1.00E+09	0	2.02E+03	2.02E+03	0	2.06E+03	2056.765	0	1.19E+02	118.8
b4258_fold_KJE_3	b4258_m folding: KJE mediated	1 ValS_DnaK_GrpE_complex + 1 atp --> 1 DnaK_mono.ATP_inact + 1 GrpE_dim_inact + 1 ValS_mono	irreversible	Protein Folding	0.00E+00	1.00E+09	0	2.02E+03	2.02E+03	0	2.06E+03	2056.765	0	1.19E+02	118.8
b4258_fold_spon	b4258_m folding: spontanous	1 b4258_m --> 1 ValS_mono	irreversible	Protein Folding	0.00E+00	1.00E+09	0	2.02E+03	2.02E+03	0	2.06E+03	2056.765	0	1.19E+02	118.8
b4258_mRNA_CONV	convsersion of mRNA to mRNA_1 (synthetic rxn)	1 b4258_mRNA --> 1 b4258_mRNA_1	irreversible	Translation	0.00E+00	1.00E+09	0	4.21E+01	4.21E+01	0	7.43E+01	74.28142	0	4.90E-01	0.49
b4258_mRNA_degr1	Degradation of b4258_mRNA I	1 Orn_dim + 714 atp + 1 b4258_mRNA_2 + 1 degradosome + 714 h2o <==> 714 adp + 1 b4258_mRNA_2_degr + 714 h + 714 pi	reversible	mRNA degradation	-1.00E+09	1.00E+09	0	4.21E+01	4.21E+01	0	7.43E+01	74.28142	0	4.90E-01	0.49
b4258_mRNA_degr2	Degradation of b4258_mRNA II	1 b4258_mRNA_2_degr + 2855 h2o --> 1 Orn_dim_inact + 707 amp 1 atp + 759 cmp + 1 degradosome_inact + 800 gmp + 2855 h + 589 ump	irreversible	mRNA degradation	0.00E+00	4.90E-01	0	4.21E+01	4.21E+01	0	7.43E+01	74.28142	0	4.90E-01	0.49
b4258_maturation1	Polypeptide b4258 peptide deformylas complex	1 Def_mono + 1 b4258_aa <==> 1 b4258_def_cplx	reversible	Protein Maturation	-1.00E+09	1.00E+09	0	2.02E+03	2.02E+03	0	2.06E+03	2056.765	0	1.19E+02	118.8
b4258_maturation2	b4258 formation	1 b4258_def_cplx + 1 h2o --> 1 Def_mono_inact + 1 b4258_m + 1 for	irreversible	Protein Maturation	0.00E+00	1.00E+09	0	2.02E+03	2.02E+03	0	2.06E+03	2056.765	0	1.19E+02	118.8
b4258_v1_mRNA_CONV	convsersion of mRNA to mRNA_1 (synthetic rxn)	1 b4258_v1_mRNA --> 1 b4258_mRNA_1	irreversible	Translation	0.00E+00	1.00E+09	0	4.21E+01	4.21E+01	0	7.43E+01	74.28142	0	4.90E-01	0.49
b4270_tRNA_16_MODIF_1	modification of position 16 nt (D) in leuX by Dus_gen	1 Dus_gen + 1 b4270_tRNA_Mg2 + 1 h + 1 nadph <==> 1 b4270_tRNA_Mg2_Dus_gen_cplx	reversible	tRNA modification	-1.00E+09	1.00E+09	0	1.45E+03	1.45E+03	0	1.12E+01	11.2	0	1.12E+01	11.2
b4270_tRNA_16_MODIF_2	modification of position 16 nt (D) in leuX by Dus_gen	1 b4270_tRNA_Mg2_Dus_gen_cplx --> 1 Dus_gen_inact + 1 b4270_tRNA_1 + 1 nadp	irreversible	tRNA modification	0.00E+00	1.00E+09	0	1.45E+03	1.45E+03	0	1.12E+01	11.2	0	1.12E+01	11.2
b4270_tRNA_18_MODIF_1	modification of position 18 nt (Gm) in leuX by TrmH_dim	1 TrmH_dim + 1 amet + 1 b4270_tRNA_1 <==> 1 b4270_tRNA_1_TrmH_dim_cplx	reversible	tRNA modification	-1.00E+09	1.00E+09	0	1.45E+03	1.45E+03	0	1.12E+01	11.2	0	1.12E+01	11.2
b4270_tRNA_18_MODIF_2	modification of position 18 nt (Gm) in leuX by TrmH_dim	1 b4270_tRNA_1_TrmH_dim_cplx --> 1 TrmH_dim_inact + 1 ahcys + 1 b4270_tRNA_2 + 1 h	irreversible	tRNA modification	0.00E+00	1.00E+09	0	1.45E+03	1.45E+03	0	1.12E+01	11.2	0	1.12E+01	11.2
b4270_tRNA_20_MODIF_1	modification of position 20 nt (D) in leuX by Dus_gen	1 Dus_gen + 1 b4270_tRNA_2 + 1 h + 1 nadph <==> 1 b4270_tRNA_2_Dus_gen_cplx	reversible	tRNA modification	-1.00E+09	1.00E+09	0	1.45E+03	1.45E+03	0	1.12E+01	11.2	0	1.12E+01	11.2
b4270_tRNA_20_MODIF_2	modification of position 20 nt (D) in leuX by Dus_gen	1 b4270_tRNA_2_Dus_gen_cplx --> 1 Dus_gen_inact + 1 b4270_tRNA_3 + 1 nadp	irreversible	tRNA modification	0.00E+00	1.00E+09	0	1.45E+03	1.45E+03	0	1.12E+01	11.2	0	1.12E+01	11.2
b4270_tRNA_37_MODIF_1	modification of position 37 nt (ms2i6A) in leuX by MiaA_dim-MiaB_mono	1 IscS_dim_S-SH + 1 MiaA_dim + 1 MiaB_mono + 2 amet + 1 b4270_tRNA_3 + 1 dmpp + 1 fldrd <==> 1 b4270_tRNA_3_MiaA_dim MiaB_mono_cplx	reversible	tRNA modification	-1.00E+09	1.00E+09	0	1.45E+03	1.45E+03	0	1.12E+01	11.2	0	1.12E+01	11.2
b4270_tRNA_37_MODIF_2	modification of position 37 nt (ms2i6A) in leuX by MiaA_dim-MiaB_mono	1 b4270_tRNA_3_MiaA_dim-MiaB_mono_cplx --> 1 IscS_dim_S-SH + 1 MiaA_dim_inact + 1 MiaB_mono_inact + 1 ahcys + 1 b4270_tRNA_4 + 1 dad-5 + 1 fldox + 2 h + 1 met-L + 1 ppi	irreversible	tRNA modification	0.00E+00	1.00E+09	0	1.45E+03	1.45E+03	0	1.12E+01	11.2	0	1.12E+01	11.2
b4270_tRNA_39_MODIF_1	modification of position 39 nt (Y) in leuX by TruA_dim	1 TruA_dim + 1 b4270_tRNA_4 <==> 1 b4270_tRNA_4_TruA_dim_cplx	reversible	tRNA modification	-1.00E+09	1.00E+09	0	1.45E+03	1.45E+03	0	1.12E+01	11.2	0	1.12E+01	11.2
b4270_tRNA_39_MODIF_2	modification of position 39 nt (Y) in leuX by TruA_dim	1 b4270_tRNA_4_TruA_dim_cplx --> 1 TruA_dim_inact + 1 b4270_tRNA_5	irreversible	tRNA modification	0.00E+00	1.00E+09	0	1.45E+03	1.45E+03	0	1.12E+01	11.2	0	1.12E+01	11.2
b4270_tRNA_55_MODIF_1	modification of position 55 nt (Y) in leuX by TruB_mono	1 TruB_mono + 1 b4270_tRNA_5 <==> 1 b4270_tRNA_5_TruB_mono_cplx	reversible	tRNA modification	-1.00E+09	1.00E+09	0	1.45E+03	1.45E+03	0	1.12E+01	11.2	0	1.12E+01	11.2
b4270_tRNA_55_MODIF_2	modification of position 55 nt (Y) in leuX by TruB_mono	1 b4270_tRNA_5_TruB_mono_cplx --> 1 TruB_mono_inact + 1 leuX_tRNA	irreversible	tRNA modification	0.00E+00	1.00E+09	0	1.45E+03	1.45E+03	0	1.12E+01	11.2	0	1.12E+01	11.2
b4270_tRNA_Mg_BIND	Binding of two Mg2 to b4270_tRNA	1 b4270_RNA + 2 mg2 <==> 1 b4270_tRNA_Mg2	reversible	Metallo-ion binding	-1.00E+09	1.00E+09	0	1.45E+03	1.45E+03	0	1.12E+01	11.2	0	1.12E+01	11.2
b4292_fold_spon	b4292_m folding: spontanous	1 b4292_m --> 1 FecR_mono	irreversible	Protein Folding	0.00E+00	1.00E+09	0	9.00E+01	9.00E+01	0	9.00E+01	90	0	9.00E+01	90

b4292_mRNA_CONV	convsersion of mRNA to mRNA_1 (synthetic rxn)	1 b4292_mRNA --> 1 b4292_mRNA_1	irreversible	Translation	0.00E+00	1.00E+09	0	0	0.00E+00	0	0	0	0	0	0
b4292_mRNA_degr1	Degradation of b4292_mRNA I	1 Orn_dim + 237 atp + 1 b4292_mRNA_2 + 1 degradosome + 237 h2o <==> 237 adp + 1 b4292_mRNA_2_degr + 237 h + 237 pi	reversible	mRNA degradation	-1.00E+09	1.00E+09	0	0	0.00E+00	0	0	0	0	0	0
b4292_mRNA_degr2	Degradation of b4292_mRNA II	1 b4292_mRNA_2_degr + 949 h2o --> 1 Orn_dim_inact + 205 amp + 286 cmp + 1 degradosome_inact + 276 gmp + 949 h + 183 ump	irreversible	mRNA degradation	0.00E+00	2.07E-01	0	0	0.00E+00	0	0	0	0	0	0
b4292_maturation1	Polypeptide b4292 peptide deformylase and methionine aminopeptidase complex	1 Def_mono + 1 Map_mono + 1 b4292_aa <==> 1 b4292_def_map_cplx	reversible	Protein Maturation	-1.00E+09	1.00E+09	0	9.00E+01	9.00E+01	0	9.00E+01	90	0	9.00E+01	90
b4292_maturation2	b4292 formation	1 b4292_def_map_cplx + 2 h2o --> 1 Def_mono_inact + 1 Map_mono_inact + 1 b4292_m + 1 for + 1 met-L	irreversible	Protein Maturation	0.00E+00	1.00E+09	0	9.00E+01	9.00E+01	0	9.00E+01	90	0	9.00E+01	90
b4293_fold_spon	b4293_m folding: spontanous	1 b4293_m --> 1 FecI_mono	irreversible	Protein Folding	0.00E+00	1.00E+09	0	0	0.00E+00	0	0	0	0	0	0
b4293_mRNA_CONV	convsersion of mRNA to mRNA_1 (synthetic rxn)	1 b4293_mRNA --> 1 b4293_mRNA_1	irreversible	Translation	0.00E+00	1.00E+09	0	0	0.00E+00	0	0	0	0	0	0
b4293_mRNA_degr1	Degradation of b4293_mRNA I	1 Orn_dim + 130 atp + 1 b4293_mRNA_2 + 1 degradosome + 130 h2o <==> 130 adp + 1 b4293_mRNA_2_degr + 130 h + 130 pi	reversible	mRNA degradation	-1.00E+09	1.00E+09	0	0	0.00E+00	0	0	0	0	0	0
b4293_mRNA_degr2	Degradation of b4293_mRNA II	1 b4293_mRNA_2_degr + 521 h2o --> 1 Orn_dim_inact + 119 amp + 1 atp + 145 cmp + 1 degradosome_inact + 140 gmp + 521 h + 117 ump	irreversible	mRNA degradation	0.00E+00	1.46E-01	0	0	0.00E+00	0	0	0	0	0	0
b4293_maturation1	Polypeptide b4293 peptide deformylase and methionine aminopeptidase complex	1 Def_mono + 1 Map_mono + 1 b4293_aa <==> 1 b4293_def_map_cplx	reversible	Protein Maturation	-1.00E+09	1.00E+09	0	0	0.00E+00	0	0	0	0	0	0
b4293_maturation2	b4293 formation	1 b4293_def_map_cplx + 2 h2o --> 1 Def_mono_inact + 1 Map_mono_inact + 1 b4293_m + 1 for + 1 met-L	irreversible	Protein Maturation	0.00E+00	1.00E+09	0	0	0.00E+00	0	0	0	0	0	0
b4368_tRNA_16_MODIF_1	modification of position 16 nt (D) in leuV by Dus_gen	1 Dus_gen + 1 b4368_tRNA_Mg2 + 1 h + 1 nadph <==> 1 b4368_tRNA_Mg2_Dus_gen_cplx	reversible	tRNA modification	-1.00E+09	1.00E+09	0	3.89E+02	3.89E+02	0	1.07E+01	10.7	0	1.07E+01	10.7
b4368_tRNA_16_MODIF_2	modification of position 16 nt (D) in leuV by Dus_gen	1 b4368_tRNA_Mg2_Dus_gen_cplx --> 1 Dus_gen_inact + 1 b4368_tRNA_1 + 1 nadp	irreversible	tRNA modification	0.00E+00	1.00E+09	0	3.89E+02	3.89E+02	0	1.07E+01	10.7	0	1.07E+01	10.7
b4368_tRNA_17_MODIF_1	modification of position 17 nt (D) in leuV by Dus_gen	1 Dus_gen + 1 b4368_tRNA_1 + 1 h + 1 nadph <==> 1 b4368_tRNA_1_Dus_gen_cplx	reversible	tRNA modification	-1.00E+09	1.00E+09	0	3.89E+02	3.89E+02	0	1.07E+01	10.7	0	1.07E+01	10.7
b4368_tRNA_17_MODIF_2	modification of position 17 nt (D) in leuV by Dus_gen	1 b4368_tRNA_1_Dus_gen_cplx --> 1 Dus_gen_inact + 1 b4368_tRNA_2 + 1 nadp	irreversible	tRNA modification	0.00E+00	1.00E+09	0	3.89E+02	3.89E+02	0	1.07E+01	10.7	0	1.07E+01	10.7
b4368_tRNA_18_MODIF_1	modification of position 18 nt (Gm) in leuV by TrmH_dim	1 TrmH_dim + 1 amet + 1 b4368_tRNA_2 <==> 1 b4368_tRNA_2_TrmH_dim_cplx	reversible	tRNA modification	-1.00E+09	1.00E+09	0	3.89E+02	3.89E+02	0	1.07E+01	10.7	0	1.07E+01	10.7
b4368_tRNA_18_MODIF_2	modification of position 18 nt (Gm) in leuV by TrmH_dim	1 b4368_tRNA_2_TrmH_dim_cplx --> 1 TrmH_dim_inact + 1 ahcys + 1 b4368_tRNA_3 + 1 h	irreversible	tRNA modification	0.00E+00	1.00E+09	0	3.89E+02	3.89E+02	0	1.07E+01	10.7	0	1.07E+01	10.7
b4368_tRNA_20_MODIF_1	modification of position 20 nt (D) in leuV by Dus_gen	1 Dus_gen + 1 b4368_tRNA_3 + 1 h + 1 nadph <==> 1 b4368_tRNA_3_Dus_gen_cplx	reversible	tRNA modification	-1.00E+09	1.00E+09	0	3.89E+02	3.89E+02	0	1.07E+01	10.7	0	1.07E+01	10.7
b4368_tRNA_20_MODIF_2	modification of position 20 nt (D) in leuV by Dus_gen	1 b4368_tRNA_3_Dus_gen_cplx --> 1 Dus_gen_inact + 1 b4368_tRNA_4 + 1 nadp	irreversible	tRNA modification	0.00E+00	1.00E+09	0	3.89E+02	3.89E+02	0	1.07E+01	10.7	0	1.07E+01	10.7
b4368_tRNA_38_MODIF_1	modification of position 38 nt (Y) in leuV by TruA_dim	1 TruA_dim + 1 b4368_tRNA_4 <==> 1 b4368_tRNA_4_TruA_dim_cplx	reversible	tRNA modification	-1.00E+09	1.00E+09	0	3.89E+02	3.89E+02	0	1.07E+01	10.7	0	1.07E+01	10.7
b4368_tRNA_38_MODIF_2	modification of position 38 nt (Y) in leuV by TruA_dim	1 b4368_tRNA_4_TruA_dim_cplx --> 1 TruA_dim_inact + 1 b4368_tRNA_5	irreversible	tRNA modification	0.00E+00	1.00E+09	0	3.89E+02	3.89E+02	0	1.07E+01	10.7	0	1.07E+01	10.7
b4368_tRNA_40_MODIF_1	modification of position 40 nt (Y) in leuV by TruA_dim	1 TruA_dim + 1 b4368_tRNA_5 <==> 1 b4368_tRNA_5_TruA_dim_cplx	reversible	tRNA modification	-1.00E+09	1.00E+09	0	3.89E+02	3.89E+02	0	1.07E+01	10.7	0	1.07E+01	10.7
b4368_tRNA_40_MODIF_2	modification of position 40 nt (Y) in leuV by TruA_dim	1 b4368_tRNA_5_TruA_dim_cplx --> 1 TruA_dim_inact + 1 b4368_tRNA_6	irreversible	tRNA modification	0.00E+00	1.00E+09	0	3.89E+02	3.89E+02	0	1.07E+01	10.7	0	1.07E+01	10.7
b4368_tRNA_54_MODIF_1	modification of position 54 nt (m5U) in leuV by TrmA_mono	1 TrmA_mono + 1 amet + 1 b4368_tRNA_6 <==> 1 b4368_tRNA_6_TrmA_mono_cplx	reversible	tRNA modification	-1.00E+09	1.00E+09	0	3.89E+02	3.89E+02	0	1.07E+01	10.7	0	1.07E+01	10.7
b4368_tRNA_54_MODIF_2	modification of position 54 nt (m5U) in leuV by TrmA_mono	1 b4368_tRNA_6_TrmA_mono_cplx --> 1 TrmA_mono_inact + 1 ahcys + 1 b4368_tRNA_7 + 1 h	irreversible	tRNA modification	0.00E+00	1.00E+09	0	3.89E+02	3.89E+02	0	1.07E+01	10.7	0	1.07E+01	10.7
b4368_tRNA_55_MODIF_1	modification of position 55 nt (Y) in leuV by TruB_mono	1 TruB_mono + 1 b4368_tRNA_7 <==> 1 b4368_tRNA_7_TruB_mono_cplx	reversible	tRNA modification	-1.00E+09	1.00E+09	0	3.89E+02	3.89E+02	0	1.07E+01	10.7	0	1.07E+01	10.7
b4368_tRNA_55_MODIF_2	modification of position 55 nt (Y) in leuV by TruB_mono	1 b4368_tRNA_7_TruB_mono_cplx --> 1 TruB_mono_inact + 1 leuV_tRNA	irreversible	tRNA modification	0.00E+00	1.00E+09	0	3.89E+02	3.89E+02	0	1.07E+01	10.7	0	1.07E+01	10.7
b4368_tRNA_Mg_BIND	Binding of two Mg2 to b4368_tRNA	1 b4368_RNA + 2 mg2 <==> 1 b4368_tRNA_Mg2	reversible	Metallo-ion binding	-1.00E+09	1.00E+09	0	3.89E+02	3.89E+02	0	1.07E+01	10.7	0	1.07E+01	10.7
b4369_tRNA_16_MODIF_1	modification of position 16 nt (D) in leuP by Dus_gen	1 Dus_gen + 1 b4369_tRNA_Mg2 + 1 h + 1 nadph <==> 1 b4369_tRNA_Mg2_Dus_gen_cplx	reversible	tRNA modification	-1.00E+09	1.00E+09	0	3.89E+02	3.89E+02	0	1.07E+01	10.7	0	1.07E+01	10.7
b4369_tRNA_16_MODIF_2	modification of position 16 nt (D) in leuP by Dus_gen	1 b4369_tRNA_Mg2_Dus_gen_cplx --> 1 Dus_gen_inact + 1 b4369_tRNA_1 + 1 nadp	irreversible	tRNA modification	0.00E+00	1.00E+09	0	3.89E+02	3.89E+02	0	1.07E+01	10.7	0	1.07E+01	10.7
b4369_tRNA_17_MODIF_1	modification of position 17 nt (D) in leuP by Dus_gen	1 Dus_gen + 1 b4369_tRNA_1 + 1 h + 1 nadph <==> 1 b4369_tRNA_1_Dus_gen_cplx	reversible	tRNA modification	-1.00E+09	1.00E+09	0	3.89E+02	3.89E+02	0	1.07E+01	10.7	0	1.07E+01	10.7
b4369_tRNA_17_MODIF_2	modification of position 17 nt (D) in leuP by Dus_gen	1 b4369_tRNA_1_Dus_gen_cplx --> 1 Dus_gen_inact + 1 b4369_tRNA_2 + 1 nadp	irreversible	tRNA modification	0.00E+00	1.00E+09	0	3.89E+02	3.89E+02	0	1.07E+01	10.7	0	1.07E+01	10.7
b4369_tRNA_18_MODIF_1	modification of position 18 nt (Gm) in leuP by TrmH_dim	1 TrmH_dim + 1 amet + 1 b4369_tRNA_2 <==> 1 b4369_tRNA_2_TrmH_dim_cplx	reversible	tRNA modification	-1.00E+09	1.00E+09	0	3.89E+02	3.89E+02	0	1.07E+01	10.7	0	1.07E+01	10.7
b4369_tRNA_18_MODIF_2	modification of position 18 nt (Gm) in leuP by TrmH_dim	1 b4369_tRNA_2_TrmH_dim_cplx --> 1 TrmH_dim_inact + 1 ahcys + 1 b4369_tRNA_3 + 1 h	irreversible	tRNA modification	0.00E+00	1.00E+09	0	3.89E+02	3.89E+02	0	1.07E+01	10.7	0	1.07E+01	10.7
b4369_tRNA_20_MODIF_1	modification of position 20 nt (D) in leuP by Dus_gen	1 Dus_gen + 1 b4369_tRNA_3 + 1 h + 1 nadph <==> 1 b4369_tRNA_3_Dus_gen_cplx	reversible	tRNA modification	-1.00E+09	1.00E+09	0	3.89E+02	3.89E+02	0	1.07E+01	10.7	0	1.07E+01	10.7
b4369_tRNA_20_MODIF_2	modification of position 20 nt (D) in leuP by Dus_gen	1 b4369_tRNA_3_Dus_gen_cplx --> 1 Dus_gen_inact + 1 b4369_tRNA_4 + 1 nadp	irreversible	tRNA modification	0.00E+00	1.00E+09	0	3.89E+02	3.89E+02	0	1.07E+01	10.7	0	1.07E+01	10.7
b4369_tRNA_38_MODIF_1	modification of position 38 nt (Y) in leuP by TruA_dim	1 TruA_dim + 1 b4369_tRNA_4 <==> 1 b4369_tRNA_4_TruA_dim_cplx	reversible	tRNA modification	-1.00E+09	1.00E+09	0	3.89E+02	3.89E+02	0	1.07E+01	10.7	0	1.07E+01	10.7
b4369_tRNA_38_MODIF_2	modification of position 38 nt (Y) in leuP by TruA_dim	1 b4369_tRNA_4_TruA_dim_cplx --> 1 TruA_dim_inact + 1 b4369_tRNA_5	irreversible	tRNA modification	0.00E+00	1.00E+09	0	3.89E+02	3.89E+02	0	1.07E+01	10.7	0	1.07E+01	10.7
b4369_tRNA_40_MODIF_1	modification of position 40 nt (Y) in leuP by TruA_dim	1 TruA_dim + 1 b4369_tRNA_5 <==> 1 b4369_tRNA_5_TruA_dim_cplx	reversible	tRNA modification	-1.00E+09	1.00E+09	0	3.89E+02	3.89E+02	0	1.07E+01	10.7	0	1.07E+01	10.7
b4369_tRNA_40_MODIF_2	modification of position 40 nt (Y) in leuP by TruA_dim	1 b4369_tRNA_5_TruA_dim_cplx --> 1 TruA_dim_inact + 1 b4369_tRNA_6	irreversible	tRNA modification	0.00E+00	1.00E+09	0	3.89E+02	3.89E+02	0	1.07E+01	10.7	0	1.07E+01	10.7
b4369_tRNA_54_MODIF_1	modification of position 54 nt (m5U) in leuP by TrmA_mono	1 TrmA_mono + 1 amet + 1 b4369_tRNA_6 <==> 1 b4369_tRNA_6_TrmA_mono_cplx	reversible	tRNA modification	-1.00E+09	1.00E+09	0	3.89E+02	3.89E+02	0	1.07E+01	10.7	0	1.07E+01	10.7
b4369_tRNA_54_MODIF_2	modification of position 54 nt (m5U) in leuP by TrmA_mono	1 b4369_tRNA_6_TrmA_mono_cplx --> 1 TrmA_mono_inact + 1 ahcys + 1 b4369_tRNA_7 + 1 h	irreversible	tRNA modification	0.00E+00	1.00E+09	0	3.89E+02	3.89E+02	0	1.07E+01	10.7	0	1.07E+01	10.7
b4369_tRNA_55_MODIF_1	modification of position 55 nt (Y) in leuP by TruB_mono	1 TruB_mono + 1 b4369_tRNA_7 <==> 1 b4369_tRNA_7_TruB_mono_cplx	reversible	tRNA modification	-1.00E+09	1.00E+09	0	3.89E+02	3.89E+02	0	1.07E+01	10.7	0	1.07E+01	10.7
b4369_tRNA_55_MODIF_2	modification of position 55 nt (Y) in leuP by TruB_mono	1 b4369_tRNA_7_TruB_mono_cplx --> 1 TruB_mono_inact + 1 leuP_tRNA	irreversible	tRNA modification	0.00E+00	1.00E+09	0	3.89E+02	3.89E+02	0	1.07E+01	10.7	0	1.07E+01	10.7
b4369_tRNA_Mg_BIND	Binding of two Mg2 to b4369_tRNA	1 b4369_RNA + 2 mg2 <==> 1 b4369_tRNA_Mg2	reversible	Metallo-ion binding	-1.00E+09	1.00E+09	0	3.89E+02	3.89E+02	0	1.07E+01	10.7	0	1.07E+01	10.7
b4370_tRNA_16_MODIF_1	modification of position 16 nt (D) in leuQ by Dus_gen	1 Dus_gen + 1 b4370_tRNA_Mg2 + 1 h + 1 nadph <==> 1 b4370_tRNA_Mg2_Dus_gen_cplx	reversible	tRNA modification	-1.00E+09	1.00E+09	0	3.89E+02	3.89E+02	0	1.07E+01	10.7	0	1.07E+01	10.7
b4370_tRNA_16_MODIF_2	modification of position 16 nt (D) in leuQ by Dus_gen	1 b4370_tRNA_Mg2_Dus_gen_cplx --> 1 Dus_gen_inact + 1 b4370_tRNA_1 + 1 nadp	irreversible	tRNA modification	0.00E+00	1.00E+09	0	3.89E+02	3.89E+02	0	1.07E+01	10.7	0	1.07E+01	10.7
b4370_tRNA_17_MODIF_1	modification of position 17 nt (D) in leuQ by Dus_gen	1 Dus_gen + 1 b4370_tRNA_1 + 1 h + 1 nadph <==> 1 b4370_tRNA_1_Dus_gen_cplx	reversible	tRNA modification	-1.00E+09	1.00E+09	0	3.89E+02	3.89E+02	0	1.07E+01	10.7	0	1.07E+01	10.7
b4370_tRNA_17_MODIF_2	modification of position 17 nt (D) in leuQ by Dus_gen	1 b4370_tRNA_1_Dus_gen_cplx --> 1 Dus_gen_inact + 1 b4370_tRNA_2 + 1 nadp	irreversible	tRNA modification	0.00E+00	1.00E+09	0	3.89E+02	3.89E+02	0	1.07E+01	10.7	0	1.07E+01	10.7
b4370_tRNA_18_MODIF_1	modification of position 18 nt (Gm) in leuQ by TrmH_dim	1 TrmH_dim + 1 amet + 1 b4370_tRNA_2 <==> 1 b4370_tRNA_2_TrmH_dim_cplx	reversible	tRNA modification	-1.00E+09	1.00E+09	0	3.89E+02	3.89E+02	0	1.07E+01	10.7	0	1.07E+01	10.7
b4370_tRNA_18_MODIF_2	modification of position 18 nt (Gm) in leuQ by TrmH_dim	1 b4370_tRNA_2_TrmH_dim_cplx --> 1 TrmH_dim_inact + 1 ahcys + 1 b4370_tRNA_3 + 1 h	irreversible	tRNA modification	0.00E+00	1.00E+09	0	3.89E+02	3.89E+02	0	1.07E+01	10.7	0	1.07E+01	10.7
b4370_tRNA_20_MODIF_1	modification of position 20 nt (D) in leuQ by Dus_gen	1 Dus_gen + 1 b4370_tRNA_3 + 1 h + 1 nadph <==> 1 b4370_tRNA_3_Dus_gen_cplx	reversible	tRNA modification	-1.00E+09	1.00E+09	0	3.89E+02	3.89E+02	0	1.07E+01	10.7	0	1.07E+01	10.7
b4370_tRNA_20_MODIF_2	modification of position 20 nt (D) in leuQ by Dus_gen	1 b4370_tRNA_3_Dus_gen_cplx --> 1 Dus_gen_inact + 1 b4370_tRNA_4 + 1 nadp	irreversible	tRNA modification	0.00E+00	1.00E+09	0	3.89E+02	3.89E+02	0	1.07E+01	10.7	0	1.07E+01	10.7
b4370_tRNA_38_MODIF_1	modification of position 38 nt (Y) in leuQ by TruA_dim	1 TruA_dim + 1 b4370_tRNA_4 <==> 1 b4370_tRNA_4_TruA_dim_cplx	reversible	tRNA modification	-1.00E+09	1.00E+09	0	3.89E+02	3.89E+02	0	1.07E+01	10.7	0	1.07E+01	10.7

b4370_tRNA_38_MODIF_2	modification of position 38 nt (Y) in leuQ by TruA_dim	1 b4370_tRNA_4_TruA_dim_cplx --> 1 TruA_dim_inact + 1 b4370_tRNA_5	irreversible	tRNA modification	0.00E+00	1.00E+09	0	3.89E+02	3.89E+02	0	1.07E+01	10.7	0	1.07E+01	10.7
b4370_tRNA_40_MODIF_1	modification of position 40 nt (Y) in leuQ by TruA_dim	1 TruA_dim + 1 b4370_tRNA_5 <==> 1 b4370_tRNA_5_TruA_dim_cplx	reversible	tRNA modification	-1.00E+09	1.00E+09	0	3.89E+02	3.89E+02	0	1.07E+01	10.7	0	1.07E+01	10.7
b4370_tRNA_40_MODIF_2	modification of position 40 nt (Y) in leuQ by TruA_dim	1 b4370_tRNA_5_TruA_dim_cplx --> 1 TruA_dim_inact + 1 b4370_tRNA_6	irreversible	tRNA modification	0.00E+00	1.00E+09	0	3.89E+02	3.89E+02	0	1.07E+01	10.7	0	1.07E+01	10.7
b4370_tRNA_54_MODIF_1	modification of position 54 nt (m5U) in leuQ by TrmA_mono	1 TrmA_mono + 1 amet + 1 b4370_tRNA_6 <==> 1 b4370_tRNA_6_TrmA_mono_cplx	reversible	tRNA modification	-1.00E+09	1.00E+09	0	3.89E+02	3.89E+02	0	1.07E+01	10.7	0	1.07E+01	10.7
b4370_tRNA_54_MODIF_2	modification of position 54 nt (m5U) in leuQ by TrmA_mono	1 b4370_tRNA_6_TrmA_mono_cplx --> 1 TrmA_mono_inact + 1 ahcys + 1 b4370_tRNA_7 + 1 h	irreversible	tRNA modification	0.00E+00	1.00E+09	0	3.89E+02	3.89E+02	0	1.07E+01	10.7	0	1.07E+01	10.7
b4370_tRNA_55_MODIF_1	modification of position 55 nt (Y) in leuQ by TruB_mono	1 TruB_mono + 1 b4370_tRNA_7 <==> 1 b4370_tRNA_7_TruB_mono_cplx	reversible	tRNA modification	-1.00E+09	1.00E+09	0	3.89E+02	3.89E+02	0	1.07E+01	10.7	0	1.07E+01	10.7
b4370_tRNA_55_MODIF_2	modification of position 55 nt (Y) in leuQ by TruB_mono	1 b4370_tRNA_7_TruB_mono_cplx --> 1 TruB_mono_inact + 1 leuQ_tRNA	irreversible	tRNA modification	0.00E+00	1.00E+09	0	3.89E+02	3.89E+02	0	1.07E+01	10.7	0	1.07E+01	10.7
b4370_tRNA_Mg_BIND	Binding of two Mg2 to b4370_tRNA	1 b4370_RNA + 2 mg2 <==> 1 b4370_tRNA_Mg2	reversible	Metallo-ion binding	-1.00E+09	1.00E+09	0	3.89E+02	3.89E+02	0	1.07E+01	10.7	0	1.07E+01	10.7
b4371_fold_GroEL/ES_1	b4371_m folding: GroEL/ES mediated: polypeptide is going in GroEL/ES complex	1 GroEL.(7)ADP.cisGroES + 7 atp + 1 b4371_m + 1 transGroES_hepta --> 7 adp + 1 b4371_m_GroEL.(7)ATP.transGroES + 1 cisGroES_hepta	irreversible	Protein Folding	0.00E+00	1.00E+09	0	2.44E+03	2.44E+03	0	4.24E+03	4237.958	0	9.91E+01	99.14
b4371_fold_GroEL/ES_2	b4371_m folding: GroEL/ES mediated: folding of polypeptide under ATP hydrolysis	1 b4371_m_GroEL.(7)ATP.transGroES + 7 h2o --> 1 RsmC_GroEL.(7)ADP.transGroES + 7 h + 7 pi	irreversible	Protein Folding	0.00E+00	1.00E+09	0	2.44E+03	2.44E+03	0	4.24E+03	4237.958	0	9.91E+01	99.14
b4371_fold_GroEL/ES_3	b4371_m folding: GroEL/ES mediated: release of native protein	1 RsmC_GroEL.(7)ADP.transGroES --> 1 GroEL.(7)ADP.transGroES + 1 RsmC_mono	irreversible	Protein Folding	0.00E+00	1.00E+09	0	2.44E+03	2.44E+03	0	4.24E+03	4237.958	0	9.91E+01	99.14
b4371_fold_KJE_1	b4371_m folding: KJE mediated	1 DnaJ_dim + 1 DnaK_mono.ATP + 1 b4371_m <==> 1 b4371_m_DnaKJ_complex	reversible	Protein Folding	-1.00E+09	1.00E+09	0	2.44E+03	2.44E+03	0	4.24E+03	4237.958	0	9.91E+01	99.14
b4371_fold_KJE_2	b4371_m folding: KJE mediated	1 GrpE_dim + 1 b4371_m_DnaKJ_complex + 1 h2o --> 1 DnaJ_dim_inact + 1 RsmC_DnaK_GrpE_complex + 1 adp + 1 h + 1 pi	irreversible	Protein Folding	0.00E+00	1.00E+09	0	2.44E+03	2.44E+03	0	4.24E+03	4237.958	0	9.91E+01	99.14
b4371_fold_KJE_3	b4371_m folding: KJE mediated	1 RsmC_DnaK_GrpE_complex + 1 atp --> 1 DnaK_mono.ATP_inact + 1 GrpE_dim_inact + 1 RsmC_mono	irreversible	Protein Folding	0.00E+00	1.00E+09	0	2.44E+03	2.44E+03	0	4.24E+03	4237.958	0	9.91E+01	99.14
b4371_mRNA_CONV	convsersion of mRNA to mRNA_1 (synthetic rxn)	1 b4371_mRNA --> 1 b4371_mRNA_1	irreversible	Translation	0.00E+00	1.00E+09	0	1.17E+02	1.17E+02	0	2.07E+02	207.2779	0	3.37E-01	0.337
b4371_mRNA_degr1	Degradation of b4371_mRNA I	1 Orn_dim + 258 atp + 1 b4371_mRNA_2 + 1 degradosome + 258 h2o <==> 258 adp + 1 b4371_mRNA_2_degr + 258 h + 258 pi	reversible	mRNA degradation	-1.00E+09	1.00E+09	0	1.17E+02	1.17E+02	0	2.07E+02	207.2779	0	3.37E-01	0.337
b4371_mRNA_degr2	Degradation of b4371_mRNA II	1 b4371_mRNA_2_degr + 1031 h2o --> 1 Orn_dim_inact + 215 amp + 1 atp + 272 cmp + 1 degradosome_inact + 303 gmp + 1031 h + 241 ump	irreversible	mRNA degradation	0.00E+00	3.37E-01	0	1.17E+02	1.17E+02	0	2.07E+02	207.2779	0	3.37E-01	0.337
b4371_maturation1	Polypeptide b4371 peptide deformylas and methionine aminopeptidase complex	1 Def_mono + 1 Map_mono + 1 b4371_aa <==> 1 b4371_def_map_cplx	reversible	Protein Maturation	-1.00E+09	1.00E+09	0	2.44E+03	2.44E+03	0	4.24E+03	4237.958	0	9.91E+01	99.14
b4371_maturation2	b4371 formation	1 b4371_def_map_cplx + 2 h2o --> 1 Def_mono_inact + 1 Map_mono_inact + 1 b4371_m + 1 for + 1 met-L	irreversible	Protein Maturation	0.00E+00	1.00E+09	0	2.44E+03	2.44E+03	0	4.24E+03	4237.958	0	9.91E+01	99.14
b4372_fold_spon	b4372_m folding: spontanous	1 b4372_m --> 1 HolD_mono	irreversible	Protein Folding	0.00E+00	1.00E+09	0	6.91E+02	6.91E+02	0	1.17E+03	1165.549	0	7.15E+01	71.5432
b4372_mRNA_CONV	convsersion of mRNA to mRNA_1 (synthetic rxn)	1 b4372_mRNA --> 1 b4372_mRNA_1	irreversible	Translation	0.00E+00	1.00E+09	0	7.75E+01	7.75E+01	0	1.37E+02	136.8437	0	9.29E-02	0.0929
b4372_mRNA_degr1	Degradation of b4372_mRNA I	1 Orn_dim + 103 atp + 1 b4372_mRNA_2 + 1 degradosome + 103 h2o <==> 103 adp + 1 b4372_mRNA_2_degr + 103 h + 103 pi	reversible	mRNA degradation	-1.00E+09	1.00E+09	0	7.75E+01	7.75E+01	0	1.37E+02	136.8437	0	9.29E-02	0.0929
b4372_mRNA_degr2	Degradation of b4372_mRNA II	1 b4372_mRNA_2_degr + 413 h2o --> 1 Orn_dim_inact + 92 amp + 1 atp + 124 cmp + 1 degradosome_inact + 115 gmp + 413 h + 82 ump	irreversible	mRNA degradation	0.00E+00	1.04E-01	0	7.75E+01	7.75E+01	0	1.37E+02	136.8437	0	9.29E-02	0.0929
b4372_maturation1	Polypeptide b4372 peptide deformylas and methionine aminopeptidase complex	1 Def_mono + 1 Map_mono + 1 b4372_aa <==> 1 b4372_def_map_cplx	reversible	Protein Maturation	-1.00E+09	1.00E+09	0	6.91E+02	6.91E+02	0	1.17E+03	1165.549	0	7.15E+01	71.5432
b4372_maturation2	b4372 formation	1 b4372_def_map_cplx + 2 h2o --> 1 Def_mono_inact + 1 Map_mono_inact + 1 b4372_m + 1 for + 1 met-L	irreversible	Protein Maturation	0.00E+00	1.00E+09	0	6.91E+02	6.91E+02	0	1.17E+03	1165.549	0	7.15E+01	71.5432
b4373_fold_spon	b4373_m folding: spontanous	1 b4373_m --> 1 RimI_mono	irreversible	Protein Folding	0.00E+00	1.00E+09	0	6.96E+02	6.96E+02	0	1.17E+03	1170.349	0	7.63E+01	76.3432
b4373_mRNA_CONV	convsersion of mRNA to mRNA_1 (synthetic rxn)	1 b4373_mRNA --> 1 b4373_mRNA_1	irreversible	Translation	0.00E+00	1.00E+09	0	7.75E+01	7.75E+01	0	1.37E+02	136.8437	0	9.29E-02	0.0929
b4373_mRNA_degr1	Degradation of b4373_mRNA I	1 Orn_dim + 103 atp + 1 b4373_mRNA_2 + 1 degradosome + 103 h2o <==> 103 adp + 1 b4373_mRNA_2_degr + 103 h + 103 pi	reversible	mRNA degradation	-1.00E+09	1.00E+09	0	7.75E+01	7.75E+01	0	1.37E+02	136.8437	0	9.29E-02	0.0929
b4373_mRNA_degr2	Degradation of b4373_mRNA II	1 b4373_mRNA_2_degr + 414 h2o --> 1 Orn_dim_inact + 105 amp + 108 cmp + 1 degradosome_inact + 112 gmp + 414 h + 90 ump	irreversible	mRNA degradation	0.00E+00	1.58E-01	0	7.75E+01	7.75E+01	0	1.37E+02	136.8437	0	9.29E-02	0.0929
b4373_maturation1	Polypeptide b4373 peptide deformylas and methionine aminopeptidase complex	1 Def_mono + 1 Map_mono + 1 b4373_aa <==> 1 b4373_def_map_cplx	reversible	Protein Maturation	-1.00E+09	1.00E+09	0	6.96E+02	6.96E+02	0	1.17E+03	1170.349	0	7.63E+01	76.3432
b4373_maturation2	b4373 formation	1 b4373_def_map_cplx + 2 h2o --> 1 Def_mono_inact + 1 Map_mono_inact + 1 b4373_m + 1 for + 1 met-L	irreversible	Protein Maturation	0.00E+00	1.00E+09	0	6.96E+02	6.96E+02	0	1.17E+03	1170.349	0	7.63E+01	76.3432
b4374_fold_spon	b4374_m folding: spontanous	1 b4374_m --> 1 YjjG_mono	irreversible	Protein Folding	0.00E+00	1.00E+09	0	1.08E+03	1.08E+03	0	1.85E+03	1854.368	0	7.66E+01	76.6077
b4374_mRNA_CONV	convsersion of mRNA to mRNA_1 (synthetic rxn)	1 b4374_mRNA --> 1 b4374_mRNA_1	irreversible	Translation	0.00E+00	1.00E+09	0	7.75E+01	7.75E+01	0	1.37E+02	136.8437	0	9.29E-02	0.0929
b4374_mRNA_degr1	Degradation of b4374_mRNA I	1 Orn_dim + 169 atp + 1 b4374_mRNA_2 + 1 degradosome + 169 h2o <==> 169 adp + 1 b4374_mRNA_2_degr + 169 h + 169 pi	reversible	mRNA degradation	-1.00E+09	1.00E+09	0	7.75E+01	7.75E+01	0	1.37E+02	136.8437	0	9.29E-02	0.0929
b4374_mRNA_degr2	Degradation of b4374_mRNA II	1 b4374_mRNA_2_degr + 677 h2o --> 1 Orn_dim_inact + 152 amp + 179 cmp + 1 degradosome_inact + 184 gmp + 677 h + 163 ump	irreversible	mRNA degradation	0.00E+00	9.29E-02	0	7.75E+01	7.75E+01	0	1.37E+02	136.8437	0	9.29E-02	0.0929
b4374_maturation1	Polypeptide b4374 peptide deformylas and methionine aminopeptidase complex	1 Def_mono + 1 Map_mono + 1 b4374_aa <==> 1 b4374_def_map_cplx	reversible	Protein Maturation	-1.00E+09	1.00E+09	0	1.08E+03	1.08E+03	0	1.85E+03	1854.368	0	7.66E+01	76.6077
b4374_maturation2	b4374 formation	1 b4374_def_map_cplx + 2 h2o --> 1 Def_mono_inact + 1 Map_mono_inact + 1 b4374_m + 1 for + 1 met-L	irreversible	Protein Maturation	0.00E+00	1.00E+09	0	1.08E+03	1.08E+03	0	1.85E+03	1854.368	0	7.66E+01	76.6077
b4375_fold_KJE_1	b4375_m folding: KJE mediated	1 DnaJ_dim + 1 DnaK_mono.ATP + 1 b4375_m <==> 1 b4375_m_DnaKJ_complex	reversible	Protein Folding	-1.00E+09	1.00E+09	0	2.49E+03	2.49E+03	0	4.33E+03	4334.315	0	9.14E+01	91.388
b4375_fold_KJE_2	b4375_m folding: KJE mediated	1 GrpE_dim + 1 b4375_m_DnaKJ_complex + 1 h2o --> 1 DnaJ_dim_inact + 1 PrfC_DnaK_GrpE_complex + 1 adp + 1 h + 1 pi	irreversible	Protein Folding	0.00E+00	1.00E+09	0	2.49E+03	2.49E+03	0	4.33E+03	4334.315	0	9.14E+01	91.388
b4375_fold_KJE_3	b4375_m folding: KJE mediated	1 PrfC_DnaK_GrpE_complex + 1 atp --> 1 DnaK_mono.ATP_inact + 1 GrpE_dim_inact + 1 PrfC_mono	irreversible	Protein Folding	0.00E+00	1.00E+09	0	2.49E+03	2.49E+03	0	4.33E+03	4334.315	0	9.14E+01	91.388
b4375_fold_spon	b4375_m folding: spontanous	1 b4375_m --> 1 PrfC_mono	irreversible	Protein Folding	0.00E+00	1.00E+09	0	2.49E+03	2.49E+03	0	4.33E+03	4334.315	0	9.14E+01	91.388
b4375_mRNA_CONV	convsersion of mRNA to mRNA_1 (synthetic rxn)	1 b4375_mRNA --> 1 b4375_mRNA_1	irreversible	Translation	0.00E+00	1.00E+09	0	7.77E+01	7.77E+01	0	1.37E+02	137.1766	0	3.08E-01	0.308
b4375_mRNA_degr1	Degradation of b4375_mRNA I	1 Orn_dim + 397 atp + 1 b4375_mRNA_2 + 1 degradosome + 397 h2o <==> 397 adp + 1 b4375_mRNA_2_degr + 397 h + 397 pi	reversible	mRNA degradation	-1.00E+09	1.00E+09	0	7.77E+01	7.77E+01	0	1.37E+02	137.1766	0	3.08E-01	0.308
b4375_mRNA_degr2	Degradation of b4375_mRNA II	1 b4375_mRNA_2_degr + 1589 h2o --> 1 Orn_dim_inact + 383 amp + 1 atp + 411 cmp + 1 degradosome_inact + 448 gmp + 1589 h + 347 ump	irreversible	mRNA degradation	0.00E+00	3.08E-01	0	7.77E+01	7.77E+01	0	1.37E+02	137.1766	0	3.08E-01	0.308
b4375_maturation1	Polypeptide b4375 peptide deformylas and methionine aminopeptidase complex	1 Def_mono + 1 Map_mono + 1 b4375_aa <==> 1 b4375_def_map_cplx	reversible	Protein Maturation	-1.00E+09	1.00E+09	0	2.49E+03	2.49E+03	0	4.33E+03	4334.315	0	9.14E+01	91.388
b4375_maturation2	b4375 formation	1 b4375_def_map_cplx + 2 h2o --> 1 Def_mono_inact + 1 Map_mono_inact + 1 b4375_m + 1 for + 1 met-L	irreversible	Protein Maturation	0.00E+00	1.00E+09	0	2.49E+03	2.49E+03	0	4.33E+03	4334.315	0	9.14E+01	91.388
cdp_exch	cdp transport	1 cdp[e] <==> 1 cdp	reversible	Transport	-1.00E+09	1.00E+09	0	0	0.00E+00	0	0	0	0	0	0
chort	transport of chor	1 chor <==> 1 chor[e]	reversible	Transport	-1.00E+09	1.00E+09	-2.76E+03	0	2.76E+03	-1.88E+02	-2.78E+01	159.66	-1.88E+02	-2.78E+01	159.66

cis/transGroES_CONV	conversion of cisGroES to transGroES	1 cisGroES_hepta <==> 1 transGroES_hepta	reversible	Protein Folding	-1.00E+09	1.00E+09	-1.49E+02	7.94E+03	8.09E+03	-2.45E+02	9.80E+03	10048.1279	-1.67E+01	5.83E+03	5842.3344
cisGroES_FORM	formation of cis GroES	1 GroS_hepta --> 1 cisGroES_hepta	irreversible	Protein complex formation	0.00E+00	1.00E+09	0	4.23E+02	4.23E+02	0	7.14E+02	714.4714	0	4.37E+01	43.73
cmp_exch	cmp transport	1 cmp[e] <==> 1 cmp	reversible	Transport	-1.00E+09	1.00E+09	-3.75E+04	-5.49E+03	3.20E+04	-6.08E+04	-4.17E+03	56611.87	-2.62E+04	-4.17E+03	22046.26
coat	transport of coa	1 coa <==> 1 coa[e]	reversible	Transport	-1.00E+09	1.00E+09	3.38E+02	5.24E+03	4.90E+03	2.49E+02	5.59E+03	5343.775	2.49E+02	2.91E+03	2664.524
ctp_exch	ctp transport	1 ctp[e] <==> 1 ctp	reversible	Transport	-1.00E+09	1.00E+09	9.45E+04	1.26E+05	3.19E+04	7.00E+04	1.26E+05	56379.6	7.00E+04	1.26E+05	56379.6
cysT_tRNA_CHARG	charging cysT_tRNA	1 Cys_RS_cys_amp + 1 cysT_tRNA --> 1 CysS_mono_inact + 1 amp + 1 cysT_tRNA_cys	irreversible	tRNA charging	0.00E+00	1.00E+09	3.38E+03	6.85E+04	6.51E+04	2.49E+03	7.80E+04	75550.35	2.49E+03	5.61E+04	53616.26
dad-5t	transport of dad-5	1 dad-5 <==> 1 dad-5[e]	reversible	Transport	-1.00E+09	1.00E+09	0	1.52E+03	1.52E+03	0	7.95E+02	795.0555	0	8.59E+01	85.864
dmppt	transport of dmpp	1 dmpp <==> 1 dmpp[e]	reversible	Transport	-1.00E+09	1.00E+09	-1.52E+03	0	1.52E+03	-8.03E+02	0	803.4932	-9.96E+01	0	99.564
fadh2t	transport of fadh2	1 fadh2 <==> 1 fadh2[e]	reversible	Transport	-1.00E+09	1.00E+09	0	0	0.00E+00	0	0	0	0	0	0
fadt	transport of fad	1 fad <==> 1 fad[e]	reversible	Transport	-1.00E+09	1.00E+09	0	0	0.00E+00	0	0	0	0	0	0
fldoxt	transport of fldox	1 fldox <==> 1 fldox[e]	reversible	Transport	-1.00E+09	1.00E+09	0	1.52E+03	1.52E+03	0	7.95E+02	795.0555	0	8.59E+01	85.864
fldrdt	transport of fldrd	1 fldrd <==> 1 fldrd[e]	reversible	Transport	-1.00E+09	1.00E+09	-1.52E+03	0	1.52E+03	-7.95E+02	0	795.0555	-8.59E+01	0	85.864
fmet_tRNA_CHARGa	charging fmet_tRNA	1 Met_RS_2met_2amp + 2 fmet_tRNA --> 1 Met_RS_dim_inact + 2 amp + 2 fmet_tRNA_met_1	irreversible	tRNA charging	0.00E+00	1.00E+09	0	1.15E+04	1.15E+04	0	1.19E+04	11853.42	0	1.07E+04	10749.04
fmet_tRNA_CHARGb	charging fmet_tRNA	1 Met_RS_met_amp + 1 fmet_tRNA --> 1 Met_RS_dim_inact + 1 amp + 1 fmet_tRNA_met_1	irreversible	tRNA charging	0.00E+00	1.00E+09	0	2.30E+04	2.30E+04	0	2.37E+04	23706.84	0	2.15E+04	21498.08
fmet_tRNA_TF_1	methionyl-tRNA formyltransferase 1	1 10fthf + 1 Fmt_mono + 1 fmet_tRNA_met_1 --> 1 Fmt_fmet_tRNA_met_1_cplx	irreversible	tRNA charging	0.00E+00	1.00E+09	5.23E+03	2.30E+04	1.78E+04	3.85E+03	2.37E+04	19852.92	3.85E+03	2.15E+04	17644.16
fmet_tRNA_TF_2	methionyl-tRNA formyltransferase 2	1 Fmt_fmet_tRNA_met_1_cplx --> 1 Fmt_mono_inact + 1 fmet_tRNA_met + 1 h + 1 thf	irreversible	tRNA charging	0.00E+00	1.00E+09	5.23E+03	2.30E+04	1.78E+04	3.85E+03	2.37E+04	19852.92	3.85E+03	2.15E+04	17644.16
gdp_exch	gdp transport	1 gdp[e] <==> 1 gdp	reversible	Transport	-1.00E+09	1.00E+09	-8.51E+06	-1.54E+06	6.97E+06	-8.64E+06	-1.13E+06	7509213	-8.04E+06	-1.13E+06	6911881
gln1_tRNA_CHARG	charging gln1_tRNA	1 Gln_RS_gln_amp + 1 gln1_tRNA --> 1 GlnS_mono_inact + 1 amp + 1 gln1_tRNA_gln	irreversible	tRNA charging	0.00E+00	1.00E+09	1.95E+04	1.61E+05	1.42E+05	1.44E+04	1.74E+05	159569.24	1.44E+04	1.48E+05	133738.94
gln2_tRNA_CHARG	charging gln2_tRNA	1 Gln_RS_gln_amp + 1 gln2_tRNA --> 1 GlnS_mono_inact + 1 amp + 1 gln2_tRNA_gln	irreversible	tRNA charging	0.00E+00	1.00E+09	6.92E+03	1.10E+05	1.03E+05	5.10E+03	1.25E+05	119982.98	5.10E+03	8.11E+04	76038.87
glnU_to_gln1	glnU_to_gln1	1 glnU_tRNA --> 1 gln1_tRNA	irreversible	tRNA charging	0.00E+00	1.00E+09	0	1.70E+02	1.70E+02	0	7.79E+00	7.79	0	7.79E+00	7.79
glnV_to_gln2	glnV_to_gln2	1 glnV_tRNA --> 1 gln2_tRNA	irreversible	tRNA charging	0.00E+00	1.00E+09	0	1.70E+02	1.70E+02	0	7.79E+00	7.79	0	7.79E+00	7.79
glnW_to_gln1	glnW_to_gln1	1 glnW_tRNA --> 1 gln1_tRNA	irreversible	tRNA charging	0.00E+00	1.00E+09	0	1.70E+02	1.70E+02	0	7.79E+00	7.79	0	7.79E+00	7.79
glnX_to_gln2	glnX_to_gln2	1 glnX_tRNA --> 1 gln2_tRNA	irreversible	tRNA charging	0.00E+00	1.00E+09	0	1.70E+02	1.70E+02	0	7.79E+00	7.79	0	7.79E+00	7.79
glu1_tRNA_CHARG	charging glu1_tRNA	1 Glu_RS_glu_amp + 1 glu1_tRNA --> 1 GltX_mono_inact + 1 amp + 1 glu1_tRNA_glu	irreversible	tRNA charging	0.00E+00	1.00E+09	5.91E+04	2.44E+05	1.85E+05	4.35E+04	2.46E+05	202239.9	4.35E+04	2.41E+05	197591.3
gluT_to_glu1	gluT_to_glu1	1 gltT_tRNA --> 1 glu1_tRNA	irreversible	tRNA charging	0.00E+00	1.00E+09	0	1.11E+02	1.11E+02	0	1.29E+01	12.9	0	1.29E+01	12.9
gluU_to_glu1	gluU_to_glu1	1 gltU_tRNA --> 1 glu1_tRNA	irreversible	tRNA charging	0.00E+00	1.00E+09	0	1.12E+02	1.12E+02	0	1.42E+01	14.2	0	1.42E+01	14.2
gluV_to_glu1	gluV_to_glu1	1 gltV_tRNA --> 1 glu1_tRNA	irreversible	tRNA charging	0.00E+00	1.00E+09	0	1.13E+02	1.13E+02	0	1.26E+01	12.6	0	1.26E+01	12.6
gluW_to_glu1	gluW_to_glu1	1 gltW_tRNA --> 1 glu1_tRNA	irreversible	tRNA charging	0.00E+00	1.00E+09	0	1.11E+02	1.11E+02	0	8.54E+00	8.54	0	8.54E+00	8.54
glxt	transport of glx	1 glx <==> 1 glx[e]	reversible	Transport	-1.00E+09	1.00E+09	0	1.86E+03	1.86E+03	2.75E+01	1.09E+02	81.96	2.75E+01	1.09E+02	81.96
gly1_tRNA_CHARGa	charging gly1_tRNA	1 Gly_RS_2gly_2amp + 2 gly1_tRNA --> 1 Gly_RS_tetra_inact + 2 amp + 2 gly1_tRNA_gly	irreversible	tRNA charging	0.00E+00	1.00E+09	0	1.61E+05	1.61E+05	0	1.71E+05	170733.1	0	1.45E+05	144524
gly1_tRNA_CHARGb	charging gly1_tRNA	1 Gly_RS_gly_amp + 1 gly1_tRNA --> 1 Gly_RS_tetra_inact + 1 amp + 1 gly1_tRNA_gly	irreversible	tRNA charging	0.00E+00	1.00E+09	0	3.22E+05	3.22E+05	0	3.41E+05	341466.1	0	2.89E+05	289047.9
glyT_tRNA_CHARGa	charging glyT_tRNA	1 Gly_RS_2gly_2amp + 2 glyT_tRNA --> 1 Gly_RS_tetra_inact + 2 amp + 2 glyT_tRNA_gly	irreversible	tRNA charging	0.00E+00	1.00E+09	0	2.07E+04	2.07E+04	0	2.35E+04	23529.24	0	1.80E+04	18036.03
glyT_tRNA_CHARGb	charging glyT_tRNA	1 Gly_RS_gly_amp + 1 glyT_tRNA --> 1 Gly_RS_tetra_inact + 1 amp + 1 glyT_tRNA_gly	irreversible	tRNA charging	0.00E+00	1.00E+09	0	4.14E+04	4.14E+04	0	4.71E+04	47058.48	0	3.61E+04	36072.07
glyU_tRNA_CHARGa	charging gly2_tRNA	1 Gly_RS_2gly_2amp + 2 glyU_tRNA --> 1 Gly_RS_tetra_inact + 2 amp + 2 glyU_tRNA_gly	irreversible	tRNA charging	0.00E+00	1.00E+09	0	2.69E+04	2.69E+04	0	3.13E+04	31269.22	0	2.30E+04	22994.44
glyU_tRNA_CHARGb	charging gly2_tRNA	1 Gly_RS_gly_amp + 1 glyU_tRNA --> 1 Gly_RS_tetra_inact + 1 amp + 1 glyU_tRNA_gly	irreversible	tRNA charging	0.00E+00	1.00E+09	0	5.39E+04	5.39E+04	0	6.25E+04	62538.44	0	4.60E+04	45988.88
glyV_to_gly1	glyV_to_gly1	1 glyV_tRNA --> 1 gly1_tRNA	irreversible	tRNA charging	0.00E+00	1.00E+09	0	3.99E+02	3.99E+02	0	1.17E+01	11.7	0	1.17E+01	11.7
glyW_to_gly1	glyW_to_gly1	1 glyW_tRNA --> 1 gly1_tRNA	irreversible	tRNA charging	0.00E+00	1.00E+09	0	3.99E+02	3.99E+02	0	6.22E+00	6.22	0	6.22E+00	6.22
glyX_to_gly1	glyX_to_gly1	1 glyX_tRNA --> 1 gly1_tRNA	irreversible	tRNA charging	0.00E+00	1.00E+09	0	3.99E+02	3.99E+02	0	1.17E+01	11.7	0	1.17E+01	11.7
glyY_to_gly1	glyY_to_gly1	1 glyY_tRNA --> 1 gly1_tRNA	irreversible	tRNA charging	0.00E+00	1.00E+09	0	3.99E+02	3.99E+02	0	1.17E+01	11.7	0	1.17E+01	11.7
gmp_exch	gmp transport	1 gmp[e] <==> 1 gmp	reversible	Transport	-1.00E+09	1.00E+09	-5.42E+04	-6.84E+03	4.73E+04	-8.85E+04	-5.26E+03	83260.94	-2.99E+04	-5.26E+03	24655.18
gtp_exch	gtp transport	1 gtp[e] <==> 1 gtp	reversible	Transport	-1.00E+09	1.00E+09	1.67E+06	8.68E+06	7.01E+06	1.23E+06	8.80E+06	7573145	1.23E+06	8.20E+06	6970628
guat	transport of gua	1 gua <==> 1 gua[e]	reversible	Transport	-1.00E+09	1.00E+09	0	1.77E+03	1.77E+03	0	7.90E+02	789.686	0	8.58E+01	85.794
h2st	transport of h2s	1 h2s <==> 1 h2s[e]	reversible	Transport	-1.00E+09	1.00E+09	0	1.86E+03	1.86E+03	0	9.36E+01	93.62	0	9.36E+01	93.62
hRNAP_FORM	holo RNA polymerase formation	2 RpoA_mono + 1 RpoB_mono + 1 RpoC_mono <==> 1 hRNAP	reversible	Protein complex formation	-1.00E+09	1.00E+09	0	4.82E+02	4.82E+02	0	6.51E+02	650.6377	0	1.02E+02	102.418
hco3t	transport of hco3	1 hco3 <==> 1 hco3[e]	reversible	Transport	-1.00E+09	1.00E+09	-1.81E+03	0	1.81E+03	-2.12E+02	-1.39E+01	197.71	-2.12E+02	-1.39E+01	197.71
hisR_tRNA_CHARGa	charging hisR_tRNA	1 His_RS_his_amp + 1 hisR_tRNA --> 1 His_RS_dim_inact + 1 amp + 1 hisR_tRNA_his	irreversible	tRNA charging	0.00E+00	1.00E+09	0	8.97E+04	8.97E+04	0	9.17E+04	91685.86	0	8.70E+04	87033.61
hisR_tRNA_CHARGb	charging hisR_tRNA	1 His_RS_2his_2amp + 2 hisR_tRNA --> 1 His_RS_dim_inact + 2 amp + 2 hisR_tRNA_his	irreversible	tRNA charging	0.00E+00	1.00E+09	0	4.48E+04	4.48E+04	0	4.58E+04	45842.93	0	4.35E+04	43516.8
ile1_tRNA_CHARG	charging ile1_tRNA	1 Ile_RS_ile_Amp + 1 ile1_tRNA --> 1 IleS_mono_inact + 1 amp + 1 ile1_tRNA_ile	irreversible	tRNA charging	0.00E+00	1.00E+09	4.22E+04	2.66E+05	2.24E+05	3.11E+04	2.68E+05	237268.6	3.11E+04	2.44E+05	212773.4
ile2_tRNA_CHARG	charging ile2_tRNA	1 Ile_RS_ile_Amp + 1 ile2_tRNA --> 1 IleS_mono_inact + 1 amp + 1 ile2_tRNA_ile	irreversible	tRNA charging	0.00E+00	1.00E+09	2.53E+02	2.35E+04	2.32E+04	1.86E+02	2.88E+04	28643.74	1.86E+02	1.69E+04	16706.63
ileT_to_ile1	ileT_to_ile1	1 ileT_tRNA --> 1 ile1_tRNA	irreversible	tRNA charging	0.00E+00	1.00E+09	0	1.12E+02	1.12E+02	0	1.36E+01	13.6	0	1.36E+01	13.6
ileU_to_ile1	ileU_to_ile1	1 ileU_tRNA --> 1 ile1_tRNA	irreversible	tRNA charging	0.00E+00	1.00E+09	0	1.06E+02	1.06E+02	0	1.15E+01	11.5	0	1.15E+01	11.5
ileV_to_ile1	ileV_to_ile1	1 ileV_tRNA --> 1 ile1_tRNA	irreversible	tRNA charging	0.00E+00	1.00E+09	0	1.11E+02	1.11E+02	0	9.54E+00	9.54	0	9.54E+00	9.54
ileX_to_ile2	ileX_to_ile2	1 ileX_tRNA --> 1 ile2_tRNA	irreversible	tRNA charging	0.00E+00	1.00E+09	0	1.45E+03	1.45E+03	0	1.05E+01	10.5	0	1.05E+01	10.5
ileY_to_ile2	ileY_to_ile2	1 ileY_tRNA --> 1 ile2_tRNA	irreversible	tRNA charging	0.00E+00	1.00E+09	0	1.52E+03	1.52E+03	0	8.74E+00	8.740001	0	8.74E+00	8.740001
leu1_tRNA_CHARG	charging leu1_tRNA	1 Leu_RS_leu_amp + 1 leu1_tRNA --> 1 LeuS_mono_inact + 1 amp 1 leu1_tRNA_leu	irreversible	tRNA charging	0.00E+00	1.00E+09	4.42E+04	2.86E+05	2.42E+05	3.26E+04	2.97E+05	264787.16	3.26E+04	2.51E+05	218188.56
leu2_tRNA_CHARG	charging leu2_tRNA	1 Leu_RS_leu_amp + 1 leu2_tRNA --> 1 LeuS_mono_inact + 1 amp 1 leu2_tRNA_leu	irreversible	tRNA charging	0.00E+00	1.00E+09	2.11E+03	7.59E+04	7.38E+04	1.55E+03	8.47E+04	83125.76	1.55E+03	6.29E+04	61390.68
leuP_to_leu1	leuP_to_leu1	1 leuP_tRNA --> 1 leu1_tRNA	irreversible	tRNA charging	0.00E+00	1.00E+09	0	3.89E+02	3.89E+02	0	1.07E+01	10.7	0	1.07E+01	10.7
leuQ_to_leu1	leuQ_to_leu1	1 leuQ_tRNA --> 1 leu1_tRNA	irreversible	tRNA charging	0.00E+00	1.00E+09	0	3.89E+02	3.89E+02	0	1.07E+01	10.7	0	1.07E+01	10.7
leuT_to_leu1	leuT_to_leu1	1 leuT_tRNA --> 1 leu1_tRNA	irreversible	tRNA charging	0.00E+00	1.00E+09	0	3.01E+02	3.01E+02	0	1.39E+01	13.9	0	1.39E+01	13.9
leuU_tRNA_CHARG	charging leuU_tRNA	1 Leu_RS_leu_amp + 1 leuU_tRNA --> 1 LeuS_mono_inact + 1 amp + 1 leuU_tRNA_leu	irreversible	tRNA charging	0.00E+00	1.00E+09	5.23E+03	1.07E+05	1.02E+05	3.85E+03	1.18E+05	113903.98	3.85E+03	9.56E+04	91727.67
leuV_to_leu1	leuV_to_leu1	1 leuV_tRNA --> 1 leu1_tRNA	irreversible	tRNA charging	0.00E+00	1.00E+09	0	3.89E+02	3.89E+02	0	1.07E+01	10.7	0	1.07E+01	10.7
leuW_tRNA_CHARG	charging leuW_tRNA	1 Leu_RS_leu_amp + 1 leuW_tRNA --> 1 LeuS_mono_inact + 1 amp + 1 leuW_tRNA_leu	irreversible	tRNA charging	0.00E+00	1.00E+09	8.44E+01	2.16E+04	2.15E+04	6.22E+01	2.54E+04	25300.64	6.22E+01	1.72E+04	17178.58
leuW_to_leu1	leuW_to_leu1	1 leuW_tRNA --> 1 leu1_tRNA	irreversible	tRNA charging	0.00E+00	1.00E+09	0	1.70E+02	1.70E+02	0	7.79E+00	7.79	0	7.79E+00	7.79
leuX_to_leu2	leuX_to_leu2	1 leuX_tRNA --> 1 leu2_tRNA	irreversible	tRNA charging	0.00E+00	1.00E+09	0	1.45E+03	1.45E+03	0	1.12E+01	11.2	0	1.12E+01	11.2
leuZ_tRNA_CHARG	charging leuZ_tRNA	1 Leu_RS_leu_amp + 1 leuZ_tRNA --> 1 LeuS_mono_inact + 1 amp 1 leuZ_tRNA_leu	irreversible	tRNA charging	0.00E+00	1.00E+09	1.52E+03	8.27E+04	8.12E+04	1.12E+03	9.15E+04	90351.38	1.12E+03	5.88E+04	57667.91

leuZ_to_leu2	leuZ_to_leu2	1 leuZ_tRNA --> 1 leu2_tRNA	irreversible	tRNA charging	0.00E+00	1.00E+09	0	3.99E+02	3.99E+02	0	6.22E+00	6.22	0	6.22E+00	6.22
lys1_tRNA1_CHARGa	charging lys1_tRNA (by lysI_RS)	1 LysI_RS_lys_amp + 1 lys1_tRNA --> 1 LysI_RS_dim_inact + 1 amp + 1 lys1_tRNA_lys	irreversible	tRNA charging	0.00E+00	1.00E+09	0	2.81E+05	2.81E+05	0	3.00E+05	299732.5	0	2.46E+05	246432.4
lys1_tRNA1_CHARGb	charging lys1_tRNA (by lysI_RS)	1 LysI_RS_2lys_2amp + 2 lys1_tRNA --> 1 LysI_RS_dim_inact + 2 amp + 2 lys1_tRNA_lys	irreversible	tRNA charging	0.00E+00	1.00E+09	0	1.40E+05	1.40E+05	0	1.50E+05	149866.2	0	1.23E+05	123216.2
lys1_tRNA2_CHARGa	charging lys1_tRNA (by lysII_RS)	1 LysII_RS_lys_amp + 1 lys1_tRNA --> 1 LysII_RS_dim_inact + 1 amp + 1 lys1_tRNA_lys	irreversible	tRNA charging	0.00E+00	1.00E+09	0	2.81E+05	2.81E+05	0	3.00E+05	299732.5	0	2.46E+05	246432.4
lys1_tRNA2_CHARGb	charging lys1_tRNA (by lysII_RS)	1 LysII_RS_2lys_2amp + 2 lys1_tRNA --> 1 LysII_RS_dim_inact + 2 amp + 2 lys1_tRNA_lys	irreversible	tRNA charging	0.00E+00	1.00E+09	0	1.40E+05	1.40E+05	0	1.50E+05	149866.2	0	1.23E+05	123216.2
lysQ_to_lys1	lysQ_to_lys1	1 lysQ_tRNA --> 1 lys1_tRNA	irreversible	tRNA charging	0.00E+00	1.00E+09	0	1.77E+03	1.77E+03	0	7.51E+00	7.51	0	7.51E+00	7.51
lysT_to_lys1	lysT_to_lys1	1 lysT_tRNA --> 1 lys1_tRNA	irreversible	tRNA charging	0.00E+00	1.00E+09	0	3.51E+02	3.51E+02	0	7.52E+00	7.52	0	7.52E+00	7.52
lysV_to_lys1	lysV_to_lys1	1 lysV_tRNA --> 1 lys1_tRNA	irreversible	tRNA charging	0.00E+00	1.00E+09	0	3.01E+02	3.01E+02	0	7.81E+00	7.81	0	7.81E+00	7.81
lysW_to_lys1	lysW_to_lys1	1 lysW_tRNA --> 1 lys1_tRNA	irreversible	tRNA charging	0.00E+00	1.00E+09	0	3.51E+02	3.51E+02	0	7.52E+00	7.52	0	7.52E+00	7.52
lysY_to_lys1	lysY_to_lys1	1 lysY_tRNA --> 1 lys1_tRNA	irreversible	tRNA charging	0.00E+00	1.00E+09	0	7.43E+02	7.43E+02	0	7.51E+00	7.51	0	7.51E+00	7.51
lysZ_to_lys1	lysZ_to_lys1	1 lysZ_tRNA --> 1 lys1_tRNA	irreversible	tRNA charging	0.00E+00	1.00E+09	0	1.77E+03	1.77E+03	0	7.51E+00	7.51	0	7.51E+00	7.51
met1_tRNA_CHARG	charging met1_tRNA	1 Met_RS_2met_2amp + 2 met1_tRNA --> 1 Met_RS_dim_inact + 2 amp + 2 met1_tRNA_met	irreversible	tRNA charging	0.00E+00	1.00E+09	7.18E+03	6.48E+04	5.76E+04	5.28E+03	7.07E+04	65408.41	5.28E+03	5.65E+04	51194.18
metT_to_met1	metT_to_met1	1 metT_tRNA --> 1 met1_tRNA	irreversible	tRNA charging	0.00E+00	1.00E+09	0	1.70E+02	1.70E+02	0	7.79E+00	7.79	0	7.79E+00	7.79
metU_to_met1	metU_to_met1	1 metU_tRNA --> 1 met1_tRNA	irreversible	tRNA charging	0.00E+00	1.00E+09	0	1.70E+02	1.70E+02	0	7.79E+00	7.79	0	7.79E+00	7.79
metV_to_fmet	metV_to_fmet	1 metV_tRNA --> 1 fmet_tRNA	irreversible	tRNA charging	0.00E+00	1.00E+09	0	3.85E+02	3.85E+02	0	9.37E+00	9.37	0	9.37E+00	9.37
metW_to_fmet	metW_to_fmet	1 metW_tRNA --> 1 fmet_tRNA	irreversible	tRNA charging	0.00E+00	1.00E+09	0	3.85E+02	3.85E+02	0	9.37E+00	9.37	0	9.37E+00	9.37
metY_to_fmet	metY_to_fmet	1 metY_tRNA --> 1 fmet_tRNA	irreversible	tRNA charging	0.00E+00	1.00E+09	0	1.23E+03	1.23E+03	0	6.95E+01	69.53628	0	2.22E+01	22.15
metZ_to_fmet	metZ_to_fmet	1 metZ_tRNA --> 1 fmet_tRNA	irreversible	tRNA charging	0.00E+00	1.00E+09	0	3.85E+02	3.85E+02	0	9.37E+00	9.37	0	9.37E+00	9.37
nadht	transport of nadh	1 nadh <==> 1 nadh[e]	reversible	Transport	-1.00E+09	1.00E+09	-1.86E+03	0	1.86E+03	-8.52E+02	0	851.846	-1.69E+02	0	168.674
nadpht	transport of nadph	1 nadph <==> 1 nadph[e]	reversible	Transport	-1.00E+09	1.00E+09	-8.00E+03	0	8.00E+03	-9.61E+03	-4.18E+01	9564.167	-2.37E+03	-4.18E+01	2326.953
nadpt	transport of nadp	1 nadp <==> 1 nadp[e]	reversible	Transport	-1.00E+09	1.00E+09	0	8.00E+03	8.00E+03	4.18E+01	9.61E+03	9564.167	4.18E+01	2.37E+03	2326.953
nadt	transport of nad	1 nad <==> 1 nad[e]	reversible	Transport	-1.00E+09	1.00E+09	0	1.86E+03	1.86E+03	0	8.52E+02	851.846	0	1.69E+02	168.674
nh3t	transport of nh3	1 nh3 <==> 1 nh3[e]	reversible	Transport	-1.00E+09	1.00E+09	0	5.86E+02	5.86E+02	0	3.55E+01	35.48	0	3.55E+01	35.48
phe1_tRNA_CHARG	charging phe1_tRNA	1 Phe_RS_phe_amp + 1 phe1_tRNA --> 1 Phe_RS_tetra_inact + 1 amp + 1 phe1_tRNA_phe	irreversible	tRNA charging	0.00E+00	1.00E+09	2.17E+04	1.73E+05	1.52E+05	1.60E+04	1.76E+05	159664.48	1.60E+04	1.63E+05	147228.38
pheU_to_phe1	pheU_to_phe1	1 pheU_tRNA --> 1 phe1_tRNA	irreversible	tRNA charging	0.00E+00	1.00E+09	0	1.52E+03	1.52E+03	0	1.18E+01	11.8	0	1.18E+01	11.8
pheV_to_phe1	pheV_to_phe1	1 pheV_tRNA --> 1 phe1_tRNA	irreversible	tRNA charging	0.00E+00	1.00E+09	0	1.52E+03	1.52E+03	0	10	10	0	10	10
ppit	transport of ppi	1 ppi <==> 1 ppi[e]	reversible	Transport	-1.00E+09	1.00E+09	1.19E+06	8.43E+06	7.24E+06	8.81E+05	8.44E+06	7556292.8	8.81E+05	8.38E+06	7498028.8
pre_Q1_FORMa	7-aminomethyl-7-deazaguanine formation from 7-cyano-7-deazaguanine by QueF	1 QueF_dim + 3 h + 2 nadph + 1 pre_Q0 <==> 1 pre_Q1_QueF_dim_cplx	reversible	tRNA modification	-1.00E+09	1.00E+09	0	1.77E+03	1.77E+03	0	7.90E+02	789.686	0	8.58E+01	85.794
pre_Q1_FORMb	7-aminomethyl-7-deazaguanine formation from 7-cyano-7-deazaguanine by QueF	1 pre_Q1_QueF_dim_cplx --> 1 QueF_dim_inact + 2 nadp + 1 pre_Q1	irreversible	tRNA modification	0.00E+00	1.00E+09	0	1.77E+03	1.77E+03	0	7.90E+02	789.686	0	8.58E+01	85.794
pro1_tRNA_CHARGa	charging pro1_tRNA	1 Pro_RS_2pro_2amp + 2 pro1_tRNA --> 1 Pro_RS_dim_inact + 2 amp + 2 pro1_tRNA_pro	irreversible	tRNA charging	0.00E+00	1.00E+09	0	6.88E+04	6.88E+04	0	7.54E+04	75430.08	0	6.39E+04	63854.35
pro1_tRNA_CHARGb	charging pro1_tRNA	1 Pro_RS_pro_amp + 1 pro1_tRNA --> 1 Pro_RS_dim_inact + 1 amp + 1 pro1_tRNA_pro	irreversible	tRNA charging	0.00E+00	1.00E+09	0	1.38E+05	1.38E+05	0	1.51E+05	150860.2	0	1.28E+05	127708.7
pro2_tRNA_CHARGa	charging pro2_tRNA	1 Pro_RS_2pro_2amp + 2 pro2_tRNA --> 1 Pro_RS_dim_inact + 2 amp + 2 pro2_tRNA_pro	irreversible	tRNA charging	0.00E+00	1.00E+09	0	2.69E+04	2.69E+04	0	3.09E+04	30917.33	0	2.10E+04	20972.82
pro2_tRNA_CHARGb	charging pro2_tRNA	1 Pro_RS_pro_amp + 1 pro2_tRNA --> 1 Pro_RS_dim_inact + 1 amp + 1 pro2_tRNA_pro	irreversible	tRNA charging	0.00E+00	1.00E+09	0	5.39E+04	5.39E+04	0	6.18E+04	61834.66	0	4.19E+04	41945.65
proK_to_pro1	proK_to_pro1	1 proK_tRNA --> 1 pro1_tRNA	irreversible	tRNA charging	0.00E+00	1.00E+09	0	1.52E+03	1.52E+03	0	13	13	0	13	13
proL_tRNA_CHARGa	charging proL_tRNA	1 Pro_RS_2pro_2amp + 2 proL_tRNA --> 1 Pro_RS_dim_inact + 2 amp + 2 proL_tRNA_pro	irreversible	tRNA charging	0.00E+00	1.00E+09	0	1.93E+04	1.93E+04	0	2.28E+04	22836.58	0	1.40E+04	14028.38
proL_tRNA_CHARGb	charging proL_tRNA	1 Pro_RS_pro_amp + 1 proL_tRNA --> 1 Pro_RS_dim_inact + 1 amp + 1 proL_tRNA_pro	irreversible	tRNA charging	0.00E+00	1.00E+09	0	3.86E+04	3.86E+04	0	4.57E+04	45673.17	0	2.81E+04	28056.76
proL_to_pro2	proL_to_pro2	1 proL_tRNA --> 1 pro2_tRNA	irreversible	tRNA charging	0.00E+00	1.00E+09	0	1.39E+03	1.39E+03	0	7.06E+00	7.06	0	7.06E+00	7.06
proM_tRNA_CHARGa	charging proM_tRNA	1 Pro_RS_2pro_2amp + 2 proM_tRNA --> 1 Pro_RS_dim_inact + 2 amp + 2 proM_tRNA_pro	irreversible	tRNA charging	0.00E+00	1.00E+09	0	2.62E+04	2.62E+04	0	3.07E+04	30666.14	0	2.22E+04	22229.43
proM_tRNA_CHARGb	charging proM_tRNA	1 Pro_RS_pro_amp + 1 proM_tRNA --> 1 Pro_RS_dim_inact + 1 amp + 1 proM_tRNA_pro	irreversible	tRNA charging	0.00E+00	1.00E+09	0	5.23E+04	5.23E+04	0	6.13E+04	61332.29	0	4.45E+04	44458.87
proM_to_pro1	proM_to_pro1	1 proM_tRNA --> 1 pro1_tRNA	irreversible	tRNA charging	0.00E+00	1.00E+09	0	3.01E+02	3.01E+02	0	1.39E+01	13.9	0	1.39E+01	13.9
proM_to_pro2	proM_to_pro2	1 proM_tRNA --> 1 pro2_tRNA	irreversible	tRNA charging	0.00E+00	1.00E+09	0	3.01E+02	3.01E+02	0	1.39E+01	13.9	0	1.39E+01	13.9
pydx5pt	transport of pydx5p	1 pydx5p <==> 1 pydx5p[e]	reversible	Transport	-1.00E+09	1.00E+09	-1.30E+03	0	1.30E+03	-2.19E+03	0	2188.119	-1.43E+02	0	142.705
rrfA_RENAME	TEMP_rrfA	1 b3855_RNA --> 1 rrfA	irreversible	rRNA formation	0.00E+00	1.00E+09	0	1.12E+02	1.12E+02	0	1.36E+01	13.6	0	1.36E+01	13.6
rrfBa_RENAME	TEMP_rrfBa	1 b3971_RNA --> 1 rrfB	irreversible	rRNA formation	0.00E+00	1.00E+09	0	1.11E+02	1.11E+02	0	0	0	0	0	0
rrfBb_RENAME	TEMP_rrfBb	1 b3971_v1_RNA --> 1 rrfB	irreversible	rRNA formation	0.00E+00	1.00E+09	0	1.11E+02	1.11E+02	0	1.29E+01	12.9	0	1.29E+01	12.9
rrfCa_RENAME	TEMP_rrfCa	1 b3759_RNA --> 1 rrfC	irreversible	rRNA formation	0.00E+00	1.00E+09	0	1.12E+02	1.12E+02	0	0	0	0	0	0
rrfCb_RENAME	TEMP_rrfCb	1 b3759_v1_RNA --> 1 rrfC	irreversible	rRNA formation	0.00E+00	1.00E+09	0	1.12E+02	1.12E+02	0	1.42E+01	14.2	0	1.42E+01	14.2
rrfDa_RENAME	TEMP_rrfDa	1 b3274_RNA --> 1 rrfD	irreversible	rRNA formation	0.00E+00	1.00E+09	0	1.06E+02	1.06E+02	0	0	0	0	0	0
rrfDb_RENAME	TEMP_rrfDb	1 b3274_v1_RNA --> 1 rrfD	irreversible	rRNA formation	0.00E+00	1.00E+09	0	1.06E+02	1.06E+02	0	1.15E+01	11.5	0	1.15E+01	11.5
rrfE_RENAME	TEMP_rrfE	1 b4010_RNA --> 1 rrfE	irreversible	rRNA formation	0.00E+00	1.00E+09	0	1.13E+02	1.13E+02	0	1.26E+01	12.6	0	1.26E+01	12.6
rrfFa_RENAME	TEMP_rrfFa	1 b3272_RNA --> 1 rrfF	irreversible	rRNA formation	0.00E+00	1.00E+09	0	1.06E+02	1.06E+02	0	0	0	0	0	0
rrfFb_RENAME	TEMP_rrfFb	1 b3272_v1_RNA --> 1 rrfF	irreversible	rRNA formation	0.00E+00	1.00E+09	0	1.06E+02	1.06E+02	0	1.15E+01	11.5	0	1.15E+01	11.5
rrfGa_RENAME	TEMP_rrfGa	1 b2588_RNA --> 1 rrfG	irreversible	rRNA formation	0.00E+00	1.00E+09	0	1.11E+02	1.11E+02	0	0	0	0	0	0
rrfGb_RENAME	TEMP_rrfGb	1 b2588_v1_RNA --> 1 rrfG	irreversible	rRNA formation	0.00E+00	1.00E+09	0	1.11E+02	1.11E+02	0	8.54E+00	8.54	0	8.54E+00	8.54
rrfHa_RENAME	TEMP_rrfHa	1 b0205_RNA --> 1 rrfH	irreversible	rRNA formation	0.00E+00	1.00E+09	0	1.11E+02	1.11E+02	0	0	0	0	0	0
rrfHb_RENAME	TEMP_rrfHb	1 b0205_v1_RNA --> 1 rrfH	irreversible	rRNA formation	0.00E+00	1.00E+09	0	1.11E+02	1.11E+02	0	9.54E+00	9.54	0	9.54E+00	9.54
sec_tRNA_CHARG1a	charging sec_tRNA	1 Ser_RS_2ser_2amp + 2 selC_tRNA --> 1 Ser_RS_dim_inact + 2 amp + 2 sec_tRNA_ser	irreversible	tRNA charging	0.00E+00	1.00E+09	0	3.19E+02	3.19E+02	0	6.85E+00	6.85	0	6.85E+00	6.85
sec_tRNA_CHARG1b	charging sec_tRNA	1 Ser_RS_ser_amp + 1 selC_tRNA --> 1 Ser_RS_dim_inact + 1 amp + 1 sec_tRNA_ser	irreversible	tRNA charging	0.00E+00	1.00E+09	0	6.39E+02	6.39E+02	0	1.37E+01	13.7	0	1.37E+01	13.7
sec_tRNA_CHARG2a	conversion of 5 sec_tRNA_ser to 5 sec_tRNA_sec	1 SelA_deca + 5 sec_tRNA_ser --> 1 5_sec_tRNA_ser_SelA_deca_cplx + 5 h2o	irreversible	tRNA charging	0.00E+00	1.00E+09	0	1.28E+02	1.28E+02	0	2.74E+00	2.74	0	2.74E+00	2.74
sec_tRNA_CHARG2b	conversion of 5 sec_tRNA_ser to 5 sec_tRNA_sec	1 5_sec_tRNA_ser_SelA_deca_cplx + 5 h2o + 5 selnp --> 1 SelA_deca_inact + 5 h + 5 pi + 5 sec_tRNA_sec	irreversible	tRNA charging	0.00E+00	1.00E+09	0	1.28E+02	1.28E+02	0	2.74E+00	2.74	0	2.74E+00	2.74

sec_tRNA_CHARG3a	conversion of 4 sec_tRNA_ser to 4 sec_tRNA_sec	1 SelA_deca + 4 sec_tRNA_ser --> 1 4_sec_tRNA_ser_SelA_deca_cplx + 4 h2o	irreversible	tRNA charging	0.00E+00	1.00E+09	0	1.60E+02	1.60E+02	0	3.43E+00	3.425	0	3.43E+00	3.425
sec_tRNA_CHARG3b	conversion of 4 sec_tRNA_ser to 4 sec_tRNA_sec	1 4_sec_tRNA_ser_SelA_deca_cplx + 4 h2o + 4 selnp --> 1 SelA_deca_inact + 4 h + 4 pi + 4 sec_tRNA_sec	irreversible	tRNA charging	0.00E+00	1.00E+09	0	1.60E+02	1.60E+02	0	3.43E+00	3.425	0	3.43E+00	3.425
sec_tRNA_CHARG4a	conversion of 3 sec_tRNA_ser to 3 sec_tRNA_sec	1 SelA_deca + 3 sec_tRNA_ser --> 1 3_sec_tRNA_ser_SelA_deca_cplx + 3 h2o	irreversible	tRNA charging	0.00E+00	1.00E+09	0	2.13E+02	2.13E+02	0	4.57E+00	4.566667	0	4.57E+00	4.566667
sec_tRNA_CHARG4b	conversion of 3 sec_tRNA_ser to 3 sec_tRNA_sec	1 3_sec_tRNA_ser_SelA_deca_cplx + 3 h2o + 3 selnp --> 1 SelA_deca_inact + 3 h + 3 pi + 3 sec_tRNA_sec	irreversible	tRNA charging	0.00E+00	1.00E+09	0	2.13E+02	2.13E+02	0	4.57E+00	4.566667	0	4.57E+00	4.566667
sec_tRNA_CHARG5a	conversion of 2 sec_tRNA_ser to 2 sec_tRNA_sec	1 SelA_deca + 2 sec_tRNA_ser --> 1 2_sec_tRNA_ser_SelA_deca_cplx + 2 h2o	irreversible	tRNA charging	0.00E+00	1.00E+09	0	3.19E+02	3.19E+02	0	6.85E+00	6.85	0	6.85E+00	6.85
sec_tRNA_CHARG5b	conversion of 2 sec_tRNA_ser to 2 sec_tRNA_sec	1 2_sec_tRNA_ser_SelA_deca_cplx + 2 h2o + 2 selnp --> 1 SelA_deca_inact + 2 h + 2 pi + 2 sec_tRNA_sec	irreversible	tRNA charging	0.00E+00	1.00E+09	0	3.19E+02	3.19E+02	0	6.85E+00	6.85	0	6.85E+00	6.85
sec_tRNA_CHARG6a	conversion of 1 sec_tRNA_ser to 1 sec_tRNA_sec	1 SelA_deca + 1 sec_tRNA_ser --> 1 1_sec_tRNA_ser_SelA_deca_cplx + 1 h2o	irreversible	tRNA charging	0.00E+00	1.00E+09	0	6.39E+02	6.39E+02	0	1.37E+01	13.7	0	1.37E+01	13.7
sec_tRNA_CHARG6b	conversion of 1 sec_tRNA_ser to 1 sec_tRNA_sec	1 1_sec_tRNA_ser_SelA_deca_cplx + 1 h2o + 1 selnp --> 1 SelA_deca_inact + 1 h + 1 pi + 1 sec_tRNA_sec	irreversible	tRNA charging	0.00E+00	1.00E+09	0	6.39E+02	6.39E+02	0	1.37E+01	13.7	0	1.37E+01	13.7
ser1_tRNA_CHARGa	charging ser1_tRNA	1 Ser_RS_2ser_2amp + 2 ser1_tRNA --> 1 Ser_RS_dim_inact + 2 amp + 2 ser1_tRNA_ser	irreversible	tRNA charging	0.00E+00	1.00E+09	0	2.73E+04	2.73E+04	0	2.94E+04	29393.06	0	2.41E+04	24136.89
ser1_tRNA_CHARGb	charging ser1_tRNA	1 Ser_RS_ser_amp + 1 ser1_tRNA --> 1 Ser_RS_dim_inact + 1 amp + 1 ser1_tRNA_ser	irreversible	tRNA charging	0.00E+00	1.00E+09	0	5.45E+04	5.45E+04	0	5.88E+04	58786.13	0	4.83E+04	48273.77
ser2_tRNA_CHARGa	charging ser2_tRNA	1 Ser_RS_2ser_2amp + 2 ser2_tRNA --> 1 Ser_RS_dim_inact + 2 amp + 2 ser2_tRNA_ser	irreversible	tRNA charging	0.00E+00	1.00E+09	0	3.59E+04	3.59E+04	0	3.75E+04	37459.52	0	3.26E+04	32571.01
ser2_tRNA_CHARGb	charging ser2_tRNA	1 Ser_RS_ser_amp + 1 ser2_tRNA --> 1 Ser_RS_dim_inact + 1 amp + 1 ser2_tRNA_ser	irreversible	tRNA charging	0.00E+00	1.00E+09	0	7.19E+04	7.19E+04	0	7.49E+04	74919.04	0	6.51E+04	65142.03
ser3_tRNA_CHARGa	charging ser3_tRNA	1 Ser_RS_2ser_2amp + 2 ser3_tRNA --> 1 Ser_RS_dim_inact + 2 amp + 2 ser3_tRNA_ser	irreversible	tRNA charging	0.00E+00	1.00E+09	0	2.19E+04	2.19E+04	0	2.55E+04	25471.68	0	1.80E+04	18002.98
ser3_tRNA_CHARGb	charging ser3_tRNA	1 Ser_RS_ser_amp + 1 ser3_tRNA --> 1 Ser_RS_dim_inact + 1 amp + 1 ser3_tRNA_ser	irreversible	tRNA charging	0.00E+00	1.00E+09	0	4.39E+04	4.39E+04	0	5.09E+04	50943.37	0	3.60E+04	36005.97
serT_tRNA_CHARGa	charging serT_tRNA	1 Ser_RS_2ser_2amp + 2 serT_tRNA --> 1 Ser_RS_dim_inact + 2 amp + 2 serT_tRNA_ser	irreversible	tRNA charging	0.00E+00	1.00E+09	0	1.70E+04	1.70E+04	0	1.97E+04	19717.82	0	1.41E+04	14092.78
serT_tRNA_CHARGb	charging serT_tRNA	1 Ser_RS_ser_amp + 1 serT_tRNA --> 1 Ser_RS_dim_inact + 1 amp + 1 serT_tRNA_ser	irreversible	tRNA charging	0.00E+00	1.00E+09	0	3.41E+04	3.41E+04	0	3.94E+04	39435.64	0	2.82E+04	28185.57
serT_to_ser2	serT_to_ser2	1 serT_tRNA --> 1 ser2_tRNA	irreversible	tRNA charging	0.00E+00	1.00E+09	0	1.33E+03	1.33E+03	0	6.75E+00	6.75	0	6.75E+00	6.75
serT_to_ser3	serT_to_ser3	1 serT_tRNA --> 1 ser3_tRNA	irreversible	tRNA charging	0.00E+00	1.00E+09	0	1.33E+03	1.33E+03	0	6.75E+00	6.75	0	6.75E+00	6.75
serU_to_ser3	serU_to_ser3	1 serU_tRNA --> 1 ser3_tRNA	irreversible	tRNA charging	0.00E+00	1.00E+09	0	1.14E+03	1.14E+03	0	6.36E+00	6.36	0	6.36E+00	6.36
serV_tRNA_CHARGa	charging serV_tRNA	1 Ser_RS_2ser_2amp + 2 serV_tRNA --> 1 Ser_RS_dim_inact + 2 amp + 2 serV_tRNA_ser	irreversible	tRNA charging	0.00E+00	1.00E+09	0	5.70E+04	5.70E+04	0	6.20E+04	62013.6	0	5.20E+04	52042.52
serV_tRNA_CHARGb	charging serV_tRNA	1 Ser_RS_ser_amp + 1 serV_tRNA --> 1 Ser_RS_dim_inact + 1 amp + 1 serV_tRNA_ser	irreversible	tRNA charging	0.00E+00	1.00E+09	0	1.14E+05	1.14E+05	0	1.24E+05	124027.2	0	1.04E+05	104085
serW_to_ser1	serW_to_ser1	1 serW_tRNA --> 1 ser1_tRNA	irreversible	tRNA charging	0.00E+00	1.00E+09	0	1.33E+03	1.33E+03	0	7.06E+00	7.06	0	7.06E+00	7.06
serW_to_ser2	serW_to_ser2	1 serW_tRNA --> 1 ser2_tRNA	irreversible	tRNA charging	0.00E+00	1.00E+09	0	1.33E+03	1.33E+03	0	7.06E+00	7.06	0	7.06E+00	7.06
serX_to_ser1	serX_to_ser1	1 serX_tRNA --> 1 ser1_tRNA	irreversible	tRNA charging	0.00E+00	1.00E+09	0	1.33E+03	1.33E+03	0	6.56E+00	6.56	0	6.56E+00	6.56
serX_to_ser2	serX_to_ser2	1 serX_tRNA --> 1 ser2_tRNA	irreversible	tRNA charging	0.00E+00	1.00E+09	0	1.33E+03	1.33E+03	0	6.56E+00	6.56	0	6.56E+00	6.56
sink_AcT_EF-TU	sink_AcT_EF-TU	1 AcT_EF-TU <==>	reversible	Sinks	-1.00E+09	1.00E+09	0	0	0.00E+00	0	0	0	0	0	0
sink_AcT_tRNA_pos_34_ac4C	sink_AcT_tRNA_pos_34_ac4C	1 AcT_tRNA_pos_34_ac4C <==>	reversible	Sinks	-1.00E+09	1.00E+09	0	0	0.00E+00	0	0	0	0	0	0
sink_AcpT_tRNA_pos_47_acp3U	sink_AcpT_tRNA_pos_47_acp3U	1 AcpT_tRNA_pos_47_acp3U <==>	reversible	Sinks	-1.00E+09	1.00E+09	0	0	0.00E+00	0	0	0	0	0	0
sink_DU_23S_2449	sink_DU_23S_2449	1 DU_23S_2449 <==>	reversible	Sinks	-1.00E+09	1.00E+09	0	0	0.00E+00	0	0	0	0	0	0
sink_EoR_tRNA_pos34_Q	sink_EoR_tRNA_pos34_Q	1 EoR_tRNA_pos34_Q <==>	reversible	Sinks	-1.00E+09	1.00E+09	0	0	0.00E+00	0	0	0	0	0	0
sink_HyL_tRNA_pos_34_ho5U	sink_HyL_tRNA_pos_34_ho5U	1 HyL_tRNA_pos_34_ho5U <==>	reversible	Sinks	-1.00E+09	1.00E+09	0	0	0.00E+00	0	0	0	0	0	0
sink_MeST_S12	sink_MeST_S12	1 MeST_S12 <==>	reversible	Sinks	-1.00E+09	1.00E+09	0	0	0.00E+00	0	0	0	0	0	0
sink_MeT_16S_1402	sink_MeT_16S_1402	1 MeT_16S_1402 <==>	reversible	Sinks	-1.00E+09	1.00E+09	0	0	0.00E+00	0	0	0	0	0	0
sink_MeT_16S_1407	sink_MeT_16S_1407	1 MeT_16S_1407 <==>	reversible	Sinks	-1.00E+09	1.00E+09	0	0	0.00E+00	0	0	0	0	0	0
sink_MeT_16S_1516	sink_MeT_16S_1516	1 MeT_16S_1516 <==>	reversible	Sinks	-1.00E+09	1.00E+09	0	0	0.00E+00	0	0	0	0	0	0
sink_MeT_16S_527	sink_MeT_16S_527	1 MeT_16S_527 <==>	reversible	Sinks	-1.00E+09	1.00E+09	0	0	0.00E+00	0	0	0	0	0	0
sink_MeT_16S_966	sink_MeT_16S_966	1 MeT_16S_966 <==>	reversible	Sinks	-1.00E+09	1.00E+09	0	0	0.00E+00	0	0	0	0	0	0
sink_MeT_23S_1618	sink_MeT_23S_1618	1 MeT_23S_1618 <==>	reversible	Sinks	-1.00E+09	1.00E+09	0	0	0.00E+00	0	0	0	0	0	0
sink_MeT_23S_1835	sink_MeT_23S_1835	1 MeT_23S_1835 <==>	reversible	Sinks	-1.00E+09	1.00E+09	0	0	0.00E+00	0	0	0	0	0	0
sink_MeT_23S_1962	sink_MeT_23S_1962	1 MeT_23S_1962 <==>	reversible	Sinks	-1.00E+09	1.00E+09	0	0	0.00E+00	0	0	0	0	0	0
sink_MeT_23S_2030	sink_MeT_23S_2030	1 MeT_23S_2030 <==>	reversible	Sinks	-1.00E+09	1.00E+09	0	0	0.00E+00	0	0	0	0	0	0
sink_MeT_23S_2069	sink_MeT_23S_2069	1 MeT_23S_2069 <==>	reversible	Sinks	-1.00E+09	1.00E+09	0	0	0.00E+00	0	0	0	0	0	0
sink_MeT_23S_2445	sink_MeT_23S_2445	1 MeT_23S_2445 <==>	reversible	Sinks	-1.00E+09	1.00E+09	0	0	0.00E+00	0	0	0	0	0	0
sink_MeT_23S_2498	sink_MeT_23S_2498	1 MeT_23S_2498 <==>	reversible	Sinks	-1.00E+09	1.00E+09	0	0	0.00E+00	0	0	0	0	0	0
sink_MeT_23S_2503	sink_MeT_23S_2503	1 MeT_23S_2503 <==>	reversible	Sinks	-1.00E+09	1.00E+09	0	0	0.00E+00	0	0	0	0	0	0
sink_MeT_EF-TU	sink_MeT_EF-TU	1 MeT_EF-TU <==>	reversible	Sinks	-1.00E+09	1.00E+09	0	0	0.00E+00	0	0	0	0	0	0
sink_MeT_L16	sink_MeT_L16	1 MeT_L16 <==>	reversible	Sinks	-1.00E+09	1.00E+09	0	0	0.00E+00	0	0	0	0	0	0
sink_MeT_L33	sink_MeT_L33	1 MeT_L33 <==>	reversible	Sinks	-1.00E+09	1.00E+09	0	0	0.00E+00	0	0	0	0	0	0
sink_MeT_L7/L12	sink_MeT_L7/L12	1 MeT_L7/L12 <==>	reversible	Sinks	-1.00E+09	1.00E+09	0	0	0.00E+00	0	0	0	0	0	0
sink_MeT_S11	sink_MeT_S11	1 MeT_S11 <==>	reversible	Sinks	-1.00E+09	1.00E+09	0	0	0.00E+00	0	0	0	0	0	0
sink_MeT_tRNA_pos_32_Cm	sink_MeT_tRNA_pos_32_Cm	1 MeT_tRNA_pos_32_Cm <==>	reversible	Sinks	-1.00E+09	1.00E+09	0	0	0.00E+00	0	0	0	0	0	0
sink_MeT_tRNA_pos_32_Um	sink_MeT_tRNA_pos_32_Um	1 MeT_tRNA_pos_32_Um <==>	reversible	Sinks	-1.00E+09	1.00E+09	0	0	0.00E+00	0	0	0	0	0	0
sink_MeT_tRNA_pos_37_m2A	sink_MeT_tRNA_pos_37_m2A	1 MeT_tRNA_pos_37_m2A <==>	reversible	Sinks	-1.00E+09	1.00E+09	0	0	0.00E+00	0	0	0	0	0	0
sink_MeT_tRNA_pos_37_m6A	sink_MeT_tRNA_pos_37_m6A	1 MeT_tRNA_pos_37_m6A <==>	reversible	Sinks	-1.00E+09	1.00E+09	0	0	0.00E+00	0	0	0	0	0	0
sink_MeT_tRNA_pos_37_m6t6A	sink_MeT_tRNA_pos_37_m6t6A	1 MeT_tRNA_pos_37_m6t6A <==>	reversible	Sinks	-1.00E+09	1.00E+09	0	0	0.00E+00	0	0	0	0	0	0
sink_RNase_m16	sink_RNase_m16	1 RNase_m16 <==>	reversible	Sinks	-1.00E+09	1.00E+09	-1000000000	0	1.00E+09	-1000000000	0	1000000000	-1000000000	0	1E+09
sink_RNase_m23	sink_RNase_m23	1 RNase_m23 <==>	reversible	Sinks	-1.00E+09	1.00E+09	-1000000000	0	1.00E+09	-1000000000	0	1000000000	-1000000000	0	1E+09
sink_RNase_m5	sink_RNase_m5	1 RNase_m5 <==>	reversible	Sinks	-1.00E+09	1.00E+09	-1000000000	0	1.00E+09	-1000000000	0	1000000000	-1000000000	0	1E+09
sink_Up_tRNA_pos_37_t6A	sink_Up_tRNA_pos_37_t6A	1 Up_tRNA_pos_37_t6A <==>	reversible	Sinks	-1.00E+09	1.00E+09	0	0	0.00E+00	0	0	0	0	0	0
sink_preQ0	sink_preQ0	1 pre_Q0 <==>	reversible	Sinks	-1.00E+09	1.00E+09	-1.77E+03	0	1.77E+03	-7.90E+02	0	789.686	-8.58E+01	0	85.794
thr1_tRNA_CHARGa	charging thr1_tRNA	1 Thr_RS_2thr_2amp + 2 thr1_tRNA --> 1 Thr_RS_dim_inact + 2 amp + 2 thr1_tRNA_thr	irreversible	tRNA charging	0.00E+00	1.00E+09	0	6.35E+04	6.35E+04	0	6.87E+04	68679.42	0	5.77E+04	57688.46
thr1_tRNA_CHARGb	charging thr1_tRNA	1 Thr_RS_thr_amp + 1 thr1_tRNA --> 1 Thr_RS_dim_inact + 1 amp - 1 thr1_tRNA_thr	irreversible	tRNA charging	0.00E+00	1.00E+09	0	1.27E+05	1.27E+05	0	1.37E+05	137358.8	0	1.15E+05	115376.9
thr2_tRNA_CHARGa	charging thr2_tRNA	1 Thr_RS_2thr_2amp + 2 thr2_tRNA --> 1 Thr_RS_dim_inact + 2 amp + 2 thr2_tRNA_thr	irreversible	tRNA charging	0.00E+00	1.00E+09	0	4.23E+04	4.23E+04	0	4.33E+04	43343.43	0	3.90E+04	39021.08
thr2_tRNA_CHARGb	charging thr2_tRNA	1 Thr_RS_thr_amp + 1 thr2_tRNA --> 1 Thr_RS_dim_inact + 1 amp - 1 thr2_tRNA_thr	irreversible	tRNA charging	0.00E+00	1.00E+09	0	8.45E+04	8.45E+04	0	8.67E+04	86686.86	0	7.80E+04	78042.17
thr3_tRNA_CHARGB	charging thr3_tRNA	1 Thr_RS_thr_amp + 1 thr3_tRNA --> 1 Thr_RS_dim_inact + 1 amp - 1 thr3_tRNA_thr	irreversible	tRNA charging	0.00E+00	1.00E+09	0	7.89E+04	7.89E+04	0	8.73E+04	87280.08	0	6.83E+04	68345.4
thr3_tRNA_CHARGa	charging thr3_tRNA	1 Thr_RS_2thr_2amp + 2 thr3_tRNA --> 1 Thr_RS_dim_inact + 2 amp + 2 thr3_tRNA_thr	irreversible	tRNA charging	0.00E+00	1.00E+09	0	3.95E+04	3.95E+04	0	4.36E+04	43640.04	0	3.42E+04	34172.7
thrT_to_thr1	thrT_to_thr1	1 thrT_tRNA --> 1 thr1_tRNA	irreversible	tRNA charging	0.00E+00	1.00E+09	0	2.71E+02	2.71E+02	0	1.28E+01	12.8	0	1.28E+01	12.8
thrT_to_thr2	thrT_to_thr2	1 thrT_tRNA --> 1 thr2_tRNA	irreversible	tRNA charging	0.00E+00	1.00E+09	0	2.71E+02	2.71E+02	0	1.28E+01	12.8	0	1.28E+01	12.8
thrU_tRNA_CHARGB	charging thrU_tRNA	1 Thr_RS_thr_amp + 1 thrU_tRNA --> 1 Thr_RS_dim_inact + 1 amp + 1 thrU_tRNA_thr	irreversible	tRNA charging	0.00E+00	1.00E+09	0	3.87E+04	3.87E+04	0	4.61E+04	46101.37	0	2.97E+04	29660.2
thrU_tRNA_CHARGa	charging thrU_tRNA	1 Thr_RS_2thr_2amp + 2 thrU_tRNA --> 1 Thr_RS_dim_inact + 2 amp + 2 thrU_tRNA_thr	irreversible	tRNA charging	0.00E+00	1.00E+09	0	1.93E+04	1.93E+04	0	2.31E+04	23050.69	0	1.48E+04	14830.1

thrU_to_thr2	thrU_to_thr2	1 thrU_tRNA --> 1 thr2_tRNA	irreversible	tRNA charging	0.00E+00	1.00E+09	0	2.71E+02	2.71E+02	0	1.28E+01	12.8	0	1.28E+01	12.8
thrU_to_thr3	thrU_to_thr3	1 thrU_tRNA --> 1 thr3_tRNA	irreversible	tRNA charging	0.00E+00	1.00E+09	0	2.71E+02	2.71E+02	0	1.28E+01	12.8	0	1.28E+01	12.8
thrV_to_thr1	thrV_to_thr1	1 thrV_tRNA --> 1 thr1_tRNA	irreversible	tRNA charging	0.00E+00	1.00E+09	0	1.06E+02	1.06E+02	0	1.15E+01	11.5	0	1.15E+01	11.5
thrV_to_thr2	thrV_to_thr2	1 thrV_tRNA --> 1 thr2_tRNA	irreversible	tRNA charging	0.00E+00	1.00E+09	0	1.06E+02	1.06E+02	0	1.15E+01	11.5	0	1.15E+01	11.5
thrW_to_thr3	thrW_to_thr3	1 thrW_tRNA --> 1 thr3_tRNA	irreversible	tRNA charging	0.00E+00	1.00E+09	0	1.77E+03	1.77E+03	0	9.38E+00	9.38	0	9.38E+00	9.38
tl_elo_b0014_18_rib1	Translation elongation 1 b0014 18 ribosome(s)	11466 EF-G.GTP + 1134 EF-Tu.GTP.ala1-tRNA + 126 EF-Tu.GTP.ala2-tRNA + 450 EF-Tu.GTP.arg1-tRNA + 468 EF-Tu.GTP.asn1-tRNA + 990 EF-Tu.GTP.asp1-tRNA + 18 EF-Tu.GTP.cysT-tRNA + 630 EF-Tu.GTP.gln1-tRNA + 36 EF-Tu.GTP.gln2-tRNA + 900 EF-Tu.GTP.glu1-tRNA + 828 EF-Tu.GTP.gly1-tRNA + 18 EF-Tu.GTP.glyU-tRNA + 144 EF-Tu.GTP.hisR-tRNA + 774 EF-Tu.GTP.ile1-tRNA + 18 EF-Tu.GTP.ile2-tRNA + 810 EF-Tu.GTP.leu1-tRNA + 18 EF-Tu.GTP.leuU-tRNA + 36 EF-Tu.GTP.leuW-tRNA + 900 EF-Tu.GTP.lys1-tRNA + 252 EF-Tu.GTP.met1-tRNA + 270 EF-Tu.GTP.phe1-tRNA + 342 EF-Tu.GTP.pro1-tRNA + 36 EF-Tu.GTP.pro2-tRNA + 36 EF-Tu.GTP.proM-tRNA + 108 EF-Tu.GTP.ser1-tRNA + 252 EF-Tu.GTP.ser2-tRNA + 90 EF-Tu.GTP.serV-tRNA + 396 EF-Tu.GTP.thr1-tRNA + 324 EF-Tu.GTP.thr2-tRNA + 72 EF-Tu.GTP.thr3-tRNA + 18 EF-Tu.GTP.trpT-tRNA + 126 EF-Tu.GTP.tyr1-tRNA + 360 EF-Tu.GTP.val1-tRNA + 54 EF-Tu.GTP.val2-tRNA + 432 EF-Tu.GTP.val3-tRNA + 57330 mg2 + 1 rib_ini_b0014_18 --> 1 rib_70_elo1_b0014_18_cplx	irreversible	Translation	0.00E+00	1.00E+09	0	4.77E+01	4.77E+01	0	7.93E+01	79.27185	0	6.86E+00	6.861
tl_elo_b0014_18_rib2	Translation elongation 2 b0014 18 ribosome(s)	11448 h2o + 1 rib_70_elo1_b0014_18_cplx --> 11448 EF-G.GDP + 11448 EF-Tu.GDP + 1134 ala1_tRNA + 126 ala2_tRNA + 450 arg1_tRNA + 468 asn1_tRNA + 990 asp1_tRNA + 18 cysT_tRNA + 18 fmet_tRNA + 630 gln1_tRNA + 36 gln2_tRNA + 900 glu1_tRNA + 828 gly1_tRNA + 18 glyU_tRNA + 22896 h + 144 hisR_tRNA + 774 ile1_tRNA + 18 ile2_tRNA + 810 leu1_tRNA + 18 leuU_tRNA + 36 leuW_tRNA + 882 lys1_tRNA + 252 met1_tRNA + 57330 mg2 + 270 phe1_tRNA + 22896 pi + 342 pro1_tRNA + 36 pro2_tRNA + 36 proM_tRNA + 1 rib_70_elo2_b0014_18_cplx + 108 ser1_tRNA + 252 ser2_tRNA + 90 serV_tRNA + 396 thr1_tRNA + 324 thr2_tRNA + 72 thr3_tRNA + 18 trpT_tRNA + 126 tyr1_tRNA + 360 val1_tRNA + 54 val2_tRNA + 432 val3_tRNA	irreversible	Translation	0.00E+00	1.00E+09	0	4.77E+01	4.77E+01	0	7.93E+01	79.27185	0	6.86E+00	6.861
tl_elo_b0014_1_rib1	Translation elongation 1 b0014 1 ribosome(s)	637 EF-G.GTP + 63 EF-Tu.GTP.ala1-tRNA + 7 EF-Tu.GTP.ala2-tRNA + 25 EF-Tu.GTP.arg1-tRNA + 26 EF-Tu.GTP.asn1-tRNA + 55 EF-Tu.GTP.asp1-tRNA + 1 EF-Tu.GTP.cysT-tRNA + 35 EF-Tu.GTP.gln1-tRNA + 2 EF-Tu.GTP.gln2-tRNA + 50 EF-Tu.GTP.glu1-tRNA + 46 EF-Tu.GTP.gly1-tRNA + 1 EF-Tu.GTP.glyU-tRNA + 8 EF-Tu.GTP.hisR-tRNA + 43 EF-Tu.GTP.ile1-tRNA + 1 EF-Tu.GTP.ile2-tRNA + 45 EF-Tu.GTP.leu1-tRNA + 1 EF-Tu.GTP.leuU-tRNA + 2 EF-Tu.GTP.leuW-tRNA + 50 EF-Tu.GTP.lys1-tRNA + 14 EF-Tu.GTP.met1-tRNA + 15 EF-Tu.GTP.phe1-tRNA + 19 EF-Tu.GTP.pro1-tRNA + 2 EF-Tu.GTP.pro2-tRNA + 2 EF-Tu.GTP.proM-tRNA + 6 EF-Tu.GTP.ser1-tRNA + 14 EF-Tu.GTP.ser2-tRNA + 5 EF-Tu.GTP.serV-tRNA + 22 EF-Tu.GTP.thr1-tRNA + 18 EF-Tu.GTP.thr2 tRNA + 4 EF-Tu.GTP.thr3-tRNA + 1 EF-Tu.GTP.trpT-tRNA + 7 EF-Tu.GTP.tyr1-tRNA + 20 EF-Tu.GTP.val1-tRNA + 3 EF-Tu.GTP.val2-tRNA + 24 EF-Tu.GTP.val3-tRNA + 3185 mg2 + 1 rib_ini_b0014_1 --> 1 rib_70_elo1_b0014_1_cplx	irreversible	Translation	0.00E+00	1.00E+09	0	4.77E+01	4.77E+01	0	7.93E+01	79.27185	0	6.86E+00	6.861
tl_elo_b0014_1_rib2	Translation elongation 2 b0014 1 ribosome(s)	636 h2o + 1 rib_70_elo1_b0014_1_cplx --> 636 EF-G.GDP + 636 EF-Tu.GDP + 63 ala1_tRNA + 7 ala2_tRNA + 25 arg1_tRNA + 26 asn1_tRNA + 55 asp1_tRNA + 1 cysT_tRNA + 1 fmet_tRNA + 35 gln1_tRNA + 2 gln2_tRNA + 50 glu1_tRNA + 46 gly1_tRNA + 1 glyU_tRNA + 1272 h + 8 hisR_tRNA + 43 ile1_tRNA + 1 ile2_tRNA + 45 leu1_tRNA + 1 leuU_tRNA + 2 leuW_tRNA + 49 lys1_tRNA + 14 met1_tRNA + 3185 mg2 + 15 phe1_tRNA + 1272 pi + 19 pro1_tRNA + 2 pro2_tRNA + 2 proM_tRNA + 1 rib_70_elo2_b0014_1_cplx + 6 ser1_tRNA + 14 ser2_tRNA + 5 serV_tRNA + 22 thr1_tRNA + 18 thr2_tRNA + 4 thr3_tRNA + 1 trpT_tRNA + 7 tyr1_tRNA + 20 val1_tRNA + 3 val2_tRNA + 24 val3_tRNA	irreversible	Translation	0.00E+00	1.00E+09	0	4.77E+01	4.77E+01	0	7.93E+01	79.27185	0	6.86E+00	6.861
tl_elo_b0014_37_rib1	Translation elongation 1 b0014 37 ribosome(s)	23569 EF-G.GTP + 2331 EF-Tu.GTP.ala1-tRNA + 259 EF-Tu.GTP.ala2-tRNA + 925 EF-Tu.GTP.arg1-tRNA + 962 EF-Tu.GTP.asn1-tRNA + 2035 EF-Tu.GTP.asp1-tRNA + 37 EF-Tu.GTP.cysT-tRNA + 1295 EF-Tu.GTP.gln1-tRNA + 74 EF-Tu.GTP.gln2-tRNA + 1850 EF-Tu.GTP.glu1-tRNA + 1702 EF-Tu.GTP.gly1-tRNA + 37 EF-Tu.GTP.glyU-tRNA + 296 EF-Tu.GTP.hisR-tRNA + 1591 EF-Tu.GTP.ile1-tRNA + 37 EF-Tu.GTP.ile2-tRNA + 1665 EF-Tu.GTP.leu1-tRNA + 37 EF-Tu.GTP.leuU-tRNA + 74 EF-Tu.GTP.leuW-tRNA + 1850 EF-Tu.GTP.lys1-tRNA + 518 EF-Tu.GTP.met1-tRNA + 555 EF-Tu.GTP.phe1-tRNA + 703 EF-Tu.GTP.pro1-tRNA + 74 EF-Tu.GTP.pro2-tRNA + 74 EF-Tu.GTP.proM-tRNA + 222 EF-Tu.GTP.ser1-tRNA + 518 EF-Tu.GTP.ser2-tRNA + 185 EF-Tu.GTP.serV-tRNA + 814 EF-Tu.GTP.thr1-tRNA + 666 EF-Tu.GTP.thr2-tRNA + 148 EF-Tu.GTP.thr3-tRNA + 37 EF-Tu.GTP.trpT-tRNA + 259 EF-Tu.GTP.tyr1-tRNA + 740 EF-Tu.GTP.val1-tRNA + 111 EF-Tu.GTP.val2-tRNA + 888 EF-Tu.GTP.val3-tRNA + 117845 mg2 + 1 rib_ini_b0014_37 --> 1 rib_70_elo1_b0014_37_cplx	irreversible	Translation	0.00E+00	1.00E+09	0	4.77E+01	4.77E+01	0	7.93E+01	79.27185	0	6.86E+00	6.861
tl_elo_b0014_37_rib2	Translation elongation 2 b0014 37 ribosome(s)	23532 h2o + 1 rib_70_elo1_b0014_37_cplx --> 23532 EF-G.GDP + 23532 EF-Tu.GDP + 2331 ala1_tRNA + 259 ala2_tRNA + 925 arg1_tRNA + 962 asn1_tRNA + 2035 asp1_tRNA + 37 cysT_tRNA + 37 fmet_tRNA + 1295 gln1_tRNA + 74 gln2_tRNA + 1850 glu1_tRNA + 1702 gly1_tRNA + 37 glyU_tRNA + 47064 h + 296 hisR_tRNA + 1591 ile1_tRNA + 37 ile2_tRNA + 1665 leu1_tRNA + 37 leuU_tRNA + 74 leuW_tRNA + 1813 lys1_tRNA + 518 met1_tRNA + 117845 mg2 + 555 phe1_tRNA + 47064 pi + 703 pro1_tRNA + 74 pro2_tRNA + 74 proM_tRNA + 1 rib_70_elo2_b0014_37_cplx + 222 ser1_tRNA + 518 ser2_tRNA + 185 serV_tRNA + 814 thr1_tRNA + 666 thr2_tRNA + 148 thr3_tRNA + 37 trpT_tRNA + 259 tyr1_tRNA + 740 val1_tRNA + 111 val2_tRNA + 888 val3_tRNA	irreversible	Translation	0.00E+00	1.00E+09	0	4.77E+01	4.77E+01	0	7.93E+01	79.27185	0	6.86E+00	6.861

tl_elo_b0015_11_rib1	Translation elongation 1 b0015 11 ribosome(s)	4125 EF-G.GTP + 264 EF-Tu.GTP.ala1-tRNA + 33 EF-Tu.GTP.ala2-tRNA + 275 EF-Tu.GTP.arg1-tRNA + 11 EF-Tu.GTP.argU-tRNA + 11 EF-Tu.GTP.argW-tRNA + 88 EF-Tu.GTP.asn1-tRNA + 220 EF-Tu.GTP.asp1-tRNA + 110 EF-Tu.GTP.cysT-tRNA + 187 EF-Tu.GTP.gln1-tRNA + 66 EF-Tu.GTP.gln2-tRNA + 341 EF-Tu.GTP.glu1-tRNA + 539 EF-Tu.GTP.gly1-tRNA + 33 EF-Tu.GTP.glyT-tRNA + 11 EF-Tu.GTP.glyU-tRNA + 110 EF-Tu.GTP.hisR-tRNA + 143 EF-Tu.GTP.ile1-tRNA + 165 EF-Tu.GTP.leu1-tRNA + 11 EF-Tu.GTP.leu2-tRNA + 44 EF-Tu.GTP.leuU-tRNA + 11 EF-Tu.GTP.leuW-tRNA + 22 EF-Tu.GTP.leuZ-tRNA + 286 EF-Tu.GTP.lys1-tRNA + 66 EF-Tu.GTP.met1-tRNA + 187 EF-Tu.GTP.phe1-tRNA + 132 EF-Tu.GTP.pro1-tRNA + 11 EF-Tu.GTP.pro2-tRNA + 33 EF-Tu.GTP.proM-tRNA + 22 EF-Tu.GTP.ser1-tRNA + 22 EF-Tu.GTP.ser2-tRNA + 11 EF-Tu.GTP.ser3-tRNA + 11 EF-Tu.GTP.serT-tRNA + 66 EF-Tu.GTP.serV-tRNA + 110 EF-Tu.GTP.thr1-tRNA + 33 EF-Tu.GTP.thr2-tRNA + 22 EF-Tu.GTP.thr3-tRNA + 33 EF-Tu.GTP.thrU-tRNA + 110 EF-Tu.GTP.tyr1-tRNA + 99 EF-Tu.GTP.val1-tRNA + 66 EF-Tu.GTP.val2-tRNA + 110 EF-Tu.GTP.val3-tRNA + 20625 mg2 + 1 rib_ini_b0015_11 --> 1 rib_70_elo1_b0015_11_cplx	irreversible	Translation	0.00E+00	1.00E+09	0	4.52E+01	4.52E+01	0	7.68E+01	76.81185	0	4.40E+00	4.401
tl_elo_b0015_11_rib2	Translation elongation 2 b0015 11 ribosome(s)	4114 h2o + 1 rib_70_elo1_b0015_11_cplx --> 4114 EF-G.GDP + 4114 EF-Tu.GDP + 264 ala1_tRNA + 33 ala2_tRNA + 264 arg1_tRNA + 11 argU_tRNA + 11 argW_tRNA + 88 asn1_tRNA + 220 asp1_tRNA + 110 cysT_tRNA + 11 fmet_tRNA + 187 gln1_tRNA + 66 gln2_tRNA + 341 glu1_tRNA + 539 gly1_tRNA + 33 glyT_tRNA + 11 glyU_tRNA + 8228 h + 110 hisR_tRNA + 143 ile1_tRNA + 165 leu1_tRNA + 11 leu2_tRNA + 44 leuU_tRNA + 11 leuW_tRNA + 22 leuZ_tRNA + 286 lys1_tRNA + 66 met1_tRNA + 20625 mg2 + 187 phe1_tRNA + 8228 pi + 132 pro1_tRNA + 11 pro2_tRNA + 33 proM_tRNA + 1 rib_70_elo2_b0015_11_cplx + 22 ser1_tRNA + 22 ser2_tRNA + 11 ser3_tRNA + 11 serT_tRNA + 66 serV_tRNA + 110 thr1_tRNA + 33 thr2_tRNA + 22 thr3_tRNA + 33 thrU_tRNA + 110 tyr1_tRNA + 99 val1_tRNA + 66 val2_tRNA + 110 val3_tRNA	irreversible	Translation	0.00E+00	1.00E+09	0	4.52E+01	4.52E+01	0	7.68E+01	76.81185	0	4.40E+00	4.401
tl_elo_b0015_1_rib1	Translation elongation 1 b0015 1 ribosome(s)	375 EF-G.GTP + 24 EF-Tu.GTP.ala1-tRNA + 3 EF-Tu.GTP.ala2-tRNA + 25 EF-Tu.GTP.arg1-tRNA + 1 EF-Tu.GTP.argU-tRNA + 1 EF Tu.GTP.argW-tRNA + 8 EF-Tu.GTP.asn1-tRNA + 20 EF-Tu.GTP.asp1-tRNA + 10 EF-Tu.GTP.cysT-tRNA + 17 EF-Tu.GTP.gln1-tRNA + 6 EF-Tu.GTP.gln2-tRNA + 31 EF-Tu.GTP.glu1-tRNA + 49 EF-Tu.GTP.gly1-tRNA + 3 EF-Tu.GTP.glyT-tRNA + 1 EF-Tu.GTP.glyU-tRNA + 10 EF-Tu.GTP.hisR-tRNA + 13 EF-Tu.GTP.ile1 tRNA + 15 EF-Tu.GTP.leu1-tRNA + 1 EF-Tu.GTP.leu2-tRNA + 4 EF-Tu.GTP.leuU-tRNA + 1 EF-Tu.GTP.leuW-tRNA + 2 EF-Tu.GTP.leuZ-tRNA + 26 EF-Tu.GTP.lys1-tRNA + 6 EF-Tu.GTP.met1-tRNA + 17 EF-Tu.GTP.phe1-tRNA + 12 EF-Tu.GTP.pro1-tRNA + 1 EF-Tu.GTP.pro2-tRNA + 3 EF-Tu.GTP.proM-tRNA + 2 EF-Tu.GTP.ser1-tRNA + 2 EF-Tu.GTP.ser2-tRNA + 1 EF-Tu.GTP.ser3-tRNA + 1 EF-Tu.GTP.serT-tRNA + 6 EF-Tu.GTP.serV-tRNA + 10 EF-Tu.GTP.thr1-tRNA + 3 EF-Tu.GTP.thr2-tRNA + 2 EF-Tu.GTP.thr3-tRNA + 3 EF-Tu.GTP.thrU-tRNA + 10 EF-Tu.GTP.tyr1-tRNA + 9 EF-Tu.GTP.val1-tRNA + 6 EF-Tu.GTP.val2-tRNA + 10 EF-Tu.GTP.val3-tRNA + 1875 mg2 + 1 rib_ini_b0015_1 --> 1 rib_70_elo1_b0015_1_cplx	irreversible	Translation	0.00E+00	1.00E+09	0	4.52E+01	4.52E+01	0	7.68E+01	76.81185	0	4.40E+00	4.401
tl_elo_b0015_1_rib2	Translation elongation 2 b0015 1 ribosome(s)	374 h2o + 1 rib_70_elo1_b0015_1_cplx --> 374 EF-G.GDP + 374 EF-Tu.GDP + 24 ala1_tRNA + 3 ala2_tRNA + 24 arg1_tRNA + 1 argU_tRNA + 1 argW_tRNA + 8 asn1_tRNA + 20 asp1_tRNA + 10 cysT_tRNA + 1 fmet_tRNA + 17 gln1_tRNA + 6 gln2_tRNA + 31 glu1_tRNA + 49 gly1_tRNA + 3 glyT_tRNA + 1 glyU_tRNA + 748 h + 10 hisR_tRNA + 13 ile1_tRNA + 15 leu1_tRNA + 1 leu2_tRNA + 4 leuU_tRNA + 1 leuW_tRNA + 2 leuZ_tRNA + 26 lys1_tRNA + 6 met1_tRNA + 1875 mg2 + 17 phe1_tRNA + 748 pi + 12 pro1_tRNA + 1 pro2_tRNA + 3 proM_tRNA + 1 rib_70_elo2_b0015_1_cplx + 2 ser1_tRNA + 2 ser2_tRNA + 1 ser3_tRNA + 1 serT_tRNA + 6 serV_tRNA + 10 thr1_tRNA + 3 thr2_tRNA + 2 thr3_tRNA + 3 thrU_tRNA + 10 tyr1_tRNA + 9 val1_tRNA + 6 val2_tRNA + 10 val3_tRNA	irreversible	Translation	0.00E+00	1.00E+09	0	4.52E+01	4.52E+01	0	7.68E+01	76.81185	0	4.40E+00	4.401
tl_elo_b0015_22_rib1	Translation elongation 1 b0015 22 ribosome(s)	8250 EF-G.GTP + 528 EF-Tu.GTP.ala1-tRNA + 66 EF-Tu.GTP.ala2-tRNA + 550 EF-Tu.GTP.arg1-tRNA + 22 EF-Tu.GTP.argU-tRNA + 22 EF-Tu.GTP.argW-tRNA + 176 EF-Tu.GTP.asn1-tRNA + 440 EF-Tu.GTP.asp1-tRNA + 220 EF-Tu.GTP.cysT-tRNA + 374 EF-Tu.GTP.gln1-tRNA + 132 EF-Tu.GTP.gln2-tRNA + 682 EF-Tu.GTP.glu1-tRNA + 1078 EF-Tu.GTP.gly1-tRNA + 66 EF-Tu.GTP.glyT-tRNA + 22 EF-Tu.GTP.glyU-tRNA + 220 EF-Tu.GTP.hisR-tRNA + 286 EF-Tu.GTP.ile1-tRNA + 330 EF-Tu.GTP.leu1-tRNA + 22 EF-Tu.GTP.leu2-tRNA + 88 EF-Tu.GTP.leuU-tRNA + 22 EF-Tu.GTP.leuW-tRNA + 44 EF-Tu.GTP.leuZ-tRNA + 572 EF-Tu.GTP.lys1-tRNA + 132 EF-Tu.GTP.met1-tRNA + 374 EF-Tu.GTP.phe1-tRNA + 264 EF-Tu.GTP.pro1-tRNA + 22 EF-Tu.GTP.pro2-tRNA + 66 EF-Tu.GTP.proM-tRNA + 44 EF-Tu.GTP.ser1-tRNA + 44 EF-Tu.GTP.ser2-tRNA + 22 EF-Tu.GTP.ser3-tRNA + 22 EF-Tu.GTP.serT-tRNA + 132 EF-Tu.GTP.serV-tRNA + 220 EF-Tu.GTP.thr1-tRNA + 66 EF-Tu.GTP.thr2-tRNA + 44 EF-Tu.GTP.thr3-tRNA + 66 EF-Tu.GTP.thrU-tRNA + 220 EF-Tu.GTP.tyr1-tRNA + 198 EF-Tu.GTP.val1-tRNA + 132 EF-Tu.GTP.val2-tRNA + 220 EF-Tu.GTP.val3-tRNA + 41250 mg2 + 1 rib_ini_b0015_22 --> 1 rib_70_elo1_b0015_22_cplx	irreversible	Translation	0.00E+00	1.00E+09	0	4.52E+01	4.52E+01	0	7.68E+01	76.81185	0	4.40E+00	4.401
tl_elo_b0015_22_rib2	Translation elongation 2 b0015 22 ribosome(s)	8228 h2o + 1 rib_70_elo1_b0015_22_cplx --> 8228 EF-G.GDP + 8228 EF-Tu.GDP + 528 ala1_tRNA + 66 ala2_tRNA + 528 arg1_tRNA + 22 argU_tRNA + 22 argW_tRNA + 176 asn1_tRNA + 440 asp1_tRNA + 220 cysT_tRNA + 22 fmet_tRNA + 374 gln1_tRNA + 132 gln2_tRNA + 682 glu1_tRNA + 1078 gly1_tRNA + 66 glyT_tRNA + 22 glyU_tRNA + 16456 h + 220 hisR_tRNA + 286 ile1_tRNA + 330 leu1_tRNA + 22 leu2_tRNA + 88 leuU_tRNA + 22 leuW_tRNA + 44 leuZ_tRNA + 572 lys1_tRNA + 132 met1_tRNA + 41250 mg2 + 374 phe1_tRNA + 16456 pi + 264 pro1_tRNA + 22 pro2_tRNA + 66 proM_tRNA + 1 rib_70_elo2_b0015_22_cplx + 44 ser1_tRNA + 44 ser2_tRNA + 22 ser3_tRNA + 22 serT_tRNA + 132 serV_tRNA + 220 thr1_tRNA + 66 thr2_tRNA + 44 thr3_tRNA + 66 thrU_tRNA + 220 tyr1_tRNA + 198 val1_tRNA + 132 val2_tRNA + 22 val3_tRNA	irreversible	Translation	0.00E+00	1.00E+09	0	4.52E+01	4.52E+01	0	7.68E+01	76.81185	0	4.40E+00	4.401

tl_elo_b0023_1_rib1	Translation elongation 1 b0023 1 ribosome(s)	86 EF-G.GTP + 18 EF-Tu.GTP.ala1-tRNA + 1 EF-Tu.GTP.ala2-tRNA + 7 EF-Tu.GTP.arg1-tRNA + 6 EF-Tu.GTP.asn1-tRNA + 2 EF-Tu.GTP.asp1-tRNA + 4 EF-Tu.GTP.gln1-tRNA + 1 EF-Tu.GTP.gln2-tRNA + 3 EF-Tu.GTP.glu1-tRNA + 2 EF-Tu.GTP.gly1-tRNA + 3 EF-Tu.GTP.hisR-tRNA + 7 EF-Tu.GTP.ile1-tRNA + 3 EF-Tu.GTP.leu1-tRNA + 14 EF-Tu.GTP.lys1-tRNA + 3 EF-Tu.GTP.met1-tRNA + 2 EF-Tu.GTP.phe1-tRNA + 1 EF-Tu.GTP.pro1-tRNA + 2 EF-Tu.GTP.ser2-tRNA + 1 EF-Tu.GTP.serT-tRNA + 1 EF-Tu.GTP.serV-tRNA + 2 EF-Tu.GTP.thr2-tRNA + 1 EF-Tu.GTP.tyr1-tRNA + 2 EF-Tu.GTP.val1-tRNA + 430 mg2 + 1 rib_ini_b0023_1 --> 1 rib_70_elo1_b0023_1_cp	irreversible	Translation	0.00E+00	1.00E+09	0	1.13E+02	1.13E+02	0	8.29E+01	82.88	0	8.29E+01	82.88
tl_elo_b0023_1_rib2	Translation elongation 2 b0023 1 ribosome(s)	85 h2o + 1 rib_70_elo1_b0023_1_cplx --> 85 EF-G.GDP + 85 EF-Tu.GDP + 17 ala1_tRNA + 1 ala2_tRNA + 7 arg1_tRNA + 6 asn1_tRNA + 2 asp1_tRNA + 1 fmet_tRNA + 4 gln1_tRNA + 1 gln2_tRNA + 3 glu1_tRNA + 2 gly1_tRNA + 170 h + 3 hisR_tRNA + 7 ile1_tRNA + 3 leu1_tRNA + 14 lys1_tRNA + 3 met1_tRNA + 430 mg2 + 2 phe1_tRNA + 170 pi + 1 pro1_tRNA + 1 rib_70_elo2_b0023_1_cplx + 2 ser2_tRNA + 1 serT_tRNA + 1 serV_tRNA + 2 thr2_tRNA + 1 tyr1_tRNA + 2 val1_tRNA	irreversible	Translation	0.00E+00	1.00E+09	0	1.13E+02	1.13E+02	0	8.29E+01	82.88	0	8.29E+01	82.88
tl_elo_b0023_2_rib1	Translation elongation 1 b0023 2 ribosome(s)	172 EF-G.GTP + 36 EF-Tu.GTP.ala1-tRNA + 2 EF-Tu.GTP.ala2-tRNA + 14 EF-Tu.GTP.arg1-tRNA + 12 EF-Tu.GTP.asn1-tRNA + 4 EF-Tu.GTP.asp1-tRNA + 8 EF-Tu.GTP.gln1-tRNA + 2 EF-Tu.GTP.gln2-tRNA + 6 EF-Tu.GTP.glu1-tRNA + 4 EF-Tu.GTP.gly1-tRNA + 6 EF-Tu.GTP.hisR-tRNA + 14 EF-Tu.GTP.ile1-tRNA + 6 EF-Tu.GTP.leu1-tRNA + 28 EF-Tu.GTP.lys1-tRNA + 6 EF-Tu.GTP.met1-tRNA + 4 EF-Tu.GTP.phe1-tRNA + 2 EF-Tu.GTP.pro1-tRNA + 4 EF-Tu.GTP.ser2-tRNA + 2 EF-Tu.GTP.serT-tRNA + 2 EF-Tu.GTP.serV-tRNA + 4 EF-Tu.GTP.thr2-tRNA + 2 EF-Tu.GTP.tyr1-tRNA + 4 EF-Tu.GTP.val1-tRNA + 860 mg2 + 1 rib_ini_b0023_2 --> 1 rib_70_elo1_b0023_2_cplx	irreversible	Translation	0.00E+00	1.00E+09	0	5.63E+01	5.63E+01	0	4.14E+01	41.44	0	4.14E+01	41.44
tl_elo_b0023_2_rib2	Translation elongation 2 b0023 2 ribosome(s)	170 h2o + 1 rib_70_elo1_b0023_2_cplx --> 170 EF-G.GDP + 170 EF-Tu.GDP + 34 ala1_tRNA + 2 ala2_tRNA + 14 arg1_tRNA + 12 asn1_tRNA + 4 asp1_tRNA + 2 fmet_tRNA + 8 gln1_tRNA + 2 gln2_tRNA + 6 glu1_tRNA + 4 gly1_tRNA + 340 h + 6 hisR_tRNA + 14 ile1_tRNA + 6 leu1_tRNA + 28 lys1_tRNA + 6 met1_tRNA + 860 mg2 + 4 phe1_tRNA + 340 pi + 2 pro1_tRNA + 1 rib_70_elo2_b0023_2_cplx + 4 ser2_tRNA + 2 serT_tRNA + 2 serV_tRNA + 4 thr2_tRNA + 2 tyr1_tRNA + 4 val1_tRNA	irreversible	Translation	0.00E+00	1.00E+09	0	5.63E+01	5.63E+01	0	4.14E+01	41.44	0	4.14E+01	41.44
tl_elo_b0023_5_rib1	Translation elongation 1 b0023 5 ribosome(s)	430 EF-G.GTP + 90 EF-Tu.GTP.ala1-tRNA + 5 EF-Tu.GTP.ala2-tRNA + 35 EF-Tu.GTP.arg1-tRNA + 30 EF-Tu.GTP.asn1-tRNA + 10 EF-Tu.GTP.asp1-tRNA + 20 EF-Tu.GTP.gln1-tRNA + 5 EF-Tu.GTP.gln2-tRNA + 15 EF-Tu.GTP.glu1-tRNA + 10 EF-Tu.GTP.gly1-tRNA + 15 EF-Tu.GTP.hisR-tRNA + 35 EF-Tu.GTP.ile1-tRNA + 15 EF-Tu.GTP.leu1-tRNA + 70 EF-Tu.GTP.lys1-tRNA + 15 EF-Tu.GTP.met1-tRNA + 10 EF-Tu.GTP.phe1-tRNA + 5 EF-Tu.GTP.pro1-tRNA + 10 EF-Tu.GTP.ser2-tRNA + 5 EF-Tu.GTP.serT-tRNA + 5 EF-Tu.GTP.serV-tRNA + 10 EF-Tu.GTP.thr2-tRNA + 5 EF-Tu.GTP.tyr1-tRNA + 10 EF-Tu.GTP.val1-tRNA + 2150 mg2 + 1 rib_ini_b0023_5 --> 1 rib_70_elo1_b0023_5_cplx	irreversible	Translation	0.00E+00	1.00E+09	0	2.25E+01	2.25E+01	0	1.66E+01	16.576	0	1.66E+01	16.576
tl_elo_b0023_5_rib2	Translation elongation 2 b0023 5 ribosome(s)	425 h2o + 1 rib_70_elo1_b0023_5_cplx --> 425 EF-G.GDP + 425 EF-Tu.GDP + 85 ala1_tRNA + 5 ala2_tRNA + 35 arg1_tRNA + 30 asn1_tRNA + 10 asp1_tRNA + 5 fmet_tRNA + 20 gln1_tRNA + 5 gln2_tRNA + 15 glu1_tRNA + 10 gly1_tRNA + 850 h + 15 hisR_tRNA + 35 ile1_tRNA + 15 leu1_tRNA + 70 lys1_tRNA + 15 met1_tRNA + 2150 mg2 + 10 phe1_tRNA + 850 pi + 5 pro1_tRNA + 1 rib_70_elo2_b0023_5_cplx + 10 ser2_tRNA + 5 serT_tRNA + 5 serV_tRNA + 10 thr2_tRNA + 5 tyr1_tRNA + 10 val1_tRNA	irreversible	Translation	0.00E+00	1.00E+09	0	2.25E+01	2.25E+01	0	1.66E+01	16.576	0	1.66E+01	16.576
tl_elo_b0025_18_rib1	Translation elongation 1 b0025 18 ribosome(s)	5616 EF-G.GTP + 360 EF-Tu.GTP.ala1-tRNA + 144 EF-Tu.GTP.ala2-tRNA + 450 EF-Tu.GTP.arg1-tRNA + 18 EF-Tu.GTP.argU-tRNA + 18 EF-Tu.GTP.argW-tRNA + 36 EF-Tu.GTP.argX-tRNA + 144 EF-Tu.GTP.asn1-tRNA + 288 EF-Tu.GTP.asp1-tRNA + 72 EF-Tu.GTP.cysT-tRNA + 180 EF-Tu.GTP.gln1-tRNA + 90 EF-Tu.GTP.gln2-tRNA + 342 EF-Tu.GTP.glu1-tRNA + 378 EF-Tu.GTP.gly1-tRNA + 18 EF-Tu.GTP.glyT-tRNA + 126 EF-Tu.GTP.glyU-tRNA + 144 EF-Tu.GTP.hisR-tRNA + 144 EF-Tu.GTP.ile1-tRNA + 90 EF-Tu.GTP.ile2-tRNA + 342 EF-Tu.GTP.leu1-tRNA + 72 EF-Tu.GTP.leu2-tRNA + 144 EF-Tu.GTP.leuU-tRNA + 18 EF-Tu.GTP.leuW-tRNA + 180 EF-Tu.GTP.leuZ-tRNA + 234 EF-Tu.GTP.lys1-tRNA + 72 EF-Tu.GTP.met1-tRNA + 306 EF-Tu.GTP.phe1-tRNA + 162 EF-Tu.GTP.pro1-tRNA + 18 EF-Tu.GTP.pro2-tRNA + 18 EF-Tu.GTP.proL-tRNA + 36 EF-Tu.GTP.proM-tRNA + 36 EF-Tu.GTP.ser1-tRNA + 18 EF-Tu.GTP.ser3-tRNA + 108 EF-Tu.GTP.serV-tRNA + 108 EF-Tu.GTP.thr1-tRNA + 54 EF-Tu.GTP.thr2-tRNA + 54 EF-Tu.GTP.thr3-tRNA + 36 EF-Tu.GTP.thrU-tRNA + 90 EF-Tu.GTP.tyr1-tRNA + 360 EF-Tu.GTP.val1-tRNA + 18 EF-Tu.GTP.val2-tRNA + 90 EF-Tu.GTP.val3-tRNA + 28080 mg2 + 1 rib_ini_b0025_18 --> 1 rib_70_e	irreversible	Translation	0.00E+00	1.00E+09	0	2.86E+01	2.86E+01	0	4.50E+01	44.98865	0	7.24E+00	7.2445
tl_elo_b0025_18_rib2	Translation elongation 2 b0025 18 ribosome(s)	5598 h2o + 1 rib_70_elo1_b0025_18_cplx --> 5598 EF-G.GDP + 5598 EF-Tu.GDP + 342 ala1_tRNA + 144 ala2_tRNA + 450 arg1_tRNA + 18 argU_tRNA + 18 argW_tRNA + 36 argX_tRNA + 144 asn1_tRNA + 288 asp1_tRNA + 72 cysT_tRNA + 18 fmet_tRNA + 180 gln1_tRNA + 90 gln2_tRNA + 342 glu1_tRNA + 378 gly1_tRNA + 18 glyT_tRNA + 126 glyU_tRNA + 11196 h + 144 hisR_tRNA + 144 ile1_tRNA + 90 ile2_tRNA + 342 leu1_tRNA + 72 leu2_tRNA + 144 leuU_tRNA + 18 leuW_tRNA + 180 leuZ_tRNA + 234 lys1_tRNA + 72 met1_tRNA + 28080 mg2 + 306 phe1_tRNA + 11196 pi + 162 pro1_tRNA + 18 pro2_tRNA + 18 proL_tRNA + 36 proM_tRNA + 1 rib_70_elo2_b0025_18_cplx + 36 ser1_tRNA + 18 ser3_tRNA + 108 serV_tRNA + 108 thr1_tRNA + 54 thr2_tRNA + 54 thr3_tRNA + 36 thrU_tRNA + 90 tyr1_tRNA + 360 val1_tRNA + 18 val2_tRNA + 90 val3_tRNA	irreversible	Translation	0.00E+00	1.00E+09	0	2.86E+01	2.86E+01	0	4.50E+01	44.98865	0	7.24E+00	7.2445

tl_elo_b0025_1_rib1	Translation elongation 1 b0025 1 ribosome(s)	312 EF-G.GTP + 20 EF-Tu.GTP.ala1-tRNA + 8 EF-Tu.GTP.ala2-tRNA + 25 EF-Tu.GTP.arg1-tRNA + 1 EF-Tu.GTP.argU-tRNA + 1 EF Tu.GTP.argW-tRNA + 2 EF-Tu.GTP.argX-tRNA + 8 EF-Tu.GTP.asn1-tRNA + 16 EF-Tu.GTP.asp1-tRNA + 4 EF-Tu.GTP.cysT-tRNA + 10 EF-Tu.GTP.gln1-tRNA + 5 EF-Tu.GTP.gln2-tRNA + 19 EF-Tu.GTP.glu1-tRNA + 21 EF-Tu.GTP.gly1-tRNA + 1 EF Tu.GTP.glyT-tRNA + 7 EF-Tu.GTP.glyU-tRNA + 8 EF-Tu.GTP.hisR-tRNA + 8 EF-Tu.GTP.ile1-tRNA + 5 EF-Tu.GTP.ile2-tRNA + 19 EF-Tu.GTP.leu1-tRNA + 4 EF-Tu.GTP.leu2-tRNA + 8 EF-Tu.GTP.leuU-tRNA + 1 EF-Tu.GTP.leuW-tRNA + 10 EF-Tu.GTP.leuZ-tRNA + 13 EF-Tu.GTP.lys1-tRNA + 4 EF-Tu.GTP.met1-tRNA + 17 EF-Tu.GTP.phe1-tRNA + 9 EF-Tu.GTP.pro1-tRNA + 1 EF-Tu.GTP.pro2-tRNA + 1 EF-Tu.GTP.proL-tRNA + 2 EF-Tu.GTP.proM-tRNA + 2 EF-Tu.GTP.ser1-tRNA + 1 EF-Tu.GTP.ser3-tRNA + 6 EF-Tu.GTP.serV-tRNA + 6 EF-Tu.GTP.thr1-tRNA + 3 EF-Tu.GTP.thr2-tRNA + 3 EF-Tu.GTP.thr3-tRNA + 2 EF-Tu.GTP.thrU-tRNA + 5 EF-Tu.GTP.tyr1-tRNA + 20 EF-Tu.GTP.val1-tRNA + 1 EF-Tu.GTP.val2-tRNA + 5 EF-Tu.GTP.val3-tRNA + 1560 mg2 + 1 rib_ini_b0025_1 --> 1 rib_70_elo1_b0025_1_cplx	irreversible	Translation	0.00E+00	1.00E+09	0	2.86E+01	2.86E+01	0	4.50E+01	44.98865	0	7.24E+00	7.2445
tl_elo_b0025_1_rib2	Translation elongation 2 b0025 1 ribosome(s)	311 h2o + 1 rib_70_elo1_b0025_1_cplx --> 311 EF-G.GDP + 311 EF-Tu.GDP + 19 ala1_tRNA + 8 ala2_tRNA + 25 arg1_tRNA + 1 argU_tRNA + 1 argW_tRNA + 2 argX_tRNA + 8 asn1_tRNA + 16 asp1_tRNA + 4 cysT_tRNA + 1 fmet_tRNA + 10 gln1_tRNA + 5 gln2_tRNA + 19 glu1_tRNA + 21 gly1_tRNA + 1 glyT_tRNA + 7 glyU_tRNA + 622 h + 8 hisR_tRNA + 8 ile1_tRNA + 5 ile2_tRNA + 19 leu1_tRNA + 4 leu2_tRNA + 8 leuU_tRNA + 1 leuW_tRNA + 10 leuZ_tRNA + 13 lys1_tRNA + 4 met1_tRNA + 1560 mg2 + 17 phe1_tRNA + 622 pi + 9 pro1_tRNA + 1 pro2_tRNA + 1 proL_tRNA + 2 proM_tRNA + 1 rib_70_elo2_b0025_1_cplx + 2 ser1_tRNA + 1 ser3_tRNA + 6 serV_tRNA + 6 thr1_tRNA + 3 thr2_tRNA + 3 thr3_tRNA + 2 thrU_tRNA + 5 tyr1_tRNA + 20 val1_tRNA + 1 val2_tRNA + 5 val3_tRNA	irreversible	Translation	0.00E+00	1.00E+09	0	2.86E+01	2.86E+01	0	4.50E+01	44.98865	0	7.24E+00	7.2445
tl_elo_b0025_9_rib1	Translation elongation 1 b0025 9 ribosome(s)	2808 EF-G.GTP + 180 EF-Tu.GTP.ala1-tRNA + 72 EF-Tu.GTP.ala2-tRNA + 225 EF-Tu.GTP.arg1-tRNA + 9 EF-Tu.GTP.argU-tRNA + 9 EF-Tu.GTP.argW-tRNA + 18 EF-Tu.GTP.argX-tRNA + 72 EF-Tu.GTP.asn1-tRNA + 144 EF-Tu.GTP.asp1-tRNA + 36 EF-Tu.GTP.cysT-tRNA + 90 EF-Tu.GTP.gln1-tRNA + 45 EF-Tu.GTP.gln2-tRNA + 171 EF-Tu.GTP.glu1-tRNA + 189 EF-Tu.GTP.gly1-tRNA + 9 EF-Tu.GTP.glyT-tRNA + 63 EF-Tu.GTP.glyU-tRNA + 72 EF-Tu.GTP.hisR-tRNA + 72 EF-Tu.GTP.ile1-tRNA + 45 EF-Tu.GTP.ile2-tRNA + 171 EF-Tu.GTP.leu1-tRNA + 36 EF-Tu.GTP.leu2-tRNA + 72 EF-Tu.GTP.leuU-tRNA + 9 EF-Tu.GTP.leuW-tRNA + 90 EF-Tu.GTP.leuZ-tRNA + 117 EF-Tu.GTP.lys1-tRNA + 36 EF-Tu.GTP.met1-tRNA + 153 EF-Tu.GTP.phe1-tRNA + 81 EF-Tu.GTP.pro1-tRNA + 9 EF-Tu.GTP.pro2-tRNA + 9 EF-Tu.GTP.proL-tRNA + 18 EF-Tu.GTP.proM-tRNA + 18 EF-Tu.GTP.ser1-tRNA + 9 EF-Tu.GTP.ser3-tRNA + 54 EF-Tu.GTP.serV-tRNA + 54 EF-Tu.GTP.thr1-tRNA + 27 EF-Tu.GTP.thr2-tRNA + 27 EF-Tu.GTP.thr3-tRNA + 18 EF-Tu.GTP.thrU-tRNA + 45 EF-Tu.GTP.tyr1-tRNA + 180 EF-Tu.GTP.val1-tRNA + 9 EF-Tu.GTP.val2-tRNA + 45 EF-Tu.GTP.val3-tRNA + 14040 mg2 + 1 rib_ini_b0025_9 --> 1 rib_70_elo1_b0025_9_cplx	irreversible	Translation	0.00E+00	1.00E+09	0	2.86E+01	2.86E+01	0	4.50E+01	44.98865	0	7.24E+00	7.2445
tl_elo_b0025_9_rib2	Translation elongation 2 b0025 9 ribosome(s)	2799 h2o + 1 rib_70_elo1_b0025_9_cplx --> 2799 EF-G.GDP + 2799 EF-Tu.GDP + 171 ala1_tRNA + 72 ala2_tRNA + 225 arg1_tRNA + 9 argU_tRNA + 9 argW_tRNA + 18 argX_tRNA + 72 asn1_tRNA + 144 asp1_tRNA + 36 cysT_tRNA + 9 fmet_tRNA + 90 gln1_tRNA + 45 gln2_tRNA + 171 glu1_tRNA + 189 gly1_tRNA + 9 glyT_tRNA + 63 glyU_tRNA + 5598 h + 72 hisR_tRNA + 72 ile1_tRNA + 45 ile2_tRNA + 171 leu1_tRNA + 36 leu2_tRNA + 72 leuU_tRNA + 9 leuW_tRNA + 90 leuZ_tRNA + 117 lys1_tRNA + 36 met1_tRNA + 14040 mg2 + 153 phe1_tRNA + 5598 pi + 81 pro1_tRNA + 9 pro2_tRNA + 9 proL_tRNA + 18 proM_tRNA + 1 rib_70_elo2_b0025_9_cplx + 18 ser1_tRNA + 9 ser3_tRNA + 54 serV_tRNA + 54 thr1_tRNA + 27 thr2_tRNA + 27 thr3_tRNA + 18 thrU_tRNA + 45 tyr1_tRNA + 180 val1_tRNA + 9 val2_tRNA + 45 val3_tRNA	irreversible	Translation	0.00E+00	1.00E+09	0	2.86E+01	2.86E+01	0	4.50E+01	44.98865	0	7.24E+00	7.2445
tl_elo_b0026_1_rib1	Translation elongation 1 b0026 1 ribosome(s)	937 EF-G.GTP + 73 EF-Tu.GTP.ala1-tRNA + 19 EF-Tu.GTP.ala2-tRNA + 46 EF-Tu.GTP.arg1-tRNA + 21 EF-Tu.GTP.asn1-tRNA + 71 EF-Tu.GTP.asp1-tRNA + 14 EF-Tu.GTP.cysT-tRNA + 27 EF-Tu.GTP.gln1-tRNA + 4 EF-Tu.GTP.gln2-tRNA + 58 EF-Tu.GTP.glu1-tRNA + 70 EF-Tu.GTP.gly1-tRNA + 2 EF-Tu.GTP.glyT-tRNA + 3 EF-Tu.GTP.glyU-tRNA + 25 EF-Tu.GTP.hisR-tRNA + 46 EF-Tu.GTP.ile1-tRNA + 56 EF-Tu.GTP.leu1-tRNA + 6 EF-Tu.GTP.leu2-tRNA + 12 EF Tu.GTP.leuU-tRNA + 2 EF-Tu.GTP.leuW-tRNA + 1 EF-Tu.GTP.leuZ-tRNA + 61 EF-Tu.GTP.lys1-tRNA + 23 EF-Tu.GTP.met1-tRNA + 31 EF-Tu.GTP.phe1-tRNA + 28 EF-Tu.GTP.pro1-tRNA + 7 EF-Tu.GTP.pro2-tRNA + 1 EF-Tu.GTP.proL-tRNA + 6 EF-Tu.GTP.proM-tRNA + 12 EF-Tu.GTP.ser1-tRNA + 10 EF-Tu.GTP.ser2-tRNA + 8 EF-Tu.GTP.ser3-tRNA + 4 EF-Tu.GTP.serT-tRNA + 13 EF-Tu.GTP.serV-tRNA + 32 EF-Tu.GTP.thr1-tRNA + 7 EF-Tu.GTP.thr2-tRNA + 6 EF-Tu.GTP.thr3-tRNA + 2 EF-Tu.GTP.thrU-tRNA + 23 EF-Tu.GTP.trpT-tRNA + 33 EF-Tu.GTP.tyr1-tRNA + 39 EF-Tu.GTP.val1-tRNA + 13 EF-Tu.GTP.val2-tRNA + 22 EF-Tu.GTP.val3-tRNA + 4685 mg2 + 1 rib_ini_b0026_1 --> 1 rib_70_elo1_b0026_1_cplx	irreversible	Translation	0.00E+00	1.00E+09	0	2.32E+01	2.32E+01	0	3.96E+01	39.62865	0	1.88E+00	1.8845
tl_elo_b0026_1_rib2	Translation elongation 2 b0026 1 ribosome(s)	936 h2o + 1 rib_70_elo1_b0026_1_cplx --> 936 EF-G.GDP + 936 EF-Tu.GDP + 73 ala1_tRNA + 18 ala2_tRNA + 46 arg1_tRNA + 21 asn1_tRNA + 71 asp1_tRNA + 14 cysT_tRNA + 1 fmet_tRNA + 27 gln1_tRNA + 4 gln2_tRNA + 58 glu1_tRNA + 70 gly1_tRNA + 2 glyT_tRNA + 3 glyU_tRNA + 1872 h + 25 hisR_tRNA + 46 ile1_tRNA + 56 leu1_tRNA + 6 leu2_tRNA + 12 leuU_tRNA + 2 leuW_tRNA + 1 leuZ_tRNA + 61 lys1_tRNA + 23 met1_tRNA + 4685 mg2 + 31 phe1_tRNA + 1872 pi + 28 pro1_tRNA + 7 pro2_tRNA + 1 proL_tRNA + 6 proM_tRNA + 1 rib_70_elo2_b0026_1_cplx + 12 ser1_tRNA + 10 ser2_tRNA + 8 ser3_tRNA + 4 serT_tRNA + 13 serV_tRNA + 32 thr1_tRNA + 7 thr2_tRNA + 6 thr3_tRNA + 2 thrU_tRNA + 23 trpT_tRNA + 33 tyr1_tRNA + 39 val1_tRNA + 13 val2_tRNA + 22 val3_tRNA	irreversible	Translation	0.00E+00	1.00E+09	0	2.32E+01	2.32E+01	0	3.96E+01	39.62865	0	1.88E+00	1.8845

tl_elo_b0026_27_rib1	Translation elongation 1 b0026 27 ribosome(s)	25299 EF-G.GTP + 1971 EF-Tu.GTP.ala1-tRNA + 513 EF-Tu.GTP.ala2-tRNA + 1242 EF-Tu.GTP.arg1-tRNA + 567 EF-Tu.GTP.asn1-tRNA + 1917 EF-Tu.GTP.asp1-tRNA + 378 EF-Tu.GTP.cysT-tRNA + 729 EF-Tu.GTP.gln1-tRNA + 108 EF-Tu.GTP.gln2-tRNA + 1566 EF-Tu.GTP.glu1-tRNA + 1890 EF-Tu.GTP.gly1-tRNA + 54 EF-Tu.GTP.glyT-tRNA + 81 EF-Tu.GTP.glyU-tRNA + 675 EF-Tu.GTP.hisR-tRNA + 1242 EF-Tu.GTP.ile1-tRNA + 1512 EF-Tu.GTP.leu1-tRNA + 162 EF-Tu.GTP.leu2-tRNA + 324 EF-Tu.GTP.leuU-tRNA + 54 EF-Tu.GTP.leuW-tRNA + 27 EF-Tu.GTP.leuZ-tRNA + 1647 EF-Tu.GTP.lys1-tRNA + 621 EF-Tu.GTP.met1-tRNA + 837 EF-Tu.GTP.phe1-tRNA + 756 EF-Tu.GTP.pro1-tRNA + 189 EF-Tu.GTP.pro2-tRNA + 27 EF-Tu.GTP.proL-tRNA + 162 EF-Tu.GTP.proM-tRNA + 324 EF-Tu.GTP.ser1-tRNA + 270 EF-Tu.GTP.ser2-tRNA + 216 EF-Tu.GTP.ser3-tRNA + 108 EF-Tu.GTP.serT-tRNA + 351 EF-Tu.GTP.serV-tRNA + 864 EF-Tu.GTP.thr1-tRNA + 189 EF-Tu.GTP.thr2-tRNA + 162 EF-Tu.GTP.thr3-tRNA + 54 EF-Tu.GTP.thrU-tRNA + 621 EF-Tu.GTP.trpT-tRNA + 891 EF-Tu.GTP.tyr1-tRNA + 1053 EF-Tu.GTP.val1-tRNA + 351 EF-Tu.GTP.val2-tRNA + 594 EF-Tu.GTP.val3-tRNA + 126495 mg2 + 1 rib_ini_b0026_27 --> 1 rib_70_	irreversible	Translation	0.00E+00	1.00E+09	0	2.32E+01	2.32E+01	0	3.96E+01	39.62865	0	1.88E+00	1.8845
tl_elo_b0026_27_rib2	Translation elongation 2 b0026 27 ribosome(s)	25272 h2o + 1 rib_70_elo1_b0026_27_cplx --> 25272 EF-G.GDP + 25272 EF-Tu.GDP + 1971 ala1_tRNA + 486 ala2_tRNA + 1242 arg1_tRNA + 567 asn1_tRNA + 1917 asp1_tRNA + 378 cysT_tRNA + 27 fmet_tRNA + 729 gln1_tRNA + 108 gln2_tRNA + 1566 glu1_tRNA + 1890 gly1_tRNA + 54 glyT_tRNA + 81 glyU_tRNA + 50544 h + 675 hisR_tRNA + 1242 ile1_tRNA + 1512 leu1_tRNA + 162 leu2_tRNA + 324 leuU_tRNA + 54 leuW_tRNA + 27 leuZ_tRNA + 1647 lys1_tRNA + 621 met1_tRNA + 126495 mg2 + 837 phe1_tRNA + 50544 pi + 756 pro1_tRNA + 189 pro2_tRNA + 27 proL_tRNA + 162 proM_tRNA + 1 rib_70_elo2_b0026_27_cplx + 324 ser1_tRNA + 270 ser2_tRNA + 216 ser3_tRNA + 108 serT_tRNA + 351 serV_tRNA + 864 thr1_tRNA + 189 thr2_tRNA + 162 thr3_tRNA + 54 thrU_tRNA + 621 trpT_tRNA + 891 tyr1_tRNA + 1053 val1_tRNA + 351 val2_tRNA + 594 val3_tRNA	irreversible	Translation	0.00E+00	1.00E+09	0	2.32E+01	2.32E+01	0	3.96E+01	39.62865	0	1.88E+00	1.8845
tl_elo_b0026_55_rib1	Translation elongation 1 b0026 55 ribosome(s)	51535 EF-G.GTP + 4015 EF-Tu.GTP.ala1-tRNA + 1045 EF-Tu.GTP.ala2-tRNA + 2530 EF-Tu.GTP.arg1-tRNA + 1155 EF-Tu.GTP.asn1-tRNA + 3905 EF-Tu.GTP.asp1-tRNA + 770 EF-Tu.GTP.cysT-tRNA + 1485 EF-Tu.GTP.gln1-tRNA + 220 EF-Tu.GTP.gln2-tRNA + 3190 EF-Tu.GTP.glu1-tRNA + 3850 EF-Tu.GTP.gly1-tRNA + 110 EF-Tu.GTP.glyT-tRNA + 165 EF-Tu.GTP.glyU-tRNA + 1375 EF-Tu.GTP.hisR-tRNA + 2530 EF-Tu.GTP.ile1-tRNA + 3080 EF-Tu.GTP.leu1-tRNA + 330 EF-Tu.GTP.leu2-tRNA + 660 EF-Tu.GTP.leuU-tRNA + 110 EF-Tu.GTP.leuW-tRNA + 55 EF-Tu.GTP.leuZ-tRNA + 3355 EF-Tu.GTP.lys1-tRNA + 1265 EF-Tu.GTP.met1-tRNA + 1705 EF-Tu.GTP.phe1-tRNA + 1540 EF-Tu.GTP.pro1-tRNA + 385 EF-Tu.GTP.pro2-tRNA + 55 EF-Tu.GTP.proL-tRNA + 330 EF-Tu.GTP.proM-tRNA + 660 EF-Tu.GTP.ser1-tRNA + 550 EF-Tu.GTP.ser2-tRNA + 440 EF-Tu.GTP.ser3-tRNA + 220 EF-Tu.GTP.serT-tRNA + 715 EF-Tu.GTP.serV-tRNA + 1760 EF-Tu.GTP.thr1-tRNA + 385 EF-Tu.GTP.thr2-tRNA + 330 EF-Tu.GTP.thr3-tRNA + 110 EF-Tu.GTP.thrU-tRNA + 1265 EF-Tu.GTP.trpT-tRNA + 1815 EF-Tu.GTP.tyr1-tRNA + 2145 EF-Tu.GTP.val1-tRNA + 715 EF-Tu.GTP.val2-tRNA + 1210 EF-Tu.GTP.val3-tRNA + 257675 mg2 + 1 rib_ini_b0026_55 --> 1 rib_70_	irreversible	Translation	0.00E+00	1.00E+09	0	2.32E+01	2.32E+01	0	3.88E+01	38.78764	0	1.88E+00	1.8845
tl_elo_b0026_55_rib2	Translation elongation 2 b0026 55 ribosome(s)	51480 h2o + 1 rib_70_elo1_b0026_55_cplx --> 51480 EF-G.GDP + 51480 EF-Tu.GDP + 4015 ala1_tRNA + 990 ala2_tRNA + 2530 arg1_tRNA + 1155 asn1_tRNA + 3905 asp1_tRNA + 770 cysT_tRNA + 55 fmet_tRNA + 1485 gln1_tRNA + 220 gln2_tRNA + 3190 glu1_tRNA + 3850 gly1_tRNA + 110 glyT_tRNA + 165 glyU_tRNA + 102960 h + 1375 hisR_tRNA + 2530 ile1_tRNA + 3080 leu1_tRNA + 330 leu2_tRNA + 660 leuU_tRNA + 110 leuW_tRNA + 55 leuZ_tRNA + 3355 lys1_tRNA + 1265 met1_tRNA + 257675 mg2 + 1705 phe1_tRNA + 102960 pi + 1540 pro1_tRNA + 385 pro2_tRNA + 55 proL_tRNA + 330 proM_tRNA + 1 rib_70_elo2_b0026_55_cplx + 660 ser1_tRNA + 550 ser2_tRNA + 440 ser3_tRNA + 220 serT_tRNA + 715 serV_tRNA + 1760 thr1_tRNA + 385 thr2_tRNA + 330 thr3_tRNA + 110 thrU_tRNA + 1265 trpT_tRNA + 1815 tyr1_tRNA + 2145 val1_tRNA + 715 val2_tRNA + 1210 val3_tRNA	irreversible	Translation	0.00E+00	1.00E+09	0	2.32E+01	2.32E+01	0	3.88E+01	38.78764	0	1.88E+00	1.8845
tl_elo_b0027_1_rib1	Translation elongation 1 b0027 1 ribosome(s)	163 EF-G.GTP + 12 EF-Tu.GTP.ala1-tRNA + 6 EF-Tu.GTP.ala2-tRNA + 5 EF-Tu.GTP.arg1-tRNA + 1 EF-Tu.GTP.argU-tRNA + 7 EF-Tu.GTP.asn1-tRNA + 8 EF-Tu.GTP.asp1-tRNA + 2 EF-Tu.GTP.cysT-tRNA + 3 EF-Tu.GTP.gln1-tRNA + 2 EF-Tu.GTP.gln2-tRNA + 1 EF-Tu.GTP.glu1-tRNA + 13 EF-Tu.GTP.gly1-tRNA + 2 EF-Tu.GTP.glyU-tRNA + 3 EF-Tu.GTP.hisR-tRNA + 14 EF-Tu.GTP.ile1-tRNA + 15 EF-Tu.GTP.leu1-tRNA + 1 EF-Tu.GTP.leu2-tRNA + 4 EF-Tu.GTP.leuU-tRNA + 2 EF-Tu.GTP.leuW-tRNA + 5 EF-Tu.GTP.lys1-tRNA + 3 EF-Tu.GTP.met1-tRNA + 12 EF-Tu.GTP.phe1-tRNA + 2 EF-Tu.GTP.pro1-tRNA + 1 EF-Tu.GTP.pro2-tRNA + 1 EF-Tu.GTP.ser2-tRNA + 3 EF-Tu.GTP.ser3-tRNA + 1 EF-Tu.GTP.serT-tRNA + 5 EF-Tu.GTP.serV-tRNA + 1 EF-Tu.GTP.thr1-tRNA + 1 EF-Tu.GTP.thr2-tRNA + 2 EF-Tu.GTP.thr3-tRNA + 1 EF-Tu.GTP.thrU-tRNA + 6 EF-Tu.GTP.trpT-tRNA + 6 EF-Tu.GTP.tyr1-tRNA + 6 EF-Tu.GTP.val1-tRNA + 5 EF-Tu.GTP.val2-tRNA + 1 EF-Tu.GTP.val3-tRNA + 815 mg2 + 1 rib_ini_b0027_1 --> 1 rib_70_elo1_b0027_1_cplx	irreversible	Translation	0.00E+00	1.00E+09	0	3.11E+01	3.11E+01	0	4.75E+01	47.50865	0	9.76E+00	9.7645
tl_elo_b0027_1_rib2	Translation elongation 2 b0027 1 ribosome(s)	162 h2o + 1 rib_70_elo1_b0027_1_cplx --> 162 EF-G.GDP + 162 EF-Tu.GDP + 12 ala1_tRNA + 6 ala2_tRNA + 5 arg1_tRNA + 1 argU_tRNA + 7 asn1_tRNA + 8 asp1_tRNA + 2 cysT_tRNA + 1 fmet_tRNA + 3 gln1_tRNA + 1 gln2_tRNA + 1 glu1_tRNA + 13 gly1_tRNA + 2 glyU_tRNA + 324 h + 3 hisR_tRNA + 14 ile1_tRNA + 15 leu1_tRNA + 1 leu2_tRNA + 4 leuU_tRNA + 2 leuW_tRNA + 5 lys1_tRNA + 3 met1_tRNA + 815 mg2 + 12 phe1_tRNA + 324 pi + 2 pro1_tRNA + 1 pro2_tRNA + 1 rib_70_elo2_b0027_1_cplx + 1 ser2_tRNA + 3 ser3_tRNA + 1 serT_tRNA + 5 serV_tRNA + 1 thr1_tRNA + 1 thr2_tRNA + 2 thr3_tRNA + 1 thrU_tRNA + 6 trpT_tRNA + 6 tyr1_tRNA + 6 val1_tRNA + 5 val2_tRNA + 1 val3_tRNA	irreversible	Translation	0.00E+00	1.00E+09	0	3.11E+01	3.11E+01	0	4.75E+01	47.50865	0	9.76E+00	9.7645

tl_elo_b0027_4_rib1	Translation elongation 1 b0027 4 ribosome(s)	652 EF-G.GTP + 48 EF-Tu.GTP.ala1-tRNA + 24 EF-Tu.GTP.ala2-tRNA + 20 EF-Tu.GTP.arg1-tRNA + 4 EF-Tu.GTP.argU-tRNA + 28 EF-Tu.GTP.asn1-tRNA + 32 EF-Tu.GTP.asp1-tRNA + 8 EF-Tu.GTP.cysT-tRNA + 12 EF-Tu.GTP.gln1-tRNA + 8 EF-Tu.GTP.gln2-tRNA + 4 EF-Tu.GTP.glu1-tRNA + 52 EF-Tu.GTP.gly1-tRNA + 8 EF-Tu.GTP.glyU-tRNA + 12 EF-Tu.GTP.hisR-tRNA + 56 EF-Tu.GTP.ile1-tRNA + 60 EF-Tu.GTP.leu1-tRNA + 4 EF-Tu.GTP.leu2-tRNA + 16 EF-Tu.GTP.leuU-tRNA + 8 EF-Tu.GTP.leuW-tRNA + 20 EF-Tu.GTP.lys1-tRNA + 12 EF-Tu.GTP.met1-tRNA + 48 EF-Tu.GTP.phe1-tRNA + 8 EF-Tu.GTP.pro1-tRNA + 4 EF-Tu.GTP.pro2-tRNA + 4 EF-Tu.GTP.ser2-tRNA + 12 EF-Tu.GTP.ser3-tRNA + 4 EF-Tu.GTP.serT-tRNA + 20 EF-Tu.GTP.serV-tRNA + 4 EF-Tu.GTP.thr1-tRNA + 4 EF-Tu.GTP.thr2-tRNA + 8 EF-Tu.GTP.thr3-tRNA + 4 EF-Tu.GTP.thrU-tRNA + 24 EF-Tu.GTP.trpT-tRNA + 24 EF-Tu.GTP.tyr1-tRNA + 24 EF-Tu.GTP.val1-tRNA + 20 EF-Tu.GTP.val2-tRNA + 4 EF-Tu.GTP.val3-tRNA + 3260 mg2 + 1 rib_ini_b0027_4 --> 1 rib_70_elo1_b0027_4_cplx	irreversible	Translation	0.00E+00	1.00E+09	0	3.11E+01	3.11E+01	0	4.75E+01	47.50865	0	9.76E+00	9.7645
tl_elo_b0027_4_rib2	Translation elongation 2 b0027 4 ribosome(s)	648 h2o + 1 rib_70_elo1_b0027_4_cplx --> 648 EF-G.GDP + 648 EF-Tu.GDP + 48 ala1_tRNA + 24 ala2_tRNA + 20 arg1_tRNA + 4 argU_tRNA + 28 asn1_tRNA + 32 asp1_tRNA + 8 cysT_tRNA + 4 fmet_tRNA + 12 gln1_tRNA + 4 gln2_tRNA + 4 glu1_tRNA + 52 gly1_tRNA + 8 glyU_tRNA + 1296 h + 12 hisR_tRNA + 56 ile1_tRNA + 60 leu1_tRNA + 4 leu2_tRNA + 16 leuU_tRNA + 8 leuW_tRNA + 20 lys1_tRNA + 12 met1_tRNA + 3260 mg2 + 48 phe1_tRNA + 1296 pi + 8 pro1_tRNA + 4 pro2_tRNA + 1 rib_70_elo2_b0027_4_cplx + 4 ser2_tRNA + 12 ser3_tRNA + 4 serT_tRNA + 20 serV_tRNA + 4 thr1_tRNA + 4 thr2_tRNA + 8 thr3_tRNA + 4 thrU_tRNA + 24 trpT_tRNA + 24 tyr1_tRNA + 24 val1_tRNA + 20 val2_tRNA + 4 val3_tRNA	irreversible	Translation	0.00E+00	1.00E+09	0	3.11E+01	3.11E+01	0	4.75E+01	47.50865	0	9.76E+00	9.7645
tl_elo_b0027_9_rib1	Translation elongation 1 b0027 9 ribosome(s)	1467 EF-G.GTP + 108 EF-Tu.GTP.ala1-tRNA + 54 EF-Tu.GTP.ala2-tRNA + 45 EF-Tu.GTP.arg1-tRNA + 9 EF-Tu.GTP.argU-tRNA + 63 EF-Tu.GTP.asn1-tRNA + 72 EF-Tu.GTP.asp1-tRNA + 18 EF-Tu.GTP.cysT-tRNA + 27 EF-Tu.GTP.gln1-tRNA + 18 EF-Tu.GTP.gln2-tRNA + 9 EF-Tu.GTP.glu1-tRNA + 117 EF-Tu.GTP.gly1-tRNA + 18 EF-Tu.GTP.glyU-tRNA + 27 EF-Tu.GTP.hisR-tRNA + 126 EF-Tu.GTP.ile1-tRNA + 135 EF-Tu.GTP.leu1-tRNA + 9 EF-Tu.GTP.leu2-tRNA + 36 EF-Tu.GTP.leuU-tRNA + 18 EF-Tu.GTP.leuW-tRNA + 45 EF-Tu.GTP.lys1-tRNA + 27 EF-Tu.GTP.met1-tRNA + 108 EF-Tu.GTP.phe1-tRNA + 18 EF-Tu.GTP.pro1-tRNA + 9 EF-Tu.GTP.pro2-tRNA + 9 EF-Tu.GTP.ser2-tRNA + 27 EF-Tu.GTP.ser3-tRNA + 9 EF-Tu.GTP.serT-tRNA + 45 EF-Tu.GTP.serV-tRNA + 9 EF-Tu.GTP.thr1-tRNA + 9 EF-Tu.GTP.thr2-tRNA + 18 EF-Tu.GTP.thr3-tRNA + 9 EF-Tu.GTP.thrU-tRNA + 54 EF-Tu.GTP.trpT-tRNA + 54 EF-Tu.GTP.tyr1-tRNA + 54 EF-Tu.GTP.val1-tRNA + 45 EF-Tu.GTP.val2-tRNA + 9 EF-Tu.GTP.val3-tRNA + 7335 mg2 + 1 rib_ini_b0027_9 --> 1 rib_70_elo1_b0027_9_cplx	irreversible	Translation	0.00E+00	1.00E+09	0	3.11E+01	3.11E+01	0	4.75E+01	47.50865	0	9.76E+00	9.7645
tl_elo_b0027_9_rib2	Translation elongation 2 b0027 9 ribosome(s)	1458 h2o + 1 rib_70_elo1_b0027_9_cplx --> 1458 EF-G.GDP + 1458 EF-Tu.GDP + 108 ala1_tRNA + 54 ala2_tRNA + 45 arg1_tRNA + 9 argU_tRNA + 63 asn1_tRNA + 72 asp1_tRNA + 18 cysT_tRNA + 9 fmet_tRNA + 27 gln1_tRNA + 9 gln2_tRNA + 9 glu1_tRNA + 117 gly1_tRNA + 18 glyU_tRNA + 2916 h + 27 hisR_tRNA + 126 ile1_tRNA + 135 leu1_tRNA + 9 leu2_tRNA + 36 leuU_tRNA + 18 leuW_tRNA + 45 lys1_tRNA + 27 met1_tRNA + 7335 mg2 + 108 phe1_tRNA + 2916 pi + 18 pro1_tRNA + 9 pro2_tRNA + 1 rib_70_elo2_b0027_9_cplx + 9 ser2_tRNA + 27 ser3_tRNA + 9 serT_tRNA + 45 serV_tRNA + 9 thr1_tRNA + 9 thr2_tRNA + 18 thr3_tRNA + 9 thrU_tRNA + 54 trpT_tRNA + 54 tyr1_tRNA + 54 val1_tRNA + 45 val2_tRNA + 9 val3_tRNA	irreversible	Translation	0.00E+00	1.00E+09	0	3.11E+01	3.11E+01	0	4.75E+01	47.50865	0	9.76E+00	9.7645
tl_elo_b0028_1_rib1	Translation elongation 1 b0028 1 ribosome(s)	148 EF-G.GTP + 9 EF-Tu.GTP.ala1-tRNA + 3 EF-Tu.GTP.ala2-tRNA + 5 EF-Tu.GTP.arg1-tRNA + 5 EF-Tu.GTP.asn1-tRNA + 12 EF-Tu.GTP.asp1-tRNA + 3 EF-Tu.GTP.gln1-tRNA + 1 EF-Tu.GTP.gln2-tRNA + 13 EF-Tu.GTP.glu1-tRNA + 10 EF-Tu.GTP.gly1-tRNA + 3 EF-Tu.GTP.glyU-tRNA + 4 EF-Tu.GTP.hisR-tRNA + 8 EF-Tu.GTP.ile1-tRNA + 10 EF-Tu.GTP.leu1-tRNA + 2 EF-Tu.GTP.leu2-tRNA + 3 EF-Tu.GTP.leuU-tRNA + 1 EF-Tu.GTP.leuW-tRNA + 4 EF-Tu.GTP.lys1-tRNA + 4 EF-Tu.GTP.met1-tRNA + 9 EF-Tu.GTP.phe1-tRNA + 5 EF-Tu.GTP.pro1-tRNA + 1 EF-Tu.GTP.pro2-tRNA + 2 EF-Tu.GTP.proM-tRNA + 2 EF-Tu.GTP.ser1-tRNA + 5 EF-Tu.GTP.ser2-tRNA + 1 EF-Tu.GTP.ser3-tRNA + 1 EF-Tu.GTP.serT-tRNA + 3 EF-Tu.GTP.serV-tRNA + 8 EF-Tu.GTP.thr1-tRNA + 1 EF-Tu.GTP.thr3-tRNA + 1 EF-Tu.GTP.tyr1-tRNA + 6 EF-Tu.GTP.val1-tRNA + 1 EF-Tu.GTP.val2-tRNA + 2 EF-Tu.GTP.val3-tRNA + 740 mg2 + 1 rib_ini_b0028_1 --> 1 rib_70_elo1_b0028_1_cplx	irreversible	Translation	0.00E+00	1.00E+09	0	3.02E+01	3.02E+01	0	4.66E+01	46.59865	0	8.85E+00	8.8545
tl_elo_b0028_1_rib2	Translation elongation 2 b0028 1 ribosome(s)	147 h2o + 1 rib_70_elo1_b0028_1_cplx --> 147 EF-G.GDP + 147 EF-Tu.GDP + 8 ala1_tRNA + 3 ala2_tRNA + 5 arg1_tRNA + 5 asn1_tRNA + 12 asp1_tRNA + 1 fmet_tRNA + 3 gln1_tRNA + 1 gln2_tRNA + 13 glu1_tRNA + 10 gly1_tRNA + 3 glyU_tRNA + 294 h + 4 hisR_tRNA + 8 ile1_tRNA + 10 leu1_tRNA + 2 leu2_tRNA + 3 leuU_tRNA + 1 leuW_tRNA + 4 lys1_tRNA + 4 met1_tRNA + 740 mg2 + 9 phe1_tRNA + 294 pi + 5 pro1_tRNA + 1 pro2_tRNA + 2 proM_tRNA + 1 rib_70_elo2_b0028_1_cplx + 2 ser1_tRNA + 5 ser2_tRNA + 1 ser3_tRNA + 1 serT_tRNA + 3 serV_tRNA + 8 thr1_tRNA + 1 thr3_tRNA + 1 tyr1_tRNA + 6 val1_tRNA + 1 val2_tRNA + 2 val3_tRNA	irreversible	Translation	0.00E+00	1.00E+09	0	3.02E+01	3.02E+01	0	4.66E+01	46.59865	0	8.85E+00	8.8545
tl_elo_b0028_4_rib1	Translation elongation 1 b0028 4 ribosome(s)	592 EF-G.GTP + 36 EF-Tu.GTP.ala1-tRNA + 12 EF-Tu.GTP.ala2-tRNA + 20 EF-Tu.GTP.arg1-tRNA + 20 EF-Tu.GTP.asn1-tRNA + 48 EF-Tu.GTP.asp1-tRNA + 12 EF-Tu.GTP.gln1-tRNA + 4 EF-Tu.GTP.gln2-tRNA + 52 EF-Tu.GTP.glu1-tRNA + 40 EF-Tu.GTP.gly1-tRNA + 12 EF-Tu.GTP.glyU-tRNA + 16 EF-Tu.GTP.hisR-tRNA + 32 EF-Tu.GTP.ile1-tRNA + 40 EF-Tu.GTP.leu1-tRNA + 8 EF-Tu.GTP.leu2-tRNA + 12 EF-Tu.GTP.leuU-tRNA + 4 EF-Tu.GTP.leuW-tRNA + 16 EF-Tu.GTP.lys1-tRNA + 16 EF-Tu.GTP.met1-tRNA + 36 EF-Tu.GTP.phe1-tRNA + 20 EF-Tu.GTP.pro1-tRNA + 4 EF-Tu.GTP.pro2-tRNA + 8 EF-Tu.GTP.proM-tRNA + 8 EF-Tu.GTP.ser1-tRNA + 20 EF-Tu.GTP.ser2-tRNA + 4 EF-Tu.GTP.ser3-tRNA + 4 EF-Tu.GTP.serT-tRNA + 12 EF-Tu.GTP.serV-tRNA + 32 EF-Tu.GTP.thr1-tRNA + 4 EF-Tu.GTP.thr3-tRNA + 4 EF-Tu.GTP.tyr1-tRNA + 24 EF-Tu.GTP.val1-tRNA + 4 EF-Tu.GTP.val2-tRNA + 8 EF-Tu.GTP.val3-tRNA + 2960 mg2 + 1 rib_ini_b0028_4 --> 1 rib_70_elo1_b0028_4_cplx	irreversible	Translation	0.00E+00	1.00E+09	0	3.02E+01	3.02E+01	0	4.66E+01	46.59865	0	8.85E+00	8.8545

tl_elo_b0028_4_rib2	Translation elongation 2 b0028 4 ribosome(s)	588 h2o + 1 rib_70_elo1_b0028_4_cplx --> 588 EF-G.GDP + 588 EF-Tu.GDP + 32 ala1_tRNA + 12 ala2_tRNA + 20 arg1_tRNA + 20 asn1_tRNA + 48 asp1_tRNA + 4 fmet_tRNA + 12 gln1_tRNA + 4 gln2_tRNA + 52 glu1_tRNA + 40 gly1_tRNA + 12 glyU_tRNA + 1176 h + 16 hisR_tRNA + 32 ile1_tRNA + 40 leu1_tRNA + 8 leu2_tRNA + 12 leuU_tRNA + 4 leuW_tRNA + 16 lys1_tRNA + 16 met1_tRNA + 2960 mg2 + 36 phe1_tRNA + 1176 pi + 20 pro1_tRNA + 4 pro2_tRN + 8 proM_tRNA + 1 rib_70_elo2_b0028_4_cplx + 8 ser1_tRNA + 20 ser2_tRNA + 4 ser3_tRNA + 4 serT_tRNA + 12 serV_tRNA + 32 thr1_tRNA + 4 thr3_tRNA + 4 tyr1_tRNA + 24 val1_tRNA + 4 val2_tRNA + 8 val3_tRNA	irreversible	Translation	0.00E+00	1.00E+09	0	3.02E+01	3.02E+01	0	4.66E+01	46.59865	0	8.85E+00	8.8545
tl_elo_b0028_8_rib1	Translation elongation 1 b0028 8 ribosome(s)	1184 EF-G.GTP + 72 EF-Tu.GTP.ala1-tRNA + 24 EF-Tu.GTP.ala2-tRNA + 40 EF-Tu.GTP.arg1-tRNA + 40 EF-Tu.GTP.asn1-tRNA + 96 EF-Tu.GTP.asp1-tRNA + 24 EF-Tu.GTP.gln1-tRNA + 8 EF-Tu.GTP.gln2-tRNA + 104 EF-Tu.GTP.glu1-tRNA + 80 EF-Tu.GTP.gly1-tRNA + 24 EF-Tu.GTP.glyU-tRNA + 32 EF-Tu.GTP.hisR-tRNA + 64 EF-Tu.GTP.ile1-tRNA + 80 EF-Tu.GTP.leu1 tRNA + 16 EF-Tu.GTP.leu2-tRNA + 24 EF-Tu.GTP.leuU-tRNA + 8 EF-Tu.GTP.leuW-tRNA + 32 EF-Tu.GTP.lys1-tRNA + 32 EF-Tu.GTP.met1-tRNA + 72 EF-Tu.GTP.phe1-tRNA + 40 EF-Tu.GTP.pro1-tRNA + 8 EF-Tu.GTP.pro2-tRNA + 16 EF-Tu.GTP.proM-tRNA + 16 EF-Tu.GTP.ser1-tRNA + 40 EF-Tu.GTP.ser2-tRNA + 8 EF-Tu.GTP.ser3-tRNA + 8 EF-Tu.GTP.serT-tRNA + 24 EF-Tu.GTP.serV-tRNA + 64 EF-Tu.GTP.thr1-tRNA + 8 EF-Tu.GTP.thr3-tRNA + 8 EF-Tu.GTP.tyr1-tRNA + 48 EF-Tu.GTP.val1-tRNA + 8 EF-Tu.GTP.val2-tRNA + 16 EF-Tu.GTP.val3-tRNA + 5920 mg2 + 1 rib_ini_b0028_8 --> 1 rib_70_elo1_b0028_8_cplx	irreversible	Translation	0.00E+00	1.00E+09	0	3.02E+01	3.02E+01	0	4.66E+01	46.59865	0	8.85E+00	8.8545
tl_elo_b0028_8_rib2	Translation elongation 2 b0028 8 ribosome(s)	1176 h2o + 1 rib_70_elo1_b0028_8_cplx --> 1176 EF-G.GDP + 1176 EF-Tu.GDP + 64 ala1_tRNA + 24 ala2_tRNA + 40 arg1_tRNA + 40 asn1_tRNA + 96 asp1_tRNA + 8 fmet_tRNA + 24 gln1_tRNA + 8 gln2_tRNA + 104 glu1_tRNA + 80 gly1_tRNA + 24 glyU_tRNA + 2352 h + 32 hisR_tRNA + 64 ile1_tRNA + 80 leu1_tRNA + 16 leu2_tRNA + 24 leuU_tRNA + 8 leuW_tRNA + 32 lys1_tRNA + 32 met1_tRNA + 5920 mg2 + 72 phe1_tRNA + 2352 pi + 40 pro1_tRNA + 8 pro2_tRN + 16 proM_tRNA + 1 rib_70_elo2_b0028_8_cplx + 16 ser1_tRNA + 4 ser2_tRNA + 8 ser3_tRNA + 8 serT_tRNA + 24 serV_tRNA + 64 thr1_tRNA + 8 thr3_tRNA + 8 tyr1_tRNA + 48 val1_tRNA + 8 val2_tRNA + 16 val3_tRNA	irreversible	Translation	0.00E+00	1.00E+09	0	3.02E+01	3.02E+01	0	4.66E+01	46.59865	0	8.85E+00	8.8545
tl_elo_b0029_18_rib1	Translation elongation 1 b0029 18 ribosome(s)	5670 EF-G.GTP + 342 EF-Tu.GTP.ala1-tRNA + 198 EF-Tu.GTP.ala2 tRNA + 414 EF-Tu.GTP.arg1-tRNA + 216 EF-Tu.GTP.asn1-tRNA + 342 EF-Tu.GTP.asp1-tRNA + 72 EF-Tu.GTP.cysT-tRNA + 216 EF-Tu.GTP.gln1-tRNA + 522 EF-Tu.GTP.glu1-tRNA + 288 EF-Tu.GTP.gly1-tRNA + 18 EF-Tu.GTP.glyT-tRNA + 90 EF-Tu.GTP.glyU-tRNA + 108 EF-Tu.GTP.hisR-tRNA + 360 EF-Tu.GTP.ile1-tRNA + 18 EF-Tu.GTP.ile2-tRNA + 270 EF-Tu.GTP.leu1 tRNA + 108 EF-Tu.GTP.leu2-tRNA + 36 EF-Tu.GTP.leuU-tRNA + 252 EF-Tu.GTP.lys1-tRNA + 90 EF-Tu.GTP.met1-tRNA + 144 EF-Tu.GTP.phe1-tRNA + 216 EF-Tu.GTP.pro1-tRNA + 90 EF-Tu.GTP.ser1-tRNA + 54 EF-Tu.GTP.ser2-tRNA + 72 EF-Tu.GTP.ser3-tRNA + 108 EF-Tu.GTP.serV-tRNA + 72 EF-Tu.GTP.thr1-tRNA + 36 EF-Tu.GTP.thr2-tRNA + 90 EF-Tu.GTP.thr3-tRNA + 18 EF-Tu.GTP.thrU-tRNA + 36 EF-Tu.GTP.trpT-tRNA + 108 EF-Tu.GTP.tyr1-tRNA + 396 EF-Tu.GTP.val1-tRNA + 180 EF-Tu.GTP.val2-tRNA + 90 EF-Tu.GTP.val3-tRNA + 28350 mg2 + 1 rib_ini_b0029_18 --> 1 rib_70_elo1_b0029_18_cplx	irreversible	Translation	0.00E+00	1.00E+09	0	2.65E+01	2.65E+01	0	4.29E+01	42.85865	0	5.11E+00	5.1145
tl_elo_b0029_18_rib2	Translation elongation 2 b0029 18 ribosome(s)	5652 h2o + 1 rib_70_elo1_b0029_18_cplx --> 5652 EF-G.GDP + 5652 EF-Tu.GDP + 342 ala1_tRNA + 198 ala2_tRNA + 414 arg1_tRNA + 216 asn1_tRNA + 324 asp1_tRNA + 72 cysT_tRNA + 18 fmet_tRNA + 216 gln1_tRNA + 522 glu1_tRNA + 288 gly1_tRNA + 18 glyT_tRNA + 90 glyU_tRNA + 11304 h + 108 hisR_tRNA + 360 ile1_tRNA + 18 ile2_tRNA + 270 leu1_tRNA + 108 leu2_tRNA + 36 leuU_tRNA + 252 lys1_tRNA + 90 met1_tRNA + 28350 mg2 + 144 phe1_tRNA + 11304 pi + 216 pro1_tRNA + 1 rib_70_elo2_b0029_18_cplx + 90 ser1_tRNA + 54 ser2_tRNA + 72 ser3_tRNA + 108 serV_tRNA + 72 thr1_tRNA + 36 thr2_tRNA + 90 thr3_tRNA + 18 thrU_tRNA + 36 trpT_tRNA + 108 tyr1_tRNA + 396 val1_tRNA + 180 val2_tRNA + 90 val3_tRNA	irreversible	Translation	0.00E+00	1.00E+09	0	2.65E+01	2.65E+01	0	4.29E+01	42.85865	0	5.11E+00	5.1145
tl_elo_b0029_1_rib1	Translation elongation 1 b0029 1 ribosome(s)	315 EF-G.GTP + 19 EF-Tu.GTP.ala1-tRNA + 11 EF-Tu.GTP.ala2-tRNA + 23 EF-Tu.GTP.arg1-tRNA + 12 EF-Tu.GTP.asn1-tRNA + 19 EF-Tu.GTP.asp1-tRNA + 4 EF-Tu.GTP.cysT-tRNA + 12 EF-Tu.GTP.gln1-tRNA + 29 EF-Tu.GTP.glu1-tRNA + 16 EF-Tu.GTP.gly1 tRNA + 1 EF-Tu.GTP.glyT-tRNA + 5 EF-Tu.GTP.glyU-tRNA + 6 EF-Tu.GTP.hisR-tRNA + 20 EF-Tu.GTP.ile1-tRNA + 1 EF-Tu.GTP.ile2-tRNA + 15 EF-Tu.GTP.leu1-tRNA + 6 EF-Tu.GTP.leu2-tRNA + 2 EF-Tu.GTP.leuU-tRNA + 14 EF-Tu.GTP.lys1-tRNA + 5 EF-Tu.GTP.met1 tRNA + 8 EF-Tu.GTP.phe1-tRNA + 12 EF-Tu.GTP.pro1-tRNA + 5 EF Tu.GTP.ser1-tRNA + 3 EF-Tu.GTP.ser2-tRNA + 4 EF-Tu.GTP.ser3-tRNA + 6 EF-Tu.GTP.serV-tRNA + 4 EF-Tu.GTP.thr1-tRNA + 2 EF-Tu.GTP.thr2-tRNA + 5 EF-Tu.GTP.thr3-tRNA + 1 EF-Tu.GTP.thrU-tRNA + 2 EF-Tu.GTP.trpT-tRNA + 6 EF-Tu.GTP.tyr1-tRNA + 22 EF-Tu.GTP.val1-tRNA + 10 EF-Tu.GTP.val2-tRNA + 5 EF-Tu.GTP.val3-tRNA + 1575 mg2 + 1 rib_ini_b0029_1 --> 1 rib_70_elo1_b0029_1_cplx	irreversible	Translation	0.00E+00	1.00E+09	0	2.65E+01	2.65E+01	0	4.29E+01	42.85865	0	5.11E+00	5.1145
tl_elo_b0029_1_rib2	Translation elongation 2 b0029 1 ribosome(s)	314 h2o + 1 rib_70_elo1_b0029_1_cplx --> 314 EF-G.GDP + 314 EF-Tu.GDP + 19 ala1_tRNA + 11 ala2_tRNA + 23 arg1_tRNA + 12 asn1_tRNA + 18 asp1_tRNA + 4 cysT_tRNA + 1 fmet_tRNA + 12 gln1_tRNA + 29 glu1_tRNA + 16 gly1_tRNA + 1 glyT_tRNA + 5 glyU_tRNA + 628 h + 6 hisR_tRNA + 20 ile1_tRNA + 1 ile2_tRNA + 15 leu1_tRNA + 6 leu2_tRNA + 2 leuU_tRNA + 14 lys1_tRNA + 5 met1_tRNA + 1575 mg2 + 8 phe1_tRNA + 628 pi + 12 pro1_tRNA + rib_70_elo2_b0029_1_cplx + 5 ser1_tRNA + 3 ser2_tRNA + 4 ser3_tRNA + 6 serV_tRNA + 4 thr1_tRNA + 2 thr2_tRNA + 5 thr3_tRNA + 1 thrU_tRNA + 2 trpT_tRNA + 6 tyr1_tRNA + 22 val1_tRNA + 10 val2_tRNA + 5 val3_tRNA	irreversible	Translation	0.00E+00	1.00E+09	0	2.65E+01	2.65E+01	0	4.29E+01	42.85865	0	5.11E+00	5.1145

tl_elo_b0029_9_rib1	Translation elongation 1 b0029 9 ribosome(s)	2835 EF-G.GTP + 171 EF-Tu.GTP.ala1-tRNA + 99 EF-Tu.GTP.ala2-<br>tRNA + 207 EF-Tu.GTP.arg1-tRNA + 108 EF-Tu.GTP.asn1-tRNA +<br>171 EF-Tu.GTP.asp1-tRNA + 36 EF-Tu.GTP.cysT-tRNA + 108 EF-<br>Tu.GTP.gln1-tRNA + 261 EF-Tu.GTP.glu1-tRNA + 144 EF-<br>Tu.GTP.gly1-tRNA + 9 EF-Tu.GTP.glyT-tRNA + 45 EF-Tu.GTP.glyU-<br>tRNA + 54 EF-Tu.GTP.hisR-tRNA + 180 EF-Tu.GTP.ile1-tRNA + 9<br>EF-Tu.GTP.ile2-tRNA + 135 EF-Tu.GTP.leu1-tRNA + 54 EF-<br>Tu.GTP.leu2-tRNA + 18 EF-Tu.GTP.leuU-tRNA + 126 EF-<br>Tu.GTP.lys1-tRNA + 45 EF-Tu.GTP.met1-tRNA + 72 EF-<br>Tu.GTP.phe1-tRNA + 108 EF-Tu.GTP.pro1-tRNA + 45 EF-<br>Tu.GTP.ser1-tRNA + 27 EF-Tu.GTP.ser2-tRNA + 36 EF-<br>Tu.GTP.ser3-tRNA + 54 EF-Tu.GTP.serV-tRNA + 36 EF-<br>Tu.GTP.thr1-tRNA + 18 EF-Tu.GTP.thr2-tRNA + 45 EF-Tu.GTP.thr3-<br>tRNA + 9 EF-Tu.GTP.thrU-tRNA + 18 EF-Tu.GTP.trpT-tRNA + 54 EF<br>Tu.GTP.tyr1-tRNA + 198 EF-Tu.GTP.val1-tRNA + 90 EF-<br>Tu.GTP.val2-tRNA + 45 EF-Tu.GTP.val3-tRNA + 14175 mg2 + 1<br>rib_ini_b0029_9 --> 1 rib_70_elo1_b0029_9_cplx	irreversible	Translation	0.00E+00	1.00E+09	0	2.65E+01	2.65E+01	0	4.29E+01	42.85865	0	5.11E+00	5.1145
tl_elo_b0029_9_rib2	Translation elongation 2 b0029 9 ribosome(s)	2826 h2o + 1 rib_70_elo1_b0029_9_cplx --> 2826 EF-G.GDP + 2826<br>EF-Tu.GDP + 171 ala1_tRNA + 99 ala2_tRNA + 207 arg1_tRNA +<br>108 asn1_tRNA + 162 asp1_tRNA + 36 cysT_tRNA + 9 fmet_tRNA +<br>108 gln1_tRNA + 261 glu1_tRNA + 144 gly1_tRNA + 9 glyT_tRNA +<br>45 glyU_tRNA + 5652 h + 54 hisR_tRNA + 180 ile1_tRNA + 9<br>ile2_tRNA + 135 leu1_tRNA + 54 leu2_tRNA + 18 leuU_tRNA + 126<br>lys1_tRNA + 45 met1_tRNA + 14175 mg2 + 72 phe1_tRNA + 5652 pi<br>+ 108 pro1_tRNA + 1 rib_70_elo2_b0029_9_cplx + 45 ser1_tRNA +<br>27 ser2_tRNA + 36 ser3_tRNA + 54 serV_tRNA + 36 thr1_tRNA + 18<br>thr2_tRNA + 45 thr3_tRNA + 9 thrU_tRNA + 18 trpT_tRNA + 54<br>tyr1_tRNA + 198 val1_tRNA + 90 val2_tRNA + 45 val3_tRNA	irreversible	Translation	0.00E+00	1.00E+09	0	2.65E+01	2.65E+01	0	4.29E+01	42.85865	0	5.11E+00	5.1145
tl_elo_b0049_16_rib1	Translation elongation 1 b0049 16 ribosome(s)	4464 EF-G.GTP + 256 EF-Tu.GTP.ala1-tRNA + 128 EF-Tu.GTP.ala2<br>tRNA + 176 EF-Tu.GTP.arg1-tRNA + 16 EF-Tu.GTP.argU-tRNA + 48<br>EF-Tu.GTP.argX-tRNA + 128 EF-Tu.GTP.asn1-tRNA + 320 EF-<br>Tu.GTP.asp1-tRNA + 96 EF-Tu.GTP.cysT-tRNA + 80 EF-<br>Tu.GTP.gln1-tRNA + 32 EF-Tu.GTP.gln2-tRNA + 288 EF-<br>Tu.GTP.glu1-tRNA + 272 EF-Tu.GTP.gly1-tRNA + 16 EF-<br>Tu.GTP.glyT-tRNA + 80 EF-Tu.GTP.glyU-tRNA + 112 EF-<br>Tu.GTP.hisR-tRNA + 128 EF-Tu.GTP.ile1-tRNA + 16 EF-<br>Tu.GTP.ile2-tRNA + 432 EF-Tu.GTP.leu1-tRNA + 48 EF-<br>Tu.GTP.leu2-tRNA + 64 EF-Tu.GTP.leuU-tRNA + 16 EF-<br>Tu.GTP.leuW-tRNA + 32 EF-Tu.GTP.leuZ-tRNA + 208 EF-<br>Tu.GTP.lys1-tRNA + 80 EF-Tu.GTP.met1-tRNA + 176 EF-<br>Tu.GTP.phe1-tRNA + 176 EF-Tu.GTP.pro1-tRNA + 80 EF-<br>Tu.GTP.pro2-tRNA + 16 EF-Tu.GTP.proL-tRNA + 32 EF-<br>Tu.GTP.proM-tRNA + 32 EF-Tu.GTP.ser1-tRNA + 16 EF-<br>Tu.GTP.ser2-tRNA + 80 EF-Tu.GTP.ser3-tRNA + 16 EF-<br>Tu.GTP.serT-tRNA + 80 EF-Tu.GTP.serV-tRNA + 112 EF-<br>Tu.GTP.thr1-tRNA + 48 EF-Tu.GTP.thr3-tRNA + 48 EF-Tu.GTP.thrU-<br>tRNA + 128 EF-Tu.GTP.trpT-tRNA + 144 EF-Tu.GTP.tyr1-tRNA +<br>128 EF-Tu.GTP.val1-tRNA + 48 EF-Tu.GTP.val2-tRNA + 32 EF-<br>Tu.GTP.val3-tRNA + 22320 mg2 + 1 rib_ini_b0049_16 --> 1 rib_70_e	irreversible	Translation	0.00E+00	1.00E+09	0	6.44E+01	6.44E+01	0	1.10E+02	109.5437	0	5.50E+00	5.498
tl_elo_b0049_16_rib2	Translation elongation 2 b0049 16 ribosome(s)	4448 h2o + 1 rib_70_elo1_b0049_16_cplx --> 4448 EF-G.GDP +<br>4448 EF-Tu.GDP + 256 ala1_tRNA + 128 ala2_tRNA + 176<br>arg1_tRNA + 16 argU_tRNA + 48 argX_tRNA + 128 asn1_tRNA +<br>320 asp1_tRNA + 96 cysT_tRNA + 16 fmet_tRNA + 80 gln1_tRNA +<br>32 gln2_tRNA + 288 glu1_tRNA + 272 gly1_tRNA + 16 glyT_tRNA +<br>80 glyU_tRNA + 8896 h + 112 hisR_tRNA + 128 ile1_tRNA + 16<br>ile2_tRNA + 432 leu1_tRNA + 48 leu2_tRNA + 64 leuU_tRNA + 16<br>leuW_tRNA + 32 leuZ_tRNA + 208 lys1_tRNA + 80 met1_tRNA +<br>22320 mg2 + 176 phe1_tRNA + 8896 pi + 176 pro1_tRNA + 80<br>pro2_tRNA + 16 proL_tRNA + 32 proM_tRNA + 1<br>rib_70_elo2_b0049_16_cplx + 32 ser1_tRNA + 80 ser3_tRNA + 16<br>serT_tRNA + 80 serV_tRNA + 112 thr1_tRNA + 48 thr3_tRNA + 48<br>thrU_tRNA + 128 trpT_tRNA + 144 tyr1_tRNA + 128 val1_tRNA + 48<br>val2_tRNA + 32 val3_tRNA	irreversible	Translation	0.00E+00	1.00E+09	0	6.44E+01	6.44E+01	0	1.10E+02	109.5437	0	5.50E+00	5.498
tl_elo_b0049_1_rib1	Translation elongation 1 b0049 1 ribosome(s)	279 EF-G.GTP + 16 EF-Tu.GTP.ala1-tRNA + 8 EF-Tu.GTP.ala2-<br>tRNA + 11 EF-Tu.GTP.arg1-tRNA + 1 EF-Tu.GTP.argU-tRNA + 3 EF<br>Tu.GTP.argX-tRNA + 8 EF-Tu.GTP.asn1-tRNA + 20 EF-<br>Tu.GTP.asp1-tRNA + 6 EF-Tu.GTP.cysT-tRNA + 5 EF-Tu.GTP.gln1-<br>tRNA + 2 EF-Tu.GTP.gln2-tRNA + 18 EF-Tu.GTP.glu1-tRNA + 17<br>EF-Tu.GTP.gly1-tRNA + 1 EF-Tu.GTP.glyT-tRNA + 5 EF-<br>Tu.GTP.glyU-tRNA + 7 EF-Tu.GTP.hisR-tRNA + 8 EF-Tu.GTP.ile1-<br>tRNA + 1 EF-Tu.GTP.ile2-tRNA + 27 EF-Tu.GTP.leu1-tRNA + 3 EF-<br>Tu.GTP.leu2-tRNA + 4 EF-Tu.GTP.leuU-tRNA + 1 EF-Tu.GTP.leuW-<br>tRNA + 2 EF-Tu.GTP.leuZ-tRNA + 13 EF-Tu.GTP.lys1-tRNA + 5 EF-<br>Tu.GTP.met1-tRNA + 11 EF-Tu.GTP.phe1-tRNA + 11 EF-<br>Tu.GTP.pro1-tRNA + 5 EF-Tu.GTP.pro2-tRNA + 1 EF-Tu.GTP.proL-<br>tRNA + 2 EF-Tu.GTP.proM-tRNA + 2 EF-Tu.GTP.ser1-tRNA + 1 EF-<br>Tu.GTP.ser2-tRNA + 5 EF-Tu.GTP.ser3-tRNA + 1 EF-Tu.GTP.serT-<br>tRNA + 5 EF-Tu.GTP.serV-tRNA + 7 EF-Tu.GTP.thr1-tRNA + 3 EF-<br>Tu.GTP.thr3-tRNA + 3 EF-Tu.GTP.thrU-tRNA + 8 EF-Tu.GTP.trpT-<br>tRNA + 9 EF-Tu.GTP.tyr1-tRNA + 8 EF-Tu.GTP.val1-tRNA + 3 EF-<br>Tu.GTP.val2-tRNA + 2 EF-Tu.GTP.val3-tRNA + 1395 mg2 + 1<br>rib_ini_b0049_1 --> 1 rib_70_elo1_b0049_1_cplx	irreversible	Translation	0.00E+00	1.00E+09	0	6.44E+01	6.44E+01	0	1.10E+02	109.5437	0	5.50E+00	5.498
tl_elo_b0049_1_rib2	Translation elongation 2 b0049 1 ribosome(s)	278 h2o + 1 rib_70_elo1_b0049_1_cplx --> 278 EF-G.GDP + 278 EF-<br>Tu.GDP + 16 ala1_tRNA + 8 ala2_tRNA + 11 arg1_tRNA + 1<br>argU_tRNA + 3 argX_tRNA + 8 asn1_tRNA + 20 asp1_tRNA + 6<br>cysT_tRNA + 1 fmet_tRNA + 5 gln1_tRNA + 2 gln2_tRNA + 18<br>glu1_tRNA + 17 gly1_tRNA + 1 glyT_tRNA + 5 glyU_tRNA + 556 h +<br>7 hisR_tRNA + 8 ile1_tRNA + 1 ile2_tRNA + 27 leu1_tRNA + 3<br>leu2_tRNA + 4 leuU_tRNA + 1 leuW_tRNA + 2 leuZ_tRNA + 13<br>lys1_tRNA + 5 met1_tRNA + 1395 mg2 + 11 phe1_tRNA + 556 pi +<br>11 pro1_tRNA + 5 pro2_tRNA + 1 proL_tRNA + 2 proM_tRNA + 1<br>rib_70_elo2_b0049_1_cplx + 2 ser1_tRNA + 5 ser3_tRNA + 1<br>serT_tRNA + 5 serV_tRNA + 7 thr1_tRNA + 3 thr3_tRNA + 3<br>thrU_tRNA + 8 trpT_tRNA + 9 tyr1_tRNA + 8 val1_tRNA + 3<br>val2_tRNA + 2 val3_tRNA	irreversible	Translation	0.00E+00	1.00E+09	0	6.44E+01	6.44E+01	0	1.10E+02	109.5437	0	5.50E+00	5.498

tl_elo_b0049_8_rib1	Translation elongation 1 b0049 8 ribosome(s)	2232 EF-G.GTP + 128 EF-Tu.GTP.ala1-tRNA + 64 EF-Tu.GTP.ala2-tRNA + 88 EF-Tu.GTP.arg1-tRNA + 8 EF-Tu.GTP.argU-tRNA + 24 EF-Tu.GTP.argX-tRNA + 64 EF-Tu.GTP.asn1-tRNA + 160 EF-Tu.GTP.asp1-tRNA + 48 EF-Tu.GTP.cysT-tRNA + 40 EF-Tu.GTP.gln1-tRNA + 16 EF-Tu.GTP.gln2-tRNA + 144 EF-Tu.GTP.glu1-tRNA + 136 EF-Tu.GTP.gly1-tRNA + 8 EF-Tu.GTP.glyT-tRNA + 40 EF-Tu.GTP.glyU-tRNA + 56 EF-Tu.GTP.hisR-tRNA + 64 EF-Tu.GTP.ile1-tRNA + 8 EF-Tu.GTP.ile2-tRNA + 216 EF-Tu.GTP.leu1-tRNA + 24 EF-Tu.GTP.leu2-tRNA + 32 EF-Tu.GTP.leuU-tRNA + 8 EF-Tu.GTP.leuW-tRNA + 16 EF-Tu.GTP.leuZ-tRNA + 104 EF-Tu.GTP.lys1-tRNA + 40 EF-Tu.GTP.met1-tRNA + 88 EF-Tu.GTP.phe1-tRNA + 88 EF-Tu.GTP.pro1-tRNA + 40 EF-Tu.GTP.pro2-tRNA + 8 EF-Tu.GTP.proL-tRNA + 16 EF-Tu.GTP.proM-tRNA + 16 EF-Tu.GTP.ser1-tRNA + 8 EF-Tu.GTP.ser2-tRNA + 40 EF-Tu.GTP.ser3-tRNA + 8 EF-Tu.GTP.serT-tRNA + 40 EF-Tu.GTP.serV-tRNA + 56 EF-Tu.GTP.thr1-tRNA + 24 EF-Tu.GTP.thr3-tRNA + 24 EF-Tu.GTP.thrU-tRNA + 64 EF-Tu.GTP.trpT-tRNA + 72 EF-Tu.GTP.tyr1-tRNA + 64 EF-Tu.GTP.val1-tRNA + 24 EF-Tu.GTP.val2-tRNA + 16 EF-Tu.GTP.val3-tRNA + 11160 mg2 + 1 rib_ini_b0049_8 --> 1 rib_70_elo	irreversible	Translation	0.00E+00	1.00E+09	0	6.44E+01	6.44E+01	0	1.10E+02	109.5437	0	5.50E+00	5.498
tl_elo_b0049_8_rib2	Translation elongation 2 b0049 8 ribosome(s)	2224 h2o + 1 rib_70_elo1_b0049_8_cplx --> 2224 EF-G.GDP + 2224 EF-Tu.GDP + 128 ala1_tRNA + 64 ala2_tRNA + 88 arg1_tRNA + 8 argU_tRNA + 24 argX_tRNA + 64 asn1_tRNA + 160 asp1_tRNA + 48 cysT_tRNA + 8 fmet_tRNA + 40 gln1_tRNA + 16 gln2_tRNA + 144 glu1_tRNA + 136 gly1_tRNA + 8 glyT_tRNA + 40 glyU_tRNA + 4448 h + 56 hisR_tRNA + 64 ile1_tRNA + 8 ile2_tRNA + 216 leu1_tRNA + 24 leu2_tRNA + 32 leuU_tRNA + 8 leuW_tRNA + 16 leuZ_tRNA + 104 lys1_tRNA + 40 met1_tRNA + 11160 mg2 + 88 phe1_tRNA + 4448 pi + 88 pro1_tRNA + 40 pro2_tRNA + 8 proL_tRNA + 16 proM_tRNA + 1 rib_70_elo2_b0049_8_cplx + 16 ser1_tRNA + 40 ser3_tRNA + 8 serT_tRNA + 40 serV_tRNA + 56 thr1_tRNA + 24 thr3_tRNA + 24 thrU_tRNA + 64 trpT_tRNA + 72 tyr1_tRNA + 64 val1_tRNA + 24 val2_tRNA + 16 val3_tRNA	irreversible	Translation	0.00E+00	1.00E+09	0	6.44E+01	6.44E+01	0	1.10E+02	109.5437	0	5.50E+00	5.498
tl_elo_b0050_1_rib1	Translation elongation 1 b0050 1 ribosome(s)	124 EF-G.GTP + 5 EF-Tu.GTP.ala1-tRNA + 1 EF-Tu.GTP.ala2-tRNA + 7 EF-Tu.GTP.arg1-tRNA + 6 EF-Tu.GTP.asn1-tRNA + 3 EF-Tu.GTP.asp1-tRNA + 1 EF-Tu.GTP.cysT-tRNA + 6 EF-Tu.GTP.gln1-tRNA + 2 EF-Tu.GTP.gln2-tRNA + 10 EF-Tu.GTP.glu1-tRNA + 9 EF-Tu.GTP.gly1-tRNA + 1 EF-Tu.GTP.glyT-tRNA + 2 EF-Tu.GTP.glyU-tRNA + 2 EF-Tu.GTP.hisR-tRNA + 11 EF-Tu.GTP.ile1-tRNA + 1 EF-Tu.GTP.ile2-tRNA + 3 EF-Tu.GTP.leu1-tRNA + 2 EF-Tu.GTP.leu2-tRNA + 3 EF-Tu.GTP.leuU-tRNA + 2 EF-Tu.GTP.met1-tRNA + 3 EF-Tu.GTP.phe1-tRNA + 1 EF-Tu.GTP.pro1-tRNA + 3 EF-Tu.GTP.pro2-tRNA + 3 EF-Tu.GTP.proL-tRNA + 2 EF-Tu.GTP.proM-tRNA + 1 EF-Tu.GTP.ser2-tRNA + 1 EF-Tu.GTP.ser3-tRNA + 1 EF-Tu.GTP.serT-tRNA + 3 EF-Tu.GTP.serV-tRNA + 7 EF-Tu.GTP.thr1-tRNA + 1 EF-Tu.GTP.thrU-tRNA + 1 EF-Tu.GTP.trpT-tRNA + 7 EF-Tu.GTP.tyr1-tRNA + 5 EF-Tu.GTP.val1-tRNA + 4 EF-Tu.GTP.val2-tRNA + 4 EF-Tu.GTP.val3-tRNA + 620 mg2 + 1 rib_ini_b0050_1 --> 1 rib_70_elo1_b0050_1_cplx	irreversible	Translation	0.00E+00	1.00E+09	0	7.17E+01	7.17E+01	0	1.17E+02	116.9137	0	1.29E+01	12.868
tl_elo_b0050_1_rib2	Translation elongation 2 b0050 1 ribosome(s)	123 h2o + 1 rib_70_elo1_b0050_1_cplx --> 123 EF-G.GDP + 123 EF-Tu.GDP + 5 ala1_tRNA + 1 ala2_tRNA + 7 arg1_tRNA + 6 asn1_tRNA + 3 asp1_tRNA + 1 cysT_tRNA + 1 fmet_tRNA + 6 gln1_tRNA + 2 gln2_tRNA + 10 glu1_tRNA + 9 gly1_tRNA + 1 glyT_tRNA + 2 glyU_tRNA + 246 h + 1 hisR_tRNA + 11 ile1_tRNA + 1 ile2_tRNA + 3 leu1_tRNA + 2 leu2_tRNA + 3 leuU_tRNA + 2 met1_tRNA + 620 mg2 + 3 phe1_tRNA + 246 pi + 1 pro1_tRNA + 3 pro2_tRNA + 3 proL_tRNA + 2 proM_tRNA + 1 rib_70_elo2_b0050_1_cplx + 1 ser2_tRNA + 1 ser3_tRNA + 1 serT_tRNA + 3 serV_tRNA + 7 thr1_tRNA + 1 thrU_tRNA + 1 trpT_tRNA + 7 tyr1_tRNA + 5 val1_tRNA + 4 val2_tRNA + 4 val3_tRNA	irreversible	Translation	0.00E+00	1.00E+09	0	7.17E+01	7.17E+01	0	1.17E+02	116.9137	0	1.29E+01	12.868
tl_elo_b0050_3_rib1	Translation elongation 1 b0050 3 ribosome(s)	372 EF-G.GTP + 15 EF-Tu.GTP.ala1-tRNA + 3 EF-Tu.GTP.ala2-tRNA + 21 EF-Tu.GTP.arg1-tRNA + 18 EF-Tu.GTP.asn1-tRNA + 9 EF-Tu.GTP.asp1-tRNA + 3 EF-Tu.GTP.cysT-tRNA + 18 EF-Tu.GTP.gln1-tRNA + 6 EF-Tu.GTP.gln2-tRNA + 30 EF-Tu.GTP.glu1-tRNA + 27 EF-Tu.GTP.gly1-tRNA + 3 EF-Tu.GTP.glyT-tRNA + 6 EF-Tu.GTP.glyU-tRNA + 6 EF-Tu.GTP.hisR-tRNA + 33 EF-Tu.GTP.ile1-tRNA + 3 EF-Tu.GTP.ile2-tRNA + 9 EF-Tu.GTP.leu1-tRNA + 6 EF-Tu.GTP.leu2-tRNA + 9 EF-Tu.GTP.leuU-tRNA + 6 EF-Tu.GTP.met1-tRNA + 9 EF-Tu.GTP.phe1-tRNA + 3 EF-Tu.GTP.pro1-tRNA + 9 EF-Tu.GTP.pro2-tRNA + 9 EF-Tu.GTP.proL-tRNA + 6 EF-Tu.GTP.proM-tRNA + 3 EF-Tu.GTP.ser2-tRNA + 3 EF-Tu.GTP.ser3-tRNA + 3 EF-Tu.GTP.serT-tRNA + 9 EF-Tu.GTP.serV-tRNA + 21 EF-Tu.GTP.thr1-tRNA + 3 EF-Tu.GTP.thrU-tRNA + 3 EF-Tu.GTP.trpT-tRNA + 21 EF-Tu.GTP.tyr1-tRNA + 15 EF-Tu.GTP.val1-tRNA + 12 EF-Tu.GTP.val2-tRNA + 12 EF-Tu.GTP.val3-tRNA + 1860 mg2 + 1 rib_ini_b0050_3 --> 1 rib_70_elo1_b0050_3_cplx	irreversible	Translation	0.00E+00	1.00E+09	0	7.17E+01	7.17E+01	0	1.17E+02	116.9137	0	1.29E+01	12.868
tl_elo_b0050_3_rib2	Translation elongation 2 b0050 3 ribosome(s)	369 h2o + 1 rib_70_elo1_b0050_3_cplx --> 369 EF-G.GDP + 369 EF-Tu.GDP + 15 ala1_tRNA + 3 ala2_tRNA + 21 arg1_tRNA + 18 asn1_tRNA + 9 asp1_tRNA + 3 cysT_tRNA + 3 fmet_tRNA + 18 gln1_tRNA + 6 gln2_tRNA + 30 glu1_tRNA + 27 gly1_tRNA + 3 glyT_tRNA + 6 glyU_tRNA + 738 h + 3 hisR_tRNA + 33 ile1_tRNA + 3 ile2_tRNA + 9 leu1_tRNA + 6 leu2_tRNA + 9 leuU_tRNA + 6 met1_tRNA + 1860 mg2 + 9 phe1_tRNA + 738 pi + 3 pro1_tRNA + 9 pro2_tRNA + 9 proL_tRNA + 6 proM_tRNA + 1 rib_70_elo2_b0050_3_cplx + 3 ser2_tRNA + 3 ser3_tRNA + 3 serT_tRNA + 9 serV_tRNA + 21 thr1_tRNA + 3 thrU_tRNA + 3 trpT_tRNA + 21 tyr1_tRNA + 15 val1_tRNA + 12 val2_tRNA + 12 val3_tRNA	irreversible	Translation	0.00E+00	1.00E+09	0	7.17E+01	7.17E+01	0	1.17E+02	116.9137	0	1.29E+01	12.868

tl_elo_b0050_7_rib1	Translation elongation 1 b0050 7 ribosome(s)	868 EF-G.GTP + 35 EF-Tu.GTP.ala1-tRNA + 7 EF-Tu.GTP.ala2-tRNA + 49 EF-Tu.GTP.arg1-tRNA + 42 EF-Tu.GTP.asn1-tRNA + 21 EF-Tu.GTP.asp1-tRNA + 7 EF-Tu.GTP.cysT-tRNA + 42 EF-Tu.GTP.gln1-tRNA + 14 EF-Tu.GTP.gln2-tRNA + 70 EF-Tu.GTP.glu1-tRNA + 63 EF-Tu.GTP.gly1-tRNA + 7 EF-Tu.GTP.glyT-tRNA + 14 EF-Tu.GTP.glyU-tRNA + 14 EF-Tu.GTP.hisR-tRNA + 77 EF-Tu.GTP.ile1-tRNA + 7 EF-Tu.GTP.ile2-tRNA + 21 EF-Tu.GTP.leu1-tRNA + 14 EF-Tu.GTP.leu2-tRNA + 21 EF-Tu.GTP.leuU-tRNA + 14 EF-Tu.GTP.met1-tRNA + 21 EF-Tu.GTP.phe1-tRNA + 7 EF-Tu.GTP.pro1-tRNA + 21 EF-Tu.GTP.pro2-tRNA + 21 EF-Tu.GTP.proL-tRNA + 14 EF-Tu.GTP.proM-tRNA + 7 EF-Tu.GTP.ser2-tRNA + 7 EF-Tu.GTP.ser3-tRNA + 7 EF-Tu.GTP.serT-tRNA + 21 EF-Tu.GTP.serV-tRNA + 49 EF-Tu.GTP.thr1-tRNA + 7 EF-Tu.GTP.thrU-tRNA + 7 EF-Tu.GTP.trpT-tRNA + 49 EF-Tu.GTP.tyr1-tRNA + 35 EF-Tu.GTP.val1-tRNA + 28 EF-Tu.GTP.val2-tRNA + 28 EF-Tu.GTP.val3-tRNA + 4340 mg2 + 1 rib_ini_b0050_7 --> 1 rib_70_elo1_b0050_7_cplx	irreversible	Translation	0.00E+00	1.00E+09	0	7.17E+01	7.17E+01	0	1.17E+02	116.9137	0	1.29E+01	12.868
tl_elo_b0050_7_rib2	Translation elongation 2 b0050 7 ribosome(s)	861 h2o + 1 rib_70_elo1_b0050_7_cplx --> 861 EF-G.GDP + 861 EF-Tu.GDP + 35 ala1_tRNA + 7 ala2_tRNA + 49 arg1_tRNA + 42 asn1_tRNA + 21 asp1_tRNA + 7 cysT_tRNA + 7 fmet_tRNA + 42 gln1_tRNA + 14 gln2_tRNA + 70 glu1_tRNA + 63 gly1_tRNA + 7 glyT_tRNA + 14 glyU_tRNA + 1722 h + 7 hisR_tRNA + 77 ile1_tRNA + 7 ile2_tRNA + 21 leu1_tRNA + 14 leu2_tRNA + 21 leuU_tRNA + 14 met1_tRNA + 4340 mg2 + 21 phe1_tRNA + 1722 pi + 7 pro1_tRNA + 21 pro2_tRNA + 21 proL_tRNA + 14 proM_tRNA + 1 rib_70_elo2_b0050_7_cplx + 7 ser2_tRNA + 7 ser3_tRNA + 7 serT_tRNA + 21 serV_tRNA + 49 thr1_tRNA + 7 thrU_tRNA + 7 trpT_tRNA + 49 tyr1_tRNA + 35 val1_tRNA + 28 val2_tRNA + 28 val3_tRNA	irreversible	Translation	0.00E+00	1.00E+09	0	7.17E+01	7.17E+01	0	1.17E+02	116.9137	0	1.29E+01	12.868
tl_elo_b0051_16_rib1	Translation elongation 1 b0051 16 ribosome(s)	4352 EF-G.GTP + 256 EF-Tu.GTP.ala1-tRNA + 128 EF-Tu.GTP.ala2 tRNA + 256 EF-Tu.GTP.arg1-tRNA + 272 EF-Tu.GTP.asn1-tRNA + 176 EF-Tu.GTP.asp1-tRNA + 32 EF-Tu.GTP.cysT-tRNA + 160 EF-Tu.GTP.gln1-tRNA + 96 EF-Tu.GTP.gln2-tRNA + 208 EF-Tu.GTP.glu1-tRNA + 176 EF-Tu.GTP.gly1-tRNA + 32 EF-Tu.GTP.glyT-tRNA + 32 EF-Tu.GTP.glyU-tRNA + 112 EF-Tu.GTP.hisR-tRNA + 208 EF-Tu.GTP.ile1-tRNA + 240 EF-Tu.GTP.leu1-tRNA + 80 EF-Tu.GTP.leu2-tRNA + 48 EF-Tu.GTP.leuU-tRNA + 64 EF-Tu.GTP.leuZ-tRNA + 144 EF-Tu.GTP.lys1-tRNA + 176 EF-Tu.GTP.met1-tRNA + 208 EF-Tu.GTP.phe1-tRNA + 176 EF-Tu.GTP.pro1-tRNA + 64 EF-Tu.GTP.pro2-tRNA + 32 EF-Tu.GTP.proL-tRNA + 48 EF-Tu.GTP.proM-tRNA + 32 EF-Tu.GTP.ser1-tRNA + 32 EF-Tu.GTP.ser2-tRNA + 16 EF-Tu.GTP.serT-tRNA + 128 EF-Tu.GTP.serV-tRNA + 80 EF-Tu.GTP.thr1-tRNA + 16 EF-Tu.GTP.thr2 tRNA + 96 EF-Tu.GTP.thr3-tRNA + 16 EF-Tu.GTP.thrU-tRNA + 128 EF-Tu.GTP.tyr1-tRNA + 176 EF-Tu.GTP.val1-tRNA + 128 EF-Tu.GTP.val2-tRNA + 80 EF-Tu.GTP.val3-tRNA + 21760 mg2 + 1 rib_ini_b0051_16 --> 1 rib_70_elo1_b0051_16_cplx	irreversible	Translation	0.00E+00	1.00E+09	0	6.90E+01	6.90E+01	0	1.18E+02	118.2198	0	4.95E+00	4.948
tl_elo_b0051_16_rib2	Translation elongation 2 b0051 16 ribosome(s)	4336 h2o + 1 rib_70_elo1_b0051_16_cplx --> 4336 EF-G.GDP + 4336 EF-Tu.GDP + 256 ala1_tRNA + 128 ala2_tRNA + 256 arg1_tRNA + 272 asn1_tRNA + 176 asp1_tRNA + 32 cysT_tRNA + 16 fmet_tRNA + 160 gln1_tRNA + 96 gln2_tRNA + 208 glu1_tRNA + 176 gly1_tRNA + 32 glyT_tRNA + 32 glyU_tRNA + 8672 h + 112 hisR_tRNA + 208 ile1_tRNA + 240 leu1_tRNA + 80 leu2_tRNA + 48 leuU_tRNA + 64 leuZ_tRNA + 144 lys1_tRNA + 176 met1_tRNA + 21760 mg2 + 208 phe1_tRNA + 8672 pi + 176 pro1_tRNA + 64 pro2_tRNA + 32 proL_tRNA + 48 proM_tRNA + 1 rib_70_elo2_b0051_16_cplx + 32 ser1_tRNA + 32 ser2_tRNA + 16 serT_tRNA + 112 serV_tRNA + 80 thr1_tRNA + 16 thr2_tRNA + 96 thr3_tRNA + 16 thrU_tRNA + 128 tyr1_tRNA + 176 val1_tRNA + 128 val2_tRNA + 80 val3_tRNA	irreversible	Translation	0.00E+00	1.00E+09	0	6.90E+01	6.90E+01	0	1.18E+02	118.2198	0	4.95E+00	4.948
tl_elo_b0051_1_rib1	Translation elongation 1 b0051 1 ribosome(s)	272 EF-G.GTP + 16 EF-Tu.GTP.ala1-tRNA + 8 EF-Tu.GTP.ala2-tRNA + 16 EF-Tu.GTP.arg1-tRNA + 17 EF-Tu.GTP.asn1-tRNA + 11 EF-Tu.GTP.asp1-tRNA + 2 EF-Tu.GTP.cysT-tRNA + 10 EF-Tu.GTP.gln1-tRNA + 6 EF-Tu.GTP.gln2-tRNA + 13 EF-Tu.GTP.glu1-tRNA + 11 EF-Tu.GTP.gly1-tRNA + 2 EF-Tu.GTP.glyT-tRNA + 2 EF-Tu.GTP.glyU-tRNA + 7 EF-Tu.GTP.hisR-tRNA + 13 EF-Tu.GTP.ile1-tRNA + 15 EF-Tu.GTP.leu1-tRNA + 5 EF-Tu.GTP.leu2-tRNA + 3 EF-Tu.GTP.leuU-tRNA + 4 EF-Tu.GTP.leuZ-tRNA + 9 EF-Tu.GTP.lys1-tRNA + 11 EF-Tu.GTP.met1-tRNA + 13 EF-Tu.GTP.phe1-tRNA + 11 EF-Tu.GTP.pro1-tRNA + 4 EF-Tu.GTP.pro2-tRNA + 2 EF-Tu.GTP.proL-tRNA + 3 EF-Tu.GTP.proM-tRNA + 2 EF-Tu.GTP.ser1-tRNA + 2 EF-Tu.GTP.ser2-tRNA + 1 EF-Tu.GTP.serT-tRNA + 8 EF-Tu.GTP.serV-tRNA + 5 EF-Tu.GTP.thr1-tRNA + 1 EF-Tu.GTP.thr2-tRNA + 6 EF-Tu.GTP.thr3-tRNA + 1 EF-Tu.GTP.thrU-tRNA + 8 EF-Tu.GTP.tyr1-tRNA + 11 EF-Tu.GTP.val1-tRNA + 8 EF-Tu.GTP.val2-tRNA + 5 EF-Tu.GTP.val3-tRNA + 1360 mg2 + 1 rib_ini_b0051_1 --> 1 rib_70_elo1_b0051_1_cplx	irreversible	Translation	0.00E+00	1.00E+09	0	6.90E+01	6.90E+01	0	1.18E+02	118.2198	0	4.95E+00	4.948
tl_elo_b0051_1_rib2	Translation elongation 2 b0051 1 ribosome(s)	271 h2o + 1 rib_70_elo1_b0051_1_cplx --> 271 EF-G.GDP + 271 EF-Tu.GDP + 16 ala1_tRNA + 8 ala2_tRNA + 16 arg1_tRNA + 17 asn1_tRNA + 11 asp1_tRNA + 2 cysT_tRNA + 1 fmet_tRNA + 10 gln1_tRNA + 6 gln2_tRNA + 13 glu1_tRNA + 11 gly1_tRNA + 2 glyT_tRNA + 2 glyU_tRNA + 542 h + 7 hisR_tRNA + 13 ile1_tRNA + 15 leu1_tRNA + 5 leu2_tRNA + 3 leuU_tRNA + 4 leuZ_tRNA + 9 lys1_tRNA + 11 met1_tRNA + 1360 mg2 + 13 phe1_tRNA + 542 pi + 11 pro1_tRNA + 4 pro2_tRNA + 2 proL_tRNA + 3 proM_tRNA + 1 rib_70_elo2_b0051_1_cplx + 2 ser1_tRNA + 2 ser2_tRNA + 1 serT_tRNA + 7 serV_tRNA + 5 thr1_tRNA + 1 thr2_tRNA + 6 thr3_tRNA + 1 thrU_tRNA + 8 tyr1_tRNA + 11 val1_tRNA + 8 val2_tRNA + 5 val3_tRNA	irreversible	Translation	0.00E+00	1.00E+09	0	6.90E+01	6.90E+01	0	1.18E+02	118.2198	0	4.95E+00	4.948

tl_elo_b0051_8_rib1	Translation elongation 1 b0051 8 ribosome(s)	2176 EF-G.GTP + 128 EF-Tu.GTP.ala1-tRNA + 64 EF-Tu.GTP.ala2-tRNA + 128 EF-Tu.GTP.arg1-tRNA + 136 EF-Tu.GTP.asn1-tRNA + 88 EF-Tu.GTP.asp1-tRNA + 16 EF-Tu.GTP.cysT-tRNA + 80 EF-Tu.GTP.gln1-tRNA + 48 EF-Tu.GTP.gln2-tRNA + 104 EF-Tu.GTP.glu1-tRNA + 88 EF-Tu.GTP.gly1-tRNA + 16 EF-Tu.GTP.glyT tRNA + 16 EF-Tu.GTP.glyU-tRNA + 56 EF-Tu.GTP.hisR-tRNA + 104 EF-Tu.GTP.ile1-tRNA + 120 EF-Tu.GTP.leu1-tRNA + 40 EF-Tu.GTP.leu2-tRNA + 24 EF-Tu.GTP.leuU-tRNA + 32 EF-Tu.GTP.leuZ-tRNA + 72 EF-Tu.GTP.lys1-tRNA + 88 EF-Tu.GTP.met1-tRNA + 104 EF-Tu.GTP.phe1-tRNA + 88 EF-Tu.GTP.pro1-tRNA + 32 EF-Tu.GTP.pro2-tRNA + 16 EF-Tu.GTP.proL-tRNA + 24 EF-Tu.GTP.proM-tRNA + 16 EF-Tu.GTP.ser1-tRNA + 16 EF-Tu.GTP.ser2-tRNA + 8 EF-Tu.GTP.serT-tRNA + 64 EF-Tu.GTP.serV-tRNA + 40 EF-Tu.GTP.thr1-tRNA + 8 EF-Tu.GTP.thr2-tRNA + 48 EF-Tu.GTP.thr3-tRNA + 8 EF-Tu.GTP.thrU-tRNA + 64 EF-Tu.GTP.tyr1-tRNA + 88 EF-Tu.GTP.val1-tRNA + 64 EF-Tu.GTP.val2-tRNA + 40 EF-Tu.GTP.val3-tRNA + 10880 mg2 + 1 rib_ini_b0051_8 --> 1 rib_70_elo1_b0051_8_cplx	irreversible	Translation	0.00E+00	1.00E+09	0	6.90E+01	6.90E+01	0	1.18E+02	118.2198	0	4.95E+00	4.948
tl_elo_b0051_8_rib2	Translation elongation 2 b0051 8 ribosome(s)	2168 h2o + 1 rib_70_elo1_b0051_8_cplx --> 2168 EF-G.GDP + 2168 EF-Tu.GDP + 128 ala1_tRNA + 64 ala2_tRNA + 128 arg1_tRNA + 136 asn1_tRNA + 88 asp1_tRNA + 16 cysT_tRNA + 8 fmet_tRNA + 80 gln1_tRNA + 48 gln2_tRNA + 104 glu1_tRNA + 88 gly1_tRNA + 16 glyT_tRNA + 16 glyU_tRNA + 4336 h + 56 hisR_tRNA + 104 ile1_tRNA + 120 leu1_tRNA + 40 leu2_tRNA + 24 leuU_tRNA + 32 leuZ_tRNA + 72 lys1_tRNA + 88 met1_tRNA + 10880 mg2 + 104 phe1_tRNA + 4336 pi + 88 pro1_tRNA + 32 pro2_tRNA + 16 proL_tRNA + 24 proM_tRNA + 1 rib_70_elo2_b0051_8_cplx + 16 ser1_tRNA + 16 ser2_tRNA + 8 serT_tRNA + 56 serV_tRNA + 40 thr1_tRNA + 8 thr2_tRNA + 48 thr3_tRNA + 8 thrU_tRNA + 64 tyr1_tRNA + 88 val1_tRNA + 64 val2_tRNA + 40 val3_tRNA	irreversible	Translation	0.00E+00	1.00E+09	0	6.90E+01	6.90E+01	0	1.18E+02	118.2198	0	4.95E+00	4.948
tl_elo_b0052_19_rib1	Translation elongation 1 b0052 19 ribosome(s)	6232 EF-G.GTP + 513 EF-Tu.GTP.ala1-tRNA + 190 EF-Tu.GTP.ala2 tRNA + 228 EF-Tu.GTP.arg1-tRNA + 57 EF-Tu.GTP.argX-tRNA + 228 EF-Tu.GTP.asn1-tRNA + 266 EF-Tu.GTP.asp1-tRNA + 76 EF-Tu.GTP.cysT-tRNA + 152 EF-Tu.GTP.gln1-tRNA + 76 EF-Tu.GTP.gln2-tRNA + 342 EF-Tu.GTP.glu1-tRNA + 418 EF-Tu.GTP.gly1-tRNA + 19 EF-Tu.GTP.glyT-tRNA + 133 EF-Tu.GTP.glyU-tRNA + 209 EF-Tu.GTP.hisR-tRNA + 342 EF-Tu.GTP.ile1-tRNA + 19 EF-Tu.GTP.ile2-tRNA + 304 EF-Tu.GTP.leu1 tRNA + 76 EF-Tu.GTP.leu2-tRNA + 304 EF-Tu.GTP.leuU-tRNA + 57 EF-Tu.GTP.leuW-tRNA + 76 EF-Tu.GTP.leuZ-tRNA + 190 EF-Tu.GTP.lys1-tRNA + 133 EF-Tu.GTP.met1-tRNA + 171 EF-Tu.GTP.phe1-tRNA + 209 EF-Tu.GTP.pro1-tRNA + 133 EF-Tu.GTP.pro2-tRNA + 95 EF-Tu.GTP.proL-tRNA + 38 EF-Tu.GTP.ser1-tRNA + 19 EF-Tu.GTP.ser3-tRNA + 19 EF-Tu.GTP.serT-tRNA + 19 EF-Tu.GTP.serV-tRNA + 228 EF-Tu.GTP.thr1-tRNA + 76 EF-Tu.GTP.thr2-tRNA + 133 EF-Tu.GTP.thr3-tRNA + 57 EF-Tu.GTP.thrU-tRNA + 19 EF-Tu.GTP.trpT-tRNA + 95 EF-Tu.GTP.tyr1-tRNA + 247 EF-Tu.GTP.val1-tRNA + 114 EF-Tu.GTP.val2-tRNA + 152 EF-Tu.GTP.val3-tRNA + 31160 mg2 + 1 rib_ini_b0052_19 --> 1 rib_70_elo1_b0052_19_cplx	irreversible	Translation	0.00E+00	1.00E+09	0	6.88E+01	6.88E+01	0	1.18E+02	117.9798	0	4.71E+00	4.708
tl_elo_b0052_19_rib2	Translation elongation 2 b0052 19 ribosome(s)	6213 h2o + 1 rib_70_elo1_b0052_19_cplx --> 6213 EF-G.GDP + 6213 EF-Tu.GDP + 513 ala1_tRNA + 190 ala2_tRNA + 228 arg1_tRNA + 57 argX_tRNA + 228 asn1_tRNA + 266 asp1_tRNA + 76 cysT_tRNA + 19 fmet_tRNA + 152 gln1_tRNA + 57 gln2_tRNA + 342 glu1_tRNA + 418 gly1_tRNA + 19 glyT_tRNA + 133 glyU_tRNA + 12426 h + 209 hisR_tRNA + 342 ile1_tRNA + 19 ile2_tRNA + 304 leu1_tRNA + 76 leu2_tRNA + 304 leuU_tRNA + 57 leuW_tRNA + 76 leuZ_tRNA + 190 lys1_tRNA + 133 met1_tRNA + 31160 mg2 + 171 phe1_tRNA + 12426 pi + 209 pro1_tRNA + 133 pro2_tRNA + 95 proL_tRNA + 1 rib_70_elo2_b0052_19_cplx + 38 ser1_tRNA + 19 ser3_tRNA + 19 serT_tRNA + 19 serV_tRNA + 228 thr1_tRNA + 76 thr2_tRNA + 133 thr3_tRNA + 57 thrU_tRNA + 19 trpT_tRNA + 95 tyr1_tRNA + 247 val1_tRNA + 114 val2_tRNA + 152 val3_tRNA	irreversible	Translation	0.00E+00	1.00E+09	0	6.88E+01	6.88E+01	0	1.18E+02	117.9798	0	4.71E+00	4.708
tl_elo_b0052_1_rib1	Translation elongation 1 b0052 1 ribosome(s)	328 EF-G.GTP + 27 EF-Tu.GTP.ala1-tRNA + 10 EF-Tu.GTP.ala2-tRNA + 12 EF-Tu.GTP.arg1-tRNA + 3 EF-Tu.GTP.argX-tRNA + 12 EF-Tu.GTP.asn1-tRNA + 14 EF-Tu.GTP.asp1-tRNA + 4 EF-Tu.GTP.cysT-tRNA + 8 EF-Tu.GTP.gln1-tRNA + 4 EF-Tu.GTP.gln2-tRNA + 18 EF-Tu.GTP.glu1-tRNA + 22 EF-Tu.GTP.gly1-tRNA + 1 EF Tu.GTP.glyT-tRNA + 7 EF-Tu.GTP.glyU-tRNA + 11 EF-Tu.GTP.hisR-tRNA + 18 EF-Tu.GTP.ile1-tRNA + 1 EF-Tu.GTP.ile2-tRNA + 16 EF-Tu.GTP.leu1-tRNA + 4 EF-Tu.GTP.leu2-tRNA + 16 EF-Tu.GTP.leuU-tRNA + 3 EF-Tu.GTP.leuW-tRNA + 4 EF-Tu.GTP.leuZ-tRNA + 10 EF Tu.GTP.lys1-tRNA + 7 EF-Tu.GTP.met1-tRNA + 9 EF-Tu.GTP.phe1-tRNA + 11 EF-Tu.GTP.pro1-tRNA + 7 EF-Tu.GTP.pro2-tRNA + 5 EF-Tu.GTP.proL-tRNA + 2 EF-Tu.GTP.ser1-tRNA + 1 EF-Tu.GTP.ser3-tRNA + 1 EF-Tu.GTP.serT-tRNA + 1 EF-Tu.GTP.serV-tRNA + 12 EF-Tu.GTP.thr1-tRNA + 4 EF-Tu.GTP.thr2-tRNA + 7 EF-Tu.GTP.thr3-tRNA + 3 EF-Tu.GTP.thrU-tRNA + 1 EF-Tu.GTP.trpT-tRNA + 5 EF-Tu.GTP.tyr1-tRNA + 13 EF-Tu.GTP.val1-tRNA + 6 EF-Tu.GTP.val2-tRNA + 8 EF-Tu.GTP.val3-tRNA + 1640 mg2 + 1 rib_ini_b0052_1 --> 1 rib_70_elo1_b0052_1_cplx	irreversible	Translation	0.00E+00	1.00E+09	0	6.88E+01	6.88E+01	0	1.18E+02	117.9798	0	4.71E+00	4.708
tl_elo_b0052_1_rib2	Translation elongation 2 b0052 1 ribosome(s)	327 h2o + 1 rib_70_elo1_b0052_1_cplx --> 327 EF-G.GDP + 327 EF-Tu.GDP + 27 ala1_tRNA + 10 ala2_tRNA + 12 arg1_tRNA + 3 argX_tRNA + 12 asn1_tRNA + 14 asp1_tRNA + 4 cysT_tRNA + 1 fmet_tRNA + 8 gln1_tRNA + 3 gln2_tRNA + 18 glu1_tRNA + 22 gly1_tRNA + 1 glyT_tRNA + 7 glyU_tRNA + 654 h + 11 hisR_tRNA + 18 ile1_tRNA + 1 ile2_tRNA + 16 leu1_tRNA + 4 leu2_tRNA + 16 leuU_tRNA + 3 leuW_tRNA + 4 leuZ_tRNA + 10 lys1_tRNA + 7 met1_tRNA + 1640 mg2 + 9 phe1_tRNA + 654 pi + 11 pro1_tRNA + [illegible] pro2_tRNA + 5 proL_tRNA + 1 rib_70_elo2_b0052_1_cplx + 2 ser1_tRNA + 1 ser3_tRNA + 1 serT_tRNA + 1 serV_tRNA + 12 thr1_tRNA + 4 thr2_tRNA + 7 thr3_tRNA + 3 thrU_tRNA + 1 trpT_tRNA + 5 tyr1_tRNA + 13 val1_tRNA + 6 val2_tRNA + 8 val3_tRNA	irreversible	Translation	0.00E+00	1.00E+09	0	6.88E+01	6.88E+01	0	1.18E+02	117.9798	0	4.71E+00	4.708

tl_elo_b0052_9_rib1	Translation elongation 1 b0052 9 ribosome(s)	2952 EF-G.GTP + 243 EF-Tu.GTP.ala1-tRNA + 90 EF-Tu.GTP.ala2-tRNA + 108 EF-Tu.GTP.arg1-tRNA + 27 EF-Tu.GTP.argX-tRNA + 108 EF-Tu.GTP.asn1-tRNA + 126 EF-Tu.GTP.asp1-tRNA + 36 EF-Tu.GTP.cysT-tRNA + 72 EF-Tu.GTP.gln1-tRNA + 36 EF-Tu.GTP.gln2-tRNA + 162 EF-Tu.GTP.glu1-tRNA + 198 EF-Tu.GTP.gly1-tRNA + 9 EF-Tu.GTP.glyT-tRNA + 63 EF-Tu.GTP.glyU-tRNA + 99 EF-Tu.GTP.hisR-tRNA + 162 EF-Tu.GTP.ile1-tRNA + 9 EF-Tu.GTP.ile2-tRNA + 144 EF-Tu.GTP.leu1-tRNA + 36 EF-Tu.GTP.leu2-tRNA + 144 EF-Tu.GTP.leuU-tRNA + 27 EF-Tu.GTP.leuW-tRNA + 36 EF-Tu.GTP.leuZ-tRNA + 90 EF-Tu.GTP.lys1-tRNA + 63 EF-Tu.GTP.met1-tRNA + 81 EF-Tu.GTP.phe1-tRNA + 99 EF-Tu.GTP.pro1-tRNA + 63 EF-Tu.GTP.pro2-tRNA + 45 EF-Tu.GTP.proL-tRNA + 18 EF-Tu.GTP.ser1-tRNA + 9 EF-Tu.GTP.ser3-tRNA + 9 EF-Tu.GTP.serT-tRNA + 9 EF-Tu.GTP.serV-tRNA + 108 EF-Tu.GTP.thr1-tRNA + 36 EF-Tu.GTP.thr2-tRNA + 63 EF-Tu.GTP.thr3-tRNA + 27 EF-Tu.GTP.thrU-tRNA + 9 EF-Tu.GTP.trpT-tRNA + 45 EF-Tu.GTP.tyr1-tRNA + 117 EF-Tu.GTP.val1-tRNA + 54 EF-Tu.GTP.val2-tRNA + 72 EF-Tu.GTP.val3-tRNA + 14760 mg2 + 1 rib_ini_b0052_9 --> 1 rib_70_elo1_b0052_9_cplx	irreversible	Translation	0.00E+00	1.00E+09	0	6.88E+01	6.88E+01	0	1.18E+02	117.9798	0	4.71E+00	4.708
tl_elo_b0052_9_rib2	Translation elongation 2 b0052 9 ribosome(s)	2943 h2o + 1 rib_70_elo1_b0052_9_cplx --> 2943 EF-G.GDP + 2943 EF-Tu.GDP + 243 ala1_tRNA + 90 ala2_tRNA + 108 arg1_tRNA + 27 argX_tRNA + 108 asn1_tRNA + 126 asp1_tRNA + 36 cysT_tRNA + 9 fmet_tRNA + 72 gln1_tRNA + 27 gln2_tRNA + 162 glu1_tRNA + 198 gly1_tRNA + 9 glyT_tRNA + 63 glyU_tRNA + 5886 h + 99 hisR_tRNA + 162 ile1_tRNA + 9 ile2_tRNA + 144 leu1_tRNA + 36 leu2_tRNA + 144 leuU_tRNA + 27 leuW_tRNA + 36 leuZ_tRNA + 90 lys1_tRNA + 63 met1_tRNA + 14760 mg2 + 81 phe1_tRNA + 5886 pi + 99 pro1_tRNA + 63 pro2_tRNA + 45 proL_tRNA + 1 rib_70_elo2_b0052_9_cplx + 18 ser1_tRNA + 9 ser3_tRNA + 9 serT_tRNA + 9 serV_tRNA + 108 thr1_tRNA + 36 thr2_tRNA + 63 thr3_tRNA + 27 thrU_tRNA + 9 trpT_tRNA + 45 tyr1_tRNA + 117 val1_tRNA + 54 val2_tRNA + 72 val3_tRNA	irreversible	Translation	0.00E+00	1.00E+09	0	6.88E+01	6.88E+01	0	1.18E+02	117.9798	0	4.71E+00	4.708
tl_elo_b0053_12_rib1	Translation elongation 1 b0053 12 ribosome(s)	5124 EF-G.GTP + 420 EF-Tu.GTP.ala1-tRNA + 156 EF-Tu.GTP.ala2 tRNA + 288 EF-Tu.GTP.arg1-tRNA + 12 EF-Tu.GTP.argW-tRNA + 324 EF-Tu.GTP.asn1-tRNA + 336 EF-Tu.GTP.asp1-tRNA + 336 EF-Tu.GTP.gln1-tRNA + 72 EF-Tu.GTP.gln2-tRNA + 264 EF-Tu.GTP.glu1-tRNA + 252 EF-Tu.GTP.gly1-tRNA + 36 EF-Tu.GTP.glyT-tRNA + 24 EF-Tu.GTP.glyU-tRNA + 96 EF-Tu.GTP.hisR-tRNA + 372 EF-Tu.GTP.ile1-tRNA + 336 EF-Tu.GTP.leu1-tRNA + 24 EF-Tu.GTP.leu2-tRNA + 48 EF-Tu.GTP.leuU-tRNA + 36 EF-Tu.GTP.leuZ-tRNA + 276 EF-Tu.GTP.lys1-tRNA + 180 EF-Tu.GTP.met1-tRNA + 120 EF-Tu.GTP.phe1-tRNA + 108 EF-Tu.GTP.pro1-tRNA + 12 EF-Tu.GTP.pro2-tRNA + 24 EF-Tu.GTP.proL-tRNA + 24 EF-Tu.GTP.proM-tRNA + 36 EF-Tu.GTP.ser1-tRNA + 72 EF-Tu.GTP.ser2-tRNA + 48 EF-Tu.GTP.ser3-tRNA + 12 EF-Tu.GTP.serT-tRNA + 156 EF-Tu.GTP.serV-tRNA + 96 EF-Tu.GTP.thr1-tRNA + 48 EF-Tu.GTP.thr2-tRNA + 48 EF-Tu.GTP.thr3-tRNA + 12 EF-Tu.GTP.thrU-tRNA + 60 EF-Tu.GTP.trpT-tRNA + 60 EF-Tu.GTP.tyr1-tRNA + 120 EF-Tu.GTP.val1-tRNA + 96 EF-Tu.GTP.val2-tRNA + 84 EF-Tu.GTP.val3-tRNA + 25620 mg2 + 1 rib_ini_b0053_12 --> 1 rib_70_elo1_b0053_12_cplx	irreversible	Translation	0.00E+00	1.00E+09	0	3.18E+01	3.18E+01	0	5.29E+01	52.87944	0	4.53E+00	4.528
tl_elo_b0053_12_rib2	Translation elongation 2 b0053 12 ribosome(s)	5112 h2o + 1 rib_70_elo1_b0053_12_cplx --> 5112 EF-G.GDP + 5112 EF-Tu.GDP + 420 ala1_tRNA + 156 ala2_tRNA + 288 arg1_tRNA + 12 argW_tRNA + 312 asn1_tRNA + 336 asp1_tRNA + 12 fmet_tRNA + 336 gln1_tRNA + 72 gln2_tRNA + 264 glu1_tRNA + 252 gly1_tRNA + 36 glyT_tRNA + 24 glyU_tRNA + 10224 h + 96 hisR_tRNA + 372 ile1_tRNA + 336 leu1_tRNA + 24 leu2_tRNA + 48 leuU_tRNA + 36 leuZ_tRNA + 276 lys1_tRNA + 180 met1_tRNA + 25620 mg2 + 120 phe1_tRNA + 10224 pi + 108 pro1_tRNA + 12 pro2_tRNA + 24 proL_tRNA + 24 proM_tRNA + 1 rib_70_elo2_b0053_12_cplx + 36 ser1_tRNA + 72 ser2_tRNA + 48 ser3_tRNA + 12 serT_tRNA + 156 serV_tRNA + 96 thr1_tRNA + 48 thr2_tRNA + 48 thr3_tRNA + 12 thrU_tRNA + 60 trpT_tRNA + 60 tyr1_tRNA + 120 val1_tRNA + 96 val2_tRNA + 84 val3_tRNA	irreversible	Translation	0.00E+00	1.00E+09	0	3.18E+01	3.18E+01	0	5.29E+01	52.87944	0	4.53E+00	4.528
tl_elo_b0053_1_rib1	Translation elongation 1 b0053 1 ribosome(s)	427 EF-G.GTP + 35 EF-Tu.GTP.ala1-tRNA + 13 EF-Tu.GTP.ala2-tRNA + 24 EF-Tu.GTP.arg1-tRNA + 1 EF-Tu.GTP.argW-tRNA + 27 EF-Tu.GTP.asn1-tRNA + 28 EF-Tu.GTP.asp1-tRNA + 28 EF-Tu.GTP.gln1-tRNA + 6 EF-Tu.GTP.gln2-tRNA + 22 EF-Tu.GTP.glu1-tRNA + 21 EF-Tu.GTP.gly1-tRNA + 3 EF-Tu.GTP.glyT-tRNA + 2 EF-Tu.GTP.glyU-tRNA + 8 EF-Tu.GTP.hisR-tRNA + 31 EF-Tu.GTP.ile1-tRNA + 28 EF-Tu.GTP.leu1-tRNA + 2 EF-Tu.GTP.leu2-tRNA + 4 EF-Tu.GTP.leuU-tRNA + 3 EF-Tu.GTP.leuZ-tRNA + 23 EF-Tu.GTP.lys1-tRNA + 15 EF-Tu.GTP.met1-tRNA + 10 EF-Tu.GTP.phe1-tRNA + 9 EF-Tu.GTP.pro1-tRNA + 1 EF-Tu.GTP.pro2-tRNA + 2 EF-Tu.GTP.proL-tRNA + 2 EF-Tu.GTP.proM-tRNA + 3 EF-Tu.GTP.ser1-tRNA + 6 EF-Tu.GTP.ser2-tRNA + 4 EF-Tu.GTP.ser3-tRNA + 1 EF-Tu.GTP.serT-tRNA + 13 EF-Tu.GTP.serV-tRNA + 8 EF-Tu.GTP.thr1-tRNA + 4 EF-Tu.GTP.thr2-tRNA + 4 EF-Tu.GTP.thr3-tRNA + 1 EF-Tu.GTP.thrU-tRNA + 5 EF-Tu.GTP.trpT-tRNA + 5 EF-Tu.GTP.tyr1-tRNA + 10 EF-Tu.GTP.val1-tRNA + 8 EF-Tu.GTP.val2-tRNA + 7 EF-Tu.GTP.val3-tRNA + 2135 mg2 + 1 rib_ini_b0053_1 --> 1 rib_70_elo1_b0053_1_cplx	irreversible	Translation	0.00E+00	1.00E+09	0	3.18E+01	3.18E+01	0	5.29E+01	52.87944	0	4.53E+00	4.528
tl_elo_b0053_1_rib2	Translation elongation 2 b0053 1 ribosome(s)	426 h2o + 1 rib_70_elo1_b0053_1_cplx --> 426 EF-G.GDP + 426 EF-Tu.GDP + 35 ala1_tRNA + 13 ala2_tRNA + 24 arg1_tRNA + 1 argW_tRNA + 26 asn1_tRNA + 28 asp1_tRNA + 1 fmet_tRNA + 28 gln1_tRNA + 6 gln2_tRNA + 22 glu1_tRNA + 21 gly1_tRNA + 3 glyT_tRNA + 2 glyU_tRNA + 852 h + 8 hisR_tRNA + 31 ile1_tRNA + 28 leu1_tRNA + 2 leu2_tRNA + 4 leuU_tRNA + 3 leuZ_tRNA + 23 lys1_tRNA + 15 met1_tRNA + 2135 mg2 + 10 phe1_tRNA + 852 pi + 9 pro1_tRNA + 1 pro2_tRNA + 2 proL_tRNA + 2 proM_tRNA + 1 rib_70_elo2_b0053_1_cplx + 3 ser1_tRNA + 6 ser2_tRNA + 4 ser3_tRNA + 1 serT_tRNA + 13 serV_tRNA + 8 thr1_tRNA + 4 thr2_tRNA + 4 thr3_tRNA + 1 thrU_tRNA + 5 trpT_tRNA + 5 tyr1_tRNA + 10 val1_tRNA + 8 val2_tRNA + 7 val3_tRNA	irreversible	Translation	0.00E+00	1.00E+09	0	3.18E+01	3.18E+01	0	5.29E+01	52.87944	0	4.53E+00	4.528

tl_elo_b0053_25_rib1	Translation elongation 1 b0053 25 ribosome(s)	10675 EF-G.GTP + 875 EF-Tu.GTP.ala1-tRNA + 325 EF-Tu.GTP.ala2-tRNA + 600 EF-Tu.GTP.arg1-tRNA + 25 EF-Tu.GTP.argW-tRNA + 675 EF-Tu.GTP.asn1-tRNA + 700 EF-Tu.GTP.asp1-tRNA + 700 EF-Tu.GTP.gln1-tRNA + 150 EF-Tu.GTP.gln2-tRNA + 550 EF-Tu.GTP.glu1-tRNA + 525 EF-Tu.GTP.gly1-tRNA + 75 EF-Tu.GTP.glyT-tRNA + 50 EF-Tu.GTP.glyU-tRNA + 200 EF-Tu.GTP.hisR-tRNA + 775 EF-Tu.GTP.ile1-tRNA + 700 EF-Tu.GTP.leu1-tRNA + 50 EF-Tu.GTP.leu2-tRNA + 100 EF-Tu.GTP.leuU-tRNA + 75 EF-Tu.GTP.leuZ-tRNA + 575 EF-Tu.GTP.lys1-tRNA + 375 EF-Tu.GTP.met1-tRNA + 250 EF-Tu.GTP.phe1-tRNA + 225 EF-Tu.GTP.pro1-tRNA + 25 EF-Tu.GTP.pro2-tRNA + 50 EF-Tu.GTP.proL-tRNA + 50 EF-Tu.GTP.proM-tRNA + 75 EF-Tu.GTP.ser1-tRNA + 150 EF-Tu.GTP.ser2-tRNA + 100 EF-Tu.GTP.ser3-tRNA + 25 EF-Tu.GTP.serT-tRNA + 325 EF-Tu.GTP.serV-tRNA + 200 EF-Tu.GTP.thr1-tRNA + 100 EF-Tu.GTP.thr2-tRNA + 100 EF-Tu.GTP.thr3-tRNA + 25 EF-Tu.GTP.thrU-tRNA + 125 EF-Tu.GTP.trpT-tRNA + 125 EF-Tu.GTP.tyr1-tRNA + 250 EF-Tu.GTP.val1-tRNA + 200 EF-Tu.GTP.val2-tRNA + 175 EF-Tu.GTP.val3-tRNA + 53375 mg2 + 1 rib_ini_b0053_25 --> 1 rib_70_elo1_b0053_25_cplx	irreversible	Translation	0.00E+00	1.00E+09	0	3.18E+01	3.18E+01	0	5.29E+01	52.87944	0	4.53E+00	4.528
tl_elo_b0053_25_rib2	Translation elongation 2 b0053 25 ribosome(s)	10650 h2o + 1 rib_70_elo1_b0053_25_cplx --> 10650 EF-G.GDP + 10650 EF-Tu.GDP + 875 ala1_tRNA + 325 ala2_tRNA + 600 arg1_tRNA + 25 argW_tRNA + 650 asn1_tRNA + 700 asp1_tRNA + 25 fmet_tRNA + 700 gln1_tRNA + 150 gln2_tRNA + 550 glu1_tRNA + 525 gly1_tRNA + 75 glyT_tRNA + 50 glyU_tRNA + 21300 h + 200 hisR_tRNA + 775 ile1_tRNA + 700 leu1_tRNA + 50 leu2_tRNA + 100 leuU_tRNA + 75 leuZ_tRNA + 575 lys1_tRNA + 375 met1_tRNA + 53375 mg2 + 250 phe1_tRNA + 21300 pi + 225 pro1_tRNA + 25 pro2_tRNA + 50 proL_tRNA + 50 proM_tRNA + 1 rib_70_elo2_b0053_25_cplx + 75 ser1_tRNA + 150 ser2_tRNA + 100 ser3_tRNA + 25 serT_tRNA + 325 serV_tRNA + 200 thr1_tRNA + 100 thr2_tRNA + 100 thr3_tRNA + 25 thrU_tRNA + 125 trpT_tRNA + 125 tyr1_tRNA + 250 val1_tRNA + 200 val2_tRNA + 175 val3_tRNA	irreversible	Translation	0.00E+00	1.00E+09	0	3.18E+01	3.18E+01	0	5.29E+01	52.87944	0	4.53E+00	4.528
tl_elo_b0058_12_rib1	Translation elongation 1 b0058 12 ribosome(s)	2616 EF-G.GTP + 216 EF-Tu.GTP.ala1-tRNA + 24 EF-Tu.GTP.ala2-tRNA + 156 EF-Tu.GTP.arg1-tRNA + 24 EF-Tu.GTP.argU-tRNA + 12 EF-Tu.GTP.argX-tRNA + 72 EF-Tu.GTP.asn1-tRNA + 120 EF-Tu.GTP.asp1-tRNA + 24 EF-Tu.GTP.cysT-tRNA + 96 EF-Tu.GTP.gln1-tRNA + 12 EF-Tu.GTP.gln2-tRNA + 192 EF-Tu.GTP.glu1-tRNA + 96 EF-Tu.GTP.gly1-tRNA + 36 EF-Tu.GTP.glyU-tRNA + 108 EF-Tu.GTP.hisR-tRNA + 96 EF-Tu.GTP.ile1-tRNA + 108 EF-Tu.GTP.leu1-tRNA + 84 EF-Tu.GTP.leu2-tRNA + 12 EF-Tu.GTP.leuU-tRNA + 12 EF-Tu.GTP.leuW-tRNA + 24 EF-Tu.GTP.leuZ-tRNA + 132 EF-Tu.GTP.lys1-tRNA + 96 EF-Tu.GTP.met1-tRNA + 48 EF-Tu.GTP.phe1-tRNA + 120 EF-Tu.GTP.pro1-tRNA + 12 EF-Tu.GTP.pro2-tRNA + 24 EF-Tu.GTP.proL-tRNA + 72 EF-Tu.GTP.proM-tRNA + 12 EF-Tu.GTP.ser1-tRNA + 24 EF-Tu.GTP.ser3-tRNA + 24 EF-Tu.GTP.serT-tRNA + 48 EF-Tu.GTP.serV-tRNA + 48 EF-Tu.GTP.thr1-tRNA + 72 EF-Tu.GTP.thr3-tRNA + 36 EF-Tu.GTP.trpT-tRNA + 108 EF-Tu.GTP.tyr1-tRNA + 168 EF-Tu.GTP.val1-tRNA + 36 EF-Tu.GTP.val2-tRNA + 12 EF-Tu.GTP.val3-tRNA + 13080 mg2 + 1 rib_ini_b0058_12 --> 1 rib_70_elo1_b0058_12_cplx	irreversible	Translation	0.00E+00	1.00E+09	0	4.14E+01	4.14E+01	0	6.76E+01	67.60359	0	7.43E+00	7.426
tl_elo_b0058_12_rib2	Translation elongation 2 b0058 12 ribosome(s)	2604 h2o + 1 rib_70_elo1_b0058_12_cplx --> 2604 EF-G.GDP + 2604 EF-Tu.GDP + 216 ala1_tRNA + 24 ala2_tRNA + 156 arg1_tRNA + 24 argU_tRNA + 12 argX_tRNA + 72 asn1_tRNA + 120 asp1_tRNA + 24 cysT_tRNA + 12 fmet_tRNA + 96 gln1_tRNA + 12 gln2_tRNA + 192 glu1_tRNA + 96 gly1_tRNA + 36 glyU_tRNA + 5208 h + 108 hisR_tRNA + 96 ile1_tRNA + 108 leu1_tRNA + 84 leu2_tRNA + 12 leuU_tRNA + 12 leuW_tRNA + 24 leuZ_tRNA + 132 lys1_tRNA + 96 met1_tRNA + 13080 mg2 + 36 phe1_tRNA + 5208 pi + 120 pro1_tRNA + 12 pro2_tRNA + 24 proL_tRNA + 72 proM_tRNA + 1 rib_70_elo2_b0058_12_cplx + 12 ser1_tRNA + 24 ser3_tRNA + 24 serT_tRNA + 48 serV_tRNA + 48 thr1_tRNA + 72 thr3_tRNA + 36 trpT_tRNA + 108 tyr1_tRNA + 168 val1_tRNA + 36 val2_tRNA + 12 val3_tRNA	irreversible	Translation	0.00E+00	1.00E+09	0	4.14E+01	4.14E+01	0	6.76E+01	67.60359	0	7.43E+00	7.426
tl_elo_b0058_1_rib1	Translation elongation 1 b0058 1 ribosome(s)	218 EF-G.GTP + 18 EF-Tu.GTP.ala1-tRNA + 2 EF-Tu.GTP.ala2-tRNA + 13 EF-Tu.GTP.arg1-tRNA + 2 EF-Tu.GTP.argU-tRNA + 1 EF-Tu.GTP.argX-tRNA + 6 EF-Tu.GTP.asn1-tRNA + 10 EF-Tu.GTP.asp1-tRNA + 2 EF-Tu.GTP.cysT-tRNA + 8 EF-Tu.GTP.gln1-tRNA + 1 EF-Tu.GTP.gln2-tRNA + 16 EF-Tu.GTP.glu1-tRNA + 8 EF-Tu.GTP.gly1-tRNA + 3 EF-Tu.GTP.glyU-tRNA + 9 EF-Tu.GTP.hisR-tRNA + 8 EF-Tu.GTP.ile1-tRNA + 9 EF-Tu.GTP.leu1-tRNA + 7 EF-Tu.GTP.leu2-tRNA + 1 EF-Tu.GTP.leuU-tRNA + 1 EF-Tu.GTP.leuW-tRNA + 2 EF-Tu.GTP.leuZ-tRNA + 11 EF-Tu.GTP.lys1-tRNA + 8 EF-Tu.GTP.met1-tRNA + 4 EF-Tu.GTP.phe1-tRNA + 10 EF-Tu.GTP.pro1-tRNA + 1 EF-Tu.GTP.pro2-tRNA + 2 EF-Tu.GTP.proL-tRNA + 6 EF-Tu.GTP.proM-tRNA + 1 EF-Tu.GTP.ser1-tRNA + 2 EF-Tu.GTP.ser3-tRNA + 2 EF-Tu.GTP.serT-tRNA + 4 EF-Tu.GTP.serV-tRNA + 4 EF-Tu.GTP.thr1-tRNA + 6 EF-Tu.GTP.thr3-tRNA + 3 EF-Tu.GTP.trpT-tRNA + 9 EF-Tu.GTP.tyr1-tRNA + 14 EF-Tu.GTP.val1-tRNA + 3 EF-Tu.GTP.val2-tRNA + 1 EF-Tu.GTP.val3-tRNA + 1090 mg2 + 1 rib_ini_b0058_1 --> 1 rib_70_elo1_b0058_1_cplx	irreversible	Translation	0.00E+00	1.00E+09	0	4.14E+01	4.14E+01	0	6.76E+01	67.60359	0	7.43E+00	7.426
tl_elo_b0058_1_rib2	Translation elongation 2 b0058 1 ribosome(s)	217 h2o + 1 rib_70_elo1_b0058_1_cplx --> 217 EF-G.GDP + 217 EF-Tu.GDP + 18 ala1_tRNA + 2 ala2_tRNA + 13 arg1_tRNA + 2 argU_tRNA + 1 argX_tRNA + 6 asn1_tRNA + 10 asp1_tRNA + 2 cysT_tRNA + 1 fmet_tRNA + 8 gln1_tRNA + 1 gln2_tRNA + 16 glu1_tRNA + 8 gly1_tRNA + 3 glyU_tRNA + 434 h + 9 hisR_tRNA + 8 ile1_tRNA + 9 leu1_tRNA + 7 leu2_tRNA + 1 leuU_tRNA + 1 leuW_tRNA + 2 leuZ_tRNA + 11 lys1_tRNA + 8 met1_tRNA + 1090 mg2 + 3 phe1_tRNA + 434 pi + 10 pro1_tRNA + 1 pro2_tRNA + 2 proL_tRNA + 6 proM_tRNA + 1 rib_70_elo2_b0058_1_cplx + 1 ser1_tRNA + 2 ser3_tRNA + 2 serT_tRNA + 4 serV_tRNA + 4 thr1_tRNA + 6 thr3_tRNA + 3 trpT_tRNA + 9 tyr1_tRNA + 14 val1_tRNA + 3 val2_tRNA + 1 val3_tRNA	irreversible	Translation	0.00E+00	1.00E+09	0	4.14E+01	4.14E+01	0	6.76E+01	67.60359	0	7.43E+00	7.426

tl_elo_b0058_6_rib1	Translation elongation 1 b0058 6 ribosome(s)	1308 EF-G.GTP + 108 EF-Tu.GTP.ala1-tRNA + 12 EF-Tu.GTP.ala2-tRNA + 78 EF-Tu.GTP.arg1-tRNA + 12 EF-Tu.GTP.argU-tRNA + 6 EF-Tu.GTP.argX-tRNA + 36 EF-Tu.GTP.asn1-tRNA + 60 EF-Tu.GTP.asp1-tRNA + 12 EF-Tu.GTP.cysT-tRNA + 48 EF-Tu.GTP.gln1-tRNA + 6 EF-Tu.GTP.gln2-tRNA + 96 EF-Tu.GTP.glu1-tRNA + 48 EF-Tu.GTP.gly1-tRNA + 18 EF-Tu.GTP.glyU-tRNA + 54 EF-Tu.GTP.hisR-tRNA + 48 EF-Tu.GTP.ile1-tRNA + 54 EF-Tu.GTP.leu1-tRNA + 42 EF-Tu.GTP.leu2-tRNA + 6 EF-Tu.GTP.leuU-tRNA + 6 EF-Tu.GTP.leuW-tRNA + 12 EF-Tu.GTP.leuZ-tRNA + 66 EF-Tu.GTP.lys1-tRNA + 48 EF-Tu.GTP.met1-tRNA + 24 EF-Tu.GTP.phe1-tRNA + 60 EF-Tu.GTP.pro1-tRNA + 6 EF-Tu.GTP.pro2-tRNA + 12 EF-Tu.GTP.proL-tRNA + 36 EF-Tu.GTP.proM-tRNA + 6 EF-Tu.GTP.ser1-tRNA + 12 EF-Tu.GTP.ser3-tRNA + 12 EF-Tu.GTP.serT-tRNA + 24 EF-Tu.GTP.serV-tRNA + 24 EF-Tu.GTP.thr1-tRNA + 36 EF-Tu.GTP.thr3 tRNA + 18 EF-Tu.GTP.trpT-tRNA + 54 EF-Tu.GTP.tyr1-tRNA + 84 EF-Tu.GTP.val1-tRNA + 18 EF-Tu.GTP.val2-tRNA + 6 EF-Tu.GTP.val3-tRNA + 6540 mg2 + 1 rib_ini_b0058_6 --> 1 rib_70_elo1_b0058_6_cplx	irreversible	Translation	0.00E+00	1.00E+09	0	4.14E+01	4.14E+01	0	6.76E+01	67.60359	0	7.43E+00	7.426
tl_elo_b0058_6_rib2	Translation elongation 2 b0058 6 ribosome(s)	1302 h2o + 1 rib_70_elo1_b0058_6_cplx --> 1302 EF-G.GDP + 1302 EF-Tu.GDP + 108 ala1_tRNA + 12 ala2_tRNA + 78 arg1_tRNA + 12 argU_tRNA + 6 argX_tRNA + 36 asn1_tRNA + 60 asp1_tRNA + 12 cysT_tRNA + 6 fmet_tRNA + 48 gln1_tRNA + 6 gln2_tRNA + 96 glu1_tRNA + 48 gly1_tRNA + 18 glyU_tRNA + 2604 h + 54 hisR_tRNA + 48 ile1_tRNA + 54 leu1_tRNA + 42 leu2_tRNA + 6 leuU_tRNA + 6 leuW_tRNA + 12 leuZ_tRNA + 66 lys1_tRNA + 48 met1_tRNA + 6540 mg2 + 18 phe1_tRNA + 2604 pi + 60 pro1_tRNA + 6 pro2_tRNA + 12 proL_tRNA + 36 proM_tRNA + 1 rib_70_elo2_b0058_6_cplx + 6 ser1_tRNA + 12 ser3_tRNA + 12 serT_tRNA + 24 serV_tRNA + 24 thr1_tRNA + 36 thr3_tRNA + 18 trpT_tRNA + 54 tyr1_tRNA + 84 val1_tRNA + 18 val2_tRNA + 6 val3_tRNA	irreversible	Translation	0.00E+00	1.00E+09	0	4.14E+01	4.14E+01	0	6.76E+01	67.60359	0	7.43E+00	7.426
tl_elo_b0059_1_rib1	Translation elongation 1 b0059 1 ribosome(s)	967 EF-G.GTP + 56 EF-Tu.GTP.ala1-tRNA + 29 EF-Tu.GTP.ala2-tRNA + 74 EF-Tu.GTP.arg1-tRNA + 40 EF-Tu.GTP.asn1-tRNA + 67 EF-Tu.GTP.asp1-tRNA + 6 EF-Tu.GTP.cysT-tRNA + 49 EF-Tu.GTP.gln1-tRNA + 8 EF-Tu.GTP.gln2-tRNA + 82 EF-Tu.GTP.glu1-tRNA + 45 EF-Tu.GTP.gly1-tRNA + 5 EF-Tu.GTP.glyT-tRNA + 4 EF-Tu.GTP.glyU-tRNA + 29 EF-Tu.GTP.hisR-tRNA + 50 EF-Tu.GTP.ile1 tRNA + 88 EF-Tu.GTP.leu1-tRNA + 8 EF-Tu.GTP.leu2-tRNA + 13 EF Tu.GTP.leuU-tRNA + 4 EF-Tu.GTP.leuW-tRNA + 4 EF-Tu.GTP.leuZ-tRNA + 26 EF-Tu.GTP.lys1-tRNA + 23 EF-Tu.GTP.met1-tRNA + 37 EF-Tu.GTP.phe1-tRNA + 27 EF-Tu.GTP.pro1-tRNA + 4 EF-Tu.GTP.pro2-tRNA + 1 EF-Tu.GTP.proL-tRNA + 4 EF-Tu.GTP.proM-tRNA + 6 EF-Tu.GTP.ser1-tRNA + 8 EF-Tu.GTP.ser2-tRNA + 5 EF-Tu.GTP.ser3-tRNA + 3 EF-Tu.GTP.serT-tRNA + 28 EF-Tu.GTP.serV tRNA + 28 EF-Tu.GTP.thr1-tRNA + 7 EF-Tu.GTP.thr2-tRNA + 8 EF-Tu.GTP.thr3-tRNA + 2 EF-Tu.GTP.thrU-tRNA + 12 EF-Tu.GTP.trpT-tRNA + 19 EF-Tu.GTP.tyr1-tRNA + 37 EF-Tu.GTP.val1-tRNA + 7 EF Tu.GTP.val2-tRNA + 14 EF-Tu.GTP.val3-tRNA + 4835 mg2 + 1 rib_ini_b0059_1 --> 1 rib_70_elo1_b0059_1_cplx	irreversible	Translation	0.00E+00	1.00E+09	0	3.58E+01	3.58E+01	0	6.20E+01	62.00359	0	1.83E+00	1.826
tl_elo_b0059_1_rib2	Translation elongation 2 b0059 1 ribosome(s)	966 h2o + 1 rib_70_elo1_b0059_1_cplx --> 966 EF-G.GDP + 966 EF-Tu.GDP + 56 ala1_tRNA + 29 ala2_tRNA + 74 arg1_tRNA + 40 asn1_tRNA + 67 asp1_tRNA + 6 cysT_tRNA + 1 fmet_tRNA + 48 gln1_tRNA + 8 gln2_tRNA + 82 glu1_tRNA + 45 gly1_tRNA + 5 glyT_tRNA + 4 glyU_tRNA + 1932 h + 29 hisR_tRNA + 50 ile1_tRNA + 88 leu1_tRNA + 8 leu2_tRNA + 13 leuU_tRNA + 4 leuW_tRNA + 4 leuZ_tRNA + 26 lys1_tRNA + 23 met1_tRNA + 4835 mg2 + 37 phe1_tRNA + 1932 pi + 27 pro1_tRNA + 4 pro2_tRNA + 1 proL_tRN/ + 4 proM_tRNA + 1 rib_70_elo2_b0059_1_cplx + 6 ser1_tRNA + 8 ser2_tRNA + 5 ser3_tRNA + 3 serT_tRNA + 28 serV_tRNA + 28 thr1_tRNA + 7 thr2_tRNA + 8 thr3_tRNA + 2 thrU_tRNA + 12 trpT_tRNA + 19 tyr1_tRNA + 37 val1_tRNA + 7 val2_tRNA + 14 val3_tRNA	irreversible	Translation	0.00E+00	1.00E+09	0	3.58E+01	3.58E+01	0	6.20E+01	62.00359	0	1.83E+00	1.826
tl_elo_b0059_28_rib1	Translation elongation 1 b0059 28 ribosome(s)	27076 EF-G.GTP + 1568 EF-Tu.GTP.ala1-tRNA + 812 EF-Tu.GTP.ala2-tRNA + 2072 EF-Tu.GTP.arg1-tRNA + 1120 EF-Tu.GTP.asn1-tRNA + 1876 EF-Tu.GTP.asp1-tRNA + 168 EF-Tu.GTP.cysT-tRNA + 1372 EF-Tu.GTP.gln1-tRNA + 224 EF-Tu.GTP.gln2-tRNA + 2296 EF-Tu.GTP.glu1-tRNA + 1260 EF-Tu.GTP.gly1-tRNA + 140 EF-Tu.GTP.glyT-tRNA + 112 EF-Tu.GTP.glyU-tRNA + 812 EF-Tu.GTP.hisR-tRNA + 1400 EF-Tu.GTP.ile1-tRNA + 2464 EF-Tu.GTP.leu1-tRNA + 224 EF-Tu.GTP.leu2-tRNA + 364 EF-Tu.GTP.leuU-tRNA + 112 EF-Tu.GTP.leuW-tRNA + 112 EF-Tu.GTP.leuZ-tRNA + 728 EF-Tu.GTP.lys1-tRNA + 644 EF-Tu.GTP.met1-tRNA + 1036 EF-Tu.GTP.phe1-tRNA + 756 EF-Tu.GTP.pro1-tRNA + 112 EF-Tu.GTP.pro2-tRNA + 28 EF-Tu.GTP.proL-tRNA + 112 EF-Tu.GTP.proM-tRNA + 168 EF-Tu.GTP.ser1-tRNA + 224 EF-Tu.GTP.ser2-tRNA + 140 EF-Tu.GTP.ser3-tRNA + 84 EF-Tu.GTP.serT-tRNA + 784 EF-Tu.GTP.serV-tRNA + 784 EF-Tu.GTP.thr1-tRNA + 196 EF-Tu.GTP.thr2-tRNA + 224 EF-Tu.GTP.thr3-tRNA + 56 EF-Tu.GTP.thrU-tRNA + 336 EF-Tu.GTP.trpT-tRNA + 532 EF-Tu.GTP.tyr1-tRNA + 1036 EF-Tu.GTP.val1-tRNA + 196 EF-Tu.GTP.val2-tRNA + 392 EF-Tu.GTP.val3-tRNA + 135380 mg2 + 1 rib_ini_b0059_28 --> 1 rib_70_	irreversible	Translation	0.00E+00	1.00E+09	0	3.58E+01	3.58E+01	0	6.20E+01	62.00359	0	1.83E+00	1.826
tl_elo_b0059_28_rib2	Translation elongation 2 b0059 28 ribosome(s)	27048 h2o + 1 rib_70_elo1_b0059_28_cplx --> 27048 EF-G.GDP + 27048 EF-Tu.GDP + 1568 ala1_tRNA + 812 ala2_tRNA + 2072 arg1_tRNA + 1120 asn1_tRNA + 1876 asp1_tRNA + 168 cysT_tRNA + 28 fmet_tRNA + 1344 gln1_tRNA + 224 gln2_tRNA + 2296 glu1_tRNA + 1260 gly1_tRNA + 140 glyT_tRNA + 112 glyU_tRNA + 54096 h + 812 hisR_tRNA + 1400 ile1_tRNA + 2464 leu1_tRNA + 224 leu2_tRNA + 364 leuU_tRNA + 112 leuW_tRNA + 112 leuZ_tRNA + 728 lys1_tRNA + 644 met1_tRNA + 135380 mg2 + 103 phe1_tRNA + 54096 pi + 756 pro1_tRNA + 112 pro2_tRNA + 28 proL_tRNA + 112 proM_tRNA + 1 rib_70_elo2_b0059_28_cplx + 168 ser1_tRNA + 224 ser2_tRNA + 140 ser3_tRNA + 84 serT_tRNA + 784 serV_tRNA + 784 thr1_tRNA + 196 thr2_tRNA + 224 thr3_tRNA + 56 thrU_tRNA + 336 trpT_tRNA + 532 tyr1_tRNA + 1036 val1_tRNA + 196 val2_tRNA + 392 val3_tRNA	irreversible	Translation	0.00E+00	1.00E+09	0	3.58E+01	3.58E+01	0	6.20E+01	62.00359	0	1.83E+00	1.826

tl_elo_b0059_56_rib1	Translation elongation 1 b0059 56 ribosome(s)	54152 EF-G.GTP + 3136 EF-Tu.GTP.ala1-tRNA + 1624 EF-Tu.GTP.ala2-tRNA + 4144 EF-Tu.GTP.arg1-tRNA + 2240 EF-Tu.GTP.asn1-tRNA + 3752 EF-Tu.GTP.asp1-tRNA + 336 EF-Tu.GTP.cysT-tRNA + 2744 EF-Tu.GTP.gln1-tRNA + 448 EF-Tu.GTP.gln2-tRNA + 4592 EF-Tu.GTP.glu1-tRNA + 2520 EF-Tu.GTP.gly1-tRNA + 280 EF-Tu.GTP.glyT-tRNA + 224 EF-Tu.GTP.glyU-tRNA + 1624 EF-Tu.GTP.hisR-tRNA + 2800 EF-Tu.GTP.ile1-tRNA + 4928 EF-Tu.GTP.leu1-tRNA + 448 EF-Tu.GTP.leu2-tRNA + 728 EF-Tu.GTP.leuU-tRNA + 224 EF-Tu.GTP.leuW-tRNA + 224 EF-Tu.GTP.leuZ-tRNA + 1456 EF-Tu.GTP.lys1-tRNA + 1288 EF-Tu.GTP.met1-tRNA + 2072 EF-Tu.GTP.phe1-tRNA + 1512 EF-Tu.GTP.pro1-tRNA + 224 EF-Tu.GTP.pro2-tRNA + 56 EF-Tu.GTP.proL-tRNA + 224 EF-Tu.GTP.proM-tRNA + 336 EF-Tu.GTP.ser1-tRNA + 448 EF-Tu.GTP.ser2-tRNA + 280 EF-Tu.GTP.ser3-tRNA + 168 EF-Tu.GTP.serT-tRNA + 1568 EF-Tu.GTP.serV-tRNA + 1568 EF-Tu.GTP.thr1-tRNA + 392 EF-Tu.GTP.thr2-tRNA + 448 EF-Tu.GTP.thr3-tRNA + 112 EF-Tu.GTP.thrU-tRNA + 672 EF-Tu.GTP.trpT-tRNA + 1064 EF-Tu.GTP.tyr1-tRNA + 2072 EF-Tu.GTP.val1-tRNA + 392 EF-Tu.GTP.val2-tRNA + 784 EF-Tu.GTP.val3-tRNA + 270760 mg2 + 1 rib_ini_b0059_56 --> 1 rib_70_	irreversible	Translation	0.00E+00	1.00E+09	0	3.58E+01	3.58E+01	0	4.28E+01	42.78851	0	1.83E+00	1.826
tl_elo_b0059_56_rib2	Translation elongation 2 b0059 56 ribosome(s)	54096 h2o + 1 rib_70_elo1_b0059_56_cplx --> 54096 EF-G.GDP + 54096 EF-Tu.GDP + 3136 ala1_tRNA + 1624 ala2_tRNA + 4144 arg1_tRNA + 2240 asn1_tRNA + 3752 asp1_tRNA + 336 cysT_tRNA + 56 fmet_tRNA + 2688 gln1_tRNA + 448 gln2_tRNA + 4592 glu1_tRNA + 2520 gly1_tRNA + 280 glyT_tRNA + 224 glyU_tRNA + 108192 h + 1624 hisR_tRNA + 2800 ile1_tRNA + 4928 leu1_tRNA + 448 leu2_tRNA + 728 leuU_tRNA + 224 leuW_tRNA + 224 leuZ_tRNA + 1456 lys1_tRNA + 1288 met1_tRNA + 270760 mg2 + 2072 phe1_tRNA + 108192 pi + 1512 pro1_tRNA + 224 pro2_tRNA + 56 proL_tRNA + 224 proM_tRNA + 1 rib_70_elo2_b0059_56_cplx + 336 ser1_tRNA + 448 ser2_tRNA + 280 ser3_tRNA + 168 serT_tRN + 1568 serV_tRNA + 1568 thr1_tRNA + 392 thr2_tRNA + 448 thr3_tRNA + 112 thrU_tRNA + 672 trpT_tRNA + 1064 tyr1_tRNA + 2072 val1_tRNA + 392 val2_tRNA + 784 val3_tRNA	irreversible	Translation	0.00E+00	1.00E+09	0	3.58E+01	3.58E+01	0	4.28E+01	42.78851	0	1.83E+00	1.826
tl_elo_b0144_18_rib1	Translation elongation 1 b0144 18 ribosome(s)	5526 EF-G.GTP + 342 EF-Tu.GTP.ala1-tRNA + 198 EF-Tu.GTP.ala2-tRNA + 306 EF-Tu.GTP.arg1-tRNA + 18 EF-Tu.GTP.argW-tRNA + 72 EF-Tu.GTP.argX-tRNA + 126 EF-Tu.GTP.asn1-tRNA + 324 EF-Tu.GTP.asp1-tRNA + 72 EF-Tu.GTP.cysT-tRNA + 234 EF-Tu.GTP.gln1-tRNA + 180 EF-Tu.GTP.gln2-tRNA + 270 EF-Tu.GTP.glu1-tRNA + 288 EF-Tu.GTP.gly1-tRNA + 36 EF-Tu.GTP.glyT-tRNA + 54 EF-Tu.GTP.glyU-tRNA + 270 EF-Tu.GTP.hisR-tRNA + 324 EF-Tu.GTP.ile1-tRNA + 36 EF-Tu.GTP.ile2-tRNA + 234 EF-Tu.GTP.leu1-tRNA + 90 EF-Tu.GTP.leu2-tRNA + 198 EF-Tu.GTP.leuU-tRNA + 36 EF-Tu.GTP.leuW-tRNA + 18 EF-Tu.GTP.leuZ-tRNA + 126 EF-Tu.GTP.lys1-tRNA + 18 EF-Tu.GTP.met1-tRNA + 216 EF-Tu.GTP.phe1-tRNA + 108 EF-Tu.GTP.pro1-tRNA + 72 EF-Tu.GTP.pro2-tRNA + 54 EF-Tu.GTP.proL-tRNA + 90 EF-Tu.GTP.proM-tRNA + 36 EF-Tu.GTP.ser1-tRNA + 36 EF-Tu.GTP.ser2-tRNA + 54 EF-Tu.GTP.ser3-tRNA + 72 EF-Tu.GTP.serT-tRNA + 72 EF-Tu.GTP.serV-tRNA + 36 EF-Tu.GTP.thr1-tRNA + 36 EF-Tu.GTP.thr2-tRNA + 36 EF-Tu.GTP.thr3-tRNA + 90 EF-Tu.GTP.thrU-tRNA + 126 EF-Tu.GTP.trpT-tRNA + 198 EF-Tu.GTP.tyr1-tRNA + 180 EF-Tu.GTP.val1-tRNA + 54 EF-Tu.GTP.val2-tRNA + 90 EF-Tu.GTP.val3-tRNA + 27630 mg2 + 1 rib_	irreversible	Translation	0.00E+00	1.00E+09	0	1.28E+02	1.28E+02	0	2.23E+02	222.8318	0	5.35E+00	5.353
tl_elo_b0144_18_rib2	Translation elongation 2 b0144 18 ribosome(s)	5508 h2o + 1 rib_70_elo1_b0144_18_cplx --> 5508 EF-G.GDP + 5508 EF-Tu.GDP + 342 ala1_tRNA + 198 ala2_tRNA + 306 arg1_tRNA + 18 argW_tRNA + 72 argX_tRNA + 126 asn1_tRNA + 324 asp1_tRNA + 54 cysT_tRNA + 18 fmet_tRNA + 234 gln1_tRNA + 180 gln2_tRNA + 270 glu1_tRNA + 288 gly1_tRNA + 36 glyT_tRNA + 54 glyU_tRNA + 11016 h + 270 hisR_tRNA + 324 ile1_tRNA + 36 ile2_tRNA + 234 leu1_tRNA + 90 leu2_tRNA + 198 leuU_tRNA + 36 leuW_tRNA + 18 leuZ_tRNA + 126 lys1_tRNA + 18 met1_tRNA + 27630 mg2 + 216 phe1_tRNA + 11016 pi + 108 pro1_tRNA + 72 pro2_tRNA + 54 proL_tRNA + 90 proM_tRNA + 1 rib_70_elo2_b0144_18_cplx + 36 ser1_tRNA + 36 ser2_tRNA + 54 ser3_tRNA + 72 serT_tRNA + 72 serV_tRNA + 36 thr1_tRNA + 36 thr2_tRNA + 36 thr3_tRNA + 90 thrU_tRNA + 126 trpT_tRNA + 198 tyr1_tRNA + 180 val1_tRNA + 54 val2_tRNA + 90 val3_tRNA	irreversible	Translation	0.00E+00	1.00E+09	0	1.28E+02	1.28E+02	0	2.23E+02	222.8318	0	5.35E+00	5.353
tl_elo_b0144_1_rib1	Translation elongation 1 b0144 1 ribosome(s)	307 EF-G.GTP + 19 EF-Tu.GTP.ala1-tRNA + 11 EF-Tu.GTP.ala2-tRNA + 17 EF-Tu.GTP.arg1-tRNA + 1 EF-Tu.GTP.argW-tRNA + 4 EF-Tu.GTP.argX-tRNA + 7 EF-Tu.GTP.asn1-tRNA + 18 EF-Tu.GTP.asp1-tRNA + 4 EF-Tu.GTP.cysT-tRNA + 13 EF-Tu.GTP.gln1-tRNA + 10 EF-Tu.GTP.gln2-tRNA + 15 EF-Tu.GTP.glu1-tRNA + 16 EF-Tu.GTP.gly1-tRNA + 2 EF-Tu.GTP.glyT-tRNA + 3 EF-Tu.GTP.glyU-tRNA + 15 EF-Tu.GTP.hisR-tRNA + 18 EF-Tu.GTP.ile1-tRNA + 2 EF-Tu.GTP.ile2-tRNA + 13 EF-Tu.GTP.leu1-tRNA + 5 EF-Tu.GTP.leu2-tRNA + 11 EF-Tu.GTP.leuU-tRNA + 2 EF-Tu.GTP.leuW-tRNA + 1 EF-Tu.GTP.leuZ-tRNA + 7 EF-Tu.GTP.lys1-tRNA + 1 EF-Tu.GTP.met1-tRNA + 12 EF-Tu.GTP.phe1-tRNA + 6 EF-Tu.GTP.pro1-tRNA + 4 EF-Tu.GTP.pro2-tRNA + 3 EF-Tu.GTP.proL-tRNA + 5 EF-Tu.GTP.proM-tRNA + 2 EF-Tu.GTP.ser1-tRNA + 2 EF-Tu.GTP.ser2-tRNA + 3 EF-Tu.GTP.ser3-tRNA + 4 EF-Tu.GTP.serT-tRNA + 4 EF-Tu.GTP.serV-tRNA + 2 EF-Tu.GTP.thr1-tRNA + 2 EF-Tu.GTP.thr2-tRNA + 2 EF-Tu.GTP.thr3-tRNA + 5 EF-Tu.GTP.thrU-tRNA + 7 EF-Tu.GTP.trpT-tRNA + 11 EF-Tu.GTP.tyr1-tRNA + 10 EF-Tu.GTP.val1-tRNA + 3 EF-Tu.GTP.val2-tRNA + 5 EF-Tu.GTP.val3-tRNA + 1535 mg2 + 1 rib_ini_b0144_1 -->	irreversible	Translation	0.00E+00	1.00E+09	0	1.28E+02	1.28E+02	0	2.23E+02	222.8318	0	5.35E+00	5.353
tl_elo_b0144_1_rib2	Translation elongation 2 b0144 1 ribosome(s)	306 h2o + 1 rib_70_elo1_b0144_1_cplx --> 306 EF-G.GDP + 306 EF-Tu.GDP + 19 ala1_tRNA + 11 ala2_tRNA + 17 arg1_tRNA + 1 argW_tRNA + 4 argX_tRNA + 7 asn1_tRNA + 18 asp1_tRNA + 3 cysT_tRNA + 1 fmet_tRNA + 13 gln1_tRNA + 10 gln2_tRNA + 15 glu1_tRNA + 16 gly1_tRNA + 2 glyT_tRNA + 3 glyU_tRNA + 612 h + 15 hisR_tRNA + 18 ile1_tRNA + 2 ile2_tRNA + 13 leu1_tRNA + 5 leu2_tRNA + 11 leuU_tRNA + 2 leuW_tRNA + 1 leuZ_tRNA + 7 lys1_tRNA + 1 met1_tRNA + 1535 mg2 + 12 phe1_tRNA + 612 pi + 6 pro1_tRNA + 4 pro2_tRNA + 3 proL_tRNA + 5 proM_tRNA + 1 rib_70_elo2_b0144_1_cplx + 2 ser1_tRNA + 2 ser2_tRNA + 3 ser3_tRNA + 4 serT_tRNA + 4 serV_tRNA + 2 thr1_tRNA + 2 thr2_tRNA + 2 thr3_tRNA + 5 thrU_tRNA + 7 trpT_tRNA + 11 tyr1_tRNA + 10 val1_tRNA + 3 val2_tRNA + 5 val3_tRNA	irreversible	Translation	0.00E+00	1.00E+09	0	1.28E+02	1.28E+02	0	2.23E+02	222.8318	0	5.35E+00	5.353

tl_elo_b0144_9_rib1	Translation elongation 1 b0144 9 ribosome(s)	2763 EF-G.GTP + 171 EF-Tu.GTP.ala1-tRNA + 99 EF-Tu.GTP.ala2-tRNA + 153 EF-Tu.GTP.arg1-tRNA + 9 EF-Tu.GTP.argW-tRNA + 36 EF-Tu.GTP.argX-tRNA + 63 EF-Tu.GTP.asn1-tRNA + 162 EF-Tu.GTP.asp1-tRNA + 36 EF-Tu.GTP.cysT-tRNA + 117 EF-Tu.GTP.gln1-tRNA + 90 EF-Tu.GTP.gln2-tRNA + 135 EF-Tu.GTP.glu1-tRNA + 144 EF-Tu.GTP.gly1-tRNA + 18 EF-Tu.GTP.glyT-tRNA + 27 EF-Tu.GTP.glyU-tRNA + 135 EF-Tu.GTP.hisR-tRNA + 162 EF-Tu.GTP.ile1-tRNA + 18 EF-Tu.GTP.ile2-tRNA + 117 EF-Tu.GTP.leu1-tRNA + 45 EF-Tu.GTP.leu2-tRNA + 99 EF-Tu.GTP.leuU-tRNA + 18 EF-Tu.GTP.leuW-tRNA + 9 EF-Tu.GTP.leuZ-tRNA + 63 EF-Tu.GTP.lys1 tRNA + 9 EF-Tu.GTP.met1-tRNA + 108 EF-Tu.GTP.phe1-tRNA + 54 EF-Tu.GTP.pro1-tRNA + 36 EF-Tu.GTP.pro2-tRNA + 27 EF-Tu.GTP.proL-tRNA + 45 EF-Tu.GTP.proM-tRNA + 18 EF-Tu.GTP.ser1-tRNA + 18 EF-Tu.GTP.ser2-tRNA + 27 EF-Tu.GTP.ser3-tRNA + 36 EF-Tu.GTP.serT-tRNA + 36 EF-Tu.GTP.serV-tRNA + 18 EF-Tu.GTP.thr1-tRNA + 18 EF-Tu.GTP.thr2 tRNA + 18 EF-Tu.GTP.thr3-tRNA + 45 EF-Tu.GTP.thrU-tRNA + 63 EF-Tu.GTP.trpT-tRNA + 99 EF-Tu.GTP.tyr1-tRNA + 90 EF-Tu.GTP.val1-tRNA + 27 EF-Tu.GTP.val2-tRNA + 45 EF-Tu.GTP.val3	irreversible	Translation	0.00E+00	1.00E+09	0	1.28E+02	1.28E+02	0	2.23E+02	222.8318	0	5.35E+00	5.353
tl_elo_b0144_9_rib2	Translation elongation 2 b0144 9 ribosome(s)	2754 h2o + 1 rib_70_elo1_b0144_9_cplx --> 2754 EF-G.GDP + 2754 EF-Tu.GDP + 171 ala1_tRNA + 99 ala2_tRNA + 153 arg1_tRNA + 9 argW_tRNA + 36 argX_tRNA + 63 asn1_tRNA + 162 asp1_tRNA + 27 cysT_tRNA + 9 fmet_tRNA + 117 gln1_tRNA + 90 gln2_tRNA + 135 glu1_tRNA + 144 gly1_tRNA + 18 glyT_tRNA + 27 glyU_tRNA + 5508 h + 135 hisR_tRNA + 162 ile1_tRNA + 18 ile2_tRNA + 117 leu1_tRNA + 45 leu2_tRNA + 99 leuU_tRNA + 18 leuW_tRNA + 9 leuZ_tRNA + 63 lys1_tRNA + 9 met1_tRNA + 13815 mg2 + 108 phe1_tRNA + 5508 pi + 54 pro1_tRNA + 36 pro2_tRNA + 27 proL_tRNA + 45 proM_tRNA + 1 rib_70_elo2_b0144_9_cplx + 18 ser1_tRNA + 18 ser2_tRNA + 27 ser3_tRNA + 36 serT_tRNA + 36 serV_tRNA + 18 thr1_tRNA + 18 thr2_tRNA + 18 thr3_tRNA + 45 thrU_tRNA + 63 trpT_tRNA + 99 tyr1_tRNA + 90 val1_tRNA + 27 val2_tRNA + 45 val3_tRNA	irreversible	Translation	0.00E+00	1.00E+09	0	1.28E+02	1.28E+02	0	2.23E+02	222.8318	0	5.35E+00	5.353
tl_elo_b0166_16_rib1	Translation elongation 1 b0166 16 ribosome(s)	4368 EF-G.GTP + 304 EF-Tu.GTP.ala1-tRNA + 80 EF-Tu.GTP.ala2-tRNA + 256 EF-Tu.GTP.arg1-tRNA + 192 EF-Tu.GTP.asn1-tRNA + 224 EF-Tu.GTP.asp1-tRNA + 48 EF-Tu.GTP.cysT-tRNA + 192 EF-Tu.GTP.gln1-tRNA + 304 EF-Tu.GTP.glu1-tRNA + 368 EF-Tu.GTP.gly1-tRNA + 48 EF-Tu.GTP.glyU-tRNA + 48 EF-Tu.GTP.hisR-tRNA + 352 EF-Tu.GTP.ile1-tRNA + 160 EF-Tu.GTP.leu1-tRNA + 16 EF-Tu.GTP.leu2-tRNA + 48 EF-Tu.GTP.leuU-tRNA + 16 EF-Tu.GTP.leuZ-tRNA + 208 EF-Tu.GTP.lys1-tRNA + 64 EF-Tu.GTP.met1-tRNA + 128 EF-Tu.GTP.phe1-tRNA + 80 EF-Tu.GTP.pro1-tRNA + 64 EF-Tu.GTP.proM-tRNA + 64 EF-Tu.GTP.ser1-tRNA + 80 EF-Tu.GTP.ser2-tRNA + 32 EF-Tu.GTP.serT-tRNA + 48 EF-Tu.GTP.serV-tRNA + 176 EF-Tu.GTP.thr1-tRNA + 16 EF-Tu.GTP.thr2-tRNA + 32 EF-Tu.GTP.thr3-tRNA + 48 EF-Tu.GTP.trpT-tRNA + 144 EF-Tu.GTP.tyr1-tRNA + 256 EF-Tu.GTP.val1-tRNA + 96 EF-Tu.GTP.val2-tRNA + 176 EF-Tu.GTP.val3-tRNA + 21840 mg2 + 1 rib_ini_b0166_16 --> 1 rib_70_elo1_b0166_16_cplx	irreversible	Translation	0.00E+00	1.00E+09	0	3.33E+01	3.33E+01	0	5.44E+01	54.3931	0	6.00E+00	5.997
tl_elo_b0166_16_rib2	Translation elongation 2 b0166 16 ribosome(s)	4352 h2o + 1 rib_70_elo1_b0166_16_cplx --> 4352 EF-G.GDP + 4352 EF-Tu.GDP + 304 ala1_tRNA + 80 ala2_tRNA + 256 arg1_tRNA + 192 asn1_tRNA + 208 asp1_tRNA + 48 cysT_tRNA + 16 fmet_tRNA + 192 gln1_tRNA + 304 glu1_tRNA + 368 gly1_tRNA + 48 glyU_tRNA + 8704 h + 48 hisR_tRNA + 352 ile1_tRNA + 160 leu1_tRNA + 16 leu2_tRNA + 48 leuU_tRNA + 16 leuZ_tRNA + 208 lys1_tRNA + 64 met1_tRNA + 21840 mg2 + 128 phe1_tRNA + 8704 pi + 80 pro1_tRNA + 64 proM_tRNA + 1 rib_70_elo2_b0166_16_cplx + 64 ser1_tRNA + 80 ser2_tRNA + 32 serT_tRNA + 48 serV_tRNA + 176 thr1_tRNA + 16 thr2_tRNA + 32 thr3_tRNA + 48 trpT_tRNA + 144 tyr1_tRNA + 256 val1_tRNA + 96 val2_tRNA + 176 val3_tRNA	irreversible	Translation	0.00E+00	1.00E+09	0	3.33E+01	3.33E+01	0	5.44E+01	54.3931	0	6.00E+00	5.997
tl_elo_b0166_1_rib1	Translation elongation 1 b0166 1 ribosome(s)	273 EF-G.GTP + 19 EF-Tu.GTP.ala1-tRNA + 5 EF-Tu.GTP.ala2-tRNA + 16 EF-Tu.GTP.arg1-tRNA + 12 EF-Tu.GTP.asn1-tRNA + 14 EF-Tu.GTP.asp1-tRNA + 3 EF-Tu.GTP.cysT-tRNA + 12 EF-Tu.GTP.gln1-tRNA + 19 EF-Tu.GTP.glu1-tRNA + 23 EF-Tu.GTP.gly1 tRNA + 3 EF-Tu.GTP.glyU-tRNA + 3 EF-Tu.GTP.hisR-tRNA + 22 EF-Tu.GTP.ile1-tRNA + 10 EF-Tu.GTP.leu1-tRNA + 1 EF-Tu.GTP.leu2-tRNA + 3 EF-Tu.GTP.leuU-tRNA + 1 EF-Tu.GTP.leuZ-tRNA + 13 EF-Tu.GTP.lys1-tRNA + 4 EF-Tu.GTP.met1-tRNA + 8 EF-Tu.GTP.phe1-tRNA + 5 EF-Tu.GTP.pro1-tRNA + 4 EF-Tu.GTP.proM-tRNA + 4 EF-Tu.GTP.ser1-tRNA + 5 EF-Tu.GTP.ser2-tRNA + 2 EF-Tu.GTP.serT-tRNA + 3 EF-Tu.GTP.serV-tRNA + 11 EF-Tu.GTP.thr1-tRNA + 1 EF-Tu.GTP.thr2-tRNA + 2 EF-Tu.GTP.thr3-tRNA + 3 EF-Tu.GTP.trpT-tRNA + 9 EF-Tu.GTP.tyr1-tRNA + 16 EF-Tu.GTP.val1-tRNA + 6 EF-Tu.GTP.val2-tRNA + 11 EF-Tu.GTP.val3-tRNA + 1365 mg2 + 1 rib_ini_b0166_1 --> 1 rib_70_elo1_b0166_1_cplx	irreversible	Translation	0.00E+00	1.00E+09	0	3.33E+01	3.33E+01	0	5.44E+01	54.3931	0	6.00E+00	5.997
tl_elo_b0166_1_rib2	Translation elongation 2 b0166 1 ribosome(s)	272 h2o + 1 rib_70_elo1_b0166_1_cplx --> 272 EF-G.GDP + 272 EF-Tu.GDP + 19 ala1_tRNA + 5 ala2_tRNA + 16 arg1_tRNA + 12 asn1_tRNA + 13 asp1_tRNA + 3 cysT_tRNA + 1 fmet_tRNA + 12 gln1_tRNA + 19 glu1_tRNA + 23 gly1_tRNA + 3 glyU_tRNA + 544 h + 3 hisR_tRNA + 22 ile1_tRNA + 10 leu1_tRNA + 1 leu2_tRNA + 3 leuU_tRNA + 1 leuZ_tRNA + 13 lys1_tRNA + 4 met1_tRNA + 1365 mg2 + 8 phe1_tRNA + 544 pi + 5 pro1_tRNA + 4 proM_tRNA + 1 rib_70_elo2_b0166_1_cplx + 4 ser1_tRNA + 5 ser2_tRNA + 2 serT_tRNA + 3 serV_tRNA + 11 thr1_tRNA + 1 thr2_tRNA + 2 thr3_tRNA + 3 trpT_tRNA + 9 tyr1_tRNA + 16 val1_tRNA + 6 val2_tRNA + 11 val3_tRNA	irreversible	Translation	0.00E+00	1.00E+09	0	3.33E+01	3.33E+01	0	5.44E+01	54.3931	0	6.00E+00	5.997

tl_elo_b0166_8_rib1	Translation elongation 1 b0166 8 ribosome(s)	2184 EF-G.GTP + 152 EF-Tu.GTP.ala1-tRNA + 40 EF-Tu.GTP.ala2-tRNA + 128 EF-Tu.GTP.arg1-tRNA + 96 EF-Tu.GTP.asn1-tRNA + 112 EF-Tu.GTP.asp1-tRNA + 24 EF-Tu.GTP.cysT-tRNA + 96 EF-Tu.GTP.gln1-tRNA + 152 EF-Tu.GTP.glu1-tRNA + 184 EF-Tu.GTP.gly1-tRNA + 24 EF-Tu.GTP.glyU-tRNA + 24 EF-Tu.GTP.hisR-tRNA + 176 EF-Tu.GTP.ile1-tRNA + 80 EF-Tu.GTP.leu1-tRNA + 8 EF-Tu.GTP.leu2-tRNA + 24 EF-Tu.GTP.leuU-tRNA + 8 EF-Tu.GTP.leuZ-tRNA + 104 EF-Tu.GTP.lys1-tRNA + 32 EF-Tu.GTP.met1-tRNA + 64 EF-Tu.GTP.phe1-tRNA + 40 EF-Tu.GTP.pro1-tRNA + 32 EF-Tu.GTP.proM-tRNA + 32 EF-Tu.GTP.ser1-tRNA + 40 EF-Tu.GTP.ser2-tRNA + 16 EF-Tu.GTP.serT-tRNA + 24 EF-Tu.GTP.serV-tRNA + 88 EF-Tu.GTP.thr1-tRNA + 8 EF-Tu.GTP.thr2-tRNA + 16 EF-Tu.GTP.thr3-tRNA + 24 EF-Tu.GTP.trpT-tRNA + 72 EF-Tu.GTP.tyr1-tRNA + 128 EF-Tu.GTP.val1-tRNA + 48 EF-Tu.GTP.val2-tRNA + 88 EF-Tu.GTP.val3-tRNA + 10920 mg2 + 1 rib_ini_b0166_8 --> 1 rib_70_elo1_b0166_8_cplx	irreversible	Translation	0.00E+00	1.00E+09	0	3.33E+01	3.33E+01	0	5.44E+01	54.3931	0	6.00E+00	5.997
tl_elo_b0166_8_rib2	Translation elongation 2 b0166 8 ribosome(s)	2176 h2o + 1 rib_70_elo1_b0166_8_cplx --> 2176 EF-G.GDP + 2176 EF-Tu.GDP + 152 ala1_tRNA + 40 ala2_tRNA + 128 arg1_tRNA + 96 asn1_tRNA + 104 asp1_tRNA + 24 cysT_tRNA + 8 fmet_tRNA + 96 gln1_tRNA + 152 glu1_tRNA + 184 gly1_tRNA + 24 glyU_tRNA + 4352 h + 24 hisR_tRNA + 176 ile1_tRNA + 80 leu1_tRNA + 8 leu2_tRNA + 24 leuU_tRNA + 8 leuZ_tRNA + 104 lys1_tRNA + 32 met1_tRNA + 10920 mg2 + 64 phe1_tRNA + 4352 pi + 40 pro1_tRNA + 32 proM_tRNA + 1 rib_70_elo2_b0166_8_cplx + 32 ser1_tRNA + 4 ser2_tRNA + 16 serT_tRNA + 24 serV_tRNA + 88 thr1_tRNA + 8 thr2_tRNA + 16 thr3_tRNA + 24 trpT_tRNA + 72 tyr1_tRNA + 128 val1_tRNA + 48 val2_tRNA + 88 val3_tRNA	irreversible	Translation	0.00E+00	1.00E+09	0	3.33E+01	3.33E+01	0	5.44E+01	54.3931	0	6.00E+00	5.997
tl_elo_b0167_1_rib1	Translation elongation 1 b0167 1 ribosome(s)	889 EF-G.GTP + 31 EF-Tu.GTP.ala1-tRNA + 34 EF-Tu.GTP.ala2-tRNA + 70 EF-Tu.GTP.arg1-tRNA + 7 EF-Tu.GTP.argX-tRNA + 28 EF-Tu.GTP.asn1-tRNA + 52 EF-Tu.GTP.asp1-tRNA + 9 EF-Tu.GTP.cysT-tRNA + 32 EF-Tu.GTP.gln1-tRNA + 22 EF-Tu.GTP.gln2-tRNA + 62 EF-Tu.GTP.glu1-tRNA + 29 EF-Tu.GTP.gly1 tRNA + 8 EF-Tu.GTP.glyT-tRNA + 9 EF-Tu.GTP.glyU-tRNA + 38 EF-Tu.GTP.hisR-tRNA + 43 EF-Tu.GTP.ile1-tRNA + 3 EF-Tu.GTP.ile2-tRNA + 57 EF-Tu.GTP.leu1-tRNA + 14 EF-Tu.GTP.leu2-tRNA + 24 EF-Tu.GTP.leuU-tRNA + 6 EF-Tu.GTP.leuW-tRNA + 21 EF-Tu.GTP.leuZ-tRNA + 22 EF-Tu.GTP.lys1-tRNA + 18 EF-Tu.GTP.met1-tRNA + 38 EF-Tu.GTP.phe1-tRNA + 18 EF-Tu.GTP.pro1-tRNA + 4 EF-Tu.GTP.pro2-tRNA + 8 EF-Tu.GTP.proL-tRNA + 10 EF-Tu.GTP.proM-tRNA + 5 EF-Tu.GTP.ser1-tRNA + 1 EF Tu.GTP.ser2-tRNA + 6 EF-Tu.GTP.ser3-tRNA + 5 EF-Tu.GTP.serT-tRNA + 25 EF-Tu.GTP.serV-tRNA + 23 EF-Tu.GTP.thr1-tRNA + 7 EF-Tu.GTP.thr2-tRNA + 12 EF-Tu.GTP.thr3-tRNA + 6 EF-Tu.GTP.thrU-tRNA + 16 EF-Tu.GTP.trpT-tRNA + 16 EF-Tu.GTP.tyr1-tRNA + 22 EF-Tu.GTP.val1-tRNA + 20 EF-Tu.GTP.val2-tRNA + 8 EF Tu.GTP.val3-tRNA + 4445 mg2 + 1 rib_ini_b0167_1 --> 1 rib_70_elo1	irreversible	Translation	0.00E+00	1.00E+09	0	2.95E+01	2.95E+01	0	5.06E+01	50.5931	0	2.20E+00	2.197
tl_elo_b0167_1_rib2	Translation elongation 2 b0167 1 ribosome(s)	888 h2o + 1 rib_70_elo1_b0167_1_cplx --> 888 EF-G.GDP + 888 EF-Tu.GDP + 31 ala1_tRNA + 34 ala2_tRNA + 70 arg1_tRNA + 7 argX_tRNA + 28 asn1_tRNA + 52 asp1_tRNA + 9 cysT_tRNA + 1 fmet_tRNA + 32 gln1_tRNA + 22 gln2_tRNA + 62 glu1_tRNA + 29 gly1_tRNA + 8 glyT_tRNA + 8 glyU_tRNA + 1776 h + 38 hisR_tRNA + 43 ile1_tRNA + 3 ile2_tRNA + 57 leu1_tRNA + 14 leu2_tRNA + 24 leuU_tRNA + 6 leuW_tRNA + 21 leuZ_tRNA + 22 lys1_tRNA + 18 met1_tRNA + 4445 mg2 + 38 phe1_tRNA + 1776 pi + 18 pro1_tRNA + 4 pro2_tRNA + 8 proL_tRNA + 10 proM_tRNA + 1 rib_70_elo2_b0167_1_cplx + 5 ser1_tRNA + 1 ser2_tRNA + 6 ser3_tRNA + 5 serT_tRNA + 25 serV_tRNA + 23 thr1_tRNA + 7 thr2_tRNA + 12 thr3_tRNA + 6 thrU_tRNA + 16 trpT_tRNA + 16 tyr1_tRNA + 22 val1_tRNA + 20 val2_tRNA + 8 val3_tRNA	irreversible	Translation	0.00E+00	1.00E+09	0	2.95E+01	2.95E+01	0	5.06E+01	50.5931	0	2.20E+00	2.197
tl_elo_b0167_26_rib1	Translation elongation 1 b0167 26 ribosome(s)	23114 EF-G.GTP + 806 EF-Tu.GTP.ala1-tRNA + 884 EF-Tu.GTP.ala2-tRNA + 1820 EF-Tu.GTP.arg1-tRNA + 182 EF-Tu.GTP.argX-tRNA + 728 EF-Tu.GTP.asn1-tRNA + 1352 EF-Tu.GTP.asp1-tRNA + 234 EF-Tu.GTP.cysT-tRNA + 832 EF-Tu.GTP.gln1-tRNA + 572 EF-Tu.GTP.gln2-tRNA + 1612 EF-Tu.GTP.glu1-tRNA + 754 EF-Tu.GTP.gly1-tRNA + 208 EF-Tu.GTP.glyT-tRNA + 234 EF-Tu.GTP.glyU-tRNA + 988 EF-Tu.GTP.hisR-tRNA + 1118 EF-Tu.GTP.ile1-tRNA + 78 EF-Tu.GTP.ile2-tRNA + 1482 EF-Tu.GTP.leu1-tRNA + 364 EF-Tu.GTP.leu2-tRNA + 624 EF-Tu.GTP.leuU-tRNA + 156 EF-Tu.GTP.leuW-tRNA + 546 EF-Tu.GTP.leuZ-tRNA + 572 EF-Tu.GTP.lys1-tRNA + 468 EF-Tu.GTP.met1-tRNA + 988 EF-Tu.GTP.phe1-tRNA + 468 EF-Tu.GTP.pro1-tRNA + 104 EF-Tu.GTP.pro2-tRNA + 208 EF-Tu.GTP.proL-tRNA + 260 EF-Tu.GTP.proM-tRNA + 130 EF-Tu.GTP.ser1-tRNA + 26 EF-Tu.GTP.ser2-tRNA + 156 EF-Tu.GTP.ser3-tRNA + 130 EF-Tu.GTP.serT-tRNA + 650 EF-Tu.GTP.serV-tRNA + 598 EF-Tu.GTP.thr1-tRNA + 182 EF-Tu.GTP.thr2-tRNA + 312 EF-Tu.GTP.thr3-tRNA + 156 EF-Tu.GTP.thrU-tRNA + 416 EF-Tu.GTP.trpT-tRNA + 416 EF-Tu.GTP.tyr1-tRNA + 572 EF-Tu.GTP.val1-tRNA + 520 EF-Tu.GTP.val2-tRNA + 208 EF-Tu.GTP.va	irreversible	Translation	0.00E+00	1.00E+09	0	2.95E+01	2.95E+01	0	5.06E+01	50.5931	0	2.20E+00	2.197
tl_elo_b0167_26_rib2	Translation elongation 2 b0167 26 ribosome(s)	23088 h2o + 1 rib_70_elo1_b0167_26_cplx --> 23088 EF-G.GDP + 23088 EF-Tu.GDP + 806 ala1_tRNA + 884 ala2_tRNA + 1820 arg1_tRNA + 182 argX_tRNA + 728 asn1_tRNA + 1352 asp1_tRNA + 234 cysT_tRNA + 26 fmet_tRNA + 832 gln1_tRNA + 572 gln2_tRNA + 1612 glu1_tRNA + 754 gly1_tRNA + 208 glyT_tRNA + 208 glyU_tRNA + 46176 h + 988 hisR_tRNA + 1118 ile1_tRNA + 78 ile2_tRNA + 1482 leu1_tRNA + 364 leu2_tRNA + 624 leuU_tRNA + 156 leuW_tRNA + 546 leuZ_tRNA + 572 lys1_tRNA + 468 met1_tRNA + 115570 mg2 + 988 phe1_tRNA + 46176 pi + 468 pro1_tRNA + 104 pro2_tRNA + 208 proL_tRNA + 260 proM_tRNA + rib_70_elo2_b0167_26_cplx + 130 ser1_tRNA + 26 ser2_tRNA + 156 ser3_tRNA + 130 serT_tRNA + 650 serV_tRNA + 598 thr1_tRNA + 182 thr2_tRNA + 312 thr3_tRNA + 156 thrU_tRNA + 416 trpT_tRNA + 416 tyr1_tRNA + 572 val1_tRNA + 520 val2_tRNA + 208 val3_tRNA	irreversible	Translation	0.00E+00	1.00E+09	0	2.95E+01	2.95E+01	0	5.06E+01	50.5931	0	2.20E+00	2.197

tl_elo_b0167_52_rib1	Translation elongation 1 b0167 52 ribosome(s)	46228 EF-G.GTP + 1612 EF-Tu.GTP.ala1-tRNA + 1768 EF-Tu.GTP.ala2-tRNA + 3640 EF-Tu.GTP.arg1-tRNA + 364 EF-Tu.GTP.argX-tRNA + 1456 EF-Tu.GTP.asn1-tRNA + 2704 EF-Tu.GTP.asp1-tRNA + 468 EF-Tu.GTP.cysT-tRNA + 1664 EF-Tu.GTP.gln1-tRNA + 1144 EF-Tu.GTP.gln2-tRNA + 3224 EF-Tu.GTP.glu1-tRNA + 1508 EF-Tu.GTP.gly1-tRNA + 416 EF-Tu.GTP.glyT-tRNA + 468 EF-Tu.GTP.glyU-tRNA + 1976 EF-Tu.GTP.hisR-tRNA + 2236 EF-Tu.GTP.ile1-tRNA + 156 EF-Tu.GTP.ile2-tRNA + 2964 EF-Tu.GTP.leu1-tRNA + 728 EF-Tu.GTP.leu2-tRNA + 1248 EF-Tu.GTP.leuU-tRNA + 312 EF-Tu.GTP.leuW-tRNA + 1092 EF-Tu.GTP.leuZ-tRNA + 1144 EF-Tu.GTP.lys1-tRNA + 936 EF-Tu.GTP.met1-tRNA + 1976 EF-Tu.GTP.phe1-tRNA + 936 EF-Tu.GTP.pro1-tRNA + 208 EF-Tu.GTP.pro2-tRNA + 416 EF-Tu.GTP.proL-tRNA + 520 EF-Tu.GTP.proM-tRNA + 260 EF-Tu.GTP.ser1-tRNA + 52 EF-Tu.GTP.ser2-tRNA + 312 EF-Tu.GTP.ser3-tRNA + 260 EF-Tu.GTP.serT-tRNA + 1300 EF-Tu.GTP.serV-tRNA + 1196 EF-Tu.GTP.thr1-tRNA + 364 EF-Tu.GTP.thr2-tRNA + 624 EF-Tu.GTP.thr3-tRNA + 312 EF-Tu.GTP.thrU-tRNA + 832 EF-Tu.GTP.trpT-tRNA + 832 EF-Tu.GTP.tyr1-tRNA + 1144 EF-Tu.GTP.val1-tRNA + 1040 EF-Tu.GTP.val2-tRNA + 416 EF-Tu.GTP.[illegible]	irreversible	Translation	0.00E+00	1.00E+09	0	2.95E+01	2.95E+01	0	3.83E+01	38.28969	0	2.20E+00	2.197
tl_elo_b0167_52_rib2	Translation elongation 2 b0167 52 ribosome(s)	46176 h2o + 1 rib_70_elo1_b0167_52_cplx --> 46176 EF-G.GDP + 46176 EF-Tu.GDP + 1612 ala1_tRNA + 1768 ala2_tRNA + 3640 arg1_tRNA + 364 argX_tRNA + 1456 asn1_tRNA + 2704 asp1_tRNA + 468 cysT_tRNA + 52 fmet_tRNA + 1664 gln1_tRNA + 1144 gln2_tRNA + 3224 glu1_tRNA + 1508 gly1_tRNA + 416 glyT_tRNA + 416 glyU_tRNA + 92352 h + 1976 hisR_tRNA + 2236 ile1_tRNA + 156 ile2_tRNA + 2964 leu1_tRNA + 728 leu2_tRNA + 1248 leuU_tRNA + 312 leuW_tRNA + 1092 leuZ_tRNA + 1144 lys1_tRNA + 936 met1_tRNA + 231140 mg2 + 1976 phe1_tRNA + 92352 pi + 936 pro1_tRNA + 208 pro2_tRNA + 416 proL_tRNA + 520 proM_tRNA + 1 rib_70_elo2_b0167_52_cplx + 260 ser1_tRNA + 52 ser2_tRNA + 312 ser3_tRNA + 260 serT_tRNA + 1300 serV_tRNA + 1196 thr1_tRNA + 364 thr2_tRNA + 624 thr3_tRNA + 312 thrU_tRNA + 832 trpT_tRNA + 832 tyr1_tRNA + 1144 val1_tRNA + 1040 val2_tRNA + 416 val3_tRNA	irreversible	Translation	0.00E+00	1.00E+09	0	2.95E+01	2.95E+01	0	3.83E+01	38.28969	0	2.20E+00	2.197
tl_elo_b0168_15_rib1	Translation elongation 1 b0168 15 ribosome(s)	3945 EF-G.GTP + 180 EF-Tu.GTP.ala1-tRNA + 30 EF-Tu.GTP.ala2-tRNA + 195 EF-Tu.GTP.arg1-tRNA + 120 EF-Tu.GTP.asn1-tRNA + 255 EF-Tu.GTP.asp1-tRNA + 105 EF-Tu.GTP.cysT-tRNA + 30 EF-Tu.GTP.gln1-tRNA + 45 EF-Tu.GTP.gln2-tRNA + 330 EF-Tu.GTP.glu1-tRNA + 300 EF-Tu.GTP.gly1-tRNA + 15 EF-Tu.GTP.glyT-tRNA + 15 EF-Tu.GTP.glyU-tRNA + 135 EF-Tu.GTP.hisR-tRNA + 375 EF-Tu.GTP.ile1-tRNA + 15 EF-Tu.GTP.ile2-tRNA + 180 EF-Tu.GTP.leu1-tRNA + 15 EF-Tu.GTP.leu2-tRNA + 15 EF-Tu.GTP.leuU-tRNA + 30 EF-Tu.GTP.leuW-tRNA + 270 EF-Tu.GTP.lys1-tRNA + 120 EF-Tu.GTP.met1-tRNA + 90 EF-Tu.GTP.phe1-tRNA + 105 EF-Tu.GTP.pro1-tRNA + 15 EF-Tu.GTP.pro2-tRNA + 45 EF-Tu.GTP.proM-tRNA + 45 EF-Tu.GTP.ser1-tRNA + 45 EF-Tu.GTP.ser2-tRNA + 30 EF-Tu.GTP.ser3-tRNA + 15 EF-Tu.GTP.serT-tRNA + 45 EF-Tu.GTP.serV-tRNA + 150 EF-Tu.GTP.thr1-tRNA + 30 EF-Tu.GTP.thr2-tRNA + 60 EF-Tu.GTP.thr3-tRNA + 15 EF-Tu.GTP.trpT-tRNA + 120 EF-Tu.GTP.tyr1-tRNA + 150 EF-Tu.GTP.val1-tRNA + 120 EF-Tu.GTP.val2-tRNA + 90 EF-Tu.GTP.val3-tRNA + 19725 mg2 + 1 rib_ini_b0168_15 --> 1 rib_70_elo1_b0168_15_cplx	irreversible	Translation	0.00E+00	1.00E+09	0	3.42E+01	3.42E+01	0	5.53E+01	55.3131	0	6.92E+00	6.917
tl_elo_b0168_15_rib2	Translation elongation 2 b0168 15 ribosome(s)	3930 h2o + 1 rib_70_elo1_b0168_15_cplx --> 3930 EF-G.GDP + 3930 EF-Tu.GDP + 180 ala1_tRNA + 30 ala2_tRNA + 195 arg1_tRNA + 120 asn1_tRNA + 255 asp1_tRNA + 105 cysT_tRNA + 15 fmet_tRNA + 30 gln1_tRNA + 45 gln2_tRNA + 315 glu1_tRNA + 300 gly1_tRNA + 15 glyT_tRNA + 15 glyU_tRNA + 7860 h + 135 hisR_tRNA + 375 ile1_tRNA + 15 ile2_tRNA + 180 leu1_tRNA + 15 leu2_tRNA + 15 leuU_tRNA + 30 leuW_tRNA + 270 lys1_tRNA + 12[illegible] met1_tRNA + 19725 mg2 + 90 phe1_tRNA + 7860 pi + 105 pro1_tRNA + 15 pro2_tRNA + 45 proM_tRNA + 1 rib_70_elo2_b0168_15_cplx + 45 ser1_tRNA + 45 ser2_tRNA + 30 ser3_tRNA + 15 serT_tRNA + 45 serV_tRNA + 150 thr1_tRNA + 30 thr2_tRNA + 60 thr3_tRNA + 15 trpT_tRNA + 120 tyr1_tRNA + 150 val1_tRNA + 120 val2_tRNA + 90 val3_tRNA	irreversible	Translation	0.00E+00	1.00E+09	0	3.42E+01	3.42E+01	0	5.53E+01	55.3131	0	6.92E+00	6.917
tl_elo_b0168_1_rib1	Translation elongation 1 b0168 1 ribosome(s)	263 EF-G.GTP + 12 EF-Tu.GTP.ala1-tRNA + 2 EF-Tu.GTP.ala2-tRNA + 13 EF-Tu.GTP.arg1-tRNA + 8 EF-Tu.GTP.asn1-tRNA + 17 EF-Tu.GTP.asp1-tRNA + 7 EF-Tu.GTP.cysT-tRNA + 2 EF-Tu.GTP.gln1-tRNA + 3 EF-Tu.GTP.gln2-tRNA + 22 EF-Tu.GTP.glu1-tRNA + 20 EF-Tu.GTP.gly1-tRNA + 1 EF-Tu.GTP.glyT-tRNA + 1 EF-Tu.GTP.glyU-tRNA + 9 EF-Tu.GTP.hisR-tRNA + 25 EF-Tu.GTP.ile1-tRNA + 1 EF-Tu.GTP.ile2-tRNA + 12 EF-Tu.GTP.leu1-tRNA + 1 EF-Tu.GTP.leu2-tRNA + 1 EF-Tu.GTP.leuU-tRNA + 2 EF-Tu.GTP.leuW-tRNA + 18 EF-Tu.GTP.lys1-tRNA + 8 EF-Tu.GTP.met1-tRNA + 6 EF-Tu.GTP.phe1-tRNA + 7 EF-Tu.GTP.pro1-tRNA + 1 EF-Tu.GTP.pro2-tRNA + 3 EF-Tu.GTP.proM-tRNA + 3 EF-Tu.GTP.ser1-tRNA + 3 EF-Tu.GTP.ser2-tRNA + 2 EF-Tu.GTP.ser3-tRNA + 1 EF-Tu.GTP.serT-tRNA + 3 EF-Tu.GTP.serV-tRNA + 10 EF-Tu.GTP.thr1-tRNA + 2 EF-Tu.GTP.thr2-tRNA + 4 EF-Tu.GTP.thr3-tRNA + 1 EF-Tu.GTP.trpT-tRNA + 8 EF-Tu.GTP.tyr1-tRNA + 10 EF-Tu.GTP.val1-tRNA + 8 EF-Tu.GTP.val2-tRNA + 6 EF-Tu.GTP.val3-tRNA + 1315 mg2 + 1 rib_ini_b0168_1 --> 1 rib_70_elo1_b0168_1_cplx	irreversible	Translation	0.00E+00	1.00E+09	0	3.42E+01	3.42E+01	0	5.53E+01	55.3131	0	6.92E+00	6.917
tl_elo_b0168_1_rib2	Translation elongation 2 b0168 1 ribosome(s)	262 h2o + 1 rib_70_elo1_b0168_1_cplx --> 262 EF-G.GDP + 262 EF-Tu.GDP + 12 ala1_tRNA + 2 ala2_tRNA + 13 arg1_tRNA + 8 asn1_tRNA + 17 asp1_tRNA + 7 cysT_tRNA + 1 fmet_tRNA + 2 gln1_tRNA + 3 gln2_tRNA + 21 glu1_tRNA + 20 gly1_tRNA + 1 glyT_tRNA + 1 glyU_tRNA + 524 h + 9 hisR_tRNA + 25 ile1_tRNA + 1 ile2_tRNA + 12 leu1_tRNA + 1 leu2_tRNA + 1 leuU_tRNA + 2 leuW_tRNA + 18 lys1_tRNA + 8 met1_tRNA + 1315 mg2 + 6 phe1_tRNA + 524 pi + 7 pro1_tRNA + 1 pro2_tRNA + 3 proM_tRNA + 1 rib_70_elo2_b0168_1_cplx + 3 ser1_tRNA + 3 ser2_tRNA + 2 ser3_tRNA + 1 serT_tRNA + 3 serV_tRNA + 10 thr1_tRNA + 2 thr2_tRNA + 4 thr3_tRNA + 1 trpT_tRNA + 8 tyr1_tRNA + 10 val1_tRNA + 8 val2_tRNA + 6 val3_tRNA	irreversible	Translation	0.00E+00	1.00E+09	0	3.42E+01	3.42E+01	0	5.53E+01	55.3131	0	6.92E+00	6.917

tl_elo_b0168_7_rib1	Translation elongation 1 b0168 7 ribosome(s)	1841 EF-G.GTP + 84 EF-Tu.GTP.ala1-tRNA + 14 EF-Tu.GTP.ala2-tRNA + 91 EF-Tu.GTP.arg1-tRNA + 56 EF-Tu.GTP.asn1-tRNA + 119 EF-Tu.GTP.asp1-tRNA + 49 EF-Tu.GTP.cysT-tRNA + 14 EF-Tu.GTP.gln1-tRNA + 21 EF-Tu.GTP.gln2-tRNA + 154 EF-Tu.GTP.glu1-tRNA + 140 EF-Tu.GTP.gly1-tRNA + 7 EF-Tu.GTP.glyT tRNA + 7 EF-Tu.GTP.glyU-tRNA + 63 EF-Tu.GTP.hisR-tRNA + 175 EF-Tu.GTP.ile1-tRNA + 7 EF-Tu.GTP.ile2-tRNA + 84 EF-Tu.GTP.leu1-tRNA + 7 EF-Tu.GTP.leu2-tRNA + 7 EF-Tu.GTP.leuU-tRNA + 14 EF-Tu.GTP.leuW-tRNA + 126 EF-Tu.GTP.lys1-tRNA + 56 EF-Tu.GTP.met1-tRNA + 42 EF-Tu.GTP.phe1-tRNA + 49 EF-Tu.GTP.pro1-tRNA + 7 EF-Tu.GTP.pro2-tRNA + 21 EF-Tu.GTP.proM-tRNA + 21 EF-Tu.GTP.ser1-tRNA + 21 EF-Tu.GTP.ser2-tRNA + 14 EF-Tu.GTP.ser3-tRNA + 7 EF-Tu.GTP.serT-tRNA + 21 EF-Tu.GTP.serV-tRNA + 70 EF-Tu.GTP.thr1-tRNA + 14 EF-Tu.GTP.thr2-tRNA + 28 EF-Tu.GTP.thr3-tRNA + 7 EF-Tu.GTP.trpT-tRNA + 56 EF-Tu.GTP.tyr1-tRNA + 70 EF-Tu.GTP.val1-tRNA + 56 EF-Tu.GTP.val2-tRNA + 42 EF-Tu.GTP.val3-tRNA + 9205 mg2 + 1 rib_ini_b0168_7 --> 1 rib_70_elo1_b0168_7_cplx	irreversible	Translation	0.00E+00	1.00E+09	0	3.42E+01	3.42E+01	0	5.53E+01	55.3131	0	6.92E+00	6.917
tl_elo_b0168_7_rib2	Translation elongation 2 b0168 7 ribosome(s)	1834 h2o + 1 rib_70_elo1_b0168_7_cplx --> 1834 EF-G.GDP + 1834 EF-Tu.GDP + 84 ala1_tRNA + 14 ala2_tRNA + 91 arg1_tRNA + 56 asn1_tRNA + 119 asp1_tRNA + 49 cysT_tRNA + 7 fmet_tRNA + 14 gln1_tRNA + 21 gln2_tRNA + 147 glu1_tRNA + 140 gly1_tRNA + 7 glyT_tRNA + 7 glyU_tRNA + 3668 h + 63 hisR_tRNA + 175 ile1_tRNA + 7 ile2_tRNA + 84 leu1_tRNA + 7 leu2_tRNA + 7 leuU_tRNA + 14 leuW_tRNA + 126 lys1_tRNA + 56 met1_tRNA + 9205 mg2 + 42 phe1_tRNA + 3668 pi + 49 pro1_tRNA + 7 pro2_tRN + 21 proM_tRNA + 1 rib_70_elo2_b0168_7_cplx + 21 ser1_tRNA + 2 ser2_tRNA + 14 ser3_tRNA + 7 serT_tRNA + 21 serV_tRNA + 70 thr1_tRNA + 14 thr2_tRNA + 28 thr3_tRNA + 7 trpT_tRNA + 56 tyr1_tRNA + 70 val1_tRNA + 56 val2_tRNA + 42 val3_tRNA	irreversible	Translation	0.00E+00	1.00E+09	0	3.42E+01	3.42E+01	0	5.53E+01	55.3131	0	6.92E+00	6.917
tl_elo_b0169_14_rib1	Translation elongation 1 b0169 14 ribosome(s)	3360 EF-G.GTP + 350 EF-Tu.GTP.ala1-tRNA + 182 EF-Tu.GTP.arg1-tRNA + 168 EF-Tu.GTP.asn1-tRNA + 238 EF-Tu.GTP.asp1-tRNA + 14 EF-Tu.GTP.cysT-tRNA + 98 EF-Tu.GTP.gln1-tRNA + 224 EF-Tu.GTP.glu1-tRNA + 238 EF-Tu.GTP.gly1-tRNA + 84 EF-Tu.GTP.hisR-tRNA + 196 EF-Tu.GTP.ile1-tRNA + 252 EF-Tu.GTP.leu1-tRNA + 42 EF-Tu.GTP.leuU-tRNA + 266 EF-Tu.GTP.lys1-tRNA + 98 EF-Tu.GTP.met1-tRNA + 168 EF-Tu.GTP.phe1-tRNA + 98 EF-Tu.GTP.pro1-tRNA + 42 EF-Tu.GTP.ser1-tRNA + 56 EF-Tu.GTP.ser2-tRNA + 56 EF-Tu.GTP.serV-tRNA + 84 EF-Tu.GTP.thr1-tRNA + 98 EF-Tu.GTP.thr2-tRNA + 42 EF-Tu.GTP.trpT-tRNA + 28 EF-Tu.GTP.tyr1-tRNA + 112 EF-Tu.GTP.val1-tRNA + 126 EF-Tu.GTP.val3-tRNA + 16800 mg2 + 1 rib_ini_b0169_14 --> 1 rib_70_elo1_b0169_14_cplx	irreversible	Translation	0.00E+00	1.00E+09	0	8.04E+00	8.04E+00	0	5.92E+00	5.92	0	5.92E+00	5.92
tl_elo_b0169_14_rib2	Translation elongation 2 b0169 14 ribosome(s)	3346 h2o + 1 rib_70_elo1_b0169_14_cplx --> 3346 EF-G.GDP + 3346 EF-Tu.GDP + 350 ala1_tRNA + 182 arg1_tRNA + 168 asn1_tRNA + 238 asp1_tRNA + 14 cysT_tRNA + 14 fmet_tRNA + 98 gln1_tRNA + 210 glu1_tRNA + 238 gly1_tRNA + 6692 h + 84 hisR_tRNA + 196 ile1_tRNA + 252 leu1_tRNA + 42 leuU_tRNA + 26 lys1_tRNA + 98 met1_tRNA + 16800 mg2 + 168 phe1_tRNA + 6692 pi + 98 pro1_tRNA + 1 rib_70_elo2_b0169_14_cplx + 42 ser1_tRNA 56 ser2_tRNA + 56 serV_tRNA + 84 thr1_tRNA + 98 thr2_tRNA + 42 trpT_tRNA + 28 tyr1_tRNA + 112 val1_tRNA + 126 val3_tRNA	irreversible	Translation	0.00E+00	1.00E+09	0	8.04E+00	8.04E+00	0	5.92E+00	5.92	0	5.92E+00	5.92
tl_elo_b0169_1_rib1	Translation elongation 1 b0169 1 ribosome(s)	240 EF-G.GTP + 25 EF-Tu.GTP.ala1-tRNA + 13 EF-Tu.GTP.arg1-tRNA + 12 EF-Tu.GTP.asn1-tRNA + 17 EF-Tu.GTP.asp1-tRNA + 1 EF-Tu.GTP.cysT-tRNA + 7 EF-Tu.GTP.gln1-tRNA + 16 EF-Tu.GTP.glu1-tRNA + 17 EF-Tu.GTP.gly1-tRNA + 6 EF-Tu.GTP.hisR-tRNA + 14 EF-Tu.GTP.ile1-tRNA + 18 EF-Tu.GTP.leu1-tRNA + 3 EF-Tu.GTP.leuU-tRNA + 19 EF-Tu.GTP.lys1-tRNA + 7 EF-Tu.GTP.met1 tRNA + 12 EF-Tu.GTP.phe1-tRNA + 7 EF-Tu.GTP.pro1-tRNA + 3 EF Tu.GTP.ser1-tRNA + 4 EF-Tu.GTP.ser2-tRNA + 4 EF-Tu.GTP.serV-tRNA + 6 EF-Tu.GTP.thr1-tRNA + 7 EF-Tu.GTP.thr2-tRNA + 3 EF-Tu.GTP.trpT-tRNA + 2 EF-Tu.GTP.tyr1-tRNA + 8 EF-Tu.GTP.val1-tRNA + 9 EF-Tu.GTP.val3-tRNA + 1200 mg2 + 1 rib_ini_b0169_1 --> 1 rib_70_elo1_b0169_1_cplx	irreversible	Translation	0.00E+00	1.00E+09	0	8.76E+01	8.76E+01	0	8.29E+01	82.88	0	1.83E+01	18.284
tl_elo_b0169_1_rib2	Translation elongation 2 b0169 1 ribosome(s)	239 h2o + 1 rib_70_elo1_b0169_1_cplx --> 239 EF-G.GDP + 239 EF-Tu.GDP + 25 ala1_tRNA + 13 arg1_tRNA + 12 asn1_tRNA + 17 asp1_tRNA + 1 cysT_tRNA + 1 fmet_tRNA + 7 gln1_tRNA + 15 glu1_tRNA + 17 gly1_tRNA + 478 h + 6 hisR_tRNA + 14 ile1_tRNA + 18 leu1_tRNA + 3 leuU_tRNA + 19 lys1_tRNA + 7 met1_tRNA + 120 mg2 + 12 phe1_tRNA + 478 pi + 7 pro1_tRNA + 1 rib_70_elo2_b0169_1_cplx + 3 ser1_tRNA + 4 ser2_tRNA + 4 serV_tRNA + 6 thr1_tRNA + 7 thr2_tRNA + 3 trpT_tRNA + 2 tyr1_tRNA + 8 val1_tRNA + 9 val3_tRNA	irreversible	Translation	0.00E+00	1.00E+09	0	8.76E+01	8.76E+01	0	8.29E+01	82.88	0	1.83E+01	18.284
tl_elo_b0169_7_rib1	Translation elongation 1 b0169 7 ribosome(s)	1680 EF-G.GTP + 175 EF-Tu.GTP.ala1-tRNA + 91 EF-Tu.GTP.arg1-tRNA + 84 EF-Tu.GTP.asn1-tRNA + 119 EF-Tu.GTP.asp1-tRNA + 7 EF-Tu.GTP.cysT-tRNA + 49 EF-Tu.GTP.gln1-tRNA + 112 EF-Tu.GTP.glu1-tRNA + 119 EF-Tu.GTP.gly1-tRNA + 42 EF-Tu.GTP.hisR-tRNA + 98 EF-Tu.GTP.ile1-tRNA + 126 EF-Tu.GTP.leu1-tRNA + 21 EF-Tu.GTP.leuU-tRNA + 133 EF-Tu.GTP.lys1-tRNA + 49 EF-Tu.GTP.met1-tRNA + 84 EF-Tu.GTP.phe1-tRNA + 49 EF-Tu.GTP.pro1-tRNA + 21 EF-Tu.GTP.ser1-tRNA + 28 EF-Tu.GTP.ser2-tRNA + 28 EF-Tu.GTP.serV-tRNA + 42 EF-Tu.GTP.thr1-tRNA + 49 EF-Tu.GTP.thr2 tRNA + 21 EF-Tu.GTP.trpT-tRNA + 14 EF-Tu.GTP.tyr1-tRNA + 56 EF-Tu.GTP.val1-tRNA + 63 EF-Tu.GTP.val3-tRNA + 8400 mg2 + 1 rib_ini_b0169_7 --> 1 rib_70_elo1_b0169_7_cplx	irreversible	Translation	0.00E+00	1.00E+09	0	1.61E+01	1.61E+01	0	1.18E+01	11.84	0	1.18E+01	11.84
tl_elo_b0169_7_rib2	Translation elongation 2 b0169 7 ribosome(s)	1673 h2o + 1 rib_70_elo1_b0169_7_cplx --> 1673 EF-G.GDP + 1673 EF-Tu.GDP + 175 ala1_tRNA + 91 arg1_tRNA + 84 asn1_tRNA + 119 asp1_tRNA + 7 cysT_tRNA + 7 fmet_tRNA + 49 gln1_tRNA + 105 glu1_tRNA + 119 gly1_tRNA + 3346 h + 42 hisR_tRNA + 98 ile1_tRNA + 126 leu1_tRNA + 21 leuU_tRNA + 133 lys1_tRNA + 49 met1_tRNA + 8400 mg2 + 84 phe1_tRNA + 3346 pi + 49 pro1_tRNA + 1 rib_70_elo2_b0169_7_cplx + 21 ser1_tRNA + 28 ser2_tRNA + 28 serV_tRNA + 42 thr1_tRNA + 49 thr2_tRNA + 21 trpT_tRNA + 14 tyr1_tRNA + 56 val1_tRNA + 63 val3_tRNA	irreversible	Translation	0.00E+00	1.00E+09	0	1.61E+01	1.61E+01	0	1.18E+01	11.84	0	1.18E+01	11.84

tl_elo_b0170_16_rib1	Translation elongation 1 b0170 16 ribosome(s)	4512 EF-G.GTP + 624 EF-Tu.GTP.ala1-tRNA + 144 EF-Tu.GTP.arg1-tRNA + 128 EF-Tu.GTP.asn1-tRNA + 224 EF-Tu.GTP.asp1-tRNA + 32 EF-Tu.GTP.cysT-tRNA + 160 EF-Tu.GTP.gln1-tRNA + 512 EF-Tu.GTP.glu1-tRNA + 384 EF-Tu.GTP.gly1-tRNA + 64 EF-Tu.GTP.hisR-tRNA + 304 EF-Tu.GTP.ile1-tRNA + 256 EF-Tu.GTP.leu1-tRNA + 448 EF-Tu.GTP.lys1-tRNA + 144 EF-Tu.GTP.met1-tRNA + 160 EF-Tu.GTP.phe1-tRNA + 48 EF-Tu.GTP.pro1-tRNA + 32 EF-Tu.GTP.proM-tRNA + 64 EF-Tu.GTP.ser1-tRNA + 64 EF-Tu.GTP.ser2-tRNA + 32 EF-Tu.GTP.serV-tRNA + 64 EF-Tu.GTP.thr1-tRNA + 112 EF-Tu.GTP.thr2-tRNA + 48 EF-Tu.GTP.tyr1-tRNA + 144 EF-Tu.GTP.val1-tRNA + 320 EF-Tu.GTP.val3-tRNA + 22560 mg2 + 1 rib_ini_b0170_16 --> 1 rib_70_elo1_b0170_16_cplx	irreversible	Translation	0.00E+00	1.00E+09	0	9.05E+01	9.05E+01	0	9.64E+01	96.44127	0	1.60E+01	16.018
tl_elo_b0170_16_rib2	Translation elongation 2 b0170 16 ribosome(s)	4496 h2o + 1 rib_70_elo1_b0170_16_cplx --> 4496 EF-G.GDP + 4496 EF-Tu.GDP + 624 ala1_tRNA + 144 arg1_tRNA + 128 asn1_tRNA + 224 asp1_tRNA + 32 cysT_tRNA + 16 fmet_tRNA + 160 gln1_tRNA + 512 glu1_tRNA + 384 gly1_tRNA + 8992 h + 64 hisR_tRNA + 304 ile1_tRNA + 256 leu1_tRNA + 448 lys1_tRNA + 144 met1_tRNA + 22560 mg2 + 160 phe1_tRNA + 8992 pi + 48 pro1_tRNA + 32 proM_tRNA + 1 rib_70_elo2_b0170_16_cplx + 64 ser1_tRNA + 48 ser2_tRNA + 32 serV_tRNA + 64 thr1_tRNA + 112 thr2_tRNA + 48 tyr1_tRNA + 144 val1_tRNA + 320 val3_tRNA	irreversible	Translation	0.00E+00	1.00E+09	0	9.05E+01	9.05E+01	0	9.64E+01	96.44127	0	1.60E+01	16.018
tl_elo_b0170_1_rib1	Translation elongation 1 b0170 1 ribosome(s)	282 EF-G.GTP + 39 EF-Tu.GTP.ala1-tRNA + 9 EF-Tu.GTP.arg1-tRNA + 8 EF-Tu.GTP.asn1-tRNA + 14 EF-Tu.GTP.asp1-tRNA + 2 EF-Tu.GTP.cysT-tRNA + 10 EF-Tu.GTP.gln1-tRNA + 32 EF-Tu.GTP.glu1-tRNA + 24 EF-Tu.GTP.gly1-tRNA + 4 EF-Tu.GTP.hisR-tRNA + 19 EF-Tu.GTP.ile1-tRNA + 16 EF-Tu.GTP.leu1-tRNA + 28 EF-Tu.GTP.lys1-tRNA + 9 EF-Tu.GTP.met1-tRNA + 10 EF-Tu.GTP.phe1-tRNA + 3 EF-Tu.GTP.pro1-tRNA + 2 EF-Tu.GTP.proM-tRNA + 4 EF-Tu.GTP.ser1-tRNA + 4 EF-Tu.GTP.ser2-tRNA + 2 EF-Tu.GTP.serV-tRNA + 4 EF-Tu.GTP.thr1-tRNA + 7 EF-Tu.GTP.thr2-tRNA + 3 EF-Tu.GTP.tyr1-tRNA + 9 EF-Tu.GTP.val1-tRNA + 20 EF-Tu.GTP.val3-tRNA + 1410 mg2 + 1 rib_ini_b0170_1 --> 1 rib_70_elo1_b0170_1_cplx	irreversible	Translation	0.00E+00	1.00E+09	0	9.05E+01	9.05E+01	0	9.64E+01	96.44127	0	1.60E+01	16.018
tl_elo_b0170_1_rib2	Translation elongation 2 b0170 1 ribosome(s)	281 h2o + 1 rib_70_elo1_b0170_1_cplx --> 281 EF-G.GDP + 281 EF-Tu.GDP + 39 ala1_tRNA + 9 arg1_tRNA + 8 asn1_tRNA + 14 asp1_tRNA + 2 cysT_tRNA + 1 fmet_tRNA + 10 gln1_tRNA + 32 glu1_tRNA + 24 gly1_tRNA + 562 h + 4 hisR_tRNA + 19 ile1_tRNA + 16 leu1_tRNA + 28 lys1_tRNA + 9 met1_tRNA + 1410 mg2 + 10 phe1_tRNA + 562 pi + 3 pro1_tRNA + 2 proM_tRNA + 1 rib_70_elo2_b0170_1_cplx + 4 ser1_tRNA + 3 ser2_tRNA + 2 serV_tRNA + 4 thr1_tRNA + 7 thr2_tRNA + 3 tyr1_tRNA + 9 val1_tRNA + 20 val3_tRNA	irreversible	Translation	0.00E+00	1.00E+09	0	9.05E+01	9.05E+01	0	9.64E+01	96.44127	0	1.60E+01	16.018
tl_elo_b0170_8_rib1	Translation elongation 1 b0170 8 ribosome(s)	2256 EF-G.GTP + 312 EF-Tu.GTP.ala1-tRNA + 72 EF-Tu.GTP.arg1-tRNA + 64 EF-Tu.GTP.asn1-tRNA + 112 EF-Tu.GTP.asp1-tRNA + 16 EF-Tu.GTP.cysT-tRNA + 80 EF-Tu.GTP.gln1-tRNA + 256 EF-Tu.GTP.glu1-tRNA + 192 EF-Tu.GTP.gly1-tRNA + 32 EF-Tu.GTP.hisR-tRNA + 152 EF-Tu.GTP.ile1-tRNA + 128 EF-Tu.GTP.leu1-tRNA + 224 EF-Tu.GTP.lys1-tRNA + 72 EF-Tu.GTP.met1-tRNA + 80 EF-Tu.GTP.phe1-tRNA + 24 EF-Tu.GTP.pro1-tRNA + 16 EF-Tu.GTP.proM-tRNA + 32 EF-Tu.GTP.ser1-tRNA + 32 EF-Tu.GTP.ser2-tRNA + 16 EF-Tu.GTP.serV-tRNA + 32 EF-Tu.GTP.thr1-tRNA + 56 EF-Tu.GTP.thr2 tRNA + 24 EF-Tu.GTP.tyr1-tRNA + 72 EF-Tu.GTP.val1-tRNA + 160 EF-Tu.GTP.val3-tRNA + 11280 mg2 + 1 rib_ini_b0170_8 --> 1 rib_70_elo1_b0170_8_cplx	irreversible	Translation	0.00E+00	1.00E+09	0	9.05E+01	9.05E+01	0	9.64E+01	96.44127	0	1.60E+01	16.018
tl_elo_b0170_8_rib2	Translation elongation 2 b0170 8 ribosome(s)	2248 h2o + 1 rib_70_elo1_b0170_8_cplx --> 2248 EF-G.GDP + 2248 EF-Tu.GDP + 312 ala1_tRNA + 72 arg1_tRNA + 64 asn1_tRNA + 112 asp1_tRNA + 16 cysT_tRNA + 8 fmet_tRNA + 80 gln1_tRNA + 256 glu1_tRNA + 192 gly1_tRNA + 4496 h + 32 hisR_tRNA + 152 ile1_tRNA + 128 leu1_tRNA + 224 lys1_tRNA + 72 met1_tRNA + 11280 mg2 + 80 phe1_tRNA + 4496 pi + 24 pro1_tRNA + 16 proM_tRNA + 1 rib_70_elo2_b0170_8_cplx + 32 ser1_tRNA + 24 ser2_tRNA + 16 serV_tRNA + 32 thr1_tRNA + 56 thr2_tRNA + 24 tyr1_tRNA + 72 val1_tRNA + 160 val3_tRNA	irreversible	Translation	0.00E+00	1.00E+09	0	9.05E+01	9.05E+01	0	9.64E+01	96.44127	0	1.60E+01	16.018
tl_elo_b0172_10_rib1	Translation elongation 1 b0172 10 ribosome(s)	1840 EF-G.GTP + 180 EF-Tu.GTP.ala1-tRNA + 10 EF-Tu.GTP.ala2-tRNA + 160 EF-Tu.GTP.arg1-tRNA + 10 EF-Tu.GTP.argU-tRNA + 50 EF-Tu.GTP.asn1-tRNA + 190 EF-Tu.GTP.asp1-tRNA + 10 EF-Tu.GTP.cysT-tRNA + 40 EF-Tu.GTP.gln1-tRNA + 20 EF-Tu.GTP.gln2-tRNA + 140 EF-Tu.GTP.glu1-tRNA + 60 EF-Tu.GTP.gly1-tRNA + 110 EF-Tu.GTP.ile1-tRNA + 10 EF-Tu.GTP.ile2 tRNA + 130 EF-Tu.GTP.leu1-tRNA + 10 EF-Tu.GTP.leu2-tRNA + 10 EF-Tu.GTP.leuU-tRNA + 160 EF-Tu.GTP.lys1-tRNA + 40 EF-Tu.GTP.met1-tRNA + 30 EF-Tu.GTP.phe1-tRNA + 70 EF-Tu.GTP.pro1-tRNA + 10 EF-Tu.GTP.proL-tRNA + 20 EF-Tu.GTP.ser1-tRNA + 40 EF-Tu.GTP.ser2-tRNA + 10 EF-Tu.GTP.serT-tRNA + 60 EF-Tu.GTP.serV-tRNA + 20 EF-Tu.GTP.thr1-tRNA + 10 EF-Tu.GTP.thr2-tRNA + 50 EF-Tu.GTP.thr3-tRNA + 10 EF-Tu.GTP.thrU-tRNA + 20 EF-Tu.GTP.tyr1-tRNA + 100 EF-Tu.GTP.val1-tRNA + 10 EF-Tu.GTP.val2-tRNA + 40 EF-Tu.GTP.val3-tRNA + 9200 mg2 + 1 rib_ini_b0172_10 --> 1 rib_70_elo1_b0172_10_cplx	irreversible	Translation	0.00E+00	1.00E+09	0	2.65E+02	2.65E+02	0	4.53E+02	452.8892	0	1.96E+01	19.599
tl_elo_b0172_10_rib2	Translation elongation 2 b0172 10 ribosome(s)	1830 h2o + 1 rib_70_elo1_b0172_10_cplx --> 1830 EF-G.GDP + 1830 EF-Tu.GDP + 180 ala1_tRNA + 10 ala2_tRNA + 160 arg1_tRNA + 10 argU_tRNA + 50 asn1_tRNA + 190 asp1_tRNA + 10 cysT_tRNA + 10 fmet_tRNA + 40 gln1_tRNA + 20 gln2_tRNA + 140 glu1_tRNA + 60 gly1_tRNA + 3660 h + 110 ile1_tRNA + 10 ile2_tRNA + 130 leu1_tRNA + 10 leu2_tRNA + 10 leuU_tRNA + 160 lys1_tRNA + 40 met1_tRNA + 9200 mg2 + 20 phe1_tRNA + 3660 pi + 70 pro1_tRNA + 10 proL_tRNA + 1 rib_70_elo2_b0172_10_cplx + 20 ser1_tRNA + 40 ser2_tRNA + 10 serT_tRNA + 60 serV_tRNA + 20 thr1_tRNA + 10 thr2_tRNA + 50 thr3_tRNA + 10 thrU_tRNA + 20 tyr1_tRNA + 100 val1_tRNA + 10 val2_tRNA + 40 val3_tRNA	irreversible	Translation	0.00E+00	1.00E+09	0	2.65E+02	2.65E+02	0	4.53E+02	452.8892	0	1.96E+01	19.599

tl_elo_b0172_1_rib1	Translation elongation 1 b0172 1 ribosome(s)	184 EF-G.GTP + 18 EF-Tu.GTP.ala1-tRNA + 1 EF-Tu.GTP.ala2-tRNA + 16 EF-Tu.GTP.arg1-tRNA + 1 EF-Tu.GTP.argU-tRNA + 5 EF-Tu.GTP.asn1-tRNA + 19 EF-Tu.GTP.asp1-tRNA + 1 EF-Tu.GTP.cysT-tRNA + 4 EF-Tu.GTP.gln1-tRNA + 2 EF-Tu.GTP.gln2-tRNA + 14 EF-Tu.GTP.glu1-tRNA + 6 EF-Tu.GTP.gly1-tRNA + 11 EF-Tu.GTP.ile1-tRNA + 1 EF-Tu.GTP.ile2-tRNA + 13 EF-Tu.GTP.leu1-tRNA + 1 EF-Tu.GTP.leu2-tRNA + 1 EF-Tu.GTP.leuU-tRNA + 16 EF-Tu.GTP.lys1-tRNA + 4 EF-Tu.GTP.met1-tRNA + 3 EF-Tu.GTP.phe1-tRNA + 7 EF-Tu.GTP.pro1-tRNA + 1 EF-Tu.GTP.proL-tRNA + 2 EF-Tu.GTP.ser1-tRNA + 4 EF-Tu.GTP.ser2-tRNA + 1 EF-Tu.GTP.serT-tRNA + 6 EF-Tu.GTP.serV-tRNA + 2 EF-Tu.GTP.thr1-tRNA + 1 EF-Tu.GTP.thr2-tRNA + 5 EF-Tu.GTP.thr3-tRNA + 1 EF-Tu.GTP.thrU-tRNA + 2 EF-Tu.GTP.tyr1-tRNA + 10 EF-Tu.GTP.val1-tRNA + 1 EF-Tu.GTP.val2-tRNA + 4 EF-Tu.GTP.val3-tRNA + 920 mg2 + 1 rib_ini_b0172_1 --> 1 rib_70_elo1_b0172_1_cplx	irreversible	Translation	0.00E+00	1.00E+09	0	2.65E+02	2.65E+02	0	4.53E+02	452.8892	0	1.96E+01	19.599
tl_elo_b0172_1_rib2	Translation elongation 2 b0172 1 ribosome(s)	183 h2o + 1 rib_70_elo1_b0172_1_cplx --> 183 EF-G.GDP + 183 EF-Tu.GDP + 18 ala1_tRNA + 1 ala2_tRNA + 16 arg1_tRNA + 1 argU_tRNA + 5 asn1_tRNA + 19 asp1_tRNA + 1 cysT_tRNA + 1 fmet_tRNA + 4 gln1_tRNA + 2 gln2_tRNA + 14 glu1_tRNA + 6 gly1_tRNA + 366 h + 11 ile1_tRNA + 1 ile2_tRNA + 13 leu1_tRNA + leu2_tRNA + 1 leuU_tRNA + 16 lys1_tRNA + 4 met1_tRNA + 920 mg2 + 2 phe1_tRNA + 366 pi + 7 pro1_tRNA + 1 proL_tRNA + 1 rib_70_elo2_b0172_1_cplx + 2 ser1_tRNA + 4 ser2_tRNA + 1 serT_tRNA + 6 serV_tRNA + 2 thr1_tRNA + 1 thr2_tRNA + 5 thr3_tRNA + 1 thrU_tRNA + 2 tyr1_tRNA + 10 val1_tRNA + 1 val2_tRNA + 4 val3_tRNA	irreversible	Translation	0.00E+00	1.00E+09	0	2.65E+02	2.65E+02	0	4.53E+02	452.8892	0	1.96E+01	19.599
tl_elo_b0172_5_rib1	Translation elongation 1 b0172 5 ribosome(s)	920 EF-G.GTP + 90 EF-Tu.GTP.ala1-tRNA + 5 EF-Tu.GTP.ala2-tRNA + 80 EF-Tu.GTP.arg1-tRNA + 5 EF-Tu.GTP.argU-tRNA + 25 EF-Tu.GTP.asn1-tRNA + 95 EF-Tu.GTP.asp1-tRNA + 5 EF-Tu.GTP.cysT-tRNA + 20 EF-Tu.GTP.gln1-tRNA + 10 EF-Tu.GTP.gln2-tRNA + 70 EF-Tu.GTP.glu1-tRNA + 30 EF-Tu.GTP.gly1-tRNA + 55 EF-Tu.GTP.ile1-tRNA + 5 EF-Tu.GTP.ile2-tRNA + 65 EF-Tu.GTP.leu1-tRNA + 5 EF-Tu.GTP.leu2-tRNA + 5 EF-Tu.GTP.leuU-tRNA + 80 EF-Tu.GTP.lys1-tRNA + 20 EF-Tu.GTP.met1-tRNA + 15 EF-Tu.GTP.phe1-tRNA + 35 EF-Tu.GTP.pro1-tRNA + 5 EF-Tu.GTP.proL-tRNA + 10 EF-Tu.GTP.ser1-tRNA + 20 EF-Tu.GTP.ser2-tRNA + 5 EF-Tu.GTP.serT-tRNA + 30 EF-Tu.GTP.serV-tRNA + 10 EF-Tu.GTP.thr1-tRNA + 5 EF-Tu.GTP.thr2-tRNA + 25 EF-Tu.GTP.thr3-tRNA + 5 EF-Tu.GTP.thrU-tRNA + 10 EF-Tu.GTP.tyr1-tRNA + 50 EF-Tu.GTP.val1-tRNA + 5 EF-Tu.GTP.val2-tRNA + 20 EF-Tu.GTP.val3-tRNA + 4600 mg2 + 1 rib_ini_b0172_5 --> 1 rib_70_elo1_b0172_5_cplx	irreversible	Translation	0.00E+00	1.00E+09	0	2.65E+02	2.65E+02	0	4.53E+02	452.8892	0	1.96E+01	19.599
tl_elo_b0172_5_rib2	Translation elongation 2 b0172 5 ribosome(s)	915 h2o + 1 rib_70_elo1_b0172_5_cplx --> 915 EF-G.GDP + 915 EF-Tu.GDP + 90 ala1_tRNA + 5 ala2_tRNA + 80 arg1_tRNA + 5 argU_tRNA + 25 asn1_tRNA + 95 asp1_tRNA + 5 cysT_tRNA + 5 fmet_tRNA + 20 gln1_tRNA + 10 gln2_tRNA + 70 glu1_tRNA + 30 gly1_tRNA + 1830 h + 55 ile1_tRNA + 5 ile2_tRNA + 65 leu1_tRNA + 5 leu2_tRNA + 5 leuU_tRNA + 80 lys1_tRNA + 20 met1_tRNA + 4600 mg2 + 10 phe1_tRNA + 1830 pi + 35 pro1_tRNA + 5 proL_tRNA + 1 rib_70_elo2_b0172_5_cplx + 10 ser1_tRNA + 20 ser2_tRNA + 5 serT_tRNA + 30 serV_tRNA + 10 thr1_tRNA + 5 thr2_tRNA + 25 thr3_tRNA + 5 thrU_tRNA + 10 tyr1_tRNA + 50 val1_tRNA + 5 val2_tRNA + 20 val3_tRNA	irreversible	Translation	0.00E+00	1.00E+09	0	2.65E+02	2.65E+02	0	4.53E+02	452.8892	0	1.96E+01	19.599
tl_elo_b0188_12_rib1	Translation elongation 1 b0188 12 ribosome(s)	5172 EF-G.GTP + 408 EF-Tu.GTP.ala1-tRNA + 168 EF-Tu.GTP.ala2-tRNA + 348 EF-Tu.GTP.arg1-tRNA + 12 EF-Tu.GTP.argU-tRNA + 12 EF-Tu.GTP.argW-tRNA + 60 EF-Tu.GTP.argX-tRNA + 132 EF-Tu.GTP.asn1-tRNA + 216 EF-Tu.GTP.asp1-tRNA + 72 EF-Tu.GTP.cysT-tRNA + 252 EF-Tu.GTP.gln1-tRNA + 156 EF-Tu.GTP.gln2-tRNA + 324 EF-Tu.GTP.glu1-tRNA + 180 EF-Tu.GTP.gly1-tRNA + 72 EF-Tu.GTP.glyT-tRNA + 108 EF-Tu.GTP.glyU-tRNA + 108 EF-Tu.GTP.hisR-tRNA + 192 EF-Tu.GTP.ile1-tRNA + 336 EF-Tu.GTP.leu1-tRNA + 96 EF-Tu.GTP.leu2-tRNA + 168 EF-Tu.GTP.leuU-tRNA + 36 EF-Tu.GTP.leuW-tRNA + 72 EF-Tu.GTP.leuZ-tRNA + 84 EF-Tu.GTP.lys1-tRNA + 48 EF-Tu.GTP.met1-tRNA + 132 EF-Tu.GTP.phe1-tRNA + 156 EF-Tu.GTP.pro1-tRNA + 36 EF-Tu.GTP.pro2-tRNA + 24 EF-Tu.GTP.proL-tRNA + 36 EF-Tu.GTP.proM-tRNA + 36 EF-Tu.GTP.ser1-tRNA + 48 EF-Tu.GTP.ser3-tRNA + 36 EF-Tu.GTP.serT-tRNA + 144 EF-Tu.GTP.serV-tRNA + 84 EF-Tu.GTP.thr1-tRNA + 144 EF-Tu.GTP.thr3-tRNA + 12 EF-Tu.GTP.thrU-tRNA + 180 EF-Tu.GTP.trpT-tRNA + 60 EF-Tu.GTP.tyr1-tRNA + 252 EF-Tu.GTP.val1-tRNA + 108 EF-Tu.GTP.val2-tRNA + 24 EF-Tu.GTP.val3-tRNA + 25860 mg2 + 1 rib_ini_b0188_12 --> 1 rib_70_e	irreversible	Translation	0.00E+00	1.00E+09	0	9.84E+01	9.84E+01	0	1.71E+02	170.9685	0	3.84E+00	3.836
tl_elo_b0188_12_rib2	Translation elongation 2 b0188 12 ribosome(s)	5160 h2o + 1 rib_70_elo1_b0188_12_cplx --> 5160 EF-G.GDP + 5160 EF-Tu.GDP + 408 ala1_tRNA + 168 ala2_tRNA + 348 arg1_tRNA + 12 argU_tRNA + 12 argW_tRNA + 60 argX_tRNA + 132 asn1_tRNA + 216 asp1_tRNA + 72 cysT_tRNA + 12 fmet_tRNA + 252 gln1_tRNA + 156 gln2_tRNA + 324 glu1_tRNA + 180 gly1_tRNA + 72 glyT_tRNA + 108 glyU_tRNA + 10320 h + 108 hisR_tRNA + 192 ile1_tRNA + 336 leu1_tRNA + 96 leu2_tRNA + 168 leuU_tRNA + 36 leuW_tRNA + 72 leuZ_tRNA + 84 lys1_tRNA + 48 met1_tRNA + 25860 mg2 + 132 phe1_tRNA + 10320 pi + 156 pro1_tRNA + 36 pro2_tRNA + 24 proL_tRNA + 36 proM_tRNA + 1 rib_70_elo2_b0188_12_cplx + 36 ser1_tRNA + 48 ser3_tRNA + 36 serT_tRNA + 132 serV_tRNA + 84 thr1_tRNA + 144 thr3_tRNA + 12 thrU_tRNA + 180 trpT_tRNA + 60 tyr1_tRNA + 252 val1_tRNA + 108 val2_tRNA + 24 val3_tRNA	irreversible	Translation	0.00E+00	1.00E+09	0	9.84E+01	9.84E+01	0	1.71E+02	170.9685	0	3.84E+00	3.836

tl_elo_b0188_1_rib1	Translation elongation 1 b0188 1 ribosome(s)	431 EF-G.GTP + 34 EF-Tu.GTP.ala1-tRNA + 14 EF-Tu.GTP.ala2-tRNA + 29 EF-Tu.GTP.arg1-tRNA + 1 EF-Tu.GTP.argU-tRNA + 1 EF-Tu.GTP.argW-tRNA + 5 EF-Tu.GTP.argX-tRNA + 11 EF-Tu.GTP.asn1-tRNA + 18 EF-Tu.GTP.asp1-tRNA + 6 EF-Tu.GTP.cysT-tRNA + 21 EF-Tu.GTP.gln1-tRNA + 13 EF-Tu.GTP.gln2-tRNA + 27 EF-Tu.GTP.glu1-tRNA + 15 EF-Tu.GTP.gly1-tRNA + 6 EF-Tu.GTP.glyT-tRNA + 9 EF-Tu.GTP.glyU-tRNA + 9 EF-Tu.GTP.hisR-tRNA + 16 EF-Tu.GTP.ile1-tRNA + 28 EF-Tu.GTP.leu1-tRNA + 8 EF-Tu.GTP.leu2-tRNA + 14 EF-Tu.GTP.leuU-tRNA + 3 EF-Tu.GTP.leuW-tRNA + 6 EF-Tu.GTP.leuZ-tRNA + 7 EF-Tu.GTP.lys1-tRNA + 4 EF-Tu.GTP.met1-tRNA + 11 EF-Tu.GTP.phe1-tRNA + 13 EF-Tu.GTP.pro1-tRNA + 3 EF-Tu.GTP.pro2-tRNA + 2 EF-Tu.GTP.proL-tRNA + 3 EF-Tu.GTP.proM-tRNA + 3 EF-Tu.GTP.ser1-tRNA + 4 EF-Tu.GTP.ser3-tRNA + 3 EF-Tu.GTP.serT-tRNA + 12 EF-Tu.GTP.serV-tRNA + 7 EF-Tu.GTP.thr1-tRNA + 12 EF-Tu.GTP.thr3-tRNA + 1 EF-Tu.GTP.thrU-tRNA + 15 EF-Tu.GTP.trpT-tRNA + 5 EF-Tu.GTP.tyr1-tRNA + 21 EF-Tu.GTP.val1-tRNA + 9 EF-Tu.GTP.val2-tRNA + 2 EF-Tu.GTP.val3-tRNA + 2155 mg2 + 1 rib_ini_b0188_1 --> 1 rib_70_elo1_b0188_1_cplx	irreversible	Translation	0.00E+00	1.00E+09	0	9.84E+01	9.84E+01	0	1.71E+02	170.9685	0	3.84E+00	3.836
tl_elo_b0188_1_rib2	Translation elongation 2 b0188 1 ribosome(s)	430 h2o + 1 rib_70_elo1_b0188_1_cplx --> 430 EF-G.GDP + 430 EF-Tu.GDP + 34 ala1_tRNA + 14 ala2_tRNA + 29 arg1_tRNA + 1 argU_tRNA + 1 argW_tRNA + 5 argX_tRNA + 11 asn1_tRNA + 18 asp1_tRNA + 6 cysT_tRNA + 1 fmet_tRNA + 21 gln1_tRNA + 13 gln2_tRNA + 27 glu1_tRNA + 15 gly1_tRNA + 6 glyT_tRNA + 9 glyU_tRNA + 860 h + 9 hisR_tRNA + 16 ile1_tRNA + 28 leu1_tRNA + 8 leu2_tRNA + 14 leuU_tRNA + 3 leuW_tRNA + 6 leuZ_tRNA + 7 lys1_tRNA + 4 met1_tRNA + 2155 mg2 + 11 phe1_tRNA + 860 pi + 13 pro1_tRNA + 3 pro2_tRNA + 2 proL_tRNA + 3 proM_tRNA + 1 rib_70_elo2_b0188_1_cplx + 3 ser1_tRNA + 4 ser3_tRNA + 3 serT_tRNA + 11 serV_tRNA + 7 thr1_tRNA + 12 thr3_tRNA + 1 thrU_tRNA + 15 trpT_tRNA + 5 tyr1_tRNA + 21 val1_tRNA + 9 val2_tRNA + 2 val3_tRNA	irreversible	Translation	0.00E+00	1.00E+09	0	9.84E+01	9.84E+01	0	1.71E+02	170.9685	0	3.84E+00	3.836
tl_elo_b0188_25_rib1	Translation elongation 1 b0188 25 ribosome(s)	10775 EF-G.GTP + 850 EF-Tu.GTP.ala1-tRNA + 350 EF-Tu.GTP.ala2-tRNA + 725 EF-Tu.GTP.arg1-tRNA + 25 EF-Tu.GTP.argU-tRNA + 25 EF-Tu.GTP.argW-tRNA + 125 EF-Tu.GTP.argX-tRNA + 275 EF-Tu.GTP.asn1-tRNA + 450 EF-Tu.GTP.asp1-tRNA + 150 EF-Tu.GTP.cysT-tRNA + 525 EF-Tu.GTP.gln1-tRNA + 325 EF-Tu.GTP.gln2-tRNA + 675 EF-Tu.GTP.glu1-tRNA + 375 EF-Tu.GTP.gly1-tRNA + 150 EF-Tu.GTP.glyT-tRNA + 225 EF-Tu.GTP.glyU-tRNA + 225 EF-Tu.GTP.hisR-tRNA + 400 EF-Tu.GTP.ile1-tRNA + 700 EF-Tu.GTP.leu1-tRNA + 200 EF-Tu.GTP.leu2-tRNA + 350 EF-Tu.GTP.leuU-tRNA + 75 EF-Tu.GTP.leuW-tRNA + 150 EF-Tu.GTP.leuZ-tRNA + 175 EF-Tu.GTP.lys1-tRNA + 100 EF-Tu.GTP.met1-tRNA + 275 EF-Tu.GTP.phe1-tRNA + 325 EF-Tu.GTP.pro1-tRNA + 75 EF-Tu.GTP.pro2-tRNA + 50 EF-Tu.GTP.proL-tRNA + 75 EF-Tu.GTP.proM-tRNA + 75 EF-Tu.GTP.ser1-tRNA + 100 EF-Tu.GTP.ser3-tRNA + 75 EF-Tu.GTP.serT-tRNA + 300 EF-Tu.GTP.serV-tRNA + 175 EF-Tu.GTP.thr1-tRNA + 300 EF-Tu.GTP.thr3-tRNA + 25 EF-Tu.GTP.thrU-tRNA + 375 EF-Tu.GTP.trpT-tRNA + 125 EF-Tu.GTP.tyr1-tRNA + 525 EF-Tu.GTP.val1-tRNA + 225 EF-Tu.GTP.val2-tRNA + 50 EF-Tu.GTP.val3-tRNA + 53875 mg2 + 1 rib_	irreversible	Translation	0.00E+00	1.00E+09	0	9.84E+01	9.84E+01	0	1.32E+02	131.6329	0	3.84E+00	3.836
tl_elo_b0188_25_rib2	Translation elongation 2 b0188 25 ribosome(s)	10750 h2o + 1 rib_70_elo1_b0188_25_cplx --> 10750 EF-G.GDP + 10750 EF-Tu.GDP + 850 ala1_tRNA + 350 ala2_tRNA + 725 arg1_tRNA + 25 argU_tRNA + 25 argW_tRNA + 125 argX_tRNA + 275 asn1_tRNA + 450 asp1_tRNA + 150 cysT_tRNA + 25 fmet_tRNA + 525 gln1_tRNA + 325 gln2_tRNA + 675 glu1_tRNA + 375 gly1_tRNA + 150 glyT_tRNA + 225 glyU_tRNA + 21500 h + 225 hisR_tRNA + 400 ile1_tRNA + 700 leu1_tRNA + 200 leu2_tRNA + 350 leuU_tRNA + 75 leuW_tRNA + 150 leuZ_tRNA + 175 lys1_tRNA + 100 met1_tRNA + 53875 mg2 + 275 phe1_tRNA + 21500 pi + 325 pro1_tRNA + 75 pro2_tRNA + 50 proL_tRNA + 75 proM_tRNA + 1 rib_70_elo2_b0188_25_cplx + 75 ser1_tRNA + 100 ser3_tRNA + 75 serT_tRNA + 275 serV_tRNA + 175 thr1_tRNA + 300 thr3_tRNA + 25 thrU_tRNA + 375 trpT_tRNA + 125 tyr1_tRNA + 525 val1_tRNA + 225 val2_tRNA + 50 val3_tRNA	irreversible	Translation	0.00E+00	1.00E+09	0	9.84E+01	9.84E+01	0	1.32E+02	131.6329	0	3.84E+00	3.836
tl_elo_b0194_16_rib1	Translation elongation 1 b0194 16 ribosome(s)	9136 EF-G.GTP + 608 EF-Tu.GTP.ala1-tRNA + 208 EF-Tu.GTP.ala2-tRNA + 544 EF-Tu.GTP.arg1-tRNA + 16 EF-Tu.GTP.argW-tRNA + 304 EF-Tu.GTP.asn1-tRNA + 544 EF-Tu.GTP.asp1-tRNA + 16 EF-Tu.GTP.cysT-tRNA + 336 EF-Tu.GTP.gln1-tRNA + 96 EF-Tu.GTP.gln2-tRNA + 768 EF-Tu.GTP.glu1-tRNA + 592 EF-Tu.GTP.gly1-tRNA + 64 EF-Tu.GTP.glyU-tRNA + 144 EF-Tu.GTP.hisR-tRNA + 576 EF-Tu.GTP.ile1-tRNA + 640 EF-Tu.GTP.leu1-tRNA + 16 EF-Tu.GTP.leu2-tRNA + 96 EF-Tu.GTP.leuU-tRNA + 32 EF-Tu.GTP.leuZ-tRNA + 448 EF-Tu.GTP.lys1-tRNA + 256 EF-Tu.GTP.met1-tRNA + 320 EF-Tu.GTP.phe1-tRNA + 368 EF-Tu.GTP.pro1-tRNA + 32 EF-Tu.GTP.pro2-tRNA + 48 EF-Tu.GTP.proM-tRNA + 128 EF-Tu.GTP.ser1-tRNA + 96 EF-Tu.GTP.ser2-tRNA + 16 EF-Tu.GTP.ser3-tRNA + 16 EF-Tu.GTP.serT-tRNA + 224 EF-Tu.GTP.serV-tRNA + 192 EF-Tu.GTP.thr1-tRNA + 144 EF-Tu.GTP.thr2-tRNA + 80 EF-Tu.GTP.thr3-tRNA + 16 EF-Tu.GTP.thrU-tRNA + 80 EF-Tu.GTP.trpT-tRNA + 288 EF-Tu.GTP.tyr1-tRNA + 448 EF-Tu.GTP.val1-tRNA + 80 EF-Tu.GTP.val2-tRNA + 256 EF-Tu.GTP.val3-tRNA + 45680 mg2 + 1 rib_ini_b0194_16 --> 1 rib_70_elo1_b0194_16_cplx	irreversible	Translation	0.00E+00	1.00E+09	0	7.54E+01	7.54E+01	0	1.31E+02	130.5277	0	3.47E+00	3.467
tl_elo_b0194_16_rib2	Translation elongation 2 b0194 16 ribosome(s)	9120 h2o + 1 rib_70_elo1_b0194_16_cplx --> 9120 EF-G.GDP + 9120 EF-Tu.GDP + 608 ala1_tRNA + 208 ala2_tRNA + 544 arg1_tRNA + 16 argW_tRNA + 304 asn1_tRNA + 544 asp1_tRNA + 16 cysT_tRNA + 16 fmet_tRNA + 336 gln1_tRNA + 96 gln2_tRNA + 768 glu1_tRNA + 576 gly1_tRNA + 64 glyU_tRNA + 18240 h + 144 hisR_tRNA + 576 ile1_tRNA + 640 leu1_tRNA + 16 leu2_tRNA + 96 leuU_tRNA + 32 leuZ_tRNA + 448 lys1_tRNA + 256 met1_tRNA + 45680 mg2 + 320 phe1_tRNA + 18240 pi + 368 pro1_tRNA + 32 pro2_tRNA + 48 proM_tRNA + 1 rib_70_elo2_b0194_16_cplx + 128 ser1_tRNA + 96 ser2_tRNA + 16 ser3_tRNA + 16 serT_tRNA + 224 serV_tRNA + 192 thr1_tRNA + 144 thr2_tRNA + 80 thr3_tRNA + 16 thrU_tRNA + 80 trpT_tRNA + 288 tyr1_tRNA + 448 val1_tRNA + 80 val2_tRNA + 256 val3_tRNA	irreversible	Translation	0.00E+00	1.00E+09	0	7.54E+01	7.54E+01	0	1.31E+02	130.5277	0	3.47E+00	3.467

tl_elo_b0194_1_rib1	Translation elongation 1 b0194 1 ribosome(s)	571 EF-G.GTP + 38 EF-Tu.GTP.ala1-tRNA + 13 EF-Tu.GTP.ala2-tRNA + 34 EF-Tu.GTP.arg1-tRNA + 1 EF-Tu.GTP.argW-tRNA + 19 EF-Tu.GTP.asn1-tRNA + 34 EF-Tu.GTP.asp1-tRNA + 1 EF-Tu.GTP.cysT-tRNA + 21 EF-Tu.GTP.gln1-tRNA + 6 EF-Tu.GTP.gln2-tRNA + 48 EF-Tu.GTP.glu1-tRNA + 37 EF-Tu.GTP.gly1-tRNA + 4 EF-Tu.GTP.glyU-tRNA + 9 EF-Tu.GTP.hisR-tRNA + 36 EF-Tu.GTP.ile1-tRNA + 40 EF-Tu.GTP.leu1-tRNA + 1 EF-Tu.GTP.leu2-tRNA + 6 EF-Tu.GTP.leuU-tRNA + 2 EF-Tu.GTP.leuZ-tRNA + 28 EF-Tu.GTP.lys1-tRNA + 16 EF-Tu.GTP.met1-tRNA + 20 EF-Tu.GTP.phe1-tRNA + 23 EF-Tu.GTP.pro1-tRNA + 2 EF-Tu.GTP.pro2-tRNA + 3 EF-Tu.GTP.proM-tRNA + 8 EF-Tu.GTP.ser1-tRNA + 6 EF-Tu.GTP.ser2-tRNA + 1 EF-Tu.GTP.ser3-tRNA + 1 EF-Tu.GTP.serT-tRNA + 14 EF-Tu.GTP.serV-tRNA + 12 EF-Tu.GTP.thr1-tRNA + 9 EF-Tu.GTP.thr2-tRNA + 5 EF-Tu.GTP.thr3-tRNA + 1 EF-Tu.GTP.thrU-tRNA + 5 EF-Tu.GTP.trpT-tRNA + 18 EF-Tu.GTP.tyr1-tRNA + 28 EF-Tu.GTP.val1-tRNA + 5 EF-Tu.GTP.val2-tRNA + 16 EF-Tu.GTP.val3-tRNA + 2855 mg2 + 1 rib_ini_b0194_1 --> 1 rib_70_elo1_b0194_1_cplx	irreversible	Translation	0.00E+00	1.00E+09	0	7.54E+01	7.54E+01	0	1.31E+02	130.5277	0	3.47E+00	3.467
tl_elo_b0194_1_rib2	Translation elongation 2 b0194 1 ribosome(s)	570 h2o + 1 rib_70_elo1_b0194_1_cplx --> 570 EF-G.GDP + 570 EF-Tu.GDP + 38 ala1_tRNA + 13 ala2_tRNA + 34 arg1_tRNA + 1 argW_tRNA + 19 asn1_tRNA + 34 asp1_tRNA + 1 cysT_tRNA + 1 fmet_tRNA + 21 gln1_tRNA + 6 gln2_tRNA + 48 glu1_tRNA + 36 gly1_tRNA + 4 glyU_tRNA + 1140 h + 9 hisR_tRNA + 36 ile1_tRNA + 40 leu1_tRNA + 1 leu2_tRNA + 6 leuU_tRNA + 2 leuZ_tRNA + 28 lys1_tRNA + 16 met1_tRNA + 2855 mg2 + 20 phe1_tRNA + 1140 pi + 23 pro1_tRNA + 2 pro2_tRNA + 3 proM_tRNA + 1 rib_70_elo2_b0194_1_cplx + 8 ser1_tRNA + 6 ser2_tRNA + 1 ser3_tRNA + 1 serT_tRNA + 14 serV_tRNA + 12 thr1_tRNA + 9 thr2_tRNA + 5 thr3_tRNA + 1 thrU_tRNA + 5 trpT_tRNA + 18 tyr1_tRNA + 28 val1_tRNA + 5 val2_tRNA + 16 val3_tRNA	irreversible	Translation	0.00E+00	1.00E+09	0	7.54E+01	7.54E+01	0	1.31E+02	130.5277	0	3.47E+00	3.467
tl_elo_b0194_33_rib1	Translation elongation 1 b0194 33 ribosome(s)	18843 EF-G.GTP + 1254 EF-Tu.GTP.ala1-tRNA + 429 EF-Tu.GTP.ala2-tRNA + 1122 EF-Tu.GTP.arg1-tRNA + 33 EF-Tu.GTP.argW-tRNA + 627 EF-Tu.GTP.asn1-tRNA + 1122 EF-Tu.GTP.asp1-tRNA + 33 EF-Tu.GTP.cysT-tRNA + 693 EF-Tu.GTP.gln1-tRNA + 198 EF-Tu.GTP.gln2-tRNA + 1584 EF-Tu.GTP.glu1-tRNA + 1221 EF-Tu.GTP.gly1-tRNA + 132 EF-Tu.GTP.glyU-tRNA + 297 EF-Tu.GTP.hisR-tRNA + 1188 EF-Tu.GTP.ile1-tRNA + 1320 EF-Tu.GTP.leu1-tRNA + 33 EF-Tu.GTP.leu2-tRNA + 198 EF-Tu.GTP.leuU-tRNA + 66 EF-Tu.GTP.leuZ-tRNA + 924 EF-Tu.GTP.lys1-tRNA + 528 EF-Tu.GTP.met1-tRNA + 660 EF-Tu.GTP.phe1-tRNA + 759 EF-Tu.GTP.pro1-tRNA + 66 EF-Tu.GTP.pro2-tRNA + 99 EF-Tu.GTP.proM-tRNA + 264 EF-Tu.GTP.ser1-tRNA + 198 EF-Tu.GTP.ser2-tRNA + 33 EF-Tu.GTP.ser3-tRNA + 33 EF-Tu.GTP.serT-tRNA + 462 EF-Tu.GTP.serV-tRNA + 396 EF-Tu.GTP.thr1-tRNA + 297 EF-Tu.GTP.thr2-tRNA + 165 EF-Tu.GTP.thr3-tRNA + 33 EF-Tu.GTP.thrU-tRNA + 165 EF-Tu.GTP.trpT-tRNA + 594 EF-Tu.GTP.tyr1-tRNA + 924 EF-Tu.GTP.val1-tRNA + 165 EF-Tu.GTP.val2-tRNA + 528 EF-Tu.GTP.val3-tRNA + 94215 mg2 + 1 rib_ini_b0194_33 --> 1 rib_70_elo1_b0194_33_cplx	irreversible	Translation	0.00E+00	1.00E+09	0	7.54E+01	7.54E+01	0	1.24E+02	124.4591	0	3.47E+00	3.467
tl_elo_b0194_33_rib2	Translation elongation 2 b0194 33 ribosome(s)	18810 h2o + 1 rib_70_elo1_b0194_33_cplx --> 18810 EF-G.GDP + 18810 EF-Tu.GDP + 1254 ala1_tRNA + 429 ala2_tRNA + 1122 arg1_tRNA + 33 argW_tRNA + 627 asn1_tRNA + 1122 asp1_tRNA + 33 cysT_tRNA + 33 fmet_tRNA + 693 gln1_tRNA + 198 gln2_tRNA + 1584 glu1_tRNA + 1188 gly1_tRNA + 132 glyU_tRNA + 37620 h + 297 hisR_tRNA + 1188 ile1_tRNA + 1320 leu1_tRNA + 33 leu2_tRNA + 198 leuU_tRNA + 66 leuZ_tRNA + 924 lys1_tRNA + 528 met1_tRNA + 94215 mg2 + 660 phe1_tRNA + 37620 pi + 759 pro1_tRNA + 66 pro2_tRNA + 99 proM_tRNA + 1 rib_70_elo2_b0194_33_cplx + 264 ser1_tRNA + 198 ser2_tRNA + 33 ser3_tRNA + 33 serT_tRNA + 462 serV_tRNA + 396 thr1_tRNA + 297 thr2_tRNA + 165 thr3_tRNA + 33 thrU_tRNA + 165 trpT_tRNA + 594 tyr1_tRNA + 924 val1_tRNA + 165 val2_tRNA + 528 val3_tRNA	irreversible	Translation	0.00E+00	1.00E+09	0	7.54E+01	7.54E+01	0	1.24E+02	124.4591	0	3.47E+00	3.467
tl_elo_b0405_10_rib1	Translation elongation 1 b0405 10 ribosome(s)	3550 EF-G.GTP + 290 EF-Tu.GTP.ala1-tRNA + 80 EF-Tu.GTP.ala2-tRNA + 180 EF-Tu.GTP.arg1-tRNA + 20 EF-Tu.GTP.argX-tRNA + 120 EF-Tu.GTP.asn1-tRNA + 250 EF-Tu.GTP.asp1-tRNA + 10 EF-Tu.GTP.cysT-tRNA + 50 EF-Tu.GTP.gln1-tRNA + 30 EF-Tu.GTP.gln2-tRNA + 260 EF-Tu.GTP.glu1-tRNA + 190 EF-Tu.GTP.gly1-tRNA + 30 EF-Tu.GTP.glyU-tRNA + 110 EF-Tu.GTP.hisR-tRNA + 180 EF-Tu.GTP.ile1-tRNA + 200 EF-Tu.GTP.leu1-tRNA + 50 EF-Tu.GTP.leu2-tRNA + 80 EF-Tu.GTP.leuU-tRNA + 20 EF-Tu.GTP.leuZ-tRNA + 140 EF-Tu.GTP.lys1-tRNA + 90 EF-Tu.GTP.met1-tRNA + 190 EF-Tu.GTP.phe1-tRNA + 130 EF-Tu.GTP.pro1-tRNA + 30 EF-Tu.GTP.pro2-tRNA + 30 EF-Tu.GTP.proL-tRNA + 20 EF-Tu.GTP.proM-tRNA + 20 EF-Tu.GTP.ser1-tRNA + 70 EF-Tu.GTP.ser3-tRNA + 10 EF-Tu.GTP.serT-tRNA + 80 EF-Tu.GTP.serV-tRNA + 110 EF-Tu.GTP.thr1-tRNA + 20 EF-Tu.GTP.thr2-tRNA + 60 EF-Tu.GTP.thr3-tRNA + 10 EF-Tu.GTP.thrU-tRNA + 120 EF-Tu.GTP.tyr1-tRNA + 150 EF-Tu.GTP.val1-tRNA + 40 EF-Tu.GTP.val2-tRNA + 80 EF-Tu.GTP.val3-tRNA + 17750 mg2 + 1 rib_ini_b0405_10 --> 1 rib_70_elo1_b0405_10_cplx	irreversible	Translation	0.00E+00	1.00E+09	0	1.15E+02	1.15E+02	0	2.01E+02	200.9631	0	3.17E+00	3.1739
tl_elo_b0405_10_rib2	Translation elongation 2 b0405 10 ribosome(s)	3540 h2o + 1 rib_70_elo1_b0405_10_cplx --> 3540 EF-G.GDP + 3540 EF-Tu.GDP + 290 ala1_tRNA + 80 ala2_tRNA + 180 arg1_tRNA + 20 argX_tRNA + 120 asn1_tRNA + 250 asp1_tRNA + 10 cysT_tRNA + 10 fmet_tRNA + 50 gln1_tRNA + 30 gln2_tRNA + 250 glu1_tRNA + 190 gly1_tRNA + 30 glyU_tRNA + 7080 h + 110 hisR_tRNA + 180 ile1_tRNA + 200 leu1_tRNA + 50 leu2_tRNA + 80 leuU_tRNA + 20 leuZ_tRNA + 140 lys1_tRNA + 90 met1_tRNA + 17750 mg2 + 190 phe1_tRNA + 7080 pi + 130 pro1_tRNA + 30 pro2_tRNA + 30 proL_tRNA + 20 proM_tRNA + 1 rib_70_elo2_b0405_10_cplx + 20 ser1_tRNA + 70 ser3_tRNA + 10 serT_tRNA + 80 serV_tRNA + 110 thr1_tRNA + 20 thr2_tRNA + 60 thr3_tRNA + 10 thrU_tRNA + 120 tyr1_tRNA + 150 val1_tRNA + 40 val2_tRNA + 80 val3_tRNA	irreversible	Translation	0.00E+00	1.00E+09	0	1.15E+02	1.15E+02	0	2.01E+02	200.9631	0	3.17E+00	3.1739

tl_elo_b0405_1_rib1	Translation elongation 1 b0405 1 ribosome(s)	355 EF-G.GTP + 29 EF-Tu.GTP.ala1-tRNA + 8 EF-Tu.GTP.ala2-tRNA + 18 EF-Tu.GTP.arg1-tRNA + 2 EF-Tu.GTP.argX-tRNA + 12 EF-Tu.GTP.asn1-tRNA + 25 EF-Tu.GTP.asp1-tRNA + 1 EF-Tu.GTP.cysT-tRNA + 5 EF-Tu.GTP.gln1-tRNA + 3 EF-Tu.GTP.gln2-tRNA + 26 EF-Tu.GTP.glu1-tRNA + 19 EF-Tu.GTP.gly1-tRNA + 3 EF Tu.GTP.glyU-tRNA + 11 EF-Tu.GTP.hisR-tRNA + 18 EF-Tu.GTP.ile1 tRNA + 20 EF-Tu.GTP.leu1-tRNA + 5 EF-Tu.GTP.leu2-tRNA + 8 EF-Tu.GTP.leuU-tRNA + 2 EF-Tu.GTP.leuZ-tRNA + 14 EF-Tu.GTP.lys1-tRNA + 9 EF-Tu.GTP.met1-tRNA + 19 EF-Tu.GTP.phe1-tRNA + 13 EF-Tu.GTP.pro1-tRNA + 3 EF-Tu.GTP.pro2-tRNA + 3 EF-Tu.GTP.proL-tRNA + 2 EF-Tu.GTP.proM-tRNA + 2 EF-Tu.GTP.ser1-tRNA + 7 EF-Tu.GTP.ser3-tRNA + 1 EF-Tu.GTP.serT-tRNA + 8 EF-Tu.GTP.serV-tRNA + 11 EF-Tu.GTP.thr1-tRNA + 2 EF-Tu.GTP.thr2-tRNA + 6 EF-Tu.GTP.thr3-tRNA + 1 EF-Tu.GTP.thrU-tRNA + 12 EF-Tu.GTP.tyr1-tRNA + 15 EF-Tu.GTP.val1-tRNA + 4 EF-Tu.GTP.val2-tRNA + 8 EF-Tu.GTP.val3-tRNA + 1775 mg2 + 1 rib_ini_b0405_1 --> 1 rib_70_elo1_b0405_1_cplx	irreversible	Translation	0.00E+00	1.00E+09	0	1.15E+02	1.15E+02	0	2.01E+02	200.9631	0	3.17E+00	3.1739
tl_elo_b0405_1_rib2	Translation elongation 2 b0405 1 ribosome(s)	354 h2o + 1 rib_70_elo1_b0405_1_cplx --> 354 EF-G.GDP + 354 EF-Tu.GDP + 29 ala1_tRNA + 8 ala2_tRNA + 18 arg1_tRNA + 2 argX_tRNA + 12 asn1_tRNA + 25 asp1_tRNA + 1 cysT_tRNA + 1 fmet_tRNA + 5 gln1_tRNA + 3 gln2_tRNA + 25 glu1_tRNA + 19 gly1_tRNA + 3 glyU_tRNA + 708 h + 11 hisR_tRNA + 18 ile1_tRNA + 20 leu1_tRNA + 5 leu2_tRNA + 8 leuU_tRNA + 2 leuZ_tRNA + 14 lys1_tRNA + 9 met1_tRNA + 1775 mg2 + 19 phe1_tRNA + 708 pi + 13 pro1_tRNA + 3 pro2_tRNA + 3 proL_tRNA + 2 proM_tRNA + 1 rib_70_elo2_b0405_1_cplx + 2 ser1_tRNA + 7 ser3_tRNA + 1 serT_tRNA + 8 serV_tRNA + 11 thr1_tRNA + 2 thr2_tRNA + 6 thr3_tRNA + 1 thrU_tRNA + 12 tyr1_tRNA + 15 val1_tRNA + 4 val2_tRNA + 8 val3_tRNA	irreversible	Translation	0.00E+00	1.00E+09	0	1.15E+02	1.15E+02	0	2.01E+02	200.9631	0	3.17E+00	3.1739
tl_elo_b0405_20_rib1	Translation elongation 1 b0405 20 ribosome(s)	7100 EF-G.GTP + 580 EF-Tu.GTP.ala1-tRNA + 160 EF-Tu.GTP.ala2 tRNA + 360 EF-Tu.GTP.arg1-tRNA + 40 EF-Tu.GTP.argX-tRNA + 240 EF-Tu.GTP.asn1-tRNA + 500 EF-Tu.GTP.asp1-tRNA + 20 EF-Tu.GTP.cysT-tRNA + 100 EF-Tu.GTP.gln1-tRNA + 60 EF-Tu.GTP.gln2-tRNA + 520 EF-Tu.GTP.glu1-tRNA + 380 EF-Tu.GTP.gly1-tRNA + 60 EF-Tu.GTP.glyU-tRNA + 220 EF-Tu.GTP.hisR-tRNA + 360 EF-Tu.GTP.ile1-tRNA + 400 EF-Tu.GTP.leu1-tRNA + 100 EF-Tu.GTP.leu2-tRNA + 160 EF-Tu.GTP.leuU-tRNA + 40 EF-Tu.GTP.leuZ-tRNA + 280 EF-Tu.GTP.lys1-tRNA + 180 EF-Tu.GTP.met1-tRNA + 380 EF-Tu.GTP.phe1-tRNA + 260 EF-Tu.GTP.pro1-tRNA + 60 EF-Tu.GTP.pro2-tRNA + 60 EF-Tu.GTP.proL-tRNA + 40 EF-Tu.GTP.proM-tRNA + 40 EF-Tu.GTP.ser1-tRNA + 140 EF-Tu.GTP.ser3-tRNA + 20 EF-Tu.GTP.serT-tRNA + 160 EF-Tu.GTP.serV-tRNA + 220 EF-Tu.GTP.thr1-tRNA + 40 EF-Tu.GTP.thr2-tRNA + 120 EF-Tu.GTP.thr3-tRNA + 20 EF-Tu.GTP.thrU-tRNA + 240 EF-Tu.GTP.tyr1-tRNA + 300 EF-Tu.GTP.val1-tRNA + 80 EF-Tu.GTP.val2-tRNA + 160 EF-Tu.GTP.val3-tRNA + 35500 mg2 + 1 rib_ini_b0405_20 --> 1 rib_70_elo1_b0405_20_cplx	irreversible	Translation	0.00E+00	1.00E+09	0	1.15E+02	1.15E+02	0	2.01E+02	200.9631	0	3.17E+00	3.1739
tl_elo_b0405_20_rib2	Translation elongation 2 b0405 20 ribosome(s)	7080 h2o + 1 rib_70_elo1_b0405_20_cplx --> 7080 EF-G.GDP + 7080 EF-Tu.GDP + 580 ala1_tRNA + 160 ala2_tRNA + 360 arg1_tRNA + 40 argX_tRNA + 240 asn1_tRNA + 500 asp1_tRNA + 20 cysT_tRNA + 20 fmet_tRNA + 100 gln1_tRNA + 60 gln2_tRNA + 500 glu1_tRNA + 380 gly1_tRNA + 60 glyU_tRNA + 14160 h + 220 hisR_tRNA + 360 ile1_tRNA + 400 leu1_tRNA + 100 leu2_tRNA + 160 leuU_tRNA + 40 leuZ_tRNA + 280 lys1_tRNA + 180 met1_tRNA + 35500 mg2 + 380 phe1_tRNA + 14160 pi + 260 pro1_tRNA + 60 pro2_tRNA + 60 proL_tRNA + 40 proM_tRNA + 1 rib_70_elo2_b0405_20_cplx + 40 ser1_tRNA + 140 ser3_tRNA + 20 serT_tRNA + 160 serV_tRNA + 220 thr1_tRNA + 40 thr2_tRNA + 12[illegible] thr3_tRNA + 20 thrU_tRNA + 240 tyr1_tRNA + 300 val1_tRNA + 80 val2_tRNA + 160 val3_tRNA	irreversible	Translation	0.00E+00	1.00E+09	0	1.15E+02	1.15E+02	0	2.01E+02	200.9631	0	3.17E+00	3.1739
tl_elo_b0406_11_rib1	Translation elongation 1 b0406 11 ribosome(s)	4114 EF-G.GTP + 165 EF-Tu.GTP.ala1-tRNA + 22 EF-Tu.GTP.ala2-tRNA + 330 EF-Tu.GTP.arg1-tRNA + 143 EF-Tu.GTP.asn1-tRNA + 341 EF-Tu.GTP.asp1-tRNA + 88 EF-Tu.GTP.cysT-tRNA + 99 EF-Tu.GTP.gln1-tRNA + 22 EF-Tu.GTP.gln2-tRNA + 253 EF-Tu.GTP.glu1-tRNA + 363 EF-Tu.GTP.gly1-tRNA + 11 EF-Tu.GTP.glyT-tRNA + 33 EF-Tu.GTP.glyU-tRNA + 110 EF-Tu.GTP.hisR-tRNA + 231 EF-Tu.GTP.ile1-tRNA + 11 EF-Tu.GTP.ile2-tRNA + 143 EF-Tu.GTP.leu1-tRNA + 44 EF-Tu.GTP.leu2-tRNA + 110 EF-Tu.GTP.leuU-tRNA + 44 EF-Tu.GTP.leuZ-tRNA + 198 EF-Tu.GTP.lys1-tRNA + 143 EF-Tu.GTP.met1-tRNA + 187 EF-Tu.GTP.phe1-tRNA + 110 EF-Tu.GTP.pro1-tRNA + 66 EF-Tu.GTP.pro2-tRNA + 44 EF-Tu.GTP.proM-tRNA + 22 EF-Tu.GTP.ser1-tRNA + 22 EF-Tu.GTP.ser2-tRNA + 11 EF-Tu.GTP.ser3-tRNA + 22 EF-Tu.GTP.serT-tRNA + 66 EF-Tu.GTP.serV-tRNA + 121 EF-Tu.GTP.thr1-tRNA + 33 EF-Tu.GTP.thr2-tRNA + 33 EF-Tu.GTP.thr3-tRNA + 44 EF-Tu.GTP.trpT-tRNA + 154 EF-Tu.GTP.tyr1-tRNA + 154 EF-Tu.GTP.val1-tRNA + 44 EF-Tu.GTP.val2-tRNA + 77 EF-Tu.GTP.val3-tRNA + 20570 mg2 + 1 rib_ini_b0406_11 --> 1 rib_70_elo1_b0406_11_cplx	irreversible	Translation	0.00E+00	1.00E+09	0	9.55E+01	9.55E+01	0	1.66E+02	165.5261	0	4.18E+00	4.176
tl_elo_b0406_11_rib2	Translation elongation 2 b0406 11 ribosome(s)	4103 h2o + 1 rib_70_elo1_b0406_11_cplx --> 4103 EF-G.GDP + 4103 EF-Tu.GDP + 165 ala1_tRNA + 22 ala2_tRNA + 330 arg1_tRNA + 143 asn1_tRNA + 330 asp1_tRNA + 88 cysT_tRNA + 11 fmet_tRNA + 99 gln1_tRNA + 22 gln2_tRNA + 253 glu1_tRNA + 363 gly1_tRNA + 11 glyT_tRNA + 33 glyU_tRNA + 8206 h + 110 hisR_tRNA + 231 ile1_tRNA + 11 ile2_tRNA + 143 leu1_tRNA + 44 leu2_tRNA + 110 leuU_tRNA + 44 leuZ_tRNA + 198 lys1_tRNA + 14[illegible] met1_tRNA + 20570 mg2 + 187 phe1_tRNA + 8206 pi + 110 pro1_tRNA + 66 pro2_tRNA + 44 proM_tRNA + 1 rib_70_elo2_b0406_11_cplx + 22 ser1_tRNA + 22 ser2_tRNA + 11 ser3_tRNA + 22 serT_tRNA + 66 serV_tRNA + 121 thr1_tRNA + 33 thr2_tRNA + 33 thr3_tRNA + 44 trpT_tRNA + 154 tyr1_tRNA + 154 val1_tRNA + 44 val2_tRNA + 77 val3_tRNA	irreversible	Translation	0.00E+00	1.00E+09	0	9.55E+01	9.55E+01	0	1.66E+02	165.5261	0	4.18E+00	4.176

tl_elo_b0406_1_rib1	Translation elongation 1 b0406 1 ribosome(s)	374 EF-G.GTP + 15 EF-Tu.GTP.ala1-tRNA + 2 EF-Tu.GTP.ala2-tRNA + 30 EF-Tu.GTP.arg1-tRNA + 13 EF-Tu.GTP.asn1-tRNA + 31 EF-Tu.GTP.asp1-tRNA + 8 EF-Tu.GTP.cysT-tRNA + 9 EF-Tu.GTP.gln1-tRNA + 2 EF-Tu.GTP.gln2-tRNA + 23 EF-Tu.GTP.glu1-tRNA + 33 EF-Tu.GTP.gly1-tRNA + 1 EF-Tu.GTP.glyT-tRNA + 3 EF-Tu.GTP.glyU-tRNA + 10 EF-Tu.GTP.hisR-tRNA + 21 EF-Tu.GTP.ile1 tRNA + 1 EF-Tu.GTP.ile2-tRNA + 13 EF-Tu.GTP.leu1-tRNA + 4 EF-Tu.GTP.leu2-tRNA + 10 EF-Tu.GTP.leuU-tRNA + 4 EF-Tu.GTP.leuZ-tRNA + 18 EF-Tu.GTP.lys1-tRNA + 13 EF-Tu.GTP.met1-tRNA + 17 EF-Tu.GTP.phe1-tRNA + 10 EF-Tu.GTP.pro1-tRNA + 6 EF-Tu.GTP.pro2-tRNA + 4 EF-Tu.GTP.proM-tRNA + 2 EF-Tu.GTP.ser1-tRNA + 2 EF-Tu.GTP.ser2-tRNA + 1 EF-Tu.GTP.ser3-tRNA + 2 EF-Tu.GTP.serT-tRNA + 6 EF-Tu.GTP.serV-tRNA + 11 EF-Tu.GTP.thr1-tRNA + 3 EF-Tu.GTP.thr2-tRNA + 3 EF-Tu.GTP.thr3-tRNA + 4 EF-Tu.GTP.trpT-tRNA + 14 EF-Tu.GTP.tyr1-tRNA + 14 EF-Tu.GTP.val1-tRNA + 4 EF-Tu.GTP.val2-tRNA + 7 EF-Tu.GTP.val3-tRNA + 1870 mg2 + 1 rib_ini_b0406_1 --> 1 rib_70_elo1_b0406_1_cplx	irreversible	Translation	0.00E+00	1.00E+09	0	9.55E+01	9.55E+01	0	1.66E+02	165.5261	0	4.18E+00	4.176
tl_elo_b0406_1_rib2	Translation elongation 2 b0406 1 ribosome(s)	373 h2o + 1 rib_70_elo1_b0406_1_cplx --> 373 EF-G.GDP + 373 EF-Tu.GDP + 15 ala1_tRNA + 2 ala2_tRNA + 30 arg1_tRNA + 13 asn1_tRNA + 30 asp1_tRNA + 8 cysT_tRNA + 1 fmet_tRNA + 9 gln1_tRNA + 2 gln2_tRNA + 23 glu1_tRNA + 33 gly1_tRNA + 1 glyT_tRNA + 3 glyU_tRNA + 746 h + 10 hisR_tRNA + 21 ile1_tRNA + 1 ile2_tRNA + 13 leu1_tRNA + 4 leu2_tRNA + 10 leuU_tRNA + 4 leuZ_tRNA + 18 lys1_tRNA + 13 met1_tRNA + 1870 mg2 + 17 phe1_tRNA + 746 pi + 10 pro1_tRNA + 6 pro2_tRNA + 4 proM_tRNA + 1 rib_70_elo2_b0406_1_cplx + 2 ser1_tRNA + 2 ser2_tRNA + 1 ser3_tRNA + 2 serT_tRNA + 6 serV_tRNA + 11 thr1_tRNA + 3 thr2_tRNA + 3 thr3_tRNA + 4 trpT_tRNA + 14 tyr1_tRNA + 14 val1_tRNA + 4 val2_tRNA + 7 val3_tRNA	irreversible	Translation	0.00E+00	1.00E+09	0	9.55E+01	9.55E+01	0	1.66E+02	165.5261	0	4.18E+00	4.176
tl_elo_b0406_22_rib1	Translation elongation 1 b0406 22 ribosome(s)	8228 EF-G.GTP + 330 EF-Tu.GTP.ala1-tRNA + 44 EF-Tu.GTP.ala2-tRNA + 660 EF-Tu.GTP.arg1-tRNA + 286 EF-Tu.GTP.asn1-tRNA + 682 EF-Tu.GTP.asp1-tRNA + 176 EF-Tu.GTP.cysT-tRNA + 198 EF-Tu.GTP.gln1-tRNA + 44 EF-Tu.GTP.gln2-tRNA + 506 EF-Tu.GTP.glu1-tRNA + 726 EF-Tu.GTP.gly1-tRNA + 22 EF-Tu.GTP.glyT-tRNA + 66 EF-Tu.GTP.glyU-tRNA + 220 EF-Tu.GTP.hisR-tRNA + 462 EF-Tu.GTP.ile1-tRNA + 22 EF-Tu.GTP.ile2-tRNA + 286 EF-Tu.GTP.leu1-tRNA + 88 EF-Tu.GTP.leu2-tRNA + 220 EF-Tu.GTP.leuU-tRNA + 88 EF-Tu.GTP.leuZ-tRNA + 396 EF-Tu.GTP.lys1-tRNA + 286 EF-Tu.GTP.met1-tRNA + 374 EF-Tu.GTP.phe1-tRNA + 220 EF-Tu.GTP.pro1-tRNA + 132 EF-Tu.GTP.pro2-tRNA + 88 EF-Tu.GTP.proM-tRNA + 44 EF-Tu.GTP.ser1-tRNA + 44 EF-Tu.GTP.ser2-tRNA + 22 EF-Tu.GTP.ser3-tRNA + 44 EF-Tu.GTP.serT-tRNA + 132 EF-Tu.GTP.serV-tRNA + 242 EF-Tu.GTP.thr1-tRNA + 66 EF-Tu.GTP.thr2-tRNA + 66 EF-Tu.GTP.thr3-tRNA + 88 EF-Tu.GTP.trpT-tRNA + 308 EF-Tu.GTP.tyr1-tRNA + 308 EF-Tu.GTP.val1-tRNA + 88 EF-Tu.GTP.val2-tRNA + 154 EF-Tu.GTP.val3-tRNA + 41140 mg2 + 1 rib_ini_b0406_22 --> 1 rib_70_elo1_b0406_22_cplx	irreversible	Translation	0.00E+00	1.00E+09	0	9.55E+01	9.55E+01	0	1.66E+02	165.5261	0	4.18E+00	4.176
tl_elo_b0406_22_rib2	Translation elongation 2 b0406 22 ribosome(s)	8206 h2o + 1 rib_70_elo1_b0406_22_cplx --> 8206 EF-G.GDP + 8206 EF-Tu.GDP + 330 ala1_tRNA + 44 ala2_tRNA + 660 arg1_tRNA + 286 asn1_tRNA + 660 asp1_tRNA + 176 cysT_tRNA + 22 fmet_tRNA + 198 gln1_tRNA + 44 gln2_tRNA + 506 glu1_tRNA + 726 gly1_tRNA + 22 glyT_tRNA + 66 glyU_tRNA + 16412 h + 220 hisR_tRNA + 462 ile1_tRNA + 22 ile2_tRNA + 286 leu1_tRNA + 88 leu2_tRNA + 220 leuU_tRNA + 88 leuZ_tRNA + 396 lys1_tRNA + 28[illegible] met1_tRNA + 41140 mg2 + 374 phe1_tRNA + 16412 pi + 220 pro1_tRNA + 132 pro2_tRNA + 88 proM_tRNA + 1 rib_70_elo2_b0406_22_cplx + 44 ser1_tRNA + 44 ser2_tRNA + 22 ser3_tRNA + 44 serT_tRNA + 132 serV_tRNA + 242 thr1_tRNA + 66 thr2_tRNA + 66 thr3_tRNA + 88 trpT_tRNA + 308 tyr1_tRNA + 308 val1_tRNA + 88 val2_tRNA + 154 val3_tRNA	irreversible	Translation	0.00E+00	1.00E+09	0	9.55E+01	9.55E+01	0	1.66E+02	165.5261	0	4.18E+00	4.176
tl_elo_b0407_1_rib1	Translation elongation 1 b0407 1 ribosome(s)	109 EF-G.GTP + 10 EF-Tu.GTP.ala1-tRNA + 2 EF-Tu.GTP.ala2-tRNA + 4 EF-Tu.GTP.arg1-tRNA + 3 EF-Tu.GTP.asn1-tRNA + 5 EF-Tu.GTP.asp1-tRNA + 2 EF-Tu.GTP.gln1-tRNA + 1 EF-Tu.GTP.gln2-tRNA + 4 EF-Tu.GTP.glu1-tRNA + 9 EF-Tu.GTP.gly1-tRNA + 1 EF-Tu.GTP.hisR-tRNA + 8 EF-Tu.GTP.ile1-tRNA + 9 EF-Tu.GTP.leu1-tRNA + 2 EF-Tu.GTP.leu2-tRNA + 9 EF-Tu.GTP.lys1-tRNA + 5 EF-Tu.GTP.met1-tRNA + 6 EF-Tu.GTP.phe1-tRNA + 3 EF-Tu.GTP.pro1-tRNA + 1 EF-Tu.GTP.proM-tRNA + 1 EF-Tu.GTP.ser1-tRNA + 2 EF-Tu.GTP.ser2-tRNA + 2 EF-Tu.GTP.serV-tRNA + 4 EF-Tu.GTP.thr1-tRNA + 1 EF-Tu.GTP.thr2-tRNA + 2 EF-Tu.GTP.thr3-tRNA + 2 EF-Tu.GTP.tyr1-tRNA + 7 EF-Tu.GTP.val1-tRNA + 1 EF-Tu.GTP.val2-tRNA + 3 EF-Tu.GTP.val3-tRNA + 545 mg2 + 1 rib_ini_b0407_1 --> 1 rib_70_elo1_b0407_1_cplx	irreversible	Translation	0.00E+00	1.00E+09	0	1.09E+02	1.09E+02	0	1.79E+02	179.3461	0	1.80E+01	17.996
tl_elo_b0407_1_rib2	Translation elongation 2 b0407 1 ribosome(s)	108 h2o + 1 rib_70_elo1_b0407_1_cplx --> 108 EF-G.GDP + 108 EF-Tu.GDP + 10 ala1_tRNA + 2 ala2_tRNA + 4 arg1_tRNA + 3 asn1_tRNA + 5 asp1_tRNA + 1 fmet_tRNA + 2 gln1_tRNA + 1 gln2_tRNA + 4 glu1_tRNA + 9 gly1_tRNA + 216 h + 1 hisR_tRNA + 8 ile1_tRNA + 8 leu1_tRNA + 2 leu2_tRNA + 9 lys1_tRNA + 5 met1_tRNA + 545 mg2 + 6 phe1_tRNA + 216 pi + 3 pro1_tRNA + 1 proM_tRNA + 1 rib_70_elo2_b0407_1_cplx + 1 ser1_tRNA + 2 ser2_tRNA + 2 serV_tRNA + 4 thr1_tRNA + 1 thr2_tRNA + 2 thr3_tRNA + 2 tyr1_tRNA + 7 val1_tRNA + 1 val2_tRNA + 3 val3_tRNA	irreversible	Translation	0.00E+00	1.00E+09	0	1.09E+02	1.09E+02	0	1.79E+02	179.3461	0	1.80E+01	17.996
tl_elo_b0407_3_rib1	Translation elongation 1 b0407 3 ribosome(s)	327 EF-G.GTP + 30 EF-Tu.GTP.ala1-tRNA + 6 EF-Tu.GTP.ala2-tRNA + 12 EF-Tu.GTP.arg1-tRNA + 9 EF-Tu.GTP.asn1-tRNA + 15 EF-Tu.GTP.asp1-tRNA + 6 EF-Tu.GTP.gln1-tRNA + 3 EF-Tu.GTP.gln2-tRNA + 12 EF-Tu.GTP.glu1-tRNA + 27 EF-Tu.GTP.gly1 tRNA + 3 EF-Tu.GTP.hisR-tRNA + 24 EF-Tu.GTP.ile1-tRNA + 27 EF Tu.GTP.leu1-tRNA + 6 EF-Tu.GTP.leu2-tRNA + 27 EF-Tu.GTP.lys1-tRNA + 15 EF-Tu.GTP.met1-tRNA + 18 EF-Tu.GTP.phe1-tRNA + 9 EF-Tu.GTP.pro1-tRNA + 3 EF-Tu.GTP.proM-tRNA + 3 EF-Tu.GTP.ser1-tRNA + 6 EF-Tu.GTP.ser2-tRNA + 6 EF-Tu.GTP.serV-tRNA + 12 EF-Tu.GTP.thr1-tRNA + 3 EF-Tu.GTP.thr2-tRNA + 6 EF-Tu.GTP.thr3-tRNA + 6 EF-Tu.GTP.tyr1-tRNA + 21 EF-Tu.GTP.val1-tRNA + 3 EF-Tu.GTP.val2-tRNA + 9 EF-Tu.GTP.val3-tRNA + 1635 mg2 + 1 rib_ini_b0407_3 --> 1 rib_70_elo1_b0407_3_cplx	irreversible	Translation	0.00E+00	1.00E+09	0	1.09E+02	1.09E+02	0	1.79E+02	179.3461	0	1.80E+01	17.996

tl_elo_b0407_3_rib2	Translation elongation 2 b0407 3 ribosome(s)	324 h2o + 1 rib_70_elo1_b0407_3_cplx --> 324 EF-G.GDP + 324 EF-Tu.GDP + 30 ala1_tRNA + 6 ala2_tRNA + 12 arg1_tRNA + 9 asn1_tRNA + 15 asp1_tRNA + 3 fmet_tRNA + 6 gln1_tRNA + 3 gln2_tRNA + 12 glu1_tRNA + 27 gly1_tRNA + 648 h + 3 hisR_tRNA + 24 ile1_tRNA + 24 leu1_tRNA + 6 leu2_tRNA + 27 lys1_tRNA + 15 met1_tRNA + 1635 mg2 + 18 phe1_tRNA + 648 pi + 9 pro1_tRNA + [illegible] proM_tRNA + 1 rib_70_elo2_b0407_3_cplx + 3 ser1_tRNA + 6 ser2_tRNA + 6 serV_tRNA + 12 thr1_tRNA + 3 thr2_tRNA + 6 thr3_tRNA + 6 tyr1_tRNA + 21 val1_tRNA + 3 val2_tRNA + 9 val3_tRNA	irreversible	Translation	0.00E+00	1.00E+09	0	1.09E+02	1.09E+02	0	1.79E+02	179.3461	0	1.80E+01	17.996
tl_elo_b0407_6_rib1	Translation elongation 1 b0407 6 ribosome(s)	654 EF-G.GTP + 60 EF-Tu.GTP.ala1-tRNA + 12 EF-Tu.GTP.ala2-tRNA + 24 EF-Tu.GTP.arg1-tRNA + 18 EF-Tu.GTP.asn1-tRNA + 30 EF-Tu.GTP.asp1-tRNA + 12 EF-Tu.GTP.gln1-tRNA + 6 EF-Tu.GTP.gln2-tRNA + 24 EF-Tu.GTP.glu1-tRNA + 54 EF-Tu.GTP.gly1-tRNA + 6 EF-Tu.GTP.hisR-tRNA + 48 EF-Tu.GTP.ile1-tRNA + 54 EF-Tu.GTP.leu1-tRNA + 12 EF-Tu.GTP.leu2-tRNA + 54 EF-Tu.GTP.lys1-tRNA + 30 EF-Tu.GTP.met1-tRNA + 36 EF-Tu.GTP.phe1-tRNA + 18 EF-Tu.GTP.pro1-tRNA + 6 EF-Tu.GTP.proM-tRNA + 6 EF-Tu.GTP.ser1-tRNA + 12 EF-Tu.GTP.ser2-tRNA + 12 EF-Tu.GTP.serV-tRNA + 24 EF-Tu.GTP.thr1-tRNA + 6 EF-Tu.GTP.thr2-tRNA + 12 EF-Tu.GTP.thr3-tRNA + 12 EF-Tu.GTP.tyr1-tRNA + 42 EF-Tu.GTP.val1-tRNA + 6 EF-Tu.GTP.val2-tRNA + 18 EF-Tu.GTP.val3-tRNA + 3270 mg2 + 1 rib_ini_b0407_6 --> 1 rib_70_elo1_b0407_6_cplx	irreversible	Translation	0.00E+00	1.00E+09	0	1.09E+02	1.09E+02	0	1.79E+02	179.3461	0	1.80E+01	17.996
tl_elo_b0407_6_rib2	Translation elongation 2 b0407 6 ribosome(s)	648 h2o + 1 rib_70_elo1_b0407_6_cplx --> 648 EF-G.GDP + 648 EF-Tu.GDP + 60 ala1_tRNA + 12 ala2_tRNA + 24 arg1_tRNA + 18 asn1_tRNA + 30 asp1_tRNA + 6 fmet_tRNA + 12 gln1_tRNA + 6 gln2_tRNA + 24 glu1_tRNA + 54 gly1_tRNA + 1296 h + 6 hisR_tRNA + 48 ile1_tRNA + 48 leu1_tRNA + 12 leu2_tRNA + 54 lys1_tRNA + 3[illegible] met1_tRNA + 3270 mg2 + 36 phe1_tRNA + 1296 pi + 18 pro1_tRNA + 6 proM_tRNA + 1 rib_70_elo2_b0407_6_cplx + 6 ser1_tRNA + 12 ser2_tRNA + 12 serV_tRNA + 24 thr1_tRNA + 6 thr2_tRNA + 12 thr3_tRNA + 12 tyr1_tRNA + 42 val1_tRNA + 6 val2_tRNA + 18 val3_tRNA	irreversible	Translation	0.00E+00	1.00E+09	0	1.09E+02	1.09E+02	0	1.79E+02	179.3461	0	1.80E+01	17.996
tl_elo_b0413_1_rib1	Translation elongation 1 b0413 1 ribosome(s)	148 EF-G.GTP + 4 EF-Tu.GTP.ala1-tRNA + 4 EF-Tu.GTP.ala2-tRNA + 14 EF-Tu.GTP.arg1-tRNA + 2 EF-Tu.GTP.argX-tRNA + 5 EF-Tu.GTP.asn1-tRNA + 8 EF-Tu.GTP.asp1-tRNA + 4 EF-Tu.GTP.cysT-tRNA + 2 EF-Tu.GTP.gln1-tRNA + 1 EF-Tu.GTP.gln2-tRNA + 17 EF-Tu.GTP.glu1-tRNA + 5 EF-Tu.GTP.gly1-tRNA + 1 EF-Tu.GTP.glyT-tRNA + 2 EF-Tu.GTP.hisR-tRNA + 7 EF-Tu.GTP.ile1-tRNA + 7 EF-Tu.GTP.leu1-tRNA + 2 EF-Tu.GTP.leu2-tRNA + 2 EF-Tu.GTP.leuU-tRNA + 10 EF-Tu.GTP.lys1-tRNA + 5 EF-Tu.GTP.met1-tRNA + 8 EF-Tu.GTP.phe1-tRNA + 4 EF-Tu.GTP.pro1-tRNA + 1 EF-Tu.GTP.proM-tRNA + 2 EF-Tu.GTP.ser1-tRNA + 2 EF-Tu.GTP.ser2-tRNA + 1 EF-Tu.GTP.ser3-tRNA + 1 EF-Tu.GTP.serT-tRNA + 5 EF-Tu.GTP.serV-tRNA + 3 EF-Tu.GTP.thr1-tRNA + 1 EF-Tu.GTP.thr2-tRNA + 2 EF-Tu.GTP.tyr1-tRNA + 11 EF-Tu.GTP.val1-tRNA + 3 EF-Tu.GTP.val2-tRNA + 2 EF-Tu.GTP.val3-tRNA + 740 mg2 + 1 rib_ini_b0413_1 --> 1 rib_70_elo1_b0413_1_cplx	irreversible	Translation	0.00E+00	1.00E+09	0	5.77E+01	5.77E+01	0	9.57E+01	95.68595	0	8.23E+00	8.2313
tl_elo_b0413_1_rib2	Translation elongation 2 b0413 1 ribosome(s)	147 h2o + 1 rib_70_elo1_b0413_1_cplx --> 147 EF-G.GDP + 147 EF-Tu.GDP + 4 ala1_tRNA + 4 ala2_tRNA + 14 arg1_tRNA + 2 argX_tRNA + 5 asn1_tRNA + 7 asp1_tRNA + 4 cysT_tRNA + 1 fmet_tRNA + 2 gln1_tRNA + 1 gln2_tRNA + 17 glu1_tRNA + 5 gly1_tRNA + 1 glyT_tRNA + 294 h + 2 hisR_tRNA + 7 ile1_tRNA + 7 leu1_tRNA + 2 leu2_tRNA + 2 leuU_tRNA + 10 lys1_tRNA + 5 met1_tRNA + 740 mg2 + 8 phe1_tRNA + 294 pi + 4 pro1_tRNA + 1 proM_tRNA + 1 rib_70_elo2_b0413_1_cplx + 2 ser1_tRNA + 2 ser2_tRNA + 1 ser3_tRNA + 1 serT_tRNA + 5 serV_tRNA + 3 thr1_tRNA + 1 thr2_tRNA + 2 tyr1_tRNA + 11 val1_tRNA + 3 val2_tRNA + 2 val3_tRNA	irreversible	Translation	0.00E+00	1.00E+09	0	5.77E+01	5.77E+01	0	9.57E+01	95.68595	0	8.23E+00	8.2313
tl_elo_b0413_4_rib1	Translation elongation 1 b0413 4 ribosome(s)	592 EF-G.GTP + 16 EF-Tu.GTP.ala1-tRNA + 16 EF-Tu.GTP.ala2-tRNA + 56 EF-Tu.GTP.arg1-tRNA + 8 EF-Tu.GTP.argX-tRNA + 20 EF-Tu.GTP.asn1-tRNA + 32 EF-Tu.GTP.asp1-tRNA + 16 EF-Tu.GTP.cysT-tRNA + 8 EF-Tu.GTP.gln1-tRNA + 4 EF-Tu.GTP.gln2-tRNA + 68 EF-Tu.GTP.glu1-tRNA + 20 EF-Tu.GTP.gly1-tRNA + 4 EF-Tu.GTP.glyT-tRNA + 8 EF-Tu.GTP.hisR-tRNA + 28 EF-Tu.GTP.ile1-tRNA + 28 EF-Tu.GTP.leu1-tRNA + 8 EF-Tu.GTP.leu2-tRNA + 8 EF-Tu.GTP.leuU-tRNA + 40 EF-Tu.GTP.lys1-tRNA + 20 EF-Tu.GTP.met1-tRNA + 32 EF-Tu.GTP.phe1-tRNA + 16 EF-Tu.GTP.pro1-tRNA + 4 EF-Tu.GTP.proM-tRNA + 8 EF-Tu.GTP.ser1-tRNA + 8 EF-Tu.GTP.ser2-tRNA + 4 EF-Tu.GTP.ser3-tRNA + 4 EF-Tu.GTP.serT-tRNA + 20 EF-Tu.GTP.serV-tRNA + 12 EF-Tu.GTP.thr1-tRNA + 4 EF-Tu.GTP.thr2-tRNA + 8 EF-Tu.GTP.tyr1-tRNA + 44 EF-Tu.GTP.val1-tRNA + 12 EF-Tu.GTP.val2-tRNA + 8 EF-Tu.GTP.val3-tRNA + 2960 mg2 + 1 rib_ini_b0413_4 --> 1 rib_70_elo1_b0413_4_cplx	irreversible	Translation	0.00E+00	1.00E+09	0	5.77E+01	5.77E+01	0	9.57E+01	95.68595	0	8.23E+00	8.2313
tl_elo_b0413_4_rib2	Translation elongation 2 b0413 4 ribosome(s)	588 h2o + 1 rib_70_elo1_b0413_4_cplx --> 588 EF-G.GDP + 588 EF-Tu.GDP + 16 ala1_tRNA + 16 ala2_tRNA + 56 arg1_tRNA + 8 argX_tRNA + 20 asn1_tRNA + 28 asp1_tRNA + 16 cysT_tRNA + 4 fmet_tRNA + 8 gln1_tRNA + 4 gln2_tRNA + 68 glu1_tRNA + 20 gly1_tRNA + 4 glyT_tRNA + 1176 h + 8 hisR_tRNA + 28 ile1_tRNA + 28 leu1_tRNA + 8 leu2_tRNA + 8 leuU_tRNA + 40 lys1_tRNA + 20 met1_tRNA + 2960 mg2 + 32 phe1_tRNA + 1176 pi + 16 pro1_tRNA + 4 proM_tRNA + 1 rib_70_elo2_b0413_4_cplx + 8 ser1_tRNA + 8 ser2_tRNA + 4 ser3_tRNA + 4 serT_tRNA + 20 serV_tRNA + 12 thr1_tRNA + 4 thr2_tRNA + 8 tyr1_tRNA + 44 val1_tRNA + 12 val2_tRNA + 8 val3_tRNA	irreversible	Translation	0.00E+00	1.00E+09	0	5.77E+01	5.77E+01	0	9.57E+01	95.68595	0	8.23E+00	8.2313
tl_elo_b0413_8_rib1	Translation elongation 1 b0413 8 ribosome(s)	1184 EF-G.GTP + 32 EF-Tu.GTP.ala1-tRNA + 32 EF-Tu.GTP.ala2-tRNA + 112 EF-Tu.GTP.arg1-tRNA + 16 EF-Tu.GTP.argX-tRNA + 40 EF-Tu.GTP.asn1-tRNA + 64 EF-Tu.GTP.asp1-tRNA + 32 EF-Tu.GTP.cysT-tRNA + 16 EF-Tu.GTP.gln1-tRNA + 8 EF-Tu.GTP.gln2-tRNA + 136 EF-Tu.GTP.glu1-tRNA + 40 EF-Tu.GTP.gly1-tRNA + 8 EF-Tu.GTP.glyT-tRNA + 16 EF-Tu.GTP.hisR-tRNA + 56 EF-Tu.GTP.ile1-tRNA + 56 EF-Tu.GTP.leu1-tRNA + 16 EF-Tu.GTP.leu2-tRNA + 16 EF-Tu.GTP.leuU-tRNA + 80 EF-Tu.GTP.lys1-tRNA + 40 EF-Tu.GTP.met1-tRNA + 64 EF-Tu.GTP.phe1-tRNA + 32 EF-Tu.GTP.pro1-tRNA + 8 EF-Tu.GTP.proM-tRNA + 16 EF-Tu.GTP.ser1-tRNA + 16 EF-Tu.GTP.ser2-tRNA + 8 EF-Tu.GTP.ser3-tRNA + 8 EF-Tu.GTP.serT-tRNA + 40 EF-Tu.GTP.serV-tRNA + 24 EF-Tu.GTP.thr1-tRNA + 8 EF-Tu.GTP.thr2-tRNA + 16 EF-Tu.GTP.tyr1-tRNA + 88 EF-Tu.GTP.val1-tRNA + 24 EF-Tu.GTP.val2-tRNA + 16 EF-Tu.GTP.val3-tRNA + 5920 mg2 + 1 rib_ini_b0413_8 --> 1 rib_70_elo1_b0413_8_cplx	irreversible	Translation	0.00E+00	1.00E+09	0	5.77E+01	5.77E+01	0	9.57E+01	95.68595	0	8.23E+00	8.2313

tl_elo_b0413_8_rib2	Translation elongation 2 b0413 8 ribosome(s)	1176 h2o + 1 rib_70_elo1_b0413_8_cplx --> 1176 EF-G.GDP + 1176 EF-Tu.GDP + 32 ala1_tRNA + 32 ala2_tRNA + 112 arg1_tRNA + 16 argX_tRNA + 40 asn1_tRNA + 56 asp1_tRNA + 32 cysT_tRNA + 8 fmet_tRNA + 16 gln1_tRNA + 8 gln2_tRNA + 136 glu1_tRNA + 40 gly1_tRNA + 8 glyT_tRNA + 2352 h + 16 hisR_tRNA + 56 ile1_tRNA + 56 leu1_tRNA + 16 leu2_tRNA + 16 leuU_tRNA + 80 lys1_tRNA + 40 met1_tRNA + 5920 mg2 + 64 phe1_tRNA + 2352 pi + 32 pro1_tRNA + 8 proM_tRNA + 1 rib_70_elo2_b0413_8_cplx + 16 ser1_tRNA + 16 ser2_tRNA + 8 ser3_tRNA + 8 serT_tRNA + 40 serV_tRNA + 24 thr1_tRNA + 8 thr2_tRNA + 16 tyr1_tRNA + 88 val1_tRNA + 24 val2_tRNA + 16 val3_tRNA	irreversible	Translation	0.00E+00	1.00E+09	0	5.77E+01	5.77E+01	0	9.57E+01	95.68595	0	8.23E+00	8.2313
tl_elo_b0414_10_rib1	Translation elongation 1 b0414 10 ribosome(s)	3660 EF-G.GTP + 260 EF-Tu.GTP.ala1-tRNA + 120 EF-Tu.GTP.ala2 tRNA + 220 EF-Tu.GTP.arg1-tRNA + 30 EF-Tu.GTP.argX-tRNA + 70 EF-Tu.GTP.asn1-tRNA + 170 EF-Tu.GTP.asp1-tRNA + 60 EF-Tu.GTP.cysT-tRNA + 130 EF-Tu.GTP.gln1-tRNA + 140 EF-Tu.GTP.gln2-tRNA + 210 EF-Tu.GTP.glu1-tRNA + 230 EF-Tu.GTP.gly1-tRNA + 30 EF-Tu.GTP.glyT-tRNA + 40 EF-Tu.GTP.glyU-tRNA + 130 EF-Tu.GTP.hisR-tRNA + 150 EF-Tu.GTP.ile1-tRNA + 20 EF-Tu.GTP.ile2-tRNA + 150 EF-Tu.GTP.leu1 tRNA + 40 EF-Tu.GTP.leu2-tRNA + 100 EF-Tu.GTP.leuU-tRNA + 30 EF-Tu.GTP.leuW-tRNA + 80 EF-Tu.GTP.leuZ-tRNA + 130 EF-Tu.GTP.lys1-tRNA + 90 EF-Tu.GTP.met1-tRNA + 60 EF-Tu.GTP.phe1-tRNA + 80 EF-Tu.GTP.pro1-tRNA + 40 EF-Tu.GTP.pro2-tRNA + 50 EF-Tu.GTP.proL-tRNA + 50 EF-Tu.GTP.proM-tRNA + 20 EF-Tu.GTP.ser2-tRNA + 30 EF-Tu.GTP.ser3-tRNA + 110 EF-Tu.GTP.serV-tRNA + 60 EF-Tu.GTP.thr1-tRNA + 20 EF-Tu.GTP.thr2-tRNA + 100 EF-Tu.GTP.thr3-tRNA + 10 EF-Tu.GTP.thrU-tRNA + 50 EF-Tu.GTP.trpT tRNA + 80 EF-Tu.GTP.tyr1-tRNA + 150 EF-Tu.GTP.val1-tRNA + 90 EF-Tu.GTP.val2-tRNA + 30 EF-Tu.GTP.val3-tRNA + 18300 mg2 + 1 rib_ini_b0414_10 --> 1 rib_70_elo1_b0414_10_cplx	irreversible	Translation	0.00E+00	1.00E+09	0	5.39E+01	5.39E+01	0	9.19E+01	91.86595	0	4.41E+00	4.4113
tl_elo_b0414_10_rib2	Translation elongation 2 b0414 10 ribosome(s)	3650 h2o + 1 rib_70_elo1_b0414_10_cplx --> 3650 EF-G.GDP + 3650 EF-Tu.GDP + 250 ala1_tRNA + 120 ala2_tRNA + 220 arg1_tRNA + 30 argX_tRNA + 70 asn1_tRNA + 170 asp1_tRNA + 60 cysT_tRNA + 10 fmet_tRNA + 130 gln1_tRNA + 140 gln2_tRNA + 210 glu1_tRNA + 230 gly1_tRNA + 30 glyT_tRNA + 40 glyU_tRNA + 7300 h + 130 hisR_tRNA + 150 ile1_tRNA + 20 ile2_tRNA + 150 leu1_tRNA + 40 leu2_tRNA + 100 leuU_tRNA + 30 leuW_tRNA + 80 leuZ_tRNA + 130 lys1_tRNA + 90 met1_tRNA + 18300 mg2 + 60 phe1_tRNA + 7300 pi + 80 pro1_tRNA + 40 pro2_tRNA + 50 proL_tRNA + 50 proM_tRNA + 1 rib_70_elo2_b0414_10_cplx + 20 ser2_tRNA + 30 ser3_tRNA + 110 serV_tRNA + 60 thr1_tRNA + 20 thr2_tRNA + 100 thr3_tRNA + 10 thrU_tRNA + 50 trpT_tRNA + 80 tyr1_tRNA + 150 val1_tRNA + 90 val2_tRNA + 30 val3_tRNA	irreversible	Translation	0.00E+00	1.00E+09	0	5.39E+01	5.39E+01	0	9.19E+01	91.86595	0	4.41E+00	4.4113
tl_elo_b0414_1_rib1	Translation elongation 1 b0414 1 ribosome(s)	366 EF-G.GTP + 26 EF-Tu.GTP.ala1-tRNA + 12 EF-Tu.GTP.ala2-tRNA + 22 EF-Tu.GTP.arg1-tRNA + 3 EF-Tu.GTP.argX-tRNA + 7 EF Tu.GTP.asn1-tRNA + 17 EF-Tu.GTP.asp1-tRNA + 6 EF-Tu.GTP.cysT-tRNA + 13 EF-Tu.GTP.gln1-tRNA + 14 EF-Tu.GTP.gln2-tRNA + 21 EF-Tu.GTP.glu1-tRNA + 23 EF-Tu.GTP.gly1 tRNA + 3 EF-Tu.GTP.glyT-tRNA + 4 EF-Tu.GTP.glyU-tRNA + 13 EF-Tu.GTP.hisR-tRNA + 15 EF-Tu.GTP.ile1-tRNA + 2 EF-Tu.GTP.ile2-tRNA + 15 EF-Tu.GTP.leu1-tRNA + 4 EF-Tu.GTP.leu2-tRNA + 10 EF Tu.GTP.leuU-tRNA + 3 EF-Tu.GTP.leuW-tRNA + 8 EF-Tu.GTP.leuZ-tRNA + 13 EF-Tu.GTP.lys1-tRNA + 9 EF-Tu.GTP.met1-tRNA + 6 EF-Tu.GTP.phe1-tRNA + 8 EF-Tu.GTP.pro1-tRNA + 4 EF-Tu.GTP.pro2-tRNA + 5 EF-Tu.GTP.proL-tRNA + 5 EF-Tu.GTP.proM-tRNA + 2 EF-Tu.GTP.ser2-tRNA + 3 EF-Tu.GTP.ser3-tRNA + 11 EF-Tu.GTP.serV tRNA + 6 EF-Tu.GTP.thr1-tRNA + 2 EF-Tu.GTP.thr2-tRNA + 10 EF-Tu.GTP.thr3-tRNA + 1 EF-Tu.GTP.thrU-tRNA + 5 EF-Tu.GTP.trpT-tRNA + 8 EF-Tu.GTP.tyr1-tRNA + 15 EF-Tu.GTP.val1-tRNA + 9 EF-Tu.GTP.val2-tRNA + 3 EF-Tu.GTP.val3-tRNA + 1830 mg2 + 1 rib_ini_b0414_1 --> 1 rib_70_elo1_b0414_1_cplx	irreversible	Translation	0.00E+00	1.00E+09	0	5.39E+01	5.39E+01	0	9.19E+01	91.86595	0	4.41E+00	4.4113
tl_elo_b0414_1_rib2	Translation elongation 2 b0414 1 ribosome(s)	365 h2o + 1 rib_70_elo1_b0414_1_cplx --> 365 EF-G.GDP + 365 EF-Tu.GDP + 25 ala1_tRNA + 12 ala2_tRNA + 22 arg1_tRNA + 3 argX_tRNA + 7 asn1_tRNA + 17 asp1_tRNA + 6 cysT_tRNA + 1 fmet_tRNA + 13 gln1_tRNA + 14 gln2_tRNA + 21 glu1_tRNA + 23 gly1_tRNA + 3 glyT_tRNA + 4 glyU_tRNA + 730 h + 13 hisR_tRNA + 15 ile1_tRNA + 2 ile2_tRNA + 15 leu1_tRNA + 4 leu2_tRNA + 10 leuU_tRNA + 3 leuW_tRNA + 8 leuZ_tRNA + 13 lys1_tRNA + 9 met1_tRNA + 1830 mg2 + 6 phe1_tRNA + 730 pi + 8 pro1_tRNA + 4 pro2_tRNA + 5 proL_tRNA + 5 proM_tRNA + 1 rib_70_elo2_b0414_1_cplx + 2 ser2_tRNA + 3 ser3_tRNA + 11 serV_tRNA + 6 thr1_tRNA + 2 thr2_tRNA + 10 thr3_tRNA + 1 thrU_tRNA + 5 trpT_tRNA + 8 tyr1_tRNA + 15 val1_tRNA + 9 val2_tRNA + 3 val3_tRNA	irreversible	Translation	0.00E+00	1.00E+09	0	5.39E+01	5.39E+01	0	9.19E+01	91.86595	0	4.41E+00	4.4113
tl_elo_b0414_21_rib1	Translation elongation 1 b0414 21 ribosome(s)	7686 EF-G.GTP + 546 EF-Tu.GTP.ala1-tRNA + 252 EF-Tu.GTP.ala2 tRNA + 462 EF-Tu.GTP.arg1-tRNA + 63 EF-Tu.GTP.argX-tRNA + 147 EF-Tu.GTP.asn1-tRNA + 357 EF-Tu.GTP.asp1-tRNA + 126 EF-Tu.GTP.cysT-tRNA + 273 EF-Tu.GTP.gln1-tRNA + 294 EF-Tu.GTP.gln2-tRNA + 441 EF-Tu.GTP.glu1-tRNA + 483 EF-Tu.GTP.gly1-tRNA + 63 EF-Tu.GTP.glyT-tRNA + 84 EF-Tu.GTP.glyU-tRNA + 273 EF-Tu.GTP.hisR-tRNA + 315 EF-Tu.GTP.ile1-tRNA + 42 EF-Tu.GTP.ile2-tRNA + 315 EF-Tu.GTP.leu1 tRNA + 84 EF-Tu.GTP.leu2-tRNA + 210 EF-Tu.GTP.leuU-tRNA + 63 EF-Tu.GTP.leuW-tRNA + 168 EF-Tu.GTP.leuZ-tRNA + 273 EF-Tu.GTP.lys1-tRNA + 189 EF-Tu.GTP.met1-tRNA + 126 EF-Tu.GTP.phe1-tRNA + 168 EF-Tu.GTP.pro1-tRNA + 84 EF-Tu.GTP.pro2-tRNA + 105 EF-Tu.GTP.proL-tRNA + 105 EF-Tu.GTP.proM-tRNA + 42 EF-Tu.GTP.ser2-tRNA + 63 EF-Tu.GTP.ser3-tRNA + 231 EF-Tu.GTP.serV-tRNA + 126 EF-Tu.GTP.thr1-tRNA + 42 EF-Tu.GTP.thr2-tRNA + 210 EF-Tu.GTP.thr3-tRNA + 21 EF-Tu.GTP.thrU-tRNA + 105 EF-Tu.GTP.trpT-tRNA + 168 EF-Tu.GTP.tyr1-tRNA + 315 EF-Tu.GTP.val1-tRNA + 189 EF-Tu.GTP.val2-tRNA + 63 EF-Tu.GTP.val3-tRNA + 38430 mg2 + 1 rib_ini_b0414_21 --> 1 rib_70_e	irreversible	Translation	0.00E+00	1.00E+09	0	5.39E+01	5.39E+01	0	9.19E+01	91.86595	0	4.41E+00	4.4113

tl_elo_b0414_21_rib2	Translation elongation 2 b0414 21 ribosome(s)	7665 h2o + 1 rib_70_elo1_b0414_21_cplx --> 7665 EF-G.GDP + 7665 EF-Tu.GDP + 525 ala1_tRNA + 252 ala2_tRNA + 462 arg1_tRNA + 63 argX_tRNA + 147 asn1_tRNA + 357 asp1_tRNA + 126 cysT_tRNA + 21 fmet_tRNA + 273 gln1_tRNA + 294 gln2_tRNA + 441 glu1_tRNA + 483 gly1_tRNA + 63 glyT_tRNA + 84 glyU_tRNA + 15330 h + 273 hisR_tRNA + 315 ile1_tRNA + 42 ile2_tRNA + 315 leu1_tRNA + 84 leu2_tRNA + 210 leuU_tRNA + 63 leuW_tRNA + 168 leuZ_tRNA + 273 lys1_tRNA + 189 met1_tRNA + 38430 mg2 + 126 phe1_tRNA + 15330 pi + 168 pro1_tRNA + 84 pro2_tRNA + 105 proL_tRNA + 105 proM_tRNA + 1 rib_70_elo2_b0414_21_cplx + 42 ser2_tRNA + 63 ser3_tRNA + 231 serV_tRNA + 126 thr1_tRNA + 42 thr2_tRNA + 210 thr3_tRNA + 21 thrU_tRNA + 105 trpT_tRNA + 168 tyr1_tRNA + 315 val1_tRNA + 189 val2_tRNA + 63 val3_tRNA	irreversible	Translation	0.00E+00	1.00E+09	0	5.39E+01	5.39E+01	0	9.19E+01	91.86595	0	4.41E+00	4.4113
tl_elo_b0415_1_rib1	Translation elongation 1 b0415 1 ribosome(s)	155 EF-G.GTP + 23 EF-Tu.GTP.ala1-tRNA + 6 EF-Tu.GTP.ala2-tRNA + 5 EF-Tu.GTP.arg1-tRNA + 9 EF-Tu.GTP.asn1-tRNA + 6 EF-Tu.GTP.asp1-tRNA + 2 EF-Tu.GTP.gln1-tRNA + 1 EF-Tu.GTP.gln2-tRNA + 11 EF-Tu.GTP.glu1-tRNA + 14 EF-Tu.GTP.gly1-tRNA + 1 EF-Tu.GTP.glyU-tRNA + 2 EF-Tu.GTP.hisR-tRNA + 15 EF-Tu.GTP.ile1-tRNA + 10 EF-Tu.GTP.leu1-tRNA + 1 EF-Tu.GTP.leu2-tRNA + 1 EF-Tu.GTP.leuU-tRNA + 8 EF-Tu.GTP.lys1-tRNA + 1 EF-Tu.GTP.met1-tRNA + 4 EF-Tu.GTP.phe1-tRNA + 3 EF-Tu.GTP.pro1-tRNA + 1 EF-Tu.GTP.pro2-tRNA + 4 EF-Tu.GTP.serV-tRNA + 7 EF-Tu.GTP.thr1-tRNA + 2 EF-Tu.GTP.thr2-tRNA + 1 EF-Tu.GTP.thr3-tRNA + 1 EF-Tu.GTP.trpT-tRNA + 3 EF-Tu.GTP.tyr1-tRNA + 4 EF-Tu.GTP.val1-tRNA + 2 EF-Tu.GTP.val2-tRNA + 7 EF-Tu.GTP.val3-tRNA + 775 mg2 + 1 rib_ini_b0415_1 --> 1 rib_70_elo1_b0415_1_cplx	irreversible	Translation	0.00E+00	1.00E+09	0	6.10E+01	6.10E+01	0	9.89E+01	98.94595	0	1.15E+01	11.4913
tl_elo_b0415_1_rib2	Translation elongation 2 b0415 1 ribosome(s)	154 h2o + 1 rib_70_elo1_b0415_1_cplx --> 154 EF-G.GDP + 154 EF-Tu.GDP + 23 ala1_tRNA + 5 ala2_tRNA + 5 arg1_tRNA + 9 asn1_tRNA + 6 asp1_tRNA + 1 fmet_tRNA + 2 gln1_tRNA + 1 gln2_tRNA + 11 glu1_tRNA + 14 gly1_tRNA + 1 glyU_tRNA + 308 h + 2 hisR_tRNA + 15 ile1_tRNA + 10 leu1_tRNA + 1 leu2_tRNA + 1 leuU_tRNA + 8 lys1_tRNA + 1 met1_tRNA + 775 mg2 + 4 phe1_tRNA + 308 pi + 3 pro1_tRNA + 1 pro2_tRNA + 1 rib_70_elo2_b0415_1_cplx + 4 serV_tRNA + 7 thr1_tRNA + 2 thr2_tRNA + 1 thr3_tRNA + 1 trpT_tRNA + 3 tyr1_tRNA + 4 val1_tRNA + 2 val2_tRNA + 7 val3_tRNA	irreversible	Translation	0.00E+00	1.00E+09	0	6.10E+01	6.10E+01	0	9.89E+01	98.94595	0	1.15E+01	11.4913
tl_elo_b0415_4_rib1	Translation elongation 1 b0415 4 ribosome(s)	620 EF-G.GTP + 92 EF-Tu.GTP.ala1-tRNA + 24 EF-Tu.GTP.ala2-tRNA + 20 EF-Tu.GTP.arg1-tRNA + 36 EF-Tu.GTP.asn1-tRNA + 24 EF-Tu.GTP.asp1-tRNA + 8 EF-Tu.GTP.gln1-tRNA + 4 EF-Tu.GTP.gln2-tRNA + 44 EF-Tu.GTP.glu1-tRNA + 56 EF-Tu.GTP.gly1-tRNA + 4 EF-Tu.GTP.glyU-tRNA + 8 EF-Tu.GTP.hisR-tRNA + 60 EF-Tu.GTP.ile1-tRNA + 40 EF-Tu.GTP.leu1-tRNA + 4 EF-Tu.GTP.leu2-tRNA + 4 EF-Tu.GTP.leuU-tRNA + 32 EF-Tu.GTP.lys1-tRNA + 4 EF-Tu.GTP.met1-tRNA + 16 EF-Tu.GTP.phe1-tRNA + 12 EF-Tu.GTP.pro1-tRNA + 4 EF-Tu.GTP.pro2-tRNA + 16 EF-Tu.GTP.serV-tRNA + 28 EF-Tu.GTP.thr1-tRNA + 8 EF-Tu.GTP.thr2-tRNA + 4 EF-Tu.GTP.thr3-tRNA + 4 EF-Tu.GTP.trpT-tRNA + 12 EF-Tu.GTP.tyr1-tRNA + 16 EF-Tu.GTP.val1-tRNA + 8 EF-Tu.GTP.val2-tRNA + 28 EF-Tu.GTP.val3-tRNA + 3100 mg2 + 1 rib_ini_b0415_4 --> 1 rib_70_elo1_b0415_4_cplx	irreversible	Translation	0.00E+00	1.00E+09	0	6.10E+01	6.10E+01	0	9.89E+01	98.94595	0	1.15E+01	11.4913
tl_elo_b0415_4_rib2	Translation elongation 2 b0415 4 ribosome(s)	616 h2o + 1 rib_70_elo1_b0415_4_cplx --> 616 EF-G.GDP + 616 EF-Tu.GDP + 92 ala1_tRNA + 20 ala2_tRNA + 20 arg1_tRNA + 36 asn1_tRNA + 24 asp1_tRNA + 4 fmet_tRNA + 8 gln1_tRNA + 4 gln2_tRNA + 44 glu1_tRNA + 56 gly1_tRNA + 4 glyU_tRNA + 1232 h + 8 hisR_tRNA + 60 ile1_tRNA + 40 leu1_tRNA + 4 leu2_tRNA + 4 leuU_tRNA + 32 lys1_tRNA + 4 met1_tRNA + 3100 mg2 + 16 phe1_tRNA + 1232 pi + 12 pro1_tRNA + 4 pro2_tRNA + 1 rib_70_elo2_b0415_4_cplx + 16 serV_tRNA + 28 thr1_tRNA + 8 thr2_tRNA + 4 thr3_tRNA + 4 trpT_tRNA + 12 tyr1_tRNA + 16 val1_tRNA + 8 val2_tRNA + 28 val3_tRNA	irreversible	Translation	0.00E+00	1.00E+09	0	6.10E+01	6.10E+01	0	9.89E+01	98.94595	0	1.15E+01	11.4913
tl_elo_b0415_9_rib1	Translation elongation 1 b0415 9 ribosome(s)	1395 EF-G.GTP + 207 EF-Tu.GTP.ala1-tRNA + 54 EF-Tu.GTP.ala2-tRNA + 45 EF-Tu.GTP.arg1-tRNA + 81 EF-Tu.GTP.asn1-tRNA + 54 EF-Tu.GTP.asp1-tRNA + 18 EF-Tu.GTP.gln1-tRNA + 9 EF-Tu.GTP.gln2-tRNA + 99 EF-Tu.GTP.glu1-tRNA + 126 EF-Tu.GTP.gly1-tRNA + 9 EF-Tu.GTP.glyU-tRNA + 18 EF-Tu.GTP.hisR-tRNA + 135 EF-Tu.GTP.ile1-tRNA + 90 EF-Tu.GTP.leu1-tRNA + 9 EF-Tu.GTP.leu2-tRNA + 9 EF-Tu.GTP.leuU-tRNA + 72 EF-Tu.GTP.lys1-tRNA + 9 EF-Tu.GTP.met1-tRNA + 36 EF-Tu.GTP.phe1-tRNA + 27 EF-Tu.GTP.pro1-tRNA + 9 EF-Tu.GTP.pro2-tRNA + 36 EF-Tu.GTP.serV-tRNA + 63 EF-Tu.GTP.thr1-tRNA + 18 EF-Tu.GTP.thr2-tRNA + 9 EF-Tu.GTP.thr3-tRNA + 9 EF-Tu.GTP.trpT-tRNA + 27 EF-Tu.GTP.tyr1-tRNA + 36 EF-Tu.GTP.val1-tRNA + 18 EF-Tu.GTP.val2-tRNA + 63 EF-Tu.GTP.val3-tRNA + 6975 mg2 + 1 rib_ini_b0415_9 --> 1 rib_70_elo1_b0415_9_cplx	irreversible	Translation	0.00E+00	1.00E+09	0	6.10E+01	6.10E+01	0	9.89E+01	98.94595	0	1.15E+01	11.4913
tl_elo_b0415_9_rib2	Translation elongation 2 b0415 9 ribosome(s)	1386 h2o + 1 rib_70_elo1_b0415_9_cplx --> 1386 EF-G.GDP + 1386 EF-Tu.GDP + 207 ala1_tRNA + 45 ala2_tRNA + 45 arg1_tRNA + 81 asn1_tRNA + 54 asp1_tRNA + 9 fmet_tRNA + 18 gln1_tRNA + 9 gln2_tRNA + 99 glu1_tRNA + 126 gly1_tRNA + 9 glyU_tRNA + 2772 h + 18 hisR_tRNA + 135 ile1_tRNA + 90 leu1_tRNA + 9 leu2_tRNA + 9 leuU_tRNA + 72 lys1_tRNA + 9 met1_tRNA + 6975 mg2 + 36 phe1_tRNA + 2772 pi + 27 pro1_tRNA + 9 pro2_tRNA + 1 rib_70_elo2_b0415_9_cplx + 36 serV_tRNA + 63 thr1_tRNA + 18 thr2_tRNA + 9 thr3_tRNA + 9 trpT_tRNA + 27 tyr1_tRNA + 36 val1_tRNA + 18 val2_tRNA + 63 val3_tRNA	irreversible	Translation	0.00E+00	1.00E+09	0	6.10E+01	6.10E+01	0	9.89E+01	98.94595	0	1.15E+01	11.4913
tl_elo_b0416_1_rib1	Translation elongation 1 b0416 1 ribosome(s)	138 EF-G.GTP + 15 EF-Tu.GTP.ala1-tRNA + 3 EF-Tu.GTP.ala2-tRNA + 9 EF-Tu.GTP.arg1-tRNA + 5 EF-Tu.GTP.asn1-tRNA + 9 EF-Tu.GTP.asp1-tRNA + 1 EF-Tu.GTP.cysT-tRNA + 6 EF-Tu.GTP.gln1-tRNA + 11 EF-Tu.GTP.glu1-tRNA + 2 EF-Tu.GTP.gly1-tRNA + 2 EF-Tu.GTP.glyT-tRNA + 1 EF-Tu.GTP.glyU-tRNA + 1 EF-Tu.GTP.hisR-tRNA + 5 EF-Tu.GTP.ile1-tRNA + 13 EF-Tu.GTP.leu1-tRNA + 1 EF-Tu.GTP.leu2-tRNA + 3 EF-Tu.GTP.leuU-tRNA + 11 EF-Tu.GTP.lys1-tRNA + 1 EF-Tu.GTP.met1-tRNA + 4 EF-Tu.GTP.phe1-tRNA + 3 EF-Tu.GTP.pro2-tRNA + 2 EF-Tu.GTP.proM-tRNA + 3 EF-Tu.GTP.ser1-tRNA + 1 EF-Tu.GTP.ser2-tRNA + 1 EF-Tu.GTP.ser3-tRNA + 2 EF-Tu.GTP.serV-tRNA + 1 EF-Tu.GTP.thr1-tRNA + 1 EF-Tu.GTP.thr2-tRNA + 1 EF-Tu.GTP.trpT-tRNA + 7 EF-Tu.GTP.tyr1-tRNA + 8 EF-Tu.GTP.val1-tRNA + 3 EF-Tu.GTP.val2-tRNA + 2 EF-Tu.GTP.val3-tRNA + 690 mg2 + 1 rib_ini_b0416_1 --> 1 rib_70_elo1_b0416_1_cp	irreversible	Translation	0.00E+00	1.00E+09	0	6.23E+01	6.23E+01	0	1.00E+02	100.246	0	1.28E+01	12.7913

tl_elo_b0416_1_rib2	Translation elongation 2 b0416 1 ribosome(s)	137 h2o + 1 rib_70_elo1_b0416_1_cplx --> 137 EF-G.GDP + 137 EF-Tu.GDP + 15 ala1_tRNA + 3 ala2_tRNA + 9 arg1_tRNA + 5 asn1_tRNA + 9 asp1_tRNA + 1 cysT_tRNA + 1 fmet_tRNA + 6 gln1_tRNA + 11 glu1_tRNA + 2 gly1_tRNA + 2 glyT_tRNA + 1 glyU_tRNA + 274 h + 1 hisR_tRNA + 5 ile1_tRNA + 13 leu1_tRNA + 1 leu2_tRNA + 3 leuU_tRNA + 10 lys1_tRNA + 1 met1_tRNA + 690 mg2 + 4 phe1_tRNA + 274 pi + 3 pro2_tRNA + 2 proM_tRNA + 1 rib_70_elo2_b0416_1_cplx + 3 ser1_tRNA + 1 ser2_tRNA + 1 ser3_tRNA + 2 serV_tRNA + 1 thr1_tRNA + 1 thr2_tRNA + 1 trpT_tRNA + 7 tyr1_tRNA + 8 val1_tRNA + 3 val2_tRNA + 2 val3_tRNA	irreversible	Translation	0.00E+00	1.00E+09	0	6.23E+01	6.23E+01	0	1.00E+02	100.246	0	1.28E+01	12.7913
tl_elo_b0416_4_rib1	Translation elongation 1 b0416 4 ribosome(s)	552 EF-G.GTP + 60 EF-Tu.GTP.ala1-tRNA + 12 EF-Tu.GTP.ala2-tRNA + 36 EF-Tu.GTP.arg1-tRNA + 20 EF-Tu.GTP.asn1-tRNA + 36 EF-Tu.GTP.asp1-tRNA + 4 EF-Tu.GTP.cysT-tRNA + 24 EF-Tu.GTP.gln1-tRNA + 44 EF-Tu.GTP.glu1-tRNA + 8 EF-Tu.GTP.gly1-tRNA + 8 EF-Tu.GTP.glyT-tRNA + 4 EF-Tu.GTP.glyU-tRNA + 4 EF-Tu.GTP.hisR-tRNA + 20 EF-Tu.GTP.ile1-tRNA + 52 EF-Tu.GTP.leu1 tRNA + 4 EF-Tu.GTP.leu2-tRNA + 12 EF-Tu.GTP.leuU-tRNA + 44 EF-Tu.GTP.lys1-tRNA + 4 EF-Tu.GTP.met1-tRNA + 16 EF-Tu.GTP.phe1-tRNA + 12 EF-Tu.GTP.pro2-tRNA + 8 EF-Tu.GTP.proM-tRNA + 12 EF-Tu.GTP.ser1-tRNA + 4 EF-Tu.GTP.ser2-tRNA + 4 EF-Tu.GTP.ser3-tRNA + 8 EF-Tu.GTP.serV-tRNA + 4 EF-Tu.GTP.thr1-tRNA + 4 EF-Tu.GTP.thr2-tRNA + 4 EF-Tu.GTP.trpT-tRNA + 28 EF-Tu.GTP.tyr1-tRNA + 32 EF-Tu.GTP.val1-tRNA + 12 EF-Tu.GTP.val2-tRNA + 8 EF-Tu.GTP.val3-tRNA + 2760 mg2 + 1 rib_ini_b0416_4 --> 1 rib_70_elo1_b0416_4_cplx	irreversible	Translation	0.00E+00	1.00E+09	0	6.23E+01	6.23E+01	0	1.00E+02	100.246	0	1.28E+01	12.7913
tl_elo_b0416_4_rib2	Translation elongation 2 b0416 4 ribosome(s)	548 h2o + 1 rib_70_elo1_b0416_4_cplx --> 548 EF-G.GDP + 548 EF-Tu.GDP + 60 ala1_tRNA + 12 ala2_tRNA + 36 arg1_tRNA + 20 asn1_tRNA + 36 asp1_tRNA + 4 cysT_tRNA + 4 fmet_tRNA + 24 gln1_tRNA + 44 glu1_tRNA + 8 gly1_tRNA + 8 glyT_tRNA + 4 glyU_tRNA + 1096 h + 4 hisR_tRNA + 20 ile1_tRNA + 52 leu1_tRNA + 4 leu2_tRNA + 12 leuU_tRNA + 40 lys1_tRNA + 4 met1_tRNA + 2760 mg2 + 16 phe1_tRNA + 1096 pi + 12 pro2_tRNA + 8 proM_tRNA + 1 rib_70_elo2_b0416_4_cplx + 12 ser1_tRNA + 4 ser2_tRNA + 4 ser3_tRNA + 8 serV_tRNA + 4 thr1_tRNA + 4 thr2_tRNA + 4 trpT_tRNA + 28 tyr1_tRNA + 32 val1_tRNA + 12 val2_tRNA + 8 val3_tRNA	irreversible	Translation	0.00E+00	1.00E+09	0	6.23E+01	6.23E+01	0	1.00E+02	100.246	0	1.28E+01	12.7913
tl_elo_b0416_8_rib1	Translation elongation 1 b0416 8 ribosome(s)	1104 EF-G.GTP + 120 EF-Tu.GTP.ala1-tRNA + 24 EF-Tu.GTP.ala2-tRNA + 72 EF-Tu.GTP.arg1-tRNA + 40 EF-Tu.GTP.asn1-tRNA + 72 EF-Tu.GTP.asp1-tRNA + 8 EF-Tu.GTP.cysT-tRNA + 48 EF-Tu.GTP.gln1-tRNA + 88 EF-Tu.GTP.glu1-tRNA + 16 EF-Tu.GTP.gly1 tRNA + 16 EF-Tu.GTP.glyT-tRNA + 8 EF-Tu.GTP.glyU-tRNA + 8 EF-Tu.GTP.hisR-tRNA + 40 EF-Tu.GTP.ile1-tRNA + 104 EF-Tu.GTP.leu1-tRNA + 8 EF-Tu.GTP.leu2-tRNA + 24 EF-Tu.GTP.leuU-tRNA + 88 EF-Tu.GTP.lys1-tRNA + 8 EF-Tu.GTP.met1-tRNA + 32 EF-Tu.GTP.phe1-tRNA + 24 EF-Tu.GTP.pro2-tRNA + 16 EF-Tu.GTP.proM-tRNA + 24 EF-Tu.GTP.ser1-tRNA + 8 EF-Tu.GTP.ser2-tRNA + 8 EF-Tu.GTP.ser3-tRNA + 16 EF-Tu.GTP.serV tRNA + 8 EF-Tu.GTP.thr1-tRNA + 8 EF-Tu.GTP.thr2-tRNA + 8 EF-Tu.GTP.trpT-tRNA + 56 EF-Tu.GTP.tyr1-tRNA + 64 EF-Tu.GTP.val1-tRNA + 24 EF-Tu.GTP.val2-tRNA + 16 EF-Tu.GTP.val3-tRNA + 5520 mg2 + 1 rib_ini_b0416_8 --> 1 rib_70_elo1_b0416_8_cplx	irreversible	Translation	0.00E+00	1.00E+09	0	6.23E+01	6.23E+01	0	1.00E+02	100.246	0	1.28E+01	12.7913
tl_elo_b0416_8_rib2	Translation elongation 2 b0416 8 ribosome(s)	1096 h2o + 1 rib_70_elo1_b0416_8_cplx --> 1096 EF-G.GDP + 1096 EF-Tu.GDP + 120 ala1_tRNA + 24 ala2_tRNA + 72 arg1_tRNA + 40 asn1_tRNA + 72 asp1_tRNA + 8 cysT_tRNA + 8 fmet_tRNA + 48 gln1_tRNA + 88 glu1_tRNA + 16 gly1_tRNA + 16 glyT_tRNA + 8 glyU_tRNA + 2192 h + 8 hisR_tRNA + 40 ile1_tRNA + 104 leu1_tRNA + 8 leu2_tRNA + 24 leuU_tRNA + 80 lys1_tRNA + 8 met1_tRNA + 5520 mg2 + 32 phe1_tRNA + 2192 pi + 24 pro2_tRNA + 16 proM_tRNA + 1 rib_70_elo2_b0416_8_cplx + 24 ser1_tRNA + 8 ser2_tRNA + 8 ser3_tRNA + 16 serV_tRNA + 8 thr1_tRNA + 8 thr2_tRNA + 8 trpT_tRNA + 56 tyr1_tRNA + 64 val1_tRNA + 24 val2_tRNA + 16 val3_tRNA	irreversible	Translation	0.00E+00	1.00E+09	0	6.23E+01	6.23E+01	0	1.00E+02	100.246	0	1.28E+01	12.7913
tl_elo_b0423_14_rib1	Translation elongation 1 b0423 14 ribosome(s)	6734 EF-G.GTP + 266 EF-Tu.GTP.ala1-tRNA + 42 EF-Tu.GTP.ala2-tRNA + 504 EF-Tu.GTP.arg1-tRNA + 266 EF-Tu.GTP.asn1-tRNA + 434 EF-Tu.GTP.asp1-tRNA + 70 EF-Tu.GTP.cysT-tRNA + 252 EF-Tu.GTP.gln1-tRNA + 14 EF-Tu.GTP.gln2-tRNA + 546 EF-Tu.GTP.glu1-tRNA + 420 EF-Tu.GTP.gly1-tRNA + 56 EF-Tu.GTP.glyU-tRNA + 182 EF-Tu.GTP.hisR-tRNA + 532 EF-Tu.GTP.ile1-tRNA + 14 EF-Tu.GTP.ile2-tRNA + 336 EF-Tu.GTP.leu1 tRNA + 56 EF-Tu.GTP.leu2-tRNA + 182 EF-Tu.GTP.leuU-tRNA + 28 EF-Tu.GTP.leuZ-tRNA + 392 EF-Tu.GTP.lys1-tRNA + 112 EF-Tu.GTP.met1-tRNA + 294 EF-Tu.GTP.phe1-tRNA + 168 EF-Tu.GTP.pro1-tRNA + 14 EF-Tu.GTP.proM-tRNA + 112 EF-Tu.GTP.ser1-tRNA + 70 EF-Tu.GTP.ser2-tRNA + 42 EF-Tu.GTP.ser3-tRNA + 126 EF-Tu.GTP.serV-tRNA + 196 EF-Tu.GTP.thr1-tRNA + 14 EF-Tu.GTP.thr2-tRNA + 70 EF-Tu.GTP.thr3-tRNA + 56 EF-Tu.GTP.trpT-tRNA + 182 EF-Tu.GTP.tyr1-tRNA + 392 EF-Tu.GTP.val1-tRNA + 168 EF-Tu.GTP.val2-tRNA + 126 EF-Tu.GTP.val3-tRNA + 33670 mg2 + 1 rib_ini_b0423_14 --> 1 rib_70_elo1_b0423_14_cplx	irreversible	Translation	0.00E+00	1.00E+09	0	9.25E+01	9.25E+01	0	1.61E+02	160.7749	0	3.48E+00	3.476
tl_elo_b0423_14_rib2	Translation elongation 2 b0423 14 ribosome(s)	6720 h2o + 1 rib_70_elo1_b0423_14_cplx --> 6720 EF-G.GDP + 6720 EF-Tu.GDP + 266 ala1_tRNA + 42 ala2_tRNA + 504 arg1_tRNA + 266 asn1_tRNA + 434 asp1_tRNA + 70 cysT_tRNA + 14 fmet_tRNA + 252 gln1_tRNA + 14 gln2_tRNA + 546 glu1_tRNA + 420 gly1_tRNA + 56 glyU_tRNA + 13440 h + 182 hisR_tRNA + 532 ile1_tRNA + 14 ile2_tRNA + 336 leu1_tRNA + 56 leu2_tRNA + 182 leuU_tRNA + 28 leuZ_tRNA + 392 lys1_tRNA + 112 met1_tRNA + 33670 mg2 + 294 phe1_tRNA + 13440 pi + 154 pro1_tRNA + 14 proM_tRNA + 1 rib_70_elo2_b0423_14_cplx + 112 ser1_tRNA + 70 ser2_tRNA + 42 ser3_tRNA + 126 serV_tRNA + 196 thr1_tRNA + 14 thr2_tRNA + 70 thr3_tRNA + 56 trpT_tRNA + 182 tyr1_tRNA + 392 val1_tRNA + 168 val2_tRNA + 126 val3_tRNA	irreversible	Translation	0.00E+00	1.00E+09	0	9.25E+01	9.25E+01	0	1.61E+02	160.7749	0	3.48E+00	3.476

tl_elo_b0423_1_rib1	Translation elongation 1 b0423 1 ribosome(s)	481 EF-G.GTP + 19 EF-Tu.GTP.ala1-tRNA + 3 EF-Tu.GTP.ala2-tRNA + 36 EF-Tu.GTP.arg1-tRNA + 19 EF-Tu.GTP.asn1-tRNA + 31 EF-Tu.GTP.asp1-tRNA + 5 EF-Tu.GTP.cysT-tRNA + 18 EF-Tu.GTP.gln1-tRNA + 1 EF-Tu.GTP.gln2-tRNA + 39 EF-Tu.GTP.glu1-tRNA + 30 EF-Tu.GTP.gly1-tRNA + 4 EF-Tu.GTP.glyU-tRNA + 13 EF-Tu.GTP.hisR-tRNA + 38 EF-Tu.GTP.ile1-tRNA + 1 EF-Tu.GTP.ile2-tRNA + 24 EF-Tu.GTP.leu1-tRNA + 4 EF-Tu.GTP.leu2-tRNA + 13 EF-Tu.GTP.leuU-tRNA + 2 EF-Tu.GTP.leuZ-tRNA + 28 EF-Tu.GTP.lys1-tRNA + 8 EF-Tu.GTP.met1-tRNA + 21 EF-Tu.GTP.phe1-tRNA + 12 EF-Tu.GTP.pro1-tRNA + 1 EF-Tu.GTP.proM-tRNA + 8 EF-Tu.GTP.ser1-tRNA + 5 EF-Tu.GTP.ser2-tRNA + 3 EF-Tu.GTP.ser3-tRNA + 9 EF-Tu.GTP.serV-tRNA + 14 EF-Tu.GTP.thr1-tRNA + 1 EF-Tu.GTP.thr2-tRNA + 5 EF-Tu.GTP.thr3-tRNA + 4 EF-Tu.GTP.trpT-tRNA + 13 EF-Tu.GTP.tyr1-tRNA + 28 EF-Tu.GTP.val1-tRNA + 12 EF-Tu.GTP.val2-tRNA + 9 EF-Tu.GTP.val3-tRNA + 2405 mg2 + 1 rib_ini_b0423_1 --> 1 rib_70_elo1_b0423_1_cplx	irreversible	Translation	0.00E+00	1.00E+09	0	9.25E+01	9.25E+01	0	1.61E+02	160.7749	0	3.48E+00	3.476
tl_elo_b0423_1_rib2	Translation elongation 2 b0423 1 ribosome(s)	480 h2o + 1 rib_70_elo1_b0423_1_cplx --> 480 EF-G.GDP + 480 EF-Tu.GDP + 19 ala1_tRNA + 3 ala2_tRNA + 36 arg1_tRNA + 19 asn1_tRNA + 31 asp1_tRNA + 5 cysT_tRNA + 1 fmet_tRNA + 18 gln1_tRNA + 1 gln2_tRNA + 39 glu1_tRNA + 30 gly1_tRNA + 4 glyU_tRNA + 960 h + 13 hisR_tRNA + 38 ile1_tRNA + 1 ile2_tRNA + 24 leu1_tRNA + 4 leu2_tRNA + 13 leuU_tRNA + 2 leuZ_tRNA + 28 lys1_tRNA + 8 met1_tRNA + 2405 mg2 + 21 phe1_tRNA + 960 pi + 11 pro1_tRNA + 1 proM_tRNA + 1 rib_70_elo2_b0423_1_cplx + 8 ser1_tRNA + 5 ser2_tRNA + 3 ser3_tRNA + 9 serV_tRNA + 14 thr1_tRNA + 1 thr2_tRNA + 5 thr3_tRNA + 4 trpT_tRNA + 13 tyr1_tRNA + 28 val1_tRNA + 12 val2_tRNA + 9 val3_tRNA	irreversible	Translation	0.00E+00	1.00E+09	0	9.25E+01	9.25E+01	0	1.61E+02	160.7749	0	3.48E+00	3.476
tl_elo_b0423_28_rib1	Translation elongation 1 b0423 28 ribosome(s)	13468 EF-G.GTP + 532 EF-Tu.GTP.ala1-tRNA + 84 EF-Tu.GTP.ala2-tRNA + 1008 EF-Tu.GTP.arg1-tRNA + 532 EF-Tu.GTP.asn1-tRNA + 868 EF-Tu.GTP.asp1-tRNA + 140 EF-Tu.GTP.cysT-tRNA + 504 EF-Tu.GTP.gln1-tRNA + 28 EF-Tu.GTP.gln2-tRNA + 1092 EF-Tu.GTP.glu1-tRNA + 840 EF-Tu.GTP.gly1-tRNA + 112 EF-Tu.GTP.glyU-tRNA + 364 EF-Tu.GTP.hisR-tRNA + 1064 EF-Tu.GTP.ile1-tRNA + 28 EF-Tu.GTP.ile2-tRNA + 672 EF-Tu.GTP.leu1-tRNA + 112 EF-Tu.GTP.leu2-tRNA + 364 EF-Tu.GTP.leuU-tRNA + 56 EF-Tu.GTP.leuZ-tRNA + 784 EF-Tu.GTP.lys1-tRNA + 224 EF-Tu.GTP.met1-tRNA + 588 EF-Tu.GTP.phe1-tRNA + 336 EF-Tu.GTP.pro1-tRNA + 28 EF-Tu.GTP.proM-tRNA + 224 EF-Tu.GTP.ser1-tRNA + 140 EF-Tu.GTP.ser2-tRNA + 84 EF-Tu.GTP.ser3-tRNA + 252 EF-Tu.GTP.serV-tRNA + 392 EF-Tu.GTP.thr1-tRNA + 28 EF-Tu.GTP.thr2-tRNA + 140 EF-Tu.GTP.thr3-tRNA + 112 EF-Tu.GTP.trpT-tRNA + 364 EF-Tu.GTP.tyr1-tRNA + 784 EF-Tu.GTP.val1-tRNA + 336 EF-Tu.GTP.val2-tRNA + 252 EF-Tu.GTP.val3-tRNA + 67340 mg2 + 1 rib_ini_b0423_28 --> 1 rib_70_elo1_b0423_28_cplx	irreversible	Translation	0.00E+00	1.00E+09	0	9.25E+01	9.25E+01	0	1.61E+02	160.7749	0	3.48E+00	3.476
tl_elo_b0423_28_rib2	Translation elongation 2 b0423 28 ribosome(s)	13440 h2o + 1 rib_70_elo1_b0423_28_cplx --> 13440 EF-G.GDP + 13440 EF-Tu.GDP + 532 ala1_tRNA + 84 ala2_tRNA + 1008 arg1_tRNA + 532 asn1_tRNA + 868 asp1_tRNA + 140 cysT_tRNA + 28 fmet_tRNA + 504 gln1_tRNA + 28 gln2_tRNA + 1092 glu1_tRNA + 840 gly1_tRNA + 112 glyU_tRNA + 26880 h + 364 hisR_tRNA + 1064 ile1_tRNA + 28 ile2_tRNA + 672 leu1_tRNA + 112 leu2_tRNA + 364 leuU_tRNA + 56 leuZ_tRNA + 784 lys1_tRNA + 224 met1_tRNA + 67340 mg2 + 588 phe1_tRNA + 26880 pi + 308 pro1_tRNA + 28 proM_tRNA + 1 rib_70_elo2_b0423_28_cplx + 224 ser1_tRNA + 140 ser2_tRNA + 84 ser3_tRNA + 252 serV_tRNA + 392 thr1_tRNA + 28 thr2_tRNA + 140 thr3_tRNA + 112 trpT_tRNA + 364 tyr1_tRNA + 784 val1_tRNA + 336 val2_tRNA + 252 val3_tRNA	irreversible	Translation	0.00E+00	1.00E+09	0	9.25E+01	9.25E+01	0	1.61E+02	160.7749	0	3.48E+00	3.476
tl_elo_b0436_12_rib1	Translation elongation 1 b0436 12 ribosome(s)	5172 EF-G.GTP + 468 EF-Tu.GTP.ala1-tRNA + 24 EF-Tu.GTP.ala2-tRNA + 312 EF-Tu.GTP.arg1-tRNA + 192 EF-Tu.GTP.asn1-tRNA + 300 EF-Tu.GTP.asp1-tRNA + 156 EF-Tu.GTP.gln1-tRNA + 36 EF-Tu.GTP.gln2-tRNA + 708 EF-Tu.GTP.glu1-tRNA + 384 EF-Tu.GTP.gly1-tRNA + 24 EF-Tu.GTP.hisR-tRNA + 300 EF-Tu.GTP.ile1-tRNA + 360 EF-Tu.GTP.leu1-tRNA + 12 EF-Tu.GTP.leuU-tRNA + 420 EF-Tu.GTP.lys1-tRNA + 120 EF-Tu.GTP.met1-tRNA + 204 EF-Tu.GTP.phe1-tRNA + 132 EF-Tu.GTP.pro1-tRNA + 12 EF-Tu.GTP.proM-tRNA + 12 EF-Tu.GTP.ser1-tRNA + 72 EF-Tu.GTP.ser2-tRNA + 12 EF-Tu.GTP.serT-tRNA + 72 EF-Tu.GTP.serV-tRNA + 120 EF-Tu.GTP.thr1-tRNA + 96 EF-Tu.GTP.thr2-tRNA + 12 EF-Tu.GTP.thr3-tRNA + 12 EF-Tu.GTP.trpT-tRNA + 96 EF-Tu.GTP.tyr1-tRNA + 216 EF-Tu.GTP.val1-tRNA + 12 EF-Tu.GTP.val2-tRNA + 276 EF-Tu.GTP.val3-tRNA + 25860 mg2 + 1 rib_ini_b0436_12 --> 1 rib_70_elo1_b0436_12_cplx	irreversible	Translation	0.00E+00	1.00E+09	0	9.28E+00	9.28E+00	0	6.91E+00	6.906667	0	2.87E+00	2.868462
tl_elo_b0436_12_rib2	Translation elongation 2 b0436 12 ribosome(s)	5160 h2o + 1 rib_70_elo1_b0436_12_cplx --> 5160 EF-G.GDP + 5160 EF-Tu.GDP + 456 ala1_tRNA + 24 ala2_tRNA + 312 arg1_tRNA + 192 asn1_tRNA + 300 asp1_tRNA + 12 fmet_tRNA + 156 gln1_tRNA + 36 gln2_tRNA + 708 glu1_tRNA + 384 gly1_tRNA + 10320 h + 24 hisR_tRNA + 300 ile1_tRNA + 360 leu1_tRNA + 12 leuU_tRNA + 420 lys1_tRNA + 120 met1_tRNA + 25860 mg2 + 204 phe1_tRNA + 10320 pi + 132 pro1_tRNA + 12 proM_tRNA + 1 rib_70_elo2_b0436_12_cplx + 12 ser1_tRNA + 72 ser2_tRNA + 12 serT_tRNA + 72 serV_tRNA + 120 thr1_tRNA + 96 thr2_tRNA + 12 thr3_tRNA + 12 trpT_tRNA + 96 tyr1_tRNA + 216 val1_tRNA + 12 val2_tRNA + 276 val3_tRNA	irreversible	Translation	0.00E+00	1.00E+09	0	9.28E+00	9.28E+00	0	6.91E+00	6.906667	0	2.87E+00	2.868462
tl_elo_b0436_1_rib1	Translation elongation 1 b0436 1 ribosome(s)	431 EF-G.GTP + 39 EF-Tu.GTP.ala1-tRNA + 2 EF-Tu.GTP.ala2-tRNA + 26 EF-Tu.GTP.arg1-tRNA + 16 EF-Tu.GTP.asn1-tRNA + 25 EF-Tu.GTP.asp1-tRNA + 13 EF-Tu.GTP.gln1-tRNA + 3 EF-Tu.GTP.gln2-tRNA + 59 EF-Tu.GTP.glu1-tRNA + 32 EF-Tu.GTP.gly1-tRNA + 2 EF-Tu.GTP.hisR-tRNA + 25 EF-Tu.GTP.ile1-tRNA + 30 EF-Tu.GTP.leu1-tRNA + 1 EF-Tu.GTP.leuU-tRNA + 35 EF-Tu.GTP.lys1-tRNA + 10 EF-Tu.GTP.met1-tRNA + 17 EF-Tu.GTP.phe1-tRNA + 11 EF-Tu.GTP.pro1-tRNA + 1 EF-Tu.GTP.proM-tRNA + 1 EF-Tu.GTP.ser1-tRNA + 6 EF-Tu.GTP.ser2-tRNA + 1 EF-Tu.GTP.serT-tRNA + 6 EF-Tu.GTP.serV-tRNA + 10 EF-Tu.GTP.thr1-tRNA + 8 EF-Tu.GTP.thr2-tRNA + 1 EF-Tu.GTP.thr3-tRNA + 1 EF-Tu.GTP.trpT-tRNA + 8 EF-Tu.GTP.tyr1-tRNA + 18 EF-Tu.GTP.val1-tRNA + 1 EF-Tu.GTP.val2-tRNA + 23 EF-Tu.GTP.val3-tRNA + 2155 mg2 + 1 rib_ini_b0436_1 --> 1 rib_70_elo1_b0436_1_cplx	irreversible	Translation	0.00E+00	1.00E+09	0	9.00E+01	9.00E+01	0	8.29E+01	82.88	0	1.55E+00	1.55375

tl_elo_b0436_1_rib2	Translation elongation 2 b0436 1 ribosome(s)	430 h2o + 1 rib_70_elo1_b0436_1_cplx --> 430 EF-G.GDP + 430 EF-Tu.GDP + 38 ala1_tRNA + 2 ala2_tRNA + 26 arg1_tRNA + 16 asn1_tRNA + 25 asp1_tRNA + 1 fmet_tRNA + 13 gln1_tRNA + 3 gln2_tRNA + 59 glu1_tRNA + 32 gly1_tRNA + 860 h + 2 hisR_tRNA + 25 ile1_tRNA + 30 leu1_tRNA + 1 leuU_tRNA + 35 lys1_tRNA + 10 met1_tRNA + 2155 mg2 + 17 phe1_tRNA + 860 pi + 11 pro1_tRNA + 1 proM_tRNA + 1 rib_70_elo2_b0436_1_cplx + 1 ser1_tRNA + 6 ser2_tRNA + 1 serT_tRNA + 6 serV_tRNA + 10 thr1_tRNA + 8 thr2_tRNA + 1 thr3_tRNA + 1 trpT_tRNA + 8 tyr1_tRNA + 18 val1_tRNA + 1 val2_tRNA + 23 val3_tRNA	irreversible	Translation	0.00E+00	1.00E+09	0	9.00E+01	9.00E+01	0	8.29E+01	82.88	0	1.55E+00	1.55375
tl_elo_b0436_25_rib1	Translation elongation 1 b0436 25 ribosome(s)	10775 EF-G.GTP + 975 EF-Tu.GTP.ala1-tRNA + 50 EF-Tu.GTP.ala2-tRNA + 650 EF-Tu.GTP.arg1-tRNA + 400 EF-Tu.GTP.asn1-tRNA + 625 EF-Tu.GTP.asp1-tRNA + 325 EF-Tu.GTP.gln1-tRNA + 75 EF-Tu.GTP.gln2-tRNA + 1475 EF-Tu.GTP.glu1-tRNA + 800 EF-Tu.GTP.gly1-tRNA + 50 EF-Tu.GTP.hisR-tRNA + 625 EF-Tu.GTP.ile1-tRNA + 750 EF-Tu.GTP.leu1-tRNA + 25 EF-Tu.GTP.leuU-tRNA + 875 EF-Tu.GTP.lys1-tRNA + 250 EF-Tu.GTP.met1-tRNA + 425 EF-Tu.GTP.phe1-tRNA + 275 EF-Tu.GTP.pro1-tRNA + 25 EF-Tu.GTP.proM-tRNA + 25 EF-Tu.GTP.ser1-tRNA + 150 EF-Tu.GTP.ser2-tRNA + 25 EF-Tu.GTP.serT-tRNA + 150 EF-Tu.GTP.serV-tRNA + 250 EF-Tu.GTP.thr1-tRNA + 200 EF-Tu.GTP.thr2-tRNA + 25 EF-Tu.GTP.thr3-tRNA + 25 EF-Tu.GTP.trpT-tRNA + 200 EF-Tu.GTP.tyr1-tRNA + 450 EF-Tu.GTP.val1-tRNA + 25 EF-Tu.GTP.val2-tRNA + 575 EF-Tu.GTP.val3-tRNA + 53875 mg2 + 1 rib_ini_b0436_25 --> 1 rib_70_elo1_b0436_25_cplx	irreversible	Translation	0.00E+00	1.00E+09	0	4.50E+00	4.50E+00	0	3.32E+00	3.3152	1.11E+00	3.32E+00	2.205662
tl_elo_b0436_25_rib2	Translation elongation 2 b0436 25 ribosome(s)	10750 h2o + 1 rib_70_elo1_b0436_25_cplx --> 10750 EF-G.GDP + 10750 EF-Tu.GDP + 950 ala1_tRNA + 50 ala2_tRNA + 650 arg1_tRNA + 400 asn1_tRNA + 625 asp1_tRNA + 25 fmet_tRNA + 325 gln1_tRNA + 75 gln2_tRNA + 1475 glu1_tRNA + 800 gly1_tRNA + 21500 h + 50 hisR_tRNA + 625 ile1_tRNA + 750 leu1_tRNA + 25 leuU_tRNA + 875 lys1_tRNA + 250 met1_tRNA + 53875 mg2 + 425 phe1_tRNA + 21500 pi + 275 pro1_tRNA + 25 proM_tRNA + 1 rib_70_elo2_b0436_25_cplx + 25 ser1_tRNA + 150 ser2_tRNA + 25 serT_tRNA + 150 serV_tRNA + 250 thr1_tRNA + 200 thr2_tRNA + 25 thr3_tRNA + 25 trpT_tRNA + 200 tyr1_tRNA + 450 val1_tRNA + 25 val2_tRNA + 575 val3_tRNA	irreversible	Translation	0.00E+00	1.00E+09	0	4.50E+00	4.50E+00	0	3.32E+00	3.3152	1.11E+00	3.32E+00	2.205662
tl_elo_b0503_10_rib1	Translation elongation 1 b0503 10 ribosome(s)	3630 EF-G.GTP + 320 EF-Tu.GTP.ala1-tRNA + 100 EF-Tu.GTP.ala2-tRNA + 220 EF-Tu.GTP.arg1-tRNA + 10 EF-Tu.GTP.argU-tRNA + 10 EF-Tu.GTP.argW-tRNA + 40 EF-Tu.GTP.argX-tRNA + 100 EF-Tu.GTP.asn1-tRNA + 160 EF-Tu.GTP.asp1-tRNA + 70 EF-Tu.GTP.cysT-tRNA + 160 EF-Tu.GTP.gln1-tRNA + 100 EF-Tu.GTP.gln2-tRNA + 310 EF-Tu.GTP.glu1-tRNA + 220 EF-Tu.GTP.gly1-tRNA + 20 EF-Tu.GTP.glyT-tRNA + 30 EF-Tu.GTP.glyU-tRNA + 130 EF-Tu.GTP.hisR-tRNA + 160 EF-Tu.GTP.ile1-tRNA + 10 EF-Tu.GTP.ile2-tRNA + 270 EF-Tu.GTP.leu1-tRNA + 40 EF-Tu.GTP.leu2-tRNA + 70 EF-Tu.GTP.leuU-tRNA + 20 EF-Tu.GTP.leuW-tRNA + 40 EF-Tu.GTP.leuZ-tRNA + 110 EF-Tu.GTP.lys1-tRNA + 80 EF-Tu.GTP.met1-tRNA + 70 EF-Tu.GTP.phe1-tRNA + 90 EF-Tu.GTP.pro1-tRNA + 10 EF-Tu.GTP.pro2-tRNA + 20 EF-Tu.GTP.proL-tRNA + 10 EF-Tu.GTP.proM-tRNA + 30 EF-Tu.GTP.ser3-tRNA + 20 EF-Tu.GTP.serT-tRNA + 60 EF-Tu.GTP.serV-tRNA + 80 EF-Tu.GTP.thr1-tRNA + 20 EF-Tu.GTP.thr2-tRNA + 50 EF-Tu.GTP.thr3-tRNA + 10 EF-Tu.GTP.thrU-tRNA + 70 EF-Tu.GTP.trpT-tRNA + 130 EF-Tu.GTP.tyr1-tRNA + 100 EF-Tu.GTP.val1-tRNA + 10 EF-Tu.GTP.val2-tRNA + 50 EF-Tu.GTP.val3-tRNA + 18150 mg2 + 1 rib_	irreversible	Translation	0.00E+00	1.00E+09	0	1.21E+02	1.21E+02	0	2.11E+02	210.8787	0	4.50E+00	4.496
tl_elo_b0503_10_rib2	Translation elongation 2 b0503 10 ribosome(s)	3620 h2o + 1 rib_70_elo1_b0503_10_cplx --> 3620 EF-G.GDP + 3620 EF-Tu.GDP + 320 ala1_tRNA + 100 ala2_tRNA + 220 arg1_tRNA + 10 argU_tRNA + 10 argW_tRNA + 30 argX_tRNA + 100 asn1_tRNA + 160 asp1_tRNA + 70 cysT_tRNA + 10 fmet_tRNA + 160 gln1_tRNA + 100 gln2_tRNA + 310 glu1_tRNA + 220 gly1_tRNA + 20 glyT_tRNA + 30 glyU_tRNA + 7240 h + 130 hisR_tRNA + 160 ile1_tRNA + 10 ile2_tRNA + 270 leu1_tRNA + 40 leu2_tRNA + 70 leuU_tRNA + 20 leuW_tRNA + 40 leuZ_tRNA + 110 lys1_tRNA + 80 met1_tRNA + 18150 mg2 + 70 phe1_tRNA + 7240 pi + 90 pro1_tRNA + 10 pro2_tRNA + 20 proL_tRNA + 10 proM_tRNA + 1 rib_70_elo2_b0503_10_cplx + 30 ser3_tRNA + 20 serT_tRNA + 60 serV_tRNA + 80 thr1_tRNA + 20 thr2_tRNA + 50 thr3_tRNA + 10 thrU_tRNA + 70 trpT_tRNA + 130 tyr1_tRNA + 100 val1_tRNA + 10 val2_tRNA + 50 val3_tRNA	irreversible	Translation	0.00E+00	1.00E+09	0	1.21E+02	1.21E+02	0	2.11E+02	210.8787	0	4.50E+00	4.496
tl_elo_b0503_1_rib1	Translation elongation 1 b0503 1 ribosome(s)	363 EF-G.GTP + 32 EF-Tu.GTP.ala1-tRNA + 10 EF-Tu.GTP.ala2-tRNA + 22 EF-Tu.GTP.arg1-tRNA + 1 EF-Tu.GTP.argU-tRNA + 1 EF-Tu.GTP.argW-tRNA + 4 EF-Tu.GTP.argX-tRNA + 10 EF-Tu.GTP.asn1-tRNA + 16 EF-Tu.GTP.asp1-tRNA + 7 EF-Tu.GTP.cysT-tRNA + 16 EF-Tu.GTP.gln1-tRNA + 10 EF-Tu.GTP.gln2-tRNA + 31 EF-Tu.GTP.glu1-tRNA + 22 EF-Tu.GTP.gly1-tRNA + 2 EF-Tu.GTP.glyT-tRNA + 3 EF-Tu.GTP.glyU-tRNA + 13 EF-Tu.GTP.hisR-tRNA + 16 EF-Tu.GTP.ile1-tRNA + 1 EF-Tu.GTP.ile2-tRNA + 27 EF-Tu.GTP.leu1-tRNA + 4 EF-Tu.GTP.leu2-tRNA + 7 EF-Tu.GTP.leuU-tRNA + 2 EF-Tu.GTP.leuW-tRNA + 4 EF-Tu.GTP.leuZ-tRNA + 11 EF-Tu.GTP.lys1-tRNA + 8 EF-Tu.GTP.met1-tRNA + 7 EF-Tu.GTP.phe1-tRNA + 9 EF-Tu.GTP.pro1-tRNA + 1 EF-Tu.GTP.pro2-tRNA + 2 EF-Tu.GTP.proL-tRNA + 1 EF-Tu.GTP.proM-tRNA + 3 EF-Tu.GTP.ser3-tRNA + 2 EF-Tu.GTP.serT-tRNA + 6 EF-Tu.GTP.serV-tRNA + 8 EF-Tu.GTP.thr1-tRNA + 2 EF-Tu.GTP.thr2-tRNA + 5 EF-Tu.GTP.thr3-tRNA + 1 EF-Tu.GTP.thrU-tRNA + 7 EF-Tu.GTP.trpT-tRNA + 13 EF-Tu.GTP.tyr1-tRNA + 10 EF-Tu.GTP.val1-tRNA + 1 EF-Tu.GTP.val2-tRNA + 5 EF-Tu.GTP.val3-tRNA + 1815 mg2 + 1 rib_ini_b0503_1 --> 1 rib_70_elo1_b0503_1_cplx	irreversible	Translation	0.00E+00	1.00E+09	0	1.21E+02	1.21E+02	0	2.11E+02	210.8787	0	4.50E+00	4.496
tl_elo_b0503_1_rib2	Translation elongation 2 b0503 1 ribosome(s)	362 h2o + 1 rib_70_elo1_b0503_1_cplx --> 362 EF-G.GDP + 362 EF-Tu.GDP + 32 ala1_tRNA + 10 ala2_tRNA + 22 arg1_tRNA + 1 argU_tRNA + 1 argW_tRNA + 3 argX_tRNA + 10 asn1_tRNA + 16 asp1_tRNA + 7 cysT_tRNA + 1 fmet_tRNA + 16 gln1_tRNA + 10 gln2_tRNA + 31 glu1_tRNA + 22 gly1_tRNA + 2 glyT_tRNA + 3 glyU_tRNA + 724 h + 13 hisR_tRNA + 16 ile1_tRNA + 1 ile2_tRNA + 27 leu1_tRNA + 4 leu2_tRNA + 7 leuU_tRNA + 2 leuW_tRNA + 4 leuZ_tRNA + 11 lys1_tRNA + 8 met1_tRNA + 1815 mg2 + 7 phe1_tRNA + 724 pi + 9 pro1_tRNA + 1 pro2_tRNA + 2 proL_tRNA + 1 proM_tRNA + 1 rib_70_elo2_b0503_1_cplx + 3 ser3_tRNA + 2 serT_tRNA + 6 serV_tRNA + 8 thr1_tRNA + 2 thr2_tRNA + 5 thr3_tRNA + 1 thrU_tRNA + 7 trpT_tRNA + 13 tyr1_tRNA + 10 val1_tRNA + 1 val2_tRNA + 5 val3_tRNA	irreversible	Translation	0.00E+00	1.00E+09	0	1.21E+02	1.21E+02	0	2.11E+02	210.8787	0	4.50E+00	4.496

tl_elo_b0503_21_rib1	Translation elongation 1 b0503 21 ribosome(s)	7623 EF-G.GTP + 672 EF-Tu.GTP.ala1-tRNA + 210 EF-Tu.GTP.ala2 tRNA + 462 EF-Tu.GTP.arg1-tRNA + 21 EF-Tu.GTP.argU-tRNA + 21 EF-Tu.GTP.argW-tRNA + 84 EF-Tu.GTP.argX-tRNA + 210 EF-Tu.GTP.asn1-tRNA + 336 EF-Tu.GTP.asp1-tRNA + 147 EF-Tu.GTP.cysT-tRNA + 336 EF-Tu.GTP.gln1-tRNA + 210 EF-Tu.GTP.gln2-tRNA + 651 EF-Tu.GTP.glu1-tRNA + 462 EF-Tu.GTP.gly1-tRNA + 42 EF-Tu.GTP.glyT-tRNA + 63 EF-Tu.GTP.glyU-tRNA + 273 EF-Tu.GTP.hisR-tRNA + 336 EF-Tu.GTP.ile1-tRNA + 21 EF-Tu.GTP.ile2-tRNA + 567 EF-Tu.GTP.leu1 tRNA + 84 EF-Tu.GTP.leu2-tRNA + 147 EF-Tu.GTP.leuU-tRNA + 42 EF-Tu.GTP.leuW-tRNA + 84 EF-Tu.GTP.leuZ-tRNA + 231 EF-Tu.GTP.lys1-tRNA + 168 EF-Tu.GTP.met1-tRNA + 147 EF-Tu.GTP.phe1-tRNA + 189 EF-Tu.GTP.pro1-tRNA + 21 EF-Tu.GTP.pro2-tRNA + 42 EF-Tu.GTP.proL-tRNA + 21 EF-Tu.GTP.proM-tRNA + 63 EF-Tu.GTP.ser3-tRNA + 42 EF-Tu.GTP.serT-tRNA + 126 EF-Tu.GTP.serV-tRNA + 168 EF-Tu.GTP.thr1-tRNA + 42 EF-Tu.GTP.thr2-tRNA + 105 EF-Tu.GTP.thr3-tRNA + 21 EF-Tu.GTP.thrU-tRNA + 147 EF-Tu.GTP.trpT-tRNA + 273 EF-Tu.GTP.tyr1-tRNA + 210 EF-Tu.GTP.val1-tRNA + 21 EF-Tu.GTP.val2-tRNA + 105 EF-Tu.GTP.val3-tRNA + 38115 mg2 + 1 rib_ini_b0503_21 --> 1 rib_70_e[illegible]	irreversible	Translation	0.00E+00	1.00E+09	0	1.21E+02	1.21E+02	0	2.11E+02	210.8787	0	4.50E+00	4.496
tl_elo_b0503_21_rib2	Translation elongation 2 b0503 21 ribosome(s)	7602 h2o + 1 rib_70_elo1_b0503_21_cplx --> 7602 EF-G.GDP + 7602 EF-Tu.GDP + 672 ala1_tRNA + 210 ala2_tRNA + 462 arg1_tRNA + 21 argU_tRNA + 21 argW_tRNA + 63 argX_tRNA + 21[illegible] asn1_tRNA + 336 asp1_tRNA + 147 cysT_tRNA + 21 fmet_tRNA + 336 gln1_tRNA + 210 gln2_tRNA + 651 glu1_tRNA + 462 gly1_tRNA + 42 glyT_tRNA + 63 glyU_tRNA + 15204 h + 273 hisR_tRNA + 336 ile1_tRNA + 21 ile2_tRNA + 567 leu1_tRNA + 84 leu2_tRNA + 147 leuU_tRNA + 42 leuW_tRNA + 84 leuZ_tRNA + 231 lys1_tRNA + 16[illegible] met1_tRNA + 38115 mg2 + 147 phe1_tRNA + 15204 pi + 189 pro1_tRNA + 21 pro2_tRNA + 42 proL_tRNA + 21 proM_tRNA + 1 rib_70_elo2_b0503_21_cplx + 63 ser3_tRNA + 42 serT_tRNA + 126 serV_tRNA + 168 thr1_tRNA + 42 thr2_tRNA + 105 thr3_tRNA + 21 thrU_tRNA + 147 trpT_tRNA + 273 tyr1_tRNA + 210 val1_tRNA + 21 val2_tRNA + 105 val3_tRNA	irreversible	Translation	0.00E+00	1.00E+09	0	1.21E+02	1.21E+02	0	2.11E+02	210.8787	0	4.50E+00	4.496
tl_elo_b0526_13_rib1	Translation elongation 1 b0526 13 ribosome(s)	5980 EF-G.GTP + 533 EF-Tu.GTP.ala1-tRNA + 104 EF-Tu.GTP.ala2 tRNA + 390 EF-Tu.GTP.arg1-tRNA + 234 EF-Tu.GTP.asn1-tRNA + 494 EF-Tu.GTP.asp1-tRNA + 65 EF-Tu.GTP.cysT-tRNA + 130 EF-Tu.GTP.gln1-tRNA + 78 EF-Tu.GTP.gln2-tRNA + 442 EF-Tu.GTP.glu1-tRNA + 325 EF-Tu.GTP.gly1-tRNA + 13 EF-Tu.GTP.glyT-tRNA + 65 EF-Tu.GTP.glyU-tRNA + 208 EF-Tu.GTP.hisR-tRNA + 273 EF-Tu.GTP.ile1-tRNA + 312 EF-Tu.GTP.leu1-tRNA + 39 EF-Tu.GTP.leu2-tRNA + 91 EF-Tu.GTP.leuU-tRNA + 13 EF-Tu.GTP.leuW-tRNA + 13 EF-Tu.GTP.leuZ-tRNA + 299 EF-Tu.GTP.lys1-tRNA + 260 EF-Tu.GTP.met1-tRNA + 247 EF-Tu.GTP.phe1-tRNA + 130 EF-Tu.GTP.pro1-tRNA + 39 EF-Tu.GTP.pro2-tRNA + 26 EF-Tu.GTP.proM-tRNA + 52 EF-Tu.GTP.ser1-tRNA + 26 EF-Tu.GTP.ser2-tRNA + 78 EF-Tu.GTP.ser3-tRNA + 13 EF-Tu.GTP.serT-tRNA + 78 EF-Tu.GTP.serV-tRNA + 143 EF-Tu.GTP.thr1-tRNA + 52 EF-Tu.GTP.thr2-tRNA + 13 EF-Tu.GTP.thr3-tRNA + 26 EF-Tu.GTP.thrU-tRNA + 91 EF-Tu.GTP.trpT-tRNA + 208 EF-Tu.GTP.tyr1-tRNA + 247 EF-Tu.GTP.val1-tRNA + 39 EF-Tu.GTP.val2-tRNA + 91 EF-Tu.GTP.val3-tRNA + 29900 mg2 + 1 rib_ini_b0526_13 --> 1 rib_70_elo1_b0526_13_cplx	irreversible	Translation	0.00E+00	1.00E+09	0	9.50E+01	9.50E+01	0	1.65E+02	165.2503	0	3.52E+00	3.516
tl_elo_b0526_13_rib2	Translation elongation 2 b0526 13 ribosome(s)	5967 h2o + 1 rib_70_elo1_b0526_13_cplx --> 5967 EF-G.GDP + 5967 EF-Tu.GDP + 533 ala1_tRNA + 104 ala2_tRNA + 390 arg1_tRNA + 234 asn1_tRNA + 494 asp1_tRNA + 65 cysT_tRNA + 13 fmet_tRNA + 130 gln1_tRNA + 78 gln2_tRNA + 442 glu1_tRNA + 325 gly1_tRNA + 13 glyT_tRNA + 65 glyU_tRNA + 11934 h + 208 hisR_tRNA + 273 ile1_tRNA + 312 leu1_tRNA + 39 leu2_tRNA + 91 leuU_tRNA + 13 leuW_tRNA + 13 leuZ_tRNA + 286 lys1_tRNA + 26[illegible] met1_tRNA + 29900 mg2 + 247 phe1_tRNA + 11934 pi + 130 pro1_tRNA + 39 pro2_tRNA + 26 proM_tRNA + 1 rib_70_elo2_b0526_13_cplx + 52 ser1_tRNA + 26 ser2_tRNA + 78 ser3_tRNA + 13 serT_tRNA + 78 serV_tRNA + 143 thr1_tRNA + 52 thr2_tRNA + 13 thr3_tRNA + 26 thrU_tRNA + 91 trpT_tRNA + 208 tyr1_tRNA + 247 val1_tRNA + 39 val2_tRNA + 91 val3_tRNA	irreversible	Translation	0.00E+00	1.00E+09	0	9.50E+01	9.50E+01	0	1.65E+02	165.2503	0	3.52E+00	3.516
tl_elo_b0526_1_rib1	Translation elongation 1 b0526 1 ribosome(s)	460 EF-G.GTP + 41 EF-Tu.GTP.ala1-tRNA + 8 EF-Tu.GTP.ala2-tRNA + 30 EF-Tu.GTP.arg1-tRNA + 18 EF-Tu.GTP.asn1-tRNA + 38 EF-Tu.GTP.asp1-tRNA + 5 EF-Tu.GTP.cysT-tRNA + 10 EF-Tu.GTP.gln1-tRNA + 6 EF-Tu.GTP.gln2-tRNA + 34 EF-Tu.GTP.glu1-tRNA + 25 EF-Tu.GTP.gly1-tRNA + 1 EF-Tu.GTP.glyT-tRNA + 5 EF-Tu.GTP.glyU-tRNA + 16 EF-Tu.GTP.hisR-tRNA + 21 EF-Tu.GTP.ile1 tRNA + 24 EF-Tu.GTP.leu1-tRNA + 3 EF-Tu.GTP.leu2-tRNA + 7 EF-Tu.GTP.leuU-tRNA + 1 EF-Tu.GTP.leuW-tRNA + 1 EF-Tu.GTP.leuZ-tRNA + 23 EF-Tu.GTP.lys1-tRNA + 20 EF-Tu.GTP.met1-tRNA + 19 EF-Tu.GTP.phe1-tRNA + 10 EF-Tu.GTP.pro1-tRNA + 3 EF-Tu.GTP.pro2-tRNA + 2 EF-Tu.GTP.proM-tRNA + 4 EF-Tu.GTP.ser1-tRNA + 2 EF-Tu.GTP.ser2-tRNA + 6 EF-Tu.GTP.ser3-tRNA + 1 EF-Tu.GTP.serT-tRNA + 6 EF-Tu.GTP.serV-tRNA + 11 EF-Tu.GTP.thr1-tRNA + 4 EF-Tu.GTP.thr2-tRNA + 1 EF-Tu.GTP.thr3-tRNA + 2 EF-Tu.GTP.thrU-tRNA + 7 EF-Tu.GTP.trpT-tRNA + 16 EF-Tu.GTP.tyr1-tRNA + 19 EF-Tu.GTP.val1-tRNA + 3 EF-Tu.GTP.val2-tRNA + 7 EF-Tu.GTP.val3-tRNA + 2300 mg2 + 1 rib_ini_b0526_1 --> 1 rib_70_elo1_b0526_1_cplx	irreversible	Translation	0.00E+00	1.00E+09	0	9.50E+01	9.50E+01	0	1.65E+02	165.2503	0	3.52E+00	3.516
tl_elo_b0526_1_rib2	Translation elongation 2 b0526 1 ribosome(s)	459 h2o + 1 rib_70_elo1_b0526_1_cplx --> 459 EF-G.GDP + 459 EF-Tu.GDP + 41 ala1_tRNA + 8 ala2_tRNA + 30 arg1_tRNA + 18 asn1_tRNA + 38 asp1_tRNA + 5 cysT_tRNA + 1 fmet_tRNA + 10 gln1_tRNA + 6 gln2_tRNA + 34 glu1_tRNA + 25 gly1_tRNA + 1 glyT_tRNA + 5 glyU_tRNA + 918 h + 16 hisR_tRNA + 21 ile1_tRNA + 24 leu1_tRNA + 3 leu2_tRNA + 7 leuU_tRNA + 1 leuW_tRNA + 1 leuZ_tRNA + 22 lys1_tRNA + 20 met1_tRNA + 2300 mg2 + 19 phe1_tRNA + 918 pi + 10 pro1_tRNA + 3 pro2_tRNA + 2 proM_tRNA + 1 rib_70_elo2_b0526_1_cplx + 4 ser1_tRNA + 2 ser2_tRNA + 6 ser3_tRNA + 1 serT_tRNA + 6 serV_tRNA + 11 thr1_tRNA + 4 thr2_tRNA + 1 thr3_tRNA + 2 thrU_tRNA + 7 trpT_tRNA + 16 tyr1_tRNA + 19 val1_tRNA + 3 val2_tRNA + 7 val3_tRNA	irreversible	Translation	0.00E+00	1.00E+09	0	9.50E+01	9.50E+01	0	1.65E+02	165.2503	0	3.52E+00	3.516

tl_elo_b0526_27_rib1	Translation elongation 1 b0526 27 ribosome(s)	12420 EF-G.GTP + 1107 EF-Tu.GTP.ala1-tRNA + 216 EF-Tu.GTP.ala2-tRNA + 810 EF-Tu.GTP.arg1-tRNA + 486 EF-Tu.GTP.asn1-tRNA + 1026 EF-Tu.GTP.asp1-tRNA + 135 EF-Tu.GTP.cysT-tRNA + 270 EF-Tu.GTP.gln1-tRNA + 162 EF-Tu.GTP.gln2-tRNA + 918 EF-Tu.GTP.glu1-tRNA + 675 EF-Tu.GTP.gly1-tRNA + 27 EF-Tu.GTP.glyT-tRNA + 135 EF-Tu.GTP.glyU-tRNA + 432 EF-Tu.GTP.hisR-tRNA + 567 EF-Tu.GTP.ile1-tRNA + 648 EF-Tu.GTP.leu1-tRNA + 81 EF-Tu.GTP.leu2-tRNA + 189 EF-Tu.GTP.leuU-tRNA + 27 EF-Tu.GTP.leuW-tRNA + 27 EF-Tu.GTP.leuZ-tRNA + 621 EF-Tu.GTP.lys1-tRNA + 540 EF-Tu.GTP.met1-tRNA + 513 EF-Tu.GTP.phe1-tRNA + 270 EF-Tu.GTP.pro1-tRNA + 81 EF-Tu.GTP.pro2-tRNA + 54 EF-Tu.GTP.proM-tRNA + 108 EF-Tu.GTP.ser1-tRNA + 54 EF-Tu.GTP.ser2-tRNA + 162 EF-Tu.GTP.ser3-tRNA + 27 EF-Tu.GTP.serT-tRNA + 162 EF-Tu.GTP.serV-tRNA + 297 EF-Tu.GTP.thr1-tRNA + 108 EF-Tu.GTP.thr2-tRNA + 27 EF-Tu.GTP.thr3-tRNA + 54 EF-Tu.GTP.thrU-tRNA + 189 EF-Tu.GTP.trpT-tRNA + 432 EF-Tu.GTP.tyr1-tRNA + 513 EF-Tu.GTP.val1-tRNA + 81 EF-Tu.GTP.val2-tRNA + 189 EF-Tu.GTP.val3-tRNA + 62100 mg2 + 1 rib_ini_b0526_27 --> 1 rib_70_elo1_b0526_27_cplx	irreversible	Translation	0.00E+00	1.00E+09	0	9.50E+01	9.50E+01	0	1.65E+02	165.2503	0	3.52E+00	3.516
tl_elo_b0526_27_rib2	Translation elongation 2 b0526 27 ribosome(s)	12393 h2o + 1 rib_70_elo1_b0526_27_cplx --> 12393 EF-G.GDP + 12393 EF-Tu.GDP + 1107 ala1_tRNA + 216 ala2_tRNA + 810 arg1_tRNA + 486 asn1_tRNA + 1026 asp1_tRNA + 135 cysT_tRNA + 27 fmet_tRNA + 270 gln1_tRNA + 162 gln2_tRNA + 918 glu1_tRNA + 675 gly1_tRNA + 27 glyT_tRNA + 135 glyU_tRNA + 24786 h + 432 hisR_tRNA + 567 ile1_tRNA + 648 leu1_tRNA + 81 leu2_tRNA + 189 leuU_tRNA + 27 leuW_tRNA + 27 leuZ_tRNA + 594 lys1_tRNA + 540 met1_tRNA + 62100 mg2 + 513 phe1_tRNA + 24786 pi + 270 pro1_tRNA + 81 pro2_tRNA + 54 proM_tRNA + 1 rib_70_elo2_b0526_27_cplx + 108 ser1_tRNA + 54 ser2_tRNA + 162 ser3_tRNA + 27 serT_tRNA + 162 serV_tRNA + 297 thr1_tRNA + 108 thr2_tRNA + 27 thr3_tRNA + 54 thrU_tRNA + 189 trpT_tRNA + 432 tyr1_tRNA + 513 val1_tRNA + 81 val2_tRNA + 189 val3_tRNA	irreversible	Translation	0.00E+00	1.00E+09	0	9.50E+01	9.50E+01	0	1.65E+02	165.2503	0	3.52E+00	3.516
tl_elo_b0638_11_rib1	Translation elongation 1 b0638 11 ribosome(s)	2222 EF-G.GTP + 143 EF-Tu.GTP.ala1-tRNA + 55 EF-Tu.GTP.ala2-tRNA + 143 EF-Tu.GTP.arg1-tRNA + 11 EF-Tu.GTP.argW-tRNA + 11 EF-Tu.GTP.argX-tRNA + 110 EF-Tu.GTP.asn1-tRNA + 99 EF-Tu.GTP.asp1-tRNA + 33 EF-Tu.GTP.cysT-tRNA + 88 EF-Tu.GTP.gln1-tRNA + 77 EF-Tu.GTP.gln2-tRNA + 154 EF-Tu.GTP.glu1-tRNA + 99 EF-Tu.GTP.gly1-tRNA + 11 EF-Tu.GTP.glyT-tRNA + 22 EF-Tu.GTP.glyU-tRNA + 121 EF-Tu.GTP.hisR-tRNA + 132 EF-Tu.GTP.ile1-tRNA + 11 EF-Tu.GTP.ile2-tRNA + 99 EF-Tu.GTP.leu1-tRNA + 77 EF-Tu.GTP.leuU-tRNA + 22 EF-Tu.GTP.leuW-tRNA + 55 EF-Tu.GTP.leuZ-tRNA + 11 EF-Tu.GTP.lys1-tRNA + 55 EF-Tu.GTP.met1-tRNA + 99 EF-Tu.GTP.phe1-tRNA + 22 EF-Tu.GTP.pro2-tRNA + 44 EF-Tu.GTP.proL-tRNA + 11 EF-Tu.GTP.ser1-tRNA + 11 EF-Tu.GTP.ser3-tRNA + 99 EF-Tu.GTP.serV-tRNA + 33 EF-Tu.GTP.thr1-tRNA + 44 EF-Tu.GTP.thr3-tRNA + 66 EF-Tu.GTP.trpT-tRNA + 33 EF-Tu.GTP.tyr1-tRNA + 33 EF-Tu.GTP.val1-tRNA + 44 EF-Tu.GTP.val2-tRNA + 44 EF-Tu.GTP.val3-tRNA + 11110 mg2 + 1 rib_ini_b0638_11 --> 1 rib_70_elo1_b0638_11_cplx	irreversible	Translation	0.00E+00	1.00E+09	0	2.84E+01	2.84E+01	0	4.59E+01	45.89653	0	5.66E+00	5.656
tl_elo_b0638_11_rib2	Translation elongation 2 b0638 11 ribosome(s)	2211 h2o + 1 rib_70_elo1_b0638_11_cplx --> 2211 EF-G.GDP + 2211 EF-Tu.GDP + 132 ala1_tRNA + 55 ala2_tRNA + 143 arg1_tRNA + 11 argW_tRNA + 11 argX_tRNA + 110 asn1_tRNA + 99 asp1_tRNA + 33 cysT_tRNA + 11 fmet_tRNA + 88 gln1_tRNA + 77 gln2_tRNA + 154 glu1_tRNA + 99 gly1_tRNA + 11 glyT_tRNA + 22 glyU_tRNA + 4422 h + 121 hisR_tRNA + 132 ile1_tRNA + 11 ile2_tRNA + 99 leu1_tRNA + 77 leuU_tRNA + 22 leuW_tRNA + 55 leuZ_tRNA + 11 lys1_tRNA + 55 met1_tRNA + 11110 mg2 + 99 phe1_tRNA + 4422 pi + 22 pro2_tRNA + 44 proL_tRNA + 1 rib_70_elo2_b0638_11_cplx + 11 ser1_tRNA + 11 ser3_tRNA + 99 serV_tRNA + 33 thr1_tRNA + 44 thr3_tRNA + 66 trpT_tRNA + 33 tyr1_tRNA + 33 val1_tRNA + 44 val2_tRNA + 44 val3_tRNA	irreversible	Translation	0.00E+00	1.00E+09	0	2.84E+01	2.84E+01	0	4.59E+01	45.89653	0	5.66E+00	5.656
tl_elo_b0638_1_rib1	Translation elongation 1 b0638 1 ribosome(s)	202 EF-G.GTP + 13 EF-Tu.GTP.ala1-tRNA + 5 EF-Tu.GTP.ala2-tRNA + 13 EF-Tu.GTP.arg1-tRNA + 1 EF-Tu.GTP.argW-tRNA + 1 EF-Tu.GTP.argX-tRNA + 10 EF-Tu.GTP.asn1-tRNA + 9 EF-Tu.GTP.asp1-tRNA + 3 EF-Tu.GTP.cysT-tRNA + 8 EF-Tu.GTP.gln1-tRNA + 7 EF-Tu.GTP.gln2-tRNA + 14 EF-Tu.GTP.glu1-tRNA + 9 EF-Tu.GTP.gly1-tRNA + 1 EF-Tu.GTP.glyT-tRNA + 2 EF-Tu.GTP.glyU-tRNA + 11 EF-Tu.GTP.hisR-tRNA + 12 EF-Tu.GTP.ile1-tRNA + 1 EF-Tu.GTP.ile2-tRNA + 9 EF-Tu.GTP.leu1-tRNA + 7 EF-Tu.GTP.leuU-tRNA + 2 EF-Tu.GTP.leuW-tRNA + 5 EF-Tu.GTP.leuZ-tRNA + 1 EF-Tu.GTP.lys1-tRNA + 5 EF-Tu.GTP.met1-tRNA + 9 EF-Tu.GTP.phe1-tRNA + 2 EF-Tu.GTP.pro2-tRNA + 4 EF-Tu.GTP.proL-tRNA + 1 EF-Tu.GTP.ser1-tRNA + 1 EF-Tu.GTP.ser3-tRNA + 9 EF-Tu.GTP.serV-tRNA + 3 EF-Tu.GTP.thr1-tRNA + 4 EF-Tu.GTP.thr3-tRNA + 6 EF-Tu.GTP.trpT-tRNA + 3 EF-Tu.GTP.tyr1-tRNA + 3 EF-Tu.GTP.val1-tRNA + 4 EF-Tu.GTP.val2-tRNA + 4 EF-Tu.GTP.val3-tRNA + 1010 mg2 + 1 rib_ini_b0638_1 --> 1 rib_70_elo1_b0638_1_cplx	irreversible	Translation	0.00E+00	1.00E+09	0	2.84E+01	2.84E+01	0	4.59E+01	45.89653	0	5.66E+00	5.656
tl_elo_b0638_1_rib2	Translation elongation 2 b0638 1 ribosome(s)	201 h2o + 1 rib_70_elo1_b0638_1_cplx --> 201 EF-G.GDP + 201 EF-Tu.GDP + 12 ala1_tRNA + 5 ala2_tRNA + 13 arg1_tRNA + 1 argW_tRNA + 1 argX_tRNA + 10 asn1_tRNA + 9 asp1_tRNA + 3 cysT_tRNA + 1 fmet_tRNA + 8 gln1_tRNA + 7 gln2_tRNA + 14 glu1_tRNA + 9 gly1_tRNA + 1 glyT_tRNA + 2 glyU_tRNA + 402 h + 11 hisR_tRNA + 12 ile1_tRNA + 1 ile2_tRNA + 9 leu1_tRNA + 7 leuU_tRNA + 2 leuW_tRNA + 5 leuZ_tRNA + 1 lys1_tRNA + 5 met1_tRNA + 1010 mg2 + 9 phe1_tRNA + 402 pi + 2 pro2_tRNA + 4 proL_tRNA + 1 rib_70_elo2_b0638_1_cplx + 1 ser1_tRNA + 1 ser3_tRNA + 9 serV_tRNA + 3 thr1_tRNA + 4 thr3_tRNA + 6 trpT_tRNA + 3 tyr1_tRNA + 3 val1_tRNA + 4 val2_tRNA + 4 val3_tRNA	irreversible	Translation	0.00E+00	1.00E+09	0	2.84E+01	2.84E+01	0	4.59E+01	45.89653	0	5.66E+00	5.656

tl_elo_b0638_5_rib1	Translation elongation 1 b0638 5 ribosome(s)	1010 EF-G.GTP + 65 EF-Tu.GTP.ala1-tRNA + 25 EF-Tu.GTP.ala2-tRNA + 65 EF-Tu.GTP.arg1-tRNA + 5 EF-Tu.GTP.argW-tRNA + 5 EF-Tu.GTP.argX-tRNA + 50 EF-Tu.GTP.asn1-tRNA + 45 EF-Tu.GTP.asp1-tRNA + 15 EF-Tu.GTP.cysT-tRNA + 40 EF-Tu.GTP.gln1-tRNA + 35 EF-Tu.GTP.gln2-tRNA + 70 EF-Tu.GTP.glu1-tRNA + 45 EF-Tu.GTP.gly1-tRNA + 5 EF-Tu.GTP.glyT-tRNA + 10 EF-Tu.GTP.glyU-tRNA + 55 EF-Tu.GTP.hisR-tRNA + 60 EF-Tu.GTP.ile1-tRNA + 5 EF-Tu.GTP.ile2-tRNA + 45 EF-Tu.GTP.leu1-tRNA + 35 EF-Tu.GTP.leuU-tRNA + 10 EF-Tu.GTP.leuW-tRNA + 25 EF-Tu.GTP.leuZ-tRNA + 5 EF-Tu.GTP.lys1 tRNA + 25 EF-Tu.GTP.met1-tRNA + 45 EF-Tu.GTP.phe1-tRNA + 10 EF-Tu.GTP.pro2-tRNA + 20 EF-Tu.GTP.proL-tRNA + 5 EF-Tu.GTP.ser1-tRNA + 5 EF-Tu.GTP.ser3-tRNA + 45 EF-Tu.GTP.serV tRNA + 15 EF-Tu.GTP.thr1-tRNA + 20 EF-Tu.GTP.thr3-tRNA + 30 EF-Tu.GTP.trpT-tRNA + 15 EF-Tu.GTP.tyr1-tRNA + 15 EF-Tu.GTP.val1-tRNA + 20 EF-Tu.GTP.val2-tRNA + 20 EF-Tu.GTP.val3 tRNA + 5050 mg2 + 1 rib_ini_b0638_5 --> 1 rib_70_elo1_b0638_5_cplx	irreversible	Translation		0.00E+00	1.00E+09	0	2.84E+01	2.84E+01	0	4.59E+01	45.89653	0	5.66E+00	5.656
tl_elo_b0638_5_rib2	Translation elongation 2 b0638 5 ribosome(s)	1005 h2o + 1 rib_70_elo1_b0638_5_cplx --> 1005 EF-G.GDP + 1005 EF-Tu.GDP + 60 ala1_tRNA + 25 ala2_tRNA + 65 arg1_tRNA + 5 argW_tRNA + 5 argX_tRNA + 50 asn1_tRNA + 45 asp1_tRNA + 15 cysT_tRNA + 5 fmet_tRNA + 40 gln1_tRNA + 35 gln2_tRNA + 70 glu1_tRNA + 45 gly1_tRNA + 5 glyT_tRNA + 10 glyU_tRNA + 2010 h + 55 hisR_tRNA + 60 ile1_tRNA + 5 ile2_tRNA + 45 leu1_tRNA + 35 leuU_tRNA + 10 leuW_tRNA + 25 leuZ_tRNA + 5 lys1_tRNA + 25 met1_tRNA + 5050 mg2 + 45 phe1_tRNA + 2010 pi + 10 pro2_tRNA + 20 proL_tRNA + 1 rib_70_elo2_b0638_5_cplx + 5 ser1_tRNA + 5 ser3_tRNA + 45 serV_tRNA + 15 thr1_tRNA + 20 thr3_tRNA + 30 trpT_tRNA + 15 tyr1_tRNA + 15 val1_tRNA + 20 val2_tRNA + 20 val3_tRNA	irreversible	Translation		0.00E+00	1.00E+09	0	2.84E+01	2.84E+01	0	4.59E+01	45.89653	0	5.66E+00	5.656
tl_elo_b0639_12_rib1	Translation elongation 1 b0639 12 ribosome(s)	2544 EF-G.GTP + 120 EF-Tu.GTP.ala1-tRNA + 36 EF-Tu.GTP.ala2-tRNA + 96 EF-Tu.GTP.arg1-tRNA + 36 EF-Tu.GTP.argX-tRNA + 120 EF-Tu.GTP.asn1-tRNA + 108 EF-Tu.GTP.asp1-tRNA + 24 EF-Tu.GTP.cysT-tRNA + 84 EF-Tu.GTP.gln1-tRNA + 96 EF-Tu.GTP.gln2-tRNA + 204 EF-Tu.GTP.glu1-tRNA + 108 EF-Tu.GTP.gly1-tRNA + 12 EF-Tu.GTP.glyT-tRNA + 96 EF-Tu.GTP.hisR-tRNA + 144 EF-Tu.GTP.ile1-tRNA + 12 EF-Tu.GTP.ile2-tRNA + 132 EF-Tu.GTP.leu1-tRNA + 72 EF-Tu.GTP.leu2-tRNA + 72 EF-Tu.GTP.leuU-tRNA + 24 EF-Tu.GTP.leuW-tRNA + 36 EF-Tu.GTP.leuZ-tRNA + 84 EF-Tu.GTP.lys1-tRNA + 24 EF-Tu.GTP.met1-tRNA + 72 EF-Tu.GTP.phe1-tRNA + 132 EF-Tu.GTP.pro1-tRNA + 36 EF-Tu.GTP.pro2-tRNA + 36 EF-Tu.GTP.proL-tRNA + 36 EF-Tu.GTP.proM-tRNA + 24 EF-Tu.GTP.ser2-tRNA + 12 EF-Tu.GTP.ser3-tRNA + 24 EF-Tu.GTP.serT-tRNA + 12 EF-Tu.GTP.serV-tRNA + 48 EF-Tu.GTP.thr1-tRNA + 36 EF-Tu.GTP.thr2 tRNA + 48 EF-Tu.GTP.thr3-tRNA + 36 EF-Tu.GTP.thrU-tRNA + 48 EF-Tu.GTP.trpT-tRNA + 108 EF-Tu.GTP.tyr1-tRNA + 60 EF-Tu.GTP.val1-tRNA + 24 EF-Tu.GTP.val2-tRNA + 12 EF-Tu.GTP.val3 tRNA + 12720 mg2 + 1 rib_ini_b0639_12 --> 1 rib_70_elo1_b0639_12_cplx	irreversible	Translation		0.00E+00	1.00E+09	0	2.90E+01	2.90E+01	0	4.65E+01	46.51653	0	6.28E+00	6.276
tl_elo_b0639_12_rib2	Translation elongation 2 b0639 12 ribosome(s)	2532 h2o + 1 rib_70_elo1_b0639_12_cplx --> 2532 EF-G.GDP + 2532 EF-Tu.GDP + 120 ala1_tRNA + 36 ala2_tRNA + 84 arg1_tRNA + 36 argX_tRNA + 120 asn1_tRNA + 108 asp1_tRNA + 24 cysT_tRNA + 12 fmet_tRNA + 84 gln1_tRNA + 96 gln2_tRNA + 204 glu1_tRNA + 108 gly1_tRNA + 12 glyT_tRNA + 5064 h + 96 hisR_tRNA + 144 ile1_tRNA + 12 ile2_tRNA + 132 leu1_tRNA + 72 leu2_tRNA + 72 leuU_tRNA + 24 leuW_tRNA + 36 leuZ_tRNA + 84 lys1_tRNA + 24 met1_tRNA + 12720 mg2 + 72 phe1_tRNA + 5064 pi + 132 pro1_tRNA + 36 pro2_tRNA + 36 proL_tRNA + 36 proM_tRNA + 1 rib_70_elo2_b0639_12_cplx + 24 ser2_tRNA + 12 ser3_tRNA + 24 serT_tRNA + 12 serV_tRNA + 48 thr1_tRNA + 36 thr2_tRNA + 48 thr3_tRNA + 36 thrU_tRNA + 48 trpT_tRNA + 108 tyr1_tRNA + 60 val1_tRNA + 24 val2_tRNA + 12 val3_tRNA	irreversible	Translation		0.00E+00	1.00E+09	0	2.90E+01	2.90E+01	0	4.65E+01	46.51653	0	6.28E+00	6.276
tl_elo_b0639_1_rib1	Translation elongation 1 b0639 1 ribosome(s)	212 EF-G.GTP + 10 EF-Tu.GTP.ala1-tRNA + 3 EF-Tu.GTP.ala2-tRNA + 8 EF-Tu.GTP.arg1-tRNA + 3 EF-Tu.GTP.argX-tRNA + 10 EF Tu.GTP.asn1-tRNA + 9 EF-Tu.GTP.asp1-tRNA + 2 EF-Tu.GTP.cysT-tRNA + 7 EF-Tu.GTP.gln1-tRNA + 8 EF-Tu.GTP.gln2-tRNA + 17 EF-Tu.GTP.glu1-tRNA + 9 EF-Tu.GTP.gly1-tRNA + 1 EF-Tu.GTP.glyT-tRNA + 8 EF-Tu.GTP.hisR-tRNA + 12 EF-Tu.GTP.ile1-tRNA + 1 EF-Tu.GTP.ile2-tRNA + 11 EF-Tu.GTP.leu1-tRNA + 6 EF-Tu.GTP.leu2-tRNA + 6 EF-Tu.GTP.leuU-tRNA + 2 EF-Tu.GTP.leuW-tRNA + 3 EF-Tu.GTP.leuZ-tRNA + 7 EF-Tu.GTP.lys1-tRNA + 2 EF-Tu.GTP.met1-tRNA + 6 EF-Tu.GTP.phe1-tRNA + 11 EF-Tu.GTP.pro1-tRNA + 3 EF Tu.GTP.pro2-tRNA + 3 EF-Tu.GTP.proL-tRNA + 3 EF-Tu.GTP.proM-tRNA + 2 EF-Tu.GTP.ser2-tRNA + 1 EF-Tu.GTP.ser3-tRNA + 2 EF-Tu.GTP.serT-tRNA + 1 EF-Tu.GTP.serV-tRNA + 4 EF-Tu.GTP.thr1-tRNA + 3 EF-Tu.GTP.thr2-tRNA + 4 EF-Tu.GTP.thr3-tRNA + 3 EF-Tu.GTP.thrU-tRNA + 4 EF-Tu.GTP.trpT-tRNA + 9 EF-Tu.GTP.tyr1-tRNA + 5 EF-Tu.GTP.val1-tRNA + 2 EF-Tu.GTP.val2-tRNA + 1 EF-Tu.GTP.val3-tRNA + 1060 mg2 + 1 rib_ini_b0639_1 --> 1 rib_70_elo1_b0639_1_cplx	irreversible	Translation		0.00E+00	1.00E+09	0	2.90E+01	2.90E+01	0	4.65E+01	46.51653	0	6.28E+00	6.276
tl_elo_b0639_1_rib2	Translation elongation 2 b0639 1 ribosome(s)	211 h2o + 1 rib_70_elo1_b0639_1_cplx --> 211 EF-G.GDP + 211 EF-Tu.GDP + 10 ala1_tRNA + 3 ala2_tRNA + 7 arg1_tRNA + 3 argX_tRNA + 10 asn1_tRNA + 9 asp1_tRNA + 2 cysT_tRNA + 1 fmet_tRNA + 7 gln1_tRNA + 8 gln2_tRNA + 17 glu1_tRNA + 9 gly1_tRNA + 1 glyT_tRNA + 422 h + 8 hisR_tRNA + 12 ile1_tRNA + 1 ile2_tRNA + 11 leu1_tRNA + 6 leu2_tRNA + 6 leuU_tRNA + 2 leuW_tRNA + 3 leuZ_tRNA + 7 lys1_tRNA + 2 met1_tRNA + 1060 mg2 + 6 phe1_tRNA + 422 pi + 11 pro1_tRNA + 3 pro2_tRNA + 3 proL_tRNA + 3 proM_tRNA + 1 rib_70_elo2_b0639_1_cplx + 2 ser2_tRNA + 1 ser3_tRNA + 2 serT_tRNA + 1 serV_tRNA + 4 thr1_tRNA + 3 thr2_tRNA + 4 thr3_tRNA + 3 thrU_tRNA + 4 trpT_tRNA + 9 tyr1_tRNA + 5 val1_tRNA + 2 val2_tRNA + 1 val3_tRNA	irreversible	Translation		0.00E+00	1.00E+09	0	2.90E+01	2.90E+01	0	4.65E+01	46.51653	0	6.28E+00	6.276

tl_elo_b0639_6_rib1	Translation elongation 1 b0639 6 ribosome(s)	1272 EF-G.GTP + 60 EF-Tu.GTP.ala1-tRNA + 18 EF-Tu.GTP.ala2-tRNA + 48 EF-Tu.GTP.arg1-tRNA + 18 EF-Tu.GTP.argX-tRNA + 60 EF-Tu.GTP.asn1-tRNA + 54 EF-Tu.GTP.asp1-tRNA + 12 EF-Tu.GTP.cysT-tRNA + 42 EF-Tu.GTP.gln1-tRNA + 48 EF-Tu.GTP.gln2-tRNA + 102 EF-Tu.GTP.glu1-tRNA + 54 EF-Tu.GTP.gly1-tRNA + 6 EF-Tu.GTP.glyT-tRNA + 48 EF-Tu.GTP.hisR-tRNA + 72 EF-Tu.GTP.ile1-tRNA + 6 EF-Tu.GTP.ile2-tRNA + 66 EF-Tu.GTP.leu1-tRNA + 36 EF-Tu.GTP.leu2-tRNA + 36 EF-Tu.GTP.leuU-tRNA + 12 EF-Tu.GTP.leuW-tRNA + 18 EF-Tu.GTP.leuZ-tRNA + 42 EF-Tu.GTP.lys1-tRNA + 12 EF-Tu.GTP.met1-tRNA + 36 EF-Tu.GTP.phe1-tRNA + 66 EF-Tu.GTP.pro1-tRNA + 18 EF-Tu.GTP.pro2-tRNA + 18 EF-Tu.GTP.proL-tRNA + 18 EF-Tu.GTP.proM-tRNA + 12 EF-Tu.GTP.ser2-tRNA + 6 EF-Tu.GTP.ser3-tRNA + 12 EF-Tu.GTP.serT-tRNA + 6 EF-Tu.GTP.serV-tRNA + 24 EF-Tu.GTP.thr1-tRNA + 18 EF-Tu.GTP.thr2-tRNA + 24 EF-Tu.GTP.thr3-tRNA + 18 EF-Tu.GTP.thrU-tRNA + 24 EF-Tu.GTP.trpT-tRNA + 54 EF-Tu.GTP.tyr1-tRNA + 30 EF-Tu.GTP.val1-tRNA + 12 EF-Tu.GTP.val2-tRNA + 6 EF Tu.GTP.val3-tRNA + 6360 mg2 + 1 rib_ini_b0639_6 --> 1 rib_70_elo1_b0639_6_cplx	irreversible	Translation	0.00E+00	1.00E+09	0	2.90E+01	2.90E+01	0	4.65E+01	46.51653	0	6.28E+00	6.276
tl_elo_b0639_6_rib2	Translation elongation 2 b0639 6 ribosome(s)	1266 h2o + 1 rib_70_elo1_b0639_6_cplx --> 1266 EF-G.GDP + 1266 EF-Tu.GDP + 60 ala1_tRNA + 18 ala2_tRNA + 42 arg1_tRNA + 18 argX_tRNA + 60 asn1_tRNA + 54 asp1_tRNA + 12 cysT_tRNA + 6 fmet_tRNA + 42 gln1_tRNA + 48 gln2_tRNA + 102 glu1_tRNA + 54 gly1_tRNA + 6 glyT_tRNA + 2532 h + 48 hisR_tRNA + 72 ile1_tRNA + 6 ile2_tRNA + 66 leu1_tRNA + 36 leu2_tRNA + 36 leuU_tRNA + 12 leuW_tRNA + 18 leuZ_tRNA + 42 lys1_tRNA + 12 met1_tRNA + 6360 mg2 + 36 phe1_tRNA + 2532 pi + 66 pro1_tRNA + 18 pro2_tRNA + 18 proL_tRNA + 18 proM_tRNA + 1 rib_70_elo2_b0639_6_cplx + 12 ser2_tRNA + 6 ser3_tRNA + 12 serT_tRNA + 6 serV_tRNA + 24 thr1_tRNA + 18 thr2_tRNA + 24 thr3_tRNA + 18 thrU_tRNA + 24 trpT_tRNA + 54 tyr1_tRNA + 30 val1_tRNA + 12 val2_tRNA + 6 val3_tRNA	irreversible	Translation	0.00E+00	1.00E+09	0	2.90E+01	2.90E+01	0	4.65E+01	46.51653	0	6.28E+00	6.276
tl_elo_b0640_10_rib1	Translation elongation 1 b0640 10 ribosome(s)	3420 EF-G.GTP + 310 EF-Tu.GTP.ala1-tRNA + 60 EF-Tu.GTP.ala2-tRNA + 200 EF-Tu.GTP.arg1-tRNA + 30 EF-Tu.GTP.argX-tRNA + 170 EF-Tu.GTP.asn1-tRNA + 150 EF-Tu.GTP.asp1-tRNA + 50 EF-Tu.GTP.cysT-tRNA + 200 EF-Tu.GTP.gln1-tRNA + 90 EF-Tu.GTP.gln2-tRNA + 200 EF-Tu.GTP.glu1-tRNA + 90 EF-Tu.GTP.gly1-tRNA + 40 EF-Tu.GTP.glyT-tRNA + 20 EF-Tu.GTP.glyU-tRNA + 90 EF-Tu.GTP.hisR-tRNA + 80 EF-Tu.GTP.ile1 tRNA + 230 EF-Tu.GTP.leu1-tRNA + 140 EF-Tu.GTP.leu2-tRNA + 180 EF-Tu.GTP.leuU-tRNA + 10 EF-Tu.GTP.leuW-tRNA + 140 EF-Tu.GTP.leuZ-tRNA + 100 EF-Tu.GTP.lys1-tRNA + 40 EF-Tu.GTP.met1-tRNA + 90 EF-Tu.GTP.phe1-tRNA + 50 EF-Tu.GTP.pro1-tRNA + 20 EF-Tu.GTP.pro2-tRNA + 30 EF-Tu.GTP.proL-tRNA + 30 EF-Tu.GTP.proM-tRNA + 10 EF-Tu.GTP.ser1-tRNA + 20 EF-Tu.GTP.ser2-tRNA + 20 EF-Tu.GTP.ser3-tRNA + 10 EF-Tu.GTP.serT-tRNA + 80 EF-Tu.GTP.serV-tRNA + 40 EF-Tu.GTP.thr1-tRNA + 30 EF-Tu.GTP.thr2 tRNA + 40 EF-Tu.GTP.thr3-tRNA + 50 EF-Tu.GTP.thrU-tRNA + 70 EF-Tu.GTP.trpT-tRNA + 50 EF-Tu.GTP.tyr1-tRNA + 90 EF-Tu.GTP.val1-tRNA + 10 EF-Tu.GTP.val2-tRNA + 60 EF-Tu.GTP.val3 tRNA + 17100 mg2 + 1 rib_ini_b0640_10 --> 1 rib_70_elo1_b0640_1	irreversible	Translation	0.00E+00	1.00E+09	0	2.70E+01	2.70E+01	0	4.45E+01	44.46653	0	4.23E+00	4.226
tl_elo_b0640_10_rib2	Translation elongation 2 b0640 10 ribosome(s)	3410 h2o + 1 rib_70_elo1_b0640_10_cplx --> 3410 EF-G.GDP + 3410 EF-Tu.GDP + 310 ala1_tRNA + 60 ala2_tRNA + 200 arg1_tRNA + 30 argX_tRNA + 170 asn1_tRNA + 150 asp1_tRNA + 50 cysT_tRNA + 10 fmet_tRNA + 200 gln1_tRNA + 90 gln2_tRNA + 200 glu1_tRNA + 80 gly1_tRNA + 40 glyT_tRNA + 20 glyU_tRNA + 6820 h + 90 hisR_tRNA + 80 ile1_tRNA + 230 leu1_tRNA + 140 leu2_tRNA + 180 leuU_tRNA + 10 leuW_tRNA + 140 leuZ_tRNA + 100 lys1_tRNA + 40 met1_tRNA + 17100 mg2 + 90 phe1_tRNA + 6820 pi + 50 pro1_tRNA + 20 pro2_tRNA + 30 proL_tRNA + 30 proM_tRNA + 1 rib_70_elo2_b0640_10_cplx + 10 ser1_tRNA + 20 ser2_tRNA + 20 ser3_tRNA + 10 serT_tRNA + 80 serV_tRNA + 40 thr1_tRNA + 30 thr2_tRNA + 40 thr3_tRNA + 50 thrU_tRNA + 70 trpT_tRNA + 50 tyr1_tRNA + 90 val1_tRNA + 10 val2_tRNA + 60 val3_tRNA	irreversible	Translation	0.00E+00	1.00E+09	0	2.70E+01	2.70E+01	0	4.45E+01	44.46653	0	4.23E+00	4.226
tl_elo_b0640_1_rib1	Translation elongation 1 b0640 1 ribosome(s)	342 EF-G.GTP + 31 EF-Tu.GTP.ala1-tRNA + 6 EF-Tu.GTP.ala2-tRNA + 20 EF-Tu.GTP.arg1-tRNA + 3 EF-Tu.GTP.argX-tRNA + 17 EF-Tu.GTP.asn1-tRNA + 15 EF-Tu.GTP.asp1-tRNA + 5 EF-Tu.GTP.cysT-tRNA + 20 EF-Tu.GTP.gln1-tRNA + 9 EF-Tu.GTP.gln2-tRNA + 20 EF-Tu.GTP.glu1-tRNA + 9 EF-Tu.GTP.gly1-tRNA + 4 EF-Tu.GTP.glyT-tRNA + 2 EF-Tu.GTP.glyU-tRNA + 9 EF-Tu.GTP.hisR-tRNA + 8 EF-Tu.GTP.ile1-tRNA + 23 EF-Tu.GTP.leu1-tRNA + 14 EF-Tu.GTP.leu2-tRNA + 18 EF-Tu.GTP.leuU-tRNA + 1 EF-Tu.GTP.leuW-tRNA + 14 EF-Tu.GTP.leuZ-tRNA + 10 EF-Tu.GTP.lys1-tRNA + 4 EF-Tu.GTP.met1-tRNA + 9 EF-Tu.GTP.phe1-tRNA + 5 EF-Tu.GTP.pro1-tRNA + 2 EF-Tu.GTP.pro2-tRNA + 3 EF-Tu.GTP.proL-tRNA + 3 EF-Tu.GTP.proM-tRNA + 1 EF-Tu.GTP.ser1-tRNA + 2 EF-Tu.GTP.ser2-tRNA + 2 EF-Tu.GTP.ser3-tRNA + 1 EF-Tu.GTP.serT-tRNA + 8 EF-Tu.GTP.serV-tRNA + 4 EF-Tu.GTP.thr1-tRNA + 3 EF-Tu.GTP.thr2-tRNA + 4 EF-Tu.GTP.thr3-tRNA + 5 EF-Tu.GTP.thrU-tRNA + 7 EF-Tu.GTP.trpT-tRNA + 5 EF-Tu.GTP.tyr1-tRNA + 9 EF-Tu.GTP.val1-tRNA + 1 EF-Tu.GTP.val2-tRNA + 6 EF-Tu.GTP.val3-tRNA + 1710 mg2 + 1 rib_ini_b0640_1 --> 1 rib_70_elo1_b0640_1_cplx	irreversible	Translation	0.00E+00	1.00E+09	0	2.70E+01	2.70E+01	0	4.45E+01	44.46653	0	4.23E+00	4.226
tl_elo_b0640_1_rib2	Translation elongation 2 b0640 1 ribosome(s)	341 h2o + 1 rib_70_elo1_b0640_1_cplx --> 341 EF-G.GDP + 341 EF-Tu.GDP + 31 ala1_tRNA + 6 ala2_tRNA + 20 arg1_tRNA + 3 argX_tRNA + 17 asn1_tRNA + 15 asp1_tRNA + 5 cysT_tRNA + 1 fmet_tRNA + 20 gln1_tRNA + 9 gln2_tRNA + 20 glu1_tRNA + 8 gly1_tRNA + 4 glyT_tRNA + 2 glyU_tRNA + 682 h + 9 hisR_tRNA + 8 ile1_tRNA + 23 leu1_tRNA + 14 leu2_tRNA + 18 leuU_tRNA + 1 leuW_tRNA + 14 leuZ_tRNA + 10 lys1_tRNA + 4 met1_tRNA + 1710 mg2 + 9 phe1_tRNA + 682 pi + 5 pro1_tRNA + 2 pro2_tRNA + 3 proL_tRNA + 3 proM_tRNA + 1 rib_70_elo2_b0640_1_cplx + 1 ser1_tRNA + 2 ser2_tRNA + 2 ser3_tRNA + 1 serT_tRNA + 8 serV_tRNA + 4 thr1_tRNA + 3 thr2_tRNA + 4 thr3_tRNA + 5 thrU_tRNA + 7 trpT_tRNA + 5 tyr1_tRNA + 9 val1_tRNA + 1 val2_tRNA + 6 val3_tRNA	irreversible	Translation	0.00E+00	1.00E+09	0	2.70E+01	2.70E+01	0	4.45E+01	44.46653	0	4.23E+00	4.226

tl_elo_b0640_20_rib1	Translation elongation 1 b0640 20 ribosome(s)	6840 EF-G.GTP + 620 EF-Tu.GTP.ala1-tRNA + 120 EF-Tu.GTP.ala2 tRNA + 400 EF-Tu.GTP.arg1-tRNA + 60 EF-Tu.GTP.argX-tRNA + 340 EF-Tu.GTP.asn1-tRNA + 300 EF-Tu.GTP.asp1-tRNA + 100 EF-Tu.GTP.cysT-tRNA + 400 EF-Tu.GTP.gln1-tRNA + 180 EF-Tu.GTP.gln2-tRNA + 400 EF-Tu.GTP.glu1-tRNA + 180 EF-Tu.GTP.gly1-tRNA + 80 EF-Tu.GTP.glyT-tRNA + 40 EF-Tu.GTP.glyU-tRNA + 180 EF-Tu.GTP.hisR-tRNA + 160 EF-Tu.GTP.ile1-tRNA + 460 EF-Tu.GTP.leu1-tRNA + 280 EF-Tu.GTP.leu2-tRNA + 360 EF-Tu.GTP.leuU-tRNA + 20 EF-Tu.GTP.leuW-tRNA + 280 EF-Tu.GTP.leuZ-tRNA + 200 EF-Tu.GTP.lys1-tRNA + 80 EF-Tu.GTP.met1-tRNA + 180 EF-Tu.GTP.phe1-tRNA + 100 EF-Tu.GTP.pro1-tRNA + 40 EF-Tu.GTP.pro2-tRNA + 60 EF-Tu.GTP.proL-tRNA + 60 EF-Tu.GTP.proM-tRNA + 20 EF-Tu.GTP.ser1-tRNA + 40 EF-Tu.GTP.ser2-tRNA + 40 EF-Tu.GTP.ser3-tRNA + 20 EF-Tu.GTP.serT-tRNA + 160 EF-Tu.GTP.serV-tRNA + 80 EF-Tu.GTP.thr1-tRNA + 60 EF-Tu.GTP.thr2-tRNA + 80 EF-Tu.GTP.thr3-tRNA + 100 EF-Tu.GTP.thrU-tRNA + 140 EF-Tu.GTP.trpT-tRNA + 100 EF-Tu.GTP.tyr1-tRNA + 180 EF-Tu.GTP.val1-tRNA + 20 EF-Tu.GTP.val2-tRNA + 120 EF-Tu.GTP.val3-tRNA + 34200 mg2 + 1 rib_ini_b0640_20 --> 1 rib_70_elo1_b0640_20_cplx	irreversible	Translation	0.00E+00	1.00E+09	0	2.70E+01	2.70E+01	0	4.45E+01	44.46653	0	4.23E+00	4.226
tl_elo_b0640_20_rib2	Translation elongation 2 b0640 20 ribosome(s)	6820 h2o + 1 rib_70_elo1_b0640_20_cplx --> 6820 EF-G.GDP + 6820 EF-Tu.GDP + 620 ala1_tRNA + 120 ala2_tRNA + 400 arg1_tRNA + 60 argX_tRNA + 340 asn1_tRNA + 300 asp1_tRNA + 100 cysT_tRNA + 20 fmet_tRNA + 400 gln1_tRNA + 180 gln2_tRNA + 400 glu1_tRNA + 160 gly1_tRNA + 80 glyT_tRNA + 40 glyU_tRNA + 13640 h + 180 hisR_tRNA + 160 ile1_tRNA + 460 leu1_tRNA + 280 leu2_tRNA + 360 leuU_tRNA + 20 leuW_tRNA + 280 leuZ_tRNA + 200 lys1_tRNA + 80 met1_tRNA + 34200 mg2 + 180 phe1_tRNA + 13640 pi + 100 pro1_tRNA + 40 pro2_tRNA + 60 proL_tRNA + 60 proM_tRNA + 1 rib_70_elo2_b0640_20_cplx + 20 ser1_tRNA + 40 ser2_tRNA + 40 ser3_tRNA + 20 serT_tRNA + 160 serV_tRNA + 80 thr1_tRNA + 60 thr2_tRNA + 80 thr3_tRNA + 100 thrU_tRNA + 140 trpT_tRNA + 100 tyr1_tRNA + 180 val1_tRNA + 20 val2_tRNA + 120 val3_tRNA	irreversible	Translation	0.00E+00	1.00E+09	0	2.70E+01	2.70E+01	0	4.45E+01	44.46653	0	4.23E+00	4.226
tl_elo_b0641_11_rib1	Translation elongation 1 b0641 11 ribosome(s)	2112 EF-G.GTP + 154 EF-Tu.GTP.ala1-tRNA + 33 EF-Tu.GTP.ala2-tRNA + 165 EF-Tu.GTP.arg1-tRNA + 88 EF-Tu.GTP.asn1-tRNA + 154 EF-Tu.GTP.asp1-tRNA + 11 EF-Tu.GTP.cysT-tRNA + 55 EF-Tu.GTP.gln1-tRNA + 33 EF-Tu.GTP.gln2-tRNA + 88 EF-Tu.GTP.glu1-tRNA + 77 EF-Tu.GTP.gly1-tRNA + 22 EF-Tu.GTP.glyU-tRNA + 11 EF-Tu.GTP.hisR-tRNA + 121 EF-Tu.GTP.ile1-tRNA + 88 EF-Tu.GTP.leu1-tRNA + 66 EF-Tu.GTP.leu2-tRNA + 11 EF-Tu.GTP.leuU-tRNA + 55 EF-Tu.GTP.leuZ-tRNA + 99 EF-Tu.GTP.lys1-tRNA + 77 EF-Tu.GTP.met1-tRNA + 44 EF-Tu.GTP.phe1-tRNA + 44 EF-Tu.GTP.pro1-tRNA + 22 EF-Tu.GTP.pro2-tRNA + 11 EF-Tu.GTP.proL-tRNA + 33 EF-Tu.GTP.proM-tRNA + 44 EF-Tu.GTP.ser1-tRNA + 11 EF-Tu.GTP.ser2-tRNA + 33 EF-Tu.GTP.ser3-tRNA + 11 EF-Tu.GTP.serT-tRNA + 44 EF-Tu.GTP.serV-tRNA + 77 EF-Tu.GTP.thr1-tRNA + 22 EF-Tu.GTP.thr2-tRNA + 77 EF-Tu.GTP.thr3-tRNA + 33 EF-Tu.GTP.thrU-tRNA + 11 EF-Tu.GTP.trpT-tRNA + 44 EF-Tu.GTP.tyr1-tRNA + 66 EF-Tu.GTP.val1-tRNA + 44 EF-Tu.GTP.val2-tRNA + 33 EF-Tu.GTP.val3-tRNA + 10560 mg2 + 1 rib_ini_b0641_11 --> 1 rib_70_elo1_b0641_11_cplx	irreversible	Translation	0.00E+00	1.00E+09	0	3.11E+01	3.11E+01	0	4.86E+01	48.59653	0	8.36E+00	8.356
tl_elo_b0641_11_rib2	Translation elongation 2 b0641 11 ribosome(s)	2101 h2o + 1 rib_70_elo1_b0641_11_cplx --> 2101 EF-G.GDP + 2101 EF-Tu.GDP + 154 ala1_tRNA + 33 ala2_tRNA + 165 arg1_tRNA + 77 asn1_tRNA + 154 asp1_tRNA + 11 cysT_tRNA + 11 fmet_tRNA + 55 gln1_tRNA + 33 gln2_tRNA + 88 glu1_tRNA + 77 gly1_tRNA + 22 glyU_tRNA + 4202 h + 11 hisR_tRNA + 121 ile1_tRNA + 88 leu1_tRNA + 66 leu2_tRNA + 11 leuU_tRNA + 55 leuZ_tRNA + 99 lys1_tRNA + 77 met1_tRNA + 10560 mg2 + 44 phe1_tRNA + 4202 pi + 44 pro1_tRNA + 22 pro2_tRNA + 11 proL_tRNA + 33 proM_tRNA + 1 rib_70_elo2_b0641_11_cplx + 44 ser1_tRNA + 11 ser2_tRNA + 33 ser3_tRNA + 11 serT_tRNA + 44 serV_tRNA + 77 thr1_tRNA + 22 thr2_tRNA + 77 thr3_tRNA + 33 thrU_tRNA + 11 trpT_tRNA + 44 tyr1_tRNA + 66 val1_tRNA + 44 val2_tRNA + 33 val3_tRNA	irreversible	Translation	0.00E+00	1.00E+09	0	3.11E+01	3.11E+01	0	4.86E+01	48.59653	0	8.36E+00	8.356
tl_elo_b0641_1_rib1	Translation elongation 1 b0641 1 ribosome(s)	192 EF-G.GTP + 14 EF-Tu.GTP.ala1-tRNA + 3 EF-Tu.GTP.ala2-tRNA + 15 EF-Tu.GTP.arg1-tRNA + 8 EF-Tu.GTP.asn1-tRNA + 14 EF-Tu.GTP.asp1-tRNA + 1 EF-Tu.GTP.cysT-tRNA + 5 EF-Tu.GTP.gln1-tRNA + 3 EF-Tu.GTP.gln2-tRNA + 8 EF-Tu.GTP.glu1-tRNA + 7 EF-Tu.GTP.gly1-tRNA + 2 EF-Tu.GTP.glyU-tRNA + 1 EF-Tu.GTP.hisR-tRNA + 11 EF-Tu.GTP.ile1-tRNA + 8 EF-Tu.GTP.leu1-tRNA + 6 EF-Tu.GTP.leu2-tRNA + 1 EF-Tu.GTP.leuU-tRNA + 5 EF-Tu.GTP.leuZ-tRNA + 9 EF-Tu.GTP.lys1-tRNA + 7 EF-Tu.GTP.met1-tRNA + 4 EF-Tu.GTP.phe1-tRNA + 4 EF-Tu.GTP.pro1-tRNA + 2 EF-Tu.GTP.pro2-tRNA + 1 EF-Tu.GTP.proL-tRNA + 3 EF-Tu.GTP.proM-tRNA + 4 EF-Tu.GTP.ser1-tRNA + 1 EF-Tu.GTP.ser2-tRNA + 3 EF-Tu.GTP.ser3-tRNA + 1 EF-Tu.GTP.serT-tRNA + 4 EF-Tu.GTP.serV-tRNA + 7 EF-Tu.GTP.thr1-tRNA + 2 EF-Tu.GTP.thr2-tRNA + 7 EF-Tu.GTP.thr3-tRNA + 3 EF-Tu.GTP.thrU-tRNA + 1 EF-Tu.GTP.trpT-tRNA + 4 EF-Tu.GTP.tyr1-tRNA + 6 EF-Tu.GTP.val1-tRNA + 4 EF-Tu.GTP.val2-tRNA + 3 EF-Tu.GTP.val3-tRNA + 960 mg2 + 1 rib_ini_b0641_1 --> 1 rib_70_elo1_b0641_1_cplx	irreversible	Translation	0.00E+00	1.00E+09	0	3.11E+01	3.11E+01	0	4.86E+01	48.59653	0	8.36E+00	8.356
tl_elo_b0641_1_rib2	Translation elongation 2 b0641 1 ribosome(s)	191 h2o + 1 rib_70_elo1_b0641_1_cplx --> 191 EF-G.GDP + 191 EF-Tu.GDP + 14 ala1_tRNA + 3 ala2_tRNA + 15 arg1_tRNA + 7 asn1_tRNA + 14 asp1_tRNA + 1 cysT_tRNA + 1 fmet_tRNA + 5 gln1_tRNA + 3 gln2_tRNA + 8 glu1_tRNA + 7 gly1_tRNA + 2 glyU_tRNA + 382 h + 1 hisR_tRNA + 11 ile1_tRNA + 8 leu1_tRNA + 6 leu2_tRNA + 1 leuU_tRNA + 5 leuZ_tRNA + 9 lys1_tRNA + 7 met1_tRNA + 960 mg2 + 4 phe1_tRNA + 382 pi + 4 pro1_tRNA + 2 pro2_tRNA + 1 proL_tRNA + 3 proM_tRNA + 1 rib_70_elo2_b0641_1_cplx + 4 ser1_tRNA + 1 ser2_tRNA + 3 ser3_tRNA + 1 serT_tRNA + 4 serV_tRNA + 7 thr1_tRNA + 2 thr2_tRNA + 7 thr3_tRNA + 3 thrU_tRNA + 1 trpT_tRNA + 4 tyr1_tRNA + 6 val1_tRNA + 4 val2_tRNA + 3 val3_tRNA	irreversible	Translation	0.00E+00	1.00E+09	0	3.11E+01	3.11E+01	0	4.86E+01	48.59653	0	8.36E+00	8.356

tl_elo_b0641_5_rib1	Translation elongation 1 b0641 5 ribosome(s)	960 EF-G.GTP + 70 EF-Tu.GTP.ala1-tRNA + 15 EF-Tu.GTP.ala2-tRNA + 75 EF-Tu.GTP.arg1-tRNA + 40 EF-Tu.GTP.asn1-tRNA + 70 EF-Tu.GTP.asp1-tRNA + 5 EF-Tu.GTP.cysT-tRNA + 25 EF-Tu.GTP.gln1-tRNA + 15 EF-Tu.GTP.gln2-tRNA + 40 EF-Tu.GTP.glu1-tRNA + 35 EF-Tu.GTP.gly1-tRNA + 10 EF-Tu.GTP.glyU-tRNA + 5 EF-Tu.GTP.hisR-tRNA + 55 EF-Tu.GTP.ile1-tRNA + 40 EF-Tu.GTP.leu1-tRNA + 30 EF-Tu.GTP.leu2-tRNA + 5 EF-Tu.GTP.leuU-tRNA + 25 EF-Tu.GTP.leuZ-tRNA + 45 EF-Tu.GTP.lys1-tRNA + 35 EF-Tu.GTP.met1-tRNA + 20 EF-Tu.GTP.phe1-tRNA + 20 EF-Tu.GTP.pro1-tRNA + 10 EF-Tu.GTP.pro2-tRNA + 5 EF-Tu.GTP.proL-tRNA + 15 EF-Tu.GTP.proM-tRNA + 20 EF-Tu.GTP.ser1-tRNA + 5 EF-Tu.GTP.ser2-tRNA + 15 EF-Tu.GTP.ser3-tRNA + 5 EF-Tu.GTP.serT-tRNA + 20 EF-Tu.GTP.serV-tRNA + 35 EF-Tu.GTP.thr1-tRNA + 10 EF-Tu.GTP.thr2-tRNA + 35 EF-Tu.GTP.thr3-tRNA + 15 EF-Tu.GTP.thrU-tRNA + 5 EF-Tu.GTP.trpT-tRNA + 20 EF-Tu.GTP.tyr1-tRNA + 30 EF-Tu.GTP.val1-tRNA + 20 EF-Tu.GTP.val2-tRNA + 15 EF-Tu.GTP.val3-tRNA + 4800 mg2 + 1 rib_ini_b0641_5 --> 1 rib_70_elo1_b0641_5_cplx	irreversible	Translation	0.00E+00	1.00E+09	0	3.11E+01	3.11E+01	0	4.86E+01	48.59653	0	8.36E+00	8.356
tl_elo_b0641_5_rib2	Translation elongation 2 b0641 5 ribosome(s)	955 h2o + 1 rib_70_elo1_b0641_5_cplx --> 955 EF-G.GDP + 955 EF-Tu.GDP + 70 ala1_tRNA + 15 ala2_tRNA + 75 arg1_tRNA + 35 asn1_tRNA + 70 asp1_tRNA + 5 cysT_tRNA + 5 fmet_tRNA + 25 gln1_tRNA + 15 gln2_tRNA + 40 glu1_tRNA + 35 gly1_tRNA + 10 glyU_tRNA + 1910 h + 5 hisR_tRNA + 55 ile1_tRNA + 40 leu1_tRNA + 30 leu2_tRNA + 5 leuU_tRNA + 25 leuZ_tRNA + 45 lys1_tRNA + 35 met1_tRNA + 4800 mg2 + 20 phe1_tRNA + 1910 pi + 20 pro1_tRNA + 10 pro2_tRNA + 5 proL_tRNA + 15 proM_tRNA + 1 rib_70_elo2_b0641_5_cplx + 20 ser1_tRNA + 5 ser2_tRNA + 15 ser3_tRNA + 5 serT_tRNA + 20 serV_tRNA + 35 thr1_tRNA + 10 thr2_tRNA + 35 thr3_tRNA + 15 thrU_tRNA + 5 trpT_tRNA + 20 tyr1_tRNA + 30 val1_tRNA + 20 val2_tRNA + 15 val3_tRNA	irreversible	Translation	0.00E+00	1.00E+09	0	3.11E+01	3.11E+01	0	4.86E+01	48.59653	0	8.36E+00	8.356
tl_elo_b0642_1_rib1	Translation elongation 1 b0642 1 ribosome(s)	859 EF-G.GTP + 67 EF-Tu.GTP.ala1-tRNA + 12 EF-Tu.GTP.ala2-tRNA + 41 EF-Tu.GTP.arg1-tRNA + 39 EF-Tu.GTP.asn1-tRNA + 58 EF-Tu.GTP.asp1-tRNA + 11 EF-Tu.GTP.cysT-tRNA + 26 EF-Tu.GTP.gln1-tRNA + 4 EF-Tu.GTP.gln2-tRNA + 66 EF-Tu.GTP.glu1-tRNA + 55 EF-Tu.GTP.gly1-tRNA + 2 EF-Tu.GTP.glyU-tRNA + 16 EF-Tu.GTP.hisR-tRNA + 34 EF-Tu.GTP.ile1-tRNA + 1 EF-Tu.GTP.ile2-tRNA + 49 EF-Tu.GTP.leu1-tRNA + 2 EF-Tu.GTP.leu2-tRNA + 12 EF-Tu.GTP.leuU-tRNA + 1 EF-Tu.GTP.leuW-tRNA + 1 EF-Tu.GTP.leuZ-tRNA + 54 EF-Tu.GTP.lys1-tRNA + 33 EF-Tu.GTP.met1-tRNA + 27 EF-Tu.GTP.phe1-tRNA + 29 EF-Tu.GTP.pro1-tRNA + 3 EF-Tu.GTP.pro2-tRNA + 2 EF-Tu.GTP.proL-tRNA + 7 EF-Tu.GTP.proM-tRNA + 10 EF-Tu.GTP.ser1-tRNA + 9 EF-Tu.GTP.ser2-tRNA + 1 EF-Tu.GTP.ser3-tRNA + 4 EF-Tu.GTP.serV-tRNA + 29 EF-Tu.GTP.thr1-tRNA + 12 EF-Tu.GTP.thr2-tRNA + 11 EF-Tu.GTP.thr3-tRNA + 2 EF-Tu.GTP.thrU-tRNA + 21 EF-Tu.GTP.trpT-tRNA + 36 EF-Tu.GTP.tyr1-tRNA + 34 EF-Tu.GTP.val1-tRNA + 8 EF-Tu.GTP.val2-tRNA + 30 EF-Tu.GTP.val3-tRNA + 4295 mg2 + 1 rib_ini_b0642_1 --> 1 rib_70_elo1_b0642_1_cplx	irreversible	Translation	0.00E+00	1.00E+09	0	2.50E+01	2.50E+01	0	4.26E+01	42.55653	0	2.32E+00	2.316
tl_elo_b0642_1_rib2	Translation elongation 2 b0642 1 ribosome(s)	858 h2o + 1 rib_70_elo1_b0642_1_cplx --> 858 EF-G.GDP + 858 EF-Tu.GDP + 67 ala1_tRNA + 12 ala2_tRNA + 41 arg1_tRNA + 39 asn1_tRNA + 58 asp1_tRNA + 11 cysT_tRNA + 1 fmet_tRNA + 26 gln1_tRNA + 4 gln2_tRNA + 66 glu1_tRNA + 54 gly1_tRNA + 2 glyU_tRNA + 1716 h + 16 hisR_tRNA + 34 ile1_tRNA + 1 ile2_tRNA + 49 leu1_tRNA + 2 leu2_tRNA + 12 leuU_tRNA + 1 leuW_tRNA + 1 leuZ_tRNA + 54 lys1_tRNA + 33 met1_tRNA + 4295 mg2 + 27 phe1_tRNA + 1716 pi + 29 pro1_tRNA + 3 pro2_tRNA + 2 proL_tRNA + 7 proM_tRNA + 1 rib_70_elo2_b0642_1_cplx + 10 ser1_tRNA + 9 ser2_tRNA + 1 ser3_tRNA + 4 serV_tRNA + 29 thr1_tRNA + 12 thr2_tRNA + 11 thr3_tRNA + 2 thrU_tRNA + 21 trpT_tRNA + 36 tyr1_tRNA + 34 val1_tRNA + 8 val2_tRNA + 30 val3_tRNA	irreversible	Translation	0.00E+00	1.00E+09	0	2.50E+01	2.50E+01	0	4.26E+01	42.55653	0	2.32E+00	2.316
tl_elo_b0642_25_rib1	Translation elongation 1 b0642 25 ribosome(s)	21475 EF-G.GTP + 1675 EF-Tu.GTP.ala1-tRNA + 300 EF-Tu.GTP.ala2-tRNA + 1025 EF-Tu.GTP.arg1-tRNA + 975 EF-Tu.GTP.asn1-tRNA + 1450 EF-Tu.GTP.asp1-tRNA + 275 EF-Tu.GTP.cysT-tRNA + 650 EF-Tu.GTP.gln1-tRNA + 100 EF-Tu.GTP.gln2-tRNA + 1650 EF-Tu.GTP.glu1-tRNA + 1375 EF-Tu.GTP.gly1-tRNA + 50 EF-Tu.GTP.glyU-tRNA + 400 EF-Tu.GTP.hisR-tRNA + 850 EF-Tu.GTP.ile1-tRNA + 25 EF-Tu.GTP.ile2-tRNA + 1225 EF-Tu.GTP.leu1-tRNA + 50 EF-Tu.GTP.leu2-tRNA + 300 EF-Tu.GTP.leuU-tRNA + 25 EF-Tu.GTP.leuW-tRNA + 25 EF-Tu.GTP.leuZ-tRNA + 1350 EF-Tu.GTP.lys1-tRNA + 825 EF-Tu.GTP.met1-tRNA + 675 EF-Tu.GTP.phe1-tRNA + 725 EF-Tu.GTP.pro1-tRNA + 75 EF-Tu.GTP.pro2-tRNA + 50 EF-Tu.GTP.proL-tRNA + 175 EF-Tu.GTP.proM-tRNA + 250 EF-Tu.GTP.ser1-tRNA + 225 EF-Tu.GTP.ser2-tRNA + 25 EF-Tu.GTP.ser3-tRNA + 100 EF-Tu.GTP.serV-tRNA + 725 EF-Tu.GTP.thr1-tRNA + 300 EF-Tu.GTP.thr2-tRNA + 275 EF-Tu.GTP.thr3-tRNA + 50 EF-Tu.GTP.thrU-tRNA + 525 EF-Tu.GTP.trpT-tRNA + 900 EF-Tu.GTP.tyr1-tRNA + 850 EF-Tu.GTP.val1-tRNA + 200 EF-Tu.GTP.val2-tRNA + 750 EF-Tu.GTP.val3-tRNA + 107375 mg2 + 1 rib_ini_b0642_25 --> 1 rib_70_elo1_b0642_25_cplx	irreversible	Translation	0.00E+00	1.00E+09	0	2.50E+01	2.50E+01	0	4.26E+01	42.55653	0	2.32E+00	2.316
tl_elo_b0642_25_rib2	Translation elongation 2 b0642 25 ribosome(s)	21450 h2o + 1 rib_70_elo1_b0642_25_cplx --> 21450 EF-G.GDP + 21450 EF-Tu.GDP + 1675 ala1_tRNA + 300 ala2_tRNA + 1025 arg1_tRNA + 975 asn1_tRNA + 1450 asp1_tRNA + 275 cysT_tRNA + 25 fmet_tRNA + 650 gln1_tRNA + 100 gln2_tRNA + 1650 glu1_tRNA + 1350 gly1_tRNA + 50 glyU_tRNA + 42900 h + 400 hisR_tRNA + 850 ile1_tRNA + 25 ile2_tRNA + 1225 leu1_tRNA + 50 leu2_tRNA + 300 leuU_tRNA + 25 leuW_tRNA + 25 leuZ_tRNA + 1350 lys1_tRNA + 825 met1_tRNA + 107375 mg2 + 675 phe1_tRNA + 42900 pi + 725 pro1_tRNA + 75 pro2_tRNA + 50 proL_tRNA + 175 proM_tRNA + 1 rib_70_elo2_b0642_25_cplx + 250 ser1_tRNA + 225 ser2_tRNA + 25 ser3_tRNA + 100 serV_tRNA + 725 thr1_tRNA + 300 thr2_tRNA + 275 thr3_tRNA + 50 thrU_tRNA + 525 trpT_tRNA + 900 tyr1_tRNA + 850 val1_tRNA + 200 val2_tRNA + 750 val3_tRNA	irreversible	Translation	0.00E+00	1.00E+09	0	2.50E+01	2.50E+01	0	4.26E+01	42.55653	0	2.32E+00	2.316

tl_elo_b0642_50_rib1	Translation elongation 1 b0642 50 ribosome(s)	42950 EF-G.GTP + 3350 EF-Tu.GTP.ala1-tRNA + 600 EF-Tu.GTP.ala2-tRNA + 2050 EF-Tu.GTP.arg1-tRNA + 1950 EF-Tu.GTP.asn1-tRNA + 2900 EF-Tu.GTP.asp1-tRNA + 550 EF-Tu.GTP.cysT-tRNA + 1300 EF-Tu.GTP.gln1-tRNA + 200 EF-Tu.GTP.gln2-tRNA + 3300 EF-Tu.GTP.glu1-tRNA + 2750 EF-Tu.GTP.gly1-tRNA + 100 EF-Tu.GTP.glyU-tRNA + 800 EF-Tu.GTP.hisR-tRNA + 1700 EF-Tu.GTP.ile1-tRNA + 50 EF-Tu.GTP.ile2-tRNA + 2450 EF-Tu.GTP.leu1-tRNA + 100 EF-Tu.GTP.leu2-tRNA + 600 EF-Tu.GTP.leuU-tRNA + 50 EF-Tu.GTP.leuW-tRNA + 50 EF-Tu.GTP.leuZ-tRNA + 2700 EF-Tu.GTP.lys1-tRNA + 1650 EF-Tu.GTP.met1-tRNA + 1350 EF-Tu.GTP.phe1-tRNA + 1450 EF-Tu.GTP.pro1-tRNA + 150 EF-Tu.GTP.pro2-tRNA + 100 EF-Tu.GTP.proL-tRNA + 350 EF-Tu.GTP.proM-tRNA + 500 EF-Tu.GTP.ser1-tRNA + 450 EF-Tu.GTP.ser2-tRNA + 50 EF-Tu.GTP.ser3-tRNA + 200 EF-Tu.GTP.serV-tRNA + 1450 EF-Tu.GTP.thr1-tRNA + 600 EF-Tu.GTP.thr2-tRNA + 550 EF-Tu.GTP.thr3-tRNA + 100 EF-Tu.GTP.thrU-tRNA + 1050 EF-Tu.GTP.trpT-tRNA + 1800 EF-Tu.GTP.tyr1-tRNA + 1700 EF-Tu.GTP.val1-tRNA + 400 EF-Tu.GTP.val2-tRNA + 1500 EF-Tu.GTP.val3-tRNA + 214750 mg2 + 1 rib_ini_b0642_50 --> 1 rib_70_elo1_b0642_50_cplx	irreversible	Translation		0.00E+00	1.00E+09	0	2.50E+01	2.50E+01	0	4.26E+01	42.55653	0	2.32E+00	2.316
tl_elo_b0642_50_rib2	Translation elongation 2 b0642 50 ribosome(s)	42900 h2o + 1 rib_70_elo1_b0642_50_cplx --> 42900 EF-G.GDP + 42900 EF-Tu.GDP + 3350 ala1_tRNA + 600 ala2_tRNA + 2050 arg1_tRNA + 1950 asn1_tRNA + 2900 asp1_tRNA + 550 cysT_tRNA + 50 fmet_tRNA + 1300 gln1_tRNA + 200 gln2_tRNA + 3300 glu1_tRNA + 2700 gly1_tRNA + 100 glyU_tRNA + 85800 h + 800 hisR_tRNA + 1700 ile1_tRNA + 50 ile2_tRNA + 2450 leu1_tRNA + 100 leu2_tRNA + 600 leuU_tRNA + 50 leuW_tRNA + 50 leuZ_tRNA + 2700 lys1_tRNA + 1650 met1_tRNA + 214750 mg2 + 1350 phe1_tRNA + 85800 pi + 1450 pro1_tRNA + 150 pro2_tRNA + 100 proL_tRNA + 350 proM_tRNA + 1 rib_70_elo2_b0642_50_cplx + 500 ser1_tRNA + 450 ser2_tRNA + 50 ser3_tRNA + 200 serV_tRNA + 1450 thr1_tRNA + 600 thr2_tRNA + 550 thr3_tRNA + 100 thrU_tRNA + 1050 trpT_tRNA + 1800 tyr1_tRNA + 1700 val1_tRNA + 400 val2_tRNA + 1500 val3_tRNA	irreversible	Translation		0.00E+00	1.00E+09	0	2.50E+01	2.50E+01	0	4.26E+01	42.55653	0	2.32E+00	2.316
tl_elo_b0661_13_rib1	Translation elongation 1 b0661 13 ribosome(s)	6149 EF-G.GTP + 221 EF-Tu.GTP.ala1-tRNA + 143 EF-Tu.GTP.ala2-tRNA + 455 EF-Tu.GTP.arg1-tRNA + 13 EF-Tu.GTP.argX-tRNA + 221 EF-Tu.GTP.asn1-tRNA + 429 EF-Tu.GTP.asp1-tRNA + 78 EF-Tu.GTP.cysT-tRNA + 169 EF-Tu.GTP.gln1-tRNA + 65 EF-Tu.GTP.gln2-tRNA + 520 EF-Tu.GTP.glu1-tRNA + 351 EF-Tu.GTP.gly1-tRNA + 13 EF-Tu.GTP.glyT-tRNA + 52 EF-Tu.GTP.glyU-tRNA + 117 EF-Tu.GTP.hisR-tRNA + 442 EF-Tu.GTP.ile1-tRNA + 364 EF-Tu.GTP.leu1-tRNA + 13 EF-Tu.GTP.leu2-tRNA + 78 EF-Tu.GTP.leuU-tRNA + 13 EF-Tu.GTP.leuW-tRNA + 13 EF-Tu.GTP.leuZ-tRNA + 273 EF-Tu.GTP.lys1-tRNA + 169 EF-Tu.GTP.met1-tRNA + 234 EF-Tu.GTP.phe1-tRNA + 221 EF-Tu.GTP.pro1-tRNA + 26 EF-Tu.GTP.pro2-tRNA + 39 EF-Tu.GTP.proM-tRNA + 65 EF-Tu.GTP.ser1-tRNA + 39 EF-Tu.GTP.ser2-tRNA + 52 EF-Tu.GTP.ser3-tRNA + 39 EF-Tu.GTP.serT-tRNA + 143 EF-Tu.GTP.serV-tRNA + 260 EF-Tu.GTP.thr1-tRNA + 13 EF-Tu.GTP.thr2-tRNA + 78 EF-Tu.GTP.thr3-tRNA + 39 EF-Tu.GTP.thrU-tRNA + 52 EF-Tu.GTP.trpT-tRNA + 182 EF-Tu.GTP.tyr1-tRNA + 273 EF-Tu.GTP.val1-tRNA + 117 EF-Tu.GTP.val2-tRNA + 65 EF-Tu.GTP.val3-tRNA + 30745 mg2 + 1 rib_ini_b0661_13 --> 1 rib_70_elo1_b0661_13_cplx	irreversible	Translation		0.00E+00	1.00E+09	0	8.87E+01	8.87E+01	0	1.54E+02	154.0876	0	3.53E+00	3.532
tl_elo_b0661_13_rib2	Translation elongation 2 b0661 13 ribosome(s)	6136 h2o + 1 rib_70_elo1_b0661_13_cplx --> 6136 EF-G.GDP + 6136 EF-Tu.GDP + 221 ala1_tRNA + 143 ala2_tRNA + 455 arg1_tRNA + 13 argX_tRNA + 221 asn1_tRNA + 429 asp1_tRNA + 78 cysT_tRNA + 13 fmet_tRNA + 169 gln1_tRNA + 65 gln2_tRNA + 520 glu1_tRNA + 351 gly1_tRNA + 13 glyT_tRNA + 52 glyU_tRNA + 12272 h + 117 hisR_tRNA + 442 ile1_tRNA + 364 leu1_tRNA + 13 leu2_tRNA + 78 leuU_tRNA + 13 leuW_tRNA + 13 leuZ_tRNA + 273 lys1_tRNA + 169 met1_tRNA + 30745 mg2 + 234 phe1_tRNA + 12272 pi + 208 pro1_tRNA + 26 pro2_tRNA + 39 proM_tRNA + 1 rib_70_elo2_b0661_13_cplx + 65 ser1_tRNA + 39 ser2_tRNA + 52 ser3_tRNA + 39 serT_tRNA + 143 serV_tRNA + 260 thr1_tRNA + 13 thr2_tRNA + 78 thr3_tRNA + 39 thrU_tRNA + 52 trpT_tRNA + 182 tyr1_tRNA + 273 val1_tRNA + 117 val2_tRNA + 65 val3_tRNA	irreversible	Translation		0.00E+00	1.00E+09	0	8.87E+01	8.87E+01	0	1.54E+02	154.0876	0	3.53E+00	3.532
tl_elo_b0661_1_rib1	Translation elongation 1 b0661 1 ribosome(s)	473 EF-G.GTP + 17 EF-Tu.GTP.ala1-tRNA + 11 EF-Tu.GTP.ala2-tRNA + 35 EF-Tu.GTP.arg1-tRNA + 1 EF-Tu.GTP.argX-tRNA + 17 EF-Tu.GTP.asn1-tRNA + 33 EF-Tu.GTP.asp1-tRNA + 6 EF-Tu.GTP.cysT-tRNA + 13 EF-Tu.GTP.gln1-tRNA + 5 EF-Tu.GTP.gln2-tRNA + 40 EF-Tu.GTP.glu1-tRNA + 27 EF-Tu.GTP.gly1-tRNA + 1 EF-Tu.GTP.glyT-tRNA + 4 EF-Tu.GTP.glyU-tRNA + 9 EF-Tu.GTP.hisR-tRNA + 34 EF-Tu.GTP.ile1-tRNA + 28 EF-Tu.GTP.leu1-tRNA + 1 EF-Tu.GTP.leu2-tRNA + 6 EF-Tu.GTP.leuU-tRNA + 1 EF-Tu.GTP.leuW-tRNA + 1 EF-Tu.GTP.leuZ-tRNA + 21 EF-Tu.GTP.lys1-tRNA + 13 EF-Tu.GTP.met1-tRNA + 18 EF-Tu.GTP.phe1-tRNA + 17 EF-Tu.GTP.pro1-tRNA + 2 EF-Tu.GTP.pro2-tRNA + 3 EF-Tu.GTP.proM-tRNA + 5 EF-Tu.GTP.ser1-tRNA + 3 EF-Tu.GTP.ser2-tRNA + 4 EF-Tu.GTP.ser3-tRNA + 3 EF-Tu.GTP.serT-tRNA + 11 EF-Tu.GTP.serV-tRNA + 20 EF-Tu.GTP.thr1-tRNA + 1 EF-Tu.GTP.thr2-tRNA + 6 EF-Tu.GTP.thr3-tRNA + 3 EF-Tu.GTP.thrU-tRNA + 4 EF-Tu.GTP.trpT-tRNA + 14 EF-Tu.GTP.tyr1-tRNA + 21 EF-Tu.GTP.val1-tRNA + 9 EF-Tu.GTP.val2-tRNA + 5 EF-Tu.GTP.val3-tRNA + 2365 mg2 + 1 rib_ini_b0661_1 --> 1 rib_70_elo1_b0661_1_cplx	irreversible	Translation		0.00E+00	1.00E+09	0	8.87E+01	8.87E+01	0	1.54E+02	154.0876	0	3.53E+00	3.532
tl_elo_b0661_1_rib2	Translation elongation 2 b0661 1 ribosome(s)	472 h2o + 1 rib_70_elo1_b0661_1_cplx --> 472 EF-G.GDP + 472 EF-Tu.GDP + 17 ala1_tRNA + 11 ala2_tRNA + 35 arg1_tRNA + 1 argX_tRNA + 17 asn1_tRNA + 33 asp1_tRNA + 6 cysT_tRNA + 1 fmet_tRNA + 13 gln1_tRNA + 5 gln2_tRNA + 40 glu1_tRNA + 27 gly1_tRNA + 1 glyT_tRNA + 4 glyU_tRNA + 944 h + 9 hisR_tRNA + 34 ile1_tRNA + 28 leu1_tRNA + 1 leu2_tRNA + 6 leuU_tRNA + 1 leuW_tRNA + 1 leuZ_tRNA + 21 lys1_tRNA + 13 met1_tRNA + 2365 mg2 + 18 phe1_tRNA + 944 pi + 16 pro1_tRNA + 2 pro2_tRNA + 3 proM_tRNA + 1 rib_70_elo2_b0661_1_cplx + 5 ser1_tRNA + 3 ser2_tRNA + 4 ser3_tRNA + 3 serT_tRNA + 11 serV_tRNA + 20 thr1_tRNA + 1 thr2_tRNA + 6 thr3_tRNA + 3 thrU_tRNA + 4 trpT_tRNA + 14 tyr1_tRNA + 21 val1_tRNA + 9 val2_tRNA + 5 val3_tRNA	irreversible	Translation		0.00E+00	1.00E+09	0	8.87E+01	8.87E+01	0	1.54E+02	154.0876	0	3.53E+00	3.532

tl_elo_b0661_27_rib1	Translation elongation 1 b0661 27 ribosome(s)	12771 EF-G.GTP + 459 EF-Tu.GTP.ala1-tRNA + 297 EF-Tu.GTP.ala2-tRNA + 945 EF-Tu.GTP.arg1-tRNA + 27 EF-Tu.GTP.argX-tRNA + 459 EF-Tu.GTP.asn1-tRNA + 891 EF-Tu.GTP.asp1-tRNA + 162 EF-Tu.GTP.cysT-tRNA + 351 EF-Tu.GTP.gln1-tRNA + 135 EF-Tu.GTP.gln2-tRNA + 1080 EF-Tu.GTP.glu1-tRNA + 729 EF-Tu.GTP.gly1-tRNA + 27 EF-Tu.GTP.glyT-tRNA + 108 EF-Tu.GTP.glyU-tRNA + 243 EF-Tu.GTP.hisR-tRNA + 918 EF-Tu.GTP.ile1-tRNA + 756 EF-Tu.GTP.leu1-tRNA + 27 EF-Tu.GTP.leu2-tRNA + 162 EF-Tu.GTP.leuU-tRNA + 27 EF-Tu.GTP.leuW-tRNA + 27 EF-Tu.GTP.leuZ-tRNA + 567 EF-Tu.GTP.lys1-tRNA + 351 EF-Tu.GTP.met1-tRNA + 486 EF-Tu.GTP.phe1-tRNA + 459 EF-Tu.GTP.pro1-tRNA + 54 EF-Tu.GTP.pro2-tRNA + 81 EF-Tu.GTP.proM-tRNA + 135 EF-Tu.GTP.ser1-tRNA + 81 EF-Tu.GTP.ser2-tRNA + 108 EF-Tu.GTP.ser3-tRNA + 81 EF-Tu.GTP.serT-tRNA + 297 EF-Tu.GTP.serV-tRNA + 540 EF-Tu.GTP.thr1-tRNA + 27 EF-Tu.GTP.thr2-tRNA + 162 EF-Tu.GTP.thr3-tRNA + 81 EF-Tu.GTP.thrU-tRNA + 108 EF-Tu.GTP.trpT-tRNA + 378 EF-Tu.GTP.tyr1-tRNA + 567 EF-Tu.GTP.val1-tRNA + 243 EF-Tu.GTP.val2-tRNA + 135 EF-Tu.GTP.val3-tRNA + 63855 mg2 + 1 rib_ini_b0661_27 --> 1 rib_70_e	irreversible	Translation	0.00E+00	1.00E+09	0	8.87E+01	8.87E+01	0	1.54E+02	154.0876	0	3.53E+00	3.532
tl_elo_b0661_27_rib2	Translation elongation 2 b0661 27 ribosome(s)	12744 h2o + 1 rib_70_elo1_b0661_27_cplx --> 12744 EF-G.GDP + 12744 EF-Tu.GDP + 459 ala1_tRNA + 297 ala2_tRNA + 945 arg1_tRNA + 27 argX_tRNA + 459 asn1_tRNA + 891 asp1_tRNA + 162 cysT_tRNA + 27 fmet_tRNA + 351 gln1_tRNA + 135 gln2_tRNA + 1080 glu1_tRNA + 729 gly1_tRNA + 27 glyT_tRNA + 108 glyU_tRNA + 25488 h + 243 hisR_tRNA + 918 ile1_tRNA + 756 leu1_tRNA + 27 leu2_tRNA + 162 leuU_tRNA + 27 leuW_tRNA + 27 leuZ_tRNA + 567 lys1_tRNA + 351 met1_tRNA + 63855 mg2 + 486 phe1_tRNA + 25488 pi + 432 pro1_tRNA + 54 pro2_tRNA + 81 proM_tRNA + 1 rib_70_elo2_b0661_27_cplx + 135 ser1_tRNA + 81 ser2_tRNA + 108 ser3_tRNA + 81 serT_tRNA + 297 serV_tRNA + 540 thr1_tRNA + 27 thr2_tRNA + 162 thr3_tRNA + 81 thrU_tRNA + 108 trpT_tRNA + 378 tyr1_tRNA + 567 val1_tRNA + 243 val2_tRNA + 135 val3_tRNA	irreversible	Translation	0.00E+00	1.00E+09	0	8.87E+01	8.87E+01	0	1.54E+02	154.0876	0	3.53E+00	3.532
tl_elo_b0680_16_rib1	Translation elongation 1 b0680 16 ribosome(s)	8848 EF-G.GTP + 464 EF-Tu.GTP.ala1-tRNA + 160 EF-Tu.GTP.ala2 tRNA + 640 EF-Tu.GTP.arg1-tRNA + 432 EF-Tu.GTP.asn1-tRNA + 592 EF-Tu.GTP.asp1-tRNA + 160 EF-Tu.GTP.cysT-tRNA + 224 EF-Tu.GTP.gln1-tRNA + 16 EF-Tu.GTP.gln2-tRNA + 736 EF-Tu.GTP.glu1-tRNA + 496 EF-Tu.GTP.gly1-tRNA + 32 EF-Tu.GTP.glyU-tRNA + 256 EF-Tu.GTP.hisR-tRNA + 560 EF-Tu.GTP.ile1-tRNA + 512 EF-Tu.GTP.leu1-tRNA + 32 EF-Tu.GTP.leu2-tRNA + 64 EF-Tu.GTP.leuU-tRNA + 32 EF-Tu.GTP.leuZ-tRNA + 512 EF-Tu.GTP.lys1-tRNA + 176 EF-Tu.GTP.met1-tRNA + 384 EF-Tu.GTP.phe1-tRNA + 400 EF-Tu.GTP.pro1-tRNA + 32 EF-Tu.GTP.pro2-tRNA + 16 EF-Tu.GTP.proM-tRNA + 80 EF-Tu.GTP.ser1-tRNA + 80 EF-Tu.GTP.ser2-tRNA + 96 EF-Tu.GTP.ser3-tRNA + 16 EF-Tu.GTP.serT-tRNA + 176 EF-Tu.GTP.serV-tRNA + 240 EF-Tu.GTP.thr1-tRNA + 80 EF-Tu.GTP.thr2-tRNA + 96 EF-Tu.GTP.thr3-tRNA + 16 EF-Tu.GTP.thrU-tRNA + 128 EF-Tu.GTP.trpT-tRNA + 352 EF-Tu.GTP.tyr1-tRNA + 320 EF-Tu.GTP.val1-tRNA + 48 EF-Tu.GTP.val2-tRNA + 192 EF-Tu.GTP.val3-tRNA + 44240 mg2 + 1 rib_ini_b0680_16 --> 1 rib_70_elo1_b0680_16_cplx	irreversible	Translation	0.00E+00	1.00E+09	0	8.14E+01	8.14E+01	0	1.39E+02	139.1751	0	6.30E+00	6.304
tl_elo_b0680_16_rib2	Translation elongation 2 b0680 16 ribosome(s)	8832 h2o + 1 rib_70_elo1_b0680_16_cplx --> 8832 EF-G.GDP + 8832 EF-Tu.GDP + 464 ala1_tRNA + 160 ala2_tRNA + 640 arg1_tRNA + 432 asn1_tRNA + 592 asp1_tRNA + 160 cysT_tRNA + 16 fmet_tRNA + 224 gln1_tRNA + 16 gln2_tRNA + 720 glu1_tRNA + 496 gly1_tRNA + 32 glyU_tRNA + 17664 h + 256 hisR_tRNA + 560 ile1_tRNA + 512 leu1_tRNA + 32 leu2_tRNA + 64 leuU_tRNA + 32 leuZ_tRNA + 512 lys1_tRNA + 176 met1_tRNA + 44240 mg2 + 384 phe1_tRNA + 17664 pi + 400 pro1_tRNA + 32 pro2_tRNA + 16 proM_tRNA + 1 rib_70_elo2_b0680_16_cplx + 80 ser1_tRNA + 80 ser2_tRNA + 96 ser3_tRNA + 16 serT_tRNA + 176 serV_tRNA + 240 thr1_tRNA + 80 thr2_tRNA + 96 thr3_tRNA + 16 thrU_tRNA + 128 trpT_tRNA + 352 tyr1_tRNA + 320 val1_tRNA + 48 val2_tRNA + 192 val3_tRNA	irreversible	Translation	0.00E+00	1.00E+09	0	8.14E+01	8.14E+01	0	1.39E+02	139.1751	0	6.30E+00	6.304
tl_elo_b0680_1_rib1	Translation elongation 1 b0680 1 ribosome(s)	553 EF-G.GTP + 29 EF-Tu.GTP.ala1-tRNA + 10 EF-Tu.GTP.ala2-tRNA + 40 EF-Tu.GTP.arg1-tRNA + 27 EF-Tu.GTP.asn1-tRNA + 37 EF-Tu.GTP.asp1-tRNA + 10 EF-Tu.GTP.cysT-tRNA + 14 EF-Tu.GTP.gln1-tRNA + 1 EF-Tu.GTP.gln2-tRNA + 46 EF-Tu.GTP.glu1-tRNA + 31 EF-Tu.GTP.gly1-tRNA + 2 EF-Tu.GTP.glyU-tRNA + 16 EF-Tu.GTP.hisR-tRNA + 35 EF-Tu.GTP.ile1-tRNA + 32 EF-Tu.GTP.leu1-tRNA + 2 EF-Tu.GTP.leu2-tRNA + 4 EF-Tu.GTP.leuU-tRNA + 2 EF-Tu.GTP.leuZ-tRNA + 32 EF-Tu.GTP.lys1-tRNA + 11 EF Tu.GTP.met1-tRNA + 24 EF-Tu.GTP.phe1-tRNA + 25 EF-Tu.GTP.pro1-tRNA + 2 EF-Tu.GTP.pro2-tRNA + 1 EF-Tu.GTP.proM-tRNA + 5 EF-Tu.GTP.ser1-tRNA + 5 EF-Tu.GTP.ser2-tRNA + 6 EF-Tu.GTP.ser3-tRNA + 1 EF-Tu.GTP.serT-tRNA + 11 EF-Tu.GTP.serV tRNA + 15 EF-Tu.GTP.thr1-tRNA + 5 EF-Tu.GTP.thr2-tRNA + 6 EF-Tu.GTP.thr3-tRNA + 1 EF-Tu.GTP.thrU-tRNA + 8 EF-Tu.GTP.trpT-tRNA + 22 EF-Tu.GTP.tyr1-tRNA + 20 EF-Tu.GTP.val1-tRNA + 3 EF-Tu.GTP.val2-tRNA + 12 EF-Tu.GTP.val3-tRNA + 2765 mg2 + 1 rib_ini_b0680_1 --> 1 rib_70_elo1_b0680_1_cplx	irreversible	Translation	0.00E+00	1.00E+09	0	8.14E+01	8.14E+01	0	1.39E+02	139.1751	0	6.30E+00	6.304
tl_elo_b0680_1_rib2	Translation elongation 2 b0680 1 ribosome(s)	552 h2o + 1 rib_70_elo1_b0680_1_cplx --> 552 EF-G.GDP + 552 EF-Tu.GDP + 29 ala1_tRNA + 10 ala2_tRNA + 40 arg1_tRNA + 27 asn1_tRNA + 37 asp1_tRNA + 10 cysT_tRNA + 1 fmet_tRNA + 14 gln1_tRNA + 1 gln2_tRNA + 45 glu1_tRNA + 31 gly1_tRNA + 2 glyU_tRNA + 1104 h + 16 hisR_tRNA + 35 ile1_tRNA + 32 leu1_tRNA + 2 leu2_tRNA + 4 leuU_tRNA + 2 leuZ_tRNA + 32 lys1_tRNA + 11 met1_tRNA + 2765 mg2 + 24 phe1_tRNA + 1104 pi + 25 pro1_tRNA + 2 pro2_tRNA + 1 proM_tRNA + 1 rib_70_elo2_b0680_1_cplx + 5 ser1_tRNA + 5 ser2_tRNA + 6 ser3_tRNA + 1 serT_tRNA + 11 serV_tRNA + 15 thr1_tRNA + 5 thr2_tRNA + 6 thr3_tRNA + 1 thrU_tRNA + 8 trpT_tRNA + 22 tyr1_tRNA + 20 val1_tRNA + 3 val2_tRNA + 12 val3_tRNA	irreversible	Translation	0.00E+00	1.00E+09	0	8.14E+01	8.14E+01	0	1.39E+02	139.1751	0	6.30E+00	6.304

tl_elo_b0680_32_rib1	Translation elongation 1 b0680 32 ribosome(s)	17696 EF-G.GTP + 928 EF-Tu.GTP.ala1-tRNA + 320 EF-Tu.GTP.ala2-tRNA + 1280 EF-Tu.GTP.arg1-tRNA + 864 EF-Tu.GTP.asn1-tRNA + 1184 EF-Tu.GTP.asp1-tRNA + 320 EF-Tu.GTP.cysT-tRNA + 448 EF-Tu.GTP.gln1-tRNA + 32 EF-Tu.GTP.gln2-tRNA + 1472 EF-Tu.GTP.glu1-tRNA + 992 EF-Tu.GTP.gly1-tRNA + 64 EF-Tu.GTP.glyU-tRNA + 512 EF-Tu.GTP.hisR-tRNA + 1120 EF-Tu.GTP.ile1-tRNA + 1024 EF-Tu.GTP.leu1-tRNA + 64 EF-Tu.GTP.leu2-tRNA + 128 EF-Tu.GTP.leuU-tRNA + 64 EF-Tu.GTP.leuZ-tRNA + 1024 EF-Tu.GTP.lys1-tRNA + 352 EF-Tu.GTP.met1-tRNA + 768 EF-Tu.GTP.phe1-tRNA + 800 EF-Tu.GTP.pro1-tRNA + 64 EF-Tu.GTP.pro2-tRNA + 32 EF-Tu.GTP.proM-tRNA + 160 EF-Tu.GTP.ser1-tRNA + 160 EF-Tu.GTP.ser2-tRNA + 192 EF-Tu.GTP.ser3-tRNA + 32 EF-Tu.GTP.serT-tRNA + 352 EF-Tu.GTP.serV-tRNA + 480 EF-Tu.GTP.thr1-tRNA + 160 EF-Tu.GTP.thr2-tRNA + 192 EF-Tu.GTP.thr3-tRNA + 32 EF-Tu.GTP.thrU-tRNA + 256 EF-Tu.GTP.trpT-tRNA + 704 EF-Tu.GTP.tyr1-tRNA + 640 EF-Tu.GTP.val1-tRNA + 96 EF-Tu.GTP.val2-tRNA + 384 EF-Tu.GTP.val3-tRNA + 88480 mg2 + 1 rib_ini_b0680_32 --> 1 rib_70_elo1_b0680_32_cplx	irreversible	Translation	0.00E+00	1.00E+09	0	8.14E+01	8.14E+01	0	1.34E+02	133.9288	0	6.30E+00	6.304
tl_elo_b0680_32_rib2	Translation elongation 2 b0680 32 ribosome(s)	17664 h2o + 1 rib_70_elo1_b0680_32_cplx --> 17664 EF-G.GDP + 17664 EF-Tu.GDP + 928 ala1_tRNA + 320 ala2_tRNA + 1280 arg1_tRNA + 864 asn1_tRNA + 1184 asp1_tRNA + 320 cysT_tRNA + 32 fmet_tRNA + 448 gln1_tRNA + 32 gln2_tRNA + 1440 glu1_tRNA + 992 gly1_tRNA + 64 glyU_tRNA + 35328 h + 512 hisR_tRNA + 1120 ile1_tRNA + 1024 leu1_tRNA + 64 leu2_tRNA + 128 leuU_tRNA + 64 leuZ_tRNA + 1024 lys1_tRNA + 352 met1_tRNA + 88480 mg2 + 768 phe1_tRNA + 35328 pi + 800 pro1_tRNA + 64 pro2_tRNA + 32 proM_tRNA + 1 rib_70_elo2_b0680_32_cplx + 160 ser1_tRNA + 160 ser2_tRNA + 192 ser3_tRNA + 32 serT_tRNA + 352 serV_tRNA + 480 thr1_tRNA + 160 thr2_tRNA + 192 thr3_tRNA + 32 thrU_tRNA + 256 trpT_tRNA + 704 tyr1_tRNA + 640 val1_tRNA + 96 val2_tRNA + 384 val3_tRNA	irreversible	Translation	0.00E+00	1.00E+09	0	8.14E+01	8.14E+01	0	1.34E+02	133.9288	0	6.30E+00	6.304
tl_elo_b0850_1_rib1	Translation elongation 1 b0850 1 ribosome(s)	94 EF-G.GTP + 6 EF-Tu.GTP.ala1-tRNA + 2 EF-Tu.GTP.ala2-tRNA + 4 EF-Tu.GTP.arg1-tRNA + 1 EF-Tu.GTP.argU-tRNA + 1 EF-Tu.GTP.argX-tRNA + 3 EF-Tu.GTP.asn1-tRNA + 3 EF-Tu.GTP.asp1-tRNA + 3 EF-Tu.GTP.gln1-tRNA + 1 EF-Tu.GTP.gln2-tRNA + 5 EF-Tu.GTP.glu1-tRNA + 3 EF-Tu.GTP.gly1-tRNA + 1 EF-Tu.GTP.glyT-tRNA + 1 EF-Tu.GTP.hisR-tRNA + 5 EF-Tu.GTP.ile1-tRNA + 1 EF-Tu.GTP.ile2-tRNA + 8 EF-Tu.GTP.leu1-tRNA + 3 EF-Tu.GTP.leu2-tRNA + 5 EF-Tu.GTP.leuU-tRNA + 1 EF-Tu.GTP.leuW-tRNA + 4 EF-Tu.GTP.lys1-tRNA + 5 EF-Tu.GTP.met1-tRNA + 3 EF-Tu.GTP.phe1-tRNA + 1 EF-Tu.GTP.pro1-tRNA + 2 EF-Tu.GTP.pro2-tRNA + 1 EF-Tu.GTP.proL-tRNA + 4 EF-Tu.GTP.proM-tRNA + 1 EF-Tu.GTP.ser1-tRNA + 1 EF-Tu.GTP.ser2-tRNA + 2 EF-Tu.GTP.ser3-tRNA + 2 EF-Tu.GTP.serT-tRNA + 1 EF-Tu.GTP.serV-tRNA + 1 EF-Tu.GTP.tyr1-tRNA + 7 EF-Tu.GTP.val1-tRNA + 2 EF-Tu.GTP.val3-tRNA + 470 mg2 + 1 rib_ini_b0850_1 --> 1 rib_70_elo1_b0850_1_cplx	irreversible	Translation	0.00E+00	1.00E+09	0	6.91E+01	6.91E+01	0	1.09E+02	109.2773	0	1.69E+01	16.907
tl_elo_b0850_1_rib2	Translation elongation 2 b0850 1 ribosome(s)	93 h2o + 1 rib_70_elo1_b0850_1_cplx --> 93 EF-G.GDP + 93 EF-Tu.GDP + 6 ala1_tRNA + 2 ala2_tRNA + 4 arg1_tRNA + 1 argU_tRNA + 1 argX_tRNA + 3 asn1_tRNA + 3 asp1_tRNA + 1 fmet_tRNA + 3 gln1_tRNA + 1 gln2_tRNA + 5 glu1_tRNA + 3 gly1_tRNA + 1 glyT_tRNA + 186 h + 5 ile1_tRNA + 1 ile2_tRNA + 8 leu1_tRNA + 3 leu2_tRNA + 5 leuU_tRNA + 1 leuW_tRNA + 4 lys1_tRNA + 5 met1_tRNA + 470 mg2 + 3 phe1_tRNA + 186 pi + 1 pro1_tRNA + 2 pro2_tRNA + 1 proL_tRNA + 4 proM_tRNA + 1 rib_70_elo2_b0850_1_cplx + 1 ser1_tRNA + 1 ser2_tRNA + 2 ser3_tRNA + 2 serT_tRNA + 1 serV_tRNA + 1 tyr1_tRNA + 7 val1_tRNA + 2 val3_tRNA	irreversible	Translation	0.00E+00	1.00E+09	0	6.91E+01	6.91E+01	0	1.09E+02	109.2773	0	1.69E+01	16.907
tl_elo_b0850_2_rib1	Translation elongation 1 b0850 2 ribosome(s)	188 EF-G.GTP + 12 EF-Tu.GTP.ala1-tRNA + 4 EF-Tu.GTP.ala2-tRNA + 8 EF-Tu.GTP.arg1-tRNA + 2 EF-Tu.GTP.argU-tRNA + 2 EF-Tu.GTP.argX-tRNA + 6 EF-Tu.GTP.asn1-tRNA + 6 EF-Tu.GTP.asp1-tRNA + 6 EF-Tu.GTP.gln1-tRNA + 2 EF-Tu.GTP.gln2-tRNA + 10 EF-Tu.GTP.glu1-tRNA + 6 EF-Tu.GTP.gly1-tRNA + 2 EF-Tu.GTP.glyT-tRNA + 2 EF-Tu.GTP.hisR-tRNA + 10 EF-Tu.GTP.ile1-tRNA + 2 EF-Tu.GTP.ile2-tRNA + 16 EF-Tu.GTP.leu1-tRNA + 6 EF-Tu.GTP.leu2-tRNA + 10 EF-Tu.GTP.leuU-tRNA + 2 EF-Tu.GTP.leuW-tRNA + 8 EF-Tu.GTP.lys1-tRNA + 10 EF-Tu.GTP.met1-tRNA + 6 EF-Tu.GTP.phe1-tRNA + 2 EF-Tu.GTP.pro1-tRNA + 4 EF-Tu.GTP.pro2-tRNA + 2 EF-Tu.GTP.proL-tRNA + 8 EF-Tu.GTP.proM-tRNA + 2 EF-Tu.GTP.ser1-tRNA + 2 EF-Tu.GTP.ser2-tRNA + 4 EF-Tu.GTP.ser3-tRNA + 4 EF-Tu.GTP.serT-tRNA + 2 EF-Tu.GTP.serV-tRNA + 2 EF-Tu.GTP.tyr1-tRNA + 14 EF-Tu.GTP.val1-tRNA + 4 EF-Tu.GTP.val3-tRNA + 940 mg2 + 1 rib_ini_b0850_2 --> 1 rib_70_elo1_b0850_2_cp	irreversible	Translation	0.00E+00	1.00E+09	0	6.91E+01	6.91E+01	0	1.09E+02	109.2773	0	1.69E+01	16.907
tl_elo_b0850_2_rib2	Translation elongation 2 b0850 2 ribosome(s)	186 h2o + 1 rib_70_elo1_b0850_2_cplx --> 186 EF-G.GDP + 186 EF-Tu.GDP + 12 ala1_tRNA + 4 ala2_tRNA + 8 arg1_tRNA + 2 argU_tRNA + 2 argX_tRNA + 6 asn1_tRNA + 6 asp1_tRNA + 2 fmet_tRNA + 6 gln1_tRNA + 2 gln2_tRNA + 10 glu1_tRNA + 6 gly1_tRNA + 2 glyT_tRNA + 372 h + 10 ile1_tRNA + 2 ile2_tRNA + 1[illegible] leu1_tRNA + 6 leu2_tRNA + 10 leuU_tRNA + 2 leuW_tRNA + 8 lys1_tRNA + 10 met1_tRNA + 940 mg2 + 6 phe1_tRNA + 372 pi + 2 pro1_tRNA + 4 pro2_tRNA + 2 proL_tRNA + 8 proM_tRNA + 1 rib_70_elo2_b0850_2_cplx + 2 ser1_tRNA + 2 ser2_tRNA + 4 ser3_tRNA + 4 serT_tRNA + 2 serV_tRNA + 2 tyr1_tRNA + 14 val1_tRNA + 4 val3_tRNA	irreversible	Translation	0.00E+00	1.00E+09	0	6.91E+01	6.91E+01	0	1.09E+02	109.2773	0	1.69E+01	16.907

[truncated: 3,681,478 more chars]
